# Exposure-Dependent Control of Malaria-Induced Inflammation in Children

**DOI:** 10.1371/journal.ppat.1004079

**Published:** 2014-04-17

**Authors:** Silvia Portugal, Jacqueline Moebius, Jeff Skinner, Safiatou Doumbo, Didier Doumtabe, Younoussou Kone, Seydou Dia, Kishore Kanakabandi, Daniel E. Sturdevant, Kimmo Virtaneva, Stephen F. Porcella, Shanping Li, Ogobara K. Doumbo, Kassoum Kayentao, Aissata Ongoiba, Boubacar Traore, Peter D. Crompton

**Affiliations:** 1 Laboratory of Immunogenetics, National Institute of Allergy and Infectious Diseases, National Institutes of Health, Rockville, Maryland, United States of America; 2 Mali International Center of Excellence in Research, University of Sciences, Techniques and Technologies of Bamako, Bamako, Mali; 3 Rocky Mountain Laboratory Research Technologies Section, Genomics Unit, National Institute of Allergy and Infectious Diseases, National Institutes of Health, Hamilton, Montana, United States of America; Case Western Reserve University, United States of America

## Abstract

In malaria-naïve individuals, *Plasmodium falciparum* infection results in high levels of parasite-infected red blood cells (iRBCs) that trigger systemic inflammation and fever. Conversely, individuals in endemic areas who are repeatedly infected are often asymptomatic and have low levels of iRBCs, even young children. We hypothesized that febrile malaria alters the immune system such that *P. falciparum* re-exposure results in reduced production of pro-inflammatory cytokines/chemokines and enhanced anti-parasite effector responses compared to responses induced before malaria. To test this hypothesis we used a systems biology approach to analyze PBMCs sampled from healthy children before the six-month malaria season and the same children seven days after treatment of their first febrile malaria episode of the ensuing season. PBMCs were stimulated with iRBC *in vitro* and various immune parameters were measured. Before the malaria season, children's immune cells responded to iRBCs by producing pro-inflammatory mediators such as IL-1β, IL-6 and IL-8. Following malaria there was a marked shift in the response to iRBCs with the same children's immune cells producing lower levels of pro-inflammatory cytokines and higher levels of anti-inflammatory cytokines (IL-10, TGF-β). In addition, molecules involved in phagocytosis and activation of adaptive immunity were upregulated after malaria as compared to before. This shift was accompanied by an increase in *P. falciparum-*specific CD4^+^Foxp3^−^ T cells that co-produce IL-10, IFN-γ and TNF; however, after the subsequent six-month dry season, a period of markedly reduced malaria transmission, *P. falciparum*–inducible IL-10 production remained partially upregulated only in children with persistent asymptomatic infections. These findings suggest that in the face of *P. falciparum* re-exposure, children acquire exposure-dependent *P. falciparum*–specific immunoregulatory responses that dampen pathogenic inflammation while enhancing anti-parasite effector mechanisms. These data provide mechanistic insight into the observation that *P. falciparum*–infected children in endemic areas are often afebrile and tend to control parasite replication.

## Author Summary

Malaria remains a major cause of disease and death worldwide. When mosquitoes infect people with malaria parasites for the first time, the parasite rapidly multiplies in the blood and the body responds by producing molecules that cause inflammation and fever, and sometimes the infection progresses to life-threatening disease. However, in regions where people are repeatedly infected with malaria parasites, most infections do not cause fever and parasites often do not multiply uncontrollably. For example, in Mali where this study was conducted, children are infected with malaria parasites ≥100 times/year but only get malaria fever ∼2 times/year and often manage to control parasite numbers in the blood. To understand these observations we collected immune cells from the blood of healthy children before the malaria season and 7 days after malaria fever. We simulated malaria infection at these time points by exposing the immune cells to malaria parasites in a test-tube. We found that re-exposing immune cells to parasites after malaria fever results in reduced expression of molecules that cause fever and enhanced expression of molecules involved in parasite killing. These findings help explain how the immune system prevents fever and controls malaria parasite growth in children who are repeatedly infected with malaria parasites.

## Introduction

In previously unexposed individuals, blood-stage *Plasmodium falciparum* parasites rapidly replicate and almost invariably induce fever and other symptoms of malaria [1] through the production of pro-inflammatory cytokines and chemokines [2]–[4]. Although the initial systemic inflammatory response is crucial for setting in motion the innate and adaptive immune effector mechanisms that control blood-stage parasites [5], [6], dysregulated inflammation has been linked to severe malaria [7], [8] which only occurs in a minority of individuals with infrequent or no prior malaria exposure [9]. Conversely, in malaria endemic areas where individuals are repeatedly exposed, *P. falciparum* infections more commonly cause a mild febrile illness or no symptoms at all, and parasite numbers in the blood are generally kept in check, even in young children [10]–[12] who have yet to acquire a fully protective antibody repertoire [13]. The nature of the immune response that enables most children to restrain *P. falciparum*-induced inflammation while maintaining control of parasite replication remains elusive [6], [14].

The notion of malaria ‘tolerance’ has long been invoked to explain the common finding of low-level, asymptomatic blood-stage infection in endemic areas [15], particularly among children, as antibodies that reliably protect against febrile malaria are only acquired after many years of exposure to genetically diverse and clonally variant *P. falciparum* antigens [13]. Several mechanisms have been proposed to explain malaria tolerance or ‘anti-disease’ immunity [14], [16] including antibody-mediated neutralization of *P. falciparum* pathogen-associated molecular pattern (PAMP) molecules such as GPI anchors [14], [17], [18]; desensitization of pattern-recognition receptor (PRR)-mediated signaling as a result of repeated stimulation [16]; and the production of anti-inflammatory mediators such as IL-10 [2], [19]–[21] and TGF-β [21]–[23] that suppress inflammation-driven anti-parasite effector mechanisms once parasite replication has been controlled [14].

Interestingly, it has long been speculated that parallels exist between malarial tolerance and bacterial endotoxin tolerance (reviewed in [16]). This speculation is based in part on early studies in humans that showed that malaria induces cross-tolerance to the febrile response normally induced by bacterial endotoxin [24], [25]. The traditional view of endotoxin tolerance holds that immune cells that are exposed to endotoxin have an altered response when re-challenged with endotoxin, such that the production of pro-inflammatory cytokines and chemokines is attenuated relative to the response induced at homeostasis [26]. Whether *P. falciparum* infection in humans ‘tolerizes’ immune cells in an analogous manner is unknown. More specifically, there is no direct evidence that febrile malaria in humans induces regulatory responses that limit the production of pro-inflammatory cytokines and chemokines upon re-exposure to *P. falciparum* parasites relative to responses induced at homeostasis before malaria in the same individual. Also, it is unclear how current views of malaria-induced tolerance/regulatory responses account for the observation that most children in endemic areas are able to restrain *P. falciparum*-induced inflammation *and* simultaneously control parasite replication, since generalized suppression of *P. falciparum*-triggered immune responses predicts that parasite replication would proceed unhindered and cause severe disease.

A potential solution to this problem is that febrile malaria temporarily alters immune cells such that the host responds to *P. falciparum* re-exposure by downregulating the production of acute phase pro-inflammatory mediators that contribute to fever and other malaria symptoms while enhancing anti-parasite effector mechanisms that control parasite replication, such as phagocytosis-mediated clearance of blood-stage parasites. This hypothesis is supported by a recent study by Foster et al. who used an *in vitro* model of lipopolysaccharide (LPS) tolerance in murine macrophages to show that the regulation of LPS-triggered inflammation is component-specific, such that pro-inflammatory mediators are transiently silenced or ‘tolerized’ while antimicrobial effectors are primed or enhanced upon re-challenge with LPS [27]. A similar phenotype was observed in a human model of LPS tolerance [28], but whether these observations reflect events induced by natural infections in humans is unknown.

Here we tested the hypothesis that febrile malaria alters children's immune cells such that pro-inflammatory mediators are downregulated and anti-parasite effector responses are upregulated upon re-exposure to *P. falciparum* parasites compared to that induced at homeostasis before malaria in the same children. In addition, given the pivotal role of IL-10 in regulating *Plasmodium*-induced inflammation in murine models [19], [29], we sought to elucidate the identity, function and kinetics of *P. falciparum*-specific IL-10-producing cells. We further asked if *P. falciparum*-inducible regulatory responses that limit inflammation are maintained in untreated children who harbor chronic asymptomatic infections, and whether these responses can be recalled in children who have not been exposed to *P. falciparum* for extended periods of time. To address these questions we applied a systems biology approach [30] to a longitudinal analysis of peripheral blood mononuclear cells (PBMCs) sampled from Malian children over a 12-month period—starting at their healthy baseline before the six-month malaria season; seven days after treatment of their first febrile malaria episode of the ensuing malaria season (when malaria symptoms had resolved); and after the subsequent six-month dry season, a period of little to no *P. falciparum* transmission.

We found that febrile malaria induces a marked shift in the response to *P. falciparum* re-exposure with cells producing lower levels of pro-inflammatory cytokines and chemokines and higher levels of anti-inflammatory cytokines compared to responses induced at homeostasis before malaria. Re-exposure was also associated with enhanced expression of pathways involved in phagocytosis and activation of adaptive immunity. This shift was accompanied by a marked increase in *P. falciparum-*specific CD4^+^Foxp3^−^ T cells that co-produce IL-10, IFN-γ and TNF. IL-10 remained partially inducible in untreated children with chronic asymptomatic infections whereas IL-10 was no longer inducible in children whose infections had been cleared by treatment.

## Results

### *P. falciparum*-inducible inflammation is downregulated and anti-parasitic effectors are upregulated after febrile malaria relative to responses induced at the healthy baseline

To obtain a global view of transcriptional changes that persist in children's PBMCs after the clinical resolution of febrile malaria, compared to each child's own healthy baseline, we profiled RNA expression of PBMCs collected from 34 healthy Malian children before the six-month malaria season, when blood smears were negative for *P. falciparum* parasites, and 7 days after treatment of their first febrile malaria episode of the ensuing malaria season, when malaria symptoms had resolved. The average age of these children was 8.5 years and 32% were female. At their first febrile malaria episode of the season, children had an axillary temperature of >37.5°C (or their parents reported fever within 24 hours), were infected with *P. falciparum* (geometric mean density 17,817 asexual parasites/µl of blood) and had no other cause of fever discernible on physical examination. The average incidence of febrile malaria during the six-month malaria season was similar for the 34 children in this study compared to children in this age group in the larger cohort (1.6 and 1.5 episodes, respectively) [31]. By definition, these malaria-susceptible children had yet to acquire *P. falciparum*-specific antibodies that reliably protect from febrile malaria. Individual demographic and clinical data are shown in Table S1. Malaria was effectively treated in all subjects with a standard 3-day course of artemether/lumefantrine.

**Figure 1:**
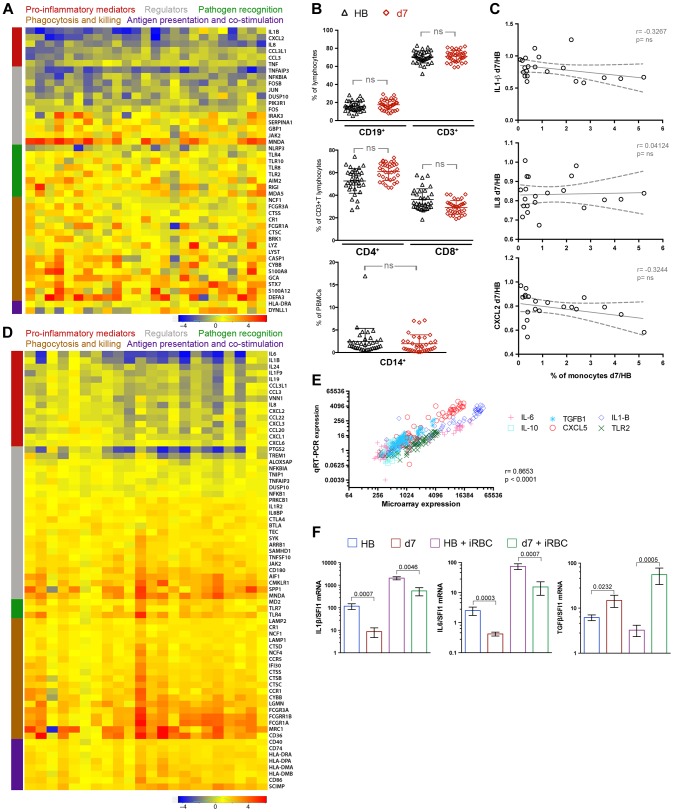
A molecular pattern of restrained inflammation and enhanced anti-parasite effector function upon *P. falciparum* re-exposure. (**A**) PBMCs were collected from 34 healthy children with blood smears negative for *P. falciparum* infection before the malaria season (HB) and 7 days after treatment of their first febrile malaria episode of the ensuing malaria season when malaria symptoms had resolved (d7). RNA was extracted from PBMCs immediately after thawing and hybridized onto Affymetrix GeneChip Human 1.0 ST arrays. RNA from all 68 PBMC samples was of sufficient quantity and quality for microarray analysis. Nine of 68 samples did not pass the microarray quality assessment and were removed from further analysis (see Supplemental Figure 1A) such that 25 children with paired RNA samples at the healthy baseline and 7 days after malaria were analyzed. The heat map shows *ex vivo* RMA-normalized log_2_ ratios (d7/HB) of differentially expressed genes (rows) for each child (columns). Genes are grouped and color-coded by function as indicated. (**B**) PBMCs analyzed by FACS for B cells (CD19^+^), T cells (CD3^+^), CD3^+^CD4^+^ T cells, CD3^+^CD8^+^ T cells, and monocytes (CD14^+^) at the healthy baseline and after malaria. (n = 34 children; except CD14^+^ monocytes, n = 30). (**C**) Ratio of monocyte percentage (d7/HB) versus the ratio of the expression level of monocyte-derived pro-inflammatory cytokines and chemokines (d7/HB). Each point represents an individual subject (n = 21 children with paired samples). (**D**) RNA was extracted from PBMCs of the same 34 children after 18 h of *in vitro* stimulation with *P. falciparum*-infected red blood cell (iRBC) lysate. After stimulation with iRBC lysate, 22 of the 34 children had RNA samples from both time points of sufficient quantity and quality for microarray analysis and also passed the microarray quality assessment. The heat map shows RMA-normalized log_2_ ratios (d7/HB) of differentially expressed genes (rows) for each child (columns) in response to *in vitro* iRBC lysate stimulation. Genes are grouped and color-coded by function as indicated. (**E**) q-RT-PCR confirmation of the microarray data. The data represent the results of one experiment with 6 genes (*IL1B*, *IL6*, *IL10*, *TGFB1*, *TLR2*, *CXCL5*) from 17 subjects at two time points (d7 and HB) from both the *ex vivo* unstimulated and *in vitro* iRBC-stimulated datasets. Each symbol represents a single gene at a given time point. PCR expression computed as antilog_2_ –dCT. *n* = 497 XY pairs. (**F**) q-RT-PCR expression of genes encoding the pro-inflammatory cytokines IL1-β and IL-6 and the anti-inflammatory cytokine TGF-β in PBMCs of children (n = 17) collected at the healthy baseline (HB) and after resolution of febrile malaria (d7), either directly *ex vivo* (unstimulated) or after *in vitro* stimulation with iRBCs for 18 h. ns, not significant (*P*≥0.05), *P* values determined by the paired *t*test (B), Pearson's (C), Spearman's (E) or paired Wilcoxon rank sum test (F). Data are shown as the means ± s.d. (B) or means ± s.e.m. (F).

PBMCs were first analyzed directly ex vivo (not re-stimulated). Principal components analysis of the microarray data showed segregation of transcription profiles based on time-point (healthy baseline vs. day 7 after malaria), but not age, gender or batch effects (Figure S1A). Within-subject gene-expression changes were computed and resulted in 1497 differentially expressed genes (DEGs)—1,351 increased and 146 decreased after the resolution of febrile malaria relative to baseline (Table S2). Ingenuity Pathway Analysis (IPA) identified “Infectious Disease”, “Immunological Disease” and “Inflammatory Disease” among the top five functional categories enriched with DEGs. Notably, all differentially expressed genes encoding cytokines and chemokines that have been associated with *P. falciparum*-induced fever and inflammation [2]–[4], [32], including the canonical pyrogenic cytokines *IL1B* and *TNF* as well as the pro-inflammatory chemokines *IL8*, *CCL3* (*MIP-1α*) and *CXCL2* (*MIP-2α*), were suppressed after the resolution of malaria to lower levels than observed at baseline (Figure 1A). Conversely, genes expressing molecules directly involved in microbial killing and activation of adaptive immunity were significantly upregulated after the resolution of febrile malaria relative to the healthy baseline (Figure 1A). Specifically, IPA identified the following canonical pathways as significantly upregulated: “Toll-like receptor signaling” (*P* = 2.15e-6), “Fcγ receptor-mediated phagocytosis in macrophages and monocytes” (*P* = 1.8e-4), “Production of nitric oxide and reactive species in macrophages” (*P* = 1.09e-7), “Antigen presentation pathway” (*P* = 0.0463), “T cell receptor signaling” (*P* = 1.19e-5) and “Interferon signaling” (*P* = 5.08e-6) (Figure S1B). The expression of several PRRs including Toll-like receptor (*TLR*)*2* and *TLR4* was increased after malaria relative to baseline (Figure 1A), consistent with recent *in vivo* exposure to *P. falciparum* PAMPs such as GPI anchors [33], hemozoin [34], CpG-containing DNA motifs bound to hemozoin [35] and AT-rich DNA motifs [36], but of note, *NLRP3*, a putative receptor for *P. falciparum* hemozoin-induced IL-1β production [37], was the only PRR to be downregulated after malaria relative to homeostasis (Figure 1A).

Because changes in mRNA levels in PBMCs can reflect an altered cell composition of the PBMC compartment or changes in gene expression in discrete cell populations, we analyzed the PBMCs used for gene-expression profiling by FACS and found no significant differences in the percentage of CD4^+^ or CD8^+^ T cells, B cells or monocytes after the resolution of febrile malaria relative to the healthy baseline (Figure 1B). Moreover, at the individual subject level we found that genes encoding myeloid-expressed pro-inflammatory mediators were downregulated after resolution of malaria relative to baseline irrespective of changes in the percentage of monocytes (Figure 1C and Figure S1C), and even as mRNA levels of other genes identified as myeloid-specific [38] were unchanged or increased after malaria relative to baseline (Figure S1D). Taken together, these data indicate that the changes in mRNA levels that persist after the resolution of febrile malaria relative to the healthy baseline reflect differential regulation of gene expression rather than gross alterations in the composition of the PBMC compartment.

These data indicate that febrile malaria induces transcriptional changes in PBMCs that persist after the resolution of febrile malaria; namely, we observed that the expression of genes encoding pro-inflammatory mediators is suppressed while the expression of molecules involved in microbial killing and activation of adaptive immunity is enhanced after malaria relative to baseline. On the basis of these findings we hypothesized that re-exposure to *P. falciparum* parasites soon after the resolution of febrile malaria would induce a qualitatively different immune response relative to that induced at the healthy baseline. To test this hypothesis and to investigate the molecular and cellular basis of regulatory responses induced upon *P. falciparum* re-exposure, we analyzed the same PBMCs from the same 34 children (collected at the healthy baseline before the malaria season and seven days after treatment of the first febrile malaria episode) following *in vitro* stimulation with *P. falciparum*-infected red-blood cell (iRBC) lysate. iRBC-inducible gene expression as well as secreted and intracellular cytokine production were examined with each child serving as his or her own healthy baseline control.

Within-subject gene expression changes induced by iRBC stimulation at both time points were computed and resulted in 456 DEGs—148 decreased and 308 increased after the resolution of febrile malaria relative to that induced at the healthy baseline (Table S3). IPA identified “Inflammatory Response” as the functional category with the highest enrichment score (*P* = 3.36e-34). Within this functional category, all differentially expressed pro-inflammatory cytokines and chemokines (*IL1B*, *IL6*, *IL8*, *IL19*, *IL24*, *CCL3*, *CCL3L1*, *CCL19*, *CCL20*, *CCL22*, *CXCL1*, *CXCL2*, *CXCL3*, and *CXCL6*) were downregulated in response to iRBC stimulation after the resolution of malaria relative to the iRBC-induced response at baseline (Figure 1D). In line with this result, IPA identified the following canonical pathways as significantly downregulated: “Acute phase response signaling” (P = 5.94e-4), “Role of hypercytokinemia/hyperchemokinemia in pathogenesis of Influenza (P = 1.19e-3), “IL-6 signaling” (P = 1.18e-3), “Agranulocyte adhesion and diapedesis” (P = 8.82e-6), “Granulocyte adhesion and diapedesis” (P = 6.55e-9) and “Role of cytokines in mediating communication between immune cells” (P = 0.017) (Figure S1E). Consistent with the downregulation of pro-inflammatory responses, *NFKB1* and *TREM-1*, a positive regulator of inflammation [39], were downregulated, while several negative regulators of inflammation were upregulated including *IL18BP*, *IL1R2*, *BTLA* and *SAMHD1* (Figure 1D).

In contrast to the downregulation of pro-inflammatory cytokine and chemokine responses, genes encoding molecules involved in microbial killing and activation of adaptive immunity were upregulated by iRBC stimulation after malaria relative to responses induced by iRBC stimulation at baseline. These included molecules involved in opsonic and non-opsonic phagocytosis, phagolysosome maturation, antigen presentation and co-stimulation (Figure 1D). IPA identified the following canonical pathways as significantly upregulated: “iNOS signaling” (*P* = 0.0012), “Antigen presentation pathway” (P = 6.87e-5), “T helper cell differentiation” (P = 1.88e-3) and “T cell receptor signaling” (*P* = 0.035) (Figure S1E). Together these data suggest that re-exposure to *P. falciparum* parasites after a recent episode of febrile malaria induces the differential expression of functionally distinct components of the immune response, whereby acute phase pro-inflammatory cytokines and chemokines that drive the initial systemic inflammatory response are restrained, while pathways involved in microbial killing and activation of adaptive immunity are upregulated.

To validate the expression of selected immune-related genes, we used quantitative real time (qRT)-PCR to analyze PBMCs from the 17 children who had microarray data from the *ex vivo* and iRBC-stimulated experiments at both time points (before and after malaria). We found a positive correlation (*r* = 0.8653; *P*<0.0001) for gene expression as detected by microarray and qRT-PCR (Figure 1E and Table S4). The qRT-PCR data confirmed decreased expression of the canonical fever-inducing cytokines *IL1B* and *IL6* and increased expression of the anti-inflammatory cytokine *TGFB* after resolution of febrile malaria relative to the healthy pre-malaria baseline, in both the unstimulated and iRBC-stimulated experiments (Figure 1F). Therefore, the qRT-PCR data confirmed a molecular pattern of restrained *P. falciparum*-inducible inflammation in children who had recently recovered from febrile malaria.

### *P. falciparum*-inducible IL-10 is upregulated after the resolution of febrile malaria and partially maintained in children with persistent asymptomatic infection

**Figure 2:**
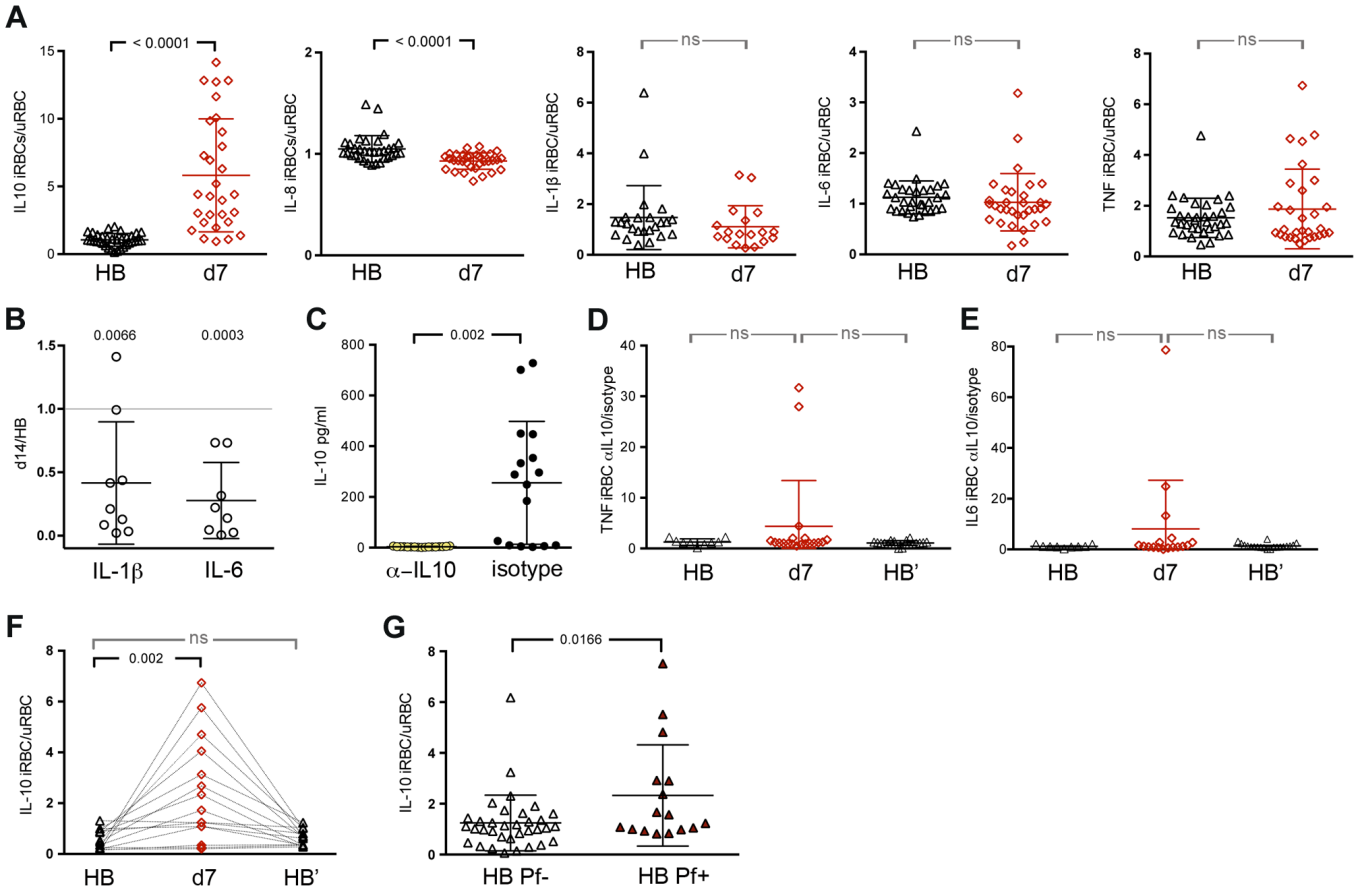
*P. falciparum*-inducible IL-10 production is upregulated upon re-exposure and partially maintained by persistent asymptomatic infection. (**A**) Production of IL-10, IL-8, IL1-β, IL-6 and TNF by PBMCs in response to *in vitro* stimulation with iRBC lysate at the healthy baseline before the malaria season (HB) and 7 days after malaria (d7) (n = 28 children with paired samples). (**B**) Production of IL-1β and IL-6 by isolated monocytes/macrophages after 6 h of *in vitro* stimulation with iRBC lysate. Results are shown as the ratio of cytokines produced 14 days after malaria (d14) versus the healthy baseline before the malaria season (HB) (n = 9 children with paired samples) (*P* = 0.0066 and P = 0.0003 for IL-1β and IL-6 respectively). (**C**) A positive control showing IL-10 production by PBMCs in response to *in vitro* stimulation with iRBC lysate in the presence of blocking antibodies specific for IL-10 and the IL-10 receptor or the isotype control (n = 17). (**D**,**E**) TNF and IL-6 production by PBMCs in response to *in vitro* stimulation with iRBC lysate in the presence of blocking antibodies specific for IL-10 and the IL-10 receptor at the healthy baseline (HB), 7 days after malaria (d7) and at the healthy baseline after the subsequent 6-month dry season (HB′) (n = 20 children, 9 paired in the 3 conditions). (**F**) IL-10 production by PBMCs in response to *in vitro* stimulation with iRBC lysate at the healthy baseline before the malaria season (HB), 7 days after malaria (d7) and at the healthy baseline after the subsequent 6-month dry season (HB′), a period of little to no P. *falciparum* transmission (n = 15 children). (**G**) IL-10 production by PBMCs in response to *in vitro* stimulation with iRBC lysate among children with asymptomatic *P. falciparum* infection at the end of the dry season (HB *Pf*+, n = 16) versus aged-matched, healthy uninfected children at the same time point (HB *Pf*−, n = 34). Data are presented as fold change relative to PBMCs stimulated with uninfected RBC (uRBC) lysate (A, F, G). ns, not significant (*P*≥0.05), *P* values were determined by a paired *t*test (A, C), one-sample Student's T-tests comparing the mean ratio against a 1∶1 ratio (B), paired *t*test followed by Bonferroni's test (D–F) or unpaired *t*test (G). Data are shown as the means ± s.d.

IL-10 plays a critical role in controlling and resolving inflammation by limiting the production of pro-inflammatory cytokines and chemokines [40], yet we did not observe differential expression of *IL10* in the microarray data. Given the known temporal dissociation between *IL-10* transcription and translation [41], we assayed supernatants of iRBC-stimulated PBMCs for secreted IL-10 using a multiplex assay that also measured IL-1β, IL-6, IL-8 and TNF. We observed that *P. falciparum*-inducible IL-10 production was higher after the resolution of febrile malaria relative to the healthy baseline of the same children before the malaria season (*P*<0.0001; Figure 2A), while *P. falciparum*-inducible production of the pro-inflammatory chemokine IL-8 was lower after the resolution of febrile malaria relative to baseline (*P*<0.0001; Figure 2A), in agreement with the microarray data. *P. falciparum*-inducible production of IL-1β and IL-6 trended toward lower levels after the resolution of febrile malaria relative to baseline, but the decrease was not statistically significant (Figure 2A). Because IL-1β and IL-6 are primarily produced by monocytes/macrophages, we isolated monocytes/macrophages (Figure S2A) from 9 additional children who had PBMCs available at their healthy baseline before the malaria season and 14 days after their first febrile malaria episode of the ensuing malaria season (Table S1). We stimulated these monocytes/macrophages with iRBCs for 6 hours and measured IL-1β and IL-6 in the supernatants. We found that *P. falciparum*-inducible production of IL-1β and IL-6 by monocytes/macrophages was lower after the resolution of febrile malaria relative to that induced at baseline (*P* = 0.0066 and P = 0.0003 for IL-1β and IL-6 respectively; Figure 2B), consistent with a reduced risk of fever in children who are exposed to ongoing *P. falciparum* transmission during the malaria season.

In an independent experiment, we sought to determine if the upregulation of *P. falciparum*-inducible IL-10 production after malaria influences the production of pro-inflammatory cytokines. We observed that blocking IL-10 activity with antibodies specific for IL-10 and the IL-10 receptor (Figure 2C) enhanced iRBC-inducible TNF and IL-6 production in some but not all children after the resolution of febrile malaria compared to baseline (Figures 2D and E).

We next asked if *P. falciparum*-inducible IL-10 responses could be recalled in children who had not been exposed to *P. falciparum* transmission for an extended period of time. We performed iRBC stimulation of PBMCs collected from 18 additional children (Table S1) at their healthy baseline before the malaria season, 7 days after treatment of their first malaria episode of the ensuing 6-month malaria season, and after the following 6-month dry season, a period of little to no *P. falciparum* transmission. This independent experiment confirmed that *P. falciparum*-inducible IL-10 is upregulated after the resolution of febrile malaria relative to baseline (*P* = 0.0082; Figure 2F). However, in the absence of ongoing malaria exposure, children reverted to an apparent homeostatic baseline in which IL-10 production was no longer inducible (Figure 2F), suggesting that ongoing malaria exposure is required to maintain P. *falciparum*-inducible IL-10 production capacity. To test this hypothesis we identified 16 untreated children (Table S1) whose asymptomatic *P. falciparum* infections persisted through the six-month dry season, and compared their *P. falciparum*-inducible IL-10 response to age-matched children who were uninfected at the same time point at the end of the dry season. We observed that *P. falciparum*-inducible IL-10 responses of persistently infected asymptomatic children were higher than responses of age-matched uninfected children (*P* = 0.0166; Figure 2G), suggesting that *P. falciparum*-inducible IL-10 upregulation is partially maintained by ongoing *P. falciparum* exposure and that IL-10 upregulation may contribute to protection from febrile malaria in the context of ongoing *P. falciparum* exposure.

### *P. falciparum*-inducible IL-10 is produced by CD4^+^CD25^+^Foxp3^−^ T cells that co-produce IFN-γ and TNF

**Figure 3:**
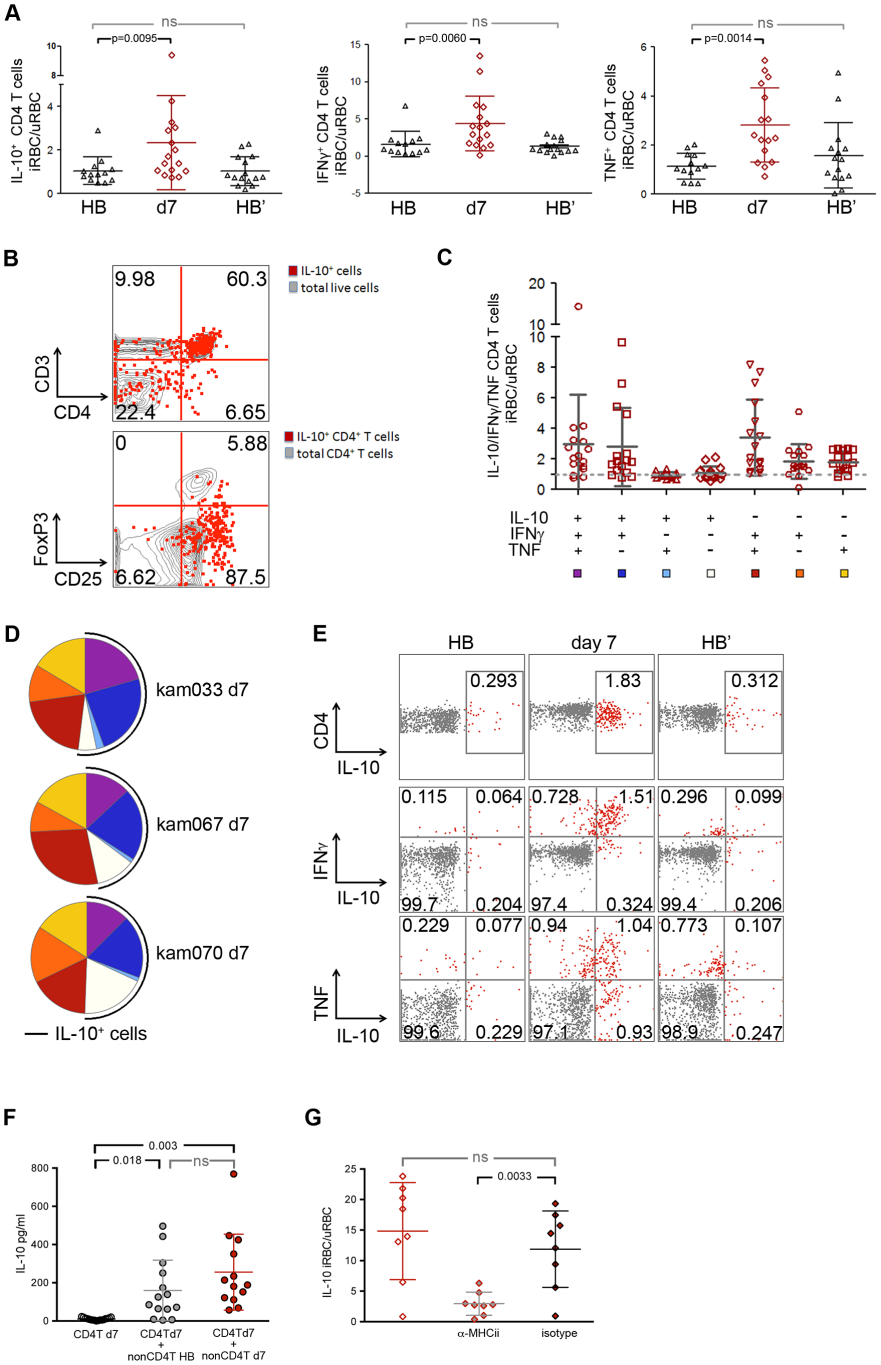
*P. falciparum*-inducible IL-10 is mainly produced by CD4^+^CD25^+^Foxp3^−^ T cells that co-produce IFNγ and TNF. (**A**) PBMCs from the healthy baseline (HB), 7 days after malaria (d7), and at the healthy baseline at the end of the subsequent dry-season (HB′) were stimulated for 18 h with iRBC lysate and assayed for the production of IL-10, IFNγ and TNF by intra-cellular FACS. Results are shown as the ratio of live CD3^+^ CD4^+^ antigen-experienced cells (CD45RO^+^ CD27^+^, CD45RO^+^ CD27^−^, and CD45RO^−^ CD27^−^) producing IL-10, IFN-γ or TNF in response to stimulation with iRBC lysate vs. uninfected RBC (uRBC) lysate (n = 16, 13 paired samples). (**B**) Overlay of IL-10-producing cells (red) among all live cells (gray) in a CD3 vs. CD4 dot plot (top) (n = 14), and IL-10-producing CD4^+^ T cells (red) with all CD4^+^ T cells (gray) in CD25 vs. FoxP3 dot plot (bottom) (n = 9; representative subject shown). (**C**) Using SPICE analysis, cytokine-producing CD4^+^ T cells were divided into 7 distinct subpopulations producing any combination of IL-10, IFNγ and TNF (n = 16). (**D**) Pie chart representation of the combination of cytokines produced by CD4^+^ T cells after iRBC stimulation for 3 representative donors 7 days after malaria (d7). The black arcs indicate the IL-10-producing CD4^+^ T cells. (**E**) Representative FACS plots of live CD3^+^ CD4^+^ antigen-experienced cells producing IL-10, IFNγ and TNF after iRBC stimulation of PBMCs collected at the healthy baseline (HB), 7 days after malaria (d7) and at the healthy baseline at the end of the subsequent dry-season (HB′). (**F**) CD4^+^ T cells were isolated from PBMCs which had been collected from children 7 days after malaria and were then stimulated for 18 h with iRBC or uRBC lysate in the absence (CD4^+^T d7) or presence of non-CD4^+^T cells isolated from PBMCs of the same individuals collected at either the healthy baseline (CD4^+^T d7 + nonCD4^+^T HB) or 7 days after malaria (CD4^+^T d7 + nonCD4^+^T d7) (n = 8 paired samples). (**G**) PBMCs collected from children 7 days after malaria were stimulated for 18 h with iRBC lysate and assayed for the production of IL-10 in the presence (αMHC-II) or absence (isotype) of antibodies specific for HLA-DR, -DQ and -DP (n = 8). ns, not significant (*P*≥0.05), *P* values determined by a linear mixed model for repeated measures ANOVA with Tukey HSD post hoc tests (A) and permutation re-sampling tests (F, G). Data are shown as the means ± s.d.

Despite evidence that IL-10 plays a critical role in regulating *Plasmodium*-induced inflammation in murine models, the cellular sources of IL-10 and the functionality and kinetics of IL-10-producing cells in the context of human malaria remain unclear [42]. To identify the predominant cellular source of *P. falciparum*-inducible IL-10 and to investigate longitudinally the functionality and kinetics of IL-10-producing cells in children exposed to intense seasonal malaria, we analyzed PBMCs by FACS with intracellular staining for IL-10, IFN-γ and TNF after *in vitro* iRBC stimulation at the healthy baseline before the malaria season, 7 days after malaria treatment (when symptoms had resolved), and after the 6-month dry season. Consistent with the kinetics of *P. falciparum*-inducible secreted IL-10 production described above (Figure 2F), we found that *P. falciparum*-inducible IL-10 production by CD4^+^ T cells increased significantly after the resolution of febrile malaria relative to the healthy baseline (*P* = 0.0095; Figure 3A), and then reverted to a state in which IL-10 production was no longer inducible by the end of the following six-month dry season (Figure 3A). Interestingly, the majority of *P. falciparum*-inducible IL-10-producing PBMCs following febrile malaria were CD3^+^CD4^+^ T cells (Figure 3B; mean 53.1%, 95%CI: 44.8–61.5) and most of these were CD25^+^FOXP3^−^ (Figure 3B; mean 78.8%, 95%CI: 72.2–85.5), while FOXP3^+^CD4^+^ T cells (regulatory T cells) represented only a small percentage of IL-10-producing T cells. *P. falciparum*-inducible IFN-γ- and TNF-producing CD4^+^ T cells also increased after the resolution of malaria compared to baseline, and like IL-10, reverted to homeostasis after the dry season (Figure 3A). At the single-cell level the majority of IL-10-producing *P. falciparum*-inducible CD4^+^ T cells also produced IFN-γ, or IFN-γ plus TNF (Figure 3C–E), thus identifying these cells as ‘self-regulating’ Th1 effector cells [43]. From these results and the microarray data emerges a consistent theme whereby re-exposure to *P. falciparum* parasites after recent febrile malaria induces exposure-dependent regulatory mechanisms that limit the production of pro-inflammatory mediators that drive systemic inflammation while enhancing effector mechanisms that control parasite replication.

Because whole microbe stimulation with *P. falciparum* iRBCs involves a complex mixture of antigens and stimuli for innate receptors, we asked whether *P. falciparum*-inducible IL-10 production by CD4^+^ T cells requires antigen presenting cells (APCs) and T cell receptor engagement. We magnetically isolated CD4^+^ T cells that had been collected after the resolution of febrile malaria and found that they failed to produce IL-10 in response to iRBC stimulation in the absence of antigen-presenting cells (Figure 3F). Moreover, iRBC-induced IL-10 production by CD4^+^ T cells was abrogated in PBMC cultures in the presence of antibodies that block major histocompatibility complex (MHC) class II molecules (Figure 3G). Together these data demonstrate that *P. falciparum*-inducible IL-10 production by CD4^+^ T cells is T cell receptor-dependent.

Having established that iRBC-inducible IL-10 production by CD4^+^ T cells requires APCs and T cell receptor engagement, we sought to understand the role that *in vivo* conditioning of APCs plays in modulating IL-10 production by *P. falciparum*-specific CD4^+^ T cells. We magnetically isolated CD4^+^ T cells collected after the resolution of febrile malaria and cultured these cells with autologous APCs collected at the healthy baseline before the malaria season or after the resolution of febrile malaria. Under both conditions iRBC-inducible IL-10 production by CD4^+^ T cells was restored to similar levels (Figures 3F), suggesting that the *in vivo* conditions during acute febrile malaria shape the functional response of CD4^+^ T cells in a manner that is independent of the *in vivo* conditioning of APCs.

## Discussion

In our previous investigations at this study site we observed that the risk of febrile malaria slowly decreases over years as individuals are exposed to intense seasonal *P. falciparum* transmission such that adults rarely experience febrile malaria when infected with blood-stage parasites [44]. The gradual acquisition of blood-stage immunity that reliably protects from the onset of febrile malaria likely reflects the need for repeated infections over years to achieve levels of broadly reactive antibodies that exceed a protective threshold [13], [45]. However, even malaria-susceptible children at this study site (who by definition have yet to acquire reliably protective antibodies) experience only 1 to 2 febrile malaria episodes per six-month malaria season despite ≥100 infective mosquito bites per person each season, and generally these children manage to keep parasite numbers in the blood in check [44]. These observations prompted us to investigate immune mechanisms beyond antibody responses that might contribute to protection from febrile malaria and parasite replication in children who are exposed to repeated *P. falciparum* infections, and also to investigate how children become susceptible again to febrile malaria after a period of decreased *P. falciparum* exposure.

**Figure 4:**
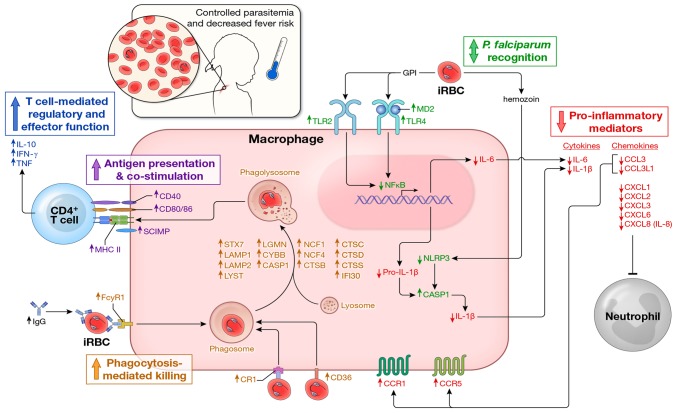
Proposed model by which children remain asymptomatic and control parasitemia upon *P. falciparum* re-exposure. In children without prior or recent malaria exposure, *P. falciparum* infection induces a robust pro-inflammatory cytokine and chemokine response (e.g. IL-1β, IL-6, IL-8) whereas effector mechanisms that mediate parasite clearance (phagocytosis, phagolysosome activation, antigen presentation, T cell co-stimulation and IFN-**γ** production by CD4^+^ T cells) are not readily inducible, leaving children susceptible to fever and other systemic symptoms of malaria as well as poorly controlled parasite replication. In contrast, febrile malaria induces an exposure-dependent regulatory state (shown here) whereby re-exposure to *P. falciparum* results in reduced production of pro-inflammatory cytokines and chemokines and enhanced expression of regulatory cytokines (e.g. IL-10 production by CD4^+^ T cells) and pathways involved in phagocytosis-mediated clearance of infected red blood cells and activation of adaptive immunity, thus enabling children to remain asymptomatic and control parasite replication in the face of ongoing *P. falciparum* exposure. In addition, *P. falciparum*-specific IgG levels are low in children who have not been recently exposed to malaria, but transiently increase in response to *P. falciparum* infection [44], [45], further enhancing exposure-dependent parasite clearance through opsonization and phagocytosis of infected erythrocytes. Arrows indicate the direction of expression observed in this study of molecules at the mRNA and/or protein levels induced by *P. falciparum* re-exposure after febrile malaria relative to responses induced by *P. falciparum* exposure at the healthy baseline. Molecules are color-coded by biological function.

We found that acute febrile malaria alters children's PBMCs such that *P. falciparum* re-exposure results in downregulation of acute phase pro-inflammatory cytokines that drive fever and systemic inflammation (e.g. IL-1β and IL-6 from monocytes/macrophages), and upregulation of immune mechanisms involved in control of inflammation (e.g. IL-10-producing CD4^+^ T cells) and parasite clearance (e.g. IFN-γ-producing CD4^+^ T cells, phagocytosis and phagolysosome maturation) (Figure 4). The maintenance of this regulatory state appears to depend on recent or ongoing *P. falciparum* exposure as children revert to a homeostatic baseline in the absence of ongoing *P. falciparum* exposure during the six-month dry season. The short-lived, exposure-dependent nature of this response mirrors the kinetics of *P. falciparum*-specific antibody responses in children [44], [45], suggesting that these responses work in concert to protect children as long as *P. falciparum* exposure is ongoing. These data offer mechanistic insights into how children who are repeatedly infected with *P. falciparum* commonly manage to remain afebrile and control parasite replication, and how they become susceptible again to febrile malaria after a period of reduced *P. falciparum* exposure. The possibility that treatment with artemether/lumefantrine contributed to these findings cannot be excluded.

These data shed light on the long-standing and enigmatic clinical notion of ‘premunition’—a partially effective, exposure-dependent immune response that protects against illness and high numbers of parasites in the blood without completely eliminating the infection [12], [46], [47]. Although premunition is often viewed as a state of immune dysregulation or suppression [12], [46], [47], we speculate that it evolved as an appropriate immune response in the face of unrelenting exposure to genetically and antigenically diverse parasites such that young children are at least partially protected from potentially life-threatening inflammation and unchecked parasite replication before they acquire durable, broadly reactive antibodies that reliably protect against the onset of malaria symptoms. Although we did not study severe malaria per se—an overlapping set of syndromes [48] which have been linked to excessive inflammation [49]—it is conceivable that the ability to rapidly downregulate *P. falciparum*-inducible inflammation in early life contributes to the rapid acquisition of strain-transcendent immunity to severe malaria which may occur after only one or two symptomatic infections [50], and conversely, that the small percentage of children who develop severe malaria are those whose genetic background, environment (e.g. co-infection history, microbiota, nutritional status) or specific interaction with parasite virulence factors [51]–[54] tips them toward dysregulated pathologic inflammatory responses.

The prospective design of this study, in which each subject served as their own healthy control, provides a rare view of the regulation and functional plasticity of innate and adaptive immune cells in response to a natural infection in humans. In general, innate immune cells such as monocytes/macrophages first detect pathogens through PRRs such as TLRs and NOD-like receptors (NLRs) which recognize highly conserved PAMPs [55]. Through these initial host-pathogen interactions, innate immune cells provide the first line of defense against pathogen invasion and also direct the quality of antigen-specific B and T cell responses. To date, only a handful of *P. falciparum* PAMPs and their respective PRRs have been identified. These include GPI anchors (TLR2>TLR4) [33], hemozoin (NLRP3) [34], CpG-containing DNA motifs bound to hemozoin (TLR9) [35] and AT-rich DNA motifs (unknown cytosolic receptor) [36]. Studies *in vitro* and in animal models show that these PAMPs drive monocytes/macrophages to produce pro-inflammatory cytokines and chemokines such as IL-1β, IL-6, IL-8 and TNF [33]–[35]. These observations are consistent with studies in humans that show these cytokines and chemokines rise and fall in the serum of individuals treated for febrile malaria [2]–[4]. However, prior to this study, the nature of the inflammatory response induced by *P. falciparum* re-exposure relative to that induced at the healthy baseline of the same individuals was unknown. Here we show that the capacity of monocytes/macrophages to produce the canonical pyrogenic cytokines IL-1β and IL-6 is reduced upon re-exposure to *P. falciparum* parasites relative to that induced at the healthy baseline of the same individuals—a finding we observed at the mRNA level by microarray and qRT-PCR in PBMCs, and at the protein level in isolated monocytes/macrophages.

Although the precise molecular mechanisms that regulate *P. falciparum*-inducible inflammation remain to be fully elucidated, this study offers insight into the multiple levels at which this regulation might occur. For example, we observed that *NFKB1* expression was downregulated after malaria relative to baseline. *NFKB1* encodes the p50 component of the canonical p65/p50 NF-κB heterodimeric transcription factor that stimulates the expression of pro-inflammatory cytokines such as IL-1β and IL-6 [56]. We also observed decreased expression of *NLRP3* after malaria relative to baseline. *NLRP3* encodes a component of the NALP3 inflammasome which is expressed in myeloid cells and activates caspase-1, thereby promoting the maturation and secretion of IL-1β [57]. Other differentially expressed PRRs such as *TLR2* and *TRL4* were upregulated after malaria relative to baseline, suggesting that the regulation of *P. falciparum*-inducible inflammation does not occur at the level of TLR expression. Interestingly, TLR expression is also upregulated in the context of tolerance induced by gram positive bacteria [58], whereas tolerance induced by gram negative bacteria is associated with reduced expression of TLR2 and TLR4 [59], underscoring the microbe-specific nature of immune-regulation.

After the resolution of febrile malaria we also observed increased expression of genes encoding proteins that limit the inflammatory response including *IL18BP*, *IL1R2*, *CTLA4*, *BTLA*, *SAMHD1 and TNFSF10*, as well as decreased expression of genes encoding proteins that promote inflammation including *PTGS2* and *TREM1*. Further studies are needed to more clearly elucidate the signaling networks involved in regulating the immune response to *P. falciparum* infection and to fully understand the relationships between perturbations to these networks and the variability in malaria clinical outcomes.

The gene expression microarray data also shed light on the regulation of chemotactic responses [60] in malaria. Relative to the response induced at the healthy baseline, re-exposure to *P. falciparum* parasites was associated with downregulated expression of chemokines that recruit macrophages (*CCL3*, *CCL3L1*; Figures 1A, 1D and 4) and neutrophils (*CXCL1*, *CXCL2*, *CXCL3*, *CXCL6*; Figures 1A, 1D and 4), but upregulated expression of monocyte/macrophage-specific chemokine receptors (*CCR1*, *CCR5*; Figure 4). We postulate that this pattern reflects the fine-tuning of chemotactic responses in the face of ongoing or repeated *P. falciparum* exposure, whereby systemic chemokine release is restrained to decrease the potential for tissue damage caused by aberrant trafficking and accumulation of effector cells such as neutrophils, whereas the reciprocal regulation of monocyte/macrophage-specific chemokines (repressed) and chemokine receptors (increased) enhances the sensitivity of monocyte/macrophages to detect decreased concentrations of chemokines.

Murine models clearly demonstrate that IL-10 and TGF-β play critical roles in regulating *Plasmodium*-induced inflammation [19], [22]. In humans, IL-10 and TGF-β levels increase in serum during acute febrile malaria and then fall after treatment [2], [20], consistent with a role for these cytokines in restraining and resolving *P. falciparum*-induced inflammation during a single malaria episode. However, whether febrile malaria conditions the immune system to modify the production of IL-10 and TGF-β upon subsequent exposure to *P. falciparum* within the same individual remained an open question. Here we show that *P. falciparum*-inducible IL-10 and TGF-β production/expression is upregulated after the resolution of febrile malaria relative to that which is inducible at the healthy baseline of the same individuals. In addition, we observed that IL-10 blockade *in vitro* enhanced IL-6 and TNF production in some but not all children, consistent with a role for IL-10 in controlling inflammation in the setting of *P. falciparum* re-exposure, but also highlighting the complexity of regulatory responses that restrain *P. falciparum*-induced inflammation.

Given the role of IL-10 in regulating *Plasmodium-*induced inflammation, we sought to illuminate the identity, function and kinetics of *P. falciparum*-specific IL-10-producing cells. Previous studies in humans have shown that total FOXP3^+^ T regulatory cells (Tregs) increase in response to experimental [23] and natural [61] *P. falciparum* infection (reviewed in [42]), which suggested that Treg-generated anti-inflammatory cytokines play an important role in controlling *P. falciparum*-inducible inflammation. However, subsequent cross-sectional studies failed to conclusively show significant differences in Treg responses between individuals with mild and severe malaria [62], [63]. Here we show that CD4^+^CD25^+^Foxp3^−^ T cells are the predominant source of *P. falciparum*-inducible IL-10, whereas Tregs contributed minimally to the overall IL-10 response. We demonstrate that IL-10 producing CD4^+^CD25^+^Foxp3^−^ T cells are *P. falciparum*-specific, in that they require APCs and T cell receptor engagement to produce IL-10. We also found that *P. falciparum*-inducible IL-10 production by CD4^+^ T cells isolated after malaria did not change significantly when these cells were co-cultured with homologous APCs collected before malaria, although there was a trend toward enhanced IL-10 production by these CD4^+^ T cells when cultured with APCs collected after malaria.

Interestingly, we observed that a significant proportion of *P. falciparum*-specific IL-10 producing CD4^+^CD25^+^Foxp3^−^ T cells co-produced the Th1 cytokines IFN-γ and/or TNF. Similar ‘self-regulating’ Th1 cells that co-produce IL-10 and IFN-γ were first identified in the lungs of patients with active pulmonary tuberculosis [64] and have since been observed in mice infected with *Toxoplasma gondii* [65] and *Leishmania major* [66] as well as in humans with visceral leishmaniasis [67]. Intriguingly, we observed that *P. falciparum*-specific IL-10 was only inducible in activated Th1 cells after recent febrile malaria, and that after the dry season IL-10 and IFN-γ were no longer inducible through *P. falciparum* stimulation. This is consistent with a recent study of Ugandan children by Jagannathan et al. in which the frequencies of *P. falciparum*-specific CD4^+^ T cells co-producing IFN-γ and IL-10 were inversely associated with days since last malaria episode [68]. Together these data support the hypothesis that IL-10 production by antigen-specific Th1 cells represents a normal phase of their differentiation program which is reached after full activation in order to restrain the inflammatory response while still allowing an efficacious immune response [43], [65], namely, IFN-γ production that promotes phagocytosis-mediated clearance of blood-stage parasites.

Importantly, we show that *P. falciparum*-specific IL-10 production remains inducible in some but not all untreated children whose low-level asymptomatic *P. falciparum* infections persisted through the six-month dry season, suggesting that the production of IL-10 and IFN-γ is finely tuned such that parasitemia is controlled without inducing clinically overt inflammation—potentially explaining the long-standing clinical observation that most individuals, if left untreated after their initial bout of febrile malaria, become afebrile and maintain control of parasitemia for months before the infection is finally cleared. The exposure-dependent inducibility of IL-10 production by Th1 cells may also explain our previous observation at the same study site that children with asymptomatic *P. falciparum* infection at the end of the dry season are at lower risk of febrile malaria during the ensuing malaria season [31], whereas uninfected children at the end of the dry season are at increased risk—corresponding temporally with their return to a homeostatic baseline in which *P. falciparum* exposure induces a pro-inflammatory phenotype. The exposure-dependent inducibility of IL-10 is also consistent with anecdotal reports of rapidly waning clinical immunity to febrile malaria in those who emigrate from malaria endemic areas [69]. Taken together these data point toward a protective effect of *P. falciparum*-specific IL-10 producing Th1 cells in malaria, a hypothesis supported by a cross-sectional study in The Gambia which showed a higher frequency of total IL-10 producing Th1 cells in children with mild versus severe malaria [62]. In contrast, a study in Uganda recently reported that frequencies of CD4^+^ T cells co-producing IFN-γ and IL-10 were not associated with protection from future malaria, although imprecise measures of malaria exposure may have led to spurious associations with protection [68]. More studies are needed to define the potential role of these cells in protection from malaria and to elucidate the molecular basis of their remarkable functional plasticity [70]—information that could define ways in which these cells could be safely induced and maintained through vaccination. Further studies are also needed to disentangle the relative contributions of IL-10 upregulation and antibodies to protection from malaria.

Malaria-induced regulatory responses that control inflammation are often viewed as globally immunosuppressive, which predicts that parasites would grow unimpeded in individuals residing in areas of ongoing *P. falciparum* transmission. However, this model is at odds with the common finding of low-level, asymptomatic infection among children in endemic areas. Therefore a key finding of this study is that despite the downregulation of *P. falciparum*-inducible inflammation after the resolution of malaria, pathways involved in clearance of blood-stage parasites and activation of adaptive immunity were upregulated. Specifically, we observed *P. falciparum*-inducible upregulation of genes encoding proteins that mediate opsonic (e.g. *FCGR1*) and non-opsonic (e.g. *CR1*, *CD36*) phagocytosis, phagolysosome maturation, antigen processing and presentation, and T cell co-stimulation (Figure 4). The limited blood volume available from children enrolled in this study precluded concomitant functional confirmation of these observations; however, the observed gene expression pattern of enhanced anti-microbial activity after the resolution of febrile malaria is consistent with the results of a study in which malaria-naïve adults, who were experimentally infected with *P. falciparum*, showed enhanced macrophage phagocytic activity after treatment relative to baseline [71]. Non-opsonic phagocytosis of iRBCs is considered to be an important first line of defense in non-immune or partially immune hosts who have yet to acquire *P. falciparum*-specific opsonizing antibodies [72]. Indeed, others have shown that the scavenger receptor CD36 mediates phagocytosis of non-opsonized iRBCs [73], and interestingly, does so without inducing pro-inflammatory cytokines [73], [74].

This study reveals several intriguing parallels between the regulation of *P. falciparum*-triggered inflammation and endotoxin tolerance [75], a link that is particularly germane in light of earlier studies in humans that showed that malaria induces cross-tolerance to the febrile response normally induced by bacterial endotoxin [24], [25], suggesting at least partial overlap of regulatory pathways induced by *Plasmodium* and gram negative bacteria. Indeed, similar to what has been described in an *in vitro* model of LPS tolerance in murine macrophages [27], we observed that the regulation of *P. falciparum*-triggered responses is component-specific, such that acute phase pro-inflammatory mediators such as IL-1β and IL-6 are transiently downregulated or ‘tolerized’, while anti-parasitic effector pathways are primed or enhanced upon re-challenge with *P. falciparum* parasites. Further reductionist studies are needed to define the molecular mechanisms by which this regulation occurs including the potential role of chromatin modification [76] and microRNAs [77]. It will be of interest to understand how malaria-induced epigenetic reprogramming of innate immune cells—or “trained immunity”—differs from that induced by other pathogens [78], [79].

To our knowledge, no human study has evaluated the genome-wide transcriptional response to a natural infection in which each subject serves as his or her own healthy control. Nearly all individuals at the study site become infected with *P. falciparum* within a predictable window of time each year [80], which enabled us to compare intra-individual changes in PBMC gene expression at the healthy baseline before the malaria season and after the resolution of febrile malaria—both directly *ex vivo* and after re-exposing PBMCs to *P. falciparum* parasites *in vitro*. An important limitation of blood transcriptome analysis is that changes in mRNA levels can be driven by *de novo* transcriptional regulation or changes in the composition of PBMCs in peripheral blood [81]. Three lines of evidence indicate that the observed changes in mRNA levels in this study are driven by *de novo* transcriptional regulation. First, by flow cytometry we did not observe gross changes in the composition of the study subjects' PBMCs from before to after malaria. This is consistent with the observation that immune cells traffic out of the peripheral circulation during acute malaria but then return to the peripheral circulation after the infection has resolved [82]. Second, at the individual subject level we found that genes encoding myeloid-expressed pro-inflammatory mediators were downregulated after malaria relative to baseline, irrespective of changes in the percentage of monocytes, even as mRNA levels of other genes identified as myeloid-specific [38] were unchanged or increased relative to baseline. And finally, we observed that *in vitro* stimulation of fixed populations of cells (PBMCs and isolated monocytes/macrophages) induces *de novo* expression of immune-related genes.

In summary, this longitudinal study of Malian children shows that febrile malaria induces exposure-dependent *P. falciparum*-specific regulatory responses that limit pathogenic inflammation and enhance anti-parasite effector responses upon *P. falciparum* re-exposure. These findings offer mechanistic insights into several long-standing clinical observations in malaria including the high incidence of asymptomatic *P. falciparum* infection in endemic areas [69], reduced fever with repeated experimental *Plasmodium* infections in humans [83], the rapid acquisition of immunity to severe malaria [50], the rapid loss of clinical immunity to febrile malaria in the absence of ongoing *P. falciparum* exposure [6] and *Plasmodium*-induced hetero-tolerance to endotoxin challenge [25]. Longitudinal studies of symptomatic and asymptomatic individuals who are repeatedly exposed to *P. falciparum* will refine our understanding of the mechanisms underlying the regulation and dysregulation of *Plasmodium*-induced inflammation and may help define the potential for interventions that safely prevent or mitigate *Plasmodium*-induced immunopathology without compromising control of parasite replication.

## Materials and Methods

### Ethics statement

The Ethics Committee of the Faculty of Medicine, Pharmacy, and Dentistry at the University of Sciences, Techniques, and Technologies of Bamako, and the Institutional Review Board of the National Institute of Allergy and Infectious Diseases, National Institutes of Health approved this study. Written informed consent was obtained from the parents or guardians of participating children.

### Study subjects

Study subjects were enrolled in an observational cohort study conducted in Kambila, Mali, a rural village of ∼1500 inhabitants where intense seasonal *P. falciparum* transmission occurs from July through December. The cohort is an age-stratified random sample of the entire village population. A detailed description of the study site and design of the cohort study has been published elsewhere [31]. The present study focused on children aged 5–13 years who had PBMCs collected at their healthy baseline before the malaria season, and 7 or 14 days after treatment of their first malaria episode of the ensuing malaria season, as well as a subset of children who also had PBMCs collected after the following six-month dry season, a period of little to no *P. falciparum* transmission. Individual demographic and clinical data are given in Table S1. Febrile malaria episodes were detected prospectively by self-referral to the study clinic, which was staffed by a physician 24 hours/day. Malaria episodes were treated with a standard 3-day course of artemether/lumefantrine.

### Detection of *P. falciparum* infection

Thick blood smears were stained with Giemsa and counted against 300 leukocytes, and *P. falciparum* densities were recorded as the number of asexual parasites/µl of whole blood based on an average leukocyte count of 7500/µl. Each smear was evaluated separately by at least two expert microscopists. *P. falciparum* was detected by PCR from dried blood spots preserved on 903 Protein Saver filter paper (Whatman) as previously described [80].

### Processing of PBMCs

Blood samples (8 ml) were drawn by venipuncture into sodium citrate-containing cell preparation tubes (BD, Vacutainer CPT Tubes) and transported 20 km to the laboratory where PBMCs were isolated and frozen within three hours according to the manufacturer's instructions. PBMCs were frozen in fetal bovine serum (FBS) (Gibco, Grand Island, NY) containing 7.5% dimethyl sulfoxide (DMSO; Sigma-Aldrich, St. Louis, MO), kept at −80°C for 24 hours, and then stored at −196°C in liquid nitrogen. For each individual, PBMCs from all time points were thawed and assayed at the same time. The trypan blue dye exclusion assay consistently demonstrated >80% viability of PBMCs after thawing.

### Microarray chip processing and data analysis

RNA extraction, cDNA amplification, synthesis and labeling was performed as previously described [84]. Hybridization, fluidics and scanning were performed according to standard Affymetrix protocols. GeneChip Operating Software GCOS v1.4 was used to convert the image files to cell intensity data (cel files). All cel files, representing individual samples were normalized using the Robust Multiarray Average (RMA) method from the *affy* package library in the R project for Statistical Computing (R Core Team 2013). Nine outlier chips were identified among the unstimulated samples using quality control plots from Partek Genomics Suite software (Partek, inc. St. Louis, Mo., v6.5 6.11.310) and principal components analyses (PCA) computed using R. An empirical Bayes moderated paired T-test was computed using the *limma* package library in R to obtain false discovery rate (FDR) adjusted p-values and fold changes. Probes were considered statistically significant if their FDR-adjusted P values were <0.05 and their absolute fold change was >1.25. Heatmaps were generated with the *gplots* package library in R. Log fold change ratios, p-values and false discovery rates from the empirical Bayes T-tests were imported into Ingenuity Pathways Analysis to examine enrichment of pathways and functional groups.

### Quantitative real-time PCR

Human yeast Sfi1 homolog spindle assembly associated gene (Sfi1) was selected as a reference gene based on its low coefficient of variation (CV) across DNA microarray analysis. Seven mRNAs were analyzed by q-RT-PCR to validate DNA microarray findings: chemokine (C-X-C motif) ligand 5 (*CXCL5*), interleukin-1 beta (*IL1B*), interleukin-6 (*IL6*), interleukin-10 (*IL10*), toll-like receptor 2 (*TLR2*), and transforming growth factor, beta 1 (*TGFB1*). All six probe and primer sets were designed using Primer Express version 3.0 (ThermoFisher Scientific, Waltham, MA) and are listed inTable S5. Seventeen out of 34 patients were selected for q-RT-PCR validation. Four RNAs were analyzed from each patient representing the two time points HB and d7 and two experimental conditions (‘*ex vivo* unstimulated’ and ‘*in vitro* stimulated with iRBC’). Template preparation and q-RT-PCR analysis was performed as described previously [84].

### Preparing *P. falciparum*-infected red blood cell lysate for *in vitro* stimulation of PBMCs

3D7 *P. falciparum* parasites were maintained in fresh human O^Rh+^erythrocytes at 3% hematocrit in RPMI 1640 medium (KD Medical) supplemented with 10% heat-inactivated O^Rh+^ human serum (Interstate Blood Bank, Memphis, Tennessee), 7.4% Sodium Bicarbonate (GIBCO, Invitrogen) and 25 µg/ml of gentamycin (GIBCO, invitrogen), at 37°C in the presence of a gas mixture containing 5% O_2_, 5% CO_2_ and 90% N_2_. Parasite cultures were shown to be free of mycoplasma and acholeplasma using an ELISA-based Mycoplasma Detection Kit (Roche) which contains polyclonal antibodies specific for *M. arginini*, *M. hyorhinis*, *A. laidlawii* and *M. orale*. *P. falciparum* schizont iRBCs were isolated in RPMI 1640 medium supplemented with 0.25% Albumax (GIBCO, Invitrogen) and 7.4% Sodium Bicarbonate (GIBCO, Invitrogen) using magnetic columns (LD MACS Separation Columns, Miltenyi Biotec). Control preparations of uninfected red blood cells (uRBC) from the same blood donor were obtained and tested in all experiments. Lysates of *P. falciparum*-infected and uninfected RBCs were obtained by three freeze-thaw cycles in liquid nitrogen and 37°C water bath.

### *In vitro* stimulation of PBMCs with *P. falciparum*-infected red blood cell lysate

PBMCs were cultured in complete RPMI (RPMI 1640 plus 10% fetal calf serum, 1% penicillin/streptomycin, 2-mercaptoethanol) in flat-bottom 96 well plates, at 37°C in a 5% CO_2_ atmosphere. 500,000 PBMCs were stimulated with lysate of infected red blood cells (iRBCs) or uninfected RBCs (uRBCs) in a ratio of 3 RBCs per PBMC for 18 h, with or without 1.25 µg/ml Brefeldin A (BFA) (Sigma-Aldrich) for the last 15 h of stimulation. A 3∶1 ratio of RBC to PBMC was used on the basis of titration experiments (from 5∶1 to 1∶1) and is consistent with previous reports [85]. PBMCs stimulated with 1.18 µg/ml Staphylococcal enterotoxin B from *Staphylococcus aureus* (SEB) (Sigma-Aldrich) was used as a positive control for cytokine production in supernatants and within cells. Following stimulation, cells were centrifuged and supernatants were recovered and frozen at −80°C for cytokine analysis. Cells stimulated in the presence of BFA were centrifuged, washed and recovered for intracellular staining and flow cytometry analysis.

### Isolation of monocytes/macrophages and *in vitro* stimulation with *P. falciparum*-infected red blood cell lysate

Monocyte/macrophages were isolated from PBMCs of Malian children by negative selection using the MACS Pan Monocyte Cell Negative Isolation kit II (Miltenyi Biotec), an indirect magnetic labeling system for the isolation of untouched monocytes/macrophages. Non-monocyte/macrophage cells were directly depleted by using a cocktail of biotin-conjugated antibodies followed by magnetic removal of labeled cells. Monocyte/macrophage purity was verified by flow cytometry using fluorescently labeled antibodies specific for CD3 PE (UCHT1), CD4 APC (RPA-T4), CD8 APC-Cy7 (SK1), CD14 FITC (M5E), CD16 Pacific blue (3G8) (BD Biosciences), CD19 PerCP-Cy5.5 (SJ25C1) (eBioscience), and 7-Aminoactinomycin D (7-AAD) viability staining (BD Biosciences). FACS analysis was performed on a BD LSR II Table flow cytometer (BD Bioscience) and analyzed using FlowJo software (Tree Star). Purified monocytes/macrophages were then stimulated in a ratio of 30 RBCs per monocyte for 6 h with lysate of *P. falciparum*-infected RBCs and cytokines were measured in supernatants.

### Flow cytometry

PBMCs were washed in PBS with 4% heat-inactivated FCS and cells were incubated for 30 min at 4°C with fluorescently labeled antibodies specific for CD3 PE (UCHT1), CD4 APC (RPA-T4), CD8 APC-Cy7 (SK1), CD14 FITC (M5E) and CD16 Pacific blue (3G8) purchased from BD Biosciences; and CD19 PerCP-Cy5.5 (SJ25C1) purchased from eBioscience. All phenotypic analyses were performed using mouse mAbs specific for human markers conjugated to fluorophores. FACS analyses were performed on a BD LSR II Table flow cytometer (BD Biosciences) and analyzed using FlowJo software (Tree Star, Inc).

### Measurement of cytokines in supernatants of stimulated PBMCs

Supernatants were thawed and immediately analyzed with Bio-plex human cytokine assays (Bio-Rad Laboratories, Inc.) as recommended by the manufacturer. The following cytokines were measured: IL-1β, IL-6, IL-8, IL-10 and TNF. Briefly, 25 µL of supernatant was diluted 1∶2 in medium and incubated with anti-cytokine antibody-coupled magnetic beads for 30 min at room temperature shaking at 300 RPM in the dark. Between each step the complexes were washed three times in wash buffer, using a vacuum manifold. The beads were then incubated with a biotinylated detector antibody for 30 min before incubation with streptavidin-phycoerythrin for 30 minutes. Finally, the complexes were resuspended in 125 µL of detection buffer and 100 beads were counted with a Luminex 200 device (Bio-Rad Laboratories, Inc.). Final concentrations were calculated from the mean fluorescence intensity and expressed in pg/mL using standard curves with known concentrations of each cytokine.

### IL-10 blocking

PBMCs were cultured in complete RPMI in flat-bottom 96 well plates, at 37°C in a 5% CO_2_ atmosphere. 500,000 PBMCs were stimulated for 18 h with lysate of infected (iRBCs) or uninfected RBCs (uRBCs) in a ratio of 3 RBCs per PBMC in the presence of anti-IL-10 (BD Pharmigen, USA) and anti-IL-10R (R&D Systems, Inc.) or in the presence of the respective isotype controls. Following stimulation cells were centrifuged and supernatants were recovered for cytokine analysis.

### Intracellular cytokine staining

After stimulation a total of 1×10^6^ PBMCs were sequentially stained for surface and intracellular markers in round-bottom 96-well plates at room temperature. To exclude dead cells, PBMCs were stained for 30 min using the LIVE/DEAD Fixable Violet Dead Cell Stain Kit (Invitrogen) followed by a surface staining with PerCP-Cy5.5 anti-human CD27 (M-T271) and APC-H7 anti-human CD45RO (UCHL1) for 20 min. After fixing and permeabilizing the cells according to the manufacturer's protocol using the FoxP3 Staining Buffer Set (eBioscience), the cells were stained with BD Horizon V500 anti-human CD3 (UCHT1), PerCP anti-human CD4 (SK3), Alexa Fluor 700 anti-human IFNγ (B27), FITC anti-human TNF (MAb11), APC anti-human IL-10 (JES3-19F1), PE-Cy7 anti-human CD25 (BC96) and PE anti-human FoxP3 (236A/E7) for 30 min. Fluorescently labeled antibodies against TNF, CD25 and FoxP3 were purchased from eBioscience, the remaining antibodies were purchased form BD Biosciences. Cells were acquired using a BD LSR II Table flow cytometer (BD) and analyzed using FlowJo software (Tree Star) and SPICE software [86].

### Isolation of CD4^+^ T cells and *in vitro* stimulation with *P. falciparum* infected red blood cell lysate

CD4^+^ T cells were isolated from PBMCs of Malian children by negative selection using the MACS CD4^+^ T Cell Negative Isolation kit II (Miltenyi Biotec), an indirect magnetic labeling system for the isolation of untouched CD4^+^ T helper cells. Non-CD4^+^ T cells were directly depleted by using a cocktail of biotin-conjugated antibodies against CD8, CD14, CD16, CD19, CD36, CD56, CD123, TCR g/d and Glycophorin A and anti-Biotin microbeads, followed by magnetic removal of labeled cells. CD4^+^ T cells purity was verified by flow cytometry, using fluorescently labeled antibodies specific for CD3 (PE) and CD4 (APC) (BD Bioscience) and a BD LSR II Table flow cytometer (BD Bioscience, USA) and analyzed using FlowJo software (Tree Star, Inc). Purified CD4^+^ T cells were then incubated either in the presence or absence of non-CD4^+^ cells and stimulated for 18 h with lysate of infected (iRBCs) or uninfected RBCs (uRBCs), for cytokine analysis of supernatants.

### MHC II blocking

PBMCs were cultured in complete RPMI in flat-bottom 96 well plates, at 37°C in a 5% CO_2_ atmosphere. 500,000 PBMCs were stimulated for 18 h with lysate of infected (iRBCs) or uninfected RBCs (uRBCs) in a ratio of 3 RBCs per PBMC, in the presence of anti-human leukocyte antigen HLA-DQ (SPVL-3; Beckmann Coulter, USA), anti-HLA-DP (B7/21; abcam, USA) and anti-HLA-DR (L243; Biolegend, USA), or in the presence of respective isotype controls. Following stimulation, cells were centrifuged and supernatants were recovered for cytokine analysis.

### Statistical analysis

Continuous data were compared using the paired or unpaired Student's T-test, paired Wilcoxon rank sum test or permutation tests of mean paired differences as appropriate. Bonferroni adjustments were applied to correct for multiple comparisons when appropriate. A linear mixed model for repeated measures ANOVA with Tukey HSD post hoc tests was also used to compare continuous variables. Pearson correlation coefficients and linear regressions with 95% confidence bands were used to examine the correlation between continuous variables. Fisher's exact test was used for contingency table analyses. The statistical test used is specified in the figure legends. Statistical significance was defined as a 2-tailed *P* value of ≤.05. Statistical tests were computed using R version 2.13.2 (http://www.R-project.org), GraphPad Prism version 5.0d (http://www.graphpad.com/scientific-software/prism/) or JMP 10.0 (www.jmp.com).

## Supporting Information

Figure S1 (A) Principal components analysis of the microarray data showed that transcription profiles of the unstimulated PBMCs segregated on the basis of time-point (healthy baseline vs 7 days after malaria), but not age, gender or batch effects. The samples of the nine individuals that did not pass the microarray quality assessment are indicated in gray. (B) Ingenuity Pathway Analysis (IPA) summary showing canonical pathways that remained affected after the resolution of febrile malaria relative to the healthy pre-malaria baseline in the unstimulated PBMC microarray experiments. The graphs show the BH adjusted p values (yellow line) of the enrichment of canonical pathways. The bars indicate the percentage of genes in a given pathway that are differentially expressed with the total number of genes in each pathway shown on the right Y-axis. The red and green portions of the bars indicate the percentage of genes within each pathway that were upregulated or downregulated, respectively. (C) Ratio of monocyte percentage (day 7 after malaria/healthy baseline) vs the ratio of the expression level of monocyte-derived mediators of the inflammatory response (day 7 after malaria/healthy baseline). Each point represents an individual subject. (D) Heat map showing RMA-normalized log2 ratios (day 7 after malaria/healthy baseline) of genes identified as myeloid-specific (Chaussabel et al., 2008) (rows) in unstimulated PBMCs for each child (columns). Statistically significant differentially expressed genes are indicated with an asterisk (n = 50 paired samples). (E) IPA summary showing canonical pathways that remained affected after the resolution of febrile malaria relative to the healthy pre-malaria baseline in the *P. falciparum* iRBC stimulated PBMC microarray experiments. The graphical elements are as described in (B).
(TIF)

Figure S2 (A) Flow cytometry gating strategy to detect monocyte/macrophage enrichment. FACS plots of PBMCs of a representative Malian child. Within the total PBMC gate the monocyte population is defined by live CD14^+^ before and after monocyte/macrophage enrichment, shown in pink. (B) Percentage of live monocyte/macrophages of total PBMCs before (PBMCs) and after (M0) monocyte/macrophage enrichment in PBMCs collected at healthy baseline (HB) and 14 days after the first malaria episode of the season (d14) (n = 9, P<0.0001). P values determined by ANOVA with Sidak's multiple comparisons test.
(TIF)

Table S1 Demographic and clinical data of study subjects and assays in which PBMC samples were used.
(PDF)

Table S2 *Ex vivo* differentially expressed genes from before to after malaria. Transcripts are significant if FDR-adjusted p-value<0.05 and absolute fold change >1.25. Transcript ID is the Affymetrix accession number.
(PDF)

Table S3 Differentially expressed genes in response to *in vitro P. falciparum* stimulation from before to after malaria. Transcripts are significant if DR-adjusted p-value<0.05 and absolute fold change >1.25. Transcript ID is the Affymetrix accession number.
(PDF)

Table S4 Microarray Expression and q-RT-PCR values of selected genes from 18 individuals at healthy baseline and day 7 after the first malaria episode with and without *P. falciparum in vitro* stimulation.
(PDF)

Table S5 Sequences of primers and probes used for q-RT-PCR validation.
(PDF)
