# Supplementary figures and images for: Exposure-Dependent Control of Malaria-Induced Inflammation in Children
Source: PLoS Pathog. 2014 Apr 17;10(4):e1004079. doi: 10.1371/journal.ppat.1004079 (PMC3990727; doi:10.1371/journal.ppat.1004079)

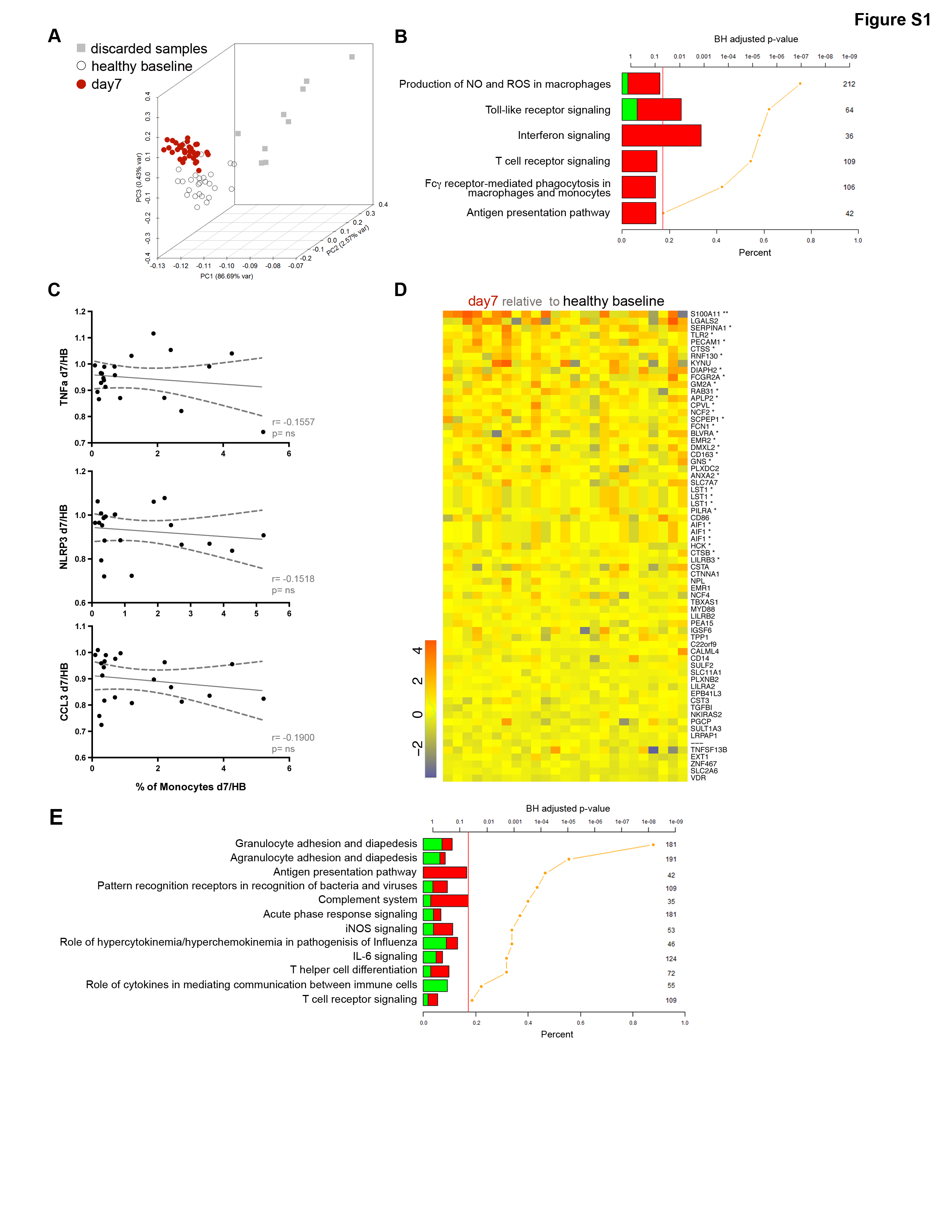

Supplement: Figure S1 — (A) Principal components analysis of the microarray data showed that transcription profiles of the unstimulated PBMCs segregated on the basis of time-point (healthy baseline vs 7 days after malaria), but not age, gender or batch effects. The samples of the nine individuals that did not pass the microarray quality assessment are indicated in gray. (B) Ingenuity Pathway Analysis (IPA) summary showing canonical pathways that remained affected after the resolution of febrile malaria relative to the healthy pre-malaria baseline in the unstimulated PBMC microarray experiments. The graphs show the BH adjusted p values (yellow line) of the enrichment of canonical pathways. The bars indicate the percentage of genes in a given pathway that are differentially expressed with the total number of genes in each pathway shown on the right Y-axis. The red and green portions of the bars indicate the percentage of genes within each pathway that were upregulated or downregulated, respectively. (C) Ratio of monocyte percentage (day 7 after malaria/healthy baseline) vs the ratio of the expression level of monocyte-derived mediators of the inflammatory response (day 7 after malaria/healthy baseline). Each point represents an individual subject. (D) Heat map showing RMA-normalized log2 ratios (day 7 after malaria/healthy baseline) of genes identified as myeloid-specific (Chaussabel et al., 2008) (rows) in unstimulated PBMCs for each child (columns). Statistically significant differentially expressed genes are indicated with an asterisk (n = 50 paired samples). (E) IPA summary showing canonical pathways that remained affected after the resolution of febrile malaria relative to the healthy pre-malaria baseline in the P. falciparum iRBC stimulated PBMC microarray experiments. The graphical elements are as described in (B). (TIF) [file ppat.1004079.s001.tif]

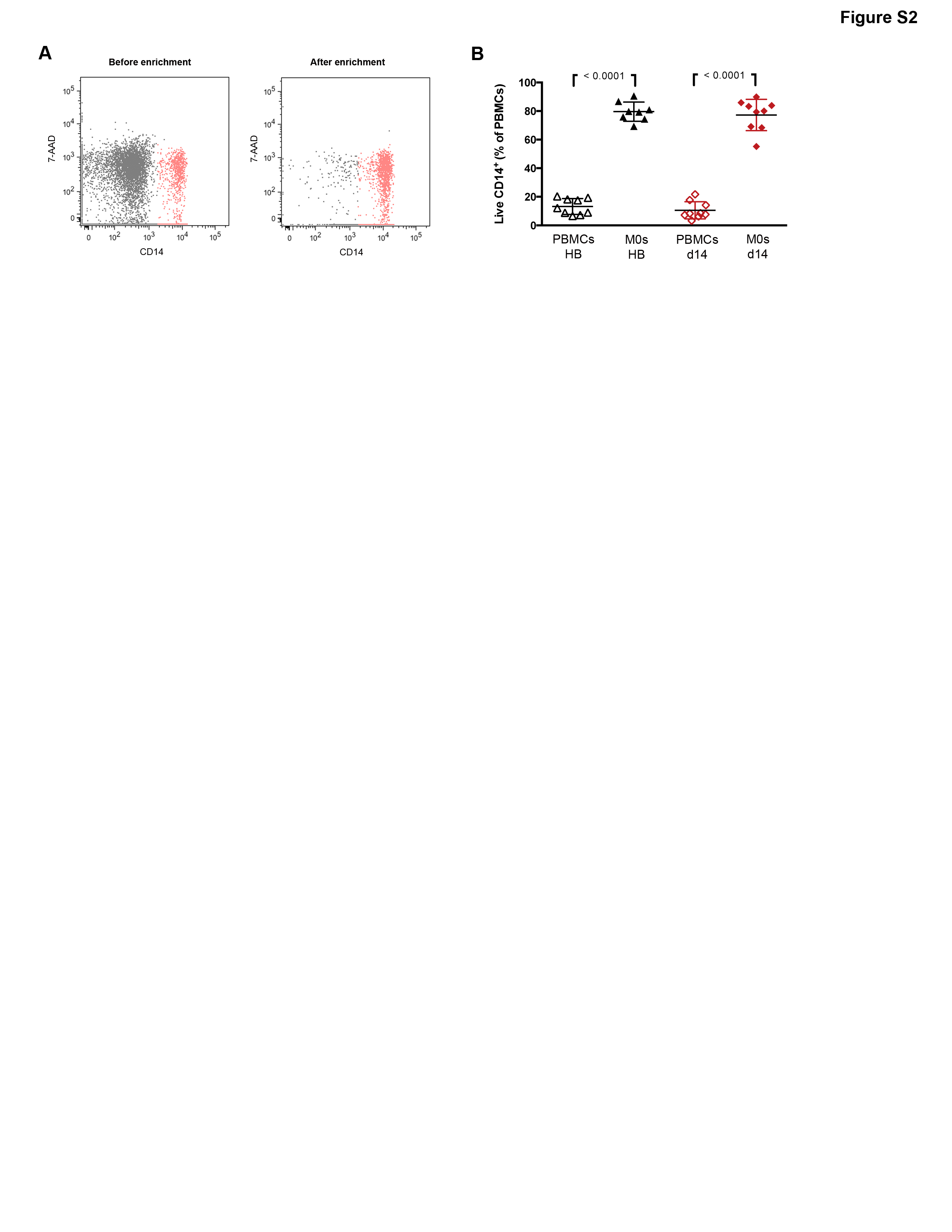

Supplement: Figure S2 — (A) Flow cytometry gating strategy to detect monocyte/macrophage enrichment. FACS plots of PBMCs of a representative Malian child. Within the total PBMC gate the monocyte population is defined by live CD14+ before and after monocyte/macrophage enrichment, shown in pink. (B) Percentage of live monocyte/macrophages of total PBMCs before (PBMCs) and after (M0) monocyte/macrophage enrichment in PBMCs collected at healthy baseline (HB) and 14 days after the first malaria episode of the season (d14) (n = 9, P<0.0001). P values determined by ANOVA with Sidak's multiple comparisons test. (TIF) [file ppat.1004079.s002.tif]
