# Supplementary material for: Exposure-Dependent Control of Malaria-Induced Inflammation in Children
Source: PLoS Pathog. 2014 Apr 17;10(4):e1004079. doi: 10.1371/journal.ppat.1004079 (PMC3990727; doi:10.1371/journal.ppat.1004079)
Supplement: Table S1 — Demographic and clinical data of study subjects and assays in which PBMC samples were used. (PDF) [file ppat.1004079.s003.pdf]

Table S1. Demographic and clinical data of study subjects and assays in which PBMC samples were used.

Subject ID	Age (yrs)	Gender	Pf PCR+ at enrollment	Temperature, 1st malaria episode (°C)	Parasitaemia, 1st malaria episode (asexual parasites/ul blood)	Microarray before vs. after malaria directly *ex vivo*	Microarray before vs. after malaria following iRBC stimulation	FACS phenotyping	Supernatant cytokine by Luminex	Monocyte/macrophage isolation	Intracellular cytokine staining	sorted CD4 T	MHCii blockade
kam004	7	m		38.7	78300				+				+
kam007	7	m		38.4	46900							+	
kam008	7	m		39.7	155700				+			+	+
kam009	6	m		39.4	422250				+				+
Kam014	6	f		38.4	120750							+	
kam016	8	f		38.7	278250							+	
kam019	6	f		38.2	675				+				+
kam021	6	f		38.2	13900				+				+
kam023	6	f		38.6	120600							+	
kam027	5	f		38.6	167550				+				+
kam028	7	f		38.2	28200							+	
kam030	6	f		38.4	68250	+	+	+	+				
kam031	6	m		38.1	24150						+	+	
kam032	7	m		38.7	112500						+		
kam033	6	f		38.3	41400						+		
kam035	7	f		39.9	283450				+		+		
kam037	5	m		39.4	22200	+	+	+	+	+			
kam042	6	f	+	39.1	4800	+	+	+	+				
kam047	7	m		38.8	43200	+		+	+				
kam050	6	m		39.4	28350	+		+	+				
kam051	6	f		39.6	6900								
kam054	5	m		38.6	38750	+	+	+	+				
kam055	6	f		39	36250	+		+	+				
kam057	6	f		37.7	42525	+	+	+	+				
kam058	5	f	+	38	23950	+	+	+	+			+	
kam059	7	f		40.1	132550				+				
kam060	6	f		37.8	57075				+				
kam062	6	m	+	NA	NA				+				
kam064	7	m		38.4	71250	+	+	+	+				
kam067	7	m		36.9	77525						+		
kam068	7	m		37.3	120300	+	+	+	+				
kam070	7	f		35.9	53400					+	+		
kam071	7	f		39.3	100050	+		+	+	+			
kam075	8	m		37.7	35938						+		
kam076	8	m		37.8	32250				+	'+	+		
kam077	9	f		38.8	173000							+	
kam078	8	f		39.1	4800				+		+	+	
kam079	9	m		38.7	5625				+				+
kam080	8	m		37.8	6000				+				+
kam081	8	m	+	38.8	57350	+ (discarded d7)	+	+	+				+
kam082	8	f		37.9	75	+	+	+	+				
kam084	8	f		40	8425	+ (discarded HB)	+	+	+				
kam085	9	m		37.7	6300				+				+
kam087	8	m		39.8	268250	+ (discarded HB)		+	+				+
kam088	10	m	+	38.4	114750				+				
kam094	8	m		38.2	72600				+		+		
kam096	8	f		40.4	481500						+		
kam097	9	f		38.5	100650	+ (discarded HB)		+	+				+
kam100	8	f		38.6	31925						+		
kam101	9	m		38.9	5550	+ (discarded HB)		+	+				
kam102	8	m		40	50	+ (discarded HB)	+	+	+	+			
kam104	10	m		38.3	9575					+			
kam105	10	f		38.3	4000						+		
kam107	10	f		37.7	13200	+ (discarded HB)	+	+	+	+			
kam108	8	f	+	39.1	122075				+				
kam109	10	m		37.8	734000	+		+	+				
kam112	9	m		39.1	4650				+		+		
kam113	10	m	+	38.4	37075	+ (discarded HB)	+	+	+	+			
kam114	8	f	+	39.2	225	+	+	+	+				
Kam117	8	m	+	NA	NA				+				
kam118	9	f		37.8	20400							+	
kam119	8	m		38.9	52250	+	+	+	+	+			
kam120	9	f		39.3	54750				+		+		
kam123	8	m		39.7	2325	+	+	+	+				
kam125	11	m	+	35.8	11450	+	+	+	+				
kam129	11	m		36.3	2075	+	+	+	+				
kam130	11	m		36.5	93320	+	+	+	+				
kam132	13	m		38.6	35000				+		+		
kam134	12	f		38.2	18975				+				
kam136	13	m		37.3	48150	+	+	+	+				
Kam137	11	m	+	37.9	47600				+				
kam138	13	m		37.9	1075				+		+		
kam139	11	f		37.9	1025						+		
Kam140	11	m	+	36.2	14400				+				
kam144	13	m		37.1	3750	+ (discarded HB)		+	+				
Kam147	13	m	+	NA	NA				+				
kam150	11	m		39.7	51675				+				
kam151	13	m		38.5	59700				+				
kam152	12	m		38.8	38475				+				
kam153	12	m		39.1	18750	+		+	+				
kam154	12	m	+	35.4	39150	+		+	+				
kam156	11	m		36.6	22800				+				
kam157	11	m		37.1	26625	+		+	+				
Kam159	13	m	+	37.8	68550				+				
Kam160	12	f	+	NA	NA				+				
kam168	11	m		37.6	14800	+	+	+	+				
kam171	11	f		38	10100				+				
kam172	13	m		38.1	60000				+				
kam173	12	m		38.2	6525				+				

NA: Subject only analyzed at healthy baseline, at the end of dry season.
