# Supplementary material for: Exposure-Dependent Control of Malaria-Induced Inflammation in Children
Source: PLoS Pathog. 2014 Apr 17;10(4):e1004079. doi: 10.1371/journal.ppat.1004079 (PMC3990727; doi:10.1371/journal.ppat.1004079)
Supplement: Table S2 — Ex vivo differentially expressed genes from before to after malaria. Transcripts are significant if FDR-adjusted p-value<0.05 and absolute fold change >1.25. Transcript ID is the Affymetrix accession number. (PDF) [file ppat.1004079.s004.pdf]

**Table S2**. *Ex vivo* differentially expressed genes from before to after malaria. Transcripts are significant if FDR-adjusted p-value < 0.05 and absolute fold change > 1.25. Transcript ID is the Affymetrix accession number.

| Transcript ID | Acession ID | Gene Symbol | Gene Description | $log^2$ Fold Change | Fold Change | p-value | FDR-adjusted p-value | Regulation relative to baseline | Figure 1A | Figure S1 |
|---|---|---|---|---|---|---|---|---|---|---|
| 8097468 | --- | --- | --- | 3.409 | 10.624 | 2.97E-03 | 2.97E-01 | Up | | |
| 7896706 | --- | --- | --- | 3.345 | 10.158 | 3.38E-09 | 3.38E-07 | Up | | |
| 7906377 | NM_002432 | MNDA | myeloid cell nuclear differentiation a | 3.174 | 9.028 | 3.14E-09 | 3.14E-07 | Up | Regulator | |
| 8149116 | NM_005217 | DEFA3 | defensin, alpha 3, neutrophil-specific | 2.567 | 5.924 | 3.53E-04 | 3.53E-02 | Up | Phagocytosis/killing | |
| 8149126 | NM_005217 | DEFA3 | defensin, alpha 3, neutrophil-specific | 2.567 | 5.924 | 3.53E-04 | 3.53E-02 | Up | Phagocytosis/killing | |
| 8149137 | NM_005217 | DEFA3 | defensin, alpha 3, neutrophil-specific | 2.567 | 5.924 | 3.53E-04 | 3.53E-02 | Up | Phagocytosis/killing | |
| 7895734 | --- | --- | --- | 2.460 | 5.500 | 1.50E-02 | 1.50E+00 | Up | | |
| 7895711 | --- | --- | --- | 2.113 | 4.327 | 2.54E-02 | 2.54E+00 | Up | | |
| 7895246 | --- | --- | --- | 2.109 | 4.313 | 2.45E-02 | 2.45E+00 | Up | | |
| 7980923 | NM_004239 | TRIP11 | thyroid hormone receptor interactor | 2.018 | 4.050 | 9.19E-06 | 9.19E-04 | Up | | |
| 7954810 | NM_198578 | LRRK2 | leucine-rich repeat kinase 2 | 2.016 | 4.043 | 1.23E-06 | 1.23E-04 | Up | | |
| 7920238 | NM_005621 | S100A12 | S100 calcium binding protein A12 | 2.009 | 4.024 | 7.24E-04 | 7.24E-02 | Up | Phagocytosis/killing | |
| 7893339 | --- | --- | --- | 1.955 | 3.877 | 4.39E-02 | 4.39E+00 | Up | | |
| 7892539 | --- | --- | --- | 1.930 | 3.810 | 3.54E-02 | 3.54E+00 | Up | | |
| 8129590 | NM_003569 | STX7 | syntaxin 7 | 1.903 | 3.740 | 6.76E-05 | 6.76E-03 | Up | Phagocytosis/killing | |
| 7968295 | --- | --- | --- | 1.846 | 3.594 | 8.48E-03 | 8.48E-01 | Up | | |
| 8051462 | --- | --- | --- | 1.838 | 3.575 | 4.93E-02 | 4.93E+00 | Up | | |
| 8046003 | NM_012198 | GCA | grancalcin, EF-hand calcium binding | 1.837 | 3.572 | 4.82E-05 | 4.82E-03 | Up | Phagocytosis/killing | |
| 7895729 | --- | --- | --- | 1.831 | 3.558 | 2.26E-02 | 2.26E+00 | Up | | |
| 7951435 | --- | --- | --- | 1.826 | 3.546 | 1.04E-02 | 1.04E+00 | Up | | |
| 7896708 | --- | --- | --- | 1.808 | 3.502 | 1.65E-02 | 1.65E+00 | Up | | |
| 8112478 | NM_004536 | NAIP | NLR family, apoptosis inhibitory prot | 1.802 | 3.488 | 4.82E-05 | 4.82E-03 | Up | | |
| 8100827 | NM_144646 | IGJ | immunoglobulin J polypeptide, linker | 1.785 | 3.445 | 5.31E-03 | 5.31E-01 | Up | | |
| 7895288 | --- | --- | --- | 1.749 | 3.361 | 5.05E-02 | 5.05E+00 | | | |
| 7896724 | --- | --- | --- | 1.748 | 3.358 | 2.53E-02 | 2.53E+00 | Up | | |
| 8017162 | NM_016125 | RNFT1 | ring finger protein, transmembrane 1 | 1.739 | 3.338 | 4.63E-04 | 4.63E-02 | Up | | |
| 7895848 | --- | --- | --- | 1.735 | 3.329 | 1.97E-02 | 1.97E+00 | Up | | |
| 7927669 | NM_003201 | TFAM | transcription factor A, mitochondrial | 1.730 | 3.318 | 1.22E-04 | 1.22E-02 | Up | | |
| 8105970 | NM_001515 | GTF2H2 | general transcription factor IIH, poly | 1.706 | 3.263 | 6.82E-04 | 6.82E-02 | Up | | |
| 8152863 | --- | --- | --- | 1.704 | 3.257 | 2.51E-02 | 2.51E+00 | Up | | |
| 8147883 | NM_020189 | ENY2 | enhancer of yellow 2 homolog (Dros | 1.699 | 3.248 | 1.26E-03 | 1.26E-01 | Up | | |
| 7894746 | --- | --- | --- | 1.692 | 3.232 | 5.91E-02 | 5.91E+00 | | | |
| 7894315 | --- | --- | --- | 1.686 | 3.217 | 1.28E-02 | 1.28E+00 | Up | | |
| 7977775 | NM_001344 | DAD1 | defender against cell death 1 | 1.679 | 3.202 | 2.07E-02 | 2.07E+00 | Up | | |
| 7978739 | NM_00107953 | TRAPPC6B | trafficking protein particle complex 6 | 1.671 | 3.184 | 4.01E-04 | 4.01E-02 | Up | | |
| 7960553 | NM_016497 | MRPL51 | mitochondrial ribosomal protein L51 | 1.660 | 3.161 | 1.66E-02 | 1.66E+00 | Up | | |
| 7895252 | --- | --- | --- | 1.655 | 3.149 | 4.24E-02 | 4.24E+00 | Up | | |
| 7893437 | --- | --- | --- | 1.645 | 3.127 | 4.14E-02 | 4.14E+00 | Up | | |
| 7896020 | --- | --- | --- | 1.641 | 3.118 | 1.43E-01 | 1.43E+01 | | | |
| 7893554 | --- | --- | --- | 1.616 | 3.066 | 4.14E-02 | 4.14E+00 | Up | | |
| 7991501 | --- | --- | --- | 1.614 | 3.061 | 2.20E-02 | 2.20E+00 | Up | | |
| 7892663 | --- | --- | --- | 1.600 | 3.031 | 2.65E-02 | 2.65E+00 | Up | | |
| 7895351 | --- | --- | --- | 1.599 | 3.030 | 2.42E-02 | 2.42E+00 | Up | | |
| 8091048 | NR_023350 | COPB2 | coatomer protein complex, subunit b | 1.582 | 2.993 | 2.74E-04 | 2.74E-02 | Up | | |
| 7896729 | --- | --- | --- | 1.559 | 2.947 | 2.86E-02 | 2.86E+00 | Up | | |
| 7895271 | --- | --- | --- | 1.559 | 2.946 | 5.94E-03 | 5.94E-01 | Up | | |
| 8112521 | NM_004536 | NAIP | NLR family, apoptosis inhibitory prot | 1.551 | 2.930 | 4.87E-05 | 4.87E-03 | Up | | |
| 8177527 | 0 | 0 | 0 | 1.551 | 2.930 | 4.87E-05 | 4.87E-03 | Up | | |
| 7893235 | --- | --- | --- | 1.549 | 2.926 | 3.17E-02 | 3.17E+00 | Up | | |
| 7896653 | --- | --- | --- | 1.545 | 2.918 | 2.25E-02 | 2.25E+00 | Up | | |
| 7895221 | --- | --- | --- | 1.532 | 2.891 | 1.72E-02 | 1.72E+00 | Up | | |
| 7895647 | --- | --- | --- | 1.527 | 2.882 | 1.42E-02 | 1.42E+00 | Up | | |
| 7896468 | --- | --- | --- | 1.526 | 2.880 | 4.60E-03 | 4.60E-01 | Up | | |
| 7893552 | --- | --- | --- | 1.511 | 2.850 | 3.22E-02 | 3.22E+00 | Up | | |
| 8072015 | NM_005160 | ADRBK2 | adrenergic, beta, receptor kinase 2 | 1.506 | 2.840 | 4.87E-05 | 4.87E-03 | Up | | |
| 7893372 | --- | --- | --- | 1.501 | 2.831 | 5.32E-02 | 5.32E+00 | | | |
| 7896340 | --- | --- | --- | 1.501 | 2.830 | 9.03E-02 | 9.03E+00 | | | |
| 8137244 | NM_018326 | GIMAP4 | GTPase, IMAP family member 4 | 1.492 | 2.812 | 4.63E-04 | 4.63E-02 | Up | | |
| 7892704 | --- | --- | --- | 1.491 | 2.811 | 4.71E-02 | 4.71E+00 | Up | | |
| 7892599 | --- | --- | --- | 1.489 | 2.807 | 1.63E-01 | 1.63E+01 | | | |
| 8082003 | NM_018456 | EAF2 | ELL associated factor 2 | 1.488 | 2.806 | 1.12E-04 | 1.12E-02 | Up | | |
| 8089926 | --- | --- | --- | 1.484 | 2.797 | 1.53E-02 | 1.53E+00 | Up | | |
| 7920244 | NM_002964 | S100A8 | S100 calcium binding protein A8 | 1.483 | 2.796 | 7.52E-03 | 7.52E-01 | Up | Phagocytosis/killing | |
| 7998117 | NM_207366 | 40435 | septin 14 | 1.481 | 2.792 | 6.62E-02 | 6.62E+00 | | | |
| 8166730 | NM_000397 | CYBB | cytochrome b-245, beta polypeptide | 1.478 | 2.786 | 1.13E-03 | 1.13E-01 | Up | Phagocytosis/killing | |
| 8092169 | NM_003810 | TNFSF10 | tumor necrosis factor (ligand) superf | 1.469 | 2.768 | 4.06E-03 | 4.06E-01 | Up | | |
| 7893576 | --- | --- | --- | 1.466 | 2.762 | 1.72E-01 | 1.72E+01 | | | |
| 7893462 | --- | --- | --- | 1.464 | 2.758 | 3.38E-02 | 3.38E+00 | Up | | |
| 8105918 | NM_001515 | GTF2H2 | general transcription factor IIH, poly | 1.449 | 2.731 | 3.49E-04 | 3.49E-02 | Up | | |
| 7974303 | NM_030755 | TMX1 | thioredoxin-related transmembrane | 1.448 | 2.728 | 3.54E-02 | 3.54E+00 | Up | | |
| 8055702 | NM_004688 | NMI | N-myc (and STAT) interactor | 1.440 | 2.713 | 1.03E-02 | 1.03E+00 | Up | | |

| | | | | | | | | | | |
|---|---|---|---|---|---|---|---|---|---|---|
| 7894304 | --- | --- | --- | 1.438 | 2.709 | 5.78E-02 | 5.78E+00 | | | |
| 7895276 | --- | --- | --- | 1.425 | 2.686 | 2.88E-02 | 2.88E+00 | Up | | |
| 7893966 | --- | --- | --- | 1.425 | 2.685 | 3.35E-02 | 3.35E+00 | Up | | |
| 7920128 | NM_005620 | S100A11 | S100 calcium binding protein A11 | 1.424 | 2.683 | 4.86E-03 | 4.86E-01 | Up | | mono |
| 7895419 | --- | --- | --- | 1.421 | 2.678 | 1.59E-02 | 1.59E+00 | Up | | |
| 7892953 | --- | --- | --- | 1.421 | 2.677 | 2.21E-02 | 2.21E+00 | Up | | |
| 8136446 | --- | --- | --- | 1.418 | 2.673 | 5.22E-02 | 5.22E+00 | | | |
| 7895088 | --- | --- | --- | 1.418 | 2.672 | 4.02E-02 | 4.02E+00 | Up | | |
| 8157691 | NM_182620 | SKA2 | spindle and kinetochore associated c | 1.411 | 2.659 | 3.26E-02 | 3.26E+00 | Up | | |
| 7895903 | --- | --- | --- | 1.409 | 2.655 | 6.89E-02 | 6.89E+00 | | | |
| 7895965 | --- | --- | --- | 1.408 | 2.653 | 8.88E-03 | 8.88E-01 | Up | | |
| 8107532 | NM_000414 | HSD17B4 | hydroxysteroid (17-beta) dehydroge | 1.405 | 2.648 | 2.56E-04 | 2.56E-02 | Up | | |
| 8056201 | NM_016836 | RBMS1 | RNA binding motif, single stranded ir | 1.401 | 2.642 | 3.16E-02 | 3.16E+00 | Up | | |
| 7892802 | --- | --- | --- | 1.401 | 2.641 | 1.09E-01 | 1.09E+01 | | | |
| 7895911 | --- | --- | --- | 1.399 | 2.637 | 1.36E-02 | 1.36E+00 | Up | | |
| 7894725 | --- | --- | --- | 1.398 | 2.635 | 3.37E-02 | 3.37E+00 | Up | | |
| 8110666 | NM_032765 | TRIM52 | tripartite motif-containing 52 | 1.396 | 2.633 | 4.97E-04 | 4.97E-02 | Up | | |
| 7935776 | NM_005063 | SCD | stearoyl-CoA desaturase (delta-9-des | 1.395 | 2.630 | 3.87E-02 | 3.87E+00 | Up | | |
| 7895101 | --- | --- | --- | 1.392 | 2.625 | 8.69E-02 | 8.69E+00 | | | |
| 8095773 | NM_003715 | USO1 | USO1 homolog, vesicle docking prot | 1.389 | 2.620 | 1.00E-03 | 1.00E-01 | Up | | |
| 7894661 | --- | --- | --- | 1.387 | 2.616 | 1.21E-01 | 1.21E+01 | | | |
| 7893806 | --- | --- | --- | 1.385 | 2.612 | 1.29E-02 | 1.29E+00 | Up | | |
| 8177674 | NM_001515 | GTF2H2 | general transcription factor IIH, poly | 1.370 | 2.585 | 4.01E-04 | 4.01E-02 | Up | | |
| 7894069 | --- | --- | --- | 1.369 | 2.583 | 1.31E-01 | 1.31E+01 | | | |
| 7892555 | --- | --- | --- | 1.364 | 2.575 | 6.12E-02 | 6.12E+00 | | | |
| 8112538 | NM_001515 | GTF2H2 | general transcription factor IIH, poly | 1.364 | 2.574 | 5.29E-04 | 5.29E-02 | Up | | |
| 8177507 | NM_001515 | GTF2H2 | general transcription factor IIH, poly | 1.364 | 2.574 | 5.29E-04 | 5.29E-02 | Up | | |
| 7893470 | --- | --- | --- | 1.364 | 2.574 | 1.60E-01 | 1.60E+01 | | | |
| 8056285 | NM_022168 | IFIH1 | interferon induced with helicase C do | 1.362 | 2.570 | 7.24E-04 | 7.24E-02 | Up | Anti-microbial/Pathogen recogn. | |
| 7933947 | NM_022079 | HERC4 | hect domain and RLD 4 | 1.359 | 2.566 | 1.23E-06 | 1.23E-04 | Up | | |
| 7895873 | --- | --- | --- | 1.359 | 2.565 | 3.64E-02 | 3.64E+00 | Up | | |
| 8160559 | NM_014314 | DDX58 | DEAD (Asp-Glu-Ala-Asp) box polypep | 1.343 | 2.537 | 6.23E-04 | 6.23E-02 | Up | Anti-microbial/Pathogen recogn. | |
| 7924549 | BC032332 | PCMTD2 | protein-L-isoaspartate (D-aspartate) | 1.343 | 2.537 | 7.98E-02 | 7.98E+00 | | | |
| 8049963 | BC032332 | PCMTD2 | protein-L-isoaspartate (D-aspartate) | 1.343 | 2.537 | 7.98E-02 | 7.98E+00 | | | |
| 8148333 | NM_015137 | EFR3A | EFR3 homolog A (S. cerevisiae) | 1.340 | 2.532 | 3.22E-04 | 3.22E-02 | Up | | |
| 7893952 | --- | --- | --- | 1.338 | 2.528 | 3.29E-03 | 3.29E-01 | Up | | |
| 7951397 | NM_033292 | CASP1 | caspase 1, apoptosis-related cystein | 1.334 | 2.522 | 3.34E-03 | 3.34E-01 | Up | Phagocytosis/killing | |
| 7893173 | --- | --- | --- | 1.332 | 2.517 | 1.79E-02 | 1.79E+00 | Up | | |
| 8114193 | NM_00103350 | SAR1B | SAR1 homolog B (S. cerevisiae) | 1.332 | 2.517 | 2.27E-02 | 2.27E+00 | Up | | |
| 7892894 | --- | --- | --- | 1.330 | 2.514 | 2.11E-01 | 2.11E+01 | | | |
| 7894827 | --- | --- | --- | 1.329 | 2.513 | 1.50E-01 | 1.50E+01 | | | |
| 7894515 | --- | --- | --- | 1.323 | 2.502 | 1.65E-01 | 1.65E+01 | | | |
| 7894386 | --- | --- | --- | 1.322 | 2.501 | 1.30E-02 | 1.30E+00 | Up | | |
| 7895032 | --- | --- | --- | 1.314 | 2.486 | 4.26E-02 | 4.26E+00 | Up | | |
| 7894836 | --- | --- | --- | 1.312 | 2.483 | 2.26E-01 | 2.26E+01 | | | |
| 7969796 | NM_004800 | TM9SF2 | transmembrane 9 superfamily mem | 1.311 | 2.480 | 4.71E-04 | 4.71E-02 | Up | | |
| 7971820 | NM_016075 | VPS36 | vacuolar protein sorting 36 homolog | 1.310 | 2.480 | 4.19E-03 | 4.19E-01 | Up | | |
| 7919580 | AK125737 // A | 0570 // LOC | hypothetical LOC440570 // hypothe | 1.310 | 2.480 | 3.28E-02 | 3.28E+00 | Up | | |
| 7919596 | AK125737 // A | 0570 // LOC | hypothetical LOC440570 // hypothe | 1.310 | 2.480 | 3.28E-02 | 3.28E+00 | Up | | |
| 8011826 | AY358809 | C17orf87 | chromosome 17 open reading frame | 1.308 | 2.476 | 6.65E-03 | 6.65E-01 | Up | | |
| 7979131 | NM_053064 | GNG2 | guanine nucleotide binding protein ( | 1.305 | 2.470 | 3.14E-02 | 3.14E+00 | Up | | |
| 7894512 | --- | --- | --- | 1.303 | 2.467 | 5.71E-02 | 5.71E+00 | | | |
| 7982531 | NM_006305 /// | // ANP32A / | acidic (leucine-rich) nuclear phospho | 1.303 | 2.467 | 2.95E-02 | 2.95E+00 | Up | | |
| 8068238 | NM_207585 | IFNAR2 | interferon (alpha, beta and omega) r | 1.301 | 2.463 | 3.25E-04 | 3.25E-02 | Up | | |
| 7896422 | --- | --- | --- | 1.298 | 2.459 | 6.00E-03 | 6.00E-01 | Up | | |
| 7893732 | --- | --- | --- | 1.295 | 2.453 | 2.09E-02 | 2.09E+00 | Up | | |
| 7999752 | NM_020466 | LYRM2 | LYR motif containing 2 | 1.294 | 2.451 | 1.91E-01 | 1.91E+01 | | | |
| 7892668 | --- | --- | --- | 1.294 | 2.451 | 1.50E-01 | 1.50E+01 | | | |
| 7892954 | --- | --- | --- | 1.292 | 2.448 | 5.95E-02 | 5.95E+00 | | | |
| 8140463 | NM_006682 | FGL2 | fibrinogen-like 2 | 1.291 | 2.446 | 1.27E-02 | 1.27E+00 | Up | | |
| 7903308 | NM_019083 | CCDC76 | coiled-coil domain containing 76 | 1.289 | 2.444 | 2.94E-03 | 2.94E-01 | Up | | |
| 8154785 | NM_016410 | CHMP5 | chromatin modifying protein 5 | 1.289 | 2.443 | 4.10E-03 | 4.10E-01 | Up | | |
| 8154305 | NM_016275 | SELT | selenoprotein T | 1.289 | 2.443 | 8.56E-02 | 8.56E+00 | | | |
| 7997520 | NM_001537 | HSBP1 | heat shock factor binding protein 1 | 1.288 | 2.442 | 4.73E-03 | 4.73E-01 | Up | | |
| 7895697 | --- | --- | --- | 1.287 | 2.441 | 3.94E-02 | 3.94E+00 | Up | | |
| 7895786 | --- | --- | --- | 1.281 | 2.431 | 1.91E-01 | 1.91E+01 | | | |
| 7931081 | NM_021622 | PLEKHA1 | pleckstrin homology domain contain | 1.277 | 2.424 | 7.57E-04 | 7.57E-02 | Up | | |
| 7896044 | --- | --- | --- | 1.275 | 2.420 | 1.02E-01 | 1.02E+01 | | | |
| 7996947 | NM_030579 | CYB5B | cytochrome b5 type B (outer mitoch | 1.273 | 2.417 | 1.34E-02 | 1.34E+00 | Up | | |
| 7895298 | --- | --- | --- | 1.271 | 2.413 | 5.55E-02 | 5.55E+00 | | | |
| 7893181 | --- | --- | --- | 1.268 | 2.409 | 3.68E-02 | 3.68E+00 | Up | | |
| 8090509 | NM_004637 | RAB7A | RAB7A, member RAS oncogene fami | 1.266 | 2.406 | 8.34E-02 | 8.34E+00 | | | |
| 7970426 | --- | --- | --- | 1.266 | 2.405 | 4.79E-02 | 4.79E+00 | Up | | |
| 7896258 | --- | --- | --- | 1.264 | 2.402 | 4.64E-02 | 4.64E+00 | Up | | |
| 8131263 | X58060 | RNU13P2 | RNA, U13 small nuclear pseudogene | 1.262 | 2.399 | 2.66E-02 | 2.66E+00 | Up | | |
| 7895548 | --- | --- | --- | 1.261 | 2.397 | 6.03E-02 | 6.03E+00 | | | |
| 7893485 | --- | --- | --- | 1.255 | 2.387 | 8.66E-02 | 8.66E+00 | | | |

| | | | | | | | | | | |
|---|---|---|---|---|---|---|---|---|---|---|
| 8004175 | NM_032731 | TXNDC17 | thioredoxin domain containing 17 | 1.254 | 2.385 | 8.92E-04 | 8.92E-02 | Up | | |
| 7896742 | NM_207366 | 40435 | septin 14 | 1.254 | 2.385 | 8.97E-02 | 8.97E+00 | | | |
| 7911347 | NM_207366 | 40435 | septin 14 | 1.254 | 2.385 | 8.97E-02 | 8.97E+00 | | | |
| 7945342 | NM_207366 | 40435 | septin 14 | 1.254 | 2.385 | 8.97E-02 | 8.97E+00 | | | |
| 8031990 | NM_207366 | 40435 | septin 14 | 1.254 | 2.385 | 8.97E-02 | 8.97E+00 | | | |
| 8085054 | NM_207366 | 40435 | septin 14 | 1.254 | 2.385 | 8.97E-02 | 8.97E+00 | | | |
| 8110670 | NM_207366 | 40435 | septin 14 | 1.254 | 2.385 | 8.97E-02 | 8.97E+00 | | | |
| 8137666 | NM_207366 | 40435 | septin 14 | 1.254 | 2.385 | 8.97E-02 | 8.97E+00 | | | |
| 8148964 | NM_207366 | 40435 | septin 14 | 1.254 | 2.385 | 8.97E-02 | 8.97E+00 | | | |
| 8112607 | --- | --- | --- | 1.252 | 2.381 | 6.20E-02 | 6.20E+00 | | | |
| 7989037 | NM_004748 | CCPG1 | cell cycle progression 1 | 1.251 | 2.380 | 1.42E-04 | 1.42E-02 | Up | | |
| 7895151 | --- | --- | --- | 1.250 | 2.379 | 5.11E-02 | 5.11E+00 | | | |
| 7964937 | NM_144982 | ZFC3H1 | zinc finger, C3H1-type containing | 1.250 | 2.378 | 1.23E-06 | 1.23E-04 | Up | | |
| 7985202 | NM_002789 | PSMA4 | proteasome (prosome, macropain) s | 1.249 | 2.376 | 2.13E-02 | 2.13E+00 | Up | | |
| 8051670 | NM_005633 | SOS1 | son of sevenless homolog 1 (Drosopł | 1.248 | 2.376 | 3.03E-03 | 3.03E-01 | Up | | |
| 7946569 | NM_016422 | RNF141 | ring finger protein 141 | 1.245 | 2.370 | 2.33E-02 | 2.33E+00 | Up | | |
| 7918345 | NM_002790 | PSMA5 | proteasome (prosome, macropain) s | 1.244 | 2.369 | 1.57E-02 | 1.57E+00 | Up | | |
| 7893539 | --- | --- | --- | 1.239 | 2.360 | 3.81E-02 | 3.81E+00 | Up | | |
| 7896450 | --- | --- | --- | 1.237 | 2.358 | 2.08E-01 | 2.08E+01 | | | |
| 7894579 | --- | --- | --- | 1.235 | 2.354 | 2.44E-02 | 2.44E+00 | Up | | |
| 7971296 | NM_00100226 | EPSTI1 | epithelial stromal interaction 1 (brea | 1.235 | 2.353 | 8.12E-04 | 8.12E-02 | Up | | |
| 8090277 | NM_022776 | OSBPL11 | oxysterol binding protein-like 11 | 1.232 | 2.348 | 2.05E-03 | 2.05E-01 | Up | | |
| 7953901 | NM_138337 | CLEC12A | C-type lectin domain family 12, mem | 1.229 | 2.345 | 6.39E-04 | 6.39E-02 | Up | | |
| 7954492 | NM_015633 | FGFR1OP2 | FGFR1 oncogene partner 2 | 1.229 | 2.344 | 1.87E-03 | 1.87E-01 | Up | | |
| 7921873 | NM_000569 | FCGR3A | Fc fragment of IgG, low affinity IIIa, r | 1.228 | 2.342 | 1.78E-03 | 1.78E-01 | Up | Phagocytosis/killing | |
| 8084488 | NM_006232 | POLR2H | polymerase (RNA) II (DNA directed) p | 1.226 | 2.339 | 8.46E-03 | 8.46E-01 | Up | | |
| 7894562 | --- | --- | --- | 1.226 | 2.339 | 5.92E-02 | 5.92E+00 | | | |
| 7895211 | --- | --- | --- | 1.225 | 2.338 | 2.14E-01 | 2.14E+01 | | | |
| 8162147 | NM_024617 | ZCCHC6 | zinc finger, CCHC domain containing | 1.222 | 2.333 | 1.26E-03 | 1.26E-01 | Up | | |
| 7895133 | --- | --- | --- | 1.222 | 2.333 | 2.48E-02 | 2.48E+00 | Up | | |
| 7893883 | --- | --- | --- | 1.218 | 2.326 | 3.20E-02 | 3.20E+00 | Up | | |
| 7893980 | --- | --- | --- | 1.214 | 2.320 | 4.38E-02 | 4.38E+00 | Up | | |
| 8042601 | NM_014497 | ZNF638 | zinc finger protein 638 | 1.213 | 2.318 | 1.74E-04 | 1.74E-02 | Up | | |
| 8154207 | --- | --- | --- | 1.213 | 2.318 | 2.53E-02 | 2.53E+00 | Up | | |
| 7921434 | NM_004833 | AIM2 | absent in melanoma 2 | 1.209 | 2.312 | 5.67E-04 | 5.67E-02 | Up | Anti-microbial/Pathogen recogn. | |
| 7895291 | --- | --- | --- | 1.208 | 2.310 | 1.24E-01 | 1.24E+01 | | | |
| 7976243 | NM_017437 | CPSF2 | cleavage and polyadenylation specifi | 1.205 | 2.306 | 9.85E-04 | 9.85E-02 | Up | | |
| 8180392 | --- | --- | --- | 1.205 | 2.306 | 1.22E-02 | 1.22E+00 | Up | | |
| 7911329 | NM_207366 | 40435 | septin 14 | 1.205 | 2.305 | 8.85E-02 | 8.85E+00 | | | |
| 8008870 | NM_030938 | TMEM49 | transmembrane protein 49 | 1.204 | 2.304 | 6.34E-03 | 6.34E-01 | Up | | |
| 7894072 | --- | --- | --- | 1.203 | 2.303 | 7.60E-02 | 7.60E+00 | | | |
| 8050695 | NM_016047 | SF3B14 | splicing factor 3B, 14 kDa subunit | 1.200 | 2.297 | 8.33E-03 | 8.33E-01 | Up | | |
| 7894698 | --- | --- | --- | 1.199 | 2.295 | 2.22E-01 | 2.22E+01 | | | |
| 8030360 | NR_000021 | SNORD32A | small nucleolar RNA, C/D box 32A | 1.196 | 2.291 | 3.72E-02 | 3.72E+00 | Up | | |
| 7895694 | --- | --- | --- | 1.189 | 2.280 | 1.44E-01 | 1.44E+01 | | | |
| 7922646 | NM_022347 | IFRG15 | interferon responsive gene 15 | 1.189 | 2.280 | 8.14E-02 | 8.14E+00 | | | |
| 8165684 | --- | --- | --- | 1.187 | 2.276 | 2.51E-01 | 2.51E+01 | | | |
| 7893314 | --- | --- | --- | 1.186 | 2.275 | 3.08E-01 | 3.08E+01 | | | |
| 8090018 | NM_031458 | PARP9 | poly (ADP-ribose) polymerase family | 1.186 | 2.275 | 2.86E-04 | 2.86E-02 | Up | | |
| 7893933 | --- | --- | --- | 1.185 | 2.273 | 1.58E-01 | 1.58E+01 | | | |
| 8083092 | NM_00108041 | ZBTB38 | zinc finger and BTB domain containir | 1.184 | 2.272 | 2.88E-03 | 2.88E-01 | Up | | |
| 7895547 | --- | --- | --- | 1.184 | 2.272 | 5.20E-02 | 5.20E+00 | | | |
| 7894419 | --- | --- | --- | 1.183 | 2.271 | 1.95E-01 | 1.95E+01 | | | |
| 7894314 | --- | --- | --- | 1.180 | 2.266 | 2.19E-01 | 2.19E+01 | | | |
| 7896041 | --- | --- | --- | 1.179 | 2.264 | 5.43E-02 | 5.43E+00 | | | |
| 8161242 | NM_016042 | EXOSC3 | exosome component 3 | 1.178 | 2.262 | 5.87E-03 | 5.87E-01 | Up | | |
| 7895820 | --- | --- | --- | 1.176 | 2.260 | 1.54E-01 | 1.54E+01 | | | |
| 7896318 | --- | --- | --- | 1.176 | 2.259 | 1.91E-01 | 1.91E+01 | | | |
| 7902367 | NM_000016 | ACADM | acyl-Coenzyme A dehydrogenase, C- | 1.169 | 2.249 | 1.37E-02 | 1.37E+00 | Up | | |
| 8089988 | NM_00101792 | CCDC58 | coiled-coil domain containing 58 | 1.169 | 2.248 | 5.74E-03 | 5.74E-01 | Up | | |
| 7894280 | --- | --- | --- | 1.168 | 2.248 | 4.54E-02 | 4.54E+00 | Up | | |
| 8043197 | NM_003761 | VAMP8 | vesicle-associated membrane protei | 1.168 | 2.247 | 6.83E-03 | 6.83E-01 | Up | | |
| 7894409 | --- | --- | --- | 1.166 | 2.244 | 1.22E-01 | 1.22E+01 | | | |
| 7893778 | --- | --- | --- | 1.164 | 2.241 | 1.01E-01 | 1.01E+01 | | | |
| 7894993 | --- | --- | --- | 1.164 | 2.241 | 2.13E-01 | 2.13E+01 | | | |
| 7894433 | --- | --- | --- | 1.158 | 2.231 | 9.14E-02 | 9.14E+00 | | | |
| 7896533 | --- | --- | --- | 1.157 | 2.230 | 1.53E-02 | 1.53E+00 | Up | | |
| 8146934 | NM_015364 | LY96 | lymphocyte antigen 96 | 1.156 | 2.229 | 1.70E-02 | 1.70E+00 | Up | | |
| 7976152 | --- | --- | --- | 1.155 | 2.227 | 1.24E-01 | 1.24E+01 | | | |
| 8099027 | --- | --- | --- | 1.153 | 2.224 | 8.87E-02 | 8.87E+00 | | | |
| 7895247 | --- | --- | --- | 1.152 | 2.223 | 1.17E-01 | 1.17E+01 | | | |
| 8040386 | NM_004939 | DDX1 | DEAD (Asp-Glu-Ala-Asp) box polypep | 1.146 | 2.213 | 6.74E-03 | 6.74E-01 | Up | | |
| 7893721 | --- | --- | --- | 1.145 | 2.212 | 9.34E-03 | 9.34E-01 | Up | | |
| 8088718 | NM_003968 | UBA3 | ubiquitin-like modifier activating enz | 1.143 | 2.209 | 1.59E-02 | 1.59E+00 | Up | | |
| 7987892 | NM_022473 | ZFP106 | zinc finger protein 106 homolog (mo | 1.139 | 2.202 | 7.79E-04 | 7.79E-02 | Up | | |
| 8096675 | NM_00112720 | TET2 | tet oncogene family member 2 | 1.137 | 2.200 | 6.32E-03 | 6.32E-01 | Up | | |
| 8139458 | AK096229 | OC10012836 | hypothetical LOC100128364 | 1.136 | 2.197 | 1.71E-02 | 1.71E+00 | Up | | |

| | | | | | | | | | | |
|---|---|---|---|---|---|---|---|---|---|---|
| 7964745 | NM_016056 | TMBIM4 | transmembrane BAX inhibitor motif | 1.132 | 2.192 | 1.26E-04 | 1.26E-02 | Up | | |
| 8021015 | NM_002647 | PIK3C3 | phosphoinositide-3-kinase, class 3 | 1.130 | 2.189 | 1.29E-02 | 1.29E+00 | Up | | |
| 7894171 | --- | --- | --- | 1.129 | 2.186 | 1.16E-01 | 1.16E+01 | | | |
| 7894946 | --- | --- | --- | 1.128 | 2.185 | 1.64E-01 | 1.64E+01 | | | |
| 7892565 | --- | --- | --- | 1.126 | 2.183 | 6.79E-02 | 6.79E+00 | | | |
| 7894712 | --- | --- | --- | 1.125 | 2.182 | 1.12E-01 | 1.12E+01 | | | |
| 7895774 | --- | --- | --- | 1.121 | 2.174 | 1.09E-01 | 1.09E+01 | | | |
| 7967420 | NM_018183 | SBNO1 | strawberry notch homolog 1 (Drosop | 1.120 | 2.174 | 3.29E-03 | 3.29E-01 | Up | | |
| 7971184 | NM_005830 | MRPS31 | mitochondrial ribosomal protein S31 | 1.120 | 2.173 | 1.79E-03 | 1.79E-01 | Up | | |
| 7896067 | --- | --- | --- | 1.120 | 2.173 | 1.41E-01 | 1.41E+01 | | | |
| 8047161 | NM_00103171( | OBFC2A | oligonucleotide/oligosaccharide-binc | 1.118 | 2.170 | 5.22E-03 | 5.22E-01 | Up | | |
| 8047839 | NM_004379 | CREB1 | cAMP responsive element binding pr | 1.117 | 2.169 | 3.47E-03 | 3.47E-01 | Up | | |
| 8072153 | NM_173510 | CCDC117 | coiled-coil domain containing 117 | 1.115 | 2.166 | 2.67E-04 | 2.67E-02 | Up | | |
| 7926596 | NM_012071 | COMMD3 | COMM domain containing 3 | 1.114 | 2.164 | 7.90E-03 | 7.90E-01 | Up | | |
| 7980535 | NM_00103749 | DYNLL1 | dynein, light chain, LC8-type 1 | 1.113 | 2.164 | 4.64E-02 | 4.64E+00 | Up | Antigen present./co-stim. | |
| 8127629 | NM_001865 | COX7A2 | cytochrome c oxidase subunit VIIa po | 1.112 | 2.161 | 3.38E-02 | 3.38E+00 | Up | | |
| 8004237 | NM_00100433 | RNASEK | ribonuclease, RNase K | 1.110 | 2.159 | 5.47E-03 | 5.47E-01 | Up | | |
| 7980438 | NM_004863 | SPTLC2 | serine palmitoyltransferase, long cha | 1.104 | 2.150 | 1.26E-03 | 1.26E-01 | Up | | |
| 7895588 | --- | --- | --- | 1.104 | 2.150 | 1.53E-01 | 1.53E+01 | | | |
| 7896664 | --- | --- | --- | 1.103 | 2.148 | 6.78E-02 | 6.78E+00 | | | |
| 8017235 | NM_006380 | APPBP2 | amyloid beta precursor protein (cyto | 1.103 | 2.147 | 4.52E-03 | 4.52E-01 | Up | | |
| 7924733 | NM_001618 | PARP1 | poly (ADP-ribose) polymerase 1 | 1.102 | 2.146 | 9.49E-04 | 9.49E-02 | Up | | |
| 7969794 | AY358798 | UNQ1829 | FRSS1829 | 1.099 | 2.142 | 2.04E-01 | 2.04E+01 | | | |
| 7893217 | --- | --- | --- | 1.099 | 2.142 | 1.59E-02 | 1.59E+00 | Up | | |
| 7971550 | NM_014166 | MED4 | mediator complex subunit 4 | 1.098 | 2.141 | 1.27E-02 | 1.27E+00 | Up | | |
| 7896406 | --- | --- | --- | 1.098 | 2.141 | 2.46E-01 | 2.46E+01 | | | |
| 8042270 | NM_00100152 | UGP2 | UDP-glucose pyrophosphorylase 2 | 1.098 | 2.141 | 1.52E-02 | 1.52E+00 | Up | | |
| 8082075 | NM_138287 | DTX3L | deltex 3-like (Drosophila) | 1.098 | 2.141 | 6.33E-03 | 6.33E-01 | Up | | |
| 7951408 | NM_052889 | CARD16 | caspase recruitment domain family, | 1.097 | 2.139 | 8.12E-03 | 8.12E-01 | Up | | |
| 7893593 | --- | --- | --- | 1.096 | 2.138 | 8.09E-02 | 8.09E+00 | | | |
| 8103011 | NM_199324 | OTUD4 | OTU domain containing 4 | 1.096 | 2.137 | 6.00E-03 | 6.00E-01 | Up | | |
| 8099449 | NM_033535 | FBXL5 | F-box and leucine-rich repeat protei | 1.094 | 2.135 | 1.61E-03 | 1.61E-01 | Up | | |
| 7893318 | --- | --- | --- | 1.094 | 2.135 | 2.11E-01 | 2.11E+01 | | | |
| 8103601 | NM_00101296 | DDX60L | DEAD (Asp-Glu-Ala-Asp) box polypep | 1.094 | 2.134 | 2.74E-02 | 2.74E+00 | Up | | |
| 7893333 | --- | --- | --- | 1.092 | 2.132 | 2.10E-01 | 2.10E+01 | | | |
| 7895447 | --- | --- | --- | 1.091 | 2.130 | 7.19E-02 | 7.19E+00 | | | |
| 7895985 | --- | --- | --- | 1.090 | 2.129 | 4.01E-02 | 4.01E+00 | Up | | |
| 8100298 | NM_00101444( | OCIAD2 | OCIA domain containing 2 | 1.090 | 2.129 | 3.08E-02 | 3.08E+00 | Up | | |
| 8155883 | NM_012383 | OSTF1 | osteoclast stimulating factor 1 | 1.084 | 2.120 | 3.32E-03 | 3.32E-01 | Up | | |
| 8070269 | NM_006052 | DSCR3 | Down syndrome critical region gene | 1.083 | 2.118 | 1.35E-02 | 1.35E+00 | Up | | |
| 8140814 | NM_018843 | SLC25A40 | solute carrier family 25, member 40 | 1.082 | 2.118 | 2.61E-02 | 2.61E+00 | Up | | |
| 7892905 | --- | --- | --- | 1.081 | 2.116 | 1.95E-01 | 1.95E+01 | | | |
| 7894527 | --- | --- | --- | 1.080 | 2.115 | 3.35E-02 | 3.35E+00 | Up | | |
| 7985134 | NM_005530 | IDH3A | isocitrate dehydrogenase 3 (NAD+) a | 1.079 | 2.112 | 3.28E-03 | 3.28E-01 | Up | | |
| 8150862 | --- | --- | --- | 1.077 | 2.110 | 6.19E-03 | 6.19E-01 | Up | | |
| 8137240 | NM_153236 | GIMAP7 | GTPase, IMAP family member 7 | 1.077 | 2.110 | 4.87E-05 | 4.87E-03 | Up | | |
| 7925257 | NM_000081 | LYST | lysosomal trafficking regulator | 1.076 | 2.108 | 1.46E-05 | 1.46E-03 | Up | Phagocytosis/killing | |
| 7929562 | NM_014803 | ZNF518A | zinc finger protein 518A | 1.075 | 2.106 | 4.36E-03 | 4.36E-01 | Up | | |
| 7979367 | NM_006544 | EXOC5 | exocyst complex component 5 | 1.074 | 2.105 | 1.16E-02 | 1.16E+00 | Up | | |
| 7894465 | --- | --- | --- | 1.073 | 2.103 | 6.39E-02 | 6.39E+00 | | | |
| 8180402 | --- | --- | --- | 1.073 | 2.103 | 1.52E-02 | 1.52E+00 | Up | | |
| 7958262 | NM_152772 | TCP11L2 | t-complex 11 (mouse)-like 2 | 1.072 | 2.103 | 9.07E-03 | 9.07E-01 | Up | | |
| 7895237 | --- | --- | --- | 1.072 | 2.102 | 4.55E-02 | 4.55E+00 | Up | | |
| 7906720 | NM_004106 | FCER1G | Fc fragment of IgE, high affinity I, rec | 1.070 | 2.100 | 4.01E-02 | 4.01E+00 | Up | | |
| 7896295 | --- | --- | --- | 1.070 | 2.100 | 2.49E-01 | 2.49E+01 | | | |
| 8139033 | NM_001637 | AOAH | acyloxyacyl hydrolase (neutrophil) | 1.070 | 2.099 | 3.70E-02 | 3.70E+00 | Up | | |
| 7929288 | NM_019053 | EXOC6 | exocyst complex component 6 | 1.069 | 2.097 | 6.19E-03 | 6.19E-01 | Up | | |
| 8106743 | NM_004385 | VCAN | versican | 1.068 | 2.097 | 7.71E-03 | 7.71E-01 | Up | | |
| 8109597 | NM_145049 | UBLCP1 | ubiquitin-like domain containing CTD | 1.068 | 2.096 | 6.43E-03 | 6.43E-01 | Up | | |
| 7971565 | NM_005767 | LPAR6 | lysophosphatidic acid receptor 6 | 1.068 | 2.096 | 2.72E-04 | 2.72E-02 | Up | | |
| 7970793 | NM_181785 | SLC46A3 | solute carrier family 46, member 3 | 1.067 | 2.095 | 1.38E-02 | 1.38E+00 | Up | | |
| 7894011 | --- | --- | --- | 1.065 | 2.093 | 1.17E-01 | 1.17E+01 | | | |
| 7926836 | NM_021252 | RAB18 | RAB18, member RAS oncogene famil | 1.065 | 2.092 | 2.29E-02 | 2.29E+00 | Up | | |
| 8064790 | NM_014737 | RASSF2 | Ras association (RalGDS/AF-6) doma | 1.062 | 2.088 | 1.53E-04 | 1.53E-02 | Up | | |
| 7957023 | NM_000239 | LYZ | lysozyme (renal amyloidosis) | 1.060 | 2.085 | 7.76E-03 | 7.76E-01 | Up | Phagocytosis/killing | |
| 8039010 | NM_018260 | ZNF701 | zinc finger protein 701 | 1.059 | 2.084 | 2.25E-02 | 2.25E+00 | Up | | |
| 7924603 | NM_002296 | LBR | lamin B receptor | 1.059 | 2.083 | 5.90E-04 | 5.90E-02 | Up | | |
| 7896684 | --- | --- | --- | 1.058 | 2.083 | 1.60E-02 | 1.60E+00 | Up | | |
| 8085287 | NM_018462 | C3orf10 | chromosome 3 open reading frame 1 | 1.058 | 2.081 | 1.18E-02 | 1.18E+00 | Up | Phagocytosis/killing | |
| 8129637 | NM_004665 | VNN2 | vanin 2 | 1.057 | 2.081 | 8.92E-03 | 8.92E-01 | Up | | |
| 8006812 | NM_002795 | PSMB3 | proteasome (prosome, macropain) s | 1.056 | 2.079 | 7.21E-03 | 7.21E-01 | Up | | |
| 7928119 | NM_004096 | EIF4EBP2 | eukaryotic translation initiation facto | 1.055 | 2.078 | 1.14E-02 | 1.14E+00 | Up | | |
| 8140967 | NM_017654 | SAMD9 | sterile alpha motif domain containin | 1.054 | 2.077 | 7.89E-03 | 7.89E-01 | Up | | |
| 7893319 | --- | --- | --- | 1.052 | 2.074 | 3.72E-01 | 3.72E+01 | | | |
| 7894693 | --- | --- | --- | 1.052 | 2.073 | 7.75E-02 | 7.75E+00 | | | |
| 8121429 | NM_014845 | FIG4 | FIG4 homolog (S. cerevisiae) | 1.052 | 2.073 | 1.35E-03 | 1.35E-01 | Up | | |
| 7894755 | --- | --- | --- | 1.051 | 2.073 | 2.31E-01 | 2.31E+01 | | | |

| | | | | | | | | | | |
|---|---|---|---|---|---|---|---|---|---|---|
| 7896567 | --- | --- | --- | 1.051 | 2.072 | 4.04E-02 | 4.04E+00 | Up | | |
| 7958884 | NM_016816 | OAS1 | 2',5'-oligoadenylate synthetase 1, 40 | 1.051 | 2.072 | 4.52E-03 | 4.52E-01 | Up | | |
| 7896622 | --- | --- | --- | 1.050 | 2.071 | 3.88E-02 | 3.88E+00 | Up | | |
| 8147724 | NM_001695 | ATP6V1C1 | ATPase, H+ transporting, lysosomal 4 | 1.048 | 2.068 | 4.52E-03 | 4.52E-01 | Up | | |
| 7896601 | --- | --- | --- | 1.046 | 2.065 | 1.07E-01 | 1.07E+01 | | | |
| 7893674 | --- | --- | --- | 1.045 | 2.064 | 2.05E-01 | 2.05E+01 | | | |
| 7927799 | ENST00000298 | REEP3 | receptor accessory protein 3 | 1.045 | 2.063 | 4.43E-02 | 4.43E+00 | Up | | |
| 8007976 | NM_006310 | NPEPPS | aminopeptidase puromycin sensitive | 1.043 | 2.061 | 3.31E-03 | 3.31E-01 | Up | | |
| 8078008 | NM_014463 | LSM3 | LSM3 homolog, U6 small nuclear RN | 1.043 | 2.061 | 6.77E-02 | 6.77E+00 | | | |
| 7896142 | --- | --- | --- | 1.043 | 2.060 | 1.14E-01 | 1.14E+01 | | | |
| 7896108 | --- | --- | --- | 1.042 | 2.059 | 3.60E-02 | 3.60E+00 | Up | | |
| 8005955 | NR_000014 | SNORD42A | small nucleolar RNA, C/D box 42A | 1.040 | 2.056 | 1.91E-01 | 1.91E+01 | | | |
| 8012000 | NM_00100433 | RNASEK | ribonuclease, RNase K | 1.039 | 2.055 | 7.65E-03 | 7.65E-01 | Up | | |
| 8041015 | NM_018158 | SLC4A1AP | solute carrier family 4 (anion exchan | 1.039 | 2.054 | 1.57E-02 | 1.57E+00 | Up | | |
| 8083445 | --- | --- | --- | 1.038 | 2.054 | 8.83E-02 | 8.83E+00 | | | |
| 8156579 | --- | --- | --- | 1.038 | 2.053 | 7.76E-02 | 7.76E+00 | | | |
| 7893028 | --- | --- | --- | 1.037 | 2.052 | 1.19E-01 | 1.19E+01 | | | |
| 8019521 | NM_004514 | FOXK2 | forkhead box K2 | 1.036 | 2.051 | 3.36E-02 | 3.36E+00 | Up | | |
| 8105908 | NM_002538 | OCLN | occludin | 1.036 | 2.050 | 2.43E-02 | 2.43E+00 | Up | | |
| 7895428 | --- | --- | --- | 1.036 | 2.050 | 1.59E-01 | 1.59E+01 | | | |
| 8129390 | --- | --- | --- | 1.034 | 2.047 | 1.11E-01 | 1.11E+01 | | | |
| 8046604 | NM_003659 | AGPS | alkylglycerone phosphate synthase | 1.033 | 2.046 | 2.71E-02 | 2.71E+00 | Up | | |
| 7892769 | --- | --- | --- | 1.033 | 2.046 | 1.44E-01 | 1.44E+01 | | | |
| 8000409 | NM_005517 | HMGN2 | high-mobility group nucleosomal bin | 1.032 | 2.045 | 9.77E-03 | 9.77E-01 | Up | | |
| 7896531 | --- | --- | --- | 1.032 | 2.045 | 3.96E-02 | 3.96E+00 | Up | | |
| 7892836 | --- | --- | --- | 1.029 | 2.040 | 1.09E-01 | 1.09E+01 | | | |
| 7895348 | --- | --- | --- | 1.027 | 2.038 | 1.54E-01 | 1.54E+01 | | | |
| 7896721 | --- | --- | --- | 1.027 | 2.038 | 2.95E-02 | 2.95E+00 | Up | | |
| 7894552 | --- | --- | --- | 1.027 | 2.037 | 5.19E-02 | 5.19E+00 | | | |
| 7896405 | --- | --- | --- | 1.025 | 2.035 | 7.48E-02 | 7.48E+00 | | | |
| 7962590 | NM_024604 | RPAP3 | RNA polymerase II associated protei | 1.024 | 2.033 | 2.23E-02 | 2.23E+00 | Up | | |
| 7998823 | NM_002613 | PDPK1 | 3-phosphoinositide dependent prote | 1.023 | 2.032 | 2.80E-02 | 2.80E+00 | Up | | |
| 7998841 | NM_002613 | PDPK1 | 3-phosphoinositide dependent prote | 1.023 | 2.032 | 2.80E-02 | 2.80E+00 | Up | | |
| 7943162 | BC012298 | C11orf54 | chromosome 11 open reading frame | 1.023 | 2.032 | 1.27E-02 | 1.27E+00 | Up | | |
| 8104449 | NM_012073 | CCT5 | chaperonin containing TCP1, subunit | 1.023 | 2.032 | 2.37E-03 | 2.37E-01 | Up | | |
| 7894638 | --- | --- | --- | 1.022 | 2.031 | 1.70E-01 | 1.70E+01 | | | |
| 7980146 | NM_006432 | NPC2 | Niemann-Pick disease, type C2 | 1.022 | 2.031 | 3.73E-03 | 3.73E-01 | Up | | |
| 8105481 | NM_152687 | GAPT | GRB2-binding adaptor protein, trans | 1.020 | 2.028 | 5.29E-04 | 5.29E-02 | Up | | |
| 7899774 | NM_004964 | HDAC1 | histone deacetylase 1 | 1.019 | 2.027 | 1.03E-02 | 1.03E+00 | Up | | |
| 7896726 | --- | --- | --- | 1.018 | 2.025 | 3.00E-02 | 3.00E+00 | Up | | |
| 8161509 | NM_004477 | FRG1 | FSHD region gene 1 | 1.018 | 2.025 | 6.27E-02 | 6.27E+00 | | | |
| 7988838 | NM_138792 | LEO1 | Leo1, Paf1/RNA polymerase II compl | 1.017 | 2.024 | 1.18E-02 | 1.18E+00 | Up | | |
| 7895666 | --- | --- | --- | 1.017 | 2.024 | 1.71E-01 | 1.71E+01 | | | |
| 8180366 | --- | --- | --- | 1.015 | 2.021 | 5.31E-02 | 5.31E+00 | | | |
| 8180367 | --- | --- | --- | 1.015 | 2.021 | 5.31E-02 | 5.31E+00 | | | |
| 7896439 | --- | --- | --- | 1.015 | 2.021 | 4.05E-02 | 4.05E+00 | Up | | |
| 7999834 | NM_015161 | ARL6IP1 | ADP-ribosylation factor-like 6 interac | 1.015 | 2.021 | 4.37E-02 | 4.37E+00 | Up | | |
| 7982204 | NM_005517 | HMGN2 | high-mobility group nucleosomal bin | 1.015 | 2.020 | 1.10E-02 | 1.10E+00 | Up | | |
| 7895016 | --- | --- | --- | 1.014 | 2.020 | 8.27E-02 | 8.27E+00 | | | |
| 7892901 | --- | --- | --- | 1.014 | 2.019 | 1.41E-01 | 1.41E+01 | | | |
| 7965627 | NM_000895 | LTA4H | leukotriene A4 hydrolase | 1.013 | 2.018 | 1.91E-03 | 1.91E-01 | Up | | |
| 7896343 | --- | --- | --- | 1.011 | 2.015 | 2.43E-01 | 2.43E+01 | | | |
| 8010078 | NR_004397 | SNORD1C | small nucleolar RNA, C/D box 1C | 1.011 | 2.015 | 8.27E-02 | 8.27E+00 | | | |
| 8056102 | NM_014880 | CD302 | CD302 molecule | 1.010 | 2.015 | 4.07E-02 | 4.07E+00 | Up | | |
| 7902400 | NR_002748 | SNORD45B | small nucleolar RNA, C/D box 45B | 1.010 | 2.015 | 2.18E-01 | 2.18E+01 | | | |
| 8043502 | --- | --- | --- | 1.010 | 2.013 | 8.82E-02 | 8.82E+00 | | | |
| 7894737 | --- | --- | --- | 1.009 | 2.013 | 6.47E-02 | 6.47E+00 | | | |
| 7940287 | NM_152866 | MS4A1 | membrane-spanning 4-domains, sub | 1.008 | 2.012 | 2.12E-02 | 2.12E+00 | Up | | |
| 7893419 | --- | --- | --- | 1.008 | 2.011 | 2.10E-01 | 2.10E+01 | | | |
| 7894826 | --- | --- | --- | 1.008 | 2.011 | 7.90E-03 | 7.90E-01 | Up | | |
| 7927095 | BC032332 | PCMTD2 | protein-L-isoaspartate (D-aspartate) | 1.007 | 2.010 | 2.11E-01 | 2.11E+01 | | | |
| 7964640 | --- | --- | --- | 1.007 | 2.010 | 2.65E-01 | 2.65E+01 | | | |
| 8143341 | NM_030647 | JHDM1D | jumonji C domain containing histone | 1.007 | 2.010 | 6.46E-03 | 6.46E-01 | Up | | |
| 8100310 | --- | --- | --- | 1.007 | 2.009 | 1.02E-01 | 1.02E+01 | | | |
| 8133876 | NM_00100154 | CD36 | CD36 molecule (thrombospondin rec | 1.006 | 2.009 | 1.05E-02 | 1.05E+00 | Up | | |
| 8075956 | NM_006498 | LGALS2 | lectin, galactoside-binding, soluble, 2 | 1.005 | 2.006 | 6.62E-02 | 6.62E+00 | | | mono |
| 7895818 | --- | --- | --- | 1.003 | 2.004 | 1.14E-01 | 1.14E+01 | | | |
| 8150714 | NM_052937 | PCMTD1 | protein-L-isoaspartate (D-aspartate) | 1.002 | 2.002 | 6.88E-03 | 6.88E-01 | Up | | |
| 8036710 | NM_004877 | GMFG | glia maturation factor, gamma | 1.001 | 2.002 | 1.29E-02 | 1.29E+00 | Up | | |
| 7893982 | --- | --- | --- | 1.001 | 2.002 | 2.15E-01 | 2.15E+01 | | | |
| 7894938 | --- | --- | --- | 1.001 | 2.001 | 2.88E-01 | 2.88E+01 | | | |
| 7965123 | NM_00114388 | PPP1R12A | protein phosphatase 1, regulatory (in | 1.000 | 2.000 | 6.50E-03 | 6.50E-01 | Up | | |
| 8072979 | NM_021974 | POLR2F | polymerase (RNA) II (DNA directed) p | 0.998 | 1.997 | 1.52E-02 | 1.52E+00 | Up | | |
| 8096463 | NM_00112842 | SMARCAD1 | SWI/SNF-related, matrix-associated a | 0.998 | 1.997 | 5.74E-03 | 5.74E-01 | Up | | |
| 7978542 | NM_020696 | KIAA1143 | KIAA1143 | 0.997 | 1.996 | 9.40E-02 | 9.40E+00 | | | |
| 8097064 | BC032332 | PCMTD2 | protein-L-isoaspartate (D-aspartate) | 0.997 | 1.995 | 2.05E-01 | 2.05E+01 | | | |
| 7948606 | NM_014206 | C11orf10 | chromosome 11 open reading frame | 0.994 | 1.992 | 4.81E-02 | 4.81E+00 | Up | | |

| | | | | | | | | | | |
|---|---|---|---|---|---|---|---|---|---|---|
| 8051501 | NM_002759 | EIF2AK2 | eukaryotic translation initiation facto | 0.993 | 1.991 | 4.34E-02 | 4.34E+00 | Up | | |
| 7893808 | --- | --- | --- | 0.993 | 1.990 | 5.75E-02 | 5.75E+00 | | | |
| 8122705 | NM_005389 | PCMT1 | protein-L-isoaspartate (D-aspartate) | 0.992 | 1.989 | 2.42E-02 | 2.42E+00 | Up | | |
| 8154178 | NM_004972 | JAK2 | Janus kinase 2 | 0.992 | 1.989 | 8.06E-04 | 8.06E-02 | Up | Regulator | |
| 7940688 | NM_002696 | POLR2G | polymerase (RNA) II (DNA directed) p | 0.991 | 1.987 | 6.32E-02 | 6.32E+00 | | | |
| 7895091 | --- | --- | --- | 0.991 | 1.987 | 1.05E-01 | 1.05E+01 | | | |
| 7963263 | --- | --- | --- | 0.991 | 1.987 | 2.21E-01 | 2.21E+01 | | | |
| 7892561 | --- | --- | --- | 0.990 | 1.986 | 1.71E-01 | 1.71E+01 | | | |
| 8113006 | --- | --- | --- | 0.989 | 1.985 | 6.42E-02 | 6.42E+00 | | | |
| 8102619 | NM_001154 | ANXA5 | annexin A5 | 0.989 | 1.985 | 1.54E-02 | 1.54E+00 | Up | | |
| 7895320 | --- | --- | --- | 0.989 | 1.985 | 1.80E-01 | 1.80E+01 | | | |
| 7983744 | NM_014547 | TMOD3 | tropomodulin 3 (ubiquitous) | 0.987 | 1.982 | 1.21E-02 | 1.21E+00 | Up | | |
| 7917516 | NM_002053 | GBP1 | guanylate binding protein 1, interfer | 0.985 | 1.979 | 2.01E-03 | 2.01E-01 | Up | Regulator | |
| 7895570 | --- | --- | --- | 0.983 | 1.976 | 2.44E-01 | 2.44E+01 | | | |
| 8150870 | NM_002806 | PSMC6 | proteasome (prosome, macropain) 2 | 0.982 | 1.975 | 2.56E-02 | 2.56E+00 | Up | | |
| 8180298 | --- | --- | --- | 0.981 | 1.973 | 1.71E-01 | 1.71E+01 | | | |
| 8079153 | NM_016006 | ABHD5 | abhydrolase domain containing 5 | 0.980 | 1.973 | 1.73E-03 | 1.73E-01 | Up | | |
| 7896297 | --- | --- | --- | 0.980 | 1.973 | 1.81E-01 | 1.81E+01 | | | |
| 8113358 | NM_005668 | ST8SIA4 | ST8 alpha-N-acetyl-neuraminide alph | 0.980 | 1.972 | 3.97E-03 | 3.97E-01 | Up | | |
| 7981068 | NM_00100223( | SERPINA1 | serpin peptidase inhibitor, clade A (a | 0.980 | 1.972 | 2.34E-03 | 2.34E-01 | Up | Regulator | mono |
| 8180311 | --- | --- | --- | 0.979 | 1.971 | 3.25E-04 | 3.25E-02 | Up | | |
| 7975224 | NM_004094 | EIF2S1 | eukaryotic translation initiation facto | 0.978 | 1.970 | 4.89E-02 | 4.89E+00 | Up | | |
| 7896001 | --- | --- | --- | 0.978 | 1.970 | 1.46E-01 | 1.46E+01 | | | |
| 7978932 | NM_006939 | SOS2 | son of sevenless homolog 2 (Drosopl | 0.978 | 1.969 | 1.18E-02 | 1.18E+00 | Up | | |
| 8180212 | --- | --- | --- | 0.976 | 1.967 | 2.06E-01 | 2.06E+01 | | | |
| 7893840 | --- | --- | --- | 0.975 | 1.966 | 6.78E-02 | 6.78E+00 | | | |
| 8005765 | NM_015626 | WSB1 | WD repeat and SOCS box-containing | 0.975 | 1.966 | 1.27E-02 | 1.27E+00 | Up | | |
| 7895055 | --- | --- | --- | 0.974 | 1.965 | 1.04E-01 | 1.04E+01 | | | |
| 8113591 | NM_005023 | PGGT1B | protein geranylgeranyltransferase ty | 0.973 | 1.963 | 3.58E-02 | 3.58E+00 | Up | | |
| 8016578 | NM_005827 | SLC35B1 | solute carrier family 35, member B1 | 0.972 | 1.962 | 1.16E-02 | 1.16E+00 | Up | | |
| 7991562 | --- | --- | --- | 0.972 | 1.962 | 1.91E-01 | 1.91E+01 | | | |
| 7893086 | --- | --- | --- | 0.972 | 1.961 | 1.31E-01 | 1.31E+01 | | | |
| 8048014 | NM_199229 | RPE | ribulose-5-phosphate-3-epimerase | 0.972 | 1.961 | 2.31E-02 | 2.31E+00 | Up | | |
| 8119076 | --- | --- | --- | 0.971 | 1.961 | 1.63E-02 | 1.63E+00 | Up | | |
| 8082688 | NM_015268 | DNAJC13 | DnaJ (Hsp40) homolog, subfamily C, | 0.970 | 1.959 | 1.23E-03 | 1.23E-01 | Up | | |
| 8094460 | NM_005349 | RBPJ | recombination signal binding proteir | 0.970 | 1.959 | 5.28E-03 | 5.28E-01 | Up | | |
| 7988753 | NM_032802 | SPPL2A | signal peptide peptidase-like 2A | 0.969 | 1.958 | 1.38E-02 | 1.38E+00 | Up | | |
| 8083850 | --- | --- | --- | 0.968 | 1.956 | 1.83E-02 | 1.83E+00 | Up | | |
| 7892675 | --- | --- | --- | 0.967 | 1.955 | 2.80E-01 | 2.80E+01 | | | |
| 8162490 | NM_032558 | HIATL1 | hippocampus abundant transcript-lik | 0.967 | 1.954 | 6.11E-02 | 6.11E+00 | | | |
| 8040618 | NM_016131 | RAB10 | RAB10, member RAS oncogene famil | 0.966 | 1.953 | 1.06E-03 | 1.06E-01 | Up | | |
| 8168531 | NM_032553 | GPR174 | G protein-coupled receptor 174 | 0.966 | 1.953 | 8.15E-02 | 8.15E+00 | | | |
| 8050443 | NM_00114228( | SMC6 | structural maintenance of chromoso | 0.965 | 1.952 | 3.57E-02 | 3.57E+00 | Up | | |
| 7914940 | NM_031280 | MRPS15 | mitochondrial ribosomal protein S15 | 0.964 | 1.951 | 2.86E-02 | 2.86E+00 | Up | | |
| 7895361 | --- | --- | --- | 0.964 | 1.951 | 2.83E-01 | 2.83E+01 | | | |
| 8133309 | NR_027775 | PMS2L5 | postmeiotic segregation increased 2- | 0.963 | 1.950 | 9.05E-02 | 9.05E+00 | | | |
| 7903980 | BC017973 | C1orf162 | chromosome 1 open reading frame 1 | 0.962 | 1.948 | 3.56E-03 | 3.56E-01 | Up | | |
| 8101699 | --- | --- | --- | 0.962 | 1.948 | 6.80E-02 | 6.80E+00 | | | |
| 7903703 | NM_006496 | GNAI3 | guanine nucleotide binding protein ( | 0.962 | 1.948 | 9.69E-03 | 9.69E-01 | Up | | |
| 7895294 | --- | --- | --- | 0.961 | 1.947 | 1.91E-01 | 1.91E+01 | | | |
| 7946275 | NR_003945 | GVIN1 | GTPase, very large interferon inducit | 0.960 | 1.945 | 1.58E-04 | 1.58E-02 | Up | | |
| 8147172 | NM_003909 | CPNE3 | copine III | 0.960 | 1.945 | 4.52E-03 | 4.52E-01 | Up | | |
| 8165682 | --- | --- | --- | 0.959 | 1.944 | 2.75E-01 | 2.75E+01 | | | |
| 7892830 | --- | --- | --- | 0.959 | 1.944 | 1.15E-01 | 1.15E+01 | | | |
| 7893763 | --- | --- | --- | 0.958 | 1.943 | 2.69E-01 | 2.69E+01 | | | |
| 8098177 | NM_007246 | KLHL2 | kelch-like 2, Mayven (Drosophila) | 0.958 | 1.942 | 5.59E-03 | 5.59E-01 | Up | | |
| 8145586 | NM_018091 | ELP3 | elongation protein 3 homolog (S. cer | 0.957 | 1.941 | 2.15E-02 | 2.15E+00 | Up | | |
| 7892630 | --- | --- | --- | 0.957 | 1.941 | 4.29E-02 | 4.29E+00 | Up | | |
| 8084128 | NM_133462 | TTC14 | tetratricopeptide repeat domain 14 | 0.955 | 1.938 | 9.83E-04 | 9.83E-02 | Up | | |
| 7939120 | NM_002901 | RCN1 | reticulocalbin 1, EF-hand calcium bin | 0.954 | 1.938 | 9.22E-03 | 9.22E-01 | Up | | |
| 7935692 | NM_00110062( | ERLIN1 | ER lipid raft associated 1 | 0.954 | 1.937 | 7.09E-02 | 7.09E+00 | | | |
| 8114829 | NM_00102494 | YIPF5 | Yip1 domain family, member 5 | 0.954 | 1.937 | 1.54E-03 | 1.54E-01 | Up | | |
| 7938313 | NM_006391 | IPO7 | importin 7 | 0.954 | 1.937 | 8.37E-03 | 8.37E-01 | Up | | |
| 8091941 | NM_007217 | PDCD10 | programmed cell death 10 | 0.953 | 1.937 | 9.69E-03 | 9.69E-01 | Up | | |
| 8140971 | NM_152703 | SAMD9L | sterile alpha motif domain containin | 0.953 | 1.936 | 7.07E-03 | 7.07E-01 | Up | | |
| 7922823 | NM_025191 | EDEM3 | ER degradation enhancer, mannosid | 0.953 | 1.936 | 3.63E-04 | 3.63E-02 | Up | | |
| 8011832 | NM_002532 | NUP88 | nucleoporin 88kDa | 0.952 | 1.935 | 4.91E-03 | 4.91E-01 | Up | | |
| 7983663 | NM_005154 | USP8 | ubiquitin specific peptidase 8 | 0.951 | 1.933 | 2.77E-02 | 2.77E+00 | Up | | |
| 7981949 | NR_003316 | SNORD116-1 | small nucleolar RNA, C/D box 116-1 | 0.951 | 1.933 | 9.50E-02 | 9.50E+00 | | | |
| 8021716 | NM_014177 | C18orf55 | chromosome 18 open reading frame | 0.951 | 1.933 | 1.38E-02 | 1.38E+00 | Up | | |
| 7961173 | --- | --- | --- | 0.950 | 1.932 | 1.64E-01 | 1.64E+01 | | | |
| 8043820 | NM_014044 | UNC50 | unc-50 homolog (C. elegans) | 0.949 | 1.931 | 4.19E-03 | 4.19E-01 | Up | | |
| 7929047 | NM_001547 | IFIT2 | interferon-induced protein with tetra | 0.949 | 1.930 | 1.94E-02 | 1.94E+00 | Up | | |
| 7956878 | NM_007199 | IRAK3 | interleukin-1 receptor-associated kin | 0.948 | 1.929 | 2.41E-02 | 2.41E+00 | Up | Regulator | |
| 7917528 | --- | --- | --- | 0.945 | 1.926 | 1.71E-01 | 1.71E+01 | | | |
| 7894736 | --- | --- | --- | 0.945 | 1.925 | 2.01E-01 | 2.01E+01 | | | |
| 7895929 | --- | --- | --- | 0.944 | 1.924 | 1.44E-01 | 1.44E+01 | | | |

| | | | | | | | | | | |
|---|---|---|---|---|---|---|---|---|---|---|
| 7971422 | NM_015070 | ZC3H13 | zinc finger CCCH-type containing 13 | 0.944 | 1.924 | 1.23E-02 | 1.23E+00 | Up | | |
| 7895409 | --- | --- | --- | 0.943 | 1.922 | 3.34E-01 | 3.34E+01 | | | |
| 8045919 | NM_022826 | 40244 | membrane-associated ring finger (C3 | 0.942 | 1.922 | 2.33E-02 | 2.33E+00 | Up | | |
| 7895490 | --- | --- | --- | 0.942 | 1.921 | 1.82E-01 | 1.82E+01 | | | |
| 8144931 | NM_001693 | ATP6V1B2 | ATPase, H+ transporting, lysosomal 5 | 0.941 | 1.920 | 4.84E-03 | 4.84E-01 | Up | | |
| 8151890 | NM_033285 | TP53INP1 | tumor protein p53 inducible nuclear | 0.940 | 1.919 | 1.48E-02 | 1.48E+00 | Up | | |
| 8003758 | NM_000430 | PAFAH1B1 | platelet-activating factor acetylhydro | 0.939 | 1.918 | 2.94E-03 | 2.94E-01 | Up | | |
| 7894295 | --- | --- | --- | 0.939 | 1.917 | 8.55E-02 | 8.55E+00 | | | |
| 7893940 | --- | --- | --- | 0.939 | 1.917 | 2.20E-01 | 2.20E+01 | | | |
| 7893619 | --- | --- | --- | 0.939 | 1.917 | 2.57E-01 | 2.57E+01 | | | |
| 8043861 | NM_015904 | EIF5B | eukaryotic translation initiation facto | 0.938 | 1.916 | 5.32E-03 | 5.32E-01 | Up | | |
| 7896383 | --- | --- | --- | 0.938 | 1.916 | 1.61E-01 | 1.61E+01 | | | |
| 8074969 | NM_001355 | DDT | D-dopachrome tautomerase | 0.938 | 1.915 | 1.88E-01 | 1.88E+01 | | | |
| 8055476 | NR_024058 | YWHAE | tyrosine 3-monooxygenase/tryptoph | 0.936 | 1.913 | 1.06E-01 | 1.06E+01 | | | |
| 8077770 | NM_018462 | C3orf10 | chromosome 3 open reading frame 1 | 0.936 | 1.913 | 5.87E-03 | 5.87E-01 | Up | Phagocytosis/killing | |
| 7894182 | --- | --- | --- | 0.935 | 1.912 | 2.70E-01 | 2.70E+01 | | | |
| 7930882 | NM_207009 | FAM45A | family with sequence similarity 45, m | 0.935 | 1.911 | 7.17E-03 | 7.17E-01 | Up | | |
| 7929065 | NM_001548 | IFIT1 | interferon-induced protein with tetra | 0.933 | 1.909 | 2.64E-02 | 2.64E+00 | Up | | |
| 8110415 | NM_022491 | SUDS3 | suppressor of defective silencing 3 h | 0.932 | 1.907 | 5.24E-03 | 5.24E-01 | Up | | |
| 7896575 | --- | --- | --- | 0.931 | 1.906 | 3.82E-01 | 3.82E+01 | | | |
| 7967473 | NM_001414 | EIF2B1 | eukaryotic translation initiation facto | 0.930 | 1.906 | 1.48E-02 | 1.48E+00 | Up | | |
| 7895661 | --- | --- | --- | 0.930 | 1.905 | 1.93E-01 | 1.93E+01 | | | |
| 7939465 | NM_016142 | HSD17B12 | hydroxysteroid (17-beta) dehydroger | 0.929 | 1.904 | 5.75E-02 | 5.75E+00 | | | |
| 8087951 | NM_018165 | PBRM1 | polybromo 1 | 0.929 | 1.904 | 2.00E-04 | 2.00E-02 | Up | | |
| 8128716 | NM_006016 | CD164 | CD164 molecule, sialomucin | 0.928 | 1.903 | 8.47E-03 | 8.47E-01 | Up | | |
| 7950578 | NM_00112862( | PAK1 | p21 protein (Cdc42/Rac)-activated ki | 0.927 | 1.902 | 6.94E-03 | 6.94E-01 | Up | | |
| 7894252 | --- | --- | --- | 0.927 | 1.902 | 1.90E-01 | 1.90E+01 | | | |
| 7893562 | --- | --- | --- | 0.927 | 1.902 | 2.38E-01 | 2.38E+01 | | | |
| 8001149 | NM_018206 | VPS35 | vacuolar protein sorting 35 homolog | 0.926 | 1.900 | 2.06E-02 | 2.06E+00 | Up | | |
| 7892864 | --- | --- | --- | 0.926 | 1.900 | 3.37E-01 | 3.37E+01 | | | |
| 7892644 | --- | --- | --- | 0.925 | 1.899 | 7.68E-02 | 7.68E+00 | | | |
| 7895161 | --- | --- | --- | 0.925 | 1.898 | 2.71E-01 | 2.71E+01 | | | |
| 7893156 | --- | --- | --- | 0.924 | 1.897 | 3.16E-01 | 3.16E+01 | | | |
| 8127534 | NM_138441 | C6orf150 | chromosome 6 open reading frame 1 | 0.923 | 1.897 | 3.72E-02 | 3.72E+00 | Up | | |
| 7895357 | --- | --- | --- | 0.923 | 1.896 | 3.99E-02 | 3.99E+00 | Up | | |
| 8069574 | NM_00110042( | C21orf91 | chromosome 21 open reading frame | 0.922 | 1.895 | 2.37E-02 | 2.37E+00 | Up | | |
| 8121031 | NM_006416 | SLC35A1 | solute carrier family 35 (CMP-sialic a | 0.921 | 1.893 | 3.55E-03 | 3.55E-01 | Up | | |
| 7894959 | --- | --- | --- | 0.921 | 1.893 | 1.92E-01 | 1.92E+01 | | | |
| 8102751 | NM_144643 | SCLT1 | sodium channel and clathrin linker 1 | 0.920 | 1.893 | 1.81E-03 | 1.81E-01 | Up | | |
| 8097903 | NM_003264 | TLR2 | toll-like receptor 2 | 0.920 | 1.892 | 2.11E-04 | 2.11E-02 | Up | Anti-microbial/Pathogen recogn. | mono |
| 7961440 | NM_024829 | PLBD1 | phospholipase B domain containing 1 | 0.920 | 1.892 | 3.45E-03 | 3.45E-01 | Up | | |
| 7894395 | --- | --- | --- | 0.920 | 1.892 | 2.29E-02 | 2.29E+00 | Up | | |
| 8048703 | NM_032338 | LLPH | LLP homolog, long-term synaptic faci | 0.919 | 1.891 | 2.31E-01 | 2.31E+01 | | | |
| 7971124 | NM_016617 | UFM1 | ubiquitin-fold modifier 1 | 0.919 | 1.890 | 9.07E-02 | 9.07E+00 | | | |
| 7896498 | --- | --- | --- | 0.918 | 1.890 | 1.90E-02 | 1.90E+00 | Up | | |
| 8180345 | --- | --- | --- | 0.916 | 1.887 | 2.35E-02 | 2.35E+00 | Up | | |
| 8020650 | --- | --- | --- | 0.915 | 1.885 | 1.06E-02 | 1.06E+00 | Up | | |
| 8120943 | NM_016230 | CYB5R4 | cytochrome b5 reductase 4 | 0.914 | 1.885 | 2.71E-02 | 2.71E+00 | Up | | |
| 8076463 | BC014647 | RRP7B | ribosomal RNA processing 7 homolog | 0.914 | 1.884 | 1.57E-01 | 1.57E+01 | | | |
| 8046573 | NR_027850 | MTX2 | metaxin 2 | 0.913 | 1.883 | 4.60E-03 | 4.60E-01 | Up | | |
| 8058201 | --- | --- | --- | 0.913 | 1.883 | 2.26E-01 | 2.26E+01 | | | |
| 7896035 | --- | --- | --- | 0.913 | 1.883 | 3.01E-01 | 3.01E+01 | | | |
| 7906819 | NM_007348 | ATF6 | activating transcription factor 6 | 0.913 | 1.883 | 1.22E-02 | 1.22E+00 | Up | | |
| 7901852 | --- | --- | --- | 0.912 | 1.882 | 3.69E-02 | 3.69E+00 | Up | | |
| 7915882 | NM_014774 | KIAA0494 | KIAA0494 | 0.912 | 1.882 | 4.52E-03 | 4.52E-01 | Up | | |
| 8107998 | NM_014402 | UQCRQ | ubiquinol-cytochrome c reductase, c | 0.912 | 1.881 | 5.32E-03 | 5.32E-01 | Up | | |
| 7895445 | --- | --- | --- | 0.911 | 1.880 | 1.27E-01 | 1.27E+01 | | | |
| 7896190 | --- | --- | --- | 0.910 | 1.880 | 1.62E-01 | 1.62E+01 | | | |
| 8095295 | --- | --- | --- | 0.910 | 1.880 | 1.74E-01 | 1.74E+01 | | | |
| 8092933 | NM_012287 | ACAP2 | ArfGAP with coiled-coil, ankyrin repe | 0.908 | 1.877 | 4.36E-03 | 4.36E-01 | Up | | |
| 8086498 | NM_020696 | KIAA1143 | KIAA1143 | 0.908 | 1.876 | 8.21E-02 | 8.21E+00 | | | |
| 7892625 | --- | --- | --- | 0.908 | 1.876 | 1.09E-01 | 1.09E+01 | | | |
| 7957404 | BC029120 | C12orf26 | chromosome 12 open reading frame | 0.907 | 1.876 | 1.59E-02 | 1.59E+00 | Up | | |
| 7894548 | --- | --- | --- | 0.907 | 1.875 | 1.89E-01 | 1.89E+01 | | | |
| 7961182 | NM_002260 | KLRC2 | killer cell lectin-like receptor subfam | 0.906 | 1.874 | 9.29E-02 | 9.29E+00 | | | |
| 8030007 | NM_001425 | EMP3 | epithelial membrane protein 3 | 0.906 | 1.873 | 1.14E-02 | 1.14E+00 | Up | | |
| 7896287 | --- | --- | --- | 0.905 | 1.872 | 7.06E-02 | 7.06E+00 | | | |
| 7925561 | NM_198076 | FAM36A | family with sequence similarity 36, m | 0.905 | 1.872 | 3.06E-03 | 3.06E-01 | Up | | |
| 7896263 | --- | --- | --- | 0.904 | 1.872 | 1.78E-01 | 1.78E+01 | | | |
| 7907657 | NM_152663 | RALGPS2 | Ral GEF with PH domain and SH3 bin | 0.904 | 1.871 | 4.50E-03 | 4.50E-01 | Up | | |
| 7910630 | BC116455 | C1orf31 | chromosome 1 open reading frame 3 | 0.903 | 1.870 | 2.32E-02 | 2.32E+00 | Up | | |
| 7893819 | --- | --- | --- | 0.903 | 1.870 | 2.94E-02 | 2.94E+00 | Up | | |
| 8050278 | NM_005742 | PDIA6 | protein disulfide isomerase family A, | 0.902 | 1.868 | 4.84E-03 | 4.84E-01 | Up | | |
| 7895234 | --- | --- | --- | 0.901 | 1.868 | 2.91E-01 | 2.91E+01 | | | |
| 7981249 | NM_032233 | SETD3 | SET domain containing 3 | 0.901 | 1.867 | 2.56E-03 | 2.56E-01 | Up | | |
| 8017210 | NM_003916 | AP1S2 | adaptor-related protein complex 1, s | 0.899 | 1.865 | 4.47E-02 | 4.47E+00 | Up | | |
| 7893632 | --- | --- | --- | 0.899 | 1.864 | 5.34E-02 | 5.34E+00 | | | |

| | | | | | | | | | | |
|---|---|---|---|---|---|---|---|---|---|---|
| 7960933 | NM_002355 | M6PR | mannose-6-phosphate receptor (cati | 0.898 | 1.863 | 3.32E-03 | 3.32E-01 | Up | | |
| 8174509 | NM_005274 | GNG5 | guanine nucleotide binding protein ( | 0.898 | 1.863 | 1.73E-02 | 1.73E+00 | Up | | |
| 8052698 | NM_006333 | C1D | C1D nuclear receptor co-repressor | 0.897 | 1.862 | 4.83E-02 | 4.83E+00 | Up | | |
| 8088803 | NM_173359 | EIF4E3 | eukaryotic translation initiation facto | 0.896 | 1.861 | 7.21E-03 | 7.21E-01 | Up | | |
| 7895259 | --- | --- | --- | 0.896 | 1.861 | 3.92E-01 | 3.92E+01 | | | |
| 8052526 | NM_003400 | XPO1 | exportin 1 (CRM1 homolog, yeast) | 0.896 | 1.861 | 1.62E-02 | 1.62E+00 | Up | | |
| 7896356 | --- | --- | --- | 0.895 | 1.860 | 4.79E-02 | 4.79E+00 | Up | | |
| 7969428 | NM_006002 | UCHL3 | ubiquitin carboxyl-terminal esterase | 0.895 | 1.860 | 6.76E-02 | 6.76E+00 | | | |
| 7893097 | --- | --- | --- | 0.895 | 1.859 | 3.58E-01 | 3.58E+01 | | | |
| 7950906 | NM_001814 | CTSC | cathepsin C | 0.894 | 1.858 | 1.62E-03 | 1.62E-01 | Up | Phagocytosis/killing | |
| 7895263 | --- | --- | --- | 0.894 | 1.858 | 1.15E-02 | 1.15E+00 | Up | | |
| 7893592 | --- | --- | --- | 0.894 | 1.858 | 7.84E-02 | 7.84E+00 | | | |
| 8137250 | NM_015660 | GIMAP2 | GTPase, IMAP family member 2 | 0.893 | 1.856 | 3.45E-02 | 3.45E+00 | Up | | |
| 7894763 | --- | --- | --- | 0.890 | 1.854 | 1.90E-01 | 1.90E+01 | | | |
| 7947396 | NM_001326 | CSTF3 | cleavage stimulation factor, 3' pre-RI | 0.890 | 1.853 | 2.01E-02 | 2.01E+00 | Up | | |
| 8172717 | --- | --- | --- | 0.890 | 1.853 | 1.13E-01 | 1.13E+01 | | | |
| 7894544 | --- | --- | --- | 0.890 | 1.853 | 2.73E-01 | 2.73E+01 | | | |
| 8133788 | NM_002835 | PTPN12 | protein tyrosine phosphatase, non-re | 0.888 | 1.851 | 1.01E-02 | 1.01E+00 | Up | | |
| 8051928 | NM_006036 | PREPL | prolyl endopeptidase-like | 0.888 | 1.850 | 2.75E-03 | 2.75E-01 | Up | | |
| 8027429 | --- | --- | --- | 0.887 | 1.849 | 3.54E-01 | 3.54E+01 | | | |
| 8107066 | NM_005575 | LNPEP | leucyl/cystinyl aminopeptidase | 0.887 | 1.849 | 1.26E-03 | 1.26E-01 | Up | | |
| 8042391 | NM_002664 | PLEK | pleckstrin | 0.886 | 1.848 | 2.05E-02 | 2.05E+00 | Up | | |
| 8017599 | NM_000442 | PECAM1 | platelet/endothelial cell adhesion mo | 0.886 | 1.848 | 6.65E-03 | 6.65E-01 | Up | | mono |
| 8110055 | NM_030627 | CPEB4 | cytoplasmic polyadenylation elemen | 0.886 | 1.848 | 7.90E-04 | 7.90E-02 | Up | | |
| 7952022 | NM_00109852( | AMICA1 | adhesion molecule, interacts with CX | 0.885 | 1.847 | 1.56E-02 | 1.56E+00 | Up | | |
| 7894110 | --- | --- | --- | 0.884 | 1.845 | 7.26E-02 | 7.26E+00 | | | |
| 8160531 | NM_018325 | C9orf72 | chromosome 9 open reading frame 7 | 0.883 | 1.844 | 6.47E-03 | 6.47E-01 | Up | | |
| 7928821 | --- | --- | --- | 0.883 | 1.844 | 3.06E-01 | 3.06E+01 | | | |
| 7968872 | NM_013238 | DNAJC15 | DnaJ (Hsp40) homolog, subfamily C, | 0.883 | 1.844 | 5.67E-02 | 5.67E+00 | | | |
| 7921625 | NM_052931 | SLAMF6 | SLAM family member 6 | 0.883 | 1.844 | 3.85E-02 | 3.85E+00 | Up | | |
| 8150112 | NM_000637 | GSR | glutathione reductase | 0.881 | 1.842 | 1.35E-02 | 1.35E+00 | Up | | |
| 7895995 | --- | --- | --- | 0.881 | 1.842 | 1.99E-01 | 1.99E+01 | | | |
| 8002301 | --- | --- | --- | 0.881 | 1.841 | 2.91E-01 | 2.91E+01 | | | |
| 8044919 | NM_002881 | RALB | v-ral simian leukemia viral oncogene | 0.881 | 1.841 | 1.03E-02 | 1.03E+00 | Up | | |
| 7915286 | NM_000310 | PPT1 | palmitoyl-protein thioesterase 1 | 0.879 | 1.840 | 4.81E-02 | 4.81E+00 | Up | | |
| 8172305 | --- | --- | --- | 0.879 | 1.839 | 3.35E-02 | 3.35E+00 | Up | | |
| 8135625 | NM_006136 | CAPZA2 | capping protein (actin filament) mus | 0.879 | 1.839 | 1.22E-02 | 1.22E+00 | Up | | |
| 7920492 | NM_00109861( | C1orf43 | chromosome 1 open reading frame 4 | 0.878 | 1.838 | 2.13E-02 | 2.13E+00 | Up | | |
| 8095230 | NM_006947 | SRP72 | signal recognition particle 72kDa | 0.877 | 1.837 | 4.03E-02 | 4.03E+00 | Up | | |
| 8054192 | NM_138798 | MITD1 | MIT, microtubule interacting and tra | 0.874 | 1.833 | 4.69E-02 | 4.69E+00 | Up | | |
| 8048940 | NM_00108039: | SP100 | SP100 nuclear antigen | 0.874 | 1.833 | 5.87E-03 | 5.87E-01 | Up | | |
| 7905047 | NM_000566 | FCGR1A | Fc fragment of IgG, high affinity Ia, re | 0.874 | 1.832 | 3.11E-02 | 3.11E+00 | Up | Phagocytosis/killing | |
| 7989224 | NM_001110 | ADAM10 | ADAM metallopeptidase domain 10 | 0.873 | 1.832 | 7.20E-03 | 7.20E-01 | Up | | |
| 7952484 | NM_00108054( | TMEM218 | transmembrane protein 218 | 0.872 | 1.831 | 4.07E-03 | 4.07E-01 | Up | | |
| 8041508 | NM_012413 | QPCT | glutaminyl-peptide cyclotransferase | 0.872 | 1.831 | 1.25E-02 | 1.25E+00 | Up | | |
| 7894411 | --- | --- | --- | 0.872 | 1.831 | 2.59E-01 | 2.59E+01 | | | |
| 7894341 | --- | --- | --- | 0.870 | 1.828 | 1.29E-01 | 1.29E+01 | | | |
| 8041236 | NM_014946 | SPAST | spastin | 0.870 | 1.828 | 4.84E-03 | 4.84E-01 | Up | | |
| 8045514 | NM_00100166 | SPOPL | speckle-type POZ protein-like | 0.870 | 1.827 | 2.66E-02 | 2.66E+00 | Up | | |
| 8092218 | --- | --- | --- | 0.869 | 1.826 | 1.86E-01 | 1.86E+01 | | | |
| 7939298 | NM_001752 | CAT | catalase | 0.868 | 1.826 | 2.73E-03 | 2.73E-01 | Up | | |
| 8146225 | NM_00113567 | C8orf40 | chromosome 8 open reading frame 4 | 0.866 | 1.823 | 1.38E-01 | 1.38E+01 | | | |
| 8023672 | NM_004869 | VPS4B | vacuolar protein sorting 4 homolog B | 0.865 | 1.822 | 2.71E-03 | 2.71E-01 | Up | | |
| 7894093 | --- | --- | --- | 0.865 | 1.821 | 2.13E-01 | 2.13E+01 | | | |
| 7895090 | --- | --- | --- | 0.864 | 1.820 | 3.07E-01 | 3.07E+01 | | | |
| 8079869 | NM_005778 | RBM5 | RNA binding motif protein 5 | 0.863 | 1.819 | 1.26E-03 | 1.26E-01 | Up | | |
| 8070083 | NM_006134 | TMEM50B | transmembrane protein 50B | 0.863 | 1.819 | 9.13E-03 | 9.13E-01 | Up | | |
| 7893496 | --- | --- | --- | 0.863 | 1.819 | 3.73E-01 | 3.73E+01 | | | |
| 7893958 | --- | --- | --- | 0.863 | 1.819 | 3.52E-01 | 3.52E+01 | | | |
| 7895954 | --- | --- | --- | 0.862 | 1.818 | 2.61E-01 | 2.61E+01 | | | |
| 7903407 | NM_020978 | AMY2B | amylase, alpha 2B (pancreatic) | 0.862 | 1.817 | 8.93E-02 | 8.93E+00 | | | |
| 8102171 | NM_033115 | TBCK | TBC1 domain containing kinase | 0.861 | 1.817 | 1.03E-02 | 1.03E+00 | Up | | |
| 7935359 | --- | --- | --- | 0.861 | 1.816 | 2.17E-01 | 2.17E+01 | | | |
| 7961654 | NM_002907 | RECQL | RecQ protein-like (DNA helicase Q1-l | 0.861 | 1.816 | 9.92E-03 | 9.92E-01 | Up | | |
| 8174893 | NM_00108155( | THOC2 | THO complex 2 | 0.860 | 1.815 | 1.68E-02 | 1.68E+00 | Up | | |
| 8137466 | --- | --- | --- | 0.859 | 1.814 | 5.07E-02 | 5.07E+00 | | | |
| 7997904 | AK295122 | ZNF778 | zinc finger protein 778 | 0.858 | 1.813 | 2.25E-01 | 2.25E+01 | | | |
| 7919780 | NM_018178 | GOLPH3L | golgi phosphoprotein 3-like | 0.858 | 1.812 | 2.51E-02 | 2.51E+00 | Up | | |
| 8089652 | NM_025146 | NAT13 | N-acetyltransferase 13 (GCN5-relate | 0.858 | 1.812 | 3.64E-02 | 3.64E+00 | Up | | |
| 7894665 | --- | --- | --- | 0.857 | 1.812 | 4.03E-02 | 4.03E+00 | Up | | |
| 7916969 | NM_005455 | ZRANB2 | zinc finger, RAN-binding domain con | 0.857 | 1.811 | 6.29E-03 | 6.29E-01 | Up | | |
| 8006030 | NM_020791 | TAOK1 | TAO kinase 1 | 0.857 | 1.811 | 4.11E-03 | 4.11E-01 | Up | | |
| 8129482 | NM_00101737 | SAMD3 | sterile alpha motif domain containin | 0.856 | 1.810 | 2.19E-02 | 2.19E+00 | Up | | |
| 8108620 | NM_144723 | ZMAT2 | zinc finger, matrin type 2 | 0.855 | 1.809 | 4.51E-02 | 4.51E+00 | Up | | |
| 7965436 | NM_003566 | EEA1 | early endosome antigen 1 | 0.855 | 1.809 | 5.94E-03 | 5.94E-01 | Up | | |
| 8014037 | NM_015986 | CRLF3 | cytokine receptor-like factor 3 | 0.855 | 1.809 | 5.63E-02 | 5.63E+00 | | | |
| 8062117 | --- | --- | --- | 0.855 | 1.809 | 3.99E-02 | 3.99E+00 | Up | | |

| | | | | | | | | | | |
|---|---|---|---|---|---|---|---|---|---|---|
| 7923974 | --- | --- | --- | 0.855 | 1.809 | 5.67E-02 | 5.67E+00 | | | |
| 7982935 | --- | --- | --- | 0.854 | 1.808 | 1.16E-01 | 1.16E+01 | | | |
| 8165692 | --- | --- | --- | 0.854 | 1.807 | 3.34E-01 | 3.34E+01 | | | |
| 8176865 | BC032332 | PCMTD2 | protein-L-isoaspartate (D-aspartate) | 0.853 | 1.807 | 3.04E-01 | 3.04E+01 | | | |
| 8177460 | BC032332 | PCMTD2 | protein-L-isoaspartate (D-aspartate) | 0.853 | 1.807 | 3.04E-01 | 3.04E+01 | | | |
| 8057744 | NM_007315 | STAT1 | signal transducer and activator of tra | 0.853 | 1.807 | 2.87E-03 | 2.87E-01 | Up | | |
| 8113050 | NM_004365 | CETN3 | centrin, EF-hand protein, 3 (CDC31 h | 0.853 | 1.806 | 3.02E-02 | 3.02E+00 | Up | | |
| 7896076 | --- | --- | --- | 0.853 | 1.806 | 1.94E-01 | 1.94E+01 | | | |
| 8060734 | --- | --- | --- | 0.852 | 1.805 | 7.63E-02 | 7.63E+00 | | | |
| 8115666 | NM_145266 | NUDCD2 | NudC domain containing 2 | 0.851 | 1.803 | 2.99E-02 | 2.99E+00 | Up | | |
| 7954419 | NM_018638 | ETNK1 | ethanolamine kinase 1 | 0.849 | 1.801 | 1.52E-04 | 1.52E-02 | Up | | |
| 8090577 | NM_003925 | MBD4 | methyl-CpG binding domain protein | 0.849 | 1.801 | 2.42E-02 | 2.42E+00 | Up | | |
| 8151471 | NM_014018 | MRPS28 | mitochondrial ribosomal protein S28 | 0.849 | 1.801 | 7.24E-02 | 7.24E+00 | | | |
| 7999562 | NM_002582 | PARN | poly(A)-specific ribonuclease (deade | 0.849 | 1.801 | 1.36E-02 | 1.36E+00 | Up | | |
| 7894400 | --- | --- | --- | 0.849 | 1.801 | 2.37E-01 | 2.37E+01 | | | |
| 7995583 | NM_025134 | CHD9 | chromodomain helicase DNA binding | 0.848 | 1.801 | 1.11E-03 | 1.11E-01 | Up | | |
| 7975989 | NM_031210 | C14orf156 | chromosome 14 open reading frame | 0.847 | 1.798 | 1.38E-01 | 1.38E+01 | | | |
| 7963923 | NR_026723 | SARNP | SAP domain containing ribonucleopr | 0.847 | 1.798 | 7.03E-02 | 7.03E+00 | | | |
| 8006325 | NM_015355 | SUZ12 | suppressor of zeste 12 homolog (Dro | 0.847 | 1.798 | 5.01E-02 | 5.01E+00 | | | |
| 7893421 | --- | --- | --- | 0.846 | 1.798 | 4.87E-01 | 4.87E+01 | | | |
| 8047401 | --- | --- | --- | 0.844 | 1.795 | 6.76E-02 | 6.76E+00 | | | |
| 8071049 | 0 | 0 | 0 | 0.844 | 1.794 | 1.27E-01 | 1.27E+01 | | | |
| 7932041 | NM_080599 | UPF2 | UPF2 regulator of nonsense transcrip | 0.843 | 1.793 | 1.36E-02 | 1.36E+00 | Up | | |
| 8136078 | --- | --- | --- | 0.843 | 1.793 | 3.56E-02 | 3.56E+00 | Up | | |
| 7974587 | NM_018477 | ACTR10 | actin-related protein 10 homolog (S. | 0.841 | 1.792 | 1.00E-02 | 1.00E+00 | Up | | |
| 8066117 | NM_015474 | SAMHD1 | SAM domain and HD domain 1 | 0.840 | 1.791 | 1.83E-04 | 1.83E-02 | Up | | |
| 7921583 | NM_00109839 | COPA | coatomer protein complex, subunit a | 0.840 | 1.790 | 1.06E-02 | 1.06E+00 | Up | | |
| 7894801 | --- | --- | --- | 0.840 | 1.790 | 2.54E-01 | 2.54E+01 | | | |
| 7895691 | --- | --- | --- | 0.840 | 1.790 | 7.43E-03 | 7.43E-01 | Up | | |
| 7983111 | NM_003825 | SNAP23 | synaptosomal-associated protein, 23 | 0.839 | 1.789 | 4.73E-02 | 4.73E+00 | Up | | |
| 8014197 | --- | --- | --- | 0.839 | 1.789 | 1.57E-01 | 1.57E+01 | | | |
| 8127943 | NM_153816 | SNX14 | sorting nexin 14 | 0.839 | 1.788 | 2.16E-02 | 2.16E+00 | Up | | |
| 7995697 | NM_017839 | LPCAT2 | lysophosphatidylcholine acyltransfer | 0.838 | 1.788 | 1.70E-02 | 1.70E+00 | Up | | |
| 7902771 | NM_016009 | SH3GLB1 | SH3-domain GRB2-like endophilin B1 | 0.838 | 1.788 | 9.69E-03 | 9.69E-01 | Up | | |
| 7892621 | --- | --- | --- | 0.838 | 1.788 | 5.90E-02 | 5.90E+00 | | | |
| 7909898 | NM_198551 | MIA3 | melanoma inhibitory activity family, | 0.837 | 1.787 | 1.23E-02 | 1.23E+00 | Up | | |
| 8083826 | NM_003262 | SEC62 | SEC62 homolog (S. cerevisiae) | 0.836 | 1.786 | 3.81E-03 | 3.81E-01 | Up | | |
| 7896605 | --- | --- | --- | 0.836 | 1.785 | 1.65E-01 | 1.65E+01 | | | |
| 8154128 | DQ246450 | DSERG1 | Down syndrome encephalopathy rel | 0.835 | 1.784 | 2.50E-02 | 2.50E+00 | Up | | |
| 8049544 | NM_00113755 | LRRFIP1 | leucine rich repeat (in FLII) interactin | 0.835 | 1.784 | 1.06E-02 | 1.06E+00 | Up | | |
| 8100292 | NM_015030 | FRYL | FRY-like | 0.835 | 1.783 | 9.31E-02 | 9.31E+00 | | | |
| 7917707 | NM_005665 | EVI5 | ecotropic viral integration site 5 | 0.834 | 1.783 | 1.60E-02 | 1.60E+00 | Up | | |
| 7894316 | --- | --- | --- | 0.833 | 1.782 | 1.12E-01 | 1.12E+01 | | | |
| 8119239 | --- | --- | --- | 0.833 | 1.782 | 4.51E-01 | 4.51E+01 | | | |
| 8129045 | NM_001527 | HDAC2 | histone deacetylase 2 | 0.832 | 1.781 | 1.81E-03 | 1.81E-01 | Up | | |
| 8035144 | --- | --- | --- | 0.832 | 1.780 | 1.51E-01 | 1.51E+01 | | | |
| 7944991 | NM_152713 | STT3A | STT3, subunit of the oligosaccharyltr | 0.831 | 1.778 | 1.73E-03 | 1.73E-01 | Up | | |
| 7893782 | --- | --- | --- | 0.830 | 1.778 | 3.77E-01 | 3.77E+01 | | | |
| 7906400 | NM_005531 | IFI16 | interferon, gamma-inducible protein | 0.830 | 1.777 | 4.84E-03 | 4.84E-01 | Up | | |
| 8065032 | NM_016649 | ESF1 | ESF1, nucleolar pre-rRNA processing | 0.829 | 1.776 | 1.40E-02 | 1.40E+00 | Up | | |
| 7894421 | --- | --- | --- | 0.828 | 1.775 | 3.23E-02 | 3.23E+00 | Up | | |
| 8052845 | NM_022173 | TIA1 | TIA1 cytotoxic granule-associated RN | 0.828 | 1.775 | 2.23E-02 | 2.23E+00 | Up | | |
| 8017421 | NM_020198 | CCDC47 | coiled-coil domain containing 47 | 0.828 | 1.775 | 1.18E-02 | 1.18E+00 | Up | | |
| 7894001 | --- | --- | --- | 0.827 | 1.774 | 2.43E-01 | 2.43E+01 | | | |
| 7909371 | NM_000573 | CR1 | complement component (3b/4b) rec | 0.827 | 1.774 | 8.67E-04 | 8.67E-02 | Up | Phagocytosis/killing | |
| 7932885 | NM_018287 | ARHGAP12 | Rho GTPase activating protein 12 | 0.826 | 1.773 | 2.26E-03 | 2.26E-01 | Up | | |
| 7895632 | --- | --- | --- | 0.826 | 1.773 | 2.77E-01 | 2.77E+01 | | | |
| 7894859 | --- | --- | --- | 0.826 | 1.773 | 3.12E-01 | 3.12E+01 | | | |
| 7894581 | --- | --- | --- | 0.826 | 1.773 | 3.48E-01 | 3.48E+01 | | | |
| 7945371 | NM_021034 | IFITM3 | interferon induced transmembrane p | 0.825 | 1.772 | 1.23E-02 | 1.23E+00 | Up | | |
| 8049016 | NM_002807 | PSMD1 | proteasome (prosome, macropain) 2 | 0.825 | 1.771 | 7.13E-03 | 7.13E-01 | Up | | |
| 7953100 | NM_002014 | FKBP4 | FK506 binding protein 4, 59kDa | 0.825 | 1.771 | 1.15E-02 | 1.15E+00 | Up | | |
| 8026520 | NM_005370 | RAB8A | RAB8A, member RAS oncogene fami | 0.825 | 1.771 | 1.20E-02 | 1.20E+00 | Up | | |
| 7893287 | --- | --- | --- | 0.824 | 1.770 | 4.12E-01 | 4.12E+01 | | | |
| 7944375 | NM_016146 | TRAPPC4 | trafficking protein particle complex 4 | 0.824 | 1.770 | 9.92E-02 | 9.92E+00 | | | |
| 7895755 | --- | --- | --- | 0.821 | 1.767 | 3.36E-01 | 3.36E+01 | | | |
| 7968199 | NM_001260 | CDK8 | cyclin-dependent kinase 8 | 0.820 | 1.766 | 7.66E-02 | 7.66E+00 | | | |
| 8030171 | NM_000146 | FTL | ferritin, light polypeptide | 0.820 | 1.765 | 1.14E-02 | 1.14E+00 | Up | | |
| 7982287 | NM_00103984 | ARHGAP11B | Rho GTPase activating protein 11B | 0.820 | 1.765 | 6.25E-02 | 6.25E+00 | | | |
| 8021453 | NM_033280 | SEC11C | SEC11 homolog C (S. cerevisiae) | 0.820 | 1.765 | 9.12E-02 | 9.12E+00 | | | |
| 8020382 | NM_005406 | ROCK1 | Rho-associated, coiled-coil containin | 0.820 | 1.765 | 4.71E-02 | 4.71E+00 | Up | | |
| 8067113 | NM_006526 | ZNF217 | zinc finger protein 217 | 0.820 | 1.765 | 4.19E-03 | 4.19E-01 | Up | | |
| 8078110 | NM_014296 | CAPN7 | calpain 7 | 0.819 | 1.765 | 3.68E-03 | 3.68E-01 | Up | | |
| 7893880 | --- | --- | --- | 0.819 | 1.764 | 1.34E-01 | 1.34E+01 | | | |
| 8138689 | NM_003930 | SKAP2 | src kinase associated phosphoprotei | 0.818 | 1.763 | 2.26E-02 | 2.26E+00 | Up | | |
| 7926207 | NM_006023 | CDC123 | cell division cycle 123 homolog (S. ce | 0.818 | 1.763 | 3.68E-02 | 3.68E+00 | Up | | |
| 8173444 | NM_000206 | IL2RG | interleukin 2 receptor, gamma (seve | 0.818 | 1.763 | 1.44E-01 | 1.44E+01 | | | |

| | | | | | | | | | | |
|---|---|---|---|---|---|---|---|---|---|---|
| 8086125 | NM_014831 | LBA1 | lupus brain antigen 1 | 0.818 | 1.763 | 2.88E-03 | 2.88E-01 | Up | | |
| 7895682 | --- | --- | --- | 0.818 | 1.762 | 2.11E-01 | 2.11E+01 | | | |
| 7935123 | NM_145246 | C10orf4 | chromosome 10 open reading frame | 0.818 | 1.762 | 1.23E-02 | 1.23E+00 | Up | | |
| 7923007 | NM_015984 | UCHL5 | ubiquitin carboxyl-terminal hydrolas | 0.817 | 1.762 | 2.36E-02 | 2.36E+00 | Up | | |
| 8061986 | NM_031483 | ITCH | itchy E3 ubiquitin protein ligase hom | 0.816 | 1.761 | 9.69E-03 | 9.69E-01 | Up | | |
| 8140258 | NR_027775 | PMS2L5 | postmeiotic segregation increased 2- | 0.815 | 1.760 | 7.76E-02 | 7.76E+00 | | | |
| 7999476 | --- | --- | --- | 0.815 | 1.759 | 3.05E-01 | 3.05E+01 | | | |
| 8137257 | NM_018384 | GIMAP5 | GTPase, IMAP family member 5 | 0.814 | 1.759 | 6.97E-04 | 6.97E-02 | Up | | |
| 7924351 | NM_004446 | EPRS | glutamyl-prolyl-tRNA synthetase | 0.814 | 1.758 | 6.29E-03 | 6.29E-01 | Up | | |
| 8150576 | --- | --- | --- | 0.814 | 1.758 | 1.42E-01 | 1.42E+01 | | | |
| 8084541 | NM_00100992 | VPS8 | vacuolar protein sorting 8 homolog ( | 0.814 | 1.758 | 1.47E-03 | 1.47E-01 | Up | | |
| 8128886 | --- | --- | --- | 0.814 | 1.758 | 7.21E-02 | 7.21E+00 | | | |
| 8173162 | --- | --- | --- | 0.812 | 1.756 | 3.54E-02 | 3.54E+00 | Up | | |
| 7919800 | NM_004079 | CTSS | cathepsin S | 0.812 | 1.756 | 1.79E-04 | 1.79E-02 | Up | Phagocytosis/killing | mono |
| 8154333 | NM_015061 | KDM4C | lysine (K)-specific demethylase 4C | 0.811 | 1.755 | 9.02E-03 | 9.02E-01 | Up | | |
| 8014650 | NM_006310 | NPEPPS | aminopeptidase puromycin sensitive | 0.809 | 1.752 | 1.04E-02 | 1.04E+00 | Up | | |
| 7892972 | --- | --- | --- | 0.809 | 1.752 | 3.62E-01 | 3.62E+01 | | | |
| 8088535 | NM_014814 | PSMD6 | proteasome (prosome, macropain) 2 | 0.809 | 1.752 | 4.03E-02 | 4.03E+00 | Up | | |
| 8103563 | NM_017631 | DDX60 | DEAD (Asp-Glu-Ala-Asp) box polypep | 0.809 | 1.751 | 8.00E-03 | 8.00E-01 | Up | | |
| 7981953 | NR_003318 | SNORD116-3 | small nucleolar RNA, C/D box 116-3 | 0.808 | 1.751 | 1.91E-01 | 1.91E+01 | | | |
| 7981966 | NR_003318 | SNORD116-3 | small nucleolar RNA, C/D box 116-3 | 0.808 | 1.751 | 1.91E-01 | 1.91E+01 | | | |
| 8068266 | NM_000629 | IFNAR1 | interferon (alpha, beta and omega) r | 0.808 | 1.751 | 1.39E-02 | 1.39E+00 | Up | | |
| 8156982 | NM_00104255 | SMC2 | structural maintenance of chromoso | 0.808 | 1.751 | 4.06E-03 | 4.06E-01 | Up | | |
| 7892598 | --- | --- | --- | 0.808 | 1.750 | 2.72E-01 | 2.72E+01 | | | |
| 7966517 | NM_00110966 | C12orf51 | chromosome 12 open reading frame | 0.807 | 1.749 | 2.37E-02 | 2.37E+00 | Up | | |
| 8140828 | NM_003130 | SRI | sorcin | 0.807 | 1.749 | 1.37E-01 | 1.37E+01 | | | |
| 7925550 | NM_001126 | ADSS | adenylosuccinate synthase | 0.807 | 1.749 | 1.76E-02 | 1.76E+00 | Up | | |
| 8107044 | NM_022350 | ERAP2 | endoplasmic reticulum aminopeptid | 0.806 | 1.749 | 4.49E-02 | 4.49E+00 | Up | | |
| 7989243 | --- | --- | --- | 0.804 | 1.746 | 2.58E-01 | 2.58E+01 | | | |
| 7896324 | --- | --- | --- | 0.802 | 1.744 | 1.76E-01 | 1.76E+01 | | | |
| 8095333 | NM_006544 | EXOC5 | exocyst complex component 5 | 0.802 | 1.743 | 2.08E-02 | 2.08E+00 | Up | | |
| 8097513 | NM_002413 | MGST2 | microsomal glutathione S-transferas | 0.801 | 1.742 | 7.97E-02 | 7.97E+00 | | | |
| 7902594 | NM_182948 | PRKACB | protein kinase, cAMP-dependent, ca | 0.800 | 1.741 | 8.48E-03 | 8.48E-01 | Up | | |
| 7895410 | --- | --- | --- | 0.800 | 1.741 | 1.43E-01 | 1.43E+01 | | | |
| 8143387 | NM_013446 | MKRN1 | makorin ring finger protein 1 | 0.799 | 1.740 | 1.05E-02 | 1.05E+00 | Up | | |
| 8136179 | --- | --- | --- | 0.799 | 1.740 | 1.43E-01 | 1.43E+01 | | | |
| 8166065 | NM_138636 | TLR8 | toll-like receptor 8 | 0.798 | 1.739 | 7.21E-03 | 7.21E-01 | Up | Anti-microbial/Pathogen recogn. | |
| 7896529 | --- | --- | --- | 0.798 | 1.738 | 2.89E-01 | 2.89E+01 | | | |
| 7978558 | NM_018453 | EAPP | E2F-associated phosphoprotein | 0.797 | 1.738 | 7.99E-02 | 7.99E+00 | | | |
| 7893362 | --- | --- | --- | 0.797 | 1.738 | 3.83E-01 | 3.83E+01 | | | |
| 8060736 | --- | --- | --- | 0.797 | 1.737 | 1.72E-01 | 1.72E+01 | | | |
| 7944285 | NM_001655 | ARCN1 | archain 1 | 0.797 | 1.737 | 3.05E-02 | 3.05E+00 | Up | | |
| 8013581 | NM_174887 | IFT20 | intraflagellar transport 20 homolog ( | 0.797 | 1.737 | 2.15E-02 | 2.15E+00 | Up | | |
| 8068460 | NM_015358 | MORC3 | MORC family CW-type zinc finger 3 | 0.797 | 1.737 | 1.59E-02 | 1.59E+00 | Up | | |
| 8180344 | --- | --- | --- | 0.796 | 1.737 | 5.03E-02 | 5.03E+00 | | | |
| 7894024 | --- | --- | --- | 0.796 | 1.736 | 1.36E-01 | 1.36E+01 | | | |
| 7894700 | --- | --- | --- | 0.795 | 1.736 | 1.75E-01 | 1.75E+01 | | | |
| 7916077 | NM_002525 | NRD1 | nardilysin (N-arginine dibasic conver | 0.795 | 1.735 | 3.62E-03 | 3.62E-01 | Up | | |
| 8098512 | NM_021942 | C4orf41 | chromosome 4 open reading frame 4 | 0.795 | 1.735 | 4.21E-03 | 4.21E-01 | Up | | |
| 7893206 | --- | --- | --- | 0.795 | 1.735 | 2.08E-01 | 2.08E+01 | | | |
| 8175755 | NM_004344 | CETN2 | centrin, EF-hand protein, 2 | 0.794 | 1.734 | 3.97E-02 | 3.97E+00 | Up | | |
| 8145291 | AF495725 | SLC25A37 | solute carrier family 25, member 37 | 0.794 | 1.733 | 4.84E-03 | 4.84E-01 | Up | | |
| 8096538 | NM_00110042 | RAP1GDS1 | RAP1, GTP-GDP dissociation stimulat | 0.793 | 1.733 | 4.15E-02 | 4.15E+00 | Up | | |
| 7896039 | --- | --- | --- | 0.792 | 1.732 | 6.01E-02 | 6.01E+00 | | | |
| 7893458 | --- | --- | --- | 0.792 | 1.732 | 3.51E-01 | 3.51E+01 | | | |
| 8163882 | NM_016322 | RAB14 | RAB14, member RAS oncogene famil | 0.792 | 1.731 | 2.54E-02 | 2.54E+00 | Up | | |
| 8008493 | NM_016424 | LUC7L3 | LUC7-like 3 (S. cerevisiae) | 0.791 | 1.731 | 4.26E-03 | 4.26E-01 | Up | | |
| 8096602 | NM_014395 | DAPP1 | dual adaptor of phosphotyrosine and | 0.791 | 1.730 | 7.52E-03 | 7.52E-01 | Up | | |
| 7922309 | NM_003762 | VAMP4 | vesicle-associated membrane protei | 0.791 | 1.730 | 3.28E-02 | 3.28E+00 | Up | | |
| 7910014 | NM_014184 | CNIH4 | cornichon homolog 4 (Drosophila) | 0.791 | 1.730 | 5.10E-02 | 5.10E+00 | | | |
| 8168466 | NM_032121 | MAGT1 | magnesium transporter 1 | 0.790 | 1.729 | 1.53E-01 | 1.53E+01 | | | |
| 7895916 | --- | --- | --- | 0.790 | 1.729 | 2.21E-01 | 2.21E+01 | | | |
| 7956795 | NM_013254 | TBK1 | TANK-binding kinase 1 | 0.790 | 1.729 | 2.35E-02 | 2.35E+00 | Up | | |
| 7955361 | NM_052879 | LARP4 | La ribonucleoprotein domain family, | 0.790 | 1.729 | 1.38E-02 | 1.38E+00 | Up | | |
| 8040985 | NM_032434 | ZNF512 | zinc finger protein 512 | 0.790 | 1.729 | 7.15E-03 | 7.15E-01 | Up | | |
| 8014063 | NM_006495 | EVI2B | ecotropic viral integration site 2B | 0.788 | 1.726 | 4.51E-02 | 4.51E+00 | Up | | |
| 7897370 | NM_004781 | VAMP3 | vesicle-associated membrane protei | 0.787 | 1.725 | 1.45E-02 | 1.45E+00 | Up | | |
| 8140500 | NM_032936 | TMEM60 | transmembrane protein 60 | 0.786 | 1.724 | 9.09E-03 | 9.09E-01 | Up | | |
| 7894294 | --- | --- | --- | 0.786 | 1.724 | 1.65E-01 | 1.65E+01 | | | |
| 8061324 | NM_012255 | XRN2 | 5'-3' exoribonuclease 2 | 0.785 | 1.723 | 1.32E-02 | 1.32E+00 | Up | | |
| 7895344 | --- | --- | --- | 0.783 | 1.721 | 2.70E-01 | 2.70E+01 | | | |
| 8119661 | BC011933 | C6orf153 | chromosome 6 open reading frame 1 | 0.783 | 1.721 | 1.26E-02 | 1.26E+00 | Up | | |
| 7895470 | --- | --- | --- | 0.783 | 1.720 | 2.72E-01 | 2.72E+01 | | | |
| 8014100 | NM_022344 | C17orf75 | chromosome 17 open reading frame | 0.782 | 1.720 | 1.34E-01 | 1.34E+01 | | | |
| 8106252 | NM_000521 | HEXB | hexosaminidase B (beta polypeptide | 0.782 | 1.720 | 3.45E-03 | 3.45E-01 | Up | | |
| 7950983 | NM_012124 | CHORDC1 | cysteine and histidine-rich domain (C | 0.782 | 1.720 | 5.81E-02 | 5.81E+00 | | | |
| 8042468 | NM_001153 | ANXA4 | annexin A4 | 0.782 | 1.720 | 3.46E-02 | 3.46E+00 | Up | | |

| | | | | | | | | | | | |
|---|---|---|---|---|---|---|---|---|---|---|---|
| 7894797 | --- | --- | --- | 0.782 | 1.719 | 3.02E-01 | 3.02E+01 | | | |
| 7980906 | --- | --- | --- | 0.782 | 1.719 | 1.76E-01 | 1.76E+01 | | | |
| 8026971 | NM_006332 | IFI30 | interferon, gamma-inducible protein | 0.782 | 1.719 | 3.77E-03 | 3.77E-01 | Up | | |
| 7893581 | --- | --- | --- | 0.782 | 1.719 | 2.79E-01 | 2.79E+01 | | | |
| 7939839 | NM_002843 | PTPRJ | protein tyrosine phosphatase, recept | 0.782 | 1.719 | 1.40E-03 | 1.40E-01 | Up | | |
| 7985166 | NM_004136 | IREB2 | iron-responsive element binding pro | 0.780 | 1.718 | 1.98E-02 | 1.98E+00 | Up | | |
| 8123985 | NM_016167 | NOL7 | nucleolar protein 7, 27kDa | 0.780 | 1.717 | 4.58E-02 | 4.58E+00 | Up | | |
| 8091503 | NM_013308 | GPR171 | G protein-coupled receptor 171 | 0.779 | 1.716 | 2.18E-02 | 2.18E+00 | Up | | |
| 7900382 | NM_006367 | CAP1 | CAP, adenylate cyclase-associated pr | 0.779 | 1.716 | 1.10E-02 | 1.10E+00 | Up | | |
| 7896673 | --- | --- | --- | 0.779 | 1.716 | 1.96E-01 | 1.96E+01 | | | |
| 7984155 | NM_003099 | SNX1 | sorting nexin 1 | 0.779 | 1.716 | 8.48E-03 | 8.48E-01 | Up | | |
| 8133531 | NR_027775 | PMS2L5 | postmeiotic segregation increased 2- | 0.779 | 1.716 | 9.89E-02 | 9.89E+00 | | | |
| 8121002 | NM_00104249: | C6orf162 | chromosome 6 open reading frame 1 | 0.779 | 1.716 | 7.68E-02 | 7.68E+00 | | | |
| 8041867 | NM_000251 | MSH2 | mutS homolog 2, colon cancer, nonp | 0.778 | 1.714 | 5.76E-02 | 5.76E+00 | | | |
| 8095269 | NM_000938 | POLR2B | polymerase (RNA) II (DNA directed) p | 0.777 | 1.714 | 4.42E-03 | 4.42E-01 | Up | | |
| 7893991 | --- | --- | --- | 0.777 | 1.714 | 5.59E-01 | 5.59E+01 | | | |
| 8093294 | NM_00112304: | CCR2 | chemokine (C-C motif) receptor 2 | 0.776 | 1.713 | 5.64E-03 | 5.64E-01 | Up | | |
| 7894837 | --- | --- | --- | 0.776 | 1.712 | 2.93E-01 | 2.93E+01 | | | |
| 7975976 | NM_012111 | AHSA1 | AHA1, activator of heat shock 90kDa | 0.776 | 1.712 | 1.28E-02 | 1.28E+00 | Up | | |
| 8102979 | --- | --- | --- | 0.776 | 1.712 | 4.88E-02 | 4.88E+00 | Up | | |
| 8080487 | NM_006254 | PRKCD | protein kinase C, delta | 0.774 | 1.710 | 3.50E-04 | 3.50E-02 | Up | | |
| 7955896 | NM_016057 | COPZ1 | coatomer protein complex, subunit z | 0.774 | 1.710 | 6.43E-02 | 6.43E+00 | | | |
| 8006123 | NM_001304 | CPD | carboxypeptidase D | 0.773 | 1.709 | 3.60E-03 | 3.60E-01 | Up | | |
| 8146914 | NM_017489 | TERF1 | telomeric repeat binding factor (NIM | 0.772 | 1.708 | 1.17E-01 | 1.17E+01 | | | |
| 7892757 | --- | --- | --- | 0.772 | 1.707 | 1.87E-01 | 1.87E+01 | | | |
| 7893779 | --- | --- | --- | 0.771 | 1.707 | 3.12E-01 | 3.12E+01 | | | |
| 8110022 | NM_003945 | ATP6V0E1 | ATPase, H+ transporting, lysosomal 9 | 0.771 | 1.707 | 9.07E-02 | 9.07E+00 | | | |
| 8141795 | NM_00109761! | POLR2J3 | polymerase (RNA) II (DNA directed) p | 0.771 | 1.706 | 2.95E-02 | 2.95E+00 | Up | | |
| 7894771 | --- | --- | --- | 0.770 | 1.706 | 2.11E-01 | 2.11E+01 | | | |
| 7893424 | --- | --- | --- | 0.770 | 1.705 | 1.13E-01 | 1.13E+01 | | | |
| 8082086 | NM_00111352: | PARP15 | poly (ADP-ribose) polymerase family | 0.770 | 1.705 | 1.86E-02 | 1.86E+00 | Up | | |
| 8016733 | NM_016424 | LUC7L3 | LUC7-like 3 (S. cerevisiae) | 0.770 | 1.705 | 9.25E-03 | 9.25E-01 | Up | | |
| 7894074 | --- | --- | --- | 0.770 | 1.705 | 3.64E-02 | 3.64E+00 | Up | | |
| 7978766 | NM_002013 | FKBP3 | FK506 binding protein 3, 25kDa | 0.769 | 1.704 | 8.37E-03 | 8.37E-01 | Up | | |
| 8031825 | AK299091 | OC10028832 | similar to zinc finger protein 587 | 0.769 | 1.704 | 1.39E-01 | 1.39E+01 | | | |
| 8040630 | --- | --- | --- | 0.769 | 1.704 | 7.39E-02 | 7.39E+00 | | | |
| 7894657 | --- | --- | --- | 0.768 | 1.703 | 2.62E-01 | 2.62E+01 | | | |
| 8116372 | NM_018434 | RNF130 | ring finger protein 130 | 0.767 | 1.702 | 4.46E-02 | 4.46E+00 | Up | | mono |
| 8096385 | NM_014606 | HERC3 | hect domain and RLD 3 | 0.767 | 1.702 | 1.50E-02 | 1.50E+00 | Up | | |
| 8152553 | NM_003184 | TAF2 | TAF2 RNA polymerase II, TATA box b | 0.766 | 1.701 | 6.33E-03 | 6.33E-01 | Up | | |
| 8046502 | NM_024583 | SCRN3 | secernin 3 | 0.766 | 1.701 | 5.77E-02 | 5.77E+00 | | | |
| 7918223 | NM_144584 | C1orf59 | chromosome 1 open reading frame 5 | 0.766 | 1.701 | 2.69E-03 | 2.69E-01 | Up | | |
| 8180342 | --- | --- | --- | 0.766 | 1.701 | 4.06E-02 | 4.06E+00 | Up | | |
| 8086706 | NM_014159 | SETD2 | SET domain containing 2 | 0.765 | 1.699 | 9.59E-03 | 9.59E-01 | Up | | |
| 8152845 | BC017297 | FAM49B | family with sequence similarity 49, m | 0.765 | 1.699 | 1.66E-02 | 1.66E+00 | Up | | |
| 8082100 | NM_017554 | PARP14 | poly (ADP-ribose) polymerase family | 0.765 | 1.699 | 4.61E-03 | 4.61E-01 | Up | | |
| 7895155 | --- | --- | --- | 0.764 | 1.698 | 8.19E-02 | 8.19E+00 | | | |
| 7893524 | --- | --- | --- | 0.764 | 1.698 | 2.65E-01 | 2.65E+01 | | | |
| 8163185 | NM_003329 | TXN | thioredoxin | 0.764 | 1.698 | 2.45E-01 | 2.45E+01 | | | |
| 7893654 | --- | --- | --- | 0.763 | 1.697 | 4.54E-01 | 4.54E+01 | | | |
| 8132843 | NM_017645 | HAUS6 | HAUS augmin-like complex, subunit 6 | 0.763 | 1.697 | 1.71E-01 | 1.71E+01 | | | |
| 7921987 | NM_019026 | TMCO1 | transmembrane and coiled-coil dom | 0.763 | 1.697 | 7.39E-02 | 7.39E+00 | | | |
| 8131475 | NM_020156 | C1GALT1 | core 1 synthase, glycoprotein-N-acet | 0.763 | 1.697 | 2.40E-02 | 2.40E+00 | Up | | |
| 8174103 | NM_00112812 | GK | glycerol kinase | 0.762 | 1.696 | 1.85E-01 | 1.85E+01 | | | |
| 7906015 | --- | --- | --- | 0.762 | 1.696 | 9.56E-02 | 9.56E+00 | | | |
| 7893012 | --- | --- | --- | 0.761 | 1.695 | 1.21E-01 | 1.21E+01 | | | |
| 8008834 | NM_004859 | CLTC | clathrin, heavy chain (Hc) | 0.761 | 1.695 | 5.33E-03 | 5.33E-01 | Up | | |
| 7938331 | NM_003442 | ZNF143 | zinc finger protein 143 | 0.761 | 1.694 | 2.86E-02 | 2.86E+00 | Up | | |
| 8004144 | NM_024039 | MIS12 | MIS12, MIND kinetochore complex c | 0.761 | 1.694 | 3.35E-02 | 3.35E+00 | Up | | |
| 8180397 | --- | --- | --- | 0.761 | 1.694 | 2.01E-01 | 2.01E+01 | | | |
| 7893441 | --- | --- | --- | 0.761 | 1.694 | 1.78E-01 | 1.78E+01 | | | |
| 8085852 | NM_018297 | NGLY1 | N-glycanase 1 | 0.760 | 1.693 | 6.23E-04 | 6.23E-02 | Up | | |
| 7953569 | NM_080549 | PTPN6 | protein tyrosine phosphatase, non-re | 0.759 | 1.693 | 7.07E-05 | 7.07E-03 | Up | | |
| 8050176 | NM_004763 | ITGB1BP1 | integrin beta 1 binding protein 1 | 0.759 | 1.692 | 3.27E-02 | 3.27E+00 | Up | | |
| 8132819 | NM_006060 | IKZF1 | IKAROS family zinc finger 1 (Ikaros) | 0.759 | 1.692 | 3.75E-03 | 3.75E-01 | Up | | |
| 8055021 | NM_017969 | IWS1 | IWS1 homolog (S. cerevisiae) | 0.759 | 1.692 | 3.29E-03 | 3.29E-01 | Up | | |
| 7935146 | NM_022451 | NOC3L | nucleolar complex associated 3 hom | 0.758 | 1.691 | 6.83E-03 | 6.83E-01 | Up | | |
| 8169361 | --- | --- | --- | 0.757 | 1.690 | 3.08E-02 | 3.08E+00 | Up | | |
| 7893908 | --- | --- | --- | 0.757 | 1.690 | 2.05E-01 | 2.05E+01 | | | |
| 7951372 | NM_033306 | CASP4 | caspase 4, apoptosis-related cysteine | 0.756 | 1.689 | 1.36E-02 | 1.36E+00 | Up | | |
| 8142540 | NM_014888 | FAM3C | family with sequence similarity 3, me | 0.756 | 1.689 | 7.23E-03 | 7.23E-01 | Up | | |
| 7893115 | --- | --- | --- | 0.756 | 1.689 | 2.05E-01 | 2.05E+01 | | | |
| 8140239 | NR_003613 | PMS2L1 | postmeiotic segregation increased 2- | 0.756 | 1.689 | 9.82E-02 | 9.82E+00 | | | |
| 8169541 | NM_144658 | DOCK11 | dedicator of cytokinesis 11 | 0.756 | 1.688 | 1.25E-02 | 1.25E+00 | Up | | |
| 7896011 | --- | --- | --- | 0.755 | 1.688 | 2.87E-01 | 2.87E+01 | | | |
| 7894272 | --- | --- | --- | 0.755 | 1.688 | 1.79E-01 | 1.79E+01 | | | |
| 8014903 | NM_00104247 | GSDMB | gasdermin B | 0.755 | 1.687 | 1.32E-02 | 1.32E+00 | Up | | |

| | | | | | | | | | | |
|---|---|---|---|---|---|---|---|---|---|---|
| 7893644 | --- | --- | --- | 0.754 | 1.686 | 3.83E-01 | 3.83E+01 | | | |
| 8107942 | NM_005732 | RAD50 | RAD50 homolog (S. cerevisiae) | 0.754 | 1.686 | 2.17E-02 | 2.17E+00 | Up | | |
| 7968270 | NR_002162 // | / ATP5EP2 / | ATP synthase, H+ transporting, mitoc | 0.753 | 1.685 | 3.37E-02 | 3.37E+00 | Up | | |
| 8108422 | --- | --- | --- | 0.752 | 1.685 | 3.71E-01 | 3.71E+01 | | | |
| 8149475 | NM_013354 | CNOT7 | CCR4-NOT transcription complex, su | 0.752 | 1.685 | 1.70E-01 | 1.70E+01 | | | |
| 7896320 | --- | --- | --- | 0.752 | 1.684 | 4.90E-01 | 4.90E+01 | | | |
| 7978201 | NM_006156 | NEDD8 | neural precursor cell expressed, deve | 0.752 | 1.684 | 9.94E-02 | 9.94E+00 | | | |
| 8120698 | NM_133645 | MTO1 | mitochondrial translation optimizatio | 0.751 | 1.683 | 9.25E-03 | 9.25E-01 | Up | | |
| 8055261 | NM_032144 | RAB6C | RAB6C, member RAS oncogene famil | 0.750 | 1.682 | 3.05E-02 | 3.05E+00 | Up | | |
| 8093191 | NM_00109842 | DLG1 | discs, large homolog 1 (Drosophila) | 0.750 | 1.681 | 1.18E-02 | 1.18E+00 | Up | | |
| 7958826 | --- | --- | --- | 0.750 | 1.681 | 1.04E-01 | 1.04E+01 | | | |
| 7895946 | --- | --- | --- | 0.750 | 1.681 | 5.21E-01 | 5.21E+01 | | | |
| 8166442 | NM_014888 | FAM3C | family with sequence similarity 3, me | 0.750 | 1.681 | 8.91E-03 | 8.91E-01 | Up | | |
| 8099721 | NM_015187 | KIAA0746 | KIAA0746 protein | 0.749 | 1.681 | 1.71E-02 | 1.71E+00 | Up | | |
| 8109620 | NM_003314 | TTC1 | tetratricopeptide repeat domain 1 | 0.749 | 1.681 | 8.13E-02 | 8.13E+00 | | | |
| 7948455 | NM_152852 | MS4A6A | membrane-spanning 4-domains, sub | 0.749 | 1.680 | 1.92E-03 | 1.92E-01 | Up | | |
| 7979732 | NM_006370 | VTI1B | vesicle transport through interaction | 0.748 | 1.680 | 1.05E-02 | 1.05E+00 | Up | | |
| 8172358 | NM_153477 | UXT | ubiquitously-expressed transcript | 0.748 | 1.680 | 8.15E-02 | 8.15E+00 | | | |
| 7948995 | ENST0000039 | ATL3 | atlastin GTPase 3 | 0.748 | 1.679 | 7.13E-02 | 7.13E+00 | | | |
| 7954436 | NM_006152 | LRMP | lymphoid-restricted membrane prote | 0.748 | 1.679 | 2.17E-02 | 2.17E+00 | Up | | |
| 7895947 | --- | --- | --- | 0.747 | 1.679 | 9.93E-02 | 9.93E+00 | | | |
| 7922504 | NM_022457 | RFWD2 | ring finger and WD repeat domain 2 | 0.747 | 1.678 | 2.20E-02 | 2.20E+00 | Up | | |
| 8048898 | NM_007237 | SP140 | SP140 nuclear body protein | 0.747 | 1.678 | 1.61E-02 | 1.61E+00 | Up | | |
| 7974483 | NM_182926 | KTN1 | kinectin 1 (kinesin receptor) | 0.746 | 1.678 | 9.14E-03 | 9.14E-01 | Up | | |
| 8107474 | NM_005509 | DMXL1 | Dmx-like 1 | 0.746 | 1.677 | 2.52E-03 | 2.52E-01 | Up | | |
| 7982878 | NM_007236 | CHP | calcium binding protein P22 | 0.746 | 1.677 | 3.71E-02 | 3.71E+00 | Up | | |
| 8050302 | NM_004850 | ROCK2 | Rho-associated, coiled-coil containin | 0.745 | 1.676 | 1.44E-02 | 1.44E+00 | Up | | |
| 8057554 | NM_014412 | CACYBP | calcyclin binding protein | 0.745 | 1.676 | 1.27E-01 | 1.27E+01 | | | |
| 7962013 | NM_016570 | ERGIC2 | ERGIC and golgi 2 | 0.745 | 1.676 | 1.94E-02 | 1.94E+00 | Up | | |
| 8103684 | NM_017867 | C4orf27 | chromosome 4 open reading frame 2 | 0.745 | 1.676 | 4.65E-02 | 4.65E+00 | Up | | |
| 8095163 | NM_00102492 | EXOC1 | exocyst complex component 1 | 0.745 | 1.676 | 6.67E-03 | 6.67E-01 | Up | | |
| 8028186 | NM_007145 | ZNF146 | zinc finger protein 146 | 0.744 | 1.675 | 1.49E-02 | 1.49E+00 | Up | | |
| 8014081 | NM_018428 | UTP6 | UTP6, small subunit (SSU) processom | 0.744 | 1.675 | 4.09E-03 | 4.09E-01 | Up | | |
| 7972888 | BC008975 | PCID2 | PCI domain containing 2 | 0.744 | 1.675 | 8.52E-02 | 8.52E+00 | | | |
| 7965064 | NM_020841 | OSBPL8 | oxysterol binding protein-like 8 | 0.744 | 1.675 | 1.54E-02 | 1.54E+00 | Up | | |
| 7896120 | --- | --- | --- | 0.744 | 1.675 | 4.62E-01 | 4.62E+01 | | | |
| 8110618 | NM_006628 | ARPP19 | cAMP-regulated phosphoprotein, 19 | 0.743 | 1.674 | 3.22E-01 | 3.22E+01 | | | |
| 8158404 | --- | --- | --- | 0.743 | 1.674 | 1.18E-01 | 1.18E+01 | | | |
| 7968331 | NM_003347 | UBE2L3 | ubiquitin-conjugating enzyme E2L 3 | 0.743 | 1.673 | 1.10E-01 | 1.10E+01 | | | |
| 7893266 | --- | --- | --- | 0.742 | 1.673 | 2.87E-01 | 2.87E+01 | | | |
| 7974380 | NM_002806 | PSMC6 | proteasome (prosome, macropain) 2 | 0.742 | 1.673 | 1.05E-02 | 1.05E+00 | Up | | |
| 8115022 | NM_00102510 | CSNK1A1 | casein kinase 1, alpha 1 | 0.742 | 1.673 | 2.13E-04 | 2.13E-02 | Up | | |
| 7900576 | NM_006347 | PPIH | peptidylprolyl isomerase H (cyclophi | 0.742 | 1.672 | 1.46E-01 | 1.46E+01 | | | |
| 8047381 | NM_003879 | CFLAR | CASP8 and FADD-like apoptosis regu | 0.741 | 1.672 | 9.57E-03 | 9.57E-01 | Up | | |
| 8045539 | NM_003937 | KYNU | kynureninase (L-kynurenine hydrolas | 0.741 | 1.671 | 9.22E-02 | 9.22E+00 | | | mono |
| 8021208 | NM_002396 | ME2 | malic enzyme 2, NAD(+)-dependent, | 0.741 | 1.671 | 2.75E-03 | 2.75E-01 | Up | | |
| 8045933 | NM_004180 | TANK | TRAF family member-associated NFK | 0.741 | 1.671 | 4.82E-02 | 4.82E+00 | Up | | |
| 7897078 | NM_007033 | RER1 | RER1 retention in endoplasmic reticu | 0.740 | 1.670 | 3.68E-02 | 3.68E+00 | Up | | |
| 8166500 | NM_003410 | ZFX | zinc finger protein, X-linked | 0.740 | 1.670 | 9.84E-02 | 9.84E+00 | | | |
| 7953977 | --- | --- | --- | 0.740 | 1.670 | 1.35E-01 | 1.35E+01 | | | |
| 7894903 | --- | --- | --- | 0.739 | 1.669 | 2.08E-01 | 2.08E+01 | | | |
| 7893948 | --- | --- | --- | 0.738 | 1.668 | 2.58E-01 | 2.58E+01 | | | |
| 7981964 | NR_003323 | SNORD116-8 | small nucleolar RNA, C/D box 116-8 | 0.738 | 1.668 | 2.06E-01 | 2.06E+01 | | | |
| 8030362 | NR_000020 | SNORD33 | small nucleolar RNA, C/D box 33 | 0.737 | 1.667 | 3.00E-01 | 3.00E+01 | | | |
| 7894532 | --- | --- | --- | 0.737 | 1.667 | 1.63E-01 | 1.63E+01 | | | |
| 8077450 | NM_018184 | ARL8B | ADP-ribosylation factor-like 8B | 0.737 | 1.667 | 1.14E-01 | 1.14E+01 | | | |
| 8001496 | NM_007006 | NUDT21 | nudix (nucleoside diphosphate linked | 0.736 | 1.665 | 1.78E-01 | 1.78E+01 | | | |
| 8168691 | NM_006729 | DIAPH2 | diaphanous homolog 2 (Drosophila) | 0.736 | 1.665 | 1.11E-02 | 1.11E+00 | Up | | mono |
| 7964119 | NM_005419 | STAT2 | signal transducer and activator of tra | 0.735 | 1.664 | 2.13E-04 | 2.13E-02 | Up | | |
| 7983910 | NM_020980 | AQP9 | aquaporin 9 | 0.735 | 1.664 | 1.14E-01 | 1.14E+01 | | | |
| 7896688 | --- | --- | --- | 0.734 | 1.663 | 1.23E-01 | 1.23E+01 | | | |
| 7901447 | NM_032864 | PRPF38A | PRP38 pre-mRNA processing factor 3 | 0.734 | 1.663 | 5.99E-02 | 5.99E+00 | | | |
| 7915758 | NM_021639 | GPBP1L1 | GC-rich promoter binding protein 1-l | 0.734 | 1.663 | 4.84E-03 | 4.84E-01 | Up | | |
| 7961767 | AK295862 | KIAA0528 | KIAA0528 | 0.733 | 1.663 | 3.97E-03 | 3.97E-01 | Up | | |
| 7977077 | NM_00112891 | MARK3 | MAP/microtubule affinity-regulating | 0.733 | 1.662 | 5.57E-02 | 5.57E+00 | | | |
| 8166632 | NM_00112812 | GK | glycerol kinase | 0.733 | 1.662 | 6.09E-02 | 6.09E+00 | | | |
| 7893558 | --- | --- | --- | 0.733 | 1.662 | 1.51E-01 | 1.51E+01 | | | |
| 7946655 | NR_002207 | CSNK2A1P | casein kinase 2, alpha 1 polypeptide | 0.733 | 1.662 | 7.08E-02 | 7.08E+00 | | | |
| 7904853 | NM_00109761 | GPR89A | G protein-coupled receptor 89A | 0.732 | 1.661 | 6.68E-02 | 6.68E+00 | | | |
| 7909782 | NM_016052 | RRP15 | ribosomal RNA processing 15 homolo | 0.732 | 1.661 | 1.72E-02 | 1.72E+00 | Up | | |
| 7984103 | NM_032857 | LACTB | lactamase, beta | 0.732 | 1.661 | 4.91E-03 | 4.91E-01 | Up | | |
| 7896329 | --- | --- | --- | 0.732 | 1.660 | 2.33E-01 | 2.33E+01 | | | |
| 7894734 | --- | --- | --- | 0.731 | 1.660 | 4.70E-01 | 4.70E+01 | | | |
| 8067903 | NM_013396 | USP25 | ubiquitin specific peptidase 25 | 0.731 | 1.660 | 2.91E-02 | 2.91E+00 | Up | | |
| 8058221 | NM_015049 | TRAK2 | trafficking protein, kinesin binding 2 | 0.730 | 1.659 | 6.84E-03 | 6.84E-01 | Up | | |
| 8092328 | NM_020166 | MCCC1 | methylcrotonoyl-Coenzyme A carbox | 0.730 | 1.658 | 1.14E-02 | 1.14E+00 | Up | | |
| 8161829 | BC034033 | C9orf41 | chromosome 9 open reading frame 4 | 0.728 | 1.657 | 2.21E-02 | 2.21E+00 | Up | | |

| | | | | | | | | | | |
|---|---|---|---|---|---|---|---|---|---|---|
| 7977736 | NM_032846 | RAB2B | RAB2B, member RAS oncogene famil | 0.728 | 1.657 | 2.58E-02 | 2.58E+00 | Up | | |
| 8012852 | --- | --- | --- | 0.728 | 1.657 | 2.53E-01 | 2.53E+01 | | | |
| 7914887 | NM_014408 | TRAPPC3 | trafficking protein particle complex 3 | 0.728 | 1.656 | 1.22E-02 | 1.22E+00 | Up | | |
| 7905571 | NM_002965 | S100A9 | S100 calcium binding protein A9 | 0.727 | 1.655 | 8.12E-04 | 8.12E-02 | Up | | |
| 8071194 | --- | --- | --- | 0.727 | 1.655 | 2.46E-01 | 2.46E+01 | | | |
| 8165642 | NM_053045 | TMEM203 | transmembrane protein 203 | 0.727 | 1.655 | 1.57E-01 | 1.57E+01 | | | |
| 7906757 | NM_00113621 | FCGR2A | Fc fragment of IgG, low affinity IIa, re | 0.726 | 1.655 | 2.51E-02 | 2.51E+00 | Up | | mono |
| 8132943 | NM_001762 | CCT6A | chaperonin containing TCP1, subunit | 0.726 | 1.654 | 1.88E-03 | 1.88E-01 | Up | | |
| 8151991 | --- | --- | --- | 0.725 | 1.653 | 7.84E-02 | 7.84E+00 | | | |
| 7893113 | --- | --- | --- | 0.725 | 1.653 | 1.33E-01 | 1.33E+01 | | | |
| 7895303 | --- | --- | --- | 0.725 | 1.653 | 2.13E-01 | 2.13E+01 | | | |
| 7952984 | --- | --- | --- | 0.725 | 1.653 | 3.82E-01 | 3.82E+01 | | | |
| 8039961 | NR_024080 | ACP1 | acid phosphatase 1, soluble | 0.725 | 1.653 | 1.96E-02 | 1.96E+00 | Up | | |
| 7893630 | --- | --- | --- | 0.725 | 1.653 | 4.14E-01 | 4.14E+01 | | | |
| 7970546 | NM_152726 | EFHA1 | EF-hand domain family, member A1 | 0.725 | 1.653 | 3.22E-02 | 3.22E+00 | Up | | |
| 8095343 | NM_012108 | STAP1 | signal transducing adaptor family me | 0.724 | 1.652 | 1.72E-01 | 1.72E+01 | | | |
| 8155248 | --- | --- | --- | 0.724 | 1.651 | 3.55E-01 | 3.55E+01 | | | |
| 8113035 | --- | --- | --- | 0.724 | 1.651 | 9.20E-02 | 9.20E+00 | | | |
| 7953383 | NR_004387 | SCARNA10 | small Cajal body-specific RNA 10 | 0.723 | 1.651 | 1.11E-01 | 1.11E+01 | | | |
| 8038899 | NM_002029 | FPR1 | formyl peptide receptor 1 | 0.723 | 1.651 | 7.89E-03 | 7.89E-01 | Up | | |
| 7894107 | --- | --- | --- | 0.722 | 1.650 | 1.41E-01 | 1.41E+01 | | | |
| 8092321 | NM_020640 | DCUN1D1 | DCN1, defective in cullin neddylation | 0.722 | 1.650 | 1.83E-01 | 1.83E+01 | | | |
| 8147439 | NM_024613 | PLEKHF2 | pleckstrin homology domain contain | 0.722 | 1.650 | 1.22E-02 | 1.22E+00 | Up | | |
| 8090533 | NM_020701 | ISY1 | ISY1 splicing factor homolog (S. cerev | 0.722 | 1.649 | 5.89E-02 | 5.89E+00 | | | |
| 8180343 | --- | --- | --- | 0.722 | 1.649 | 3.48E-02 | 3.48E+00 | Up | | |
| 7951032 | NR_003026 | SNORA1 | small nucleolar RNA, H/ACA box 1 | 0.721 | 1.649 | 1.25E-01 | 1.25E+01 | | | |
| 7957570 | NM_005761 | PLXNC1 | plexin C1 | 0.721 | 1.649 | 7.65E-03 | 7.65E-01 | Up | | |
| 7989659 | --- | --- | --- | 0.721 | 1.648 | 1.47E-01 | 1.47E+01 | | | |
| 8091637 | NM_004733 | SLC33A1 | solute carrier family 33 (acetyl-CoA t | 0.721 | 1.648 | 3.99E-02 | 3.99E+00 | Up | | |
| 8000323 | NM_005003 | NDUFAB1 | NADH dehydrogenase (ubiquinone) 1 | 0.721 | 1.648 | 1.45E-01 | 1.45E+01 | | | |
| 7905444 | NM_030918 | SNX27 | sorting nexin family member 27 | 0.721 | 1.648 | 1.29E-02 | 1.29E+00 | Up | | |
| 7906767 | NM_201563 | FCGR2C | Fc fragment of IgG, low affinity IIc, re | 0.720 | 1.648 | 9.46E-03 | 9.46E-01 | Up | | |
| 8044686 | NM_005721 | ACTR3 | ARP3 actin-related protein 3 homolo | 0.720 | 1.647 | 4.52E-04 | 4.52E-02 | Up | | |
| 7961865 | NM_033360 | KRAS | v-Ki-ras2 Kirsten rat sarcoma viral on | 0.719 | 1.647 | 6.33E-03 | 6.33E-01 | Up | | |
| 8174474 | NM_022977 | ACSL4 | acyl-CoA synthetase long-chain famil | 0.719 | 1.646 | 1.62E-02 | 1.62E+00 | Up | | |
| 7939197 | NM_005734 | HIPK3 | homeodomain interacting protein ki | 0.719 | 1.646 | 5.45E-03 | 5.45E-01 | Up | | |
| 7930276 | NM_014720 | SLK | STE20-like kinase (yeast) | 0.719 | 1.646 | 1.46E-02 | 1.46E+00 | Up | | |
| 8052269 | NM_00113559 | CCDC88A | coiled-coil domain containing 88A | 0.718 | 1.645 | 2.97E-03 | 2.97E-01 | Up | | |
| 7895118 | --- | --- | --- | 0.718 | 1.644 | 1.11E-01 | 1.11E+01 | | | |
| 8164701 | NM_015046 | SETX | senataxin | 0.717 | 1.644 | 5.18E-03 | 5.18E-01 | Up | | |
| 7930956 | NM_007190 | SEC23IP | SEC23 interacting protein | 0.717 | 1.644 | 6.82E-04 | 6.82E-02 | Up | | |
| 7924405 | NM_012414 | RAB3GAP2 | RAB3 GTPase activating protein subu | 0.717 | 1.643 | 1.28E-02 | 1.28E+00 | Up | | |
| 7899346 | NM_015933 | CCDC72 | coiled-coil domain containing 72 | 0.716 | 1.642 | 6.42E-02 | 6.42E+00 | | | |
| 8030946 | NM_00103988 | ZNF808 | zinc finger protein 808 | 0.716 | 1.642 | 2.72E-02 | 2.72E+00 | Up | | |
| 7894284 | --- | --- | --- | 0.716 | 1.642 | 2.54E-01 | 2.54E+01 | | | |
| 8048980 | NM_016289 | CAB39 | calcium binding protein 39 | 0.715 | 1.642 | 2.03E-02 | 2.03E+00 | Up | | |
| 8051387 | NM_032574 | DPY30 | dpy-30 homolog (C. elegans) | 0.715 | 1.642 | 4.03E-02 | 4.03E+00 | Up | | |
| 8140269 | NR_027775 | PMS2L5 | postmeiotic segregation increased 2- | 0.715 | 1.642 | 1.07E-01 | 1.07E+01 | | | |
| 7904695 | NM_004892 | SEC22B | SEC22 vesicle trafficking protein hom | 0.715 | 1.641 | 4.02E-02 | 4.02E+00 | Up | | |
| 7895172 | --- | --- | --- | 0.714 | 1.640 | 1.55E-01 | 1.55E+01 | | | |
| 7895005 | --- | --- | --- | 0.714 | 1.640 | 2.46E-01 | 2.46E+01 | | | |
| 8080685 | NM_007159 | SLMAP | sarcolemma associated protein | 0.714 | 1.640 | 3.00E-02 | 3.00E+00 | Up | | |
| 8172035 | NM_006520 | DYNLT3 | dynein, light chain, Tctex-type 3 | 0.714 | 1.640 | 5.80E-02 | 5.80E+00 | | | |
| 8107164 | NM_015216 | HISPPD1 | histidine acid phosphatase domain c | 0.714 | 1.640 | 1.61E-02 | 1.61E+00 | Up | | |
| 8099826 | NM_030956 | TLR10 | toll-like receptor 10 | 0.714 | 1.640 | 9.07E-03 | 9.07E-01 | Up | Anti-microbial/Pathogen recogn. | |
| 7903404 | NM_017619 | RNPC3 | RNA-binding region (RNP1, RRM) cor | 0.713 | 1.640 | 1.11E-01 | 1.11E+01 | | | |
| 7896491 | --- | --- | --- | 0.713 | 1.639 | 1.38E-01 | 1.38E+01 | | | |
| 8180341 | --- | --- | --- | 0.712 | 1.638 | 3.80E-02 | 3.80E+00 | Up | | |
| 7965956 | NM_006166 | NFYB | nuclear transcription factor Y, beta | 0.712 | 1.638 | 3.73E-03 | 3.73E-01 | Up | | |
| 7993167 | NM_024997 | ATF7IP2 | activating transcription factor 7 inter | 0.712 | 1.638 | 8.05E-02 | 8.05E+00 | | | |
| 8052762 | NM_002056 | GFPT1 | glutamine-fructose-6-phosphate tran | 0.712 | 1.638 | 1.23E-02 | 1.23E+00 | Up | | |
| 8041913 | NR_024188 | KLRAQ1 | KLRAQ motif containing 1 | 0.711 | 1.637 | 1.12E-02 | 1.12E+00 | Up | | |
| 7966368 | NM_002710 | PPP1CC | protein phosphatase 1, catalytic subu | 0.711 | 1.637 | 2.86E-02 | 2.86E+00 | Up | | |
| 8082607 | NM_014382 | ATP2C1 | ATPase, Ca++ transporting, type 2C, | 0.710 | 1.636 | 1.30E-02 | 1.30E+00 | Up | | |
| 7892947 | --- | --- | --- | 0.710 | 1.636 | 1.76E-01 | 1.76E+01 | | | |
| 8151711 | NM_002485 | NBN | nibrin | 0.710 | 1.636 | 1.57E-02 | 1.57E+00 | Up | | |
| 8163795 | NM_005047 | PSMD5 | proteasome (prosome, macropain) 2 | 0.709 | 1.635 | 3.07E-02 | 3.07E+00 | Up | | |
| 7893516 | --- | --- | --- | 0.709 | 1.635 | 9.59E-02 | 9.59E+00 | | | |
| 8109344 | NM_000405 | GM2A | GM2 ganglioside activator | 0.709 | 1.635 | 1.04E-02 | 1.04E+00 | Up | | mono |
| 8170166 | NM_014500 | HTATSF1 | HIV-1 Tat specific factor 1 | 0.709 | 1.634 | 8.48E-03 | 8.48E-01 | Up | | |
| 8136177 | AF277177 | OC10028755 | PNAS-119 | 0.708 | 1.634 | 1.06E-01 | 1.06E+01 | | | |
| 8049574 | NM_080678 | UBE2F | ubiquitin-conjugating enzyme E2F (p | 0.708 | 1.634 | 3.99E-02 | 3.99E+00 | Up | | |
| 7893776 | --- | --- | --- | 0.708 | 1.634 | 2.41E-01 | 2.41E+01 | | | |
| 8022441 | NM_005406 | ROCK1 | Rho-associated, coiled-coil containin | 0.708 | 1.633 | 3.64E-02 | 3.64E+00 | Up | | |
| 7979698 | NM_015994 | ATP6V1D | ATPase, H+ transporting, lysosomal 3 | 0.708 | 1.633 | 1.27E-01 | 1.27E+01 | | | |
| 7973371 | BC009645 | C14orf119 | chromosome 14 open reading frame | 0.708 | 1.633 | 5.56E-02 | 5.56E+00 | | | |
| 8069620 | NM_080794 | MRPL39 | mitochondrial ribosomal protein L39 | 0.707 | 1.633 | 7.24E-02 | 7.24E+00 | | | |

| | | | | | | | | | | | |
|---|---|---|---|---|---|---|---|---|---|---|---|
| 8135576 | NM_015641 | TES | testis derived transcript (3 LIM doma | 0.707 | 1.632 | 5.48E-02 | 5.48E+00 | | | |
| 7895810 | --- | --- | --- | 0.706 | 1.632 | 7.87E-02 | 7.87E+00 | | | |
| 7893297 | --- | --- | --- | 0.706 | 1.632 | 2.31E-01 | 2.31E+01 | | | |
| 7905099 | NM_007259 | VPS45 | vacuolar protein sorting 45 homolog | 0.706 | 1.631 | 2.30E-02 | 2.30E+00 | Up | | |
| 7951046 | NM_005591 | MRE11A | MRE11 meiotic recombination 11 ho | 0.706 | 1.631 | 1.25E-02 | 1.25E+00 | Up | | |
| 8051882 | NM_133259 | LRPPRC | leucine-rich PPR-motif containing | 0.705 | 1.631 | 9.77E-03 | 9.77E-01 | Up | | |
| 8094719 | NM_018177 | N4BP2 | NEDD4 binding protein 2 | 0.705 | 1.631 | 4.24E-02 | 4.24E+00 | Up | | |
| 7917576 | NM_052942 | GBP5 | guanylate binding protein 5 | 0.705 | 1.630 | 6.02E-02 | 6.02E+00 | | | |
| 8107129 | NM_180991 | SLCO4C1 | solute carrier organic anion transpor | 0.704 | 1.629 | 8.53E-02 | 8.53E+00 | | | |
| 7922219 | NM_000655 | SELL | selectin L | 0.704 | 1.629 | 1.84E-02 | 1.84E+00 | Up | | |
| 7904478 | AF172850 | LOC51152 | melanoma antigen | 0.704 | 1.629 | 2.16E-01 | 2.16E+01 | | | |
| 7892699 | --- | --- | --- | 0.704 | 1.629 | 3.79E-01 | 3.79E+01 | | | |
| 8089527 | NM_022488 | ATG3 | ATG3 autophagy related 3 homolog ( | 0.703 | 1.628 | 2.69E-02 | 2.69E+00 | Up | | |
| 8045090 | NM_020120 | UGGT1 | UDP-glucose glycoprotein glucosyltra | 0.703 | 1.628 | 2.69E-03 | 2.69E-01 | Up | | |
| 8131479 | NM_019005 | MIOS | missing oocyte, meiosis regulator, ho | 0.703 | 1.628 | 2.35E-02 | 2.35E+00 | Up | | |
| 8083656 | NM_022736 | MFSD1 | major facilitator superfamily domain | 0.703 | 1.627 | 6.10E-02 | 6.10E+00 | | | |
| 8099807 | AF220235 | GAFA3 | FGF-2 activity-associated protein 3 | 0.702 | 1.627 | 1.98E-01 | 1.98E+01 | | | |
| 7936578 | NM_022063 | C10orf84 | chromosome 10 open reading frame | 0.702 | 1.627 | 2.61E-02 | 2.61E+00 | Up | | |
| 8172876 | NM_006306 | SMC1A | structural maintenance of chromoso | 0.701 | 1.626 | 5.31E-03 | 5.31E-01 | Up | | |
| 8124430 | NM_005320 | HIST1H1D | histone cluster 1, H1d | 0.701 | 1.625 | 3.01E-02 | 3.01E+00 | Up | | |
| 7895202 | --- | --- | --- | 0.699 | 1.623 | 1.08E-01 | 1.08E+01 | | | |
| 7918424 | --- | --- | --- | 0.698 | 1.623 | 1.45E-01 | 1.45E+01 | | | |
| 7893079 | --- | --- | --- | 0.698 | 1.623 | 1.62E-01 | 1.62E+01 | | | |
| 8084016 | NM_006218 | PIK3CA | phosphoinositide-3-kinase, catalytic, | 0.698 | 1.622 | 2.51E-02 | 2.51E+00 | Up | | |
| 7949931 | NM_017635 | SUV420H1 | suppressor of variegation 4-20 homo | 0.697 | 1.622 | 9.46E-03 | 9.46E-01 | Up | | |
| 7977693 | NM_020920 | CHD8 | chromodomain helicase DNA binding | 0.697 | 1.622 | 5.19E-03 | 5.19E-01 | Up | | |
| 8017133 | NM_182620 | SKA2 | spindle and kinetochore associated c | 0.697 | 1.621 | 2.14E-01 | 2.14E+01 | | | |
| 7954997 | NM_00102535( | ANO6 | anoctamin 6 | 0.696 | 1.621 | 4.84E-02 | 4.84E+00 | Up | | |
| 8044804 | NM_020548 | DBI | diazepam binding inhibitor (GABA re | 0.695 | 1.619 | 7.26E-03 | 7.26E-01 | Up | | |
| 8097679 | NM_007080 | LSM6 | LSM6 homolog, U6 small nuclear RN. | 0.695 | 1.619 | 1.26E-01 | 1.26E+01 | | | |
| 8153959 | NM_203447 | DOCK8 | dedicator of cytokinesis 8 | 0.695 | 1.619 | 1.31E-03 | 1.31E-01 | Up | | |
| 8178199 | 0 | 0 | 0 | 0.694 | 1.618 | 4.27E-02 | 4.27E+00 | Up | | |
| 8179489 | 0 | 0 | 0 | 0.694 | 1.618 | 4.27E-02 | 4.27E+00 | Up | | |
| 8095539 | NM_020368 | UTP3 | UTP3, small subunit (SSU) processom | 0.694 | 1.618 | 7.31E-03 | 7.31E-01 | Up | | |
| 8007919 | NR_002940 | LRRC37A4 | leucine rich repeat containing 37, me | 0.694 | 1.618 | 4.88E-02 | 4.88E+00 | Up | | |
| 8151296 | NM_016027 | LACTB2 | lactamase, beta 2 | 0.694 | 1.618 | 4.15E-02 | 4.15E+00 | Up | | |
| 7982100 | --- | --- | --- | 0.693 | 1.617 | 2.75E-01 | 2.75E+01 | | | |
| 7896050 | --- | --- | --- | 0.693 | 1.617 | 3.77E-02 | 3.77E+00 | Up | | |
| 7979085 | NM_002863 | PYGL | phosphorylase, glycogen, liver | 0.693 | 1.617 | 8.46E-03 | 8.46E-01 | Up | | |
| 8139165 | NM_014396 | VPS41 | vacuolar protein sorting 41 homolog | 0.693 | 1.617 | 1.51E-02 | 1.51E+00 | Up | | |
| 8084963 | NM_002577 | PAK2 | p21 protein (Cdc42/Rac)-activated ki | 0.693 | 1.617 | 4.80E-02 | 4.80E+00 | Up | | |
| 7971027 | NM_013338 | ALG5 | asparagine-linked glycosylation 5, do | 0.693 | 1.617 | 9.51E-02 | 9.51E+00 | | | |
| 7971482 | --- | --- | --- | 0.693 | 1.617 | 1.39E-01 | 1.39E+01 | | | |
| 7990020 | NM_006305 | ANP32A | acidic (leucine-rich) nuclear phospho | 0.693 | 1.617 | 3.32E-03 | 3.32E-01 | Up | | |
| 7971653 | NR_002612 | DLEU2 | deleted in lymphocytic leukemia 2 (n | 0.693 | 1.617 | 8.95E-02 | 8.95E+00 | | | |
| 7894416 | --- | --- | --- | 0.692 | 1.616 | 2.39E-01 | 2.39E+01 | | | |
| 8091432 | NM_016094 | COMMD2 | COMM domain containing 2 | 0.691 | 1.615 | 1.51E-01 | 1.51E+01 | | | |
| 7913415 | NM_032236 | USP48 | ubiquitin specific peptidase 48 | 0.691 | 1.615 | 1.18E-02 | 1.18E+00 | Up | | |
| 7895754 | --- | --- | --- | 0.691 | 1.614 | 4.44E-01 | 4.44E+01 | | | |
| 8127145 | NM_021814 | ELOVL5 | ELOVL family member 5, elongation | 0.691 | 1.614 | 1.33E-01 | 1.33E+01 | | | |
| 7895952 | --- | --- | --- | 0.691 | 1.614 | 4.57E-01 | 4.57E+01 | | | |
| 7986010 | NM_003870 | IQGAP1 | IQ motif containing GTPase activatin | 0.690 | 1.614 | 1.61E-02 | 1.61E+00 | Up | | |
| 8128111 | NM_016021 | UBE2J1 | ubiquitin-conjugating enzyme E2, J1 | 0.690 | 1.614 | 1.69E-02 | 1.69E+00 | Up | | |
| 7963750 | NM_012117 | CBX5 | chromobox homolog 5 (HP1 alpha ho | 0.690 | 1.614 | 2.30E-01 | 2.30E+01 | | | |
| 7999718 | AY507846 | C16orf63 | chromosome 16 open reading frame | 0.690 | 1.614 | 1.42E-01 | 1.42E+01 | | | |
| 8152628 | NM_024295 | DERL1 | Der1-like domain family, member 1 | 0.690 | 1.613 | 5.33E-02 | 5.33E+00 | | | |
| 8100308 | --- | --- | --- | 0.689 | 1.613 | 1.58E-01 | 1.58E+01 | | | |
| 8041902 | NM_002158 | FOXN2 | forkhead box N2 | 0.689 | 1.613 | 1.83E-02 | 1.83E+00 | Up | | |
| 8006112 | NM_032141 | CCDC55 | coiled-coil domain containing 55 | 0.689 | 1.612 | 7.68E-02 | 7.68E+00 | | | |
| 8137232 | NM_175571 | GIMAP8 | GTPase, IMAP family member 8 | 0.689 | 1.612 | 5.98E-04 | 5.98E-02 | Up | | |
| 8094717 | --- | --- | --- | 0.689 | 1.612 | 3.10E-01 | 3.10E+01 | | | |
| 8156575 | NM_015251 | ATMIN | ATM interactor | 0.688 | 1.611 | 1.60E-01 | 1.60E+01 | | | |
| 8145281 | NM_016612 | SLC25A37 | solute carrier family 25, member 37 | 0.688 | 1.611 | 4.74E-03 | 4.74E-01 | Up | | |
| 8121118 | NM_012115 | CASP8AP2 | caspase 8 associated protein 2 | 0.687 | 1.610 | 1.06E-01 | 1.06E+01 | | | |
| 7974725 | NM_016475 | JKAMP | JNK1/MAPK8-associated membrane | 0.686 | 1.609 | 4.57E-02 | 4.57E+00 | Up | | |
| 7989696 | NM_00112788! | SPG21 | spastic paraplegia 21 (autosomal rec | 0.686 | 1.609 | 2.23E-02 | 2.23E+00 | Up | | |
| 7959772 | NM_006312 | NCOR2 | nuclear receptor co-repressor 2 | 0.686 | 1.609 | 4.66E-02 | 4.66E+00 | Up | | |
| 8012110 | NM_007278 | GABARAP | GABA(A) receptor-associated protein | 0.685 | 1.608 | 2.50E-01 | 2.50E+01 | | | |
| 7894350 | --- | --- | --- | 0.685 | 1.608 | 3.99E-02 | 3.99E+00 | Up | | |
| 7978776 | NM_018353 | C14orf106 | chromosome 14 open reading frame | 0.685 | 1.608 | 1.18E-02 | 1.18E+00 | Up | | |
| 7973936 | NM_002791 | PSMA6 | proteasome (prosome, macropain) s | 0.684 | 1.607 | 7.99E-02 | 7.99E+00 | | | |
| 8101376 | NM_014933 | SEC31A | SEC31 homolog A (S. cerevisiae) | 0.684 | 1.607 | 5.36E-03 | 5.36E-01 | Up | | |
| 7896619 | --- | --- | --- | 0.684 | 1.606 | 2.80E-01 | 2.80E+01 | | | |
| 7893733 | --- | --- | --- | 0.684 | 1.606 | 4.38E-01 | 4.38E+01 | | | |
| 7893700 | --- | --- | --- | 0.683 | 1.606 | 2.98E-01 | 2.98E+01 | | | |
| 8096109 | NM_016067 | MRPS18C | mitochondrial ribosomal protein S18 | 0.683 | 1.606 | 1.32E-01 | 1.32E+01 | | | |
| 7893894 | --- | --- | --- | 0.683 | 1.606 | 3.24E-01 | 3.24E+01 | | | |

| | | | | | | | | | | | |
|---|---|---|---|---|---|---|---|---|---|---|---|
| 8053064 | NM_018221 | MOBKL1B | MOB1, Mps One Binder kinase activa | 0.683 | 1.605 | 4.73E-02 | 4.73E+00 | Up | | | |
| 7895693 | --- | --- | --- | 0.683 | 1.605 | 1.09E-01 | 1.09E+01 | | | | |
| 7926786 | NM_019043 | APBB1IP | amyloid beta (A4) precursor protein- | 0.682 | 1.605 | 3.46E-03 | 3.46E-01 | Up | | | |
| 8174779 | NM_013995 | LAMP2 | lysosomal-associated membrane pro | 0.682 | 1.604 | 2.88E-03 | 2.88E-01 | Up | | | |
| 7895177 | --- | --- | --- | 0.681 | 1.604 | 1.86E-01 | 1.86E+01 | | | | |
| 8048926 | NM_138402 | SP140L | SP140 nuclear body protein-like | 0.681 | 1.603 | 2.27E-02 | 2.27E+00 | Up | | | |
| 7989735 | NM_006660 | CLPX | ClpX caseinolytic peptidase X homolo | 0.681 | 1.603 | 4.34E-02 | 4.34E+00 | Up | | | |
| 8160756 | NM_015397 | DCAF12 | DDB1 and CUL4 associated factor 12 | 0.681 | 1.603 | 2.29E-02 | 2.29E+00 | Up | | | |
| 7896205 | --- | --- | --- | 0.680 | 1.602 | 1.49E-01 | 1.49E+01 | | | | |
| 8120428 | NM_000801 | FKBP1A | FK506 binding protein 1A, 12kDa | 0.680 | 1.602 | 3.69E-02 | 3.69E+00 | Up | | | |
| 7984289 | NM_004663 | RAB11A | RAB11A, member RAS oncogene fam | 0.680 | 1.602 | 1.79E-01 | 1.79E+01 | | | | |
| 7981290 | NM_004184 | WARS | tryptophanyl-tRNA synthetase | 0.680 | 1.602 | 8.63E-03 | 8.63E-01 | Up | | | |
| 7893487 | --- | --- | --- | 0.680 | 1.602 | 4.23E-01 | 4.23E+01 | | | | |
| 7932964 | NM_006333 | C1D | C1D nuclear receptor co-repressor | 0.679 | 1.601 | 3.76E-01 | 3.76E+01 | | | | |
| 8120758 | NM_015571 | SENP6 | SUMO1/sentrin specific peptidase 6 | 0.679 | 1.601 | 5.02E-02 | 5.02E+00 | | | | |
| 8100251 | NM_015030 | FRYL | FRY-like | 0.679 | 1.601 | 2.16E-02 | 2.16E+00 | Up | | | |
| 7904726 | NM_006472 | TXNIP | thioredoxin interacting protein | 0.679 | 1.601 | 8.04E-05 | 8.04E-03 | Up | | | |
| 7930304 | NM_004832 | GSTO1 | glutathione S-transferase omega 1 | 0.679 | 1.601 | 2.50E-02 | 2.50E+00 | Up | | | |
| 7895069 | --- | --- | --- | 0.679 | 1.601 | 5.09E-01 | 5.09E+01 | | | | |
| 7927536 | NM_005437 | NCOA4 | nuclear receptor coactivator 4 | 0.678 | 1.600 | 2.08E-02 | 2.08E+00 | Up | | | |
| 8085081 | NM_016302 | CRBN | cereblon | 0.678 | 1.600 | 6.23E-02 | 6.23E+00 | | | | |
| 7896687 | --- | --- | --- | 0.678 | 1.599 | 2.35E-02 | 2.35E+00 | Up | | | |
| 7999553 | NM_018340 | CPPED1 | calcineurin-like phosphoesterase dor | 0.678 | 1.599 | 6.50E-03 | 6.50E-01 | Up | | | |
| 8031815 | NM_173632 | ZNF776 | zinc finger protein 776 | 0.677 | 1.599 | 5.59E-03 | 5.59E-01 | Up | | | |
| 8023195 | NM_005901 | SMAD2 | SMAD family member 2 | 0.677 | 1.599 | 1.78E-02 | 1.78E+00 | Up | | | |
| 7914550 | NM_178547 | ZBTB8OS | zinc finger and BTB domain containir | 0.677 | 1.599 | 5.11E-02 | 5.11E+00 | | | | |
| 8127425 | NM_018368 | LMBRD1 | LMBR1 domain containing 1 | 0.677 | 1.599 | 5.99E-02 | 5.99E+00 | | | | |
| 7895669 | --- | --- | --- | 0.676 | 1.598 | 1.14E-01 | 1.14E+01 | | | | |
| 7927106 | --- | --- | --- | 0.675 | 1.597 | 3.77E-01 | 3.77E+01 | | | | |
| 8057441 | NM_020943 | CWC22 | CWC22 spliceosome-associated prot | 0.675 | 1.596 | 2.50E-02 | 2.50E+00 | Up | | | |
| 7894566 | --- | --- | --- | 0.675 | 1.596 | 2.81E-01 | 2.81E+01 | | | | |
| 8062880 | NM_003404 | YWHAB | tyrosine 3-monooxygenase/tryptoph | 0.674 | 1.596 | 1.51E-02 | 1.51E+00 | Up | | | |
| 8146500 | NM_002350 | LYN | v-yes-1 Yamaguchi sarcoma viral rela | 0.674 | 1.596 | 6.54E-02 | 6.54E+00 | | | | |
| 7896319 | --- | --- | --- | 0.674 | 1.595 | 2.67E-01 | 2.67E+01 | | | | |
| 7972180 | NM_024546 | RNF219 | ring finger protein 219 | 0.674 | 1.595 | 1.68E-02 | 1.68E+00 | Up | | | |
| 7897404 | NM_007262 | PARK7 | Parkinson disease (autosomal recess | 0.673 | 1.595 | 8.69E-03 | 8.69E-01 | Up | | | |
| 7892637 | --- | --- | --- | 0.672 | 1.594 | 2.84E-01 | 2.84E+01 | | | | |
| 8150962 | NM_014729 | TOX | thymocyte selection-associated high | 0.672 | 1.593 | 1.27E-02 | 1.27E+00 | Up | | | |
| 7896659 | --- | --- | --- | 0.671 | 1.592 | 4.40E-01 | 4.40E+01 | | | | |
| 8079742 | --- | --- | --- | 0.670 | 1.591 | 4.74E-01 | 4.74E+01 | | | | |
| 7896271 | --- | --- | --- | 0.670 | 1.591 | 2.93E-01 | 2.93E+01 | | | | |
| 8122013 | NM_032438 | L3MBTL3 | l(3)mbt-like 3 (Drosophila) | 0.670 | 1.591 | 4.66E-02 | 4.66E+00 | Up | | | |
| 8058197 | --- | --- | --- | 0.669 | 1.590 | 7.63E-02 | 7.63E+00 | | | | |
| 7896493 | --- | --- | --- | 0.669 | 1.590 | 2.84E-01 | 2.84E+01 | | | | |
| 7894761 | --- | --- | --- | 0.669 | 1.590 | 1.83E-01 | 1.83E+01 | | | | |
| 8029845 | --- | --- | --- | 0.669 | 1.590 | 3.45E-01 | 3.45E+01 | | | | |
| 7977749 | NM_019852 | METTL3 | methyltransferase like 3 | 0.669 | 1.589 | 1.60E-03 | 1.60E-01 | Up | | | |
| 8047372 | NM_002491 | NDUFB3 | NADH dehydrogenase (ubiquinone) 1 | 0.668 | 1.589 | 3.85E-02 | 3.85E+00 | Up | | | |
| 7974912 | --- | --- | --- | 0.668 | 1.589 | 9.31E-02 | 9.31E+00 | | | | |
| 7981192 | NM_018036 | ATG2B | ATG2 autophagy related 2 homolog I | 0.668 | 1.589 | 2.74E-02 | 2.74E+00 | Up | | | |
| 8058182 | NM_173822 | FAM126B | family with sequence similarity 126, | 0.668 | 1.589 | 4.34E-02 | 4.34E+00 | Up | | | |
| 7896250 | --- | --- | --- | 0.667 | 1.588 | 2.76E-01 | 2.76E+01 | | | | |
| 8126428 | NM_033502 | TRERF1 | transcriptional regulating factor 1 | 0.667 | 1.588 | 5.82E-02 | 5.82E+00 | | | | |
| 7999841 | NM_015092 | SMG1 | SMG1 homolog, phosphatidylinosito | 0.666 | 1.587 | 6.38E-03 | 6.38E-01 | Up | | | |
| 7936706 | NM_024834 | C10orf119 | chromosome 10 open reading frame | 0.665 | 1.586 | 3.05E-02 | 3.05E+00 | Up | | | |
| 7896283 | --- | --- | --- | 0.665 | 1.586 | 4.01E-01 | 4.01E+01 | | | | |
| 8162833 | NM_015051 | ERP44 | endoplasmic reticulum protein 44 | 0.665 | 1.585 | 2.34E-02 | 2.34E+00 | Up | | | |
| 7896483 | --- | --- | --- | 0.665 | 1.585 | 4.79E-01 | 4.79E+01 | | | | |
| 8110522 | NM_001746 | CANX | calnexin | 0.664 | 1.584 | 2.33E-02 | 2.33E+00 | Up | | | |
| 7895227 | --- | --- | --- | 0.663 | 1.584 | 8.14E-02 | 8.14E+00 | | | | |
| 8097373 | BC034253 | C4orf29 | chromosome 4 open reading frame 2 | 0.663 | 1.584 | 9.53E-02 | 9.53E+00 | | | | |
| 8073645 | NM_015380 | SAMM50 | sorting and assembly machinery com | 0.663 | 1.584 | 7.20E-02 | 7.20E+00 | | | | |
| 8134454 | NM_015379 | BRI3 | brain protein I3 | 0.663 | 1.584 | 1.79E-03 | 1.79E-01 | Up | | | |
| 7893798 | --- | --- | --- | 0.663 | 1.583 | 2.46E-01 | 2.46E+01 | | | | |
| 8133914 | NR_024549 | DMTF1 | cyclin D binding myb-like transcriptio | 0.663 | 1.583 | 3.23E-02 | 3.23E+00 | Up | | | |
| 7956593 | NM_006812 | OS9 | osteosarcoma amplified 9, endoplasi | 0.662 | 1.583 | 1.26E-03 | 1.26E-01 | Up | | | |
| 7929132 | NM_032373 | PCGF5 | polycomb group ring finger 5 | 0.662 | 1.582 | 6.70E-02 | 6.70E+00 | | | | |
| 7979906 | NM_016468 | COX16 | COX16 cytochrome c oxidase assemb | 0.662 | 1.582 | 2.43E-01 | 2.43E+01 | | | | |
| 8163452 | NM_015258 | FKBP15 | FK506 binding protein 15, 133kDa | 0.662 | 1.582 | 6.01E-04 | 6.01E-02 | Up | | | |
| 7896181 | --- | --- | --- | 0.662 | 1.582 | 5.31E-01 | 5.31E+01 | | | | |
| 8130499 | NM_006519 | DYNLT1 | dynein, light chain, Tctex-type 1 | 0.661 | 1.581 | 1.58E-01 | 1.58E+01 | | | | |
| 7918157 | NM_006113 | VAV3 | vav 3 guanine nucleotide exchange f | 0.661 | 1.581 | 2.55E-02 | 2.55E+00 | Up | | | |
| 8140478 | NM_017439 | PION | pigeon homolog (Drosophila) | 0.661 | 1.581 | 5.38E-02 | 5.38E+00 | | | | |
| 8083183 | NM_00108041 | SR140 | U2-associated SR140 protein | 0.660 | 1.581 | 1.85E-02 | 1.85E+00 | Up | | | |
| 8115734 | NM_005565 | LCP2 | lymphocyte cytosolic protein 2 (SH2 | 0.660 | 1.580 | 2.47E-02 | 2.47E+00 | Up | | | |
| 7894270 | --- | --- | --- | 0.660 | 1.580 | 2.07E-01 | 2.07E+01 | | | | |
| 7892594 | --- | --- | --- | 0.659 | 1.579 | 3.93E-01 | 3.93E+01 | | | | |

| | | | | | | | | | | |
|---|---|---|---|---|---|---|---|---|---|---|
| 7995379 | BC013044 | DNAJA2 | DnaJ (Hsp40) homolog, subfamily A, | 0.659 | 1.579 | 3.28E-01 | 3.28E+01 | | | |
| 8142878 | NM_139286 | CDC26 | cell division cycle 26 homolog (S. cer | 0.659 | 1.579 | 2.00E-01 | 2.00E+01 | | | |
| 7957806 | NM_017988 | SCYL2 | SCY1-like 2 (S. cerevisiae) | 0.659 | 1.579 | 4.06E-02 | 4.06E+00 | Up | | |
| 7899005 | NM_014313 | TMEM50A | transmembrane protein 50A | 0.658 | 1.578 | 5.11E-02 | 5.11E+00 | | | |
| 7921868 | NM_000569 | FCGR3A | Fc fragment of IgG, low affinity IIIa, r | 0.658 | 1.577 | 1.65E-02 | 1.65E+00 | Up | Phagocytosis/killing | |
| 8145470 | NM_001386 | DPYSL2 | dihydropyrimidinase-like 2 | 0.657 | 1.577 | 9.13E-04 | 9.13E-02 | Up | | |
| 7896101 | --- | --- | --- | 0.657 | 1.577 | 2.70E-01 | 2.70E+01 | | | |
| 8123315 | NM_206855 | QKI | quaking homolog, KH domain RNA bi | 0.657 | 1.577 | 3.28E-03 | 3.28E-01 | Up | | |
| 7971039 | NM_00101428( | FAM48A | family with sequence similarity 48, n | 0.657 | 1.576 | 1.46E-02 | 1.46E+00 | Up | | |
| 7937866 | --- | --- | --- | 0.656 | 1.576 | 9.76E-02 | 9.76E+00 | | | |
| 8016562 | NM_00100723( | SPOP | speckle-type POZ protein | 0.656 | 1.576 | 1.58E-02 | 1.58E+00 | Up | | |
| 7945132 | NM_002017 | FLI1 | Friend leukemia virus integration 1 | 0.656 | 1.576 | 1.81E-03 | 1.81E-01 | Up | | |
| 7955908 | NM_005337 | NCKAP1L | NCK-associated protein 1-like | 0.656 | 1.576 | 1.38E-02 | 1.38E+00 | Up | | |
| 8122348 | NM_016485 | VTA1 | Vps20-associated 1 homolog (S. cere | 0.656 | 1.576 | 2.56E-02 | 2.56E+00 | Up | | |
| 8084146 | NM_00101343( | FXR1 | fragile X mental retardation, autosor | 0.656 | 1.576 | 7.25E-02 | 7.25E+00 | | | |
| 7993341 | NM_016561 | BFAR | bifunctional apoptosis regulator | 0.656 | 1.575 | 2.10E-02 | 2.10E+00 | Up | | |
| 8046759 | NM_018981 | DNAJC10 | DnaJ (Hsp40) homolog, subfamily C, | 0.656 | 1.575 | 3.71E-02 | 3.71E+00 | Up | | |
| 8020804 | --- | --- | --- | 0.655 | 1.575 | 2.02E-01 | 2.02E+01 | | | |
| 8140280 | NR_003614 | PMS2L2 | postmeiotic segregation increased 2- | 0.655 | 1.575 | 9.51E-02 | 9.51E+00 | | | |
| 8162313 | NM_013417 | IARS | isoleucyl-tRNA synthetase | 0.655 | 1.574 | 8.48E-03 | 8.48E-01 | Up | | |
| 7895039 | --- | --- | --- | 0.655 | 1.574 | 1.27E-01 | 1.27E+01 | | | |
| 7893460 | --- | --- | --- | 0.655 | 1.574 | 1.34E-01 | 1.34E+01 | | | |
| 8052331 | NM_033109 | PNPT1 | polyribonucleotide nucleotidyltransf | 0.654 | 1.574 | 1.01E-01 | 1.01E+01 | | | |
| 7894908 | --- | --- | --- | 0.654 | 1.574 | 2.82E-01 | 2.82E+01 | | | |
| 8005200 | NR_003043 | SNORD49B | small nucleolar RNA, C/D box 49B | 0.654 | 1.574 | 3.71E-01 | 3.71E+01 | | | |
| 7969271 | NM_00113091 | SUGT1 | SGT1, suppressor of G2 allele of SKP1 | 0.654 | 1.573 | 2.17E-01 | 2.17E+01 | | | |
| 7894914 | --- | --- | --- | 0.654 | 1.573 | 2.25E-01 | 2.25E+01 | | | |
| 8009205 | NM_007372 | DDX42 | DEAD (Asp-Glu-Ala-Asp) box polypep | 0.654 | 1.573 | 5.31E-03 | 5.31E-01 | Up | | |
| 7951140 | AY358248 | UNQ6228 | hypothetical LOC100131541 | 0.654 | 1.573 | 8.82E-03 | 8.82E-01 | Up | | |
| 8164013 | NM_018387 | STRBP | spermatid perinuclear RNA binding p | 0.653 | 1.573 | 6.13E-02 | 6.13E+00 | | | |
| 7978626 | --- | --- | --- | 0.653 | 1.572 | 3.48E-01 | 3.48E+01 | | | |
| 8138128 | NM_015622 | C7orf28A | chromosome 7 open reading frame 2 | 0.652 | 1.572 | 2.51E-02 | 2.51E+00 | Up | | |
| 8050190 | NM_003183 | ADAM17 | ADAM metallopeptidase domain 17 | 0.652 | 1.571 | 1.74E-02 | 1.74E+00 | Up | | |
| 7893736 | --- | --- | --- | 0.652 | 1.571 | 3.62E-01 | 3.62E+01 | | | |
| 7897620 | NM_002631 | PGD | phosphogluconate dehydrogenase | 0.652 | 1.571 | 7.88E-02 | 7.88E+00 | | | |
| 8052024 | NM_025133 | FBXO11 | F-box protein 11 | 0.652 | 1.571 | 1.86E-02 | 1.86E+00 | Up | | |
| 7893474 | --- | --- | --- | 0.652 | 1.571 | 1.38E-01 | 1.38E+01 | | | |
| 8001693 | NM_016284 | CNOT1 | CCR4-NOT transcription complex, su | 0.651 | 1.571 | 1.27E-02 | 1.27E+00 | Up | | |
| 8124166 | NM_00108048( | MBOAT1 | membrane bound O-acyltransferase | 0.651 | 1.570 | 3.35E-02 | 3.35E+00 | Up | | |
| 7896225 | --- | --- | --- | 0.651 | 1.570 | 2.09E-01 | 2.09E+01 | | | |
| 7895034 | --- | --- | --- | 0.651 | 1.570 | 9.59E-02 | 9.59E+00 | | | |
| 7924107 | NM_014873 | LPGAT1 | lysophosphatidylglycerol acyltransfe | 0.651 | 1.570 | 1.91E-02 | 1.91E+00 | Up | | |
| 8120251 | NM_033480 | FBXO9 | F-box protein 9 | 0.651 | 1.570 | 6.06E-03 | 6.06E-01 | Up | | |
| 8018966 | NM_003255 | TIMP2 | TIMP metallopeptidase inhibitor 2 | 0.651 | 1.570 | 1.04E-02 | 1.04E+00 | Up | | |
| 7932510 | --- | --- | --- | 0.651 | 1.570 | 4.67E-02 | 4.67E+00 | Up | | |
| 8020110 | NM_006868 | RAB31 | RAB31, member RAS oncogene famil | 0.650 | 1.569 | 2.01E-02 | 2.01E+00 | Up | | mono |
| 8117368 | NM_003542 | HIST1H4C | histone cluster 1, H4c | 0.650 | 1.569 | 2.88E-01 | 2.88E+01 | | | |
| 7951752 | NM_020886 | USP28 | ubiquitin specific peptidase 28 | 0.649 | 1.568 | 8.05E-03 | 8.05E-01 | Up | | |
| 7893550 | --- | --- | --- | 0.649 | 1.568 | 5.10E-01 | 5.10E+01 | | | |
| 7895131 | --- | --- | --- | 0.649 | 1.568 | 2.20E-01 | 2.20E+01 | | | |
| 8007885 | NM_006178 | NSF | N-ethylmaleimide-sensitive factor | 0.648 | 1.567 | 1.53E-01 | 1.53E+01 | | | |
| 7969017 | NM_000321 | RB1 | retinoblastoma 1 | 0.648 | 1.567 | 8.17E-02 | 8.17E+00 | | | |
| 7965515 | NM_018838 | NDUFA12 | NADH dehydrogenase (ubiquinone) 1 | 0.648 | 1.567 | 2.06E-01 | 2.06E+01 | | | |
| 8120022 | NM_001253 | CDC5L | CDC5 cell division cycle 5-like (S. pon | 0.648 | 1.567 | 6.32E-02 | 6.32E+00 | | | |
| 8109576 | NM_017872 | THG1L | tRNA-histidine guanylyltransferase 1 | 0.648 | 1.567 | 4.27E-02 | 4.27E+00 | Up | | |
| 7983512 | NM_021199 | SQRDL | sulfide quinone reductase-like (yeast | 0.648 | 1.567 | 8.48E-03 | 8.48E-01 | Up | | |
| 8084067 | NM_178042 | ACTL6A | actin-like 6A | 0.648 | 1.566 | 6.74E-02 | 6.74E+00 | | | |
| 8044417 | NM_032824 | TMEM87B | transmembrane protein 87B | 0.647 | 1.566 | 1.80E-02 | 1.80E+00 | Up | | |
| 8148293 | NM_173685 | NSMCE2 | non-SMC element 2, MMS21 homolo | 0.647 | 1.566 | 1.87E-01 | 1.87E+01 | | | |
| 8105436 | NM_005921 | MAP3K1 | mitogen-activated protein kinase kin | 0.646 | 1.565 | 1.56E-02 | 1.56E+00 | Up | | |
| 8114861 | NM_020117 | LARS | leucyl-tRNA synthetase | 0.646 | 1.565 | 1.27E-02 | 1.27E+00 | Up | | |
| 7894560 | --- | --- | --- | 0.645 | 1.564 | 2.94E-01 | 2.94E+01 | | | |
| 8087874 | NM_025222 | WDR82 | WD repeat domain 82 | 0.645 | 1.564 | 3.15E-02 | 3.15E+00 | Up | | |
| 7896705 | --- | --- | --- | 0.645 | 1.563 | 1.05E-01 | 1.05E+01 | | | |
| 7999419 | NM_014015 | DEXI | Dexi homolog (mouse) | 0.645 | 1.563 | 1.69E-01 | 1.69E+01 | | | |
| 8173437 | NM_00102526 | CXorf65 | chromosome X open reading frame 6 | 0.645 | 1.563 | 9.93E-02 | 9.93E+00 | | | |
| 7976307 | NM_005113 | GOLGA5 | golgi autoantigen, golgin subfamily a | 0.644 | 1.563 | 3.82E-02 | 3.82E+00 | Up | | |
| 8030978 | NM_138374 | ZNF845 | zinc finger protein 845 | 0.644 | 1.563 | 1.97E-01 | 1.97E+01 | | | |
| 7909601 | NR_004389 | SNORA16B | small nucleolar RNA, H/ACA box 16B | 0.644 | 1.562 | 3.40E-01 | 3.40E+01 | | | |
| 8099631 | --- | --- | --- | 0.643 | 1.562 | 8.76E-02 | 8.76E+00 | | | |
| 7927353 | NM_00114400( | AGAP5 | ArfGAP with GTPase domain, ankyrin | 0.642 | 1.561 | 2.86E-02 | 2.86E+00 | Up | | |
| 7933331 | NM_00114400( | AGAP5 | ArfGAP with GTPase domain, ankyrin | 0.642 | 1.561 | 2.86E-02 | 2.86E+00 | Up | | |
| 8171762 | NM_004586 | RPS6KA3 | ribosomal protein S6 kinase, 90kDa, | 0.642 | 1.560 | 5.61E-02 | 5.61E+00 | | | |
| 7893777 | --- | --- | --- | 0.642 | 1.560 | 1.28E-01 | 1.28E+01 | | | |
| 7939087 | BC047775 | C11orf46 | chromosome 11 open reading frame | 0.641 | 1.560 | 1.44E-01 | 1.44E+01 | | | |
| 7896331 | --- | --- | --- | 0.641 | 1.559 | 4.29E-01 | 4.29E+01 | | | |
| 8122689 | AY358952 | C6orf72 | chromosome 6 open reading frame 7 | 0.640 | 1.558 | 2.50E-01 | 2.50E+01 | | | |

| | | | | | | | | | | |
|---|---|---|---|---|---|---|---|---|---|---|
| 7912385 | NM_00100199 | EXOSC10 | exosome component 10 | 0.640 | 1.558 | 1.08E-02 | 1.08E+00 | Up | | |
| 8023735 | NM_019022 | TMX3 | thioredoxin-related transmembrane | 0.640 | 1.558 | 1.62E-02 | 1.62E+00 | Up | | |
| 7937465 | NM_006755 | TALDO1 | transaldolase 1 | 0.640 | 1.558 | 2.72E-02 | 2.72E+00 | Up | | |
| 7892744 | --- | --- | --- | 0.640 | 1.558 | 4.76E-01 | 4.76E+01 | | | |
| 7908861 | AF314543 | OCR1 | ovarian cancer-related protein 1 | 0.639 | 1.557 | 1.75E-01 | 1.75E+01 | | | |
| 7961166 | NM_013431 | KLRC4 | killer cell lectin-like receptor subfam | 0.639 | 1.557 | 1.94E-01 | 1.94E+01 | | | |
| 7912762 | --- | --- | --- | 0.637 | 1.555 | 3.53E-01 | 3.53E+01 | | | |
| 7909283 | --- | --- | --- | 0.637 | 1.555 | 1.39E-01 | 1.39E+01 | | | |
| 8056113 | NM_002349 | LY75 | lymphocyte antigen 75 | 0.637 | 1.555 | 4.50E-02 | 4.50E+00 | Up | | |
| 8112746 | NM_018268 | WDR41 | WD repeat domain 41 | 0.637 | 1.555 | 1.62E-02 | 1.62E+00 | Up | | |
| 8016245 | NR_002940 | LRRC37A4 | leucine rich repeat containing 37, me | 0.636 | 1.554 | 4.95E-02 | 4.95E+00 | Up | | |
| 7989023 | NM_004580 | RAB27A | RAB27A, member RAS oncogene fam | 0.636 | 1.554 | 7.56E-03 | 7.56E-01 | Up | | |
| 7892619 | --- | --- | --- | 0.636 | 1.554 | 2.84E-01 | 2.84E+01 | | | |
| 7896542 | --- | --- | --- | 0.636 | 1.553 | 2.84E-01 | 2.84E+01 | | | |
| 7968344 | NM_001629 | ALOX5AP | arachidonate 5-lipoxygenase-activati | 0.635 | 1.553 | 8.90E-02 | 8.90E+00 | | | |
| 7978905 | --- | --- | --- | 0.635 | 1.553 | 4.76E-02 | 4.76E+00 | Up | | |
| 8149793 | NM_004901 | ENTPD4 | ectonucleoside triphosphate diphosp | 0.635 | 1.553 | 3.37E-02 | 3.37E+00 | Up | | |
| 7893279 | --- | --- | --- | 0.635 | 1.553 | 4.69E-01 | 4.69E+01 | | | |
| 8114618 | NM_002488 | NDUFA2 | NADH dehydrogenase (ubiquinone) 1 | 0.635 | 1.552 | 1.60E-01 | 1.60E+01 | | | |
| 7962085 | NM_006390 | IPO8 | importin 8 | 0.634 | 1.552 | 1.11E-01 | 1.11E+01 | | | |
| 8154394 | NM_00103969 | SNAPC3 | small nuclear RNA activating comple | 0.633 | 1.551 | 1.36E-03 | 1.36E-01 | Up | | |
| 7917088 | NM_005482 | PIGK | phosphatidylinositol glycan anchor b | 0.633 | 1.551 | 1.11E-01 | 1.11E+01 | | | |
| 7892575 | --- | --- | --- | 0.633 | 1.551 | 5.15E-01 | 5.15E+01 | | | |
| 7984190 | NM_016213 | TRIP4 | thyroid hormone receptor interactor | 0.633 | 1.551 | 6.43E-03 | 6.43E-01 | Up | | |
| 7893345 | --- | --- | --- | 0.633 | 1.551 | 4.41E-01 | 4.41E+01 | | | |
| 7964868 | ENST00000451 | OC10013161 | PRO1454 | 0.633 | 1.551 | 4.24E-01 | 4.24E+01 | | | |
| 8120378 | NM_020931 | KIAA1586 | KIAA1586 | 0.633 | 1.550 | 1.63E-02 | 1.63E+00 | Up | | |
| 7895040 | --- | --- | --- | 0.633 | 1.550 | 2.78E-01 | 2.78E+01 | | | |
| 7959014 | --- | --- | --- | 0.632 | 1.550 | 5.16E-01 | 5.16E+01 | | | |
| 8043431 | AF113887 // A | GKC // IGKC | immunoglobulin kappa constant // i | 0.632 | 1.550 | 6.21E-03 | 6.21E-01 | Up | | |
| 8043251 | NM_017952 | PTCD3 | Pentatricopeptide repeat domain 3 | 0.631 | 1.549 | 5.58E-02 | 5.58E+00 | | | |
| 7938422 | NM_014633 | CTR9 | Ctr9, Paf1/RNA polymerase II comple | 0.631 | 1.549 | 1.62E-02 | 1.62E+00 | Up | | |
| 7917103 | NM_015534 | ZZZ3 | zinc finger, ZZ-type containing 3 | 0.631 | 1.549 | 1.04E-02 | 1.04E+00 | Up | | |
| 8143957 | NM_005614 | RHEB | Ras homolog enriched in brain | 0.631 | 1.549 | 1.22E-01 | 1.22E+01 | | | |
| 7893160 | --- | --- | --- | 0.631 | 1.549 | 6.89E-02 | 6.89E+00 | | | |
| 7899737 | NM_003757 | EIF3I | eukaryotic translation initiation facto | 0.630 | 1.548 | 9.15E-02 | 9.15E+00 | | | |
| 8169750 | NM_00104275 | STAG2 | stromal antigen 2 | 0.630 | 1.548 | 3.53E-02 | 3.53E+00 | Up | | |
| 8154951 | NM_002065 | GLUL | glutamate-ammonia ligase (glutamin | 0.629 | 1.547 | 1.65E-01 | 1.65E+01 | | | |
| 8014825 | NM_032875 | FBXL20 | F-box and leucine-rich repeat protei | 0.629 | 1.547 | 3.87E-02 | 3.87E+00 | Up | | |
| 8052443 | NM_014709 | USP34 | ubiquitin specific peptidase 34 | 0.629 | 1.547 | 8.46E-03 | 8.46E-01 | Up | | |
| 8178050 | NM_001320 | CSNK2B | casein kinase 2, beta polypeptide | 0.629 | 1.547 | 1.44E-01 | 1.44E+01 | | | |
| 8141133 | NM_006304 | SHFM1 | split hand/foot malformation (ectroc | 0.629 | 1.546 | 1.69E-01 | 1.69E+01 | | | |
| 7953723 | NM_016184 | CLEC4A | C-type lectin domain family 4, memb | 0.628 | 1.545 | 6.83E-03 | 6.83E-01 | Up | | |
| 7925821 | --- | --- | --- | 0.628 | 1.545 | 4.81E-01 | 4.81E+01 | | | |
| 7964579 | NM_005730 | CTDSP2 | CTD (carboxy-terminal domain, RNA | 0.628 | 1.545 | 2.97E-03 | 2.97E-01 | Up | | |
| 7900009 | NM_017629 | EIF2C4 | eukaryotic translation initiation facto | 0.628 | 1.545 | 1.04E-02 | 1.04E+00 | Up | | |
| 8104838 | NM_194283 | DNAJC21 | DnaJ (Hsp40) homolog, subfamily C, | 0.627 | 1.544 | 2.65E-02 | 2.65E+00 | Up | | |
| 7929072 | NM_012420 | IFIT5 | interferon-induced protein with tetra | 0.627 | 1.544 | 2.35E-02 | 2.35E+00 | Up | | |
| 8112687 | NM_005713 | COL4A3BP | collagen, type IV, alpha 3 (Goodpastu | 0.627 | 1.544 | 3.64E-02 | 3.64E+00 | Up | | |
| 7919888 | NM_00103870 | CDC42SE1 | CDC42 small effector 1 | 0.626 | 1.544 | 9.92E-03 | 9.92E-01 | Up | | |
| 8046346 | NM_003642 | HAT1 | histone acetyltransferase 1 | 0.626 | 1.544 | 1.85E-01 | 1.85E+01 | | | |
| 8005501 | NM_00113503 | FAM18B2 | family with sequence similarity 18, m | 0.626 | 1.543 | 8.72E-02 | 8.72E+00 | | | |
| 7945182 | NM_001642 | APLP2 | amyloid beta (A4) precursor-like prot | 0.625 | 1.542 | 7.13E-03 | 7.13E-01 | Up | | mono |
| 8084045 | NM_033540 | MFN1 | mitofusin 1 | 0.625 | 1.542 | 8.85E-02 | 8.85E+00 | | | |
| 7894950 | --- | --- | --- | 0.625 | 1.542 | 4.89E-01 | 4.89E+01 | | | |
| 7979269 | NM_000161 | GCH1 | GTP cyclohydrolase 1 | 0.625 | 1.542 | 3.16E-02 | 3.16E+00 | Up | | |
| 7953949 | NM_002262 | KLRD1 | killer cell lectin-like receptor subfam | 0.625 | 1.542 | 4.76E-02 | 4.76E+00 | Up | | |
| 8017651 | NM_022739 | SMURF2 | SMAD specific E3 ubiquitin protein li | 0.625 | 1.542 | 1.02E-02 | 1.02E+00 | Up | | |
| 7896291 | --- | --- | --- | 0.624 | 1.542 | 3.57E-01 | 3.57E+01 | | | |
| 8088700 | NM_007114 | TMF1 | TATA element modulatory factor 1 | 0.624 | 1.541 | 2.14E-02 | 2.14E+00 | Up | | |
| 7903619 | NM_006513 | SARS | seryl-tRNA synthetase | 0.624 | 1.541 | 1.76E-02 | 1.76E+00 | Up | | |
| 7893921 | --- | --- | --- | 0.623 | 1.540 | 2.07E-01 | 2.07E+01 | | | |
| 7902398 | NR_002749 | SNORD45A | small nucleolar RNA, C/D box 45A | 0.623 | 1.540 | 4.91E-01 | 4.91E+01 | | | |
| 8104825 | NM_018321 | BXDC2 | brix domain containing 2 | 0.622 | 1.539 | 2.56E-01 | 2.56E+01 | | | |
| 7921900 | NM_053282 | SH2D1B | SH2 domain containing 1B | 0.622 | 1.539 | 3.33E-02 | 3.33E+00 | Up | | |
| 8069998 | AF282851 | C21orf59 | chromosome 21 open reading frame | 0.622 | 1.539 | 4.26E-02 | 4.26E+00 | Up | | |
| 8103106 | NM_006726 | LRBA | LPS-responsive vesicle trafficking, be | 0.622 | 1.539 | 5.79E-02 | 5.79E+00 | | | |
| 7959205 | NM_014730 | MLEC | malectin | 0.621 | 1.538 | 3.28E-03 | 3.28E-01 | Up | | |
| 7981724 | BC021276 | IGHD | immunoglobulin heavy constant delt | 0.621 | 1.538 | 3.51E-02 | 3.51E+00 | Up | | |
| 8104944 | NM_015384 | NIPBL | Nipped-B homolog (Drosophila) | 0.621 | 1.538 | 2.53E-02 | 2.53E+00 | Up | | |
| 7973902 | NM_003136 | SRP54 | signal recognition particle 54kDa | 0.621 | 1.538 | 6.06E-02 | 6.06E+00 | | | |
| 7978760 | NM_017658 | KLHL28 | kelch-like 28 (Drosophila) | 0.621 | 1.538 | 9.78E-02 | 9.78E+00 | | | |
| 8020254 | NM_031216 | SEH1L | SEH1-like (S. cerevisiae) | 0.620 | 1.537 | 1.72E-02 | 1.72E+00 | Up | | |
| 8067017 | NM_003859 | DPM1 | dolichyl-phosphate mannosyltransfe | 0.620 | 1.537 | 1.16E-01 | 1.16E+01 | | | |
| 7894832 | --- | --- | --- | 0.620 | 1.537 | 4.82E-01 | 4.82E+01 | | | |
| 7969703 | NM_002271 | IPO5 | importin 5 | 0.620 | 1.537 | 7.20E-04 | 7.20E-02 | Up | | |
| 8007715 | NM_021079 | NMT1 | N-myristoyltransferase 1 | 0.620 | 1.537 | 1.02E-02 | 1.02E+00 | Up | | |

| | | | | | | | | | | |
|---|---|---|---|---|---|---|---|---|---|---|
| 7988286 | NM_025137 | SPG11 | spastic paraplegia 11 (autosomal rec | 0.620 | 1.537 | 3.35E-03 | 3.35E-01 | Up | | |
| 7892605 | --- | --- | --- | 0.620 | 1.537 | 3.82E-01 | 3.82E+01 | | | |
| 7895865 | --- | --- | --- | 0.620 | 1.537 | 1.94E-01 | 1.94E+01 | | | |
| 8085660 | NM_206831 | DPH3 | DPH3, KTI11 homolog (S. cerevisiae) | 0.620 | 1.537 | 2.87E-03 | 2.87E-01 | Up | | |
| 7893062 | --- | --- | --- | 0.620 | 1.537 | 3.67E-01 | 3.67E+01 | | | |
| 7934706 | --- | --- | --- | 0.619 | 1.536 | 2.20E-01 | 2.20E+01 | | | |
| 7934717 | 0 | 0 | 0 | 0.619 | 1.536 | 2.20E-01 | 2.20E+01 | | | |
| 7942626 | NM_003369 | UVRAG | UV radiation resistance associated ge | 0.619 | 1.536 | 2.27E-02 | 2.27E+00 | Up | | |
| 7942594 | NR_000025 | SNORD15B | small nucleolar RNA, C/D box 15B | 0.618 | 1.534 | 2.75E-01 | 2.75E+01 | | | |
| 7987325 | NM_014691 | AQR | aquarius homolog (mouse) | 0.618 | 1.534 | 4.54E-02 | 4.54E+00 | Up | | |
| 8007990 | --- | --- | --- | 0.618 | 1.534 | 5.62E-01 | 5.62E+01 | | | |
| 8056045 | --- | --- | --- | 0.617 | 1.533 | 1.80E-02 | 1.80E+00 | Up | | |
| 8035304 | NM_004335 | BST2 | bone marrow stromal cell antigen 2 | 0.617 | 1.533 | 5.94E-02 | 5.94E+00 | | | |
| 7892861 | --- | --- | --- | 0.617 | 1.533 | 1.12E-01 | 1.12E+01 | | | |
| 7942914 | NM_152991 | EED | embryonic ectoderm development | 0.616 | 1.533 | 3.23E-02 | 3.23E+00 | Up | | |
| 7893258 | --- | --- | --- | 0.616 | 1.533 | 2.68E-01 | 2.68E+01 | | | |
| 7928401 | NM_203298 | CHCHD1 | coiled-coil-helix-coiled-coil-helix dom | 0.616 | 1.533 | 9.34E-02 | 9.34E+00 | | | |
| 7917561 | NM_052941 | GBP4 | guanylate binding protein 4 | 0.616 | 1.533 | 2.26E-02 | 2.26E+00 | Up | | |
| 7894691 | --- | --- | --- | 0.616 | 1.532 | 1.72E-01 | 1.72E+01 | | | |
| 7892615 | --- | --- | --- | 0.615 | 1.532 | 3.35E-01 | 3.35E+01 | | | |
| 8095148 | NM_018475 | TMEM165 | transmembrane protein 165 | 0.615 | 1.532 | 8.47E-03 | 8.47E-01 | Up | | |
| 8169580 | NM_001560 | IL13RA1 | interleukin 13 receptor, alpha 1 | 0.615 | 1.531 | 2.01E-02 | 2.01E+00 | Up | | |
| 8081069 | NM_018293 | ZNF654 | zinc finger protein 654 | 0.615 | 1.531 | 9.85E-02 | 9.85E+00 | | | |
| 7921752 | NM_00102559 | ARHGAP30 | Rho GTPase activating protein 30 | 0.614 | 1.531 | 1.74E-04 | 1.74E-02 | Up | | |
| 7893081 | --- | --- | --- | 0.614 | 1.531 | 1.63E-01 | 1.63E+01 | | | |
| 8012304 | NM_021210 | TRAPPC1 | trafficking protein particle complex 1 | 0.614 | 1.531 | 1.74E-01 | 1.74E+01 | | | |
| 8085374 | NM_002880 | RAF1 | v-raf-1 murine leukemia viral oncoge | 0.614 | 1.530 | 2.61E-02 | 2.61E+00 | Up | | |
| 8086008 | NM_016141 | DYNC1LI1 | dynein, cytoplasmic 1, light intermed | 0.614 | 1.530 | 1.93E-02 | 1.93E+00 | Up | | |
| 7893264 | --- | --- | --- | 0.614 | 1.530 | 1.75E-01 | 1.75E+01 | | | |
| 7896136 | --- | --- | --- | 0.614 | 1.530 | 1.11E-01 | 1.11E+01 | | | |
| 7944493 | NM_005188 | CBL | Cas-Br-M (murine) ecotropic retrovir | 0.613 | 1.529 | 9.13E-04 | 9.13E-02 | Up | | |
| 7968297 | NM_015932 | POMP | proteasome maturation protein | 0.613 | 1.529 | 3.60E-02 | 3.60E+00 | Up | | |
| 8095574 | NM_000788 | DCK | deoxycytidine kinase | 0.613 | 1.529 | 3.03E-02 | 3.03E+00 | Up | | |
| 7902553 | NM_006417 | IFI44 | interferon-induced protein 44 | 0.612 | 1.529 | 1.65E-01 | 1.65E+01 | | | |
| 8175052 | NM_00113084 | AIFM1 | apoptosis-inducing factor, mitochond | 0.612 | 1.529 | 1.51E-02 | 1.51E+00 | Up | | |
| 8135321 | --- | --- | --- | 0.612 | 1.528 | 2.74E-02 | 2.74E+00 | Up | | |
| 8139706 | NM_014302 | SEC61G | Sec61 gamma subunit | 0.612 | 1.528 | 1.02E-01 | 1.02E+01 | | | |
| 8064351 | NM_177559 | CSNK2A1 | casein kinase 2, alpha 1 polypeptide | 0.612 | 1.528 | 7.91E-02 | 7.91E+00 | | | |
| 7938592 | NM_032228 | FAR1 | fatty acyl CoA reductase 1 | 0.612 | 1.528 | 1.26E-03 | 1.26E-01 | Up | | |
| 8015607 | NM_139276 | STAT3 | signal transducer and activator of tra | 0.611 | 1.527 | 2.23E-02 | 2.23E+00 | Up | | |
| 8138805 | NM_019029 | CPVL | carboxypeptidase, vitellogenic-like | 0.611 | 1.527 | 4.80E-03 | 4.80E-01 | Up | | mono |
| 8106534 | NM_00111439 | PAPD4 | PAP associated domain containing 4 | 0.611 | 1.527 | 8.78E-02 | 8.78E+00 | | | |
| 8095159 | --- | --- | --- | 0.610 | 1.527 | 1.56E-01 | 1.56E+01 | | | |
| 8049180 | NM_004846 | EIF4E2 | eukaryotic translation initiation facto | 0.610 | 1.527 | 1.70E-01 | 1.70E+01 | | | |
| 8116622 | NM_003804 | RIPK1 | receptor (TNFRSF)-interacting serine | 0.610 | 1.526 | 7.08E-02 | 7.08E+00 | | | |
| 7901418 | NM_152265 | BTF3L4 | basic transcription factor 3-like 4 | 0.610 | 1.526 | 1.91E-01 | 1.91E+01 | | | |
| 7903803 | NM_006621 | AHCYL1 | adenosylhomocysteinase-like 1 | 0.610 | 1.526 | 3.91E-03 | 3.91E-01 | Up | | |
| 7943158 | NR_002569 | SCARNA9 | small Cajal body-specific RNA 9 | 0.610 | 1.526 | 3.25E-01 | 3.25E+01 | | | |
| 7930996 | NM_018117 | BRWD2 | bromodomain and WD repeat doma | 0.610 | 1.526 | 5.94E-03 | 5.94E-01 | Up | | |
| 8118571 | NM_002800 | PSMB9 | proteasome (prosome, macropain) s | 0.610 | 1.526 | 6.52E-02 | 6.52E+00 | | | |
| 8178211 | NM_002800 | PSMB9 | proteasome (prosome, macropain) s | 0.610 | 1.526 | 6.52E-02 | 6.52E+00 | | | |
| 8179495 | NM_002800 | PSMB9 | proteasome (prosome, macropain) s | 0.610 | 1.526 | 6.52E-02 | 6.52E+00 | | | |
| 7980005 | NM_00100574 | NUMB | numb homolog (Drosophila) | 0.609 | 1.526 | 1.62E-03 | 1.62E-01 | Up | | |
| 7922773 | NM_000433 | NCF2 | neutrophil cytosolic factor 2 | 0.609 | 1.526 | 5.94E-03 | 5.94E-01 | Up | | mono |
| 8007228 | NM_00113002 | ATP6V0A1 | ATPase, H+ transporting, lysosomal V | 0.609 | 1.525 | 1.44E-02 | 1.44E+00 | Up | | |
| 8103437 | NM_005038 | PPID | peptidylprolyl isomerase D | 0.609 | 1.525 | 2.11E-02 | 2.11E+00 | Up | | |
| 8044913 | --- | --- | --- | 0.609 | 1.525 | 3.50E-02 | 3.50E+00 | Up | | |
| 8119529 | NM_015255 | UBR2 | ubiquitin protein ligase E3 componer | 0.609 | 1.525 | 4.45E-02 | 4.45E+00 | Up | | |
| 8163930 | NM_014222 | NDUFA8 | NADH dehydrogenase (ubiquinone) 1 | 0.609 | 1.525 | 1.45E-01 | 1.45E+01 | | | |
| 7895277 | --- | --- | --- | 0.608 | 1.524 | 1.76E-01 | 1.76E+01 | | | |
| 7893816 | --- | --- | --- | 0.608 | 1.524 | 1.85E-01 | 1.85E+01 | | | |
| 7958216 | NM_015275 | KIAA1033 | KIAA1033 | 0.608 | 1.524 | 5.72E-02 | 5.72E+00 | | | |
| 8019807 | NM_005151 | USP14 | ubiquitin specific peptidase 14 (tRNA | 0.608 | 1.524 | 1.07E-01 | 1.07E+01 | | | |
| 7895475 | --- | --- | --- | 0.608 | 1.524 | 1.35E-01 | 1.35E+01 | | | |
| 8008646 | NM_021626 | SCPEP1 | serine carboxypeptidase 1 | 0.607 | 1.523 | 2.06E-02 | 2.06E+00 | Up | | mono |
| 8146427 | NM_052937 | PCMTD1 | protein-L-isoaspartate (D-aspartate) | 0.607 | 1.523 | 4.25E-02 | 4.25E+00 | Up | | |
| 8075130 | NM_012399 | PITPNB | phosphatidylinositol transfer protein | 0.606 | 1.522 | 2.54E-01 | 2.54E+01 | | | |
| 8105340 | NM_006144 | GZMA | granzyme A (granzyme 1, cytotoxic T | 0.606 | 1.522 | 1.36E-01 | 1.36E+01 | | | |
| 8170963 | AY168775 | NCRNA0020 | non-protein coding RNA 204 | 0.605 | 1.522 | 1.95E-01 | 1.95E+01 | | | |
| 8006081 | --- | --- | --- | 0.605 | 1.521 | 3.45E-01 | 3.45E+01 | | | |
| 7912802 | BC036435 | OC10013214 | hypothetical LOC100132147 | 0.605 | 1.521 | 1.27E-01 | 1.27E+01 | | | |
| 8079630 | NM_015933 | CCDC72 | coiled-coil domain containing 72 | 0.605 | 1.521 | 7.61E-02 | 7.61E+00 | | | |
| 8052125 | NM_016115 | ASB3 | ankyrin repeat and SOCS box-contair | 0.605 | 1.521 | 3.01E-02 | 3.01E+00 | Up | | |
| 8089519 | NM_181780 | BTLA | B and T lymphocyte associated | 0.605 | 1.521 | 1.66E-02 | 1.66E+00 | Up | | |
| 7895312 | --- | --- | --- | 0.605 | 1.521 | 3.75E-01 | 3.75E+01 | | | |
| 7959164 | NM_00103749 | DYNLL1 | dynein, light chain, LC8-type 1 | 0.604 | 1.520 | 1.49E-02 | 1.49E+00 | Up | Antigen present./co-stim. | |
| 8034097 | NR_024333 | LOC147727 | hypothetical LOC147727 | 0.604 | 1.520 | 1.82E-01 | 1.82E+01 | | | |

| | | | | | | | | | | |
|---|---|---|---|---|---|---|---|---|---|---|
| 7905339 | NM_144618 | GABPB2 | GA binding protein transcription fact | 0.604 | 1.520 | 4.74E-02 | 4.74E+00 | Up | | |
| 7893647 | --- | --- | --- | 0.604 | 1.520 | 8.81E-02 | 8.81E+00 | | | |
| 7927323 | NM_018232 | FAM21B | family with sequence similarity 21, m | 0.604 | 1.520 | 1.23E-02 | 1.23E+00 | Up | | |
| 8113157 | NM_014639 | TTC37 | tetratricopeptide repeat domain 37 | 0.604 | 1.519 | 2.95E-02 | 2.95E+00 | Up | | |
| 8083090 | NM_00108041 | ZBTB38 | zinc finger and BTB domain containir | 0.603 | 1.519 | 1.06E-02 | 1.06E+00 | Up | | |
| 8099897 | NM_003359 | UGDH | UDP-glucose dehydrogenase | 0.603 | 1.519 | 6.58E-02 | 6.58E+00 | | | |
| 8123714 | --- | --- | --- | 0.603 | 1.519 | 3.13E-01 | 3.13E+01 | | | |
| 7930179 | --- | --- | --- | 0.603 | 1.519 | 2.54E-01 | 2.54E+01 | | | |
| 8014551 | NM_007247 | SYNRG | synergin, gamma | 0.602 | 1.518 | 3.41E-02 | 3.41E+00 | Up | | |
| 8176709 | NM_032576 | CYorf15B | chromosome Y open reading frame 1 | 0.602 | 1.518 | 2.06E-01 | 2.06E+01 | | | |
| 8114489 | --- | --- | --- | 0.602 | 1.518 | 1.09E-01 | 1.09E+01 | | | |
| 7938364 | BX641032 | WEE1 | WEE1 homolog (S. pombe) | 0.602 | 1.517 | 7.06E-02 | 7.06E+00 | | | |
| 7895467 | --- | --- | --- | 0.601 | 1.517 | 3.17E-01 | 3.17E+01 | | | |
| 8094259 | NM_015907 | LAP3 | leucine aminopeptidase 3 | 0.601 | 1.517 | 2.99E-02 | 2.99E+00 | Up | | |
| 8152988 | NM_00104555 | SLA | Src-like-adaptor | 0.601 | 1.516 | 8.27E-02 | 8.27E+00 | | | |
| 7938370 | NM_015055 | SWAP70 | SWAP switching B-cell complex 70kD | 0.600 | 1.516 | 1.71E-02 | 1.71E+00 | Up | | |
| 7989387 | NM_020821 | VPS13C | vacuolar protein sorting 13 homolog | 0.600 | 1.516 | 2.45E-02 | 2.45E+00 | Up | | |
| 8113250 | NM_00104045 | ERAP1 | endoplasmic reticulum aminopeptida | 0.600 | 1.516 | 1.84E-02 | 1.84E+00 | Up | | |
| 8179827 | NM_001288 | CLIC1 | chloride intracellular channel 1 | 0.600 | 1.516 | 1.18E-02 | 1.18E+00 | Up | | |
| 8046279 | --- | --- | --- | 0.599 | 1.515 | 4.22E-03 | 4.22E-01 | Up | | |
| 8095187 | NM_025009 | CEP135 | centrosomal protein 135kDa | 0.599 | 1.515 | 1.10E-01 | 1.10E+01 | | | |
| 8176155 | AY168775 | NCRNA0020 | non-protein coding RNA 204 | 0.599 | 1.515 | 2.02E-01 | 2.02E+01 | | | |
| 8165011 | NM_002003 | FCN1 | ficolin (collagen/fibrinogen domain c | 0.599 | 1.515 | 3.77E-03 | 3.77E-01 | Up | | mono |
| 8144894 | NM_018142 | INTS10 | integrator complex subunit 10 | 0.599 | 1.514 | 5.02E-02 | 5.02E+00 | | | |
| 8106730 | NM_022550 | XRCC4 | X-ray repair complementing defectiv | 0.599 | 1.514 | 3.26E-02 | 3.26E+00 | Up | | |
| 7987361 | NM_014106 | ZNF770 | zinc finger protein 770 | 0.598 | 1.514 | 2.40E-02 | 2.40E+00 | Up | | |
| 8164644 | NM_033387 | FAM78A | family with sequence similarity 78, m | 0.598 | 1.513 | 6.62E-04 | 6.62E-02 | Up | | |
| 8107691 | NM_032177 | PHAX | phosphorylated adaptor for RNA exp | 0.597 | 1.513 | 6.00E-02 | 6.00E+00 | | | |
| 8078153 | --- | --- | --- | 0.597 | 1.513 | 3.14E-02 | 3.14E+00 | Up | | |
| 8006542 | NM_00103000 | AP2B1 | adaptor-related protein complex 2, b | 0.597 | 1.513 | 6.69E-03 | 6.69E-01 | Up | | |
| 7922887 | --- | --- | --- | 0.597 | 1.512 | 1.63E-01 | 1.63E+01 | | | |
| 8046213 | NM_172070 | UBR3 | ubiquitin protein ligase E3 componer | 0.597 | 1.512 | 7.92E-02 | 7.92E+00 | | | |
| 8022473 | NM_052911 | ESCO1 | establishment of cohesion 1 homolo | 0.597 | 1.512 | 7.53E-02 | 7.53E+00 | | | |
| 8052307 | NM_00112296 | SMEK2 | SMEK homolog 2, suppressor of mek | 0.597 | 1.512 | 2.56E-02 | 2.56E+00 | Up | | |
| 8132515 | NM_000712 | BLVRA | biliverdin reductase A | 0.596 | 1.512 | 4.57E-02 | 4.57E+00 | Up | | mono |
| 7896522 | --- | --- | --- | 0.596 | 1.512 | 3.65E-01 | 3.65E+01 | | | |
| 8023757 | NM_006566 | CD226 | CD226 molecule | 0.596 | 1.511 | 1.86E-01 | 1.86E+01 | | | |
| 8102311 | NM_001226 | CASP6 | caspase 6, apoptosis-related cysteine | 0.595 | 1.511 | 3.17E-02 | 3.17E+00 | Up | | |
| 8004510 | NM_001251 | CD68 | CD68 molecule | 0.595 | 1.510 | 2.94E-03 | 2.94E-01 | Up | | |
| 7893111 | --- | --- | --- | 0.595 | 1.510 | 4.41E-01 | 4.41E+01 | | | |
| 8178193 | NM_019111 | HLA-DRA | major histocompatibility complex, cla | 0.595 | 1.510 | 8.58E-04 | 8.58E-02 | Up | Antigen present./co-stim. | |
| 8061772 | NM_012325 | MAPRE1 | microtubule-associated protein, RP/E | 0.595 | 1.510 | 8.60E-02 | 8.60E+00 | | | |
| 8101934 | NM_00103172 | DNAJB14 | DnaJ (Hsp40) homolog, subfamily B, | 0.594 | 1.510 | 1.33E-02 | 1.33E+00 | Up | | |
| 8006531 | NM_144975 | SLFN5 | schlafen family member 5 | 0.594 | 1.510 | 7.26E-03 | 7.26E-01 | Up | | |
| 7925792 | NM_006624 | ZMYND11 | zinc finger, MYND domain containing | 0.594 | 1.509 | 6.23E-02 | 6.23E+00 | | | |
| 8096938 | NM_016648 | LARP7 | La ribonucleoprotein domain family, | 0.594 | 1.509 | 4.58E-02 | 4.58E+00 | Up | | |
| 7954940 | NM_016488 | PPHLN1 | periphilin 1 | 0.594 | 1.509 | 4.78E-02 | 4.78E+00 | Up | | |
| 7983335 | NM_016396 | CTDSPL2 | CTD (carboxy-terminal domain, RNA | 0.593 | 1.508 | 1.64E-01 | 1.64E+01 | | | |
| 7918474 | NM_178454 | DRAM2 | DNA-damage regulated autophagy m | 0.593 | 1.508 | 3.04E-02 | 3.04E+00 | Up | | |
| 7916372 | NM_004872 | TMEM59 | transmembrane protein 59 | 0.593 | 1.508 | 3.64E-02 | 3.64E+00 | Up | | |
| 8041967 | NM_015701 | ERLEC1 | endoplasmic reticulum lectin 1 | 0.592 | 1.508 | 3.34E-02 | 3.34E+00 | Up | | |
| 8102730 | NM_152778 | MFSD8 | major facilitator superfamily domain | 0.592 | 1.508 | 3.75E-03 | 3.75E-01 | Up | | |
| 7895996 | --- | --- | --- | 0.592 | 1.508 | 3.26E-01 | 3.26E+01 | | | |
| 8175177 | NM_018388 | MBNL3 | muscleblind-like 3 (Drosophila) | 0.592 | 1.507 | 1.23E-01 | 1.23E+01 | | | |
| 7978123 | NM_002818 | PSME2 | proteasome (prosome, macropain) a | 0.592 | 1.507 | 3.73E-03 | 3.73E-01 | Up | | |
| 8015712 | NM_00104043 | CCDC56 | coiled-coil domain containing 56 | 0.592 | 1.507 | 1.70E-02 | 1.70E+00 | Up | | |
| 8004184 | NM_017523 | XAF1 | XIAP associated factor 1 | 0.592 | 1.507 | 6.83E-02 | 6.83E+00 | | | |
| 8133030 | NM_002040 | GABPA | GA binding protein transcription fact | 0.592 | 1.507 | 2.24E-01 | 2.24E+01 | | | |
| 7927786 | NM_00100133 | REEP3 | receptor accessory protein 3 | 0.592 | 1.507 | 3.87E-02 | 3.87E+00 | Up | | |
| 7955277 | NM_003217 | TMBIM6 | transmembrane BAX inhibitor motif | 0.591 | 1.507 | 3.78E-02 | 3.78E+00 | Up | | |
| 7909011 | NM_002393 | MDM4 | Mdm4 p53 binding protein homolog | 0.591 | 1.506 | 1.48E-02 | 1.48E+00 | Up | | |
| 7929247 | NM_017824 | 40242 | membrane-associated ring finger (C3 | 0.591 | 1.506 | 2.66E-02 | 2.66E+00 | Up | | |
| 8139896 | NR_022007 | PMS2L4 | postmeiotic segregation increased 2 | 0.590 | 1.505 | 2.31E-01 | 2.31E+01 | | | |
| 8117435 | NM_007047 | BTN3A2 | butyrophilin, subfamily 3, member A | 0.590 | 1.505 | 1.23E-02 | 1.23E+00 | Up | | |
| 8111339 | NM_00104044 | MTMR12 | myotubularin related protein 12 | 0.590 | 1.505 | 1.19E-02 | 1.19E+00 | Up | | |
| 7935058 | NM_013451 | MYOF | myoferlin | 0.590 | 1.505 | 1.06E-03 | 1.06E-01 | Up | | |
| 8178598 | NM_001288 | CLIC1 | chloride intracellular channel 1 | 0.590 | 1.505 | 1.52E-02 | 1.52E+00 | Up | | |
| 7930470 | NM_007373 | SHOC2 | soc-2 suppressor of clear homolog (C | 0.590 | 1.505 | 3.45E-02 | 3.45E+00 | Up | | |
| 8163383 | NM_022486 | SUSD1 | sushi domain containing 1 | 0.589 | 1.505 | 4.50E-02 | 4.50E+00 | Up | | |
| 8047174 | NM_00112725 | SLC39A10 | solute carrier family 39 (zinc transpo | 0.589 | 1.505 | 9.30E-02 | 9.30E+00 | | | |
| 8118661 | NM_014260 | PFDN6 | prefoldin subunit 6 | 0.589 | 1.505 | 4.66E-02 | 4.66E+00 | Up | | |
| 8178270 | NM_014260 | PFDN6 | prefoldin subunit 6 | 0.589 | 1.505 | 4.66E-02 | 4.66E+00 | Up | | |
| 8179559 | NM_014260 | PFDN6 | prefoldin subunit 6 | 0.589 | 1.505 | 4.66E-02 | 4.66E+00 | Up | | |
| 7893046 | --- | --- | --- | 0.589 | 1.504 | 5.93E-01 | 5.93E+01 | | | |
| 7923503 | NM_015999 | ADIPOR1 | adiponectin receptor 1 | 0.588 | 1.504 | 4.72E-02 | 4.72E+00 | Up | | |
| 7952325 | NM_006597 | HSPA8 | heat shock 70kDa protein 8 | 0.588 | 1.503 | 4.74E-03 | 4.74E-01 | Up | | |
| 8180373 | --- | --- | --- | 0.588 | 1.503 | 4.11E-01 | 4.11E+01 | | | |

| | | | | | | | | | | |
|---|---|---|---|---|---|---|---|---|---|---|
| 8098714 | NM_004477 | FRG1 | FSHD region gene 1 | 0.588 | 1.503 | 7.51E-02 | 7.51E+00 | | | |
| 7895629 | --- | --- | --- | 0.587 | 1.502 | 2.71E-01 | 2.71E+01 | | | |
| 8069768 | --- | --- | --- | 0.587 | 1.502 | 1.23E-01 | 1.23E+01 | | | |
| 8122327 | NM_015439 | CCDC28A | coiled-coil domain containing 28A | 0.587 | 1.502 | 3.91E-02 | 3.91E+00 | Up | | |
| 8071649 | BC043345 | BMS1 | BMS1 homolog, ribosome assembly | 0.586 | 1.502 | 3.57E-01 | 3.57E+01 | | | |
| 7946635 | NM_021211 | ZBED5 | zinc finger, BED-type containing 5 | 0.586 | 1.501 | 5.71E-03 | 5.71E-01 | Up | | |
| 7904469 | NM_015326 | SRGAP2 | SLIT-ROBO Rho GTPase activating pro | 0.586 | 1.501 | 1.33E-01 | 1.33E+01 | | | |
| 7981111 | NM_177438 | DICER1 | dicer 1, ribonuclease type III | 0.586 | 1.501 | 1.43E-02 | 1.43E+00 | Up | | |
| 7936346 | NM_022494 | ZDHHC6 | zinc finger, DHHC-type containing 6 | 0.586 | 1.501 | 7.84E-02 | 7.84E+00 | | | |
| 8168432 | AF086467 | RLIM | ring finger protein, LIM domain inter | 0.585 | 1.500 | 9.43E-02 | 9.43E+00 | | | |
| 7895019 | --- | --- | --- | 0.585 | 1.500 | 3.40E-01 | 3.40E+01 | | | |
| 8154856 | NM_017811 | UBE2R2 | ubiquitin-conjugating enzyme E2R 2 | 0.585 | 1.500 | 3.12E-02 | 3.12E+00 | Up | | |
| 8109802 | NM_002887 | RARS | arginyl-tRNA synthetase | 0.585 | 1.500 | 5.24E-02 | 5.24E+00 | | | |
| 7950838 | NM_007166 | PICALM | phosphatidylinositol binding clathrin | 0.585 | 1.500 | 2.47E-02 | 2.47E+00 | Up | | |
| 7947694 | NM_00100893 | CKAP5 | cytoskeleton associated protein 5 | 0.585 | 1.500 | 7.52E-03 | 7.52E-01 | Up | | |
| 7930498 | NM_016234 | ACSL5 | acyl-CoA synthetase long-chain famil | 0.584 | 1.499 | 1.02E-02 | 1.02E+00 | Up | | |
| 8113073 | NM_020801 | ARRDC3 | arrestin domain containing 3 | 0.584 | 1.499 | 1.62E-01 | 1.62E+01 | | | |
| 7893380 | --- | --- | --- | 0.583 | 1.498 | 2.39E-01 | 2.39E+01 | | | |
| 7993774 | --- | --- | --- | 0.583 | 1.498 | 1.45E-01 | 1.45E+01 | | | |
| 7920531 | NM_001111 | ADAR | adenosine deaminase, RNA-specific | 0.583 | 1.498 | 1.40E-02 | 1.40E+00 | Up | | |
| 7893257 | --- | --- | --- | 0.582 | 1.497 | 2.70E-01 | 2.70E+01 | | | |
| 7893690 | --- | --- | --- | 0.582 | 1.497 | 1.91E-01 | 1.91E+01 | | | |
| 7894449 | --- | --- | --- | 0.582 | 1.497 | 2.33E-01 | 2.33E+01 | | | |
| 7895168 | --- | --- | --- | 0.581 | 1.496 | 3.13E-01 | 3.13E+01 | | | |
| 8059650 | NM_080424 | SP110 | SP110 nuclear body protein | 0.581 | 1.496 | 1.95E-02 | 1.95E+00 | Up | | |
| 8179481 | NM_019111 | HLA-DRA | major histocompatibility complex, cl | 0.581 | 1.496 | 1.03E-03 | 1.03E-01 | Up | Antigen present./co-stim. | |
| 8013307 | NR_026718 | FOXO3B | forkhead box O3B pseudogene | 0.581 | 1.495 | 2.27E-02 | 2.27E+00 | Up | | |
| 7974387 | NM_145251 | STYX | serine/threonine/tyrosine interacting | 0.580 | 1.495 | 1.40E-01 | 1.40E+01 | | | |
| 7979260 | NM_004124 | GMFB | glia maturation factor, beta | 0.580 | 1.495 | 9.28E-02 | 9.28E+00 | | | |
| 8098328 | NM_017423 | GALNT7 | UDP-N-acetyl-alpha-D-galactosamine | 0.580 | 1.495 | 2.52E-02 | 2.52E+00 | Up | | |
| 8089954 | NM_00102357 | IQCB1 | IQ motif containing B1 | 0.580 | 1.495 | 5.75E-02 | 5.75E+00 | | | |
| 8050687 | --- | --- | --- | 0.580 | 1.495 | 1.55E-01 | 1.55E+01 | | | |
| 7958379 | NM_007062 | PWP1 | PWP1 homolog (S. cerevisiae) | 0.580 | 1.495 | 9.24E-02 | 9.24E+00 | | | |
| 8101489 | NM_139076 | FAM175A | family with sequence similarity 175, | 0.580 | 1.495 | 1.39E-01 | 1.39E+01 | | | |
| 7976101 | NM_024824 | ZC3H14 | zinc finger CCCH-type containing 14 | 0.580 | 1.495 | 5.06E-03 | 5.06E-01 | Up | | |
| 8122440 | NM_032860 | LTV1 | LTV1 homolog (S. cerevisiae) | 0.579 | 1.494 | 1.21E-01 | 1.21E+01 | | | |
| 8068697 | NM_002463 | MX2 | myxovirus (influenza virus) resistanc | 0.579 | 1.494 | 3.36E-02 | 3.36E+00 | Up | | |
| 8092957 | --- | --- | --- | 0.579 | 1.494 | 8.66E-02 | 8.66E+00 | | | |
| 7958913 | NM_002535 | OAS2 | 2'-5'-oligoadenylate synthetase 2, 69 | 0.579 | 1.494 | 1.44E-02 | 1.44E+00 | Up | | |
| 7896310 | --- | --- | --- | 0.579 | 1.494 | 9.15E-02 | 9.15E+00 | | | |
| 8020903 | NM_020474 | GALNT1 | UDP-N-acetyl-alpha-D-galactosamine | 0.579 | 1.493 | 6.19E-02 | 6.19E+00 | | | |
| 7893363 | --- | --- | --- | 0.578 | 1.493 | 3.92E-01 | 3.92E+01 | | | |
| 8161004 | NM_001782 | CD72 | CD72 molecule | 0.578 | 1.493 | 1.26E-02 | 1.26E+00 | Up | | |
| 7910520 | NM_014236 | GNPAT | glyceronephosphate O-acyltransfera | 0.578 | 1.493 | 4.36E-02 | 4.36E+00 | Up | | |
| 7895967 | --- | --- | --- | 0.578 | 1.493 | 1.70E-01 | 1.70E+01 | | | |
| 8089000 | NM_00100839 | CGGBP1 | CGG triplet repeat binding protein 1 | 0.577 | 1.492 | 7.06E-02 | 7.06E+00 | | | |
| 7972561 | --- | --- | --- | 0.577 | 1.492 | 9.90E-02 | 9.90E+00 | | | |
| 7966127 | NM_003006 | SELPLG | selectin P ligand | 0.577 | 1.492 | 4.70E-03 | 4.70E-01 | Up | | |
| 8105353 | NM_015360 | SKIV2L2 | superkiller viralicidic activity 2-like 2 | 0.577 | 1.492 | 1.40E-01 | 1.40E+01 | | | |
| 7966332 | NM_016226 | VPS29 | vacuolar protein sorting 29 homolog | 0.577 | 1.492 | 3.77E-02 | 3.77E+00 | Up | | |
| 8148057 | --- | --- | --- | 0.577 | 1.492 | 6.10E-02 | 6.10E+00 | | | |
| 8063607 | NM_020673 | RAB22A | RAB22A, member RAS oncogene fam | 0.577 | 1.491 | 9.92E-03 | 9.92E-01 | Up | | |
| 7894675 | --- | --- | --- | 0.577 | 1.491 | 3.59E-01 | 3.59E+01 | | | |
| 7893494 | --- | --- | --- | 0.576 | 1.491 | 3.50E-01 | 3.50E+01 | | | |
| 8101158 | NM_005506 | SCARB2 | scavenger receptor class B, member | 0.576 | 1.491 | 3.68E-02 | 3.68E+00 | Up | | |
| 7991762 | NM_000558 | HBA1 | hemoglobin, alpha 1 | 0.576 | 1.491 | 2.25E-01 | 2.25E+01 | | | |
| 7991766 | NM_000558 | HBA1 | hemoglobin, alpha 1 | 0.576 | 1.491 | 2.25E-01 | 2.25E+01 | | | |
| 8057700 | NM_016467 | ORMDL1 | ORM1-like 1 (S. cerevisiae) | 0.576 | 1.491 | 9.22E-02 | 9.22E+00 | | | |
| 8016429 | --- | --- | --- | 0.575 | 1.490 | 1.39E-01 | 1.39E+01 | | | |
| 8094948 | NM_020846 | SLAIN2 | SLAIN motif family, member 2 | 0.575 | 1.490 | 1.36E-02 | 1.36E+00 | Up | | |
| 7933252 | NM_133446 | AGAP4 | ArfGAP with GTPase domain, ankyrin | 0.575 | 1.490 | 2.66E-02 | 2.66E+00 | Up | | |
| 7936064 | NM_012229 | NT5C2 | 5'-nucleotidase, cytosolic II | 0.575 | 1.489 | 1.58E-01 | 1.58E+01 | | | |
| 8054217 | NM_005783 | TXNDC9 | thioredoxin domain containing 9 | 0.575 | 1.489 | 1.83E-02 | 1.83E+00 | Up | | |
| 8003953 | NM_002798 | PSMB6 | proteasome (prosome, macropain) s | 0.574 | 1.489 | 4.87E-02 | 4.87E+00 | Up | | |
| 8114658 | NM_005219 | DIAPH1 | diaphanous homolog 1 (Drosophila) | 0.574 | 1.489 | 3.70E-03 | 3.70E-01 | Up | | |
| 8100902 | NM_032217 | ANKRD17 | ankyrin repeat domain 17 | 0.574 | 1.489 | 7.79E-04 | 7.79E-02 | Up | | |
| 8059596 | NM_004238 | TRIP12 | thyroid hormone receptor interactor | 0.574 | 1.488 | 1.31E-02 | 1.31E+00 | Up | | |
| 8023191 | NM_005901 | SMAD2 | SMAD family member 2 | 0.574 | 1.488 | 1.54E-01 | 1.54E+01 | | | |
| 8080621 | NR_024460 | CCDC66 | coiled-coil domain containing 66 | 0.574 | 1.488 | 5.35E-02 | 5.35E+00 | | | |
| 7966098 | NM_014706 | SART3 | squamous cell carcinoma antigen rec | 0.573 | 1.488 | 4.62E-02 | 4.62E+00 | Up | | |
| 8094556 | NM_018290 | PGM2 | phosphoglucomutase 2 | 0.573 | 1.488 | 6.12E-02 | 6.12E+00 | | | |
| 7943620 | NM_000051 | ATM | ataxia telangiectasia mutated | 0.573 | 1.488 | 1.59E-02 | 1.59E+00 | Up | | |
| 8180306 | --- | --- | --- | 0.573 | 1.488 | 2.88E-01 | 2.88E+01 | | | |
| 8016847 | NM_005082 | TRIM25 | tripartite motif-containing 25 | 0.573 | 1.487 | 3.50E-04 | 3.50E-02 | Up | | |
| 8147228 | NM_001359 | DECR1 | 2,4-dienoyl CoA reductase 1, mitoch | 0.573 | 1.487 | 1.95E-02 | 1.95E+00 | Up | | |
| 7927513 | NM_015262 | FAM21C | family with sequence similarity 21, m | 0.572 | 1.487 | 4.20E-02 | 4.20E+00 | Up | | |
| 7990528 | NM_005701 | SNUPN | snurportin 1 | 0.572 | 1.487 | 1.82E-02 | 1.82E+00 | Up | | |

| | | | | | | | | | | |
|---|---|---|---|---|---|---|---|---|---|---|
| 8080781 | NM_017771 | PXK | PX domain containing serine/threoni | 0.572 | 1.486 | 1.20E-01 | 1.20E+01 | | | |
| 7955787 | NM_138473 | SP1 | Sp1 transcription factor | 0.571 | 1.486 | 1.47E-03 | 1.47E-01 | Up | | |
| 8107655 | NM_004384 | CSNK1G3 | casein kinase 1, gamma 3 | 0.571 | 1.485 | 5.34E-02 | 5.34E+00 | | | |
| 7934870 | NM_032810 | ATAD1 | ATPase family, AAA domain containi | 0.571 | 1.485 | 4.37E-02 | 4.37E+00 | Up | | |
| 7909931 | NM_144695 | C1orf58 | chromosome 1 open reading frame 5 | 0.570 | 1.485 | 9.60E-02 | 9.60E+00 | | | |
| 8152867 | NM_018482 | ASAP1 | ArfGAP with SH3 domain, ankyrin re | 0.570 | 1.485 | 2.17E-02 | 2.17E+00 | Up | | |
| 7957260 | NM_006851 | GLIPR1 | GLI pathogenesis-related 1 | 0.569 | 1.484 | 1.02E-01 | 1.02E+01 | | | |
| 8091656 | NM_00111352 | METT5D1 | methyltransferase 5 domain contain | 0.569 | 1.484 | 2.18E-01 | 2.18E+01 | | | |
| 8089062 | NM_019895 | CLDND1 | claudin domain containing 1 | 0.569 | 1.484 | 7.31E-02 | 7.31E+00 | | | |
| 7925622 | NM_015446 | AHCTF1 | AT hook containing transcription fac | 0.569 | 1.484 | 1.37E-02 | 1.37E+00 | Up | | |
| 8079346 | NM_014016 | SACM1L | SAC1 suppressor of actin mutations 1 | 0.569 | 1.484 | 1.02E-01 | 1.02E+01 | | | |
| 7896707 | --- | --- | --- | 0.569 | 1.483 | 4.98E-02 | 4.98E+00 | Up | | |
| 8026339 | NM_003096 | SNRPG | small nuclear ribonucleoprotein poly | 0.569 | 1.483 | 1.18E-01 | 1.18E+01 | | | |
| 7991171 | --- | --- | --- | 0.569 | 1.483 | 2.69E-01 | 2.69E+01 | | | |
| 8166455 | NM_006406 | PRDX4 | peroxiredoxin 4 | 0.568 | 1.483 | 1.02E-01 | 1.02E+01 | | | |
| 8065730 | NM_003908 | EIF2S2 | eukaryotic translation initiation facto | 0.568 | 1.483 | 1.01E-02 | 1.01E+00 | Up | | |
| 8147262 | NM_016023 | OTUD6B | OTU domain containing 6B | 0.568 | 1.482 | 8.85E-02 | 8.85E+00 | | | |
| 8180371 | --- | --- | --- | 0.568 | 1.482 | 6.43E-03 | 6.43E-01 | Up | | |
| 7892922 | --- | --- | --- | 0.568 | 1.482 | 5.79E-01 | 5.79E+01 | | | |
| 7958846 | NM_002834 | PTPN11 | protein tyrosine phosphatase, non-re | 0.567 | 1.482 | 4.59E-02 | 4.59E+00 | Up | | |
| 7902541 | NM_006820 | IFI44L | interferon-induced protein 44-like | 0.567 | 1.482 | 2.75E-01 | 2.75E+01 | | | |
| 8088958 | NM_000158 | GBE1 | glucan (1,4-alpha-), branching enzym | 0.566 | 1.480 | 1.87E-02 | 1.87E+00 | Up | | |
| 8025964 | NM_152262 | ZNF439 | zinc finger protein 439 | 0.566 | 1.480 | 2.45E-02 | 2.45E+00 | Up | | |
| 8096617 | NM_017935 | BANK1 | B-cell scaffold protein with ankyrin re | 0.566 | 1.480 | 1.14E-01 | 1.14E+01 | | | |
| 8084904 | NR_003265 | SDHALP2 | succinate dehydrogenase complex, s | 0.565 | 1.480 | 4.24E-03 | 4.24E-01 | Up | | |
| 7917455 | NM_004261 | 40436 | 15 kDa selenoprotein | 0.565 | 1.480 | 1.92E-01 | 1.92E+01 | | | |
| 8059393 | NM_003590 | CUL3 | cullin 3 | 0.565 | 1.479 | 1.75E-02 | 1.75E+00 | Up | | |
| 7929052 | NM_00103168 | IFIT3 | interferon-induced protein with tetra | 0.565 | 1.479 | 1.99E-02 | 1.99E+00 | Up | | |
| 8014664 | --- | --- | --- | 0.565 | 1.479 | 5.73E-01 | 5.73E+01 | | | |
| 8019649 | --- | --- | --- | 0.565 | 1.479 | 5.73E-01 | 5.73E+01 | | | |
| 7893641 | --- | --- | --- | 0.565 | 1.479 | 1.04E-01 | 1.04E+01 | | | |
| 8078738 | NM_005109 | OXSR1 | oxidative-stress responsive 1 | 0.565 | 1.479 | 6.45E-02 | 6.45E+00 | | | |
| 7978428 | NM_00108389 | STRN3 | striatin, calmodulin binding protein 3 | 0.564 | 1.478 | 3.17E-02 | 3.17E+00 | Up | | |
| 7956785 | NM_007235 | XPOT | exportin, tRNA (nuclear export recep | 0.564 | 1.478 | 7.48E-02 | 7.48E+00 | | | |
| 8014891 | NM_012481 | IKZF3 | IKAROS family zinc finger 3 (Aiolos) | 0.564 | 1.478 | 6.43E-02 | 6.43E+00 | | | |
| 7951497 | NM_002519 | NPAT | nuclear protein, ataxia-telangiectasia | 0.564 | 1.478 | 3.35E-02 | 3.35E+00 | Up | | |
| 8079842 | NM_005777 | RBM6 | RNA binding motif protein 6 | 0.564 | 1.478 | 4.03E-02 | 4.03E+00 | Up | | |
| 8127637 | NM_018247 | TMEM30A | transmembrane protein 30A | 0.564 | 1.478 | 5.94E-03 | 5.94E-01 | Up | | |
| 7922669 | NM_005819 | STX6 | syntaxin 6 | 0.564 | 1.478 | 4.06E-03 | 4.06E-01 | Up | | |
| 7892661 | --- | --- | --- | 0.563 | 1.478 | 3.11E-01 | 3.11E+01 | | | |
| 7909455 | NM_025228 | TRAF3IP3 | TRAF3 interacting protein 3 | 0.563 | 1.477 | 4.86E-03 | 4.86E-01 | Up | | |
| 7972069 | NM_015057 | MYCBP2 | MYC binding protein 2 | 0.563 | 1.477 | 1.08E-03 | 1.08E-01 | Up | | |
| 7962312 | NM_005164 | ABCD2 | ATP-binding cassette, sub-family D (A | 0.562 | 1.476 | 6.03E-02 | 6.03E+00 | | | |
| 7894061 | --- | --- | --- | 0.562 | 1.476 | 1.60E-01 | 1.60E+01 | | | |
| 8156945 | NM_019592 | RNF20 | ring finger protein 20 | 0.562 | 1.476 | 8.48E-03 | 8.48E-01 | Up | | |
| 7895442 | --- | --- | --- | 0.562 | 1.476 | 1.40E-01 | 1.40E+01 | | | |
| 7927233 | NM_015262 | FAM21C | family with sequence similarity 21, m | 0.561 | 1.476 | 1.45E-02 | 1.45E+00 | Up | | |
| 8134180 | NM_019004 | ANKIB1 | ankyrin repeat and IBR domain conta | 0.561 | 1.476 | 3.84E-02 | 3.84E+00 | Up | | |
| 8144717 | --- | --- | --- | 0.561 | 1.476 | 1.66E-01 | 1.66E+01 | | | |
| 7928752 | NM_014394 | GHITM | growth hormone inducible transmem | 0.561 | 1.476 | 1.05E-01 | 1.05E+01 | | | |
| 7988713 | NM_017672 | TRPM7 | transient receptor potential cation c | 0.561 | 1.475 | 4.97E-02 | 4.97E+00 | Up | | |
| 7894812 | --- | --- | --- | 0.561 | 1.475 | 4.20E-01 | 4.20E+01 | | | |
| 8084717 | NM_173216 | ST6GAL1 | ST6 beta-galactosamide alpha-2,6-sia | 0.560 | 1.475 | 8.61E-02 | 8.61E+00 | | | |
| 7977674 | NM_007192 | SUPT16H | suppressor of Ty 16 homolog (S. cere | 0.560 | 1.475 | 1.27E-01 | 1.27E+01 | | | |
| 8102848 | NM_030648 | SETD7 | SET domain containing (lysine methy | 0.560 | 1.474 | 3.50E-02 | 3.50E+00 | Up | | |
| 7893667 | --- | --- | --- | 0.560 | 1.474 | 2.43E-01 | 2.43E+01 | | | |
| 7952056 | NM_000732 | CD3D | CD3d molecule, delta (CD3-TCR com | 0.560 | 1.474 | 4.05E-02 | 4.05E+00 | Up | | |
| 7896294 | --- | --- | --- | 0.560 | 1.474 | 5.08E-01 | 5.08E+01 | | | |
| 8056220 | NM_015446 | AHCTF1 | AT hook containing transcription fac | 0.560 | 1.474 | 1.86E-01 | 1.86E+01 | | | |
| 7995382 | NM_00103183 | PHKB | phosphorylase kinase, beta | 0.559 | 1.474 | 7.53E-03 | 7.53E-01 | Up | | |
| 7987192 | NM_00104249 | SLC12A6 | solute carrier family 12 (potassium/c | 0.559 | 1.474 | 8.37E-03 | 8.37E-01 | Up | | |
| 8103226 | NM_152680 | TMEM154 | transmembrane protein 154 | 0.559 | 1.474 | 2.39E-01 | 2.39E+01 | | | |
| 7987225 | NM_018648 | NOP10 | NOP10 ribonucleoprotein homolog ( | 0.559 | 1.473 | 2.53E-01 | 2.53E+01 | | | |
| 8048847 | NM_00113518 | AGFG1 | ArfGAP with FG repeats 1 | 0.558 | 1.472 | 7.69E-03 | 7.69E-01 | Up | | |
| 7938035 | NM_006074 | TRIM22 | tripartite motif-containing 22 | 0.558 | 1.472 | 1.69E-02 | 1.69E+00 | Up | | |
| 8131957 | NM_013322 | SNX10 | sorting nexin 10 | 0.558 | 1.472 | 4.88E-02 | 4.88E+00 | Up | | |
| 8134429 | --- | --- | --- | 0.557 | 1.471 | 2.34E-01 | 2.34E+01 | | | |
| 7910640 | NM_004837 | GGPS1 | geranylgeranyl diphosphate synthase | 0.557 | 1.471 | 1.09E-01 | 1.09E+01 | | | |
| 7893500 | --- | --- | --- | 0.557 | 1.471 | 3.84E-01 | 3.84E+01 | | | |
| 7966135 | NM_014325 | CORO1C | coronin, actin binding protein, 1C | 0.556 | 1.471 | 1.74E-01 | 1.74E+01 | | | |
| 7900336 | --- | --- | --- | 0.556 | 1.470 | 7.90E-02 | 7.90E+00 | | | |
| 8025500 | NM_024292 | UBL5 | ubiquitin-like 5 | 0.556 | 1.470 | 1.52E-01 | 1.52E+01 | | | |
| 7979473 | NM_016029 | DHRS7 | dehydrogenase/reductase (SDR fami | 0.556 | 1.470 | 8.52E-02 | 8.52E+00 | | | |
| 8154836 | NR_003573 // | / ANXA2P2 | annexin A2 pseudogene 2 // annexin | 0.556 | 1.470 | 1.11E-02 | 1.11E+00 | Up | | |
| 8036820 | NM_00114257 | ZNF780A | zinc finger protein 780A | 0.555 | 1.470 | 9.04E-02 | 9.04E+00 | | | |
| 7895928 | --- | --- | --- | 0.555 | 1.470 | 2.89E-01 | 2.89E+01 | | | |
| 7892549 | --- | --- | --- | 0.555 | 1.469 | 4.00E-01 | 4.00E+01 | | | |

| | | | | | | | | | | |
|---|---|---|---|---|---|---|---|---|---|---|
| 8041283 | NM_016252 | BIRC6 | baculoviral IAP repeat-containing 6 | 0.555 | 1.469 | 6.32E-03 | 6.32E-01 | Up | | |
| 7893075 | --- | --- | --- | 0.555 | 1.469 | 2.27E-01 | 2.27E+01 | | | |
| 7894154 | --- | --- | --- | 0.555 | 1.469 | 3.58E-01 | 3.58E+01 | | | |
| 7917482 | NM_00100866: | CCBL2 | cysteine conjugate-beta lyase 2 | 0.554 | 1.469 | 1.07E-01 | 1.07E+01 | | | |
| 7940996 | NM_012094 | PRDX5 | peroxiredoxin 5 | 0.554 | 1.468 | 4.90E-02 | 4.90E+00 | Up | | |
| 7927560 | NM_00100575: | FAM21A | family with sequence similarity 21, m | 0.554 | 1.468 | 1.39E-02 | 1.39E+00 | Up | | |
| 7892514 | --- | --- | --- | 0.554 | 1.468 | 8.93E-02 | 8.93E+00 | | | |
| 7894940 | --- | --- | --- | 0.554 | 1.468 | 5.06E-01 | 5.06E+01 | | | |
| 7993148 | NM_000303 | PMM2 | phosphomannomutase 2 | 0.554 | 1.468 | 4.31E-02 | 4.31E+00 | Up | | |
| 8123767 | NM_003144 | SSR1 | signal sequence receptor, alpha | 0.554 | 1.468 | 1.94E-02 | 1.94E+00 | Up | | |
| 7901513 | NM_002979 | SCP2 | sterol carrier protein 2 | 0.554 | 1.468 | 2.02E-02 | 2.02E+00 | Up | | |
| 8088219 | NM_00111273( | C3orf63 | chromosome 3 open reading frame 6 | 0.554 | 1.468 | 6.17E-04 | 6.17E-02 | Up | | |
| 8157231 | --- | --- | --- | 0.553 | 1.468 | 1.92E-01 | 1.92E+01 | | | |
| 7938880 | NM_00109852( | HTATIP2 | HIV-1 Tat interactive protein 2, 30kD | 0.553 | 1.467 | 9.09E-02 | 9.09E+00 | | | |
| 8155301 | NM_144964 | RG9MTD3 | RNA (guanine-9-) methyltransferase | 0.553 | 1.467 | 3.89E-02 | 3.89E+00 | Up | | |
| 7973056 | NM_001641 | APEX1 | APEX nuclease (multifunctional DNA | 0.553 | 1.467 | 3.91E-02 | 3.91E+00 | Up | | |
| 7895283 | --- | --- | --- | 0.552 | 1.467 | 1.73E-01 | 1.73E+01 | | | |
| 7980742 | ENST0000043: | LOC283588 | hypothetical LOC283588 | 0.552 | 1.466 | 7.44E-02 | 7.44E+00 | | | |
| 8128052 | NM_003800 | RNGTT | RNA guanylyltransferase and 5'-phos | 0.552 | 1.466 | 8.85E-02 | 8.85E+00 | | | |
| 8116534 | NM_032765 | TRIM52 | tripartite motif-containing 52 | 0.552 | 1.466 | 1.09E-02 | 1.09E+00 | Up | | |
| 8174119 | NM_00101165: | ZMAT1 | zinc finger, matrin type 1 | 0.552 | 1.466 | 2.34E-02 | 2.34E+00 | Up | | |
| 7895829 | --- | --- | --- | 0.552 | 1.466 | 3.05E-01 | 3.05E+01 | | | |
| 7960221 | NM_005056 | KDM5A | lysine (K)-specific demethylase 5A | 0.551 | 1.466 | 1.39E-02 | 1.39E+00 | Up | | |
| 8157021 | NM_015469 | NIPSNAP3A | nipsnap homolog 3A (C. elegans) | 0.551 | 1.465 | 5.50E-02 | 5.50E+00 | | | |
| 7896014 | --- | --- | --- | 0.551 | 1.465 | 6.40E-01 | 6.40E+01 | | | |
| 8120102 | NM_012120 | CD2AP | CD2-associated protein | 0.551 | 1.465 | 1.37E-01 | 1.37E+01 | | | |
| 8034873 | NM_013447 | EMR2 | egf-like module containing, mucin-lik | 0.551 | 1.465 | 3.25E-04 | 3.25E-02 | Up | | mono |
| 7895066 | --- | --- | --- | 0.551 | 1.465 | 5.03E-01 | 5.03E+01 | | | |
| 8118548 | NM_019111 | HLA-DRA | major histocompatibility complex, cla | 0.551 | 1.465 | 2.18E-03 | 2.18E-01 | Up | Antigen present./co-stim. | |
| 7895724 | --- | --- | --- | 0.550 | 1.464 | 6.69E-01 | 6.69E+01 | | | |
| 7919095 | NM_024408 | NOTCH2 | Notch homolog 2 (Drosophila) | 0.550 | 1.464 | 6.59E-04 | 6.59E-02 | Up | | |
| 8157216 | NM_003358 | UGCG | UDP-glucose ceramide glucosyltrans | 0.550 | 1.464 | 7.31E-02 | 7.31E+00 | | | |
| 7927972 | NM_004896 | VPS26A | vacuolar protein sorting 26 homolog | 0.550 | 1.464 | 1.80E-01 | 1.80E+01 | | | |
| 7963575 | NM_001417 | EIF4B | eukaryotic translation initiation facto | 0.550 | 1.464 | 6.35E-02 | 6.35E+00 | | | |
| 7946703 | NM_016451 | COPB1 | coatomer protein complex, subunit b | 0.549 | 1.463 | 2.86E-02 | 2.86E+00 | Up | | |
| 8162352 | NR_024020 | NOL8 | nucleolar protein 8 | 0.549 | 1.463 | 3.81E-02 | 3.81E+00 | Up | | |
| 7985488 | BC112329 | FAM103A1 | family with sequence similarity 103, | 0.549 | 1.463 | 2.62E-01 | 2.62E+01 | | | |
| 7934133 | NM_021129 | PPA1 | pyrophosphatase (inorganic) 1 | 0.549 | 1.463 | 4.98E-02 | 4.98E+00 | Up | | |
| 8085689 | NM_014744 | TBC1D5 | TBC1 domain family, member 5 | 0.548 | 1.462 | 3.90E-02 | 3.90E+00 | Up | | |
| 7892522 | --- | --- | --- | 0.548 | 1.462 | 2.17E-01 | 2.17E+01 | | | |
| 7896460 | --- | --- | --- | 0.548 | 1.462 | 4.08E-01 | 4.08E+01 | | | |
| 7918255 | NM_00104821( | CLCC1 | chloride channel CLIC-like 1 | 0.547 | 1.462 | 6.43E-03 | 6.43E-01 | Up | | |
| 7988789 | NM_015263 | DMXL2 | Dmx-like 2 | 0.547 | 1.461 | 4.05E-02 | 4.05E+00 | Up | | mono |
| 8139100 | M30894 | TARP | TCR gamma alternate reading frame | 0.547 | 1.461 | 1.88E-01 | 1.88E+01 | | | |
| 8123137 | NM_005891 | ACAT2 | acetyl-Coenzyme A acetyltransferase | 0.547 | 1.461 | 7.51E-02 | 7.51E+00 | | | |
| 7930422 | NM_005445 | SMC3 | structural maintenance of chromoso | 0.547 | 1.461 | 1.04E-01 | 1.04E+01 | | | |
| 7895630 | --- | --- | --- | 0.545 | 1.459 | 4.29E-01 | 4.29E+01 | | | |
| 7934297 | --- | --- | --- | 0.545 | 1.459 | 1.57E-01 | 1.57E+01 | | | |
| 7895105 | --- | --- | --- | 0.544 | 1.458 | 4.70E-01 | 4.70E+01 | | | |
| 8096556 | NM_015143 | METAP1 | methionyl aminopeptidase 1 | 0.544 | 1.458 | 1.14E-01 | 1.14E+01 | | | |
| 8008113 | NM_005831 | CALCOCO2 | calcium binding and coiled-coil doma | 0.544 | 1.458 | 7.53E-02 | 7.53E+00 | | | |
| 7892890 | --- | --- | --- | 0.543 | 1.457 | 2.64E-01 | 2.64E+01 | | | |
| 7969228 | NM_00100412: | ALG11 | asparagine-linked glycosylation 11, a | 0.543 | 1.457 | 9.20E-02 | 9.20E+00 | | | |
| 7895710 | --- | --- | --- | 0.543 | 1.457 | 1.61E-01 | 1.61E+01 | | | |
| 8180411 | --- | --- | --- | 0.543 | 1.457 | 1.71E-01 | 1.71E+01 | | | |
| 8005689 | NR_003554 | LOC220594 | TL132 protein | 0.543 | 1.457 | 3.37E-01 | 3.37E+01 | | | |
| 7893390 | --- | --- | --- | 0.542 | 1.456 | 1.05E-01 | 1.05E+01 | | | |
| 8053763 | NM_031902 | MRPS5 | mitochondrial ribosomal protein S5 | 0.542 | 1.456 | 3.51E-02 | 3.51E+00 | Up | | |
| 8085263 | NM_018447 | TMEM111 | transmembrane protein 111 | 0.542 | 1.456 | 1.70E-01 | 1.70E+01 | | | |
| 7998952 | NM_033208 | TIGD7 | tigger transposable element derived | 0.542 | 1.456 | 1.41E-02 | 1.41E+00 | Up | | |
| 7939137 | NM_006360 | EIF3M | eukaryotic translation initiation facto | 0.542 | 1.456 | 1.25E-01 | 1.25E+01 | | | |
| 7962367 | NM_033114 | ZCRB1 | zinc finger CCHC-type and RNA bindi | 0.542 | 1.456 | 1.29E-01 | 1.29E+01 | | | |
| 7957177 | NM_014999 | RAB21 | RAB21, member RAS oncogene famil | 0.542 | 1.456 | 2.06E-01 | 2.06E+01 | | | |
| 7978570 | NM_021249 | SNX6 | sorting nexin 6 | 0.542 | 1.456 | 5.42E-02 | 5.42E+00 | | | |
| 7913319 | NM_003760 | EIF4G3 | eukaryotic translation initiation facto | 0.541 | 1.455 | 1.26E-03 | 1.26E-01 | Up | | |
| 8141843 | NM_006989 | RASA4 | RAS p21 protein activator 4 | 0.541 | 1.455 | 3.65E-01 | 3.65E+01 | | | |
| 8059799 | --- | --- | --- | 0.541 | 1.455 | 4.62E-01 | 4.62E+01 | | | |
| 7927889 | NM_018237 | CCAR1 | cell division cycle and apoptosis regu | 0.541 | 1.455 | 5.52E-02 | 5.52E+00 | | | |
| 7896246 | --- | --- | --- | 0.540 | 1.454 | 3.96E-01 | 3.96E+01 | | | |
| 7931873 | NM_001494 | GDI2 | GDP dissociation inhibitor 2 | 0.540 | 1.454 | 2.15E-02 | 2.15E+00 | Up | | |
| 8115886 | NM_032361 | THOC3 | THO complex 3 | 0.540 | 1.454 | 1.88E-03 | 1.88E-01 | Up | | |
| 8004111 | NM_004703 | RABEP1 | rabaptin, RAB GTPase binding effecto | 0.540 | 1.454 | 5.87E-02 | 5.87E+00 | | | |
| 8006345 | NM_00103356: | RHOT1 | ras homolog gene family, member T1 | 0.540 | 1.454 | 2.34E-01 | 2.34E+01 | | | |
| 8016898 | NM_00107816( | SFRS1 | splicing factor, arginine/serine-rich 1 | 0.540 | 1.454 | 7.30E-03 | 7.30E-01 | Up | | |
| 8113469 | NM_014819 | PJA2 | praja ring finger 2 | 0.540 | 1.454 | 4.18E-02 | 4.18E+00 | Up | | |
| 8047215 | --- | --- | --- | 0.540 | 1.454 | 2.61E-01 | 2.61E+01 | | | |
| 7894318 | --- | --- | --- | 0.540 | 1.454 | 5.49E-01 | 5.49E+01 | | | |

| | | | | | | | | | | |
|---|---|---|---|---|---|---|---|---|---|---|
| 8097867 | NM_015196 | KIAA0922 | KIAA0922 | 0.540 | 1.453 | 1.66E-02 | 1.66E+00 | Up | | |
| 8097480 | NM_057175 | NARG1 | NMDA receptor regulated 1 | 0.539 | 1.453 | 1.67E-01 | 1.67E+01 | | | |
| 8094408 | NM_013367 | ANAPC4 | anaphase promoting complex subun | 0.539 | 1.453 | 5.62E-02 | 5.62E+00 | | | |
| 7892787 | --- | --- | --- | 0.539 | 1.453 | 5.10E-01 | 5.10E+01 | | | |
| 7895149 | --- | --- | --- | 0.539 | 1.453 | 2.49E-01 | 2.49E+01 | | | |
| 7896430 | --- | --- | --- | 0.538 | 1.452 | 2.20E-01 | 2.20E+01 | | | |
| 7971541 | NM_003850 | SUCLA2 | succinate-CoA ligase, ADP-forming, b | 0.538 | 1.452 | 9.16E-02 | 9.16E+00 | | | |
| 8180246 | --- | --- | --- | 0.538 | 1.452 | 1.67E-02 | 1.67E+00 | Up | | |
| 8022009 | NM_022840 | METTL4 | methyltransferase like 4 | 0.538 | 1.452 | 3.59E-02 | 3.59E+00 | Up | | |
| 8169920 | NM_016024 | RBMX2 | RNA binding motif protein, X-linked 2 | 0.538 | 1.452 | 1.12E-01 | 1.12E+01 | | | |
| 8170971 | NM_001363 | DKC1 | dyskeratosis congenita 1, dyskerin | 0.537 | 1.451 | 3.36E-02 | 3.36E+00 | Up | | |
| 7980403 | BC015054 | C14orf133 | chromosome 14 open reading frame | 0.537 | 1.451 | 8.22E-02 | 8.22E+00 | | | |
| 8124273 | NM_030939 | C6orf62 | chromosome 6 open reading frame 6 | 0.537 | 1.451 | 1.26E-01 | 1.26E+01 | | | |
| 7894355 | --- | --- | --- | 0.537 | 1.451 | 5.23E-01 | 5.23E+01 | | | |
| 7893969 | --- | --- | --- | 0.537 | 1.451 | 2.39E-01 | 2.39E+01 | | | |
| 7892855 | --- | --- | --- | 0.536 | 1.450 | 2.56E-01 | 2.56E+01 | | | |
| 7957793 | NM_022496 | ACTR6 | ARP6 actin-related protein 6 homolo | 0.536 | 1.450 | 2.09E-01 | 2.09E+01 | | | |
| 8074791 | NM_002745 | MAPK1 | mitogen-activated protein kinase 1 | 0.536 | 1.450 | 3.98E-02 | 3.98E+00 | Up | | |
| 8127698 | NM_017934 | PHIP | pleckstrin homology domain interact | 0.536 | 1.450 | 9.17E-03 | 9.17E-01 | Up | | |
| 7970907 | NM_014887 | N4BP2L2 | NEDD4 binding protein 2-like 2 | 0.536 | 1.450 | 4.08E-03 | 4.08E-01 | Up | | |
| 8115524 | NM_014666 | CLINT1 | clathrin interactor 1 | 0.536 | 1.450 | 3.70E-02 | 3.70E+00 | Up | | |
| 7970602 | NM_006437 | PARP4 | poly (ADP-ribose) polymerase family | 0.536 | 1.449 | 2.77E-02 | 2.77E+00 | Up | | |
| 7973384 | NM_004643 | PABPN1 | poly(A) binding protein, nuclear 1 | 0.535 | 1.449 | 1.07E-01 | 1.07E+01 | | | |
| 8128472 | NM_006828 | ASCC3 | activating signal cointegrator 1 comp | 0.535 | 1.449 | 1.23E-02 | 1.23E+00 | Up | | |
| 7980463 | NM_012245 | SNW1 | SNW domain containing 1 | 0.535 | 1.449 | 2.64E-01 | 2.64E+01 | | | |
| 7894583 | --- | --- | --- | 0.535 | 1.449 | 3.32E-01 | 3.32E+01 | | | |
| 7896323 | --- | --- | --- | 0.534 | 1.448 | 5.70E-01 | 5.70E+01 | | | |
| 7895657 | --- | --- | --- | 0.534 | 1.448 | 1.52E-01 | 1.52E+01 | | | |
| 7893642 | --- | --- | --- | 0.534 | 1.448 | 1.40E-01 | 1.40E+01 | | | |
| 8179298 | NM_001320 | CSNK2B | casein kinase 2, beta polypeptide | 0.534 | 1.448 | 1.57E-01 | 1.57E+01 | | | |
| 8005814 | NM_016231 | NLK | nemo-like kinase | 0.534 | 1.448 | 2.23E-02 | 2.23E+00 | Up | | |
| 8142852 | NM_012470 | TNPO3 | transportin 3 | 0.534 | 1.448 | 1.45E-02 | 1.45E+00 | Up | | |
| 7903893 | NM_000560 | CD53 | CD53 molecule | 0.534 | 1.448 | 4.38E-02 | 4.38E+00 | Up | | |
| 7906056 | NR_024117 | MSTO2P | misato homolog 2 pseudogene | 0.534 | 1.448 | 2.62E-02 | 2.62E+00 | Up | | |
| 7971996 | --- | --- | --- | 0.533 | 1.447 | 3.70E-01 | 3.70E+01 | | | |
| 7932530 | NM_005028 | PIP4K2A | phosphatidylinositol-5-phosphate 4- | 0.533 | 1.447 | 1.20E-01 | 1.20E+01 | | | |
| 7895175 | --- | --- | --- | 0.533 | 1.447 | 2.92E-01 | 2.92E+01 | | | |
| 7893416 | --- | --- | --- | 0.533 | 1.447 | 5.06E-01 | 5.06E+01 | | | |
| 7911155 | NM_016002 | SCCPDH | saccharopine dehydrogenase (putati | 0.533 | 1.447 | 8.70E-02 | 8.70E+00 | | | |
| 8051443 | NM_003162 | STRN | striatin, calmodulin binding protein | 0.533 | 1.447 | 5.26E-02 | 5.26E+00 | | | |
| 7997099 | NM_012426 | SF3B3 | splicing factor 3b, subunit 3, 130kDa | 0.533 | 1.447 | 6.30E-02 | 6.30E+00 | | | |
| 8130038 | NM_00104268 | SHPRH | SNF2 histone linker PHD RING helica | 0.533 | 1.447 | 1.36E-02 | 1.36E+00 | Up | | |
| 7946439 | NM_020644 | TMEM9B | TMEM9 domain family, member B | 0.533 | 1.447 | 2.10E-02 | 2.10E+00 | Up | | |
| 8095048 | NM_030917 | FIP1L1 | FIP1 like 1 (S. cerevisiae) | 0.532 | 1.446 | 1.16E-01 | 1.16E+01 | | | |
| 7981943 | NR_022008 | PAR5 | Prader-Willi/Angelman syndrome-5 | 0.532 | 1.446 | 5.69E-01 | 5.69E+01 | | | |
| 7990657 | NM_144572 | TBC1D2B | TBC1 domain family, member 2B | 0.532 | 1.446 | 5.51E-03 | 5.51E-01 | Up | | |
| 8147396 | NM_017864 | INTS8 | integrator complex subunit 8 | 0.532 | 1.446 | 1.01E-02 | 1.01E+00 | Up | | |
| 7918487 | NM_024901 | DENND2D | DENN/MADD domain containing 2D | 0.532 | 1.445 | 8.51E-03 | 8.51E-01 | Up | | |
| 8116760 | NM_031480 | RIOK1 | RIO kinase 1 (yeast) | 0.532 | 1.445 | 1.38E-01 | 1.38E+01 | | | |
| 7975344 | NM_018375 | SLC39A9 | solute carrier family 39 (zinc transpo | 0.531 | 1.445 | 9.00E-02 | 9.00E+00 | | | |
| 8089584 | NM_015412 | C3orf17 | chromosome 3 open reading frame 1 | 0.531 | 1.445 | 7.28E-02 | 7.28E+00 | | | |
| 8046201 | NM_003142 | SSB | Sjogren syndrome antigen B (autoan | 0.531 | 1.445 | 1.23E-01 | 1.23E+01 | | | |
| 8116245 | --- | --- | --- | 0.531 | 1.445 | 3.46E-01 | 3.46E+01 | | | |
| 8078360 | NM_178862 | STT3B | STT3, subunit of the oligosaccharyltr | 0.530 | 1.444 | 4.34E-02 | 4.34E+00 | Up | | |
| 8123520 | BC118988 | C20orf69 | chromosome 20 open reading frame | 0.530 | 1.444 | 6.28E-02 | 6.28E+00 | | | |
| 8093683 | --- | --- | --- | 0.530 | 1.444 | 2.29E-02 | 2.29E+00 | Up | | |
| 8123181 | NM_000876 | IGF2R | insulin-like growth factor 2 receptor | 0.530 | 1.444 | 5.06E-03 | 5.06E-01 | Up | | |
| 7893350 | --- | --- | --- | 0.529 | 1.443 | 3.54E-01 | 3.54E+01 | | | |
| 8106633 | NM_002439 | MSH3 | mutS homolog 3 (E. coli) | 0.529 | 1.443 | 3.48E-02 | 3.48E+00 | Up | | |
| 7932227 | NM_004808 | NMT2 | N-myristoyltransferase 2 | 0.529 | 1.443 | 6.44E-02 | 6.44E+00 | | | |
| 7896059 | --- | --- | --- | 0.529 | 1.443 | 4.36E-01 | 4.36E+01 | | | |
| 7981730 | AB001736 | IGLJ3 | immunoglobulin lambda joining 3 | 0.528 | 1.442 | 1.38E-02 | 1.38E+00 | Up | | |
| 8128260 | NM_145331 | MAP3K7 | mitogen-activated protein kinase kin | 0.528 | 1.442 | 3.84E-02 | 3.84E+00 | Up | | |
| 8042576 | NM_017567 | NAGK | N-acetylglucosamine kinase | 0.528 | 1.442 | 8.76E-03 | 8.76E-01 | Up | | |
| 7994102 | NM_032486 | DCTN5 | dynactin 5 (p25) | 0.527 | 1.441 | 1.39E-01 | 1.39E+01 | | | |
| 8112772 | NM_003664 | AP3B1 | adaptor-related protein complex 3, b | 0.527 | 1.441 | 3.87E-02 | 3.87E+00 | Up | | |
| 7895405 | --- | --- | --- | 0.527 | 1.441 | 4.45E-01 | 4.45E+01 | | | |
| 7893122 | --- | --- | --- | 0.527 | 1.441 | 3.16E-01 | 3.16E+01 | | | |
| 7981958 | NR_003320 | SNORD116-5 | small nucleolar RNA, C/D box 116-5 | 0.526 | 1.440 | 3.86E-01 | 3.86E+01 | | | |
| 7981962 | NR_003320 | SNORD116-5 | small nucleolar RNA, C/D box 116-5 | 0.526 | 1.440 | 3.86E-01 | 3.86E+01 | | | |
| 7894300 | --- | --- | --- | 0.526 | 1.440 | 2.50E-01 | 2.50E+01 | | | |
| 8166805 | NM_005765 | ATP6AP2 | ATPase, H+ transporting, lysosomal a | 0.526 | 1.440 | 6.08E-02 | 6.08E+00 | | | |
| 8160622 | NM_018225 | SMU1 | smu-1 suppressor of mec-8 and unc- | 0.526 | 1.440 | 3.36E-02 | 3.36E+00 | Up | | |
| 8129181 | NM_020399 | GOPC | golgi associated PDZ and coiled-coil r | 0.526 | 1.440 | 8.86E-02 | 8.86E+00 | | | |
| 8050658 | NM_017552 | ATAD2B | ATPase family, AAA domain containi | 0.526 | 1.439 | 4.33E-02 | 4.33E+00 | Up | | |
| 8097813 | NM_00102559 | ARFIP1 | ADP-ribosylation factor interacting p | 0.525 | 1.439 | 2.33E-02 | 2.33E+00 | Up | | |
| 7893394 | --- | --- | --- | 0.525 | 1.439 | 5.35E-01 | 5.35E+01 | | | |

| | | | | | | | | | | |
|---|---|---|---|---|---|---|---|---|---|---|
| 7907790 | NM_014810 | CEP350 | centrosomal protein 350kDa | 0.525 | 1.439 | 1.27E-02 | 1.27E+00 | Up | | |
| 7960794 | NM_004244 | CD163 | CD163 molecule | 0.525 | 1.439 | 4.21E-03 | 4.21E-01 | Up | | mono |
| 8170428 | NM_000252 | MTM1 | myotubularin 1 | 0.525 | 1.439 | 3.38E-02 | 3.38E+00 | Up | | |
| 7977657 | NM_031314 | HNRNPC | heterogeneous nuclear ribonucleopr | 0.525 | 1.439 | 4.81E-02 | 4.81E+00 | Up | | |
| 7947540 | NM_145803 | TRAF6 | TNF receptor-associated factor 6 | 0.525 | 1.439 | 8.22E-03 | 8.22E-01 | Up | | |
| 7939368 | NM_017583 | TRIM44 | tripartite motif-containing 44 | 0.524 | 1.438 | 3.22E-02 | 3.22E+00 | Up | | |
| 8128013 | NM_020320 | RARS2 | arginyl-tRNA synthetase 2, mitochon | 0.524 | 1.438 | 5.63E-02 | 5.63E+00 | | | |
| 8043468 | --- | --- | --- | 0.524 | 1.438 | 5.70E-02 | 5.70E+00 | | | |
| 7894083 | --- | --- | --- | 0.524 | 1.438 | 1.47E-01 | 1.47E+01 | | | |
| 8122426 | NM_014721 | PHACTR2 | phosphatase and actin regulator 2 | 0.524 | 1.438 | 1.58E-01 | 1.58E+01 | | | |
| 8168366 | NM_006223 | PIN4 | protein (peptidylprolyl cis/trans isom | 0.524 | 1.438 | 2.36E-01 | 2.36E+01 | | | |
| 8010139 | NM_00114399 | SEC14L1 | SEC14-like 1 (S. cerevisiae) | 0.524 | 1.438 | 1.74E-02 | 1.74E+00 | Up | | |
| 8072577 | NM_003405 | YWHAH | tyrosine 3-monooxygenase/tryptoph | 0.524 | 1.438 | 1.95E-02 | 1.95E+00 | Up | | |
| 7892562 | --- | --- | --- | 0.524 | 1.438 | 3.11E-01 | 3.11E+01 | | | |
| 8052721 | NM_000945 | PPP3R1 | protein phosphatase 3 (formerly 2B) | 0.523 | 1.437 | 1.73E-01 | 1.73E+01 | | | |
| 7981728 | ENST00000390 | OC10029321 | similar to hCG2042717 | 0.523 | 1.437 | 1.19E-01 | 1.19E+01 | | | |
| 7960900 | NM_014358 | CLEC4E | C-type lectin domain family 4, memb | 0.523 | 1.437 | 2.46E-01 | 2.46E+01 | | | |
| 7894504 | --- | --- | --- | 0.523 | 1.437 | 4.04E-01 | 4.04E+01 | | | |
| 8101844 | NM_000671 | ADH5 | alcohol dehydrogenase 5 (class III), c | 0.522 | 1.436 | 1.67E-02 | 1.67E+00 | Up | | |
| 8017312 | NM_005121 | MED13 | mediator complex subunit 13 | 0.522 | 1.436 | 5.45E-02 | 5.45E+00 | | | |
| 7935180 | NM_020992 | PDLIM1 | PDZ and LIM domain 1 | 0.522 | 1.436 | 9.22E-02 | 9.22E+00 | | | |
| 8112458 | NM_003187 | TAF9 | TAF9 RNA polymerase II, TATA box b | 0.522 | 1.436 | 9.76E-02 | 9.76E+00 | | | |
| 8152764 | NM_014751 | MTSS1 | metastasis suppressor 1 | 0.522 | 1.436 | 9.31E-03 | 9.31E-01 | Up | | |
| 7916570 | NM_145243 | OMA1 | OMA1 homolog, zinc metallopeptida | 0.522 | 1.436 | 1.55E-01 | 1.55E+01 | | | |
| 7895599 | --- | --- | --- | 0.522 | 1.436 | 2.46E-01 | 2.46E+01 | | | |
| 7896611 | --- | --- | --- | 0.522 | 1.436 | 5.43E-01 | 5.43E+01 | | | |
| 8078600 | NM_006756 | TCEA1 | transcription elongation factor A (SII | 0.522 | 1.435 | 1.32E-01 | 1.32E+01 | | | |
| 8073799 | NM_013236 | ATXN10 | ataxin 10 | 0.521 | 1.435 | 6.10E-02 | 6.10E+00 | | | |
| 8166335 | NM_000284 | PDHA1 | pyruvate dehydrogenase (lipoamide) | 0.521 | 1.435 | 3.29E-02 | 3.29E+00 | Up | | |
| 7957759 | NM_181861 | APAF1 | apoptotic peptidase activating factor | 0.521 | 1.435 | 3.29E-03 | 3.29E-01 | Up | | |
| 7896553 | --- | --- | --- | 0.521 | 1.435 | 3.76E-01 | 3.76E+01 | | | |
| 8000413 | NM_145080 | NSMCE1 | non-SMC element 1 homolog (S. cere | 0.521 | 1.435 | 2.57E-01 | 2.57E+01 | | | |
| 8171024 | --- | --- | --- | 0.521 | 1.435 | 3.49E-03 | 3.49E-01 | Up | | |
| 7896651 | --- | --- | --- | 0.520 | 1.434 | 4.94E-01 | 4.94E+01 | | | |
| 8175492 | NM_173694 | ATP11C | ATPase, class VI, type 11C | 0.520 | 1.434 | 1.94E-02 | 1.94E+00 | Up | | |
| 8069561 | NM_152265 | BTF3L4 | basic transcription factor 3-like 4 | 0.520 | 1.434 | 2.69E-01 | 2.69E+01 | | | |
| 7893141 | --- | --- | --- | 0.520 | 1.434 | 5.52E-01 | 5.52E+01 | | | |
| 7896171 | --- | --- | --- | 0.520 | 1.434 | 3.86E-01 | 3.86E+01 | | | |
| 7971461 | NM_002298 | LCP1 | lymphocyte cytosolic protein 1 (L-pla | 0.520 | 1.434 | 8.78E-04 | 8.78E-02 | Up | | |
| 7893797 | --- | --- | --- | 0.520 | 1.434 | 3.37E-01 | 3.37E+01 | | | |
| 8150928 | NM_003580 | NSMAF | neutral sphingomyelinase (N-SMase) | 0.519 | 1.433 | 4.93E-02 | 4.93E+00 | Up | | |
| 8125512 | NM_000593 | TAP1 | transporter 1, ATP-binding cassette, | 0.519 | 1.433 | 2.27E-02 | 2.27E+00 | Up | | |
| 8178867 | NM_000593 | TAP1 | transporter 1, ATP-binding cassette, | 0.519 | 1.433 | 2.27E-02 | 2.27E+00 | Up | | |
| 8180061 | NM_000593 | TAP1 | transporter 1, ATP-binding cassette, | 0.519 | 1.433 | 2.27E-02 | 2.27E+00 | Up | | |
| 8082478 | NM_016128 | COPG | coatomer protein complex, subunit g | 0.519 | 1.433 | 3.89E-03 | 3.89E-01 | Up | | |
| 7906386 | NM_152501 | PYHIN1 | pyrin and HIN domain family, membe | 0.519 | 1.433 | 1.22E-01 | 1.22E+01 | | | |
| 8156826 | NM_004612 | TGFBR1 | transforming growth factor, beta rec | 0.519 | 1.433 | 4.84E-03 | 4.84E-01 | Up | | |
| 8091327 | NM_021105 | PLSCR1 | phospholipid scramblase 1 | 0.518 | 1.432 | 3.79E-02 | 3.79E+00 | Up | | |
| 7907702 | NM_003101 | SOAT1 | sterol O-acyltransferase 1 | 0.518 | 1.432 | 6.04E-02 | 6.04E+00 | | | |
| 8163775 | NM_00108049 | MEGF9 | multiple EGF-like-domains 9 | 0.518 | 1.432 | 4.18E-02 | 4.18E+00 | Up | | |
| 7920335 | --- | --- | --- | 0.518 | 1.432 | 2.53E-01 | 2.53E+01 | | | |
| 8168470 | NM_001866 | COX7B | cytochrome c oxidase subunit VIIb | 0.518 | 1.432 | 5.32E-01 | 5.32E+01 | | | |
| 7954711 | NM_018169 | C12orf35 | chromosome 12 open reading frame | 0.518 | 1.432 | 1.90E-02 | 1.90E+00 | Up | | |
| 8001876 | NM_00101815 | NAE1 | NEDD8 activating enzyme E1 subunit | 0.518 | 1.431 | 2.52E-01 | 2.52E+01 | | | |
| 7947991 | NM_015231 | NUP160 | nucleoporin 160kDa | 0.517 | 1.431 | 1.30E-02 | 1.30E+00 | Up | | |
| 8146564 | NM_002865 | RAB2A | RAB2A, member RAS oncogene fami | 0.517 | 1.431 | 2.18E-01 | 2.18E+01 | | | |
| 8013908 | NM_020772 | NUFIP2 | nuclear fragile X mental retardation | 0.517 | 1.431 | 1.00E-01 | 1.00E+01 | | | |
| 7999496 | NM_014153 | ZC3H7A | zinc finger CCCH-type containing 7A | 0.517 | 1.431 | 2.28E-01 | 2.28E+01 | | | |
| 8009164 | NM_005828 | DCAF7 | DDB1 and CUL4 associated factor 7 | 0.516 | 1.430 | 1.97E-02 | 1.97E+00 | Up | | |
| 7984112 | NM_016530 | RAB8B | RAB8B, member RAS oncogene famil | 0.516 | 1.430 | 2.61E-01 | 2.61E+01 | | | |
| 8173206 | --- | --- | --- | 0.516 | 1.430 | 2.00E-01 | 2.00E+01 | | | |
| 8112302 | NM_00104824 | C5orf43 | chromosome 5 open reading frame 4 | 0.515 | 1.429 | 6.20E-02 | 6.20E+00 | | | |
| 7892574 | --- | --- | --- | 0.515 | 1.429 | 2.83E-01 | 2.83E+01 | | | |
| 8107706 | NM_005573 | LMNB1 | lamin B1 | 0.515 | 1.429 | 3.89E-02 | 3.89E+00 | Up | | |
| 8050128 | NM_020738 | KIDINS220 | kinase D-interacting substrate, 220kI | 0.515 | 1.429 | 2.94E-02 | 2.94E+00 | Up | | |
| 8164607 | NM_015033 | FNBP1 | formin binding protein 1 | 0.515 | 1.429 | 2.08E-03 | 2.08E-01 | Up | | |
| 7952739 | NM_014155 | ZBTB44 | zinc finger and BTB domain containin | 0.515 | 1.429 | 1.23E-01 | 1.23E+01 | | | |
| 7920766 | NM_018489 | ASH1L | ash1 (absent, small, or homeotic)-lik | 0.515 | 1.429 | 1.16E-01 | 1.16E+01 | | | |
| 8125059 | NM_001288 | CLIC1 | chloride intracellular channel 1 | 0.514 | 1.428 | 1.63E-02 | 1.63E+00 | Up | | |
| 7935002 | NM_003133 | SRP9 | signal recognition particle 9kDa | 0.514 | 1.428 | 8.75E-02 | 8.75E+00 | | | |
| 7950544 | NM_004705 | PRKRIR | protein-kinase, interferon-inducible | 0.513 | 1.428 | 2.66E-01 | 2.66E+01 | | | |
| 7964701 | NM_002076 | GNS | glucosamine (N-acetyl)-6-sulfatase | 0.513 | 1.427 | 1.70E-02 | 1.70E+00 | Up | | mono |
| 8007865 | --- | --- | --- | 0.513 | 1.427 | 1.15E-01 | 1.15E+01 | | | |
| 8007883 | --- | --- | --- | 0.513 | 1.427 | 1.15E-01 | 1.15E+01 | | | |
| 8101945 | NM_002106 | H2AFZ | H2A histone family, member Z | 0.513 | 1.427 | 2.43E-01 | 2.43E+01 | | | |
| 7934278 | NM_000917 | P4HA1 | prolyl 4-hydroxylase, alpha polypept | 0.513 | 1.427 | 8.72E-02 | 8.72E+00 | | | |
| 8023228 | NM_017653 | DYM | dymeclin | 0.513 | 1.427 | 4.14E-02 | 4.14E+00 | Up | | |

| | | | | | | | | | | | |
|---|---|---|---|---|---|---|---|---|---|---|---|
| 7995258 | NM_003414 | ZNF267 | zinc finger protein 267 | 0.513 | 1.427 | 3.73E-01 | 3.73E+01 | | | | |
| 7997168 | NM_014761 | KIAA0174 | KIAA0174 | 0.513 | 1.427 | 1.71E-01 | 1.71E+01 | | | | |
| 7961983 | NM_016551 | TM7SF3 | transmembrane 7 superfamily memb | 0.513 | 1.427 | 4.86E-03 | 4.86E-01 | Up | | | |
| 8030980 | NR_003699 | ZNF525 | zinc finger protein 525 | 0.513 | 1.427 | 1.85E-01 | 1.85E+01 | | | | |
| 7940066 | NM_015959 | TMX2 | thioredoxin-related transmembrane | 0.513 | 1.427 | 2.40E-01 | 2.40E+01 | | | | |
| 7893128 | --- | --- | --- | 0.513 | 1.427 | 6.10E-01 | 6.10E+01 | | | | |
| 7978260 | NM_00113605( | DHRS1 | dehydrogenase/reductase (SDR fami | 0.512 | 1.426 | 3.46E-02 | 3.46E+00 | Up | | | |
| 8002865 | NM_006324 | CFDP1 | craniofacial development protein 1 | 0.512 | 1.426 | 1.01E-01 | 1.01E+01 | | | | |
| 7971563 | NM_020357 | PCNP | PEST proteolytic signal containing nu | 0.512 | 1.426 | 4.05E-01 | 4.05E+01 | | | | |
| 7911017 | NM_006642 | SDCCAG8 | serologically defined colon cancer ar | 0.512 | 1.426 | 9.00E-03 | 9.00E-01 | Up | | | |
| 7919251 | NM_016334 | GPR89B | G protein-coupled receptor 89B | 0.512 | 1.426 | 1.75E-01 | 1.75E+01 | | | | |
| 7954382 | NM_024854 | PYROXD1 | pyridine nucleotide-disulphide oxido | 0.511 | 1.426 | 1.92E-01 | 1.92E+01 | | | | |
| 7895739 | --- | --- | --- | 0.511 | 1.425 | 4.68E-01 | 4.68E+01 | | | | |
| 7989253 | NM_024755 | SLTM | SAFB-like, transcription modulator | 0.511 | 1.425 | 4.02E-02 | 4.02E+00 | Up | | | |
| 7954021 | NM_001310 | CREBL2 | cAMP responsive element binding pr | 0.511 | 1.425 | 2.66E-02 | 2.66E+00 | Up | | | |
| 7942032 | NM_018312 | SAPS3 | SAPS domain family, member 3 | 0.511 | 1.425 | 1.29E-02 | 1.29E+00 | Up | | | |
| 7895482 | --- | --- | --- | 0.511 | 1.425 | 4.71E-01 | 4.71E+01 | | | | |
| 8112728 | NM_00108970 | CG_175733 | RAP1B, member of RAS oncogene fa | 0.511 | 1.425 | 5.96E-02 | 5.96E+00 | | | | |
| 8143132 | NM_00112861 | LUZP6 | leucine zipper protein 6 | 0.511 | 1.425 | 2.78E-02 | 2.78E+00 | Up | | | |
| 8077528 | NM_00108051 | SETD5 | SET domain containing 5 | 0.511 | 1.425 | 1.06E-02 | 1.06E+00 | Up | | | |
| 7893833 | --- | --- | --- | 0.511 | 1.425 | 5.04E-01 | 5.04E+01 | | | | |
| 7896308 | --- | --- | --- | 0.511 | 1.425 | 7.48E-02 | 7.48E+00 | | | | |
| 8098291 | NM_173872 | CLCN3 | chloride channel 3 | 0.511 | 1.425 | 2.82E-02 | 2.82E+00 | Up | | | |
| 8175393 | NM_004840 | ARHGEF6 | Rac/Cdc42 guanine nucleotide excha | 0.510 | 1.424 | 1.03E-01 | 1.03E+01 | | | | |
| 7978595 | NM_013448 | BAZ1A | bromodomain adjacent to zinc finger | 0.510 | 1.424 | 1.79E-02 | 1.79E+00 | Up | | | |
| 7980891 | NM_00112859( | TC2N | tandem C2 domains, nuclear | 0.510 | 1.424 | 1.41E-01 | 1.41E+01 | | | | |
| 8015490 | NM_003315 | DNAJC7 | DnaJ (Hsp40) homolog, subfamily C, | 0.510 | 1.424 | 1.17E-01 | 1.17E+01 | | | | |
| 8100382 | NM_012110 | CHIC2 | cysteine-rich hydrophobic domain 2 | 0.510 | 1.424 | 1.95E-01 | 1.95E+01 | | | | |
| 7926545 | NM_032812 | PLXDC2 | plexin domain containing 2 | 0.510 | 1.424 | 5.91E-02 | 5.91E+00 | | | | mono |
| 8089203 | NM_020654 | SENP7 | SUMO1/sentrin specific peptidase 7 | 0.510 | 1.424 | 1.31E-01 | 1.31E+01 | | | | |
| 8127660 | --- | --- | --- | 0.509 | 1.423 | 2.08E-01 | 2.08E+01 | | | | |
| 7894185 | --- | --- | --- | 0.509 | 1.423 | 4.23E-01 | 4.23E+01 | | | | |
| 7946089 | NM_033034 | TRIM5 | tripartite motif-containing 5 | 0.509 | 1.423 | 5.80E-03 | 5.80E-01 | Up | | | |
| 8174379 | NM_002814 | PSMD10 | proteasome (prosome, macropain) 2 | 0.508 | 1.423 | 9.05E-02 | 9.05E+00 | | | | |
| 7968516 | NM_015032 | PDS5B | PDS5, regulator of cohesion mainten | 0.508 | 1.422 | 5.24E-02 | 5.24E+00 | | | | |
| 8100495 | NM_002703 | PPAT | phosphoribosyl pyrophosphate amid | 0.508 | 1.422 | 3.52E-02 | 3.52E+00 | Up | | | |
| 7894915 | --- | --- | --- | 0.508 | 1.422 | 2.72E-01 | 2.72E+01 | | | | |
| 7995128 | NM_000887 | ITGAX | integrin, alpha X (complement comp | 0.508 | 1.422 | 9.88E-04 | 9.88E-02 | Up | | | |
| 7900119 | NM_005119 | THRAP3 | thyroid hormone receptor associated | 0.508 | 1.422 | 6.15E-02 | 6.15E+00 | | | | |
| 7894372 | --- | --- | --- | 0.507 | 1.422 | 1.88E-01 | 1.88E+01 | | | | |
| 8112020 | NM_176806 | MOCS2 | molybdenum cofactor synthesis 2 | 0.507 | 1.421 | 3.64E-02 | 3.64E+00 | Up | | | |
| 7893468 | --- | --- | --- | 0.507 | 1.421 | 4.12E-01 | 4.12E+01 | | | | |
| 8159961 | --- | --- | --- | 0.507 | 1.421 | 1.25E-01 | 1.25E+01 | | | | |
| 7892688 | --- | --- | --- | 0.507 | 1.421 | 4.13E-01 | 4.13E+01 | | | | |
| 8094271 | NM_025205 | MED28 | mediator complex subunit 28 | 0.507 | 1.421 | 1.91E-01 | 1.91E+01 | | | | |
| 7990810 | NM_006441 | MTHFS | 5,10-methenyltetrahydrofolate synth | 0.507 | 1.421 | 7.74E-02 | 7.74E+00 | | | | |
| 8070160 | NM_001697 | ATP5O | ATP synthase, H+ transporting, mitoc | 0.507 | 1.421 | 1.60E-01 | 1.60E+01 | | | | |
| 8169419 | BC117377 | ALG13 | asparagine-linked glycosylation 13 h | 0.507 | 1.421 | 2.27E-01 | 2.27E+01 | | | | |
| 8109368 | NM_005754 | G3BP1 | GTPase activating protein (SH3 doma | 0.506 | 1.420 | 2.23E-01 | 2.23E+01 | | | | |
| 8173713 | NM_032121 | MAGT1 | magnesium transporter 1 | 0.506 | 1.420 | 1.03E-01 | 1.03E+01 | | | | |
| 8047059 | NM_031314 | HNRNPC | heterogeneous nuclear ribonucleopr | 0.506 | 1.420 | 1.83E-02 | 1.83E+00 | Up | | | |
| 8083063 | NM_00110464 | SLC25A36 | solute carrier family 25, member 36 | 0.506 | 1.420 | 8.66E-02 | 8.66E+00 | | | | |
| 8112967 | NM_174909 | TMEM167A | transmembrane protein 167A | 0.506 | 1.420 | 3.23E-01 | 3.23E+01 | | | | |
| 7893060 | --- | --- | --- | 0.506 | 1.420 | 2.31E-01 | 2.31E+01 | | | | |
| 7896519 | --- | --- | --- | 0.506 | 1.420 | 1.98E-01 | 1.98E+01 | | | | |
| 8040080 | NM_080657 | RSAD2 | radical S-adenosyl methionine doma | 0.506 | 1.420 | 2.58E-01 | 2.58E+01 | | | | |
| 8131831 | NM_007342 | NUPL2 | nucleoporin like 2 | 0.505 | 1.419 | 2.80E-02 | 2.80E+00 | Up | | | |
| 8173607 | --- | --- | --- | 0.505 | 1.419 | 3.00E-01 | 3.00E+01 | | | | |
| 8042830 | NR_027405 | MTHFD2 | methylenetetrahydrofolate dehydro | 0.505 | 1.419 | 1.67E-01 | 1.67E+01 | | | | |
| 8133809 | NM_198467 | RSBN1L | round spermatid basic protein 1-like | 0.505 | 1.419 | 4.96E-02 | 4.96E+00 | Up | | | |
| 7927202 | NM_006963 | ZNF22 | zinc finger protein 22 (KOX 15) | 0.505 | 1.419 | 1.66E-02 | 1.66E+00 | Up | | | |
| 7895048 | --- | --- | --- | 0.504 | 1.418 | 6.36E-01 | 6.36E+01 | | | | |
| 8005512 | NM_002767 | PRPSAP2 | phosphoribosyl pyrophosphate synth | 0.504 | 1.418 | 3.21E-02 | 3.21E+00 | Up | | | |
| 7933405 | NM_133446 | AGAP4 | ArfGAP with GTPase domain, ankyrin | 0.504 | 1.418 | 2.35E-02 | 2.35E+00 | Up | | | |
| 8163481 | NM_139286 | CDC26 | cell division cycle 26 homolog (S. cer | 0.504 | 1.418 | 4.52E-01 | 4.52E+01 | | | | |
| 7981718 | BC020240 // E | GHM // IGHM | immunoglobulin heavy constant mu | 0.503 | 1.418 | 4.84E-03 | 4.84E-01 | Up | | | |
| 8001666 | NM_001896 | CSNK2A2 | casein kinase 2, alpha prime polypep | 0.503 | 1.418 | 2.87E-01 | 2.87E+01 | | | | |
| 8084092 | NM_002492 | NDUFB5 | NADH dehydrogenase (ubiquinone) 1 | 0.503 | 1.417 | 2.14E-01 | 2.14E+01 | | | | |
| 7976148 | NR_024620 | PRO1768 | PRO1768 | 0.503 | 1.417 | 5.23E-02 | 5.23E+00 | | | | |
| 8101624 | NM_020803 | KLHL8 | kelch-like 8 (Drosophila) | 0.503 | 1.417 | 1.66E-01 | 1.66E+01 | | | | |
| 8106354 | NM_006633 | IQGAP2 | IQ motif containing GTPase activatin | 0.503 | 1.417 | 8.01E-02 | 8.01E+00 | | | | |
| 8093336 | NM_003441 | ZNF141 | zinc finger protein 141 | 0.503 | 1.417 | 4.87E-02 | 4.87E+00 | Up | | | |
| 7980523 | NM_015859 | GTF2A1 | general transcription factor IIA, 1, 19 | 0.502 | 1.417 | 4.20E-02 | 4.20E+00 | Up | | | |
| 7917359 | NM_017953 | ZNHIT6 | zinc finger, HIT type 6 | 0.502 | 1.417 | 3.83E-01 | 3.83E+01 | | | | |
| 8055639 | --- | --- | --- | 0.502 | 1.417 | 3.41E-01 | 3.41E+01 | | | | |
| 7956910 | NM_018448 | CAND1 | cullin-associated and neddylation-dis | 0.502 | 1.417 | 2.86E-02 | 2.86E+00 | Up | | | |
| 7970428 | NM_00103965( | ZMYM5 | zinc finger, MYM-type 5 | 0.501 | 1.415 | 1.95E-01 | 1.95E+01 | | | | |

| | | | | | | | | | | |
|---|---|---|---|---|---|---|---|---|---|---|
| 7895204 | --- | --- | --- | 0.501 | 1.415 | 5.51E-01 | 5.51E+01 | | | |
| 8054131 | BC047722 | C2orf64 | chromosome 2 open reading frame 6 | 0.500 | 1.414 | 1.48E-02 | 1.48E+00 | Up | | |
| 8166264 | --- | --- | --- | 0.500 | 1.414 | 1.43E-01 | 1.43E+01 | | | |
| 8058552 | NM_005896 | IDH1 | isocitrate dehydrogenase 1 (NADP+), | 0.500 | 1.414 | 8.63E-03 | 8.63E-01 | Up | | |
| 7934812 | NM_015045 | WAPAL | wings apart-like homolog (Drosophila | 0.500 | 1.414 | 1.57E-02 | 1.57E+00 | Up | | |
| 7894607 | --- | --- | --- | 0.500 | 1.414 | 4.88E-01 | 4.88E+01 | | | |
| 8084173 | NM_014616 | ATP11B | ATPase, class VI, type 11B | 0.500 | 1.414 | 1.07E-01 | 1.07E+01 | | | |
| 7965846 | NM_016053 | CCDC53 | coiled-coil domain containing 53 | 0.500 | 1.414 | 1.14E-01 | 1.14E+01 | | | |
| 8042337 | NM_00100538 | ACTR2 | ARP2 actin-related protein 2 homolo | 0.500 | 1.414 | 3.26E-02 | 3.26E+00 | Up | | |
| 7908421 | NM_004600 | TROVE2 | TROVE domain family, member 2 | 0.499 | 1.413 | 2.01E-01 | 2.01E+01 | | | |
| 8144516 | NM_153332 | ERI1 | exoribonuclease 1 | 0.499 | 1.413 | 4.66E-02 | 4.66E+00 | Up | | |
| 8086150 | NM_006309 | LRRFIP2 | leucine rich repeat (in FLII) interactin | 0.499 | 1.413 | 2.70E-02 | 2.70E+00 | Up | | |
| 7895846 | --- | --- | --- | 0.499 | 1.413 | 1.36E-01 | 1.36E+01 | | | |
| 8111552 | NM_00108541 | C5orf33 | chromosome 5 open reading frame 3 | 0.498 | 1.412 | 2.18E-01 | 2.18E+01 | | | |
| 8132243 | --- | --- | --- | 0.498 | 1.412 | 3.71E-01 | 3.71E+01 | | | |
| 7957540 | NM_014050 | MRPL42 | mitochondrial ribosomal protein L42 | 0.498 | 1.412 | 3.05E-01 | 3.05E+01 | | | |
| 7959597 | NM_006815 | TMED2 | transmembrane emp24 domain traff | 0.498 | 1.412 | 1.06E-01 | 1.06E+01 | | | |
| 8087380 | NM_003363 | USP4 | ubiquitin specific peptidase 4 (proto- | 0.498 | 1.412 | 9.56E-02 | 9.56E+00 | | | |
| 8138224 | NM_002489 | NDUFA4 | NADH dehydrogenase (ubiquinone) | 0.497 | 1.412 | 7.33E-02 | 7.33E+00 | | | |
| 7933872 | NM_000399 | EGR2 | early growth response 2 | 0.497 | 1.412 | 7.34E-02 | 7.34E+00 | | | |
| 7895507 | --- | --- | --- | 0.497 | 1.411 | 3.82E-01 | 3.82E+01 | | | |
| 7896277 | --- | --- | --- | 0.497 | 1.411 | 2.53E-01 | 2.53E+01 | | | |
| 7993371 | NM_00100406 | NOMO3 | NODAL modulator 3 | 0.496 | 1.410 | 1.08E-01 | 1.08E+01 | | | |
| 7993511 | NM_00100406 | NOMO3 | NODAL modulator 3 | 0.496 | 1.410 | 1.08E-01 | 1.08E+01 | | | |
| 7999791 | NM_00100406 | NOMO3 | NODAL modulator 3 | 0.496 | 1.410 | 1.08E-01 | 1.08E+01 | | | |
| 8023561 | NM_005570 | LMAN1 | lectin, mannose-binding, 1 | 0.496 | 1.410 | 2.53E-01 | 2.53E+01 | | | |
| 8010454 | NM_020914 | RNF213 | ring finger protein 213 | 0.496 | 1.410 | 9.08E-03 | 9.08E-01 | Up | | |
| 7895110 | --- | --- | --- | 0.496 | 1.410 | 1.98E-01 | 1.98E+01 | | | |
| 7892966 | --- | --- | --- | 0.495 | 1.410 | 3.96E-01 | 3.96E+01 | | | |
| 7904340 | NM_006699 | MAN1A2 | mannosidase, alpha, class 1A, memb | 0.495 | 1.409 | 3.44E-02 | 3.44E+00 | Up | | |
| 7927552 | NM_00107766 | AGAP6 | ArfGAP with GTPase domain, ankyrin | 0.495 | 1.409 | 3.58E-02 | 3.58E+00 | Up | | |
| 7932285 | NM_024948 | FAM188A | family with sequence similarity 188, | 0.495 | 1.409 | 1.25E-01 | 1.25E+01 | | | |
| 8091141 | NM_019001 | XRN1 | 5'-3' exoribonuclease 1 | 0.495 | 1.409 | 2.13E-02 | 2.13E+00 | Up | | |
| 7893444 | --- | --- | --- | 0.494 | 1.409 | 4.75E-01 | 4.75E+01 | | | |
| 8146544 | NM_00107761 | UBXN2B | UBX domain protein 2B | 0.494 | 1.408 | 2.27E-01 | 2.27E+01 | | | |
| 8121461 | NM_015891 | CDC40 | cell division cycle 40 homolog (S. cer | 0.494 | 1.408 | 6.10E-02 | 6.10E+00 | | | |
| 7967386 | NM_022782 | MPHOSPH9 | M-phase phosphoprotein 9 | 0.494 | 1.408 | 7.18E-02 | 7.18E+00 | | | |
| 7902345 | NM_138467 | TYW3 | tRNA-yW synthesizing protein 3 hom | 0.494 | 1.408 | 4.40E-01 | 4.40E+01 | | | |
| 7980265 | NM_203488 | ACYP1 | acylphosphatase 1, erythrocyte (com | 0.494 | 1.408 | 9.31E-02 | 9.31E+00 | | | |
| 7896528 | --- | --- | --- | 0.493 | 1.408 | 2.50E-02 | 2.50E+00 | Up | | |
| 8080645 | NM_012096 | APPL1 | adaptor protein, phosphotyrosine in | 0.493 | 1.407 | 1.22E-01 | 1.22E+01 | | | |
| 7895399 | --- | --- | --- | 0.492 | 1.407 | 6.07E-01 | 6.07E+01 | | | |
| 7962869 | NM_004818 | DDX23 | DEAD (Asp-Glu-Ala-Asp) box polypep | 0.492 | 1.407 | 5.82E-02 | 5.82E+00 | | | |
| 7968234 | NR_002575 | SNORA27 | small nucleolar RNA, H/ACA box 27 | 0.492 | 1.407 | 4.97E-01 | 4.97E+01 | | | |
| 7893788 | --- | --- | --- | 0.492 | 1.406 | 2.56E-01 | 2.56E+01 | | | |
| 8135211 | NR_026879 | FAM185A | family with sequence similarity 185, | 0.492 | 1.406 | 1.93E-01 | 1.93E+01 | | | |
| 8035779 | NM_00107667 | ZNF626 | zinc finger protein 626 | 0.492 | 1.406 | 5.84E-01 | 5.84E+01 | | | |
| 8030908 | NM_144684 | ZNF480 | zinc finger protein 480 | 0.491 | 1.406 | 4.71E-02 | 4.71E+00 | Up | | |
| 8166195 | NM_005089 | ZRSR2 | zinc finger (CCCH type), RNA-binding | 0.491 | 1.406 | 2.57E-02 | 2.57E+00 | Up | | |
| 8105937 | AK289851 | SMA5 | glucuronidase, beta pseudogene | 0.491 | 1.405 | 6.64E-02 | 6.64E+00 | | | |
| 7894846 | --- | --- | --- | 0.491 | 1.405 | 3.66E-01 | 3.66E+01 | | | |
| 7904254 | NM_000701 | ATP1A1 | ATPase, Na+/K+ transporting, alpha | 0.491 | 1.405 | 5.85E-02 | 5.85E+00 | | | |
| 8036813 | NM_00100585 | ZNF780B | zinc finger protein 780B | 0.490 | 1.405 | 1.35E-01 | 1.35E+01 | | | |
| 8060772 | NM_003818 | CDS2 | CDP-diacylglycerol synthase (phosph | 0.490 | 1.405 | 1.14E-01 | 1.14E+01 | | | |
| 8146171 | NM_001556 | IKBKB | inhibitor of kappa light polypeptide g | 0.490 | 1.405 | 3.68E-03 | 3.68E-01 | Up | | |
| 7942551 | --- | --- | --- | 0.490 | 1.404 | 4.48E-01 | 4.48E+01 | | | |
| 8112007 | NM_198449 | EMB | embigin homolog (mouse) | 0.490 | 1.404 | 4.77E-02 | 4.77E+00 | Up | | |
| 7894911 | --- | --- | --- | 0.489 | 1.404 | 5.51E-01 | 5.51E+01 | | | |
| 8157947 | NM_00109927 | ZBTB34 | zinc finger and BTB domain containin | 0.489 | 1.404 | 4.49E-03 | 4.49E-01 | Up | | |
| 8091190 | NM_001184 | ATR | ataxia telangiectasia and Rad3 relate | 0.489 | 1.404 | 5.46E-02 | 5.46E+00 | | | |
| 8050761 | --- | --- | --- | 0.489 | 1.404 | 2.40E-01 | 2.40E+01 | | | |
| 8041273 | NM_032312 | YIPF4 | Yip1 domain family, member 4 | 0.489 | 1.403 | 9.74E-03 | 9.74E-01 | Up | | |
| 7893518 | --- | --- | --- | 0.489 | 1.403 | 4.37E-01 | 4.37E+01 | | | |
| 7904755 | NM_003846 | PEX11B | peroxisomal biogenesis factor 11 bet | 0.489 | 1.403 | 2.41E-02 | 2.41E+00 | Up | | |
| 8085815 | NM_001068 | TOP2B | topoisomerase (DNA) II beta 180kDa | 0.489 | 1.403 | 3.45E-02 | 3.45E+00 | Up | | |
| 8084213 | --- | --- | --- | 0.489 | 1.403 | 3.53E-01 | 3.53E+01 | | | |
| 7956937 | NM_015646 | RAP1B | RAP1B, member of RAS oncogene fa | 0.488 | 1.403 | 3.99E-02 | 3.99E+00 | Up | | |
| 7953243 | NM_005002 | NDUFA9 | NADH dehydrogenase (ubiquinone) | 0.487 | 1.402 | 6.01E-02 | 6.01E+00 | | | |
| 7902822 | NM_006256 | PKN2 | protein kinase N2 | 0.487 | 1.402 | 1.87E-01 | 1.87E+01 | | | |
| 7958211 | NM_152318 | C12orf45 | chromosome 12 open reading frame | 0.487 | 1.402 | 2.16E-01 | 2.16E+01 | | | |
| 8121161 | NM_015323 | KIAA0776 | KIAA0776 | 0.487 | 1.402 | 2.01E-01 | 2.01E+01 | | | |
| 8118218 | NM_001320 | CSNK2B | casein kinase 2, beta polypeptide | 0.487 | 1.402 | 1.92E-01 | 1.92E+01 | | | |
| 8129410 | NM_00101092 | THEMIS | thymocyte selection associated | 0.487 | 1.401 | 1.78E-01 | 1.78E+01 | | | |
| 7922846 | NM_052966 | FAM129A | family with sequence similarity 129, | 0.487 | 1.401 | 1.83E-02 | 1.83E+00 | Up | | |
| 7957715 | NM_00113517 | NEDD1 | neural precursor cell expressed, deve | 0.487 | 1.401 | 8.54E-02 | 8.54E+00 | | | |
| 8157524 | NR_024168 | TLR4 | toll-like receptor 4 | 0.487 | 1.401 | 2.05E-02 | 2.05E+00 | Up | Anti-microbial/Pathogen recogn. | |
| 8073088 | NM_021822 | APOBEC3G | apolipoprotein B mRNA editing enzy | 0.486 | 1.401 | 1.48E-01 | 1.48E+01 | | | |

| | | | | | | | | | | |
|---|---|---|---|---|---|---|---|---|---|---|
| 7892933 | --- | --- | --- | 0.486 | 1.401 | 5.18E-01 | 5.18E+01 | | | |
| 7894888 | --- | --- | --- | 0.486 | 1.401 | 4.38E-01 | 4.38E+01 | | | |
| 8105463 | NM_022913 | GPBP1 | GC-rich promoter binding protein 1 | 0.486 | 1.401 | 8.14E-02 | 8.14E+00 | | | |
| 8093976 | NM_020773 | TBC1D14 | TBC1 domain family, member 14 | 0.486 | 1.401 | 7.88E-02 | 7.88E+00 | | | |
| 7968274 | NM_175854 | PAN3 | PAN3 poly(A) specific ribonuclease su | 0.486 | 1.400 | 1.16E-01 | 1.16E+01 | | | |
| 8130071 | NM_024713 | C15orf29 | chromosome 15 open reading frame | 0.486 | 1.400 | 7.68E-02 | 7.68E+00 | | | |
| 8113369 | NM_180991 | SLCO4C1 | solute carrier organic anion transpor | 0.486 | 1.400 | 3.62E-02 | 3.62E+00 | Up | | |
| 8170891 | NM_001493 | GDI1 | GDP dissociation inhibitor 1 | 0.485 | 1.400 | 1.04E-01 | 1.04E+01 | | | |
| 7896508 | --- | --- | --- | 0.485 | 1.400 | 1.86E-01 | 1.86E+01 | | | |
| 8138912 | NR_024466 | LSM5 | LSM5 homolog, U6 small nuclear RN | 0.485 | 1.400 | 1.40E-01 | 1.40E+01 | | | |
| 8066716 | NM_133171 | ELMO2 | engulfment and cell motility 2 | 0.485 | 1.400 | 3.73E-03 | 3.73E-01 | Up | | |
| 7894605 | --- | --- | --- | 0.485 | 1.400 | 7.45E-02 | 7.45E+00 | | | |
| 7933574 | NM_00107768 | AGAP7 | ArfGAP with GTPase domain, ankyrin | 0.485 | 1.399 | 2.23E-02 | 2.23E+00 | Up | | |
| 7976598 | NM_032632 | PAPOLA | poly(A) polymerase alpha | 0.484 | 1.399 | 1.35E-02 | 1.35E+00 | Up | | |
| 8013431 | NM_007202 | AKAP10 | A kinase (PRKA) anchor protein 10 | 0.484 | 1.398 | 1.18E-02 | 1.18E+00 | Up | | |
| 8042119 | NM_022894 | PAPOLG | poly(A) polymerase gamma | 0.484 | 1.398 | 1.06E-01 | 1.06E+01 | | | |
| 8133074 | NR_027393 | INTS4L1 | integrator complex subunit 4-like 1 | 0.484 | 1.398 | 1.05E-01 | 1.05E+01 | | | |
| 8130765 | BC112329 | FAM103A1 | family with sequence similarity 103, | 0.483 | 1.398 | 3.69E-01 | 3.69E+01 | | | |
| 7963289 | NM_016293 | BIN2 | bridging integrator 2 | 0.483 | 1.398 | 5.70E-02 | 5.70E+00 | | | |
| 7969114 | NM_031915 | SETDB2 | SET domain, bifurcated 2 | 0.483 | 1.398 | 2.64E-01 | 2.64E+01 | | | |
| 7987172 | NM_020154 | C15orf24 | chromosome 15 open reading frame | 0.483 | 1.398 | 3.45E-01 | 3.45E+01 | | | |
| 7989144 | --- | --- | --- | 0.483 | 1.398 | 2.77E-01 | 2.77E+01 | | | |
| 8119858 | NM_006502 | POLH | polymerase (DNA directed), eta | 0.483 | 1.397 | 2.13E-02 | 2.13E+00 | Up | | |
| 7896367 | --- | --- | --- | 0.483 | 1.397 | 1.99E-01 | 1.99E+01 | | | |
| 8157534 | NM_007018 | CEP110 | centrosomal protein 110kDa | 0.483 | 1.397 | 6.84E-02 | 6.84E+00 | | | |
| 8007867 | NM_00100660 | LRRC37A2 | leucine rich repeat containing 37, me | 0.482 | 1.397 | 5.00E-02 | 5.00E+00 | Up | | |
| 7920317 | NM_004515 | ILF2 | interleukin enhancer binding factor 2 | 0.482 | 1.397 | 1.78E-01 | 1.78E+01 | | | |
| 7956894 | NM_033647 | HELB | helicase (DNA) B | 0.482 | 1.397 | 5.20E-02 | 5.20E+00 | | | |
| 8136614 | NM_018238 | AGK | acylglycerol kinase | 0.482 | 1.397 | 1.01E-01 | 1.01E+01 | | | |
| 8129861 | NM_000416 | IFNGR1 | interferon gamma receptor 1 | 0.482 | 1.397 | 4.73E-02 | 4.73E+00 | Up | | |
| 8009255 | NM_138363 | CCDC45 | coiled-coil domain containing 45 | 0.482 | 1.396 | 8.48E-02 | 8.48E+00 | | | |
| 8090893 | NM_018133 | MSL2 | male-specific lethal 2 homolog (Dros | 0.482 | 1.396 | 2.31E-01 | 2.31E+01 | | | |
| 8000482 | NM_015171 | XPO6 | exportin 6 | 0.482 | 1.396 | 3.40E-02 | 3.40E+00 | Up | | |
| 7895574 | --- | --- | --- | 0.481 | 1.396 | 4.09E-02 | 4.09E+00 | Up | | |
| 8114326 | NM_016603 | FAM13B | family with sequence similarity 13, m | 0.481 | 1.395 | 5.11E-02 | 5.11E+00 | | | |
| 7894188 | --- | --- | --- | 0.481 | 1.395 | 3.92E-01 | 3.92E+01 | | | |
| 8073056 | NM_145699 | APOBEC3A | apolipoprotein B mRNA editing enzy | 0.481 | 1.395 | 3.89E-02 | 3.89E+00 | Up | | |
| 8092392 | NM_130446 | KLHL6 | kelch-like 6 (Drosophila) | 0.481 | 1.395 | 4.02E-02 | 4.02E+00 | Up | | |
| 8127787 | NM_015525 | IBTK | inhibitor of Bruton agammaglobuline | 0.480 | 1.395 | 4.03E-02 | 4.03E+00 | Up | | |
| 8152477 | NM_006265 | RAD21 | RAD21 homolog (S. pombe) | 0.480 | 1.395 | 4.28E-02 | 4.28E+00 | Up | | |
| 7896210 | --- | --- | --- | 0.480 | 1.395 | 2.45E-01 | 2.45E+01 | | | |
| 7969060 | NM_00107967 | FNDC3A | fibronectin type III domain containin | 0.480 | 1.395 | 1.23E-02 | 1.23E+00 | Up | | |
| 8170326 | NM_002024 | FMR1 | fragile X mental retardation 1 | 0.479 | 1.394 | 2.30E-02 | 2.30E+00 | Up | | |
| 7989335 | NM_00100285 | ANXA2 | annexin A2 | 0.479 | 1.394 | 1.31E-02 | 1.31E+00 | Up | | mono |
| 7895761 | --- | --- | --- | 0.479 | 1.394 | 5.25E-02 | 5.25E+00 | | | |
| 8089801 | NM_002093 | GSK3B | glycogen synthase kinase 3 beta | 0.479 | 1.393 | 1.97E-01 | 1.97E+01 | | | |
| 8078014 | NM_003043 | SLC6A6 | solute carrier family 6 (neurotransmi | 0.479 | 1.393 | 3.10E-02 | 3.10E+00 | Up | | |
| 8050875 | NM_018263 | ASXL2 | additional sex combs like 2 (Drosoph | 0.478 | 1.393 | 2.75E-03 | 2.75E-01 | Up | | |
| 8173135 | NM_000032 | ALAS2 | aminolevulinate, delta-, synthase 2 | 0.478 | 1.393 | 3.91E-02 | 3.91E+00 | Up | | |
| 7896241 | --- | --- | --- | 0.478 | 1.393 | 3.42E-01 | 3.42E+01 | | | |
| 7974653 | NM_014749 | KIAA0586 | KIAA0586 | 0.478 | 1.392 | 9.69E-03 | 9.69E-01 | Up | | |
| 8109428 | NR_024084 | SAP30L | SAP30-like | 0.478 | 1.392 | 9.24E-02 | 9.24E+00 | | | |
| 8116402 | NM_139068 | MAPK9 | mitogen-activated protein kinase 9 | 0.477 | 1.392 | 1.83E-01 | 1.83E+01 | | | |
| 8088247 | NM_00112861 | ARHGEF3 | Rho guanine nucleotide exchange fa | 0.477 | 1.392 | 3.01E-02 | 3.01E+00 | Up | | |
| 8056792 | --- | --- | --- | 0.477 | 1.392 | 1.78E-01 | 1.78E+01 | | | |
| 7946041 | NM_000519 | HBD | hemoglobin, delta | 0.477 | 1.392 | 4.81E-02 | 4.81E+00 | Up | | |
| 7951554 | NM_002906 | RDX | radixin | 0.477 | 1.392 | 4.19E-02 | 4.19E+00 | Up | | |
| 7917472 | NM_001514 | GTF2B | general transcription factor IIB | 0.477 | 1.391 | 2.89E-01 | 2.89E+01 | | | |
| 7910099 | NM_00113044 | SRP9 | signal recognition particle 9kDa | 0.477 | 1.391 | 3.81E-02 | 3.81E+00 | Up | | |
| 7942839 | NM_015885 | PCF11 | PCF11, cleavage and polyadenylation | 0.476 | 1.391 | 3.41E-02 | 3.41E+00 | Up | | |
| 7947138 | NM_022725 | FANCF | Fanconi anemia, complementation g | 0.476 | 1.391 | 1.73E-02 | 1.73E+00 | Up | | |
| 7893392 | --- | --- | --- | 0.476 | 1.390 | 4.37E-01 | 4.37E+01 | | | |
| 7977786 | NM_003982 | SLC7A7 | solute carrier family 7 (cationic amin | 0.475 | 1.390 | 7.07E-02 | 7.07E+00 | | | mono |
| 7899957 | NM_005095 | ZMYM4 | zinc finger, MYM-type 4 | 0.475 | 1.390 | 9.65E-03 | 9.65E-01 | Up | | |
| 8020508 | NM_003831 | RIOK3 | RIO kinase 3 (yeast) | 0.475 | 1.390 | 8.78E-02 | 8.78E+00 | | | |
| 8033754 | NM_006631 | ZNF266 | zinc finger protein 266 | 0.475 | 1.390 | 1.22E-01 | 1.22E+01 | | | |
| 8086451 | NM_00109966 | HIGD1A | HIG1 hypoxia inducible domain famil | 0.475 | 1.389 | 4.05E-01 | 4.05E+01 | | | |
| 7981481 | NM_004894 | C14orf2 | chromosome 14 open reading frame | 0.475 | 1.389 | 1.03E-01 | 1.03E+01 | | | |
| 8047865 | NM_015040 | PIKFYVE | phosphoinositide kinase, FYVE finger | 0.474 | 1.389 | 2.58E-02 | 2.58E+00 | Up | | |
| 8002237 | NM_032178 | SLC7A6OS | solute carrier family 7, member 6 op | 0.474 | 1.389 | 5.72E-03 | 5.72E-01 | Up | | |
| 8160587 | NM_002493 | NDUFB6 | NADH dehydrogenase (ubiquinone) 1 | 0.474 | 1.389 | 4.35E-01 | 4.35E+01 | | | |
| 8139264 | NR_003655 | POLR2J4 | polymerase (RNA) II (DNA directed) p | 0.474 | 1.389 | 2.17E-01 | 2.17E+01 | | | |
| 8148358 | NM_016018 | PHF20L1 | PHD finger protein 20-like 1 | 0.474 | 1.389 | 1.84E-02 | 1.84E+00 | Up | | |
| 8140878 | NM_194455 | KRIT1 | KRIT1, ankyrin repeat containing | 0.474 | 1.389 | 1.58E-02 | 1.58E+00 | Up | | |
| 8105153 | NM_012343 | NNT | nicotinamide nucleotide transhydrog | 0.474 | 1.389 | 1.69E-02 | 1.69E+00 | Up | | |
| 7904482 | NM_015326 | SRGAP2 | SLIT-ROBO Rho GTPase activating pr | 0.473 | 1.388 | 2.82E-01 | 2.82E+01 | | | |
| 7942932 | NM_016401 | C11orf73 | chromosome 11 open reading frame | 0.473 | 1.388 | 1.09E-02 | 1.09E+00 | Up | | |

| | | | | | | | | | | |
|---|---|---|---|---|---|---|---|---|---|---|
| 7893304 | --- | --- | --- | 0.473 | 1.388 | 3.62E-01 | 3.62E+01 | | | |
| 7901385 | NM_024586 | OSBPL9 | oxysterol binding protein-like 9 | 0.473 | 1.388 | 2.20E-02 | 2.20E+00 | Up | | |
| 7904930 | NM_016334 | GPR89B | G protein-coupled receptor 89B | 0.473 | 1.388 | 1.90E-01 | 1.90E+01 | | | |
| 8002919 | NM_00113008 | KARS | lysyl-tRNA synthetase | 0.473 | 1.388 | 9.65E-02 | 9.65E+00 | | | |
| 7967881 | NM_017520 | MPHOSPH8 | M-phase phosphoprotein 8 | 0.473 | 1.388 | 9.72E-02 | 9.72E+00 | | | |
| 8007850 | NM_014834 | LRRC37A | leucine rich repeat containing 37A | 0.473 | 1.388 | 4.96E-02 | 4.96E+00 | Up | | |
| 8021496 | NM_020854 | KIAA1468 | KIAA1468 | 0.473 | 1.388 | 2.92E-02 | 2.92E+00 | Up | | |
| 8142143 | NM_006348 | COG5 | component of oligomeric golgi comp | 0.472 | 1.387 | 5.76E-02 | 5.76E+00 | | | |
| 8130032 | NM_032145 | FBXO30 | F-box protein 30 | 0.472 | 1.387 | 2.95E-02 | 2.95E+00 | Up | | |
| 8166104 | NM_003611 | OFD1 | oral-facial-digital syndrome 1 | 0.472 | 1.387 | 8.40E-02 | 8.40E+00 | | | |
| 7951131 | --- | --- | --- | 0.472 | 1.387 | 1.67E-01 | 1.67E+01 | | | |
| 8096905 | NM_018569 | C4orf16 | chromosome 4 open reading frame 1 | 0.472 | 1.387 | 2.27E-02 | 2.27E+00 | Up | | |
| 7914194 | NM_014280 | DNAJC8 | DnaJ (Hsp40) homolog, subfamily C, | 0.472 | 1.387 | 2.02E-01 | 2.02E+01 | | | |
| 7894776 | --- | --- | --- | 0.472 | 1.387 | 3.19E-01 | 3.19E+01 | | | |
| 8047641 | NM_177538 | CYP20A1 | cytochrome P450, family 20, subfam | 0.471 | 1.387 | 1.75E-01 | 1.75E+01 | | | |
| 7952319 | --- | --- | --- | 0.471 | 1.386 | 5.21E-01 | 5.21E+01 | | | |
| 7896523 | --- | --- | --- | 0.471 | 1.386 | 4.88E-01 | 4.88E+01 | | | |
| 7896701 | --- | --- | --- | 0.471 | 1.386 | 1.28E-01 | 1.28E+01 | | | |
| 7983679 | NM_007347 | AP4E1 | adaptor-related protein complex 4, e | 0.470 | 1.386 | 8.32E-02 | 8.32E+00 | | | |
| 8134122 | NM_005751 | AKAP9 | A kinase (PRKA) anchor protein (yoti | 0.470 | 1.385 | 3.31E-03 | 3.31E-01 | Up | | |
| 7989347 | NM_024611 | NARG2 | NMDA receptor regulated 2 | 0.470 | 1.385 | 6.29E-02 | 6.29E+00 | | | |
| 8105104 | NM_175921 | C5orf51 | chromosome 5 open reading frame 5 | 0.470 | 1.385 | 1.27E-01 | 1.27E+01 | | | |
| 7895072 | --- | --- | --- | 0.470 | 1.385 | 2.53E-01 | 2.53E+01 | | | |
| 7970347 | NM_00107864 | CDC16 | cell division cycle 16 homolog (S. cer | 0.470 | 1.385 | 3.03E-02 | 3.03E+00 | Up | | |
| 8000687 | NM_015092 | SMG1 | SMG1 homolog, phosphatidylinosito | 0.470 | 1.385 | 1.61E-01 | 1.61E+01 | | | |
| 7997712 | NM_002163 | IRF8 | interferon regulatory factor 8 | 0.469 | 1.384 | 1.78E-02 | 1.78E+00 | Up | | |
| 7893519 | --- | --- | --- | 0.469 | 1.384 | 4.83E-01 | 4.83E+01 | | | |
| 7894469 | --- | --- | --- | 0.469 | 1.384 | 3.10E-01 | 3.10E+01 | | | |
| 8180305 | --- | --- | --- | 0.469 | 1.384 | 6.75E-02 | 6.75E+00 | | | |
| 7989493 | NM_015920 | RPS27L | ribosomal protein S27-like | 0.469 | 1.384 | 1.05E-01 | 1.05E+01 | | | |
| 8093086 | NM_005017 | PCYT1A | phosphate cytidylyltransferase 1, cho | 0.468 | 1.383 | 2.06E-01 | 2.06E+01 | | | |
| 8168557 | NM_003022 | SH3BGRL | SH3 domain binding glutamic acid-ric | 0.468 | 1.383 | 9.72E-02 | 9.72E+00 | | | |
| 8105136 | NM_003432 | ZNF131 | zinc finger protein 131 | 0.468 | 1.383 | 3.46E-01 | 3.46E+01 | | | |
| 7916045 | NM_001981 | EPS15 | epidermal growth factor receptor pa | 0.468 | 1.383 | 6.08E-02 | 6.08E+00 | | | |
| 7963046 | NM_006082 | TUBA1B | tubulin, alpha 1b | 0.468 | 1.383 | 3.18E-02 | 3.18E+00 | Up | | |
| 8135363 | NM_002649 | PIK3CG | phosphoinositide-3-kinase, catalytic, | 0.468 | 1.383 | 8.85E-02 | 8.85E+00 | | | |
| 7894674 | --- | --- | --- | 0.467 | 1.382 | 1.76E-02 | 1.76E+00 | Up | | |
| 8092251 | NM_021629 | GNB4 | guanine nucleotide binding protein ( | 0.467 | 1.382 | 4.20E-02 | 4.20E+00 | Up | | |
| 7893898 | --- | --- | --- | 0.467 | 1.382 | 5.45E-02 | 5.45E+00 | | | |
| 7894269 | --- | --- | --- | 0.467 | 1.382 | 3.10E-01 | 3.10E+01 | | | |
| 8048146 | NM_021141 | XRCC5 | X-ray repair complementing defectiv | 0.467 | 1.382 | 3.07E-02 | 3.07E+00 | Up | | |
| 8133690 | NM_005918 | MDH2 | malate dehydrogenase 2, NAD (mito | 0.467 | 1.382 | 7.86E-02 | 7.86E+00 | | | |
| 7920123 | NM_002966 | S100A10 | S100 calcium binding protein A10 | 0.467 | 1.382 | 3.57E-01 | 3.57E+01 | | | |
| 7965508 | --- | --- | --- | 0.466 | 1.382 | 2.06E-01 | 2.06E+01 | | | |
| 8105647 | NM_00109375 | C5orf44 | chromosome 5 open reading frame 4 | 0.466 | 1.381 | 1.92E-01 | 1.92E+01 | | | |
| 8150906 | NM_017813 | IMPAD1 | inositol monophosphatase domain c | 0.466 | 1.381 | 5.21E-01 | 5.21E+01 | | | |
| 7935707 | NM_001278 | CHUK | conserved helix-loop-helix ubiquitou | 0.466 | 1.381 | 2.16E-01 | 2.16E+01 | | | |
| 8150287 | NM_023034 | WHSC1L1 | Wolf-Hirschhorn syndrome candidat | 0.466 | 1.381 | 4.39E-02 | 4.39E+00 | Up | | |
| 7987926 | --- | --- | --- | 0.465 | 1.381 | 2.66E-01 | 2.66E+01 | | | |
| 7946033 | NM_000518 | HBB | hemoglobin, beta | 0.465 | 1.381 | 1.44E-01 | 1.44E+01 | | | |
| 7984263 | NM_016395 | PTPLAD1 | protein tyrosine phosphatase-like A | 0.465 | 1.380 | 1.65E-01 | 1.65E+01 | | | |
| 7927082 | NR_003086 | HSD17B7P2 | hydroxysteroid (17-beta) dehydroge | 0.465 | 1.380 | 3.03E-01 | 3.03E+01 | | | |
| 8006028 | --- | --- | --- | 0.465 | 1.380 | 9.90E-02 | 9.90E+00 | | | |
| 8035787 | --- | --- | --- | 0.465 | 1.380 | 2.29E-01 | 2.29E+01 | | | |
| 8162117 | NM_016548 | GOLM1 | golgi membrane protein 1 | 0.464 | 1.379 | 1.46E-02 | 1.46E+00 | Up | | |
| 7950606 | NM_016578 | RSF1 | remodeling and spacing factor 1 | 0.464 | 1.379 | 1.52E-01 | 1.52E+01 | | | |
| 7892683 | --- | --- | --- | 0.464 | 1.379 | 6.36E-01 | 6.36E+01 | | | |
| 8081503 | NM_014648 | DZIP3 | DAZ interacting protein 3, zinc finger | 0.463 | 1.379 | 3.79E-02 | 3.79E+00 | Up | | |
| 7896222 | --- | --- | --- | 0.463 | 1.379 | 2.09E-01 | 2.09E+01 | | | |
| 8121312 | NM_016487 | C6orf203 | chromosome 6 open reading frame 2 | 0.463 | 1.379 | 3.86E-01 | 3.86E+01 | | | |
| 7969651 | NM_006260 | DNAJC3 | DnaJ (Hsp40) homolog, subfamily C, | 0.463 | 1.379 | 2.16E-01 | 2.16E+01 | | | |
| 8092035 | NM_024947 | PHC3 | polyhomeotic homolog 3 (Drosophila | 0.463 | 1.379 | 1.87E-01 | 1.87E+01 | | | |
| 8059319 | NM_005687 | FARSB | phenylalanyl-tRNA synthetase, beta | 0.463 | 1.378 | 6.41E-02 | 6.41E+00 | | | |
| 8113542 | NM_005669 | REEP5 | receptor accessory protein 5 | 0.463 | 1.378 | 4.98E-02 | 4.98E+00 | Up | | |
| 7894683 | --- | --- | --- | 0.462 | 1.378 | 6.13E-01 | 6.13E+01 | | | |
| 7895347 | --- | --- | --- | 0.462 | 1.378 | 3.96E-01 | 3.96E+01 | | | |
| 8053890 | NM_020151 | STARD7 | StAR-related lipid transfer (START) d | 0.462 | 1.378 | 1.82E-02 | 1.82E+00 | Up | | |
| 7895056 | --- | --- | --- | 0.462 | 1.377 | 4.37E-01 | 4.37E+01 | | | |
| 7896647 | --- | --- | --- | 0.462 | 1.377 | 1.84E-01 | 1.84E+01 | | | |
| 7896690 | --- | --- | --- | 0.462 | 1.377 | 5.93E-02 | 5.93E+00 | | | |
| 7966851 | NM_016281 | TAOK3 | TAO kinase 3 | 0.462 | 1.377 | 1.19E-01 | 1.19E+01 | | | |
| 7934196 | NM_002778 | PSAP | prosaposin | 0.461 | 1.377 | 4.36E-03 | 4.36E-01 | Up | | |
| 7895645 | --- | --- | --- | 0.461 | 1.377 | 6.03E-01 | 6.03E+01 | | | |
| 7894650 | --- | --- | --- | 0.461 | 1.377 | 2.75E-01 | 2.75E+01 | | | |
| 8176174 | NM_002436 | MPP1 | membrane protein, palmitoylated 1, | 0.461 | 1.376 | 7.03E-02 | 7.03E+00 | | | |
| 7905171 | NM_004698 | PRPF3 | PRP3 pre-mRNA processing factor 3 | 0.461 | 1.376 | 1.13E-01 | 1.13E+01 | | | |
| 8170097 | NM_00104253 | SLC9A6 | solute carrier family 9 (sodium/hydr | 0.461 | 1.376 | 2.57E-01 | 2.57E+01 | | | |

| | | | | | | | | | | |
|---|---|---|---|---|---|---|---|---|---|---|
| 7899016 | NM_003002 | SDHD | succinate dehydrogenase complex, s | 0.460 | 1.376 | 1.15E-01 | 1.15E+01 | | | |
| 7961900 | NM_002223 | ITPR2 | inositol 1,4,5-triphosphate receptor, | 0.460 | 1.376 | 2.55E-02 | 2.55E+00 | Up | | |
| 7966462 | NM_024953 | C12orf30 | chromosome 12 open reading frame | 0.460 | 1.376 | 3.99E-02 | 3.99E+00 | Up | | |
| 7895857 | --- | --- | --- | 0.460 | 1.375 | 1.79E-01 | 1.79E+01 | | | |
| 7932703 | NM_145698 | ACBD5 | acyl-Coenzyme A binding domain co | 0.459 | 1.375 | 2.56E-01 | 2.56E+01 | | | |
| 8130952 | NM_002793 | PSMB1 | proteasome (prosome, macropain) s | 0.459 | 1.375 | 3.37E-01 | 3.37E+01 | | | |
| 7976621 | NM_003384 | VRK1 | vaccinia related kinase 1 | 0.459 | 1.375 | 3.92E-01 | 3.92E+01 | | | |
| 8070819 | NM_004339 | PTTG1IP | pituitary tumor-transforming 1 inter | 0.458 | 1.374 | 1.45E-02 | 1.45E+00 | Up | | |
| 8158714 | NM_014285 | EXOSC2 | exosome component 2 | 0.458 | 1.374 | 1.50E-01 | 1.50E+01 | | | |
| 8155192 | NM_022343 | GLIPR2 | GLI pathogenesis-related 2 | 0.458 | 1.374 | 2.18E-02 | 2.18E+00 | Up | | |
| 7896714 | --- | --- | --- | 0.458 | 1.374 | 1.20E-01 | 1.20E+01 | | | |
| 7895805 | --- | --- | --- | 0.458 | 1.374 | 6.78E-01 | 6.78E+01 | | | |
| 7894511 | --- | --- | --- | 0.458 | 1.373 | 2.61E-01 | 2.61E+01 | | | |
| 7940857 | NM_006819 | STIP1 | stress-induced-phosphoprotein 1 | 0.458 | 1.373 | 2.90E-02 | 2.90E+00 | Up | | |
| 8020241 | NM_020232 | PSMG2 | proteasome (prosome, macropain) a | 0.457 | 1.373 | 6.32E-02 | 6.32E+00 | | | |
| 7969179 | NM_024570 | RNASEH2B | ribonuclease H2, subunit B | 0.457 | 1.373 | 6.57E-02 | 6.57E+00 | | | |
| 8079021 | NM_001904 | CTNNB1 | catenin (cadherin-associated protein | 0.457 | 1.373 | 1.39E-01 | 1.39E+01 | | | |
| 7979725 | NM_004569 | PIGH | phosphatidylinositol glycan anchor b | 0.457 | 1.373 | 3.82E-01 | 3.82E+01 | | | |
| 8151842 | NM_015496 | KIAA1429 | KIAA1429 | 0.457 | 1.373 | 4.00E-02 | 4.00E+00 | Up | | |
| 7965723 | NM_015054 | UHRF1BP1L | UHRF1 binding protein 1-like | 0.457 | 1.373 | 4.05E-02 | 4.05E+00 | Up | | |
| 8114396 | NM_004661 | CDC23 | cell division cycle 23 homolog (S. cer | 0.457 | 1.372 | 5.91E-02 | 5.91E+00 | | | |
| 8040362 | XR_038318 | LOC729992 | similar to heat shock 70kD protein bi | 0.457 | 1.372 | 4.01E-02 | 4.01E+00 | Up | | |
| 7934393 | NM_00114235 | PPP3CB | protein phosphatase 3 (formerly 2B) | 0.457 | 1.372 | 5.99E-02 | 5.99E+00 | | | |
| 8114778 | NM_004483 | GCSH | glycine cleavage system protein H (a | 0.456 | 1.372 | 3.30E-01 | 3.30E+01 | | | |
| 7892569 | --- | --- | --- | 0.456 | 1.372 | 4.33E-01 | 4.33E+01 | | | |
| 8122261 | NM_000416 | IFNGR1 | interferon gamma receptor 1 | 0.456 | 1.372 | 1.28E-01 | 1.28E+01 | | | |
| 8074640 | --- | --- | --- | 0.456 | 1.372 | 1.95E-01 | 1.95E+01 | | | |
| 8169701 | NM_014060 | MCTS1 | malignant T cell amplified sequence | 0.456 | 1.372 | 1.75E-01 | 1.75E+01 | | | |
| 7892766 | --- | --- | --- | 0.455 | 1.371 | 4.79E-01 | 4.79E+01 | | | |
| 8056829 | NM_004882 | CIR1 | corepressor interacting with RBPJ, 1 | 0.455 | 1.371 | 2.51E-01 | 2.51E+01 | | | |
| 8163275 | NM_00108039 | KIAA0368 | KIAA0368 | 0.455 | 1.371 | 1.96E-02 | 1.96E+00 | Up | | |
| 8047097 | NM_014905 | GLS | glutaminase | 0.455 | 1.371 | 1.62E-02 | 1.62E+00 | Up | | |
| 8092409 | NM_018622 | PARL | presenilin associated, rhomboid-like | 0.455 | 1.370 | 1.44E-01 | 1.44E+01 | | | |
| 7918657 | NM_015967 | PTPN22 | protein tyrosine phosphatase, non-re | 0.454 | 1.369 | 1.08E-01 | 1.08E+01 | | | |
| 8107356 | NM_152624 | DCP2 | DCP2 decapping enzyme homolog (S | 0.454 | 1.369 | 3.75E-02 | 3.75E+00 | Up | | |
| 8139057 | NM_014800 | ELMO1 | engulfment and cell motility 1 | 0.454 | 1.369 | 8.55E-02 | 8.55E+00 | | | |
| 8166511 | NM_005391 | PDK3 | pyruvate dehydrogenase kinase, isoz | 0.453 | 1.369 | 1.13E-01 | 1.13E+01 | | | |
| 8043218 | NM_006590 | USP39 | ubiquitin specific peptidase 39 | 0.453 | 1.369 | 2.32E-02 | 2.32E+00 | Up | | |
| 7896700 | --- | --- | --- | 0.453 | 1.369 | 1.84E-01 | 1.84E+01 | | | |
| 8161988 | NM_013438 | UBQLN1 | ubiquilin 1 | 0.453 | 1.369 | 8.28E-02 | 8.28E+00 | | | |
| 8176255 | NM_018196 | TMLHE | trimethyllysine hydroxylase, epsilon | 0.453 | 1.368 | 1.98E-02 | 1.98E+00 | Up | | |
| 7917148 | NM_022831 | AIDA | axin interactor, dorsalization associa | 0.452 | 1.368 | 3.12E-01 | 3.12E+01 | | | |
| 7981732 | ENST0000033 | VSIG6 | V-set and immunoglobulin domain co | 0.452 | 1.368 | 7.77E-02 | 7.77E+00 | | | |
| 7895900 | --- | --- | --- | 0.452 | 1.368 | 6.74E-01 | 6.74E+01 | | | |
| 7896671 | --- | --- | --- | 0.452 | 1.368 | 6.10E-01 | 6.10E+01 | | | |
| 8075585 | NM_014306 | C22orf28 | chromosome 22 open reading frame | 0.452 | 1.368 | 1.89E-01 | 1.89E+01 | | | |
| 7983298 | NM_016400 | C15orf63 | chromosome 15 open reading frame | 0.452 | 1.368 | 8.99E-02 | 8.99E+00 | | | |
| 7989806 | NM_197960 | DPP8 | dipeptidyl-peptidase 8 | 0.452 | 1.368 | 4.47E-02 | 4.47E+00 | Up | | |
| 7930537 | NM_00114627 | TCF7L2 | transcription factor 7-like 2 (T-cell sp | 0.451 | 1.367 | 7.30E-03 | 7.30E-01 | Up | | |
| 7898833 | NM_00100999 | KDM1 | lysine (K)-specific demethylase 1 | 0.451 | 1.367 | 2.63E-01 | 2.63E+01 | | | |
| 7944223 | NM_005933 | MLL | myeloid/lymphoid or mixed-lineage | 0.451 | 1.367 | 3.86E-02 | 3.86E+00 | Up | | |
| 7974247 | --- | --- | --- | 0.451 | 1.367 | 3.82E-01 | 3.82E+01 | | | |
| 8171041 | NM_005638 | VAMP7 | vesicle-associated membrane protei | 0.451 | 1.367 | 2.77E-01 | 2.77E+01 | | | |
| 8176962 | NM_005638 | VAMP7 | vesicle-associated membrane protei | 0.451 | 1.367 | 2.77E-01 | 2.77E+01 | | | |
| 7966321 | NM_016301 | GPN3 | GPN-loop GTPase 3 | 0.451 | 1.367 | 1.13E-01 | 1.13E+01 | | | |
| 8041149 | NM_015131 | WDR43 | WD repeat domain 43 | 0.450 | 1.366 | 2.44E-01 | 2.44E+01 | | | |
| 7958414 | NM_014301 | ISCU | iron-sulfur cluster scaffold homolog | 0.450 | 1.366 | 2.36E-01 | 2.36E+01 | | | |
| 7987048 | NM_017762 | MTMR10 | myotubularin related protein 10 | 0.450 | 1.366 | 2.77E-02 | 2.77E+00 | Up | | |
| 8164177 | NM_00100661 | MAPKAP1 | mitogen-activated protein kinase ass | 0.449 | 1.366 | 6.65E-02 | 6.65E+00 | | | |
| 8103911 | NM_002199 | IRF2 | interferon regulatory factor 2 | 0.449 | 1.365 | 1.79E-01 | 1.79E+01 | | | |
| 8118149 | NM_007161 | LST1 | leukocyte specific transcript 1 | 0.449 | 1.365 | 2.15E-02 | 2.15E+00 | Up | | mono |
| 8177988 | NM_007161 | LST1 | leukocyte specific transcript 1 | 0.449 | 1.365 | 2.15E-02 | 2.15E+00 | Up | | mono |
| 8179268 | NM_007161 | LST1 | leukocyte specific transcript 1 | 0.449 | 1.365 | 2.15E-02 | 2.15E+00 | Up | | mono |
| 8049827 | NM_00100849 | 40423 | septin 2 | 0.449 | 1.365 | 3.11E-02 | 3.11E+00 | Up | | |
| 8020527 | NM_013326 | C18orf8 | chromosome 18 open reading frame | 0.449 | 1.365 | 1.31E-01 | 1.31E+01 | | | |
| 8082911 | NM_006153 | NCK1 | NCK adaptor protein 1 | 0.449 | 1.365 | 2.21E-01 | 2.21E+01 | | | |
| 8039796 | NM_014453 | CHMP2A | chromatin modifying protein 2A | 0.449 | 1.365 | 2.18E-02 | 2.18E+00 | Up | | |
| 8081710 | NM_017699 | SIDT1 | SID1 transmembrane family, membe | 0.449 | 1.365 | 5.81E-02 | 5.81E+00 | | | |
| 8096917 | --- | --- | --- | 0.448 | 1.365 | 4.67E-01 | 4.67E+01 | | | |
| 8095802 | NM_018115 | SDAD1 | SDA1 domain containing 1 | 0.448 | 1.364 | 6.02E-01 | 6.02E+01 | | | |
| 8115147 | NM_00102515 | CD74 | CD74 molecule, major histocompatib | 0.448 | 1.364 | 4.90E-04 | 4.90E-02 | Up | | |
| 8102912 | NM_015130 | TBC1D9 | TBC1 domain family, member 9 (with | 0.448 | 1.364 | 1.97E-02 | 1.97E+00 | Up | | |
| 8126259 | AJ420538 | C6orf130 | chromosome 6 open reading frame 1 | 0.448 | 1.364 | 1.55E-01 | 1.55E+01 | | | |
| 8117653 | --- | --- | --- | 0.448 | 1.364 | 3.46E-01 | 3.46E+01 | | | |
| 7913571 | NM_00110239 | HNRNPR | heterogeneous nuclear ribonucleopr | 0.448 | 1.364 | 1.64E-01 | 1.64E+01 | | | |
| 7909400 | NM_002389 | CD46 | CD46 molecule, complement regulat | 0.448 | 1.364 | 1.84E-02 | 1.84E+00 | Up | | |
| 7894878 | --- | --- | --- | 0.447 | 1.364 | 1.57E-01 | 1.57E+01 | | | |

| | | | | | | | | | | |
|---|---|---|---|---|---|---|---|---|---|---|
| 8088151 | NM_022899 | ACTR8 | ARP8 actin-related protein 8 homolo | 0.447 | 1.364 | 1.18E-01 | 1.18E+01 | | | |
| 8092239 | --- | --- | --- | 0.447 | 1.363 | 6.69E-01 | 6.69E+01 | | | |
| 8086953 | NM_016479 | SHISA5 | shisa homolog 5 (Xenopus laevis) | 0.447 | 1.363 | 9.39E-03 | 9.39E-01 | Up | | |
| 7997230 | NM_002811 | PSMD7 | proteasome (prosome, macropain) 2 | 0.447 | 1.363 | 4.00E-01 | 4.00E+01 | | | |
| 8044127 | NM_024093 | C2orf49 | chromosome 2 open reading frame 4 | 0.447 | 1.363 | 3.99E-01 | 3.99E+01 | | | |
| 8098150 | NM_018352 | C4orf43 | chromosome 4 open reading frame 4 | 0.447 | 1.363 | 1.72E-01 | 1.72E+01 | | | |
| 8009382 | NM_004459 | BPTF | bromodomain PHD finger transcripti | 0.446 | 1.363 | 9.74E-03 | 9.74E-01 | Up | | |
| 7921298 | NM_030764 | FCRL2 | Fc receptor-like 2 | 0.446 | 1.363 | 4.74E-03 | 4.74E-01 | Up | | |
| 8077929 | --- | --- | --- | 0.446 | 1.363 | 1.25E-01 | 1.25E+01 | | | |
| 8009457 | NM_212472 | PRKAR1A | protein kinase, cAMP-dependent, re | 0.446 | 1.362 | 2.58E-02 | 2.58E+00 | Up | | |
| 8040142 | NM_016207 | CPSF3 | cleavage and polyadenylation specifi | 0.445 | 1.362 | 9.51E-02 | 9.51E+00 | | | |
| 7919133 | NM_00101798 | FCGR1B | Fc fragment of IgG, high affinity Ib, re | 0.445 | 1.362 | 1.24E-01 | 1.24E+01 | | | |
| 7956670 | NM_006313 | USP15 | ubiquitin specific peptidase 15 | 0.445 | 1.362 | 9.07E-02 | 9.07E+00 | | | |
| 8157270 | NM_001859 | SLC31A1 | solute carrier family 31 (copper trans | 0.445 | 1.361 | 1.51E-01 | 1.51E+01 | | | |
| 7914218 | NM_00113521 | TAF12 | TAF12 RNA polymerase II, TATA box | 0.445 | 1.361 | 7.72E-02 | 7.72E+00 | | | |
| 8027439 | NM_00113615 | ZNF507 | zinc finger protein 507 | 0.445 | 1.361 | 1.19E-01 | 1.19E+01 | | | |
| 7926565 | NM_004641 | MLLT10 | myeloid/lymphoid or mixed-lineage | 0.445 | 1.361 | 3.78E-02 | 3.78E+00 | Up | | |
| 7915485 | NM_006824 | EBNA1BP2 | EBNA1 binding protein 2 | 0.444 | 1.361 | 5.74E-02 | 5.74E+00 | | | |
| 7895592 | --- | --- | --- | 0.444 | 1.361 | 5.32E-01 | 5.32E+01 | | | |
| 7893823 | --- | --- | --- | 0.444 | 1.361 | 6.79E-01 | 6.79E+01 | | | |
| 8083282 | NM_032383 | HPS3 | Hermansky-Pudlak syndrome 3 | 0.444 | 1.361 | 1.60E-01 | 1.60E+01 | | | |
| 8045846 | --- | --- | --- | 0.444 | 1.361 | 1.42E-01 | 1.42E+01 | | | |
| 8040338 | --- | --- | --- | 0.444 | 1.360 | 5.63E-02 | 5.63E+00 | | | |
| 7991587 | NM_203472 | SELS | selenoprotein S | 0.444 | 1.360 | 3.17E-02 | 3.17E+00 | Up | | |
| 7895945 | --- | --- | --- | 0.444 | 1.360 | 1.77E-02 | 1.77E+00 | Up | | |
| 8142770 | ENST0000042 | MGC27345 | hypothetical protein MGC27345 | 0.443 | 1.359 | 7.12E-02 | 7.12E+00 | | | |
| 8133610 | NM_00101373 | STAG3L3 | stromal antigen 3-like 3 | 0.443 | 1.359 | 7.82E-02 | 7.82E+00 | | | |
| 8074237 | NM_017424 | CECR1 | cat eye syndrome chromosome regio | 0.443 | 1.359 | 7.33E-03 | 7.33E-01 | Up | | |
| 7895578 | --- | --- | --- | 0.442 | 1.359 | 5.72E-01 | 5.72E+01 | | | |
| 8112491 | AK289851 | SMA5 | glucuronidase, beta pseudogene | 0.442 | 1.358 | 8.30E-02 | 8.30E+00 | | | |
| 8048234 | NM_152862 | ARPC2 | actin related protein 2/3 complex, su | 0.442 | 1.358 | 1.05E-04 | 1.05E-02 | Up | | |
| 7894877 | --- | --- | --- | 0.442 | 1.358 | 1.92E-01 | 1.92E+01 | | | |
| 8161906 | NM_002072 | GNAQ | guanine nucleotide binding protein ( | 0.442 | 1.358 | 2.04E-01 | 2.04E+01 | | | |
| 8180289 | --- | --- | --- | 0.441 | 1.358 | 1.34E-02 | 1.34E+00 | Up | | |
| 7892701 | --- | --- | --- | 0.441 | 1.358 | 1.85E-01 | 1.85E+01 | | | |
| 8104760 | NM_152295 | TARS | threonyl-tRNA synthetase | 0.441 | 1.357 | 7.35E-02 | 7.35E+00 | | | |
| 8180199 | --- | --- | --- | 0.441 | 1.357 | 1.09E-02 | 1.09E+00 | Up | | |
| 7895062 | --- | --- | --- | 0.441 | 1.357 | 2.49E-01 | 2.49E+01 | | | |
| 8038343 | --- | --- | --- | 0.441 | 1.357 | 4.30E-02 | 4.30E+00 | Up | | |
| 8054614 | NM_022662 | ANAPC1 | anaphase promoting complex subun | 0.440 | 1.357 | 1.94E-02 | 1.94E+00 | Up | | |
| 8079950 | NM_002070 | GNAI2 | guanine nucleotide binding protein ( | 0.440 | 1.357 | 5.31E-03 | 5.31E-01 | Up | | |
| 8171802 | NM_00103717 | ACOT9 | acyl-CoA thioesterase 9 | 0.440 | 1.357 | 4.67E-02 | 4.67E+00 | Up | | |
| 8180356 | --- | --- | --- | 0.440 | 1.357 | 3.64E-01 | 3.64E+01 | | | |
| 8017702 | --- | --- | --- | 0.440 | 1.357 | 9.62E-02 | 9.62E+00 | | | |
| 8095005 | --- | --- | --- | 0.440 | 1.357 | 2.70E-01 | 2.70E+01 | | | |
| 8099410 | NM_148894 | BOD1L | biorientation of chromosomes in cell | 0.440 | 1.356 | 6.23E-02 | 6.23E+00 | | | |
| 8159815 | NM_00114535 | CBWD1 | COBW domain containing 1 | 0.440 | 1.356 | 7.62E-02 | 7.62E+00 | | | |
| 8150797 | NM_015941 | ATP6V1H | ATPase, H+ transporting, lysosomal 5 | 0.440 | 1.356 | 7.13E-02 | 7.13E+00 | | | |
| 7905060 | NM_000566 | FCGR1A | Fc fragment of IgG, high affinity Ia, re | 0.440 | 1.356 | 1.22E-01 | 1.22E+01 | | Phagocytosis/killing | |
| 7957008 | NM_007007 | CPSF6 | cleavage and polyadenylation specifi | 0.439 | 1.356 | 1.16E-01 | 1.16E+01 | | | |
| 8022251 | NM_00104238 | PPP4R1 | protein phosphatase 4, regulatory su | 0.439 | 1.356 | 5.52E-02 | 5.52E+00 | | | |
| 7911335 | --- | --- | --- | 0.439 | 1.356 | 4.86E-01 | 4.86E+01 | | | |
| 8165694 | --- | --- | --- | 0.439 | 1.356 | 4.86E-01 | 4.86E+01 | | | |
| 8134814 | NM_013439 | PILRA | paired immunoglobin-like type 2 rec | 0.439 | 1.356 | 2.85E-02 | 2.85E+00 | Up | | mono |
| 7904974 | NR_003242 | C1orf152 | profilin 1 pseudogene | 0.439 | 1.356 | 6.91E-02 | 6.91E+00 | | | |
| 7896718 | --- | --- | --- | 0.439 | 1.356 | 2.08E-01 | 2.08E+01 | | | |
| 7922870 | NM_030934 | C1orf25 | chromosome 1 open reading frame 2 | 0.439 | 1.355 | 4.51E-02 | 4.51E+00 | Up | | |
| 8180194 | --- | --- | --- | 0.439 | 1.355 | 7.21E-03 | 7.21E-01 | Up | | |
| 8180288 | --- | --- | --- | 0.439 | 1.355 | 7.21E-03 | 7.21E-01 | Up | | |
| 8180293 | --- | --- | --- | 0.439 | 1.355 | 7.21E-03 | 7.21E-01 | Up | | |
| 7896197 | --- | --- | --- | 0.438 | 1.355 | 4.06E-01 | 4.06E+01 | | | |
| 8061114 | NM_00101154 | DSTN | destrin (actin depolymerizing factor) | 0.438 | 1.355 | 1.30E-01 | 1.30E+01 | | | |
| 8124280 | NM_014722 | FAM65B | family with sequence similarity 65, m | 0.438 | 1.355 | 1.63E-02 | 1.63E+00 | Up | | |
| 8159854 | NM_014878 | KIAA0020 | KIAA0020 | 0.438 | 1.354 | 9.66E-02 | 9.66E+00 | | | |
| 8067295 | NM_016045 | SLMO2 | slowmo homolog 2 (Drosophila) | 0.438 | 1.354 | 4.97E-02 | 4.97E+00 | Up | | |
| 8090133 | NM_022757 | CCDC14 | coiled-coil domain containing 14 | 0.438 | 1.354 | 3.98E-02 | 3.98E+00 | Up | | |
| 8081740 | NM_001690 | ATP6V1A | ATPase, H+ transporting, lysosomal 7 | 0.438 | 1.354 | 1.22E-01 | 1.22E+01 | | | |
| 7894009 | --- | --- | --- | 0.437 | 1.354 | 5.02E-01 | 5.02E+01 | | | |
| 8046695 | NM_000885 | ITGA4 | integrin, alpha 4 (antigen CD49D, alp | 0.437 | 1.354 | 7.26E-03 | 7.26E-01 | Up | | |
| 7895890 | --- | --- | --- | 0.437 | 1.354 | 2.80E-01 | 2.80E+01 | | | |
| 8135688 | NM_016200 | LSM8 | LSM8 homolog, U6 small nuclear RN | 0.437 | 1.354 | 1.27E-01 | 1.27E+01 | | | |
| 7894430 | --- | --- | --- | 0.437 | 1.354 | 3.41E-01 | 3.41E+01 | | | |
| 7923712 | NM_005057 | RBBP5 | retinoblastoma binding protein 5 | 0.437 | 1.354 | 7.02E-02 | 7.02E+00 | | | |
| 8119067 | NM_173562 | KCTD20 | potassium channel tetramerisation d | 0.437 | 1.353 | 9.28E-02 | 9.28E+00 | | | |
| 7893098 | --- | --- | --- | 0.436 | 1.353 | 4.19E-01 | 4.19E+01 | | | |
| 8016891 | NM_007146 | VEZF1 | vascular endothelial zinc finger 1 | 0.436 | 1.353 | 2.13E-02 | 2.13E+00 | Up | | |
| 8059413 | NM_014689 | DOCK10 | dedicator of cytokinesis 10 | 0.436 | 1.353 | 7.71E-02 | 7.71E+00 | | | |

| | | | | | | | | | | |
|---|---|---|---|---|---|---|---|---|---|---|
| 7958273 | --- | --- | --- | 0.436 | 1.353 | 4.27E-01 | 4.27E+01 | | | |
| 7917532 | NM_004120 | GBP2 | guanylate binding protein 2, interfer | 0.436 | 1.353 | 3.95E-02 | 3.95E+00 | Up | | |
| 8068583 | NM_002243 | KCNJ15 | potassium inwardly-rectifying chann | 0.436 | 1.353 | 6.27E-02 | 6.27E+00 | | | |
| 8138401 | NM_015132 | SNX13 | sorting nexin 13 | 0.436 | 1.353 | 3.88E-02 | 3.88E+00 | Up | | |
| 8020630 | NM_153211 | TTC39C | tetratricopeptide repeat domain 39C | 0.436 | 1.353 | 2.76E-01 | 2.76E+01 | | | |
| 7894642 | --- | --- | --- | 0.436 | 1.353 | 4.68E-01 | 4.68E+01 | | | |
| 7962112 | NM_00100225 | CAPRIN2 | caprin family member 2 | 0.436 | 1.353 | 7.09E-02 | 7.09E+00 | | | |
| 7895141 | --- | --- | --- | 0.435 | 1.352 | 3.53E-01 | 3.53E+01 | | | |
| 7950248 | NM_014824 | FCHSD2 | FCH and double SH3 domains 2 | 0.435 | 1.352 | 1.83E-01 | 1.83E+01 | | | |
| 7979802 | --- | --- | --- | 0.435 | 1.352 | 3.23E-01 | 3.23E+01 | | | |
| 8160718 | NR_023917 | PTENP1 | phosphatase and tensin homolog ps | 0.435 | 1.352 | 2.78E-02 | 2.78E+00 | Up | | |
| 8178991 | NM_00114196 | DAXX | death-domain associated protein | 0.435 | 1.352 | 1.04E-02 | 1.04E+00 | Up | | |
| 7895349 | --- | --- | --- | 0.435 | 1.352 | 6.39E-01 | 6.39E+01 | | | |
| 8119609 | NM_006586 | CNPY3 | canopy 3 homolog (zebrafish) | 0.435 | 1.351 | 1.16E-02 | 1.16E+00 | Up | | |
| 8042291 | NM_203437 | AFTPH | aftiphilin | 0.434 | 1.351 | 3.35E-02 | 3.35E+00 | Up | | |
| 7894081 | --- | --- | --- | 0.434 | 1.351 | 3.15E-01 | 3.15E+01 | | | |
| 7896081 | --- | --- | --- | 0.434 | 1.351 | 5.17E-01 | 5.17E+01 | | | |
| 7921970 | NM_000696 | ALDH9A1 | aldehyde dehydrogenase 9 family, m | 0.434 | 1.351 | 1.60E-02 | 1.60E+00 | Up | | |
| 8007397 | NM_176863 | PSME3 | proteasome (prosome, macropain) a | 0.434 | 1.351 | 4.38E-02 | 4.38E+00 | Up | | |
| 8102789 | NM_017489 | TERF1 | telomeric repeat binding factor (NIM | 0.433 | 1.350 | 2.53E-01 | 2.53E+01 | | | |
| 8068168 | NM_000454 | SOD1 | superoxide dismutase 1, soluble | 0.433 | 1.350 | 8.25E-02 | 8.25E+00 | | | |
| 8064218 | NM_012469 | PRPF6 | PRP6 pre-mRNA processing factor 6 | 0.433 | 1.350 | 1.15E-02 | 1.15E+00 | Up | | |
| 7893340 | --- | --- | --- | 0.433 | 1.350 | 2.03E-01 | 2.03E+01 | | | |
| 7893118 | --- | --- | --- | 0.433 | 1.350 | 1.84E-01 | 1.84E+01 | | | |
| 7900922 | NM_004047 | ATP6V0B | ATPase, H+ transporting, lysosomal 2 | 0.433 | 1.350 | 1.39E-01 | 1.39E+01 | | | |
| 8119000 | NM_001315 | MAPK14 | mitogen-activated protein kinase 14 | 0.433 | 1.350 | 3.99E-02 | 3.99E+00 | Up | | |
| 8151993 | NM_004374 | COX6C | cytochrome c oxidase subunit VIc | 0.433 | 1.350 | 1.62E-01 | 1.62E+01 | | | |
| 7895037 | --- | --- | --- | 0.433 | 1.350 | 5.67E-01 | 5.67E+01 | | | |
| 7895257 | --- | --- | --- | 0.432 | 1.350 | 2.75E-01 | 2.75E+01 | | | |
| 8104298 | NM_004553 | NDUFS6 | NADH dehydrogenase (ubiquinone) F | 0.432 | 1.349 | 2.53E-01 | 2.53E+01 | | | |
| 8092534 | NM_080652 | TMEM41A | transmembrane protein 41A | 0.432 | 1.349 | 9.93E-02 | 9.93E+00 | | | |
| 8043105 | NM_020122 | KCMF1 | potassium channel modulatory facto | 0.432 | 1.349 | 3.19E-01 | 3.19E+01 | | | |
| 8112428 | NM_005582 | CD180 | CD180 molecule | 0.432 | 1.349 | 1.23E-02 | 1.23E+00 | Up | | |
| 8138045 | NM_014413 | EIF2AK1 | eukaryotic translation initiation facto | 0.432 | 1.349 | 1.14E-01 | 1.14E+01 | | | |
| 7892610 | --- | --- | --- | 0.432 | 1.349 | 5.47E-01 | 5.47E+01 | | | |
| 8117237 | --- | --- | --- | 0.432 | 1.349 | 4.02E-02 | 4.02E+00 | Up | | |
| 7893477 | --- | --- | --- | 0.432 | 1.349 | 4.48E-01 | 4.48E+01 | | | |
| 8082229 | NM_000373 | UMPS | uridine monophosphate synthetase | 0.432 | 1.349 | 1.07E-01 | 1.07E+01 | | | |
| 8067288 | NM_00100197 | ATP5E | ATP synthase, H+ transporting, mitoc | 0.432 | 1.349 | 8.34E-02 | 8.34E+00 | | | |
| 7988921 | NM_000259 | MYO5A | myosin VA (heavy chain 12, myoxin) | 0.432 | 1.349 | 5.00E-02 | 5.00E+00 | | | |
| 8090490 | NM_002950 | RPN1 | ribophorin I | 0.432 | 1.349 | 4.39E-02 | 4.39E+00 | Up | | |
| 8031732 | NM_173631 | ZNF547 | zinc finger protein 547 | 0.432 | 1.349 | 1.14E-02 | 1.14E+00 | Up | | |
| 7957649 | NM_003095 | SNRPF | small nuclear ribonucleoprotein poly | 0.432 | 1.349 | 3.66E-01 | 3.66E+01 | | | |
| 7974603 | NM_002788 | PSMA3 | proteasome (prosome, macropain) s | 0.431 | 1.349 | 1.82E-01 | 1.82E+01 | | | |
| 8013094 | NM_003653 | COPS3 | COP9 constitutive photomorphogeni | 0.431 | 1.349 | 2.53E-01 | 2.53E+01 | | | |
| 8020267 | NM_032142 | CEP192 | centrosomal protein 192kDa | 0.431 | 1.348 | 3.98E-02 | 3.98E+00 | Up | | |
| 7937330 | NM_006435 | IFITM2 | interferon induced transmembrane p | 0.431 | 1.348 | 3.54E-02 | 3.54E+00 | Up | | |
| 7982527 | NM_014106 | ZNF770 | zinc finger protein 770 | 0.431 | 1.348 | 2.61E-01 | 2.61E+01 | | | |
| 8070701 | NM_000100 | CSTB | cystatin B (stefin B) | 0.431 | 1.348 | 2.49E-01 | 2.49E+01 | | | |
| 8133300 | NR_003614 | PMS2L2 | postmeiotic segregation increased 2- | 0.431 | 1.348 | 3.02E-01 | 3.02E+01 | | | |
| 7903908 | NM_006090 | CEPT1 | choline/ethanolamine phosphotrans | 0.431 | 1.348 | 1.29E-01 | 1.29E+01 | | | |
| 8149534 | NM_004315 | ASAH1 | N-acylsphingosine amidohydrolase (a | 0.431 | 1.348 | 8.79E-02 | 8.79E+00 | | | |
| 8078834 | NM_020839 | WDR48 | WD repeat domain 48 | 0.431 | 1.348 | 1.14E-01 | 1.14E+01 | | | |
| 8130408 | NM_00113070 | IPCEF1 | interaction protein for cytohesin exc | 0.431 | 1.348 | 8.47E-02 | 8.47E+00 | | | |
| 8104570 | NM_019018 | FAM105A | family with sequence similarity 105, | 0.430 | 1.348 | 2.42E-01 | 2.42E+01 | | | |
| 8121734 | NM_014034 | ASF1A | ASF1 anti-silencing function 1 homol | 0.430 | 1.348 | 4.68E-01 | 4.68E+01 | | | |
| 8163948 | NR_027125 | RBM18 | RNA binding motif protein 18 | 0.430 | 1.347 | 1.69E-02 | 1.69E+00 | Up | | |
| 7948997 | NM_015459 | ATL3 | atlastin GTPase 3 | 0.430 | 1.347 | 2.82E-02 | 2.82E+00 | Up | | |
| 8105633 | NM_015342 | PPWD1 | peptidylprolyl isomerase domain and | 0.430 | 1.347 | 2.44E-01 | 2.44E+01 | | | |
| 8096361 | NM_016323 | HERC5 | hect domain and RLD 5 | 0.430 | 1.347 | 2.44E-01 | 2.44E+01 | | | |
| 8109475 | NM_014180 | MRPL22 | mitochondrial ribosomal protein L22 | 0.430 | 1.347 | 2.49E-01 | 2.49E+01 | | | |
| 7903414 | NM_000699 | AMY2A | amylase, alpha 2A (pancreatic) | 0.430 | 1.347 | 3.38E-01 | 3.38E+01 | | | |
| 7896163 | --- | --- | --- | 0.429 | 1.347 | 5.17E-01 | 5.17E+01 | | | |
| 7944006 | NM_016090 | RBM7 | RNA binding motif protein 7 | 0.429 | 1.347 | 2.67E-01 | 2.67E+01 | | | |
| 7894895 | --- | --- | --- | 0.429 | 1.347 | 2.95E-01 | 2.95E+01 | | | |
| 7935270 | NM_013314 | BLNK | B-cell linker | 0.429 | 1.347 | 6.93E-02 | 6.93E+00 | | | |
| 8022488 | NM_138340 | ABHD3 | abhydrolase domain containing 3 | 0.429 | 1.347 | 1.55E-01 | 1.55E+01 | | | |
| 7963244 | NM_005653 | TFCP2 | transcription factor CP2 | 0.429 | 1.346 | 2.43E-01 | 2.43E+01 | | | |
| 8180396 | --- | --- | --- | 0.429 | 1.346 | 3.69E-01 | 3.69E+01 | | | |
| 8180292 | --- | --- | --- | 0.429 | 1.346 | 6.32E-03 | 6.32E-01 | Up | | |
| 8042161 | NM_002618 | PEX13 | peroxisomal biogenesis factor 13 | 0.429 | 1.346 | 2.01E-01 | 2.01E+01 | | | |
| 7967072 | NM_032314 | COQ5 | coenzyme Q5 homolog, methyltrans | 0.429 | 1.346 | 1.54E-01 | 1.54E+01 | | | |
| 7969129 | NM_00104044 | PHF11 | PHD finger protein 11 | 0.429 | 1.346 | 2.37E-01 | 2.37E+01 | | | |
| 7950654 | NM_024079 | ALG8 | asparagine-linked glycosylation 8, alp | 0.429 | 1.346 | 1.68E-01 | 1.68E+01 | | | |
| 7934451 | NM_00114400 | AGAP5 | ArfGAP with GTPase domain, ankyrin | 0.428 | 1.346 | 1.63E-02 | 1.63E+00 | Up | | |
| 8150565 | NM_00116022 | RNF170 | ring finger protein 170 | 0.428 | 1.345 | 1.64E-01 | 1.64E+01 | | | |
| 8050908 | NM_000182 | HADHA | hydroxyacyl-Coenzyme A dehydroge | 0.428 | 1.345 | 4.48E-02 | 4.48E+00 | Up | | |

| | | | | | | | | | | |
|---|---|---|---|---|---|---|---|---|---|---|
| 7938293 | NR_002977 | SNORA45 | small nucleolar RNA, H/ACA box 45 | 0.428 | 1.345 | 5.83E-01 | 5.83E+01 | | | |
| 8139879 | --- | --- | --- | 0.428 | 1.345 | 2.53E-01 | 2.53E+01 | | | |
| 8006170 | NR_015341 | LRRC37B2 | leucine rich repeat containing 37, me | 0.427 | 1.345 | 5.89E-02 | 5.89E+00 | | | |
| 8058161 | NM_006190 | ORC2L | origin recognition complex, subunit 2 | 0.427 | 1.345 | 2.34E-02 | 2.34E+00 | Up | | |
| 7900235 | NM_012090 | MACF1 | microtubule-actin crosslinking factor | 0.427 | 1.345 | 3.97E-03 | 3.97E-01 | Up | | |
| 7968915 | NM_004128 | GTF2F2 | general transcription factor IIF, polyp | 0.427 | 1.345 | 1.89E-01 | 1.89E+01 | | | |
| 8112081 | NM_019030 | DHX29 | DEAH (Asp-Glu-Ala-His) box polypept | 0.427 | 1.345 | 1.58E-01 | 1.58E+01 | | | |
| 8101648 | NM_016245 | HSD17B11 | hydroxysteroid (17-beta) dehydroge | 0.427 | 1.345 | 3.54E-01 | 3.54E+01 | | | |
| 7894362 | --- | --- | --- | 0.427 | 1.344 | 6.34E-01 | 6.34E+01 | | | |
| 7983502 | NM_012388 | PLDN | pallidin homolog (mouse) | 0.427 | 1.344 | 1.26E-01 | 1.26E+01 | | | |
| 7913300 | NM_016287 | HP1BP3 | heterochromatin protein 1, binding p | 0.427 | 1.344 | 4.73E-02 | 4.73E+00 | Up | | |
| 8081612 | NM_018394 | ABHD10 | abhydrolase domain containing 10 | 0.427 | 1.344 | 1.18E-01 | 1.18E+01 | | | |
| 8103755 | NM_012180 | FBXO8 | F-box protein 8 | 0.426 | 1.344 | 7.80E-02 | 7.80E+00 | | | |
| 8053057 | --- | --- | --- | 0.426 | 1.344 | 2.38E-01 | 2.38E+01 | | | |
| 8146243 | NM_032410 | HOOK3 | hook homolog 3 (Drosophila) | 0.426 | 1.344 | 3.16E-01 | 3.16E+01 | | | |
| 7995096 | NM_00114580 | ITGAM | integrin, alpha M (complement comp | 0.426 | 1.344 | 3.87E-03 | 3.87E-01 | Up | | |
| 8165700 | --- | --- | --- | 0.426 | 1.343 | 3.26E-01 | 3.26E+01 | | | |
| 8136849 | NM_00114367 | GSTK1 | glutathione S-transferase kappa 1 | 0.426 | 1.343 | 1.09E-02 | 1.09E+00 | Up | | |
| 7956908 | --- | --- | --- | 0.426 | 1.343 | 1.98E-01 | 1.98E+01 | | | |
| 8113064 | NM_198273 | LYSMD3 | LysM, putative peptidoglycan-bindin | 0.425 | 1.343 | 2.77E-01 | 2.77E+01 | | | |
| 8113083 | NM_032042 | FAM172A | family with sequence similarity 172, | 0.425 | 1.343 | 2.01E-01 | 2.01E+01 | | | |
| 8162696 | NR_027302 | XPA | xeroderma pigmentosum, compleme | 0.425 | 1.343 | 6.38E-02 | 6.38E+00 | | | |
| 8160487 | NM_00103168 | PLAA | phospholipase A2-activating protein | 0.425 | 1.342 | 1.21E-01 | 1.21E+01 | | | |
| 7895769 | --- | --- | --- | 0.425 | 1.342 | 5.84E-01 | 5.84E+01 | | | |
| 8124183 | --- | --- | --- | 0.425 | 1.342 | 1.44E-01 | 1.44E+01 | | | |
| 7893834 | --- | --- | --- | 0.424 | 1.342 | 3.61E-01 | 3.61E+01 | | | |
| 8083032 | --- | --- | --- | 0.424 | 1.342 | 6.50E-02 | 6.50E+00 | | | |
| 8125919 | NM_00114577 | FKBP5 | FK506 binding protein 5 | 0.424 | 1.342 | 1.54E-01 | 1.54E+01 | | | |
| 8059776 | NM_002242 | KCNJ13 | potassium inwardly-rectifying chann | 0.424 | 1.341 | 5.08E-02 | 5.08E+00 | | | |
| 8066051 | NM_032013 | NDRG3 | NDRG family member 3 | 0.424 | 1.341 | 1.14E-01 | 1.14E+01 | | | |
| 7900051 | NM_024852 | EIF2C3 | eukaryotic translation initiation facto | 0.424 | 1.341 | 8.19E-02 | 8.19E+00 | | | |
| 7970473 | NM_022459 | XPO4 | exportin 4 | 0.423 | 1.341 | 4.71E-02 | 4.71E+00 | Up | | |
| 8053562 | NM_016079 | VPS24 | vacuolar protein sorting 24 homolog | 0.423 | 1.341 | 1.02E-01 | 1.02E+01 | | | |
| 8146216 | NM_00113569 | VDAC3 | voltage-dependent anion channel 3 | 0.423 | 1.341 | 1.86E-01 | 1.86E+01 | | | |
| 8027592 | NM_00111409 | LSM14A | LSM14A, SCD6 homolog A (S. cerevis | 0.423 | 1.341 | 8.25E-02 | 8.25E+00 | | | |
| 7974471 | --- | --- | --- | 0.423 | 1.341 | 6.26E-01 | 6.26E+01 | | | |
| 8051464 | NM_019024 | HEATR5B | HEAT repeat containing 5B | 0.423 | 1.341 | 3.35E-02 | 3.35E+00 | Up | | |
| 7967900 | NM_003453 | ZMYM2 | zinc finger, MYM-type 2 | 0.423 | 1.341 | 1.49E-01 | 1.49E+01 | | | |
| 8115606 | NM_006425 | SLU7 | SLU7 splicing factor homolog (S. cere | 0.423 | 1.341 | 2.83E-01 | 2.83E+01 | | | |
| 8104738 | NM_006713 | SUB1 | SUB1 homolog (S. cerevisiae) | 0.423 | 1.340 | 1.35E-03 | 1.35E-01 | Up | | |
| 7967084 | NM_015918 | POP5 | processing of precursor 5, ribonuclea | 0.422 | 1.340 | 2.43E-01 | 2.43E+01 | | | |
| 8021113 | NM_145055 | C18orf25 | chromosome 18 open reading frame | 0.422 | 1.340 | 4.79E-02 | 4.79E+00 | Up | | |
| 8140915 | NM_000466 | PEX1 | peroxisomal biogenesis factor 1 | 0.422 | 1.340 | 2.23E-02 | 2.23E+00 | Up | | |
| 8012581 | NM_004853 | STX8 | syntaxin 8 | 0.422 | 1.340 | 2.65E-01 | 2.65E+01 | | | |
| 8123524 | NM_018303 | EXOC2 | exocyst complex component 2 | 0.422 | 1.340 | 9.74E-02 | 9.74E+00 | | | |
| 8177137 | NM_007125 | UTY | ubiquitously transcribed tetratricope | 0.422 | 1.339 | 1.95E-01 | 1.95E+01 | | | |
| 7895480 | --- | --- | --- | 0.422 | 1.339 | 5.60E-01 | 5.60E+01 | | | |
| 8135235 | NM_004279 | PMPCB | peptidase (mitochondrial processing | 0.421 | 1.339 | 4.02E-01 | 4.02E+01 | | | |
| 7893439 | --- | --- | --- | 0.421 | 1.339 | 3.71E-01 | 3.71E+01 | | | |
| 8069450 | NM_206962 | PRMT2 | protein arginine methyltransferase 2 | 0.421 | 1.339 | 1.74E-02 | 1.74E+00 | Up | | |
| 7894598 | --- | --- | --- | 0.421 | 1.339 | 4.70E-01 | 4.70E+01 | | | |
| 7956152 | NM_006191 | PA2G4 | proliferation-associated 2G4, 38kDa | 0.421 | 1.339 | 1.95E-01 | 1.95E+01 | | | |
| 8165995 | NM_005333 | HCCS | holocytochrome c synthase (cytochro | 0.421 | 1.339 | 2.61E-02 | 2.61E+00 | Up | | |
| 8059578 | --- | --- | --- | 0.421 | 1.339 | 2.62E-01 | 2.62E+01 | | | |
| 7971373 | --- | --- | --- | 0.421 | 1.338 | 5.57E-01 | 5.57E+01 | | | |
| 8047441 | --- | --- | --- | 0.421 | 1.338 | 1.05E-01 | 1.05E+01 | | | |
| 7894242 | --- | --- | --- | 0.420 | 1.338 | 6.90E-01 | 6.90E+01 | | | |
| 7966441 | NR_015404 | C12orf47 | chromosome 12 open reading frame | 0.420 | 1.338 | 2.13E-01 | 2.13E+01 | | | |
| 8011759 | NM_005022 | PFN1 | profilin 1 | 0.420 | 1.338 | 2.58E-02 | 2.58E+00 | Up | | |
| 8112331 | NM_030940 | ISCA1 | iron-sulfur cluster assembly 1 homol | 0.420 | 1.338 | 4.60E-01 | 4.60E+01 | | | |
| 8180286 | --- | --- | --- | 0.420 | 1.338 | 2.63E-01 | 2.63E+01 | | | |
| 7924701 | NM_022735 | ACBD3 | acyl-Coenzyme A binding domain co | 0.420 | 1.338 | 2.93E-02 | 2.93E+00 | Up | | |
| 7895937 | --- | --- | --- | 0.420 | 1.338 | 4.38E-01 | 4.38E+01 | | | |
| 7986394 | NM_144598 | LRRC28 | leucine rich repeat containing 28 | 0.419 | 1.337 | 4.71E-02 | 4.71E+00 | Up | | |
| 7894883 | --- | --- | --- | 0.419 | 1.337 | 3.56E-01 | 3.56E+01 | | | |
| 8001211 | NM_030790 | ITFG1 | integrin alpha FG-GAP repeat contai | 0.419 | 1.337 | 3.64E-01 | 3.64E+01 | | | |
| 8068713 | NM_002462 | MX1 | myxovirus (influenza virus) resistanc | 0.419 | 1.337 | 1.92E-01 | 1.92E+01 | | | |
| 7921076 | NM_182679 | GPATCH4 | G patch domain containing 4 | 0.419 | 1.337 | 5.65E-02 | 5.65E+00 | | | |
| 8038962 | NM_00110265 | // ZNF836 // | zinc finger protein 836 // zinc finger | 0.419 | 1.337 | 2.77E-02 | 2.77E+00 | Up | | |
| 7904751 | BC017770 | RBM8A | RNA binding motif protein 8A | 0.418 | 1.336 | 2.86E-01 | 2.86E+01 | | | |
| 7893751 | --- | --- | --- | 0.418 | 1.336 | 6.19E-01 | 6.19E+01 | | | |
| 8180301 | --- | --- | --- | 0.418 | 1.336 | 8.56E-02 | 8.56E+00 | | | |
| 7896302 | --- | --- | --- | 0.418 | 1.336 | 2.77E-01 | 2.77E+01 | | | |
| 8048120 | NM_004044 | ATIC | 5-aminoimidazole-4-carboxamide rib | 0.418 | 1.336 | 2.19E-01 | 2.19E+01 | | | |
| 8096808 | NM_017918 | CCDC109B | coiled-coil domain containing 109B | 0.417 | 1.336 | 2.36E-01 | 2.36E+01 | | | |
| 7922912 | NM_003292 | TPR | translocated promoter region (to act | 0.417 | 1.335 | 3.39E-02 | 3.39E+00 | Up | | |
| 8035773 | NM_00109926 | ZNF506 | zinc finger protein 506 | 0.417 | 1.335 | 2.85E-01 | 2.85E+01 | | | |

| | | | | | | | | | | |
|---|---|---|---|---|---|---|---|---|---|---|
| 8158372 | NM_003011 | SET | SET nuclear oncogene | 0.417 | 1.335 | 1.02E-01 | 1.02E+01 | | | |
| 8162276 | NM_005384 | NFIL3 | nuclear factor, interleukin 3 regulate | 0.417 | 1.335 | 1.97E-01 | 1.97E+01 | | | |
| 8139977 | NM_00101373 | STAG3L3 | stromal antigen 3-like 3 | 0.417 | 1.335 | 9.61E-02 | 9.61E+00 | | | |
| 8108161 | --- | --- | --- | 0.417 | 1.335 | 3.02E-01 | 3.02E+01 | | | |
| 8124610 | NM_006510 | TRIM27 | tripartite motif-containing 27 | 0.417 | 1.335 | 5.47E-03 | 5.47E-01 | Up | | |
| 8179575 | NM_006510 | TRIM27 | tripartite motif-containing 27 | 0.417 | 1.335 | 5.47E-03 | 5.47E-01 | Up | | |
| 8068280 | NM_005534 | IFNGR2 | interferon gamma receptor 2 (interfe | 0.416 | 1.334 | 8.81E-03 | 8.81E-01 | Up | | |
| 8151074 | NM_002603 | PDE7A | phosphodiesterase 7A | 0.416 | 1.334 | 1.56E-01 | 1.56E+01 | | | |
| 8043476 | ENST00000390 | IGKC | immunoglobulin kappa constant | 0.416 | 1.334 | 1.60E-01 | 1.60E+01 | | | |
| 8022767 | NM_014939 | KIAA1012 | KIAA1012 | 0.416 | 1.334 | 7.65E-02 | 7.65E+00 | | | |
| 8001350 | NM_013263 | BRD7 | bromodomain containing 7 | 0.416 | 1.334 | 5.32E-02 | 5.32E+00 | | | |
| 8107375 | NM_022828 | YTHDC2 | YTH domain containing 2 | 0.416 | 1.334 | 1.01E-01 | 1.01E+01 | | | |
| 8123598 | NM_030666 | SERPINB1 | serpin peptidase inhibitor, clade B (o | 0.416 | 1.334 | 2.74E-01 | 2.74E+01 | | | |
| 8173673 | NM_000489 | ATRX | alpha thalassemia/mental retardatio | 0.416 | 1.334 | 4.87E-02 | 4.87E+00 | Up | | |
| 8092201 | NM_024665 | TBL1XR1 | transducin (beta)-like 1 X-linked rece | 0.416 | 1.334 | 1.99E-01 | 1.99E+01 | | | |
| 8180302 | --- | --- | --- | 0.415 | 1.334 | 8.85E-02 | 8.85E+00 | | | |
| 7927215 | NM_000698 | ALOX5 | arachidonate 5-lipoxygenase | 0.415 | 1.334 | 1.30E-02 | 1.30E+00 | Up | | |
| 7933561 | NM_003631 | PARG | poly (ADP-ribose) glycohydrolase | 0.415 | 1.334 | 1.08E-01 | 1.08E+01 | | | |
| 7893622 | --- | --- | --- | 0.415 | 1.333 | 5.43E-01 | 5.43E+01 | | | |
| 7892807 | --- | --- | --- | 0.415 | 1.333 | 3.65E-01 | 3.65E+01 | | | |
| 7895180 | --- | --- | --- | 0.415 | 1.333 | 5.47E-01 | 5.47E+01 | | | |
| 8041206 | NM_030915 | LBH | limb bud and heart development hor | 0.415 | 1.333 | 3.24E-03 | 3.24E-01 | Up | | |
| 7898574 | NM_00103236 | C1orf151 | chromosome 1 open reading frame 1 | 0.414 | 1.333 | 8.64E-02 | 8.64E+00 | | | |
| 7948399 | NM_152716 | PATL1 | protein associated with topoisomera | 0.414 | 1.333 | 4.82E-02 | 4.82E+00 | Up | | |
| 8165680 | --- | --- | --- | 0.414 | 1.333 | 5.61E-01 | 5.61E+01 | | | |
| 7968711 | NR_026745 | COG6 | component of oligomeric golgi comp | 0.414 | 1.332 | 1.09E-01 | 1.09E+01 | | | |
| 8082431 | NM_004637 | RAB7A | RAB7A, member RAS oncogene famil | 0.414 | 1.332 | 2.47E-01 | 2.47E+01 | | | |
| 7976080 | NM_003608 | GPR65 | G protein-coupled receptor 65 | 0.414 | 1.332 | 2.36E-01 | 2.36E+01 | | | |
| 7894234 | --- | --- | --- | 0.413 | 1.332 | 3.76E-01 | 3.76E+01 | | | |
| 7968835 | NM_016248 | AKAP11 | A kinase (PRKA) anchor protein 11 | 0.413 | 1.332 | 2.46E-01 | 2.46E+01 | | | |
| 8063369 | NM_018683 | RNF114 | ring finger protein 114 | 0.413 | 1.332 | 1.55E-01 | 1.55E+01 | | | |
| 8142415 | NM_152556 | C7orf60 | chromosome 7 open reading frame 6 | 0.413 | 1.332 | 3.11E-01 | 3.11E+01 | | | |
| 8107005 | NM_001750 | CAST | calpastatin | 0.413 | 1.332 | 6.87E-02 | 6.87E+00 | | | |
| 7958158 | NM_013320 | HCFC2 | host cell factor C2 | 0.413 | 1.332 | 8.27E-02 | 8.27E+00 | | | |
| 7893942 | --- | --- | --- | 0.413 | 1.331 | 5.45E-01 | 5.45E+01 | | | |
| 8049199 | NM_00110314 | GIGYF2 | GRB10 interacting GYF protein 2 | 0.413 | 1.331 | 3.52E-02 | 3.52E+00 | Up | | |
| 7895674 | --- | --- | --- | 0.413 | 1.331 | 2.39E-01 | 2.39E+01 | | | |
| 7920515 | NM_017582 | UBE2Q1 | ubiquitin-conjugating enzyme E2Q fa | 0.412 | 1.331 | 1.11E-01 | 1.11E+01 | | | |
| 8043937 | NM_017546 | C2orf29 | chromosome 2 open reading frame 2 | 0.412 | 1.331 | 1.44E-02 | 1.44E+00 | Up | | |
| 7920839 | NM_006912 | RIT1 | Ras-like without CAAX 1 | 0.412 | 1.331 | 2.64E-01 | 2.64E+01 | | | |
| 7896432 | --- | --- | --- | 0.412 | 1.331 | 2.87E-01 | 2.87E+01 | | | |
| 8169659 | NM_004541 | NDUFA1 | NADH dehydrogenase (ubiquinone) 1 | 0.412 | 1.331 | 2.61E-01 | 2.61E+01 | | | |
| 7989323 | NM_004330 | BNIP2 | BCL2/adenovirus E1B 19kDa interact | 0.412 | 1.331 | 2.93E-01 | 2.93E+01 | | | |
| 8177478 | NM_133338 | RAD17 | RAD17 homolog (S. pombe) | 0.412 | 1.331 | 2.01E-01 | 2.01E+01 | | | |
| 8113214 | NM_002064 | GLRX | glutaredoxin (thioltransferase) | 0.412 | 1.330 | 3.17E-01 | 3.17E+01 | | | |
| 8180290 | --- | --- | --- | 0.412 | 1.330 | 1.03E-02 | 1.03E+00 | Up | | |
| 8180291 | --- | --- | --- | 0.412 | 1.330 | 1.03E-02 | 1.03E+00 | Up | | |
| 8156167 | NM_024635 | MAK10 | MAK10 homolog, amino-acid N-acety | 0.412 | 1.330 | 1.23E-01 | 1.23E+01 | | | |
| 8050719 | NM_006277 | ITSN2 | intersectin 2 | 0.412 | 1.330 | 1.98E-02 | 1.98E+00 | Up | | |
| 8147101 | NM_001951 | E2F5 | E2F transcription factor 5, p130-bind | 0.412 | 1.330 | 3.20E-01 | 3.20E+01 | | | |
| 8103622 | NM_032783 | CBR4 | carbonyl reductase 4 | 0.411 | 1.330 | 3.81E-02 | 3.81E+00 | Up | | |
| 7984453 | NM_016166 | PIAS1 | protein inhibitor of activated STAT, 1 | 0.411 | 1.330 | 1.26E-01 | 1.26E+01 | | | |
| 8121927 | NM_030963 | RNF146 | ring finger protein 146 | 0.411 | 1.330 | 5.67E-02 | 5.67E+00 | | | |
| 8005029 | NM_003010 | MAP2K4 | mitogen-activated protein kinase kin | 0.411 | 1.330 | 7.76E-02 | 7.76E+00 | | | |
| 7956697 | NM_015026 | MON2 | MON2 homolog (S. cerevisiae) | 0.411 | 1.330 | 4.67E-02 | 4.67E+00 | Up | | |
| 8162294 | NM_006415 | SPTLC1 | serine palmitoyltransferase, long cha | 0.411 | 1.330 | 6.87E-02 | 6.87E+00 | | | |
| 8180239 | --- | --- | --- | 0.411 | 1.330 | 2.19E-01 | 2.19E+01 | | | |
| 7894729 | --- | --- | --- | 0.411 | 1.330 | 8.40E-02 | 8.40E+00 | | | |
| 8006183 | NR_024187 | SUZ12P | suppressor of zeste 12 homolog pseu | 0.410 | 1.329 | 1.18E-01 | 1.18E+01 | | | |
| 7928959 | NM_000314 | PTEN | phosphatase and tensin homolog | 0.410 | 1.329 | 4.77E-02 | 4.77E+00 | Up | | |
| 8014233 | NM_00110458 | SLFN11 | schlafen family member 11 | 0.410 | 1.329 | 1.18E-01 | 1.18E+01 | | | |
| 8143108 | --- | --- | --- | 0.410 | 1.329 | 2.31E-01 | 2.31E+01 | | | |
| 8051762 | NM_021097 | SLC8A1 | solute carrier family 8 (sodium/calciu | 0.410 | 1.329 | 2.29E-02 | 2.29E+00 | Up | | |
| 7995263 | AF067420 | IGHA1 | immunoglobulin heavy constant alph | 0.410 | 1.329 | 4.55E-02 | 4.55E+00 | Up | | |
| 8082827 | NM_025180 | CEP63 | centrosomal protein 63kDa | 0.410 | 1.328 | 1.01E-01 | 1.01E+01 | | | |
| 8099841 | NM_006068 | TLR6 | toll-like receptor 6 | 0.410 | 1.328 | 7.49E-02 | 7.49E+00 | | | |
| 8109062 | NM_205836 | FBXO38 | F-box protein 38 | 0.410 | 1.328 | 4.70E-02 | 4.70E+00 | Up | | |
| 8052669 | NM_014755 | SERTAD2 | SERTA domain containing 2 | 0.410 | 1.328 | 9.53E-03 | 9.53E-01 | Up | | |
| 7894374 | --- | --- | --- | 0.410 | 1.328 | 2.78E-01 | 2.78E+01 | | | |
| 7963911 | NM_001780 | CD63 | CD63 molecule | 0.410 | 1.328 | 2.13E-04 | 2.13E-02 | Up | | |
| 7893240 | --- | --- | --- | 0.409 | 1.328 | 4.84E-01 | 4.84E+01 | | | |
| 8045425 | NM_015361 | R3HDM1 | R3H domain containing 1 | 0.409 | 1.328 | 1.07E-01 | 1.07E+01 | | | |
| 7893718 | --- | --- | --- | 0.409 | 1.328 | 5.10E-01 | 5.10E+01 | | | |
| 7896594 | --- | --- | --- | 0.409 | 1.328 | 1.47E-01 | 1.47E+01 | | | |
| 8082035 | NM_175862 | CD86 | CD86 molecule | 0.409 | 1.328 | 1.07E-01 | 1.07E+01 | | | mono |
| 7893108 | --- | --- | --- | 0.409 | 1.328 | 4.72E-01 | 4.72E+01 | | | |
| 8035765 | NM_021030 | ZNF14 | zinc finger protein 14 | 0.409 | 1.327 | 2.11E-01 | 2.11E+01 | | | |

| | | | | | | | | | | |
|---|---|---|---|---|---|---|---|---|---|---|
| 8105061 | NM_001465 | FYB | FYN binding protein (FYB-120/130) | 0.408 | 1.327 | 6.92E-02 | 6.92E+00 | | | |
| 8128867 | NM_015076 | CDC2L6 | cell division cycle 2-like 6 (CDK8-like) | 0.408 | 1.327 | 8.86E-02 | 8.86E+00 | | | |
| 8052010 | NM_001743 | CALM2 | calmodulin 2 (phosphorylase kinase, | 0.408 | 1.327 | 3.49E-03 | 3.49E-01 | Up | | |
| 7895540 | --- | --- | --- | 0.408 | 1.327 | 5.82E-01 | 5.82E+01 | | | |
| 8140196 | NM_00102520 | STAG3L2 | stromal antigen 3-like 2 | 0.408 | 1.327 | 1.14E-01 | 1.14E+01 | | | |
| 8155422 | NM_201453 | CBWD3 | COBW domain containing 3 | 0.408 | 1.327 | 1.44E-01 | 1.44E+01 | | | |
| 8154153 | NM_017913 | CDC37L1 | cell division cycle 37 homolog (S. cer | 0.408 | 1.327 | 1.97E-01 | 1.97E+01 | | | |
| 7907439 | NM_004905 | PRDX6 | peroxiredoxin 6 | 0.408 | 1.327 | 3.21E-01 | 3.21E+01 | | | |
| 8094704 | NM_005339 | UBE2K | ubiquitin-conjugating enzyme E2K (U | 0.408 | 1.327 | 3.63E-01 | 3.63E+01 | | | |
| 8161575 | ENST00000377 | CBWD5 | COBW domain containing 5 | 0.408 | 1.327 | 1.82E-01 | 1.82E+01 | | | |
| 7895085 | --- | --- | --- | 0.408 | 1.327 | 4.83E-01 | 4.83E+01 | | | |
| 8108847 | NM_183401 | RNF14 | ring finger protein 14 | 0.408 | 1.327 | 2.01E-01 | 2.01E+01 | | | |
| 7894561 | --- | --- | --- | 0.408 | 1.326 | 3.14E-01 | 3.14E+01 | | | |
| 8096533 | --- | --- | --- | 0.407 | 1.326 | 6.16E-02 | 6.16E+00 | | | |
| 7992877 | NM_153028 | ZNF75A | zinc finger protein 75a | 0.407 | 1.326 | 1.24E-01 | 1.24E+01 | | | |
| 7969003 | NM_021999 | ITM2B | integral membrane protein 2B | 0.407 | 1.326 | 1.30E-02 | 1.30E+00 | Up | | |
| 7893371 | --- | --- | --- | 0.407 | 1.326 | 1.78E-01 | 1.78E+01 | | | |
| 7909815 | NM_018060 | IARS2 | isoleucyl-tRNA synthetase 2, mitocho | 0.407 | 1.326 | 2.72E-02 | 2.72E+00 | Up | | |
| 8054329 | NM_173647 | RNF149 | ring finger protein 149 | 0.407 | 1.326 | 6.16E-02 | 6.16E+00 | | | |
| 7897890 | NM_015378 | VPS13D | vacuolar protein sorting 13 homolog | 0.407 | 1.326 | 2.54E-02 | 2.54E+00 | Up | | |
| 7953385 | NM_002046 | GAPDH | glyceraldehyde-3-phosphate dehydro | 0.407 | 1.326 | 2.30E-02 | 2.30E+00 | Up | | |
| 8131927 | NM_016447 | MPP6 | membrane protein, palmitoylated 6 | 0.406 | 1.325 | 6.94E-02 | 6.94E+00 | | | |
| 7934920 | NM_00112760 | LIPA | lipase A, lysosomal acid, cholesterol | 0.406 | 1.325 | 1.04E-01 | 1.04E+01 | | | |
| 7894380 | --- | --- | --- | 0.406 | 1.325 | 3.94E-01 | 3.94E+01 | | | |
| 8083854 | NM_002740 | PRKCI | protein kinase C, iota | 0.406 | 1.325 | 8.82E-02 | 8.82E+00 | | | |
| 8030899 | NM_00101085 | ZNF766 | zinc finger protein 766 | 0.406 | 1.325 | 1.82E-01 | 1.82E+01 | | | |
| 8052382 | NM_00111463 | FANCL | Fanconi anemia, complementation g | 0.406 | 1.325 | 2.17E-01 | 2.17E+01 | | | |
| 7915640 | NM_020365 | EIF2B3 | eukaryotic translation initiation facto | 0.406 | 1.325 | 3.79E-02 | 3.79E+00 | Up | | |
| 8145636 | NM_024567 | HMBOX1 | homeobox containing 1 | 0.406 | 1.325 | 2.58E-01 | 2.58E+01 | | | |
| 7959282 | NM_194271 | RNF34 | ring finger protein 34 | 0.406 | 1.325 | 1.55E-01 | 1.55E+01 | | | |
| 7894964 | --- | --- | --- | 0.406 | 1.325 | 1.55E-01 | 1.55E+01 | | | |
| 8043349 | NM_022662 | ANAPC1 | anaphase promoting complex subun | 0.405 | 1.324 | 1.92E-01 | 1.92E+01 | | | |
| 7971222 | NM_004294 | MTRF1 | mitochondrial translational release f | 0.405 | 1.324 | 1.02E-01 | 1.02E+01 | | | |
| 8072407 | NR_002323 | TUG1 | taurine upregulated 1 (non-protein c | 0.405 | 1.324 | 1.85E-01 | 1.85E+01 | | | |
| 8146517 | NM_00101166 | CHCHD7 | coiled-coil-helix-coiled-coil-helix dom | 0.405 | 1.324 | 3.39E-01 | 3.39E+01 | | | |
| 7955376 | NM_173602 | DIP2B | DIP2 disco-interacting protein 2 hom | 0.405 | 1.324 | 5.87E-03 | 5.87E-01 | Up | | |
| 7927267 | NM_019054 | FAM35A | family with sequence similarity 35, m | 0.405 | 1.324 | 4.18E-02 | 4.18E+00 | Up | | |
| 7892909 | --- | --- | --- | 0.405 | 1.324 | 7.02E-01 | 7.02E+01 | | | |
| 8025429 | NM_005968 | HNRNPM | heterogeneous nuclear ribonucleopr | 0.404 | 1.324 | 3.79E-02 | 3.79E+00 | Up | | |
| 8011713 | NM_022059 | CXCL16 | chemokine (C-X-C motif) ligand 16 | 0.404 | 1.324 | 7.91E-02 | 7.91E+00 | | | |
| 7895772 | --- | --- | --- | 0.404 | 1.323 | 6.52E-01 | 6.52E+01 | | | |
| 8023382 | NM_003927 | MBD2 | methyl-CpG binding domain protein | 0.404 | 1.323 | 4.87E-02 | 4.87E+00 | Up | | |
| 7979196 | NM_198066 | GNPNAT1 | glucosamine-phosphate N-acetyltrar | 0.404 | 1.323 | 1.04E-02 | 1.04E+00 | Up | | |
| 7896169 | --- | --- | --- | 0.404 | 1.323 | 3.97E-01 | 3.97E+01 | | | |
| 7948814 | NM_00107955 | HNRNPUL2 | heterogeneous nuclear ribonucleopr | 0.404 | 1.323 | 4.51E-02 | 4.51E+00 | Up | | |
| 8169683 | NM_006777 | ZBTB33 | zinc finger and BTB domain containir | 0.403 | 1.323 | 1.69E-01 | 1.69E+01 | | | |
| 7960896 | NM_00104007 | LOC650293 | seven transmembrane helix receptor | 0.403 | 1.322 | 1.60E-01 | 1.60E+01 | | | |
| 8053690 | BC032451 | IGK@ | immunoglobulin kappa locus | 0.403 | 1.322 | 1.26E-03 | 1.26E-01 | Up | | |
| 7954752 | NM_012062 | DNM1L | dynamin 1-like | 0.403 | 1.322 | 1.37E-01 | 1.37E+01 | | | |
| 8113491 | NM_139164 | STARD4 | StAR-related lipid transfer (START) d | 0.403 | 1.322 | 6.46E-02 | 6.46E+00 | | | |
| 8180418 | --- | --- | --- | 0.403 | 1.322 | 2.85E-01 | 2.85E+01 | | | |
| 7894977 | --- | --- | --- | 0.403 | 1.322 | 5.33E-01 | 5.33E+01 | | | |
| 8135162 | NM_024653 | PRKRIP1 | PRKR interacting protein 1 (IL11 indu | 0.403 | 1.322 | 4.01E-02 | 4.01E+00 | Up | | |
| 7892846 | --- | --- | --- | 0.402 | 1.322 | 1.04E-01 | 1.04E+01 | | | |
| 7918550 | --- | --- | --- | 0.402 | 1.322 | 5.20E-01 | 5.20E+01 | | | |
| 7893034 | --- | --- | --- | 0.402 | 1.321 | 2.58E-01 | 2.58E+01 | | | |
| 7896289 | --- | --- | --- | 0.402 | 1.321 | 4.20E-01 | 4.20E+01 | | | |
| 8028248 | NM_144694 | ZNF570 | zinc finger protein 570 | 0.402 | 1.321 | 5.83E-02 | 5.83E+00 | | | |
| 8101086 | NM_014435 | NAAA | N-acylethanolamine acid amidase | 0.402 | 1.321 | 3.15E-02 | 3.15E+00 | Up | | |
| 8067382 | NM_002792 | PSMA7 | proteasome (prosome, macropain) s | 0.402 | 1.321 | 9.03E-02 | 9.03E+00 | | | |
| 7894212 | --- | --- | --- | 0.401 | 1.321 | 5.24E-01 | 5.24E+01 | | | |
| 8020825 | --- | --- | --- | 0.401 | 1.321 | 5.85E-01 | 5.85E+01 | | | |
| 8101828 | NM_005723 | TSPAN5 | tetraspanin 5 | 0.401 | 1.321 | 1.47E-01 | 1.47E+01 | | | |
| 8136163 | NM_014997 | KLHDC10 | kelch domain containing 10 | 0.401 | 1.321 | 3.48E-01 | 3.48E+01 | | | |
| 8117476 | NM_006994 | BTN3A3 | butyrophilin, subfamily 3, member A | 0.401 | 1.320 | 2.02E-01 | 2.02E+01 | | | |
| 8160914 | NM_007126 | VCP | valosin-containing protein | 0.401 | 1.320 | 5.27E-02 | 5.27E+00 | | | |
| 7894285 | --- | --- | --- | 0.401 | 1.320 | 4.02E-01 | 4.02E+01 | | | |
| 8077652 | NM_005718 | ARPC4 | actin related protein 2/3 complex, su | 0.400 | 1.320 | 8.95E-02 | 8.95E+00 | | | |
| 8094533 | AK304357 | FLJ16686 | FLJ16686 protein | 0.400 | 1.320 | 1.85E-01 | 1.85E+01 | | | |
| 8127743 | NM_004242 | HMGN3 | high mobility group nucleosomal bin | 0.400 | 1.320 | 1.09E-01 | 1.09E+01 | | | |
| 8180295 | --- | --- | --- | 0.400 | 1.320 | 7.69E-03 | 7.69E-01 | Up | | |
| 8142098 | NM_020725 | ATXN7L1 | ataxin 7-like 1 | 0.400 | 1.320 | 5.73E-01 | 5.73E+01 | | | |
| 8085914 | NM_003615 | SLC4A7 | solute carrier family 4, sodium bicarb | 0.400 | 1.319 | 1.17E-01 | 1.17E+01 | | | |
| 8045381 | NM_058241 | CCNT2 | cyclin T2 | 0.400 | 1.319 | 9.12E-02 | 9.12E+00 | | | |
| 7933427 | NM_00114400 | AGAP5 | ArfGAP with GTPase domain, ankyrir | 0.400 | 1.319 | 2.54E-02 | 2.54E+00 | Up | | |
| 7912852 | NM_001412 | EIF1AX | eukaryotic translation initiation facto | 0.400 | 1.319 | 1.72E-01 | 1.72E+01 | | | |
| 7997164 | --- | --- | --- | 0.400 | 1.319 | 3.39E-01 | 3.39E+01 | | | |

| | | | | | | | | | | | |
|---|---|---|---|---|---|---|---|---|---|---|---|
| 7899604 | NM_016505 | ZCCHC17 | zinc finger, CCHC domain containing | 0.400 | 1.319 | 3.20E-01 | 3.20E+01 | | | |
| 7979044 | NM_020921 | NIN | ninein (GSK3B interacting protein) | 0.400 | 1.319 | 8.96E-03 | 8.96E-01 | Up | | |
| 7892729 | --- | --- | --- | 0.399 | 1.319 | 6.36E-01 | 6.36E+01 | | | |
| 7893514 | --- | --- | --- | 0.399 | 1.319 | 4.71E-01 | 4.71E+01 | | | |
| 7988970 | NM_019600 | KIAA1370 | KIAA1370 | 0.399 | 1.319 | 1.81E-01 | 1.81E+01 | | | |
| 7894694 | --- | --- | --- | 0.399 | 1.318 | 5.60E-01 | 5.60E+01 | | | |
| 7981722 | AK128476 | IGHA1 | immunoglobulin heavy constant alph | 0.399 | 1.318 | 5.54E-02 | 5.54E+00 | | | |
| 8180296 | --- | --- | --- | 0.399 | 1.318 | 6.63E-03 | 6.63E-01 | Up | | |
| 7895808 | --- | --- | --- | 0.398 | 1.318 | 1.15E-01 | 1.15E+01 | | | |
| 8038919 | NM_021632 | ZNF350 | zinc finger protein 350 | 0.398 | 1.318 | 3.15E-01 | 3.15E+01 | | | |
| 7896438 | --- | --- | --- | 0.398 | 1.318 | 7.57E-04 | 7.57E-02 | Up | | |
| 8105191 | NM_024615 | PARP8 | poly (ADP-ribose) polymerase family | 0.398 | 1.318 | 1.51E-01 | 1.51E+01 | | | |
| 8090639 | NM_014602 | PIK3R4 | phosphoinositide-3-kinase, regulator | 0.398 | 1.318 | 9.25E-03 | 9.25E-01 | Up | | |
| 7995994 | NM_133368 | RSPRY1 | ring finger and SPRY domain contain | 0.398 | 1.318 | 1.49E-02 | 1.49E+00 | Up | | |
| 8001185 | NM_005880 | DNAJA2 | DnaJ (Hsp40) homolog, subfamily A, | 0.398 | 1.318 | 1.56E-01 | 1.56E+01 | | | |
| 8171001 | NM_023934 | FUNDC2 | FUN14 domain containing 2 | 0.398 | 1.318 | 1.14E-01 | 1.14E+01 | | | |
| 7903119 | NM_002858 | ABCD3 | ATP-binding cassette, sub-family D (A | 0.398 | 1.318 | 3.60E-01 | 3.60E+01 | | | |
| 7896395 | --- | --- | --- | 0.398 | 1.318 | 4.24E-01 | 4.24E+01 | | | |
| 7930614 | NM_198514 | NHLRC2 | NHL repeat containing 2 | 0.398 | 1.317 | 1.80E-02 | 1.80E+00 | Up | | |
| 8111892 | NM_000436 | OXCT1 | 3-oxoacid CoA transferase 1 | 0.398 | 1.317 | 1.05E-01 | 1.05E+01 | | | |
| 7895198 | --- | --- | --- | 0.398 | 1.317 | 4.88E-01 | 4.88E+01 | | | |
| 7935746 | NM_00100134 | BLOC1S2 | biogenesis of lysosomal organelles co | 0.398 | 1.317 | 1.02E-01 | 1.02E+01 | | | |
| 7894226 | --- | --- | --- | 0.398 | 1.317 | 5.18E-01 | 5.18E+01 | | | |
| 7893856 | --- | --- | --- | 0.397 | 1.317 | 4.38E-01 | 4.38E+01 | | | |
| 7919825 | NM_001668 | ARNT | aryl hydrocarbon receptor nuclear tr | 0.397 | 1.317 | 4.09E-02 | 4.09E+00 | Up | | |
| 8019885 | NM_015295 | SMCHD1 | structural maintenance of chromoso | 0.397 | 1.317 | 1.74E-01 | 1.74E+01 | | | |
| 8001477 | NM_001144 | AMFR | autocrine motility factor receptor | 0.397 | 1.317 | 3.53E-02 | 3.53E+00 | Up | | |
| 7906348 | NM_001765 | CD1C | CD1c molecule | 0.397 | 1.317 | 2.07E-01 | 2.07E+01 | | | |
| 8016546 | NM_014897 | ZNF652 | zinc finger protein 652 | 0.397 | 1.317 | 1.97E-02 | 1.97E+00 | Up | | |
| 7978174 | NM_014169 | CHMP4A | chromatin modifying protein 4A | 0.397 | 1.317 | 1.17E-01 | 1.17E+01 | | | |
| 8110169 | NM_014613 | FAF2 | Fas associated factor family member | 0.397 | 1.317 | 1.79E-01 | 1.79E+01 | | | |
| 7900413 | NM_005857 | ZMPSTE24 | zinc metallopeptidase (STE24 homolo | 0.397 | 1.316 | 3.74E-01 | 3.74E+01 | | | |
| 8016285 | NM_00111373 | ARL17P1 | ADP-ribosylation factor-like 17 pseud | 0.396 | 1.316 | 4.29E-03 | 4.29E-01 | Up | | |
| 8083901 | NM_022763 | FNDC3B | fibronectin type III domain containin | 0.396 | 1.316 | 1.64E-02 | 1.64E+00 | Up | | |
| 8110318 | NM_013237 | PRELID1 | PRELI domain containing 1 | 0.396 | 1.316 | 1.72E-01 | 1.72E+01 | | | |
| 7893177 | --- | --- | --- | 0.396 | 1.316 | 5.63E-01 | 5.63E+01 | | | |
| 7956949 | NM_020401 | NUP107 | nucleoporin 107kDa | 0.396 | 1.316 | 2.20E-01 | 2.20E+01 | | | |
| 8180394 | --- | --- | --- | 0.396 | 1.316 | 4.24E-01 | 4.24E+01 | | | |
| 7894118 | --- | --- | --- | 0.396 | 1.316 | 2.08E-01 | 2.08E+01 | | | |
| 7909967 | NM_001748 | CAPN2 | calpain 2, (m/II) large subunit | 0.396 | 1.316 | 8.27E-02 | 8.27E+00 | | | |
| 7940259 | NM_021201 | MS4A7 | membrane-spanning 4-domains, sub | 0.396 | 1.315 | 1.23E-02 | 1.23E+00 | Up | | |
| 7916564 | --- | --- | --- | 0.396 | 1.315 | 1.80E-01 | 1.80E+01 | | | |
| 8136938 | --- | --- | --- | 0.395 | 1.315 | 3.11E-01 | 3.11E+01 | | | |
| 8143599 | --- | --- | --- | 0.395 | 1.315 | 3.11E-01 | 3.11E+01 | | | |
| 8068254 | NM_000628 | IL10RB | interleukin 10 receptor, beta | 0.395 | 1.315 | 1.79E-02 | 1.79E+00 | Up | | |
| 8079392 | NM_00112339 | CCR2 | chemokine (C-C motif) receptor 2 | 0.395 | 1.315 | 1.65E-02 | 1.65E+00 | Up | | |
| 7995755 | NM_018233 | OGFOD1 | 2-oxoglutarate and iron-dependent o | 0.395 | 1.315 | 1.42E-01 | 1.42E+01 | | | |
| 7912196 | --- | --- | --- | 0.395 | 1.315 | 4.75E-01 | 4.75E+01 | | | |
| 8076077 | NM_006386 | DDX17 | DEAD (Asp-Glu-Ala-Asp) box polypep | 0.395 | 1.315 | 1.52E-02 | 1.52E+00 | Up | | |
| 7895187 | --- | --- | --- | 0.395 | 1.315 | 5.26E-01 | 5.26E+01 | | | |
| 8122672 | NM_015093 | MAP3K7IP2 | mitogen-activated protein kinase kin | 0.395 | 1.315 | 1.20E-01 | 1.20E+01 | | | |
| 7962935 | NM_212461 | PRKAG1 | protein kinase, AMP-activated, gamr | 0.395 | 1.315 | 2.41E-01 | 2.41E+01 | | | |
| 8174051 | NM_000061 | BTK | Bruton agammaglobulinemia tyrosin | 0.395 | 1.315 | 5.20E-02 | 5.20E+00 | | | |
| 8180179 | NM_00114196 | DAXX | death-domain associated protein | 0.395 | 1.314 | 1.54E-02 | 1.54E+00 | Up | | |
| 8059708 | NR_002921 | SNORA75 | small nucleolar RNA, H/ACA box 75 | 0.394 | 1.314 | 4.08E-01 | 4.08E+01 | | | |
| 8054092 | NM_015348 | TMEM131 | transmembrane protein 131 | 0.394 | 1.314 | 1.86E-02 | 1.86E+00 | Up | | |
| 8050427 | NM_030797 | FAM49A | family with sequence similarity 49, m | 0.394 | 1.314 | 4.86E-02 | 4.86E+00 | Up | | |
| 7985695 | NM_006738 | AKAP13 | A kinase (PRKA) anchor protein 13 | 0.394 | 1.314 | 3.29E-03 | 3.29E-01 | Up | | |
| 7896703 | --- | --- | --- | 0.394 | 1.314 | 1.11E-01 | 1.11E+01 | | | |
| 8108134 | NM_014829 | DDX46 | DEAD (Asp-Glu-Ala-Asp) box polypep | 0.394 | 1.314 | 4.03E-02 | 4.03E+00 | Up | | |
| 7954104 | NM_018179 | ATF7IP | activating transcription factor 7 inter | 0.394 | 1.314 | 2.98E-02 | 2.98E+00 | Up | | |
| 7917120 | NM_015017 | USP33 | ubiquitin specific peptidase 33 | 0.394 | 1.314 | 8.60E-02 | 8.60E+00 | | | |
| 7895610 | --- | --- | --- | 0.394 | 1.314 | 5.54E-01 | 5.54E+01 | | | |
| 7892742 | --- | --- | --- | 0.394 | 1.314 | 5.36E-01 | 5.36E+01 | | | |
| 7968232 | NR_002574 | SNORD102 | small nucleolar RNA, C/D box 102 | 0.394 | 1.314 | 5.34E-01 | 5.34E+01 | | | |
| 8025973 | NM_144566 | ZNF700 | zinc finger protein 700 | 0.394 | 1.314 | 1.63E-02 | 1.63E+00 | Up | | |
| 8055624 | NM_014795 | ZEB2 | zinc finger E-box binding homeobox | 0.394 | 1.314 | 8.56E-02 | 8.56E+00 | | | |
| 7892983 | --- | --- | --- | 0.393 | 1.313 | 3.74E-01 | 3.74E+01 | | | |
| 8131519 | NM_014660 | PHF14 | PHD finger protein 14 | 0.393 | 1.313 | 5.01E-02 | 5.01E+00 | | | |
| 7895866 | --- | --- | --- | 0.393 | 1.313 | 3.28E-01 | 3.28E+01 | | | |
| 8146000 | NM_003816 | ADAM9 | ADAM metallopeptidase domain 9 (m | 0.393 | 1.313 | 6.68E-02 | 6.68E+00 | | | |
| 8134318 | NM_022900 | CASD1 | CAS1 domain containing 1 | 0.393 | 1.313 | 7.39E-02 | 7.39E+00 | | | |
| 8071426 | NM_004782 | SNAP29 | synaptosomal-associated protein, 29 | 0.393 | 1.313 | 3.64E-02 | 3.64E+00 | Up | | |
| 8095870 | NM_004354 | CCNG2 | cyclin G2 | 0.392 | 1.313 | 5.71E-03 | 5.71E-01 | Up | | |
| 8128429 | NM_005190 | CCNC | cyclin C | 0.392 | 1.312 | 2.22E-01 | 2.22E+01 | | | |
| 7893497 | --- | --- | --- | 0.392 | 1.312 | 6.44E-01 | 6.44E+01 | | | |
| 7990839 | NM_181900 | STARD5 | StAR-related lipid transfer (START) d | 0.392 | 1.312 | 1.58E-01 | 1.58E+01 | | | |

| | | | | | | | | | | |
|---|---|---|---|---|---|---|---|---|---|---|
| 7896394 | --- | --- | --- | 0.392 | 1.312 | 5.60E-01 | 5.60E+01 | | | |
| 8044613 | NM_00114535! | CBWD1 | COBW domain containing 1 | 0.392 | 1.312 | 1.52E-01 | 1.52E+01 | | | |
| 7988245 | NM_005926 | MFAP1 | microfibrillar-associated protein 1 | 0.392 | 1.312 | 1.78E-01 | 1.78E+01 | | | |
| 7898910 | NM_017761 | PNRC2 | proline-rich nuclear receptor coactiv | 0.391 | 1.312 | 1.74E-01 | 1.74E+01 | | | |
| 8039933 | NM_017761 | PNRC2 | proline-rich nuclear receptor coactiv | 0.391 | 1.312 | 1.74E-01 | 1.74E+01 | | | |
| 7893815 | --- | --- | --- | 0.391 | 1.312 | 4.66E-02 | 4.66E+00 | Up | | |
| 8099926 | NM_00110039 | PDS5A | PDS5, regulator of cohesion mainten | 0.391 | 1.311 | 9.53E-02 | 9.53E+00 | | | |
| 7917604 | NM_201269 | ZNF644 | zinc finger protein 644 | 0.391 | 1.311 | 2.79E-01 | 2.79E+01 | | | |
| 8010924 | NM_00112815! | VPS53 | vacuolar protein sorting 53 homolog | 0.391 | 1.311 | 1.54E-01 | 1.54E+01 | | | |
| 8006336 | NM_052888 | LRRC37B | leucine rich repeat containing 37B | 0.390 | 1.311 | 1.48E-01 | 1.48E+01 | | | |
| 8113504 | NM_004772 | C5orf13 | chromosome 5 open reading frame 1 | 0.390 | 1.311 | 1.87E-01 | 1.87E+01 | | | |
| 7893663 | --- | --- | --- | 0.390 | 1.311 | 3.90E-01 | 3.90E+01 | | | |
| 7907861 | NM_004736 | XPR1 | xenotropic and polytropic retrovirus | 0.390 | 1.311 | 8.17E-02 | 8.17E+00 | | | |
| 8180359 | --- | --- | --- | 0.390 | 1.310 | 8.84E-02 | 8.84E+00 | | | |
| 7907135 | NM_199344 | SFT2D2 | SFT2 domain containing 2 | 0.390 | 1.310 | 3.75E-01 | 3.75E+01 | | | |
| 8073062 | NM_004900 | APOBEC3B | apolipoprotein B mRNA editing enzy | 0.390 | 1.310 | 4.94E-02 | 4.94E+00 | Up | | |
| 8116859 | NM_016462 | TMEM14C | transmembrane protein 14C | 0.390 | 1.310 | 4.27E-02 | 4.27E+00 | Up | | |
| 7948364 | NM_00103939 | MPEG1 | macrophage expressed 1 | 0.390 | 1.310 | 1.79E-01 | 1.79E+01 | | | |
| 8042503 | NM_002357 | MXD1 | MAX dimerization protein 1 | 0.390 | 1.310 | 1.64E-01 | 1.64E+01 | | | |
| 8177628 | NM_176816 | CCDC125 | coiled-coil domain containing 125 | 0.389 | 1.310 | 1.45E-01 | 1.45E+01 | | | |
| 7896442 | --- | --- | --- | 0.389 | 1.310 | 4.41E-01 | 4.41E+01 | | | |
| 8117458 | NM_00114500 | BTN3A1 | butyrophilin, subfamily 3, member A | 0.389 | 1.310 | 9.10E-02 | 9.10E+00 | | | |
| 8106271 | NM_014886 | TINP1 | TGF beta-inducible nuclear protein 1 | 0.389 | 1.310 | 3.17E-01 | 3.17E+01 | | | |
| 7982957 | NM_00108054 | MGA | MAX gene associated | 0.389 | 1.310 | 1.59E-01 | 1.59E+01 | | | |
| 7954969 | NM_00111418 | IRAK4 | interleukin-1 receptor-associated kin | 0.389 | 1.309 | 2.48E-01 | 2.48E+01 | | | |
| 8020423 | NM_020774 | MIB1 | mindbomb homolog 1 (Drosophila) | 0.389 | 1.309 | 9.80E-02 | 9.80E+00 | | | |
| 7895948 | --- | --- | --- | 0.388 | 1.309 | 3.32E-01 | 3.32E+01 | | | |
| 8122182 | NM_004865 | TBPL1 | TBP-like 1 | 0.388 | 1.309 | 2.35E-01 | 2.35E+01 | | | |
| 7942544 | NM_00109863 | RNF169 | ring finger protein 169 | 0.388 | 1.309 | 4.55E-02 | 4.55E+00 | Up | | |
| 7933723 | NM_152230 | IPMK | inositol polyphosphate multikinase | 0.388 | 1.309 | 1.66E-01 | 1.66E+01 | | | |
| 8116346 | --- | --- | --- | 0.388 | 1.308 | 3.62E-01 | 3.62E+01 | | | |
| 8139232 | NM_002787 | PSMA2 | proteasome (prosome, macropain) s | 0.387 | 1.308 | 3.30E-01 | 3.30E+01 | | | |
| 8050503 | NM_00100823 | TTC32 | tetratricopeptide repeat domain 32 | 0.387 | 1.308 | 3.82E-01 | 3.82E+01 | | | |
| 8172905 | NM_004493 | HSD17B10 | hydroxysteroid (17-beta) dehydroge | 0.387 | 1.308 | 8.05E-02 | 8.05E+00 | | | |
| 7893308 | --- | --- | --- | 0.387 | 1.308 | 4.85E-01 | 4.85E+01 | | | |
| 8058147 | NM_131916 | PPIL3 | peptidylprolyl isomerase (cyclophilin | 0.387 | 1.308 | 3.54E-01 | 3.54E+01 | | | |
| 8103951 | NM_001995 | ACSL1 | acyl-CoA synthetase long-chain famil | 0.387 | 1.308 | 3.82E-01 | 3.82E+01 | | | |
| 8151788 | NM_203390 | RBM12B | RNA binding motif protein 12B | 0.387 | 1.307 | 9.74E-03 | 9.74E-01 | Up | | |
| 8155946 | NM_033305 | VPS13A | vacuolar protein sorting 13 homolog | 0.386 | 1.307 | 5.19E-02 | 5.19E+00 | | | |
| 8108015 | NM_002154 | HSPA4 | heat shock 70kDa protein 4 | 0.386 | 1.307 | 2.97E-02 | 2.97E+00 | Up | | |
| 7892682 | --- | --- | --- | 0.386 | 1.307 | 3.92E-01 | 3.92E+01 | | | |
| 8112622 | NM_032380 | GFM2 | G elongation factor, mitochondrial 2 | 0.386 | 1.307 | 1.03E-01 | 1.03E+01 | | | |
| 8144584 | --- | --- | --- | 0.386 | 1.307 | 6.07E-01 | 6.07E+01 | | | |
| 7916836 | NM_00101806 | SERBP1 | SERPINE1 mRNA binding protein 1 | 0.386 | 1.307 | 1.39E-01 | 1.39E+01 | | | |
| 7932019 | ENST00000379 | C10orf31 | chromosome 10 open reading frame | 0.386 | 1.307 | 2.33E-01 | 2.33E+01 | | | |
| 7893508 | --- | --- | --- | 0.386 | 1.307 | 3.89E-01 | 3.89E+01 | | | |
| 7942503 | NM_016147 | PPME1 | protein phosphatase methylesterase | 0.386 | 1.307 | 4.90E-02 | 4.90E+00 | Up | | |
| 7985809 | NM_007011 | ABHD2 | abhydrolase domain containing 2 | 0.386 | 1.306 | 1.57E-02 | 1.57E+00 | Up | | |
| 8130102 | NM_007044 | KATNA1 | katanin p60 (ATPase-containing) sub | 0.386 | 1.306 | 9.71E-02 | 9.71E+00 | | | |
| 8069532 | NM_006948 | HSPA13 | heat shock protein 70kDa family, me | 0.386 | 1.306 | 3.57E-01 | 3.57E+01 | | | |
| 7980189 | NM_00103947 | KIAA0317 | KIAA0317 | 0.385 | 1.306 | 5.54E-02 | 5.54E+00 | | | |
| 8155234 | NM_032226 | ZCCHC7 | zinc finger, CCHC domain containing | 0.385 | 1.306 | 1.16E-01 | 1.16E+01 | | | |
| 7938448 | NM_017944 | USP47 | ubiquitin specific peptidase 47 | 0.385 | 1.306 | 1.54E-01 | 1.54E+01 | | | |
| 8106776 | NM_001867 | COX7C | cytochrome c oxidase subunit VIIc | 0.385 | 1.306 | 1.71E-01 | 1.71E+01 | | | |
| 7928171 | NM_003901 | SGPL1 | sphingosine-1-phosphate lyase 1 | 0.385 | 1.306 | 2.34E-01 | 2.34E+01 | | | |
| 8021372 | --- | --- | --- | 0.385 | 1.306 | 3.01E-01 | 3.01E+01 | | | |
| 7896321 | --- | --- | --- | 0.385 | 1.305 | 4.04E-01 | 4.04E+01 | | | |
| 8097148 | NM_015312 | KIAA1109 | KIAA1109 | 0.384 | 1.305 | 1.40E-01 | 1.40E+01 | | | |
| 8133049 | NM_016220 | ZNF107 | zinc finger protein 107 | 0.384 | 1.305 | 1.74E-01 | 1.74E+01 | | | |
| 8102817 | NM_201999 | ELF2 | E74-like factor 2 (ets domain transcr | 0.384 | 1.305 | 1.01E-01 | 1.01E+01 | | | |
| 7972190 | NM_022118 | RBM26 | RNA binding motif protein 26 | 0.384 | 1.305 | 1.19E-01 | 1.19E+01 | | | |
| 7893822 | --- | --- | --- | 0.384 | 1.305 | 6.35E-01 | 6.35E+01 | | | |
| 7896689 | --- | --- | --- | 0.384 | 1.305 | 1.12E-02 | 1.12E+00 | Up | | |
| 8124022 | NM_183040 | DTNBP1 | dystrobrevin binding protein 1 | 0.384 | 1.305 | 1.65E-02 | 1.65E+00 | Up | | |
| 7906764 | NM_002155 | HSPA6 | heat shock 70kDa protein 6 (HSP70B | 0.384 | 1.305 | 4.84E-02 | 4.84E+00 | Up | | |
| 7895038 | --- | --- | --- | 0.383 | 1.304 | 5.68E-01 | 5.68E+01 | | | |
| 8129254 | NM_005907 | MAN1A1 | mannosidase, alpha, class 1A, memb | 0.383 | 1.304 | 4.40E-02 | 4.40E+00 | Up | | |
| 7969048 | --- | --- | --- | 0.383 | 1.304 | 1.58E-01 | 1.58E+01 | | | |
| 7935462 | NM_016046 | EXOSC1 | exosome component 1 | 0.383 | 1.304 | 2.69E-01 | 2.69E+01 | | | |
| 8142407 | NM_022484 | TMEM168 | transmembrane protein 168 | 0.383 | 1.304 | 7.13E-02 | 7.13E+00 | | | |
| 7971388 | NM_00101087 | SLC25A30 | solute carrier family 25, member 30 | 0.383 | 1.304 | 1.56E-01 | 1.56E+01 | | | |
| 7978866 | NM_004713 | SDCCAG1 | serologically defined colon cancer ar | 0.383 | 1.304 | 1.83E-01 | 1.83E+01 | | | |
| 8123802 | NM_201280 | MUTED | muted homolog (mouse) | 0.383 | 1.304 | 2.10E-01 | 2.10E+01 | | | |
| 8177560 | NM_018429 | BDP1 | B double prime 1, subunit of RNA po | 0.383 | 1.304 | 4.13E-02 | 4.13E+00 | Up | | |
| 7956819 | NM_178169 | RASSF3 | Ras association (RalGDS/AF-6) doma | 0.383 | 1.304 | 3.50E-02 | 3.50E+00 | Up | | |
| 8041257 | NM_017964 | SLC30A6 | solute carrier family 30 (zinc transpo | 0.383 | 1.304 | 1.15E-01 | 1.15E+01 | | | |
| 7967685 | NM_194356 | STX2 | syntaxin 2 | 0.383 | 1.304 | 1.27E-01 | 1.27E+01 | | | |

| | | | | | | | | | | |
|---|---|---|---|---|---|---|---|---|---|---|
| 8130436 | --- | --- | --- | 0.383 | 1.304 | 1.17E-01 | 1.17E+01 | | | |
| 7974771 | NM_022495 | C14orf135 | chromosome 14 open reading frame | 0.382 | 1.303 | 2.09E-01 | 2.09E+01 | | | |
| 8118158 | NM_004847 | AIF1 | allograft inflammatory factor 1 | 0.382 | 1.303 | 3.73E-02 | 3.73E+00 | Up | | mono |
| 8177996 | NM_004847 | AIF1 | allograft inflammatory factor 1 | 0.382 | 1.303 | 3.73E-02 | 3.73E+00 | Up | | mono |
| 8179276 | NM_004847 | AIF1 | allograft inflammatory factor 1 | 0.382 | 1.303 | 3.73E-02 | 3.73E+00 | Up | | mono |
| 8040552 | NM_147223 | NCOA1 | nuclear receptor coactivator 1 | 0.382 | 1.303 | 9.77E-02 | 9.77E+00 | | | |
| 7959473 | NM_003677 | DENR | density-regulated protein | 0.382 | 1.303 | 4.18E-03 | 4.18E-01 | Up | | |
| 7943413 | NM_001165 | BIRC3 | baculoviral IAP repeat-containing 3 | 0.382 | 1.303 | 1.96E-01 | 1.96E+01 | | | |
| 7954511 | NM_015000 | STK38L | serine/threonine kinase 38 like | 0.382 | 1.303 | 3.37E-01 | 3.37E+01 | | | |
| 8096669 | NM_017628 | TET2 | tet oncogene family member 2 | 0.381 | 1.302 | 2.18E-02 | 2.18E+00 | Up | | |
| 7894716 | --- | --- | --- | 0.381 | 1.302 | 8.59E-02 | 8.59E+00 | | | |
| 7899160 | NM_001803 | CD52 | CD52 molecule | 0.381 | 1.302 | 1.99E-01 | 1.99E+01 | | | |
| 7934367 | NM_004034 | ANXA7 | annexin A7 | 0.381 | 1.302 | 5.62E-02 | 5.62E+00 | | | |
| 8126018 | NM_007271 | STK38 | serine/threonine kinase 38 | 0.381 | 1.302 | 4.20E-02 | 4.20E+00 | Up | | |
| 7921275 | NM_052939 | FCRL3 | Fc receptor-like 3 | 0.381 | 1.302 | 7.43E-03 | 7.43E-01 | Up | | |
| 7933237 | NM_174890 | ANUBL1 | AN1, ubiquitin-like, homolog (Xenop | 0.381 | 1.302 | 5.02E-02 | 5.02E+00 | | | |
| 7893139 | --- | --- | --- | 0.381 | 1.302 | 5.98E-01 | 5.98E+01 | | | |
| 7896311 | --- | --- | --- | 0.381 | 1.302 | 6.20E-01 | 6.20E+01 | | | |
| 7895153 | --- | --- | --- | 0.381 | 1.302 | 7.21E-01 | 7.21E+01 | | | |
| 8125500 | NM_004159 | PSMB8 | proteasome (prosome, macropain) s | 0.380 | 1.302 | 3.58E-02 | 3.58E+00 | Up | | |
| 8178855 | NM_004159 | PSMB8 | proteasome (prosome, macropain) s | 0.380 | 1.302 | 3.58E-02 | 3.58E+00 | Up | | |
| 8180049 | NM_004159 | PSMB8 | proteasome (prosome, macropain) s | 0.380 | 1.302 | 3.58E-02 | 3.58E+00 | Up | | |
| 7942889 | NM_032273 | TMEM126A | transmembrane protein 126A | 0.380 | 1.302 | 4.40E-01 | 4.40E+01 | | | |
| 8089234 | NM_014415 | ZBTB11 | zinc finger and BTB domain containir | 0.380 | 1.302 | 8.76E-02 | 8.76E+00 | | | |
| 7899829 | NM_022753 | S100PBP | S100P binding protein | 0.380 | 1.301 | 9.21E-02 | 9.21E+00 | | | |
| 7893089 | --- | --- | --- | 0.380 | 1.301 | 1.86E-01 | 1.86E+01 | | | |
| 8045619 | NM_015630 | EPC2 | enhancer of polycomb homolog 2 (D | 0.380 | 1.301 | 1.18E-01 | 1.18E+01 | | | |
| 7945962 | NM_003141 | TRIM21 | tripartite motif-containing 21 | 0.380 | 1.301 | 9.66E-02 | 9.66E+00 | | | |
| 7894306 | --- | --- | --- | 0.380 | 1.301 | 3.34E-01 | 3.34E+01 | | | |
| 8143781 | NM_024711 | GIMAP6 | GTPase, IMAP family member 6 | 0.379 | 1.301 | 1.63E-02 | 1.63E+00 | Up | | |
| 8016982 | NM_003168 | SUPT4H1 | suppressor of Ty 4 homolog 1 (S. cer | 0.379 | 1.301 | 1.45E-01 | 1.45E+01 | | | |
| 8035842 | NM_003430 | ZNF91 | zinc finger protein 91 | 0.379 | 1.301 | 1.18E-01 | 1.18E+01 | | | |
| 7940775 | NM_004585 | RARRES3 | retinoic acid receptor responder (taz | 0.379 | 1.301 | 1.16E-01 | 1.16E+01 | | | |
| 7956989 | NM_002392 | MDM2 | Mdm2 p53 binding protein homolog | 0.379 | 1.300 | 1.65E-01 | 1.65E+01 | | | |
| 8059852 | NR_024322 | MSL3L2 | male-specific lethal 3-like 2 (Drosoph | 0.379 | 1.300 | 3.61E-01 | 3.61E+01 | | | |
| 8097647 | NM_002940 | ABCE1 | ATP-binding cassette, sub-family E (O | 0.379 | 1.300 | 4.72E-01 | 4.72E+01 | | | |
| 7942912 | --- | --- | --- | 0.379 | 1.300 | 2.48E-01 | 2.48E+01 | | | |
| 7954185 | NM_015954 | DERA | 2-deoxyribose-5-phosphate aldolase | 0.379 | 1.300 | 4.81E-02 | 4.81E+00 | Up | | |
| 7918847 | NM_00110239( | SIKE1 | suppressor of IKBKE 1 | 0.378 | 1.300 | 2.86E-02 | 2.86E+00 | Up | | |
| 7893874 | --- | --- | --- | 0.378 | 1.300 | 4.82E-01 | 4.82E+01 | | | |
| 8017840 | ENST00000443 | LRRC37A4 | leucine rich repeat containing 37, me | 0.378 | 1.300 | 1.37E-01 | 1.37E+01 | | | |
| 7895901 | --- | --- | --- | 0.378 | 1.300 | 6.14E-01 | 6.14E+01 | | | |
| 7961083 | NM_005127 | CLEC2B | C-type lectin domain family 2, memb | 0.378 | 1.300 | 1.13E-01 | 1.13E+01 | | | |
| 7958644 | NM_170665 | ATP2A2 | ATPase, Ca++ transporting, cardiac m | 0.378 | 1.300 | 1.22E-01 | 1.22E+01 | | | |
| 8180202 | --- | --- | --- | 0.378 | 1.300 | 9.30E-02 | 9.30E+00 | | | |
| 8180258 | --- | --- | --- | 0.378 | 1.300 | 9.30E-02 | 9.30E+00 | | | |
| 7894274 | --- | --- | --- | 0.378 | 1.299 | 7.19E-01 | 7.19E+01 | | | |
| 8011011 | NR_024058 | YWHAE | tyrosine 3-monooxygenase/tryptoph | 0.378 | 1.299 | 6.15E-02 | 6.15E+00 | | | |
| 7952339 | NR_001453 | SNORD14C | small nucleolar RNA, C/D box 14C | 0.378 | 1.299 | 7.11E-01 | 7.11E+01 | | | |
| 7910550 | NM_005999 | TSNAX | translin-associated factor X | 0.378 | 1.299 | 2.39E-01 | 2.39E+01 | | | |
| 7947221 | NM_018362 | LIN7C | lin-7 homolog C (C. elegans) | 0.377 | 1.299 | 3.38E-01 | 3.38E+01 | | | |
| 7894542 | --- | --- | --- | 0.377 | 1.299 | 4.25E-01 | 4.25E+01 | | | |
| 8105612 | NM_005869 | SDCCAG10 | serologically defined colon cancer ar | 0.377 | 1.299 | 2.97E-01 | 2.97E+01 | | | |
| 7892501 | --- | --- | --- | 0.377 | 1.299 | 6.17E-01 | 6.17E+01 | | | |
| 7908978 | NM_014827 | ZC3H11A | zinc finger CCCH-type containing 11A | 0.377 | 1.299 | 3.18E-01 | 3.18E+01 | | | |
| 8072610 | NM_012179 | FBXO7 | F-box protein 7 | 0.377 | 1.299 | 1.10E-01 | 1.10E+01 | | | |
| 8018364 | NM_002086 | GRB2 | growth factor receptor-bound protei | 0.377 | 1.299 | 4.96E-02 | 4.96E+00 | Up | | |
| 7961285 | NM_176889 | TAS2R20 | taste receptor, type 2, member 20 | 0.377 | 1.299 | 4.00E-01 | 4.00E+01 | | | |
| 8180349 | --- | --- | --- | 0.377 | 1.299 | 4.37E-02 | 4.37E+00 | Up | | |
| 7907124 | NM_152902 | TIPRL | TIP41, TOR signaling pathway regulat | 0.377 | 1.299 | 3.26E-01 | 3.26E+01 | | | |
| 8072710 | NM_030641 | APOL6 | apolipoprotein L, 6 | 0.377 | 1.299 | 3.38E-02 | 3.38E+00 | Up | | |
| 7967149 | NM_016237 | ANAPC5 | anaphase promoting complex subun | 0.377 | 1.298 | 7.57E-02 | 7.57E+00 | | | |
| 7994343 | --- | --- | --- | 0.377 | 1.298 | 6.17E-01 | 6.17E+01 | | | |
| 7963646 | NM_015665 | AAAS | achalasia, adrenocortical insufficienc | 0.377 | 1.298 | 6.28E-02 | 6.28E+00 | | | |
| 8093112 | NM_015562 | UBXN7 | UBX domain protein 7 | 0.377 | 1.298 | 2.74E-01 | 2.74E+01 | | | |
| 7918703 | NM_006594 | AP4B1 | adaptor-related protein complex 4, b | 0.376 | 1.298 | 6.23E-02 | 6.23E+00 | | | |
| 7936419 | NM_018017 | C10orf118 | chromosome 10 open reading frame | 0.376 | 1.298 | 3.49E-01 | 3.49E+01 | | | |
| 8056060 | NM_013450 | BAZ2B | bromodomain adjacent to zinc finger | 0.376 | 1.298 | 8.82E-02 | 8.82E+00 | | | |
| 7957613 | NM_017599 | VEZT | vezatin, adherens junctions transme | 0.376 | 1.298 | 9.68E-02 | 9.68E+00 | | | |
| 7892678 | --- | --- | --- | 0.376 | 1.298 | 4.22E-01 | 4.22E+01 | | | |
| 7894687 | --- | --- | --- | 0.376 | 1.298 | 1.65E-01 | 1.65E+01 | | | |
| 7958130 | NM_003299 | HSP90B1 | heat shock protein 90kDa beta (Grp9 | 0.376 | 1.298 | 4.28E-02 | 4.28E+00 | Up | | |
| 8144669 | NM_004462 | FDFT1 | farnesyl-diphosphate farnesyltransfe | 0.376 | 1.297 | 2.52E-01 | 2.52E+01 | | | |
| 8101260 | NM_058172 | ANTXR2 | anthrax toxin receptor 2 | 0.376 | 1.297 | 2.17E-01 | 2.17E+01 | | | |
| 8152865 | --- | --- | --- | 0.375 | 1.297 | 7.01E-02 | 7.01E+00 | | | |
| 8110894 | NM_017755 | NSUN2 | NOL1/NOP2/Sun domain family, mer | 0.375 | 1.297 | 9.31E-02 | 9.31E+00 | | | |
| 8132830 | --- | --- | --- | 0.375 | 1.297 | 4.46E-01 | 4.46E+01 | | | |

| | | | | | | | | | | |
|---|---|---|---|---|---|---|---|---|---|---|
| 8038809 | NM_005601 | NKG7 | natural killer cell group 7 sequence | 0.375 | 1.297 | 1.35E-01 | 1.35E+01 | | | |
| 7894310 | --- | --- | --- | 0.375 | 1.297 | 4.48E-01 | 4.48E+01 | | | |
| 8053417 | NM_001747 | CAPG | capping protein (actin filament), gels | 0.375 | 1.297 | 1.26E-03 | 1.26E-01 | Up | | |
| 7903032 | NM_007358 | MTF2 | metal response element binding tran | 0.375 | 1.297 | 3.72E-01 | 3.72E+01 | | | |
| 7895525 | --- | --- | --- | 0.375 | 1.297 | 3.78E-01 | 3.78E+01 | | | |
| 8051119 | NM_177983 | PPM1G | protein phosphatase 1G (formerly 2C | 0.374 | 1.296 | 4.73E-02 | 4.73E+00 | Up | | |
| 8078312 | NM_182523 | CMC1 | COX assembly mitochondrial protein | 0.374 | 1.296 | 5.49E-02 | 5.49E+00 | | | |
| 8052250 | NM_00100536 | MTIF2 | mitochondrial translational initiation | 0.374 | 1.296 | 1.02E-01 | 1.02E+01 | | | |
| 8021312 | NM_015285 | WDR7 | WD repeat domain 7 | 0.374 | 1.296 | 1.45E-01 | 1.45E+01 | | | |
| 8029615 | --- | --- | --- | 0.374 | 1.296 | 6.77E-01 | 6.77E+01 | | | |
| 7911870 | NM_014704 | KIAA0562 | KIAA0562 | 0.374 | 1.296 | 2.27E-02 | 2.27E+00 | Up | | |
| 8061668 | NM_002110 | HCK | hemopoietic cell kinase | 0.374 | 1.296 | 2.74E-02 | 2.74E+00 | Up | | mono |
| 7971967 | NM_014953 | DIS3 | DIS3 mitotic control homolog (S. cere | 0.374 | 1.296 | 7.71E-02 | 7.71E+00 | | | |
| 7914270 | NM_006762 | LAPTM5 | lysosomal protein transmembrane 5 | 0.374 | 1.296 | 8.11E-02 | 8.11E+00 | | | |
| 8120992 | NM_015021 | ZNF292 | zinc finger protein 292 | 0.374 | 1.296 | 1.19E-01 | 1.19E+01 | | | |
| 7896715 | --- | --- | --- | 0.374 | 1.296 | 2.05E-01 | 2.05E+01 | | | |
| 8016552 | NM_002634 | PHB | prohibitin | 0.373 | 1.295 | 1.19E-01 | 1.19E+01 | | | |
| 8116664 | NM_003913 | PRPF4B | PRP4 pre-mRNA processing factor 4 | 0.373 | 1.295 | 3.39E-01 | 3.39E+01 | | | |
| 7892835 | --- | --- | --- | 0.373 | 1.295 | 3.21E-01 | 3.21E+01 | | | |
| 8133818 | NM_00112735 | PHTF2 | putative homeodomain transcription | 0.373 | 1.295 | 5.91E-03 | 5.91E-01 | Up | | |
| 7937798 | --- | --- | --- | 0.373 | 1.295 | 3.26E-01 | 3.26E+01 | | | |
| 8019762 | --- | --- | --- | 0.373 | 1.295 | 1.50E-01 | 1.50E+01 | | | |
| 7976333 | NM_015676 | C14orf109 | chromosome 14 open reading frame | 0.373 | 1.295 | 8.04E-02 | 8.04E+00 | | | |
| 8151136 | NM_006837 | COPS5 | COP9 constitutive photomorphogeni | 0.372 | 1.294 | 5.34E-02 | 5.34E+00 | | | |
| 7936683 | NM_00103392 | TIAL1 | TIA1 cytotoxic granule-associated RN | 0.372 | 1.294 | 5.00E-02 | 5.00E+00 | Up | | |
| 7893590 | --- | --- | --- | 0.372 | 1.294 | 3.57E-01 | 3.57E+01 | | | |
| 7958152 | NM_002629 | PGAM1 | phosphoglycerate mutase 1 (brain) | 0.372 | 1.294 | 3.50E-02 | 3.50E+00 | Up | | |
| 8143279 | NM_020119 | ZC3HAV1 | zinc finger CCCH-type, antiviral 1 | 0.372 | 1.294 | 8.82E-02 | 8.82E+00 | | | |
| 8116571 | --- | --- | --- | 0.372 | 1.294 | 5.06E-01 | 5.06E+01 | | | |
| 7964271 | NM_000946 | PRIM1 | primase, DNA, polypeptide 1 (49kDa | 0.372 | 1.294 | 3.17E-01 | 3.17E+01 | | | |
| 8161587 | NM_201453 | CBWD3 | COBW domain containing 3 | 0.371 | 1.294 | 9.06E-02 | 9.06E+00 | | | |
| 8057719 | NM_014362 | HIBCH | 3-hydroxyisobutyryl-Coenzyme A hyd | 0.371 | 1.294 | 2.46E-01 | 2.46E+01 | | | |
| 8102232 | NM_016269 | LEF1 | lymphoid enhancer-binding factor 1 | 0.371 | 1.294 | 1.62E-01 | 1.62E+01 | | | |
| 8058335 | NM_003352 | SUMO1 | SMT3 suppressor of mif two 3 homo | 0.371 | 1.294 | 2.46E-01 | 2.46E+01 | | | |
| 8156861 | NM_017919 | STX17 | syntaxin 17 | 0.371 | 1.294 | 1.92E-01 | 1.92E+01 | | | |
| 7973214 | NM_014828 | TOX4 | TOX high mobility group box family r | 0.371 | 1.293 | 2.19E-01 | 2.19E+01 | | | |
| 7895112 | --- | --- | --- | 0.371 | 1.293 | 7.02E-02 | 7.02E+00 | | | |
| 8130720 | NM_145169 | SFT2D1 | SFT2 domain containing 1 | 0.371 | 1.293 | 2.82E-01 | 2.82E+01 | | | |
| 7955462 | --- | --- | --- | 0.371 | 1.293 | 5.59E-01 | 5.59E+01 | | | |
| 8149330 | NM_147780 | CTSB | cathepsin B | 0.371 | 1.293 | 1.33E-02 | 1.33E+00 | Up | | mono |
| 7922432 | NM_172071 | RC3H1 | ring finger and CCCH-type zinc finger | 0.371 | 1.293 | 1.36E-01 | 1.36E+01 | | | |
| 8054064 | NM_025190 | ANKRD36B | ankyrin repeat domain 36B | 0.371 | 1.293 | 1.83E-01 | 1.83E+01 | | | |
| 7896421 | --- | --- | --- | 0.370 | 1.293 | 4.23E-01 | 4.23E+01 | | | |
| 7894013 | --- | --- | --- | 0.370 | 1.292 | 6.37E-01 | 6.37E+01 | | | |
| 7896221 | --- | --- | --- | 0.370 | 1.292 | 9.85E-02 | 9.85E+00 | | | |
| 7929511 | NM_001776 | ENTPD1 | ectonucleoside triphosphate diphosp | 0.370 | 1.292 | 7.79E-02 | 7.79E+00 | | | |
| 7974621 | NM_002892 | ARID4A | AT rich interactive domain 4A (RBP1- | 0.369 | 1.292 | 2.70E-01 | 2.70E+01 | | | |
| 8136401 | NM_015135 | NUP205 | nucleoporin 205kDa | 0.369 | 1.292 | 1.54E-01 | 1.54E+01 | | | |
| 8134730 | NM_152755 | CNPY4 | canopy 4 homolog (zebrafish) | 0.369 | 1.292 | 1.57E-02 | 1.57E+00 | Up | | |
| 7990417 | NM_005697 | SCAMP2 | secretory carrier membrane protein | 0.369 | 1.292 | 2.27E-02 | 2.27E+00 | Up | | |
| 8103389 | NM_001334 | CTSO | cathepsin O | 0.369 | 1.292 | 1.15E-01 | 1.15E+01 | | | |
| 7893814 | --- | --- | --- | 0.369 | 1.292 | 5.65E-01 | 5.65E+01 | | | |
| 7962904 | NM_001659 | ARF3 | ADP-ribosylation factor 3 | 0.369 | 1.291 | 9.56E-02 | 9.56E+00 | | | |
| 7956211 | NM_021019 | MYL6 | myosin, light chain 6, alkali, smooth | 0.369 | 1.291 | 1.31E-03 | 1.31E-01 | Up | | |
| 7954279 | NM_153207 | AEBP2 | AE binding protein 2 | 0.368 | 1.291 | 1.47E-01 | 1.47E+01 | | | |
| 8107282 | NM_139281 | WDR36 | WD repeat domain 36 | 0.368 | 1.291 | 1.26E-01 | 1.26E+01 | | | |
| 8110408 | NM_032361 | THOC3 | THO complex 3 | 0.368 | 1.291 | 4.74E-03 | 4.74E-01 | Up | | |
| 8080926 | NM_006407 | ARL6IP5 | ADP-ribosylation-like factor 6 interac | 0.368 | 1.291 | 2.17E-01 | 2.17E+01 | | | |
| 7975416 | NM_014982 | PCNX | pecanex homolog (Drosophila) | 0.368 | 1.291 | 6.01E-02 | 6.01E+00 | | | |
| 8128683 | NM_003795 | SNX3 | sorting nexin 3 | 0.368 | 1.290 | 6.12E-02 | 6.12E+00 | | | |
| 8110166 | NM_138820 | HIGD2A | HIG1 hypoxia inducible domain famil | 0.368 | 1.290 | 2.46E-01 | 2.46E+01 | | | |
| 7895287 | --- | --- | --- | 0.368 | 1.290 | 6.73E-01 | 6.73E+01 | | | |
| 8030991 | NR_003148 // 7804 // LOC | | tropomyosin 3 pseudogene // tropo | 0.367 | 1.290 | 8.10E-03 | 8.10E-01 | Up | | |
| 7925996 | NM_032807 | FBXO18 | F-box protein, helicase, 18 | 0.367 | 1.290 | 7.22E-03 | 7.22E-01 | Up | | |
| 7981217 | NM_018036 | ATG2B | ATG2 autophagy related 2 homolog | 0.367 | 1.290 | 1.09E-01 | 1.09E+01 | | | |
| 8111286 | NM_013235 | RNASEN | ribonuclease type III, nuclear | 0.367 | 1.290 | 1.67E-01 | 1.67E+01 | | | |
| 8013965 | NM_033389 | SSH2 | slingshot homolog 2 (Drosophila) | 0.367 | 1.289 | 2.58E-02 | 2.58E+00 | Up | | |
| 8103834 | NM_000027 | AGA | aspartylglucosaminidase | 0.367 | 1.289 | 8.08E-02 | 8.08E+00 | | | |
| 7929256 | --- | --- | --- | 0.367 | 1.289 | 3.44E-01 | 3.44E+01 | | | |
| 7987981 | NM_174916 | UBR1 | ubiquitin protein ligase E3 componer | 0.367 | 1.289 | 9.45E-02 | 9.45E+00 | | | |
| 7989611 | NM_032231 | FAM96A | family with sequence similarity 96, m | 0.366 | 1.289 | 1.31E-01 | 1.31E+01 | | | |
| 7928300 | NM_173473 | C10orf104 | chromosome 10 open reading frame | 0.366 | 1.289 | 1.86E-01 | 1.86E+01 | | | |
| 7981740 | BC073771 // BHA1 // IGHA | | immunoglobulin heavy constant alph | 0.366 | 1.289 | 7.00E-02 | 7.00E+00 | | | |
| 8115490 | NM_033274 | ADAM19 | ADAM metallopeptidase domain 19 | 0.366 | 1.289 | 3.14E-03 | 3.14E-01 | Up | | |
| 7990620 | NM_005724 | TSPAN3 | tetraspanin 3 | 0.366 | 1.289 | 9.13E-02 | 9.13E+00 | | | |
| 7899504 | NM_006582 | GMEB1 | glucocorticoid modulatory element b | 0.366 | 1.289 | 2.67E-01 | 2.67E+01 | | | |
| 7895704 | --- | --- | --- | 0.365 | 1.288 | 3.27E-01 | 3.27E+01 | | | |

| | | | | | | | | | | | |
|---|---|---|---|---|---|---|---|---|---|---|---|
| 7974473 | NM_017943 | FBXO34 | F-box protein 34 | 0.365 | 1.288 | 2.79E-01 | 2.79E+01 | | | | |
| 7967624 | NM_145648 | SLC15A4 | solute carrier family 15, member 4 | 0.365 | 1.288 | 6.90E-02 | 6.90E+00 | | | | |
| 8115997 | NM_00103167 | RAB24 | RAB24, member RAS oncogene famil | 0.365 | 1.288 | 2.51E-02 | 2.51E+00 | Up | | | |
| 8151421 | --- | --- | --- | 0.365 | 1.288 | 5.34E-01 | 5.34E+01 | | | | |
| 8148208 | NM_144963 | FAM91A1 | family with sequence similarity 91, m | 0.365 | 1.288 | 3.21E-01 | 3.21E+01 | | | | |
| 7893574 | --- | --- | --- | 0.365 | 1.288 | 3.11E-01 | 3.11E+01 | | | | |
| 8041168 | NR_002741 | SNORD53 | small nucleolar RNA, C/D box 53 | 0.365 | 1.288 | 2.33E-01 | 2.33E+01 | | | | |
| 7906904 | NM_016371 | HSD17B7 | hydroxysteroid (17-beta) dehydroger | 0.365 | 1.288 | 4.23E-01 | 4.23E+01 | | | | |
| 7949482 | NM_032193 | RNASEH2C | ribonuclease H2, subunit C | 0.364 | 1.287 | 1.92E-02 | 1.92E+00 | Up | | | |
| 8120602 | NM_024576 | OGFRL1 | opioid growth factor receptor-like 1 | 0.364 | 1.287 | 1.57E-01 | 1.57E+01 | | | | |
| 7965480 | NM_002629 | PGAM1 | phosphoglycerate mutase 1 (brain) | 0.363 | 1.287 | 4.20E-02 | 4.20E+00 | Up | | | |
| 7915130 | NM_006802 | SF3A3 | splicing factor 3a, subunit 3, 60kDa | 0.363 | 1.286 | 1.45E-01 | 1.45E+01 | | | | |
| 7938669 | NM_014267 | C11orf58 | chromosome 11 open reading frame | 0.363 | 1.286 | 4.91E-01 | 4.91E+01 | | | | |
| 8076272 | NM_003932 | ST13 | suppression of tumorigenicity 13 (co | 0.363 | 1.286 | 7.94E-02 | 7.94E+00 | | | | |
| 8052139 | --- | --- | --- | 0.363 | 1.286 | 2.08E-01 | 2.08E+01 | | | | |
| 8089867 | NM_173825 | RABL3 | RAB, member of RAS oncogene famil | 0.363 | 1.286 | 4.73E-01 | 4.73E+01 | | | | |
| 8019842 | NM_001071 | TYMS | thymidylate synthetase | 0.363 | 1.286 | 1.88E-01 | 1.88E+01 | | | | |
| 8035793 | NM_00115929 | ZNF737 | zinc finger protein 737 | 0.363 | 1.286 | 5.73E-01 | 5.73E+01 | | | | |
| 7996593 | NM_006565 | CTCF | CCCTC-binding factor (zinc finger pro | 0.363 | 1.286 | 1.94E-01 | 1.94E+01 | | | | |
| 8140730 | NM_024315 | C7orf23 | chromosome 7 open reading frame 2 | 0.362 | 1.286 | 2.60E-01 | 2.60E+01 | | | | |
| 8116227 | NM_020666 | CLK4 | CDC-like kinase 4 | 0.362 | 1.285 | 4.36E-01 | 4.36E+01 | | | | |
| 7966397 | NM_002973 | ATXN2 | ataxin 2 | 0.362 | 1.285 | 3.58E-02 | 3.58E+00 | Up | | | |
| 7939158 | NM_00107678 | QSER1 | glutamine and serine rich 1 | 0.362 | 1.285 | 1.07E-01 | 1.07E+01 | | | | |
| 8144699 | --- | --- | --- | 0.361 | 1.285 | 5.08E-01 | 5.08E+01 | | | | |
| 8161537 | NM_201453 | CBWD3 | COBW domain containing 3 | 0.361 | 1.284 | 1.60E-01 | 1.60E+01 | | | | |
| 7922268 | NM_014970 | KIFAP3 | kinesin-associated protein 3 | 0.361 | 1.284 | 1.67E-01 | 1.67E+01 | | | | |
| 8096116 | NM_032717 | AGPAT9 | 1-acylglycerol-3-phosphate O-acyltra | 0.361 | 1.284 | 1.03E-02 | 1.03E+00 | Up | | | |
| 8180263 | --- | --- | --- | 0.361 | 1.284 | 1.89E-01 | 1.89E+01 | | | | |
| 7935647 | NM_078470 | COX15 | COX15 homolog, cytochrome c oxida | 0.361 | 1.284 | 2.13E-02 | 2.13E+00 | Up | | | |
| 8016300 | NM_00111373 | ARL17P1 | ADP-ribosylation factor-like 17 pseud | 0.361 | 1.284 | 3.22E-02 | 3.22E+00 | Up | | | |
| 8114113 | NM_017665 | ZCCHC10 | zinc finger, CCHC domain containing | 0.361 | 1.284 | 5.79E-02 | 5.79E+00 | | | | |
| 8010983 | NM_021962 | ABR | active BCR-related gene | 0.360 | 1.284 | 4.50E-02 | 4.50E+00 | Up | | | |
| 7895814 | --- | --- | --- | 0.360 | 1.284 | 3.39E-01 | 3.39E+01 | | | | |
| 8137596 | NM_014671 | UBE3C | ubiquitin protein ligase E3C | 0.360 | 1.284 | 2.89E-02 | 2.89E+00 | Up | | | |
| 8125941 | NM_003137 | SRPK1 | SFRS protein kinase 1 | 0.360 | 1.284 | 1.01E-01 | 1.01E+01 | | | | |
| 8083630 | NM_024996 | GFM1 | G elongation factor, mitochondrial 1 | 0.360 | 1.284 | 2.28E-01 | 2.28E+01 | | | | |
| 7992917 | NM_015041 | CLUAP1 | clusterin associated protein 1 | 0.360 | 1.283 | 1.72E-01 | 1.72E+01 | | | | |
| 8011850 | NM_001212 | C1QBP | complement component 1, q subcon | 0.360 | 1.283 | 8.91E-02 | 8.91E+00 | | | | |
| 8166989 | NM_00112989 | ZNF673 | zinc finger family member 673 | 0.359 | 1.283 | 3.11E-02 | 3.11E+00 | Up | | | |
| 7935337 | NM_152309 | PIK3AP1 | phosphoinositide-3-kinase adaptor p | 0.359 | 1.283 | 3.92E-02 | 3.92E+00 | Up | | | |
| 7963935 | NM_032364 | DNAJC14 | DnaJ (Hsp40) homolog, subfamily C, | 0.359 | 1.283 | 2.65E-01 | 2.65E+01 | | | | |
| 8128698 | NM_014454 | SESN1 | sestrin 1 | 0.359 | 1.283 | 3.95E-02 | 3.95E+00 | Up | | | |
| 8155930 | NM_001490 | GCNT1 | glucosaminyl (N-acetyl) transferase 1 | 0.359 | 1.283 | 4.80E-02 | 4.80E+00 | Up | | | |
| 7895017 | --- | --- | --- | 0.359 | 1.283 | 2.52E-01 | 2.52E+01 | | | | |
| 7909586 | NM_006243 | PPP2R5A | protein phosphatase 2, regulatory su | 0.359 | 1.282 | 2.93E-01 | 2.93E+01 | | | | |
| 8125734 | NM_00114196 | DAXX | death-domain associated protein | 0.359 | 1.282 | 2.16E-02 | 2.16E+00 | Up | | | |
| 8010949 | NM_016080 | GLOD4 | glyoxalase domain containing 4 | 0.359 | 1.282 | 1.22E-01 | 1.22E+01 | | | | |
| 7979179 | NM_014584 | ERO1L | ERO1-like (S. cerevisiae) | 0.359 | 1.282 | 3.06E-01 | 3.06E+01 | | | | |
| 8177635 | NM_003187 | TAF9 | TAF9 RNA polymerase II, TATA box b | 0.359 | 1.282 | 1.42E-01 | 1.42E+01 | | | | |
| 8132376 | NM_032016 | STARD3NL | STARD3 N-terminal like | 0.359 | 1.282 | 2.37E-01 | 2.37E+01 | | | | |
| 8119184 | NM_003958 | RNF8 | ring finger protein 8 | 0.358 | 1.282 | 5.85E-02 | 5.85E+00 | | | | |
| 8066905 | NM_021035 | ZNFX1 | zinc finger, NFX1-type containing 1 | 0.358 | 1.282 | 3.89E-02 | 3.89E+00 | Up | | | |
| 8164155 | NM_00112335 | PPP6C | protein phosphatase 6, catalytic sub | 0.358 | 1.281 | 3.59E-01 | 3.59E+01 | | | | |
| 7895035 | --- | --- | --- | 0.357 | 1.281 | 5.97E-01 | 5.97E+01 | | | | |
| 7894664 | --- | --- | --- | 0.357 | 1.281 | 4.17E-01 | 4.17E+01 | | | | |
| 7999766 | NM_006985 | NPIP | nuclear pore complex interacting pro | 0.357 | 1.281 | 2.47E-02 | 2.47E+00 | Up | | | |
| 8126860 | NM_000255 | MUT | methylmalonyl Coenzyme A mutase | 0.357 | 1.281 | 1.02E-01 | 1.02E+01 | | | | |
| 7895544 | --- | --- | --- | 0.357 | 1.281 | 4.88E-01 | 4.88E+01 | | | | |
| 8123507 | NM_003194 | TBP | TATA box binding protein | 0.357 | 1.280 | 2.08E-01 | 2.08E+01 | | | | |
| 7927548 | NM_006327 | TIMM23 | translocase of inner mitochondrial m | 0.357 | 1.280 | 5.21E-01 | 5.21E+01 | | | | |
| 7893125 | --- | --- | --- | 0.356 | 1.280 | 5.31E-01 | 5.31E+01 | | | | |
| 8123936 | NM_00114239 | NEDD9 | neural precursor cell expressed, deve | 0.356 | 1.280 | 5.04E-02 | 5.04E+00 | | | | |
| 8136658 | --- | --- | --- | 0.356 | 1.280 | 5.24E-01 | 5.24E+01 | | | | |
| 8133754 | NR_023383 | PMS2L11 | postmeiotic segregation increased 2- | 0.356 | 1.280 | 2.23E-01 | 2.23E+01 | | | | |
| 7961151 | NM_007360 | KLRK1 | killer cell lectin-like receptor subfam | 0.355 | 1.279 | 8.67E-02 | 8.67E+00 | | | | |
| 7892726 | --- | --- | --- | 0.355 | 1.279 | 2.64E-01 | 2.64E+01 | | | | |
| 8137054 | NM_003592 | CUL1 | cullin 1 | 0.355 | 1.279 | 1.04E-01 | 1.04E+01 | | | | |
| 8063509 | NM_016407 | C20orf43 | chromosome 20 open reading frame | 0.355 | 1.279 | 1.04E-02 | 1.04E+00 | Up | | | |
| 8073733 | NM_153645 | NUP50 | nucleoporin 50kDa | 0.355 | 1.279 | 1.01E-02 | 1.01E+00 | Up | | | |
| 7892814 | --- | --- | --- | 0.354 | 1.278 | 2.28E-01 | 2.28E+01 | | | | |
| 8168472 | NM_000052 | ATP7A | ATPase, Cu++ transporting, alpha po | 0.354 | 1.278 | 3.99E-02 | 3.99E+00 | Up | | | |
| 8139228 | NM_00109985 | C7orf25 | chromosome 7 open reading frame 2 | 0.354 | 1.278 | 4.15E-01 | 4.15E+01 | | | | |
| 8065569 | NM_138578 | BCL2L1 | BCL2-like 1 | 0.354 | 1.278 | 1.02E-01 | 1.02E+01 | | | | |
| 8097600 | NM_003601 | SMARCA5 | SWI/SNF related, matrix associated, | 0.354 | 1.278 | 1.24E-01 | 1.24E+01 | | | | |
| 7905986 | NM_002004 | FDPS | farnesyl diphosphate synthase (farne | 0.354 | 1.278 | 1.07E-01 | 1.07E+01 | | | | |
| 7950753 | NM_021825 | CCDC90B | coiled-coil domain containing 90B | 0.354 | 1.278 | 4.40E-01 | 4.40E+01 | | | | |
| 8130438 | NM_016020 | TFB1M | transcription factor B1, mitochondria | 0.354 | 1.278 | 2.07E-02 | 2.07E+00 | Up | | | |

| | | | | | | | | | | |
|---|---|---|---|---|---|---|---|---|---|---|
| 8167161 | --- | --- | --- | 0.354 | 1.278 | 4.00E-02 | 4.00E+00 | Up | | |
| 8112439 | NM_176816 | CCDC125 | coiled-coil domain containing 125 | 0.353 | 1.278 | 1.53E-01 | 1.53E+01 | | | |
| 8148280 | NM_003129 | SQLE | squalene epoxidase | 0.353 | 1.278 | 2.86E-02 | 2.86E+00 | Up | | |
| 8104350 | NM_015325 | KIAA0947 | KIAA0947 | 0.353 | 1.278 | 7.76E-02 | 7.76E+00 | | | |
| 7964522 | NM_000075 | CDK4 | cyclin-dependent kinase 4 | 0.353 | 1.278 | 2.35E-01 | 2.35E+01 | | | |
| 8006638 | NM_024835 | GGNBP2 | gametogenetin binding protein 2 | 0.353 | 1.278 | 1.24E-01 | 1.24E+01 | | | |
| 7893168 | --- | --- | --- | 0.353 | 1.277 | 4.27E-01 | 4.27E+01 | | | |
| 7928444 | NM_014000 | VCL | vinculin | 0.353 | 1.277 | 2.62E-02 | 2.62E+00 | Up | | |
| 8108873 | NM_015071 | ARHGAP26 | Rho GTPase activating protein 26 | 0.353 | 1.277 | 4.76E-02 | 4.76E+00 | Up | | |
| 7894772 | --- | --- | --- | 0.353 | 1.277 | 4.57E-02 | 4.57E+00 | Up | | |
| 8117955 | NM_014046 | MRPS18B | mitochondrial ribosomal protein S18 | 0.352 | 1.277 | 1.17E-01 | 1.17E+01 | | | |
| 8177824 | NM_014046 | MRPS18B | mitochondrial ribosomal protein S18 | 0.352 | 1.277 | 1.17E-01 | 1.17E+01 | | | |
| 8179139 | NM_014046 | MRPS18B | mitochondrial ribosomal protein S18 | 0.352 | 1.277 | 1.17E-01 | 1.17E+01 | | | |
| 8033987 | NM_002162 | ICAM3 | intercellular adhesion molecule 3 | 0.352 | 1.276 | 6.00E-02 | 6.00E+00 | | | |
| 8045398 | NM_012233 | RAB3GAP1 | RAB3 GTPase activating protein subu | 0.352 | 1.276 | 1.58E-01 | 1.58E+01 | | | |
| 7894930 | --- | --- | --- | 0.352 | 1.276 | 4.81E-01 | 4.81E+01 | | | |
| 8105878 | NM_133338 | RAD17 | RAD17 homolog (S. pombe) | 0.352 | 1.276 | 1.86E-01 | 1.86E+01 | | | |
| 8106025 | NM_018429 | BDP1 | B double prime 1, subunit of RNA po | 0.352 | 1.276 | 5.14E-02 | 5.14E+00 | | | |
| 8127031 | NM_002388 | MCM3 | minichromosome maintenance com | 0.352 | 1.276 | 3.35E-02 | 3.35E+00 | Up | | |
| 8137414 | NM_016118 | NUB1 | negative regulator of ubiquitin-like p | 0.352 | 1.276 | 2.72E-01 | 2.72E+01 | | | |
| 8101429 | NM_016619 | PLAC8 | placenta-specific 8 | 0.352 | 1.276 | 1.36E-01 | 1.36E+01 | | | |
| 7951325 | NM_032299 | DCUN1D5 | DCN1, defective in cullin neddylation | 0.352 | 1.276 | 4.02E-01 | 4.02E+01 | | | |
| 8157858 | NM_015635 | GAPVD1 | GTPase activating protein and VPS9 d | 0.352 | 1.276 | 1.58E-01 | 1.58E+01 | | | |
| 8016099 | NM_004247 | EFTUD2 | elongation factor Tu GTP binding dor | 0.352 | 1.276 | 8.71E-02 | 8.71E+00 | | | |
| 8002381 | NM_015386 | COG4 | component of oligomeric golgi comp | 0.351 | 1.276 | 4.57E-02 | 4.57E+00 | Up | | |
| 8041888 | NM_000179 | MSH6 | mutS homolog 6 (E. coli) | 0.351 | 1.276 | 2.93E-01 | 2.93E+01 | | | |
| 8151101 | NM_00108041( | MYBL1 | v-myb myeloblastosis viral oncogene | 0.351 | 1.275 | 3.93E-01 | 3.93E+01 | | | |
| 8078544 | NM_000249 | MLH1 | mutL homolog 1, colon cancer, nonp | 0.351 | 1.275 | 1.96E-01 | 1.96E+01 | | | |
| 7944560 | NM_015313 | ARHGEF12 | Rho guanine nucleotide exchange fa | 0.351 | 1.275 | 2.18E-02 | 2.18E+00 | Up | | |
| 8128034 | NM_018064 | AKIRIN2 | akirin 2 | 0.351 | 1.275 | 2.19E-01 | 2.19E+01 | | | |
| 8176719 | NM_004681 | EIF1AY | eukaryotic translation initiation facto | 0.351 | 1.275 | 7.10E-01 | 7.10E+01 | | | |
| 7908543 | NM_133494 | NEK7 | NIMA (never in mitosis gene a)-relat | 0.351 | 1.275 | 3.65E-01 | 3.65E+01 | | | |
| 8051396 | NM_021209 | NLRC4 | NLR family, CARD domain containing | 0.350 | 1.275 | 6.83E-03 | 6.83E-01 | Up | | |
| 8003848 | --- | --- | --- | 0.350 | 1.274 | 4.81E-01 | 4.81E+01 | | | |
| 8019081 | --- | --- | --- | 0.350 | 1.274 | 1.18E-01 | 1.18E+01 | | | |
| 7896259 | --- | --- | --- | 0.350 | 1.274 | 6.58E-01 | 6.58E+01 | | | |
| 7931728 | NM_015155 | LARP4B | La ribonucleoprotein domain family, | 0.350 | 1.274 | 4.03E-02 | 4.03E+00 | Up | | |
| 8173729 | NM_00102989 | PGAM4 | phosphoglycerate mutase family me | 0.349 | 1.274 | 3.44E-02 | 3.44E+00 | Up | | |
| 8176282 | NM_00102989 | PGAM4 | phosphoglycerate mutase family me | 0.349 | 1.274 | 3.44E-02 | 3.44E+00 | Up | | |
| 7923221 | NM_00103172 | DDX59 | DEAD (Asp-Glu-Ala-Asp) box polypep | 0.349 | 1.274 | 3.99E-02 | 3.99E+00 | Up | | |
| 8051926 | --- | --- | --- | 0.349 | 1.274 | 4.26E-01 | 4.26E+01 | | | |
| 8085000 | NM_032773 | LRCH3 | leucine-rich repeats and calponin ho | 0.349 | 1.274 | 8.84E-02 | 8.84E+00 | | | |
| 8052680 | NM_004161 | RAB1A | RAB1A, member RAS oncogene fami | 0.349 | 1.274 | 4.04E-01 | 4.04E+01 | | | |
| 7899394 | NM_00110555 | C1orf38 | chromosome 1 open reading frame 3 | 0.349 | 1.274 | 1.53E-02 | 1.53E+00 | Up | | |
| 7983940 | NM_00104045 | FAM63B | family with sequence similarity 63, m | 0.348 | 1.273 | 4.59E-01 | 4.59E+01 | | | |
| 7895787 | --- | --- | --- | 0.348 | 1.273 | 2.65E-01 | 2.65E+01 | | | |
| 8023252 | NM_000985 | RPL17 | ribosomal protein L17 | 0.348 | 1.273 | 3.90E-01 | 3.90E+01 | | | |
| 7914139 | NM_014110 | PPP1R8 | protein phosphatase 1, regulatory (in | 0.348 | 1.273 | 6.58E-01 | 6.58E+01 | | | |
| 8062213 | NM_016436 | PHF20 | PHD finger protein 20 | 0.348 | 1.273 | 1.75E-01 | 1.75E+01 | | | |
| 7939424 | NR_024625 | API5 | apoptosis inhibitor 5 | 0.348 | 1.273 | 2.59E-01 | 2.59E+01 | | | |
| 8053610 | NM_022662 | ANAPC1 | anaphase promoting complex subun | 0.348 | 1.273 | 2.14E-01 | 2.14E+01 | | | |
| 8094599 | NM_016531 | KLF3 | Kruppel-like factor 3 (basic) | 0.348 | 1.273 | 3.16E-01 | 3.16E+01 | | | |
| 7936762 | NM_00100197 | ATE1 | arginyltransferase 1 | 0.347 | 1.272 | 2.45E-01 | 2.45E+01 | | | |
| 7906613 | NM_021181 | SLAMF7 | SLAM family member 7 | 0.347 | 1.272 | 1.53E-01 | 1.53E+01 | | | |
| 7984298 | NM_133375 | DIS3L | DIS3 mitotic control homolog (S. cere | 0.347 | 1.272 | 5.62E-02 | 5.62E+00 | | | |
| 7893890 | --- | --- | --- | 0.347 | 1.272 | 5.07E-01 | 5.07E+01 | | | |
| 8108238 | NM_00100141 | SMAD5 | SMAD family member 5 | 0.347 | 1.272 | 3.83E-02 | 3.83E+00 | Up | | |
| 7961059 | NM_002258 | KLRB1 | killer cell lectin-like receptor subfam | 0.347 | 1.272 | 5.16E-01 | 5.16E+01 | | | |
| 8003903 | NM_004313 | ARRB2 | arrestin, beta 2 | 0.347 | 1.272 | 2.78E-02 | 2.78E+00 | Up | | |
| 7927658 | NM_003338 | UBE2D1 | ubiquitin-conjugating enzyme E2D 1 | 0.347 | 1.272 | 2.00E-01 | 2.00E+01 | | | |
| 7896188 | --- | --- | --- | 0.347 | 1.272 | 4.04E-01 | 4.04E+01 | | | |
| 7990582 | NM_020843 | SCAPER | S-phase cyclin A-associated protein i | 0.347 | 1.272 | 1.03E-01 | 1.03E+01 | | | |
| 7932311 | NM_012425 | RSU1 | Ras suppressor protein 1 | 0.347 | 1.272 | 1.77E-01 | 1.77E+01 | | | |
| 7952953 | NM_018979 | WNK1 | WNK lysine deficient protein kinase 1 | 0.347 | 1.272 | 1.08E-02 | 1.08E+00 | Up | | |
| 7894654 | --- | --- | --- | 0.347 | 1.272 | 5.81E-01 | 5.81E+01 | | | |
| 7968931 | NM_031431 | COG3 | component of oligomeric golgi comp | 0.347 | 1.272 | 8.60E-02 | 8.60E+00 | | | |
| 7895558 | --- | --- | --- | 0.347 | 1.272 | 6.84E-01 | 6.84E+01 | | | |
| 8095854 | NM_018243 | 40432 | septin 11 | 0.346 | 1.271 | 4.00E-02 | 4.00E+00 | Up | | |
| 7947624 | NM_00110180 | PHF21A | PHD finger protein 21A | 0.346 | 1.271 | 9.46E-02 | 9.46E+00 | | | |
| 7917240 | NM_004388 | CTBS | chitobiase, di-N-acetyl- | 0.346 | 1.271 | 2.58E-01 | 2.58E+01 | | | |
| 7969693 | NM_021033 | RAP2A | RAP2A, member of RAS oncogene fa | 0.346 | 1.271 | 1.89E-01 | 1.89E+01 | | | |
| 7893153 | --- | --- | --- | 0.346 | 1.271 | 6.73E-01 | 6.73E+01 | | | |
| 7895414 | --- | --- | --- | 0.346 | 1.271 | 3.26E-01 | 3.26E+01 | | | |
| 7937287 | NM_002817 | PSMD13 | proteasome (prosome, macropain) 2 | 0.346 | 1.271 | 1.95E-01 | 1.95E+01 | | | |
| 7914809 | NM_024874 | KIAA0319L | KIAA0319-like | 0.346 | 1.271 | 3.72E-02 | 3.72E+00 | Up | | |
| 7942879 | NM_018480 | TMEM126B | transmembrane protein 126B | 0.346 | 1.271 | 3.18E-01 | 3.18E+01 | | | |
| 7970999 | NM_015087 | SPG20 | spastic paraplegia 20 (Troyer syndro | 0.345 | 1.270 | 2.53E-01 | 2.53E+01 | | | |

| | | | | | | | | | | | |
|---|---|---|---|---|---|---|---|---|---|---|---|
| 7894927 | --- | --- | --- | 0.345 | 1.270 | 4.98E-01 | 4.98E+01 | | | | |
| 7977803 | NM_00107735 | RBM23 | RNA binding motif protein 23 | 0.345 | 1.270 | 2.66E-01 | 2.66E+01 | | | | |
| 7901479 | NM_024646 | ZYG11B | zyg-11 homolog B (C. elegans) | 0.345 | 1.270 | 3.73E-02 | 3.73E+00 | Up | | | |
| 7902382 | NM_004582 | RABGGTB | Rab geranylgeranyltransferase, beta | 0.345 | 1.270 | 2.25E-01 | 2.25E+01 | | | | |
| 8031097 | NM_004542 | NDUFA3 | NADH dehydrogenase (ubiquinone) 1 | 0.345 | 1.270 | 1.40E-01 | 1.40E+01 | | | | |
| 8163402 | NM_005156 | ROD1 | ROD1 regulator of differentiation 1 ( | 0.345 | 1.270 | 9.59E-02 | 9.59E+00 | | | | |
| 7915261 | NM_017646 | TRIT1 | tRNA isopentenyltransferase 1 | 0.345 | 1.270 | 1.13E-01 | 1.13E+01 | | | | |
| 8017212 | NM_032582 | USP32 | ubiquitin specific peptidase 32 | 0.344 | 1.270 | 2.66E-01 | 2.66E+01 | | | | |
| 7971602 | NM_018191 | RCBTB1 | regulator of chromosome condensat | 0.344 | 1.270 | 3.29E-02 | 3.29E+00 | Up | | | |
| 7969979 | NM_032859 | ABHD13 | abhydrolase domain containing 13 | 0.344 | 1.270 | 7.79E-02 | 7.79E+00 | | | | |
| 8079772 | NM_001640 | APEH | N-acylaminoacyl-peptide hydrolase | 0.344 | 1.269 | 4.06E-02 | 4.06E+00 | Up | | | |
| 8172119 | NM_004229 | MED14 | mediator complex subunit 14 | 0.344 | 1.269 | 8.69E-02 | 8.69E+00 | | | | |
| 7893187 | --- | --- | --- | 0.344 | 1.269 | 2.89E-01 | 2.89E+01 | | | | |
| 7952988 | NR_027948 | ERC1 | ELKS/RAB6-interacting/CAST family r | 0.344 | 1.269 | 4.27E-02 | 4.27E+00 | Up | | | |
| 8059770 | NM_145702 | TIGD1 | tigger transposable element derived | 0.344 | 1.269 | 1.85E-01 | 1.85E+01 | | | | |
| 8099649 | NM_001358 | DHX15 | DEAH (Asp-Glu-Ala-His) box polypept | 0.344 | 1.269 | 2.92E-02 | 2.92E+00 | Up | | | |
| 8086148 | NM_000992 | RPL29 | ribosomal protein L29 | 0.343 | 1.269 | 2.63E-01 | 2.63E+01 | | | | |
| 8054771 | NM_025181 | SLC35F5 | solute carrier family 35, member F5 | 0.343 | 1.269 | 4.47E-02 | 4.47E+00 | Up | | | |
| 8006820 | NM_006148 | LASP1 | LIM and SH3 protein 1 | 0.343 | 1.269 | 4.99E-02 | 4.99E+00 | Up | | | |
| 8081348 | NM_020357 | PCNP | PEST proteolytic signal containing nu | 0.343 | 1.269 | 3.71E-01 | 3.71E+01 | | | | |
| 8128394 | NM_032870 | SFRS18 | splicing factor, arginine/serine-rich 1 | 0.343 | 1.268 | 9.28E-02 | 9.28E+00 | | | | |
| 8164596 | NM_016520 | C9orf78 | chromosome 9 open reading frame 7 | 0.343 | 1.268 | 3.12E-01 | 3.12E+01 | | | | |
| 8146550 | NM_005625 | SDCBP | syndecan binding protein (syntenin) | 0.343 | 1.268 | 3.72E-01 | 3.72E+01 | | | | |
| 7929624 | NM_002629 | PGAM1 | phosphoglycerate mutase 1 (brain) | 0.343 | 1.268 | 4.14E-02 | 4.14E+00 | Up | | | |
| 7893517 | --- | --- | --- | 0.343 | 1.268 | 3.20E-01 | 3.20E+01 | | | | |
| 8022625 | NM_00100755 | SS18 | synovial sarcoma translocation, chro | 0.342 | 1.268 | 1.49E-01 | 1.49E+01 | | | | |
| 8081362 | NM_024548 | CEP97 | centrosomal protein 97kDa | 0.342 | 1.268 | 1.22E-01 | 1.22E+01 | | | | |
| 8016858 | NM_004645 | COIL | coilin | 0.342 | 1.268 | 2.37E-01 | 2.37E+01 | | | | |
| 8063242 | NM_006420 | ARFGEF2 | ADP-ribosylation factor guanine nucl | 0.342 | 1.268 | 3.17E-02 | 3.17E+00 | Up | | | |
| 8155636 | NM_201453 | CBWD3 | COBW domain containing 3 | 0.342 | 1.268 | 1.44E-01 | 1.44E+01 | | | | |
| 7964250 | NM_006601 | PTGES3 | prostaglandin E synthase 3 (cytosolic | 0.342 | 1.267 | 3.95E-01 | 3.95E+01 | | | | |
| 8039196 | NM_00108145 | LILRB3 | leukocyte immunoglobulin-like recep | 0.342 | 1.267 | 9.88E-04 | 9.88E-02 | Up | | | mono |
| 7986433 | NM_198243 | ASB7 | ankyrin repeat and SOCS box-contair | 0.342 | 1.267 | 1.04E-01 | 1.04E+01 | | | | |
| 7956876 | NM_032338 | LLPH | LLP homolog, long-term synaptic fac | 0.341 | 1.267 | 6.46E-01 | 6.46E+01 | | | | |
| 7903765 | NM_000561 | GSTM1 | glutathione S-transferase mu 1 | 0.341 | 1.267 | 3.15E-01 | 3.15E+01 | | | | |
| 7933084 | NM_005746 | NAMPT | nicotinamide phosphoribosyltransfer | 0.341 | 1.267 | 3.71E-01 | 3.71E+01 | | | | |
| 8013696 | NM_014680 | KIAA0100 | KIAA0100 | 0.341 | 1.267 | 4.28E-02 | 4.28E+00 | Up | | | |
| 7925609 | --- | --- | --- | 0.341 | 1.267 | 4.56E-01 | 4.56E+01 | | | | |
| 7896709 | --- | --- | --- | 0.341 | 1.267 | 4.49E-01 | 4.49E+01 | | | | |
| 8046306 | NM_015530 | GORASP2 | golgi reassembly stacking protein 2, | 0.341 | 1.267 | 1.09E-01 | 1.09E+01 | | | | |
| 8140037 | NM_032408 | BAZ1B | bromodomain adjacent to zinc finger | 0.341 | 1.267 | 4.81E-02 | 4.81E+00 | Up | | | |
| 7895231 | --- | --- | --- | 0.341 | 1.267 | 6.38E-01 | 6.38E+01 | | | | |
| 7894471 | --- | --- | --- | 0.341 | 1.267 | 1.69E-03 | 1.69E-01 | Up | | | |
| 8089249 | NM_000986 | RPL24 | ribosomal protein L24 | 0.341 | 1.266 | 2.23E-01 | 2.23E+01 | | | | |
| 7893196 | --- | --- | --- | 0.341 | 1.266 | 2.43E-01 | 2.43E+01 | | | | |
| 8014066 | NM_00100392 | EVI2A | ecotropic viral integration site 2A | 0.341 | 1.266 | 7.49E-02 | 7.49E+00 | | | | |
| 8060839 | NM_019095 | CRLS1 | cardiolipin synthase 1 | 0.341 | 1.266 | 2.85E-01 | 2.85E+01 | | | | |
| 7895334 | --- | --- | --- | 0.340 | 1.266 | 3.99E-01 | 3.99E+01 | | | | |
| 7952986 | NM_213655 | HSN2 | hereditary sensory neuropathy, type | 0.340 | 1.266 | 1.49E-01 | 1.49E+01 | | | | |
| 7927288 | NM_019054 | FAM35A | family with sequence similarity 35, m | 0.340 | 1.266 | 8.02E-02 | 8.02E+00 | | | | |
| 7966268 | NM_057169 | GIT2 | G protein-coupled receptor kinase in | 0.340 | 1.266 | 2.11E-01 | 2.11E+01 | | | | |
| 8056860 | NM_003387 | WIPF1 | WAS/WASL interacting protein famil | 0.340 | 1.266 | 5.97E-02 | 5.97E+00 | | | | |
| 7892973 | --- | --- | --- | 0.340 | 1.266 | 4.34E-01 | 4.34E+01 | | | | |
| 8001507 | NM_031885 | BBS2 | Bardet-Biedl syndrome 2 | 0.340 | 1.266 | 1.84E-01 | 1.84E+01 | | | | |
| 8125556 | NM_033554 | HLA-DPA1 | major histocompatibility complex, cl | 0.340 | 1.266 | 3.52E-01 | 3.52E+01 | | | | |
| 8178891 | NM_033554 | HLA-DPA1 | major histocompatibility complex, cl | 0.340 | 1.266 | 3.52E-01 | 3.52E+01 | | | | |
| 7905733 | NM_006118 | HAX1 | HCLS1 associated protein X-1 | 0.340 | 1.266 | 4.62E-01 | 4.62E+01 | | | | |
| 7893471 | --- | --- | --- | 0.339 | 1.265 | 7.31E-01 | 7.31E+01 | | | | |
| 7932512 | NM_022365 | DNAJC1 | DnaJ (Hsp40) homolog, subfamily C, | 0.339 | 1.265 | 4.06E-01 | 4.06E+01 | | | | |
| 8055672 | NM_015702 | MMADHC | methylmalonic aciduria (cobalamin d | 0.339 | 1.265 | 4.28E-01 | 4.28E+01 | | | | |
| 8053533 | NM_006839 | IMMT | inner membrane protein, mitochond | 0.339 | 1.265 | 4.05E-02 | 4.05E+00 | Up | | | |
| 8103859 | NM_00101273 | DCTD | dCMP deaminase | 0.339 | 1.265 | 2.16E-01 | 2.16E+01 | | | | |
| 8138773 | NM_022170 | EIF4H | eukaryotic translation initiation facto | 0.339 | 1.265 | 3.57E-01 | 3.57E+01 | | | | |
| 7948088 | NM_032315 | SLC25A33 | solute carrier family 25, member 33 | 0.339 | 1.265 | 2.17E-01 | 2.17E+01 | | | | |
| 7904702 | NM_203458 | NOTCH2NL | Notch homolog 2 (Drosophila) N-terr | 0.339 | 1.265 | 1.31E-01 | 1.31E+01 | | | | |
| 7893896 | --- | --- | --- | 0.339 | 1.265 | 3.88E-01 | 3.88E+01 | | | | |
| 8023415 | NM_00108396 | TCF4 | transcription factor 4 | 0.339 | 1.265 | 2.17E-02 | 2.17E+00 | Up | | | |
| 8156718 | NM_002486 | NCBP1 | nuclear cap binding protein subunit 1 | 0.339 | 1.265 | 1.95E-01 | 1.95E+01 | | | | |
| 7927775 | NM_030759 | NRBF2 | nuclear receptor binding factor 2 | 0.339 | 1.265 | 6.71E-02 | 6.71E+00 | | | | |
| 8092691 | NM_001706 | BCL6 | B-cell CLL/lymphoma 6 | 0.339 | 1.265 | 6.37E-02 | 6.37E+00 | | | | |
| 7893466 | --- | --- | --- | 0.339 | 1.265 | 5.95E-01 | 5.95E+01 | | | | |
| 8043848 | NM_145212 | MRPL30 | mitochondrial ribosomal protein L30 | 0.339 | 1.265 | 8.93E-02 | 8.93E+00 | | | | |
| 8015685 | NM_001991 | EZH1 | enhancer of zeste homolog 1 (Droso | 0.339 | 1.264 | 1.51E-01 | 1.51E+01 | | | | |
| 7945101 | NM_014026 | DCPS | decapping enzyme, scavenger | 0.338 | 1.264 | 8.51E-02 | 8.51E+00 | | | | |
| 7894136 | --- | --- | --- | 0.338 | 1.264 | 5.70E-01 | 5.70E+01 | | | | |
| 8141490 | NR_003613 | PMS2L1 | postmeiotic segregation increased 2- | 0.338 | 1.264 | 1.64E-01 | 1.64E+01 | | | | |
| 7910398 | NM_004578 | RAB4A | RAB4A, member RAS oncogene fami | 0.338 | 1.264 | 1.07E-01 | 1.07E+01 | | | | |

| | | | | | | | | | | |
|---|---|---|---|---|---|---|---|---|---|---|
| 8112070 | --- | --- | --- | 0.338 | 1.264 | 3.29E-01 | 3.29E+01 | | | |
| 7894963 | --- | --- | --- | 0.338 | 1.264 | 6.31E-01 | 6.31E+01 | | | |
| 7957549 | --- | --- | --- | 0.338 | 1.264 | 3.25E-01 | 3.25E+01 | | | |
| 8036324 | NM_00101275( | ZNF260 | zinc finger protein 260 | 0.338 | 1.264 | 1.45E-02 | 1.45E+00 | Up | | |
| 8111584 | BC144069 | C5orf42 | chromosome 5 open reading frame 4 | 0.338 | 1.264 | 1.96E-02 | 1.96E+00 | Up | | |
| 7895932 | --- | --- | --- | 0.338 | 1.264 | 5.77E-01 | 5.77E+01 | | | |
| 7893070 | --- | --- | --- | 0.338 | 1.264 | 4.89E-01 | 4.89E+01 | | | |
| 7896630 | --- | --- | --- | 0.338 | 1.264 | 7.28E-01 | 7.28E+01 | | | |
| 8051573 | NM_006449 | CDC42EP3 | CDC42 effector protein (Rho GTPase | 0.338 | 1.264 | 2.06E-02 | 2.06E+00 | Up | | |
| 8147848 | NM_181354 | OXR1 | oxidation resistance 1 | 0.337 | 1.264 | 1.60E-01 | 1.60E+01 | | | |
| 8081316 | NM_006070 | TFG | TRK-fused gene | 0.337 | 1.263 | 2.15E-01 | 2.15E+01 | | | |
| 7942287 | --- | --- | --- | 0.337 | 1.263 | 2.87E-01 | 2.87E+01 | | | |
| 7896112 | --- | --- | --- | 0.337 | 1.263 | 4.09E-01 | 4.09E+01 | | | |
| 8151066 | NM_018120 | ARMC1 | armadillo repeat containing 1 | 0.337 | 1.263 | 3.68E-02 | 3.68E+00 | Up | | |
| 8060813 | NM_032485 | MCM8 | minichromosome maintenance comp | 0.337 | 1.263 | 1.51E-01 | 1.51E+01 | | | |
| 7903425 | NM_004038 | AMY1A | amylase, alpha 1A (salivary) | 0.337 | 1.263 | 3.11E-01 | 3.11E+01 | | | |
| 7903440 | NM_004038 | AMY1A | amylase, alpha 1A (salivary) | 0.337 | 1.263 | 3.11E-01 | 3.11E+01 | | | |
| 7918134 | NM_004038 | AMY1A | amylase, alpha 1A (salivary) | 0.337 | 1.263 | 3.11E-01 | 3.11E+01 | | | |
| 8022996 | NM_020964 | KIAA1632 | KIAA1632 | 0.337 | 1.263 | 7.26E-03 | 7.26E-01 | Up | | |
| 7961962 | --- | --- | --- | 0.337 | 1.263 | 3.16E-01 | 3.16E+01 | | | |
| 7893346 | --- | --- | --- | 0.337 | 1.263 | 5.00E-01 | 5.00E+01 | | | |
| 7918188 | NM_013386 | SLC25A24 | solute carrier family 25 (mitochondri | 0.336 | 1.263 | 3.69E-02 | 3.69E+00 | Up | | |
| 7948696 | NM_004739 | MTA2 | metastasis associated 1 family, mem | 0.336 | 1.263 | 6.90E-02 | 6.90E+00 | | | |
| 8156263 | NM_006717 | SPIN1 | spindlin 1 | 0.336 | 1.262 | 2.54E-01 | 2.54E+01 | | | |
| 7892776 | --- | --- | --- | 0.336 | 1.262 | 2.42E-01 | 2.42E+01 | | | |
| 7989195 | --- | --- | --- | 0.336 | 1.262 | 4.85E-01 | 4.85E+01 | | | |
| 8047403 | NM_032977 | CASP10 | caspase 10, apoptosis-related cystei | 0.336 | 1.262 | 7.21E-03 | 7.21E-01 | Up | | |
| 7896426 | --- | --- | --- | 0.336 | 1.262 | 4.71E-01 | 4.71E+01 | | | |
| 8022557 | --- | --- | --- | 0.336 | 1.262 | 5.95E-01 | 5.95E+01 | | | |
| 7893575 | --- | --- | --- | 0.336 | 1.262 | 5.44E-01 | 5.44E+01 | | | |
| 7911897 | NM_207356 | C1orf174 | chromosome 1 open reading frame 1 | 0.335 | 1.262 | 1.83E-01 | 1.83E+01 | | | |
| 7940486 | BC002331 | SDHAF2 | succinate dehydrogenase complex a | 0.335 | 1.261 | 2.30E-01 | 2.30E+01 | | | |
| 8042801 | NM_080916 | DGUOK | deoxyguanosine kinase | 0.335 | 1.261 | 1.18E-01 | 1.18E+01 | | | |
| 8145793 | NR_003041 | SNORD13 | small nucleolar RNA, C/D box 13 | 0.335 | 1.261 | 5.59E-01 | 5.59E+01 | | | |
| 7894098 | --- | --- | --- | 0.335 | 1.261 | 3.10E-01 | 3.10E+01 | | | |
| 8055890 | NM_005843 | STAM2 | signal transducing adaptor molecule | 0.335 | 1.261 | 1.80E-01 | 1.80E+01 | | | |
| 7978997 | NM_198794 | MAP4K5 | mitogen-activated protein kinase kin | 0.335 | 1.261 | 2.44E-01 | 2.44E+01 | | | |
| 7892793 | --- | --- | --- | 0.335 | 1.261 | 7.24E-01 | 7.24E+01 | | | |
| 7953981 | NM_001987 | ETV6 | ets variant 6 | 0.335 | 1.261 | 7.97E-02 | 7.97E+00 | | | |
| 7932616 | NM_005470 | ABI1 | abl-interactor 1 | 0.334 | 1.261 | 8.65E-02 | 8.65E+00 | | | |
| 8136259 | NM_013255 | MKLN1 | muskelin 1, intracellular mediator co | 0.334 | 1.261 | 2.97E-01 | 2.97E+01 | | | |
| 8063211 | NM_181659 | NCOA3 | nuclear receptor coactivator 3 | 0.334 | 1.261 | 5.35E-02 | 5.35E+00 | | | |
| 7911341 | --- | --- | --- | 0.334 | 1.261 | 4.84E-01 | 4.84E+01 | | | |
| 7953892 | NM_016523 | KLRF1 | killer cell lectin-like receptor subfam | 0.334 | 1.261 | 5.49E-01 | 5.49E+01 | | | |
| 8133095 | NR_027392 | INTS4L2 | integrator complex subunit 4-like 2 | 0.334 | 1.261 | 1.79E-01 | 1.79E+01 | | | |
| 7960518 | NM_001065 | TNFRSF1A | tumor necrosis factor receptor super | 0.334 | 1.261 | 4.84E-03 | 4.84E-01 | Up | | |
| 8077815 | NR_026829 | C3orf42 | chromosome 3 open reading frame 4 | 0.334 | 1.260 | 2.92E-01 | 2.92E+01 | | | |
| 7931216 | NM_032182 | FAM175B | family with sequence similarity 175, | 0.334 | 1.260 | 4.36E-01 | 4.36E+01 | | | |
| 8156688 | NM_014290 | TDRD7 | tudor domain containing 7 | 0.334 | 1.260 | 9.26E-03 | 9.26E-01 | Up | | |
| 7892850 | --- | --- | --- | 0.333 | 1.260 | 7.21E-01 | 7.21E+01 | | | |
| 7895839 | --- | --- | --- | 0.333 | 1.260 | 7.34E-01 | 7.34E+01 | | | |
| 8157038 | NM_080546 | SLC44A1 | solute carrier family 44, member 1 | 0.333 | 1.260 | 5.72E-02 | 5.72E+00 | | | |
| 8155268 | NM_022490 | POLR1E | polymerase (RNA) I polypeptide E, 53 | 0.333 | 1.260 | 2.33E-01 | 2.33E+01 | | | |
| 7892749 | --- | --- | --- | 0.333 | 1.260 | 5.81E-01 | 5.81E+01 | | | |
| 7993580 | NM_006985 | NPIP | nuclear pore complex interacting pro | 0.333 | 1.260 | 2.77E-02 | 2.77E+00 | Up | | |
| 7894781 | --- | --- | --- | 0.333 | 1.259 | 5.08E-01 | 5.08E+01 | | | |
| 8168416 | NM_032747 | USMG5 | up-regulated during skeletal muscle | 0.333 | 1.259 | 5.77E-01 | 5.77E+01 | | | |
| 8000375 | NM_00100663 | ARHGAP17 | Rho GTPase activating protein 17 | 0.332 | 1.259 | 8.49E-03 | 8.49E-01 | Up | | |
| 8099395 | NM_004249 | RAB28 | RAB28, member RAS oncogene famil | 0.332 | 1.259 | 4.05E-01 | 4.05E+01 | | | |
| 7934513 | NM_207012 | AP3M1 | adaptor-related protein complex 3, n | 0.332 | 1.259 | 1.83E-02 | 1.83E+00 | Up | | |
| 7893047 | --- | --- | --- | 0.332 | 1.259 | 5.53E-01 | 5.53E+01 | | | |
| 8089128 | NM_014820 | TOMM70A | translocase of outer mitochondrial m | 0.332 | 1.258 | 3.52E-02 | 3.52E+00 | Up | | |
| 8049784 | NM_002712 | PPP1R7 | protein phosphatase 1, regulatory (ir | 0.331 | 1.258 | 2.38E-01 | 2.38E+01 | | | |
| 8138776 | NM_152740 | HIBADH | 3-hydroxyisobutyrate dehydrogenase | 0.331 | 1.258 | 1.75E-01 | 1.75E+01 | | | |
| 7928937 | NM_004897 | MINPP1 | multiple inositol polyphosphate histi | 0.331 | 1.258 | 1.39E-01 | 1.39E+01 | | | |
| 7892564 | --- | --- | --- | 0.331 | 1.258 | 2.80E-01 | 2.80E+01 | | | |
| 7993359 | NM_006985 | NPIP | nuclear pore complex interacting pro | 0.331 | 1.258 | 7.32E-02 | 7.32E+00 | | | |
| 7948420 | NM_001444 | FABP5 | fatty acid binding protein 5 (psoriasis | 0.331 | 1.258 | 5.00E-01 | 5.00E+01 | | | |
| 7980309 | NM_007176 | C14orf1 | chromosome 14 open reading frame | 0.331 | 1.258 | 2.86E-01 | 2.86E+01 | | | |
| 7978187 | NM_014169 | CHMP4A | chromatin modifying protein 4A | 0.331 | 1.258 | 7.88E-02 | 7.88E+00 | | | |
| 8156538 | NM_032558 | HIATL1 | hippocampus abundant transcript-lik | 0.331 | 1.258 | 4.86E-01 | 4.86E+01 | | | |
| 7895006 | --- | --- | --- | 0.331 | 1.257 | 4.79E-01 | 4.79E+01 | | | |
| 8096004 | NM_198892 | BMP2K | BMP2 inducible kinase | 0.330 | 1.257 | 3.92E-02 | 3.92E+00 | Up | | |
| 7936614 | NM_003750 | EIF3A | eukaryotic translation initiation facto | 0.330 | 1.257 | 1.71E-01 | 1.71E+01 | | | |
| 8094625 | NM_015990 | KLHL5 | kelch-like 5 (Drosophila) | 0.330 | 1.257 | 2.39E-01 | 2.39E+01 | | | |
| 7945829 | --- | --- | --- | 0.330 | 1.257 | 3.91E-01 | 3.91E+01 | | | |
| 7978449 | NM_015382 | HECTD1 | HECT domain containing 1 | 0.330 | 1.257 | 1.12E-01 | 1.12E+01 | | | |

| | | | | | | | | | | |
|---|---|---|---|---|---|---|---|---|---|---|
| 8002041 | NM_004691 | ATP6V0D1 | ATPase, H+ transporting, lysosomal 3 | 0.330 | 1.257 | 4.82E-02 | 4.82E+00 | Up | | |
| 8046680 | NM_019091 | PLEKHA3 | pleckstrin homology domain contain | 0.330 | 1.257 | 3.96E-01 | 3.96E+01 | | | |
| 8058670 | NM_016260 | IKZF2 | IKAROS family zinc finger 2 (Helios) | 0.330 | 1.257 | 8.93E-02 | 8.93E+00 | | | |
| 7894901 | --- | --- | --- | 0.330 | 1.257 | 6.16E-01 | 6.16E+01 | | | |
| 7894711 | --- | --- | --- | 0.330 | 1.257 | 3.51E-01 | 3.51E+01 | | | |
| 8145365 | NM_024940 | DOCK5 | dedicator of cytokinesis 5 | 0.329 | 1.257 | 6.91E-02 | 6.91E+00 | | | |
| 7893923 | --- | --- | --- | 0.329 | 1.257 | 3.19E-01 | 3.19E+01 | | | |
| 7903474 | --- | --- | --- | 0.329 | 1.256 | 1.52E-01 | 1.52E+01 | | | |
| 7971513 | NM_001984 | ESD | esterase D/formylglutathione hydrol | 0.329 | 1.256 | 3.00E-01 | 3.00E+01 | | | |
| 8111136 | NM_00103485( | FAM134B | family with sequence similarity 134, | 0.329 | 1.256 | 1.34E-01 | 1.34E+01 | | | |
| 8011462 | NM_018553 | C17orf85 | chromosome 17 open reading frame | 0.329 | 1.256 | 1.32E-01 | 1.32E+01 | | | |
| 8126450 | NM_000986 | RPL24 | ribosomal protein L24 | 0.329 | 1.256 | 2.78E-01 | 2.78E+01 | | | |
| 7893794 | --- | --- | --- | 0.328 | 1.256 | 6.11E-01 | 6.11E+01 | | | |
| 8042416 | NM_014882 | ARHGAP25 | Rho GTPase activating protein 25 | 0.328 | 1.255 | 5.36E-02 | 5.36E+00 | | | |
| 7895094 | --- | --- | --- | 0.328 | 1.255 | 7.25E-01 | 7.25E+01 | | | |
| 7896391 | --- | --- | --- | 0.328 | 1.255 | 5.48E-01 | 5.48E+01 | | | |
| 7942679 | NM_018367 | ACER3 | alkaline ceramidase 3 | 0.328 | 1.255 | 3.49E-01 | 3.49E+01 | | | |
| 8069553 | NM_003489 | NRIP1 | nuclear receptor interacting protein | 0.328 | 1.255 | 3.15E-01 | 3.15E+01 | | | |
| 8162086 | NM_015239 | AGTPBP1 | ATP/GTP binding protein 1 | 0.328 | 1.255 | 2.73E-01 | 2.73E+01 | | | |
| 8133434 | NM_022170 | EIF4H | eukaryotic translation initiation facto | 0.328 | 1.255 | 3.73E-01 | 3.73E+01 | | | |
| 8124940 | NR_003140 | SNORD117 | small nucleolar RNA, C/D box 117 | 0.328 | 1.255 | 6.04E-01 | 6.04E+01 | | | |
| 7933760 | NM_005436 | CCDC6 | coiled-coil domain containing 6 | 0.328 | 1.255 | 3.57E-01 | 3.57E+01 | | | |
| 7973303 | X01403 | TRA@ | T cell receptor alpha locus | 0.327 | 1.255 | 5.08E-01 | 5.08E+01 | | | |
| 7896697 | --- | --- | --- | 0.327 | 1.255 | 1.20E-01 | 1.20E+01 | | | |
| 7894757 | --- | --- | --- | 0.327 | 1.255 | 5.15E-01 | 5.15E+01 | | | |
| 8086752 | NR_003041 | SNORD13 | small nucleolar RNA, C/D box 13 | 0.327 | 1.255 | 5.48E-01 | 5.48E+01 | | | |
| 8120756 | --- | --- | --- | 0.327 | 1.255 | 5.82E-01 | 5.82E+01 | | | |
| 7960259 | --- | --- | --- | 0.327 | 1.254 | 4.23E-01 | 4.23E+01 | | | |
| 7896232 | --- | --- | --- | 0.327 | 1.254 | 2.43E-01 | 2.43E+01 | | | |
| 8070330 | NM_003720 | PSMG1 | proteasome (prosome, macropain) a | 0.327 | 1.254 | 1.60E-01 | 1.60E+01 | | | |
| 8036224 | NM_003332 | TYROBP | TYRO protein tyrosine kinase binding | 0.327 | 1.254 | 4.24E-02 | 4.24E+00 | Up | | |
| 7894783 | --- | --- | --- | 0.327 | 1.254 | 7.30E-01 | 7.30E+01 | | | |
| 7894885 | --- | --- | --- | 0.327 | 1.254 | 1.80E-01 | 1.80E+01 | | | |
| 8059712 | NR_004398 | SNORD82 | small nucleolar RNA, C/D box 82 | 0.327 | 1.254 | 7.27E-01 | 7.27E+01 | | | |
| 8043465 | BC029444 // B | GKC // IGKC | immunoglobulin kappa constant // i | 0.327 | 1.254 | 4.10E-02 | 4.10E+00 | Up | | |
| 8099340 | NM_017491 | WDR1 | WD repeat domain 1 | 0.326 | 1.254 | 1.60E-01 | 1.60E+01 | | | |
| 7973067 | NM_000270 | NP | nucleoside phosphorylase | 0.326 | 1.254 | 3.20E-01 | 3.20E+01 | | | |
| 7894405 | --- | --- | --- | 0.326 | 1.254 | 7.38E-01 | 7.38E+01 | | | |
| 8168316 | NM_181672 | OGT | O-linked N-acetylglucosamine (GlcNA | 0.326 | 1.254 | 5.36E-02 | 5.36E+00 | | | |
| 8100834 | NM_002092 | GRSF1 | G-rich RNA sequence binding factor 1 | 0.326 | 1.253 | 4.27E-02 | 4.27E+00 | Up | | |
| 7896154 | --- | --- | --- | 0.326 | 1.253 | 6.53E-01 | 6.53E+01 | | | |
| 7993349 | NM_006985 | NPIP | nuclear pore complex interacting pro | 0.326 | 1.253 | 8.50E-02 | 8.50E+00 | | | |
| 7957737 | NM_00103228: | TMPO | thymopoietin | 0.325 | 1.253 | 9.37E-02 | 9.37E+00 | | | |
| 8029273 | NM_001410 | MEGF8 | multiple EGF-like-domains 8 | 0.325 | 1.253 | 3.76E-01 | 3.76E+01 | | | |
| 7985224 | NM_144572 | TBC1D2B | TBC1 domain family, member 2B | 0.325 | 1.253 | 3.41E-01 | 3.41E+01 | | | |
| 7894282 | --- | --- | --- | 0.325 | 1.253 | 2.25E-01 | 2.25E+01 | | | |
| 7934852 | NM_005271 | GLUD1 | glutamate dehydrogenase 1 | 0.325 | 1.253 | 2.54E-01 | 2.54E+01 | | | |
| 7908097 | NM_173156 | SMG7 | Smg-7 homolog, nonsense mediated | 0.325 | 1.253 | 1.27E-02 | 1.27E+00 | Up | | |
| 7895831 | --- | --- | --- | 0.325 | 1.253 | 7.47E-01 | 7.47E+01 | | | |
| 7893792 | --- | --- | --- | 0.324 | 1.252 | 3.31E-01 | 3.31E+01 | | | |
| 7892981 | --- | --- | --- | 0.324 | 1.252 | 3.35E-01 | 3.35E+01 | | | |
| 8084844 | NM_130837 | OPA1 | optic atrophy 1 (autosomal dominan | 0.324 | 1.252 | 1.91E-01 | 1.91E+01 | | | |
| 7895299 | --- | --- | --- | 0.324 | 1.252 | 5.84E-01 | 5.84E+01 | | | |
| 8092265 | NM_020409 | MRPL47 | mitochondrial ribosomal protein L47 | 0.324 | 1.252 | 4.75E-01 | 4.75E+01 | | | |
| 8131356 | NM_015622 | C7orf28A | chromosome 7 open reading frame 2 | 0.324 | 1.252 | 4.50E-02 | 4.50E+00 | Up | | |
| 8082058 | NM_005213 | CSTA | cystatin A (stefin A) | 0.324 | 1.252 | 3.44E-01 | 3.44E+01 | | | mono |
| 7991566 | NM_018148 | LINS1 | lines homolog 1 (Drosophila) | 0.324 | 1.251 | 1.11E-01 | 1.11E+01 | | | |
| 8061075 | NM_003092 | SNRPB2 | small nuclear ribonucleoprotein poly | 0.324 | 1.251 | 3.40E-01 | 3.40E+01 | | | |
| 8150757 | NM_014781 | RB1CC1 | RB1-inducible coiled-coil 1 | 0.323 | 1.251 | 3.31E-01 | 3.31E+01 | | | |
| 7959322 | NM_002813 | PSMD9 | proteasome (prosome, macropain) 2 | 0.323 | 1.251 | 1.02E-01 | 1.02E+01 | | | |
| 7896580 | --- | --- | --- | 0.323 | 1.251 | 3.91E-01 | 3.91E+01 | | | |
| 8029377 | NM_013398 | ZNF224 | zinc finger protein 224 | 0.323 | 1.251 | 9.07E-02 | 9.07E+00 | | | |
| 8060611 | NM_033453 | ITPA | inosine triphosphatase (nucleoside t | 0.323 | 1.251 | 1.04E-02 | 1.04E+00 | Up | | |
| 8131975 | NM_006024 | TAX1BP1 | Tax1 (human T-cell leukemia virus ty | 0.322 | 1.250 | 2.52E-01 | 2.52E+01 | | | |
| 7994131 | NM_002738 | PRKCB | protein kinase C, beta | 0.322 | 1.250 | 4.35E-02 | 4.35E+00 | Up | | |
| 7923889 | NM_006893 | LGTN | ligatin | 0.322 | 1.250 | 2.41E-02 | 2.41E+00 | Up | | |
| 7894479 | --- | --- | --- | 0.322 | 1.250 | 7.20E-01 | 7.20E+01 | | | |
| 8097470 | --- | --- | --- | 0.322 | 1.250 | 1.30E-01 | 1.30E+01 | | | |
| 7893547 | --- | --- | --- | 0.322 | 1.250 | 4.73E-01 | 4.73E+01 | | | |
| 8147049 | NM_001444 | FABP5 | fatty acid binding protein 5 (psoriasis | 0.322 | 1.250 | 5.15E-01 | 5.15E+01 | | | |
| 8116969 | NM_016167 | NOL7 | nucleolar protein 7, 27kDa | 0.322 | 1.250 | 1.40E-01 | 1.40E+01 | | | |
| 7894679 | --- | --- | --- | 0.322 | 1.250 | 6.37E-01 | 6.37E+01 | | | |
| 7893738 | --- | --- | --- | 0.322 | 1.250 | 3.81E-01 | 3.81E+01 | | | |
| 7895054 | --- | --- | --- | 0.321 | 1.250 | 5.04E-01 | 5.04E+01 | | | |
| 8071061 | AK097082 | psiTPTE22 | TPTE pseudogene | 0.321 | 1.249 | 1.99E-01 | 1.99E+01 | | | |
| 8110589 | NM_015455 | CNOT6 | CCR4-NOT transcription complex, su | 0.321 | 1.249 | 2.33E-01 | 2.33E+01 | | | |
| 7955719 | NM_00109966 | HIGD1A | HIG1 hypoxia inducible domain famil | 0.321 | 1.249 | 6.24E-01 | 6.24E+01 | | | |

| | | | | | | | | | | |
|---|---|---|---|---|---|---|---|---|---|---|
| 8094848 | NM_006345 | SLC30A9 | solute carrier family 30 (zinc transpo | 0.321 | 1.249 | 2.96E-01 | 2.96E+01 | | | |
| 8103979 | NM_020827 | KIAA1430 | KIAA1430 | 0.321 | 1.249 | 3.15E-01 | 3.15E+01 | | | |
| 7893578 | --- | --- | --- | 0.321 | 1.249 | 6.04E-01 | 6.04E+01 | | | |
| 7896156 | --- | --- | --- | 0.321 | 1.249 | 5.02E-01 | 5.02E+01 | | | |
| 8091009 | NM_006219 | PIK3CB | phosphoinositide-3-kinase, catalytic, | 0.321 | 1.249 | 1.62E-01 | 1.62E+01 | | | |
| 8064302 | NM_018257 | PCMTD2 | protein-L-isoaspartate (D-aspartate) | 0.321 | 1.249 | 4.55E-01 | 4.55E+01 | | | |
| 7894404 | --- | --- | --- | 0.320 | 1.249 | 6.03E-02 | 6.03E+00 | | | |
| 7983843 | NM_207036 | TCF12 | transcription factor 12 | 0.320 | 1.248 | 1.31E-01 | 1.31E+01 | | | |
| 8093126 | --- | --- | --- | 0.320 | 1.248 | 4.63E-01 | 4.63E+01 | | | |
| 7893715 | --- | --- | --- | 0.320 | 1.248 | 6.23E-01 | 6.23E+01 | | | |
| 7956658 | NM_004731 | SLC16A7 | solute carrier family 16, member 7 (r | 0.320 | 1.248 | 5.33E-02 | 5.33E+00 | | | |
| 7937802 | NM_004356 | CD81 | CD81 molecule | 0.319 | 1.248 | 1.56E-01 | 1.56E+01 | | | |
| 8165709 | --- | --- | --- | 0.319 | 1.248 | 4.97E-01 | 4.97E+01 | | | |
| 8055913 | NM_017892 | PRPF40A | PRP40 pre-mRNA processing factor 4 | 0.319 | 1.248 | 1.28E-01 | 1.28E+01 | | | |
| 8017810 | NM_002816 | PSMD12 | proteasome (prosome, macropain) 2 | 0.319 | 1.248 | 9.19E-02 | 9.19E+00 | | | |
| 7919940 | NM_005997 | VPS72 | vacuolar protein sorting 72 homolog | 0.319 | 1.247 | 1.05E-01 | 1.05E+01 | | | |
| 8095009 | NM_00104040 | DCUN1D4 | DCN1, defective in cullin neddylation | 0.319 | 1.247 | 3.36E-01 | 3.36E+01 | | | |
| 7994826 | NM_002209 | ITGAL | integrin, alpha L (antigen CD11A (p1 | 0.319 | 1.247 | 2.90E-02 | 2.90E+00 | | | |
| 7928705 | NM_030927 | TSPAN14 | tetraspanin 14 | 0.319 | 1.247 | 2.50E-02 | 2.50E+00 | | | |
| 8088664 | NM_003848 | SUCLG2 | succinate-CoA ligase, GDP-forming, b | 0.319 | 1.247 | 2.78E-01 | 2.78E+01 | | | |
| 8174195 | --- | --- | --- | 0.319 | 1.247 | 2.05E-01 | 2.05E+01 | | | |
| 8102006 | NM_005908 | MANBA | mannosidase, beta A, lysosomal | 0.319 | 1.247 | 2.03E-01 | 2.03E+01 | | | |
| 8144812 | NM_006197 | PCM1 | pericentriolar material 1 | 0.319 | 1.247 | 3.61E-02 | 3.61E+00 | | | |
| 7895290 | --- | --- | --- | 0.319 | 1.247 | 5.92E-01 | 5.92E+01 | | | |
| 7981976 | NR_003329 | SNORD116-1 | small nucleolar RNA, C/D box 116-14 | 0.318 | 1.247 | 7.38E-01 | 7.38E+01 | | | |
| 8115765 | NM_033644 | FBXW11 | F-box and WD repeat domain contai | 0.318 | 1.247 | 4.85E-02 | 4.85E+00 | | | |
| 7896482 | --- | --- | --- | 0.318 | 1.247 | 7.40E-01 | 7.40E+01 | | | |
| 8017688 | NM_199340 | LRRC37A3 | leucine rich repeat containing 37, me | 0.318 | 1.247 | 1.13E-01 | 1.13E+01 | | | |
| 7985053 | NM_147188 | FBXO22 | F-box protein 22 | 0.318 | 1.247 | 1.04E-02 | 1.04E+00 | | | |
| 7894343 | --- | --- | --- | 0.318 | 1.247 | 4.39E-01 | 4.39E+01 | | | |
| 7933228 | NM_00100226 | 40245 | membrane-associated ring finger (C3 | 0.318 | 1.246 | 3.73E-01 | 3.73E+01 | | | |
| 8107613 | NM_003100 | SNX2 | sorting nexin 2 | 0.318 | 1.246 | 1.70E-01 | 1.70E+01 | | | |
| 7893583 | --- | --- | --- | 0.318 | 1.246 | 7.19E-01 | 7.19E+01 | | | |
| 8028162 | NM_001281 | TBCB | tubulin folding cofactor B | 0.317 | 1.246 | 8.61E-03 | 8.61E-01 | | | |
| 8166826 | NM_00103959 | USP9X | ubiquitin specific peptidase 9, X-linke | 0.317 | 1.246 | 1.15E-01 | 1.15E+01 | | | |
| 8106280 | NM_000859 | HMGCR | 3-hydroxy-3-methylglutaryl-Coenzym | 0.317 | 1.246 | 2.87E-01 | 2.87E+01 | | | |
| 7925229 | NM_152490 | B3GALNT2 | beta-1,3-N-acetylgalactosaminyltran | 0.317 | 1.246 | 5.15E-02 | 5.15E+00 | | | |
| 8139421 | NM_012412 | H2AFV | H2A histone family, member V | 0.317 | 1.246 | 1.37E-01 | 1.37E+01 | | | |
| 8083523 | NM_003875 | GMPS | guanine monphosphate synthetase | 0.317 | 1.246 | 2.93E-01 | 2.93E+01 | | | |
| 7962489 | NM_015899 | PLEKHA9 | pleckstrin homology domain contain | 0.317 | 1.246 | 4.26E-02 | 4.26E+00 | | | |
| 8087409 | NM_001664 | RHOA | ras homolog gene family, member A | 0.317 | 1.246 | 3.51E-02 | 3.51E+00 | | | |
| 8115074 | --- | --- | --- | 0.317 | 1.245 | 7.33E-01 | 7.33E+01 | | | |
| 8012961 | NM_006311 | NCOR1 | nuclear receptor co-repressor 1 | 0.316 | 1.245 | 2.13E-02 | 2.13E+00 | | | |
| 7893369 | --- | --- | --- | 0.316 | 1.245 | 2.00E-01 | 2.00E+01 | | | |
| 7942527 | NM_006591 | POLD3 | polymerase (DNA-directed), delta 3, | 0.316 | 1.245 | 2.43E-01 | 2.43E+01 | | | |
| 8108378 | NM_001903 | CTNNA1 | catenin (cadherin-associated protein | 0.316 | 1.245 | 2.18E-02 | 2.18E+00 | | | mono |
| 8016257 | --- | --- | --- | 0.316 | 1.245 | 1.62E-01 | 1.62E+01 | | | |
| 7999889 | NM_016641 | GDE1 | glycerophosphodiester phosphodies | 0.316 | 1.245 | 1.47E-01 | 1.47E+01 | | | |
| 8057561 | NM_018471 | ZC3H15 | zinc finger CCCH-type containing 15 | 0.316 | 1.245 | 6.05E-01 | 6.05E+01 | | | |
| 8083709 | NM_005496 | SMC4 | structural maintenance of chromoso | 0.316 | 1.245 | 1.28E-01 | 1.28E+01 | | | |
| 7985044 | NM_173469 | UBE2Q2 | ubiquitin-conjugating enzyme E2Q fa | 0.316 | 1.245 | 7.06E-02 | 7.06E+00 | | | |
| 8103881 | NM_152682 | RWDD4A | RWD domain containing 4A | 0.316 | 1.245 | 3.92E-01 | 3.92E+01 | | | |
| 7928489 | --- | --- | --- | 0.316 | 1.245 | 5.97E-01 | 5.97E+01 | | | |
| 7980001 | --- | --- | --- | 0.316 | 1.244 | 2.74E-01 | 2.74E+01 | | | |
| 8096098 | NM_016129 | COPS4 | COP9 constitutive photomorphogeni | 0.315 | 1.244 | 4.97E-01 | 4.97E+01 | | | |
| 7895365 | --- | --- | --- | 0.315 | 1.244 | 1.51E-01 | 1.51E+01 | | | |
| 8056837 | NM_00103304 | GPR155 | G protein-coupled receptor 155 | 0.315 | 1.244 | 1.08E-01 | 1.08E+01 | | | |
| 7917912 | NM_000110 | DPYD | dihydropyrimidine dehydrogenase | 0.315 | 1.244 | 1.78E-01 | 1.78E+01 | | | |
| 8150818 | NM_006756 | TCEA1 | transcription elongation factor A (SII | 0.315 | 1.244 | 1.57E-01 | 1.57E+01 | | | |
| 8121300 | NM_018292 | QRSL1 | glutaminyl-tRNA synthase (glutamine | 0.315 | 1.244 | 1.35E-01 | 1.35E+01 | | | |
| 7895439 | --- | --- | --- | 0.315 | 1.244 | 3.39E-01 | 3.39E+01 | | | |
| 7908003 | NM_030769 | NPL | N-acetylneuraminate pyruvate lyase | 0.315 | 1.244 | 9.20E-02 | 9.20E+00 | | | mono |
| 8043504 | NM_002371 | MAL | mal, T-cell differentiation protein | 0.315 | 1.244 | 1.67E-01 | 1.67E+01 | | | |
| 8133504 | NM_032999 | GTF2I | general transcription factor IIi | 0.315 | 1.244 | 3.25E-01 | 3.25E+01 | | | |
| 8108166 | NM_024715 | TXNDC15 | thioredoxin domain containing 15 | 0.315 | 1.244 | 8.30E-02 | 8.30E+00 | | | |
| 8041360 | NM_017735 | TTC27 | tetratricopeptide repeat domain 27 | 0.315 | 1.244 | 2.92E-01 | 2.92E+01 | | | |
| 8124262 | NM_016614 | TTRAP | TRAF and TNF receptor associated pr | 0.314 | 1.244 | 2.54E-01 | 2.54E+01 | | | |
| 8090448 | NM_003707 | RUVBL1 | RuvB-like 1 (E. coli) | 0.314 | 1.244 | 3.53E-01 | 3.53E+01 | | | |
| 7896150 | --- | --- | --- | 0.314 | 1.244 | 7.14E-01 | 7.14E+01 | | | |
| 8014452 | --- | --- | --- | 0.314 | 1.243 | 3.82E-01 | 3.82E+01 | | | |
| 7939434 | NM_018259 | TTC17 | tetratricopeptide repeat domain 17 | 0.314 | 1.243 | 1.48E-01 | 1.48E+01 | | | |
| 7893005 | --- | --- | --- | 0.314 | 1.243 | 6.82E-01 | 6.82E+01 | | | |
| 7902476 | BX537792 | FAM73A | family with sequence similarity 73, m | 0.314 | 1.243 | 1.57E-01 | 1.57E+01 | | | |
| 7946742 | NM_024514 | CYP2R1 | cytochrome P450, family 2, subfamil | 0.314 | 1.243 | 2.67E-01 | 2.67E+01 | | | |
| 7999634 | NM_006985 | NPIP | nuclear pore complex interacting pro | 0.314 | 1.243 | 3.54E-01 | 3.54E+01 | | | |
| 8139947 | NM_032158 | NSUN5C | NOL1/NOP2/Sun domain family, mer | 0.314 | 1.243 | 1.43E-02 | 1.43E+00 | | | |
| 8111974 | NM_006451 | PAIP1 | poly(A) binding protein interacting p | 0.314 | 1.243 | 1.78E-01 | 1.78E+01 | | | |

| | | | | | | | | | | | |
|---|---|---|---|---|---|---|---|---|---|---|---|
| 8085999 | NM_017801 | CMTM6 | CKLF-like MARVEL transmembrane d | 0.314 | 1.243 | 2.29E-01 | 2.29E+01 | | | | |
| 8043480 | --- | --- | --- | 0.313 | 1.243 | 3.01E-02 | 3.01E+00 | | | | |
| 8130087 | NM_139126 | PPIL4 | peptidylprolyl isomerase (cyclophilin | 0.313 | 1.242 | 4.46E-01 | 4.46E+01 | | | | |
| 7919715 | NM_030920 | ANP32E | acidic (leucine-rich) nuclear phospho | 0.313 | 1.242 | 3.96E-01 | 3.96E+01 | | | | |
| 8049042 | --- | --- | --- | 0.313 | 1.242 | 1.54E-01 | 1.54E+01 | | | | |
| 7895275 | --- | --- | --- | 0.313 | 1.242 | 5.02E-02 | 5.02E+00 | | | | |
| 7994769 | NM_007074 | CORO1A | coronin, actin binding protein, 1A | 0.313 | 1.242 | 2.34E-02 | 2.34E+00 | | | | |
| 7922104 | NM_004483 | GCSH | glycine cleavage system protein H (a | 0.313 | 1.242 | 2.22E-01 | 2.22E+01 | | | | |
| 8008132 | NM_005175 | ATP5G1 | ATP synthase, H+ transporting, mito | 0.313 | 1.242 | 1.53E-01 | 1.53E+01 | | | | |
| 7892985 | --- | --- | --- | 0.313 | 1.242 | 2.27E-01 | 2.27E+01 | | | | |
| 8062492 | NM_020336 | KIAA1219 | KIAA1219 | 0.313 | 1.242 | 1.68E-01 | 1.68E+01 | | | | |
| 8131709 | NM_003112 | SP4 | Sp4 transcription factor | 0.313 | 1.242 | 4.39E-02 | 4.39E+00 | | | | |
| 8135229 | NM_031905 | ARMC10 | armadillo repeat containing 10 | 0.313 | 1.242 | 7.90E-02 | 7.90E+00 | | | | |
| 8116548 | NM_020185 | DUSP22 | dual specificity phosphatase 22 | 0.313 | 1.242 | 6.15E-02 | 6.15E+00 | | | | |
| 7911578 | NM_024011 | CDC2L2 | cell division cycle 2-like 2 (PITSLRE pr | 0.313 | 1.242 | 1.14E-01 | 1.14E+01 | | | | |
| 8072279 | NM_153050 | MTMR3 | myotubularin related protein 3 | 0.312 | 1.242 | 1.21E-01 | 1.21E+01 | | | | |
| 8114211 | --- | --- | --- | 0.312 | 1.241 | 5.57E-01 | 5.57E+01 | | | | |
| 8162880 | NM_019051 | MRPL50 | mitochondrial ribosomal protein L50 | 0.312 | 1.241 | 1.93E-01 | 1.93E+01 | | | | |
| 8094911 | NM_020453 | ATP10D | ATPase, class V, type 10D | 0.312 | 1.241 | 1.22E-02 | 1.22E+00 | | | | |
| 8152222 | NM_015878 | AZIN1 | antizyme inhibitor 1 | 0.312 | 1.241 | 3.11E-01 | 3.11E+01 | | | | |
| 7969626 | NM_180989 | GPR180 | G protein-coupled receptor 180 | 0.311 | 1.241 | 7.08E-02 | 7.08E+00 | | | | |
| 7893272 | --- | --- | --- | 0.311 | 1.241 | 1.45E-01 | 1.45E+01 | | | | |
| 8045347 | --- | --- | --- | 0.311 | 1.241 | 6.31E-01 | 6.31E+01 | | | | |
| 7968898 | BC039586 | KIAA1704 | KIAA1704 | 0.311 | 1.241 | 1.54E-01 | 1.54E+01 | | | | |
| 8019924 | NM_006471 | MYL12A | myosin, light chain 12A, regulatory, r | 0.311 | 1.241 | 1.27E-01 | 1.27E+01 | | | | |
| 7893243 | --- | --- | --- | 0.311 | 1.241 | 6.52E-01 | 6.52E+01 | | | | |
| 7979223 | NM_00116014 | DDHD1 | DDHD domain containing 1 | 0.311 | 1.240 | 3.23E-01 | 3.23E+01 | | | | |
| 8049961 | NM_183421 | FBXO25 | F-box protein 25 | 0.311 | 1.240 | 8.00E-01 | 8.00E+01 | | | | |
| 8050548 | NM_014713 | LAPTM4A | lysosomal protein transmembrane 4 | 0.311 | 1.240 | 5.87E-01 | 5.87E+01 | | | | |
| 7906662 | NM_016406 | UFC1 | ubiquitin-fold modifier conjugating e | 0.311 | 1.240 | 5.31E-01 | 5.31E+01 | | | | |
| 7930008 | NM_004741 | NOLC1 | nucleolar and coiled-body phosphop | 0.311 | 1.240 | 4.67E-02 | 4.67E+00 | | | | |
| 8138108 | NM_006854 | KDELR2 | KDEL (Lys-Asp-Glu-Leu) endoplasmic | 0.310 | 1.240 | 2.45E-01 | 2.45E+01 | | | | |
| 7893356 | --- | --- | --- | 0.310 | 1.240 | 5.23E-01 | 5.23E+01 | | | | |
| 8174794 | NM_00107987 | CUL4B | cullin 4B | 0.310 | 1.240 | 7.64E-02 | 7.64E+00 | | | | |
| 8150101 | --- | --- | --- | 0.310 | 1.240 | 4.56E-01 | 4.56E+01 | | | | |
| 8063716 | NM_030773 | TUBB1 | tubulin, beta 1 | 0.310 | 1.240 | 1.59E-01 | 1.59E+01 | | | | |
| 8121502 | NM_138408 | GTF3C6 | general transcription factor IIIC, poly | 0.310 | 1.240 | 4.44E-01 | 4.44E+01 | | | | |
| 8180393 | --- | --- | --- | 0.310 | 1.240 | 5.40E-01 | 5.40E+01 | | | | |
| 8154531 | NM_017925 | DENND4C | DENN/MADD domain containing 4C | 0.310 | 1.239 | 2.93E-01 | 2.93E+01 | | | | |
| 7893734 | --- | --- | --- | 0.309 | 1.239 | 4.14E-01 | 4.14E+01 | | | | |
| 8056222 | NM_001935 | DPP4 | dipeptidyl-peptidase 4 | 0.309 | 1.239 | 4.54E-01 | 4.54E+01 | | | | |
| 8022279 | --- | --- | --- | 0.309 | 1.239 | 7.15E-01 | 7.15E+01 | | | | |
| 7896401 | --- | --- | --- | 0.309 | 1.239 | 7.20E-01 | 7.20E+01 | | | | |
| 8180236 | --- | --- | --- | 0.309 | 1.239 | 2.19E-01 | 2.19E+01 | | | | |
| 8180265 | --- | --- | --- | 0.309 | 1.239 | 2.19E-01 | 2.19E+01 | | | | |
| 7924558 | NM_002533 | NVL | nuclear VCP-like | 0.309 | 1.239 | 1.21E-01 | 1.21E+01 | | | | |
| 8013741 | NM_006923 | SDF2 | stromal cell-derived factor 2 | 0.309 | 1.239 | 4.14E-01 | 4.14E+01 | | | | |
| 7991735 | NM_024571 | SNRNP25 | small nuclear ribonucleoprotein 25kI | 0.309 | 1.239 | 2.14E-01 | 2.14E+01 | | | | |
| 8099696 | NM_153825 | SEPSECS | Sep (O-phosphoserine) tRNA:Sec (sel | 0.309 | 1.238 | 3.19E-01 | 3.19E+01 | | | | |
| 8009784 | NM_015971 | MRPS7 | mitochondrial ribosomal protein S7 | 0.308 | 1.238 | 3.55E-01 | 3.55E+01 | | | | |
| 8013915 | --- | --- | --- | 0.308 | 1.238 | 4.32E-02 | 4.32E+00 | | | | |
| 8109528 | NM_00103733 | CYFIP2 | cytoplasmic FMR1 interacting protei | 0.308 | 1.238 | 1.16E-01 | 1.16E+01 | | | | |
| 7933164 | NM_004966 | HNRNPF | heterogeneous nuclear ribonucleopr | 0.308 | 1.238 | 1.61E-02 | 1.61E+00 | | | | |
| 8089999 | NR_026698 | KPNA1 | karyopherin alpha 1 (importin alpha | 0.308 | 1.238 | 2.45E-01 | 2.45E+01 | | | | |
| 8155453 | --- | --- | --- | 0.308 | 1.238 | 3.84E-01 | 3.84E+01 | | | | |
| 8161375 | --- | --- | --- | 0.308 | 1.238 | 3.84E-01 | 3.84E+01 | | | | |
| 7894649 | --- | --- | --- | 0.308 | 1.238 | 7.86E-01 | 7.86E+01 | | | | |
| 8114691 | NM_003883 | HDAC3 | histone deacetylase 3 | 0.308 | 1.238 | 7.68E-02 | 7.68E+00 | | | | |
| 8081337 | --- | --- | --- | 0.308 | 1.238 | 5.94E-01 | 5.94E+01 | | | | |
| 8050071 | NM_018269 | ADI1 | acireductone dioxygenase 1 | 0.308 | 1.238 | 7.04E-02 | 7.04E+00 | | | | |
| 7943956 | --- | --- | --- | 0.308 | 1.238 | 5.66E-01 | 5.66E+01 | | | | |
| 7892882 | --- | --- | --- | 0.307 | 1.237 | 3.06E-01 | 3.06E+01 | | | | |
| 8043187 | NM_005911 | MAT2A | methionine adenosyltransferase II, a | 0.307 | 1.237 | 7.68E-02 | 7.68E+00 | | | | |
| 8075306 | --- | --- | --- | 0.307 | 1.237 | 1.30E-01 | 1.30E+01 | | | | |
| 7998129 | NM_016310 | POLR3K | polymerase (RNA) III (DNA directed) | 0.307 | 1.237 | 2.39E-01 | 2.39E+01 | | | | |
| 7978718 | NM_006364 | SEC23A | Sec23 homolog A (S. cerevisiae) | 0.307 | 1.237 | 8.14E-02 | 8.14E+00 | | | | |
| 7926851 | NR_024557 | WAC | WW domain containing adaptor with | 0.307 | 1.237 | 1.31E-01 | 1.31E+01 | | | | |
| 8154254 | NM_020829 | KIAA1432 | KIAA1432 | 0.307 | 1.237 | 1.60E-01 | 1.60E+01 | | | | |
| 8069508 | ENST0000033 | CCDC29 | coiled-coil domain containing 29 | 0.307 | 1.237 | 6.56E-01 | 6.56E+01 | | | | |
| 8100125 | --- | --- | --- | 0.306 | 1.237 | 4.15E-01 | 4.15E+01 | | | | |
| 8095894 | NM_020236 | MRPL1 | mitochondrial ribosomal protein L1 | 0.306 | 1.236 | 5.42E-01 | 5.42E+01 | | | | |
| 8033605 | NM_012335 | MYO1F | myosin IF | 0.306 | 1.236 | 6.89E-02 | 6.89E+00 | | | | |
| 8001099 | NR_002827 | HERC2P4 | hect domain and RLD 2 pseudogene | 0.306 | 1.236 | 3.46E-01 | 3.46E+01 | | | | |
| 8004219 | --- | --- | --- | 0.306 | 1.236 | 5.34E-01 | 5.34E+01 | | | | |
| 8064432 | NM_000801 | FKBP1A | FK506 binding protein 1A, 12kDa | 0.306 | 1.236 | 3.65E-02 | 3.65E+00 | | | | |
| 8147580 | NM_017890 | VPS13B | vacuolar protein sorting 13 homolog | 0.306 | 1.236 | 7.42E-02 | 7.42E+00 | | | | |
| 7893604 | --- | --- | --- | 0.306 | 1.236 | 6.74E-01 | 6.74E+01 | | | | |

| | | | | | | | | | | | | |
|---|---|---|---|---|---|---|---|---|---|---|---|---|
| 8129706 | NM_006620 | HBS1L | HBS1-like (S. cerevisiae) | 0.306 | 1.236 | 3.93E-01 | 3.93E+01 | | | | | |
| 7930380 | NM_016824 | ADD3 | adducin 3 (gamma) | 0.306 | 1.236 | 2.21E-02 | 2.21E+00 | | | | | |
| 7979864 | NM_004450 | ERH | enhancer of rudimentary homolog (D | 0.306 | 1.236 | 3.90E-01 | 3.90E+01 | | | | | |
| 7920341 | NM_020699 | GATAD2B | GATA zinc finger domain containing 2 | 0.306 | 1.236 | 1.94E-01 | 1.94E+01 | | | | | |
| 7898894 | NM_020362 | C1orf128 | chromosome 1 open reading frame 1 | 0.305 | 1.236 | 3.24E-01 | 3.24E+01 | | | | | |
| 7896752 | --- | --- | --- | 0.305 | 1.236 | 5.22E-01 | 5.22E+01 | | | | | |
| 8165667 | --- | --- | --- | 0.305 | 1.236 | 5.22E-01 | 5.22E+01 | | | | | |
| 7893697 | --- | --- | --- | 0.305 | 1.236 | 7.27E-01 | 7.27E+01 | | | | | |
| 7901048 | NR_000024 | SNORD46 | small nucleolar RNA, C/D box 46 | 0.305 | 1.235 | 5.76E-01 | 5.76E+01 | | | | | |
| 7912412 | NM_004958 | MTOR | mechanistic target of rapamycin (ser | 0.305 | 1.235 | 4.66E-02 | 4.66E+00 | | | | | |
| 7967287 | NM_017612 | ZCCHC8 | zinc finger, CCHC domain containing | 0.305 | 1.235 | 2.54E-01 | 2.54E+01 | | | | | |
| 8089299 | NM_001777 | CD47 | CD47 molecule | 0.305 | 1.235 | 5.02E-02 | 5.02E+00 | | | | | |
| 7914563 | NM_003680 | YARS | tyrosyl-tRNA synthetase | 0.305 | 1.235 | 2.78E-01 | 2.78E+01 | | | | | |
| 7967240 | NM_022916 | VPS33A | vacuolar protein sorting 33 homolog | 0.305 | 1.235 | 2.75E-01 | 2.75E+01 | | | | | |
| 7905631 | NM_023015 | INTS3 | integrator complex subunit 3 | 0.304 | 1.235 | 2.61E-02 | 2.61E+00 | | | | | |
| 7893594 | --- | --- | --- | 0.304 | 1.235 | 6.68E-01 | 6.68E+01 | | | | | |
| 7914555 | --- | --- | --- | 0.304 | 1.235 | 5.02E-01 | 5.02E+01 | | | | | |
| 8054054 | NM_025190 | ANKRD36B | ankyrin repeat domain 36B | 0.304 | 1.235 | 3.14E-01 | 3.14E+01 | | | | | |
| 7980547 | NM_005065 | SEL1L | sel-1 suppressor of lin-12-like (C. ele | 0.304 | 1.234 | 1.67E-01 | 1.67E+01 | | | | | |
| 8022393 | NM_002828 | PTPN2 | protein tyrosine phosphatase, non-r | 0.303 | 1.234 | 1.98E-01 | 1.98E+01 | | | | | |
| 8163105 | --- | --- | --- | 0.303 | 1.234 | 5.84E-01 | 5.84E+01 | | | | | |
| 8124756 | NM_002714 | PPP1R10 | protein phosphatase 1, regulatory (in | 0.303 | 1.234 | 7.28E-02 | 7.28E+00 | | | | | |
| 8178358 | NM_002714 | PPP1R10 | protein phosphatase 1, regulatory (in | 0.303 | 1.234 | 7.28E-02 | 7.28E+00 | | | | | |
| 8179664 | NM_002714 | PPP1R10 | protein phosphatase 1, regulatory (in | 0.303 | 1.234 | 7.28E-02 | 7.28E+00 | | | | | |
| 8145894 | NM_004674 | ASH2L | ash2 (absent, small, or homeotic)-lik | 0.303 | 1.234 | 7.13E-02 | 7.13E+00 | | | | | |
| 7893892 | --- | --- | --- | 0.303 | 1.234 | 4.83E-01 | 4.83E+01 | | | | | |
| 8149849 | NM_000825 | GNRH1 | gonadotropin-releasing hormone 1 ( | 0.303 | 1.234 | 2.17E-01 | 2.17E+01 | | | | | |
| 8088911 | NM_00112822 | ZNF717 | zinc finger protein 717 | 0.303 | 1.234 | 4.08E-01 | 4.08E+01 | | | | | |
| 8160284 | NM_017645 | HAUS6 | HAUS augmin-like complex, subunit | 0.303 | 1.234 | 3.71E-01 | 3.71E+01 | | | | | |
| 8064471 | NM_006065 | SIRPB1 | signal-regulatory protein beta 1 | 0.303 | 1.234 | 8.61E-03 | 8.61E-01 | | | | | |
| 7956842 | NM_014319 | LEMD3 | LEM domain containing 3 | 0.303 | 1.234 | 1.80E-01 | 1.80E+01 | | | | | |
| 8082940 | NM_015396 | ARMC8 | armadillo repeat containing 8 | 0.303 | 1.234 | 3.20E-01 | 3.20E+01 | | | | | |
| 7929201 | NM_003972 | BTAF1 | BTAF1 RNA polymerase II, B-TFIID tra | 0.303 | 1.233 | 8.87E-02 | 8.87E+00 | | | | | |
| 8171013 | NM_003372 | VBP1 | von Hippel-Lindau binding protein 1 | 0.303 | 1.233 | 3.31E-01 | 3.31E+01 | | | | | |
| 8006850 | NM_016507 | CRKRS | Cdc2-related kinase, arginine/serine- | 0.303 | 1.233 | 3.08E-01 | 3.08E+01 | | | | | |
| 8020149 | NM_003826 | NAPG | N-ethylmaleimide-sensitive factor at | 0.302 | 1.233 | 4.79E-01 | 4.79E+01 | | | | | |
| 7938762 | NM_005316 | GTF2H1 | general transcription factor IIH, poly | 0.302 | 1.233 | 1.29E-01 | 1.29E+01 | | | | | |
| 8009380 | NR_003706 | SNORA38B | small nucleolar RNA, H/ACA box 38B | 0.302 | 1.233 | 3.30E-01 | 3.30E+01 | | | | | |
| 8180395 | --- | --- | --- | 0.302 | 1.233 | 5.84E-01 | 5.84E+01 | | | | | |
| 7909689 | NM_020197 | SMYD2 | SET and MYND domain containing 2 | 0.302 | 1.233 | 1.03E-01 | 1.03E+01 | | | | | |
| 7895356 | --- | --- | --- | 0.302 | 1.233 | 6.06E-01 | 6.06E+01 | | | | | |
| 7984188 | --- | --- | --- | 0.302 | 1.233 | 5.17E-01 | 5.17E+01 | | | | | |
| 8083272 | NM_004130 | GYG1 | glycogenin 1 | 0.302 | 1.233 | 4.10E-01 | 4.10E+01 | | | | | |
| 7913169 | NM_004930 | CAPZB | capping protein (actin filament) mus | 0.302 | 1.232 | 3.18E-01 | 3.18E+01 | | | | | |
| 8133633 | NM_00103957 | NSUN5B | NOL1/NOP2/Sun domain family, mem | 0.302 | 1.232 | 2.62E-02 | 2.62E+00 | | | | | |
| 7925003 | NM_024525 | TTC13 | tetratricopeptide repeat domain 13 | 0.301 | 1.232 | 1.15E-01 | 1.15E+01 | | | | | |
| 7962499 | NM_004719 | SFRS2IP | splicing factor, arginine/serine-rich 2 | 0.301 | 1.232 | 1.58E-01 | 1.58E+01 | | | | | |
| 8121861 | NM_181782 | NCOA7 | nuclear receptor coactivator 7 | 0.301 | 1.232 | 6.05E-02 | 6.05E+00 | | | | | |
| 8005155 | --- | --- | --- | 0.301 | 1.232 | 2.10E-01 | 2.10E+01 | | | | | |
| 8095262 | NM_005612 | REST | RE1-silencing transcription factor | 0.300 | 1.231 | 1.72E-02 | 1.72E+00 | | | | | |
| 8171747 | NM_001412 | EIF1AX | eukaryotic translation initiation facto | 0.300 | 1.231 | 1.80E-01 | 1.80E+01 | | | | | |
| 8128356 | NM_012160 | FBXL4 | F-box and leucine-rich repeat protein | 0.300 | 1.231 | 1.07E-01 | 1.07E+01 | | | | | |
| 8027233 | NM_033204 | ZNF101 | zinc finger protein 101 | 0.300 | 1.231 | 7.06E-02 | 7.06E+00 | | | | | |
| 8038382 | NM_017916 | PIH1D1 | PIH1 domain containing 1 | 0.300 | 1.231 | 1.50E-01 | 1.50E+01 | | | | | |
| 8002778 | NM_152649 | MLKL | mixed lineage kinase domain-like | 0.300 | 1.231 | 2.02E-01 | 2.02E+01 | | | | | |
| 7951068 | NM_016403 | CWC15 | CWC15 spliceosome-associated prot | 0.300 | 1.231 | 5.18E-01 | 5.18E+01 | | | | | |
| 8071547 | NM_198157 | UBE2L3 | ubiquitin-conjugating enzyme E2L 3 | 0.300 | 1.231 | 1.63E-02 | 1.63E+00 | | | | | |
| 7894648 | --- | --- | --- | 0.300 | 1.231 | 6.19E-01 | 6.19E+01 | | | | | |
| 8070102 | NM_000819 | GART | phosphoribosylglycinamide formyltra | 0.300 | 1.231 | 5.31E-02 | 5.31E+00 | | | | | |
| 8055060 | NM_018383 | WDR33 | WD repeat domain 33 | 0.299 | 1.231 | 4.47E-01 | 4.47E+01 | | | | | |
| 8160405 | NM_018847 | KLHL9 | kelch-like 9 (Drosophila) | 0.299 | 1.231 | 2.34E-02 | 2.34E+00 | | | | | |
| 8035880 | NM_006003 | UQCRFS1 | ubiquinol-cytochrome c reductase, R | 0.299 | 1.231 | 7.13E-02 | 7.13E+00 | | | | | |
| 8072798 | NM_013385 | CYTH4 | cytohesin 4 | 0.299 | 1.230 | 1.67E-01 | 1.67E+01 | | | | | |
| 7910001 | NM_003676 | DEGS1 | degenerative spermatocyte homolog | 0.299 | 1.230 | 1.87E-01 | 1.87E+01 | | | | | |
| 8171481 | NM_003916 | AP1S2 | adaptor-related protein complex 1, s | 0.299 | 1.230 | 2.62E-01 | 2.62E+01 | | | | | |
| 8121214 | --- | --- | --- | 0.299 | 1.230 | 5.65E-01 | 5.65E+01 | | | | | |
| 7990231 | NR_023318 | ADPGK | ADP-dependent glucokinase | 0.299 | 1.230 | 1.41E-01 | 1.41E+01 | | | | | |
| 8148317 | NM_002467 | MYC | v-myc myelocytomatosis viral oncog | 0.299 | 1.230 | 6.86E-02 | 6.86E+00 | | | | | |
| 7894865 | --- | --- | --- | 0.299 | 1.230 | 3.92E-01 | 3.92E+01 | | | | | |
| 8021228 | NM_005359 | SMAD4 | SMAD family member 4 | 0.299 | 1.230 | 3.93E-01 | 3.93E+01 | | | | | |
| 8147994 | NM_032334 | UTP23 | UTP23, small subunit (SSU) processo | 0.299 | 1.230 | 7.16E-02 | 7.16E+00 | | | | | |
| 7916274 | NM_002370 | MAGOH | mago-nashi homolog, proliferation-a | 0.299 | 1.230 | 5.03E-01 | 5.03E+01 | | | | | |
| 7972840 | NM_006322 | TUBGCP3 | tubulin, gamma complex associated | 0.299 | 1.230 | 8.08E-02 | 8.08E+00 | | | | | |
| 8071332 | NM_002882 | RANBP1 | RAN binding protein 1 | 0.299 | 1.230 | 3.26E-01 | 3.26E+01 | | | | | |
| 7974781 | NM_021003 | PPM1A | protein phosphatase 1A (formerly 2C | 0.299 | 1.230 | 2.72E-01 | 2.72E+01 | | | | | |
| 7938128 | NM_144666 | DNHD1 | dynein heavy chain domain 1 | 0.299 | 1.230 | 3.61E-01 | 3.61E+01 | | | | | |
| 7905185 | NM_015203 | RPRD2 | regulation of nuclear pre-mRNA dom | 0.298 | 1.230 | 3.23E-01 | 3.23E+01 | | | | | |

| | | | | | | | | | | |
|---|---|---|---|---|---|---|---|---|---|---|
| 7892968 | --- | --- | --- | 0.298 | 1.230 | 4.57E-01 | 4.57E+01 | | | |
| 8154868 | --- | --- | --- | 0.298 | 1.230 | 5.16E-01 | 5.16E+01 | | | |
| 8173629 | NM_004299 | ABCB7 | ATP-binding cassette, sub-family B (M | 0.298 | 1.230 | 2.47E-01 | 2.47E+01 | | | |
| 8150103 | NM_002095 | GTF2E2 | general transcription factor IIE, polyp | 0.298 | 1.229 | 4.42E-01 | 4.42E+01 | | | |
| 7934731 | NM_006333 | C1D | C1D nuclear receptor co-repressor | 0.298 | 1.229 | 6.32E-01 | 6.32E+01 | | | |
| 8025076 | NM_005428 | VAV1 | vav 1 guanine nucleotide exchange fa | 0.298 | 1.229 | 6.12E-02 | 6.12E+00 | | | |
| 8119078 | --- | --- | --- | 0.298 | 1.229 | 4.27E-01 | 4.27E+01 | | | |
| 7995421 | NM_031490 | LONP2 | lon peptidase 2, peroxisomal | 0.298 | 1.229 | 8.27E-02 | 8.27E+00 | | | |
| 7922250 | NM_181093 | SCYL3 | SCY1-like 3 (S. cerevisiae) | 0.297 | 1.229 | 1.65E-01 | 1.65E+01 | | | |
| 8038993 | NM_006969 | ZNF28 | zinc finger protein 28 | 0.297 | 1.229 | 9.54E-02 | 9.54E+00 | | | |
| 7983761 | --- | --- | --- | 0.297 | 1.228 | 2.72E-01 | 2.72E+01 | | | |
| 8016088 | NM_144609 | CCDC43 | coiled-coil domain containing 43 | 0.297 | 1.228 | 1.70E-01 | 1.70E+01 | | | |
| 8149273 | --- | --- | --- | 0.297 | 1.228 | 1.15E-01 | 1.15E+01 | | | |
| 7909140 | --- | --- | --- | 0.297 | 1.228 | 6.07E-01 | 6.07E+01 | | | |
| 7895790 | --- | --- | --- | 0.297 | 1.228 | 2.01E-01 | 2.01E+01 | | | |
| 7985119 | NM_018200 | HMG20A | high-mobility group 20A | 0.297 | 1.228 | 4.49E-01 | 4.49E+01 | | | |
| 8146482 | NM_024831 | TGS1 | trimethylguanosine synthase homolo | 0.297 | 1.228 | 2.08E-01 | 2.08E+01 | | | |
| 8113039 | NM_002397 | MEF2C | myocyte enhancer factor 2C | 0.296 | 1.228 | 2.65E-01 | 2.65E+01 | | | |
| 8042519 | NM_016297 | PCYOX1 | prenylcysteine oxidase 1 | 0.296 | 1.228 | 1.39E-01 | 1.39E+01 | | | |
| 7970569 | NM_014363 | SACS | spastic ataxia of Charlevoix-Saguena | 0.296 | 1.228 | 1.86E-01 | 1.86E+01 | | | |
| 7993339 | ENST00000391 | OC10012903 | PRO2812 | 0.296 | 1.228 | 1.33E-01 | 1.33E+01 | | | |
| 8151281 | NM_014294 | TRAM1 | translocation associated membrane | 0.296 | 1.228 | 1.53E-01 | 1.53E+01 | | | |
| 8152750 | NM_194291 | TMEM65 | transmembrane protein 65 | 0.296 | 1.228 | 1.97E-01 | 1.97E+01 | | | |
| 7893796 | --- | --- | --- | 0.296 | 1.228 | 5.73E-01 | 5.73E+01 | | | |
| 8156321 | NM_003177 | SYK | spleen tyrosine kinase | 0.295 | 1.227 | 1.75E-02 | 1.75E+00 | | | |
| 7894873 | --- | --- | --- | 0.295 | 1.227 | 5.88E-01 | 5.88E+01 | | | |
| 7948534 | NM_001923 | DDB1 | damage-specific DNA binding protein | 0.295 | 1.227 | 9.43E-02 | 9.43E+00 | | | |
| 7966356 | NM_00104010 | HVCN1 | hydrogen voltage-gated channel 1 | 0.295 | 1.227 | 1.10E-02 | 1.10E+00 | | | |
| 7953765 | NM_020734 | RIMKLB | ribosomal modification protein rimK | 0.295 | 1.227 | 1.05E-01 | 1.05E+01 | | | |
| 8127977 | NM_00115967 | SYNCRIP | synaptotagmin binding, cytoplasmic | 0.295 | 1.227 | 1.38E-01 | 1.38E+01 | | | |
| 8090237 | NM_021964 | ZNF148 | zinc finger protein 148 | 0.295 | 1.227 | 2.37E-01 | 2.37E+01 | | | |
| 8073345 | NM_001429 | EP300 | E1A binding protein p300 | 0.294 | 1.226 | 5.87E-02 | 5.87E+00 | | | |
| 7916928 | NM_030816 | ANKRD13C | ankyrin repeat domain 13C | 0.294 | 1.226 | 1.33E-01 | 1.33E+01 | | | |
| 7980296 | NM_006827 | TMED10 | transmembrane emp24-like traffickin | 0.294 | 1.226 | 2.77E-01 | 2.77E+01 | | | |
| 7908409 | NM_002923 | RGS2 | regulator of G-protein signaling 2, 24 | 0.294 | 1.226 | 4.67E-01 | 4.67E+01 | | | |
| 8097543 | NM_014487 | ZNF330 | zinc finger protein 330 | 0.294 | 1.226 | 6.02E-01 | 6.02E+01 | | | |
| 8042195 | NM_152392 | AHSA2 | AHA1, activator of heat shock 90kDa | 0.294 | 1.226 | 1.16E-01 | 1.16E+01 | | | |
| 7951447 | NM_152434 | CWF19L2 | CWF19-like 2, cell cycle control (S. po | 0.294 | 1.226 | 3.24E-01 | 3.24E+01 | | | |
| 8168018 | NM_173834 | YIPF6 | Yip1 domain family, member 6 | 0.293 | 1.226 | 7.00E-02 | 7.00E+00 | | | |
| 8008074 | NM_176096 | CDK5RAP3 | CDK5 regulatory subunit associated p | 0.293 | 1.226 | 1.16E-01 | 1.16E+01 | | | |
| 7920984 | NM_005998 | CCT3 | chaperonin containing TCP1, subunit | 0.293 | 1.225 | 7.72E-02 | 7.72E+00 | | | |
| 7949496 | NM_005507 | CFL1 | cofilin 1 (non-muscle) | 0.293 | 1.225 | 1.21E-01 | 1.21E+01 | | | |
| 7959080 | NM_022491 | SUDS3 | suppressor of defective silencing 3 h | 0.293 | 1.225 | 4.47E-02 | 4.47E+00 | | | |
| 8097262 | NM_145207 | SPATA5 | spermatogenesis associated 5 | 0.293 | 1.225 | 1.29E-01 | 1.29E+01 | | | |
| 7892774 | --- | --- | --- | 0.293 | 1.225 | 5.66E-01 | 5.66E+01 | | | |
| 8144378 | NM_018361 | AGPAT5 | 1-acylglycerol-3-phosphate O-acyltra | 0.293 | 1.225 | 1.58E-01 | 1.58E+01 | | | |
| 8075089 | NM_00100869 | TFIP11 | tuftelin interacting protein 11 | 0.293 | 1.225 | 2.52E-01 | 2.52E+01 | | | |
| 7970162 | NM_015205 | ATP11A | ATPase, class VI, type 11A | 0.293 | 1.225 | 6.25E-02 | 6.25E+00 | | | |
| 7938687 | NM_005013 | NUCB2 | nucleobindin 2 | 0.293 | 1.225 | 5.07E-01 | 5.07E+01 | | | |
| 8109462 | NM_004779 | CNOT8 | CCR4-NOT transcription complex, su | 0.292 | 1.225 | 4.36E-01 | 4.36E+01 | | | |
| 8118322 | NR_002742 | SNORD52 | small nucleolar RNA, C/D box 52 | 0.292 | 1.225 | 6.48E-01 | 6.48E+01 | | | |
| 8139244 | NM_018224 | C7orf44 | chromosome 7 open reading frame 4 | 0.292 | 1.225 | 3.70E-01 | 3.70E+01 | | | |
| 8101701 | NM_152542 | PPM1K | protein phosphatase 1K (PP2C doma | 0.292 | 1.225 | 1.40E-01 | 1.40E+01 | | | |
| 8090934 | --- | --- | --- | 0.292 | 1.224 | 5.85E-01 | 5.85E+01 | | | |
| 7935951 | NM_024541 | C10orf76 | chromosome 10 open reading frame | 0.292 | 1.224 | 7.49E-02 | 7.49E+00 | | | |
| 8002999 | NM_004483 | GCSH | glycine cleavage system protein H (a | 0.292 | 1.224 | 2.80E-01 | 2.80E+01 | | | |
| 8078412 | NM_015442 | CNOT10 | CCR4-NOT transcription complex, su | 0.292 | 1.224 | 1.39E-01 | 1.39E+01 | | | |
| 8137979 | NM_001101 | ACTB | actin, beta | 0.292 | 1.224 | 2.56E-03 | 2.56E-01 | | | |
| 8180269 | --- | --- | --- | 0.292 | 1.224 | 6.10E-01 | 6.10E+01 | | | |
| 8043278 | NM_016622 | MRPL35 | mitochondrial ribosomal protein L35 | 0.292 | 1.224 | 5.30E-01 | 5.30E+01 | | | |
| 8008887 | NM_003161 | RPS6KB1 | ribosomal protein S6 kinase, 70kDa, | 0.291 | 1.224 | 8.47E-02 | 8.47E+00 | | | |
| 7895626 | --- | --- | --- | 0.291 | 1.224 | 4.04E-01 | 4.04E+01 | | | |
| 7985493 | NM_023003 | TM6SF1 | transmembrane 6 superfamily memb | 0.291 | 1.224 | 1.37E-01 | 1.37E+01 | | | |
| 8148728 | NM_001916 | CYC1 | cytochrome c-1 | 0.291 | 1.223 | 5.89E-02 | 5.89E+00 | | | |
| 8129804 | NM_005923 | MAP3K5 | mitogen-activated protein kinase kin | 0.291 | 1.223 | 2.86E-02 | 2.86E+00 | | | |
| 8147221 | NM_004337 | OSGIN2 | oxidative stress induced growth inhib | 0.290 | 1.223 | 1.95E-01 | 1.95E+01 | | | |
| 7915160 | NM_022157 | RRAGC | Ras-related GTP binding C | 0.290 | 1.223 | 1.70E-01 | 1.70E+01 | | | |
| 7954503 | NM_004264 | MED21 | mediator complex subunit 21 | 0.290 | 1.223 | 5.16E-01 | 5.16E+01 | | | |
| 8044961 | NR_023343 | RNU4ATAC | RNA, U4atac small nuclear (U12-dep | 0.290 | 1.223 | 7.31E-01 | 7.31E+01 | | | |
| 8025103 | NM_001974 | EMR1 | egf-like module containing, mucin-lik | 0.290 | 1.223 | 4.80E-02 | 4.80E+00 | | | mono |
| 7947934 | NM_014342 | MTCH2 | mitochondrial carrier homolog 2 (C. e | 0.290 | 1.223 | 3.84E-01 | 3.84E+01 | | | |
| 7939242 | NM_005898 | CAPRIN1 | cell cycle associated protein 1 | 0.290 | 1.223 | 3.10E-02 | 3.10E+00 | | | |
| 7977571 | NM_017807 | OSGEP | O-sialoglycoprotein endopeptidase | 0.290 | 1.223 | 1.88E-01 | 1.88E+01 | | | |
| 8047538 | NM_001204 | BMPR2 | bone morphogenetic protein recepto | 0.290 | 1.223 | 1.07E-01 | 1.07E+01 | | | |
| 7898192 | NM_015291 | DNAJC16 | DnaJ (Hsp40) homolog, subfamily C, | 0.290 | 1.222 | 2.38E-02 | 2.38E+00 | | | |
| 7906185 | NM_144772 | APOA1BP | apolipoprotein A-I binding protein | 0.290 | 1.222 | 1.93E-01 | 1.93E+01 | | | |
| 7997453 | NM_002661 | PLCG2 | phospholipase C, gamma 2 (phospha | 0.289 | 1.222 | 6.87E-02 | 6.87E+00 | | | |

| | | | | | | | | | | |
|---|---|---|---|---|---|---|---|---|---|---|
| 7985089 | NM_002902 | RCN2 | reticulocalbin 2, EF-hand calcium bin | 0.289 | 1.222 | 2.57E-01 | 2.57E+01 | | | |
| 8016789 | NM_017643 | MBTD1 | mbt domain containing 1 | 0.289 | 1.222 | 3.49E-01 | 3.49E+01 | | | |
| 8176375 | NM_001008 | RPS4Y1 | ribosomal protein S4, Y-linked 1 | 0.289 | 1.222 | 7.64E-01 | 7.64E+01 | | | |
| 8070010 | NM_003895 | SYNJ1 | synaptojanin 1 | 0.289 | 1.222 | 1.80E-01 | 1.80E+01 | | | |
| 8072744 | NM_013416 | NCF4 | neutrophil cytosolic factor 4, 40kDa | 0.289 | 1.222 | 2.12E-01 | 2.12E+01 | | | mono |
| 8022882 | NM_006965 | ZNF24 | zinc finger protein 24 | 0.289 | 1.222 | 2.13E-01 | 2.13E+01 | | | |
| 8178205 | NM_020056 | HLA-DQA2 | major histocompatibility complex, cla | 0.289 | 1.222 | 1.37E-01 | 1.37E+01 | | | |
| 8180257 | --- | --- | --- | 0.289 | 1.222 | 1.67E-01 | 1.67E+01 | | | |
| 7976646 | --- | --- | --- | 0.289 | 1.221 | 5.03E-01 | 5.03E+01 | | | |
| 8136557 | NM_00113096 | TBXAS1 | thromboxane A synthase 1 (platelet) | 0.289 | 1.221 | 2.06E-02 | 2.06E+00 | | | mono |
| 7902592 | NM_006465 | ARID3B | AT rich interactive domain 3B (BRIGH | 0.288 | 1.221 | 2.21E-01 | 2.21E+01 | | | |
| 7895422 | --- | --- | --- | 0.288 | 1.221 | 4.40E-01 | 4.40E+01 | | | |
| 7895074 | --- | --- | --- | 0.288 | 1.221 | 6.79E-01 | 6.79E+01 | | | |
| 8164907 | NM_020385 | REXO4 | REX4, RNA exonuclease 4 homolog (S | 0.288 | 1.221 | 6.27E-02 | 6.27E+00 | | | |
| 8091778 | NR_003001 | SCARNA7 | small Cajal body-specific RNA 7 | 0.288 | 1.221 | 6.26E-01 | 6.26E+01 | | | |
| 8018814 | --- | --- | --- | 0.288 | 1.221 | 6.91E-01 | 6.91E+01 | | | |
| 8149927 | NM_001831 | CLU | clusterin | 0.288 | 1.221 | 1.66E-01 | 1.66E+01 | | | |
| 8130916 | NM_018288 | PHF10 | PHD finger protein 10 | 0.288 | 1.221 | 1.80E-01 | 1.80E+01 | | | |
| 7892791 | --- | --- | --- | 0.287 | 1.220 | 6.92E-01 | 6.92E+01 | | | |
| 8092640 | NM_002916 | RFC4 | replication factor C (activator 1) 4, 37 | 0.287 | 1.220 | 1.45E-01 | 1.45E+01 | | | |
| 8050367 | NM_015909 | NBAS | neuroblastoma amplified sequence | 0.287 | 1.220 | 9.82E-02 | 9.82E+00 | | | |
| 8113008 | --- | --- | --- | 0.287 | 1.220 | 6.59E-01 | 6.59E+01 | | | |
| 8111739 | NM_001465 | FYB | FYN binding protein (FYB-120/130) | 0.287 | 1.220 | 1.08E-01 | 1.08E+01 | | | |
| 7918323 | NM_002959 | SORT1 | sortilin 1 | 0.287 | 1.220 | 9.69E-03 | 9.69E-01 | | | |
| 8042588 | NM_005791 | MPHOSPH10 | M-phase phosphoprotein 10 (U3 sma | 0.287 | 1.220 | 3.69E-01 | 3.69E+01 | | | |
| 8063636 | NM_00100143 | STX16 | syntaxin 16 | 0.287 | 1.220 | 2.12E-01 | 2.12E+01 | | | |
| 7894926 | --- | --- | --- | 0.287 | 1.220 | 7.06E-01 | 7.06E+01 | | | |
| 8054978 | NM_000122 | ERCC3 | excision repair cross-complementing | 0.286 | 1.220 | 3.86E-02 | 3.86E+00 | | | |
| 7990700 | NM_025234 | WDR61 | WD repeat domain 61 | 0.286 | 1.219 | 4.35E-01 | 4.35E+01 | | | |
| 8002592 | NM_00103000 | AP1G1 | adaptor-related protein complex 1, g | 0.286 | 1.219 | 3.39E-01 | 3.39E+01 | | | |
| 8114814 | NM_000176 | NR3C1 | nuclear receptor subfamily 3, group | 0.286 | 1.219 | 1.02E-01 | 1.02E+01 | | | |
| 8092849 | NM_024524 | ATP13A3 | ATPase type 13A3 | 0.286 | 1.219 | 3.73E-01 | 3.73E+01 | | | |
| 7975725 | NM_019589 | YLPM1 | YLP motif containing 1 | 0.286 | 1.219 | 2.06E-01 | 2.06E+01 | | | |
| 7895907 | --- | --- | --- | 0.286 | 1.219 | 3.96E-01 | 3.96E+01 | | | |
| 8027416 | NM_003796 | C19orf2 | chromosome 19 open reading frame | 0.286 | 1.219 | 3.89E-01 | 3.89E+01 | | | |
| 7895888 | --- | --- | --- | 0.286 | 1.219 | 5.38E-01 | 5.38E+01 | | | |
| 7896464 | --- | --- | --- | 0.286 | 1.219 | 6.89E-01 | 6.89E+01 | | | |
| 8160033 | NM_003094 | SNRPE | small nuclear ribonucleoprotein poly | 0.286 | 1.219 | 5.05E-01 | 5.05E+01 | | | |
| 8112469 | BC171739 | GUSBL1 | glucuronidase, beta-like 1 | 0.286 | 1.219 | 2.92E-01 | 2.92E+01 | | | |
| 8108435 | NM_181838 | UBE2D2 | ubiquitin-conjugating enzyme E2D 2 | 0.286 | 1.219 | 3.45E-01 | 3.45E+01 | | | |
| 8135488 | NM_00109966 | LRRN3 | leucine rich repeat neuronal 3 | 0.285 | 1.219 | 9.28E-02 | 9.28E+00 | | | |
| 7991630 | NM_078474 | TM2D3 | TM2 domain containing 3 | 0.285 | 1.219 | 1.20E-01 | 1.20E+01 | | | |
| 8008819 | NM_00100540 | YPEL2 | yippee-like 2 (Drosophila) | 0.285 | 1.219 | 3.60E-02 | 3.60E+00 | | | |
| 7902992 | NM_024813 | RPAP2 | RNA polymerase II associated protei | 0.285 | 1.219 | 2.72E-01 | 2.72E+01 | | | |
| 7894546 | --- | --- | --- | 0.285 | 1.219 | 3.32E-02 | 3.32E+00 | | | |
| 8006477 | NM_052857 | ZNF830 | zinc finger protein 830 | 0.285 | 1.218 | 3.75E-01 | 3.75E+01 | | | |
| 7895147 | --- | --- | --- | 0.285 | 1.218 | 4.48E-01 | 4.48E+01 | | | |
| 8112182 | NM_152622 | MIER3 | mesoderm induction early response | 0.285 | 1.218 | 3.24E-01 | 3.24E+01 | | | |
| 8069910 | NM_020706 | SFRS15 | splicing factor, arginine/serine-rich 1 | 0.284 | 1.218 | 2.69E-02 | 2.69E+00 | | | |
| 7933632 | --- | --- | --- | 0.284 | 1.218 | 8.52E-02 | 8.52E+00 | | | |
| 8052784 | NM_015700 | NFU1 | NFU1 iron-sulfur cluster scaffold hon | 0.284 | 1.218 | 3.46E-01 | 3.46E+01 | | | |
| 8012247 | NM_133491 | SAT2 | spermidine/spermine N1-acetyltrans | 0.284 | 1.218 | 1.81E-01 | 1.81E+01 | | | |
| 7978272 | NM_014430 | CIDEB | cell death-inducing DFFA-like effecto | 0.284 | 1.218 | 2.69E-01 | 2.69E+01 | | | |
| 7978208 | NM_012461 | TINF2 | TERF1 (TRF1)-interacting nuclear fact | 0.284 | 1.218 | 2.79E-01 | 2.79E+01 | | | |
| 8180100 | NM_033554 | HLA-DPA1 | major histocompatibility complex, cla | 0.284 | 1.218 | 3.99E-01 | 3.99E+01 | | | |
| 7984641 | NM_005744 | ARIH1 | ariadne homolog, ubiquitin-conjugat | 0.284 | 1.218 | 5.39E-01 | 5.39E+01 | | | |
| 7895883 | --- | --- | --- | 0.284 | 1.217 | 1.10E-01 | 1.10E+01 | | | |
| 7968670 | NM_016617 | UFM1 | ubiquitin-fold modifier 1 | 0.284 | 1.217 | 2.39E-01 | 2.39E+01 | | | |
| 8132218 | NM_198428 | BBS9 | Bardet-Biedl syndrome 9 | 0.284 | 1.217 | 2.48E-01 | 2.48E+01 | | | |
| 8140170 | NM_173537 | GTF2IRD2 | GTF2I repeat domain containing 2 | 0.284 | 1.217 | 3.17E-02 | 3.17E+00 | | | |
| 7911078 | NM_016076 | PPPDE1 | PPPDE peptidase domain containing | 0.284 | 1.217 | 1.59E-01 | 1.59E+01 | | | |
| 8148985 | NM_207332 | ERICH1 | glutamate-rich 1 | 0.283 | 1.217 | 2.40E-01 | 2.40E+01 | | | |
| 8022110 | NM_00114382 | ZFP161 | zinc finger protein 161 homolog (mo | 0.283 | 1.217 | 2.53E-01 | 2.53E+01 | | | |
| 8006573 | NM_139215 | TAF15 | TAF15 RNA polymerase II, TATA box | 0.283 | 1.217 | 7.81E-02 | 7.81E+00 | | | |
| 8143988 | NM_170606 | MLL3 | myeloid/lymphoid or mixed-lineage | 0.283 | 1.217 | 1.11E-01 | 1.11E+01 | | | |
| 8125825 | NM_006703 | NUDT3 | nudix (nucleoside diphosphate linked | 0.283 | 1.217 | 3.81E-02 | 3.81E+00 | | | |
| 8136095 | NM_015328 | AHCYL2 | adenosylhomocysteinase-like 2 | 0.283 | 1.217 | 1.01E-01 | 1.01E+01 | | | |
| 7992887 | AK093979 | ZNF75A | zinc finger protein 75a | 0.283 | 1.217 | 2.54E-01 | 2.54E+01 | | | |
| 8056545 | NM_013233 | STK39 | serine threonine kinase 39 (STE20/SP | 0.283 | 1.217 | 3.48E-01 | 3.48E+01 | | | |
| 7936891 | NM_212554 | METTL10 | methyltransferase like 10 | 0.283 | 1.217 | 3.63E-01 | 3.63E+01 | | | |
| 7980990 | NM_032490 | C14orf142 | chromosome 14 open reading frame | 0.283 | 1.217 | 6.48E-02 | 6.48E+00 | | | |
| 7906703 | NM_004550 | NDUFS2 | NADH dehydrogenase (ubiquinone) F | 0.283 | 1.217 | 2.10E-01 | 2.10E+01 | | | |
| 8027621 | NM_000175 | GPI | glucose phosphate isomerase | 0.283 | 1.217 | 7.51E-02 | 7.51E+00 | | | |
| 7943176 | NM_004268 | MED17 | mediator complex subunit 17 | 0.283 | 1.217 | 3.07E-01 | 3.07E+01 | | | |
| 8094240 | NM_001775 | CD38 | CD38 molecule | 0.283 | 1.216 | 1.52E-01 | 1.52E+01 | | | |
| 8068375 | NM_058182 | FAM165B | family with sequence similarity 165, | 0.283 | 1.216 | 7.39E-02 | 7.39E+00 | | | |
| 8160478 | BC071953 | C9orf82 | chromosome 9 open reading frame 8 | 0.282 | 1.216 | 1.57E-01 | 1.57E+01 | | | |

| | | | | | | | | | | |
|---|---|---|---|---|---|---|---|---|---|---|
| 7894518 | --- | --- | --- | 0.282 | 1.216 | 1.73E-02 | 1.73E+00 | | | |
| 8160647 | NM_004323 | BAG1 | BCL2-associated athanogene | 0.282 | 1.216 | 2.19E-01 | 2.19E+01 | | | |
| 7982154 | NR_002824 | HERC2P2 | hect domain and RLD 2 pseudogene | 0.282 | 1.216 | 9.85E-02 | 9.85E+00 | | | |
| 8066889 | NM_017453 | STAU1 | staufen, RNA binding protein, homol | 0.282 | 1.216 | 1.54E-01 | 1.54E+01 | | | |
| 7893374 | --- | --- | --- | 0.282 | 1.216 | 2.01E-01 | 2.01E+01 | | | |
| 8135464 | NM_000108 | DLD | dihydrolipoamide dehydrogenase | 0.282 | 1.216 | 3.95E-01 | 3.95E+01 | | | |
| 7894167 | --- | --- | --- | 0.281 | 1.215 | 7.29E-01 | 7.29E+01 | | | |
| 8165486 | NM_053045 | TMEM203 | transmembrane protein 203 | 0.281 | 1.215 | 1.44E-01 | 1.44E+01 | | | |
| 8101762 | NM_000345 | SNCA | synuclein, alpha (non A4 component | 0.281 | 1.215 | 1.64E-01 | 1.64E+01 | | | |
| 8117194 | NM_020662 | MRS2 | MRS2 magnesium homeostasis facto | 0.281 | 1.215 | 3.53E-01 | 3.53E+01 | | | |
| 7936307 | NM_005871 | SMNDC1 | survival motor neuron domain conta | 0.281 | 1.215 | 4.21E-01 | 4.21E+01 | | | |
| 8075182 | NM_005080 | XBP1 | X-box binding protein 1 | 0.281 | 1.215 | 1.87E-01 | 1.87E+01 | | | |
| 7895726 | --- | --- | --- | 0.281 | 1.215 | 6.79E-01 | 6.79E+01 | | | |
| 8127158 | NM_001498 | GCLC | glutamate-cysteine ligase, catalytic s | 0.281 | 1.215 | 2.93E-01 | 2.93E+01 | | | |
| 7996744 | NM_173163 | NFATC3 | nuclear factor of activated T-cells, cy | 0.281 | 1.215 | 1.38E-01 | 1.38E+01 | | | |
| 7916443 | NM_015306 | USP24 | ubiquitin specific peptidase 24 | 0.281 | 1.215 | 1.00E-01 | 1.00E+01 | | | |
| 8123494 | NM_032448 | FAM120B | family with sequence similarity 120B | 0.281 | 1.215 | 8.79E-02 | 8.79E+00 | | | |
| 8146717 | NM_013257 | SGK3 | serum/glucocorticoid regulated kina | 0.280 | 1.215 | 3.74E-01 | 3.74E+01 | | | |
| 8167006 | NM_006915 | RP2 | retinitis pigmentosa 2 (X-linked rece | 0.280 | 1.215 | 4.24E-01 | 4.24E+01 | | | |
| 7958800 | NM_003668 | MAPKAPK5 | mitogen-activated protein kinase-act | 0.280 | 1.214 | 2.09E-01 | 2.09E+01 | | | |
| 7975545 | NM_000021 | PSEN1 | presenilin 1 | 0.280 | 1.214 | 1.37E-01 | 1.37E+01 | | | |
| 8078729 | NM_002468 | MYD88 | myeloid differentiation primary resp | 0.280 | 1.214 | 3.68E-02 | 3.68E+00 | | | mono |
| 7953021 | NM_024551 | ADIPOR2 | adiponectin receptor 2 | 0.280 | 1.214 | 1.61E-01 | 1.61E+01 | | | |
| 8062908 | NM_006282 | STK4 | serine/threonine kinase 4 | 0.280 | 1.214 | 5.63E-02 | 5.63E+00 | | | |
| 7921121 | NM_145729 | MRPL24 | mitochondrial ribosomal protein L24 | 0.280 | 1.214 | 2.27E-01 | 2.27E+01 | | | |
| 7956166 | NM_015292 | ESYT1 | extended synaptotagmin-like protein | 0.280 | 1.214 | 1.63E-01 | 1.63E+01 | | | |
| 8039212 | NM_005874 | LILRB2 | leukocyte immunoglobulin-like recep | 0.280 | 1.214 | 1.40E-02 | 1.40E+00 | | | mono |
| 7895743 | --- | --- | --- | 0.280 | 1.214 | 7.31E-01 | 7.31E+01 | | | |
| 7979931 | NM_005466 | MED6 | mediator complex subunit 6 | 0.280 | 1.214 | 5.47E-01 | 5.47E+01 | | | |
| 8106784 | NM_002890 | RASA1 | RAS p21 protein activator (GTPase ac | 0.280 | 1.214 | 2.10E-01 | 2.10E+01 | | | |
| 8079662 | NM_006321 | ARIH2 | ariadne homolog 2 (Drosophila) | 0.280 | 1.214 | 2.39E-01 | 2.39E+01 | | | |
| 7914489 | NM_080391 | PTP4A2 | protein tyrosine phosphatase type IV | 0.279 | 1.214 | 5.50E-03 | 5.50E-01 | | | |
| 8142886 | NM_003344 | UBE2H | ubiquitin-conjugating enzyme E2H (U | 0.279 | 1.214 | 2.68E-01 | 2.68E+01 | | | |
| 8078227 | NM_003884 | KAT2B | K(lysine) acetyltransferase 2B | 0.279 | 1.214 | 3.00E-01 | 3.00E+01 | | | |
| 8157941 | NM_00109927( | ZBTB34 | zinc finger and BTB domain containir | 0.279 | 1.214 | 4.71E-03 | 4.71E-01 | | | |
| 8062206 | NM_080748 | ROMO1 | reactive oxygen species modulator 1 | 0.279 | 1.213 | 3.44E-01 | 3.44E+01 | | | |
| 7986701 | NR_002824 | HERC2P2 | hect domain and RLD 2 pseudogene | 0.279 | 1.213 | 7.95E-02 | 7.95E+00 | | | |
| 7895619 | --- | --- | --- | 0.279 | 1.213 | 8.10E-01 | 8.10E+01 | | | |
| 7894637 | --- | --- | --- | 0.279 | 1.213 | 7.13E-01 | 7.13E+01 | | | |
| 7900833 | NM_014663 | KDM4A | lysine (K)-specific demethylase 4A | 0.279 | 1.213 | 7.68E-02 | 7.68E+00 | | | |
| 8058373 | NM_018256 | WDR12 | WD repeat domain 12 | 0.279 | 1.213 | 4.06E-01 | 4.06E+01 | | | |
| 7925691 | NM_003431 | ZNF124 | zinc finger protein 124 | 0.279 | 1.213 | 2.40E-01 | 2.40E+01 | | | |
| 7903972 | NM_001688 | ATP5F1 | ATP synthase, H+ transporting, mitoc | 0.278 | 1.213 | 1.01E-01 | 1.01E+01 | | | |
| 8045287 | --- | --- | --- | 0.278 | 1.213 | 5.72E-01 | 5.72E+01 | | | |
| 8080438 | NM_014041 | SPCS1 | signal peptidase complex subunit 1 h | 0.278 | 1.213 | 2.70E-01 | 2.70E+01 | | | |
| 8082408 | NM_013336 | SEC61A1 | Sec61 alpha 1 subunit (S. cerevisiae) | 0.278 | 1.213 | 6.39E-02 | 6.39E+00 | | | |
| 7973611 | --- | --- | --- | 0.278 | 1.213 | 3.69E-01 | 3.69E+01 | | | |
| 7948037 | --- | --- | --- | 0.278 | 1.213 | 6.05E-01 | 6.05E+01 | | | |
| 7916727 | NM_014288 | ITGB3BP | integrin beta 3 binding protein (beta | 0.278 | 1.213 | 2.16E-01 | 2.16E+01 | | | |
| 7946516 | NM_030962 | SBF2 | SET binding factor 2 | 0.278 | 1.213 | 2.84E-02 | 2.84E+00 | | | |
| 8119198 | NM_015050 | FTSJD2 | FtsJ methyltransferase domain conta | 0.278 | 1.212 | 3.26E-01 | 3.26E+01 | | | |
| 7946478 | NM_015213 | DENND5A | DENN/MADD domain containing 5A | 0.278 | 1.212 | 1.24E-01 | 1.24E+01 | | | |
| 7929282 | NM_002729 | HHEX | hematopoietically expressed homeo | 0.278 | 1.212 | 3.84E-01 | 3.84E+01 | | | |
| 8053735 | --- | --- | --- | 0.278 | 1.212 | 2.86E-02 | 2.86E+00 | | | |
| 8110289 | NM_022455 | NSD1 | nuclear receptor binding SET domair | 0.278 | 1.212 | 4.58E-02 | 4.58E+00 | | | |
| 8107520 | NM_014350 | TNFAIP8 | tumor necrosis factor, alpha-inducec | 0.278 | 1.212 | 1.06E-02 | 1.06E+00 | | | |
| 8144758 | NM_016353 | ZDHHC2 | zinc finger, DHHC-type containing 2 | 0.277 | 1.212 | 1.74E-01 | 1.74E+01 | | | |
| 8131374 | NM_006303 | AIMP2 | aminoacyl tRNA synthetase complex | 0.277 | 1.212 | 1.38E-01 | 1.38E+01 | | | |
| 8160036 | AK292632 | C9orf123 | chromosome 9 open reading frame 1 | 0.277 | 1.212 | 3.86E-01 | 3.86E+01 | | | |
| 7932023 | NM_014688 | USP6NL | USP6 N-terminal like | 0.277 | 1.212 | 2.96E-01 | 2.96E+01 | | | |
| 7922460 | --- | --- | --- | 0.277 | 1.212 | 4.47E-01 | 4.47E+01 | | | |
| 8140227 | NM_000265 | NCF1 | neutrophil cytosolic factor 1 | 0.277 | 1.212 | 2.23E-02 | 2.23E+00 | | Phagocytosis/killing | |
| 7951034 | NR_002920 | SNORA8 | small nucleolar RNA, H/ACA box 8 | 0.277 | 1.212 | 6.40E-01 | 6.40E+01 | | | |
| 7905163 | NM_018997 | MRPS21 | mitochondrial ribosomal protein S21 | 0.277 | 1.212 | 5.30E-01 | 5.30E+01 | | | |
| 7894104 | --- | --- | --- | 0.277 | 1.211 | 6.09E-01 | 6.09E+01 | | | |
| 8124448 | NM_003543 | HIST1H4H | histone cluster 1, H4h | 0.277 | 1.211 | 2.94E-01 | 2.94E+01 | | | |
| 7903827 | NM_033088 | FAM40A | family with sequence similarity 40, m | 0.277 | 1.211 | 8.41E-02 | 8.41E+00 | | | |
| 8151149 | NM_006421 | ARFGEF1 | ADP-ribosylation factor guanine nucl | 0.276 | 1.211 | 1.91E-01 | 1.91E+01 | | | |
| 8029136 | NM_001783 | CD79A | CD79a molecule, immunoglobulin-as | 0.276 | 1.211 | 1.09E-01 | 1.09E+01 | | | |
| 7978833 | NM_001001 | RPL36AL | ribosomal protein L36a-like | 0.276 | 1.211 | 2.29E-01 | 2.29E+01 | | | |
| 8108330 | NM_016604 | KDM3B | lysine (K)-specific demethylase 3B | 0.276 | 1.211 | 1.68E-01 | 1.68E+01 | | | |
| 7898407 | BC036435 | OC10013214 | hypothetical LOC100132147 | 0.276 | 1.211 | 3.02E-01 | 3.02E+01 | | | |
| 7973036 | NM_005484 | PARP2 | poly (ADP-ribose) polymerase 2 | 0.276 | 1.211 | 1.78E-01 | 1.78E+01 | | | |
| 8064007 | NM_017896 | C20orf11 | chromosome 20 open reading frame | 0.276 | 1.211 | 4.33E-01 | 4.33E+01 | | | |
| 8152782 | NM_014846 | KIAA0196 | KIAA0196 | 0.276 | 1.211 | 1.34E-01 | 1.34E+01 | | | |
| 7967841 | NM_00116134 | CHFR | checkpoint with forkhead and ring fi | 0.276 | 1.211 | 2.99E-02 | 2.99E+00 | | | |
| 8112767 | NM_004607 | TBCA | tubulin folding cofactor A | 0.276 | 1.211 | 3.16E-01 | 3.16E+01 | | | |

| | | | | | | | | | | |
|---|---|---|---|---|---|---|---|---|---|---|
| 8124008 | NM_00103171 | CCDC90A | coiled-coil domain containing 90A | 0.276 | 1.210 | 4.54E-02 | 4.54E+00 | | | |
| 8133314 | NM_000265 | NCF1 | neutrophil cytosolic factor 1 | 0.275 | 1.210 | 2.27E-02 | 2.27E+00 | | Phagocytosis/killing | |
| 7990151 | NM_182470 | PKM2 | pyruvate kinase, muscle | 0.275 | 1.210 | 3.60E-02 | 3.60E+00 | | | |
| 8002133 | NM_002801 | PSMB10 | proteasome (prosome, macropain) s | 0.275 | 1.210 | 8.49E-02 | 8.49E+00 | | | |
| 8045697 | NM_018151 | RIF1 | RAP1 interacting factor homolog (ye | 0.275 | 1.210 | 1.88E-01 | 1.88E+01 | | | |
| 7989516 | NM_003922 | HERC1 | hect (homologous to the E6-AP (UBE | 0.275 | 1.210 | 1.20E-01 | 1.20E+01 | | | |
| 8169868 | NM_006649 | UTP14A | UTP14, U3 small nucleolar ribonucle | 0.275 | 1.210 | 1.21E-01 | 1.21E+01 | | | |
| 7969153 | NM_213590 | TRIM13 | tripartite motif-containing 13 | 0.274 | 1.210 | 3.74E-02 | 3.74E+00 | | | |
| 7986665 | NM_030922 | NIPA2 | non imprinted in Prader-Willi/Angelr | 0.274 | 1.210 | 1.59E-01 | 1.59E+01 | | | |
| 7903988 | NM_00101093 | RAP1A | RAP1A, member of RAS oncogene fa | 0.274 | 1.209 | 4.35E-01 | 4.35E+01 | | | |
| 7936949 | NM_018180 | DHX32 | DEAH (Asp-Glu-Ala-His) box polypept | 0.274 | 1.209 | 1.08E-01 | 1.08E+01 | | | |
| 8152111 | NM_00104251 | ZNF706 | zinc finger protein 706 | 0.274 | 1.209 | 1.93E-01 | 1.93E+01 | | | |
| 8129379 | NM_00100203 | ECHDC1 | enoyl Coenzyme A hydratase domair | 0.274 | 1.209 | 6.19E-01 | 6.19E+01 | | | |
| 8162850 | NM_017746 | TEX10 | testis expressed 10 | 0.274 | 1.209 | 1.71E-01 | 1.71E+01 | | | |
| 7896085 | --- | --- | --- | 0.274 | 1.209 | 6.58E-01 | 6.58E+01 | | | |
| 7957043 | NM_006654 | FRS2 | fibroblast growth factor receptor sub | 0.274 | 1.209 | 2.68E-01 | 2.68E+01 | | | |
| 7894478 | --- | --- | --- | 0.273 | 1.209 | 4.25E-01 | 4.25E+01 | | | |
| 8007446 | NM_005533 | IFI35 | interferon-induced protein 35 | 0.273 | 1.209 | 1.20E-01 | 1.20E+01 | | | |
| 7945156 | --- | --- | --- | 0.273 | 1.209 | 6.32E-01 | 6.32E+01 | | | |
| 8109843 | NM_004946 | DOCK2 | dedicator of cytokinesis 2 | 0.273 | 1.209 | 5.31E-02 | 5.31E+00 | | | |
| 8097657 | NM_005900 | SMAD1 | SMAD family member 1 | 0.273 | 1.209 | 9.62E-03 | 9.62E-01 | | | |
| 7893699 | --- | --- | --- | 0.273 | 1.209 | 6.07E-01 | 6.07E+01 | | | |
| 7960362 | AY358109 | UNQ3104 | ACAH3104 | 0.273 | 1.208 | 1.06E-01 | 1.06E+01 | | | |
| 8128669 | NM_014028 | OSTM1 | osteopetrosis associated transmemb | 0.273 | 1.208 | 1.09E-01 | 1.09E+01 | | | |
| 8125360 | NM_002586 | PBX2 | pre-B-cell leukemia homeobox 2 | 0.273 | 1.208 | 1.14E-01 | 1.14E+01 | | | |
| 7958253 | NM_00114519 | C12orf75 | chromosome 12 open reading frame | 0.273 | 1.208 | 4.22E-01 | 4.22E+01 | | | |
| 8107096 | --- | --- | --- | 0.273 | 1.208 | 5.45E-02 | 5.45E+00 | | | |
| 8109732 | NM_013283 | MAT2B | methionine adenosyltransferase II, b | 0.273 | 1.208 | 4.35E-01 | 4.35E+01 | | | |
| 8017143 | NM_016077 | PTRH2 | peptidyl-tRNA hydrolase 2 | 0.272 | 1.208 | 2.41E-01 | 2.41E+01 | | | |
| 7893956 | --- | --- | --- | 0.272 | 1.208 | 7.07E-02 | 7.07E+00 | | | |
| 8105681 | NM_018695 | ERBB2IP | erbb2 interacting protein | 0.272 | 1.208 | 1.10E-01 | 1.10E+01 | | | |
| 8069711 | NM_015565 | RNF160 | ring finger protein 160 | 0.272 | 1.208 | 1.85E-01 | 1.85E+01 | | | |
| 7906564 | NM_003768 | PEA15 | phosphoprotein enriched in astrocyt | 0.272 | 1.208 | 1.04E-01 | 1.04E+01 | | | mono |
| 8066136 | NM_002895 | RBL1 | retinoblastoma-like 1 (p107) | 0.272 | 1.208 | 1.71E-01 | 1.71E+01 | | | |
| 7896137 | --- | --- | --- | 0.272 | 1.208 | 1.90E-01 | 1.90E+01 | | | |
| 7893542 | --- | --- | --- | 0.272 | 1.207 | 5.35E-01 | 5.35E+01 | | | |
| 7894557 | --- | --- | --- | 0.272 | 1.207 | 5.29E-01 | 5.29E+01 | | | |
| 8094378 | NM_018323 | PI4K2B | phosphatidylinositol 4-kinase type 2 | 0.272 | 1.207 | 2.44E-01 | 2.44E+01 | | | |
| 8043375 | --- | --- | --- | 0.272 | 1.207 | 5.48E-01 | 5.48E+01 | | | |
| 8058428 | NM_005006 | NDUFS1 | NADH dehydrogenase (ubiquinone) | 0.272 | 1.207 | 1.88E-01 | 1.88E+01 | | | |
| 7996393 | NM_001755 | CBFB | core-binding factor, beta subunit | 0.272 | 1.207 | 1.63E-01 | 1.63E+01 | | | |
| 8021183 | NR_003003 | SCARNA17 | small Cajal body-specific RNA 17 | 0.271 | 1.207 | 7.45E-01 | 7.45E+01 | | | |
| 7893950 | --- | --- | --- | 0.271 | 1.207 | 6.97E-01 | 6.97E+01 | | | |
| 8150599 | NM_006904 | PRKDC | protein kinase, DNA-activated, cataly | 0.271 | 1.207 | 1.82E-01 | 1.82E+01 | | | |
| 8082118 | --- | --- | --- | 0.271 | 1.207 | 5.46E-01 | 5.46E+01 | | | |
| 7896275 | --- | --- | --- | 0.271 | 1.207 | 8.17E-01 | 8.17E+01 | | | |
| 8058118 | NM_152387 | KCTD18 | potassium channel tetramerisation d | 0.271 | 1.207 | 6.01E-02 | 6.01E+00 | | | |
| 8112564 | BC171739 | GUSBL1 | glucuronidase, beta-like 1 | 0.271 | 1.207 | 3.20E-01 | 3.20E+01 | | | |
| 8028172 | NM_001749 | CAPNS1 | calpain, small subunit 1 | 0.271 | 1.207 | 1.31E-01 | 1.31E+01 | | | |
| 8023656 | NM_002035 | KDSR | 3-ketodihydrosphingosine reductase | 0.271 | 1.207 | 2.13E-01 | 2.13E+01 | | | |
| 8008965 | --- | --- | --- | 0.271 | 1.206 | 3.64E-01 | 3.64E+01 | | | |
| 8126474 | NM_014623 | MEA1 | male-enhanced antigen 1 | 0.271 | 1.206 | 2.82E-01 | 2.82E+01 | | | |
| 8051226 | NM_017910 | TRMT61B | tRNA methyltransferase 61 homolog | 0.271 | 1.206 | 3.77E-01 | 3.77E+01 | | | |
| 8108424 | NM_00103311 | PAIP2 | poly(A) binding protein interacting p | 0.270 | 1.206 | 2.36E-01 | 2.36E+01 | | | |
| 8168280 | NM_00114540 | NONO | non-POU domain containing, octame | 0.270 | 1.206 | 2.75E-01 | 2.75E+01 | | | |
| 7939265 | NM_024662 | NAT10 | N-acetyltransferase 10 (GCN5-relate | 0.270 | 1.206 | 2.84E-02 | 2.84E+00 | | | |
| 7964360 | NM_003153 | STAT6 | signal transducer and activator of tra | 0.270 | 1.206 | 2.30E-01 | 2.30E+01 | | | |
| 7905258 | NM_00114541 | SETDB1 | SET domain, bifurcated 1 | 0.270 | 1.206 | 5.91E-02 | 5.91E+00 | | | |
| 7941460 | NM_006442 | DRAP1 | DR1-associated protein 1 (negative c | 0.270 | 1.206 | 3.86E-01 | 3.86E+01 | | | |
| 7894331 | --- | --- | --- | 0.270 | 1.205 | 7.82E-01 | 7.82E+01 | | | |
| 7896138 | --- | --- | --- | 0.270 | 1.205 | 5.48E-02 | 5.48E+00 | | | |
| 7899187 | NM_005517 | HMGN2 | high-mobility group nucleosomal bin | 0.269 | 1.205 | 1.62E-01 | 1.62E+01 | | | |
| 8094974 | NM_00107983 | OCIAD1 | OCIA domain containing 1 | 0.269 | 1.205 | 4.88E-01 | 4.88E+01 | | | |
| 7992402 | NM_004548 | NDUFB10 | NADH dehydrogenase (ubiquinone) | 0.269 | 1.205 | 1.40E-01 | 1.40E+01 | | | |
| 7893202 | --- | --- | --- | 0.269 | 1.205 | 7.87E-01 | 7.87E+01 | | | |
| 8026106 | NM_004343 | CALR | calreticulin | 0.269 | 1.205 | 2.61E-01 | 2.61E+01 | | | |
| 7893261 | --- | --- | --- | 0.269 | 1.205 | 6.89E-01 | 6.89E+01 | | | |
| 7895134 | --- | --- | --- | 0.269 | 1.205 | 7.31E-01 | 7.31E+01 | | | |
| 8140311 | NM_005395 | PMS2L3 | postmeiotic segregation increased 2 | 0.269 | 1.205 | 2.20E-01 | 2.20E+01 | | | |
| 7983469 | NR_027635 | SPATA5L1 | spermatogenesis associated 5-like 1 | 0.269 | 1.205 | 1.00E-01 | 1.00E+01 | | | |
| 8162047 | NM_002140 | HNRNPK | heterogeneous nuclear ribonucleopr | 0.268 | 1.205 | 2.63E-01 | 2.63E+01 | | | |
| 8094574 | NM_015173 | TBC1D1 | TBC1 (tre-2/USP6, BUB2, cdc16) dom | 0.268 | 1.204 | 1.63E-01 | 1.63E+01 | | | |
| 8090256 | NM_003794 | SNX4 | sorting nexin 4 | 0.268 | 1.204 | 4.45E-01 | 4.45E+01 | | | |
| 8128138 | NM_014611 | MDN1 | MDN1, midasin homolog (yeast) | 0.268 | 1.204 | 2.51E-02 | 2.51E+00 | | | |
| 7970524 | NM_153251 | ZDHHC20 | zinc finger, DHHC-type containing 20 | 0.268 | 1.204 | 8.93E-02 | 8.93E+00 | | | |
| 8054437 | NM_022662 | ANAPC1 | anaphase promoting complex subun | 0.268 | 1.204 | 6.02E-01 | 6.02E+01 | | | |
| 8016077 | NM_00100290 | GPATCH8 | G patch domain containing 8 | 0.268 | 1.204 | 2.67E-01 | 2.67E+01 | | | |

| | | | | | | | | | | |
|---|---|---|---|---|---|---|---|---|---|---|
| 8001067 | NR_002827 | HERC2P4 | hect domain and RLD 2 pseudogene | 0.268 | 1.204 | 4.03E-01 | 4.03E+01 | | | |
| 8056005 | NM_001105 | ACVR1 | activin A receptor, type I | 0.268 | 1.204 | 1.07E-01 | 1.07E+01 | | | |
| 7938301 | NM_020642 | C11orf17 | chromosome 11 open reading frame | 0.267 | 1.204 | 2.52E-01 | 2.52E+01 | | | |
| 8102860 | NM_002107 // | 3F3A // H3F3 | H3 histone, family 3A // H3 histone, | 0.267 | 1.203 | 1.29E-01 | 1.29E+01 | | | |
| 8067007 | NM_021109 | TMSB4X | thymosin beta 4, X-linked | 0.267 | 1.203 | 2.17E-02 | 2.17E+00 | | | |
| 8080419 | NM_206825 | GNL3 | guanine nucleotide binding protein-l | 0.267 | 1.203 | 3.99E-01 | 3.99E+01 | | | |
| 7935320 | NM_020123 | TM9SF3 | transmembrane 9 superfamily memb | 0.267 | 1.203 | 4.60E-01 | 4.60E+01 | | | |
| 8075239 | NM_00100287 | THOC5 | THO complex 5 | 0.267 | 1.203 | 5.67E-02 | 5.67E+00 | | | |
| 7965812 | NM_024312 | GNPTAB | N-acetylglucosamine-1-phosphate tr | 0.267 | 1.203 | 3.93E-01 | 3.93E+01 | | | |
| 8029856 | NM_004491 | GRLF1 | glucocorticoid receptor DNA binding | 0.267 | 1.203 | 1.25E-01 | 1.25E+01 | | | |
| 7953351 | NM_014865 | NCAPD2 | non-SMC condensin I complex, subu | 0.267 | 1.203 | 8.49E-02 | 8.49E+00 | | | |
| 7923698 | --- | --- | --- | 0.267 | 1.203 | 2.93E-01 | 2.93E+01 | | | |
| 8082816 | NM_021203 | SRPRB | signal recognition particle receptor, | 0.267 | 1.203 | 3.63E-01 | 3.63E+01 | | | |
| 7892979 | --- | --- | --- | 0.267 | 1.203 | 4.77E-01 | 4.77E+01 | | | |
| 7895963 | --- | --- | --- | 0.267 | 1.203 | 6.83E-01 | 6.83E+01 | | | |
| 7995580 | --- | --- | --- | 0.267 | 1.203 | 3.44E-02 | 3.44E+00 | | | |
| 7944667 | NM_003105 | SORL1 | sortilin-related receptor, L(DLR class) | 0.266 | 1.203 | 2.29E-01 | 2.29E+01 | | | |
| 7953428 | NM_000616 | CD4 | CD4 molecule | 0.266 | 1.203 | 9.30E-02 | 9.30E+00 | | | |
| 7964234 | NM_001686 | ATP5B | ATP synthase, H+ transporting, mitoc | 0.266 | 1.203 | 3.46E-02 | 3.46E+00 | | | |
| 7981183 | NM_021966 | TCL1A | T-cell leukemia/lymphoma 1A | 0.266 | 1.203 | 6.07E-02 | 6.07E+00 | | | |
| 8118556 | NM_002122 | HLA-DQA1 | major histocompatibility complex, cl | 0.266 | 1.203 | 3.72E-01 | 3.72E+01 | | | |
| 8047243 | NM_138395 | MARS2 | methionyl-tRNA synthetase 2, mitocl | 0.266 | 1.202 | 1.43E-02 | 1.43E+00 | | | |
| 7896643 | --- | --- | --- | 0.266 | 1.202 | 7.36E-01 | 7.36E+01 | | | |
| 8145259 | NM_152272 | CHMP7 | CHMP family, member 7 | 0.266 | 1.202 | 1.19E-01 | 1.19E+01 | | | |
| 8167965 | NM_002444 | MSN | moesin | 0.266 | 1.202 | 5.71E-02 | 5.71E+00 | | | |
| 7942232 | NM_018161 | NADSYN1 | NAD synthetase 1 | 0.265 | 1.202 | 6.14E-02 | 6.14E+00 | | | |
| 7959657 | NM_012463 | ATP6V0A2 | ATPase, H+ transporting, lysosomal V | 0.265 | 1.202 | 2.13E-01 | 2.13E+01 | | | |
| 8139943 | NR_003666 | SPDYE7P | speedy homolog E7 (Xenopus laevis) | 0.265 | 1.202 | 3.77E-01 | 3.77E+01 | | | |
| 7973564 | NM_176783 | PSME1 | proteasome (prosome, macropain) a | 0.265 | 1.202 | 4.63E-02 | 4.63E+00 | | | |
| 8121483 | --- | --- | --- | 0.265 | 1.202 | 4.56E-01 | 4.56E+01 | | | |
| 7973867 | --- | --- | --- | 0.265 | 1.201 | 4.62E-01 | 4.62E+01 | | | |
| 8128894 | NM_002912 | REV3L | REV3-like, catalytic subunit of DNA p | 0.265 | 1.201 | 3.41E-02 | 3.41E+00 | | | |
| 8038998 | NM_199132 | ZNF468 | zinc finger protein 468 | 0.265 | 1.201 | 1.97E-01 | 1.97E+01 | | | |
| 7948192 | NM_003146 | SSRP1 | structure specific recognition protein | 0.265 | 1.201 | 1.37E-01 | 1.37E+01 | | | |
| 7977127 | NM_00113010 | KLC1 | kinesin light chain 1 | 0.265 | 1.201 | 1.62E-01 | 1.62E+01 | | | |
| 8151475 | NM_00102525 | TPD52 | tumor protein D52 | 0.264 | 1.201 | 2.99E-02 | 2.99E+00 | | | |
| 7953409 | NM_002824 | PTMS | parathymosin | 0.264 | 1.201 | 1.83E-01 | 1.83E+01 | | | |
| 7895981 | --- | --- | --- | 0.264 | 1.201 | 7.80E-01 | 7.80E+01 | | | |
| 8007023 | NM_00101224 | MSL1 | male-specific lethal 1 homolog (Dros | 0.264 | 1.201 | 4.49E-01 | 4.49E+01 | | | |
| 8162529 | --- | --- | --- | 0.264 | 1.201 | 4.17E-01 | 4.17E+01 | | | |
| 8038029 | NM_014959 | CARD8 | caspase recruitment domain family, | 0.264 | 1.201 | 4.82E-02 | 4.82E+00 | | | |
| 7894838 | --- | --- | --- | 0.264 | 1.201 | 4.13E-01 | 4.13E+01 | | | |
| 7972743 | --- | --- | --- | 0.264 | 1.201 | 1.86E-01 | 1.86E+01 | | | |
| 7991357 | NR_023361 | AP3S2 | adaptor-related protein complex 3, s | 0.264 | 1.201 | 5.42E-01 | 5.42E+01 | | | |
| 8010426 | NM_020954 | KIAA1618 | KIAA1618 | 0.264 | 1.201 | 5.45E-02 | 5.45E+00 | | | |
| 7910446 | NM_007357 | COG2 | component of oligomeric golgi comp | 0.264 | 1.201 | 3.17E-01 | 3.17E+01 | | | |
| 7894617 | --- | --- | --- | 0.264 | 1.201 | 5.99E-01 | 5.99E+01 | | | |
| 8030931 | NM_032423 | ZNF528 | zinc finger protein 528 | 0.264 | 1.200 | 2.58E-02 | 2.58E+00 | | | |
| 8158560 | NM_014506 | TOR1B | torsin family 1, member B (torsin B) | 0.264 | 1.200 | 8.78E-02 | 8.78E+00 | | | |
| 8011077 | NM_006224 | PITPNA | phosphatidylinositol transfer protein | 0.263 | 1.200 | 4.02E-01 | 4.02E+01 | | | |
| 8034454 | NM_001930 | DHPS | deoxyhypusine synthase | 0.263 | 1.200 | 2.36E-01 | 2.36E+01 | | | |
| 8010184 | --- | --- | --- | 0.263 | 1.200 | 5.35E-01 | 5.35E+01 | | | |
| 7896007 | --- | --- | --- | 0.263 | 1.200 | 7.03E-01 | 7.03E+01 | | | |
| 7946728 | NM_148976 | PSMA1 | proteasome (prosome, macropain) s | 0.263 | 1.200 | 4.65E-01 | 4.65E+01 | | | |
| 8005733 | NR_003678 | C20orf191 | nuclear receptor co-repressor 1 pseu | 0.263 | 1.200 | 2.62E-01 | 2.62E+01 | | | |
| 7892856 | --- | --- | --- | 0.263 | 1.200 | 8.07E-01 | 8.07E+01 | | | |
| 8042335 | NM_003375 | VDAC2 | voltage-dependent anion channel 2 | 0.263 | 1.200 | 3.12E-01 | 3.12E+01 | | | |
| 8116532 | NR_002591 | SNORD95 | small nucleolar RNA, C/D box 95 | 0.263 | 1.200 | 2.07E-01 | 2.07E+01 | | | |
| 8126588 | NM_020750 | XPO5 | exportin 5 | 0.263 | 1.200 | 1.89E-01 | 1.89E+01 | | | |
| 8041422 | NM_170672 | RASGRP3 | RAS guanyl releasing protein 3 (calciu | 0.263 | 1.200 | 1.71E-01 | 1.71E+01 | | | |
| 7893063 | --- | --- | --- | 0.263 | 1.200 | 3.01E-01 | 3.01E+01 | | | |
| 7981737 | AF067420 // | AHA1 // IGHA | immunoglobulin heavy constant alph | 0.262 | 1.199 | 2.23E-01 | 2.23E+01 | | | |
| 8154523 | NM_006570 | RRAGA | Ras-related GTP binding A | 0.262 | 1.199 | 1.76E-01 | 1.76E+01 | | | |
| 7959153 | NM_004373 | COX6A1 | cytochrome c oxidase subunit VIa po | 0.262 | 1.199 | 1.27E-01 | 1.27E+01 | | | |
| 7999769 | NM_006985 | NPIP | nuclear pore complex interacting pro | 0.262 | 1.199 | 6.15E-02 | 6.15E+00 | | | |
| 8124798 | NM_133471 | KIAA1949 | KIAA1949 | 0.262 | 1.199 | 2.01E-02 | 2.01E+00 | | | |
| 7893658 | --- | --- | --- | 0.262 | 1.199 | 8.49E-01 | 8.49E+01 | | | |
| 8023165 | NM_032124 | HDHD2 | haloacid dehalogenase-like hydrolase | 0.262 | 1.199 | 4.81E-01 | 4.81E+01 | | | |
| 8146285 | NM_152419 | HGSNAT | heparan-alpha-glucosaminide N-acet | 0.262 | 1.199 | 2.17E-01 | 2.17E+01 | | | |
| 7896089 | --- | --- | --- | 0.261 | 1.199 | 3.53E-01 | 3.53E+01 | | | |
| 7895457 | --- | --- | --- | 0.261 | 1.199 | 6.96E-01 | 6.96E+01 | | | |
| 8075430 | NM_014941 | MORC2 | MORC family CW-type zinc finger 2 | 0.261 | 1.198 | 1.91E-01 | 1.91E+01 | | | |
| 8053427 | --- | --- | --- | 0.261 | 1.198 | 2.13E-01 | 2.13E+01 | | | |
| 7982620 | NM_00101370 | EIF2AK4 | eukaryotic translation initiation facto | 0.261 | 1.198 | 4.82E-02 | 4.82E+00 | | | |
| 7892880 | --- | --- | --- | 0.261 | 1.198 | 7.50E-01 | 7.50E+01 | | | |
| 8118564 | NM_020056 | HLA-DQA2 | major histocompatibility complex, cl | 0.261 | 1.198 | 1.52E-01 | 1.52E+01 | | | |
| 8114365 | NM_139199 | BRD8 | bromodomain containing 8 | 0.261 | 1.198 | 6.99E-02 | 6.99E+00 | | | |

| | | | | | | | | | | |
|---|---|---|---|---|---|---|---|---|---|---|
| 7894740 | --- | --- | --- | 0.261 | 1.198 | 2.17E-01 | 2.17E+01 | | | |
| 7927285 | NM_005614 | RHEB | Ras homolog enriched in brain | 0.261 | 1.198 | 9.45E-02 | 9.45E+00 | | | |
| 7981955 | NR_003319 | SNORD116-4 | small nucleolar RNA, C/D box 116-4 | 0.260 | 1.198 | 4.43E-01 | 4.43E+01 | | | |
| 8073194 | NM_004810 | GRAP2 | GRB2-related adaptor protein 2 | 0.260 | 1.198 | 3.94E-01 | 3.94E+01 | | | |
| 8025478 | NM_032497 | ZNF559 | zinc finger protein 559 | 0.260 | 1.198 | 2.09E-01 | 2.09E+01 | | | |
| 7932938 | NM_025209 | EPC1 | enhancer of polycomb homolog 1 (D | 0.260 | 1.198 | 1.57E-01 | 1.57E+01 | | | |
| 8178811 | 0 | 0 | 0 | 0.260 | 1.198 | 1.00E-01 | 1.00E+01 | | | |
| 7980580 | NM_000153 | GALC | galactosylceramidase | 0.260 | 1.198 | 3.40E-01 | 3.40E+01 | | | |
| 8087746 | --- | --- | --- | 0.260 | 1.197 | 3.64E-01 | 3.64E+01 | | | |
| 8178377 | NM_003587 | DHX16 | DEAH (Asp-Glu-Ala-His) box polypept | 0.260 | 1.197 | 2.54E-02 | 2.54E+00 | | | |
| 8168580 | NM_198450 | APOOL | apolipoprotein O-like | 0.260 | 1.197 | 3.29E-01 | 3.29E+01 | | | |
| 7895030 | --- | --- | --- | 0.260 | 1.197 | 6.28E-01 | 6.28E+01 | | | |
| 8148982 | AK128318 | LOC389607 | hypothetical LOC389607 | 0.260 | 1.197 | 1.91E-01 | 1.91E+01 | | | |
| 7938366 | BX641032 | WEE1 | WEE1 homolog (S. pombe) | 0.260 | 1.197 | 7.42E-01 | 7.42E+01 | | | |
| 7896279 | --- | --- | --- | 0.260 | 1.197 | 8.07E-01 | 8.07E+01 | | | |
| 7894375 | --- | --- | --- | 0.260 | 1.197 | 4.86E-01 | 4.86E+01 | | | |
| 8065280 | NM_020343 | C20orf74 | chromosome 20 open reading frame | 0.260 | 1.197 | 6.78E-02 | 6.78E+00 | | | |
| 7918034 | NM_00107739 | DPH5 | DPH5 homolog (S. cerevisiae) | 0.260 | 1.197 | 4.38E-01 | 4.38E+01 | | | |
| 7961390 | NM_015987 | HEBP1 | heme binding protein 1 | 0.260 | 1.197 | 3.28E-01 | 3.28E+01 | | | |
| 8122554 | NM_006834 | RAB32 | RAB32, member RAS oncogene famil | 0.259 | 1.197 | 3.93E-01 | 3.93E+01 | | | |
| 8075481 | --- | --- | --- | 0.259 | 1.197 | 7.02E-01 | 7.02E+01 | | | |
| 7893172 | --- | --- | --- | 0.259 | 1.197 | 5.90E-01 | 5.90E+01 | | | |
| 8099570 | BC050697 | DCAF16 | DDB1 and CUL4 associated factor 16 | 0.259 | 1.197 | 2.34E-01 | 2.34E+01 | | | |
| 8101061 | --- | --- | --- | 0.259 | 1.197 | 3.38E-01 | 3.38E+01 | | | |
| 8058927 | NM_022152 | TMBIM1 | transmembrane BAX inhibitor motif | 0.259 | 1.197 | 6.64E-01 | 6.64E+01 | | | |
| 7992021 | NM_005861 | STUB1 | STIP1 homology and U-box containin | 0.259 | 1.197 | 5.71E-02 | 5.71E+00 | | | |
| 7925480 | NM_000143 | FH | fumarate hydratase | 0.259 | 1.197 | 1.58E-01 | 1.58E+01 | | | |
| 8146647 | --- | --- | --- | 0.259 | 1.197 | 2.34E-01 | 2.34E+01 | | | |
| 7935054 | --- | --- | --- | 0.259 | 1.197 | 3.68E-02 | 3.68E+00 | | | |
| 8062603 | NM_003286 | TOP1 | topoisomerase (DNA) I | 0.259 | 1.197 | 7.61E-02 | 7.61E+00 | | | |
| 8071069 | NM_014339 | IL17RA | interleukin 17 receptor A | 0.259 | 1.197 | 2.16E-02 | 2.16E+00 | | | |
| 8098121 | NM_014247 | RAPGEF2 | Rap guanine nucleotide exchange fac | 0.259 | 1.197 | 1.23E-01 | 1.23E+01 | | | |
| 7964739 | NM_032338 | LLPH | LLP homolog, long-term synaptic faci | 0.259 | 1.196 | 2.59E-01 | 2.59E+01 | | | |
| 7922416 | NR_003941 | SNORD75 | small nucleolar RNA, C/D box 75 | 0.259 | 1.196 | 7.68E-01 | 7.68E+01 | | | |
| 8121727 | NR_002730 | BRD7P3 | bromodomain containing 7 pseudog | 0.259 | 1.196 | 4.19E-01 | 4.19E+01 | | | |
| 7895554 | --- | --- | --- | 0.258 | 1.196 | 5.67E-02 | 5.67E+00 | | | |
| 8023063 | NM_00100193 | ATP5A1 | ATP synthase, H+ transporting, mito | 0.258 | 1.196 | 8.51E-02 | 8.51E+00 | | | |
| 8155062 | NM_006285 | TESK1 | testis-specific kinase 1 | 0.258 | 1.196 | 7.18E-02 | 7.18E+00 | | | |
| 7893388 | --- | --- | --- | 0.258 | 1.196 | 5.05E-01 | 5.05E+01 | | | |
| 7958600 | NM_033121 | ANKRD13A | ankyrin repeat domain 13A | 0.258 | 1.196 | 3.74E-01 | 3.74E+01 | | | |
| 8074647 | NM_058004 | PI4KA | phosphatidylinositol 4-kinase, cataly | 0.258 | 1.196 | 1.95E-01 | 1.95E+01 | | | |
| 7896720 | --- | --- | --- | 0.258 | 1.196 | 5.12E-01 | 5.12E+01 | | | |
| 8157700 | NM_012197 | RABGAP1 | RAB GTPase activating protein 1 | 0.258 | 1.196 | 3.26E-01 | 3.26E+01 | | | |
| 7911989 | NM_000983 | RPL22 | ribosomal protein L22 | 0.258 | 1.196 | 2.87E-01 | 2.87E+01 | | | |
| 7916185 | NM_00100988 | ZCCHC11 | zinc finger, CCHC domain containing | 0.258 | 1.196 | 1.59E-01 | 1.59E+01 | | | |
| 8152148 | NM_015902 | UBR5 | ubiquitin protein ligase E3 componer | 0.258 | 1.196 | 2.58E-01 | 2.58E+01 | | | |
| 8061186 | NM_006363 | SEC23B | Sec23 homolog B (S. cerevisiae) | 0.258 | 1.196 | 2.54E-01 | 2.54E+01 | | | |
| 7920810 | NM_00103753 | GON4L | gon-4-like (C. elegans) | 0.258 | 1.196 | 5.38E-02 | 5.38E+00 | | | |
| 8001104 | ENST00000390 | OC10029014 | hypothetical protein LOC100290146 | 0.258 | 1.196 | 1.25E-01 | 1.25E+01 | | | |
| 7918437 | NM_006402 | HBXIP | hepatitis B virus x interacting protein | 0.258 | 1.195 | 3.89E-01 | 3.89E+01 | | | |
| 7932554 | NM_020824 | ARHGAP21 | Rho GTPase activating protein 21 | 0.258 | 1.195 | 6.05E-02 | 6.05E+00 | | | |
| 8009366 | NM_015462 | NOL11 | nucleolar protein 11 | 0.258 | 1.195 | 5.40E-01 | 5.40E+01 | | | |
| 7896029 | --- | --- | --- | 0.257 | 1.195 | 6.25E-01 | 6.25E+01 | | | |
| 7895226 | --- | --- | --- | 0.257 | 1.195 | 6.17E-01 | 6.17E+01 | | | |
| 8021727 | NM_018235 | CNDP2 | CNDP dipeptidase 2 (metallopeptida | 0.257 | 1.195 | 1.45E-01 | 1.45E+01 | | | |
| 7903334 | NM_003672 | CDC14A | CDC14 cell division cycle 14 homolog | 0.257 | 1.195 | 1.77E-01 | 1.77E+01 | | | |
| 8154572 | --- | --- | --- | 0.257 | 1.195 | 6.46E-01 | 6.46E+01 | | | |
| 7895650 | --- | --- | --- | 0.257 | 1.195 | 7.68E-01 | 7.68E+01 | | | |
| 7959251 | NM_002562 | P2RX7 | purinergic receptor P2X, ligand-gate | 0.257 | 1.195 | 1.09E-01 | 1.09E+01 | | | |
| 8171493 | NM_175859 | CTPS2 | CTP synthase II | 0.257 | 1.195 | 2.52E-02 | 2.52E+00 | | | |
| 8169085 | NM_207318 | CXorf39 | chromosome X open reading frame | 0.257 | 1.195 | 1.17E-01 | 1.17E+01 | | | |
| 7907773 | NM_015602 | TOR1AIP1 | torsin A interacting protein 1 | 0.257 | 1.195 | 1.22E-01 | 1.22E+01 | | | |
| 8116867 | NM_030969 | TMEM14B | transmembrane protein 14B | 0.257 | 1.195 | 3.07E-01 | 3.07E+01 | | | |
| 8009353 | NM_181671 | PITPNC1 | phosphatidylinositol transfer protein | 0.257 | 1.195 | 5.51E-01 | 5.51E+01 | | | |
| 7894681 | --- | --- | --- | 0.257 | 1.195 | 7.70E-01 | 7.70E+01 | | | |
| 8165794 | NM_002414 | CD99 | CD99 molecule | 0.256 | 1.195 | 3.12E-01 | 3.12E+01 | | | |
| 8176360 | NM_002414 | CD99 | CD99 molecule | 0.256 | 1.195 | 3.12E-01 | 3.12E+01 | | | |
| 7924190 | NM_144567 | ANGEL2 | angel homolog 2 (Drosophila) | 0.256 | 1.195 | 3.72E-01 | 3.72E+01 | | | |
| 7896021 | --- | --- | --- | 0.256 | 1.194 | 8.22E-01 | 8.22E+01 | | | |
| 7896351 | --- | --- | --- | 0.256 | 1.194 | 6.51E-01 | 6.51E+01 | | | |
| 7931268 | NM_016567 | BCCIP | BRCA2 and CDKN1A interacting prot | 0.256 | 1.194 | 4.54E-01 | 4.54E+01 | | | |
| 7894100 | --- | --- | --- | 0.256 | 1.194 | 3.93E-01 | 3.93E+01 | | | |
| 8175360 | NM_173470 | MMGT1 | membrane magnesium transporter 1 | 0.256 | 1.194 | 2.57E-01 | 2.57E+01 | | | |
| 8133518 | NM_000265 | NCF1 | neutrophil cytosolic factor 1 | 0.256 | 1.194 | 2.35E-02 | 2.35E+00 | | Phagocytosis/killing | |
| 7998664 | NR_002327 | SNORA10 | small nucleolar RNA, H/ACA box 10 | 0.256 | 1.194 | 5.38E-01 | 5.38E+01 | | | |
| 8041204 | NR_002327 | SNORA10 | small nucleolar RNA, H/ACA box 10 | 0.256 | 1.194 | 5.38E-01 | 5.38E+01 | | | |
| 7909175 | NM_015326 | SRGAP2 | SLIT-ROBO Rho GTPase activating pr | 0.256 | 1.194 | 6.70E-02 | 6.70E+00 | | | |

| | | | | | | | | | | |
|---|---|---|---|---|---|---|---|---|---|---|
| 7940822 | --- | --- | --- | 0.256 | 1.194 | 4.41E-01 | 4.41E+01 | | | |
| 7923810 | --- | --- | --- | 0.256 | 1.194 | 6.92E-01 | 6.92E+01 | | | |
| 7894820 | --- | --- | --- | 0.255 | 1.194 | 2.97E-01 | 2.97E+01 | | | |
| 8011875 | NM_016041 | DERL2 | Der1-like domain family, member 2 | 0.255 | 1.194 | 2.61E-01 | 2.61E+01 | | | |
| 8146198 | NM_002690 | POLB | polymerase (DNA directed), beta | 0.255 | 1.194 | 3.20E-01 | 3.20E+01 | | | |
| 7902396 | NR_003042 | SNORD45C | small nucleolar RNA, C/D box 45C | 0.255 | 1.194 | 7.74E-01 | 7.74E+01 | | | |
| 7933290 | NR_003611 | BMS1P5 | BMS1 pseudogene 5 | 0.255 | 1.194 | 6.55E-01 | 6.55E+01 | | | |
| 8089911 | NM_005335 | HCLS1 | hematopoietic cell-specific Lyn subst | 0.255 | 1.193 | 2.03E-01 | 2.03E+01 | | | |
| 7894866 | --- | --- | --- | 0.255 | 1.193 | 2.51E-01 | 2.51E+01 | | | |
| 8090960 | NM_016216 | DBR1 | debranching enzyme homolog 1 (S. c | 0.255 | 1.193 | 3.82E-01 | 3.82E+01 | | | |
| 7987449 | NM_003134 | SRP14 | signal recognition particle 14kDa (ho | 0.255 | 1.193 | 3.92E-01 | 3.92E+01 | | | |
| 7893493 | --- | --- | --- | 0.255 | 1.193 | 8.11E-01 | 8.11E+01 | | | |
| 8140424 | NM_175064 | SPDYE1 | speedy homolog E1 (Xenopus laevis) | 0.254 | 1.193 | 2.53E-01 | 2.53E+01 | | | |
| 8041982 | NM_138448 | ACYP2 | acylphosphatase 2, muscle type | 0.254 | 1.193 | 2.91E-01 | 2.91E+01 | | | |
| 8027837 | NM_001771 | CD22 | CD22 molecule | 0.254 | 1.193 | 2.43E-01 | 2.43E+01 | | | |
| 7893884 | --- | --- | --- | 0.254 | 1.193 | 4.17E-01 | 4.17E+01 | | | |
| 8099051 | NM_003703 | NOP14 | NOP14 nucleolar protein homolog (y | 0.254 | 1.193 | 1.38E-01 | 1.38E+01 | | | |
| 8104998 | NM_018034 | WDR70 | WD repeat domain 70 | 0.254 | 1.193 | 2.53E-01 | 2.53E+01 | | | |
| 7948565 | NM_00116145 | CYBASC3 | cytochrome b, ascorbate dependent | 0.254 | 1.193 | 1.96E-02 | 1.96E+00 | | | |
| 7894017 | --- | --- | --- | 0.254 | 1.193 | 5.54E-01 | 5.54E+01 | | | |
| 8107518 | --- | --- | --- | 0.254 | 1.193 | 7.08E-01 | 7.08E+01 | | | |
| 7893032 | --- | --- | --- | 0.254 | 1.193 | 5.18E-01 | 5.18E+01 | | | |
| 8151254 | NM_006540 | NCOA2 | nuclear receptor coactivator 2 | 0.254 | 1.192 | 3.48E-01 | 3.48E+01 | | | |
| 7941936 | NM_000852 | GSTP1 | glutathione S-transferase pi 1 | 0.254 | 1.192 | 3.26E-01 | 3.26E+01 | | | |
| 7968761 | NM_024561 | NARG1L | NMDA receptor regulated 1-like | 0.254 | 1.192 | 2.80E-01 | 2.80E+01 | | | |
| 7895219 | --- | --- | --- | 0.254 | 1.192 | 5.73E-01 | 5.73E+01 | | | |
| 8020411 | NM_006938 | SNRPD1 | small nuclear ribonucleoprotein D1 p | 0.254 | 1.192 | 2.44E-01 | 2.44E+01 | | | |
| 7925201 | NM_016374 | ARID4B | AT rich interactive domain 4B (RBP1- | 0.254 | 1.192 | 2.98E-01 | 2.98E+01 | | | |
| 7991374 | NM_002168 | IDH2 | isocitrate dehydrogenase 2 (NADP+), | 0.254 | 1.192 | 1.53E-01 | 1.53E+01 | | | |
| 8109639 | NM_004219 | PTTG1 | pituitary tumor-transforming 1 | 0.253 | 1.192 | 4.40E-01 | 4.40E+01 | | | |
| 7900468 | NM_00114258 | NFYC | nuclear transcription factor Y, gamm | 0.253 | 1.192 | 2.69E-02 | 2.69E+00 | | | |
| 7984408 | NM_145160 | MAP2K5 | mitogen-activated protein kinase kin | 0.253 | 1.192 | 4.96E-01 | 4.96E+01 | | | |
| 7895003 | --- | --- | --- | 0.253 | 1.192 | 4.52E-01 | 4.52E+01 | | | |
| 8131349 | NM_00109762 | OCM | oncomodulin | 0.253 | 1.192 | 4.61E-01 | 4.61E+01 | | | |
| 8117034 | NM_006877 | GMPR | guanosine monophosphate reductas | 0.253 | 1.192 | 1.86E-01 | 1.86E+01 | | | |
| 8140454 | NM_175064 | SPDYE1 | speedy homolog E1 (Xenopus laevis) | 0.253 | 1.192 | 2.47E-01 | 2.47E+01 | | | |
| 8044909 | --- | --- | --- | 0.253 | 1.192 | 6.05E-01 | 6.05E+01 | | | |
| 8111796 | NM_206907 | PRKAA1 | protein kinase, AMP-activated, alpha | 0.253 | 1.192 | 3.22E-01 | 3.22E+01 | | | |
| 7896175 | --- | --- | --- | 0.253 | 1.191 | 1.12E-01 | 1.12E+01 | | | |
| 8118979 | --- | --- | --- | 0.253 | 1.191 | 8.41E-01 | 8.41E+01 | | | |
| 8164006 | --- | --- | --- | 0.253 | 1.191 | 7.01E-01 | 7.01E+01 | | | |
| 7894203 | --- | --- | --- | 0.253 | 1.191 | 7.08E-01 | 7.08E+01 | | | |
| 8168602 | --- | --- | --- | 0.253 | 1.191 | 4.84E-01 | 4.84E+01 | | | |
| 7892696 | --- | --- | --- | 0.253 | 1.191 | 4.42E-01 | 4.42E+01 | | | |
| 8169984 | NM_000194 | HPRT1 | hypoxanthine phosphoribosyltransfe | 0.253 | 1.191 | 5.62E-01 | 5.62E+01 | | | |
| 8061262 | NM_016100 | NAT5 | N-acetyltransferase 5 (GCN5-related | 0.252 | 1.191 | 3.54E-01 | 3.54E+01 | | | |
| 7980080 | NM_001249 | ENTPD5 | ectonucleoside triphosphate diphosp | 0.252 | 1.191 | 1.27E-01 | 1.27E+01 | | | |
| 7938348 | NM_003390 | WEE1 | WEE1 homolog (S. pombe) | 0.252 | 1.191 | 2.92E-02 | 2.92E+00 | | | |
| 8113881 | NM_016340 | RAPGEF6 | Rap guanine nucleotide exchange fac | 0.252 | 1.191 | 1.70E-01 | 1.70E+01 | | | |
| 7919856 | NM_181746 | LASS2 | LAG1 homolog, ceramide synthase 2 | 0.252 | 1.190 | 3.82E-01 | 3.82E+01 | | | |
| 7908940 | NM_00100139 | ATP2B4 | ATPase, Ca++ transporting, plasma m | 0.251 | 1.190 | 3.28E-01 | 3.28E+01 | | | |
| 7899023 | NM_015627 | LDLRAP1 | low density lipoprotein receptor ada | 0.251 | 1.190 | 6.28E-02 | 6.28E+00 | | | |
| 7935027 | NM_004969 | IDE | insulin-degrading enzyme | 0.251 | 1.190 | 8.38E-02 | 8.38E+00 | | | |
| 7892521 | --- | --- | --- | 0.251 | 1.190 | 4.42E-01 | 4.42E+01 | | | |
| 8172914 | NM_031407 | HUWE1 | HECT, UBA and WWE domain contai | 0.251 | 1.190 | 5.77E-02 | 5.77E+00 | | | |
| 8044766 | NM_016133 | INSIG2 | insulin induced gene 2 | 0.251 | 1.190 | 4.79E-01 | 4.79E+01 | | | |
| 7893025 | --- | --- | --- | 0.251 | 1.190 | 7.24E-01 | 7.24E+01 | | | |
| 7895494 | --- | --- | --- | 0.251 | 1.190 | 7.83E-01 | 7.83E+01 | | | |
| 7914917 | NM_032881 | LSM10 | LSM10, U7 small nuclear RNA associa | 0.251 | 1.190 | 2.57E-01 | 2.57E+01 | | | |
| 7899534 | NM_203342 | EPB41 | erythrocyte membrane protein band | 0.251 | 1.190 | 1.26E-01 | 1.26E+01 | | | |
| 8045946 | NM_005805 | PSMD14 | proteasome (prosome, macropain) 2 | 0.251 | 1.190 | 4.20E-01 | 4.20E+01 | | | |
| 8036557 | --- | --- | --- | 0.251 | 1.190 | 6.79E-01 | 6.79E+01 | | | |
| 7923582 | --- | --- | --- | 0.251 | 1.190 | 5.38E-01 | 5.38E+01 | | | |
| 8161044 | NM_003289 | TPM2 | tropomyosin 2 (beta) | 0.250 | 1.190 | 1.75E-01 | 1.75E+01 | | | |
| 8148158 | NM_145647 | WDR67 | WD repeat domain 67 | 0.250 | 1.189 | 2.64E-01 | 2.64E+01 | | | |
| 7895264 | --- | --- | --- | 0.250 | 1.189 | 7.49E-01 | 7.49E+01 | | | |
| 8142468 | NM_153649 | TPM3 | tropomyosin 3 | 0.250 | 1.189 | 1.51E-02 | 1.51E+00 | | | |
| 8080923 | NM_002802 | PSMC1 | proteasome (prosome, macropain) 2 | 0.250 | 1.189 | 4.70E-01 | 4.70E+01 | | | |
| 8052562 | NM_006430 | CCT4 | chaperonin containing TCP1, subunit | 0.250 | 1.189 | 1.47E-01 | 1.47E+01 | | | |
| 7997226 | --- | --- | --- | 0.250 | 1.189 | 3.56E-01 | 3.56E+01 | | | |
| 8103005 | NM_014885 | ANAPC10 | anaphase promoting complex subun | 0.250 | 1.189 | 6.52E-01 | 6.52E+01 | | | |
| 7892803 | --- | --- | --- | 0.250 | 1.189 | 1.02E-01 | 1.02E+01 | | | |
| 7907445 | NM_014458 | KLHL20 | kelch-like 20 (Drosophila) | 0.250 | 1.189 | 1.22E-01 | 1.22E+01 | | | |
| 8167069 | NM_003334 | UBA1 | ubiquitin-like modifier activating enz | 0.250 | 1.189 | 5.73E-02 | 5.73E+00 | | | |
| 7956031 | NM_014182 | ORMDL2 | ORM1-like 2 (S. cerevisiae) | 0.250 | 1.189 | 4.34E-01 | 4.34E+01 | | | |
| 8093685 | NM_002111 | HTT | huntingtin | 0.250 | 1.189 | 1.87E-02 | 1.87E+00 | | | |
| 8024436 | NM_004152 | OAZ1 | ornithine decarboxylase antizyme 1 | 0.249 | 1.189 | 7.76E-02 | 7.76E+00 | | | |

| | | | | | | | | | | |
|---|---|---|---|---|---|---|---|---|---|---|
| 7984319 | NM_002755 | MAP2K1 | mitogen-activated protein kinase kin | 0.249 | 1.189 | 1.77E-01 | 1.77E+01 | | | |
| 8025672 | NM_020428 | SLC44A2 | solute carrier family 44, member 2 | 0.249 | 1.189 | 1.15E-01 | 1.15E+01 | | | |
| 8180229 | --- | --- | --- | 0.249 | 1.189 | 2.57E-01 | 2.57E+01 | | | |
| 7895758 | --- | --- | --- | 0.249 | 1.189 | 5.10E-01 | 5.10E+01 | | | |
| 7906576 | NM_015331 | NCSTN | nicastrin | 0.249 | 1.189 | 1.02E-01 | 1.02E+01 | | | |
| 8106475 | --- | --- | --- | 0.249 | 1.188 | 4.47E-02 | 4.47E+00 | | | |
| 7894086 | --- | --- | --- | 0.249 | 1.188 | 4.39E-01 | 4.39E+01 | | | |
| 7914112 | NM_005248 | FGR | Gardner-Rasheed feline sarcoma vira | 0.249 | 1.188 | 2.62E-02 | 2.62E+00 | | | |
| 8019250 | NM_000918 | P4HB | prolyl 4-hydroxylase, beta polypeptid | 0.249 | 1.188 | 1.49E-01 | 1.49E+01 | | | |
| 8082788 | NM_00113442 | CDV3 | CDV3 homolog (mouse) | 0.249 | 1.188 | 1.67E-01 | 1.67E+01 | | | |
| 7904287 | NM_001767 | CD2 | CD2 molecule | 0.249 | 1.188 | 3.70E-01 | 3.70E+01 | | | |
| 8000791 | NM_031477 | YPEL3 | yippee-like 3 (Drosophila) | 0.249 | 1.188 | 2.01E-01 | 2.01E+01 | | | |
| 8118207 | NR_002971 | SNORA38 | small nucleolar RNA, H/ACA box 38 | 0.249 | 1.188 | 3.21E-01 | 3.21E+01 | | | |
| 7972828 | NM_017664 | ANKRD10 | ankyrin repeat domain 10 | 0.249 | 1.188 | 1.46E-01 | 1.46E+01 | | | |
| 8088092 | NM_052859 | RFT1 | RFT1 homolog (S. cerevisiae) | 0.249 | 1.188 | 5.25E-02 | 5.25E+00 | | | |
| 7978628 | NM_017917 | PPP2R3C | protein phosphatase 2 (formerly 2A) | 0.249 | 1.188 | 2.39E-01 | 2.39E+01 | | | |
| 8150491 | NM_00109941 | MYST3 | MYST histone acetyltransferase (mor | 0.248 | 1.188 | 3.19E-01 | 3.19E+01 | | | |
| 8165309 | NM_003792 | EDF1 | endothelial differentiation-related fa | 0.248 | 1.188 | 3.95E-01 | 3.95E+01 | | | |
| 8148309 | --- | --- | --- | 0.248 | 1.188 | 4.87E-01 | 4.87E+01 | | | |
| 8152626 | --- | --- | --- | 0.248 | 1.188 | 7.06E-01 | 7.06E+01 | | | |
| 8055445 | NM_001349 | DARS | aspartyl-tRNA synthetase | 0.248 | 1.188 | 5.07E-01 | 5.07E+01 | | | |
| 8157125 | NM_002874 | RAD23B | RAD23 homolog B (S. cerevisiae) | 0.248 | 1.188 | 1.63E-01 | 1.63E+01 | | | |
| 8054804 | NM_019044 | CCDC93 | coiled-coil domain containing 93 | 0.248 | 1.188 | 3.83E-01 | 3.83E+01 | | | |
| 8047565 | NM_173511 | FAM117B | family with sequence similarity 117, | 0.248 | 1.187 | 6.08E-02 | 6.08E+00 | | | |
| 8172156 | --- | --- | --- | 0.247 | 1.187 | 5.00E-01 | 5.00E+01 | | | |
| 7948424 | NM_017840 | MRPL16 | mitochondrial ribosomal protein L16 | 0.247 | 1.187 | 1.34E-01 | 1.34E+01 | | | |
| 8001178 | BC056676 | C16orf87 | chromosome 16 open reading frame | 0.247 | 1.187 | 3.71E-01 | 3.71E+01 | | | |
| 8019183 | NM_001614 | ACTG1 | actin, gamma 1 | 0.247 | 1.187 | 1.84E-02 | 1.84E+00 | | | |
| 7991335 | NM_001150 | ANPEP | alanyl (membrane) aminopeptidase | 0.247 | 1.187 | 1.06E-01 | 1.06E+01 | | | |
| 8135268 | NM_001417 | EIF4B | eukaryotic translation initiation facto | 0.247 | 1.187 | 5.13E-01 | 5.13E+01 | | | |
| 7920000 | NM_015100 | POGZ | pogo transposable element with ZNF | 0.247 | 1.187 | 1.35E-01 | 1.35E+01 | | | |
| 8026729 | NM_024050 | DDA1 | DET1 and DDB1 associated 1 | 0.247 | 1.187 | 8.74E-02 | 8.74E+00 | | | |
| 8000184 | NM_005849 | IGSF6 | immunoglobulin superfamily, memb | 0.247 | 1.187 | 4.06E-01 | 4.06E+01 | | | mono |
| 7986569 | NR_002824 | HERC2P2 | hect domain and RLD 2 pseudogene | 0.246 | 1.186 | 2.05E-01 | 2.05E+01 | | | |
| 8058342 | --- | --- | --- | 0.246 | 1.186 | 3.36E-01 | 3.36E+01 | | | |
| 8150844 | --- | --- | --- | 0.246 | 1.186 | 2.72E-01 | 2.72E+01 | | | |
| 7975459 | NM_015556 | SIPA1L1 | signal-induced proliferation-associat | 0.246 | 1.186 | 1.86E-01 | 1.86E+01 | | | |
| 7973116 | NM_00102999 | METT11D1 | methyltransferase 11 domain contai | 0.246 | 1.186 | 1.97E-01 | 1.97E+01 | | | |
| 8077353 | NM_182916 | TRNT1 | tRNA nucleotidyl transferase, CCA-ad | 0.246 | 1.186 | 3.33E-01 | 3.33E+01 | | | |
| 8015031 | NM_001838 | CCR7 | chemokine (C-C motif) receptor 7 | 0.246 | 1.186 | 3.49E-01 | 3.49E+01 | | | |
| 8044236 | NM_181453 | GCC2 | GRIP and coiled-coil domain containi | 0.246 | 1.186 | 4.65E-01 | 4.65E+01 | | | |
| 8168303 | NM_004606 | TAF1 | TAF1 RNA polymerase II, TATA box b | 0.245 | 1.185 | 2.60E-01 | 2.60E+01 | | | |
| 8157092 | NM_018112 | TMEM38B | transmembrane protein 38B | 0.245 | 1.185 | 1.55E-01 | 1.55E+01 | | | |
| 7895052 | --- | --- | --- | 0.245 | 1.185 | 5.35E-01 | 5.35E+01 | | | |
| 7925978 | NM_017782 | C10orf18 | chromosome 10 open reading frame | 0.245 | 1.185 | 2.75E-01 | 2.75E+01 | | | |
| 7983616 | NM_00100155 | GALK2 | galactokinase 2 | 0.245 | 1.185 | 3.69E-02 | 3.69E+00 | | | |
| 8057933 | NM_012086 | GTF3C3 | general transcription factor IIIC, poly | 0.245 | 1.185 | 4.12E-01 | 4.12E+01 | | | |
| 7969341 | NM_030794 | TDRD3 | tudor domain containing 3 | 0.245 | 1.185 | 2.24E-01 | 2.24E+01 | | | |
| 8141363 | NM_033017 | TRIM4 | tripartite motif-containing 4 | 0.245 | 1.185 | 8.09E-03 | 8.09E-01 | | | |
| 7920852 | BC062637 | KIAA0907 | KIAA0907 | 0.245 | 1.185 | 7.07E-02 | 7.07E+00 | | | |
| 7893073 | --- | --- | --- | 0.245 | 1.185 | 7.65E-01 | 7.65E+01 | | | |
| 8159945 | NM_016282 | AK3 | adenylate kinase 3 | 0.245 | 1.185 | 3.14E-02 | 3.14E+00 | | | |
| 8018558 | NM_004035 | ACOX1 | acyl-Coenzyme A oxidase 1, palmitoy | 0.245 | 1.185 | 8.56E-02 | 8.56E+00 | | | |
| 7905365 | NM_00113563 | PIP5K1A | phosphatidylinositol-4-phosphate 5- | 0.244 | 1.185 | 3.94E-01 | 3.94E+01 | | | |
| 8174340 | NM_018301 | RBM41 | RNA binding motif protein 41 | 0.244 | 1.185 | 1.99E-01 | 1.99E+01 | | | |
| 7971134 | NM_025138 | C13orf23 | chromosome 13 open reading frame | 0.244 | 1.185 | 1.77E-01 | 1.77E+01 | | | |
| 8133549 | NM_00100379 | GTF2IRD2B | GTF2I repeat domain containing 2B | 0.244 | 1.185 | 6.60E-02 | 6.60E+00 | | | |
| 7911343 | AF284753 | UIMC1 | ubiquitin interaction motif containin | 0.244 | 1.185 | 4.82E-02 | 4.82E+00 | | | |
| 8165703 | AF284753 | UIMC1 | ubiquitin interaction motif containin | 0.244 | 1.185 | 4.82E-02 | 4.82E+00 | | | |
| 7893978 | --- | --- | --- | 0.244 | 1.184 | 7.33E-01 | 7.33E+01 | | | |
| 8054664 | NM_032494 | ZC3H8 | zinc finger CCCH-type containing 8 | 0.244 | 1.184 | 1.30E-01 | 1.30E+01 | | | |
| 7983811 | NM_004855 | PIGB | phosphatidylinositol glycan anchor b | 0.244 | 1.184 | 3.80E-01 | 3.80E+01 | | | |
| 7894194 | --- | --- | --- | 0.244 | 1.184 | 3.67E-01 | 3.67E+01 | | | |
| 8095566 | NM_173468 | MOBKL1A | MOB1, Mps One Binder kinase activa | 0.244 | 1.184 | 1.53E-01 | 1.53E+01 | | | |
| 7903321 | NM_00113084 | RTCD1 | RNA terminal phosphate cyclase dom | 0.244 | 1.184 | 5.42E-01 | 5.42E+01 | | | |
| 7895449 | --- | --- | --- | 0.243 | 1.184 | 6.98E-01 | 6.98E+01 | | | |
| 7958207 | --- | --- | --- | 0.243 | 1.184 | 5.21E-01 | 5.21E+01 | | | |
| 8113120 | BC028919 | TOB2 | transducer of ERBB2, 2 | 0.243 | 1.184 | 3.20E-01 | 3.20E+01 | | | |
| 8165707 | BC028919 | TOB2 | transducer of ERBB2, 2 | 0.243 | 1.184 | 3.20E-01 | 3.20E+01 | | | |
| 7948211 | AB209132 | SSRP1 | structure specific recognition protein | 0.243 | 1.184 | 6.75E-01 | 6.75E+01 | | | |
| 8061129 | BC016869 | C20orf72 | chromosome 20 open reading frame | 0.243 | 1.184 | 1.54E-01 | 1.54E+01 | | | |
| 7946228 | NM_000391 | TPP1 | tripeptidyl peptidase I | 0.243 | 1.183 | 2.48E-01 | 2.48E+01 | | | mono |
| 7896336 | --- | --- | --- | 0.243 | 1.183 | 7.16E-01 | 7.16E+01 | | | |
| 7925823 | NM_012341 | GTPBP4 | GTP binding protein 4 | 0.243 | 1.183 | 3.44E-01 | 3.44E+01 | | | |
| 7927008 | NM_181698 | CCNY | cyclin Y | 0.243 | 1.183 | 2.52E-02 | 2.52E+00 | | | |
| 8079187 | AK131507 | ZNF852 | zinc finger protein 852 | 0.243 | 1.183 | 5.03E-01 | 5.03E+01 | | | |
| 8084439 | NM_182917 | EIF4G1 | eukaryotic translation initiation facto | 0.243 | 1.183 | 1.56E-02 | 1.56E+00 | | | |

| | | | | | | | | | | |
|---|---|---|---|---|---|---|---|---|---|---|
| 7990092 | NM_006901 | MYO9A | myosin IXA | 0.242 | 1.183 | 7.26E-02 | 7.26E+00 | | | |
| 7933071 | --- | --- | --- | 0.242 | 1.183 | 4.23E-01 | 4.23E+01 | | | |
| 8146379 | NM_003350 | UBE2V2 | ubiquitin-conjugating enzyme E2 var | 0.242 | 1.183 | 4.41E-01 | 4.41E+01 | | | |
| 8115076 | NM_005211 | CSF1R | colony stimulating factor 1 receptor | 0.242 | 1.183 | 4.71E-02 | 4.71E+00 | | | |
| 8103483 | --- | --- | --- | 0.242 | 1.183 | 5.98E-01 | 5.98E+01 | | | |
| 8113344 | --- | --- | --- | 0.242 | 1.183 | 5.98E-01 | 5.98E+01 | | | |
| 8114207 | --- | --- | --- | 0.242 | 1.183 | 5.98E-01 | 5.98E+01 | | | |
| 7943126 | NM_033395 | KIAA1731 | KIAA1731 | 0.242 | 1.183 | 1.65E-01 | 1.65E+01 | | | |
| 7976876 | NM_001376 | DYNC1H1 | dynein, cytoplasmic 1, heavy chain 1 | 0.242 | 1.183 | 6.78E-02 | 6.78E+00 | | | |
| 7895042 | --- | --- | --- | 0.242 | 1.183 | 5.06E-01 | 5.06E+01 | | | |
| 8085770 | --- | --- | --- | 0.242 | 1.182 | 4.02E-01 | 4.02E+01 | | | |
| 7906777 | NM_004001 | FCGR2B | Fc fragment of IgG, low affinity IIb, re | 0.242 | 1.182 | 1.48E-01 | 1.48E+01 | | | |
| 8106006 | NM_000344 | SMN1 | survival of motor neuron 1, telomeri | 0.242 | 1.182 | 3.54E-01 | 3.54E+01 | | | |
| 8048370 | NM_004328 | BCS1L | BCS1-like (yeast) | 0.242 | 1.182 | 1.57E-01 | 1.57E+01 | | | |
| 8146425 | --- | --- | --- | 0.241 | 1.182 | 2.12E-01 | 2.12E+01 | | | |
| 8142912 | NM_032842 | TMEM209 | transmembrane protein 209 | 0.241 | 1.182 | 2.92E-01 | 2.92E+01 | | | |
| 8021368 | --- | --- | --- | 0.241 | 1.182 | 5.87E-01 | 5.87E+01 | | | |
| 8125447 | NM_002123 | HLA-DQB1 | major histocompatibility complex, cla | 0.241 | 1.182 | 3.12E-01 | 3.12E+01 | | | |
| 8051413 | NM_015475 | FAM98A | family with sequence similarity 98, m | 0.241 | 1.182 | 2.88E-01 | 2.88E+01 | | | |
| 7970262 | NM_00100889 | CUL4A | cullin 4A | 0.241 | 1.182 | 5.49E-02 | 5.49E+00 | | | |
| 7905938 | NM_018845 | RAG1AP1 | recombination activating gene 1 acti | 0.241 | 1.182 | 9.08E-02 | 9.08E+00 | | | |
| 8059097 | NM_015680 | C2orf24 | chromosome 2 open reading frame 2 | 0.241 | 1.182 | 2.10E-01 | 2.10E+01 | | | |
| 7896170 | --- | --- | --- | 0.241 | 1.182 | 2.39E-01 | 2.39E+01 | | | |
| 7916590 | NM_001625 | AK2 | adenylate kinase 2 | 0.241 | 1.182 | 7.50E-01 | 7.50E+01 | | | |
| 7903519 | NM_018061 | PRPF38B | PRP38 pre-mRNA processing factor 3 | 0.241 | 1.182 | 1.25E-01 | 1.25E+01 | | | |
| 8150036 | NM_015254 | KIF13B | kinesin family member 13B | 0.241 | 1.182 | 1.31E-01 | 1.31E+01 | | | |
| 8128572 | NM_002726 | PREP | prolyl endopeptidase | 0.241 | 1.181 | 2.41E-01 | 2.41E+01 | | | |
| 8099860 | NM_002913 | RFC1 | replication factor C (activator 1) 1, 14 | 0.241 | 1.181 | 4.11E-01 | 4.11E+01 | | | |
| 7950370 | --- | --- | --- | 0.240 | 1.181 | 5.81E-01 | 5.81E+01 | | | |
| 8040908 | NM_014748 | SNX17 | sorting nexin 17 | 0.240 | 1.181 | 7.90E-02 | 7.90E+00 | | | |
| 8141829 | NM_032959 | POLR2J2 | polymerase (RNA) II (DNA directed) p | 0.240 | 1.181 | 1.23E-01 | 1.23E+01 | | | |
| 8070811 | NM_006936 | SUMO3 | SMT3 suppressor of mif two 3 homo | 0.240 | 1.181 | 1.27E-01 | 1.27E+01 | | | |
| 8124775 | NM_003587 | DHX16 | DEAH (Asp-Glu-Ala-His) box polypept | 0.240 | 1.181 | 3.64E-02 | 3.64E+00 | | | |
| 8143088 | NM_013316 | CNOT4 | CCR4-NOT transcription complex, su | 0.240 | 1.181 | 1.38E-01 | 1.38E+01 | | | |
| 7893698 | --- | --- | --- | 0.240 | 1.181 | 4.61E-01 | 4.61E+01 | | | |
| 7895171 | --- | --- | --- | 0.240 | 1.181 | 4.83E-01 | 4.83E+01 | | | |
| 7943231 | NM_017704 | ANKRD49 | ankyrin repeat domain 49 | 0.240 | 1.181 | 5.11E-01 | 5.11E+01 | | | |
| 7999598 | NM_173474 | NTAN1 | N-terminal asparagine amidase | 0.240 | 1.181 | 5.59E-01 | 5.59E+01 | | | |
| 8114030 | NM_007054 | KIF3A | kinesin family member 3A | 0.240 | 1.181 | 2.27E-01 | 2.27E+01 | | | |
| 8178826 | 0 | 0 | 0 | 0.239 | 1.181 | 3.15E-01 | 3.15E+01 | | | |
| 8180022 | 0 | 0 | 0 | 0.239 | 1.181 | 3.15E-01 | 3.15E+01 | | | |
| 8145136 | NM_005605 | PPP3CC | protein phosphatase 3 (formerly 2B) | 0.239 | 1.180 | 4.21E-01 | 4.21E+01 | | | |
| 8112894 | --- | --- | --- | 0.239 | 1.180 | 5.93E-01 | 5.93E+01 | | | |
| 7982514 | --- | --- | --- | 0.239 | 1.180 | 5.98E-01 | 5.98E+01 | | | |
| 7894809 | --- | --- | --- | 0.239 | 1.180 | 3.95E-01 | 3.95E+01 | | | |
| 7934301 | NM_007265 | ECD | ecdysoneless homolog (Drosophila) | 0.239 | 1.180 | 2.45E-01 | 2.45E+01 | | | |
| 7919600 | --- | --- | --- | 0.239 | 1.180 | 2.31E-01 | 2.31E+01 | | | |
| 7992987 | NM_00112720 | HMOX2 | heme oxygenase (decycling) 2 | 0.239 | 1.180 | 2.12E-01 | 2.12E+01 | | | |
| 8132503 | NM_004760 | STK17A | serine/threonine kinase 17a | 0.239 | 1.180 | 4.22E-01 | 4.22E+01 | | | |
| 8146122 | NM_016099 | GOLGA7 | golgi autoantigen, golgin subfamily a | 0.239 | 1.180 | 4.68E-01 | 4.68E+01 | | | |
| 8103922 | NM_004346 | CASP3 | caspase 3, apoptosis-related cysteine | 0.238 | 1.180 | 4.37E-01 | 4.37E+01 | | | |
| 7893657 | --- | --- | --- | 0.238 | 1.180 | 7.51E-01 | 7.51E+01 | | | |
| 7895944 | --- | --- | --- | 0.238 | 1.179 | 3.97E-01 | 3.97E+01 | | | |
| 8091546 | NM_00112322 | TMEM14E | transmembrane protein 14E | 0.238 | 1.179 | 5.83E-01 | 5.83E+01 | | | |
| 7894220 | --- | --- | --- | 0.238 | 1.179 | 5.45E-01 | 5.45E+01 | | | |
| 8048835 | NM_020194 | MFF | mitochondrial fission factor | 0.238 | 1.179 | 2.95E-01 | 2.95E+01 | | | |
| 7896748 | --- | --- | --- | 0.238 | 1.179 | 8.37E-01 | 8.37E+01 | | | |
| 8045423 | NR_002973 | SNORA40 | small nucleolar RNA, H/ACA box 40 | 0.238 | 1.179 | 7.62E-01 | 7.62E+01 | | | |
| 8098904 | NM_006527 | SLBP | stem-loop binding protein | 0.238 | 1.179 | 1.58E-01 | 1.58E+01 | | | |
| 8020349 | NR_003366 | ANKRD20B | ankyrin repeat domain 20B | 0.237 | 1.179 | 7.62E-01 | 7.62E+01 | | | |
| 8180003 | 0 | 0 | 0 | 0.237 | 1.179 | 1.19E-01 | 1.19E+01 | | | |
| 7945902 | NM_016320 | NUP98 | nucleoporin 98kDa | 0.237 | 1.179 | 1.81E-01 | 1.81E+01 | | | |
| 8003180 | NM_00114554 | ZDHHC7 | zinc finger, DHHC-type containing 7 | 0.237 | 1.179 | 8.27E-02 | 8.27E+00 | | | |
| 8133176 | NM_014504 | RABGEF1 | RAB guanine nucleotide exchange fa | 0.237 | 1.179 | 3.12E-01 | 3.12E+01 | | | |
| 7947894 | NM_198700 | CUGBP1 | CUG triplet repeat, RNA binding prot | 0.237 | 1.179 | 7.76E-02 | 7.76E+00 | | | |
| 7997633 | NM_005153 | USP10 | ubiquitin specific peptidase 10 | 0.237 | 1.179 | 5.93E-01 | 5.93E+01 | | | |
| 8061138 | NM_020536 | CSRP2BP | CSRP2 binding protein | 0.237 | 1.179 | 9.07E-02 | 9.07E+00 | | | |
| 8114083 | NM_014423 | AFF4 | AF4/FMR2 family, member 4 | 0.237 | 1.179 | 8.94E-02 | 8.94E+00 | | | |
| 8121277 | NM_001624 | AIM1 | absent in melanoma 1 | 0.237 | 1.179 | 2.24E-01 | 2.24E+01 | | | |
| 8104166 | NM_004168 | SDHA | succinate dehydrogenase complex, s | 0.237 | 1.178 | 3.22E-01 | 3.22E+01 | | | |
| 8136602 | NM_004546 | NDUFB2 | NADH dehydrogenase (ubiquinone) 1 | 0.237 | 1.178 | 1.50E-01 | 1.50E+01 | | | |
| 7979250 | NM_005776 | CNIH | cornichon homolog (Drosophila) | 0.237 | 1.178 | 2.89E-01 | 2.89E+01 | | | |
| 7916403 | NM_145716 | SSBP3 | single stranded DNA binding protein | 0.237 | 1.178 | 2.12E-01 | 2.12E+01 | | | |
| 7947434 | NM_033406 | FBXO3 | F-box protein 3 | 0.237 | 1.178 | 3.73E-01 | 3.73E+01 | | | |
| 7915718 | NM_007170 | TESK2 | testis-specific kinase 2 | 0.236 | 1.178 | 4.79E-02 | 4.79E+00 | | | |
| 7893968 | --- | --- | --- | 0.236 | 1.178 | 7.02E-01 | 7.02E+01 | | | |
| 8007212 | NM_003152 | STAT5A | signal transducer and activator of tra | 0.236 | 1.178 | 5.46E-02 | 5.46E+00 | | | |

| | | | | | | | | | | |
|---|---|---|---|---|---|---|---|---|---|---|
| 7893286 | --- | --- | --- | 0.236 | 1.178 | 8.38E-01 | 8.38E+01 | | | |
| 7895960 | --- | --- | --- | 0.236 | 1.178 | 7.28E-01 | 7.28E+01 | | | |
| 8041212 | NM_182551 | LCLAT1 | lysocardiolipin acyltransferase 1 | 0.236 | 1.178 | 1.91E-01 | 1.91E+01 | | | |
| 7892813 | --- | --- | --- | 0.236 | 1.178 | 7.01E-01 | 7.01E+01 | | | |
| 7931778 | NM_014889 | PITRM1 | pitrilysin metallopeptidase 1 | 0.236 | 1.178 | 1.58E-01 | 1.58E+01 | | | |
| 8008517 | NM_198175 | NME1 | non-metastatic cells 1, protein (NM2 | 0.236 | 1.178 | 2.87E-01 | 2.87E+01 | | | |
| 8105958 | NM_000344 | SMN1 | survival of motor neuron 1, telomeri | 0.236 | 1.177 | 3.09E-01 | 3.09E+01 | | | |
| 8177647 | NM_000344 | SMN1 | survival of motor neuron 1, telomeri | 0.236 | 1.177 | 3.09E-01 | 3.09E+01 | | | |
| 7960594 | NM_001273 | CHD4 | chromodomain helicase DNA binding | 0.236 | 1.177 | 1.90E-01 | 1.90E+01 | | | |
| 8130988 | NM_018452 | C6orf35 | chromosome 6 open reading frame 3 | 0.236 | 1.177 | 1.55E-01 | 1.55E+01 | | | |
| 8021301 | NM_004163 | RAB27B | RAB27B, member RAS oncogene fam | 0.236 | 1.177 | 4.22E-01 | 4.22E+01 | | | |
| 8021365 | NM_005603 | ATP8B1 | ATPase, class I, type 8B, member 1 | 0.236 | 1.177 | 5.49E-01 | 5.49E+01 | | | |
| 7904965 | AB042555 | PDE4DIP | phosphodiesterase 4D interacting pr | 0.236 | 1.177 | 3.87E-01 | 3.87E+01 | | | |
| 7970696 | NM_182488 | USP12 | ubiquitin specific peptidase 12 | 0.235 | 1.177 | 3.44E-01 | 3.44E+01 | | | |
| 8085608 | NM_012260 | HACL1 | 2-hydroxyacyl-CoA lyase 1 | 0.235 | 1.177 | 1.39E-01 | 1.39E+01 | | | |
| 8075057 | --- | --- | --- | 0.235 | 1.177 | 6.32E-01 | 6.32E+01 | | | |
| 7916120 | NM_015913 | TXNDC12 | thioredoxin domain containing 12 (e | 0.235 | 1.177 | 3.23E-01 | 3.23E+01 | | | |
| 8047419 | NM_001228 | CASP8 | caspase 8, apoptosis-related cysteine | 0.235 | 1.177 | 1.22E-01 | 1.22E+01 | | | |
| 7899273 | NM_006600 | NUDC | nuclear distribution gene C homolog | 0.235 | 1.177 | 2.74E-01 | 2.74E+01 | | | |
| 8141898 | NM_014377 | DNAJC2 | DnaJ (Hsp40) homolog, subfamily C, | 0.235 | 1.177 | 3.31E-01 | 3.31E+01 | | | |
| 8085531 | NM_022497 | MRPS25 | mitochondrial ribosomal protein S25 | 0.235 | 1.177 | 3.96E-02 | 3.96E+00 | | | |
| 8147483 | NM_178812 | MTDH | metadherin | 0.235 | 1.177 | 1.08E-01 | 1.08E+01 | | | |
| 7974245 | --- | --- | --- | 0.235 | 1.177 | 6.05E-01 | 6.05E+01 | | | |
| 7922689 | NM_002065 | GLUL | glutamate-ammonia ligase (glutamin | 0.234 | 1.176 | 5.45E-02 | 5.45E+00 | | | |
| 8134552 | NM_005720 | ARPC1B | actin related protein 2/3 complex, su | 0.234 | 1.176 | 3.73E-02 | 3.73E+00 | | | |
| 8069644 | NM_000484 | APP | amyloid beta (A4) precursor protein | 0.234 | 1.176 | 1.07E-01 | 1.07E+01 | | | |
| 7943580 | NM_003478 | CUL5 | cullin 5 | 0.234 | 1.176 | 3.97E-01 | 3.97E+01 | | | |
| 7953878 | NM_00100441 | CLEC2D | C-type lectin domain family 2, memb | 0.234 | 1.176 | 4.01E-01 | 4.01E+01 | | | |
| 7901299 | NM_016308 | CMPK1 | cytidine monophosphate (UMP-CMP | 0.234 | 1.176 | 3.49E-01 | 3.49E+01 | | | |
| 7908867 | NM_138391 | TMEM183A | transmembrane protein 183A | 0.234 | 1.176 | 3.29E-01 | 3.29E+01 | | | |
| 8129087 | --- | --- | --- | 0.234 | 1.176 | 6.75E-01 | 6.75E+01 | | | |
| 8152668 | NM_014109 | ATAD2 | ATPase family, AAA domain containi | 0.234 | 1.176 | 1.96E-01 | 1.96E+01 | | | |
| 7892762 | --- | --- | --- | 0.234 | 1.176 | 6.23E-01 | 6.23E+01 | | | |
| 7944049 | NM_00104045 | SIDT2 | SID1 transmembrane family, membe | 0.234 | 1.176 | 1.12E-01 | 1.12E+01 | | | |
| 7922474 | NM_014656 | KIAA0040 | KIAA0040 | 0.234 | 1.176 | 8.03E-02 | 8.03E+00 | | | |
| 7894974 | --- | --- | --- | 0.234 | 1.176 | 2.30E-01 | 2.30E+01 | | | |
| 8036908 | NM_013368 | SERTAD3 | SERTA domain containing 3 | 0.234 | 1.176 | 1.60E-01 | 1.60E+01 | | | |
| 8116020 | NM_006816 | LMAN2 | lectin, mannose-binding 2 | 0.234 | 1.176 | 2.75E-01 | 2.75E+01 | | | |
| 7954591 | NM_021821 | MRPS35 | mitochondrial ribosomal protein S35 | 0.234 | 1.176 | 3.97E-01 | 3.97E+01 | | | |
| 7895853 | --- | --- | --- | 0.233 | 1.176 | 7.50E-01 | 7.50E+01 | | | |
| 8134257 | NM_004126 | GNG11 | guanine nucleotide binding protein ( | 0.233 | 1.176 | 6.00E-01 | 6.00E+01 | | | |
| 8086077 | NM_015097 | CLASP2 | cytoplasmic linker associated protein | 0.233 | 1.176 | 1.42E-01 | 1.42E+01 | | | |
| 8124144 | NM_003472 | DEK | DEK oncogene | 0.233 | 1.175 | 2.51E-01 | 2.51E+01 | | | |
| 8103508 | NM_017923 | 40238 | membrane-associated ring finger (C3 | 0.233 | 1.175 | 2.27E-02 | 2.27E+00 | | | |
| 8056047 | NM_00112821 | WDSUB1 | WD repeat, sterile alpha motif and U | 0.233 | 1.175 | 5.59E-01 | 5.59E+01 | | | |
| 8076690 | NM_00100988 | C22orf9 | chromosome 22 open reading frame | 0.233 | 1.175 | 1.12E-02 | 1.12E+00 | | | mono |
| 7930031 | NM_004193 | GBF1 | golgi-specific brefeldin A resistant gu | 0.233 | 1.175 | 1.54E-01 | 1.54E+01 | | | |
| 8022170 | NM_00102466 | RPL6 | ribosomal protein L6 | 0.233 | 1.175 | 5.27E-02 | 5.27E+00 | | | |
| 8180078 | NM_002118 | HLA-DMB | major histocompatibility complex, cl | 0.233 | 1.175 | 3.72E-01 | 3.72E+01 | | | |
| 8130464 | NM_018452 | C6orf35 | chromosome 6 open reading frame 3 | 0.233 | 1.175 | 2.19E-01 | 2.19E+01 | | | |
| 7970892 | NM_052818 | N4BP2L1 | NEDD4 binding protein 2-like 1 | 0.233 | 1.175 | 1.80E-01 | 1.80E+01 | | | |
| 7975687 | NM_00102467 | LIN52 | lin-52 homolog (C. elegans) | 0.233 | 1.175 | 2.54E-01 | 2.54E+01 | | | |
| 8102214 | NM_005443 | PAPSS1 | 3'-phosphoadenosine 5'-phosphosul | 0.233 | 1.175 | 1.47E-01 | 1.47E+01 | | | |
| 7896596 | --- | --- | --- | 0.233 | 1.175 | 6.89E-01 | 6.89E+01 | | | |
| 8075910 | NM_002872 | RAC2 | ras-related C3 botulinum toxin subst | 0.232 | 1.175 | 8.30E-02 | 8.30E+00 | | | |
| 8096661 | --- | --- | --- | 0.232 | 1.175 | 2.26E-01 | 2.26E+01 | | | |
| 7927062 | NM_006954 | ZNF33A | zinc finger protein 33A | 0.232 | 1.175 | 4.01E-01 | 4.01E+01 | | | |
| 8177544 | AK289851 | SMA5 | glucuronidase, beta pseudogene | 0.232 | 1.175 | 3.52E-01 | 3.52E+01 | | | |
| 7948775 | NM_024099 | C11orf48 | chromosome 11 open reading frame | 0.232 | 1.175 | 2.70E-01 | 2.70E+01 | | | |
| 7892579 | --- | --- | --- | 0.232 | 1.175 | 5.13E-01 | 5.13E+01 | | | |
| 8066212 | --- | --- | --- | 0.232 | 1.175 | 3.92E-01 | 3.92E+01 | | | |
| 7937335 | NM_003641 | IFITM1 | interferon induced transmembrane p | 0.232 | 1.175 | 5.42E-01 | 5.42E+01 | | | |
| 8119223 | AK127725 | FLJ45825 | hypothetical protein LOC646888 | 0.232 | 1.175 | 5.52E-01 | 5.52E+01 | | | |
| 7894183 | --- | --- | --- | 0.232 | 1.174 | 7.59E-01 | 7.59E+01 | | | |
| 8043791 | NM_00113422 | INPP4A | inositol polyphosphate-4-phosphatas | 0.232 | 1.174 | 2.25E-01 | 2.25E+01 | | | |
| 7894180 | --- | --- | --- | 0.232 | 1.174 | 7.21E-01 | 7.21E+01 | | | |
| 8122457 | NM_003764 | STX11 | syntaxin 11 | 0.232 | 1.174 | 1.89E-01 | 1.89E+01 | | | |
| 8117106 | NM_182757 | RNF144B | ring finger protein 144B | 0.232 | 1.174 | 2.94E-02 | 2.94E+00 | | | |
| 7978114 | BC002491 | FAM158A | family with sequence similarity 158, | 0.232 | 1.174 | 1.65E-01 | 1.65E+01 | | | |
| 7893393 | --- | --- | --- | 0.232 | 1.174 | 3.11E-02 | 3.11E+00 | | | |
| 8109697 | NM_004060 | CCNG1 | cyclin G1 | 0.231 | 1.174 | 4.79E-01 | 4.79E+01 | | | |
| 7916316 | NM_018087 | TMEM48 | transmembrane protein 48 | 0.231 | 1.174 | 1.61E-01 | 1.61E+01 | | | |
| 7960716 | NM_00114483 | PHB2 | prohibitin 2 | 0.231 | 1.174 | 2.03E-01 | 2.03E+01 | | | |
| 8007594 | NM_177441 | TMUB2 | transmembrane and ubiquitin-like do | 0.231 | 1.174 | 6.76E-02 | 6.76E+00 | | | |
| 8064939 | NM_021156 | TMX4 | thioredoxin-related transmembrane | 0.231 | 1.174 | 4.42E-01 | 4.42E+01 | | | |
| 8042720 | NM_006429 | CCT7 | chaperonin containing TCP1, subunit | 0.231 | 1.174 | 3.87E-01 | 3.87E+01 | | | |
| 7924388 | NM_006085 | BPNT1 | 3'(2'), 5'-bisphosphate nucleotidase | 0.231 | 1.174 | 1.39E-01 | 1.39E+01 | | | |

| | | | | | | | | | | |
|---|---|---|---|---|---|---|---|---|---|---|
| 7976189 | NM_002802 | PSMC1 | proteasome (prosome, macropain) 2 | 0.231 | 1.174 | 5.15E-01 | 5.15E+01 | | | |
| 8041027 | NM_004891 | MRPL33 | mitochondrial ribosomal protein L33 | 0.231 | 1.174 | 5.59E-01 | 5.59E+01 | | | |
| 7912198 | NM_001428 | ENO1 | enolase 1, (alpha) | 0.231 | 1.174 | 3.74E-01 | 3.74E+01 | | | |
| 7894281 | --- | --- | --- | 0.231 | 1.174 | 5.51E-01 | 5.51E+01 | | | |
| 7892871 | --- | --- | --- | 0.231 | 1.173 | 7.87E-01 | 7.87E+01 | | | |
| 8034851 | NM_032571 | EMR3 | egf-like module containing, mucin-lik | 0.231 | 1.173 | 4.03E-02 | 4.03E+00 | | | |
| 8020100 | NM_006788 | RALBP1 | ralA binding protein 1 | 0.231 | 1.173 | 4.82E-02 | 4.82E+00 | | | |
| 8132531 | NM_175064 | SPDYE1 | speedy homolog E1 (Xenopus laevis) | 0.231 | 1.173 | 2.42E-01 | 2.42E+01 | | | |
| 8156581 | NM_00101089 | C9orf102 | chromosome 9 open reading frame 1 | 0.230 | 1.173 | 3.39E-01 | 3.39E+01 | | | |
| 7909214 | NM_182663 | RASSF5 | Ras association (RalGDS/AF-6) doma | 0.230 | 1.173 | 1.21E-01 | 1.21E+01 | | | |
| 8120833 | NM_031469 | SH3BGRL2 | SH3 domain binding glutamic acid-ric | 0.230 | 1.173 | 4.30E-01 | 4.30E+01 | | | |
| 7901110 | NM_006066 | AKR1A1 | aldo-keto reductase family 1, membe | 0.230 | 1.173 | 3.30E-01 | 3.30E+01 | | | |
| 8016508 | NM_007241 | SNF8 | SNF8, ESCRT-II complex subunit, hom | 0.230 | 1.173 | 2.95E-01 | 2.95E+01 | | | |
| 8096771 | NM_021227 | OSTC | oligosaccharyltransferase complex su | 0.230 | 1.173 | 4.29E-01 | 4.29E+01 | | | |
| 7892944 | --- | --- | --- | 0.230 | 1.173 | 5.18E-01 | 5.18E+01 | | | |
| 7893936 | --- | --- | --- | 0.230 | 1.173 | 5.90E-01 | 5.90E+01 | | | |
| 7963261 | AK292677 | LOC494150 | prohibitin pseudogene | 0.230 | 1.173 | 1.75E-01 | 1.75E+01 | | | |
| 7892819 | --- | --- | --- | 0.230 | 1.173 | 8.17E-01 | 8.17E+01 | | | |
| 7955019 | NM_152641 | ARID2 | AT rich interactive domain 2 (ARID, R | 0.230 | 1.173 | 3.06E-01 | 3.06E+01 | | | |
| 8060063 | NM_004544 | NDUFA10 | NADH dehydrogenase (ubiquinone) 1 | 0.230 | 1.173 | 2.54E-01 | 2.54E+01 | | | |
| 7999642 | NM_014647 | KIAA0430 | KIAA0430 | 0.230 | 1.172 | 1.97E-01 | 1.97E+01 | | | |
| 8050215 | NM_006826 | YWHAQ | tyrosine 3-monooxygenase/tryptoph | 0.229 | 1.172 | 2.99E-01 | 2.99E+01 | | | |
| 8138789 | NM_175061 | JAZF1 | JAZF zinc finger 1 | 0.229 | 1.172 | 2.42E-01 | 2.42E+01 | | | |
| 8043459 | ENST00000390 | IGKC | immunoglobulin kappa constant | 0.229 | 1.172 | 1.02E-01 | 1.02E+01 | | | |
| 7959604 | NM_020936 | DDX55 | DEAD (Asp-Glu-Ala-Asp) box polypep | 0.229 | 1.172 | 5.38E-02 | 5.38E+00 | | | |
| 8103240 | NR_001562 | ANXA2P1 | annexin A2 pseudogene 1 | 0.229 | 1.172 | 7.19E-02 | 7.19E+00 | | | |
| 7953395 | NM_016319 | COPS7A | COP9 constitutive photomorphogeni | 0.229 | 1.172 | 4.81E-02 | 4.81E+00 | | | |
| 7892990 | --- | --- | --- | 0.229 | 1.172 | 8.29E-01 | 8.29E+01 | | | |
| 7982290 | --- | --- | --- | 0.229 | 1.172 | 4.24E-01 | 4.24E+01 | | | |
| 8167654 | NM_018094 | GSPT2 | G1 to S phase transition 2 | 0.229 | 1.172 | 2.17E-01 | 2.17E+01 | | | |
| 7912928 | NM_003000 | SDHB | succinate dehydrogenase complex, s | 0.228 | 1.172 | 5.03E-01 | 5.03E+01 | | | |
| 8035456 | NM_145256 | LRRC25 | leucine rich repeat containing 25 | 0.228 | 1.171 | 3.47E-02 | 3.47E+00 | | | |
| 8122600 | NM_00112771 | STXBP5 | syntaxin binding protein 5 (tomosyn) | 0.228 | 1.171 | 2.61E-01 | 2.61E+01 | | | |
| 8148149 | NM_014943 | ZHX2 | zinc fingers and homeoboxes 2 | 0.228 | 1.171 | 2.76E-01 | 2.76E+01 | | | |
| 8078270 | NM_002948 | RPL15 | ribosomal protein L15 | 0.228 | 1.171 | 1.80E-01 | 1.80E+01 | | | |
| 7893449 | --- | --- | --- | 0.228 | 1.171 | 1.89E-01 | 1.89E+01 | | | |
| 7894767 | --- | --- | --- | 0.228 | 1.171 | 6.58E-01 | 6.58E+01 | | | |
| 7968333 | NM_005800 | USPL1 | ubiquitin specific peptidase like 1 | 0.228 | 1.171 | 3.54E-01 | 3.54E+01 | | | |
| 8080013 | NM_004635 | MAPKAPK3 | mitogen-activated protein kinase-act | 0.228 | 1.171 | 1.07E-01 | 1.07E+01 | | | |
| 8041281 | --- | --- | --- | 0.228 | 1.171 | 1.23E-01 | 1.23E+01 | | | |
| 8112312 | NM_014473 | DIMT1L | DIM1 dimethyladenosine transferase | 0.228 | 1.171 | 2.42E-01 | 2.42E+01 | | | |
| 7987405 | NM_005739 | RASGRP1 | RAS guanyl releasing protein 1 (calciu | 0.228 | 1.171 | 3.77E-01 | 3.77E+01 | | | |
| 7893672 | --- | --- | --- | 0.228 | 1.171 | 2.97E-01 | 2.97E+01 | | | |
| 7894461 | --- | --- | --- | 0.227 | 1.171 | 8.34E-01 | 8.34E+01 | | | |
| 7895762 | --- | --- | --- | 0.227 | 1.171 | 1.73E-01 | 1.73E+01 | | | |
| 7933976 | --- | --- | --- | 0.227 | 1.170 | 3.94E-01 | 3.94E+01 | | | |
| 7933413 | NR_003611 | BMS1P5 | BMS1 pseudogene 5 | 0.227 | 1.170 | 6.41E-01 | 6.41E+01 | | | |
| 8091458 | NM_014445 | SERP1 | stress-associated endoplasmic reticu | 0.227 | 1.170 | 9.89E-02 | 9.89E+00 | | | |
| 8115783 | NM_005990 | STK10 | serine/threonine kinase 10 | 0.227 | 1.170 | 1.78E-01 | 1.78E+01 | | | |
| 8111698 | NM_152756 | RICTOR | RPTOR independent companion of M | 0.227 | 1.170 | 4.05E-01 | 4.05E+01 | | | |
| 8160011 | NM_00101796 | KIAA2026 | KIAA2026 | 0.227 | 1.170 | 4.40E-01 | 4.40E+01 | | | |
| 7934178 | NM_000281 | PCBD1 | pterin-4 alpha-carbinolamine dehydr | 0.227 | 1.170 | 1.10E-01 | 1.10E+01 | | | |
| 8030064 | NM_017457 | CYTH2 | cytohesin 2 | 0.227 | 1.170 | 4.75E-01 | 4.75E+01 | | | |
| 7895770 | --- | --- | --- | 0.227 | 1.170 | 6.55E-01 | 6.55E+01 | | | |
| 7996954 | NM_138714 | NFAT5 | nuclear factor of activated T-cells 5, | 0.227 | 1.170 | 3.89E-02 | 3.89E+00 | | | |
| 8009176 | NM_016360 | TACO1 | translational activator of mitochondr | 0.226 | 1.170 | 9.32E-02 | 9.32E+00 | | | |
| 7898278 | NM_015001 | SPEN | spen homolog, transcriptional regula | 0.226 | 1.170 | 4.65E-02 | 4.65E+00 | | | |
| 8105801 | NM_022902 | SLC30A5 | solute carrier family 30 (zinc transpo | 0.226 | 1.170 | 1.24E-01 | 1.24E+01 | | | |
| 8041487 | NM_174931 | CCDC75 | coiled-coil domain containing 75 | 0.226 | 1.170 | 2.72E-01 | 2.72E+01 | | | |
| 7895955 | --- | --- | --- | 0.226 | 1.170 | 8.09E-01 | 8.09E+01 | | | |
| 8166469 | NR_027783 | SAT1 | spermidine/spermine N1-acetyltrans | 0.226 | 1.170 | 2.55E-01 | 2.55E+01 | | | |
| 7894879 | --- | --- | --- | 0.226 | 1.170 | 7.26E-01 | 7.26E+01 | | | |
| 8046975 | NM_032168 | WDR75 | WD repeat domain 75 | 0.226 | 1.170 | 6.42E-01 | 6.42E+01 | | | |
| 7936777 | NM_017615 | NSMCE4A | non-SMC element 4 homolog A (S. ce | 0.226 | 1.170 | 2.87E-01 | 2.87E+01 | | | |
| 8124059 | NM_005124 | NUP153 | nucleoporin 153kDa | 0.226 | 1.169 | 2.11E-01 | 2.11E+01 | | | |
| 8069633 | NM_00100370 | ATP5J | ATP synthase, H+ transporting, mitoc | 0.226 | 1.169 | 7.13E-02 | 7.13E+00 | | | |
| 8131614 | NM_001621 | AHR | aryl hydrocarbon receptor | 0.226 | 1.169 | 4.08E-01 | 4.08E+01 | | | |
| 7910752 | NM_000254 | MTR | 5-methyltetrahydrofolate-homocyste | 0.226 | 1.169 | 1.01E-01 | 1.01E+01 | | | |
| 8078033 | NM_016474 | C3orf19 | chromosome 3 open reading frame 1 | 0.226 | 1.169 | 3.44E-01 | 3.44E+01 | | | |
| 7896130 | --- | --- | --- | 0.225 | 1.169 | 7.66E-01 | 7.66E+01 | | | |
| 8083223 | NM_173552 | C3orf58 | chromosome 3 open reading frame 5 | 0.225 | 1.169 | 9.56E-02 | 9.56E+00 | | | |
| 7892643 | --- | --- | --- | 0.225 | 1.169 | 5.84E-01 | 5.84E+01 | | | |
| 7975521 | NM_021239 | RBM25 | RNA binding motif protein 25 | 0.225 | 1.169 | 2.46E-01 | 2.46E+01 | | | |
| 7892733 | --- | --- | --- | 0.225 | 1.169 | 6.52E-01 | 6.52E+01 | | | |
| 8091511 | NM_014879 | P2RY14 | purinergic receptor P2Y, G-protein co | 0.225 | 1.169 | 1.01E-01 | 1.01E+01 | | | |
| 7893784 | --- | --- | --- | 0.225 | 1.169 | 7.99E-01 | 7.99E+01 | | | |
| 8128522 | NM_020771 | HACE1 | HECT domain and ankyrin repeat cor | 0.225 | 1.168 | 2.57E-01 | 2.57E+01 | | | |

| | | | | | | | | | | |
|---|---|---|---|---|---|---|---|---|---|---|
| 8166072 | NM_021109 | TMSB4X | thymosin beta 4, X-linked | 0.225 | 1.168 | 6.66E-03 | 6.66E-01 | | | |
| 7976863 | NM_00116172! | PPP2R5C | protein phosphatase 2, regulatory su | 0.224 | 1.168 | 5.63E-02 | 5.63E+00 | | | |
| 8163896 | NM_004099 | STOM | stomatin | 0.224 | 1.168 | 2.68E-01 | 2.68E+01 | | | |
| 8132292 | NM_001788 | 40428 | septin 7 | 0.224 | 1.168 | 1.55E-01 | 1.55E+01 | | | |
| 7949765 | NM_00100870! | PPP1CA | protein phosphatase 1, catalytic subu | 0.224 | 1.168 | 4.01E-01 | 4.01E+01 | | | |
| 7893103 | --- | --- | --- | 0.224 | 1.168 | 6.31E-01 | 6.31E+01 | | | |
| 8112409 | NM_019072 | SGTB | small glutamine-rich tetratricopeptid | 0.224 | 1.168 | 5.55E-01 | 5.55E+01 | | | |
| 8022927 | NM_012319 | SLC39A6 | solute carrier family 39 (zinc transpo | 0.224 | 1.168 | 3.06E-01 | 3.06E+01 | | | |
| 8107750 | NM_130809 | PRRC1 | proline-rich coiled-coil 1 | 0.224 | 1.168 | 1.30E-01 | 1.30E+01 | | | |
| 7895913 | --- | --- | --- | 0.224 | 1.168 | 7.07E-01 | 7.07E+01 | | | |
| 8017283 | NR_026641 | INTS2 | integrator complex subunit 2 | 0.224 | 1.168 | 2.57E-01 | 2.57E+01 | | | |
| 8054395 | NM_025076 | UXS1 | UDP-glucuronate decarboxylase 1 | 0.224 | 1.168 | 4.98E-01 | 4.98E+01 | | | |
| 8077931 | NM_014160 | MKRN2 | makorin ring finger protein 2 | 0.224 | 1.168 | 3.64E-02 | 3.64E+00 | | | |
| 8116494 | NM_152283 | ZFP62 | zinc finger protein 62 homolog (mou | 0.224 | 1.168 | 3.47E-01 | 3.47E+01 | | | |
| 7951144 | NM_024725 | CCDC82 | coiled-coil domain containing 82 | 0.224 | 1.168 | 5.09E-01 | 5.09E+01 | | | |
| 7899703 | NM_175852 | TXLNA | taxilin alpha | 0.224 | 1.168 | 4.04E-02 | 4.04E+00 | | | |
| 7999433 | --- | --- | --- | 0.224 | 1.168 | 2.78E-02 | 2.78E+00 | | | |
| 7959586 | NM_180699 | SNRNP35 | small nuclear ribonucleoprotein 35kI | 0.224 | 1.168 | 1.49E-01 | 1.49E+01 | | | |
| 8169240 | NM_002764 | PRPS1 | phosphoribosyl pyrophosphate synth | 0.223 | 1.167 | 7.30E-01 | 7.30E+01 | | | |
| 7895723 | --- | --- | --- | 0.223 | 1.167 | 7.61E-01 | 7.61E+01 | | | |
| 8169928 | NR_027141 | FAM45B | family with sequence similarity 45, m | 0.223 | 1.167 | 2.49E-01 | 2.49E+01 | | | |
| 7896094 | --- | --- | --- | 0.223 | 1.167 | 1.64E-01 | 1.64E+01 | | | |
| 7908732 | NM_018085 | IPO9 | importin 9 | 0.223 | 1.167 | 2.03E-01 | 2.03E+01 | | | |
| 7986383 | NM_000875 | IGF1R | insulin-like growth factor 1 receptor | 0.223 | 1.167 | 4.33E-01 | 4.33E+01 | | | |
| 7989661 | NM_002537 | OAZ2 | ornithine decarboxylase antizyme 2 | 0.223 | 1.167 | 4.73E-02 | 4.73E+00 | | | |
| 8173430 | NM_013346 | SNX12 | sorting nexin 12 | 0.223 | 1.167 | 2.89E-01 | 2.89E+01 | | | |
| 7912086 | NM_024654 | NOL9 | nucleolar protein 9 | 0.223 | 1.167 | 2.27E-01 | 2.27E+01 | | | |
| 7929596 | NM_032440 | LCOR | ligand dependent nuclear receptor c | 0.223 | 1.167 | 4.77E-01 | 4.77E+01 | | | |
| 7921786 | NM_012394 | PFDN2 | prefoldin subunit 2 | 0.223 | 1.167 | 1.22E-01 | 1.22E+01 | | | |
| 8081945 | NM_004547 | NDUFB4 | NADH dehydrogenase (ubiquinone) 1 | 0.223 | 1.167 | 4.41E-01 | 4.41E+01 | | | |
| 8093314 | NM_004547 | NDUFB4 | NADH dehydrogenase (ubiquinone) 1 | 0.223 | 1.167 | 4.41E-01 | 4.41E+01 | | | |
| 7895638 | --- | --- | --- | 0.222 | 1.167 | 7.36E-01 | 7.36E+01 | | | |
| 8142464 | --- | --- | --- | 0.222 | 1.167 | 5.63E-01 | 5.63E+01 | | | |
| 7917674 | NM_00112282 | SET | SET nuclear oncogene | 0.222 | 1.167 | 5.49E-01 | 5.49E+01 | | | |
| 7893492 | --- | --- | --- | 0.222 | 1.167 | 7.04E-01 | 7.04E+01 | | | |
| 7909545 | NM_145759 | TRAF5 | TNF receptor-associated factor 5 | 0.222 | 1.166 | 1.43E-01 | 1.43E+01 | | | |
| 8168852 | NM_019597 | HNRNPH2 | heterogeneous nuclear ribonucleopr | 0.222 | 1.166 | 3.74E-01 | 3.74E+01 | | | |
| 7895444 | --- | --- | --- | 0.222 | 1.166 | 7.59E-01 | 7.59E+01 | | | |
| 8140955 | NM_001259 | CDK6 | cyclin-dependent kinase 6 | 0.222 | 1.166 | 3.10E-01 | 3.10E+01 | | | |
| 8030277 | NM_001774 | CD37 | CD37 molecule | 0.222 | 1.166 | 1.15E-01 | 1.15E+01 | | | |
| 8056343 | NM_014900 | COBLL1 | COBL-like 1 | 0.222 | 1.166 | 6.79E-02 | 6.79E+00 | | | |
| 7935910 | NM_012215 | MGEA5 | meningioma expressed antigen 5 (hy | 0.222 | 1.166 | 1.42E-01 | 1.42E+01 | | | |
| 7970392 | --- | --- | --- | 0.222 | 1.166 | 7.80E-01 | 7.80E+01 | | | |
| 7970413 | NR_003272 | PSPC1 | paraspeckle component 1 | 0.221 | 1.166 | 3.87E-01 | 3.87E+01 | | | |
| 8126542 | NM_014345 | ZNF318 | zinc finger protein 318 | 0.221 | 1.166 | 3.12E-02 | 3.12E+00 | | | |
| 7957052 | NM_006431 | CCT2 | chaperonin containing TCP1, subunit | 0.221 | 1.166 | 4.59E-01 | 4.59E+01 | | | |
| 8084986 | NM_032288 | FYTTD1 | forty-two-three domain containing 1 | 0.221 | 1.166 | 4.27E-01 | 4.27E+01 | | | |
| 7895767 | --- | --- | --- | 0.221 | 1.165 | 6.90E-01 | 6.90E+01 | | | |
| 8165658 | U18810 | VIPR2 | vasoactive intestinal peptide recepto | 0.221 | 1.165 | 8.11E-01 | 8.11E+01 | | | |
| 8006392 | NM_002815 | PSMD11 | proteasome (prosome, macropain) 2 | 0.221 | 1.165 | 4.48E-01 | 4.48E+01 | | | |
| 7893091 | --- | --- | --- | 0.221 | 1.165 | 7.53E-02 | 7.53E+00 | | | |
| 7925565 | NM_031844 | HNRNPU | heterogeneous nuclear ribonucleopr | 0.221 | 1.165 | 9.61E-03 | 9.61E-01 | | | |
| 7896474 | --- | --- | --- | 0.220 | 1.165 | 5.74E-01 | 5.74E+01 | | | |
| 7918725 | NM_015906 | TRIM33 | tripartite motif-containing 33 | 0.220 | 1.165 | 1.05E-01 | 1.05E+01 | | | |
| 8018482 | NM_012478 | WBP2 | WW domain binding protein 2 | 0.220 | 1.165 | 2.75E-01 | 2.75E+01 | | | |
| 8008991 | --- | --- | --- | 0.220 | 1.165 | 8.18E-01 | 8.18E+01 | | | |
| 8013641 | NM_033198 | PIGS | phosphatidylinositol glycan anchor b | 0.220 | 1.165 | 1.97E-01 | 1.97E+01 | | | |
| 8056426 | NM_024753 | TTC21B | tetratricopeptide repeat domain 21B | 0.220 | 1.165 | 4.05E-01 | 4.05E+01 | | | |
| 7893218 | --- | --- | --- | 0.220 | 1.164 | 4.98E-01 | 4.98E+01 | | | |
| 8180123 | NM_022553 | VPS52 | vacuolar protein sorting 52 homolog | 0.220 | 1.164 | 1.95E-01 | 1.95E+01 | | | |
| 7946211 | NM_015324 | RRP8 | ribosomal RNA processing 8, methylt | 0.220 | 1.164 | 1.86E-01 | 1.86E+01 | | | |
| 7896010 | --- | --- | --- | 0.220 | 1.164 | 7.73E-01 | 7.73E+01 | | | |
| 8135197 | NM_00103161 | SPDYE2 | speedy homolog E2 (Xenopus laevis) | 0.220 | 1.164 | 1.62E-01 | 1.62E+01 | | | |
| 8134470 | NM_003496 | TRRAP | transformation/transcription domain | 0.220 | 1.164 | 4.81E-02 | 4.81E+00 | | | |
| 8169740 | NM_002107 | H3F3A | H3 histone, family 3A | 0.219 | 1.164 | 6.79E-02 | 6.79E+00 | | | |
| 8069766 | --- | --- | --- | 0.219 | 1.164 | 2.93E-01 | 2.93E+01 | | | |
| 7896063 | --- | --- | --- | 0.219 | 1.164 | 6.61E-01 | 6.61E+01 | | | |
| 7951077 | NM_144665 | SESN3 | sestrin 3 | 0.219 | 1.164 | 3.39E-01 | 3.39E+01 | | | |
| 7899602 | --- | --- | --- | 0.219 | 1.164 | 6.54E-01 | 6.54E+01 | | | |
| 7943605 | NM_000019 | ACAT1 | acetyl-Coenzyme A acetyltransferase | 0.219 | 1.164 | 3.22E-01 | 3.22E+01 | | | |
| 8126394 | NM_018141 | MRPS10 | mitochondrial ribosomal protein S10 | 0.219 | 1.164 | 6.45E-01 | 6.45E+01 | | | |
| 8100428 | NM_004898 | CLOCK | clock homolog (mouse) | 0.219 | 1.164 | 4.23E-01 | 4.23E+01 | | | |
| 8054045 | NM_199336 | FAHD2B | fumarylacetoacetate hydrolase doma | 0.219 | 1.164 | 7.77E-02 | 7.77E+00 | | | |
| 8158240 | NM_021109 | TMSB4X | thymosin beta 4, X-linked | 0.219 | 1.164 | 2.35E-02 | 2.35E+00 | | | |
| 8020919 | NM_031446 | C18orf21 | chromosome 18 open reading frame | 0.219 | 1.164 | 5.62E-01 | 5.62E+01 | | | |
| 8051394 | --- | --- | --- | 0.219 | 1.164 | 4.15E-01 | 4.15E+01 | | | |
| 7895499 | --- | --- | --- | 0.219 | 1.164 | 7.05E-01 | 7.05E+01 | | | |

| | | | | | | | | | | |
|---|---|---|---|---|---|---|---|---|---|---|
| 8155096 | NM_006368 | CREB3 | cAMP responsive element binding pr | 0.219 | 1.164 | 2.15E-01 | 2.15E+01 | | | |
| 8108006 | NM_052971 | LEAP2 | liver expressed antimicrobial peptide | 0.218 | 1.163 | 2.41E-01 | 2.41E+01 | | | |
| 8043718 | NM_001862 | COX5B | cytochrome c oxidase subunit Vb | 0.218 | 1.163 | 5.28E-01 | 5.28E+01 | | | |
| 8060225 | NM_203346 | HDLBP | high density lipoprotein binding prot | 0.218 | 1.163 | 2.41E-01 | 2.41E+01 | | | |
| 8146268 | NM_002027 | FNTA | farnesyltransferase, CAAX box, alpha | 0.218 | 1.163 | 1.03E-01 | 1.03E+01 | | | |
| 7940660 | --- | --- | --- | 0.218 | 1.163 | 2.36E-01 | 2.36E+01 | | | |
| 7966534 | NM_00102466 | RPL6 | ribosomal protein L6 | 0.218 | 1.163 | 6.09E-02 | 6.09E+00 | | | |
| 7979916 | NM_018373 | SYNJ2BP | synaptojanin 2 binding protein | 0.218 | 1.163 | 4.56E-01 | 4.56E+01 | | | |
| 8042515 | NM_006196 | PCBP1 | poly(rC) binding protein 1 | 0.218 | 1.163 | 1.87E-01 | 1.87E+01 | | | |
| 8111360 | NM_016107 | ZFR | zinc finger RNA binding protein | 0.218 | 1.163 | 1.65E-01 | 1.65E+01 | | | |
| 8081128 | NM_022072 | NSUN3 | NOL1/NOP2/Sun domain family, mer | 0.218 | 1.163 | 6.13E-01 | 6.13E+01 | | | |
| 7966448 | NM_138341 | TMEM116 | transmembrane protein 116 | 0.218 | 1.163 | 1.06E-01 | 1.06E+01 | | | |
| 7917503 | NM_018284 | GBP3 | guanylate binding protein 3 | 0.218 | 1.163 | 3.93E-01 | 3.93E+01 | | | |
| 8102362 | NM_052864 | TIFA | TRAF-interacting protein with forkhe | 0.218 | 1.163 | 6.89E-02 | 6.89E+00 | | | |
| 8095221 | NM_00107952 | PAICS | phosphoribosylaminoimidazole carbo | 0.218 | 1.163 | 1.58E-01 | 1.58E+01 | | | |
| 7986293 | NM_018349 | MCTP2 | multiple C2 domains, transmembran | 0.218 | 1.163 | 3.35E-01 | 3.35E+01 | | | |
| 8037474 | NM_013256 | ZNF180 | zinc finger protein 180 | 0.218 | 1.163 | 9.22E-02 | 9.22E+00 | | | |
| 8055151 | NM_00109977 | POTEF | POTE ankyrin domain family, membe | 0.218 | 1.163 | 8.36E-02 | 8.36E+00 | | | |
| 7984132 | NM_006537 | USP3 | ubiquitin specific peptidase 3 | 0.218 | 1.163 | 3.60E-01 | 3.60E+01 | | | |
| 7907353 | NM_015935 | METTL13 | methyltransferase like 13 | 0.218 | 1.163 | 7.41E-02 | 7.41E+00 | | | |
| 8128712 | --- | --- | --- | 0.218 | 1.163 | 7.24E-02 | 7.24E+00 | | | |
| 7896285 | --- | --- | --- | 0.218 | 1.163 | 3.26E-01 | 3.26E+01 | | | |
| 8115375 | NM_018691 | FAM114A2 | family with sequence similarity 114, | 0.218 | 1.163 | 2.27E-01 | 2.27E+01 | | | |
| 7894634 | --- | --- | --- | 0.217 | 1.163 | 1.15E-01 | 1.15E+01 | | | |
| 7918634 | NM_006608 | PHTF1 | putative homeodomain transcription | 0.217 | 1.163 | 2.45E-01 | 2.45E+01 | | | |
| 7904574 | NM_00103970 | NBPF10 | neuroblastoma breakpoint family, m | 0.217 | 1.163 | 3.33E-02 | 3.33E+00 | | | |
| 8150988 | NM_004318 | ASPH | aspartate beta-hydroxylase | 0.217 | 1.162 | 6.04E-02 | 6.04E+00 | | | |
| 8123364 | NM_031409 | CCR6 | chemokine (C-C motif) receptor 6 | 0.217 | 1.162 | 9.22E-02 | 9.22E+00 | | | |
| 8103206 | NM_033632 | FBXW7 | F-box and WD repeat domain contai | 0.217 | 1.162 | 3.76E-01 | 3.76E+01 | | | |
| 8155203 | NM_007096 | CLTA | clathrin, light chain (Lca) | 0.217 | 1.162 | 1.56E-01 | 1.56E+01 | | | |
| 7968703 | NM_00101275 | NHLRC3 | NHL repeat containing 3 | 0.217 | 1.162 | 2.73E-01 | 2.73E+01 | | | |
| 7995252 | NM_00113091 | ZNF720 | zinc finger protein 720 | 0.217 | 1.162 | 4.72E-01 | 4.72E+01 | | | |
| 8094501 | NM_020860 | STIM2 | stromal interaction molecule 2 | 0.217 | 1.162 | 3.05E-01 | 3.05E+01 | | | |
| 8029029 | NM_007040 | HNRNPUL1 | heterogeneous nuclear ribonucleopr | 0.217 | 1.162 | 2.02E-01 | 2.02E+01 | | | |
| 8075477 | NR_024209 | RNF185 | ring finger protein 185 | 0.216 | 1.162 | 6.51E-01 | 6.51E+01 | | | |
| 8120441 | NM_015153 | PHF3 | PHD finger protein 3 | 0.216 | 1.161 | 3.91E-01 | 3.91E+01 | | | |
| 8073682 | NM_022141 | PARVG | parvin, gamma | 0.216 | 1.161 | 2.87E-01 | 2.87E+01 | | | |
| 7894937 | --- | --- | --- | 0.216 | 1.161 | 8.38E-01 | 8.38E+01 | | | |
| 7893475 | --- | --- | --- | 0.216 | 1.161 | 4.30E-01 | 4.30E+01 | | | |
| 8145782 | NM_032509 | MAK16 | MAK16 homolog (S. cerevisiae) | 0.216 | 1.161 | 3.13E-01 | 3.13E+01 | | | |
| 8105506 | NM_020928 | ZSWIM6 | zinc finger, SWIM-type containing 6 | 0.216 | 1.161 | 5.52E-01 | 5.52E+01 | | | |
| 8108180 | --- | --- | --- | 0.216 | 1.161 | 4.15E-01 | 4.15E+01 | | | |
| 8156060 | NM_007005 | TLE4 | transducin-like enhancer of split 4 (E | 0.216 | 1.161 | 3.37E-01 | 3.37E+01 | | | |
| 8145922 | NM_015214 | DDHD2 | DDHD domain containing 2 | 0.216 | 1.161 | 5.66E-01 | 5.66E+01 | | | |
| 8010686 | NM_00100224 | ANAPC11 | anaphase promoting complex subun | 0.215 | 1.161 | 2.27E-01 | 2.27E+01 | | | |
| 8083704 | NM_013263 | BRD7 | bromodomain containing 7 | 0.215 | 1.161 | 2.86E-01 | 2.86E+01 | | | |
| 8006148 | NM_004871 | GOSR1 | golgi SNAP receptor complex membe | 0.215 | 1.161 | 5.09E-01 | 5.09E+01 | | | |
| 7932160 | BC072452 | FAM107B | family with sequence similarity 107, | 0.215 | 1.161 | 2.51E-01 | 2.51E+01 | | | |
| 8053834 | NM_025190 | ANKRD36B | ankyrin repeat domain 36B | 0.215 | 1.161 | 5.98E-01 | 5.98E+01 | | | |
| 7983306 | NM_024908 | WDR76 | WD repeat domain 76 | 0.215 | 1.161 | 2.08E-01 | 2.08E+01 | | | |
| 8014241 | NM_018042 | SLFN12 | schlafen family member 12 | 0.215 | 1.161 | 3.42E-01 | 3.42E+01 | | | |
| 7965652 | NM_002595 | PCTK2 | PCTAIRE protein kinase 2 | 0.215 | 1.160 | 4.74E-01 | 4.74E+01 | | | |
| 8007992 | NM_002265 | KPNB1 | karyopherin (importin) beta 1 | 0.215 | 1.160 | 2.51E-01 | 2.51E+01 | | | |
| 7956088 | NM_002868 | RAB5B | RAB5B, member RAS oncogene famil | 0.215 | 1.160 | 2.13E-01 | 2.13E+01 | | | |
| 7994187 | NM_014494 | TNRC6A | trinucleotide repeat containing 6A | 0.214 | 1.160 | 2.58E-01 | 2.58E+01 | | | |
| 8004497 | NM_001416 | EIF4A1 | eukaryotic translation initiation facto | 0.214 | 1.160 | 3.11E-01 | 3.11E+01 | | | |
| 8021546 | NM_017742 | ZCCHC2 | zinc finger, CCHC domain containing | 0.214 | 1.160 | 1.56E-01 | 1.56E+01 | | | |
| 7894516 | --- | --- | --- | 0.214 | 1.160 | 8.85E-01 | 8.85E+01 | | | |
| 8173269 | NM_031206 | LAS1L | LAS1-like (S. cerevisiae) | 0.214 | 1.160 | 2.15E-01 | 2.15E+01 | | | |
| 7897527 | NM_00110556 | UBE4B | ubiquitination factor E4B (UFD2 hom | 0.214 | 1.160 | 1.62E-01 | 1.62E+01 | | | |
| 7892639 | --- | --- | --- | 0.214 | 1.160 | 3.31E-01 | 3.31E+01 | | | |
| 8008087 | NM_003204 | NFE2L1 | nuclear factor (erythroid-derived 2)- | 0.214 | 1.160 | 2.54E-01 | 2.54E+01 | | | |
| 8121269 | --- | --- | --- | 0.214 | 1.160 | 4.33E-01 | 4.33E+01 | | | |
| 8139758 | NM_016139 | CHCHD2 | coiled-coil-helix-coiled-coil-helix dom | 0.214 | 1.160 | 2.34E-01 | 2.34E+01 | | | |
| 7894214 | --- | --- | --- | 0.214 | 1.160 | 2.41E-01 | 2.41E+01 | | | |
| 7896719 | --- | --- | --- | 0.214 | 1.160 | 7.40E-01 | 7.40E+01 | | | |
| 8002729 | NR_027264 | GLG1 | golgi apparatus protein 1 | 0.214 | 1.160 | 1.61E-01 | 1.61E+01 | | | |
| 8013262 | NM_032582 | USP32 | ubiquitin specific peptidase 32 | 0.214 | 1.160 | 3.84E-01 | 3.84E+01 | | | |
| 7895665 | --- | --- | --- | 0.214 | 1.160 | 7.76E-01 | 7.76E+01 | | | |
| 8002660 | NM_017853 | TXNL4B | thioredoxin-like 4B | 0.214 | 1.160 | 6.81E-01 | 6.81E+01 | | | |
| 7989968 | NM_033429 | CALML4 | calmodulin-like 4 | 0.214 | 1.160 | 1.80E-01 | 1.80E+01 | | | mono |
| 8142100 | NM_020725 | ATXN7L1 | ataxin 7-like 1 | 0.214 | 1.160 | 7.44E-01 | 7.44E+01 | | | |
| 7895813 | --- | --- | --- | 0.213 | 1.159 | 6.36E-01 | 6.36E+01 | | | |
| 7893785 | --- | --- | --- | 0.213 | 1.159 | 6.85E-01 | 6.85E+01 | | | |
| 7918813 | NM_002524 | NRAS | neuroblastoma RAS viral (v-ras) onco | 0.213 | 1.159 | 5.44E-01 | 5.44E+01 | | | |
| 8097655 | --- | --- | --- | 0.213 | 1.159 | 1.41E-01 | 1.41E+01 | | | |
| 7962557 | --- | --- | --- | 0.213 | 1.159 | 3.33E-01 | 3.33E+01 | | | |

| | | | | | | | | | | |
|---|---|---|---|---|---|---|---|---|---|---|
| 7893683 | --- | --- | --- | 0.213 | 1.159 | 4.55E-01 | 4.55E+01 | | | |
| 7921667 | NM_001778 | CD48 | CD48 molecule | 0.213 | 1.159 | 4.33E-01 | 4.33E+01 | | | |
| 7918593 | NM_175744 | RHOC | ras homolog gene family, member C | 0.213 | 1.159 | 6.59E-02 | 6.59E+00 | | | |
| 7961208 | NM_018048 | MAGOHB | mago-nashi homolog B (Drosophila) | 0.213 | 1.159 | 1.96E-01 | 1.96E+01 | | | |
| 7895775 | --- | --- | --- | 0.213 | 1.159 | 7.18E-01 | 7.18E+01 | | | |
| 7895044 | --- | --- | --- | 0.213 | 1.159 | 8.38E-01 | 8.38E+01 | | | |
| 8018315 | NM_006937 | SUMO2 | SMT3 suppressor of mif two 3 homo | 0.213 | 1.159 | 4.62E-01 | 4.62E+01 | | | |
| 7929858 | NM_018121 | FAM178A | family with sequence similarity 178, | 0.213 | 1.159 | 3.57E-02 | 3.57E+00 | | | |
| 7967486 | NM_025140 | CCDC92 | coiled-coil domain containing 92 | 0.213 | 1.159 | 1.24E-01 | 1.24E+01 | | | |
| 8070046 | NR_027873 | C21orf66 | chromosome 21 open reading frame | 0.213 | 1.159 | 1.23E-01 | 1.23E+01 | | | |
| 8006715 | NM_001488 | TADA2L | transcriptional adaptor 2 (ADA2 hom | 0.213 | 1.159 | 1.18E-01 | 1.18E+01 | | | |
| 8025998 | NM_003437 | ZNF136 | zinc finger protein 136 | 0.213 | 1.159 | 1.83E-01 | 1.83E+01 | | | |
| 8111788 | NM_012382 | TTC33 | tetratricopeptide repeat domain 33 | 0.213 | 1.159 | 4.05E-01 | 4.05E+01 | | | |
| 7969202 | --- | --- | --- | 0.213 | 1.159 | 5.51E-01 | 5.51E+01 | | | |
| 7895404 | --- | --- | --- | 0.212 | 1.159 | 3.95E-01 | 3.95E+01 | | | |
| 8017621 | NM_007215 | POLG2 | polymerase (DNA directed), gamma | 0.212 | 1.159 | 5.86E-01 | 5.86E+01 | | | |
| 7940153 | NM_022074 | FAM111A | family with sequence similarity 111, | 0.212 | 1.159 | 2.66E-01 | 2.66E+01 | | | |
| 8079274 | NM_144638 | TMEM42 | transmembrane protein 42 | 0.212 | 1.159 | 4.38E-02 | 4.38E+00 | | | |
| 8040163 | NM_00103961 | IAH1 | isoamyl acetate-hydrolyzing esterase | 0.212 | 1.159 | 6.81E-01 | 6.81E+01 | | | |
| 8129193 | NM_00104247 | C6orf204 | chromosome 6 open reading frame 2 | 0.212 | 1.159 | 3.84E-01 | 3.84E+01 | | | |
| 7965357 | NM_003774 | GALNT4 | UDP-N-acetyl-alpha-D-galactosamine | 0.212 | 1.159 | 2.51E-01 | 2.51E+01 | | | |
| 8096251 | NM_024047 | NUDT9 | nudix (nucleoside diphosphate linked | 0.212 | 1.158 | 5.67E-02 | 5.67E+00 | | | |
| 7912808 | NM_00110266 | NBPF16 | neuroblastoma breakpoint family, m | 0.212 | 1.158 | 3.26E-02 | 3.26E+00 | | | |
| 7896074 | --- | --- | --- | 0.212 | 1.158 | 1.89E-01 | 1.89E+01 | | | |
| 7984871 | NM_004383 | CSK | c-src tyrosine kinase | 0.212 | 1.158 | 9.06E-02 | 9.06E+00 | | | |
| 8002347 | NM_001605 | AARS | alanyl-tRNA synthetase | 0.212 | 1.158 | 1.23E-01 | 1.23E+01 | | | |
| 7979862 | --- | --- | --- | 0.212 | 1.158 | 5.06E-01 | 5.06E+01 | | | |
| 8101774 | NM_183049 | TMSL3 | thymosin-like 3 | 0.211 | 1.158 | 4.94E-03 | 4.94E-01 | | | |
| 7993546 | NM_006985 | NPIP | nuclear pore complex interacting pro | 0.211 | 1.158 | 7.43E-02 | 7.43E+00 | | | |
| 8080980 | AY036895 | FLJ10213 | hypothetical protein FLJ10213 | 0.211 | 1.158 | 6.11E-01 | 6.11E+01 | | | |
| 8104422 | NM_002454 | MTRR | 5-methyltetrahydrofolate-homocyste | 0.211 | 1.158 | 3.77E-01 | 3.77E+01 | | | |
| 8016324 | NM_00111409 | CDC27 | cell division cycle 27 homolog (S. cer | 0.211 | 1.158 | 3.64E-01 | 3.64E+01 | | | |
| 8138581 | NM_013293 | TRA2A | transformer 2 alpha homolog (Droso | 0.211 | 1.158 | 4.14E-01 | 4.14E+01 | | | |
| 7895551 | --- | --- | --- | 0.211 | 1.158 | 2.92E-03 | 2.92E-01 | | | |
| 8121525 | NM_153369 | KIAA1919 | KIAA1919 | 0.211 | 1.158 | 3.37E-01 | 3.37E+01 | | | |
| 7949518 | NM_198897 | FIBP | fibroblast growth factor (acidic) intra | 0.211 | 1.157 | 1.48E-01 | 1.48E+01 | | | |
| 7968254 | NM_152705 | POLR1D | polymerase (RNA) I polypeptide D, 1 | 0.211 | 1.157 | 2.63E-01 | 2.63E+01 | | | |
| 7894551 | --- | --- | --- | 0.211 | 1.157 | 5.85E-01 | 5.85E+01 | | | |
| 8098581 | NM_031953 | SNX25 | sorting nexin 25 | 0.211 | 1.157 | 4.89E-01 | 4.89E+01 | | | |
| 8067011 | NM_015339 | ADNP | activity-dependent neuroprotector h | 0.211 | 1.157 | 5.12E-01 | 5.12E+01 | | | |
| 8084064 | NM_006636 | MTHFD2 | methylenetetrahydrofolate dehydro | 0.211 | 1.157 | 3.99E-01 | 3.99E+01 | | | |
| 8130129 | NM_198887 | NUP43 | nucleoporin 43kDa | 0.211 | 1.157 | 4.72E-01 | 4.72E+01 | | | |
| 8078262 | NM_003341 | UBE2E1 | ubiquitin-conjugating enzyme E2E 1 | 0.211 | 1.157 | 1.95E-01 | 1.95E+01 | | | |
| 7995456 | NM_182922 | HEATR3 | HEAT repeat containing 3 | 0.211 | 1.157 | 2.87E-01 | 2.87E+01 | | | |
| 7931353 | NM_006504 | PTPRE | protein tyrosine phosphatase, recept | 0.211 | 1.157 | 1.31E-01 | 1.31E+01 | | | |
| 7896727 | --- | --- | --- | 0.210 | 1.157 | 6.16E-01 | 6.16E+01 | | | |
| 7962516 | NM_030674 | SLC38A1 | solute carrier family 38, member 1 | 0.210 | 1.157 | 5.72E-01 | 5.72E+01 | | | |
| 8162019 | NM_017576 | KIF27 | kinesin family member 27 | 0.210 | 1.157 | 1.74E-01 | 1.74E+01 | | | |
| 7922121 | NR_026844 | ANKRD36BL1 | ankyrin repeat domain 36B-like 1 (ps | 0.210 | 1.157 | 6.16E-01 | 6.16E+01 | | | |
| 7895376 | --- | --- | --- | 0.210 | 1.157 | 1.57E-01 | 1.57E+01 | | | |
| 8073242 | NM_000026 | ADSL | adenylosuccinate lyase | 0.210 | 1.157 | 3.50E-01 | 3.50E+01 | | | |
| 7970655 | NM_004685 | MTMR6 | myotubularin related protein 6 | 0.210 | 1.157 | 5.13E-01 | 5.13E+01 | | | |
| 7896269 | --- | --- | --- | 0.210 | 1.157 | 7.07E-01 | 7.07E+01 | | | |
| 8007799 | NR_026680 | MGC57346 | hypothetical LOC401884 | 0.210 | 1.157 | 3.15E-01 | 3.15E+01 | | | |
| 8085372 | --- | --- | --- | 0.210 | 1.157 | 6.49E-01 | 6.49E+01 | | | |
| 8018097 | NM_00109883 | FAM104A | family with sequence similarity 104, | 0.210 | 1.157 | 1.43E-01 | 1.43E+01 | | | |
| 7979437 | NM_144581 | C14orf149 | chromosome 14 open reading frame | 0.210 | 1.157 | 2.58E-02 | 2.58E+00 | | | |
| 7895160 | --- | --- | --- | 0.210 | 1.157 | 8.29E-01 | 8.29E+01 | | | |
| 8088384 | NM_000925 | PDHB | pyruvate dehydrogenase (lipoamide) | 0.210 | 1.157 | 6.10E-01 | 6.10E+01 | | | |
| 8178833 | NM_002120 | HLA-DOB | major histocompatibility complex, cl | 0.210 | 1.156 | 4.11E-01 | 4.11E+01 | | | |
| 8008629 | NM_003647 | DGKE | diacylglycerol kinase, epsilon 64kDa | 0.210 | 1.156 | 1.99E-01 | 1.99E+01 | | | |
| 8110803 | NM_030782 | CLPTM1L | CLPTM1-like | 0.210 | 1.156 | 2.39E-01 | 2.39E+01 | | | |
| 7908553 | NM_002838 | PTPRC | protein tyrosine phosphatase, recept | 0.210 | 1.156 | 2.01E-02 | 2.01E+00 | | | |
| 8127109 | NM_016513 | ICK | intestinal cell (MAK-like) kinase | 0.209 | 1.156 | 2.13E-01 | 2.13E+01 | | | |
| 7896254 | --- | --- | --- | 0.209 | 1.156 | 7.70E-01 | 7.70E+01 | | | |
| 8077376 | NM_00109995 | ITPR1 | inositol 1,4,5-triphosphate receptor, | 0.209 | 1.156 | 8.66E-02 | 8.66E+00 | | | |
| 7926170 | NM_018706 | DHTKD1 | dehydrogenase E1 and transketolase | 0.209 | 1.156 | 1.42E-01 | 1.42E+01 | | | |
| 8008598 | NM_00110240 | PCTP | phosphatidylcholine transfer protein | 0.209 | 1.156 | 4.81E-02 | 4.81E+00 | | | |
| 8009761 | NM_024844 | NUP85 | nucleoporin 85kDa | 0.209 | 1.156 | 1.99E-01 | 1.99E+01 | | | |
| 8121578 | NM_152729 | NT5DC1 | 5'-nucleotidase domain containing 1 | 0.209 | 1.156 | 2.81E-01 | 2.81E+01 | | | |
| 7983350 | NM_003758 | EIF3J | eukaryotic translation initiation facto | 0.209 | 1.156 | 4.48E-01 | 4.48E+01 | | | |
| 7941946 | NM_007103 | NDUFV1 | NADH dehydrogenase (ubiquinone) f | 0.209 | 1.156 | 2.48E-01 | 2.48E+01 | | | |
| 7991057 | NM_025238 | BTBD1 | BTB (POZ) domain containing 1 | 0.209 | 1.156 | 1.79E-01 | 1.79E+01 | | | |
| 7895850 | --- | --- | --- | 0.209 | 1.156 | 6.91E-01 | 6.91E+01 | | | |
| 8163733 | NM_018249 | CDK5RAP2 | CDK5 regulatory subunit associated p | 0.209 | 1.156 | 2.09E-01 | 2.09E+01 | | | |
| 7980271 | NM_033116 | NEK9 | NIMA (never in mitosis gene a)- relat | 0.209 | 1.156 | 3.15E-01 | 3.15E+01 | | | |
| 7999326 | NM_003470 | USP7 | ubiquitin specific peptidase 7 (herpe | 0.209 | 1.156 | 4.63E-01 | 4.63E+01 | | | |

| | | | | | | | | | | |
|---|---|---|---|---|---|---|---|---|---|---|
| 8170027 | NM_182540 | DDX26B | DEAD/H (Asp-Glu-Ala-Asp/His) box p | 0.209 | 1.156 | 2.23E-01 | 2.23E+01 | | | |
| 7954407 | NM_018686 | CMAS | cytidine monophosphate N-acetylne | 0.208 | 1.155 | 1.85E-01 | 1.85E+01 | | | |
| 8150126 | NM_00100955 | PPP2CB | protein phosphatase 2 (formerly 2A) | 0.208 | 1.155 | 2.79E-01 | 2.79E+01 | | | |
| 7892576 | --- | --- | --- | 0.208 | 1.155 | 5.46E-01 | 5.46E+01 | | | |
| 7893761 | --- | --- | --- | 0.208 | 1.155 | 8.16E-01 | 8.16E+01 | | | |
| 8043100 | NM_021103 | TMSB10 | thymosin beta 10 | 0.208 | 1.155 | 8.82E-02 | 8.82E+00 | | | |
| 8007794 | NM_00103908 | ARL17 | ADP-ribosylation factor-like 17 | 0.208 | 1.155 | 1.85E-01 | 1.85E+01 | | | |
| 8112709 | NM_00109927 | C5orf37 | chromosome 5 open reading frame 3 | 0.208 | 1.155 | 5.17E-01 | 5.17E+01 | | | |
| 8102950 | NM_003866 | INPP4B | inositol polyphosphate-4-phosphata | 0.208 | 1.155 | 2.61E-01 | 2.61E+01 | | | |
| 8057394 | NM_178123 | SESTD1 | SEC14 and spectrin domains 1 | 0.208 | 1.155 | 4.35E-01 | 4.35E+01 | | | |
| 8094169 | NM_182485 | CPEB2 | cytoplasmic polyadenylation elemen | 0.208 | 1.155 | 2.51E-01 | 2.51E+01 | | | |
| 7893270 | --- | --- | --- | 0.208 | 1.155 | 5.62E-01 | 5.62E+01 | | | |
| 7946048 | NM_000559 | HBG1 | hemoglobin, gamma A | 0.208 | 1.155 | 3.43E-01 | 3.43E+01 | | | |
| 7946054 | NM_000559 | HBG1 | hemoglobin, gamma A | 0.208 | 1.155 | 3.43E-01 | 3.43E+01 | | | |
| 8094876 | NM_021927 | GUF1 | GUF1 GTPase homolog (S. cerevisiae | 0.208 | 1.155 | 4.98E-01 | 4.98E+01 | | | |
| 7923453 | NM_006618 | KDM5B | lysine (K)-specific demethylase 5B | 0.208 | 1.155 | 1.88E-01 | 1.88E+01 | | | |
| 7895918 | --- | --- | --- | 0.207 | 1.155 | 5.00E-01 | 5.00E+01 | | | |
| 8096224 | NM_005935 | AFF1 | AF4/FMR2 family, member 1 | 0.207 | 1.155 | 9.70E-02 | 9.70E+00 | | | |
| 8142059 | --- | --- | --- | 0.207 | 1.154 | 3.60E-02 | 3.60E+00 | | | |
| 8169792 | NM_002351 | SH2D1A | SH2 domain protein 1A | 0.207 | 1.154 | 5.57E-01 | 5.57E+01 | | | |
| 8135378 | NM_002736 | PRKAR2B | protein kinase, cAMP-dependent, re | 0.207 | 1.154 | 5.01E-01 | 5.01E+01 | | | |
| 8078916 | NR_002325 | SNORA6 | small nucleolar RNA, H/ACA box 6 | 0.207 | 1.154 | 7.83E-01 | 7.83E+01 | | | |
| 8180312 | --- | --- | --- | 0.207 | 1.154 | 4.12E-01 | 4.12E+01 | | | |
| 8138370 | NM_020319 | ANKMY2 | ankyrin repeat and MYND domain co | 0.207 | 1.154 | 1.60E-01 | 1.60E+01 | | | |
| 8089295 | --- | --- | --- | 0.207 | 1.154 | 3.74E-01 | 3.74E+01 | | | |
| 7893727 | --- | --- | --- | 0.207 | 1.154 | 1.56E-01 | 1.56E+01 | | | |
| 7895677 | --- | --- | --- | 0.207 | 1.154 | 7.76E-01 | 7.76E+01 | | | |
| 7896604 | --- | --- | --- | 0.207 | 1.154 | 4.30E-01 | 4.30E+01 | | | |
| 7946559 | NM_00101799 | GNG10 | guanine nucleotide binding protein ( | 0.207 | 1.154 | 5.45E-01 | 5.45E+01 | | | |
| 8151927 | --- | --- | --- | 0.206 | 1.154 | 6.50E-01 | 6.50E+01 | | | |
| 7894669 | --- | --- | --- | 0.206 | 1.154 | 7.98E-01 | 7.98E+01 | | | |
| 8089261 | NM_170662 | CBLB | Cas-Br-M (murine) ecotropic retrovir | 0.206 | 1.154 | 2.49E-01 | 2.49E+01 | | | |
| 7946221 | NM_006284 | TAF10 | TAF10 RNA polymerase II, TATA box | 0.206 | 1.154 | 4.59E-01 | 4.59E+01 | | | |
| 8027604 | NM_014686 | KIAA0355 | KIAA0355 | 0.206 | 1.154 | 4.24E-02 | 4.24E+00 | | | |
| 8104901 | NM_002185 | IL7R | interleukin 7 receptor | 0.206 | 1.154 | 5.97E-01 | 5.97E+01 | | | |
| 8149248 | --- | --- | --- | 0.206 | 1.154 | 7.60E-01 | 7.60E+01 | | | |
| 8052204 | NM_020532 | RTN4 | reticulon 4 | 0.206 | 1.154 | 1.22E-02 | 1.22E+00 | | | |
| 7946426 | --- | --- | --- | 0.206 | 1.153 | 4.78E-01 | 4.78E+01 | | | |
| 7894094 | --- | --- | --- | 0.206 | 1.153 | 7.59E-01 | 7.59E+01 | | | |
| 7972487 | NM_015296 | DOCK9 | dedicator of cytokinesis 9 | 0.206 | 1.153 | 2.46E-01 | 2.46E+01 | | | |
| 8154793 | NM_002504 | NFX1 | nuclear transcription factor, X-box bi | 0.206 | 1.153 | 3.69E-01 | 3.69E+01 | | | |
| 8163424 | --- | --- | --- | 0.206 | 1.153 | 4.24E-01 | 4.24E+01 | | | |
| 7977841 | NM_017815 | HAUS4 | HAUS augmin-like complex, subunit | 0.206 | 1.153 | 1.06E-01 | 1.06E+01 | | | |
| 7894810 | --- | --- | --- | 0.206 | 1.153 | 8.36E-01 | 8.36E+01 | | | |
| 8088813 | NM_00112612 | PROK2 | prokineticin 2 | 0.206 | 1.153 | 1.59E-01 | 1.59E+01 | | | |
| 7957633 | NM_006838 | METAP2 | methionyl aminopeptidase 2 | 0.206 | 1.153 | 5.74E-01 | 5.74E+01 | | | |
| 7971573 | NM_001268 | RCBTB2 | regulator of chromosome condensat | 0.205 | 1.153 | 9.08E-02 | 9.08E+00 | | | |
| 7894389 | --- | --- | --- | 0.205 | 1.153 | 2.58E-01 | 2.58E+01 | | | |
| 7945579 | NM_00114267 | CHID1 | chitinase domain containing 1 | 0.205 | 1.153 | 5.79E-02 | 5.79E+00 | | | |
| 7950990 | NM_152313 | SLC36A4 | solute carrier family 36 (proton/amir | 0.205 | 1.153 | 6.22E-01 | 6.22E+01 | | | |
| 8147654 | NM_005034 | POLR2K | polymerase (RNA) II (DNA directed) p | 0.205 | 1.153 | 6.48E-01 | 6.48E+01 | | | |
| 8098326 | --- | --- | --- | 0.205 | 1.153 | 2.35E-01 | 2.35E+01 | | | |
| 8019930 | NM_033546 | MYL12B | myosin, light chain 12B, regulatory | 0.205 | 1.153 | 2.17E-01 | 2.17E+01 | | | |
| 7893628 | --- | --- | --- | 0.205 | 1.153 | 6.32E-01 | 6.32E+01 | | | |
| 8142110 | NM_006754 | SYPL1 | synaptophysin-like 1 | 0.205 | 1.153 | 2.24E-01 | 2.24E+01 | | | |
| 8108174 | NM_032151 | PCBD2 | pterin-4 alpha-carbinolamine dehydr | 0.205 | 1.153 | 1.43E-01 | 1.43E+01 | | | |
| 8053366 | NM_003849 | SUCLG1 | succinate-CoA ligase, alpha subunit | 0.205 | 1.153 | 2.26E-01 | 2.26E+01 | | | |
| 7991465 | --- | --- | --- | 0.205 | 1.153 | 5.22E-01 | 5.22E+01 | | | |
| 8041713 | NM_177968 | PPM1B | protein phosphatase 1B (formerly 2C | 0.205 | 1.152 | 3.81E-01 | 3.81E+01 | | | |
| 7892732 | --- | --- | --- | 0.205 | 1.152 | 8.08E-01 | 8.08E+01 | | | |
| 8168500 | NM_000291 | PGK1 | phosphoglycerate kinase 1 | 0.205 | 1.152 | 1.28E-01 | 1.28E+01 | | | |
| 8022326 | NM_023075 | MPPE1 | metallophosphoesterase 1 | 0.205 | 1.152 | 3.51E-01 | 3.51E+01 | | | |
| 7899955 | --- | --- | --- | 0.205 | 1.152 | 8.54E-03 | 8.54E-01 | | | |
| 7986411 | NM_005587 | MEF2A | myocyte enhancer factor 2A | 0.204 | 1.152 | 2.69E-02 | 2.69E+00 | | | |
| 8017843 | NM_004694 | SLC16A6 | solute carrier family 16, member 6 (r | 0.204 | 1.152 | 6.13E-01 | 6.13E+01 | | | |
| 8163784 | NM_012164 | FBXW2 | F-box and WD repeat domain contai | 0.204 | 1.152 | 3.54E-01 | 3.54E+01 | | | |
| 8083119 | NM_014245 | RNF7 | ring finger protein 7 | 0.204 | 1.152 | 3.01E-01 | 3.01E+01 | | | |
| 8084912 | NR_003265 | SDHALP2 | succinate dehydrogenase complex, s | 0.204 | 1.152 | 5.17E-01 | 5.17E+01 | | | |
| 7896434 | --- | --- | --- | 0.204 | 1.152 | 3.70E-01 | 3.70E+01 | | | |
| 8036602 | NM_001398 | ECH1 | enoyl Coenzyme A hydratase 1, pero | 0.204 | 1.152 | 3.56E-01 | 3.56E+01 | | | |
| 8173766 | NM_153252 | BRWD3 | bromodomain and WD repeat doma | 0.204 | 1.152 | 1.96E-01 | 1.96E+01 | | | |
| 7896456 | --- | --- | --- | 0.204 | 1.152 | 5.96E-01 | 5.96E+01 | | | |
| 8107934 | --- | --- | --- | 0.204 | 1.152 | 1.21E-02 | 1.21E+00 | | | |
| 7893322 | --- | --- | --- | 0.204 | 1.152 | 4.03E-01 | 4.03E+01 | | | |
| 8022506 | --- | --- | --- | 0.204 | 1.152 | 3.51E-01 | 3.51E+01 | | | |
| 7920333 | --- | --- | --- | 0.204 | 1.152 | 4.32E-01 | 4.32E+01 | | | |
| 8021147 | NM_016097 | IER3IP1 | immediate early response 3 interacti | 0.203 | 1.151 | 8.16E-01 | 8.16E+01 | | | |

| | | | | | | | | | | |
|---|---|---|---|---|---|---|---|---|---|---|
| 7893461 | --- | --- | --- | 0.203 | 1.151 | 7.37E-01 | 7.37E+01 | | | |
| 7896552 | --- | --- | --- | 0.203 | 1.151 | 7.95E-01 | 7.95E+01 | | | |
| 7952601 | NM_00114382( | ETS1 | v-ets erythroblastosis virus E26 onco | 0.203 | 1.151 | 1.21E-01 | 1.21E+01 | | | |
| 7951093 | NM_144664 | FAM76B | family with sequence similarity 76, n | 0.203 | 1.151 | 3.10E-01 | 3.10E+01 | | | |
| 8122464 | NM_007124 | UTRN | utrophin | 0.203 | 1.151 | 1.59E-01 | 1.59E+01 | | | |
| 8030982 | NM_00104018! | ZNF765 | zinc finger protein 765 | 0.203 | 1.151 | 8.31E-02 | 8.31E+00 | | | |
| 8095545 | NM_00103744: | RUFY3 | RUN and FYVE domain containing 3 | 0.203 | 1.151 | 1.89E-01 | 1.89E+01 | | | |
| 8044333 | NM_004987 | LIMS1 | LIM and senescent cell antigen-like d | 0.203 | 1.151 | 4.54E-01 | 4.54E+01 | | | |
| 8054519 | NM_004987 | LIMS1 | LIM and senescent cell antigen-like d | 0.203 | 1.151 | 4.54E-01 | 4.54E+01 | | | |
| 8125134 | NM_005527 | HSPA1L | heat shock 70kDa protein 1-like | 0.203 | 1.151 | 1.72E-01 | 1.72E+01 | | | |
| 8178650 | NM_005527 | HSPA1L | heat shock 70kDa protein 1-like | 0.203 | 1.151 | 1.72E-01 | 1.72E+01 | | | |
| 8179848 | NM_005527 | HSPA1L | heat shock 70kDa protein 1-like | 0.203 | 1.151 | 1.72E-01 | 1.72E+01 | | | |
| 7920472 | NM_152263 | TPM3 | tropomyosin 3 | 0.203 | 1.151 | 3.46E-02 | 3.46E+00 | | | |
| 7974816 | NM_153811 | SLC38A6 | solute carrier family 38, member 6 | 0.202 | 1.151 | 4.53E-01 | 4.53E+01 | | | |
| 8044049 | NM_003853 | IL18RAP | interleukin 18 receptor accessory pro | 0.202 | 1.151 | 5.26E-01 | 5.26E+01 | | | |
| 7903753 | NM_000848 | GSTM2 | glutathione S-transferase mu 2 (mus | 0.202 | 1.151 | 2.79E-01 | 2.79E+01 | | | |
| 7904976 | NM_00110266: | NBPF16 | neuroblastoma breakpoint family, m | 0.202 | 1.151 | 1.79E-02 | 1.79E+00 | | | |
| 7892635 | --- | --- | --- | 0.202 | 1.150 | 2.49E-01 | 2.49E+01 | | | |
| 8043470 | --- | --- | --- | 0.202 | 1.150 | 3.93E-01 | 3.93E+01 | | | |
| 7993825 | NM_00101239: | RUNDC2B | RUN domain containing 2B | 0.202 | 1.150 | 6.03E-01 | 6.03E+01 | | | |
| 7956401 | NM_005412 | SHMT2 | serine hydroxymethyltransferase 2 (r | 0.202 | 1.150 | 3.28E-01 | 3.28E+01 | | | |
| 7948744 | NM_198335 | GANAB | glucosidase, alpha; neutral AB | 0.202 | 1.150 | 1.99E-01 | 1.99E+01 | | | |
| 8051820 | NM_022065 | THADA | thyroid adenoma associated | 0.202 | 1.150 | 1.78E-01 | 1.78E+01 | | | |
| 7918911 | --- | --- | --- | 0.202 | 1.150 | 5.12E-01 | 5.12E+01 | | | |
| 7970467 | NM_174928 | N6AMT2 | N-6 adenine-specific DNA methyltrar | 0.202 | 1.150 | 5.24E-01 | 5.24E+01 | | | |
| 8131815 | NM_018846 | KLHL7 | kelch-like 7 (Drosophila) | 0.202 | 1.150 | 4.98E-01 | 4.98E+01 | | | |
| 7894462 | --- | --- | --- | 0.202 | 1.150 | 8.31E-01 | 8.31E+01 | | | |
| 7892633 | --- | --- | --- | 0.202 | 1.150 | 5.85E-01 | 5.85E+01 | | | |
| 7911138 | NM_152609 | C1orf71 | chromosome 1 open reading frame 7 | 0.201 | 1.150 | 4.78E-01 | 4.78E+01 | | | |
| 7930880 | --- | --- | --- | 0.201 | 1.150 | 6.92E-01 | 6.92E+01 | | | |
| 7895460 | --- | --- | --- | 0.201 | 1.150 | 7.92E-01 | 7.92E+01 | | | |
| 7894744 | --- | --- | --- | 0.201 | 1.150 | 2.73E-01 | 2.73E+01 | | | |
| 8108510 | NM_020690 | KHD1-EIF4EE | ANKHD1-EIF4EBP3 readthrough tran | 0.201 | 1.150 | 5.97E-02 | 5.97E+00 | | | |
| 8072946 | NM_016091 | EIF3L | eukaryotic translation initiation facto | 0.201 | 1.150 | 6.18E-01 | 6.18E+01 | | | |
| 7957242 | NM_00113626: | LOC552889 | hypothetical protein LOC552889 | 0.201 | 1.149 | 3.34E-01 | 3.34E+01 | | | |
| 7996290 | NM_052999 | CMTM1 | CKLF-like MARVEL transmembrane d | 0.201 | 1.149 | 1.20E-01 | 1.20E+01 | | | |
| 7998940 | NM_000243 | MEFV | Mediterranean fever | 0.201 | 1.149 | 2.11E-02 | 2.11E+00 | | | |
| 8061685 | NM_014742 | TM9SF4 | transmembrane 9 superfamily prote | 0.201 | 1.149 | 2.43E-01 | 2.43E+01 | | | |
| 7906852 | NM_175866 | UHMK1 | U2AF homology motif (UHM) kinase | 0.201 | 1.149 | 3.39E-01 | 3.39E+01 | | | |
| 8070826 | NM_000211 | ITGB2 | integrin, beta 2 (complement compo | 0.201 | 1.149 | 1.05E-01 | 1.05E+01 | | | |
| 8114138 | NM_020199 | C5orf15 | chromosome 5 open reading frame 1 | 0.201 | 1.149 | 8.56E-02 | 8.56E+00 | | | |
| 7896520 | --- | --- | --- | 0.201 | 1.149 | 6.89E-01 | 6.89E+01 | | | |
| 8027674 | NM_018443 | ZNF302 | zinc finger protein 302 | 0.200 | 1.149 | 3.89E-02 | 3.89E+00 | | | |
| 7984517 | NM_015554 | GLCE | glucuronic acid epimerase | 0.200 | 1.149 | 1.14E-01 | 1.14E+01 | | | |
| 8180237 | --- | --- | --- | 0.200 | 1.149 | 4.78E-01 | 4.78E+01 | | | |
| 7901915 | NM_013339 | ALG6 | asparagine-linked glycosylation 6, alp | 0.200 | 1.149 | 4.96E-01 | 4.96E+01 | | | |
| 8044965 | NM_004622 | TSN | translin | 0.200 | 1.149 | 4.10E-01 | 4.10E+01 | | | |
| 8171516 | NM_002893 | RBBP7 | retinoblastoma binding protein 7 | 0.200 | 1.149 | 4.90E-01 | 4.90E+01 | | | |
| 7902891 | NM_182976 | ZNF326 | zinc finger protein 326 | 0.200 | 1.149 | 3.32E-01 | 3.32E+01 | | | |
| 8119132 | NM_173558 | FGD2 | FYVE, RhoGEF and PH domain contai | 0.200 | 1.149 | 2.10E-02 | 2.10E+00 | | | |
| 8069744 | NM_016940 | RWDD2B | RWD domain containing 2B | 0.200 | 1.149 | 4.97E-01 | 4.97E+01 | | | |
| 8084947 | NM_00110557: | FBXO45 | F-box protein 45 | 0.200 | 1.149 | 1.21E-01 | 1.21E+01 | | | |
| 8134581 | NM_006409 | ARPC1A | actin related protein 2/3 complex, su | 0.200 | 1.149 | 2.42E-01 | 2.42E+01 | | | |
| 8092177 | NM_00114627( | NCEH1 | neutral cholesterol ester hydrolase 1 | 0.200 | 1.149 | 3.37E-02 | 3.37E+00 | | | |
| 8125123 | --- | --- | --- | 0.200 | 1.149 | 3.01E-01 | 3.01E+01 | | | |
| 7945058 | NM_024556 | FAM118B | family with sequence similarity 118, | 0.200 | 1.149 | 3.14E-01 | 3.14E+01 | | | |
| 7893226 | --- | --- | --- | 0.200 | 1.148 | 4.34E-01 | 4.34E+01 | | | |
| 8101971 | NM_000944 | PPP3CA | protein phosphatase 3 (formerly 2B) | 0.200 | 1.148 | 2.98E-01 | 2.98E+01 | | | |
| 7988963 | NM_006628 | ARPP19 | cAMP-regulated phosphoprotein, 19 | 0.200 | 1.148 | 2.44E-01 | 2.44E+01 | | | |
| 7896095 | --- | --- | --- | 0.200 | 1.148 | 4.15E-01 | 4.15E+01 | | | |
| 8157264 | NM_001860 | SLC31A2 | solute carrier family 31 (copper trans | 0.200 | 1.148 | 2.15E-01 | 2.15E+01 | | | |
| 7937915 | NM_001033 | RRM1 | ribonucleotide reductase M1 | 0.200 | 1.148 | 5.16E-01 | 5.16E+01 | | | |
| 8020129 | NM_003574 | VAPA | VAMP (vesicle-associated membrane | 0.200 | 1.148 | 4.88E-01 | 4.88E+01 | | | |
| 7980998 | NM_00100286( | BTBD7 | BTB (POZ) domain containing 7 | 0.200 | 1.148 | 1.08E-01 | 1.08E+01 | | | |
| 7906021 | NM_018116 | MSTO1 | misato homolog 1 (Drosophila) | 0.199 | 1.148 | 3.11E-01 | 3.11E+01 | | | |
| 8087748 | NM_014703 | VPRBP | Vpr (HIV-1) binding protein | 0.199 | 1.148 | 1.92E-01 | 1.92E+01 | | | |
| 8081135 | NM_00113442: | CDV3 | CDV3 homolog (mouse) | 0.199 | 1.148 | 4.26E-01 | 4.26E+01 | | | |
| 8074632 | NM_032775 | KLHL22 | kelch-like 22 (Drosophila) | 0.199 | 1.148 | 8.63E-03 | 8.63E-01 | | | |
| 7961532 | NM_001175 | ARHGDIB | Rho GDP dissociation inhibitor (GDI) | 0.199 | 1.148 | 5.77E-03 | 5.77E-01 | | | |
| 8042487 | NM_178439 | GMCL1 | germ cell-less homolog 1 (Drosophila | 0.199 | 1.148 | 2.96E-01 | 2.96E+01 | | | |
| 8115464 | NM_032782 | HAVCR2 | hepatitis A virus cellular receptor 2 | 0.199 | 1.148 | 1.52E-01 | 1.52E+01 | | | |
| 7951873 | NM_025164 | QSK | serine/threonine-protein kinase QSK | 0.199 | 1.148 | 1.49E-01 | 1.49E+01 | | | |
| 7990632 | NM_024776 | SGK269 | NKF3 kinase family member | 0.199 | 1.148 | 5.95E-01 | 5.95E+01 | | | |
| 7942328 | NM_000804 | FOLR3 | folate receptor 3 (gamma) | 0.199 | 1.148 | 1.57E-01 | 1.57E+01 | | | |
| 8180299 | --- | --- | --- | 0.199 | 1.148 | 5.26E-01 | 5.26E+01 | | | |
| 7976512 | NR_015415 | FLJ45244 | hypothetical locus FLJ45244 | 0.198 | 1.147 | 1.48E-01 | 1.48E+01 | | | |
| 7892932 | --- | --- | --- | 0.198 | 1.147 | 8.54E-01 | 8.54E+01 | | | |

| | | | | | | | | | | | |
|---|---|---|---|---|---|---|---|---|---|---|---|
| 8117321 | NM_006355 | TRIM38 | tripartite motif-containing 38 | 0.198 | 1.147 | 5.23E-01 | 5.23E+01 | | | | |
| 7924923 | NM_018230 | NUP133 | nucleoporin 133kDa | 0.198 | 1.147 | 4.64E-01 | 4.64E+01 | | | | |
| 7973221 | AK301287 | TRAJ17 | T cell receptor alpha joining 17 | 0.198 | 1.147 | 4.11E-03 | 4.11E-01 | | | | |
| 8151709 | --- | --- | --- | 0.198 | 1.147 | 6.43E-01 | 6.43E+01 | | | | |
| 7895881 | --- | --- | --- | 0.198 | 1.147 | 6.88E-01 | 6.88E+01 | | | | |
| 7896161 | --- | --- | --- | 0.198 | 1.147 | 6.55E-01 | 6.55E+01 | | | | |
| 8043322 | NM_022662 | ANAPC1 | anaphase promoting complex subun | 0.198 | 1.147 | 6.59E-01 | 6.59E+01 | | | | |
| 7894513 | --- | --- | --- | 0.198 | 1.147 | 3.96E-01 | 3.96E+01 | | | | |
| 7893548 | --- | --- | --- | 0.198 | 1.147 | 7.83E-01 | 7.83E+01 | | | | |
| 8106660 | NM_006909 | RASGRF2 | Ras protein-specific guanine nucleot | 0.198 | 1.147 | 1.56E-01 | 1.56E+01 | | | | |
| 7895215 | --- | --- | --- | 0.198 | 1.147 | 3.62E-01 | 3.62E+01 | | | | |
| 8062409 | NM_030877 | CTNNBL1 | catenin, beta like 1 | 0.197 | 1.146 | 3.68E-01 | 3.68E+01 | | | | |
| 8114653 | NM_005642 | TAF7 | TAF7 RNA polymerase II, TATA box b | 0.197 | 1.146 | 4.47E-01 | 4.47E+01 | | | | |
| 8110090 | NM_022754 | SFXN1 | sideroflexin 1 | 0.197 | 1.146 | 2.36E-01 | 2.36E+01 | | | | |
| 8174692 | NM_145799 | 40427 | septin 6 | 0.197 | 1.146 | 2.62E-01 | 2.62E+01 | | | | |
| 8075217 | NM_001127 | AP1B1 | adaptor-related protein complex 1, b | 0.197 | 1.146 | 4.00E-02 | 4.00E+00 | | | | |
| 7892941 | --- | --- | --- | 0.197 | 1.146 | 3.13E-01 | 3.13E+01 | | | | |
| 7980955 | NM_004545 | NDUFB1 | NADH dehydrogenase (ubiquinone) 1 | 0.197 | 1.146 | 4.31E-01 | 4.31E+01 | | | | |
| 7974090 | NM_005930 | CTAGE5 | CTAGE family, member 5 | 0.197 | 1.146 | 2.42E-01 | 2.42E+01 | | | | |
| 7903188 | NM_021190 | PTBP2 | polypyrimidine tract binding protein | 0.197 | 1.146 | 4.88E-01 | 4.88E+01 | | | | |
| 8103728 | NM_00113068 | HMGB2 | high-mobility group box 2 | 0.197 | 1.146 | 3.92E-01 | 3.92E+01 | | | | |
| 8089743 | NM_212543 | B4GALT4 | UDP-Gal:betaGlcNAc beta 1,4- galact | 0.196 | 1.146 | 3.00E-01 | 3.00E+01 | | | | |
| 7942520 | --- | --- | --- | 0.196 | 1.146 | 1.39E-01 | 1.39E+01 | | | | |
| 8062174 | NM_198398 | ERGIC3 | ERGIC and golgi 3 | 0.196 | 1.146 | 2.57E-01 | 2.57E+01 | | | | |
| 7919197 | NM_032305 | POLR3GL | polymerase (RNA) III (DNA directed) | 0.196 | 1.146 | 1.64E-01 | 1.64E+01 | | | | |
| 8047443 | NM_018571 | STRADB | STE20-related kinase adaptor beta | 0.196 | 1.145 | 6.51E-01 | 6.51E+01 | | | | |
| 7900979 | NM_018150 | RNF220 | ring finger protein 220 | 0.196 | 1.145 | 1.64E-01 | 1.64E+01 | | | | |
| 7990165 | NM_020214 | PARP6 | poly (ADP-ribose) polymerase family | 0.196 | 1.145 | 4.35E-01 | 4.35E+01 | | | | |
| 7894765 | --- | --- | --- | 0.196 | 1.145 | 3.63E-01 | 3.63E+01 | | | | |
| 7995448 | NM_153261 | TMEM188 | transmembrane protein 188 | 0.196 | 1.145 | 1.79E-01 | 1.79E+01 | | | | |
| 8059687 | --- | --- | --- | 0.196 | 1.145 | 7.01E-02 | 7.01E+00 | | | | |
| 7893665 | --- | --- | --- | 0.195 | 1.145 | 1.71E-01 | 1.71E+01 | | | | |
| 8125470 | NM_002120 | HLA-DOB | major histocompatibility complex, cl | 0.195 | 1.145 | 4.32E-01 | 4.32E+01 | | | | |
| 8060086 | AF487338 | MYEOV2 | myeloma overexpressed 2 | 0.195 | 1.145 | 3.42E-01 | 3.42E+01 | | | | |
| 8112994 | --- | --- | --- | 0.195 | 1.145 | 3.08E-01 | 3.08E+01 | | | | |
| 8163972 | NM_00110058 | RC3H2 | ring finger and CCCH-type zinc finger | 0.195 | 1.145 | 4.53E-01 | 4.53E+01 | | | | |
| 8081256 | NM_018309 | TBC1D23 | TBC1 domain family, member 23 | 0.195 | 1.145 | 5.31E-01 | 5.31E+01 | | | | |
| 7894191 | --- | --- | --- | 0.195 | 1.145 | 4.18E-01 | 4.18E+01 | | | | |
| 8097570 | NM_032557 | USP38 | ubiquitin specific peptidase 38 | 0.195 | 1.145 | 4.03E-01 | 4.03E+01 | | | | |
| 8016725 | NM_052855 | ANKRD40 | ankyrin repeat domain 40 | 0.195 | 1.145 | 3.38E-01 | 3.38E+01 | | | | |
| 7892996 | --- | --- | --- | 0.195 | 1.145 | 3.43E-01 | 3.43E+01 | | | | |
| 7901253 | BC144026 | C1orf223 | chromosome 1 open reading frame 2 | 0.195 | 1.145 | 3.12E-01 | 3.12E+01 | | | | |
| 7919584 | NM_00102459 | HIST2H2BF | histone cluster 2, H2bf | 0.195 | 1.144 | 6.33E-01 | 6.33E+01 | | | | |
| 7905299 | NM_021222 | PRUNE | prune homolog (Drosophila) | 0.195 | 1.144 | 2.32E-01 | 2.32E+01 | | | | |
| 7896249 | --- | --- | --- | 0.195 | 1.144 | 7.70E-01 | 7.70E+01 | | | | |
| 8169645 | NM_003336 | UBE2A | ubiquitin-conjugating enzyme E2A (R | 0.194 | 1.144 | 3.92E-01 | 3.92E+01 | | | | |
| 8056930 | NM_001689 | ATP5G3 | ATP synthase, H+ transporting, mito | 0.194 | 1.144 | 6.29E-03 | 6.29E-01 | | | | |
| 8168438 | NM_145052 | UPRT | uracil phosphoribosyltransferase (FU | 0.194 | 1.144 | 4.17E-01 | 4.17E+01 | | | | |
| 7958532 | NM_130466 | UBE3B | ubiquitin protein ligase E3B | 0.194 | 1.144 | 3.17E-01 | 3.17E+01 | | | | |
| 7919438 | NM_00110266 | NBPF16 | neuroblastoma breakpoint family, m | 0.194 | 1.144 | 2.02E-02 | 2.02E+00 | | | | |
| 7894116 | --- | --- | --- | 0.194 | 1.144 | 2.69E-01 | 2.69E+01 | | | | |
| 7951734 | NM_004724 | ZW10 | ZW10, kinetochore associated, hom | 0.194 | 1.144 | 1.75E-01 | 1.75E+01 | | | | |
| 8029399 | NM_00114622 | ZNF226 | zinc finger protein 226 | 0.194 | 1.144 | 2.23E-02 | 2.23E+00 | | | | |
| 8076417 | NM_002490 | NDUFA6 | NADH dehydrogenase (ubiquinone) 1 | 0.194 | 1.144 | 5.31E-01 | 5.31E+01 | | | | |
| 7894596 | --- | --- | --- | 0.194 | 1.144 | 6.18E-01 | 6.18E+01 | | | | |
| 8028524 | NM_004924 | ACTN4 | actinin, alpha 4 | 0.194 | 1.144 | 1.70E-01 | 1.70E+01 | | | | |
| 8180323 | --- | --- | --- | 0.194 | 1.144 | 5.10E-01 | 5.10E+01 | | | | |
| 8166230 | NM_018360 | CXorf15 | chromosome X open reading frame 1 | 0.194 | 1.144 | 3.00E-01 | 3.00E+01 | | | | |
| 8003859 | --- | --- | --- | 0.194 | 1.144 | 8.34E-01 | 8.34E+01 | | | | |
| 7996860 | NM_024562 | TMCO7 | transmembrane and coiled-coil dom | 0.194 | 1.144 | 1.63E-01 | 1.63E+01 | | | | |
| 8100615 | NM_018227 | UBA6 | ubiquitin-like modifier activating enz | 0.194 | 1.144 | 4.82E-01 | 4.82E+01 | | | | |
| 7927108 | NM_014753 | BMS1 | BMS1 homolog, ribosome assembly | 0.194 | 1.144 | 4.46E-01 | 4.46E+01 | | | | |
| 7975863 | NM_017926 | C14orf118 | chromosome 14 open reading frame | 0.193 | 1.144 | 5.96E-01 | 5.96E+01 | | | | |
| 8141150 | NM_133436 | ASNS | asparagine synthetase | 0.193 | 1.143 | 3.06E-01 | 3.06E+01 | | | | |
| 7892540 | --- | --- | --- | 0.193 | 1.143 | 3.58E-01 | 3.58E+01 | | | | |
| 7893631 | --- | --- | --- | 0.193 | 1.143 | 5.60E-01 | 5.60E+01 | | | | |
| 8101511 | NM_014991 | WDFY3 | WD repeat and FYVE domain contain | 0.193 | 1.143 | 2.13E-02 | 2.13E+00 | | | | |
| 7895537 | --- | --- | --- | 0.193 | 1.143 | 8.54E-01 | 8.54E+01 | | | | |
| 8049246 | NM_00101791 | INPP5D | inositol polyphosphate-5-phosphata | 0.193 | 1.143 | 2.23E-01 | 2.23E+01 | | | | |
| 7965048 | NM_004537 | NAP1L1 | nucleosome assembly protein 1-like | 0.192 | 1.143 | 9.31E-02 | 9.31E+00 | | | | |
| 8167449 | NM_002668 | PLP2 | proteolipid protein 2 (colonic epithel | 0.192 | 1.143 | 6.71E-01 | 6.71E+01 | | | | |
| 7966427 | NM_006768 | BRAP | BRCA1 associated protein | 0.192 | 1.143 | 5.73E-01 | 5.73E+01 | | | | |
| 7956443 | NM_004990 | MARS | methionyl-tRNA synthetase | 0.192 | 1.143 | 2.79E-01 | 2.79E+01 | | | | |
| 8146637 | NM_152758 | YTHDF3 | YTH domain family, member 3 | 0.192 | 1.143 | 5.56E-01 | 5.56E+01 | | | | |
| 8042207 | NM_152516 | COMMD1 | copper metabolism (Murr1) domain | 0.192 | 1.143 | 6.57E-01 | 6.57E+01 | | | | |
| 8014702 | NM_005937 | MLLT6 | myeloid/lymphoid or mixed-lineage | 0.192 | 1.142 | 6.56E-01 | 6.56E+01 | | | | |
| 8170187 | NM_000074 | CD40LG | CD40 ligand | 0.192 | 1.142 | 3.17E-01 | 3.17E+01 | | | | |

| | | | | | | | | | | |
|---|---|---|---|---|---|---|---|---|---|---|
| 8052834 | BC005079 | C2orf42 | chromosome 2 open reading frame 4 | 0.192 | 1.142 | 2.64E-01 | 2.64E+01 | | | |
| 7986323 | NM_015710 | GLTSCR2 | glioma tumor suppressor candidate | 0.192 | 1.142 | 6.16E-01 | 6.16E+01 | | | |
| 8069448 | --- | --- | --- | 0.192 | 1.142 | 7.56E-01 | 7.56E+01 | | | |
| 8063078 | NM_00112769 | CTSA | cathepsin A | 0.192 | 1.142 | 1.98E-01 | 1.98E+01 | | | |
| 7895297 | --- | --- | --- | 0.192 | 1.142 | 5.17E-01 | 5.17E+01 | | | |
| 7975268 | NM_001172 | ARG2 | arginase, type II | 0.192 | 1.142 | 7.81E-02 | 7.81E+00 | | | |
| 8099364 | NM_053042 | ZNF518B | zinc finger protein 518B | 0.192 | 1.142 | 3.69E-01 | 3.69E+01 | | | |
| 7913256 | NM_005216 | DDOST | dolichyl-diphosphooligosaccharide-p | 0.192 | 1.142 | 3.91E-01 | 3.91E+01 | | | |
| 8150866 | --- | --- | --- | 0.192 | 1.142 | 7.43E-01 | 7.43E+01 | | | |
| 7994415 | NM_00103780 | EIF3C | eukaryotic translation initiation facto | 0.192 | 1.142 | 3.79E-01 | 3.79E+01 | | | |
| 8000507 | NM_00103780 | EIF3C | eukaryotic translation initiation facto | 0.192 | 1.142 | 3.79E-01 | 3.79E+01 | | | |
| 8019563 | NR_003682 | MGC70870 | C-terminal binding protein 2 pseudo | 0.191 | 1.142 | 1.44E-01 | 1.44E+01 | | | |
| 7932966 | NM_033666 | ITGB1 | integrin, beta 1 (fibronectin receptor | 0.191 | 1.142 | 4.66E-01 | 4.66E+01 | | | |
| 7921552 | NM_015726 | DCAF8 | DDB1 and CUL4 associated factor 8 | 0.191 | 1.142 | 2.82E-01 | 2.82E+01 | | | |
| 8131494 | --- | --- | --- | 0.191 | 1.142 | 4.05E-01 | 4.05E+01 | | | |
| 7892780 | --- | --- | --- | 0.191 | 1.142 | 6.43E-01 | 6.43E+01 | | | |
| 8139198 | NM_152682 | RWDD4A | RWD domain containing 4A | 0.191 | 1.142 | 7.50E-01 | 7.50E+01 | | | |
| 8117081 | NM_153042 | AOF1 | amine oxidase (flavin containing) do | 0.191 | 1.141 | 3.01E-01 | 3.01E+01 | | | |
| 7987475 | NM_004573 | PLCB2 | phospholipase C, beta 2 | 0.191 | 1.141 | 4.67E-02 | 4.67E+00 | | | |
| 8134699 | NM_006833 | COPS6 | COP9 constitutive photomorphogeni | 0.191 | 1.141 | 5.82E-01 | 5.82E+01 | | | |
| 8147156 | NM_007013 | WWP1 | WW domain containing E3 ubiquitin | 0.191 | 1.141 | 4.47E-01 | 4.47E+01 | | | |
| 8114612 | NM_000591 | CD14 | CD14 molecule | 0.191 | 1.141 | 4.04E-01 | 4.04E+01 | | | mono |
| 7914180 | NM_014752 | SPCS2 | signal peptidase complex subunit 2 h | 0.191 | 1.141 | 7.57E-01 | 7.57E+01 | | | |
| 8065165 | NM_152227 | SNX5 | sorting nexin 5 | 0.191 | 1.141 | 1.24E-01 | 1.24E+01 | | | |
| 7915101 | NM_005540 | INPP5B | inositol polyphosphate-5-phosphata | 0.190 | 1.141 | 3.09E-01 | 3.09E+01 | | | |
| 7894258 | --- | --- | --- | 0.190 | 1.141 | 6.78E-01 | 6.78E+01 | | | |
| 7894536 | --- | --- | --- | 0.190 | 1.141 | 2.40E-02 | 2.40E+00 | | | |
| 8112043 | --- | --- | --- | 0.190 | 1.141 | 6.29E-01 | 6.29E+01 | | | |
| 8133326 | NM_172020 | POM121 | POM121 membrane glycoprotein (ra | 0.190 | 1.141 | 6.83E-01 | 6.83E+01 | | | |
| 8113023 | NM_153354 | TMEM161B | transmembrane protein 161B | 0.190 | 1.141 | 4.93E-01 | 4.93E+01 | | | |
| 8000869 | NM_052838 | 40422 | septin 1 | 0.190 | 1.141 | 2.20E-01 | 2.20E+01 | | | |
| 8077458 | NM_014674 | EDEM1 | ER degradation enhancer, mannosid | 0.190 | 1.141 | 4.12E-01 | 4.12E+01 | | | |
| 8143327 | NM_022750 | PARP12 | poly (ADP-ribose) polymerase family | 0.190 | 1.141 | 2.52E-01 | 2.52E+01 | | | |
| 7950294 | NM_016565 | CHCHD8 | coiled-coil-helix-coiled-coil-helix dom | 0.190 | 1.141 | 4.29E-02 | 4.29E+00 | | | |
| 8014603 | NM_00112339 | TBC1D3 | TBC1 domain family, member 3 | 0.190 | 1.141 | 6.27E-02 | 6.27E+00 | | | |
| 8143417 | NM_004333 | BRAF | v-raf murine sarcoma viral oncogene | 0.190 | 1.141 | 3.39E-01 | 3.39E+01 | | | |
| 7896060 | --- | --- | --- | 0.190 | 1.141 | 1.36E-02 | 1.36E+00 | | | |
| 8125530 | NM_002118 | HLA-DMB | major histocompatibility complex, cl | 0.190 | 1.141 | 5.07E-01 | 5.07E+01 | | | |
| 8110499 | NM_025158 | RUFY1 | RUN and FYVE domain containing 1 | 0.190 | 1.141 | 1.96E-01 | 1.96E+01 | | | |
| 7919351 | NM_183372 | RP11-94I2.2 | neuroblastoma breakpoint family, m | 0.190 | 1.141 | 2.33E-02 | 2.33E+00 | | | |
| 8068218 | --- | --- | --- | 0.190 | 1.141 | 3.32E-01 | 3.32E+01 | | | |
| 7933115 | NR_027269 | CDC10L | CDC10 cell division cycle 10 homolog | 0.190 | 1.141 | 3.81E-01 | 3.81E+01 | | | |
| 7896140 | --- | --- | --- | 0.190 | 1.141 | 5.20E-01 | 5.20E+01 | | | |
| 8026007 | NM_153358 | ZNF791 | zinc finger protein 791 | 0.190 | 1.141 | 5.55E-01 | 5.55E+01 | | | |
| 7919747 | --- | --- | --- | 0.190 | 1.141 | 7.97E-01 | 7.97E+01 | | | |
| 8168447 | BC001220 | CXorf26 | chromosome X open reading frame 2 | 0.190 | 1.141 | 2.96E-01 | 2.96E+01 | | | |
| 8135876 | NM_014390 | SND1 | staphylococcal nuclease and tudor d | 0.190 | 1.141 | 3.00E-01 | 3.00E+01 | | | |
| 8052399 | NM_022893 | BCL11A | B-cell CLL/lymphoma 11A (zinc finge | 0.190 | 1.140 | 8.66E-02 | 8.66E+00 | | | |
| 8071920 | NM_004175 | SNRPD3 | small nuclear ribonucleoprotein D3 p | 0.190 | 1.140 | 1.99E-01 | 1.99E+01 | | | |
| 7895687 | --- | --- | --- | 0.189 | 1.140 | 2.37E-02 | 2.37E+00 | | | |
| 7958000 | NM_020244 | CHPT1 | choline phosphotransferase 1 | 0.189 | 1.140 | 4.68E-01 | 4.68E+01 | | | |
| 7935863 | --- | --- | --- | 0.189 | 1.140 | 4.43E-01 | 4.43E+01 | | | |
| 8093039 | NR_003264 | SDHALP1 | succinate dehydrogenase complex, s | 0.189 | 1.140 | 2.34E-01 | 2.34E+01 | | | |
| 7894774 | --- | --- | --- | 0.189 | 1.140 | 4.32E-01 | 4.32E+01 | | | |
| 8066417 | NM_006811 | SERINC3 | serine incorporator 3 | 0.189 | 1.140 | 4.00E-01 | 4.00E+01 | | | |
| 7963698 | NM_00113005 | ATF7 | activating transcription factor 7 | 0.189 | 1.140 | 1.38E-01 | 1.38E+01 | | | |
| 8099760 | NM_015230 | ARAP2 | ArfGAP with RhoGAP domain, ankyri | 0.189 | 1.140 | 5.13E-01 | 5.13E+01 | | | |
| 7892924 | --- | --- | --- | 0.189 | 1.140 | 1.91E-01 | 1.91E+01 | | | |
| 7950469 | --- | --- | --- | 0.189 | 1.140 | 9.16E-02 | 9.16E+00 | | | |
| 7893868 | --- | --- | --- | 0.189 | 1.140 | 4.73E-01 | 4.73E+01 | | | |
| 8000636 | NM_130464 | NPIPL3 | nuclear pore complex interacting pro | 0.188 | 1.140 | 5.40E-01 | 5.40E+01 | | | |
| 7959267 | NM_002560 | P2RX4 | purinergic receptor P2X, ligand-gate | 0.188 | 1.140 | 3.35E-01 | 3.35E+01 | | | |
| 7893074 | --- | --- | --- | 0.188 | 1.139 | 8.31E-01 | 8.31E+01 | | | |
| 8013272 | NM_014695 | CCDC144A | coiled-coil domain containing 144A | 0.188 | 1.139 | 4.04E-01 | 4.04E+01 | | | |
| 8131871 | NM_138771 | CCDC126 | coiled-coil domain containing 126 | 0.188 | 1.139 | 9.49E-02 | 9.49E+00 | | | |
| 7936661 | NM_006793 | PRDX3 | peroxiredoxin 3 | 0.188 | 1.139 | 2.70E-01 | 2.70E+01 | | | |
| 7951679 | NM_012459 | TIMM8B | translocase of inner mitochondrial m | 0.188 | 1.139 | 2.29E-01 | 2.29E+01 | | | |
| 7964466 | NM_006400 | DCTN2 | dynactin 2 (p50) | 0.188 | 1.139 | 5.85E-01 | 5.85E+01 | | | |
| 7895561 | --- | --- | --- | 0.188 | 1.139 | 3.77E-01 | 3.77E+01 | | | |
| 7894999 | --- | --- | --- | 0.187 | 1.139 | 9.93E-02 | 9.93E+00 | | | |
| 7942553 | NM_014752 | SPCS2 | signal peptidase complex subunit 2 h | 0.187 | 1.139 | 7.38E-01 | 7.38E+01 | | | |
| 7920409 | NM_006694 | JTB | jumping translocation breakpoint | 0.187 | 1.139 | 3.51E-01 | 3.51E+01 | | | |
| 7895827 | --- | --- | --- | 0.187 | 1.139 | 4.49E-02 | 4.49E+00 | | | |
| 7941769 | NM_012308 | KDM2A | lysine (K)-specific demethylase 2A | 0.187 | 1.138 | 3.15E-01 | 3.15E+01 | | | |
| 8093961 | NM_014743 | KIAA0232 | KIAA0232 | 0.187 | 1.138 | 7.24E-02 | 7.24E+00 | | | |
| 8044669 | NM_013412 | RABL2A | RAB, member of RAS oncogene fami | 0.187 | 1.138 | 2.93E-01 | 2.93E+01 | | | |
| 8046997 | NM_019048 | ASNSD1 | asparagine synthetase domain conta | 0.187 | 1.138 | 2.94E-01 | 2.94E+01 | | | |

| | | | | | | | | | | |
|---|---|---|---|---|---|---|---|---|---|---|
| 8027205 | NM_015965 | NDUFA13 | NADH dehydrogenase (ubiquinone) 1 | 0.187 | 1.138 | 4.91E-01 | 4.91E+01 | | | |
| 8163023 | NM_003640 | IKBKAP | inhibitor of kappa light polypeptide g | 0.187 | 1.138 | 3.98E-01 | 3.98E+01 | | | |
| 8066822 | NM_018837 | SULF2 | sulfatase 2 | 0.187 | 1.138 | 8.12E-02 | 8.12E+00 | | | mono |
| 8011141 | NM_006445 | PRPF8 | PRP8 pre-mRNA processing factor 8 I | 0.187 | 1.138 | 2.55E-01 | 2.55E+01 | | | |
| 8136654 | --- | --- | --- | 0.187 | 1.138 | 6.32E-01 | 6.32E+01 | | | |
| 7904999 | NM_00110266: | NBPF16 | neuroblastoma breakpoint family, m | 0.186 | 1.138 | 1.63E-02 | 1.63E+00 | | | |
| 8100971 | NM_002704 | PPBP | pro-platelet basic protein (chemokin | 0.186 | 1.138 | 6.55E-01 | 6.55E+01 | | | |
| 8090898 | NM_005862 | STAG1 | stromal antigen 1 | 0.186 | 1.138 | 5.82E-01 | 5.82E+01 | | | |
| 8144667 | --- | --- | --- | 0.186 | 1.138 | 4.83E-02 | 4.83E+00 | | | |
| 7895528 | --- | --- | --- | 0.186 | 1.138 | 6.94E-01 | 6.94E+01 | | | |
| 7895107 | --- | --- | --- | 0.186 | 1.138 | 4.33E-01 | 4.33E+01 | | | |
| 7929012 | NM_020799 | STAMBPL1 | STAM binding protein-like 1 | 0.186 | 1.138 | 5.62E-01 | 5.62E+01 | | | |
| 7895817 | --- | --- | --- | 0.186 | 1.138 | 7.18E-01 | 7.18E+01 | | | |
| 8112865 | NM_178276 | SERINC5 | serine incorporator 5 | 0.186 | 1.138 | 4.37E-01 | 4.37E+01 | | | |
| 8004485 | NM_015670 | SENP3 | SUMO1/sentrin/SMT3 specific peptic | 0.186 | 1.138 | 1.68E-01 | 1.68E+01 | | | |
| 8015955 | NM_020218 | ATXN7L3 | ataxin 7-like 3 | 0.186 | 1.138 | 7.75E-02 | 7.75E+00 | | | |
| 7893184 | --- | --- | --- | 0.186 | 1.138 | 9.10E-02 | 9.10E+00 | | | |
| 8021756 | NM_017757 | ZNF407 | zinc finger protein 407 | 0.186 | 1.138 | 3.80E-01 | 3.80E+01 | | | |
| 7892512 | --- | --- | --- | 0.186 | 1.137 | 4.73E-01 | 4.73E+01 | | | |
| 7967898 | --- | --- | --- | 0.186 | 1.137 | 1.71E-01 | 1.71E+01 | | | |
| 7957167 | NM_018279 | TMEM19 | transmembrane protein 19 | 0.186 | 1.137 | 5.40E-01 | 5.40E+01 | | | |
| 8126646 | NM_018135 | MRPS18A | mitochondrial ribosomal protein S18 | 0.186 | 1.137 | 2.92E-01 | 2.92E+01 | | | |
| 8083221 | NM_002586 | PBX2 | pre-B-cell leukemia homeobox 2 | 0.186 | 1.137 | 3.79E-01 | 3.79E+01 | | | |
| 8014008 | NM_000386 | BLMH | bleomycin hydrolase | 0.186 | 1.137 | 4.13E-01 | 4.13E+01 | | | |
| 7946933 | NM_012139 | SERGEF | secretion regulating guanine nucleot | 0.186 | 1.137 | 4.20E-01 | 4.20E+01 | | | |
| 8011884 | NM_033004 | NLRP1 | NLR family, pyrin domain containing | 0.186 | 1.137 | 4.84E-02 | 4.84E+00 | | | |
| 8085628 | NM_015199 | ANKRD28 | ankyrin repeat domain 28 | 0.186 | 1.137 | 5.17E-01 | 5.17E+01 | | | |
| 7999614 | NM_006985 | NPIP | nuclear pore complex interacting prc | 0.185 | 1.137 | 3.82E-01 | 3.82E+01 | | | |
| 8156750 | NM_006401 | ANP32B | acidic (leucine-rich) nuclear phospho | 0.185 | 1.137 | 1.85E-01 | 1.85E+01 | | | |
| 8046515 | NM_002107 // | 3F3A // H3F3 | H3 histone, family 3A // H3 histone, | 0.185 | 1.137 | 6.00E-02 | 6.00E+00 | | | |
| 7910124 | NM_002107 // | 3F3A // H3F3 | H3 histone, family 3A // H3 histone, | 0.185 | 1.137 | 3.94E-02 | 3.94E+00 | | | |
| 7961489 | NM_016312 | WBP11 | WW domain binding protein 11 | 0.185 | 1.137 | 6.08E-01 | 6.08E+01 | | | |
| 7988644 | NM_024837 | ATP8B4 | ATPase, class I, type 8B, member 4 | 0.185 | 1.137 | 1.10E-01 | 1.10E+01 | | | |
| 7911619 | NM_002074 | GNB1 | guanine nucleotide binding protein ( | 0.185 | 1.137 | 4.41E-01 | 4.41E+01 | | | |
| 8124469 | BC171739 | GUSBL1 | glucuronidase, beta-like 1 | 0.185 | 1.137 | 4.58E-01 | 4.58E+01 | | | |
| 8006746 | NM_00112339 | TBC1D3 | TBC1 domain family, member 3 | 0.185 | 1.137 | 7.84E-02 | 7.84E+00 | | | |
| 8027685 | NM_00102999 | ZNF181 | zinc finger protein 181 | 0.185 | 1.137 | 3.46E-01 | 3.46E+01 | | | |
| 8138613 | NM_015550 | OSBPL3 | oxysterol binding protein-like 3 | 0.185 | 1.137 | 1.75E-01 | 1.75E+01 | | | |
| 8129974 | NM_032020 | FUCA2 | fucosidase, alpha-L- 2, plasma | 0.185 | 1.136 | 4.46E-01 | 4.46E+01 | | | |
| 8108475 | NM_00100718 | C5orf53 | chromosome 5 open reading frame 5 | 0.184 | 1.136 | 5.01E-01 | 5.01E+01 | | | |
| 7995655 | NM_00108043 | FTO | fat mass and obesity associated | 0.184 | 1.136 | 2.77E-01 | 2.77E+01 | | | |
| 8103289 | NM_002669 | PLRG1 | pleiotropic regulator 1 (PRL1 homolc | 0.184 | 1.136 | 4.94E-01 | 4.94E+01 | | | |
| 8103646 | NM_012224 | NEK1 | NIMA (never in mitosis gene a)-relate | 0.184 | 1.136 | 6.17E-01 | 6.17E+01 | | | |
| 8142120 | NM_005746 | NAMPT | nicotinamide phosphoribosyltransfer | 0.184 | 1.136 | 5.60E-01 | 5.60E+01 | | | |
| 7896511 | --- | --- | --- | 0.184 | 1.136 | 1.33E-03 | 1.33E-01 | | | |
| 7931187 | NM_004725 | BUB3 | budding uninhibited by benzimidazo | 0.184 | 1.136 | 5.79E-01 | 5.79E+01 | | | |
| 8138857 | NM_024051 | GGCT | gamma-glutamyl cyclotransferase | 0.184 | 1.136 | 1.78E-01 | 1.78E+01 | | | |
| 8097128 | NM_00103419 | EXOSC9 | exosome component 9 | 0.184 | 1.136 | 5.07E-01 | 5.07E+01 | | | |
| 8135204 | NM_00103161 | SPDYE2 | speedy homolog E2 (Xenopus laevis) | 0.184 | 1.136 | 2.22E-01 | 2.22E+01 | | | |
| 8043443 | --- | --- | --- | 0.184 | 1.136 | 2.89E-01 | 2.89E+01 | | | |
| 7928695 | NM_032333 | C10orf58 | chromosome 10 open reading frame | 0.184 | 1.136 | 3.30E-01 | 3.30E+01 | | | |
| 8110392 | NM_017510 | TMED9 | transmembrane emp24 protein trans | 0.184 | 1.136 | 3.08E-01 | 3.08E+01 | | | |
| 8046380 | NM_000210 | ITGA6 | integrin, alpha 6 | 0.184 | 1.136 | 5.02E-01 | 5.02E+01 | | | |
| 7895737 | --- | --- | --- | 0.184 | 1.136 | 4.15E-01 | 4.15E+01 | | | |
| 8138824 | NM_014766 | SCRN1 | secernin 1 | 0.184 | 1.136 | 2.72E-01 | 2.72E+01 | | | |
| 7907531 | NM_005684 | GPR52 | G protein-coupled receptor 52 | 0.183 | 1.136 | 2.01E-01 | 2.01E+01 | | | |
| 8025788 | NM_00112884 | SMARCA4 | SWI/SNF related, matrix associated, | 0.183 | 1.135 | 6.27E-02 | 6.27E+00 | | | |
| 8145134 | --- | --- | --- | 0.183 | 1.135 | 3.83E-01 | 3.83E+01 | | | |
| 8079079 | NM_005385 | NKTR | natural killer-tumor recognition sequ | 0.183 | 1.135 | 4.02E-01 | 4.02E+01 | | | |
| 8152323 | NM_001568 | EIF3E | eukaryotic translation initiation facto | 0.183 | 1.135 | 5.25E-01 | 5.25E+01 | | | |
| 7923917 | NM_005449 | FAIM3 | Fas apoptotic inhibitory molecule 3 | 0.183 | 1.135 | 3.99E-01 | 3.99E+01 | | | |
| 8001537 | NM_024946 | FAM192A | family with sequence similarity 192, | 0.183 | 1.135 | 4.43E-01 | 4.43E+01 | | | |
| 8159992 | NM_024896 | ERMP1 | endoplasmic reticulum metallopepti | 0.183 | 1.135 | 1.64E-01 | 1.64E+01 | | | |
| 8160346 | NM_00101091 | PTPLAD2 | protein tyrosine phosphatase-like A | 0.183 | 1.135 | 6.92E-01 | 6.92E+01 | | | |
| 8105989 | AK302597 | POM121L8P | POM121 membrane glycoprotein-lik | 0.183 | 1.135 | 4.25E-01 | 4.25E+01 | | | |
| 8096955 | --- | --- | --- | 0.183 | 1.135 | 1.23E-01 | 1.23E+01 | | | |
| 7894957 | --- | --- | --- | 0.183 | 1.135 | 8.38E-01 | 8.38E+01 | | | |
| 7940914 | NM_004470 | FKBP2 | FK506 binding protein 2, 13kDa | 0.182 | 1.135 | 2.43E-01 | 2.43E+01 | | | |
| 7893830 | --- | --- | --- | 0.182 | 1.135 | 7.60E-01 | 7.60E+01 | | | |
| 8155770 | NM_015110 | SMC5 | structural maintenance of chromoso | 0.182 | 1.135 | 3.82E-01 | 3.82E+01 | | | |
| 8064003 | BC046933 | TCFL5 | transcription factor-like 5 (basic helix | 0.182 | 1.134 | 4.38E-01 | 4.38E+01 | | | |
| 8173549 | --- | --- | --- | 0.182 | 1.134 | 2.54E-01 | 2.54E+01 | | | |
| 8180364 | --- | --- | --- | 0.182 | 1.134 | 4.16E-01 | 4.16E+01 | | | |
| 8180365 | --- | --- | --- | 0.182 | 1.134 | 4.16E-01 | 4.16E+01 | | | |
| 7973840 | NM_00103005 | ARHGAP5 | Rho GTPase activating protein 5 | 0.182 | 1.134 | 1.29E-01 | 1.29E+01 | | | |
| 7893627 | --- | --- | --- | 0.182 | 1.134 | 6.78E-01 | 6.78E+01 | | | |
| 8104201 | NM_007277 | EXOC3 | exocyst complex component 3 | 0.182 | 1.134 | 1.93E-01 | 1.93E+01 | | | |

| | | | | | | | | | | |
|---|---|---|---|---|---|---|---|---|---|---|
| 8117485 | NM_078476 | BTN2A1 | butyrophilin, subfamily 2, member A | 0.182 | 1.134 | 1.46E-01 | 1.46E+01 | | | |
| 8070988 | NM_003906 | MCM3AP | minichromosome maintenance comp | 0.182 | 1.134 | 1.41E-01 | 1.41E+01 | | | |
| 8093398 | NM_006315 | PCGF3 | polycomb group ring finger 3 | 0.182 | 1.134 | 1.46E-01 | 1.46E+01 | | | |
| 8167790 | NM_058163 | TSR2 | TSR2, 20S rRNA accumulation, homo | 0.182 | 1.134 | 4.75E-01 | 4.75E+01 | | | |
| 8045136 | NM_032144 | RAB6C | RAB6C, member RAS oncogene famil | 0.182 | 1.134 | 1.25E-01 | 1.25E+01 | | | |
| 8033801 | NM_00113003 | ZNF562 | zinc finger protein 562 | 0.182 | 1.134 | 3.27E-01 | 3.27E+01 | | | |
| 7939072 | NM_152636 | METT5D1 | methyltransferase 5 domain contain | 0.182 | 1.134 | 3.10E-01 | 3.10E+01 | | | |
| 7964089 | NM_014871 | PAN2 | PAN2 poly(A) specific ribonuclease s | 0.181 | 1.134 | 8.44E-02 | 8.44E+00 | | | |
| 8113130 | NM_024717 | MCTP1 | multiple C2 domains, transmembran | 0.181 | 1.134 | 3.53E-01 | 3.53E+01 | | | |
| 7915091 | NM_005955 | MTF1 | metal-regulatory transcription factor | 0.181 | 1.134 | 4.83E-01 | 4.83E+01 | | | |
| 8011823 | NM_032530 | ZNF594 | zinc finger protein 594 | 0.181 | 1.134 | 3.30E-01 | 3.30E+01 | | | |
| 7983734 | NM_014548 | TMOD2 | tropomodulin 2 (neuronal) | 0.181 | 1.134 | 4.01E-01 | 4.01E+01 | | | |
| 8064833 | NM_00100992 | C20orf30 | chromosome 20 open reading frame | 0.181 | 1.134 | 5.92E-01 | 5.92E+01 | | | |
| 8135277 | NM_182931 | MLL5 | myeloid/lymphoid or mixed-lineage | 0.181 | 1.134 | 2.67E-01 | 2.67E+01 | | | |
| 7959834 | NM_144669 | GLT1D1 | glycosyltransferase 1 domain contai | 0.181 | 1.134 | 2.98E-02 | 2.98E+00 | | | |
| 7973770 | NM_016106 | SCFD1 | sec1 family domain containing 1 | 0.181 | 1.134 | 7.00E-01 | 7.00E+01 | | | |
| 7895908 | --- | --- | --- | 0.181 | 1.134 | 4.01E-01 | 4.01E+01 | | | |
| 8124828 | NM_005803 | FLOT1 | flotillin 1 | 0.181 | 1.134 | 4.74E-01 | 4.74E+01 | | | |
| 8178419 | NM_005803 | FLOT1 | flotillin 1 | 0.181 | 1.134 | 4.74E-01 | 4.74E+01 | | | |
| 8179688 | NM_005803 | FLOT1 | flotillin 1 | 0.181 | 1.134 | 4.74E-01 | 4.74E+01 | | | |
| 7896124 | --- | --- | --- | 0.181 | 1.134 | 2.85E-01 | 2.85E+01 | | | |
| 7919271 | NM_183372 | RP11-94I2.2 | neuroblastoma breakpoint family, m | 0.181 | 1.134 | 2.53E-02 | 2.53E+00 | | | |
| 8076339 | NM_032758 | PHF5A | PHD finger protein 5A | 0.181 | 1.133 | 3.05E-01 | 3.05E+01 | | | |
| 7949948 | NM_022338 | C11orf24 | chromosome 11 open reading frame | 0.181 | 1.133 | 1.66E-01 | 1.66E+01 | | | |
| 8172088 | NM_00112338 | BCOR | BCL6 co-repressor | 0.181 | 1.133 | 1.19E-01 | 1.19E+01 | | | |
| 7973352 | NM_014045 | LRP10 | low density lipoprotein receptor-rela | 0.180 | 1.133 | 1.01E-01 | 1.01E+01 | | | |
| 7942174 | NM_003626 | PPFIA1 | protein tyrosine phosphatase, recep | 0.180 | 1.133 | 2.23E-01 | 2.23E+01 | | | |
| 8176698 | NM_00100585 | CYorf15A | chromosome Y open reading frame 1 | 0.180 | 1.133 | 7.01E-01 | 7.01E+01 | | | |
| 8112519 | --- | --- | --- | 0.180 | 1.133 | 1.45E-01 | 1.45E+01 | | | |
| 8131111 | NM_00103728 | EIF3B | eukaryotic translation initiation facto | 0.180 | 1.133 | 2.36E-01 | 2.36E+01 | | | |
| 8136388 | NM_018295 | TMEM140 | transmembrane protein 140 | 0.180 | 1.133 | 1.05E-01 | 1.05E+01 | | | |
| 7893587 | --- | --- | --- | 0.180 | 1.133 | 6.62E-01 | 6.62E+01 | | | |
| 7936567 | NM_014904 | RAB11FIP2 | RAB11 family interacting protein 2 (c | 0.180 | 1.133 | 5.36E-01 | 5.36E+01 | | | |
| 7993281 | NM_00108053 | SNX29 | sorting nexin 29 | 0.180 | 1.133 | 2.84E-01 | 2.84E+01 | | | |
| 8138670 | NM_031243 | HNRNPA2B1 | heterogeneous nuclear ribonucleopr | 0.180 | 1.133 | 1.37E-02 | 1.37E+00 | | | |
| 8158918 | NM_012204 | GTF3C4 | general transcription factor IIIC, poly | 0.180 | 1.133 | 2.75E-01 | 2.75E+01 | | | |
| 8125649 | NM_022553 | VPS52 | vacuolar protein sorting 52 homolog | 0.180 | 1.133 | 2.62E-01 | 2.62E+01 | | | |
| 8178917 | NM_022553 | VPS52 | vacuolar protein sorting 52 homolog | 0.180 | 1.133 | 2.62E-01 | 2.62E+01 | | | |
| 8143459 | --- | --- | --- | 0.180 | 1.133 | 5.98E-01 | 5.98E+01 | | | |
| 7947969 | NM_015308 | FNBP4 | formin binding protein 4 | 0.180 | 1.133 | 4.17E-01 | 4.17E+01 | | | |
| 7895899 | --- | --- | --- | 0.180 | 1.133 | 7.22E-03 | 7.22E-01 | | | |
| 7893058 | --- | --- | --- | 0.179 | 1.132 | 4.55E-01 | 4.55E+01 | | | |
| 7972810 | NM_024537 | CARS2 | cysteinyl-tRNA synthetase 2, mitoch | 0.179 | 1.132 | 1.74E-01 | 1.74E+01 | | | |
| 8180200 | --- | --- | --- | 0.179 | 1.132 | 6.20E-01 | 6.20E+01 | | | |
| 7893682 | --- | --- | --- | 0.179 | 1.132 | 8.66E-01 | 8.66E+01 | | | |
| 8135250 | NM_002803 | PSMC2 | proteasome (prosome, macropain) 2 | 0.179 | 1.132 | 5.89E-01 | 5.89E+01 | | | |
| 8004521 | NR_024603 | MPDU1 | mannose-P-dolichol utilization defec | 0.179 | 1.132 | 4.14E-01 | 4.14E+01 | | | |
| 7993433 | NM_015027 | PDXDC1 | pyridoxal-dependent decarboxylase | 0.179 | 1.132 | 3.33E-01 | 3.33E+01 | | | |
| 8088106 | NM_00113505 | TKT | transketolase | 0.179 | 1.132 | 7.91E-02 | 7.91E+00 | | | |
| 8169519 | NM_019045 | WDR44 | WD repeat domain 44 | 0.179 | 1.132 | 5.93E-01 | 5.93E+01 | | | |
| 8043283 | NM_018433 | KDM3A | lysine (K)-specific demethylase 3A | 0.179 | 1.132 | 5.44E-01 | 5.44E+01 | | | |
| 7941511 | NM_006842 | SF3B2 | splicing factor 3b, subunit 2, 145kDa | 0.179 | 1.132 | 2.27E-01 | 2.27E+01 | | | |
| 8179519 | NM_002121 | HLA-DPB1 | major histocompatibility complex, cla | 0.179 | 1.132 | 4.98E-01 | 4.98E+01 | | | |
| 8105987 | --- | --- | --- | 0.179 | 1.132 | 9.49E-02 | 9.49E+00 | | | |
| 8166493 | NM_001415 | EIF2S3 | eukaryotic translation initiation facto | 0.179 | 1.132 | 4.61E-01 | 4.61E+01 | | | |
| 7892952 | --- | --- | --- | 0.179 | 1.132 | 6.53E-01 | 6.53E+01 | | | |
| 7900454 | NM_022774 | DEM1 | defects in morphology 1 homolog (S. | 0.178 | 1.132 | 3.81E-01 | 3.81E+01 | | | |
| 8081465 | NM_00114256 | BBX | bobby sox homolog (Drosophila) | 0.178 | 1.132 | 4.27E-01 | 4.27E+01 | | | |
| 8144089 | NM_022458 | LMBR1 | limb region 1 homolog (mouse) | 0.178 | 1.132 | 4.13E-01 | 4.13E+01 | | | |
| 7936011 | NM_024040 | CUEDC2 | CUE domain containing 2 | 0.178 | 1.132 | 2.10E-01 | 2.10E+01 | | | |
| 8138116 | NM_016265 | ZNF12 | zinc finger protein 12 | 0.178 | 1.132 | 4.03E-01 | 4.03E+01 | | | |
| 8108127 | NM_001745 | CAMLG | calcium modulating ligand | 0.178 | 1.132 | 5.17E-01 | 5.17E+01 | | | |
| 7985662 | NM_002605 | PDE8A | phosphodiesterase 8A | 0.178 | 1.132 | 5.27E-01 | 5.27E+01 | | | |
| 7896148 | --- | --- | --- | 0.178 | 1.131 | 3.92E-01 | 3.92E+01 | | | |
| 7922637 | NM_145034 | TOR1AIP2 | torsin A interacting protein 2 | 0.178 | 1.131 | 1.53E-01 | 1.53E+01 | | | |
| 8018288 | NM_006356 | ATP5H | ATP synthase, H+ transporting, mitoc | 0.178 | 1.131 | 3.58E-01 | 3.58E+01 | | | |
| 8156228 | NM_001912 | CTSL1 | cathepsin L1 | 0.178 | 1.131 | 4.31E-01 | 4.31E+01 | | | |
| 8178802 | 0 | 0 | 0 | 0.178 | 1.131 | 2.83E-01 | 2.83E+01 | | | |
| 7894211 | --- | --- | --- | 0.178 | 1.131 | 7.40E-01 | 7.40E+01 | | | |
| 8155073 | NM_174923 | CCDC107 | coiled-coil domain containing 107 | 0.178 | 1.131 | 2.37E-01 | 2.37E+01 | | | |
| 8006214 | NM_018404 | ADAP2 | ArfGAP with dual PH domains 2 | 0.178 | 1.131 | 2.97E-01 | 2.97E+01 | | | |
| 7896362 | --- | --- | --- | 0.178 | 1.131 | 4.71E-01 | 4.71E+01 | | | |
| 7893482 | --- | --- | --- | 0.178 | 1.131 | 7.99E-01 | 7.99E+01 | | | |
| 8016018 | NM_00114378 | SLC25A39 | solute carrier family 25, member 39 | 0.178 | 1.131 | 1.35E-01 | 1.35E+01 | | | |
| 7963614 | NM_000889 | ITGB7 | integrin, beta 7 | 0.178 | 1.131 | 1.26E-02 | 1.26E+00 | | | |
| 8092627 | NM_018138 | TBCCD1 | TBCC domain containing 1 | 0.177 | 1.131 | 1.62E-01 | 1.62E+01 | | | |
| 8107578 | NM_152546 | SRFBP1 | serum response factor binding prote | 0.177 | 1.131 | 5.74E-01 | 5.74E+01 | | | |

| | | | | | | | | | | |
|---|---|---|---|---|---|---|---|---|---|---|
| 7911038 | NM_205768 | ZNF238 | zinc finger protein 238 | 0.177 | 1.131 | 4.20E-01 | 4.20E+01 | | | |
| 7893123 | --- | --- | --- | 0.177 | 1.131 | 8.21E-01 | 8.21E+01 | | | |
| 7898337 | NM_018090 | NECAP2 | NECAP endocytosis associated 2 | 0.177 | 1.131 | 4.19E-01 | 4.19E+01 | | | |
| 7938890 | NM_005788 | PRMT3 | protein arginine methyltransferase 3 | 0.177 | 1.131 | 4.95E-01 | 4.95E+01 | | | |
| 8084423 | NM_002808 | PSMD2 | proteasome (prosome, macropain) 2 | 0.177 | 1.131 | 2.75E-01 | 2.75E+01 | | | |
| 7957536 | uc001tcn.1 | NUDT4 | nudix (nucleoside diphosphate linked | 0.177 | 1.131 | 2.45E-01 | 2.45E+01 | | | |
| 8117834 | NM_021959 | PPP1R11 | protein phosphatase 1, regulatory (in | 0.177 | 1.130 | 4.92E-01 | 4.92E+01 | | | |
| 8177744 | NM_021959 | PPP1R11 | protein phosphatase 1, regulatory (in | 0.177 | 1.130 | 4.92E-01 | 4.92E+01 | | | |
| 7927595 | --- | --- | --- | 0.177 | 1.130 | 4.21E-01 | 4.21E+01 | | | |
| 7943853 | NM_003002 | SDHD | succinate dehydrogenase complex, s | 0.177 | 1.130 | 1.40E-01 | 1.40E+01 | | | |
| 7893913 | --- | --- | --- | 0.177 | 1.130 | 7.41E-01 | 7.41E+01 | | | |
| 8130962 | NM_002598 | PDCD2 | programmed cell death 2 | 0.177 | 1.130 | 8.62E-02 | 8.62E+00 | | | |
| 8132642 | NM_021130 | PPIA | peptidylprolyl isomerase A (cyclophil | 0.176 | 1.130 | 1.19E-01 | 1.19E+01 | | | |
| 7893955 | --- | --- | --- | 0.176 | 1.130 | 8.44E-01 | 8.44E+01 | | | |
| 7958828 | NM_00114390( | TRAFD1 | TRAF-type zinc finger domain contai | 0.176 | 1.130 | 1.67E-01 | 1.67E+01 | | | |
| 7895722 | --- | --- | --- | 0.176 | 1.130 | 8.94E-01 | 8.94E+01 | | | |
| 8047659 | NM_005759 | ABI2 | abl-interactor 2 | 0.176 | 1.130 | 1.29E-01 | 1.29E+01 | | | |
| 8056909 | NM_001880 | ATF2 | activating transcription factor 2 | 0.176 | 1.130 | 3.48E-01 | 3.48E+01 | | | |
| 7917906 | NM_000971 | RPL7 | ribosomal protein L7 | 0.176 | 1.130 | 2.80E-01 | 2.80E+01 | | | |
| 7976648 | NM_00109940: | CCNK | cyclin K | 0.176 | 1.130 | 3.83E-01 | 3.83E+01 | | | |
| 7895575 | --- | --- | --- | 0.176 | 1.130 | 7.57E-01 | 7.57E+01 | | | |
| 7893556 | --- | --- | --- | 0.176 | 1.130 | 7.57E-01 | 7.57E+01 | | | |
| 8033587 | NM_032152 | PRAM1 | PML-RARA regulated adaptor molecu | 0.176 | 1.130 | 6.11E-02 | 6.11E+00 | | | |
| 8049512 | NM_00113755: | LRRFIP1 | leucine rich repeat (in FLII) interactin | 0.176 | 1.130 | 2.31E-01 | 2.31E+01 | | | |
| 8134680 | NM_003439 | ZKSCAN1 | zinc finger with KRAB and SCAN dom | 0.176 | 1.130 | 2.54E-01 | 2.54E+01 | | | |
| 7894807 | --- | --- | --- | 0.176 | 1.130 | 6.51E-01 | 6.51E+01 | | | |
| 8164067 | NM_002799 | PSMB7 | proteasome (prosome, macropain) s | 0.176 | 1.130 | 6.13E-01 | 6.13E+01 | | | |
| 8005903 | NM_003170 | SUPT6H | suppressor of Ty 6 homolog (S. cerev | 0.176 | 1.130 | 3.87E-01 | 3.87E+01 | | | |
| 7895119 | --- | --- | --- | 0.176 | 1.129 | 3.55E-01 | 3.55E+01 | | | |
| 7895656 | --- | --- | --- | 0.176 | 1.129 | 3.71E-01 | 3.71E+01 | | | |
| 8134211 | NM_032120 | C7orf64 | chromosome 7 open reading frame 6 | 0.176 | 1.129 | 7.13E-01 | 7.13E+01 | | | |
| 8038078 | NM_006801 | KDELR1 | KDEL (Lys-Asp-Glu-Leu) endoplasmic | 0.175 | 1.129 | 1.02E-01 | 1.02E+01 | | | |
| 7906194 | AK055377 | GPATCH4 | G patch domain containing 4 | 0.175 | 1.129 | 2.88E-01 | 2.88E+01 | | | |
| 8160722 | NM_018449 | UBAP2 | ubiquitin associated protein 2 | 0.175 | 1.129 | 1.01E-01 | 1.01E+01 | | | |
| 7986068 | NM_000057 | BLM | Bloom syndrome, RecQ helicase-like | 0.175 | 1.129 | 2.29E-01 | 2.29E+01 | | | |
| 8106393 | NM_001992 | F2R | coagulation factor II (thrombin) rece | 0.175 | 1.129 | 1.96E-01 | 1.96E+01 | | | |
| 8160839 | NM_024348 | DCTN3 | dynactin 3 (p22) | 0.175 | 1.129 | 4.63E-01 | 4.63E+01 | | | |
| 8038515 | NM_016440 | VRK3 | vaccinia related kinase 3 | 0.175 | 1.129 | 9.22E-02 | 9.22E+00 | | | |
| 8043945 | NM_145686 | MAP4K4 | mitogen-activated protein kinase kin | 0.175 | 1.129 | 2.86E-01 | 2.86E+01 | | | |
| 8154765 | NM_001539 | DNAJA1 | DnaJ (Hsp40) homolog, subfamily A, | 0.175 | 1.129 | 2.93E-01 | 2.93E+01 | | | |
| 8098547 | --- | --- | --- | 0.174 | 1.129 | 3.81E-01 | 3.81E+01 | | | |
| 8055862 | NM_012097 | ARL5A | ADP-ribosylation factor-like 5A | 0.174 | 1.128 | 1.60E-01 | 1.60E+01 | | | |
| 8109222 | NM_000971 | RPL7 | ribosomal protein L7 | 0.174 | 1.128 | 3.23E-01 | 3.23E+01 | | | |
| 7989619 | NM_000942 | PPIB | peptidylprolyl isomerase B (cyclophil | 0.174 | 1.128 | 3.61E-01 | 3.61E+01 | | | |
| 8172369 | NM_006962 | ZNF182 | zinc finger protein 182 | 0.174 | 1.128 | 2.64E-01 | 2.64E+01 | | | |
| 8065963 | NM_152930 | CPNE1 | copine I | 0.173 | 1.128 | 1.37E-01 | 1.37E+01 | | | |
| 7894558 | --- | --- | --- | 0.173 | 1.128 | 7.02E-01 | 7.02E+01 | | | |
| 8139121 | ENST00000390 | TRGV3 | T cell receptor gamma variable 3 | 0.173 | 1.128 | 3.87E-01 | 3.87E+01 | | | |
| 8078187 | NM_015184 | PLCL2 | phospholipase C-like 2 | 0.173 | 1.128 | 5.85E-01 | 5.85E+01 | | | |
| 7895375 | --- | --- | --- | 0.173 | 1.128 | 6.40E-01 | 6.40E+01 | | | |
| 7970681 | NM_005977 | RNF6 | ring finger protein (C3H2C3 type) 6 | 0.173 | 1.127 | 5.06E-01 | 5.06E+01 | | | |
| 8119153 | NM_004373 | COX6A1 | cytochrome c oxidase subunit VIa po | 0.173 | 1.127 | 1.43E-01 | 1.43E+01 | | | |
| 8086607 | NM_002343 | LTF | lactotransferrin | 0.173 | 1.127 | 4.85E-02 | 4.85E+00 | | | |
| 8030374 | NM_004107 | FCGRT | Fc fragment of IgG, receptor, transpo | 0.173 | 1.127 | 1.18E-01 | 1.18E+01 | | | |
| 7942783 | BC002752 | C11orf67 | chromosome 11 open reading frame | 0.173 | 1.127 | 3.44E-01 | 3.44E+01 | | | |
| 8083794 | NM_018657 | MYNN | myoneurin | 0.173 | 1.127 | 4.71E-01 | 4.71E+01 | | | |
| 7961175 | NM_002261 | KLRC3 | killer cell lectin-like receptor subfam | 0.173 | 1.127 | 6.89E-01 | 6.89E+01 | | | |
| 7965842 | --- | --- | --- | 0.173 | 1.127 | 3.55E-01 | 3.55E+01 | | | |
| 8119109 | NM_152734 | C6orf89 | chromosome 6 open reading frame 8 | 0.173 | 1.127 | 5.28E-01 | 5.28E+01 | | | |
| 8127549 | NM_012434 | SLC17A5 | solute carrier family 17 (anion/sugar | 0.172 | 1.127 | 3.21E-01 | 3.21E+01 | | | |
| 8091120 | NM_00103954 | GK5 | glycerol kinase 5 (putative) | 0.172 | 1.127 | 1.43E-01 | 1.43E+01 | | | |
| 7904486 | AK294414 | NBPF20 | neuroblastoma breakpoint family, m | 0.172 | 1.127 | 1.66E-03 | 1.66E-01 | | | |
| 7960898 | --- | --- | --- | 0.172 | 1.127 | 1.62E-01 | 1.62E+01 | | | |
| 8049088 | NM_022730 | COPS7B | COP9 constitutive photomorphogeni | 0.172 | 1.127 | 3.01E-01 | 3.01E+01 | | | |
| 8154211 | --- | --- | --- | 0.172 | 1.127 | 5.60E-01 | 5.60E+01 | | | |
| 8117447 | NM_006995 | BTN2A2 | butyrophilin, subfamily 2, member A | 0.172 | 1.127 | 9.98E-02 | 9.98E+00 | | | |
| 7955721 | NM_00100430 | ZNF740 | zinc finger protein 740 | 0.172 | 1.126 | 1.20E-01 | 1.20E+01 | | | |
| 7902883 | NM_00113447 | LRRC8D | leucine rich repeat containing 8 fami | 0.172 | 1.126 | 1.48E-01 | 1.48E+01 | | | |
| 8068494 | --- | --- | --- | 0.172 | 1.126 | 1.56E-01 | 1.56E+01 | | | |
| 7893288 | --- | --- | --- | 0.172 | 1.126 | 4.29E-01 | 4.29E+01 | | | |
| 8141750 | NM_00103161 | SPDYE2 | speedy homolog E2 (Xenopus laevis) | 0.171 | 1.126 | 2.66E-01 | 2.66E+01 | | | |
| 8151512 | NM_018440 | PAG1 | phosphoprotein associated with glyc | 0.171 | 1.126 | 2.39E-01 | 2.39E+01 | | | |
| 8085556 | NM_004844 | SH3BP5 | SH3-domain binding protein 5 (BTK-a | 0.171 | 1.126 | 1.08E-01 | 1.08E+01 | | | |
| 8102720 | NM_020337 | ANKRD50 | ankyrin repeat domain 50 | 0.171 | 1.126 | 1.36E-01 | 1.36E+01 | | | |
| 7915408 | NM_014947 | FOXJ3 | forkhead box J3 | 0.171 | 1.126 | 3.35E-01 | 3.35E+01 | | | |
| 8103518 | NM_012403 | ANP32C | acidic (leucine-rich) nuclear phospho | 0.171 | 1.126 | 9.85E-02 | 9.85E+00 | | | |
| 7895690 | --- | --- | --- | 0.171 | 1.126 | 4.65E-01 | 4.65E+01 | | | |

| | | | | | | | | | | |
|---|---|---|---|---|---|---|---|---|---|---|
| 7895552 | --- | --- | --- | 0.171 | 1.126 | 6.14E-01 | 6.14E+01 | | | |
| 8163002 | NM_004235 | KLF4 | Kruppel-like factor 4 (gut) | 0.171 | 1.126 | 2.55E-01 | 2.55E+01 | | | |
| 7953812 | NM_004426 | PHC1 | polyhomeotic homolog 1 (Drosophila | 0.171 | 1.126 | 1.10E-01 | 1.10E+01 | | | |
| 7922040 | NM_198053 | CD247 | CD247 molecule | 0.171 | 1.126 | 3.26E-01 | 3.26E+01 | | | |
| 8048195 | NM_000998 | RPL37A | ribosomal protein L37a | 0.171 | 1.126 | 1.37E-01 | 1.37E+01 | | | |
| 7938683 | NR_002822 | MGC72080 | MGC72080 pseudogene | 0.171 | 1.126 | 4.38E-01 | 4.38E+01 | | | |
| 8031867 | --- | --- | --- | 0.170 | 1.125 | 4.16E-01 | 4.16E+01 | | | |
| 8031827 | NM_032828 | ZNF587 | zinc finger protein 587 | 0.170 | 1.125 | 2.24E-01 | 2.24E+01 | | | |
| 8070215 | NM_017438 | SETD4 | SET domain containing 4 | 0.170 | 1.125 | 3.51E-01 | 3.51E+01 | | | |
| 7937735 | NM_002339 | LSP1 | lymphocyte-specific protein 1 | 0.170 | 1.125 | 1.03E-01 | 1.03E+01 | | | |
| 7989159 | NM_017661 | ZNF280D | zinc finger protein 280D | 0.170 | 1.125 | 3.07E-01 | 3.07E+01 | | | |
| 8132592 | NM_002541 | OGDH | oxoglutarate (alpha-ketoglutarate) d | 0.170 | 1.125 | 1.88E-01 | 1.88E+01 | | | |
| 8100603 | NM_001812 | CENPC1 | centromere protein C 1 | 0.170 | 1.125 | 5.38E-01 | 5.38E+01 | | | |
| 8112807 | NM_000046 | ARSB | arylsulfatase B | 0.170 | 1.125 | 1.88E-02 | 1.88E+00 | | | |
| 7892857 | --- | --- | --- | 0.170 | 1.125 | 6.34E-01 | 6.34E+01 | | | |
| 7895641 | --- | --- | --- | 0.170 | 1.125 | 6.98E-01 | 6.98E+01 | | | |
| 7894438 | --- | --- | --- | 0.170 | 1.125 | 8.10E-01 | 8.10E+01 | | | |
| 7941927 | NM_003977 | AIP | aryl hydrocarbon receptor interactin | 0.170 | 1.125 | 3.78E-01 | 3.78E+01 | | | |
| 7942381 | NM_033388 | ATG16L2 | ATG16 autophagy related 16-like 2 (S | 0.170 | 1.125 | 1.13E-01 | 1.13E+01 | | | |
| 8133654 | NM_00109943 | SPDYE5 | speedy homolog E5 (Xenopus laevis) | 0.169 | 1.125 | 3.97E-01 | 3.97E+01 | | | |
| 7893993 | --- | --- | --- | 0.169 | 1.125 | 4.01E-03 | 4.01E-01 | | | |
| 7895521 | --- | --- | --- | 0.169 | 1.125 | 1.19E-01 | 1.19E+01 | | | |
| 8083757 | NM_015938 | NMD3 | NMD3 homolog (S. cerevisiae) | 0.169 | 1.124 | 6.82E-01 | 6.82E+01 | | | |
| 8154209 | --- | --- | --- | 0.169 | 1.124 | 6.83E-01 | 6.83E+01 | | | |
| 7895931 | --- | --- | --- | 0.169 | 1.124 | 6.85E-01 | 6.85E+01 | | | |
| 7914996 | BX538212 | MEAF6 | MYST/Esa1-associated factor 6 | 0.169 | 1.124 | 4.72E-01 | 4.72E+01 | | | |
| 8099967 | NM_00109863 | RBM47 | RNA binding motif protein 47 | 0.169 | 1.124 | 4.29E-02 | 4.29E+00 | | | |
| 7894451 | --- | --- | --- | 0.169 | 1.124 | 5.24E-01 | 5.24E+01 | | | |
| 7929032 | NM_000043 | FAS | Fas (TNF receptor superfamily, mem | 0.169 | 1.124 | 4.75E-01 | 4.75E+01 | | | |
| 8043902 | NM_024065 | PDCL3 | phosducin-like 3 | 0.169 | 1.124 | 6.23E-01 | 6.23E+01 | | | |
| 7893194 | --- | --- | --- | 0.169 | 1.124 | 2.13E-02 | 2.13E+00 | | | |
| 8165648 | AK290098 | C7orf11 | chromosome 7 open reading frame 1 | 0.168 | 1.124 | 4.28E-01 | 4.28E+01 | | | |
| 7963988 | NM_003075 | SMARCC2 | SWI/SNF related, matrix associated, | 0.168 | 1.124 | 2.33E-01 | 2.33E+01 | | | |
| 8101718 | NM_032906 | PIGY | phosphatidylinositol glycan anchor b | 0.168 | 1.124 | 5.46E-01 | 5.46E+01 | | | |
| 8044584 | NM_012455 | PSD4 | pleckstrin and Sec7 domain containi | 0.168 | 1.124 | 1.44E-02 | 1.44E+00 | | | |
| 8155312 | NM_024345 | DCAF10 | DDB1 and CUL4 associated factor 10 | 0.168 | 1.124 | 1.86E-01 | 1.86E+01 | | | |
| 7893472 | --- | --- | --- | 0.168 | 1.124 | 8.34E-01 | 8.34E+01 | | | |
| 8106122 | NM_002270 | TNPO1 | transportin 1 | 0.168 | 1.124 | 4.17E-01 | 4.17E+01 | | | |
| 7919412 | NM_00110266 | NBPF16 | neuroblastoma breakpoint family, m | 0.168 | 1.124 | 1.40E-02 | 1.40E+00 | | | |
| 8091648 | NM_007107 | SSR3 | signal sequence receptor, gamma (tr | 0.168 | 1.124 | 6.23E-01 | 6.23E+01 | | | |
| 7933509 | NM_000124 | ERCC6 | excision repair cross-complementing | 0.168 | 1.123 | 2.72E-01 | 2.72E+01 | | | |
| 7918825 | NM_00100755 | CSDE1 | cold shock domain containing E1, RN | 0.168 | 1.123 | 1.78E-01 | 1.78E+01 | | | |
| 8107234 | NM_002372 | MAN2A1 | mannosidase, alpha, class 2A, memb | 0.168 | 1.123 | 4.02E-01 | 4.02E+01 | | | |
| 8133038 | --- | --- | --- | 0.168 | 1.123 | 6.93E-01 | 6.93E+01 | | | |
| 8029413 | NM_182490 | ZNF227 | zinc finger protein 227 | 0.168 | 1.123 | 3.05E-01 | 3.05E+01 | | | |
| 8120600 | --- | --- | --- | 0.167 | 1.123 | 3.68E-01 | 3.68E+01 | | | |
| 7964203 | NM_013449 | BAZ2A | bromodomain adjacent to zinc finger | 0.167 | 1.123 | 1.77E-01 | 1.77E+01 | | | |
| 8055426 | NM_005915 | MCM6 | minichromosome maintenance comp | 0.167 | 1.123 | 6.09E-01 | 6.09E+01 | | | |
| 7995552 | NM_015247 | CYLD | cylindromatosis (turban tumor syndr | 0.167 | 1.123 | 3.33E-01 | 3.33E+01 | | | |
| 8036808 | NM_018457 | PRR13 | proline rich 13 | 0.167 | 1.123 | 1.43E-01 | 1.43E+01 | | | |
| 7968928 | NM_002901 | RCN1 | reticulocalbin 1, EF-hand calcium bin | 0.167 | 1.123 | 5.13E-01 | 5.13E+01 | | | |
| 8016628 | NM_032595 | PPP1R9B | protein phosphatase 1, regulatory (ir | 0.167 | 1.123 | 1.12E-01 | 1.12E+01 | | | |
| 7915870 | NM_022745 | ATPAF1 | ATP synthase mitochondrial F1 comp | 0.167 | 1.123 | 4.30E-01 | 4.30E+01 | | | |
| 7994576 | NR_002939 | RUNDC2C | RUN domain containing 2C | 0.167 | 1.123 | 5.82E-01 | 5.82E+01 | | | |
| 8066848 | NM_020820 | PREX1 | phosphatidylinositol-3,4,5-trisphospl | 0.167 | 1.122 | 2.55E-01 | 2.55E+01 | | | |
| 8165911 | NM_005647 | TBL1X | transducin (beta)-like 1X-linked | 0.167 | 1.122 | 2.50E-01 | 2.50E+01 | | | |
| 8034055 | NM_023008 | KRI1 | KRI1 homolog (S. cerevisiae) | 0.167 | 1.122 | 3.38E-01 | 3.38E+01 | | | |
| 8022022 | NM_014646 | LPIN2 | lipin 2 | 0.167 | 1.122 | 9.69E-02 | 9.69E+00 | | | |
| 8055220 | NM_00108353 | POTEE | POTE ankyrin domain family, membe | 0.166 | 1.122 | 2.06E-01 | 2.06E+01 | | | |
| 7929634 | NM_198046 | ZDHHC16 | zinc finger, DHHC-type containing 16 | 0.166 | 1.122 | 2.27E-01 | 2.27E+01 | | | |
| 8009014 | NM_006852 | TLK2 | tousled-like kinase 2 | 0.166 | 1.122 | 4.48E-01 | 4.48E+01 | | | |
| 8178220 | NM_002121 | HLA-DPB1 | major histocompatibility complex, cla | 0.166 | 1.122 | 4.50E-01 | 4.50E+01 | | | |
| 8075720 | NM_030882 | APOL2 | apolipoprotein L, 2 | 0.166 | 1.122 | 4.75E-02 | 4.75E+00 | | | |
| 8009227 | NM_002805 | PSMC5 | proteasome (prosome, macropain) 2 | 0.166 | 1.122 | 4.61E-01 | 4.61E+01 | | | |
| 8078252 | NM_152653 | UBE2E2 | ubiquitin-conjugating enzyme E2E 2 | 0.166 | 1.122 | 4.02E-01 | 4.02E+01 | | | |
| 7896446 | --- | --- | --- | 0.166 | 1.122 | 9.59E-02 | 9.59E+00 | | | |
| 7932678 | NM_139312 | YME1L1 | YME1-like 1 (S. cerevisiae) | 0.166 | 1.122 | 6.73E-01 | 6.73E+01 | | | |
| 7892541 | --- | --- | --- | 0.166 | 1.122 | 4.41E-01 | 4.41E+01 | | | |
| 7911136 | --- | --- | --- | 0.166 | 1.122 | 7.54E-01 | 7.54E+01 | | | |
| 8158446 | NM_015354 | NUP188 | nucleoporin 188kDa | 0.166 | 1.122 | 1.90E-01 | 1.90E+01 | | | |
| 7956524 | NM_024779 | PIP4K2C | phosphatidylinositol-5-phosphate 4- | 0.166 | 1.122 | 3.07E-01 | 3.07E+01 | | | |
| 7927964 | NM_002727 | SRGN | serglycin | 0.166 | 1.122 | 5.74E-01 | 5.74E+01 | | | |
| 8068974 | NM_003274 | TRAPPC10 | trafficking protein particle complex 1 | 0.166 | 1.122 | 2.89E-01 | 2.89E+01 | | | |
| 8093643 | NM_176801 | ADD1 | adducin 1 (alpha) | 0.165 | 1.122 | 4.09E-01 | 4.09E+01 | | | |
| 7974214 | NM_172193 | KLHDC1 | kelch domain containing 1 | 0.165 | 1.122 | 6.46E-01 | 6.46E+01 | | | |
| 8115234 | NM_001155 | ANXA6 | annexin A6 | 0.165 | 1.121 | 4.37E-01 | 4.37E+01 | | | |
| 8085714 | --- | --- | --- | 0.165 | 1.121 | 3.89E-01 | 3.89E+01 | | | |

| | | | | | | | | | | |
|---|---|---|---|---|---|---|---|---|---|---|
| 7923501 | --- | --- | --- | 0.165 | 1.121 | 5.80E-01 | 5.80E+01 | | | |
| 8161056 | NM_006289 | TLN1 | talin 1 | 0.165 | 1.121 | 1.00E-01 | 1.00E+01 | | | |
| 8099541 | NM_000320 | QDPR | quinoid dihydropteridine reductase | 0.165 | 1.121 | 2.44E-01 | 2.44E+01 | | | |
| 8052866 | NM_032822 | FAM136A | family with sequence similarity 136, | 0.165 | 1.121 | 3.50E-01 | 3.50E+01 | | | |
| 8138030 | NR_003085 | PMS2 | PMS2 postmeiotic segregation increa | 0.165 | 1.121 | 4.40E-01 | 4.40E+01 | | | |
| 8150276 | NM_00110255 | PPAPDC1B | phosphatidic acid phosphatase type | 0.165 | 1.121 | 2.24E-01 | 2.24E+01 | | | |
| 8077663 | NM_032492 | JAGN1 | jagunal homolog 1 (Drosophila) | 0.165 | 1.121 | 3.88E-01 | 3.88E+01 | | | |
| 7903294 | NM_033055 | HIAT1 | hippocampus abundant transcript 1 | 0.165 | 1.121 | 7.37E-01 | 7.37E+01 | | | |
| 8151376 | NM_000971 | RPL7 | ribosomal protein L7 | 0.165 | 1.121 | 2.86E-01 | 2.86E+01 | | | |
| 7936134 | NM_024928 | OBFC1 | oligonucleotide/oligosaccharide-binc | 0.165 | 1.121 | 6.06E-01 | 6.06E+01 | | | |
| 7974341 | NM_053064 | GNG2 | guanine nucleotide binding protein ( | 0.165 | 1.121 | 4.64E-01 | 4.64E+01 | | | |
| 7994161 | NM_006910 | RBBP6 | retinoblastoma binding protein 6 | 0.165 | 1.121 | 5.07E-01 | 5.07E+01 | | | |
| 8016745 | NM_00113052 | SPAG9 | sperm associated antigen 9 | 0.165 | 1.121 | 4.79E-01 | 4.79E+01 | | | |
| 8038967 | NM_00110554 | ZNF83 | zinc finger protein 83 | 0.165 | 1.121 | 5.11E-01 | 5.11E+01 | | | |
| 8014487 | NM_198839 | ACACA | acetyl-Coenzyme A carboxylase alph | 0.165 | 1.121 | 5.62E-02 | 5.62E+00 | | | |
| 8128123 | NM_021244 | RRAGD | Ras-related GTP binding D | 0.164 | 1.121 | 3.88E-01 | 3.88E+01 | | | |
| 8096079 | --- | --- | --- | 0.164 | 1.121 | 3.97E-01 | 3.97E+01 | | | |
| 8100026 | NM_006095 | ATP8A1 | ATPase, aminophospholipid transpor | 0.164 | 1.121 | 4.59E-01 | 4.59E+01 | | | |
| 8147864 | NM_014673 | TTC35 | tetratricopeptide repeat domain 35 | 0.164 | 1.120 | 7.86E-01 | 7.86E+01 | | | |
| 8043433 | --- | --- | --- | 0.164 | 1.120 | 3.91E-01 | 3.91E+01 | | | |
| 8009653 | NM_007261 | CD300A | CD300a molecule | 0.164 | 1.120 | 1.96E-01 | 1.96E+01 | | | |
| 8129522 | NM_004830 | MED23 | mediator complex subunit 23 | 0.164 | 1.120 | 4.72E-01 | 4.72E+01 | | | |
| 8030860 | NM_001462 | FPR2 | formyl peptide receptor 2 | 0.164 | 1.120 | 3.17E-01 | 3.17E+01 | | | |
| 7896576 | --- | --- | --- | 0.164 | 1.120 | 5.71E-01 | 5.71E+01 | | | |
| 8169009 | NM_00108042 | BEX4 | brain expressed, X-linked 4 | 0.164 | 1.120 | 5.87E-01 | 5.87E+01 | | | |
| 7896662 | --- | --- | --- | 0.164 | 1.120 | 6.60E-01 | 6.60E+01 | | | |
| 8173615 | NM_183353 | RLIM | ring finger protein, LIM domain inter | 0.164 | 1.120 | 5.83E-01 | 5.83E+01 | | | |
| 7964575 | AK022448 | AVIL | advillin | 0.164 | 1.120 | 6.15E-01 | 6.15E+01 | | | |
| 7894470 | --- | --- | --- | 0.163 | 1.120 | 6.10E-01 | 6.10E+01 | | | |
| 8171352 | NM_00101165 | TRAPPC2 | trafficking protein particle complex 2 | 0.163 | 1.120 | 4.24E-01 | 4.24E+01 | | | |
| 8146898 | --- | --- | --- | 0.163 | 1.120 | 6.98E-01 | 6.98E+01 | | | |
| 8068105 | NR_027655 | BACH1 | BTB and CNC homology 1, basic leuc | 0.163 | 1.120 | 2.98E-01 | 2.98E+01 | | | |
| 8039034 | NM_00110260 | ZNF160 | zinc finger protein 160 | 0.163 | 1.120 | 1.65E-01 | 1.65E+01 | | | |
| 7903079 | NM_001938 | DR1 | down-regulator of transcription 1, TB | 0.163 | 1.120 | 1.91E-01 | 1.91E+01 | | | |
| 7895621 | --- | --- | --- | 0.163 | 1.120 | 8.75E-02 | 8.75E+00 | | | |
| 7944765 | --- | --- | --- | 0.163 | 1.120 | 5.43E-01 | 5.43E+01 | | | |
| 7901336 | NM_018571 | STRADB | STE20-related kinase adaptor beta | 0.163 | 1.120 | 7.64E-01 | 7.64E+01 | | | |
| 7938756 | AF512499 | FAM10A5 | family with sequence similarity 10, m | 0.163 | 1.120 | 3.69E-01 | 3.69E+01 | | | |
| 7896387 | --- | --- | --- | 0.163 | 1.119 | 6.32E-02 | 6.32E+00 | | | |
| 8063283 | NM_001316 | CSE1L | CSE1 chromosome segregation 1-like | 0.163 | 1.119 | 5.59E-01 | 5.59E+01 | | | |
| 8163616 | --- | --- | --- | 0.162 | 1.119 | 6.18E-01 | 6.18E+01 | | | |
| 7998174 | NM_018032 | LUC7L | LUC7-like (S. cerevisiae) | 0.162 | 1.119 | 3.59E-01 | 3.59E+01 | | | |
| 8064485 | NM_018556 | SIRPG | signal-regulatory protein gamma | 0.162 | 1.119 | 1.50E-01 | 1.50E+01 | | | |
| 7979813 | NM_004926 | ZFP36L1 | zinc finger protein 36, C3H type-like | 0.162 | 1.119 | 9.33E-02 | 9.33E+00 | | | |
| 7896416 | --- | --- | --- | 0.162 | 1.119 | 4.55E-01 | 4.55E+01 | | | |
| 8128956 | NM_002037 | FYN | FYN oncogene related to SRC, FGR, Y | 0.162 | 1.119 | 4.79E-01 | 4.79E+01 | | | |
| 7935230 | NM_002860 | ALDH18A1 | aldehyde dehydrogenase 18 family, | 0.162 | 1.119 | 1.22E-01 | 1.22E+01 | | | |
| 8068353 | NM_006933 | SLC5A3 | solute carrier family 5 (sodium/myo- | 0.162 | 1.119 | 1.19E-01 | 1.19E+01 | | | |
| 8043484 | --- | --- | --- | 0.162 | 1.119 | 8.32E-02 | 8.32E+00 | | | |
| 7916747 | NM_002227 | JAK1 | Janus kinase 1 | 0.162 | 1.119 | 5.64E-01 | 5.64E+01 | | | |
| 8174134 | NM_00100693 | TCEAL6 | transcription elongation factor A (SII | 0.162 | 1.118 | 2.17E-01 | 2.17E+01 | | | |
| 8130116 | NM_004690 | LATS1 | LATS, large tumor suppressor, homo | 0.161 | 1.118 | 3.31E-01 | 3.31E+01 | | | |
| 7906475 | NM_00100431 | FCRL6 | Fc receptor-like 6 | 0.161 | 1.118 | 5.93E-01 | 5.93E+01 | | | |
| 8072302 | NM_153050 | MTMR3 | myotubularin related protein 3 | 0.161 | 1.118 | 4.04E-01 | 4.04E+01 | | | |
| 8090091 | NM_198402 | PTPLB | protein tyrosine phosphatase-like (p | 0.161 | 1.118 | 7.24E-01 | 7.24E+01 | | | |
| 8010188 | NM_00114264 | TNRC6C | trinucleotide repeat containing 6C | 0.161 | 1.118 | 2.76E-01 | 2.76E+01 | | | |
| 7895256 | --- | --- | --- | 0.161 | 1.118 | 7.22E-01 | 7.22E+01 | | | |
| 8080991 | NM_194247 | HNRNPA3 | heterogeneous nuclear ribonucleopr | 0.161 | 1.118 | 1.88E-01 | 1.88E+01 | | | |
| 8039947 | NM_006625 | FUSIP1 | FUS interacting protein (serine/argin | 0.161 | 1.118 | 5.96E-01 | 5.96E+01 | | | |
| 7999827 | NM_001019 | RPS15A | ribosomal protein S15a | 0.161 | 1.118 | 3.51E-01 | 3.51E+01 | | | |
| 8167797 | NM_019067 | GNL3L | guanine nucleotide binding protein-l | 0.161 | 1.118 | 1.86E-01 | 1.86E+01 | | | |
| 8171684 | NM_031892 | SH3KBP1 | SH3-domain kinase binding protein 1 | 0.161 | 1.118 | 3.62E-01 | 3.62E+01 | | | |
| 7909142 | NM_022731 | NUCKS1 | nuclear casein kinase and cyclin-depe | 0.161 | 1.118 | 6.31E-01 | 6.31E+01 | | | |
| 8015590 | NM_012448 | STAT5B | signal transducer and activator of tra | 0.161 | 1.118 | 3.99E-01 | 3.99E+01 | | | |
| 8086600 | NM_001295 | CCR1 | chemokine (C-C motif) receptor 1 | 0.160 | 1.118 | 3.73E-01 | 3.73E+01 | | | |
| 8019687 | NM_00100224 | ANAPC11 | anaphase promoting complex subun | 0.160 | 1.118 | 4.02E-01 | 4.02E+01 | | | |
| 7977454 | NM_00108353 | POTEE | POTE ankyrin domain family, membe | 0.160 | 1.117 | 1.37E-01 | 1.37E+01 | | | |
| 8067040 | NM_012340 | NFATC2 | nuclear factor of activated T-cells, cy | 0.160 | 1.117 | 4.73E-01 | 4.73E+01 | | | |
| 7952768 | NM_014758 | SNX19 | sorting nexin 19 | 0.160 | 1.117 | 2.92E-01 | 2.92E+01 | | | |
| 7893843 | --- | --- | --- | 0.160 | 1.117 | 8.30E-01 | 8.30E+01 | | | |
| 7894120 | --- | --- | --- | 0.160 | 1.117 | 8.61E-01 | 8.61E+01 | | | |
| 8054393 | --- | --- | --- | 0.160 | 1.117 | 7.86E-01 | 7.86E+01 | | | |
| 7989883 | --- | --- | --- | 0.160 | 1.117 | 8.79E-01 | 8.79E+01 | | | |
| 8040365 | NM_021643 | TRIB2 | tribbles homolog 2 (Drosophila) | 0.160 | 1.117 | 2.52E-01 | 2.52E+01 | | | |
| 7994237 | NM_016309 | LCMT1 | leucine carboxyl methyltransferase 1 | 0.160 | 1.117 | 5.30E-01 | 5.30E+01 | | | |
| 8180346 | --- | --- | --- | 0.160 | 1.117 | 5.94E-01 | 5.94E+01 | | | |
| 8001102 | --- | --- | --- | 0.160 | 1.117 | 9.19E-02 | 9.19E+00 | | | |

| | | | | | | | | | | |
|---|---|---|---|---|---|---|---|---|---|---|
| 7895305 | --- | --- | --- | 0.160 | 1.117 | 3.62E-01 | 3.62E+01 | | | |
| 8063351 | NM_015266 | SLC9A8 | solute carrier family 9 (sodium/hydro | 0.159 | 1.117 | 4.90E-01 | 4.90E+01 | | | |
| 7895216 | --- | --- | --- | 0.159 | 1.117 | 8.25E-03 | 8.25E-01 | | | |
| 7898739 | NM_044472 | CDC42 | cell division cycle 42 (GTP binding pro | 0.159 | 1.117 | 4.65E-01 | 4.65E+01 | | | |
| 8047038 | NM_000534 | PMS1 | PMS1 postmeiotic segregation increa | 0.159 | 1.117 | 2.01E-01 | 2.01E+01 | | | |
| 8027352 | --- | --- | --- | 0.159 | 1.117 | 1.16E-01 | 1.16E+01 | | | |
| 7896040 | --- | --- | --- | 0.159 | 1.117 | 6.21E-01 | 6.21E+01 | | | |
| 8034299 | NM_032377 | ELOF1 | elongation factor 1 homolog (S. cere | 0.159 | 1.117 | 1.30E-01 | 1.30E+01 | | | |
| 8180417 | --- | --- | --- | 0.159 | 1.117 | 5.04E-01 | 5.04E+01 | | | |
| 8024936 | NM_002967 | SAFB | scaffold attachment factor B | 0.159 | 1.117 | 3.11E-01 | 3.11E+01 | | | |
| 8039226 | NM_006865 | LILRA3 | leukocyte immunoglobulin-like recep | 0.159 | 1.117 | 1.46E-01 | 1.46E+01 | | | |
| 8040831 | NM_016085 | C2orf28 | chromosome 2 open reading frame 2 | 0.159 | 1.116 | 5.59E-01 | 5.59E+01 | | | |
| 7894391 | --- | --- | --- | 0.159 | 1.116 | 8.03E-01 | 8.03E+01 | | | |
| 8057034 | NM_003690 | PRKRA | protein kinase, interferon-inducible d | 0.159 | 1.116 | 4.78E-01 | 4.78E+01 | | | |
| 7948987 | NM_007069 | PLA2G16 | phospholipase A2, group XVI | 0.159 | 1.116 | 4.57E-01 | 4.57E+01 | | | |
| 8002211 | NM_018380 | DDX28 | DEAD (Asp-Glu-Ala-Asp) box polypep | 0.159 | 1.116 | 4.00E-01 | 4.00E+01 | | | |
| 8171901 | NM_152787 | MAP3K7IP3 | mitogen-activated protein kinase kin | 0.159 | 1.116 | 1.64E-01 | 1.64E+01 | | | |
| 7965855 | NM_024057 | NUP37 | nucleoporin 37kDa | 0.158 | 1.116 | 7.09E-01 | 7.09E+01 | | | |
| 8088830 | NM_018130 | SHQ1 | SHQ1 homolog (S. cerevisiae) | 0.158 | 1.116 | 1.22E-01 | 1.22E+01 | | | |
| 7976515 | NM_016417 | GLRX5 | glutaredoxin 5 | 0.158 | 1.116 | 7.55E-02 | 7.55E+00 | | | |
| 7893973 | --- | --- | --- | 0.158 | 1.116 | 7.31E-01 | 7.31E+01 | | | |
| 7920271 | NM_019554 | S100A4 | S100 calcium binding protein A4 | 0.158 | 1.116 | 2.78E-01 | 2.78E+01 | | | |
| 8043697 | NM_025190 | ANKRD36B | ankyrin repeat domain 36B | 0.158 | 1.116 | 5.02E-01 | 5.02E+01 | | | |
| 7906948 | --- | --- | --- | 0.158 | 1.116 | 2.82E-01 | 2.82E+01 | | | |
| 8138596 | NR_002711 // | LK2P // CLK2 | CDC-like kinase 2, pseudogene // CD | 0.158 | 1.116 | 3.66E-01 | 3.66E+01 | | | |
| 7894501 | --- | --- | --- | 0.158 | 1.116 | 7.91E-01 | 7.91E+01 | | | |
| 8155696 | NM_138333 | FAM122A | family with sequence similarity 122A | 0.158 | 1.116 | 2.16E-01 | 2.16E+01 | | | |
| 7895057 | --- | --- | --- | 0.158 | 1.116 | 8.11E-01 | 8.11E+01 | | | |
| 8007757 | NM_005892 | FMNL1 | formin-like 1 | 0.158 | 1.116 | 2.24E-01 | 2.24E+01 | | | |
| 8040036 | NM_001011 | RPS7 | ribosomal protein S7 | 0.158 | 1.116 | 2.57E-01 | 2.57E+01 | | | |
| 8100227 | --- | --- | --- | 0.158 | 1.116 | 4.55E-01 | 4.55E+01 | | | |
| 8088526 | NM_025075 | THOC7 | THO complex 7 homolog (Drosophila | 0.158 | 1.116 | 8.34E-01 | 8.34E+01 | | | |
| 8033069 | NM_175614 | NDUFA11 | NADH dehydrogenase (ubiquinone) 1 | 0.158 | 1.116 | 2.67E-01 | 2.67E+01 | | | |
| 8029831 | NM_005184 | CALM3 | calmodulin 3 (phosphorylase kinase, | 0.158 | 1.115 | 2.20E-01 | 2.20E+01 | | | |
| 8148572 | NM_002346 | LY6E | lymphocyte antigen 6 complex, locus | 0.158 | 1.115 | 3.19E-01 | 3.19E+01 | | | |
| 7968976 | NM_015116 | LRCH1 | leucine-rich repeats and calponin ho | 0.158 | 1.115 | 4.21E-01 | 4.21E+01 | | | |
| 7994280 | NM_000418 | IL4R | interleukin 4 receptor | 0.157 | 1.115 | 3.00E-01 | 3.00E+01 | | | |
| 7942465 | NM_016055 | MRPL48 | mitochondrial ribosomal protein L48 | 0.157 | 1.115 | 3.30E-01 | 3.30E+01 | | | |
| 7905589 | ENST00000368 | C1orf77 | chromosome 1 open reading frame 7 | 0.157 | 1.115 | 1.95E-01 | 1.95E+01 | | | |
| 8023920 | NM_006701 | TXNL4A | thioredoxin-like 4A | 0.157 | 1.115 | 5.68E-01 | 5.68E+01 | | | |
| 8180285 | --- | --- | --- | 0.157 | 1.115 | 4.03E-01 | 4.03E+01 | | | |
| 7925851 | NM_014023 | WDR37 | WD repeat domain 37 | 0.157 | 1.115 | 1.40E-01 | 1.40E+01 | | | |
| 7950473 | NM_004041 | ARRB1 | arrestin, beta 1 | 0.157 | 1.115 | 1.89E-01 | 1.89E+01 | | | |
| 7896084 | --- | --- | --- | 0.157 | 1.115 | 3.62E-01 | 3.62E+01 | | | |
| 8004241 | NM_00100433 | RNASEK | ribonuclease, RNase K | 0.157 | 1.115 | 1.62E-01 | 1.62E+01 | | | |
| 8060722 | NM_153638 | PANK2 | pantothenate kinase 2 | 0.157 | 1.115 | 3.71E-01 | 3.71E+01 | | | |
| 7944525 | --- | --- | --- | 0.157 | 1.115 | 5.74E-01 | 5.74E+01 | | | |
| 7958147 | NM_003211 | TDG | thymine-DNA glycosylase | 0.157 | 1.115 | 5.92E-01 | 5.92E+01 | | | |
| 7970704 | NM_152912 | MTIF3 | mitochondrial translational initiation | 0.157 | 1.115 | 3.88E-01 | 3.88E+01 | | | |
| 7993872 | NM_003366 | UQCRC2 | ubiquinol-cytochrome c reductase co | 0.157 | 1.115 | 5.60E-01 | 5.60E+01 | | | |
| 8035808 | NM_173531 | ZNF100 | zinc finger protein 100 | 0.157 | 1.115 | 2.83E-01 | 2.83E+01 | | | |
| 8065832 | NM_015638 | TRPC4AP | transient receptor potential cation ch | 0.156 | 1.115 | 4.03E-01 | 4.03E+01 | | | |
| 7893360 | --- | --- | --- | 0.156 | 1.115 | 7.19E-01 | 7.19E+01 | | | |
| 8044849 | NM_002830 | PTPN4 | protein tyrosine phosphatase, non-re | 0.156 | 1.114 | 2.88E-01 | 2.88E+01 | | | |
| 7945873 | NR_002822 | MGC72080 | MGC72080 pseudogene | 0.156 | 1.114 | 7.05E-01 | 7.05E+01 | | | |
| 7986520 | AK302717 | C15orf51 | dynamin 1 pseudogene | 0.156 | 1.114 | 6.01E-01 | 6.01E+01 | | | |
| 7940781 | NM_201428 | RTN3 | reticulon 3 | 0.156 | 1.114 | 7.76E-02 | 7.76E+00 | | | |
| 8001841 | NM_006141 | DYNC1LI2 | dynein, cytoplasmic 1, light intermed | 0.156 | 1.114 | 3.58E-01 | 3.58E+01 | | | |
| 7918955 | NM_00113558 | GDAP2 | ganglioside induced differentiation a | 0.156 | 1.114 | 5.98E-01 | 5.98E+01 | | | |
| 8068397 | --- | --- | --- | 0.156 | 1.114 | 4.25E-01 | 4.25E+01 | | | |
| 8048283 | NM_000578 | SLC11A1 | solute carrier family 11 (proton-coup | 0.156 | 1.114 | 8.97E-02 | 8.97E+00 | | | mono |
| 7896201 | --- | --- | --- | 0.156 | 1.114 | 8.32E-01 | 8.32E+01 | | | |
| 7896490 | --- | --- | --- | 0.156 | 1.114 | 8.11E-01 | 8.11E+01 | | | |
| 7893483 | --- | --- | --- | 0.156 | 1.114 | 3.91E-01 | 3.91E+01 | | | |
| 7968800 | NM_178009 | DGKH | diacylglycerol kinase, eta | 0.156 | 1.114 | 2.68E-01 | 2.68E+01 | | | |
| 8081758 | NM_017577 | GRAMD1C | GRAM domain containing 1C | 0.156 | 1.114 | 3.28E-01 | 3.28E+01 | | | |
| 8180196 | --- | --- | --- | 0.156 | 1.114 | 4.48E-02 | 4.48E+00 | | | |
| 8012423 | NM_025099 | C17orf68 | chromosome 17 open reading frame | 0.156 | 1.114 | 8.45E-02 | 8.45E+00 | | | |
| 8000799 | NM_024307 | GDPD3 | glycerophosphodiester phosphodiest | 0.156 | 1.114 | 1.46E-01 | 1.46E+01 | | | |
| 8088468 | AF134979 | NPCDR1 | nasopharyngeal carcinoma, down-re | 0.156 | 1.114 | 4.47E-01 | 4.47E+01 | | | |
| 7996772 | NM_003983 | SLC7A6 | solute carrier family 7 (cationic amin | 0.156 | 1.114 | 4.98E-01 | 4.98E+01 | | | |
| 7970317 | NR_026580 | TFDP1 | transcription factor Dp-1 | 0.155 | 1.114 | 4.97E-01 | 4.97E+01 | | | |
| 8086141 | NM_014805 | EPM2AIP1 | EPM2A (laforin) interacting protein 1 | 0.155 | 1.114 | 6.05E-01 | 6.05E+01 | | | |
| 8024194 | NM_002085 | GPX4 | glutathione peroxidase 4 (phospholip | 0.155 | 1.114 | 5.65E-01 | 5.65E+01 | | | |
| 8073022 | NM_00100288 | CBY1 | chibby homolog 1 (Drosophila) | 0.155 | 1.114 | 2.74E-01 | 2.74E+01 | | | |
| 7903010 | NM_000969 | RPL5 | ribosomal protein L5 | 0.155 | 1.114 | 1.02E-01 | 1.02E+01 | | | |
| 8078905 | NM_002295 | RPSA | ribosomal protein SA | 0.155 | 1.114 | 2.47E-01 | 2.47E+01 | | | |

| | | | | | | | | | | |
|---|---|---|---|---|---|---|---|---|---|---|
| 8032410 | NM_130807 | MOBKL2A | MOB1, Mps One Binder kinase activa | 0.155 | 1.113 | 7.13E-02 | 7.13E+00 | | | |
| 7897460 | NM_032315 | SLC25A33 | solute carrier family 25, member 33 | 0.155 | 1.113 | 4.13E-01 | 4.13E+01 | | | |
| 8046726 | NM_00113044 | SSFA2 | sperm specific antigen 2 | 0.155 | 1.113 | 3.49E-01 | 3.49E+01 | | | |
| 8002987 | NM_020188 | C16orf61 | chromosome 16 open reading frame | 0.155 | 1.113 | 7.45E-01 | 7.45E+01 | | | |
| 7901038 | NM_001012 | RPS8 | ribosomal protein S8 | 0.155 | 1.113 | 5.11E-02 | 5.11E+00 | | | |
| 8023481 | NM_004539 | NARS | asparaginyl-tRNA synthetase | 0.155 | 1.113 | 5.38E-01 | 5.38E+01 | | | |
| 7974352 | NM_016039 | C14orf166 | chromosome 14 open reading frame | 0.155 | 1.113 | 7.07E-01 | 7.07E+01 | | | |
| 7903389 | --- | --- | --- | 0.155 | 1.113 | 6.78E-01 | 6.78E+01 | | | |
| 7938681 | --- | --- | --- | 0.155 | 1.113 | 4.68E-01 | 4.68E+01 | | | |
| 7986509 | ENST00000423 | C15orf51 | dynamin 1 pseudogene | 0.155 | 1.113 | 7.18E-01 | 7.18E+01 | | | |
| 7986512 | ENST00000426 | OC10028966 | similar to dynamin 1 | 0.155 | 1.113 | 7.18E-01 | 7.18E+01 | | | |
| 7986527 | AK125787 | C15orf51 | dynamin 1 pseudogene | 0.155 | 1.113 | 7.18E-01 | 7.18E+01 | | | |
| 7917283 | NM_153259 | MCOLN2 | mucolipin 2 | 0.155 | 1.113 | 4.58E-01 | 4.58E+01 | | | |
| 8077550 | NM_00107752 | MTMR14 | myotubularin related protein 14 | 0.154 | 1.113 | 4.45E-01 | 4.45E+01 | | | |
| 7966266 | --- | --- | --- | 0.154 | 1.113 | 5.14E-01 | 5.14E+01 | | | |
| 7960131 | --- | --- | --- | 0.154 | 1.113 | 2.41E-01 | 2.41E+01 | | | |
| 8081171 | NM_153605 | CRYBG3 | beta-gamma crystallin domain conta | 0.154 | 1.113 | 1.61E-01 | 1.61E+01 | | | |
| 8033780 | NM_024106 | ZNF426 | zinc finger protein 426 | 0.154 | 1.113 | 4.95E-01 | 4.95E+01 | | | |
| 7957271 | --- | --- | --- | 0.154 | 1.113 | 3.89E-01 | 3.89E+01 | | | |
| 8118644 | NM_022551 | RPS18 | ribosomal protein S18 | 0.154 | 1.113 | 2.06E-01 | 2.06E+01 | | | |
| 8178253 | NM_022551 | RPS18 | ribosomal protein S18 | 0.154 | 1.113 | 2.06E-01 | 2.06E+01 | | | |
| 8179544 | NM_022551 | RPS18 | ribosomal protein S18 | 0.154 | 1.113 | 2.06E-01 | 2.06E+01 | | | |
| 8064191 | NM_199360 | TPD52L2 | tumor protein D52-like 2 | 0.154 | 1.113 | 1.74E-01 | 1.74E+01 | | | |
| 8089285 | --- | --- | --- | 0.154 | 1.113 | 6.94E-01 | 6.94E+01 | | | |
| 7930213 | NM_006951 | TAF5 | TAF5 RNA polymerase II, TATA box b | 0.154 | 1.113 | 2.72E-01 | 2.72E+01 | | | |
| 7895152 | --- | --- | --- | 0.154 | 1.113 | 8.10E-01 | 8.10E+01 | | | |
| 8053386 | --- | --- | --- | 0.154 | 1.113 | 1.98E-01 | 1.98E+01 | | | |
| 7969204 | NM_052950 | WDFY2 | WD repeat and FYVE domain contair | 0.154 | 1.113 | 2.05E-01 | 2.05E+01 | | | |
| 8146336 | AK301677 | KIAA0146 | KIAA0146 | 0.154 | 1.113 | 2.96E-01 | 2.96E+01 | | | |
| 7894563 | --- | --- | --- | 0.154 | 1.113 | 8.15E-01 | 8.15E+01 | | | |
| 8045455 | NM_014607 | UBXN4 | UBX domain protein 4 | 0.154 | 1.112 | 5.76E-01 | 5.76E+01 | | | |
| 7920873 | NR_002974 | SNORA42 | small nucleolar RNA, H/ACA box 42 | 0.153 | 1.112 | 6.66E-01 | 6.66E+01 | | | |
| 8156199 | NM_004938 | DAPK1 | death-associated protein kinase 1 | 0.153 | 1.112 | 3.08E-01 | 3.08E+01 | | | |
| 7988093 | NM_00114198 | TP53BP1 | tumor protein p53 binding protein 1 | 0.153 | 1.112 | 1.99E-01 | 1.99E+01 | | | |
| 8115562 | NM_144726 | RNF145 | ring finger protein 145 | 0.153 | 1.112 | 1.99E-01 | 1.99E+01 | | | |
| 8035581 | NM_00101739 | SFRS14 | splicing factor, arginine/serine-rich 1 | 0.153 | 1.112 | 1.78E-01 | 1.78E+01 | | | |
| 8052703 | NM_138458 | WDR92 | WD repeat domain 92 | 0.153 | 1.112 | 2.52E-01 | 2.52E+01 | | | |
| 7902611 | --- | --- | --- | 0.153 | 1.112 | 3.22E-01 | 3.22E+01 | | | |
| 7904506 | AK095030 | NBPF20 | neuroblastoma breakpoint family, m | 0.153 | 1.112 | 3.26E-03 | 3.26E-01 | | | |
| 7917591 | --- | --- | --- | 0.153 | 1.112 | 5.41E-01 | 5.41E+01 | | | |
| 8150559 | NM_018105 | THAP1 | THAP domain containing, apoptosis a | 0.153 | 1.112 | 3.05E-01 | 3.05E+01 | | | |
| 7905598 | NM_012437 | SNAPIN | SNAP-associated protein | 0.153 | 1.112 | 6.74E-01 | 6.74E+01 | | | |
| 7961693 | NM_002300 | LDHB | lactate dehydrogenase B | 0.153 | 1.112 | 2.88E-01 | 2.88E+01 | | | |
| 8125204 | NM_002904 | RDBP | RD RNA binding protein | 0.153 | 1.112 | 2.36E-01 | 2.36E+01 | | | |
| 8178686 | NM_002904 | RDBP | RD RNA binding protein | 0.153 | 1.112 | 2.36E-01 | 2.36E+01 | | | |
| 8179913 | NM_002904 | RDBP | RD RNA binding protein | 0.153 | 1.112 | 2.36E-01 | 2.36E+01 | | | |
| 8110417 | NM_00107952 | FAM153C | family with sequence similarity 153, | 0.153 | 1.112 | 2.77E-01 | 2.77E+01 | | | |
| 7916262 | BC010908 | C1orf123 | chromosome 1 open reading frame 1 | 0.153 | 1.112 | 6.29E-01 | 6.29E+01 | | | |
| 7894453 | --- | --- | --- | 0.153 | 1.112 | 5.74E-01 | 5.74E+01 | | | |
| 8054354 | --- | --- | --- | 0.153 | 1.112 | 4.43E-01 | 4.43E+01 | | | |
| 8078569 | NM_002078 | GOLGA4 | golgi autoantigen, golgin subfamily a | 0.153 | 1.112 | 5.76E-01 | 5.76E+01 | | | |
| 7892570 | --- | --- | --- | 0.153 | 1.112 | 8.91E-01 | 8.91E+01 | | | |
| 8096335 | NM_017912 | HERC6 | hect domain and RLD 6 | 0.153 | 1.112 | 5.06E-01 | 5.06E+01 | | | |
| 7990211 | NM_000520 | HEXA | hexosaminidase A (alpha polypeptide | 0.152 | 1.111 | 4.09E-01 | 4.09E+01 | | | |
| 8149399 | NM_152271 | LONRF1 | LON peptidase N-terminal domain ar | 0.152 | 1.111 | 3.35E-01 | 3.35E+01 | | | |
| 7919305 | NM_005399 | PRKAB2 | protein kinase, AMP-activated, beta | 0.152 | 1.111 | 5.07E-01 | 5.07E+01 | | | |
| 7898570 | --- | --- | --- | 0.152 | 1.111 | 6.94E-01 | 6.94E+01 | | | |
| 8014974 | NM_001067 | TOP2A | topoisomerase (DNA) II alpha 170kD | 0.152 | 1.111 | 7.63E-02 | 7.63E+00 | | | |
| 7962895 | NM_016594 | FKBP11 | FK506 binding protein 11, 19 kDa | 0.152 | 1.111 | 3.99E-01 | 3.99E+01 | | | |
| 8132070 | NM_002047 | GARS | glycyl-tRNA synthetase | 0.152 | 1.111 | 6.51E-01 | 6.51E+01 | | | |
| 7914834 | NM_002794 | PSMB2 | proteasome (prosome, macropain) s | 0.152 | 1.111 | 5.05E-01 | 5.05E+01 | | | |
| 7893902 | --- | --- | --- | 0.152 | 1.111 | 8.75E-01 | 8.75E+01 | | | |
| 8114225 | NM_004893 | H2AFY | H2A histone family, member Y | 0.152 | 1.111 | 1.83E-01 | 1.83E+01 | | | |
| 8036525 | NM_00104260 | MAP4K1 | mitogen-activated protein kinase kin | 0.152 | 1.111 | 1.92E-01 | 1.92E+01 | | | |
| 7950284 | NM_002869 | RAB6A | RAB6A, member RAS oncogene fami | 0.152 | 1.111 | 5.03E-01 | 5.03E+01 | | | |
| 7983054 | NR_027911 | CAPN3 | calpain 3, (p94) | 0.152 | 1.111 | 1.50E-01 | 1.50E+01 | | | |
| 8054227 | NM_016316 | REV1 | REV1 homolog (S. cerevisiae) | 0.152 | 1.111 | 4.65E-01 | 4.65E+01 | | | |
| 8118974 | NM_007104 | RPL10A | ribosomal protein L10a | 0.152 | 1.111 | 3.57E-01 | 3.57E+01 | | | |
| 8136341 | NM_199186 | BPGM | 2,3-bisphosphoglycerate mutase | 0.152 | 1.111 | 1.65E-01 | 1.65E+01 | | | |
| 7896698 | --- | --- | --- | 0.152 | 1.111 | 5.08E-01 | 5.08E+01 | | | |
| 7893857 | --- | --- | --- | 0.152 | 1.111 | 6.26E-01 | 6.26E+01 | | | |
| 8025255 | NM_006949 | STXBP2 | syntaxin binding protein 2 | 0.152 | 1.111 | 2.18E-01 | 2.18E+01 | | | |
| 8013450 | NM_00104268 | LGALS9B | lectin, galactoside-binding, soluble, 9 | 0.151 | 1.111 | 4.59E-01 | 4.59E+01 | | | |
| 7981720 | --- | --- | --- | 0.151 | 1.111 | 3.44E-01 | 3.44E+01 | | | |
| 8065607 | NM_002657 | PLAGL2 | pleiomorphic adenoma gene-like 2 | 0.151 | 1.111 | 3.16E-01 | 3.16E+01 | | | |
| 7923141 | NM_144977 | DENND1B | DENN/MADD domain containing 1B | 0.151 | 1.111 | 6.66E-01 | 6.66E+01 | | | |
| 7964413 | NM_014925 | R3HDM2 | R3H domain containing 2 | 0.151 | 1.111 | 2.33E-01 | 2.33E+01 | | | |

| | | | | | | | | | | |
|---|---|---|---|---|---|---|---|---|---|---|
| 7991581 | NM_014918 | CHSY1 | chondroitin sulfate synthase 1 | 0.151 | 1.111 | 5.67E-02 | 5.67E+00 | | | |
| 7896427 | --- | --- | --- | 0.151 | 1.111 | 1.49E-01 | 1.49E+01 | | | |
| 7896128 | --- | --- | --- | 0.151 | 1.111 | 5.26E-01 | 5.26E+01 | | | |
| 8026698 | NM_00103354 | C19orf62 | chromosome 19 open reading frame | 0.151 | 1.110 | 2.96E-01 | 2.96E+01 | | | |
| 8074642 | --- | --- | --- | 0.151 | 1.110 | 5.30E-01 | 5.30E+01 | | | |
| 8179049 | NR_024240 | HLA-J | major histocompatibility complex, cl | 0.151 | 1.110 | 9.14E-02 | 9.14E+00 | | | |
| 8072735 | NM_145343 | APOL1 | apolipoprotein L, 1 | 0.151 | 1.110 | 3.39E-01 | 3.39E+01 | | | |
| 7979516 | NM_020810 | TRMT5 | TRM5 tRNA methyltransferase 5 hon | 0.151 | 1.110 | 4.75E-02 | 4.75E+00 | | | |
| 7896227 | --- | --- | --- | 0.151 | 1.110 | 6.65E-03 | 6.65E-01 | | | |
| 7894384 | --- | --- | --- | 0.151 | 1.110 | 8.13E-01 | 8.13E+01 | | | |
| 8067593 | NM_017798 | YTHDF1 | YTH domain family, member 1 | 0.150 | 1.110 | 6.03E-02 | 6.03E+00 | | | |
| 8034512 | NR_002751 | SNORD41 | small nucleolar RNA, C/D box 41 | 0.150 | 1.110 | 9.01E-01 | 9.01E+01 | | | |
| 8116929 | NM_002948 | RPL15 | ribosomal protein L15 | 0.150 | 1.110 | 3.82E-01 | 3.82E+01 | | | |
| 8131496 | NM_138426 | GLCCI1 | glucocorticoid induced transcript 1 | 0.150 | 1.110 | 2.19E-01 | 2.19E+01 | | | |
| 7907049 | --- | --- | --- | 0.150 | 1.109 | 1.18E-01 | 1.18E+01 | | | |
| 8124911 | NM_005514 | HLA-B | major histocompatibility complex, cl | 0.150 | 1.109 | 9.39E-02 | 9.39E+00 | | | |
| 8124055 | --- | --- | --- | 0.150 | 1.109 | 4.04E-01 | 4.04E+01 | | | |
| 7952557 | NM_003139 | SRPR | signal recognition particle receptor ( | 0.150 | 1.109 | 4.25E-01 | 4.25E+01 | | | |
| 7972867 | NM_00112720 | PCID2 | PCI domain containing 2 | 0.150 | 1.109 | 6.01E-01 | 6.01E+01 | | | |
| 8011542 | NM_015113 | ZZEF1 | zinc finger, ZZ-type with EF-hand do | 0.149 | 1.109 | 1.91E-01 | 1.91E+01 | | | |
| 7894756 | --- | --- | --- | 0.149 | 1.109 | 5.25E-01 | 5.25E+01 | | | |
| 7904562 | AK294944 | NBPF14 | neuroblastoma breakpoint family, m | 0.149 | 1.109 | 4.47E-03 | 4.47E-01 | | | |
| 7981601 | AK301335 | IGHV4-31 | immunoglobulin heavy variable 4-31 | 0.149 | 1.109 | 2.10E-02 | 2.10E+00 | | | |
| 8045816 | NM_00108311 | GPD2 | glycerol-3-phosphate dehydrogenase | 0.149 | 1.109 | 5.43E-01 | 5.43E+01 | | | |
| 7956417 | --- | --- | --- | 0.149 | 1.109 | 7.92E-01 | 7.92E+01 | | | |
| 8179041 | 0 | 0 | 0 | 0.149 | 1.109 | 1.23E-01 | 1.23E+01 | | | |
| 8176230 | NM_171998 | RAB39B | RAB39B, member RAS oncogene fam | 0.149 | 1.109 | 2.00E-01 | 2.00E+01 | | | |
| 8004030 | NM_015528 | RNF167 | ring finger protein 167 | 0.149 | 1.109 | 2.34E-01 | 2.34E+01 | | | |
| 8074464 | NM_005659 | UFD1L | ubiquitin fusion degradation 1 like (y | 0.149 | 1.109 | 7.38E-01 | 7.38E+01 | | | |
| 7894238 | --- | --- | --- | 0.149 | 1.109 | 8.19E-01 | 8.19E+01 | | | |
| 8013191 | NM_002018 | FLII | flightless I homolog (Drosophila) | 0.149 | 1.109 | 2.17E-01 | 2.17E+01 | | | |
| 8105935 | AK302597 | POM121L8P | POM121 membrane glycoprotein-lik | 0.149 | 1.109 | 5.52E-01 | 5.52E+01 | | | |
| 7895557 | --- | --- | --- | 0.149 | 1.109 | 7.72E-03 | 7.72E-01 | | | |
| 7910494 | NM_022786 | ARV1 | ARV1 homolog (S. cerevisiae) | 0.149 | 1.108 | 7.38E-01 | 7.38E+01 | | | |
| 8070341 | NM_033656 | BRWD1 | bromodomain and WD repeat doma | 0.148 | 1.108 | 4.72E-01 | 4.72E+01 | | | |
| 7902282 | NM_00103664 | HHLA3 | HERV-H LTR-associating 3 | 0.148 | 1.108 | 3.03E-01 | 3.03E+01 | | | |
| 7905374 | NM_002810 | PSMD4 | proteasome (prosome, macropain) 2 | 0.148 | 1.108 | 2.29E-01 | 2.29E+01 | | | |
| 8175369 | NM_024597 | MAP7D3 | MAP7 domain containing 3 | 0.148 | 1.108 | 2.51E-01 | 2.51E+01 | | | |
| 7934459 | NM_003635 | NDST2 | N-deacetylase/N-sulfotransferase (h | 0.148 | 1.108 | 3.90E-01 | 3.90E+01 | | | |
| 7894806 | --- | --- | --- | 0.148 | 1.108 | 4.10E-01 | 4.10E+01 | | | |
| 8053388 | NM_006464 | TGOLN2 | trans-golgi network protein 2 | 0.148 | 1.108 | 2.59E-01 | 2.59E+01 | | | |
| 8082570 | --- | --- | --- | 0.148 | 1.108 | 6.42E-01 | 6.42E+01 | | | |
| 8012464 | NR_024447 | OC10012828 | hypothetical protein LOC100128288 | 0.148 | 1.108 | 2.73E-01 | 2.73E+01 | | | |
| 7967255 | NM_002956 | CLIP1 | CAP-GLY domain containing linker pr | 0.148 | 1.108 | 4.62E-01 | 4.62E+01 | | | |
| 8065372 | NM_022080 | NAPB | N-ethylmaleimide-sensitive factor at | 0.148 | 1.108 | 7.83E-02 | 7.83E+00 | | | |
| 8008038 | --- | --- | --- | 0.148 | 1.108 | 3.51E-01 | 3.51E+01 | | | |
| 8015460 | NM_001096 | ACLY | ATP citrate lyase | 0.148 | 1.108 | 2.08E-01 | 2.08E+01 | | | |
| 7892620 | --- | --- | --- | 0.148 | 1.108 | 8.38E-01 | 8.38E+01 | | | |
| 7895841 | --- | --- | --- | 0.148 | 1.108 | 6.89E-01 | 6.89E+01 | | | |
| 8045768 | NM_152522 | ARL6IP6 | ADP-ribosylation-like factor 6 intera | 0.147 | 1.108 | 3.97E-01 | 3.97E+01 | | | |
| 8180354 | --- | --- | --- | 0.147 | 1.108 | 6.63E-01 | 6.63E+01 | | | |
| 8056766 | NM_003705 | SLC25A12 | solute carrier family 25 (mitochondri | 0.147 | 1.107 | 1.79E-01 | 1.79E+01 | | | |
| 7934733 | NM_145869 | ANXA11 | annexin A11 | 0.147 | 1.107 | 1.67E-01 | 1.67E+01 | | | |
| 7944722 | NM_032873 | UBASH3B | ubiquitin associated and SH3 domair | 0.147 | 1.107 | 2.10E-01 | 2.10E+01 | | | |
| 7952869 | NM_014174 | THYN1 | thymocyte nuclear protein 1 | 0.147 | 1.107 | 4.16E-01 | 4.16E+01 | | | |
| 7993680 | BC050464 | C16orf62 | chromosome 16 open reading frame | 0.147 | 1.107 | 4.42E-01 | 4.42E+01 | | | |
| 8024358 | NM_001319 | CSNK1G2 | casein kinase 1, gamma 2 | 0.147 | 1.107 | 3.79E-01 | 3.79E+01 | | | |
| 8048752 | NM_022915 | MRPL44 | mitochondrial ribosomal protein L44 | 0.147 | 1.107 | 6.84E-01 | 6.84E+01 | | | |
| 7893739 | --- | --- | --- | 0.147 | 1.107 | 1.27E-01 | 1.27E+01 | | | |
| 7928551 | NM_033022 | RPS24 | ribosomal protein S24 | 0.147 | 1.107 | 2.84E-01 | 2.84E+01 | | | |
| 8047086 | NM_005966 | NAB1 | NGFI-A binding protein 1 (EGR1 bind | 0.147 | 1.107 | 5.02E-01 | 5.02E+01 | | | |
| 7918050 | NM_002295 | RPSA | ribosomal protein SA | 0.147 | 1.107 | 2.69E-01 | 2.69E+01 | | | |
| 7896159 | --- | --- | --- | 0.147 | 1.107 | 7.49E-01 | 7.49E+01 | | | |
| 8085145 | NM_020165 | RAD18 | RAD18 homolog (S. cerevisiae) | 0.147 | 1.107 | 2.04E-01 | 2.04E+01 | | | |
| 7941563 | --- | --- | --- | 0.147 | 1.107 | 8.50E-01 | 8.50E+01 | | | |
| 8180351 | --- | --- | --- | 0.147 | 1.107 | 2.94E-01 | 2.94E+01 | | | |
| 7989309 | --- | --- | --- | 0.146 | 1.107 | 5.98E-01 | 5.98E+01 | | | |
| 8123463 | NM_018288 | PHF10 | PHD finger protein 10 | 0.146 | 1.107 | 2.81E-01 | 2.81E+01 | | | |
| 7914141 | NM_002946 | RPA2 | replication protein A2, 32kDa | 0.146 | 1.107 | 5.49E-01 | 5.49E+01 | | | |
| 8177046 | --- | --- | --- | 0.146 | 1.107 | 4.81E-01 | 4.81E+01 | | | |
| 8115476 | NM_004270 | MED7 | mediator complex subunit 7 | 0.146 | 1.107 | 6.52E-01 | 6.52E+01 | | | |
| 8071881 | NM_015330 | CYTSA | cytospin A | 0.146 | 1.107 | 3.33E-01 | 3.33E+01 | | | |
| 8146142 | NM_178819 | AGPAT6 | 1-acylglycerol-3-phosphate O-acyltra | 0.146 | 1.107 | 5.41E-01 | 5.41E+01 | | | |
| 8167749 | NM_014138 | FAM156A | family with sequence similarity 156, | 0.146 | 1.107 | 3.23E-01 | 3.23E+01 | | | |
| 7943218 | NM_015368 | PANX1 | pannexin 1 | 0.146 | 1.107 | 4.81E-01 | 4.81E+01 | | | |
| 8131996 | NM_182898 | CREB5 | cAMP responsive element binding pr | 0.146 | 1.106 | 6.45E-02 | 6.45E+00 | | | |
| 8161563 | --- | --- | --- | 0.146 | 1.106 | 9.54E-02 | 9.54E+00 | | | |

| | | | | | | | | | | |
|---|---|---|---|---|---|---|---|---|---|---|
| 7991143 | NM_014300 | SEC11A | SEC11 homolog A (S. cerevisiae) | 0.146 | 1.106 | 1.08E-01 | 1.08E+01 | | | |
| 7980970 | NM_014216 | ITPK1 | inositol 1,3,4-triphosphate 5/6 kinase | 0.146 | 1.106 | 1.78E-01 | 1.78E+01 | | | |
| 8100347 | NM_152540 | SCFD2 | sec1 family domain containing 2 | 0.146 | 1.106 | 5.75E-01 | 5.75E+01 | | | |
| 7947681 | NM_004308 | ARHGAP1 | Rho GTPase activating protein 1 | 0.146 | 1.106 | 3.69E-01 | 3.69E+01 | | | |
| 7904500 | ENST00000436 | NBPF12 | neuroblastoma breakpoint family, m | 0.146 | 1.106 | 5.72E-03 | 5.72E-01 | | | |
| 7971013 | --- | --- | --- | 0.146 | 1.106 | 5.15E-01 | 5.15E+01 | | | |
| 7892662 | --- | --- | --- | 0.146 | 1.106 | 7.84E-01 | 7.84E+01 | | | |
| 8006239 | NM_00104249 | NF1 | neurofibromin 1 | 0.146 | 1.106 | 2.12E-01 | 2.12E+01 | | | |
| 8072488 | NM_004147 | DRG1 | developmentally regulated GTP bind | 0.146 | 1.106 | 8.01E-01 | 8.01E+01 | | | |
| 8003089 | NM_003791 | MBTPS1 | membrane-bound transcription facto | 0.146 | 1.106 | 3.50E-01 | 3.50E+01 | | | |
| 8076403 | NM_000262 | NAGA | N-acetylgalactosaminidase, alpha- | 0.145 | 1.106 | 7.97E-02 | 7.97E+00 | | | |
| 8003116 | NM_031463 | HSDL1 | hydroxysteroid dehydrogenase like 1 | 0.145 | 1.106 | 2.37E-01 | 2.37E+01 | | | |
| 7976642 | --- | --- | --- | 0.145 | 1.106 | 6.89E-01 | 6.89E+01 | | | |
| 8177732 | NM_002116 | HLA-A | major histocompatibility complex, cl | 0.145 | 1.106 | 2.25E-01 | 2.25E+01 | | | |
| 7899486 | NR_003109 | TRNAU1AP | tRNA selenocysteine 1 associated pr | 0.145 | 1.106 | 3.65E-01 | 3.65E+01 | | | |
| 8125713 | NM_172208 | TAPBP | TAP binding protein (tapasin) | 0.145 | 1.106 | 1.27E-01 | 1.27E+01 | | | |
| 8168875 | NM_016607 | ARMCX3 | armadillo repeat containing, X-linked | 0.145 | 1.106 | 4.29E-01 | 4.29E+01 | | | |
| 7902166 | NM_020948 | MIER1 | mesoderm induction early response | 0.145 | 1.106 | 5.98E-01 | 5.98E+01 | | | |
| 8111941 | NM_00109827 | HMGCS1 | 3-hydroxy-3-methylglutaryl-Coenzym | 0.145 | 1.106 | 4.96E-01 | 4.96E+01 | | | |
| 8178977 | NM_172208 | TAPBP | TAP binding protein (tapasin) | 0.145 | 1.106 | 1.44E-01 | 1.44E+01 | | | |
| 7912994 | NM_00113626 | IFFO2 | intermediate filament family orphan | 0.145 | 1.106 | 3.22E-01 | 3.22E+01 | | | |
| 7894538 | --- | --- | --- | 0.145 | 1.106 | 8.38E-01 | 8.38E+01 | | | |
| 7993404 | NM_006985 | NPIP | nuclear pore complex interacting pro | 0.145 | 1.106 | 1.91E-01 | 1.91E+01 | | | |
| 8009561 | NM_000999 | RPL38 | ribosomal protein L38 | 0.145 | 1.106 | 1.60E-01 | 1.60E+01 | | | |
| 8046590 | NM_194247 | HNRNPA3 | heterogeneous nuclear ribonucleopr | 0.145 | 1.106 | 2.38E-01 | 2.38E+01 | | | |
| 7892587 | --- | --- | --- | 0.145 | 1.106 | 8.70E-01 | 8.70E+01 | | | |
| 8048171 | AK125369 | KFZp434H14 | hypothetical protein DKFZp434H141 | 0.145 | 1.106 | 3.30E-01 | 3.30E+01 | | | |
| 8107204 | --- | --- | --- | 0.145 | 1.106 | 4.47E-01 | 4.47E+01 | | | |
| 8164587 | NM_000113 | TOR1A | torsin family 1, member A (torsin A) | 0.145 | 1.106 | 1.53E-01 | 1.53E+01 | | | |
| 7975760 | NM_014239 | EIF2B2 | eukaryotic translation initiation facto | 0.145 | 1.106 | 4.83E-01 | 4.83E+01 | | | |
| 8142307 | NM_015723 | PNPLA8 | patatin-like phospholipase domain c | 0.145 | 1.106 | 5.59E-01 | 5.59E+01 | | | |
| 7899220 | NM_006015 | ARID1A | AT rich interactive domain 1A (SWI-li | 0.145 | 1.106 | 8.61E-02 | 8.61E+00 | | | |
| 8034448 | NM_016145 | C19orf56 | chromosome 19 open reading frame | 0.145 | 1.105 | 5.89E-01 | 5.89E+01 | | | |
| 8179731 | 0 | 0 | 0 | 0.145 | 1.105 | 1.22E-01 | 1.22E+01 | | | |
| 8176578 | NM_004654 | USP9Y | ubiquitin specific peptidase 9, Y-linke | 0.145 | 1.105 | 7.53E-01 | 7.53E+01 | | | |
| 7944216 | NM_006476 | ATP5L | ATP synthase, H+ transporting, mito | 0.145 | 1.105 | 3.84E-01 | 3.84E+01 | | | |
| 7925043 | NM_175876 | EXOC8 | exocyst complex component 8 | 0.144 | 1.105 | 3.21E-01 | 3.21E+01 | | | |
| 7997272 | NM_007285 | GABARAPL2 | GABA(A) receptor-associated protein | 0.144 | 1.105 | 7.20E-01 | 7.20E+01 | | | |
| 7913869 | NM_203401 | STMN1 | stathmin 1 | 0.144 | 1.105 | 3.19E-01 | 3.19E+01 | | | |
| 8124134 | NM_000367 | TPMT | thiopurine S-methyltransferase | 0.144 | 1.105 | 1.59E-01 | 1.59E+01 | | | |
| 8082120 | NM_032839 | DIRC2 | disrupted in renal carcinoma 2 | 0.144 | 1.105 | 6.45E-01 | 6.45E+01 | | | |
| 8077526 | --- | --- | --- | 0.144 | 1.105 | 3.38E-01 | 3.38E+01 | | | |
| 7923489 | NM_021633 | KLHL12 | kelch-like 12 (Drosophila) | 0.144 | 1.105 | 2.92E-01 | 2.92E+01 | | | |
| 7999468 | NR_024320 | LITAF | lipopolysaccharide-induced TNF facto | 0.144 | 1.105 | 4.98E-01 | 4.98E+01 | | | |
| 8078479 | NM_013374 | PDCD6IP | programmed cell death 6 interacting | 0.144 | 1.105 | 3.52E-01 | 3.52E+01 | | | |
| 8112883 | NR_004845 | LOC644936 | cytoplasmic beta-actin pseudogene | 0.144 | 1.105 | 2.57E-02 | 2.57E+00 | | | |
| 8009685 | NM_004252 | SLC9A3R1 | solute carrier family 9 (sodium/hydro | 0.144 | 1.105 | 9.52E-02 | 9.52E+00 | | | |
| 8083144 | --- | --- | --- | 0.144 | 1.105 | 8.03E-01 | 8.03E+01 | | | |
| 7905700 | NM_014847 | UBAP2L | ubiquitin associated protein 2-like | 0.144 | 1.105 | 3.87E-01 | 3.87E+01 | | | |
| 7894708 | --- | --- | --- | 0.144 | 1.105 | 7.30E-01 | 7.30E+01 | | | |
| 7991214 | --- | --- | --- | 0.144 | 1.105 | 7.18E-01 | 7.18E+01 | | | |
| 7895511 | --- | --- | --- | 0.144 | 1.105 | 8.62E-01 | 8.62E+01 | | | |
| 7919436 | NM_178230 | PPIAL4A | peptidylprolyl isomerase A (cyclophi | 0.144 | 1.105 | 2.14E-01 | 2.14E+01 | | | |
| 8093141 | NM_182627 | WDR53 | WD repeat domain 53 | 0.144 | 1.105 | 3.36E-01 | 3.36E+01 | | | |
| 8096733 | NM_00113625 | SGMS2 | sphingomyelin synthase 2 | 0.144 | 1.105 | 3.80E-01 | 3.80E+01 | | | |
| 7999233 | NM_032569 | GLYR1 | glyoxylate reductase 1 homolog (Ara | 0.144 | 1.105 | 2.79E-01 | 2.79E+01 | | | |
| 8010804 | NM_00103861 | NARF | nuclear prelamin A recognition facto | 0.144 | 1.105 | 2.65E-01 | 2.65E+01 | | | |
| 8092314 | NM_145261 | DNAJC19 | DnaJ (Hsp40) homolog, subfamily C, | 0.143 | 1.105 | 6.97E-01 | 6.97E+01 | | | |
| 7904881 | NR_002305 | PDIA3P | protein disulfide isomerase family A, | 0.143 | 1.104 | 4.50E-01 | 4.50E+01 | | | |
| 7938286 | NM_000990 | RPL27A | ribosomal protein L27a | 0.143 | 1.104 | 2.97E-01 | 2.97E+01 | | | |
| 7898881 | NM_003198 | TCEB3 | transcription elongation factor B (SII | 0.143 | 1.104 | 3.12E-01 | 3.12E+01 | | | |
| 7930226 | NM_014976 | PDCD11 | programmed cell death 11 | 0.143 | 1.104 | 1.63E-01 | 1.63E+01 | | | |
| 8154725 | NM_000224 | KRT18 | keratin 18 | 0.143 | 1.104 | 4.06E-01 | 4.06E+01 | | | |
| 8150872 | NM_001023 | RPS20 | ribosomal protein S20 | 0.143 | 1.104 | 2.27E-01 | 2.27E+01 | | | |
| 8127419 | --- | --- | --- | 0.143 | 1.104 | 4.14E-01 | 4.14E+01 | | | |
| 8105331 | NM_002104 | GZMK | granzyme K (granzyme 3; tryptase II) | 0.143 | 1.104 | 7.93E-01 | 7.93E+01 | | | |
| 7969058 | --- | --- | --- | 0.143 | 1.104 | 2.28E-01 | 2.28E+01 | | | |
| 7983594 | NM_00102524 | DUT | deoxyuridine triphosphatase | 0.143 | 1.104 | 1.48E-01 | 1.48E+01 | | | |
| 7944185 | NM_000073 | CD3G | CD3g molecule, gamma (CD3-TCR co | 0.143 | 1.104 | 7.34E-01 | 7.34E+01 | | | |
| 8076998 | NM_012401 | PLXNB2 | plexin B2 | 0.143 | 1.104 | 9.59E-02 | 9.59E+00 | | | mono |
| 8005202 | NR_002744 | SNORD49A | small nucleolar RNA, C/D box 49A | 0.143 | 1.104 | 8.80E-01 | 8.80E+01 | | | |
| 8021418 | NM_006785 | MALT1 | mucosa associated lymphoid tissue l | 0.143 | 1.104 | 5.11E-01 | 5.11E+01 | | | |
| 8145702 | NM_000553 | WRN | Werner syndrome, RecQ helicase-lik | 0.143 | 1.104 | 5.67E-01 | 5.67E+01 | | | |
| 7953508 | NM_000365 | TPI1 | triosephosphate isomerase 1 | 0.143 | 1.104 | 1.59E-01 | 1.59E+01 | | | |
| 8041000 | NM_007266 | GPN1 | GPN-loop GTPase 1 | 0.142 | 1.104 | 7.47E-01 | 7.47E+01 | | | |
| 8081241 | BC006512 | C3orf26 | chromosome 3 open reading frame 2 | 0.142 | 1.104 | 6.07E-01 | 6.07E+01 | | | |
| 7963869 | NM_004426 | PHC1 | polyhomeotic homolog 1 (Drosophila | 0.142 | 1.104 | 1.87E-01 | 1.87E+01 | | | |

| | | | | | | | | | | |
|---|---|---|---|---|---|---|---|---|---|---|
| 7932911 | NM_004521 | KIF5B | kinesin family member 5B | 0.142 | 1.104 | 4.28E-01 | 4.28E+01 | | | |
| 8064438 | NM_016143 | NSFL1C | NSFL1 (p97) cofactor (p47) | 0.142 | 1.104 | 2.44E-01 | 2.44E+01 | | | |
| 7960654 | NM_016162 | ING4 | inhibitor of growth family, member 4 | 0.142 | 1.103 | 4.98E-01 | 4.98E+01 | | | |
| 8100378 | NR_027153 | RPL21P44 | ribosomal protein L21 pseudogene 4 | 0.142 | 1.103 | 6.89E-01 | 6.89E+01 | | | |
| 8050565 | NM_015317 | PUM2 | pumilio homolog 2 (Drosophila) | 0.142 | 1.103 | 5.01E-01 | 5.01E+01 | | | |
| 7962893 | --- | --- | --- | 0.142 | 1.103 | 5.25E-01 | 5.25E+01 | | | |
| 8102518 | NM_00100170 | C4orf3 | chromosome 4 open reading frame 3 | 0.142 | 1.103 | 2.31E-01 | 2.31E+01 | | | |
| 8180317 | --- | --- | --- | 0.142 | 1.103 | 7.73E-01 | 7.73E+01 | | | |
| 8019716 | NM_00104028 | TBC1D3G | TBC1 domain family, member 3G | 0.142 | 1.103 | 1.38E-01 | 1.38E+01 | | | |
| 8090866 | NR_024400 | ANAPC13 | anaphase promoting complex subun | 0.141 | 1.103 | 4.55E-01 | 4.55E+01 | | | |
| 8030368 | NM_001015 | RPS11 | ribosomal protein S11 | 0.141 | 1.103 | 1.37E-01 | 1.37E+01 | | | |
| 8017106 | NM_015294 | TRIM37 | tripartite motif-containing 37 | 0.141 | 1.103 | 4.84E-01 | 4.84E+01 | | | |
| 8031207 | NM_00113091 | LILRA2 | leukocyte immunoglobulin-like recep | 0.141 | 1.103 | 1.57E-01 | 1.57E+01 | | | mono |
| 8152656 | NM_00101792 | ZHX1 | zinc fingers and homeoboxes 1 | 0.141 | 1.103 | 1.02E-01 | 1.02E+01 | | | |
| 7894205 | --- | --- | --- | 0.141 | 1.103 | 9.62E-02 | 9.62E+00 | | | |
| 7917741 | NM_016040 | TMED5 | transmembrane emp24 protein trans | 0.141 | 1.103 | 4.33E-01 | 4.33E+01 | | | |
| 8172056 | NM_000328 | RPGR | retinitis pigmentosa GTPase regulato | 0.141 | 1.103 | 2.27E-01 | 2.27E+01 | | | |
| 8048340 | NM_005444 | RQCD1 | RCD1 required for cell differentiation | 0.141 | 1.103 | 4.02E-01 | 4.02E+01 | | | |
| 8078350 | NM_00102484 | TGFBR2 | transforming growth factor, beta rec | 0.141 | 1.103 | 4.05E-01 | 4.05E+01 | | | |
| 7987526 | NM_00108079 | C15orf57 | chromosome 15 open reading frame | 0.141 | 1.103 | 2.51E-01 | 2.51E+01 | | | |
| 8107348 | --- | --- | --- | 0.141 | 1.103 | 3.01E-01 | 3.01E+01 | | | |
| 7893305 | --- | --- | --- | 0.141 | 1.102 | 3.82E-01 | 3.82E+01 | | | |
| 7924526 | NM_005426 | TP53BP2 | tumor protein p53 binding protein, 2 | 0.141 | 1.102 | 3.92E-01 | 3.92E+01 | | | |
| 8080084 | NM_006010 | MANF | mesencephalic astrocyte-derived neu | 0.141 | 1.102 | 5.11E-01 | 5.11E+01 | | | |
| 8050213 | --- | --- | --- | 0.141 | 1.102 | 6.82E-01 | 6.82E+01 | | | |
| 7965359 | NM_00100132 | ATP2B1 | ATPase, Ca++ transporting, plasma m | 0.141 | 1.102 | 5.48E-01 | 5.48E+01 | | | |
| 8174005 | NM_024917 | TRMT2B | TRM2 tRNA methyltransferase 2 hon | 0.140 | 1.102 | 9.39E-02 | 9.39E+00 | | | |
| 7926127 | NM_00102507 | CUGBP2 | CUG triplet repeat, RNA binding prot | 0.140 | 1.102 | 2.66E-01 | 2.66E+01 | | | |
| 8082750 | NM_024818 | UBA5 | ubiquitin-like modifier activating enz | 0.140 | 1.102 | 6.82E-01 | 6.82E+01 | | | |
| 8026638 | NM_004145 | MYO9B | myosin IXB | 0.140 | 1.102 | 1.78E-01 | 1.78E+01 | | | |
| 7943297 | NM_014679 | CEP57 | centrosomal protein 57kDa | 0.140 | 1.102 | 5.78E-01 | 5.78E+01 | | | |
| 7894939 | --- | --- | --- | 0.140 | 1.102 | 8.64E-01 | 8.64E+01 | | | |
| 8077993 | NM_024334 | TMEM43 | transmembrane protein 43 | 0.140 | 1.102 | 2.78E-01 | 2.78E+01 | | | |
| 8043236 | NM_012483 | GNLY | granulysin | 0.140 | 1.102 | 5.63E-01 | 5.63E+01 | | | |
| 8027473 | NM_004708 | PDCD5 | programmed cell death 5 | 0.140 | 1.102 | 3.81E-01 | 3.81E+01 | | | |
| 7896642 | --- | --- | --- | 0.140 | 1.102 | 9.57E-02 | 9.57E+00 | | | |
| 8158317 | NM_00113043 | SPTAN1 | spectrin, alpha, non-erythrocytic 1 (a | 0.140 | 1.102 | 4.23E-01 | 4.23E+01 | | | |
| 7928907 | --- | --- | --- | 0.140 | 1.102 | 8.80E-01 | 8.80E+01 | | | |
| 8061958 | NM_176812 | CHMP4B | chromatin modifying protein 4B | 0.139 | 1.101 | 5.57E-02 | 5.57E+00 | | | |
| 7893321 | --- | --- | --- | 0.139 | 1.101 | 1.22E-01 | 1.22E+01 | | | |
| 7897663 | NM_007375 | TARDBP | TAR DNA binding protein | 0.139 | 1.101 | 4.03E-01 | 4.03E+01 | | | |
| 7945573 | NM_021128 | POLR2L | polymerase (RNA) II (DNA directed) p | 0.139 | 1.101 | 2.90E-01 | 2.90E+01 | | | |
| 7896728 | --- | --- | --- | 0.139 | 1.101 | 5.35E-01 | 5.35E+01 | | | |
| 7894971 | --- | --- | --- | 0.139 | 1.101 | 6.93E-02 | 6.93E+00 | | | |
| 8134613 | NM_213603 | ZNF789 | zinc finger protein 789 | 0.139 | 1.101 | 3.00E-01 | 3.00E+01 | | | |
| 7914235 | NM_005626 | SFRS4 | splicing factor, arginine/serine-rich 4 | 0.139 | 1.101 | 4.96E-01 | 4.96E+01 | | | |
| 8002322 | NR_003610 | PDXDC2 | pyridoxal-dependent decarboxylase | 0.139 | 1.101 | 5.39E-01 | 5.39E+01 | | | |
| 8166607 | --- | --- | --- | 0.139 | 1.101 | 4.11E-01 | 4.11E+01 | | | |
| 8164129 | --- | --- | --- | 0.139 | 1.101 | 8.31E-01 | 8.31E+01 | | | |
| 7905116 | NM_016274 | PLEKHO1 | pleckstrin homology domain contain | 0.139 | 1.101 | 2.81E-01 | 2.81E+01 | | | |
| 7904569 | --- | --- | --- | 0.139 | 1.101 | 3.90E-03 | 3.90E-01 | | | |
| 8154733 | NM_002197 | ACO1 | aconitase 1, soluble | 0.139 | 1.101 | 2.58E-01 | 2.58E+01 | | | |
| 8179595 | NM_001470 | GABBR1 | gamma-aminobutyric acid (GABA) B | 0.139 | 1.101 | 9.28E-02 | 9.28E+00 | | | |
| 7943552 | NM_015423 | AASDHPPT | aminoadipate-semialdehyde dehydro | 0.139 | 1.101 | 2.73E-01 | 2.73E+01 | | | |
| 7895232 | --- | --- | --- | 0.139 | 1.101 | 8.79E-01 | 8.79E+01 | | | |
| 8065668 | NM_016408 | CDK5RAP1 | CDK5 regulatory subunit associated p | 0.139 | 1.101 | 2.02E-01 | 2.02E+01 | | | |
| 8149574 | NM_018371 | CSGALNACT1 | chondroitin sulfate N-acetylgalactosa | 0.139 | 1.101 | 2.14E-01 | 2.14E+01 | | | |
| 7993846 | --- | --- | --- | 0.138 | 1.101 | 5.99E-01 | 5.99E+01 | | | |
| 8014633 | NM_00112339 | TBC1D3D | TBC1 domain family, member 3D | 0.138 | 1.101 | 1.27E-01 | 1.27E+01 | | | |
| 7945786 | NM_005969 | NAP1L4 | nucleosome assembly protein 1-like | 0.138 | 1.101 | 4.88E-01 | 4.88E+01 | | | |
| 8123606 | BC025340 | MGC39372 | hypothetical protein MGC39372 | 0.138 | 1.101 | 4.57E-01 | 4.57E+01 | | | |
| 8102350 | --- | --- | --- | 0.138 | 1.101 | 3.31E-01 | 3.31E+01 | | | |
| 8131335 | --- | --- | --- | 0.138 | 1.101 | 8.38E-01 | 8.38E+01 | | | |
| 8013521 | --- | --- | --- | 0.138 | 1.101 | 6.99E-01 | 6.99E+01 | | | |
| 8094342 | NM_145048 | PACRGL | PARK2 co-regulated-like | 0.138 | 1.101 | 4.58E-01 | 4.58E+01 | | | |
| 8089596 | ENST0000039 | WDR52 | WD repeat domain 52 | 0.138 | 1.101 | 4.11E-01 | 4.11E+01 | | | |
| 8084345 | NM_004068 | AP2M1 | adaptor-related protein complex 2, r | 0.138 | 1.101 | 3.71E-01 | 3.71E+01 | | | |
| 8035803 | NM_021269 | ZNF708 | zinc finger protein 708 | 0.138 | 1.100 | 1.04E-01 | 1.04E+01 | | | |
| 7965789 | NM_001177 | ARL1 | ADP-ribosylation factor-like 1 | 0.138 | 1.100 | 6.69E-01 | 6.69E+01 | | | |
| 8063028 | NM_052951 | DNTTIP1 | deoxynucleotidyltransferase, termin | 0.138 | 1.100 | 2.65E-01 | 2.65E+01 | | | |
| 8119525 | AY465895 | HCRP1 | hepatocellular carcinoma-related HC | 0.138 | 1.100 | 7.18E-01 | 7.18E+01 | | | |
| 8112857 | NM_00101089 | MTX3 | metaxin 3 | 0.138 | 1.100 | 6.26E-01 | 6.26E+01 | | | |
| 8091954 | NM_014498 | GOLIM4 | golgi integral membrane protein 4 | 0.138 | 1.100 | 2.65E-01 | 2.65E+01 | | | |
| 8089647 | NM_00100989 | KIAA2018 | KIAA2018 | 0.138 | 1.100 | 6.14E-01 | 6.14E+01 | | | |
| 8051427 | NM_005102 | FEZ2 | fasciculation and elongation protein | 0.138 | 1.100 | 3.55E-01 | 3.55E+01 | | | |
| 8165046 | NM_015447 | CAMSAP1 | calmodulin regulated spectrin-associ | 0.138 | 1.100 | 2.64E-01 | 2.64E+01 | | | |
| 8121588 | NM_013352 | DSE | dermatan sulfate epimerase | 0.138 | 1.100 | 3.07E-01 | 3.07E+01 | | | |

| | | | | | | | | | | |
|---|---|---|---|---|---|---|---|---|---|---|
| 8122222 | NM_018945 | PDE7B | phosphodiesterase 7B | 0.138 | 1.100 | 3.30E-01 | 3.30E+01 | | | |
| 8077914 | NM_025265 | TSEN2 | tRNA splicing endonuclease 2 homol | 0.138 | 1.100 | 2.21E-01 | 2.21E+01 | | | |
| 7907680 | NM_014864 | FAM20B | family with sequence similarity 20, m | 0.137 | 1.100 | 6.31E-01 | 6.31E+01 | | | |
| 7927936 | NM_004728 | DDX21 | DEAD (Asp-Glu-Ala-Asp) box polypep | 0.137 | 1.100 | 7.10E-01 | 7.10E+01 | | | |
| 8145954 | NM_006283 | TACC1 | transforming, acidic coiled-coil conta | 0.137 | 1.100 | 4.41E-01 | 4.41E+01 | | | |
| 7893262 | --- | --- | --- | 0.137 | 1.100 | 5.96E-01 | 5.96E+01 | | | |
| 8143040 | NM_032826 | SLC35B4 | solute carrier family 35, member B4 | 0.137 | 1.100 | 4.81E-01 | 4.81E+01 | | | |
| 7896339 | --- | --- | --- | 0.137 | 1.100 | 5.61E-01 | 5.61E+01 | | | |
| 7961187 | NM_002259 | KLRC1 | killer cell lectin-like receptor subfam | 0.137 | 1.100 | 3.71E-01 | 3.71E+01 | | | |
| 8053484 | NM_003896 | ST3GAL5 | ST3 beta-galactoside alpha-2,3-sialyl | 0.137 | 1.100 | 5.47E-01 | 5.47E+01 | | | |
| 7920337 | NM_023015 | INTS3 | integrator complex subunit 3 | 0.137 | 1.100 | 3.32E-01 | 3.32E+01 | | | |
| 7913712 | NM_006625 | FUSIP1 | FUS interacting protein (serine/argin | 0.137 | 1.100 | 5.71E-01 | 5.71E+01 | | | |
| 8093330 | --- | --- | --- | 0.137 | 1.100 | 3.29E-01 | 3.29E+01 | | | |
| 8047339 | NR_026584 | BZW1L1 | basic leucine zipper and W2 domains | 0.137 | 1.100 | 6.83E-01 | 6.83E+01 | | | |
| 8026300 | NM_078481 | CD97 | CD97 molecule | 0.137 | 1.099 | 2.76E-01 | 2.76E+01 | | | |
| 7921806 | NM_003779 | B4GALT3 | UDP-Gal:betaGlcNAc beta 1,4- galact | 0.137 | 1.099 | 2.08E-01 | 2.08E+01 | | | |
| 7927926 | NM_024045 | DDX50 | DEAD (Asp-Glu-Ala-Asp) box polypep | 0.137 | 1.099 | 5.35E-01 | 5.35E+01 | | | |
| 7989132 | NM_022841 | RFX7 | regulatory factor X, 7 | 0.137 | 1.099 | 4.42E-01 | 4.42E+01 | | | |
| 8033912 | NM_00113082 | DNMT1 | DNA (cytosine-5-)-methyltransferase | 0.137 | 1.099 | 1.19E-01 | 1.19E+01 | | | |
| 8121416 | --- | --- | --- | 0.137 | 1.099 | 3.92E-01 | 3.92E+01 | | | |
| 8174737 | NM_024528 | NKAP | NFKB activating protein | 0.137 | 1.099 | 5.63E-01 | 5.63E+01 | | | |
| 8049317 | NM_152879 | DGKD | diacylglycerol kinase, delta 130kDa | 0.137 | 1.099 | 4.72E-01 | 4.72E+01 | | | |
| 7956220 | NM_024068 | OBFC2B | oligonucleotide/oligosaccharide-bind | 0.136 | 1.099 | 5.24E-01 | 5.24E+01 | | | |
| 7964064 | NM_004077 | CS | citrate synthase | 0.136 | 1.099 | 4.37E-01 | 4.37E+01 | | | |
| 8129901 | NM_031922 | REPS1 | RALBP1 associated Eps domain conta | 0.136 | 1.099 | 5.98E-01 | 5.98E+01 | | | |
| 7931393 | NM_006541 | GLRX3 | glutaredoxin 3 | 0.136 | 1.099 | 4.00E-01 | 4.00E+01 | | | |
| 8151384 | NM_014393 | STAU2 | staufen, RNA binding protein, homol | 0.136 | 1.099 | 2.88E-01 | 2.88E+01 | | | |
| 8157253 | NM_00101299 | SNX30 | sorting nexin family member 30 | 0.136 | 1.099 | 1.42E-01 | 1.42E+01 | | | |
| 8007931 | NM_000212 | ITGB3 | integrin, beta 3 (platelet glycoprotei | 0.136 | 1.099 | 5.10E-01 | 5.10E+01 | | | |
| 8047217 | NM_025147 | COQ10B | coenzyme Q10 homolog B (S. cerevis | 0.136 | 1.099 | 6.38E-01 | 6.38E+01 | | | |
| 7893536 | --- | --- | --- | 0.136 | 1.099 | 1.12E-02 | 1.12E+00 | | | |
| 7987145 | NM_00110318 | FMN1 | formin 1 | 0.136 | 1.099 | 5.13E-01 | 5.13E+01 | | | |
| 7914665 | --- | --- | --- | 0.136 | 1.099 | 5.57E-01 | 5.57E+01 | | | |
| 7894029 | --- | --- | --- | 0.136 | 1.099 | 2.29E-01 | 2.29E+01 | | | |
| 7893050 | --- | --- | --- | 0.136 | 1.099 | 4.28E-02 | 4.28E+00 | | | |
| 7895752 | --- | --- | --- | 0.136 | 1.099 | 6.58E-01 | 6.58E+01 | | | |
| 7896554 | --- | --- | --- | 0.136 | 1.099 | 1.38E-01 | 1.38E+01 | | | |
| 8167305 | NM_006579 | EBP | emopamil binding protein (sterol iso | 0.136 | 1.099 | 6.55E-01 | 6.55E+01 | | | |
| 8099506 | NM_153365 | TAPT1 | transmembrane anterior posterior tr | 0.136 | 1.099 | 5.72E-01 | 5.72E+01 | | | |
| 8001818 | NM_004614 | TK2 | thymidine kinase 2, mitochondrial | 0.136 | 1.099 | 4.05E-01 | 4.05E+01 | | | |
| 7964021 | NM_194358 | RNF41 | ring finger protein 41 | 0.136 | 1.099 | 5.70E-01 | 5.70E+01 | | | |
| 7940051 | NM_015457 | ZDHHC5 | zinc finger, DHHC-type containing 5 | 0.136 | 1.099 | 2.58E-01 | 2.58E+01 | | | |
| 8135323 | NM_021930 | RINT1 | RAD50 interactor 1 | 0.136 | 1.099 | 2.24E-01 | 2.24E+01 | | | |
| 8073081 | NM_145298 | APOBEC3F | apolipoprotein B mRNA editing enzy | 0.136 | 1.099 | 3.81E-01 | 3.81E+01 | | | |
| 8075828 | NM_006860 | RABL4 | RAB, member of RAS oncogene fami | 0.136 | 1.099 | 3.83E-01 | 3.83E+01 | | | |
| 7966706 | NM_015335 | MED13L | mediator complex subunit 13-like | 0.136 | 1.099 | 5.47E-01 | 5.47E+01 | | | |
| 8178322 | NM_006937 | SUMO2 | SMT3 suppressor of mif two 3 homo | 0.136 | 1.099 | 6.10E-01 | 6.10E+01 | | | |
| 7901601 | NM_016491 | MRPL37 | mitochondrial ribosomal protein L37 | 0.135 | 1.098 | 3.48E-01 | 3.48E+01 | | | |
| 8020183 | NM_014214 | IMPA2 | inositol(myo)-1(or 4)-monophosphat | 0.135 | 1.098 | 1.22E-01 | 1.22E+01 | | | |
| 8011112 | --- | --- | --- | 0.135 | 1.098 | 7.28E-01 | 7.28E+01 | | | |
| 7989834 | AK296134 | C15orf44 | chromosome 15 open reading frame | 0.135 | 1.098 | 5.50E-01 | 5.50E+01 | | | |
| 8019655 | NM_00100141 | TBC1D3B | TBC1 domain family, member 3B | 0.135 | 1.098 | 1.40E-01 | 1.40E+01 | | | |
| 8169859 | NM_018990 | SASH3 | SAM and SH3 domain containing 3 | 0.135 | 1.098 | 1.84E-01 | 1.84E+01 | | | |
| 8141273 | NM_014891 | PDAP1 | PDGFA associated protein 1 | 0.135 | 1.098 | 3.35E-01 | 3.35E+01 | | | |
| 8180353 | --- | --- | --- | 0.135 | 1.098 | 6.69E-01 | 6.69E+01 | | | |
| 8139392 | NM_019082 | DDX56 | DEAD (Asp-Glu-Ala-Asp) box polypep | 0.135 | 1.098 | 1.31E-01 | 1.31E+01 | | | |
| 7997852 | NM_016209 | TRAPPC2L | trafficking protein particle complex 2 | 0.135 | 1.098 | 3.61E-01 | 3.61E+01 | | | |
| 8167897 | NM_016656 | RRAGB | Ras-related GTP binding B | 0.135 | 1.098 | 7.46E-01 | 7.46E+01 | | | |
| 8167270 | NM_177439 | FTSJ1 | FtsJ homolog 1 (E. coli) | 0.135 | 1.098 | 2.19E-01 | 2.19E+01 | | | |
| 8017019 | NM_004687 | MTMR4 | myotubularin related protein 4 | 0.135 | 1.098 | 1.80E-01 | 1.80E+01 | | | |
| 8134992 | NM_015908 | SRRT | serrate RNA effector molecule homo | 0.135 | 1.098 | 5.19E-01 | 5.19E+01 | | | |
| 8160602 | NM_175069 | APTX | aprataxin | 0.135 | 1.098 | 3.98E-01 | 3.98E+01 | | | |
| 7982309 | NM_014967 | MTMR15 | myotubularin related protein 15 | 0.135 | 1.098 | 2.87E-01 | 2.87E+01 | | | |
| 8151549 | NM_005536 | IMPA1 | inositol(myo)-1(or 4)-monophosphat | 0.134 | 1.098 | 6.25E-01 | 6.25E+01 | | | |
| 7999478 | NM_015914 | TXNDC11 | thioredoxin domain containing 11 | 0.134 | 1.098 | 2.62E-01 | 2.62E+01 | | | |
| 8014437 | NM_00104028 | TBC1D3G | TBC1 domain family, member 3G | 0.134 | 1.098 | 1.33E-01 | 1.33E+01 | | | |
| 8101212 | NM_006835 | CCNI | cyclin I | 0.134 | 1.098 | 4.04E-01 | 4.04E+01 | | | |
| 8135275 | --- | --- | --- | 0.134 | 1.098 | 7.96E-01 | 7.96E+01 | | | |
| 8053713 | --- | --- | --- | 0.134 | 1.098 | 6.70E-01 | 6.70E+01 | | | |
| 8172471 | NM_006875 | PIM2 | pim-2 oncogene | 0.134 | 1.097 | 2.53E-01 | 2.53E+01 | | | |
| 7896603 | --- | --- | --- | 0.134 | 1.097 | 9.61E-02 | 9.61E+00 | | | |
| 8045208 | NM_00108353 | POTEE | POTE ankyrin domain family, membe | 0.134 | 1.097 | 4.10E-02 | 4.10E+00 | | | |
| 8170009 | NM_00107817 | FAM127A | family with sequence similarity 127, | 0.134 | 1.097 | 6.14E-01 | 6.14E+01 | | | |
| 8007797 | NM_001029 // | PS26 // RPS2 | ribosomal protein S26 // ribosomal | 0.134 | 1.097 | 6.55E-01 | 6.55E+01 | | | |
| 8147988 | --- | --- | --- | 0.134 | 1.097 | 7.67E-01 | 7.67E+01 | | | |
| 7921834 | NM_001643 | APOA2 | apolipoprotein A-II | 0.134 | 1.097 | 3.99E-01 | 3.99E+01 | | | |
| 8174717 | NM_080632 | UPF3B | UPF3 regulator of nonsense transcrip | 0.134 | 1.097 | 4.89E-01 | 4.89E+01 | | | |

| | | | | | | | | | | |
|---|---|---|---|---|---|---|---|---|---|---|
| 8000501 | NR_003610 | PDXDC2 | pyridoxal-dependent decarboxylase | 0.134 | 1.097 | 4.92E-01 | 4.92E+01 | | | |
| 7893014 | --- | --- | --- | 0.134 | 1.097 | 8.95E-01 | 8.95E+01 | | | |
| 8038395 | NM_015953 | NOSIP | nitric oxide synthase interacting prot | 0.134 | 1.097 | 3.70E-01 | 3.70E+01 | | | |
| 7894102 | --- | --- | --- | 0.134 | 1.097 | 5.54E-02 | 5.54E+00 | | | |
| 8126360 | NM_004275 | MED20 | mediator complex subunit 20 | 0.134 | 1.097 | 1.13E-01 | 1.13E+01 | | | |
| 7920047 | NM_031420 | MRPL9 | mitochondrial ribosomal protein L9 | 0.134 | 1.097 | 7.28E-01 | 7.28E+01 | | | |
| 8007028 | NM_007359 | CASC3 | cancer susceptibility candidate 3 | 0.133 | 1.097 | 4.20E-01 | 4.20E+01 | | | |
| 7932796 | NM_021738 | SVIL | supervillin | 0.133 | 1.097 | 1.01E-01 | 1.01E+01 | | | |
| 7983321 | NM_138423 | CASC4 | cancer susceptibility candidate 4 | 0.133 | 1.097 | 3.82E-01 | 3.82E+01 | | | |
| 7926084 | NM_00100197 | ATP5C1 | ATP synthase, H+ transporting, mitoc | 0.133 | 1.097 | 7.28E-01 | 7.28E+01 | | | |
| 8166059 | NM_016562 | TLR7 | toll-like receptor 7 | 0.133 | 1.097 | 1.61E-01 | 1.61E+01 | | | |
| 7900228 | NM_004552 | NDUFS5 | NADH dehydrogenase (ubiquinone) I | 0.133 | 1.097 | 7.36E-01 | 7.36E+01 | | | |
| 7895538 | --- | --- | --- | 0.133 | 1.097 | 5.33E-02 | 5.33E+00 | | | |
| 7892778 | --- | --- | --- | 0.133 | 1.097 | 2.46E-01 | 2.46E+01 | | | |
| 7958346 | NM_152261 | C12orf23 | chromosome 12 open reading frame | 0.133 | 1.097 | 5.22E-01 | 5.22E+01 | | | |
| 7894640 | --- | --- | --- | 0.133 | 1.097 | 1.35E-02 | 1.35E+00 | | | |
| 7927854 | NM_012207 | HNRNPH3 | heterogeneous nuclear ribonucleopr | 0.133 | 1.097 | 4.48E-01 | 4.48E+01 | | | |
| 7895393 | --- | --- | --- | 0.133 | 1.097 | 6.74E-01 | 6.74E+01 | | | |
| 8112335 | --- | --- | --- | 0.133 | 1.097 | 5.53E-01 | 5.53E+01 | | | |
| 8134079 | NM_033107 | GTPBP10 | GTP-binding protein 10 (putative) | 0.133 | 1.096 | 7.26E-01 | 7.26E+01 | | | |
| 8086810 | NM_003074 | SMARCC1 | SWI/SNF related, matrix associated, | 0.133 | 1.096 | 5.48E-01 | 5.48E+01 | | | |
| 8105714 | NM_139168 | SFRS12 | splicing factor, arginine/serine-rich 1 | 0.132 | 1.096 | 6.29E-01 | 6.29E+01 | | | |
| 8127107 | --- | --- | --- | 0.132 | 1.096 | 6.68E-01 | 6.68E+01 | | | |
| 7933619 | NM_147156 | SGMS1 | sphingomyelin synthase 1 | 0.132 | 1.096 | 5.04E-01 | 5.04E+01 | | | |
| 7893052 | --- | --- | --- | 0.132 | 1.096 | 8.36E-01 | 8.36E+01 | | | |
| 7957277 | NM_015336 | ZDHHC17 | zinc finger, DHHC-type containing 17 | 0.132 | 1.096 | 5.63E-01 | 5.63E+01 | | | |
| 7989849 | NM_00114482 | DENND4A | DENN/MADD domain containing 4A | 0.132 | 1.096 | 6.55E-01 | 6.55E+01 | | | |
| 7936559 | NM_173791 | PDZD8 | PDZ domain containing 8 | 0.132 | 1.096 | 2.79E-01 | 2.79E+01 | | | |
| 7894904 | --- | --- | --- | 0.132 | 1.096 | 8.90E-01 | 8.90E+01 | | | |
| 8065359 | NM_012072 | CD93 | CD93 molecule | 0.132 | 1.096 | 8.75E-02 | 8.75E+00 | | | |
| 8051372 | NM_015955 | MEMO1 | mediator of cell motility 1 | 0.132 | 1.096 | 3.41E-01 | 3.41E+01 | | | |
| 7893212 | --- | --- | --- | 0.132 | 1.096 | 6.82E-01 | 6.82E+01 | | | |
| 8016870 | NM_016070 | MRPS23 | mitochondrial ribosomal protein S23 | 0.132 | 1.095 | 7.06E-01 | 7.06E+01 | | | |
| 7977445 | NR_026800 | KIAA0125 | KIAA0125 | 0.132 | 1.095 | 2.15E-01 | 2.15E+01 | | | |
| 7894036 | --- | --- | --- | 0.132 | 1.095 | 9.66E-02 | 9.66E+00 | | | |
| 7894973 | --- | --- | --- | 0.132 | 1.095 | 9.07E-01 | 9.07E+01 | | | |
| 8102781 | ENST00000330 | OC10029230 | similar to hCG1993567 | 0.131 | 1.095 | 3.50E-01 | 3.50E+01 | | | |
| 7893664 | --- | --- | --- | 0.131 | 1.095 | 8.38E-01 | 8.38E+01 | | | |
| 8045321 | NM_00109977 | POTEF | POTE ankyrin domain family, membe | 0.131 | 1.095 | 4.65E-02 | 4.65E+00 | | | |
| 7937913 | --- | --- | --- | 0.131 | 1.095 | 7.21E-01 | 7.21E+01 | | | |
| 7896702 | --- | --- | --- | 0.131 | 1.095 | 6.25E-01 | 6.25E+01 | | | |
| 8060758 | NM_000311 | PRNP | prion protein | 0.131 | 1.095 | 2.67E-01 | 2.67E+01 | | | |
| 7996759 | --- | --- | --- | 0.131 | 1.095 | 5.38E-01 | 5.38E+01 | | | |
| 8004331 | NM_014716 | ACAP1 | ArfGAP with coiled-coil, ankyrin repe | 0.131 | 1.095 | 5.43E-02 | 5.43E+00 | | | |
| 7893983 | --- | --- | --- | 0.131 | 1.095 | 7.30E-01 | 7.30E+01 | | | |
| 7953111 | NM_018463 | ITFG2 | integrin alpha FG-GAP repeat contair | 0.131 | 1.095 | 3.04E-01 | 3.04E+01 | | | |
| 8106429 | NM_018046 | AGGF1 | angiogenic factor with G patch and F | 0.131 | 1.095 | 4.58E-01 | 4.58E+01 | | | |
| 7892986 | --- | --- | --- | 0.131 | 1.095 | 5.84E-01 | 5.84E+01 | | | |
| 8056993 | --- | --- | --- | 0.131 | 1.095 | 6.33E-01 | 6.33E+01 | | | |
| 8084323 | NM_004423 | DVL3 | dishevelled, dsh homolog 3 (Drosoph | 0.131 | 1.095 | 9.59E-02 | 9.59E+00 | | | |
| 7913185 | --- | --- | --- | 0.131 | 1.095 | 6.68E-01 | 6.68E+01 | | | |
| 7909992 | NM_015176 | FBXO28 | F-box protein 28 | 0.131 | 1.095 | 6.35E-01 | 6.35E+01 | | | |
| 8080198 | NM_144641 | PPM1M | protein phosphatase 1M (PP2C doma | 0.131 | 1.095 | 2.98E-01 | 2.98E+01 | | | |
| 7971920 | NM_194247 | HNRNPA3 | heterogeneous nuclear ribonucleopr | 0.130 | 1.095 | 3.35E-01 | 3.35E+01 | | | |
| 8046848 | NM_018471 | ZC3H15 | zinc finger CCCH-type containing 15 | 0.130 | 1.095 | 5.67E-01 | 5.67E+01 | | | |
| 8108099 | NM_021982 | SEC24A | SEC24 family, member A (S. cerevisia | 0.130 | 1.095 | 2.66E-01 | 2.66E+01 | | | |
| 8180294 | --- | --- | --- | 0.130 | 1.095 | 6.79E-01 | 6.79E+01 | | | |
| 8141107 | NM_00116021 | SLC25A13 | solute carrier family 25, member 13 | 0.130 | 1.094 | 2.36E-01 | 2.36E+01 | | | |
| 7896115 | --- | --- | --- | 0.130 | 1.094 | 4.09E-02 | 4.09E+00 | | | |
| 8040927 | NM_013392 | NRBP1 | nuclear receptor binding protein 1 | 0.130 | 1.094 | 5.82E-01 | 5.82E+01 | | | |
| 8119503 | NM_138572 | TAF8 | TAF8 RNA polymerase II, TATA box b | 0.130 | 1.094 | 3.90E-01 | 3.90E+01 | | | |
| 8178498 | NM_005514 | HLA-B | major histocompatibility complex, cl | 0.130 | 1.094 | 1.82E-01 | 1.82E+01 | | | |
| 8027932 | NM_001863 | COX6B1 | cytochrome c oxidase subunit Vib po | 0.130 | 1.094 | 6.45E-01 | 6.45E+01 | | | |
| 8129953 | NM_006734 | HIVEP2 | human immunodeficiency virus type | 0.130 | 1.094 | 6.65E-01 | 6.65E+01 | | | |
| 8058486 | NM_145280 | FAM119A | family with sequence similarity 119, | 0.130 | 1.094 | 3.45E-01 | 3.45E+01 | | | |
| 7950796 | NM_00103961 | CREBZF | CREB/ATF bZIP transcription factor | 0.130 | 1.094 | 5.38E-01 | 5.38E+01 | | | |
| 8090737 | NM_153240 | NPHP3 | nephronophthisis 3 (adolescent) | 0.130 | 1.094 | 3.36E-01 | 3.36E+01 | | | |
| 8128638 | NM_198081 | SCML4 | sex comb on midleg-like 4 (Drosophi | 0.130 | 1.094 | 1.12E-01 | 1.12E+01 | | | |
| 8083429 | NM_021038 | MBNL1 | muscleblind-like (Drosophila) | 0.130 | 1.094 | 3.02E-01 | 3.02E+01 | | | |
| 7896443 | --- | --- | --- | 0.130 | 1.094 | 2.27E-01 | 2.27E+01 | | | |
| 7896247 | --- | --- | --- | 0.130 | 1.094 | 2.35E-01 | 2.35E+01 | | | |
| 8133582 | NR_003664 | SPDYE8P | speedy homolog E8 (Xenopus laevis) | 0.130 | 1.094 | 3.63E-01 | 3.63E+01 | | | |
| 7976726 | NM_016337 | EVL | Enah/Vasp-like | 0.130 | 1.094 | 1.60E-01 | 1.60E+01 | | | |
| 7896526 | --- | --- | --- | 0.130 | 1.094 | 7.73E-01 | 7.73E+01 | | | |
| 7894043 | --- | --- | --- | 0.129 | 1.094 | 3.11E-01 | 3.11E+01 | | | |
| 8063873 | NM_144498 | OSBPL2 | oxysterol binding protein-like 2 | 0.129 | 1.094 | 4.48E-01 | 4.48E+01 | | | |
| 7896397 | --- | --- | --- | 0.129 | 1.094 | 3.35E-01 | 3.35E+01 | | | |

| | | | | | | | | | | |
|---|---|---|---|---|---|---|---|---|---|---|
| 7936937 | NM_000375 | UROS | uroporphyrinogen III synthase | 0.129 | 1.094 | 5.95E-01 | 5.95E+01 | | | |
| 8018694 | NM_002766 | PRPSAP1 | phosphoribosyl pyrophosphate synth | 0.129 | 1.094 | 5.66E-01 | 5.66E+01 | | | |
| 7944850 | NM_032811 | TBRG1 | transforming growth factor beta regu | 0.129 | 1.094 | 4.67E-01 | 4.67E+01 | | | |
| 7948332 | NM_004811 | LPXN | leupaxin | 0.129 | 1.094 | 5.40E-01 | 5.40E+01 | | | |
| 8014420 | NM_00112339 | TBC1D3D | TBC1 domain family, member 3D | 0.129 | 1.094 | 1.59E-01 | 1.59E+01 | | | |
| 8102839 | NM_002494 | NDUFC1 | NADH dehydrogenase (ubiquinone) 1 | 0.129 | 1.094 | 5.98E-01 | 5.98E+01 | | | |
| 7926239 | NM_00100821 | OPTN | optineurin | 0.129 | 1.094 | 4.79E-01 | 4.79E+01 | | | |
| 7914153 | NM_001990 | EYA3 | eyes absent homolog 3 (Drosophila) | 0.129 | 1.094 | 2.80E-01 | 2.80E+01 | | | |
| 7892884 | --- | --- | --- | 0.129 | 1.094 | 8.56E-01 | 8.56E+01 | | | |
| 7905826 | NR_024163 | CKS1B | CDC28 protein kinase regulatory sub | 0.129 | 1.094 | 4.78E-01 | 4.78E+01 | | | |
| 7892831 | --- | --- | --- | 0.129 | 1.094 | 1.02E-01 | 1.02E+01 | | | |
| 8066275 | --- | --- | --- | 0.129 | 1.093 | 8.51E-01 | 8.51E+01 | | | |
| 8148022 | NM_080651 | MED30 | mediator complex subunit 30 | 0.129 | 1.093 | 5.62E-01 | 5.62E+01 | | | |
| 8151561 | NM_024699 | ZFAND1 | zinc finger, AN1-type domain 1 | 0.129 | 1.093 | 8.46E-01 | 8.46E+01 | | | |
| 8144239 | NM_183421 | FBXO25 | F-box protein 25 | 0.129 | 1.093 | 3.47E-01 | 3.47E+01 | | | |
| 8007441 | NM_000988 | RPL27 | ribosomal protein L27 | 0.129 | 1.093 | 4.90E-01 | 4.90E+01 | | | |
| 8042737 | NM_015120 | ALMS1 | Alstrom syndrome 1 | 0.128 | 1.093 | 2.35E-01 | 2.35E+01 | | | |
| 8161632 | NM_00109966 | PTAR1 | protein prenyltransferase alpha subu | 0.128 | 1.093 | 5.88E-01 | 5.88E+01 | | | |
| 8142096 | NM_020725 | ATXN7L1 | ataxin 7-like 1 | 0.128 | 1.093 | 8.51E-01 | 8.51E+01 | | | |
| 8153039 | --- | --- | --- | 0.128 | 1.093 | 7.32E-01 | 7.32E+01 | | | |
| 7934154 | --- | --- | --- | 0.128 | 1.093 | 4.86E-01 | 4.86E+01 | | | |
| 8165674 | AY423734 | SH3KBP1 | SH3-domain kinase binding protein 1 | 0.128 | 1.093 | 2.06E-01 | 2.06E+01 | | | |
| 7960947 | NM_000014 | A2M | alpha-2-macroglobulin | 0.128 | 1.093 | 3.28E-01 | 3.28E+01 | | | |
| 8137008 | AK290098 | C7orf11 | chromosome 7 open reading frame 1 | 0.128 | 1.093 | 5.65E-01 | 5.65E+01 | | | |
| 7894699 | --- | --- | --- | 0.128 | 1.093 | 5.80E-01 | 5.80E+01 | | | |
| 8122336 | NM_021243 | C6orf115 | chromosome 6 open reading frame 1 | 0.128 | 1.093 | 5.71E-01 | 5.71E+01 | | | |
| 7969770 | --- | --- | --- | 0.128 | 1.093 | 8.01E-01 | 8.01E+01 | | | |
| 7989013 | NM_016304 | RSL24D1 | ribosomal L24 domain containing 1 | 0.128 | 1.092 | 6.43E-01 | 6.43E+01 | | | |
| 7899719 | NM_024296 | CCDC28B | coiled-coil domain containing 28B | 0.128 | 1.092 | 1.37E-01 | 1.37E+01 | | | |
| 7896155 | --- | --- | --- | 0.128 | 1.092 | 7.60E-01 | 7.60E+01 | | | |
| 8158783 | NM_005085 | NUP214 | nucleoporin 214kDa | 0.128 | 1.092 | 4.70E-01 | 4.70E+01 | | | |
| 8023646 | NM_000633 | BCL2 | B-cell CLL/lymphoma 2 | 0.127 | 1.092 | 2.97E-01 | 2.97E+01 | | | |
| 8083310 | NM_007282 | RNF13 | ring finger protein 13 | 0.127 | 1.092 | 3.84E-01 | 3.84E+01 | | | |
| 7921677 | NM_016382 | CD244 | CD244 molecule, natural killer cell re | 0.127 | 1.092 | 3.57E-01 | 3.57E+01 | | | |
| 8078091 | NM_003298 | NR2C2 | nuclear receptor subfamily 2, group | 0.127 | 1.092 | 3.42E-01 | 3.42E+01 | | | |
| 8152666 | --- | --- | --- | 0.127 | 1.092 | 7.92E-01 | 7.92E+01 | | | |
| 7904874 | NM_183372 | RP11-94I2.2 | neuroblastoma breakpoint family, m | 0.127 | 1.092 | 1.32E-02 | 1.32E+00 | | | |
| 8079149 | --- | --- | --- | 0.127 | 1.092 | 5.46E-01 | 5.46E+01 | | | |
| 7981773 | --- | --- | --- | 0.127 | 1.092 | 4.57E-01 | 4.57E+01 | | | |
| 8106068 | NM_022132 | MCCC2 | methylcrotonoyl-Coenzyme A carbox | 0.127 | 1.092 | 5.23E-01 | 5.23E+01 | | | |
| 8177601 | NM_022132 | MCCC2 | methylcrotonoyl-Coenzyme A carbox | 0.127 | 1.092 | 5.23E-01 | 5.23E+01 | | | |
| 8112388 | NM_001656 | TRIM23 | tripartite motif-containing 23 | 0.127 | 1.092 | 7.25E-01 | 7.25E+01 | | | |
| 7893962 | --- | --- | --- | 0.127 | 1.092 | 6.69E-01 | 6.69E+01 | | | |
| 7909603 | NM_013349 | NENF | neuron derived neurotrophic factor | 0.127 | 1.092 | 6.93E-01 | 6.93E+01 | | | |
| 8012605 | NM_201433 | GAS7 | growth arrest-specific 7 | 0.127 | 1.092 | 1.65E-01 | 1.65E+01 | | | |
| 7894796 | --- | --- | --- | 0.127 | 1.092 | 8.38E-01 | 8.38E+01 | | | |
| 7997048 | NM_007242 | DDX19B | DEAD (Asp-Glu-Ala-As) box polypept | 0.127 | 1.092 | 3.46E-01 | 3.46E+01 | | | |
| 7894745 | --- | --- | --- | 0.127 | 1.092 | 6.50E-01 | 6.50E+01 | | | |
| 8047272 | NM_015535 | SPATS2L | spermatogenesis associated, serine- | 0.127 | 1.092 | 3.88E-01 | 3.88E+01 | | | |
| 7970565 | --- | --- | --- | 0.126 | 1.092 | 1.74E-01 | 1.74E+01 | | | |
| 7959927 | NM_004592 | SFRS8 | splicing factor, arginine/serine-rich 8 | 0.126 | 1.092 | 4.55E-01 | 4.55E+01 | | | |
| 7894824 | --- | --- | --- | 0.126 | 1.092 | 6.67E-01 | 6.67E+01 | | | |
| 7936284 | NM_020383 | XPNPEP1 | X-prolyl aminopeptidase (aminopept | 0.126 | 1.091 | 3.64E-01 | 3.64E+01 | | | |
| 8013788 | NM_004475 | FLOT2 | flotillin 2 | 0.126 | 1.091 | 5.47E-01 | 5.47E+01 | | | |
| 7907492 | NM_014857 | RABGAP1L | RAB GTPase activating protein 1-like | 0.126 | 1.091 | 4.14E-01 | 4.14E+01 | | | |
| 7935403 | NM_032900 | ARHGAP19 | Rho GTPase activating protein 19 | 0.126 | 1.091 | 3.58E-01 | 3.58E+01 | | | |
| 7987960 | NM_173500 | TTBK2 | tau tubulin kinase 2 | 0.126 | 1.091 | 2.19E-01 | 2.19E+01 | | | |
| 7964260 | --- | --- | --- | 0.126 | 1.091 | 7.67E-01 | 7.67E+01 | | | |
| 8141173 | NM_006188 | OCM2 | oncomodulin 2 | 0.126 | 1.091 | 6.27E-01 | 6.27E+01 | | | |
| 7921319 | NM_052938 | FCRL1 | Fc receptor-like 1 | 0.126 | 1.091 | 6.51E-01 | 6.51E+01 | | | |
| 8087669 | NM_006545 | TUSC4 | tumor suppressor candidate 4 | 0.126 | 1.091 | 4.79E-01 | 4.79E+01 | | | |
| 7895609 | --- | --- | --- | 0.126 | 1.091 | 6.67E-01 | 6.67E+01 | | | |
| 7896370 | --- | --- | --- | 0.126 | 1.091 | 7.26E-01 | 7.26E+01 | | | |
| 8095360 | --- | --- | --- | 0.126 | 1.091 | 6.59E-01 | 6.59E+01 | | | |
| 7894027 | --- | --- | --- | 0.126 | 1.091 | 7.09E-01 | 7.09E+01 | | | |
| 8052622 | NR_003131 | LOC388955 | PX19 protein pseudogene | 0.126 | 1.091 | 3.24E-01 | 3.24E+01 | | | |
| 7895392 | --- | --- | --- | 0.126 | 1.091 | 1.95E-02 | 1.95E+00 | | | |
| 7895300 | --- | --- | --- | 0.126 | 1.091 | 8.96E-01 | 8.96E+01 | | | |
| 8020419 | --- | --- | --- | 0.126 | 1.091 | 4.80E-01 | 4.80E+01 | | | |
| 8024111 | NM_004368 | CNN2 | calponin 2 | 0.126 | 1.091 | 7.43E-01 | 7.43E+01 | | | |
| 8078832 | --- | --- | --- | 0.126 | 1.091 | 2.91E-01 | 2.91E+01 | | | |
| 8040018 | NM_016030 | TTC15 | tetratricopeptide repeat domain 15 | 0.125 | 1.091 | 2.36E-01 | 2.36E+01 | | | |
| 8018600 | NM_014230 | SRP68 | signal recognition particle 68kDa | 0.125 | 1.091 | 6.19E-01 | 6.19E+01 | | | |
| 8041553 | NM_024775 | GEMIN6 | gem (nuclear organelle) associated p | 0.125 | 1.091 | 6.09E-01 | 6.09E+01 | | | |
| 7894982 | --- | --- | --- | 0.125 | 1.091 | 7.69E-03 | 7.69E-01 | | | |
| 7938111 | NM_012192 | FXC1 | fracture callus 1 homolog (rat) | 0.125 | 1.091 | 2.77E-01 | 2.77E+01 | | | |
| 8033460 | --- | --- | --- | 0.125 | 1.091 | 5.74E-01 | 5.74E+01 | | | |

| | | | | | | | | | | |
|---|---|---|---|---|---|---|---|---|---|---|
| 8166956 | NM_021140 | KDM6A | lysine (K)-specific demethylase 6A | 0.125 | 1.091 | 5.45E-01 | 5.45E+01 | | | |
| 8144953 | NM_00110016 | XPO7 | exportin 7 | 0.125 | 1.091 | 4.35E-01 | 4.35E+01 | | | |
| 8007355 | NM_032353 | VPS25 | vacuolar protein sorting 25 homolog | 0.125 | 1.091 | 4.14E-01 | 4.14E+01 | | | |
| 8134631 | NM_138494 | ZNF655 | zinc finger protein 655 | 0.125 | 1.091 | 4.23E-01 | 4.23E+01 | | | |
| 8075507 | NM_019843 | EIF4ENIF1 | eukaryotic translation initiation facto | 0.125 | 1.091 | 3.16E-01 | 3.16E+01 | | | |
| 7912257 | NM_00100956 | CLSTN1 | calsyntenin 1 | 0.125 | 1.090 | 1.90E-01 | 1.90E+01 | | | |
| 8014574 | NM_007010 | DDX52 | DEAD (Asp-Glu-Ala-Asp) box polypep | 0.125 | 1.090 | 7.46E-01 | 7.46E+01 | | | |
| 8091452 | NM_00107980 | TMEM183B | transmembrane protein 183B | 0.125 | 1.090 | 4.68E-01 | 4.68E+01 | | | |
| 8151118 | NM_025054 | VCPIP1 | valosin containing protein (p97)/p47 | 0.125 | 1.090 | 3.62E-01 | 3.62E+01 | | | |
| 8099246 | NM_025196 | GRPEL1 | GrpE-like 1, mitochondrial (E. coli) | 0.125 | 1.090 | 4.05E-01 | 4.05E+01 | | | |
| 7910492 | --- | --- | --- | 0.125 | 1.090 | 6.94E-01 | 6.94E+01 | | | |
| 7895780 | --- | --- | --- | 0.124 | 1.090 | 8.09E-02 | 8.09E+00 | | | |
| 7923119 | NM_194314 | ZBTB41 | zinc finger and BTB domain containin | 0.124 | 1.090 | 6.54E-01 | 6.54E+01 | | | |
| 7967794 | NM_015114 | ANKLE2 | ankyrin repeat and LEM domain cont | 0.124 | 1.090 | 3.83E-01 | 3.83E+01 | | | |
| 7979565 | NM_080666 | WDR89 | WD repeat domain 89 | 0.124 | 1.090 | 7.14E-01 | 7.14E+01 | | | |
| 8130578 | NR_002960 | SNORA20 | small nucleolar RNA, H/ACA box 20 | 0.124 | 1.090 | 8.57E-01 | 8.57E+01 | | | |
| 8115261 | NM_015621 | CCDC69 | coiled-coil domain containing 69 | 0.124 | 1.090 | 4.48E-01 | 4.48E+01 | | | |
| 8076209 | NM_000967 | RPL3 | ribosomal protein L3 | 0.124 | 1.090 | 1.75E-01 | 1.75E+01 | | | |
| 8112327 | NM_001826 | CKS1B | CDC28 protein kinase regulatory sub | 0.124 | 1.090 | 8.37E-01 | 8.37E+01 | | | |
| 8136140 | NM_005011 | NRF1 | nuclear respiratory factor 1 | 0.124 | 1.090 | 2.40E-01 | 2.40E+01 | | | |
| 8011018 | NM_016823 | CRK | v-crk sarcoma virus CT10 oncogene h | 0.124 | 1.090 | 2.09E-01 | 2.09E+01 | | | |
| 7893817 | --- | --- | --- | 0.124 | 1.090 | 7.72E-01 | 7.72E+01 | | | |
| 8168968 | NM_014710 | GPRASP1 | G protein-coupled receptor associate | 0.124 | 1.090 | 3.96E-01 | 3.96E+01 | | | |
| 7999384 | --- | --- | --- | 0.124 | 1.090 | 7.70E-01 | 7.70E+01 | | | |
| 8025728 | NM_031209 | QTRT1 | queuine tRNA-ribosyltransferase 1 | 0.124 | 1.090 | 1.80E-01 | 1.80E+01 | | | |
| 7955777 | NM_021640 | C12orf10 | chromosome 12 open reading frame | 0.124 | 1.090 | 2.35E-01 | 2.35E+01 | | | |
| 8104378 | NM_006999 | POLS | polymerase (DNA directed) sigma | 0.124 | 1.090 | 7.22E-01 | 7.22E+01 | | | |
| 8180287 | --- | --- | --- | 0.124 | 1.090 | 2.45E-01 | 2.45E+01 | | | |
| 7944382 | NM_021729 | VPS11 | vacuolar protein sorting 11 homolog | 0.124 | 1.090 | 4.42E-01 | 4.42E+01 | | | |
| 7895170 | --- | --- | --- | 0.124 | 1.090 | 2.57E-01 | 2.57E+01 | | | |
| 8167412 | NM_005710 | PQBP1 | polyglutamine binding protein 1 | 0.124 | 1.090 | 3.42E-01 | 3.42E+01 | | | |
| 8158829 | NM_013318 | BAT2L | HLA-B associated transcript 2-like | 0.124 | 1.090 | 7.43E-01 | 7.43E+01 | | | |
| 8110123 | NM_00107952 | FAM153B | family with sequence similarity 153, | 0.124 | 1.089 | 2.55E-01 | 2.55E+01 | | | |
| 8133590 | NR_003664 | SPDYE8P | speedy homolog E8 (Xenopus laevis) | 0.124 | 1.089 | 3.08E-01 | 3.08E+01 | | | |
| 7936856 | NM_015892 | CHST15 | carbohydrate (N-acetylgalactosamin | 0.123 | 1.089 | 2.38E-01 | 2.38E+01 | | | |
| 8118622 | NM_014234 | HSD17B8 | hydroxysteroid (17-beta) dehydroge | 0.123 | 1.089 | 4.84E-01 | 4.84E+01 | | | |
| 8178234 | NM_014234 | HSD17B8 | hydroxysteroid (17-beta) dehydroge | 0.123 | 1.089 | 4.84E-01 | 4.84E+01 | | | |
| 8179534 | NM_014234 | HSD17B8 | hydroxysteroid (17-beta) dehydroge | 0.123 | 1.089 | 4.84E-01 | 4.84E+01 | | | |
| 7893545 | --- | --- | --- | 0.123 | 1.089 | 3.83E-01 | 3.83E+01 | | | |
| 7895358 | --- | --- | --- | 0.123 | 1.089 | 6.13E-01 | 6.13E+01 | | | |
| 7945283 | NM_014384 | ACAD8 | acyl-Coenzyme A dehydrogenase fam | 0.123 | 1.089 | 3.32E-01 | 3.32E+01 | | | |
| 8022320 | ENST0000044 | NPIPL3 | nuclear pore complex interacting pro | 0.123 | 1.089 | 5.01E-01 | 5.01E+01 | | | |
| 7962537 | NM_018976 | SLC38A2 | solute carrier family 38, member 2 | 0.123 | 1.089 | 7.01E-01 | 7.01E+01 | | | |
| 8171587 | NM_000292 | PHKA2 | phosphorylase kinase, alpha 2 (liver) | 0.123 | 1.089 | 2.44E-01 | 2.44E+01 | | | |
| 8143629 | NR_002157 | OR2A9P | olfactory receptor, family 2, subfami | 0.123 | 1.089 | 2.69E-01 | 2.69E+01 | | | |
| 8171865 | --- | --- | --- | 0.123 | 1.089 | 1.88E-01 | 1.88E+01 | | | |
| 8027297 | NR_027130 | ZNF738 | zinc finger protein 738 | 0.123 | 1.089 | 4.51E-01 | 4.51E+01 | | | |
| 8099912 | BC008502 | C4orf34 | chromosome 4 open reading frame 3 | 0.123 | 1.089 | 7.07E-01 | 7.07E+01 | | | |
| 8176276 | NM_000489 | ATRX | alpha thalassemia/mental retardatio | 0.123 | 1.089 | 8.06E-01 | 8.06E+01 | | | |
| 7965343 | NM_172240 | WDR51B | WD repeat domain 51B | 0.123 | 1.089 | 7.34E-01 | 7.34E+01 | | | |
| 7921652 | NM_003037 | SLAMF1 | signaling lymphocytic activation mol | 0.123 | 1.089 | 5.62E-01 | 5.62E+01 | | | |
| 8172538 | NM_007075 | WDR45 | WD repeat domain 45 | 0.123 | 1.089 | 4.23E-01 | 4.23E+01 | | | |
| 8165856 | --- | --- | --- | 0.123 | 1.089 | 7.17E-01 | 7.17E+01 | | | |
| 8039131 | NM_013342 | TFPT | TCF3 (E2A) fusion partner (in childho | 0.122 | 1.089 | 5.18E-01 | 5.18E+01 | | | |
| 8157828 | NM_030978 | ARPC5L | actin related protein 2/3 complex, su | 0.122 | 1.089 | 2.39E-01 | 2.39E+01 | | | |
| 8020668 | NM_018439 | IMPACT | Impact homolog (mouse) | 0.122 | 1.089 | 6.88E-01 | 6.88E+01 | | | |
| 7912750 | NM_018994 | FBXO42 | F-box protein 42 | 0.122 | 1.088 | 5.60E-01 | 5.60E+01 | | | |
| 8053059 | NM_212552 | BOLA3 | bolA homolog 3 (E. coli) | 0.122 | 1.088 | 3.20E-01 | 3.20E+01 | | | |
| 7977732 | NR_002916 | SNORD8 | small nucleolar RNA, C/D box 8 | 0.122 | 1.088 | 7.45E-01 | 7.45E+01 | | | |
| 8032284 | NM_006830 | UQCR | ubiquinol-cytochrome c reductase, 6 | 0.122 | 1.088 | 5.53E-01 | 5.53E+01 | | | |
| 8081537 | NM_016388 | TRAT1 | T cell receptor associated transmem | 0.122 | 1.088 | 7.59E-01 | 7.59E+01 | | | |
| 8118310 | NM_005345 | HSPA1A | heat shock 70kDa protein 1A | 0.122 | 1.088 | 6.70E-01 | 6.70E+01 | | | |
| 7997059 | NM_018332 | DDX19A | DEAD (Asp-Glu-Ala-As) box polypept | 0.122 | 1.088 | 4.14E-01 | 4.14E+01 | | | |
| 8165064 | NM_016172 | UBAC1 | UBA domain containing 1 | 0.122 | 1.088 | 3.42E-01 | 3.42E+01 | | | |
| 8123407 | NM_00104000 | MLLT4 | myeloid/lymphoid or mixed-lineage | 0.122 | 1.088 | 1.90E-01 | 1.90E+01 | | | |
| 7959574 | NM_020382 | SETD8 | SET domain containing (lysine methy | 0.122 | 1.088 | 7.04E-01 | 7.04E+01 | | | |
| 8134589 | NM_003910 | BUD31 | BUD31 homolog (S. cerevisiae) | 0.122 | 1.088 | 7.49E-01 | 7.49E+01 | | | |
| 8087224 | NM_000387 | SLC25A20 | solute carrier family 25 (carnitine/ac | 0.122 | 1.088 | 6.31E-01 | 6.31E+01 | | | |
| 7943293 | NM_015036 | ENDOD1 | endonuclease domain containing 1 | 0.122 | 1.088 | 2.07E-01 | 2.07E+01 | | | |
| 8055135 | --- | --- | --- | 0.122 | 1.088 | 6.84E-01 | 6.84E+01 | | | |
| 8082012 | NM_021082 | SLC15A2 | solute carrier family 15 (H+/peptide | 0.122 | 1.088 | 1.38E-01 | 1.38E+01 | | | |
| 7894978 | --- | --- | --- | 0.122 | 1.088 | 6.78E-01 | 6.78E+01 | | | |
| 7895060 | --- | --- | --- | 0.121 | 1.088 | 8.41E-01 | 8.41E+01 | | | |
| 8144047 | NM_007349 | PAXIP1 | PAX interacting (with transcription-a | 0.121 | 1.088 | 3.02E-01 | 3.02E+01 | | | |
| 7921930 | --- | --- | --- | 0.121 | 1.088 | 4.52E-01 | 4.52E+01 | | | |
| 8091118 | --- | --- | --- | 0.121 | 1.088 | 3.46E-01 | 3.46E+01 | | | |
| 8141762 | NM_006234 | POLR2J | polymerase (RNA) II (DNA directed) | 0.121 | 1.088 | 4.53E-01 | 4.53E+01 | | | |

| | | | | | | | | | | |
|---|---|---|---|---|---|---|---|---|---|---|
| 8139256 | NM_017920 | URG4 | up-regulated gene 4 | 0.121 | 1.088 | 2.52E-01 | 2.52E+01 | | | |
| 7894540 | --- | --- | --- | 0.121 | 1.088 | 3.11E-01 | 3.11E+01 | | | |
| 8133114 | NM_173517 | VKORC1L1 | vitamin K epoxide reductase comple: | 0.121 | 1.088 | 4.71E-01 | 4.71E+01 | | | |
| 8064868 | NM_019593 | RP5-1022P6.: | hypothetical protein KIAA1434 | 0.121 | 1.088 | 6.36E-01 | 6.36E+01 | | | |
| 8138361 | NM_021029 | RPL36A | ribosomal protein L36a | 0.121 | 1.087 | 7.66E-01 | 7.66E+01 | | | |
| 8139113 | --- | --- | --- | 0.121 | 1.087 | 2.40E-01 | 2.40E+01 | | | |
| 7895476 | --- | --- | --- | 0.121 | 1.087 | 3.69E-01 | 3.69E+01 | | | |
| 7925492 | NM_014322 | OPN3 | opsin 3 | 0.121 | 1.087 | 3.31E-01 | 3.31E+01 | | | |
| 7894159 | --- | --- | --- | 0.121 | 1.087 | 9.18E-01 | 9.18E+01 | | | |
| 8143733 | NR_027788 | ZNF767 | zinc finger family member 767 | 0.121 | 1.087 | 5.45E-01 | 5.45E+01 | | | |
| 7994655 | NM_024516 | C16orf53 | chromosome 16 open reading frame | 0.120 | 1.087 | 4.24E-01 | 4.24E+01 | | | |
| 8027642 | NM_032346 | PDCD2L | programmed cell death 2-like | 0.120 | 1.087 | 6.08E-01 | 6.08E+01 | | | |
| 7968787 | NR_002822 | MGC72080 | MGC72080 pseudogene | 0.120 | 1.087 | 7.81E-01 | 7.81E+01 | | | |
| 8007917 | --- | --- | --- | 0.120 | 1.087 | 5.79E-01 | 5.79E+01 | | | |
| 7987869 | NM_015497 | TMEM87A | transmembrane protein 87A | 0.120 | 1.087 | 7.33E-01 | 7.33E+01 | | | |
| 7962792 | NM_00100413 | OR10AD1 | olfactory receptor, family 10, subfam | 0.120 | 1.087 | 5.01E-01 | 5.01E+01 | | | |
| 8014376 | NM_00100141 | TBC1D3B | TBC1 domain family, member 3B | 0.120 | 1.087 | 1.76E-01 | 1.76E+01 | | | |
| 8152096 | NM_145690 | YWHAZ | tyrosine 3-monooxygenase/tryptoph | 0.120 | 1.087 | 4.89E-01 | 4.89E+01 | | | |
| 7925184 | NM_015014 | RBM34 | RNA binding motif protein 34 | 0.120 | 1.087 | 7.70E-01 | 7.70E+01 | | | |
| 8164896 | NM_003172 | SURF1 | surfeit 1 | 0.120 | 1.087 | 4.17E-01 | 4.17E+01 | | | |
| 8139433 | NM_033054 | MYO1G | myosin IG | 0.120 | 1.087 | 4.87E-01 | 4.87E+01 | | | |
| 7967091 | NM_139015 | UNQ1887 | signal peptide peptidase 3 | 0.120 | 1.086 | 4.59E-01 | 4.59E+01 | | | |
| 7892622 | --- | --- | --- | 0.120 | 1.086 | 8.46E-01 | 8.46E+01 | | | |
| 7908169 | NM_007212 | RNF2 | ring finger protein 2 | 0.120 | 1.086 | 3.63E-01 | 3.63E+01 | | | |
| 8115918 | NM_007097 | CLTB | clathrin, light chain (Lcb) | 0.120 | 1.086 | 2.77E-01 | 2.77E+01 | | | |
| 7925033 | NM_152379 | C1orf131 | chromosome 1 open reading frame 1 | 0.119 | 1.086 | 3.78E-01 | 3.78E+01 | | | |
| 8167125 | NM_004651 | USP11 | ubiquitin specific peptidase 11 | 0.119 | 1.086 | 2.22E-01 | 2.22E+01 | | | |
| 8053214 | NM_181575 | AUP1 | ancient ubiquitous protein 1 | 0.119 | 1.086 | 3.39E-01 | 3.39E+01 | | | |
| 8154059 | NM_003070 | SMARCA2 | SWI/SNF related, matrix associated, | 0.119 | 1.086 | 4.09E-01 | 4.09E+01 | | | |
| 7920903 | NM_003145 | SSR2 | signal sequence receptor, beta (trans | 0.119 | 1.086 | 8.13E-01 | 8.13E+01 | | | |
| 8030993 | NM_00100840 | ZNF761 | zinc finger protein 761 | 0.119 | 1.086 | 5.26E-01 | 5.26E+01 | | | |
| 8084360 | NM_018358 | ABCF3 | ATP-binding cassette, sub-family F (G | 0.119 | 1.086 | 4.37E-01 | 4.37E+01 | | | |
| 7893090 | --- | --- | --- | 0.119 | 1.086 | 3.89E-01 | 3.89E+01 | | | |
| 7994683 | NM_00108361 | TMEM219 | transmembrane protein 219 | 0.119 | 1.086 | 2.32E-01 | 2.32E+01 | | | |
| 7930927 | NM_014937 | INPP5F | inositol polyphosphate-5-phosphatas | 0.119 | 1.086 | 3.52E-01 | 3.52E+01 | | | |
| 8091806 | NM_000984 | RPL23A | ribosomal protein L23a | 0.119 | 1.086 | 2.48E-01 | 2.48E+01 | | | |
| 7938563 | NM_001178 | ARNTL | aryl hydrocarbon receptor nuclear tr | 0.119 | 1.086 | 6.56E-01 | 6.56E+01 | | | |
| 7901123 | NM_172164 | NASP | nuclear autoantigenic sperm protein | 0.119 | 1.086 | 5.13E-01 | 5.13E+01 | | | |
| 7895373 | --- | --- | --- | 0.119 | 1.086 | 1.75E-01 | 1.75E+01 | | | |
| 8064208 | NM_025219 | DNAJC5 | DnaJ (Hsp40) homolog, subfamily C, | 0.119 | 1.086 | 3.17E-01 | 3.17E+01 | | | |
| 7894934 | --- | --- | --- | 0.119 | 1.086 | 2.67E-01 | 2.67E+01 | | | |
| 8015969 | NM_014233 | UBTF | upstream binding transcription facto | 0.119 | 1.086 | 2.54E-01 | 2.54E+01 | | | |
| 8083605 | NM_016625 | RSRC1 | arginine/serine-rich coiled-coil 1 | 0.119 | 1.086 | 7.13E-01 | 7.13E+01 | | | |
| 8171111 | NM_001636 | SLC25A6 | solute carrier family 25 (mitochondri | 0.119 | 1.086 | 3.80E-01 | 3.80E+01 | | | |
| 8177003 | NM_001636 | SLC25A6 | solute carrier family 25 (mitochondri | 0.119 | 1.086 | 3.80E-01 | 3.80E+01 | | | |
| 8136918 | NM_003461 | ZYX | zyxin | 0.119 | 1.086 | 2.16E-01 | 2.16E+01 | | | |
| 8168087 | NM_001551 | IGBP1 | immunoglobulin (CD79A) binding pro | 0.119 | 1.086 | 6.66E-01 | 6.66E+01 | | | |
| 7996041 | NM_020312 | COQ9 | coenzyme Q9 homolog (S. cerevisiae | 0.119 | 1.086 | 1.68E-01 | 1.68E+01 | | | |
| 8031213 | NM_006863 | LILRA1 | leukocyte immunoglobulin-like recep | 0.119 | 1.086 | 1.08E-01 | 1.08E+01 | | | |
| 7955810 | NM_018457 | PRR13 | proline rich 13 | 0.119 | 1.086 | 2.38E-01 | 2.38E+01 | | | |
| 7990379 | NM_00114244 | EDC3 | enhancer of mRNA decapping 3 hom | 0.119 | 1.086 | 3.68E-01 | 3.68E+01 | | | |
| 7894854 | --- | --- | --- | 0.119 | 1.086 | 2.06E-01 | 2.06E+01 | | | |
| 8121704 | NM_138459 | NUS1 | nuclear undecaprenyl pyrophosphate | 0.119 | 1.086 | 2.51E-01 | 2.51E+01 | | | |
| 8054997 | NM_006609 | MAP3K2 | mitogen-activated protein kinase kin | 0.118 | 1.086 | 7.69E-01 | 7.69E+01 | | | |
| 7954613 | NM_018318 | CCDC91 | coiled-coil domain containing 91 | 0.118 | 1.085 | 7.95E-01 | 7.95E+01 | | | |
| 8084219 | NM_017644 | KLHL24 | kelch-like 24 (Drosophila) | 0.118 | 1.085 | 6.96E-01 | 6.96E+01 | | | |
| 8149857 | NM_017634 | KCTD9 | potassium channel tetramerisation d | 0.118 | 1.085 | 3.37E-01 | 3.37E+01 | | | |
| 8156604 | BC022957 | C9orf102 | chromosome 9 open reading frame 1 | 0.118 | 1.085 | 7.24E-01 | 7.24E+01 | | | |
| 8090772 | NM_007027 | TOPBP1 | topoisomerase (DNA) II binding prot | 0.118 | 1.085 | 5.70E-01 | 5.70E+01 | | | |
| 7895387 | --- | --- | --- | 0.118 | 1.085 | 6.86E-01 | 6.86E+01 | | | |
| 8101210 | --- | --- | --- | 0.118 | 1.085 | 3.85E-01 | 3.85E+01 | | | |
| 8086505 | NM_016598 | ZDHHC3 | zinc finger, DHHC-type containing 3 | 0.118 | 1.085 | 3.88E-01 | 3.88E+01 | | | |
| 8180316 | --- | --- | --- | 0.118 | 1.085 | 8.22E-01 | 8.22E+01 | | | |
| 8143441 | BC012493 | KIAA1147 | KIAA1147 | 0.118 | 1.085 | 7.02E-01 | 7.02E+01 | | | |
| 8022342 | NM_006796 | AFG3L2 | AFG3 ATPase family gene 3-like 2 (ye | 0.118 | 1.085 | 2.75E-01 | 2.75E+01 | | | |
| 8041995 | NM_003128 | SPTBN1 | spectrin, beta, non-erythrocytic 1 | 0.118 | 1.085 | 5.74E-01 | 5.74E+01 | | | |
| 8176191 | NM_004965 | HMGN1 | high-mobility group nucleosome bin | 0.118 | 1.085 | 7.05E-01 | 7.05E+01 | | | |
| 7962760 | NM_014554 | SENP1 | SUMO1/sentrin specific peptidase 1 | 0.118 | 1.085 | 5.00E-01 | 5.00E+01 | | | |
| 8139118 | --- | --- | --- | 0.118 | 1.085 | 4.45E-01 | 4.45E+01 | | | |
| 7908766 | NM_006335 | TIMM17A | translocase of inner mitochondrial m | 0.117 | 1.085 | 8.40E-01 | 8.40E+01 | | | |
| 7896149 | --- | --- | --- | 0.117 | 1.085 | 1.80E-01 | 1.80E+01 | | | |
| 8168644 | --- | --- | --- | 0.117 | 1.085 | 1.05E-01 | 1.05E+01 | | | |
| 8143065 | NR_024185 | C7orf49 | chromosome 7 open reading frame 4 | 0.117 | 1.085 | 4.73E-01 | 4.73E+01 | | | |
| 8018343 | NM_020679 | MIF4GD | MIF4G domain containing | 0.117 | 1.085 | 1.78E-01 | 1.78E+01 | | | |
| 8139125 | BC030554 | TARP | TCR gamma alternate reading frame | 0.117 | 1.085 | 5.96E-01 | 5.96E+01 | | | |
| 7941243 | NM_006268 | DPF2 | D4, zinc and double PHD fingers fam | 0.117 | 1.085 | 6.65E-01 | 6.65E+01 | | | |
| 8106702 | NM_032280 | ZCCHC9 | zinc finger, CHCC domain containing | 0.117 | 1.085 | 3.73E-01 | 3.73E+01 | | | |

| | | | | | | | | | | |
|---|---|---|---|---|---|---|---|---|---|---|
| 8120061 | NM_014936 | ENPP4 | ectonucleotide pyrophosphatase/ph | 0.117 | 1.085 | 3.19E-01 | 3.19E+01 | | | |
| 7936062 | --- | --- | --- | 0.117 | 1.085 | 3.22E-01 | 3.22E+01 | | | |
| 7974066 | NM_002687 | PNN | pinin, desmosome associated protei | 0.117 | 1.085 | 3.08E-01 | 3.08E+01 | | | |
| 7896200 | --- | --- | --- | 0.117 | 1.084 | 7.98E-01 | 7.98E+01 | | | |
| 8045887 | NR_002822 | MGC72080 | MGC72080 pseudogene | 0.117 | 1.084 | 7.87E-01 | 7.87E+01 | | | |
| 8096919 | NM_025144 | ALPK1 | alpha-kinase 1 | 0.117 | 1.084 | 2.09E-01 | 2.09E+01 | | | |
| 7985402 | NR_024474 | OC10013314 | hypothetical protein LOC100133144 | 0.117 | 1.084 | 6.14E-01 | 6.14E+01 | | | |
| 8060379 | NM_178578 | PSMF1 | proteasome (prosome, macropain) i | 0.117 | 1.084 | 4.36E-01 | 4.36E+01 | | | |
| 8101224 | --- | --- | --- | 0.117 | 1.084 | 5.87E-01 | 5.87E+01 | | | |
| 8083333 | NM_032025 | EIF2A | eukaryotic translation initiation facto | 0.117 | 1.084 | 6.88E-01 | 6.88E+01 | | | |
| 7898161 | NM_024329 | EFHD2 | EF-hand domain family, member D2 | 0.117 | 1.084 | 3.79E-01 | 3.79E+01 | | | |
| 8118111 | NM_006674 | HCP5 | HLA complex P5 | 0.117 | 1.084 | 6.44E-01 | 6.44E+01 | | | |
| 8177717 | NM_018950 | HLA-F | major histocompatibility complex, cl | 0.117 | 1.084 | 3.34E-01 | 3.34E+01 | | | |
| 8081375 | NM_145037 | FAM55C | family with sequence similarity 55, m | 0.117 | 1.084 | 6.73E-01 | 6.73E+01 | | | |
| 7956076 | NM_001798 | CDK2 | cyclin-dependent kinase 2 | 0.117 | 1.084 | 2.59E-01 | 2.59E+01 | | | |
| 8162502 | NM_000507 | FBP1 | fructose-1,6-bisphosphatase 1 | 0.116 | 1.084 | 5.96E-01 | 5.96E+01 | | | |
| 8090546 | NM_00112719 | CNBP | CCHC-type zinc finger, nucleic acid b | 0.116 | 1.084 | 3.71E-01 | 3.71E+01 | | | |
| 7893725 | --- | --- | --- | 0.116 | 1.084 | 6.11E-02 | 6.11E+00 | | | |
| 7894264 | --- | --- | --- | 0.116 | 1.084 | 7.83E-01 | 7.83E+01 | | | |
| 7987584 | NM_017553 | INO80 | INO80 homolog (S. cerevisiae) | 0.116 | 1.084 | 2.86E-01 | 2.86E+01 | | | |
| 8152759 | NR_027427 | TATDN1 | TatD DNase domain containing 1 | 0.116 | 1.084 | 7.61E-01 | 7.61E+01 | | | |
| 7960874 | NM_004054 | C3AR1 | complement component 3a receptor | 0.116 | 1.084 | 6.53E-01 | 6.53E+01 | | | |
| 8028219 | NM_144689 | ZNF420 | zinc finger protein 420 | 0.116 | 1.084 | 5.98E-01 | 5.98E+01 | | | |
| 8103244 | NM_173662 | RNF175 | ring finger protein 175 | 0.116 | 1.084 | 3.95E-01 | 3.95E+01 | | | |
| 7895296 | --- | --- | --- | 0.116 | 1.084 | 8.27E-01 | 8.27E+01 | | | |
| 8151252 | --- | --- | --- | 0.116 | 1.084 | 7.06E-01 | 7.06E+01 | | | |
| 7895676 | --- | --- | --- | 0.116 | 1.084 | 7.26E-01 | 7.26E+01 | | | |
| 7909628 | NM_014053 | FLVCR1 | feline leukemia virus subgroup C cell | 0.116 | 1.084 | 3.81E-01 | 3.81E+01 | | | |
| 7978192 | NM_138476 | MDP1 | magnesium-dependent phosphatase | 0.116 | 1.084 | 5.35E-01 | 5.35E+01 | | | |
| 7893506 | --- | --- | --- | 0.116 | 1.084 | 9.07E-01 | 9.07E+01 | | | |
| 7969935 | NM_000123 | ERCC5 | excision repair cross-complementing | 0.116 | 1.084 | 6.98E-01 | 6.98E+01 | | | |
| 8073334 | NM_014248 | RBX1 | ring-box 1 | 0.116 | 1.084 | 8.48E-01 | 8.48E+01 | | | |
| 7971241 | NR_002822 | MGC72080 | MGC72080 pseudogene | 0.116 | 1.083 | 7.86E-01 | 7.86E+01 | | | |
| 7991598 | BC067846 | SNRPA1 | small nuclear ribonucleoprotein poly | 0.116 | 1.083 | 8.46E-01 | 8.46E+01 | | | |
| 8149638 | NM_003974 | DOK2 | docking protein 2, 56kDa | 0.116 | 1.083 | 3.26E-01 | 3.26E+01 | | | |
| 7893773 | --- | --- | --- | 0.116 | 1.083 | 1.61E-02 | 1.61E+00 | | | |
| 8125835 | NM_001014 | RPS10 | ribosomal protein S10 | 0.116 | 1.083 | 4.95E-01 | 4.95E+01 | | | |
| 8129458 | NM_033515 | ARHGAP18 | Rho GTPase activating protein 18 | 0.116 | 1.083 | 6.62E-01 | 6.62E+01 | | | |
| 7937217 | NM_004092 | ECHS1 | enoyl Coenzyme A hydratase, short c | 0.115 | 1.083 | 4.38E-01 | 4.38E+01 | | | |
| 8028940 | NM_080732 | EGLN2 | egl nine homolog 2 (C. elegans) | 0.115 | 1.083 | 9.54E-02 | 9.54E+00 | | | |
| 8008933 | NM_00109943 | BCAS3 | breast carcinoma amplified sequence | 0.115 | 1.083 | 4.31E-01 | 4.31E+01 | | | |
| 8123621 | NM_004568 | SERPINB6 | serpin peptidase inhibitor, clade B (o | 0.115 | 1.083 | 5.56E-01 | 5.56E+01 | | | |
| 8111629 | NM_153485 | NUP155 | nucleoporin 155kDa | 0.115 | 1.083 | 6.21E-01 | 6.21E+01 | | | |
| 8098289 | --- | --- | --- | 0.115 | 1.083 | 3.74E-01 | 3.74E+01 | | | |
| 7934255 | NM_006077 | CBARA1 | calcium binding atopy-related autoa | 0.115 | 1.083 | 7.08E-01 | 7.08E+01 | | | |
| 7899350 | NM_00114391 | FAM76A | family with sequence similarity 76, m | 0.115 | 1.083 | 5.34E-01 | 5.34E+01 | | | |
| 7902789 | NM_012262 | HS2ST1 | heparan sulfate 2-O-sulfotransferase | 0.115 | 1.083 | 4.86E-01 | 4.86E+01 | | | |
| 8112376 | NM_022145 | CENPK | centromere protein K | 0.115 | 1.083 | 7.80E-01 | 7.80E+01 | | | |
| 8105663 | NM_020726 | NLN | neurolysin (metallopeptidase M3 fa | 0.115 | 1.083 | 2.48E-01 | 2.48E+01 | | | |
| 8150186 | NM_024787 | RNF122 | ring finger protein 122 | 0.115 | 1.083 | 3.51E-01 | 3.51E+01 | | | |
| 8001317 | NM_153029 | N4BP1 | NEDD4 binding protein 1 | 0.115 | 1.083 | 4.25E-01 | 4.25E+01 | | | |
| 7894965 | --- | --- | --- | 0.115 | 1.083 | 5.21E-01 | 5.21E+01 | | | |
| 7969677 | NM_144778 | MBNL2 | muscleblind-like 2 (Drosophila) | 0.115 | 1.083 | 6.14E-01 | 6.14E+01 | | | |
| 8053775 | NM_032788 | ZNF514 | zinc finger protein 514 | 0.115 | 1.083 | 2.37E-01 | 2.37E+01 | | | |
| 8045289 | NM_012249 | RHOQ | ras homolog gene family, member Q | 0.115 | 1.083 | 3.27E-01 | 3.27E+01 | | | |
| 8155110 | NM_00108049 | RGP1 | RGP1 retrograde golgi transport hom | 0.115 | 1.083 | 3.15E-01 | 3.15E+01 | | | |
| 7926356 | NM_00100148 | PTER | phosphotriesterase related | 0.115 | 1.083 | 3.93E-01 | 3.93E+01 | | | |
| 8124531 | NM_003533 | HIST1H3I | histone cluster 1, H3i | 0.115 | 1.083 | 8.11E-01 | 8.11E+01 | | | |
| 8105311 | NM_002495 | NDUFS4 | NADH dehydrogenase (ubiquinone) F | 0.115 | 1.083 | 8.50E-01 | 8.50E+01 | | | |
| 8152133 | NM_015713 | RRM2B | ribonucleotide reductase M2 B (TP53 | 0.115 | 1.083 | 4.03E-01 | 4.03E+01 | | | |
| 8170443 | NM_003828 | MTMR1 | myotubularin related protein 1 | 0.115 | 1.083 | 6.76E-01 | 6.76E+01 | | | |
| 8079590 | NM_004345 | CAMP | cathelicidin antimicrobial peptide | 0.115 | 1.083 | 2.55E-01 | 2.55E+01 | | | |
| 8016259 | NM_00100660 | LRRC37A2 | leucine rich repeat containing 37, m | 0.114 | 1.083 | 5.69E-01 | 5.69E+01 | | | |
| 8074748 | NR_003700 | PI4KAP2 | phosphatidylinositol 4-kinase, cataly | 0.114 | 1.082 | 4.57E-01 | 4.57E+01 | | | |
| 7988344 | --- | --- | --- | 0.114 | 1.082 | 5.55E-01 | 5.55E+01 | | | |
| 7894485 | --- | --- | --- | 0.114 | 1.082 | 2.25E-01 | 2.25E+01 | | | |
| 8124307 | NR_002174 | CMAH | cytidine monophosphate-N-acetylne | 0.114 | 1.082 | 2.39E-01 | 2.39E+01 | | | |
| 8096663 | NM_00100838 | CISD2 | CDGSH iron sulfur domain 2 | 0.114 | 1.082 | 7.39E-01 | 7.39E+01 | | | |
| 8169995 | AK295485 | FAM122C | family with sequence similarity 122C | 0.114 | 1.082 | 3.66E-01 | 3.66E+01 | | | |
| 7893862 | --- | --- | --- | 0.114 | 1.082 | 8.74E-01 | 8.74E+01 | | | |
| 7894042 | --- | --- | --- | 0.114 | 1.082 | 7.44E-01 | 7.44E+01 | | | |
| 7986501 | --- | --- | --- | 0.114 | 1.082 | 8.96E-01 | 8.96E+01 | | | |
| 7895627 | --- | --- | --- | 0.114 | 1.082 | 4.26E-01 | 4.26E+01 | | | |
| 7914750 | BC034598 | C1orf212 | chromosome 1 open reading frame 2 | 0.114 | 1.082 | 1.72E-01 | 1.72E+01 | | | |
| 8015545 | NM_201434 | RAB5C | RAB5C, member RAS oncogene fami | 0.114 | 1.082 | 7.26E-01 | 7.26E+01 | | | |
| 8161580 | --- | --- | --- | 0.114 | 1.082 | 1.77E-01 | 1.77E+01 | | | |
| 7913380 | NM_032264 | NBPF3 | neuroblastoma breakpoint family, m | 0.114 | 1.082 | 1.41E-02 | 1.41E+00 | | | |

| | | | | | | | | | | |
|---|---|---|---|---|---|---|---|---|---|---|
| 8002523 | NM_018348 | FTSJD1 | FtsJ methyltransferase domain conta | 0.114 | 1.082 | 2.61E-01 | 2.61E+01 | | | |
| 8073072 | NM_152426 | APOBEC3D | apolipoprotein B mRNA editing enzy | 0.114 | 1.082 | 2.52E-01 | 2.52E+01 | | | |
| 7977511 | NM_007110 | TEP1 | telomerase-associated protein 1 | 0.114 | 1.082 | 6.96E-02 | 6.96E+00 | | | |
| 8037389 | NM_019108 | C19orf61 | chromosome 19 open reading frame | 0.114 | 1.082 | 1.41E-01 | 1.41E+01 | | | |
| 7983393 | NM_003104 | SORD | sorbitol dehydrogenase | 0.114 | 1.082 | 1.83E-01 | 1.83E+01 | | | |
| 7895045 | --- | --- | --- | 0.113 | 1.082 | 8.57E-01 | 8.57E+01 | | | |
| 8170418 | --- | --- | --- | 0.113 | 1.082 | 5.98E-01 | 5.98E+01 | | | |
| 7966089 | NM_00114234: | CMKLR1 | chemokine-like receptor 1 | 0.113 | 1.082 | 2.03E-01 | 2.03E+01 | | | |
| 8007462 | NR_003108 | NBR2 | neighbor of BRCA1 gene 2 (non-prot | 0.113 | 1.082 | 3.85E-01 | 3.85E+01 | | | |
| 8157516 | NM_012210 | TRIM32 | tripartite motif-containing 32 | 0.113 | 1.082 | 2.60E-01 | 2.60E+01 | | | |
| 8000537 | NR_003610 | PDXDC2 | pyridoxal-dependent decarboxylase | 0.113 | 1.082 | 5.58E-01 | 5.58E+01 | | | |
| 8072678 | NM_002133 | HMOX1 | heme oxygenase (decycling) 1 | 0.113 | 1.082 | 5.52E-01 | 5.52E+01 | | | |
| 8166015 | NM_006800 | MSL3 | male-specific lethal 3 homolog (Dros | 0.113 | 1.082 | 6.02E-01 | 6.02E+01 | | | |
| 7895819 | --- | --- | --- | 0.113 | 1.082 | 7.26E-03 | 7.26E-01 | | | |
| 7944096 | NM_207343 | RNF214 | ring finger protein 214 | 0.113 | 1.082 | 4.78E-01 | 4.78E+01 | | | |
| 7901867 | NM_003368 | USP1 | ubiquitin specific peptidase 1 | 0.113 | 1.081 | 3.75E-01 | 3.75E+01 | | | |
| 7942168 | NM_003824 | FADD | Fas (TNFRSF6)-associated via death d | 0.113 | 1.081 | 1.58E-01 | 1.58E+01 | | | |
| 7915015 | NM_013285 | GNL2 | guanine nucleotide binding protein-l | 0.113 | 1.081 | 6.47E-01 | 6.47E+01 | | | |
| 7895273 | --- | --- | --- | 0.113 | 1.081 | 2.92E-01 | 2.92E+01 | | | |
| 7909102 | --- | --- | --- | 0.113 | 1.081 | 3.96E-01 | 3.96E+01 | | | |
| 7972297 | NM_005845 | ABCC4 | ATP-binding cassette, sub-family C (C | 0.113 | 1.081 | 5.59E-01 | 5.59E+01 | | | |
| 7963054 | NM_006009 | TUBA1A | tubulin, alpha 1a | 0.113 | 1.081 | 3.43E-01 | 3.43E+01 | | | |
| 7928491 | NM_012330 | MYST4 | MYST histone acetyltransferase (mor | 0.113 | 1.081 | 7.65E-01 | 7.65E+01 | | | |
| 8153684 | NM_012079 | DGAT1 | diacylglycerol O-acyltransferase hom | 0.112 | 1.081 | 2.15E-01 | 2.15E+01 | | | |
| 8153939 | NM_012079 | DGAT1 | diacylglycerol O-acyltransferase hom | 0.112 | 1.081 | 2.15E-01 | 2.15E+01 | | | |
| 7956301 | NM_002332 | LRP1 | low density lipoprotein-related prote | 0.112 | 1.081 | 4.39E-02 | 4.39E+00 | | | |
| 7939477 | NM_139178 | ALKBH3 | alkB, alkylation repair homolog 3 (E. | 0.112 | 1.081 | 6.93E-01 | 6.93E+01 | | | |
| 7895301 | --- | --- | --- | 0.112 | 1.081 | 8.99E-01 | 8.99E+01 | | | |
| 8095341 | --- | --- | --- | 0.112 | 1.081 | 2.75E-01 | 2.75E+01 | | | |
| 8088339 | NM_001660 | ARF4 | ADP-ribosylation factor 4 | 0.112 | 1.081 | 4.88E-01 | 4.88E+01 | | | |
| 7932765 | NM_173496 | MPP7 | membrane protein, palmitoylated 7 | 0.112 | 1.081 | 6.48E-01 | 6.48E+01 | | | |
| 7907213 | NM_152281 | GORAB | golgin, RAB6-interacting | 0.112 | 1.081 | 5.83E-01 | 5.83E+01 | | | |
| 7925130 | NM_005646 | TARBP1 | TAR (HIV-1) RNA binding protein 1 | 0.112 | 1.081 | 6.02E-01 | 6.02E+01 | | | |
| 7926807 | NM_014317 | PDSS1 | prenyl (decaprenyl) diphosphate syn | 0.112 | 1.081 | 4.88E-01 | 4.88E+01 | | | |
| 8124397 | NM_005319 | HIST1H1C | histone cluster 1, H1c | 0.112 | 1.081 | 7.14E-01 | 7.14E+01 | | | |
| 8156519 | --- | --- | --- | 0.112 | 1.081 | 6.76E-01 | 6.76E+01 | | | |
| 8071768 | NM_003073 | SMARCB1 | SWI/SNF related, matrix associated, | 0.112 | 1.081 | 4.89E-01 | 4.89E+01 | | | |
| 7895336 | --- | --- | --- | 0.112 | 1.081 | 2.72E-01 | 2.72E+01 | | | |
| 7893603 | --- | --- | --- | 0.112 | 1.081 | 4.01E-01 | 4.01E+01 | | | |
| 7892891 | --- | --- | --- | 0.112 | 1.081 | 1.00E-01 | 1.00E+01 | | | |
| 8016414 | NM_003726 | SKAP1 | src kinase associated phosphoprotei | 0.112 | 1.081 | 6.77E-01 | 6.77E+01 | | | |
| 7922330 | NM_153747 | PIGC | phosphatidylinositol glycan anchor b | 0.112 | 1.080 | 5.18E-01 | 5.18E+01 | | | |
| 8148501 | NM_032611 | PTP4A3 | protein tyrosine phosphatase type IV | 0.112 | 1.080 | 2.31E-01 | 2.31E+01 | | | |
| 8078214 | NM_004162 | RAB5A | RAB5A, member RAS oncogene fami | 0.112 | 1.080 | 7.24E-01 | 7.24E+01 | | | |
| 8179019 | NM_018950 | HLA-F | major histocompatibility complex, cl | 0.112 | 1.080 | 3.45E-01 | 3.45E+01 | | | |
| 8137584 | NM_138400 | NOM1 | nucleolar protein with MIF4G domai | 0.112 | 1.080 | 4.24E-01 | 4.24E+01 | | | |
| 7997239 | NR_003610 | PDXDC2 | pyridoxal-dependent decarboxylase | 0.112 | 1.080 | 5.58E-01 | 5.58E+01 | | | |
| 8119408 | NM_002505 | NFYA | nuclear transcription factor Y, alpha | 0.111 | 1.080 | 3.77E-01 | 3.77E+01 | | | |
| 8169709 | NM_016417 | GLRX5 | glutaredoxin 5 | 0.111 | 1.080 | 5.28E-01 | 5.28E+01 | | | |
| 8123062 | NM_020823 | TMEM181 | transmembrane protein 181 | 0.111 | 1.080 | 5.46E-01 | 5.46E+01 | | | |
| 8126402 | NM_033502 | TRERF1 | transcriptional regulating factor 1 | 0.111 | 1.080 | 2.30E-01 | 2.30E+01 | | | |
| 7996012 | NM_012106 | ARL2BP | ADP-ribosylation factor-like 2 binding | 0.111 | 1.080 | 8.37E-01 | 8.37E+01 | | | |
| 8125750 | NM_000976 | RPL12 | ribosomal protein L12 | 0.111 | 1.080 | 4.83E-01 | 4.83E+01 | | | |
| 8100541 | NM_001553 | IGFBP7 | insulin-like growth factor binding pro | 0.111 | 1.080 | 1.17E-01 | 1.17E+01 | | | |
| 8071206 | NM_003776 | MRPL40 | mitochondrial ribosomal protein L40 | 0.111 | 1.080 | 5.93E-01 | 5.93E+01 | | | |
| 8025741 | NM_00100536( | DNM2 | dynamin 2 | 0.111 | 1.080 | 5.16E-01 | 5.16E+01 | | | |
| 8031981 | NR_003659 | WASH3P | WAS protein family homolog 3 pseud | 0.111 | 1.080 | 1.63E-01 | 1.63E+01 | | | |
| 8094830 | NM_018126 | TMEM33 | transmembrane protein 33 | 0.111 | 1.080 | 4.13E-01 | 4.13E+01 | | | |
| 8133209 | NR_003666 | SPDYE7P | speedy homolog E7 (Xenopus laevis) | 0.111 | 1.080 | 4.23E-01 | 4.23E+01 | | | |
| 8174253 | NM_00114241{ | MORF4L2 | mortality factor 4 like 2 | 0.111 | 1.080 | 7.08E-01 | 7.08E+01 | | | |
| 8026440 | NM_000984 | RPL23A | ribosomal protein L23a | 0.111 | 1.080 | 2.82E-01 | 2.82E+01 | | | |
| 8105321 | --- | --- | --- | 0.111 | 1.080 | 7.04E-01 | 7.04E+01 | | | |
| 8016607 | --- | --- | --- | 0.111 | 1.080 | 4.51E-01 | 4.51E+01 | | | |
| 7892992 | --- | --- | --- | 0.111 | 1.080 | 4.51E-02 | 4.51E+00 | | | |
| 7896525 | --- | --- | --- | 0.111 | 1.080 | 7.59E-01 | 7.59E+01 | | | |
| 8081055 | NM_014043 | CHMP2B | chromatin modifying protein 2B | 0.111 | 1.080 | 8.48E-01 | 8.48E+01 | | | |
| 7935639 | NM_031212 | SLC25A28 | solute carrier family 25, member 28 | 0.111 | 1.080 | 2.62E-01 | 2.62E+01 | | | |
| 7909271 | NM_006850 | IL24 | interleukin 24 | 0.111 | 1.080 | 2.48E-01 | 2.48E+01 | | | |
| 8154130 | --- | --- | --- | 0.111 | 1.080 | 3.98E-01 | 3.98E+01 | | | |
| 8131292 | NM_021163 | RBAK | RB-associated KRAB zinc finger | 0.111 | 1.080 | 5.61E-01 | 5.61E+01 | | | |
| 7894499 | --- | --- | --- | 0.111 | 1.080 | 1.16E-01 | 1.16E+01 | | | |
| 8117995 | NM_178014 | TUBB | tubulin, beta | 0.111 | 1.080 | 6.99E-01 | 6.99E+01 | | | |
| 8177858 | NM_178014 | TUBB | tubulin, beta | 0.111 | 1.080 | 6.99E-01 | 6.99E+01 | | | |
| 8179174 | NM_178014 | TUBB | tubulin, beta | 0.111 | 1.080 | 6.99E-01 | 6.99E+01 | | | |
| 7947867 | NM_002804 | PSMC3 | proteasome (prosome, macropain) 2 | 0.111 | 1.080 | 5.24E-01 | 5.24E+01 | | | |
| 8043900 | --- | --- | --- | 0.111 | 1.080 | 6.41E-01 | 6.41E+01 | | | |
| 7973545 | NM_025230 | DCAF11 | DDB1 and CUL4 associated factor 11 | 0.110 | 1.080 | 3.80E-01 | 3.80E+01 | | | |

| | | | | | | | | | | |
|---|---|---|---|---|---|---|---|---|---|---|
| 8166098 | NM_004251 | RAB9A | RAB9A, member RAS oncogene fami | 0.110 | 1.080 | 4.71E-01 | 4.71E+01 | | | |
| 8042972 | --- | --- | --- | 0.110 | 1.080 | 4.85E-01 | 4.85E+01 | | | |
| 8018082 | NM_00115977( | SLC39A11 | solute carrier family 39 (metal ion tra | 0.110 | 1.079 | 4.09E-01 | 4.09E+01 | | | |
| 8118667 | --- | --- | --- | 0.110 | 1.079 | 5.52E-01 | 5.52E+01 | | | |
| 7894869 | --- | --- | --- | 0.110 | 1.079 | 9.17E-01 | 9.17E+01 | | | |
| 7893564 | --- | --- | --- | 0.110 | 1.079 | 6.23E-01 | 6.23E+01 | | | |
| 7895847 | --- | --- | --- | 0.110 | 1.079 | 8.98E-01 | 8.98E+01 | | | |
| 8018922 | NM_004762 | CYTH1 | cytohesin 1 | 0.110 | 1.079 | 6.76E-01 | 6.76E+01 | | | |
| 7896049 | --- | --- | --- | 0.110 | 1.079 | 8.55E-01 | 8.55E+01 | | | |
| 7924491 | BC043142 | AIDA | axin interactor, dorsalization associa | 0.109 | 1.079 | 1.67E-01 | 1.67E+01 | | | |
| 8134740 | NM_00100839 | C7orf59 | chromosome 7 open reading frame 5 | 0.109 | 1.079 | 5.47E-01 | 5.47E+01 | | | |
| 7997489 | --- | --- | --- | 0.109 | 1.079 | 6.58E-02 | 6.58E+00 | | | |
| 7896501 | --- | --- | --- | 0.109 | 1.079 | 2.31E-01 | 2.31E+01 | | | |
| 7906235 | NM_005973 | PRCC | papillary renal cell carcinoma (transl | 0.109 | 1.079 | 3.51E-01 | 3.51E+01 | | | |
| 7915563 | --- | --- | --- | 0.109 | 1.079 | 8.37E-01 | 8.37E+01 | | | |
| 7895063 | --- | --- | --- | 0.109 | 1.079 | 4.98E-01 | 4.98E+01 | | | |
| 7896534 | --- | --- | --- | 0.109 | 1.079 | 8.32E-01 | 8.32E+01 | | | |
| 8125850 | NM_024294 | C6orf106 | chromosome 6 open reading frame 1 | 0.109 | 1.079 | 4.84E-01 | 4.84E+01 | | | |
| 7981439 | NM_00101504 | BAG5 | BCL2-associated athanogene 5 | 0.109 | 1.079 | 5.28E-01 | 5.28E+01 | | | |
| 7893744 | --- | --- | --- | 0.109 | 1.078 | 6.88E-01 | 6.88E+01 | | | |
| 8090662 | --- | --- | --- | 0.109 | 1.078 | 7.49E-01 | 7.49E+01 | | | |
| 8160557 | --- | --- | --- | 0.109 | 1.078 | 7.71E-01 | 7.71E+01 | | | |
| 8053733 | NM_020382 | SETD8 | SET domain containing (lysine methy | 0.109 | 1.078 | 8.01E-01 | 8.01E+01 | | | |
| 7947015 | NM_006292 | TSG101 | tumor susceptibility gene 101 | 0.109 | 1.078 | 6.92E-01 | 6.92E+01 | | | |
| 8142671 | NM_003941 | WASL | Wiskott-Aldrich syndrome-like | 0.109 | 1.078 | 3.38E-01 | 3.38E+01 | | | |
| 8103535 | NR_026575 | GK3P | glycerol kinase 3 pseudogene | 0.109 | 1.078 | 6.38E-01 | 6.38E+01 | | | |
| 8149942 | NM_018246 | CCDC25 | coiled-coil domain containing 25 | 0.109 | 1.078 | 5.08E-01 | 5.08E+01 | | | |
| 7896559 | --- | --- | --- | 0.109 | 1.078 | 8.80E-01 | 8.80E+01 | | | |
| 8162827 | NR_024532 | ALG2 | asparagine-linked glycosylation 2, alp | 0.109 | 1.078 | 4.34E-01 | 4.34E+01 | | | |
| 8140443 | NR_003262 // | SL2A // FDP | MGC44478 // MGC44478 // MGC44 | 0.108 | 1.078 | 3.93E-01 | 3.93E+01 | | | |
| 8097521 | NM_032547 | SCOC | short coiled-coil protein | 0.108 | 1.078 | 4.21E-01 | 4.21E+01 | | | |
| 7993833 | NM_016025 | METTL9 | methyltransferase like 9 | 0.108 | 1.078 | 4.10E-01 | 4.10E+01 | | | |
| 7916219 | BC015313 | C1orf163 | chromosome 1 open reading frame 1 | 0.108 | 1.078 | 3.78E-01 | 3.78E+01 | | | |
| 8077513 | NM_015453 | THUMPD3 | THUMP domain containing 3 | 0.108 | 1.078 | 8.12E-01 | 8.12E+01 | | | |
| 7945875 | NM_00109965 | 86C // FAM | family with sequence similarity 86, m | 0.108 | 1.078 | 3.78E-01 | 3.78E+01 | | | |
| 7894574 | --- | --- | --- | 0.108 | 1.078 | 8.05E-01 | 8.05E+01 | | | |
| 7999044 | NM_004380 | CREBBP | CREB binding protein | 0.108 | 1.078 | 4.61E-01 | 4.61E+01 | | | |
| 7983274 | NM_005313 | PDIA3 | protein disulfide isomerase family A, | 0.108 | 1.078 | 5.29E-01 | 5.29E+01 | | | |
| 8137252 | NM_130759 | GIMAP1 | GTPase, IMAP family member 1 | 0.108 | 1.078 | 1.88E-01 | 1.88E+01 | | | |
| 8009513 | --- | --- | --- | 0.108 | 1.078 | 2.19E-01 | 2.19E+01 | | | |
| 7896683 | --- | --- | --- | 0.108 | 1.078 | 2.23E-01 | 2.23E+01 | | | |
| 7914617 | NM_013411 | AK2 | adenylate kinase 2 | 0.108 | 1.078 | 6.34E-01 | 6.34E+01 | | | |
| 7958819 | NM_006817 | ERP29 | endoplasmic reticulum protein 29 | 0.108 | 1.078 | 2.53E-01 | 2.53E+01 | | | |
| 8005166 | NM_018955 | UBB | ubiquitin B | 0.108 | 1.077 | 6.93E-01 | 6.93E+01 | | | |
| 7917156 | NM_003902 | FUBP1 | far upstream element (FUSE) binding | 0.108 | 1.077 | 6.92E-01 | 6.92E+01 | | | |
| 7893786 | --- | --- | --- | 0.108 | 1.077 | 8.46E-01 | 8.46E+01 | | | |
| 8117140 | NM_017774 | CDKAL1 | CDK5 regulatory subunit associated p | 0.108 | 1.077 | 5.04E-01 | 5.04E+01 | | | |
| 8145201 | NM_021174 | KIAA1967 | KIAA1967 | 0.107 | 1.077 | 4.44E-01 | 4.44E+01 | | | |
| 7983360 | NM_004048 | B2M | beta-2-microglobulin | 0.107 | 1.077 | 9.46E-03 | 9.46E-01 | | | |
| 7895100 | --- | --- | --- | 0.107 | 1.077 | 7.22E-03 | 7.22E-01 | | | |
| 7997525 | NM_012213 | MLYCD | malonyl-CoA decarboxylase | 0.107 | 1.077 | 3.07E-01 | 3.07E+01 | | | |
| 7993467 | NM_00114397 | NDE1 | nudE nuclear distribution gene E hor | 0.107 | 1.077 | 5.57E-01 | 5.57E+01 | | | |
| 8087485 | NM_003335 | UBA7 | ubiquitin-like modifier activating enz | 0.107 | 1.077 | 4.12E-01 | 4.12E+01 | | | |
| 8091260 | NM_173653 | SLC9A9 | solute carrier family 9 (sodium/hydro | 0.107 | 1.077 | 4.20E-01 | 4.20E+01 | | | |
| 8054888 | NM_015282 | CLASP1 | cytoplasmic linker associated protein | 0.107 | 1.077 | 6.17E-01 | 6.17E+01 | | | |
| 7994265 | --- | --- | --- | 0.107 | 1.077 | 7.91E-01 | 7.91E+01 | | | |
| 8022118 | NM_012307 | EPB41L3 | erythrocyte membrane protein band | 0.107 | 1.077 | 1.95E-01 | 1.95E+01 | | | mono |
| 7974617 | --- | --- | --- | 0.107 | 1.077 | 5.64E-01 | 5.64E+01 | | | |
| 8156761 | NM_018946 | NANS | N-acetylneuraminic acid synthase | 0.107 | 1.077 | 7.27E-01 | 7.27E+01 | | | |
| 8005943 | NM_000984 | RPL23A | ribosomal protein L23a | 0.107 | 1.077 | 3.33E-01 | 3.33E+01 | | | |
| 8145490 | NM_173174 | PTK2B | PTK2B protein tyrosine kinase 2 beta | 0.107 | 1.077 | 4.31E-01 | 4.31E+01 | | | |
| 8133600 | NR_003664 | SPDYE8P | speedy homolog E8 (Xenopus laevis) | 0.107 | 1.077 | 3.28E-01 | 3.28E+01 | | | |
| 8140009 | NR_003664 | SPDYE8P | speedy homolog E8 (Xenopus laevis) | 0.107 | 1.077 | 3.28E-01 | 3.28E+01 | | | |
| 8116130 | NM_00107952 | FAM153B | family with sequence similarity 153, | 0.107 | 1.077 | 2.97E-01 | 2.97E+01 | | | |
| 8179055 | NM_170783 | ZNRD1 | zinc ribbon domain containing 1 | 0.107 | 1.077 | 8.12E-01 | 8.12E+01 | | | |
| 8023246 | BC093004 | C18orf32 | chromosome 18 open reading frame | 0.107 | 1.077 | 6.17E-01 | 6.17E+01 | | | |
| 7968514 | --- | --- | --- | 0.107 | 1.077 | 6.15E-01 | 6.15E+01 | | | |
| 8004699 | NM_00100527 | CHD3 | chromodomain helicase DNA binding | 0.107 | 1.077 | 3.51E-01 | 3.51E+01 | | | |
| 8180318 | --- | --- | --- | 0.107 | 1.077 | 8.42E-01 | 8.42E+01 | | | |
| 8150356 | NR_003129 | RNF5P1 | ring finger protein 5 pseudogene 1 | 0.107 | 1.077 | 5.35E-01 | 5.35E+01 | | | |
| 8065403 | NM_000099 | CST3 | cystatin C | 0.107 | 1.077 | 5.17E-01 | 5.17E+01 | | | mono |
| 8015715 | NM_003766 | BECN1 | beclin 1, autophagy related | 0.107 | 1.077 | 6.40E-01 | 6.40E+01 | | | |
| 7936809 | NM_152644 | FAM24B | family with sequence similarity 24, m | 0.107 | 1.077 | 7.56E-01 | 7.56E+01 | | | |
| 7977820 | NM_00103961 | PRMT5 | protein arginine methyltransferase 5 | 0.107 | 1.077 | 5.72E-01 | 5.72E+01 | | | |
| 8077123 | NR_027928 | CHKB-CPT1B | choline kinase-like, carnitine palmito | 0.107 | 1.077 | 2.45E-01 | 2.45E+01 | | | |
| 7894020 | --- | --- | --- | 0.107 | 1.077 | 6.89E-01 | 6.89E+01 | | | |
| 7896057 | --- | --- | --- | 0.107 | 1.077 | 5.73E-01 | 5.73E+01 | | | |

| | | | | | | | | | | |
|---|---|---|---|---|---|---|---|---|---|---|
| 7995330 | --- | --- | --- | 0.106 | 1.077 | 2.14E-01 | 2.14E+01 | | | |
| 7973584 | NM_017999 | RNF31 | ring finger protein 31 | 0.106 | 1.077 | 2.19E-01 | 2.19E+01 | | | |
| 8063903 | NM_001024 | RPS21 | ribosomal protein S21 | 0.106 | 1.077 | 2.41E-01 | 2.41E+01 | | | |
| 8045171 | NM_033416 | IMP4 | IMP4, U3 small nucleolar ribonucleo | 0.106 | 1.077 | 6.75E-01 | 6.75E+01 | | | |
| 7916643 | NM_032027 | TM2D1 | TM2 domain containing 1 | 0.106 | 1.076 | 3.27E-01 | 3.27E+01 | | | |
| 7907466 | NM_018122 | DARS2 | aspartyl-tRNA synthetase 2, mitocho | 0.106 | 1.076 | 2.95E-01 | 2.95E+01 | | | |
| 7894682 | --- | --- | --- | 0.106 | 1.076 | 1.95E-01 | 1.95E+01 | | | |
| 8017582 | NM_018469 | TEX2 | testis expressed 2 | 0.106 | 1.076 | 1.56E-01 | 1.56E+01 | | | |
| 8160151 | NM_178566 | ZDHHC21 | zinc finger, DHHC-type containing 21 | 0.106 | 1.076 | 6.82E-01 | 6.82E+01 | | | |
| 8051066 | NM_002437 | MPV17 | MpV17 mitochondrial inner membra | 0.106 | 1.076 | 7.70E-01 | 7.70E+01 | | | |
| 7893422 | --- | --- | --- | 0.106 | 1.076 | 8.37E-01 | 8.37E+01 | | | |
| 7931239 | NM_015608 | C10orf137 | chromosome 10 open reading frame | 0.106 | 1.076 | 3.76E-01 | 3.76E+01 | | | |
| 7895595 | --- | --- | --- | 0.106 | 1.076 | 1.44E-01 | 1.44E+01 | | | |
| 8026533 | NM_032855 | HSH2D | hematopoietic SH2 domain containir | 0.106 | 1.076 | 4.04E-01 | 4.04E+01 | | | |
| 8180224 | --- | --- | --- | 0.106 | 1.076 | 3.05E-01 | 3.05E+01 | | | |
| 7893343 | --- | --- | --- | 0.106 | 1.076 | 6.83E-01 | 6.83E+01 | | | |
| 8168362 | AY572224 | FLJ44635 | TPT1-like protein | 0.106 | 1.076 | 5.05E-02 | 5.05E+00 | | | |
| 7914764 | NM_007167 | ZMYM6 | zinc finger, MYM-type 6 | 0.106 | 1.076 | 3.02E-01 | 3.02E+01 | | | |
| 7954631 | NM_018099 | FAR2 | fatty acyl CoA reductase 2 | 0.105 | 1.076 | 4.66E-01 | 4.66E+01 | | | |
| 8088893 | --- | --- | --- | 0.105 | 1.076 | 6.35E-01 | 6.35E+01 | | | |
| 7979824 | NM_00113000 | ACTN1 | actinin, alpha 1 | 0.105 | 1.076 | 5.46E-01 | 5.46E+01 | | | |
| 7936596 | NM_153810 | C10orf46 | chromosome 10 open reading frame | 0.105 | 1.076 | 3.39E-01 | 3.39E+01 | | | |
| 8150225 | NM_00100281 | RAB11FIP1 | RAB11 family interacting protein 1 (c | 0.105 | 1.076 | 2.29E-01 | 2.29E+01 | | | |
| 7899484 | --- | --- | --- | 0.105 | 1.076 | 8.35E-01 | 8.35E+01 | | | |
| 7894647 | --- | --- | --- | 0.105 | 1.076 | 1.81E-02 | 1.81E+00 | | | |
| 7978911 | NM_00101270 | C14orf182 | chromosome 14 open reading frame | 0.105 | 1.076 | 5.79E-01 | 5.79E+01 | | | |
| 7958019 | NM_018370 | DRAM1 | DNA-damage regulated autophagy m | 0.105 | 1.076 | 4.95E-01 | 4.95E+01 | | | |
| 7946563 | --- | --- | --- | 0.105 | 1.076 | 6.62E-01 | 6.62E+01 | | | |
| 7892868 | --- | --- | --- | 0.105 | 1.076 | 4.09E-01 | 4.09E+01 | | | |
| 8179322 | NM_005345 | HSPA1A | heat shock 70kDa protein 1A | 0.105 | 1.076 | 6.90E-01 | 6.90E+01 | | | |
| 7950336 | NM_015531 | C2CD3 | C2 calcium-dependent domain conta | 0.105 | 1.076 | 1.34E-01 | 1.34E+01 | | | |
| 8059071 | NM_024782 | NHEJ1 | nonhomologous end-joining factor 1 | 0.105 | 1.075 | 6.44E-01 | 6.44E+01 | | | |
| 8033135 | NM_002096 | GTF2F1 | general transcription factor IIF, polyp | 0.105 | 1.075 | 3.15E-01 | 3.15E+01 | | | |
| 7894446 | --- | --- | --- | 0.105 | 1.075 | 5.64E-01 | 5.64E+01 | | | |
| 7893722 | --- | --- | --- | 0.105 | 1.075 | 3.13E-01 | 3.13E+01 | | | |
| 7929168 | NM_025235 | TNKS2 | tankyrase, TRF1-interacting ankyrin- | 0.105 | 1.075 | 6.10E-01 | 6.10E+01 | | | |
| 8076547 | NR_027779 | TTLL1 | tubulin tyrosine ligase-like family, me | 0.105 | 1.075 | 4.35E-01 | 4.35E+01 | | | |
| 7911559 | NM_017971 | MRPL20 | mitochondrial ribosomal protein L20 | 0.105 | 1.075 | 7.05E-01 | 7.05E+01 | | | |
| 8039939 | NM_017971 | MRPL20 | mitochondrial ribosomal protein L20 | 0.105 | 1.075 | 7.05E-01 | 7.05E+01 | | | |
| 8139780 | --- | --- | --- | 0.105 | 1.075 | 7.89E-01 | 7.89E+01 | | | |
| 8161852 | --- | --- | --- | 0.104 | 1.075 | 7.04E-01 | 7.04E+01 | | | |
| 7896549 | --- | --- | --- | 0.104 | 1.075 | 3.71E-02 | 3.71E+00 | | | |
| 8172656 | --- | --- | --- | 0.104 | 1.075 | 5.16E-01 | 5.16E+01 | | | |
| 8140707 | --- | --- | --- | 0.104 | 1.075 | 5.38E-01 | 5.38E+01 | | | |
| 8036351 | BC052603 | ZNF850P | zinc finger protein 850 pseudogene | 0.104 | 1.075 | 2.00E-01 | 2.00E+01 | | | |
| 7951207 | NM_052932 | TMEM123 | transmembrane protein 123 | 0.104 | 1.075 | 5.45E-01 | 5.45E+01 | | | |
| 7967067 | NM_00103749 | DYNLL1 | dynein, light chain, LC8-type 1 | 0.104 | 1.075 | 4.00E-01 | 4.00E+01 | | Antigen present./co-stim. | |
| 8063835 | NM_144703 | LSM14B | LSM14B, SCD6 homolog B (S. cerevis | 0.104 | 1.075 | 4.34E-01 | 4.34E+01 | | | |
| 7994620 | NM_007317 | KIF22 | kinesin family member 22 | 0.104 | 1.075 | 3.42E-01 | 3.42E+01 | | | |
| 7907104 | NM_018442 | DCAF6 | DDB1 and CUL4 associated factor 6 | 0.104 | 1.075 | 6.33E-01 | 6.33E+01 | | | |
| 8108861 | NM_030571 | NDFIP1 | Nedd4 family interacting protein 1 | 0.104 | 1.075 | 5.59E-01 | 5.59E+01 | | | |
| 7893812 | --- | --- | --- | 0.104 | 1.075 | 8.99E-01 | 8.99E+01 | | | |
| 7986517 | ENST00000423 | C15orf51 | dynamin 1 pseudogene | 0.104 | 1.075 | 6.24E-01 | 6.24E+01 | | | |
| 7986522 | ENST00000423 | C15orf51 | dynamin 1 pseudogene | 0.104 | 1.075 | 6.24E-01 | 6.24E+01 | | | |
| 8016239 | NR_027774 | PLEKHM1 | pleckstrin homology domain contain | 0.104 | 1.075 | 3.74E-01 | 3.74E+01 | | | |
| 7990566 | NM_000126 | ETFA | electron-transfer-flavoprotein, alpha | 0.104 | 1.075 | 7.53E-01 | 7.53E+01 | | | |
| 7892745 | --- | --- | --- | 0.104 | 1.075 | 9.07E-01 | 9.07E+01 | | | |
| 8047356 | NM_021824 | NIF3L1 | NIF3 NGG1 interacting factor 3-like 1 | 0.104 | 1.075 | 7.30E-01 | 7.30E+01 | | | |
| 7900585 | NM_004559 | YBX1 | Y box binding protein 1 | 0.104 | 1.075 | 7.92E-02 | 7.92E+00 | | | |
| 7899057 | NM_019557 | FAM54B | family with sequence similarity 54, m | 0.104 | 1.075 | 6.04E-01 | 6.04E+01 | | | |
| 8003771 | --- | --- | --- | 0.104 | 1.075 | 3.84E-01 | 3.84E+01 | | | |
| 7896026 | --- | --- | --- | 0.104 | 1.075 | 6.10E-01 | 6.10E+01 | | | |
| 7990029 | --- | --- | --- | 0.104 | 1.075 | 5.67E-01 | 5.67E+01 | | | |
| 7896244 | --- | --- | --- | 0.104 | 1.074 | 8.31E-01 | 8.31E+01 | | | |
| 7894439 | --- | --- | --- | 0.104 | 1.074 | 8.49E-01 | 8.49E+01 | | | |
| 8161433 | --- | --- | --- | 0.104 | 1.074 | 5.99E-01 | 5.99E+01 | | | |
| 8072757 | NM_000395 | CSF2RB | colony stimulating factor 2 receptor, | 0.104 | 1.074 | 2.15E-01 | 2.15E+01 | | | |
| 8081799 | NM_173799 | TIGIT | T cell immunoreceptor with Ig and IT | 0.103 | 1.074 | 3.12E-01 | 3.12E+01 | | | |
| 8068522 | NM_003316 | TTC3 | tetratricopeptide repeat domain 3 | 0.103 | 1.074 | 6.53E-01 | 6.53E+01 | | | |
| 7894186 | --- | --- | --- | 0.103 | 1.074 | 8.38E-01 | 8.38E+01 | | | |
| 7896398 | --- | --- | --- | 0.103 | 1.074 | 7.13E-01 | 7.13E+01 | | | |
| 7926609 | NM_005180 | BMI1 | BMI1 polycomb ring finger oncogene | 0.103 | 1.074 | 7.94E-01 | 7.94E+01 | | | |
| 8084303 | NM_003907 | EIF2B5 | eukaryotic translation initiation facto | 0.103 | 1.074 | 4.79E-01 | 4.79E+01 | | | |
| 8045860 | NM_003628 | PKP4 | plakophilin 4 | 0.103 | 1.074 | 3.29E-01 | 3.29E+01 | | | |
| 7984203 | NM_015042 | ZNF609 | zinc finger protein 609 | 0.103 | 1.074 | 7.07E-01 | 7.07E+01 | | | |
| 8050763 | ENST00000380 | CENPO | centromere protein O | 0.103 | 1.074 | 5.61E-01 | 5.61E+01 | | | |
| 7894722 | --- | --- | --- | 0.103 | 1.074 | 1.14E-01 | 1.14E+01 | | | |

| | | | | | | | | | | |
|---|---|---|---|---|---|---|---|---|---|---|
| 7935810 | NM_005004 | NDUFB8 | NADH dehydrogenase (ubiquinone) 1 | 0.103 | 1.074 | 6.15E-01 | 6.15E+01 | | | |
| 8067864 | NR_003366 | ANKRD20B | ankyrin repeat domain 20B | 0.103 | 1.074 | 4.39E-01 | 4.39E+01 | | | |
| 8046169 | NM_004792 | PPIG | peptidylprolyl isomerase G (cyclophi | 0.103 | 1.074 | 7.93E-01 | 7.93E+01 | | | |
| 8137448 | NM_022087 | GALNT11 | UDP-N-acetyl-alpha-D-galactosamine | 0.103 | 1.074 | 5.65E-01 | 5.65E+01 | | | |
| 8002102 | --- | --- | --- | 0.103 | 1.074 | 6.48E-01 | 6.48E+01 | | | |
| 7895123 | --- | --- | --- | 0.103 | 1.074 | 3.99E-02 | 3.99E+00 | | | |
| 8042086 | NM_006296 | VRK2 | vaccinia related kinase 2 | 0.103 | 1.074 | 6.68E-01 | 6.68E+01 | | | |
| 8097116 | --- | --- | --- | 0.103 | 1.074 | 8.83E-01 | 8.83E+01 | | | |
| 7976160 | NM_018319 | TDP1 | tyrosyl-DNA phosphodiesterase 1 | 0.103 | 1.074 | 6.24E-01 | 6.24E+01 | | | |
| 8160308 | NM_001010 | RPS6 | ribosomal protein S6 | 0.103 | 1.074 | 6.94E-02 | 6.94E+00 | | | |
| 8070141 | NM_145858 | CRYZL1 | crystallin, zeta (quinone reductase)-l | 0.103 | 1.074 | 7.87E-01 | 7.87E+01 | | | |
| 8117800 | NM_002116 | HLA-A | major histocompatibility complex, cla | 0.103 | 1.074 | 5.02E-01 | 5.02E+01 | | | |
| 7895395 | --- | --- | --- | 0.103 | 1.074 | 7.87E-01 | 7.87E+01 | | | |
| 7893015 | --- | --- | --- | 0.103 | 1.074 | 6.00E-01 | 6.00E+01 | | | |
| 7955043 | --- | --- | --- | 0.103 | 1.074 | 7.30E-01 | 7.30E+01 | | | |
| 8030351 | NM_012423 | RPL13A | ribosomal protein L13a | 0.102 | 1.074 | 1.67E-01 | 1.67E+01 | | | |
| 8145454 | NM_004331 | BNIP3L | BCL2/adenovirus E1B 19kDa interact | 0.102 | 1.074 | 7.31E-01 | 7.31E+01 | | | |
| 8090637 | --- | --- | --- | 0.102 | 1.074 | 6.89E-01 | 6.89E+01 | | | |
| 7901856 | --- | --- | --- | 0.102 | 1.074 | 6.15E-01 | 6.15E+01 | | | |
| 8158952 | NM_001402 | EEF1A1 | eukaryotic translation elongation fac | 0.102 | 1.074 | 3.39E-02 | 3.39E+00 | | | |
| 7975813 | --- | --- | --- | 0.102 | 1.073 | 5.78E-01 | 5.78E+01 | | | |
| 7894412 | --- | --- | --- | 0.102 | 1.073 | 5.84E-01 | 5.84E+01 | | | |
| 8143367 | NM_207113 | SLC37A3 | solute carrier family 37 (glycerol-3-p | 0.102 | 1.073 | 6.64E-01 | 6.64E+01 | | | |
| 8012416 | NR_026951 | C17orf44 | chromosome 17 open reading frame | 0.102 | 1.073 | 5.89E-01 | 5.89E+01 | | | |
| 8131406 | NM_018890 | RAC1 | ras-related C3 botulinum toxin subst | 0.102 | 1.073 | 5.57E-01 | 5.57E+01 | | | |
| 7896301 | --- | --- | --- | 0.102 | 1.073 | 6.28E-01 | 6.28E+01 | | | |
| 8023450 | NR_024546 | TXNL1 | thioredoxin-like 1 | 0.102 | 1.073 | 7.35E-01 | 7.35E+01 | | | |
| 7971246 | NM_015058 | KIAA0564 | KIAA0564 | 0.102 | 1.073 | 5.00E-01 | 5.00E+01 | | | |
| 8166525 | NM_016937 | POLA1 | polymerase (DNA directed), alpha 1, | 0.102 | 1.073 | 6.45E-01 | 6.45E+01 | | | |
| 7895001 | --- | --- | --- | 0.102 | 1.073 | 6.48E-01 | 6.48E+01 | | | |
| 7909661 | NM_012424 | RPS6KC1 | ribosomal protein S6 kinase, 52kDa, | 0.102 | 1.073 | 5.74E-01 | 5.74E+01 | | | |
| 8107769 | NM_001046 | SLC12A2 | solute carrier family 12 (sodium/pota | 0.102 | 1.073 | 7.13E-01 | 7.13E+01 | | | |
| 8003583 | NM_007317 | KIF22 | kinesin family member 22 | 0.102 | 1.073 | 3.63E-01 | 3.63E+01 | | | |
| 8011245 | NM_018128 | TSR1 | TSR1, 20S rRNA accumulation, homo | 0.101 | 1.073 | 5.83E-01 | 5.83E+01 | | | |
| 8171758 | NR_023358 | SCARNA9L | small Cajal body-specific RNA 9-like ( | 0.101 | 1.073 | 9.28E-01 | 9.28E+01 | | | |
| 7899192 | NM_002953 | RPS6KA1 | ribosomal protein S6 kinase, 90kDa, | 0.101 | 1.073 | 2.03E-01 | 2.03E+01 | | | |
| 8019517 | --- | --- | --- | 0.101 | 1.073 | 7.58E-01 | 7.58E+01 | | | |
| 8050591 | NM_174889 | NDUFAF2 | NADH dehydrogenase (ubiquinone) 1 | 0.101 | 1.073 | 6.91E-01 | 6.91E+01 | | | |
| 7903582 | NM_00103500( | RPL17 | ribosomal protein L17 | 0.101 | 1.073 | 4.33E-01 | 4.33E+01 | | | |
| 8073015 | NM_006855 | KDELR3 | KDEL (Lys-Asp-Glu-Leu) endoplasmic | 0.101 | 1.072 | 4.97E-01 | 4.97E+01 | | | |
| 8163428 | AK000637 | C9orf80 | chromosome 9 open reading frame 8 | 0.101 | 1.072 | 4.60E-01 | 4.60E+01 | | | |
| 7896639 | --- | --- | --- | 0.101 | 1.072 | 2.68E-02 | 2.68E+00 | | | |
| 8148949 | NR_002807 | TMED10P | transmembrane emp24-like traffickir | 0.101 | 1.072 | 6.70E-01 | 6.70E+01 | | | |
| 7963064 | NM_006337 | MCRS1 | microspherule protein 1 | 0.101 | 1.072 | 1.65E-01 | 1.65E+01 | | | |
| 7951036 | NR_003033 | SNORD5 | small nucleolar RNA, C/D box 5 | 0.101 | 1.072 | 9.28E-01 | 9.28E+01 | | | |
| 7892786 | --- | --- | --- | 0.101 | 1.072 | 8.54E-01 | 8.54E+01 | | | |
| 8164870 | --- | --- | --- | 0.101 | 1.072 | 7.56E-01 | 7.56E+01 | | | |
| 7894758 | --- | --- | --- | 0.101 | 1.072 | 7.68E-01 | 7.68E+01 | | | |
| 7917037 | NM_00113004: | CRYZ | crystallin, zeta (quinone reductase) | 0.100 | 1.072 | 6.74E-01 | 6.74E+01 | | | |
| 8158298 | NM_00100372: | GLE1 | GLE1 RNA export mediator homolog | 0.100 | 1.072 | 3.97E-01 | 3.97E+01 | | | |
| 7893861 | --- | --- | --- | 0.100 | 1.072 | 4.23E-01 | 4.23E+01 | | | |
| 7915567 | NM_024066 | ERI3 | exoribonuclease 3 | 0.100 | 1.072 | 6.16E-01 | 6.16E+01 | | | |
| 7894653 | --- | --- | --- | 0.100 | 1.072 | 1.32E-01 | 1.32E+01 | | | |
| 8130211 | NM_182961 | SYNE1 | spectrin repeat containing, nuclear e | 0.100 | 1.072 | 5.61E-01 | 5.61E+01 | | | |
| 7981142 | NM_024734 | CLMN | calmin (calponin-like, transmembran | 0.100 | 1.072 | 4.46E-01 | 4.46E+01 | | | |
| 7893182 | --- | --- | --- | 0.100 | 1.072 | 4.80E-01 | 4.80E+01 | | | |
| 8093601 | NM_003704 | C4orf8 | chromosome 4 open reading frame 8 | 0.100 | 1.072 | 4.86E-01 | 4.86E+01 | | | |
| 7948741 | NM_012200 | B3GAT3 | beta-1,3-glucuronyltransferase 3 (glu | 0.100 | 1.072 | 7.24E-01 | 7.24E+01 | | | |
| 7905968 | NM_002455 | MTX1 | metaxin 1 | 0.100 | 1.072 | 4.98E-01 | 4.98E+01 | | | |
| 7955156 | NM_033124 | CCDC65 | coiled-coil domain containing 65 | 0.100 | 1.072 | 5.46E-01 | 5.46E+01 | | | |
| 8128767 | NM_014797 | ZBTB24 | zinc finger and BTB domain containir | 0.100 | 1.072 | 5.63E-01 | 5.63E+01 | | | |
| 7951004 | NM_020179 | C11orf75 | chromosome 11 open reading frame | 0.100 | 1.072 | 3.59E-01 | 3.59E+01 | | | |
| 8117760 | NM_00109847! | HLA-F | major histocompatibility complex, cla | 0.100 | 1.072 | 3.34E-01 | 3.34E+01 | | | |
| 8107094 | --- | --- | --- | 0.100 | 1.071 | 3.59E-01 | 3.59E+01 | | | |
| 7935011 | NM_014912 | CPEB3 | cytoplasmic polyadenylation elemen | 0.100 | 1.071 | 2.14E-01 | 2.14E+01 | | | |
| 8180230 | --- | --- | --- | 0.100 | 1.071 | 6.54E-01 | 6.54E+01 | | | |
| 8156452 | NM_014612 | FAM120A | family with sequence similarity 120A | 0.100 | 1.071 | 2.19E-01 | 2.19E+01 | | | |
| 8115397 | NM_032385 | C5orf4 | chromosome 5 open reading frame 4 | 0.100 | 1.071 | 2.25E-01 | 2.25E+01 | | | |
| 7905127 | --- | --- | --- | 0.099 | 1.071 | 4.51E-01 | 4.51E+01 | | | |
| 7892959 | --- | --- | --- | 0.099 | 1.071 | 7.82E-01 | 7.82E+01 | | | |
| 8158862 | NR_002914 | SNORD62A | small nucleolar RNA, C/D box 62A | 0.099 | 1.071 | 7.64E-01 | 7.64E+01 | | | |
| 8158864 | NR_002914 | SNORD62A | small nucleolar RNA, C/D box 62A | 0.099 | 1.071 | 7.64E-01 | 7.64E+01 | | | |
| 7895484 | --- | --- | --- | 0.099 | 1.071 | 7.82E-01 | 7.82E+01 | | | |
| 8138950 | NM_203288 | RP9 | retinitis pigmentosa 9 (autosomal do | 0.099 | 1.071 | 7.43E-01 | 7.43E+01 | | | |
| 8042953 | NM_019896 | POLE4 | polymerase (DNA-directed), epsilon 4 | 0.099 | 1.071 | 5.63E-01 | 5.63E+01 | | | |
| 8002312 | NM_014062 | NOB1 | NIN1/RPN12 binding protein 1 homo | 0.099 | 1.071 | 7.62E-01 | 7.62E+01 | | | |
| 8067201 | --- | --- | --- | 0.099 | 1.071 | 8.44E-01 | 8.44E+01 | | | |

| | | | | | | | | | | |
|---|---|---|---|---|---|---|---|---|---|---|
| 7894010 | --- | --- | --- | 0.099 | 1.071 | 9.18E-01 | 9.18E+01 | | | |
| 7892847 | --- | --- | --- | 0.099 | 1.071 | 9.30E-01 | 9.30E+01 | | | |
| 7971644 | NM_020456 | C13orf1 | chromosome 13 open reading frame | 0.099 | 1.071 | 3.59E-01 | 3.59E+01 | | | |
| 7933484 | NM_00100693 | LRRC18 | leucine rich repeat containing 18 | 0.099 | 1.071 | 4.25E-01 | 4.25E+01 | | | |
| 7920114 | NM_053055 | THEM4 | thioesterase superfamily member 4 | 0.099 | 1.071 | 4.24E-01 | 4.24E+01 | | | |
| 8009417 | NM_002266 | KPNA2 | karyopherin alpha 2 (RAG cohort 1, i | 0.099 | 1.071 | 5.35E-01 | 5.35E+01 | | | |
| 8091103 | NM_006286 | TFDP2 | transcription factor Dp-2 (E2F dimeri | 0.099 | 1.071 | 7.53E-01 | 7.53E+01 | | | |
| 8180319 | --- | --- | --- | 0.099 | 1.071 | 8.43E-01 | 8.43E+01 | | | |
| 8057990 | NM_153697 | ANKRD44 | ankyrin repeat domain 44 | 0.099 | 1.071 | 6.78E-01 | 6.78E+01 | | | |
| 8121298 | --- | --- | --- | 0.099 | 1.071 | 6.85E-01 | 6.85E+01 | | | |
| 8029884 | NR_027280 | SAE1 | SUMO1 activating enzyme subunit 1 | 0.099 | 1.071 | 6.22E-01 | 6.22E+01 | | | |
| 8055104 | NM_00114592 | SAP130 | Sin3A-associated protein, 130kDa | 0.099 | 1.071 | 3.46E-01 | 3.46E+01 | | | |
| 8157203 | NM_00101588 | DNAJC25 | DnaJ (Hsp40) homolog, subfamily C , | 0.099 | 1.071 | 2.15E-01 | 2.15E+01 | | | |
| 7988031 | NM_001006 | RPS3A | ribosomal protein S3A | 0.099 | 1.071 | 6.39E-02 | 6.39E+00 | | | |
| 7895874 | --- | --- | --- | 0.099 | 1.071 | 8.79E-01 | 8.79E+01 | | | |
| 7895514 | --- | --- | --- | 0.099 | 1.071 | 2.87E-02 | 2.87E+00 | | | |
| 8066668 | NM_020967 | NCOA5 | nuclear receptor coactivator 5 | 0.099 | 1.071 | 5.78E-01 | 5.78E+01 | | | |
| 7992973 | NM_005147 | DNAJA3 | DnaJ (Hsp40) homolog, subfamily A, | 0.098 | 1.071 | 4.17E-01 | 4.17E+01 | | | |
| 8078984 | NM_003973 | RPL14 | ribosomal protein L14 | 0.098 | 1.071 | 3.26E-01 | 3.26E+01 | | | |
| 8032909 | NM_005817 | PLIN3 | perilipin 3 | 0.098 | 1.071 | 2.76E-01 | 2.76E+01 | | | |
| 8073192 | NM_006003 | UQCRFS1 | ubiquinol-cytochrome c reductase, R | 0.098 | 1.070 | 7.51E-01 | 7.51E+01 | | | |
| 8177725 | NM_002127 | HLA-G | major histocompatibility complex, cl | 0.098 | 1.070 | 2.95E-01 | 2.95E+01 | | | |
| 8179034 | NM_002127 | HLA-G | major histocompatibility complex, cl | 0.098 | 1.070 | 2.95E-01 | 2.95E+01 | | | |
| 7894367 | --- | --- | --- | 0.098 | 1.070 | 3.36E-01 | 3.36E+01 | | | |
| 7893633 | --- | --- | --- | 0.098 | 1.070 | 2.71E-01 | 2.71E+01 | | | |
| 7896078 | --- | --- | --- | 0.098 | 1.070 | 1.22E-01 | 1.22E+01 | | | |
| 7895481 | --- | --- | --- | 0.098 | 1.070 | 7.85E-01 | 7.85E+01 | | | |
| 8082605 | --- | --- | --- | 0.098 | 1.070 | 2.02E-01 | 2.02E+01 | | | |
| 8117128 | NM_001949 | E2F3 | E2F transcription factor 3 | 0.098 | 1.070 | 2.63E-01 | 2.63E+01 | | | |
| 7895886 | --- | --- | --- | 0.098 | 1.070 | 8.14E-01 | 8.14E+01 | | | |
| 7894641 | --- | --- | --- | 0.098 | 1.070 | 7.81E-01 | 7.81E+01 | | | |
| 8039698 | NM_133460 | ZNF418 | zinc finger protein 418 | 0.098 | 1.070 | 3.11E-01 | 3.11E+01 | | | |
| 8176395 | --- | --- | --- | 0.098 | 1.070 | 6.29E-02 | 6.29E+00 | | | |
| 8130567 | NM_030752 | TCP1 | t-complex 1 | 0.098 | 1.070 | 7.31E-01 | 7.31E+01 | | | |
| 7911339 | --- | --- | --- | 0.098 | 1.070 | 8.95E-01 | 8.95E+01 | | | |
| 8165698 | --- | --- | --- | 0.098 | 1.070 | 8.95E-01 | 8.95E+01 | | | |
| 8139482 | NR_002919 | SNORA5A | small nucleolar RNA, H/ACA box 5A | 0.098 | 1.070 | 9.17E-01 | 9.17E+01 | | | |
| 8014397 | NM_00100141 | TBC1D3C | TBC1 domain family, member 3C | 0.098 | 1.070 | 2.56E-01 | 2.56E+01 | | | |
| 7893838 | --- | --- | --- | 0.098 | 1.070 | 1.44E-01 | 1.44E+01 | | | |
| 7916562 | NM_002136 | HNRNPA1 | heterogeneous nuclear ribonucleopr | 0.098 | 1.070 | 3.73E-01 | 3.73E+01 | | | |
| 7901219 | NM_199044 | NSUN4 | NOL1/NOP2/Sun domain family, mer | 0.098 | 1.070 | 2.94E-01 | 2.94E+01 | | | |
| 7896485 | --- | --- | --- | 0.098 | 1.070 | 2.72E-01 | 2.72E+01 | | | |
| 8063408 | --- | --- | --- | 0.098 | 1.070 | 8.90E-01 | 8.90E+01 | | | |
| 8155458 | D14041 | RBPJ | recombination signal binding protein | 0.098 | 1.070 | 7.61E-01 | 7.61E+01 | | | |
| 8139250 | NM_032014 | MRPS24 | mitochondrial ribosomal protein S24 | 0.098 | 1.070 | 6.79E-01 | 6.79E+01 | | | |
| 8176469 | --- | --- | --- | 0.097 | 1.070 | 8.48E-01 | 8.48E+01 | | | |
| 7948667 | NM_001620 | AHNAK | AHNAK nucleoprotein | 0.097 | 1.070 | 2.39E-01 | 2.39E+01 | | | |
| 8130732 | NM_016098 | BRP44L | brain protein 44-like | 0.097 | 1.070 | 7.44E-01 | 7.44E+01 | | | |
| 7905043 | NR_027002 | LOC388692 | hypothetical LOC388692 | 0.097 | 1.070 | 2.62E-01 | 2.62E+01 | | | |
| 8136539 | NM_016019 | LUC7L2 | LUC7-like 2 (S. cerevisiae) | 0.097 | 1.070 | 6.73E-01 | 6.73E+01 | | | |
| 8109350 | NM_078483 | SLC36A1 | solute carrier family 36 (proton/amir | 0.097 | 1.070 | 4.52E-01 | 4.52E+01 | | | |
| 7893056 | --- | --- | --- | 0.097 | 1.070 | 8.18E-01 | 8.18E+01 | | | |
| 7926368 | NM_003380 | VIM | vimentin | 0.097 | 1.070 | 1.84E-01 | 1.84E+01 | | | |
| 7939383 | NM_024841 | PRR5L | proline rich 5 like | 0.097 | 1.070 | 2.12E-01 | 2.12E+01 | | | |
| 8164698 | NM_004269 | MED27 | mediator complex subunit 27 | 0.097 | 1.070 | 7.90E-01 | 7.90E+01 | | | |
| 7978824 | NM_00103000 | RPS29 | ribosomal protein S29 | 0.097 | 1.070 | 5.99E-01 | 5.99E+01 | | | |
| 7904830 | NM_014455 | RNF115 | ring finger protein 115 | 0.097 | 1.070 | 7.93E-01 | 7.93E+01 | | | |
| 8066200 | BC013755 | KIAA0406 | KIAA0406 | 0.097 | 1.070 | 2.93E-01 | 2.93E+01 | | | |
| 8038824 | NM_033130 | SIGLEC10 | sialic acid binding Ig-like lectin 10 | 0.097 | 1.070 | 1.94E-01 | 1.94E+01 | | | |
| 8060599 | NM_030811 | MRPS26 | mitochondrial ribosomal protein S26 | 0.097 | 1.070 | 4.37E-01 | 4.37E+01 | | | |
| 8090840 | NM_00100586 | RYK | RYK receptor-like tyrosine kinase | 0.097 | 1.070 | 7.79E-01 | 7.79E+01 | | | |
| 7926059 | --- | --- | --- | 0.097 | 1.069 | 7.49E-01 | 7.49E+01 | | | |
| 7976216 | NM_00110236 | C14orf159 | chromosome 14 open reading frame | 0.097 | 1.069 | 2.41E-01 | 2.41E+01 | | | |
| 7892840 | --- | --- | --- | 0.097 | 1.069 | 8.33E-01 | 8.33E+01 | | | |
| 7946504 | NM_015012 | TMEM41B | transmembrane protein 41B | 0.097 | 1.069 | 5.02E-01 | 5.02E+01 | | | |
| 7945864 | NM_00113052 | ZNF195 | zinc finger protein 195 | 0.097 | 1.069 | 4.12E-01 | 4.12E+01 | | | |
| 7894068 | --- | --- | --- | 0.097 | 1.069 | 8.20E-01 | 8.20E+01 | | | |
| 8103188 | NM_004564 | PET112L | PET112-like (yeast) | 0.097 | 1.069 | 4.48E-01 | 4.48E+01 | | | |
| 8107920 | NM_003060 | SLC22A5 | solute carrier family 22 (organic catio | 0.097 | 1.069 | 3.88E-01 | 3.88E+01 | | | |
| 8071119 | NM_015367 | BCL2L13 | BCL2-like 13 (apoptosis facilitator) | 0.097 | 1.069 | 7.67E-02 | 7.67E+00 | | | |
| 7928471 | NM_006721 | ADK | adenosine kinase | 0.097 | 1.069 | 6.91E-01 | 6.91E+01 | | | |
| 7894577 | --- | --- | --- | 0.097 | 1.069 | 9.38E-01 | 9.38E+01 | | | |
| 7895631 | --- | --- | --- | 0.096 | 1.069 | 8.34E-01 | 8.34E+01 | | | |
| 7895235 | --- | --- | --- | 0.096 | 1.069 | 6.96E-01 | 6.96E+01 | | | |
| 8138531 | NM_001402 | EEF1A1 | eukaryotic translation elongation fac | 0.096 | 1.069 | 3.65E-02 | 3.65E+00 | | | |
| 8009727 | NM_001545 | ICT1 | immature colon carcinoma transcrip | 0.096 | 1.069 | 7.20E-01 | 7.20E+01 | | | |
| 8056966 | --- | --- | --- | 0.096 | 1.069 | 4.37E-01 | 4.37E+01 | | | |

| | | | | | | | | | | |
|---|---|---|---|---|---|---|---|---|---|---|
| 7901804 | NM_176877 | INADL | InaD-like (Drosophila) | 0.096 | 1.069 | 5.46E-01 | 5.46E+01 | | | |
| 8180404 | --- | --- | --- | 0.096 | 1.069 | 5.47E-01 | 5.47E+01 | | | |
| 7974125 | NM_015091 | FAM179B | family with sequence similarity 179, | 0.096 | 1.069 | 2.95E-01 | 2.95E+01 | | | |
| 7921713 | NM_016946 | F11R | F11 receptor | 0.096 | 1.069 | 2.14E-01 | 2.14E+01 | | | |
| 8053046 | NM_003584 | DUSP11 | dual specificity phosphatase 11 (RNA | 0.096 | 1.069 | 7.57E-01 | 7.57E+01 | | | |
| 8061416 | NM_003650 | CST7 | cystatin F (leukocystatin) | 0.096 | 1.069 | 6.57E-01 | 6.57E+01 | | | |
| 7904997 | NM_00114388 | PPIAL4B | peptidylprolyl isomerase A (cyclophil | 0.096 | 1.069 | 3.14E-01 | 3.14E+01 | | | |
| 7905016 | NM_00114388 | PPIAL4B | peptidylprolyl isomerase A (cyclophil | 0.096 | 1.069 | 3.14E-01 | 3.14E+01 | | | |
| 7896631 | --- | --- | --- | 0.096 | 1.069 | 4.43E-03 | 4.43E-01 | | | |
| 7895717 | --- | --- | --- | 0.096 | 1.069 | 4.00E-01 | 4.00E+01 | | | |
| 7894916 | --- | --- | --- | 0.096 | 1.069 | 4.70E-01 | 4.70E+01 | | | |
| 8078173 | NM_138381 | OXNAD1 | oxidoreductase NAD-binding domain | 0.096 | 1.069 | 6.39E-01 | 6.39E+01 | | | |
| 7908330 | AY854248 | C1orf27 | chromosome 1 open reading frame 2 | 0.096 | 1.069 | 8.20E-01 | 8.20E+01 | | | |
| 8043449 | BC030813 | IGK@ | immunoglobulin kappa locus | 0.096 | 1.069 | 5.56E-01 | 5.56E+01 | | | |
| 8138489 | NM_018719 | CDCA7L | cell division cycle associated 7-like | 0.096 | 1.069 | 8.43E-01 | 8.43E+01 | | | |
| 8133571 | NR_003664 | SPDYE8P | speedy homolog E8 (Xenopus laevis) | 0.096 | 1.069 | 3.96E-01 | 3.96E+01 | | | |
| 8000692 | NM_00103182 | BOLA2 | bolA homolog 2 (E. coli) | 0.096 | 1.069 | 2.97E-01 | 2.97E+01 | | | |
| 7894813 | --- | --- | --- | 0.096 | 1.069 | 1.27E-01 | 1.27E+01 | | | |
| 8177669 | BC171739 | GUSBL1 | glucuronidase, beta-like 1 | 0.096 | 1.069 | 7.44E-01 | 7.44E+01 | | | |
| 7945803 | NM_00101443 | CARS | cysteinyl-tRNA synthetase | 0.096 | 1.069 | 5.59E-01 | 5.59E+01 | | | |
| 7937275 | NM_021932 | RIC8A | resistance to inhibitors of cholineste | 0.096 | 1.069 | 4.91E-01 | 4.91E+01 | | | |
| 8072274 | NM_00100368 | UCRC | ubiquinol-cytochrome c reductase co | 0.096 | 1.069 | 7.08E-01 | 7.08E+01 | | | |
| 8132417 | NM_003718 | CDC2L5 | cell division cycle 2-like 5 (cholineste | 0.096 | 1.069 | 5.07E-01 | 5.07E+01 | | | |
| 7952445 | NM_024631 | C11orf61 | chromosome 11 open reading frame | 0.096 | 1.069 | 4.57E-01 | 4.57E+01 | | | |
| 8169742 | NM_001167 | XIAP | X-linked inhibitor of apoptosis | 0.096 | 1.069 | 3.82E-01 | 3.82E+01 | | | |
| 7899688 | NM_012316 | KPNA6 | karyopherin alpha 6 (importin alpha | 0.096 | 1.069 | 7.46E-01 | 7.46E+01 | | | |
| 8015642 | NM_016556 | PSMC3IP | PSMC3 interacting protein | 0.096 | 1.069 | 4.19E-01 | 4.19E+01 | | | |
| 7984124 | NM_031301 | APH1B | anterior pharynx defective 1 homolo | 0.096 | 1.069 | 5.68E-01 | 5.68E+01 | | | |
| 7903541 | NM_007269 | STXBP3 | syntaxin binding protein 3 | 0.096 | 1.069 | 8.58E-01 | 8.58E+01 | | | |
| 8009666 | NM_175738 | RAB37 | RAB37, member RAS oncogene famil | 0.096 | 1.069 | 3.86E-01 | 3.86E+01 | | | |
| 8052861 | NM_003096 | SNRPG | small nuclear ribonucleoprotein poly | 0.096 | 1.069 | 7.20E-01 | 7.20E+01 | | | |
| 8093993 | NM_152293 | TADA2B | transcriptional adaptor 2 (ADA2 hom | 0.096 | 1.069 | 5.59E-01 | 5.59E+01 | | | |
| 8108927 | NM_018989 | RBM27 | RNA binding motif protein 27 | 0.096 | 1.069 | 7.16E-01 | 7.16E+01 | | | |
| 8063668 | NM_000516 | GNAS | GNAS complex locus | 0.096 | 1.069 | 1.72E-01 | 1.72E+01 | | | |
| 8067978 | NM_021130 | PPIA | peptidylprolyl isomerase A (cyclophil | 0.096 | 1.069 | 2.77E-01 | 2.77E+01 | | | |
| 7960730 | NM_005768 | LPCAT3 | lysophosphatidylcholine acyltransfer | 0.096 | 1.069 | 7.56E-01 | 7.56E+01 | | | |
| 7895258 | --- | --- | --- | 0.095 | 1.068 | 9.29E-01 | 9.29E+01 | | | |
| 7920697 | NM_000157 | GBA | glucosidase, beta; acid (includes gluc | 0.095 | 1.068 | 4.44E-01 | 4.44E+01 | | | |
| 8075728 | NM_002473 | MYH9 | myosin, heavy chain 9, non-muscle | 0.095 | 1.068 | 4.48E-01 | 4.48E+01 | | | |
| 8063473 | NM_000976 | RPL12 | ribosomal protein L12 | 0.095 | 1.068 | 4.42E-01 | 4.42E+01 | | | |
| 8168079 | --- | --- | --- | 0.095 | 1.068 | 7.71E-01 | 7.71E+01 | | | |
| 7975787 | NM_00113504 | JDP2 | Jun dimerization protein 2 | 0.095 | 1.068 | 2.00E-01 | 2.00E+01 | | | |
| 8020068 | NM_015208 | ANKRD12 | ankyrin repeat domain 12 | 0.095 | 1.068 | 6.67E-01 | 6.67E+01 | | | |
| 8088820 | NM_012234 | RYBP | RING1 and YY1 binding protein | 0.095 | 1.068 | 7.18E-01 | 7.18E+01 | | | |
| 7894227 | --- | --- | --- | 0.095 | 1.068 | 8.41E-01 | 8.41E+01 | | | |
| 7896621 | --- | --- | --- | 0.095 | 1.068 | 7.73E-01 | 7.73E+01 | | | |
| 8093156 | NM_007362 | NCBP2 | nuclear cap binding protein subunit | 0.095 | 1.068 | 6.78E-01 | 6.78E+01 | | | |
| 7961371 | NM_030640 | DUSP16 | dual specificity phosphatase 16 | 0.095 | 1.068 | 5.27E-01 | 5.27E+01 | | | |
| 8143661 | --- | --- | --- | 0.095 | 1.068 | 8.51E-01 | 8.51E+01 | | | |
| 8115895 | NM_020444 | KIAA1191 | KIAA1191 | 0.095 | 1.068 | 6.16E-01 | 6.16E+01 | | | |
| 7992670 | NM_002613 | PDPK1 | 3-phosphoinositide dependent prote | 0.095 | 1.068 | 3.70E-01 | 3.70E+01 | | | |
| 7944623 | NM_152715 | TBCEL | tubulin folding cofactor E-like | 0.095 | 1.068 | 7.53E-01 | 7.53E+01 | | | |
| 8090630 | NM_00101739 | TMCC1 | transmembrane and coiled-coil dom | 0.095 | 1.068 | 6.71E-01 | 6.71E+01 | | | |
| 8012197 | NM_020360 | PLSCR3 | phospholipid scramblase 3 | 0.095 | 1.068 | 1.78E-01 | 1.78E+01 | | | |
| 8066786 | NM_183047 | ZMYND8 | zinc finger, MYND-type containing 8 | 0.095 | 1.068 | 5.27E-01 | 5.27E+01 | | | |
| 8070629 | BC005107 | C21orf105 | chromosome 21 open reading frame | 0.095 | 1.068 | 6.17E-01 | 6.17E+01 | | | |
| 8037794 | NM_016457 | PRKD2 | protein kinase D2 | 0.095 | 1.068 | 2.65E-01 | 2.65E+01 | | | |
| 7975292 | NM_002877 | RAD51L1 | RAD51-like 1 (S. cerevisiae) | 0.095 | 1.068 | 5.69E-01 | 5.69E+01 | | | |
| 7909188 | NM_014002 | IKBKE | inhibitor of kappa light polypeptide g | 0.095 | 1.068 | 2.48E-01 | 2.48E+01 | | | |
| 7959623 | NM_001516 | GTF2H3 | general transcription factor IIH, poly | 0.095 | 1.068 | 5.65E-01 | 5.65E+01 | | | |
| 7895779 | --- | --- | --- | 0.095 | 1.068 | 1.45E-01 | 1.45E+01 | | | |
| 8017634 | NM_004396 | DDX5 | DEAD (Asp-Glu-Ala-Asp) box polypep | 0.094 | 1.068 | 1.92E-01 | 1.92E+01 | | | |
| 8097782 | NM_001006 | RPS3A | ribosomal protein S3A | 0.094 | 1.068 | 9.27E-02 | 9.27E+00 | | | |
| 7937476 | NM_001004 | RPLP2 | ribosomal protein, large, P2 | 0.094 | 1.068 | 5.20E-01 | 5.20E+01 | | | |
| 7910229 | NM_00102422 | ARF1 | ADP-ribosylation factor 1 | 0.094 | 1.068 | 3.30E-01 | 3.30E+01 | | | |
| 8062576 | NM_021931 | DHX35 | DEAH (Asp-Glu-Ala-His) box polypept | 0.094 | 1.067 | 6.13E-01 | 6.13E+01 | | | |
| 7892906 | --- | --- | --- | 0.094 | 1.067 | 7.95E-01 | 7.95E+01 | | | |
| 8082248 | NR_002822 | MGC72080 | MGC72080 pseudogene | 0.094 | 1.067 | 8.24E-01 | 8.24E+01 | | | |
| 7930682 | NM_020940 | FAM160B1 | family with sequence similarity 160, | 0.094 | 1.067 | 7.41E-01 | 7.41E+01 | | | |
| 7985777 | NM_002201 | ISG20 | interferon stimulated exonuclease ge | 0.094 | 1.067 | 4.56E-01 | 4.56E+01 | | | |
| 7970376 | NM_032436 | ZNF828 | zinc finger protein 828 | 0.094 | 1.067 | 4.56E-01 | 4.56E+01 | | | |
| 8093500 | NM_006342 | TACC3 | transforming, acidic coiled-coil conta | 0.094 | 1.067 | 6.14E-01 | 6.14E+01 | | | |
| 8076515 | NM_014570 | ARFGAP3 | ADP-ribosylation factor GTPase activ | 0.094 | 1.067 | 7.05E-01 | 7.05E+01 | | | |
| 7893151 | --- | --- | --- | 0.094 | 1.067 | 4.62E-01 | 4.62E+01 | | | |
| 7998604 | NM_005326 | HAGH | hydroxyacylglutathione hydrolase | 0.094 | 1.067 | 4.73E-01 | 4.73E+01 | | | |
| 7985757 | NM_022839 | MRPS11 | mitochondrial ribosomal protein S11 | 0.094 | 1.067 | 5.58E-01 | 5.58E+01 | | | |
| 8029385 | NM_013362 | ZNF225 | zinc finger protein 225 | 0.094 | 1.067 | 4.10E-01 | 4.10E+01 | | | |

| | | | | | | | | | | |
|---|---|---|---|---|---|---|---|---|---|---|
| 8178298 | NM_001470 | GABBR1 | gamma-aminobutyric acid (GABA) B | 0.094 | 1.067 | 2.18E-01 | 2.18E+01 | | | |
| 7896364 | --- | --- | --- | 0.094 | 1.067 | 8.26E-01 | 8.26E+01 | | | |
| 7948113 | NR_027688 | OR7E5P | olfactory receptor, family 7, subfami | 0.094 | 1.067 | 7.28E-01 | 7.28E+01 | | | |
| 8043360 | BC030813 | IGK@ | immunoglobulin kappa locus | 0.094 | 1.067 | 4.44E-01 | 4.44E+01 | | | |
| 8135458 | NM_024814 | CBLL1 | Cas-Br-M (murine) ecotropic retrovir | 0.094 | 1.067 | 8.38E-01 | 8.38E+01 | | | |
| 7979328 | NM_014924 | KIAA0831 | KIAA0831 | 0.094 | 1.067 | 7.85E-01 | 7.85E+01 | | | |
| 8173261 | NM_018684 | ZC4H2 | zinc finger, C4H2 domain containing | 0.094 | 1.067 | 3.84E-01 | 3.84E+01 | | | |
| 8076355 | NM_002676 | PMM1 | phosphomannomutase 1 | 0.094 | 1.067 | 5.09E-01 | 5.09E+01 | | | |
| 7895026 | --- | --- | --- | 0.094 | 1.067 | 6.57E-02 | 6.57E+00 | | | |
| 8043564 | NM_016044 | FAHD2A | fumarylacetoacetate hydrolase doma | 0.094 | 1.067 | 4.03E-01 | 4.03E+01 | | | |
| 7894195 | --- | --- | --- | 0.094 | 1.067 | 7.47E-01 | 7.47E+01 | | | |
| 7893119 | --- | --- | --- | 0.093 | 1.067 | 6.84E-01 | 6.84E+01 | | | |
| 8180166 | NM_003190 | TAPBP | TAP binding protein (tapasin) | 0.093 | 1.067 | 2.57E-01 | 2.57E+01 | | | |
| 8044745 | NM_006773 | DDX18 | DEAD (Asp-Glu-Ala-Asp) box polypep | 0.093 | 1.067 | 7.69E-01 | 7.69E+01 | | | |
| 7894307 | --- | --- | --- | 0.093 | 1.067 | 1.91E-01 | 1.91E+01 | | | |
| 8118932 | NM_022047 | DEF6 | differentially expressed in FDCP 6 ho | 0.093 | 1.067 | 3.21E-01 | 3.21E+01 | | | |
| 7894790 | --- | --- | --- | 0.093 | 1.067 | 8.97E-01 | 8.97E+01 | | | |
| 8117402 | NM_003545 | HIST1H4E | histone cluster 1, H4e | 0.093 | 1.067 | 7.69E-01 | 7.69E+01 | | | |
| 7892889 | --- | --- | --- | 0.093 | 1.067 | 9.48E-02 | 9.48E+00 | | | |
| 8140356 | --- | --- | --- | 0.093 | 1.067 | 5.80E-01 | 5.80E+01 | | | |
| 7893407 | --- | --- | --- | 0.093 | 1.067 | 7.45E-01 | 7.45E+01 | | | |
| 7971163 | --- | --- | --- | 0.093 | 1.067 | 7.02E-01 | 7.02E+01 | | | |
| 8116835 | NM_145649 | GCNT2 | glucosaminyl (N-acetyl) transferase 2 | 0.093 | 1.067 | 3.85E-01 | 3.85E+01 | | | |
| 7976967 | NM_018335 | ZNF839 | zinc finger protein 839 | 0.093 | 1.067 | 5.38E-01 | 5.38E+01 | | | |
| 7926299 | NM_016299 | HSPA14 | heat shock 70kDa protein 14 | 0.093 | 1.067 | 4.67E-01 | 4.67E+01 | | | |
| 7990511 | NM_002833 | PTPN9 | protein tyrosine phosphatase, non-re | 0.093 | 1.067 | 4.43E-01 | 4.43E+01 | | | |
| 8082444 | NM_014049 | ACAD9 | acyl-Coenzyme A dehydrogenase fan | 0.093 | 1.066 | 4.86E-01 | 4.86E+01 | | | |
| 8115681 | NM_024594 | PANK3 | pantothenate kinase 3 | 0.093 | 1.066 | 5.10E-01 | 5.10E+01 | | | |
| 7895317 | --- | --- | --- | 0.093 | 1.066 | 5.51E-01 | 5.51E+01 | | | |
| 8152976 | NM_144649 | TMEM71 | transmembrane protein 71 | 0.093 | 1.066 | 7.89E-01 | 7.89E+01 | | | |
| 7904742 | NM_153713 | LIX1L | Lix1 homolog (mouse)-like | 0.093 | 1.066 | 2.78E-01 | 2.78E+01 | | | |
| 7898939 | NM_020448 | NIPAL3 | NIPA-like domain containing 3 | 0.093 | 1.066 | 5.35E-01 | 5.35E+01 | | | |
| 7893040 | --- | --- | --- | 0.093 | 1.066 | 7.80E-01 | 7.80E+01 | | | |
| 8115806 | NM_152277 | UBTD2 | ubiquitin domain containing 2 | 0.093 | 1.066 | 5.07E-01 | 5.07E+01 | | | |
| 8124813 | NM_014641 | MDC1 | mediator of DNA-damage checkpoin | 0.092 | 1.066 | 3.26E-01 | 3.26E+01 | | | |
| 8178404 | NM_014641 | MDC1 | mediator of DNA-damage checkpoin | 0.092 | 1.066 | 3.26E-01 | 3.26E+01 | | | |
| 7895145 | --- | --- | --- | 0.092 | 1.066 | 9.09E-01 | 9.09E+01 | | | |
| 8014140 | --- | --- | --- | 0.092 | 1.066 | 5.62E-01 | 5.62E+01 | | | |
| 7896345 | --- | --- | --- | 0.092 | 1.066 | 8.38E-01 | 8.38E+01 | | | |
| 8091764 | NM_002268 | KPNA4 | karyopherin alpha 4 (importin alpha | 0.092 | 1.066 | 7.99E-01 | 7.99E+01 | | | |
| 7894251 | --- | --- | --- | 0.092 | 1.066 | 6.37E-01 | 6.37E+01 | | | |
| 7986863 | NM_004667 | HERC2 | hect domain and RLD 2 | 0.092 | 1.066 | 2.38E-01 | 2.38E+01 | | | |
| 8057056 | NM_133378 | TTN | titin | 0.092 | 1.066 | 2.48E-01 | 2.48E+01 | | | |
| 8149380 | NR_003668 | DEFB109P1B | defensin, beta 109, pseudogene 1B | 0.092 | 1.066 | 7.41E-01 | 7.41E+01 | | | |
| 8051547 | NM_005813 | PRKD3 | protein kinase D3 | 0.092 | 1.066 | 3.89E-01 | 3.89E+01 | | | |
| 8110982 | NM_004394 | DAP | death-associated protein | 0.092 | 1.066 | 4.50E-01 | 4.50E+01 | | | |
| 8116724 | NM_006567 | FARS2 | phenylalanyl-tRNA synthetase 2, mit | 0.092 | 1.066 | 8.08E-01 | 8.08E+01 | | | |
| 8119161 | NM_002648 | PIM1 | pim-1 oncogene | 0.092 | 1.066 | 3.09E-01 | 3.09E+01 | | | |
| 7893382 | --- | --- | --- | 0.092 | 1.066 | 1.22E-01 | 1.22E+01 | | | |
| 7892875 | --- | --- | --- | 0.092 | 1.066 | 4.45E-01 | 4.45E+01 | | | |
| 8129095 | --- | --- | --- | 0.092 | 1.066 | 6.73E-01 | 6.73E+01 | | | |
| 7977879 | NM_00114493 | PSMB5 | proteasome (prosome, macropain) s | 0.092 | 1.066 | 4.45E-01 | 4.45E+01 | | | |
| 7893281 | --- | --- | --- | 0.092 | 1.066 | 1.60E-01 | 1.60E+01 | | | |
| 7945262 | NM_032801 | JAM3 | junctional adhesion molecule 3 | 0.092 | 1.066 | 3.80E-01 | 3.80E+01 | | | |
| 8005458 | NM_00104007 | LGALS9C | lectin, galactoside-binding, soluble, 9 | 0.092 | 1.066 | 5.50E-01 | 5.50E+01 | | | |
| 8077171 | NM_00113092 | RABL2B | RAB, member of RAS oncogene fami | 0.092 | 1.066 | 5.32E-01 | 5.32E+01 | | | |
| 8103222 | --- | --- | --- | 0.092 | 1.066 | 2.00E-01 | 2.00E+01 | | | |
| 7893914 | --- | --- | --- | 0.091 | 1.065 | 2.61E-01 | 2.61E+01 | | | |
| 8068361 | NM_006933 | SLC5A3 | solute carrier family 5 (sodium/myo- | 0.091 | 1.065 | 7.25E-01 | 7.25E+01 | | | |
| 7933999 | NM_017987 | RUFY2 | RUN and FYVE domain containing 2 | 0.091 | 1.065 | 5.88E-01 | 5.88E+01 | | | |
| 7894376 | --- | --- | --- | 0.091 | 1.065 | 9.68E-02 | 9.68E+00 | | | |
| 8106096 | --- | --- | --- | 0.091 | 1.065 | 8.89E-02 | 8.89E+00 | | | |
| 8125483 | NM_000544 | TAP2 | transporter 2, ATP-binding cassette, | 0.091 | 1.065 | 3.46E-01 | 3.46E+01 | | | |
| 8064601 | NM_023935 | DDRGK1 | DDRGK domain containing 1 | 0.091 | 1.065 | 6.74E-01 | 6.74E+01 | | | |
| 7922414 | NR_003942 | SNORD76 | small nucleolar RNA, C/D box 76 | 0.091 | 1.065 | 7.44E-01 | 7.44E+01 | | | |
| 8123695 | NM_206836 | PECI | peroxisomal D3,D2-enoyl-CoA isome | 0.091 | 1.065 | 7.56E-01 | 7.56E+01 | | | |
| 7898998 | NM_016124 | RHD | Rh blood group, D antigen | 0.091 | 1.065 | 3.56E-01 | 3.56E+01 | | | |
| 8074251 | NM_001696 | ATP6V1E1 | ATPase, H+ transporting, lysosomal 3 | 0.091 | 1.065 | 7.31E-01 | 7.31E+01 | | | |
| 8066195 | NM_006698 | BLCAP | bladder cancer associated protein | 0.091 | 1.065 | 2.58E-01 | 2.58E+01 | | | |
| 7951896 | NM_004716 | PCSK7 | proprotein convertase subtilisin/kexi | 0.091 | 1.065 | 4.54E-01 | 4.54E+01 | | | |
| 7896606 | --- | --- | --- | 0.091 | 1.065 | 5.68E-01 | 5.68E+01 | | | |
| 8127822 | --- | --- | --- | 0.091 | 1.065 | 7.50E-01 | 7.50E+01 | | | |
| 7912481 | NM_00112732 | MAD2L2 | MAD2 mitotic arrest deficient-like 2 | 0.091 | 1.065 | 5.35E-01 | 5.35E+01 | | | |
| 7905395 | NM_002796 | PSMB4 | proteasome (prosome, macropain) s | 0.090 | 1.065 | 8.44E-01 | 8.44E+01 | | | |
| 7900354 | NM_006112 | PPIE | peptidylprolyl isomerase E (cyclophil | 0.090 | 1.065 | 6.49E-01 | 6.49E+01 | | | |
| 7924636 | NM_014698 | TMEM63A | transmembrane protein 63A | 0.090 | 1.065 | 5.44E-01 | 5.44E+01 | | | |
| 7949490 | NM_138368 | KFZp761E19 | DKFZp761E198 protein | 0.090 | 1.065 | 2.56E-01 | 2.56E+01 | | | |

| | | | | | | | | | | |
|---|---|---|---|---|---|---|---|---|---|---|
| 7892525 | --- | --- | --- | 0.090 | 1.065 | 6.65E-01 | 6.65E+01 | | | |
| 7895343 | --- | --- | --- | 0.090 | 1.065 | 1.27E-01 | 1.27E+01 | | | |
| 8151413 | NM_005648 | TCEB1 | transcription elongation factor B (SIII | 0.090 | 1.065 | 4.43E-01 | 4.43E+01 | | | |
| 8033789 | NM_00100872 | ZNF121 | zinc finger protein 121 | 0.090 | 1.065 | 4.29E-01 | 4.29E+01 | | | |
| 7913814 | NM_015484 | SYF2 | SYF2 homolog, RNA splicing factor (S | 0.090 | 1.065 | 7.34E-01 | 7.34E+01 | | | |
| 7905664 | NM_024330 | SLC27A3 | solute carrier family 27 (fatty acid tra | 0.090 | 1.064 | 1.91E-01 | 1.91E+01 | | | |
| 8144586 | NM_015458 | MTMR9 | myotubularin related protein 9 | 0.090 | 1.064 | 3.73E-01 | 3.73E+01 | | | |
| 7952830 | NM_015261 | NCAPD3 | non-SMC condensin II complex, subu | 0.090 | 1.064 | 4.41E-01 | 4.41E+01 | | | |
| 7956419 | --- | --- | --- | 0.090 | 1.064 | 6.23E-01 | 6.23E+01 | | | |
| 8160238 | NM_033222 | PSIP1 | PC4 and SFRS1 interacting protein 1 | 0.090 | 1.064 | 6.61E-01 | 6.61E+01 | | | |
| 7994559 | NR_003610 | PDXDC2 | pyridoxal-dependent decarboxylase | 0.090 | 1.064 | 6.28E-01 | 6.28E+01 | | | |
| 8111101 | NM_054027 | ANKH | ankylosis, progressive homolog (mou | 0.090 | 1.064 | 4.64E-01 | 4.64E+01 | | | |
| 7985117 | --- | --- | --- | 0.090 | 1.064 | 2.96E-01 | 2.96E+01 | | | |
| 8134433 | --- | --- | --- | 0.090 | 1.064 | 3.15E-01 | 3.15E+01 | | | |
| 7958582 | NM_032300 | TCHP | trichoplein, keratin filament binding | 0.090 | 1.064 | 1.33E-01 | 1.33E+01 | | | |
| 8118535 | NM_006913 | RNF5 | ring finger protein 5 | 0.090 | 1.064 | 5.93E-01 | 5.93E+01 | | | |
| 8179472 | NM_006913 | RNF5 | ring finger protein 5 | 0.090 | 1.064 | 5.93E-01 | 5.93E+01 | | | |
| 7932326 | NM_001081 | CUBN | cubilin (intrinsic factor-cobalamin re | 0.090 | 1.064 | 1.23E-01 | 1.23E+01 | | | |
| 8073470 | NM_00114296 | CTA-216E10.( | hypothetical FLJ23584 | 0.090 | 1.064 | 2.98E-01 | 2.98E+01 | | | |
| 7892764 | --- | --- | --- | 0.090 | 1.064 | 8.82E-01 | 8.82E+01 | | | |
| 7896371 | --- | --- | --- | 0.089 | 1.064 | 6.15E-01 | 6.15E+01 | | | |
| 7956159 | NM_00103526 | RPL41 | ribosomal protein L41 | 0.089 | 1.064 | 8.11E-01 | 8.11E+01 | | | |
| 8062623 | NM_002660 | PLCG1 | phospholipase C, gamma 1 | 0.089 | 1.064 | 5.62E-01 | 5.62E+01 | | | |
| 7893241 | --- | --- | --- | 0.089 | 1.064 | 9.12E-01 | 9.12E+01 | | | |
| 8093258 | NM_032263 | IQCG | IQ motif containing G | 0.089 | 1.064 | 4.07E-01 | 4.07E+01 | | | |
| 7893507 | --- | --- | --- | 0.089 | 1.064 | 1.77E-02 | 1.77E+00 | | | |
| 8009008 | NM_181725 | METTL2A | methyltransferase like 2A | 0.089 | 1.064 | 6.36E-01 | 6.36E+01 | | | |
| 8131303 | NM_015610 | WIPI2 | WD repeat domain, phosphoinositid | 0.089 | 1.064 | 4.95E-01 | 4.95E+01 | | | |
| 7892902 | --- | --- | --- | 0.089 | 1.064 | 3.77E-01 | 3.77E+01 | | | |
| 7960381 | CR627161 | EFCAB4B | EF-hand calcium binding domain 4B | 0.089 | 1.064 | 7.08E-01 | 7.08E+01 | | | |
| 8047635 | NM_000976 | RPL12 | ribosomal protein L12 | 0.089 | 1.064 | 4.80E-01 | 4.80E+01 | | | |
| 7933877 | NM_004241 | JMJD1C | jumonji domain containing 1C | 0.089 | 1.064 | 7.42E-01 | 7.42E+01 | | | |
| 7955646 | NM_001417 | EIF4B | eukaryotic translation initiation facto | 0.089 | 1.064 | 5.43E-01 | 5.43E+01 | | | |
| 8175924 | NM_003491 | ARD1A | ARD1 homolog A, N-acetyltransferas | 0.089 | 1.064 | 7.04E-01 | 7.04E+01 | | | |
| 7894406 | --- | --- | --- | 0.089 | 1.064 | 1.79E-01 | 1.79E+01 | | | |
| 7893133 | --- | --- | --- | 0.089 | 1.064 | 4.72E-01 | 4.72E+01 | | | |
| 8000411 | NM_00114534 | JMJD5 | jumonji domain containing 5 | 0.089 | 1.063 | 4.59E-01 | 4.59E+01 | | | |
| 8179638 | NM_003449 | TRIM26 | tripartite motif-containing 26 | 0.089 | 1.063 | 4.05E-01 | 4.05E+01 | | | |
| 7969828 | --- | --- | --- | 0.089 | 1.063 | 2.58E-01 | 2.58E+01 | | | |
| 7983157 | NM_024956 | TMEM62 | transmembrane protein 62 | 0.089 | 1.063 | 6.99E-01 | 6.99E+01 | | | |
| 7895909 | --- | --- | --- | 0.089 | 1.063 | 7.57E-01 | 7.57E+01 | | | |
| 7895224 | --- | --- | --- | 0.089 | 1.063 | 5.35E-01 | 5.35E+01 | | | |
| 8062349 | NM_002951 | RPN2 | ribophorin II | 0.089 | 1.063 | 6.74E-01 | 6.74E+01 | | | |
| 7902448 | NM_00103500 | RPL17 | ribosomal protein L17 | 0.088 | 1.063 | 4.74E-01 | 4.74E+01 | | | |
| 7986350 | NM_183376 | ARRDC4 | arrestin domain containing 4 | 0.088 | 1.063 | 4.68E-01 | 4.68E+01 | | | |
| 8038877 | NM_003830 | SIGLEC5 | sialic acid binding Ig-like lectin 5 | 0.088 | 1.063 | 3.31E-01 | 3.31E+01 | | | |
| 8169352 | NM_018698 | NXT2 | nuclear transport factor 2-like expor | 0.088 | 1.063 | 6.22E-01 | 6.22E+01 | | | |
| 7985444 | NR_003661 | FLJ43276 | similar to ubiquitin-conjugating enzy | 0.088 | 1.063 | 6.96E-01 | 6.96E+01 | | | |
| 7997281 | NM_018975 | TERF2IP | telomeric repeat binding factor 2, int | 0.088 | 1.063 | 3.82E-01 | 3.82E+01 | | | |
| 8142087 | NM_020725 | ATXN7L1 | ataxin 7-like 1 | 0.088 | 1.063 | 6.46E-01 | 6.46E+01 | | | |
| 8122909 | NM_014892 | RBM16 | RNA binding motif protein 16 | 0.088 | 1.063 | 7.35E-01 | 7.35E+01 | | | |
| 8139203 | NM_138701 | C7orf11 | chromosome 7 open reading frame 1 | 0.088 | 1.063 | 5.02E-01 | 5.02E+01 | | | |
| 8137404 | NM_019015 | CHPF2 | chondroitin polymerizing factor 2 | 0.088 | 1.063 | 3.91E-01 | 3.91E+01 | | | |
| 7895188 | --- | --- | --- | 0.088 | 1.063 | 8.81E-01 | 8.81E+01 | | | |
| 7905355 | NM_024041 | SCNM1 | sodium channel modifier 1 | 0.088 | 1.063 | 7.21E-01 | 7.21E+01 | | | |
| 7922793 | NM_005717 | ARPC5 | actin related protein 2/3 complex, su | 0.088 | 1.063 | 4.80E-01 | 4.80E+01 | | | |
| 7990830 | NM_015154 | MESDC2 | mesoderm development candidate 2 | 0.088 | 1.063 | 4.02E-01 | 4.02E+01 | | | |
| 7966301 | NM_016238 | ANAPC7 | anaphase promoting complex subun | 0.088 | 1.063 | 4.71E-01 | 4.71E+01 | | | |
| 8099107 | NM_017816 | LYAR | Ly1 antibody reactive homolog (mou | 0.088 | 1.063 | 8.29E-01 | 8.29E+01 | | | |
| 8151935 | NM_000989 | RPL30 | ribosomal protein L30 | 0.088 | 1.063 | 5.63E-01 | 5.63E+01 | | | |
| 7982904 | NM_015138 | RTF1 | Rtf1, Paf1/RNA polymerase II comple | 0.088 | 1.063 | 7.32E-01 | 7.32E+01 | | | |
| 7894951 | --- | --- | --- | 0.088 | 1.063 | 8.70E-01 | 8.70E+01 | | | |
| 7995017 | NM_004604 | STX4 | syntaxin 4 | 0.088 | 1.063 | 6.88E-01 | 6.88E+01 | | | |
| 7895705 | --- | --- | --- | 0.087 | 1.063 | 2.27E-01 | 2.27E+01 | | | |
| 7959123 | NM_006253 | PRKAB1 | protein kinase, AMP-activated, beta | 0.087 | 1.062 | 5.35E-01 | 5.35E+01 | | | |
| 8075542 | NM_173566 | C22orf30 | chromosome 22 open reading frame | 0.087 | 1.062 | 3.81E-01 | 3.81E+01 | | | |
| 8172154 | NM_002952 | RPS2 | ribosomal protein S2 | 0.087 | 1.062 | 2.64E-01 | 2.64E+01 | | | |
| 8127526 | NM_001000 // | PL39 // RPL3 | ribosomal protein L39 // ribosomal | 0.087 | 1.062 | 4.98E-01 | 4.98E+01 | | | |
| 7892753 | --- | --- | --- | 0.087 | 1.062 | 1.17E-01 | 1.17E+01 | | | |
| 8150149 | --- | --- | --- | 0.087 | 1.062 | 7.56E-01 | 7.56E+01 | | | |
| 8111331 | NM_022130 | GOLPH3 | golgi phosphoprotein 3 (coat-protein | 0.087 | 1.062 | 4.41E-01 | 4.41E+01 | | | |
| 8085412 | NM_00113438 | IQSEC1 | IQ motif and Sec7 domain 1 | 0.087 | 1.062 | 5.15E-01 | 5.15E+01 | | | |
| 7926096 | NM_031923 | TAF3 | TAF3 RNA polymerase II, TATA box b | 0.087 | 1.062 | 4.38E-01 | 4.38E+01 | | | |
| 7975747 | NM_001933 | DLST | dihydrolipoamide S-succinyltransfera | 0.087 | 1.062 | 5.55E-01 | 5.55E+01 | | | |
| 8138977 | NM_015283 | DPY19L1 | dpy-19-like 1 (C. elegans) | 0.087 | 1.062 | 5.31E-01 | 5.31E+01 | | | |
| 8022914 | NM_018170 | RPRD1A | regulation of nuclear pre-mRNA dom | 0.087 | 1.062 | 7.73E-01 | 7.73E+01 | | | |
| 7962951 | NM_003482 | MLL2 | myeloid/lymphoid or mixed-lineage | 0.087 | 1.062 | 5.54E-02 | 5.54E+00 | | | |

| | | | | | | | | | | |
|---|---|---|---|---|---|---|---|---|---|---|
| 8071434 | NM_005207 | CRKL | v-crk sarcoma virus CT10 oncogene h | 0.087 | 1.062 | 4.35E-01 | 4.35E+01 | | | |
| 8027024 | NM_00103393( | UBA52 | ubiquitin A-52 residue ribosomal pro | 0.087 | 1.062 | 7.04E-01 | 7.04E+01 | | | |
| 8124654 | NM_001470 | GABBR1 | gamma-aminobutyric acid (GABA) B | 0.087 | 1.062 | 1.61E-01 | 1.61E+01 | | | |
| 7896380 | --- | --- | --- | 0.087 | 1.062 | 2.67E-01 | 2.67E+01 | | | |
| 8045182 | NM_014369 | PTPN18 | protein tyrosine phosphatase, non-re | 0.087 | 1.062 | 2.45E-01 | 2.45E+01 | | | |
| 8000823 | NM_015092 | SMG1 | SMG1 homolog, phosphatidylinosito | 0.087 | 1.062 | 6.22E-01 | 6.22E+01 | | | |
| 7934326 | NM_016065 | MRPS16 | mitochondrial ribosomal protein S16 | 0.087 | 1.062 | 5.29E-01 | 5.29E+01 | | | |
| 7894481 | --- | --- | --- | 0.087 | 1.062 | 6.51E-02 | 6.51E+00 | | | |
| 7940191 | NM_004177 | STX3 | syntaxin 3 | 0.086 | 1.062 | 2.39E-01 | 2.39E+01 | | | |
| 8118594 | NM_002121 | HLA-DPB1 | major histocompatibility complex, cl | 0.086 | 1.062 | 6.98E-01 | 6.98E+01 | | | |
| 8115410 | NM_015465 | GEMIN5 | gem (nuclear organelle) associated p | 0.086 | 1.062 | 3.99E-01 | 3.99E+01 | | | |
| 8140211 | NR_003664 | SPDYE8P | speedy homolog E8 (Xenopus laevis) | 0.086 | 1.062 | 3.44E-01 | 3.44E+01 | | | |
| 8000638 | NM_015092 | SMG1 | SMG1 homolog, phosphatidylinosito | 0.086 | 1.062 | 6.23E-01 | 6.23E+01 | | | |
| 7992255 | NM_032520 | GNPTG | N-acetylglucosamine-1-phosphate tr | 0.086 | 1.062 | 5.63E-01 | 5.63E+01 | | | |
| 7970831 | NM_007106 | UBL3 | ubiquitin-like 3 | 0.086 | 1.062 | 7.03E-01 | 7.03E+01 | | | |
| 7894263 | --- | --- | --- | 0.086 | 1.062 | 1.82E-01 | 1.82E+01 | | | |
| 7895562 | --- | --- | --- | 0.086 | 1.061 | 9.02E-01 | 9.02E+01 | | | |
| 7947989 | --- | --- | --- | 0.086 | 1.061 | 8.15E-01 | 8.15E+01 | | | |
| 7922889 | NM_006469 | IVNS1ABP | influenza virus NS1A binding protein | 0.086 | 1.061 | 7.82E-01 | 7.82E+01 | | | |
| 8083749 | NM_139245 | PPM1L | protein phosphatase 1 (formerly 2C) | 0.086 | 1.061 | 5.77E-01 | 5.77E+01 | | | |
| 8124994 | NM_021184 | C6orf47 | chromosome 6 open reading frame 4 | 0.086 | 1.061 | 3.42E-01 | 3.42E+01 | | | |
| 7921487 | NM_003564 | TAGLN2 | transgelin 2 | 0.086 | 1.061 | 7.01E-01 | 7.01E+01 | | | |
| 7896366 | --- | --- | --- | 0.086 | 1.061 | 6.94E-02 | 6.94E+00 | | | |
| 8000217 | NM_015092 | SMG1 | SMG1 homolog, phosphatidylinosito | 0.086 | 1.061 | 2.55E-01 | 2.55E+01 | | | |
| 7937079 | NM_004052 | BNIP3 | BCL2/adenovirus E1B 19kDa interact | 0.086 | 1.061 | 8.31E-01 | 8.31E+01 | | | |
| 8035905 | NM_032139 | ANKRD27 | ankyrin repeat domain 27 (VPS9 dom | 0.086 | 1.061 | 3.41E-01 | 3.41E+01 | | | |
| 8089467 | NM_024508 | ZBED2 | zinc finger, BED-type containing 2 | 0.086 | 1.061 | 3.11E-01 | 3.11E+01 | | | |
| 8096781 | NM_006323 | SEC24B | SEC24 family, member B (S. cerevisia | 0.086 | 1.061 | 7.65E-01 | 7.65E+01 | | | |
| 7895324 | --- | --- | --- | 0.086 | 1.061 | 7.84E-01 | 7.84E+01 | | | |
| 8015039 | NM_003079 | SMARCE1 | SWI/SNF related, matrix associated, | 0.086 | 1.061 | 5.70E-01 | 5.70E+01 | | | |
| 7928411 | NM_015037 | KIAA0913 | KIAA0913 | 0.086 | 1.061 | 3.18E-01 | 3.18E+01 | | | |
| 7956200 | NM_002475 | MYL6B | myosin, light chain 6B, alkali, smooth | 0.086 | 1.061 | 3.10E-01 | 3.10E+01 | | | |
| 8116910 | NM_002114 | HIVEP1 | human immunodeficiency virus type | 0.086 | 1.061 | 7.15E-01 | 7.15E+01 | | | |
| 7995354 | NM_014321 | ORC6L | origin recognition complex, subunit 6 | 0.085 | 1.061 | 5.97E-01 | 5.97E+01 | | | |
| 7916356 | NM_016126 | HSPB11 | heat shock protein family B (small), r | 0.085 | 1.061 | 8.71E-01 | 8.71E+01 | | | |
| 8130626 | --- | --- | --- | 0.085 | 1.061 | 8.82E-01 | 8.82E+01 | | | |
| 8094228 | NM_004334 | BST1 | bone marrow stromal cell antigen 1 | 0.085 | 1.061 | 2.77E-01 | 2.77E+01 | | | |
| 8042283 | NM_014181 | HSPC159 | galectin-related protein | 0.085 | 1.061 | 8.56E-01 | 8.56E+01 | | | |
| 7913001 | NM_020765 | UBR4 | ubiquitin protein ligase E3 componer | 0.085 | 1.061 | 6.24E-01 | 6.24E+01 | | | |
| 8029814 | NM_006247 | PPP5C | protein phosphatase 5, catalytic subu | 0.085 | 1.061 | 2.52E-01 | 2.52E+01 | | | |
| 7941537 | NM_018026 | PACS1 | phosphofurin acidic cluster sorting p | 0.085 | 1.061 | 7.01E-01 | 7.01E+01 | | | |
| 8150489 | --- | --- | --- | 0.085 | 1.061 | 5.91E-01 | 5.91E+01 | | | |
| 8033075 | NM_003624 | RANBP3 | RAN binding protein 3 | 0.085 | 1.061 | 4.89E-01 | 4.89E+01 | | | |
| 8165644 | --- | --- | --- | 0.085 | 1.061 | 6.26E-01 | 6.26E+01 | | | |
| 8102482 | NM_014822 | SEC24D | SEC24 family, member D (S. cerevisia | 0.085 | 1.061 | 5.02E-01 | 5.02E+01 | | | |
| 7950626 | BC071734 | RPS20P27 | ribosomal protein S20 pseudogene 2 | 0.085 | 1.061 | 8.54E-01 | 8.54E+01 | | | |
| 7893606 | --- | --- | --- | 0.085 | 1.061 | 7.37E-01 | 7.37E+01 | | | |
| 7920799 | NM_139118 | YY1AP1 | YY1 associated protein 1 | 0.085 | 1.061 | 7.34E-01 | 7.34E+01 | | | |
| 7895824 | --- | --- | --- | 0.085 | 1.061 | 7.69E-01 | 7.69E+01 | | | |
| 8050089 | BC113076 | TMSL2 | thymosin-like 2 (pseudogene) | 0.085 | 1.061 | 1.26E-01 | 1.26E+01 | | | |
| 8115939 | --- | --- | --- | 0.085 | 1.061 | 5.33E-01 | 5.33E+01 | | | |
| 8036045 | NM_175872 | ZNF792 | zinc finger protein 792 | 0.085 | 1.060 | 2.75E-01 | 2.75E+01 | | | |
| 8034420 | NM_000528 | MAN2B1 | mannosidase, alpha, class 2B, memb | 0.085 | 1.060 | 2.97E-01 | 2.97E+01 | | | |
| 7908841 | NM_032105 | PPP1R12B | protein phosphatase 1, regulatory (ir | 0.085 | 1.060 | 4.49E-01 | 4.49E+01 | | | |
| 7894392 | --- | --- | --- | 0.084 | 1.060 | 8.64E-02 | 8.64E+00 | | | |
| 8142663 | NM_005000 | NDUFA5 | NADH dehydrogenase (ubiquinone) 1 | 0.084 | 1.060 | 8.65E-01 | 8.65E+01 | | | |
| 8082442 | --- | --- | --- | 0.084 | 1.060 | 8.29E-01 | 8.29E+01 | | | |
| 7892820 | --- | --- | --- | 0.084 | 1.060 | 5.63E-02 | 5.63E+00 | | | |
| 7955450 | NM_015416 | LETMD1 | LETM1 domain containing 1 | 0.084 | 1.060 | 4.05E-01 | 4.05E+01 | | | |
| 8137526 | NM_005542 | INSIG1 | insulin induced gene 1 | 0.084 | 1.060 | 6.74E-01 | 6.74E+01 | | | |
| 7932186 | NM_00103385 | DCLRE1C | DNA cross-link repair 1C (PSO2 homo | 0.084 | 1.060 | 5.09E-01 | 5.09E+01 | | | |
| 8010243 | NM_004710 | SYNGR2 | synaptogyrin 2 | 0.084 | 1.060 | 5.42E-01 | 5.42E+01 | | | |
| 8108163 | NM_152409 | C5orf24 | chromosome 5 open reading frame 2 | 0.084 | 1.060 | 3.97E-01 | 3.97E+01 | | | |
| 7896151 | --- | --- | --- | 0.084 | 1.060 | 3.51E-02 | 3.51E+00 | | | |
| 8141380 | NM_032924 | ZNF3 | zinc finger protein 3 | 0.084 | 1.060 | 4.45E-01 | 4.45E+01 | | | |
| 8070655 | NM_00102520 | U2AF1 | U2 small nuclear RNA auxiliary factor | 0.084 | 1.060 | 6.89E-01 | 6.89E+01 | | | |
| 8116559 | NM_002460 | IRF4 | interferon regulatory factor 4 | 0.084 | 1.060 | 5.82E-01 | 5.82E+01 | | | |
| 7944435 | NM_015517 | HINFP | histone H4 transcription factor | 0.084 | 1.060 | 3.69E-01 | 3.69E+01 | | | |
| 8036389 | NM_152279 | ZNF585B | zinc finger protein 585B | 0.084 | 1.060 | 5.76E-01 | 5.76E+01 | | | |
| 7965467 | ENST0000035 | RPL41 | ribosomal protein L41 | 0.084 | 1.060 | 9.90E-02 | 9.90E+00 | | | |
| 7982129 | ENST0000035 | RPL41 | ribosomal protein L41 | 0.084 | 1.060 | 9.90E-02 | 9.90E+00 | | | |
| 8013222 | NM_004618 | TOP3A | topoisomerase (DNA) III alpha | 0.084 | 1.060 | 5.01E-01 | 5.01E+01 | | | |
| 8036777 | NM_001436 | FBL | fibrillarin | 0.083 | 1.060 | 7.76E-01 | 7.76E+01 | | | |
| 8139840 | NM_00100725 | ERV3 | endogenous retroviral sequence 3 (ir | 0.083 | 1.059 | 3.46E-01 | 3.46E+01 | | | |
| 8090678 | NM_007208 | MRPL3 | mitochondrial ribosomal protein L3 | 0.083 | 1.059 | 6.91E-01 | 6.91E+01 | | | |
| 8174710 | NM_001000 // | PL39 // RPL3 | ribosomal protein L39 // ribosomal | 0.083 | 1.059 | 5.12E-01 | 5.12E+01 | | | |
| 7893981 | --- | --- | --- | 0.083 | 1.059 | 7.33E-01 | 7.33E+01 | | | |

| | | | | | | | | | | |
|---|---|---|---|---|---|---|---|---|---|---|
| 7916808 | NM_015139 | SLC35D1 | solute carrier family 35 (UDP-glucuro | 0.083 | 1.059 | 7.49E-01 | 7.49E+01 | | | |
| 8156935 | NM_003452 | ZNF189 | zinc finger protein 189 | 0.083 | 1.059 | 4.75E-01 | 4.75E+01 | | | |
| 7894535 | --- | --- | --- | 0.083 | 1.059 | 7.61E-01 | 7.61E+01 | | | |
| 7896646 | --- | --- | --- | 0.083 | 1.059 | 5.95E-01 | 5.95E+01 | | | |
| 8174983 | --- | --- | --- | 0.083 | 1.059 | 6.02E-01 | 6.02E+01 | | | |
| 8066407 | NM_00108047 | FITM2 | fat storage-inducing transmembrane | 0.083 | 1.059 | 6.02E-01 | 6.02E+01 | | | |
| 8175811 | NM_152274 | FAM58A | family with sequence similarity 58, m | 0.083 | 1.059 | 6.35E-01 | 6.35E+01 | | | |
| 8180029 | NR_003937 | HLA-DQB2 | major histocompatibility complex, cla | 0.083 | 1.059 | 4.42E-01 | 4.42E+01 | | | |
| 8092230 | NM_022470 | ZMAT3 | zinc finger, matrin type 3 | 0.083 | 1.059 | 4.10E-01 | 4.10E+01 | | | |
| 7968242 | NM_002097 | GTF3A | general transcription factor IIIA | 0.083 | 1.059 | 5.78E-01 | 5.78E+01 | | | |
| 7896578 | --- | --- | --- | 0.083 | 1.059 | 8.90E-01 | 8.90E+01 | | | |
| 8038792 | NM_001985 | ETFB | electron-transfer-flavoprotein, beta | 0.083 | 1.059 | 6.64E-01 | 6.64E+01 | | | |
| 7972062 | NM_012158 | FBXL3 | F-box and leucine-rich repeat protei | 0.083 | 1.059 | 7.86E-01 | 7.86E+01 | | | |
| 8071559 | NM_022044 | SDF2L1 | stromal cell-derived factor 2-like 1 | 0.083 | 1.059 | 3.77E-01 | 3.77E+01 | | | |
| 7931951 | NM_00102988 | SFMBT2 | Scm-like with four mbt domains 2 | 0.083 | 1.059 | 5.93E-01 | 5.93E+01 | | | |
| 7893357 | --- | --- | --- | 0.083 | 1.059 | 8.19E-01 | 8.19E+01 | | | |
| 7894947 | --- | --- | --- | 0.083 | 1.059 | 8.02E-01 | 8.02E+01 | | | |
| 7946957 | NM_138421 | SAAL1 | serum amyloid A-like 1 | 0.083 | 1.059 | 7.66E-01 | 7.66E+01 | | | |
| 8180398 | --- | --- | --- | 0.083 | 1.059 | 3.00E-01 | 3.00E+01 | | | |
| 8000236 | NM_001802 | CDR2 | cerebellar degeneration-related prot | 0.082 | 1.059 | 7.40E-01 | 7.40E+01 | | | |
| 7947861 | NM_00108054 | SPI1 | spleen focus forming virus (SFFV) pro | 0.082 | 1.059 | 4.02E-01 | 4.02E+01 | | | |
| 8007272 | NM_025233 | COASY | Coenzyme A synthase | 0.082 | 1.059 | 2.27E-01 | 2.27E+01 | | | |
| 8171879 | --- | --- | --- | 0.082 | 1.059 | 6.64E-01 | 6.64E+01 | | | |
| 8165705 | --- | --- | --- | 0.082 | 1.059 | 3.10E-02 | 3.10E+00 | | | |
| 8001410 | NM_00101239 | AKTIP | AKT interacting protein | 0.082 | 1.059 | 8.43E-01 | 8.43E+01 | | | |
| 7915516 | NM_201542 | MED8 | mediator complex subunit 8 | 0.082 | 1.059 | 6.05E-01 | 6.05E+01 | | | |
| 8175319 | NM_007131 | ZNF75D | zinc finger protein 75D | 0.082 | 1.059 | 2.65E-01 | 2.65E+01 | | | |
| 7896223 | --- | --- | --- | 0.082 | 1.059 | 9.07E-01 | 9.07E+01 | | | |
| 8134435 | NM_014916 | LMTK2 | lemur tyrosine kinase 2 | 0.082 | 1.059 | 6.15E-01 | 6.15E+01 | | | |
| 8132458 | NM_031903 | MRPL32 | mitochondrial ribosomal protein L32 | 0.082 | 1.059 | 7.45E-01 | 7.45E+01 | | | |
| 7895354 | --- | --- | --- | 0.082 | 1.059 | 4.77E-02 | 4.77E+00 | | | |
| 8122202 | NM_00113017 | MYB | v-myb myeloblastosis viral oncogene | 0.082 | 1.058 | 4.46E-01 | 4.46E+01 | | | |
| 8141222 | NM_001006 | RPS3A | ribosomal protein S3A | 0.082 | 1.058 | 1.62E-01 | 1.62E+01 | | | |
| 8091550 | NM_020776 | KIAA1328 | KIAA1328 | 0.082 | 1.058 | 8.90E-01 | 8.90E+01 | | | |
| 8173338 | --- | --- | --- | 0.082 | 1.058 | 7.82E-01 | 7.82E+01 | | | |
| 8026122 | NM_005053 | RAD23A | RAD23 homolog A (S. cerevisiae) | 0.082 | 1.058 | 3.47E-01 | 3.47E+01 | | | |
| 8007745 | NM_006460 | HEXIM1 | hexamethylene bis-acetamide induci | 0.082 | 1.058 | 5.97E-01 | 5.97E+01 | | | |
| 8085665 | NM_015150 | RFTN1 | raftlin, lipid raft linker 1 | 0.082 | 1.058 | 5.73E-01 | 5.73E+01 | | | |
| 8146685 | NM_015169 | RRS1 | RRS1 ribosome biogenesis regulator | 0.082 | 1.058 | 6.10E-01 | 6.10E+01 | | | |
| 7895520 | --- | --- | --- | 0.082 | 1.058 | 1.29E-01 | 1.29E+01 | | | |
| 7893886 | --- | --- | --- | 0.082 | 1.058 | 9.08E-01 | 9.08E+01 | | | |
| 7915733 | NM_002574 | PRDX1 | peroxiredoxin 1 | 0.082 | 1.058 | 8.02E-01 | 8.02E+01 | | | |
| 8017547 | NM_00109978 | ICAM2 | intercellular adhesion molecule 2 | 0.081 | 1.058 | 5.93E-01 | 5.93E+01 | | | |
| 8076511 | NM_000969 | RPL5 | ribosomal protein L5 | 0.081 | 1.058 | 2.34E-01 | 2.34E+01 | | | |
| 7974461 | NR_003225 | LGALS3 | lectin, galactoside-binding, soluble, 3 | 0.081 | 1.058 | 3.85E-01 | 3.85E+01 | | | |
| 8170775 | NM_006280 | SSR4 | signal sequence receptor, delta (tran | 0.081 | 1.058 | 5.69E-01 | 5.69E+01 | | | |
| 7930894 | NM_005308 | GRK5 | G protein-coupled receptor kinase 5 | 0.081 | 1.058 | 3.73E-01 | 3.73E+01 | | | |
| 7955425 | NM_005171 | ATF1 | activating transcription factor 1 | 0.081 | 1.058 | 8.44E-01 | 8.44E+01 | | | |
| 8172504 | NM_020137 | GRIPAP1 | GRIP1 associated protein 1 | 0.081 | 1.058 | 5.27E-01 | 5.27E+01 | | | |
| 8117890 | NM_005516 | HLA-E | major histocompatibility complex, cla | 0.081 | 1.058 | 5.51E-01 | 5.51E+01 | | | |
| 7895920 | --- | --- | --- | 0.081 | 1.058 | 1.64E-02 | 1.64E+00 | | | |
| 8134098 | NM_012395 | PFTK1 | PFTAIRE protein kinase 1 | 0.081 | 1.058 | 7.33E-01 | 7.33E+01 | | | |
| 8056734 | NM_012290 | TLK1 | tousled-like kinase 1 | 0.081 | 1.058 | 8.44E-01 | 8.44E+01 | | | |
| 8177232 | NM_00114670 | KDM5D | lysine (K)-specific demethylase 5D | 0.081 | 1.058 | 7.01E-01 | 7.01E+01 | | | |
| 8069943 | NM_014825 | URB1 | URB1 ribosome biogenesis 1 homolo | 0.081 | 1.058 | 3.65E-01 | 3.65E+01 | | | |
| 8021768 | NM_005786 | TSHZ1 | teashirt zinc finger homeobox 1 | 0.081 | 1.058 | 3.64E-01 | 3.64E+01 | | | |
| 8078248 | NM_002128 | HMGB1 | high-mobility group box 1 | 0.081 | 1.058 | 6.53E-01 | 6.53E+01 | | | |
| 7893283 | --- | --- | --- | 0.081 | 1.058 | 2.22E-01 | 2.22E+01 | | | |
| 8138862 | --- | --- | --- | 0.081 | 1.058 | 5.43E-01 | 5.43E+01 | | | |
| 8141050 | NM_001006 | RPS3A | ribosomal protein S3A | 0.081 | 1.058 | 1.14E-01 | 1.14E+01 | | | |
| 8016540 | NM_00114380 | PHOSPHO1 | phosphatase, orphan 1 | 0.081 | 1.058 | 3.79E-01 | 3.79E+01 | | | |
| 7894770 | --- | --- | --- | 0.081 | 1.058 | 3.16E-02 | 3.16E+00 | | | |
| 8075691 | NM_021104 | RPL41 | ribosomal protein L41 | 0.081 | 1.058 | 4.16E-02 | 4.16E+00 | | | |
| 7894324 | --- | --- | --- | 0.081 | 1.057 | 5.10E-01 | 5.10E+01 | | | |
| 8083352 | NM_016275 | SELT | selenoprotein T | 0.080 | 1.057 | 5.73E-01 | 5.73E+01 | | | |
| 7894016 | --- | --- | --- | 0.080 | 1.057 | 9.44E-01 | 9.44E+01 | | | |
| 7919157 | NM_00114388 | PPIAL4B | peptidylprolyl isomerase A (cyclophil | 0.080 | 1.057 | 2.23E-01 | 2.23E+01 | | | |
| 8051812 | --- | --- | --- | 0.080 | 1.057 | 8.15E-01 | 8.15E+01 | | | |
| 8004940 | NM_020233 | C17orf48 | chromosome 17 open reading frame | 0.080 | 1.057 | 7.11E-01 | 7.11E+01 | | | |
| 7896280 | --- | --- | --- | 0.080 | 1.057 | 6.90E-01 | 6.90E+01 | | | |
| 8102050 | NM_178833 | NHEDC2 | Na+/H+ exchanger domain containin | 0.080 | 1.057 | 5.42E-01 | 5.42E+01 | | | |
| 8136983 | NR_002158 | OR2A20P | olfactory receptor, family 2, subfami | 0.080 | 1.057 | 5.43E-01 | 5.43E+01 | | | |
| 8066557 | --- | --- | --- | 0.080 | 1.057 | 4.79E-01 | 4.79E+01 | | | |
| 7895157 | --- | --- | --- | 0.080 | 1.057 | 1.77E-01 | 1.77E+01 | | | |
| 8125752 | NM_015921 | CUTA | cutA divalent cation tolerance homo | 0.080 | 1.057 | 6.60E-01 | 6.60E+01 | | | |
| 8083164 | --- | --- | --- | 0.080 | 1.057 | 4.64E-01 | 4.64E+01 | | | |
| 7892617 | --- | --- | --- | 0.080 | 1.057 | 3.72E-02 | 3.72E+00 | | | |

| | | | | | | | | | | |
|---|---|---|---|---|---|---|---|---|---|---|
| 8121782 | --- | --- | --- | 0.080 | 1.057 | 4.58E-01 | 4.58E+01 | | | |
| 7956046 | NM_201444 | DGKA | diacylglycerol kinase, alpha 80kDa | 0.080 | 1.057 | 7.34E-01 | 7.34E+01 | | | |
| 7995055 | NM_182958 | MYST1 | MYST histone acetyltransferase 1 | 0.080 | 1.057 | 6.32E-01 | 6.32E+01 | | | |
| 8043438 | BC093097 | IGKC | immunoglobulin kappa constant | 0.080 | 1.057 | 7.82E-01 | 7.82E+01 | | | |
| 8058203 | NM_139163 | ALS2CR12 | amyotrophic lateral sclerosis 2 (juver | 0.080 | 1.057 | 3.65E-01 | 3.65E+01 | | | |
| 8105432 | NM_021104 | RPL41 | ribosomal protein L41 | 0.080 | 1.057 | 4.40E-02 | 4.40E+00 | | | |
| 8117813 | NR_024240 | HLA-J | major histocompatibility complex, cla | 0.080 | 1.057 | 3.91E-01 | 3.91E+01 | | | |
| 7893701 | --- | --- | --- | 0.080 | 1.057 | 3.57E-01 | 3.57E+01 | | | |
| 8027279 | NM_025189 | ZNF430 | zinc finger protein 430 | 0.080 | 1.057 | 6.22E-01 | 6.22E+01 | | | |
| 8114145 | NM_003374 | VDAC1 | voltage-dependent anion channel 1 | 0.080 | 1.057 | 3.82E-01 | 3.82E+01 | | | |
| 7892719 | --- | --- | --- | 0.080 | 1.057 | 5.91E-01 | 5.91E+01 | | | |
| 8006690 | NM_012138 | AATF | apoptosis antagonizing transcription | 0.080 | 1.057 | 7.04E-01 | 7.04E+01 | | | |
| 7983173 | NM_012142 | CCNDBP1 | cyclin D-type binding-protein 1 | 0.080 | 1.057 | 6.79E-01 | 6.79E+01 | | | |
| 7949603 | NM_015399 | BRMS1 | breast cancer metastasis suppressor | 0.080 | 1.057 | 6.69E-01 | 6.69E+01 | | | |
| 7997025 | NM_017990 | PDPR | pyruvate dehydrogenase phosphatas | 0.080 | 1.057 | 7.61E-01 | 7.61E+01 | | | |
| 7908437 | NM_024529 | CDC73 | cell division cycle 73, Paf1/RNA polyr | 0.080 | 1.057 | 7.92E-01 | 7.92E+01 | | | |
| 7925531 | NM_181690 | AKT3 | v-akt murine thymoma viral oncoger | 0.080 | 1.057 | 8.61E-01 | 8.61E+01 | | | |
| 7971375 | NM_003295 | TPT1 | tumor protein, translationally-contro | 0.079 | 1.057 | 5.12E-01 | 5.12E+01 | | | |
| 7905505 | NM_014357 | LCE2B | late cornified envelope 2B | 0.079 | 1.057 | 2.34E-01 | 2.34E+01 | | | |
| 8076260 | NM_006358 | SLC25A17 | solute carrier family 25 (mitochondri | 0.079 | 1.056 | 6.96E-01 | 6.96E+01 | | | |
| 7936641 | NM_213649 | SFXN4 | sideroflexin 4 | 0.079 | 1.056 | 3.84E-01 | 3.84E+01 | | | |
| 8161484 | ENST00000367 | MTHFD1L | methylenetetrahydrofolate dehydro | 0.079 | 1.056 | 7.03E-01 | 7.03E+01 | | | |
| 8155455 | AK126863 | LOC441426 | hypothetical gene supported by AK1 | 0.079 | 1.056 | 5.17E-01 | 5.17E+01 | | | |
| 8125671 | NM_005452 | WDR46 | WD repeat domain 46 | 0.079 | 1.056 | 4.17E-01 | 4.17E+01 | | | |
| 8178939 | NM_005452 | WDR46 | WD repeat domain 46 | 0.079 | 1.056 | 4.17E-01 | 4.17E+01 | | | |
| 7950307 | NM_003355 | UCP2 | uncoupling protein 2 (mitochondrial, | 0.079 | 1.056 | 6.46E-01 | 6.46E+01 | | | |
| 7893219 | --- | --- | --- | 0.079 | 1.056 | 8.31E-01 | 8.31E+01 | | | |
| 8087405 | NM_201397 | GPX1 | glutathione peroxidase 1 | 0.079 | 1.056 | 1.56E-01 | 1.56E+01 | | | |
| 8156319 | NR_002822 | MGC72080 | MGC72080 pseudogene | 0.079 | 1.056 | 8.36E-01 | 8.36E+01 | | | |
| 8180262 | --- | --- | --- | 0.079 | 1.056 | 4.61E-01 | 4.61E+01 | | | |
| 8054308 | NM_00110242( | TBC1D8 | TBC1 domain family, member 8 (with | 0.079 | 1.056 | 4.75E-01 | 4.75E+01 | | | |
| 8147344 | NM_00116177( | PDP1 | pyruvate dehyrogenase phosphatase | 0.079 | 1.056 | 5.59E-01 | 5.59E+01 | | | |
| 8031011 | --- | --- | --- | 0.079 | 1.056 | 5.88E-01 | 5.88E+01 | | | |
| 7953518 | U72518 | LOC171220 | destrin-2 pseudogene | 0.079 | 1.056 | 7.44E-01 | 7.44E+01 | | | |
| 8139996 | NR_003664 | SPDYE8P | speedy homolog E8 (Xenopus laevis) | 0.079 | 1.056 | 3.94E-01 | 3.94E+01 | | | |
| 8039340 | NM_003283 | TNNT1 | troponin T type 1 (skeletal, slow) | 0.079 | 1.056 | 3.14E-01 | 3.14E+01 | | | |
| 7964262 | NM_00111320: | NACA | nascent polypeptide-associated com | 0.079 | 1.056 | 3.60E-01 | 3.60E+01 | | | |
| 7907966 | --- | --- | --- | 0.079 | 1.056 | 7.29E-01 | 7.29E+01 | | | |
| 7994362 | NM_138414 | CCDC101 | coiled-coil domain containing 101 | 0.079 | 1.056 | 6.16E-01 | 6.16E+01 | | | |
| 8107259 | NM_138773 | SLC25A46 | solute carrier family 25, member 46 | 0.079 | 1.056 | 7.39E-01 | 7.39E+01 | | | |
| 7932209 | --- | --- | --- | 0.078 | 1.056 | 8.04E-01 | 8.04E+01 | | | |
| 8163326 | --- | --- | --- | 0.078 | 1.056 | 8.16E-01 | 8.16E+01 | | | |
| 7958784 | NM_000690 | ALDH2 | aldehyde dehydrogenase 2 family (m | 0.078 | 1.056 | 4.43E-01 | 4.43E+01 | | | |
| 7895397 | --- | --- | --- | 0.078 | 1.056 | 7.44E-01 | 7.44E+01 | | | |
| 8053801 | NM_025190 | ANKRD36B | ankyrin repeat domain 36B | 0.078 | 1.056 | 8.22E-01 | 8.22E+01 | | | |
| 8072206 | NM_005243 | EWSR1 | Ewing sarcoma breakpoint region 1 | 0.078 | 1.056 | 4.71E-01 | 4.71E+01 | | | |
| 8139411 | NM_182547 | TMED4 | transmembrane emp24 protein trans | 0.078 | 1.056 | 4.81E-01 | 4.81E+01 | | | |
| 8021563 | --- | --- | --- | 0.078 | 1.056 | 7.61E-01 | 7.61E+01 | | | |
| 7936826 | NM_022466 | IKZF5 | IKAROS family zinc finger 5 (Pegasus) | 0.078 | 1.056 | 8.83E-01 | 8.83E+01 | | | |
| 7894819 | --- | --- | --- | 0.078 | 1.056 | 8.44E-01 | 8.44E+01 | | | |
| 8093829 | NM_145291 | ZNF509 | zinc finger protein 509 | 0.078 | 1.055 | 3.94E-01 | 3.94E+01 | | | |
| 8119492 | NM_004053 | BYSL | bystin-like | 0.078 | 1.055 | 5.93E-01 | 5.93E+01 | | | |
| 7969792 | --- | --- | --- | 0.078 | 1.055 | 1.22E-01 | 1.22E+01 | | | |
| 7894122 | --- | --- | --- | 0.078 | 1.055 | 2.58E-02 | 2.58E+00 | | | |
| 7901135 | NM_016486 | TMEM69 | transmembrane protein 69 | 0.078 | 1.055 | 6.33E-01 | 6.33E+01 | | | |
| 8140420 | NR_003262 // | SL2A // FDP! | MGC44478 // MGC44478 // MGC44 | 0.078 | 1.055 | 5.15E-01 | 5.15E+01 | | | |
| 7926021 | NM_00114554: | RBM17 | RNA binding motif protein 17 | 0.077 | 1.055 | 4.64E-01 | 4.64E+01 | | | |
| 8180251 | --- | --- | --- | 0.077 | 1.055 | 2.65E-01 | 2.65E+01 | | | |
| 8180261 | --- | --- | --- | 0.077 | 1.055 | 1.98E-01 | 1.98E+01 | | | |
| 8035193 | NM_024104 | C19orf42 | chromosome 19 open reading frame | 0.077 | 1.055 | 7.90E-01 | 7.90E+01 | | | |
| 7894604 | --- | --- | --- | 0.077 | 1.055 | 8.99E-01 | 8.99E+01 | | | |
| 7930162 | NM_017787 | C10orf26 | chromosome 10 open reading frame | 0.077 | 1.055 | 4.83E-01 | 4.83E+01 | | | |
| 7972577 | NM_001029 | RPS26 | ribosomal protein S26 | 0.077 | 1.055 | 7.22E-01 | 7.22E+01 | | | |
| 7972003 | NM_007249 | KLF12 | Kruppel-like factor 12 | 0.077 | 1.055 | 8.10E-01 | 8.10E+01 | | | |
| 7895262 | --- | --- | --- | 0.077 | 1.055 | 7.34E-01 | 7.34E+01 | | | |
| 8023133 | NM_004671 | PIAS2 | protein inhibitor of activated STAT, 2 | 0.077 | 1.055 | 7.72E-01 | 7.72E+01 | | | |
| 7991126 | NM_032856 | WDR73 | WD repeat domain 73 | 0.077 | 1.055 | 4.92E-01 | 4.92E+01 | | | |
| 7984540 | NM_138555 | KIF23 | kinesin family member 23 | 0.077 | 1.055 | 4.17E-01 | 4.17E+01 | | | |
| 8112959 | --- | --- | --- | 0.077 | 1.055 | 5.18E-01 | 5.18E+01 | | | |
| 7958197 | NR_001593 | RPL18AP3 | ribosomal protein L18a pseudogene | 0.077 | 1.055 | 4.26E-01 | 4.26E+01 | | | |
| 7922174 | NM_000130 | F5 | coagulation factor V (proaccelerin, la | 0.077 | 1.055 | 3.80E-01 | 3.80E+01 | | | |
| 8107353 | BC104811 | ZRSR1 | zinc finger (CCCH type), RNA-binding | 0.077 | 1.055 | 8.04E-01 | 8.04E+01 | | | |
| 8007620 | NM_002087 | GRN | granulin | 0.077 | 1.055 | 3.55E-01 | 3.55E+01 | | | |
| 8082886 | NM_000532 | PCCB | propionyl Coenzyme A carboxylase, l | 0.077 | 1.055 | 7.03E-01 | 7.03E+01 | | | |
| 7942476 | NM_025155 | PAAF1 | proteasomal ATPase-associated facto | 0.077 | 1.055 | 6.70E-01 | 6.70E+01 | | | |
| 8072529 | NM_014662 | DEPDC5 | DEP domain containing 5 | 0.077 | 1.055 | 3.77E-01 | 3.77E+01 | | | |
| 7951633 | NM_024740 | ALG9 | asparagine-linked glycosylation 9, alp | 0.077 | 1.055 | 4.94E-01 | 4.94E+01 | | | |

| | | | | | | | | | | |
|---|---|---|---|---|---|---|---|---|---|---|
| 8119582 | NM_015349 | KIAA0240 | KIAA0240 | 0.077 | 1.055 | 2.36E-01 | 2.36E+01 | | | |
| 8027268 | BC067843 | ZNF66 | zinc finger protein 66 | 0.077 | 1.055 | 5.75E-01 | 5.75E+01 | | | |
| 7895323 | --- | --- | --- | 0.077 | 1.055 | 2.82E-01 | 2.82E+01 | | | |
| 7946680 | NM_032320 | BTBD10 | BTB (POZ) domain containing 10 | 0.077 | 1.055 | 6.92E-01 | 6.92E+01 | | | |
| 7998931 | NM_198088 | ZNF200 | zinc finger protein 200 | 0.077 | 1.055 | 4.05E-01 | 4.05E+01 | | | |
| 8047778 | NR_002589 | SNORD51 | small nucleolar RNA, C/D box 51 | 0.077 | 1.055 | 7.86E-01 | 7.86E+01 | | | |
| 8066939 | NM_004776 | B4GALT5 | UDP-Gal:betaGlcNAc beta 1,4- galact | 0.077 | 1.055 | 6.80E-01 | 6.80E+01 | | | |
| 7923967 | NM_018566 | YOD1 | YOD1 OTU deubiquinating enzyme 1 | 0.077 | 1.055 | 3.27E-01 | 3.27E+01 | | | |
| 7894447 | --- | --- | --- | 0.077 | 1.055 | 9.30E-01 | 9.30E+01 | | | |
| 7893670 | --- | --- | --- | 0.077 | 1.054 | 3.27E-01 | 3.27E+01 | | | |
| 8061364 | NM_021104 | RPL41 | ribosomal protein L41 | 0.077 | 1.054 | 5.30E-02 | 5.30E+00 | | | |
| 7988581 | NM_014701 | SECISBP2L | SECIS binding protein 2-like | 0.076 | 1.054 | 7.45E-01 | 7.45E+01 | | | |
| 7969574 | NM_000224 | KRT18 | keratin 18 | 0.076 | 1.054 | 6.47E-01 | 6.47E+01 | | | |
| 8079598 | NM_016089 | ZNF589 | zinc finger protein 589 | 0.076 | 1.054 | 4.02E-01 | 4.02E+01 | | | |
| 7894522 | --- | --- | --- | 0.076 | 1.054 | 4.13E-01 | 4.13E+01 | | | |
| 8075695 | NR_027833 | APOL3 | apolipoprotein L, 3 | 0.076 | 1.054 | 5.55E-01 | 5.55E+01 | | | |
| 8180193 | --- | --- | --- | 0.076 | 1.054 | 4.76E-02 | 4.76E+00 | | | |
| 8021653 | NM_002640 | SERPINB8 | serpin peptidase inhibitor, clade B (o | 0.076 | 1.054 | 7.49E-01 | 7.49E+01 | | | |
| 8180389 | --- | --- | --- | 0.076 | 1.054 | 8.53E-01 | 8.53E+01 | | | |
| 8157933 | NM_014007 | ZBTB43 | zinc finger and BTB domain containir | 0.076 | 1.054 | 6.75E-01 | 6.75E+01 | | | |
| 8173340 | NM_145119 | PJA1 | praja ring finger 1 | 0.076 | 1.054 | 2.93E-01 | 2.93E+01 | | | |
| 8052925 | NM_144582 | TEX261 | testis expressed 261 | 0.076 | 1.054 | 3.18E-01 | 3.18E+01 | | | |
| 7958895 | NM_006187 | OAS3 | 2'-5'-oligoadenylate synthetase 3, 10 | 0.076 | 1.054 | 5.70E-01 | 5.70E+01 | | | |
| 7974542 | NM_017799 | C14orf101 | chromosome 14 open reading frame | 0.076 | 1.054 | 5.85E-01 | 5.85E+01 | | | |
| 7894460 | --- | --- | --- | 0.076 | 1.054 | 1.17E-01 | 1.17E+01 | | | |
| 8034021 | NM_007065 | CDC37 | cell division cycle 37 homolog (S. cer | 0.076 | 1.054 | 6.85E-01 | 6.85E+01 | | | |
| 8109438 | NM_033551 | LARP1 | La ribonucleoprotein domain family, | 0.076 | 1.054 | 5.94E-01 | 5.94E+01 | | | |
| 8041179 | NM_024692 | CLIP4 | CAP-GLY domain containing linker pr | 0.076 | 1.054 | 8.47E-01 | 8.47E+01 | | | |
| 8117777 | NM_002127 | HLA-G | major histocompatibility complex, cla | 0.076 | 1.054 | 3.28E-01 | 3.28E+01 | | | |
| 7920057 | NM_00108396 | TDRKH | tudor and KH domain containing | 0.076 | 1.054 | 4.83E-01 | 4.83E+01 | | | |
| 7973427 | NM_024328 | THTPA | thiamine triphosphatase | 0.076 | 1.054 | 7.30E-01 | 7.30E+01 | | | |
| 7958174 | NM_003330 | TXNRD1 | thioredoxin reductase 1 | 0.076 | 1.054 | 6.06E-01 | 6.06E+01 | | | |
| 8018652 | NM_052916 | RNF157 | ring finger protein 157 | 0.075 | 1.054 | 7.79E-01 | 7.79E+01 | | | |
| 7926979 | --- | --- | --- | 0.075 | 1.054 | 8.39E-01 | 8.39E+01 | | | |
| 7948574 | NM_024811 | CPSF7 | cleavage and polyadenylation specifi | 0.075 | 1.054 | 5.55E-01 | 5.55E+01 | | | |
| 7946567 | --- | --- | --- | 0.075 | 1.054 | 7.46E-01 | 7.46E+01 | | | |
| 7976957 | NM_181291 | WDR20 | WD repeat domain 20 | 0.075 | 1.054 | 3.82E-01 | 3.82E+01 | | | |
| 7979694 | --- | --- | --- | 0.075 | 1.054 | 3.82E-01 | 3.82E+01 | | | |
| 8136067 | NM_178562 | TSPAN33 | tetraspanin 33 | 0.075 | 1.054 | 2.38E-01 | 2.38E+01 | | | |
| 7896481 | --- | --- | --- | 0.075 | 1.054 | 3.54E-01 | 3.54E+01 | | | |
| 8180375 | --- | --- | --- | 0.075 | 1.054 | 7.54E-01 | 7.54E+01 | | | |
| 7925364 | NM_018072 | HEATR1 | HEAT repeat containing 1 | 0.075 | 1.053 | 6.73E-01 | 6.73E+01 | | | |
| 8108403 | NM_199189 | MATR3 | matrin 3 | 0.075 | 1.053 | 4.44E-01 | 4.44E+01 | | | |
| 8002533 | NM_145911 | ZNF23 | zinc finger protein 23 (KOX 16) | 0.075 | 1.053 | 5.72E-01 | 5.72E+01 | | | |
| 8099668 | --- | --- | --- | 0.075 | 1.053 | 8.62E-01 | 8.62E+01 | | | |
| 7896403 | --- | --- | --- | 0.075 | 1.053 | 1.09E-01 | 1.09E+01 | | | |
| 8022531 | NM_000271 | NPC1 | Niemann-Pick disease, type C1 | 0.075 | 1.053 | 6.55E-01 | 6.55E+01 | | | |
| 8114363 | --- | --- | --- | 0.075 | 1.053 | 8.77E-01 | 8.77E+01 | | | |
| 8047379 | --- | --- | --- | 0.075 | 1.053 | 4.83E-01 | 4.83E+01 | | | |
| 7987248 | NR_027410 | GOLGA8B | golgi autoantigen, golgin subfamily a | 0.075 | 1.053 | 6.40E-01 | 6.40E+01 | | | |
| 8142774 | NM_018077 | RBM28 | RNA binding motif protein 28 | 0.075 | 1.053 | 7.10E-01 | 7.10E+01 | | | |
| 8010271 | NM_024419 | PGS1 | phosphatidylglycerophosphate synth | 0.075 | 1.053 | 4.59E-01 | 4.59E+01 | | | |
| 8164742 | NM_022779 | DDX31 | DEAD (Asp-Glu-Ala-Asp) box polypep | 0.075 | 1.053 | 3.67E-01 | 3.67E+01 | | | |
| 8086698 | NM_144716 | CCDC12 | coiled-coil domain containing 12 | 0.075 | 1.053 | 7.42E-01 | 7.42E+01 | | | |
| 7895280 | --- | --- | --- | 0.075 | 1.053 | 4.23E-01 | 4.23E+01 | | | |
| 7937175 | NM_006659 | TUBGCP2 | tubulin, gamma complex associated | 0.075 | 1.053 | 3.65E-01 | 3.65E+01 | | | |
| 8166402 | NM_004595 | SMS | spermine synthase | 0.075 | 1.053 | 7.11E-01 | 7.11E+01 | | | |
| 7895228 | --- | --- | --- | 0.075 | 1.053 | 8.92E-01 | 8.92E+01 | | | |
| 8144392 | NR_003668 | DEFB109P1B | defensin, beta 109, pseudogene 1B | 0.075 | 1.053 | 7.66E-01 | 7.66E+01 | | | |
| 8149245 | NR_003668 | DEFB109P1B | defensin, beta 109, pseudogene 1B | 0.075 | 1.053 | 7.66E-01 | 7.66E+01 | | | |
| 7893905 | --- | --- | --- | 0.075 | 1.053 | 8.38E-01 | 8.38E+01 | | | |
| 7921133 | NM_004494 | HDGF | hepatoma-derived growth factor (hig | 0.075 | 1.053 | 4.88E-01 | 4.88E+01 | | | |
| 8062545 | NM_024855 | ACTR5 | ARP5 actin-related protein 5 homolo | 0.075 | 1.053 | 5.24E-01 | 5.24E+01 | | | |
| 8047223 | NM_002157 | HSPE1 | heat shock 10kDa protein 1 (chaperc | 0.075 | 1.053 | 7.45E-01 | 7.45E+01 | | | |
| 7894834 | --- | --- | --- | 0.075 | 1.053 | 8.68E-01 | 8.68E+01 | | | |
| 8011765 | NM_004890 | SPAG7 | sperm associated antigen 7 | 0.075 | 1.053 | 5.19E-01 | 5.19E+01 | | | |
| 7943721 | NM_004109 | FDX1 | ferredoxin 1 | 0.075 | 1.053 | 5.86E-01 | 5.86E+01 | | | |
| 8110450 | NM_031266 | HNRNPAB | heterogeneous nuclear ribonucleopr | 0.075 | 1.053 | 5.43E-01 | 5.43E+01 | | | |
| 7893077 | --- | --- | --- | 0.075 | 1.053 | 2.30E-02 | 2.30E+00 | | | |
| 8169811 | NM_000276 | OCRL | oculocerebrorenal syndrome of Lowe | 0.075 | 1.053 | 4.65E-01 | 4.65E+01 | | | |
| 7917080 | S72422 // S72 | LSTP // DLST | dihydrolipoamide S-succinyltransfera | 0.075 | 1.053 | 5.40E-01 | 5.40E+01 | | | |
| 7907310 | NM_015172 | BAT2D1 | BAT2 domain containing 1 | 0.074 | 1.053 | 7.19E-01 | 7.19E+01 | | | |
| 7893152 | --- | --- | --- | 0.074 | 1.053 | 2.75E-01 | 2.75E+01 | | | |
| 8175102 | NM_182314 | ENOX2 | ecto-NOX disulfide-thiol exchanger 2 | 0.074 | 1.053 | 6.95E-01 | 6.95E+01 | | | |
| 7895680 | --- | --- | --- | 0.074 | 1.053 | 1.34E-01 | 1.34E+01 | | | |
| 7906863 | NM_003115 | UAP1 | UDP-N-acteylglucosamine pyrophosp | 0.074 | 1.053 | 6.89E-01 | 6.89E+01 | | | |
| 8161169 | --- | --- | --- | 0.074 | 1.053 | 2.45E-01 | 2.45E+01 | | | |

| | | | | | | | | | | |
|---|---|---|---|---|---|---|---|---|---|---|
| 8080184 | NM_000688 | ALAS1 | aminolevulinate, delta-, synthase 1 | 0.074 | 1.053 | 5.19E-01 | 5.19E+01 | | | |
| 7990916 | NM_001021 | RPS17 | ribosomal protein S17 | 0.074 | 1.053 | 1.55E-01 | 1.55E+01 | | | |
| 7990965 | NM_001021 | RPS17 | ribosomal protein S17 | 0.074 | 1.053 | 1.55E-01 | 1.55E+01 | | | |
| 7943424 | NM_001166 | BIRC2 | baculoviral IAP repeat-containing 2 | 0.074 | 1.053 | 8.19E-01 | 8.19E+01 | | | |
| 8002266 | NM_00103969( | CHTF8 | CTF8, chromosome transmission fide | 0.074 | 1.053 | 5.85E-01 | 5.85E+01 | | | |
| 8122136 | NM_001016 | RPS12 | ribosomal protein S12 | 0.074 | 1.053 | 8.15E-02 | 8.15E+00 | | | |
| 7894590 | --- | --- | --- | 0.074 | 1.053 | 7.82E-01 | 7.82E+01 | | | |
| 7894320 | --- | --- | --- | 0.074 | 1.053 | 4.20E-01 | 4.20E+01 | | | |
| 7895587 | --- | --- | --- | 0.074 | 1.052 | 7.68E-02 | 7.68E+00 | | | |
| 7893731 | --- | --- | --- | 0.074 | 1.052 | 2.62E-01 | 2.62E+01 | | | |
| 7893900 | --- | --- | --- | 0.074 | 1.052 | 8.74E-01 | 8.74E+01 | | | |
| 7906017 | AF268613 | POU5F1P4 | POU class 5 homeobox 1 pseudogen | 0.074 | 1.052 | 4.34E-01 | 4.34E+01 | | | |
| 7920258 | NM_014624 | S100A6 | S100 calcium binding protein A6 | 0.074 | 1.052 | 5.98E-01 | 5.98E+01 | | | |
| 7907092 | NM_003953 | MPZL1 | myelin protein zero-like 1 | 0.074 | 1.052 | 3.90E-01 | 3.90E+01 | | | |
| 8162631 | NM_014930 | ZNF510 | zinc finger protein 510 | 0.074 | 1.052 | 5.54E-01 | 5.54E+01 | | | |
| 8163964 | NM_005388 | PDCL | phosducin-like | 0.074 | 1.052 | 7.86E-01 | 7.86E+01 | | | |
| 8039674 | NM_00108538 | ZNF154 | zinc finger protein 154 | 0.074 | 1.052 | 6.41E-01 | 6.41E+01 | | | |
| 7914214 | NR_002976 | SNORA44 | small nucleolar RNA, H/ACA box 44 | 0.074 | 1.052 | 8.03E-01 | 8.03E+01 | | | |
| 7896424 | --- | --- | --- | 0.074 | 1.052 | 6.32E-01 | 6.32E+01 | | | |
| 7986769 | NM_130839 | UBE3A | ubiquitin protein ligase E3A | 0.073 | 1.052 | 7.63E-01 | 7.63E+01 | | | |
| 7895342 | --- | --- | --- | 0.073 | 1.052 | 1.07E-01 | 1.07E+01 | | | |
| 8058664 | --- | --- | --- | 0.073 | 1.052 | 8.91E-01 | 8.91E+01 | | | |
| 8119842 | NM_203290 | POLR1C | polymerase (RNA) I polypeptide C, 3 | 0.073 | 1.052 | 8.25E-01 | 8.25E+01 | | | |
| 7894496 | --- | --- | --- | 0.073 | 1.052 | 9.08E-01 | 9.08E+01 | | | |
| 8097586 | NM_207123 | GAB1 | GRB2-associated binding protein 1 | 0.073 | 1.052 | 2.83E-01 | 2.83E+01 | | | |
| 8180192 | --- | --- | --- | 0.073 | 1.052 | 2.15E-02 | 2.15E+00 | | | |
| 8084206 | NM_032047 | B3GNT5 | UDP-GlcNAc:betaGal beta-1,3-N-ace | 0.073 | 1.052 | 8.79E-01 | 8.79E+01 | | | |
| 7941272 | NR_002819 | MALAT1 | metastasis associated lung adenoca | 0.073 | 1.052 | 2.64E-02 | 2.64E+00 | | | |
| 7945084 | --- | --- | --- | 0.073 | 1.052 | 8.77E-01 | 8.77E+01 | | | |
| 8042381 | NM_020143 | PNO1 | partner of NOB1 homolog (S. cerevis | 0.073 | 1.052 | 7.17E-01 | 7.17E+01 | | | |
| 7895124 | --- | --- | --- | 0.073 | 1.052 | 8.71E-01 | 8.71E+01 | | | |
| 7991835 | --- | --- | --- | 0.073 | 1.052 | 5.25E-01 | 5.25E+01 | | | |
| 8172813 | NM_014138 | FAM156A | family with sequence similarity 156, | 0.073 | 1.052 | 4.88E-01 | 4.88E+01 | | | |
| 8089040 | NM_00104253 | MINA | MYC induced nuclear antigen | 0.073 | 1.052 | 7.66E-01 | 7.66E+01 | | | |
| 8104139 | NR_027420 // | 9834 // LOC | ankyrin repeat domain 57 pseudoge | 0.073 | 1.052 | 6.34E-01 | 6.34E+01 | | | |
| 7929593 | NR_026712 | RPL13AP5 | ribosomal protein L13a pseudogene | 0.073 | 1.052 | 6.03E-01 | 6.03E+01 | | | |
| 8140151 | NM_181471 | RFC2 | replication factor C (activator 1) 2, 4 | 0.073 | 1.052 | 6.64E-01 | 6.64E+01 | | | |
| 7892838 | --- | --- | --- | 0.073 | 1.052 | 6.05E-01 | 6.05E+01 | | | |
| 8152453 | NM_014112 | TRPS1 | trichorhinophalangeal syndrome I | 0.073 | 1.052 | 5.13E-01 | 5.13E+01 | | | |
| 8136293 | NM_021807 | EXOC4 | exocyst complex component 4 | 0.073 | 1.052 | 5.36E-01 | 5.36E+01 | | | |
| 7894685 | --- | --- | --- | 0.073 | 1.052 | 6.09E-01 | 6.09E+01 | | | |
| 7894431 | --- | --- | --- | 0.073 | 1.052 | 9.17E-01 | 9.17E+01 | | | |
| 8041813 | NM_014171 | CRIPT | cysteine-rich PDZ-binding protein | 0.073 | 1.052 | 8.43E-01 | 8.43E+01 | | | |
| 7900426 | NM_022733 | SMAP2 | small ArfGAP2 | 0.073 | 1.052 | 7.71E-01 | 7.71E+01 | | | |
| 7894385 | --- | --- | --- | 0.073 | 1.052 | 8.37E-01 | 8.37E+01 | | | |
| 7895210 | --- | --- | --- | 0.073 | 1.052 | 3.71E-01 | 3.71E+01 | | | |
| 8088001 | NM_003157 | NEK4 | NIMA (never in mitosis gene a)-relat | 0.073 | 1.052 | 4.25E-01 | 4.25E+01 | | | |
| 8023526 | ENST00000446 | OC10013299 | similar to hCG1979072 | 0.073 | 1.052 | 8.57E-01 | 8.57E+01 | | | |
| 7893101 | --- | --- | --- | 0.073 | 1.052 | 3.54E-02 | 3.54E+00 | | | |
| 7894867 | --- | --- | --- | 0.073 | 1.052 | 8.64E-01 | 8.64E+01 | | | |
| 8141024 | NM_005868 | BET1 | blocked early in transport 1 homolog | 0.072 | 1.051 | 4.16E-01 | 4.16E+01 | | | |
| 7902930 | NR_003130 | HSP90B3P | heat shock protein 90kDa beta (Grp | 0.072 | 1.051 | 5.45E-01 | 5.45E+01 | | | |
| 8002760 | ENST00000412 | OC10013234 | similar to heat shock 10 kDa protein | 0.072 | 1.051 | 8.20E-01 | 8.20E+01 | | | |
| 7997332 | NM_00110566 | NUDT7 | nudix (nucleoside diphosphate linke | 0.072 | 1.051 | 7.33E-01 | 7.33E+01 | | | |
| 7894732 | --- | --- | --- | 0.072 | 1.051 | 7.76E-01 | 7.76E+01 | | | |
| 7967987 | NM_024026 | MRP63 | mitochondrial ribosomal protein 63 | 0.072 | 1.051 | 4.83E-01 | 4.83E+01 | | | |
| 7894124 | --- | --- | --- | 0.072 | 1.051 | 8.98E-01 | 8.98E+01 | | | |
| 7949410 | BC018448 | MALAT1 | metastasis associated lung adenoca | 0.072 | 1.051 | 4.47E-02 | 4.47E+00 | | | |
| 8035714 | NM_016573 | GMIP | GEM interacting protein | 0.072 | 1.051 | 3.70E-01 | 3.70E+01 | | | |
| 8097955 | --- | --- | --- | 0.072 | 1.051 | 8.61E-01 | 8.61E+01 | | | |
| 7895604 | --- | --- | --- | 0.072 | 1.051 | 8.96E-01 | 8.96E+01 | | | |
| 7901951 | NM_002633 | PGM1 | phosphoglucomutase 1 | 0.072 | 1.051 | 5.10E-01 | 5.10E+01 | | | |
| 8040090 | NM_014746 | RNF144A | ring finger protein 144A | 0.072 | 1.051 | 5.13E-01 | 5.13E+01 | | | |
| 8026787 | NM_173544 | FAM129C | family with sequence similarity 129, | 0.072 | 1.051 | 4.70E-01 | 4.70E+01 | | | |
| 8052380 | --- | --- | --- | 0.072 | 1.051 | 4.76E-01 | 4.76E+01 | | | |
| 8037621 | NM_012155 | EML2 | echinoderm microtubule associated | 0.072 | 1.051 | 3.91E-01 | 3.91E+01 | | | |
| 7986765 | NM_000969 | RPL5 | ribosomal protein L5 | 0.072 | 1.051 | 3.72E-01 | 3.72E+01 | | | |
| 7896064 | --- | --- | --- | 0.072 | 1.051 | 2.20E-01 | 2.20E+01 | | | |
| 8160769 | --- | --- | --- | 0.071 | 1.051 | 6.34E-01 | 6.34E+01 | | | |
| 8143684 | NM_004911 | PDIA4 | protein disulfide isomerase family A, | 0.071 | 1.051 | 7.58E-01 | 7.58E+01 | | | |
| 7980680 | NM_00108547 | FOXN3 | forkhead box N3 | 0.071 | 1.051 | 6.65E-01 | 6.65E+01 | | | |
| 7894146 | --- | --- | --- | 0.071 | 1.051 | 9.00E-01 | 9.00E+01 | | | |
| 7943288 | NM_032102 | SFRS2B | splicing factor, arginine/serine-rich 2 | 0.071 | 1.051 | 4.78E-01 | 4.78E+01 | | | |
| 8071566 | NM_014337 | PPIL2 | peptidylprolyl isomerase (cyclophilin | 0.071 | 1.051 | 3.94E-01 | 3.94E+01 | | | |
| 7893549 | --- | --- | --- | 0.071 | 1.051 | 9.18E-01 | 9.18E+01 | | | |
| 8137044 | --- | --- | --- | 0.071 | 1.051 | 6.69E-01 | 6.69E+01 | | | |
| 8145529 | --- | --- | --- | 0.071 | 1.050 | 6.11E-01 | 6.11E+01 | | | |

| | | | | | | | | | | |
|---|---|---|---|---|---|---|---|---|---|---|
| 8083792 | --- | --- | --- | 0.071 | 1.050 | 4.79E-01 | 4.79E+01 | | | |
| 8063427 | NM_014484 | MOCS3 | molybdenum cofactor synthesis 3 | 0.071 | 1.050 | 5.98E-01 | 5.98E+01 | | | |
| 7919971 | NM_000449 | RFX5 | regulatory factor X, 5 (influences HLA | 0.071 | 1.050 | 7.73E-01 | 7.73E+01 | | | |
| 7899519 | NM_016258 | YTHDF2 | YTH domain family, member 2 | 0.071 | 1.050 | 5.49E-01 | 5.49E+01 | | | |
| 8056716 | NM_014168 | METTL5 | methyltransferase like 5 | 0.071 | 1.050 | 6.87E-01 | 6.87E+01 | | | |
| 8093130 | NM_152617 | RNF168 | ring finger protein 168 | 0.071 | 1.050 | 7.99E-01 | 7.99E+01 | | | |
| 8031792 | NM_006385 | ZNF211 | zinc finger protein 211 | 0.071 | 1.050 | 7.31E-01 | 7.31E+01 | | | |
| 8079334 | NM_014240 | LIMD1 | LIM domains containing 1 | 0.070 | 1.050 | 3.93E-01 | 3.93E+01 | | | |
| 7940079 | NM_00108545 | CTNND1 | catenin (cadherin-associated protein | 0.070 | 1.050 | 5.59E-01 | 5.59E+01 | | | |
| 7964076 | NM_014255 | CNPY2 | canopy 2 homolog (zebrafish) | 0.070 | 1.050 | 4.94E-01 | 4.94E+01 | | | |
| 7924327 | NM_018040 | GPATCH2 | G patch domain containing 2 | 0.070 | 1.050 | 5.86E-01 | 5.86E+01 | | | |
| 8120043 | NM_00102463 | RUNX2 | runt-related transcription factor 2 | 0.070 | 1.050 | 5.09E-01 | 5.09E+01 | | | |
| 7896384 | --- | --- | --- | 0.070 | 1.050 | 1.14E-01 | 1.14E+01 | | | |
| 7993035 | NM_016936 | UBN1 | ubinuclein 1 | 0.070 | 1.050 | 5.10E-01 | 5.10E+01 | | | |
| 8167601 | NM_00114507 | USP27X | ubiquitin specific peptidase 27, X-link | 0.070 | 1.050 | 6.73E-01 | 6.73E+01 | | | |
| 7995477 | --- | --- | --- | 0.070 | 1.050 | 7.81E-01 | 7.81E+01 | | | |
| 8053107 | NM_004082 | DCTN1 | dynactin 1 (p150, glued homolog, Dr | 0.070 | 1.050 | 5.21E-01 | 5.21E+01 | | | |
| 8065992 | NM_021100 | NFS1 | NFS1 nitrogen fixation 1 homolog (S. | 0.070 | 1.050 | 1.98E-01 | 1.98E+01 | | | |
| 7919751 | NM_021960 | MCL1 | myeloid cell leukemia sequence 1 (B | 0.070 | 1.050 | 5.58E-01 | 5.58E+01 | | | |
| 8034349 | NM_016264 | ZNF44 | zinc finger protein 44 | 0.070 | 1.050 | 5.95E-01 | 5.95E+01 | | | |
| 8110463 | NM_058230 | ZNF354B | zinc finger protein 354B | 0.070 | 1.050 | 6.54E-01 | 6.54E+01 | | | |
| 8036357 | NM_206894 | ZNF790 | zinc finger protein 790 | 0.070 | 1.050 | 4.35E-01 | 4.35E+01 | | | |
| 7935819 | NM_032112 | MRPL43 | mitochondrial ribosomal protein L43 | 0.070 | 1.050 | 6.13E-01 | 6.13E+01 | | | |
| 8157650 | NM_000962 | PTGS1 | prostaglandin-endoperoxide synthas | 0.070 | 1.050 | 4.44E-01 | 4.44E+01 | | | |
| 8005736 | --- | --- | --- | 0.070 | 1.050 | 7.46E-01 | 7.46E+01 | | | |
| 7913110 | BC034589 | KIAA0090 | KIAA0090 | 0.070 | 1.050 | 5.82E-01 | 5.82E+01 | | | |
| 8005171 | NM_016113 | TRPV2 | transient receptor potential cation c | 0.070 | 1.050 | 4.69E-01 | 4.69E+01 | | | |
| 7894506 | --- | --- | --- | 0.070 | 1.050 | 9.09E-01 | 9.09E+01 | | | |
| 7930170 | NM_00113620 | C10orf32 | chromosome 10 open reading frame | 0.070 | 1.050 | 6.46E-01 | 6.46E+01 | | | |
| 8001782 | NM_002954 | RPS27A | ribosomal protein S27a | 0.070 | 1.050 | 1.72E-01 | 1.72E+01 | | | |
| 7986132 | NM_006122 | MAN2A2 | mannosidase, alpha, class 2A, memb | 0.070 | 1.049 | 4.31E-01 | 4.31E+01 | | | |
| 7894239 | --- | --- | --- | 0.070 | 1.049 | 5.28E-01 | 5.28E+01 | | | |
| 8117207 | NM_170740 | ALDH5A1 | aldehyde dehydrogenase 5 family, m | 0.070 | 1.049 | 4.83E-01 | 4.83E+01 | | | |
| 7993159 | NM_014117 | C16orf72 | chromosome 16 open reading frame | 0.070 | 1.049 | 6.59E-01 | 6.59E+01 | | | |
| 7894991 | --- | --- | --- | 0.070 | 1.049 | 8.97E-02 | 8.97E+00 | | | |
| 7986687 | NR_003521 | WHAMML1 | WAS protein homolog associated wit | 0.070 | 1.049 | 7.57E-01 | 7.57E+01 | | | |
| 8071314 | NM_022720 | DGCR8 | DiGeorge syndrome critical region ge | 0.070 | 1.049 | 4.43E-01 | 4.43E+01 | | | |
| 8132539 | NM_014063 | DBNL | drebrin-like | 0.070 | 1.049 | 6.42E-01 | 6.42E+01 | | | |
| 8092095 | NM_015028 | TNIK | TRAF2 and NCK interacting kinase | 0.070 | 1.049 | 8.32E-01 | 8.32E+01 | | | |
| 7894457 | --- | --- | --- | 0.069 | 1.049 | 9.15E-01 | 9.15E+01 | | | |
| 8031669 | NM_00100166 | ZNF470 | zinc finger protein 470 | 0.069 | 1.049 | 5.51E-01 | 5.51E+01 | | | |
| 7892650 | --- | --- | --- | 0.069 | 1.049 | 8.83E-01 | 8.83E+01 | | | |
| 7942586 | NM_001005 | RPS3 | ribosomal protein S3 | 0.069 | 1.049 | 2.78E-01 | 2.78E+01 | | | |
| 8140445 | NR_003664 | SPDYE8P | speedy homolog E8 (Xenopus laevis) | 0.069 | 1.049 | 3.99E-01 | 3.99E+01 | | | |
| 8117377 | NM_005321 | HIST1H1E | histone cluster 1, H1e | 0.069 | 1.049 | 7.79E-01 | 7.79E+01 | | | |
| 8144422 | NM_00104007 | LOC650293 | seven transmembrane helix receptor | 0.069 | 1.049 | 4.21E-01 | 4.21E+01 | | | |
| 7974835 | NM_006255 | PRKCH | protein kinase C, eta | 0.069 | 1.049 | 6.77E-01 | 6.77E+01 | | | |
| 8063156 | NM_001250 | CD40 | CD40 molecule, TNF receptor superfa | 0.069 | 1.049 | 4.55E-01 | 4.55E+01 | | | |
| 7921621 | --- | --- | --- | 0.069 | 1.049 | 7.59E-01 | 7.59E+01 | | | |
| 8051637 | NM_198963 | DHX57 | DEAH (Asp-Glu-Ala-Asp/His) box poly | 0.069 | 1.049 | 6.37E-01 | 6.37E+01 | | | |
| 7895924 | --- | --- | --- | 0.069 | 1.049 | 3.41E-01 | 3.41E+01 | | | |
| 8031047 | NM_00102081 | MYADM | myeloid-associated differentiation m | 0.069 | 1.049 | 7.74E-01 | 7.74E+01 | | | |
| 7934906 | NM_00114194 | ACTA2 | actin, alpha 2, smooth muscle, aorta | 0.069 | 1.049 | 7.05E-01 | 7.05E+01 | | | |
| 7952126 | NR_003040 | RPL23AP64 | ribosomal protein L23a pseudogene | 0.069 | 1.049 | 8.46E-01 | 8.46E+01 | | | |
| 7929609 | BC024315 | C10orf12 | chromosome 10 open reading frame | 0.069 | 1.049 | 6.79E-01 | 6.79E+01 | | | |
| 7895622 | --- | --- | --- | 0.069 | 1.049 | 8.81E-01 | 8.81E+01 | | | |
| 8051963 | NM_018079 | SRBD1 | S1 RNA binding domain 1 | 0.069 | 1.049 | 7.01E-01 | 7.01E+01 | | | |
| 8007505 | NM_004941 | DHX8 | DEAH (Asp-Glu-Ala-His) box polypept | 0.069 | 1.049 | 7.40E-01 | 7.40E+01 | | | |
| 7893809 | --- | --- | --- | 0.069 | 1.049 | 8.45E-01 | 8.45E+01 | | | |
| 8131631 | NM_178423 | HDAC9 | histone deacetylase 9 | 0.069 | 1.049 | 2.85E-01 | 2.85E+01 | | | |
| 8133012 | --- | --- | --- | 0.069 | 1.049 | 7.78E-01 | 7.78E+01 | | | |
| 8067206 | NM_00100873 | HMGB1L1 | high-mobility group box 1-like 1 | 0.069 | 1.049 | 7.71E-01 | 7.71E+01 | | | |
| 8143035 | --- | --- | --- | 0.069 | 1.049 | 4.80E-01 | 4.80E+01 | | | |
| 8089029 | NM_176815 | DHFRL1 | dihydrofolate reductase-like 1 | 0.068 | 1.049 | 5.57E-01 | 5.57E+01 | | | |
| 8168215 | NM_005120 | MED12 | mediator complex subunit 12 | 0.068 | 1.049 | 4.42E-01 | 4.42E+01 | | | |
| 7896496 | --- | --- | --- | 0.068 | 1.049 | 9.43E-02 | 9.43E+00 | | | |
| 7966299 | --- | --- | --- | 0.068 | 1.049 | 6.07E-01 | 6.07E+01 | | | |
| 8177788 | NM_005516 | HLA-E | major histocompatibility complex, cla | 0.068 | 1.049 | 6.55E-01 | 6.55E+01 | | | |
| 8179103 | NM_005516 | HLA-E | major histocompatibility complex, cla | 0.068 | 1.049 | 6.55E-01 | 6.55E+01 | | | |
| 8087283 | NM_005051 | QARS | glutaminyl-tRNA synthetase | 0.068 | 1.049 | 7.10E-01 | 7.10E+01 | | | |
| 7896174 | --- | --- | --- | 0.068 | 1.049 | 6.57E-01 | 6.57E+01 | | | |
| 8126629 | NM_019096 | GTPBP2 | GTP binding protein 2 | 0.068 | 1.049 | 6.36E-01 | 6.36E+01 | | | |
| 8117372 | NM_003512 | HIST1H2AC | histone cluster 1, H2ac | 0.068 | 1.048 | 8.19E-01 | 8.19E+01 | | | |
| 7895165 | --- | --- | --- | 0.068 | 1.048 | 3.14E-01 | 3.14E+01 | | | |
| 7894076 | --- | --- | --- | 0.068 | 1.048 | 8.29E-01 | 8.29E+01 | | | |
| 7942791 | NR_003287 | OC10000858 | 28S ribosomal RNA | 0.068 | 1.048 | 3.31E-02 | 3.31E+00 | | | |
| 7893036 | --- | --- | --- | 0.068 | 1.048 | 8.18E-01 | 8.18E+01 | | | |

| | | | | | | | | | | |
|---|---|---|---|---|---|---|---|---|---|---|
| 8023868 | NR_024484 | LOC400657 | hypothetical LOC400657 | 0.068 | 1.048 | 6.24E-01 | 6.24E+01 | | | |
| 8117822 | NM_170783 | ZNRD1 | zinc ribbon domain containing 1 | 0.068 | 1.048 | 8.03E-01 | 8.03E+01 | | | |
| 8001776 | --- | --- | --- | 0.068 | 1.048 | 4.57E-01 | 4.57E+01 | | | |
| 8112476 | --- | --- | --- | 0.068 | 1.048 | 7.47E-01 | 7.47E+01 | | | |
| 7892592 | --- | --- | --- | 0.068 | 1.048 | 8.21E-01 | 8.21E+01 | | | |
| 8116530 | NR_002592 | SNORD96A | small nucleolar RNA, C/D box 96A | 0.068 | 1.048 | 9.01E-01 | 9.01E+01 | | | |
| 8116610 | NM_000904 | NQO2 | NAD(P)H dehydrogenase, quinone 2 | 0.068 | 1.048 | 6.93E-01 | 6.93E+01 | | | |
| 8156770 | NM_024642 | GALNT12 | UDP-N-acetyl-alpha-D-galactosamine | 0.068 | 1.048 | 6.38E-01 | 6.38E+01 | | | |
| 8040639 | NM_000183 | HADHB | hydroxyacyl-Coenzyme A dehydroge | 0.068 | 1.048 | 8.48E-01 | 8.48E+01 | | | |
| 7894418 | --- | --- | --- | 0.068 | 1.048 | 4.61E-01 | 4.61E+01 | | | |
| 7955768 | NM_002624 | PFDN5 | prefoldin subunit 5 | 0.068 | 1.048 | 7.53E-01 | 7.53E+01 | | | |
| 7896425 | --- | --- | --- | 0.067 | 1.048 | 5.55E-01 | 5.55E+01 | | | |
| 8122966 | NM_00100134 | CLDN20 | claudin 20 | 0.067 | 1.048 | 7.71E-01 | 7.71E+01 | | | |
| 8107321 | AB048207 | C5orf26 | chromosome 5 open reading frame 2 | 0.067 | 1.048 | 8.04E-01 | 8.04E+01 | | | |
| 7893677 | --- | --- | --- | 0.067 | 1.048 | 5.03E-02 | 5.03E+00 | | | |
| 8125172 | NM_006709 | EHMT2 | euchromatic histone-lysine N-methy | 0.067 | 1.048 | 4.17E-01 | 4.17E+01 | | | |
| 8179884 | NM_006709 | EHMT2 | euchromatic histone-lysine N-methy | 0.067 | 1.048 | 4.17E-01 | 4.17E+01 | | | |
| 8061136 | NM_002823 | PTMA | prothymosin, alpha | 0.067 | 1.048 | 8.32E-01 | 8.32E+01 | | | |
| 8031690 | NM_003417 | ZNF264 | zinc finger protein 264 | 0.067 | 1.048 | 4.14E-01 | 4.14E+01 | | | |
| 7924760 | NM_002221 | ITPKB | inositol 1,4,5-trisphosphate 3-kinase | 0.067 | 1.048 | 5.82E-01 | 5.82E+01 | | | |
| 7946807 | NM_021029 | RPL36A | ribosomal protein L36a | 0.067 | 1.048 | 7.95E-01 | 7.95E+01 | | | |
| 8005473 | NM_006451 | PAIP1 | poly(A) binding protein interacting p | 0.067 | 1.048 | 7.93E-01 | 7.93E+01 | | | |
| 8135922 | NM_018396 | METTL2B | methyltransferase like 2B | 0.067 | 1.048 | 6.31E-01 | 6.31E+01 | | | |
| 8063394 | NM_002827 | PTPN1 | protein tyrosine phosphatase, non-re | 0.067 | 1.048 | 6.79E-01 | 6.79E+01 | | | |
| 7896111 | --- | --- | --- | 0.067 | 1.047 | 1.06E-01 | 1.06E+01 | | | |
| 8015655 | NM_178126 | FAM134C | family with sequence similarity 134, | 0.067 | 1.047 | 7.09E-01 | 7.09E+01 | | | |
| 7895156 | --- | --- | --- | 0.067 | 1.047 | 5.20E-01 | 5.20E+01 | | | |
| 7974481 | --- | --- | --- | 0.067 | 1.047 | 7.71E-01 | 7.71E+01 | | | |
| 8144496 | NM_00104007 | LOC650293 | seven transmembrane helix receptor | 0.067 | 1.047 | 4.54E-01 | 4.54E+01 | | | |
| 7918365 | --- | --- | --- | 0.067 | 1.047 | 5.78E-01 | 5.78E+01 | | | |
| 8108568 | NM_006083 | IK | IK cytokine, down-regulator of HLA II | 0.066 | 1.047 | 8.13E-01 | 8.13E+01 | | | |
| 7967456 | NM_145058 | RILPL2 | Rab interacting lysosomal protein-lik | 0.066 | 1.047 | 7.75E-01 | 7.75E+01 | | | |
| 7894290 | --- | --- | --- | 0.066 | 1.047 | 6.26E-01 | 6.26E+01 | | | |
| 8134201 | NM_021167 | GATAD1 | GATA zinc finger domain containing | 0.066 | 1.047 | 3.01E-01 | 3.01E+01 | | | |
| 8119896 | --- | --- | --- | 0.066 | 1.047 | 6.64E-01 | 6.64E+01 | | | |
| 8052803 | NM_014911 | AAK1 | AP2 associated kinase 1 | 0.066 | 1.047 | 6.76E-01 | 6.76E+01 | | | |
| 7893754 | --- | --- | --- | 0.066 | 1.047 | 9.20E-01 | 9.20E+01 | | | |
| 7892977 | --- | --- | --- | 0.066 | 1.047 | 5.97E-01 | 5.97E+01 | | | |
| 8044111 | NM_182640 | MRPS9 | mitochondrial ribosomal protein S9 | 0.066 | 1.047 | 7.43E-01 | 7.43E+01 | | | |
| 8148476 | NM_014957 | DENND3 | DENN/MADD domain containing 3 | 0.066 | 1.047 | 4.91E-01 | 4.91E+01 | | | |
| 7894321 | --- | --- | --- | 0.066 | 1.047 | 8.46E-01 | 8.46E+01 | | | |
| 8118147 | --- | --- | --- | 0.066 | 1.047 | 4.91E-01 | 4.91E+01 | | | |
| 7892606 | --- | --- | --- | 0.066 | 1.047 | 2.27E-01 | 2.27E+01 | | | |
| 8029050 | NM_052848 | CCDC97 | coiled-coil domain containing 97 | 0.066 | 1.047 | 5.32E-01 | 5.32E+01 | | | |
| 7895391 | --- | --- | --- | 0.066 | 1.047 | 3.90E-01 | 3.90E+01 | | | |
| 7959078 | --- | --- | --- | 0.066 | 1.047 | 4.13E-01 | 4.13E+01 | | | |
| 7895333 | --- | --- | --- | 0.066 | 1.047 | 2.08E-01 | 2.08E+01 | | | |
| 7893201 | --- | --- | --- | 0.066 | 1.047 | 8.18E-01 | 8.18E+01 | | | |
| 7941714 | NM_024650 | C11orf80 | chromosome 11 open reading frame | 0.066 | 1.047 | 5.47E-01 | 5.47E+01 | | | |
| 8000156 | ENST00000446 | SMG1 | SMG1 homolog, phosphatidylinosito | 0.066 | 1.047 | 7.31E-01 | 7.31E+01 | | | |
| 7894909 | --- | --- | --- | 0.066 | 1.047 | 1.84E-02 | 1.84E+00 | | | |
| 8047771 | NM_001959 | EEF1B2 | eukaryotic translation elongation fac | 0.066 | 1.047 | 5.65E-01 | 5.65E+01 | | | |
| 7977409 | NM_001311 | CRIP1 | cysteine-rich protein 1 (intestinal) | 0.066 | 1.047 | 5.80E-01 | 5.80E+01 | | | |
| 7939184 | NM_018393 | TCP11L1 | t-complex 11 (mouse)-like 1 | 0.066 | 1.047 | 7.03E-01 | 7.03E+01 | | | |
| 7894236 | --- | --- | --- | 0.066 | 1.047 | 2.09E-02 | 2.09E+00 | | | |
| 7896458 | --- | --- | --- | 0.066 | 1.047 | 8.67E-01 | 8.67E+01 | | | |
| 8068289 | NM_138927 | SON | SON DNA binding protein | 0.066 | 1.047 | 6.54E-01 | 6.54E+01 | | | |
| 8068410 | NM_015955 | MEMO1 | mediator of cell motility 1 | 0.066 | 1.047 | 5.77E-01 | 5.77E+01 | | | |
| 8087201 | NM_016291 | IP6K2 | inositol hexakisphosphate kinase 2 | 0.066 | 1.046 | 6.62E-01 | 6.62E+01 | | | |
| 8117510 | NM_006353 | HMGN4 | high mobility group nucleosomal bin | 0.065 | 1.046 | 4.61E-01 | 4.61E+01 | | | |
| 8120249 | NR_001445 // | N7SK // RN7 | RNA, 7SK small nuclear // RNA, 7SK | 0.065 | 1.046 | 1.22E-01 | 1.22E+01 | | | |
| 7893906 | --- | --- | --- | 0.065 | 1.046 | 7.58E-01 | 7.58E+01 | | | |
| 7893820 | --- | --- | --- | 0.065 | 1.046 | 4.96E-01 | 4.96E+01 | | | |
| 7892907 | --- | --- | --- | 0.065 | 1.046 | 4.66E-01 | 4.66E+01 | | | |
| 7894369 | --- | --- | --- | 0.065 | 1.046 | 5.90E-01 | 5.90E+01 | | | |
| 8038904 | NM_032679 | ZNF577 | zinc finger protein 577 | 0.065 | 1.046 | 6.48E-01 | 6.48E+01 | | | |
| 8027368 | NM_203282 | ZNF254 | zinc finger protein 254 | 0.065 | 1.046 | 7.61E-01 | 7.61E+01 | | | |
| 7993608 | NM_016138 | COQ7 | coenzyme Q7 homolog, ubiquinone | 0.065 | 1.046 | 5.68E-01 | 5.68E+01 | | | |
| 8079462 | NM_015175 | NBEAL2 | neurobeachin-like 2 | 0.065 | 1.046 | 2.09E-01 | 2.09E+01 | | | |
| 8149122 | AF355799 | DEFT1P | defensin, theta 1 pseudogene | 0.065 | 1.046 | 2.66E-01 | 2.66E+01 | | | |
| 8149132 | AF355799 | DEFT1P | defensin, theta 1 pseudogene | 0.065 | 1.046 | 2.66E-01 | 2.66E+01 | | | |
| 7991640 | NM_152334 | TARSL2 | threonyl-tRNA synthetase-like 2 | 0.065 | 1.046 | 7.29E-01 | 7.29E+01 | | | |
| 7895398 | --- | --- | --- | 0.065 | 1.046 | 5.07E-01 | 5.07E+01 | | | |
| 7982507 | NM_016454 | TMEM85 | transmembrane protein 85 | 0.065 | 1.046 | 8.00E-01 | 8.00E+01 | | | |
| 7893710 | --- | --- | --- | 0.065 | 1.046 | 3.71E-01 | 3.71E+01 | | | |
| 7942650 | NM_020193 | C11orf30 | chromosome 11 open reading frame | 0.065 | 1.046 | 4.80E-01 | 4.80E+01 | | | |
| 7949717 | --- | --- | --- | 0.065 | 1.046 | 3.57E-01 | 3.57E+01 | | | |

| | | | | | | | | | | |
|---|---|---|---|---|---|---|---|---|---|---|
| 7934959 | --- | --- | --- | 0.065 | 1.046 | 5.97E-01 | 5.97E+01 | | | |
| 7944401 | NM_000190 | HMBS | hydroxymethylbilane synthase | 0.065 | 1.046 | 4.60E-01 | 4.60E+01 | | | |
| 7894666 | --- | --- | --- | 0.065 | 1.046 | 7.50E-01 | 7.50E+01 | | | |
| 8027876 | NM_032635 | TMEM147 | transmembrane protein 147 | 0.065 | 1.046 | 7.54E-01 | 7.54E+01 | | | |
| 8118863 | NM_015245 | ANKS1A | ankyrin repeat and sterile alpha mot | 0.065 | 1.046 | 4.53E-01 | 4.53E+01 | | | |
| 7898070 | NM_012231 | PRDM2 | PR domain containing 2, with ZNF do | 0.065 | 1.046 | 7.47E-01 | 7.47E+01 | | | |
| 7972548 | NM_005292 | GPR18 | G protein-coupled receptor 18 | 0.064 | 1.046 | 8.33E-01 | 8.33E+01 | | | |
| 8075063 | NM_022081 | HPS4 | Hermansky-Pudlak syndrome 4 | 0.064 | 1.046 | 5.96E-01 | 5.96E+01 | | | |
| 8063444 | ENST00000371 | TSHZ2 | teashirt zinc finger homeobox 2 | 0.064 | 1.046 | 6.74E-01 | 6.74E+01 | | | |
| 8085486 | NM_004628 | XPC | xeroderma pigmentosum, compleme | 0.064 | 1.046 | 6.81E-01 | 6.81E+01 | | | |
| 7932834 | NM_018109 | MTPAP | mitochondrial poly(A) polymerase | 0.064 | 1.045 | 4.64E-01 | 4.64E+01 | | | |
| 8022941 | NM_015476 | C18orf10 | chromosome 18 open reading frame | 0.064 | 1.045 | 7.57E-01 | 7.57E+01 | | | |
| 7923131 | NM_00114279 | DENND1B | DENN/MADD domain containing 1B | 0.064 | 1.045 | 8.93E-01 | 8.93E+01 | | | |
| 7894332 | --- | --- | --- | 0.064 | 1.045 | 7.59E-01 | 7.59E+01 | | | |
| 7950810 | NM_206927 | SYTL2 | synaptotagmin-like 2 | 0.064 | 1.045 | 7.25E-01 | 7.25E+01 | | | |
| 8086538 | BC047037 | LOC644714 | hypothetical protein LOC644714 | 0.064 | 1.045 | 9.19E-01 | 9.19E+01 | | | |
| 8047815 | NM_00113619 | FASTKD2 | FAST kinase domains 2 | 0.064 | 1.045 | 8.54E-01 | 8.54E+01 | | | |
| 7926896 | NM_001826 | CKS1B | CDC28 protein kinase regulatory sub | 0.064 | 1.045 | 9.06E-01 | 9.06E+01 | | | |
| 7893709 | --- | --- | --- | 0.064 | 1.045 | 9.14E-01 | 9.14E+01 | | | |
| 7956743 | NM_00103499 | RPL14 | ribosomal protein L14 | 0.064 | 1.045 | 6.30E-01 | 6.30E+01 | | | |
| 7973797 | NM_00113505 | COCH | coagulation factor C homolog, cochli | 0.064 | 1.045 | 2.59E-01 | 2.59E+01 | | | |
| 8014248 | NM_144682 | SLFN13 | schlafen family member 13 | 0.064 | 1.045 | 7.82E-01 | 7.82E+01 | | | |
| 8118116 | NM_005931 | MICB | MHC class I polypeptide-related sequ | 0.064 | 1.045 | 4.54E-01 | 4.54E+01 | | | |
| 7899071 | --- | --- | --- | 0.064 | 1.045 | 6.47E-01 | 6.47E+01 | | | |
| 7894217 | --- | --- | --- | 0.064 | 1.045 | 8.65E-01 | 8.65E+01 | | | |
| 8009792 | --- | --- | --- | 0.064 | 1.045 | 8.76E-01 | 8.76E+01 | | | |
| 8092968 | --- | --- | --- | 0.064 | 1.045 | 7.63E-01 | 7.63E+01 | | | |
| 7902874 | NM_032270 | LRRC8C | leucine rich repeat containing 8 fami | 0.064 | 1.045 | 5.27E-01 | 5.27E+01 | | | |
| 8156043 | NM_058179 | PSAT1 | phosphoserine aminotransferase 1 | 0.064 | 1.045 | 6.43E-01 | 6.43E+01 | | | |
| 7895602 | --- | --- | --- | 0.064 | 1.045 | 9.04E-01 | 9.04E+01 | | | |
| 8139128 | --- | --- | --- | 0.064 | 1.045 | 8.59E-01 | 8.59E+01 | | | |
| 7892930 | --- | --- | --- | 0.064 | 1.045 | 4.59E-01 | 4.59E+01 | | | |
| 8150014 | NM_018250 | INTS9 | integrator complex subunit 9 | 0.064 | 1.045 | 3.36E-01 | 3.36E+01 | | | |
| 7969177 | NR_002183 | FAM10A4 | ST13-like tumor suppressor | 0.064 | 1.045 | 6.36E-01 | 6.36E+01 | | | |
| 8026024 | NM_004317 | ASNA1 | arsA arsenite transporter, ATP-bindir | 0.063 | 1.045 | 7.32E-01 | 7.32E+01 | | | |
| 8028514 | NM_013234 | EIF3K | eukaryotic translation initiation facto | 0.063 | 1.045 | 8.39E-01 | 8.39E+01 | | | |
| 8177955 | NM_005931 | MICB | MHC class I polypeptide-related sequ | 0.063 | 1.045 | 3.53E-01 | 3.53E+01 | | | |
| 7895012 | --- | --- | --- | 0.063 | 1.045 | 2.97E-01 | 2.97E+01 | | | |
| 8072461 | NM_016733 | LIMK2 | LIM domain kinase 2 | 0.063 | 1.045 | 4.48E-01 | 4.48E+01 | | | |
| 7895230 | --- | --- | --- | 0.063 | 1.045 | 1.91E-01 | 1.91E+01 | | | |
| 7936673 | NM_00100533 | RGS10 | regulator of G-protein signaling 10 | 0.063 | 1.045 | 7.59E-01 | 7.59E+01 | | | |
| 8096489 | NM_006457 | PDLIM5 | PDZ and LIM domain 5 | 0.063 | 1.045 | 5.05E-01 | 5.05E+01 | | | |
| 7957551 | NM_003877 | SOCS2 | suppressor of cytokine signaling 2 | 0.063 | 1.045 | 4.40E-01 | 4.40E+01 | | | |
| 7994371 | ENST0000044 | NPIPL3 | nuclear pore complex interacting pro | 0.063 | 1.045 | 6.90E-01 | 6.90E+01 | | | |
| 7957530 | NM_021104 | RPL41 | ribosomal protein L41 | 0.063 | 1.045 | 1.10E-01 | 1.10E+01 | | | |
| 8117929 | NM_00102509 | ABCF1 | ATP-binding cassette, sub-family F (G | 0.063 | 1.045 | 7.42E-01 | 7.42E+01 | | | |
| 8177797 | NM_00102509 | ABCF1 | ATP-binding cassette, sub-family F (G | 0.063 | 1.045 | 7.42E-01 | 7.42E+01 | | | |
| 8179112 | NM_00102509 | ABCF1 | ATP-binding cassette, sub-family F (G | 0.063 | 1.045 | 7.42E-01 | 7.42E+01 | | | |
| 7927186 | NM_032023 | RASSF4 | Ras association (RalGDS/AF-6) doma | 0.063 | 1.045 | 5.57E-01 | 5.57E+01 | | | |
| 7993310 | NM_014048 | MKL2 | MKL/myocardin-like 2 | 0.063 | 1.045 | 3.42E-01 | 3.42E+01 | | | |
| 7894192 | --- | --- | --- | 0.063 | 1.045 | 3.12E-01 | 3.12E+01 | | | |
| 8173531 | NM_018486 | HDAC8 | histone deacetylase 8 | 0.063 | 1.045 | 7.73E-01 | 7.73E+01 | | | |
| 8057771 | NM_003151 | STAT4 | signal transducer and activator of tra | 0.063 | 1.045 | 7.75E-01 | 7.75E+01 | | | |
| 8076533 | NM_007229 | PACSIN2 | protein kinase C and casein kinase su | 0.063 | 1.045 | 6.83E-01 | 6.83E+01 | | | |
| 7892827 | --- | --- | --- | 0.063 | 1.044 | 1.96E-01 | 1.96E+01 | | | |
| 7975167 | NM_020806 | GPHN | gephyrin | 0.063 | 1.044 | 6.01E-01 | 6.01E+01 | | | |
| 7895982 | --- | --- | --- | 0.063 | 1.044 | 9.27E-01 | 9.27E+01 | | | |
| 7970287 | NM_005561 | LAMP1 | lysosomal-associated membrane pro | 0.063 | 1.044 | 5.80E-01 | 5.80E+01 | | | |
| 8131811 | --- | --- | --- | 0.063 | 1.044 | 6.25E-01 | 6.25E+01 | | | |
| 8178489 | 0 | 0 | 0 | 0.063 | 1.044 | 5.72E-01 | 5.72E+01 | | | |
| 7996403 | NM_025187 | C16orf70 | chromosome 16 open reading frame | 0.063 | 1.044 | 6.16E-01 | 6.16E+01 | | | |
| 8036242 | NM_032878 | ALKBH6 | alkB, alkylation repair homolog 6 (E. | 0.062 | 1.044 | 7.51E-01 | 7.51E+01 | | | |
| 7919637 | NM_003528 | HIST2H2BE | histone cluster 2, H2be | 0.062 | 1.044 | 8.44E-01 | 8.44E+01 | | | |
| 8072876 | NM_002305 | LGALS1 | lectin, galactoside-binding, soluble, 1 | 0.062 | 1.044 | 6.57E-01 | 6.57E+01 | | | |
| 7893420 | --- | --- | --- | 0.062 | 1.044 | 5.59E-01 | 5.59E+01 | | | |
| 7948679 | NM_001404 | EEF1G | eukaryotic translation elongation fac | 0.062 | 1.044 | 5.46E-01 | 5.46E+01 | | | |
| 8143663 | NM_004456 | EZH2 | enhancer of zeste homolog 2 (Droso | 0.062 | 1.044 | 7.91E-01 | 7.91E+01 | | | |
| 8085206 | NM_003656 | CAMK1 | calcium/calmodulin-dependent prote | 0.062 | 1.044 | 5.70E-01 | 5.70E+01 | | | |
| 7896028 | --- | --- | --- | 0.062 | 1.044 | 8.66E-01 | 8.66E+01 | | | |
| 7893228 | --- | --- | --- | 0.062 | 1.044 | 9.19E-01 | 9.19E+01 | | | |
| 7894138 | --- | --- | --- | 0.062 | 1.044 | 6.69E-01 | 6.69E+01 | | | |
| 7895979 | --- | --- | --- | 0.062 | 1.044 | 8.28E-01 | 8.28E+01 | | | |
| 7894495 | --- | --- | --- | 0.062 | 1.044 | 6.01E-01 | 6.01E+01 | | | |
| 8084945 | --- | --- | --- | 0.062 | 1.044 | 7.78E-01 | 7.78E+01 | | | |
| 7892796 | --- | --- | --- | 0.062 | 1.044 | 1.72E-01 | 1.72E+01 | | | |
| 7896614 | --- | --- | --- | 0.062 | 1.044 | 7.62E-01 | 7.62E+01 | | | |
| 8087236 | NM_00100999 | DALRD3 | DALR anticodon binding domain con | 0.061 | 1.044 | 4.59E-01 | 4.59E+01 | | | |

| | | | | | | | | | | |
|---|---|---|---|---|---|---|---|---|---|---|
| 7905051 | NM_00114388 | PPIAL4B | peptidylprolyl isomerase A (cyclophil | 0.061 | 1.043 | 2.94E-01 | 2.94E+01 | | | |
| 7919162 | NM_00114388 | PPIAL4B | peptidylprolyl isomerase A (cyclophil | 0.061 | 1.043 | 2.94E-01 | 2.94E+01 | | | |
| 7939676 | NR_024587 | KIAA0652 | KIAA0652 | 0.061 | 1.043 | 6.83E-01 | 6.83E+01 | | | |
| 8074878 | --- | --- | --- | 0.061 | 1.043 | 5.16E-01 | 5.16E+01 | | | |
| 7908779 | NM_020216 | RNPEP | arginyl aminopeptidase (aminopepti | 0.061 | 1.043 | 6.32E-01 | 6.32E+01 | | | |
| 7941795 | --- | --- | --- | 0.061 | 1.043 | 6.29E-01 | 6.29E+01 | | | |
| 7948476 | NM_014502 | PRPF19 | PRP19/PSO4 pre-mRNA processing fa | 0.061 | 1.043 | 5.10E-01 | 5.10E+01 | | | |
| 7914500 | AF258548 | C1orf91 | chromosome 1 open reading frame 9 | 0.061 | 1.043 | 3.99E-01 | 3.99E+01 | | | |
| 7893875 | --- | --- | --- | 0.061 | 1.043 | 5.25E-01 | 5.25E+01 | | | |
| 8074890 | --- | --- | --- | 0.061 | 1.043 | 8.54E-01 | 8.54E+01 | | | |
| 8114511 | NM_016459 | MGC29506 | hypothetical protein MGC29506 | 0.061 | 1.043 | 6.88E-01 | 6.88E+01 | | | |
| 7974870 | NM_003082 | SNAPC1 | small nuclear RNA activating comple | 0.061 | 1.043 | 6.92E-01 | 6.92E+01 | | | |
| 8045148 | ENST0000042 | FAM128A | family with sequence similarity 128, | 0.061 | 1.043 | 5.24E-01 | 5.24E+01 | | | |
| 8055284 | ENST0000042 | FAM128A | family with sequence similarity 128, | 0.061 | 1.043 | 5.24E-01 | 5.24E+01 | | | |
| 7972055 | NM_138444 | KCTD12 | potassium channel tetramerisation d | 0.061 | 1.043 | 3.41E-01 | 3.41E+01 | | | |
| 8065762 | NM_080476 | PIGU | phosphatidylinositol glycan anchor b | 0.061 | 1.043 | 7.12E-01 | 7.12E+01 | | | |
| 8042052 | NM_002954 | RPS27A | ribosomal protein S27a | 0.061 | 1.043 | 2.85E-01 | 2.85E+01 | | | |
| 8152053 | NM_198401 | ANKRD46 | ankyrin repeat domain 46 | 0.061 | 1.043 | 6.14E-01 | 6.14E+01 | | | |
| 8120883 | NM_015018 | DOPEY1 | dopey family member 1 | 0.061 | 1.043 | 6.75E-01 | 6.75E+01 | | | |
| 8026155 | NM_052876 | NACC1 | nucleus accumbens associated 1, BE | 0.061 | 1.043 | 6.65E-01 | 6.65E+01 | | | |
| 8155332 | NM_00103716 | C7orf70 | chromosome 7 open reading frame 7 | 0.061 | 1.043 | 6.18E-01 | 6.18E+01 | | | |
| 7992205 | NM_194259 | UBE2I | ubiquitin-conjugating enzyme E2I (U | 0.061 | 1.043 | 5.36E-01 | 5.36E+01 | | | |
| 7898875 | NM_000975 | RPL11 | ribosomal protein L11 | 0.061 | 1.043 | 6.04E-01 | 6.04E+01 | | | |
| 8162706 | NM_016481 | C9orf156 | chromosome 9 open reading frame 1 | 0.061 | 1.043 | 7.46E-01 | 7.46E+01 | | | |
| 7995631 | NM_005611 | RBL2 | retinoblastoma-like 2 (p130) | 0.061 | 1.043 | 7.97E-01 | 7.97E+01 | | | |
| 7895489 | --- | --- | --- | 0.061 | 1.043 | 6.73E-01 | 6.73E+01 | | | |
| 8130768 | NM_003730 | RNASET2 | ribonuclease T2 | 0.061 | 1.043 | 6.65E-01 | 6.65E+01 | | | |
| 8063484 | NM_00103352 | CSTF1 | cleavage stimulation factor, 3' pre-R | 0.061 | 1.043 | 6.40E-01 | 6.40E+01 | | | |
| 7938777 | NM_005566 | LDHA | lactate dehydrogenase A | 0.061 | 1.043 | 8.34E-01 | 8.34E+01 | | | |
| 8092418 | NM_005688 | ABCC5 | ATP-binding cassette, sub-family C (C | 0.061 | 1.043 | 3.60E-01 | 3.60E+01 | | | |
| 8115847 | --- | --- | --- | 0.061 | 1.043 | 7.03E-01 | 7.03E+01 | | | |
| 8180388 | --- | --- | --- | 0.061 | 1.043 | 8.77E-01 | 8.77E+01 | | | |
| 8164649 | NM_013318 | BAT2L | HLA-B associated transcript 2-like | 0.060 | 1.043 | 5.52E-01 | 5.52E+01 | | | |
| 7906128 | NM_014655 | SLC25A44 | solute carrier family 25, member 44 | 0.060 | 1.043 | 6.93E-01 | 6.93E+01 | | | |
| 8024062 | NM_001928 | CFD | complement factor D (adipsin) | 0.060 | 1.043 | 4.38E-01 | 4.38E+01 | | | |
| 7945275 | NM_052875 | VPS26B | vacuolar protein sorting 26 homolog | 0.060 | 1.043 | 6.54E-01 | 6.54E+01 | | | |
| 8161919 | NM_005077 | TLE1 | transducin-like enhancer of split 1 (E | 0.060 | 1.043 | 6.20E-01 | 6.20E+01 | | | |
| 7910241 | NM_000858 | GUK1 | guanylate kinase 1 | 0.060 | 1.043 | 7.64E-01 | 7.64E+01 | | | |
| 7923885 | NM_014002 | IKBKE | inhibitor of kappa light polypeptide g | 0.060 | 1.043 | 5.63E-01 | 5.63E+01 | | | |
| 7896472 | --- | --- | --- | 0.060 | 1.043 | 9.23E-01 | 9.23E+01 | | | |
| 7896218 | --- | --- | --- | 0.060 | 1.043 | 7.55E-01 | 7.55E+01 | | | |
| 7894277 | --- | --- | --- | 0.060 | 1.043 | 7.20E-01 | 7.20E+01 | | | |
| 8046628 | NM_152945 | RBM45 | RNA binding motif protein 45 | 0.060 | 1.043 | 8.48E-01 | 8.48E+01 | | | |
| 7892987 | --- | --- | --- | 0.060 | 1.043 | 2.10E-01 | 2.10E+01 | | | |
| 8073135 | NM_019008 | SMCR7L | Smith-Magenis syndrome chromoso | 0.060 | 1.043 | 4.58E-01 | 4.58E+01 | | | |
| 8111455 | NR_003504 | GUSBL1 | glucuronidase, beta-like 1 | 0.060 | 1.043 | 8.98E-01 | 8.98E+01 | | | |
| 7893445 | --- | --- | --- | 0.060 | 1.043 | 9.63E-01 | 9.63E+01 | | | |
| 7962579 | NM_00114366 | AMIGO2 | adhesion molecule with Ig-like doma | 0.060 | 1.043 | 5.80E-01 | 5.80E+01 | | | |
| 8084607 | NM_021627 | SENP2 | SUMO1/sentrin/SMT3 specific pepti | 0.060 | 1.043 | 8.36E-01 | 8.36E+01 | | | |
| 8111814 | NM_000997 | RPL37 | ribosomal protein L37 | 0.060 | 1.042 | 6.44E-01 | 6.44E+01 | | | |
| 7924582 | NM_025160 | WDR26 | WD repeat domain 26 | 0.060 | 1.042 | 8.18E-01 | 8.18E+01 | | | |
| 7994353 | NM_018690 | APOB48R | apolipoprotein B48 receptor | 0.060 | 1.042 | 5.23E-01 | 5.23E+01 | | | |
| 7894278 | --- | --- | --- | 0.060 | 1.042 | 3.92E-01 | 3.92E+01 | | | |
| 8168976 | NM_00100405 | GPRASP2 | G protein-coupled receptor associate | 0.060 | 1.042 | 5.17E-01 | 5.17E+01 | | | |
| 7937696 | NM_00100432 | KRTAP5-2 | keratin associated protein 5-2 | 0.060 | 1.042 | 5.34E-01 | 5.34E+01 | | | |
| 8060539 | NM_002836 | PTPRA | protein tyrosine phosphatase, recept | 0.060 | 1.042 | 5.25E-01 | 5.25E+01 | | | |
| 7894078 | --- | --- | --- | 0.060 | 1.042 | 6.85E-01 | 6.85E+01 | | | |
| 8043932 | NM_00109969 | RPL31 | ribosomal protein L31 | 0.060 | 1.042 | 8.31E-01 | 8.31E+01 | | | |
| 8146649 | NM_014637 | MTFR1 | mitochondrial fission regulator 1 | 0.060 | 1.042 | 6.54E-01 | 6.54E+01 | | | |
| 7980098 | NM_005589 | ALDH6A1 | aldehyde dehydrogenase 6 family, m | 0.060 | 1.042 | 5.53E-01 | 5.53E+01 | | | |
| 7893759 | --- | --- | --- | 0.059 | 1.042 | 9.11E-01 | 9.11E+01 | | | |
| 8147970 | NM_198120 | EBAG9 | estrogen receptor binding site assoc | 0.059 | 1.042 | 8.51E-01 | 8.51E+01 | | | |
| 8121729 | NM_002667 | PLN | phospholamban | 0.059 | 1.042 | 7.67E-01 | 7.67E+01 | | | |
| 7905058 | NR_027002 | LOC388692 | hypothetical LOC388692 | 0.059 | 1.042 | 8.43E-01 | 8.43E+01 | | | |
| 7929976 | NM_015448 | P11-529I10. | deleted in a mouse model of primary | 0.059 | 1.042 | 4.84E-01 | 4.84E+01 | | | |
| 8145085 | NM_001722 | POLR3D | polymerase (RNA) III (DNA directed) | 0.059 | 1.042 | 6.86E-01 | 6.86E+01 | | | |
| 7908407 | NM_002954 | RPS27A | ribosomal protein S27a | 0.059 | 1.042 | 1.79E-01 | 1.79E+01 | | | |
| 8064637 | NM_00100998 | C20orf194 | chromosome 20 open reading frame | 0.059 | 1.042 | 5.86E-01 | 5.86E+01 | | | |
| 8076137 | NM_015374 | UNC84B | unc-84 homolog B (C. elegans) | 0.059 | 1.042 | 5.38E-01 | 5.38E+01 | | | |
| 8175169 | NM_021183 | RAP2C | RAP2C, member of RAS oncogene fa | 0.059 | 1.042 | 7.80E-01 | 7.80E+01 | | | |
| 8158544 | NM_014064 | METTL11A | methyltransferase like 11A | 0.059 | 1.042 | 5.53E-01 | 5.53E+01 | | | |
| 8167201 | NM_007137 | ZNF81 | zinc finger protein 81 | 0.059 | 1.042 | 4.12E-01 | 4.12E+01 | | | |
| 8066303 | NM_032221 | CHD6 | chromodomain helicase DNA binding | 0.059 | 1.042 | 7.90E-01 | 7.90E+01 | | | |
| 7897172 | NM_182752 | TPRG1L | tumor protein p63 regulated 1-like | 0.059 | 1.042 | 7.36E-01 | 7.36E+01 | | | |
| 8006237 | ENST0000043 | LOC400590 | hypothetical LOC400590 | 0.059 | 1.042 | 7.62E-01 | 7.62E+01 | | | |
| 7893231 | --- | --- | --- | 0.059 | 1.042 | 5.32E-01 | 5.32E+01 | | | |
| 8142554 | NM_005763 | AASS | aminoadipate-semialdehyde synthas | 0.059 | 1.042 | 4.99E-01 | 4.99E+01 | | | |

| | | | | | | | | | | |
|---|---|---|---|---|---|---|---|---|---|---|
| 8031871 | NM_133502 | ZNF274 | zinc finger protein 274 | 0.059 | 1.042 | 4.07E-01 | 4.07E+01 | | | |
| 8133215 | NM_022906 | STAG3L4 | stromal antigen 3-like 4 | 0.059 | 1.042 | 8.66E-01 | 8.66E+01 | | | |
| 8018305 | NM_016185 | HN1 | hematological and neurological expr | 0.059 | 1.042 | 6.90E-01 | 6.90E+01 | | | |
| 8019532 | NM_006822 | RAB40B | RAB40B, member RAS oncogene fam | 0.059 | 1.042 | 4.04E-01 | 4.04E+01 | | | |
| 8162449 | NM_031486 | ZNF484 | zinc finger protein 484 | 0.059 | 1.042 | 8.95E-01 | 8.95E+01 | | | |
| 7959070 | NM_002567 | PEBP1 | phosphatidylethanolamine binding p | 0.059 | 1.042 | 6.89E-01 | 6.89E+01 | | | |
| 8030569 | NM_015428 | ZNF473 | zinc finger protein 473 | 0.059 | 1.042 | 6.01E-01 | 6.01E+01 | | | |
| 8125687 | NM_004761 | RGL2 | ral guanine nucleotide dissociation st | 0.059 | 1.042 | 4.81E-01 | 4.81E+01 | | | |
| 7896392 | --- | --- | --- | 0.059 | 1.042 | 9.01E-01 | 9.01E+01 | | | |
| 8017437 | NM_017647 | FTSJ3 | FtsJ homolog 3 (E. coli) | 0.059 | 1.042 | 6.88E-01 | 6.88E+01 | | | |
| 8106479 | NM_004866 | SCAMP1 | secretory carrier membrane protein | 0.059 | 1.041 | 8.14E-01 | 8.14E+01 | | | |
| 7928800 | NM_018999 | FAM190B | family with sequence similarity 190, | 0.059 | 1.041 | 8.18E-01 | 8.18E+01 | | | |
| 8060949 | NM_022096 | ANKRD5 | ankyrin repeat domain 5 | 0.059 | 1.041 | 6.76E-01 | 6.76E+01 | | | |
| 7895964 | --- | --- | --- | 0.058 | 1.041 | 7.79E-01 | 7.79E+01 | | | |
| 8178955 | NM_004761 | RGL2 | ral guanine nucleotide dissociation st | 0.058 | 1.041 | 5.93E-01 | 5.93E+01 | | | |
| 8180144 | NM_004761 | RGL2 | ral guanine nucleotide dissociation st | 0.058 | 1.041 | 5.93E-01 | 5.93E+01 | | | |
| 8102643 | NM_001237 | CCNA2 | cyclin A2 | 0.058 | 1.041 | 5.84E-01 | 5.84E+01 | | | |
| 8021275 | NM_007195 | POLI | polymerase (DNA directed) iota | 0.058 | 1.041 | 8.14E-01 | 8.14E+01 | | | |
| 8060427 | NM_080836 | STK35 | serine/threonine kinase 35 | 0.058 | 1.041 | 7.54E-01 | 7.54E+01 | | | |
| 7944262 | --- | --- | --- | 0.058 | 1.041 | 5.23E-01 | 5.23E+01 | | | |
| 7953715 | NR_024260 | NECAP1 | NECAP endocytosis associated 1 | 0.058 | 1.041 | 8.34E-01 | 8.34E+01 | | | |
| 7892900 | --- | --- | --- | 0.058 | 1.041 | 6.10E-01 | 6.10E+01 | | | |
| 8058415 | NM_017759 | INO80D | INO80 complex subunit D | 0.058 | 1.041 | 8.02E-01 | 8.02E+01 | | | |
| 8155169 | NM_021111 | RECK | reversion-inducing-cysteine-rich prot | 0.058 | 1.041 | 4.67E-01 | 4.67E+01 | | | |
| 8156026 | NM_00109880 | CEP78 | centrosomal protein 78kDa | 0.058 | 1.041 | 9.03E-01 | 9.03E+01 | | | |
| 7919153 | --- | --- | --- | 0.058 | 1.041 | 9.32E-01 | 9.32E+01 | | | |
| 7919403 | --- | --- | --- | 0.058 | 1.041 | 9.32E-01 | 9.32E+01 | | | |
| 7974146 | NM_017922 | PRPF39 | PRP39 pre-mRNA processing factor 3 | 0.058 | 1.041 | 8.70E-01 | 8.70E+01 | | | |
| 8043687 | AK304740 | ANKRD36 | ankyrin repeat domain 36 | 0.058 | 1.041 | 7.70E-01 | 7.70E+01 | | | |
| 7971620 | NM_002267 | KPNA3 | karyopherin alpha 3 (importin alpha | 0.058 | 1.041 | 8.62E-01 | 8.62E+01 | | | |
| 7956114 | NM_001029 | RPS26 | ribosomal protein S26 | 0.058 | 1.041 | 7.47E-01 | 7.47E+01 | | | |
| 8151987 | --- | --- | --- | 0.058 | 1.041 | 8.83E-01 | 8.83E+01 | | | |
| 7895501 | --- | --- | --- | 0.058 | 1.041 | 8.69E-01 | 8.69E+01 | | | |
| 7895753 | --- | --- | --- | 0.058 | 1.041 | 9.32E-01 | 9.32E+01 | | | |
| 7893783 | --- | --- | --- | 0.058 | 1.041 | 5.43E-01 | 5.43E+01 | | | |
| 7895778 | --- | --- | --- | 0.057 | 1.041 | 8.39E-01 | 8.39E+01 | | | |
| 7894224 | --- | --- | --- | 0.057 | 1.041 | 7.40E-01 | 7.40E+01 | | | |
| 7992998 | NM_00114229 | MGRN1 | mahogunin, ring finger 1 | 0.057 | 1.041 | 3.46E-01 | 3.46E+01 | | | |
| 8032455 | NM_018049 | PLEKHJ1 | pleckstrin homology domain contain | 0.057 | 1.041 | 3.80E-01 | 3.80E+01 | | | |
| 8069026 | NM_004649 | C21orf33 | chromosome 21 open reading frame | 0.057 | 1.041 | 4.85E-01 | 4.85E+01 | | | |
| 8019737 | NM_002266 | KPNA2 | karyopherin alpha 2 (RAG cohort 1, i | 0.057 | 1.041 | 7.25E-01 | 7.25E+01 | | | |
| 7897561 | NM_015074 | KIF1B | kinesin family member 1B | 0.057 | 1.041 | 4.54E-01 | 4.54E+01 | | | |
| 8132055 | NM_152793 | C7orf41 | chromosome 7 open reading frame 4 | 0.057 | 1.041 | 5.77E-01 | 5.77E+01 | | | |
| 7917645 | NR_003287 | OC10000858 | 28S ribosomal RNA | 0.057 | 1.041 | 9.62E-02 | 9.62E+00 | | | |
| 8107470 | NM_002823 | PTMA | prothymosin, alpha | 0.057 | 1.041 | 1.55E-01 | 1.55E+01 | | | |
| 7892853 | --- | --- | --- | 0.057 | 1.040 | 2.31E-01 | 2.31E+01 | | | |
| 8048761 | --- | --- | --- | 0.057 | 1.040 | 8.20E-01 | 8.20E+01 | | | |
| 8013985 | --- | --- | --- | 0.057 | 1.040 | 7.87E-01 | 7.87E+01 | | | |
| 7894954 | --- | --- | --- | 0.057 | 1.040 | 7.31E-01 | 7.31E+01 | | | |
| 7933582 | NM_006327 | TIMM23 | translocase of inner mitochondrial m | 0.057 | 1.040 | 8.99E-01 | 8.99E+01 | | | |
| 8180034 | NM_000544 | TAP2 | transporter 2, ATP-binding cassette, | 0.057 | 1.040 | 5.35E-01 | 5.35E+01 | | | |
| 7946565 | --- | --- | --- | 0.057 | 1.040 | 5.46E-02 | 5.46E+00 | | | |
| 8043131 | NM_00113502 | ELMOD3 | ELMO/CED-12 domain containing 3 | 0.057 | 1.040 | 3.39E-01 | 3.39E+01 | | | |
| 8096160 | NM_00102561 | ARHGAP24 | Rho GTPase activating protein 24 | 0.057 | 1.040 | 5.89E-01 | 5.89E+01 | | | |
| 8091809 | --- | --- | --- | 0.057 | 1.040 | 6.79E-01 | 6.79E+01 | | | |
| 7950016 | NM_153451 | ORAOV1 | oral cancer overexpressed 1 | 0.057 | 1.040 | 7.24E-01 | 7.24E+01 | | | |
| 7894041 | --- | --- | --- | 0.057 | 1.040 | 8.15E-01 | 8.15E+01 | | | |
| 7954961 | --- | --- | --- | 0.057 | 1.040 | 4.95E-01 | 4.95E+01 | | | |
| 8131600 | NM_014399 | TSPAN13 | tetraspanin 13 | 0.057 | 1.040 | 5.77E-01 | 5.77E+01 | | | |
| 8180327 | --- | --- | --- | 0.057 | 1.040 | 7.69E-01 | 7.69E+01 | | | |
| 8178552 | NM_021184 | C6orf47 | chromosome 6 open reading frame 4 | 0.057 | 1.040 | 5.23E-01 | 5.23E+01 | | | |
| 8179808 | NM_021184 | C6orf47 | chromosome 6 open reading frame 4 | 0.057 | 1.040 | 5.23E-01 | 5.23E+01 | | | |
| 8026868 | NM_000980 | RPL18A | ribosomal protein L18a | 0.057 | 1.040 | 5.24E-01 | 5.24E+01 | | | |
| 8112916 | --- | --- | --- | 0.057 | 1.040 | 6.03E-02 | 6.03E+00 | | | |
| 8174970 | --- | --- | --- | 0.057 | 1.040 | 6.03E-02 | 6.03E+00 | | | |
| 8114709 | NM_033449 | FCHSD1 | FCH and double SH3 domains 1 | 0.057 | 1.040 | 5.26E-01 | 5.26E+01 | | | |
| 7892707 | --- | --- | --- | 0.057 | 1.040 | 9.00E-01 | 9.00E+01 | | | |
| 7942592 | NR_000005 | SNORD15A | small nucleolar RNA, C/D box 15A | 0.057 | 1.040 | 9.05E-01 | 9.05E+01 | | | |
| 8086048 | NM_00103977 | TMPPE | transmembrane protein with metallo | 0.057 | 1.040 | 5.03E-01 | 5.03E+01 | | | |
| 8143307 | NM_022740 | HIPK2 | homeodomain interacting protein ki | 0.057 | 1.040 | 6.17E-01 | 6.17E+01 | | | |
| 8077858 | NM_006395 | ATG7 | ATG7 autophagy related 7 homolog ( | 0.057 | 1.040 | 8.18E-01 | 8.18E+01 | | | |
| 8063453 | NM_002623 | PFDN4 | prefoldin subunit 4 | 0.057 | 1.040 | 9.11E-01 | 9.11E+01 | | | |
| 8066697 | NM_173179 | SLC35C2 | solute carrier family 35, member C2 | 0.056 | 1.040 | 5.58E-01 | 5.58E+01 | | | |
| 7975815 | NM_015072 | TTLL5 | tubulin tyrosine ligase-like family, me | 0.056 | 1.040 | 6.18E-01 | 6.18E+01 | | | |
| 7893515 | --- | --- | --- | 0.056 | 1.040 | 6.23E-01 | 6.23E+01 | | | |
| 7892706 | --- | --- | --- | 0.056 | 1.040 | 9.33E-01 | 9.33E+01 | | | |
| 8031223 | NM_006669 | LILRB1 | leukocyte immunoglobulin-like recep | 0.056 | 1.040 | 5.31E-01 | 5.31E+01 | | | |

| | | | | | | | | | | |
|---|---|---|---|---|---|---|---|---|---|---|
| 8165650 | --- | --- | --- | 0.056 | 1.040 | 1.16E-01 | 1.16E+01 | | | |
| 8026513 | NM_00114516( | TPM4 | tropomyosin 4 | 0.056 | 1.040 | 3.97E-01 | 3.97E+01 | | | |
| 7973652 | NM_174944 | TSSK4 | testis-specific serine kinase 4 | 0.056 | 1.040 | 6.64E-01 | 6.64E+01 | | | |
| 7948274 | NM_004223 | UBE2L6 | ubiquitin-conjugating enzyme E2L 6 | 0.056 | 1.040 | 5.40E-01 | 5.40E+01 | | | |
| 7894578 | --- | --- | --- | 0.056 | 1.040 | 5.95E-01 | 5.95E+01 | | | |
| 7970973 | --- | --- | --- | 0.056 | 1.040 | 8.96E-01 | 8.96E+01 | | | |
| 7895640 | --- | --- | --- | 0.056 | 1.040 | 8.98E-01 | 8.98E+01 | | | |
| 7971167 | NR_024507 | LOC646982 | twelve-thirteen translocation leuken | 0.056 | 1.040 | 3.66E-01 | 3.66E+01 | | | |
| 7933180 | NM_00109928: | ZNF239 | zinc finger protein 239 | 0.056 | 1.040 | 3.97E-01 | 3.97E+01 | | | |
| 8149208 | ENST00000420 | OR7E125P | olfactory receptor, family 7, subfami | 0.056 | 1.040 | 6.78E-01 | 6.78E+01 | | | |
| 7944365 | NM_198489 | CCDC84 | coiled-coil domain containing 84 | 0.056 | 1.040 | 7.97E-01 | 7.97E+01 | | | |
| 8084794 | NM_002182 | IL1RAP | interleukin 1 receptor accessory prot | 0.056 | 1.040 | 5.37E-01 | 5.37E+01 | | | |
| 7892967 | --- | --- | --- | 0.056 | 1.040 | 9.62E-01 | 9.62E+01 | | | |
| 7894970 | --- | --- | --- | 0.056 | 1.039 | 1.85E-01 | 1.85E+01 | | | |
| 8017850 | NM_017983 | WIPI1 | WD repeat domain, phosphoinositide | 0.056 | 1.039 | 5.57E-01 | 5.57E+01 | | | |
| 8085052 | --- | --- | --- | 0.056 | 1.039 | 8.16E-01 | 8.16E+01 | | | |
| 8117888 | NM_006325 | RAN | RAN, member RAS oncogene family | 0.056 | 1.039 | 8.37E-01 | 8.37E+01 | | | |
| 7928369 | NM_004922 | SEC24C | SEC24 family, member C (S. cerevisia | 0.056 | 1.039 | 6.56E-01 | 6.56E+01 | | | |
| 7894815 | --- | --- | --- | 0.056 | 1.039 | 9.11E-01 | 9.11E+01 | | | |
| 7949896 | ENST00000454 | / OR7E87P | olfactory receptor, family 7, subfami | 0.056 | 1.039 | 6.78E-01 | 6.78E+01 | | | |
| 7935968 | NM_003893 | LDB1 | LIM domain binding 1 | 0.055 | 1.039 | 8.78E-01 | 8.78E+01 | | | |
| 7894415 | --- | --- | --- | 0.055 | 1.039 | 7.68E-01 | 7.68E+01 | | | |
| 8074606 | NM_017414 | USP18 | ubiquitin specific peptidase 18 | 0.055 | 1.039 | 8.45E-01 | 8.45E+01 | | | |
| 7895196 | --- | --- | --- | 0.055 | 1.039 | 2.54E-01 | 2.54E+01 | | | |
| 7893147 | --- | --- | --- | 0.055 | 1.039 | 2.33E-01 | 2.33E+01 | | | |
| 8005157 | NM_004278 | PIGL | phosphatidylinositol glycan anchor b | 0.055 | 1.039 | 6.91E-01 | 6.91E+01 | | | |
| 7894886 | --- | --- | --- | 0.055 | 1.039 | 8.45E-01 | 8.45E+01 | | | |
| 7929116 | NM_006413 | RPP30 | ribonuclease P/MRP 30kDa subunit | 0.055 | 1.039 | 8.53E-01 | 8.53E+01 | | | |
| 8044462 | NM_153712 | TTL | tubulin tyrosine ligase | 0.055 | 1.039 | 5.86E-01 | 5.86E+01 | | | |
| 8094499 | --- | --- | --- | 0.055 | 1.039 | 8.82E-01 | 8.82E+01 | | | |
| 7894889 | --- | --- | --- | 0.055 | 1.039 | 6.99E-01 | 6.99E+01 | | | |
| 8043203 | NM_006634 | VAMP5 | vesicle-associated membrane protei | 0.055 | 1.039 | 7.51E-01 | 7.51E+01 | | | |
| 7950332 | BC035599 | C2CD3 | C2 calcium-dependent domain conta | 0.055 | 1.039 | 5.93E-01 | 5.93E+01 | | | |
| 7919407 | NM_00114388: | PPIAL4B | peptidylprolyl isomerase A (cyclophil | 0.055 | 1.039 | 3.42E-01 | 3.42E+01 | | | |
| 8060418 | NM_00104002: | SIRPA | signal-regulatory protein alpha | 0.055 | 1.039 | 5.79E-01 | 5.79E+01 | | | |
| 8091554 | --- | --- | --- | 0.055 | 1.039 | 5.74E-01 | 5.74E+01 | | | |
| 8079753 | NM_004393 | DAG1 | dystroglycan 1 (dystrophin-associate | 0.055 | 1.039 | 4.20E-01 | 4.20E+01 | | | |
| 8173745 | NM_006639 | CYSLTR1 | cysteinyl leukotriene receptor 1 | 0.055 | 1.039 | 9.22E-01 | 9.22E+01 | | | |
| 8061715 | NM_004798 | KIF3B | kinesin family member 3B | 0.055 | 1.039 | 6.48E-01 | 6.48E+01 | | | |
| 8161114 | NM_020944 | GBA2 | glucosidase, beta (bile acid) 2 | 0.055 | 1.039 | 6.79E-01 | 6.79E+01 | | | |
| 7893866 | --- | --- | --- | 0.054 | 1.038 | 3.18E-01 | 3.18E+01 | | | |
| 7893285 | --- | --- | --- | 0.054 | 1.038 | 6.90E-01 | 6.90E+01 | | | |
| 7896541 | --- | --- | --- | 0.054 | 1.038 | 1.59E-01 | 1.59E+01 | | | |
| 8003217 | NM_006067 | COX4NB | COX4 neighbor | 0.054 | 1.038 | 7.77E-01 | 7.77E+01 | | | |
| 8132964 | NM_015411 | SUMF2 | sulfatase modifying factor 2 | 0.054 | 1.038 | 6.83E-01 | 6.83E+01 | | | |
| 7968417 | NM_023037 | FRY | furry homolog (Drosophila) | 0.054 | 1.038 | 4.94E-01 | 4.94E+01 | | | |
| 8033479 | NM_001419 | ELAVL1 | ELAV (embryonic lethal, abnormal vi: | 0.054 | 1.038 | 5.59E-01 | 5.59E+01 | | | |
| 8048772 | NM_032276 | RHBDD1 | rhomboid domain containing 1 | 0.054 | 1.038 | 7.66E-01 | 7.66E+01 | | | |
| 8157945 | NM_00109927( | ZBTB34 | zinc finger and BTB domain containir | 0.054 | 1.038 | 7.33E-01 | 7.33E+01 | | | |
| 8180328 | --- | --- | --- | 0.054 | 1.038 | 7.87E-01 | 7.87E+01 | | | |
| 7907024 | NM_017542 | POGK | pogo transposable element with KRA | 0.054 | 1.038 | 7.66E-01 | 7.66E+01 | | | |
| 8146955 | --- | --- | --- | 0.054 | 1.038 | 7.20E-01 | 7.20E+01 | | | |
| 8175621 | NM_032508 | TMEM185A | transmembrane protein 185A | 0.054 | 1.038 | 7.01E-01 | 7.01E+01 | | | |
| 7967056 | NM_016399 | TRIAP1 | TP53 regulated inhibitor of apoptosis | 0.054 | 1.038 | 6.63E-01 | 6.63E+01 | | | |
| 8173009 | NM_015107 | PHF8 | PHD finger protein 8 | 0.054 | 1.038 | 7.49E-01 | 7.49E+01 | | | |
| 8008682 | NM_138962 | MSI2 | musashi homolog 2 (Drosophila) | 0.054 | 1.038 | 7.44E-01 | 7.44E+01 | | | |
| 8144267 | NM_018941 | CLN8 | ceroid-lipofuscinosis, neuronal 8 (ep | 0.054 | 1.038 | 3.09E-01 | 3.09E+01 | | | |
| 7916229 | NM_018281 | ECHDC2 | enoyl Coenzyme A hydratase domair | 0.054 | 1.038 | 5.52E-01 | 5.52E+01 | | | |
| 8152041 | NM_183419 | RNF19A | ring finger protein 19A | 0.054 | 1.038 | 8.48E-01 | 8.48E+01 | | | |
| 7934477 | NM_172171 | CAMK2G | calcium/calmodulin-dependent prote | 0.054 | 1.038 | 5.93E-01 | 5.93E+01 | | | |
| 7904907 | NM_004326 | BCL9 | B-cell CLL/lymphoma 9 | 0.054 | 1.038 | 6.15E-01 | 6.15E+01 | | | |
| 8119227 | NM_021943 | ZFAND3 | zinc finger, AN1-type domain 3 | 0.054 | 1.038 | 6.45E-01 | 6.45E+01 | | | |
| 8015835 | NM_004090 | DUSP3 | dual specificity phosphatase 3 | 0.054 | 1.038 | 4.45E-01 | 4.45E+01 | | | |
| 7935474 | NM_022362 | MMS19 | MMS19 nucleotide excision repair ho | 0.054 | 1.038 | 5.59E-01 | 5.59E+01 | | | |
| 8106722 | NM_00101797: | ATP6AP1L | ATPase, H+ transporting, lysosomal a | 0.054 | 1.038 | 6.39E-01 | 6.39E+01 | | | |
| 7934161 | NM_005041 | PRF1 | perforin 1 (pore forming protein) | 0.054 | 1.038 | 7.03E-01 | 7.03E+01 | | | |
| 7896459 | --- | --- | --- | 0.054 | 1.038 | 9.45E-01 | 9.45E+01 | | | |
| 7977103 | --- | --- | --- | 0.054 | 1.038 | 7.83E-01 | 7.83E+01 | | | |
| 7893998 | --- | --- | --- | 0.054 | 1.038 | 9.31E-01 | 9.31E+01 | | | |
| 8180357 | --- | --- | --- | 0.053 | 1.038 | 9.04E-01 | 9.04E+01 | | | |
| 7893298 | --- | --- | --- | 0.053 | 1.038 | 6.69E-01 | 6.69E+01 | | | |
| 7892785 | --- | --- | --- | 0.053 | 1.038 | 9.50E-01 | 9.50E+01 | | | |
| 8152465 | NM_003756 | EIF3H | eukaryotic translation initiation facto | 0.053 | 1.038 | 8.38E-01 | 8.38E+01 | | | |
| 8110914 | --- | --- | --- | 0.053 | 1.038 | 4.69E-01 | 4.69E+01 | | | |
| 7902474 | --- | --- | --- | 0.053 | 1.038 | 8.77E-01 | 8.77E+01 | | | |
| 7959549 | NM_00100225: | ARL6IP4 | ADP-ribosylation-like factor 6 interac | 0.053 | 1.038 | 5.94E-01 | 5.94E+01 | | | |
| 8079693 | NM_018031 | WDR6 | WD repeat domain 6 | 0.053 | 1.038 | 5.28E-01 | 5.28E+01 | | | |

| | | | | | | | | | | |
|---|---|---|---|---|---|---|---|---|---|---|
| 7981387 | NM_006035 | CDC42BPB | CDC42 binding protein kinase beta (I | 0.053 | 1.038 | 5.09E-01 | 5.09E+01 | | | |
| 7975136 | NM_178155 | FUT8 | fucosyltransferase 8 (alpha (1,6) fuc | 0.053 | 1.038 | 8.17E-01 | 8.17E+01 | | | |
| 8013633 | NM_054035 | UNC119 | unc-119 homolog (C. elegans) | 0.053 | 1.037 | 7.43E-01 | 7.43E+01 | | | |
| 7895605 | --- | --- | --- | 0.053 | 1.037 | 8.86E-01 | 8.86E+01 | | | |
| 8025488 | NM_003451 | ZNF177 | zinc finger protein 177 | 0.053 | 1.037 | 3.97E-01 | 3.97E+01 | | | |
| 8155630 | ENST00000367 | MTHFD1L | methylenetetrahydrofolate dehydro | 0.053 | 1.037 | 7.48E-01 | 7.48E+01 | | | |
| 8161377 | ENST00000367 | MTHFD1L | methylenetetrahydrofolate dehydro | 0.053 | 1.037 | 7.48E-01 | 7.48E+01 | | | |
| 8118209 | NM_019101 | APOM | apolipoprotein M | 0.053 | 1.037 | 6.32E-01 | 6.32E+01 | | | |
| 8178043 | NM_019101 | APOM | apolipoprotein M | 0.053 | 1.037 | 6.32E-01 | 6.32E+01 | | | |
| 8179291 | NM_019101 | APOM | apolipoprotein M | 0.053 | 1.037 | 6.32E-01 | 6.32E+01 | | | |
| 8133062 | NR_003099 | ZNF273 | zinc finger protein 273 | 0.053 | 1.037 | 7.77E-01 | 7.77E+01 | | | |
| 7907404 | NM_014283 | C1orf9 | chromosome 1 open reading frame 9 | 0.053 | 1.037 | 7.61E-01 | 7.61E+01 | | | |
| 7896494 | --- | --- | --- | 0.053 | 1.037 | 5.75E-01 | 5.75E+01 | | | |
| 8136473 | NM_015905 | TRIM24 | tripartite motif-containing 24 | 0.053 | 1.037 | 6.42E-01 | 6.42E+01 | | | |
| 8107996 | --- | --- | --- | 0.053 | 1.037 | 7.85E-01 | 7.85E+01 | | | |
| 8031640 | NM_152478 | ZNF583 | zinc finger protein 583 | 0.053 | 1.037 | 6.90E-01 | 6.90E+01 | | | |
| 7893469 | --- | --- | --- | 0.053 | 1.037 | 3.49E-01 | 3.49E+01 | | | |
| 8024582 | NM_003775 | S1PR4 | sphingosine-1-phosphate receptor 4 | 0.053 | 1.037 | 5.40E-01 | 5.40E+01 | | | |
| 8039166 | NM_024298 | MBOAT7 | membrane bound O-acyltransferase | 0.053 | 1.037 | 4.31E-01 | 4.31E+01 | | | |
| 7956013 | NM_001487 | BLOC1S1 | biogenesis of lysosomal organelles c | 0.053 | 1.037 | 8.63E-01 | 8.63E+01 | | | |
| 8063697 | NM_198976 | TH1L | TH1-like (Drosophila) | 0.053 | 1.037 | 7.50E-01 | 7.50E+01 | | | |
| 8022972 | NM_000972 | RPL7A | ribosomal protein L7a | 0.053 | 1.037 | 8.31E-01 | 8.31E+01 | | | |
| 8149258 | NM_004225 | MFHAS1 | malignant fibrous histiocytoma ampl | 0.053 | 1.037 | 6.93E-01 | 6.93E+01 | | | |
| 8149847 | --- | --- | --- | 0.053 | 1.037 | 6.35E-01 | 6.35E+01 | | | |
| 7986639 | ENST00000338 | VSIG6 | V-set and immunoglobulin domain c | 0.053 | 1.037 | 6.46E-01 | 6.46E+01 | | | |
| 7894356 | --- | --- | --- | 0.052 | 1.037 | 7.98E-01 | 7.98E+01 | | | |
| 7949674 | NM_031492 | RBM4B | RNA binding motif protein 4B | 0.052 | 1.037 | 7.27E-01 | 7.27E+01 | | | |
| 8165156 | NM_00103970 | SDCCAG3 | serologically defined colon cancer ar | 0.052 | 1.037 | 6.19E-01 | 6.19E+01 | | | |
| 7927981 | NM_003171 | SUPV3L1 | suppressor of var1, 3-like 1 (S. cerevi | 0.052 | 1.037 | 8.65E-01 | 8.65E+01 | | | |
| 8042574 | NR_002822 | MGC72080 | MGC72080 pseudogene | 0.052 | 1.037 | 8.79E-01 | 8.79E+01 | | | |
| 8034313 | NR_002944 | HNRPA1L-2 | heterogeneous nuclear ribonucleopr | 0.052 | 1.037 | 6.06E-01 | 6.06E+01 | | | |
| 7959173 | NM_014868 | RNF10 | ring finger protein 10 | 0.052 | 1.037 | 6.65E-01 | 6.65E+01 | | | |
| 7970370 | NM_023011 | UPF3A | UPF3 regulator of nonsense transcri | 0.052 | 1.037 | 6.27E-01 | 6.27E+01 | | | |
| 8089230 | --- | --- | --- | 0.052 | 1.037 | 7.36E-01 | 7.36E+01 | | | |
| 7908147 | NR_023349 | TSEN15 | tRNA splicing endonuclease 15 homo | 0.052 | 1.037 | 7.14E-01 | 7.14E+01 | | | |
| 8104506 | NM_007118 | TRIO | triple functional domain (PTPRF inte | 0.052 | 1.037 | 5.16E-01 | 5.16E+01 | | | |
| 7895842 | --- | --- | --- | 0.052 | 1.037 | 8.97E-01 | 8.97E+01 | | | |
| 7996313 | NM_144673 | CMTM2 | CKLF-like MARVEL transmembrane d | 0.052 | 1.037 | 6.42E-01 | 6.42E+01 | | | |
| 8043585 | NM_004804 | CIAO1 | cytosolic iron-sulfur protein assembl | 0.052 | 1.037 | 7.68E-01 | 7.68E+01 | | | |
| 8084818 | NM_178335 | CCDC50 | coiled-coil domain containing 50 | 0.052 | 1.037 | 7.93E-01 | 7.93E+01 | | | |
| 8165646 | --- | --- | --- | 0.052 | 1.037 | 8.51E-01 | 8.51E+01 | | | |
| 8018189 | NM_181449 | CD300E | CD300e molecule | 0.052 | 1.036 | 4.58E-01 | 4.58E+01 | | | |
| 7963590 | NM_015989 | CSAD | cysteine sulfinic acid decarboxylase | 0.052 | 1.036 | 6.62E-01 | 6.62E+01 | | | |
| 7954717 | NM_001714 | BICD1 | bicaudal D homolog 1 (Drosophila) | 0.052 | 1.036 | 6.70E-01 | 6.70E+01 | | | |
| 7893660 | --- | --- | --- | 0.052 | 1.036 | 5.93E-01 | 5.93E+01 | | | |
| 8126371 | NM_001760 | CCND3 | cyclin D3 | 0.052 | 1.036 | 5.57E-01 | 5.57E+01 | | | |
| 7901376 | NM_014372 | RNF11 | ring finger protein 11 | 0.052 | 1.036 | 8.03E-01 | 8.03E+01 | | | |
| 8120382 | NM_00103162 | ZNF451 | zinc finger protein 451 | 0.052 | 1.036 | 8.59E-01 | 8.59E+01 | | | |
| 7981872 | --- | --- | --- | 0.052 | 1.036 | 7.24E-01 | 7.24E+01 | | | |
| 7896186 | --- | --- | --- | 0.052 | 1.036 | 9.36E-01 | 9.36E+01 | | | |
| 8150087 | --- | --- | --- | 0.052 | 1.036 | 5.80E-01 | 5.80E+01 | | | |
| 8145272 | NM_00113610 | R3HCC1 | R3H domain and coiled-coil containi | 0.051 | 1.036 | 7.25E-01 | 7.25E+01 | | | |
| 7990253 | NM_012428 | NPTN | neuroplastin | 0.051 | 1.036 | 8.14E-01 | 8.14E+01 | | | |
| 8156748 | --- | --- | --- | 0.051 | 1.036 | 9.37E-01 | 9.37E+01 | | | |
| 7970542 | --- | --- | --- | 0.051 | 1.036 | 8.37E-01 | 8.37E+01 | | | |
| 8108217 | NM_000358 | TGFBI | transforming growth factor, beta-ind | 0.051 | 1.036 | 5.73E-01 | 5.73E+01 | | | mono |
| 7896461 | --- | --- | --- | 0.051 | 1.036 | 9.38E-01 | 9.38E+01 | | | |
| 7896266 | --- | --- | --- | 0.051 | 1.036 | 4.15E-01 | 4.15E+01 | | | |
| 7893676 | --- | --- | --- | 0.051 | 1.036 | 9.54E-01 | 9.54E+01 | | | |
| 7947490 | NM_015957 | APIP | APAF1 interacting protein | 0.051 | 1.036 | 6.36E-01 | 6.36E+01 | | | |
| 8180387 | --- | --- | --- | 0.051 | 1.036 | 9.03E-01 | 9.03E+01 | | | |
| 7953844 | NM_004400 | DDX12 | DEAD/H (Asp-Glu-Ala-Asp/His) box p | 0.051 | 1.036 | 3.61E-01 | 3.61E+01 | | | |
| 8044263 | NM_006267 | RANBP2 | RAN binding protein 2 | 0.051 | 1.036 | 8.31E-01 | 8.31E+01 | | | |
| 7894900 | --- | --- | --- | 0.051 | 1.036 | 8.18E-01 | 8.18E+01 | | | |
| 8102410 | NM_001029 | RPS26 | ribosomal protein S26 | 0.051 | 1.036 | 7.96E-01 | 7.96E+01 | | | |
| 7893448 | --- | --- | --- | 0.051 | 1.036 | 6.40E-01 | 6.40E+01 | | | |
| 8132406 | NM_005402 | RALA | v-ral simian leukemia viral oncogene | 0.051 | 1.036 | 7.34E-01 | 7.34E+01 | | | |
| 8080714 | NM_001457 | FLNB | filamin B, beta | 0.051 | 1.036 | 6.62E-01 | 6.62E+01 | | | |
| 8158022 | NM_007135 | ZNF79 | zinc finger protein 79 | 0.051 | 1.036 | 5.98E-01 | 5.98E+01 | | | |
| 7895994 | --- | --- | --- | 0.051 | 1.036 | 8.41E-01 | 8.41E+01 | | | |
| 8084232 | NM_018023 | YEATS2 | YEATS domain containing 2 | 0.051 | 1.036 | 5.75E-01 | 5.75E+01 | | | |
| 7894153 | --- | --- | --- | 0.051 | 1.036 | 9.55E-01 | 9.55E+01 | | | |
| 7947358 | NM_002901 | RCN1 | reticulocalbin 1, EF-hand calcium bin | 0.051 | 1.036 | 7.86E-01 | 7.86E+01 | | | |
| 7996891 | NM_032830 | CIRH1A | cirrhosis, autosomal recessive 1A (ci | 0.051 | 1.036 | 8.70E-01 | 8.70E+01 | | | |
| 8060675 | NM_021873 | CDC25B | cell division cycle 25 homolog B (S. p | 0.051 | 1.036 | 5.53E-01 | 5.53E+01 | | | |
| 8165735 | NM_00116153 | CSF2RA | colony stimulating factor 2 receptor, | 0.051 | 1.036 | 5.94E-01 | 5.94E+01 | | | |
| 8176306 | NM_00116153 | CSF2RA | colony stimulating factor 2 receptor, | 0.051 | 1.036 | 5.94E-01 | 5.94E+01 | | | |

| | | | | | | | | | | |
|---|---|---|---|---|---|---|---|---|---|---|
| 8037387 | --- | --- | --- | 0.051 | 1.036 | 9.60E-01 | 9.60E+01 | | | |
| 7958275 | NM_018082 | POLR3B | polymerase (RNA) III (DNA directed) | 0.051 | 1.036 | 7.35E-01 | 7.35E+01 | | | |
| 8104463 | NM_005885 | 40243 | membrane-associated ring finger (C3 | 0.050 | 1.036 | 7.88E-01 | 7.88E+01 | | | |
| 8059689 | NM_005381 | NCL | nucleolin | 0.050 | 1.036 | 5.53E-01 | 5.53E+01 | | | |
| 7894614 | --- | --- | --- | 0.050 | 1.036 | 9.01E-01 | 9.01E+01 | | | |
| 8112731 | NM_004101 | F2RL2 | coagulation factor II (thrombin) rece | 0.050 | 1.035 | 7.67E-01 | 7.67E+01 | | | |
| 7895374 | --- | --- | --- | 0.050 | 1.035 | 4.34E-01 | 4.34E+01 | | | |
| 7904883 | NM_004284 | CHD1L | chromodomain helicase DNA binding | 0.050 | 1.035 | 8.60E-01 | 8.60E+01 | | | |
| 8165665 | --- | --- | --- | 0.050 | 1.035 | 4.94E-01 | 4.94E+01 | | | |
| 8014073 | NM_018405 | C17orf79 | chromosome 17 open reading frame | 0.050 | 1.035 | 6.44E-01 | 6.44E+01 | | | |
| 8176219 | NM_00101802 | MTCP1 | mature T-cell proliferation 1 | 0.050 | 1.035 | 5.32E-01 | 5.32E+01 | | | |
| 8096025 | --- | --- | --- | 0.050 | 1.035 | 7.43E-01 | 7.43E+01 | | | |
| 7892530 | --- | --- | --- | 0.050 | 1.035 | 4.49E-01 | 4.49E+01 | | | |
| 7975595 | NM_024644 | C14orf169 | chromosome 14 open reading frame | 0.050 | 1.035 | 6.92E-01 | 6.92E+01 | | | |
| 8075106 | NM_003595 | TPST2 | tyrosylprotein sulfotransferase 2 | 0.050 | 1.035 | 5.79E-01 | 5.79E+01 | | | |
| 7986214 | NM_013272 | SLCO3A1 | solute carrier organic anion transpor | 0.050 | 1.035 | 6.09E-01 | 6.09E+01 | | | |
| 8169949 | NM_016542 | RP6-213H19. | serine/threonine protein kinase MST | 0.050 | 1.035 | 8.83E-01 | 8.83E+01 | | | |
| 8116807 | NM_152551 | SNRNP48 | small nuclear ribonucleoprotein 48k | 0.050 | 1.035 | 8.91E-01 | 8.91E+01 | | | |
| 8033899 | NM_003755 | EIF3G | eukaryotic translation initiation facto | 0.050 | 1.035 | 7.59E-01 | 7.59E+01 | | | |
| 7993973 | NM_018119 | POLR3E | polymerase (RNA) III (DNA directed) | 0.050 | 1.035 | 5.86E-01 | 5.86E+01 | | | |
| 8180259 | --- | --- | --- | 0.050 | 1.035 | 9.34E-01 | 9.34E+01 | | | |
| 8032484 | NM_012458 | TIMM13 | translocase of inner mitochondrial m | 0.050 | 1.035 | 7.83E-01 | 7.83E+01 | | | |
| 7929719 | NM_014472 | C10orf28 | chromosome 10 open reading frame | 0.050 | 1.035 | 8.51E-01 | 8.51E+01 | | | |
| 7936884 | NM_014661 | FAM53B | family with sequence similarity 53, m | 0.050 | 1.035 | 7.11E-01 | 7.11E+01 | | | |
| 8022814 | NM_002136 | HNRNPA1 | heterogeneous nuclear ribonucleopr | 0.050 | 1.035 | 6.74E-01 | 6.74E+01 | | | |
| 8124088 | NM_022113 | KIF13A | kinesin family member 13A | 0.050 | 1.035 | 5.08E-01 | 5.08E+01 | | | |
| 8174677 | AK298877 | CXorf56 | chromosome X open reading frame 5 | 0.050 | 1.035 | 6.46E-01 | 6.46E+01 | | | |
| 8097066 | NM_020961 | METTL14 | methyltransferase like 14 | 0.050 | 1.035 | 8.68E-01 | 8.68E+01 | | | |
| 7996174 | NM_024598 | C16orf57 | chromosome 16 open reading frame | 0.050 | 1.035 | 5.39E-01 | 5.39E+01 | | | |
| 7946149 | NM_001164 | APBB1 | amyloid beta (A4) precursor protein- | 0.050 | 1.035 | 6.46E-01 | 6.46E+01 | | | |
| 7892747 | --- | --- | --- | 0.050 | 1.035 | 8.48E-01 | 8.48E+01 | | | |
| 8143905 | NM_007189 | ABCF2 | ATP-binding cassette, sub-family F (G | 0.050 | 1.035 | 7.04E-01 | 7.04E+01 | | | |
| 7952129 | NM_001028 | RPS25 | ribosomal protein S25 | 0.050 | 1.035 | 8.40E-01 | 8.40E+01 | | | |
| 7894096 | --- | --- | --- | 0.050 | 1.035 | 9.66E-01 | 9.66E+01 | | | |
| 7950671 | NM_080491 | GAB2 | GRB2-associated binding protein 2 | 0.049 | 1.035 | 6.80E-01 | 6.80E+01 | | | |
| 7896194 | --- | --- | --- | 0.049 | 1.035 | 6.42E-01 | 6.42E+01 | | | |
| 8175835 | NM_00113945 | BCAP31 | B-cell receptor-associated protein 31 | 0.049 | 1.035 | 7.75E-01 | 7.75E+01 | | | |
| 7926661 | NM_012228 | MSRB2 | methionine sulfoxide reductase B2 | 0.049 | 1.035 | 5.53E-01 | 5.53E+01 | | | |
| 8107855 | NR_023344 | RNU6ATAC | RNA, U6atac small nuclear (U12-dep | 0.049 | 1.035 | 7.44E-01 | 7.44E+01 | | | |
| 7910427 | NM_004481 | GALNT2 | UDP-N-acetyl-alpha-D-galactosamine | 0.049 | 1.035 | 4.72E-01 | 4.72E+01 | | | |
| 8094456 | NM_00114543 | LOC389203 | hypothetical protein LOC389203 | 0.049 | 1.035 | 8.51E-01 | 8.51E+01 | | | |
| 7896616 | --- | --- | --- | 0.049 | 1.035 | 9.07E-01 | 9.07E+01 | | | |
| 8045453 | --- | --- | --- | 0.049 | 1.035 | 7.84E-01 | 7.84E+01 | | | |
| 8027330 | NM_00101166 | PCGF6 | polycomb group ring finger 6 | 0.049 | 1.035 | 8.68E-01 | 8.68E+01 | | | |
| 8088458 | NM_002012 | FHIT | fragile histidine triad gene | 0.049 | 1.035 | 7.03E-01 | 7.03E+01 | | | |
| 8052731 | --- | --- | --- | 0.049 | 1.035 | 8.00E-01 | 8.00E+01 | | | |
| 7896404 | --- | --- | --- | 0.049 | 1.035 | 9.28E-01 | 9.28E+01 | | | |
| 8125461 | AK097297 | HLA-DQB1 | major histocompatibility complex, cl | 0.049 | 1.035 | 9.43E-01 | 9.43E+01 | | | |
| 8115978 | NM_016290 | UIMC1 | ubiquitin interaction motif containin | 0.049 | 1.035 | 8.00E-01 | 8.00E+01 | | | |
| 7996455 | NM_001950 | E2F4 | E2F transcription factor 4, p107/p13 | 0.049 | 1.035 | 5.73E-01 | 5.73E+01 | | | |
| 7986186 | NM_033544 | RCCD1 | RCC1 domain containing 1 | 0.049 | 1.035 | 7.45E-01 | 7.45E+01 | | | |
| 7975632 | NM_152444 | PTGR2 | prostaglandin reductase 2 | 0.049 | 1.035 | 6.77E-01 | 6.77E+01 | | | |
| 8178676 | NM_000434 | NEU1 | sialidase 1 (lysosomal sialidase) | 0.049 | 1.034 | 7.92E-01 | 7.92E+01 | | | |
| 7895713 | --- | --- | --- | 0.049 | 1.034 | 8.67E-01 | 8.67E+01 | | | |
| 8089038 | --- | --- | --- | 0.049 | 1.034 | 8.62E-01 | 8.62E+01 | | | |
| 8112914 | --- | --- | --- | 0.049 | 1.034 | 8.62E-01 | 8.62E+01 | | | |
| 8054930 | NM_032390 | MKI67IP | MKI67 (FHA domain) interacting nuc | 0.049 | 1.034 | 8.75E-01 | 8.75E+01 | | | |
| 7990815 | NM_00110088 | ST20 | suppressor of tumorigenicity 20 | 0.049 | 1.034 | 8.18E-01 | 8.18E+01 | | | |
| 7954602 | --- | --- | --- | 0.049 | 1.034 | 9.00E-01 | 9.00E+01 | | | |
| 8172478 | NM_017602 | OTUD5 | OTU domain containing 5 | 0.049 | 1.034 | 6.68E-01 | 6.68E+01 | | | |
| 7988082 | NM_152455 | ZSCAN29 | zinc finger and SCAN domain contain | 0.049 | 1.034 | 7.07E-01 | 7.07E+01 | | | |
| 7967452 | --- | --- | --- | 0.049 | 1.034 | 9.01E-01 | 9.01E+01 | | | |
| 7991049 | NM_144597 | C15orf40 | chromosome 15 open reading frame | 0.049 | 1.034 | 8.46E-01 | 8.46E+01 | | | |
| 7894498 | --- | --- | --- | 0.049 | 1.034 | 9.40E-01 | 9.40E+01 | | | |
| 7892993 | --- | --- | --- | 0.049 | 1.034 | 5.79E-01 | 5.79E+01 | | | |
| 7898607 | --- | --- | --- | 0.049 | 1.034 | 6.53E-01 | 6.53E+01 | | | |
| 8178841 | NM_018833 | TAP2 | transporter 2, ATP-binding cassette, | 0.049 | 1.034 | 6.53E-01 | 6.53E+01 | | | |
| 7913918 | NM_183008 | UBXN11 | UBX domain protein 11 | 0.048 | 1.034 | 7.18E-01 | 7.18E+01 | | | |
| 8081564 | NM_198196 | CD96 | CD96 molecule | 0.048 | 1.034 | 8.65E-01 | 8.65E+01 | | | |
| 7966315 | NM_005719 | ARPC3 | actin related protein 2/3 complex, su | 0.048 | 1.034 | 9.30E-01 | 9.30E+01 | | | |
| 7899377 | NM_138558 | PPP1R8 | protein phosphatase 1, regulatory (in | 0.048 | 1.034 | 5.64E-01 | 5.64E+01 | | | |
| 7955464 | NM_014764 | DAZAP2 | DAZ associated protein 2 | 0.048 | 1.034 | 8.42E-01 | 8.42E+01 | | | |
| 7894789 | --- | --- | --- | 0.048 | 1.034 | 3.49E-01 | 3.49E+01 | | | |
| 7913705 | NM_001841 | CNR2 | cannabinoid receptor 2 (macrophage | 0.048 | 1.034 | 6.19E-01 | 6.19E+01 | | | |
| 7926916 | NR_024285 | ZEB1 | zinc finger E-box binding homeobox | 0.048 | 1.034 | 7.37E-01 | 7.37E+01 | | | |
| 8000603 | NM_003321 | TUFM | Tu translation elongation factor, mit | 0.048 | 1.034 | 6.80E-01 | 6.80E+01 | | | |
| 8148196 | --- | --- | --- | 0.048 | 1.034 | 8.53E-01 | 8.53E+01 | | | |

| | | | | | | | | | | |
|---|---|---|---|---|---|---|---|---|---|---|
| 7908988 | NM_003094 | SNRPE | small nuclear ribonucleoprotein poly | 0.048 | 1.034 | 8.78E-01 | 8.78E+01 | | | |
| 7895624 | --- | --- | --- | 0.048 | 1.034 | 8.74E-01 | 8.74E+01 | | | |
| 7911309 | NR_024540 | WASH5P | WAS protein family homolog 5 pseud | 0.048 | 1.034 | 4.33E-01 | 4.33E+01 | | | |
| 7946610 | NM_001418 | EIF4G2 | eukaryotic translation initiation facto | 0.048 | 1.034 | 8.47E-01 | 8.47E+01 | | | |
| 8124901 | NM_002117 // | LA-C // HLA- | major histocompatibility complex, cl | 0.048 | 1.034 | 6.39E-01 | 6.39E+01 | | | |
| 8163426 | --- | --- | --- | 0.048 | 1.034 | 7.89E-01 | 7.89E+01 | | | |
| 8055606 | NM_00100663 | GTDC1 | glycosyltransferase-like domain cont | 0.048 | 1.034 | 8.13E-01 | 8.13E+01 | | | |
| 7936100 | NM_015916 | CALHM2 | calcium homeostasis modulator 2 | 0.048 | 1.034 | 5.01E-01 | 5.01E+01 | | | |
| 7893414 | --- | --- | --- | 0.048 | 1.034 | 3.63E-01 | 3.63E+01 | | | |
| 8108472 | NM_005859 | PURA | purine-rich element binding protein | 0.048 | 1.034 | 6.26E-01 | 6.26E+01 | | | |
| 7893208 | --- | --- | --- | 0.048 | 1.034 | 6.25E-01 | 6.25E+01 | | | |
| 8151929 | --- | --- | --- | 0.048 | 1.034 | 7.80E-01 | 7.80E+01 | | | |
| 7892556 | --- | --- | --- | 0.048 | 1.034 | 2.54E-01 | 2.54E+01 | | | |
| 8136516 | NM_173569 | UBN2 | ubinuclein 2 | 0.048 | 1.034 | 7.46E-01 | 7.46E+01 | | | |
| 8037856 | NM_015168 | ZC3H4 | zinc finger CCCH-type containing 4 | 0.048 | 1.034 | 5.18E-01 | 5.18E+01 | | | |
| 8050336 | NR_003094 | E2F6 | E2F transcription factor 6 | 0.048 | 1.034 | 8.39E-01 | 8.39E+01 | | | |
| 7897044 | NM_002744 | PRKCZ | protein kinase C, zeta | 0.048 | 1.034 | 6.67E-01 | 6.67E+01 | | | |
| 8093256 | --- | --- | --- | 0.048 | 1.034 | 7.71E-01 | 7.71E+01 | | | |
| 8031277 | NM_015868 | KIR2DL3 | killer cell immunoglobulin-like recept | 0.048 | 1.034 | 8.38E-01 | 8.38E+01 | | | |
| 7959751 | NM_152437 | ZNF664 | zinc finger protein 664 | 0.048 | 1.034 | 5.31E-01 | 5.31E+01 | | | |
| 8043474 | ENST00000390 | LOC652493 | similar to Ig kappa chain V-I region H | 0.048 | 1.034 | 7.79E-01 | 7.79E+01 | | | |
| 7896213 | --- | --- | --- | 0.048 | 1.034 | 8.68E-01 | 8.68E+01 | | | |
| 7895370 | --- | --- | --- | 0.048 | 1.034 | 9.24E-01 | 9.24E+01 | | | |
| 8001147 | NM_198490 | RAB43 | RAB43, member RAS oncogene famil | 0.048 | 1.034 | 7.53E-01 | 7.53E+01 | | | |
| 8068866 | NM_004571 | PKNOX1 | PBX/knotted 1 homeobox 1 | 0.047 | 1.033 | 5.90E-01 | 5.90E+01 | | | |
| 7894328 | --- | --- | --- | 0.047 | 1.033 | 9.32E-01 | 9.32E+01 | | | |
| 7991258 | NM_002693 | POLG | polymerase (DNA directed), gamma | 0.047 | 1.033 | 6.36E-01 | 6.36E+01 | | | |
| 8018761 | NM_006456 | ST6GALNAC2 | ST6 (alpha-N-acetyl-neuraminyl-2,3- | 0.047 | 1.033 | 4.61E-01 | 4.61E+01 | | | |
| 8180372 | --- | --- | --- | 0.047 | 1.033 | 4.61E-01 | 4.61E+01 | | | |
| 8116520 | NM_006098 | GNB2L1 | guanine nucleotide binding protein ( | 0.047 | 1.033 | 7.49E-01 | 7.49E+01 | | | |
| 7978360 | NM_033423 | GZMH | granzyme H (cathepsin G-like 2, prot | 0.047 | 1.033 | 7.92E-01 | 7.92E+01 | | | |
| 7952805 | NR_024344 | LOC283174 | hypothetical LOC283174 | 0.047 | 1.033 | 5.93E-01 | 5.93E+01 | | | |
| 8012397 | NM_017622 | C17orf59 | chromosome 17 open reading frame | 0.047 | 1.033 | 4.25E-01 | 4.25E+01 | | | |
| 8172086 | --- | --- | --- | 0.047 | 1.033 | 6.10E-01 | 6.10E+01 | | | |
| 8031933 | NM_001009 | RPS5 | ribosomal protein S5 | 0.047 | 1.033 | 8.10E-01 | 8.10E+01 | | | |
| 8129924 | NM_153235 | TXLNB | taxilin beta | 0.047 | 1.033 | 6.35E-01 | 6.35E+01 | | | |
| 7974455 | NM_144578 | MAPK1IP1L | mitogen-activated protein kinase 1 i | 0.047 | 1.033 | 8.35E-01 | 8.35E+01 | | | |
| 7893251 | --- | --- | --- | 0.047 | 1.033 | 9.37E-01 | 9.37E+01 | | | |
| 8074157 | NR_026982 | RPL23AP82 | ribosomal protein L23a pseudogene | 0.047 | 1.033 | 6.96E-01 | 6.96E+01 | | | |
| 7977046 | NM_006291 | TNFAIP2 | tumor necrosis factor, alpha-induced | 0.047 | 1.033 | 3.46E-01 | 3.46E+01 | | | |
| 8005785 | NM_014238 | KSR1 | kinase suppressor of ras 1 | 0.047 | 1.033 | 5.89E-01 | 5.89E+01 | | | |
| 8072883 | NM_024313 | NOL12 | nucleolar protein 12 | 0.047 | 1.033 | 6.71E-01 | 6.71E+01 | | | |
| 8065325 | --- | --- | --- | 0.047 | 1.033 | 8.57E-01 | 8.57E+01 | | | |
| 8060738 | --- | --- | --- | 0.047 | 1.033 | 5.72E-01 | 5.72E+01 | | | |
| 7974814 | --- | --- | --- | 0.047 | 1.033 | 8.75E-01 | 8.75E+01 | | | |
| 8112558 | NR_003504 | GUSBL1 | glucuronidase, beta-like 1 | 0.047 | 1.033 | 9.22E-01 | 9.22E+01 | | | |
| 7996100 | NM_170776 | GPR97 | G protein-coupled receptor 97 | 0.047 | 1.033 | 6.74E-01 | 6.74E+01 | | | |
| 8115210 | NM_006058 | TNIP1 | TNFAIP3 interacting protein 1 | 0.047 | 1.033 | 7.28E-01 | 7.28E+01 | | | |
| 7896018 | --- | --- | --- | 0.047 | 1.033 | 8.02E-01 | 8.02E+01 | | | |
| 7916592 | NM_00108548 | MYSM1 | Myb-like, SWIRM and MPN domains | 0.047 | 1.033 | 8.77E-01 | 8.77E+01 | | | |
| 8064336 | NM_144628 | TBC1D20 | TBC1 domain family, member 20 | 0.047 | 1.033 | 5.71E-01 | 5.71E+01 | | | |
| 8009995 | NM_00108041 | UNK | unkempt homolog (Drosophila) | 0.047 | 1.033 | 5.10E-01 | 5.10E+01 | | | |
| 8028756 | NM_006503 | PSMC4 | proteasome (prosome, macropain) 2 | 0.047 | 1.033 | 7.62E-01 | 7.62E+01 | | | |
| 8099887 | NM_00102492 | RPL9 | ribosomal protein L9 | 0.047 | 1.033 | 6.82E-01 | 6.82E+01 | | | |
| 8135422 | NM_00100840 | BCAP29 | B-cell receptor-associated protein 29 | 0.047 | 1.033 | 7.08E-01 | 7.08E+01 | | | |
| 7979849 | NM_003861 | DCAF5 | DDB1 and CUL4 associated factor 5 | 0.047 | 1.033 | 8.52E-01 | 8.52E+01 | | | |
| 8085340 | NM_014667 | VGLL4 | vestigial like 4 (Drosophila) | 0.046 | 1.033 | 6.46E-01 | 6.46E+01 | | | |
| 8130660 | --- | --- | --- | 0.046 | 1.033 | 8.82E-01 | 8.82E+01 | | | |
| 8084982 | NR_024388 | LOC152217 | hypothetical LOC152217 | 0.046 | 1.033 | 6.73E-01 | 6.73E+01 | | | |
| 8162533 | NM_00108360 | PTCH1 | patched homolog 1 (Drosophila) | 0.046 | 1.033 | 6.65E-01 | 6.65E+01 | | | |
| 8166179 | NR_026551 | CA5BP | carbonic anhydrase VB pseudogene | 0.046 | 1.033 | 7.49E-01 | 7.49E+01 | | | |
| 7942255 | NM_00101250 | KRTAP5-7 | keratin associated protein 5-7 | 0.046 | 1.033 | 6.07E-01 | 6.07E+01 | | | |
| 8134789 | NM_013440 | PILRB | paired immunoglobin-like type 2 rec | 0.046 | 1.032 | 6.20E-01 | 6.20E+01 | | | |
| 8159609 | NM_003731 | SSNA1 | Sjogren syndrome nuclear autoantig | 0.046 | 1.032 | 7.73E-01 | 7.73E+01 | | | |
| 8103413 | --- | --- | --- | 0.046 | 1.032 | 8.19E-01 | 8.19E+01 | | | |
| 7992893 | --- | --- | --- | 0.046 | 1.032 | 9.39E-01 | 9.39E+01 | | | |
| 8008029 | NM_013351 | TBX21 | T-box 21 | 0.046 | 1.032 | 5.80E-01 | 5.80E+01 | | | |
| 8078650 | NM_00100839 | CTDSPL | CTD (carboxy-terminal domain, RNA | 0.046 | 1.032 | 5.09E-01 | 5.09E+01 | | | |
| 7899813 | NM_005610 | RBBP4 | retinoblastoma binding protein 4 | 0.046 | 1.032 | 9.05E-01 | 9.05E+01 | | | |
| 8072113 | NM_00101369 | SRRD | SRR1 domain containing | 0.046 | 1.032 | 8.69E-01 | 8.69E+01 | | | |
| 8101449 | NM_00109854 | HPSE | heparanase | 0.046 | 1.032 | 6.24E-01 | 6.24E+01 | | | |
| 8037005 | NM_000660 | TGFB1 | transforming growth factor, beta 1 | 0.046 | 1.032 | 8.52E-01 | 8.52E+01 | | | |
| 8015796 | --- | --- | --- | 0.046 | 1.032 | 9.43E-01 | 9.43E+01 | | | |
| 7926670 | --- | --- | --- | 0.046 | 1.032 | 8.38E-01 | 8.38E+01 | | | |
| 8161017 | NM_014450 | SIT1 | signaling threshold regulating transm | 0.046 | 1.032 | 5.57E-01 | 5.57E+01 | | | |
| 7893131 | --- | --- | --- | 0.046 | 1.032 | 9.40E-01 | 9.40E+01 | | | |
| 8023871 | NM_175907 | ZADH2 | zinc binding alcohol dehydrogenase | 0.046 | 1.032 | 8.06E-01 | 8.06E+01 | | | |

| | | | | | | | | | | |
|---|---|---|---|---|---|---|---|---|---|---|
| 8025414 | NM_004218 | RAB11B | RAB11B, member RAS oncogene fam | 0.046 | 1.032 | 8.59E-01 | 8.59E+01 | | | |
| 8165661 | --- | --- | --- | 0.046 | 1.032 | 2.68E-01 | 2.68E+01 | | | |
| 7892940 | --- | --- | --- | 0.046 | 1.032 | 4.63E-01 | 4.63E+01 | | | |
| 7978066 | NM_003917 | AP1G2 | adaptor-related protein complex 1, g | 0.045 | 1.032 | 7.01E-01 | 7.01E+01 | | | |
| 8106278 | --- | --- | --- | 0.045 | 1.032 | 9.27E-01 | 9.27E+01 | | | |
| 7925954 | NM_00104716 | NET1 | neuroepithelial cell transforming 1 | 0.045 | 1.032 | 8.43E-01 | 8.43E+01 | | | |
| 7928558 | NM_020338 | ZMIZ1 | zinc finger, MIZ-type containing 1 | 0.045 | 1.032 | 6.03E-01 | 6.03E+01 | | | |
| 8156897 | BC008993 | C9orf30 | chromosome 9 open reading frame 3 | 0.045 | 1.032 | 8.22E-01 | 8.22E+01 | | | |
| 7892656 | --- | --- | --- | 0.045 | 1.032 | 9.18E-01 | 9.18E+01 | | | |
| 7990949 | NM_000661 | RPL9 | ribosomal protein L9 | 0.045 | 1.032 | 6.92E-01 | 6.92E+01 | | | |
| 8166184 | NM_007220 | CA5B | carbonic anhydrase VB, mitochondri | 0.045 | 1.032 | 8.90E-01 | 8.90E+01 | | | |
| 7916343 | NM_018982 | YIPF1 | Yip1 domain family, member 1 | 0.045 | 1.032 | 8.30E-01 | 8.30E+01 | | | |
| 8113660 | --- | --- | --- | 0.045 | 1.032 | 8.20E-01 | 8.20E+01 | | | |
| 8009432 | NM_016627 | AMZ2 | archaelysin family metallopeptidase | 0.045 | 1.032 | 8.19E-01 | 8.19E+01 | | | |
| 8017675 | NR_024386 | PLEKHM1P | pleckstrin homology domain contain | 0.045 | 1.032 | 7.81E-01 | 7.81E+01 | | | |
| 8000003 | NM_017736 | THUMPD1 | THUMP domain containing 1 | 0.045 | 1.032 | 8.16E-01 | 8.16E+01 | | | |
| 7936902 | --- | --- | --- | 0.045 | 1.032 | 9.23E-01 | 9.23E+01 | | | |
| 8042306 | --- | --- | --- | 0.045 | 1.032 | 9.45E-01 | 9.45E+01 | | | |
| 8109693 | --- | --- | --- | 0.045 | 1.032 | 5.77E-01 | 5.77E+01 | | | |
| 7911085 | NM_198076 | FAM36A | family with sequence similarity 36, m | 0.045 | 1.032 | 7.57E-01 | 7.57E+01 | | | |
| 7995574 | NM_002136 | HNRNPA1 | heterogeneous nuclear ribonucleopr | 0.045 | 1.032 | 5.96E-01 | 5.96E+01 | | | |
| 8027566 | NM_001806 | CEBPG | CCAAT/enhancer binding protein (C/ | 0.045 | 1.032 | 7.85E-01 | 7.85E+01 | | | |
| 7892837 | --- | --- | --- | 0.045 | 1.032 | 9.53E-01 | 9.53E+01 | | | |
| 8145440 | NM_002717 | PPP2R2A | protein phosphatase 2 (formerly 2A) | 0.045 | 1.032 | 8.83E-01 | 8.83E+01 | | | |
| 7995843 | NM_014669 | NUP93 | nucleoporin 93kDa | 0.045 | 1.032 | 8.53E-01 | 8.53E+01 | | | |
| 8034843 | NM_004146 | NDUFB7 | NADH dehydrogenase (ubiquinone) 1 | 0.045 | 1.032 | 7.41E-01 | 7.41E+01 | | | |
| 7966938 | NM_006836 | GCN1L1 | GCN1 general control of amino-acid | 0.045 | 1.031 | 6.88E-01 | 6.88E+01 | | | |
| 8133442 | NM_032464 | LAT2 | linker for activation of T cells family, | 0.045 | 1.031 | 5.50E-01 | 5.50E+01 | | | |
| 7907700 | --- | --- | --- | 0.045 | 1.031 | 8.36E-01 | 8.36E+01 | | | |
| 8029392 | NM_006630 | ZNF234 | zinc finger protein 234 | 0.045 | 1.031 | 8.20E-01 | 8.20E+01 | | | |
| 8053599 | NM_012477 | WBP1 | WW domain binding protein 1 | 0.045 | 1.031 | 8.18E-01 | 8.18E+01 | | | |
| 7893762 | --- | --- | --- | 0.045 | 1.031 | 6.90E-01 | 6.90E+01 | | | |
| 7893689 | --- | --- | --- | 0.045 | 1.031 | 4.09E-01 | 4.09E+01 | | | |
| 7979551 | NM_006246 | PPP2R5E | protein phosphatase 2, regulatory su | 0.045 | 1.031 | 7.01E-01 | 7.01E+01 | | | |
| 7918275 | NM_00114255 | WDR47 | WD repeat domain 47 | 0.045 | 1.031 | 7.98E-01 | 7.98E+01 | | | |
| 8014841 | NM_004774 | MED1 | mediator complex subunit 1 | 0.045 | 1.031 | 8.44E-01 | 8.44E+01 | | | |
| 8122684 | NM_00100225 | SUMO4 | SMT3 suppressor of mif two 3 homo | 0.045 | 1.031 | 7.07E-01 | 7.07E+01 | | | |
| 7942858 | NM_182603 | ANKRD42 | ankyrin repeat domain 42 | 0.045 | 1.031 | 6.32E-01 | 6.32E+01 | | | |
| 7903878 | NM_022768 | RBM15 | RNA binding motif protein 15 | 0.045 | 1.031 | 7.45E-01 | 7.45E+01 | | | |
| 7934850 | --- | --- | --- | 0.044 | 1.031 | 8.64E-01 | 8.64E+01 | | | |
| 8002289 | NM_005652 | TERF2 | telomeric repeat binding factor 2 | 0.044 | 1.031 | 7.82E-01 | 7.82E+01 | | | |
| 8158028 | NM_138361 | LRSAM1 | leucine rich repeat and sterile alpha | 0.044 | 1.031 | 5.26E-01 | 5.26E+01 | | | |
| 8131949 | NM_016587 | CBX3 | chromobox homolog 3 (HP1 gamma | 0.044 | 1.031 | 7.87E-01 | 7.87E+01 | | | |
| 7894550 | --- | --- | --- | 0.044 | 1.031 | 8.34E-01 | 8.34E+01 | | | |
| 7894525 | --- | --- | --- | 0.044 | 1.031 | 9.53E-01 | 9.53E+01 | | | |
| 7894354 | --- | --- | --- | 0.044 | 1.031 | 8.49E-01 | 8.49E+01 | | | |
| 7950197 | NM_015242 | ARAP1 | ArfGAP with RhoGAP domain, ankyri | 0.044 | 1.031 | 6.03E-01 | 6.03E+01 | | | |
| 8177011 | NM_004192 | ASMTL | acetylserotonin O-methyltransferase | 0.044 | 1.031 | 7.44E-01 | 7.44E+01 | | | |
| 8033207 | NM_024898 | DENND1C | DENN/MADD domain containing 1C | 0.044 | 1.031 | 6.38E-01 | 6.38E+01 | | | |
| 7896633 | --- | --- | --- | 0.044 | 1.031 | 9.62E-01 | 9.62E+01 | | | |
| 8079294 | NM_015004 | EXOSC7 | exosome component 7 | 0.044 | 1.031 | 9.22E-01 | 9.22E+01 | | | |
| 7989596 | NM_014326 | DAPK2 | death-associated protein kinase 2 | 0.044 | 1.031 | 5.97E-01 | 5.97E+01 | | | |
| 7971361 | NM_012345 | NUFIP1 | nuclear fragile X mental retardation | 0.044 | 1.031 | 8.18E-01 | 8.18E+01 | | | |
| 8122699 | BC071678 | RPS18P9 | ribosomal protein S18 pseudogene 9 | 0.044 | 1.031 | 7.30E-01 | 7.30E+01 | | | |
| 8125436 | NM_002125 | HLA-DRB5 | major histocompatibility complex, cl | 0.044 | 1.031 | 8.71E-01 | 8.71E+01 | | | |
| 7893165 | --- | --- | --- | 0.044 | 1.031 | 9.26E-01 | 9.26E+01 | | | |
| 7923662 | NM_002646 | PIK3C2B | phosphoinositide-3-kinase, class 2, b | 0.044 | 1.031 | 5.78E-01 | 5.78E+01 | | | |
| 8018439 | NM_005324 | H3F3B | H3 histone, family 3B (H3.3B) | 0.044 | 1.031 | 7.03E-01 | 7.03E+01 | | | |
| 8161857 | NM_018339 | RFK | riboflavin kinase | 0.044 | 1.031 | 5.31E-01 | 5.31E+01 | | | |
| 7941214 | NM_002689 | POLA2 | polymerase (DNA directed), alpha 2 | 0.044 | 1.031 | 7.29E-01 | 7.29E+01 | | | |
| 7894361 | --- | --- | --- | 0.044 | 1.031 | 8.71E-01 | 8.71E+01 | | | |
| 8000706 | NM_006319 | CDIPT | CDP-diacylglycerol--inositol 3-phosp | 0.044 | 1.031 | 6.62E-01 | 6.62E+01 | | | |
| 7968796 | --- | --- | --- | 0.044 | 1.031 | 8.00E-01 | 8.00E+01 | | | |
| 7910706 | NM_006499 | LGALS8 | lectin, galactoside-binding, soluble, 8 | 0.044 | 1.031 | 8.65E-01 | 8.65E+01 | | | |
| 8086057 | NM_014517 | UBP1 | upstream binding protein 1 (LBP-1a) | 0.044 | 1.031 | 8.02E-01 | 8.02E+01 | | | |
| 8037197 | NM_198477 | CXCL17 | chemokine (C-X-C motif) ligand 17 | 0.043 | 1.031 | 5.65E-01 | 5.65E+01 | | | |
| 8096635 | NM_003998 | NFKB1 | nuclear factor of kappa light polypep | 0.043 | 1.031 | 8.85E-01 | 8.85E+01 | | | |
| 8154885 | NM_001161 | NUDT2 | nudix (nucleoside diphosphate linked | 0.043 | 1.031 | 6.65E-01 | 6.65E+01 | | | |
| 7893498 | --- | --- | --- | 0.043 | 1.031 | 4.25E-01 | 4.25E+01 | | | |
| 7994006 | NM_015092 | SMG1 | SMG1 homolog, phosphatidylinosito | 0.043 | 1.031 | 6.83E-01 | 6.83E+01 | | | |
| 8180254 | --- | --- | --- | 0.043 | 1.030 | 5.53E-01 | 5.53E+01 | | | |
| 8124950 | NM_002341 | LTB | lymphotoxin beta (TNF superfamily, | 0.043 | 1.030 | 3.73E-01 | 3.73E+01 | | | |
| 8178512 | NM_002341 | LTB | lymphotoxin beta (TNF superfamily, | 0.043 | 1.030 | 3.73E-01 | 3.73E+01 | | | |
| 8179768 | NM_002341 | LTB | lymphotoxin beta (TNF superfamily, | 0.043 | 1.030 | 3.73E-01 | 3.73E+01 | | | |
| 8059969 | NM_030768 | ILKAP | integrin-linked kinase-associated ser | 0.043 | 1.030 | 8.11E-01 | 8.11E+01 | | | |
| 7894387 | --- | --- | --- | 0.043 | 1.030 | 9.65E-01 | 9.65E+01 | | | |
| 8098637 | NM_207352 | CYP4V2 | cytochrome P450, family 4, subfamil | 0.043 | 1.030 | 7.87E-01 | 7.87E+01 | | | |

| | | | | | | | | | | |
|---|---|---|---|---|---|---|---|---|---|---|
| 7896623 | --- | --- | --- | 0.043 | 1.030 | 8.68E-01 | 8.68E+01 | | | |
| 7983206 | NM_014444 | TUBGCP4 | tubulin, gamma complex associated | 0.043 | 1.030 | 7.81E-01 | 7.81E+01 | | | |
| 8154973 | NM_203299 | C9orf131 | chromosome 9 open reading frame 1 | 0.043 | 1.030 | 6.46E-01 | 6.46E+01 | | | |
| 7978527 | NM_080664 | C14orf126 | chromosome 14 open reading frame | 0.043 | 1.030 | 6.21E-01 | 6.21E+01 | | | |
| 8074925 | NR_024448 | LOC91316 | glucuronidase, beta/ immunoglobuli | 0.043 | 1.030 | 9.28E-01 | 9.28E+01 | | | |
| 8114900 | NM_004576 | PPP2R2B | protein phosphatase 2 (formerly 2A) | 0.043 | 1.030 | 6.24E-01 | 6.24E+01 | | | |
| 8101340 | NR_003249 | HNRPDL | heterogeneous nuclear ribonucleopr | 0.043 | 1.030 | 6.24E-01 | 6.24E+01 | | | |
| 8152119 | NM_00104062 | NCALD | neurocalcin delta | 0.043 | 1.030 | 7.28E-01 | 7.28E+01 | | | |
| 7892543 | --- | --- | --- | 0.043 | 1.030 | 8.64E-01 | 8.64E+01 | | | |
| 8013622 | NM_144610 | FLJ25006 | uncharacterized serine/threonine-pr | 0.043 | 1.030 | 6.75E-01 | 6.75E+01 | | | |
| 7947027 | NM_00104069 | UEVLD | UEV and lactate/malate dehyrogena | 0.042 | 1.030 | 8.45E-01 | 8.45E+01 | | | |
| 7969096 | NM_030911 | CDADC1 | cytidine and dCMP deaminase doma | 0.042 | 1.030 | 7.97E-01 | 7.97E+01 | | | |
| 7945894 | --- | --- | --- | 0.042 | 1.030 | 8.50E-01 | 8.50E+01 | | | |
| 8180408 | --- | --- | --- | 0.042 | 1.030 | 5.59E-01 | 5.59E+01 | | | |
| 8043413 | NM_144563 | RPIA | ribose 5-phosphate isomerase A | 0.042 | 1.030 | 7.68E-01 | 7.68E+01 | | | |
| 8065018 | NM_017714 | TASP1 | taspase, threonine aspartase, 1 | 0.042 | 1.030 | 7.61E-01 | 7.61E+01 | | | |
| 8132188 | NM_015060 | AVL9 | AVL9 homolog (S. cerevisiase) | 0.042 | 1.030 | 8.10E-01 | 8.10E+01 | | | |
| 8166243 | NM_004726 | REPS2 | RALBP1 associated Eps domain conta | 0.042 | 1.030 | 6.94E-01 | 6.94E+01 | | | |
| 8029996 | BC043386 | C19orf68 | chromosome 19 open reading frame | 0.042 | 1.030 | 5.65E-01 | 5.65E+01 | | | |
| 8097056 | NR_002963 | SNORA24 | small nucleolar RNA, H/ACA box 24 | 0.042 | 1.030 | 9.42E-01 | 9.42E+01 | | | |
| 7978838 | NM_018139 | C14orf104 | chromosome 14 open reading frame | 0.042 | 1.030 | 8.46E-01 | 8.46E+01 | | | |
| 8065612 | NM_080616 | C20orf112 | chromosome 20 open reading frame | 0.042 | 1.030 | 6.54E-01 | 6.54E+01 | | | |
| 7930454 | NM_145341 | PDCD4 | programmed cell death 4 (neoplastic | 0.042 | 1.030 | 7.25E-01 | 7.25E+01 | | | |
| 7945944 | NM_001665 | RHOG | ras homolog gene family, member G | 0.042 | 1.030 | 7.31E-01 | 7.31E+01 | | | |
| 8007290 | NM_170607 | MLX | MAX-like protein X | 0.042 | 1.030 | 8.14E-01 | 8.14E+01 | | | |
| 7902435 | --- | --- | --- | 0.042 | 1.029 | 6.89E-01 | 6.89E+01 | | | |
| 7990618 | --- | --- | --- | 0.042 | 1.029 | 6.46E-01 | 6.46E+01 | | | |
| 8155563 | ENST0000036 | MTHFD1L | methylenetetrahydrofolate dehydro | 0.042 | 1.029 | 8.23E-01 | 8.23E+01 | | | |
| 7999520 | NM_015659 | RSL1D1 | ribosomal L1 domain containing 1 | 0.042 | 1.029 | 8.84E-01 | 8.84E+01 | | | |
| 8180386 | --- | --- | --- | 0.042 | 1.029 | 9.18E-01 | 9.18E+01 | | | |
| 8062041 | NM_018677 | ACSS2 | acyl-CoA synthetase short-chain fam | 0.042 | 1.029 | 5.77E-01 | 5.77E+01 | | | |
| 7946849 | --- | --- | --- | 0.042 | 1.029 | 9.10E-01 | 9.10E+01 | | | |
| 8032974 | NM_014649 | SAFB2 | scaffold attachment factor B2 | 0.042 | 1.029 | 8.12E-01 | 8.12E+01 | | | |
| 8089036 | --- | --- | --- | 0.042 | 1.029 | 7.12E-01 | 7.12E+01 | | | |
| 8088478 | --- | --- | --- | 0.042 | 1.029 | 7.68E-01 | 7.68E+01 | | | |
| 8009183 | NM_203351 | MAP3K3 | mitogen-activated protein kinase kin | 0.042 | 1.029 | 6.22E-01 | 6.22E+01 | | | |
| 8122317 | NM_014320 | HEBP2 | heme binding protein 2 | 0.042 | 1.029 | 9.18E-01 | 9.18E+01 | | | |
| 7894573 | --- | --- | --- | 0.042 | 1.029 | 6.62E-01 | 6.62E+01 | | | |
| 8069880 | NM_003253 | TIAM1 | T-cell lymphoma invasion and metas | 0.042 | 1.029 | 7.47E-01 | 7.47E+01 | | | |
| 8068551 | NM_101395 | DYRK1A | dual-specificity tyrosine-(Y)-phospho | 0.041 | 1.029 | 8.79E-01 | 8.79E+01 | | | |
| 8105995 | AK289851 | SMA5 | glucuronidase, beta pseudogene | 0.041 | 1.029 | 9.30E-01 | 9.30E+01 | | | |
| 8160213 | NM_152574 | TTC39B | tetratricopeptide repeat domain 39B | 0.041 | 1.029 | 9.15E-01 | 9.15E+01 | | | |
| 8152812 | NM_174911 | FAM84B | family with sequence similarity 84, m | 0.041 | 1.029 | 6.04E-01 | 6.04E+01 | | | |
| 7918749 | BC063894 | DENND2C | DENN/MADD domain containing 2C | 0.041 | 1.029 | 6.29E-01 | 6.29E+01 | | | |
| 7914094 | NM_006990 | WASF2 | WAS protein family, member 2 | 0.041 | 1.029 | 8.80E-01 | 8.80E+01 | | | |
| 7971998 | --- | --- | --- | 0.041 | 1.029 | 7.89E-01 | 7.89E+01 | | | |
| 7992458 | --- | --- | --- | 0.041 | 1.029 | 6.66E-01 | 6.66E+01 | | | |
| 8031297 | NM_014218 | KIR2DL1 | killer cell immunoglobulin-like recept | 0.041 | 1.029 | 8.86E-01 | 8.86E+01 | | | |
| 7961031 | NM_004400 | DDX12 | DEAD/H (Asp-Glu-Ala-Asp/His) box p | 0.041 | 1.029 | 4.41E-01 | 4.41E+01 | | | |
| 8004802 | NM_017622 | C17orf59 | chromosome 17 open reading frame | 0.041 | 1.029 | 7.84E-01 | 7.84E+01 | | | |
| 8175039 | NM_001421 | ELF4 | E74-like factor 4 (ets domain transcr | 0.041 | 1.029 | 5.92E-01 | 5.92E+01 | | | |
| 7892848 | --- | --- | --- | 0.041 | 1.029 | 9.03E-01 | 9.03E+01 | | | |
| 8037847 | NM_004069 | AP2S1 | adaptor-related protein complex 2, s | 0.041 | 1.029 | 7.80E-01 | 7.80E+01 | | | |
| 8135114 | NM_181552 | CUX1 | cut-like homeobox 1 | 0.041 | 1.029 | 4.39E-01 | 4.39E+01 | | | |
| 7950269 | --- | --- | --- | 0.041 | 1.029 | 9.39E-01 | 9.39E+01 | | | |
| 8171834 | NM_000661 | RPL9 | ribosomal protein L9 | 0.041 | 1.029 | 7.27E-01 | 7.27E+01 | | | |
| 7893926 | --- | --- | --- | 0.041 | 1.029 | 6.26E-01 | 6.26E+01 | | | |
| 7893316 | --- | --- | --- | 0.041 | 1.029 | 5.47E-01 | 5.47E+01 | | | |
| 7894919 | --- | --- | --- | 0.041 | 1.029 | 6.70E-01 | 6.70E+01 | | | |
| 8180409 | --- | --- | --- | 0.041 | 1.028 | 6.77E-01 | 6.77E+01 | | | |
| 8066009 | NM_184234 | RBM39 | RNA binding motif protein 39 | 0.041 | 1.028 | 7.16E-01 | 7.16E+01 | | | |
| 7894464 | --- | --- | --- | 0.041 | 1.028 | 8.19E-01 | 8.19E+01 | | | |
| 8096314 | NM_000297 | PKD2 | polycystic kidney disease 2 (autosom | 0.040 | 1.028 | 5.65E-01 | 5.65E+01 | | | |
| 7938485 | NM_014632 | MICAL2 | microtubule associated monoxygena | 0.040 | 1.028 | 5.87E-01 | 5.87E+01 | | | |
| 8111666 | --- | --- | --- | 0.040 | 1.028 | 9.46E-01 | 9.46E+01 | | | |
| 8031122 | NM_014516 | CNOT3 | CCR4-NOT transcription complex, su | 0.040 | 1.028 | 5.99E-01 | 5.99E+01 | | | |
| 7951614 | NM_002716 | PPP2R1B | protein phosphatase 2 (formerly 2A) | 0.040 | 1.028 | 7.47E-01 | 7.47E+01 | | | |
| 8067554 | NM_006602 | TCFL5 | transcription factor-like 5 (basic helix | 0.040 | 1.028 | 6.31E-01 | 6.31E+01 | | | |
| 8172110 | BC025334 | CXorf38 | chromosome X open reading frame 3 | 0.040 | 1.028 | 9.21E-01 | 9.21E+01 | | | |
| 7952341 | NM_024769 | ASAM | adipocyte-specific adhesion molecul | 0.040 | 1.028 | 6.45E-01 | 6.45E+01 | | | |
| 8108399 | --- | --- | --- | 0.040 | 1.028 | 8.88E-01 | 8.88E+01 | | | |
| 8141688 | NM_001084 | PLOD3 | procollagen-lysine, 2-oxoglutarate 5- | 0.040 | 1.028 | 6.36E-01 | 6.36E+01 | | | |
| 7973530 | NM_004563 | PCK2 | phosphoenolpyruvate carboxykinase | 0.040 | 1.028 | 6.96E-01 | 6.96E+01 | | | |
| 8104621 | NR_003660 | GUSBL2 | glucuronidase, beta-like 2 | 0.040 | 1.028 | 9.17E-01 | 9.17E+01 | | | |
| 8039842 | NM_012312 | KIR2DS2 | killer cell immunoglobulin-like recept | 0.040 | 1.028 | 8.55E-01 | 8.55E+01 | | | |
| 8160805 | NM_147202 | C9orf25 | chromosome 9 open reading frame 2 | 0.040 | 1.028 | 5.44E-01 | 5.44E+01 | | | |
| 8153776 | NM_183057 | VPS28 | vacuolar protein sorting 28 homolog | 0.040 | 1.028 | 7.39E-01 | 7.39E+01 | | | |

| | | | | | | | | | | |
|---|---|---|---|---|---|---|---|---|---|---|
| 8132646 | NM_031443 | CCM2 | cerebral cavernous malformation 2 | 0.040 | 1.028 | 6.50E-01 | 6.50E+01 | | | |
| 7896679 | --- | --- | --- | 0.040 | 1.028 | 5.91E-01 | 5.91E+01 | | | |
| 8118086 | NM_007109 | TCF19 | transcription factor 19 | 0.040 | 1.028 | 6.17E-01 | 6.17E+01 | | | |
| 8177947 | NM_007109 | TCF19 | transcription factor 19 | 0.040 | 1.028 | 6.17E-01 | 6.17E+01 | | | |
| 8178790 | NM_022107 | GPSM3 | G-protein signaling modulator 3 (AG | 0.040 | 1.028 | 8.25E-01 | 8.25E+01 | | | |
| 8094156 | NM_000798 | DRD5 | dopamine receptor D5 | 0.040 | 1.028 | 5.62E-01 | 5.62E+01 | | | |
| 8138941 | NM_00100200 | NT5C3 | 5'-nucleotidase, cytosolic III | 0.040 | 1.028 | 8.99E-01 | 8.99E+01 | | | |
| 7982284 | ENST00000314 | LOC728047 | similar to Golgin subfamily A membe | 0.040 | 1.028 | 7.34E-01 | 7.34E+01 | | | |
| 8180374 | --- | --- | --- | 0.040 | 1.028 | 8.86E-01 | 8.86E+01 | | | |
| 7895707 | --- | --- | --- | 0.040 | 1.028 | 7.33E-01 | 7.33E+01 | | | |
| 8028241 | NM_032453 | ZNF527 | zinc finger protein 527 | 0.040 | 1.028 | 6.46E-01 | 6.46E+01 | | | |
| 8032392 | NM_199054 | MKNK2 | MAP kinase interacting serine/threo | 0.040 | 1.028 | 7.77E-01 | 7.77E+01 | | | |
| 8016412 | --- | --- | --- | 0.040 | 1.028 | 8.88E-01 | 8.88E+01 | | | |
| 8167185 | NM_003254 | TIMP1 | TIMP metallopeptidase inhibitor 1 | 0.040 | 1.028 | 8.79E-01 | 8.79E+01 | | | |
| 7974255 | --- | --- | --- | 0.040 | 1.028 | 7.98E-01 | 7.98E+01 | | | |
| 7902074 | NM_002303 | LEPR | leptin receptor | 0.040 | 1.028 | 6.56E-01 | 6.56E+01 | | | |
| 7894267 | --- | --- | --- | 0.040 | 1.028 | 5.88E-01 | 5.88E+01 | | | |
| 7905147 | BC017761 | C1orf54 | chromosome 1 open reading frame 5 | 0.040 | 1.028 | 8.61E-01 | 8.61E+01 | | | |
| 7894787 | --- | --- | --- | 0.040 | 1.028 | 6.41E-01 | 6.41E+01 | | | |
| 8141425 | NM_005641 | TAF6 | TAF6 RNA polymerase II, TATA box b | 0.040 | 1.028 | 5.72E-01 | 5.72E+01 | | | |
| 8093053 | NM_003234 | TFRC | transferrin receptor (p90, CD71) | 0.040 | 1.028 | 9.24E-01 | 9.24E+01 | | | |
| 7987840 | NM_015289 | VPS39 | vacuolar protein sorting 39 homolog | 0.040 | 1.028 | 8.33E-01 | 8.33E+01 | | | |
| 7987536 | NM_018145 | FAM82A2 | family with sequence similarity 82, m | 0.040 | 1.028 | 6.67E-01 | 6.67E+01 | | | |
| 8085984 | NM_017784 | OSBPL10 | oxysterol binding protein-like 10 | 0.040 | 1.028 | 6.09E-01 | 6.09E+01 | | | |
| 7972737 | NM_002312 | LIG4 | ligase IV, DNA, ATP-dependent | 0.039 | 1.028 | 6.17E-01 | 6.17E+01 | | | |
| 7950597 | NM_001293 | CLNS1A | chloride channel, nucleotide-sensitiv | 0.039 | 1.028 | 8.61E-01 | 8.61E+01 | | | |
| 7894784 | --- | --- | --- | 0.039 | 1.028 | 8.50E-01 | 8.50E+01 | | | |
| 7894070 | --- | --- | --- | 0.039 | 1.028 | 8.95E-01 | 8.95E+01 | | | |
| 8066256 | NR_002910 | SNORA71B | small nucleolar RNA, H/ACA box 71B | 0.039 | 1.028 | 9.24E-01 | 9.24E+01 | | | |
| 7892720 | --- | --- | --- | 0.039 | 1.028 | 8.91E-01 | 8.91E+01 | | | |
| 7954008 | --- | --- | --- | 0.039 | 1.028 | 8.44E-01 | 8.44E+01 | | | |
| 8180407 | --- | --- | --- | 0.039 | 1.028 | 6.27E-01 | 6.27E+01 | | | |
| 7983606 | NM_014335 | EID1 | EP300 interacting inhibitor of differe | 0.039 | 1.028 | 8.58E-01 | 8.58E+01 | | | |
| 7945734 | AB029488 | C11orf21 | chromosome 11 open reading frame | 0.039 | 1.028 | 7.13E-01 | 7.13E+01 | | | |
| 7975066 | NM_004857 | AKAP5 | A kinase (PRKA) anchor protein 5 | 0.039 | 1.028 | 8.39E-01 | 8.39E+01 | | | |
| 8100210 | NM_003328 | TXK | TXK tyrosine kinase | 0.039 | 1.028 | 9.38E-01 | 9.38E+01 | | | |
| 8087830 | NM_000992 | RPL29 | ribosomal protein L29 | 0.039 | 1.028 | 8.18E-01 | 8.18E+01 | | | |
| 8041542 | NM_138801 | GALM | galactose mutarotase (aldose 1-epim | 0.039 | 1.028 | 7.30E-01 | 7.30E+01 | | | |
| 8046461 | NM_133646 | ZAK | sterile alpha motif and leucine zippe | 0.039 | 1.027 | 6.45E-01 | 6.45E+01 | | | |
| 8114567 | NM_002622 | PFDN1 | prefoldin subunit 1 | 0.039 | 1.027 | 8.60E-01 | 8.60E+01 | | | |
| 8135089 | NM_006349 | ZNHIT1 | zinc finger, HIT type 1 | 0.039 | 1.027 | 6.35E-01 | 6.35E+01 | | | |
| 8054611 | NR_024204 | NCRNA0015 | non-protein coding RNA 152 | 0.039 | 1.027 | 9.44E-01 | 9.44E+01 | | | |
| 7896497 | --- | --- | --- | 0.039 | 1.027 | 6.94E-01 | 6.94E+01 | | | |
| 8058024 | NM_012433 | SF3B1 | splicing factor 3b, subunit 1, 155kDa | 0.039 | 1.027 | 7.82E-01 | 7.82E+01 | | | |
| 8065194 | --- | --- | --- | 0.039 | 1.027 | 8.64E-01 | 8.64E+01 | | | |
| 7892782 | --- | --- | --- | 0.039 | 1.027 | 5.45E-01 | 5.45E+01 | | | |
| 7895463 | --- | --- | --- | 0.039 | 1.027 | 7.11E-01 | 7.11E+01 | | | |
| 8067279 | NM_001336 | CTSZ | cathepsin Z | 0.039 | 1.027 | 6.13E-01 | 6.13E+01 | | | |
| 7894610 | --- | --- | --- | 0.039 | 1.027 | 9.62E-01 | 9.62E+01 | | | |
| 7947917 | NM_016506 | KBTBD4 | kelch repeat and BTB (POZ) domain c | 0.039 | 1.027 | 6.14E-01 | 6.14E+01 | | | |
| 7923753 | NM_030952 | NUAK2 | NUAK family, SNF1-like kinase, 2 | 0.039 | 1.027 | 6.16E-01 | 6.16E+01 | | | |
| 7896415 | --- | --- | --- | 0.039 | 1.027 | 9.38E-01 | 9.38E+01 | | | |
| 7954388 | NM_016072 | GOLT1B | golgi transport 1 homolog B (S. cerev | 0.038 | 1.027 | 9.38E-01 | 9.38E+01 | | | |
| 7892536 | --- | --- | --- | 0.038 | 1.027 | 9.52E-01 | 9.52E+01 | | | |
| 7912112 | NM_018198 | DNAJC11 | DnaJ (Hsp40) homolog, subfamily C, | 0.038 | 1.027 | 8.66E-01 | 8.66E+01 | | | |
| 7993185 | NM_002484 | NUBP1 | nucleotide binding protein 1 (MinD h | 0.038 | 1.027 | 9.15E-01 | 9.15E+01 | | | |
| 8086482 | NM_181489 | ZNF445 | zinc finger protein 445 | 0.038 | 1.027 | 6.41E-01 | 6.41E+01 | | | |
| 8031784 | NM_003435 | ZNF134 | zinc finger protein 134 | 0.038 | 1.027 | 7.61E-01 | 7.61E+01 | | | |
| 8164810 | NM_006266 | RALGDS | ral guanine nucleotide dissociation s | 0.038 | 1.027 | 6.40E-01 | 6.40E+01 | | | |
| 7896369 | --- | --- | --- | 0.038 | 1.027 | 7.80E-01 | 7.80E+01 | | | |
| 7980246 | NM_00104010 | MLH3 | mutL homolog 3 (E. coli) | 0.038 | 1.027 | 8.30E-01 | 8.30E+01 | | | |
| 8009241 | NR_004380 | SNORD104 | small nucleolar RNA, C/D box 104 | 0.038 | 1.027 | 9.61E-01 | 9.61E+01 | | | |
| 7932867 | NM_00114377 | ZNF438 | zinc finger protein 438 | 0.038 | 1.027 | 8.19E-01 | 8.19E+01 | | | |
| 7893841 | --- | --- | --- | 0.038 | 1.027 | 8.82E-01 | 8.82E+01 | | | |
| 8155849 | NM_000700 | ANXA1 | annexin A1 | 0.038 | 1.027 | 9.30E-01 | 9.30E+01 | | | |
| 8069200 | AF426268 | C21orf89 | chromosome 21 open reading frame | 0.038 | 1.027 | 7.53E-01 | 7.53E+01 | | | |
| 7990898 | NM_000661 | RPL9 | ribosomal protein L9 | 0.038 | 1.027 | 7.48E-01 | 7.48E+01 | | | |
| 7896252 | --- | --- | --- | 0.038 | 1.027 | 6.53E-01 | 6.53E+01 | | | |
| 8043436 | BC073772 // B | GKC // IGK | immunoglobulin kappa constant // i | 0.038 | 1.027 | 9.39E-01 | 9.39E+01 | | | |
| 7896505 | --- | --- | --- | 0.038 | 1.027 | 9.65E-01 | 9.65E+01 | | | |
| 7929026 | NM_00114194 | ACTA2 | actin, alpha 2, smooth muscle, aorta | 0.038 | 1.027 | 6.11E-01 | 6.11E+01 | | | |
| 7987279 | NR_027410 | GOLGA8B | golgi autoantigen, golgin subfamily a | 0.038 | 1.027 | 8.12E-01 | 8.12E+01 | | | |
| 7893781 | --- | --- | --- | 0.038 | 1.027 | 6.68E-01 | 6.68E+01 | | | |
| 8176253 | --- | --- | --- | 0.038 | 1.027 | 8.96E-01 | 8.96E+01 | | | |
| 8007084 | NM_000964 | RARA | retinoic acid receptor, alpha | 0.038 | 1.027 | 6.57E-01 | 6.57E+01 | | | |
| 8131013 | NM_00113096 | UNC84A | unc-84 homolog A (C. elegans) | 0.038 | 1.026 | 8.43E-01 | 8.43E+01 | | | |
| 8146456 | NR_004385 | RNU105C | RNA, U105C small nucleolar | 0.038 | 1.026 | 8.16E-01 | 8.16E+01 | | | |

| | | | | | | | | | | |
|---|---|---|---|---|---|---|---|---|---|---|
| 8127542 | --- | --- | --- | 0.038 | 1.026 | 8.80E-01 | 8.80E+01 | | | |
| 8073293 | --- | --- | --- | 0.038 | 1.026 | 7.87E-01 | 7.87E+01 | | | |
| 8002194 | NM_022355 | DPEP2 | dipeptidase 2 | 0.038 | 1.026 | 7.39E-01 | 7.39E+01 | | | |
| 7894037 | --- | --- | --- | 0.038 | 1.026 | 8.66E-01 | 8.66E+01 | | | |
| 8174636 | NM_006937 | SUMO2 | SMT3 suppressor of mif two 3 homo | 0.037 | 1.026 | 9.28E-01 | 9.28E+01 | | | |
| 7898988 | NM_013943 | CLIC4 | chloride intracellular channel 4 | 0.037 | 1.026 | 8.43E-01 | 8.43E+01 | | | |
| 7893651 | --- | --- | --- | 0.037 | 1.026 | 4.59E-01 | 4.59E+01 | | | |
| 8165686 | --- | --- | --- | 0.037 | 1.026 | 4.66E-01 | 4.66E+01 | | | |
| 8014189 | NM_057178 | RFFL | ring finger and FYVE-like domain con | 0.037 | 1.026 | 7.71E-01 | 7.71E+01 | | | |
| 7953737 | NM_00100703 | CLEC6A | C-type lectin domain family 6, memb | 0.037 | 1.026 | 7.70E-01 | 7.70E+01 | | | |
| 8027381 | --- | --- | --- | 0.037 | 1.026 | 8.39E-01 | 8.39E+01 | | | |
| 8172158 | NM_003688 | CASK | calcium/calmodulin-dependent serin | 0.037 | 1.026 | 8.57E-01 | 8.57E+01 | | | |
| 8138545 | --- | --- | --- | 0.037 | 1.026 | 9.07E-01 | 9.07E+01 | | | |
| 7996341 | NM_020786 | PDP2 | pyruvate dehyrogenase phosphatase | 0.037 | 1.026 | 6.56E-01 | 6.56E+01 | | | |
| 7961339 | NM_002336 | LRP6 | low density lipoprotein receptor-rela | 0.037 | 1.026 | 6.88E-01 | 6.88E+01 | | | |
| 7894218 | --- | --- | --- | 0.037 | 1.026 | 6.60E-01 | 6.60E+01 | | | |
| 7895745 | --- | --- | --- | 0.037 | 1.026 | 7.33E-01 | 7.33E+01 | | | |
| 8161147 | NM_032593 | HINT2 | histidine triad nucleotide binding pro | 0.037 | 1.026 | 9.18E-01 | 9.18E+01 | | | |
| 8142036 | NM_182691 | SRPK2 | SFRS protein kinase 2 | 0.037 | 1.026 | 8.58E-01 | 8.58E+01 | | | |
| 7996919 | NM_013245 | VPS4A | vacuolar protein sorting 4 homolog A | 0.037 | 1.026 | 8.49E-01 | 8.49E+01 | | | |
| 7896073 | --- | --- | --- | 0.037 | 1.026 | 6.65E-01 | 6.65E+01 | | | |
| 8009533 | NM_018714 | COG1 | component of oligomeric golgi comp | 0.037 | 1.026 | 6.76E-01 | 6.76E+01 | | | |
| 8089930 | NM_004487 | GOLGB1 | golgin B1, golgi integral membrane p | 0.037 | 1.026 | 8.63E-01 | 8.63E+01 | | | |
| 8005753 | --- | --- | --- | 0.037 | 1.026 | 9.27E-01 | 9.27E+01 | | | |
| 8158597 | NM_00113655 | GPR107 | G protein-coupled receptor 107 | 0.037 | 1.026 | 6.00E-01 | 6.00E+01 | | | |
| 8086572 | NM_024513 | FYCO1 | FYVE and coiled-coil domain contain | 0.037 | 1.026 | 5.43E-01 | 5.43E+01 | | | |
| 7893764 | --- | --- | --- | 0.037 | 1.026 | 8.35E-01 | 8.35E+01 | | | |
| 7971122 | --- | --- | --- | 0.037 | 1.026 | 8.11E-01 | 8.11E+01 | | | |
| 8125859 | NM_005643 | TAF11 | TAF11 RNA polymerase II, TATA box | 0.037 | 1.026 | 8.78E-01 | 8.78E+01 | | | |
| 7896656 | --- | --- | --- | 0.037 | 1.026 | 8.40E-01 | 8.40E+01 | | | |
| 8167912 | NM_007250 | KLF8 | Kruppel-like factor 8 | 0.037 | 1.026 | 7.88E-01 | 7.88E+01 | | | |
| 7904448 | --- | --- | --- | 0.036 | 1.026 | 7.26E-01 | 7.26E+01 | | | |
| 8112961 | NM_001025 | RPS23 | ribosomal protein S23 | 0.036 | 1.026 | 7.83E-01 | 7.83E+01 | | | |
| 7952950 | ENST00000318 | OC10004971 | hypothetical protein LOC100049716 | 0.036 | 1.026 | 7.59E-01 | 7.59E+01 | | | |
| 8138145 | NR_002822 | MGC72080 | MGC72080 pseudogene | 0.036 | 1.026 | 9.19E-01 | 9.19E+01 | | | |
| 8039829 | NM_014219 | KIR2DL2 | killer cell immunoglobulin-like recep | 0.036 | 1.026 | 8.84E-01 | 8.84E+01 | | | |
| 8122933 | NM_012454 | TIAM2 | T-cell lymphoma invasion and metas | 0.036 | 1.026 | 5.61E-01 | 5.61E+01 | | | |
| 8151436 | NM_000318 | PXMP3 | peroxisomal membrane protein 3, 35 | 0.036 | 1.026 | 8.61E-01 | 8.61E+01 | | | |
| 8058458 | --- | --- | --- | 0.036 | 1.026 | 8.44E-01 | 8.44E+01 | | | |
| 7982535 | NM_032499 | C15orf41 | chromosome 15 open reading frame | 0.036 | 1.026 | 8.25E-01 | 8.25E+01 | | | |
| 7937533 | NM_012305 | AP2A2 | adaptor-related protein complex 2, a | 0.036 | 1.025 | 6.10E-01 | 6.10E+01 | | | |
| 7945069 | --- | --- | --- | 0.036 | 1.025 | 8.44E-01 | 8.44E+01 | | | |
| 7895274 | --- | --- | --- | 0.036 | 1.025 | 9.05E-01 | 9.05E+01 | | | |
| 8125463 | NR_003937 | HLA-DQB2 | major histocompatibility complex, cl | 0.036 | 1.025 | 7.53E-01 | 7.53E+01 | | | |
| 7999356 | --- | --- | --- | 0.036 | 1.025 | 9.32E-01 | 9.32E+01 | | | |
| 8003087 | --- | --- | --- | 0.036 | 1.025 | 9.22E-01 | 9.22E+01 | | | |
| 7985918 | --- | --- | --- | 0.036 | 1.025 | 8.84E-01 | 8.84E+01 | | | |
| 7906753 | --- | --- | --- | 0.036 | 1.025 | 8.70E-01 | 8.70E+01 | | | |
| 8154574 | NM_017794 | KIAA1797 | KIAA1797 | 0.036 | 1.025 | 6.90E-01 | 6.90E+01 | | | |
| 7893248 | --- | --- | --- | 0.036 | 1.025 | 9.40E-01 | 9.40E+01 | | | |
| 7983953 | NM_017610 | RNF111 | ring finger protein 111 | 0.036 | 1.025 | 7.82E-01 | 7.82E+01 | | | |
| 8125328 | NM_006411 | AGPAT1 | 1-acylglycerol-3-phosphate O-acyltra | 0.036 | 1.025 | 6.89E-01 | 6.89E+01 | | | |
| 8178762 | NM_006411 | AGPAT1 | 1-acylglycerol-3-phosphate O-acyltra | 0.036 | 1.025 | 6.89E-01 | 6.89E+01 | | | |
| 8179958 | NM_006411 | AGPAT1 | 1-acylglycerol-3-phosphate O-acyltra | 0.036 | 1.025 | 6.89E-01 | 6.89E+01 | | | |
| 8176163 | NM_00108157 | GAB3 | GRB2-associated binding protein 3 | 0.036 | 1.025 | 6.63E-01 | 6.63E+01 | | | |
| 7896694 | --- | --- | --- | 0.036 | 1.025 | 8.37E-01 | 8.37E+01 | | | |
| 7944152 | NM_001558 | IL10RA | interleukin 10 receptor, alpha | 0.036 | 1.025 | 8.37E-01 | 8.37E+01 | | | |
| 8005089 | NM_001303 | COX10 | COX10 homolog, cytochrome c oxida | 0.036 | 1.025 | 7.56E-01 | 7.56E+01 | | | |
| 7962194 | NM_00101369 | H3F3C | H3 histone, family 3C | 0.036 | 1.025 | 8.90E-01 | 8.90E+01 | | | |
| 8018708 | NM_022066 | UBE2O | ubiquitin-conjugating enzyme E2O | 0.036 | 1.025 | 6.83E-01 | 6.83E+01 | | | |
| 8123737 | --- | --- | --- | 0.036 | 1.025 | 7.53E-01 | 7.53E+01 | | | |
| 8021966 | NM_017512 | ENOSF1 | enolase superfamily member 1 | 0.035 | 1.025 | 7.80E-01 | 7.80E+01 | | | |
| 7953200 | NM_001759 | CCND2 | cyclin D2 | 0.035 | 1.025 | 8.05E-01 | 8.05E+01 | | | |
| 7906746 | NM_003001 | SDHC | succinate dehydrogenase complex, s | 0.035 | 1.025 | 7.76E-01 | 7.76E+01 | | | |
| 8121510 | NM_032194 | BXDC1 | brix domain containing 1 | 0.035 | 1.025 | 9.40E-01 | 9.40E+01 | | | |
| 8163999 | NM_006626 | ZBTB6 | zinc finger and BTB domain containi | 0.035 | 1.025 | 7.64E-01 | 7.64E+01 | | | |
| 8008096 | NM_152244 | SNX11 | sorting nexin 11 | 0.035 | 1.025 | 7.18E-01 | 7.18E+01 | | | |
| 8012212 | NM_020899 | ZBTB4 | zinc finger and BTB domain containi | 0.035 | 1.025 | 6.40E-01 | 6.40E+01 | | | |
| 7941179 | NM_005186 | CAPN1 | calpain 1, (mu/I) large subunit | 0.035 | 1.025 | 7.11E-01 | 7.11E+01 | | | |
| 8054945 | NM_139343 | BIN1 | bridging integrator 1 | 0.035 | 1.025 | 7.42E-01 | 7.42E+01 | | | |
| 7921344 | NM_012081 | ELL2 | elongation factor, RNA polymerase II | 0.035 | 1.025 | 9.32E-01 | 9.32E+01 | | | |
| 8083876 | NM_005414 | SKIL | SKI-like oncogene | 0.035 | 1.025 | 9.39E-01 | 9.39E+01 | | | |
| 8042843 | NM_032118 | WDR54 | WD repeat domain 54 | 0.035 | 1.025 | 8.63E-01 | 8.63E+01 | | | |
| 8014749 | NM_000978 | RPL23 | ribosomal protein L23 | 0.035 | 1.025 | 6.53E-01 | 6.53E+01 | | | |
| 8008213 | NM_007067 | MYST2 | MYST histone acetyltransferase 2 | 0.035 | 1.025 | 8.33E-01 | 8.33E+01 | | | |
| 7907370 | NM_015569 | DNM3 | dynamin 3 | 0.035 | 1.025 | 8.23E-01 | 8.23E+01 | | | |
| 8074577 | NR_003700 | PI4KAP2 | phosphatidylinositol 4-kinase, catal | 0.035 | 1.025 | 6.76E-01 | 6.76E+01 | | | |

| | | | | | | | | | | |
|---|---|---|---|---|---|---|---|---|---|---|
| 7987027 | NR_024074 | GOLGA9P | golgi autoantigen, golgin subfamily a | 0.035 | 1.025 | 7.50E-01 | 7.50E+01 | | | |
| 7908635 | --- | --- | --- | 0.035 | 1.025 | 6.22E-01 | 6.22E+01 | | | |
| 7895571 | --- | --- | --- | 0.035 | 1.025 | 9.70E-01 | 9.70E+01 | | | |
| 7927167 | --- | --- | --- | 0.035 | 1.024 | 8.03E-01 | 8.03E+01 | | | |
| 7894308 | --- | --- | --- | 0.035 | 1.024 | 7.02E-01 | 7.02E+01 | | | |
| 7974257 | NM_015684 | ATP5S | ATP synthase, H+ transporting, mitoc | 0.035 | 1.024 | 8.51E-01 | 8.51E+01 | | | |
| 8099471 | NM_031950 | FGFBP2 | fibroblast growth factor binding prot | 0.035 | 1.024 | 8.68E-01 | 8.68E+01 | | | |
| 7951826 | NM_032725 | BUD13 | BUD13 homolog (S. cerevisiae) | 0.035 | 1.024 | 8.37E-01 | 8.37E+01 | | | |
| 8117667 | NM_00102356( | ZNF187 | zinc finger protein 187 | 0.035 | 1.024 | 7.72E-01 | 7.72E+01 | | | |
| 7894491 | --- | --- | --- | 0.035 | 1.024 | 9.46E-01 | 9.46E+01 | | | |
| 8169365 | NM_032227 | TMEM164 | transmembrane protein 164 | 0.034 | 1.024 | 5.95E-01 | 5.95E+01 | | | |
| 7920600 | NM_183001 | SHC1 | SHC (Src homology 2 domain contair | 0.034 | 1.024 | 6.74E-01 | 6.74E+01 | | | |
| 7974533 | NM_021255 | PELI2 | pellino homolog 2 (Drosophila) | 0.034 | 1.024 | 7.35E-01 | 7.35E+01 | | | |
| 8044882 | NM_020909 | EPB41L5 | erythrocyte membrane protein band | 0.034 | 1.024 | 7.80E-01 | 7.80E+01 | | | |
| 7923812 | NM_003929 | RAB7L1 | RAB7, member RAS oncogene family | 0.034 | 1.024 | 7.53E-01 | 7.53E+01 | | | |
| 8080762 | NM_020676 | ABHD6 | abhydrolase domain containing 6 | 0.034 | 1.024 | 7.85E-01 | 7.85E+01 | | | |
| 8006229 | NM_032322 | RNF135 | ring finger protein 135 | 0.034 | 1.024 | 7.03E-01 | 7.03E+01 | | | |
| 8172307 | NM_007130 | ZNF41 | zinc finger protein 41 | 0.034 | 1.024 | 5.97E-01 | 5.97E+01 | | | |
| 8005847 | NM_021137 | TNFAIP1 | tumor necrosis factor, alpha-induced | 0.034 | 1.024 | 6.84E-01 | 6.84E+01 | | | |
| 7955078 | NM_000289 | PFKM | phosphofructokinase, muscle | 0.034 | 1.024 | 6.51E-01 | 6.51E+01 | | | |
| 7989725 | NM_005707 | PDCD7 | programmed cell death 7 | 0.034 | 1.024 | 8.20E-01 | 8.20E+01 | | | |
| 7986428 | AK127420 | LOC400464 | similar to FLJ43276 protein | 0.034 | 1.024 | 7.15E-01 | 7.15E+01 | | | |
| 7926319 | NM_024670 | SUV39H2 | suppressor of variegation 3-9 homol | 0.034 | 1.024 | 6.63E-01 | 6.63E+01 | | | |
| 7994659 | NM_017458 | MVP | major vault protein | 0.034 | 1.024 | 7.79E-01 | 7.79E+01 | | | |
| 8082244 | NR_002822 | MGC72080 | MGC72080 pseudogene | 0.034 | 1.024 | 9.35E-01 | 9.35E+01 | | | |
| 8106761 | --- | --- | --- | 0.034 | 1.024 | 8.34E-01 | 8.34E+01 | | | |
| 7893769 | --- | --- | --- | 0.034 | 1.024 | 6.44E-01 | 6.44E+01 | | | |
| 7893464 | --- | --- | --- | 0.034 | 1.024 | 7.74E-01 | 7.74E+01 | | | |
| 8170298 | NM_00101298! | E2NL // UBE | ubiquitin-conjugating enzyme E2N-li | 0.034 | 1.024 | 8.60E-01 | 8.60E+01 | | | |
| 8017346 | NR_027486 | TBC1D3P2 | TBC1 domain family, member 3 pseu | 0.034 | 1.024 | 6.44E-01 | 6.44E+01 | | | |
| 8063074 | NM_080603 | ZSWIM1 | zinc finger, SWIM-type containing 1 | 0.034 | 1.024 | 7.64E-01 | 7.64E+01 | | | |
| 7894941 | --- | --- | --- | 0.034 | 1.024 | 5.55E-01 | 5.55E+01 | | | |
| 7896346 | --- | --- | --- | 0.034 | 1.024 | 5.45E-01 | 5.45E+01 | | | |
| 7920567 | NM_006556 | PMVK | phosphomevalonate kinase | 0.034 | 1.024 | 7.82E-01 | 7.82E+01 | | | |
| 8085350 | NM_138807 | C3orf31 | chromosome 3 open reading frame 3 | 0.034 | 1.024 | 8.39E-01 | 8.39E+01 | | | |
| 8180260 | --- | --- | --- | 0.034 | 1.024 | 8.43E-01 | 8.43E+01 | | | |
| 8016402 | NM_00112722 | CBX1 | chromobox homolog 1 (HP1 beta ho | 0.034 | 1.024 | 6.58E-01 | 6.58E+01 | | | |
| 8099918 | AK291454 | UBE2K | ubiquitin-conjugating enzyme E2K (U | 0.034 | 1.024 | 8.15E-01 | 8.15E+01 | | | |
| 8142730 | NM_176814 | ZNF800 | zinc finger protein 800 | 0.034 | 1.024 | 8.44E-01 | 8.44E+01 | | | |
| 7960666 | NM_133476 | ZNF384 | zinc finger protein 384 | 0.034 | 1.024 | 6.57E-01 | 6.57E+01 | | | |
| 7967870 | --- | --- | --- | 0.034 | 1.024 | 9.08E-01 | 9.08E+01 | | | |
| 7896385 | --- | --- | --- | 0.034 | 1.024 | 9.57E-01 | 9.57E+01 | | | |
| 7920354 | NM_014856 | DENND4B | DENN/MADD domain containing 4B | 0.034 | 1.024 | 6.53E-01 | 6.53E+01 | | | |
| 7948267 | NM_012456 | TIMM10 | translocase of inner mitochondrial m | 0.034 | 1.024 | 9.03E-01 | 9.03E+01 | | | |
| 8117580 | NM_003509 | HIST1H2AI | histone cluster 1, H2ai | 0.034 | 1.024 | 8.00E-01 | 8.00E+01 | | | |
| 8162260 | --- | --- | --- | 0.033 | 1.023 | 9.30E-01 | 9.30E+01 | | | |
| 7975661 | NM_182476 | COQ6 | coenzyme Q6 homolog, monooxyger | 0.033 | 1.023 | 8.38E-01 | 8.38E+01 | | | |
| 8119034 | NM_015695 | BRPF3 | bromodomain and PHD finger contai | 0.033 | 1.023 | 6.96E-01 | 6.96E+01 | | | |
| 7944011 | NM_015523 | REXO2 | REX2, RNA exonuclease 2 homolog (S | 0.033 | 1.023 | 9.51E-01 | 9.51E+01 | | | |
| 7946676 | --- | --- | --- | 0.033 | 1.023 | 9.18E-01 | 9.18E+01 | | | |
| 7961960 | --- | --- | --- | 0.033 | 1.023 | 8.97E-01 | 8.97E+01 | | | |
| 7894620 | --- | --- | --- | 0.033 | 1.023 | 9.73E-01 | 9.73E+01 | | | |
| 7895712 | --- | --- | --- | 0.033 | 1.023 | 7.59E-01 | 7.59E+01 | | | |
| 7950142 | NM_030813 | CLPB | ClpB caseinolytic peptidase B homol | 0.033 | 1.023 | 6.58E-01 | 6.58E+01 | | | |
| 7895150 | --- | --- | --- | 0.033 | 1.023 | 9.72E-01 | 9.72E+01 | | | |
| 8009443 | NM_014960 | ARSG | arylsulfatase G | 0.033 | 1.023 | 7.21E-01 | 7.21E+01 | | | |
| 7911337 | --- | --- | --- | 0.033 | 1.023 | 9.51E-01 | 9.51E+01 | | | |
| 7973871 | --- | --- | --- | 0.033 | 1.023 | 9.51E-01 | 9.51E+01 | | | |
| 8165696 | --- | --- | --- | 0.033 | 1.023 | 9.51E-01 | 9.51E+01 | | | |
| 7985226 | NM_206839 | MORF4L1 | mortality factor 4 like 1 | 0.033 | 1.023 | 8.61E-01 | 8.61E+01 | | | |
| 7894207 | --- | --- | --- | 0.033 | 1.023 | 7.39E-01 | 7.39E+01 | | | |
| 8100068 | --- | --- | --- | 0.033 | 1.023 | 7.40E-01 | 7.40E+01 | | | |
| 7906330 | NM_001766 | CD1D | CD1d molecule | 0.033 | 1.023 | 6.80E-01 | 6.80E+01 | | | |
| 8036473 | NM_033256 | PPP1R14A | protein phosphatase 1, regulatory (ir | 0.033 | 1.023 | 9.20E-01 | 9.20E+01 | | | |
| 7971239 | --- | --- | --- | 0.033 | 1.023 | 9.53E-01 | 9.53E+01 | | | |
| 7893329 | --- | --- | --- | 0.033 | 1.023 | 8.81E-01 | 8.81E+01 | | | |
| 8041820 | NM_014011 | SOCS5 | suppressor of cytokine signaling 5 | 0.033 | 1.023 | 7.82E-01 | 7.82E+01 | | | |
| 8161520 | NR_002836 | PGM5P2 | phosphoglucomutase 5 pseudogene | 0.033 | 1.023 | 7.34E-01 | 7.34E+01 | | | |
| 8097553 | NM_172174 | IL15 | interleukin 15 | 0.033 | 1.023 | 7.69E-01 | 7.69E+01 | | | |
| 7924150 | NM_018252 | TMEM206 | transmembrane protein 206 | 0.033 | 1.023 | 8.15E-01 | 8.15E+01 | | | |
| 8004081 | NM_153018 | ZFP3 | zinc finger protein 3 homolog (mous | 0.033 | 1.023 | 7.33E-01 | 7.33E+01 | | | |
| 8085431 | NM_024923 | NUP210 | nucleoporin 210kDa | 0.033 | 1.023 | 7.34E-01 | 7.34E+01 | | | |
| 8044258 | NM_004987 | LIMS1 | LIM and senescent cell antigen-like d | 0.032 | 1.023 | 8.52E-01 | 8.52E+01 | | | |
| 7973448 | NM_021004 | DHRS4 | dehydrogenase/reductase (SDR fami | 0.032 | 1.023 | 8.77E-01 | 8.77E+01 | | | |
| 7927732 | NM_032199 | ARID5B | AT rich interactive domain 5B (MRF1 | 0.032 | 1.023 | 9.06E-01 | 9.06E+01 | | | |
| 8173189 | NM_00100668 | SPIN2B | spindlin family, member 2B | 0.032 | 1.023 | 8.70E-01 | 8.70E+01 | | | |
| 8110253 | NM_012279 | ZNF346 | zinc finger protein 346 | 0.032 | 1.023 | 7.76E-01 | 7.76E+01 | | | |

| | | | | | | | | | | |
|---|---|---|---|---|---|---|---|---|---|---|
| 8030437 | NM_198319 | PRMT1 | protein arginine methyltransferase 1 | 0.032 | 1.023 | 8.82E-01 | 8.82E+01 | | | |
| 7900446 | NM_198494 | ZNF642 | zinc finger protein 642 | 0.032 | 1.023 | 8.04E-01 | 8.04E+01 | | | |
| 8149071 | NM_001147 | ANGPT2 | angiopoietin 2 | 0.032 | 1.023 | 7.65E-01 | 7.65E+01 | | | |
| 8004293 | NM_203413 | C17orf81 | chromosome 17 open reading frame | 0.032 | 1.022 | 7.69E-01 | 7.69E+01 | | | |
| 8146334 | --- | --- | --- | 0.032 | 1.022 | 6.75E-01 | 6.75E+01 | | | |
| 7961252 | NM_007244 | PRR4 | proline rich 4 (lacrimal) | 0.032 | 1.022 | 8.13E-01 | 8.13E+01 | | | |
| 8004271 | NM_000018 | ACADVL | acyl-Coenzyme A dehydrogenase, ve | 0.032 | 1.022 | 8.78E-01 | 8.78E+01 | | | |
| 8050240 | NM_002539 | ODC1 | ornithine decarboxylase 1 | 0.032 | 1.022 | 8.69E-01 | 8.69E+01 | | | |
| 8148955 | NM_023080 | C8orf33 | chromosome 8 open reading frame 3 | 0.032 | 1.022 | 7.82E-01 | 7.82E+01 | | | |
| 7893313 | --- | --- | --- | 0.032 | 1.022 | 9.46E-01 | 9.46E+01 | | | |
| 8006984 | NM_002809 | PSMD3 | proteasome (prosome, macropain) 2 | 0.032 | 1.022 | 7.50E-01 | 7.50E+01 | | | |
| 8126705 | --- | --- | --- | 0.032 | 1.022 | 9.18E-01 | 9.18E+01 | | | |
| 8062016 | NM_014183 | DYNLRB1 | dynein, light chain, roadblock-type 1 | 0.032 | 1.022 | 8.84E-01 | 8.84E+01 | | | |
| 7895659 | --- | --- | --- | 0.032 | 1.022 | 7.36E-01 | 7.36E+01 | | | |
| 8154635 | NM_002451 | MTAP | methylthioadenosine phosphorylase | 0.032 | 1.022 | 5.24E-01 | 5.24E+01 | | | |
| 7902205 | NM_001559 | IL12RB2 | interleukin 12 receptor, beta 2 | 0.032 | 1.022 | 8.83E-01 | 8.83E+01 | | | |
| 7899153 | NM_031286 | SH3BGRL3 | SH3 domain binding glutamic acid-ric | 0.032 | 1.022 | 7.13E-01 | 7.13E+01 | | | |
| 7893684 | --- | --- | --- | 0.032 | 1.022 | 9.52E-01 | 9.52E+01 | | | |
| 7892684 | --- | --- | --- | 0.031 | 1.022 | 9.71E-01 | 9.71E+01 | | | |
| 7948229 | NM_017611 | SLC43A3 | solute carrier family 43, member 3 | 0.031 | 1.022 | 7.37E-01 | 7.37E+01 | | | |
| 7985240 | NM_007364 | TMED3 | transmembrane emp24 protein trans | 0.031 | 1.022 | 7.07E-01 | 7.07E+01 | | | |
| 8138189 | NM_002947 | RPA3 | replication protein A3, 14kDa | 0.031 | 1.022 | 8.15E-01 | 8.15E+01 | | | |
| 7963721 | NM_020898 | CALCOCO1 | calcium binding and coiled-coil doma | 0.031 | 1.022 | 8.44E-01 | 8.44E+01 | | | |
| 7893189 | --- | --- | --- | 0.031 | 1.022 | 4.39E-01 | 4.39E+01 | | | |
| 8046086 | NM_203463 | LASS6 | LAG1 homolog, ceramide synthase 6 | 0.031 | 1.022 | 9.24E-01 | 9.24E+01 | | | |
| 7896342 | --- | --- | --- | 0.031 | 1.022 | 9.21E-01 | 9.21E+01 | | | |
| 8157454 | NM_004888 | ATP6V1G1 | ATPase, H+ transporting, lysosomal 1 | 0.031 | 1.022 | 8.79E-01 | 8.79E+01 | | | |
| 7896712 | --- | --- | --- | 0.031 | 1.022 | 9.36E-01 | 9.36E+01 | | | |
| 7993606 | --- | --- | --- | 0.031 | 1.022 | 9.25E-01 | 9.25E+01 | | | |
| 8017173 | NR_027486 | TBC1D3P2 | TBC1 domain family, member 3 pseu | 0.031 | 1.022 | 7.11E-01 | 7.11E+01 | | | |
| 8155707 | NM_004817 | TJP2 | tight junction protein 2 (zona occlud | 0.031 | 1.022 | 7.13E-01 | 7.13E+01 | | | |
| 8054135 | NM_012214 | MGAT4A | mannosyl (alpha-1,3-)-glycoprotein b | 0.031 | 1.022 | 9.42E-01 | 9.42E+01 | | | |
| 7912496 | NM_005957 | MTHFR | 5,10-methylenetetrahydrofolate red | 0.031 | 1.022 | 7.02E-01 | 7.02E+01 | | | |
| 7893640 | --- | --- | --- | 0.031 | 1.022 | 8.35E-01 | 8.35E+01 | | | |
| 7895193 | --- | --- | --- | 0.031 | 1.022 | 8.90E-01 | 8.90E+01 | | | |
| 8143054 | NM_001628 | AKR1B1 | aldo-keto reductase family 1, memb | 0.031 | 1.022 | 9.32E-01 | 9.32E+01 | | | |
| 8152211 | --- | --- | --- | 0.031 | 1.022 | 9.74E-01 | 9.74E+01 | | | |
| 7990487 | NM_015477 | SIN3A | SIN3 homolog A, transcription regula | 0.031 | 1.022 | 8.51E-01 | 8.51E+01 | | | |
| 8168357 | NR_002309 | RPS26P11 | ribosomal protein S26 pseudogene 1 | 0.031 | 1.022 | 8.78E-01 | 8.78E+01 | | | |
| 8093539 | NM_133330 | WHSC1 | Wolf-Hirschhorn syndrome candidat | 0.031 | 1.022 | 6.56E-01 | 6.56E+01 | | | |
| 8124726 | NM_003449 | TRIM26 | tripartite motif-containing 26 | 0.031 | 1.022 | 7.66E-01 | 7.66E+01 | | | |
| 8107133 | NM_000919 | PAM | peptidylglycine alpha-amidating mon | 0.031 | 1.022 | 7.77E-01 | 7.77E+01 | | | |
| 8023261 | NM_006111 | ACAA2 | acetyl-Coenzyme A acyltransferase 2 | 0.031 | 1.021 | 9.00E-01 | 9.00E+01 | | | |
| 8059012 | --- | --- | --- | 0.031 | 1.021 | 9.15E-01 | 9.15E+01 | | | |
| 7896192 | --- | --- | --- | 0.031 | 1.021 | 7.43E-01 | 7.43E+01 | | | |
| 8172244 | NM_173794 | FUNDC1 | FUN14 domain containing 1 | 0.031 | 1.021 | 8.78E-01 | 8.78E+01 | | | |
| 8180355 | --- | --- | --- | 0.031 | 1.021 | 8.26E-01 | 8.26E+01 | | | |
| 7964810 | NM_017440 | MDM1 | Mdm1 nuclear protein homolog (mo | 0.030 | 1.021 | 8.38E-01 | 8.38E+01 | | | |
| 7895326 | --- | --- | --- | 0.030 | 1.021 | 7.06E-01 | 7.06E+01 | | | |
| 8067199 | --- | --- | --- | 0.030 | 1.021 | 9.60E-01 | 9.60E+01 | | | |
| 8170992 | NR_002984 | SNORA56 | small nucleolar RNA, H/ACA box 56 | 0.030 | 1.021 | 9.19E-01 | 9.19E+01 | | | |
| 8176460 | NM_002760 | PRKY | protein kinase, Y-linked | 0.030 | 1.021 | 8.78E-01 | 8.78E+01 | | | |
| 8045736 | NM_052905 | FMNL2 | formin-like 2 | 0.030 | 1.021 | 6.58E-01 | 6.58E+01 | | | |
| 7895097 | --- | --- | --- | 0.030 | 1.021 | 9.61E-01 | 9.61E+01 | | | |
| 7936028 | NM_005736 | ACTR1A | ARP1 actin-related protein 1 homolo | 0.030 | 1.021 | 8.46E-01 | 8.46E+01 | | | |
| 7896036 | --- | --- | --- | 0.030 | 1.021 | 7.80E-01 | 7.80E+01 | | | |
| 7996883 | NM_005329 | HAS3 | hyaluronan synthase 3 | 0.030 | 1.021 | 7.71E-01 | 7.71E+01 | | | |
| 8065122 | --- | --- | --- | 0.030 | 1.021 | 7.05E-01 | 7.05E+01 | | | |
| 8167854 | NM_014481 | APEX2 | APEX nuclease (apurinic/apyrimidini | 0.030 | 1.021 | 7.71E-01 | 7.71E+01 | | | |
| 8106769 | XR_019029 | LOC645181 | similar to PDGFA associated protein | 0.030 | 1.021 | 8.67E-01 | 8.67E+01 | | | |
| 8022572 | NM_080597 | OSBPL1A | oxysterol binding protein-like 1A | 0.030 | 1.021 | 7.20E-01 | 7.20E+01 | | | |
| 8050869 | --- | --- | --- | 0.030 | 1.021 | 8.78E-01 | 8.78E+01 | | | |
| 8163086 | NM_032012 | C9orf5 | chromosome 9 open reading frame 5 | 0.030 | 1.021 | 8.82E-01 | 8.82E+01 | | | |
| 7894709 | --- | --- | --- | 0.030 | 1.021 | 8.87E-01 | 8.87E+01 | | | |
| 7899087 | NR_026686 | PDIK1L | PDLIM1 interacting kinase 1 like | 0.030 | 1.021 | 8.38E-01 | 8.38E+01 | | | |
| 7895579 | --- | --- | --- | 0.030 | 1.021 | 8.19E-01 | 8.19E+01 | | | |
| 7895896 | --- | --- | --- | 0.030 | 1.021 | 8.91E-01 | 8.91E+01 | | | |
| 7893222 | --- | --- | --- | 0.030 | 1.021 | 9.63E-01 | 9.63E+01 | | | |
| 7895267 | --- | --- | --- | 0.030 | 1.021 | 8.77E-01 | 8.77E+01 | | | |
| 7974566 | NM_018229 | MUDENG | MU-2/AP1M2 domain containing, de | 0.030 | 1.021 | 9.01E-01 | 9.01E+01 | | | |
| 7893653 | --- | --- | --- | 0.030 | 1.021 | 9.51E-01 | 9.51E+01 | | | |
| 8020321 | --- | --- | --- | 0.029 | 1.021 | 7.73E-01 | 7.73E+01 | | | |
| 8130783 | AK000385 | RNASET2 | ribonuclease T2 | 0.029 | 1.021 | 8.00E-01 | 8.00E+01 | | | |
| 7986515 | ENST00000426 | OC10028966 | similar to dynamin 1 | 0.029 | 1.021 | 8.86E-01 | 8.86E+01 | | | |
| 7986525 | ENST00000426 | OC10028966 | similar to dynamin 1 | 0.029 | 1.021 | 8.86E-01 | 8.86E+01 | | | |
| 8093230 | NM_00114564 | KIAA0226 | KIAA0226 | 0.029 | 1.021 | 7.59E-01 | 7.59E+01 | | | |
| 8058837 | NM_018441 | PECR | peroxisomal trans-2-enoyl-CoA redu | 0.029 | 1.021 | 9.35E-01 | 9.35E+01 | | | |

| | | | | | | | | | | |
|---|---|---|---|---|---|---|---|---|---|---|
| 8028102 | --- | --- | --- | 0.029 | 1.021 | 9.08E-01 | 9.08E+01 | | | |
| 8146921 | NM_172037 | RDH10 | retinol dehydrogenase 10 (all-trans) | 0.029 | 1.021 | 9.18E-01 | 9.18E+01 | | | |
| 7971711 | --- | --- | --- | 0.029 | 1.020 | 9.06E-01 | 9.06E+01 | | | |
| 8027650 | NM_005499 | UBA2 | ubiquitin-like modifier activating enz | 0.029 | 1.020 | 9.20E-01 | 9.20E+01 | | | |
| 7953341 | NM_018009 | TAPBPL | TAP binding protein-like | 0.029 | 1.020 | 7.69E-01 | 7.69E+01 | | | |
| 8019211 | NM_017921 | NPLOC4 | nuclear protein localization 4 homolo | 0.029 | 1.020 | 8.94E-01 | 8.94E+01 | | | |
| 7913249 | NM_032409 | PINK1 | PTEN induced putative kinase 1 | 0.029 | 1.020 | 8.47E-01 | 8.47E+01 | | | |
| 7899289 | NM_015023 | WDTC1 | WD and tetratricopeptide repeats 1 | 0.029 | 1.020 | 7.84E-01 | 7.84E+01 | | | |
| 8038815 | NM_030657 | LIM2 | lens intrinsic membrane protein 2, 1! | 0.029 | 1.020 | 8.47E-01 | 8.47E+01 | | | |
| 8007197 | NM_017595 | NKIRAS2 | NFKB inhibitor interacting Ras-like 2 | 0.029 | 1.020 | 8.30E-01 | 8.30E+01 | | | mono |
| 8150217 | NR_003671 | hCG_164017 | hCG1640171 | 0.029 | 1.020 | 8.96E-01 | 8.96E+01 | | | |
| 8138091 | NM_139179 | DAGLB | diacylglycerol lipase, beta | 0.029 | 1.020 | 8.13E-01 | 8.13E+01 | | | |
| 8170479 | NM_00101798 | VMA21 | VMA21 vacuolar H+-ATPase homolog | 0.029 | 1.020 | 7.25E-01 | 7.25E+01 | | | |
| 8176133 | NM_000402 | G6PD | glucose-6-phosphate dehydrogenase | 0.029 | 1.020 | 7.22E-01 | 7.22E+01 | | | |
| 8173627 | --- | --- | --- | 0.028 | 1.020 | 9.51E-01 | 9.51E+01 | | | |
| 7984079 | NM_000366 | TPM1 | tropomyosin 1 (alpha) | 0.028 | 1.020 | 8.07E-01 | 8.07E+01 | | | |
| 8105229 | NM_015946 | PELO | pelota homolog (Drosophila) | 0.028 | 1.020 | 7.83E-01 | 7.83E+01 | | | |
| 8160332 | NM_004529 | MLLT3 | myeloid/lymphoid or mixed-lineage | 0.028 | 1.020 | 9.37E-01 | 9.37E+01 | | | |
| 7997827 | NM_00114286 | FAM38A | family with sequence similarity 38, m | 0.028 | 1.020 | 8.29E-01 | 8.29E+01 | | | |
| 8139430 | NM_033224 | PURB | purine-rich element binding protein | 0.028 | 1.020 | 7.77E-01 | 7.77E+01 | | | |
| 8016213 | --- | --- | --- | 0.028 | 1.020 | 9.23E-01 | 9.23E+01 | | | |
| 8107814 | NM_016048 | ISOC1 | isochorismatase domain containing 1 | 0.028 | 1.020 | 9.21E-01 | 9.21E+01 | | | |
| 7946201 | NM_012402 | ARFIP2 | ADP-ribosylation factor interacting p | 0.028 | 1.020 | 8.19E-01 | 8.19E+01 | | | |
| 8153911 | NM_014066 | COMMD5 | COMM domain containing 5 | 0.028 | 1.020 | 6.52E-01 | 6.52E+01 | | | |
| 8089928 | --- | --- | --- | 0.028 | 1.020 | 9.23E-01 | 9.23E+01 | | | |
| 8112918 | --- | --- | --- | 0.028 | 1.020 | 8.88E-01 | 8.88E+01 | | | |
| 8117219 | NM_018473 | ACOT13 | acyl-CoA thioesterase 13 | 0.028 | 1.020 | 9.40E-01 | 9.40E+01 | | | |
| 7961483 | NM_175054 | HIST4H4 | histone cluster 4, H4 | 0.028 | 1.020 | 8.70E-01 | 8.70E+01 | | | |
| 8149673 | NM_025232 | REEP4 | receptor accessory protein 4 | 0.028 | 1.020 | 8.67E-01 | 8.67E+01 | | | |
| 7899448 | NM_017638 | MED18 | mediator complex subunit 18 | 0.028 | 1.020 | 7.47E-01 | 7.47E+01 | | | |
| 7951422 | NM_032424 | KIAA1826 | KIAA1826 | 0.028 | 1.020 | 9.39E-01 | 9.39E+01 | | | |
| 8083893 | --- | --- | --- | 0.028 | 1.020 | 8.70E-01 | 8.70E+01 | | | |
| 8019376 | NM_022156 | DUS1L | dihydrouridine synthase 1-like (S. cer | 0.028 | 1.020 | 8.17E-01 | 8.17E+01 | | | |
| 8037331 | NM_006297 | XRCC1 | X-ray repair complementing defectiv | 0.028 | 1.019 | 8.18E-01 | 8.18E+01 | | | |
| 8073379 | NM_031488 | L3MBTL2 | l(3)mbt-like 2 (Drosophila) | 0.028 | 1.019 | 6.92E-01 | 6.92E+01 | | | |
| 7894656 | --- | --- | --- | 0.028 | 1.019 | 9.63E-01 | 9.63E+01 | | | |
| 8058340 | --- | --- | --- | 0.028 | 1.019 | 6.96E-01 | 6.96E+01 | | | |
| 7991989 | NM_138769 | RHOT2 | ras homolog gene family, member T2 | 0.028 | 1.019 | 7.03E-01 | 7.03E+01 | | | |
| 8069332 | NR_002776 | MCM3APAS | MCM3AP antisense RNA (non-protei | 0.028 | 1.019 | 7.36E-01 | 7.36E+01 | | | |
| 8062339 | BC130646 | C20orf118 | chromosome 20 open reading frame | 0.028 | 1.019 | 6.47E-01 | 6.47E+01 | | | |
| 8146239 | --- | --- | --- | 0.028 | 1.019 | 8.00E-01 | 8.00E+01 | | | |
| 7935780 | NM_015490 | SEC31B | SEC31 homolog B (S. cerevisiae) | 0.027 | 1.019 | 7.78E-01 | 7.78E+01 | | | |
| 7895801 | --- | --- | --- | 0.027 | 1.019 | 9.21E-01 | 9.21E+01 | | | |
| 8116128 | NR_026921 | LOC202181 | hypothetical protein LOC202181 | 0.027 | 1.019 | 9.23E-01 | 9.23E+01 | | | |
| 7895143 | --- | --- | --- | 0.027 | 1.019 | 8.80E-01 | 8.80E+01 | | | |
| 8165630 | NM_032937 | C9orf37 | chromosome 9 open reading frame 3 | 0.027 | 1.019 | 9.07E-01 | 9.07E+01 | | | |
| 7893039 | --- | --- | --- | 0.027 | 1.019 | 7.51E-01 | 7.51E+01 | | | |
| 8020878 | NM_014268 | MAPRE2 | microtubule-associated protein, RP/I | 0.027 | 1.019 | 9.22E-01 | 9.22E+01 | | | |
| 8057439 | --- | --- | --- | 0.027 | 1.019 | 8.86E-01 | 8.86E+01 | | | |
| 7892978 | --- | --- | --- | 0.027 | 1.019 | 9.53E-01 | 9.53E+01 | | | |
| 8089478 | NM_00100875 | GCET2 | germinal center expressed transcript | 0.027 | 1.019 | 8.91E-01 | 8.91E+01 | | | |
| 8178526 | NM_004639 | BAT3 | HLA-B associated transcript 3 | 0.027 | 1.019 | 8.16E-01 | 8.16E+01 | | | |
| 8179782 | NM_004639 | BAT3 | HLA-B associated transcript 3 | 0.027 | 1.019 | 8.16E-01 | 8.16E+01 | | | |
| 8107421 | NM_001284 | AP3S1 | adaptor-related protein complex 3, s | 0.027 | 1.019 | 8.84E-01 | 8.84E+01 | | | |
| 8169634 | --- | --- | --- | 0.027 | 1.019 | 9.56E-01 | 9.56E+01 | | | |
| 7896349 | --- | --- | --- | 0.027 | 1.019 | 8.46E-01 | 8.46E+01 | | | |
| 7897482 | NM_005026 | PIK3CD | phosphoinositide-3-kinase, catalytic, | 0.027 | 1.019 | 7.44E-01 | 7.44E+01 | | | |
| 8136289 | --- | --- | --- | 0.027 | 1.019 | 7.84E-01 | 7.84E+01 | | | |
| 8170865 | NM_000116 | TAZ | tafazzin | 0.027 | 1.019 | 8.04E-01 | 8.04E+01 | | | |
| 7951701 | --- | --- | --- | 0.027 | 1.019 | 8.70E-01 | 8.70E+01 | | | |
| 8117655 | NM_006299 | ZNF193 | zinc finger protein 193 | 0.027 | 1.019 | 7.63E-01 | 7.63E+01 | | | |
| 8110916 | ENST0000382 | LOC442132 | similar to hypothetical protein FLJ36 | 0.027 | 1.019 | 8.16E-01 | 8.16E+01 | | | |
| 7919763 | NM_207042 | ENSA | endosulfine alpha | 0.027 | 1.019 | 8.19E-01 | 8.19E+01 | | | |
| 7925904 | NM_00104017 | AKR1CL2 | aldo-keto reductase family 1, membe | 0.027 | 1.019 | 8.59E-01 | 8.59E+01 | | | |
| 8050689 | NM_025203 | C2orf44 | chromosome 2 open reading frame 4 | 0.027 | 1.019 | 7.92E-01 | 7.92E+01 | | | |
| 8125139 | NM_000434 | NEU1 | sialidase 1 (lysosomal sialidase) | 0.027 | 1.019 | 8.80E-01 | 8.80E+01 | | | |
| 8179851 | NM_000434 | NEU1 | sialidase 1 (lysosomal sialidase) | 0.027 | 1.019 | 8.80E-01 | 8.80E+01 | | | |
| 7953291 | NM_001769 | CD9 | CD9 molecule | 0.027 | 1.019 | 8.18E-01 | 8.18E+01 | | | |
| 7933634 | --- | --- | --- | 0.027 | 1.019 | 9.30E-01 | 9.30E+01 | | | |
| 7908968 | NM_017773 | LAX1 | lymphocyte transmembrane adaptor | 0.027 | 1.019 | 8.43E-01 | 8.43E+01 | | | |
| 7895517 | --- | --- | --- | 0.027 | 1.019 | 8.30E-01 | 8.30E+01 | | | |
| 8030881 | NM_014225 | PPP2R1A | protein phosphatase 2 (formerly 2A) | 0.026 | 1.019 | 8.57E-01 | 8.57E+01 | | | |
| 7894135 | --- | --- | --- | 0.026 | 1.019 | 7.85E-01 | 7.85E+01 | | | |
| 7894000 | --- | --- | --- | 0.026 | 1.018 | 5.55E-01 | 5.55E+01 | | | |
| 7941501 | NM_003860 | BANF1 | barrier to autointegration factor 1 | 0.026 | 1.018 | 7.37E-01 | 7.37E+01 | | | |
| 8044189 | --- | --- | --- | 0.026 | 1.018 | 6.80E-01 | 6.80E+01 | | | |
| 7893249 | --- | --- | --- | 0.026 | 1.018 | 9.30E-01 | 9.30E+01 | | | |

| | | | | | | | | | | |
|---|---|---|---|---|---|---|---|---|---|---|
| 8040249 | NM_005742 | PDIA6 | protein disulfide isomerase family A, | 0.026 | 1.018 | 7.46E-01 | 7.46E+01 | | | |
| 8106473 | --- | --- | --- | 0.026 | 1.018 | 8.70E-01 | 8.70E+01 | | | |
| 7917232 | NM_005274 | GNG5 | guanine nucleotide binding protein ( | 0.026 | 1.018 | 7.40E-01 | 7.40E+01 | | | |
| 8154245 | NM_025239 | PDCD1LG2 | programmed cell death 1 ligand 2 | 0.026 | 1.018 | 8.12E-01 | 8.12E+01 | | | |
| 7927305 | NM_00114400( | AGAP5 | ArfGAP with GTPase domain, ankyrin | 0.026 | 1.018 | 8.96E-01 | 8.96E+01 | | | |
| 8131881 | NM_199136 | C7orf46 | chromosome 7 open reading frame 4 | 0.026 | 1.018 | 8.18E-01 | 8.18E+01 | | | |
| 8006540 | NR_003037 | SNORD7 | small nucleolar RNA, C/D box 7 | 0.026 | 1.018 | 8.19E-01 | 8.19E+01 | | | |
| 8050102 | NM_207315 | CMPK2 | cytidine monophosphate (UMP-CMP | 0.026 | 1.018 | 7.98E-01 | 7.98E+01 | | | |
| 8123230 | NR_003288 | LOC729603 | calcium binding protein P22 pseudog | 0.026 | 1.018 | 8.05E-01 | 8.05E+01 | | | |
| 8067380 | NM_144703 | LSM14B | LSM14B, SCD6 homolog B (S. cerevis | 0.026 | 1.018 | 8.39E-01 | 8.39E+01 | | | |
| 8102415 | NM_001221 | CAMK2D | calcium/calmodulin-dependent prot | 0.026 | 1.018 | 9.42E-01 | 9.42E+01 | | | |
| 7894276 | --- | --- | --- | 0.026 | 1.018 | 9.78E-01 | 9.78E+01 | | | |
| 7892829 | --- | --- | --- | 0.026 | 1.018 | 9.22E-01 | 9.22E+01 | | | |
| 8126312 | NM_007162 | TFEB | transcription factor EB | 0.026 | 1.018 | 7.04E-01 | 7.04E+01 | | | |
| 8115158 | NM_00102507 | RPS14 | ribosomal protein S14 | 0.026 | 1.018 | 8.62E-01 | 8.62E+01 | | | |
| 7939341 | NM_000610 | CD44 | CD44 molecule (Indian blood group) | 0.026 | 1.018 | 8.51E-01 | 8.51E+01 | | | |
| 8083457 | NM_002886 | RAP2B | RAP2B, member of RAS oncogene fa | 0.026 | 1.018 | 7.97E-01 | 7.97E+01 | | | |
| 7986741 | NM_00100141 | LOC283767 | golgi autoantigen, golgin subfamily a | 0.026 | 1.018 | 7.98E-01 | 7.98E+01 | | | |
| 7893087 | --- | --- | --- | 0.026 | 1.018 | 9.62E-01 | 9.62E+01 | | | |
| 8148941 | NM_003416 | ZNF7 | zinc finger protein 7 | 0.026 | 1.018 | 7.37E-01 | 7.37E+01 | | | |
| 7999754 | NM_022166 | XYLT1 | xylosyltransferase I | 0.026 | 1.018 | 6.81E-01 | 6.81E+01 | | | |
| 8008922 | NM_003620 | PPM1D | protein phosphatase 1D magnesium- | 0.026 | 1.018 | 8.81E-01 | 8.81E+01 | | | |
| 7960559 | NM_080730 | IFFO1 | intermediate filament family orphan | 0.026 | 1.018 | 7.14E-01 | 7.14E+01 | | | |
| 8087985 | NM_00101098 | GLT8D1 | glycosyltransferase 8 domain contair | 0.026 | 1.018 | 8.89E-01 | 8.89E+01 | | | |
| 7893953 | --- | --- | --- | 0.026 | 1.018 | 8.52E-01 | 8.52E+01 | | | |
| 8012918 | NM_173622 | CDRT4 | CMT1A duplicated region transcript | 0.026 | 1.018 | 8.98E-01 | 8.98E+01 | | | |
| 8157463 | NM_153045 | C9orf91 | chromosome 9 open reading frame 9 | 0.026 | 1.018 | 7.53E-01 | 7.53E+01 | | | |
| 7959777 | NM_080626 | BRI3BP | BRI3 binding protein | 0.026 | 1.018 | 8.60E-01 | 8.60E+01 | | | |
| 8106107 | NM_024754 | PTCD2 | pentatricopeptide repeat domain 2 | 0.026 | 1.018 | 7.45E-01 | 7.45E+01 | | | |
| 8144619 | --- | --- | --- | 0.025 | 1.018 | 7.59E-01 | 7.59E+01 | | | |
| 7999025 | NM_016292 | TRAP1 | TNF receptor-associated protein 1 | 0.025 | 1.018 | 8.55E-01 | 8.55E+01 | | | |
| 7911289 | NM_024836 | ZNF672 | zinc finger protein 672 | 0.025 | 1.018 | 7.56E-01 | 7.56E+01 | | | |
| 8027377 | --- | --- | --- | 0.025 | 1.018 | 8.43E-01 | 8.43E+01 | | | |
| 7984405 | NM_00114393 | C15orf61 | chromosome 15 open reading frame | 0.025 | 1.018 | 8.68E-01 | 8.68E+01 | | | |
| 8065817 | NM_000178 | GSS | glutathione synthetase | 0.025 | 1.018 | 8.94E-01 | 8.94E+01 | | | |
| 7924956 | NM_012089 | ABCB10 | ATP-binding cassette, sub-family B (I | 0.025 | 1.018 | 8.16E-01 | 8.16E+01 | | | |
| 8162264 | NM_001698 | AUH | AU RNA binding protein/enoyl-Coen | 0.025 | 1.018 | 9.02E-01 | 9.02E+01 | | | |
| 8007008 | NM_003250 | THRA | thyroid hormone receptor, alpha (er | 0.025 | 1.017 | 8.38E-01 | 8.38E+01 | | | |
| 8179228 | NM_007109 | TCF19 | transcription factor 19 | 0.025 | 1.017 | 7.46E-01 | 7.46E+01 | | | |
| 7987012 | NM_139320 | CHRFAM7A | CHRNA7 (cholinergic receptor, nicoti | 0.025 | 1.017 | 7.83E-01 | 7.83E+01 | | | |
| 7894293 | --- | --- | --- | 0.025 | 1.017 | 9.69E-01 | 9.69E+01 | | | |
| 8023766 | NM_173630 | RTTN | rotatin | 0.025 | 1.017 | 8.14E-01 | 8.14E+01 | | | |
| 7964757 | NM_016836 | RBMS1 | RNA binding motif, single stranded ir | 0.025 | 1.017 | 9.70E-01 | 9.70E+01 | | | |
| 8172296 | NR_024234 | NDUFB11 | NADH dehydrogenase (ubiquinone) 1 | 0.025 | 1.017 | 8.70E-01 | 8.70E+01 | | | |
| 7970922 | --- | --- | --- | 0.025 | 1.017 | 9.66E-01 | 9.66E+01 | | | |
| 8079796 | NM_022064 | RNF123 | ring finger protein 123 | 0.025 | 1.017 | 7.56E-01 | 7.56E+01 | | | |
| 7914904 | NM_032017 | STK40 | serine/threonine kinase 40 | 0.025 | 1.017 | 7.82E-01 | 7.82E+01 | | | |
| 7893289 | --- | --- | --- | 0.025 | 1.017 | 7.94E-01 | 7.94E+01 | | | |
| 7990033 | NM_005078 | TLE3 | transducin-like enhancer of split 3 (E | 0.025 | 1.017 | 7.70E-01 | 7.70E+01 | | | |
| 8051204 | NM_014860 | SUPT7L | suppressor of Ty 7 (S. cerevisiae)-like | 0.025 | 1.017 | 9.41E-01 | 9.41E+01 | | | |
| 8042257 | --- | --- | --- | 0.025 | 1.017 | 8.24E-01 | 8.24E+01 | | | |
| 8169028 | NM_206917 | NGFRAP1 | nerve growth factor receptor (TNFRS | 0.025 | 1.017 | 8.59E-01 | 8.59E+01 | | | |
| 8049635 | NM_015650 | TRAF3IP1 | TNF receptor-associated factor 3 inte | 0.025 | 1.017 | 8.15E-01 | 8.15E+01 | | | |
| 8140859 | NM_006980 | MTERF | mitochondrial transcription terminat | 0.025 | 1.017 | 9.36E-01 | 9.36E+01 | | | |
| 7947425 | NM_203330 | CD59 | CD59 molecule, complement regulat | 0.025 | 1.017 | 8.91E-01 | 8.91E+01 | | | |
| 8133736 | NM_020892 | DTX2 | deltex homolog 2 (Drosophila) | 0.025 | 1.017 | 8.47E-01 | 8.47E+01 | | | |
| 8038416 | NM_001571 | IRF3 | interferon regulatory factor 3 | 0.025 | 1.017 | 7.99E-01 | 7.99E+01 | | | |
| 8065756 | --- | --- | --- | 0.025 | 1.017 | 9.46E-01 | 9.46E+01 | | | |
| 8159702 | NM_024757 | EHMT1 | euchromatic histone-lysine N-methy | 0.024 | 1.017 | 7.00E-01 | 7.00E+01 | | | |
| 7952069 | NM_020153 | C11orf60 | chromosome 11 open reading frame | 0.024 | 1.017 | 8.95E-01 | 8.95E+01 | | | |
| 8035829 | NM_000995 | RPL34 | ribosomal protein L34 | 0.024 | 1.017 | 7.73E-01 | 7.73E+01 | | | |
| 7894689 | --- | --- | --- | 0.024 | 1.017 | 9.69E-01 | 9.69E+01 | | | |
| 7988208 | NR_002318 | CATSPER2P1 | cation channel, sperm associated 2 p | 0.024 | 1.017 | 9.09E-01 | 9.09E+01 | | | |
| 8064522 | NM_174856 | IDH3B | isocitrate dehydrogenase 3 (NAD+) b | 0.024 | 1.017 | 9.27E-01 | 9.27E+01 | | | |
| 8080773 | NM_00109878 | RPP14 | ribonuclease P/MRP 14kDa subunit | 0.024 | 1.017 | 9.57E-01 | 9.57E+01 | | | |
| 8072009 | BC047380 | CRYBB2P1 | crystallin, beta B2 pseudogene 1 | 0.024 | 1.017 | 8.77E-01 | 8.77E+01 | | | |
| 7895437 | --- | --- | --- | 0.024 | 1.017 | 9.73E-01 | 9.73E+01 | | | |
| 8055202 | --- | --- | --- | 0.024 | 1.017 | 9.51E-01 | 9.51E+01 | | | |
| 8137693 | NM_00103161 | COX19 | COX19 cytochrome c oxidase assemb | 0.024 | 1.017 | 8.87E-01 | 8.87E+01 | | | |
| 8012099 | NM_024297 | PHF23 | PHD finger protein 23 | 0.024 | 1.017 | 8.46E-01 | 8.46E+01 | | | |
| 8147469 | NM_016134 | PGCP | plasma glutamate carboxypeptidase | 0.024 | 1.017 | 9.35E-01 | 9.35E+01 | | | mono |
| 8015757 | --- | --- | --- | 0.024 | 1.017 | 9.47E-01 | 9.47E+01 | | | |
| 8093624 | NM_00114585 | SH3BP2 | SH3-domain binding protein 2 | 0.024 | 1.017 | 7.44E-01 | 7.44E+01 | | | |
| 8136985 | NM_00100528 | OR2A1 | olfactory receptor, family 2, subfami | 0.024 | 1.017 | 8.35E-01 | 8.35E+01 | | | |
| 7999173 | NM_145253 | FAM100A | family with sequence similarity 100, | 0.024 | 1.017 | 8.03E-01 | 8.03E+01 | | | |
| 8037835 | NM_005628 | SLC1A5 | solute carrier family 1 (neutral amin | 0.024 | 1.017 | 7.74E-01 | 7.74E+01 | | | |
| 7988227 | NM_00103351 | SERINC4 | serine incorporator 4 | 0.024 | 1.017 | 7.71E-01 | 7.71E+01 | | | |

| | | | | | | | | | | | |
|---|---|---|---|---|---|---|---|---|---|---|---|
| 7893180 | --- | --- | --- | 0.024 | 1.017 | 8.95E-01 | 8.95E+01 | | | | |
| 7906728 | NM_032174 | TOMM40L | translocase of outer mitochondrial m | 0.024 | 1.017 | 7.50E-01 | 7.50E+01 | | | | |
| 8025142 | NM_00113095 | ARHGEF18 | Rho/Rac guanine nucleotide exchang | 0.024 | 1.017 | 8.44E-01 | 8.44E+01 | | | | |
| 7905349 | NM_024575 | TNFAIP8L2 | tumor necrosis factor, alpha-induced | 0.024 | 1.017 | 8.17E-01 | 8.17E+01 | | | | |
| 8138454 | NM_00100292 | TWISTNB | TWIST neighbor | 0.024 | 1.017 | 9.29E-01 | 9.29E+01 | | | | |
| 7979878 | NM_003049 | SLC10A1 | solute carrier family 10 (sodium/bile | 0.024 | 1.017 | 8.43E-01 | 8.43E+01 | | | | |
| 7970111 | NM_003899 | ARHGEF7 | Rho guanine nucleotide exchange fa | 0.024 | 1.017 | 9.13E-01 | 9.13E+01 | | | | |
| 8003465 | --- | --- | --- | 0.024 | 1.017 | 9.28E-01 | 9.28E+01 | | | | |
| 8180363 | --- | --- | --- | 0.024 | 1.017 | 7.95E-01 | 7.95E+01 | | | | |
| 7893236 | --- | --- | --- | 0.024 | 1.017 | 8.56E-01 | 8.56E+01 | | | | |
| 7992293 | NM_024600 | TMEM204 | transmembrane protein 204 | 0.024 | 1.017 | 7.66E-01 | 7.66E+01 | | | | |
| 8050255 | NM_024894 | NOL10 | nucleolar protein 10 | 0.024 | 1.017 | 9.54E-01 | 9.54E+01 | | | | |
| 7895914 | --- | --- | --- | 0.024 | 1.017 | 9.56E-01 | 9.56E+01 | | | | |
| 8026679 | NM_018467 | USE1 | unconventional SNARE in the ER 1 ho | 0.024 | 1.016 | 9.34E-01 | 9.34E+01 | | | | |
| 7895000 | --- | --- | --- | 0.024 | 1.016 | 8.55E-01 | 8.55E+01 | | | | |
| 8135718 | NM_019071 | ING3 | inhibitor of growth family, member 3 | 0.024 | 1.016 | 9.51E-01 | 9.51E+01 | | | | |
| 7894858 | --- | --- | --- | 0.024 | 1.016 | 8.54E-01 | 8.54E+01 | | | | |
| 7903393 | NM_001400 | S1PR1 | sphingosine-1-phosphate receptor 1 | 0.024 | 1.016 | 8.63E-01 | 8.63E+01 | | | | |
| 8154357 | --- | --- | --- | 0.023 | 1.016 | 8.08E-01 | 8.08E+01 | | | | |
| 8034615 | NM_017722 | TRMT1 | TRM1 tRNA methyltransferase 1 hom | 0.023 | 1.016 | 7.99E-01 | 7.99E+01 | | | | |
| 8050115 | AK127578 | C2orf46 | chromosome 2 open reading frame 4 | 0.023 | 1.016 | 8.98E-01 | 8.98E+01 | | | | |
| 7895760 | --- | --- | --- | 0.023 | 1.016 | 8.19E-01 | 8.19E+01 | | | | |
| 7904713 | --- | --- | --- | 0.023 | 1.016 | 7.99E-01 | 7.99E+01 | | | | |
| 7919129 | --- | --- | --- | 0.023 | 1.016 | 7.99E-01 | 7.99E+01 | | | | |
| 7914334 | NM_004814 | SNRNP40 | small nuclear ribonucleoprotein 40k | 0.023 | 1.016 | 9.56E-01 | 9.56E+01 | | | | |
| 7991386 | NM_006384 | CIB1 | calcium and integrin binding 1 (calmy | 0.023 | 1.016 | 9.01E-01 | 9.01E+01 | | | | |
| 8064976 | NM_015190 | DNAJC9 | DnaJ (Hsp40) homolog, subfamily C, | 0.023 | 1.016 | 8.27E-01 | 8.27E+01 | | | | |
| 8077370 | NM_006515 | SETMAR | SET domain and mariner transposase | 0.023 | 1.016 | 8.08E-01 | 8.08E+01 | | | | |
| 7991546 | NM_178842 | LASS3 | LAG1 homolog, ceramide synthase 3 | 0.023 | 1.016 | 7.36E-01 | 7.36E+01 | | | | |
| 8043377 | NM_012477 | WBP1 | WW domain binding protein 1 | 0.023 | 1.016 | 9.06E-01 | 9.06E+01 | | | | |
| 8089082 | NM_080927 | DCBLD2 | discoidin, CUB and LCCL domain cont | 0.023 | 1.016 | 7.67E-01 | 7.67E+01 | | | | |
| 7905754 | NM_020452 | ATP8B2 | ATPase, class I, type 8B, member 2 | 0.023 | 1.016 | 8.00E-01 | 8.00E+01 | | | | |
| 8020760 | --- | --- | --- | 0.023 | 1.016 | 8.61E-01 | 8.61E+01 | | | | |
| 7896198 | --- | --- | --- | 0.023 | 1.016 | 8.85E-01 | 8.85E+01 | | | | |
| 8174228 | NM_153333 | TCEAL8 | transcription elongation factor A (SII | 0.023 | 1.016 | 8.90E-01 | 8.90E+01 | | | | |
| 7895889 | --- | --- | --- | 0.023 | 1.016 | 9.80E-01 | 9.80E+01 | | | | |
| 8080100 | NM_015106 | RAD54L2 | RAD54-like 2 (S. cerevisiae) | 0.023 | 1.016 | 9.15E-01 | 9.15E+01 | | | | |
| 7928944 | NM_004670 | PAPSS2 | 3'-phosphoadenosine 5'-phosphosul | 0.023 | 1.016 | 8.82E-01 | 8.82E+01 | | | | |
| 7892960 | --- | --- | --- | 0.023 | 1.016 | 8.51E-01 | 8.51E+01 | | | | |
| 8090030 | NM_024610 | HSPBAP1 | HSPB (heat shock 27kDa) associated | 0.023 | 1.016 | 8.77E-01 | 8.77E+01 | | | | |
| 7996725 | NM_017803 | DUS2L | dihydrouridine synthase 2-like, SMM | 0.023 | 1.016 | 8.63E-01 | 8.63E+01 | | | | |
| 8028380 | NM_002812 | PSMD8 | proteasome (prosome, macropain) 2 | 0.023 | 1.016 | 9.20E-01 | 9.20E+01 | | | | |
| 8081115 | NM_182896 | ARL13B | ADP-ribosylation factor-like 13B | 0.023 | 1.016 | 8.77E-01 | 8.77E+01 | | | | |
| 7892554 | --- | --- | --- | 0.023 | 1.016 | 9.77E-01 | 9.77E+01 | | | | |
| 8016600 | NM_170685 | TAC4 | tachykinin 4 (hemokinin) | 0.023 | 1.016 | 7.69E-01 | 7.69E+01 | | | | |
| 7913156 | NM_003689 | AKR7A2 | aldo-keto reductase family 7, membe | 0.023 | 1.016 | 8.56E-01 | 8.56E+01 | | | | |
| 7896282 | --- | --- | --- | 0.023 | 1.016 | 8.33E-01 | 8.33E+01 | | | | |
| 8165285 | NM_178469 | LCN8 | lipocalin 8 | 0.023 | 1.016 | 7.64E-01 | 7.64E+01 | | | | |
| 7896178 | --- | --- | --- | 0.023 | 1.016 | 9.50E-01 | 9.50E+01 | | | | |
| 7908692 | NR_026667 | RPS10P7 | ribosomal protein S10 pseudogene 7 | 0.022 | 1.016 | 9.01E-01 | 9.01E+01 | | | | |
| 8031726 | NM_020657 | ZNF304 | zinc finger protein 304 | 0.022 | 1.016 | 9.11E-01 | 9.11E+01 | | | | |
| 8047248 | NM_00111466 | PLCL1 | phospholipase C-like 1 | 0.022 | 1.016 | 9.48E-01 | 9.48E+01 | | | | |
| 7998655 | NM_002952 | RPS2 | ribosomal protein S2 | 0.022 | 1.016 | 8.13E-01 | 8.13E+01 | | | | |
| 7954310 | NR_003286 | OC10000858 | 18S ribosomal RNA | 0.022 | 1.016 | 6.93E-01 | 6.93E+01 | | | | |
| 7903183 | --- | --- | --- | 0.022 | 1.016 | 7.84E-01 | 7.84E+01 | | | | |
| 7892899 | --- | --- | --- | 0.022 | 1.016 | 7.56E-01 | 7.56E+01 | | | | |
| 7893949 | --- | --- | --- | 0.022 | 1.016 | 8.78E-01 | 8.78E+01 | | | | |
| 8137091 | NM_170686 | ZNF398 | zinc finger protein 398 | 0.022 | 1.016 | 8.19E-01 | 8.19E+01 | | | | |
| 8011599 | NM_016376 | ANKFY1 | ankyrin repeat and FYVE domain con | 0.022 | 1.016 | 8.51E-01 | 8.51E+01 | | | | |
| 8031884 | NM_014480 | ZNF544 | zinc finger protein 544 | 0.022 | 1.015 | 8.55E-01 | 8.55E+01 | | | | |
| 8052141 | --- | --- | --- | 0.022 | 1.015 | 9.59E-01 | 9.59E+01 | | | | |
| 7918367 | NM_005272 | GNAT2 | guanine nucleotide binding protein ( | 0.022 | 1.015 | 8.02E-01 | 8.02E+01 | | | | |
| 8103043 | NM_00102999 | SLC10A7 | solute carrier family 10 (sodium/bile | 0.022 | 1.015 | 8.65E-01 | 8.65E+01 | | | | |
| 7973377 | NM_004050 | BCL2L2 | BCL2-like 2 | 0.022 | 1.015 | 8.34E-01 | 8.34E+01 | | | | |
| 8165622 | NM_138462 | ZMYND19 | zinc finger, MYND-type containing 19 | 0.022 | 1.015 | 8.51E-01 | 8.51E+01 | | | | |
| 8164105 | NM_002077 | GOLGA1 | golgi autoantigen, golgin subfamily a | 0.022 | 1.015 | 8.38E-01 | 8.38E+01 | | | | |
| 8005661 | NM_00103355 | CYTSB | cytospin B | 0.022 | 1.015 | 7.80E-01 | 7.80E+01 | | | | |
| 7998055 | NM_002386 | MC1R | melanocortin 1 receptor (alpha mela | 0.022 | 1.015 | 7.38E-01 | 7.38E+01 | | | | |
| 8079037 | NM_00104264 | TRAK1 | trafficking protein, kinesin binding 1 | 0.022 | 1.015 | 8.17E-01 | 8.17E+01 | | | | |
| 8053668 | NM_004836 | EIF2AK3 | eukaryotic translation initiation facto | 0.022 | 1.015 | 9.26E-01 | 9.26E+01 | | | | |
| 8108593 | NM_017706 | WDR55 | WD repeat domain 55 | 0.022 | 1.015 | 8.71E-01 | 8.71E+01 | | | | |
| 8097973 | NM_000857 | GUCY1B3 | guanylate cyclase 1, soluble, beta 3 | 0.021 | 1.015 | 8.47E-01 | 8.47E+01 | | | | |
| 7986598 | NM_00100141 | LOC283767 | golgi autoantigen, golgin subfamily a | 0.021 | 1.015 | 8.38E-01 | 8.38E+01 | | | | |
| 7974339 | --- | --- | --- | 0.021 | 1.015 | 8.35E-01 | 8.35E+01 | | | | |
| 7926283 | NM_003675 | PRPF18 | PRP18 pre-mRNA processing factor 1 | 0.021 | 1.015 | 9.74E-01 | 9.74E+01 | | | | |
| 8068363 | NR_027267 | C21orf82 | chromosome 21 open reading frame | 0.021 | 1.015 | 7.77E-01 | 7.77E+01 | | | | |
| 8124967 | NM_004639 | BAT3 | HLA-B associated transcript 3 | 0.021 | 1.015 | 8.55E-01 | 8.55E+01 | | | | |

| | | | | | | | | | | |
|---|---|---|---|---|---|---|---|---|---|---|
| 7997396 | NM_015251 | ATMIN | ATM interactor | 0.021 | 1.015 | 9.03E-01 | 9.03E+01 | | | |
| 7892654 | --- | --- | --- | 0.021 | 1.015 | 8.27E-01 | 8.27E+01 | | | |
| 7893768 | --- | --- | --- | 0.021 | 1.015 | 9.75E-01 | 9.75E+01 | | | |
| 7935660 | NM_015221 | DNMBP | dynamin binding protein | 0.021 | 1.015 | 8.42E-01 | 8.42E+01 | | | |
| 7893191 | --- | --- | --- | 0.021 | 1.015 | 8.55E-01 | 8.55E+01 | | | |
| 7900438 | NM_023070 | ZNF643 | zinc finger protein 643 | 0.021 | 1.015 | 8.77E-01 | 8.77E+01 | | | |
| 8047854 | NM_00114230( | CCNYL1 | cyclin Y-like 1 | 0.021 | 1.015 | 9.13E-01 | 9.13E+01 | | | |
| 7974576 | NM_00101171: | NAT12 | N-acetyltransferase 12 (GCN5-relatec | 0.021 | 1.015 | 8.71E-01 | 8.71E+01 | | | |
| 8076449 | NR_002184 | RRP7B | ribosomal RNA processing 7 homolog | 0.021 | 1.015 | 8.70E-01 | 8.70E+01 | | | |
| 7963741 | NM_014311 | SMUG1 | single-strand-selective monofunction | 0.021 | 1.015 | 8.31E-01 | 8.31E+01 | | | |
| 7895330 | --- | --- | --- | 0.021 | 1.015 | 9.49E-01 | 9.49E+01 | | | |
| 8084715 | --- | --- | --- | 0.021 | 1.015 | 8.51E-01 | 8.51E+01 | | | |
| 7895279 | --- | --- | --- | 0.021 | 1.015 | 9.84E-01 | 9.84E+01 | | | |
| 7927146 | NM_018590 | CSGALNACT2 | chondroitin sulfate N-acetylgalactosa | 0.021 | 1.014 | 9.58E-01 | 9.58E+01 | | | |
| 8119648 | NM_057161 | KLHDC3 | kelch domain containing 3 | 0.021 | 1.014 | 8.56E-01 | 8.56E+01 | | | |
| 8131860 | BC012331 | C7orf30 | chromosome 7 open reading frame 3 | 0.021 | 1.014 | 9.32E-01 | 9.32E+01 | | | |
| 7916135 | NM_032449 | CC2D1B | coiled-coil and C2 domain containing | 0.021 | 1.014 | 7.35E-01 | 7.35E+01 | | | |
| 7930205 | NM_00114390! | CG_202441( | rcRPE | 0.021 | 1.014 | 9.19E-01 | 9.19E+01 | | | |
| 8125545 | NM_002119 | HLA-DOA | major histocompatibility complex, cla | 0.020 | 1.014 | 8.27E-01 | 8.27E+01 | | | |
| 8011093 | NM_152346 | SLC43A2 | solute carrier family 43, member 2 | 0.020 | 1.014 | 7.53E-01 | 7.53E+01 | | | |
| 7951091 | --- | --- | --- | 0.020 | 1.014 | 9.41E-01 | 9.41E+01 | | | |
| 8121757 | NM_004506 | HSF2 | heat shock transcription factor 2 | 0.020 | 1.014 | 9.33E-01 | 9.33E+01 | | | |
| 8047078 | NM_017694 | MFSD6 | major facilitator superfamily domain | 0.020 | 1.014 | 8.83E-01 | 8.83E+01 | | | |
| 7924092 | NM_021194 | SLC30A1 | solute carrier family 30 (zinc transpo | 0.020 | 1.014 | 8.21E-01 | 8.21E+01 | | | |
| 8003840 | NM_00101476 | TMEM93 | transmembrane protein 93 | 0.020 | 1.014 | 8.06E-01 | 8.06E+01 | | | |
| 7952036 | NM_198275 | MPZL3 | myelin protein zero-like 3 | 0.020 | 1.014 | 8.98E-01 | 8.98E+01 | | | |
| 8132909 | --- | --- | --- | 0.020 | 1.014 | 8.65E-01 | 8.65E+01 | | | |
| 8078958 | --- | --- | --- | 0.020 | 1.014 | 8.19E-01 | 8.19E+01 | | | |
| 7895556 | --- | --- | --- | 0.020 | 1.014 | 8.86E-01 | 8.86E+01 | | | |
| 8148474 | --- | --- | --- | 0.020 | 1.014 | 8.68E-01 | 8.68E+01 | | | |
| 7892547 | --- | --- | --- | 0.020 | 1.014 | 9.35E-01 | 9.35E+01 | | | |
| 7997197 | NM_014003 | DHX38 | DEAH (Asp-Glu-Ala-His) box polypept | 0.020 | 1.014 | 9.14E-01 | 9.14E+01 | | | |
| 8019988 | NM_00110524 | PTPRM | protein tyrosine phosphatase, recept | 0.020 | 1.014 | 8.00E-01 | 8.00E+01 | | | |
| 7931888 | NM_019046 | ANKRD16 | ankyrin repeat domain 16 | 0.020 | 1.014 | 8.25E-01 | 8.25E+01 | | | |
| 8039695 | NM_152475 | ZNF417 | zinc finger protein 417 | 0.020 | 1.014 | 9.20E-01 | 9.20E+01 | | | |
| 8138547 | NM_019059 | TOMM7 | translocase of outer mitochondrial m | 0.020 | 1.014 | 9.07E-01 | 9.07E+01 | | | |
| 8001030 | NM_013258 | PYCARD | PYD and CARD domain containing | 0.020 | 1.014 | 8.58E-01 | 8.58E+01 | | | |
| 8013860 | NM_078471 | MYO18A | myosin XVIIIA | 0.020 | 1.014 | 7.39E-01 | 7.39E+01 | | | |
| 8170906 | NM_004699 | FAM50A | family with sequence similarity 50, m | 0.020 | 1.014 | 9.28E-01 | 9.28E+01 | | | |
| 7893000 | --- | --- | --- | 0.020 | 1.014 | 9.14E-01 | 9.14E+01 | | | |
| 8086201 | NM_001607 | ACAA1 | acetyl-Coenzyme A acyltransferase 1 | 0.020 | 1.014 | 8.45E-01 | 8.45E+01 | | | |
| 7909144 | AK094426 | LOC284581 | hypothetical protein LOC284581 | 0.020 | 1.014 | 8.36E-01 | 8.36E+01 | | | |
| 7928524 | NM_003375 | VDAC2 | voltage-dependent anion channel 2 | 0.020 | 1.014 | 8.56E-01 | 8.56E+01 | | | |
| 7893051 | --- | --- | --- | 0.020 | 1.014 | 9.39E-01 | 9.39E+01 | | | |
| 7984624 | --- | --- | --- | 0.020 | 1.014 | 9.24E-01 | 9.24E+01 | | | |
| 8173179 | NR_002308 | P11-278E11. | ubiquinol-cytochrome c reductase bi | 0.020 | 1.014 | 9.22E-01 | 9.22E+01 | | | |
| 7895815 | --- | --- | --- | 0.019 | 1.014 | 8.54E-01 | 8.54E+01 | | | |
| 8180268 | --- | --- | --- | 0.019 | 1.014 | 9.53E-01 | 9.53E+01 | | | |
| 7923778 | NM_001973 | ELK4 | ELK4, ETS-domain protein (SRF acces | 0.019 | 1.014 | 8.43E-01 | 8.43E+01 | | | |
| 8064879 | NM_015939 | TRMT6 | tRNA methyltransferase 6 homolog ( | 0.019 | 1.014 | 8.91E-01 | 8.91E+01 | | | |
| 8004221 | NM_000697 | ALOX12 | arachidonate 12-lipoxygenase | 0.019 | 1.013 | 8.32E-01 | 8.32E+01 | | | |
| 8035175 | --- | --- | --- | 0.019 | 1.013 | 9.24E-01 | 9.24E+01 | | | |
| 8133331 | NM_172020 | POM121 | POM121 membrane glycoprotein (ra | 0.019 | 1.013 | 8.66E-01 | 8.66E+01 | | | |
| 7895714 | --- | --- | --- | 0.019 | 1.013 | 8.07E-01 | 8.07E+01 | | | |
| 7974920 | NM_182914 | SYNE2 | spectrin repeat containing, nuclear e | 0.019 | 1.013 | 9.16E-01 | 9.16E+01 | | | |
| 7994582 | NM_003166 | SULT1A3 | sulfotransferase family, cytosolic, 1A | 0.019 | 1.013 | 8.55E-01 | 8.55E+01 | | | mono |
| 8142324 | NM_00113047! | THAP5 | THAP domain containing 5 | 0.019 | 1.013 | 8.95E-01 | 8.95E+01 | | | |
| 8009502 | NM_000891 | KCNJ2 | potassium inwardly-rectifying chann | 0.019 | 1.013 | 8.09E-01 | 8.09E+01 | | | |
| 8065196 | --- | --- | --- | 0.019 | 1.013 | 8.97E-01 | 8.97E+01 | | | |
| 8164464 | NM_006336 | ZER1 | zer-1 homolog (C. elegans) | 0.019 | 1.013 | 8.42E-01 | 8.42E+01 | | | |
| 8021222 | NM_018696 | ELAC1 | elaC homolog 1 (E. coli) | 0.019 | 1.013 | 8.42E-01 | 8.42E+01 | | | |
| 8087374 | NM_198562 | C3orf62 | chromosome 3 open reading frame 6 | 0.019 | 1.013 | 8.82E-01 | 8.82E+01 | | | |
| 7986736 | NM_00100141: | LOC283767 | golgi autoantigen, golgin subfamily a | 0.019 | 1.013 | 8.51E-01 | 8.51E+01 | | | |
| 8007058 | NM_133264 | WIPF2 | WAS/WASL interacting protein famil | 0.019 | 1.013 | 8.66E-01 | 8.66E+01 | | | |
| 8153258 | BC033223 | SLC45A4 | solute carrier family 45, member 4 | 0.019 | 1.013 | 9.20E-01 | 9.20E+01 | | | |
| 7911568 | NM_014188 | SSU72 | SSU72 RNA polymerase II CTD phosp | 0.019 | 1.013 | 8.90E-01 | 8.90E+01 | | | |
| 8027018 | BC001080 | C19orf50 | chromosome 19 open reading frame | 0.019 | 1.013 | 8.41E-01 | 8.41E+01 | | | |
| 8077099 | NM_005138 | SCO2 | SCO cytochrome oxidase deficient ho | 0.019 | 1.013 | 8.44E-01 | 8.44E+01 | | | |
| 7894360 | --- | --- | --- | 0.019 | 1.013 | 9.82E-01 | 9.82E+01 | | | |
| 8166585 | AK057304 | FLJ32742 | hypothetical locus FLJ32742 | 0.019 | 1.013 | 8.95E-01 | 8.95E+01 | | | |
| 8130003 | NM_031287 | SF3B5 | splicing factor 3b, subunit 5, 10kDa | 0.019 | 1.013 | 9.44E-01 | 9.44E+01 | | | |
| 7894639 | --- | --- | --- | 0.019 | 1.013 | 9.52E-01 | 9.52E+01 | | | |
| 7894748 | --- | --- | --- | 0.019 | 1.013 | 9.61E-01 | 9.61E+01 | | | |
| 7895240 | --- | --- | --- | 0.019 | 1.013 | 9.53E-01 | 9.53E+01 | | | |
| 8010841 | NM_022158 | FN3K | fructosamine 3 kinase | 0.019 | 1.013 | 9.35E-01 | 9.35E+01 | | | |
| 8016787 | --- | --- | --- | 0.019 | 1.013 | 9.33E-01 | 9.33E+01 | | | |
| 7958455 | NM_003362 | UNG | uracil-DNA glycosylase | 0.019 | 1.013 | 8.82E-01 | 8.82E+01 | | | |

| | | | | | | | | | | |
|---|---|---|---|---|---|---|---|---|---|---|
| 8093462 | NM_00101740! | MAEA | macrophage erythroblast attacher | 0.019 | 1.013 | 9.06E-01 | 9.06E+01 | | | |
| 8090193 | NM_020733 | HEG1 | HEG homolog 1 (zebrafish) | 0.019 | 1.013 | 8.63E-01 | 8.63E+01 | | | |
| 8031293 | NM_014511 | KIR2DL3 | killer cell immunoglobulin-like recept | 0.018 | 1.013 | 9.77E-01 | 9.77E+01 | | | |
| 8023344 | NM_00110165 | CXXC1 | CXXC finger 1 (PHD domain) | 0.018 | 1.013 | 8.86E-01 | 8.86E+01 | | | |
| 8089771 | NM_005191 | CD80 | CD80 molecule | 0.018 | 1.013 | 8.51E-01 | 8.51E+01 | | | |
| 8060627 | NM_139321 | ATRN | attractin | 0.018 | 1.013 | 8.59E-01 | 8.59E+01 | | | |
| 7898750 | NM_00108362 | ZBTB40 | zinc finger and BTB domain containin | 0.018 | 1.013 | 9.04E-01 | 9.04E+01 | | | |
| 7964300 | NM_014830 | ZBTB39 | zinc finger and BTB domain containin | 0.018 | 1.013 | 8.51E-01 | 8.51E+01 | | | |
| 8076465 | NM_032311 | POLDIP3 | polymerase (DNA-directed), delta int | 0.018 | 1.013 | 9.00E-01 | 9.00E+01 | | | |
| 8154916 | NM_000155 | GALT | galactose-1-phosphate uridylyltransf | 0.018 | 1.013 | 8.91E-01 | 8.91E+01 | | | |
| 8016832 | NM_012329 | MMD | monocyte to macrophage differentia | 0.018 | 1.013 | 9.39E-01 | 9.39E+01 | | | |
| 8021824 | --- | --- | --- | 0.018 | 1.013 | 9.73E-01 | 9.73E+01 | | | |
| 8020058 | NM_021074 | NDUFV2 | NADH dehydrogenase (ubiquinone) f | 0.018 | 1.013 | 9.57E-01 | 9.57E+01 | | | |
| 7920664 | NM_007112 | THBS3 | thrombospondin 3 | 0.018 | 1.013 | 8.80E-01 | 8.80E+01 | | | |
| 8031358 | NM_012314 | KIR2DS4 | killer cell immunoglobulin-like recept | 0.018 | 1.013 | 9.36E-01 | 9.36E+01 | | | |
| 7997702 | NM_001861 | COX4I1 | cytochrome c oxidase subunit IV isof | 0.018 | 1.013 | 9.27E-01 | 9.27E+01 | | | |
| 7892969 | --- | --- | --- | 0.018 | 1.013 | 8.38E-01 | 8.38E+01 | | | |
| 8096753 | NM_005327 | HADH | hydroxyacyl-Coenzyme A dehydroge | 0.018 | 1.012 | 8.99E-01 | 8.99E+01 | | | |
| 8005809 | NM_009587 | LGALS9 | lectin, galactoside-binding, soluble, 9 | 0.018 | 1.012 | 9.54E-01 | 9.54E+01 | | | |
| 7954434 | --- | --- | --- | 0.018 | 1.012 | 8.48E-01 | 8.48E+01 | | | |
| 7979658 | NM_002083 | GPX2 | glutathione peroxidase 2 (gastrointe | 0.018 | 1.012 | 8.71E-01 | 8.71E+01 | | | |
| 8013348 | NM_002952 | RPS2 | ribosomal protein S2 | 0.018 | 1.012 | 8.62E-01 | 8.62E+01 | | | |
| 8171846 | --- | --- | --- | 0.018 | 1.012 | 9.53E-01 | 9.53E+01 | | | |
| 8162236 | NM_006378 | SEMA4D | sema domain, immunoglobulin doma | 0.018 | 1.012 | 9.60E-01 | 9.60E+01 | | | |
| 7942342 | NM_001567 | INPPL1 | inositol polyphosphate phosphatase- | 0.018 | 1.012 | 8.63E-01 | 8.63E+01 | | | |
| 8129099 | NM_003309 | TSPYL1 | TSPY-like 1 | 0.018 | 1.012 | 9.34E-01 | 9.34E+01 | | | |
| 7894007 | --- | --- | --- | 0.018 | 1.012 | 8.66E-01 | 8.66E+01 | | | |
| 7998825 | NM_002613 | PDPK1 | 3-phosphoinositide dependent prote | 0.017 | 1.012 | 9.63E-01 | 9.63E+01 | | | |
| 7896292 | --- | --- | --- | 0.017 | 1.012 | 9.58E-01 | 9.58E+01 | | | |
| 7893130 | --- | --- | --- | 0.017 | 1.012 | 8.15E-01 | 8.15E+01 | | | |
| 7893242 | --- | --- | --- | 0.017 | 1.012 | 8.16E-01 | 8.16E+01 | | | |
| 8168843 | NM_021029 | RPL36A | ribosomal protein L36a | 0.017 | 1.012 | 9.30E-01 | 9.30E+01 | | | |
| 7906786 | NM_032738 | FCRLA | Fc receptor-like A | 0.017 | 1.012 | 8.86E-01 | 8.86E+01 | | | |
| 8012218 | --- | --- | --- | 0.017 | 1.012 | 9.12E-01 | 9.12E+01 | | | |
| 8053379 | AK296608 | LOC129293 | hypothetical protein LOC129293 | 0.017 | 1.012 | 9.31E-01 | 9.31E+01 | | | |
| 7998774 | NM_080594 | RNPS1 | RNA binding protein S1, serine-rich d | 0.017 | 1.012 | 8.97E-01 | 8.97E+01 | | | |
| 7895239 | --- | --- | --- | 0.017 | 1.012 | 8.95E-01 | 8.95E+01 | | | |
| 8042772 | NM_213622 | STAMBP | STAM binding protein | 0.017 | 1.012 | 9.26E-01 | 9.26E+01 | | | |
| 7896045 | --- | --- | --- | 0.017 | 1.012 | 8.50E-01 | 8.50E+01 | | | |
| 8003991 | NM_153827 | MINK1 | misshapen-like kinase 1 (zebrafish) | 0.017 | 1.012 | 8.71E-01 | 8.71E+01 | | | |
| 8155598 | --- | --- | --- | 0.017 | 1.012 | 8.91E-01 | 8.91E+01 | | | |
| 8155600 | --- | --- | --- | 0.017 | 1.012 | 8.91E-01 | 8.91E+01 | | | |
| 8109750 | NM_001002 | RPLP0 | ribosomal protein, large, P0 | 0.017 | 1.012 | 9.30E-01 | 9.30E+01 | | | |
| 8046804 | NM_138285 | NUP35 | nucleoporin 35kDa | 0.017 | 1.012 | 8.94E-01 | 8.94E+01 | | | |
| 7978064 | --- | --- | --- | 0.017 | 1.012 | 8.70E-01 | 8.70E+01 | | | |
| 7896488 | --- | --- | --- | 0.017 | 1.012 | 8.79E-01 | 8.79E+01 | | | |
| 8027414 | --- | --- | --- | 0.017 | 1.012 | 9.18E-01 | 9.18E+01 | | | |
| 8039680 | NM_024833 | ZNF671 | zinc finger protein 671 | 0.017 | 1.012 | 9.36E-01 | 9.36E+01 | | | |
| 8030804 | NM_001772 | CD33 | CD33 molecule | 0.017 | 1.012 | 8.72E-01 | 8.72E+01 | | | |
| 8016994 | NM_017763 | RNF43 | ring finger protein 43 | 0.017 | 1.012 | 7.57E-01 | 7.57E+01 | | | |
| 8012891 | --- | --- | --- | 0.017 | 1.012 | 9.17E-01 | 9.17E+01 | | | |
| 7930577 | NM_033338 | CASP7 | caspase 7, apoptosis-related cysteine | 0.017 | 1.012 | 8.66E-01 | 8.66E+01 | | | |
| 8076374 | NM_005008 | NHP2L1 | NHP2 non-histone chromosome prot | 0.017 | 1.012 | 9.29E-01 | 9.29E+01 | | | |
| 8065752 | --- | --- | --- | 0.017 | 1.012 | 9.84E-01 | 9.84E+01 | | | |
| 7982230 | NR_024074 | GOLGA9P | golgi autoantigen, golgin subfamily a | 0.017 | 1.012 | 8.55E-01 | 8.55E+01 | | | |
| 8043512 | NM_021088 | ZNF2 | zinc finger protein 2 | 0.017 | 1.012 | 8.34E-01 | 8.34E+01 | | | |
| 7895926 | --- | --- | --- | 0.017 | 1.012 | 8.81E-01 | 8.81E+01 | | | |
| 7919929 | NM_013353 | TMOD4 | tropomodulin 4 (muscle) | 0.016 | 1.011 | 8.49E-01 | 8.49E+01 | | | |
| 7942274 | ENST00000329 | OR7E87P | olfactory receptor, family 7, subfami | 0.016 | 1.011 | 9.57E-01 | 9.57E+01 | | | |
| 8176026 | NM_001456 | FLNA | filamin A, alpha | 0.016 | 1.011 | 9.06E-01 | 9.06E+01 | | | |
| 7902472 | --- | --- | --- | 0.016 | 1.011 | 9.46E-01 | 9.46E+01 | | | |
| 8085026 | NM_000996 | RPL35A | ribosomal protein L35a | 0.016 | 1.011 | 8.18E-01 | 8.18E+01 | | | |
| 7895766 | --- | --- | --- | 0.016 | 1.011 | 8.58E-01 | 8.58E+01 | | | |
| 7943954 | --- | --- | --- | 0.016 | 1.011 | 8.66E-01 | 8.66E+01 | | | |
| 8169350 | --- | --- | --- | 0.016 | 1.011 | 7.42E-01 | 7.42E+01 | | | |
| 8034408 | NM_020714 | ZNF490 | zinc finger protein 490 | 0.016 | 1.011 | 8.65E-01 | 8.65E+01 | | | |
| 7978917 | NM_00101483( | LOC196913 | hypothetical protein LOC196913 | 0.016 | 1.011 | 8.58E-01 | 8.58E+01 | | | |
| 7893396 | --- | --- | --- | 0.016 | 1.011 | 9.64E-01 | 9.64E+01 | | | |
| 7892740 | --- | --- | --- | 0.016 | 1.011 | 9.89E-01 | 9.89E+01 | | | |
| 7907090 | AY194294 | OC10012875 | INM04 | 0.016 | 1.011 | 8.90E-01 | 8.90E+01 | | | |
| 8180410 | --- | --- | --- | 0.016 | 1.011 | 9.48E-01 | 9.48E+01 | | | |
| 7967060 | NM_003769 | SFRS9 | splicing factor, arginine/serine-rich 9 | 0.016 | 1.011 | 9.27E-01 | 9.27E+01 | | | |
| 8069565 | NM_00113091 | BTG3 | BTG family, member 3 | 0.016 | 1.011 | 9.64E-01 | 9.64E+01 | | | |
| 8065889 | NM_018244 | UQCC | ubiquinol-cytochrome c reductase co | 0.016 | 1.011 | 8.49E-01 | 8.49E+01 | | | |
| 7895491 | --- | --- | --- | 0.016 | 1.011 | 9.81E-01 | 9.81E+01 | | | |
| 8047262 | NM_153689 | C2orf69 | chromosome 2 open reading frame 6 | 0.016 | 1.011 | 9.21E-01 | 9.21E+01 | | | |
| 7978492 | NM_015473 | HEATR5A | HEAT repeat containing 5A | 0.016 | 1.011 | 8.35E-01 | 8.35E+01 | | | |

| | | | | | | | | | | |
|---|---|---|---|---|---|---|---|---|---|---|
| 8138151 | NR_002822 | MGC72080 | MGC72080 pseudogene | 0.016 | 1.011 | 9.65E-01 | 9.65E+01 | | | |
| 8018982 | NM_138793 | CANT1 | calcium activated nucleotidase 1 | 0.016 | 1.011 | 8.74E-01 | 8.74E+01 | | | |
| 8071392 | NM_00100389: | MED15 | mediator complex subunit 15 | 0.016 | 1.011 | 9.15E-01 | 9.15E+01 | | | |
| 7893836 | --- | --- | --- | 0.016 | 1.011 | 8.43E-01 | 8.43E+01 | | | |
| 7952795 | --- | --- | --- | 0.016 | 1.011 | 8.66E-01 | 8.66E+01 | | | |
| 8068857 | NM_021075 | NDUFV3 | NADH dehydrogenase (ubiquinone) f | 0.016 | 1.011 | 8.88E-01 | 8.88E+01 | | | |
| 7959039 | NM_153348 | FBXW8 | F-box and WD repeat domain contai | 0.015 | 1.011 | 8.43E-01 | 8.43E+01 | | | |
| 7933080 | --- | --- | --- | 0.015 | 1.011 | 9.47E-01 | 9.47E+01 | | | |
| 8131512 | --- | --- | --- | 0.015 | 1.011 | 8.43E-01 | 8.43E+01 | | | |
| 8059361 | NM_020830 | WDFY1 | WD repeat and FYVE domain contair | 0.015 | 1.011 | 9.36E-01 | 9.36E+01 | | | |
| 7927425 | NM_020945 | WDFY4 | WDFY family member 4 | 0.015 | 1.011 | 9.07E-01 | 9.07E+01 | | | |
| 8069178 | NR_027673 | ADARB1 | adenosine deaminase, RNA-specific, | 0.015 | 1.011 | 8.62E-01 | 8.62E+01 | | | |
| 7937892 | NR_027015 | PGAP2 | post-GPI attachment to proteins 2 | 0.015 | 1.011 | 8.85E-01 | 8.85E+01 | | | |
| 8050079 | NM_002936 | RNASEH1 | ribonuclease H1 | 0.015 | 1.011 | 9.54E-01 | 9.54E+01 | | | |
| 7942779 | --- | --- | --- | 0.015 | 1.011 | 9.65E-01 | 9.65E+01 | | | |
| 8078260 | --- | --- | --- | 0.015 | 1.011 | 9.39E-01 | 9.39E+01 | | | |
| 8173812 | NM_030763 | NSBP1 | nucleosomal binding protein 1 | 0.015 | 1.011 | 8.97E-01 | 8.97E+01 | | | |
| 8158961 | NM_00112282: | GTF3C5 | general transcription factor IIIC, poly | 0.015 | 1.011 | 9.07E-01 | 9.07E+01 | | | |
| 7895559 | --- | --- | --- | 0.015 | 1.011 | 9.55E-01 | 9.55E+01 | | | |
| 7984989 | NM_017828 | COMMD4 | COMM domain containing 4 | 0.015 | 1.011 | 8.86E-01 | 8.86E+01 | | | |
| 7929689 | NR_026753 | MARVELD1 | MARVEL domain containing 1 | 0.015 | 1.011 | 8.81E-01 | 8.81E+01 | | | |
| 7899560 | --- | --- | --- | 0.015 | 1.011 | 9.51E-01 | 9.51E+01 | | | |
| 7943124 | --- | --- | --- | 0.015 | 1.010 | 8.67E-01 | 8.67E+01 | | | |
| 8037355 | NM_182498 | ZNF428 | zinc finger protein 428 | 0.015 | 1.010 | 8.95E-01 | 8.95E+01 | | | |
| 7901477 | --- | --- | --- | 0.015 | 1.010 | 8.99E-01 | 8.99E+01 | | | |
| 8117685 | NM_024493 | ZKSCAN3 | zinc finger with KRAB and SCAN dom | 0.015 | 1.010 | 8.96E-01 | 8.96E+01 | | | |
| 7896716 | --- | --- | --- | 0.015 | 1.010 | 9.49E-01 | 9.49E+01 | | | |
| 7999317 | NM_015421 | TMEM186 | transmembrane protein 186 | 0.015 | 1.010 | 9.42E-01 | 9.42E+01 | | | |
| 7892759 | --- | --- | --- | 0.015 | 1.010 | 9.78E-01 | 9.78E+01 | | | |
| 8171193 | --- | --- | --- | 0.015 | 1.010 | 8.90E-01 | 8.90E+01 | | | |
| 8113914 | NM_133372 | FNIP1 | folliculin interacting protein 1 | 0.015 | 1.010 | 9.66E-01 | 9.66E+01 | | | |
| 8145293 | NM_014265 | ADAM28 | ADAM metallopeptidase domain 28 | 0.015 | 1.010 | 9.58E-01 | 9.58E+01 | | | |
| 8068612 | NM_004627 | WRB | tryptophan rich basic protein | 0.015 | 1.010 | 9.59E-01 | 9.59E+01 | | | |
| 7907537 | NM_014412 | CACYBP | calcyclin binding protein | 0.015 | 1.010 | 8.77E-01 | 8.77E+01 | | | |
| 8144184 | NM_020728 | ESYT2 | extended synaptotagmin-like proteir | 0.015 | 1.010 | 9.01E-01 | 9.01E+01 | | | |
| 8176669 | --- | --- | --- | 0.015 | 1.010 | 8.91E-01 | 8.91E+01 | | | |
| 8177189 | --- | --- | --- | 0.015 | 1.010 | 8.91E-01 | 8.91E+01 | | | |
| 7953333 | NM_001242 | CD27 | CD27 molecule | 0.015 | 1.010 | 8.74E-01 | 8.74E+01 | | | |
| 8156164 | NM_017576 | KIF27 | kinesin family member 27 | 0.014 | 1.010 | 9.83E-01 | 9.83E+01 | | | |
| 7993274 | NM_032167 | RUNDC2A | RUN domain containing 2A | 0.014 | 1.010 | 9.10E-01 | 9.10E+01 | | | |
| 7964177 | NM_012064 | MIP | major intrinsic protein of lens fiber | 0.014 | 1.010 | 8.75E-01 | 8.75E+01 | | | |
| 8002057 | NM_00108248( | ACD | adrenocortical dysplasia homolog (m | 0.014 | 1.010 | 8.52E-01 | 8.52E+01 | | | |
| 7962151 | NM_144973 | DENND5B | DENN/MADD domain containing 5B | 0.014 | 1.010 | 8.86E-01 | 8.86E+01 | | | |
| 7895486 | --- | --- | --- | 0.014 | 1.010 | 9.23E-01 | 9.23E+01 | | | |
| 8143575 | NM_005232 | EPHA1 | EPH receptor A1 | 0.014 | 1.010 | 8.56E-01 | 8.56E+01 | | | |
| 7900030 | NM_012199 | EIF2C1 | eukaryotic translation initiation facto | 0.014 | 1.010 | 9.04E-01 | 9.04E+01 | | | |
| 8180338 | --- | --- | --- | 0.014 | 1.010 | 9.30E-01 | 9.30E+01 | | | |
| 7895738 | --- | --- | --- | 0.014 | 1.010 | 9.64E-01 | 9.64E+01 | | | |
| 8015900 | NM_032376 | TMEM101 | transmembrane protein 101 | 0.014 | 1.010 | 8.99E-01 | 8.99E+01 | | | |
| 8025132 | NM_024341 | ZNF557 | zinc finger protein 557 | 0.014 | 1.010 | 9.16E-01 | 9.16E+01 | | | |
| 7959361 | NM_014938 | MLXIP | MLX interacting protein | 0.014 | 1.010 | 9.21E-01 | 9.21E+01 | | | |
| 8032544 | NM_021217 | ZNF77 | zinc finger protein 77 | 0.014 | 1.010 | 8.62E-01 | 8.62E+01 | | | |
| 8134349 | --- | --- | --- | 0.014 | 1.010 | 8.64E-01 | 8.64E+01 | | | |
| 7922648 | NM_015602 | TOR1AIP1 | torsin A interacting protein 1 | 0.014 | 1.010 | 8.63E-01 | 8.63E+01 | | | |
| 8111129 | NM_033414 | ZNF622 | zinc finger protein 622 | 0.014 | 1.009 | 9.73E-01 | 9.73E+01 | | | |
| 7897424 | --- | --- | --- | 0.014 | 1.009 | 9.08E-01 | 9.08E+01 | | | |
| 7979980 | --- | --- | --- | 0.014 | 1.009 | 9.43E-01 | 9.43E+01 | | | |
| 8100376 | --- | --- | --- | 0.013 | 1.009 | 8.81E-01 | 8.81E+01 | | | |
| 7965335 | NM_001946 | DUSP6 | dual specificity phosphatase 6 | 0.013 | 1.009 | 9.51E-01 | 9.51E+01 | | | |
| 8145854 | NM_007198 | PROSC | proline synthetase co-transcribed ho | 0.013 | 1.009 | 9.03E-01 | 9.03E+01 | | | |
| 8093950 | NM_005980 | S100P | S100 calcium binding protein P | 0.013 | 1.009 | 9.38E-01 | 9.38E+01 | | | |
| 7929589 | --- | --- | --- | 0.013 | 1.009 | 9.32E-01 | 9.32E+01 | | | |
| 8153223 | NM_153831 | PTK2 | PTK2 protein tyrosine kinase 2 | 0.013 | 1.009 | 9.26E-01 | 9.26E+01 | | | |
| 8137826 | NM_013393 | FTSJ2 | FtsJ homolog 2 (E. coli) | 0.013 | 1.009 | 8.87E-01 | 8.87E+01 | | | |
| 7911600 | NM_023018 | NADK | NAD kinase | 0.013 | 1.009 | 8.59E-01 | 8.59E+01 | | | |
| 8112596 | NM_023039 | ANKRA2 | ankyrin repeat, family A (RFXANK-lik | 0.013 | 1.009 | 9.77E-01 | 9.77E+01 | | | |
| 7978653 | NM_014990 | GARNL1 | GTPase activating Rap/RanGAP dom | 0.013 | 1.009 | 9.68E-01 | 9.68E+01 | | | |
| 8170590 | NM_015922 | NSDHL | NAD(P) dependent steroid dehydrog | 0.013 | 1.009 | 9.17E-01 | 9.17E+01 | | | |
| 8130009 | --- | --- | --- | 0.013 | 1.009 | 9.55E-01 | 9.55E+01 | | | |
| 7947481 | NM_198381 | ELF5 | E74-like factor 5 (ets domain transcr | 0.013 | 1.009 | 8.70E-01 | 8.70E+01 | | | |
| 7894213 | --- | --- | --- | 0.013 | 1.009 | 8.99E-01 | 8.99E+01 | | | |
| 8172827 | NM_004187 | KDM5C | lysine (K)-specific demethylase 5C | 0.013 | 1.009 | 9.11E-01 | 9.11E+01 | | | |
| 7980496 | NM_152446 | C14orf145 | chromosome 14 open reading frame | 0.013 | 1.009 | 9.30E-01 | 9.30E+01 | | | |
| 7928019 | NM_033500 | HK1 | hexokinase 1 | 0.013 | 1.009 | 8.81E-01 | 8.81E+01 | | | |
| 7939818 | NM_175732 | PTPMT1 | protein tyrosine phosphatase, mitoc | 0.013 | 1.009 | 9.25E-01 | 9.25E+01 | | | |
| 7896600 | --- | --- | --- | 0.013 | 1.009 | 9.84E-01 | 9.84E+01 | | | |
| 7893766 | --- | --- | --- | 0.013 | 1.009 | 9.59E-01 | 9.59E+01 | | | |

| | | | | | | | | | | |
|---|---|---|---|---|---|---|---|---|---|---|
| 8037970 | NM_003706 | PLA2G4C | phospholipase A2, group IVC (cytoso | 0.013 | 1.009 | 9.11E-01 | 9.11E+01 | | | |
| 7948685 | NM_022830 | TUT1 | terminal uridylyl transferase 1, U6 sn | 0.013 | 1.009 | 8.29E-01 | 8.29E+01 | | | |
| 7973983 | --- | --- | --- | 0.013 | 1.009 | 8.91E-01 | 8.91E+01 | | | |
| 7961891 | NM_030762 | BHLHE41 | basic helix-loop-helix family, membe | 0.013 | 1.009 | 8.88E-01 | 8.88E+01 | | | |
| 7893582 | --- | --- | --- | 0.013 | 1.009 | 9.79E-01 | 9.79E+01 | | | |
| 8081431 | NM_001627 | ALCAM | activated leukocyte cell adhesion mo | 0.013 | 1.009 | 9.41E-01 | 9.41E+01 | | | |
| 8083011 | NM_020191 | MRPS22 | mitochondrial ribosomal protein S22 | 0.013 | 1.009 | 9.56E-01 | 9.56E+01 | | | |
| 8022606 | --- | --- | --- | 0.012 | 1.009 | 9.40E-01 | 9.40E+01 | | | |
| 8073430 | NM_001098 | ACO2 | aconitase 2, mitochondrial | 0.012 | 1.009 | 9.22E-01 | 9.22E+01 | | | |
| 8009075 | NM_025185 | TANC2 | tetratricopeptide repeat, ankyrin rep | 0.012 | 1.009 | 9.22E-01 | 9.22E+01 | | | |
| 8087473 | NM_153273 | IP6K1 | inositol hexakisphosphate kinase 1 | 0.012 | 1.009 | 8.94E-01 | 8.94E+01 | | | |
| 7959100 | --- | --- | --- | 0.012 | 1.009 | 9.00E-01 | 9.00E+01 | | | |
| 7956639 | NM_005726 | TSFM | Ts translation elongation factor, mito | 0.012 | 1.009 | 9.35E-01 | 9.35E+01 | | | |
| 8068788 | NM_018961 | UBASH3A | ubiquitin associated and SH3 domair | 0.012 | 1.008 | 9.22E-01 | 9.22E+01 | | | |
| 7894103 | --- | --- | --- | 0.012 | 1.008 | 9.91E-01 | 9.91E+01 | | | |
| 8013179 | NM_145691 | ATPAF2 | ATP synthase mitochondrial F1 comp | 0.012 | 1.008 | 9.11E-01 | 9.11E+01 | | | |
| 7911331 | --- | --- | --- | 0.012 | 1.008 | 9.81E-01 | 9.81E+01 | | | |
| 7924463 | --- | --- | --- | 0.012 | 1.008 | 9.81E-01 | 9.81E+01 | | | |
| 7927089 | --- | --- | --- | 0.012 | 1.008 | 9.81E-01 | 9.81E+01 | | | |
| 7945347 | --- | --- | --- | 0.012 | 1.008 | 9.81E-01 | 9.81E+01 | | | |
| 7998115 | --- | --- | --- | 0.012 | 1.008 | 9.81E-01 | 9.81E+01 | | | |
| 8031997 | --- | --- | --- | 0.012 | 1.008 | 9.81E-01 | 9.81E+01 | | | |
| 8102530 | --- | --- | --- | 0.012 | 1.008 | 9.81E-01 | 9.81E+01 | | | |
| 8137668 | --- | --- | --- | 0.012 | 1.008 | 9.81E-01 | 9.81E+01 | | | |
| 8078898 | NM_017875 | SLC25A38 | solute carrier family 25, member 38 | 0.012 | 1.008 | 9.51E-01 | 9.51E+01 | | | |
| 7949104 | NM_00109867 | RASGRP2 | RAS guanyl releasing protein 2 (calciu | 0.012 | 1.008 | 9.27E-01 | 9.27E+01 | | | |
| 7970301 | NM_017905 | TMCO3 | transmembrane and coiled-coil doma | 0.012 | 1.008 | 8.82E-01 | 8.82E+01 | | | |
| 8085358 | --- | --- | --- | 0.012 | 1.008 | 9.35E-01 | 9.35E+01 | | | |
| 8105181 | NM_016640 | MRPS30 | mitochondrial ribosomal protein S30 | 0.012 | 1.008 | 9.37E-01 | 9.37E+01 | | | |
| 8180326 | --- | --- | --- | 0.012 | 1.008 | 9.56E-01 | 9.56E+01 | | | |
| 8039068 | NM_021074 | NDUFV2 | NADH dehydrogenase (ubiquinone) f | 0.012 | 1.008 | 9.73E-01 | 9.73E+01 | | | |
| 8168179 | NM_021120 | DLG3 | discs, large homolog 3 (Drosophila) | 0.012 | 1.008 | 9.15E-01 | 9.15E+01 | | | |
| 8168391 | NR_026594 | CXorf50B | chromosome X open reading frame 5 | 0.012 | 1.008 | 9.57E-01 | 9.57E+01 | | | |
| 8020930 | NM_018255 | ELP2 | elongation protein 2 homolog (S. cer | 0.012 | 1.008 | 9.70E-01 | 9.70E+01 | | | |
| 8157139 | --- | --- | --- | 0.012 | 1.008 | 9.53E-01 | 9.53E+01 | | | |
| 8159654 | NM_015456 | COBRA1 | cofactor of BRCA1 | 0.012 | 1.008 | 8.98E-01 | 8.98E+01 | | | |
| 7974253 | --- | --- | --- | 0.012 | 1.008 | 9.47E-01 | 9.47E+01 | | | |
| 8180283 | --- | --- | --- | 0.012 | 1.008 | 9.05E-01 | 9.05E+01 | | | |
| 7912956 | NM_00113620 | RCC2 | regulator of chromosome condensat | 0.011 | 1.008 | 9.21E-01 | 9.21E+01 | | | |
| 8065136 | NM_00104257 | RRBP1 | ribosome binding protein 1 homolog | 0.011 | 1.008 | 8.85E-01 | 8.85E+01 | | | |
| 8024572 | NM_002068 | GNA15 | guanine nucleotide binding protein ( | 0.011 | 1.008 | 9.60E-01 | 9.60E+01 | | | |
| 7936833 | --- | --- | --- | 0.011 | 1.008 | 9.63E-01 | 9.63E+01 | | | |
| 8135856 | NM_001662 | ARF5 | ADP-ribosylation factor 5 | 0.011 | 1.008 | 8.77E-01 | 8.77E+01 | | | |
| 8087860 | NM_007284 | TWF2 | twinfilin, actin-binding protein, hom | 0.011 | 1.008 | 8.98E-01 | 8.98E+01 | | | |
| 7893059 | --- | --- | --- | 0.011 | 1.008 | 9.47E-01 | 9.47E+01 | | | |
| 7916743 | --- | --- | --- | 0.011 | 1.008 | 9.20E-01 | 9.20E+01 | | | |
| 8170360 | NM_002032 | FTH1 | ferritin, heavy polypeptide 1 | 0.011 | 1.008 | 9.43E-01 | 9.43E+01 | | | |
| 7966183 | NM_00114537 | ALKBH2 | alkB, alkylation repair homolog 2 (E. | 0.011 | 1.008 | 9.44E-01 | 9.44E+01 | | | |
| 7893244 | --- | --- | --- | 0.011 | 1.008 | 9.11E-01 | 9.11E+01 | | | |
| 7893835 | --- | --- | --- | 0.011 | 1.008 | 9.18E-01 | 9.18E+01 | | | |
| 8000399 | NM_00101298 | ZKSCAN2 | zinc finger with KRAB and SCAN dom | 0.011 | 1.008 | 9.48E-01 | 9.48E+01 | | | |
| 7999023 | --- | --- | --- | 0.011 | 1.008 | 9.16E-01 | 9.16E+01 | | | |
| 7980958 | NM_005606 | LGMN | legumain | 0.011 | 1.008 | 9.40E-01 | 9.40E+01 | | | |
| 8164560 | --- | --- | --- | 0.011 | 1.008 | 8.80E-01 | 8.80E+01 | | | |
| 7895225 | --- | --- | --- | 0.011 | 1.008 | 9.10E-01 | 9.10E+01 | | | |
| 8105991 | BC171739 | GUSBL1 | glucuronidase, beta-like 1 | 0.011 | 1.008 | 9.77E-01 | 9.77E+01 | | | |
| 8109407 | NM_198321 | GALNT10 | UDP-N-acetyl-alpha-D-galactosamine | 0.011 | 1.008 | 8.92E-01 | 8.92E+01 | | | |
| 7928395 | NM_173540 | FUT11 | fucosyltransferase 11 (alpha (1,3) fu | 0.011 | 1.008 | 9.52E-01 | 9.52E+01 | | | |
| 7894196 | --- | --- | --- | 0.011 | 1.008 | 9.90E-01 | 9.90E+01 | | | |
| 7994737 | NM_000034 | ALDOA | aldolase A, fructose-bisphosphate | 0.011 | 1.008 | 9.26E-01 | 9.26E+01 | | | |
| 7892627 | --- | --- | --- | 0.011 | 1.007 | 9.50E-01 | 9.50E+01 | | | |
| 8173513 | NM_001007 | RPS4X | ribosomal protein S4, X-linked | 0.011 | 1.007 | 9.30E-01 | 9.30E+01 | | | |
| 7977273 | NM_152328 | ADSSL1 | adenylosuccinate synthase like 1 | 0.011 | 1.007 | 9.02E-01 | 9.02E+01 | | | |
| 7893353 | --- | --- | --- | 0.011 | 1.007 | 9.41E-01 | 9.41E+01 | | | |
| 7912166 | NM_012102 | RERE | arginine-glutamic acid dipeptide (RE) | 0.011 | 1.007 | 9.03E-01 | 9.03E+01 | | | |
| 7895248 | --- | --- | --- | 0.011 | 1.007 | 9.67E-01 | 9.67E+01 | | | |
| 8083075 | NM_152282 | ACPL2 | acid phosphatase-like 2 | 0.011 | 1.007 | 8.56E-01 | 8.56E+01 | | | |
| 8178188 | NR_003129 | RNF5P1 | ring finger protein 5 pseudogene 1 | 0.010 | 1.007 | 9.62E-01 | 9.62E+01 | | | |
| 7906061 | NM_152280 | SYT11 | synaptotagmin XI | 0.010 | 1.007 | 9.42E-01 | 9.42E+01 | | | |
| 8108954 | NM_006706 | TCERG1 | transcription elongation regulator 1 | 0.010 | 1.007 | 9.59E-01 | 9.59E+01 | | | |
| 8161229 | NM_012166 | FBXO10 | F-box protein 10 | 0.010 | 1.007 | 9.41E-01 | 9.41E+01 | | | |
| 8098103 | NM_020840 | FNIP2 | folliculin interacting protein 2 | 0.010 | 1.007 | 9.30E-01 | 9.30E+01 | | | |
| 8100456 | --- | --- | --- | 0.010 | 1.007 | 9.20E-01 | 9.20E+01 | | | |
| 8028916 | NM_004596 | SNRPA | small nuclear ribonucleoprotein poly | 0.010 | 1.007 | 9.43E-01 | 9.43E+01 | | | |
| 8136869 | NM_032982 | CASP2 | caspase 2, apoptosis-related cystein | 0.010 | 1.007 | 9.56E-01 | 9.56E+01 | | | |
| 7918517 | NM_024102 | WDR77 | WD repeat domain 77 | 0.010 | 1.007 | 9.48E-01 | 9.48E+01 | | | |
| 8041031 | NM_199193 | BRE | brain and reproductive organ-expres | 0.010 | 1.007 | 9.59E-01 | 9.59E+01 | | | |

| | | | | | | | | | | |
|---|---|---|---|---|---|---|---|---|---|---|
| 7941478 | NM_005146 | SART1 | squamous cell carcinoma antigen rec | 0.010 | 1.007 | 9.53E-01 | 9.53E+01 | | | |
| 7981494 | NM_00101443 | AKT1 | v-akt murine thymoma viral oncoger | 0.010 | 1.007 | 9.18E-01 | 9.18E+01 | | | |
| 7893085 | --- | --- | --- | 0.010 | 1.007 | 9.24E-01 | 9.24E+01 | | | |
| 8126574 | NM_015388 | YIPF3 | Yip1 domain family, member 3 | 0.010 | 1.007 | 9.60E-01 | 9.60E+01 | | | |
| 7897824 | NM_014874 | MFN2 | mitofusin 2 | 0.010 | 1.007 | 9.46E-01 | 9.46E+01 | | | |
| 7897685 | NM_013319 | UBIAD1 | UbiA prenyltransferase domain cont | 0.010 | 1.007 | 9.26E-01 | 9.26E+01 | | | |
| 8113623 | NM_021649 | TICAM2 | toll-like receptor adaptor molecule 2 | 0.010 | 1.007 | 9.37E-01 | 9.37E+01 | | | |
| 8130628 | NM_020133 | AGPAT4 | 1-acylglycerol-3-phosphate O-acyltra | 0.010 | 1.007 | 8.98E-01 | 8.98E+01 | | | |
| 8030470 | NM_014203 | AP2A1 | adaptor-related protein complex 2, a | 0.010 | 1.007 | 9.14E-01 | 9.14E+01 | | | |
| 7946128 | NM_032127 | FAM160A2 | family with sequence similarity 160, | 0.010 | 1.007 | 9.03E-01 | 9.03E+01 | | | |
| 8140782 | NM_000927 | ABCB1 | ATP-binding cassette, sub-family B (M | 0.010 | 1.007 | 9.53E-01 | 9.53E+01 | | | |
| 7894768 | --- | --- | --- | 0.010 | 1.007 | 8.91E-01 | 8.91E+01 | | | |
| 8009476 | NM_002758 | MAP2K6 | mitogen-activated protein kinase kin | 0.010 | 1.007 | 9.67E-01 | 9.67E+01 | | | |
| 8130403 | --- | --- | --- | 0.010 | 1.007 | 9.01E-01 | 9.01E+01 | | | |
| 7988426 | NM_013309 | SLC30A4 | solute carrier family 30 (zinc transpo | 0.010 | 1.007 | 9.29E-01 | 9.29E+01 | | | |
| 8036737 | NM_001020 | RPS16 | ribosomal protein S16 | 0.010 | 1.007 | 9.71E-01 | 9.71E+01 | | | |
| 8118826 | NM_003093 | SNRPC | small nuclear ribonucleoprotein poly | 0.010 | 1.007 | 9.62E-01 | 9.62E+01 | | | |
| 8088167 | NM_021237 | SELK | selenoprotein K | 0.010 | 1.007 | 9.75E-01 | 9.75E+01 | | | |
| 8167815 | NM_014599 | MAGED2 | melanoma antigen family D, 2 | 0.010 | 1.007 | 9.20E-01 | 9.20E+01 | | | |
| 8082252 | --- | --- | --- | 0.010 | 1.007 | 9.68E-01 | 9.68E+01 | | | |
| 8115865 | NM_138369 | BOD1 | biorientation of chromosomes in cell | 0.010 | 1.007 | 9.56E-01 | 9.56E+01 | | | |
| 7910146 | NM_000447 | PSEN2 | presenilin 2 (Alzheimer disease 4) | 0.010 | 1.007 | 9.01E-01 | 9.01E+01 | | | |
| 8166049 | NM_00103909 | PRPS2 | phosphoribosyl pyrophosphate synth | 0.010 | 1.007 | 9.55E-01 | 9.55E+01 | | | |
| 8035494 | NM_012181 | FKBP8 | FK506 binding protein 8, 38kDa | 0.010 | 1.007 | 9.30E-01 | 9.30E+01 | | | |
| 7919815 | NM_000396 | CTSK | cathepsin K | 0.009 | 1.007 | 9.60E-01 | 9.60E+01 | | | |
| 8066459 | --- | --- | --- | 0.009 | 1.007 | 9.33E-01 | 9.33E+01 | | | |
| 8061483 | NR_004846 | CTD-2514C3. | hypothetical LOC100134868 | 0.009 | 1.007 | 9.17E-01 | 9.17E+01 | | | |
| 8066983 | --- | --- | --- | 0.009 | 1.007 | 9.81E-01 | 9.81E+01 | | | |
| 8178011 | NM_080686 | BAT2 | HLA-B associated transcript 2 | 0.009 | 1.007 | 9.11E-01 | 9.11E+01 | | | |
| 8068919 | NM_003681 | PDXK | pyridoxal (pyridoxine, vitamin B6) kin | 0.009 | 1.007 | 9.12E-01 | 9.12E+01 | | | |
| 8106181 | NM_00103763 | BTF3 | basic transcription factor 3 | 0.009 | 1.007 | 9.32E-01 | 9.32E+01 | | | |
| 7996807 | NM_133458 | ZFP90 | zinc finger protein 90 homolog (mou | 0.009 | 1.007 | 9.45E-01 | 9.45E+01 | | | |
| 8124040 | NM_000332 | ATXN1 | ataxin 1 | 0.009 | 1.007 | 9.33E-01 | 9.33E+01 | | | |
| 7984985 | NM_00103864 | GOLGA6 | golgi autoantigen, golgin subfamily a | 0.009 | 1.007 | 9.54E-01 | 9.54E+01 | | | |
| 8020029 | NM_00102530 | RAB12 | RAB12, member RAS oncogene famil | 0.009 | 1.007 | 9.57E-01 | 9.57E+01 | | | |
| 7977955 | NR_026862 | PPP1R3E | protein phosphatase 1, regulatory (in | 0.009 | 1.006 | 9.08E-01 | 9.08E+01 | | | |
| 7940711 | NM_199337 | TMEM179B | transmembrane protein 179B | 0.009 | 1.006 | 9.45E-01 | 9.45E+01 | | | |
| 8069753 | NM_006585 | CCT8 | chaperonin containing TCP1, subunit | 0.009 | 1.006 | 9.48E-01 | 9.48E+01 | | | |
| 8038407 | NM_006270 | RRAS | related RAS viral (r-ras) oncogene ho | 0.009 | 1.006 | 9.18E-01 | 9.18E+01 | | | |
| 8128247 | NM_021813 | BACH2 | BTB and CNC homology 1, basic leuc | 0.009 | 1.006 | 9.26E-01 | 9.26E+01 | | | |
| 7913694 | NM_000147 | FUCA1 | fucosidase, alpha-L- 1, tissue | 0.009 | 1.006 | 9.31E-01 | 9.31E+01 | | | |
| 8157729 | --- | --- | --- | 0.009 | 1.006 | 9.89E-01 | 9.89E+01 | | | |
| 7947652 | NM_017749 | AMBRA1 | autophagy/beclin-1 regulator 1 | 0.009 | 1.006 | 8.96E-01 | 8.96E+01 | | | |
| 7987675 | NM_015540 | RPAP1 | RNA polymerase II associated protei | 0.009 | 1.006 | 8.96E-01 | 8.96E+01 | | | |
| 7990429 | NM_020447 | C15orf17 | chromosome 15 open reading frame | 0.009 | 1.006 | 9.62E-01 | 9.62E+01 | | | |
| 8148467 | NM_017444 | CHRAC1 | chromatin accessibility complex 1 | 0.009 | 1.006 | 9.41E-01 | 9.41E+01 | | | |
| 8083000 | NM_00103303 | FAIM | Fas apoptotic inhibitory molecule | 0.009 | 1.006 | 9.50E-01 | 9.50E+01 | | | |
| 7940798 | NM_017490 | MARK2 | MAP/microtubule affinity-regulating | 0.009 | 1.006 | 9.78E-01 | 9.78E+01 | | | |
| 7967709 | --- | --- | --- | 0.009 | 1.006 | 9.30E-01 | 9.30E+01 | | | |
| 8011430 | NM_002208 | ITGAE | integrin, alpha E (antigen CD103, hur | 0.009 | 1.006 | 9.07E-01 | 9.07E+01 | | | |
| 8102745 | NM_006320 | PGRMC2 | progesterone receptor membrane co | 0.009 | 1.006 | 9.50E-01 | 9.50E+01 | | | |
| 8031152 | NM_001013 | RPS9 | ribosomal protein S9 | 0.009 | 1.006 | 9.41E-01 | 9.41E+01 | | | |
| 7898568 | AK096102 | OC10013019 | hypothetical protein LOC100130193 | 0.009 | 1.006 | 9.67E-01 | 9.67E+01 | | | |
| 8158183 | NM_004408 | DNM1 | dynamin 1 | 0.009 | 1.006 | 9.16E-01 | 9.16E+01 | | | |
| 8114111 | --- | --- | --- | 0.009 | 1.006 | 9.66E-01 | 9.66E+01 | | | |
| 8153043 | NM_020863 | ZFAT | zinc finger and AT hook domain cont | 0.009 | 1.006 | 9.30E-01 | 9.30E+01 | | | |
| 8156116 | NM_00100155 | C9orf103 | chromosome 9 open reading frame 1 | 0.009 | 1.006 | 9.57E-01 | 9.57E+01 | | | |
| 8055980 | NM_004288 | CYTIP | cytohesin 1 interacting protein | 0.009 | 1.006 | 9.78E-01 | 9.78E+01 | | | |
| 8097717 | NM_024605 | ARHGAP10 | Rho GTPase activating protein 10 | 0.009 | 1.006 | 9.42E-01 | 9.42E+01 | | | |
| 7951112 | NR_023356 | MTMR2 | myotubularin related protein 2 | 0.009 | 1.006 | 9.57E-01 | 9.57E+01 | | | |
| 8160016 | NM_012416 | RANBP6 | RAN binding protein 6 | 0.008 | 1.006 | 9.76E-01 | 9.76E+01 | | | |
| 7892989 | --- | --- | --- | 0.008 | 1.006 | 9.47E-01 | 9.47E+01 | | | |
| 8000676 | NM_130464 | NPIPL3 | nuclear pore complex interacting pro | 0.008 | 1.006 | 9.54E-01 | 9.54E+01 | | | |
| 8128133 | NM_020466 | LYRM2 | LYR motif containing 2 | 0.008 | 1.006 | 9.59E-01 | 9.59E+01 | | | |
| 8131042 | AK126112 | FLJ44124 | hypothetical LOC641737 | 0.008 | 1.006 | 9.61E-01 | 9.61E+01 | | | |
| 8031821 | NM_017652 | ZNF586 | zinc finger protein 586 | 0.008 | 1.006 | 9.58E-01 | 9.58E+01 | | | |
| 7988281 | --- | --- | --- | 0.008 | 1.006 | 9.46E-01 | 9.46E+01 | | | |
| 8118319 | --- | --- | --- | 0.008 | 1.006 | 9.78E-01 | 9.78E+01 | | | |
| 8180390 | --- | --- | --- | 0.008 | 1.006 | 9.36E-01 | 9.36E+01 | | | |
| 8008664 | NM_003488 | AKAP1 | A kinase (PRKA) anchor protein 1 | 0.008 | 1.006 | 9.60E-01 | 9.60E+01 | | | |
| 8151099 | --- | --- | --- | 0.008 | 1.006 | 9.56E-01 | 9.56E+01 | | | |
| 7977003 | NM_015156 | RCOR1 | REST corepressor 1 | 0.008 | 1.006 | 9.71E-01 | 9.71E+01 | | | |
| 8157761 | NM_00114500 | NEK6 | NIMA (never in mitosis gene a)-relate | 0.008 | 1.006 | 9.21E-01 | 9.21E+01 | | | |
| 7959882 | NM_006325 | RAN | RAN, member RAS oncogene family | 0.008 | 1.006 | 9.39E-01 | 9.39E+01 | | | |
| 8030199 | NM_003089 | SNRNP70 | small nuclear ribonucleoprotein 70kI | 0.008 | 1.006 | 9.55E-01 | 9.55E+01 | | | |
| 8143383 | --- | --- | --- | 0.008 | 1.006 | 9.93E-01 | 9.93E+01 | | | |
| 7984174 | NM_024798 | SNX22 | sorting nexin 22 | 0.008 | 1.006 | 9.20E-01 | 9.20E+01 | | | |

| | | | | | | | | | | |
|---|---|---|---|---|---|---|---|---|---|---|
| 8136647 | NM_018980 | TAS2R5 | taste receptor, type 2, member 5 | 0.008 | 1.006 | 9.10E-01 | 9.10E+01 | | | |
| 8004195 | NM_153230 | FBXO39 | F-box protein 39 | 0.008 | 1.006 | 9.22E-01 | 9.22E+01 | | | |
| 8023955 | NM_001728 | BSG | basigin (Ok blood group) | 0.008 | 1.006 | 9.14E-01 | 9.14E+01 | | | |
| 7945730 | NM_005170 | ASCL2 | achaete-scute complex homolog 2 (D | 0.008 | 1.006 | 9.50E-01 | 9.50E+01 | | | |
| 8146930 | NM_017866 | TMEM70 | transmembrane protein 70 | 0.008 | 1.006 | 9.60E-01 | 9.60E+01 | | | |
| 8003448 | NM_013275 | ANKRD11 | ankyrin repeat domain 11 | 0.008 | 1.005 | 9.45E-01 | 9.45E+01 | | | |
| 8141791 | NM_00109761 | POLR2J3 | polymerase (RNA) II (DNA directed) p | 0.008 | 1.005 | 9.76E-01 | 9.76E+01 | | | |
| 7895451 | --- | --- | --- | 0.008 | 1.005 | 9.21E-01 | 9.21E+01 | | | |
| 8099073 | NM_002337 | LRPAP1 | low density lipoprotein receptor-rela | 0.008 | 1.005 | 9.37E-01 | 9.37E+01 | | | mono |
| 8139592 | NM_004507 | HUS1 | HUS1 checkpoint homolog (S. pombe | 0.008 | 1.005 | 9.81E-01 | 9.81E+01 | | | |
| 7976976 | NM_014844 | TECPR2 | tectonin beta-propeller repeat conta | 0.008 | 1.005 | 9.24E-01 | 9.24E+01 | | | |
| 8067221 | NM_030776 | ZBP1 | Z-DNA binding protein 1 | 0.008 | 1.005 | 9.30E-01 | 9.30E+01 | | | |
| 7904394 | --- | --- | --- | 0.008 | 1.005 | 9.65E-01 | 9.65E+01 | | | |
| 8122194 | --- | --- | --- | 0.007 | 1.005 | 9.38E-01 | 9.38E+01 | | | |
| 7894992 | --- | --- | --- | 0.007 | 1.005 | 9.32E-01 | 9.32E+01 | | | |
| 7948379 | NM_002556 | OSBP | oxysterol binding protein | 0.007 | 1.005 | 9.45E-01 | 9.45E+01 | | | |
| 7973629 | NM_00104820 | REC8 | REC8 homolog (yeast) | 0.007 | 1.005 | 9.29E-01 | 9.29E+01 | | | |
| 8088846 | --- | --- | --- | 0.007 | 1.005 | 9.74E-01 | 9.74E+01 | | | |
| 8126121 | NM_052893 | BTBD9 | BTB (POZ) domain containing 9 | 0.007 | 1.005 | 9.78E-01 | 9.78E+01 | | | |
| 7928064 | NM_145306 | C10orf35 | chromosome 10 open reading frame | 0.007 | 1.005 | 9.21E-01 | 9.21E+01 | | | |
| 8066964 | NM_199129 | TMEM189 | transmembrane protein 189 | 0.007 | 1.005 | 8.98E-01 | 8.98E+01 | | | |
| 7931479 | NM_005539 | INPP5A | inositol polyphosphate-5-phosphata | 0.007 | 1.005 | 9.72E-01 | 9.72E+01 | | | |
| 8124391 | NM_003513 | HIST1H2AB | histone cluster 1, H2ab | 0.007 | 1.005 | 9.75E-01 | 9.75E+01 | | | |
| 7894089 | --- | --- | --- | 0.007 | 1.005 | 9.81E-01 | 9.81E+01 | | | |
| 8098508 | NM_001564 | ING2 | inhibitor of growth family, member 2 | 0.007 | 1.005 | 9.91E-01 | 9.91E+01 | | | |
| 7893355 | --- | --- | --- | 0.007 | 1.005 | 9.83E-01 | 9.83E+01 | | | |
| 8140468 | NM_017439 | PION | pigeon homolog (Drosophila) | 0.007 | 1.005 | 9.85E-01 | 9.85E+01 | | | |
| 7921571 | NM_002857 | PEX19 | peroxisomal biogenesis factor 19 | 0.007 | 1.005 | 9.57E-01 | 9.57E+01 | | | |
| 8068039 | NM_002040 | GABPA | GA binding protein transcription fact | 0.007 | 1.005 | 9.38E-01 | 9.38E+01 | | | |
| 7894055 | --- | --- | --- | 0.007 | 1.005 | 9.62E-01 | 9.62E+01 | | | |
| 8127051 | NM_012288 | TRAM2 | translocation associated membrane | 0.007 | 1.005 | 9.45E-01 | 9.45E+01 | | | |
| 7897632 | NM_198544 | APITD1 | apoptosis-inducing, TAF9-like domai | 0.007 | 1.005 | 9.25E-01 | 9.25E+01 | | | |
| 7952185 | NM_001382 | DPAGT1 | dolichyl-phosphate (UDP-N-acetylglu | 0.007 | 1.005 | 9.60E-01 | 9.60E+01 | | | |
| 7893140 | --- | --- | --- | 0.007 | 1.005 | 9.20E-01 | 9.20E+01 | | | |
| 8035232 | --- | --- | --- | 0.007 | 1.005 | 9.46E-01 | 9.46E+01 | | | |
| 8160767 | --- | --- | --- | 0.007 | 1.005 | 9.96E-01 | 9.96E+01 | | | |
| 8074939 | NM_213720 | CHCHD10 | coiled-coil-helix-coiled-coil-helix dom | 0.007 | 1.005 | 9.49E-01 | 9.49E+01 | | | |
| 8101728 | NM_014883 | FAM13A | family with sequence similarity 13, m | 0.007 | 1.005 | 9.18E-01 | 9.18E+01 | | | |
| 7972442 | --- | --- | --- | 0.007 | 1.005 | 9.22E-01 | 9.22E+01 | | | |
| 8164653 | NM_031432 | UCK1 | uridine-cytidine kinase 1 | 0.006 | 1.004 | 9.66E-01 | 9.66E+01 | | | |
| 7896711 | --- | --- | --- | 0.006 | 1.004 | 9.81E-01 | 9.81E+01 | | | |
| 7981383 | --- | --- | --- | 0.006 | 1.004 | 9.55E-01 | 9.55E+01 | | | |
| 8070389 | NM_004965 | HMGN1 | high-mobility group nucleosome bin | 0.006 | 1.004 | 9.46E-01 | 9.46E+01 | | | |
| 8076241 | NM_020831 | MKL1 | megakaryoblastic leukemia (transloc | 0.006 | 1.004 | 9.62E-01 | 9.62E+01 | | | |
| 8112139 | NM_002184 | IL6ST | interleukin 6 signal transducer (gp13 | 0.006 | 1.004 | 9.90E-01 | 9.90E+01 | | | |
| 8043981 | NM_004633 | IL1R2 | interleukin 1 receptor, type II | 0.006 | 1.004 | 9.51E-01 | 9.51E+01 | | | |
| 8081953 | NM_005513 | GTF2E1 | general transcription factor IIE, polyp | 0.006 | 1.004 | 9.83E-01 | 9.83E+01 | | | |
| 8013753 | NM_031934 | RAB34 | RAB34, member RAS oncogene famil | 0.006 | 1.004 | 9.63E-01 | 9.63E+01 | | | |
| 7949060 | NM_138689 // | PPP1R14B | protein phosphatase 1, regulatory (in | 0.006 | 1.004 | 9.62E-01 | 9.62E+01 | | | |
| 8079005 | NM_198484 | ZNF621 | zinc finger protein 621 | 0.006 | 1.004 | 9.37E-01 | 9.37E+01 | | | |
| 8116620 | NM_00101298 | 136B // FAM | family with sequence similarity 136, | 0.006 | 1.004 | 9.35E-01 | 9.35E+01 | | | |
| 7892712 | --- | --- | --- | 0.006 | 1.004 | 9.72E-01 | 9.72E+01 | | | |
| 8099091 | --- | --- | --- | 0.006 | 1.004 | 9.49E-01 | 9.49E+01 | | | |
| 7938388 | --- | --- | --- | 0.006 | 1.004 | 9.62E-01 | 9.62E+01 | | | |
| 7985983 | NM_016645 | NGRN | neugrin, neurite outgrowth associate | 0.006 | 1.004 | 9.68E-01 | 9.68E+01 | | | |
| 8031238 | NM_006847 | LILRB4 | leukocyte immunoglobulin-like recep | 0.006 | 1.004 | 9.32E-01 | 9.32E+01 | | | |
| 7963713 | NM_005176 | ATP5G2 | ATP synthase, H+ transporting, mitoc | 0.006 | 1.004 | 9.42E-01 | 9.42E+01 | | | |
| 7893904 | --- | --- | --- | 0.006 | 1.004 | 9.89E-01 | 9.89E+01 | | | |
| 8069700 | NM_013240 | N6AMT1 | N-6 adenine-specific DNA methyltran | 0.006 | 1.004 | 9.59E-01 | 9.59E+01 | | | |
| 8063174 | ENST0000044 | ZNF840P | zinc finger protein 840 (pseudogene) | 0.006 | 1.004 | 9.84E-01 | 9.84E+01 | | | |
| 7975793 | NM_006399 | BATF | basic leucine zipper transcription fac | 0.006 | 1.004 | 9.52E-01 | 9.52E+01 | | | |
| 7948367 | --- | --- | --- | 0.006 | 1.004 | 9.56E-01 | 9.56E+01 | | | |
| 7915659 | NM_024602 | HECTD3 | HECT domain containing 3 | 0.006 | 1.004 | 9.65E-01 | 9.65E+01 | | | |
| 8042962 | NM_014763 | MRPL19 | mitochondrial ribosomal protein L19 | 0.006 | 1.004 | 9.69E-01 | 9.69E+01 | | | |
| 8118824 | --- | --- | --- | 0.006 | 1.004 | 9.76E-01 | 9.76E+01 | | | |
| 7964832 | --- | --- | --- | 0.006 | 1.004 | 9.90E-01 | 9.90E+01 | | | |
| 7895956 | --- | --- | --- | 0.006 | 1.004 | 9.17E-01 | 9.17E+01 | | | |
| 8158418 | NM_004435 | ENDOG | endonuclease G | 0.006 | 1.004 | 9.38E-01 | 9.38E+01 | | | |
| 7893870 | --- | --- | --- | 0.006 | 1.004 | 9.74E-01 | 9.74E+01 | | | |
| 7967622 | --- | --- | --- | 0.006 | 1.004 | 9.73E-01 | 9.73E+01 | | | |
| 8094870 | NM_00108050 | SHISA3 | shisa homolog 3 (Xenopus laevis) | 0.006 | 1.004 | 9.80E-01 | 9.80E+01 | | | |
| 7998817 | NM_00104821 | CEMP1 | cementum protein 1 | 0.006 | 1.004 | 9.53E-01 | 9.53E+01 | | | |
| 8005231 | NR_026809 | FAM106A | family with sequence similarity 106, | 0.006 | 1.004 | 9.43E-01 | 9.43E+01 | | | |
| 7952145 | NM_006389 | HYOU1 | hypoxia up-regulated 1 | 0.006 | 1.004 | 9.73E-01 | 9.73E+01 | | | |
| 8164572 | NM_017873 | ASB6 | ankyrin repeat and SOCS box-contain | 0.006 | 1.004 | 9.59E-01 | 9.59E+01 | | | |
| 8173613 | NM_016120 | RLIM | ring finger protein, LIM domain inter | 0.006 | 1.004 | 9.89E-01 | 9.89E+01 | | | |
| 8061211 | NM_080820 | DTD1 | D-tyrosyl-tRNA deacylase 1 homolog | 0.005 | 1.004 | 9.79E-01 | 9.79E+01 | | | |

| | | | | | | | | | | |
|---|---|---|---|---|---|---|---|---|---|---|
| 8167042 | NM_005676 | RBM10 | RNA binding motif protein 10 | 0.005 | 1.004 | 9.57E-01 | 9.57E+01 | | | |
| 7924893 | --- | --- | --- | 0.005 | 1.004 | 9.60E-01 | 9.60E+01 | | | |
| 8000899 | NM_024671 | ZNF768 | zinc finger protein 768 | 0.005 | 1.004 | 9.65E-01 | 9.65E+01 | | | |
| 8028674 | NM_003169 | SUPT5H | suppressor of Ty 5 homolog (S. cerev | 0.005 | 1.004 | 9.78E-01 | 9.78E+01 | | | |
| 8035789 | NM_00103988 | ZNF826 | zinc finger protein 826 | 0.005 | 1.004 | 9.53E-01 | 9.53E+01 | | | |
| 7899654 | NM_006559 | KHDRBS1 | KH domain containing, RNA binding, | 0.005 | 1.004 | 9.75E-01 | 9.75E+01 | | | |
| 7963965 | NM_032345 | WIBG | within bgcn homolog (Drosophila) | 0.005 | 1.004 | 9.54E-01 | 9.54E+01 | | | |
| 8033300 | NM_00108045 | GPR108 | G protein-coupled receptor 108 | 0.005 | 1.004 | 9.56E-01 | 9.56E+01 | | | |
| 7947674 | --- | --- | --- | 0.005 | 1.004 | 9.81E-01 | 9.81E+01 | | | |
| 7893161 | --- | --- | --- | 0.005 | 1.004 | 9.96E-01 | 9.96E+01 | | | |
| 7963212 | NM_000617 | SLC11A2 | solute carrier family 11 (proton-coup | 0.005 | 1.004 | 9.74E-01 | 9.74E+01 | | | |
| 7905581 | NM_006271 | S100A1 | S100 calcium binding protein A1 | 0.005 | 1.004 | 9.28E-01 | 9.28E+01 | | | |
| 8116177 | NM_153373 | AGXT2L2 | alanine-glyoxylate aminotransferase | 0.005 | 1.004 | 9.53E-01 | 9.53E+01 | | | |
| 8023889 | NM_00102510 | MBP | myelin basic protein | 0.005 | 1.004 | 9.57E-01 | 9.57E+01 | | | |
| 8173156 | --- | --- | --- | 0.005 | 1.004 | 9.94E-01 | 9.94E+01 | | | |
| 7945666 | NM_001909 | CTSD | cathepsin D | 0.005 | 1.004 | 9.48E-01 | 9.48E+01 | | | |
| 8016390 | NM_016429 | COPZ2 | coatomer protein complex, subunit z | 0.005 | 1.004 | 9.52E-01 | 9.52E+01 | | | |
| 8058512 | NM_00108047 | PLEKHM3 | pleckstrin homology domain contain | 0.005 | 1.004 | 9.65E-01 | 9.65E+01 | | | |
| 8151747 | NM_00100849 | TMEM64 | transmembrane protein 64 | 0.005 | 1.003 | 9.56E-01 | 9.56E+01 | | | |
| 7893300 | --- | --- | --- | 0.005 | 1.003 | 9.93E-01 | 9.93E+01 | | | |
| 7894844 | --- | --- | --- | 0.005 | 1.003 | 9.73E-01 | 9.73E+01 | | | |
| 8149365 | NR_003494 | FAM86B1 | family with sequence similarity 86, m | 0.005 | 1.003 | 9.55E-01 | 9.55E+01 | | | |
| 8116297 | NM_005520 | HNRNPH1 | heterogeneous nuclear ribonucleopr | 0.005 | 1.003 | 9.41E-01 | 9.41E+01 | | | |
| 8038861 | NM_001245 | SIGLEC6 | sialic acid binding Ig-like lectin 6 | 0.005 | 1.003 | 9.44E-01 | 9.44E+01 | | | |
| 7901858 | --- | --- | --- | 0.005 | 1.003 | 9.57E-01 | 9.57E+01 | | | |
| 7983938 | --- | --- | --- | 0.005 | 1.003 | 9.77E-01 | 9.77E+01 | | | |
| 7896184 | --- | --- | --- | 0.005 | 1.003 | 9.81E-01 | 9.81E+01 | | | |
| 7941985 | NM_006019 | TCIRG1 | T-cell, immune regulator 1, ATPase, I | 0.005 | 1.003 | 9.44E-01 | 9.44E+01 | | | |
| 7894312 | --- | --- | --- | 0.005 | 1.003 | 9.82E-01 | 9.82E+01 | | | |
| 8055911 | ENST00000428 | OC10012944 | PRO2055 | 0.005 | 1.003 | 9.73E-01 | 9.73E+01 | | | |
| 8070782 | NM_198694 | KRTAP10-5 | keratin associated protein 10-5 | 0.005 | 1.003 | 9.66E-01 | 9.66E+01 | | | |
| 8058052 | NM_002156 | HSPD1 | heat shock 60kDa protein 1 (chaperc | 0.005 | 1.003 | 9.83E-01 | 9.83E+01 | | | |
| 8174086 | NM_019007 | ARMCX6 | armadillo repeat containing, X-linked | 0.005 | 1.003 | 9.76E-01 | 9.76E+01 | | | |
| 8074934 | NM_013378 | VPREB3 | pre-B lymphocyte 3 | 0.005 | 1.003 | 9.68E-01 | 9.68E+01 | | | |
| 7910198 | BC007286 | ZNF678 | zinc finger protein 678 | 0.005 | 1.003 | 9.85E-01 | 9.85E+01 | | | |
| 8057463 | NM_201548 | CERKL | ceramide kinase-like | 0.005 | 1.003 | 9.57E-01 | 9.57E+01 | | | |
| 7894986 | --- | --- | --- | 0.005 | 1.003 | 9.91E-01 | 9.91E+01 | | | |
| 7980940 | NM_004993 | ATXN3 | ataxin 3 | 0.005 | 1.003 | 9.93E-01 | 9.93E+01 | | | |
| 8016266 | NM_015443 | KIAA1267 | KIAA1267 | 0.005 | 1.003 | 9.73E-01 | 9.73E+01 | | | |
| 7994781 | NM_003166 | SULT1A3 | sulfotransferase family, cytosolic, 1A | 0.004 | 1.003 | 9.62E-01 | 9.62E+01 | | | |
| 8005839 | NM_014573 | TMEM97 | transmembrane protein 97 | 0.004 | 1.003 | 9.64E-01 | 9.64E+01 | | | |
| 7943760 | NM_015191 | SIK2 | salt-inducible kinase 2 | 0.004 | 1.003 | 9.70E-01 | 9.70E+01 | | | |
| 7907830 | NM_002826 | QSOX1 | quiescin Q6 sulfhydryl oxidase 1 | 0.004 | 1.003 | 9.59E-01 | 9.59E+01 | | | |
| 7903171 | NM_015485 | RWDD3 | RWD domain containing 3 | 0.004 | 1.003 | 9.83E-01 | 9.83E+01 | | | |
| 7987114 | NR_024074 | GOLGA9P | golgi autoantigen, golgin subfamily a | 0.004 | 1.003 | 9.62E-01 | 9.62E+01 | | | |
| 8123044 | NM_020245 | TULP4 | tubby like protein 4 | 0.004 | 1.003 | 9.63E-01 | 9.63E+01 | | | |
| 8139242 | --- | --- | --- | 0.004 | 1.003 | 9.79E-01 | 9.79E+01 | | | |
| 8075969 | --- | --- | --- | 0.004 | 1.003 | 9.86E-01 | 9.86E+01 | | | |
| 8017402 | NM_153335 | STRADA | STE20-related kinase adaptor alpha | 0.004 | 1.003 | 9.59E-01 | 9.59E+01 | | | |
| 8066567 | --- | --- | --- | 0.004 | 1.003 | 9.52E-01 | 9.52E+01 | | | |
| 7918457 | NM_002232 | KCNA3 | potassium voltage-gated channel, sh | 0.004 | 1.003 | 9.89E-01 | 9.89E+01 | | | |
| 8149809 | --- | --- | --- | 0.004 | 1.003 | 9.65E-01 | 9.65E+01 | | | |
| 7985605 | NM_014630 | ZNF592 | zinc finger protein 592 | 0.004 | 1.003 | 9.70E-01 | 9.70E+01 | | | |
| 8021478 | --- | --- | --- | 0.004 | 1.003 | 9.73E-01 | 9.73E+01 | | | |
| 8121596 | BC146842 | FAM26F | family with sequence similarity 26, m | 0.004 | 1.003 | 9.77E-01 | 9.77E+01 | | | |
| 8095260 | --- | --- | --- | 0.004 | 1.003 | 9.73E-01 | 9.73E+01 | | | |
| 7893540 | --- | --- | --- | 0.004 | 1.003 | 9.53E-01 | 9.53E+01 | | | |
| 8000131 | NM_130464 | NPIPL3 | nuclear pore complex interacting pro | 0.004 | 1.003 | 9.79E-01 | 9.79E+01 | | | |
| 7962831 | NM_001240 | CCNT1 | cyclin T1 | 0.004 | 1.003 | 9.92E-01 | 9.92E+01 | | | |
| 8010212 | NM_152468 | TMC8 | transmembrane channel-like 8 | 0.004 | 1.003 | 9.62E-01 | 9.62E+01 | | | |
| 7999217 | NM_024589 | ROGDI | rogdi homolog (Drosophila) | 0.004 | 1.003 | 9.55E-01 | 9.55E+01 | | | |
| 8026926 | NM_015016 | MAST3 | microtubule associated serine/threo | 0.004 | 1.003 | 9.57E-01 | 9.57E+01 | | | |
| 8005305 | NM_024052 | C17orf39 | chromosome 17 open reading frame | 0.004 | 1.003 | 9.66E-01 | 9.66E+01 | | | |
| 7895616 | --- | --- | --- | 0.004 | 1.003 | 9.93E-01 | 9.93E+01 | | | |
| 8020086 | --- | --- | --- | 0.004 | 1.003 | 9.81E-01 | 9.81E+01 | | | |
| 8026315 | NM_213560 | PKN1 | protein kinase N1 | 0.004 | 1.003 | 9.77E-01 | 9.77E+01 | | | |
| 7940112 | NM_00100528 | OR9Q2 | olfactory receptor, family 9, subfami | 0.004 | 1.003 | 9.57E-01 | 9.57E+01 | | | |
| 8027272 | NM_003429 | ZNF85 | zinc finger protein 85 | 0.004 | 1.003 | 9.73E-01 | 9.73E+01 | | | |
| 8056812 | NM_013341 | OLA1 | Obg-like ATPase 1 | 0.004 | 1.002 | 9.81E-01 | 9.81E+01 | | | |
| 7900888 | NM_014652 | IPO13 | importin 13 | 0.004 | 1.002 | 9.60E-01 | 9.60E+01 | | | |
| 7896009 | --- | --- | --- | 0.004 | 1.002 | 9.97E-01 | 9.97E+01 | | | |
| 7968323 | ENST00000400 | LOC440131 | similar to bA90M5.1 (novel protein) | 0.003 | 1.002 | 9.56E-01 | 9.56E+01 | | | |
| 8108716 | NM_020957 | PCDHB16 | protocadherin beta 16 | 0.003 | 1.002 | 9.73E-01 | 9.73E+01 | | | |
| 7980765 | NM_003485 | GPR68 | G protein-coupled receptor 68 | 0.003 | 1.002 | 9.76E-01 | 9.76E+01 | | | |
| 8016708 | NM_018509 | LRRC59 | leucine rich repeat containing 59 | 0.003 | 1.002 | 9.69E-01 | 9.69E+01 | | | |
| 7893546 | --- | --- | --- | 0.003 | 1.002 | 9.82E-01 | 9.82E+01 | | | |
| 7895184 | --- | --- | --- | 0.003 | 1.002 | 9.90E-01 | 9.90E+01 | | | |

| | | | | | | | | | | |
|---|---|---|---|---|---|---|---|---|---|---|
| 8040614 | --- | --- | --- | 0.003 | 1.002 | 9.76E-01 | 9.76E+01 | | | |
| 8032212 | AK127759 | NDUFS7 | NADH dehydrogenase (ubiquinone) F | 0.003 | 1.002 | 9.76E-01 | 9.76E+01 | | | |
| 8038913 | NM_023074 | ZNF649 | zinc finger protein 649 | 0.003 | 1.002 | 9.89E-01 | 9.89E+01 | | | |
| 7981824 | NM_014608 | CYFIP1 | cytoplasmic FMR1 interacting protei | 0.003 | 1.002 | 9.69E-01 | 9.69E+01 | | | |
| 8180362 | --- | --- | --- | 0.003 | 1.002 | 9.75E-01 | 9.75E+01 | | | |
| 8057689 | NM_022353 | OSGEPL1 | O-sialoglycoprotein endopeptidase-l | 0.003 | 1.002 | 9.89E-01 | 9.89E+01 | | | |
| 8058940 | NM_020935 | USP37 | ubiquitin specific peptidase 37 | 0.003 | 1.002 | 9.90E-01 | 9.90E+01 | | | |
| 7928890 | NM_133447 | AGAP11 | ankyrin repeat and GTPase domain A | 0.003 | 1.002 | 9.73E-01 | 9.73E+01 | | | |
| 7934185 | NM_022153 | C10orf54 | chromosome 10 open reading frame | 0.003 | 1.002 | 9.68E-01 | 9.68E+01 | | | |
| 7911566 | --- | --- | --- | 0.003 | 1.002 | 9.96E-01 | 9.96E+01 | | | |
| 8039945 | --- | --- | --- | 0.003 | 1.002 | 9.96E-01 | 9.96E+01 | | | |
| 8107868 | NM_020240 | CDC42SE2 | CDC42 small effector 2 | 0.003 | 1.002 | 9.90E-01 | 9.90E+01 | | | |
| 7896270 | --- | --- | --- | 0.003 | 1.002 | 9.90E-01 | 9.90E+01 | | | |
| 7895082 | --- | --- | --- | 0.003 | 1.002 | 9.97E-01 | 9.97E+01 | | | |
| 7944867 | NM_170601 | SIAE | sialic acid acetylesterase | 0.003 | 1.002 | 9.93E-01 | 9.93E+01 | | | |
| 8024019 | NM_002819 | PTBP1 | polypyrimidine tract binding protein | 0.003 | 1.002 | 9.88E-01 | 9.88E+01 | | | |
| 7966345 | NM_139283 | PPTC7 | PTC7 protein phosphatase homolog | 0.003 | 1.002 | 9.91E-01 | 9.91E+01 | | | |
| 8076813 | BC041833 | FLJ32756 | hypothetical LOC642757 | 0.003 | 1.002 | 9.79E-01 | 9.79E+01 | | | |
| 8032418 | NM_003938 | AP3D1 | adaptor-related protein complex 3, d | 0.003 | 1.002 | 9.78E-01 | 9.78E+01 | | | |
| 7979982 | --- | --- | --- | 0.003 | 1.002 | 9.79E-01 | 9.79E+01 | | | |
| 7995069 | NM_004960 | FUS | fusion (involved in t(12;16) in malign | 0.003 | 1.002 | 9.80E-01 | 9.80E+01 | | | |
| 7927071 | NM_00100709 | ZNF37A | zinc finger protein 37A | 0.003 | 1.002 | 9.78E-01 | 9.78E+01 | | | |
| 8039896 | NM_014512 | KIR2DS1 | killer cell immunoglobulin-like recept | 0.003 | 1.002 | 9.89E-01 | 9.89E+01 | | | |
| 8076307 | NM_002883 | RANGAP1 | Ran GTPase activating protein 1 | 0.003 | 1.002 | 9.73E-01 | 9.73E+01 | | | |
| 7894382 | --- | --- | --- | 0.003 | 1.002 | 9.71E-01 | 9.71E+01 | | | |
| 7941685 | NM_005125 | CCS | copper chaperone for superoxide dis | 0.003 | 1.002 | 9.84E-01 | 9.84E+01 | | | |
| 8170576 | NM_153478 | CSAG1 | chondrosarcoma associated gene 1 | 0.003 | 1.002 | 9.78E-01 | 9.78E+01 | | | |
| 8113097 | NM_00114567 | C5orf36 | chromosome 5 open reading frame 3 | 0.003 | 1.002 | 9.94E-01 | 9.94E+01 | | | |
| 8029340 | NM_003445 | ZNF155 | zinc finger protein 155 | 0.003 | 1.002 | 9.82E-01 | 9.82E+01 | | | |
| 7956930 | NM_006482 | DYRK2 | dual-specificity tyrosine-(Y)-phospho | 0.003 | 1.002 | 9.76E-01 | 9.76E+01 | | | |
| 8093494 | NM_175918 | CRIPAK | cysteine-rich PAK1 inhibitor | 0.003 | 1.002 | 9.92E-01 | 9.92E+01 | | | |
| 8092514 | NM_022149 | MAGEF1 | melanoma antigen family F, 1 | 0.003 | 1.002 | 9.69E-01 | 9.69E+01 | | | |
| 8000284 | NM_015044 | GGA2 | golgi associated, gamma adaptin ear | 0.003 | 1.002 | 9.90E-01 | 9.90E+01 | | | |
| 8098789 | NM_005255 | GAK | cyclin G associated kinase | 0.003 | 1.002 | 9.77E-01 | 9.77E+01 | | | |
| 8078397 | NM_178868 | CMTM8 | CKLF-like MARVEL transmembrane d | 0.003 | 1.002 | 9.83E-01 | 9.83E+01 | | | |
| 7893842 | --- | --- | --- | 0.002 | 1.002 | 9.95E-01 | 9.95E+01 | | | |
| 7973458 | NM_198083 | DHRS4L2 | dehydrogenase/reductase (SDR fami | 0.002 | 1.002 | 9.90E-01 | 9.90E+01 | | | |
| 7912535 | --- | --- | --- | 0.002 | 1.002 | 9.92E-01 | 9.92E+01 | | | |
| 8039692 | NM_00114498 | ZNF814 | zinc finger protein 814 | 0.002 | 1.002 | 9.98E-01 | 9.98E+01 | | | |
| 8118174 | NM_080686 | BAT2 | HLA-B associated transcript 2 | 0.002 | 1.002 | 9.78E-01 | 9.78E+01 | | | |
| 8030831 | NM_007147 | ZNF175 | zinc finger protein 175 | 0.002 | 1.002 | 9.90E-01 | 9.90E+01 | | | |
| 7996081 | NM_201524 | GPR56 | G protein-coupled receptor 56 | 0.002 | 1.002 | 9.87E-01 | 9.87E+01 | | | |
| 8061073 | --- | --- | --- | 0.002 | 1.002 | 9.89E-01 | 9.89E+01 | | | |
| 8149148 | ENST0000042 | OR7E125P | olfactory receptor, family 7, subfami | 0.002 | 1.002 | 9.83E-01 | 9.83E+01 | | | |
| 8144082 | NR_026865 | C7orf13 | chromosome 7 open reading frame 1 | 0.002 | 1.002 | 9.87E-01 | 9.87E+01 | | | |
| 8009351 | --- | --- | --- | 0.002 | 1.002 | 9.91E-01 | 9.91E+01 | | | |
| 8132305 | NM_030636 | EEPD1 | endonuclease/exonuclease/phospha | 0.002 | 1.001 | 9.77E-01 | 9.77E+01 | | | |
| 8121743 | --- | --- | --- | 0.002 | 1.001 | 9.80E-01 | 9.80E+01 | | | |
| 8022356 | NM_00112862 | SPIRE1 | spire homolog 1 (Drosophila) | 0.002 | 1.001 | 9.82E-01 | 9.82E+01 | | | |
| 8145977 | NM_021623 | PLEKHA2 | pleckstrin homology domain contain | 0.002 | 1.001 | 9.96E-01 | 9.96E+01 | | | |
| 7893938 | --- | --- | --- | 0.002 | 1.001 | 9.90E-01 | 9.90E+01 | | | |
| 8105189 | BC137195 | OC10028767 | hypothetical protein LOC100287671 | 0.002 | 1.001 | 9.88E-01 | 9.88E+01 | | | |
| 7959621 | --- | --- | --- | 0.002 | 1.001 | 9.90E-01 | 9.90E+01 | | | |
| 8053975 | NM_00114229 | LMAN2L | lectin, mannose-binding 2-like | 0.002 | 1.001 | 9.79E-01 | 9.79E+01 | | | |
| 7896099 | --- | --- | --- | 0.002 | 1.001 | 9.88E-01 | 9.88E+01 | | | |
| 8058855 | --- | --- | --- | 0.002 | 1.001 | 9.85E-01 | 9.85E+01 | | | |
| 7956539 | NM_178502 | DTX3 | deltex homolog 3 (Drosophila) | 0.002 | 1.001 | 9.86E-01 | 9.86E+01 | | | |
| 7953626 | NM_014718 | CLSTN3 | calsyntenin 3 | 0.002 | 1.001 | 9.84E-01 | 9.84E+01 | | | |
| 7895496 | --- | --- | --- | 0.002 | 1.001 | 9.87E-01 | 9.87E+01 | | | |
| 8159337 | NM_015160 | PMPCA | peptidase (mitochondrial processing | 0.002 | 1.001 | 9.90E-01 | 9.90E+01 | | | |
| 8017776 | NM_014877 | HELZ | helicase with zinc finger | 0.002 | 1.001 | 9.94E-01 | 9.94E+01 | | | |
| 8081818 | --- | --- | --- | 0.002 | 1.001 | 9.87E-01 | 9.87E+01 | | | |
| 7976744 | NM_003403 | YY1 | YY1 transcription factor | 0.002 | 1.001 | 9.87E-01 | 9.87E+01 | | | |
| 8135638 | NM_018412 | ST7 | suppression of tumorigenicity 7 | 0.002 | 1.001 | 9.89E-01 | 9.89E+01 | | | |
| 7918153 | --- | --- | --- | 0.001 | 1.001 | 9.81E-01 | 9.81E+01 | | | |
| 7895802 | --- | --- | --- | 0.001 | 1.001 | 9.92E-01 | 9.92E+01 | | | |
| 7893692 | --- | --- | --- | 0.001 | 1.001 | 9.84E-01 | 9.84E+01 | | | |
| 8160981 | BC004406 | KIAA1539 | KIAA1539 | 0.001 | 1.001 | 9.82E-01 | 9.82E+01 | | | |
| 7997414 | NM_022041 | GAN | gigaxonin | 0.001 | 1.001 | 9.91E-01 | 9.91E+01 | | | |
| 8018324 | NM_138619 | GGA3 | golgi associated, gamma adaptin ear | 0.001 | 1.001 | 9.91E-01 | 9.91E+01 | | | |
| 8077612 | NM_00102593 | TTLL3 | tubulin tyrosine ligase-like family, m | 0.001 | 1.001 | 9.90E-01 | 9.90E+01 | | | |
| 8003922 | NM_00100168 | MED11 | mediator complex subunit 11 | 0.001 | 1.001 | 9.90E-01 | 9.90E+01 | | | |
| 8023466 | NM_00101251 | FECH | ferrochelatase (protoporphyria) | 0.001 | 1.001 | 9.89E-01 | 9.89E+01 | | | |
| 8031629 | ENST0000037 | ZSCAN5C | zinc finger and SCAN domain contain | 0.001 | 1.001 | 9.93E-01 | 9.93E+01 | | | |
| 7989759 | NM_017851 | PARP16 | poly (ADP-ribose) polymerase family | 0.001 | 1.001 | 9.89E-01 | 9.89E+01 | | | |
| 8012843 | NM_144680 | ZNF18 | zinc finger protein 18 | 0.001 | 1.001 | 9.87E-01 | 9.87E+01 | | | |
| 7997808 | ENST00000435 | MGC23284 | hypothetical LOC197187 | 0.001 | 1.001 | 9.93E-01 | 9.93E+01 | | | |

| | | | | | | | | | | |
|---|---|---|---|---|---|---|---|---|---|---|
| 8000480 | --- | --- | --- | 0.001 | 1.001 | 9.95E-01 | 9.95E+01 | | | |
| 8071806 | NM_001355 | DDT | D-dopachrome tautomerase | 0.001 | 1.001 | 9.93E-01 | 9.93E+01 | | | |
| 8112570 | NM_015084 | MRPS27 | mitochondrial ribosomal protein S27 | 0.001 | 1.001 | 9.92E-01 | 9.92E+01 | | | |
| 8006433 | NM_002982 | CCL2 | chemokine (C-C motif) ligand 2 | 0.001 | 1.001 | 9.96E-01 | 9.96E+01 | | | |
| 7901212 | ENST0000031 | UQCRH | ubiquinol-cytochrome c reductase hi | 0.001 | 1.001 | 9.98E-01 | 9.98E+01 | | | |
| 8012931 | NM_006382 | CDRT1 | CMT1A duplicated region transcript | 0.001 | 1.001 | 9.94E-01 | 9.94E+01 | | | |
| 8165690 | --- | --- | --- | 0.001 | 1.001 | 9.90E-01 | 9.90E+01 | | | |
| 8075343 | NM_005877 | SF3A1 | splicing factor 3a, subunit 1, 120kDa | 0.001 | 1.000 | 9.97E-01 | 9.97E+01 | | | |
| 8015445 | NM_052935 | NT5C3L | 5'-nucleotidase, cytosolic III-like | 0.001 | 1.000 | 9.94E-01 | 9.94E+01 | | | |
| 8141169 | NR_002822 | MGC72080 | MGC72080 pseudogene | 0.001 | 1.000 | 9.97E-01 | 9.97E+01 | | | |
| 7894760 | --- | --- | --- | 0.001 | 1.000 | 9.99E-01 | 9.99E+01 | | | |
| 8011774 | NM_015099 | CAMTA2 | calmodulin binding transcription acti | 0.000 | 1.000 | 9.95E-01 | 9.95E+01 | | | |
| 8075406 | NM_014303 | PES1 | pescadillo homolog 1, containing BR | 0.000 | 1.000 | 9.96E-01 | 9.96E+01 | | | |
| 8055592 | --- | --- | --- | 0.000 | 1.000 | 9.98E-01 | 9.98E+01 | | | |
| 7895208 | --- | --- | --- | 0.000 | 1.000 | 9.99E-01 | 9.99E+01 | | | |
| 8070961 | NM_002340 | LSS | lanosterol synthase (2,3-oxidosquale | 0.000 | 1.000 | 9.96E-01 | 9.96E+01 | | | |
| 7897236 | NM_003636 | KCNAB2 | potassium voltage-gated channel, sh | 0.000 | 1.000 | 9.97E-01 | 9.97E+01 | | | |
| 8129497 | NM_001431 | EPB41L2 | erythrocyte membrane protein band | 0.000 | 1.000 | 9.97E-01 | 9.97E+01 | | | |
| 8036830 | NM_152479 | TTC9B | tetratricopeptide repeat domain 9B | 0.000 | 1.000 | 9.97E-01 | 9.97E+01 | | | |
| 8139055 | --- | --- | --- | 0.000 | 1.000 | 9.99E-01 | 9.99E+01 | | | |
| 8033392 | NM_020196 | XAB2 | XPA binding protein 2 | 0.000 | 1.000 | 9.98E-01 | 9.98E+01 | | | |
| 8019463 | NM_139062 | CSNK1D | casein kinase 1, delta | 0.000 | 1.000 | 9.99E-01 | 9.99E+01 | | | |
| 8165676 | --- | --- | --- | 0.000 | 1.000 | 9.97E-01 | 9.97E+01 | | | |
| 8112560 | AK289851 | SMA5 | glucuronidase, beta pseudogene | 0.000 | 1.000 | 1.00E+00 | 1.00E+02 | | | |
| 8161211 | NM_016734 | PAX5 | paired box 5 | 0.000 | 1.000 | 9.99E-01 | 9.99E+01 | | | |
| 8000748 | NM_003609 | HIRIP3 | HIRA interacting protein 3 | 0.000 | 1.000 | 9.99E-01 | 9.99E+01 | | | |
| 8075709 | NM_030643 | APOL4 | apolipoprotein L, 4 | 0.000 | 1.000 | 9.99E-01 | 9.99E+01 | | | |
| 8060854 | NM_182734 | PLCB1 | phospholipase C, beta 1 (phosphoinc | 0.000 | 1.000 | 9.99E-01 | 9.99E+01 | | | |
| 8037679 | NM_004943 | DMWD | dystrophia myotonica, WD repeat co | 0.000 | 1.000 | 9.99E-01 | 9.99E+01 | | | |
| 7950391 | NM_173582 | PGM2L1 | phosphoglucomutase 2-like 1 | 0.000 | 1.000 | 1.00E+00 | 1.00E+02 | | | |
| 8128737 | NM_022765 | MICAL1 | microtubule associated monoxygena | 0.000 | 1.000 | 9.99E-01 | 9.99E+01 | | | |
| 8171170 | --- | --- | --- | 0.000 | 1.000 | 9.99E-01 | 9.99E+01 | | | |
| 8176921 | --- | --- | --- | 0.000 | 1.000 | 1.00E+00 | 1.00E+02 | | | |
| 8177424 | --- | --- | --- | 0.000 | 1.000 | 1.00E+00 | 1.00E+02 | | | |
| 8110032 | NM_153607 | C5orf41 | chromosome 5 open reading frame 4 | 0.000 | 1.000 | 1.00E+00 | 1.00E+02 | | | |
| 8084742 | NM_005578 | LPP | LIM domain containing preferred tra | 0.000 | 1.000 | 1.00E+00 | 1.00E+02 | | | |
| 8059565 | NM_017933 | PID1 | phosphotyrosine interaction domain | 0.000 | 1.000 | 1.00E+00 | 1.00E+02 | | | |
| 7894611 | --- | --- | --- | 0.000 | 1.000 | 9.98E-01 | 9.98E+01 | | | |
| 7964436 | NM_032496 | ARHGAP9 | Rho GTPase activating protein 9 | 0.000 | 1.000 | 9.99E-01 | 9.99E+01 | | | |
| 7917752 | --- | --- | --- | 0.000 | 1.000 | 9.99E-01 | 9.99E+01 | | | |
| 8022426 | ENST00000342 | LOC646359 | similar to telomeric repeat binding fa | 0.000 | 1.000 | 9.99E-01 | 9.99E+01 | | | |
| 8129618 | NM_004666 | VNN1 | vanin 1 | 0.000 | 1.000 | 9.99E-01 | 9.99E+01 | | | |
| 7899790 | NM_052841 | TSSK3 | testis-specific serine kinase 3 | 0.000 | 1.000 | 9.99E-01 | 9.99E+01 | | | |
| 8073662 | NM_00100382 | PARVB | parvin, beta | 0.000 | 1.000 | 9.98E-01 | 9.98E+01 | | | |
| 8136589 | --- | --- | --- | 0.000 | 1.000 | 9.98E-01 | 9.98E+01 | | | |
| 8074057 | --- | --- | --- | 0.000 | 1.000 | 9.99E-01 | 9.99E+01 | | | |
| 7894336 | --- | --- | --- | 0.000 | 1.000 | 9.99E-01 | 9.99E+01 | | | |
| 7927767 | NM_032804 | ADO | 2-aminoethanethiol (cysteamine) dic | 0.000 | 1.000 | 9.95E-01 | 9.95E+01 | | | |
| 8078603 | --- | --- | --- | 0.000 | 1.000 | 9.99E-01 | 9.99E+01 | | | |
| 8111989 | --- | --- | --- | -0.001 | 1.000 | 9.97E-01 | 9.97E+01 | | | |
| 7947649 | NM_000741 | CHRM4 | cholinergic receptor, muscarinic 4 | -0.001 | 1.000 | 9.94E-01 | 9.94E+01 | | | |
| 8011291 | --- | --- | --- | -0.001 | 0.999 | 9.95E-01 | 9.95E+01 | | | |
| 7926936 | NM_024688 | C10orf68 | chromosome 10 open reading frame | -0.001 | 0.999 | 9.94E-01 | 9.94E+01 | | | |
| 7908022 | NM_001357 | DHX9 | DEAH (Asp-Glu-Ala-His) box polypept | -0.001 | 0.999 | 9.97E-01 | 9.97E+01 | | | |
| 8008530 | NM_016001 | UTP18 | UTP18, small subunit (SSU) processo | -0.001 | 0.999 | 9.97E-01 | 9.97E+01 | | | |
| 7923905 | --- | --- | --- | -0.001 | 0.999 | 9.99E-01 | 9.99E+01 | | | |
| 7963157 | NM_013277 | RACGAP1 | Rac GTPase activating protein 1 | -0.001 | 0.999 | 9.96E-01 | 9.96E+01 | | | |
| 8042905 | NM_013247 | HTRA2 | HtrA serine peptidase 2 | -0.001 | 0.999 | 9.94E-01 | 9.94E+01 | | | |
| 8102862 | NM_018717 | MAML3 | mastermind-like 3 (Drosophila) | -0.001 | 0.999 | 9.89E-01 | 9.89E+01 | | | |
| 7959144 | --- | --- | --- | -0.001 | 0.999 | 9.92E-01 | 9.92E+01 | | | |
| 8080911 | NM_032505 | KBTBD8 | kelch repeat and BTB (POZ) domain c | -0.001 | 0.999 | 9.89E-01 | 9.89E+01 | | | |
| 7893878 | --- | --- | --- | -0.001 | 0.999 | 9.95E-01 | 9.95E+01 | | | |
| 8167887 | NM_014061 | MAGEH1 | melanoma antigen family H, 1 | -0.001 | 0.999 | 9.92E-01 | 9.92E+01 | | | |
| 8158214 | NM_016035 | COQ4 | coenzyme Q4 homolog (S. cerevisiae | -0.001 | 0.999 | 9.90E-01 | 9.90E+01 | | | |
| 7943160 | NR_002569 | SCARNA9 | small Cajal body-specific RNA 9 | -0.001 | 0.999 | 9.88E-01 | 9.88E+01 | | | |
| 8081343 | NM_017819 | RG9MTD1 | RNA (guanine-9-) methyltransferase | -0.001 | 0.999 | 9.95E-01 | 9.95E+01 | | | |
| 8031750 | NM_00109849 | ZNF419 | zinc finger protein 419 | -0.001 | 0.999 | 9.92E-01 | 9.92E+01 | | | |
| 7982350 | NR_024074 | GOLGA9P | golgi autoantigen, golgin subfamily a | -0.001 | 0.999 | 9.96E-01 | 9.96E+01 | | | |
| 7987139 | NR_024074 | GOLGA9P | golgi autoantigen, golgin subfamily a | -0.001 | 0.999 | 9.96E-01 | 9.96E+01 | | | |
| 7894497 | --- | --- | --- | -0.001 | 0.999 | 9.96E-01 | 9.96E+01 | | | |
| 7896236 | --- | --- | --- | -0.001 | 0.999 | 9.99E-01 | 9.99E+01 | | | |
| 8024497 | NM_003249 | THOP1 | thimet oligopeptidase 1 | -0.001 | 0.999 | 9.86E-01 | 9.86E+01 | | | |
| 8099797 | NM_00108539 | RELL1 | RELT-like 1 | -0.001 | 0.999 | 9.91E-01 | 9.91E+01 | | | |
| 7924669 | NM_013328 | PYCR2 | pyrroline-5-carboxylate reductase fa | -0.001 | 0.999 | 9.85E-01 | 9.85E+01 | | | |
| 7981951 | NR_003317 | SNORD116-2 | small nucleolar RNA, C/D box 116-2 | -0.001 | 0.999 | 9.94E-01 | 9.94E+01 | | | |
| 8092564 | NM_004593 | TRA2B | transformer 2 beta homolog (Drosop | -0.002 | 0.999 | 9.95E-01 | 9.95E+01 | | | |
| 8012953 | NM_006470 | TRIM16 | tripartite motif-containing 16 | -0.002 | 0.999 | 9.89E-01 | 9.89E+01 | | | |

| | | | | | | | | | | |
|---|---|---|---|---|---|---|---|---|---|---|
| 7989975 | NM_017882 | CLN6 | ceroid-lipofuscinosis, neuronal 6, lat | -0.002 | 0.999 | 9.82E-01 | 9.82E+01 | | | |
| 8089072 | NM_000097 | CPOX | coproporphyrinogen oxidase | -0.002 | 0.999 | 9.92E-01 | 9.92E+01 | | | |
| 7945436 | NM_176795 | HRAS | v-Ha-ras Harvey rat sarcoma viral on | -0.002 | 0.999 | 9.90E-01 | 9.90E+01 | | | |
| 8046333 | NM_024843 | CYBRD1 | cytochrome b reductase 1 | -0.002 | 0.999 | 9.86E-01 | 9.86E+01 | | | |
| 7896337 | --- | --- | --- | -0.002 | 0.999 | 9.98E-01 | 9.98E+01 | | | |
| 7972723 | NM_018011 | ARGLU1 | arginine and glutamate rich 1 | -0.002 | 0.999 | 9.90E-01 | 9.90E+01 | | | |
| 7936096 | NM_014976 | PDCD11 | programmed cell death 11 | -0.002 | 0.999 | 9.94E-01 | 9.94E+01 | | | |
| 7893821 | --- | --- | --- | -0.002 | 0.999 | 9.96E-01 | 9.96E+01 | | | |
| 8129985 | NM_006718 | PLAGL1 | pleiomorphic adenoma gene-like 1 | -0.002 | 0.999 | 9.83E-01 | 9.83E+01 | | | |
| 7923824 | NM_173854 | SLC41A1 | solute carrier family 41, member 1 | -0.002 | 0.999 | 9.83E-01 | 9.83E+01 | | | |
| 8168387 | NR_026594 | CXorf50B | chromosome X open reading frame 5 | -0.002 | 0.999 | 9.89E-01 | 9.89E+01 | | | |
| 8098576 | NM_001151 | SLC25A4 | solute carrier family 25 (mitochondri | -0.002 | 0.999 | 9.83E-01 | 9.83E+01 | | | |
| 8114320 | NM_006805 | HNRNPA0 | heterogeneous nuclear ribonucleopr | -0.002 | 0.999 | 9.90E-01 | 9.90E+01 | | | |
| 7959157 | NM_176818 | GATC | glutamyl-tRNA(Gln) amidotransferas | -0.002 | 0.999 | 9.81E-01 | 9.81E+01 | | | |
| 7992737 | NM_138439 | FLYWCH2 | FLYWCH family member 2 | -0.002 | 0.999 | 9.78E-01 | 9.78E+01 | | | |
| 7936041 | NM_004311 | ARL3 | ADP-ribosylation factor-like 3 | -0.002 | 0.999 | 9.95E-01 | 9.95E+01 | | | |
| 8138067 | NM_004227 | CYTH3 | cytohesin 3 | -0.002 | 0.999 | 9.81E-01 | 9.81E+01 | | | |
| 8082250 | --- | --- | --- | -0.002 | 0.999 | 9.89E-01 | 9.89E+01 | | | |
| 7896144 | --- | --- | --- | -0.002 | 0.999 | 9.78E-01 | 9.78E+01 | | | |
| 8148642 | NM_024736 | GSDMD | gasdermin D | -0.002 | 0.999 | 9.84E-01 | 9.84E+01 | | | |
| 8098924 | NM_012318 | LETM1 | leucine zipper-EF-hand containing tr | -0.002 | 0.999 | 9.83E-01 | 9.83E+01 | | | |
| 8035628 | NM_176880 | NR2C2AP | nuclear receptor 2C2-associated pro | -0.002 | 0.999 | 9.91E-01 | 9.91E+01 | | | |
| 7894990 | --- | --- | --- | -0.002 | 0.999 | 9.94E-01 | 9.94E+01 | | | |
| 7895440 | --- | --- | --- | -0.002 | 0.999 | 9.80E-01 | 9.80E+01 | | | |
| 7934320 | NM_015190 | DNAJC9 | DnaJ (Hsp40) homolog, subfamily C, | -0.002 | 0.999 | 9.81E-01 | 9.81E+01 | | | |
| 8026272 | NM_004843 | IL27RA | interleukin 27 receptor, alpha | -0.002 | 0.999 | 9.81E-01 | 9.81E+01 | | | |
| 8067563 | NM_033081 | DIDO1 | death inducer-obliterator 1 | -0.002 | 0.998 | 9.84E-01 | 9.84E+01 | | | |
| 7893831 | --- | --- | --- | -0.002 | 0.998 | 9.91E-01 | 9.91E+01 | | | |
| 7998222 | NM_006428 | MRPL28 | mitochondrial ribosomal protein L28 | -0.002 | 0.998 | 9.91E-01 | 9.91E+01 | | | |
| 7903920 | NM_00102519 | CHI3L2 | chitinase 3-like 2 | -0.002 | 0.998 | 9.86E-01 | 9.86E+01 | | | |
| 8034210 | NM_198536 | TMEM205 | transmembrane protein 205 | -0.002 | 0.998 | 9.93E-01 | 9.93E+01 | | | |
| 8032730 | NM_001961 | EEF2 | eukaryotic translation elongation fac | -0.002 | 0.998 | 9.90E-01 | 9.90E+01 | | | |
| 7977119 | NM_032374 | C14orf153 | chromosome 14 open reading frame | -0.002 | 0.998 | 9.92E-01 | 9.92E+01 | | | |
| 8100179 | NM_152995 | NFXL1 | nuclear transcription factor, X-box bi | -0.002 | 0.998 | 9.90E-01 | 9.90E+01 | | | |
| 7907058 | NM_002697 | POU2F1 | POU class 2 homeobox 1 | -0.002 | 0.998 | 9.77E-01 | 9.77E+01 | | | |
| 8180297 | --- | --- | --- | -0.002 | 0.998 | 9.86E-01 | 9.86E+01 | | | |
| 8114152 | NM_006930 | SKP1 | S-phase kinase-associated protein 1 | -0.002 | 0.998 | 9.91E-01 | 9.91E+01 | | | |
| 8122701 | --- | --- | --- | -0.002 | 0.998 | 9.96E-01 | 9.96E+01 | | | |
| 8100532 | NM_032313 | C4orf14 | chromosome 4 open reading frame 1 | -0.002 | 0.998 | 9.89E-01 | 9.89E+01 | | | |
| 8148658 | NM_014789 | ZNF623 | zinc finger protein 623 | -0.002 | 0.998 | 9.81E-01 | 9.81E+01 | | | |
| 7993017 | --- | --- | --- | -0.002 | 0.998 | 9.90E-01 | 9.90E+01 | | | |
| 8168399 | NM_00103984 | CHIC1 | cysteine-rich hydrophobic domain 1 | -0.002 | 0.998 | 9.82E-01 | 9.82E+01 | | | |
| 8143110 | NM_012450 | SLC13A4 | solute carrier family 13 (sodium/sulf | -0.002 | 0.998 | 9.74E-01 | 9.74E+01 | | | |
| 8012469 | NM_000987 | RPL26 | ribosomal protein L26 | -0.003 | 0.998 | 9.69E-01 | 9.69E+01 | | | |
| 7974585 | --- | --- | --- | -0.003 | 0.998 | 9.77E-01 | 9.77E+01 | | | |
| 7894305 | --- | --- | --- | -0.003 | 0.998 | 9.95E-01 | 9.95E+01 | | | |
| 7917470 | --- | --- | --- | -0.003 | 0.998 | 9.79E-01 | 9.79E+01 | | | |
| 7894145 | --- | --- | --- | -0.003 | 0.998 | 9.77E-01 | 9.77E+01 | | | |
| 7939507 | NM_032592 | ACCS | 1-aminocyclopropane-1-carboxylate | -0.003 | 0.998 | 9.73E-01 | 9.73E+01 | | | |
| 8025927 | NM_145295 | ZNF627 | zinc finger protein 627 | -0.003 | 0.998 | 9.78E-01 | 9.78E+01 | | | |
| 7934384 | NM_00102459 | ZMYND17 | zinc finger, MYND-type containing 17 | -0.003 | 0.998 | 9.72E-01 | 9.72E+01 | | | |
| 8002333 | NM_015092 | SMG1 | SMG1 homolog, phosphatidylinosito | -0.003 | 0.998 | 9.89E-01 | 9.89E+01 | | | |
| 8136934 | NM_176883 | TAS2R41 | taste receptor, type 2, member 41 | -0.003 | 0.998 | 9.91E-01 | 9.91E+01 | | | |
| 8051605 | NM_138394 | HNRPLL | heterogeneous nuclear ribonucleopr | -0.003 | 0.998 | 9.89E-01 | 9.89E+01 | | | |
| 7896412 | --- | --- | --- | -0.003 | 0.998 | 9.74E-01 | 9.74E+01 | | | |
| 7920737 | NM_003993 | CLK2 | CDC-like kinase 2 | -0.003 | 0.998 | 9.84E-01 | 9.84E+01 | | | |
| 7898679 | NM_032264 | NBPF3 | neuroblastoma breakpoint family, m | -0.003 | 0.998 | 9.76E-01 | 9.76E+01 | | | |
| 8095214 | --- | --- | --- | -0.003 | 0.998 | 9.92E-01 | 9.92E+01 | | | |
| 7999196 | NM_021646 | ZNF500 | zinc finger protein 500 | -0.003 | 0.998 | 9.60E-01 | 9.60E+01 | | | |
| 7895004 | --- | --- | --- | -0.003 | 0.998 | 9.77E-01 | 9.77E+01 | | | |
| 7970810 | NM_003045 | SLC7A1 | solute carrier family 7 (cationic amin | -0.003 | 0.998 | 9.77E-01 | 9.77E+01 | | | |
| 8166630 | ENST0000037 | LOC652904 | CDC28 protein kinase regulatory sub | -0.003 | 0.998 | 9.73E-01 | 9.73E+01 | | | |
| 8062794 | --- | --- | --- | -0.004 | 0.998 | 9.81E-01 | 9.81E+01 | | | |
| 8180217 | --- | --- | --- | -0.004 | 0.998 | 9.94E-01 | 9.94E+01 | | | |
| 7949904 | NM_030930 | UNC93B1 | unc-93 homolog B1 (C. elegans) | -0.004 | 0.998 | 9.62E-01 | 9.62E+01 | | | |
| 8071595 | --- | --- | --- | -0.004 | 0.998 | 9.94E-01 | 9.94E+01 | | | |
| 7895966 | --- | --- | --- | -0.004 | 0.998 | 9.89E-01 | 9.89E+01 | | | |
| 8168723 | NM_013347 | RPA4 | replication protein A4, 34kDa | -0.004 | 0.997 | 9.78E-01 | 9.78E+01 | | | |
| 8074458 | BC062599 | C22orf39 | chromosome 22 open reading frame | -0.004 | 0.997 | 9.65E-01 | 9.65E+01 | | | |
| 8037152 | NM_019884 | GSK3A | glycogen synthase kinase 3 alpha | -0.004 | 0.997 | 9.78E-01 | 9.78E+01 | | | |
| 8061154 | NM_003434 | ZNF133 | zinc finger protein 133 | -0.004 | 0.997 | 9.64E-01 | 9.64E+01 | | | |
| 8000205 | NM_130464 | NPIPL3 | nuclear pore complex interacting pro | -0.004 | 0.997 | 9.80E-01 | 9.80E+01 | | | |
| 7957604 | --- | --- | --- | -0.004 | 0.997 | 9.64E-01 | 9.64E+01 | | | |
| 8093343 | NM_00112717 | PIGG | phosphatidylinositol glycan anchor b | -0.004 | 0.997 | 9.73E-01 | 9.73E+01 | | | |
| 8160968 | NM_013442 | STOML2 | stomatin (EPB72)-like 2 | -0.004 | 0.997 | 9.85E-01 | 9.85E+01 | | | |
| 8043347 | --- | --- | --- | -0.004 | 0.997 | 9.77E-01 | 9.77E+01 | | | |
| 7934411 | NM_152586 | USP54 | ubiquitin specific peptidase 54 | -0.004 | 0.997 | 9.61E-01 | 9.61E+01 | | | |

| | | | | | | | | | | |
|---|---|---|---|---|---|---|---|---|---|---|
| 7987230 | NM_153613 | LPCAT4 | lysophosphatidylcholine acyltransfer | -0.004 | 0.997 | 9.53E-01 | 9.53E+01 | | | |
| 8076481 | NM_000398 | CYB5R3 | cytochrome b5 reductase 3 | -0.004 | 0.997 | 9.56E-01 | 9.56E+01 | | | |
| 7895797 | --- | --- | --- | -0.004 | 0.997 | 9.89E-01 | 9.89E+01 | | | |
| 8120269 | AK095315 | FBXO9 | F-box protein 9 | -0.004 | 0.997 | 9.87E-01 | 9.87E+01 | | | |
| 8118544 | --- | --- | --- | -0.004 | 0.997 | 9.69E-01 | 9.69E+01 | | | |
| 7981960 | NR_003321 | SNORD116-6 | small nucleolar RNA, C/D box 116-6 | -0.004 | 0.997 | 9.90E-01 | 9.90E+01 | | | |
| 8161513 | NR_002836 | PGM5P2 | phosphoglucomutase 5 pseudogene | -0.004 | 0.997 | 9.66E-01 | 9.66E+01 | | | |
| 8091095 | --- | --- | --- | -0.004 | 0.997 | 9.81E-01 | 9.81E+01 | | | |
| 7940667 | NR_004390 | SNORA57 | small nucleolar RNA, H/ACA box 57 | -0.004 | 0.997 | 9.89E-01 | 9.89E+01 | | | |
| 8115871 | AK131247 | FLJ16171 | FLJ16171 protein | -0.004 | 0.997 | 9.77E-01 | 9.77E+01 | | | |
| 8084694 | NM_001967 | EIF4A2 | eukaryotic translation initiation facto | -0.004 | 0.997 | 9.73E-01 | 9.73E+01 | | | |
| 8062569 | --- | --- | --- | -0.004 | 0.997 | 9.84E-01 | 9.84E+01 | | | |
| 8041617 | NM_020744 | MTA3 | metastasis associated 1 family, mem | -0.004 | 0.997 | 9.80E-01 | 9.80E+01 | | | |
| 8104725 | --- | --- | --- | -0.004 | 0.997 | 9.81E-01 | 9.81E+01 | | | |
| 8075555 | NM_00101085 | C22orf42 | chromosome 22 open reading frame | -0.004 | 0.997 | 9.60E-01 | 9.60E+01 | | | |
| 7995479 | NM_00104028 | PAPD5 | PAP associated domain containing 5 | -0.004 | 0.997 | 9.94E-01 | 9.94E+01 | | | |
| 7893893 | --- | --- | --- | -0.004 | 0.997 | 9.65E-01 | 9.65E+01 | | | |
| 8165947 | NM_015691 | WWC3 | WWC family member 3 | -0.004 | 0.997 | 9.50E-01 | 9.50E+01 | | | |
| 8122598 | --- | --- | --- | -0.004 | 0.997 | 9.61E-01 | 9.61E+01 | | | |
| 8143627 | NM_00100528 | OR2A1 | olfactory receptor, family 2, subfami | -0.004 | 0.997 | 9.68E-01 | 9.68E+01 | | | |
| 7928342 | NM_015901 | NUDT13 | nudix (nucleoside diphosphate linked | -0.004 | 0.997 | 9.73E-01 | 9.73E+01 | | | |
| 8088292 | NM_130387 | ASB14 | ankyrin repeat and SOCS box-contair | -0.004 | 0.997 | 9.79E-01 | 9.79E+01 | | | |
| 7926817 | --- | --- | --- | -0.004 | 0.997 | 9.77E-01 | 9.77E+01 | | | |
| 7895652 | --- | --- | --- | -0.005 | 0.997 | 9.68E-01 | 9.68E+01 | | | |
| 8064613 | NM_032034 | SLC4A11 | solute carrier family 4, sodium borat | -0.005 | 0.997 | 9.45E-01 | 9.45E+01 | | | |
| 7927231 | NM_00114400 | AGAP5 | ArfGAP with GTPase domain, ankyrir | -0.005 | 0.997 | 9.88E-01 | 9.88E+01 | | | |
| 8093839 | NM_014392 | D4S234E | DNA segment on chromosome 4 (un | -0.005 | 0.997 | 9.58E-01 | 9.58E+01 | | | |
| 7945989 | --- | --- | --- | -0.005 | 0.997 | 9.66E-01 | 9.66E+01 | | | |
| 7957746 | NM_213611 | SLC25A3 | solute carrier family 25 (mitochondri | -0.005 | 0.997 | 9.77E-01 | 9.77E+01 | | | |
| 8139158 | --- | --- | --- | -0.005 | 0.997 | 9.62E-01 | 9.62E+01 | | | |
| 7989628 | NM_022048 | CSNK1G1 | casein kinase 1, gamma 1 | -0.005 | 0.997 | 9.70E-01 | 9.70E+01 | | | |
| 7950669 | NM_00102985 | KCTD21 | potassium channel tetramerisation d | -0.005 | 0.997 | 9.58E-01 | 9.58E+01 | | | |
| 7982753 | NM_152260 | RPUSD2 | RNA pseudouridylate synthase doma | -0.005 | 0.997 | 9.57E-01 | 9.57E+01 | | | |
| 7985147 | NM_018602 | DNAJA4 | DnaJ (Hsp40) homolog, subfamily A, | -0.005 | 0.997 | 9.69E-01 | 9.69E+01 | | | |
| 7896378 | --- | --- | --- | -0.005 | 0.997 | 9.93E-01 | 9.93E+01 | | | |
| 8157605 | NM_004099 | STOM | stomatin | -0.005 | 0.997 | 9.95E-01 | 9.95E+01 | | | |
| 7978335 | NM_020195 | SDR39U1 | short chain dehydrogenase/reductas | -0.005 | 0.997 | 9.84E-01 | 9.84E+01 | | | |
| 7929243 | NM_017838 | NHP2 | NHP2 ribonucleoprotein homolog (ye | -0.005 | 0.997 | 9.87E-01 | 9.87E+01 | | | |
| 7899253 | NM_032283 | ZDHHC18 | zinc finger, DHHC-type containing 18 | -0.005 | 0.997 | 9.62E-01 | 9.62E+01 | | | |
| 7964834 | NM_001874 | CPM | carboxypeptidase M | -0.005 | 0.997 | 9.61E-01 | 9.61E+01 | | | |
| 7894777 | --- | --- | --- | -0.005 | 0.997 | 9.87E-01 | 9.87E+01 | | | |
| 7903742 | NM_000850 | GSTM4 | glutathione S-transferase mu 4 | -0.005 | 0.997 | 9.90E-01 | 9.90E+01 | | | |
| 8073522 | NM_004599 | SREBF2 | sterol regulatory element binding tra | -0.005 | 0.997 | 9.55E-01 | 9.55E+01 | | | |
| 8167575 | NM_00112734 | GAGE12B | G antigen 12B | -0.005 | 0.997 | 9.77E-01 | 9.77E+01 | | | |
| 8093590 | NM_002938 | RNF4 | ring finger protein 4 | -0.005 | 0.996 | 9.75E-01 | 9.75E+01 | | | |
| 8042495 | NM_006857 | SNRNP27 | small nuclear ribonucleoprotein 27kI | -0.005 | 0.996 | 9.89E-01 | 9.89E+01 | | | |
| 8084891 | NM_153690 | FAM43A | family with sequence similarity 43, n | -0.005 | 0.996 | 9.32E-01 | 9.32E+01 | | | |
| 8171373 | NM_017856 | GEMIN8 | gem (nuclear organelle) associated p | -0.005 | 0.996 | 9.75E-01 | 9.75E+01 | | | |
| 8119627 | NM_006245 | PPP2R5D | protein phosphatase 2, regulatory su | -0.005 | 0.996 | 9.74E-01 | 9.74E+01 | | | |
| 8021707 | NM_004232 | SOCS6 | suppressor of cytokine signaling 6 | -0.005 | 0.996 | 9.44E-01 | 9.44E+01 | | | |
| 8049542 | NM_00113755 | LRRFIP1 | leucine rich repeat (in FLII) interactin | -0.005 | 0.996 | 9.96E-01 | 9.96E+01 | | | |
| 7993071 | NM_019109 | ALG1 | asparagine-linked glycosylation 1, be | -0.005 | 0.996 | 9.51E-01 | 9.51E+01 | | | |
| 7894635 | --- | --- | --- | -0.005 | 0.996 | 9.95E-01 | 9.95E+01 | | | |
| 8000263 | NM_153603 | COG7 | component of oligomeric golgi comp | -0.005 | 0.996 | 9.60E-01 | 9.60E+01 | | | |
| 8024304 | --- | --- | --- | -0.005 | 0.996 | 9.28E-01 | 9.28E+01 | | | |
| 8171760 | NR_023358 | SCARNA9L | small Cajal body-specific RNA 9-like ( | -0.005 | 0.996 | 9.90E-01 | 9.90E+01 | | | |
| 7958331 | NM_018157 | RIC8B | resistance to inhibitors of cholineste | -0.005 | 0.996 | 9.58E-01 | 9.58E+01 | | | |
| 8045086 | --- | --- | --- | -0.006 | 0.996 | 9.42E-01 | 9.42E+01 | | | |
| 7940582 | NM_004183 | BEST1 | bestrophin 1 | -0.006 | 0.996 | 9.41E-01 | 9.41E+01 | | | |
| 7894420 | --- | --- | --- | -0.006 | 0.996 | 9.94E-01 | 9.94E+01 | | | |
| 8083094 | NM_006506 | RASA2 | RAS p21 protein activator 2 | -0.006 | 0.996 | 9.90E-01 | 9.90E+01 | | | |
| 7999291 | NM_152459 | C16orf89 | chromosome 16 open reading frame | -0.006 | 0.996 | 9.30E-01 | 9.30E+01 | | | |
| 8128939 | NM_147686 | TRAF3IP2 | TRAF3 interacting protein 2 | -0.006 | 0.996 | 9.42E-01 | 9.42E+01 | | | |
| 8092621 | NM_017541 | CRYGS | crystallin, gamma S | -0.006 | 0.996 | 9.75E-01 | 9.75E+01 | | | |
| 7954655 | NM_030653 | DDX11 | DEAD/H (Asp-Glu-Ala-Asp/His) box p | -0.006 | 0.996 | 9.31E-01 | 9.31E+01 | | | |
| 7963187 | NM_00111354 | LIMA1 | LIM domain and actin binding 1 | -0.006 | 0.996 | 9.59E-01 | 9.59E+01 | | | |
| 8039909 | NM_00103957 | CCNL2 | cyclin L2 | -0.006 | 0.996 | 9.73E-01 | 9.73E+01 | | | |
| 7994026 | NM_130464 | NPIPL3 | nuclear pore complex interacting pro | -0.006 | 0.996 | 9.68E-01 | 9.68E+01 | | | |
| 7896133 | --- | --- | --- | -0.006 | 0.996 | 9.76E-01 | 9.76E+01 | | | |
| 8142945 | NM_012133 | COPG2 | coatomer protein complex, subunit g | -0.006 | 0.996 | 9.35E-01 | 9.35E+01 | | | |
| 7912646 | NM_001229 | CASP9 | caspase 9, apoptosis-related cysteine | -0.006 | 0.996 | 9.54E-01 | 9.54E+01 | | | |
| 7935730 | NM_018294 | CWF19L1 | CWF19-like 1, cell cycle control (S. po | -0.006 | 0.996 | 9.87E-01 | 9.87E+01 | | | |
| 8155550 | AK292642 | LOC554249 | hypothetical LOC554249 | -0.006 | 0.996 | 9.30E-01 | 9.30E+01 | | | |
| 7940679 | NM_173810 | TTC9C | tetratricopeptide repeat domain 9C | -0.006 | 0.996 | 9.56E-01 | 9.56E+01 | | | |
| 7991917 | NM_021168 | RAB40C | RAB40C, member RAS oncogene fam | -0.006 | 0.996 | 9.27E-01 | 9.27E+01 | | | |
| 8075963 | AY364241 | SH3BP1 | SH3-domain binding protein 1 | -0.006 | 0.996 | 9.51E-01 | 9.51E+01 | | | |
| 7912040 | NM_148965 | TNFRSF25 | tumor necrosis factor receptor super | -0.006 | 0.996 | 9.22E-01 | 9.22E+01 | | | |

| | | | | | | | | | | |
|---|---|---|---|---|---|---|---|---|---|---|
| 8071414 | NR_026815 | TMEM191A | transmembrane protein 191A | -0.006 | 0.996 | 9.59E-01 | 9.59E+01 | | | |
| 8069057 | NR_024108 | PFKL | phosphofructokinase, liver | -0.006 | 0.996 | 9.32E-01 | 9.32E+01 | | | |
| 7972546 | --- | --- | --- | -0.006 | 0.996 | 9.59E-01 | 9.59E+01 | | | |
| 7893646 | --- | --- | --- | -0.006 | 0.996 | 9.63E-01 | 9.63E+01 | | | |
| 7986838 | NM_000275 | OCA2 | oculocutaneous albinism II | -0.006 | 0.996 | 9.09E-01 | 9.09E+01 | | | |
| 7973813 | NM_007077 | AP4S1 | adaptor-related protein complex 4, s | -0.006 | 0.996 | 9.73E-01 | 9.73E+01 | | | |
| 8012257 | NM_000546 | TP53 | tumor protein p53 | -0.006 | 0.996 | 9.53E-01 | 9.53E+01 | | | |
| 7892908 | --- | --- | --- | -0.006 | 0.996 | 9.89E-01 | 9.89E+01 | | | |
| 7895959 | --- | --- | --- | -0.006 | 0.996 | 9.95E-01 | 9.95E+01 | | | |
| 8049847 | NM_014808 | FARP2 | FERM, RhoGEF and pleckstrin domai | -0.006 | 0.996 | 9.51E-01 | 9.51E+01 | | | |
| 7960359 | --- | --- | --- | -0.006 | 0.996 | 9.74E-01 | 9.74E+01 | | | |
| 7935572 | NM_032709 | PYROXD2 | pyridine nucleotide-disulphide oxido | -0.006 | 0.996 | 9.47E-01 | 9.47E+01 | | | |
| 7962783 | NM_024095 | ASB8 | ankyrin repeat and SOCS box-contair | -0.007 | 0.996 | 9.70E-01 | 9.70E+01 | | | |
| 8021984 | NM_005433 | YES1 | v-yes-1 Yamaguchi sarcoma viral onc | -0.007 | 0.995 | 9.70E-01 | 9.70E+01 | | | |
| 8146756 | NM_00107720 | CSPP1 | centrosome and spindle pole associa | -0.007 | 0.995 | 9.87E-01 | 9.87E+01 | | | |
| 8159965 | --- | --- | --- | -0.007 | 0.995 | 9.84E-01 | 9.84E+01 | | | |
| 7925062 | NM_020808 | SIPA1L2 | signal-induced proliferation-associat | -0.007 | 0.995 | 9.40E-01 | 9.40E+01 | | | |
| 8087308 | NM_006677 | USP19 | ubiquitin specific peptidase 19 | -0.007 | 0.995 | 9.20E-01 | 9.20E+01 | | | |
| 7896047 | --- | --- | --- | -0.007 | 0.995 | 9.78E-01 | 9.78E+01 | | | |
| 8078405 | NM_138410 | CMTM7 | CKLF-like MARVEL transmembrane d | -0.007 | 0.995 | 9.55E-01 | 9.55E+01 | | | |
| 7939767 | NM_003682 | MADD | MAP-kinase activating death domain | -0.007 | 0.995 | 9.77E-01 | 9.77E+01 | | | |
| 8058985 | NM_022453 | RNF25 | ring finger protein 25 | -0.007 | 0.995 | 9.61E-01 | 9.61E+01 | | | |
| 7966202 | NM_031954 | KCTD10 | potassium channel tetramerisation d | -0.007 | 0.995 | 9.64E-01 | 9.64E+01 | | | |
| 8018209 | NM_015654 | NAT9 | N-acetyltransferase 9 (GCN5-related | -0.007 | 0.995 | 9.54E-01 | 9.54E+01 | | | |
| 7933772 | NM_020987 | ANK3 | ankyrin 3, node of Ranvier (ankyrin G | -0.007 | 0.995 | 9.58E-01 | 9.58E+01 | | | |
| 7981895 | NM_00101242 | GOLGA8E | golgi autoantigen, golgin subfamily a | -0.007 | 0.995 | 9.45E-01 | 9.45E+01 | | | |
| 8102695 | --- | --- | --- | -0.007 | 0.995 | 9.27E-01 | 9.27E+01 | | | |
| 7963502 | NM_175078 | KRT77 | keratin 77 | -0.007 | 0.995 | 9.28E-01 | 9.28E+01 | | | |
| 8164336 | NM_003863 | DPM2 | dolichyl-phosphate mannosyltransfe | -0.007 | 0.995 | 9.51E-01 | 9.51E+01 | | | |
| 8066279 | NM_015035 | ZHX3 | zinc fingers and homeoboxes 3 | -0.007 | 0.995 | 9.27E-01 | 9.27E+01 | | | |
| 7924897 | NM_145257 | C1orf96 | chromosome 1 open reading frame 9 | -0.007 | 0.995 | 9.27E-01 | 9.27E+01 | | | |
| 8011396 | NM_013276 | SHPK | sedoheptulokinase | -0.007 | 0.995 | 9.45E-01 | 9.45E+01 | | | |
| 8175664 | --- | --- | --- | -0.007 | 0.995 | 9.44E-01 | 9.44E+01 | | | |
| 7894018 | --- | --- | --- | -0.007 | 0.995 | 9.70E-01 | 9.70E+01 | | | |
| 8153920 | NM_021061 | ZNF250 | zinc finger protein 250 | -0.007 | 0.995 | 9.21E-01 | 9.21E+01 | | | |
| 8154359 | AY779046 | RPL18A | ribosomal protein L18a | -0.007 | 0.995 | 9.35E-01 | 9.35E+01 | | | |
| 8105523 | NM_004520 | KIF2A | kinesin heavy chain member 2A | -0.007 | 0.995 | 9.71E-01 | 9.71E+01 | | | |
| 7992854 | NM_004220 | ZNF213 | zinc finger protein 213 | -0.007 | 0.995 | 8.91E-01 | 8.91E+01 | | | |
| 7962794 | NM_152320 | ZNF641 | zinc finger protein 641 | -0.007 | 0.995 | 9.48E-01 | 9.48E+01 | | | |
| 8087372 | NM_022903 | CCDC71 | coiled-coil domain containing 71 | -0.007 | 0.995 | 9.33E-01 | 9.33E+01 | | | |
| 7896627 | --- | --- | --- | -0.008 | 0.995 | 9.64E-01 | 9.64E+01 | | | |
| 7896333 | --- | --- | --- | -0.008 | 0.995 | 9.44E-01 | 9.44E+01 | | | |
| 7892844 | --- | --- | --- | -0.008 | 0.995 | 9.92E-01 | 9.92E+01 | | | |
| 7895719 | --- | --- | --- | -0.008 | 0.995 | 9.87E-01 | 9.87E+01 | | | |
| 8175269 | NM_145284 | FAM122B | family with sequence similarity 122B | -0.008 | 0.995 | 9.57E-01 | 9.57E+01 | | | |
| 8169904 | NM_003951 | SLC25A14 | solute carrier family 25 (mitochondri | -0.008 | 0.995 | 9.60E-01 | 9.60E+01 | | | |
| 8010050 | NM_182565 | FAM100B | family with sequence similarity 100, | -0.008 | 0.995 | 9.77E-01 | 9.77E+01 | | | |
| 8101437 | NM_015697 | COQ2 | coenzyme Q2 homolog, prenyltransf | -0.008 | 0.995 | 9.61E-01 | 9.61E+01 | | | |
| 7982504 | NM_152595 | PGBD4 | piggyBac transposable element deriv | -0.008 | 0.995 | 9.41E-01 | 9.41E+01 | | | |
| 7992795 | NM_024339 | THOC6 | THO complex 6 homolog (Drosophila | -0.008 | 0.995 | 9.57E-01 | 9.57E+01 | | | |
| 7914603 | NM_153341 | RNF19B | ring finger protein 19B | -0.008 | 0.995 | 9.73E-01 | 9.73E+01 | | | |
| 7892524 | --- | --- | --- | -0.008 | 0.995 | 9.90E-01 | 9.90E+01 | | | |
| 8097030 | --- | --- | --- | -0.008 | 0.995 | 9.67E-01 | 9.67E+01 | | | |
| 7914296 | NM_00102065 | PUM1 | pumilio homolog 1 (Drosophila) | -0.008 | 0.994 | 9.70E-01 | 9.70E+01 | | | |
| 7941797 | NM_001619 | ADRBK1 | adrenergic, beta, receptor kinase 1 | -0.008 | 0.994 | 9.55E-01 | 9.55E+01 | | | |
| 7995629 | --- | --- | --- | -0.008 | 0.994 | 9.73E-01 | 9.73E+01 | | | |
| 8165653 | AK293612 | HNRNPM | heterogeneous nuclear ribonucleopr | -0.008 | 0.994 | 9.29E-01 | 9.29E+01 | | | |
| 7900624 | NM_015911 | ZNF691 | zinc finger protein 691 | -0.008 | 0.994 | 9.40E-01 | 9.40E+01 | | | |
| 7919578 | --- | --- | --- | -0.008 | 0.994 | 9.91E-01 | 9.91E+01 | | | |
| 8123148 | NM_014161 | MRPL18 | mitochondrial ribosomal protein L18 | -0.008 | 0.994 | 9.88E-01 | 9.88E+01 | | | |
| 7907788 | --- | --- | --- | -0.008 | 0.994 | 9.79E-01 | 9.79E+01 | | | |
| 7906469 | NM_017823 | DUSP23 | dual specificity phosphatase 23 | -0.008 | 0.994 | 9.28E-01 | 9.28E+01 | | | |
| 7895784 | --- | --- | --- | -0.009 | 0.994 | 9.62E-01 | 9.62E+01 | | | |
| 8049990 | NM_00100291 | FAM150B | family with sequence similarity 150, | -0.009 | 0.994 | 8.86E-01 | 8.86E+01 | | | |
| 8164398 | NM_004486 | GOLGA2 | golgi autoantigen, golgin subfamily a | -0.009 | 0.994 | 9.16E-01 | 9.16E+01 | | | |
| 8038942 | NM_014650 | ZNF432 | zinc finger protein 432 | -0.009 | 0.994 | 9.81E-01 | 9.81E+01 | | | |
| 8145669 | NM_00100871 | RBPMS | RNA binding protein with multiple sp | -0.009 | 0.994 | 8.91E-01 | 8.91E+01 | | | |
| 7937876 | --- | --- | --- | -0.009 | 0.994 | 9.18E-01 | 9.18E+01 | | | |
| 7928600 | NM_00109969 | EIF5AL1 | eukaryotic translation initiation facto | -0.009 | 0.994 | 9.87E-01 | 9.87E+01 | | | |
| 7983773 | --- | --- | --- | -0.009 | 0.994 | 9.20E-01 | 9.20E+01 | | | |
| 7940824 | NM_024771 | NAT11 | N-acetyltransferase 11 (GCN5-relate | -0.009 | 0.994 | 9.56E-01 | 9.56E+01 | | | |
| 8117739 | --- | --- | --- | -0.009 | 0.994 | 9.13E-01 | 9.13E+01 | | | |
| 8040467 | --- | --- | --- | -0.009 | 0.994 | 9.51E-01 | 9.51E+01 | | | |
| 7968972 | NM_005694 | COX17 | COX17 cytochrome c oxidase assemb | -0.009 | 0.994 | 9.85E-01 | 9.85E+01 | | | |
| 7896517 | --- | --- | --- | -0.009 | 0.994 | 9.68E-01 | 9.68E+01 | | | |
| 8110858 | AK295082 | PDCD6 | programmed cell death 6 | -0.009 | 0.994 | 9.65E-01 | 9.65E+01 | | | |
| 8005191 | BC027986 | NCRNA0018 | non-protein coding RNA 188 | -0.009 | 0.994 | 9.55E-01 | 9.55E+01 | | | |

| | | | | | | | | | | |
|---|---|---|---|---|---|---|---|---|---|---|
| 8032863 | NM_019107 | C19orf10 | chromosome 19 open reading frame | -0.009 | 0.994 | 9.52E-01 | 9.52E+01 | | | |
| 8061414 | --- | --- | --- | -0.009 | 0.994 | 9.17E-01 | 9.17E+01 | | | |
| 8178074 | NM_138277 | C6orf25 | chromosome 6 open reading frame 2 | -0.009 | 0.994 | 9.19E-01 | 9.19E+01 | | | |
| 8180284 | --- | --- | --- | -0.009 | 0.994 | 9.42E-01 | 9.42E+01 | | | |
| 8010768 | --- | --- | --- | -0.009 | 0.994 | 9.79E-01 | 9.79E+01 | | | |
| 7949465 | NM_021975 | RELA | v-rel reticuloendotheliosis viral onco | -0.009 | 0.994 | 9.55E-01 | 9.55E+01 | | | |
| 7893116 | --- | --- | --- | -0.009 | 0.994 | 9.01E-01 | 9.01E+01 | | | |
| 7912670 | NM_006004 | UQCRH | ubiquinol-cytochrome c reductase hi | -0.009 | 0.994 | 9.88E-01 | 9.88E+01 | | | |
| 7893561 | --- | --- | --- | -0.009 | 0.994 | 9.91E-01 | 9.91E+01 | | | |
| 8085537 | NM_022340 | ZFYVE20 | zinc finger, FYVE domain containing 2 | -0.009 | 0.994 | 9.39E-01 | 9.39E+01 | | | |
| 7928318 | NM_138357 | CCDC109A | coiled-coil domain containing 109A | -0.009 | 0.994 | 9.48E-01 | 9.48E+01 | | | |
| 7952914 | NM_032358 | CCDC77 | coiled-coil domain containing 77 | -0.009 | 0.994 | 9.67E-01 | 9.67E+01 | | | |
| 7980381 | NM_213601 | TMED8 | transmembrane emp24 protein trans | -0.009 | 0.994 | 9.35E-01 | 9.35E+01 | | | |
| 8002802 | NM_030581 | WDR59 | WD repeat domain 59 | -0.009 | 0.994 | 9.51E-01 | 9.51E+01 | | | |
| 7940118 | NR_024091 | ZFP91-CNTF | ZFP91-CNTF readthrough transcript | -0.009 | 0.994 | 9.61E-01 | 9.61E+01 | | | |
| 8149389 | NR_003494 | FAM86B1 | family with sequence similarity 86, m | -0.009 | 0.994 | 9.10E-01 | 9.10E+01 | | | |
| 8053187 | NM_032673 | PCGF1 | polycomb group ring finger 1 | -0.009 | 0.994 | 9.65E-01 | 9.65E+01 | | | |
| 8123006 | NM_003898 | SYNJ2 | synaptojanin 2 | -0.009 | 0.994 | 9.09E-01 | 9.09E+01 | | | |
| 7892613 | --- | --- | --- | -0.009 | 0.994 | 9.32E-01 | 9.32E+01 | | | |
| 7993807 | NM_020422 | TMEM159 | transmembrane protein 159 | -0.009 | 0.994 | 9.46E-01 | 9.46E+01 | | | |
| 7925737 | NM_00100195 | OR11L1 | olfactory receptor, family 11, subfam | -0.009 | 0.993 | 8.85E-01 | 8.85E+01 | | | |
| 7914178 | --- | --- | --- | -0.009 | 0.993 | 9.43E-01 | 9.43E+01 | | | |
| 8013354 | --- | --- | --- | -0.009 | 0.993 | 9.77E-01 | 9.77E+01 | | | |
| 8133145 | NM_014478 | CRCP | CGRP receptor component | -0.009 | 0.993 | 9.65E-01 | 9.65E+01 | | | |
| 8011516 | NM_174954 | ATP2A3 | ATPase, Ca++ transporting, ubiquitou | -0.009 | 0.993 | 9.18E-01 | 9.18E+01 | | | |
| 8012924 | NM_145301 | FAM18B2 | family with sequence similarity 18, m | -0.010 | 0.993 | 9.55E-01 | 9.55E+01 | | | |
| 7973314 | NM_005015 | OXA1L | oxidase (cytochrome c) assembly 1-li | -0.010 | 0.993 | 9.63E-01 | 9.63E+01 | | | |
| 8039257 | NM_002287 | LAIR1 | leukocyte-associated immunoglobuli | -0.010 | 0.993 | 9.41E-01 | 9.41E+01 | | | |
| 7911539 | NM_00103957 | CCNL2 | cyclin L2 | -0.010 | 0.993 | 9.40E-01 | 9.40E+01 | | | |
| 7984276 | NM_004727 | SLC24A1 | solute carrier family 24 (sodium/pota | -0.010 | 0.993 | 9.10E-01 | 9.10E+01 | | | |
| 7988623 | --- | --- | --- | -0.010 | 0.993 | 9.13E-01 | 9.13E+01 | | | |
| 7984562 | NM_001003 | RPLP1 | ribosomal protein, large, P1 | -0.010 | 0.993 | 9.64E-01 | 9.64E+01 | | | |
| 7892689 | --- | --- | --- | -0.010 | 0.993 | 9.82E-01 | 9.82E+01 | | | |
| 8006005 | NM_016518 | PIPOX | pipecolic acid oxidase | -0.010 | 0.993 | 8.79E-01 | 8.79E+01 | | | |
| 8005638 | NM_00103180 | ALDH3A2 | aldehyde dehydrogenase 3 family, m | -0.010 | 0.993 | 9.52E-01 | 9.52E+01 | | | |
| 8078605 | NM_178339 | C3orf35 | chromosome 3 open reading frame 3 | -0.010 | 0.993 | 9.09E-01 | 9.09E+01 | | | |
| 7978754 | NM_014267 | C11orf58 | chromosome 11 open reading frame | -0.010 | 0.993 | 9.87E-01 | 9.87E+01 | | | |
| 8149296 | NM_017884 | PINX1 | PIN2-interacting protein 1 | -0.010 | 0.993 | 9.71E-01 | 9.71E+01 | | | |
| 8010770 | NM_00104242 | SLC16A3 | solute carrier family 16, member 3 (r | -0.010 | 0.993 | 8.91E-01 | 8.91E+01 | | | |
| 7968890 | NM_003646 | DGKZ | diacylglycerol kinase, zeta 104kDa | -0.010 | 0.993 | 9.33E-01 | 9.33E+01 | | | |
| 7968035 | NM_153023 | SPATA13 | spermatogenesis associated 13 | -0.010 | 0.993 | 9.24E-01 | 9.24E+01 | | | |
| 7915392 | NM_024503 | HIVEP3 | human immunodeficiency virus type | -0.010 | 0.993 | 8.77E-01 | 8.77E+01 | | | |
| 8038086 | NM_000979 | RPL18 | ribosomal protein L18 | -0.010 | 0.993 | 9.50E-01 | 9.50E+01 | | | |
| 7997245 | --- | --- | --- | -0.010 | 0.993 | 9.49E-01 | 9.49E+01 | | | |
| 7963235 | NM_030809 | CSRNP2 | cysteine-serine-rich nuclear protein 2 | -0.010 | 0.993 | 9.57E-01 | 9.57E+01 | | | |
| 7956269 | --- | --- | --- | -0.010 | 0.993 | 9.87E-01 | 9.87E+01 | | | |
| 8074192 | --- | --- | --- | -0.010 | 0.993 | 9.26E-01 | 9.26E+01 | | | |
| 7894599 | --- | --- | --- | -0.010 | 0.993 | 9.29E-01 | 9.29E+01 | | | |
| 7976053 | --- | --- | --- | -0.010 | 0.993 | 9.42E-01 | 9.42E+01 | | | |
| 7893967 | --- | --- | --- | -0.010 | 0.993 | 9.58E-01 | 9.58E+01 | | | |
| 7903586 | NM_020141 | TMEM167B | transmembrane protein 167B | -0.010 | 0.993 | 9.68E-01 | 9.68E+01 | | | |
| 8039905 | NM_020141 | TMEM167B | transmembrane protein 167B | -0.010 | 0.993 | 9.68E-01 | 9.68E+01 | | | |
| 8089795 | NM_005694 | COX17 | COX17 cytochrome c oxidase assemb | -0.010 | 0.993 | 9.82E-01 | 9.82E+01 | | | |
| 7895260 | --- | --- | --- | -0.010 | 0.993 | 9.81E-01 | 9.81E+01 | | | |
| 8087254 | NM_000884 | IMPDH2 | IMP (inosine monophosphate) dehyd | -0.010 | 0.993 | 9.62E-01 | 9.62E+01 | | | |
| 8021924 | NM_005131 | THOC1 | THO complex 1 | -0.010 | 0.993 | 9.66E-01 | 9.66E+01 | | | |
| 7906223 | NM_015997 | C1orf66 | chromosome 1 open reading frame 6 | -0.010 | 0.993 | 8.87E-01 | 8.87E+01 | | | |
| 8165656 | --- | --- | --- | -0.010 | 0.993 | 9.88E-01 | 9.88E+01 | | | |
| 7956522 | AF063608 | KIF5A | kinesin family member 5A | -0.010 | 0.993 | 9.01E-01 | 9.01E+01 | | | |
| 8078450 | NM_006371 | CRTAP | cartilage associated protein | -0.010 | 0.993 | 9.06E-01 | 9.06E+01 | | | |
| 7894223 | --- | --- | --- | -0.010 | 0.993 | 9.08E-01 | 9.08E+01 | | | |
| 8108447 | NM_016463 | CXXC5 | CXXC finger 5 | -0.010 | 0.993 | 9.09E-01 | 9.09E+01 | | | |
| 8097476 | --- | --- | --- | -0.010 | 0.993 | 9.10E-01 | 9.10E+01 | | | |
| 8162825 | --- | --- | --- | -0.010 | 0.993 | 9.60E-01 | 9.60E+01 | | | |
| 7893747 | --- | --- | --- | -0.010 | 0.993 | 9.74E-01 | 9.74E+01 | | | |
| 7892817 | --- | --- | --- | -0.010 | 0.993 | 9.44E-01 | 9.44E+01 | | | |
| 8005441 | NM_144775 | SMCR8 | Smith-Magenis syndrome chromoso | -0.011 | 0.993 | 9.29E-01 | 9.29E+01 | | | |
| 7975045 | NM_005956 | MTHFD1 | methylenetetrahydrofolate dehydro | -0.011 | 0.993 | 9.45E-01 | 9.45E+01 | | | |
| 8180270 | --- | --- | --- | -0.011 | 0.993 | 9.23E-01 | 9.23E+01 | | | |
| 8098414 | NM_021928 | SPCS3 | signal peptidase complex subunit 3 h | -0.011 | 0.993 | 9.73E-01 | 9.73E+01 | | | |
| 8117773 | AK128290 | LOC554223 | hypothetical LOC554223 | -0.011 | 0.993 | 8.90E-01 | 8.90E+01 | | | |
| 8171139 | NM_004729 | ZBED1 | zinc finger, BED-type containing 1 | -0.011 | 0.993 | 8.51E-01 | 8.51E+01 | | | |
| 8177029 | NM_004729 | ZBED1 | zinc finger, BED-type containing 1 | -0.011 | 0.993 | 8.51E-01 | 8.51E+01 | | | |
| 8159687 | NM_032477 | MRPL41 | mitochondrial ribosomal protein L41 | -0.011 | 0.993 | 9.53E-01 | 9.53E+01 | | | |
| 7917974 | --- | --- | --- | -0.011 | 0.993 | 9.87E-01 | 9.87E+01 | | | |
| 7988424 | --- | --- | --- | -0.011 | 0.993 | 9.69E-01 | 9.69E+01 | | | |
| 7903592 | NM_020775 | KIAA1324 | KIAA1324 | -0.011 | 0.993 | 9.03E-01 | 9.03E+01 | | | |

| | | | | | | | | | | |
|---|---|---|---|---|---|---|---|---|---|---|
| 7896675 | --- | --- | --- | -0.011 | 0.993 | 9.37E-01 | 9.37E+01 | | | |
| 7923707 | NM_203376 | TMEM81 | transmembrane protein 81 | -0.011 | 0.993 | 9.21E-01 | 9.21E+01 | | | |
| 8114536 | NM_198282 | TMEM173 | transmembrane protein 173 | -0.011 | 0.993 | 9.16E-01 | 9.16E+01 | | | |
| 8149833 | --- | --- | --- | -0.011 | 0.993 | 9.08E-01 | 9.08E+01 | | | |
| 8088054 | NM_198563 | TMEM110 | transmembrane protein 110 | -0.011 | 0.992 | 9.33E-01 | 9.33E+01 | | | |
| 7932094 | NM_006214 | PHYH | phytanoyl-CoA 2-hydroxylase | -0.011 | 0.992 | 9.18E-01 | 9.18E+01 | | | |
| 8166797 | NM_021242 | MID1IP1 | MID1 interacting protein 1 (gastrulat | -0.011 | 0.992 | 9.19E-01 | 9.19E+01 | | | |
| 8025895 | NM_002743 | PRKCSH | protein kinase C substrate 80K-H | -0.011 | 0.992 | 9.29E-01 | 9.29E+01 | | | |
| 8106250 | --- | --- | --- | -0.011 | 0.992 | 9.21E-01 | 9.21E+01 | | | |
| 8153021 | NM_003033 | ST3GAL1 | ST3 beta-galactoside alpha-2,3-sialyl | -0.011 | 0.992 | 9.51E-01 | 9.51E+01 | | | |
| 7973660 | NM_016576 | GMPR2 | guanosine monophosphate reductas | -0.011 | 0.992 | 9.57E-01 | 9.57E+01 | | | |
| 8019912 | NM_032048 | EMILIN2 | elastin microfibril interfacer 2 | -0.011 | 0.992 | 8.88E-01 | 8.88E+01 | | | |
| 8046685 | NM_182678 | UBE2E3 | ubiquitin-conjugating enzyme E2E 3 | -0.011 | 0.992 | 9.78E-01 | 9.78E+01 | | | |
| 8001924 | NR_024525 | FAM96B | family with sequence similarity 96, m | -0.011 | 0.992 | 9.60E-01 | 9.60E+01 | | | |
| 8113059 | NM_203406 | MBLAC2 | metallo-beta-lactamase domain cont | -0.011 | 0.992 | 8.80E-01 | 8.80E+01 | | | |
| 8130173 | NM_130900 | RAET1L | retinoic acid early transcript 1L | -0.011 | 0.992 | 8.98E-01 | 8.98E+01 | | | |
| 7895205 | --- | --- | --- | -0.011 | 0.992 | 9.73E-01 | 9.73E+01 | | | |
| 8031536 | NM_007279 | U2AF2 | U2 small nuclear RNA auxiliary factor | -0.011 | 0.992 | 9.21E-01 | 9.21E+01 | | | |
| 8046861 | NM_002210 | ITGAV | integrin, alpha V (vitronectin recepto | -0.011 | 0.992 | 9.42E-01 | 9.42E+01 | | | |
| 8022902 | NM_00109881 | INO80C | INO80 complex subunit C | -0.011 | 0.992 | 9.58E-01 | 9.58E+01 | | | |
| 8054077 | NM_005735 | ACTR1B | ARP1 actin-related protein 1 homolo | -0.011 | 0.992 | 9.35E-01 | 9.35E+01 | | | |
| 7927814 | NM_012238 | SIRT1 | sirtuin (silent mating type informatio | -0.011 | 0.992 | 9.75E-01 | 9.75E+01 | | | |
| 8013465 | NM_005557 | KRT16 | keratin 16 | -0.011 | 0.992 | 8.51E-01 | 8.51E+01 | | | |
| 8036365 | NM_00103723 | ZNF829 | zinc finger protein 829 | -0.011 | 0.992 | 9.56E-01 | 9.56E+01 | | | |
| 8123342 | NM_007045 | FGFR1OP | FGFR1 oncogene partner | -0.011 | 0.992 | 9.57E-01 | 9.57E+01 | | | |
| 8031483 | NM_00113613 | RPL28 | ribosomal protein L28 | -0.011 | 0.992 | 9.56E-01 | 9.56E+01 | | | |
| 7893807 | --- | --- | --- | -0.011 | 0.992 | 9.73E-01 | 9.73E+01 | | | |
| 7985930 | --- | --- | --- | -0.012 | 0.992 | 9.02E-01 | 9.02E+01 | | | |
| 8048304 | NM_021198 | CTDSP1 | CTD (carboxy-terminal domain, RNA | -0.012 | 0.992 | 8.99E-01 | 8.99E+01 | | | |
| 7963313 | NM_007210 | GALNT6 | UDP-N-acetyl-alpha-D-galactosamine | -0.012 | 0.992 | 8.73E-01 | 8.73E+01 | | | |
| 7895936 | --- | --- | --- | -0.012 | 0.992 | 8.70E-01 | 8.70E+01 | | | |
| 8114209 | --- | --- | --- | -0.012 | 0.992 | 9.61E-01 | 9.61E+01 | | | |
| 8121489 | NM_001634 | AMD1 | adenosylmethionine decarboxylase 1 | -0.012 | 0.992 | 9.71E-01 | 9.71E+01 | | | |
| 8117646 | NM_006298 | ZNF192 | zinc finger protein 192 | -0.012 | 0.992 | 9.78E-01 | 9.78E+01 | | | |
| 8000884 | NM_024096 | DCTPP1 | dCTP pyrophosphatase 1 | -0.012 | 0.992 | 9.16E-01 | 9.16E+01 | | | |
| 7914342 | NM_004102 | FABP3 | fatty acid binding protein 3, muscle a | -0.012 | 0.992 | 9.16E-01 | 9.16E+01 | | | |
| 8002762 | NM_018124 | RFWD3 | ring finger and WD repeat domain 3 | -0.012 | 0.992 | 9.33E-01 | 9.33E+01 | | | |
| 7895047 | --- | --- | --- | -0.012 | 0.992 | 9.21E-01 | 9.21E+01 | | | |
| 7960177 | NM_003044 | SLC6A12 | solute carrier family 6 (neurotransmi | -0.012 | 0.992 | 8.58E-01 | 8.58E+01 | | | |
| 8081214 | NM_005290 | GPR15 | G protein-coupled receptor 15 | -0.012 | 0.992 | 9.49E-01 | 9.49E+01 | | | |
| 8113616 | NM_020177 | FEM1C | fem-1 homolog c (C. elegans) | -0.012 | 0.992 | 9.33E-01 | 9.33E+01 | | | |
| 8123763 | --- | --- | --- | -0.012 | 0.992 | 8.82E-01 | 8.82E+01 | | | |
| 8005110 | NM_020652 | ZNF286A | zinc finger protein 286A | -0.012 | 0.992 | 9.64E-01 | 9.64E+01 | | | |
| 7895173 | --- | --- | --- | -0.012 | 0.992 | 9.46E-01 | 9.46E+01 | | | |
| 7952426 | NM_014312 | VSIG2 | V-set and immunoglobulin domain co | -0.012 | 0.992 | 8.74E-01 | 8.74E+01 | | | |
| 8028309 | --- | --- | --- | -0.012 | 0.992 | 9.62E-01 | 9.62E+01 | | | |
| 8068350 | AK127913 | FLJ46020 | FLJ46020 protein | -0.012 | 0.992 | 8.94E-01 | 8.94E+01 | | | |
| 8019238 | BC090923 | C17orf90 | chromosome 17 open reading frame | -0.012 | 0.992 | 8.80E-01 | 8.80E+01 | | | |
| 8139737 | NM_004577 | PSPH | phosphoserine phosphatase | -0.012 | 0.992 | 9.02E-01 | 9.02E+01 | | | |
| 7955729 | NM_032889 | MFSD5 | major facilitator superfamily domain | -0.012 | 0.992 | 9.72E-01 | 9.72E+01 | | | |
| 7893278 | --- | --- | --- | -0.012 | 0.992 | 9.47E-01 | 9.47E+01 | | | |
| 8093476 | NM_020894 | KIAA1530 | KIAA1530 | -0.012 | 0.992 | 8.75E-01 | 8.75E+01 | | | |
| 8088474 | --- | --- | --- | -0.012 | 0.992 | 9.48E-01 | 9.48E+01 | | | |
| 8073457 | NM_001469 | XRCC6 | X-ray repair complementing defectiv | -0.012 | 0.992 | 9.52E-01 | 9.52E+01 | | | |
| 8165669 | AB064665 | LOC440552 | OK/SW-cl.16 | -0.012 | 0.992 | 8.89E-01 | 8.89E+01 | | | |
| 7954061 | --- | --- | --- | -0.012 | 0.992 | 8.77E-01 | 8.77E+01 | | | |
| 8142084 | CR601484 | YBX1P2 | Y box binding protein 1 pseudogene | -0.012 | 0.992 | 8.96E-01 | 8.96E+01 | | | |
| 7975956 | NM_001513 | GSTZ1 | glutathione transferase zeta 1 | -0.012 | 0.992 | 8.82E-01 | 8.82E+01 | | | |
| 8162247 | --- | --- | --- | -0.012 | 0.992 | 8.82E-01 | 8.82E+01 | | | |
| 8158523 | NM_178001 | PPP2R4 | protein phosphatase 2A activator, re | -0.012 | 0.992 | 8.94E-01 | 8.94E+01 | | | |
| 7896634 | --- | --- | --- | -0.012 | 0.992 | 9.73E-01 | 9.73E+01 | | | |
| 8109086 | NM_000024 | ADRB2 | adrenergic, beta-2-, receptor, surfac | -0.012 | 0.992 | 9.35E-01 | 9.35E+01 | | | |
| 8178590 | NM_013974 | DDAH2 | dimethylarginine dimethylaminohyd | -0.012 | 0.992 | 8.91E-01 | 8.91E+01 | | | |
| 8179819 | NM_013974 | DDAH2 | dimethylarginine dimethylaminohyd | -0.012 | 0.992 | 8.91E-01 | 8.91E+01 | | | |
| 8125527 | AF275684 // A | R2P1 // PPP1 | protein phosphatase 1, regulatory (in | -0.012 | 0.992 | 9.21E-01 | 9.21E+01 | | | |
| 8178882 | AF275684 // A | R2P1 // PPP1 | protein phosphatase 1, regulatory (in | -0.012 | 0.992 | 9.21E-01 | 9.21E+01 | | | |
| 8180076 | AF275684 // A | R2P1 // PPP1 | protein phosphatase 1, regulatory (in | -0.012 | 0.992 | 9.21E-01 | 9.21E+01 | | | |
| 7919854 | --- | --- | --- | -0.012 | 0.991 | 9.63E-01 | 9.63E+01 | | | |
| 8029129 | NM_001022 | RPS19 | ribosomal protein S19 | -0.012 | 0.991 | 9.35E-01 | 9.35E+01 | | | |
| 7953135 | NM_003324 | TULP3 | tubby like protein 3 | -0.012 | 0.991 | 9.22E-01 | 9.22E+01 | | | |
| 7896069 | --- | --- | --- | -0.012 | 0.991 | 9.82E-01 | 9.82E+01 | | | |
| 7895253 | --- | --- | --- | -0.012 | 0.991 | 9.42E-01 | 9.42E+01 | | | |
| 8060837 | --- | --- | --- | -0.012 | 0.991 | 9.01E-01 | 9.01E+01 | | | |
| 7895495 | --- | --- | --- | -0.012 | 0.991 | 9.50E-01 | 9.50E+01 | | | |
| 7969263 | NM_00101172 | HNRNPA1L2 | heterogeneous nuclear ribonucleopr | -0.012 | 0.991 | 9.32E-01 | 9.32E+01 | | | |
| 8024843 | NM_005483 | CHAF1A | chromatin assembly factor 1, subuni | -0.012 | 0.991 | 8.98E-01 | 8.98E+01 | | | |
| 8159127 | NM_002957 | RXRA | retinoid X receptor, alpha | -0.012 | 0.991 | 8.76E-01 | 8.76E+01 | | | |

| | | | | | | | | | | |
|---|---|---|---|---|---|---|---|---|---|---|
| 7947528 | NM_014186 | COMMD9 | COMM domain containing 9 | -0.012 | 0.991 | 9.20E-01 | 9.20E+01 | | | |
| 8085481 | NM_144636 | CHCHD4 | coiled-coil-helix-coiled-coil-helix dom | -0.013 | 0.991 | 8.93E-01 | 8.93E+01 | | | |
| 7893164 | --- | --- | --- | -0.013 | 0.991 | 9.65E-01 | 9.65E+01 | | | |
| 7904303 | NM_004258 | IGSF2 | immunoglobulin superfamily, memb | -0.013 | 0.991 | 8.79E-01 | 8.79E+01 | | | |
| 7995926 | NM_032206 | NLRC5 | NLR family, CARD domain containing | -0.013 | 0.991 | 8.71E-01 | 8.71E+01 | | | |
| 7904050 | NM_020963 | MOV10 | Mov10, Moloney leukemia virus 10, | -0.013 | 0.991 | 8.65E-01 | 8.65E+01 | | | |
| 8024282 | NM_170711 | DAZAP1 | DAZ associated protein 1 | -0.013 | 0.991 | 8.71E-01 | 8.71E+01 | | | |
| 7914000 | NM_021969 | NR0B2 | nuclear receptor subfamily 0, group | -0.013 | 0.991 | 8.71E-01 | 8.71E+01 | | | |
| 7895359 | --- | --- | --- | -0.013 | 0.991 | 9.34E-01 | 9.34E+01 | | | |
| 8089659 | AK302488 | KIAA1407 | KIAA1407 | -0.013 | 0.991 | 9.18E-01 | 9.18E+01 | | | |
| 8098240 | --- | --- | --- | -0.013 | 0.991 | 8.77E-01 | 8.77E+01 | | | |
| 8126452 | NM_000287 | PEX6 | peroxisomal biogenesis factor 6 | -0.013 | 0.991 | 8.88E-01 | 8.88E+01 | | | |
| 8000651 | NM_015092 | SMG1 | SMG1 homolog, phosphatidylinosito | -0.013 | 0.991 | 8.95E-01 | 8.95E+01 | | | |
| 8021832 | NM_198531 | ATP9B | ATPase, class II, type 9B | -0.013 | 0.991 | 9.35E-01 | 9.35E+01 | | | |
| 7952361 | NM_003455 | ZNF202 | zinc finger protein 202 | -0.013 | 0.991 | 8.75E-01 | 8.75E+01 | | | |
| 8121087 | NM_00101085 | PM20D2 | peptidase M20 domain containing 2 | -0.013 | 0.991 | 9.60E-01 | 9.60E+01 | | | |
| 7895601 | --- | --- | --- | -0.013 | 0.991 | 9.20E-01 | 9.20E+01 | | | |
| 8005959 | NM_178170 | NEK8 | NIMA (never in mitosis gene a)- relat | -0.013 | 0.991 | 8.57E-01 | 8.57E+01 | | | |
| 7900546 | NM_024664 | PPCS | phosphopantothenoylcysteine synth | -0.013 | 0.991 | 9.51E-01 | 9.51E+01 | | | |
| 8143534 | NM_000420 | KEL | Kell blood group, metallo-endopepti | -0.013 | 0.991 | 8.37E-01 | 8.37E+01 | | | |
| 8066579 | NM_080614 | WFDC3 | WAP four-disulfide core domain 3 | -0.013 | 0.991 | 8.68E-01 | 8.68E+01 | | | |
| 8116710 | NR_026590 | CDYL | chromodomain protein, Y-like | -0.013 | 0.991 | 9.20E-01 | 9.20E+01 | | | |
| 8134621 | NM_014569 | ZKSCAN5 | zinc finger with KRAB and SCAN dom | -0.013 | 0.991 | 8.90E-01 | 8.90E+01 | | | |
| 8143984 | --- | --- | --- | -0.013 | 0.991 | 9.12E-01 | 9.12E+01 | | | |
| 8005679 | NR_023380 | CCDC144C | coiled-coil domain containing 144C | -0.013 | 0.991 | 8.67E-01 | 8.67E+01 | | | |
| 8156873 | NM_014425 | INVS | inversin | -0.013 | 0.991 | 8.98E-01 | 8.98E+01 | | | |
| 7929711 | NM_00101091 | GOLGA7B | golgi autoantigen, golgin subfamily a | -0.013 | 0.991 | 8.38E-01 | 8.38E+01 | | | |
| 7969370 | --- | --- | --- | -0.013 | 0.991 | 8.99E-01 | 8.99E+01 | | | |
| 7893033 | --- | --- | --- | -0.013 | 0.991 | 9.93E-01 | 9.93E+01 | | | |
| 8064857 | --- | --- | --- | -0.013 | 0.991 | 9.55E-01 | 9.55E+01 | | | |
| 7897774 | NM_001286 | CLCN6 | chloride channel 6 | -0.013 | 0.991 | 9.28E-01 | 9.28E+01 | | | |
| 8062981 | NM_015937 | PIGT | phosphatidylinositol glycan anchor b | -0.013 | 0.991 | 8.99E-01 | 8.99E+01 | | | |
| 8099235 | NM_203462 | MRFAP1L1 | Morf4 family associated protein 1-lik | -0.013 | 0.991 | 9.33E-01 | 9.33E+01 | | | |
| 8083777 | ENST00000407 | LOC131055 | peptidylprolyl isomerase A pseudoge | -0.013 | 0.991 | 8.40E-01 | 8.40E+01 | | | |
| 8048995 | NM_030926 | ITM2C | integral membrane protein 2C | -0.013 | 0.991 | 8.99E-01 | 8.99E+01 | | | |
| 8157843 | NM_005833 | RABEPK | Rab9 effector protein with kelch mot | -0.013 | 0.991 | 9.02E-01 | 9.02E+01 | | | |
| 8070169 | --- | --- | --- | -0.013 | 0.991 | 9.15E-01 | 9.15E+01 | | | |
| 8158686 | NM_003934 | FUBP3 | far upstream element (FUSE) binding | -0.014 | 0.991 | 9.07E-01 | 9.07E+01 | | | |
| 8063566 | NM_003610 | RAE1 | RAE1 RNA export 1 homolog (S. pom | -0.014 | 0.991 | 9.53E-01 | 9.53E+01 | | | |
| 7895070 | --- | --- | --- | -0.014 | 0.991 | 8.69E-01 | 8.69E+01 | | | |
| 7996976 | NM_007014 | WWP2 | WW domain containing E3 ubiquitin | -0.014 | 0.991 | 8.97E-01 | 8.97E+01 | | | |
| 7893273 | --- | --- | --- | -0.014 | 0.991 | 9.65E-01 | 9.65E+01 | | | |
| 8064042 | NM_175609 | ARFGAP1 | ADP-ribosylation factor GTPase activ | -0.014 | 0.990 | 8.46E-01 | 8.46E+01 | | | |
| 7944791 | --- | --- | --- | -0.014 | 0.990 | 9.02E-01 | 9.02E+01 | | | |
| 7959052 | NM_181578 | RFC5 | replication factor C (activator 1) 5, 3 | -0.014 | 0.990 | 8.82E-01 | 8.82E+01 | | | |
| 7894960 | --- | --- | --- | -0.014 | 0.990 | 8.98E-01 | 8.98E+01 | | | |
| 7951824 | --- | --- | --- | -0.014 | 0.990 | 9.63E-01 | 9.63E+01 | | | |
| 7996064 | NM_153837 | GPR114 | G protein-coupled receptor 114 | -0.014 | 0.990 | 8.20E-01 | 8.20E+01 | | | |
| 7995492 | NM_001114 | ADCY7 | adenylate cyclase 7 | -0.014 | 0.990 | 8.79E-01 | 8.79E+01 | | | |
| 7929768 | NM_015960 | CUTC | cutC copper transporter homolog (E. | -0.014 | 0.990 | 9.73E-01 | 9.73E+01 | | | |
| 8131387 | NM_032172 | USP42 | ubiquitin specific peptidase 42 | -0.014 | 0.990 | 8.78E-01 | 8.78E+01 | | | |
| 7910416 | NM_014777 | URB2 | URB2 ribosome biogenesis 2 homolo | -0.014 | 0.990 | 8.58E-01 | 8.58E+01 | | | |
| 8157582 | NM_000177 | GSN | gelsolin (amyloidosis, Finnish type) | -0.014 | 0.990 | 8.02E-01 | 8.02E+01 | | | |
| 7896670 | --- | --- | --- | -0.014 | 0.990 | 8.90E-01 | 8.90E+01 | | | |
| 7986359 | NM_000875 | IGF1R | insulin-like growth factor 1 receptor | -0.014 | 0.990 | 9.46E-01 | 9.46E+01 | | | |
| 7973918 | NM_173607 | FAM177A1 | family with sequence similarity 177, | -0.014 | 0.990 | 9.81E-01 | 9.81E+01 | | | |
| 8117572 | NM_00107678 | ZNF391 | zinc finger protein 391 | -0.014 | 0.990 | 8.57E-01 | 8.57E+01 | | | |
| 8065444 | NM_032501 | ACSS1 | acyl-CoA synthetase short-chain fam | -0.014 | 0.990 | 8.57E-01 | 8.57E+01 | | | |
| 8072729 | --- | --- | --- | -0.014 | 0.990 | 8.95E-01 | 8.95E+01 | | | |
| 7936463 | NM_002313 | ABLIM1 | actin binding LIM protein 1 | -0.014 | 0.990 | 9.18E-01 | 9.18E+01 | | | |
| 7893937 | --- | --- | --- | -0.014 | 0.990 | 9.18E-01 | 9.18E+01 | | | |
| 8145624 | NM_001440 | EXTL3 | exostoses (multiple)-like 3 | -0.014 | 0.990 | 8.78E-01 | 8.78E+01 | | | |
| 7982812 | NM_00107726 | ZFYVE19 | zinc finger, FYVE domain containing | -0.014 | 0.990 | 8.66E-01 | 8.66E+01 | | | |
| 8000998 | NM_024006 | VKORC1 | vitamin K epoxide reductase comple | -0.014 | 0.990 | 9.14E-01 | 9.14E+01 | | | |
| 8178090 | NM_00104043 | C6orf48 | chromosome 6 open reading frame 4 | -0.015 | 0.990 | 9.68E-01 | 9.68E+01 | | | |
| 8179326 | NM_00104043 | C6orf48 | chromosome 6 open reading frame 4 | -0.015 | 0.990 | 9.68E-01 | 9.68E+01 | | | |
| 8164521 | NM_020145 | SH3GLB2 | SH3-domain GRB2-like endophilin B2 | -0.015 | 0.990 | 8.62E-01 | 8.62E+01 | | | |
| 8172280 | NM_032591 | SLC9A7 | solute carrier family 9 (sodium/hydr | -0.015 | 0.990 | 9.00E-01 | 9.00E+01 | | | |
| 7899849 | --- | --- | --- | -0.015 | 0.990 | 8.92E-01 | 8.92E+01 | | | |
| 7960331 | NM_031474 | NRIP2 | nuclear receptor interacting protein | -0.015 | 0.990 | 8.98E-01 | 8.98E+01 | | | |
| 7980744 | NM_004755 | RPS6KA5 | ribosomal protein S6 kinase, 90kDa, | -0.015 | 0.990 | 9.75E-01 | 9.75E+01 | | | |
| 8157905 | NM_033446 | FAM125B | family with sequence similarity 125, | -0.015 | 0.990 | 8.59E-01 | 8.59E+01 | | | |
| 7953130 | NR_027363 | C12orf32 | chromosome 12 open reading frame | -0.015 | 0.990 | 8.68E-01 | 8.68E+01 | | | |
| 7933204 | NM_007021 | C10orf10 | chromosome 10 open reading frame | -0.015 | 0.990 | 8.51E-01 | 8.51E+01 | | | |
| 8143790 | NM_014020 | TMEM176B | transmembrane protein 176B | -0.015 | 0.990 | 9.11E-01 | 9.11E+01 | | | |
| 8004416 | NM_000747 | CHRNB1 | cholinergic receptor, nicotinic, beta | -0.015 | 0.990 | 8.22E-01 | 8.22E+01 | | | |
| 7954243 | --- | --- | --- | -0.015 | 0.990 | 9.29E-01 | 9.29E+01 | | | |

| | | | | | | | | | | |
|---|---|---|---|---|---|---|---|---|---|---|
| 7898211 | NM_032341 | DDI2 | DDI1, DNA-damage inducible 1, hom | -0.015 | 0.990 | 9.30E-01 | 9.30E+01 | | | |
| 8006736 | NM_007026 | DUSP14 | dual specificity phosphatase 14 | -0.015 | 0.990 | 8.36E-01 | 8.36E+01 | | | |
| 8055249 | NM_00100999 | FAM168B | family with sequence similarity 168, | -0.015 | 0.990 | 8.88E-01 | 8.88E+01 | | | |
| 8000848 | NM_006110 | CD2BP2 | CD2 (cytoplasmic tail) binding protei | -0.015 | 0.990 | 8.85E-01 | 8.85E+01 | | | |
| 7898276 | --- | --- | --- | -0.015 | 0.990 | 9.14E-01 | 9.14E+01 | | | |
| 7895453 | --- | --- | --- | -0.015 | 0.990 | 9.68E-01 | 9.68E+01 | | | |
| 8125341 | NM_001136 | AGER | advanced glycosylation end product- | -0.015 | 0.990 | 8.62E-01 | 8.62E+01 | | | |
| 8165271 | NM_00100171 | LCN10 | lipocalin 10 | -0.015 | 0.990 | 8.59E-01 | 8.59E+01 | | | |
| 7993478 | NM_004996 | ABCC1 | ATP-binding cassette, sub-family C (C | -0.015 | 0.990 | 8.83E-01 | 8.83E+01 | | | |
| 7997779 | NM_144604 | ZC3H18 | zinc finger CCCH-type containing 18 | -0.015 | 0.990 | 9.10E-01 | 9.10E+01 | | | |
| 7977249 | NM_022489 | INF2 | inverted formin, FH2 and WH2 doma | -0.015 | 0.990 | 8.31E-01 | 8.31E+01 | | | |
| 8066536 | NM_080827 | WFDC6 | WAP four-disulfide core domain 6 | -0.015 | 0.989 | 8.58E-01 | 8.58E+01 | | | |
| 7894112 | --- | --- | --- | -0.015 | 0.989 | 9.61E-01 | 9.61E+01 | | | |
| 8113761 | NM_020747 | ZNF608 | zinc finger protein 608 | -0.015 | 0.989 | 8.31E-01 | 8.31E+01 | | | |
| 7901460 | NM_015696 | GPX7 | glutathione peroxidase 7 | -0.015 | 0.989 | 8.62E-01 | 8.62E+01 | | | |
| 8150206 | NM_152344 | LSM12 | LSM12 homolog (S. cerevisiae) | -0.015 | 0.989 | 9.53E-01 | 9.53E+01 | | | |
| 7950119 | BC001706 | C11orf59 | chromosome 11 open reading frame | -0.015 | 0.989 | 8.89E-01 | 8.89E+01 | | | |
| 7942453 | NM_021200 | PLEKHB1 | pleckstrin homology domain contain | -0.015 | 0.989 | 8.96E-01 | 8.96E+01 | | | |
| 8099817 | NR_026804 | FLJ13197 | hypothetical FLJ13197 | -0.015 | 0.989 | 8.81E-01 | 8.81E+01 | | | |
| 7985364 | NM_172217 | IL16 | interleukin 16 (lymphocyte chemoat | -0.015 | 0.989 | 8.20E-01 | 8.20E+01 | | | |
| 8035187 | NM_004831 | MED26 | mediator complex subunit 26 | -0.015 | 0.989 | 8.93E-01 | 8.93E+01 | | | |
| 8033352 | NM_144614 | MBD3L2 | methyl-CpG binding domain protein | -0.015 | 0.989 | 8.30E-01 | 8.30E+01 | | | |
| 8092404 | NM_024871 | MAP6D1 | MAP6 domain containing 1 | -0.016 | 0.989 | 8.51E-01 | 8.51E+01 | | | |
| 7997680 | NM_014615 | KIAA0182 | KIAA0182 | -0.016 | 0.989 | 8.49E-01 | 8.49E+01 | | | |
| 7983123 | NM_018097 | HAUS2 | HAUS augmin-like complex, subunit | -0.016 | 0.989 | 9.28E-01 | 9.28E+01 | | | |
| 7961022 | BC051265 | PTMA | prothymosin, alpha | -0.016 | 0.989 | 8.58E-01 | 8.58E+01 | | | |
| 8143385 | --- | --- | --- | -0.016 | 0.989 | 8.85E-01 | 8.85E+01 | | | |
| 8051998 | NM_139279 | MCFD2 | multiple coagulation factor deficienc | -0.016 | 0.989 | 9.48E-01 | 9.48E+01 | | | |
| 8146278 | NM_032237 | SGK196 | protein kinase-like protein SgK196 | -0.016 | 0.989 | 8.94E-01 | 8.94E+01 | | | |
| 7978389 | --- | --- | --- | -0.016 | 0.989 | 8.95E-01 | 8.95E+01 | | | |
| 7960984 | NM_002864 | PZP | pregnancy-zone protein | -0.016 | 0.989 | 8.65E-01 | 8.65E+01 | | | |
| 8072989 | NM_012407 | PICK1 | protein interacting with PRKCA 1 | -0.016 | 0.989 | 8.35E-01 | 8.35E+01 | | | |
| 7895515 | --- | --- | --- | -0.016 | 0.989 | 9.38E-01 | 9.38E+01 | | | |
| 8090505 | --- | --- | --- | -0.016 | 0.989 | 8.03E-01 | 8.03E+01 | | | |
| 7967127 | NM_006549 | CAMKK2 | calcium/calmodulin-dependent prot | -0.016 | 0.989 | 8.86E-01 | 8.86E+01 | | | |
| 7974198 | NM_152329 | PPIL5 | peptidylprolyl isomerase (cyclophilin | -0.016 | 0.989 | 9.36E-01 | 9.36E+01 | | | |
| 7964145 | NM_003920 | TIMELESS | timeless homolog (Drosophila) | -0.016 | 0.989 | 8.63E-01 | 8.63E+01 | | | |
| 8140020 | NM_018044 | NSUN5 | NOL1/NOP2/Sun domain family, mer | -0.016 | 0.989 | 8.77E-01 | 8.77E+01 | | | |
| 7977615 | NM_198232 | RNASE1 | ribonuclease, RNase A family, 1 (pan | -0.016 | 0.989 | 8.28E-01 | 8.28E+01 | | | |
| 7988124 | NM_014659 | HISPPD2A | histidine acid phosphatase domain c | -0.016 | 0.989 | 9.18E-01 | 9.18E+01 | | | |
| 8028705 | NM_00100156 | TIMM50 | translocase of inner mitochondrial m | -0.016 | 0.989 | 9.26E-01 | 9.26E+01 | | | |
| 7892928 | --- | --- | --- | -0.016 | 0.989 | 8.29E-01 | 8.29E+01 | | | |
| 7966213 | NM_052845 | MMAB | methylmalonic aciduria (cobalamin c | -0.016 | 0.989 | 8.88E-01 | 8.88E+01 | | | |
| 8128312 | --- | --- | --- | -0.016 | 0.989 | 8.70E-01 | 8.70E+01 | | | |
| 7893121 | --- | --- | --- | -0.016 | 0.989 | 8.92E-01 | 8.92E+01 | | | |
| 8043034 | --- | --- | --- | -0.016 | 0.989 | 8.88E-01 | 8.88E+01 | | | |
| 7896176 | --- | --- | --- | -0.016 | 0.989 | 8.51E-01 | 8.51E+01 | | | |
| 8143188 | NM_194071 | CREB3L2 | cAMP responsive element binding pr | -0.016 | 0.989 | 8.43E-01 | 8.43E+01 | | | |
| 8021905 | BC014195 | C18orf22 | chromosome 18 open reading frame | -0.016 | 0.989 | 8.74E-01 | 8.74E+01 | | | |
| 8126387 | NM_002098 | GUCA1B | guanylate cyclase activator 1B (retin | -0.016 | 0.989 | 8.49E-01 | 8.49E+01 | | | |
| 8164034 | NM_020946 | DENND1A | DENN/MADD domain containing 1A | -0.016 | 0.989 | 9.20E-01 | 9.20E+01 | | | |
| 7981032 | NM_020414 | DDX24 | DEAD (Asp-Glu-Ala-Asp) box polypep | -0.016 | 0.989 | 9.55E-01 | 9.55E+01 | | | |
| 7940561 | NM_004111 | FEN1 | flap structure-specific endonuclease | -0.016 | 0.989 | 9.15E-01 | 9.15E+01 | | | |
| 7997662 | NM_014732 | KIAA0513 | KIAA0513 | -0.016 | 0.989 | 8.29E-01 | 8.29E+01 | | | |
| 7991209 | NM_022163 | MRPL46 | mitochondrial ribosomal protein L46 | -0.016 | 0.989 | 9.52E-01 | 9.52E+01 | | | |
| 8000702 | NM_175900 | C16orf54 | chromosome 16 open reading frame | -0.016 | 0.989 | 8.07E-01 | 8.07E+01 | | | |
| 8003601 | NM_175900 | C16orf54 | chromosome 16 open reading frame | -0.016 | 0.989 | 8.07E-01 | 8.07E+01 | | | |
| 7984470 | NM_015322 | FEM1B | fem-1 homolog b (C. elegans) | -0.016 | 0.989 | 8.70E-01 | 8.70E+01 | | | |
| 7938396 | NM_000480 | AMPD3 | adenosine monophosphate deamina | -0.016 | 0.989 | 8.37E-01 | 8.37E+01 | | | |
| 8012274 | NM_032356 | LSMD1 | LSM domain containing 1 | -0.016 | 0.989 | 9.01E-01 | 9.01E+01 | | | |
| 8061542 | NM_178580 | HM13 | histocompatibility (minor) 13 | -0.016 | 0.989 | 7.97E-01 | 7.97E+01 | | | |
| 7900654 | NM_144626 | TMEM125 | transmembrane protein 125 | -0.016 | 0.989 | 7.84E-01 | 7.84E+01 | | | |
| 8100782 | --- | --- | --- | -0.016 | 0.989 | 8.00E-01 | 8.00E+01 | | | |
| 8140398 | NM_012479 | YWHAG | tyrosine 3-monooxygenase/tryptoph | -0.016 | 0.989 | 8.65E-01 | 8.65E+01 | | | |
| 7990879 | NM_024580 | EFTUD1 | elongation factor Tu GTP binding dor | -0.016 | 0.989 | 9.53E-01 | 9.53E+01 | | | |
| 8061497 | NR_003579 | FRG1B | FSHD region gene 1 family, member | -0.016 | 0.989 | 9.14E-01 | 9.14E+01 | | | |
| 8149345 | --- | --- | --- | -0.016 | 0.989 | 8.99E-01 | 8.99E+01 | | | mono |
| 8002245 | --- | --- | --- | -0.017 | 0.989 | 9.55E-01 | 9.55E+01 | | | |
| 7969986 | NM_006573 | TNFSF13B | tumor necrosis factor (ligand) superf | -0.017 | 0.989 | 9.67E-01 | 9.67E+01 | | | mono |
| 7906305 | --- | --- | --- | -0.017 | 0.989 | 9.22E-01 | 9.22E+01 | | | |
| 8114300 | NM_017415 | KLHL3 | kelch-like 3 (Drosophila) | -0.017 | 0.989 | 8.72E-01 | 8.72E+01 | | | |
| 7896065 | --- | --- | --- | -0.017 | 0.988 | 9.87E-01 | 9.87E+01 | | | |
| 8038954 | NM_178523 | ZNF616 | zinc finger protein 616 | -0.017 | 0.988 | 9.18E-01 | 9.18E+01 | | | |
| 8048175 | NM_014140 | SMARCAL1 | SWI/SNF related, matrix associated, | -0.017 | 0.988 | 8.46E-01 | 8.46E+01 | | | |
| 8031778 | NM_020880 | ZNF530 | zinc finger protein 530 | -0.017 | 0.988 | 8.00E-01 | 8.00E+01 | | | |
| 7892597 | --- | --- | --- | -0.017 | 0.988 | 9.22E-01 | 9.22E+01 | | | |
| 8043018 | --- | --- | --- | -0.017 | 0.988 | 8.49E-01 | 8.49E+01 | | | |

| | | | | | | | | | | |
|---|---|---|---|---|---|---|---|---|---|---|
| 8130539 | NM_054114 | TAGAP | T-cell activation RhoGTPase activatin | -0.017 | 0.988 | 9.40E-01 | 9.40E+01 | | | |
| 8125048 | NM_013974 | DDAH2 | dimethylarginine dimethylaminohyd | -0.017 | 0.988 | 8.39E-01 | 8.39E+01 | | | |
| 7892824 | --- | --- | --- | -0.017 | 0.988 | 8.98E-01 | 8.98E+01 | | | |
| 7958439 | NM_032663 | USP30 | ubiquitin specific peptidase 30 | -0.017 | 0.988 | 8.97E-01 | 8.97E+01 | | | |
| 7915594 | --- | --- | --- | -0.017 | 0.988 | 9.24E-01 | 9.24E+01 | | | |
| 8063955 | NM_007346 | OGFR | opioid growth factor receptor | -0.017 | 0.988 | 8.27E-01 | 8.27E+01 | | | |
| 8121370 | NM_032131 | ARMC2 | armadillo repeat containing 2 | -0.017 | 0.988 | 7.79E-01 | 7.79E+01 | | | |
| 7892551 | --- | --- | --- | -0.017 | 0.988 | 8.10E-01 | 8.10E+01 | | | |
| 7895733 | --- | --- | --- | -0.017 | 0.988 | 9.42E-01 | 9.42E+01 | | | |
| 8041225 | NM_014600 | EHD3 | EH-domain containing 3 | -0.017 | 0.988 | 8.36E-01 | 8.36E+01 | | | |
| 8016482 | --- | --- | --- | -0.017 | 0.988 | 8.80E-01 | 8.80E+01 | | | |
| 7977319 | NM_138790 | PLD4 | phospholipase D family, member 4 | -0.017 | 0.988 | 8.00E-01 | 8.00E+01 | | | |
| 7984846 | NM_00113002 | CLK3 | CDC-like kinase 3 | -0.017 | 0.988 | 8.78E-01 | 8.78E+01 | | | |
| 8035795 | NM_145297 | ZNF626 | zinc finger protein 626 | -0.017 | 0.988 | 9.35E-01 | 9.35E+01 | | | |
| 8133540 | NM_00114506 | GATSL1 | GATS protein-like 1 | -0.017 | 0.988 | 8.10E-01 | 8.10E+01 | | | |
| 8140249 | NM_00114506 | GATSL1 | GATS protein-like 1 | -0.017 | 0.988 | 8.10E-01 | 8.10E+01 | | | |
| 8028323 | --- | --- | --- | -0.017 | 0.988 | 8.33E-01 | 8.33E+01 | | | |
| 7953390 | NM_00103991 | ZNF384 | zinc finger protein 384 | -0.017 | 0.988 | 9.07E-01 | 9.07E+01 | | | |
| 8175330 | --- | --- | --- | -0.017 | 0.988 | 8.85E-01 | 8.85E+01 | | | |
| 7896624 | --- | --- | --- | -0.017 | 0.988 | 8.83E-01 | 8.83E+01 | | | |
| 7990027 | NR_026808 | C15orf28 | chromosome 15 open reading frame | -0.017 | 0.988 | 8.73E-01 | 8.73E+01 | | | |
| 8054796 | --- | --- | --- | -0.017 | 0.988 | 8.33E-01 | 8.33E+01 | | | |
| 8171119 | NM_004192 | ASMTL | acetylserotonin O-methyltransferase | -0.017 | 0.988 | 8.79E-01 | 8.79E+01 | | | |
| 7952549 | NM_032795 | RPUSD4 | RNA pseudouridylate synthase doma | -0.017 | 0.988 | 9.35E-01 | 9.35E+01 | | | |
| 8075705 | --- | --- | --- | -0.017 | 0.988 | 8.97E-01 | 8.97E+01 | | | |
| 7930703 | NM_139169 | TRUB1 | TruB pseudouridine (psi) synthase ho | -0.017 | 0.988 | 9.20E-01 | 9.20E+01 | | | |
| 8180382 | --- | --- | --- | -0.017 | 0.988 | 7.67E-01 | 7.67E+01 | | | |
| 8124327 | --- | --- | --- | -0.018 | 0.988 | 8.51E-01 | 8.51E+01 | | | |
| 8110734 | NR_027633 | BRD9 | bromodomain containing 9 | -0.018 | 0.988 | 9.11E-01 | 9.11E+01 | | | |
| 7898975 | NM_005839 | SRRM1 | serine/arginine repetitive matrix 1 | -0.018 | 0.988 | 9.21E-01 | 9.21E+01 | | | |
| 7996785 | NM_019023 | PRMT7 | protein arginine methyltransferase 7 | -0.018 | 0.988 | 8.26E-01 | 8.26E+01 | | | |
| 8118820 | --- | --- | --- | -0.018 | 0.988 | 9.88E-01 | 9.88E+01 | | | |
| 7925677 | NM_024804 | ZNF669 | zinc finger protein 669 | -0.018 | 0.988 | 8.29E-01 | 8.29E+01 | | | |
| 8064716 | NM_023068 | SIGLEC1 | sialic acid binding Ig-like lectin 1, sial | -0.018 | 0.988 | 7.91E-01 | 7.91E+01 | | | |
| 8025458 | NM_020933 | ZNF317 | zinc finger protein 317 | -0.018 | 0.988 | 8.87E-01 | 8.87E+01 | | | |
| 8060895 | NR_004386 | RNU105B | RNA, U105B small nucleolar | -0.018 | 0.988 | 9.09E-01 | 9.09E+01 | | | |
| 7913558 | NM_033631 | LUZP1 | leucine zipper protein 1 | -0.018 | 0.988 | 8.36E-01 | 8.36E+01 | | | |
| 7983677 | --- | --- | --- | -0.018 | 0.988 | 8.64E-01 | 8.64E+01 | | | |
| 7898902 | NM_007260 | LYPLA2 | lysophospholipase II | -0.018 | 0.988 | 8.56E-01 | 8.56E+01 | | | |
| 7907849 | NM_033343 | LHX4 | LIM homeobox 4 | -0.018 | 0.988 | 7.76E-01 | 7.76E+01 | | | |
| 8087825 | NM_032750 | ABHD14B | abhydrolase domain containing 14B | -0.018 | 0.988 | 8.02E-01 | 8.02E+01 | | | |
| 7995438 | --- | --- | --- | -0.018 | 0.988 | 8.15E-01 | 8.15E+01 | | | |
| 8143919 | NM_00100380 | SMARCD3 | SWI/SNF related, matrix associated, | -0.018 | 0.988 | 7.99E-01 | 7.99E+01 | | | |
| 7935627 | NM_002079 | GOT1 | glutamic-oxaloacetic transaminase 1 | -0.018 | 0.988 | 9.13E-01 | 9.13E+01 | | | |
| 7998927 | --- | --- | --- | -0.018 | 0.988 | 9.12E-01 | 9.12E+01 | | | |
| 8051075 | NM_001521 | GTF3C2 | general transcription factor IIIC, poly | -0.018 | 0.988 | 8.17E-01 | 8.17E+01 | | | |
| 8124691 | AY358246 | HCG8 | HLA complex group 8 | -0.018 | 0.988 | 9.01E-01 | 9.01E+01 | | | |
| 7921031 | --- | --- | --- | -0.018 | 0.988 | 9.57E-01 | 9.57E+01 | | | |
| 7991120 | NR_003661 | FLJ43276 | similar to ubiquitin-conjugating enzy | -0.018 | 0.988 | 9.30E-01 | 9.30E+01 | | | |
| 7934753 | NM_00109969 | EIF5AL1 | eukaryotic translation initiation facto | -0.018 | 0.988 | 9.73E-01 | 9.73E+01 | | | |
| 8144397 | NM_201402 | USP17L2 | ubiquitin specific peptidase 17-like 2 | -0.018 | 0.988 | 8.39E-01 | 8.39E+01 | | | |
| 8149241 | NM_201402 | USP17L2 | ubiquitin specific peptidase 17-like 2 | -0.018 | 0.988 | 8.39E-01 | 8.39E+01 | | | |
| 8174338 | NM_024657 | MORC4 | MORC family CW-type zinc finger 4 | -0.018 | 0.988 | 7.84E-01 | 7.84E+01 | | | |
| 7977906 | NM_014977 | ACIN1 | apoptotic chromatin condensation in | -0.018 | 0.988 | 9.13E-01 | 9.13E+01 | | | |
| 7951668 | --- | --- | --- | -0.018 | 0.988 | 9.39E-01 | 9.39E+01 | | | |
| 7949792 | NM_005608 | PTPRCAP | protein tyrosine phosphatase, recept | -0.018 | 0.988 | 8.32E-01 | 8.32E+01 | | | |
| 8057377 | NM_173648 | CCDC141 | coiled-coil domain containing 141 | -0.018 | 0.988 | 9.36E-01 | 9.36E+01 | | | |
| 8076056 | NM_001894 | CSNK1E | casein kinase 1, epsilon | -0.018 | 0.988 | 8.17E-01 | 8.17E+01 | | | |
| 7903358 | NM_001078 | VCAM1 | vascular cell adhesion molecule 1 | -0.018 | 0.988 | 8.13E-01 | 8.13E+01 | | | |
| 8016366 | NM_145255 | MRPL10 | mitochondrial ribosomal protein L10 | -0.018 | 0.987 | 8.65E-01 | 8.65E+01 | | | |
| 7982723 | NM_002225 | IVD | isovaleryl Coenzyme A dehydrogenas | -0.018 | 0.987 | 8.34E-01 | 8.34E+01 | | | |
| 8049657 | NM_00104044 | ASB1 | ankyrin repeat and SOCS box-contair | -0.018 | 0.987 | 8.97E-01 | 8.97E+01 | | | |
| 8156373 | NM_00108353 | FGD3 | FYVE, RhoGEF and PH domain contai | -0.018 | 0.987 | 8.51E-01 | 8.51E+01 | | | |
| 7947676 | NM_173811 | HARBI1 | harbinger transposase derived 1 | -0.018 | 0.987 | 8.23E-01 | 8.23E+01 | | | |
| 8026821 | NM_018174 | MAP1S | microtubule-associated protein 1S | -0.018 | 0.987 | 7.78E-01 | 7.78E+01 | | | |
| 8079167 | NM_00114503 | C3orf77 | chromosome 3 open reading frame 7 | -0.018 | 0.987 | 7.98E-01 | 7.98E+01 | | | |
| 7938295 | NM_000990 | RPL27A | ribosomal protein L27a | -0.018 | 0.987 | 8.28E-01 | 8.28E+01 | | | |
| 7959232 | --- | --- | --- | -0.018 | 0.987 | 8.70E-01 | 8.70E+01 | | | |
| 8129363 | NM_016063 | HDDC2 | HD domain containing 2 | -0.018 | 0.987 | 9.60E-01 | 9.60E+01 | | | |
| 8064156 | NM_032527 | ZGPAT | zinc finger, CCCH-type with G patch d | -0.018 | 0.987 | 7.61E-01 | 7.61E+01 | | | |
| 8097417 | NM_199320 | PHF17 | PHD finger protein 17 | -0.018 | 0.987 | 8.64E-01 | 8.64E+01 | | | |
| 7970842 | --- | --- | --- | -0.019 | 0.987 | 9.08E-01 | 9.08E+01 | | | |
| 7981335 | NM_00101796 | HSP90AA1 | heat shock protein 90kDa alpha (cyto | -0.019 | 0.987 | 9.00E-01 | 9.00E+01 | | | |
| 7909510 | NM_018194 | HHAT | hedgehog acyltransferase | -0.019 | 0.987 | 8.05E-01 | 8.05E+01 | | | |
| 7998510 | NM_001287 | CLCN7 | chloride channel 7 | -0.019 | 0.987 | 8.07E-01 | 8.07E+01 | | | |
| 8131444 | NR_002217 | PMS2CL | PMS2 C-terminal like pseudogene | -0.019 | 0.987 | 9.03E-01 | 9.03E+01 | | | |
| 7960544 | NM_199245 | VAMP1 | vesicle-associated membrane protei | -0.019 | 0.987 | 9.41E-01 | 9.41E+01 | | | |

| | | | | | | | | | | |
|---|---|---|---|---|---|---|---|---|---|---|
| 8063814 | NM_144703 | LSM14B | LSM14B, SCD6 homolog B (S. cerevis | -0.019 | 0.987 | 7.11E-01 | 7.11E+01 | | | |
| 8100362 | NM_00112632 | LNX1 | ligand of numb-protein X 1 | -0.019 | 0.987 | 7.52E-01 | 7.52E+01 | | | |
| 8071301 | NM_152906 | C22orf25 | chromosome 22 open reading frame | -0.019 | 0.987 | 8.59E-01 | 8.59E+01 | | | |
| 7944179 | NM_000733 | CD3E | CD3e molecule, epsilon (CD3-TCR co | -0.019 | 0.987 | 9.53E-01 | 9.53E+01 | | | |
| 8169882 | NM_021946 | BCORL1 | BCL6 co-repressor-like 1 | -0.019 | 0.987 | 7.92E-01 | 7.92E+01 | | | |
| 7972921 | --- | --- | --- | -0.019 | 0.987 | 9.67E-01 | 9.67E+01 | | | |
| 8073949 | NM_00113510 | CRELD2 | cysteine-rich with EGF-like domains | -0.019 | 0.987 | 9.01E-01 | 9.01E+01 | | | |
| 8089759 | NM_018266 | TMEM39A | transmembrane protein 39A | -0.019 | 0.987 | 9.36E-01 | 9.36E+01 | | | |
| 7893415 | --- | --- | --- | -0.019 | 0.987 | 9.57E-01 | 9.57E+01 | | | |
| 8065855 | NR_026728 | EDEM2 | ER degradation enhancer, mannosid | -0.019 | 0.987 | 9.17E-01 | 9.17E+01 | | | |
| 8024532 | NM_173480 | ZNF57 | zinc finger protein 57 | -0.019 | 0.987 | 8.06E-01 | 8.06E+01 | | | |
| 7974207 | NM_002408 | MGAT2 | mannosyl (alpha-1,6-)-glycoprotein b | -0.019 | 0.987 | 9.46E-01 | 9.46E+01 | | | |
| 7963304 | NM_001971 | CELA1 | chymotrypsin-like elastase family, m | -0.019 | 0.987 | 8.25E-01 | 8.25E+01 | | | |
| 7895662 | --- | --- | --- | -0.019 | 0.987 | 8.78E-01 | 8.78E+01 | | | |
| 8063315 | NM_017895 | DDX27 | DEAD (Asp-Glu-Ala-Asp) box polypep | -0.019 | 0.987 | 9.33E-01 | 9.33E+01 | | | |
| 8051985 | --- | --- | --- | -0.019 | 0.987 | 8.00E-01 | 8.00E+01 | | | |
| 8141717 | NM_016068 | FIS1 | fission 1 (mitochondrial outer memb | -0.019 | 0.987 | 9.45E-01 | 9.45E+01 | | | |
| 8173524 | NM_00114488 | CITED1 | Cbp/p300-interacting transactivator, | -0.019 | 0.987 | 8.10E-01 | 8.10E+01 | | | |
| 8178903 | NM_021976 | RXRB | retinoid X receptor, beta | -0.019 | 0.987 | 8.15E-01 | 8.15E+01 | | | |
| 7895542 | --- | --- | --- | -0.019 | 0.987 | 9.73E-01 | 9.73E+01 | | | |
| 8169898 | NM_004794 | RAB33A | RAB33A, member RAS oncogene fam | -0.019 | 0.987 | 8.29E-01 | 8.29E+01 | | | |
| 7892955 | --- | --- | --- | -0.019 | 0.987 | 8.67E-01 | 8.67E+01 | | | |
| 7920642 | NM_00101801 | MUC1 | mucin 1, cell surface associated | -0.019 | 0.987 | 8.29E-01 | 8.29E+01 | | | |
| 8115840 | NM_004387 | NKX2-5 | NK2 transcription factor related, locu | -0.019 | 0.987 | 7.82E-01 | 7.82E+01 | | | |
| 8120402 | NM_004282 | BAG2 | BCL2-associated athanogene 2 | -0.019 | 0.987 | 8.95E-01 | 8.95E+01 | | | |
| 8045142 | NM_025029 | FAM128B | family with sequence similarity 128, | -0.019 | 0.987 | 8.96E-01 | 8.96E+01 | | | |
| 8175998 | NM_004992 | MECP2 | methyl CpG binding protein 2 (Rett s | -0.019 | 0.987 | 8.27E-01 | 8.27E+01 | | | |
| 7948782 | NM_015853 | UBXN1 | UBX domain protein 1 | -0.019 | 0.987 | 9.24E-01 | 9.24E+01 | | | |
| 8079019 | NM_001030 | RPS27 | ribosomal protein S27 | -0.019 | 0.987 | 8.93E-01 | 8.93E+01 | | | |
| 7949746 | NM_021173 | POLD4 | polymerase (DNA-directed), delta 4 | -0.019 | 0.987 | 8.82E-01 | 8.82E+01 | | | |
| 8157074 | NM_00107980 | FKTN | fukutin | -0.019 | 0.987 | 8.38E-01 | 8.38E+01 | | | |
| 7982712 | NM_033286 | C15orf23 | chromosome 15 open reading frame | -0.019 | 0.987 | 8.78E-01 | 8.78E+01 | | | |
| 8050007 | NM_012293 | PXDN | peroxidasin homolog (Drosophila) | -0.019 | 0.987 | 7.56E-01 | 7.56E+01 | | | |
| 8007904 | NM_004287 | GOSR2 | golgi SNAP receptor complex membe | -0.019 | 0.987 | 8.91E-01 | 8.91E+01 | | | |
| 8148808 | NM_032450 | HEATR7A | HEAT repeat containing 7A | -0.019 | 0.987 | 8.24E-01 | 8.24E+01 | | | |
| 7894428 | --- | --- | --- | -0.019 | 0.987 | 9.07E-01 | 9.07E+01 | | | |
| 7969559 | --- | --- | --- | -0.019 | 0.987 | 7.85E-01 | 7.85E+01 | | | |
| 8062137 | NM_007186 | CEP250 | centrosomal protein 250kDa | -0.019 | 0.987 | 7.42E-01 | 7.42E+01 | | | |
| 8029969 | NM_003009 | SEPW1 | selenoprotein W, 1 | -0.020 | 0.987 | 8.99E-01 | 8.99E+01 | | | |
| 8088905 | ENST00000442 | OC10028729 | similar to hCG2036843 | -0.020 | 0.987 | 7.81E-01 | 7.81E+01 | | | |
| 8138749 | NM_152739 | HOXA9 | homeobox A9 | -0.020 | 0.987 | 7.87E-01 | 7.87E+01 | | | |
| 8139015 | --- | --- | --- | -0.020 | 0.987 | 7.40E-01 | 7.40E+01 | | | |
| 7940473 | NM_016464 | TMEM138 | transmembrane protein 138 | -0.020 | 0.986 | 9.01E-01 | 9.01E+01 | | | |
| 8108370 | NM_001964 | EGR1 | early growth response 1 | -0.020 | 0.986 | 8.96E-01 | 8.96E+01 | | | |
| 7915995 | NM_007051 | FAF1 | Fas (TNFRSF6) associated factor 1 | -0.020 | 0.986 | 9.42E-01 | 9.42E+01 | | | |
| 8072687 | NM_006739 | MCM5 | minichromosome maintenance comp | -0.020 | 0.986 | 8.79E-01 | 8.79E+01 | | | |
| 7915277 | NM_005376 | MYCL1 | v-myc myelocytomatosis viral oncog | -0.020 | 0.986 | 8.36E-01 | 8.36E+01 | | | |
| 8169044 | NM_00100693 | TCEAL3 | transcription elongation factor A (SII | -0.020 | 0.986 | 8.69E-01 | 8.69E+01 | | | |
| 8151411 | --- | --- | --- | -0.020 | 0.986 | 8.36E-01 | 8.36E+01 | | | |
| 7998427 | NM_012467 | TPSG1 | tryptase gamma 1 | -0.020 | 0.986 | 7.36E-01 | 7.36E+01 | | | |
| 8051097 | NM_00103411 | EIF2B4 | eukaryotic translation initiation facto | -0.020 | 0.986 | 8.37E-01 | 8.37E+01 | | | |
| 8121563 | NM_002356 | MARCKS | myristoylated alanine-rich protein ki | -0.020 | 0.986 | 7.67E-01 | 7.67E+01 | | | |
| 7910111 | NM_000120 | EPHX1 | epoxide hydrolase 1, microsomal (xe | -0.020 | 0.986 | 8.29E-01 | 8.29E+01 | | | |
| 8117395 | NM_003522 | HIST1H2BF | histone cluster 1, H2bf | -0.020 | 0.986 | 9.66E-01 | 9.66E+01 | | | |
| 7906954 | NM_002585 | PBX1 | pre-B-cell leukemia homeobox 1 | -0.020 | 0.986 | 8.10E-01 | 8.10E+01 | | | |
| 8120967 | NM_002526 | NT5E | 5'-nucleotidase, ecto (CD73) | -0.020 | 0.986 | 8.99E-01 | 8.99E+01 | | | |
| 8152079 | NM_002568 | PABPC1 | poly(A) binding protein, cytoplasmic | -0.020 | 0.986 | 8.38E-01 | 8.38E+01 | | | |
| 7894804 | --- | --- | --- | -0.020 | 0.986 | 9.36E-01 | 9.36E+01 | | | |
| 7975361 | NM_014734 | KIAA0247 | KIAA0247 | -0.020 | 0.986 | 8.71E-01 | 8.71E+01 | | | |
| 8062119 | AF348994 // A | T1JP // MT1 | metallothionein 1J (pseudogene) // | -0.020 | 0.986 | 9.30E-01 | 9.30E+01 | | | |
| 8122840 | X74439 | ESR1 | estrogen receptor 1 | -0.020 | 0.986 | 7.72E-01 | 7.72E+01 | | | |
| 8167942 | NM_007157 | ZXDB | zinc finger, X-linked, duplicated B | -0.020 | 0.986 | 8.85E-01 | 8.85E+01 | | | |
| 8009552 | NM_017941 | C17orf80 | chromosome 17 open reading frame | -0.020 | 0.986 | 8.80E-01 | 8.80E+01 | | | |
| 8079074 | NM_016305 | SS18L2 | synovial sarcoma translocation gene | -0.020 | 0.986 | 9.53E-01 | 9.53E+01 | | | |
| 8032871 | NM_139159 | DPP9 | dipeptidyl-peptidase 9 | -0.020 | 0.986 | 8.43E-01 | 8.43E+01 | | | |
| 8072494 | NM_00100746 | SFI1 | Sfi1 homolog, spindle assembly asso | -0.020 | 0.986 | 7.57E-01 | 7.57E+01 | | | |
| 8092839 | NM_00113505 | LRRC15 | leucine rich repeat containing 15 | -0.020 | 0.986 | 6.70E-01 | 6.70E+01 | | | |
| 8074688 | NM_030573 | THAP7 | THAP domain containing 7 | -0.020 | 0.986 | 7.69E-01 | 7.69E+01 | | | |
| 7901565 | NM_000792 | DIO1 | deiodinase, iodothyronine, type I | -0.020 | 0.986 | 8.38E-01 | 8.38E+01 | | | |
| 8028311 | NM_021102 | SPINT2 | serine peptidase inhibitor, Kunitz typ | -0.020 | 0.986 | 8.47E-01 | 8.47E+01 | | | |
| 7893269 | --- | --- | --- | -0.020 | 0.986 | 9.14E-01 | 9.14E+01 | | | |
| 8050713 | NM_199346 | PFN4 | profilin family, member 4 | -0.020 | 0.986 | 8.51E-01 | 8.51E+01 | | | |
| 8178771 | NM_001136 | AGER | advanced glycosylation end product- | -0.021 | 0.986 | 7.92E-01 | 7.92E+01 | | | |
| 7894645 | --- | --- | --- | -0.021 | 0.986 | 8.39E-01 | 8.39E+01 | | | |
| 8064188 | --- | --- | --- | -0.021 | 0.986 | 8.38E-01 | 8.38E+01 | | | |
| 8017711 | NM_006572 | GNA13 | guanine nucleotide binding protein ( | -0.021 | 0.986 | 9.37E-01 | 9.37E+01 | | | |
| 7924996 | NM_032800 | C1orf198 | chromosome 1 open reading frame 1 | -0.021 | 0.986 | 8.25E-01 | 8.25E+01 | | | |

| | | | | | | | | | | |
|---|---|---|---|---|---|---|---|---|---|---|
| 7920757 | NM_002004 | FDPS | farnesyl diphosphate synthase (farne | -0.021 | 0.986 | 8.01E-01 | 8.01E+01 | | | |
| 8051012 | NM_013388 | PREB | prolactin regulatory element binding | -0.021 | 0.986 | 7.53E-01 | 7.53E+01 | | | |
| 7941382 | NM_182710 | KAT5 | K(lysine) acetyltransferase 5 | -0.021 | 0.986 | 9.07E-01 | 9.07E+01 | | | |
| 8125638 | NM_021976 | RXRB | retinoid X receptor, beta | -0.021 | 0.986 | 8.13E-01 | 8.13E+01 | | | |
| 7899407 | NM_014474 | SMPDL3B | sphingomyelin phosphodiesterase, a | -0.021 | 0.986 | 7.36E-01 | 7.36E+01 | | | |
| 8136061 | NR_002187 | tcag7.1015 | triosephosphate isomerase 1 pseudo | -0.021 | 0.986 | 7.98E-01 | 7.98E+01 | | | |
| 8019486 | NM_003004 | SECTM1 | secreted and transmembrane 1 | -0.021 | 0.986 | 8.18E-01 | 8.18E+01 | | | |
| 7950628 | NM_033547 | INTS4 | integrator complex subunit 4 | -0.021 | 0.986 | 8.94E-01 | 8.94E+01 | | | |
| 8152117 | --- | --- | --- | -0.021 | 0.986 | 7.92E-01 | 7.92E+01 | | | |
| 8170364 | NM_002025 | AFF2 | AF4/FMR2 family, member 2 | -0.021 | 0.986 | 7.64E-01 | 7.64E+01 | | | |
| 7992897 | NM_003450 | ZNF174 | zinc finger protein 174 | -0.021 | 0.986 | 8.37E-01 | 8.37E+01 | | | |
| 8024896 | --- | --- | --- | -0.021 | 0.986 | 7.90E-01 | 7.90E+01 | | | |
| 8074432 | NM_003325 | HIRA | HIR histone cell cycle regulation defe | -0.021 | 0.986 | 7.70E-01 | 7.70E+01 | | | |
| 7942957 | NM_007173 | PRSS23 | protease, serine, 23 | -0.021 | 0.986 | 9.10E-01 | 9.10E+01 | | | |
| 8015410 | --- | --- | --- | -0.021 | 0.986 | 8.45E-01 | 8.45E+01 | | | |
| 8110106 | NM_00113105 | HRH2 | histamine receptor H2 | -0.021 | 0.986 | 7.80E-01 | 7.80E+01 | | | |
| 8027908 | NM_024321 | RBM42 | RNA binding motif protein 42 | -0.021 | 0.986 | 8.70E-01 | 8.70E+01 | | | |
| 8180315 | --- | --- | --- | -0.021 | 0.986 | 8.56E-01 | 8.56E+01 | | | |
| 8079637 | NM_130384 | ATRIP | ATR interacting protein | -0.021 | 0.986 | 7.53E-01 | 7.53E+01 | | | |
| 8126524 | NM_199184 | C6orf108 | chromosome 6 open reading frame 1 | -0.021 | 0.986 | 8.55E-01 | 8.55E+01 | | | |
| 7977507 | NR_002312 | RPPH1 | ribonuclease P RNA component H1 | -0.021 | 0.986 | 9.63E-01 | 9.63E+01 | | | |
| 8174141 | NM_00101297 | BEX5 | brain expressed, X-linked 5 | -0.021 | 0.986 | 9.32E-01 | 9.32E+01 | | | |
| 7978795 | NM_080746 | RPL10L | ribosomal protein L10-like | -0.021 | 0.986 | 7.57E-01 | 7.57E+01 | | | |
| 8058388 | --- | --- | --- | -0.021 | 0.985 | 9.47E-01 | 9.47E+01 | | | |
| 7921179 | NM_198236 | ARHGEF11 | Rho guanine nucleotide exchange fa | -0.021 | 0.985 | 7.35E-01 | 7.35E+01 | | | |
| 7895411 | --- | --- | --- | -0.021 | 0.985 | 9.81E-01 | 9.81E+01 | | | |
| 8042917 | NM_001381 | DOK1 | docking protein 1, 62kDa (downstrea | -0.021 | 0.985 | 7.98E-01 | 7.98E+01 | | | |
| 7893637 | --- | --- | --- | -0.021 | 0.985 | 9.73E-01 | 9.73E+01 | | | |
| 8082517 | NR_026991 | C3orf47 | chromosome 3 open reading frame 4 | -0.021 | 0.985 | 8.81E-01 | 8.81E+01 | | | |
| 7980773 | NM_00108041 | CCDC88C | coiled-coil domain containing 88C | -0.021 | 0.985 | 8.68E-01 | 8.68E+01 | | | |
| 7893567 | --- | --- | --- | -0.021 | 0.985 | 9.45E-01 | 9.45E+01 | | | |
| 7895036 | --- | --- | --- | -0.021 | 0.985 | 9.72E-01 | 9.72E+01 | | | |
| 8143850 | NM_004935 | CDK5 | cyclin-dependent kinase 5 | -0.021 | 0.985 | 8.99E-01 | 8.99E+01 | | | |
| 7896157 | --- | --- | --- | -0.021 | 0.985 | 9.81E-01 | 9.81E+01 | | | |
| 7912254 | NR_027045 | C1orf200 | chromosome 1 open reading frame 2 | -0.021 | 0.985 | 7.75E-01 | 7.75E+01 | | | |
| 8136591 | NM_052853 | ADCK2 | aarF domain containing kinase 2 | -0.021 | 0.985 | 8.70E-01 | 8.70E+01 | | | |
| 7959212 | NM_00108053 | UNC119B | unc-119 homolog B (C. elegans) | -0.021 | 0.985 | 8.77E-01 | 8.77E+01 | | | |
| 7951781 | BC071695 | C11orf71 | chromosome 11 open reading frame | -0.021 | 0.985 | 7.66E-01 | 7.66E+01 | | | |
| 8125748 | NR_001444 | LYPLA2P1 | lysophospholipase II pseudogene 1 | -0.021 | 0.985 | 8.70E-01 | 8.70E+01 | | | |
| 7896191 | --- | --- | --- | -0.021 | 0.985 | 9.39E-01 | 9.39E+01 | | | |
| 8123951 | NM_00114394 | C6orf105 | chromosome 6 open reading frame 1 | -0.022 | 0.985 | 7.70E-01 | 7.70E+01 | | | |
| 8147371 | --- | --- | --- | -0.022 | 0.985 | 8.35E-01 | 8.35E+01 | | | |
| 8059864 | NM_001485 | GBX2 | gastrulation brain homeobox 2 | -0.022 | 0.985 | 7.44E-01 | 7.44E+01 | | | |
| 8137874 | NM_032415 | CARD11 | caspase recruitment domain family, | -0.022 | 0.985 | 8.78E-01 | 8.78E+01 | | | |
| 8082163 | --- | --- | --- | -0.022 | 0.985 | 8.34E-01 | 8.34E+01 | | | |
| 8068938 | NM_003683 | RRP1 | ribosomal RNA processing 1 homolo | -0.022 | 0.985 | 7.59E-01 | 7.59E+01 | | | |
| 8037537 | NM_000400 | ERCC2 | excision repair cross-complementing | -0.022 | 0.985 | 8.11E-01 | 8.11E+01 | | | |
| 7893928 | --- | --- | --- | -0.022 | 0.985 | 8.68E-01 | 8.68E+01 | | | |
| 8024001 | --- | --- | --- | -0.022 | 0.985 | 8.34E-01 | 8.34E+01 | | | |
| 7970084 | NM_018210 | CARKD | carbohydrate kinase domain contain | -0.022 | 0.985 | 7.91E-01 | 7.91E+01 | | | |
| 7986049 | NM_022769 | CRTC3 | CREB regulated transcription coactiv | -0.022 | 0.985 | 9.32E-01 | 9.32E+01 | | | |
| 8037991 | NM_000234 | LIG1 | ligase I, DNA, ATP-dependent | -0.022 | 0.985 | 7.40E-01 | 7.40E+01 | | | |
| 8123080 | NM_00100999 | SYTL3 | synaptotagmin-like 3 | -0.022 | 0.985 | 9.31E-01 | 9.31E+01 | | | |
| 7948092 | NR_003034 | LOC441601 | septin 7 pseudogene | -0.022 | 0.985 | 8.95E-01 | 8.95E+01 | | | |
| 8101324 | NM_031370 | HNRNPD | heterogeneous nuclear ribonucleopr | -0.022 | 0.985 | 8.77E-01 | 8.77E+01 | | | |
| 8021047 | NM_015559 | SETBP1 | SET binding protein 1 | -0.022 | 0.985 | 7.36E-01 | 7.36E+01 | | | |
| 8053158 | NM_006302 | MOGS | mannosyl-oligosaccharide glucosidas | -0.022 | 0.985 | 7.98E-01 | 7.98E+01 | | | |
| 7973306 | NM_022060 | ABHD4 | abhydrolase domain containing 4 | -0.022 | 0.985 | 8.00E-01 | 8.00E+01 | | | |
| 8123929 | NM_207582 | HERV-FRD | HERV-FRD provirus ancestral Env pol | -0.022 | 0.985 | 7.85E-01 | 7.85E+01 | | | |
| 7898300 | NM_004070 | CLCNKA | chloride channel Ka | -0.022 | 0.985 | 7.96E-01 | 7.96E+01 | | | |
| 8013035 | NM_020787 | ZNF624 | zinc finger protein 624 | -0.022 | 0.985 | 8.10E-01 | 8.10E+01 | | | |
| 8158112 | NM_001261 | CDK9 | cyclin-dependent kinase 9 | -0.022 | 0.985 | 7.50E-01 | 7.50E+01 | | | |
| 8035801 | --- | --- | --- | -0.022 | 0.985 | 8.93E-01 | 8.93E+01 | | | |
| 7933186 | NM_006973 | ZNF32 | zinc finger protein 32 | -0.022 | 0.985 | 9.60E-01 | 9.60E+01 | | | |
| 8067495 | NM_031215 | CABLES2 | Cdk5 and Abl enzyme substrate 2 | -0.022 | 0.985 | 7.74E-01 | 7.74E+01 | | | |
| 8025551 | NM_018381 | C19orf66 | chromosome 19 open reading frame | -0.022 | 0.985 | 8.76E-01 | 8.76E+01 | | | |
| 8058914 | NM_001087 | AAMP | angio-associated, migratory cell prot | -0.022 | 0.985 | 7.64E-01 | 7.64E+01 | | | |
| 7927801 | NR_001446 | ANXA2P3 | annexin A2 pseudogene 3 | -0.022 | 0.985 | 7.78E-01 | 7.78E+01 | | | |
| 7967810 | NM_005895 | GOLGA3 | golgi autoantigen, golgin subfamily a | -0.022 | 0.985 | 7.47E-01 | 7.47E+01 | | | |
| 8148597 | NM_173832 | ZFP41 | zinc finger protein 41 homolog (mou | -0.022 | 0.985 | 7.51E-01 | 7.51E+01 | | | |
| 8066574 | NM_172006 | WFDC10B | WAP four-disulfide core domain 10B | -0.022 | 0.985 | 8.16E-01 | 8.16E+01 | | | |
| 7914557 | NM_030786 | SYNC | syncoilin, intermediate filament prot | -0.022 | 0.985 | 8.38E-01 | 8.38E+01 | | | |
| 7899134 | NM_022778 | CCDC21 | coiled-coil domain containing 21 | -0.022 | 0.985 | 8.46E-01 | 8.46E+01 | | | |
| 8033043 | NM_000150 | FUT6 | fucosyltransferase 6 (alpha (1,3) fuco | -0.022 | 0.985 | 7.03E-01 | 7.03E+01 | | | |
| 8053901 | NM_017849 | TMEM127 | transmembrane protein 127 | -0.022 | 0.985 | 7.77E-01 | 7.77E+01 | | | |
| 7915466 | --- | --- | --- | -0.022 | 0.985 | 9.01E-01 | 9.01E+01 | | | |
| 8053036 | NM_016058 | TPRKB | TP53RK binding protein | -0.022 | 0.985 | 9.70E-01 | 9.70E+01 | | | |

| | | | | | | | | | | |
|---|---|---|---|---|---|---|---|---|---|---|
| 8164131 | NM_173690 | SCAI | suppressor of cancer cell invasion | -0.022 | 0.985 | 9.21E-01 | 9.21E+01 | | | |
| 8029423 | NM_181756 | ZNF233 | zinc finger protein 233 | -0.022 | 0.985 | 7.62E-01 | 7.62E+01 | | | |
| 8164100 | NM_007209 | RPL35 | ribosomal protein L35 | -0.022 | 0.985 | 9.24E-01 | 9.24E+01 | | | |
| 7981490 | NM_207379 | TMEM179 | transmembrane protein 179 | -0.022 | 0.985 | 7.40E-01 | 7.40E+01 | | | |
| 8029701 | NM_00108040 | FLJ40125 | protein phosphatase 1B-like | -0.023 | 0.985 | 8.71E-01 | 8.71E+01 | | | |
| 8124551 | NR_002936 | LOC222699 | transducer of ERBB2, 2 pseudogene | -0.023 | 0.985 | 7.79E-01 | 7.79E+01 | | | |
| 7946812 | NM_001017 | RPS13 | ribosomal protein S13 | -0.023 | 0.984 | 8.90E-01 | 8.90E+01 | | | |
| 7896121 | --- | --- | --- | -0.023 | 0.984 | 7.70E-01 | 7.70E+01 | | | |
| 8108199 | NM_145282 | LOC153328 | mitochondrial carrier protein-like | -0.023 | 0.984 | 6.71E-01 | 6.71E+01 | | | |
| 7893138 | --- | --- | --- | -0.023 | 0.984 | 9.67E-01 | 9.67E+01 | | | |
| 8033159 | NM_004158 | PSPN | persephin | -0.023 | 0.984 | 7.41E-01 | 7.41E+01 | | | |
| 7895598 | --- | --- | --- | -0.023 | 0.984 | 8.61E-01 | 8.61E+01 | | | |
| 7894876 | --- | --- | --- | -0.023 | 0.984 | 9.88E-01 | 9.88E+01 | | | |
| 8101622 | NM_138501 | TECR | trans-2,3-enoyl-CoA reductase | -0.023 | 0.984 | 8.85E-01 | 8.85E+01 | | | |
| 8095451 | NM_214711 | C4orf40 | chromosome 4 open reading frame 4 | -0.023 | 0.984 | 7.14E-01 | 7.14E+01 | | | |
| 7933707 | NM_032997 | ZWINT | ZW10 interactor | -0.023 | 0.984 | 7.85E-01 | 7.85E+01 | | | |
| 8010915 | NM_182705 | FAM101B | family with sequence similarity 101, | -0.023 | 0.984 | 7.78E-01 | 7.78E+01 | | | |
| 7917338 | NM_003921 | BCL10 | B-cell CLL/lymphoma 10 | -0.023 | 0.984 | 9.32E-01 | 9.32E+01 | | | |
| 7921538 | NM_052868 | IGSF8 | immunoglobulin superfamily, memb | -0.023 | 0.984 | 6.94E-01 | 6.94E+01 | | | |
| 8048699 | --- | --- | --- | -0.023 | 0.984 | 7.62E-01 | 7.62E+01 | | | |
| 8013776 | NM_018182 | C17orf63 | chromosome 17 open reading frame | -0.023 | 0.984 | 7.16E-01 | 7.16E+01 | | | |
| 7982738 | NM_014952 | BAHD1 | bromo adjacent homology domain c | -0.023 | 0.984 | 7.43E-01 | 7.43E+01 | | | |
| 8131970 | NM_001989 | EVX1 | even-skipped homeobox 1 | -0.023 | 0.984 | 7.49E-01 | 7.49E+01 | | | |
| 8085429 | AY358240 | UNQ6487 | LMNE6487 | -0.023 | 0.984 | 8.52E-01 | 8.52E+01 | | | |
| 7895203 | --- | --- | --- | -0.023 | 0.984 | 9.81E-01 | 9.81E+01 | | | |
| 7911486 | NM_017871 | CPSF3L | cleavage and polyadenylation specifi | -0.023 | 0.984 | 7.31E-01 | 7.31E+01 | | | |
| 8091444 | --- | --- | --- | -0.023 | 0.984 | 8.40E-01 | 8.40E+01 | | | |
| 7940160 | NM_015177 | DTX4 | deltex homolog 4 (Drosophila) | -0.023 | 0.984 | 7.55E-01 | 7.55E+01 | | | |
| 8037962 | NM_019855 | CABP5 | calcium binding protein 5 | -0.023 | 0.984 | 8.42E-01 | 8.42E+01 | | | |
| 7914530 | NM_00114388 | BSDC1 | BSD domain containing 1 | -0.023 | 0.984 | 7.71E-01 | 7.71E+01 | | | |
| 8065433 | NM_020531 | C20orf3 | chromosome 20 open reading frame | -0.023 | 0.984 | 9.06E-01 | 9.06E+01 | | | |
| 8159790 | NR_024077 | WASH2P | WAS protein family homolog 2 pseu | -0.023 | 0.984 | 8.18E-01 | 8.18E+01 | | | |
| 8096176 | NM_080683 | PTPN13 | protein tyrosine phosphatase, non-r | -0.023 | 0.984 | 7.59E-01 | 7.59E+01 | | | |
| 7989277 | NM_004998 | MYO1E | myosin IE | -0.023 | 0.984 | 8.51E-01 | 8.51E+01 | | | |
| 7904226 | NM_018420 | SLC22A15 | solute carrier family 22, member 15 | -0.023 | 0.984 | 6.77E-01 | 6.77E+01 | | | |
| 8063893 | NM_007002 | ADRM1 | adhesion regulating molecule 1 | -0.023 | 0.984 | 7.97E-01 | 7.97E+01 | | | |
| 8063478 | NM_080821 | C20orf108 | chromosome 20 open reading frame | -0.023 | 0.984 | 7.78E-01 | 7.78E+01 | | | |
| 8003171 | NM_021149 | COTL1 | coactosin-like 1 (Dictyostelium) | -0.023 | 0.984 | 8.44E-01 | 8.44E+01 | | | |
| 7894872 | --- | --- | --- | -0.023 | 0.984 | 7.83E-01 | 7.83E+01 | | | |
| 8137558 | NM_053043 | RBM33 | RNA binding motif protein 33 | -0.023 | 0.984 | 9.09E-01 | 9.09E+01 | | | |
| 7938263 | NM_003754 | EIF3F | eukaryotic translation initiation facto | -0.023 | 0.984 | 9.21E-01 | 9.21E+01 | | | |
| 7982688 | NM_020168 | PAK6 | p21 protein (Cdc42/Rac)-activated ki | -0.024 | 0.984 | 7.11E-01 | 7.11E+01 | | | |
| 8027390 | NM_006627 | POP4 | processing of precursor 4, ribonucle | -0.024 | 0.984 | 9.01E-01 | 9.01E+01 | | | |
| 8034075 | NM_001800 | CDKN2D | cyclin-dependent kinase inhibitor 2D | -0.024 | 0.984 | 7.21E-01 | 7.21E+01 | | | |
| 7892693 | --- | --- | --- | -0.024 | 0.984 | 8.31E-01 | 8.31E+01 | | | |
| 8000834 | NM_015092 | SMG1 | SMG1 homolog, phosphatidylinosito | -0.024 | 0.984 | 7.97E-01 | 7.97E+01 | | | |
| 8153457 | AY358690 | EEF1D | eukaryotic translation elongation fac | -0.024 | 0.984 | 8.86E-01 | 8.86E+01 | | | |
| 8055183 | NM_017751 | SMPD4 | sphingomyelin phosphodiesterase 4, | -0.024 | 0.984 | 8.89E-01 | 8.89E+01 | | | |
| 8066776 | NM_033550 | TP53RK | TP53 regulating kinase | -0.024 | 0.984 | 7.93E-01 | 7.93E+01 | | | |
| 7898663 | NM_032409 | PINK1 | PTEN induced putative kinase 1 | -0.024 | 0.984 | 8.70E-01 | 8.70E+01 | | | |
| 8044649 | NR_024077 | WASH2P | WAS protein family homolog 2 pseu | -0.024 | 0.984 | 8.25E-01 | 8.25E+01 | | | |
| 8096777 | --- | --- | --- | -0.024 | 0.984 | 9.66E-01 | 9.66E+01 | | | |
| 7912374 | NM_003132 | SRM | spermidine synthase | -0.024 | 0.984 | 8.28E-01 | 8.28E+01 | | | |
| 7895845 | --- | --- | --- | -0.024 | 0.984 | 9.68E-01 | 9.68E+01 | | | |
| 8059026 | --- | --- | --- | -0.024 | 0.984 | 8.30E-01 | 8.30E+01 | | | |
| 8003484 | NM_004913 | C16orf7 | chromosome 16 open reading frame | -0.024 | 0.984 | 7.45E-01 | 7.45E+01 | | | |
| 7934114 | NM_173555 | TYSND1 | trypsin domain containing 1 | -0.024 | 0.984 | 7.12E-01 | 7.12E+01 | | | |
| 8134460 | AK054626 | FLJ30064 | hypothetical protein LOC644975 | -0.024 | 0.984 | 7.90E-01 | 7.90E+01 | | | |
| 8118734 | NM_002224 | ITPR3 | inositol 1,4,5-triphosphate receptor, | -0.024 | 0.984 | 7.72E-01 | 7.72E+01 | | | |
| 8024255 | NR_024247 | MUM1 | melanoma associated antigen (muta | -0.024 | 0.984 | 7.41E-01 | 7.41E+01 | | | |
| 8055085 | --- | --- | --- | -0.024 | 0.984 | 9.38E-01 | 9.38E+01 | | | |
| 8119169 | NM_017772 | TBC1D22B | TBC1 domain family, member 22B | -0.024 | 0.984 | 8.62E-01 | 8.62E+01 | | | |
| 8025968 | NM_021915 | ZNF69 | zinc finger protein 69 | -0.024 | 0.983 | 8.17E-01 | 8.17E+01 | | | |
| 8065738 | NM_000687 | AHCY | adenosylhomocysteinase | -0.024 | 0.983 | 7.63E-01 | 7.63E+01 | | | |
| 7976667 | --- | --- | --- | -0.024 | 0.983 | 8.58E-01 | 8.58E+01 | | | |
| 7894474 | --- | --- | --- | -0.024 | 0.983 | 7.97E-01 | 7.97E+01 | | | |
| 7951977 | NM_022003 | FXYD6 | FXYD domain containing ion transpo | -0.024 | 0.983 | 7.15E-01 | 7.15E+01 | | | |
| 8092065 | --- | --- | --- | -0.024 | 0.983 | 8.04E-01 | 8.04E+01 | | | |
| 7901611 | ENST00000361 | C1orf191 | chromosome 1 open reading frame 1 | -0.024 | 0.983 | 7.74E-01 | 7.74E+01 | | | |
| 7893341 | --- | --- | --- | -0.024 | 0.983 | 8.82E-01 | 8.82E+01 | | | |
| 8167165 | NM_001654 | ARAF | v-raf murine sarcoma 3611 viral onc | -0.024 | 0.983 | 8.23E-01 | 8.23E+01 | | | |
| 8012150 | NM_004489 | GPS2 | G protein pathway suppressor 2 | -0.024 | 0.983 | 8.39E-01 | 8.39E+01 | | | |
| 7928630 | 0 | 0 | 0 | -0.024 | 0.983 | 9.68E-01 | 9.68E+01 | | | |
| 8124524 | NM_003510 | HIST1H2AK | histone cluster 1, H2ak | -0.024 | 0.983 | 8.87E-01 | 8.87E+01 | | | |
| 7896620 | --- | --- | --- | -0.024 | 0.983 | 7.82E-01 | 7.82E+01 | | | |
| 7893391 | --- | --- | --- | -0.024 | 0.983 | 8.17E-01 | 8.17E+01 | | | |
| 8117522 | NM_013375 | ABT1 | activator of basal transcription 1 | -0.024 | 0.983 | 7.89E-01 | 7.89E+01 | | | |

| | | | | | | | | | | |
|---|---|---|---|---|---|---|---|---|---|---|
| 8045349 | NM_002410 | MGAT5 | mannosyl (alpha-1,6-)-glycoprotein b | -0.024 | 0.983 | 9.25E-01 | 9.25E+01 | | | |
| 8059576 | --- | --- | --- | -0.024 | 0.983 | 6.62E-01 | 6.62E+01 | | | |
| 8144361 | NM_024596 | MCPH1 | microcephalin 1 | -0.024 | 0.983 | 8.86E-01 | 8.86E+01 | | | |
| 8041172 | NM_199280 | FAM179A | family with sequence similarity 179, | -0.024 | 0.983 | 7.89E-01 | 7.89E+01 | | | |
| 8025321 | NR_002931 | CLEC4GP1 | C-type lectin domain family 4, memb | -0.024 | 0.983 | 7.10E-01 | 7.10E+01 | | | |
| 8039923 | NM_017900 | AURKAIP1 | aurora kinase A interacting protein 1 | -0.024 | 0.983 | 7.81E-01 | 7.81E+01 | | | |
| 8164362 | NM_025072 | PTGES2 | prostaglandin E synthase 2 | -0.024 | 0.983 | 6.44E-01 | 6.44E+01 | | | |
| 8015914 | NM_005474 | HDAC5 | histone deacetylase 5 | -0.024 | 0.983 | 6.75E-01 | 6.75E+01 | | | |
| 8030925 | NM_00114543 | LOC400713 | zinc finger protein LOC400713 | -0.024 | 0.983 | 9.09E-01 | 9.09E+01 | | | |
| 8073875 | NM_018006 | TRMU | tRNA 5-methylaminomethyl-2-thiour | -0.025 | 0.983 | 8.82E-01 | 8.82E+01 | | | |
| 7986541 | NR_003659 | WASH3P | WAS protein family homolog 3 pseud | -0.025 | 0.983 | 8.02E-01 | 8.02E+01 | | | |
| 8024273 | NM_024407 | NDUFS7 | NADH dehydrogenase (ubiquinone) F | -0.025 | 0.983 | 7.74E-01 | 7.74E+01 | | | |
| 8079217 | NM_003420 | ZNF35 | zinc finger protein 35 | -0.025 | 0.983 | 8.08E-01 | 8.08E+01 | | | |
| 7894799 | --- | --- | --- | -0.025 | 0.983 | 6.60E-01 | 6.60E+01 | | | |
| 8160935 | NM_004629 | FANCG | Fanconi anemia, complementation g | -0.025 | 0.983 | 7.44E-01 | 7.44E+01 | | | |
| 7892886 | --- | --- | --- | -0.025 | 0.983 | 9.37E-01 | 9.37E+01 | | | |
| 7929750 | NM_020354 | ENTPD7 | ectonucleoside triphosphate diphosp | -0.025 | 0.983 | 6.91E-01 | 6.91E+01 | | | |
| 7930181 | NM_020682 | AS3MT | arsenic (+3 oxidation state) methyltr | -0.025 | 0.983 | 7.69E-01 | 7.69E+01 | | | |
| 8174684 | NM_017544 | NKRF | NFKB repressing factor | -0.025 | 0.983 | 9.19E-01 | 9.19E+01 | | | |
| 8161362 | NR_003670 | KGFLP2 | keratinocyte growth factor-like prote | -0.025 | 0.983 | 7.31E-01 | 7.31E+01 | | | |
| 7997933 | NM_033251 | RPL13 | ribosomal protein L13 | -0.025 | 0.983 | 7.80E-01 | 7.80E+01 | | | |
| 7937782 | NM_139022 | TSPAN32 | tetraspanin 32 | -0.025 | 0.983 | 7.03E-01 | 7.03E+01 | | | |
| 7996571 | NM_024519 | FAM65A | family with sequence similarity 65, m | -0.025 | 0.983 | 7.83E-01 | 7.83E+01 | | | |
| 8082504 | NM_00100610 | C3orf37 | chromosome 3 open reading frame 3 | -0.025 | 0.983 | 9.07E-01 | 9.07E+01 | | | |
| 8060977 | NM_00100960 | C20orf94 | chromosome 20 open reading frame | -0.025 | 0.983 | 9.26E-01 | 9.26E+01 | | | |
| 7963670 | NM_006301 | MAP3K12 | mitogen-activated protein kinase kin | -0.025 | 0.983 | 7.83E-01 | 7.83E+01 | | | |
| 7948900 | NR_002561 | SNORD30 | small nucleolar RNA, C/D box 30 | -0.025 | 0.983 | 9.81E-01 | 9.81E+01 | | | |
| 7975926 | NM_033426 | KIAA1737 | KIAA1737 | -0.025 | 0.983 | 6.80E-01 | 6.80E+01 | | | |
| 7895606 | --- | --- | --- | -0.025 | 0.983 | 8.77E-01 | 8.77E+01 | | | |
| 8035958 | NM_032816 | CCDC123 | coiled-coil domain containing 123 | -0.025 | 0.983 | 7.54E-01 | 7.54E+01 | | | |
| 8150842 | --- | --- | --- | -0.025 | 0.983 | 7.40E-01 | 7.40E+01 | | | |
| 7952488 | --- | --- | --- | -0.025 | 0.983 | 8.02E-01 | 8.02E+01 | | | |
| 8171203 | --- | --- | --- | -0.025 | 0.983 | 8.79E-01 | 8.79E+01 | | | |
| 8090938 | NM_173543 | DZIP1L | DAZ interacting protein 1-like | -0.025 | 0.983 | 6.97E-01 | 6.97E+01 | | | |
| 7927643 | --- | --- | --- | -0.025 | 0.983 | 8.63E-01 | 8.63E+01 | | | |
| 8174731 | NM_006978 | RNF113A | ring finger protein 113A | -0.025 | 0.983 | 8.91E-01 | 8.91E+01 | | | |
| 7951596 | NM_006235 | POU2AF1 | POU class 2 associating factor 1 | -0.025 | 0.983 | 7.19E-01 | 7.19E+01 | | | |
| 7896448 | --- | --- | --- | -0.025 | 0.983 | 7.07E-01 | 7.07E+01 | | | |
| 8028104 | NM_014266 | HCST | hematopoietic cell signal transducer | -0.025 | 0.983 | 9.32E-01 | 9.32E+01 | | | |
| 7894256 | --- | --- | --- | -0.025 | 0.983 | 7.89E-01 | 7.89E+01 | | | |
| 8022145 | NM_173464 | L3MBTL4 | l(3)mbt-like 4 (Drosophila) | -0.025 | 0.983 | 8.17E-01 | 8.17E+01 | | | |
| 7935421 | NM_012083 | FRAT2 | frequently rearranged in advanced T | -0.025 | 0.983 | 7.99E-01 | 7.99E+01 | | | |
| 8020162 | NM_054028 | AMAC1L2 | acyl-malonyl condensing enzyme 1-li | -0.025 | 0.983 | 6.92E-01 | 6.92E+01 | | | |
| 7945652 | NM_00101270 | KRTAP5-3 | keratin associated protein 5-3 | -0.025 | 0.983 | 7.28E-01 | 7.28E+01 | | | |
| 7946380 | NM_014818 | TRIM66 | tripartite motif-containing 66 | -0.025 | 0.983 | 6.84E-01 | 6.84E+01 | | | |
| 7968329 | --- | --- | --- | -0.025 | 0.983 | 7.60E-01 | 7.60E+01 | | | |
| 7991453 | NM_207446 | FAM174B | family with sequence similarity 174, | -0.025 | 0.983 | 6.55E-01 | 6.55E+01 | | | |
| 8150830 | NM_006330 | LYPLA1 | lysophospholipase I | -0.025 | 0.983 | 9.39E-01 | 9.39E+01 | | | |
| 7953993 | NM_030766 | BCL2L14 | BCL2-like 14 (apoptosis facilitator) | -0.025 | 0.983 | 7.88E-01 | 7.88E+01 | | | |
| 8071691 | NM_004327 | BCR | breakpoint cluster region | -0.025 | 0.983 | 7.82E-01 | 7.82E+01 | | | |
| 8081028 | --- | --- | --- | -0.025 | 0.983 | 7.65E-01 | 7.65E+01 | | | |
| 8079060 | NM_004624 | VIPR1 | vasoactive intestinal peptide recepto | -0.025 | 0.983 | 6.84E-01 | 6.84E+01 | | | |
| 7965471 | NM_003348 | UBE2N | ubiquitin-conjugating enzyme E2N (U | -0.025 | 0.983 | 9.18E-01 | 9.18E+01 | | | |
| 8108603 | NM_012208 | HARS2 | histidyl-tRNA synthetase 2, mitochor | -0.025 | 0.983 | 8.90E-01 | 8.90E+01 | | | |
| 7975705 | NM_194279 | ISCA2 | iron-sulfur cluster assembly 2 homol | -0.025 | 0.982 | 7.88E-01 | 7.88E+01 | | | |
| 7925589 | NM_022743 | SMYD3 | SET and MYND domain containing 3 | -0.026 | 0.982 | 9.10E-01 | 9.10E+01 | | | |
| 8027819 | NM_021175 | HAMP | hepcidin antimicrobial peptide | -0.026 | 0.982 | 7.37E-01 | 7.37E+01 | | | |
| 8099121 | NM_016930 | STX18 | syntaxin 18 | -0.026 | 0.982 | 9.00E-01 | 9.00E+01 | | | |
| 8042211 | NM_006577 | B3GNT2 | UDP-GlcNAc:betaGal beta-1,3-N-ace | -0.026 | 0.982 | 9.24E-01 | 9.24E+01 | | | |
| 8016806 | --- | --- | --- | -0.026 | 0.982 | 8.93E-01 | 8.93E+01 | | | |
| 8128371 | NM_032511 | C6orf168 | chromosome 6 open reading frame 1 | -0.026 | 0.982 | 6.90E-01 | 6.90E+01 | | | |
| 8139500 | NM_022748 | TNS3 | tensin 3 | -0.026 | 0.982 | 6.63E-01 | 6.63E+01 | | | |
| 7914921 | --- | --- | --- | -0.026 | 0.982 | 7.03E-01 | 7.03E+01 | | | |
| 8054762 | NR_024528 | RPL23AP7 | ribosomal protein L23a pseudogene | -0.026 | 0.982 | 8.39E-01 | 8.39E+01 | | | |
| 7980454 | NM_006020 | ALKBH1 | alkB, alkylation repair homolog 1 (E. | -0.026 | 0.982 | 8.57E-01 | 8.57E+01 | | | |
| 8167150 | --- | --- | --- | -0.026 | 0.982 | 8.23E-01 | 8.23E+01 | | | |
| 8032057 | NM_005035 | POLRMT | polymerase (RNA) mitochondrial (DN | -0.026 | 0.982 | 7.69E-01 | 7.69E+01 | | | |
| 8027521 | NM_018025 | GPATCH1 | G patch domain containing 1 | -0.026 | 0.982 | 8.03E-01 | 8.03E+01 | | | |
| 8031387 | NM_004829 | NCR1 | natural cytotoxicity triggering recept | -0.026 | 0.982 | 7.77E-01 | 7.77E+01 | | | |
| 7966035 | NM_006825 | CKAP4 | cytoskeleton-associated protein 4 | -0.026 | 0.982 | 7.02E-01 | 7.02E+01 | | | |
| 8130553 | AK130765 | FLJ27255 | hypothetical LOC401281 | -0.026 | 0.982 | 7.71E-01 | 7.71E+01 | | | |
| 8109999 | NM_00103171 | ERGIC1 | endoplasmic reticulum-golgi interme | -0.026 | 0.982 | 8.71E-01 | 8.71E+01 | | | |
| 8143070 | NM_014149 | WDR91 | WD repeat domain 91 | -0.026 | 0.982 | 6.84E-01 | 6.84E+01 | | | |
| 8024882 | --- | --- | --- | -0.026 | 0.982 | 7.89E-01 | 7.89E+01 | | | |
| 8004464 | NM_172089 | FSF12-TNFSF | TNFSF12-TNFSF13 readthrough trans | -0.026 | 0.982 | 7.65E-01 | 7.65E+01 | | | |
| 8140291 | NM_003602 | FKBP6 | FK506 binding protein 6, 36kDa | -0.026 | 0.982 | 8.90E-01 | 8.90E+01 | | | |
| 8152819 | --- | --- | --- | -0.026 | 0.982 | 8.85E-01 | 8.85E+01 | | | |

| | | | | | | | | | | |
|---|---|---|---|---|---|---|---|---|---|---|
| 8054477 | CR590757 | RPL22P15 | ribosomal protein L22 pseudogene 1 | -0.026 | 0.982 | 7.30E-01 | 7.30E+01 | | | |
| 7958692 | NM_00108253 | TCTN1 | tectonic family member 1 | -0.026 | 0.982 | 7.33E-01 | 7.33E+01 | | | |
| 8162586 | NM_007001 | SLC35D2 | solute carrier family 35, member D2 | -0.026 | 0.982 | 8.32E-01 | 8.32E+01 | | | |
| 8030391 | --- | --- | --- | -0.026 | 0.982 | 8.56E-01 | 8.56E+01 | | | |
| 7893559 | --- | --- | --- | -0.026 | 0.982 | 9.51E-01 | 9.51E+01 | | | |
| 8038547 | NR_002798 | NAPSB | napsin B aspartic peptidase pseudog | -0.026 | 0.982 | 7.82E-01 | 7.82E+01 | | | |
| 8084035 | NM_016331 | ZNF639 | zinc finger protein 639 | -0.026 | 0.982 | 9.28E-01 | 9.28E+01 | | | |
| 8068902 | NM_015056 | RRP1B | ribosomal RNA processing 1 homolog | -0.026 | 0.982 | 8.54E-01 | 8.54E+01 | | | |
| 7950409 | NM_005472 | KCNE3 | potassium voltage-gated channel, Isk | -0.026 | 0.982 | 7.11E-01 | 7.11E+01 | | | |
| 7893094 | --- | --- | --- | -0.026 | 0.982 | 9.40E-01 | 9.40E+01 | | | |
| 7963134 | NM_00103780 | NCKAP5L | NCK-associated protein 5-like | -0.026 | 0.982 | 7.56E-01 | 7.56E+01 | | | |
| 7896087 | --- | --- | --- | -0.026 | 0.982 | 7.79E-01 | 7.79E+01 | | | |
| 8006779 | NM_020876 | ARHGAP23 | Rho GTPase activating protein 23 | -0.026 | 0.982 | 7.91E-01 | 7.91E+01 | | | |
| 8163729 | --- | --- | --- | -0.026 | 0.982 | 9.13E-01 | 9.13E+01 | | | |
| 8039413 | NM_014931 | SAPS1 | SAPS domain family, member 1 | -0.026 | 0.982 | 7.14E-01 | 7.14E+01 | | | |
| 7966259 | NM_016433 | GLTP | glycolipid transfer protein | -0.026 | 0.982 | 8.69E-01 | 8.69E+01 | | | |
| 7894115 | --- | --- | --- | -0.026 | 0.982 | 9.37E-01 | 9.37E+01 | | | |
| 8082066 | NM_014367 | FAM162A | family with sequence similarity 162, | -0.026 | 0.982 | 9.38E-01 | 9.38E+01 | | | |
| 8175647 | NM_031462 | CD99L2 | CD99 molecule-like 2 | -0.026 | 0.982 | 6.77E-01 | 6.77E+01 | | | |
| 8157153 | NM_007203 | ALM2-AKAP | PALM2-AKAP2 readthrough transcrip | -0.026 | 0.982 | 6.34E-01 | 6.34E+01 | | | |
| 7985099 | NM_003978 | PSTPIP1 | proline-serine-threonine phosphatas | -0.026 | 0.982 | 8.47E-01 | 8.47E+01 | | | |
| 7994386 | NM_007245 | ATXN2L | ataxin 2-like | -0.026 | 0.982 | 7.75E-01 | 7.75E+01 | | | |
| 7991017 | BC020234 | LOC283693 | actin, gamma pseudogene | -0.026 | 0.982 | 6.66E-01 | 6.66E+01 | | | |
| 8037123 | NM_002698 | POU2F2 | POU class 2 homeobox 2 | -0.026 | 0.982 | 7.36E-01 | 7.36E+01 | | | |
| 7895721 | --- | --- | --- | -0.026 | 0.982 | 8.85E-01 | 8.85E+01 | | | |
| 8161026 | NM_174923 | CCDC107 | coiled-coil domain containing 107 | -0.026 | 0.982 | 6.55E-01 | 6.55E+01 | | | |
| 8144685 | --- | --- | --- | -0.026 | 0.982 | 8.08E-01 | 8.08E+01 | | | |
| 8072133 | NR_026963 | LOC284900 | hypothetical LOC284900 | -0.026 | 0.982 | 7.20E-01 | 7.20E+01 | | | |
| 7969815 | NM_206808 | CLYBL | citrate lyase beta like | -0.026 | 0.982 | 7.97E-01 | 7.97E+01 | | | |
| 8079993 | NM_007022 | CYB561D2 | cytochrome b-561 domain containin | -0.026 | 0.982 | 7.27E-01 | 7.27E+01 | | | |
| 7893271 | --- | --- | --- | -0.026 | 0.982 | 9.65E-01 | 9.65E+01 | | | |
| 8001816 | ENST0000045 | FLJ27243 | FLJ27243 protein | -0.026 | 0.982 | 8.04E-01 | 8.04E+01 | | | |
| 7978956 | NM_024884 | L2HGDH | L-2-hydroxyglutarate dehydrogenase | -0.026 | 0.982 | 7.30E-01 | 7.30E+01 | | | |
| 7898655 | NM_001785 | CDA | cytidine deaminase | -0.027 | 0.982 | 7.31E-01 | 7.31E+01 | | | |
| 7981460 | NM_015316 | PPP1R13B | protein phosphatase 1, regulatory (i | -0.027 | 0.982 | 7.39E-01 | 7.39E+01 | | | |
| 7893142 | --- | --- | --- | -0.027 | 0.982 | 9.77E-01 | 9.77E+01 | | | |
| 7912361 | NM_006610 | MASP2 | mannan-binding lectin serine peptid | -0.027 | 0.982 | 7.78E-01 | 7.78E+01 | | | |
| 7910096 | --- | --- | --- | -0.027 | 0.982 | 8.45E-01 | 8.45E+01 | | | |
| 8147756 | NM_024812 | BAALC | brain and acute leukemia, cytoplasm | -0.027 | 0.982 | 7.27E-01 | 7.27E+01 | | | |
| 8058969 | --- | --- | --- | -0.027 | 0.982 | 8.98E-01 | 8.98E+01 | | | |
| 7978054 | --- | --- | --- | -0.027 | 0.982 | 8.54E-01 | 8.54E+01 | | | |
| 8107090 | --- | --- | --- | -0.027 | 0.982 | 8.77E-01 | 8.77E+01 | | | |
| 7932792 | --- | --- | --- | -0.027 | 0.982 | 7.94E-01 | 7.94E+01 | | | |
| 8133122 | NM_00102494 | ASL | argininosuccinate lyase | -0.027 | 0.982 | 7.25E-01 | 7.25E+01 | | | |
| 7893481 | --- | --- | --- | -0.027 | 0.982 | 9.18E-01 | 9.18E+01 | | | |
| 8023882 | NM_014643 | ZNF516 | zinc finger protein 516 | -0.027 | 0.982 | 6.35E-01 | 6.35E+01 | | | |
| 7894624 | --- | --- | --- | -0.027 | 0.982 | 8.34E-01 | 8.34E+01 | | | |
| 8056753 | NM_024770 | METTL8 | methyltransferase like 8 | -0.027 | 0.982 | 8.22E-01 | 8.22E+01 | | | |
| 8005687 | NR_026809 | FAM106A | family with sequence similarity 106, | -0.027 | 0.982 | 7.34E-01 | 7.34E+01 | | | |
| 8141094 | NM_002612 | PDK4 | pyruvate dehydrogenase kinase, isoz | -0.027 | 0.982 | 6.24E-01 | 6.24E+01 | | | |
| 7907079 | NM_052862 | RCSD1 | RCSD domain containing 1 | -0.027 | 0.982 | 7.73E-01 | 7.73E+01 | | | |
| 7897803 | NM_000302 | PLOD1 | procollagen-lysine 1, 2-oxoglutarate | -0.027 | 0.982 | 7.42E-01 | 7.42E+01 | | | |
| 8156506 | NM_152422 | PTPDC1 | protein tyrosine phosphatase domai | -0.027 | 0.982 | 6.92E-01 | 6.92E+01 | | | |
| 7900597 | BC001508 | C1orf50 | chromosome 1 open reading frame 5 | -0.027 | 0.982 | 9.10E-01 | 9.10E+01 | | | |
| 7974916 | --- | --- | --- | -0.027 | 0.982 | 8.51E-01 | 8.51E+01 | | | |
| 8167322 | NM_017883 | WDR13 | WD repeat domain 13 | -0.027 | 0.981 | 7.53E-01 | 7.53E+01 | | | |
| 7957611 | --- | --- | --- | -0.027 | 0.981 | 7.38E-01 | 7.38E+01 | | | |
| 8006590 | --- | --- | --- | -0.027 | 0.981 | 9.13E-01 | 9.13E+01 | | | |
| 7949377 | NM_001997 | FAU | Finkel-Biskis-Reilly murine sarcoma v | -0.027 | 0.981 | 7.86E-01 | 7.86E+01 | | | |
| 7954481 | NM_005086 | SSPN | sarcospan (Kras oncogene-associated | -0.027 | 0.981 | 6.65E-01 | 6.65E+01 | | | |
| 8073582 | BC039496 | LOC388906 | hypothetical protein LOC388906 | -0.027 | 0.981 | 6.44E-01 | 6.44E+01 | | | |
| 7896350 | --- | --- | --- | -0.027 | 0.981 | 9.20E-01 | 9.20E+01 | | | |
| 7987163 | ENST00000414 | FMN1 | formin 1 | -0.027 | 0.981 | 8.91E-01 | 8.91E+01 | | | |
| 7998083 | NR_003228 | AFG3L1 | AFG3 ATPase family gene 3-like 1 (S. | -0.027 | 0.981 | 6.88E-01 | 6.88E+01 | | | |
| 7896390 | --- | --- | --- | -0.027 | 0.981 | 8.49E-01 | 8.49E+01 | | | |
| 7932860 | --- | --- | --- | -0.027 | 0.981 | 8.23E-01 | 8.23E+01 | | | |
| 8133192 | NM_017994 | C7orf42 | chromosome 7 open reading frame 4 | -0.027 | 0.981 | 9.14E-01 | 9.14E+01 | | | |
| 7967230 | NM_019887 | DIABLO | diablo homolog (Drosophila) | -0.027 | 0.981 | 7.69E-01 | 7.69E+01 | | | |
| 8034565 | NM_001375 | DNASE2 | deoxyribonuclease II, lysosomal | -0.027 | 0.981 | 6.88E-01 | 6.88E+01 | | | |
| 7995352 | --- | --- | --- | -0.027 | 0.981 | 7.44E-01 | 7.44E+01 | | | |
| 8170602 | NM_007150 | ZNF185 | zinc finger protein 185 (LIM domain) | -0.027 | 0.981 | 6.70E-01 | 6.70E+01 | | | |
| 8138155 | --- | --- | --- | -0.027 | 0.981 | 8.21E-01 | 8.21E+01 | | | |
| 8092532 | --- | --- | --- | -0.027 | 0.981 | 8.41E-01 | 8.41E+01 | | | |
| 7892504 | --- | --- | --- | -0.027 | 0.981 | 7.90E-01 | 7.90E+01 | | | |
| 8140070 | NM_012453 | TBL2 | transducin (beta)-like 2 | -0.027 | 0.981 | 6.67E-01 | 6.67E+01 | | | |
| 8092358 | NM_015078 | MCF2L2 | MCF.2 cell line derived transforming | -0.027 | 0.981 | 5.95E-01 | 5.95E+01 | | | |
| 7903719 | NM_004037 | AMPD2 | adenosine monophosphate deamina | -0.027 | 0.981 | 7.56E-01 | 7.56E+01 | | | |

| | | | | | | | | | | |
|---|---|---|---|---|---|---|---|---|---|---|
| 8026724 | NM_023937 | MRPL34 | mitochondrial ribosomal protein L34 | -0.027 | 0.981 | 7.01E-01 | 7.01E+01 | | | |
| 8003322 | NM_000101 | CYBA | cytochrome b-245, alpha polypeptid | -0.028 | 0.981 | 7.64E-01 | 7.64E+01 | | | |
| 7905789 | NM_000565 | IL6R | interleukin 6 receptor | -0.028 | 0.981 | 8.03E-01 | 8.03E+01 | | | |
| 7895898 | --- | --- | --- | -0.028 | 0.981 | 9.06E-01 | 9.06E+01 | | | |
| 8115884 | --- | --- | --- | -0.028 | 0.981 | 8.47E-01 | 8.47E+01 | | | |
| 8171066 | NR_024540 | WASH5P | WAS protein family homolog 5 pseud | -0.028 | 0.981 | 8.12E-01 | 8.12E+01 | | | |
| 8020449 | --- | --- | --- | -0.028 | 0.981 | 7.37E-01 | 7.37E+01 | | | |
| 8141419 | --- | --- | --- | -0.028 | 0.981 | 6.78E-01 | 6.78E+01 | | | |
| 7982985 | NM_014994 | MAPKBP1 | mitogen-activated protein kinase bin | -0.028 | 0.981 | 5.96E-01 | 5.96E+01 | | | |
| 8035896 | NM_020856 | TSHZ3 | teashirt zinc finger homeobox 3 | -0.028 | 0.981 | 7.64E-01 | 7.64E+01 | | | |
| 7938519 | NM_032867 | MICALCL | MICAL C-terminal like | -0.028 | 0.981 | 6.92E-01 | 6.92E+01 | | | |
| 8147132 | NM_000067 | CA2 | carbonic anhydrase II | -0.028 | 0.981 | 7.96E-01 | 7.96E+01 | | | |
| 7998033 | NM_014972 | TCF25 | transcription factor 25 (basic helix-lo | -0.028 | 0.981 | 8.95E-01 | 8.95E+01 | | | |
| 8149358 | NR_003668 | DEFB109P1B | defensin, beta 109, pseudogene 1B | -0.028 | 0.981 | 8.88E-01 | 8.88E+01 | | | |
| 7925728 | NM_00100528( | OR6F1 | olfactory receptor, family 6, subfami | -0.028 | 0.981 | 7.18E-01 | 7.18E+01 | | | |
| 7988605 | NM_004236 | COPS2 | COP9 constitutive photomorphogeni | -0.028 | 0.981 | 9.64E-01 | 9.64E+01 | | | |
| 8040655 | NM_033505 | SELI | selenoprotein I | -0.028 | 0.981 | 8.77E-01 | 8.77E+01 | | | |
| 7909896 | --- | --- | --- | -0.028 | 0.981 | 6.28E-01 | 6.28E+01 | | | |
| 8041886 | AK026773 | LOC440863 | hypothetical gene supported by AK0 | -0.028 | 0.981 | 6.23E-01 | 6.23E+01 | | | |
| 8079311 | NM_015340 | LARS2 | leucyl-tRNA synthetase 2, mitochond | -0.028 | 0.981 | 8.94E-01 | 8.94E+01 | | | |
| 7954006 | --- | --- | --- | -0.028 | 0.981 | 7.47E-01 | 7.47E+01 | | | |
| 7991406 | NM_003981 | PRC1 | protein regulator of cytokinesis 1 | -0.028 | 0.981 | 8.17E-01 | 8.17E+01 | | | |
| 8073890 | NM_015124 | GRAMD4 | GRAM domain containing 4 | -0.028 | 0.981 | 7.75E-01 | 7.75E+01 | | | |
| 7892670 | --- | --- | --- | -0.028 | 0.981 | 9.37E-01 | 9.37E+01 | | | |
| 8131155 | NM_152558 | IQCE | IQ motif containing E | -0.028 | 0.981 | 6.56E-01 | 6.56E+01 | | | |
| 7894151 | --- | --- | --- | -0.028 | 0.981 | 9.54E-01 | 9.54E+01 | | | |
| 7941425 | NM_025128 | MUS81 | MUS81 endonuclease homolog (S. ce | -0.028 | 0.981 | 6.76E-01 | 6.76E+01 | | | |
| 8096718 | NM_004757 | AIMP1 | aminoacyl tRNA synthetase complex | -0.028 | 0.981 | 9.11E-01 | 9.11E+01 | | | |
| 7921793 | NM_032998 | DEDD | death effector domain containing | -0.028 | 0.981 | 8.38E-01 | 8.38E+01 | | | |
| 8006655 | NM_024308 | DHRS11 | dehydrogenase/reductase (SDR fami | -0.028 | 0.981 | 6.52E-01 | 6.52E+01 | | | |
| 8119874 | NM_014628 | MAD2L1BP | MAD2L1 binding protein | -0.028 | 0.981 | 7.91E-01 | 7.91E+01 | | | |
| 7899173 | NM_024887 | DHDDS | dehydrodolichyl diphosphate syntha | -0.028 | 0.981 | 8.70E-01 | 8.70E+01 | | | |
| 7955469 | NM_00103996( | SLC4A8 | solute carrier family 4, sodium bicarl | -0.028 | 0.981 | 7.39E-01 | 7.39E+01 | | | |
| 7923584 | NR_027902 | C1orf157 | chromosome 1 open reading frame 1 | -0.028 | 0.981 | 7.02E-01 | 7.02E+01 | | | |
| 8123989 | NM_005493 | RANBP9 | RAN binding protein 9 | -0.028 | 0.981 | 9.46E-01 | 9.46E+01 | | | |
| 7977452 | ENST00000400 | FLJ39632 | hypothetical LOC642477 | -0.028 | 0.981 | 7.35E-01 | 7.35E+01 | | | |
| 8089694 | NM_007136 | ZNF80 | zinc finger protein 80 | -0.028 | 0.981 | 7.51E-01 | 7.51E+01 | | | |
| 7893351 | --- | --- | --- | -0.028 | 0.981 | 7.69E-01 | 7.69E+01 | | | |
| 7894853 | --- | --- | --- | -0.028 | 0.981 | 8.34E-01 | 8.34E+01 | | | |
| 7978312 | NM_006871 | RIPK3 | receptor-interacting serine-threonine | -0.028 | 0.981 | 6.57E-01 | 6.57E+01 | | | |
| 7893639 | --- | --- | --- | -0.028 | 0.981 | 9.47E-01 | 9.47E+01 | | | |
| 8157246 | BC105048 | KIAA1958 | KIAA1958 | -0.028 | 0.981 | 6.99E-01 | 6.99E+01 | | | |
| 8006845 | NM_000981 | RPL19 | ribosomal protein L19 | -0.028 | 0.981 | 6.67E-01 | 6.67E+01 | | | |
| 8173181 | NR_027139 | SPIN3 | spindlin family, member 3 | -0.028 | 0.981 | 8.31E-01 | 8.31E+01 | | | |
| 8171837 | NM_030624 | KLHL15 | kelch-like 15 (Drosophila) | -0.028 | 0.980 | 8.37E-01 | 8.37E+01 | | | |
| 7938133 | NM_144666 | DNHD1 | dynein heavy chain domain 1 | -0.028 | 0.980 | 8.11E-01 | 8.11E+01 | | | |
| 8122240 | --- | --- | --- | -0.028 | 0.980 | 9.65E-01 | 9.65E+01 | | | |
| 8165398 | NM_004479 | FUT7 | fucosyltransferase 7 (alpha (1,3) fuco | -0.028 | 0.980 | 6.51E-01 | 6.51E+01 | | | |
| 8032804 | NM_003025 | SH3GL1 | SH3-domain GRB2-like 1 | -0.029 | 0.980 | 7.08E-01 | 7.08E+01 | | | |
| 8174972 | NM_178470 | DCAF12L1 | DDB1 and CUL4 associated factor 12 | -0.029 | 0.980 | 7.24E-01 | 7.24E+01 | | | |
| 8175308 | NR_024493 | NCRNA00087 | non-protein coding RNA 87 | -0.029 | 0.980 | 6.63E-01 | 6.63E+01 | | | |
| 8086467 | NM_018075 | ANO10 | anoctamin 10 | -0.029 | 0.980 | 8.28E-01 | 8.28E+01 | | | |
| 7933659 | NM_015235 | CSTF2T | cleavage stimulation factor, 3' pre-RI | -0.029 | 0.980 | 7.78E-01 | 7.78E+01 | | | |
| 7894668 | --- | --- | --- | -0.029 | 0.980 | 8.29E-01 | 8.29E+01 | | | |
| 8110681 | NM_138464 | C5orf55 | chromosome 5 open reading frame 5 | -0.029 | 0.980 | 6.77E-01 | 6.77E+01 | | | |
| 7964033 | NM_173595 | ANKRD52 | ankyrin repeat domain 52 | -0.029 | 0.980 | 6.33E-01 | 6.33E+01 | | | |
| 7892956 | --- | --- | --- | -0.029 | 0.980 | 6.90E-01 | 6.90E+01 | | | |
| 7896235 | --- | --- | --- | -0.029 | 0.980 | 7.88E-01 | 7.88E+01 | | | |
| 7894795 | --- | --- | --- | -0.029 | 0.980 | 9.07E-01 | 9.07E+01 | | | |
| 8176076 | NM_00100993 | DNASE1L1 | deoxyribonuclease I-like 1 | -0.029 | 0.980 | 6.53E-01 | 6.53E+01 | | | |
| 7894402 | --- | --- | --- | -0.029 | 0.980 | 9.51E-01 | 9.51E+01 | | | |
| 8150253 | NM_000349 | STAR | steroidogenic acute regulatory prote | -0.029 | 0.980 | 6.83E-01 | 6.83E+01 | | | |
| 8142345 | NM_014705 | DOCK4 | dedicator of cytokinesis 4 | -0.029 | 0.980 | 6.48E-01 | 6.48E+01 | | | |
| 8145887 | --- | --- | --- | -0.029 | 0.980 | 7.64E-01 | 7.64E+01 | | | |
| 8040045 | NM_199235 | COLEC11 | collectin sub-family member 11 | -0.029 | 0.980 | 6.04E-01 | 6.04E+01 | | | |
| 8091780 | NM_00103862 | B3GALNT1 | beta-1,3-N-acetylgalactosaminyltran | -0.029 | 0.980 | 6.05E-01 | 6.05E+01 | | | |
| 8002262 | --- | --- | --- | -0.029 | 0.980 | 8.78E-01 | 8.78E+01 | | | |
| 7896183 | --- | --- | --- | -0.029 | 0.980 | 8.01E-01 | 8.01E+01 | | | |
| 7896878 | NM_080605 | B3GALT6 | UDP-Gal:betaGal beta 1,3-galactosyl | -0.029 | 0.980 | 7.28E-01 | 7.28E+01 | | | |
| 8017102 | --- | --- | --- | -0.029 | 0.980 | 7.21E-01 | 7.21E+01 | | | |
| 8085393 | NM_018306 | TMEM40 | transmembrane protein 40 | -0.029 | 0.980 | 7.72E-01 | 7.72E+01 | | | |
| 8167407 | NM_181532 | ERAS | ES cell expressed Ras | -0.029 | 0.980 | 6.23E-01 | 6.23E+01 | | | |
| 8034631 | NM_003765 | STX10 | syntaxin 10 | -0.029 | 0.980 | 7.74E-01 | 7.74E+01 | | | |
| 7968658 | NM_181503 | EXOSC8 | exosome component 8 | -0.029 | 0.980 | 9.61E-01 | 9.61E+01 | | | |
| 8019296 | NM_016538 | SIRT7 | sirtuin (silent mating type informatio | -0.029 | 0.980 | 7.30E-01 | 7.30E+01 | | | |
| 8081645 | NM_024616 | C3orf52 | chromosome 3 open reading frame 5 | -0.029 | 0.980 | 6.80E-01 | 6.80E+01 | | | |
| 7973732 | NM_015299 | KIAA0323 | KIAA0323 | -0.029 | 0.980 | 6.68E-01 | 6.68E+01 | | | |

| | | | | | | | | | | |
|---|---|---|---|---|---|---|---|---|---|---|
| 7974363 | NM_000953 | PTGDR | prostaglandin D2 receptor (DP) | -0.029 | 0.980 | 7.66E-01 | 7.66E+01 | | | |
| 8180325 | --- | --- | --- | -0.029 | 0.980 | 8.59E-01 | 8.59E+01 | | | |
| 8017186 | NM_022070 | HEATR6 | HEAT repeat containing 6 | -0.029 | 0.980 | 7.63E-01 | 7.63E+01 | | | |
| 7931455 | NM_00114375 | LRRC27 | leucine rich repeat containing 27 | -0.029 | 0.980 | 6.73E-01 | 6.73E+01 | | | |
| 7922752 | --- | --- | --- | -0.029 | 0.980 | 8.93E-01 | 8.93E+01 | | | |
| 8110043 | NM_013979 | BNIP1 | BCL2/adenovirus E1B 19kDa interact | -0.029 | 0.980 | 8.13E-01 | 8.13E+01 | | | |
| 8080960 | --- | --- | --- | -0.029 | 0.980 | 7.49E-01 | 7.49E+01 | | | |
| 8050813 | NM_175629 | DNMT3A | DNA (cytosine-5-)-methyltransferase | -0.029 | 0.980 | 5.90E-01 | 5.90E+01 | | | |
| 8134689 | NM_145914 | ZSCAN21 | zinc finger and SCAN domain contain | -0.029 | 0.980 | 7.05E-01 | 7.05E+01 | | | |
| 8121886 | NM_138571 | HINT3 | histidine triad nucleotide binding pro | -0.029 | 0.980 | 9.18E-01 | 9.18E+01 | | | |
| 8013268 | NR_026809 | FAM106A | family with sequence similarity 106, | -0.029 | 0.980 | 6.92E-01 | 6.92E+01 | | | |
| 8005603 | NM_018242 | SLC47A1 | solute carrier family 47, member 1 | -0.029 | 0.980 | 5.38E-01 | 5.38E+01 | | | |
| 8180111 | NM_021976 | RXRB | retinoid X receptor, beta | -0.029 | 0.980 | 7.22E-01 | 7.22E+01 | | | |
| 8070182 | NM_004414 | RCAN1 | regulator of calcineurin 1 | -0.029 | 0.980 | 7.23E-01 | 7.23E+01 | | | |
| 7942409 | NM_176796 | P2RY6 | pyrimidinergic receptor P2Y, G-prote | -0.029 | 0.980 | 6.69E-01 | 6.69E+01 | | | |
| 8042942 | NM_000189 | HK2 | hexokinase 2 | -0.029 | 0.980 | 7.45E-01 | 7.45E+01 | | | |
| 8053949 | NM_017991 | KIAA1310 | KIAA1310 | -0.030 | 0.980 | 8.04E-01 | 8.04E+01 | | | |
| 7942279 | NM_018172 | FAM86C | family with sequence similarity 86, n | -0.030 | 0.980 | 7.15E-01 | 7.15E+01 | | | |
| 8113724 | --- | --- | --- | -0.030 | 0.980 | 6.92E-01 | 6.92E+01 | | | |
| 8179967 | NM_001136 | AGER | advanced glycosylation end product- | -0.030 | 0.980 | 7.02E-01 | 7.02E+01 | | | |
| 8029958 | NM_015710 | GLTSCR2 | glioma tumor suppressor candidate r | -0.030 | 0.980 | 7.91E-01 | 7.91E+01 | | | |
| 7950067 | NM_001360 | DHCR7 | 7-dehydrocholesterol reductase | -0.030 | 0.980 | 6.96E-01 | 6.96E+01 | | | |
| 7940698 | NM_006473 | TAF6L | TAF6-like RNA polymerase II, p300/C | -0.030 | 0.980 | 7.24E-01 | 7.24E+01 | | | |
| 8140319 | NM_005338 | HIP1 | huntingtin interacting protein 1 | -0.030 | 0.980 | 6.73E-01 | 6.73E+01 | | | |
| 8042867 | NM_012477 | WBP1 | WW domain binding protein 1 | -0.030 | 0.980 | 7.63E-01 | 7.63E+01 | | | |
| 8079964 | --- | --- | --- | -0.030 | 0.980 | 8.00E-01 | 8.00E+01 | | | |
| 8078147 | NM_000060 | BTD | biotinidase | -0.030 | 0.980 | 7.28E-01 | 7.28E+01 | | | |
| 7986442 | --- | --- | --- | -0.030 | 0.980 | 8.82E-01 | 8.82E+01 | | | |
| 8176384 | NM_003411 | ZFY | zinc finger protein, Y-linked | -0.030 | 0.980 | 8.86E-01 | 8.86E+01 | | | |
| 8113445 | BC126144 | FBXL17 | F-box and leucine-rich repeat protein | -0.030 | 0.979 | 6.55E-01 | 6.55E+01 | | | |
| 8024373 | NM_00103984 | C19orf36 | chromosome 19 open reading frame | -0.030 | 0.979 | 5.55E-01 | 5.55E+01 | | | |
| 8049959 | AK123321 | FLJ41327 | FLJ41327 protein | -0.030 | 0.979 | 8.91E-01 | 8.91E+01 | | | |
| 7952394 | NM_00100291 | OR8D2 | olfactory receptor, family 8, subfami | -0.030 | 0.979 | 7.08E-01 | 7.08E+01 | | | |
| 7895777 | --- | --- | --- | -0.030 | 0.979 | 7.72E-01 | 7.72E+01 | | | |
| 8175254 | --- | --- | --- | -0.030 | 0.979 | 7.31E-01 | 7.31E+01 | | | |
| 7963513 | NM_015848 | KRT76 | keratin 76 | -0.030 | 0.979 | 6.67E-01 | 6.67E+01 | | | |
| 7953749 | NM_080387 | CLEC4D | C-type lectin domain family 4, memb | -0.030 | 0.979 | 8.90E-01 | 8.90E+01 | | | |
| 7970735 | --- | --- | --- | -0.030 | 0.979 | 7.35E-01 | 7.35E+01 | | | |
| 8060103 | NM_016552 | ANKMY1 | ankyrin repeat and MYND domain co | -0.030 | 0.979 | 7.04E-01 | 7.04E+01 | | | |
| 7921738 | NM_007122 | USF1 | upstream transcription factor 1 | -0.030 | 0.979 | 8.43E-01 | 8.43E+01 | | | |
| 8041763 | NM_005400 | PRKCE | protein kinase C, epsilon | -0.030 | 0.979 | 7.39E-01 | 7.39E+01 | | | |
| 8160024 | NM_000170 | GLDC | glycine dehydrogenase (decarboxyla | -0.030 | 0.979 | 8.08E-01 | 8.08E+01 | | | |
| 8131414 | NM_00113438 | ZDHHC4 | zinc finger, DHHC-type containing 4 | -0.030 | 0.979 | 8.16E-01 | 8.16E+01 | | | |
| 8037505 | NM_024108 | TRAPPC6A | trafficking protein particle complex 6 | -0.030 | 0.979 | 8.25E-01 | 8.25E+01 | | | |
| 7949798 | NM_025124 | TMEM134 | transmembrane protein 134 | -0.030 | 0.979 | 6.79E-01 | 6.79E+01 | | | |
| 7920185 | NM_032563 | LCE3D | late cornified envelope 3D | -0.030 | 0.979 | 6.65E-01 | 6.65E+01 | | | |
| 7947423 | --- | --- | --- | -0.030 | 0.979 | 8.59E-01 | 8.59E+01 | | | |
| 7894085 | --- | --- | --- | -0.030 | 0.979 | 8.23E-01 | 8.23E+01 | | | |
| 8160912 | NM_203299 | C9orf131 | chromosome 9 open reading frame 1 | -0.030 | 0.979 | 9.30E-01 | 9.30E+01 | | | |
| 7940835 | NM_004074 | COX8A | cytochrome c oxidase subunit 8A (ub | -0.030 | 0.979 | 8.74E-01 | 8.74E+01 | | | |
| 7986250 | NM_001271 | CHD2 | chromodomain helicase DNA binding | -0.030 | 0.979 | 8.30E-01 | 8.30E+01 | | | |
| 8134965 | NM_020246 | SLC12A9 | solute carrier family 12 (potassium/c | -0.030 | 0.979 | 7.62E-01 | 7.62E+01 | | | |
| 7906552 | NM_001231 | CASQ1 | calsequestrin 1 (fast-twitch, skeletal | -0.030 | 0.979 | 7.10E-01 | 7.10E+01 | | | |
| 7991731 | BC032901 | OC10013216 | similar to hCG1742852 | -0.031 | 0.979 | 6.98E-01 | 6.98E+01 | | | |
| 7896561 | --- | --- | --- | -0.031 | 0.979 | 9.39E-01 | 9.39E+01 | | | |
| 7893828 | --- | --- | --- | -0.031 | 0.979 | 8.18E-01 | 8.18E+01 | | | |
| 7982206 | NR_024074 | GOLGA9P | golgi autoantigen, golgin subfamily a | -0.031 | 0.979 | 6.31E-01 | 6.31E+01 | | | |
| 8025126 | NM_144614 | MBD3L2 | methyl-CpG binding domain protein | -0.031 | 0.979 | 6.79E-01 | 6.79E+01 | | | |
| 8110688 | NM_004174 | SLC9A3 | solute carrier family 9 (sodium/hydro | -0.031 | 0.979 | 5.23E-01 | 5.23E+01 | | | |
| 7943827 | NM_001931 | DLAT | dihydrolipoamide S-acetyltransferase | -0.031 | 0.979 | 7.85E-01 | 7.85E+01 | | | |
| 8038989 | NM_198457 | ZNF600 | zinc finger protein 600 | -0.031 | 0.979 | 8.29E-01 | 8.29E+01 | | | |
| 8118249 | NM_138277 | C6orf25 | chromosome 6 open reading frame 2 | -0.031 | 0.979 | 7.04E-01 | 7.04E+01 | | | |
| 7915991 | NM_032110 | DMRTA2 | DMRT-like family A2 | -0.031 | 0.979 | 5.40E-01 | 5.40E+01 | | | |
| 8139356 | NM_015332 | NUDCD3 | NudC domain containing 3 | -0.031 | 0.979 | 7.82E-01 | 7.82E+01 | | | |
| 7923731 | NM_015375 | DSTYK | dual serine/threonine and tyrosine p | -0.031 | 0.979 | 7.10E-01 | 7.10E+01 | | | |
| 7924553 | ENST00000415 | DEGS1 | degenerative spermatocyte homolog | -0.031 | 0.979 | 8.03E-01 | 8.03E+01 | | | |
| 7988283 | ENST0000313 | LOC645212 | hypothetical LOC645212 | -0.031 | 0.979 | 7.10E-01 | 7.10E+01 | | | |
| 8139907 | --- | --- | --- | -0.031 | 0.979 | 8.07E-01 | 8.07E+01 | | | |
| 8148261 | --- | --- | --- | -0.031 | 0.979 | 8.21E-01 | 8.21E+01 | | | |
| 7938231 | NM_003621 | PPFIBP2 | PTPRF interacting protein, binding pr | -0.031 | 0.979 | 7.63E-01 | 7.63E+01 | | | |
| 7969243 | NM_018204 | CKAP2 | cytoskeleton associated protein 2 | -0.031 | 0.979 | 8.91E-01 | 8.91E+01 | | | |
| 8093943 | AK026375 | LOC93622 | hypothetical LOC93622 | -0.031 | 0.979 | 7.06E-01 | 7.06E+01 | | | |
| 8148737 | NM_032272 | MAF1 | MAF1 homolog (S. cerevisiae) | -0.031 | 0.979 | 7.43E-01 | 7.43E+01 | | | |
| 7993195 | NM_000246 | CIITA | class II, major histocompatibility com | -0.031 | 0.979 | 6.19E-01 | 6.19E+01 | | | |
| 8035939 | --- | --- | --- | -0.031 | 0.979 | 7.84E-01 | 7.84E+01 | | | |
| 7895435 | --- | --- | --- | -0.031 | 0.979 | 7.50E-01 | 7.50E+01 | | | |
| 8040456 | NM_00110556 | MSGN1 | mesogenin 1 | -0.031 | 0.979 | 6.13E-01 | 6.13E+01 | | | |

| | | | | | | | | | | |
|---|---|---|---|---|---|---|---|---|---|---|
| 7982326 | NM_015995 | KLF13 | Kruppel-like factor 13 | -0.031 | 0.979 | 5.91E-01 | 5.91E+01 | | | |
| 7968126 | NM_002339 | LSP1 | lymphocyte-specific protein 1 | -0.031 | 0.979 | 8.80E-01 | 8.80E+01 | | | |
| 8013068 | NM_178836 | PLD6 | phospholipase D family, member 6 | -0.031 | 0.979 | 5.82E-01 | 5.82E+01 | | | |
| 8011110 | --- | --- | --- | -0.031 | 0.978 | 9.45E-01 | 9.45E+01 | | | |
| 7895144 | --- | --- | --- | -0.031 | 0.978 | 6.54E-01 | 6.54E+01 | | | |
| 8078138 | NM_033083 | EAF1 | ELL associated factor 1 | -0.031 | 0.978 | 8.40E-01 | 8.40E+01 | | | |
| 8031807 | NM_138347 | ZNF551 | zinc finger protein 551 | -0.031 | 0.978 | 8.00E-01 | 8.00E+01 | | | |
| 8002854 | NM_014567 | BCAR1 | breast cancer anti-estrogen resistanc | -0.032 | 0.978 | 6.06E-01 | 6.06E+01 | | | |
| 8081590 | NM_00113443 | PHLDB2 | pleckstrin homology-like domain, fan | -0.032 | 0.978 | 7.52E-01 | 7.52E+01 | | | |
| 8167334 | NM_000377 | WAS | Wiskott-Aldrich syndrome (eczema-t | -0.032 | 0.978 | 8.03E-01 | 8.03E+01 | | | |
| 8086842 | NM_002375 | MAP4 | microtubule-associated protein 4 | -0.032 | 0.978 | 5.82E-01 | 5.82E+01 | | | |
| 7917433 | NM_020729 | ODF2L | outer dense fiber of sperm tails 2-lik | -0.032 | 0.978 | 9.07E-01 | 9.07E+01 | | | |
| 8097670 | NM_172250 | MMAA | methylmalonic aciduria (cobalamin d | -0.032 | 0.978 | 8.03E-01 | 8.03E+01 | | | |
| 7892754 | --- | --- | --- | -0.032 | 0.978 | 9.80E-01 | 9.80E+01 | | | |
| 8034700 | BC119719 | C19orf57 | chromosome 19 open reading frame | -0.032 | 0.978 | 5.77E-01 | 5.77E+01 | | | |
| 8035477 | NM_006532 | ELL | elongation factor RNA polymerase II | -0.032 | 0.978 | 5.72E-01 | 5.72E+01 | | | |
| 8013507 | --- | --- | --- | -0.032 | 0.978 | 8.14E-01 | 8.14E+01 | | | |
| 8180336 | --- | --- | --- | -0.032 | 0.978 | 7.81E-01 | 7.81E+01 | | | |
| 8096899 | NM_152400 | C4orf32 | chromosome 4 open reading frame 3 | -0.032 | 0.978 | 5.47E-01 | 5.47E+01 | | | |
| 8029560 | NM_001294 | CLPTM1 | cleft lip and palate associated transm | -0.032 | 0.978 | 6.77E-01 | 6.77E+01 | | | |
| 8045974 | NM_022058 | SLC4A10 | solute carrier family 4, sodium bicarb | -0.032 | 0.978 | 7.50E-01 | 7.50E+01 | | | |
| 8119423 | NR_026938 | LOC221442 | adenylate cyclase 10 pseudogene | -0.032 | 0.978 | 9.43E-01 | 9.43E+01 | | | |
| 8102037 | --- | --- | --- | -0.032 | 0.978 | 7.89E-01 | 7.89E+01 | | | |
| 8110147 | NM_198567 | C5orf25 | chromosome 5 open reading frame 2 | -0.032 | 0.978 | 8.70E-01 | 8.70E+01 | | | |
| 7994858 | NM_024031 | PRR14 | proline rich 14 | -0.032 | 0.978 | 5.23E-01 | 5.23E+01 | | | |
| 7893247 | --- | --- | --- | -0.032 | 0.978 | 6.88E-01 | 6.88E+01 | | | |
| 8028791 | NM_012268 | PLD3 | phospholipase D family, member 3 | -0.032 | 0.978 | 5.41E-01 | 5.41E+01 | | | |
| 8074168 | ENST00000400 | FLJ39632 | hypothetical LOC642477 | -0.032 | 0.978 | 6.84E-01 | 6.84E+01 | | | |
| 8163825 | NM_005658 | TRAF1 | TNF receptor-associated factor 1 | -0.032 | 0.978 | 6.36E-01 | 6.36E+01 | | | |
| 8080162 | NM_015407 | ABHD14A | abhydrolase domain containing 14A | -0.032 | 0.978 | 6.96E-01 | 6.96E+01 | | | |
| 8024444 | AY358234 | UNQ6493 | EPWW6493 | -0.032 | 0.978 | 7.01E-01 | 7.01E+01 | | | |
| 8050846 | NM_021907 | DTNB | dystrobrevin, beta | -0.032 | 0.978 | 7.22E-01 | 7.22E+01 | | | |
| 8148694 | NM_000837 | GRINA | glutamate receptor, ionotropic, N-m | -0.032 | 0.978 | 7.25E-01 | 7.25E+01 | | | |
| 7948612 | NM_013402 | FADS1 | fatty acid desaturase 1 | -0.032 | 0.978 | 7.08E-01 | 7.08E+01 | | | |
| 8144498 | AK304567 | hCG_199054 | family with sequence similarity 86, m | -0.032 | 0.978 | 7.69E-01 | 7.69E+01 | | | |
| 8028924 | NM_006533 | MIA | melanoma inhibitory activity | -0.032 | 0.978 | 6.44E-01 | 6.44E+01 | | | |
| 8114491 | NM_005847 | SLC23A1 | solute carrier family 23 (nucleobase | -0.032 | 0.978 | 5.24E-01 | 5.24E+01 | | | |
| 8101992 | NM_022154 | SLC39A8 | solute carrier family 39 (zinc transpo | -0.032 | 0.978 | 8.55E-01 | 8.55E+01 | | | |
| 7895498 | --- | --- | --- | -0.032 | 0.978 | 7.56E-01 | 7.56E+01 | | | |
| 8037018 | NM_030578 | B9D2 | B9 protein domain 2 | -0.032 | 0.978 | 6.56E-01 | 6.56E+01 | | | |
| 7896228 | --- | --- | --- | -0.032 | 0.978 | 6.47E-01 | 6.47E+01 | | | |
| 8115732 | AY358216 | UNQ9374 | VCEW9374 | -0.032 | 0.978 | 8.74E-01 | 8.74E+01 | | | |
| 8048075 | --- | --- | --- | -0.032 | 0.978 | 8.90E-01 | 8.90E+01 | | | |
| 8007561 | NM_138387 | G6PC3 | glucose 6 phosphatase, catalytic, 3 | -0.032 | 0.978 | 6.24E-01 | 6.24E+01 | | | |
| 8061222 | --- | --- | --- | -0.032 | 0.978 | 7.80E-01 | 7.80E+01 | | | |
| 7903688 | NM_182580 | CYB561D1 | cytochrome b-561 domain containin | -0.032 | 0.978 | 6.95E-01 | 6.95E+01 | | | |
| 8058477 | NM_003709 | KLF7 | Kruppel-like factor 7 (ubiquitous) | -0.032 | 0.978 | 9.10E-01 | 9.10E+01 | | | |
| 8140709 | NM_00114274 | KIAA1324L | KIAA1324-like | -0.032 | 0.978 | 7.83E-01 | 7.83E+01 | | | |
| 8000425 | NM_001520 | GTF3C1 | general transcription factor IIIC, poly | -0.032 | 0.978 | 7.18E-01 | 7.18E+01 | | | |
| 7893148 | --- | --- | --- | -0.032 | 0.978 | 7.57E-01 | 7.57E+01 | | | |
| 8049195 | --- | --- | --- | -0.032 | 0.978 | 6.42E-01 | 6.42E+01 | | | |
| 8074286 | NM_015241 | MICAL3 | microtubule associated monoxygena | -0.033 | 0.978 | 7.34E-01 | 7.34E+01 | | | |
| 7895759 | --- | --- | --- | -0.033 | 0.978 | 9.44E-01 | 9.44E+01 | | | |
| 7893810 | --- | --- | --- | -0.033 | 0.978 | 8.80E-01 | 8.80E+01 | | | |
| 8011861 | NM_020162 | DHX33 | DEAH (Asp-Glu-Ala-His) box polypept | -0.033 | 0.978 | 8.31E-01 | 8.31E+01 | | | |
| 8123362 | BX647686 | CCR6 | chemokine (C-C motif) receptor 6 | -0.033 | 0.978 | 6.54E-01 | 6.54E+01 | | | |
| 7894673 | --- | --- | --- | -0.033 | 0.978 | 7.87E-01 | 7.87E+01 | | | |
| 8049299 | NR_003006 | SCARNA6 | small Cajal body-specific RNA 6 | -0.033 | 0.978 | 8.77E-01 | 8.77E+01 | | | |
| 7971216 | --- | --- | --- | -0.033 | 0.978 | 6.62E-01 | 6.62E+01 | | | |
| 7895658 | --- | --- | --- | -0.033 | 0.978 | 8.91E-01 | 8.91E+01 | | | |
| 8052872 | NM_003236 | TGFA | transforming growth factor, alpha | -0.033 | 0.978 | 6.18E-01 | 6.18E+01 | | | |
| 7897210 | NM_004402 | DFFB | DNA fragmentation factor, 40kDa, be | -0.033 | 0.978 | 6.17E-01 | 6.17E+01 | | | |
| 8031893 | NM_021089 | ZNF8 | zinc finger protein 8 | -0.033 | 0.978 | 7.24E-01 | 7.24E+01 | | | |
| 7945620 | NM_019009 | TOLLIP | toll interacting protein | -0.033 | 0.977 | 6.21E-01 | 6.21E+01 | | | |
| 7982564 | NM_152594 | SPRED1 | sprouty-related, EVH1 domain conta | -0.033 | 0.977 | 6.23E-01 | 6.23E+01 | | | |
| 8141477 | NM_178831 | GATS | GATS, stromal antigen 3 opposite str | -0.033 | 0.977 | 5.57E-01 | 5.57E+01 | | | |
| 8071671 | NM_002073 | GNAZ | guanine nucleotide binding protein ( | -0.033 | 0.977 | 5.96E-01 | 5.96E+01 | | | |
| 8043745 | ENST00000429 | TMEM131 | transmembrane protein 131 | -0.033 | 0.977 | 6.78E-01 | 6.78E+01 | | | |
| 8116740 | NM_00100369 | RREB1 | ras responsive element binding prote | -0.033 | 0.977 | 7.30E-01 | 7.30E+01 | | | |
| 8139712 | NM_030796 | VOPP1 | vesicular, overexpressed in cancer, p | -0.033 | 0.977 | 5.98E-01 | 5.98E+01 | | | |
| 8149356 | NM_201402 // | 17L2 // USP | ubiquitin specific peptidase 17-like 2 | -0.033 | 0.977 | 6.55E-01 | 6.55E+01 | | | |
| 7997896 | NM_182531 | ZNF778 | zinc finger protein 778 | -0.033 | 0.977 | 7.24E-01 | 7.24E+01 | | | |
| 8097146 | --- | --- | --- | -0.033 | 0.977 | 7.45E-01 | 7.45E+01 | | | |
| 7950641 | NM_023930 | KCTD14 | potassium channel tetramerisation d | -0.033 | 0.977 | 6.21E-01 | 6.21E+01 | | | |
| 7900510 | NM_001905 | CTPS | CTP synthase | -0.033 | 0.977 | 8.93E-01 | 8.93E+01 | | | |
| 8097827 | --- | --- | --- | -0.033 | 0.977 | 8.74E-01 | 8.74E+01 | | | |
| 8154163 | NM_005772 | RCL1 | RNA terminal phosphate cyclase-like | -0.033 | 0.977 | 9.07E-01 | 9.07E+01 | | | |

| | | | | | | | | | | |
|---|---|---|---|---|---|---|---|---|---|---|
| 7990757 | NM_004390 | CTSH | cathepsin H | -0.033 | 0.977 | 8.76E-01 | 8.76E+01 | | | |
| 7894352 | --- | --- | --- | -0.033 | 0.977 | 8.82E-01 | 8.82E+01 | | | |
| 8057931 | --- | --- | --- | -0.033 | 0.977 | 7.12E-01 | 7.12E+01 | | | |
| 7997498 | --- | --- | --- | -0.033 | 0.977 | 7.89E-01 | 7.89E+01 | | | |
| 7928547 | --- | --- | --- | -0.033 | 0.977 | 6.85E-01 | 6.85E+01 | | | |
| 7928408 | NM_015037 | KIAA0913 | KIAA0913 | -0.033 | 0.977 | 6.54E-01 | 6.54E+01 | | | |
| 7999279 | NM_016256 | NAGPA | N-acetylglucosamine-1-phosphodies | -0.033 | 0.977 | 6.73E-01 | 6.73E+01 | | | |
| 8095826 | NM_003943 | STBD1 | starch binding domain 1 | -0.033 | 0.977 | 6.84E-01 | 6.84E+01 | | | |
| 8149387 | NR_003494 | FAM86B1 | family with sequence similarity 86, m | -0.033 | 0.977 | 9.32E-01 | 9.32E+01 | | | |
| 7895539 | --- | --- | --- | -0.033 | 0.977 | 7.37E-01 | 7.37E+01 | | | |
| 8050031 | NM_015025 | MYT1L | myelin transcription factor 1-like | -0.033 | 0.977 | 5.34E-01 | 5.34E+01 | | | |
| 8139468 | NM_004749 | TBRG4 | transforming growth factor beta regu | -0.033 | 0.977 | 7.22E-01 | 7.22E+01 | | | |
| 7953594 | NM_006331 | EMG1 | EMG1 nucleolar protein homolog (S. | -0.033 | 0.977 | 9.43E-01 | 9.43E+01 | | | |
| 8136807 | 0 | 0 | 0 | -0.033 | 0.977 | 7.71E-01 | 7.71E+01 | | | |
| 7893920 | --- | --- | --- | -0.033 | 0.977 | 9.42E-01 | 9.42E+01 | | | |
| 8158167 | NM_005564 | LCN2 | lipocalin 2 | -0.033 | 0.977 | 6.98E-01 | 6.98E+01 | | | |
| 8036143 | NM_00104042! | U2AF1L4 | U2 small nuclear RNA auxiliary factor | -0.033 | 0.977 | 9.32E-01 | 9.32E+01 | | | |
| 7967304 | NM_198261 | RSRC2 | arginine/serine-rich coiled-coil 2 | -0.033 | 0.977 | 9.03E-01 | 9.03E+01 | | | |
| 8178470 | NM_002701 | POU5F1 | POU class 5 homeobox 1 | -0.033 | 0.977 | 7.11E-01 | 7.11E+01 | | | |
| 7892548 | --- | --- | --- | -0.033 | 0.977 | 8.90E-01 | 8.90E+01 | | | |
| 7908777 | --- | --- | --- | -0.033 | 0.977 | 7.73E-01 | 7.73E+01 | | | |
| 7917000 | --- | --- | --- | -0.033 | 0.977 | 6.07E-01 | 6.07E+01 | | | |
| 8061184 | --- | --- | --- | -0.033 | 0.977 | 7.61E-01 | 7.61E+01 | | | |
| 7966738 | AK298857 | C12orf49 | chromosome 12 open reading frame | -0.034 | 0.977 | 7.49E-01 | 7.49E+01 | | | |
| 8124742 | NM_005275 | GNL1 | guanine nucleotide binding protein-l | -0.034 | 0.977 | 7.35E-01 | 7.35E+01 | | | |
| 7911993 | NM_012405 | ICMT | isoprenylcysteine carboxyl methyltra | -0.034 | 0.977 | 6.21E-01 | 6.21E+01 | | | |
| 8000941 | NM_00108041 | ZNF629 | zinc finger protein 629 | -0.034 | 0.977 | 5.57E-01 | 5.57E+01 | | | |
| 7901592 | BC035374 | C1orf83 | chromosome 1 open reading frame 8 | -0.034 | 0.977 | 8.71E-01 | 8.71E+01 | | | |
| 7911532 | NM_017900 | AURKAIP1 | aurora kinase A interacting protein 1 | -0.034 | 0.977 | 6.47E-01 | 6.47E+01 | | | |
| 8145942 | NM_144652 | LETM2 | leucine zipper-EF-hand containing tra | -0.034 | 0.977 | 6.88E-01 | 6.88E+01 | | | |
| 7931930 | NM_006257 | PRKCQ | protein kinase C, theta | -0.034 | 0.977 | 9.30E-01 | 9.30E+01 | | | |
| 7894456 | --- | --- | --- | -0.034 | 0.977 | 7.03E-01 | 7.03E+01 | | | |
| 7972444 | NM_003576 | STK24 | serine/threonine kinase 24 (STE20 ho | -0.034 | 0.977 | 8.09E-01 | 8.09E+01 | | | |
| 7941457 | NM_006848 | CCDC85B | coiled-coil domain containing 85B | -0.034 | 0.977 | 6.40E-01 | 6.40E+01 | | | |
| 8077712 | NM_00103171 | CRELD1 | cysteine-rich with EGF-like domains 1 | -0.034 | 0.977 | 6.83E-01 | 6.83E+01 | | | |
| 7963880 | NM_00114499 | ITGA7 | integrin, alpha 7 | -0.034 | 0.977 | 5.47E-01 | 5.47E+01 | | | |
| 7963817 | NM_144594 | GTSF1 | gametocyte specific factor 1 | -0.034 | 0.977 | 7.74E-01 | 7.74E+01 | | | |
| 8028004 | NM_019104 | LIN37 | lin-37 homolog (C. elegans) | -0.034 | 0.977 | 7.34E-01 | 7.34E+01 | | | |
| 7959330 | NM_144668 | WDR66 | WD repeat domain 66 | -0.034 | 0.977 | 5.55E-01 | 5.55E+01 | | | |
| 8174105 | NM_032946 | NXF5 | nuclear RNA export factor 5 | -0.034 | 0.977 | 7.41E-01 | 7.41E+01 | | | |
| 8169624 | NM_145305 | SLC25A43 | solute carrier family 25, member 43 | -0.034 | 0.977 | 6.06E-01 | 6.06E+01 | | | |
| 8048432 | NM_000784 | CYP27A1 | cytochrome P450, family 27, subfam | -0.034 | 0.977 | 6.41E-01 | 6.41E+01 | | | |
| 7946340 | NM_024557 | RIC3 | resistance to inhibitors of cholineste | -0.034 | 0.977 | 7.19E-01 | 7.19E+01 | | | |
| 7934810 | --- | --- | --- | -0.034 | 0.977 | 6.73E-01 | 6.73E+01 | | | |
| 7895855 | --- | --- | --- | -0.034 | 0.977 | 9.53E-01 | 9.53E+01 | | | |
| 7906671 | NM_00101444 | USP21 | ubiquitin specific peptidase 21 | -0.034 | 0.977 | 8.11E-01 | 8.11E+01 | | | |
| 8123232 | NM_003057 | SLC22A1 | solute carrier family 22 (organic catio | -0.034 | 0.977 | 6.30E-01 | 6.30E+01 | | | |
| 7893428 | --- | --- | --- | -0.034 | 0.977 | 8.64E-01 | 8.64E+01 | | | |
| 7984008 | --- | --- | --- | -0.034 | 0.977 | 7.72E-01 | 7.72E+01 | | | |
| 7892876 | --- | --- | --- | -0.034 | 0.977 | 7.26E-01 | 7.26E+01 | | | |
| 8141445 | BC015722 | C7orf43 | chromosome 7 open reading frame 4 | -0.034 | 0.977 | 6.50E-01 | 6.50E+01 | | | |
| 8080212 | NM_145262 | GLYCTK | glycerate kinase | -0.034 | 0.977 | 6.40E-01 | 6.40E+01 | | | |
| 8018786 | NM_00100852 | MXRA7 | matrix-remodelling associated 7 | -0.034 | 0.977 | 7.31E-01 | 7.31E+01 | | | |
| 7894475 | --- | --- | --- | -0.034 | 0.977 | 9.06E-01 | 9.06E+01 | | | |
| 8071107 | NM_031481 | SLC25A18 | solute carrier family 25 (mitochondri | -0.034 | 0.976 | 5.88E-01 | 5.88E+01 | | | |
| 7894026 | --- | --- | --- | -0.034 | 0.976 | 9.36E-01 | 9.36E+01 | | | |
| 8163892 | AF220263 | C9orf31 | chromosome 9 open reading frame 3 | -0.034 | 0.976 | 6.85E-01 | 6.85E+01 | | | |
| 8108376 | --- | --- | --- | -0.034 | 0.976 | 8.24E-01 | 8.24E+01 | | | |
| 8061447 | NM_002862 | PYGB | phosphorylase, glycogen; brain | -0.034 | 0.976 | 7.34E-01 | 7.34E+01 | | | |
| 7894058 | --- | --- | --- | -0.034 | 0.976 | 9.49E-01 | 9.49E+01 | | | |
| 7992905 | NM_00108360 | NAT15 | N-acetyltransferase 15 (GCN5-relate | -0.034 | 0.976 | 7.28E-01 | 7.28E+01 | | | |
| 8061368 | NM_013248 | NXT1 | NTF2-like export factor 1 | -0.034 | 0.976 | 6.17E-01 | 6.17E+01 | | | |
| 7893704 | --- | --- | --- | -0.034 | 0.976 | 9.08E-01 | 9.08E+01 | | | |
| 7914245 | NM_00102473 | MECR | mitochondrial trans-2-enoyl-CoA red | -0.034 | 0.976 | 6.13E-01 | 6.13E+01 | | | |
| 7893096 | --- | --- | --- | -0.034 | 0.976 | 7.08E-01 | 7.08E+01 | | | |
| 7999382 | --- | --- | --- | -0.034 | 0.976 | 7.31E-01 | 7.31E+01 | | | |
| 7901046 | NR_000015 | SNORD55 | small nucleolar RNA, C/D box 55 | -0.034 | 0.976 | 8.61E-01 | 8.61E+01 | | | |
| 8138930 | NR_003500 | RP9P | retinitis pigmentosa 9 pseudogene | -0.035 | 0.976 | 6.52E-01 | 6.52E+01 | | | |
| 8159501 | NM_178536 | LCN12 | lipocalin 12 | -0.035 | 0.976 | 5.59E-01 | 5.59E+01 | | | |
| 8052979 | NM_144579 | SFXN5 | sideroflexin 5 | -0.035 | 0.976 | 5.63E-01 | 5.63E+01 | | | |
| 8073513 | NM_024821 | CCDC134 | coiled-coil domain containing 134 | -0.035 | 0.976 | 6.05E-01 | 6.05E+01 | | | |
| 8063115 | NM_004994 | MMP9 | matrix metallopeptidase 9 (gelatinas | -0.035 | 0.976 | 6.24E-01 | 6.24E+01 | | | |
| 8065637 | NM_053041 | COMMD7 | COMM domain containing 7 | -0.035 | 0.976 | 7.49E-01 | 7.49E+01 | | | |
| 7997192 | NM_020995 | HPR | haptoglobin-related protein | -0.035 | 0.976 | 6.85E-01 | 6.85E+01 | | | |
| 8134599 | NM_006693 | CPSF4 | cleavage and polyadenylation specifi | -0.035 | 0.976 | 7.36E-01 | 7.36E+01 | | | |
| 8135955 | NM_001219 | CALU | calumenin | -0.035 | 0.976 | 9.44E-01 | 9.44E+01 | | | |
| 7940869 | NM_178443 | FERMT3 | fermitin family homolog 3 (Drosophi | -0.035 | 0.976 | 6.86E-01 | 6.86E+01 | | | |

| | | | | | | | | | | |
|---|---|---|---|---|---|---|---|---|---|---|
| 8059680 | NM_000867 | HTR2B | 5-hydroxytryptamine (serotonin) rec | -0.035 | 0.976 | 6.26E-01 | 6.26E+01 | | | |
| 7894427 | --- | --- | --- | -0.035 | 0.976 | 9.57E-01 | 9.57E+01 | | | |
| 8149733 | NM_003842 | TNFRSF10B | tumor necrosis factor receptor super | -0.035 | 0.976 | 7.47E-01 | 7.47E+01 | | | |
| 7939492 | NM_00114503 | LOC387763 | hypothetical protein LOC387763 | -0.035 | 0.976 | 5.18E-01 | 5.18E+01 | | | |
| 8016187 | NM_152343 | C17orf46 | chromosome 17 open reading frame | -0.035 | 0.976 | 5.97E-01 | 5.97E+01 | | | |
| 8122660 | NM_005715 | UST | uronyl-2-sulfotransferase | -0.035 | 0.976 | 5.21E-01 | 5.21E+01 | | | |
| 8041826 | NM_020458 | TTC7A | tetratricopeptide repeat domain 7A | -0.035 | 0.976 | 5.67E-01 | 5.67E+01 | | | |
| 7893413 | --- | --- | --- | -0.035 | 0.976 | 6.99E-01 | 6.99E+01 | | | |
| 7914354 | NM_012392 | PEF1 | penta-EF-hand domain containing 1 | -0.035 | 0.976 | 7.81E-01 | 7.81E+01 | | | |
| 8013042 | NM_000422 | KRT17 | keratin 17 | -0.035 | 0.976 | 5.45E-01 | 5.45E+01 | | | |
| 8149315 | BC132953 | C8orf15 | chromosome 8 open reading frame 1 | -0.035 | 0.976 | 7.37E-01 | 7.37E+01 | | | |
| 7940135 | NM_080661 | GLYATL1 | glycine-N-acyltransferase-like 1 | -0.035 | 0.976 | 6.01E-01 | 6.01E+01 | | | |
| 7966749 | NM_017899 | TESC | tescalcin | -0.035 | 0.976 | 8.51E-01 | 8.51E+01 | | | |
| 7898594 | NM_000871 | HTR6 | 5-hydroxytryptamine (serotonin) rec | -0.035 | 0.976 | 6.06E-01 | 6.06E+01 | | | |
| 7897449 | NM_025106 | SPSB1 | splA/ryanodine receptor domain and | -0.035 | 0.976 | 6.38E-01 | 6.38E+01 | | | |
| 7908041 | NM_002293 | LAMC1 | laminin, gamma 1 (formerly LAMB2) | -0.035 | 0.976 | 5.82E-01 | 5.82E+01 | | | |
| 8180308 | --- | --- | --- | -0.035 | 0.976 | 6.04E-01 | 6.04E+01 | | | |
| 8144395 | NM_201402 // | 17L2 // USP | ubiquitin specific peptidase 17-like 2 | -0.035 | 0.976 | 6.24E-01 | 6.24E+01 | | | |
| 8126484 | --- | --- | --- | -0.035 | 0.976 | 6.54E-01 | 6.54E+01 | | | |
| 7944769 | NM_020716 | GRAMD1B | GRAM domain containing 1B | -0.035 | 0.976 | 6.94E-01 | 6.94E+01 | | | |
| 7955110 | ENST00000454 | KFZP779L18 | hypothetical LOC643162 | -0.035 | 0.976 | 6.60E-01 | 6.60E+01 | | | |
| 8104731 | --- | --- | --- | -0.035 | 0.976 | 8.88E-01 | 8.88E+01 | | | |
| 7992409 | NM_174903 | RNF151 | ring finger protein 151 | -0.035 | 0.976 | 6.07E-01 | 6.07E+01 | | | |
| 7893599 | --- | --- | --- | -0.035 | 0.976 | 7.21E-01 | 7.21E+01 | | | |
| 7944882 | NM_022370 | ROBO3 | roundabout, axon guidance receptor | -0.035 | 0.976 | 5.35E-01 | 5.35E+01 | | | |
| 7960512 | --- | --- | --- | -0.035 | 0.976 | 7.18E-01 | 7.18E+01 | | | |
| 7893605 | --- | --- | --- | -0.035 | 0.976 | 5.85E-01 | 5.85E+01 | | | |
| 7997427 | NM_198390 | CMIP | c-Maf-inducing protein | -0.035 | 0.976 | 6.83E-01 | 6.83E+01 | | | |
| 7906775 | NR_024151 | HSPA7 | heat shock 70kDa protein 7 (HSP70B | -0.035 | 0.976 | 7.75E-01 | 7.75E+01 | | | |
| 8060082 | NM_00108083 | PRR21 | proline rich 21 | -0.035 | 0.976 | 6.17E-01 | 6.17E+01 | | | |
| 8017511 | NM_000626 | CD79B | CD79b molecule, immunoglobulin-as | -0.035 | 0.976 | 6.70E-01 | 6.70E+01 | | | |
| 8048647 | NM_052902 | STK11IP | serine/threonine kinase 11 interactir | -0.035 | 0.976 | 4.99E-01 | 4.99E+01 | | | |
| 7895577 | --- | --- | --- | -0.035 | 0.976 | 7.75E-01 | 7.75E+01 | | | |
| 8007435 | NM_173079 | RUNDC1 | RUN domain containing 1 | -0.035 | 0.976 | 7.00E-01 | 7.00E+01 | | | |
| 8033795 | NM_152289 | ZNF561 | zinc finger protein 561 | -0.035 | 0.976 | 6.98E-01 | 6.98E+01 | | | |
| 7959148 | NM_012240 | SIRT4 | sirtuin (silent mating type informatio | -0.035 | 0.976 | 7.05E-01 | 7.05E+01 | | | |
| 8126839 | NM_014452 | TNFRSF21 | tumor necrosis factor receptor super | -0.035 | 0.976 | 6.55E-01 | 6.55E+01 | | | |
| 7895506 | --- | --- | --- | -0.035 | 0.976 | 5.97E-01 | 5.97E+01 | | | |
| 7996345 | NM_003869 | CES2 | carboxylesterase 2 (intestine, liver) | -0.035 | 0.976 | 6.48E-01 | 6.48E+01 | | | |
| 7920633 | NM_018973 | DPM3 | dolichyl-phosphate mannosyltransfe | -0.035 | 0.976 | 6.64E-01 | 6.64E+01 | | | |
| 8119403 | NM_006789 | APOBEC2 | apolipoprotein B mRNA editing enzy | -0.035 | 0.976 | 7.07E-01 | 7.07E+01 | | | |
| 8156358 | --- | --- | --- | -0.036 | 0.976 | 9.57E-01 | 9.57E+01 | | | |
| 8064394 | ENST00000217 | RPS10P5 | ribosomal protein S10 pseudogene 5 | -0.036 | 0.976 | 6.21E-01 | 6.21E+01 | | | |
| 8109093 | NM_014945 | ABLIM3 | actin binding LIM protein family, me | -0.036 | 0.976 | 6.33E-01 | 6.33E+01 | | | |
| 7895174 | --- | --- | --- | -0.036 | 0.976 | 8.03E-01 | 8.03E+01 | | | |
| 8120210 | NM_002190 | IL17A | interleukin 17A | -0.036 | 0.976 | 6.30E-01 | 6.30E+01 | | | |
| 8152491 | NM_000127 | EXT1 | exostoses (multiple) 1 | -0.036 | 0.976 | 8.08E-01 | 8.08E+01 | | | mono |
| 8107673 | NM_023927 | GRAMD3 | GRAM domain containing 3 | -0.036 | 0.976 | 5.96E-01 | 5.96E+01 | | | |
| 8109326 | NM_032947 | MST150 | MSTP150 | -0.036 | 0.976 | 5.81E-01 | 5.81E+01 | | | |
| 7892915 | --- | --- | --- | -0.036 | 0.975 | 9.59E-01 | 9.59E+01 | | | |
| 7980024 | NM_203309 | HEATR4 | HEAT repeat containing 4 | -0.036 | 0.975 | 6.57E-01 | 6.57E+01 | | | |
| 8088348 | NM_152678 | FAM116A | family with sequence similarity 116, | -0.036 | 0.975 | 9.41E-01 | 9.41E+01 | | | |
| 7894621 | --- | --- | --- | -0.036 | 0.975 | 6.90E-01 | 6.90E+01 | | | |
| 7912347 | NM_00107984 | CASZ1 | castor zinc finger 1 | -0.036 | 0.975 | 7.98E-01 | 7.98E+01 | | | |
| 8021442 | NM_018181 | ZNF532 | zinc finger protein 532 | -0.036 | 0.975 | 6.52E-01 | 6.52E+01 | | | |
| 7895116 | --- | --- | --- | -0.036 | 0.975 | 9.27E-01 | 9.27E+01 | | | |
| 8070867 | ENST00000416 | C21orf122 | chromosome 21 open reading frame | -0.036 | 0.975 | 6.67E-01 | 6.67E+01 | | | |
| 8146908 | NM_004770 | KCNB2 | potassium voltage-gated channel, Sh | -0.036 | 0.975 | 5.79E-01 | 5.79E+01 | | | |
| 7956978 | NM_018656 | SLC35E3 | solute carrier family 35, member E3 | -0.036 | 0.975 | 4.77E-01 | 4.77E+01 | | | |
| 8008702 | --- | --- | --- | -0.036 | 0.975 | 6.56E-01 | 6.56E+01 | | | |
| 8003401 | NM_000485 | APRT | adenine phosphoribosyltransferase | -0.036 | 0.975 | 7.61E-01 | 7.61E+01 | | | |
| 8076298 | --- | --- | --- | -0.036 | 0.975 | 7.02E-01 | 7.02E+01 | | | |
| 7892677 | --- | --- | --- | -0.036 | 0.975 | 5.93E-01 | 5.93E+01 | | | |
| 7895434 | --- | --- | --- | -0.036 | 0.975 | 9.21E-01 | 9.21E+01 | | | |
| 8119974 | NM_00107817 | SLC29A1 | solute carrier family 29 (nucleoside t | -0.036 | 0.975 | 5.75E-01 | 5.75E+01 | | | |
| 7894791 | --- | --- | --- | -0.036 | 0.975 | 8.80E-01 | 8.80E+01 | | | |
| 7912659 | NM_024758 | AGMAT | agmatine ureohydrolase (agmatinase | -0.036 | 0.975 | 7.13E-01 | 7.13E+01 | | | |
| 8180235 | --- | --- | --- | -0.036 | 0.975 | 5.36E-01 | 5.36E+01 | | | |
| 8172333 | NM_002621 | CFP | complement factor properdin | -0.036 | 0.975 | 5.94E-01 | 5.94E+01 | | | |
| 7911730 | NM_018216 | PANK4 | pantothenate kinase 4 | -0.036 | 0.975 | 6.90E-01 | 6.90E+01 | | | |
| 8034320 | NM_00108041 | ZNF433 | zinc finger protein 433 | -0.036 | 0.975 | 6.73E-01 | 6.73E+01 | | | |
| 8135568 | NM_199072 | MDFIC | MyoD family inhibitor domain contai | -0.036 | 0.975 | 5.48E-01 | 5.48E+01 | | | |
| 7896027 | --- | --- | --- | -0.036 | 0.975 | 9.58E-01 | 9.58E+01 | | | |
| 8069541 | NM_022136 | SAMSN1 | SAM domain, SH3 domain and nucle | -0.036 | 0.975 | 8.97E-01 | 8.97E+01 | | | |
| 8036923 | NM_004756 | NUMBL | numb homolog (Drosophila)-like | -0.036 | 0.975 | 4.16E-01 | 4.16E+01 | | | |
| 7942596 | NM_001235 | SERPINH1 | serpin peptidase inhibitor, clade H (h | -0.036 | 0.975 | 6.26E-01 | 6.26E+01 | | | |
| 7937709 | NM_00101241 | KRTAP5-6 | keratin associated protein 5-6 | -0.036 | 0.975 | 5.76E-01 | 5.76E+01 | | | |

| | | | | | | | | | | |
|---|---|---|---|---|---|---|---|---|---|---|
| 7908125 | NM_015149 | RGL1 | ral guanine nucleotide dissociation st | -0.036 | 0.975 | 7.35E-01 | 7.35E+01 | | | |
| 7916130 | NM_138417 | KTI12 | KTI12 homolog, chromatin associate | -0.036 | 0.975 | 7.05E-01 | 7.05E+01 | | | |
| 7960575 | NM_00103371 | NOP2 | NOP2 nucleolar protein homolog (ye | -0.036 | 0.975 | 6.55E-01 | 6.55E+01 | | | |
| 7892777 | --- | --- | --- | -0.036 | 0.975 | 8.95E-01 | 8.95E+01 | | | |
| 7983951 | --- | --- | --- | -0.037 | 0.975 | 9.20E-01 | 9.20E+01 | | | |
| 7957126 | NM_014505 | KCNMB4 | potassium large conductance calcium | -0.037 | 0.975 | 4.86E-01 | 4.86E+01 | | | |
| 8053200 | NM_133637 | DQX1 | DEAQ box RNA-dependent ATPase 1 | -0.037 | 0.975 | 4.84E-01 | 4.84E+01 | | | |
| 8139107 | --- | --- | --- | -0.037 | 0.975 | 8.99E-01 | 8.99E+01 | | | |
| 8090364 | AK093796 | ZXDC | ZXD family zinc finger C | -0.037 | 0.975 | 8.83E-01 | 8.83E+01 | | | |
| 8177344 | NR_001541 | TTTY5 | testis-specific transcript, Y-linked 5 (r | -0.037 | 0.975 | 4.50E-01 | 4.50E+01 | | | |
| 8123728 | NM_020408 | LYRM4 | LYR motif containing 4 | -0.037 | 0.975 | 8.63E-01 | 8.63E+01 | | | |
| 8014916 | NM_139280 | ORMDL3 | ORM1-like 3 (S. cerevisiae) | -0.037 | 0.975 | 6.42E-01 | 6.42E+01 | | | |
| 8061373 | NM_022482 | GZF1 | GDNF-inducible zinc finger protein 1 | -0.037 | 0.975 | 7.76E-01 | 7.76E+01 | | | |
| 8165438 | NM_013379 | DPP7 | dipeptidyl-peptidase 7 | -0.037 | 0.975 | 5.38E-01 | 5.38E+01 | | | |
| 7894995 | --- | --- | --- | -0.037 | 0.975 | 8.30E-01 | 8.30E+01 | | | |
| 8001651 | NM_020807 | ZNF319 | zinc finger protein 319 | -0.037 | 0.975 | 5.95E-01 | 5.95E+01 | | | |
| 7944418 | NM_014807 | C2CD2L | C2CD2-like | -0.037 | 0.975 | 4.87E-01 | 4.87E+01 | | | |
| 8170630 | NM_032882 | PNMA6A | paraneoplastic antigen like 6A | -0.037 | 0.975 | 7.15E-01 | 7.15E+01 | | | |
| 7893238 | --- | --- | --- | -0.037 | 0.975 | 9.16E-01 | 9.16E+01 | | | |
| 8071655 | --- | --- | --- | -0.037 | 0.975 | 6.07E-01 | 6.07E+01 | | | |
| 8067167 | NM_198433 | AURKA | aurora kinase A | -0.037 | 0.975 | 7.33E-01 | 7.33E+01 | | | |
| 8000738 | NM_178863 | KCTD13 | potassium channel tetramerisation d | -0.037 | 0.975 | 5.60E-01 | 5.60E+01 | | | |
| 8003857 | --- | --- | --- | -0.037 | 0.975 | 8.78E-01 | 8.78E+01 | | | |
| 7893600 | --- | --- | --- | -0.037 | 0.975 | 8.91E-01 | 8.91E+01 | | | |
| 7907008 | --- | --- | --- | -0.037 | 0.975 | 7.97E-01 | 7.97E+01 | | | |
| 7954029 | NM_004064 | CDKN1B | cyclin-dependent kinase inhibitor 1B | -0.037 | 0.975 | 8.49E-01 | 8.49E+01 | | | |
| 7973618 | NM_006084 | IRF9 | interferon regulatory factor 9 | -0.037 | 0.975 | 8.43E-01 | 8.43E+01 | | | |
| 8065868 | NM_002212 | EIF6 | eukaryotic translation initiation facto | -0.037 | 0.975 | 7.65E-01 | 7.65E+01 | | | |
| 7977440 | NR_026800 | KIAA0125 | KIAA0125 | -0.037 | 0.975 | 8.29E-01 | 8.29E+01 | | | |
| 8018646 | NM_001454 | FOXJ1 | forkhead box J1 | -0.037 | 0.975 | 5.98E-01 | 5.98E+01 | | | |
| 8166124 | NM_006357 | UBE2E3 | ubiquitin-conjugating enzyme E2E 3 | -0.037 | 0.975 | 7.53E-01 | 7.53E+01 | | | |
| 7994095 | NM_019116 | UBFD1 | ubiquitin family domain containing 1 | -0.037 | 0.975 | 6.08E-01 | 6.08E+01 | | | |
| 8180383 | --- | --- | --- | -0.037 | 0.975 | 5.63E-01 | 5.63E+01 | | | |
| 7896204 | --- | --- | --- | -0.037 | 0.975 | 6.41E-01 | 6.41E+01 | | | |
| 8148662 | NM_173831 | ZNF707 | zinc finger protein 707 | -0.037 | 0.975 | 5.98E-01 | 5.98E+01 | | | |
| 8065370 | --- | --- | --- | -0.037 | 0.975 | 7.26E-01 | 7.26E+01 | | | |
| 7899462 | NM_00104819 | RCC1 | regulator of chromosome condensat | -0.037 | 0.975 | 6.92E-01 | 6.92E+01 | | | |
| 7981512 | --- | --- | --- | -0.037 | 0.975 | 6.03E-01 | 6.03E+01 | | | |
| 7964759 | NM_021150 | GRIP1 | glutamate receptor interacting prote | -0.037 | 0.974 | 5.81E-01 | 5.81E+01 | | | |
| 8014199 | NM_002878 | RAD51L3 | RAD51-like 3 (S. cerevisiae) | -0.037 | 0.974 | 6.89E-01 | 6.89E+01 | | | |
| 7970727 | NM_001265 | CDX2 | caudal type homeobox 2 | -0.037 | 0.974 | 4.93E-01 | 4.93E+01 | | | |
| 8008185 | NM_016428 | ABI3 | ABI family, member 3 | -0.037 | 0.974 | 6.13E-01 | 6.13E+01 | | | |
| 7895009 | --- | --- | --- | -0.037 | 0.974 | 6.48E-01 | 6.48E+01 | | | |
| 8069003 | NM_005049 | PWP2 | PWP2 periodic tryptophan protein ho | -0.037 | 0.974 | 6.24E-01 | 6.24E+01 | | | |
| 8051799 | NM_012205 | HAAO | 3-hydroxyanthranilate 3,4-dioxygena | -0.037 | 0.974 | 5.17E-01 | 5.17E+01 | | | |
| 8165903 | --- | --- | --- | -0.037 | 0.974 | 5.76E-01 | 5.76E+01 | | | |
| 8094872 | --- | --- | --- | -0.037 | 0.974 | 7.59E-01 | 7.59E+01 | | | |
| 8126681 | NM_020745 | AARS2 | alanyl-tRNA synthetase 2, mitochond | -0.037 | 0.974 | 5.53E-01 | 5.53E+01 | | | |
| 8074980 | NM_000853 | GSTT1 | glutathione S-transferase theta 1 | -0.037 | 0.974 | 7.69E-01 | 7.69E+01 | | | |
| 8071717 | NM_153615 | RGL4 | ral guanine nucleotide dissociation st | -0.038 | 0.974 | 7.24E-01 | 7.24E+01 | | | |
| 8086754 | NM_012235 | SCAP | SREBF chaperone | -0.038 | 0.974 | 5.26E-01 | 5.26E+01 | | | |
| 7901748 | NM_00111341 | FGGY | FGGY carbohydrate kinase domain co | -0.038 | 0.974 | 6.50E-01 | 6.50E+01 | | | |
| 8067248 | NR_003505 | PPP4R1L | protein phosphatase 4, regulatory su | -0.038 | 0.974 | 7.41E-01 | 7.41E+01 | | | |
| 8152291 | NM_139166 | ABRA | actin-binding Rho activating protein | -0.038 | 0.974 | 6.08E-01 | 6.08E+01 | | | |
| 8064502 | NM_003091 | SNRPB | small nuclear ribonucleoprotein poly | -0.038 | 0.974 | 8.67E-01 | 8.67E+01 | | | |
| 8073311 | NM_022098 | XPNPEP3 | X-prolyl aminopeptidase (aminopept | -0.038 | 0.974 | 6.03E-01 | 6.03E+01 | | | |
| 8100328 | NM_022832 | USP46 | ubiquitin specific peptidase 46 | -0.038 | 0.974 | 7.33E-01 | 7.33E+01 | | | |
| 8159415 | NM_024718 | C9orf86 | chromosome 9 open reading frame 8 | -0.038 | 0.974 | 6.23E-01 | 6.23E+01 | | | |
| 8009277 | NM_003835 | RGS9 | regulator of G-protein signaling 9 | -0.038 | 0.974 | 4.75E-01 | 4.75E+01 | | | |
| 8040486 | --- | --- | --- | -0.038 | 0.974 | 6.96E-01 | 6.96E+01 | | | |
| 7895255 | --- | --- | --- | -0.038 | 0.974 | 9.45E-01 | 9.45E+01 | | | |
| 8039871 | NM_00108353 | KIR3DS1 | killer cell immunoglobulin-like recept | -0.038 | 0.974 | 8.18E-01 | 8.18E+01 | | | |
| 7895322 | --- | --- | --- | -0.038 | 0.974 | 9.31E-01 | 9.31E+01 | | | |
| 8132589 | --- | --- | --- | -0.038 | 0.974 | 7.31E-01 | 7.31E+01 | | | |
| 8101839 | NM_001968 | EIF4E | eukaryotic translation initiation facto | -0.038 | 0.974 | 6.53E-01 | 6.53E+01 | | | |
| 7977653 | --- | --- | --- | -0.038 | 0.974 | 6.70E-01 | 6.70E+01 | | | |
| 7907146 | NM_005149 | TBX19 | T-box 19 | -0.038 | 0.974 | 5.95E-01 | 5.95E+01 | | | |
| 8077103 | NM_00111375 | TYMP | thymidine phosphorylase | -0.038 | 0.974 | 5.49E-01 | 5.49E+01 | | | |
| 8081548 | NM_015480 | PVRL3 | poliovirus receptor-related 3 | -0.038 | 0.974 | 5.77E-01 | 5.77E+01 | | | |
| 8009349 | --- | --- | --- | -0.038 | 0.974 | 7.22E-01 | 7.22E+01 | | | |
| 7951157 | AK313893 | CCDC82 | coiled-coil domain containing 82 | -0.038 | 0.974 | 8.13E-01 | 8.13E+01 | | | |
| 8090351 | NM_025112 | ZXDC | ZXD family zinc finger C | -0.038 | 0.974 | 6.97E-01 | 6.97E+01 | | | |
| 7915170 | NM_012333 | MYCBP | c-myc binding protein | -0.038 | 0.974 | 7.05E-01 | 7.05E+01 | | | |
| 8153002 | NM_00113524 | NDRG1 | N-myc downstream regulated 1 | -0.038 | 0.974 | 6.89E-01 | 6.89E+01 | | | |
| 8114002 | ENST0000041€ | P4HA2 | prolyl 4-hydroxylase, alpha polypept | -0.038 | 0.974 | 7.94E-01 | 7.94E+01 | | | |
| 8172443 | NM_002536 | TBC1D25 | TBC1 domain family, member 25 | -0.038 | 0.974 | 8.67E-01 | 8.67E+01 | | | |
| 8019962 | --- | --- | --- | -0.038 | 0.974 | 6.64E-01 | 6.64E+01 | | | |

| | | | | | | | | | | |
|---|---|---|---|---|---|---|---|---|---|---|
| 7894063 | --- | --- | --- | -0.038 | 0.974 | 8.74E-01 | 8.74E+01 | | | |
| 8137474 | NM_020445 | ACTR3B | ARP3 actin-related protein 3 homolo | -0.038 | 0.974 | 8.63E-01 | 8.63E+01 | | | |
| 7909418 | NR_026817 | LOC148696 | hypothetical LOC148696 | -0.038 | 0.974 | 5.84E-01 | 5.84E+01 | | | |
| 7962238 | --- | --- | --- | -0.038 | 0.974 | 5.71E-01 | 5.71E+01 | | | |
| 8165752 | NM_002183 | IL3RA | interleukin 3 receptor, alpha (low aff | -0.038 | 0.974 | 7.90E-01 | 7.90E+01 | | | |
| 8176323 | NM_002183 | IL3RA | interleukin 3 receptor, alpha (low aff | -0.038 | 0.974 | 7.90E-01 | 7.90E+01 | | | |
| 8160835 | NM_148179 | C9orf23 | chromosome 9 open reading frame 2 | -0.038 | 0.974 | 6.53E-01 | 6.53E+01 | | | |
| 8129675 | --- | --- | --- | -0.038 | 0.974 | 6.46E-01 | 6.46E+01 | | | |
| 8008139 | NM_023079 | UBE2Z | ubiquitin-conjugating enzyme E2Z | -0.038 | 0.974 | 8.44E-01 | 8.44E+01 | | | |
| 8037775 | NM_000960 | PTGIR | prostaglandin I2 (prostacyclin) recep | -0.038 | 0.974 | 5.96E-01 | 5.96E+01 | | | |
| 8154363 | NM_001029 | RPS26 | ribosomal protein S26 | -0.038 | 0.974 | 8.94E-01 | 8.94E+01 | | | |
| 7994756 | NM_002720 | PPP4C | protein phosphatase 4 (formerly X), | -0.038 | 0.974 | 8.62E-01 | 8.62E+01 | | | |
| 8070171 | --- | --- | --- | -0.038 | 0.974 | 6.79E-01 | 6.79E+01 | | | |
| 8091087 | NM_178177 | NMNAT3 | nicotinamide nucleotide adenylyltrar | -0.038 | 0.974 | 6.22E-01 | 6.22E+01 | | | |
| 7942073 | NM_002180 | IGHMBP2 | immunoglobulin mu binding protein | -0.038 | 0.974 | 5.27E-01 | 5.27E+01 | | | |
| 8118915 | NM_003427 | ZNF76 | zinc finger protein 76 (expressed in t | -0.038 | 0.974 | 5.85E-01 | 5.85E+01 | | | |
| 8180267 | --- | --- | --- | -0.038 | 0.974 | 5.80E-01 | 5.80E+01 | | | |
| 8000244 | NM_020718 | USP31 | ubiquitin specific peptidase 31 | -0.038 | 0.974 | 5.91E-01 | 5.91E+01 | | | |
| 8179750 | NM_004640 | BAT1 | HLA-B associated transcript 1 | -0.038 | 0.974 | 8.70E-01 | 8.70E+01 | | | |
| 8026895 | NM_138442 | CCDC124 | coiled-coil domain containing 124 | -0.038 | 0.974 | 5.83E-01 | 5.83E+01 | | | |
| 8005699 | NM_015510 | DHRS7B | dehydrogenase/reductase (SDR fami | -0.038 | 0.974 | 6.73E-01 | 6.73E+01 | | | |
| 8035345 | NM_005543 | INSL3 | insulin-like 3 (Leydig cell) | -0.039 | 0.974 | 7.37E-01 | 7.37E+01 | | | |
| 7928909 | NM_019054 | FAM35A | family with sequence similarity 35, n | -0.039 | 0.974 | 8.55E-01 | 8.55E+01 | | | |
| 7914084 | NM_207397 | CD164L2 | CD164 sialomucin-like 2 | -0.039 | 0.974 | 5.46E-01 | 5.46E+01 | | | |
| 8158145 | --- | --- | --- | -0.039 | 0.974 | 5.63E-01 | 5.63E+01 | | | |
| 8061966 | NM_016732 | RALY | RNA binding protein, autoantigenic ( | -0.039 | 0.974 | 7.00E-01 | 7.00E+01 | | | |
| 8164665 | NM_005312 | RAPGEF1 | Rap guanine nucleotide exchange fac | -0.039 | 0.974 | 7.95E-01 | 7.95E+01 | | | |
| 8125537 | NM_006120 | HLA-DMA | major histocompatibility complex, cl | -0.039 | 0.974 | 8.47E-01 | 8.47E+01 | | | |
| 8063449 | --- | --- | --- | -0.039 | 0.974 | 7.26E-01 | 7.26E+01 | | | |
| 8079426 | NM_147196 | TMIE | transmembrane inner ear | -0.039 | 0.974 | 5.82E-01 | 5.82E+01 | | | |
| 7944931 | NM_198277 | SLC37A2 | solute carrier family 37 (glycerol-3-p | -0.039 | 0.974 | 6.42E-01 | 6.42E+01 | | | |
| 8151869 | --- | --- | --- | -0.039 | 0.974 | 8.41E-01 | 8.41E+01 | | | |
| 7894779 | --- | --- | --- | -0.039 | 0.974 | 5.66E-01 | 5.66E+01 | | | |
| 8013026 | NM_020653 | ZNF287 | zinc finger protein 287 | -0.039 | 0.974 | 7.21E-01 | 7.21E+01 | | | |
| 8116595 | NM_020135 | WRNIP1 | Werner helicase interacting protein 1 | -0.039 | 0.974 | 6.20E-01 | 6.20E+01 | | | |
| 8120501 | NM_001858 | COL19A1 | collagen, type XIX, alpha 1 | -0.039 | 0.974 | 5.24E-01 | 5.24E+01 | | | |
| 7895115 | --- | --- | --- | -0.039 | 0.974 | 6.51E-01 | 6.51E+01 | | | |
| 8113274 | --- | --- | --- | -0.039 | 0.974 | 9.38E-01 | 9.38E+01 | | | |
| 7911110 | --- | --- | --- | -0.039 | 0.974 | 7.07E-01 | 7.07E+01 | | | |
| 8131583 | NM_00115976 | BZW2 | basic leucine zipper and W2 domains | -0.039 | 0.974 | 8.99E-01 | 8.99E+01 | | | |
| 7896640 | --- | --- | --- | -0.039 | 0.973 | 9.65E-01 | 9.65E+01 | | | |
| 8123562 | NM_001500 | GMDS | GDP-mannose 4,6-dehydratase | -0.039 | 0.973 | 6.24E-01 | 6.24E+01 | | | |
| 8126658 | NM_178148 | SLC35B2 | solute carrier family 35, member B2 | -0.039 | 0.973 | 6.62E-01 | 6.62E+01 | | | |
| 8087210 | NM_004157 | PRKAR2A | protein kinase, cAMP-dependent, re | -0.039 | 0.973 | 7.79E-01 | 7.79E+01 | | | |
| 8082422 | NM_021937 | EEFSEC | eukaryotic elongation factor, selenoc | -0.039 | 0.973 | 5.43E-01 | 5.43E+01 | | | |
| 8125766 | NM_001188 | BAK1 | BCL2-antagonist/killer 1 | -0.039 | 0.973 | 8.59E-01 | 8.59E+01 | | | |
| 7947450 | NM_005574 | LMO2 | LIM domain only 2 (rhombotin-like 1 | -0.039 | 0.973 | 5.61E-01 | 5.61E+01 | | | |
| 8064557 | AK293638 | FAM113A | family with sequence similarity 113, | -0.039 | 0.973 | 5.50E-01 | 5.50E+01 | | | |
| 8118100 | NM_000247 | MICA | MHC class I polypeptide-related sequ | -0.039 | 0.973 | 7.99E-01 | 7.99E+01 | | | |
| 8087780 | --- | --- | --- | -0.039 | 0.973 | 7.26E-01 | 7.26E+01 | | | |
| 8037061 | NM_173506 | LYPD4 | LY6/PLAUR domain containing 4 | -0.039 | 0.973 | 6.33E-01 | 6.33E+01 | | | |
| 8165319 | NM_018998 | FBXW5 | F-box and WD repeat domain contai | -0.039 | 0.973 | 5.51E-01 | 5.51E+01 | | | |
| 7941059 | ENST00000316 | LOC439914 | hypothetical gene supported by NM_ | -0.039 | 0.973 | 5.80E-01 | 5.80E+01 | | | |
| 8031759 | NM_198542 | ZNF773 | zinc finger protein 773 | -0.039 | 0.973 | 6.84E-01 | 6.84E+01 | | | |
| 8099233 | ENST00000446 | OC10013313 | hypothetical protein LOC100133131 | -0.039 | 0.973 | 5.83E-01 | 5.83E+01 | | | |
| 7892738 | --- | --- | --- | -0.039 | 0.973 | 8.13E-01 | 8.13E+01 | | | |
| 7896126 | --- | --- | --- | -0.039 | 0.973 | 8.58E-01 | 8.58E+01 | | | |
| 7915207 | NM_00113565 | PABPC4 | poly(A) binding protein, cytoplasmic | -0.039 | 0.973 | 7.71E-01 | 7.71E+01 | | | |
| 7912292 | NM_032368 | LZIC | leucine zipper and CTNNBIP1 domair | -0.039 | 0.973 | 9.40E-01 | 9.40E+01 | | | |
| 7914127 | NM_002038 | IFI6 | interferon, alpha-inducible protein 6 | -0.039 | 0.973 | 7.82E-01 | 7.82E+01 | | | |
| 8019018 | NM_003655 | CBX4 | chromobox homolog 4 (Pc class hom | -0.039 | 0.973 | 5.35E-01 | 5.35E+01 | | | |
| 8034379 | NM_030824 | ZNF442 | zinc finger protein 442 | -0.039 | 0.973 | 5.41E-01 | 5.41E+01 | | | |
| 8159667 | NM_017723 | C9orf167 | chromosome 9 open reading frame 1 | -0.039 | 0.973 | 5.58E-01 | 5.58E+01 | | | |
| 8085094 | NM_182760 | SUMF1 | sulfatase modifying factor 1 | -0.039 | 0.973 | 6.82E-01 | 6.82E+01 | | | |
| 8130470 | --- | --- | --- | -0.039 | 0.973 | 5.40E-01 | 5.40E+01 | | | |
| 8018449 | NM_199242 | UNC13D | unc-13 homolog D (C. elegans) | -0.039 | 0.973 | 3.73E-01 | 3.73E+01 | | | |
| 8034202 | NM_004283 | RAB3D | RAB3D, member RAS oncogene fami | -0.039 | 0.973 | 6.24E-01 | 6.24E+01 | | | |
| 8162848 | --- | --- | --- | -0.039 | 0.973 | 7.59E-01 | 7.59E+01 | | | |
| 8125843 | NM_012391 | SPDEF | SAM pointed domain containing ets | -0.040 | 0.973 | 4.90E-01 | 4.90E+01 | | | |
| 8084215 | --- | --- | --- | -0.040 | 0.973 | 6.46E-01 | 6.46E+01 | | | |
| 7925929 | NM_003739 | AKR1C3 | aldo-keto reductase family 1, membe | -0.040 | 0.973 | 6.79E-01 | 6.79E+01 | | | |
| 7894600 | --- | --- | --- | -0.040 | 0.973 | 9.36E-01 | 9.36E+01 | | | |
| 8169459 | NR_002993 | SNORA35 | small nucleolar RNA, H/ACA box 35 | -0.040 | 0.973 | 6.55E-01 | 6.55E+01 | | | |
| 8161824 | NM_017998 | C9orf40 | chromosome 9 open reading frame 4 | -0.040 | 0.973 | 5.09E-01 | 5.09E+01 | | | |
| 7956038 | NM_002429 | MMP19 | matrix metallopeptidase 19 | -0.040 | 0.973 | 6.03E-01 | 6.03E+01 | | | |
| 8096765 | NM_033625 | RPL34 | ribosomal protein L34 | -0.040 | 0.973 | 8.00E-01 | 8.00E+01 | | | |
| 8001750 | NM_018231 | SLC38A7 | solute carrier family 38, member 7 | -0.040 | 0.973 | 4.97E-01 | 4.97E+01 | | | |

| | | | | | | | | | | |
|---|---|---|---|---|---|---|---|---|---|---|
| 7942289 | NM_018320 | RNF121 | ring finger protein 121 | -0.040 | 0.973 | 7.79E-01 | 7.79E+01 | | | |
| 7929919 | NM_030971 | SFXN3 | sideroflexin 3 | -0.040 | 0.973 | 6.28E-01 | 6.28E+01 | | | |
| 8032755 | NM_015898 | ZBTB7A | zinc finger and BTB domain containir | -0.040 | 0.973 | 5.03E-01 | 5.03E+01 | | | |
| 7932985 | NM_003873 | NRP1 | neuropilin 1 | -0.040 | 0.973 | 5.43E-01 | 5.43E+01 | | | |
| 7922462 | NM_022100 | MRPS14 | mitochondrial ribosomal protein S14 | -0.040 | 0.973 | 9.08E-01 | 9.08E+01 | | | |
| 7983365 | NM_182985 | TRIM69 | tripartite motif-containing 69 | -0.040 | 0.973 | 5.04E-01 | 5.04E+01 | | | |
| 7896051 | --- | --- | --- | -0.040 | 0.973 | 7.99E-01 | 7.99E+01 | | | |
| 7895130 | --- | --- | --- | -0.040 | 0.973 | 7.36E-01 | 7.36E+01 | | | |
| 7911634 | NM_178545 | TMEM52 | transmembrane protein 52 | -0.040 | 0.973 | 5.35E-01 | 5.35E+01 | | | |
| 8002152 | NM_005072 | SLC12A4 | solute carrier family 12 (potassium/c | -0.040 | 0.973 | 5.68E-01 | 5.68E+01 | | | |
| 8002667 | NM_031293 | PMFBP1 | polyamine modulated factor 1 bindir | -0.040 | 0.973 | 4.80E-01 | 4.80E+01 | | | |
| 8033241 | NM_001252 | CD70 | CD70 molecule | -0.040 | 0.973 | 5.13E-01 | 5.13E+01 | | | |
| 8071390 | --- | --- | --- | -0.040 | 0.973 | 9.51E-01 | 9.51E+01 | | | |
| 7967636 | NR_026666 | MGC16384 | hypothetical LOC114130 | -0.040 | 0.973 | 8.95E-01 | 8.95E+01 | | | |
| 8070863 | BC004343 | C21orf122 | chromosome 21 open reading frame | -0.040 | 0.973 | 5.39E-01 | 5.39E+01 | | | |
| 8038261 | NM_002103 | GYS1 | glycogen synthase 1 (muscle) | -0.040 | 0.973 | 5.25E-01 | 5.25E+01 | | | |
| 7896756 | BC037297 | FAM87A | family with sequence similarity 87, n | -0.040 | 0.973 | 6.53E-01 | 6.53E+01 | | | |
| 7895969 | --- | --- | --- | -0.040 | 0.973 | 6.67E-01 | 6.67E+01 | | | |
| 8117071 | NM_016255 | FAM8A1 | family with sequence similarity 8, me | -0.040 | 0.973 | 6.43E-01 | 6.43E+01 | | | |
| 8124920 | --- | --- | --- | -0.040 | 0.973 | 8.52E-01 | 8.52E+01 | | | |
| 8026193 | NM_014047 | C19orf53 | chromosome 19 open reading frame | -0.040 | 0.973 | 7.98E-01 | 7.98E+01 | | | |
| 8162759 | NM_018421 | TBC1D2 | TBC1 domain family, member 2 | -0.040 | 0.973 | 5.62E-01 | 5.62E+01 | | | |
| 8052598 | NM_015910 | C2orf86 | chromosome 2 open reading frame 8 | -0.040 | 0.973 | 7.30E-01 | 7.30E+01 | | | |
| 7981283 | NM_00103935 | SLC25A29 | solute carrier family 25, member 29 | -0.040 | 0.972 | 4.75E-01 | 4.75E+01 | | | |
| 7899909 | --- | --- | --- | -0.040 | 0.972 | 9.16E-01 | 9.16E+01 | | | |
| 8005576 | NM_139033 | MAPK7 | mitogen-activated protein kinase 7 | -0.040 | 0.972 | 5.68E-01 | 5.68E+01 | | | |
| 8044773 | NM_006770 | MARCO | macrophage receptor with collagenc | -0.040 | 0.972 | 4.88E-01 | 4.88E+01 | | | |
| 8168604 | NM_053281 | DACH2 | dachshund homolog 2 (Drosophila) | -0.040 | 0.972 | 4.23E-01 | 4.23E+01 | | | |
| 7898549 | NM_016183 | MRTO4 | mRNA turnover 4 homolog (S. cerevi | -0.040 | 0.972 | 8.49E-01 | 8.49E+01 | | | |
| 8001764 | NM_002080 | GOT2 | glutamic-oxaloacetic transaminase 2 | -0.040 | 0.972 | 7.04E-01 | 7.04E+01 | | | |
| 8068192 | NM_178817 | MRAP | melanocortin 2 receptor accessory p | -0.040 | 0.972 | 5.23E-01 | 5.23E+01 | | | |
| 8164373 | NM_012127 | CIZ1 | CDKN1A interacting zinc finger prote | -0.040 | 0.972 | 6.05E-01 | 6.05E+01 | | | |
| 7975311 | NM_018199 | EXD2 | exonuclease 3'-5' domain containing | -0.040 | 0.972 | 7.59E-01 | 7.59E+01 | | | |
| 8078818 | NM_005107 | EXOG | endo/exonuclease (5'-3'), endonucle | -0.040 | 0.972 | 8.47E-01 | 8.47E+01 | | | |
| 7892758 | --- | --- | --- | -0.040 | 0.972 | 9.41E-01 | 9.41E+01 | | | |
| 8015908 | NM_152344 | LSM12 | LSM12 homolog (S. cerevisiae) | -0.040 | 0.972 | 8.72E-01 | 8.72E+01 | | | |
| 7957606 | --- | --- | --- | -0.040 | 0.972 | 7.55E-01 | 7.55E+01 | | | |
| 7960757 | NM_016546 | C1RL | complement component 1, r subcom | -0.040 | 0.972 | 6.48E-01 | 6.48E+01 | | | |
| 7947189 | NM_030771 | CCDC34 | coiled-coil domain containing 34 | -0.040 | 0.972 | 5.06E-01 | 5.06E+01 | | | |
| 7979127 | --- | --- | --- | -0.041 | 0.972 | 7.35E-01 | 7.35E+01 | | | |
| 8161164 | NM_00100448 | OR13J1 | olfactory receptor, family 13, subfam | -0.041 | 0.972 | 5.10E-01 | 5.10E+01 | | | |
| 8011692 | NM_014389 | PELP1 | proline, glutamate and leucine rich p | -0.041 | 0.972 | 5.98E-01 | 5.98E+01 | | | |
| 8164269 | NM_000118 | ENG | endoglin | -0.041 | 0.972 | 4.42E-01 | 4.42E+01 | | | |
| 7927502 | NM_003055 | SLC18A3 | solute carrier family 18 (vesicular ace | -0.041 | 0.972 | 4.30E-01 | 4.30E+01 | | | |
| 7949776 | NM_020441 | CORO1B | coronin, actin binding protein, 1B | -0.041 | 0.972 | 4.45E-01 | 4.45E+01 | | | |
| 8035023 | NM_014371 | AKAP8L | A kinase (PRKA) anchor protein 8-like | -0.041 | 0.972 | 7.24E-01 | 7.24E+01 | | | |
| 8040278 | NM_152391 | PQLC3 | PQ loop repeat containing 3 | -0.041 | 0.972 | 7.73E-01 | 7.73E+01 | | | |
| 7926223 | NM_153498 | CAMK1D | calcium/calmodulin-dependent prot | -0.041 | 0.972 | 9.01E-01 | 9.01E+01 | | | |
| 8070930 | --- | --- | --- | -0.041 | 0.972 | 6.03E-01 | 6.03E+01 | | | |
| 8118833 | NM_017754 | UHRF1BP1 | UHRF1 binding protein 1 | -0.041 | 0.972 | 6.98E-01 | 6.98E+01 | | | |
| 8038839 | NM_014442 | SIGLEC8 | sialic acid binding Ig-like lectin 8 | -0.041 | 0.972 | 5.17E-01 | 5.17E+01 | | | |
| 8103094 | NM_000901 | NR3C2 | nuclear receptor subfamily 3, group | -0.041 | 0.972 | 7.91E-01 | 7.91E+01 | | | |
| 7905854 | NM_015872 | ZBTB7B | zinc finger and BTB domain containir | -0.041 | 0.972 | 5.41E-01 | 5.41E+01 | | | |
| 8009526 | NM_001050 | SSTR2 | somatostatin receptor 2 | -0.041 | 0.972 | 5.55E-01 | 5.55E+01 | | | |
| 7893695 | --- | --- | --- | -0.041 | 0.972 | 8.18E-01 | 8.18E+01 | | | |
| 8044351 | CR590757 | RPL22P15 | ribosomal protein L22 pseudogene 1 | -0.041 | 0.972 | 5.75E-01 | 5.75E+01 | | | |
| 8133167 | NM_153033 | KCTD7 | potassium channel tetramerisation d | -0.041 | 0.972 | 6.48E-01 | 6.48E+01 | | | |
| 8026806 | NM_024656 | GLT25D1 | glycosyltransferase 25 domain conta | -0.041 | 0.972 | 5.52E-01 | 5.52E+01 | | | |
| 7986463 | NM_024652 | LRRK1 | leucine-rich repeat kinase 1 | -0.041 | 0.972 | 4.46E-01 | 4.46E+01 | | | |
| 7917331 | NR_024113 | C1orf52 | chromosome 1 open reading frame 5 | -0.041 | 0.972 | 6.99E-01 | 6.99E+01 | | | |
| 7901357 | --- | --- | --- | -0.041 | 0.972 | 5.68E-01 | 5.68E+01 | | | |
| 8156523 | --- | --- | --- | -0.041 | 0.972 | 7.09E-01 | 7.09E+01 | | | |
| 8178508 | NM_130463 | ATP6V1G2 | ATPase, H+ transporting, lysosomal 1 | -0.041 | 0.972 | 6.23E-01 | 6.23E+01 | | | |
| 7922686 | NM_00100999 | ZNF648 | zinc finger protein 648 | -0.041 | 0.972 | 4.98E-01 | 4.98E+01 | | | |
| 8002121 | NM_001907 | CTRL | chymotrypsin-like | -0.041 | 0.972 | 7.60E-01 | 7.60E+01 | | | |
| 7918426 | NM_004696 | SLC16A4 | solute carrier family 16, member 4 (r | -0.041 | 0.972 | 7.60E-01 | 7.60E+01 | | | |
| 7896477 | --- | --- | --- | -0.041 | 0.972 | 8.59E-01 | 8.59E+01 | | | |
| 8056810 | AK095037 | OC10012931 | hypothetical LOC100129312 | -0.041 | 0.972 | 5.51E-01 | 5.51E+01 | | | |
| 8144279 | NM_018941 | CLN8 | ceroid-lipofuscinosis, neuronal 8 (ep | -0.041 | 0.972 | 6.18E-01 | 6.18E+01 | | | |
| 7998211 | NM_003502 | AXIN1 | axin 1 | -0.041 | 0.972 | 5.82E-01 | 5.82E+01 | | | |
| 8043114 | NM_031283 | TCF7L1 | transcription factor 7-like 1 (T-cell sp | -0.041 | 0.972 | 5.16E-01 | 5.16E+01 | | | |
| 7893616 | --- | --- | --- | -0.041 | 0.972 | 8.37E-01 | 8.37E+01 | | | |
| 7986110 | NM_002005 | FES | feline sarcoma oncogene | -0.041 | 0.972 | 4.71E-01 | 4.71E+01 | | | |
| 8162142 | NM_030940 | ISCA1 | iron-sulfur cluster assembly 1 homol | -0.041 | 0.972 | 7.19E-01 | 7.19E+01 | | | |
| 7895527 | --- | --- | --- | -0.041 | 0.972 | 9.03E-01 | 9.03E+01 | | | |
| 7898805 | NM_000491 | C1QB | complement component 1, q subcon | -0.041 | 0.972 | 5.04E-01 | 5.04E+01 | | | |
| 8174496 | NM_015365 | AMMECR1 | Alport syndrome, mental retardation | -0.041 | 0.972 | 5.26E-01 | 5.26E+01 | | | |

| | | | | | | | | | | |
|---|---|---|---|---|---|---|---|---|---|---|
| 8030525 | NM_017432 | PTOV1 | prostate tumor overexpressed 1 | -0.041 | 0.972 | 5.34E-01 | 5.34E+01 | | | |
| 7900395 | NM_012421 | RLF | rearranged L-myc fusion | -0.041 | 0.972 | 9.03E-01 | 9.03E+01 | | | |
| 8094154 | AF073924 | OR7E35P | olfactory receptor, family 7, subfami | -0.041 | 0.972 | 6.59E-01 | 6.59E+01 | | | |
| 8077595 | NM_00100369 | BRPF1 | bromodomain and PHD finger contai | -0.041 | 0.972 | 6.50E-01 | 6.50E+01 | | | |
| 8063857 | NM_015666 | GTPBP5 | GTP binding protein 5 (putative) | -0.041 | 0.972 | 5.26E-01 | 5.26E+01 | | | |
| 8148329 | --- | --- | --- | -0.041 | 0.972 | 5.85E-01 | 5.85E+01 | | | |
| 8087419 | NM_000481 | AMT | aminomethyltransferase | -0.041 | 0.972 | 6.66E-01 | 6.66E+01 | | | |
| 7894808 | --- | --- | --- | -0.041 | 0.972 | 8.43E-01 | 8.43E+01 | | | |
| 8137964 | NM_024963 | FBXL18 | F-box and leucine-rich repeat protei | -0.041 | 0.972 | 4.58E-01 | 4.58E+01 | | | |
| 7981278 | NM_206918 | DEGS2 | degenerative spermatocyte homolog | -0.041 | 0.972 | 5.19E-01 | 5.19E+01 | | | |
| 7941074 | NM_144585 | SLC22A12 | solute carrier family 22 (organic anio | -0.042 | 0.972 | 4.07E-01 | 4.07E+01 | | | |
| 8064762 | NM_001810 | CENPB | centromere protein B, 80kDa | -0.042 | 0.972 | 4.21E-01 | 4.21E+01 | | | |
| 8060344 | NM_021158 | TRIB3 | tribbles homolog 3 (Drosophila) | -0.042 | 0.972 | 5.08E-01 | 5.08E+01 | | | |
| 7919314 | NM_001461 | FMO5 | flavin containing monooxygenase 5 | -0.042 | 0.972 | 6.47E-01 | 6.47E+01 | | | |
| 8085220 | NM_133480 | TADA3L | transcriptional adaptor 3 (NGG1 hom | -0.042 | 0.972 | 6.42E-01 | 6.42E+01 | | | |
| 8025199 | NM_006702 | PNPLA6 | patatin-like phospholipase domain c | -0.042 | 0.972 | 4.57E-01 | 4.57E+01 | | | |
| 8114158 | NM_002715 | PPP2CA | protein phosphatase 2 (formerly 2A) | -0.042 | 0.972 | 8.98E-01 | 8.98E+01 | | | |
| 7925918 | NM_001353 | AKR1C1 | aldo-keto reductase family 1, memb | -0.042 | 0.972 | 6.24E-01 | 6.24E+01 | | | |
| 7955535 | NM_004302 | ACVR1B | activin A receptor, type IB | -0.042 | 0.972 | 4.89E-01 | 4.89E+01 | | | |
| 8000811 | NM_00104005 | MAPK3 | mitogen-activated protein kinase 3 | -0.042 | 0.971 | 6.79E-01 | 6.79E+01 | | | |
| 7936507 | BC021737 | C10orf82 | chromosome 10 open reading frame | -0.042 | 0.971 | 6.07E-01 | 6.07E+01 | | | |
| 8068202 | NM_058187 | C21orf63 | chromosome 21 open reading frame | -0.042 | 0.971 | 5.41E-01 | 5.41E+01 | | | |
| 7977435 | --- | --- | --- | -0.042 | 0.971 | 5.26E-01 | 5.26E+01 | | | |
| 7984843 | NM_006465 | ARID3B | AT rich interactive domain 3B (BRIGH | -0.042 | 0.971 | 7.83E-01 | 7.83E+01 | | | |
| 8034983 | NM_058243 | BRD4 | bromodomain containing 4 | -0.042 | 0.971 | 5.16E-01 | 5.16E+01 | | | |
| 7910901 | NR_027247 | OC10013033 | actin, gamma-like | -0.042 | 0.971 | 7.97E-01 | 7.97E+01 | | | |
| 7939005 | --- | --- | --- | -0.042 | 0.971 | 6.73E-01 | 6.73E+01 | | | |
| 8033356 | NM_144614 | MBD3L2 | methyl-CpG binding domain protein | -0.042 | 0.971 | 5.70E-01 | 5.70E+01 | | | |
| 8033359 | NM_144614 | MBD3L2 | methyl-CpG binding domain protein | -0.042 | 0.971 | 5.70E-01 | 5.70E+01 | | | |
| 7998542 | NM_014714 | IFT140 | intraflagellar transport 140 homolog | -0.042 | 0.971 | 4.98E-01 | 4.98E+01 | | | |
| 8114010 | NM_002198 | IRF1 | interferon regulatory factor 1 | -0.042 | 0.971 | 8.62E-01 | 8.62E+01 | | | |
| 7895917 | --- | --- | --- | -0.042 | 0.971 | 6.48E-01 | 6.48E+01 | | | |
| 7970251 | NM_003891 | PROZ | protein Z, vitamin K-dependent plasr | -0.042 | 0.971 | 5.97E-01 | 5.97E+01 | | | |
| 7894189 | --- | --- | --- | -0.042 | 0.971 | 7.57E-01 | 7.57E+01 | | | |
| 8082350 | NM_004526 | MCM2 | minichromosome maintenance comp | -0.042 | 0.971 | 4.16E-01 | 4.16E+01 | | | |
| 7948864 | NM_003164 | STX5 | syntaxin 5 | -0.042 | 0.971 | 8.47E-01 | 8.47E+01 | | | |
| 7906995 | NM_012474 | UCK2 | uridine-cytidine kinase 2 | -0.042 | 0.971 | 5.53E-01 | 5.53E+01 | | | |
| 8044133 | NM_003581 | NCK2 | NCK adaptor protein 2 | -0.042 | 0.971 | 8.70E-01 | 8.70E+01 | | | |
| 8029375 | --- | --- | --- | -0.042 | 0.971 | 6.58E-01 | 6.58E+01 | | | |
| 7900857 | NM_174963 | ST3GAL3 | ST3 beta-galactoside alpha-2,3-sialyl | -0.042 | 0.971 | 6.02E-01 | 6.02E+01 | | | |
| 8018177 | NM_006678 | CD300C | CD300c molecule | -0.042 | 0.971 | 5.98E-01 | 5.98E+01 | | | |
| 8039530 | NM_203374 | ZNF784 | zinc finger protein 784 | -0.042 | 0.971 | 3.86E-01 | 3.86E+01 | | | |
| 7992870 | NM_005741 | ZNF263 | zinc finger protein 263 | -0.042 | 0.971 | 6.79E-01 | 6.79E+01 | | | |
| 8116956 | NM_031244 | SIRT5 | sirtuin (silent mating type informatic | -0.042 | 0.971 | 7.18E-01 | 7.18E+01 | | | |
| 7895362 | --- | --- | --- | -0.042 | 0.971 | 8.22E-01 | 8.22E+01 | | | |
| 8022410 | --- | --- | --- | -0.042 | 0.971 | 7.27E-01 | 7.27E+01 | | | |
| 8040698 | NM_017877 | C2orf18 | chromosome 2 open reading frame 1 | -0.042 | 0.971 | 6.24E-01 | 6.24E+01 | | | |
| 7919642 | NM_175065 | HIST2H2AB | histone cluster 2, H2ab | -0.042 | 0.971 | 8.72E-01 | 8.72E+01 | | | |
| 8153424 | NM_00110087 | C8orf73 | chromosome 8 open reading frame 7 | -0.042 | 0.971 | 7.98E-01 | 7.98E+01 | | | |
| 7962185 | NR_004854 | AMN1 | antagonist of mitotic exit network 1 | -0.042 | 0.971 | 8.94E-01 | 8.94E+01 | | | |
| 7992692 | NM_016333 | SRRM2 | serine/arginine repetitive matrix 2 | -0.042 | 0.971 | 6.50E-01 | 6.50E+01 | | | |
| 8008588 | NM_002126 | HLF | hepatic leukemia factor | -0.042 | 0.971 | 4.90E-01 | 4.90E+01 | | | |
| 8043363 | NR_024204 | NCRNA0015 | non-protein coding RNA 152 | -0.042 | 0.971 | 9.24E-01 | 9.24E+01 | | | |
| 8098877 | NR_024569 | OC10013087 | hypothetical LOC100130872 | -0.042 | 0.971 | 4.78E-01 | 4.78E+01 | | | |
| 7981161 | NM_152592 | C14orf49 | chromosome 14 open reading frame | -0.042 | 0.971 | 4.43E-01 | 4.43E+01 | | | |
| 7952408 | NM_170601 | SIAE | sialic acid acetylesterase | -0.042 | 0.971 | 4.18E-01 | 4.18E+01 | | | |
| 8171229 | NM_004650 | PNPLA4 | patatin-like phospholipase domain c | -0.043 | 0.971 | 6.66E-01 | 6.66E+01 | | | |
| 7919208 | NR_002328 | GNRHR2 | gonadotropin-releasing hormone (ty | -0.043 | 0.971 | 7.60E-01 | 7.60E+01 | | | |
| 8013479 | NM_00100430 | CCDC144NL | coiled-coil domain containing 144 fa | -0.043 | 0.971 | 4.32E-01 | 4.32E+01 | | | |
| 7924969 | NM_014409 | TAF5L | TAF5-like RNA polymerase II, p300/C | -0.043 | 0.971 | 6.37E-01 | 6.37E+01 | | | |
| 8145047 | NM_003018 | SFTPC | surfactant protein C | -0.043 | 0.971 | 5.08E-01 | 5.08E+01 | | | |
| 8012837 | NM_00110138 | CG_177601 | phosphoinositide-interacting regulat | -0.043 | 0.971 | 5.24E-01 | 5.24E+01 | | | |
| 7892984 | --- | --- | --- | -0.043 | 0.971 | 8.78E-01 | 8.78E+01 | | | |
| 8125125 | NM_021177 | LSM2 | LSM2 homolog, U6 small nuclear RN | -0.043 | 0.971 | 8.51E-01 | 8.51E+01 | | | |
| 8178641 | NM_021177 | LSM2 | LSM2 homolog, U6 small nuclear RN | -0.043 | 0.971 | 8.51E-01 | 8.51E+01 | | | |
| 8179839 | NM_021177 | LSM2 | LSM2 homolog, U6 small nuclear RN | -0.043 | 0.971 | 8.51E-01 | 8.51E+01 | | | |
| 8035551 | BC004943 | MGC10814 | hypothetical protein MGC10814 | -0.043 | 0.971 | 5.99E-01 | 5.99E+01 | | | |
| 7984743 | NM_00102473 | CD276 | CD276 molecule | -0.043 | 0.971 | 5.93E-01 | 5.93E+01 | | | |
| 8038735 | NM_144947 | KLK11 | kallikrein-related peptidase 11 | -0.043 | 0.971 | 5.79E-01 | 5.79E+01 | | | |
| 7978553 | NM_138288 | C14orf147 | chromosome 14 open reading frame | -0.043 | 0.971 | 7.92E-01 | 7.92E+01 | | | |
| 8136954 | NM_00113002 | FAM115C | family with sequence similarity 115, | -0.043 | 0.971 | 6.41E-01 | 6.41E+01 | | | |
| 8031865 | --- | --- | --- | -0.043 | 0.971 | 6.55E-01 | 6.55E+01 | | | |
| 8143788 | --- | --- | --- | -0.043 | 0.971 | 6.31E-01 | 6.31E+01 | | | |
| 8048257 | NM_015488 | PNKD | paroxysmal nonkinesigenic dyskinesi | -0.043 | 0.971 | 5.17E-01 | 5.17E+01 | | | |
| 7895675 | --- | --- | --- | -0.043 | 0.971 | 8.91E-01 | 8.91E+01 | | | |
| 7994109 | NM_005030 | PLK1 | polo-like kinase 1 (Drosophila) | -0.043 | 0.971 | 6.64E-01 | 6.64E+01 | | | |
| 8011415 | NM_002561 | P2RX5 | purinergic receptor P2X, ligand-gate | -0.043 | 0.971 | 8.47E-01 | 8.47E+01 | | | |

| | | | | | | | | | | |
|---|---|---|---|---|---|---|---|---|---|---|
| 8015526 | NM_021078 | KAT2A | K(lysine) acetyltransferase 2A | -0.043 | 0.971 | 6.07E-01 | 6.07E+01 | | | |
| 8011747 | NM_003562 | SLC25A11 | solute carrier family 25 (mitochondri | -0.043 | 0.971 | 7.75E-01 | 7.75E+01 | | | |
| 7911657 | AK094642 | hCG_164728 | hypothetical protein LOC728690 | -0.043 | 0.971 | 4.38E-01 | 4.38E+01 | | | |
| 7894044 | --- | --- | --- | -0.043 | 0.971 | 9.50E-01 | 9.50E+01 | | | |
| 8174837 | NM_00108014 | CT47A1 | cancer/testis antigen family 47, mem | -0.043 | 0.971 | 4.47E-01 | 4.47E+01 | | | |
| 8174841 | NM_00108014 | CT47A1 | cancer/testis antigen family 47, mem | -0.043 | 0.971 | 4.47E-01 | 4.47E+01 | | | |
| 8174845 | NM_00108014 | CT47A1 | cancer/testis antigen family 47, mem | -0.043 | 0.971 | 4.47E-01 | 4.47E+01 | | | |
| 8174849 | NM_00108014 | CT47A1 | cancer/testis antigen family 47, mem | -0.043 | 0.971 | 4.47E-01 | 4.47E+01 | | | |
| 8174862 | NM_00108013 | CT47A10 | cancer/testis antigen family 47, mem | -0.043 | 0.971 | 4.47E-01 | 4.47E+01 | | | |
| 8174867 | NM_00108014 | CT47A1 | cancer/testis antigen family 47, mem | -0.043 | 0.971 | 4.47E-01 | 4.47E+01 | | | |
| 8174872 | NM_00108014 | CT47A1 | cancer/testis antigen family 47, mem | -0.043 | 0.971 | 4.47E-01 | 4.47E+01 | | | |
| 8174876 | NM_00108014 | CT47A1 | cancer/testis antigen family 47, mem | -0.043 | 0.971 | 4.47E-01 | 4.47E+01 | | | |
| 8174882 | NM_00108014 | CT47A1 | cancer/testis antigen family 47, mem | -0.043 | 0.971 | 4.47E-01 | 4.47E+01 | | | |
| 8032588 | NM_00113650 | LOC284422 | similar to HSPC323 | -0.043 | 0.971 | 5.78E-01 | 5.78E+01 | | | |
| 8065469 | NM_00104247 | ABHD12 | abhydrolase domain containing 12 | -0.043 | 0.971 | 6.36E-01 | 6.36E+01 | | | |
| 8056972 | --- | --- | --- | -0.043 | 0.971 | 5.83E-01 | 5.83E+01 | | | |
| 8068401 | NM_001757 | CBR1 | carbonyl reductase 1 | -0.043 | 0.971 | 7.03E-01 | 7.03E+01 | | | |
| 8094056 | NM_053044 | HTRA3 | HtrA serine peptidase 3 | -0.043 | 0.971 | 3.94E-01 | 3.94E+01 | | | |
| 7945475 | NM_021924 | MUPCDH | mucin-like protocadherin | -0.043 | 0.971 | 4.50E-01 | 4.50E+01 | | | |
| 8014925 | NM_014815 | MED24 | mediator complex subunit 24 | -0.043 | 0.971 | 6.31E-01 | 6.31E+01 | | | |
| 8040469 | AY189285 | OC10013137 | hypothetical LOC100131373 | -0.043 | 0.971 | 5.59E-01 | 5.59E+01 | | | |
| 8179313 | NM_138277 | C6orf25 | chromosome 6 open reading frame 2 | -0.043 | 0.971 | 5.74E-01 | 5.74E+01 | | | |
| 8084951 | NM_198565 | LRRC33 | leucine rich repeat containing 33 | -0.043 | 0.971 | 5.50E-01 | 5.50E+01 | | | |
| 7923991 | NM_025179 | PLXNA2 | plexin A2 | -0.043 | 0.971 | 4.27E-01 | 4.27E+01 | | | |
| 7971862 | --- | --- | --- | -0.043 | 0.971 | 6.17E-01 | 6.17E+01 | | | |
| 8034754 | NM_138352 | SAMD1 | sterile alpha motif domain containin | -0.043 | 0.971 | 5.39E-01 | 5.39E+01 | | | |
| 8030866 | NM_002030 | FPR3 | formyl peptide receptor 3 | -0.043 | 0.970 | 5.65E-01 | 5.65E+01 | | | |
| 7950471 | NM_00100528 | OR2AT4 | olfactory receptor, family 2, subfami | -0.043 | 0.970 | 6.54E-01 | 6.54E+01 | | | |
| 7963137 | --- | --- | --- | -0.043 | 0.970 | 9.19E-01 | 9.19E+01 | | | |
| 8178346 | NM_005275 | GNL1 | guanine nucleotide binding protein-l | -0.043 | 0.970 | 6.32E-01 | 6.32E+01 | | | |
| 8080001 | NM_016173 | HEMK1 | HemK methyltransferase family mem | -0.043 | 0.970 | 7.27E-01 | 7.27E+01 | | | |
| 7964577 | --- | --- | --- | -0.043 | 0.970 | 5.96E-01 | 5.96E+01 | | | |
| 8061912 | --- | --- | --- | -0.043 | 0.970 | 5.98E-01 | 5.98E+01 | | | |
| 8162610 | NM_033331 | CDC14B | CDC14 cell division cycle 14 homolog | -0.043 | 0.970 | 6.39E-01 | 6.39E+01 | | | |
| 8024898 | NM_018708 | FEM1A | fem-1 homolog a (C. elegans) | -0.043 | 0.970 | 7.61E-01 | 7.61E+01 | | | |
| 7896859 | --- | --- | --- | -0.043 | 0.970 | 5.13E-01 | 5.13E+01 | | | |
| 7991742 | NM_002434 | MPG | N-methylpurine-DNA glycosylase | -0.043 | 0.970 | 2.97E-01 | 2.97E+01 | | | |
| 7993248 | NM_152308 | C16orf75 | chromosome 16 open reading frame | -0.043 | 0.970 | 5.11E-01 | 5.11E+01 | | | |
| 8144658 | NM_145043 | NEIL2 | nei like 2 (E. coli) | -0.043 | 0.970 | 5.03E-01 | 5.03E+01 | | | |
| 7923319 | NM_016456 | TMEM9 | transmembrane protein 9 | -0.043 | 0.970 | 5.77E-01 | 5.77E+01 | | | |
| 8033892 | NM_031917 | ANGPTL6 | angiopoietin-like 6 | -0.043 | 0.970 | 5.25E-01 | 5.25E+01 | | | |
| 7998119 | NR_024540 | WASH5P | WAS protein family homolog 5 pseud | -0.043 | 0.970 | 7.46E-01 | 7.46E+01 | | | |
| 8164781 | NM_000368 | TSC1 | tuberous sclerosis 1 | -0.043 | 0.970 | 7.97E-01 | 7.97E+01 | | | |
| 8094026 | --- | --- | --- | -0.043 | 0.970 | 6.17E-01 | 6.17E+01 | | | |
| 8004534 | NM_001040 | SHBG | sex hormone-binding globulin | -0.044 | 0.970 | 4.97E-01 | 4.97E+01 | | | |
| 8032996 | NM_205767 | C19orf70 | chromosome 19 open reading frame | -0.044 | 0.970 | 6.27E-01 | 6.27E+01 | | | |
| 8035236 | NM_033417 | HAUS8 | HAUS augmin-like complex, subunit 8 | -0.044 | 0.970 | 7.74E-01 | 7.74E+01 | | | |
| 8132725 | NM_003364 | UPP1 | uridine phosphorylase 1 | -0.044 | 0.970 | 7.31E-01 | 7.31E+01 | | | |
| 7997321 | NM_014940 | MON1B | MON1 homolog B (yeast) | -0.044 | 0.970 | 5.54E-01 | 5.54E+01 | | | |
| 8071951 | --- | --- | --- | -0.044 | 0.970 | 6.65E-01 | 6.65E+01 | | | |
| 7940884 | NM_032344 | NUDT22 | nudix (nucleoside diphosphate linked | -0.044 | 0.970 | 5.49E-01 | 5.49E+01 | | | |
| 7962327 | NM_052885 | SLC2A13 | solute carrier family 2 (facilitated glu | -0.044 | 0.970 | 4.43E-01 | 4.43E+01 | | | |
| 8051773 | --- | --- | --- | -0.044 | 0.970 | 7.24E-01 | 7.24E+01 | | | |
| 8003357 | NM_00114286 | FAM38A | family with sequence similarity 38, m | -0.044 | 0.970 | 4.17E-01 | 4.17E+01 | | | |
| 7922727 | NM_00110245 | RGS8 | regulator of G-protein signaling 8 | -0.044 | 0.970 | 4.83E-01 | 4.83E+01 | | | |
| 7936322 | NM_020918 | GPAM | glycerol-3-phosphate acyltransferase | -0.044 | 0.970 | 6.72E-01 | 6.72E+01 | | | |
| 7905691 | NM_001030 | RPS27 | ribosomal protein S27 | -0.044 | 0.970 | 7.34E-01 | 7.34E+01 | | | |
| 8015798 | NR_027413 | OC10013058 | hypothetical LOC100130581 | -0.044 | 0.970 | 7.45E-01 | 7.45E+01 | | | |
| 7900157 | NM_001030 | RPS27 | ribosomal protein S27 | -0.044 | 0.970 | 7.69E-01 | 7.69E+01 | | | |
| 7939805 | NM_00112822 | SLC39A13 | solute carrier family 39 (zinc transpo | -0.044 | 0.970 | 3.62E-01 | 3.62E+01 | | | |
| 7893634 | --- | --- | --- | -0.044 | 0.970 | 6.15E-01 | 6.15E+01 | | | |
| 7955250 | NM_012272 | PRPF40B | PRP40 pre-mRNA processing factor 4 | -0.044 | 0.970 | 4.59E-01 | 4.59E+01 | | | |
| 8168393 | NM_00104250 | PABPC1L2B | poly(A) binding protein, cytoplasmic | -0.044 | 0.970 | 4.97E-01 | 4.97E+01 | | | |
| 8042825 | --- | --- | --- | -0.044 | 0.970 | 4.91E-01 | 4.91E+01 | | | |
| 8103079 | NM_138364 | PRMT10 | protein arginine methyltransferase 1 | -0.044 | 0.970 | 8.91E-01 | 8.91E+01 | | | |
| 7953675 | NM_024865 | NANOG | Nanog homeobox | -0.044 | 0.970 | 4.42E-01 | 4.42E+01 | | | |
| 8171182 | NM_005044 | PRKX | protein kinase, X-linked | -0.044 | 0.970 | 6.50E-01 | 6.50E+01 | | | |
| 7900216 | NM_024595 | AKIRIN1 | akirin 1 | -0.044 | 0.970 | 5.48E-01 | 5.48E+01 | | | |
| 8085716 | NM_002971 | SATB1 | SATB homeobox 1 | -0.044 | 0.970 | 8.52E-01 | 8.52E+01 | | | |
| 8045637 | NM_004522 | KIF5C | kinesin family member 5C | -0.044 | 0.970 | 6.74E-01 | 6.74E+01 | | | |
| 7954460 | NM_00100166 | LYRM5 | LYR motif containing 5 | -0.044 | 0.970 | 7.38E-01 | 7.38E+01 | | | |
| 8025183 | NM_020533 | MCOLN1 | mucolipin 1 | -0.044 | 0.970 | 5.16E-01 | 5.16E+01 | | | |
| 8129069 | --- | --- | --- | -0.044 | 0.970 | 8.59E-01 | 8.59E+01 | | | |
| 8013015 | NM_181716 | CENPV | centromere protein V | -0.044 | 0.970 | 6.60E-01 | 6.60E+01 | | | |
| 7892634 | --- | --- | --- | -0.044 | 0.970 | 6.70E-01 | 6.70E+01 | | | |
| 7980327 | NM_015305 | ANGEL1 | angel homolog 1 (Drosophila) | -0.044 | 0.970 | 4.44E-01 | 4.44E+01 | | | |
| 8053165 | NM_053050 | MRPL53 | mitochondrial ribosomal protein L53 | -0.044 | 0.970 | 8.69E-01 | 8.69E+01 | | | |

| | | | | | | | | | | |
|---|---|---|---|---|---|---|---|---|---|---|
| 8040753 | NM_017727 | TMEM214 | transmembrane protein 214 | -0.044 | 0.970 | 5.10E-01 | 5.10E+01 | | | |
| 8043666 | NM_017623 | CNNM3 | cyclin M3 | -0.044 | 0.970 | 6.65E-01 | 6.65E+01 | | | |
| 8149555 | NM_015310 | PSD3 | pleckstrin and Sec7 domain containi | -0.044 | 0.970 | 5.65E-01 | 5.65E+01 | | | |
| 8124553 | NM_019110 | ZKSCAN4 | zinc finger with KRAB and SCAN dom | -0.044 | 0.970 | 5.84E-01 | 5.84E+01 | | | |
| 7895382 | --- | --- | --- | -0.044 | 0.970 | 9.46E-01 | 9.46E+01 | | | |
| 8036291 | NM_00104247 | ZNF565 | zinc finger protein 565 | -0.044 | 0.970 | 5.00E-01 | 5.00E+01 | | | |
| 7955179 | NM_032704 | TUBA1C | tubulin, alpha 1c | -0.044 | 0.970 | 8.21E-01 | 8.21E+01 | | | |
| 8155393 | AK292642 | LOC554249 | hypothetical LOC554249 | -0.044 | 0.970 | 4.24E-01 | 4.24E+01 | | | |
| 8161451 | AK292642 | LOC554249 | hypothetical LOC554249 | -0.044 | 0.970 | 4.24E-01 | 4.24E+01 | | | |
| 7919984 | NM_003944 | SELENBP1 | selenium binding protein 1 | -0.044 | 0.970 | 5.39E-01 | 5.39E+01 | | | |
| 8143749 | NM_207336 | ZNF467 | zinc finger protein 467 | -0.044 | 0.970 | 5.21E-01 | 5.21E+01 | | | mono |
| 8030539 | NM_024682 | TBC1D17 | TBC1 domain family, member 17 | -0.044 | 0.970 | 4.65E-01 | 4.65E+01 | | | |
| 8003621 | NM_018146 | RNMTL1 | RNA methyltransferase like 1 | -0.044 | 0.970 | 6.10E-01 | 6.10E+01 | | | |
| 8119801 | NM_033450 | ABCC10 | ATP-binding cassette, sub-family C (( | -0.044 | 0.970 | 4.36E-01 | 4.36E+01 | | | |
| 7992867 | AK096958 | FLJ39639 | hypothetical protein FLJ39639 | -0.044 | 0.970 | 6.63E-01 | 6.63E+01 | | | |
| 8171435 | NM_003662 | PIR | pirin (iron-binding nuclear protein) | -0.044 | 0.970 | 5.02E-01 | 5.02E+01 | | | |
| 7893134 | --- | --- | --- | -0.044 | 0.970 | 9.46E-01 | 9.46E+01 | | | |
| 7972946 | NM_007368 | RASA3 | RAS p21 protein activator 3 | -0.044 | 0.970 | 8.67E-01 | 8.67E+01 | | | |
| 7992685 | NM_018992 | KCTD5 | potassium channel tetramerisation d | -0.044 | 0.970 | 4.74E-01 | 4.74E+01 | | | |
| 8122198 | --- | --- | --- | -0.044 | 0.970 | 8.61E-01 | 8.61E+01 | | | |
| 8100231 | NM_003215 | TEC | tec protein tyrosine kinase | -0.045 | 0.970 | 5.92E-01 | 5.92E+01 | | | |
| 8030133 | NM_006184 | NUCB1 | nucleobindin 1 | -0.045 | 0.970 | 7.00E-01 | 7.00E+01 | | | |
| 8108483 | NM_031467 | SLC4A9 | solute carrier family 4, sodium bicarl | -0.045 | 0.970 | 5.23E-01 | 5.23E+01 | | | |
| 8108977 | --- | --- | --- | -0.045 | 0.970 | 6.94E-01 | 6.94E+01 | | | |
| 8091595 | AK128002 | FLJ46120 | hypothetical LOC647008 | -0.045 | 0.970 | 5.51E-01 | 5.51E+01 | | | |
| 8127778 | NM_017633 | FAM46A | family with sequence similarity 46, m | -0.045 | 0.970 | 6.19E-01 | 6.19E+01 | | | |
| 8129963 | NM_182503 | ADAT2 | adenosine deaminase, tRNA-specific | -0.045 | 0.970 | 8.59E-01 | 8.59E+01 | | | |
| 8135064 | NM_030961 | TRIM56 | tripartite motif-containing 56 | -0.045 | 0.969 | 5.77E-01 | 5.77E+01 | | | |
| 8143433 | NM_016071 | MRPS33 | mitochondrial ribosomal protein S33 | -0.045 | 0.969 | 8.80E-01 | 8.80E+01 | | | |
| 8028397 | NM_174905 | FAM98C | family with sequence similarity 98, m | -0.045 | 0.969 | 4.17E-01 | 4.17E+01 | | | |
| 7894609 | --- | --- | --- | -0.045 | 0.969 | 7.53E-01 | 7.53E+01 | | | |
| 7992863 | NM_012360 | OR1F1 | olfactory receptor, family 1, subfami | -0.045 | 0.969 | 5.59E-01 | 5.59E+01 | | | |
| 8004266 | NM_201566 | SLC16A13 | solute carrier family 16, member 13 | -0.045 | 0.969 | 7.26E-01 | 7.26E+01 | | | |
| 8099082 | --- | --- | --- | -0.045 | 0.969 | 7.73E-01 | 7.73E+01 | | | |
| 7910680 | NM_003272 | GPR137B | G protein-coupled receptor 137B | -0.045 | 0.969 | 7.47E-01 | 7.47E+01 | | | |
| 8043685 | --- | --- | --- | -0.045 | 0.969 | 6.42E-01 | 6.42E+01 | | | |
| 8177782 | NM_024839 | RPP21 | ribonuclease P/MRP 21kDa subunit | -0.045 | 0.969 | 8.82E-01 | 8.82E+01 | | | |
| 8179097 | NM_024839 | RPP21 | ribonuclease P/MRP 21kDa subunit | -0.045 | 0.969 | 8.82E-01 | 8.82E+01 | | | |
| 8008700 | AK021772 | FLJ11710 | hypothetical protein FLJ11710 | -0.045 | 0.969 | 7.18E-01 | 7.18E+01 | | | |
| 8092959 | NM_006241 | PPP1R2 | protein phosphatase 1, regulatory (ir | -0.045 | 0.969 | 8.71E-01 | 8.71E+01 | | | |
| 7941260 | NM_031904 | FRMD8 | FERM domain containing 8 | -0.045 | 0.969 | 5.86E-01 | 5.86E+01 | | | |
| 7895132 | --- | --- | --- | -0.045 | 0.969 | 9.48E-01 | 9.48E+01 | | | |
| 7959995 | NM_015409 | EP400 | E1A binding protein p400 | -0.045 | 0.969 | 5.81E-01 | 5.81E+01 | | | |
| 8028645 | NM_017592 | MED29 | mediator complex subunit 29 | -0.045 | 0.969 | 6.42E-01 | 6.42E+01 | | | |
| 8137986 | NM_207111 | RNF216 | ring finger protein 216 | -0.045 | 0.969 | 7.74E-01 | 7.74E+01 | | | |
| 8005475 | NM_00103733 | TRIM16L | tripartite motif-containing 16-like | -0.045 | 0.969 | 6.76E-01 | 6.76E+01 | | | |
| 7964499 | NM_00112277 | AGAP2 | ArfGAP with GTPase domain, ankyrin | -0.045 | 0.969 | 4.13E-01 | 4.13E+01 | | | |
| 8031737 | NM_152909 | ZNF548 | zinc finger protein 548 | -0.045 | 0.969 | 6.92E-01 | 6.92E+01 | | | |
| 8028656 | NM_022835 | PLEKHG2 | pleckstrin homology domain contain | -0.045 | 0.969 | 4.61E-01 | 4.61E+01 | | | |
| 8165808 | NM_00114191 | XG | Xg blood group | -0.045 | 0.969 | 5.79E-01 | 5.79E+01 | | | |
| 8003806 | NM_003552 | OR1D4 | olfactory receptor, family 1, subfami | -0.045 | 0.969 | 6.81E-01 | 6.81E+01 | | | |
| 7894201 | --- | --- | --- | -0.045 | 0.969 | 8.79E-01 | 8.79E+01 | | | |
| 8000167 | NM_015092 | SMG1 | SMG1 homolog, phosphatidylinosito | -0.045 | 0.969 | 6.27E-01 | 6.27E+01 | | | |
| 7894459 | --- | --- | --- | -0.045 | 0.969 | 9.21E-01 | 9.21E+01 | | | |
| 8043597 | NM_178495 | ITPRIPL1 | inositol 1,4,5-triphosphate receptor | -0.045 | 0.969 | 4.88E-01 | 4.88E+01 | | | |
| 8072316 | NM_00101743 | CCDC157 | coiled-coil domain containing 157 | -0.045 | 0.969 | 4.73E-01 | 4.73E+01 | | | |
| 7893237 | --- | --- | --- | -0.045 | 0.969 | 5.64E-01 | 5.64E+01 | | | |
| 7930398 | NM_005962 | MXI1 | MAX interactor 1 | -0.045 | 0.969 | 7.46E-01 | 7.46E+01 | | | |
| 7951838 | NM_003904 | ZNF259 | zinc finger protein 259 | -0.045 | 0.969 | 8.38E-01 | 8.38E+01 | | | |
| 7893572 | --- | --- | --- | -0.045 | 0.969 | 9.34E-01 | 9.34E+01 | | | |
| 7893383 | --- | --- | --- | -0.045 | 0.969 | 7.82E-01 | 7.82E+01 | | | |
| 8005267 | NM_030665 | RAI1 | retinoic acid induced 1 | -0.045 | 0.969 | 4.37E-01 | 4.37E+01 | | | |
| 7945645 | NM_00100592 | KRTAP5-1 | keratin associated protein 5-1 | -0.045 | 0.969 | 4.65E-01 | 4.65E+01 | | | |
| 8032491 | NM_032737 | LMNB2 | lamin B2 | -0.045 | 0.969 | 5.55E-01 | 5.55E+01 | | | |
| 7922051 | NM_003851 | CREG1 | cellular repressor of E1A-stimulated | -0.045 | 0.969 | 6.37E-01 | 6.37E+01 | | | |
| 8180247 | --- | --- | --- | -0.045 | 0.969 | 5.27E-01 | 5.27E+01 | | | |
| 8149146 | --- | --- | --- | -0.045 | 0.969 | 6.32E-01 | 6.32E+01 | | | |
| 7924853 | NM_181462 | MRPL55 | mitochondrial ribosomal protein L55 | -0.045 | 0.969 | 6.38E-01 | 6.38E+01 | | | |
| 8025058 | NM_004240 | TRIP10 | thyroid hormone receptor interactor | -0.045 | 0.969 | 5.62E-01 | 5.62E+01 | | | |
| 8097443 | --- | --- | --- | -0.045 | 0.969 | 6.12E-01 | 6.12E+01 | | | |
| 7955358 | BC007849 | C12orf62 | chromosome 12 open reading frame | -0.045 | 0.969 | 8.99E-01 | 8.99E+01 | | | |
| 7896212 | --- | --- | --- | -0.045 | 0.969 | 6.52E-01 | 6.52E+01 | | | |
| 8123340 | --- | --- | --- | -0.045 | 0.969 | 5.89E-01 | 5.89E+01 | | | |
| 7947915 | --- | --- | --- | -0.045 | 0.969 | 6.77E-01 | 6.77E+01 | | | |
| 8115327 | NM_003118 | SPARC | secreted protein, acidic, cysteine-ricl | -0.046 | 0.969 | 8.83E-01 | 8.83E+01 | | | |
| 7984217 | NM_025201 | PLEKHO2 | pleckstrin homology domain contain | -0.046 | 0.969 | 5.05E-01 | 5.05E+01 | | | |
| 8079131 | NM_00112990 | LOC729085 | hypothetical protein LOC729085 | -0.046 | 0.969 | 4.71E-01 | 4.71E+01 | | | |

| | | | | | | | | | | | |
|---|---|---|---|---|---|---|---|---|---|---|---|
| 8066444 | --- | --- | --- | -0.046 | 0.969 | 6.47E-01 | 6.47E+01 | | | | |
| 7977497 | NM_021178 | CCNB1IP1 | cyclin B1 interacting protein 1 | -0.046 | 0.969 | 7.25E-01 | 7.25E+01 | | | | |
| 8180391 | --- | --- | --- | -0.046 | 0.969 | 3.33E-01 | 3.33E+01 | | | | |
| 8127484 | NM_080742 | B3GAT2 | beta-1,3-glucuronyltransferase 2 (glu | -0.046 | 0.969 | 5.66E-01 | 5.66E+01 | | | | |
| 8125373 | NM_022107 | GPSM3 | G-protein signaling modulator 3 (AGS | -0.046 | 0.969 | 7.11E-01 | 7.11E+01 | | | | |
| 8179987 | NM_022107 | GPSM3 | G-protein signaling modulator 3 (AGS | -0.046 | 0.969 | 7.11E-01 | 7.11E+01 | | | | |
| 7998852 | NM_007108 | TCEB2 | transcription elongation factor B (SIII | -0.046 | 0.969 | 7.61E-01 | 7.61E+01 | | | | |
| 8104115 | --- | --- | --- | -0.046 | 0.969 | 8.42E-01 | 8.42E+01 | | | | |
| 8118007 | NM_001517 | GTF2H4 | general transcription factor IIH, poly | -0.046 | 0.969 | 4.63E-01 | 4.63E+01 | | | | |
| 8177885 | NM_001517 | GTF2H4 | general transcription factor IIH, poly | -0.046 | 0.969 | 4.63E-01 | 4.63E+01 | | | | |
| 8179205 | NM_001517 | GTF2H4 | general transcription factor IIH, poly | -0.046 | 0.969 | 4.63E-01 | 4.63E+01 | | | | |
| 7949146 | NM_004630 | SF1 | splicing factor 1 | -0.046 | 0.969 | 7.11E-01 | 7.11E+01 | | | | |
| 8072353 | AY358802 | UNQ1945 | VLGN1945 | -0.046 | 0.969 | 4.71E-01 | 4.71E+01 | | | | |
| 8077351 | --- | --- | --- | -0.046 | 0.969 | 6.08E-01 | 6.08E+01 | | | | |
| 8048411 | NM_014640 | TTLL4 | tubulin tyrosine ligase-like family, me | -0.046 | 0.969 | 6.65E-01 | 6.65E+01 | | | | |
| 7933008 | --- | --- | --- | -0.046 | 0.969 | 6.37E-01 | 6.37E+01 | | | | |
| 7993946 | NM_013302 | EEF2K | eukaryotic elongation factor-2 kinase | -0.046 | 0.969 | 4.55E-01 | 4.55E+01 | | | | |
| 8011945 | NM_014804 | KIAA0753 | KIAA0753 | -0.046 | 0.969 | 6.40E-01 | 6.40E+01 | | | | |
| 8026687 | NM_024578 | OCEL1 | occludin/ELL domain containing 1 | -0.046 | 0.969 | 5.97E-01 | 5.97E+01 | | | | |
| 7922652 | --- | --- | --- | -0.046 | 0.969 | 5.79E-01 | 5.79E+01 | | | | |
| 7988687 | NM_005254 | GABPB1 | GA binding protein transcription fact | -0.046 | 0.969 | 8.51E-01 | 8.51E+01 | | | | |
| 7912145 | NM_001561 | TNFRSF9 | tumor necrosis factor receptor super | -0.046 | 0.969 | 6.54E-01 | 6.54E+01 | | | | |
| 7892935 | --- | --- | --- | -0.046 | 0.969 | 8.14E-01 | 8.14E+01 | | | | |
| 8119712 | NM_003131 | SRF | serum response factor (c-fos serum r | -0.046 | 0.969 | 6.82E-01 | 6.82E+01 | | | | |
| 8129454 | --- | --- | --- | -0.046 | 0.969 | 6.42E-01 | 6.42E+01 | | | | |
| 7906652 | NM_005600 | NIT1 | nitrilase 1 | -0.046 | 0.969 | 7.34E-01 | 7.34E+01 | | | | |
| 7941444 | NM_001335 | CTSW | cathepsin W | -0.046 | 0.969 | 8.87E-01 | 8.87E+01 | | | | |
| 8159078 | NM_017586 | C9orf7 | chromosome 9 open reading frame 7 | -0.046 | 0.969 | 4.82E-01 | 4.82E+01 | | | | |
| 8153474 | NM_003313 | TSTA3 | tissue specific transplantation antige | -0.046 | 0.969 | 6.69E-01 | 6.69E+01 | | | | |
| 7995292 | NM_005629 | SLC6A8 | solute carrier family 6 (neurotransmi | -0.046 | 0.969 | 4.44E-01 | 4.44E+01 | | | | |
| 8014264 | NM_000286 | PEX12 | peroxisomal biogenesis factor 12 | -0.046 | 0.969 | 5.93E-01 | 5.93E+01 | | | | |
| 8180310 | --- | --- | --- | -0.046 | 0.968 | 8.79E-01 | 8.79E+01 | | | | |
| 7956470 | NM_052897 | MBD6 | methyl-CpG binding domain protein | -0.046 | 0.968 | 4.43E-01 | 4.43E+01 | | | | |
| 8161727 | NM_016014 | FAM108B1 | family with sequence similarity 108, | -0.046 | 0.968 | 5.88E-01 | 5.88E+01 | | | | |
| 7920877 | NM_004723 | ARHGEF2 | Rho/Rac guanine nucleotide exchang | -0.046 | 0.968 | 4.76E-01 | 4.76E+01 | | | | |
| 8015759 | NM_006373 | VAT1 | vesicle amine transport protein 1 ho | -0.046 | 0.968 | 5.83E-01 | 5.83E+01 | | | | |
| 7949340 | NM_138456 | BATF2 | basic leucine zipper transcription fac | -0.046 | 0.968 | 4.54E-01 | 4.54E+01 | | | | |
| 8110685 | NR_024158 | LOC25845 | hypothetical LOC25845 | -0.046 | 0.968 | 7.18E-01 | 7.18E+01 | | | | |
| 7985259 | NM_019006 | ZFAND6 | zinc finger, AN1-type domain 6 | -0.046 | 0.968 | 8.70E-01 | 8.70E+01 | | | | |
| 8040815 | NM_032604 | ABHD1 | abhydrolase domain containing 1 | -0.046 | 0.968 | 5.12E-01 | 5.12E+01 | | | | |
| 7950447 | NM_182969 | XRRA1 | X-ray radiation resistance associated | -0.046 | 0.968 | 4.76E-01 | 4.76E+01 | | | | |
| 7894848 | --- | --- | --- | -0.046 | 0.968 | 7.74E-01 | 7.74E+01 | | | | |
| 8161964 | NM_174938 | FRMD3 | FERM domain containing 3 | -0.046 | 0.968 | 4.47E-01 | 4.47E+01 | | | | |
| 8013947 | NM_032854 | CORO6 | coronin 6 | -0.046 | 0.968 | 3.55E-01 | 3.55E+01 | | | | |
| 8109179 | NM_133263 | PPARGC1B | peroxisome proliferator-activated re | -0.046 | 0.968 | 3.67E-01 | 3.67E+01 | | | | |
| 8109426 | AK090581 | GALNT10 | UDP-N-acetyl-alpha-D-galactosamine | -0.046 | 0.968 | 5.80E-01 | 5.80E+01 | | | | |
| 7981771 | --- | --- | --- | -0.046 | 0.968 | 6.59E-01 | 6.59E+01 | | | | |
| 7981893 | --- | --- | --- | -0.046 | 0.968 | 6.59E-01 | 6.59E+01 | | | | |
| 7960764 | NM_031491 | RBP5 | retinol binding protein 5, cellular | -0.046 | 0.968 | 5.80E-01 | 5.80E+01 | | | | |
| 7893752 | --- | --- | --- | -0.046 | 0.968 | 9.22E-01 | 9.22E+01 | | | | |
| 8003410 | NM_000512 | GALNS | galactosamine (N-acetyl)-6-sulfate su | -0.047 | 0.968 | 4.72E-01 | 4.72E+01 | | | | |
| 7949971 | NM_001876 | CPT1A | carnitine palmitoyltransferase 1A (liv | -0.047 | 0.968 | 7.28E-01 | 7.28E+01 | | | | |
| 8017718 | NM_004655 | AXIN2 | axin 2 | -0.047 | 0.968 | 4.60E-01 | 4.60E+01 | | | | |
| 7896090 | --- | --- | --- | -0.047 | 0.968 | 7.22E-01 | 7.22E+01 | | | | |
| 8110841 | NM_024830 | LPCAT1 | lysophosphatidylcholine acyltransfer | -0.047 | 0.968 | 6.50E-01 | 6.50E+01 | | | | |
| 8102869 | --- | --- | --- | -0.047 | 0.968 | 6.77E-01 | 6.77E+01 | | | | |
| 8180250 | --- | --- | --- | -0.047 | 0.968 | 4.62E-01 | 4.62E+01 | | | | |
| 7987916 | NM_153260 | LRRC57 | leucine rich repeat containing 57 | -0.047 | 0.968 | 5.44E-01 | 5.44E+01 | | | | |
| 8112433 | --- | --- | --- | -0.047 | 0.968 | 5.36E-01 | 5.36E+01 | | | | |
| 8044499 | NM_005415 | SLC20A1 | solute carrier family 20 (phosphate t | -0.047 | 0.968 | 8.58E-01 | 8.58E+01 | | | | |
| 8067361 | NM_003185 | TAF4 | TAF4 RNA polymerase II, TATA box b | -0.047 | 0.968 | 4.54E-01 | 4.54E+01 | | | | |
| 8071564 | --- | --- | --- | -0.047 | 0.968 | 6.17E-01 | 6.17E+01 | | | | |
| 8158212 | --- | --- | --- | -0.047 | 0.968 | 7.31E-01 | 7.31E+01 | | | | |
| 7982356 | --- | --- | --- | -0.047 | 0.968 | 7.71E-01 | 7.71E+01 | | | | |
| 7987046 | --- | --- | --- | -0.047 | 0.968 | 7.71E-01 | 7.71E+01 | | | | |
| 7987137 | --- | --- | --- | -0.047 | 0.968 | 7.71E-01 | 7.71E+01 | | | | |
| 8050766 | NM_004036 | ADCY3 | adenylate cyclase 3 | -0.047 | 0.968 | 4.89E-01 | 4.89E+01 | | | | |
| 8154151 | NM_203453 | PPAPDC2 | phosphatidic acid phosphatase type | -0.047 | 0.968 | 6.31E-01 | 6.31E+01 | | | | |
| 8158177 | NM_024112 | C9orf16 | chromosome 9 open reading frame 1 | -0.047 | 0.968 | 3.19E-01 | 3.19E+01 | | | | |
| 8074388 | NM_005984 | SLC25A1 | solute carrier family 25 (mitochondri | -0.047 | 0.968 | 4.51E-01 | 4.51E+01 | | | | |
| 8167650 | BC046248 | CXorf67 | chromosome X open reading frame 6 | -0.047 | 0.968 | 4.04E-01 | 4.04E+01 | | | | |
| 8078933 | NM_015460 | MYRIP | myosin VIIA and Rab interacting prot | -0.047 | 0.968 | 3.71E-01 | 3.71E+01 | | | | |
| 7991837 | NM_005009 | NME4 | non-metastatic cells 4, protein expre | -0.047 | 0.968 | 4.18E-01 | 4.18E+01 | | | | |
| 7935139 | NR_002319 | PIPSL | PIP5K1A and PSMD4-like | -0.047 | 0.968 | 7.36E-01 | 7.36E+01 | | | | |
| 8073032 | NM_020243 | TOMM22 | translocase of outer mitochondrial m | -0.047 | 0.968 | 8.18E-01 | 8.18E+01 | | | | |
| 7941062 | NM_018484 | SLC22A11 | solute carrier family 22 (organic anio | -0.047 | 0.968 | 4.21E-01 | 4.21E+01 | | | | |
| 8031260 | NM_153443 | KIR3DL3 | killer cell immunoglobulin-like recept | -0.047 | 0.968 | 5.69E-01 | 5.69E+01 | | | | |

| | | | | | | | | | | |
|---|---|---|---|---|---|---|---|---|---|---|
| 8147206 | NM_003821 | RIPK2 | receptor-interacting serine-threonine | -0.047 | 0.968 | 8.69E-01 | 8.69E+01 | | | |
| 8000310 | NM_00108361 | EARS2 | glutamyl-tRNA synthetase 2, mitocho | -0.047 | 0.968 | 4.35E-01 | 4.35E+01 | | | |
| 7930139 | NM_030912 | TRIM8 | tripartite motif-containing 8 | -0.047 | 0.968 | 6.10E-01 | 6.10E+01 | | | |
| 7942284 | NM_00100203 | DEFB108B | defensin, beta 108B | -0.047 | 0.968 | 7.80E-01 | 7.80E+01 | | | |
| 8094136 | NM_00100203 | DEFB108B | defensin, beta 108B | -0.047 | 0.968 | 7.80E-01 | 7.80E+01 | | | |
| 8174853 | NM_00108014 | CT47A6 | cancer/testis antigen family 47, mem | -0.047 | 0.968 | 3.50E-01 | 3.50E+01 | | | |
| 8173583 | NM_033053 | DMRTC1 | DMRT-like family C1 | -0.047 | 0.968 | 5.19E-01 | 5.19E+01 | | | |
| 8037045 | NM_001817 | CEACAM4 | carcinoembryonic antigen-related ce | -0.047 | 0.968 | 4.43E-01 | 4.43E+01 | | | |
| 7897066 | NR_024445 | OC10012800 | hypothetical protein LOC100128003 | -0.047 | 0.968 | 5.20E-01 | 5.20E+01 | | | |
| 7972567 | NM_033132 | ZIC5 | Zic family member 5 (odd-paired hor | -0.047 | 0.968 | 4.51E-01 | 4.51E+01 | | | |
| 7949440 | NM_00109940 | EHBP1L1 | EH domain binding protein 1-like 1 | -0.047 | 0.968 | 5.07E-01 | 5.07E+01 | | | |
| 8132929 | NM_001483 | GBAS | glioblastoma amplified sequence | -0.047 | 0.968 | 8.49E-01 | 8.49E+01 | | | |
| 8041122 | NM_002709 | PPP1CB | protein phosphatase 1, catalytic sub | -0.047 | 0.968 | 7.59E-01 | 7.59E+01 | | | |
| 7967463 | NM_178314 | RILPL1 | Rab interacting lysosomal protein-lik | -0.047 | 0.968 | 6.15E-01 | 6.15E+01 | | | |
| 7904737 | NM_00103988 | ANKRD34A | ankyrin repeat domain 34A | -0.047 | 0.968 | 4.07E-01 | 4.07E+01 | | | |
| 8034806 | NM_005804 | DDX39 | DEAD (Asp-Glu-Ala-Asp) box polypep | -0.047 | 0.968 | 8.19E-01 | 8.19E+01 | | | |
| 7896107 | --- | --- | --- | -0.047 | 0.968 | 6.36E-01 | 6.36E+01 | | | |
| 8034544 | NM_005809 | PRDX2 | peroxiredoxin 2 | -0.047 | 0.968 | 6.31E-01 | 6.31E+01 | | | |
| 8045009 | NM_002101 | GYPC | glycophorin C (Gerbich blood group) | -0.048 | 0.968 | 7.27E-01 | 7.27E+01 | | | |
| 7896492 | --- | --- | --- | -0.048 | 0.968 | 9.35E-01 | 9.35E+01 | | | |
| 8003773 | NM_015085 | GARNL4 | GTPase activating Rap/RanGAP doma | -0.048 | 0.968 | 5.58E-01 | 5.58E+01 | | | |
| 7915245 | NM_016257 | HPCAL4 | hippocalcin like 4 | -0.048 | 0.968 | 4.54E-01 | 4.54E+01 | | | |
| 7950762 | --- | --- | --- | -0.048 | 0.968 | 6.73E-01 | 6.73E+01 | | | |
| 8065353 | NM_000361 | THBD | thrombomodulin | -0.048 | 0.968 | 4.59E-01 | 4.59E+01 | | | |
| 8099362 | --- | --- | --- | -0.048 | 0.968 | 8.18E-01 | 8.18E+01 | | | |
| 8142343 | --- | --- | --- | -0.048 | 0.968 | 9.29E-01 | 9.29E+01 | | | |
| 7898328 | NM_00111460 | C1orf144 | chromosome 1 open reading frame 1 | -0.048 | 0.968 | 8.61E-01 | 8.61E+01 | | | |
| 8093219 | NM_203314 | BDH1 | 3-hydroxybutyrate dehydrogenase, t | -0.048 | 0.968 | 5.59E-01 | 5.59E+01 | | | |
| 8034783 | NM_00100870 | LPHN1 | latrophilin 1 | -0.048 | 0.968 | 3.28E-01 | 3.28E+01 | | | |
| 7976158 | --- | --- | --- | -0.048 | 0.967 | 5.34E-01 | 5.34E+01 | | | |
| 8124889 | NM_002701 | POU5F1 | POU class 5 homeobox 1 | -0.048 | 0.967 | 4.80E-01 | 4.80E+01 | | | |
| 8179719 | NM_002701 | POU5F1 | POU class 5 homeobox 1 | -0.048 | 0.967 | 4.80E-01 | 4.80E+01 | | | |
| 8080909 | --- | --- | --- | -0.048 | 0.967 | 8.08E-01 | 8.08E+01 | | | |
| 8078857 | NM_00110551 | TTC21A | tetratricopeptide repeat domain 21A | -0.048 | 0.967 | 4.24E-01 | 4.24E+01 | | | |
| 7899675 | NM_018056 | TMEM39B | transmembrane protein 39B | -0.048 | 0.967 | 4.99E-01 | 4.99E+01 | | | |
| 8012450 | NM_201520 | SLC25A35 | solute carrier family 25, member 35 | -0.048 | 0.967 | 5.39E-01 | 5.39E+01 | | | |
| 7893480 | --- | --- | --- | -0.048 | 0.967 | 7.03E-01 | 7.03E+01 | | | |
| 8028341 | NM_021185 | CATSPERG | cation channel, sperm-associated, ga | -0.048 | 0.967 | 4.25E-01 | 4.25E+01 | | | |
| 7955055 | NM_017842 | SLC48A1 | solute carrier family 48 (heme transp | -0.048 | 0.967 | 4.46E-01 | 4.46E+01 | | | |
| 7928208 | NM_018344 | SLC29A3 | solute carrier family 29 (nucleoside t | -0.048 | 0.967 | 3.14E-01 | 3.14E+01 | | | |
| 7894742 | --- | --- | --- | -0.048 | 0.967 | 6.60E-01 | 6.60E+01 | | | |
| 8149907 | NM_171982 | TRIM35 | tripartite motif-containing 35 | -0.048 | 0.967 | 3.19E-01 | 3.19E+01 | | | |
| 8162868 | --- | --- | --- | -0.048 | 0.967 | 5.68E-01 | 5.68E+01 | | | |
| 8101228 | NM_144571 | CNOT6L | CCR4-NOT transcription complex, su | -0.048 | 0.967 | 9.01E-01 | 9.01E+01 | | | |
| 8134890 | NM_005273 | GNB2 | guanine nucleotide binding protein ( | -0.048 | 0.967 | 3.17E-01 | 3.17E+01 | | | |
| 8121275 | --- | --- | --- | -0.048 | 0.967 | 8.07E-01 | 8.07E+01 | | | |
| 7896253 | --- | --- | --- | -0.048 | 0.967 | 9.01E-01 | 9.01E+01 | | | |
| 8117187 | --- | --- | --- | -0.048 | 0.967 | 6.69E-01 | 6.69E+01 | | | |
| 8061780 | NM_025227 | BPIL1 | bactericidal/permeability-increasing | -0.048 | 0.967 | 3.20E-01 | 3.20E+01 | | | |
| 8031659 | NM_020828 | ZFP28 | zinc finger protein 28 homolog (mou | -0.048 | 0.967 | 4.83E-01 | 4.83E+01 | | | |
| 8008064 | NM_018129 | PNPO | pyridoxamine 5'-phosphate oxidase | -0.048 | 0.967 | 5.50E-01 | 5.50E+01 | | | |
| 8048518 | NR_003063 | TUBA4B | tubulin, alpha 4b (pseudogene) | -0.048 | 0.967 | 4.21E-01 | 4.21E+01 | | | |
| 7893320 | --- | --- | --- | -0.048 | 0.967 | 9.39E-01 | 9.39E+01 | | | |
| 8087271 | NM_017730 | QRICH1 | glutamine-rich 1 | -0.048 | 0.967 | 8.71E-01 | 8.71E+01 | | | |
| 7938390 | NM_001124 | ADM | adrenomedullin | -0.048 | 0.967 | 3.14E-01 | 3.14E+01 | | | |
| 7895220 | --- | --- | --- | -0.048 | 0.967 | 5.08E-01 | 5.08E+01 | | | |
| 8110755 | NM_006598 | SLC12A7 | solute carrier family 12 (potassium/c | -0.048 | 0.967 | 5.17E-01 | 5.17E+01 | | | |
| 7913883 | NM_000437 | PAFAH2 | platelet-activating factor acetylhydro | -0.048 | 0.967 | 4.42E-01 | 4.42E+01 | | | |
| 8071044 | 0 | 0 | 0 | -0.048 | 0.967 | 5.23E-01 | 5.23E+01 | | | |
| 8130013 | NM_005670 | EPM2A | epilepsy, progressive myoclonus type | -0.048 | 0.967 | 5.82E-01 | 5.82E+01 | | | |
| 7998103 | NM_001481 | GAS8 | growth arrest-specific 8 | -0.048 | 0.967 | 5.19E-01 | 5.19E+01 | | | |
| 8161353 | AK292642 | LOC554249 | hypothetical LOC554249 | -0.048 | 0.967 | 3.15E-01 | 3.15E+01 | | | |
| 7968563 | NM_002915 | RFC3 | replication factor C (activator 1) 3, 38 | -0.048 | 0.967 | 8.54E-01 | 8.54E+01 | | | |
| 8000906 | NM_033410 | ZNF764 | zinc finger protein 764 | -0.048 | 0.967 | 5.03E-01 | 5.03E+01 | | | |
| 7984922 | NM_002435 | MPI | mannose phosphate isomerase | -0.048 | 0.967 | 6.45E-01 | 6.45E+01 | | | |
| 8168674 | --- | --- | --- | -0.048 | 0.967 | 7.70E-01 | 7.70E+01 | | | |
| 8139484 | NR_002991 | SNORA5C | small nucleolar RNA, H/ACA box 5C | -0.048 | 0.967 | 5.17E-01 | 5.17E+01 | | | |
| 8126512 | NM_015950 | MRPL2 | mitochondrial ribosomal protein L2 | -0.048 | 0.967 | 4.60E-01 | 4.60E+01 | | | |
| 8110882 | BC132707 | FLJ33360 | FLJ33360 protein | -0.048 | 0.967 | 5.39E-01 | 5.39E+01 | | | |
| 8174767 | NM_017938 | FAM70A | family with sequence similarity 70, m | -0.048 | 0.967 | 4.05E-01 | 4.05E+01 | | | |
| 8006019 | NM_005208 | CRYBA1 | crystallin, beta A1 | -0.048 | 0.967 | 4.34E-01 | 4.34E+01 | | | |
| 7929822 | NM_003393 | WNT8B | wingless-type MMTV integration site | -0.048 | 0.967 | 4.88E-01 | 4.88E+01 | | | |
| 7984227 | NM_182703 | ANKDD1A | ankyrin repeat and death domain co | -0.048 | 0.967 | 3.79E-01 | 3.79E+01 | | | |
| 8109333 | NM_002084 | GPX3 | glutathione peroxidase 3 (plasma) | -0.048 | 0.967 | 4.59E-01 | 4.59E+01 | | | |
| 8029728 | NM_000164 | GIPR | gastric inhibitory polypeptide recept | -0.048 | 0.967 | 2.84E-01 | 2.84E+01 | | | |
| 7971197 | NM_172373 | ELF1 | E74-like factor 1 (ets domain transcr | -0.049 | 0.967 | 9.12E-01 | 9.12E+01 | | | |
| 8040334 | --- | --- | --- | -0.049 | 0.967 | 6.50E-01 | 6.50E+01 | | | |

| | | | | | | | | | | |
|---|---|---|---|---|---|---|---|---|---|---|
| 8176098 | NM_014235 | UBL4A | ubiquitin-like 4A | -0.049 | 0.967 | 4.16E-01 | 4.16E+01 | | | |
| 7894894 | --- | --- | --- | -0.049 | 0.967 | 7.78E-01 | 7.78E+01 | | | |
| 7937415 | NM_020901 | PHRF1 | PHD and ring finger domains 1 | -0.049 | 0.967 | 5.35E-01 | 5.35E+01 | | | |
| 7892681 | --- | --- | --- | -0.049 | 0.967 | 6.51E-01 | 6.51E+01 | | | |
| 8013061 | NM_012452 | TNFRSF13B | tumor necrosis factor receptor super | -0.049 | 0.967 | 3.61E-01 | 3.61E+01 | | | |
| 7963092 | NM_175736 | FMNL3 | formin-like 3 | -0.049 | 0.967 | 5.57E-01 | 5.57E+01 | | | |
| 7900999 | NM_00114563( | C1orf228 | chromosome 1 open reading frame 2 | -0.049 | 0.967 | 4.98E-01 | 4.98E+01 | | | |
| 7926410 | NM_002438 | MRC1 | mannose receptor, C type 1 | -0.049 | 0.967 | 3.87E-01 | 3.87E+01 | | | |
| 7926451 | NM_002438 | MRC1 | mannose receptor, C type 1 | -0.049 | 0.967 | 3.87E-01 | 3.87E+01 | | | |
| 8001108 | AK128823 // A | 5121 // FLJ4 | similar to Ig heavy chain V-I region V | -0.049 | 0.967 | 4.86E-01 | 4.86E+01 | | | |
| 8072796 | NM_153609 | TMPRSS6 | transmembrane protease, serine 6 | -0.049 | 0.967 | 5.15E-01 | 5.15E+01 | | | |
| 8063240 | --- | --- | --- | -0.049 | 0.967 | 6.98E-01 | 6.98E+01 | | | |
| 7970388 | --- | --- | --- | -0.049 | 0.967 | 6.08E-01 | 6.08E+01 | | | |
| 8012856 | NM_018127 | ELAC2 | elaC homolog 2 (E. coli) | -0.049 | 0.967 | 6.30E-01 | 6.30E+01 | | | |
| 7920082 | NM_005060 | RORC | RAR-related orphan receptor C | -0.049 | 0.967 | 3.42E-01 | 3.42E+01 | | | |
| 8133413 | NM_002314 | LIMK1 | LIM domain kinase 1 | -0.049 | 0.967 | 3.26E-01 | 3.26E+01 | | | |
| 8082368 | NM_015720 | PODXL2 | podocalyxin-like 2 | -0.049 | 0.967 | 3.76E-01 | 3.76E+01 | | | |
| 7923394 | NM_138795 | ARL8A | ADP-ribosylation factor-like 8A | -0.049 | 0.967 | 5.92E-01 | 5.92E+01 | | | |
| 8090433 | NM_007283 | MGLL | monoglyceride lipase | -0.049 | 0.967 | 4.42E-01 | 4.42E+01 | | | |
| 7919394 | NR_024510 | LOC728855 | hypothetical LOC728855 | -0.049 | 0.967 | 5.62E-01 | 5.62E+01 | | | |
| 8031617 | NM_018337 | ZNF444 | zinc finger protein 444 | -0.049 | 0.967 | 3.92E-01 | 3.92E+01 | | | |
| 7993223 | NM_015226 | CLEC16A | C-type lectin domain family 16, mem | -0.049 | 0.967 | 5.80E-01 | 5.80E+01 | | | |
| 8052624 | AK094167 | FLJ36848 | hypothetical LOC647115 | -0.049 | 0.967 | 4.21E-01 | 4.21E+01 | | | |
| 8031090 | NM_145814 | CACNG6 | calcium channel, voltage-dependent | -0.049 | 0.967 | 4.43E-01 | 4.43E+01 | | | |
| 8149762 | NM_003844 | TNFRSF10A | tumor necrosis factor receptor super | -0.049 | 0.967 | 7.26E-01 | 7.26E+01 | | | |
| 8075921 | NM_052906 | ELFN2 | extracellular leucine-rich repeat and | -0.049 | 0.967 | 4.46E-01 | 4.46E+01 | | | |
| 7960143 | NM_003428 | ZNF84 | zinc finger protein 84 | -0.049 | 0.967 | 5.75E-01 | 5.75E+01 | | | |
| 8164937 | BC146946 | FAM163B | family with sequence similarity 163, | -0.049 | 0.967 | 3.30E-01 | 3.30E+01 | | | |
| 8036503 | NM_170604 | RASGRP4 | RAS guanyl releasing protein 4 | -0.049 | 0.967 | 4.85E-01 | 4.85E+01 | | | |
| 7988091 | --- | --- | --- | -0.049 | 0.967 | 6.80E-01 | 6.80E+01 | | | |
| 8162466 | NM_198841 | FAM120AOS | family with sequence similarity 120A | -0.049 | 0.967 | 5.86E-01 | 5.86E+01 | | | |
| 8062211 | NM_032194 | BXDC1 | brix domain containing 1 | -0.049 | 0.967 | 9.39E-01 | 9.39E+01 | | | |
| 7981266 | NM_00114499 | CCDC85C | coiled-coil domain containing 85C | -0.049 | 0.967 | 4.83E-01 | 4.83E+01 | | | |
| 8093957 | NM_018366 | CNO | cappuccino homolog (mouse) | -0.049 | 0.967 | 4.61E-01 | 4.61E+01 | | | |
| 8019031 | NM_019020 | TBC1D16 | TBC1 domain family, member 16 | -0.049 | 0.967 | 3.21E-01 | 3.21E+01 | | | |
| 7897426 | NM_001215 | CA6 | carbonic anhydrase VI | -0.049 | 0.967 | 3.77E-01 | 3.77E+01 | | | |
| 7894980 | --- | --- | --- | -0.049 | 0.967 | 7.34E-01 | 7.34E+01 | | | |
| 7894033 | --- | --- | --- | -0.049 | 0.967 | 7.26E-01 | 7.26E+01 | | | |
| 8077779 | --- | --- | --- | -0.049 | 0.966 | 7.73E-01 | 7.73E+01 | | | |
| 8095585 | NM_00109848 | SLC4A4 | solute carrier family 4, sodium bicarl | -0.049 | 0.966 | 4.88E-01 | 4.88E+01 | | | |
| 7981787 | NM_00100141 | LOC283767 | golgi autoantigen, golgin subfamily a | -0.049 | 0.966 | 4.23E-01 | 4.23E+01 | | | |
| 8018620 | NM_00114529 | EXOC7 | exocyst complex component 7 | -0.049 | 0.966 | 6.62E-01 | 6.62E+01 | | | |
| 7892623 | --- | --- | --- | -0.049 | 0.966 | 9.21E-01 | 9.21E+01 | | | |
| 7916422 | NM_152268 | PARS2 | prolyl-tRNA synthetase 2, mitochonc | -0.049 | 0.966 | 4.04E-01 | 4.04E+01 | | | |
| 7941111 | NM_013306 | SNX15 | sorting nexin 15 | -0.049 | 0.966 | 5.40E-01 | 5.40E+01 | | | |
| 7992388 | NM_012225 | NUBP2 | nucleotide binding protein 2 (MinD h | -0.049 | 0.966 | 4.71E-01 | 4.71E+01 | | | |
| 7915200 | --- | --- | --- | -0.049 | 0.966 | 5.22E-01 | 5.22E+01 | | | |
| 8180307 | --- | --- | --- | -0.049 | 0.966 | 4.24E-01 | 4.24E+01 | | | |
| 7967175 | NM_032590 | KDM2B | lysine (K)-specific demethylase 2B | -0.049 | 0.966 | 5.74E-01 | 5.74E+01 | | | |
| 8137635 | --- | --- | --- | -0.049 | 0.966 | 4.22E-01 | 4.22E+01 | | | |
| 8102024 | NM_181886 | UBE2D3 | ubiquitin-conjugating enzyme E2D 3 | -0.049 | 0.966 | 7.44E-01 | 7.44E+01 | | | |
| 8179258 | NM_00115974 | LTA | lymphotoxin alpha (TNF superfamily, | -0.049 | 0.966 | 5.75E-01 | 5.75E+01 | | | |
| 8010161 | NM_006640 | 40430 | septin 9 | -0.050 | 0.966 | 6.69E-01 | 6.69E+01 | | | |
| 8135392 | NM_012257 | HBP1 | HMG-box transcription factor 1 | -0.050 | 0.966 | 8.83E-01 | 8.83E+01 | | | |
| 8110437 | NM_022762 | RMND5B | required for meiotic nuclear division | -0.050 | 0.966 | 6.04E-01 | 6.04E+01 | | | |
| 8005707 | NM_002756 | MAP2K3 | mitogen-activated protein kinase kin | -0.050 | 0.966 | 5.02E-01 | 5.02E+01 | | | |
| 8102783 | --- | --- | --- | -0.050 | 0.966 | 5.29E-01 | 5.29E+01 | | | |
| 8175900 | NM_001666 | ARHGAP4 | Rho GTPase activating protein 4 | -0.050 | 0.966 | 5.91E-01 | 5.91E+01 | | | |
| 8009737 | NM_015353 | KCTD2 | potassium channel tetramerisation d | -0.050 | 0.966 | 5.36E-01 | 5.36E+01 | | | |
| 7997319 | --- | --- | --- | -0.050 | 0.966 | 5.64E-01 | 5.64E+01 | | | |
| 8047702 | NM_012092 | ICOS | inducible T-cell co-stimulator | -0.050 | 0.966 | 8.02E-01 | 8.02E+01 | | | |
| 8004832 | NM_201520 | SLC25A35 | solute carrier family 25, member 35 | -0.050 | 0.966 | 6.41E-01 | 6.41E+01 | | | |
| 7897439 | --- | --- | --- | -0.050 | 0.966 | 7.28E-01 | 7.28E+01 | | | |
| 8161431 | --- | --- | --- | -0.050 | 0.966 | 4.45E-01 | 4.45E+01 | | | |
| 8040843 | NM_004341 | CAD | carbamoyl-phosphate synthetase 2, | -0.050 | 0.966 | 3.85E-01 | 3.85E+01 | | | |
| 7941104 | NM_001667 | ARL2 | ADP-ribosylation factor-like 2 | -0.050 | 0.966 | 6.60E-01 | 6.60E+01 | | | |
| 8147479 | --- | --- | --- | -0.050 | 0.966 | 5.92E-01 | 5.92E+01 | | | |
| 8126066 | NM_014341 | MTCH1 | mitochondrial carrier homolog 1 (C. | -0.050 | 0.966 | 7.11E-01 | 7.11E+01 | | | |
| 8088065 | NM_00100515 | SFMBT1 | Scm-like with four mbt domains 1 | -0.050 | 0.966 | 7.34E-01 | 7.34E+01 | | | |
| 8135497 | NM_021994 | ZNF277 | zinc finger protein 277 | -0.050 | 0.966 | 8.98E-01 | 8.98E+01 | | | |
| 8146448 | NM_014175 | MRPL15 | mitochondrial ribosomal protein L15 | -0.050 | 0.966 | 9.06E-01 | 9.06E+01 | | | |
| 7911793 | NR_015440 | FLJ42875 | hypothetical LOC440556 | -0.050 | 0.966 | 7.11E-01 | 7.11E+01 | | | |
| 8049934 | NM_022134 | GAL3ST2 | galactose-3-O-sulfotransferase 2 | -0.050 | 0.966 | 3.02E-01 | 3.02E+01 | | | |
| 8070891 | AF426264 | C21orf86 | chromosome 21 open reading frame | -0.050 | 0.966 | 4.45E-01 | 4.45E+01 | | | |
| 8109283 | NM_001543 | NDST1 | N-deacetylase/N-sulfotransferase (h | -0.050 | 0.966 | 4.38E-01 | 4.38E+01 | | | |
| 8088889 | --- | --- | --- | -0.050 | 0.966 | 5.48E-01 | 5.48E+01 | | | |
| 7934719 | NM_003019 | SFTPD | surfactant protein D | -0.050 | 0.966 | 4.24E-01 | 4.24E+01 | | | |

| | | | | | | | | | | |
|---|---|---|---|---|---|---|---|---|---|---|
| 7915529 | NM_031207 | HYI | hydroxypyruvate isomerase homolog | -0.050 | 0.966 | 4.45E-01 | 4.45E+01 | | | |
| 7929990 | NM_015062 | PPRC1 | peroxisome proliferator-activated re | -0.050 | 0.966 | 5.45E-01 | 5.45E+01 | | | |
| 8170390 | NM_000202 | IDS | iduronate 2-sulfatase | -0.050 | 0.966 | 7.17E-01 | 7.17E+01 | | | |
| 8095694 | NM_002620 | PF4V1 | platelet factor 4 variant 1 | -0.050 | 0.966 | 6.48E-01 | 6.48E+01 | | | |
| 7893551 | --- | --- | --- | -0.050 | 0.966 | 9.18E-01 | 9.18E+01 | | | |
| 7910591 | NM_032324 | C1orf57 | chromosome 1 open reading frame 5 | -0.050 | 0.966 | 8.51E-01 | 8.51E+01 | | | |
| 8010260 | NM_001168 | BIRC5 | baculoviral IAP repeat-containing 5 | -0.050 | 0.966 | 4.53E-01 | 4.53E+01 | | | |
| 8066461 | NM_006809 | TOMM34 | translocase of outer mitochondrial m | -0.050 | 0.966 | 8.13E-01 | 8.13E+01 | | | |
| 8167623 | --- | --- | --- | -0.050 | 0.966 | 4.86E-01 | 4.86E+01 | | | |
| 7898353 | NR_027002 | LOC388692 | hypothetical LOC388692 | -0.050 | 0.966 | 8.15E-01 | 8.15E+01 | | | |
| 7975626 | NM_194278 | C14orf43 | chromosome 14 open reading frame | -0.050 | 0.966 | 7.50E-01 | 7.50E+01 | | | |
| 7956007 | NM_206899 | OR10P1 | olfactory receptor, family 10, subfam | -0.050 | 0.966 | 4.76E-01 | 4.76E+01 | | | |
| 8148263 | NM_017956 | TRMT12 | tRNA methyltransferase 12 homolog | -0.050 | 0.966 | 7.68E-01 | 7.68E+01 | | | |
| 8006479 | NM_013975 | LIG3 | ligase III, DNA, ATP-dependent | -0.050 | 0.966 | 6.09E-01 | 6.09E+01 | | | |
| 8165711 | NM_018390 | PLCXD1 | phosphatidylinositol-specific phosph | -0.050 | 0.966 | 4.87E-01 | 4.87E+01 | | | |
| 7997982 | NM_052988 | CDK10 | cyclin-dependent kinase 10 | -0.050 | 0.966 | 7.22E-01 | 7.22E+01 | | | |
| 8121515 | NM_018593 | SLC16A10 | solute carrier family 16, member 10 | -0.050 | 0.966 | 4.97E-01 | 4.97E+01 | | | |
| 7982271 | NR_024074 | GOLGA9P | golgi autoantigen, golgin subfamily a | -0.050 | 0.966 | 6.48E-01 | 6.48E+01 | | | |
| 8068651 | NM_006198 | PCP4 | Purkinje cell protein 4 | -0.050 | 0.966 | 4.26E-01 | 4.26E+01 | | | |
| 8013399 | NM_014683 | ULK2 | unc-51-like kinase 2 (C. elegans) | -0.050 | 0.966 | 7.67E-01 | 7.67E+01 | | | |
| 8038458 | NM_007254 | PNKP | polynucleotide kinase 3'-phosphatas | -0.050 | 0.966 | 4.24E-01 | 4.24E+01 | | | |
| 8026133 | NM_152654 | DAND5 | DAN domain family, member 5 | -0.050 | 0.966 | 4.30E-01 | 4.30E+01 | | | |
| 8026182 | NM_00103172 | MRI1 | methylthioribose-1-phosphate isome | -0.050 | 0.966 | 5.25E-01 | 5.25E+01 | | | |
| 7963459 | NM_080747 | KRT72 | keratin 72 | -0.050 | 0.966 | 7.50E-01 | 7.50E+01 | | | |
| 8173428 | --- | --- | --- | -0.050 | 0.966 | 6.82E-01 | 6.82E+01 | | | |
| 8063723 | NM_178457 | ZNF831 | zinc finger protein 831 | -0.051 | 0.966 | 5.95E-01 | 5.95E+01 | | | |
| 7987439 | NM_007223 | GPR176 | G protein-coupled receptor 176 | -0.051 | 0.966 | 2.88E-01 | 2.88E+01 | | | |
| 8036004 | NM_004364 | CEBPA | CCAAT/enhancer binding protein (C/ | -0.051 | 0.966 | 4.74E-01 | 4.74E+01 | | | |
| 7937508 | NM_004357 | CD151 | CD151 molecule (Raph blood group) | -0.051 | 0.966 | 4.58E-01 | 4.58E+01 | | | |
| 8120239 | NM_014051 | TMEM14A | transmembrane protein 14A | -0.051 | 0.965 | 9.13E-01 | 9.13E+01 | | | |
| 8070791 | NM_181684 | KRTAP12-2 | keratin associated protein 12-2 | -0.051 | 0.965 | 5.30E-01 | 5.30E+01 | | | |
| 7981859 | NR_024074 | GOLGA9P | golgi autoantigen, golgin subfamily a | -0.051 | 0.965 | 5.32E-01 | 5.32E+01 | | | |
| 8034583 | NM_00110557 | SYCE2 | synaptonemal complex central elem | -0.051 | 0.965 | 4.44E-01 | 4.44E+01 | | | |
| 7893696 | --- | --- | --- | -0.051 | 0.965 | 6.66E-01 | 6.66E+01 | | | |
| 7943690 | NM_004398 | DDX10 | DEAD (Asp-Glu-Ala-Asp) box polypep | -0.051 | 0.965 | 8.56E-01 | 8.56E+01 | | | |
| 7956623 | NM_138396 | 40246 | membrane-associated ring finger (C3 | -0.051 | 0.965 | 4.34E-01 | 4.34E+01 | | | |
| 8063839 | NM_198935 | SS18L1 | synovial sarcoma translocation gene | -0.051 | 0.965 | 2.93E-01 | 2.93E+01 | | | |
| 8110461 | --- | --- | --- | -0.051 | 0.965 | 5.30E-01 | 5.30E+01 | | | |
| 7895497 | --- | --- | --- | -0.051 | 0.965 | 4.34E-01 | 4.34E+01 | | | |
| 7993114 | NM_024109 | C16orf68 | chromosome 16 open reading frame | -0.051 | 0.965 | 5.35E-01 | 5.35E+01 | | | |
| 7892545 | --- | --- | --- | -0.051 | 0.965 | 7.69E-01 | 7.69E+01 | | | |
| 8032576 | NM_198969 | AES | amino-terminal enhancer of split | -0.051 | 0.965 | 7.56E-01 | 7.56E+01 | | | |
| 7972748 | --- | --- | --- | -0.051 | 0.965 | 6.92E-01 | 6.92E+01 | | | |
| 7937438 | NM_174940 | TMEM80 | transmembrane protein 80 | -0.051 | 0.965 | 3.77E-01 | 3.77E+01 | | | |
| 8068478 | NM_005441 | CHAF1B | chromatin assembly factor 1, subuni | -0.051 | 0.965 | 6.42E-01 | 6.42E+01 | | | |
| 8094874 | --- | --- | --- | -0.051 | 0.965 | 7.37E-01 | 7.37E+01 | | | |
| 8130939 | NM_005618 | DLL1 | delta-like 1 (Drosophila) | -0.051 | 0.965 | 2.40E-01 | 2.40E+01 | | | |
| 7976808 | --- | --- | --- | -0.051 | 0.965 | 3.26E-01 | 3.26E+01 | | | |
| 8038023 | --- | --- | --- | -0.051 | 0.965 | 5.52E-01 | 5.52E+01 | | | |
| 8137566 | NM_030936 | RNF32 | ring finger protein 32 | -0.051 | 0.965 | 4.26E-01 | 4.26E+01 | | | |
| 7975598 | NM_00103716 | ACOT1 | acyl-CoA thioesterase 1 | -0.051 | 0.965 | 6.32E-01 | 6.32E+01 | | | |
| 7895651 | --- | --- | --- | -0.051 | 0.965 | 5.96E-01 | 5.96E+01 | | | |
| 8167573 | NM_00112734 | GAGE12B | G antigen 12B | -0.051 | 0.965 | 6.79E-01 | 6.79E+01 | | | |
| 7927645 | --- | --- | --- | -0.051 | 0.965 | 6.90E-01 | 6.90E+01 | | | |
| 8148930 | --- | --- | --- | -0.051 | 0.965 | 6.20E-01 | 6.20E+01 | | | |
| 7893358 | --- | --- | --- | -0.051 | 0.965 | 9.32E-01 | 9.32E+01 | | | |
| 8073214 | NM_00102484 | TNRC6B | trinucleotide repeat containing 6B | -0.051 | 0.965 | 6.89E-01 | 6.89E+01 | | | |
| 7955606 | NM_021934 | C12orf44 | chromosome 12 open reading frame | -0.051 | 0.965 | 6.40E-01 | 6.40E+01 | | | |
| 7990361 | NM_032907 | UBL7 | ubiquitin-like 7 (bone marrow strom | -0.051 | 0.965 | 5.51E-01 | 5.51E+01 | | | |
| 8147785 | NM_015420 | DCAF13 | DDB1 and CUL4 associated factor 13 | -0.051 | 0.965 | 6.04E-01 | 6.04E+01 | | | |
| 8173217 | NM_015185 | ARHGEF9 | Cdc42 guanine nucleotide exchange | -0.051 | 0.965 | 7.61E-01 | 7.61E+01 | | | |
| 8001455 | --- | --- | --- | -0.051 | 0.965 | 6.03E-01 | 6.03E+01 | | | |
| 8055281 | NR_002826 | LOC401010 | nucleolar complex associated 2 hom | -0.051 | 0.965 | 5.12E-01 | 5.12E+01 | | | |
| 8006466 | NM_207313 | TMEM132E | transmembrane protein 132E | -0.051 | 0.965 | 4.10E-01 | 4.10E+01 | | | |
| 8081838 | NM_020754 | CDGAP | Cdc42 GTPase-activating protein | -0.051 | 0.965 | 6.47E-01 | 6.47E+01 | | | |
| 7892583 | --- | --- | --- | -0.051 | 0.965 | 7.51E-01 | 7.51E+01 | | | |
| 7958948 | NM_00114487 | CCDC42B | coiled-coil domain containing 42B | -0.051 | 0.965 | 5.35E-01 | 5.35E+01 | | | |
| 8055089 | NM_004805 | POLR2D | polymerase (RNA) II (DNA directed) p | -0.051 | 0.965 | 7.91E-01 | 7.91E+01 | | | |
| 7895400 | --- | --- | --- | -0.051 | 0.965 | 7.55E-01 | 7.55E+01 | | | |
| 8033818 | NM_058164 | OLFM2 | olfactomedin 2 | -0.052 | 0.965 | 4.02E-01 | 4.02E+01 | | | |
| 7939613 | NM_005456 | MAPK8IP1 | mitogen-activated protein kinase 8 ir | -0.052 | 0.965 | 4.37E-01 | 4.37E+01 | | | |
| 7981538 | NM_002226 | JAG2 | jagged 2 | -0.052 | 0.965 | 3.39E-01 | 3.39E+01 | | | |
| 7952641 | NM_00114268 | RICS | Rho GTPase-activating protein | -0.052 | 0.965 | 4.90E-01 | 4.90E+01 | | | |
| 8058390 | NM_213589 | RAPH1 | Ras association (RalGDS/AF-6) and pl | -0.052 | 0.965 | 5.46E-01 | 5.46E+01 | | | |
| 8099130 | --- | --- | --- | -0.052 | 0.965 | 5.44E-01 | 5.44E+01 | | | |
| 7894125 | --- | --- | --- | -0.052 | 0.965 | 6.89E-01 | 6.89E+01 | | | |
| 8133275 | NM_172020 | POM121 | POM121 membrane glycoprotein (ra | -0.052 | 0.965 | 6.09E-01 | 6.09E+01 | | | |

| | | | | | | | | | | |
|---|---|---|---|---|---|---|---|---|---|---|
| 8132617 | NM_031449 | ZMIZ2 | zinc finger, MIZ-type containing 2 | -0.052 | 0.965 | 3.12E-01 | 3.12E+01 | | | |
| 8108453 | NM_032289 | PSD2 | pleckstrin and Sec7 domain containin | -0.052 | 0.965 | 3.83E-01 | 3.83E+01 | | | |
| 8060370 | NM_031424 | FAM110A | family with sequence similarity 110, | -0.052 | 0.965 | 5.21E-01 | 5.21E+01 | | | |
| 7894626 | --- | --- | --- | -0.052 | 0.965 | 6.85E-01 | 6.85E+01 | | | |
| 8104119 | NM_020040 | TUBB4Q | tubulin, beta polypeptide 4, member | -0.052 | 0.965 | 5.20E-01 | 5.20E+01 | | | |
| 7980069 | NM_182480 | COQ6 | coenzyme Q6 homolog, monooxygen | -0.052 | 0.965 | 4.60E-01 | 4.60E+01 | | | |
| 8073698 | NM_00101298 | RP3-474I12.5 | hypothetical LOC388910 | -0.052 | 0.965 | 4.18E-01 | 4.18E+01 | | | |
| 7912839 | NR_026752 | CROCCL1 | ciliary rootlet coiled-coil, rootletin-lik | -0.052 | 0.965 | 5.32E-01 | 5.32E+01 | | | |
| 8017499 | NM_022579 | CSHL1 | chorionic somatomammotropin horn | -0.052 | 0.965 | 4.28E-01 | 4.28E+01 | | | |
| 7918681 | NM_018364 | RSBN1 | round spermatid basic protein 1 | -0.052 | 0.965 | 8.32E-01 | 8.32E+01 | | | |
| 7903717 | --- | --- | --- | -0.052 | 0.965 | 6.50E-01 | 6.50E+01 | | | |
| 8015511 | NM_024119 | DHX58 | DEXH (Asp-Glu-X-His) box polypeptid | -0.052 | 0.965 | 4.13E-01 | 4.13E+01 | | | |
| 7998967 | NM_017810 | ZNF434 | zinc finger protein 434 | -0.052 | 0.965 | 4.55E-01 | 4.55E+01 | | | |
| 7906797 | NM_00100290 | FCRLB | Fc receptor-like B | -0.052 | 0.965 | 4.39E-01 | 4.39E+01 | | | |
| 8055291 | BC043584 | C2orf27B | chromosome 2 open reading frame 2 | -0.052 | 0.965 | 4.55E-01 | 4.55E+01 | | | |
| 8176286 | NM_018390 | PLCXD1 | phosphatidylinositol-specific phosph | -0.052 | 0.965 | 5.19E-01 | 5.19E+01 | | | |
| 8053840 | --- | --- | --- | -0.052 | 0.965 | 6.83E-01 | 6.83E+01 | | | |
| 7898483 | NM_018125 | ARHGEF10L | Rho guanine nucleotide exchange fa | -0.052 | 0.964 | 4.22E-01 | 4.22E+01 | | | |
| 8145795 | XM_00234609 | OC10029353 | similar to ribosomal protein 10 | -0.052 | 0.964 | 6.56E-01 | 6.56E+01 | | | |
| 7914791 | NM_005066 | SFPQ | splicing factor proline/glutamine-rich | -0.052 | 0.964 | 5.97E-01 | 5.97E+01 | | | |
| 8017253 | --- | --- | --- | -0.052 | 0.964 | 5.94E-01 | 5.94E+01 | | | |
| 8078567 | --- | --- | --- | -0.052 | 0.964 | 3.86E-01 | 3.86E+01 | | | |
| 8157949 | NM_014636 | RALGPS1 | Ral GEF with PH domain and SH3 bin | -0.052 | 0.964 | 2.42E-01 | 2.42E+01 | | | |
| 7957467 | NM_00100989 | C12orf29 | chromosome 12 open reading frame | -0.052 | 0.964 | 8.28E-01 | 8.28E+01 | | | |
| 8035682 | --- | --- | --- | -0.052 | 0.964 | 4.00E-01 | 4.00E+01 | | | |
| 7981566 | NM_177533 | NUDT14 | nudix (nucleoside diphosphate linked | -0.052 | 0.964 | 4.70E-01 | 4.70E+01 | | | |
| 8042040 | NM_00103975 | EML6 | echinoderm microtubule associated | -0.052 | 0.964 | 6.15E-01 | 6.15E+01 | | | |
| 8131813 | --- | --- | --- | -0.052 | 0.964 | 6.94E-01 | 6.94E+01 | | | |
| 7895692 | --- | --- | --- | -0.052 | 0.964 | 5.85E-01 | 5.85E+01 | | | |
| 8023323 | NM_015846 | MBD1 | methyl-CpG binding domain protein | -0.052 | 0.964 | 5.97E-01 | 5.97E+01 | | | |
| 8062557 | NM_015568 | PPP1R16B | protein phosphatase 1, regulatory (in | -0.052 | 0.964 | 6.57E-01 | 6.57E+01 | | | |
| 8033330 | --- | --- | --- | -0.052 | 0.964 | 9.39E-01 | 9.39E+01 | | | |
| 7946180 | NM_006458 | TRIM3 | tripartite motif-containing 3 | -0.052 | 0.964 | 3.49E-01 | 3.49E+01 | | | |
| 8072626 | NM_000362 | TIMP3 | TIMP metallopeptidase inhibitor 3 | -0.052 | 0.964 | 4.34E-01 | 4.34E+01 | | | |
| 8045882 | NM_00101792 | DAPL1 | death associated protein-like 1 | -0.052 | 0.964 | 4.08E-01 | 4.08E+01 | | | |
| 7893385 | --- | --- | --- | -0.053 | 0.964 | 7.53E-01 | 7.53E+01 | | | |
| 7903970 | --- | --- | --- | -0.053 | 0.964 | 8.63E-01 | 8.63E+01 | | | |
| 8159873 | --- | --- | --- | -0.053 | 0.964 | 7.70E-01 | 7.70E+01 | | | |
| 8019507 | NM_00110040 | C17orf62 | chromosome 17 open reading frame | -0.053 | 0.964 | 3.13E-01 | 3.13E+01 | | | |
| 7977761 | NM_005407 | SALL2 | sal-like 2 (Drosophila) | -0.053 | 0.964 | 4.70E-01 | 4.70E+01 | | | |
| 8113220 | NM_012081 | ELL2 | elongation factor, RNA polymerase II | -0.053 | 0.964 | 7.22E-01 | 7.22E+01 | | | |
| 8110218 | NM_00109940 | EIF4E1B | eukaryotic translation initiation facto | -0.053 | 0.964 | 3.50E-01 | 3.50E+01 | | | |
| 8049097 | NM_152383 | DIS3L2 | DIS3 mitotic control homolog (S. cere | -0.053 | 0.964 | 5.88E-01 | 5.88E+01 | | | |
| 7950271 | EF363480 | FAM168A | family with sequence similarity 168, | -0.053 | 0.964 | 4.36E-01 | 4.36E+01 | | | |
| 7982810 | NM_00113044 | C15orf62 | chromosome 15 open reading frame | -0.053 | 0.964 | 3.32E-01 | 3.32E+01 | | | |
| 7896586 | --- | --- | --- | -0.053 | 0.964 | 8.01E-01 | 8.01E+01 | | | |
| 8013588 | NM_015584 | POLDIP2 | polymerase (DNA-directed), delta int | -0.053 | 0.964 | 8.55E-01 | 8.55E+01 | | | |
| 7945657 | NM_00101270 | KRTAP5-4 | keratin associated protein 5-4 | -0.053 | 0.964 | 5.24E-01 | 5.24E+01 | | | |
| 8092081 | --- | --- | --- | -0.053 | 0.964 | 5.88E-01 | 5.88E+01 | | | |
| 8167656 | NM_00100533 | MAGED1 | melanoma antigen family D, 1 | -0.053 | 0.964 | 5.93E-01 | 5.93E+01 | | | |
| 8009746 | NM_004695 | SLC16A5 | solute carrier family 16, member 5 (r | -0.053 | 0.964 | 3.74E-01 | 3.74E+01 | | | |
| 8069399 | NM_015151 | DIP2A | DIP2 disco-interacting protein 2 hom | -0.053 | 0.964 | 6.03E-01 | 6.03E+01 | | | |
| 7994615 | NM_152338 | ZG16 | zymogen granule protein 16 homolo | -0.053 | 0.964 | 3.24E-01 | 3.24E+01 | | | |
| 8156633 | BC144521 | KIAA1529 | KIAA1529 | -0.053 | 0.964 | 3.94E-01 | 3.94E+01 | | | |
| 8060286 | NM_012145 | DTYMK | deoxythymidylate kinase (thymidylat | -0.053 | 0.964 | 5.57E-01 | 5.57E+01 | | | |
| 8077262 | NM_012145 | DTYMK | deoxythymidylate kinase (thymidylat | -0.053 | 0.964 | 5.57E-01 | 5.57E+01 | | | |
| 7941822 | NM_207354 | ANKRD13D | ankyrin repeat domain 13 family, me | -0.053 | 0.964 | 5.80E-01 | 5.80E+01 | | | |
| 7940046 | NM_006831 | CLP1 | CLP1, cleavage and polyadenylation | -0.053 | 0.964 | 6.07E-01 | 6.07E+01 | | | |
| 8027128 | NM_178526 | SLC25A42 | solute carrier family 25, member 42 | -0.053 | 0.964 | 2.10E-01 | 2.10E+01 | | | |
| 7949264 | NM_006795 | EHD1 | EH-domain containing 1 | -0.053 | 0.964 | 4.44E-01 | 4.44E+01 | | | |
| 8065566 | NM_00103750 | DEFB124 | defensin, beta 124 | -0.053 | 0.964 | 5.02E-01 | 5.02E+01 | | | |
| 7966876 | --- | --- | --- | -0.053 | 0.964 | 6.34E-01 | 6.34E+01 | | | |
| 8007152 | --- | --- | --- | -0.053 | 0.964 | 6.59E-01 | 6.59E+01 | | | |
| 7904084 | NR_002796 | AFARP1 | AKR7 family pseudogene | -0.053 | 0.964 | 5.47E-01 | 5.47E+01 | | | |
| 7931159 | NM_153336 | PSTK | phosphoseryl-tRNA kinase | -0.053 | 0.964 | 6.39E-01 | 6.39E+01 | | | |
| 8163731 | --- | --- | --- | -0.053 | 0.964 | 5.94E-01 | 5.94E+01 | | | |
| 8175871 | NM_000425 | L1CAM | L1 cell adhesion molecule | -0.053 | 0.964 | 4.11E-01 | 4.11E+01 | | | |
| 8016590 | NM_030802 | FAM117A | family with sequence similarity 117, | -0.053 | 0.964 | 3.72E-01 | 3.72E+01 | | | |
| 7959957 | NM_003565 | ULK1 | unc-51-like kinase 1 (C. elegans) | -0.053 | 0.964 | 4.04E-01 | 4.04E+01 | | | |
| 8130383 | NM_019041 | MTRF1L | mitochondrial translational release f | -0.053 | 0.964 | 8.57E-01 | 8.57E+01 | | | |
| 7892865 | --- | --- | --- | -0.053 | 0.964 | 7.69E-01 | 7.69E+01 | | | |
| 7975924 | --- | --- | --- | -0.053 | 0.964 | 5.53E-01 | 5.53E+01 | | | |
| 8000932 | NM_00101497 | C16orf93 | chromosome 16 open reading frame | -0.053 | 0.964 | 4.58E-01 | 4.58E+01 | | | |
| 7946860 | NM_000352 | ABCC8 | ATP-binding cassette, sub-family C (C | -0.053 | 0.964 | 2.91E-01 | 2.91E+01 | | | |
| 8029854 | NM_005628 | SLC1A5 | solute carrier family 1 (neutral amino | -0.053 | 0.964 | 4.51E-01 | 4.51E+01 | | | |
| 7897339 | NM_015215 | CAMTA1 | calmodulin binding transcription acti | -0.053 | 0.964 | 3.95E-01 | 3.95E+01 | | | |
| 7963139 | NM_181708 | BCDIN3D | BCDIN3 domain containing | -0.053 | 0.964 | 5.60E-01 | 5.60E+01 | | | |

| | | | | | | | | | | | | |
|---|---|---|---|---|---|---|---|---|---|---|---|---|
| 8117965 | NM_024909 | C6orf134 | chromosome 6 open reading frame 1 | -0.053 | 0.964 | 3.66E-01 | 3.66E+01 | | | | |
| 8179149 | NM_024909 | C6orf134 | chromosome 6 open reading frame 1 | -0.053 | 0.964 | 3.66E-01 | 3.66E+01 | | | | |
| 8087100 | NM_003365 | UQCRC1 | ubiquinol-cytochrome c reductase co | -0.053 | 0.964 | 5.66E-01 | 5.66E+01 | | | | |
| 8044793 | NM_182915 | STEAP3 | STEAP family member 3 | -0.053 | 0.964 | 2.65E-01 | 2.65E+01 | | | | |
| 8053984 | NM_144994 | ANKRD23 | ankyrin repeat domain 23 | -0.053 | 0.964 | 4.85E-01 | 4.85E+01 | | | | |
| 8138745 | NM_006896 | HOXA7 | homeobox A7 | -0.053 | 0.964 | 3.51E-01 | 3.51E+01 | | | | |
| 7967025 | --- | --- | --- | -0.053 | 0.964 | 5.80E-01 | 5.80E+01 | | | | |
| 8171079 | NM_012227 | GTPBP6 | GTP binding protein 6 (putative) | -0.053 | 0.964 | 7.00E-01 | 7.00E+01 | | | | |
| 8138065 | --- | --- | --- | -0.053 | 0.964 | 5.14E-01 | 5.14E+01 | | | | |
| 8180255 | --- | --- | --- | -0.053 | 0.964 | 8.08E-01 | 8.08E+01 | | | | |
| 8180321 | --- | --- | --- | -0.053 | 0.964 | 8.08E-01 | 8.08E+01 | | | | |
| 8030782 | NM_014441 | SIGLEC9 | sialic acid binding Ig-like lectin 9 | -0.053 | 0.964 | 5.23E-01 | 5.23E+01 | | | | |
| 7952132 | NM_001467 | SLC37A4 | solute carrier family 37 (glucose-6-ph | -0.053 | 0.964 | 3.33E-01 | 3.33E+01 | | | | |
| 7923438 | NM_001030 | RPS27 | ribosomal protein S27 | -0.053 | 0.964 | 6.65E-01 | 6.65E+01 | | | | |
| 7932788 | --- | --- | --- | -0.053 | 0.964 | 4.42E-01 | 4.42E+01 | | | | |
| 8147548 | NM_00114586 | POP1 | processing of precursor 1, ribonuclea | -0.053 | 0.964 | 5.34E-01 | 5.34E+01 | | | | |
| 7934533 | NM_00100727 | DUSP13 | dual specificity phosphatase 13 | -0.053 | 0.964 | 3.80E-01 | 3.80E+01 | | | | |
| 8103520 | NM_00101241 | TRIM61 | tripartite motif-containing 61 | -0.053 | 0.964 | 5.83E-01 | 5.83E+01 | | | | |
| 7949364 | NM_080668 | CDCA5 | cell division cycle associated 5 | -0.053 | 0.964 | 4.16E-01 | 4.16E+01 | | | | |
| 8178476 | NM_004640 | BAT1 | HLA-B associated transcript 1 | -0.054 | 0.964 | 8.21E-01 | 8.21E+01 | | | | |
| 8129120 | NM_00108548 | FAM162B | family with sequence similarity 162, | -0.054 | 0.964 | 4.03E-01 | 4.03E+01 | | | | |
| 8020220 | NM_032525 | TUBB6 | tubulin, beta 6 | -0.054 | 0.964 | 4.36E-01 | 4.36E+01 | | | | |
| 8110224 | NM_012171 | TSPAN17 | tetraspanin 17 | -0.054 | 0.964 | 3.62E-01 | 3.62E+01 | | | | |
| 8050113 | AK125905 | OC10012958 | hypothetical LOC100129581 | -0.054 | 0.964 | 3.29E-01 | 3.29E+01 | | | | |
| 7996490 | NM_014187 | TMEM208 | transmembrane protein 208 | -0.054 | 0.964 | 8.71E-01 | 8.71E+01 | | | | |
| 8038770 | NM_022046 | KLK14 | kallikrein-related peptidase 14 | -0.054 | 0.964 | 3.92E-01 | 3.92E+01 | | | | |
| 8169049 | NM_004780 | TCEAL1 | transcription elongation factor A (SII | -0.054 | 0.964 | 6.69E-01 | 6.69E+01 | | | | |
| 7948912 | NM_000738 | CHRM1 | cholinergic receptor, muscarinic 1 | -0.054 | 0.963 | 4.15E-01 | 4.15E+01 | | | | |
| 8069666 | --- | --- | --- | -0.054 | 0.963 | 4.32E-01 | 4.32E+01 | | | | |
| 7993165 | --- | --- | --- | -0.054 | 0.963 | 7.44E-01 | 7.44E+01 | | | | |
| 8148841 | NM_024531 | GPR172A | G protein-coupled receptor 172A | -0.054 | 0.963 | 4.35E-01 | 4.35E+01 | | | | |
| 8088768 | --- | --- | --- | -0.054 | 0.963 | 8.32E-01 | 8.32E+01 | | | | |
| 8038139 | NM_145807 | NTN5 | netrin 5 | -0.054 | 0.963 | 4.91E-01 | 4.91E+01 | | | | |
| 7941639 | NM_024649 | BBS1 | Bardet-Biedl syndrome 1 | -0.054 | 0.963 | 5.29E-01 | 5.29E+01 | | | | |
| 7960357 | --- | --- | --- | -0.054 | 0.963 | 3.31E-01 | 3.31E+01 | | | | |
| 7898585 | NM_182744 | NBL1 | neuroblastoma, suppression of tumo | -0.054 | 0.963 | 3.21E-01 | 3.21E+01 | | | | |
| 8048717 | NM_152386 | SGPP2 | sphingosine-1-phosphate phosphota | -0.054 | 0.963 | 4.89E-01 | 4.89E+01 | | | | |
| 8004428 | NM_00110261 | AMAC1L3 | acyl-malonyl condensing enzyme 1-li | -0.054 | 0.963 | 3.75E-01 | 3.75E+01 | | | | |
| 7945241 | AK096377 | C11orf44 | chromosome 11 open reading frame | -0.054 | 0.963 | 4.74E-01 | 4.74E+01 | | | | |
| 7967660 | NM_015347 | RIMBP2 | RIMS binding protein 2 | -0.054 | 0.963 | 4.24E-01 | 4.24E+01 | | | | |
| 7977773 | --- | --- | --- | -0.054 | 0.963 | 5.20E-01 | 5.20E+01 | | | | |
| 8019046 | NM_014740 | EIF4A3 | eukaryotic translation initiation facto | -0.054 | 0.963 | 8.55E-01 | 8.55E+01 | | | | |
| 8076072 | NM_152868 | KCNJ4 | potassium inwardly-rectifying chann | -0.054 | 0.963 | 2.24E-01 | 2.24E+01 | | | | |
| 8132992 | --- | --- | --- | -0.054 | 0.963 | 6.79E-01 | 6.79E+01 | | | | |
| 8086683 | NM_182702 | TESSP2 | testis serine protease 2 | -0.054 | 0.963 | 2.88E-01 | 2.88E+01 | | | | |
| 7892763 | --- | --- | --- | -0.054 | 0.963 | 5.07E-01 | 5.07E+01 | | | | |
| 8144643 | NM_002052 | GATA4 | GATA binding protein 4 | -0.054 | 0.963 | 3.05E-01 | 3.05E+01 | | | | |
| 8082574 | NM_007117 | TRH | thyrotropin-releasing hormone | -0.054 | 0.963 | 4.07E-01 | 4.07E+01 | | | | |
| 8049478 | NM_198189 | COPS8 | COP9 constitutive photomorphogeni | -0.054 | 0.963 | 7.68E-01 | 7.68E+01 | | | | |
| 7919749 | --- | --- | --- | -0.054 | 0.963 | 9.43E-01 | 9.43E+01 | | | | |
| 8073068 | NM_014508 | APOBEC3C | apolipoprotein B mRNA editing enzy | -0.054 | 0.963 | 8.87E-01 | 8.87E+01 | | | | |
| 7941255 | NM_145719 | TIGD3 | tigger transposable element derived | -0.054 | 0.963 | 3.43E-01 | 3.43E+01 | | | | |
| 8158269 | NM_002540 | ODF2 | outer dense fiber of sperm tails 2 | -0.054 | 0.963 | 6.84E-01 | 6.84E+01 | | | | |
| 8163535 | NM_001633 | AMBP | alpha-1-microglobulin/bikunin precu | -0.054 | 0.963 | 3.69E-01 | 3.69E+01 | | | | |
| 8180406 | --- | --- | --- | -0.054 | 0.963 | 4.08E-01 | 4.08E+01 | | | | |
| 8153727 | NM_013291 | CPSF1 | cleavage and polyadenylation specifi | -0.054 | 0.963 | 4.61E-01 | 4.61E+01 | | | | |
| 8104180 | NM_020731 | AHRR | aryl-hydrocarbon receptor repressor | -0.054 | 0.963 | 4.27E-01 | 4.27E+01 | | | | |
| 8015257 | NM_004138 | KRT33A | keratin 33A | -0.054 | 0.963 | 4.73E-01 | 4.73E+01 | | | | |
| 7901687 | NM_182532 | TMEM61 | transmembrane protein 61 | -0.054 | 0.963 | 3.12E-01 | 3.12E+01 | | | | |
| 8116445 | NM_182925 | FLT4 | fms-related tyrosine kinase 4 | -0.054 | 0.963 | 3.42E-01 | 3.42E+01 | | | | |
| 8037103 | NM_002088 | GRIK5 | glutamate receptor, ionotropic, kain | -0.054 | 0.963 | 2.80E-01 | 2.80E+01 | | | | |
| 7925089 | NM_014801 | PCNXL2 | pecanex-like 2 (Drosophila) | -0.054 | 0.963 | 4.32E-01 | 4.32E+01 | | | | |
| 7892870 | --- | --- | --- | -0.054 | 0.963 | 8.99E-01 | 8.99E+01 | | | | |
| 8164060 | --- | --- | --- | -0.054 | 0.963 | 4.60E-01 | 4.60E+01 | | | | |
| 7899528 | NM_000911 | OPRD1 | opioid receptor, delta 1 | -0.054 | 0.963 | 4.49E-01 | 4.49E+01 | | | | |
| 7934527 | NM_00100389 | DUPD1 | dual specificity phosphatase and pro | -0.054 | 0.963 | 3.97E-01 | 3.97E+01 | | | | |
| 8074227 | NM_033070 | CECR5 | cat eye syndrome chromosome regio | -0.054 | 0.963 | 3.50E-01 | 3.50E+01 | | | | |
| 8027100 | NM_019070 | DDX49 | DEAD (Asp-Glu-Ala-Asp) box polypep | -0.054 | 0.963 | 2.68E-01 | 2.68E+01 | | | | |
| 8026051 | NM_006397 | RNASEH2A | ribonuclease H2, subunit A | -0.054 | 0.963 | 7.28E-01 | 7.28E+01 | | | | |
| 8154285 | NM_005511 | MLANA | melan-A | -0.055 | 0.963 | 4.91E-01 | 4.91E+01 | | | | |
| 8084895 | NM_152673 | MUC20 | mucin 20, cell surface associated | -0.055 | 0.963 | 4.75E-01 | 4.75E+01 | | | | |
| 7979671 | NM_145113 | MAX | MYC associated factor X | -0.055 | 0.963 | 7.45E-01 | 7.45E+01 | | | | |
| 8148923 | NM_014665 | LRRC14 | leucine rich repeat containing 14 | -0.055 | 0.963 | 2.89E-01 | 2.89E+01 | | | | |
| 7980390 | NM_00111347 | C14orf148 | chromosome 14 open reading frame | -0.055 | 0.963 | 4.00E-01 | 4.00E+01 | | | | |
| 8013747 | NM_152465 | PROCA1 | protein interacting with cyclin A1 | -0.055 | 0.963 | 3.83E-01 | 3.83E+01 | | | | |
| 7914202 | AY277594 | SNHG12 | small nucleolar RNA host gene 12 (no | -0.055 | 0.963 | 8.58E-01 | 8.58E+01 | | | | |
| 8045664 | NM_177964 | LYPD6B | LY6/PLAUR domain containing 6B | -0.055 | 0.963 | 3.82E-01 | 3.82E+01 | | | | |

| | | | | | | | | | | |
|---|---|---|---|---|---|---|---|---|---|---|
| 8180322 | --- | --- | --- | -0.055 | 0.963 | 5.03E-01 | 5.03E+01 | | | |
| 8101366 | NM_00103758: | SCD5 | stearoyl-CoA desaturase 5 | -0.055 | 0.963 | 3.45E-01 | 3.45E+01 | | | |
| 7899093 | NM_024869 | GRRP1 | glycine/arginine rich protein 1 | -0.055 | 0.963 | 2.19E-01 | 2.19E+01 | | | |
| 8058514 | --- | --- | --- | -0.055 | 0.963 | 7.05E-01 | 7.05E+01 | | | |
| 7956026 | NM_005811 | GDF11 | growth differentiation factor 11 | -0.055 | 0.963 | 3.38E-01 | 3.38E+01 | | | |
| 7969881 | NM_003291 | TPP2 | tripeptidyl peptidase II | -0.055 | 0.963 | 8.48E-01 | 8.48E+01 | | | |
| 7942091 | NM_139075 | TPCN2 | two pore segment channel 2 | -0.055 | 0.963 | 3.17E-01 | 3.17E+01 | | | |
| 7894523 | --- | --- | --- | -0.055 | 0.963 | 6.53E-01 | 6.53E+01 | | | |
| 7977933 | NM_012244 | SLC7A8 | solute carrier family 7 (cationic amin | -0.055 | 0.963 | 4.80E-01 | 4.80E+01 | | | |
| 7893463 | --- | --- | --- | -0.055 | 0.963 | 6.50E-01 | 6.50E+01 | | | |
| 7929901 | NM_021830 | C10orf2 | chromosome 10 open reading frame | -0.055 | 0.963 | 6.88E-01 | 6.88E+01 | | | |
| 7894262 | --- | --- | --- | -0.055 | 0.963 | 7.45E-01 | 7.45E+01 | | | |
| 8040712 | NM_001809 | CENPA | centromere protein A | -0.055 | 0.963 | 3.25E-01 | 3.25E+01 | | | |
| 8037408 | NM_002250 | KCNN4 | potassium intermediate/small condu | -0.055 | 0.963 | 2.60E-01 | 2.60E+01 | | | |
| 8152938 | NM_00114509! | HHLA1 | HERV-H LTR-associating 1 | -0.055 | 0.963 | 3.26E-01 | 3.26E+01 | | | |
| 7895596 | --- | --- | --- | -0.055 | 0.963 | 6.59E-01 | 6.59E+01 | | | |
| 7941136 | NM_013265 | C11orf2 | chromosome 11 open reading frame | -0.055 | 0.963 | 6.68E-01 | 6.68E+01 | | | |
| 7893932 | --- | --- | --- | -0.055 | 0.963 | 6.10E-01 | 6.10E+01 | | | |
| 7934271 | NM_032562 | PLA2G12B | phospholipase A2, group XIIB | -0.055 | 0.963 | 4.13E-01 | 4.13E+01 | | | |
| 7892896 | --- | --- | --- | -0.055 | 0.963 | 6.24E-01 | 6.24E+01 | | | |
| 8082523 | NM_052985 | IFT122 | intraflagellar transport 122 homolog | -0.055 | 0.963 | 6.07E-01 | 6.07E+01 | | | |
| 8059475 | --- | --- | --- | -0.055 | 0.963 | 8.69E-01 | 8.69E+01 | | | |
| 7976200 | NM_006888 | CALM1 | calmodulin 1 (phosphorylase kinase, | -0.055 | 0.963 | 5.01E-01 | 5.01E+01 | | | |
| 7970864 | NM_006644 | HSPH1 | heat shock 105kDa/110kDa protein 1 | -0.055 | 0.963 | 8.53E-01 | 8.53E+01 | | | |
| 7895233 | --- | --- | --- | -0.055 | 0.963 | 6.24E-01 | 6.24E+01 | | | |
| 8054209 | NM_174898 | LYG1 | lysozyme G-like 1 | -0.055 | 0.963 | 7.87E-01 | 7.87E+01 | | | |
| 7956287 | NM_005967 | NAB2 | NGFI-A binding protein 2 (EGR1 bind | -0.055 | 0.963 | 4.34E-01 | 4.34E+01 | | | |
| 7895620 | --- | --- | --- | -0.055 | 0.963 | 8.08E-01 | 8.08E+01 | | | |
| 8053909 | NM_014014 | SNRNP200 | small nuclear ribonucleoprotein 200 | -0.055 | 0.963 | 8.09E-01 | 8.09E+01 | | | |
| 8161192 | NM_194328 | RNF38 | ring finger protein 38 | -0.055 | 0.963 | 8.76E-01 | 8.76E+01 | | | |
| 7896417 | --- | --- | --- | -0.055 | 0.963 | 6.75E-01 | 6.75E+01 | | | |
| 8130739 | NM_021135 | RPS6KA2 | ribosomal protein S6 kinase, 90kDa, | -0.055 | 0.963 | 3.01E-01 | 3.01E+01 | | | |
| 8047062 | NM_00104251! | C2orf88 | chromosome 2 open reading frame 8 | -0.055 | 0.963 | 5.82E-01 | 5.82E+01 | | | |
| 8086949 | NM_024661 | CCDC51 | coiled-coil domain containing 51 | -0.055 | 0.963 | 4.71E-01 | 4.71E+01 | | | |
| 8053496 | NM_015425 | POLR1A | polymerase (RNA) I polypeptide A, 1! | -0.055 | 0.963 | 4.25E-01 | 4.25E+01 | | | |
| 7939068 | NM_001028 | RPS25 | ribosomal protein S25 | -0.055 | 0.963 | 7.55E-01 | 7.55E+01 | | | |
| 7901443 | --- | --- | --- | -0.055 | 0.963 | 5.30E-01 | 5.30E+01 | | | |
| 7923483 | NM_002871 | RABIF | RAB interacting factor | -0.055 | 0.963 | 4.95E-01 | 4.95E+01 | | | |
| 7897997 | NM_00101369: | PRAMEF3 | PRAME family member 3 | -0.055 | 0.963 | 3.37E-01 | 3.37E+01 | | | |
| 7911218 | NM_015431 | TRIM58 | tripartite motif-containing 58 | -0.055 | 0.963 | 3.13E-01 | 3.13E+01 | | | |
| 7895126 | --- | --- | --- | -0.055 | 0.963 | 5.91E-01 | 5.91E+01 | | | |
| 8009705 | NM_178160 | OTOP2 | otopetrin 2 | -0.055 | 0.962 | 3.95E-01 | 3.95E+01 | | | |
| 7999120 | NM_024535 | CORO7 | coronin 7 | -0.055 | 0.962 | 3.99E-01 | 3.99E+01 | | | |
| 8036483 | NM_033557 | YIF1B | Yip1 interacting factor homolog B (S. | -0.055 | 0.962 | 3.78E-01 | 3.78E+01 | | | |
| 7945767 | NM_000076 | CDKN1C | cyclin-dependent kinase inhibitor 1C | -0.055 | 0.962 | 2.82E-01 | 2.82E+01 | | | |
| 8078962 | NM_005875 | EIF1B | eukaryotic translation initiation facto | -0.055 | 0.962 | 8.58E-01 | 8.58E+01 | | | |
| 8063549 | --- | --- | --- | -0.055 | 0.962 | 7.21E-01 | 7.21E+01 | | | |
| 7925048 | NM_022051 | EGLN1 | egl nine homolog 1 (C. elegans) | -0.055 | 0.962 | 4.93E-01 | 4.93E+01 | | | |
| 8107115 | --- | --- | --- | -0.055 | 0.962 | 5.58E-01 | 5.58E+01 | | | |
| 8058498 | NM_003468 | FZD5 | frizzled homolog 5 (Drosophila) | -0.055 | 0.962 | 3.35E-01 | 3.35E+01 | | | |
| 8047963 | --- | --- | --- | -0.055 | 0.962 | 4.51E-01 | 4.51E+01 | | | |
| 7974190 | NM_145261 | DNAJC19 | DnaJ (Hsp40) homolog, subfamily C, | -0.055 | 0.962 | 9.47E-01 | 9.47E+01 | | | |
| 8035666 | NM_172231 | SF4 | splicing factor 4 | -0.055 | 0.962 | 6.01E-01 | 6.01E+01 | | | |
| 8055139 | AK056598 | LOC151121 | hypothetical LOC151121 | -0.055 | 0.962 | 3.64E-01 | 3.64E+01 | | | |
| 8029236 | NM_001410 | MEGF8 | multiple EGF-like-domains 8 | -0.055 | 0.962 | 3.15E-01 | 3.15E+01 | | | |
| 7930194 | NM_017649 | CNNM2 | cyclin M2 | -0.055 | 0.962 | 5.01E-01 | 5.01E+01 | | | |
| 8039740 | NM_024620 | ZNF329 | zinc finger protein 329 | -0.055 | 0.962 | 7.58E-01 | 7.58E+01 | | | |
| 8019061 | NM_000199 | SGSH | N-sulfoglucosamine sulfohydrolase | -0.055 | 0.962 | 4.11E-01 | 4.11E+01 | | | |
| 7897877 | NM_001066 | TNFRSF1B | tumor necrosis factor receptor super | -0.055 | 0.962 | 4.38E-01 | 4.38E+01 | | | |
| 7927173 | NM_145312 | ZNF485 | zinc finger protein 485 | -0.055 | 0.962 | 6.57E-01 | 6.57E+01 | | | |
| 7894403 | --- | --- | --- | -0.056 | 0.962 | 9.08E-01 | 9.08E+01 | | | |
| 8016094 | NM_005497 | GJC1 | gap junction protein, gamma 1, 45kD | -0.056 | 0.962 | 3.71E-01 | 3.71E+01 | | | |
| 7902911 | --- | --- | --- | -0.056 | 0.962 | 3.41E-01 | 3.41E+01 | | | |
| 7981752 | NR_027407 | GOLGA8D | golgi autoantigen, golgin subfamily a | -0.056 | 0.962 | 2.97E-01 | 2.97E+01 | | | |
| 7899361 | NM_177424 | STX12 | syntaxin 12 | -0.056 | 0.962 | 7.37E-01 | 7.37E+01 | | | |
| 8037814 | --- | --- | --- | -0.056 | 0.962 | 5.04E-01 | 5.04E+01 | | | |
| 8013159 | NM_00108296: | TOM1L2 | target of myb1-like 2 (chicken) | -0.056 | 0.962 | 5.34E-01 | 5.34E+01 | | | |
| 7895139 | --- | --- | --- | -0.056 | 0.962 | 8.55E-02 | 8.55E+00 | | | |
| 8064430 | --- | --- | --- | -0.056 | 0.962 | 3.99E-01 | 3.99E+01 | | | |
| 7930790 | NM_006229 | PNLIPRP1 | pancreatic lipase-related protein 1 | -0.056 | 0.962 | 3.01E-01 | 3.01E+01 | | | |
| 7986767 | BC069077 | C15orf49 | chromosome 15 open reading frame | -0.056 | 0.962 | 5.73E-01 | 5.73E+01 | | | |
| 8092000 | NR_001566 | TERC | telomerase RNA component | -0.056 | 0.962 | 6.92E-01 | 6.92E+01 | | | |
| 7893919 | --- | --- | --- | -0.056 | 0.962 | 7.74E-01 | 7.74E+01 | | | |
| 8126288 | NM_024807 | TREML2 | triggering receptor expressed on my | -0.056 | 0.962 | 3.58E-01 | 3.58E+01 | | | |
| 8162940 | NM_005502 | ABCA1 | ATP-binding cassette, sub-family A (A | -0.056 | 0.962 | 4.71E-01 | 4.71E+01 | | | |
| 7893598 | --- | --- | --- | -0.056 | 0.962 | 6.04E-01 | 6.04E+01 | | | |
| 7906622 | NM_002348 | LY9 | lymphocyte antigen 9 | -0.056 | 0.962 | 6.27E-01 | 6.27E+01 | | | |

| | | | | | | | | | | |
|---|---|---|---|---|---|---|---|---|---|---|
| 7971692 | NM_012141 | INTS6 | integrator complex subunit 6 | -0.056 | 0.962 | 5.41E-01 | 5.41E+01 | | | |
| 8164217 | NM_022833 | FAM129B | family with sequence similarity 129, | -0.056 | 0.962 | 3.93E-01 | 3.93E+01 | | | |
| 8137680 | NM_006869 | ADAP1 | ArfGAP with dual PH domains 1 | -0.056 | 0.962 | 4.31E-01 | 4.31E+01 | | | |
| 7952116 | NM_182557 | BCL9L | B-cell CLL/lymphoma 9-like | -0.056 | 0.962 | 4.55E-01 | 4.55E+01 | | | |
| 8085283 | NM_173472 | C3orf24 | chromosome 3 open reading frame 2 | -0.056 | 0.962 | 4.35E-01 | 4.35E+01 | | | |
| 7892736 | --- | --- | --- | -0.056 | 0.962 | 7.47E-01 | 7.47E+01 | | | |
| 8006445 | NM_002986 | CCL11 | chemokine (C-C motif) ligand 11 | -0.056 | 0.962 | 4.45E-01 | 4.45E+01 | | | |
| 8108478 | NM_032412 | C5orf32 | chromosome 5 open reading frame 3 | -0.056 | 0.962 | 5.39E-01 | 5.39E+01 | | | |
| 8034286 | NM_016581 | ECSIT | ECSIT homolog (Drosophila) | -0.056 | 0.962 | 3.36E-01 | 3.36E+01 | | | |
| 8072894 | NM_00103914 | TRIOBP | TRIO and F-actin binding protein | -0.056 | 0.962 | 1.99E-01 | 1.99E+01 | | | |
| 7919038 | NM_201263 | WARS2 | tryptophanyl tRNA synthetase 2, mit | -0.056 | 0.962 | 6.50E-01 | 6.50E+01 | | | |
| 8045604 | NM_018328 | MBD5 | methyl-CpG binding domain protein | -0.056 | 0.962 | 4.79E-01 | 4.79E+01 | | | |
| 7994235 | ENST00000426 | LOC554206 | hypothetical LOC554206 | -0.056 | 0.962 | 5.71E-01 | 5.71E+01 | | | |
| 8159549 | NM_004479 | FUT7 | fucosyltransferase 7 (alpha (1,3) fuco | -0.056 | 0.962 | 2.41E-01 | 2.41E+01 | | | |
| 8115812 | --- | --- | --- | -0.056 | 0.962 | 4.24E-01 | 4.24E+01 | | | |
| 8079746 | NM_022171 | TCTA | T-cell leukemia translocation altered | -0.056 | 0.962 | 6.84E-01 | 6.84E+01 | | | |
| 8116579 | NM_033260 | FOXQ1 | forkhead box Q1 | -0.056 | 0.962 | 4.11E-01 | 4.11E+01 | | | |
| 8035449 | NM_012321 | LSM4 | LSM4 homolog, U6 small nuclear RN | -0.056 | 0.962 | 3.94E-01 | 3.94E+01 | | | |
| 8092073 | NM_020390 | EIF5A2 | eukaryotic translation initiation facto | -0.056 | 0.962 | 4.50E-01 | 4.50E+01 | | | |
| 8148020 | --- | --- | --- | -0.056 | 0.962 | 5.58E-01 | 5.58E+01 | | | |
| 7971713 | NM_024705 | DHRS12 | dehydrogenase/reductase (SDR fami | -0.056 | 0.962 | 5.35E-01 | 5.35E+01 | | | |
| 7947270 | NM_002233 | KCNA4 | potassium voltage-gated channel, sh | -0.056 | 0.962 | 3.62E-01 | 3.62E+01 | | | |
| 8137352 | NM_003040 | SLC4A2 | solute carrier family 4, anion exchan | -0.056 | 0.962 | 3.62E-01 | 3.62E+01 | | | |
| 8148710 | NM_019037 | EXOSC4 | exosome component 4 | -0.056 | 0.962 | 5.49E-01 | 5.49E+01 | | | |
| 8000856 | NM_015527 | TBC1D10B | TBC1 domain family, member 10B | -0.056 | 0.962 | 4.88E-01 | 4.88E+01 | | | |
| 7913864 | NM_00114545 | STMN1 | stathmin 1 | -0.056 | 0.962 | 3.37E-01 | 3.37E+01 | | | |
| 8030403 | NM_020719 | PRR12 | proline rich 12 | -0.056 | 0.962 | 5.12E-01 | 5.12E+01 | | | |
| 8059716 | BC033054 | C2orf52 | chromosome 2 open reading frame 5 | -0.056 | 0.962 | 4.17E-01 | 4.17E+01 | | | |
| 8002216 | --- | --- | --- | -0.056 | 0.962 | 9.35E-01 | 9.35E+01 | | | |
| 7921110 | NM_030980 | ISG20L2 | interferon stimulated exonuclease ge | -0.056 | 0.962 | 7.61E-01 | 7.61E+01 | | | |
| 8002571 | NM_015020 | PHLPP2 | PH domain and leucine rich repeat p | -0.056 | 0.962 | 4.97E-01 | 4.97E+01 | | | |
| 8175537 | AK093505 | CXorf18 | chromosome X open reading frame 1 | -0.056 | 0.962 | 3.00E-01 | 3.00E+01 | | | |
| 7921773 | NM_030916 | PVRL4 | poliovirus receptor-related 4 | -0.056 | 0.962 | 3.67E-01 | 3.67E+01 | | | |
| 8039820 | NM_153443 | KIR3DL3 | killer cell immunoglobulin-like recept | -0.056 | 0.962 | 5.57E-01 | 5.57E+01 | | | |
| 8029065 | NM_000709 | BCKDHA | branched chain keto acid dehydroge | -0.056 | 0.962 | 4.96E-01 | 4.96E+01 | | | |
| 7927784 | NM_032776 | JMJD1C | jumonji domain containing 1C | -0.056 | 0.962 | 5.56E-01 | 5.56E+01 | | | |
| 7990457 | NM_006715 | MAN2C1 | mannosidase, alpha, class 2C, memb | -0.056 | 0.962 | 4.40E-01 | 4.40E+01 | | | |
| 7967117 | NM_003733 | OASL | 2'-5'-oligoadenylate synthetase-like | -0.056 | 0.962 | 4.28E-01 | 4.28E+01 | | | |
| 7971150 | NM_005780 | LHFP | lipoma HMGIC fusion partner | -0.057 | 0.962 | 2.33E-01 | 2.33E+01 | | | |
| 8129082 | NM_000493 | COL10A1 | collagen, type X, alpha 1 | -0.057 | 0.962 | 4.10E-01 | 4.10E+01 | | | |
| 7949075 | NM_016404 | TRMT112 | tRNA methyltransferase 11-2 homolo | -0.057 | 0.962 | 9.27E-01 | 9.27E+01 | | | |
| 8084266 | NM_182537 | HTR3D | 5-hydroxytryptamine (serotonin) rec | -0.057 | 0.962 | 4.19E-01 | 4.19E+01 | | | |
| 8062293 | NM_014902 | DLGAP4 | discs, large (Drosophila) homolog-as | -0.057 | 0.962 | 2.68E-01 | 2.68E+01 | | | |
| 7915202 | --- | --- | --- | -0.057 | 0.962 | 7.05E-01 | 7.05E+01 | | | |
| 8036460 | NM_004647 | DPF1 | D4, zinc and double PHD fingers fam | -0.057 | 0.962 | 3.83E-01 | 3.83E+01 | | | |
| 7990452 | --- | --- | --- | -0.057 | 0.962 | 4.69E-01 | 4.69E+01 | | | |
| 8167103 | NM_006201 | PCTK1 | PCTAIRE protein kinase 1 | -0.057 | 0.962 | 7.60E-01 | 7.60E+01 | | | |
| 8006367 | NM_138328 | RHBDL3 | rhomboid, veinlet-like 3 (Drosophila) | -0.057 | 0.962 | 3.55E-01 | 3.55E+01 | | | |
| 8028286 | NM_015073 | SIPA1L3 | signal-induced proliferation-associat | -0.057 | 0.962 | 3.30E-01 | 3.30E+01 | | | |
| 8094070 | BC035655 | C4orf23 | chromosome 4 open reading frame 2 | -0.057 | 0.962 | 6.69E-01 | 6.69E+01 | | | |
| 7894435 | --- | --- | --- | -0.057 | 0.962 | 7.75E-01 | 7.75E+01 | | | |
| 7995976 | NM_152727 | CPNE2 | copine II | -0.057 | 0.961 | 3.19E-01 | 3.19E+01 | | | |
| 7908650 | NM_178275 | IGFN1 | immunoglobulin-like and fibronectin | -0.057 | 0.961 | 4.52E-01 | 4.52E+01 | | | |
| 7966046 | NM_00103305 | MTERFD3 | MTERF domain containing 3 | -0.057 | 0.961 | 6.93E-01 | 6.93E+01 | | | |
| 8060325 | NM_207469 | DEFB132 | defensin, beta 132 | -0.057 | 0.961 | 4.47E-01 | 4.47E+01 | | | |
| 8036989 | --- | --- | --- | -0.057 | 0.961 | 4.21E-01 | 4.21E+01 | | | |
| 7960261 | NM_134424 | RAD52 | RAD52 homolog (S. cerevisiae) | -0.057 | 0.961 | 5.97E-01 | 5.97E+01 | | | |
| 8045216 | NM_207364 | GPR148 | G protein-coupled receptor 148 | -0.057 | 0.961 | 3.49E-01 | 3.49E+01 | | | |
| 8136936 | NM_178561 | CTAGE6 | CTAGE family, member 6 | -0.057 | 0.961 | 4.90E-01 | 4.90E+01 | | | |
| 8071086 | NM_031413 | CECR2 | cat eye syndrome chromosome regio | -0.057 | 0.961 | 3.33E-01 | 3.33E+01 | | | |
| 8039719 | NM_025027 | ZNF606 | zinc finger protein 606 | -0.057 | 0.961 | 2.57E-01 | 2.57E+01 | | | |
| 8004360 | NM_00100291 | KCTD11 | potassium channel tetramerisation d | -0.057 | 0.961 | 4.41E-01 | 4.41E+01 | | | |
| 8143961 | NM_016203 | PRKAG2 | protein kinase, AMP-activated, gamr | -0.057 | 0.961 | 5.54E-01 | 5.54E+01 | | | |
| 8071823 | NM_012295 | CABIN1 | calcineurin binding protein 1 | -0.057 | 0.961 | 4.28E-01 | 4.28E+01 | | | |
| 8041808 | NM_012249 | RHOQ | ras homolog gene family, member Q | -0.057 | 0.961 | 5.36E-01 | 5.36E+01 | | | |
| 7900603 | AK299874 | C1orf50 | chromosome 1 open reading frame 5 | -0.057 | 0.961 | 3.47E-01 | 3.47E+01 | | | |
| 8077728 | AK092352 | LOC442075 | hypothetical LOC442075 | -0.057 | 0.961 | 4.44E-01 | 4.44E+01 | | | |
| 7919923 | NM_212551 | LYSMD1 | LysM, putative peptidoglycan-bindin | -0.057 | 0.961 | 4.57E-01 | 4.57E+01 | | | |
| 8108066 | NM_003337 | UBE2B | ubiquitin-conjugating enzyme E2B (R | -0.057 | 0.961 | 8.60E-01 | 8.60E+01 | | | |
| 8124650 | NM_006398 | UBD | ubiquitin D | -0.057 | 0.961 | 3.23E-01 | 3.23E+01 | | | |
| 8123760 | NR_026970 | RP3-398D13. | hypothetical LOC285780 | -0.057 | 0.961 | 6.70E-01 | 6.70E+01 | | | |
| 8054364 | NM_004257 | TGFBRAP1 | transforming growth factor, beta rec | -0.057 | 0.961 | 8.49E-01 | 8.49E+01 | | | |
| 8029688 | NM_012099 | CD3EAP | CD3e molecule, epsilon associated p | -0.057 | 0.961 | 3.66E-01 | 3.66E+01 | | | |
| 8066820 | --- | --- | --- | -0.057 | 0.961 | 6.77E-01 | 6.77E+01 | | | |
| 8064396 | NM_015985 | ANGPT4 | angiopoietin 4 | -0.057 | 0.961 | 2.38E-01 | 2.38E+01 | | | |
| 8177834 | NM_024909 | C6orf134 | chromosome 6 open reading frame 1 | -0.057 | 0.961 | 3.32E-01 | 3.32E+01 | | | |
| 7893136 | --- | --- | --- | -0.057 | 0.961 | 9.60E-01 | 9.60E+01 | | | |

| | | | | | | | | | | |
|---|---|---|---|---|---|---|---|---|---|---|
| 7964828 | --- | --- | --- | -0.057 | 0.961 | 7.28E-01 | 7.28E+01 | | | |
| 8112803 | NM_005779 | LHFPL2 | lipoma HMGIC fusion partner-like 2 | -0.057 | 0.961 | 2.74E-01 | 2.74E+01 | | | |
| 8064415 | ENST0000038 | C20orf46 | chromosome 20 open reading frame | -0.057 | 0.961 | 4.45E-01 | 4.45E+01 | | | |
| 8029056 | NM_00104259 | TMEM91 | transmembrane protein 91 | -0.057 | 0.961 | 5.13E-01 | 5.13E+01 | | | |
| 8167347 | NM_003173 | SUV39H1 | suppressor of variegation 3-9 homolo | -0.057 | 0.961 | 3.59E-01 | 3.59E+01 | | | |
| 8111913 | --- | --- | --- | -0.057 | 0.961 | 6.28E-01 | 6.28E+01 | | | |
| 8119396 | NM_00101087 | BZRPL1 | benzodiazapine receptor (peripheral | -0.057 | 0.961 | 2.79E-01 | 2.79E+01 | | | |
| 8154316 | NM_152896 | UHRF2 | ubiquitin-like with PHD and ring fing | -0.057 | 0.961 | 8.67E-01 | 8.67E+01 | | | |
| 7914212 | NR_002987 | SNORA61 | small nucleolar RNA, H/ACA box 61 | -0.057 | 0.961 | 9.42E-01 | 9.42E+01 | | | |
| 7940028 | NM_000062 | SERPING1 | serpin peptidase inhibitor, clade G ( | -0.057 | 0.961 | 5.62E-01 | 5.62E+01 | | | |
| 8034698 | --- | --- | --- | -0.057 | 0.961 | 5.82E-01 | 5.82E+01 | | | |
| 7893100 | --- | --- | --- | -0.057 | 0.961 | 6.27E-01 | 6.27E+01 | | | |
| 8033554 | NM_016579 | CD320 | CD320 molecule | -0.057 | 0.961 | 2.64E-01 | 2.64E+01 | | | |
| 7919326 | NM_016361 | ACP6 | acid phosphatase 6, lysophosphatidi | -0.057 | 0.961 | 4.93E-01 | 4.93E+01 | | | |
| 7988444 | NM_016132 | MYEF2 | myelin expression factor 2 | -0.057 | 0.961 | 7.59E-01 | 7.59E+01 | | | |
| 8063650 | NM_024663 | NPEPL1 | aminopeptidase-like 1 | -0.057 | 0.961 | 3.95E-01 | 3.95E+01 | | | |
| 7970624 | NM_018451 | CENPJ | centromere protein J | -0.057 | 0.961 | 6.04E-01 | 6.04E+01 | | | |
| 7925244 | --- | --- | --- | -0.057 | 0.961 | 6.29E-01 | 6.29E+01 | | | |
| 8062404 | --- | --- | --- | -0.057 | 0.961 | 3.72E-01 | 3.72E+01 | | | |
| 8032518 | NM_145173 | DIRAS1 | DIRAS family, GTP-binding RAS-like 1 | -0.057 | 0.961 | 3.71E-01 | 3.71E+01 | | | |
| 8049166 | NM_005199 | CHRNG | cholinergic receptor, nicotinic, gamm | -0.057 | 0.961 | 3.04E-01 | 3.04E+01 | | | |
| 8015741 | NM_00113604 | AARSD1 | alanyl-tRNA synthetase domain cont | -0.058 | 0.961 | 5.87E-01 | 5.87E+01 | | | |
| 7994252 | NM_001169 | AQP8 | aquaporin 8 | -0.058 | 0.961 | 3.26E-01 | 3.26E+01 | | | |
| 8153568 | NM_201384 | PLEC1 | plectin 1, intermediate filament bind | -0.058 | 0.961 | 1.81E-01 | 1.81E+01 | | | |
| 8132062 | NM_147128 | ZNRF2 | zinc and ring finger 2 | -0.058 | 0.961 | 5.34E-01 | 5.34E+01 | | | |
| 8010092 | NM_024311 | MFSD11 | major facilitator superfamily domain | -0.058 | 0.961 | 5.34E-01 | 5.34E+01 | | | |
| 8159318 | NM_00114563 | GPSM1 | G-protein signaling modulator 1 (AG | -0.058 | 0.961 | 3.42E-01 | 3.42E+01 | | | |
| 8160857 | NM_005866 | SIGMAR1 | sigma non-opioid intracellular recept | -0.058 | 0.961 | 3.69E-01 | 3.69E+01 | | | |
| 7999725 | --- | --- | --- | -0.058 | 0.961 | 6.12E-01 | 6.12E+01 | | | |
| 7895446 | --- | --- | --- | -0.058 | 0.961 | 6.99E-01 | 6.99E+01 | | | |
| 8068422 | NM_005128 | DOPEY2 | dopey family member 2 | -0.058 | 0.961 | 3.94E-01 | 3.94E+01 | | | |
| 7894764 | --- | --- | --- | -0.058 | 0.961 | 8.39E-01 | 8.39E+01 | | | |
| 7995320 | --- | --- | --- | -0.058 | 0.961 | 4.88E-01 | 4.88E+01 | | | |
| 8049722 | NM_005301 | GPR35 | G protein-coupled receptor 35 | -0.058 | 0.961 | 3.35E-01 | 3.35E+01 | | | |
| 7912585 | NM_00101369 | PRAMEF3 | PRAME family member 3 | -0.058 | 0.961 | 2.90E-01 | 2.90E+01 | | | |
| 8143939 | AK127717 | LOC401433 | hypothetical LOC401433 | -0.058 | 0.961 | 4.93E-01 | 4.93E+01 | | | |
| 8015252 | NM_031964 | KRTAP17-1 | keratin associated protein 17-1 | -0.058 | 0.961 | 3.56E-01 | 3.56E+01 | | | |
| 8034304 | NM_00111103 | ACP5 | acid phosphatase 5, tartrate resistan | -0.058 | 0.961 | 3.60E-01 | 3.60E+01 | | | |
| 8128592 | NM_004849 | ATG5 | ATG5 autophagy related 5 homolog | -0.058 | 0.961 | 8.83E-01 | 8.83E+01 | | | |
| 8085293 | NR_024138 | GHRL | ghrelin/obestatin prepropeptide | -0.058 | 0.961 | 4.66E-01 | 4.66E+01 | | | |
| 8110886 | NM_032286 | MED10 | mediator complex subunit 10 | -0.058 | 0.961 | 8.89E-01 | 8.89E+01 | | | |
| 8155048 | NM_014806 | RUSC2 | RUN and SH3 domain containing 2 | -0.058 | 0.961 | 3.83E-01 | 3.83E+01 | | | |
| 8124942 | NM_130463 | ATP6V1G2 | ATPase, H+ transporting, lysosomal 1 | -0.058 | 0.961 | 4.42E-01 | 4.42E+01 | | | |
| 8179762 | NM_130463 | ATP6V1G2 | ATPase, H+ transporting, lysosomal 1 | -0.058 | 0.961 | 4.42E-01 | 4.42E+01 | | | |
| 8113938 | NM_015256 | ACSL6 | acyl-CoA synthetase long-chain famil | -0.058 | 0.961 | 3.21E-01 | 3.21E+01 | | | |
| 7982131 | NM_00101242 | GOLGA8G | golgi autoantigen, golgin subfamily a | -0.058 | 0.961 | 3.05E-01 | 3.05E+01 | | | |
| 7986922 | NM_00101242 | GOLGA8G | golgi autoantigen, golgin subfamily a | -0.058 | 0.961 | 3.05E-01 | 3.05E+01 | | | |
| 8096002 | --- | --- | --- | -0.058 | 0.961 | 5.59E-01 | 5.59E+01 | | | |
| 7911422 | NM_016547 | SDF4 | stromal cell derived factor 4 | -0.058 | 0.961 | 5.52E-01 | 5.52E+01 | | | |
| 8034034 | NM_203500 | KEAP1 | kelch-like ECH-associated protein 1 | -0.058 | 0.961 | 3.98E-01 | 3.98E+01 | | | |
| 8008627 | NM_005450 | NOG | noggin | -0.058 | 0.961 | 5.20E-01 | 5.20E+01 | | | |
| 7992474 | NM_000548 | TSC2 | tuberous sclerosis 2 | -0.058 | 0.961 | 3.01E-01 | 3.01E+01 | | | |
| 8009094 | ENST0000041 | LOC342541 | similar to TRIMCyp | -0.058 | 0.961 | 4.61E-01 | 4.61E+01 | | | |
| 8137470 | --- | --- | --- | -0.058 | 0.961 | 7.39E-01 | 7.39E+01 | | | |
| 7915472 | NM_006516 | SLC2A1 | solute carrier family 2 (facilitated glu | -0.058 | 0.961 | 4.54E-01 | 4.54E+01 | | | |
| 8015827 | NM_025237 | SOST | sclerosteosis | -0.058 | 0.961 | 2.97E-01 | 2.97E+01 | | | |
| 7964460 | NM_004083 | DDIT3 | DNA-damage-inducible transcript 3 | -0.058 | 0.961 | 6.52E-01 | 6.52E+01 | | | |
| 7945678 | ENST0000039 | C11orf89 | chromosome 11 open reading frame | -0.058 | 0.961 | 3.21E-01 | 3.21E+01 | | | |
| 7994570 | --- | --- | --- | -0.058 | 0.960 | 4.75E-01 | 4.75E+01 | | | |
| 8024299 | NM_001018 | RPS15 | ribosomal protein S15 | -0.058 | 0.960 | 6.90E-01 | 6.90E+01 | | | |
| 8045229 | NM_032995 | ARHGEF4 | Rho guanine nucleotide exchange fa | -0.058 | 0.960 | 3.17E-01 | 3.17E+01 | | | |
| 8010021 | NM_001258 | CDK3 | cyclin-dependent kinase 3 | -0.058 | 0.960 | 3.37E-01 | 3.37E+01 | | | |
| 8111210 | --- | --- | --- | -0.058 | 0.960 | 6.32E-01 | 6.32E+01 | | | |
| 8172548 | NM_015698 | GPKOW | G patch domain and KOW motifs | -0.058 | 0.960 | 6.74E-01 | 6.74E+01 | | | |
| 7991503 | NM_152449 | LYSMD4 | LysM, putative peptidoglycan-bindin | -0.058 | 0.960 | 3.74E-01 | 3.74E+01 | | | |
| 8155661 | NM_199135 | FOXD4L3 | forkhead box D4-like 3 | -0.058 | 0.960 | 1.96E-01 | 1.96E+01 | | | |
| 7896143 | --- | --- | --- | -0.058 | 0.960 | 8.11E-01 | 8.11E+01 | | | |
| 8169640 | NM_001152 | SLC25A5 | solute carrier family 25 (mitochondri | -0.058 | 0.960 | 5.63E-01 | 5.63E+01 | | | |
| 7984779 | NM_033240 | PML | promyelocytic leukemia | -0.058 | 0.960 | 3.43E-01 | 3.43E+01 | | | |
| 7913226 | NM_012400 | PLA2G2D | phospholipase A2, group IID | -0.058 | 0.960 | 3.19E-01 | 3.19E+01 | | | |
| 7945648 | NM_00100432 | KRTAP5-2 | keratin associated protein 5-2 | -0.058 | 0.960 | 3.32E-01 | 3.32E+01 | | | |
| 7978970 | NM_004196 | CDKL1 | cyclin-dependent kinase-like 1 (CDC2 | -0.058 | 0.960 | 3.28E-01 | 3.28E+01 | | | |
| 8099200 | NM_144720 | JAKMIP1 | janus kinase and microtubule interac | -0.058 | 0.960 | 4.49E-01 | 4.49E+01 | | | |
| 7893504 | --- | --- | --- | -0.058 | 0.960 | 9.10E-01 | 9.10E+01 | | | |
| 7987454 | NM_00100394 | BMF | Bcl2 modifying factor | -0.058 | 0.960 | 4.02E-01 | 4.02E+01 | | | |
| 8162231 | NM_00114228 | SEMA4D | sema domain, immunoglobulin doma | -0.058 | 0.960 | 5.35E-01 | 5.35E+01 | | | |
| 8030339 | NM_001459 | FLT3LG | fms-related tyrosine kinase 3 ligand | -0.058 | 0.960 | 3.21E-01 | 3.21E+01 | | | |

| | | | | | | | | | | |
|---|---|---|---|---|---|---|---|---|---|---|
| 8001932 | NM_033309 | B3GNT9 | UDP-GlcNAc:betaGal beta-1,3-N-acet | -0.059 | 0.960 | 3.51E-01 | 3.51E+01 | | | |
| 7909478 | NM_014388 | C1orf107 | chromosome 1 open reading frame 1 | -0.059 | 0.960 | 4.73E-01 | 4.73E+01 | | | |
| 7999412 | NM_00107951 | FAM18A | family with sequence similarity 18, m | -0.059 | 0.960 | 2.96E-01 | 2.96E+01 | | | |
| 7931582 | NM_152911 | PAOX | polyamine oxidase (exo-N4-amino) | -0.059 | 0.960 | 2.02E-01 | 2.02E+01 | | | |
| 7899424 | NM_178191 | ATPIF1 | ATPase inhibitory factor 1 | -0.059 | 0.960 | 6.46E-01 | 6.46E+01 | | | |
| 7959563 | NM_152269 | C12orf65 | chromosome 12 open reading frame | -0.059 | 0.960 | 4.70E-01 | 4.70E+01 | | | |
| 8024966 | NM_015414 | RPL36 | ribosomal protein L36 | -0.059 | 0.960 | 4.09E-01 | 4.09E+01 | | | |
| 8023862 | --- | --- | --- | -0.059 | 0.960 | 4.60E-01 | 4.60E+01 | | | |
| 8086406 | NM_004206 | SEC22C | SEC22 vesicle trafficking protein hom | -0.059 | 0.960 | 7.87E-01 | 7.87E+01 | | | |
| 8028233 | NM_181786 | HKR1 | GLI-Kruppel family member HKR1 | -0.059 | 0.960 | 6.48E-01 | 6.48E+01 | | | |
| 8138741 | NM_024014 | HOXA6 | homeobox A6 | -0.059 | 0.960 | 3.50E-01 | 3.50E+01 | | | |
| 7930872 | NM_199461 | NANOS1 | nanos homolog 1 (Drosophila) | -0.059 | 0.960 | 3.12E-01 | 3.12E+01 | | | |
| 7933010 | NM_019619 | PARD3 | par-3 partitioning defective 3 homolo | -0.059 | 0.960 | 3.62E-01 | 3.62E+01 | | | |
| 7898227 | NM_015164 | PLEKHM2 | pleckstrin homology domain contain | -0.059 | 0.960 | 4.14E-01 | 4.14E+01 | | | |
| 8144600 | NM_054028 | AMAC1L2 | acyl-malonyl condensing enzyme 1-li | -0.059 | 0.960 | 5.88E-01 | 5.88E+01 | | | |
| 7954077 | NM_020853 | KIAA1467 | KIAA1467 | -0.059 | 0.960 | 4.13E-01 | 4.13E+01 | | | |
| 7939996 | --- | --- | --- | -0.059 | 0.960 | 4.71E-01 | 4.71E+01 | | | |
| 8037853 | NM_017854 | TMEM160 | transmembrane protein 160 | -0.059 | 0.960 | 2.44E-01 | 2.44E+01 | | | |
| 7992632 | NM_006181 | NTN3 | netrin 3 | -0.059 | 0.960 | 2.45E-01 | 2.45E+01 | | | |
| 7893596 | --- | --- | --- | -0.059 | 0.960 | 8.79E-01 | 8.79E+01 | | | |
| 7934945 | NM_148977 | PANK1 | pantothenate kinase 1 | -0.059 | 0.960 | 2.00E-01 | 2.00E+01 | | | |
| 7938076 | NM_00100517 | OR52W1 | olfactory receptor, family 52, subfam | -0.059 | 0.960 | 3.16E-01 | 3.16E+01 | | | |
| 7939902 | NR_024504 | LOC646813 | hypothetical LOC646813 | -0.059 | 0.960 | 7.88E-01 | 7.88E+01 | | | |
| 8041223 | --- | --- | --- | -0.059 | 0.960 | 6.11E-01 | 6.11E+01 | | | |
| 7906107 | NM_022367 | SEMA4A | sema domain, immunoglobulin doma | -0.059 | 0.960 | 2.75E-01 | 2.75E+01 | | | |
| 7931726 | --- | --- | --- | -0.059 | 0.960 | 5.84E-01 | 5.84E+01 | | | |
| 8127544 | NM_001402 | EEF1A1 | eukaryotic translation elongation fac | -0.059 | 0.960 | 7.71E-01 | 7.71E+01 | | | |
| 8039655 | NM_00103965 | ZNF550 | zinc finger protein 550 | -0.059 | 0.960 | 6.06E-01 | 6.06E+01 | | | |
| 8137271 | NM_001091 | ABP1 | amiloride binding protein 1 (amine o | -0.059 | 0.960 | 3.37E-01 | 3.37E+01 | | | |
| 8069161 | NM_198697 | KRTAP12-3 | keratin associated protein 12-3 | -0.059 | 0.960 | 3.92E-01 | 3.92E+01 | | | |
| 8081657 | NM_00100419 | CD200 | CD200 molecule | -0.059 | 0.960 | 3.49E-01 | 3.49E+01 | | | |
| 7998679 | NM_178167 | ZNF598 | zinc finger protein 598 | -0.059 | 0.960 | 2.27E-01 | 2.27E+01 | | | |
| 8043725 | NM_001079 | ZAP70 | zeta-chain (TCR) associated protein k | -0.059 | 0.960 | 6.26E-01 | 6.26E+01 | | | |
| 7990540 | NM_018285 | IMP3 | IMP3, U3 small nucleolar ribonucleo | -0.059 | 0.960 | 3.94E-01 | 3.94E+01 | | | |
| 8144917 | NM_000237 | LPL | lipoprotein lipase | -0.059 | 0.960 | 3.92E-01 | 3.92E+01 | | | |
| 8049998 | NM_152834 | TMEM18 | transmembrane protein 18 | -0.059 | 0.960 | 7.01E-01 | 7.01E+01 | | | |
| 8157101 | --- | --- | --- | -0.059 | 0.960 | 6.20E-01 | 6.20E+01 | | | |
| 8169006 | NM_00103183 | RAB40AL | RAB40A, member RAS oncogene fam | -0.059 | 0.960 | 4.13E-01 | 4.13E+01 | | | |
| 8021712 | --- | --- | --- | -0.059 | 0.960 | 4.81E-01 | 4.81E+01 | | | |
| 8116655 | NM_00112859 | PSMG4 | proteasome (prosome, macropain) a | -0.059 | 0.960 | 4.82E-01 | 4.82E+01 | | | |
| 8121095 | NM_014942 | ANKRD6 | ankyrin repeat domain 6 | -0.059 | 0.960 | 4.53E-01 | 4.53E+01 | | | |
| 7969372 | --- | --- | --- | -0.059 | 0.960 | 3.38E-01 | 3.38E+01 | | | |
| 7944113 | NM_014956 | CEP164 | centrosomal protein 164kDa | -0.059 | 0.960 | 3.94E-01 | 3.94E+01 | | | |
| 8001457 | NM_00102519 | CES1 | carboxylesterase 1 (monocyte/macro | -0.059 | 0.960 | 6.47E-01 | 6.47E+01 | | | |
| 8137700 | NM_032350 | C7orf50 | chromosome 7 open reading frame 5 | -0.059 | 0.960 | 2.90E-01 | 2.90E+01 | | | |
| 8016452 | NM_024015 | HOXB4 | homeobox B4 | -0.059 | 0.960 | 2.70E-01 | 2.70E+01 | | | |
| 7951159 | --- | --- | --- | -0.059 | 0.960 | 6.27E-01 | 6.27E+01 | | | |
| 7912863 | NR_002729 | MSTP9 | macrophage stimulating, pseudogen | -0.059 | 0.960 | 2.31E-01 | 2.31E+01 | | | |
| 8116096 | NM_016222 | DDX41 | DEAD (Asp-Glu-Ala-Asp) box polypep | -0.059 | 0.960 | 7.59E-01 | 7.59E+01 | | | |
| 8124305 | --- | --- | --- | -0.059 | 0.960 | 6.50E-01 | 6.50E+01 | | | |
| 7893976 | --- | --- | --- | -0.059 | 0.960 | 7.71E-01 | 7.71E+01 | | | |
| 7941709 | NM_002896 | RBM4 | RNA binding motif protein 4 | -0.059 | 0.960 | 7.51E-01 | 7.51E+01 | | | |
| 7972977 | --- | --- | --- | -0.059 | 0.960 | 5.96E-01 | 5.96E+01 | | | |
| 8179224 | NM_00101090 | MUC21 | mucin 21, cell surface associated | -0.059 | 0.960 | 4.42E-01 | 4.42E+01 | | | |
| 8014454 | NM_025109 | MYO19 | myosin XIX | -0.059 | 0.960 | 4.21E-01 | 4.21E+01 | | | |
| 7953532 | NM_001975 | ENO2 | enolase 2 (gamma, neuronal) | -0.059 | 0.960 | 7.65E-01 | 7.65E+01 | | | |
| 7913593 | NM_003196 | TCEA3 | transcription elongation factor A (SII | -0.059 | 0.960 | 6.11E-01 | 6.11E+01 | | | |
| 8031495 | NM_020378 | NAT14 | N-acetyltransferase 14 (GCN5-relate | -0.059 | 0.960 | 3.90E-01 | 3.90E+01 | | | |
| 8070712 | --- | --- | --- | -0.059 | 0.960 | 8.56E-01 | 8.56E+01 | | | |
| 8072124 | NM_001886 | CRYBA4 | crystallin, beta A4 | -0.059 | 0.960 | 3.62E-01 | 3.62E+01 | | | |
| 7963359 | NM_002282 | KRT83 | keratin 83 | -0.059 | 0.960 | 3.49E-01 | 3.49E+01 | | | |
| 8149243 | NM_201402 // | 17L2 // USP | ubiquitin specific peptidase 17-like 2 | -0.059 | 0.960 | 3.74E-01 | 3.74E+01 | | | |
| 7894005 | --- | --- | --- | -0.059 | 0.960 | 4.50E-01 | 4.50E+01 | | | |
| 7944510 | NM_032015 | RNF26 | ring finger protein 26 | -0.059 | 0.960 | 4.47E-01 | 4.47E+01 | | | |
| 8013906 | NM_002822 | TWF1 | twinfilin, actin-binding protein, hom | -0.059 | 0.960 | 8.50E-01 | 8.50E+01 | | | |
| 8059139 | NM_00107719 | ATG9A | ATG9 autophagy related 9 homolog A | -0.059 | 0.960 | 6.15E-01 | 6.15E+01 | | | |
| 7995322 | --- | --- | --- | -0.060 | 0.960 | 4.79E-01 | 4.79E+01 | | | |
| 8075263 | NM_003634 | NIPSNAP1 | nipsnap homolog 1 (C. elegans) | -0.060 | 0.960 | 7.58E-01 | 7.58E+01 | | | |
| 8099924 | --- | --- | --- | -0.060 | 0.960 | 1.64E-01 | 1.64E+01 | | | |
| 8134834 | NM_006076 | AGFG2 | ArfGAP with FG repeats 2 | -0.060 | 0.960 | 2.92E-01 | 2.92E+01 | | | |
| 8168762 | NM_001325 | CSTF2 | cleavage stimulation factor, 3' pre-R | -0.060 | 0.960 | 4.63E-01 | 4.63E+01 | | | |
| 7977105 | NM_152307 | TRMT61A | tRNA methyltransferase 61 homolog | -0.060 | 0.960 | 2.01E-01 | 2.01E+01 | | | |
| 8180313 | --- | --- | --- | -0.060 | 0.960 | 5.41E-01 | 5.41E+01 | | | |
| 7892628 | --- | --- | --- | -0.060 | 0.960 | 9.01E-01 | 9.01E+01 | | | |
| 8140297 | NM_00109941 | POM121C | POM121 membrane glycoprotein C | -0.060 | 0.959 | 6.03E-01 | 6.03E+01 | | | |
| 7896257 | --- | --- | --- | -0.060 | 0.959 | 8.89E-01 | 8.89E+01 | | | |
| 8003425 | NM_005187 | CBFA2T3 | core-binding factor, runt domain, alp | -0.060 | 0.959 | 2.87E-01 | 2.87E+01 | | | |

| | | | | | | | | | | |
|---|---|---|---|---|---|---|---|---|---|---|
| 7895583 | --- | --- | --- | -0.060 | 0.959 | 9.34E-01 | 9.34E+01 | | | |
| 7936928 | NM_147191 | MMP21 | matrix metallopeptidase 21 | -0.060 | 0.959 | 3.20E-01 | 3.20E+01 | | | |
| 8035941 | NM_014270 | SLC7A9 | solute carrier family 7 (cationic amin | -0.060 | 0.959 | 2.32E-01 | 2.32E+01 | | | |
| 8157890 | NM_006195 | PBX3 | pre-B-cell leukemia homeobox 3 | -0.060 | 0.959 | 5.03E-01 | 5.03E+01 | | | |
| 8141659 | --- | --- | --- | -0.060 | 0.959 | 3.73E-01 | 3.73E+01 | | | |
| 8167603 | NM_00112789 | CLCN5 | chloride channel 5 | -0.060 | 0.959 | 3.81E-01 | 3.81E+01 | | | |
| 8022653 | NR_024259 | LOC728606 | hypothetical LOC728606 | -0.060 | 0.959 | 3.20E-01 | 3.20E+01 | | | |
| 8108050 | NM_003202 | TCF7 | transcription factor 7 (T-cell specific, | -0.060 | 0.959 | 6.35E-01 | 6.35E+01 | | | |
| 8076002 | NM_013356 | SLC16A8 | solute carrier family 16, member 8 (r | -0.060 | 0.959 | 3.08E-01 | 3.08E+01 | | | |
| 7956613 | NM_005981 | TSPAN31 | tetraspanin 31 | -0.060 | 0.959 | 4.84E-01 | 4.84E+01 | | | |
| 8069511 | NR_027270 | C21orf81 | ankyrin repeat domain 20 family, me | -0.060 | 0.959 | 6.16E-01 | 6.16E+01 | | | |
| 7972021 | NM_014832 | TBC1D4 | TBC1 domain family, member 4 | -0.060 | 0.959 | 4.58E-01 | 4.58E+01 | | | |
| 7994341 | --- | --- | --- | -0.060 | 0.959 | 8.51E-01 | 8.51E+01 | | | |
| 7952671 | --- | --- | --- | -0.060 | 0.959 | 4.06E-01 | 4.06E+01 | | | |
| 7960283 | NM_172364 | CACNA2D4 | calcium channel, voltage-dependent | -0.060 | 0.959 | 2.27E-01 | 2.27E+01 | | | |
| 7960689 | NR_026581 | MLF2 | myeloid leukemia factor 2 | -0.060 | 0.959 | 4.26E-01 | 4.26E+01 | | | |
| 7902913 | NM_003503 | CDC7 | cell division cycle 7 homolog (S. cere | -0.060 | 0.959 | 8.27E-01 | 8.27E+01 | | | |
| 7974249 | NM_001663 | ARF6 | ADP-ribosylation factor 6 | -0.060 | 0.959 | 4.64E-01 | 4.64E+01 | | | |
| 8032480 | NM_016199 | LSM7 | LSM7 homolog, U6 small nuclear RN | -0.060 | 0.959 | 8.62E-01 | 8.62E+01 | | | |
| 8135763 | NM_057168 | WNT16 | wingless-type MMTV integration site | -0.060 | 0.959 | 3.26E-01 | 3.26E+01 | | | |
| 8090070 | NM_183357 | ADCY5 | adenylate cyclase 5 | -0.060 | 0.959 | 4.40E-01 | 4.40E+01 | | | |
| 8148512 | NM_207414 | FLJ43860 | FLJ43860 protein | -0.060 | 0.959 | 4.97E-01 | 4.97E+01 | | | |
| 7941364 | NM_032223 | PCNXL3 | pecanex-like 3 (Drosophila) | -0.060 | 0.959 | 2.85E-01 | 2.85E+01 | | | |
| 7989953 | NM_024666 | AAGAB | alpha- and gamma-adaptin binding p | -0.060 | 0.959 | 8.16E-01 | 8.16E+01 | | | |
| 7936091 | NM_032747 | USMG5 | up-regulated during skeletal muscle | -0.060 | 0.959 | 9.01E-01 | 9.01E+01 | | | |
| 8153536 | NM_00113603 | PUF60 | poly-U binding splicing factor 60KDa | -0.060 | 0.959 | 3.05E-01 | 3.05E+01 | | | |
| 8000192 | ENST00000198 | RRN3 | RRN3 RNA polymerase I transcriptio | -0.060 | 0.959 | 8.98E-01 | 8.98E+01 | | | |
| 8067183 | --- | --- | --- | -0.060 | 0.959 | 4.47E-01 | 4.47E+01 | | | |
| 8083775 | --- | --- | --- | -0.060 | 0.959 | 3.16E-01 | 3.16E+01 | | | |
| 7985066 | NM_152335 | C15orf27 | chromosome 15 open reading frame | -0.060 | 0.959 | 3.30E-01 | 3.30E+01 | | | |
| 8063000 | NM_006103 | WFDC2 | WAP four-disulfide core domain 2 | -0.060 | 0.959 | 2.69E-01 | 2.69E+01 | | | |
| 7913978 | NM_018066 | GPN2 | GPN-loop GTPase 2 | -0.060 | 0.959 | 3.25E-01 | 3.25E+01 | | | |
| 8007363 | NM_032387 | WNK4 | WNK lysine deficient protein kinase | -0.060 | 0.959 | 2.68E-01 | 2.68E+01 | | | |
| 8015240 | NM_033187 | KRTAP4-3 | keratin associated protein 4-3 | -0.060 | 0.959 | 4.96E-01 | 4.96E+01 | | | |
| 8004385 | NM_020795 | NLGN2 | neuroligin 2 | -0.060 | 0.959 | 2.72E-01 | 2.72E+01 | | | |
| 8126486 | NM_014780 | CUL7 | cullin 7 | -0.060 | 0.959 | 3.78E-01 | 3.78E+01 | | | |
| 7979757 | NM_015346 | ZFYVE26 | zinc finger, FYVE domain containing | -0.060 | 0.959 | 2.66E-01 | 2.66E+01 | | | |
| 7966996 | NM_053275 | RPLP0 | ribosomal protein, large, P0 | -0.060 | 0.959 | 6.72E-01 | 6.72E+01 | | | |
| 8084739 | NR_024413 | FLJ42393 | hypothetical LOC401105 | -0.060 | 0.959 | 3.11E-01 | 3.11E+01 | | | |
| 8054254 | NM_002285 | AFF3 | AF4/FMR2 family, member 3 | -0.060 | 0.959 | 5.42E-01 | 5.42E+01 | | | |
| 7958202 | NM_018413 | CHST11 | carbohydrate (chondroitin 4) sulfotra | -0.060 | 0.959 | 7.51E-01 | 7.51E+01 | | | |
| 8165255 | --- | --- | --- | -0.060 | 0.959 | 2.81E-01 | 2.81E+01 | | | |
| 8008339 | NM_018346 | RSAD1 | radical S-adenosyl methionine doma | -0.060 | 0.959 | 3.87E-01 | 3.87E+01 | | | |
| 8037878 | NM_020160 | MEIS3 | Meis homeobox 3 | -0.060 | 0.959 | 3.79E-01 | 3.79E+01 | | | |
| 7893386 | --- | --- | --- | -0.060 | 0.959 | 7.31E-01 | 7.31E+01 | | | |
| 8132922 | NM_015969 | MRPS17 | mitochondrial ribosomal protein S17 | -0.060 | 0.959 | 5.35E-01 | 5.35E+01 | | | |
| 8142017 | --- | --- | --- | -0.060 | 0.959 | 3.74E-01 | 3.74E+01 | | | |
| 8048468 | NM_024293 | FAM134A | family with sequence similarity 134, | -0.061 | 0.959 | 4.28E-01 | 4.28E+01 | | | |
| 8052667 | --- | --- | --- | -0.061 | 0.959 | 5.21E-01 | 5.21E+01 | | | |
| 8027996 | NM_172341 | PSENEN | presenilin enhancer 2 homolog (C. el | -0.061 | 0.959 | 4.09E-01 | 4.09E+01 | | | |
| 8029521 | NM_00112891 | TOMM40 | translocase of outer mitochondrial m | -0.061 | 0.959 | 3.84E-01 | 3.84E+01 | | | |
| 8154727 | AF091236 | LOC138412 | solute carrier family 25 (mitochondri | -0.061 | 0.959 | 6.99E-01 | 6.99E+01 | | | |
| 8130474 | NM_032861 | SERAC1 | serine active site containing 1 | -0.061 | 0.959 | 4.06E-01 | 4.06E+01 | | | |
| 7956097 | NM_000456 | SUOX | sulfite oxidase | -0.061 | 0.959 | 4.15E-01 | 4.15E+01 | | | |
| 7913644 | NM_004091 | E2F2 | E2F transcription factor 2 | -0.061 | 0.959 | 4.01E-01 | 4.01E+01 | | | |
| 8003844 | NM_031965 | GSG2 | germ cell associated 2 (haspin) | -0.061 | 0.959 | 3.26E-01 | 3.26E+01 | | | |
| 8039809 | NM_198055 | MZF1 | myeloid zinc finger 1 | -0.061 | 0.959 | 3.19E-01 | 3.19E+01 | | | |
| 8143697 | NM_152411 | ZNF786 | zinc finger protein 786 | -0.061 | 0.959 | 3.34E-01 | 3.34E+01 | | | |
| 7893294 | --- | --- | --- | -0.061 | 0.959 | 5.15E-01 | 5.15E+01 | | | |
| 7894286 | --- | --- | --- | -0.061 | 0.959 | 8.61E-01 | 8.61E+01 | | | |
| 8088020 | NM_002218 | ITIH4 | inter-alpha (globulin) inhibitor H4 (p | -0.061 | 0.959 | 2.35E-01 | 2.35E+01 | | | |
| 8052583 | --- | --- | --- | -0.061 | 0.959 | 5.28E-01 | 5.28E+01 | | | |
| 8010295 | NM_00104257 | ENGASE | endo-beta-N-acetylglucosaminidase | -0.061 | 0.959 | 3.44E-01 | 3.44E+01 | | | |
| 7940530 | NM_00112739 | C11orf9 | chromosome 11 open reading frame | -0.061 | 0.959 | 2.69E-01 | 2.69E+01 | | | |
| 7920182 | NM_178435 | LCE3E | late cornified envelope 3E | -0.061 | 0.959 | 3.88E-01 | 3.88E+01 | | | |
| 8039664 | NM_017879 | ZNF416 | zinc finger protein 416 | -0.061 | 0.959 | 6.62E-01 | 6.62E+01 | | | |
| 8073909 | NM_014346 | TBC1D22A | TBC1 domain family, member 22A | -0.061 | 0.959 | 7.27E-01 | 7.27E+01 | | | |
| 8155521 | NR_024060 | FAM27A | family with sequence similarity 27, n | -0.061 | 0.959 | 5.42E-01 | 5.42E+01 | | | |
| 8130176 | NM_024518 | ULBP3 | UL16 binding protein 3 | -0.061 | 0.959 | 3.36E-01 | 3.36E+01 | | | |
| 7942674 | NM_015516 | TSKU | tsukushin | -0.061 | 0.959 | 3.18E-01 | 3.18E+01 | | | |
| 7922328 | --- | --- | --- | -0.061 | 0.959 | 3.87E-01 | 3.87E+01 | | | |
| 8173596 | NM_00101297 | PABPC1L2A | poly(A) binding protein, cytoplasmic | -0.061 | 0.959 | 2.97E-01 | 2.97E+01 | | | |
| 7893745 | --- | --- | --- | -0.061 | 0.959 | 6.36E-01 | 6.36E+01 | | | |
| 7987099 | NM_130901 | OTUD7A | OTU domain containing 7A | -0.061 | 0.959 | 3.20E-01 | 3.20E+01 | | | |
| 7910414 | --- | --- | --- | -0.061 | 0.959 | 4.98E-01 | 4.98E+01 | | | |
| 8104617 | XM_936262 // | 1764 // LOC | similar to TBP-associated factor 11 / | -0.061 | 0.959 | 4.21E-01 | 4.21E+01 | | | |
| 8074261 | NM_197966 | BID | BH3 interacting domain death agoni | -0.061 | 0.959 | 4.22E-01 | 4.22E+01 | | | |

| | | | | | | | | | | |
|---|---|---|---|---|---|---|---|---|---|---|
| 8065693 | NM_031232 | NECAB3 | N-terminal EF-hand calcium binding | -0.061 | 0.959 | 2.51E-01 | 2.51E+01 | | | |
| 8036702 | NM_172139 | IL28B | interleukin 28B (interferon, lambda | -0.061 | 0.959 | 5.07E-01 | 5.07E+01 | | | |
| 8043657 | NM_020184 | CNNM4 | cyclin M4 | -0.061 | 0.959 | 4.64E-01 | 4.64E+01 | | | |
| 8137336 | NM_004769 | ACCN3 | amiloride-sensitive cation channel 3 | -0.061 | 0.959 | 3.12E-01 | 3.12E+01 | | | |
| 7966003 | NM_018171 | APPL2 | adaptor protein, phosphotyrosine in | -0.061 | 0.958 | 6.44E-01 | 6.44E+01 | | | |
| 7958761 | NM_00113653 | ACAD10 | acyl-Coenzyme A dehydrogenase fan | -0.061 | 0.958 | 4.91E-01 | 4.91E+01 | | | |
| 7896038 | --- | --- | --- | -0.061 | 0.958 | 6.84E-01 | 6.84E+01 | | | |
| 7996377 | NM_173815 | CES8 | carboxylesterase 8 (putative) | -0.061 | 0.958 | 2.77E-01 | 2.77E+01 | | | |
| 8100964 | --- | --- | --- | -0.061 | 0.958 | 5.94E-01 | 5.94E+01 | | | |
| 8171653 | NM_00100167 | MAP3K15 | mitogen-activated protein kinase kin | -0.061 | 0.958 | 2.60E-01 | 2.60E+01 | | | |
| 8010820 | NM_004514 | FOXK2 | forkhead box K2 | -0.061 | 0.958 | 3.31E-01 | 3.31E+01 | | | |
| 7892735 | --- | --- | --- | -0.061 | 0.958 | 9.56E-01 | 9.56E+01 | | | |
| 7995674 | NM_024335 | IRX6 | iroquois homeobox 6 | -0.061 | 0.958 | 2.67E-01 | 2.67E+01 | | | |
| 8035993 | NM_019849 | SLC7A10 | solute carrier family 7, (neutral amin | -0.062 | 0.958 | 1.84E-01 | 1.84E+01 | | | |
| 8025028 | NM_006012 | CLPP | ClpP caseinolytic peptidase, ATP-dep | -0.062 | 0.958 | 3.62E-01 | 3.62E+01 | | | |
| 8159243 | NM_014811 | KIAA0649 | KIAA0649 | -0.062 | 0.958 | 2.37E-01 | 2.37E+01 | | | |
| 8072266 | NM_182527 | CABP7 | calcium binding protein 7 | -0.062 | 0.958 | 3.00E-01 | 3.00E+01 | | | |
| 8034712 | NM_024825 | PODNL1 | podocan-like 1 | -0.062 | 0.958 | 4.01E-01 | 4.01E+01 | | | |
| 7895217 | --- | --- | --- | -0.062 | 0.958 | 7.35E-01 | 7.35E+01 | | | |
| 8180210 | --- | --- | --- | -0.062 | 0.958 | 3.43E-01 | 3.43E+01 | | | |
| 7973865 | --- | --- | --- | -0.062 | 0.958 | 3.79E-01 | 3.79E+01 | | | |
| 8039180 | NM_00108144 | LILRB5 | leukocyte immunoglobulin-like recep | -0.062 | 0.958 | 2.59E-01 | 2.59E+01 | | | |
| 7979808 | --- | --- | --- | -0.062 | 0.958 | 5.14E-01 | 5.14E+01 | | | |
| 8002143 | NM_000229 | LCAT | lecithin-cholesterol acyltransferase | -0.062 | 0.958 | 3.39E-01 | 3.39E+01 | | | |
| 7897378 | NM_016831 | PER3 | period homolog 3 (Drosophila) | -0.062 | 0.958 | 1.31E-01 | 1.31E+01 | | | |
| 7977482 | NM_138376 | TTC5 | tetratricopeptide repeat domain 5 | -0.062 | 0.958 | 5.89E-01 | 5.89E+01 | | | |
| 8060339 | NM_024958 | NRSN2 | neurensin 2 | -0.062 | 0.958 | 2.67E-01 | 2.67E+01 | | | |
| 7896572 | --- | --- | --- | -0.062 | 0.958 | 8.50E-01 | 8.50E+01 | | | |
| 7959220 | NM_000017 | ACADS | acyl-Coenzyme A dehydrogenase, C- | -0.062 | 0.958 | 2.22E-01 | 2.22E+01 | | | |
| 7892517 | --- | --- | --- | -0.062 | 0.958 | 9.27E-01 | 9.27E+01 | | | |
| 7920944 | NM_015327 | SMG5 | Smg-5 homolog, nonsense mediated | -0.062 | 0.958 | 4.64E-01 | 4.64E+01 | | | |
| 8104220 | NM_018140 | CEP72 | centrosomal protein 72kDa | -0.062 | 0.958 | 3.46E-01 | 3.46E+01 | | | |
| 7902036 | --- | --- | --- | -0.062 | 0.958 | 3.91E-01 | 3.91E+01 | | | |
| 7980720 | NM_00101085 | TTC7B | tetratricopeptide repeat domain 7B | -0.062 | 0.958 | 2.53E-01 | 2.53E+01 | | | |
| 8103431 | NM_00100839 | C4orf46 | chromosome 4 open reading frame 4 | -0.062 | 0.958 | 5.66E-01 | 5.66E+01 | | | |
| 8015262 | NM_002279 | KRT33B | keratin 33B | -0.062 | 0.958 | 3.78E-01 | 3.78E+01 | | | |
| 8065256 | NM_016652 | CRNKL1 | crooked neck pre-mRNA splicing fact | -0.062 | 0.958 | 6.38E-01 | 6.38E+01 | | | |
| 7955184 | NM_006262 | PRPH | peripherin | -0.062 | 0.958 | 3.79E-01 | 3.79E+01 | | | |
| 8048141 | NM_00114231 | TMEM169 | transmembrane protein 169 | -0.062 | 0.958 | 3.53E-01 | 3.53E+01 | | | |
| 8073623 | NM_00104437 | MPPED1 | metallophosphoesterase domain con | -0.062 | 0.958 | 2.53E-01 | 2.53E+01 | | | |
| 8049582 | NM_016510 | SCLY | selenocysteine lyase | -0.062 | 0.958 | 2.80E-01 | 2.80E+01 | | | |
| 8004804 | NM_012393 | PFAS | phosphoribosylformylglycinamidine | -0.062 | 0.958 | 2.59E-01 | 2.59E+01 | | | |
| 8166712 | --- | --- | --- | -0.062 | 0.958 | 3.25E-01 | 3.25E+01 | | | |
| 8090972 | NM_178130 | TXNDC6 | thioredoxin domain containing 6 | -0.062 | 0.958 | 2.55E-01 | 2.55E+01 | | | |
| 8063739 | NM_080672 | PHACTR3 | phosphatase and actin regulator 3 | -0.062 | 0.958 | 1.72E-01 | 1.72E+01 | | | |
| 8046555 | NM_014621 | HOXD4 | homeobox D4 | -0.062 | 0.958 | 2.77E-01 | 2.77E+01 | | | |
| 7995340 | --- | --- | --- | -0.062 | 0.958 | 5.23E-01 | 5.23E+01 | | | |
| 7905324 | BC002469 | C1orf56 | chromosome 1 open reading frame 5 | -0.062 | 0.958 | 4.03E-01 | 4.03E+01 | | | |
| 8077042 | NM_002972 | SBF1 | SET binding factor 1 | -0.062 | 0.958 | 3.13E-01 | 3.13E+01 | | | |
| 8064842 | --- | --- | --- | -0.062 | 0.958 | 1.44E-01 | 1.44E+01 | | | |
| 7896420 | --- | --- | --- | -0.062 | 0.958 | 8.91E-01 | 8.91E+01 | | | |
| 7918552 | NM_019099 | C1orf183 | chromosome 1 open reading frame 1 | -0.062 | 0.958 | 3.27E-01 | 3.27E+01 | | | |
| 8163491 | NM_00101236 | WDR31 | WD repeat domain 31 | -0.062 | 0.958 | 3.05E-01 | 3.05E+01 | | | |
| 8011328 | --- | --- | --- | -0.062 | 0.958 | 4.60E-01 | 4.60E+01 | | | |
| 8019136 | BC064483 | C17orf56 | chromosome 17 open reading frame | -0.062 | 0.958 | 3.59E-01 | 3.59E+01 | | | |
| 8048712 | --- | --- | --- | -0.062 | 0.958 | 2.72E-01 | 2.72E+01 | | | |
| 8067709 | NM_012384 | GMEB2 | glucocorticoid modulatory element b | -0.062 | 0.958 | 4.03E-01 | 4.03E+01 | | | |
| 7914592 | NM_033504 | TMEM54 | transmembrane protein 54 | -0.062 | 0.958 | 2.04E-01 | 2.04E+01 | | | |
| 7952884 | NM_054025 | B3GAT1 | beta-1,3-glucuronyltransferase 1 (glu | -0.062 | 0.958 | 3.50E-01 | 3.50E+01 | | | |
| 8155246 | --- | --- | --- | -0.062 | 0.958 | 8.34E-01 | 8.34E+01 | | | |
| 7900087 | NM_017825 | ADPRHL2 | ADP-ribosylhydrolase like 2 | -0.062 | 0.958 | 4.58E-01 | 4.58E+01 | | | |
| 8153937 | NM_031309 | SCRT1 | scratch homolog 1, zinc finger protei | -0.062 | 0.958 | 3.92E-01 | 3.92E+01 | | | |
| 7895510 | --- | --- | --- | -0.062 | 0.958 | 7.71E-01 | 7.71E+01 | | | |
| 8069517 | --- | --- | --- | -0.062 | 0.958 | 5.14E-01 | 5.14E+01 | | | |
| 8175947 | NM_005334 | HCFC1 | host cell factor C1 (VP16-accessory p | -0.062 | 0.958 | 5.45E-01 | 5.45E+01 | | | |
| 8027345 | NM_020855 | ZNF492 | zinc finger protein 492 | -0.062 | 0.958 | 6.90E-01 | 6.90E+01 | | | |
| 7925701 | --- | --- | --- | -0.062 | 0.958 | 7.77E-01 | 7.77E+01 | | | |
| 7945958 | NM_00100516 | OR52B4 | olfactory receptor, family 52, subfam | -0.062 | 0.958 | 4.62E-01 | 4.62E+01 | | | |
| 8087576 | NM_006764 | IFRD2 | interferon-related developmental re | -0.063 | 0.958 | 2.03E-01 | 2.03E+01 | | | |
| 8087119 | NM_022911 | SLC26A6 | solute carrier family 26, member 6 | -0.063 | 0.958 | 3.01E-01 | 3.01E+01 | | | |
| 8175299 | NM_00107817 | FAM127C | family with sequence similarity 127, | -0.063 | 0.958 | 4.74E-01 | 4.74E+01 | | | |
| 8178295 | NM_006398 | UBD | ubiquitin D | -0.063 | 0.958 | 2.90E-01 | 2.90E+01 | | | |
| 7893805 | --- | --- | --- | -0.063 | 0.958 | 6.36E-01 | 6.36E+01 | | | |
| 7923426 | NM_014176 | UBE2T | ubiquitin-conjugating enzyme E2T (p | -0.063 | 0.958 | 3.07E-01 | 3.07E+01 | | | |
| 7923762 | NM_018203 | KLHDC8A | kelch domain containing 8A | -0.063 | 0.958 | 2.70E-01 | 2.70E+01 | | | |
| 7895585 | --- | --- | --- | -0.063 | 0.958 | 5.79E-01 | 5.79E+01 | | | |
| 8097472 | --- | --- | --- | -0.063 | 0.958 | 7.99E-01 | 7.99E+01 | | | |

| | | | | | | | | | | |
|---|---|---|---|---|---|---|---|---|---|---|
| 7895872 | --- | --- | --- | -0.063 | 0.958 | 5.44E-01 | 5.44E+01 | | | |
| 7920193 | NM_178351 | LCE1C | late cornified envelope 1C | -0.063 | 0.958 | 3.58E-01 | 3.58E+01 | | | |
| 8036938 | NM_024876 | ADCK4 | aarF domain containing kinase 4 | -0.063 | 0.958 | 2.51E-01 | 2.51E+01 | | | |
| 8087634 | NM_007275 | TUSC2 | tumor suppressor candidate 2 | -0.063 | 0.957 | 3.70E-01 | 3.70E+01 | | | |
| 7977149 | NM_024071 | ZFYVE21 | zinc finger, FYVE domain containing 2 | -0.063 | 0.957 | 2.79E-01 | 2.79E+01 | | | |
| 8043055 | NM_001370 | DNAH6 | dynein, axonemal, heavy chain 6 | -0.063 | 0.957 | 2.61E-01 | 2.61E+01 | | | |
| 7970498 | NM_014572 | LATS2 | LATS, large tumor suppressor, homo | -0.063 | 0.957 | 1.74E-01 | 1.74E+01 | | | |
| 7951807 | NM_014333 | CADM1 | cell adhesion molecule 1 | -0.063 | 0.957 | 3.32E-01 | 3.32E+01 | | | |
| 8128620 | NM_00108045 | BEND3 | BEN domain containing 3 | -0.063 | 0.957 | 1.93E-01 | 1.93E+01 | | | |
| 7948599 | ENST0000024 | KFZP434K02 | hypothetical LOC26070 | -0.063 | 0.957 | 3.19E-01 | 3.19E+01 | | | |
| 8137264 | NM_018487 | TMEM176A | transmembrane protein 176A | -0.063 | 0.957 | 6.52E-01 | 6.52E+01 | | | |
| 8092682 | NM_001048 | SST | somatostatin | -0.063 | 0.957 | 3.90E-01 | 3.90E+01 | | | |
| 7988033 | NM_000119 | EPB42 | erythrocyte membrane protein band | -0.063 | 0.957 | 4.75E-01 | 4.75E+01 | | | |
| 8132290 | --- | --- | --- | -0.063 | 0.957 | 5.79E-01 | 5.79E+01 | | | |
| 7941296 | NM_006396 | SSSCA1 | Sjogren syndrome/scleroderma auto | -0.063 | 0.957 | 4.59E-01 | 4.59E+01 | | | |
| 8138088 | NM_00103716 | C7orf70 | chromosome 7 open reading frame 7 | -0.063 | 0.957 | 4.33E-01 | 4.33E+01 | | | |
| 8014969 | NM_152219 | GJD3 | gap junction protein, delta 3, 31.9kD | -0.063 | 0.957 | 3.59E-01 | 3.59E+01 | | | |
| 8137112 | NM_012256 | ZNF212 | zinc finger protein 212 | -0.063 | 0.957 | 4.35E-01 | 4.35E+01 | | | |
| 7907351 | --- | --- | --- | -0.063 | 0.957 | 5.12E-01 | 5.12E+01 | | | |
| 8061647 | NM_00101171 | XKR7 | XK, Kell blood group complex subuni | -0.063 | 0.957 | 2.33E-01 | 2.33E+01 | | | |
| 8030789 | NM_014385 | SIGLEC7 | sialic acid binding Ig-like lectin 7 | -0.063 | 0.957 | 2.49E-01 | 2.49E+01 | | | |
| 7945014 | NM_001274 | CHEK1 | CHK1 checkpoint homolog (S. pombe | -0.063 | 0.957 | 7.52E-01 | 7.52E+01 | | | |
| 7896929 | NM_022834 | VWA1 | von Willebrand factor A domain cont | -0.063 | 0.957 | 2.82E-01 | 2.82E+01 | | | |
| 8042333 | --- | --- | --- | -0.063 | 0.957 | 4.42E-01 | 4.42E+01 | | | |
| 7919217 | NM_00101275 | NUDT17 | nudix (nucleoside diphosphate linked | -0.063 | 0.957 | 4.78E-01 | 4.78E+01 | | | |
| 8010287 | NM_030968 | C1QTNF1 | C1q and tumor necrosis factor relate | -0.063 | 0.957 | 2.78E-01 | 2.78E+01 | | | |
| 8035886 | NM_031448 | C19orf12 | chromosome 19 open reading frame | -0.063 | 0.957 | 3.64E-01 | 3.64E+01 | | | |
| 7926105 | NM_00100229 | GATA3 | GATA binding protein 3 | -0.063 | 0.957 | 5.53E-01 | 5.53E+01 | | | |
| 8021783 | NM_007345 | ZNF236 | zinc finger protein 236 | -0.063 | 0.957 | 3.02E-01 | 3.02E+01 | | | |
| 7901549 | NM_000098 | CPT2 | carnitine palmitoyltransferase 2 | -0.063 | 0.957 | 3.81E-01 | 3.81E+01 | | | |
| 7971350 | NM_183422 | TSC22D1 | TSC22 domain family, member 1 | -0.063 | 0.957 | 6.98E-01 | 6.98E+01 | | | |
| 8094960 | NM_152679 | SLC10A4 | solute carrier family 10 (sodium/bile | -0.063 | 0.957 | 3.21E-01 | 3.21E+01 | | | |
| 8113790 | NM_178450 | 40240 | membrane-associated ring finger (C3 | -0.063 | 0.957 | 7.92E-01 | 7.92E+01 | | | |
| 7994506 | NM_032815 | NFATC2IP | nuclear factor of activated T-cells, cy | -0.063 | 0.957 | 3.93E-01 | 3.93E+01 | | | |
| 7952179 | NM_002105 | H2AFX | H2A histone family, member X | -0.063 | 0.957 | 3.48E-01 | 3.48E+01 | | | |
| 8014794 | NM_199247 | CACNB1 | calcium channel, voltage-dependent | -0.063 | 0.957 | 2.69E-01 | 2.69E+01 | | | |
| 8082305 | NM_032343 | CHCHD6 | coiled-coil-helix-coiled-coil-helix dom | -0.063 | 0.957 | 5.01E-01 | 5.01E+01 | | | |
| 7923534 | NM_004997 | MYBPH | myosin binding protein H | -0.063 | 0.957 | 2.69E-01 | 2.69E+01 | | | |
| 7894505 | --- | --- | --- | -0.063 | 0.957 | 9.09E-01 | 9.09E+01 | | | |
| 7892713 | --- | --- | --- | -0.063 | 0.957 | 5.11E-01 | 5.11E+01 | | | |
| 7966638 | NM_016196 | RBM19 | RNA binding motif protein 19 | -0.063 | 0.957 | 2.51E-01 | 2.51E+01 | | | |
| 7949015 | NM_00114493 | LOC65998 | hypothetical protein LOC65998 | -0.063 | 0.957 | 2.12E-01 | 2.12E+01 | | | |
| 8140534 | NM_006379 | SEMA3C | sema domain, immunoglobulin doma | -0.063 | 0.957 | 3.90E-01 | 3.90E+01 | | | |
| 8132557 | NM_001129 | AEBP1 | AE binding protein 1 | -0.063 | 0.957 | 3.23E-01 | 3.23E+01 | | | |
| 8114647 | NM_031947 | SLC25A2 | solute carrier family 25 (mitochondri | -0.063 | 0.957 | 4.71E-01 | 4.71E+01 | | | |
| 8001271 | NM_033151 | ABCC11 | ATP-binding cassette, sub-family C (C | -0.063 | 0.957 | 2.69E-01 | 2.69E+01 | | | |
| 8029907 | NM_001736 | C5AR1 | complement component 5a receptor | -0.063 | 0.957 | 6.46E-01 | 6.46E+01 | | | |
| 7958931 | NM_004416 | DTX1 | deltex homolog 1 (Drosophila) | -0.063 | 0.957 | 3.01E-01 | 3.01E+01 | | | |
| 8100754 | --- | --- | --- | -0.063 | 0.957 | 4.75E-01 | 4.75E+01 | | | |
| 8125887 | NM_003214 | TEAD3 | TEA domain family member 3 | -0.063 | 0.957 | 1.80E-01 | 1.80E+01 | | | |
| 8137542 | NM_053043 | RBM33 | RNA binding motif protein 33 | -0.063 | 0.957 | 6.83E-01 | 6.83E+01 | | | |
| 7903369 | NM_133496 | SLC30A7 | solute carrier family 30 (zinc transpo | -0.063 | 0.957 | 8.35E-01 | 8.35E+01 | | | |
| 8074780 | NM_013313 | YPEL1 | yippee-like 1 (Drosophila) | -0.063 | 0.957 | 6.61E-01 | 6.61E+01 | | | |
| 8053406 | NM_017750 | RETSAT | retinol saturase (all-trans-retinol 13, | -0.063 | 0.957 | 2.53E-01 | 2.53E+01 | | | |
| 8126653 | NM_032111 | MRPL14 | mitochondrial ribosomal protein L14 | -0.063 | 0.957 | 6.22E-01 | 6.22E+01 | | | |
| 8026991 | NM_017712 | PGPEP1 | pyroglutamyl-peptidase I | -0.063 | 0.957 | 3.44E-01 | 3.44E+01 | | | |
| 8079165 | --- | --- | --- | -0.064 | 0.957 | 4.54E-01 | 4.54E+01 | | | |
| 8136889 | --- | --- | --- | -0.064 | 0.957 | 5.00E-01 | 5.00E+01 | | | |
| 8004309 | NM_001042 | SLC2A4 | solute carrier family 2 (facilitated glu | -0.064 | 0.957 | 2.43E-01 | 2.43E+01 | | | |
| 7949916 | NM_001277 | CHKA | choline kinase alpha | -0.064 | 0.957 | 5.84E-01 | 5.84E+01 | | | |
| 8038556 | NM_004851 | NAPSA | napsin A aspartic peptidase | -0.064 | 0.957 | 3.33E-01 | 3.33E+01 | | | |
| 8118098 | --- | --- | --- | -0.064 | 0.957 | 5.32E-01 | 5.32E+01 | | | |
| 8172573 | NM_003179 | SYP | synaptophysin | -0.064 | 0.957 | 3.58E-01 | 3.58E+01 | | | |
| 8033813 | NM_017703 | FBXL12 | F-box and leucine-rich repeat protein | -0.064 | 0.957 | 3.04E-01 | 3.04E+01 | | | |
| 8119722 | NM_015089 | CUL9 | cullin 9 | -0.064 | 0.957 | 2.29E-01 | 2.29E+01 | | | |
| 8049448 | NM_00103713 | AGAP1 | ArfGAP with GTPase domain, ankyrin | -0.064 | 0.957 | 3.49E-01 | 3.49E+01 | | | |
| 7934244 | NM_00100276 | DNAJB12 | DnaJ (Hsp40) homolog, subfamily B, | -0.064 | 0.957 | 4.05E-01 | 4.05E+01 | | | |
| 8035050 | NM_022904 | RASAL3 | RAS protein activator like 3 | -0.064 | 0.957 | 4.27E-01 | 4.27E+01 | | | |
| 7920912 | NM_020131 | UBQLN4 | ubiquilin 4 | -0.064 | 0.957 | 3.62E-01 | 3.62E+01 | | | |
| 7893527 | --- | --- | --- | -0.064 | 0.957 | 8.59E-01 | 8.59E+01 | | | |
| 8025301 | NR_026692 | CD209 | CD209 molecule | -0.064 | 0.957 | 7.01E-01 | 7.01E+01 | | | |
| 8124437 | NM_021018 | HIST1H3F | histone cluster 1, H3f | -0.064 | 0.957 | 7.79E-01 | 7.79E+01 | | | |
| 8089830 | NM_00109967 | LRRC58 | leucine rich repeat containing 58 | -0.064 | 0.957 | 5.95E-01 | 5.95E+01 | | | |
| 7905559 | NM_00101085 | LELP1 | late cornified envelope-like proline-r | -0.064 | 0.957 | 3.16E-01 | 3.16E+01 | | | |
| 8010737 | NM_005052 | RAC3 | ras-related C3 botulinum toxin subst | -0.064 | 0.957 | 2.13E-01 | 2.13E+01 | | | |
| 8061605 | NM_033118 | MYLK2 | myosin light chain kinase 2 | -0.064 | 0.957 | 2.66E-01 | 2.66E+01 | | | |
| 8105499 | NM_174889 | NDUFAF2 | NADH dehydrogenase (ubiquinone) 1 | -0.064 | 0.957 | 7.98E-01 | 7.98E+01 | | | |

| | | | | | | | | | | |
|---|---|---|---|---|---|---|---|---|---|---|
| 7912211 | NM_207420 | SLC2A7 | solute carrier family 2 (facilitated glu | -0.064 | 0.957 | 2.33E-01 | 2.33E+01 | | | |
| 7939150 | NM_024081 | PRRG4 | proline rich Gla (G-carboxyglutamic a | -0.064 | 0.957 | 4.23E-01 | 4.23E+01 | | | |
| 8029458 | NM_00103921 | CEACAM16 | carcinoembryonic antigen-related ce | -0.064 | 0.957 | 2.32E-01 | 2.32E+01 | | | |
| 7941311 | NM_153253 | SIPA1 | signal-induced proliferation-associat | -0.064 | 0.957 | 1.51E-01 | 1.51E+01 | | | |
| 7897263 | NM_207396 | RNF207 | ring finger protein 207 | -0.064 | 0.957 | 2.39E-01 | 2.39E+01 | | | |
| 7933469 | NM_021226 | ARHGAP22 | Rho GTPase activating protein 22 | -0.064 | 0.957 | 3.25E-01 | 3.25E+01 | | | |
| 8010017 | NM_001258 | CDK3 | cyclin-dependent kinase 3 | -0.064 | 0.957 | 2.65E-01 | 2.65E+01 | | | |
| 7994603 | NM_00103028 | SPN | sialophorin | -0.064 | 0.957 | 2.85E-01 | 2.85E+01 | | | |
| 8167314 | NM_002536 | TBC1D25 | TBC1 domain family, member 25 | -0.064 | 0.957 | 5.58E-01 | 5.58E+01 | | | |
| 7896312 | --- | --- | --- | -0.064 | 0.957 | 6.73E-01 | 6.73E+01 | | | |
| 7979426 | NM_022571 | GPR135 | G protein-coupled receptor 135 | -0.064 | 0.957 | 2.08E-01 | 2.08E+01 | | | |
| 7960397 | NM_020638 | FGF23 | fibroblast growth factor 23 | -0.064 | 0.957 | 3.30E-01 | 3.30E+01 | | | |
| 7979548 | NM_145171 | GPHB5 | glycoprotein hormone beta 5 | -0.064 | 0.957 | 2.97E-01 | 2.97E+01 | | | |
| 7984952 | NM_015492 | C15orf39 | chromosome 15 open reading frame | -0.064 | 0.957 | 2.14E-01 | 2.14E+01 | | | |
| 7992656 | NM_015944 | AMDHD2 | amidohydrolase domain containing 2 | -0.064 | 0.957 | 2.98E-01 | 2.98E+01 | | | |
| 7941879 | NM_198517 | TBC1D10C | TBC1 domain family, member 10C | -0.064 | 0.957 | 3.21E-01 | 3.21E+01 | | | |
| 8074399 | NM_007098 | CLTCL1 | clathrin, heavy chain-like 1 | -0.064 | 0.957 | 2.58E-01 | 2.58E+01 | | | |
| 7937200 | NM_015722 | CALY | calcyon neuron-specific vesicular pro | -0.064 | 0.957 | 2.75E-01 | 2.75E+01 | | | |
| 8129377 | --- | --- | --- | -0.064 | 0.957 | 2.37E-01 | 2.37E+01 | | | |
| 8123558 | NM_148959 | HUS1B | HUS1 checkpoint homolog b (S. pom | -0.064 | 0.957 | 3.49E-01 | 3.49E+01 | | | |
| 8118945 | NM_006238 | PPARD | peroxisome proliferator-activated re | -0.064 | 0.957 | 5.16E-01 | 5.16E+01 | | | |
| 8175444 | NM_004114 | FGF13 | fibroblast growth factor 13 | -0.064 | 0.957 | 2.50E-01 | 2.50E+01 | | | |
| 8137942 | NM_00108049 | TNRC18 | trinucleotide repeat containing 18 | -0.064 | 0.956 | 4.70E-01 | 4.70E+01 | | | |
| 8132130 | NM_000823 | GHRHR | growth hormone releasing hormone | -0.064 | 0.956 | 2.82E-01 | 2.82E+01 | | | |
| 7988763 | NM_207381 | TNFAIP8L3 | tumor necrosis factor, alpha-induced | -0.064 | 0.956 | 2.35E-01 | 2.35E+01 | | | |
| 8062490 | NR_002986 | SNORA60 | small nucleolar RNA, H/ACA box 60 | -0.064 | 0.956 | 4.17E-01 | 4.17E+01 | | | |
| 7895942 | --- | --- | --- | -0.064 | 0.956 | 6.38E-01 | 6.38E+01 | | | |
| 8056798 | NM_003111 | SP3 | Sp3 transcription factor | -0.064 | 0.956 | 3.99E-01 | 3.99E+01 | | | |
| 8005555 | AK026312 | LOC79999 | hypothetical LOC79999 | -0.064 | 0.956 | 2.81E-01 | 2.81E+01 | | | |
| 8013327 | AK026312 | LOC79999 | hypothetical LOC79999 | -0.064 | 0.956 | 2.81E-01 | 2.81E+01 | | | |
| 7942267 | NM_00101271 | KRTAP5-10 | keratin associated protein 5-10 | -0.064 | 0.956 | 2.74E-01 | 2.74E+01 | | | |
| 8019778 | NM_002861 | PCYT2 | phosphate cytidylyltransferase 2, eth | -0.064 | 0.956 | 3.38E-01 | 3.38E+01 | | | |
| 8111443 | NM_181435 | C1QTNF3 | C1q and tumor necrosis factor relate | -0.064 | 0.956 | 3.67E-01 | 3.67E+01 | | | |
| 7955195 | NM_005480 | TROAP | trophinin associated protein (tastin) | -0.064 | 0.956 | 2.62E-01 | 2.62E+01 | | | |
| 8029219 | NM_173633 | TMEM145 | transmembrane protein 145 | -0.064 | 0.956 | 2.95E-01 | 2.95E+01 | | | |
| 8044563 | NM_032556 | IL1F10 | interleukin 1 family, member 10 (the | -0.064 | 0.956 | 2.01E-01 | 2.01E+01 | | | |
| 7893166 | --- | --- | --- | -0.064 | 0.956 | 9.18E-01 | 9.18E+01 | | | |
| 7975799 | NM_017791 | FLVCR2 | feline leukemia virus subgroup C cell | -0.064 | 0.956 | 3.04E-01 | 3.04E+01 | | | |
| 8007069 | --- | --- | --- | -0.064 | 0.956 | 5.80E-01 | 5.80E+01 | | | |
| 7949568 | AK090761 | OC10013123 | hypothetical LOC100131232 | -0.064 | 0.956 | 3.15E-01 | 3.15E+01 | | | |
| 8033025 | NM_020175 | DUS3L | dihydrouridine synthase 3-like (S. cer | -0.064 | 0.956 | 2.15E-01 | 2.15E+01 | | | |
| 8036636 | NM_012237 | SIRT2 | sirtuin (silent mating type informatio | -0.064 | 0.956 | 3.38E-01 | 3.38E+01 | | | |
| 8164862 | NM_006753 | SURF6 | surfeit 6 | -0.065 | 0.956 | 4.67E-01 | 4.67E+01 | | | |
| 8153201 | NM_012154 | EIF2C2 | eukaryotic translation initiation facto | -0.065 | 0.956 | 6.04E-01 | 6.04E+01 | | | |
| 8072374 | NM_00100147 | SLC35E4 | solute carrier family 35, member E4 | -0.065 | 0.956 | 2.70E-01 | 2.70E+01 | | | |
| 8068810 | NM_018964 | SLC37A1 | solute carrier family 37 (glycerol-3-p | -0.065 | 0.956 | 4.00E-01 | 4.00E+01 | | | |
| 8004400 | NM_175734 | C17orf74 | chromosome 17 open reading frame | -0.065 | 0.956 | 3.16E-01 | 3.16E+01 | | | |
| 8068610 | --- | --- | --- | -0.065 | 0.956 | 9.22E-01 | 9.22E+01 | | | |
| 8113981 | NM_004199 | P4HA2 | prolyl 4-hydroxylase, alpha polypept | -0.065 | 0.956 | 2.01E-01 | 2.01E+01 | | | |
| 8040101 | --- | --- | --- | -0.065 | 0.956 | 5.45E-01 | 5.45E+01 | | | |
| 8125017 | NM_021160 | BAT5 | HLA-B associated transcript 5 | -0.065 | 0.956 | 3.42E-01 | 3.42E+01 | | | |
| 7918792 | BC063894 | DENND2C | DENN/MADD domain containing 2C | -0.065 | 0.956 | 2.77E-01 | 2.77E+01 | | | |
| 8092596 | NM_001346 | DGKG | diacylglycerol kinase, gamma 90kDa | -0.065 | 0.956 | 2.20E-01 | 2.20E+01 | | | |
| 8035612 | NM_00114578 | MEF2B | myocyte enhancer factor 2B | -0.065 | 0.956 | 3.50E-01 | 3.50E+01 | | | |
| 8086908 | NM_00113008 | PLXNB1 | plexin B1 | -0.065 | 0.956 | 1.62E-01 | 1.62E+01 | | | |
| 8100001 | --- | --- | --- | -0.065 | 0.956 | 5.61E-01 | 5.61E+01 | | | |
| 7952707 | NM_020228 | PRDM10 | PR domain containing 10 | -0.065 | 0.956 | 2.98E-01 | 2.98E+01 | | | |
| 8000924 | NM_138447 | ZNF689 | zinc finger protein 689 | -0.065 | 0.956 | 2.32E-01 | 2.32E+01 | | | |
| 8116484 | NM_00111461 | MGAT1 | mannosyl (alpha-1,3-)-glycoprotein b | -0.065 | 0.956 | 4.23E-01 | 4.23E+01 | | | |
| 8045563 | NM_018460 | ARHGAP15 | Rho GTPase activating protein 15 | -0.065 | 0.956 | 8.88E-01 | 8.88E+01 | | | |
| 7894723 | --- | --- | --- | -0.065 | 0.956 | 5.42E-01 | 5.42E+01 | | | |
| 7893960 | --- | --- | --- | -0.065 | 0.956 | 5.08E-01 | 5.08E+01 | | | |
| 8004201 | NR_002710 | ALOX12P2 | arachidonate 12-lipoxygenase pseud | -0.065 | 0.956 | 3.67E-01 | 3.67E+01 | | | |
| 8125201 | NM_181842 | ZBTB12 | zinc finger and BTB domain containi | -0.065 | 0.956 | 1.82E-01 | 1.82E+01 | | | |
| 8178683 | NM_181842 | ZBTB12 | zinc finger and BTB domain containi | -0.065 | 0.956 | 1.82E-01 | 1.82E+01 | | | |
| 8070563 | NR_027243 | C21orf128 | chromosome 21 open reading frame | -0.065 | 0.956 | 3.80E-01 | 3.80E+01 | | | |
| 8053710 | --- | --- | --- | -0.065 | 0.956 | 2.68E-01 | 2.68E+01 | | | |
| 8064451 | NM_00112296 | SIRPB2 | signal-regulatory protein beta 2 | -0.065 | 0.956 | 4.17E-01 | 4.17E+01 | | | |
| 8025000 | NM_00101792 | VMAC | vimentin-type intermediate filament | -0.065 | 0.956 | 3.82E-01 | 3.82E+01 | | | |
| 7986565 | XM_00112627 | LOC648913 | similar to a disintegrin and metallope | -0.065 | 0.956 | 2.10E-01 | 2.10E+01 | | | |
| 8018297 | NM_014595 | NT5C | 5', 3'-nucleotidase, cytosolic | -0.065 | 0.956 | 2.38E-01 | 2.38E+01 | | | |
| 7995417 | --- | --- | --- | -0.065 | 0.956 | 2.78E-01 | 2.78E+01 | | | |
| 7950023 | NM_005117 | FGF19 | fibroblast growth factor 19 | -0.065 | 0.956 | 2.91E-01 | 2.91E+01 | | | |
| 7906244 | NM_00100779 | NTRK1 | neurotrophic tyrosine kinase, recept | -0.065 | 0.956 | 2.11E-01 | 2.11E+01 | | | |
| 7942315 | NM_00114530 | LRTOMT | leucine rich transmembrane and 0-m | -0.065 | 0.956 | 3.79E-01 | 3.79E+01 | | | |
| 7945071 | NM_017547 | FOXRED1 | FAD-dependent oxidoreductase dom | -0.065 | 0.956 | 3.34E-01 | 3.34E+01 | | | |
| 7937247 | NM_130784 | SYCE1 | synaptonemal complex central elem | -0.065 | 0.956 | 3.04E-01 | 3.04E+01 | | | |

| | | | | | | | | | | |
|---|---|---|---|---|---|---|---|---|---|---|
| 7910694 | NM_080738 | EDARADD | EDAR-associated death domain | -0.065 | 0.956 | 5.82E-01 | 5.82E+01 | | | |
| 8132667 | NM_021116 | ADCY1 | adenylate cyclase 1 (brain) | -0.065 | 0.956 | 1.14E-01 | 1.14E+01 | | | |
| 7906163 | NR_026549 | RHBG | Rh family, B glycoprotein (gene/pseu | -0.065 | 0.956 | 2.31E-01 | 2.31E+01 | | | |
| 8084382 | NM_138345 | VWA5B2 | von Willebrand factor A domain cont | -0.065 | 0.956 | 3.65E-01 | 3.65E+01 | | | |
| 8164967 | NM_00113439 | VAV2 | vav 2 guanine nucleotide exchange f | -0.065 | 0.956 | 5.09E-01 | 5.09E+01 | | | |
| 8156577 | --- | --- | --- | -0.065 | 0.956 | 2.39E-01 | 2.39E+01 | | | |
| 8003850 | NM_144611 | CYB5D2 | cytochrome b5 domain containing 2 | -0.065 | 0.956 | 4.50E-01 | 4.50E+01 | | | |
| 8124716 | NM_006778 | TRIM10 | tripartite motif-containing 10 | -0.065 | 0.956 | 2.99E-01 | 2.99E+01 | | | |
| 8179628 | NM_006778 | TRIM10 | tripartite motif-containing 10 | -0.065 | 0.956 | 2.99E-01 | 2.99E+01 | | | |
| 8018972 | AK057217 | TIMP2 | TIMP metallopeptidase inhibitor 2 | -0.065 | 0.956 | 4.34E-01 | 4.34E+01 | | | |
| 8006679 | --- | --- | --- | -0.065 | 0.956 | 3.71E-01 | 3.71E+01 | | | |
| 8034762 | NM_002730 | PRKACA | protein kinase, cAMP-dependent, ca | -0.065 | 0.956 | 4.10E-01 | 4.10E+01 | | | |
| 7941900 | NM_003952 | RPS6KB2 | ribosomal protein S6 kinase, 70kDa, | -0.065 | 0.956 | 4.18E-01 | 4.18E+01 | | | |
| 7966293 | BC133010 | C12orf76 | chromosome 12 open reading frame | -0.065 | 0.956 | 4.98E-01 | 4.98E+01 | | | |
| 7968734 | NM_014252 | SLC25A15 | solute carrier family 25 (mitochondri | -0.065 | 0.956 | 4.29E-01 | 4.29E+01 | | | |
| 8113551 | NM_00108537 | MCC | mutated in colorectal cancers | -0.065 | 0.956 | 3.27E-01 | 3.27E+01 | | | |
| 8066393 | NM_020433 | JPH2 | junctophilin 2 | -0.065 | 0.956 | 2.12E-01 | 2.12E+01 | | | |
| 7994518 | NM_032038 | SPNS1 | spinster homolog 1 (Drosophila) | -0.065 | 0.956 | 3.28E-01 | 3.28E+01 | | | |
| 7923440 | --- | --- | --- | -0.065 | 0.956 | 9.50E-01 | 9.50E+01 | | | |
| 8086517 | NM_022842 | CDCP1 | CUB domain containing protein 1 | -0.065 | 0.956 | 2.20E-01 | 2.20E+01 | | | |
| 7906486 | NM_020125 | SLAMF8 | SLAM family member 8 | -0.065 | 0.956 | 2.97E-01 | 2.97E+01 | | | |
| 8059387 | NM_024785 | FAM124B | family with sequence similarity 124B | -0.065 | 0.956 | 3.20E-01 | 3.20E+01 | | | |
| 8170353 | NM_152578 | FMR1NB | fragile X mental retardation 1 neighb | -0.065 | 0.956 | 4.49E-01 | 4.49E+01 | | | |
| 7959131 | NM_207311 | CCDC64 | coiled-coil domain containing 64 | -0.065 | 0.956 | 5.39E-01 | 5.39E+01 | | | |
| 7926297 | --- | --- | --- | -0.065 | 0.956 | 5.79E-01 | 5.79E+01 | | | |
| 8065248 | AK097497 | OC10013026 | hypothetical LOC100130264 | -0.065 | 0.956 | 3.74E-01 | 3.74E+01 | | | |
| 7971858 | --- | --- | --- | -0.065 | 0.956 | 5.83E-01 | 5.83E+01 | | | |
| 8019643 | 0 | 0 | 0 | -0.065 | 0.956 | 6.38E-01 | 6.38E+01 | | | |
| 8103399 | NM_016205 | PDGFC | platelet derived growth factor C | -0.065 | 0.956 | 6.24E-01 | 6.24E+01 | | | |
| 7910589 | NM_019090 | KIAA1383 | KIAA1383 | -0.065 | 0.956 | 2.36E-01 | 2.36E+01 | | | |
| 8178561 | NM_021160 | BAT5 | HLA-B associated transcript 5 | -0.065 | 0.956 | 3.29E-01 | 3.29E+01 | | | |
| 7945420 | NM_002939 | RNH1 | ribonuclease/angiogenin inhibitor 1 | -0.065 | 0.956 | 3.91E-01 | 3.91E+01 | | | |
| 7973679 | BC025332 | C14orf21 | chromosome 14 open reading frame | -0.066 | 0.956 | 5.87E-01 | 5.87E+01 | | | |
| 7950534 | NM_004626 | WNT11 | wingless-type MMTV integration site | -0.066 | 0.956 | 2.87E-01 | 2.87E+01 | | | |
| 7909642 | NM_024749 | VASH2 | vasohibin 2 | -0.066 | 0.956 | 2.54E-01 | 2.54E+01 | | | |
| 7942964 | NM_022918 | TMEM135 | transmembrane protein 135 | -0.066 | 0.956 | 6.13E-01 | 6.13E+01 | | | |
| 7989251 | --- | --- | --- | -0.066 | 0.956 | 4.25E-01 | 4.25E+01 | | | |
| 8100015 | NM_207406 | BEND4 | BEN domain containing 4 | -0.066 | 0.956 | 2.52E-01 | 2.52E+01 | | | |
| 7995825 | NM_005949 | MT1F | metallothionein 1F | -0.066 | 0.956 | 5.28E-01 | 5.28E+01 | | | |
| 8060353 | NM_031229 | RBCK1 | RanBP-type and C3HC4-type zinc fing | -0.066 | 0.956 | 2.83E-01 | 2.83E+01 | | | |
| 7895424 | --- | --- | --- | -0.066 | 0.956 | 8.39E-01 | 8.39E+01 | | | |
| 7968999 | NM_018283 | NUDT15 | nudix (nucleoside diphosphate linked | -0.066 | 0.956 | 4.25E-01 | 4.25E+01 | | | |
| 8139160 | NM_00110528 | FAM183B | acyloxyacyl hydrolase (neutrophil) | -0.066 | 0.956 | 7.36E-01 | 7.36E+01 | | | |
| 7991401 | NM_198527 | HDDC3 | HD domain containing 3 | -0.066 | 0.956 | 6.17E-01 | 6.17E+01 | | | |
| 7895938 | --- | --- | --- | -0.066 | 0.956 | 5.22E-01 | 5.22E+01 | | | |
| 7894706 | --- | --- | --- | -0.066 | 0.955 | 9.24E-01 | 9.24E+01 | | | |
| 8118890 | NM_152753 | SCUBE3 | signal peptide, CUB domain, EGF-like | -0.066 | 0.955 | 3.85E-01 | 3.85E+01 | | | |
| 7911403 | NM_004195 | TNFRSF18 | tumor necrosis factor receptor super | -0.066 | 0.955 | 2.48E-01 | 2.48E+01 | | | |
| 7916282 | NM_004631 | LRP8 | low density lipoprotein receptor-rela | -0.066 | 0.955 | 3.16E-01 | 3.16E+01 | | | |
| 8071745 | NM_182520 | C22orf15 | chromosome 22 open reading frame | -0.066 | 0.955 | 3.08E-01 | 3.08E+01 | | | |
| 7990827 | ENST0000044 | OC10013374 | hypothetical LOC100133746 | -0.066 | 0.955 | 5.19E-01 | 5.19E+01 | | | |
| 8052143 | NM_006794 | GPR75 | G protein-coupled receptor 75 | -0.066 | 0.955 | 3.53E-01 | 3.53E+01 | | | |
| 8177706 | NM_007160 | OR2H2 | olfactory receptor, family 2, subfami | -0.066 | 0.955 | 5.43E-01 | 5.43E+01 | | | |
| 8179009 | NM_007160 | OR2H2 | olfactory receptor, family 2, subfami | -0.066 | 0.955 | 5.43E-01 | 5.43E+01 | | | |
| 7941164 | NM_004927 | MRPL49 | mitochondrial ribosomal protein L49 | -0.066 | 0.955 | 5.59E-01 | 5.59E+01 | | | |
| 8166819 | --- | --- | --- | -0.066 | 0.955 | 3.05E-01 | 3.05E+01 | | | |
| 7915329 | --- | --- | --- | -0.066 | 0.955 | 5.83E-01 | 5.83E+01 | | | |
| 8022970 | --- | --- | --- | -0.066 | 0.955 | 2.63E-01 | 2.63E+01 | | | |
| 8060196 | NM_182501 | MTERFD2 | MTERF domain containing 2 | -0.066 | 0.955 | 6.58E-01 | 6.58E+01 | | | |
| 7896550 | --- | --- | --- | -0.066 | 0.955 | 9.43E-01 | 9.43E+01 | | | |
| 7981488 | AF462446 | OC10028814 | hypothetical protein LOC100288144 | -0.066 | 0.955 | 2.92E-01 | 2.92E+01 | | | |
| 8026217 | NM_017721 | CC2D1A | coiled-coil and C2 domain containing | -0.066 | 0.955 | 2.25E-01 | 2.25E+01 | | | |
| 7896613 | --- | --- | --- | -0.066 | 0.955 | 7.03E-01 | 7.03E+01 | | | |
| 8025888 | NM_175871 | C19orf39 | chromosome 19 open reading frame | -0.066 | 0.955 | 3.45E-01 | 3.45E+01 | | | |
| 8084921 | AK094115 | OC10012826 | hypothetical LOC100128262 | -0.066 | 0.955 | 3.35E-01 | 3.35E+01 | | | |
| 7930074 | NM_002502 | NFKB2 | nuclear factor of kappa light polypep | -0.066 | 0.955 | 3.78E-01 | 3.78E+01 | | | |
| 8006627 | NM_004773 | ZNHIT3 | zinc finger, HIT type 3 | -0.066 | 0.955 | 7.59E-01 | 7.59E+01 | | | |
| 8029476 | NM_012116 | CBLC | Cas-Br-M (murine) ecotropic retrovir | -0.066 | 0.955 | 2.97E-01 | 2.97E+01 | | | |
| 8093872 | NM_005750 | C4orf6 | chromosome 4 open reading frame 6 | -0.066 | 0.955 | 3.30E-01 | 3.30E+01 | | | |
| 7982267 | --- | --- | --- | -0.066 | 0.955 | 6.67E-01 | 6.67E+01 | | | |
| 8061883 | NM_130852 | PLUNC | palate, lung and nasal epithelium ass | -0.066 | 0.955 | 2.23E-01 | 2.23E+01 | | | |
| 8092883 | --- | --- | --- | -0.066 | 0.955 | 5.11E-01 | 5.11E+01 | | | |
| 8159616 | NM_00114402 | NDOR1 | NADPH dependent diflavin oxidored | -0.066 | 0.955 | 2.73E-01 | 2.73E+01 | | | |
| 7913682 | NM_000191 | HMGCL | 3-hydroxymethyl-3-methylglutaryl-C | -0.066 | 0.955 | 4.18E-01 | 4.18E+01 | | | |
| 7908917 | NM_006763 | BTG2 | BTG family, member 2 | -0.066 | 0.955 | 7.30E-01 | 7.30E+01 | | | |
| 7941127 | NM_006782 | ZFPL1 | zinc finger protein-like 1 | -0.066 | 0.955 | 4.16E-01 | 4.16E+01 | | | |
| 7898916 | NM_198173 | GRHL3 | grainyhead-like 3 (Drosophila) | -0.066 | 0.955 | 2.69E-01 | 2.69E+01 | | | |

| | | | | | | | | | | |
|---|---|---|---|---|---|---|---|---|---|---|
| 8063522 | --- | --- | --- | -0.066 | 0.955 | 8.91E-01 | 8.91E+01 | | | |
| 8019559 | AK126018 | B3GNTL1 | UDP-GlcNAc:betaGal beta-1,3-N-acet | -0.066 | 0.955 | 3.49E-01 | 3.49E+01 | | | |
| 8071783 | NM_030807 | SLC2A11 | solute carrier family 2 (facilitated glu | -0.066 | 0.955 | 2.52E-01 | 2.52E+01 | | | |
| 7938179 | NM_207186 | OR10A4 | olfactory receptor, family 10, subfam | -0.066 | 0.955 | 2.43E-01 | 2.43E+01 | | | |
| 7894931 | --- | --- | --- | -0.066 | 0.955 | 7.49E-01 | 7.49E+01 | | | |
| 8133070 | --- | --- | --- | -0.066 | 0.955 | 7.82E-01 | 7.82E+01 | | | |
| 8129880 | NM_022121 | PERP | PERP, TP53 apoptosis effector | -0.066 | 0.955 | 4.25E-01 | 4.25E+01 | | | |
| 8123274 | NM_005922 | MAP3K4 | mitogen-activated protein kinase kin | -0.066 | 0.955 | 7.36E-01 | 7.36E+01 | | | |
| 8029147 | NM_199002 | ARHGEF1 | Rho guanine nucleotide exchange fac | -0.066 | 0.955 | 4.05E-01 | 4.05E+01 | | | |
| 8016532 | NM_031498 | GNGT2 | guanine nucleotide binding protein ( | -0.066 | 0.955 | 5.56E-01 | 5.56E+01 | | | |
| 7948054 | --- | --- | --- | -0.066 | 0.955 | 4.09E-01 | 4.09E+01 | | | |
| 8068145 | NM_181605 | KRTAP6-3 | keratin associated protein 6-3 | -0.066 | 0.955 | 2.44E-01 | 2.44E+01 | | | |
| 8178884 | NM_006120 | HLA-DMA | major histocompatibility complex, cla | -0.066 | 0.955 | 7.45E-01 | 7.45E+01 | | | |
| 8180086 | NM_006120 | HLA-DMA | major histocompatibility complex, cla | -0.066 | 0.955 | 7.45E-01 | 7.45E+01 | | | |
| 7902038 | NM_00100535 | AK3L1 | adenylate kinase 3-like 1 | -0.066 | 0.955 | 5.49E-01 | 5.49E+01 | | | |
| 8044634 | NM_012184 | FOXD4L1 | forkhead box D4-like 1 | -0.066 | 0.955 | 1.03E-01 | 1.03E+01 | | | |
| 7976828 | AK123165 | FLJ41170 | hypothetical LOC440200 | -0.066 | 0.955 | 2.29E-01 | 2.29E+01 | | | |
| 8166925 | NM_000240 | MAOA | monoamine oxidase A | -0.066 | 0.955 | 2.18E-01 | 2.18E+01 | | | |
| 7898371 | ENST00000369 | 4634 // LOC | similar to UPF0627 protein ENSP000 | -0.066 | 0.955 | 6.14E-01 | 6.14E+01 | | | |
| 7912861 | ENST00000369 | 4634 // LOC | similar to UPF0627 protein ENSP000 | -0.066 | 0.955 | 6.14E-01 | 6.14E+01 | | | |
| 8167893 | --- | --- | --- | -0.066 | 0.955 | 4.68E-01 | 4.68E+01 | | | |
| 8163962 | NM_00100445 | OR1B1 | olfactory receptor, family 1, subfami | -0.066 | 0.955 | 2.60E-01 | 2.60E+01 | | | |
| 7984353 | NR_027654 | SMAD6 | SMAD family member 6 | -0.066 | 0.955 | 2.39E-01 | 2.39E+01 | | | |
| 8074771 | NM_015672 | RIMBP3 | RIMS binding protein 3 | -0.066 | 0.955 | 2.67E-01 | 2.67E+01 | | | |
| 8011499 | NM_002558 | P2RX1 | purinergic receptor P2X, ligand-gated | -0.066 | 0.955 | 3.47E-01 | 3.47E+01 | | | |
| 8076792 | NM_022766 | CERK | ceramide kinase | -0.066 | 0.955 | 5.13E-01 | 5.13E+01 | | | |
| 8007134 | NM_033191 | KRTAP9-4 | keratin associated protein 9-4 | -0.066 | 0.955 | 2.99E-01 | 2.99E+01 | | | |
| 8107985 | NM_00103978 | CCNI2 | cyclin I family, member 2 | -0.067 | 0.955 | 2.79E-01 | 2.79E+01 | | | |
| 8122756 | NM_00102988 | PLEKHG1 | pleckstrin homology domain contain | -0.067 | 0.955 | 5.76E-01 | 5.76E+01 | | | |
| 7895278 | --- | --- | --- | -0.067 | 0.955 | 8.20E-01 | 8.20E+01 | | | |
| 8136987 | NM_005435 | ARHGEF5 | Rho guanine nucleotide exchange fac | -0.067 | 0.955 | 2.65E-01 | 2.65E+01 | | | |
| 7997247 | NM_032268 | ZNRF1 | zinc and ring finger 1 | -0.067 | 0.955 | 3.46E-01 | 3.46E+01 | | | |
| 8092067 | NM_00109964 | RPL22L1 | ribosomal protein L22-like 1 | -0.067 | 0.955 | 8.98E-01 | 8.98E+01 | | | |
| 8056763 | --- | --- | --- | -0.067 | 0.955 | 6.41E-01 | 6.41E+01 | | | |
| 8026548 | NM_00113052 | AP1M1 | adaptor-related protein complex 1, r | -0.067 | 0.955 | 3.67E-01 | 3.67E+01 | | | |
| 7892998 | --- | --- | --- | -0.067 | 0.955 | 7.60E-01 | 7.60E+01 | | | |
| 8087685 | NM_007024 | TMEM115 | transmembrane protein 115 | -0.067 | 0.955 | 2.60E-01 | 2.60E+01 | | | |
| 8029188 | NM_133444 | ZNF526 | zinc finger protein 526 | -0.067 | 0.955 | 2.11E-01 | 2.11E+01 | | | |
| 7996468 | NM_024712 | ELMO3 | engulfment and cell motility 3 | -0.067 | 0.955 | 1.60E-01 | 1.60E+01 | | | |
| 8098902 | --- | --- | --- | -0.067 | 0.955 | 5.62E-01 | 5.62E+01 | | | |
| 8115580 | NM_052927 | PWWP2A | PWWP domain containing 2A | -0.067 | 0.955 | 3.62E-01 | 3.62E+01 | | | |
| 8116070 | NM_005451 | PDLIM7 | PDZ and LIM domain 7 (enigma) | -0.067 | 0.955 | 3.73E-01 | 3.73E+01 | | | |
| 8144253 | NM_004745 | DLGAP2 | discs, large (Drosophila) homolog-ass | -0.067 | 0.955 | 1.80E-01 | 1.80E+01 | | | |
| 8089849 | --- | --- | --- | -0.067 | 0.955 | 3.46E-01 | 3.46E+01 | | | |
| 7930264 | NM_004210 | NEURL | neuralized homolog (Drosophila) | -0.067 | 0.955 | 2.81E-01 | 2.81E+01 | | | |
| 7951865 | NM_000039 | APOA1 | apolipoprotein A-I | -0.067 | 0.955 | 2.58E-01 | 2.58E+01 | | | |
| 7907859 | --- | --- | --- | -0.067 | 0.955 | 8.48E-01 | 8.48E+01 | | | |
| 8003263 | NM_015144 | ZCCHC14 | zinc finger, CCHC domain containing | -0.067 | 0.955 | 3.32E-01 | 3.32E+01 | | | |
| 7992067 | --- | --- | --- | -0.067 | 0.955 | 2.05E-01 | 2.05E+01 | | | |
| 8135931 | NR_024368 | tcag7.907 | hypothetical LOC402483 | -0.067 | 0.955 | 2.13E-01 | 2.13E+01 | | | |
| 8015412 | NM_002230 | JUP | junction plakoglobin | -0.067 | 0.955 | 3.34E-01 | 3.34E+01 | | | |
| 8021461 | NM_002091 | GRP | gastrin-releasing peptide | -0.067 | 0.955 | 2.58E-01 | 2.58E+01 | | | |
| 8118093 | NR_026791 | HCG27 | HLA complex group 27 | -0.067 | 0.955 | 3.83E-01 | 3.83E+01 | | | |
| 8076331 | NM_016272 | TOB2 | transducer of ERBB2, 2 | -0.067 | 0.955 | 3.40E-01 | 3.40E+01 | | | |
| 8137537 | NM_001427 | EN2 | engrailed homeobox 2 | -0.067 | 0.955 | 1.40E-01 | 1.40E+01 | | | |
| 8104580 | NM_138348 | FAM105B | family with sequence similarity 105, | -0.067 | 0.955 | 7.97E-01 | 7.97E+01 | | | |
| 7996241 | NM_00116030 | SETD6 | SET domain containing 6 | -0.067 | 0.955 | 3.64E-01 | 3.64E+01 | | | |
| 8124859 | NM_014070 | C6orf15 | chromosome 6 open reading frame 1 | -0.067 | 0.955 | 2.00E-01 | 2.00E+01 | | | |
| 8096032 | NM_020226 | PRDM8 | PR domain containing 8 | -0.067 | 0.955 | 2.19E-01 | 2.19E+01 | | | |
| 7993126 | NM_020686 | ABAT | 4-aminobutyrate aminotransferase | -0.067 | 0.955 | 2.43E-01 | 2.43E+01 | | | |
| 8120273 | --- | --- | --- | -0.067 | 0.955 | 5.29E-01 | 5.29E+01 | | | |
| 8016044 | NM_000419 | ITGA2B | integrin, alpha 2b (platelet glycoprot | -0.067 | 0.955 | 2.78E-01 | 2.78E+01 | | | |
| 7941610 | NM_145065 | PELI3 | pellino homolog 3 (Drosophila) | -0.067 | 0.955 | 2.17E-01 | 2.17E+01 | | | |
| 7901314 | --- | --- | --- | -0.067 | 0.955 | 5.07E-01 | 5.07E+01 | | | |
| 8033996 | NM_003331 | TYK2 | tyrosine kinase 2 | -0.067 | 0.955 | 2.61E-01 | 2.61E+01 | | | |
| 8000600 | --- | --- | --- | -0.067 | 0.955 | 1.83E-01 | 1.83E+01 | | | |
| 8012466 | NM_213597 | KRBA2 | KRAB-A domain containing 2 | -0.067 | 0.955 | 2.85E-01 | 2.85E+01 | | | |
| 7976804 | --- | --- | --- | -0.067 | 0.955 | 3.97E-01 | 3.97E+01 | | | |
| 7918606 | NM_005167 | PPM1J | protein phosphatase 1J (PP2C domai | -0.067 | 0.955 | 2.73E-01 | 2.73E+01 | | | |
| 7963970 | NM_006928 | SILV | silver homolog (mouse) | -0.067 | 0.955 | 3.02E-01 | 3.02E+01 | | | |
| 8084524 | NM_004443 | EPHB3 | EPH receptor B3 | -0.067 | 0.955 | 1.70E-01 | 1.70E+01 | | | |
| 7992463 | NM_00113001 | SLC9A3R2 | solute carrier family 9 (sodium/hydro | -0.067 | 0.955 | 1.93E-01 | 1.93E+01 | | | |
| 8146435 | NM_170587 | RGS20 | regulator of G-protein signaling 20 | -0.067 | 0.954 | 1.94E-01 | 1.94E+01 | | | |
| 8112584 | NM_152625 | ZNF366 | zinc finger protein 366 | -0.067 | 0.954 | 2.77E-01 | 2.77E+01 | | | |
| 8107307 | NM_001744 | CAMK4 | calcium/calmodulin-dependent prote | -0.067 | 0.954 | 8.24E-01 | 8.24E+01 | | | |
| 8006788 | NM_005937 | MLLT6 | myeloid/lymphoid or mixed-lineage | -0.067 | 0.954 | 1.40E-01 | 1.40E+01 | | | |
| 7926403 | NM_00109884 | FAM23A | family with sequence similarity 23, m | -0.067 | 0.954 | 1.33E-01 | 1.33E+01 | | | |

| | | | | | | | | | | |
|---|---|---|---|---|---|---|---|---|---|---|
| 7926445 | NM_00109884 | FAM23A | family with sequence similarity 23, n | -0.067 | 0.954 | 1.33E-01 | 1.33E+01 | | | |
| 7985997 | --- | --- | --- | -0.067 | 0.954 | 4.31E-01 | 4.31E+01 | | | |
| 8168345 | NM_052957 | ACRC | acidic repeat containing | -0.067 | 0.954 | 4.59E-01 | 4.59E+01 | | | |
| 8074695 | NR_003608 | MGC16703 | tubulin, alpha pseudogene | -0.067 | 0.954 | 2.75E-01 | 2.75E+01 | | | |
| 8164077 | NM_004959 | NR5A1 | nuclear receptor subfamily 5, group | -0.067 | 0.954 | 2.26E-01 | 2.26E+01 | | | |
| 8028624 | NM_018028 | SAMD4B | sterile alpha motif domain containin | -0.067 | 0.954 | 1.60E-01 | 1.60E+01 | | | |
| 8061869 | NM_178466 | C20orf71 | chromosome 20 open reading frame | -0.067 | 0.954 | 1.75E-01 | 1.75E+01 | | | |
| 8174469 | NM_012282 | KCNE1L | KCNE1-like | -0.067 | 0.954 | 2.14E-01 | 2.14E+01 | | | |
| 8024816 | NM_024333 | FSD1 | fibronectin type III and SPRY domain | -0.067 | 0.954 | 1.87E-01 | 1.87E+01 | | | |
| 8117861 | NR_027822 | HLA-L | major histocompatibility complex, cl | -0.067 | 0.954 | 4.64E-01 | 4.64E+01 | | | |
| 8179080 | NR_027822 | HLA-L | major histocompatibility complex, cl | -0.067 | 0.954 | 4.64E-01 | 4.64E+01 | | | |
| 8034469 | NM_032301 | FBXW9 | F-box and WD repeat domain contai | -0.067 | 0.954 | 1.89E-01 | 1.89E+01 | | | |
| 7952227 | NM_015645 | C1QTNF5 | C1q and tumor necrosis factor relate | -0.067 | 0.954 | 1.22E-01 | 1.22E+01 | | | |
| 8144577 | NM_00104003 | C8orf74 | chromosome 8 open reading frame 7 | -0.068 | 0.954 | 2.59E-01 | 2.59E+01 | | | |
| 8021001 | NM_020776 | KIAA1328 | KIAA1328 | -0.068 | 0.954 | 2.75E-01 | 2.75E+01 | | | |
| 8066757 | NM_022829 | SLC13A3 | solute carrier family 13 (sodium-dep | -0.068 | 0.954 | 2.52E-01 | 2.52E+01 | | | |
| 8065880 | NM_178468 | FAM83C | family with sequence similarity 83, n | -0.068 | 0.954 | 3.32E-01 | 3.32E+01 | | | |
| 8132045 | NM_032639 | PLEKHA8 | pleckstrin homology domain contain | -0.068 | 0.954 | 3.26E-01 | 3.26E+01 | | | |
| 7898653 | NM_207334 | FAM43B | family with sequence similarity 43, n | -0.068 | 0.954 | 1.12E-01 | 1.12E+01 | | | |
| 8118345 | NM_001710 | CFB | complement factor B | -0.068 | 0.954 | 2.19E-01 | 2.19E+01 | | | |
| 8141757 | NM_017621 | ALKBH4 | alkB, alkylation repair homolog 4 (E. | -0.068 | 0.954 | 2.87E-01 | 2.87E+01 | | | |
| 8075016 | NM_207644 | C22orf36 | chromosome 22 open reading frame | -0.068 | 0.954 | 1.72E-01 | 1.72E+01 | | | |
| 8025035 | NM_032306 | ALKBH7 | alkB, alkylation repair homolog 7 (E. | -0.068 | 0.954 | 5.48E-01 | 5.48E+01 | | | |
| 7899562 | NM_133178 | PTPRU | protein tyrosine phosphatase, recep | -0.068 | 0.954 | 2.65E-01 | 2.65E+01 | | | |
| 8157362 | NM_133374 | ZNF618 | zinc finger protein 618 | -0.068 | 0.954 | 3.60E-01 | 3.60E+01 | | | |
| 8036720 | NM_019088 | PAF1 | Paf1, RNA polymerase II associated f | -0.068 | 0.954 | 6.62E-01 | 6.62E+01 | | | |
| 7998444 | ENST0000044 | PRSS29P | protease, serine, 29 pseudogene | -0.068 | 0.954 | 1.68E-01 | 1.68E+01 | | | |
| 8067178 | NM_080615 | GCNT7 | glucosaminyl (N-acetyl) transferase f | -0.068 | 0.954 | 5.40E-01 | 5.40E+01 | | | |
| 8157976 | NM_032293 | GARNL3 | GTPase activating Rap/RanGAP doma | -0.068 | 0.954 | 2.55E-01 | 2.55E+01 | | | |
| 7963366 | NM_002283 | KRT85 | keratin 85 | -0.068 | 0.954 | 2.46E-01 | 2.46E+01 | | | |
| 7949577 | NM_020470 | YIF1A | Yip1 interacting factor homolog A (S. | -0.068 | 0.954 | 3.83E-01 | 3.83E+01 | | | |
| 8146357 | NM_005914 | MCM4 | minichromosome maintenance comp | -0.068 | 0.954 | 4.53E-01 | 4.53E+01 | | | |
| 7957665 | NM_005230 | ELK3 | ELK3, ETS-domain protein (SRF acces | -0.068 | 0.954 | 7.39E-01 | 7.39E+01 | | | |
| 7998872 | NR_026864 | TMPRSS8 | transmembrane protease, serine 8 h | -0.068 | 0.954 | 1.95E-01 | 1.95E+01 | | | |
| 7895212 | --- | --- | --- | -0.068 | 0.954 | 7.04E-01 | 7.04E+01 | | | |
| 8029280 | NM_020406 | CD177 | CD177 molecule | -0.068 | 0.954 | 2.60E-01 | 2.60E+01 | | | |
| 8132580 | NM_006555 | YKT6 | YKT6 v-SNARE homolog (S. cerevisiae | -0.068 | 0.954 | 6.84E-01 | 6.84E+01 | | | |
| 8071051 | AK126241 // | 4253 // FLJ4 | hypothetical gene supported by AK0 | -0.068 | 0.954 | 3.54E-01 | 3.54E+01 | | | |
| 8091552 | --- | --- | --- | -0.068 | 0.954 | 3.47E-01 | 3.47E+01 | | | |
| 7971071 | NM_145203 | CSNK1A1L | casein kinase 1, alpha 1-like | -0.068 | 0.954 | 3.00E-01 | 3.00E+01 | | | |
| 7949273 | --- | --- | --- | -0.068 | 0.954 | 3.79E-01 | 3.79E+01 | | | |
| 8173503 | NM_00102445 | RGAG4 | retrotransposon gag domain contain | -0.068 | 0.954 | 3.62E-01 | 3.62E+01 | | | |
| 8088126 | --- | --- | --- | -0.068 | 0.954 | 8.85E-01 | 8.85E+01 | | | |
| 8180249 | --- | --- | --- | -0.068 | 0.954 | 3.31E-01 | 3.31E+01 | | | |
| 8071536 | NM_015094 | HIC2 | hypermethylated in cancer 2 | -0.068 | 0.954 | 3.55E-01 | 3.55E+01 | | | |
| 7992529 | NM_032271 | TRAF7 | TNF receptor-associated factor 7 | -0.068 | 0.954 | 2.76E-01 | 2.76E+01 | | | |
| 7920178 | NM_016190 | CRNN | cornulin | -0.068 | 0.954 | 2.97E-01 | 2.97E+01 | | | |
| 8005260 | NM_018019 | MED9 | mediator complex subunit 9 | -0.068 | 0.954 | 3.25E-01 | 3.25E+01 | | | |
| 8033179 | NM_173637 | SLC25A41 | solute carrier family 25, member 41 | -0.068 | 0.954 | 2.68E-01 | 2.68E+01 | | | |
| 8055309 | --- | --- | --- | -0.068 | 0.954 | 9.93E-02 | 9.93E+00 | | | |
| 8164252 | NM_170600 | SH2D3C | SH2 domain containing 3C | -0.068 | 0.954 | 1.94E-01 | 1.94E+01 | | | |
| 7907486 | NM_032522 | ZBTB37 | zinc finger and BTB domain containin | -0.068 | 0.954 | 5.03E-01 | 5.03E+01 | | | |
| 7918294 | NM_00112296 | C1orf194 | chromosome 1 open reading frame 1 | -0.068 | 0.954 | 2.46E-01 | 2.46E+01 | | | |
| 8095139 | NM_024592 | SRD5A3 | steroid 5 alpha-reductase 3 | -0.068 | 0.954 | 4.06E-01 | 4.06E+01 | | | |
| 8031511 | NM_153219 | ZNF524 | zinc finger protein 524 | -0.068 | 0.954 | 2.91E-01 | 2.91E+01 | | | |
| 8127824 | NM_198920 | UBE2CBP | ubiquitin-conjugating enzyme E2C bi | -0.068 | 0.954 | 2.35E-01 | 2.35E+01 | | | |
| 7895826 | --- | --- | --- | -0.068 | 0.954 | 4.55E-01 | 4.55E+01 | | | |
| 7941976 | NM_002496 | NDUFS8 | NADH dehydrogenase (ubiquinone) | -0.068 | 0.954 | 5.35E-01 | 5.35E+01 | | | |
| 7994637 | NM_00104253 | MAZ | MYC-associated zinc finger protein ( | -0.068 | 0.954 | 2.12E-01 | 2.12E+01 | | | |
| 8028851 | NM_138392 | SHKBP1 | SH3KBP1 binding protein 1 | -0.068 | 0.954 | 2.61E-01 | 2.61E+01 | | | |
| 8178439 | NM_014070 | C6orf15 | chromosome 6 open reading frame 1 | -0.068 | 0.954 | 1.99E-01 | 1.99E+01 | | | |
| 7894644 | --- | --- | --- | -0.068 | 0.954 | 4.80E-01 | 4.80E+01 | | | |
| 8072659 | NR_024194 | TOM1 | target of myb1 (chicken) | -0.068 | 0.954 | 4.11E-01 | 4.11E+01 | | | |
| 7949592 | NM_004292 | RIN1 | Ras and Rab interactor 1 | -0.068 | 0.954 | 2.43E-01 | 2.43E+01 | | | |
| 8062371 | NM_022077 | MANBAL | mannosidase, beta A, lysosomal-like | -0.068 | 0.954 | 5.24E-01 | 5.24E+01 | | | |
| 7984704 | NM_002499 | NEO1 | neogenin homolog 1 (chicken) | -0.068 | 0.954 | 7.17E-01 | 7.17E+01 | | | |
| 8055711 | NM_004543 | NEB | nebulin | -0.068 | 0.954 | 9.59E-02 | 9.59E+00 | | | |
| 8142765 | NM_022143 | LRRC4 | leucine rich repeat containing 4 | -0.068 | 0.954 | 3.16E-01 | 3.16E+01 | | | |
| 7899323 | NM_032872 | SYTL1 | synaptotagmin-like 1 | -0.068 | 0.954 | 2.11E-01 | 2.11E+01 | | | |
| 7898869 | ENST0000037 | MDS2 | myelodysplastic syndrome 2 translo | -0.068 | 0.954 | 5.53E-01 | 5.53E+01 | | | |
| 8023941 | NM_130760 | MADCAM1 | mucosal vascular addressin cell adhe | -0.068 | 0.954 | 1.57E-01 | 1.57E+01 | | | |
| 7959880 | --- | --- | --- | -0.068 | 0.954 | 6.77E-01 | 6.77E+01 | | | |
| 8037608 | NM_00101798 | OPA3 | optic atrophy 3 (autosomal recessive | -0.068 | 0.954 | 1.80E-01 | 1.80E+01 | | | |
| 7966122 | NM_181724 | TMEM119 | transmembrane protein 119 | -0.068 | 0.954 | 3.48E-01 | 3.48E+01 | | | |
| 8164562 | NM_199350 | C9orf50 | chromosome 9 open reading frame 5 | -0.068 | 0.954 | 2.03E-01 | 2.03E+01 | | | |
| 7892948 | --- | --- | --- | -0.068 | 0.954 | 4.37E-01 | 4.37E+01 | | | |
| 7993800 | NM_00112830 | LYRM1 | LYR motif containing 1 | -0.068 | 0.954 | 3.94E-01 | 3.94E+01 | | | |

| | | | | | | | | | | |
|---|---|---|---|---|---|---|---|---|---|---|
| 8104590 | AK129556 | OC10013074 | hypothetical LOC100130744 | -0.068 | 0.954 | 3.44E-01 | 3.44E+01 | | | |
| 7892748 | --- | --- | --- | -0.068 | 0.954 | 3.71E-01 | 3.71E+01 | | | |
| 8006415 | NM_015544 | TMEM98 | transmembrane protein 98 | -0.068 | 0.954 | 2.82E-01 | 2.82E+01 | | | |
| 7900699 | NM_001255 | CDC20 | cell division cycle 20 homolog (S. cer | -0.068 | 0.954 | 3.16E-01 | 3.16E+01 | | | |
| 7895483 | --- | --- | --- | -0.068 | 0.954 | 4.82E-01 | 4.82E+01 | | | |
| 7992722 | NM_006799 | PRSS21 | protease, serine, 21 (testisin) | -0.069 | 0.954 | 2.59E-01 | 2.59E+01 | | | |
| 7899990 | NM_014284 | NCDN | neurochondrin | -0.069 | 0.954 | 3.92E-01 | 3.92E+01 | | | |
| 8019316 | NM_006907 | PYCR1 | pyrroline-5-carboxylate reductase 1 | -0.069 | 0.954 | 1.61E-01 | 1.61E+01 | | | |
| 8015366 | NM_000526 | KRT14 | keratin 14 | -0.069 | 0.954 | 3.50E-01 | 3.50E+01 | | | |
| 7924910 | NM_001100 | ACTA1 | actin, alpha 1, skeletal muscle | -0.069 | 0.954 | 1.62E-01 | 1.62E+01 | | | |
| 8047677 | NM_006139 | CD28 | CD28 molecule | -0.069 | 0.954 | 8.43E-01 | 8.43E+01 | | | |
| 8162177 | --- | --- | --- | -0.069 | 0.954 | 6.80E-01 | 6.80E+01 | | | |
| 7949637 | NM_170739 | MRPL11 | mitochondrial ribosomal protein L11 | -0.069 | 0.954 | 5.70E-01 | 5.70E+01 | | | |
| 8028407 | NM_000540 | RYR1 | ryanodine receptor 1 (skeletal) | -0.069 | 0.953 | 8.77E-02 | 8.77E+00 | | | |
| 7948829 | NM_024784 | ZBTB3 | zinc finger and BTB domain containir | -0.069 | 0.953 | 2.99E-01 | 2.99E+01 | | | |
| 7938100 | NM_000543 | SMPD1 | sphingomyelin phosphodiesterase 1, | -0.069 | 0.953 | 2.32E-01 | 2.32E+01 | | | |
| 8179028 | AK128290 | LOC554223 | hypothetical LOC554223 | -0.069 | 0.953 | 3.72E-01 | 3.72E+01 | | | |
| 8059720 | NM_006056 | NMUR1 | neuromedin U receptor 1 | -0.069 | 0.953 | 1.65E-01 | 1.65E+01 | | | |
| 7918900 | --- | --- | --- | -0.069 | 0.953 | 3.56E-01 | 3.56E+01 | | | |
| 7909862 | NM_024709 | C1orf115 | chromosome 1 open reading frame 1 | -0.069 | 0.953 | 2.21E-01 | 2.21E+01 | | | |
| 8048489 | NM_018089 | ANKZF1 | ankyrin repeat and zinc finger domai | -0.069 | 0.953 | 6.41E-01 | 6.41E+01 | | | |
| 8063547 | --- | --- | --- | -0.069 | 0.953 | 4.96E-01 | 4.96E+01 | | | |
| 7955829 | NM_134323 | TARBP2 | TAR (HIV-1) RNA binding protein 2 | -0.069 | 0.953 | 2.03E-01 | 2.03E+01 | | | |
| 8035234 | --- | --- | --- | -0.069 | 0.953 | 5.54E-01 | 5.54E+01 | | | |
| 8002087 | NM_020850 | RANBP10 | RAN binding protein 10 | -0.069 | 0.953 | 2.45E-01 | 2.45E+01 | | | |
| 7987772 | NM_139265 | EHD4 | EH-domain containing 4 | -0.069 | 0.953 | 2.58E-01 | 2.58E+01 | | | |
| 8071516 | NM_004327 | BCR | breakpoint cluster region | -0.069 | 0.953 | 3.20E-01 | 3.20E+01 | | | |
| 8063869 | AK126744 | FLJ44790 | hypothetical FLJ44790 | -0.069 | 0.953 | 2.67E-01 | 2.67E+01 | | | |
| 8015349 | NM_002276 | KRT19 | keratin 19 | -0.069 | 0.953 | 3.69E-01 | 3.69E+01 | | | |
| 8109226 | NM_001804 | CDX1 | caudal type homeobox 1 | -0.069 | 0.953 | 1.57E-01 | 1.57E+01 | | | |
| 7894075 | --- | --- | --- | -0.069 | 0.953 | 6.68E-01 | 6.68E+01 | | | |
| 8114787 | NM_005471 | GNPDA1 | glucosamine-6-phosphate deaminase | -0.069 | 0.953 | 6.29E-01 | 6.29E+01 | | | |
| 7944803 | NM_00113014 | VWA5A | von Willebrand factor A domain cont | -0.069 | 0.953 | 3.27E-01 | 3.27E+01 | | | |
| 7912898 | NM_022089 | ATP13A2 | ATPase type 13A2 | -0.069 | 0.953 | 1.78E-01 | 1.78E+01 | | | |
| 7968015 | NM_148957 | TNFRSF19 | tumor necrosis factor receptor super | -0.069 | 0.953 | 2.27E-01 | 2.27E+01 | | | |
| 7898713 | NM_007352 | CELA3B | chymotrypsin-like elastase family, m | -0.069 | 0.953 | 2.68E-01 | 2.68E+01 | | | |
| 7897286 | AY358179 | UNQ6193 | GSQS6193 | -0.069 | 0.953 | 2.94E-01 | 2.94E+01 | | | |
| 7965976 | --- | --- | --- | -0.069 | 0.953 | 3.93E-01 | 3.93E+01 | | | |
| 8065541 | NR_003678 | C20orf191 | nuclear receptor co-repressor 1 pseu | -0.069 | 0.953 | 5.71E-01 | 5.71E+01 | | | |
| 8123467 | NM_018341 | C6orf70 | chromosome 6 open reading frame 7 | -0.069 | 0.953 | 8.62E-01 | 8.62E+01 | | | |
| 8003962 | NM_002663 | PLD2 | phospholipase D2 | -0.069 | 0.953 | 2.97E-01 | 2.97E+01 | | | |
| 8114050 | NM_015146 | 40429 | septin 8 | -0.069 | 0.953 | 2.43E-01 | 2.43E+01 | | | |
| 8065089 | NM_024704 | KIF16B | kinesin family member 16B | -0.069 | 0.953 | 6.02E-01 | 6.02E+01 | | | |
| 8028192 | --- | --- | --- | -0.069 | 0.953 | 2.98E-01 | 2.98E+01 | | | |
| 8123678 | NM_183373 | C6orf145 | chromosome 6 open reading frame 1 | -0.069 | 0.953 | 2.25E-01 | 2.25E+01 | | | |
| 8040231 | BC093999 | C2orf48 | chromosome 2 open reading frame 4 | -0.069 | 0.953 | 3.76E-01 | 3.76E+01 | | | |
| 7978538 | NR_027263 | C14orf128 | chromosome 14 open reading frame | -0.069 | 0.953 | 3.11E-01 | 3.11E+01 | | | |
| 7896381 | --- | --- | --- | -0.069 | 0.953 | 9.41E-01 | 9.41E+01 | | | |
| 8149749 | NM_003840 | TNFRSF10D | tumor necrosis factor receptor super | -0.069 | 0.953 | 2.34E-01 | 2.34E+01 | | | |
| 7892918 | --- | --- | --- | -0.069 | 0.953 | 7.72E-01 | 7.72E+01 | | | |
| 8095072 | ENST0000031 | LOC441016 | similar to COMM domain containing | -0.069 | 0.953 | 3.79E-01 | 3.79E+01 | | | |
| 8158866 | NM_00113611 | POMT1 | protein-O-mannosyltransferase 1 | -0.069 | 0.953 | 4.25E-01 | 4.25E+01 | | | |
| 8002249 | NM_018667 | SMPD3 | sphingomyelin phosphodiesterase 3, | -0.069 | 0.953 | 2.80E-01 | 2.80E+01 | | | |
| 8136811 | NM_004445 | EPHB6 | EPH receptor B6 | -0.069 | 0.953 | 3.47E-01 | 3.47E+01 | | | |
| 7996137 | NM_005886 | KATNB1 | katanin p80 (WD repeat containing) | -0.069 | 0.953 | 1.76E-01 | 1.76E+01 | | | |
| 8164481 | NM_016390 | C9orf114 | chromosome 9 open reading frame 1 | -0.069 | 0.953 | 3.20E-01 | 3.20E+01 | | | |
| 7967412 | NM_004642 | CDK2AP1 | cyclin-dependent kinase 2 associated | -0.069 | 0.953 | 2.75E-01 | 2.75E+01 | | | |
| 7894134 | --- | --- | --- | -0.069 | 0.953 | 5.37E-01 | 5.37E+01 | | | |
| 7894612 | --- | --- | --- | -0.069 | 0.953 | 4.41E-01 | 4.41E+01 | | | |
| 7946589 | NM_130385 | MRVI1 | murine retrovirus integration site 1 h | -0.069 | 0.953 | 2.05E-01 | 2.05E+01 | | | |
| 7910611 | NM_002245 | KCNK1 | potassium channel, subfamily K, mer | -0.069 | 0.953 | 1.51E-01 | 1.51E+01 | | | |
| 8100893 | NM_173827 | COX18 | COX18 cytochrome c oxidase assemb | -0.069 | 0.953 | 4.73E-01 | 4.73E+01 | | | |
| 8135915 | NM_013332 | C7orf68 | chromosome 7 open reading frame 6 | -0.069 | 0.953 | 3.14E-01 | 3.14E+01 | | | |
| 8035380 | NM_005535 | IL12RB1 | interleukin 12 receptor, beta 1 | -0.069 | 0.953 | 3.60E-01 | 3.60E+01 | | | |
| 7898673 | --- | --- | --- | -0.069 | 0.953 | 6.45E-01 | 6.45E+01 | | | |
| 8124955 | NM_00114546 | NCR3 | natural cytotoxicity triggering recept | -0.069 | 0.953 | 4.74E-01 | 4.74E+01 | | | |
| 8178517 | NM_00114546 | NCR3 | natural cytotoxicity triggering recept | -0.069 | 0.953 | 4.74E-01 | 4.74E+01 | | | |
| 8179773 | NM_00114546 | NCR3 | natural cytotoxicity triggering recept | -0.069 | 0.953 | 4.74E-01 | 4.74E+01 | | | |
| 7980044 | NM_006029 | PNMA1 | paraneoplastic antigen MA1 | -0.069 | 0.953 | 5.31E-01 | 5.31E+01 | | | |
| 7946267 | NM_022061 | MRPL17 | mitochondrial ribosomal protein L17 | -0.070 | 0.953 | 4.01E-01 | 4.01E+01 | | | |
| 7961230 | NM_003651 | CSDA | cold shock domain protein A | -0.070 | 0.953 | 8.49E-01 | 8.49E+01 | | | |
| 7963555 | NM_173352 | KRT78 | keratin 78 | -0.070 | 0.953 | 1.72E-01 | 1.72E+01 | | | |
| 7896355 | --- | --- | --- | -0.070 | 0.953 | 8.99E-01 | 8.99E+01 | | | |
| 7927519 | NM_006327 | TIMM23 | translocase of inner mitochondrial m | -0.070 | 0.953 | 8.67E-01 | 8.67E+01 | | | |
| 7956162 | NM_032786 | ZC3H10 | zinc finger CCCH-type containing 10 | -0.070 | 0.953 | 3.12E-01 | 3.12E+01 | | | |
| 8093360 | NM_000283 | PDE6B | phosphodiesterase 6B, cGMP-specifi | -0.070 | 0.953 | 2.01E-01 | 2.01E+01 | | | |
| 8173204 | --- | --- | --- | -0.070 | 0.953 | 4.42E-01 | 4.42E+01 | | | |

| | | | | | | | | | | |
|---|---|---|---|---|---|---|---|---|---|---|
| 8051197 | NM_024584 | CCDC121 | coiled-coil domain containing 121 | -0.070 | 0.953 | 5.07E-01 | 5.07E+01 | | | |
| 7930561 | NM_004132 | HABP2 | hyaluronan binding protein 2 | -0.070 | 0.953 | 2.58E-01 | 2.58E+01 | | | |
| 8064686 | NM_025220 | ADAM33 | ADAM metallopeptidase domain 33 | -0.070 | 0.953 | 1.67E-01 | 1.67E+01 | | | |
| 8109254 | NM_00113524 | TCOF1 | Treacher Collins-Franceschetti syndr | -0.070 | 0.953 | 1.78E-01 | 1.78E+01 | | | |
| 8113443 | --- | --- | --- | -0.070 | 0.953 | 4.56E-01 | 4.56E+01 | | | |
| 8180225 | --- | --- | --- | -0.070 | 0.953 | 2.52E-01 | 2.52E+01 | | | |
| 7994487 | NM_001770 | CD19 | CD19 molecule | -0.070 | 0.953 | 1.82E-01 | 1.82E+01 | | | |
| 8077688 | NM_153461 | IL17RC | interleukin 17 receptor C | -0.070 | 0.953 | 1.99E-01 | 1.99E+01 | | | |
| 7967002 | NM_00108085 | PXN | paxillin | -0.070 | 0.953 | 2.14E-01 | 2.14E+01 | | | |
| 7963946 | NM_002429 | MMP19 | matrix metallopeptidase 19 | -0.070 | 0.953 | 2.75E-01 | 2.75E+01 | | | |
| 7967032 | --- | --- | --- | -0.070 | 0.953 | 4.33E-01 | 4.33E+01 | | | |
| 7921332 | NM_005894 | CD5L | CD5 molecule-like | -0.070 | 0.953 | 1.83E-01 | 1.83E+01 | | | |
| 7892515 | --- | --- | --- | -0.070 | 0.953 | 6.55E-01 | 6.55E+01 | | | |
| 7893302 | --- | --- | --- | -0.070 | 0.953 | 6.93E-01 | 6.93E+01 | | | |
| 7975386 | --- | --- | --- | -0.070 | 0.953 | 8.12E-01 | 8.12E+01 | | | |
| 8162194 | NM_178432 | CCRK | cell cycle related kinase | -0.070 | 0.953 | 1.65E-01 | 1.65E+01 | | | |
| 8153890 | NM_138367 | ZNF251 | zinc finger protein 251 | -0.070 | 0.953 | 4.68E-01 | 4.68E+01 | | | |
| 7982282 | --- | --- | --- | -0.070 | 0.953 | 6.42E-01 | 6.42E+01 | | | |
| 7982307 | --- | --- | --- | -0.070 | 0.953 | 6.42E-01 | 6.42E+01 | | | |
| 7898967 | NM_00101098 | C1orf130 | chromosome 1 open reading frame 1 | -0.070 | 0.953 | 1.61E-01 | 1.61E+01 | | | |
| 8069131 | ENST0000044 | C21orf30 | chromosome 21 open reading frame | -0.070 | 0.953 | 2.96E-01 | 2.96E+01 | | | |
| 7893408 | --- | --- | --- | -0.070 | 0.953 | 8.44E-01 | 8.44E+01 | | | |
| 7998002 | NM_152287 | ZNF276 | zinc finger protein 276 | -0.070 | 0.953 | 3.96E-01 | 3.96E+01 | | | |
| 7911278 | NM_030904 | OR2T1 | olfactory receptor, family 2, subfami | -0.070 | 0.953 | 3.03E-01 | 3.03E+01 | | | |
| 8154838 | ENST0000033 | TRBV21OR9- | T cell receptor beta variable 21/OR9 | -0.070 | 0.953 | 4.07E-01 | 4.07E+01 | | | |
| 8052735 | NM_015463 | CNRIP1 | cannabinoid receptor interacting pro | -0.070 | 0.953 | 1.71E-01 | 1.71E+01 | | | |
| 8058614 | NM_006055 | LANCL1 | LanC lantibiotic synthetase compone | -0.070 | 0.953 | 6.31E-01 | 6.31E+01 | | | |
| 8077160 | NM_000487 | ARSA | arylsulfatase A | -0.070 | 0.953 | 2.91E-01 | 2.91E+01 | | | |
| 7943892 | NM_181351 | NCAM1 | neural cell adhesion molecule 1 | -0.070 | 0.953 | 5.27E-01 | 5.27E+01 | | | |
| 8086185 | NM_006225 | PLCD1 | phospholipase C, delta 1 | -0.070 | 0.953 | 2.77E-01 | 2.77E+01 | | | |
| 8043890 | NM_00101171 | NMS | neuromedin S | -0.070 | 0.953 | 2.62E-01 | 2.62E+01 | | | |
| 7980702 | NM_017970 | C14orf102 | chromosome 14 open reading frame | -0.070 | 0.953 | 4.65E-01 | 4.65E+01 | | | |
| 8020889 | NM_00113517 | ZNF397 | zinc finger protein 397 | -0.070 | 0.953 | 3.83E-01 | 3.83E+01 | | | |
| 8014882 | NM_032339 | C17orf37 | chromosome 17 open reading frame | -0.070 | 0.953 | 7.01E-01 | 7.01E+01 | | | |
| 7896569 | --- | --- | --- | -0.070 | 0.953 | 8.71E-01 | 8.71E+01 | | | |
| 8119080 | NM_003017 | SFRS3 | splicing factor, arginine/serine-rich 3 | -0.070 | 0.953 | 6.83E-01 | 6.83E+01 | | | |
| 8074349 | NM_005137 | DGCR2 | DiGeorge syndrome critical region ge | -0.070 | 0.953 | 2.94E-01 | 2.94E+01 | | | |
| 7953483 | NM_00109853 | USP5 | ubiquitin specific peptidase 5 (isope | -0.070 | 0.953 | 3.31E-01 | 3.31E+01 | | | |
| 7915787 | NM_003629 | PIK3R3 | phosphoinositide-3-kinase, regulator | -0.070 | 0.953 | 4.11E-01 | 4.11E+01 | | | |
| 8072108 | NM_020437 | ASPHD2 | aspartate beta-hydroxylase domain c | -0.070 | 0.953 | 3.99E-01 | 3.99E+01 | | | |
| 8053761 | --- | --- | --- | -0.070 | 0.953 | 5.17E-01 | 5.17E+01 | | | |
| 8074070 | NM_017584 | MIOX | myo-inositol oxygenase | -0.070 | 0.953 | 2.20E-01 | 2.20E+01 | | | |
| 8136801 | NR_001296 | TRY6 | trypsinogen C | -0.070 | 0.953 | 3.64E-01 | 3.64E+01 | | | |
| 7986675 | NM_144599 | NIPA1 | non imprinted in Prader-Willi/Angelr | -0.070 | 0.953 | 3.99E-01 | 3.99E+01 | | | |
| 8007701 | NM_016438 | HIGD1B | HIG1 hypoxia inducible domain famil | -0.070 | 0.953 | 4.61E-01 | 4.61E+01 | | | |
| 8048639 | NM_002191 | INHA | inhibin, alpha | -0.070 | 0.953 | 2.16E-01 | 2.16E+01 | | | |
| 7945462 | NM_004031 | IRF7 | interferon regulatory factor 7 | -0.070 | 0.953 | 1.75E-01 | 1.75E+01 | | | |
| 7915363 | NM_00103169 | SCMH1 | sex comb on midleg homolog 1 (Dros | -0.070 | 0.953 | 4.72E-01 | 4.72E+01 | | | |
| 8071411 | NR_024592 | POM121L4P | POM121 membrane glycoprotein-lik | -0.070 | 0.953 | 4.64E-01 | 4.64E+01 | | | |
| 7977270 | AK131040 | LOC388022 | hypothetical gene supported by AK1 | -0.070 | 0.953 | 8.85E-01 | 8.85E+01 | | | |
| 8037352 | NM_00100756 | IRGQ | immunity-related GTPase family, Q | -0.070 | 0.952 | 2.90E-01 | 2.90E+01 | | | |
| 8180278 | --- | --- | --- | -0.070 | 0.952 | 2.93E-01 | 2.93E+01 | | | |
| 7944195 | NM_004788 | UBE4A | ubiquitination factor E4A (UFD2 hom | -0.070 | 0.952 | 8.37E-01 | 8.37E+01 | | | |
| 8040211 | NM_003597 | KLF11 | Kruppel-like factor 11 | -0.070 | 0.952 | 6.24E-01 | 6.24E+01 | | | |
| 8111812 | --- | --- | --- | -0.070 | 0.952 | 5.91E-01 | 5.91E+01 | | | |
| 8062695 | NM_006275 | SFRS6 | splicing factor, arginine/serine-rich 6 | -0.070 | 0.952 | 5.66E-01 | 5.66E+01 | | | |
| 7899417 | NM_018053 | XKR8 | XK, Kell blood group complex subuni | -0.070 | 0.952 | 3.14E-01 | 3.14E+01 | | | |
| 7992010 | NM_003961 | RHBDL1 | rhomboid, veinlet-like 1 (Drosophila) | -0.070 | 0.952 | 1.62E-01 | 1.62E+01 | | | |
| 8029847 | NM_00103988 | FKRP | fukutin related protein | -0.070 | 0.952 | 1.24E-01 | 1.24E+01 | | | |
| 8150439 | NM_020476 | ANK1 | ankyrin 1, erythrocytic | -0.070 | 0.952 | 1.65E-01 | 1.65E+01 | | | |
| 7893708 | --- | --- | --- | -0.070 | 0.952 | 5.19E-01 | 5.19E+01 | | | |
| 7938170 | NM_00100448 | OR2AG1 | olfactory receptor, family 2, subfami | -0.070 | 0.952 | 3.77E-01 | 3.77E+01 | | | |
| 7948511 | NM_152718 | VWCE | von Willebrand factor C and EGF dor | -0.070 | 0.952 | 1.61E-01 | 1.61E+01 | | | |
| 7893511 | --- | --- | --- | -0.070 | 0.952 | 5.63E-01 | 5.63E+01 | | | |
| 7941148 | NM_003273 | TM7SF2 | transmembrane 7 superfamily memb | -0.070 | 0.952 | 2.42E-01 | 2.42E+01 | | | |
| 7897288 | NM_031475 | ESPN | espin | -0.070 | 0.952 | 2.77E-01 | 2.77E+01 | | | |
| 8040802 | NM_000221 | KHK | ketohexokinase (fructokinase) | -0.070 | 0.952 | 3.62E-01 | 3.62E+01 | | | |
| 8123488 | NR_026780 | C6orf208 | chromosome 6 open reading frame 2 | -0.070 | 0.952 | 2.84E-01 | 2.84E+01 | | | |
| 7897329 | NM_138350 | THAP3 | THAP domain containing, apoptosis a | -0.071 | 0.952 | 3.71E-01 | 3.71E+01 | | | |
| 7968212 | NM_006646 | WASF3 | WAS protein family, member 3 | -0.071 | 0.952 | 1.30E-01 | 1.30E+01 | | | |
| 8005449 | NM_000422 | KRT17 | keratin 17 | -0.071 | 0.952 | 2.07E-01 | 2.07E+01 | | | |
| 7987572 | --- | --- | --- | -0.071 | 0.952 | 6.27E-01 | 6.27E+01 | | | |
| 7937944 | NM_00100413 | OR52M1 | olfactory receptor, family 52, subfam | -0.071 | 0.952 | 3.31E-01 | 3.31E+01 | | | |
| 7908157 | --- | --- | --- | -0.071 | 0.952 | 3.69E-01 | 3.69E+01 | | | |
| 8088172 | --- | --- | --- | -0.071 | 0.952 | 3.67E-01 | 3.67E+01 | | | |
| 8085581 | NM_005677 | COLQ | collagen-like tail subunit (single stran | -0.071 | 0.952 | 2.59E-01 | 2.59E+01 | | | |
| 8061247 | NM_018993 | RIN2 | Ras and Rab interactor 2 | -0.071 | 0.952 | 2.05E-01 | 2.05E+01 | | | |

| | | | | | | | | | | |
|---|---|---|---|---|---|---|---|---|---|---|
| 8162729 | NM_014788 | TRIM14 | tripartite motif-containing 14 | -0.071 | 0.952 | 3.59E-01 | 3.59E+01 | | | |
| 7999387 | NM_001424 | EMP2 | epithelial membrane protein 2 | -0.071 | 0.952 | 2.68E-01 | 2.68E+01 | | | |
| 7895077 | --- | --- | --- | -0.071 | 0.952 | 9.31E-01 | 9.31E+01 | | | |
| 7895943 | --- | --- | --- | -0.071 | 0.952 | 4.88E-01 | 4.88E+01 | | | |
| 8163714 | --- | --- | --- | -0.071 | 0.952 | 4.09E-01 | 4.09E+01 | | | |
| 8119918 | NM_153246 | C6orf223 | chromosome 6 open reading frame 2 | -0.071 | 0.952 | 3.11E-01 | 3.11E+01 | | | |
| 8166872 | --- | --- | --- | -0.071 | 0.952 | 6.80E-01 | 6.80E+01 | | | |
| 7928915 | NM_00109933 | FAM22A | family with sequence similarity 22, m | -0.071 | 0.952 | 7.96E-02 | 7.96E+00 | | | |
| 8139889 | --- | --- | --- | -0.071 | 0.952 | 8.80E-01 | 8.80E+01 | | | |
| 7993369 | --- | --- | --- | -0.071 | 0.952 | 2.31E-01 | 2.31E+01 | | | |
| 7996051 | NM_032940 | POLR2C | polymerase (RNA) II (DNA directed) p | -0.071 | 0.952 | 6.84E-01 | 6.84E+01 | | | |
| 7952386 | ENST0000030 | OR10G6 | olfactory receptor, family 10, subfam | -0.071 | 0.952 | 2.86E-01 | 2.86E+01 | | | |
| 8076046 | NM_012264 | TMEM184B | transmembrane protein 184B | -0.071 | 0.952 | 6.04E-01 | 6.04E+01 | | | |
| 7922029 | NM_005814 | GPA33 | glycoprotein A33 (transmembrane) | -0.071 | 0.952 | 5.26E-01 | 5.26E+01 | | | |
| 7976069 | --- | --- | --- | -0.071 | 0.952 | 5.00E-01 | 5.00E+01 | | | |
| 8003193 | BC093665 | FAM92B | family with sequence similarity 92, m | -0.071 | 0.952 | 2.55E-01 | 2.55E+01 | | | |
| 8141664 | NM_003378 | VGF | VGF nerve growth factor inducible | -0.071 | 0.952 | 1.85E-01 | 1.85E+01 | | | |
| 8179451 | NM_005155 | PPT2 | palmitoyl-protein thioesterase 2 | -0.071 | 0.952 | 1.55E-01 | 1.55E+01 | | | |
| 8128606 | NM_032730 | RTN4IP1 | reticulon 4 interacting protein 1 | -0.071 | 0.952 | 8.46E-01 | 8.46E+01 | | | |
| 7924386 | --- | --- | --- | -0.071 | 0.952 | 6.63E-01 | 6.63E+01 | | | |
| 7904969 | NM_030796 | VOPP1 | vesicular, overexpressed in cancer, p | -0.071 | 0.952 | 2.42E-01 | 2.42E+01 | | | |
| 7949645 | NM_207340 | ZDHHC24 | zinc finger, DHHC-type containing 24 | -0.071 | 0.952 | 2.29E-01 | 2.29E+01 | | | |
| 7900167 | NM_018101 | CDCA8 | cell division cycle associated 8 | -0.071 | 0.952 | 2.50E-01 | 2.50E+01 | | | |
| 7995030 | NM_024706 | ZNF668 | zinc finger protein 668 | -0.071 | 0.952 | 3.82E-01 | 3.82E+01 | | | |
| 7894117 | --- | --- | --- | -0.071 | 0.952 | 6.48E-01 | 6.48E+01 | | | |
| 8110327 | NM_006480 | RGS14 | regulator of G-protein signaling 14 | -0.071 | 0.952 | 2.05E-01 | 2.05E+01 | | | |
| 7899851 | NM_052998 | ADC | arginine decarboxylase | -0.071 | 0.952 | 2.54E-01 | 2.54E+01 | | | |
| 7987365 | NM_024865 | NANOG | Nanog homeobox | -0.071 | 0.952 | 2.02E-01 | 2.02E+01 | | | |
| 8075052 | NM_182492 | LRP5L | low density lipoprotein receptor-rela | -0.071 | 0.952 | 3.91E-01 | 3.91E+01 | | | |
| 7994939 | NM_014771 | RNF40 | ring finger protein 40 | -0.071 | 0.952 | 2.72E-01 | 2.72E+01 | | | |
| 8010379 | NM_024110 | CARD14 | caspase recruitment domain family, | -0.071 | 0.952 | 1.52E-01 | 1.52E+01 | | | |
| 8009326 | NM_145811 | CACNG5 | calcium channel, voltage-dependent | -0.071 | 0.952 | 2.20E-01 | 2.20E+01 | | | |
| 7953303 | NM_018173 | PLEKHG6 | pleckstrin homology domain contain | -0.071 | 0.952 | 1.83E-01 | 1.83E+01 | | | |
| 8139778 | --- | --- | --- | -0.071 | 0.952 | 5.85E-01 | 5.85E+01 | | | |
| 7938269 | NM_003320 | TUB | tubby homolog (mouse) | -0.071 | 0.952 | 2.72E-01 | 2.72E+01 | | | |
| 8020123 | NM_00109852 | TXNDC2 | thioredoxin domain containing 2 (spe | -0.071 | 0.952 | 2.56E-01 | 2.56E+01 | | | |
| 8043393 | NM_018271 | THNSL2 | threonine synthase-like 2 (S. cerevisi | -0.071 | 0.952 | 2.25E-01 | 2.25E+01 | | | |
| 8125038 | NR_003673 | LY6G6E | lymphocyte antigen 6 complex, locus | -0.071 | 0.952 | 3.31E-01 | 3.31E+01 | | | |
| 8178582 | NR_003673 | LY6G6E | lymphocyte antigen 6 complex, locus | -0.071 | 0.952 | 3.31E-01 | 3.31E+01 | | | |
| 8179810 | NR_003673 | LY6G6E | lymphocyte antigen 6 complex, locus | -0.071 | 0.952 | 3.31E-01 | 3.31E+01 | | | |
| 8170400 | ENST0000043 | FLJ16423 | hypothetical LOC642889 | -0.071 | 0.952 | 4.75E-01 | 4.75E+01 | | | |
| 8003156 | --- | --- | --- | -0.071 | 0.952 | 5.46E-01 | 5.46E+01 | | | |
| 7927955 | NM_015634 | KIAA1279 | KIAA1279 | -0.071 | 0.952 | 6.12E-01 | 6.12E+01 | | | |
| 7894526 | --- | --- | --- | -0.071 | 0.952 | 7.00E-01 | 7.00E+01 | | | |
| 7960771 | NM_174941 | CD163L1 | CD163 molecule-like 1 | -0.071 | 0.952 | 2.29E-01 | 2.29E+01 | | | |
| 8117583 | NM_003509 | HIST1H2AI | histone cluster 1, H2ai | -0.071 | 0.952 | 4.40E-01 | 4.40E+01 | | | |
| 8015946 | NM_178542 | C17orf65 | chromosome 17 open reading frame | -0.071 | 0.952 | 3.89E-01 | 3.89E+01 | | | |
| 7894232 | --- | --- | --- | -0.071 | 0.952 | 4.96E-01 | 4.96E+01 | | | |
| 7940891 | NM_005528 | DNAJC4 | DnaJ (Hsp40) homolog, subfamily C, | -0.071 | 0.952 | 2.75E-01 | 2.75E+01 | | | |
| 7932221 | BC029034 | C10orf111 | chromosome 10 open reading frame | -0.071 | 0.952 | 2.34E-01 | 2.34E+01 | | | |
| 8084634 | NM_016306 | DNAJB11 | DnaJ (Hsp40) homolog, subfamily B, | -0.071 | 0.952 | 8.46E-01 | 8.46E+01 | | | |
| 8099551 | NM_015688 | FAM184B | family with sequence similarity 184, | -0.071 | 0.952 | 2.28E-01 | 2.28E+01 | | | |
| 7924756 | --- | --- | --- | -0.071 | 0.952 | 5.07E-01 | 5.07E+01 | | | |
| 7945146 | NM_000890 | KCNJ5 | potassium inwardly-rectifying chann | -0.071 | 0.952 | 2.15E-01 | 2.15E+01 | | | |
| 7940762 | NM_00114253 | LGALS12 | lectin, galactoside-binding, soluble, 1 | -0.071 | 0.952 | 2.82E-01 | 2.82E+01 | | | |
| 7915435 | NM_00112339 | CLDN19 | claudin 19 | -0.071 | 0.952 | 2.55E-01 | 2.55E+01 | | | |
| 8082473 | NM_000174 | GP9 | glycoprotein IX (platelet) | -0.071 | 0.952 | 2.14E-01 | 2.14E+01 | | | |
| 8148607 | NM_138465 | GLI4 | GLI family zinc finger 4 | -0.071 | 0.952 | 2.32E-01 | 2.32E+01 | | | |
| 7961569 | --- | --- | --- | -0.071 | 0.952 | 5.52E-01 | 5.52E+01 | | | |
| 8001082 | NM_005629 | SLC6A8 | solute carrier family 6 (neurotransmi | -0.071 | 0.952 | 2.11E-01 | 2.11E+01 | | | |
| 7942697 | NM_004055 | CAPN5 | calpain 5 | -0.072 | 0.952 | 1.97E-01 | 1.97E+01 | | | |
| 8079563 | NM_138615 | DHX30 | DEAH (Asp-Glu-Ala-His) box polypept | -0.072 | 0.952 | 3.99E-01 | 3.99E+01 | | | |
| 8068833 | NM_002606 | PDE9A | phosphodiesterase 9A | -0.072 | 0.952 | 4.83E-01 | 4.83E+01 | | | |
| 8148850 | NM_174922 | ADCK5 | aarF domain containing kinase 5 | -0.072 | 0.952 | 2.06E-01 | 2.06E+01 | | | |
| 8140371 | NM_031925 | TMEM120A | transmembrane protein 120A | -0.072 | 0.952 | 4.09E-01 | 4.09E+01 | | | |
| 8038487 | NM_172374 | IL4I1 | interleukin 4 induced 1 | -0.072 | 0.952 | 1.65E-01 | 1.65E+01 | | | |
| 8099084 | NR_024253 | LOC348926 | family with sequence similarity 86, m | -0.072 | 0.952 | 2.53E-01 | 2.53E+01 | | | |
| 8033674 | NM_024690 | MUC16 | mucin 16, cell surface associated | -0.072 | 0.952 | 1.90E-01 | 1.90E+01 | | | |
| 8164008 | NR_026677 | C9orf45 | chromosome 9 open reading frame 4 | -0.072 | 0.952 | 4.24E-01 | 4.24E+01 | | | |
| 8115543 | NM_024007 | EBF1 | early B-cell factor 1 | -0.072 | 0.952 | 7.66E-01 | 7.66E+01 | | | |
| 8117710 | --- | --- | --- | -0.072 | 0.952 | 1.94E-01 | 1.94E+01 | | | |
| 7922001 | BC114214 | FAM78B | family with sequence similarity 78, m | -0.072 | 0.952 | 2.41E-01 | 2.41E+01 | | | |
| 7895084 | --- | --- | --- | -0.072 | 0.952 | 5.40E-01 | 5.40E+01 | | | |
| 8024100 | NM_138690 | GRIN3B | glutamate receptor, ionotropic, N-m | -0.072 | 0.952 | 1.44E-01 | 1.44E+01 | | | |
| 7894988 | --- | --- | --- | -0.072 | 0.952 | 4.83E-01 | 4.83E+01 | | | |
| 8071642 | ENST0000039 | IGLV6-57 | immunoglobulin lambda variable 6-5 | -0.072 | 0.952 | 4.79E-01 | 4.79E+01 | | | |
| 7896375 | --- | --- | --- | -0.072 | 0.952 | 8.67E-01 | 8.67E+01 | | | |

| | | | | | | | | | | |
|---|---|---|---|---|---|---|---|---|---|---|
| 7984759 | NM_153356 | TBC1D21 | TBC1 domain family, member 21 | -0.072 | 0.952 | 2.52E-01 | 2.52E+01 | | | |
| 7958759 | --- | --- | --- | -0.072 | 0.952 | 6.20E-01 | 6.20E+01 | | | |
| 7977987 | NM_002471 | MYH6 | myosin, heavy chain 6, cardiac muscl | -0.072 | 0.951 | 1.66E-01 | 1.66E+01 | | | |
| 8163257 | NM_057159 | LPAR1 | lysophosphatidic acid receptor 1 | -0.072 | 0.951 | 3.53E-01 | 3.53E+01 | | | |
| 7930559 | --- | --- | --- | -0.072 | 0.951 | 5.95E-01 | 5.95E+01 | | | |
| 8053551 | NM_022912 | REEP1 | receptor accessory protein 1 | -0.072 | 0.951 | 2.42E-01 | 2.42E+01 | | | |
| 7913609 | NM_017707 | ASAP3 | ArfGAP with SH3 domain, ankyrin re | -0.072 | 0.951 | 1.71E-01 | 1.71E+01 | | | |
| 8151369 | NM_153225 | C8orf84 | chromosome 8 open reading frame 8 | -0.072 | 0.951 | 2.50E-01 | 2.50E+01 | | | |
| 8073826 | NM_005036 | PPARA | peroxisome proliferator-activated re | -0.072 | 0.951 | 3.33E-01 | 3.33E+01 | | | |
| 7893030 | --- | --- | --- | -0.072 | 0.951 | 9.11E-01 | 9.11E+01 | | | |
| 8147481 | --- | --- | --- | -0.072 | 0.951 | 2.00E-01 | 2.00E+01 | | | |
| 8076344 | NM_00101805 | POLR3H | polymerase (RNA) III (DNA directed) | -0.072 | 0.951 | 4.80E-01 | 4.80E+01 | | | |
| 8164808 | --- | --- | --- | -0.072 | 0.951 | 3.79E-01 | 3.79E+01 | | | |
| 8024003 | NM_002579 | PALM | paralemmin | -0.072 | 0.951 | 2.24E-01 | 2.24E+01 | | | |
| 7995539 | NM_022162 | NOD2 | nucleotide-binding oligomerization d | -0.072 | 0.951 | 3.25E-01 | 3.25E+01 | | | |
| 7892702 | --- | --- | --- | -0.072 | 0.951 | 9.17E-01 | 9.17E+01 | | | |
| 8119107 | --- | --- | --- | -0.072 | 0.951 | 2.59E-01 | 2.59E+01 | | | |
| 8159111 | NM_017588 | WDR5 | WD repeat domain 5 | -0.072 | 0.951 | 7.52E-01 | 7.52E+01 | | | |
| 8117531 | --- | --- | --- | -0.072 | 0.951 | 4.24E-01 | 4.24E+01 | | | |
| 8124467 | --- | --- | --- | -0.072 | 0.951 | 4.24E-01 | 4.24E+01 | | | |
| 8120585 | NM_00104430 | SMAP1 | small ArfGAP 1 | -0.072 | 0.951 | 5.91E-01 | 5.91E+01 | | | |
| 7955637 | NM_199187 | KRT18 | keratin 18 | -0.072 | 0.951 | 3.74E-01 | 3.74E+01 | | | |
| 8099537 | --- | --- | --- | -0.072 | 0.951 | 3.55E-01 | 3.55E+01 | | | |
| 8000716 | NM_012410 | SEZ6L2 | seizure related 6 homolog (mouse)-li | -0.072 | 0.951 | 1.75E-01 | 1.75E+01 | | | |
| 8100426 | --- | --- | --- | -0.072 | 0.951 | 6.24E-01 | 6.24E+01 | | | |
| 7906742 | NM_00110256 | PCP4L1 | Purkinje cell protein 4 like 1 | -0.072 | 0.951 | 2.55E-01 | 2.55E+01 | | | |
| 8024078 | NM_005224 | ARID3A | AT rich interactive domain 3A (BRIGH | -0.072 | 0.951 | 1.54E-01 | 1.54E+01 | | | |
| 8014230 | NM_152462 | AMAC1 | acyl-malonyl condensing enzyme 1 | -0.072 | 0.951 | 4.09E-01 | 4.09E+01 | | | |
| 8176245 | NM_012151 | F8A1 | coagulation factor VIII-associated (in | -0.072 | 0.951 | 2.14E-01 | 2.14E+01 | | | |
| 7964183 | NM_013267 | GLS2 | glutaminase 2 (liver, mitochondrial) | -0.072 | 0.951 | 3.05E-01 | 3.05E+01 | | | |
| 8077211 | NM_013325 | ATG4B | ATG4 autophagy related 4 homolog | -0.072 | 0.951 | 4.43E-01 | 4.43E+01 | | | |
| 8036103 | NM_198538 | SBSN | suprabasin | -0.072 | 0.951 | 2.27E-01 | 2.27E+01 | | | |
| 8019280 | NM_002861 | PCYT2 | phosphate cytidylyltransferase 2, eth | -0.072 | 0.951 | 2.45E-01 | 2.45E+01 | | | |
| 7909890 | NM_021958 | HLX | H2.0-like homeobox | -0.072 | 0.951 | 2.24E-01 | 2.24E+01 | | | |
| 8030838 | ENST0000030 | FLJ30403 | hypothetical protein LOC729975 | -0.072 | 0.951 | 3.99E-01 | 3.99E+01 | | | |
| 8159900 | NM_152629 | GLIS3 | GLIS family zinc finger 3 | -0.072 | 0.951 | 1.37E-01 | 1.37E+01 | | | |
| 8109821 | NM_006013 | RPL10 | ribosomal protein L10 | -0.072 | 0.951 | 6.91E-01 | 6.91E+01 | | | |
| 7895545 | --- | --- | --- | -0.072 | 0.951 | 6.12E-01 | 6.12E+01 | | | |
| 8002967 | --- | --- | --- | -0.072 | 0.951 | 6.83E-01 | 6.83E+01 | | | |
| 7914580 | NM_153756 | FNDC5 | fibronectin type III domain containin | -0.072 | 0.951 | 1.67E-01 | 1.67E+01 | | | |
| 8109505 | NR_002168 | PPP1R2P3 | protein phosphatase 1, regulatory (in | -0.072 | 0.951 | 3.91E-01 | 3.91E+01 | | | |
| 7985317 | NM_018689 | KIAA1199 | KIAA1199 | -0.072 | 0.951 | 2.22E-01 | 2.22E+01 | | | |
| 8078300 | NM_017897 | OXSM | 3-oxoacyl-ACP synthase, mitochondr | -0.072 | 0.951 | 7.33E-01 | 7.33E+01 | | | |
| 8073943 | NM_014838 | ZBED4 | zinc finger, BED-type containing 4 | -0.072 | 0.951 | 4.90E-01 | 4.90E+01 | | | |
| 8035694 | NM_025245 | PBX4 | pre-B-cell leukemia homeobox 4 | -0.072 | 0.951 | 3.57E-01 | 3.57E+01 | | | |
| 8006052 | NM_138349 | TP53I13 | tumor protein p53 inducible protein | -0.072 | 0.951 | 1.46E-01 | 1.46E+01 | | | |
| 8012043 | NM_001671 | ASGR1 | asialoglycoprotein receptor 1 | -0.072 | 0.951 | 2.15E-01 | 2.15E+01 | | | |
| 8039120 | NM_206818 | OSCAR | osteoclast associated, immunoglobu | -0.072 | 0.951 | 1.90E-01 | 1.90E+01 | | | |
| 8051024 | NM_178553 | C2orf53 | chromosome 2 open reading frame 5 | -0.072 | 0.951 | 1.27E-01 | 1.27E+01 | | | |
| 7996685 | NM_014329 | EDC4 | enhancer of mRNA decapping 4 | -0.072 | 0.951 | 2.78E-01 | 2.78E+01 | | | |
| 7893802 | --- | --- | --- | -0.072 | 0.951 | 3.12E-01 | 3.12E+01 | | | |
| 7991512 | NR_003260 | C15orf51 | dynamin 1 pseudogene | -0.072 | 0.951 | 4.80E-01 | 4.80E+01 | | | |
| 8007537 | NM_145273 | CD300LG | CD300 molecule-like family member | -0.072 | 0.951 | 1.89E-01 | 1.89E+01 | | | |
| 8080676 | NM_177966 | PDE12 | phosphodiesterase 12 | -0.072 | 0.951 | 7.24E-01 | 7.24E+01 | | | |
| 7985690 | BX648930 | LOC440300 | chondroitin sulfate proteoglycan 4 p | -0.073 | 0.951 | 2.59E-01 | 2.59E+01 | | | |
| 7944271 | NM_00108044 | TTC36 | tetratricopeptide repeat domain 36 | -0.073 | 0.951 | 2.14E-01 | 2.14E+01 | | | |
| 7949412 | NM_00113014 | LTBP3 | latent transforming growth factor be | -0.073 | 0.951 | 2.15E-01 | 2.15E+01 | | | |
| 8054758 | NR_024005 | MGC13005 | hypothetical LOC84771 | -0.073 | 0.951 | 4.83E-01 | 4.83E+01 | | | |
| 7986561 | ENST0000033 | VSIG7 | V-set and immunoglobulin domain c | -0.073 | 0.951 | 4.98E-01 | 4.98E+01 | | | |
| 8084887 | --- | --- | --- | -0.073 | 0.951 | 3.34E-01 | 3.34E+01 | | | |
| 7901662 | NM_004623 | TTC4 | tetratricopeptide repeat domain 4 | -0.073 | 0.951 | 4.23E-01 | 4.23E+01 | | | |
| 7996034 | NM_002987 | CCL17 | chemokine (C-C motif) ligand 17 | -0.073 | 0.951 | 1.80E-01 | 1.80E+01 | | | |
| 7914015 | NM_052943 | FAM46B | family with sequence similarity 46, n | -0.073 | 0.951 | 2.07E-01 | 2.07E+01 | | | |
| 7923547 | NM_001276 | CHI3L1 | chitinase 3-like 1 (cartilage glycoprot | -0.073 | 0.951 | 2.48E-01 | 2.48E+01 | | | |
| 8071597 | BC032452 | IGL@ | immunoglobulin lambda locus | -0.073 | 0.951 | 5.63E-02 | 5.63E+00 | | | |
| 7963577 | NM_032840 | SPRYD3 | SPRY domain containing 3 | -0.073 | 0.951 | 3.59E-01 | 3.59E+01 | | | |
| 7977868 | NM_021944 | C14orf93 | chromosome 14 open reading frame | -0.073 | 0.951 | 1.84E-01 | 1.84E+01 | | | |
| 7975390 | NM_00103485 | SMOC1 | SPARC related modular calcium bind | -0.073 | 0.951 | 1.25E-01 | 1.25E+01 | | | |
| 8156706 | NM_003275 | TMOD1 | tropomodulin 1 | -0.073 | 0.951 | 2.66E-01 | 2.66E+01 | | | |
| 8007641 | ENST00000434 | LOC728675 | hypothetical LOC728675 | -0.073 | 0.951 | 2.94E-01 | 2.94E+01 | | | |
| 7911529 | NM_032348 | MXRA8 | matrix-remodelling associated 8 | -0.073 | 0.951 | 3.80E-01 | 3.80E+01 | | | |
| 8131069 | NM_00103996 | GPER | G protein-coupled estrogen receptor | -0.073 | 0.951 | 1.21E-01 | 1.21E+01 | | | |
| 8024120 | NM_019112 | ABCA7 | ATP-binding cassette, sub-family A ( | -0.073 | 0.951 | 1.38E-01 | 1.38E+01 | | | |
| 7896692 | --- | --- | --- | -0.073 | 0.951 | 8.26E-02 | 8.26E+00 | | | |
| 8064111 | NM_032957 | RTEL1 | regulator of telomere elongation hel | -0.073 | 0.951 | 1.38E-01 | 1.38E+01 | | | |
| 8034101 | NM_006858 | TMED1 | transmembrane emp24 protein tran | -0.073 | 0.951 | 2.27E-01 | 2.27E+01 | | | |
| 8075316 | NM_020530 | OSM | oncostatin M | -0.073 | 0.951 | 2.36E-01 | 2.36E+01 | | | |

| | | | | | | | | | | |
|---|---|---|---|---|---|---|---|---|---|---|
| 8062326 | NR_026562 | C20orf24 | chromosome 20 open reading frame | -0.073 | 0.951 | 8.10E-01 | 8.10E+01 | | | |
| 8003125 | NM_005679 | TAF1C | TATA box binding protein (TBP)-asso | -0.073 | 0.951 | 1.91E-01 | 1.91E+01 | | | |
| 7914467 | NM_144569 | SPOCD1 | SPOC domain containing 1 | -0.073 | 0.951 | 1.61E-01 | 1.61E+01 | | | |
| 7931204 | NM_022126 | LHPP | phospholysine phosphohistidine inor | -0.073 | 0.951 | 3.07E-01 | 3.07E+01 | | | |
| 8078690 | NM_007335 | DLEC1 | deleted in lung and esophageal canc | -0.073 | 0.951 | 2.08E-01 | 2.08E+01 | | | |
| 8081335 | ENST00000426 | OC10028785 | similar to CG5323 | -0.073 | 0.951 | 3.21E-01 | 3.21E+01 | | | |
| 8154841 | ENST00000390 | TRBV23OR9-2 | T cell receptor beta variable 23/OR9- | -0.073 | 0.951 | 5.15E-01 | 5.15E+01 | | | |
| 8175539 | NM_012317 | LDOC1 | leucine zipper, down-regulated in ca | -0.073 | 0.951 | 3.07E-01 | 3.07E+01 | | | |
| 8050894 | NM_002254 | KIF3C | kinesin family member 3C | -0.073 | 0.951 | 1.10E-01 | 1.10E+01 | | | |
| 7926900 | NM_005204 | MAP3K8 | mitogen-activated protein kinase kin | -0.073 | 0.951 | 5.66E-01 | 5.66E+01 | | | |
| 8062527 | NM_00101808 | ADIG | adipogenin | -0.073 | 0.951 | 2.80E-01 | 2.80E+01 | | | |
| 7931607 | NM_138384 | MTG1 | mitochondrial GTPase 1 homolog (S. | -0.073 | 0.951 | 2.43E-01 | 2.43E+01 | | | |
| 8080344 | NM_015136 | STAB1 | stabilin 1 | -0.073 | 0.951 | 1.00E-01 | 1.00E+01 | | | |
| 7993624 | NM_016524 | SYT17 | synaptotagmin XVII | -0.073 | 0.951 | 1.98E-01 | 1.98E+01 | | | |
| 8175432 | NR_002735 | SNORD61 | small nucleolar RNA, C/D box 61 | -0.073 | 0.951 | 8.94E-01 | 8.94E+01 | | | |
| 7895706 | --- | --- | --- | -0.073 | 0.951 | 9.53E-01 | 9.53E+01 | | | |
| 7931863 | NM_024701 | ASB13 | ankyrin repeat and SOCS box-contair | -0.073 | 0.951 | 1.97E-01 | 1.97E+01 | | | |
| 7937667 | NM_003957 | BRSK2 | BR serine/threonine kinase 2 | -0.073 | 0.951 | 1.77E-01 | 1.77E+01 | | | |
| 8071554 | NM_152612 | CCDC116 | coiled-coil domain containing 116 | -0.073 | 0.951 | 1.90E-01 | 1.90E+01 | | | |
| 8025621 | NM_003259 | ICAM5 | intercellular adhesion molecule 5, te | -0.073 | 0.951 | 1.37E-01 | 1.37E+01 | | | |
| 8012887 | NM_00100753 | CDRT15 | CMT1A duplicated region transcript | -0.073 | 0.951 | 2.61E-01 | 2.61E+01 | | | |
| 8029918 | NM_014681 | DHX34 | DEAH (Asp-Glu-Ala-His) box polypept | -0.073 | 0.951 | 2.69E-01 | 2.69E+01 | | | |
| 7893901 | --- | --- | --- | -0.073 | 0.951 | 5.26E-01 | 5.26E+01 | | | |
| 7998063 | NM_006086 | TUBB3 | tubulin, beta 3 | -0.073 | 0.951 | 1.21E-01 | 1.21E+01 | | | |
| 7948798 | NM_00113070 | BSCL2 | Berardinelli-Seip congenital lipodystr | -0.073 | 0.951 | 2.55E-01 | 2.55E+01 | | | |
| 8089993 | NM_019069 | WDR5B | WD repeat domain 5B | -0.073 | 0.951 | 3.46E-01 | 3.46E+01 | | | |
| 8073013 | --- | --- | --- | -0.073 | 0.951 | 4.13E-01 | 4.13E+01 | | | |
| 7896418 | --- | --- | --- | -0.073 | 0.951 | 6.23E-01 | 6.23E+01 | | | |
| 7973363 | NM_173527 | REM2 | RAS (RAD and GEM)-like GTP binding | -0.073 | 0.951 | 2.58E-01 | 2.58E+01 | | | |
| 8063785 | AY358539 | C20orf197 | chromosome 20 open reading frame | -0.073 | 0.951 | 2.02E-01 | 2.02E+01 | | | |
| 7896106 | --- | --- | --- | -0.073 | 0.951 | 7.15E-01 | 7.15E+01 | | | |
| 8020647 | NR_024232 | TTC39C | tetratricopeptide repeat domain 39C | -0.073 | 0.951 | 4.74E-01 | 4.74E+01 | | | |
| 7928619 | NM_00109933 | FAM22A | family with sequence similarity 22, m | -0.073 | 0.951 | 9.07E-02 | 9.07E+00 | | | |
| 8061303 | NM_002196 | INSM1 | insulinoma-associated 1 | -0.073 | 0.951 | 1.86E-01 | 1.86E+01 | | | |
| 8084496 | NM_003741 | CHRD | chordin | -0.073 | 0.951 | 1.45E-01 | 1.45E+01 | | | |
| 8007803 | NR_026905 | C17orf69 | chromosome 17 open reading frame | -0.073 | 0.951 | 3.26E-01 | 3.26E+01 | | | |
| 7908907 | NM_000674 | ADORA1 | adenosine A1 receptor | -0.073 | 0.951 | 2.10E-01 | 2.10E+01 | | | |
| 8029541 | NM_001646 | APOC4 | apolipoprotein C-IV | -0.073 | 0.951 | 2.31E-01 | 2.31E+01 | | | |
| 7971386 | NR_002967 | SNORA31 | small nucleolar RNA, H/ACA box 31 | -0.073 | 0.950 | 8.64E-01 | 8.64E+01 | | | |
| 7970096 | NM_005537 | ING1 | inhibitor of growth family, member | -0.073 | 0.950 | 2.07E-01 | 2.07E+01 | | | |
| 7950235 | NM_006645 | STARD10 | StAR-related lipid transfer (START) d | -0.073 | 0.950 | 1.86E-01 | 1.86E+01 | | | |
| 7928069 | NM_005203 | COL13A1 | collagen, type XIII, alpha 1 | -0.073 | 0.950 | 2.00E-01 | 2.00E+01 | | | |
| 7980358 | NM_013382 | POMT2 | protein-O-mannosyltransferase 2 | -0.073 | 0.950 | 2.24E-01 | 2.24E+01 | | | |
| 8039280 | NM_00112725 | NLRP7 | NLR family, pyrin domain containing | -0.073 | 0.950 | 1.85E-01 | 1.85E+01 | | | |
| 7919761 | --- | --- | --- | -0.073 | 0.950 | 9.01E-01 | 9.01E+01 | | | |
| 8072272 | --- | --- | --- | -0.073 | 0.950 | 6.37E-01 | 6.37E+01 | | | |
| 8006592 | --- | --- | --- | -0.073 | 0.950 | 5.88E-01 | 5.88E+01 | | | |
| 7989315 | NM_004492 | GTF2A2 | general transcription factor IIA, 2, 12 | -0.073 | 0.950 | 9.19E-01 | 9.19E+01 | | | |
| 7926506 | NM_201596 | CACNB2 | calcium channel, voltage-dependent | -0.073 | 0.950 | 2.50E-01 | 2.50E+01 | | | |
| 7896358 | --- | --- | --- | -0.073 | 0.950 | 4.71E-01 | 4.71E+01 | | | |
| 8053022 | NM_001965 | EGR4 | early growth response 4 | -0.073 | 0.950 | 2.79E-01 | 2.79E+01 | | | |
| 7896278 | --- | --- | --- | -0.073 | 0.950 | 4.93E-01 | 4.93E+01 | | | |
| 7970716 | NM_153371 | LNX2 | ligand of numb-protein X 2 | -0.073 | 0.950 | 6.53E-01 | 6.53E+01 | | | |
| 8116348 | NM_198868 | TBC1D9B | TBC1 domain family, member 9B (wi | -0.073 | 0.950 | 1.44E-01 | 1.44E+01 | | | |
| 7919743 | NM_019032 | ADAMTSL4 | ADAMTS-like 4 | -0.073 | 0.950 | 5.49E-01 | 5.49E+01 | | | |
| 8159541 | BC002613 | C9orf142 | chromosome 9 open reading frame 1 | -0.073 | 0.950 | 2.17E-01 | 2.17E+01 | | | |
| 8135031 | ENST00000379 | MUC12 | mucin 12, cell surface associated | -0.073 | 0.950 | 5.79E-01 | 5.79E+01 | | | |
| 7906467 | --- | --- | --- | -0.073 | 0.950 | 2.68E-01 | 2.68E+01 | | | |
| 7911359 | NM_015658 | NOC2L | nucleolar complex associated 2 hom | -0.073 | 0.950 | 3.38E-01 | 3.38E+01 | | | |
| 8038890 | NM_001523 | HAS1 | hyaluronan synthase 1 | -0.073 | 0.950 | 2.53E-01 | 2.53E+01 | | | |
| 8024740 | NM_015897 | PIAS4 | protein inhibitor of activated STAT, 4 | -0.074 | 0.950 | 1.68E-01 | 1.68E+01 | | | |
| 7945394 | NM_00101230 | ANO9 | anoctamin 9 | -0.074 | 0.950 | 2.36E-01 | 2.36E+01 | | | |
| 7998053 | --- | --- | --- | -0.074 | 0.950 | 7.82E-01 | 7.82E+01 | | | |
| 8143863 | NM_006712 | FASTK | Fas-activated serine/threonine kinas | -0.074 | 0.950 | 3.08E-01 | 3.08E+01 | | | |
| 7929988 | NM_024747 | HPS6 | Hermansky-Pudlak syndrome 6 | -0.074 | 0.950 | 3.09E-01 | 3.09E+01 | | | |
| 7942439 | NM_032871 | RELT | RELT tumor necrosis factor receptor | -0.074 | 0.950 | 1.71E-01 | 1.71E+01 | | | |
| 7919568 | --- | --- | --- | -0.074 | 0.950 | 5.76E-01 | 5.76E+01 | | | |
| 8073114 | NM_006116 | MAP3K7IP1 | mitogen-activated protein kinase kin | -0.074 | 0.950 | 2.19E-01 | 2.19E+01 | | | |
| 8042326 | NM_015147 | CEP68 | centrosomal protein 68kDa | -0.074 | 0.950 | 3.12E-01 | 3.12E+01 | | | |
| 7939376 | NM_174902 | LDLRAD3 | low density lipoprotein receptor clas | -0.074 | 0.950 | 1.45E-01 | 1.45E+01 | | | |
| 8003892 | NM_00111497 | SMTNL2 | smoothelin-like 2 | -0.074 | 0.950 | 1.40E-01 | 1.40E+01 | | | |
| 8175710 | NM_00108084 | CSAG2 | CSAG family, member 2 | -0.074 | 0.950 | 2.62E-01 | 2.62E+01 | | | |
| 7922707 | NM_021133 | RNASEL | ribonuclease L (2',5'-oligoisoadenylat | -0.074 | 0.950 | 7.66E-01 | 7.66E+01 | | | |
| 8077116 | NM_00101444 | ODF3B | outer dense fiber of sperm tails 3B | -0.074 | 0.950 | 4.73E-01 | 4.73E+01 | | | |
| 7995838 | NM_005952 | MT1X | metallothionein 1X | -0.074 | 0.950 | 4.43E-01 | 4.43E+01 | | | |
| 8002692 | NM_006885 | ZFHX3 | zinc finger homeobox 3 | -0.074 | 0.950 | 9.06E-02 | 9.06E+00 | | | |
| 8110821 | NM_001044 | SLC6A3 | solute carrier family 6 (neurotransmi | -0.074 | 0.950 | 1.45E-01 | 1.45E+01 | | | |

| | | | | | | | | | | |
|---|---|---|---|---|---|---|---|---|---|---|
| 8165021 | --- | --- | --- | -0.074 | 0.950 | 3.17E-01 | 3.17E+01 | | | |
| 7981046 | NM_032036 | IFI27L2 | interferon, alpha-inducible protein 2 | -0.074 | 0.950 | 4.96E-01 | 4.96E+01 | | | |
| 8134431 | --- | --- | --- | -0.074 | 0.950 | 3.26E-01 | 3.26E+01 | | | |
| 7958565 | NM_000431 | MVK | mevalonate kinase | -0.074 | 0.950 | 1.98E-01 | 1.98E+01 | | | |
| 8137228 | NM_173680 | ZNF775 | zinc finger protein 775 | -0.074 | 0.950 | 1.96E-01 | 1.96E+01 | | | |
| 8079305 | NM_003278 | CLEC3B | C-type lectin domain family 3, memb | -0.074 | 0.950 | 1.67E-01 | 1.67E+01 | | | |
| 8080973 | NM_174907 | PPP4R2 | protein phosphatase 4, regulatory su | -0.074 | 0.950 | 8.39E-01 | 8.39E+01 | | | |
| 7955694 | NM_002178 | IGFBP6 | insulin-like growth factor binding pro | -0.074 | 0.950 | 2.14E-01 | 2.14E+01 | | | |
| 8002370 | NM_006927 | ST3GAL2 | ST3 beta-galactoside alpha-2,3-sialyl | -0.074 | 0.950 | 3.40E-01 | 3.40E+01 | | | |
| 7896657 | --- | --- | --- | -0.074 | 0.950 | 9.01E-01 | 9.01E+01 | | | |
| 8145227 | NM_00116003( | RHOBTB2 | Rho-related BTB domain containing 2 | -0.074 | 0.950 | 2.47E-01 | 2.47E+01 | | | |
| 8033487 | NM_032447 | FBN3 | fibrillin 3 | -0.074 | 0.950 | 1.19E-01 | 1.19E+01 | | | |
| 7998233 | NM_021259 | TMEM8A | transmembrane protein 8A | -0.074 | 0.950 | 2.71E-01 | 2.71E+01 | | | |
| 7943347 | --- | --- | --- | -0.074 | 0.950 | 4.09E-01 | 4.09E+01 | | | |
| 8033433 | NM_198492 | CLEC4G | C-type lectin domain family 4, memb | -0.074 | 0.950 | 2.69E-01 | 2.69E+01 | | | |
| 8083214 | NM_004267 | CHST2 | carbohydrate (N-acetylglucosamine-( | -0.074 | 0.950 | 6.72E-02 | 6.72E+00 | | | |
| 8132998 | --- | --- | --- | -0.074 | 0.950 | 8.78E-01 | 8.78E+01 | | | |
| 8165552 | NM_00113096! | NELF | nasal embryonic LHRH factor | -0.074 | 0.950 | 2.19E-01 | 2.19E+01 | | | |
| 7924508 | NM_017982 | SUSD4 | sushi domain containing 4 | -0.074 | 0.950 | 9.52E-02 | 9.52E+00 | | | |
| 8061881 | --- | --- | --- | -0.074 | 0.950 | 2.31E-01 | 2.31E+01 | | | |
| 8028583 | NM_00101483: | PAK4 | p21 protein (Cdc42/Rac)-activated ki | -0.074 | 0.950 | 1.55E-01 | 1.55E+01 | | | |
| 7905519 | NM_178353 | LCE1E | late cornified envelope 1E | -0.074 | 0.950 | 1.83E-01 | 1.83E+01 | | | |
| 8160663 | NM_001170 | AQP7 | aquaporin 7 | -0.074 | 0.950 | 4.44E-01 | 4.44E+01 | | | |
| 7920025 | --- | --- | --- | -0.074 | 0.950 | 3.63E-01 | 3.63E+01 | | | |
| 8010664 | NM_002949 | MRPL12 | mitochondrial ribosomal protein L12 | -0.074 | 0.950 | 2.58E-01 | 2.58E+01 | | | |
| 7999916 | NM_00100291: | GPR139 | G protein-coupled receptor 139 | -0.074 | 0.950 | 2.65E-01 | 2.65E+01 | | | |
| 7945349 | NM_016526 | BET1L | blocked early in transport 1 homolog | -0.074 | 0.950 | 4.86E-01 | 4.86E+01 | | | |
| 8171222 | NM_00113556! | HDHD1A | haloacid dehalogenase-like hydrolase | -0.074 | 0.950 | 3.22E-01 | 3.22E+01 | | | |
| 8080619 | --- | --- | --- | -0.074 | 0.950 | 5.28E-01 | 5.28E+01 | | | |
| 8178115 | NM_001710 | CFB | complement factor B | -0.074 | 0.950 | 2.10E-01 | 2.10E+01 | | | |
| 8090295 | NM_017836 | SLC41A3 | solute carrier family 41, member 3 | -0.074 | 0.950 | 4.68E-01 | 4.68E+01 | | | |
| 7972890 | NM_024719 | GRTP1 | growth hormone regulated TBC prot | -0.074 | 0.950 | 1.65E-01 | 1.65E+01 | | | |
| 8062123 | NM_006690 | MMP24 | matrix metallopeptidase 24 (membra | -0.074 | 0.950 | 2.26E-01 | 2.26E+01 | | | |
| 8157638 | NM_138777 | MRRF | mitochondrial ribosome recycling fac | -0.074 | 0.950 | 7.72E-01 | 7.72E+01 | | | |
| 7999447 | ENST00000344 | LOC400499 | hypothetical LOC400499 | -0.074 | 0.950 | 2.50E-01 | 2.50E+01 | | | |
| 7960642 | NM_032489 | ACRBP | acrosin binding protein | -0.074 | 0.950 | 2.22E-01 | 2.22E+01 | | | |
| 8087337 | NM_002292 | LAMB2 | laminin, beta 2 (laminin S) | -0.074 | 0.950 | 1.35E-01 | 1.35E+01 | | | |
| 8165183 | NM_014866 | SEC16A | SEC16 homolog A (S. cerevisiae) | -0.074 | 0.950 | 2.36E-01 | 2.36E+01 | | | |
| 8048478 | NM_138802 | ZFAND2B | zinc finger, AN1-type domain 2B | -0.074 | 0.950 | 3.96E-01 | 3.96E+01 | | | |
| 8002051 | NM_001138 | AGRP | agouti related protein homolog (mou | -0.074 | 0.950 | 2.44E-01 | 2.44E+01 | | | |
| 7985001 | NM_024608 | NEIL1 | nei endonuclease VIII-like 1 (E. coli) | -0.074 | 0.950 | 2.03E-01 | 2.03E+01 | | | |
| 7896677 | --- | --- | --- | -0.074 | 0.950 | 7.36E-01 | 7.36E+01 | | | |
| 8133202 | NM_018264 | TYW1 | tRNA-yW synthesizing protein 1 hom | -0.074 | 0.950 | 5.43E-01 | 5.43E+01 | | | |
| 8032290 | NM_003200 | TCF3 | transcription factor 3 (E2A immunog | -0.074 | 0.950 | 1.21E-01 | 1.21E+01 | | | |
| 8061831 | NM_182519 | C20orf186 | chromosome 20 open reading frame | -0.074 | 0.950 | 1.22E-01 | 1.22E+01 | | | |
| 8019347 | --- | --- | --- | -0.074 | 0.950 | 4.28E-01 | 4.28E+01 | | | |
| 8126212 | --- | --- | --- | -0.074 | 0.950 | 2.14E-01 | 2.14E+01 | | | |
| 8008511 | NM_175575 | WFIKKN2 | WAP, follistatin/kazal, immunoglobu | -0.074 | 0.950 | 1.54E-01 | 1.54E+01 | | | |
| 8091071 | NM_004164 | RBP2 | retinol binding protein 2, cellular | -0.074 | 0.950 | 2.87E-01 | 2.87E+01 | | | |
| 8159211 | NM_004108 | FCN2 | ficolin (collagen/fibrinogen domain c | -0.074 | 0.950 | 2.18E-01 | 2.18E+01 | | | |
| 7985934 | NM_020210 | SEMA4B | sema domain, immunoglobulin doma | -0.074 | 0.950 | 1.37E-01 | 1.37E+01 | | | |
| 8009755 | NM_024585 | ARMC7 | armadillo repeat containing 7 | -0.074 | 0.950 | 3.34E-01 | 3.34E+01 | | | |
| 8036840 | NM_001626 | AKT2 | v-akt murine thymoma viral oncogen | -0.074 | 0.950 | 4.79E-01 | 4.79E+01 | | | |
| 8100962 | --- | --- | --- | -0.074 | 0.950 | 2.99E-01 | 2.99E+01 | | | |
| 7894856 | --- | --- | --- | -0.074 | 0.950 | 5.77E-01 | 5.77E+01 | | | |
| 8106017 | --- | --- | --- | -0.074 | 0.950 | 7.83E-01 | 7.83E+01 | | | |
| 8156404 | NM_032310 | C9orf89 | chromosome 9 open reading frame 8 | -0.074 | 0.950 | 5.08E-01 | 5.08E+01 | | | |
| 8126303 | NM_018643 | TREM1 | triggering receptor expressed on my | -0.074 | 0.950 | 9.16E-01 | 9.16E+01 | | | |
| 8107720 | AK093561 | OC10013179 | hypothetical LOC100131792 | -0.075 | 0.950 | 2.19E-01 | 2.19E+01 | | | |
| 8066619 | NM_006227 | PLTP | phospholipid transfer protein | -0.075 | 0.950 | 1.40E-01 | 1.40E+01 | | | |
| 8007895 | NM_003396 | WNT9B | wingless-type MMTV integration site | -0.075 | 0.950 | 2.15E-01 | 2.15E+01 | | | |
| 8145151 | NM_005775 | SORBS3 | sorbin and SH3 domain containing 3 | -0.075 | 0.950 | 2.66E-01 | 2.66E+01 | | | |
| 8168028 | NM_00114250: | STARD8 | StAR-related lipid transfer (START) d | -0.075 | 0.950 | 2.30E-01 | 2.30E+01 | | | |
| 8035120 | NM_021235 | EPS15L1 | epidermal growth factor receptor pa | -0.075 | 0.950 | 3.20E-01 | 3.20E+01 | | | |
| 8101043 | NM_203505 | G3BP2 | GTPase activating protein (SH3 doma | -0.075 | 0.950 | 7.31E-01 | 7.31E+01 | | | |
| 8024995 | NM_004558 | NRTN | neurturin | -0.075 | 0.950 | 1.48E-01 | 1.48E+01 | | | |
| 8073296 | NM_005297 | MCHR1 | melanin-concentrating hormone rece | -0.075 | 0.950 | 1.20E-01 | 1.20E+01 | | | |
| 7897295 | NM_138697 | TAS1R1 | taste receptor, type 1, member 1 | -0.075 | 0.950 | 1.89E-01 | 1.89E+01 | | | |
| 7911263 | NM_00100469( | OR2M5 | olfactory receptor, family 2, subfami | -0.075 | 0.950 | 2.66E-01 | 2.66E+01 | | | |
| 8070194 | NM_00100189( | RUNX1 | runt-related transcription factor 1 | -0.075 | 0.950 | 4.43E-01 | 4.43E+01 | | | |
| 8020653 | NM_012189 | CABYR | calcium binding tyrosine-(Y)-phospho | -0.075 | 0.950 | 1.57E-01 | 1.57E+01 | | | |
| 8075906 | NM_001051 | SSTR3 | somatostatin receptor 3 | -0.075 | 0.950 | 2.65E-01 | 2.65E+01 | | | |
| 8071713 | AF174606 | FBXW4P1 | F-box and WD repeat domain contai | -0.075 | 0.950 | 4.23E-01 | 4.23E+01 | | | |
| 8011027 | NM_00108077! | MYO1C | myosin IC | -0.075 | 0.950 | 2.21E-01 | 2.21E+01 | | | |
| 7905406 | NM_020770 | CGN | cingulin | -0.075 | 0.949 | 2.73E-01 | 2.73E+01 | | | |
| 7892765 | --- | --- | --- | -0.075 | 0.949 | 4.36E-01 | 4.36E+01 | | | |
| 7998072 | NM_207514 | DEF8 | differentially expressed in FDCP 8 ho | -0.075 | 0.949 | 3.64E-01 | 3.64E+01 | | | |

| | | | | | | | | | | |
|---|---|---|---|---|---|---|---|---|---|---|
| 7948282 | NM_145008 | YPEL4 | yippee-like 4 (Drosophila) | -0.075 | 0.949 | 1.56E-01 | 1.56E+01 | | | |
| 8131179 | NM_025250 | TTYH3 | tweety homolog 3 (Drosophila) | -0.075 | 0.949 | 1.63E-01 | 1.63E+01 | | | |
| 8173373 | NM_016484 | PDZD11 | PDZ domain containing 11 | -0.075 | 0.949 | 7.61E-01 | 7.61E+01 | | | |
| 8021169 | NM_006033 | LIPG | lipase, endothelial | -0.075 | 0.949 | 1.55E-01 | 1.55E+01 | | | |
| 8035249 | NM_005234 | NR2F6 | nuclear receptor subfamily 2, group | -0.075 | 0.949 | 1.79E-01 | 1.79E+01 | | | |
| 8073096 | NM_181773 | APOBEC3H | apolipoprotein B mRNA editing enzy | -0.075 | 0.949 | 4.83E-01 | 4.83E+01 | | | |
| 8013804 | NM_144683 | DHRS13 | dehydrogenase/reductase (SDR fami | -0.075 | 0.949 | 2.21E-01 | 2.21E+01 | | | |
| 8002303 | NM_000903 | NQO1 | NAD(P)H dehydrogenase, quinone 1 | -0.075 | 0.949 | 2.70E-01 | 2.70E+01 | | | |
| 8027437 | --- | --- | --- | -0.075 | 0.949 | 5.77E-01 | 5.77E+01 | | | |
| 7948656 | NM_002032 | FTH1 | ferritin, heavy polypeptide 1 | -0.075 | 0.949 | 6.64E-01 | 6.64E+01 | | | |
| 8118023 | NM_001517 | GTF2H4 | general transcription factor IIH, poly | -0.075 | 0.949 | 3.62E-01 | 3.62E+01 | | | |
| 7894049 | --- | --- | --- | -0.075 | 0.949 | 5.75E-01 | 5.75E+01 | | | |
| 8082314 | NM_032242 | PLXNA1 | plexin A1 | -0.075 | 0.949 | 2.51E-01 | 2.51E+01 | | | |
| 8053073 | NM_133478 | SLC4A5 | solute carrier family 4, sodium bicarb | -0.075 | 0.949 | 2.49E-01 | 2.49E+01 | | | |
| 8153409 | NM_201589 | MAFA | v-maf musculoaponeurotic fibrosarc | -0.075 | 0.949 | 1.35E-01 | 1.35E+01 | | | |
| 8172270 | --- | --- | --- | -0.075 | 0.949 | 7.77E-01 | 7.77E+01 | | | |
| 7994889 | NM_006662 | SRCAP | Snf2-related CREBBP activator protei | -0.075 | 0.949 | 4.19E-01 | 4.19E+01 | | | |
| 7901691 | NM_057176 | BSND | Bartter syndrome, infantile, with sen | -0.075 | 0.949 | 2.89E-01 | 2.89E+01 | | | |
| 8002104 | NM_025082 | CENPT | centromere protein T | -0.075 | 0.949 | 3.94E-01 | 3.94E+01 | | | |
| 8064779 | NM_000678 | ADRA1D | adrenergic, alpha-1D-, receptor | -0.075 | 0.949 | 2.59E-01 | 2.59E+01 | | | |
| 7916489 | --- | --- | --- | -0.075 | 0.949 | 2.74E-01 | 2.74E+01 | | | |
| 8079707 | NM_199417 | NDUFAF3 | NADH dehydrogenase (ubiquinone) 1 | -0.075 | 0.949 | 2.66E-01 | 2.66E+01 | | | |
| 7951271 | NM_002421 | MMP1 | matrix metallopeptidase 1 (interstitia | -0.075 | 0.949 | 2.03E-01 | 2.03E+01 | | | |
| 7912706 | NM_004431 | EPHA2 | EPH receptor A2 | -0.075 | 0.949 | 1.28E-01 | 1.28E+01 | | | |
| 7904048 | --- | --- | --- | -0.075 | 0.949 | 9.21E-01 | 9.21E+01 | | | |
| 8066482 | NM_145652 | WFDC5 | WAP four-disulfide core domain 5 | -0.075 | 0.949 | 2.12E-01 | 2.12E+01 | | | |
| 8037071 | NM_006423 | RABAC1 | Rab acceptor 1 (prenylated) | -0.075 | 0.949 | 6.23E-01 | 6.23E+01 | | | |
| 7913850 | NM_019557 | FAM54B | family with sequence similarity 54, m | -0.075 | 0.949 | 1.48E-01 | 1.48E+01 | | | |
| 7896746 | --- | --- | --- | -0.075 | 0.949 | 9.00E-01 | 9.00E+01 | | | |
| 8092552 | NM_006548 | IGF2BP2 | insulin-like growth factor 2 mRNA bi | -0.075 | 0.949 | 3.12E-01 | 3.12E+01 | | | |
| 8110861 | NM_032479 | MRPL36 | mitochondrial ribosomal protein L36 | -0.075 | 0.949 | 2.51E-01 | 2.51E+01 | | | |
| 7960370 | NM_032680 | EFCAB4B | EF-hand calcium binding domain 4B | -0.075 | 0.949 | 4.54E-01 | 4.54E+01 | | | |
| 7893427 | --- | --- | --- | -0.075 | 0.949 | 7.60E-01 | 7.60E+01 | | | |
| 7970194 | NM_00111273 | MCF2L | MCF.2 cell line derived transforming | -0.075 | 0.949 | 1.58E-01 | 1.58E+01 | | | |
| 8012383 | NM_032354 | TMEM107 | transmembrane protein 107 | -0.075 | 0.949 | 2.72E-01 | 2.72E+01 | | | |
| 7939595 | NM_021117 | CRY2 | cryptochrome 2 (photolyase-like) | -0.075 | 0.949 | 1.94E-01 | 1.94E+01 | | | |
| 8109819 | NR_024356 | FBLL1 | fibrillarin-like 1 | -0.075 | 0.949 | 2.18E-01 | 2.18E+01 | | | |
| 7981523 | --- | --- | --- | -0.075 | 0.949 | 2.73E-01 | 2.73E+01 | | | |
| 7967620 | --- | --- | --- | -0.075 | 0.949 | 2.42E-01 | 2.42E+01 | | | |
| 8130641 | NR_024277 | C6orf59 | chromosome 6 open reading frame 5 | -0.075 | 0.949 | 6.95E-01 | 6.95E+01 | | | |
| 8027956 | NM_014727 | MLL4 | myeloid/lymphoid or mixed-lineage | -0.075 | 0.949 | 7.68E-02 | 7.68E+00 | | | |
| 7995793 | NR_001447 | MT1L | metallothionein 1L (gene/pseudogen | -0.075 | 0.949 | 3.75E-01 | 3.75E+01 | | | |
| 8082246 | --- | --- | --- | -0.076 | 0.949 | 3.72E-01 | 3.72E+01 | | | |
| 7979943 | NM_033141 | MAP3K9 | mitogen-activated protein kinase kin | -0.076 | 0.949 | 1.87E-01 | 1.87E+01 | | | |
| 8077185 | NM_000039 | APOA1 | apolipoprotein A-I | -0.076 | 0.949 | 2.15E-01 | 2.15E+01 | | | |
| 8003283 | NM_017566 | KLHDC4 | kelch domain containing 4 | -0.076 | 0.949 | 3.07E-01 | 3.07E+01 | | | |
| 7992934 | NM_005223 | DNASE1 | deoxyribonuclease I | -0.076 | 0.949 | 1.97E-01 | 1.97E+01 | | | |
| 7910559 | NM_018662 | DISC1 | disrupted in schizophrenia 1 | -0.076 | 0.949 | 6.38E-02 | 6.38E+00 | | | |
| 7940924 | NM_000932 | PLCB3 | phospholipase C, beta 3 (phosphatid | -0.076 | 0.949 | 1.18E-01 | 1.18E+01 | | | |
| 7892642 | --- | --- | --- | -0.076 | 0.949 | 4.32E-01 | 4.32E+01 | | | |
| 8002547 | NM_006961 | ZNF19 | zinc finger protein 19 | -0.076 | 0.949 | 2.96E-01 | 2.96E+01 | | | |
| 8069852 | NM_181602 | KRTAP6-1 | keratin associated protein 6-1 | -0.076 | 0.949 | 3.07E-01 | 3.07E+01 | | | |
| 7925509 | --- | --- | --- | -0.076 | 0.949 | 1.98E-01 | 1.98E+01 | | | |
| 8180203 | --- | --- | --- | -0.076 | 0.949 | 2.04E-01 | 2.04E+01 | | | |
| 8018502 | NM_173547 | TRIM65 | tripartite motif-containing 65 | -0.076 | 0.949 | 1.81E-01 | 1.81E+01 | | | |
| 7939559 | NM_130783 | TSPAN18 | tetraspanin 18 | -0.076 | 0.949 | 5.81E-01 | 5.81E+01 | | | |
| 7905553 | NM_003125 | SPRR1B | small proline-rich protein 1B (cornifi | -0.076 | 0.949 | 1.72E-01 | 1.72E+01 | | | |
| 8180266 | --- | --- | --- | -0.076 | 0.949 | 2.14E-01 | 2.14E+01 | | | |
| 8135909 | NM_000230 | LEP | leptin | -0.076 | 0.949 | 2.25E-01 | 2.25E+01 | | | |
| 8032782 | NM_144615 | TMIGD2 | transmembrane and immunoglobuli | -0.076 | 0.949 | 2.50E-01 | 2.50E+01 | | | |
| 8169443 | --- | --- | --- | -0.076 | 0.949 | 3.45E-01 | 3.45E+01 | | | |
| 7949898 | ENST00000319 | LOC645332 | family with sequence similarity 86, m | -0.076 | 0.949 | 2.62E-01 | 2.62E+01 | | | |
| 8071382 | NM_003426 | ZNF74 | zinc finger protein 74 | -0.076 | 0.949 | 8.34E-02 | 8.34E+00 | | | |
| 7967711 | NM_175066 | DDX51 | DEAD (Asp-Glu-Ala-Asp) box polypep | -0.076 | 0.949 | 1.87E-01 | 1.87E+01 | | | |
| 8139859 | NM_000181 | GUSB | glucuronidase, beta | -0.076 | 0.949 | 7.71E-01 | 7.71E+01 | | | |
| 8178177 | NM_000500 | CYP21A2 | cytochrome P450, family 21, subfam | -0.076 | 0.949 | 2.58E-01 | 2.58E+01 | | | |
| 8179440 | NM_000500 | CYP21A2 | cytochrome P450, family 21, subfam | -0.076 | 0.949 | 2.58E-01 | 2.58E+01 | | | |
| 8124868 | NM_00110556 | CCHCR1 | coiled-coil alpha-helical rod protein 1 | -0.076 | 0.949 | 1.19E-01 | 1.19E+01 | | | |
| 8094130 | NM_00110566 | USP17 | ubiquitin specific peptidase 17 | -0.076 | 0.949 | 1.95E-01 | 1.95E+01 | | | |
| 8050594 | NM_022460 | HS1BP3 | HCLS1 binding protein 3 | -0.076 | 0.949 | 1.69E-01 | 1.69E+01 | | | |
| 8013473 | AK127974 | LOC339240 | keratin pseudogene | -0.076 | 0.949 | 4.00E-01 | 4.00E+01 | | | |
| 7912194 | BC029383 | KFZp566H08 | hypothetical LOC54744 | -0.076 | 0.949 | 8.29E-01 | 8.29E+01 | | | |
| 7972579 | NM_032813 | TMTC4 | transmembrane and tetratricopeptid | -0.076 | 0.949 | 2.94E-01 | 2.94E+01 | | | |
| 8007730 | NM_00113570 | ACBD4 | acyl-Coenzyme A binding domain co | -0.076 | 0.949 | 2.03E-01 | 2.03E+01 | | | |
| 8116921 | NM_001955 | EDN1 | endothelin 1 | -0.076 | 0.949 | 2.61E-01 | 2.61E+01 | | | |
| 7947147 | NM_148893 | SVIP | small VCP/p97-interacting protein | -0.076 | 0.949 | 5.74E-01 | 5.74E+01 | | | |
| 7952103 | NM_004397 | DDX6 | DEAD (Asp-Glu-Ala-Asp) box polypep | -0.076 | 0.949 | 7.46E-01 | 7.46E+01 | | | |

| | | | | | | | | | | |
|---|---|---|---|---|---|---|---|---|---|---|
| 7905028 | CR541728 | LSP1 | lymphocyte-specific protein 1 | -0.076 | 0.949 | 7.73E-01 | 7.73E+01 | | | |
| 7908328 | --- | --- | --- | -0.076 | 0.949 | 5.52E-01 | 5.52E+01 | | | |
| 8159531 | NM_207510 | LCNL1 | lipocalin-like 1 | -0.076 | 0.949 | 1.94E-01 | 1.94E+01 | | | |
| 8037695 | NM_004819 | SYMPK | symplekin | -0.076 | 0.949 | 2.72E-01 | 2.72E+01 | | | |
| 7914040 | --- | --- | --- | -0.076 | 0.949 | 4.05E-01 | 4.05E+01 | | | |
| 8097626 | NR_003675 | LOC441046 | glucuronidase, beta pseudogene | -0.076 | 0.949 | 1.68E-01 | 1.68E+01 | | | |
| 7955624 | NM_002284 | KRT86 | keratin 86 | -0.076 | 0.949 | 9.62E-02 | 9.62E+00 | | | |
| 7914648 | NM_198040 | PHC2 | polyhomeotic homolog 2 (Drosophila | -0.076 | 0.949 | 1.64E-01 | 1.64E+01 | | | |
| 8178399 | NM_007243 | NRM | nurim (nuclear envelope membrane | -0.076 | 0.949 | 2.89E-01 | 2.89E+01 | | | |
| 8179683 | NM_007243 | NRM | nurim (nuclear envelope membrane | -0.076 | 0.949 | 2.89E-01 | 2.89E+01 | | | |
| 8178095 | NM_000063 | C2 | complement component 2 | -0.076 | 0.949 | 2.96E-01 | 2.96E+01 | | | |
| 8179331 | NM_000063 | C2 | complement component 2 | -0.076 | 0.949 | 2.96E-01 | 2.96E+01 | | | |
| 7930148 | NM_178858 | SFXN2 | sideroflexin 2 | -0.076 | 0.949 | 2.51E-01 | 2.51E+01 | | | |
| 8125798 | NM_002418 | MLN | motilin | -0.076 | 0.949 | 1.22E-01 | 1.22E+01 | | | |
| 7992828 | NM_00101263 | IL32 | interleukin 32 | -0.076 | 0.949 | 6.11E-01 | 6.11E+01 | | | |
| 8103725 | --- | --- | --- | -0.076 | 0.949 | 3.71E-01 | 3.71E+01 | | | |
| 8150165 | NM_032664 | FUT10 | fucosyltransferase 10 (alpha (1,3) fuc | -0.076 | 0.949 | 2.04E-01 | 2.04E+01 | | | |
| 8073743 | NM_006953 | UPK3A | uroplakin 3A | -0.076 | 0.949 | 2.37E-01 | 2.37E+01 | | | |
| 8179709 | NM_205854 | SFTA2 | surfactant associated 2 | -0.076 | 0.949 | 2.37E-01 | 2.37E+01 | | | |
| 8127446 | NM_001851 | COL9A1 | collagen, type IX, alpha 1 | -0.076 | 0.949 | 1.15E-01 | 1.15E+01 | | | |
| 8061112 | --- | --- | --- | -0.076 | 0.949 | 2.38E-01 | 2.38E+01 | | | |
| 8050790 | NM_016544 | DNAJC27 | DnaJ (Hsp40) homolog, subfamily C, | -0.076 | 0.948 | 6.47E-01 | 6.47E+01 | | | |
| 8031499 | AF086519 | LOC284297 | hypothetical LOC284297 | -0.076 | 0.948 | 1.56E-01 | 1.56E+01 | | | |
| 7946659 | --- | --- | --- | -0.076 | 0.948 | 2.23E-01 | 2.23E+01 | | | |
| 7986947 | NR_024074 | GOLGA9P | golgi autoantigen, golgin subfamily a | -0.076 | 0.948 | 4.10E-01 | 4.10E+01 | | | |
| 7899043 | NM_020451 | SEPN1 | selenoprotein N, 1 | -0.076 | 0.948 | 2.87E-01 | 2.87E+01 | | | |
| 8039859 | NM_002255 | KIR2DL4 | killer cell immunoglobulin-like recep | -0.076 | 0.948 | 5.01E-01 | 5.01E+01 | | | |
| 7973709 | NM_004554 | NFATC4 | nuclear factor of activated T-cells, cy | -0.076 | 0.948 | 1.40E-01 | 1.40E+01 | | | |
| 8083546 | NM_003471 | KCNAB1 | potassium voltage-gated channel, sh | -0.076 | 0.948 | 1.65E-01 | 1.65E+01 | | | |
| 7941863 | --- | --- | --- | -0.076 | 0.948 | 2.42E-01 | 2.42E+01 | | | |
| 8133372 | NM_000501 | ELN | elastin | -0.076 | 0.948 | 1.08E-01 | 1.08E+01 | | | |
| 8172520 | NM_006521 | TFE3 | transcription factor binding to IGHM | -0.076 | 0.948 | 2.28E-01 | 2.28E+01 | | | |
| 7909127 | NM_181644 | MFSD4 | major facilitator superfamily domain | -0.076 | 0.948 | 1.74E-01 | 1.74E+01 | | | |
| 7941505 | NM_001323 | CST6 | cystatin E/M | -0.076 | 0.948 | 2.72E-01 | 2.72E+01 | | | |
| 8125289 | NR_001284 | TNXA | tenascin XA pseudogene | -0.076 | 0.948 | 2.97E-01 | 2.97E+01 | | | |
| 7949540 | NM_00113563 | C11orf68 | chromosome 11 open reading frame | -0.076 | 0.948 | 3.33E-01 | 3.33E+01 | | | |
| 8035201 | NM_015692 | CPAMD8 | C3 and PZP-like, alpha-2-macroglobu | -0.076 | 0.948 | 1.18E-01 | 1.18E+01 | | | |
| 8079279 | NM_003241 | TGM4 | transglutaminase 4 (prostate) | -0.076 | 0.948 | 2.46E-01 | 2.46E+01 | | | |
| 8004247 | NM_00114279 | C17orf49 | chromosome 17 open reading frame | -0.077 | 0.948 | 6.10E-01 | 6.10E+01 | | | |
| 7911591 | NM_00111078 | RP11-345P4. | similar to solute carrier family 35, m | -0.077 | 0.948 | 3.10E-01 | 3.10E+01 | | | |
| 8053429 | NM_000821 | GGCX | gamma-glutamyl carboxylase | -0.077 | 0.948 | 1.91E-01 | 1.91E+01 | | | |
| 8133219 | --- | --- | --- | -0.077 | 0.948 | 1.33E-01 | 1.33E+01 | | | |
| 8155167 | NM_00103979 | HRCT1 | histidine rich carboxyl terminus 1 | -0.077 | 0.948 | 2.88E-01 | 2.88E+01 | | | |
| 8031522 | NM_016535 | ZNF581 | zinc finger protein 581 | -0.077 | 0.948 | 2.47E-01 | 2.47E+01 | | | |
| 7990729 | NM_000750 | CHRNB4 | cholinergic receptor, nicotinic, beta 4 | -0.077 | 0.948 | 3.00E-01 | 3.00E+01 | | | |
| 7977216 | NM_015656 | KIF26A | kinesin family member 26A | -0.077 | 0.948 | 7.60E-02 | 7.60E+00 | | | |
| 7896088 | --- | --- | --- | -0.077 | 0.948 | 5.22E-01 | 5.22E+01 | | | |
| 7971899 | --- | --- | --- | -0.077 | 0.948 | 2.47E-01 | 2.47E+01 | | | |
| 8168873 | NM_019007 | ARMCX6 | armadillo repeat containing, X-linked | -0.077 | 0.948 | 8.99E-01 | 8.99E+01 | | | |
| 8027343 | AK302042 | LOC440518 | similar to Golgin subfamily A membe | -0.077 | 0.948 | 3.31E-01 | 3.31E+01 | | | |
| 7895289 | --- | --- | --- | -0.077 | 0.948 | 8.45E-01 | 8.45E+01 | | | |
| 8015230 | NM_033059 | KRTAP4-11 | keratin associated protein 4-11 | -0.077 | 0.948 | 2.67E-01 | 2.67E+01 | | | |
| 8082667 | NM_152395 | NUDT16 | nudix (nucleoside diphosphate linked | -0.077 | 0.948 | 1.78E-01 | 1.78E+01 | | | |
| 7967493 | NM_006312 | NCOR2 | nuclear receptor co-repressor 2 | -0.077 | 0.948 | 1.13E-01 | 1.13E+01 | | | |
| 8133477 | NM_016328 | GTF2IRD1 | GTF2I repeat domain containing 1 | -0.077 | 0.948 | 2.65E-01 | 2.65E+01 | | | |
| 7894373 | --- | --- | --- | -0.077 | 0.948 | 6.15E-01 | 6.15E+01 | | | |
| 7899480 | NR_002907 | SNORA73A | small nucleolar RNA, H/ACA box 73A | -0.077 | 0.948 | 9.31E-01 | 9.31E+01 | | | |
| 8101925 | NM_021970 | MAPKSP1 | MAPK scaffold protein 1 | -0.077 | 0.948 | 9.01E-01 | 9.01E+01 | | | |
| 8115927 | NM_014901 | RNF44 | ring finger protein 44 | -0.077 | 0.948 | 2.19E-01 | 2.19E+01 | | | |
| 7952384 | NM_00100447 | OR10S1 | olfactory receptor, family 10, subfam | -0.077 | 0.948 | 2.25E-01 | 2.25E+01 | | | |
| 8009913 | NR_003587 | MYO15B | myosin XVB pseudogene | -0.077 | 0.948 | 4.27E-01 | 4.27E+01 | | | |
| 8052994 | NM_015470 | RAB11FIP5 | RAB11 family interacting protein 5 (c | -0.077 | 0.948 | 1.80E-01 | 1.80E+01 | | | |
| 8141180 | NM_015395 | TECPR1 | tectonin beta-propeller repeat conta | -0.077 | 0.948 | 2.63E-01 | 2.63E+01 | | | |
| 7949383 | NM_032431 | SYVN1 | synovial apoptosis inhibitor 1, synovi | -0.077 | 0.948 | 5.77E-01 | 5.77E+01 | | | |
| 7966488 | NM_00110966 | C12orf51 | chromosome 12 open reading frame | -0.077 | 0.948 | 2.81E-01 | 2.81E+01 | | | |
| 7915351 | NM_144990 | SLFNL1 | schlafen-like 1 | -0.077 | 0.948 | 1.65E-01 | 1.65E+01 | | | |
| 7896723 | --- | --- | --- | -0.077 | 0.948 | 2.03E-01 | 2.03E+01 | | | |
| 7941380 | --- | --- | --- | -0.077 | 0.948 | 2.85E-01 | 2.85E+01 | | | |
| 7933372 | NM_004962 | GDF10 | growth differentiation factor 10 | -0.077 | 0.948 | 1.79E-01 | 1.79E+01 | | | |
| 8059731 | NM_002601 | PDE6D | phosphodiesterase 6D, cGMP-specifi | -0.077 | 0.948 | 7.83E-01 | 7.83E+01 | | | |
| 7986503 | NR_003260 | C15orf51 | dynamin 1 pseudogene | -0.077 | 0.948 | 3.24E-01 | 3.24E+01 | | | |
| 7994675 | NM_181718 | ASPHD1 | aspartate beta-hydroxylase domain c | -0.077 | 0.948 | 1.62E-01 | 1.62E+01 | | | |
| 8164995 | NM_007371 | BRD3 | bromodomain containing 3 | -0.077 | 0.948 | 1.99E-01 | 1.99E+01 | | | |
| 8080121 | NM_015926 | TEX264 | testis expressed 264 | -0.077 | 0.948 | 1.25E-01 | 1.25E+01 | | | |
| 7995161 | NM_005353 | ITGAD | integrin, alpha D | -0.077 | 0.948 | 1.41E-01 | 1.41E+01 | | | |
| 7995336 | --- | --- | --- | -0.077 | 0.948 | 3.28E-01 | 3.28E+01 | | | |
| 8049702 | NM_023083 | CAPN10 | calpain 10 | -0.077 | 0.948 | 8.30E-02 | 8.30E+00 | | | |

| | | | | | | | | | | |
|---|---|---|---|---|---|---|---|---|---|---|
| 7977655 | NM_00100473 | OR5AU1 | olfactory receptor, family 5, subfami | -0.077 | 0.948 | 2.41E-01 | 2.41E+01 | | | |
| 7956749 | NM_014254 | TMEM5 | transmembrane protein 5 | -0.077 | 0.948 | 5.77E-01 | 5.77E+01 | | | |
| 8082916 | NM_144717 | IL20RB | interleukin 20 receptor beta | -0.077 | 0.948 | 3.74E-01 | 3.74E+01 | | | |
| 8066953 | NM_006038 | SPATA2 | spermatogenesis associated 2 | -0.077 | 0.948 | 1.60E-01 | 1.60E+01 | | | |
| 7921360 | NM_00100518 | OR6Y1 | olfactory receptor, family 6, subfami | -0.077 | 0.948 | 2.68E-01 | 2.68E+01 | | | |
| 8080093 | NM_013286 | RBM15B | RNA binding motif protein 15B | -0.077 | 0.948 | 2.68E-01 | 2.68E+01 | | | |
| 7919386 | --- | --- | --- | -0.077 | 0.948 | 4.26E-01 | 4.26E+01 | | | |
| 7963280 | NM_00103387 | SMAGP | small trans-membrane and glycosyla | -0.077 | 0.948 | 5.42E-01 | 5.42E+01 | | | |
| 8170580 | NM_00108084 | CSAG2 | CSAG family, member 2 | -0.077 | 0.948 | 2.38E-01 | 2.38E+01 | | | |
| 7971177 | NM_002015 | FOXO1 | forkhead box O1 | -0.077 | 0.948 | 1.95E-01 | 1.95E+01 | | | |
| 7997735 | NM_005250 | FOXL1 | forkhead box L1 | -0.077 | 0.948 | 1.77E-01 | 1.77E+01 | | | |
| 7895095 | --- | --- | --- | -0.077 | 0.948 | 9.10E-01 | 9.10E+01 | | | |
| 8135271 | --- | --- | --- | -0.077 | 0.948 | 3.63E-01 | 3.63E+01 | | | |
| 7937936 | --- | --- | --- | -0.077 | 0.948 | 4.14E-01 | 4.14E+01 | | | |
| 8051777 | NM_004718 | COX7A2L | cytochrome c oxidase subunit VIIa po | -0.077 | 0.948 | 3.76E-01 | 3.76E+01 | | | |
| 8023855 | NM_148923 | CYB5A | cytochrome b5 type A (microsomal) | -0.077 | 0.948 | 8.58E-01 | 8.58E+01 | | | |
| 7934615 | NM_004747 | DLG5 | discs, large homolog 5 (Drosophila) | -0.077 | 0.948 | 9.84E-02 | 9.84E+00 | | | |
| 8118069 | NM_00101090 | MUC21 | mucin 21, cell surface associated | -0.077 | 0.948 | 2.45E-01 | 2.45E+01 | | | |
| 7896431 | --- | --- | --- | -0.077 | 0.948 | 6.41E-01 | 6.41E+01 | | | |
| 7982187 | NM_005503 | APBA2 | amyloid beta (A4) precursor protein- | -0.077 | 0.948 | 4.90E-01 | 4.90E+01 | | | |
| 8003861 | NM_182538 | SPNS3 | spinster homolog 3 (Drosophila) | -0.077 | 0.948 | 1.70E-01 | 1.70E+01 | | | |
| 8113658 | AK291655 | LVRN | laeverin | -0.077 | 0.948 | 2.61E-01 | 2.61E+01 | | | |
| 7951910 | NM_012104 | BACE1 | beta-site APP-cleaving enzyme 1 | -0.077 | 0.948 | 1.78E-01 | 1.78E+01 | | | |
| 8066091 | BC113405 | C20orf117 | chromosome 20 open reading frame | -0.077 | 0.948 | 1.97E-01 | 1.97E+01 | | | |
| 8068305 | NM_003024 | ITSN1 | intersectin 1 (SH3 domain protein) | -0.077 | 0.948 | 8.76E-02 | 8.76E+00 | | | |
| 7937198 | --- | --- | --- | -0.077 | 0.948 | 3.08E-01 | 3.08E+01 | | | |
| 8051113 | NM_144631 | ZNF513 | zinc finger protein 513 | -0.077 | 0.948 | 1.14E-01 | 1.14E+01 | | | |
| 8165765 | NR_026711 | NCRNA0010 | non-protein coding RNA 105 | -0.077 | 0.948 | 2.68E-01 | 2.68E+01 | | | |
| 7992447 | NM_004209 | SYNGR3 | synaptogyrin 3 | -0.077 | 0.948 | 1.99E-01 | 1.99E+01 | | | |
| 8002272 | NM_032382 | COG8 | component of oligomeric golgi comp | -0.077 | 0.948 | 2.11E-01 | 2.11E+01 | | | |
| 7960709 | NM_00114631 | SPSB2 | splA/ryanodine receptor domain and | -0.077 | 0.948 | 1.30E-01 | 1.30E+01 | | | |
| 7898377 | NM_014675 | CROCC | ciliary rootlet coiled-coil, rootletin | -0.077 | 0.948 | 1.64E-01 | 1.64E+01 | | | |
| 8165833 | NM_00101171 | ARSH | arylsulfatase family, member H | -0.077 | 0.948 | 1.99E-01 | 1.99E+01 | | | |
| 7908758 | NM_198149 | SHISA4 | shisa homolog 4 (Xenopus laevis) | -0.077 | 0.948 | 1.66E-01 | 1.66E+01 | | | |
| 8025877 | NM_022737 | LPPR2 | lipid phosphate phosphatase-related | -0.077 | 0.948 | 2.12E-01 | 2.12E+01 | | | |
| 8069863 | NM_175857 | KRTAP8-1 | keratin associated protein 8-1 | -0.077 | 0.948 | 1.72E-01 | 1.72E+01 | | | |
| 8037657 | NM_004409 | DMPK | dystrophia myotonica-protein kinase | -0.078 | 0.948 | 1.16E-01 | 1.16E+01 | | | |
| 8073309 | --- | --- | --- | -0.078 | 0.948 | 3.65E-01 | 3.65E+01 | | | |
| 8180385 | --- | --- | --- | -0.078 | 0.948 | 3.65E-01 | 3.65E+01 | | | |
| 8072229 | NM_021076 | NEFH | neurofilament, heavy polypeptide | -0.078 | 0.948 | 3.16E-01 | 3.16E+01 | | | |
| 7970395 | NM_006001 | TUBA3C | tubulin, alpha 3c | -0.078 | 0.948 | 1.82E-01 | 1.82E+01 | | | |
| 8114856 | BC130586 | PLAC8L1 | PLAC8-like 1 | -0.078 | 0.948 | 5.16E-01 | 5.16E+01 | | | |
| 7930376 | --- | --- | --- | -0.078 | 0.948 | 4.06E-01 | 4.06E+01 | | | |
| 7933129 | NR_026777 | ZNF37B | zinc finger protein 37B (pseudogene) | -0.078 | 0.948 | 4.87E-01 | 4.87E+01 | | | |
| 8117628 | --- | --- | --- | -0.078 | 0.948 | 2.06E-01 | 2.06E+01 | | | |
| 8044548 | NM_014440 | IL1F6 | interleukin 1 family, member 6 (epsil | -0.078 | 0.948 | 2.73E-01 | 2.73E+01 | | | |
| 8058997 | NM_017431 | PRKAG3 | protein kinase, AMP-activated, gamr | -0.078 | 0.948 | 2.08E-01 | 2.08E+01 | | | |
| 8096821 | NM_018983 | GAR1 | GAR1 ribonucleoprotein homolog (ye | -0.078 | 0.948 | 3.65E-01 | 3.65E+01 | | | |
| 7979741 | --- | --- | --- | -0.078 | 0.948 | 3.12E-01 | 3.12E+01 | | | |
| 8158825 | NM_032728 | PPAPDC3 | phosphatidic acid phosphatase type | -0.078 | 0.948 | 2.39E-01 | 2.39E+01 | | | |
| 7961300 | NM_006249 | PRB3 | proline-rich protein BstNI subfamily | -0.078 | 0.948 | 3.06E-01 | 3.06E+01 | | | |
| 7998637 | NM_016332 | SEPX1 | selenoprotein X, 1 | -0.078 | 0.948 | 3.71E-01 | 3.71E+01 | | | |
| 8140433 | NM_012230 | POMZP3 | POM (POM121 homolog, rat) and ZP | -0.078 | 0.948 | 5.19E-01 | 5.19E+01 | | | |
| 8009568 | NM_032646 | TTYH2 | tweety homolog 2 (Drosophila) | -0.078 | 0.948 | 1.86E-01 | 1.86E+01 | | | |
| 8060765 | NM_012409 | PRND | prion protein 2 (dublet) | -0.078 | 0.948 | 2.50E-01 | 2.50E+01 | | | |
| 7915229 | NM_014571 | HEYL | hairy/enhancer-of-split related with | -0.078 | 0.948 | 1.40E-01 | 1.40E+01 | | | |
| 8155250 | NM_012203 | GRHPR | glyoxylate reductase/hydroxypyruva | -0.078 | 0.948 | 3.57E-01 | 3.57E+01 | | | |
| 8090388 | BC032025 | C3orf22 | chromosome 3 open reading frame 2 | -0.078 | 0.948 | 1.50E-01 | 1.50E+01 | | | |
| 7913252 | NM_032409 | PINK1 | PTEN induced putative kinase 1 | -0.078 | 0.947 | 4.72E-01 | 4.72E+01 | | | |
| 7979355 | --- | --- | --- | -0.078 | 0.947 | 3.42E-01 | 3.42E+01 | | | |
| 8025179 | NM_018083 | ZNF358 | zinc finger protein 358 | -0.078 | 0.947 | 1.31E-01 | 1.31E+01 | | | |
| 8156295 | NM_024077 | SECISBP2 | SECIS binding protein 2 | -0.078 | 0.947 | 7.54E-01 | 7.54E+01 | | | |
| 7981777 | AK127783 | LOC400968 | hypothetical LOC400968 | -0.078 | 0.947 | 2.48E-01 | 2.48E+01 | | | |
| 7953450 | NM_014262 | LEPREL2 | leprecan-like 2 | -0.078 | 0.947 | 6.32E-02 | 6.32E+00 | | | |
| 8043426 | --- | --- | --- | -0.078 | 0.947 | 3.01E-01 | 3.01E+01 | | | |
| 8180358 | --- | --- | --- | -0.078 | 0.947 | 1.82E-01 | 1.82E+01 | | | |
| 8087447 | NM_020998 | MST1 | macrophage stimulating 1 (hepatocy | -0.078 | 0.947 | 5.58E-01 | 5.58E+01 | | | |
| 8055406 | NM_002299 | LCT | lactase | -0.078 | 0.947 | 2.07E-01 | 2.07E+01 | | | |
| 8090420 | NM_016372 | TPRA1 | transmembrane protein, adipocyte a | -0.078 | 0.947 | 1.68E-01 | 1.68E+01 | | | |
| 7902861 | NM_00113447 | LRRC8B | leucine rich repeat containing 8 fami | -0.078 | 0.947 | 6.26E-01 | 6.26E+01 | | | |
| 8024584 | NM_020170 | NCLN | nicalin homolog (zebrafish) | -0.078 | 0.947 | 1.97E-01 | 1.97E+01 | | | |
| 7973702 | NM_00114391 | LTB4R | leukotriene B4 receptor | -0.078 | 0.947 | 2.33E-01 | 2.33E+01 | | | |
| 8148049 | NM_002514 | NOV | nephroblastoma overexpressed gene | -0.078 | 0.947 | 1.24E-01 | 1.24E+01 | | | |
| 8180209 | --- | --- | --- | -0.078 | 0.947 | 2.22E-01 | 2.22E+01 | | | |
| 7971864 | --- | --- | --- | -0.078 | 0.947 | 4.21E-01 | 4.21E+01 | | | |
| 8094938 | NM_207330 | NIPAL1 | NIPA-like domain containing 1 | -0.078 | 0.947 | 1.51E-01 | 1.51E+01 | | | |
| 8164200 | NM_012098 | ANGPTL2 | angiopoietin-like 2 | -0.078 | 0.947 | 1.24E-01 | 1.24E+01 | | | |

| | | | | | | | | | | |
|---|---|---|---|---|---|---|---|---|---|---|
| 7982254 | --- | --- | --- | -0.078 | 0.947 | 3.12E-01 | 3.12E+01 | | | |
| 7987110 | --- | --- | --- | -0.078 | 0.947 | 3.12E-01 | 3.12E+01 | | | |
| 8071170 | NR_024593 | POM121L10P | POM121 membrane glycoprotein-lik | -0.078 | 0.947 | 1.41E-01 | 1.41E+01 | | | |
| 8015218 | NM_031960 | KRTAP4-8 | keratin associated protein 4-8 | -0.078 | 0.947 | 3.04E-01 | 3.04E+01 | | | |
| 7894432 | --- | --- | --- | -0.078 | 0.947 | 4.94E-01 | 4.94E+01 | | | |
| 8153175 | NM_031466 | TRAPPC9 | trafficking protein particle complex 9 | -0.078 | 0.947 | 5.85E-01 | 5.85E+01 | | | |
| 8025285 | NM_174918 | C19orf59 | chromosome 19 open reading frame | -0.078 | 0.947 | 2.71E-01 | 2.71E+01 | | | |
| 8110183 | NM_017675 | PCDH24 | protocadherin 24 | -0.078 | 0.947 | 2.28E-01 | 2.28E+01 | | | |
| 8019494 | BC008897 | C17orf101 | chromosome 17 open reading frame | -0.078 | 0.947 | 3.34E-01 | 3.34E+01 | | | |
| 7999008 | NM_032444 | BTBD12 | BTB (POZ) domain containing 12 | -0.078 | 0.947 | 2.15E-01 | 2.15E+01 | | | |
| 8116952 | --- | --- | --- | -0.078 | 0.947 | 8.79E-01 | 8.79E+01 | | | |
| 8131438 | --- | --- | --- | -0.078 | 0.947 | 3.96E-01 | 3.96E+01 | | | |
| 8064322 | BC134417 | C20orf96 | chromosome 20 open reading frame | -0.078 | 0.947 | 2.67E-01 | 2.67E+01 | | | |
| 7925361 | NM_006499 | LGALS8 | lectin, galactoside-binding, soluble, 8 | -0.078 | 0.947 | 1.49E-01 | 1.49E+01 | | | |
| 8123651 | NM_178012 | TUBB2B | tubulin, beta 2B | -0.078 | 0.947 | 1.77E-01 | 1.77E+01 | | | |
| 8156112 | --- | --- | --- | -0.078 | 0.947 | 3.19E-01 | 3.19E+01 | | | |
| 8033002 | NM_004793 | LONP1 | lon peptidase 1, mitochondrial | -0.078 | 0.947 | 3.44E-01 | 3.44E+01 | | | |
| 7942769 | AL834516 | KFZp434E111 | hypothetical protein DKFZp434E1119 | -0.078 | 0.947 | 3.38E-01 | 3.38E+01 | | | |
| 7895590 | --- | --- | --- | -0.078 | 0.947 | 6.23E-01 | 6.23E+01 | | | |
| 8059081 | NM_00114488 | SLC23A3 | solute carrier family 23 (nucleobase | -0.078 | 0.947 | 1.60E-01 | 1.60E+01 | | | |
| 8169465 | --- | --- | --- | -0.078 | 0.947 | 1.53E-01 | 1.53E+01 | | | |
| 7897322 | NM_153812 | PHF13 | PHD finger protein 13 | -0.078 | 0.947 | 2.93E-01 | 2.93E+01 | | | |
| 7944339 | --- | --- | --- | -0.078 | 0.947 | 4.21E-01 | 4.21E+01 | | | |
| 7898057 | NM_006474 | PDPN | podoplanin | -0.078 | 0.947 | 1.18E-01 | 1.18E+01 | | | |
| 7910257 | NM_020435 | GJC2 | gap junction protein, gamma 2, 47kD | -0.078 | 0.947 | 1.58E-01 | 1.58E+01 | | | |
| 8162601 | NM_153695 | ZNF367 | zinc finger protein 367 | -0.078 | 0.947 | 1.86E-01 | 1.86E+01 | | | |
| 8044963 | --- | --- | --- | -0.078 | 0.947 | 1.74E-01 | 1.74E+01 | | | |
| 8093878 | NM_153717 | EVC | Ellis van Creveld syndrome | -0.078 | 0.947 | 1.72E-01 | 1.72E+01 | | | |
| 7973748 | NR_026731 | C14orf23 | chromosome 14 open reading frame | -0.078 | 0.947 | 1.99E-01 | 1.99E+01 | | | |
| 7895223 | --- | --- | --- | -0.078 | 0.947 | 6.46E-01 | 6.46E+01 | | | |
| 8154392 | --- | --- | --- | -0.078 | 0.947 | 4.11E-01 | 4.11E+01 | | | |
| 8037579 | NM_202001 | ERCC1 | excision repair cross-complementing | -0.078 | 0.947 | 2.88E-01 | 2.88E+01 | | | |
| 7895768 | --- | --- | --- | -0.078 | 0.947 | 8.86E-01 | 8.86E+01 | | | |
| 8179713 | NM_014070 | C6orf15 | chromosome 6 open reading frame 1 | -0.078 | 0.947 | 1.48E-01 | 1.48E+01 | | | |
| 8148548 | NM_005672 | PSCA | prostate stem cell antigen | -0.078 | 0.947 | 2.22E-01 | 2.22E+01 | | | |
| 7997556 | NM_178452 | LRRC50 | leucine rich repeat containing 50 | -0.078 | 0.947 | 1.97E-01 | 1.97E+01 | | | |
| 8075118 | NM_001887 | CRYBB1 | crystallin, beta B1 | -0.078 | 0.947 | 9.54E-02 | 9.54E+00 | | | |
| 8116998 | NM_004973 | JARID2 | jumonji, AT rich interactive domain 2 | -0.079 | 0.947 | 6.54E-01 | 6.54E+01 | | | |
| 8180252 | --- | --- | --- | -0.079 | 0.947 | 1.63E-01 | 1.63E+01 | | | |
| 8101757 | NM_198281 | GPRIN3 | GPRIN family member 3 | -0.079 | 0.947 | 3.48E-01 | 3.48E+01 | | | |
| 7911676 | --- | --- | --- | -0.079 | 0.947 | 1.80E-01 | 1.80E+01 | | | |
| 8072962 | NM_033386 | MICALL1 | MICAL-like 1 | -0.079 | 0.947 | 7.87E-02 | 7.87E+00 | | | |
| 8174193 | --- | --- | --- | -0.079 | 0.947 | 5.44E-01 | 5.44E+01 | | | |
| 8035737 | NM_020410 | ATP13A1 | ATPase type 13A1 | -0.079 | 0.947 | 1.54E-01 | 1.54E+01 | | | |
| 7902634 | NM_025065 | BXDC5 | brix domain containing 5 | -0.079 | 0.947 | 8.96E-01 | 8.96E+01 | | | |
| 7919606 | NM_00102459 | HIST2H2BF | histone cluster 2, H2bf | -0.079 | 0.947 | 7.49E-01 | 7.49E+01 | | | |
| 7999435 | NM_002762 | PRM2 | protamine 2 | -0.079 | 0.947 | 1.99E-01 | 1.99E+01 | | | |
| 7949619 | NM_001532 | SLC29A2 | solute carrier family 29 (nucleoside t | -0.079 | 0.947 | 2.08E-01 | 2.08E+01 | | | |
| 7935588 | NM_000195 | HPS1 | Hermansky-Pudlak syndrome 1 | -0.079 | 0.947 | 1.68E-01 | 1.68E+01 | | | |
| 8058273 | NM_033066 | MPP4 | membrane protein, palmitoylated 4 | -0.079 | 0.947 | 3.09E-01 | 3.09E+01 | | | |
| 8117243 | NM_017640 | LRRC16A | leucine rich repeat containing 16A | -0.079 | 0.947 | 3.56E-01 | 3.56E+01 | | | |
| 7950644 | NM_004549 | NDUFC2 | NADH dehydrogenase (ubiquinone) 1 | -0.079 | 0.947 | 8.97E-01 | 8.97E+01 | | | |
| 8066266 | NM_005461 | MAFB | v-maf musculoaponeurotic fibrosarc | -0.079 | 0.947 | 4.23E-01 | 4.23E+01 | | | |
| 8015665 | NM_024927 | PLEKHH3 | pleckstrin homology domain contain | -0.079 | 0.947 | 1.99E-01 | 1.99E+01 | | | |
| 7919051 | NM_00108047 | ZNF697 | zinc finger protein 697 | -0.079 | 0.947 | 1.74E-01 | 1.74E+01 | | | |
| 7994350 | --- | --- | --- | -0.079 | 0.947 | 1.08E-01 | 1.08E+01 | | | |
| 8107646 | NM_00113623 | PRDM6 | PR domain containing 6 | -0.079 | 0.947 | 2.88E-01 | 2.88E+01 | | | |
| 7998983 | NM_178844 | NLRC3 | NLR family, CARD domain containing | -0.079 | 0.947 | 2.19E-01 | 2.19E+01 | | | |
| 7970439 | NM_021954 | GJA3 | gap junction protein, alpha 3, 46kDa | -0.079 | 0.947 | 1.48E-01 | 1.48E+01 | | | |
| 8022380 | NM_024899 | CEP76 | centrosomal protein 76kDa | -0.079 | 0.947 | 4.79E-01 | 4.79E+01 | | | |
| 8025041 | NM_139161 | CRB3 | crumbs homolog 3 (Drosophila) | -0.079 | 0.947 | 1.73E-01 | 1.73E+01 | | | |
| 8154872 | NM_016525 | UBAP1 | ubiquitin associated protein 1 | -0.079 | 0.947 | 7.47E-01 | 7.47E+01 | | | |
| 8177931 | NM_00101090 | MUC21 | mucin 21, cell surface associated | -0.079 | 0.947 | 2.20E-01 | 2.20E+01 | | | |
| 8118324 | NM_000063 | C2 | complement component 2 | -0.079 | 0.947 | 2.62E-01 | 2.62E+01 | | | |
| 8028389 | NM_00104252 | SPRED3 | sprouty-related, EVH1 domain conta | -0.079 | 0.947 | 1.83E-01 | 1.83E+01 | | | |
| 8035566 | NM_004838 | HOMER3 | homer homolog 3 (Drosophila) | -0.079 | 0.947 | 1.54E-01 | 1.54E+01 | | | |
| 7932612 | --- | --- | --- | -0.079 | 0.947 | 7.31E-01 | 7.31E+01 | | | |
| 8023212 | NM_00103936 | ZBTB7C | zinc finger and BTB domain containin | -0.079 | 0.947 | 1.11E-01 | 1.11E+01 | | | |
| 7942877 | --- | --- | --- | -0.079 | 0.947 | 7.61E-01 | 7.61E+01 | | | |
| 8015221 | NM_033059 | KRTAP4-11 | keratin associated protein 4-11 | -0.079 | 0.947 | 3.40E-01 | 3.40E+01 | | | |
| 8055404 | NM_014607 | UBXN4 | UBX domain protein 4 | -0.079 | 0.947 | 3.49E-01 | 3.49E+01 | | | |
| 7980352 | NM_021257 | NGB | neuroglobin | -0.079 | 0.947 | 1.93E-01 | 1.93E+01 | | | |
| 7924823 | NM_023007 | JMJD4 | jumonji domain containing 4 | -0.079 | 0.947 | 1.93E-01 | 1.93E+01 | | | |
| 8090852 | NM_016201 | AMOTL2 | angiomotin like 2 | -0.079 | 0.947 | 1.17E-01 | 1.17E+01 | | | |
| 8025850 | NM_020812 | DOCK6 | dedicator of cytokinesis 6 | -0.079 | 0.947 | 1.29E-01 | 1.29E+01 | | | |
| 7895379 | --- | --- | --- | -0.079 | 0.947 | 8.12E-01 | 8.12E+01 | | | |
| 8085946 | NM_005442 | EOMES | eomesodermin homolog (Xenopus la | -0.079 | 0.947 | 1.10E-01 | 1.10E+01 | | | |

| | | | | | | | | | | |
|---|---|---|---|---|---|---|---|---|---|---|
| 8158839 | NM_013318 | BAT2L | HLA-B associated transcript 2-like | -0.079 | 0.947 | 6.00E-01 | 6.00E+01 | | | |
| 8095135 | --- | --- | --- | -0.079 | 0.947 | 5.64E-01 | 5.64E+01 | | | |
| 8090349 | --- | --- | --- | -0.079 | 0.947 | 1.80E-01 | 1.80E+01 | | | |
| 7955107 | NM_00101363 | C12orf68 | chromosome 12 open reading frame | -0.079 | 0.947 | 1.90E-01 | 1.90E+01 | | | |
| 8000117 | NM_001888 | CRYM | crystallin, mu | -0.079 | 0.947 | 2.38E-01 | 2.38E+01 | | | |
| 7942693 | NM_138706 | B3GNT6 | UDP-GlcNAc:betaGal beta-1,3-N-ace | -0.079 | 0.947 | 8.30E-02 | 8.30E+00 | | | |
| 8076365 | NM_015704 | PPPDE2 | PPPDE peptidase domain containing | -0.079 | 0.947 | 5.61E-01 | 5.61E+01 | | | |
| 8121365 | NM_001455 | FOXO3 | forkhead box O3 | -0.079 | 0.947 | 3.02E-01 | 3.02E+01 | | | |
| 7991070 | NM_016073 | HDGFRP3 | hepatoma-derived growth factor, rel | -0.079 | 0.947 | 5.78E-01 | 5.78E+01 | | | |
| 8079517 | NM_025010 | KLHL18 | kelch-like 18 (Drosophila) | -0.079 | 0.947 | 2.10E-01 | 2.10E+01 | | | |
| 8059339 | --- | --- | --- | -0.079 | 0.947 | 3.14E-01 | 3.14E+01 | | | |
| 7940851 | NM_013280 | FLRT1 | fibronectin leucine rich transmembra | -0.079 | 0.947 | 2.13E-01 | 2.13E+01 | | | |
| 8078305 | NM_052953 | LRRC3B | leucine rich repeat containing 3B | -0.079 | 0.947 | 1.60E-01 | 1.60E+01 | | | |
| 8032275 | NM_003926 | MBD3 | methyl-CpG binding domain protein | -0.079 | 0.947 | 1.27E-01 | 1.27E+01 | | | |
| 8016699 | NM_016504 | MRPL27 | mitochondrial ribosomal protein L27 | -0.079 | 0.947 | 3.73E-01 | 3.73E+01 | | | |
| 8131919 | NM_000905 | NPY | neuropeptide Y | -0.079 | 0.947 | 1.75E-01 | 1.75E+01 | | | |
| 7934966 | --- | --- | --- | -0.079 | 0.947 | 1.40E-01 | 1.40E+01 | | | |
| 8143518 | NM_019841 | TRPV5 | transient receptor potential cation c | -0.079 | 0.947 | 1.85E-01 | 1.85E+01 | | | |
| 8152815 | BC137383 | TMEM75 | transmembrane protein 75 | -0.079 | 0.947 | 3.07E-01 | 3.07E+01 | | | |
| 7997139 | NM_001740 | CALB2 | calbindin 2 | -0.079 | 0.947 | 2.04E-01 | 2.04E+01 | | | |
| 7963851 | NM_00109881 | KIAA0748 | KIAA0748 | -0.079 | 0.947 | 8.08E-01 | 8.08E+01 | | | |
| 7894597 | --- | --- | --- | -0.079 | 0.946 | 5.97E-01 | 5.97E+01 | | | |
| 8086876 | --- | --- | --- | -0.079 | 0.946 | 4.17E-01 | 4.17E+01 | | | |
| 7893585 | --- | --- | --- | -0.079 | 0.946 | 4.78E-01 | 4.78E+01 | | | |
| 8019669 | NM_000160 | GCGR | glucagon receptor | -0.079 | 0.946 | 8.16E-02 | 8.16E+00 | | | |
| 8118509 | NM_005155 | PPT2 | palmitoyl-protein thioesterase 2 | -0.079 | 0.946 | 1.00E-01 | 1.00E+01 | | | |
| 8049888 | NM_013325 | ATG4B | ATG4 autophagy related 4 homolog | -0.079 | 0.946 | 3.97E-01 | 3.97E+01 | | | |
| 8092888 | NM_138399 | TMEM44 | transmembrane protein 44 | -0.079 | 0.946 | 1.20E-01 | 1.20E+01 | | | |
| 8001830 | NM_181521 | CMTM4 | CKLF-like MARVEL transmembrane d | -0.079 | 0.946 | 1.60E-01 | 1.60E+01 | | | |
| 8023059 | --- | --- | --- | -0.080 | 0.946 | 2.29E-01 | 2.29E+01 | | | |
| 8032718 | NM_001348 | DAPK3 | death-associated protein kinase 3 | -0.080 | 0.946 | 2.92E-01 | 2.92E+01 | | | |
| 7892925 | --- | --- | --- | -0.080 | 0.946 | 2.67E-01 | 2.67E+01 | | | |
| 8087885 | NM_004656 | BAP1 | BRCA1 associated protein-1 (ubiquiti | -0.080 | 0.946 | 5.70E-01 | 5.70E+01 | | | |
| 7893435 | --- | --- | --- | -0.080 | 0.946 | 4.97E-01 | 4.97E+01 | | | |
| 7955331 | NM_003076 | SMARCD1 | SWI/SNF related, matrix associated, | -0.080 | 0.946 | 2.65E-01 | 2.65E+01 | | | |
| 8048350 | NM_032726 | PLCD4 | phospholipase C, delta 4 | -0.080 | 0.946 | 2.09E-01 | 2.09E+01 | | | |
| 8118127 | NM_005007 | NFKBIL1 | nuclear factor of kappa light polypep | -0.080 | 0.946 | 2.20E-01 | 2.20E+01 | | | |
| 8177967 | NM_005007 | NFKBIL1 | nuclear factor of kappa light polypep | -0.080 | 0.946 | 2.20E-01 | 2.20E+01 | | | |
| 8179249 | NM_005007 | NFKBIL1 | nuclear factor of kappa light polypep | -0.080 | 0.946 | 2.20E-01 | 2.20E+01 | | | |
| 8011407 | NM_014604 | TAX1BP3 | Tax1 (human T-cell leukemia virus ty | -0.080 | 0.946 | 2.98E-01 | 2.98E+01 | | | |
| 7950078 | NM_00100540 | KRTAP5-11 | keratin associated protein 5-11 | -0.080 | 0.946 | 1.45E-01 | 1.45E+01 | | | |
| 7899018 | NM_018202 | TMEM57 | transmembrane protein 57 | -0.080 | 0.946 | 6.66E-01 | 6.66E+01 | | | |
| 7896132 | --- | --- | --- | -0.080 | 0.946 | 3.28E-01 | 3.28E+01 | | | |
| 8163109 | NM_014334 | C9orf4 | chromosome 9 open reading frame 4 | -0.080 | 0.946 | 1.97E-01 | 1.97E+01 | | | |
| 7894613 | --- | --- | --- | -0.080 | 0.946 | 3.70E-01 | 3.70E+01 | | | |
| 8071136 | NM_00112764 | PEX26 | peroxisomal biogenesis factor 26 | -0.080 | 0.946 | 1.36E-01 | 1.36E+01 | | | |
| 7992996 | AY358225 | UNQ3118 | GRTR3118 | -0.080 | 0.946 | 7.57E-01 | 7.57E+01 | | | |
| 8126531 | NM_206922 | CRIP3 | cysteine-rich protein 3 | -0.080 | 0.946 | 3.42E-01 | 3.42E+01 | | | |
| 8086317 | NM_031899 | GORASP1 | golgi reassembly stacking protein 1, | -0.080 | 0.946 | 9.90E-02 | 9.90E+00 | | | |
| 7995346 | --- | --- | --- | -0.080 | 0.946 | 2.97E-01 | 2.97E+01 | | | |
| 8027074 | NM_002911 | UPF1 | UPF1 regulator of nonsense transcrip | -0.080 | 0.946 | 2.69E-01 | 2.69E+01 | | | |
| 8044915 | --- | --- | --- | -0.080 | 0.946 | 5.56E-01 | 5.56E+01 | | | |
| 8070789 | NM_198698 | KRTAP12-4 | keratin associated protein 12-4 | -0.080 | 0.946 | 1.71E-01 | 1.71E+01 | | | |
| 8051583 | NM_000104 | CYP1B1 | cytochrome P450, family 1, subfamil | -0.080 | 0.946 | 4.81E-01 | 4.81E+01 | | | |
| 8040792 | NM_007046 | EMILIN1 | elastin microfibril interfacer 1 | -0.080 | 0.946 | 2.01E-01 | 2.01E+01 | | | |
| 8143296 | NM_198508 | KLRG2 | killer cell lectin-like receptor subfam | -0.080 | 0.946 | 1.74E-01 | 1.74E+01 | | | |
| 8006440 | NM_006273 | CCL7 | chemokine (C-C motif) ligand 7 | -0.080 | 0.946 | 2.46E-01 | 2.46E+01 | | | |
| 7965573 | NM_021229 | NTN4 | netrin 4 | -0.080 | 0.946 | 2.26E-02 | 2.26E+00 | | | |
| 8010030 | NM_003857 | GALR2 | galanin receptor 2 | -0.080 | 0.946 | 1.64E-01 | 1.64E+01 | | | |
| 8018196 | NM_139018 | CD300LF | CD300 molecule-like family member | -0.080 | 0.946 | 4.14E-01 | 4.14E+01 | | | |
| 7947815 | NM_001610 | ACP2 | acid phosphatase 2, lysosomal | -0.080 | 0.946 | 3.10E-01 | 3.10E+01 | | | |
| 8014768 | NM_020405 | PLXDC1 | plexin domain containing 1 | -0.080 | 0.946 | 1.99E-01 | 1.99E+01 | | | |
| 8035254 | NM_031941 | USHBP1 | Usher syndrome 1C binding protein | -0.080 | 0.946 | 1.22E-01 | 1.22E+01 | | | |
| 8033667 | NM_144693 | ZNF558 | zinc finger protein 558 | -0.080 | 0.946 | 5.82E-01 | 5.82E+01 | | | |
| 8106403 | NM_005242 | F2RL1 | coagulation factor II (thrombin) rece | -0.080 | 0.946 | 1.81E-01 | 1.81E+01 | | | |
| 8039511 | NM_024710 | ISOC2 | isochorismatase domain containing | -0.080 | 0.946 | 1.44E-01 | 1.44E+01 | | | |
| 8019570 | NM_033059 | KRTAP4-11 | keratin associated protein 4-11 | -0.080 | 0.946 | 1.64E-01 | 1.64E+01 | | | |
| 8158242 | NM_030914 | URM1 | ubiquitin related modifier 1 homolog | -0.080 | 0.946 | 3.25E-01 | 3.25E+01 | | | |
| 8115346 | NM_004045 | ATOX1 | ATX1 antioxidant protein 1 homolog | -0.080 | 0.946 | 3.60E-01 | 3.60E+01 | | | |
| 8106098 | NM_005909 | MAP1B | microtubule-associated protein 1B | -0.080 | 0.946 | 1.64E-01 | 1.64E+01 | | | |
| 7944275 | NM_032780 | TMEM25 | transmembrane protein 25 | -0.080 | 0.946 | 2.15E-01 | 2.15E+01 | | | |
| 7992895 | NM_017810 | ZNF434 | zinc finger protein 434 | -0.080 | 0.946 | 6.89E-01 | 6.89E+01 | | | |
| 8032770 | NM_016539 | SIRT6 | sirtuin (silent mating type informatio | -0.080 | 0.946 | 1.39E-01 | 1.39E+01 | | | |
| 7997976 | AK303024 | C16orf55 | chromosome 16 open reading frame | -0.080 | 0.946 | 2.44E-01 | 2.44E+01 | | | |
| 8137805 | NM_003550 | MAD1L1 | MAD1 mitotic arrest deficient-like 1 | -0.080 | 0.946 | 1.02E-01 | 1.02E+01 | | | |
| 8091696 | --- | --- | --- | -0.080 | 0.946 | 2.29E-01 | 2.29E+01 | | | |
| 7954469 | NM_007211 | RASSF8 | Ras association (RalGDS/AF-6) doma | -0.080 | 0.946 | 1.16E-01 | 1.16E+01 | | | |

| | | | | | | | | | | |
|---|---|---|---|---|---|---|---|---|---|---|
| 8072784 | NM_024681 | KCTD17 | potassium channel tetramerisation d | -0.080 | 0.946 | 1.11E-01 | 1.11E+01 | | | |
| 7920264 | NM_002962 | S100A5 | S100 calcium binding protein A5 | -0.080 | 0.946 | 3.84E-01 | 3.84E+01 | | | |
| 8087116 | NM_00100826 | TMEM89 | transmembrane protein 89 | -0.080 | 0.946 | 1.80E-01 | 1.80E+01 | | | |
| 8068200 | NR_026845 | C21orf119 | chromosome 21 open reading frame | -0.080 | 0.946 | 2.30E-01 | 2.30E+01 | | | |
| 8172453 | NM_005834 | TIMM17B | translocase of inner mitochondrial m | -0.080 | 0.946 | 2.51E-01 | 2.51E+01 | | | |
| 7938748 | --- | --- | --- | -0.080 | 0.946 | 3.37E-01 | 3.37E+01 | | | |
| 8080226 | NM_015512 | DNAH1 | dynein, axonemal, heavy chain 1 | -0.080 | 0.946 | 5.25E-02 | 5.25E+00 | | | |
| 8044450 | NM_198581 | ZC3H6 | zinc finger CCCH-type containing 6 | -0.080 | 0.946 | 6.48E-01 | 6.48E+01 | | | |
| 8055992 | NM_145259 | ACVR1C | activin A receptor, type IC | -0.080 | 0.946 | 1.75E-01 | 1.75E+01 | | | |
| 8129231 | NM_024581 | FAM184A | family with sequence similarity 184, | -0.080 | 0.946 | 1.53E-01 | 1.53E+01 | | | |
| 8164848 | --- | --- | --- | -0.080 | 0.946 | 3.92E-01 | 3.92E+01 | | | |
| 8037647 | NM_00108046 | FBXO46 | F-box protein 46 | -0.080 | 0.946 | 1.57E-01 | 1.57E+01 | | | |
| 7926385 | NM_003473 | STAM | signal transducing adaptor molecule | -0.080 | 0.946 | 8.07E-01 | 8.07E+01 | | | |
| 7910385 | --- | --- | --- | -0.080 | 0.946 | 5.06E-01 | 5.06E+01 | | | |
| 8003607 | NM_00101367 | C17orf97 | chromosome 17 open reading frame | -0.080 | 0.946 | 1.80E-01 | 1.80E+01 | | | |
| 8171180 | --- | --- | --- | -0.080 | 0.946 | 4.52E-01 | 4.52E+01 | | | |
| 8043632 | NM_00111338 | FER1L5 | fer-1-like 5 (C. elegans) | -0.080 | 0.946 | 2.03E-01 | 2.03E+01 | | | |
| 8144949 | --- | --- | --- | -0.081 | 0.946 | 4.03E-01 | 4.03E+01 | | | |
| 7896359 | --- | --- | --- | -0.081 | 0.946 | 5.78E-01 | 5.78E+01 | | | |
| 8153334 | NM_005672 | PSCA | prostate stem cell antigen | -0.081 | 0.946 | 1.46E-01 | 1.46E+01 | | | |
| 8136863 | NM_153345 | TMEM139 | transmembrane protein 139 | -0.081 | 0.946 | 2.65E-01 | 2.65E+01 | | | |
| 8031857 | NM_003436 | ZNF135 | zinc finger protein 135 | -0.081 | 0.946 | 2.09E-01 | 2.09E+01 | | | |
| 8036232 | NM_00103987 | C19orf46 | chromosome 19 open reading frame | -0.081 | 0.946 | 1.67E-01 | 1.67E+01 | | | |
| 7924868 | NM_145214 | TRIM11 | tripartite motif-containing 11 | -0.081 | 0.946 | 1.62E-01 | 1.62E+01 | | | |
| 8137202 | NM_00109922 | ZNF862 | zinc finger protein 862 | -0.081 | 0.946 | 4.12E-01 | 4.12E+01 | | | |
| 7937900 | NM_003156 | STIM1 | stromal interaction molecule 1 | -0.081 | 0.946 | 6.94E-01 | 6.94E+01 | | | |
| 8165038 | NM_00101241 | SOHLH1 | spermatogenesis and oogenesis spec | -0.081 | 0.946 | 1.64E-01 | 1.64E+01 | | | |
| 7934783 | NM_015613 | LRIT1 | leucine-rich repeat, immunoglobulin | -0.081 | 0.946 | 1.69E-01 | 1.69E+01 | | | |
| 7988987 | NM_004498 | ONECUT1 | one cut homeobox 1 | -0.081 | 0.946 | 1.07E-01 | 1.07E+01 | | | |
| 8148615 | NM_030895 | ZNF696 | zinc finger protein 696 | -0.081 | 0.946 | 1.54E-01 | 1.54E+01 | | | |
| 8040949 | NM_173853 | KRTCAP3 | keratinocyte associated protein 3 | -0.081 | 0.946 | 2.50E-01 | 2.50E+01 | | | |
| 7943890 | ENST0000041 | OC10013268 | hypothetical LOC100132686 | -0.081 | 0.946 | 2.78E-01 | 2.78E+01 | | | |
| 8153678 | NM_015201 | BOP1 | block of proliferation 1 | -0.081 | 0.946 | 3.41E-01 | 3.41E+01 | | | |
| 7934653 | NM_007055 | POLR3A | polymerase (RNA) III (DNA directed) | -0.081 | 0.946 | 4.38E-01 | 4.38E+01 | | | |
| 8040221 | AF504647 | OC10013150 | hypothetical LOC100131506 | -0.081 | 0.946 | 3.30E-01 | 3.30E+01 | | | |
| 8158627 | NM_014286 | FREQ | frequenin homolog (Drosophila) | -0.081 | 0.946 | 1.78E-01 | 1.78E+01 | | | |
| 8094122 | NM_00110566 | USP17 | ubiquitin specific peptidase 17 | -0.081 | 0.946 | 1.79E-01 | 1.79E+01 | | | |
| 7922299 | --- | --- | --- | -0.081 | 0.946 | 6.44E-01 | 6.44E+01 | | | |
| 8166569 | --- | --- | --- | -0.081 | 0.946 | 1.67E-01 | 1.67E+01 | | | |
| 8032815 | NM_025241 | UBXN6 | UBX domain protein 6 | -0.081 | 0.946 | 1.99E-01 | 1.99E+01 | | | |
| 7997733 | NM_005251 | FOXC2 | forkhead box C2 (MFH-1, mesenchyr | -0.081 | 0.946 | 1.06E-01 | 1.06E+01 | | | |
| 7929315 | NM_183374 | CYP26C1 | cytochrome P450, family 26, subfam | -0.081 | 0.946 | 1.20E-01 | 1.20E+01 | | | |
| 7991313 | NM_002666 | PLIN1 | perilipin 1 | -0.081 | 0.946 | 1.39E-01 | 1.39E+01 | | | |
| 8168958 | NM_022838 | ARMCX5 | armadillo repeat containing, X-linked | -0.081 | 0.946 | 2.11E-01 | 2.11E+01 | | | |
| 7956018 | NM_002905 | RDH5 | retinol dehydrogenase 5 (11-cis/9-cis | -0.081 | 0.946 | 1.48E-01 | 1.48E+01 | | | |
| 7970737 | NM_004119 | FLT3 | fms-related tyrosine kinase 3 | -0.081 | 0.946 | 2.43E-01 | 2.43E+01 | | | |
| 8032899 | NM_182919 | TICAM1 | toll-like receptor adaptor molecule 1 | -0.081 | 0.946 | 1.70E-01 | 1.70E+01 | | | |
| 7918394 | NM_139053 | EPS8L3 | EPS8-like 3 | -0.081 | 0.946 | 7.64E-02 | 7.64E+00 | | | |
| 8045674 | NM_194317 | LYPD6 | LY6/PLAUR domain containing 6 | -0.081 | 0.945 | 1.59E-01 | 1.59E+01 | | | |
| 7971243 | --- | --- | --- | -0.081 | 0.945 | 1.72E-01 | 1.72E+01 | | | |
| 8072636 | NM_00100849 | ISX | intestine-specific homeobox | -0.081 | 0.945 | 1.97E-01 | 1.97E+01 | | | |
| 8149465 | NM_019851 | FGF20 | fibroblast growth factor 20 | -0.081 | 0.945 | 6.61E-02 | 6.61E+00 | | | |
| 8096077 | --- | --- | --- | -0.081 | 0.945 | 7.18E-01 | 7.18E+01 | | | |
| 8049187 | NM_025202 | EFHD1 | EF-hand domain family, member D1 | -0.081 | 0.945 | 2.25E-01 | 2.25E+01 | | | |
| 8035648 | NM_00100152 | TM6SF2 | transmembrane 6 superfamily memb | -0.081 | 0.945 | 1.31E-01 | 1.31E+01 | | | |
| 7989670 | NM_194272 | RBPMS2 | RNA binding protein with multiple sp | -0.081 | 0.945 | 1.81E-01 | 1.81E+01 | | | |
| 7979984 | NM_021260 | ZFYVE1 | zinc finger, FYVE domain containing | -0.081 | 0.945 | 3.06E-01 | 3.06E+01 | | | |
| 8027213 | NM_198537 | YJEFN3 | YjeF N-terminal domain containing 3 | -0.081 | 0.945 | 8.82E-02 | 8.82E+00 | | | |
| 7960088 | NM_00114264 | FBRSL1 | fibrosin-like 1 | -0.081 | 0.945 | 2.16E-01 | 2.16E+01 | | | |
| 8010832 | NM_024619 | FN3KRP | fructosamine 3 kinase related protei | -0.081 | 0.945 | 7.80E-01 | 7.80E+01 | | | |
| 8010328 | NM_017950 | CCDC40 | coiled-coil domain containing 40 | -0.081 | 0.945 | 9.13E-02 | 9.13E+00 | | | |
| 7990400 | NM_00109943 | ULK3 | unc-51-like kinase 3 (C. elegans) | -0.081 | 0.945 | 1.85E-01 | 1.85E+01 | | | |
| 8175775 | NM_004988 | MAGEA1 | melanoma antigen family A, 1 (direct | -0.081 | 0.945 | 1.42E-01 | 1.42E+01 | | | |
| 8139796 | AK128010 | LOC441233 | hypothetical LOC441233 | -0.081 | 0.945 | 5.60E-01 | 5.60E+01 | | | |
| 7941662 | NM_001104 | ACTN3 | actinin, alpha 3 | -0.081 | 0.945 | 2.68E-01 | 2.68E+01 | | | |
| 8161533 | NM_00108547 | FOXD4L6 | forkhead box D4-like 6 | -0.081 | 0.945 | 3.23E-02 | 3.23E+00 | | | |
| 7982228 | --- | --- | --- | -0.081 | 0.945 | 5.86E-01 | 5.86E+01 | | | |
| 7982250 | --- | --- | --- | -0.081 | 0.945 | 5.86E-01 | 5.86E+01 | | | |
| 7987112 | --- | --- | --- | -0.081 | 0.945 | 5.86E-01 | 5.86E+01 | | | |
| 7996675 | NM_020457 | THAP11 | THAP domain containing 11 | -0.081 | 0.945 | 2.48E-01 | 2.48E+01 | | | |
| 7893824 | --- | --- | --- | -0.081 | 0.945 | 6.46E-01 | 6.46E+01 | | | |
| 8085138 | NM_000916 | OXTR | oxytocin receptor | -0.081 | 0.945 | 1.67E-01 | 1.67E+01 | | | |
| 8021154 | NM_00114239 | KIAA0427 | KIAA0427 | -0.081 | 0.945 | 1.25E-01 | 1.25E+01 | | | |
| 7923183 | NM_012482 | ZNF281 | zinc finger protein 281 | -0.081 | 0.945 | 2.57E-01 | 2.57E+01 | | | |
| 8132151 | NM_001118 | ADCYAP1R1 | adenylate cyclase activating polypep | -0.081 | 0.945 | 1.32E-01 | 1.32E+01 | | | |
| 8110932 | NM_003966 | SEMA5A | sema domain, seven thrombospondi | -0.081 | 0.945 | 1.32E-01 | 1.32E+01 | | | |
| 8012028 | NM_080912 | ASGR2 | asialoglycoprotein receptor 2 | -0.081 | 0.945 | 2.36E-01 | 2.36E+01 | | | |

| | | | | | | | | | | |
|---|---|---|---|---|---|---|---|---|---|---|
| 7997158 | NM_00101796 | MARVELD3 | MARVEL domain containing 3 | -0.081 | 0.945 | 2.28E-01 | 2.28E+01 | | | |
| 8121685 | NM_173674 | DCBLD1 | discoidin, CUB and LCCL domain cont | -0.081 | 0.945 | 1.54E-01 | 1.54E+01 | | | |
| 7929664 | NM_138413 | DHDPSL | dihydrodipicolinate synthase-like, m | -0.081 | 0.945 | 1.66E-01 | 1.66E+01 | | | |
| 7941350 | NM_032223 | PCNXL3 | pecanex-like 3 (Drosophila) | -0.081 | 0.945 | 2.75E-01 | 2.75E+01 | | | |
| 7907962 | --- | --- | --- | -0.081 | 0.945 | 4.01E-01 | 4.01E+01 | | | |
| 7895643 | --- | --- | --- | -0.081 | 0.945 | 7.72E-01 | 7.72E+01 | | | |
| 7945521 | NM_182612 | PDDC1 | Parkinson disease 7 domain containi | -0.081 | 0.945 | 1.38E-01 | 1.38E+01 | | | |
| 7895345 | --- | --- | --- | -0.081 | 0.945 | 9.41E-01 | 9.41E+01 | | | |
| 8173106 | NM_198510 | ITIH5L | inter-alpha (globulin) inhibitor H5-lik | -0.081 | 0.945 | 1.56E-01 | 1.56E+01 | | | |
| 8038278 | NM_000894 | LHB | luteinizing hormone beta polypeptid | -0.081 | 0.945 | 2.74E-01 | 2.74E+01 | | | |
| 8051050 | NM_003459 | SLC30A3 | solute carrier family 30 (zinc transpo | -0.081 | 0.945 | 2.35E-01 | 2.35E+01 | | | |
| 8158890 | NM_032536 | NTNG2 | netrin G2 | -0.081 | 0.945 | 1.80E-01 | 1.80E+01 | | | |
| 8104321 | NM_139056 | ADAMTS16 | ADAM metallopeptidase with throm | -0.081 | 0.945 | 1.60E-01 | 1.60E+01 | | | |
| 7976407 | BC009073 | FAM181A | family with sequence similarity 181, | -0.081 | 0.945 | 2.01E-01 | 2.01E+01 | | | |
| 8025659 | NM_032885 | ATG4D | ATG4 autophagy related 4 homolog | -0.081 | 0.945 | 1.83E-01 | 1.83E+01 | | | |
| 7942793 | NM_003251 | THRSP | thyroid hormone responsive (SPOT1 | -0.082 | 0.945 | 2.02E-01 | 2.02E+01 | | | |
| 8035435 | NM_00114530 | KIAA1683 | KIAA1683 | -0.082 | 0.945 | 1.17E-01 | 1.17E+01 | | | |
| 8015232 | NM_033188 | KRTAP4-5 | keratin associated protein 4-5 | -0.082 | 0.945 | 3.30E-01 | 3.30E+01 | | | |
| 8064203 | NM_080622 | C20orf135 | chromosome 20 open reading frame | -0.082 | 0.945 | 1.78E-01 | 1.78E+01 | | | |
| 7914923 | NM_145047 | OSCP1 | organic solute carrier partner 1 | -0.082 | 0.945 | 1.49E-01 | 1.49E+01 | | | |
| 8092661 | NM_001879 | MASP1 | mannan-binding lectin serine peptid | -0.082 | 0.945 | 1.27E-01 | 1.27E+01 | | | |
| 7893146 | --- | --- | --- | -0.082 | 0.945 | 5.98E-01 | 5.98E+01 | | | |
| 8159808 | NM_207305 | FOXD4 | forkhead box D4 | -0.082 | 0.945 | 5.20E-02 | 5.20E+00 | | | |
| 8109629 | NM_00104044 | FABP6 | fatty acid binding protein 6, ileal | -0.082 | 0.945 | 2.76E-01 | 2.76E+01 | | | |
| 8137330 | ENST0000035 | ABCB8 | ATP-binding cassette, sub-family B ( | -0.082 | 0.945 | 2.01E-01 | 2.01E+01 | | | |
| 8118696 | NM_006772 | SYNGAP1 | synaptic Ras GTPase activating prote | -0.082 | 0.945 | 3.81E-02 | 3.81E+00 | | | |
| 8104253 | NM_033120 | NKD2 | naked cuticle homolog 2 (Drosophila | -0.082 | 0.945 | 1.61E-01 | 1.61E+01 | | | |
| 8132840 | NM_182595 | POM121L12 | POM121 membrane glycoprotein-lik | -0.082 | 0.945 | 2.13E-01 | 2.13E+01 | | | |
| 7962212 | NM_004572 | PKP2 | plakophilin 2 | -0.082 | 0.945 | 1.78E-01 | 1.78E+01 | | | |
| 8029640 | NM_212550 | BLOC1S3 | biogenesis of lysosomal organelles c | -0.082 | 0.945 | 1.39E-01 | 1.39E+01 | | | |
| 8092241 | NM_171830 | KCNMB3 | potassium large conductance calciun | -0.082 | 0.945 | 3.18E-01 | 3.18E+01 | | | |
| 7892652 | --- | --- | --- | -0.082 | 0.945 | 8.18E-01 | 8.18E+01 | | | |
| 7997179 | NM_001361 | DHODH | dihydroorotate dehydrogenase | -0.082 | 0.945 | 2.65E-01 | 2.65E+01 | | | |
| 8179221 | NM_080870 | DPCR1 | diffuse panbronchiolitis critical regio | -0.082 | 0.945 | 3.98E-01 | 3.98E+01 | | | |
| 8117900 | NM_013993 | DDR1 | discoidin domain receptor tyrosine k | -0.082 | 0.945 | 7.84E-02 | 7.84E+00 | | | |
| 8067233 | NM_020182 | PMEPA1 | prostate transmembrane protein, an | -0.082 | 0.945 | 1.16E-01 | 1.16E+01 | | | |
| 7976436 | NM_145249 | IFI27L1 | interferon, alpha-inducible protein 2 | -0.082 | 0.945 | 2.09E-01 | 2.09E+01 | | | |
| 8074817 | NM_003935 | TOP3B | topoisomerase (DNA) III beta | -0.082 | 0.945 | 2.62E-01 | 2.62E+01 | | | |
| 7898582 | BC072682 | RPS14P3 | ribosomal protein S14 pseudogene 3 | -0.082 | 0.945 | 6.06E-01 | 6.06E+01 | | | |
| 7896054 | --- | --- | --- | -0.082 | 0.945 | 7.87E-01 | 7.87E+01 | | | |
| 8000582 | NM_001054 | SULT1A2 | sulfotransferase family, cytosolic, 1A | -0.082 | 0.945 | 3.87E-01 | 3.87E+01 | | | |
| 8028058 | NM_199180 | KIRREL2 | kin of IRRE like 2 (Drosophila) | -0.082 | 0.945 | 1.62E-01 | 1.62E+01 | | | |
| 8067722 | NM_015894 | STMN3 | stathmin-like 3 | -0.082 | 0.945 | 1.48E-01 | 1.48E+01 | | | |
| 8001564 | NM_018110 | DOK4 | docking protein 4 | -0.082 | 0.945 | 9.28E-02 | 9.28E+00 | | | |
| 8009631 | NM_181790 | GPR142 | G protein-coupled receptor 142 | -0.082 | 0.945 | 1.23E-01 | 1.23E+01 | | | |
| 7943236 | NM_002033 | FUT4 | fucosyltransferase 4 (alpha (1,3) fuc | -0.082 | 0.945 | 6.23E-02 | 6.23E+00 | | | |
| 8090098 | NM_053025 | MYLK | myosin light chain kinase | -0.082 | 0.945 | 2.36E-01 | 2.36E+01 | | | |
| 8016468 | NM_004502 | HOXB7 | homeobox B7 | -0.082 | 0.945 | 7.68E-02 | 7.68E+00 | | | |
| 7941749 | NM_177963 | SYT12 | synaptotagmin XII | -0.082 | 0.945 | 1.68E-01 | 1.68E+01 | | | |
| 8056215 | --- | --- | --- | -0.082 | 0.945 | 7.55E-01 | 7.55E+01 | | | |
| 8039013 | NM_203307 | ZNF321 | zinc finger protein 321 | -0.082 | 0.945 | 8.05E-01 | 8.05E+01 | | | |
| 8070744 | NM_004928 | C21orf2 | chromosome 21 open reading frame | -0.082 | 0.945 | 2.57E-01 | 2.57E+01 | | | |
| 7984016 | NM_207322 | FAM148A | family with sequence similarity 148, | -0.082 | 0.945 | 1.77E-01 | 1.77E+01 | | | |
| 8132832 | AK057400 | OC10012942 | hypothetical protein LOC100129427 | -0.082 | 0.945 | 1.88E-01 | 1.88E+01 | | | |
| 7912343 | NM_00107984 | CASZ1 | castor zinc finger 1 | -0.082 | 0.945 | 3.29E-01 | 3.29E+01 | | | |
| 8131265 | NM_014855 | KIAA0415 | KIAA0415 | -0.082 | 0.945 | 1.11E-01 | 1.11E+01 | | | |
| 8150219 | NM_018310 | BRF2 | BRF2, subunit of RNA polymerase III | -0.082 | 0.945 | 3.94E-01 | 3.94E+01 | | | |
| 7941865 | NM_004584 | RAD9A | RAD9 homolog A (S. pombe) | -0.082 | 0.945 | 9.57E-02 | 9.57E+00 | | | |
| 8154848 | NM_007343 | PRSS3 | protease, serine, 3 | -0.082 | 0.945 | 2.68E-01 | 2.68E+01 | | | |
| 7939590 | NM_018389 | SLC35C1 | solute carrier family 35, member C1 | -0.082 | 0.945 | 2.71E-01 | 2.71E+01 | | | |
| 8041570 | NM_152390 | TMEM178 | transmembrane protein 178 | -0.082 | 0.945 | 1.04E-01 | 1.04E+01 | | | |
| 7916393 | NM_00103167 | CYB5RL | cytochrome b5 reductase-like | -0.082 | 0.945 | 1.86E-01 | 1.86E+01 | | | |
| 7940002 | NM_00100521 | LRRC55 | leucine rich repeat containing 55 | -0.082 | 0.945 | 2.27E-01 | 2.27E+01 | | | |
| 8163533 | AK056275 | FLJ31713 | hypothetical protein FLJ31713 | -0.082 | 0.945 | 2.43E-01 | 2.43E+01 | | | |
| 7920707 | NM_006589 | FAM189B | family with sequence similarity 189, | -0.082 | 0.945 | 1.53E-01 | 1.53E+01 | | | |
| 8048595 | NM_013335 | GMPPA | GDP-mannose pyrophosphorylase A | -0.082 | 0.945 | 1.25E-01 | 1.25E+01 | | | |
| 8165492 | NM_031297 | RNF208 | ring finger protein 208 | -0.082 | 0.945 | 1.57E-01 | 1.57E+01 | | | |
| 8165637 | NM_031297 | RNF208 | ring finger protein 208 | -0.082 | 0.945 | 1.57E-01 | 1.57E+01 | | | |
| 8176667 | --- | --- | --- | -0.082 | 0.945 | 3.28E-01 | 3.28E+01 | | | |
| 8177191 | --- | --- | --- | -0.082 | 0.945 | 3.28E-01 | 3.28E+01 | | | |
| 8065517 | NM_015655 | ZNF337 | zinc finger protein 337 | -0.082 | 0.945 | 3.58E-01 | 3.58E+01 | | | |
| 7963174 | NM_147190 | LASS5 | LAG1 homolog, ceramide synthase 5 | -0.082 | 0.945 | 7.05E-01 | 7.05E+01 | | | |
| 7906435 | NM_002036 | DARC | Duffy blood group, chemokine recep | -0.082 | 0.945 | 7.86E-02 | 7.86E+00 | | | |
| 8153862 | NM_00102467 | LRRC24 | leucine rich repeat containing 24 | -0.082 | 0.945 | 1.08E-01 | 1.08E+01 | | | |
| 8116031 | NM_00102988 | PFN3 | profilin 3 | -0.082 | 0.945 | 1.79E-01 | 1.79E+01 | | | |
| 8175732 | NM_175742 | MAGEA2 | melanoma antigen family A, 2 | -0.082 | 0.945 | 9.43E-02 | 9.43E+00 | | | |
| 8076962 | NM_002969 | MAPK12 | mitogen-activated protein kinase 12 | -0.082 | 0.945 | 1.78E-01 | 1.78E+01 | | | |

| | | | | | | | | | | |
|---|---|---|---|---|---|---|---|---|---|---|
| 8147057 | NM_152284 | CHMP4C | chromatin modifying protein 4C | -0.082 | 0.945 | 1.48E-01 | 1.48E+01 | | | |
| 7894158 | --- | --- | --- | -0.082 | 0.945 | 8.80E-01 | 8.80E+01 | | | |
| 8065633 | --- | --- | --- | -0.082 | 0.944 | 4.35E-01 | 4.35E+01 | | | |
| 8108080 | NM_015288 | PHF15 | PHD finger protein 15 | -0.082 | 0.944 | 3.93E-01 | 3.93E+01 | | | |
| 8031720 | NM_213598 | ZNF543 | zinc finger protein 543 | -0.082 | 0.944 | 3.37E-01 | 3.37E+01 | | | |
| 7969143 | --- | --- | --- | -0.082 | 0.944 | 2.22E-01 | 2.22E+01 | | | |
| 7981317 | --- | --- | --- | -0.082 | 0.944 | 2.29E-01 | 2.29E+01 | | | |
| 7923700 | NM_006338 | LRRN2 | leucine rich repeat neuronal 2 | -0.082 | 0.944 | 1.93E-01 | 1.93E+01 | | | |
| 8010012 | NM_00111332 | OC10013493 | hypothetical protein LOC100134934 | -0.082 | 0.944 | 2.50E-01 | 2.50E+01 | | | |
| 8074522 | NM_001670 | ARVCF | armadillo repeat gene deletes in velo | -0.082 | 0.944 | 1.99E-01 | 1.99E+01 | | | |
| 8031744 | NM_006959 | ZNF17 | zinc finger protein 17 | -0.082 | 0.944 | 2.74E-01 | 2.74E+01 | | | |
| 7951660 | ENST00000317 | RPL37AP8 | ribosomal protein L37a pseudogene | -0.082 | 0.944 | 3.39E-01 | 3.39E+01 | | | |
| 7940600 | NM_00104069 | INCENP | inner centromere protein antigens 1 | -0.082 | 0.944 | 1.54E-01 | 1.54E+01 | | | |
| 8075897 | NM_031910 | C1QTNF6 | C1q and tumor necrosis factor relate | -0.082 | 0.944 | 1.19E-01 | 1.19E+01 | | | |
| 7894582 | --- | --- | --- | -0.082 | 0.944 | 7.86E-01 | 7.86E+01 | | | |
| 8145989 | NM_153692 | HTRA4 | HtrA serine peptidase 4 | -0.082 | 0.944 | 1.81E-01 | 1.81E+01 | | | |
| 7894565 | --- | --- | --- | -0.082 | 0.944 | 4.89E-01 | 4.89E+01 | | | |
| 8094355 | --- | --- | --- | -0.082 | 0.944 | 4.71E-01 | 4.71E+01 | | | |
| 8071276 | NM_080647 | TBX1 | T-box 1 | -0.082 | 0.944 | 2.05E-01 | 2.05E+01 | | | |
| 7933945 | --- | --- | --- | -0.082 | 0.944 | 3.75E-01 | 3.75E+01 | | | |
| 8157731 | NM_173689 | CRB2 | crumbs homolog 2 (Drosophila) | -0.082 | 0.944 | 7.76E-02 | 7.76E+00 | | | |
| 7919950 | NM_002651 | PI4KB | phosphatidylinositol 4-kinase, cataly | -0.083 | 0.944 | 5.03E-01 | 5.03E+01 | | | |
| 7913187 | NM_181719 | TMCO4 | transmembrane and coiled-coil dom | -0.083 | 0.944 | 1.30E-01 | 1.30E+01 | | | |
| 7913450 | NM_005529 | HSPG2 | heparan sulfate proteoglycan 2 | -0.083 | 0.944 | 2.78E-02 | 2.78E+00 | | | |
| 8098942 | NM_005663 | WHSC2 | Wolf-Hirschhorn syndrome candidat | -0.083 | 0.944 | 1.85E-01 | 1.85E+01 | | | |
| 8045301 | NM_017751 | SMPD4 | sphingomyelin phosphodiesterase 4, | -0.083 | 0.944 | 2.78E-01 | 2.78E+01 | | | |
| 7985472 | NR_003246 | FLJ40113 | golgi autoantigen, golgin subfamily a | -0.083 | 0.944 | 5.38E-01 | 5.38E+01 | | | |
| 7917151 | ENST00000334 | NEXN | nexilin (F actin binding protein) | -0.083 | 0.944 | 1.33E-01 | 1.33E+01 | | | |
| 7941087 | NM_006244 | PPP2R5B | protein phosphatase 2, regulatory su | -0.083 | 0.944 | 1.51E-01 | 1.51E+01 | | | |
| 8139281 | NM_013284 | POLM | polymerase (DNA directed), mu | -0.083 | 0.944 | 1.24E-01 | 1.24E+01 | | | |
| 7943919 | NM_017868 | TTC12 | tetratricopeptide repeat domain 12 | -0.083 | 0.944 | 2.57E-01 | 2.57E+01 | | | |
| 7933498 | ENST00000311 | FAM170B | family with sequence similarity 170, | -0.083 | 0.944 | 2.02E-01 | 2.02E+01 | | | |
| 8131326 | NM_00104066 | SLC29A4 | solute carrier family 29 (nucleoside t | -0.083 | 0.944 | 3.76E-01 | 3.76E+01 | | | |
| 8144880 | NM_022071 | SH2D4A | SH2 domain containing 4A | -0.083 | 0.944 | 1.18E-01 | 1.18E+01 | | | |
| 7991668 | 0 | 0 | 0 | -0.083 | 0.944 | 1.99E-01 | 1.99E+01 | | | |
| 8157300 | NM_017688 | BSPRY | B-box and SPRY domain containing | -0.083 | 0.944 | 1.16E-01 | 1.16E+01 | | | |
| 7904137 | NM_198268 | HIPK1 | homeodomain interacting protein ki | -0.083 | 0.944 | 7.02E-01 | 7.02E+01 | | | |
| 8115814 | NM_00101799 | SH3PXD2B | SH3 and PX domains 2B | -0.083 | 0.944 | 1.28E-01 | 1.28E+01 | | | |
| 8177867 | NM_013993 | DDR1 | discoidin domain receptor tyrosine k | -0.083 | 0.944 | 9.02E-02 | 9.02E+00 | | | |
| 8180379 | --- | --- | --- | -0.083 | 0.944 | 4.61E-01 | 4.61E+01 | | | |
| 8069503 | AK125677 | LOC441956 | similar to cDNA sequence BC021523 | -0.083 | 0.944 | 3.62E-01 | 3.62E+01 | | | |
| 7937728 | NM_00114582 | TNNI2 | troponin I type 2 (skeletal, fast) | -0.083 | 0.944 | 3.23E-01 | 3.23E+01 | | | |
| 8079189 | NM_018651 | ZNF167 | zinc finger protein 167 | -0.083 | 0.944 | 2.70E-01 | 2.70E+01 | | | |
| 7981874 | NM_00101242 | GOLGA8E | golgi autoantigen, golgin subfamily a | -0.083 | 0.944 | 1.39E-01 | 1.39E+01 | | | |
| 7954063 | NR_003932 | RPL13AP20 | ribosomal protein L13a pseudogene | -0.083 | 0.944 | 4.39E-01 | 4.39E+01 | | | |
| 8079377 | NM_006564 | CXCR6 | chemokine (C-X-C motif) receptor 6 | -0.083 | 0.944 | 3.25E-01 | 3.25E+01 | | | |
| 8010405 | NM_173626 | SLC26A11 | solute carrier family 26, member 11 | -0.083 | 0.944 | 1.12E-01 | 1.12E+01 | | | |
| 8027169 | NM_017660 | GATAD2A | GATA zinc finger domain containing | -0.083 | 0.944 | 4.95E-01 | 4.95E+01 | | | |
| 8158912 | NM_020064 | BARHL1 | BarH-like homeobox 1 | -0.083 | 0.944 | 1.72E-01 | 1.72E+01 | | | |
| 8028119 | NM_00108396 | WDR62 | WD repeat domain 62 | -0.083 | 0.944 | 7.99E-02 | 7.99E+00 | | | |
| 8087145 | NM_001407 | CELSR3 | cadherin, EGF LAG seven-pass G-type | -0.083 | 0.944 | 8.16E-02 | 8.16E+00 | | | |
| 8163013 | --- | --- | --- | -0.083 | 0.944 | 2.94E-01 | 2.94E+01 | | | |
| 7892893 | --- | --- | --- | -0.083 | 0.944 | 7.77E-01 | 7.77E+01 | | | |
| 7909027 | NM_00100538 | NFASC | neurofascin homolog (chicken) | -0.083 | 0.944 | 1.04E-01 | 1.04E+01 | | | |
| 8146579 | NM_017780 | CHD7 | chromodomain helicase DNA binding | -0.083 | 0.944 | 5.59E-01 | 5.59E+01 | | | |
| 8074909 | NM_020070 | IGLL1 | immunoglobulin lambda-like polype | -0.083 | 0.944 | 1.94E-01 | 1.94E+01 | | | |
| 8156620 | NM_017561 | FAM22F | family with sequence similarity 22, m | -0.083 | 0.944 | 8.15E-02 | 8.15E+00 | | | |
| 8071289 | NM_000754 | COMT | catechol-O-methyltransferase | -0.083 | 0.944 | 1.49E-01 | 1.49E+01 | | | |
| 8131000 | NM_017802 | HEATR2 | HEAT repeat containing 2 | -0.083 | 0.944 | 1.37E-01 | 1.37E+01 | | | |
| 7996022 | NM_002990 | CCL22 | chemokine (C-C motif) ligand 22 | -0.083 | 0.944 | 8.83E-02 | 8.83E+00 | | | |
| 7934083 | NM_020999 | NEUROG3 | neurogenin 3 | -0.083 | 0.944 | 2.37E-01 | 2.37E+01 | | | |
| 8008736 | NM_006151 | LPO | lactoperoxidase | -0.083 | 0.944 | 1.18E-01 | 1.18E+01 | | | |
| 8157324 | NM_144488 | RGS3 | regulator of G-protein signaling 3 | -0.083 | 0.944 | 4.06E-02 | 4.06E+00 | | | |
| 7895024 | --- | --- | --- | -0.083 | 0.944 | 9.28E-01 | 9.28E+01 | | | |
| 7913938 | NM_00103977 | AIM1L | absent in melanoma 1-like | -0.083 | 0.944 | 8.77E-02 | 8.77E+00 | | | |
| 7946661 | NM_015881 | DKK3 | dickkopf homolog 3 (Xenopus laevis) | -0.083 | 0.944 | 6.46E-02 | 6.46E+00 | | | |
| 8027805 | NM_003367 | USF2 | upstream transcription factor 2, c-fo | -0.083 | 0.944 | 2.92E-01 | 2.92E+01 | | | |
| 8067543 | ENST00000370 | RP11-93B14. | hypothetical protein FLJ32154 | -0.083 | 0.944 | 1.82E-01 | 1.82E+01 | | | |
| 8174389 | NM_033641 | COL4A6 | collagen, type IV, alpha 6 | -0.083 | 0.944 | 5.42E-02 | 5.42E+00 | | | |
| 8123893 | NM_005906 | MAK | male germ cell-associated kinase | -0.083 | 0.944 | 1.11E-01 | 1.11E+01 | | | |
| 8061620 | NM_00100840 | TTLL9 | tubulin tyrosine ligase-like family, m | -0.083 | 0.944 | 2.03E-01 | 2.03E+01 | | | |
| 7958352 | NM_00101807 | BTBD11 | BTB (POZ) domain containing 11 | -0.083 | 0.944 | 2.70E-01 | 2.70E+01 | | | |
| 8133625 | NM_198924 | TRIM73 | tripartite motif-containing 73 | -0.083 | 0.944 | 2.23E-01 | 2.23E+01 | | | |
| 8149918 | NM_000742 | CHRNA2 | cholinergic receptor, nicotinic, alpha | -0.083 | 0.944 | 1.72E-01 | 1.72E+01 | | | |
| 8035506 | NM_004750 | CRLF1 | cytokine receptor-like factor 1 | -0.083 | 0.944 | 1.00E-01 | 1.00E+01 | | | |
| 7963786 | NM_002205 | ITGA5 | integrin, alpha 5 (fibronectin recepto | -0.083 | 0.944 | 2.78E-01 | 2.78E+01 | | | |
| 8007148 | NM_000805 | GAST | gastrin | -0.083 | 0.944 | 2.28E-01 | 2.28E+01 | | | |

| | | | | | | | | | | |
|---|---|---|---|---|---|---|---|---|---|---|
| 8148640 | --- | --- | --- | -0.083 | 0.944 | 3.82E-01 | 3.82E+01 | | | |
| 8049509 | NM_015893 | PRLH | prolactin releasing hormone | -0.083 | 0.944 | 1.79E-01 | 1.79E+01 | | | |
| 7938812 | NM_153347 | TMEM86A | transmembrane protein 86A | -0.083 | 0.944 | 1.68E-01 | 1.68E+01 | | | |
| 8125936 | NM_001832 | CLPS | colipase, pancreatic | -0.083 | 0.944 | 1.38E-01 | 1.38E+01 | | | |
| 8097461 | NM_012118 | CCRN4L | CCR4 carbon catabolite repression 4- | -0.083 | 0.944 | 1.37E-01 | 1.37E+01 | | | |
| 8085233 | NM_173659 | RPUSD3 | RNA pseudouridylate synthase doma | -0.083 | 0.944 | 1.71E-01 | 1.71E+01 | | | |
| 8051241 | NM_004304 | ALK | anaplastic lymphoma receptor tyrosi | -0.083 | 0.944 | 1.21E-01 | 1.21E+01 | | | |
| 8110347 | NM_003052 | SLC34A1 | solute carrier family 34 (sodium pho | -0.083 | 0.944 | 1.86E-01 | 1.86E+01 | | | |
| 8139314 | NM_033508 | GCK | glucokinase (hexokinase 4) | -0.083 | 0.944 | 5.58E-02 | 5.58E+00 | | | |
| 8053030 | NM_016347 | NAT8B | N-acetyltransferase 8B (GCN5-relate | -0.083 | 0.944 | 1.13E-01 | 1.13E+01 | | | |
| 8040578 | NM_024322 | CENPO | centromere protein O | -0.083 | 0.944 | 2.84E-01 | 2.84E+01 | | | |
| 7916225 | --- | --- | --- | -0.083 | 0.944 | 3.06E-01 | 3.06E+01 | | | |
| 8062796 | NM_024034 | GDAP1L1 | ganglioside-induced differentiation-a | -0.083 | 0.944 | 1.22E-01 | 1.22E+01 | | | |
| 7930099 | NM_024326 | FBXL15 | F-box and leucine-rich repeat protei | -0.083 | 0.944 | 1.13E-01 | 1.13E+01 | | | |
| 7933209 | NM_006963 | ZNF22 | zinc finger protein 22 (KOX 15) | -0.083 | 0.944 | 2.69E-01 | 2.69E+01 | | | |
| 8076668 | BC104183 | KIAA1644 | KIAA1644 | -0.083 | 0.944 | 1.14E-01 | 1.14E+01 | | | |
| 7950743 | NM_014488 | RAB30 | RAB30, member RAS oncogene famil | -0.083 | 0.944 | 8.46E-01 | 8.46E+01 | | | |
| 8028016 | NM_00103988 | C19orf55 | chromosome 19 open reading frame | -0.083 | 0.944 | 2.24E-01 | 2.24E+01 | | | |
| 8031311 | NM_013289 | KIR3DL1 | killer cell immunoglobulin-like recep | -0.083 | 0.944 | 2.84E-01 | 2.84E+01 | | | |
| 7955943 | NM_000924 | PDE1B | phosphodiesterase 1B, calmodulin-d | -0.083 | 0.944 | 2.00E-01 | 2.00E+01 | | | |
| 7999362 | --- | --- | --- | -0.083 | 0.944 | 7.82E-01 | 7.82E+01 | | | |
| 8116316 | NM_014275 | MGAT4B | mannosyl (alpha-1,3-)-glycoprotein b | -0.084 | 0.944 | 1.30E-01 | 1.30E+01 | | | |
| 8037043 | --- | --- | --- | -0.084 | 0.944 | 1.58E-01 | 1.58E+01 | | | |
| 7929932 | NM_030929 | KAZALD1 | Kazal-type serine peptidase inhibitor | -0.084 | 0.944 | 7.67E-02 | 7.67E+00 | | | |
| 8138718 | NM_006735 | HOXA2 | homeobox A2 | -0.084 | 0.944 | 2.63E-01 | 2.63E+01 | | | |
| 7904036 | NM_006135 | CAPZA1 | capping protein (actin filament) mus | -0.084 | 0.944 | 5.52E-01 | 5.52E+01 | | | |
| 7935553 | NM_032211 | LOXL4 | lysyl oxidase-like 4 | -0.084 | 0.944 | 1.23E-01 | 1.23E+01 | | | |
| 8006768 | NM_014598 | SOCS7 | suppressor of cytokine signaling 7 | -0.084 | 0.944 | 1.34E-01 | 1.34E+01 | | | |
| 7964484 | NM_001478 | B4GALNT1 | beta-1,4-N-acetyl-galactosaminyl tra | -0.084 | 0.944 | 1.52E-01 | 1.52E+01 | | | |
| 7931379 | NM_002412 | MGMT | O-6-methylguanine-DNA methyltran | -0.084 | 0.944 | 5.31E-01 | 5.31E+01 | | | |
| 7936115 | NM_014631 | SH3PXD2A | SH3 and PX domains 2A | -0.084 | 0.944 | 1.22E-01 | 1.22E+01 | | | |
| 7893438 | --- | --- | --- | -0.084 | 0.944 | 4.19E-01 | 4.19E+01 | | | |
| 7972923 | NM_000705 | ATP4B | ATPase, H+/K+ exchanging, beta poly | -0.084 | 0.944 | 2.05E-01 | 2.05E+01 | | | |
| 8159239 | BC034752 | C9orf62 | chromosome 9 open reading frame 6 | -0.084 | 0.944 | 1.91E-01 | 1.91E+01 | | | |
| 7977621 | NM_201540 | NDRG2 | NDRG family member 2 | -0.084 | 0.944 | 1.85E-01 | 1.85E+01 | | | |
| 7968913 | --- | --- | --- | -0.084 | 0.944 | 2.85E-01 | 2.85E+01 | | | |
| 8156549 | NM_032823 | C9orf3 | chromosome 9 open reading frame 3 | -0.084 | 0.944 | 3.06E-01 | 3.06E+01 | | | |
| 7897648 | NM_004565 | PEX14 | peroxisomal biogenesis factor 14 | -0.084 | 0.944 | 1.92E-01 | 1.92E+01 | | | |
| 7955142 | NM_000725 | CACNB3 | calcium channel, voltage-dependent | -0.084 | 0.944 | 1.84E-01 | 1.84E+01 | | | |
| 8073941 | --- | --- | --- | -0.084 | 0.944 | 1.64E-01 | 1.64E+01 | | | |
| 8124689 | NR_002139 | HCG4 | HLA complex group 4 | -0.084 | 0.944 | 3.30E-01 | 3.30E+01 | | | |
| 8108579 | NM_018502 | TMCO6 | transmembrane and coiled-coil dom | -0.084 | 0.944 | 2.68E-01 | 2.68E+01 | | | |
| 8055967 | --- | --- | --- | -0.084 | 0.944 | 3.20E-01 | 3.20E+01 | | | |
| 8087790 | NM_004704 | RRP9 | ribosomal RNA processing 9, small su | -0.084 | 0.944 | 3.27E-01 | 3.27E+01 | | | |
| 8070081 | --- | --- | --- | -0.084 | 0.944 | 2.66E-01 | 2.66E+01 | | | |
| 8137151 | NM_198455 | SSPO | SCO-spondin homolog (Bos taurus) | -0.084 | 0.944 | 8.26E-02 | 8.26E+00 | | | |
| 7905233 | NM_019032 | ADAMTSL4 | ADAMTS-like 4 | -0.084 | 0.944 | 1.41E-01 | 1.41E+01 | | | |
| 8136045 | NM_002200 | IRF5 | interferon regulatory factor 5 | -0.084 | 0.944 | 1.70E-01 | 1.70E+01 | | | |
| 7929550 | NM_00113437 | CCNJ | cyclin J | -0.084 | 0.944 | 2.42E-01 | 2.42E+01 | | | |
| 8099240 | --- | --- | --- | -0.084 | 0.944 | 6.87E-01 | 6.87E+01 | | | |
| 7985932 | --- | --- | --- | -0.084 | 0.944 | 1.80E-01 | 1.80E+01 | | | |
| 8070786 | NM_198688 | KRTAP10-6 | keratin associated protein 10-6 | -0.084 | 0.944 | 9.02E-02 | 9.02E+00 | | | |
| 8024446 | NM_00107723 | SPPL2B | signal peptide peptidase-like 2B | -0.084 | 0.944 | 1.16E-01 | 1.16E+01 | | | |
| 8009796 | NM_014738 | KIAA0195 | KIAA0195 | -0.084 | 0.944 | 1.65E-01 | 1.65E+01 | | | |
| 8094116 | NM_00110566 | USP17 | ubiquitin specific peptidase 17 | -0.084 | 0.944 | 1.16E-01 | 1.16E+01 | | | |
| 8094118 | NM_00110566 | USP17 | ubiquitin specific peptidase 17 | -0.084 | 0.944 | 1.16E-01 | 1.16E+01 | | | |
| 8094120 | NM_00110566 | USP17 | ubiquitin specific peptidase 17 | -0.084 | 0.944 | 1.16E-01 | 1.16E+01 | | | |
| 8094124 | NM_00110566 | USP17 | ubiquitin specific peptidase 17 | -0.084 | 0.944 | 1.16E-01 | 1.16E+01 | | | |
| 8094126 | NM_00110566 | USP17 | ubiquitin specific peptidase 17 | -0.084 | 0.944 | 1.16E-01 | 1.16E+01 | | | |
| 8094128 | NM_00110566 | USP17 | ubiquitin specific peptidase 17 | -0.084 | 0.944 | 1.16E-01 | 1.16E+01 | | | |
| 8094132 | NM_00110566 | USP17 | ubiquitin specific peptidase 17 | -0.084 | 0.944 | 1.16E-01 | 1.16E+01 | | | |
| 7943226 | NM_00108048 | FOLR4 | folate receptor 4 (delta) homolog (m | -0.084 | 0.944 | 9.93E-02 | 9.93E+00 | | | |
| 8150509 | NM_000930 | PLAT | plasminogen activator, tissue | -0.084 | 0.944 | 1.38E-01 | 1.38E+01 | | | |
| 7905492 | NM_178433 | LCE3B | late cornified envelope 3B | -0.084 | 0.944 | 1.60E-01 | 1.60E+01 | | | |
| 7994216 | NM_052944 | SLC5A11 | solute carrier family 5 (sodium/gluco | -0.084 | 0.944 | 8.56E-02 | 8.56E+00 | | | |
| 8035884 | --- | --- | --- | -0.084 | 0.944 | 1.56E-01 | 1.56E+01 | | | |
| 7973745 | NM_005249 | FOXG1 | forkhead box G1 | -0.084 | 0.943 | 1.48E-01 | 1.48E+01 | | | |
| 8099132 | NM_018659 | CYTL1 | cytokine-like 1 | -0.084 | 0.943 | 1.11E-01 | 1.11E+01 | | | |
| 7953520 | NM_201650 | LRRC23 | leucine rich repeat containing 23 | -0.084 | 0.943 | 7.96E-02 | 7.96E+00 | | | |
| 7901613 | NM_147161 | ACOT11 | acyl-CoA thioesterase 11 | -0.084 | 0.943 | 2.00E-01 | 2.00E+01 | | | |
| 8076851 | NM_024105 | ALG12 | asparagine-linked glycosylation 12, a | -0.084 | 0.943 | 2.27E-01 | 2.27E+01 | | | |
| 8168205 | NM_005938 | FOXO4 | forkhead box O4 | -0.084 | 0.943 | 1.55E-01 | 1.55E+01 | | | |
| 7923406 | NM_080588 | PTPN7 | protein tyrosine phosphatase, non-re | -0.084 | 0.943 | 2.13E-01 | 2.13E+01 | | | |
| 7951987 | NM_00107726 | TMPRSS13 | transmembrane protease, serine 13 | -0.084 | 0.943 | 1.95E-01 | 1.95E+01 | | | |
| 8001552 | NM_020313 | CIAPIN1 | cytokine induced apoptosis inhibitor | -0.084 | 0.943 | 4.54E-01 | 4.54E+01 | | | |
| 8059748 | NM_004826 | ECEL1 | endothelin converting enzyme-like 1 | -0.084 | 0.943 | 1.89E-01 | 1.89E+01 | | | |
| 8006906 | NM_00100586 | ERBB2 | v-erb-b2 erythroblastic leukemia vira | -0.084 | 0.943 | 1.18E-01 | 1.18E+01 | | | |

| | | | | | | | | | | |
|---|---|---|---|---|---|---|---|---|---|---|
| 8014160 | NM_002981 | CCL1 | chemokine (C-C motif) ligand 1 | -0.084 | 0.943 | 1.24E-01 | 1.24E+01 | | | |
| 7909446 | NM_005525 | HSD11B1 | hydroxysteroid (11-beta) dehydroge | -0.084 | 0.943 | 1.09E-01 | 1.09E+01 | | | |
| 8150318 | NM_023110 | FGFR1 | fibroblast growth factor receptor 1 | -0.084 | 0.943 | 1.41E-01 | 1.41E+01 | | | |
| 7909250 | NM_153758 | IL19 | interleukin 19 | -0.084 | 0.943 | 2.46E-01 | 2.46E+01 | | | |
| 8118498 | NM_000500 | CYP21A2 | cytochrome P450, family 21, subfam | -0.084 | 0.943 | 1.89E-01 | 1.89E+01 | | | |
| 8100070 | NM_198353 | KCTD8 | potassium channel tetramerisation d | -0.084 | 0.943 | 1.21E-01 | 1.21E+01 | | | |
| 8122703 | --- | --- | --- | -0.084 | 0.943 | 5.50E-01 | 5.50E+01 | | | |
| 7893002 | --- | --- | --- | -0.084 | 0.943 | 7.61E-01 | 7.61E+01 | | | |
| 7921840 | NM_00107748 | NR1I3 | nuclear receptor subfamily 1, group | -0.084 | 0.943 | 3.22E-01 | 3.22E+01 | | | |
| 8026564 | NM_016270 | KLF2 | Kruppel-like factor 2 (lung) | -0.084 | 0.943 | 8.46E-02 | 8.46E+00 | | | |
| 8180213 | --- | --- | --- | -0.084 | 0.943 | 1.91E-01 | 1.91E+01 | | | |
| 8119124 | NM_153370 | PI16 | peptidase inhibitor 16 | -0.084 | 0.943 | 2.54E-01 | 2.54E+01 | | | |
| 8172460 | NM_00104249 | SLC35A2 | solute carrier family 35 (UDP-galacto | -0.084 | 0.943 | 2.19E-01 | 2.19E+01 | | | |
| 8016444 | NM_002146 | HOXB3 | homeobox B3 | -0.084 | 0.943 | 1.22E-01 | 1.22E+01 | | | |
| 8031328 | NM_006737 | KIR3DL2 | killer cell immunoglobulin-like recept | -0.084 | 0.943 | 3.33E-01 | 3.33E+01 | | | |
| 7894046 | --- | --- | --- | -0.084 | 0.943 | 6.40E-01 | 6.40E+01 | | | |
| 7894288 | --- | --- | --- | -0.084 | 0.943 | 3.95E-01 | 3.95E+01 | | | |
| 8015730 | NR_027254 | LOC388387 | hypothetical LOC388387 | -0.084 | 0.943 | 2.54E-01 | 2.54E+01 | | | |
| 8025918 | NM_001299 | CNN1 | calponin 1, basic, smooth muscle | -0.084 | 0.943 | 2.46E-01 | 2.46E+01 | | | |
| 8070239 | NM_000411 | HLCS | holocarboxylase synthetase (biotin-( | -0.084 | 0.943 | 1.14E-01 | 1.14E+01 | | | |
| 8117922 | NM_025263 | PRR3 | proline rich 3 | -0.084 | 0.943 | 3.12E-01 | 3.12E+01 | | | |
| 8180403 | --- | --- | --- | -0.084 | 0.943 | 4.40E-01 | 4.40E+01 | | | |
| 7909529 | NM_00113622 | RCOR3 | REST corepressor 3 | -0.084 | 0.943 | 7.51E-01 | 7.51E+01 | | | |
| 8003344 | NM_178310 | SNAI3 | snail homolog 3 (Drosophila) | -0.084 | 0.943 | 1.48E-01 | 1.48E+01 | | | |
| 8129375 | --- | --- | --- | -0.084 | 0.943 | 2.04E-01 | 2.04E+01 | | | |
| 7995222 | NM_003041 | SLC5A2 | solute carrier family 5 (sodium/gluco | -0.084 | 0.943 | 1.78E-01 | 1.78E+01 | | | |
| 8097687 | NM_004575 | POU4F2 | POU class 4 homeobox 2 | -0.084 | 0.943 | 1.03E-01 | 1.03E+01 | | | |
| 8036363 | --- | --- | --- | -0.084 | 0.943 | 8.59E-01 | 8.59E+01 | | | |
| 7905088 | NM_003517 | HIST2H2AC | histone cluster 2, H2ac | -0.084 | 0.943 | 7.13E-01 | 7.13E+01 | | | |
| 7926150 | NM_014688 | USP6NL | USP6 N-terminal like | -0.084 | 0.943 | 1.80E-01 | 1.80E+01 | | | |
| 8033332 | NR_024075 | EMR4P | egf-like module containing, mucin-lik | -0.085 | 0.943 | 2.11E-01 | 2.11E+01 | | | |
| 7947947 | NM_024783 | AGBL2 | ATP/GTP binding protein-like 2 | -0.085 | 0.943 | 2.01E-01 | 2.01E+01 | | | |
| 7918876 | --- | --- | --- | -0.085 | 0.943 | 3.15E-01 | 3.15E+01 | | | |
| 7950444 | --- | --- | --- | -0.085 | 0.943 | 4.10E-01 | 4.10E+01 | | | |
| 7892636 | --- | --- | --- | -0.085 | 0.943 | 3.77E-01 | 3.77E+01 | | | |
| 7899753 | NM_005356 | LCK | lymphocyte-specific protein tyrosine | -0.085 | 0.943 | 4.64E-01 | 4.64E+01 | | | |
| 8078665 | NM_015873 | VILL | villin-like | -0.085 | 0.943 | 1.38E-01 | 1.38E+01 | | | |
| 8094032 | NM_018986 | SH3TC1 | SH3 domain and tetratricopeptide re | -0.085 | 0.943 | 1.22E-01 | 1.22E+01 | | | |
| 8036969 | NM_000762 | CYP2A6 | cytochrome P450, family 2, subfamil | -0.085 | 0.943 | 7.64E-02 | 7.64E+00 | | | |
| 7993848 | NM_144672 | OTOA | otoancorin | -0.085 | 0.943 | 9.18E-02 | 9.18E+00 | | | |
| 7925773 | NM_017865 | ZNF692 | zinc finger protein 692 | -0.085 | 0.943 | 5.66E-01 | 5.66E+01 | | | |
| 8149835 | NM_006158 | NEFL | neurofilament, light polypeptide | -0.085 | 0.943 | 2.50E-01 | 2.50E+01 | | | |
| 8112738 | --- | --- | --- | -0.085 | 0.943 | 3.77E-01 | 3.77E+01 | | | |
| 8067727 | NM_003224 | ARFRP1 | ADP-ribosylation factor related prote | -0.085 | 0.943 | 9.29E-02 | 9.29E+00 | | | |
| 7962659 | NM_015401 | HDAC7 | histone deacetylase 7 | -0.085 | 0.943 | 1.04E-01 | 1.04E+01 | | | |
| 7893106 | --- | --- | --- | -0.085 | 0.943 | 4.93E-01 | 4.93E+01 | | | |
| 8041447 | NM_016441 | CRIM1 | cysteine rich transmembrane BMP re | -0.085 | 0.943 | 2.54E-01 | 2.54E+01 | | | |
| 7955817 | NM_005016 | PCBP2 | poly(rC) binding protein 2 | -0.085 | 0.943 | 6.69E-01 | 6.69E+01 | | | |
| 7961887 | --- | --- | --- | -0.085 | 0.943 | 4.76E-01 | 4.76E+01 | | | |
| 7992639 | NM_020705 | TBC1D24 | TBC1 domain family, member 24 | -0.085 | 0.943 | 1.45E-01 | 1.45E+01 | | | |
| 8175638 | ENST0000043 | FLJ16423 | hypothetical LOC642889 | -0.085 | 0.943 | 4.03E-01 | 4.03E+01 | | | |
| 7985636 | NM_004213 | SLC28A1 | solute carrier family 28 (sodium-coup | -0.085 | 0.943 | 8.55E-02 | 8.55E+00 | | | |
| 8015706 | NR_024461 | OC10019093 | hypothetical LOC100190938 | -0.085 | 0.943 | 6.01E-02 | 6.01E+00 | | | |
| 8176336 | NR_026711 | NCRNA0010 | non-protein coding RNA 105 | -0.085 | 0.943 | 2.45E-01 | 2.45E+01 | | | |
| 8109563 | NM_00109928 | NIPAL4 | NIPA-like domain containing 4 | -0.085 | 0.943 | 1.77E-01 | 1.77E+01 | | | |
| 7992518 | NM_014353 | RAB26 | RAB26, member RAS oncogene famil | -0.085 | 0.943 | 1.53E-01 | 1.53E+01 | | | |
| 7968759 | --- | --- | --- | -0.085 | 0.943 | 3.47E-01 | 3.47E+01 | | | |
| 7913146 | NM_012067 | AKR7A3 | aldo-keto reductase family 7, membe | -0.085 | 0.943 | 1.69E-01 | 1.69E+01 | | | |
| 7992758 | NM_172229 | KREMEN2 | kringle containing transmembrane p | -0.085 | 0.943 | 1.01E-01 | 1.01E+01 | | | |
| 8142795 | NM_000883 | IMPDH1 | IMP (inosine monophosphate) dehyd | -0.085 | 0.943 | 1.52E-01 | 1.52E+01 | | | |
| 8103873 | --- | --- | --- | -0.085 | 0.943 | 3.57E-01 | 3.57E+01 | | | |
| 7996715 | NM_198443 | NRN1L | neuritin 1-like | -0.085 | 0.943 | 1.56E-01 | 1.56E+01 | | | |
| 7894537 | --- | --- | --- | -0.085 | 0.943 | 8.83E-01 | 8.83E+01 | | | |
| 8014706 | NM_007144 | PCGF2 | polycomb group ring finger 2 | -0.085 | 0.943 | 6.06E-02 | 6.06E+00 | | | |
| 8061529 | NM_014012 | REM1 | RAS (RAD and GEM)-like GTP-binding | -0.085 | 0.943 | 1.04E-01 | 1.04E+01 | | | |
| 8163505 | NM_031219 | HDHD3 | haloacid dehalogenase-like hydrolas | -0.085 | 0.943 | 2.19E-01 | 2.19E+01 | | | |
| 8142997 | NM_020911 | PLXNA4 | plexin A4 | -0.085 | 0.943 | 9.49E-02 | 9.49E+00 | | | |
| 8011640 | NM_014520 | MYBBP1A | MYB binding protein (P160) 1a | -0.085 | 0.943 | 9.27E-02 | 9.27E+00 | | | |
| 8040587 | NM_014971 | EFR3B | EFR3 homolog B (S. cerevisiae) | -0.085 | 0.943 | 1.42E-01 | 1.42E+01 | | | |
| 8036867 | BC027935 | C19orf47 | chromosome 19 open reading frame | -0.085 | 0.943 | 1.62E-01 | 1.62E+01 | | | |
| 8007259 | --- | --- | --- | -0.085 | 0.943 | 1.59E-01 | 1.59E+01 | | | |
| 8007675 | NM_002390 | ADAM11 | ADAM metallopeptidase domain 11 | -0.085 | 0.943 | 1.93E-01 | 1.93E+01 | | | |
| 8061305 | NM_018474 | C20orf19 | chromosome 20 open reading frame | -0.085 | 0.943 | 7.39E-01 | 7.39E+01 | | | |
| 7944603 | NM_014619 | GRIK4 | glutamate receptor, ionotropic, kain | -0.085 | 0.943 | 5.82E-02 | 5.82E+00 | | | |
| 8098782 | NM_006651 | CPLX1 | complexin 1 | -0.085 | 0.943 | 1.45E-01 | 1.45E+01 | | | |
| 8137215 | NM_00114292 | LRRC61 | leucine rich repeat containing 61 | -0.085 | 0.943 | 1.07E-01 | 1.07E+01 | | | |
| 8012475 | NM_005964 | MYH10 | myosin, heavy chain 10, non-muscle | -0.085 | 0.943 | 2.14E-01 | 2.14E+01 | | | |

| | | | | | | | | | | |
|---|---|---|---|---|---|---|---|---|---|---|
| 8025633 | NM_00111130 | PDE4A | phosphodiesterase 4A, cAMP-specifi | -0.085 | 0.943 | 1.07E-01 | 1.07E+01 | | | |
| 8148448 | NM_006558 | KHDRBS3 | KH domain containing, RNA binding, | -0.085 | 0.943 | 1.21E-01 | 1.21E+01 | | | |
| 8008706 | NM_080677 | DYNLL2 | dynein, light chain, LC8-type 2 | -0.085 | 0.943 | 3.55E-01 | 3.55E+01 | | | |
| 8077804 | NM_014760 | TATDN2 | TatD DNase domain containing 2 | -0.085 | 0.943 | 5.70E-01 | 5.70E+01 | | | |
| 8164304 | NM_013443 | ST6GALNAC6 | ST6 (alpha-N-acetyl-neuraminyl-2,3- | -0.085 | 0.943 | 3.02E-01 | 3.02E+01 | | | |
| 7902848 | --- | --- | --- | -0.085 | 0.943 | 8.14E-01 | 8.14E+01 | | | |
| 8141228 | NM_00113445 | TMEM130 | transmembrane protein 130 | -0.085 | 0.943 | 1.52E-01 | 1.52E+01 | | | |
| 8031949 | NM_207395 | ZNF324B | zinc finger protein 324B | -0.085 | 0.943 | 1.10E-01 | 1.10E+01 | | | |
| 8140113 | NM_004603 | STX1A | syntaxin 1A (brain) | -0.085 | 0.943 | 1.91E-01 | 1.91E+01 | | | |
| 8157804 | NM_182487 | OLFML2A | olfactomedin-like 2A | -0.085 | 0.943 | 1.16E-01 | 1.16E+01 | | | |
| 7984771 | NM_005576 | LOXL1 | lysyl oxidase-like 1 | -0.085 | 0.943 | 8.43E-02 | 8.43E+00 | | | |
| 7960637 | NM_020400 | LPAR5 | lysophosphatidic acid receptor 5 | -0.085 | 0.943 | 2.41E-01 | 2.41E+01 | | | |
| 8152522 | NM_006209 | ENPP2 | ectonucleotide pyrophosphatase/ph | -0.085 | 0.943 | 3.45E-01 | 3.45E+01 | | | |
| 7960253 | NM_016533 | NINJ2 | ninjurin 2 | -0.085 | 0.943 | 2.43E-01 | 2.43E+01 | | | |
| 7907847 | --- | --- | --- | -0.085 | 0.943 | 3.46E-01 | 3.46E+01 | | | |
| 8133119 | BC014249 | VKORC1L1 | vitamin K epoxide reductase comple | -0.085 | 0.943 | 1.21E-01 | 1.21E+01 | | | |
| 8170753 | NM_014370 | SRPK3 | SFRS protein kinase 3 | -0.085 | 0.943 | 6.07E-02 | 6.07E+00 | | | |
| 7951485 | NM_017515 | SLC35F2 | solute carrier family 35, member F2 | -0.085 | 0.942 | 4.80E-01 | 4.80E+01 | | | |
| 7986642 | NR_027407 | GOLGA8D | golgi autoantigen, golgin subfamily a | -0.085 | 0.942 | 1.26E-01 | 1.26E+01 | | | |
| 7895408 | --- | --- | --- | -0.085 | 0.942 | 8.46E-01 | 8.46E+01 | | | |
| 8102468 | NM_003619 | PRSS12 | protease, serine, 12 (neurotrypsin, m | -0.085 | 0.942 | 1.22E-01 | 1.22E+01 | | | |
| 8079919 | NM_144499 | GNAT1 | guanine nucleotide binding protein ( | -0.085 | 0.942 | 1.30E-01 | 1.30E+01 | | | |
| 7975889 | NM_014909 | VASH1 | vasohibin 1 | -0.085 | 0.942 | 9.81E-02 | 9.81E+00 | | | |
| 8034578 | NM_006563 | KLF1 | Kruppel-like factor 1 (erythroid) | -0.086 | 0.942 | 2.00E-01 | 2.00E+01 | | | |
| 7931097 | NM_002775 | HTRA1 | HtrA serine peptidase 1 | -0.086 | 0.942 | 6.62E-02 | 6.62E+00 | | | |
| 7986960 | NM_015307 | FAM189A1 | family with sequence similarity 189, | -0.086 | 0.942 | 2.43E-01 | 2.43E+01 | | | |
| 8149256 | --- | --- | --- | -0.086 | 0.942 | 1.96E-01 | 1.96E+01 | | | |
| 8145799 | NM_080872 | UNC5D | unc-5 homolog D (C. elegans) | -0.086 | 0.942 | 3.96E-02 | 3.96E+00 | | | |
| 7950701 | NM_00109881 | ODZ4 | odz, odd Oz/ten-m homolog 4 (Droso | -0.086 | 0.942 | 7.71E-02 | 7.71E+00 | | | |
| 8084732 | NM_022147 | RTP4 | receptor (chemosensory) transporte | -0.086 | 0.942 | 4.79E-01 | 4.79E+01 | | | |
| 8063043 | NM_181802 | UBE2C | ubiquitin-conjugating enzyme E2C | -0.086 | 0.942 | 1.74E-01 | 1.74E+01 | | | |
| 7999427 | NM_005425 | TNP2 | transition protein 2 (during histone t | -0.086 | 0.942 | 1.80E-01 | 1.80E+01 | | | |
| 7985418 | ENST0000342 | LOC440297 | chondroitin sulfate proteoglycan 4 p | -0.086 | 0.942 | 5.86E-02 | 5.86E+00 | | | |
| 7985459 | ENST0000342 | LOC440297 | chondroitin sulfate proteoglycan 4 p | -0.086 | 0.942 | 5.86E-02 | 5.86E+00 | | | |
| 7990928 | ENST0000342 | LOC440297 | chondroitin sulfate proteoglycan 4 p | -0.086 | 0.942 | 5.86E-02 | 5.86E+00 | | | |
| 7894979 | --- | --- | --- | -0.086 | 0.942 | 2.95E-01 | 2.95E+01 | | | |
| 8158224 | NM_005094 | SLC27A4 | solute carrier family 27 (fatty acid tra | -0.086 | 0.942 | 6.45E-02 | 6.45E+00 | | | |
| 7896654 | --- | --- | --- | -0.086 | 0.942 | 9.09E-01 | 9.09E+01 | | | |
| 7924840 | --- | --- | --- | -0.086 | 0.942 | 3.58E-01 | 3.58E+01 | | | |
| 8152606 | NM_021021 | SNTB1 | syntrophin, beta 1 (dystrophin-assoc | -0.086 | 0.942 | 1.83E-01 | 1.83E+01 | | | |
| 8104394 | NM_020546 | ADCY2 | adenylate cyclase 2 (brain) | -0.086 | 0.942 | 2.03E-01 | 2.03E+01 | | | |
| 8019005 | NM_00108257 | HCG_177600 | hexaribonucleotide binding protein 3 | -0.086 | 0.942 | 1.99E-01 | 1.99E+01 | | | |
| 7896102 | --- | --- | --- | -0.086 | 0.942 | 9.46E-01 | 9.46E+01 | | | |
| 8009873 | NR_003587 | MYO15B | myosin XVB pseudogene | -0.086 | 0.942 | 2.23E-01 | 2.23E+01 | | | |
| 7983553 | NM_205850 | SLC24A5 | solute carrier family 24, member 5 | -0.086 | 0.942 | 3.30E-01 | 3.30E+01 | | | |
| 8018379 | NM_020753 | CASKIN2 | CASK interacting protein 2 | -0.086 | 0.942 | 1.98E-01 | 1.98E+01 | | | |
| 7899348 | --- | --- | --- | -0.086 | 0.942 | 1.65E-01 | 1.65E+01 | | | |
| 7959234 | NM_000545 | HNF1A | HNF1 homeobox A | -0.086 | 0.942 | 1.21E-01 | 1.21E+01 | | | |
| 8077989 | NR_002223 | TPRXL | tetra-peptide repeat homeobox-like | -0.086 | 0.942 | 1.75E-01 | 1.75E+01 | | | |
| 7893105 | --- | --- | --- | -0.086 | 0.942 | 3.71E-01 | 3.71E+01 | | | |
| 8175023 | NM_016032 | ZDHHC9 | zinc finger, DHHC-type containing 9 | -0.086 | 0.942 | 1.91E-01 | 1.91E+01 | | | |
| 7904018 | NM_018704 | CTTNBP2NL | CTTNBP2 N-terminal like | -0.086 | 0.942 | 3.38E-01 | 3.38E+01 | | | |
| 8013536 | NM_000625 | NOS2 | nitric oxide synthase 2, inducible | -0.086 | 0.942 | 8.30E-02 | 8.30E+00 | | | |
| 8049528 | NM_00113755 | LRRFIP1 | leucine rich repeat (in FLII) interactin | -0.086 | 0.942 | 8.70E-01 | 8.70E+01 | | | |
| 7893158 | --- | --- | --- | -0.086 | 0.942 | 5.88E-01 | 5.88E+01 | | | |
| 7898314 | NM_000085 | CLCNKB | chloride channel Kb | -0.086 | 0.942 | 1.21E-01 | 1.21E+01 | | | |
| 8171284 | NM_000273 | GPR143 | G protein-coupled receptor 143 | -0.086 | 0.942 | 1.96E-01 | 1.96E+01 | | | |
| 7946446 | NM_020645 | NRIP3 | nuclear receptor interacting protein | -0.086 | 0.942 | 2.59E-01 | 2.59E+01 | | | |
| 8061919 | NM_005093 | CBFA2T2 | core-binding factor, runt domain, alp | -0.086 | 0.942 | 2.19E-01 | 2.19E+01 | | | |
| 8157383 | NM_032888 | COL27A1 | collagen, type XXVII, alpha 1 | -0.086 | 0.942 | 1.03E-01 | 1.03E+01 | | | |
| 7894271 | --- | --- | --- | -0.086 | 0.942 | 9.13E-01 | 9.13E+01 | | | |
| 7960744 | NM_001733 | C1R | complement component 1, r subcom | -0.086 | 0.942 | 1.60E-01 | 1.60E+01 | | | |
| 7985765 | --- | --- | --- | -0.086 | 0.942 | 2.04E-01 | 2.04E+01 | | | |
| 8147303 | --- | --- | --- | -0.086 | 0.942 | 2.89E-01 | 2.89E+01 | | | |
| 8143879 | NM_00113604 | TMUB1 | transmembrane and ubiquitin-like do | -0.086 | 0.942 | 2.17E-01 | 2.17E+01 | | | |
| 8140965 | --- | --- | --- | -0.086 | 0.942 | 2.52E-01 | 2.52E+01 | | | |
| 7962240 | --- | --- | --- | -0.086 | 0.942 | 1.44E-01 | 1.44E+01 | | | |
| 8110668 | --- | --- | --- | -0.086 | 0.942 | 6.65E-01 | 6.65E+01 | | | |
| 7970648 | NM_152704 | FAM123A | family with sequence similarity 123A | -0.086 | 0.942 | 8.75E-02 | 8.75E+00 | | | |
| 7983987 | --- | --- | --- | -0.086 | 0.942 | 4.11E-01 | 4.11E+01 | | | |
| 8100977 | NM_002994 | CXCL5 | chemokine (C-X-C motif) ligand 5 | -0.086 | 0.942 | 2.96E-01 | 2.96E+01 | | | |
| 8149966 | NM_173833 | SCARA5 | scavenger receptor class A, member | -0.086 | 0.942 | 1.86E-01 | 1.86E+01 | | | |
| 8010237 | ENST0000340 | C17orf99 | chromosome 17 open reading frame | -0.086 | 0.942 | 1.95E-01 | 1.95E+01 | | | |
| 8156196 | NM_00100170 | C9orf170 | chromosome 9 open reading frame 1 | -0.086 | 0.942 | 1.49E-01 | 1.49E+01 | | | |
| 8145622 | --- | --- | --- | -0.086 | 0.942 | 7.37E-01 | 7.37E+01 | | | |
| 8074196 | NM_014406 | CCT8L2 | chaperonin containing TCP1, subunit | -0.086 | 0.942 | 2.29E-01 | 2.29E+01 | | | |
| 7915345 | NM_00114258 | NFYC | nuclear transcription factor Y, gamm | -0.086 | 0.942 | 1.21E-01 | 1.21E+01 | | | |

| | | | | | | | | | | |
|---|---|---|---|---|---|---|---|---|---|---|
| 8051298 | NM_024572 | GALNT14 | UDP-N-acetyl-alpha-D-galactosamine | -0.086 | 0.942 | 6.92E-02 | 6.92E+00 | | | |
| 8050474 | NM_020905 | RDH14 | retinol dehydrogenase 14 (all-trans/9 | -0.086 | 0.942 | 3.00E-01 | 3.00E+01 | | | |
| 8177951 | NR_026791 | HCG27 | HLA complex group 27 | -0.086 | 0.942 | 3.13E-01 | 3.13E+01 | | | |
| 7934299 | NM_015901 | NUDT13 | nudix (nucleoside diphosphate linked | -0.086 | 0.942 | 8.24E-01 | 8.24E+01 | | | |
| 7911902 | NM_015102 | NPHP4 | nephronophthisis 4 | -0.086 | 0.942 | 1.79E-02 | 1.79E+00 | | | |
| 8148559 | NM_016647 | C8orf55 | chromosome 8 open reading frame 5 | -0.086 | 0.942 | 1.07E-01 | 1.07E+01 | | | |
| 8071155 | NM_017414 | USP18 | ubiquitin specific peptidase 18 | -0.086 | 0.942 | 4.03E-01 | 4.03E+01 | | | |
| 8005557 | NM_014964 | EPN2 | epsin 2 | -0.086 | 0.942 | 9.31E-02 | 9.31E+00 | | | |
| 7986945 | --- | --- | --- | -0.086 | 0.942 | 5.49E-01 | 5.49E+01 | | | |
| 7895782 | --- | --- | --- | -0.086 | 0.942 | 4.67E-01 | 4.67E+01 | | | |
| 8113701 | --- | --- | --- | -0.086 | 0.942 | 8.39E-01 | 8.39E+01 | | | |
| 7896457 | --- | --- | --- | -0.086 | 0.942 | 9.16E-01 | 9.16E+01 | | | |
| 8049952 | NM_173821 | C2orf85 | chromosome 2 open reading frame 8 | -0.086 | 0.942 | 1.15E-01 | 1.15E+01 | | | |
| 7940959 | NM_020155 | GPR137 | G protein-coupled receptor 137 | -0.086 | 0.942 | 1.52E-01 | 1.52E+01 | | | |
| 8002969 | NM_00103180 | MAF | v-maf musculoaponeurotic fibrosarc | -0.087 | 0.942 | 2.07E-01 | 2.07E+01 | | | |
| 8146388 | AK128232 | FLJ46365 | hypothetical LOC401459 | -0.087 | 0.942 | 1.27E-01 | 1.27E+01 | | | |
| 7982587 | NM_207444 | C15orf53 | chromosome 15 open reading frame | -0.087 | 0.942 | 1.15E-01 | 1.15E+01 | | | |
| 8086669 | NM_199183 | TESSP5 | testis serine protease 5 | -0.087 | 0.942 | 7.46E-02 | 7.46E+00 | | | |
| 7952677 | NM_006165 | NFRKB | nuclear factor related to kappaB bin | -0.087 | 0.942 | 3.47E-01 | 3.47E+01 | | | |
| 7959848 | --- | --- | --- | -0.087 | 0.942 | 3.12E-01 | 3.12E+01 | | | |
| 8011270 | ENST0000038 | LOC284009 | hypothetical protein LOC284009 | -0.087 | 0.942 | 1.02E-01 | 1.02E+01 | | | |
| 8180093 | NM_002119 | HLA-DOA | major histocompatibility complex, cl | -0.087 | 0.942 | 1.61E-01 | 1.61E+01 | | | |
| 7906898 | --- | --- | --- | -0.087 | 0.942 | 1.88E-01 | 1.88E+01 | | | |
| 7960054 | NR_003290 | EP400NL | EP400 N-terminal like | -0.087 | 0.942 | 1.30E-01 | 1.30E+01 | | | |
| 8031475 | NM_139172 | TMEM190 | transmembrane protein 190 | -0.087 | 0.942 | 1.03E-01 | 1.03E+01 | | | |
| 8027584 | NM_024076 | KCTD15 | potassium channel tetramerisation d | -0.087 | 0.942 | 1.47E-01 | 1.47E+01 | | | |
| 7914880 | NM_005202 | COL8A2 | collagen, type VIII, alpha 2 | -0.087 | 0.942 | 1.23E-01 | 1.23E+01 | | | |
| 8005865 | NM_015077 | SARM1 | sterile alpha and TIR motif containin | -0.087 | 0.942 | 1.43E-01 | 1.43E+01 | | | |
| 7925876 | NM_002627 | PFKP | phosphofructokinase, platelet | -0.087 | 0.942 | 6.15E-01 | 6.15E+01 | | | |
| 7982339 | AK097050 | OC10013085 | hypothetical protein LOC100130857 | -0.087 | 0.942 | 2.28E-01 | 2.28E+01 | | | |
| 7953150 | NM_003213 | TEAD4 | TEA domain family member 4 | -0.087 | 0.942 | 5.23E-02 | 5.23E+00 | | | |
| 7893112 | --- | --- | --- | -0.087 | 0.942 | 9.23E-01 | 9.23E+01 | | | |
| 7989473 | NM_00100759 | FAM148B | family with sequence similarity 148, | -0.087 | 0.942 | 1.84E-01 | 1.84E+01 | | | |
| 7955045 | BC008360 | FAM113B | family with sequence similarity 113, | -0.087 | 0.942 | 2.43E-01 | 2.43E+01 | | | |
| 8008825 | NM_024612 | DHX40 | DEAH (Asp-Glu-Ala-His) box polypept | -0.087 | 0.942 | 4.41E-01 | 4.41E+01 | | | |
| 7948836 | NM_00108050 | TMEM223 | transmembrane protein 223 | -0.087 | 0.942 | 4.18E-01 | 4.18E+01 | | | |
| 7923233 | NM_017596 | KIF21B | kinesin family member 21B | -0.087 | 0.942 | 1.05E-01 | 1.05E+01 | | | |
| 7893728 | --- | --- | --- | -0.087 | 0.942 | 5.89E-01 | 5.89E+01 | | | |
| 8170468 | NM_005342 | HMGB3 | high-mobility group box 3 | -0.087 | 0.942 | 3.19E-01 | 3.19E+01 | | | |
| 8063389 | AK090605 | LOC284751 | hypothetical LOC284751 | -0.087 | 0.942 | 2.55E-01 | 2.55E+01 | | | |
| 8170562 | NM_175742 | MAGEA2 | melanoma antigen family A, 2 | -0.087 | 0.942 | 7.68E-02 | 7.68E+00 | | | |
| 8133721 | NM_001540 | HSPB1 | heat shock 27kDa protein 1 | -0.087 | 0.942 | 2.15E-01 | 2.15E+01 | | | |
| 7898627 | NM_00103950 | VWA5B1 | von Willebrand factor A domain cont | -0.087 | 0.942 | 1.05E-01 | 1.05E+01 | | | |
| 7989501 | NM_001218 | CA12 | carbonic anhydrase XII | -0.087 | 0.942 | 1.37E-01 | 1.37E+01 | | | |
| 8069340 | NM_058181 | C21orf57 | chromosome 21 open reading frame | -0.087 | 0.942 | 6.11E-01 | 6.11E+01 | | | |
| 8006569 | NM_145272 | C17orf50 | chromosome 17 open reading frame | -0.087 | 0.942 | 1.87E-01 | 1.87E+01 | | | |
| 8049152 | NM_000751 | CHRND | cholinergic receptor, nicotinic, delta | -0.087 | 0.942 | 1.74E-01 | 1.74E+01 | | | |
| 8164343 | NM_00103525 | FAM102A | family with sequence similarity 102, | -0.087 | 0.942 | 7.73E-01 | 7.73E+01 | | | |
| 8075550 | AK294232 | C22orf24 | chromosome 22 open reading frame | -0.087 | 0.941 | 1.86E-01 | 1.86E+01 | | | |
| 8098470 | NM_024949 | WWC2 | WW and C2 domain containing 2 | -0.087 | 0.941 | 2.67E-01 | 2.67E+01 | | | |
| 8102636 | NM_152399 | TMEM155 | transmembrane protein 155 | -0.087 | 0.941 | 1.46E-01 | 1.46E+01 | | | |
| 8179184 | NM_013993 | DDR1 | discoidin domain receptor tyrosine k | -0.087 | 0.941 | 5.11E-02 | 5.11E+00 | | | |
| 7970674 | --- | --- | --- | -0.087 | 0.941 | 1.45E-01 | 1.45E+01 | | | |
| 8013157 | NM_00103355 | TOM1L2 | target of myb1-like 2 (chicken) | -0.087 | 0.941 | 2.26E-01 | 2.26E+01 | | | |
| 7898256 | NM_00101364 | TMEM82 | transmembrane protein 82 | -0.087 | 0.941 | 9.73E-02 | 9.73E+00 | | | |
| 7925763 | NM_030645 | SH3BP5L | SH3-binding domain protein 5-like | -0.087 | 0.941 | 1.07E-01 | 1.07E+01 | | | |
| 8064808 | NM_005116 | SLC23A2 | solute carrier family 23 (nucleobase | -0.087 | 0.941 | 3.67E-01 | 3.67E+01 | | | |
| 7896061 | --- | --- | --- | -0.087 | 0.941 | 6.79E-01 | 6.79E+01 | | | |
| 8170247 | AK093505 | CXorf18 | chromosome X open reading frame 1 | -0.087 | 0.941 | 1.61E-01 | 1.61E+01 | | | |
| 7965094 | NM_203394 | E2F7 | E2F transcription factor 7 | -0.087 | 0.941 | 1.21E-01 | 1.21E+01 | | | |
| 8030383 | NM_020650 | RCN3 | reticulocalbin 3, EF-hand calcium bin | -0.087 | 0.941 | 3.12E-01 | 3.12E+01 | | | |
| 7949275 | --- | --- | --- | -0.087 | 0.941 | 1.36E-01 | 1.36E+01 | | | |
| 7960165 | NM_003415 | ZNF268 | zinc finger protein 268 | -0.087 | 0.941 | 2.10E-01 | 2.10E+01 | | | |
| 7991478 | NM_00110261 | LOC145814 | pyroglutamyl-peptidase 1-like | -0.087 | 0.941 | 1.54E-01 | 1.54E+01 | | | |
| 8149811 | NM_006167 | NKX3-1 | NK3 homeobox 1 | -0.087 | 0.941 | 1.12E-01 | 1.12E+01 | | | |
| 7894466 | --- | --- | --- | -0.087 | 0.941 | 7.23E-01 | 7.23E+01 | | | |
| 7895282 | --- | --- | --- | -0.087 | 0.941 | 5.42E-01 | 5.42E+01 | | | |
| 8136727 | 0 | 0 | 0 | -0.087 | 0.941 | 3.36E-02 | 3.36E+00 | | | |
| 8146559 | --- | --- | --- | -0.087 | 0.941 | 1.44E-01 | 1.44E+01 | | | |
| 7893571 | --- | --- | --- | -0.087 | 0.941 | 8.28E-01 | 8.28E+01 | | | |
| 8160870 | NM_006664 | CCL27 | chemokine (C-C motif) ligand 27 | -0.087 | 0.941 | 2.06E-01 | 2.06E+01 | | | |
| 8148304 | NM_025195 | TRIB1 | tribbles homolog 1 (Drosophila) | -0.087 | 0.941 | 7.30E-01 | 7.30E+01 | | | |
| 8109746 | --- | --- | --- | -0.087 | 0.941 | 1.94E-01 | 1.94E+01 | | | |
| 7974531 | NR_004844 | RPL13AP3 | ribosomal protein L13a pseudogene | -0.087 | 0.941 | 2.19E-01 | 2.19E+01 | | | |
| 8114733 | NM_022481 | ARAP3 | ArfGAP with RhoGAP domain, ankyri | -0.087 | 0.941 | 7.84E-02 | 7.84E+00 | | | |
| 8036749 | NM_153232 | EID2 | EP300 interacting inhibitor of differe | -0.087 | 0.941 | 5.24E-02 | 5.24E+00 | | | |
| 7893003 | --- | --- | --- | -0.087 | 0.941 | 3.27E-01 | 3.27E+01 | | | |

| | | | | | | | | | | |
|---|---|---|---|---|---|---|---|---|---|---|
| 7971218 | NM_032138 | KBTBD7 | kelch repeat and BTB (POZ) domain c | -0.087 | 0.941 | 4.24E-01 | 4.24E+01 | | | |
| 7905831 | NM_025207 | FLAD1 | FAD1 flavin adenine dinucleotide syn | -0.087 | 0.941 | 1.19E-01 | 1.19E+01 | | | |
| 8139723 | NR_027342 | FKBP9L | FK506 binding protein 9-like | -0.087 | 0.941 | 1.55E-01 | 1.55E+01 | | | |
| 8054465 | ENST00000429 | hCG_173246 | hCG1732469 | -0.087 | 0.941 | 1.22E-01 | 1.22E+01 | | | |
| 8010787 | NM_173620 | HEXDC | hexosaminidase (glycosyl hydrolase f | -0.087 | 0.941 | 1.48E-01 | 1.48E+01 | | | |
| 8060609 | --- | --- | --- | -0.087 | 0.941 | 2.68E-01 | 2.68E+01 | | | |
| 7939738 | NM_000107 | DDB2 | damage-specific DNA binding protein | -0.087 | 0.941 | 4.88E-01 | 4.88E+01 | | | |
| 7973894 | CR595167 | RPL23AP71 | ribosomal protein L23a pseudogene | -0.087 | 0.941 | 1.18E-01 | 1.18E+01 | | | |
| 7998414 | --- | --- | --- | -0.087 | 0.941 | 9.23E-02 | 9.23E+00 | | | |
| 7948630 | NM_021727 | FADS3 | fatty acid desaturase 3 | -0.087 | 0.941 | 1.52E-01 | 1.52E+01 | | | |
| 8045814 | --- | --- | --- | -0.087 | 0.941 | 2.59E-01 | 2.59E+01 | | | |
| 8180414 | --- | --- | --- | -0.087 | 0.941 | 1.07E-01 | 1.07E+01 | | | |
| 8132097 | NM_032222 | FAM188B | family with sequence similarity 188, | -0.088 | 0.941 | 1.26E-01 | 1.26E+01 | | | |
| 8052956 | NM_015189 | EXOC6B | exocyst complex component 6B | -0.088 | 0.941 | 6.85E-01 | 6.85E+01 | | | |
| 8061946 | NM_032819 | ZNF341 | zinc finger protein 341 | -0.088 | 0.941 | 5.46E-02 | 5.46E+00 | | | |
| 8174239 | NM_032621 | BEX2 | brain expressed X-linked 2 | -0.088 | 0.941 | 7.39E-01 | 7.39E+01 | | | |
| 8004043 | NM_001976 | ENO3 | enolase 3 (beta, muscle) | -0.088 | 0.941 | 6.62E-01 | 6.62E+01 | | | |
| 8166876 | NM_001356 | DDX3X | DEAD (Asp-Glu-Ala-Asp) box polypep | -0.088 | 0.941 | 5.00E-01 | 5.00E+01 | | | |
| 7959298 | NM_00108082 | TMEM120B | transmembrane protein 120B | -0.088 | 0.941 | 2.68E-01 | 2.68E+01 | | | |
| 8175311 | NM_00103170 | CXorf48 | chromosome X open reading frame 4 | -0.088 | 0.941 | 3.09E-01 | 3.09E+01 | | | |
| 7894181 | --- | --- | --- | -0.088 | 0.941 | 5.38E-01 | 5.38E+01 | | | |
| 7963375 | NM_033045 | KRT84 | keratin 84 | -0.088 | 0.941 | 1.84E-01 | 1.84E+01 | | | |
| 8027556 | NM_002333 | LRP3 | low density lipoprotein receptor-rela | -0.088 | 0.941 | 1.06E-01 | 1.06E+01 | | | |
| 7998392 | --- | --- | --- | -0.088 | 0.941 | 8.97E-02 | 8.97E+00 | | | |
| 8113873 | NM_005340 | HINT1 | histidine triad nucleotide binding pro | -0.088 | 0.941 | 4.40E-01 | 4.40E+01 | | | |
| 8051133 | NR_002201 | FTHL3P | ferritin, heavy polypeptide-like 3 pse | -0.088 | 0.941 | 4.54E-01 | 4.54E+01 | | | |
| 8074944 | NM_198440 | DERL3 | Der1-like domain family, member 3 | -0.088 | 0.941 | 1.56E-01 | 1.56E+01 | | | |
| 7911458 | NM_030649 | ACAP3 | ArfGAP with coiled-coil, ankyrin repe | -0.088 | 0.941 | 1.48E-01 | 1.48E+01 | | | |
| 7958051 | NM_004316 | ASCL1 | achaete-scute complex homolog 1 (D | -0.088 | 0.941 | 1.83E-01 | 1.83E+01 | | | |
| 7919131 | --- | --- | --- | -0.088 | 0.941 | 3.14E-01 | 3.14E+01 | | | |
| 8040415 | --- | --- | --- | -0.088 | 0.941 | 2.46E-01 | 2.46E+01 | | | |
| 7950299 | NM_153614 | DNAJB13 | DnaJ (Hsp40) related, subfamily B, m | -0.088 | 0.941 | 1.12E-01 | 1.12E+01 | | | |
| 8130867 | NM_003247 | THBS2 | thrombospondin 2 | -0.088 | 0.941 | 1.20E-01 | 1.20E+01 | | | |
| 8037502 | NM_00113106 | NKPD1 | NTPase, KAP family P-loop domain co | -0.088 | 0.941 | 1.38E-01 | 1.38E+01 | | | |
| 8124806 | NM_007243 | NRM | nurim (nuclear envelope membrane | -0.088 | 0.941 | 1.95E-01 | 1.95E+01 | | | |
| 8019170 | BC108932 | C17orf55 | chromosome 17 open reading frame | -0.088 | 0.941 | 1.16E-01 | 1.16E+01 | | | |
| 8063437 | NM_173485 | TSHZ2 | teashirt zinc finger homeobox 2 | -0.088 | 0.941 | 2.41E-01 | 2.41E+01 | | | |
| 7898176 | NM_033440 | CELA2A | chymotrypsin-like elastase family, m | -0.088 | 0.941 | 2.28E-01 | 2.28E+01 | | | |
| 7920725 | NM_005698 | SCAMP3 | secretory carrier membrane protein | -0.088 | 0.941 | 3.97E-01 | 3.97E+01 | | | |
| 7894436 | --- | --- | --- | -0.088 | 0.941 | 4.97E-01 | 4.97E+01 | | | |
| 8145889 | NM_004095 | EIF4EBP1 | eukaryotic translation initiation facto | -0.088 | 0.941 | 1.52E-01 | 1.52E+01 | | | |
| 7918936 | NM_024626 | VTCN1 | V-set domain containing T cell activa | -0.088 | 0.941 | 2.59E-01 | 2.59E+01 | | | |
| 7957654 | NM_152435 | AMDHD1 | amidohydrolase domain containing 1 | -0.088 | 0.941 | 1.30E-01 | 1.30E+01 | | | |
| 8036913 | NM_000713 | BLVRB | biliverdin reductase B (flavin reducta | -0.088 | 0.941 | 4.45E-01 | 4.45E+01 | | | |
| 8141305 | NM_032164 | ZNF394 | zinc finger protein 394 | -0.088 | 0.941 | 6.78E-01 | 6.78E+01 | | | |
| 7953651 | NM_00113102 | PEX5 | peroxisomal biogenesis factor 5 | -0.088 | 0.941 | 3.38E-01 | 3.38E+01 | | | |
| 7950321 | NM_003356 | UCP3 | uncoupling protein 3 (mitochondrial, | -0.088 | 0.941 | 1.76E-01 | 1.76E+01 | | | |
| 8069301 | NM_001849 | COL6A2 | collagen, type VI, alpha 2 | -0.088 | 0.941 | 4.85E-02 | 4.85E+00 | | | |
| 7995334 | --- | --- | --- | -0.088 | 0.941 | 1.71E-01 | 1.71E+01 | | | |
| 8017488 | NM_022640 | CSH1 | chorionic somatomammotropin horm | -0.088 | 0.941 | 9.57E-02 | 9.57E+00 | | | |
| 8079401 | NM_000579 | CCR5 | chemokine (C-C motif) receptor 5 | -0.088 | 0.941 | 1.58E-01 | 1.58E+01 | | | |
| 8093298 | NM_000579 | CCR5 | chemokine (C-C motif) receptor 5 | -0.088 | 0.941 | 1.58E-01 | 1.58E+01 | | | |
| 8059783 | NM_019850 | NGEF | neuronal guanine nucleotide exchan | -0.088 | 0.941 | 7.84E-02 | 7.84E+00 | | | |
| 8002020 | NM_016140 | TPPP3 | tubulin polymerization-promoting pr | -0.088 | 0.941 | 2.11E-01 | 2.11E+01 | | | |
| 7892751 | --- | --- | --- | -0.088 | 0.941 | 3.46E-01 | 3.46E+01 | | | |
| 8163807 | NM_015651 | PHF19 | PHD finger protein 19 | -0.088 | 0.941 | 1.56E-01 | 1.56E+01 | | | |
| 8116012 | NM_031300 | MXD3 | MAX dimerization protein 3 | -0.088 | 0.941 | 9.66E-02 | 9.66E+00 | | | |
| 8037816 | NM_013403 | STRN4 | striatin, calmodulin binding protein 4 | -0.088 | 0.941 | 1.69E-01 | 1.69E+01 | | | |
| 8018823 | NM_00112719 | TMC6 | transmembrane channel-like 6 | -0.088 | 0.941 | 1.62E-01 | 1.62E+01 | | | |
| 7893544 | --- | --- | --- | -0.088 | 0.941 | 2.86E-01 | 2.86E+01 | | | |
| 8008409 | NM_018896 | CACNA1G | calcium channel, voltage-dependent | -0.088 | 0.941 | 8.72E-02 | 8.72E+00 | | | |
| 8087935 | NM_022908 | NT5DC2 | 5'-nucleotidase domain containing 2 | -0.088 | 0.941 | 1.21E-01 | 1.21E+01 | | | |
| 8080938 | NM_198159 | MITF | microphthalmia-associated transcrip | -0.088 | 0.941 | 9.03E-02 | 9.03E+00 | | | |
| 7972257 | --- | --- | --- | -0.088 | 0.941 | 6.25E-01 | 6.25E+01 | | | |
| 8033097 | NM_000635 | RFX2 | regulatory factor X, 2 (influences HLA | -0.088 | 0.941 | 9.75E-02 | 9.75E+00 | | | |
| 8031346 | NM_002255 | KIR2DL4 | killer cell immunoglobulin-like recept | -0.088 | 0.941 | 3.92E-01 | 3.92E+01 | | | |
| 8030736 | NM_033068 | ACPT | acid phosphatase, testicular | -0.088 | 0.941 | 6.31E-02 | 6.31E+00 | | | |
| 8057613 | --- | --- | --- | -0.088 | 0.941 | 4.86E-01 | 4.86E+01 | | | |
| 8164766 | NM_152572 | C9orf98 | chromosome 9 open reading frame 9 | -0.088 | 0.941 | 8.99E-02 | 8.99E+00 | | | |
| 7927363 | NM_00113755 | FAM25B | family with sequence similarity 25, m | -0.088 | 0.941 | 3.40E-01 | 3.40E+01 | | | |
| 7933327 | NM_00113755 | FAM25B | family with sequence similarity 25, m | -0.088 | 0.941 | 3.40E-01 | 3.40E+01 | | | |
| 7933423 | NM_00113755 | FAM25B | family with sequence similarity 25, m | -0.088 | 0.941 | 3.40E-01 | 3.40E+01 | | | |
| 7976292 | NM_024832 | RIN3 | Ras and Rab interactor 3 | -0.088 | 0.941 | 2.40E-01 | 2.40E+01 | | | |
| 8038225 | NM_020904 | PLEKHA4 | pleckstrin homology domain contain | -0.088 | 0.941 | 1.02E-01 | 1.02E+01 | | | |
| 7894896 | --- | --- | --- | -0.088 | 0.941 | 6.22E-01 | 6.22E+01 | | | |
| 8118669 | NM_002263 | KIFC1 | kinesin family member C1 | -0.088 | 0.941 | 1.09E-01 | 1.09E+01 | | | |
| 8074701 | NM_004173 | SLC7A4 | solute carrier family 7 (cationic amin | -0.088 | 0.941 | 1.91E-01 | 1.91E+01 | | | |

| | | | | | | | | | | | |
|---|---|---|---|---|---|---|---|---|---|---|---|
| 7994692 | NM_016151 | TAOK2 | TAO kinase 2 | -0.088 | 0.941 | 8.02E-02 | 8.02E+00 | | | |
| 7996608 | NM_00101383 | RLTPR | RGD motif, leucine rich repeats, trop | -0.088 | 0.941 | 5.67E-02 | 5.67E+00 | | | |
| 8112613 | --- | --- | --- | -0.088 | 0.941 | 2.52E-01 | 2.52E+01 | | | |
| 7918300 | NM_00103229 | PSRC1 | proline/serine-rich coiled-coil 1 | -0.088 | 0.941 | 9.72E-02 | 9.72E+00 | | | |
| 8143825 | NM_000238 | KCNH2 | potassium voltage-gated channel, su | -0.089 | 0.940 | 8.13E-02 | 8.13E+00 | | | |
| 8037032 | NM_198540 | B3GNT8 | UDP-GlcNAc:betaGal beta-1,3-N-ace | -0.089 | 0.940 | 1.08E-01 | 1.08E+01 | | | |
| 8071020 | NM_058180 | C21orf58 | chromosome 21 open reading frame | -0.089 | 0.940 | 9.64E-02 | 9.64E+00 | | | |
| 7973924 | NM_014672 | KIAA0391 | KIAA0391 | -0.089 | 0.940 | 7.62E-01 | 7.62E+01 | | | |
| 8070953 | NM_00114285 | C21orf56 | chromosome 21 open reading frame | -0.089 | 0.940 | 2.25E-01 | 2.25E+01 | | | |
| 8017555 | NM_001433 | ERN1 | endoplasmic reticulum to nucleus sig | -0.089 | 0.940 | 1.03E-01 | 1.03E+01 | | | |
| 8175690 | NM_00101154 | MAGEA10 | melanoma antigen family A, 10 | -0.089 | 0.940 | 1.85E-01 | 1.85E+01 | | | |
| 8160682 | NM_022917 | NOL6 | nucleolar protein family 6 (RNA-asso | -0.089 | 0.940 | 1.49E-01 | 1.49E+01 | | | |
| 8002975 | NM_152342 | CDYL2 | chromodomain protein, Y-like 2 | -0.089 | 0.940 | 1.83E-01 | 1.83E+01 | | | |
| 8038804 | NM_152353 | CLDND2 | claudin domain containing 2 | -0.089 | 0.940 | 1.55E-01 | 1.55E+01 | | | |
| 7937763 | NM_021134 | MRPL23 | mitochondrial ribosomal protein L23 | -0.089 | 0.940 | 1.41E-01 | 1.41E+01 | | | |
| 8026341 | NM_138501 | TECR | trans-2,3-enoyl-CoA reductase | -0.089 | 0.940 | 8.10E-01 | 8.10E+01 | | | |
| 7893259 | --- | --- | --- | -0.089 | 0.940 | 7.21E-01 | 7.21E+01 | | | |
| 8063057 | NM_033421 | SNX21 | sorting nexin family member 21 | -0.089 | 0.940 | 1.30E-01 | 1.30E+01 | | | |
| 8018774 | NM_018414 | ST6GALNAC1 | ST6 (alpha-N-acetyl-neuraminyl-2,3- | -0.089 | 0.940 | 1.16E-01 | 1.16E+01 | | | |
| 8014704 | --- | --- | --- | -0.089 | 0.940 | 3.10E-01 | 3.10E+01 | | | |
| 8025278 | NM_020415 | RETN | resistin | -0.089 | 0.940 | 1.57E-01 | 1.57E+01 | | | |
| 8087907 | NM_020163 | SEMA3G | sema domain, immunoglobulin doma | -0.089 | 0.940 | 1.25E-01 | 1.25E+01 | | | |
| 8104314 | NM_024337 | IRX1 | iroquois homeobox 1 | -0.089 | 0.940 | 6.26E-02 | 6.26E+00 | | | |
| 7940070 | NM_170746 | C11orf31 | chromosome 11 open reading frame | -0.089 | 0.940 | 5.31E-01 | 5.31E+01 | | | |
| 7920228 | NM_020393 | PGLYRP4 | peptidoglycan recognition protein 4 | -0.089 | 0.940 | 1.32E-01 | 1.32E+01 | | | |
| 8031700 | NM_00101587 | AURKC | aurora kinase C | -0.089 | 0.940 | 1.16E-01 | 1.16E+01 | | | |
| 8180273 | --- | --- | --- | -0.089 | 0.940 | 1.72E-01 | 1.72E+01 | | | |
| 7992568 | NM_004424 | E4F1 | E4F transcription factor 1 | -0.089 | 0.940 | 9.53E-02 | 9.53E+00 | | | |
| 8049670 | NM_002081 | GPC1 | glypican 1 | -0.089 | 0.940 | 7.83E-02 | 7.83E+00 | | | |
| 8017460 | NM_00109842 | SMARCD2 | SWI/SNF related, matrix associated, | -0.089 | 0.940 | 2.67E-01 | 2.67E+01 | | | |
| 7894490 | --- | --- | --- | -0.089 | 0.940 | 3.86E-01 | 3.86E+01 | | | |
| 8026170 | NM_030818 | CCDC130 | coiled-coil domain containing 130 | -0.089 | 0.940 | 3.61E-01 | 3.61E+01 | | | |
| 8139270 | NM_006989 | RASA4 | RAS p21 protein activator 4 | -0.089 | 0.940 | 3.73E-01 | 3.73E+01 | | | |
| 7963020 | NM_021044 | DHH | desert hedgehog homolog (Drosophi | -0.089 | 0.940 | 7.66E-02 | 7.66E+00 | | | |
| 8114476 | NM_00103763 | SIL1 | SIL1 homolog, endoplasmic reticulum | -0.089 | 0.940 | 1.87E-01 | 1.87E+01 | | | |
| 8171890 | --- | --- | --- | -0.089 | 0.940 | 3.71E-01 | 3.71E+01 | | | |
| 8134745 | NM_012447 | STAG3 | stromal antigen 3 | -0.089 | 0.940 | 7.80E-02 | 7.80E+00 | | | |
| 8070925 | --- | --- | --- | -0.089 | 0.940 | 8.85E-02 | 8.85E+00 | | | |
| 7938010 | NM_00100516 | OR52B6 | olfactory receptor, family 52, subfam | -0.089 | 0.940 | 2.93E-01 | 2.93E+01 | | | |
| 7939825 | NM_004551 | NDUFS3 | NADH dehydrogenase (ubiquinone) | -0.089 | 0.940 | 6.70E-01 | 6.70E+01 | | | |
| 8115584 | NM_024565 | CCNJL | cyclin J-like | -0.089 | 0.940 | 1.40E-01 | 1.40E+01 | | | |
| 7894823 | --- | --- | --- | -0.089 | 0.940 | 7.31E-01 | 7.31E+01 | | | |
| 7948176 | NM_033396 | TNKS1BP1 | tankyrase 1 binding protein 1, 182kD | -0.089 | 0.940 | 1.67E-01 | 1.67E+01 | | | |
| 8130703 | NM_003181 | T | T, brachyury homolog (mouse) | -0.089 | 0.940 | 8.93E-02 | 8.93E+00 | | | |
| 8093013 | NM_005781 | TNK2 | tyrosine kinase, non-receptor, 2 | -0.089 | 0.940 | 1.31E-01 | 1.31E+01 | | | |
| 8019908 | --- | --- | --- | -0.089 | 0.940 | 8.81E-01 | 8.81E+01 | | | |
| 7898102 | NM_201628 | RP1-21O18.1 | kazrin | -0.089 | 0.940 | 9.24E-02 | 9.24E+00 | | | |
| 7996677 | NM_005796 | NUTF2 | nuclear transport factor 2 | -0.089 | 0.940 | 7.03E-01 | 7.03E+01 | | | |
| 8130073 | NM_207360 | ZC3H12D | zinc finger CCCH-type containing 12D | -0.089 | 0.940 | 1.75E-01 | 1.75E+01 | | | |
| 7990090 | --- | --- | --- | -0.089 | 0.940 | 5.42E-01 | 5.42E+01 | | | |
| 8084397 | NM_014693 | ECE2 | endothelin converting enzyme 2 | -0.089 | 0.940 | 1.13E-01 | 1.13E+01 | | | |
| 7948772 | NM_030628 | INTS5 | integrator complex subunit 5 | -0.089 | 0.940 | 2.19E-01 | 2.19E+01 | | | |
| 8172028 | --- | --- | --- | -0.089 | 0.940 | 4.24E-01 | 4.24E+01 | | | |
| 8077238 | NM_152783 | D2HGDH | D-2-hydroxyglutarate dehydrogenase | -0.089 | 0.940 | 5.72E-02 | 5.72E+00 | | | |
| 7987097 | --- | --- | --- | -0.089 | 0.940 | 3.05E-01 | 3.05E+01 | | | |
| 8157698 | NM_080859 | OR1K1 | olfactory receptor, family 1, subfami | -0.089 | 0.940 | 1.54E-01 | 1.54E+01 | | | |
| 7933178 | --- | --- | --- | -0.089 | 0.940 | 4.12E-01 | 4.12E+01 | | | |
| 8063097 | NM_022104 | PCIF1 | PDX1 C-terminal inhibiting factor 1 | -0.089 | 0.940 | 3.36E-01 | 3.36E+01 | | | |
| 8024204 | NM_000455 | STK11 | serine/threonine kinase 11 | -0.089 | 0.940 | 1.70E-01 | 1.70E+01 | | | |
| 8010719 | NM_144999 | LRRC45 | leucine rich repeat containing 45 | -0.089 | 0.940 | 8.85E-02 | 8.85E+00 | | | |
| 7965090 | NM_001321 | CSRP2 | cysteine and glycine-rich protein 2 | -0.089 | 0.940 | 3.54E-01 | 3.54E+01 | | | |
| 7918569 | NM_017744 | ST7L | suppression of tumorigenicity 7 like | -0.089 | 0.940 | 3.45E-01 | 3.45E+01 | | | |
| 8024518 | NM_152791 | ZNF555 | zinc finger protein 555 | -0.089 | 0.940 | 1.80E-01 | 1.80E+01 | | | |
| 8118963 | NM_021922 | FANCE | Fanconi anemia, complementation g | -0.089 | 0.940 | 2.60E-01 | 2.60E+01 | | | |
| 7931977 | NM_030569 | ITIH5 | inter-alpha (globulin) inhibitor H5 | -0.089 | 0.940 | 1.59E-01 | 1.59E+01 | | | |
| 7939620 | NM_152312 | GYLTL1B | glycosyltransferase-like 1B | -0.089 | 0.940 | 4.12E-02 | 4.12E+00 | | | |
| 8006319 | --- | --- | --- | -0.089 | 0.940 | 3.93E-01 | 3.93E+01 | | | |
| 7940409 | NM_014207 | CD5 | CD5 molecule | -0.089 | 0.940 | 6.37E-01 | 6.37E+01 | | | |
| 7905308 | NM_00115964 | BNIPL | BCL2/adenovirus E1B 19kD interactin | -0.089 | 0.940 | 1.75E-01 | 1.75E+01 | | | |
| 7979927 | NM_003814 | ADAM20 | ADAM metallopeptidase domain 20 | -0.089 | 0.940 | 1.39E-01 | 1.39E+01 | | | |
| 7949956 | NM_004923 | MTL5 | metallothionein-like 5, testis-specific | -0.089 | 0.940 | 6.83E-02 | 6.83E+00 | | | |
| 7943984 | NM_006006 | ZBTB16 | zinc finger and BTB domain containin | -0.089 | 0.940 | 1.09E-01 | 1.09E+01 | | | |
| 8008277 | NM_000023 | SGCA | sarcoglycan, alpha (50kDa dystrophin | -0.089 | 0.940 | 7.73E-02 | 7.73E+00 | | | |
| 8026954 | NM_005027 | PIK3R2 | phosphoinositide-3-kinase, regulator | -0.089 | 0.940 | 1.63E-01 | 1.63E+01 | | | |
| 8005446 | AK127974 | LOC339240 | keratin pseudogene | -0.089 | 0.940 | 3.48E-01 | 3.48E+01 | | | |
| 8159521 | NM_000954 | PTGDS | prostaglandin D2 synthase 21kDa (br | -0.089 | 0.940 | 7.80E-02 | 7.80E+00 | | | |
| 7896376 | --- | --- | --- | -0.089 | 0.940 | 4.50E-01 | 4.50E+01 | | | |

| | | | | | | | | | | |
|---|---|---|---|---|---|---|---|---|---|---|
| 7927153 | --- | --- | --- | -0.089 | 0.940 | 2.72E-01 | 2.72E+01 | | | |
| 8097282 | NM_005841 | SPRY1 | sprouty homolog 1, antagonist of FG | -0.089 | 0.940 | 2.36E-01 | 2.36E+01 | | | |
| 7992197 | --- | --- | --- | -0.089 | 0.940 | 6.27E-02 | 6.27E+00 | | | |
| 8027748 | NM_021910 | FXYD3 | FXYD domain containing ion transpo | -0.089 | 0.940 | 1.33E-01 | 1.33E+01 | | | |
| 7913357 | NM_001397 | ECE1 | endothelin converting enzyme 1 | -0.089 | 0.940 | 4.73E-01 | 4.73E+01 | | | |
| 8110678 | NM_145265 | CCDC127 | coiled-coil domain containing 127 | -0.089 | 0.940 | 7.36E-01 | 7.36E+01 | | | |
| 7932109 | NM_012247 | SEPHS1 | selenophosphate synthetase 1 | -0.089 | 0.940 | 6.49E-01 | 6.49E+01 | | | |
| 7937112 | NM_017609 | C10orf92 | chromosome 10 open reading frame | -0.089 | 0.940 | 1.09E-01 | 1.09E+01 | | | |
| 8135149 | NM_020979 | SH2B2 | SH2B adaptor protein 2 | -0.089 | 0.940 | 9.43E-02 | 9.43E+00 | | | |
| 8175558 | NM_145665 | SPANXE | SPANX family, member E | -0.089 | 0.940 | 3.06E-01 | 3.06E+01 | | | |
| 7907690 | NM_022371 | TOR3A | torsin family 3, member A | -0.089 | 0.940 | 3.06E-01 | 3.06E+01 | | | |
| 8145568 | --- | --- | --- | -0.090 | 0.940 | 7.73E-02 | 7.73E+00 | | | |
| 8002904 | NM_012091 | ADAT1 | adenosine deaminase, tRNA-specific | -0.090 | 0.940 | 3.54E-01 | 3.54E+01 | | | |
| 7937707 | NR_026643 | FAM99A | family with sequence similarity 99, m | -0.090 | 0.940 | 3.11E-01 | 3.11E+01 | | | |
| 7995338 | --- | --- | --- | -0.090 | 0.940 | 2.59E-01 | 2.59E+01 | | | |
| 8034482 | NM_00113619 | TNPO2 | transportin 2 | -0.090 | 0.940 | 2.83E-01 | 2.83E+01 | | | |
| 8147693 | NR_002182 | NACAP1 | nascent-polypeptide-associated com | -0.090 | 0.940 | 1.95E-01 | 1.95E+01 | | | |
| 8143772 | NM_002889 | RARRES2 | retinoic acid receptor responder (taz | -0.090 | 0.940 | 1.15E-01 | 1.15E+01 | | | |
| 8068952 | NM_020132 | AGPAT3 | 1-acylglycerol-3-phosphate O-acyltra | -0.090 | 0.940 | 1.79E-01 | 1.79E+01 | | | |
| 8056041 | --- | --- | --- | -0.090 | 0.940 | 1.85E-01 | 1.85E+01 | | | |
| 8012528 | NM_00112807 | SPDYE4 | speedy homolog E4 (Xenopus laevis) | -0.090 | 0.940 | 1.33E-01 | 1.33E+01 | | | |
| 7905848 | NM_018655 | LENEP | lens epithelial protein | -0.090 | 0.940 | 1.85E-01 | 1.85E+01 | | | |
| 7940333 | NM_00109883 | MS4A15 | membrane-spanning 4-domains, sub | -0.090 | 0.940 | 1.77E-01 | 1.77E+01 | | | |
| 8059186 | NM_002846 | PTPRN | protein tyrosine phosphatase, recept | -0.090 | 0.940 | 1.70E-01 | 1.70E+01 | | | |
| 8021774 | ENST00000405 | FLJ44313 | FLJ44313 protein | -0.090 | 0.940 | 1.20E-01 | 1.20E+01 | | | |
| 8139935 | NM_00114544 | TYW1B | tRNA-yW synthesizing protein 1 hom | -0.090 | 0.940 | 5.60E-01 | 5.60E+01 | | | |
| 8007112 | NM_00114604 | KRTAP4-9 | keratin associated protein 4-9 | -0.090 | 0.940 | 2.60E-01 | 2.60E+01 | | | |
| 8138735 | NM_019102 | HOXA5 | homeobox A5 | -0.090 | 0.940 | 1.71E-01 | 1.71E+01 | | | |
| 7910164 | NM_020247 | CABC1 | chaperone, ABC1 activity of bc1 com | -0.090 | 0.940 | 1.69E-01 | 1.69E+01 | | | |
| 7933192 | NR_002726 | HNRNPA3P1 | heterogeneous nuclear ribonucleopr | -0.090 | 0.940 | 4.11E-01 | 4.11E+01 | | | |
| 8094134 | NR_027279 | USP17L6P | ubiquitin specific peptidase 17-like 6 | -0.090 | 0.940 | 6.49E-01 | 6.49E+01 | | | |
| 8136893 | NM_000083 | CLCN1 | chloride channel 1, skeletal muscle | -0.090 | 0.940 | 7.07E-02 | 7.07E+00 | | | |
| 8174977 | --- | --- | --- | -0.090 | 0.940 | 2.63E-01 | 2.63E+01 | | | |
| 8042513 | --- | --- | --- | -0.090 | 0.940 | 3.14E-01 | 3.14E+01 | | | |
| 7899821 | NM_020888 | KIAA1522 | KIAA1522 | -0.090 | 0.940 | 5.94E-02 | 5.94E+00 | | | |
| 8065798 | NM_178026 | GGT7 | gamma-glutamyltransferase 7 | -0.090 | 0.940 | 8.85E-02 | 8.85E+00 | | | |
| 7991224 | NM_178232 | HAPLN3 | hyaluronan and proteoglycan link pro | -0.090 | 0.940 | 1.99E-01 | 1.99E+01 | | | |
| 8007651 | NM_145663 | DBF4B | DBF4 homolog B (S. cerevisiae) | -0.090 | 0.940 | 1.24E-01 | 1.24E+01 | | | |
| 8093150 | --- | --- | --- | -0.090 | 0.940 | 3.04E-01 | 3.04E+01 | | | |
| 8018579 | NM_001988 | EVPL | envoplakin | -0.090 | 0.940 | 9.55E-02 | 9.55E+00 | | | |
| 7996164 | NM_199046 | TEPP | testis, prostate and placenta express | -0.090 | 0.940 | 1.28E-01 | 1.28E+01 | | | |
| 8071676 | NM_004914 | RAB36 | RAB36, member RAS oncogene famil | -0.090 | 0.940 | 1.67E-01 | 1.67E+01 | | | |
| 8175528 | NM_005634 | SOX3 | SRY (sex determining region Y)-box 3 | -0.090 | 0.940 | 1.27E-01 | 1.27E+01 | | | |
| 8033740 | NM_00100519 | OR7D4 | olfactory receptor, family 7, subfami | -0.090 | 0.940 | 2.57E-01 | 2.57E+01 | | | |
| 7914184 | NM_000952 | PTAFR | platelet-activating factor receptor | -0.090 | 0.940 | 4.64E-02 | 4.64E+00 | | | |
| 7960068 | NM_024078 | NOC4L | nucleolar complex associated 4 hom | -0.090 | 0.940 | 1.66E-01 | 1.66E+01 | | | |
| 8141728 | NM_022777 | RABL5 | RAB, member RAS oncogene family-l | -0.090 | 0.940 | 2.54E-01 | 2.54E+01 | | | |
| 8036787 | NM_003890 | FCGBP | Fc fragment of IgG binding protein | -0.090 | 0.940 | 2.06E-01 | 2.06E+01 | | | |
| 7978831 | NM_152329 | PPIL5 | peptidylprolyl isomerase (cyclophilin | -0.090 | 0.940 | 2.37E-01 | 2.37E+01 | | | |
| 8029592 | NM_007056 | SFRS16 | splicing factor, arginine/serine-rich 1 | -0.090 | 0.940 | 2.29E-01 | 2.29E+01 | | | |
| 8180340 | --- | --- | --- | -0.090 | 0.940 | 3.58E-02 | 3.58E+00 | | | |
| 7955845 | NM_017410 | HOXC13 | homeobox C13 | -0.090 | 0.940 | 1.58E-01 | 1.58E+01 | | | |
| 7973468 | NM_138360 | LRRC16B | leucine rich repeat containing 16B | -0.090 | 0.940 | 3.79E-02 | 3.79E+00 | | | |
| 8065776 | NM_014071 | NCOA6 | nuclear receptor coactivator 6 | -0.090 | 0.940 | 2.96E-01 | 2.96E+01 | | | |
| 7912672 | NR_024279 | hCG_200814 | hypothetical LOC729614 | -0.090 | 0.940 | 3.18E-01 | 3.18E+01 | | | |
| 8001981 | NM_013241 | FHOD1 | formin homology 2 domain containin | -0.090 | 0.940 | 1.23E-01 | 1.23E+01 | | | |
| 8144774 | NM_152415 | VPS37A | vacuolar protein sorting 37 homolog | -0.090 | 0.940 | 3.76E-01 | 3.76E+01 | | | |
| 7967586 | --- | --- | --- | -0.090 | 0.940 | 1.95E-01 | 1.95E+01 | | | |
| 8085370 | NR_003112 | GSTM1L | glutathione S-transferase mu 1-like | -0.090 | 0.939 | 2.47E-01 | 2.47E+01 | | | |
| 8038633 | NM_002257 | KLK1 | kallikrein 1 | -0.090 | 0.939 | 1.74E-01 | 1.74E+01 | | | |
| 8060257 | NM_006374 | STK25 | serine/threonine kinase 25 (STE20 ho | -0.090 | 0.939 | 7.38E-02 | 7.38E+00 | | | |
| 8044491 | NM_032309 | CHCHD5 | coiled-coil-helix-coiled-coil-helix dom | -0.090 | 0.939 | 9.07E-02 | 9.07E+00 | | | |
| 8019772 | NM_005782 | THOC4 | THO complex 4 | -0.090 | 0.939 | 7.53E-01 | 7.53E+01 | | | |
| 7945781 | NM_003311 | PHLDA2 | pleckstrin homology-like domain, fam | -0.090 | 0.939 | 1.76E-01 | 1.76E+01 | | | |
| 8063761 | NM_177980 | CDH26 | cadherin-like 26 | -0.090 | 0.939 | 8.26E-02 | 8.26E+00 | | | |
| 7944516 | --- | --- | --- | -0.090 | 0.939 | 1.27E-01 | 1.27E+01 | | | |
| 8176093 | NM_006014 | LAGE3 | L antigen family, member 3 | -0.090 | 0.939 | 2.44E-01 | 2.44E+01 | | | |
| 8101065 | NM_006239 | PPEF2 | protein phosphatase, EF-hand calciu | -0.090 | 0.939 | 1.20E-01 | 1.20E+01 | | | |
| 8074806 | NM_014634 | PPM1F | protein phosphatase 1F (PP2C doma | -0.090 | 0.939 | 8.97E-02 | 8.97E+00 | | | |
| 8001014 | NM_173502 | PRSS36 | protease, serine, 36 | -0.090 | 0.939 | 1.09E-01 | 1.09E+01 | | | |
| 8153652 | NM_030974 | SHARPIN | SHANK-associated RH domain intera | -0.090 | 0.939 | 1.66E-01 | 1.66E+01 | | | |
| 8141517 | BC026269 | C7orf47 | chromosome 7 open reading frame 4 | -0.090 | 0.939 | 3.77E-01 | 3.77E+01 | | | |
| 7914516 | NR_026850 | MTMR9L | myotubularin related protein 9-like | -0.090 | 0.939 | 9.76E-02 | 9.76E+00 | | | |
| 8071368 | NR_026815 | TMEM191A | transmembrane protein 191A | -0.090 | 0.939 | 1.91E-01 | 1.91E+01 | | | |
| 8155345 | NR_026801 | FAM74A3 | family with sequence similarity 74, m | -0.090 | 0.939 | 1.62E-01 | 1.62E+01 | | | |
| 8068671 | NM_012105 | BACE2 | beta-site APP-cleaving enzyme 2 | -0.090 | 0.939 | 3.43E-01 | 3.43E+01 | | | |
| 8073039 | NM_004286 | GTPBP1 | GTP binding protein 1 | -0.090 | 0.939 | 3.31E-01 | 3.31E+01 | | | |

| | | | | | | | | | | |
|---|---|---|---|---|---|---|---|---|---|---|
| 8017867 | NM_017565 | FAM20A | family with sequence similarity 20, m | -0.090 | 0.939 | 2.54E-01 | 2.54E+01 | | | |
| 7993259 | NM_003498 | SNN | stannin | -0.090 | 0.939 | 1.98E-01 | 1.98E+01 | | | |
| 8116932 | NM_030948 | PHACTR1 | phosphatase and actin regulator 1 | -0.090 | 0.939 | 5.74E-02 | 5.74E+00 | | | |
| 8081073 | NM_173824 | C3orf38 | chromosome 3 open reading frame 3 | -0.090 | 0.939 | 8.17E-01 | 8.17E+01 | | | |
| 8005529 | NM_152351 | SLC5A10 | solute carrier family 5 (sodium/gluco | -0.090 | 0.939 | 9.64E-02 | 9.64E+00 | | | |
| 8140061 | NM_001707 | BCL7B | B-cell CLL/lymphoma 7B | -0.090 | 0.939 | 5.15E-01 | 5.15E+01 | | | |
| 8108013 | --- | --- | --- | -0.090 | 0.939 | 7.79E-01 | 7.79E+01 | | | |
| 8100851 | --- | --- | --- | -0.090 | 0.939 | 9.61E-02 | 9.61E+00 | | | |
| 8086627 | NM_147129 | ALS2CL | ALS2 C-terminal like | -0.090 | 0.939 | 1.03E-01 | 1.03E+01 | | | |
| 8043155 | --- | --- | --- | -0.090 | 0.939 | 3.82E-01 | 3.82E+01 | | | |
| 8051793 | NM_148962 | OXER1 | oxoeicosanoid (OXE) receptor 1 | -0.090 | 0.939 | 1.84E-01 | 1.84E+01 | | | |
| 7972808 | AK093771 | CARKD | carbohydrate kinase domain contain | -0.090 | 0.939 | 1.37E-01 | 1.37E+01 | | | |
| 8046895 | NM_177454 | FAM171B | family with sequence similarity 171, | -0.090 | 0.939 | 1.57E-01 | 1.57E+01 | | | |
| 8125993 | NM_016135 | ETV7 | ets variant 7 | -0.090 | 0.939 | 6.59E-02 | 6.59E+00 | | | |
| 7957032 | NM_006530 | YEATS4 | YEATS domain containing 4 | -0.090 | 0.939 | 8.45E-01 | 8.45E+01 | | | |
| 8104912 | NM_005983 | SKP2 | S-phase kinase-associated protein 2 | -0.090 | 0.939 | 6.91E-01 | 6.91E+01 | | | |
| 8131942 | --- | --- | --- | -0.090 | 0.939 | 2.16E-01 | 2.16E+01 | | | |
| 8045291 | NM_080386 | TUBA3D | tubulin, alpha 3d | -0.090 | 0.939 | 1.10E-01 | 1.10E+01 | | | |
| 8180378 | --- | --- | --- | -0.091 | 0.939 | 1.92E-01 | 1.92E+01 | | | |
| 7927155 | NM_173160 | FXYD4 | FXYD domain containing ion transpo | -0.091 | 0.939 | 1.07E-01 | 1.07E+01 | | | |
| 8124926 | NM_004640 | BAT1 | HLA-B associated transcript 1 | -0.091 | 0.939 | 6.57E-01 | 6.57E+01 | | | |
| 8099670 | NM_00113072( | CCDC149 | coiled-coil domain containing 149 | -0.091 | 0.939 | 1.34E-01 | 1.34E+01 | | | |
| 8010248 | NM_00101098: | AFMID | arylformamidase | -0.091 | 0.939 | 1.05E-01 | 1.05E+01 | | | |
| 8167652 | AY730278 | LOC389857 | hypothetical protein | -0.091 | 0.939 | 1.13E-01 | 1.13E+01 | | | |
| 8172715 | AY730278 | LOC389857 | hypothetical protein | -0.091 | 0.939 | 1.13E-01 | 1.13E+01 | | | |
| 8153664 | NM_015201 | BOP1 | block of proliferation 1 | -0.091 | 0.939 | 1.16E-01 | 1.16E+01 | | | |
| 8085058 | --- | --- | --- | -0.091 | 0.939 | 3.45E-01 | 3.45E+01 | | | |
| 8009875 | NR_003587 | MYO15B | myosin XVB pseudogene | -0.091 | 0.939 | 5.90E-02 | 5.90E+00 | | | |
| 7937059 | NM_174937 | TCERG1L | transcription elongation regulator 1- | -0.091 | 0.939 | 9.61E-02 | 9.61E+00 | | | |
| 8075857 | NM_003312 | TST | thiosulfate sulfurtransferase (rhodar | -0.091 | 0.939 | 1.48E-01 | 1.48E+01 | | | |
| 7985268 | NM_000137 | FAH | fumarylacetoacetate hydrolase (fum | -0.091 | 0.939 | 1.94E-01 | 1.94E+01 | | | |
| 7912537 | NM_004753 | DHRS3 | dehydrogenase/reductase (SDR fami | -0.091 | 0.939 | 6.29E-01 | 6.29E+01 | | | |
| 7896453 | --- | --- | --- | -0.091 | 0.939 | 8.90E-01 | 8.90E+01 | | | |
| 8106352 | XM_00171851 | OC10012956 | hypothetical protein LOC100129566 | -0.091 | 0.939 | 3.38E-01 | 3.38E+01 | | | |
| 8097811 | AL137273 | KFZP434I071 | hypothetical protein DKFZP434I0714 | -0.091 | 0.939 | 2.62E-01 | 2.62E+01 | | | |
| 8135069 | NM_000602 | SERPINE1 | serpin peptidase inhibitor, clade E (n | -0.091 | 0.939 | 1.31E-01 | 1.31E+01 | | | |
| 8018935 | --- | --- | --- | -0.091 | 0.939 | 9.01E-01 | 9.01E+01 | | | |
| 8084726 | NM_153708 | RTP1 | receptor (chemosensory) transporte | -0.091 | 0.939 | 1.86E-01 | 1.86E+01 | | | |
| 8073612 | NM_000714 | TSPO | translocator protein (18kDa) | -0.091 | 0.939 | 2.74E-01 | 2.74E+01 | | | |
| 8122038 | NM_052913 | TMEM200A | transmembrane protein 200A | -0.091 | 0.939 | 1.41E-01 | 1.41E+01 | | | |
| 8153363 | NM_000497 | CYP11B1 | cytochrome P450, family 11, subfam | -0.091 | 0.939 | 1.22E-01 | 1.22E+01 | | | |
| 8058333 | --- | --- | --- | -0.091 | 0.939 | 6.28E-01 | 6.28E+01 | | | |
| 7944795 | NM_00100196 | OR4D5 | olfactory receptor, family 4, subfami | -0.091 | 0.939 | 2.00E-01 | 2.00E+01 | | | |
| 8114550 | NM_004883 | NRG2 | neuregulin 2 | -0.091 | 0.939 | 5.68E-02 | 5.68E+00 | | | |
| 7976766 | NM_024515 | WDR25 | WD repeat domain 25 | -0.091 | 0.939 | 1.30E-01 | 1.30E+01 | | | |
| 8024089 | NM_024100 | WDR18 | WD repeat domain 18 | -0.091 | 0.939 | 6.86E-02 | 6.86E+00 | | | |
| 8141803 | NM_006989 | RASA4 | RAS p21 protein activator 4 | -0.091 | 0.939 | 4.35E-02 | 4.35E+00 | | | |
| 8161648 | NM_001206 | KLF9 | Kruppel-like factor 9 | -0.091 | 0.939 | 1.51E-01 | 1.51E+01 | | | |
| 8026861 | NM_014256 | B3GNT3 | UDP-GlcNAc:betaGal beta-1,3-N-ace | -0.091 | 0.939 | 1.03E-01 | 1.03E+01 | | | |
| 8035956 | --- | --- | --- | -0.091 | 0.939 | 4.61E-01 | 4.61E+01 | | | |
| 7997257 | NM_153688 | ZFP1 | zinc finger protein 1 homolog (mous | -0.091 | 0.939 | 4.39E-01 | 4.39E+01 | | | |
| 7967736 | NM_006231 | POLE | polymerase (DNA directed), epsilon | -0.091 | 0.939 | 1.38E-01 | 1.38E+01 | | | |
| 8038280 | NM_000737 | CGB | chorionic gonadotropin, beta polype | -0.091 | 0.939 | 9.29E-02 | 9.29E+00 | | | |
| 8083034 | NM_022131 | CLSTN2 | calsyntenin 2 | -0.091 | 0.939 | 1.63E-01 | 1.63E+01 | | | |
| 8159017 | NM_153710 | C9orf96 | chromosome 9 open reading frame 9 | -0.091 | 0.939 | 1.63E-01 | 1.63E+01 | | | |
| 8075401 | NM_004861 | GAL3ST1 | galactose-3-O-sulfotransferase 1 | -0.091 | 0.939 | 1.34E-01 | 1.34E+01 | | | |
| 8073207 | NM_138435 | FAM83F | family with sequence similarity 83, m | -0.091 | 0.939 | 1.81E-01 | 1.81E+01 | | | |
| 8113305 | NM_001270 | CHD1 | chromodomain helicase DNA binding | -0.091 | 0.939 | 7.25E-01 | 7.25E+01 | | | |
| 8145361 | NM_005382 | NEFM | neurofilament, medium polypeptide | -0.091 | 0.939 | 1.50E-01 | 1.50E+01 | | | |
| 8034608 | NM_005583 | LYL1 | lymphoblastic leukemia derived sequ | -0.091 | 0.939 | 1.03E-01 | 1.03E+01 | | | |
| 8176570 | NM_00112738 | LOC653543 | double homeobox, 4-like | -0.091 | 0.939 | 5.39E-01 | 5.39E+01 | | | |
| 8093518 | NM_000142 | FGFR3 | fibroblast growth factor receptor 3 | -0.091 | 0.939 | 8.56E-02 | 8.56E+00 | | | |
| 8019071 | NM_020914 | RNF213 | ring finger protein 213 | -0.091 | 0.939 | 2.19E-01 | 2.19E+01 | | | |
| 7917697 | NM_005263 | GFI1 | growth factor independent 1 transcr | -0.091 | 0.939 | 1.19E-01 | 1.19E+01 | | | |
| 8089314 | NM_018010 | IFT57 | intraflagellar transport 57 homolog ( | -0.091 | 0.939 | 7.04E-01 | 7.04E+01 | | | |
| 8110649 | NM_033549 | TRIM41 | tripartite motif-containing 41 | -0.091 | 0.939 | 3.19E-01 | 3.19E+01 | | | |
| 7893491 | --- | --- | --- | -0.091 | 0.939 | 3.26E-01 | 3.26E+01 | | | |
| 8071997 | NM_000496 | CRYBB2 | crystallin, beta B2 | -0.091 | 0.939 | 1.26E-01 | 1.26E+01 | | | |
| 8180206 | --- | --- | --- | -0.091 | 0.939 | 4.24E-01 | 4.24E+01 | | | |
| 7894843 | --- | --- | --- | -0.091 | 0.939 | 9.20E-01 | 9.20E+01 | | | |
| 7893473 | --- | --- | --- | -0.091 | 0.939 | 5.07E-01 | 5.07E+01 | | | |
| 8061944 | BC137111 | C20orf134 | chromosome 20 open reading frame | -0.091 | 0.939 | 1.16E-01 | 1.16E+01 | | | |
| 8043570 | --- | --- | --- | -0.091 | 0.939 | 1.54E-01 | 1.54E+01 | | | |
| 8169617 | NM_006667 | PGRMC1 | progesterone receptor membrane co | -0.091 | 0.939 | 6.16E-01 | 6.16E+01 | | | |
| 8150266 | NM_014462 | LSM1 | LSM1 homolog, U6 small nuclear RN/ | -0.091 | 0.939 | 8.37E-01 | 8.37E+01 | | | |
| 7985248 | NM_015206 | KIAA1024 | KIAA1024 | -0.091 | 0.939 | 1.60E-01 | 1.60E+01 | | | |
| 8162438 | NM_00100380( | BICD2 | bicaudal D homolog 2 (Drosophila) | -0.091 | 0.939 | 1.66E-01 | 1.66E+01 | | | |

| | | | | | | | | | | |
|---|---|---|---|---|---|---|---|---|---|---|
| 8018975 | NM_005567 | LGALS3BP | lectin, galactoside-binding, soluble, 3 | -0.091 | 0.939 | 2.94E-01 | 2.94E+01 | | | |
| 8016086 | --- | --- | --- | -0.091 | 0.939 | 6.52E-01 | 6.52E+01 | | | |
| 7893543 | --- | --- | --- | -0.091 | 0.939 | 8.42E-01 | 8.42E+01 | | | |
| 7995776 | NM_005954 | MT3 | metallothionein 3 | -0.091 | 0.939 | 1.07E-01 | 1.07E+01 | | | |
| 7994817 | NM_016643 | ZNF771 | zinc finger protein 771 | -0.091 | 0.939 | 2.13E-01 | 2.13E+01 | | | |
| 7895884 | --- | --- | --- | -0.091 | 0.939 | 5.27E-01 | 5.27E+01 | | | |
| 8005117 | NM_178571 | TBC1D26 | TBC1 domain family, member 26 | -0.091 | 0.939 | 2.05E-01 | 2.05E+01 | | | |
| 8159981 | NM_006911 | RLN1 | relaxin 1 | -0.091 | 0.939 | 2.03E-01 | 2.03E+01 | | | |
| 7894747 | --- | --- | --- | -0.091 | 0.939 | 2.65E-01 | 2.65E+01 | | | |
| 8131091 | NM_002360 | MAFK | v-maf musculoaponeurotic fibrosarc | -0.091 | 0.939 | 1.62E-01 | 1.62E+01 | | | |
| 8164535 | NM_000755 | CRAT | carnitine acetyltransferase | -0.091 | 0.939 | 4.00E-02 | 4.00E+00 | | | |
| 8062705 | NM_032107 | L3MBTL | l(3)mbt-like (Drosophila) | -0.091 | 0.939 | 1.21E-01 | 1.21E+01 | | | |
| 8171350 | NM_152634 | TCEANC | transcription elongation factor A (SII | -0.091 | 0.939 | 1.97E-01 | 1.97E+01 | | | |
| 8003158 | NM_020947 | KIAA1609 | KIAA1609 | -0.091 | 0.939 | 1.50E-01 | 1.50E+01 | | | |
| 7963328 | NM_00101369( | FIGNL2 | fidgetin-like 2 | -0.091 | 0.939 | 2.41E-01 | 2.41E+01 | | | |
| 7895961 | --- | --- | --- | -0.091 | 0.939 | 5.58E-01 | 5.58E+01 | | | |
| 8092978 | NM_018406 | MUC4 | mucin 4, cell surface associated | -0.091 | 0.939 | 8.07E-02 | 8.07E+00 | | | |
| 8158739 | NM_006059 | LAMC3 | laminin, gamma 3 | -0.092 | 0.939 | 6.54E-02 | 6.54E+00 | | | |
| 7944530 | NM_178507 | OAF | OAF homolog (Drosophila) | -0.092 | 0.939 | 1.62E-01 | 1.62E+01 | | | |
| 8119016 | NM_002754 | MAPK13 | mitogen-activated protein kinase 13 | -0.092 | 0.939 | 5.94E-01 | 5.94E+01 | | | |
| 8172654 | NR_026742 | LOC158572 | hypothetical LOC158572 | -0.092 | 0.939 | 3.17E-01 | 3.17E+01 | | | |
| 8113421 | --- | --- | --- | -0.092 | 0.938 | 2.93E-01 | 2.93E+01 | | | |
| 7992744 | NM_032296 | FLYWCH1 | FLYWCH-type zinc finger 1 | -0.092 | 0.938 | 1.35E-01 | 1.35E+01 | | | |
| 8087653 | NM_015896 | ZMYND10 | zinc finger, MYND-type containing 10 | -0.092 | 0.938 | 8.22E-02 | 8.22E+00 | | | |
| 8121613 | NM_016104 | RWDD1 | RWD domain containing 1 | -0.092 | 0.938 | 5.52E-01 | 5.52E+01 | | | |
| 7943098 | NM_005959 | MTNR1B | melatonin receptor 1B | -0.092 | 0.938 | 1.21E-01 | 1.21E+01 | | | |
| 8151784 | --- | --- | --- | -0.092 | 0.938 | 1.65E-01 | 1.65E+01 | | | |
| 8071312 | --- | --- | --- | -0.092 | 0.938 | 1.27E-01 | 1.27E+01 | | | |
| 7985257 | --- | --- | --- | -0.092 | 0.938 | 1.61E-01 | 1.61E+01 | | | |
| 7894741 | --- | --- | --- | -0.092 | 0.938 | 3.48E-01 | 3.48E+01 | | | |
| 7911862 | NM_020710 | LRRC47 | leucine rich repeat containing 47 | -0.092 | 0.938 | 3.73E-01 | 3.73E+01 | | | |
| 7954071 | NR_002774 | HTR7P | 5-hydroxytryptamine (serotonin) rec | -0.092 | 0.938 | 1.51E-01 | 1.51E+01 | | | |
| 8135172 | NM_00112634( | ORAI2 | ORAI calcium release-activated calciu | -0.092 | 0.938 | 8.02E-02 | 8.02E+00 | | | |
| 8118613 | NM_006979 | SLC39A7 | solute carrier family 39 (zinc transpo | -0.092 | 0.938 | 1.67E-01 | 1.67E+01 | | | |
| 8178225 | NM_006979 | SLC39A7 | solute carrier family 39 (zinc transpo | -0.092 | 0.938 | 1.67E-01 | 1.67E+01 | | | |
| 8179525 | NM_006979 | SLC39A7 | solute carrier family 39 (zinc transpo | -0.092 | 0.938 | 1.67E-01 | 1.67E+01 | | | |
| 7981525 | NM_017955 | CDCA4 | cell division cycle associated 4 | -0.092 | 0.938 | 9.83E-02 | 9.83E+00 | | | |
| 8016194 | NM_003954 | MAP3K14 | mitogen-activated protein kinase kin | -0.092 | 0.938 | 4.87E-02 | 4.87E+00 | | | |
| 7941608 | --- | --- | --- | -0.092 | 0.938 | 1.60E-01 | 1.60E+01 | | | |
| 7900878 | NM_057091 | ARTN | artemin | -0.092 | 0.938 | 1.34E-01 | 1.34E+01 | | | |
| 8057887 | NM_004226 | STK17B | serine/threonine kinase 17b | -0.092 | 0.938 | 6.73E-01 | 6.73E+01 | | | |
| 7979886 | --- | --- | --- | -0.092 | 0.938 | 5.80E-01 | 5.80E+01 | | | |
| 8027184 | NM_015329 | KIAA0892 | KIAA0892 | -0.092 | 0.938 | 6.96E-02 | 6.96E+00 | | | |
| 8022514 | NM_032933 | C18orf45 | chromosome 18 open reading frame | -0.092 | 0.938 | 1.61E-01 | 1.61E+01 | | | |
| 7931681 | --- | --- | --- | -0.092 | 0.938 | 7.75E-01 | 7.75E+01 | | | |
| 7937882 | NM_004314 | ART1 | ADP-ribosyltransferase 1 | -0.092 | 0.938 | 1.60E-01 | 1.60E+01 | | | |
| 7903281 | NM_012243 | SLC35A3 | solute carrier family 35 (UDP-N-acety | -0.092 | 0.938 | 6.38E-01 | 6.38E+01 | | | |
| 8071084 | NR_024483 | CECR4 | cat eye syndrome chromosome regio | -0.092 | 0.938 | 1.22E-01 | 1.22E+01 | | | |
| 7935528 | NM_003015 | SFRP5 | secreted frizzled-related protein 5 | -0.092 | 0.938 | 8.16E-02 | 8.16E+00 | | | |
| 7931643 | NM_000773 | CYP2E1 | cytochrome P450, family 2, subfamil | -0.092 | 0.938 | 4.26E-01 | 4.26E+01 | | | |
| 7963142 | NM_012306 | FAIM2 | Fas apoptotic inhibitory molecule 2 | -0.092 | 0.938 | 1.41E-01 | 1.41E+01 | | | |
| 8117881 | NM_024839 | RPP21 | ribonuclease P/MRP 21kDa subunit | -0.092 | 0.938 | 6.26E-01 | 6.26E+01 | | | |
| 7912250 | EF592573 | MIR34A | microRNA 34a | -0.092 | 0.938 | 2.07E-01 | 2.07E+01 | | | |
| 7935865 | NM_013274 | POLL | polymerase (DNA directed), lambda | -0.092 | 0.938 | 1.26E-01 | 1.26E+01 | | | |
| 8180339 | --- | --- | --- | -0.092 | 0.938 | 2.94E-01 | 2.94E+01 | | | |
| 8064388 | NM_033409 | C20orf54 | chromosome 20 open reading frame | -0.092 | 0.938 | 6.70E-02 | 6.70E+00 | | | |
| 8134117 | NM_003505 | FZD1 | frizzled homolog 1 (Drosophila) | -0.092 | 0.938 | 1.16E-01 | 1.16E+01 | | | |
| 7940971 | NM_033310 | KCNK4 | potassium channel, subfamily K, mer | -0.092 | 0.938 | 9.80E-02 | 9.80E+00 | | | |
| 7950374 | NM_182904 | P4HA3 | prolyl 4-hydroxylase, alpha polypept | -0.092 | 0.938 | 6.75E-02 | 6.75E+00 | | | |
| 8178448 | NM_00110556 | CCHCR1 | coiled-coil alpha-helical rod protein 1 | -0.092 | 0.938 | 4.77E-02 | 4.77E+00 | | | |
| 7893029 | --- | --- | --- | -0.092 | 0.938 | 1.68E-01 | 1.68E+01 | | | |
| 7918284 | NM_005645 | TAF13 | TAF13 RNA polymerase II, TATA box | -0.092 | 0.938 | 9.24E-01 | 9.24E+01 | | | |
| 8039139 | NM_024316 | LENG1 | leukocyte receptor cluster (LRC) mer | -0.092 | 0.938 | 3.16E-01 | 3.16E+01 | | | |
| 7900192 | NM_198446 | C1orf122 | chromosome 1 open reading frame 1 | -0.092 | 0.938 | 1.68E-01 | 1.68E+01 | | | |
| 8170235 | --- | --- | --- | -0.092 | 0.938 | 3.18E-01 | 3.18E+01 | | | |
| 8040690 | NM_00110551 | C2orf70 | chromosome 2 open reading frame 7 | -0.092 | 0.938 | 1.60E-01 | 1.60E+01 | | | |
| 8165295 | ENST0000039 | LCN8 | lipocalin 8 | -0.092 | 0.938 | 3.93E-01 | 3.93E+01 | | | |
| 8076384 | --- | --- | --- | -0.092 | 0.938 | 5.26E-01 | 5.26E+01 | | | |
| 8037363 | NM_145296 | CADM4 | cell adhesion molecule 4 | -0.092 | 0.938 | 7.04E-02 | 7.04E+00 | | | |
| 8067743 | NM_025224 | ZBTB46 | zinc finger and BTB domain containin | -0.092 | 0.938 | 6.39E-02 | 6.39E+00 | | | |
| 8035156 | NM_006387 | CHERP | calcium homeostasis endoplasmic re | -0.092 | 0.938 | 1.32E-01 | 1.32E+01 | | | |
| 7947881 | NM_005055 | RAPSN | receptor-associated protein of the sy | -0.092 | 0.938 | 1.26E-01 | 1.26E+01 | | | |
| 8031157 | NM_020659 | TTYH1 | tweety homolog 1 (Drosophila) | -0.092 | 0.938 | 8.17E-02 | 8.17E+00 | | | |
| 7895403 | --- | --- | --- | -0.092 | 0.938 | 5.88E-01 | 5.88E+01 | | | |
| 8003656 | NM_000934 | SERPINF2 | serpin peptidase inhibitor, clade F (a | -0.092 | 0.938 | 1.38E-01 | 1.38E+01 | | | |
| 8060745 | NM_175839 | SMOX | spermine oxidase | -0.092 | 0.938 | 2.02E-01 | 2.02E+01 | | | |
| 7896985 | NM_080875 | MIB2 | mindbomb homolog 2 (Drosophila) | -0.092 | 0.938 | 9.57E-02 | 9.57E+00 | | | |

| | | | | | | | | | | |
|---|---|---|---|---|---|---|---|---|---|---|
| 8033455 | ENST0000032 | FLJ22184 | hypothetical protein FLJ22184 | -0.092 | 0.938 | 9.20E-02 | 9.20E+00 | | | |
| 8040340 | NM_145693 | LPIN1 | lipin 1 | -0.092 | 0.938 | 3.92E-01 | 3.92E+01 | | | |
| 8162254 | NM_017594 | DIRAS2 | DIRAS family, GTP-binding RAS-like 2 | -0.092 | 0.938 | 1.14E-01 | 1.14E+01 | | | |
| 8161499 | BC032035 | FAM27E3 | family with sequence similarity 27, m | -0.092 | 0.938 | 2.54E-01 | 2.54E+01 | | | |
| 8124194 | --- | --- | --- | -0.092 | 0.938 | 2.00E-01 | 2.00E+01 | | | |
| 8066214 | NM_004613 | TGM2 | transglutaminase 2 (C polypeptide, p | -0.092 | 0.938 | 5.55E-02 | 5.55E+00 | | | |
| 7958398 | NM_014653 | WSCD2 | WSC domain containing 2 | -0.092 | 0.938 | 1.35E-01 | 1.35E+01 | | | |
| 8065363 | AF258576 | LOC200261 | hypothetical LOC200261 | -0.092 | 0.938 | 9.64E-02 | 9.64E+00 | | | |
| 7950864 | NM_00101481 | ME3 | malic enzyme 3, NADP(+)-dependent | -0.092 | 0.938 | 1.00E-01 | 1.00E+01 | | | |
| 7928531 | NR_024421 | C10orf41 | chromosome 10 open reading frame | -0.092 | 0.938 | 3.90E-01 | 3.90E+01 | | | |
| 7981078 | NM_00108045 | SERPINA11 | serpin peptidase inhibitor, clade A (a | -0.092 | 0.938 | 1.08E-01 | 1.08E+01 | | | |
| 8007548 | NR_026770 | C17orf88 | chromosome 17 open reading frame | -0.092 | 0.938 | 2.19E-01 | 2.19E+01 | | | |
| 7989365 | NM_134260 | RORA | RAR-related orphan receptor A | -0.092 | 0.938 | 4.29E-01 | 4.29E+01 | | | |
| 7970828 | AB064667 | OK/SW-CL.58 | OK/SW-CL.58 | -0.092 | 0.938 | 2.75E-01 | 2.75E+01 | | | |
| 7925813 | NR_027152 | C10orf108 | chromosome 10 open reading frame | -0.092 | 0.938 | 6.68E-02 | 6.68E+00 | | | |
| 7954653 | NM_00108050 | TSPAN11 | tetraspanin 11 | -0.092 | 0.938 | 2.11E-01 | 2.11E+01 | | | |
| 8033956 | NM_004230 | S1PR2 | sphingosine-1-phosphate receptor 2 | -0.092 | 0.938 | 9.18E-02 | 9.18E+00 | | | |
| 8143594 | --- | --- | --- | -0.092 | 0.938 | 1.02E-01 | 1.02E+01 | | | |
| 8051215 | NM_022128 | RBKS | ribokinase | -0.093 | 0.938 | 2.87E-01 | 2.87E+01 | | | |
| 8116086 | NM_024872 | DOK3 | docking protein 3 | -0.093 | 0.938 | 1.27E-01 | 1.27E+01 | | | |
| 8046048 | NM_024969 | CSRNP3 | cysteine-serine-rich nuclear protein 3 | -0.093 | 0.938 | 1.33E-01 | 1.33E+01 | | | |
| 8092457 | NM_005787 | ALG3 | asparagine-linked glycosylation 3, alp | -0.093 | 0.938 | 1.94E-01 | 1.94E+01 | | | |
| 7966172 | NM_018711 | SVOP | SV2 related protein homolog (rat) | -0.093 | 0.938 | 1.88E-01 | 1.88E+01 | | | |
| 8161446 | BC032035 | FAM27E3 | family with sequence similarity 27, m | -0.093 | 0.938 | 1.96E-01 | 1.96E+01 | | | |
| 7898052 | NM_00109985 | PRAMEF20 | PRAME family member 20 | -0.093 | 0.938 | 1.94E-01 | 1.94E+01 | | | |
| 8002344 | NM_058219 | EXOSC6 | exosome component 6 | -0.093 | 0.938 | 1.09E-01 | 1.09E+01 | | | |
| 7985310 | NM_021214 | FAM108C1 | family with sequence similarity 108, | -0.093 | 0.938 | 9.54E-02 | 9.54E+00 | | | |
| 8075633 | --- | --- | --- | -0.093 | 0.938 | 2.32E-01 | 2.32E+01 | | | |
| 8134644 | NM_145115 | ZNF498 | zinc finger protein 498 | -0.093 | 0.938 | 1.98E-01 | 1.98E+01 | | | |
| 8074108 | NM_012324 | MAPK8IP2 | mitogen-activated protein kinase 8 in | -0.093 | 0.938 | 6.66E-02 | 6.66E+00 | | | |
| 8129695 | NM_022568 | ALDH8A1 | aldehyde dehydrogenase 8 family, m | -0.093 | 0.938 | 1.00E-01 | 1.00E+01 | | | |
| 8006377 | NM_00109850 | ZNF207 | zinc finger protein 207 | -0.093 | 0.938 | 7.56E-01 | 7.56E+01 | | | |
| 8071927 | NM_005265 | GGT1 | gamma-glutamyltransferase 1 | -0.093 | 0.938 | 5.89E-02 | 5.89E+00 | | | |
| 8116051 | NM_080881 | DBN1 | drebrin 1 | -0.093 | 0.938 | 4.73E-02 | 4.73E+00 | | | |
| 7979820 | NM_207442 | C14orf181 | chromosome 14 open reading frame | -0.093 | 0.938 | 5.34E-02 | 5.34E+00 | | | |
| 8060101 | AK092442 | PP14571 | similar to hCG1777210 | -0.093 | 0.938 | 2.09E-01 | 2.09E+01 | | | |
| 7962625 | NM_00109853 | RAPGEF3 | Rap guanine nucleotide exchange fac | -0.093 | 0.938 | 5.15E-02 | 5.15E+00 | | | |
| 7895178 | --- | --- | --- | -0.093 | 0.938 | 5.03E-01 | 5.03E+01 | | | |
| 8032473 | NM_198532 | C19orf35 | chromosome 19 open reading frame | -0.093 | 0.938 | 1.51E-01 | 1.51E+01 | | | |
| 8151457 | NM_012258 | HEY1 | hairy/enhancer-of-split related with | -0.093 | 0.938 | 1.14E-01 | 1.14E+01 | | | |
| 8090464 | NM_153330 | DNAJB8 | DnaJ (Hsp40) homolog, subfamily B, | -0.093 | 0.938 | 1.86E-01 | 1.86E+01 | | | |
| 8103822 | NM_005429 | VEGFC | vascular endothelial growth factor C | -0.093 | 0.938 | 1.61E-01 | 1.61E+01 | | | |
| 7935521 | NM_021732 | AVPI1 | arginine vasopressin-induced 1 | -0.093 | 0.938 | 2.77E-01 | 2.77E+01 | | | |
| 7962842 | NM_015270 | ADCY6 | adenylate cyclase 6 | -0.093 | 0.938 | 9.28E-02 | 9.28E+00 | | | |
| 8134915 | NM_003386 | ZAN | zonadhesin | -0.093 | 0.938 | 4.47E-02 | 4.47E+00 | | | |
| 8007123 | NM_031961 | KRTAP9-2 | keratin associated protein 9-2 | -0.093 | 0.938 | 1.57E-01 | 1.57E+01 | | | |
| 8160816 | NM_198573 | ENHO | energy homeostasis associated | -0.093 | 0.938 | 1.03E-01 | 1.03E+01 | | | |
| 8024219 | NM_001687 | ATP5D | ATP synthase, H+ transporting, mitoc | -0.093 | 0.938 | 5.89E-02 | 5.89E+00 | | | |
| 8017010 | NM_00108043 | HSF5 | heat shock transcription factor famil | -0.093 | 0.938 | 2.13E-01 | 2.13E+01 | | | |
| 8066038 | NM_032214 | SLA2 | Src-like-adaptor 2 | -0.093 | 0.938 | 2.71E-01 | 2.71E+01 | | | |
| 8162998 | --- | --- | --- | -0.093 | 0.938 | 2.11E-01 | 2.11E+01 | | | |
| 7959696 | NM_207437 | DNAH10 | dynein, axonemal, heavy chain 10 | -0.093 | 0.938 | 4.72E-02 | 4.72E+00 | | | |
| 7980233 | NM_002632 | PGF | placental growth factor | -0.093 | 0.938 | 1.19E-01 | 1.19E+01 | | | |
| 8016016 | --- | --- | --- | -0.093 | 0.938 | 2.80E-01 | 2.80E+01 | | | |
| 7978166 | NM_006405 | TM9SF1 | transmembrane 9 superfamily memb | -0.093 | 0.938 | 6.45E-01 | 6.45E+01 | | | |
| 7897991 | NM_00101227 | PRAMEF7 | PRAME family member 7 | -0.093 | 0.938 | 9.51E-02 | 9.51E+00 | | | |
| 7912591 | NM_00101227 | PRAMEF7 | PRAME family member 7 | -0.093 | 0.938 | 9.51E-02 | 9.51E+00 | | | |
| 7912606 | NM_00101227 | PRAMEF7 | PRAME family member 7 | -0.093 | 0.938 | 9.51E-02 | 9.51E+00 | | | |
| 8090214 | NM_024628 | SLC12A8 | solute carrier family 12 (potassium/c | -0.093 | 0.938 | 7.79E-02 | 7.79E+00 | | | |
| 7956488 | NM_004984 | KIF5A | kinesin family member 5A | -0.093 | 0.938 | 3.91E-02 | 3.91E+00 | | | |
| 8003679 | NM_002945 | RPA1 | replication protein A1, 70kDa | -0.093 | 0.938 | 9.03E-02 | 9.03E+00 | | | |
| 8137101 | NM_003575 | ZNF282 | zinc finger protein 282 | -0.093 | 0.938 | 1.42E-01 | 1.42E+01 | | | |
| 7954173 | NM_007178 | STRAP | serine/threonine kinase receptor ass | -0.093 | 0.938 | 5.72E-01 | 5.72E+01 | | | |
| 8045533 | --- | --- | --- | -0.093 | 0.938 | 8.85E-01 | 8.85E+01 | | | |
| 7930994 | BC108662 | C10orf85 | chromosome 10 open reading frame | -0.093 | 0.938 | 1.83E-01 | 1.83E+01 | | | |
| 8031908 | NM_181846 | ZSCAN22 | zinc finger and SCAN domain contain | -0.093 | 0.938 | 1.48E-01 | 1.48E+01 | | | |
| 8065421 | NM_001900 | CST5 | cystatin D | -0.093 | 0.938 | 5.76E-02 | 5.76E+00 | | | |
| 8159876 | NM_134428 | RFX3 | regulatory factor X, 3 (influences HLA | -0.093 | 0.938 | 4.12E-01 | 4.12E+01 | | | |
| 7900710 | NM_00101296 | C1orf84 | chromosome 1 open reading frame 8 | -0.093 | 0.938 | 1.44E-02 | 1.44E+00 | | | |
| 8115487 | NM_00100134 | C5orf40 | chromosome 5 open reading frame 4 | -0.093 | 0.938 | 5.65E-01 | 5.65E+01 | | | |
| 7952086 | NM_007180 | TREH | trehalase (brush-border membrane g | -0.093 | 0.938 | 1.28E-01 | 1.28E+01 | | | |
| 8180228 | --- | --- | --- | -0.093 | 0.938 | 9.48E-02 | 9.48E+00 | | | |
| 8048272 | BC052750 | C2orf62 | chromosome 2 open reading frame 6 | -0.093 | 0.938 | 1.10E-01 | 1.10E+01 | | | |
| 8011671 | NM_00112289 | GGT6 | gamma-glutamyltransferase 6 | -0.093 | 0.937 | 1.21E-01 | 1.21E+01 | | | |
| 8031176 | NM_052925 | LENG8 | leukocyte receptor cluster (LRC) mem | -0.093 | 0.937 | 4.97E-01 | 4.97E+01 | | | |
| 8074880 | NM_004914 | RAB36 | RAB36, member RAS oncogene famil | -0.093 | 0.937 | 1.82E-01 | 1.82E+01 | | | |
| 8098165 | --- | --- | --- | -0.093 | 0.937 | 9.67E-02 | 9.67E+00 | | | |

| | | | | | | | | | | |
|---|---|---|---|---|---|---|---|---|---|---|
| 8032127 | NM_138774 | C19orf22 | chromosome 19 open reading frame | -0.093 | 0.937 | 1.68E-01 | 1.68E+01 | | | |
| 7925587 | --- | --- | --- | -0.093 | 0.937 | 1.86E-01 | 1.86E+01 | | | |
| 8081277 | NM_020202 | NIT2 | nitrilase family, member 2 | -0.093 | 0.937 | 8.03E-01 | 8.03E+01 | | | |
| 8074892 | NR_003950 | ZDHHC8P | zinc finger, DHHC-type containing 8 p | -0.093 | 0.937 | 7.96E-02 | 7.96E+00 | | | |
| 7892845 | --- | --- | --- | -0.093 | 0.937 | 5.49E-01 | 5.49E+01 | | | |
| 7945550 | NM_018494 | LRDD | leucine-rich repeats and death doma | -0.093 | 0.937 | 5.02E-02 | 5.02E+00 | | | |
| 7950425 | NM_015424 | CHRDL2 | chordin-like 2 | -0.093 | 0.937 | 1.07E-01 | 1.07E+01 | | | |
| 8021714 | --- | --- | --- | -0.093 | 0.937 | 1.79E-01 | 1.79E+01 | | | |
| 7916667 | --- | --- | --- | -0.093 | 0.937 | 6.45E-01 | 6.45E+01 | | | |
| 8016336 | NM_145798 | OSBPL7 | oxysterol binding protein-like 7 | -0.093 | 0.937 | 1.31E-01 | 1.31E+01 | | | |
| 8143725 | NM_152557 | ZNF746 | zinc finger protein 746 | -0.093 | 0.937 | 1.56E-01 | 1.56E+01 | | | |
| 7967641 | NM_133448 | TMEM132D | transmembrane protein 132D | -0.093 | 0.937 | 1.25E-01 | 1.25E+01 | | | |
| 8180300 | --- | --- | --- | -0.093 | 0.937 | 1.59E-01 | 1.59E+01 | | | |
| 8000910 | NM_145271 | ZNF688 | zinc finger protein 688 | -0.093 | 0.937 | 9.17E-02 | 9.17E+00 | | | |
| 7948493 | NM_016582 | SLC15A3 | solute carrier family 15, member 3 | -0.093 | 0.937 | 3.23E-01 | 3.23E+01 | | | |
| 8143899 | --- | --- | --- | -0.093 | 0.937 | 3.08E-01 | 3.08E+01 | | | |
| 8153430 | NM_145201 | NAPRT1 | nicotinate phosphoribosyltransferase | -0.093 | 0.937 | 6.34E-02 | 6.34E+00 | | | |
| 8070795 | NM_181686 // | 12-1 // KRT | keratin associated protein 12-1 // ke | -0.093 | 0.937 | 1.23E-01 | 1.23E+01 | | | |
| 8086503 | NM_00113517 | ZDHHC3 | zinc finger, DHHC-type containing 3 | -0.093 | 0.937 | 2.94E-01 | 2.94E+01 | | | |
| 7918925 | NM_025188 | TRIM45 | tripartite motif-containing 45 | -0.093 | 0.937 | 6.48E-02 | 6.48E+00 | | | |
| 8129218 | NM_153255 | MCM9 | minichromosome maintenance comp | -0.093 | 0.937 | 5.53E-01 | 5.53E+01 | | | |
| 7905528 | NM_178348 | LCE1A | late cornified envelope 1A | -0.093 | 0.937 | 1.08E-01 | 1.08E+01 | | | |
| 7893503 | --- | --- | --- | -0.093 | 0.937 | 7.66E-01 | 7.66E+01 | | | |
| 8150274 | --- | --- | --- | -0.093 | 0.937 | 1.60E-01 | 1.60E+01 | | | |
| 8166817 | --- | --- | --- | -0.093 | 0.937 | 1.65E-01 | 1.65E+01 | | | |
| 8113651 | NM_004707 | ATG12 | ATG12 autophagy related 12 homolo | -0.093 | 0.937 | 5.08E-01 | 5.08E+01 | | | |
| 7897179 | NM_00112624 | TP73 | tumor protein p73 | -0.093 | 0.937 | 8.01E-02 | 8.01E+00 | | | |
| 7998251 | NM_032366 | C16orf13 | chromosome 16 open reading frame | -0.093 | 0.937 | 1.09E-01 | 1.09E+01 | | | |
| 7946697 | NM_012250 | RRAS2 | related RAS viral (r-ras) oncogene ho | -0.093 | 0.937 | 2.34E-01 | 2.34E+01 | | | |
| 8016215 | NM_199282 | ARHGAP27 | Rho GTPase activating protein 27 | -0.093 | 0.937 | 8.87E-02 | 8.87E+00 | | | |
| 8149820 | NM_00113627 | NKX2-6 | NK2 transcription factor related, locu | -0.094 | 0.937 | 1.83E-01 | 1.83E+01 | | | |
| 7895509 | --- | --- | --- | -0.094 | 0.937 | 3.55E-01 | 3.55E+01 | | | |
| 8148184 | NM_032899 | FAM83A | family with sequence similarity 83, m | -0.094 | 0.937 | 1.02E-01 | 1.02E+01 | | | |
| 8018428 | NM_000154 | GALK1 | galactokinase 1 | -0.094 | 0.937 | 9.13E-02 | 9.13E+00 | | | |
| 8177964 | NM_00101170 | MCCD1 | mitochondrial coiled-coil domain 1 | -0.094 | 0.937 | 1.31E-01 | 1.31E+01 | | | |
| 8179246 | NM_00101170 | MCCD1 | mitochondrial coiled-coil domain 1 | -0.094 | 0.937 | 1.31E-01 | 1.31E+01 | | | |
| 7967109 | BC014661 | C12orf43 | chromosome 12 open reading frame | -0.094 | 0.937 | 1.38E-01 | 1.38E+01 | | | |
| 8156919 | NM_00101811 | MURC | muscle-related coiled-coil protein | -0.094 | 0.937 | 1.17E-01 | 1.17E+01 | | | |
| 7954124 | NM_177925 | H2AFJ | H2A histone family, member J | -0.094 | 0.937 | 1.60E-01 | 1.60E+01 | | | |
| 7895688 | --- | --- | --- | -0.094 | 0.937 | 4.45E-01 | 4.45E+01 | | | |
| 7936108 | NM_00100141 | CALHM1 | calcium homeostasis modulator 1 | -0.094 | 0.937 | 8.47E-02 | 8.47E+00 | | | |
| 8008704 | --- | --- | --- | -0.094 | 0.937 | 2.22E-01 | 2.22E+01 | | | |
| 7941843 | NM_017857 | SSH3 | slingshot homolog 3 (Drosophila) | -0.094 | 0.937 | 1.29E-01 | 1.29E+01 | | | |
| 8159379 | NM_032928 | TMEM141 | transmembrane protein 141 | -0.094 | 0.937 | 7.44E-01 | 7.44E+01 | | | |
| 8075934 | NM_014550 | CARD10 | caspase recruitment domain family, | -0.094 | 0.937 | 4.84E-02 | 4.84E+00 | | | |
| 7976778 | AK094097 | C14orf70 | chromosome 14 open reading frame | -0.094 | 0.937 | 1.64E-01 | 1.64E+01 | | | |
| 8028984 | NM_000774 | CYP2F1 | cytochrome P450, family 2, subfamil | -0.094 | 0.937 | 2.85E-01 | 2.85E+01 | | | |
| 8041745 | NM_024766 | C2orf34 | chromosome 2 open reading frame 3 | -0.094 | 0.937 | 7.80E-01 | 7.80E+01 | | | |
| 8170237 | --- | --- | --- | -0.094 | 0.937 | 1.51E-01 | 1.51E+01 | | | |
| 7945680 | NR_002196 | H19 | H19, imprinted maternally expressed | -0.094 | 0.937 | 1.65E-01 | 1.65E+01 | | | |
| 7910265 | NM_00109862 | OBSCN | obscurin, cytoskeletal calmodulin an | -0.094 | 0.937 | 1.80E-02 | 1.80E+00 | | | |
| 8145175 | NM_176871 | PDLIM2 | PDZ and LIM domain 2 (mystique) | -0.094 | 0.937 | 1.06E-01 | 1.06E+01 | | | |
| 7992131 | --- | --- | --- | -0.094 | 0.937 | 1.49E-01 | 1.49E+01 | | | |
| 8001615 | NM_001297 | CNGB1 | cyclic nucleotide gated channel beta | -0.094 | 0.937 | 4.83E-02 | 4.83E+00 | | | |
| 7907611 | NM_170692 | RASAL2 | RAS protein activator like 2 | -0.094 | 0.937 | 6.03E-02 | 6.03E+00 | | | |
| 7936882 | NM_00114634 | NKX1-2 | NK1 homeobox 2 | -0.094 | 0.937 | 1.08E-01 | 1.08E+01 | | | |
| 8077879 | NM_133625 | SYN2 | synapsin II | -0.094 | 0.937 | 1.25E-01 | 1.25E+01 | | | |
| 8111457 | NM_002853 | RAD1 | RAD1 homolog (S. pombe) | -0.094 | 0.937 | 6.24E-01 | 6.24E+01 | | | |
| 8147112 | NM_198584 | CA13 | carbonic anhydrase XIII | -0.094 | 0.937 | 1.08E-01 | 1.08E+01 | | | |
| 8031999 | NM_003712 | PPAP2C | phosphatidic acid phosphatase type | -0.094 | 0.937 | 9.57E-02 | 9.57E+00 | | | |
| 7940391 | NM_006725 | CD6 | CD6 molecule | -0.094 | 0.937 | 3.74E-01 | 3.74E+01 | | | |
| 7905500 | NM_178430 | LCE2D | late cornified envelope 2D | -0.094 | 0.937 | 3.33E-01 | 3.33E+01 | | | |
| 8160670 | NM_004925 | AQP3 | aquaporin 3 (Gill blood group) | -0.094 | 0.937 | 2.29E-01 | 2.29E+01 | | | |
| 8034696 | --- | --- | --- | -0.094 | 0.937 | 1.94E-01 | 1.94E+01 | | | |
| 8004057 | NM_006612 | KIF1C | kinesin family member 1C | -0.094 | 0.937 | 1.16E-01 | 1.16E+01 | | | |
| 7941236 | NM_006779 | CDC42EP2 | CDC42 effector protein (Rho GTPase | -0.094 | 0.937 | 8.05E-02 | 8.05E+00 | | | |
| 8169035 | NM_024863 | TCEAL4 | transcription elongation factor A (SII | -0.094 | 0.937 | 3.92E-01 | 3.92E+01 | | | |
| 7937363 | NM_007183 | PKP3 | plakophilin 3 | -0.094 | 0.937 | 4.85E-02 | 4.85E+00 | | | |
| 7948973 | NM_054108 | HRASLS5 | HRAS-like suppressor family, membe | -0.094 | 0.937 | 6.07E-02 | 6.07E+00 | | | |
| 8038477 | NM_032375 | AKT1S1 | AKT1 substrate 1 (proline-rich) | -0.094 | 0.937 | 1.11E-01 | 1.11E+01 | | | |
| 8146941 | --- | --- | --- | -0.094 | 0.937 | 2.65E-01 | 2.65E+01 | | | |
| 8109141 | NM_152407 | GRPEL2 | GrpE-like 2, mitochondrial (E. coli) | -0.094 | 0.937 | 2.24E-01 | 2.24E+01 | | | |
| 7905283 | NM_003568 | ANXA9 | annexin A9 | -0.094 | 0.937 | 1.14E-01 | 1.14E+01 | | | |
| 8171136 | NM_178129 | P2RY8 | purinergic receptor P2Y, G-protein co | -0.094 | 0.937 | 6.48E-01 | 6.48E+01 | | | |
| 8177026 | NM_178129 | P2RY8 | purinergic receptor P2Y, G-protein co | -0.094 | 0.937 | 6.48E-01 | 6.48E+01 | | | |
| 7918149 | --- | --- | --- | -0.094 | 0.937 | 3.23E-01 | 3.23E+01 | | | |
| 8159476 | NM_021138 | TRAF2 | TNF receptor-associated factor 2 | -0.094 | 0.937 | 7.71E-02 | 7.71E+00 | | | |

| | | | | | | | | | | |
|---|---|---|---|---|---|---|---|---|---|---|
| 7993108 | AK094332 | LOC440337 | hypothetical LOC440337 | -0.094 | 0.937 | 1.16E-01 | 1.16E+01 | | | |
| 7967202 | NM_019034 | RHOF | ras homolog gene family, member F | -0.094 | 0.937 | 1.76E-01 | 1.76E+01 | | | |
| 7941890 | NM_020811 | ATPGD1 | ATP-grasp domain containing 1 | -0.094 | 0.937 | 9.03E-02 | 9.03E+00 | | | |
| 8168424 | NM_006517 | SLC16A2 | solute carrier family 16, member 2 (r | -0.094 | 0.937 | 7.37E-02 | 7.37E+00 | | | |
| 7938625 | --- | --- | --- | -0.094 | 0.937 | 3.80E-01 | 3.80E+01 | | | |
| 8008969 | NM_005994 | TBX2 | T-box 2 | -0.094 | 0.937 | 4.30E-02 | 4.30E+00 | | | |
| 8010499 | AK096344 | FLJ35220 | hypothetical protein FLJ35220 | -0.094 | 0.937 | 9.09E-02 | 9.09E+00 | | | |
| 7928823 | NM_00103001! | OPN4 | opsin 4 | -0.094 | 0.937 | 7.55E-02 | 7.55E+00 | | | |
| 7895254 | --- | --- | --- | -0.094 | 0.937 | 8.18E-01 | 8.18E+01 | | | |
| 7896582 | --- | --- | --- | -0.094 | 0.937 | 6.45E-01 | 6.45E+01 | | | |
| 8013243 | NM_004169 | SHMT1 | serine hydroxymethyltransferase 1 (s | -0.094 | 0.937 | 1.30E-01 | 1.30E+01 | | | |
| 8179648 | NM_005275 | GNL1 | guanine nucleotide binding protein-l | -0.094 | 0.937 | 1.24E-01 | 1.24E+01 | | | |
| 8005657 | --- | --- | --- | -0.094 | 0.937 | 2.28E-01 | 2.28E+01 | | | |
| 8059111 | NM_005689 | ABCB6 | ATP-binding cassette, sub-family B (M | -0.094 | 0.937 | 2.62E-01 | 2.62E+01 | | | |
| 8007790 | NM_003954 | MAP3K14 | mitogen-activated protein kinase kin | -0.094 | 0.937 | 6.95E-02 | 6.95E+00 | | | |
| 8175302 | NM_00107817 | FAM127B | family with sequence similarity 127, | -0.094 | 0.937 | 2.28E-01 | 2.28E+01 | | | |
| 7893850 | --- | --- | --- | -0.094 | 0.937 | 5.73E-01 | 5.73E+01 | | | |
| 8137953 | NM_00108049! | TNRC18 | trinucleotide repeat containing 18 | -0.094 | 0.937 | 8.04E-02 | 8.04E+00 | | | |
| 8106818 | --- | --- | --- | -0.094 | 0.937 | 2.15E-01 | 2.15E+01 | | | |
| 7994572 | NR_002939 | RUNDC2C | RUN domain containing 2C | -0.094 | 0.937 | 5.70E-01 | 5.70E+01 | | | |
| 8136837 | NM_00100166 | OR6V1 | olfactory receptor, family 6, subfami | -0.094 | 0.937 | 4.73E-02 | 4.73E+00 | | | |
| 8095074 | NM_133267 | GSX2 | GS homeobox 2 | -0.094 | 0.937 | 6.42E-02 | 6.42E+00 | | | |
| 8069142 | NM_198687 | KRTAP10-4 | keratin associated protein 10-4 | -0.094 | 0.937 | 2.63E-01 | 2.63E+01 | | | |
| 7999149 | NM_020677 | NMRAL1 | NmrA-like family domain containing | -0.094 | 0.937 | 5.49E-01 | 5.49E+01 | | | |
| 8103025 | NM_178835 | ZNF827 | zinc finger protein 827 | -0.094 | 0.937 | 1.26E-01 | 1.26E+01 | | | |
| 7912012 | NM_007274 | ACOT7 | acyl-CoA thioesterase 7 | -0.094 | 0.937 | 5.18E-02 | 5.18E+00 | | | |
| 7995040 | NM_00112295 | BCKDK | branched chain ketoacid dehydroger | -0.094 | 0.937 | 1.58E-01 | 1.58E+01 | | | |
| 7992439 | NM_005262 | GFER | growth factor, augmenter of liver reg | -0.094 | 0.937 | 2.23E-01 | 2.23E+01 | | | |
| 8114526 | NM_152686 | DNAJC18 | DnaJ (Hsp40) homolog, subfamily C, | -0.094 | 0.937 | 2.61E-01 | 2.61E+01 | | | |
| 8105631 | --- | --- | --- | -0.094 | 0.937 | 1.36E-01 | 1.36E+01 | | | |
| 7964718 | --- | --- | --- | -0.094 | 0.937 | 4.63E-01 | 4.63E+01 | | | |
| 8055038 | NM_017980 | LIMS2 | LIM and senescent cell antigen-like d | -0.094 | 0.937 | 9.51E-02 | 9.51E+00 | | | |
| 8053944 | NR_026875 | NEURL3 | neuralized homolog 3 (Drosophila) p | -0.094 | 0.937 | 8.72E-02 | 8.72E+00 | | | |
| 8107406 | --- | --- | --- | -0.094 | 0.937 | 2.04E-01 | 2.04E+01 | | | |
| 8109981 | NM_003862 | FGF18 | fibroblast growth factor 18 | -0.094 | 0.937 | 7.61E-02 | 7.61E+00 | | | |
| 7989680 | NM_025049 | PIF1 | PIF1 5'-to-3' DNA helicase homolog ( | -0.094 | 0.937 | 1.14E-02 | 1.14E+00 | | | |
| 8174288 | NM_00101275! | MCART6 | mitochondrial carrier triple repeat 6 | -0.094 | 0.937 | 1.73E-01 | 1.73E+01 | | | |
| 7920401 | NM_014437 | SLC39A1 | solute carrier family 39 (zinc transpo | -0.094 | 0.937 | 2.04E-01 | 2.04E+01 | | | |
| 8042413 | NM_138964 | PROKR1 | prokineticin receptor 1 | -0.094 | 0.937 | 1.21E-01 | 1.21E+01 | | | |
| 8008052 | NM_003110 | SP2 | Sp2 transcription factor | -0.095 | 0.937 | 4.72E-01 | 4.72E+01 | | | |
| 7929383 | NM_00113465 | TMEM20 | transmembrane protein 20 | -0.095 | 0.937 | 2.22E-01 | 2.22E+01 | | | |
| 8008454 | NM_003786 | ABCC3 | ATP-binding cassette, sub-family C (C | -0.095 | 0.937 | 9.55E-02 | 9.55E+00 | | | |
| 8088142 | NM_018397 | CHDH | choline dehydrogenase | -0.095 | 0.937 | 1.91E-01 | 1.91E+01 | | | |
| 7905329 | NM_006818 | MLLT11 | myeloid/lymphoid or mixed-lineage | -0.095 | 0.937 | 4.93E-01 | 4.93E+01 | | | |
| 8002444 | AK128439 | VAC14 | Vac14 homolog (S. cerevisiae) | -0.095 | 0.937 | 1.52E-01 | 1.52E+01 | | | |
| 8172580 | NM_005183 | CACNA1F | calcium channel, voltage-dependent | -0.095 | 0.937 | 5.18E-02 | 5.18E+00 | | | |
| 7949454 | NM_002419 | MAP3K11 | mitogen-activated protein kinase kin | -0.095 | 0.937 | 1.57E-01 | 1.57E+01 | | | |
| 8109969 | NM_021025 | TLX3 | T-cell leukemia homeobox 3 | -0.095 | 0.936 | 2.43E-01 | 2.43E+01 | | | |
| 8165171 | NM_019892 | INPP5E | inositol polyphosphate-5-phosphatas | -0.095 | 0.936 | 1.12E-01 | 1.12E+01 | | | |
| 8106820 | NM_006467 | POLR3G | polymerase (RNA) III (DNA directed) | -0.095 | 0.936 | 7.81E-01 | 7.81E+01 | | | |
| 7995306 | --- | --- | --- | -0.095 | 0.936 | 9.66E-02 | 9.66E+00 | | | |
| 8149590 | --- | --- | --- | -0.095 | 0.936 | 6.48E-01 | 6.48E+01 | | | |
| 8037642 | NM_177542 | SNRPD2 | small nuclear ribonucleoprotein D2 p | -0.095 | 0.936 | 7.94E-01 | 7.94E+01 | | | |
| 7960117 | NM_018663 | PXMP2 | peroxisomal membrane protein 2, 22 | -0.095 | 0.936 | 9.06E-02 | 9.06E+00 | | | |
| 8156569 | --- | --- | --- | -0.095 | 0.936 | 1.61E-01 | 1.61E+01 | | | |
| 8092836 | NM_00108051 | CPN2 | carboxypeptidase N, polypeptide 2 | -0.095 | 0.936 | 1.18E-01 | 1.18E+01 | | | |
| 7893591 | --- | --- | --- | -0.095 | 0.936 | 9.04E-01 | 9.04E+01 | | | |
| 8050497 | NM_145260 | OSR1 | odd-skipped related 1 (Drosophila) | -0.095 | 0.936 | 5.90E-02 | 5.90E+00 | | | |
| 8036699 | NM_00108046 | SYCN | syncollin | -0.095 | 0.936 | 1.34E-01 | 1.34E+01 | | | |
| 8159519 | ENST00000371 | C9orf141 | chromosome 9 open reading frame 1 | -0.095 | 0.936 | 2.25E-01 | 2.25E+01 | | | |
| 7981750 | AK127783 | LOC400968 | hypothetical LOC400968 | -0.095 | 0.936 | 2.39E-01 | 2.39E+01 | | | |
| 8068891 | AK123727 | FLJ41733 | FLJ41733 protein | -0.095 | 0.936 | 1.19E-01 | 1.19E+01 | | | |
| 7934544 | NM_144589 | COMTD1 | catechol-O-methyltransferase domai | -0.095 | 0.936 | 3.73E-02 | 3.73E+00 | | | |
| 8075493 | NM_014323 | PATZ1 | POZ (BTB) and AT hook containing zi | -0.095 | 0.936 | 2.34E-01 | 2.34E+01 | | | |
| 8063187 | NM_172111 | EYA2 | eyes absent homolog 2 (Drosophila) | -0.095 | 0.936 | 1.55E-01 | 1.55E+01 | | | |
| 8136832 | NM_178829 | C7orf34 | chromosome 7 open reading frame 3 | -0.095 | 0.936 | 1.86E-01 | 1.86E+01 | | | |
| 8153823 | NM_032687 | CYHR1 | cysteine/histidine-rich 1 | -0.095 | 0.936 | 1.30E-01 | 1.30E+01 | | | |
| 8155327 | NM_000692 | ALDH1B1 | aldehyde dehydrogenase 1 family, m | -0.095 | 0.936 | 2.10E-01 | 2.10E+01 | | | |
| 7898431 | NM_016233 | PADI3 | peptidyl arginine deiminase, type III | -0.095 | 0.936 | 1.31E-01 | 1.31E+01 | | | |
| 7924663 | NM_020997 | LEFTY1 | left-right determination factor 1 | -0.095 | 0.936 | 2.11E-01 | 2.11E+01 | | | |
| 7915238 | NM_032526 | NT5C1A | 5'-nucleotidase, cytosolic IA | -0.095 | 0.936 | 1.03E-01 | 1.03E+01 | | | |
| 8072382 | NM_030758 | OSBP2 | oxysterol binding protein 2 | -0.095 | 0.936 | 4.73E-02 | 4.73E+00 | | | |
| 8000963 | NM_052874 | STX1B | syntaxin 1B | -0.095 | 0.936 | 1.60E-01 | 1.60E+01 | | | |
| 7937407 | NM_003475 | RASSF7 | Ras association (RalGDS/AF-6) doma | -0.095 | 0.936 | 1.21E-01 | 1.21E+01 | | | |
| 7992337 | NM_144570 | HN1L | hematological and neurological expr | -0.095 | 0.936 | 1.05E-01 | 1.05E+01 | | | |
| 8153609 | NM_032789 | PARP10 | poly (ADP-ribose) polymerase family | -0.095 | 0.936 | 6.19E-02 | 6.19E+00 | | | |
| 8103023 | --- | --- | --- | -0.095 | 0.936 | 1.06E-01 | 1.06E+01 | | | |

| | | | | | | | | | | |
|---|---|---|---|---|---|---|---|---|---|---|
| 8097447 | --- | --- | --- | -0.095 | 0.936 | 3.82E-01 | 3.82E+01 | | | |
| 8143759 | NM_145230 | ATP6V0E2 | ATPase, H+ transporting V0 subunit e | -0.095 | 0.936 | 2.08E-01 | 2.08E+01 | | | |
| 8082254 | NR_024251 | OC10012555 | family with sequence similarity 86, m | -0.095 | 0.936 | 1.13E-01 | 1.13E+01 | | | |
| 7977928 | NM_001805 | CEBPE | CCAAT/enhancer binding protein (C/ | -0.095 | 0.936 | 9.49E-02 | 9.49E+00 | | | |
| 7893560 | --- | --- | --- | -0.095 | 0.936 | 7.53E-01 | 7.53E+01 | | | |
| 7973898 | --- | --- | --- | -0.095 | 0.936 | 1.62E-01 | 1.62E+01 | | | |
| 8108832 | NM_014773 | KIAA0141 | KIAA0141 | -0.095 | 0.936 | 1.27E-01 | 1.27E+01 | | | |
| 7901535 | NM_153703 | PODN | podocan | -0.095 | 0.936 | 9.15E-02 | 9.15E+00 | | | |
| 8022623 | --- | --- | --- | -0.095 | 0.936 | 3.09E-01 | 3.09E+01 | | | |
| 7940626 | NM_002407 | SCGB2A1 | secretoglobin, family 2A, member 1 | -0.095 | 0.936 | 1.33E-01 | 1.33E+01 | | | |
| 7908924 | NM_002725 | PRELP | proline/arginine-rich end leucine-ricl | -0.095 | 0.936 | 5.28E-02 | 5.28E+00 | | | |
| 8064370 | NM_004609 | TCF15 | transcription factor 15 (basic helix-lo | -0.095 | 0.936 | 5.17E-02 | 5.17E+00 | | | |
| 7989887 | NM_032445 | MEGF11 | multiple EGF-like-domains 11 | -0.095 | 0.936 | 8.00E-02 | 8.00E+00 | | | |
| 8005399 | NM_017758 | ALKBH5 | alkB, alkylation repair homolog 5 (E. | -0.095 | 0.936 | 2.36E-01 | 2.36E+01 | | | |
| 7901445 | --- | --- | --- | -0.095 | 0.936 | 1.02E-01 | 1.02E+01 | | | |
| 8049075 | NM_145236 | B3GNT7 | UDP-GlcNAc:betaGal beta-1,3-N-ace | -0.095 | 0.936 | 1.57E-01 | 1.57E+01 | | | |
| 8071989 | NM_004076 | CRYBB3 | crystallin, beta B3 | -0.095 | 0.936 | 8.92E-02 | 8.92E+00 | | | |
| 7985562 | NR_003246 | FLJ40113 | golgi autoantigen, golgin subfamily a | -0.095 | 0.936 | 4.51E-01 | 4.51E+01 | | | |
| 7942417 | NM_014786 | ARHGEF17 | Rho guanine nucleotide exchange fa | -0.095 | 0.936 | 7.26E-02 | 7.26E+00 | | | |
| 8162823 | --- | --- | --- | -0.095 | 0.936 | 5.34E-01 | 5.34E+01 | | | |
| 7992845 | NM_003456 | ZNF205 | zinc finger protein 205 | -0.095 | 0.936 | 5.08E-02 | 5.08E+00 | | | |
| 8070876 | NM_015227 | POFUT2 | protein O-fucosyltransferase 2 | -0.095 | 0.936 | 1.89E-01 | 1.89E+01 | | | |
| 8076909 | NM_020461 | TUBGCP6 | tubulin, gamma complex associated | -0.095 | 0.936 | 1.22E-01 | 1.22E+01 | | | |
| 7963438 | NM_033448 | KRT71 | keratin 71 | -0.095 | 0.936 | 1.85E-01 | 1.85E+01 | | | |
| 7961320 | NM_006248 | PRB2 | proline-rich protein BstNI subfamily | -0.095 | 0.936 | 6.12E-02 | 6.12E+00 | | | |
| 8023968 | NM_004359 | CDC34 | cell division cycle 34 homolog (S. cer | -0.095 | 0.936 | 1.23E-01 | 1.23E+01 | | | |
| 8010061 | NM_182965 | SPHK1 | sphingosine kinase 1 | -0.095 | 0.936 | 2.65E-01 | 2.65E+01 | | | |
| 8137133 | NM_032534 | KRBA1 | KRAB-A domain containing 1 | -0.095 | 0.936 | 6.81E-02 | 6.81E+00 | | | |
| 8159566 | NM_016219 | MAN1B1 | mannosidase, alpha, class 1B, memb | -0.095 | 0.936 | 9.51E-02 | 9.51E+00 | | | |
| 8038747 | NM_145894 | KLK12 | kallikrein-related peptidase 12 | -0.095 | 0.936 | 9.28E-02 | 9.28E+00 | | | |
| 8126556 | NM_023932 | DLK2 | delta-like 2 homolog (Drosophila) | -0.095 | 0.936 | 1.11E-01 | 1.11E+01 | | | |
| 8025296 | NM_174894 | TRAPPC5 | trafficking protein particle complex 5 | -0.095 | 0.936 | 3.25E-01 | 3.25E+01 | | | |
| 8133896 | --- | --- | --- | -0.095 | 0.936 | 7.92E-02 | 7.92E+00 | | | |
| 8082905 | NM_025246 | TMEM22 | transmembrane protein 22 | -0.095 | 0.936 | 7.34E-02 | 7.34E+00 | | | |
| 8126436 | NM_000322 | PRPH2 | peripherin 2 (retinal degeneration, sl | -0.095 | 0.936 | 1.19E-01 | 1.19E+01 | | | |
| 7984245 | NM_178859 | OSTbeta | organic solute transporter beta | -0.095 | 0.936 | 2.21E-01 | 2.21E+01 | | | |
| 8134452 | NM_177455 | BHLHA15 | basic helix-loop-helix family, membe | -0.095 | 0.936 | 5.95E-02 | 5.95E+00 | | | |
| 8168790 | NM_021637 | TMEM35 | transmembrane protein 35 | -0.095 | 0.936 | 1.27E-01 | 1.27E+01 | | | |
| 8180304 | --- | --- | --- | -0.096 | 0.936 | 1.54E-01 | 1.54E+01 | | | |
| 7959314 | NM_015048 | SETD1B | SET domain containing 1B | -0.096 | 0.936 | 3.08E-01 | 3.08E+01 | | | |
| 7973080 | AK093210 | LOC254028 | hypothetical LOC254028 | -0.096 | 0.936 | 1.80E-01 | 1.80E+01 | | | |
| 8034217 | NM_000121 | EPOR | erythropoietin receptor | -0.096 | 0.936 | 9.36E-02 | 9.36E+00 | | | |
| 8012823 | NM_004589 | SCO1 | SCO cytochrome oxidase deficient ho | -0.096 | 0.936 | 6.58E-01 | 6.58E+01 | | | |
| 8037301 | NM_014400 | LYPD3 | LY6/PLAUR domain containing 3 | -0.096 | 0.936 | 1.25E-01 | 1.25E+01 | | | |
| 7956395 | NM_007224 | NXPH4 | neurexophilin 4 | -0.096 | 0.936 | 1.18E-01 | 1.18E+01 | | | |
| 8026250 | NM_138353 | DCAF15 | DDB1 and CUL4 associated factor 15 | -0.096 | 0.936 | 2.21E-01 | 2.21E+01 | | | |
| 8170635 | NM_00108048 | ZNF275 | zinc finger protein 275 | -0.096 | 0.936 | 7.79E-01 | 7.79E+01 | | | |
| 7937391 | NM_198075 | LRRC56 | leucine rich repeat containing 56 | -0.096 | 0.936 | 5.81E-02 | 5.81E+00 | | | |
| 7963061 | NM_00100822 | C1QL4 | complement component 1, q subcon | -0.096 | 0.936 | 9.03E-02 | 9.03E+00 | | | |
| 8147503 | NM_018407 | LAPTM4B | lysosomal protein transmembrane 4 | -0.096 | 0.936 | 3.52E-01 | 3.52E+01 | | | |
| 8010562 | NM_006340 | BAIAP2 | BAI1-associated protein 2 | -0.096 | 0.936 | 1.07E-01 | 1.07E+01 | | | |
| 7897119 | AF425266 | C1orf93 | chromosome 1 open reading frame 9 | -0.096 | 0.936 | 1.72E-01 | 1.72E+01 | | | |
| 8055486 | --- | --- | --- | -0.096 | 0.936 | 8.76E-01 | 8.76E+01 | | | |
| 8117840 | NM_138700 | TRIM40 | tripartite motif-containing 40 | -0.096 | 0.936 | 1.62E-01 | 1.62E+01 | | | |
| 8161373 | ENST00000438 | OC10028952 | hypothetical protein LOC100289528 | -0.096 | 0.936 | 6.99E-01 | 6.99E+01 | | | |
| 8161554 | ENST00000438 | OC10028952 | hypothetical protein LOC100289528 | -0.096 | 0.936 | 6.99E-01 | 6.99E+01 | | | |
| 8023977 | NM_005317 | GZMM | granzyme M (lymphocyte met-ase 1) | -0.096 | 0.936 | 8.64E-02 | 8.64E+00 | | | |
| 8118995 | NM_182548 | LHFPL5 | lipoma HMGIC fusion partner-like 5 | -0.096 | 0.936 | 1.23E-01 | 1.23E+01 | | | |
| 8024864 | NM_00100152 | HDGF2 | hepatoma-derived growth factor-rel | -0.096 | 0.936 | 6.71E-02 | 6.71E+00 | | | |
| 7893985 | --- | --- | --- | -0.096 | 0.936 | 7.01E-01 | 7.01E+01 | | | |
| 8119898 | NM_00102536 | VEGFA | vascular endothelial growth factor A | -0.096 | 0.936 | 1.12E-01 | 1.12E+01 | | | |
| 7962226 | NM_198992 | SYT10 | synaptotagmin X | -0.096 | 0.936 | 1.38E-01 | 1.38E+01 | | | |
| 8097743 | --- | --- | --- | -0.096 | 0.936 | 3.68E-01 | 3.68E+01 | | | |
| 7922976 | NM_000963 | PTGS2 | prostaglandin-endoperoxide synthas | -0.096 | 0.936 | 8.80E-01 | 8.80E+01 | | | |
| 7992841 | --- | --- | --- | -0.096 | 0.936 | 6.57E-01 | 6.57E+01 | | | |
| 7929882 | NM_017893 | SEMA4G | sema domain, immunoglobulin doma | -0.096 | 0.936 | 1.24E-01 | 1.24E+01 | | | |
| 7996430 | NM_00104066 | HSF4 | heat shock transcription factor 4 | -0.096 | 0.936 | 6.12E-02 | 6.12E+00 | | | |
| 7908879 | NM_015053 | PPFIA4 | protein tyrosine phosphatase, recep | -0.096 | 0.936 | 4.54E-02 | 4.54E+00 | | | |
| 7921637 | NM_003874 | CD84 | CD84 molecule | -0.096 | 0.936 | 4.41E-01 | 4.41E+01 | | | |
| 8037023 | NM_020158 | EXOSC5 | exosome component 5 | -0.096 | 0.936 | 1.15E-01 | 1.15E+01 | | | |
| 7954963 | --- | --- | --- | -0.096 | 0.936 | 4.78E-01 | 4.78E+01 | | | |
| 8037931 | NM_00110141 | ZNF541 | zinc finger protein 541 | -0.096 | 0.936 | 9.51E-02 | 9.51E+00 | | | |
| 8141522 | BC031966 | C7orf61 | chromosome 7 open reading frame 6 | -0.096 | 0.936 | 1.41E-01 | 1.41E+01 | | | |
| 7940840 | NM_017670 | OTUB1 | OTU domain, ubiquitin aldehyde bin | -0.096 | 0.936 | 4.13E-01 | 4.13E+01 | | | |
| 8156529 | NM_194320 | ZNF169 | zinc finger protein 169 | -0.096 | 0.936 | 2.08E-01 | 2.08E+01 | | | |
| 8159838 | NM_152569 | C9orf66 | chromosome 9 open reading frame 6 | -0.096 | 0.936 | 2.89E-01 | 2.89E+01 | | | |
| 7966839 | NM_019086 | FLJ20674 | hypothetical protein FLJ20674 | -0.096 | 0.936 | 8.52E-02 | 8.52E+00 | | | |

| | | | | | | | | | | |
|---|---|---|---|---|---|---|---|---|---|---|
| 7992145 | NM_001053 | SSTR5 | somatostatin receptor 5 | -0.096 | 0.936 | 1.18E-01 | 1.18E+01 | | | |
| 8108683 | NM_018936 | PCDHB2 | protocadherin beta 2 | -0.096 | 0.936 | 1.79E-01 | 1.79E+01 | | | |
| 8118124 | NM_00101170( | MCCD1 | mitochondrial coiled-coil domain 1 | -0.096 | 0.936 | 1.44E-01 | 1.44E+01 | | | |
| 8011734 | NM_000080 | CHRNE | cholinergic receptor, nicotinic, epsilo | -0.096 | 0.936 | 1.10E-01 | 1.10E+01 | | | |
| 8061035 | NM_080676 | MACROD2 | MACRO domain containing 2 | -0.096 | 0.936 | 6.70E-02 | 6.70E+00 | | | |
| 7893454 | --- | --- | --- | -0.096 | 0.936 | 2.89E-01 | 2.89E+01 | | | |
| 8064831 | --- | --- | --- | -0.096 | 0.936 | 5.34E-01 | 5.34E+01 | | | |
| 7997010 | NM_182619 | CLEC18A | C-type lectin domain family 18, mem | -0.096 | 0.936 | 1.11E-01 | 1.11E+01 | | | |
| 8146462 | NM_022454 | SOX17 | SRY (sex determining region Y)-box 1 | -0.096 | 0.936 | 1.95E-01 | 1.95E+01 | | | |
| 8137332 | NM_007188 | ABCB8 | ATP-binding cassette, sub-family B (N | -0.096 | 0.936 | 9.57E-02 | 9.57E+00 | | | |
| 8143937 | NM_016118 | NUB1 | negative regulator of ubiquitin-like p | -0.096 | 0.936 | 2.40E-01 | 2.40E+01 | | | |
| 7919729 | NM_016022 | APH1A | anterior pharynx defective 1 homolo | -0.096 | 0.936 | 4.62E-01 | 4.62E+01 | | | |
| 7926677 | NM_00114537 | OTUD1 | OTU domain containing 1 | -0.096 | 0.935 | 8.25E-01 | 8.25E+01 | | | |
| 8018754 | NM_134268 | CYGB | cytoglobin | -0.096 | 0.935 | 1.28E-01 | 1.28E+01 | | | |
| 8078136 | --- | --- | --- | -0.096 | 0.935 | 4.22E-01 | 4.22E+01 | | | |
| 7948293 | NM_153450 | MED19 | mediator complex subunit 19 | -0.096 | 0.935 | 5.15E-01 | 5.15E+01 | | | |
| 7990191 | NM_052840 | BRUNOL6 | bruno-like 6, RNA binding protein (D | -0.096 | 0.935 | 5.98E-02 | 5.98E+00 | | | |
| 7967089 | --- | --- | --- | -0.096 | 0.935 | 2.29E-01 | 2.29E+01 | | | |
| 8042310 | NM_003038 | SLC1A4 | solute carrier family 1 (glutamate/ne | -0.096 | 0.935 | 1.20E-01 | 1.20E+01 | | | |
| 7893354 | --- | --- | --- | -0.096 | 0.935 | 8.15E-01 | 8.15E+01 | | | |
| 8176572 | ENST00000455 | FRG2C | FSHD region gene 2 family, member | -0.096 | 0.935 | 2.99E-01 | 2.99E+01 | | | |
| 8040530 | NM_054033 | FKBP1B | FK506 binding protein 1B, 12.6 kDa | -0.096 | 0.935 | 9.26E-02 | 9.26E+00 | | | |
| 7937774 | NM_016412 | IGF2AS | insulin-like growth factor 2 antisense | -0.096 | 0.935 | 5.71E-02 | 5.71E+00 | | | |
| 8149705 | --- | --- | --- | -0.096 | 0.935 | 2.78E-01 | 2.78E+01 | | | |
| 8063755 | BC054002 | C20orf177 | chromosome 20 open reading frame | -0.096 | 0.935 | 3.80E-01 | 3.80E+01 | | | |
| 7893338 | --- | --- | --- | -0.096 | 0.935 | 3.37E-01 | 3.37E+01 | | | |
| 7895427 | --- | --- | --- | -0.096 | 0.935 | 2.61E-01 | 2.61E+01 | | | |
| 7940349 | NM_024098 | CCDC86 | coiled-coil domain containing 86 | -0.096 | 0.935 | 1.56E-01 | 1.56E+01 | | | |
| 8151931 | NM_033512 | TSPYL5 | TSPY-like 5 | -0.096 | 0.935 | 1.71E-01 | 1.71E+01 | | | |
| 7941274 | NM_020680 | SCYL1 | SCY1-like 1 (S. cerevisiae) | -0.096 | 0.935 | 1.58E-01 | 1.58E+01 | | | |
| 8031084 | NM_031895 | CACNG8 | calcium channel, voltage-dependent | -0.096 | 0.935 | 5.63E-02 | 5.63E+00 | | | |
| 7941743 | NM_024036 | LRFN4 | leucine rich repeat and fibronectin ty | -0.096 | 0.935 | 1.22E-01 | 1.22E+01 | | | |
| 8128650 | NM_007214 | SEC63 | SEC63 homolog (S. cerevisiae) | -0.096 | 0.935 | 8.31E-01 | 8.31E+01 | | | |
| 8085606 | --- | --- | --- | -0.096 | 0.935 | 1.54E-01 | 1.54E+01 | | | |
| 7934215 | NM_014767 | SPOCK2 | sparc/osteonectin, cwcv and kazal-lik | -0.096 | 0.935 | 1.02E-01 | 1.02E+01 | | | |
| 8177750 | NM_138700 | TRIM40 | tripartite motif-containing 40 | -0.096 | 0.935 | 1.39E-01 | 1.39E+01 | | | |
| 8179060 | NM_138700 | TRIM40 | tripartite motif-containing 40 | -0.096 | 0.935 | 1.39E-01 | 1.39E+01 | | | |
| 8074593 | NM_015672 | RIMBP3 | RIMS binding protein 3 | -0.096 | 0.935 | 7.81E-02 | 7.81E+00 | | | |
| 8070129 | NM_017613 | DONSON | downstream neighbor of SON | -0.097 | 0.935 | 1.28E-01 | 1.28E+01 | | | |
| 8092931 | --- | --- | --- | -0.097 | 0.935 | 2.48E-01 | 2.48E+01 | | | |
| 8007569 | AK291924 | C17orf53 | chromosome 17 open reading frame | -0.097 | 0.935 | 1.45E-01 | 1.45E+01 | | | |
| 8176730 | NM_00103956 | RPS4Y2 | ribosomal protein S4, Y-linked 2 | -0.097 | 0.935 | 6.09E-01 | 6.09E+01 | | | |
| 8035553 | NM_007263 | COPE | coatomer protein complex, subunit e | -0.097 | 0.935 | 1.28E-01 | 1.28E+01 | | | |
| 8093035 | --- | --- | --- | -0.097 | 0.935 | 1.20E-01 | 1.20E+01 | | | |
| 7931859 | NM_017422 | CALML5 | calmodulin-like 5 | -0.097 | 0.935 | 8.09E-02 | 8.09E+00 | | | |
| 7895882 | --- | --- | --- | -0.097 | 0.935 | 5.66E-01 | 5.66E+01 | | | |
| 7995344 | --- | --- | --- | -0.097 | 0.935 | 2.41E-01 | 2.41E+01 | | | |
| 8069148 | NM_198695 | KRTAP10-8 | keratin associated protein 10-8 | -0.097 | 0.935 | 8.27E-02 | 8.27E+00 | | | |
| 7900076 | NM_014466 | TEKT2 | tektin 2 (testicular) | -0.097 | 0.935 | 1.77E-01 | 1.77E+01 | | | |
| 7941961 | NM_000694 | ALDH3B1 | aldehyde dehydrogenase 3 family, m | -0.097 | 0.935 | 6.88E-02 | 6.88E+00 | | | |
| 8084929 | NM_152672 | OSTalpha | organic solute transporter alpha | -0.097 | 0.935 | 8.87E-02 | 8.87E+00 | | | |
| 8068883 | NM_000394 | CRYAA | crystallin, alpha A | -0.097 | 0.935 | 5.80E-02 | 5.80E+00 | | | |
| 8116195 | NM_173465 | COL23A1 | collagen, type XXIII, alpha 1 | -0.097 | 0.935 | 7.16E-02 | 7.16E+00 | | | |
| 7921526 | NM_145167 | PIGM | phosphatidylinositol glycan anchor b | -0.097 | 0.935 | 2.54E-01 | 2.54E+01 | | | |
| 7913290 | NM_00110316 | SH2D5 | SH2 domain containing 5 | -0.097 | 0.935 | 7.27E-02 | 7.27E+00 | | | |
| 7984862 | NM_000761 | CYP1A2 | cytochrome P450, family 1, subfamil | -0.097 | 0.935 | 8.66E-02 | 8.66E+00 | | | |
| 8048673 | NM_201574 | SLC4A3 | solute carrier family 4, anion exchan | -0.097 | 0.935 | 1.10E-01 | 1.10E+01 | | | |
| 8028872 | NM_00104254 | LTBP4 | latent transforming growth factor be | -0.097 | 0.935 | 5.29E-02 | 5.29E+00 | | | |
| 7922008 | NM_053053 | TADA1L | transcriptional adaptor 1 (HFI1 homo | -0.097 | 0.935 | 3.51E-01 | 3.51E+01 | | | |
| 8069085 | NM_003307 | TRPM2 | transient receptor potential cation c | -0.097 | 0.935 | 6.35E-02 | 6.35E+00 | | | |
| 8154476 | NM_003026 | SH3GL2 | SH3-domain GRB2-like 2 | -0.097 | 0.935 | 6.89E-02 | 6.89E+00 | | | |
| 7997863 | NM_174917 | ACSF3 | acyl-CoA synthetase family member | -0.097 | 0.935 | 2.74E-01 | 2.74E+01 | | | |
| 8169073 | NM_194324 | TMSB15B | thymosin beta 15B | -0.097 | 0.935 | 4.05E-01 | 4.05E+01 | | | |
| 7997907 | NM_013275 | ANKRD11 | ankyrin repeat domain 11 | -0.097 | 0.935 | 4.26E-01 | 4.26E+01 | | | |
| 8170322 | NM_013275 | ANKRD11 | ankyrin repeat domain 11 | -0.097 | 0.935 | 4.26E-01 | 4.26E+01 | | | |
| 7962623 | NM_00109853 | RAPGEF3 | Rap guanine nucleotide exchange fac | -0.097 | 0.935 | 1.74E-01 | 1.74E+01 | | | |
| 8030148 | NM_014475 | DHDH | dihydrodiol dehydrogenase (dimeric) | -0.097 | 0.935 | 1.05E-01 | 1.05E+01 | | | |
| 8032380 | NM_017797 | BTBD2 | BTB (POZ) domain containing 2 | -0.097 | 0.935 | 2.72E-01 | 2.72E+01 | | | |
| 8021866 | NM_172387 | NFATC1 | nuclear factor of activated T-cells, cy | -0.097 | 0.935 | 6.78E-02 | 6.78E+00 | | | |
| 8168083 | NM_207320 | OTUD6A | OTU domain containing 6A | -0.097 | 0.935 | 2.01E-01 | 2.01E+01 | | | |
| 8007493 | NM_001661 | ARL4D | ADP-ribosylation factor-like 4D | -0.097 | 0.935 | 1.92E-01 | 1.92E+01 | | | |
| 8087925 | NM_003280 | TNNC1 | troponin C type 1 (slow) | -0.097 | 0.935 | 2.08E-01 | 2.08E+01 | | | |
| 7939835 | ENST00000356 | FAM180B | family with sequence similarity 180, | -0.097 | 0.935 | 1.33E-01 | 1.33E+01 | | | |
| 8033064 | NM_002034 | FUT5 | fucosyltransferase 5 (alpha (1,3) fuco | -0.097 | 0.935 | 1.80E-01 | 1.80E+01 | | | |
| 7930341 | NM_014978 | SORCS3 | sortilin-related VPS10 domain contai | -0.097 | 0.935 | 1.27E-01 | 1.27E+01 | | | |
| 8105328 | NM_052870 | SNX18 | sorting nexin 18 | -0.097 | 0.935 | 3.64E-01 | 3.64E+01 | | | |
| 8097773 | NM_006439 | MAB21L2 | mab-21-like 2 (C. elegans) | -0.097 | 0.935 | 2.76E-01 | 2.76E+01 | | | |

| | | | | | | | | | | |
|---|---|---|---|---|---|---|---|---|---|---|
| 8076673 | NM_032287 | LDOC1L | leucine zipper, down-regulated in ca | -0.097 | 0.935 | 5.46E-02 | 5.46E+00 | | | |
| 8089701 | NM_015642 | ZBTB20 | zinc finger and BTB domain containir | -0.097 | 0.935 | 8.15E-01 | 8.15E+01 | | | |
| 8147777 | NM_138455 | CTHRC1 | collagen triple helix repeat containin | -0.097 | 0.935 | 1.06E-01 | 1.06E+01 | | | |
| 8135273 | --- | --- | --- | -0.097 | 0.935 | 1.13E-01 | 1.13E+01 | | | |
| 7949553 | NM_053054 | CATSPER1 | cation channel, sperm associated 1 | -0.097 | 0.935 | 8.59E-02 | 8.59E+00 | | | |
| 8175844 | NM_174869 | IDH3G | isocitrate dehydrogenase 3 (NAD+) g | -0.097 | 0.935 | 3.00E-01 | 3.00E+01 | | | |
| 7899870 | NM_152493 | ZNF362 | zinc finger protein 362 | -0.097 | 0.935 | 1.68E-01 | 1.68E+01 | | | |
| 8024048 | NM_002777 | PRTN3 | proteinase 3 | -0.097 | 0.935 | 9.80E-02 | 9.80E+00 | | | |
| 8027793 | NM_205834 | LSR | lipolysis stimulated lipoprotein recep | -0.097 | 0.935 | 4.49E-02 | 4.49E+00 | | | |
| 8063337 | NR_003605 | C20orf199 | chromosome 20 open reading frame | -0.097 | 0.935 | 4.15E-01 | 4.15E+01 | | | |
| 8072436 | NM_00100283 | INPP5J | inositol polyphosphate-5-phosphatas | -0.097 | 0.935 | 5.00E-02 | 5.00E+00 | | | |
| 8032149 | NM_002695 | POLR2E | polymerase (RNA) II (DNA directed) p | -0.097 | 0.935 | 2.37E-01 | 2.37E+01 | | | |
| 7942796 | NM_00102985 | KCTD21 | potassium channel tetramerisation d | -0.097 | 0.935 | 2.47E-01 | 2.47E+01 | | | |
| 8118259 | NM_025259 | MSH5 | mutS homolog 5 (E. coli) | -0.097 | 0.935 | 2.87E-01 | 2.87E+01 | | | |
| 7954604 | NM_020782 | KLHDC5 | kelch domain containing 5 | -0.097 | 0.935 | 1.46E-01 | 1.46E+01 | | | |
| 7892529 | --- | --- | --- | -0.097 | 0.935 | 5.32E-01 | 5.32E+01 | | | |
| 8001682 | NM_00108049 | KLKBL4 | plasma kallikrein-like protein 4 | -0.097 | 0.935 | 7.12E-02 | 7.12E+00 | | | |
| 8053009 | NM_00108041 | FBXO41 | F-box protein 41 | -0.097 | 0.935 | 7.49E-02 | 7.49E+00 | | | |
| 7976795 | NR_003530 | MEG3 | maternally expressed 3 (non-protein | -0.097 | 0.935 | 1.25E-01 | 1.25E+01 | | | |
| 8139207 | NM_002192 | INHBA | inhibin, beta A | -0.097 | 0.935 | 1.54E-01 | 1.54E+01 | | | |
| 7981181 | NR_003002 | SCARNA13 | small Cajal body-specific RNA 13 | -0.097 | 0.935 | 8.11E-01 | 8.11E+01 | | | |
| 8084630 | --- | --- | --- | -0.097 | 0.935 | 1.84E-01 | 1.84E+01 | | | |
| 8132302 | ENST0000038 | PP13004 | hypothetical LOC402481 | -0.097 | 0.935 | 2.83E-01 | 2.83E+01 | | | |
| 7990269 | BC111368 | C15orf59 | chromosome 15 open reading frame | -0.097 | 0.935 | 8.96E-02 | 8.96E+00 | | | |
| 8072847 | NM_018957 | SH3BP1 | SH3-domain binding protein 1 | -0.097 | 0.935 | 6.50E-02 | 6.50E+00 | | | |
| 8007115 | NM_00114604 | KRTAP4-9 | keratin associated protein 4-9 | -0.097 | 0.935 | 1.84E-01 | 1.84E+01 | | | |
| 8085360 | NM_003256 | TIMP4 | TIMP metallopeptidase inhibitor 4 | -0.097 | 0.935 | 1.03E-01 | 1.03E+01 | | | |
| 7964535 | NM_000785 | CYP27B1 | cytochrome P450, family 27, subfam | -0.097 | 0.935 | 5.11E-02 | 5.11E+00 | | | |
| 7968265 | NM_000209 | PDX1 | pancreatic and duodenal homeobox | -0.097 | 0.935 | 5.11E-02 | 5.11E+00 | | | |
| 7991323 | NM_003847 | PEX11A | peroxisomal biogenesis factor 11 alp | -0.097 | 0.935 | 1.48E-01 | 1.48E+01 | | | |
| 8099388 | NM_005114 | HS3ST1 | heparan sulfate (glucosamine) 3-O-s | -0.097 | 0.935 | 1.54E-01 | 1.54E+01 | | | |
| 8149283 | NM_178857 | RP1L1 | retinitis pigmentosa 1-like 1 | -0.097 | 0.935 | 1.60E-01 | 1.60E+01 | | | |
| 8175438 | --- | --- | --- | -0.097 | 0.935 | 3.18E-01 | 3.18E+01 | | | |
| 7895033 | --- | --- | --- | -0.097 | 0.935 | 2.62E-01 | 2.62E+01 | | | |
| 8122971 | NM_020732 | ARID1B | AT rich interactive domain 1B (SWI1- | -0.097 | 0.935 | 4.60E-01 | 4.60E+01 | | | |
| 8171029 | NM_012151 | F8A1 | coagulation factor VIII-associated (in | -0.097 | 0.935 | 1.20E-01 | 1.20E+01 | | | |
| 8180416 | --- | --- | --- | -0.097 | 0.935 | 1.31E-01 | 1.31E+01 | | | |
| 7935006 | NM_152429 | FGFBP3 | fibroblast growth factor binding prot | -0.097 | 0.935 | 7.81E-02 | 7.81E+00 | | | |
| 8049969 | NM_015677 | SH3YL1 | SH3 domain containing, Ysc84-like 1 | -0.097 | 0.935 | 2.54E-01 | 2.54E+01 | | | |
| 7904955 | --- | --- | --- | -0.097 | 0.935 | 2.30E-01 | 2.30E+01 | | | |
| 7949650 | NM_003793 | CTSF | cathepsin F | -0.097 | 0.935 | 8.97E-02 | 8.97E+00 | | | |
| 8053449 | NM_00103173 | TMEM150A | transmembrane protein 150A | -0.097 | 0.935 | 8.38E-02 | 8.38E+00 | | | |
| 8159265 | NM_00101804 | PAEP | progestagen-associated endometrial | -0.097 | 0.935 | 9.22E-02 | 9.22E+00 | | | |
| 7998449 | NM_00100141 | C16orf42 | chromosome 16 open reading frame | -0.097 | 0.935 | 2.04E-01 | 2.04E+01 | | | |
| 7947526 | --- | --- | --- | -0.097 | 0.935 | 1.79E-01 | 1.79E+01 | | | |
| 7899422 | NM_014280 | DNAJC8 | DnaJ (Hsp40) homolog, subfamily C, | -0.097 | 0.935 | 6.44E-01 | 6.44E+01 | | | |
| 8126240 | AK123643 | FLJ41649 | hypothetical LOC401260 | -0.097 | 0.935 | 1.15E-01 | 1.15E+01 | | | |
| 7904314 | NM_003594 | TTF2 | transcription termination factor, RNA | -0.097 | 0.935 | 3.94E-01 | 3.94E+01 | | | |
| 8080168 | NM_000666 | ACY1 | aminoacylase 1 | -0.097 | 0.935 | 1.25E-01 | 1.25E+01 | | | |
| 8090936 | --- | --- | --- | -0.098 | 0.935 | 4.10E-01 | 4.10E+01 | | | |
| 7913566 | NM_000864 | HTR1D | 5-hydroxytryptamine (serotonin) rec | -0.098 | 0.935 | 2.14E-01 | 2.14E+01 | | | |
| 7995007 | NM_025193 | HSD3B7 | hydroxy-delta-5-steroid dehydrogena | -0.098 | 0.935 | 7.82E-02 | 7.82E+00 | | | |
| 7895073 | --- | --- | --- | -0.098 | 0.935 | 7.83E-01 | 7.83E+01 | | | |
| 8154100 | NM_003383 | VLDLR | very low density lipoprotein recepto | -0.098 | 0.935 | 1.96E-01 | 1.96E+01 | | | |
| 7998668 | NM_144603 | NOXO1 | NADPH oxidase organizer 1 | -0.098 | 0.935 | 7.04E-02 | 7.04E+00 | | | |
| 8144119 | --- | --- | --- | -0.098 | 0.935 | 1.21E-01 | 1.21E+01 | | | |
| 8091411 | NM_014220 | TM4SF1 | transmembrane 4 L six family memb | -0.098 | 0.935 | 1.63E-01 | 1.63E+01 | | | |
| 8075817 | --- | --- | --- | -0.098 | 0.935 | 4.39E-02 | 4.39E+00 | | | |
| 7948504 | NM_017966 | VPS37C | vacuolar protein sorting 37 homolog | -0.098 | 0.935 | 7.67E-02 | 7.67E+00 | | | |
| 8167673 | NM_00109880 | MAGED4 | melanoma antigen family D, 4 | -0.098 | 0.935 | 1.17E-01 | 1.17E+01 | | | |
| 8172722 | NM_00109880 | MAGED4 | melanoma antigen family D, 4 | -0.098 | 0.935 | 1.17E-01 | 1.17E+01 | | | |
| 7923528 | NM_002479 | MYOG | myogenin (myogenic factor 4) | -0.098 | 0.935 | 1.16E-01 | 1.16E+01 | | | |
| 8158671 | NM_000050 | ASS1 | argininosuccinate synthetase 1 | -0.098 | 0.935 | 9.82E-02 | 9.82E+00 | | | |
| 8175261 | --- | --- | --- | -0.098 | 0.934 | 1.46E-01 | 1.46E+01 | | | |
| 8066189 | NM_021081 | GHRH | growth hormone releasing hormone | -0.098 | 0.934 | 8.27E-02 | 8.27E+00 | | | |
| 8113071 | ENST0000044 | LOC729040 | hypothetical LOC729040 | -0.098 | 0.934 | 1.22E-01 | 1.22E+01 | | | |
| 8045088 | --- | --- | --- | -0.098 | 0.934 | 6.44E-01 | 6.44E+01 | | | |
| 8052087 | --- | --- | --- | -0.098 | 0.934 | 3.53E-01 | 3.53E+01 | | | |
| 7912511 | NM_138346 | KIAA2013 | KIAA2013 | -0.098 | 0.934 | 2.46E-01 | 2.46E+01 | | | |
| 7977888 | NM_022478 | CDH24 | cadherin-like 24 | -0.098 | 0.934 | 1.10E-01 | 1.10E+01 | | | |
| 8167457 | NM_014008 | CCDC22 | coiled-coil domain containing 22 | -0.098 | 0.934 | 2.12E-01 | 2.12E+01 | | | |
| 8022711 | NM_024422 | DSC2 | desmocollin 2 | -0.098 | 0.934 | 9.80E-02 | 9.80E+00 | | | |
| 7955231 | NM_012284 | KCNH3 | potassium voltage-gated channel, su | -0.098 | 0.934 | 8.82E-02 | 8.82E+00 | | | |
| 7907211 | --- | --- | --- | -0.098 | 0.934 | 2.98E-01 | 2.98E+01 | | | |
| 7918444 | NM_005549 | KCNA10 | potassium voltage-gated channel, sh | -0.098 | 0.934 | 1.21E-01 | 1.21E+01 | | | |
| 7998485 | BC092497 | C16orf91 | chromosome 16 open reading frame | -0.098 | 0.934 | 1.43E-01 | 1.43E+01 | | | |
| 7923378 | NM_004078 | CSRP1 | cysteine and glycine-rich protein 1 | -0.098 | 0.934 | 3.08E-01 | 3.08E+01 | | | |

| | | | | | | | | | | | |
|---|---|---|---|---|---|---|---|---|---|---|---|
| 7910539 | NM_00101098 | C1orf124 | chromosome 1 open reading frame 1 | -0.098 | 0.934 | 3.38E-01 | 3.38E+01 | | | | |
| 8000590 | NM_177534 | SULT1A1 | sulfotransferase family, cytosolic, 1A | -0.098 | 0.934 | 1.94E-01 | 1.94E+01 | | | | |
| 8158725 | NM_005157 | ABL1 | c-abl oncogene 1, receptor tyrosine l | -0.098 | 0.934 | 3.18E-01 | 3.18E+01 | | | | |
| 7986567 | --- | --- | --- | -0.098 | 0.934 | 2.09E-01 | 2.09E+01 | | | | |
| 7986633 | --- | --- | --- | -0.098 | 0.934 | 2.09E-01 | 2.09E+01 | | | | |
| 8137675 | NM_002735 | PRKAR1B | protein kinase, cAMP-dependent, re | -0.098 | 0.934 | 2.54E-01 | 2.54E+01 | | | | |
| 7943398 | NM_00113014 | YAP1 | Yes-associated protein 1, 65kDa | -0.098 | 0.934 | 1.45E-01 | 1.45E+01 | | | | |
| 8128079 | NM_080743 | SRrp35 | serine-arginine repressor protein (35 | -0.098 | 0.934 | 7.29E-02 | 7.29E+00 | | | | |
| 8010978 | ENST00000435 | OC10013087 | hypothetical LOC100130876 | -0.098 | 0.934 | 7.18E-01 | 7.18E+01 | | | | |
| 8077781 | NM_000551 | VHL | von Hippel-Lindau tumor suppressor | -0.098 | 0.934 | 3.98E-01 | 3.98E+01 | | | | |
| 7957560 | NM_003805 | CRADD | CASP2 and RIPK1 domain containing | -0.098 | 0.934 | 1.86E-01 | 1.86E+01 | | | | |
| 7945539 | NM_024698 | SLC25A22 | solute carrier family 25 (mitochondri | -0.098 | 0.934 | 9.23E-02 | 9.23E+00 | | | | |
| 7963406 | NM_005555 | KRT6B | keratin 6B | -0.098 | 0.934 | 3.04E-01 | 3.04E+01 | | | | |
| 8021725 | --- | --- | --- | -0.098 | 0.934 | 2.57E-01 | 2.57E+01 | | | | |
| 8019699 | NM_033059 | KRTAP4-11 | keratin associated protein 4-11 | -0.098 | 0.934 | 6.62E-02 | 6.62E+00 | | | | |
| 8015890 | NM_004160 | PYY | peptide YY | -0.098 | 0.934 | 1.34E-01 | 1.34E+01 | | | | |
| 8029193 | NM_015125 | CIC | capicua homolog (Drosophila) | -0.098 | 0.934 | 3.64E-02 | 3.64E+00 | | | | |
| 7910261 | NM_00101086 | C1orf69 | chromosome 1 open reading frame 6 | -0.098 | 0.934 | 8.60E-02 | 8.60E+00 | | | | |
| 8153167 | NM_016601 | KCNK9 | potassium channel, subfamily K, mem | -0.098 | 0.934 | 1.49E-01 | 1.49E+01 | | | | |
| 7920141 | NM_007113 | TCHH | trichohyalin | -0.098 | 0.934 | 4.27E-02 | 4.27E+00 | | | | |
| 7912622 | ENST00000376 | LRRC38 | leucine rich repeat containing 38 | -0.098 | 0.934 | 9.62E-02 | 9.62E+00 | | | | |
| 8000574 | NM_00104248 | NUPR1 | nuclear protein, transcriptional regu | -0.098 | 0.934 | 1.75E-01 | 1.75E+01 | | | | |
| 7978285 | NM_139247 | ADCY4 | adenylate cyclase 4 | -0.098 | 0.934 | 1.05E-01 | 1.05E+01 | | | | |
| 8153273 | NM_207414 | FLJ43860 | FLJ43860 protein | -0.098 | 0.934 | 6.00E-02 | 6.00E+00 | | | | |
| 8031646 | AK302988 | OC10028811 | hypothetical protein LOC100288114 | -0.098 | 0.934 | 8.89E-01 | 8.89E+01 | | | | |
| 8126173 | NM_00113510 | KCNK16 | potassium channel, subfamily K, mem | -0.098 | 0.934 | 1.11E-01 | 1.11E+01 | | | | |
| 7894008 | --- | --- | --- | -0.098 | 0.934 | 6.70E-01 | 6.70E+01 | | | | |
| 7990643 | NM_032808 | LINGO1 | leucine rich repeat and Ig domain co | -0.098 | 0.934 | 3.90E-02 | 3.90E+00 | | | | |
| 8157696 | NM_00100192 | OR5C1 | olfactory receptor, family 5, subfami | -0.098 | 0.934 | 8.59E-02 | 8.59E+00 | | | | |
| 8054479 | NM_005434 | MALL | mal, T-cell differentiation protein-lik | -0.098 | 0.934 | 1.18E-01 | 1.18E+01 | | | | |
| 8087640 | NM_007182 | RASSF1 | Ras association (RalGDS/AF-6) doma | -0.098 | 0.934 | 2.68E-01 | 2.68E+01 | | | | |
| 7893402 | --- | --- | --- | -0.098 | 0.934 | 2.61E-01 | 2.61E+01 | | | | |
| 7894050 | --- | --- | --- | -0.098 | 0.934 | 6.21E-01 | 6.21E+01 | | | | |
| 8159385 | NM_00103937 | KIAA1984 | KIAA1984 | -0.098 | 0.934 | 4.57E-02 | 4.57E+00 | | | | |
| 8001953 | NM_178516 | EXOC3L | exocyst complex component 3-like | -0.098 | 0.934 | 7.07E-02 | 7.07E+00 | | | | |
| 8035177 | NM_024881 | SLC35E1 | solute carrier family 35, member E1 | -0.098 | 0.934 | 1.21E-01 | 1.21E+01 | | | | |
| 8045857 | BC021739 | LOC554201 | hypothetical LOC554201 | -0.098 | 0.934 | 2.74E-01 | 2.74E+01 | | | | |
| 7923360 | NM_003281 | TNNI1 | troponin I type 1 (skeletal, slow) | -0.098 | 0.934 | 8.52E-02 | 8.52E+00 | | | | |
| 7919566 | --- | --- | --- | -0.098 | 0.934 | 2.98E-01 | 2.98E+01 | | | | |
| 8032682 | NM_139355 | MATK | megakaryocyte-associated tyrosine k | -0.098 | 0.934 | 6.02E-02 | 6.02E+00 | | | | |
| 8048249 | NM_00107719 | GPBAR1 | G protein-coupled bile acid receptor | -0.098 | 0.934 | 6.58E-02 | 6.58E+00 | | | | |
| 8052397 | --- | --- | --- | -0.098 | 0.934 | 1.14E-01 | 1.14E+01 | | | | |
| 7896043 | --- | --- | --- | -0.098 | 0.934 | 4.99E-01 | 4.99E+01 | | | | |
| 7949067 | NM_004322 | BAD | BCL2-associated agonist of cell death | -0.098 | 0.934 | 7.04E-02 | 7.04E+00 | | | | |
| 7984961 | NM_018652 | GOLGA6B | golgi autoantigen, golgin subfamily a | -0.098 | 0.934 | 3.84E-02 | 3.84E+00 | | | | |
| 8139966 | NM_198924 | TRIM73 | tripartite motif-containing 73 | -0.098 | 0.934 | 1.36E-01 | 1.36E+01 | | | | |
| 8131253 | NM_00103716 | FOXK1 | forkhead box K1 | -0.098 | 0.934 | 7.79E-02 | 7.79E+00 | | | | |
| 7960894 | NR_024420 | LOC389634 | hypothetical LOC389634 | -0.098 | 0.934 | 1.01E-01 | 1.01E+01 | | | | |
| 8006877 | NM_006804 | STARD3 | StAR-related lipid transfer (START) d | -0.098 | 0.934 | 2.19E-01 | 2.19E+01 | | | | |
| 8058518 | --- | --- | --- | -0.098 | 0.934 | 4.08E-01 | 4.08E+01 | | | | |
| 7998381 | NM_022773 | LMF1 | lipase maturation factor 1 | -0.098 | 0.934 | 9.23E-02 | 9.23E+00 | | | | |
| 8037179 | NM_032488 | CNFN | cornifelin | -0.098 | 0.934 | 5.48E-02 | 5.48E+00 | | | | |
| 8117869 | NM_172016 | TRIM39 | tripartite motif-containing 39 | -0.098 | 0.934 | 5.13E-01 | 5.13E+01 | | | | |
| 8177770 | NM_172016 | TRIM39 | tripartite motif-containing 39 | -0.098 | 0.934 | 5.13E-01 | 5.13E+01 | | | | |
| 8179088 | NM_172016 | TRIM39 | tripartite motif-containing 39 | -0.098 | 0.934 | 5.13E-01 | 5.13E+01 | | | | |
| 8025450 | NM_00100469 | OR2Z1 | olfactory receptor, family 2, subfami | -0.098 | 0.934 | 1.36E-01 | 1.36E+01 | | | | |
| 8043602 | NM_015341 | NCAPH | non-SMC condensin I complex, subu | -0.099 | 0.934 | 2.66E-01 | 2.66E+01 | | | | |
| 8015396 | NM_177977 | HAP1 | huntingtin-associated protein 1 | -0.099 | 0.934 | 1.14E-01 | 1.14E+01 | | | | |
| 7952388 | NM_00100446 | OR10G7 | olfactory receptor, family 10, subfam | -0.099 | 0.934 | 2.72E-01 | 2.72E+01 | | | | |
| 8114239 | NM_130848 | C5orf20 | chromosome 5 open reading frame 2 | -0.099 | 0.934 | 7.61E-02 | 7.61E+00 | | | | |
| 7994267 | NM_00114554 | C16orf82 | chromosome 16 open reading frame | -0.099 | 0.934 | 4.53E-02 | 4.53E+00 | | | | |
| 8073422 | NM_003216 | TEF | thyrotrophic embryonic factor | -0.099 | 0.934 | 5.50E-02 | 5.50E+00 | | | | |
| 7950490 | --- | --- | --- | -0.099 | 0.934 | 2.36E-01 | 2.36E+01 | | | | |
| 8010766 | --- | --- | --- | -0.099 | 0.934 | 7.02E-01 | 7.02E+01 | | | | |
| 7999431 | NM_021247 | PRM3 | protamine 3 | -0.099 | 0.934 | 1.44E-01 | 1.44E+01 | | | | |
| 7925972 | NM_005185 | CALML3 | calmodulin-like 3 | -0.099 | 0.934 | 6.43E-02 | 6.43E+00 | | | | |
| 8175391 | --- | --- | --- | -0.099 | 0.934 | 4.60E-01 | 4.60E+01 | | | | |
| 8087739 | NM_013324 | CISH | cytokine inducible SH2-containing pr | -0.099 | 0.934 | 7.21E-02 | 7.21E+00 | | | | |
| 7920928 | NM_024897 | PAQR6 | progestin and adipoQ receptor famil | -0.099 | 0.934 | 1.71E-01 | 1.71E+01 | | | | |
| 8038683 | NM_002774 | KLK6 | kallikrein-related peptidase 6 | -0.099 | 0.934 | 1.20E-01 | 1.20E+01 | | | | |
| 8159670 | NM_006647 | NOXA1 | NADPH oxidase activator 1 | -0.099 | 0.934 | 8.18E-02 | 8.18E+00 | | | | |
| 8126729 | NM_00111408 | CLIC5 | chloride intracellular channel 5 | -0.099 | 0.934 | 1.87E-01 | 1.87E+01 | | | | |
| 7988212 | NM_025165 | ELL3 | elongation factor RNA polymerase II- | -0.099 | 0.934 | 2.02E-01 | 2.02E+01 | | | | |
| 8005328 | NM_016239 | MYO15A | myosin XVA | -0.099 | 0.934 | 2.33E-02 | 2.33E+00 | | | | |
| 8027040 | NM_012109 | TMEM59L | transmembrane protein 59-like | -0.099 | 0.934 | 5.15E-02 | 5.15E+00 | | | | |
| 8033233 | NM_006087 | TUBB4 | tubulin, beta 4 | -0.099 | 0.934 | 3.84E-01 | 3.84E+01 | | | | |
| 8000757 | NM_003586 | DOC2A | double C2-like domains, alpha | -0.099 | 0.934 | 9.68E-02 | 9.68E+00 | | | | |

| | | | | | | | | | | |
|---|---|---|---|---|---|---|---|---|---|---|
| 8121257 | NM_001198 | PRDM1 | PR domain containing 1, with ZNF do | -0.099 | 0.934 | 6.87E-01 | 6.87E+01 | | | |
| 8030416 | NM_021228 | SCAF1 | SR-related CTD-associated factor 1 | -0.099 | 0.934 | 6.26E-02 | 6.26E+00 | | | |
| 8073259 | NM_015705 | SGSM3 | small G protein signaling modulator | -0.099 | 0.934 | 9.54E-02 | 9.54E+00 | | | |
| 7979663 | NM_198686 | RAB15 | RAB15, member RAS onocogene fam | -0.099 | 0.934 | 1.05E-01 | 1.05E+01 | | | |
| 7892775 | --- | --- | --- | -0.099 | 0.934 | 9.04E-01 | 9.04E+01 | | | |
| 8061725 | NM_015338 | ASXL1 | additional sex combs like 1 (Drosoph | -0.099 | 0.934 | 2.79E-01 | 2.79E+01 | | | |
| 7975410 | --- | --- | --- | -0.099 | 0.934 | 3.57E-01 | 3.57E+01 | | | |
| 8005601 | --- | --- | --- | -0.099 | 0.934 | 8.95E-02 | 8.95E+00 | | | |
| 8141768 | NM_006989 | RASA4 | RAS p21 protein activator 4 | -0.099 | 0.934 | 5.51E-02 | 5.51E+00 | | | |
| 7972438 | NM_178861 | RNF113B | ring finger protein 113B | -0.099 | 0.934 | 1.54E-01 | 1.54E+01 | | | |
| 8087624 | NM_033158 | HYAL2 | hyaluronoglucosaminidase 2 | -0.099 | 0.934 | 5.02E-02 | 5.02E+00 | | | |
| 8167645 | NM_153183 | NUDT10 | nudix (nucleoside diphosphate linked | -0.099 | 0.934 | 7.81E-02 | 7.81E+00 | | | |
| 7977234 | BC041103 | C14orf180 | chromosome 14 open reading frame | -0.099 | 0.934 | 6.89E-02 | 6.89E+00 | | | |
| 8145193 | NM_00101384 | C8orf58 | chromosome 8 open reading frame 5 | -0.099 | 0.934 | 8.43E-02 | 8.43E+00 | | | |
| 8008900 | NM_000717 | CA4 | carbonic anhydrase IV | -0.099 | 0.934 | 5.80E-02 | 5.80E+00 | | | |
| 8037186 | NM_005357 | LIPE | lipase, hormone-sensitive | -0.099 | 0.934 | 6.30E-02 | 6.30E+00 | | | |
| 8076193 | AK074445 | FLJ23865 | hypothetical protein FLJ23865 | -0.099 | 0.934 | 3.05E-01 | 3.05E+01 | | | |
| 8043209 | NM_016494 | RNF181 | ring finger protein 181 | -0.099 | 0.934 | 7.39E-01 | 7.39E+01 | | | |
| 8005134 | NM_000676 | ADORA2B | adenosine A2b receptor | -0.099 | 0.934 | 2.02E-01 | 2.02E+01 | | | |
| 8033118 | NM_005934 | MLLT1 | myeloid/lymphoid or mixed-lineage | -0.099 | 0.934 | 4.06E-02 | 4.06E+00 | | | |
| 8161024 | NR_003051 | RMRP | RNA component of mitochondrial RN | -0.099 | 0.934 | 8.40E-01 | 8.40E+01 | | | |
| 7893447 | --- | --- | --- | -0.099 | 0.934 | 3.82E-01 | 3.82E+01 | | | |
| 8117535 | NM_021064 | HIST1H2AG | histone cluster 1, H2ag | -0.099 | 0.934 | 3.41E-01 | 3.41E+01 | | | |
| 8062251 | NM_012156 | EPB41L1 | erythrocyte membrane protein band | -0.099 | 0.934 | 3.89E-02 | 3.89E+00 | | | |
| 7894296 | --- | --- | --- | -0.099 | 0.934 | 9.17E-01 | 9.17E+01 | | | |
| 8134827 | NM_173564 | C7orf51 | chromosome 7 open reading frame 5 | -0.099 | 0.934 | 5.72E-02 | 5.72E+00 | | | |
| 7961306 | NM_005039 | PRB1 | proline-rich protein BstNI subfamily | -0.099 | 0.934 | 6.60E-02 | 6.60E+00 | | | |
| 8038150 | NM_182574 | MAMSTR | MEF2 activating motif and SAP doma | -0.099 | 0.934 | 3.86E-02 | 3.86E+00 | | | |
| 7985285 | NM_014862 | ARNT2 | aryl-hydrocarbon receptor nuclear tr | -0.099 | 0.934 | 7.28E-02 | 7.28E+00 | | | |
| 8003149 | NM_172347 | KCNG4 | potassium voltage-gated channel, su | -0.099 | 0.934 | 1.01E-01 | 1.01E+01 | | | |
| 8063386 | NM_005194 | CEBPB | CCAAT/enhancer binding protein (C/ | -0.099 | 0.934 | 6.09E-02 | 6.09E+00 | | | |
| 8053263 | --- | --- | --- | -0.099 | 0.934 | 1.22E-01 | 1.22E+01 | | | |
| 7894994 | --- | --- | --- | -0.099 | 0.934 | 8.13E-01 | 8.13E+01 | | | |
| 8030557 | NM_012068 | ATF5 | activating transcription factor 5 | -0.099 | 0.934 | 1.02E-01 | 1.02E+01 | | | |
| 8167369 | NM_006044 | HDAC6 | histone deacetylase 6 | -0.099 | 0.934 | 1.09E-01 | 1.09E+01 | | | |
| 8086341 | NM_194293 | XIRP1 | xin actin-binding repeat containing 1 | -0.099 | 0.934 | 4.20E-02 | 4.20E+00 | | | |
| 7942771 | AK096213 | FLJ38894 | hypothetical protein LOC646029 | -0.099 | 0.934 | 1.74E-01 | 1.74E+01 | | | |
| 7895328 | --- | --- | --- | -0.099 | 0.934 | 3.58E-01 | 3.58E+01 | | | |
| 7895061 | --- | --- | --- | -0.099 | 0.934 | 8.56E-01 | 8.56E+01 | | | |
| 8097449 | NM_032961 | PCDH10 | protocadherin 10 | -0.099 | 0.934 | 4.57E-02 | 4.57E+00 | | | |
| 7974689 | NM_016651 | DACT1 | dapper, antagonist of beta-catenin, l | -0.099 | 0.934 | 1.10E-01 | 1.10E+01 | | | |
| 8110783 | NM_198253 | TERT | telomerase reverse transcriptase | -0.099 | 0.934 | 4.16E-02 | 4.16E+00 | | | |
| 7950899 | NM_022337 | RAB38 | RAB38, member RAS oncogene famil | -0.099 | 0.934 | 1.36E-01 | 1.36E+01 | | | |
| 7962441 | NM_002822 | TWF1 | twinfilin, actin-binding protein, hom | -0.099 | 0.934 | 5.81E-01 | 5.81E+01 | | | |
| 7895672 | --- | --- | --- | -0.099 | 0.934 | 6.24E-01 | 6.24E+01 | | | |
| 7920594 | NM_138300 | PYGO2 | pygopus homolog 2 (Drosophila) | -0.099 | 0.934 | 2.60E-01 | 2.60E+01 | | | |
| 8090891 | NM_004441 | EPHB1 | EPH receptor B1 | -0.099 | 0.934 | 8.85E-02 | 8.85E+00 | | | |
| 7956430 | NM_005269 | GLI1 | GLI family zinc finger 1 | -0.099 | 0.934 | 7.53E-02 | 7.53E+00 | | | |
| 8030094 | NM_000511 | FUT2 | fucosyltransferase 2 (secretor status | -0.099 | 0.933 | 1.33E-01 | 1.33E+01 | | | |
| 8091352 | NM_020131 | UBQLN4 | ubiquilin 4 | -0.099 | 0.933 | 3.94E-01 | 3.94E+01 | | | |
| 8062766 | NM_002466 | MYBL2 | v-myb myeloblastosis viral oncogene | -0.099 | 0.933 | 9.76E-02 | 9.76E+00 | | | |
| 8031675 | NM_021216 | ZNF71 | zinc finger protein 71 | -0.099 | 0.933 | 6.86E-02 | 6.86E+00 | | | |
| 7991846 | NM_020664 | DECR2 | 2,4-dienoyl CoA reductase 2, peroxis | -0.099 | 0.933 | 4.31E-02 | 4.31E+00 | | | |
| 7996761 | NM_012320 | PLA2G15 | phospholipase A2, group XV | -0.099 | 0.933 | 1.41E-01 | 1.41E+01 | | | |
| 7991173 | NM_022480 | KLHL25 | kelch-like 25 (Drosophila) | -0.099 | 0.933 | 1.05E-01 | 1.05E+01 | | | |
| 8117741 | NM_007160 | OR2H2 | olfactory receptor, family 2, subfami | -0.099 | 0.933 | 9.85E-02 | 9.85E+00 | | | |
| 7899643 | NM_001525 | HCRTR1 | hypocretin (orexin) receptor 1 | -0.099 | 0.933 | 7.02E-02 | 7.02E+00 | | | |
| 8137865 | NM_007353 | GNA12 | guanine nucleotide binding protein ( | -0.099 | 0.933 | 2.29E-01 | 2.29E+01 | | | |
| 8151056 | NM_004820 | CYP7B1 | cytochrome P450, family 7, subfamil | -0.099 | 0.933 | 3.64E-02 | 3.64E+00 | | | |
| 7990273 | NM_004809 | STOML1 | stomatin (EPB72)-like 1 | -0.099 | 0.933 | 1.06E-01 | 1.06E+01 | | | |
| 8161503 | NR_024060 | FAM27A | family with sequence similarity 27, m | -0.099 | 0.933 | 5.81E-02 | 5.81E+00 | | | |
| 7894087 | --- | --- | --- | -0.099 | 0.933 | 3.52E-01 | 3.52E+01 | | | |
| 8010848 | NM_005993 | TBCD | tubulin folding cofactor D | -0.099 | 0.933 | 6.39E-02 | 6.39E+00 | | | |
| 8017671 | NR_026899 | LOC146880 | hypothetical LOC146880 | -0.099 | 0.933 | 1.82E-01 | 1.82E+01 | | | |
| 7914042 | NM_004672 | MAP3K6 | mitogen-activated protein kinase kin | -0.099 | 0.933 | 6.59E-02 | 6.59E+00 | | | |
| 8170998 | NM_012151 | F8A1 | coagulation factor VIII-associated (in | -0.100 | 0.933 | 1.13E-01 | 1.13E+01 | | | |
| 8099467 | NM_005130 | FGFBP1 | fibroblast growth factor binding prot | -0.100 | 0.933 | 1.25E-01 | 1.25E+01 | | | |
| 7901311 | NM_004474 | FOXD2 | forkhead box D2 | -0.100 | 0.933 | 1.17E-01 | 1.17E+01 | | | |
| 7946428 | NM_020643 | C11orf16 | chromosome 11 open reading frame | -0.100 | 0.933 | 8.46E-02 | 8.46E+00 | | | |
| 8097415 | --- | --- | --- | -0.100 | 0.933 | 2.32E-01 | 2.32E+01 | | | |
| 7998861 | NM_152891 | PRSS33 | protease, serine, 33 | -0.100 | 0.933 | 9.10E-02 | 9.10E+00 | | | |
| 7977438 | --- | --- | --- | -0.100 | 0.933 | 2.71E-01 | 2.71E+01 | | | |
| 7896952 | NM_018188 | ATAD3A | ATPase family, AAA domain containi | -0.100 | 0.933 | 9.49E-02 | 9.49E+00 | | | |
| 8130715 | NM_175922 | PRR18 | proline rich 18 | -0.100 | 0.933 | 3.83E-02 | 3.83E+00 | | | |
| 7971015 | NM_00112721 | SMAD9 | SMAD family member 9 | -0.100 | 0.933 | 5.30E-02 | 5.30E+00 | | | |
| 8170882 | NM_001183 | ATP6AP1 | ATPase, H+ transporting, lysosomal a | -0.100 | 0.933 | 1.55E-01 | 1.55E+01 | | | |
| 8130185 | NM_020861 | ZBTB2 | zinc finger and BTB domain containi | -0.100 | 0.933 | 2.55E-01 | 2.55E+01 | | | |

| | | | | | | | | | | |
|---|---|---|---|---|---|---|---|---|---|---|
| 8170412 | NM_005364 | MAGEA8 | melanoma antigen family A, 8 | -0.100 | 0.933 | 1.15E-01 | 1.15E+01 | | | |
| 7954559 | NM_003622 | PPFIBP1 | PTPRF interacting protein, binding pr | -0.100 | 0.933 | 7.80E-02 | 7.80E+00 | | | |
| 7988833 | NM_153374 | LYSMD2 | LysM, putative peptidoglycan-bindin | -0.100 | 0.933 | 1.30E-01 | 1.30E+01 | | | |
| 8087182 | NM_016453 | NCKIPSD | NCK interacting protein with SH3 do | -0.100 | 0.933 | 5.05E-02 | 5.05E+00 | | | |
| 8097938 | NM_000910 | NPY2R | neuropeptide Y receptor Y2 | -0.100 | 0.933 | 8.22E-02 | 8.22E+00 | | | |
| 7948213 | NM_006093 | PRG3 | proteoglycan 3 | -0.100 | 0.933 | 1.70E-01 | 1.70E+01 | | | |
| 8112342 | NM_197941 | ADAMTS6 | ADAM metallopeptidase with throm | -0.100 | 0.933 | 9.02E-02 | 9.02E+00 | | | |
| 8094741 | --- | --- | --- | -0.100 | 0.933 | 7.15E-01 | 7.15E+01 | | | |
| 8019437 | NM_198082 | CCDC57 | coiled-coil domain containing 57 | -0.100 | 0.933 | 5.93E-02 | 5.93E+00 | | | |
| 7923516 | NM_016243 | CYB5R1 | cytochrome b5 reductase 1 | -0.100 | 0.933 | 5.66E-01 | 5.66E+01 | | | |
| 7931140 | NR_003570 | FLJ46361 | deleted in malignant brain tumors 1 | -0.100 | 0.933 | 1.39E-01 | 1.39E+01 | | | |
| 7972247 | BC036697 | SP3P | Sp3 transcription factor pseudogene | -0.100 | 0.933 | 3.80E-02 | 3.80E+00 | | | |
| 8012473 | NM_00114668 | RNF222 | ring finger protein 222 | -0.100 | 0.933 | 5.48E-02 | 5.48E+00 | | | |
| 8007921 | NM_00100284 | MYL4 | myosin, light chain 4, alkali; atrial, er | -0.100 | 0.933 | 2.88E-01 | 2.88E+01 | | | |
| 7919612 | NM_00112337 | HIST2H3D | histone cluster 2, H3d | -0.100 | 0.933 | 1.41E-01 | 1.41E+01 | | | |
| 8060030 | NM_006037 | HDAC4 | histone deacetylase 4 | -0.100 | 0.933 | 1.09E-01 | 1.09E+01 | | | |
| 7896479 | --- | --- | --- | -0.100 | 0.933 | 5.02E-01 | 5.02E+01 | | | |
| 8024956 | NM_198706 | HSD11B1L | hydroxysteroid (11-beta) dehydroge | -0.100 | 0.933 | 8.08E-02 | 8.08E+00 | | | |
| 8024170 | NM_012292 | HMHA1 | histocompatibility (minor) HA-1 | -0.100 | 0.933 | 9.70E-02 | 9.70E+00 | | | |
| 7895794 | --- | --- | --- | -0.100 | 0.933 | 3.15E-01 | 3.15E+01 | | | |
| 8104281 | NM_182632 | SLC6A18 | solute carrier family 6, member 18 | -0.100 | 0.933 | 7.84E-02 | 7.84E+00 | | | |
| 7932243 | NM_00101092 | FAM171A1 | family with sequence similarity 171, | -0.100 | 0.933 | 5.02E-02 | 5.02E+00 | | | |
| 8049536 | NM_00113755 | LRRFIP1 | leucine rich repeat (in FLII) interactin | -0.100 | 0.933 | 9.06E-02 | 9.06E+00 | | | |
| 8143708 | --- | --- | --- | -0.100 | 0.933 | 1.40E-01 | 1.40E+01 | | | |
| 8037732 | NM_00102986 | NANOS2 | nanos homolog 2 (Drosophila) | -0.100 | 0.933 | 1.57E-01 | 1.57E+01 | | | |
| 7929911 | NM_032429 | LZTS2 | leucine zipper, putative tumor suppr | -0.100 | 0.933 | 9.49E-02 | 9.49E+00 | | | |
| 8150249 | NM_000025 | ADRB3 | adrenergic, beta-3-, receptor | -0.100 | 0.933 | 1.69E-01 | 1.69E+01 | | | |
| 8167476 | NM_033215 | PPP1R3F | protein phosphatase 1, regulatory (ir | -0.100 | 0.933 | 8.97E-02 | 8.97E+00 | | | |
| 8113733 | NM_153223 | CEP120 | centrosomal protein 120kDa | -0.100 | 0.933 | 6.16E-01 | 6.16E+01 | | | |
| 8039526 | NM_032836 | FIZ1 | FLT3-interacting zinc finger 1 | -0.100 | 0.933 | 4.25E-02 | 4.25E+00 | | | |
| 8067121 | NR_002189 | SUMO1P1 | SUMO1 pseudogene 1 | -0.100 | 0.933 | 1.91E-01 | 1.91E+01 | | | |
| 8003503 | NM_000135 | FANCA | Fanconi anemia, complementation g | -0.100 | 0.933 | 2.54E-02 | 2.54E+00 | | | |
| 8065242 | AF161557 | HSPC072 | hypothetical LOC29075 | -0.100 | 0.933 | 5.28E-01 | 5.28E+01 | | | |
| 8087806 | NM_020418 | PCBP4 | poly(rC) binding protein 4 | -0.100 | 0.933 | 7.67E-02 | 7.67E+00 | | | |
| 8133670 | NM_000941 | POR | P450 (cytochrome) oxidoreductase | -0.100 | 0.933 | 1.11E-01 | 1.11E+01 | | | |
| 7919168 | NM_022359 | PDE4DIP | phosphodiesterase 4D interacting pr | -0.100 | 0.933 | 1.32E-01 | 1.32E+01 | | | |
| 7901744 | --- | --- | --- | -0.100 | 0.933 | 1.08E-01 | 1.08E+01 | | | |
| 8023990 | NM_020637 | FGF22 | fibroblast growth factor 22 | -0.100 | 0.933 | 1.18E-01 | 1.18E+01 | | | |
| 8124038 | --- | --- | --- | -0.100 | 0.933 | 1.56E-01 | 1.56E+01 | | | |
| 8112890 | NM_004269 | MED27 | mediator complex subunit 27 | -0.100 | 0.933 | 8.51E-01 | 8.51E+01 | | | |
| 8003804 | NM_014565 | OR1A1 | olfactory receptor, family 1, subfami | -0.100 | 0.933 | 1.56E-01 | 1.56E+01 | | | |
| 8158250 | NM_016174 | CERCAM | cerebral endothelial cell adhesion m | -0.100 | 0.933 | 1.38E-01 | 1.38E+01 | | | |
| 8011222 | NM_017575 | SMG6 | Smg-6 homolog, nonsense mediated | -0.100 | 0.933 | 3.27E-01 | 3.27E+01 | | | |
| 7931519 | NM_152643 | KNDC1 | kinase non-catalytic C-lobe domain ( | -0.100 | 0.933 | 4.15E-02 | 4.15E+00 | | | |
| 8065416 | NM_001322 | CST2 | cystatin SA | -0.100 | 0.933 | 1.20E-01 | 1.20E+01 | | | |
| 8038299 | NM_033142 | CGB7 | chorionic gonadotropin, beta polype | -0.100 | 0.933 | 2.79E-02 | 2.79E+00 | | | |
| 7945377 | NM_00113505 | SIGIRR | single immunoglobulin and toll-inter | -0.100 | 0.933 | 3.33E-02 | 3.33E+00 | | | |
| 7948643 | NM_013401 | RAB3IL1 | RAB3A interacting protein (rabin3)-li | -0.100 | 0.933 | 7.37E-02 | 7.37E+00 | | | |
| 7893104 | --- | --- | --- | -0.100 | 0.933 | 8.08E-01 | 8.08E+01 | | | |
| 8136159 | --- | --- | --- | -0.100 | 0.933 | 4.58E-01 | 4.58E+01 | | | |
| 8114185 | NM_080656 | CDKN2AIPNL | CDKN2A interacting protein N-termi | -0.100 | 0.933 | 4.37E-01 | 4.37E+01 | | | |
| 8165217 | NM_017617 | NOTCH1 | Notch homolog 1, translocation-asso | -0.100 | 0.933 | 4.35E-02 | 4.35E+00 | | | |
| 8165610 | NM_138778 | WDR85 | WD repeat domain 85 | -0.100 | 0.933 | 1.66E-01 | 1.66E+01 | | | |
| 8064168 | NM_017806 | LIME1 | Lck interacting transmembrane adap | -0.100 | 0.933 | 7.71E-02 | 7.71E+00 | | | |
| 7933312 | NM_00109884 | ANXA8L1 | annexin A8-like 1 | -0.100 | 0.933 | 6.05E-02 | 6.05E+00 | | | |
| 8067802 | NM_00103946 | RGS19 | regulator of G-protein signaling 19 | -0.100 | 0.933 | 8.85E-02 | 8.85E+00 | | | |
| 7960889 | NR_024254 | LOC653113 | family with sequence similarity 86, m | -0.100 | 0.933 | 2.75E-01 | 2.75E+01 | | | |
| 8119427 | NM_198153 | TREML4 | triggering receptor expressed on my | -0.100 | 0.933 | 1.84E-01 | 1.84E+01 | | | |
| 8004784 | NM_001141 | ALOX15B | arachidonate 15-lipoxygenase, type | -0.100 | 0.933 | 5.70E-02 | 5.70E+00 | | | |
| 8137931 | NM_00110060 | MMD2 | monocyte to macrophage differentia | -0.100 | 0.933 | 1.01E-01 | 1.01E+01 | | | |
| 7914075 | NM_003665 | FCN3 | ficolin (collagen/fibrinogen domain c | -0.100 | 0.933 | 9.18E-02 | 9.18E+00 | | | |
| 8055664 | --- | --- | --- | -0.100 | 0.933 | 2.31E-01 | 2.31E+01 | | | |
| 7999319 | NM_014316 | CARHSP1 | calcium regulated heat stable protei | -0.100 | 0.933 | 1.49E-01 | 1.49E+01 | | | |
| 8038292 | NM_033043 | CGB5 | chorionic gonadotropin, beta polype | -0.100 | 0.933 | 5.78E-02 | 5.78E+00 | | | |
| 8160786 | NM_020702 | KIAA1161 | KIAA1161 | -0.100 | 0.933 | 6.44E-02 | 6.44E+00 | | | |
| 8074020 | NM_031454 | P3-402G11. | selenoprotein O | -0.100 | 0.933 | 3.35E-02 | 3.35E+00 | | | |
| 8116384 | NM_175062 | RASGEF1C | RasGEF domain family, member 1C | -0.100 | 0.933 | 7.48E-02 | 7.48E+00 | | | |
| 7909965 | NM_152610 | C1orf65 | chromosome 1 open reading frame 6 | -0.100 | 0.933 | 7.71E-02 | 7.71E+00 | | | |
| 8067351 | NM_006242 | PPP1R3D | protein phosphatase 1, regulatory (ir | -0.100 | 0.933 | 5.82E-02 | 5.82E+00 | | | |
| 8124862 | NM_001264 | CDSN | corneodesmosin | -0.100 | 0.933 | 4.74E-02 | 4.74E+00 | | | |
| 8071466 | NM_006767 | LZTR1 | leucine-zipper-like transcription regu | -0.100 | 0.933 | 1.58E-01 | 1.58E+01 | | | |
| 8154654 | NR_024274 | C9orf53 | chromosome 9 open reading frame 5 | -0.100 | 0.933 | 1.28E-01 | 1.28E+01 | | | |
| 7992552 | NM_022372 | MLST8 | MTOR associated protein, LST8 hom | -0.100 | 0.933 | 2.09E-02 | 2.09E+00 | | | |
| 8005407 | NM_004140 | LLGL1 | lethal giant larvae homolog 1 (Droso | -0.100 | 0.933 | 6.75E-02 | 6.75E+00 | | | |
| 8164918 | NM_017585 | SLC2A6 | solute carrier family 2 (facilitated glu | -0.100 | 0.933 | 1.01E-01 | 1.01E+01 | | | mono |
| 8036676 | NM_024907 | FBXO17 | F-box protein 17 | -0.100 | 0.933 | 9.35E-02 | 9.35E+00 | | | |
| 8052018 | NM_022055 | KCNK12 | potassium channel, subfamily K, mer | -0.101 | 0.933 | 9.20E-02 | 9.20E+00 | | | |

| | | | | | | | | | | |
|---|---|---|---|---|---|---|---|---|---|---|
| 7947742 | --- | --- | --- | -0.101 | 0.933 | 2.12E-01 | 2.12E+01 | | | |
| 8107897 | NM_003687 | PDLIM4 | PDZ and LIM domain 4 | -0.101 | 0.933 | 9.82E-02 | 9.82E+00 | | | |
| 7948033 | --- | --- | --- | -0.101 | 0.933 | 2.97E-01 | 2.97E+01 | | | |
| 7986001 | ENST00000338 | LOC728477 | similar to hCG27445 | -0.101 | 0.933 | 1.63E-01 | 1.63E+01 | | | |
| 7922456 | --- | --- | --- | -0.101 | 0.933 | 3.73E-01 | 3.73E+01 | | | |
| 8023377 | NM_016626 | MEX3C | mex-3 homolog C (C. elegans) | -0.101 | 0.933 | 5.11E-01 | 5.11E+01 | | | |
| 8000903 | NM_023931 | ZNF747 | zinc finger protein 747 | -0.101 | 0.933 | 9.75E-02 | 9.75E+00 | | | |
| 7954787 | --- | --- | --- | -0.101 | 0.933 | 6.37E-02 | 6.37E+00 | | | |
| 7894864 | --- | --- | --- | -0.101 | 0.933 | 3.30E-01 | 3.30E+01 | | | |
| 8180244 | --- | --- | --- | -0.101 | 0.933 | 3.30E-01 | 3.30E+01 | | | |
| 8176910 | NR_001555 | GOLGA2LY1 | golgi autoantigen, golgin subfamily a | -0.101 | 0.933 | 6.98E-02 | 6.98E+00 | | | |
| 8177413 | NR_001555 | GOLGA2LY1 | golgi autoantigen, golgin subfamily a | -0.101 | 0.933 | 6.98E-02 | 6.98E+00 | | | |
| 8062377 | NM_005417 | SRC | v-src sarcoma (Schmidt-Ruppin A-2) | -0.101 | 0.933 | 6.35E-02 | 6.35E+00 | | | |
| 7969145 | NM_138450 | ARL11 | ADP-ribosylation factor-like 11 | -0.101 | 0.933 | 1.72E-01 | 1.72E+01 | | | |
| 7897728 | NM_018438 | FBXO6 | F-box protein 6 | -0.101 | 0.933 | 1.51E-01 | 1.51E+01 | | | |
| 7927405 | NM_020945 | WDFY4 | WDFY family member 4 | -0.101 | 0.933 | 4.43E-01 | 4.43E+01 | | | |
| 7903667 | NM_00104070 | SYPL2 | synaptophysin-like 2 | -0.101 | 0.933 | 3.64E-02 | 3.64E+00 | | | |
| 8079733 | NM_173546 | KLHDC8B | kelch domain containing 8B | -0.101 | 0.933 | 1.39E-01 | 1.39E+01 | | | |
| 8031516 | NM_016202 | ZNF580 | zinc finger protein 580 | -0.101 | 0.933 | 1.25E-01 | 1.25E+01 | | | |
| 8056206 | NM_016836 | RBMS1 | RNA binding motif, single stranded in | -0.101 | 0.933 | 3.48E-01 | 3.48E+01 | | | |
| 8138757 | NM_018951 | HOXA10 | homeobox A10 | -0.101 | 0.933 | 4.15E-02 | 4.15E+00 | | | |
| 8024391 | NM_032482 | DOT1L | DOT1-like, histone H3 methyltransfe | -0.101 | 0.933 | 1.38E-01 | 1.38E+01 | | | |
| 7930106 | NM_024789 | TMEM180 | transmembrane protein 180 | -0.101 | 0.932 | 6.13E-02 | 6.13E+00 | | | |
| 7979351 | NR_027123 | C14orf33 | chromosome 14 open reading frame | -0.101 | 0.932 | 9.69E-02 | 9.69E+00 | | | |
| 7951695 | NM_00114502 | C11orf34 | chromosome 11 open reading frame | -0.101 | 0.932 | 3.78E-01 | 3.78E+01 | | | |
| 7916022 | --- | --- | --- | -0.101 | 0.932 | 1.32E-01 | 1.32E+01 | | | |
| 7948249 | NM_003627 | SLC43A1 | solute carrier family 43, member 1 | -0.101 | 0.932 | 7.63E-02 | 7.63E+00 | | | |
| 8070503 | NM_00104042 | PRDM15 | PR domain containing 15 | -0.101 | 0.932 | 7.53E-02 | 7.53E+00 | | | |
| 7914847 | BC026909 | C1orf216 | chromosome 1 open reading frame 2 | -0.101 | 0.932 | 1.42E-01 | 1.42E+01 | | | |
| 7896661 | --- | --- | --- | -0.101 | 0.932 | 7.56E-01 | 7.56E+01 | | | |
| 8004431 | NM_000937 | POLR2A | polymerase (RNA) II (DNA directed) p | -0.101 | 0.932 | 4.86E-01 | 4.86E+01 | | | |
| 7974799 | NM_002431 | MNAT1 | menage a trois homolog 1, cyclin H a | -0.101 | 0.932 | 7.50E-01 | 7.50E+01 | | | |
| 8058849 | NM_020814 | 40241 | membrane-associated ring finger (C3 | -0.101 | 0.932 | 4.72E-02 | 4.72E+00 | | | |
| 7892573 | --- | --- | --- | -0.101 | 0.932 | 5.29E-01 | 5.29E+01 | | | |
| 8045030 | NM_00108052 | MYO7B | myosin VIIB | -0.101 | 0.932 | 3.96E-02 | 3.96E+00 | | | |
| 7994647 | NM_145239 | PRRT2 | proline-rich transmembrane protein | -0.101 | 0.932 | 9.03E-02 | 9.03E+00 | | | |
| 8052908 | NM_173535 | CLEC4F | C-type lectin domain family 4, memb | -0.101 | 0.932 | 1.58E-01 | 1.58E+01 | | | |
| 8003875 | NM_00112475 | SPNS2 | spinster homolog 2 (Drosophila) | -0.101 | 0.932 | 7.79E-02 | 7.79E+00 | | | |
| 7908612 | NM_00110551 | FAM58B | family with sequence similarity 58, m | -0.101 | 0.932 | 1.78E-01 | 1.78E+01 | | | |
| 7924874 | NM_016102 | TRIM17 | tripartite motif-containing 17 | -0.101 | 0.932 | 4.39E-02 | 4.39E+00 | | | |
| 7896325 | --- | --- | --- | -0.101 | 0.932 | 8.56E-01 | 8.56E+01 | | | |
| 8142993 | --- | --- | --- | -0.101 | 0.932 | 5.07E-01 | 5.07E+01 | | | |
| 8004556 | NM_018081 | WRAP53 | WD repeat containing, antisense to T | -0.101 | 0.932 | 6.31E-02 | 6.31E+00 | | | |
| 7928645 | NM_00109933 | FAM22A | family with sequence similarity 22, m | -0.101 | 0.932 | 5.09E-02 | 5.09E+00 | | | |
| 7985253 | BC101201 | C15orf37 | chromosome 15 open reading frame | -0.101 | 0.932 | 1.18E-01 | 1.18E+01 | | | |
| 8010313 | NM_178543 | ENPP7 | ectonucleotide pyrophosphatase/ph | -0.101 | 0.932 | 1.44E-01 | 1.44E+01 | | | |
| 8070912 | NM_194255 | SLC19A1 | solute carrier family 19 (folate transp | -0.101 | 0.932 | 6.35E-02 | 6.35E+00 | | | |
| 8148748 | NR_024207 | KIAA1875 | KIAA1875 | -0.101 | 0.932 | 8.05E-02 | 8.05E+00 | | | |
| 7938738 | NM_004976 | KCNC1 | potassium voltage-gated channel, Sh | -0.101 | 0.932 | 5.55E-02 | 5.55E+00 | | | |
| 8110265 | NM_213647 | FGFR4 | fibroblast growth factor receptor 4 | -0.101 | 0.932 | 5.23E-02 | 5.23E+00 | | | |
| 8029580 | NM_006509 | RELB | v-rel reticuloendotheliosis viral onco | -0.101 | 0.932 | 1.84E-01 | 1.84E+01 | | | |
| 8041781 | NM_001430 | EPAS1 | endothelial PAS domain protein 1 | -0.101 | 0.932 | 6.57E-02 | 6.57E+00 | | | |
| 8149898 | NM_030795 | STMN4 | stathmin-like 4 | -0.101 | 0.932 | 7.75E-02 | 7.75E+00 | | | |
| 8072004 | NM_00101361 | IGLL3 | immunoglobulin lambda-like polypep | -0.101 | 0.932 | 1.98E-01 | 1.98E+01 | | | |
| 8150433 | NM_152568 | NKX6-3 | NK6 homeobox 3 | -0.101 | 0.932 | 4.62E-02 | 4.62E+00 | | | |
| 8018445 | NM_00108041 | UNK | unkempt homolog (Drosophila) | -0.101 | 0.932 | 2.59E-01 | 2.59E+01 | | | |
| 7998351 | NM_00102519 | MSLNL | mesothelin-like | -0.101 | 0.932 | 9.86E-02 | 9.86E+00 | | | |
| 8178442 | NM_001264 | CDSN | corneodesmosin | -0.101 | 0.932 | 5.52E-02 | 5.52E+00 | | | |
| 8161458 | NR_003670 | KGFLP2 | keratinocyte growth factor-like prote | -0.101 | 0.932 | 1.14E-01 | 1.14E+01 | | | |
| 8005777 | BC112354 | LOC440419 | similar to ubiquitin specific protease | -0.101 | 0.932 | 3.54E-02 | 3.54E+00 | | | |
| 8098880 | NM_00101261 | CTBP1 | C-terminal binding protein 1 | -0.101 | 0.932 | 2.20E-01 | 2.20E+01 | | | |
| 7930120 | NM_016169 | SUFU | suppressor of fused homolog (Droso | -0.101 | 0.932 | 1.14E-01 | 1.14E+01 | | | |
| 7965673 | --- | --- | --- | -0.101 | 0.932 | 8.97E-02 | 8.97E+00 | | | |
| 8167930 | NM_174912 | FAAH2 | fatty acid amide hydrolase 2 | -0.101 | 0.932 | 5.35E-01 | 5.35E+01 | | | |
| 8145555 | NM_016240 | SCARA3 | scavenger receptor class A, member | -0.101 | 0.932 | 5.01E-02 | 5.01E+00 | | | |
| 8054449 | AK095987 | hCG_199359 | hypothetical LOC644903 | -0.101 | 0.932 | 9.10E-02 | 9.10E+00 | | | |
| 8110872 | NM_033267 | IRX2 | iroquois homeobox 2 | -0.101 | 0.932 | 6.34E-02 | 6.34E+00 | | | |
| 8014812 | NM_198993 | STAC2 | SH3 and cysteine rich domain 2 | -0.101 | 0.932 | 8.04E-02 | 8.04E+00 | | | |
| 7912520 | NM_002521 | NPPB | natriuretic peptide precursor B | -0.101 | 0.932 | 1.14E-01 | 1.14E+01 | | | |
| 8164235 | NM_00100291 | PTRH1 | peptidyl-tRNA hydrolase 1 homolog | -0.102 | 0.932 | 1.43E-01 | 1.43E+01 | | | |
| 7998367 | NM_058192 | RPUSD1 | RNA pseudouridylate synthase doma | -0.102 | 0.932 | 9.18E-02 | 9.18E+00 | | | |
| 8159036 | NM_139025 | ADAMTS13 | ADAM metallopeptidase with throm | -0.102 | 0.932 | 4.52E-02 | 4.52E+00 | | | |
| 8135514 | NM_001550 | IFRD1 | interferon-related developmental re | -0.102 | 0.932 | 7.12E-01 | 7.12E+01 | | | |
| 7895261 | --- | --- | --- | -0.102 | 0.932 | 8.52E-01 | 8.52E+01 | | | |
| 7944447 | NM_022169 | ABCG4 | ATP-binding cassette, sub-family G (V | -0.102 | 0.932 | 4.52E-02 | 4.52E+00 | | | |
| 8019083 | NR_026857 | FLJ90757 | hypothetical LOC440465 | -0.102 | 0.932 | 1.31E-01 | 1.31E+01 | | | |
| 8094968 | NM_175619 | ZAR1 | zygote arrest 1 | -0.102 | 0.932 | 6.43E-02 | 6.43E+00 | | | |

| | | | | | | | | | | |
|---|---|---|---|---|---|---|---|---|---|---|
| 7973158 | NM_018071 | FLJ10357 | hypothetical protein FLJ10357 | -0.102 | 0.932 | 9.81E-02 | 9.81E+00 | | | |
| 8025586 | NM_146388 | MRPL4 | mitochondrial ribosomal protein L4 | -0.102 | 0.932 | 6.65E-02 | 6.65E+00 | | | |
| 7994308 | NM_015202 | KIAA0556 | KIAA0556 | -0.102 | 0.932 | 1.65E-01 | 1.65E+01 | | | |
| 8148917 | NM_138431 | MFSD3 | major facilitator superfamily domain | -0.102 | 0.932 | 5.74E-02 | 5.74E+00 | | | |
| 7909064 | NM_005076 | CNTN2 | contactin 2 (axonal) | -0.102 | 0.932 | 3.99E-02 | 3.99E+00 | | | |
| 7961293 | NM_176887 | TAS2R46 | taste receptor, type 2, member 46 | -0.102 | 0.932 | 8.86E-01 | 8.86E+01 | | | |
| 8090872 | NM_178554 | KY | kyphoscoliosis peptidase | -0.102 | 0.932 | 5.63E-02 | 5.63E+00 | | | |
| 7893972 | --- | --- | --- | -0.102 | 0.932 | 3.05E-01 | 3.05E+01 | | | |
| 7915949 | AK090467 | LOC284542 | hypothetical protein LOC284542 | -0.102 | 0.932 | 5.45E-02 | 5.45E+00 | | | |
| 7958960 | NM_00114381 | TPCN1 | two pore segment channel 1 | -0.102 | 0.932 | 2.43E-01 | 2.43E+01 | | | |
| 8055204 | --- | --- | --- | -0.102 | 0.932 | 3.84E-01 | 3.84E+01 | | | |
| 8125818 | NM_178508 | C6orf1 | chromosome 6 open reading frame 1 | -0.102 | 0.932 | 1.00E-01 | 1.00E+01 | | | |
| 8037170 | NM_002573 | PAFAH1B3 | platelet-activating factor acetylhydro | -0.102 | 0.932 | 7.30E-02 | 7.30E+00 | | | |
| 8104141 | NM_052909 | PLEKHG4B | pleckstrin homology domain contain | -0.102 | 0.932 | 3.58E-02 | 3.58E+00 | | | |
| 8038362 | NM_178449 | PTH2 | parathyroid hormone 2 | -0.102 | 0.932 | 1.02E-01 | 1.02E+01 | | | |
| 7899727 | NM_00116004 | IQCC | IQ motif containing C | -0.102 | 0.932 | 1.76E-01 | 1.76E+01 | | | |
| 8141395 | NM_005916 | MCM7 | minichromosome maintenance comp | -0.102 | 0.932 | 6.49E-01 | 6.49E+01 | | | |
| 7977780 | NM_022060 | ABHD4 | abhydrolase domain containing 4 | -0.102 | 0.932 | 8.55E-02 | 8.55E+00 | | | |
| 8162803 | NM_173551 | ANKS6 | ankyrin repeat and sterile alpha mot | -0.102 | 0.932 | 7.06E-02 | 7.06E+00 | | | |
| 8036563 | NM_144691 | CAPN12 | calpain 12 | -0.102 | 0.932 | 1.06E-01 | 1.06E+01 | | | |
| 8123644 | NM_001069 | TUBB2A | tubulin, beta 2A | -0.102 | 0.932 | 3.92E-01 | 3.92E+01 | | | |
| 7897076 | --- | --- | --- | -0.102 | 0.932 | 1.40E-01 | 1.40E+01 | | | |
| 8160637 | NM_001497 | B4GALT1 | UDP-Gal:betaGlcNAc beta 1,4- galact | -0.102 | 0.932 | 5.24E-01 | 5.24E+01 | | | |
| 8006321 | --- | --- | --- | -0.102 | 0.932 | 1.60E-01 | 1.60E+01 | | | |
| 8007483 | NM_145041 | TMEM106A | transmembrane protein 106A | -0.102 | 0.932 | 1.92E-01 | 1.92E+01 | | | |
| 7945245 | NM_016522 | NTM | neurotrimin | -0.102 | 0.932 | 1.45E-01 | 1.45E+01 | | | |
| 8142899 | NM_016478 | ZC3HC1 | zinc finger, C3HC-type containing 1 | -0.102 | 0.932 | 2.38E-01 | 2.38E+01 | | | |
| 7980452 | --- | --- | --- | -0.102 | 0.932 | 6.03E-01 | 6.03E+01 | | | |
| 8072170 | NM_00103957 | KREMEN1 | kringle containing transmembrane p | -0.102 | 0.932 | 7.33E-02 | 7.33E+00 | | | |
| 7894444 | --- | --- | --- | -0.102 | 0.932 | 2.02E-01 | 2.02E+01 | | | |
| 8028613 | NM_172140 | IL29 | interleukin 29 (interferon, lambda 1) | -0.102 | 0.932 | 1.27E-01 | 1.27E+01 | | | |
| 8092765 | NM_178496 | C3orf59 | chromosome 3 open reading frame 5 | -0.102 | 0.932 | 1.03E-01 | 1.03E+01 | | | |
| 7941004 | NM_032251 | CCDC88B | coiled-coil domain containing 88B | -0.102 | 0.932 | 3.89E-02 | 3.89E+00 | | | |
| 8141459 | NM_024637 | GAL3ST4 | galactose-3-O-sulfotransferase 4 | -0.102 | 0.932 | 2.13E-01 | 2.13E+01 | | | |
| 8136940 | NM_00113002 | FAM115C | family with sequence similarity 115, | -0.102 | 0.932 | 8.05E-02 | 8.05E+00 | | | |
| 7976506 | NR_015340 | SERPINA13 | serpin peptidase inhibitor, clade A (a | -0.102 | 0.932 | 1.07E-01 | 1.07E+01 | | | |
| 8003700 | NM_001383 | DPH1 | DPH1 homolog (S. cerevisiae) | -0.102 | 0.932 | 7.68E-02 | 7.68E+00 | | | |
| 8070708 | NR_026961 | LOC284837 | hypothetical LOC284837 | -0.102 | 0.932 | 1.16E-01 | 1.16E+01 | | | |
| 7981573 | NM_145685 | BRF1 | BRF1 homolog, subunit of RNA polym | -0.102 | 0.932 | 7.03E-02 | 7.03E+00 | | | |
| 8142230 | --- | --- | --- | -0.102 | 0.932 | 5.20E-02 | 5.20E+00 | | | |
| 8035703 | NM_004720 | LPAR2 | lysophosphatidic acid receptor 2 | -0.102 | 0.932 | 1.02E-01 | 1.02E+01 | | | |
| 8019273 | NM_005782 | THOC4 | THO complex 4 | -0.102 | 0.932 | 6.09E-01 | 6.09E+01 | | | |
| 8038393 | --- | --- | --- | -0.102 | 0.932 | 2.09E-01 | 2.09E+01 | | | |
| 7896098 | --- | --- | --- | -0.102 | 0.932 | 5.26E-01 | 5.26E+01 | | | |
| 8072926 | NM_005318 | H1F0 | H1 histone family, member 0 | -0.102 | 0.932 | 1.33E-01 | 1.33E+01 | | | |
| 8025783 | BC011833 | C19orf52 | chromosome 19 open reading frame | -0.102 | 0.932 | 4.39E-02 | 4.39E+00 | | | |
| 8069122 | NM_030891 | LRRC3 | leucine rich repeat containing 3 | -0.102 | 0.931 | 3.42E-02 | 3.42E+00 | | | |
| 8059674 | NM_005683 | GPR55 | G protein-coupled receptor 55 | -0.102 | 0.931 | 1.17E-01 | 1.17E+01 | | | |
| 7945130 | --- | --- | --- | -0.102 | 0.931 | 1.26E-01 | 1.26E+01 | | | |
| 8107350 | NM_003135 | SRP19 | signal recognition particle 19kDa | -0.102 | 0.931 | 3.22E-01 | 3.22E+01 | | | |
| 7938608 | NM_006108 | SPON1 | spondin 1, extracellular matrix prote | -0.102 | 0.931 | 1.09E-01 | 1.09E+01 | | | |
| 7912724 | NM_153213 | ARHGEF19 | Rho guanine nucleotide exchange fa | -0.102 | 0.931 | 6.59E-02 | 6.59E+00 | | | |
| 8074541 | NM_022727 | TRMT2A | TRM2 tRNA methyltransferase 2 hon | -0.102 | 0.931 | 3.85E-02 | 3.85E+00 | | | |
| 7999591 | NR_023387 | ABCC6P2 | ATP-binding cassette, sub-family C, n | -0.102 | 0.931 | 1.67E-01 | 1.67E+01 | | | |
| 8032839 | NM_032108 | SEMA6B | sema domain, transmembrane doma | -0.102 | 0.931 | 4.05E-02 | 4.05E+00 | | | |
| 8113348 | --- | --- | --- | -0.102 | 0.931 | 3.95E-01 | 3.95E+01 | | | |
| 8075458 | NM_134270 | SMTN | smoothelin | -0.102 | 0.931 | 8.18E-02 | 8.18E+00 | | | |
| 7948834 | NM_006473 | TAF6L | TAF6-like RNA polymerase II, p300/C | -0.103 | 0.931 | 9.29E-02 | 9.29E+00 | | | |
| 7896160 | --- | --- | --- | -0.103 | 0.931 | 1.43E-01 | 1.43E+01 | | | |
| 8165131 | NM_003086 | SNAPC4 | small nuclear RNA activating comple | -0.103 | 0.931 | 5.24E-02 | 5.24E+00 | | | |
| 8071541 | NR_026815 | TMEM191A | transmembrane protein 191A | -0.103 | 0.931 | 1.85E-01 | 1.85E+01 | | | |
| 7997765 | NM_00112746 | ZNF469 | zinc finger protein 469 | -0.103 | 0.931 | 1.11E-01 | 1.11E+01 | | | |
| 7982341 | NM_000746 | CHRNA7 | cholinergic receptor, nicotinic, alpha | -0.103 | 0.931 | 1.21E-01 | 1.21E+01 | | | |
| 8032224 | NM_152482 | C19orf25 | chromosome 19 open reading frame | -0.103 | 0.931 | 6.96E-02 | 6.96E+00 | | | |
| 8162059 | NM_022127 | SLC28A3 | solute carrier family 28 (sodium-coup | -0.103 | 0.931 | 5.67E-02 | 5.67E+00 | | | |
| 8076677 | NM_138415 | PHF21B | PHD finger protein 21B | -0.103 | 0.931 | 5.39E-02 | 5.39E+00 | | | |
| 8011912 | NM_014336 | AIPL1 | aryl hydrocarbon receptor interactin | -0.103 | 0.931 | 5.27E-02 | 5.27E+00 | | | |
| 7946069 | NM_033180 | OR51B2 | olfactory receptor, family 51, subfam | -0.103 | 0.931 | 1.30E-01 | 1.30E+01 | | | |
| 8159354 | NM_016215 | EGFL7 | EGF-like-domain, multiple 7 | -0.103 | 0.931 | 4.81E-02 | 4.81E+00 | | | |
| 7983490 | NR_022014 | C15orf21 | Dresden prostate cancer 2 | -0.103 | 0.931 | 1.40E-01 | 1.40E+01 | | | |
| 7949679 | NM_006946 | SPTBN2 | spectrin, beta, non-erythrocytic 2 | -0.103 | 0.931 | 6.00E-02 | 6.00E+00 | | | |
| 8038288 | NM_033377 | CGB1 | chorionic gonadotropin, beta polype | -0.103 | 0.931 | 4.55E-02 | 4.55E+00 | | | |
| 8003233 | NR_027490 | MTHFSD | methenyltetrahydrofolate synthetas | -0.103 | 0.931 | 1.34E-01 | 1.34E+01 | | | |
| 7988077 | NM_014793 | LCMT2 | leucine carboxyl methyltransferase 2 | -0.103 | 0.931 | 4.65E-01 | 4.65E+01 | | | |
| 8071532 | NM_015672 | RIMBP3 | RIMS binding protein 3 | -0.103 | 0.931 | 5.62E-02 | 5.62E+00 | | | |
| 8073705 | NM_181333 | PRR5 | proline rich 5 (renal) | -0.103 | 0.931 | 4.03E-02 | 4.03E+00 | | | |
| 8087731 | BC034766 | C3orf18 | chromosome 3 open reading frame 1 | -0.103 | 0.931 | 5.18E-02 | 5.18E+00 | | | |

| | | | | | | | | | | |
|---|---|---|---|---|---|---|---|---|---|---|
| 7953699 | NM_018416 | FOXJ2 | forkhead box J2 | -0.103 | 0.931 | 5.34E-02 | 5.34E+00 | | | |
| 8146945 | NM_018972 | GDAP1 | ganglioside-induced differentiation-a | -0.103 | 0.931 | 1.44E-01 | 1.44E+01 | | | |
| 8094251 | NR_027696 | FLJ39653 | hypothetical FLJ39653 | -0.103 | 0.931 | 9.31E-02 | 9.31E+00 | | | |
| 8067521 | NM_080473 | GATA5 | GATA binding protein 5 | -0.103 | 0.931 | 2.93E-02 | 2.93E+00 | | | |
| 8008289 | NM_153229 | TMEM92 | transmembrane protein 92 | -0.103 | 0.931 | 8.61E-02 | 8.61E+00 | | | |
| 8002029 | NM_013304 | ZDHHC1 | zinc finger, DHHC-type containing 1 | -0.103 | 0.931 | 3.14E-02 | 3.14E+00 | | | |
| 8180314 | --- | --- | --- | -0.103 | 0.931 | 2.62E-01 | 2.62E+01 | | | |
| 8002403 | NM_138383 | MTSS1L | metastasis suppressor 1-like | -0.103 | 0.931 | 1.29E-01 | 1.29E+01 | | | |
| 7950372 | --- | --- | --- | -0.103 | 0.931 | 6.26E-01 | 6.26E+01 | | | |
| 7929653 | NM_020349 | ANKRD2 | ankyrin repeat domain 2 (stretch res | -0.103 | 0.931 | 6.99E-02 | 6.99E+00 | | | |
| 8037737 | NM_002516 | NOVA2 | neuro-oncological ventral antigen 2 | -0.103 | 0.931 | 3.89E-02 | 3.89E+00 | | | |
| 8035351 | NM_000215 | JAK3 | Janus kinase 3 | -0.103 | 0.931 | 4.72E-02 | 4.72E+00 | | | |
| 7895308 | --- | --- | --- | -0.103 | 0.931 | 3.07E-01 | 3.07E+01 | | | |
| 7941469 | NM_152762 | TSGA10IP | testis specific, 10 interacting protein | -0.103 | 0.931 | 5.41E-02 | 5.41E+00 | | | |
| 8013919 | NM_198147 | ABHD15 | abhydrolase domain containing 15 | -0.103 | 0.931 | 1.49E-01 | 1.49E+01 | | | |
| 8030842 | --- | --- | --- | -0.103 | 0.931 | 2.43E-01 | 2.43E+01 | | | |
| 7938208 | NM_014469 | RBMXL2 | RNA binding motif protein, X-linked- | -0.103 | 0.931 | 5.84E-02 | 5.84E+00 | | | |
| 8165881 | NM_013452 | VCX | variable charge, X-linked | -0.103 | 0.931 | 5.15E-02 | 5.15E+00 | | | |
| 8163437 | NM_033051 | SLC46A2 | solute carrier family 46, member 2 | -0.103 | 0.931 | 6.68E-02 | 6.68E+00 | | | |
| 7895642 | --- | --- | --- | -0.103 | 0.931 | 5.11E-01 | 5.11E+01 | | | |
| 8126337 | NM_006653 | FRS3 | fibroblast growth factor receptor sub | -0.103 | 0.931 | 7.32E-02 | 7.32E+00 | | | |
| 7899627 | NM_022164 | TINAGL1 | tubulointerstitial nephritis antigen-li | -0.103 | 0.931 | 9.43E-02 | 9.43E+00 | | | |
| 7957275 | --- | --- | --- | -0.103 | 0.931 | 2.18E-01 | 2.18E+01 | | | |
| 7921462 | NM_00101366 | VSIG8 | V-set and immunoglobulin domain co | -0.103 | 0.931 | 1.90E-01 | 1.90E+01 | | | |
| 8094679 | NM_175737 | KLB | klotho beta | -0.103 | 0.931 | 7.63E-02 | 7.63E+00 | | | |
| 8019392 | NM_004104 | FASN | fatty acid synthase | -0.103 | 0.931 | 4.67E-02 | 4.67E+00 | | | |
| 8044391 | NM_006343 | MERTK | c-mer proto-oncogene tyrosine kinas | -0.103 | 0.931 | 7.70E-02 | 7.70E+00 | | | |
| 8071185 | NR_002733 | DGCR5 | DiGeorge syndrome critical region ge | -0.103 | 0.931 | 5.02E-02 | 5.02E+00 | | | |
| 8038598 | NM_016148 | SHANK1 | SH3 and multiple ankyrin repeat don | -0.103 | 0.931 | 6.40E-02 | 6.40E+00 | | | |
| 8101657 | --- | --- | --- | -0.103 | 0.931 | 2.18E-01 | 2.18E+01 | | | |
| 8176650 | NM_004679 | VCY | variable charge, Y-linked | -0.103 | 0.931 | 7.53E-02 | 7.53E+00 | | | |
| 8177169 | NM_004679 | VCY | variable charge, Y-linked | -0.103 | 0.931 | 7.53E-02 | 7.53E+00 | | | |
| 8000543 | NM_000086 | CLN3 | ceroid-lipofuscinosis, neuronal 3 | -0.103 | 0.931 | 1.78E-01 | 1.78E+01 | | | |
| 8152369 | NM_014379 | KCNV1 | potassium channel, subfamily V, mer | -0.103 | 0.931 | 1.11E-01 | 1.11E+01 | | | |
| 7894370 | --- | --- | --- | -0.103 | 0.931 | 8.29E-01 | 8.29E+01 | | | |
| 8070257 | NM_153681 | PIGP | phosphatidylinositol glycan anchor b | -0.103 | 0.931 | 1.71E-01 | 1.71E+01 | | | |
| 7898350 | BC126340 // B | 2147 // LOC | hypothetical LOC100132147 // hypo | -0.103 | 0.931 | 1.95E-01 | 1.95E+01 | | | |
| 7912884 | BC126340 // B | 2147 // LOC | hypothetical LOC100132147 // hypo | -0.103 | 0.931 | 1.95E-01 | 1.95E+01 | | | |
| 8027856 | NM_005304 | FFAR3 | free fatty acid receptor 3 | -0.103 | 0.931 | 5.92E-02 | 5.92E+00 | | | |
| 8074615 | --- | --- | --- | -0.103 | 0.931 | 2.39E-01 | 2.39E+01 | | | |
| 7933366 | NM_016204 | GDF2 | growth differentiation factor 2 | -0.103 | 0.931 | 6.78E-02 | 6.78E+00 | | | |
| 7949744 | --- | --- | --- | -0.103 | 0.931 | 7.64E-01 | 7.64E+01 | | | |
| 7912162 | --- | --- | --- | -0.103 | 0.931 | 1.21E-01 | 1.21E+01 | | | |
| 8092845 | NM_004488 | GP5 | glycoprotein V (platelet) | -0.103 | 0.931 | 4.80E-02 | 4.80E+00 | | | |
| 8036656 | NM_017827 | SARS2 | seryl-tRNA synthetase 2, mitochondr | -0.103 | 0.931 | 1.60E-01 | 1.60E+01 | | | |
| 7955663 | NM_170754 | TENC1 | tensin like C1 domain containing pho | -0.103 | 0.931 | 4.15E-02 | 4.15E+00 | | | |
| 7915333 | NM_014747 | RIMS3 | regulating synaptic membrane exocy | -0.103 | 0.931 | 8.64E-02 | 8.64E+00 | | | |
| 7978748 | NM_203301 | FBXO33 | F-box protein 33 | -0.103 | 0.931 | 4.21E-01 | 4.21E+01 | | | |
| 7944955 | NM_022062 | PKNOX2 | PBX/knotted 1 homeobox 2 | -0.103 | 0.931 | 6.68E-02 | 6.68E+00 | | | |
| 8093997 | NM_020777 | SORCS2 | sortilin-related VPS10 domain contai | -0.103 | 0.931 | 2.66E-02 | 2.66E+00 | | | |
| 7997135 | NM_138383 | MTSS1L | metastasis suppressor 1-like | -0.103 | 0.931 | 9.39E-02 | 9.39E+00 | | | |
| 8094086 | --- | --- | --- | -0.103 | 0.931 | 7.98E-02 | 7.98E+00 | | | |
| 7991898 | ENST00000409 | PIGQ | phosphatidylinositol glycan anchor b | -0.103 | 0.931 | 6.38E-02 | 6.38E+00 | | | |
| 8074030 | NM_014678 | SAPS2 | SAPS domain family, member 2 | -0.103 | 0.931 | 8.98E-02 | 8.98E+00 | | | |
| 8119052 | NM_173676 | PNPLA1 | patatin-like phospholipase domain co | -0.103 | 0.931 | 8.56E-02 | 8.56E+00 | | | |
| 8126248 | NM_173561 | UNC5CL | unc-5 homolog C (C. elegans)-like | -0.103 | 0.931 | 1.28E-01 | 1.28E+01 | | | |
| 8024246 | NM_017914 | C19orf24 | chromosome 19 open reading frame | -0.103 | 0.931 | 2.79E-01 | 2.79E+01 | | | |
| 8122637 | NM_015278 | SASH1 | SAM and SH3 domain containing 1 | -0.103 | 0.931 | 1.82E-01 | 1.82E+01 | | | |
| 7937341 | NM_178537 | B4GALNT4 | beta-1,4-N-acetyl-galactosaminyl tra | -0.104 | 0.931 | 5.72E-02 | 5.72E+00 | | | |
| 8013616 | NM_080669 | SLC46A1 | solute carrier family 46 (folate transp | -0.104 | 0.931 | 1.21E-01 | 1.21E+01 | | | |
| 7921222 | NM_00100434 | ETV3L | ets variant 3-like | -0.104 | 0.931 | 4.56E-02 | 4.56E+00 | | | |
| 8072454 | NR_024210 | RNF185 | ring finger protein 185 | -0.104 | 0.931 | 5.87E-01 | 5.87E+01 | | | |
| 7937823 | NM_000218 | KCNQ1 | potassium voltage-gated channel, KQ | -0.104 | 0.931 | 1.33E-01 | 1.33E+01 | | | |
| 8070615 | NM_018669 | WDR4 | WD repeat domain 4 | -0.104 | 0.931 | 5.99E-02 | 5.99E+00 | | | |
| 8086417 | --- | --- | --- | -0.104 | 0.931 | 3.58E-01 | 3.58E+01 | | | |
| 8069150 | NM_198690 | KRTAP10-9 | keratin associated protein 10-9 | -0.104 | 0.931 | 1.31E-01 | 1.31E+01 | | | |
| 7992594 | NM_001761 | CCNF | cyclin F | -0.104 | 0.931 | 3.97E-02 | 3.97E+00 | | | |
| 8064108 | BC000912 | C20orf195 | chromosome 20 open reading frame | -0.104 | 0.931 | 4.20E-02 | 4.20E+00 | | | |
| 8009145 | NM_030779 | KCNH6 | potassium voltage-gated channel, su | -0.104 | 0.931 | 6.00E-02 | 6.00E+00 | | | |
| 7997533 | NM_013370 | OSGIN1 | oxidative stress induced growth inhil | -0.104 | 0.931 | 4.03E-02 | 4.03E+00 | | | |
| 8049487 | NM_024101 | MLPH | melanophilin | -0.104 | 0.931 | 5.37E-02 | 5.37E+00 | | | |
| 7937208 | NM_198472 | C10orf125 | chromosome 10 open reading frame | -0.104 | 0.931 | 1.78E-01 | 1.78E+01 | | | |
| 8123976 | NM_018988 | GFOD1 | glucose-fructose oxidoreductase dor | -0.104 | 0.931 | 5.24E-02 | 5.24E+00 | | | |
| 8019331 | NM_00114511 | MYADML2 | myeloid-associated differentiation m | -0.104 | 0.931 | 2.16E-02 | 2.16E+00 | | | |
| 8005857 | NM_152464 | TMEM199 | transmembrane protein 199 | -0.104 | 0.931 | 3.49E-01 | 3.49E+01 | | | |
| 8129037 | --- | --- | --- | -0.104 | 0.931 | 2.10E-01 | 2.10E+01 | | | |
| 7908376 | NM_130782 | RGS18 | regulator of G-protein signaling 18 | -0.104 | 0.931 | 8.64E-01 | 8.64E+01 | | | |

| | | | | | | | | | | |
|---|---|---|---|---|---|---|---|---|---|---|
| 7978331 | NM_00103977 | CBLN3 | cerebellin 3 precursor | -0.104 | 0.931 | 8.98E-02 | 8.98E+00 | | | |
| 8001306 | NM_00100661 | SIAH1 | seven in absentia homolog 1 (Drosop | -0.104 | 0.931 | 2.78E-01 | 2.78E+01 | | | |
| 7896476 | --- | --- | --- | -0.104 | 0.931 | 8.18E-01 | 8.18E+01 | | | |
| 8103309 | --- | --- | --- | -0.104 | 0.931 | 9.03E-01 | 9.03E+01 | | | |
| 8030429 | NM_138639 | BCL2L12 | BCL2-like 12 (proline rich) | -0.104 | 0.931 | 1.16E-01 | 1.16E+01 | | | |
| 8144995 | NM_003867 | FGF17 | fibroblast growth factor 17 | -0.104 | 0.931 | 6.12E-02 | 6.12E+00 | | | |
| 8086689 | NM_000258 | MYL3 | myosin, light chain 3, alkali; ventricu | -0.104 | 0.931 | 9.70E-02 | 9.70E+00 | | | |
| 7916364 | NM_00101097 | LDLRAD1 | low density lipoprotein receptor clas | -0.104 | 0.931 | 9.55E-02 | 9.55E+00 | | | |
| 8065185 | NM_021220 | OVOL2 | ovo-like 2 (Drosophila) | -0.104 | 0.931 | 3.04E-02 | 3.04E+00 | | | |
| 8137802 | ENST00000382 | LOC401296 | hypothetical LOC401296 | -0.104 | 0.931 | 8.97E-02 | 8.97E+00 | | | |
| 7894928 | --- | --- | --- | -0.104 | 0.931 | 7.89E-01 | 7.89E+01 | | | |
| 7917466 | NR_026988 | LOC339524 | hypothetical LOC339524 | -0.104 | 0.931 | 1.24E-01 | 1.24E+01 | | | |
| 8063014 | NM_172005 | WFDC13 | WAP four-disulfide core domain 13 | -0.104 | 0.931 | 1.38E-01 | 1.38E+01 | | | |
| 8065576 | NM_004118 | FOXS1 | forkhead box S1 | -0.104 | 0.931 | 6.65E-02 | 6.65E+00 | | | |
| 8004394 | NM_199339 | SPEM1 | spermatid maturation 1 | -0.104 | 0.931 | 9.03E-02 | 9.03E+00 | | | |
| 7898707 | NM_00101369 | LDLRAD2 | low density lipoprotein receptor clas | -0.104 | 0.931 | 3.98E-02 | 3.98E+00 | | | |
| 7936923 | M21191 | ALDOAP2 | aldolase A, fructose-bisphosphate ps | -0.104 | 0.931 | 1.65E-01 | 1.65E+01 | | | |
| 8035278 | NM_020959 | ANO8 | anoctamin 8 | -0.104 | 0.931 | 1.21E-01 | 1.21E+01 | | | |
| 8109612 | NM_000679 | ADRA1B | adrenergic, alpha-1B-, receptor | -0.104 | 0.931 | 1.15E-01 | 1.15E+01 | | | |
| 8153459 | NM_023078 | PYCRL | pyrroline-5-carboxylate reductase-lik | -0.104 | 0.931 | 6.35E-02 | 6.35E+00 | | | |
| 8012787 | NM_002470 | MYH3 | myosin, heavy chain 3, skeletal musc | -0.104 | 0.931 | 1.68E-01 | 1.68E+01 | | | |
| 7896695 | --- | --- | --- | -0.104 | 0.931 | 1.67E-01 | 1.67E+01 | | | |
| 8062286 | BC019311 | C20orf4 | chromosome 20 open reading frame | -0.104 | 0.931 | 2.73E-01 | 2.73E+01 | | | |
| 8065202 | NM_00109940 | C20orf12 | chromosome 20 open reading frame | -0.104 | 0.930 | 5.14E-02 | 5.14E+00 | | | |
| 7919604 | AK092418 | HIST2H2BF | histone cluster 2, H2bf | -0.104 | 0.930 | 9.95E-02 | 9.95E+00 | | | |
| 8002937 | --- | --- | --- | -0.104 | 0.930 | 4.85E-01 | 4.85E+01 | | | |
| 8074212 | NM_00103781 | GAB4 | GRB2-associated binding protein fam | -0.104 | 0.930 | 1.12E-01 | 1.12E+01 | | | |
| 7994609 | NM_014298 | QPRT | quinolinate phosphoribosyltransfera | -0.104 | 0.930 | 7.84E-02 | 7.84E+00 | | | |
| 7895840 | --- | --- | --- | -0.104 | 0.930 | 8.24E-01 | 8.24E+01 | | | |
| 7927091 | NR_024497 | LOC399744 | hypothetical LOC399744 | -0.104 | 0.930 | 1.19E-01 | 1.19E+01 | | | |
| 7945086 | NM_00103966 | TIRAP | toll-interleukin 1 receptor (TIR) doma | -0.104 | 0.930 | 2.39E-01 | 2.39E+01 | | | |
| 7950442 | --- | --- | --- | -0.104 | 0.930 | 1.20E-01 | 1.20E+01 | | | |
| 8141595 | NM_022574 | GIGYF1 | GRB10 interacting GYF protein 1 | -0.104 | 0.930 | 6.09E-02 | 6.09E+00 | | | |
| 7931899 | NM_002189 | IL15RA | interleukin 15 receptor, alpha | -0.104 | 0.930 | 8.64E-02 | 8.64E+00 | | | |
| 8122982 | NM_024630 | ZDHHC14 | zinc finger, DHHC-type containing 14 | -0.104 | 0.930 | 2.43E-01 | 2.43E+01 | | | |
| 8064978 | NM_000214 | JAG1 | jagged 1 (Alagille syndrome) | -0.104 | 0.930 | 9.61E-02 | 9.61E+00 | | | |
| 7964677 | NM_152440 | C12orf66 | chromosome 12 open reading frame | -0.104 | 0.930 | 1.26E-01 | 1.26E+01 | | | |
| 7991758 | NM_00100393 | HBM | hemoglobin, mu | -0.104 | 0.930 | 8.97E-02 | 8.97E+00 | | | |
| 8090485 | NM_007354 | C3orf27 | chromosome 3 open reading frame 2 | -0.104 | 0.930 | 4.15E-02 | 4.15E+00 | | | |
| 8067680 | NM_00103733 | PRIC285 | peroxisomal proliferator-activated re | -0.104 | 0.930 | 3.15E-02 | 3.15E+00 | | | |
| 8157939 | --- | --- | --- | -0.104 | 0.930 | 1.47E-01 | 1.47E+01 | | | |
| 8074223 | NM_031890 | CECR6 | cat eye syndrome chromosome regio | -0.104 | 0.930 | 7.26E-02 | 7.26E+00 | | | |
| 7910503 | NM_00100434 | TRIM67 | tripartite motif-containing 67 | -0.104 | 0.930 | 6.95E-02 | 6.95E+00 | | | |
| 8132013 | NM_004067 | CHN2 | chimerin (chimaerin) 2 | -0.104 | 0.930 | 4.51E-01 | 4.51E+01 | | | |
| 8044933 | NM_005270 | GLI2 | GLI family zinc finger 2 | -0.104 | 0.930 | 6.45E-02 | 6.45E+00 | | | |
| 7949080 | NM_015080 | NRXN2 | neurexin 2 | -0.104 | 0.930 | 2.61E-02 | 2.61E+00 | | | |
| 8004404 | NM_178518 | TMEM102 | transmembrane protein 102 | -0.104 | 0.930 | 1.10E-01 | 1.10E+01 | | | |
| 8054281 | NM_198461 | LONRF2 | LON peptidase N-terminal domain ar | -0.104 | 0.930 | 8.41E-02 | 8.41E+00 | | | |
| 8065084 | AK125594 | LOC613266 | hypothetical LOC613266 | -0.104 | 0.930 | 2.41E-01 | 2.41E+01 | | | |
| 8015572 | NM_001524 | HCRT | hypocretin (orexin) neuropeptide pre | -0.104 | 0.930 | 6.60E-02 | 6.60E+00 | | | |
| 8023121 | NM_013305 | ST8SIA5 | ST8 alpha-N-acetyl-neuraminide alph | -0.104 | 0.930 | 3.83E-02 | 3.83E+00 | | | |
| 8125091 | NM_006295 | VARS | valyl-tRNA synthetase | -0.104 | 0.930 | 1.12E-01 | 1.12E+01 | | | |
| 8178609 | NM_006295 | VARS | valyl-tRNA synthetase | -0.104 | 0.930 | 1.12E-01 | 1.12E+01 | | | |
| 7947462 | NM_145804 | ABTB2 | ankyrin repeat and BTB (POZ) domai | -0.104 | 0.930 | 3.63E-02 | 3.63E+00 | | | |
| 7929958 | NM_033637 | BTRC | beta-transducin repeat containing | -0.104 | 0.930 | 1.93E-01 | 1.93E+01 | | | |
| 7937150 | NM_001109 | ADAM8 | ADAM metallopeptidase domain 8 | -0.104 | 0.930 | 7.71E-02 | 7.71E+00 | | | |
| 8151334 | NM_005098 | MSC | musculin (activated B-cell factor-1) | -0.104 | 0.930 | 2.02E-01 | 2.02E+01 | | | |
| 7900944 | NM_152499 | CCDC24 | coiled-coil domain containing 24 | -0.104 | 0.930 | 5.18E-02 | 5.18E+00 | | | |
| 7992043 | BC001181 | FAM173A | family with sequence similarity 173, | -0.104 | 0.930 | 1.40E-01 | 1.40E+01 | | | |
| 8005433 | --- | --- | --- | -0.104 | 0.930 | 7.34E-02 | 7.34E+00 | | | |
| 8172806 | NM_133179 | XAGE3 | X antigen family, member 3 | -0.104 | 0.930 | 1.30E-01 | 1.30E+01 | | | |
| 8050425 | --- | --- | --- | -0.104 | 0.930 | 2.44E-01 | 2.44E+01 | | | |
| 8061445 | --- | --- | --- | -0.104 | 0.930 | 3.12E-01 | 3.12E+01 | | | |
| 8136039 | NM_004231 | ATP6V1F | ATPase, H+ transporting, lysosomal 1 | -0.104 | 0.930 | 5.16E-01 | 5.16E+01 | | | |
| 8164314 | NM_175039 | ST6GALNAC4 | ST6 (alpha-N-acetyl-neuraminyl-2,3- | -0.104 | 0.930 | 1.11E-01 | 1.11E+01 | | | |
| 8160472 | NM_00100412 | TUSC1 | tumor suppressor candidate 1 | -0.104 | 0.930 | 7.97E-02 | 7.97E+00 | | | |
| 8018352 | NM_00112612 | SLC25A19 | solute carrier family 25 (mitochondri | -0.104 | 0.930 | 2.08E-01 | 2.08E+01 | | | |
| 8130827 | AB016899 | C6orf54 | chromosome 6 open reading frame 5 | -0.104 | 0.930 | 1.35E-01 | 1.35E+01 | | | |
| 8050060 | NM_003310 | TSSC1 | tumor suppressing subtransferable c | -0.104 | 0.930 | 1.46E-01 | 1.46E+01 | | | |
| 8171539 | NM_021785 | RAI2 | retinoic acid induced 2 | -0.104 | 0.930 | 2.78E-02 | 2.78E+00 | | | |
| 8033248 | NM_003807 | TNFSF14 | tumor necrosis factor (ligand) superf | -0.104 | 0.930 | 8.95E-02 | 8.95E+00 | | | |
| 7897305 | NM_005341 | ZBTB48 | zinc finger and BTB domain containin | -0.104 | 0.930 | 1.21E-01 | 1.21E+01 | | | |
| 8029779 | NM_198541 | IGFL1 | IGF-like family member 1 | -0.104 | 0.930 | 7.49E-02 | 7.49E+00 | | | |
| 8083071 | NM_080862 | SPSB4 | splA/ryanodine receptor domain and | -0.104 | 0.930 | 7.49E-02 | 7.49E+00 | | | |
| 8048211 | NM_198483 | RUFY4 | RUN and FYVE domain containing 4 | -0.104 | 0.930 | 3.80E-02 | 3.80E+00 | | | |
| 8134904 | NM_005837 | POP7 | processing of precursor 7, ribonuclea | -0.104 | 0.930 | 4.65E-01 | 4.65E+01 | | | |
| 8040374 | NM_145175 | FAM84A | family with sequence similarity 84, m | -0.104 | 0.930 | 5.27E-02 | 5.27E+00 | | | |

| | | | | | | | | | | |
|---|---|---|---|---|---|---|---|---|---|---|
| 7952205 | NM_006500 | MCAM | melanoma cell adhesion molecule | -0.104 | 0.930 | 2.10E-02 | 2.10E+00 | | | |
| 8063634 | ENST00000419 | MGC4294 | hypothetical protein MGC4294 | -0.104 | 0.930 | 2.11E-01 | 2.11E+01 | | | |
| 8029754 | NM_004497 | FOXA3 | forkhead box A3 | -0.104 | 0.930 | 1.19E-01 | 1.19E+01 | | | |
| 7933149 | NM_145313 | RASGEF1A | RasGEF domain family, member 1A | -0.104 | 0.930 | 7.13E-02 | 7.13E+00 | | | |
| 8135181 | NM_152892 | LRWD1 | leucine-rich repeats and WD repeat | -0.104 | 0.930 | 4.21E-02 | 4.21E+00 | | | |
| 7894142 | --- | --- | --- | -0.104 | 0.930 | 6.77E-01 | 6.77E+01 | | | |
| 8024676 | NM_133261 | GIPC3 | GIPC PDZ domain containing family, | -0.104 | 0.930 | 1.61E-01 | 1.61E+01 | | | |
| 8067756 | NM_017859 | UCKL1 | uridine-cytidine kinase 1-like 1 | -0.104 | 0.930 | 1.06E-01 | 1.06E+01 | | | |
| 8080320 | NM_007184 | NISCH | nischarin | -0.104 | 0.930 | 3.97E-02 | 3.97E+00 | | | |
| 8081358 | NM_014415 | ZBTB11 | zinc finger and BTB domain containi | -0.104 | 0.930 | 2.79E-01 | 2.79E+01 | | | |
| 8148715 | NM_003801 | GPAA1 | glycosylphosphatidylinositol anchor | -0.104 | 0.930 | 8.21E-02 | 8.21E+00 | | | |
| 7943795 | NM_080659 | C11orf52 | chromosome 11 open reading frame | -0.104 | 0.930 | 8.51E-02 | 8.51E+00 | | | |
| 8074481 | NM_053004 | GNB1L | guanine nucleotide binding protein ( | -0.105 | 0.930 | 1.45E-02 | 1.45E+00 | | | |
| 8011331 | NM_002551 | OR3A2 | olfactory receptor, family 3, subfami | -0.105 | 0.930 | 1.94E-01 | 1.94E+01 | | | |
| 7994292 | NM_181078 | IL21R | interleukin 21 receptor | -0.105 | 0.930 | 4.33E-02 | 4.33E+00 | | | |
| 8029311 | NM_024327 | ZNF576 | zinc finger protein 576 | -0.105 | 0.930 | 5.09E-02 | 5.09E+00 | | | |
| 8005132 | NR_002211 | MEIS3P1 | Meis homeobox 3 pseudogene 1 | -0.105 | 0.930 | 8.75E-02 | 8.75E+00 | | | |
| 8023708 | --- | --- | --- | -0.105 | 0.930 | 2.30E-01 | 2.30E+01 | | | |
| 8066451 | NM_182970 | RIMS4 | regulating synaptic membrane exocy | -0.105 | 0.930 | 4.97E-02 | 4.97E+00 | | | |
| 8031253 | NR_003061 | LILRP2 | leukocyte immunoglobulin-like recep | -0.105 | 0.930 | 1.37E-01 | 1.37E+01 | | | |
| 8039246 | NM_012276 | LILRA4 | leukocyte immunoglobulin-like recep | -0.105 | 0.930 | 1.07E-01 | 1.07E+01 | | | |
| 8000978 | NM_00103950 | POL3S | polyserase 3 | -0.105 | 0.930 | 4.83E-02 | 4.83E+00 | | | |
| 7963567 | NM_002273 | KRT8 | keratin 8 | -0.105 | 0.930 | 2.54E-01 | 2.54E+01 | | | |
| 7977344 | NM_00110091 | PACS2 | phosphofurin acidic cluster sorting p | -0.105 | 0.930 | 5.41E-02 | 5.41E+00 | | | |
| 7893849 | --- | --- | --- | -0.105 | 0.930 | 9.12E-01 | 9.12E+01 | | | |
| 7998157 | NM_00107735 | C16orf35 | chromosome 16 open reading frame | -0.105 | 0.930 | 1.06E-01 | 1.06E+01 | | | |
| 7892697 | --- | --- | --- | -0.105 | 0.930 | 3.18E-01 | 3.18E+01 | | | |
| 8062480 | NR_003239 | SNHG11 | small nucleolar RNA host gene 11 (n | -0.105 | 0.930 | 6.03E-02 | 6.03E+00 | | | |
| 7896655 | --- | --- | --- | -0.105 | 0.930 | 9.20E-01 | 9.20E+01 | | | |
| 7896540 | --- | --- | --- | -0.105 | 0.930 | 5.26E-01 | 5.26E+01 | | | |
| 7998267 | NM_00100592 | JMJD8 | jumonji domain containing 8 | -0.105 | 0.930 | 1.08E-01 | 1.08E+01 | | | |
| 8074716 | NR_003267 | GGT3P | gamma-glutamyltransferase 3 pseud | -0.105 | 0.930 | 2.89E-02 | 2.89E+00 | | | |
| 8150908 | NM_017813 | IMPAD1 | inositol monophosphatase domain c | -0.105 | 0.930 | 6.84E-01 | 6.84E+01 | | | |
| 8041727 | NM_000341 | SLC3A1 | solute carrier family 3 (cystine, dibas | -0.105 | 0.930 | 7.43E-02 | 7.43E+00 | | | |
| 8030067 | NM_004605 | SULT2B1 | sulfotransferase family, cytosolic, 2B | -0.105 | 0.930 | 9.89E-02 | 9.89E+00 | | | |
| 8059222 | NM_012100 | DNPEP | aspartyl aminopeptidase | -0.105 | 0.930 | 2.35E-01 | 2.35E+01 | | | |
| 8056821 | --- | --- | --- | -0.105 | 0.930 | 2.43E-01 | 2.43E+01 | | | |
| 8032922 | BC137009 | C19orf31 | chromosome 19 open reading frame | -0.105 | 0.930 | 5.09E-02 | 5.09E+00 | | | |
| 7959146 | NR_001577 | NME2P1 | non-metastatic cells 2, protein (NM2 | -0.105 | 0.930 | 5.35E-01 | 5.35E+01 | | | |
| 7941587 | NM_182553 | CNIH2 | cornichon homolog 2 (Drosophila) | -0.105 | 0.930 | 5.02E-02 | 5.02E+00 | | | |
| 7985577 | NM_181877 | ZSCAN2 | zinc finger and SCAN domain contain | -0.105 | 0.930 | 1.04E-01 | 1.04E+01 | | | |
| 8155514 | BC032035 | FAM27E3 | family with sequence similarity 27, n | -0.105 | 0.930 | 1.56E-01 | 1.56E+01 | | | |
| 8052200 | NM_00100393 | TSPYL6 | TSPY-like 6 | -0.105 | 0.930 | 7.32E-02 | 7.32E+00 | | | |
| 8176648 | --- | --- | --- | -0.105 | 0.930 | 8.26E-02 | 8.26E+00 | | | |
| 8143144 | NM_002825 | PTN | pleiotrophin | -0.105 | 0.930 | 7.50E-02 | 7.50E+00 | | | |
| 8061428 | NM_001247 | ENTPD6 | ectonucleoside triphosphate diphos | -0.105 | 0.930 | 1.66E-01 | 1.66E+01 | | | |
| 7998921 | NM_032805 | ZSCAN10 | zinc finger and SCAN domain contain | -0.105 | 0.930 | 6.96E-02 | 6.96E+00 | | | |
| 7934698 | NM_00109866 | SFTPA2 | surfactant protein A2 | -0.105 | 0.930 | 8.96E-02 | 8.96E+00 | | | |
| 7934708 | NM_00109866 | SFTPA2 | surfactant protein A2 | -0.105 | 0.930 | 8.96E-02 | 8.96E+00 | | | |
| 8179351 | NM_001710 | CFB | complement factor B | -0.105 | 0.930 | 2.04E-01 | 2.04E+01 | | | |
| 7989785 | NM_020962 | IGDCC4 | immunoglobulin superfamily, DCC su | -0.105 | 0.930 | 9.88E-02 | 9.88E+00 | | | |
| 8152703 | NM_058229 | FBXO32 | F-box protein 32 | -0.105 | 0.930 | 6.71E-01 | 6.71E+01 | | | |
| 8137670 | NM_002607 | PDGFA | platelet-derived growth factor alpha | -0.105 | 0.930 | 3.14E-01 | 3.14E+01 | | | |
| 8040238 | NM_134421 | HPCAL1 | hippocalcin-like 1 | -0.105 | 0.930 | 8.46E-02 | 8.46E+00 | | | |
| 7945663 | --- | --- | --- | -0.105 | 0.930 | 1.25E-01 | 1.25E+01 | | | |
| 8065460 | NM_014588 | VSX1 | visual system homeobox 1 | -0.105 | 0.930 | 2.35E-02 | 2.35E+00 | | | |
| 7896193 | --- | --- | --- | -0.105 | 0.930 | 3.93E-01 | 3.93E+01 | | | |
| 8160953 | NM_032634 | PIGO | phosphatidylinositol glycan anchor b | -0.105 | 0.930 | 1.94E-01 | 1.94E+01 | | | |
| 8103853 | NR_027107 | MGC45800 | hypothetical LOC90768 | -0.105 | 0.930 | 6.37E-02 | 6.37E+00 | | | |
| 7913896 | NM_00100443 | SLC30A2 | solute carrier family 30 (zinc transpo | -0.105 | 0.930 | 9.06E-02 | 9.06E+00 | | | |
| 8012539 | NM_00101085 | PIK3R6 | phosphoinositide-3-kinase, regulator | -0.105 | 0.930 | 9.09E-02 | 9.09E+00 | | | |
| 7959535 | NM_024623 | OGFOD2 | 2-oxoglutarate and iron-dependent c | -0.105 | 0.930 | 6.08E-02 | 6.08E+00 | | | |
| 7944825 | X64981 | OR10D1P | olfactory receptor, family 10, subfam | -0.105 | 0.930 | 1.25E-01 | 1.25E+01 | | | |
| 7900365 | NM_00113649 | MFSD2 | major facilitator superfamily domain | -0.105 | 0.930 | 8.46E-02 | 8.46E+00 | | | |
| 8070716 | ENST00000430 | C21orf32 | chromosome 21 open reading frame | -0.105 | 0.930 | 2.34E-01 | 2.34E+01 | | | |
| 8099029 | NM_024309 | TNIP2 | TNFAIP3 interacting protein 2 | -0.105 | 0.930 | 1.89E-01 | 1.89E+01 | | | |
| 8144448 | NM_018088 | FAM90A1 | family with sequence similarity 90, n | -0.105 | 0.930 | 3.05E-02 | 3.05E+00 | | | |
| 7898556 | NM_00104012 | PQLC2 | PQ loop repeat containing 2 | -0.105 | 0.930 | 4.65E-02 | 4.65E+00 | | | |
| 8049538 | NM_00113755 | LRRFIP1 | leucine rich repeat (in FLII) interacti | -0.105 | 0.930 | 1.78E-01 | 1.78E+01 | | | |
| 7913235 | NM_00110557 | PLA2G2C | phospholipase A2, group IIC | -0.105 | 0.930 | 1.11E-01 | 1.11E+01 | | | |
| 8074316 | NR_003267 | GGT3P | gamma-glutamyltransferase 3 pseud | -0.105 | 0.930 | 2.72E-02 | 2.72E+00 | | | |
| 8141648 | NM_015831 | ACHE | acetylcholinesterase (Yt blood group | -0.105 | 0.930 | 7.76E-02 | 7.76E+00 | | | |
| 7896632 | --- | --- | --- | -0.105 | 0.930 | 4.70E-01 | 4.70E+01 | | | |
| 7959025 | NM_00110990 | RNFT2 | ring finger protein, transmembrane | -0.106 | 0.929 | 7.86E-02 | 7.86E+00 | | | |
| 8000890 | NM_012248 | SEPHS2 | selenophosphate synthetase 2 | -0.106 | 0.929 | 5.72E-02 | 5.72E+00 | | | |
| 7907968 | --- | --- | --- | -0.106 | 0.929 | 2.04E-01 | 2.04E+01 | | | |
| 7930921 | NM_004281 | BAG3 | BCL2-associated athanogene 3 | -0.106 | 0.929 | 1.05E-01 | 1.05E+01 | | | |

| | | | | | | | | | | |
|---|---|---|---|---|---|---|---|---|---|---|
| 7897257 | NM_207396 | RNF207 | ring finger protein 207 | -0.106 | 0.929 | 2.34E-02 | 2.34E+00 | | | |
| 8005879 | NR_027384 | SLC13A2 | solute carrier family 13 (sodium-dep | -0.106 | 0.929 | 1.14E-01 | 1.14E+01 | | | |
| 8018731 | NM_024599 | RHBDF2 | rhomboid 5 homolog 2 (Drosophila) | -0.106 | 0.929 | 5.58E-02 | 5.58E+00 | | | |
| 8085244 | NM_022094 | CIDEC | cell death-inducing DFFA-like effecto | -0.106 | 0.929 | 1.45E-01 | 1.45E+01 | | | |
| 7976443 | NM_00113008 | IFI27 | interferon, alpha-inducible protein 2 | -0.106 | 0.929 | 3.45E-01 | 3.45E+01 | | | |
| 8046922 | NM_000090 | COL3A1 | collagen, type III, alpha 1 | -0.106 | 0.929 | 4.83E-02 | 4.83E+00 | | | |
| 8139367 | NM_013389 | NPC1L1 | NPC1 (Niemann-Pick disease, type C1 | -0.106 | 0.929 | 5.67E-02 | 5.67E+00 | | | |
| 8038069 | NM_018273 | TMEM143 | transmembrane protein 143 | -0.106 | 0.929 | 8.67E-02 | 8.67E+00 | | | |
| 8037422 | NM_00103174 | LYPD5 | LY6/PLAUR domain containing 5 | -0.106 | 0.929 | 1.21E-01 | 1.21E+01 | | | |
| 7912056 | NM_198681 | PLEKHG5 | pleckstrin homology domain contain | -0.106 | 0.929 | 6.69E-02 | 6.69E+00 | | | |
| 8034589 | NM_004461 | FARSA | phenylalanyl-tRNA synthetase, alpha | -0.106 | 0.929 | 4.17E-01 | 4.17E+01 | | | |
| 7942715 | NM_006189 | OMP | olfactory marker protein | -0.106 | 0.929 | 3.98E-02 | 3.98E+00 | | | |
| 8068136 | NM_181600 | KRTAP13-4 | keratin associated protein 13-4 | -0.106 | 0.929 | 1.87E-01 | 1.87E+01 | | | |
| 8072189 | NM_133455 | EMID1 | EMI domain containing 1 | -0.106 | 0.929 | 3.73E-02 | 3.73E+00 | | | |
| 8062681 | --- | --- | --- | -0.106 | 0.929 | 5.12E-01 | 5.12E+01 | | | |
| 8165538 | NM_00103311 | ENTPD8 | ectonucleoside triphosphate diphosp | -0.106 | 0.929 | 4.00E-02 | 4.00E+00 | | | |
| 8025382 | NM_024552 | LASS4 | LAG1 homolog, ceramide synthase 4 | -0.106 | 0.929 | 1.61E-01 | 1.61E+01 | | | |
| 8040774 | NM_021831 | AGBL5 | ATP/GTP binding protein-like 5 | -0.106 | 0.929 | 1.96E-01 | 1.96E+01 | | | |
| 8088642 | NM_015541 | LRIG1 | leucine-rich repeats and immunoglol | -0.106 | 0.929 | 6.13E-02 | 6.13E+00 | | | |
| 8164872 | NM_181491 | MED22 | mediator complex subunit 22 | -0.106 | 0.929 | 1.51E-01 | 1.51E+01 | | | |
| 8085475 | NM_004625 | WNT7A | wingless-type MMTV integration site | -0.106 | 0.929 | 7.91E-02 | 7.91E+00 | | | |
| 7960407 | NM_020996 | FGF6 | fibroblast growth factor 6 | -0.106 | 0.929 | 4.79E-02 | 4.79E+00 | | | |
| 7988050 | NM_201631 | TGM5 | transglutaminase 5 | -0.106 | 0.929 | 4.52E-02 | 4.52E+00 | | | |
| 7894632 | --- | --- | --- | -0.106 | 0.929 | 6.93E-01 | 6.93E+01 | | | |
| 8131705 | NR_026673 | RPL23P8 | ribosomal protein L23 pseudogene 8 | -0.106 | 0.929 | 4.20E-01 | 4.20E+01 | | | |
| 8066521 | NM_014477 | TP53TG5 | TP53 target 5 | -0.106 | 0.929 | 6.73E-02 | 6.73E+00 | | | |
| 7976425 | NM_023112 | OTUB2 | OTU domain, ubiquitin aldehyde binc | -0.106 | 0.929 | 1.27E-01 | 1.27E+01 | | | |
| 8136641 | NM_016943 | TAS2R3 | taste receptor, type 2, member 3 | -0.106 | 0.929 | 1.38E-01 | 1.38E+01 | | | |
| 7949863 | NM_005995 | TBX10 | T-box 10 | -0.106 | 0.929 | 4.73E-02 | 4.73E+00 | | | |
| 7944341 | NM_006760 | UPK2 | uroplakin 2 | -0.106 | 0.929 | 1.05E-01 | 1.05E+01 | | | |
| 8038571 | NM_138334 | JOSD2 | Josephin domain containing 2 | -0.106 | 0.929 | 4.37E-02 | 4.37E+00 | | | |
| 8133155 | NM_003596 | TPST1 | tyrosylprotein sulfotransferase 1 | -0.106 | 0.929 | 1.50E-01 | 1.50E+01 | | | |
| 8001587 | NM_005550 | KIFC3 | kinesin family member C3 | -0.106 | 0.929 | 4.82E-02 | 4.82E+00 | | | |
| 7895122 | --- | --- | --- | -0.106 | 0.929 | 6.79E-01 | 6.79E+01 | | | |
| 8108949 | NM_002700 | POU4F3 | POU class 4 homeobox 3 | -0.106 | 0.929 | 3.72E-02 | 3.72E+00 | | | |
| 8132523 | NM_015983 | UBE2D4 | ubiquitin-conjugating enzyme E2D 4 | -0.106 | 0.929 | 1.71E-01 | 1.71E+01 | | | |
| 8125870 | NM_00109372 | TCP11 | t-complex 11 homolog (mouse) | -0.106 | 0.929 | 5.11E-02 | 5.11E+00 | | | |
| 8129562 | NM_001901 | CTGF | connective tissue growth factor | -0.106 | 0.929 | 4.58E-02 | 4.58E+00 | | | |
| 8132092 | NM_006774 | INMT | indolethylamine N-methyltransferase | -0.106 | 0.929 | 8.99E-02 | 8.99E+00 | | | |
| 8156923 | NM_207299 | RP11-35N6.1 | plasticity related gene 3 | -0.106 | 0.929 | 7.47E-02 | 7.47E+00 | | | |
| 8114068 | NM_133456 | SHROOM1 | shroom family member 1 | -0.106 | 0.929 | 6.95E-02 | 6.95E+00 | | | |
| 7977567 | BC146951 | KLHL33 | kelch-like 33 (Drosophila) | -0.106 | 0.929 | 2.43E-01 | 2.43E+01 | | | |
| 7993458 | NM_033201 | C16orf45 | chromosome 16 open reading frame | -0.106 | 0.929 | 1.39E-01 | 1.39E+01 | | | |
| 7905951 | NM_025058 | TRIM46 | tripartite motif-containing 46 | -0.106 | 0.929 | 3.04E-02 | 3.04E+00 | | | |
| 8064175 | NM_020062 | SLC2A4RG | SLC2A4 regulator | -0.106 | 0.929 | 5.31E-02 | 5.31E+00 | | | |
| 7952350 | NM_018400 | SCN3B | sodium channel, voltage-gated, type | -0.106 | 0.929 | 1.35E-01 | 1.35E+01 | | | |
| 8139299 | NM_00112721 | POLD2 | polymerase (DNA directed), delta 2, | -0.106 | 0.929 | 4.18E-01 | 4.18E+01 | | | |
| 8110631 | NM_152547 | BTNL9 | butyrophilin-like 9 | -0.106 | 0.929 | 7.71E-02 | 7.71E+00 | | | |
| 8161938 | --- | --- | --- | -0.106 | 0.929 | 3.31E-01 | 3.31E+01 | | | |
| 8127024 | NM_052872 | IL17F | interleukin 17F | -0.106 | 0.929 | 9.72E-02 | 9.72E+00 | | | |
| 7917670 | --- | --- | --- | -0.106 | 0.929 | 1.05E-01 | 1.05E+01 | | | |
| 8074569 | NR_003267 // | // GGT3P // | gamma-glutamyltransferase 3 pseud | -0.106 | 0.929 | 5.30E-02 | 5.30E+00 | | | |
| 7915910 | NM_005764 | PDZK1IP1 | PDZK1 interacting protein 1 | -0.106 | 0.929 | 9.28E-02 | 9.28E+00 | | | |
| 8115168 | NM_018047 | RBM22 | RNA binding motif protein 22 | -0.106 | 0.929 | 7.47E-01 | 7.47E+01 | | | |
| 7966829 | NM_018639 | WSB2 | WD repeat and SOCS box-containing | -0.106 | 0.929 | 4.96E-01 | 4.96E+01 | | | |
| 8040827 | NM_175769 | TCF23 | transcription factor 23 | -0.106 | 0.929 | 4.79E-02 | 4.79E+00 | | | |
| 7893767 | --- | --- | --- | -0.106 | 0.929 | 4.77E-01 | 4.77E+01 | | | |
| 7940669 | NM_00108537 | C11orf83 | chromosome 11 open reading frame | -0.106 | 0.929 | 1.05E-01 | 1.05E+01 | | | |
| 8118726 | NR_027908 | C6orf227 | chromosome 6 open reading frame 2 | -0.106 | 0.929 | 3.04E-02 | 3.04E+00 | | | |
| 8149104 | NM_001926 | DEFA6 | defensin, alpha 6, Paneth cell-specifi | -0.106 | 0.929 | 1.45E-01 | 1.45E+01 | | | |
| 8073152 | NM_021096 | CACNA1I | calcium channel, voltage-dependent | -0.106 | 0.929 | 2.43E-02 | 2.43E+00 | | | |
| 7920552 | NM_002249 | KCNN3 | potassium intermediate/small condu | -0.106 | 0.929 | 7.09E-02 | 7.09E+00 | | | |
| 7947801 | NM_016223 | PACSIN3 | protein kinase C and casein kinase su | -0.106 | 0.929 | 3.63E-02 | 3.63E+00 | | | |
| 8107530 | --- | --- | --- | -0.106 | 0.929 | 2.01E-01 | 2.01E+01 | | | |
| 7969671 | --- | --- | --- | -0.106 | 0.929 | 1.85E-01 | 1.85E+01 | | | |
| 7910913 | --- | --- | --- | -0.106 | 0.929 | 7.32E-02 | 7.32E+00 | | | |
| 7967544 | NM_005505 | SCARB1 | scavenger receptor class B, member | -0.106 | 0.929 | 6.59E-02 | 6.59E+00 | | | |
| 8091078 | NM_002899 | RBP1 | retinol binding protein 1, cellular | -0.106 | 0.929 | 9.01E-02 | 9.01E+00 | | | |
| 7893661 | --- | --- | --- | -0.106 | 0.929 | 7.53E-02 | 7.53E+00 | | | |
| 8140367 | NM_002991 | CCL24 | chemokine (C-C motif) ligand 24 | -0.106 | 0.929 | 7.52E-02 | 7.52E+00 | | | |
| 8067754 | U63828 | C20orf181 | chromosome 20 open reading frame | -0.106 | 0.929 | 4.36E-02 | 4.36E+00 | | | |
| 8067602 | NM_152864 | NKAIN4 | Na+/K+ transporting ATPase interact | -0.106 | 0.929 | 9.93E-02 | 9.93E+00 | | | |
| 8030187 | NM_033043 | CGB5 | chorionic gonadotropin, beta polype | -0.106 | 0.929 | 3.99E-02 | 3.99E+00 | | | |
| 7895089 | --- | --- | --- | -0.106 | 0.929 | 8.42E-01 | 8.42E+01 | | | |
| 8180216 | --- | --- | --- | -0.106 | 0.929 | 1.65E-01 | 1.65E+01 | | | |
| 7946853 | NM_000525 | KCNJ11 | potassium inwardly-rectifying chann | -0.106 | 0.929 | 4.42E-02 | 4.42E+00 | | | |
| 7967358 | NM_020845 | PITPNM2 | phosphatidylinositol transfer protein | -0.107 | 0.929 | 7.70E-02 | 7.70E+00 | | | |

| | | | | | | | | | | |
|---|---|---|---|---|---|---|---|---|---|---|
| 8074473 | NM_00113086: | CLDN5 | claudin 5 | -0.107 | 0.929 | 5.24E-02 | 5.24E+00 | | | |
| 7949843 | NM_016366 | CABP2 | calcium binding protein 2 | -0.107 | 0.929 | 4.43E-02 | 4.43E+00 | | | |
| 8016261 | NM_005456 | MAPK8IP1 | mitogen-activated protein kinase 8 i | -0.107 | 0.929 | 9.37E-02 | 9.37E+00 | | | |
| 8126279 | NM_018965 | TREM2 | triggering receptor expressed on my | -0.107 | 0.929 | 8.47E-02 | 8.47E+00 | | | |
| 8035311 | ENST00000341 | TMEM221 | transmembrane protein 221 | -0.107 | 0.929 | 3.84E-02 | 3.84E+00 | | | |
| 8086530 | NM_015444 | TMEM158 | transmembrane protein 158 | -0.107 | 0.929 | 4.82E-02 | 4.82E+00 | | | |
| 7992302 | NM_020825 | CRAMP1L | Crm, cramped-like (Drosophila) | -0.107 | 0.929 | 2.98E-02 | 2.98E+00 | | | |
| 8107992 | NM_175873 | ANKRD43 | ankyrin repeat domain 43 | -0.107 | 0.929 | 5.57E-02 | 5.57E+00 | | | |
| 7911283 | NM_00100549! | OR2T3 | olfactory receptor, family 2, subfami | -0.107 | 0.929 | 1.13E-01 | 1.13E+01 | | | |
| 8034151 | NM_020812 | DOCK6 | dedicator of cytokinesis 6 | -0.107 | 0.929 | 5.74E-02 | 5.74E+00 | | | |
| 7940493 | NM_145017 | C11orf66 | chromosome 11 open reading frame | -0.107 | 0.929 | 4.79E-02 | 4.79E+00 | | | |
| 8073775 | NM_006486 | FBLN1 | fibulin 1 | -0.107 | 0.929 | 7.74E-02 | 7.74E+00 | | | |
| 8027285 | NM_182515 | ZNF714 | zinc finger protein 714 | -0.107 | 0.929 | 1.61E-01 | 1.61E+01 | | | |
| 8154620 | --- | --- | --- | -0.107 | 0.929 | 1.24E-01 | 1.24E+01 | | | |
| 8013833 | NM_178860 | SEZ6 | seizure related 6 homolog (mouse) | -0.107 | 0.929 | 6.51E-02 | 6.51E+00 | | | |
| 8084478 | BC037299 | FAM131A | family with sequence similarity 131, | -0.107 | 0.929 | 5.59E-02 | 5.59E+00 | | | |
| 8001048 | NM_003041 | SLC5A2 | solute carrier family 5 (sodium/gluco | -0.107 | 0.929 | 1.58E-01 | 1.58E+01 | | | |
| 8094101 | NM_00101444 | CPZ | carboxypeptidase Z | -0.107 | 0.929 | 7.07E-02 | 7.07E+00 | | | |
| 8047952 | NM_182587 | UNC80 | unc-80 homolog (C. elegans) | -0.107 | 0.929 | 7.74E-02 | 7.74E+00 | | | |
| 8012344 | NM_032580 | HES7 | hairy and enhancer of split 7 (Drosop | -0.107 | 0.929 | 8.08E-02 | 8.08E+00 | | | |
| 8171216 | NM_016379 | VCX3A | variable charge, X-linked 3A | -0.107 | 0.929 | 4.43E-02 | 4.43E+00 | | | |
| 8013294 | NM_00103939 | TBC1D28 | TBC1 domain family, member 28 | -0.107 | 0.929 | 8.83E-02 | 8.83E+00 | | | |
| 7931665 | NM_033178 | DUX4 | double homeobox, 4 | -0.107 | 0.929 | 3.15E-02 | 3.15E+00 | | | |
| 7896167 | --- | --- | --- | -0.107 | 0.929 | 6.32E-01 | 6.32E+01 | | | |
| 7895736 | --- | --- | --- | -0.107 | 0.929 | 1.53E-01 | 1.53E+01 | | | |
| 7911720 | NM_153818 | PEX10 | peroxisomal biogenesis factor 10 | -0.107 | 0.929 | 4.90E-02 | 4.90E+00 | | | |
| 7922402 | NR_002746 | SNORD47 | small nucleolar RNA, C/D box 47 | -0.107 | 0.929 | 8.93E-01 | 8.93E+01 | | | |
| 7895121 | --- | --- | --- | -0.107 | 0.929 | 5.54E-01 | 5.54E+01 | | | |
| 7893610 | --- | --- | --- | -0.107 | 0.929 | 3.63E-01 | 3.63E+01 | | | |
| 7922525 | NM_004319 | ASTN1 | astrotactin 1 | -0.107 | 0.929 | 3.58E-02 | 3.58E+00 | | | |
| 8018169 | NM_174892 | CD300LB | CD300 molecule-like family member | -0.107 | 0.929 | 9.24E-02 | 9.24E+00 | | | |
| 8071420 | NM_000185 | SERPIND1 | serpin peptidase inhibitor, clade D (h | -0.107 | 0.929 | 1.62E-01 | 1.62E+01 | | | |
| 8040290 | --- | --- | --- | -0.107 | 0.929 | 1.02E-01 | 1.02E+01 | | | |
| 7905677 | NM_130898 | CREB3L4 | cAMP responsive element binding pr | -0.107 | 0.929 | 1.00E-01 | 1.00E+01 | | | |
| 8093171 | NM_005929 | MFI2 | antigen p97 (melanoma associated) i | -0.107 | 0.929 | 3.91E-02 | 3.91E+00 | | | |
| 7949808 | NM_004910 | PITPNM1 | phosphatidylinositol transfer protein | -0.107 | 0.929 | 7.16E-02 | 7.16E+00 | | | |
| 8071357 | AB051440 | LOC440792 | proline dehydrogenase (oxidase) 1 p | -0.107 | 0.928 | 1.12E-01 | 1.12E+01 | | | |
| 8153342 | NM_205545 | LYPD2 | LY6/PLAUR domain containing 2 | -0.107 | 0.928 | 5.55E-02 | 5.55E+00 | | | |
| 8128549 | --- | --- | --- | -0.107 | 0.928 | 4.48E-01 | 4.48E+01 | | | |
| 7931417 | NM_00110552 | JAKMIP3 | Janus kinase and microtubule interac | -0.107 | 0.928 | 1.72E-02 | 1.72E+00 | | | |
| 7933075 | NM_031866 | FZD8 | frizzled homolog 8 (Drosophila) | -0.107 | 0.928 | 1.16E-01 | 1.16E+01 | | | |
| 8053467 | NM_000542 | SFTPB | surfactant protein B | -0.107 | 0.928 | 3.58E-02 | 3.58E+00 | | | |
| 8141560 | NM_003227 | TFR2 | transferrin receptor 2 | -0.107 | 0.928 | 8.60E-02 | 8.60E+00 | | | |
| 8116696 | NM_00108540 | C6orf201 | chromosome 6 open reading frame 2 | -0.107 | 0.928 | 8.81E-02 | 8.81E+00 | | | |
| 8155501 | --- | --- | --- | -0.107 | 0.928 | 1.65E-01 | 1.65E+01 | | | |
| 8029107 | NM_001815 | CEACAM3 | carcinoembryonic antigen-related ce | -0.107 | 0.928 | 3.32E-02 | 3.32E+00 | | | |
| 7940020 | --- | --- | --- | -0.107 | 0.928 | 1.26E-01 | 1.26E+01 | | | |
| 8024637 | NM_00113619 | FZR1 | fizzy/cell division cycle 20 related 1 ( | -0.107 | 0.928 | 6.86E-02 | 6.86E+00 | | | |
| 7991939 | NM_145294 | WDR90 | WD repeat domain 90 | -0.107 | 0.928 | 2.15E-02 | 2.15E+00 | | | |
| 8055247 | --- | --- | --- | -0.107 | 0.928 | 1.69E-01 | 1.69E+01 | | | |
| 8099304 | NM_003501 | ACOX3 | acyl-Coenzyme A oxidase 3, pristano | -0.107 | 0.928 | 8.96E-02 | 8.96E+00 | | | |
| 8005313 | NM_001388 | DRG2 | developmentally regulated GTP bind | -0.107 | 0.928 | 1.85E-01 | 1.85E+01 | | | |
| 8158431 | NM_00110087 | PHYHD1 | phytanoyl-CoA dioxygenase domain | -0.107 | 0.928 | 9.53E-02 | 9.53E+00 | | | |
| 7927367 | NM_00104008 | ANXA8 | annexin A8 | -0.107 | 0.928 | 2.52E-02 | 2.52E+00 | | | |
| 8137118 | NR_015357 | ZNF783 | zinc finger family member 783 | -0.107 | 0.928 | 1.02E-01 | 1.02E+01 | | | |
| 8107671 | --- | --- | --- | -0.107 | 0.928 | 1.42E-01 | 1.42E+01 | | | |
| 7973510 | NM_006032 | CPNE6 | copine VI (neuronal) | -0.107 | 0.928 | 5.65E-02 | 5.65E+00 | | | |
| 8059900 | --- | --- | --- | -0.107 | 0.928 | 1.07E-01 | 1.07E+01 | | | |
| 8008263 | NM_002611 | PDK2 | pyruvate dehydrogenase kinase, isoz | -0.107 | 0.928 | 2.46E-01 | 2.46E+01 | | | |
| 8077082 | NM_033200 | LMF2 | lipase maturation factor 2 | -0.107 | 0.928 | 3.97E-02 | 3.97E+00 | | | |
| 8173208 | NM_00101296 | SPIN4 | spindlin family, member 4 | -0.107 | 0.928 | 9.19E-02 | 9.19E+00 | | | |
| 8143610 | NM_005435 | ARHGEF5 | Rho guanine nucleotide exchange fa | -0.108 | 0.928 | 6.30E-02 | 6.30E+00 | | | |
| 8138770 | NM_000522 | HOXA13 | homeobox A13 | -0.108 | 0.928 | 5.48E-02 | 5.48E+00 | | | |
| 8077777 | --- | --- | --- | -0.108 | 0.928 | 6.85E-01 | 6.85E+01 | | | |
| 8055194 | NM_207312 | TUBA3E | tubulin, alpha 3e | -0.108 | 0.928 | 3.59E-01 | 3.59E+01 | | | |
| 7896961 | NM_031921 | ATAD3B | ATPase family, AAA domain containi | -0.108 | 0.928 | 5.34E-02 | 5.34E+00 | | | |
| 8059158 | NM_024506 | GLB1L | galactosidase, beta 1-like | -0.108 | 0.928 | 8.44E-02 | 8.44E+00 | | | |
| 8092348 | NM_014398 | LAMP3 | lysosomal-associated membrane pro | -0.108 | 0.928 | 1.27E-01 | 1.27E+01 | | | |
| 7954012 | NM_058169 | LOH12CR1 | loss of heterozygosity, 12, chromoso | -0.108 | 0.928 | 1.30E-01 | 1.30E+01 | | | |
| 8019149 | NM_00103798 | SLC38A10 | solute carrier family 38, member 10 | -0.108 | 0.928 | 6.28E-02 | 6.28E+00 | | | |
| 8144428 | NM_018088 | FAM90A1 | family with sequence similarity 90, n | -0.108 | 0.928 | 2.23E-02 | 2.23E+00 | | | |
| 8144432 | NM_018088 | FAM90A1 | family with sequence similarity 90, n | -0.108 | 0.928 | 2.23E-02 | 2.23E+00 | | | |
| 8144436 | NM_018088 | FAM90A1 | family with sequence similarity 90, n | -0.108 | 0.928 | 2.23E-02 | 2.23E+00 | | | |
| 8144444 | NM_018088 | FAM90A1 | family with sequence similarity 90, n | -0.108 | 0.928 | 2.23E-02 | 2.23E+00 | | | |
| 7998902 | NM_017885 | HCFC1R1 | host cell factor C1 regulator 1 (XPO1 | -0.108 | 0.928 | 3.00E-02 | 3.00E+00 | | | |
| 8068496 | NM_009586 | SIM2 | single-minded homolog 2 (Drosophil | -0.108 | 0.928 | 4.55E-02 | 4.55E+00 | | | |
| 7910377 | --- | --- | --- | -0.108 | 0.928 | 6.49E-01 | 6.49E+01 | | | |

| | | | | | | | | | | |
|---|---|---|---|---|---|---|---|---|---|---|
| 7946245 | NM_003737 | DCHS1 | dachsous 1 (Drosophila) | -0.108 | 0.928 | 4.72E-02 | 4.72E+00 | | | |
| 7941302 | NM_00109878 | FAM89B | family with sequence similarity 89, m | -0.108 | 0.928 | 5.80E-02 | 5.80E+00 | | | |
| 8179814 | NM_025261 | LY6G6C | lymphocyte antigen 6 complex, locus | -0.108 | 0.928 | 9.62E-02 | 9.62E+00 | | | |
| 8035465 | NM_016368 | ISYNA1 | inositol-3-phosphate synthase 1 | -0.108 | 0.928 | 9.39E-02 | 9.39E+00 | | | |
| 8021357 | NM_004852 | ONECUT2 | one cut homeobox 2 | -0.108 | 0.928 | 2.67E-02 | 2.67E+00 | | | |
| 8046500 | --- | --- | --- | -0.108 | 0.928 | 9.56E-02 | 9.56E+00 | | | |
| 7905881 | NM_207196 | ADAM15 | ADAM metallopeptidase domain 15 | -0.108 | 0.928 | 1.10E-01 | 1.10E+01 | | | |
| 8030181 | NM_033378 | CGB2 | chorionic gonadotropin, beta polype | -0.108 | 0.928 | 2.15E-02 | 2.15E+00 | | | |
| 8109901 | NM_012188 | FOXI1 | forkhead box I1 | -0.108 | 0.928 | 7.66E-02 | 7.66E+00 | | | |
| 8052370 | --- | --- | --- | -0.108 | 0.928 | 1.90E-01 | 1.90E+01 | | | |
| 8137639 | NM_018051 | WDR60 | WD repeat domain 60 | -0.108 | 0.928 | 5.03E-01 | 5.03E+01 | | | |
| 8146645 | NM_152414 | BHLHE22 | basic helix-loop-helix family, membe | -0.108 | 0.928 | 1.85E-02 | 1.85E+00 | | | |
| 7984364 | NM_005902 | SMAD3 | SMAD family member 3 | -0.108 | 0.928 | 3.34E-01 | 3.34E+01 | | | |
| 7937518 | NM_00102523 | TSPAN4 | tetraspanin 4 | -0.108 | 0.928 | 3.19E-02 | 3.19E+00 | | | |
| 8049137 | NM_001631 | ALPI | alkaline phosphatase, intestinal | -0.108 | 0.928 | 3.13E-02 | 3.13E+00 | | | |
| 8144424 | NM_018088 | FAM90A1 | family with sequence similarity 90, m | -0.108 | 0.928 | 3.02E-02 | 3.02E+00 | | | |
| 8144440 | NM_018088 | FAM90A1 | family with sequence similarity 90, m | -0.108 | 0.928 | 3.02E-02 | 3.02E+00 | | | |
| 8011924 | NM_031220 | PITPNM3 | PITPNM family member 3 | -0.108 | 0.928 | 5.01E-02 | 5.01E+00 | | | |
| 8073573 | NM_00100203 | FAM109B | family with sequence similarity 109, | -0.108 | 0.928 | 4.50E-02 | 4.50E+00 | | | |
| 8002209 | --- | --- | --- | -0.108 | 0.928 | 1.62E-01 | 1.62E+01 | | | |
| 8075657 | --- | --- | --- | -0.108 | 0.928 | 2.07E-01 | 2.07E+01 | | | |
| 8066489 | NM_080869 | WFDC12 | WAP four-disulfide core domain 12 | -0.108 | 0.928 | 1.52E-01 | 1.52E+01 | | | |
| 7991860 | NM_014700 | RAB11FIP3 | RAB11 family interacting protein 3 (c | -0.108 | 0.928 | 7.53E-02 | 7.53E+00 | | | |
| 8118804 | NM_020804 | PACSIN1 | protein kinase C and casein kinase su | -0.108 | 0.928 | 3.46E-02 | 3.46E+00 | | | |
| 8097507 | NM_031296 | RAB33B | RAB33B, member RAS oncogene fam | -0.108 | 0.928 | 2.86E-01 | 2.86E+01 | | | |
| 8067662 | NM_005975 | PTK6 | PTK6 protein tyrosine kinase 6 | -0.108 | 0.928 | 4.30E-02 | 4.30E+00 | | | |
| 7952927 | NM_173593 | B4GALNT3 | beta-1,4-N-acetyl-galactosaminyl tra | -0.108 | 0.928 | 6.52E-02 | 6.52E+00 | | | |
| 7898124 | NM_052929 | FHAD1 | forkhead-associated (FHA) phosphop | -0.108 | 0.928 | 3.29E-02 | 3.29E+00 | | | |
| 7895360 | --- | --- | --- | -0.108 | 0.928 | 7.67E-01 | 7.67E+01 | | | |
| 7898533 | NM_152375 | KLHDC7A | kelch domain containing 7A | -0.108 | 0.928 | 7.21E-02 | 7.21E+00 | | | |
| 7911047 | --- | --- | --- | -0.108 | 0.928 | 1.47E-01 | 1.47E+01 | | | |
| 7912316 | NM_00107984 | CASZ1 | castor zinc finger 1 | -0.108 | 0.928 | 3.20E-02 | 3.20E+00 | | | |
| 8155510 | NR_024060 | FAM27A | family with sequence similarity 27, m | -0.108 | 0.928 | 4.60E-02 | 4.60E+00 | | | |
| 7973336 | NM_004995 | MMP14 | matrix metallopeptidase 14 (membra | -0.108 | 0.928 | 1.18E-01 | 1.18E+01 | | | |
| 8015016 | NM_032865 | TNS4 | tensin 4 | -0.108 | 0.928 | 6.07E-02 | 6.07E+00 | | | |
| 7915743 | NM_00111493 | CCDC17 | coiled-coil domain containing 17 | -0.108 | 0.928 | 9.30E-02 | 9.30E+00 | | | |
| 8037742 | NM_005091 | PGLYRP1 | peptidoglycan recognition protein 1 | -0.108 | 0.928 | 4.01E-02 | 4.01E+00 | | | |
| 8142814 | NM_001708 | OPN1SW | opsin 1 (cone pigments), short-wave | -0.108 | 0.928 | 2.49E-01 | 2.49E+01 | | | |
| 8148902 | NM_005309 | GPT | glutamic-pyruvate transaminase (ala | -0.108 | 0.928 | 7.33E-02 | 7.33E+00 | | | |
| 7962930 | NM_015086 | DDN | dendrin | -0.108 | 0.928 | 6.33E-02 | 6.33E+00 | | | |
| 7995332 | --- | --- | --- | -0.108 | 0.928 | 2.07E-01 | 2.07E+01 | | | |
| 8069156 | NM_198692 | KRTAP10-11 | keratin associated protein 10-11 | -0.108 | 0.928 | 8.84E-02 | 8.84E+00 | | | |
| 8065344 | NM_021784 | FOXA2 | forkhead box A2 | -0.108 | 0.928 | 7.18E-02 | 7.18E+00 | | | |
| 7906085 | NM_170707 | LMNA | lamin A/C | -0.108 | 0.928 | 4.14E-02 | 4.14E+00 | | | |
| 7915297 | NM_001852 | COL9A2 | collagen, type IX, alpha 2 | -0.108 | 0.928 | 3.46E-02 | 3.46E+00 | | | |
| 8013574 | AK097823 | FLJ40504 | keratin 18 pseudogene | -0.108 | 0.928 | 4.56E-02 | 4.56E+00 | | | |
| 8034315 | NM_00108049 | ZNF823 | zinc finger protein 823 | -0.108 | 0.928 | 1.94E-01 | 1.94E+01 | | | |
| 8108359 | NM_016606 | REEP2 | receptor accessory protein 2 | -0.108 | 0.928 | 7.28E-02 | 7.28E+00 | | | |
| 7929616 | NM_005479 | FRAT1 | frequently rearranged in advanced T | -0.108 | 0.928 | 5.61E-01 | 5.61E+01 | | | |
| 7980425 | NM_199296 | ISM2 | isthmin 2 homolog (zebrafish) | -0.108 | 0.928 | 3.57E-02 | 3.57E+00 | | | |
| 8118794 | NM_145899 | HMGA1 | high mobility group AT-hook 1 | -0.108 | 0.928 | 1.12E-01 | 1.12E+01 | | | |
| 8012226 | NM_004860 | FXR2 | fragile X mental retardation, autosor | -0.108 | 0.928 | 3.42E-02 | 3.42E+00 | | | |
| 8125788 | NM_181336 | LEMD2 | LEM domain containing 2 | -0.109 | 0.928 | 2.75E-02 | 2.75E+00 | | | |
| 8059028 | NM_194302 | CCDC108 | coiled-coil domain containing 108 | -0.109 | 0.928 | 2.24E-02 | 2.24E+00 | | | |
| 8045804 | --- | --- | --- | -0.109 | 0.928 | 8.17E-01 | 8.17E+01 | | | |
| 7993726 | NM_017888 | ACSM5 | acyl-CoA synthetase medium-chain f | -0.109 | 0.928 | 5.45E-02 | 5.45E+00 | | | |
| 8094028 | NM_198595 | AFAP1 | actin filament associated protein 1 | -0.109 | 0.928 | 1.13E-01 | 1.13E+01 | | | |
| 8062844 | NM_024331 | TTPAL | tocopherol (alpha) transfer protein-l | -0.109 | 0.928 | 3.54E-01 | 3.54E+01 | | | |
| 8030706 | NM_175063 | C19orf63 | chromosome 19 open reading frame | -0.109 | 0.928 | 5.17E-02 | 5.17E+00 | | | |
| 7892509 | --- | --- | --- | -0.109 | 0.928 | 8.34E-01 | 8.34E+01 | | | |
| 8130891 | NM_182552 | WDR27 | WD repeat domain 27 | -0.109 | 0.927 | 2.19E-01 | 2.19E+01 | | | |
| 7995772 | NM_032935 | MT4 | metallothionein 4 | -0.109 | 0.927 | 2.00E-01 | 2.00E+01 | | | |
| 8115941 | NM_052899 | GPRIN1 | G protein regulated inducer of neuri | -0.109 | 0.927 | 3.98E-02 | 3.98E+00 | | | |
| 7914350 | NM_178865 | SERINC2 | serine incorporator 2 | -0.109 | 0.927 | 1.32E-01 | 1.32E+01 | | | |
| 8009929 | NM_00116299 | LOC643008 | hypothetical protein LOC643008 | -0.109 | 0.927 | 1.49E-01 | 1.49E+01 | | | |
| 7997542 | NM_019065 | NECAB2 | N-terminal EF-hand calcium binding | -0.109 | 0.927 | 2.48E-02 | 2.48E+00 | | | |
| 8162082 | --- | --- | --- | -0.109 | 0.927 | 2.28E-01 | 2.28E+01 | | | |
| 8153304 | NM_145003 | TSNARE1 | t-SNARE domain containing 1 | -0.109 | 0.927 | 6.88E-02 | 6.88E+00 | | | |
| 8010137 | NR_003013 | SCARNA16 | small Cajal body-specific RNA 16 | -0.109 | 0.927 | 4.89E-01 | 4.89E+01 | | | |
| 8116168 | NM_017838 | NHP2 | NHP2 ribonucleoprotein homolog (ye | -0.109 | 0.927 | 4.93E-01 | 4.93E+01 | | | |
| 7894413 | --- | --- | --- | -0.109 | 0.927 | 4.85E-01 | 4.85E+01 | | | |
| 7920971 | NM_144580 | C1orf85 | chromosome 1 open reading frame 8 | -0.109 | 0.927 | 1.04E-01 | 1.04E+01 | | | |
| 8028750 | NM_203471 | LGALS14 | lectin, galactoside-binding, soluble, 1 | -0.109 | 0.927 | 1.22E-01 | 1.22E+01 | | | |
| 8056728 | --- | --- | --- | -0.109 | 0.927 | 6.10E-01 | 6.10E+01 | | | |
| 7955297 | NM_001651 | AQP5 | aquaporin 5 | -0.109 | 0.927 | 3.58E-02 | 3.58E+00 | | | |
| 8065510 | NM_152667 | NANP | N-acetylneuraminic acid phosphatas | -0.109 | 0.927 | 1.12E-01 | 1.12E+01 | | | |
| 8053004 | NM_032319 | C2orf7 | chromosome 2 open reading frame 7 | -0.109 | 0.927 | 2.75E-01 | 2.75E+01 | | | |

| | | | | | | | | | | |
|---|---|---|---|---|---|---|---|---|---|---|
| 7987650 | NM_002344 | LTK | leukocyte receptor tyrosine kinase | -0.109 | 0.927 | 4.24E-02 | 4.24E+00 | | | |
| 8165083 | NM_178138 | LHX3 | LIM homeobox 3 | -0.109 | 0.927 | 4.47E-02 | 4.47E+00 | | | |
| 7996219 | NM_020465 | NDRG4 | NDRG family member 4 | -0.109 | 0.927 | 2.33E-02 | 2.33E+00 | | | |
| 8158666 | AK128680 | HMCN2 | hemicentin 2 | -0.109 | 0.927 | 8.77E-02 | 8.77E+00 | | | |
| 8180197 | --- | --- | --- | -0.109 | 0.927 | 8.01E-02 | 8.01E+00 | | | |
| 7940643 | NM_00108392( | ASRGL1 | asparaginase like 1 | -0.109 | 0.927 | 4.93E-02 | 4.93E+00 | | | |
| 8046373 | NM_178120 | DLX1 | distal-less homeobox 1 | -0.109 | 0.927 | 5.42E-02 | 5.42E+00 | | | |
| 7958749 | NM_005475 | SH2B3 | SH2B adaptor protein 3 | -0.109 | 0.927 | 2.85E-02 | 2.85E+00 | | | |
| 7922162 | NM_006996 | SLC19A2 | solute carrier family 19 (thiamine tra | -0.109 | 0.927 | 2.09E-01 | 2.09E+01 | | | |
| 8028930 | NM_016154 | RAB4B | RAB4B, member RAS oncogene famil | -0.109 | 0.927 | 8.47E-02 | 8.47E+00 | | | |
| 7897691 | NM_020780 | PTCHD2 | patched domain containing 2 | -0.109 | 0.927 | 5.51E-02 | 5.51E+00 | | | |
| 8032761 | NM_030662 | MAP2K2 | mitogen-activated protein kinase kin | -0.109 | 0.927 | 1.06E-01 | 1.06E+01 | | | |
| 7931914 | NM_000417 | IL2RA | interleukin 2 receptor, alpha | -0.109 | 0.927 | 1.72E-01 | 1.72E+01 | | | |
| 7911699 | AK024162 | FLJ14100 | hypothetical protein FLJ14100 | -0.109 | 0.927 | 2.47E-01 | 2.47E+01 | | | |
| 7948718 | NM_153265 | EML3 | echinoderm microtubule associated | -0.109 | 0.927 | 1.15E-01 | 1.15E+01 | | | |
| 7894119 | --- | --- | --- | -0.109 | 0.927 | 5.60E-01 | 5.60E+01 | | | |
| 7990654 | --- | --- | --- | -0.109 | 0.927 | 3.37E-01 | 3.37E+01 | | | |
| 7983290 | NM_00101810( | SERF2 | small EDRK-rich factor 2 | -0.109 | 0.927 | 2.61E-01 | 2.61E+01 | | | |
| 8050702 | NM_004881 | TP53I3 | tumor protein p53 inducible protein | -0.109 | 0.927 | 2.63E-01 | 2.63E+01 | | | |
| 8164496 | NM_004059 | CCBL1 | cysteine conjugate-beta lyase, cytop | -0.109 | 0.927 | 5.24E-02 | 5.24E+00 | | | |
| 7898030 | NM_00109985 | PRAMEF20 | PRAME family member 20 | -0.109 | 0.927 | 1.55E-01 | 1.55E+01 | | | |
| 8138602 | NM_004403 | DFNA5 | deafness, autosomal dominant 5 | -0.109 | 0.927 | 6.39E-02 | 6.39E+00 | | | |
| 8053142 | NM_033046 | RTKN | rhotekin | -0.109 | 0.927 | 2.42E-02 | 2.42E+00 | | | |
| 7969772 | --- | --- | --- | -0.109 | 0.927 | 2.06E-01 | 2.06E+01 | | | |
| 8121739 | --- | --- | --- | -0.109 | 0.927 | 2.06E-02 | 2.06E+00 | | | |
| 8045332 | --- | --- | --- | -0.109 | 0.927 | 2.25E-01 | 2.25E+01 | | | |
| 7899455 | NM_00104818 | PHACTR4 | phosphatase and actin regulator 4 | -0.109 | 0.927 | 8.44E-01 | 8.44E+01 | | | |
| 8149204 | NM_018088 | FAM90A1 | family with sequence similarity 90, m | -0.109 | 0.927 | 2.59E-02 | 2.59E+00 | | | |
| 7901969 | NM_005012 | ROR1 | receptor tyrosine kinase-like orphan | -0.109 | 0.927 | 3.99E-02 | 3.99E+00 | | | |
| 8085122 | AB024705 | C3orf32 | chromosome 3 open reading frame 3 | -0.109 | 0.927 | 3.09E-02 | 3.09E+00 | | | |
| 8029939 | NM_015711 | GLTSCR1 | glioma tumor suppressor candidate r | -0.109 | 0.927 | 4.58E-02 | 4.58E+00 | | | |
| 7895390 | --- | --- | --- | -0.109 | 0.927 | 4.74E-01 | 4.74E+01 | | | |
| 8075529 | NM_014338 | PISD | phosphatidylserine decarboxylase | -0.109 | 0.927 | 1.40E-01 | 1.40E+01 | | | |
| 7994985 | NM_014712 | SETD1A | SET domain containing 1A | -0.109 | 0.927 | 3.74E-02 | 3.74E+00 | | | |
| 7959500 | NM_003959 | HIP1R | huntingtin interacting protein 1 relat | -0.109 | 0.927 | 8.05E-02 | 8.05E+00 | | | |
| 8037640 | --- | --- | --- | -0.109 | 0.927 | 5.85E-02 | 5.85E+00 | | | |
| 8031466 | NM_032701 | SUV420H2 | suppressor of variegation 4-20 homo | -0.109 | 0.927 | 7.02E-02 | 7.02E+00 | | | |
| 8087852 | NM_017442 | TLR9 | toll-like receptor 9 | -0.109 | 0.927 | 8.03E-02 | 8.03E+00 | | | |
| 8165345 | NM_001606 | ABCA2 | ATP-binding cassette, sub-family A (A | -0.109 | 0.927 | 3.45E-02 | 3.45E+00 | | | |
| 8032829 | NM_00108040( | PLIN4 | perilipin 4 | -0.109 | 0.927 | 4.82E-02 | 4.82E+00 | | | |
| 8060294 | NM_005018 | PDCD1 | programmed cell death 1 | -0.109 | 0.927 | 3.84E-02 | 3.84E+00 | | | |
| 7945232 | NM_139055 | ADAMTS15 | ADAM metallopeptidase with throm | -0.109 | 0.927 | 7.68E-02 | 7.68E+00 | | | |
| 8084012 | --- | --- | --- | -0.109 | 0.927 | 1.49E-01 | 1.49E+01 | | | |
| 7903676 | NM_153340 | ATXN7L2 | ataxin 7-like 2 | -0.109 | 0.927 | 4.65E-02 | 4.65E+00 | | | |
| 8014035 | --- | --- | --- | -0.109 | 0.927 | 2.87E-01 | 2.87E+01 | | | |
| 8026298 | --- | --- | --- | -0.109 | 0.927 | 2.87E-01 | 2.87E+01 | | | |
| 7967021 | BC052611 | PXN | paxillin | -0.109 | 0.927 | 5.92E-02 | 5.92E+00 | | | |
| 7957417 | NM_152588 | TMTC2 | transmembrane and tetratricopeptid | -0.109 | 0.927 | 2.16E-01 | 2.16E+01 | | | |
| 8175455 | --- | --- | --- | -0.109 | 0.927 | 1.27E-01 | 1.27E+01 | | | |
| 7942118 | NM_138768 | MYEOV | myeloma overexpressed (in a subset | -0.109 | 0.927 | 1.18E-01 | 1.18E+01 | | | |
| 7984819 | NM_025055 | CCDC33 | coiled-coil domain containing 33 | -0.109 | 0.927 | 2.66E-02 | 2.66E+00 | | | |
| 8064382 | AK125343 | SRXN1 | sulfiredoxin 1 homolog (S. cerevisiae | -0.109 | 0.927 | 1.28E-01 | 1.28E+01 | | | |
| 8038505 | NM_052884 | SIGLEC11 | sialic acid binding Ig-like lectin 11 | -0.109 | 0.927 | 9.24E-02 | 9.24E+00 | | | |
| 7998265 | AK097604 | OC10013028 | hypothetical LOC100130285 | -0.109 | 0.927 | 6.76E-02 | 6.76E+00 | | | |
| 7995348 | --- | --- | --- | -0.109 | 0.927 | 2.09E-01 | 2.09E+01 | | | |
| 8062890 | NM_00112475( | PABPC1L | poly(A) binding protein, cytoplasmic | -0.109 | 0.927 | 8.63E-02 | 8.63E+00 | | | |
| 8175802 | NM_080701 | TREX2 | three prime repair exonuclease 2 | -0.109 | 0.927 | 6.30E-02 | 6.30E+00 | | | |
| 8012326 | NM_021628 | ALOXE3 | arachidonate lipoxygenase 3 | -0.109 | 0.927 | 6.91E-02 | 6.91E+00 | | | |
| 7963774 | NM_015481 | ZNF385A | zinc finger protein 385A | -0.109 | 0.927 | 3.03E-02 | 3.03E+00 | | | |
| 8076387 | NM_052945 | TNFRSF13C | tumor necrosis factor receptor super | -0.109 | 0.927 | 7.46E-02 | 7.46E+00 | | | |
| 7911371 | NR_027693 | C1orf170 | chromosome 1 open reading frame 1 | -0.109 | 0.927 | 5.20E-02 | 5.20E+00 | | | |
| 8168373 | --- | --- | --- | -0.109 | 0.927 | 2.17E-01 | 2.17E+01 | | | |
| 7982497 | NM_012125 | CHRM5 | cholinergic receptor, muscarinic 5 | -0.109 | 0.927 | 1.44E-01 | 1.44E+01 | | | |
| 7928838 | NM_007078 | LDB3 | LIM domain binding 3 | -0.109 | 0.927 | 5.96E-02 | 5.96E+00 | | | |
| 8160889 | NM_002989 | CCL21 | chemokine (C-C motif) ligand 21 | -0.110 | 0.927 | 4.38E-02 | 4.38E+00 | | | |
| 8034827 | NM_005716 | GIPC1 | GIPC PDZ domain containing family, | -0.110 | 0.927 | 4.18E-02 | 4.18E+00 | | | |
| 7954036 | NM_016355 | DDX47 | DEAD (Asp-Glu-Ala-Asp) box polypep | -0.110 | 0.927 | 5.92E-01 | 5.92E+01 | | | |
| 8174761 | NR_027131 | CXorf42 | chromosome X open reading frame 4 | -0.110 | 0.927 | 2.09E-01 | 2.09E+01 | | | |
| 8106976 | NM_199243 | GPR150 | G protein-coupled receptor 150 | -0.110 | 0.927 | 3.60E-02 | 3.60E+00 | | | |
| 8065683 | NM_003098 | SNTA1 | syntrophin, alpha 1 (dystrophin-asso | -0.110 | 0.927 | 3.18E-02 | 3.18E+00 | | | |
| 8032371 | NM_031213 | FAM108A1 | family with sequence similarity 108, | -0.110 | 0.927 | 1.76E-01 | 1.76E+01 | | | |
| 8158554 | NM_016307 | PRRX2 | paired related homeobox 2 | -0.110 | 0.927 | 3.66E-02 | 3.66E+00 | | | |
| 7967792 | --- | --- | --- | -0.110 | 0.927 | 4.66E-01 | 4.66E+01 | | | |
| 8025563 | NM_00104066 | PPAN-P2RY1 | PPAN-P2RY11 readthrough transcrip | -0.110 | 0.927 | 2.99E-02 | 2.99E+00 | | | |
| 8070632 | NM_000071 | CBS | cystathionine-beta-synthase | -0.110 | 0.927 | 2.52E-02 | 2.52E+00 | | | |
| 7990873 | NM_032246 | MEX3B | mex-3 homolog B (C. elegans) | -0.110 | 0.927 | 2.44E-02 | 2.44E+00 | | | |
| 7927482 | NM_00114293 | CHAT | choline acetyltransferase | -0.110 | 0.927 | 4.61E-02 | 4.61E+00 | | | |

| | | | | | | | | | | |
|---|---|---|---|---|---|---|---|---|---|---|
| 8155537 | --- | --- | --- | -0.110 | 0.927 | 1.48E-01 | 1.48E+01 | | | |
| 8060796 | BC035800 | C20orf196 | chromosome 20 open reading frame | -0.110 | 0.927 | 2.34E-01 | 2.34E+01 | | | |
| 7926659 | --- | --- | --- | -0.110 | 0.927 | 2.36E-01 | 2.36E+01 | | | |
| 8051589 | NM_022374 | ATL2 | atlastin GTPase 2 | -0.110 | 0.927 | 7.42E-01 | 7.42E+01 | | | |
| 8076497 | NM_017436 | A4GALT | alpha 1,4-galactosyltransferase | -0.110 | 0.927 | 1.67E-02 | 1.67E+00 | | | |
| 7932881 | AK094743 | LOC390414 | hypothetical LOC390414 | -0.110 | 0.927 | 1.13E-01 | 1.13E+01 | | | |
| 7930663 | NM_198496 | VWA2 | von Willebrand factor A domain cont | -0.110 | 0.927 | 3.26E-02 | 3.26E+00 | | | |
| 8024013 | BC052236 | C19orf21 | chromosome 19 open reading frame | -0.110 | 0.927 | 6.05E-02 | 6.05E+00 | | | |
| 8075365 | NM_00101798 | RNF215 | ring finger protein 215 | -0.110 | 0.927 | 2.74E-02 | 2.74E+00 | | | |
| 7977203 | NM_00108046 | ASPG | asparaginase homolog (S. cerevisiae) | -0.110 | 0.927 | 6.70E-02 | 6.70E+00 | | | |
| 7985920 | NM_00103995 | MESP2 | mesoderm posterior 2 homolog (mo | -0.110 | 0.927 | 3.99E-02 | 3.99E+00 | | | |
| 7977397 | NM_001312 | CRIP2 | cysteine-rich protein 2 | -0.110 | 0.927 | 6.34E-02 | 6.34E+00 | | | |
| 8087611 | NM_007312 | HYAL1 | hyaluronoglucosaminidase 1 | -0.110 | 0.927 | 3.99E-02 | 3.99E+00 | | | |
| 8082165 | NM_00102466 | KALRN | kalirin, RhoGEF kinase | -0.110 | 0.927 | 4.38E-02 | 4.38E+00 | | | |
| 7919589 | NM_00112337 | HIST2H3D | histone cluster 2, H3d | -0.110 | 0.927 | 7.86E-02 | 7.86E+00 | | | |
| 7975506 | NM_015604 | DCAF4 | DDB1 and CUL4 associated factor 4 | -0.110 | 0.927 | 1.28E-01 | 1.28E+01 | | | |
| 7914630 | NM_018207 | TRIM62 | tripartite motif-containing 62 | -0.110 | 0.927 | 1.71E-02 | 1.71E+00 | | | |
| 8175724 | NM_005367 | MAGEA12 | melanoma antigen family A, 12 | -0.110 | 0.927 | 6.83E-02 | 6.83E+00 | | | |
| 8160782 | --- | --- | --- | -0.110 | 0.927 | 2.48E-01 | 2.48E+01 | | | |
| 7901696 | NM_174936 | PCSK9 | proprotein convertase subtilisin/kex | -0.110 | 0.927 | 4.70E-02 | 4.70E+00 | | | |
| 8153762 | NM_017767 | SLC39A4 | solute carrier family 39 (zinc transpo | -0.110 | 0.927 | 4.91E-02 | 4.91E+00 | | | |
| 7896608 | --- | --- | --- | -0.110 | 0.927 | 5.73E-01 | 5.73E+01 | | | |
| 7950086 | NM_006185 | NUMA1 | nuclear mitotic apparatus protein 1 | -0.110 | 0.927 | 2.82E-01 | 2.82E+01 | | | |
| 8071147 | NM_018943 | TUBA8 | tubulin, alpha 8 | -0.110 | 0.927 | 1.25E-01 | 1.25E+01 | | | |
| 7987574 | NM_133639 | RHOV | ras homolog gene family, member V | -0.110 | 0.927 | 6.94E-02 | 6.94E+00 | | | |
| 8045279 | --- | --- | --- | -0.110 | 0.927 | 3.81E-01 | 3.81E+01 | | | |
| 7931656 | NM_033178 | DUX4 | double homeobox, 4 | -0.110 | 0.927 | 2.58E-02 | 2.58E+00 | | | |
| 7931659 | NM_033178 | DUX4 | double homeobox, 4 | -0.110 | 0.927 | 2.58E-02 | 2.58E+00 | | | |
| 7931662 | NM_033178 | DUX4 | double homeobox, 4 | -0.110 | 0.927 | 2.58E-02 | 2.58E+00 | | | |
| 7931668 | NM_033178 | DUX4 | double homeobox, 4 | -0.110 | 0.927 | 2.58E-02 | 2.58E+00 | | | |
| 7931671 | NM_033178 | DUX4 | double homeobox, 4 | -0.110 | 0.927 | 2.58E-02 | 2.58E+00 | | | |
| 8058869 | NM_022648 | TNS1 | tensin 1 | -0.110 | 0.927 | 2.57E-02 | 2.57E+00 | | | |
| 8139656 | NM_00100155 | GRB10 | growth factor receptor-bound protei | -0.110 | 0.927 | 1.40E-01 | 1.40E+01 | | | |
| 8164428 | NM_015679 | TRUB2 | TruB pseudouridine (psi) synthase ho | -0.110 | 0.927 | 1.61E-01 | 1.61E+01 | | | |
| 8038725 | NM_002776 | KLK10 | kallikrein-related peptidase 10 | -0.110 | 0.927 | 8.14E-02 | 8.14E+00 | | | |
| 8115664 | --- | --- | --- | -0.110 | 0.927 | 2.29E-01 | 2.29E+01 | | | |
| 7900931 | NM_003780 | B4GALT2 | UDP-Gal:betaGlcNAc beta 1,4- galact | -0.110 | 0.927 | 3.69E-02 | 3.69E+00 | | | |
| 7967039 | NM_002442 | MSI1 | musashi homolog 1 (Drosophila) | -0.110 | 0.927 | 4.89E-02 | 4.89E+00 | | | |
| 7918203 | NM_00114398 | NBPF4 | neuroblastoma breakpoint family, m | -0.110 | 0.927 | 1.31E-02 | 1.31E+00 | | | |
| 8026013 | NM_00109973 | MORG1 | mitogen-activated protein kinase org | -0.110 | 0.927 | 1.55E-01 | 1.55E+01 | | | |
| 7963052 | --- | --- | --- | -0.110 | 0.927 | 2.17E-01 | 2.17E+01 | | | |
| 8178712 | NM_032470 | TNXB | tenascin XB | -0.110 | 0.927 | 6.33E-02 | 6.33E+00 | | | |
| 8179935 | NM_032470 | TNXB | tenascin XB | -0.110 | 0.927 | 6.33E-02 | 6.33E+00 | | | |
| 8122986 | NM_016224 | SNX9 | sorting nexin 9 | -0.110 | 0.927 | 5.55E-01 | 5.55E+01 | | | |
| 7999160 | NM_013399 | C16orf5 | chromosome 16 open reading frame | -0.110 | 0.927 | 7.95E-02 | 7.95E+00 | | | |
| 8113879 | --- | --- | --- | -0.110 | 0.927 | 2.78E-01 | 2.78E+01 | | | |
| 7894173 | --- | --- | --- | -0.110 | 0.927 | 2.53E-01 | 2.53E+01 | | | |
| 8030049 | NM_017457 | CYTH2 | cytohesin 2 | -0.110 | 0.927 | 7.26E-01 | 7.26E+01 | | | |
| 8104369 | NM_001047 | SRD5A1 | steroid-5-alpha-reductase, alpha pol | -0.110 | 0.927 | 1.68E-01 | 1.68E+01 | | | |
| 7915841 | NM_00109761 | KNCN | kinocilin | -0.110 | 0.927 | 9.66E-02 | 9.66E+00 | | | |
| 8049239 | AY358535 | C2orf82 | chromosome 2 open reading frame 8 | -0.110 | 0.927 | 2.95E-02 | 2.95E+00 | | | |
| 7900095 | NM_018067 | MAP7D1 | MAP7 domain containing 1 | -0.110 | 0.927 | 8.85E-02 | 8.85E+00 | | | |
| 8059244 | NM_024536 | CHPF | chondroitin polymerizing factor | -0.110 | 0.927 | 4.51E-02 | 4.51E+00 | | | |
| 8064575 | NM_000490 | AVP | arginine vasopressin | -0.110 | 0.926 | 8.43E-02 | 8.43E+00 | | | |
| 8019231 | NM_002602 | PDE6G | phosphodiesterase 6G, cGMP-specifi | -0.110 | 0.926 | 7.99E-02 | 7.99E+00 | | | |
| 8168779 | --- | --- | --- | -0.110 | 0.926 | 5.76E-01 | 5.76E+01 | | | |
| 8091562 | NM_020865 | DHX36 | DEAH (Asp-Glu-Ala-His) box polypept | -0.110 | 0.926 | 7.37E-01 | 7.37E+01 | | | |
| 8004255 | NM_181844 | BCL6B | B-cell CLL/lymphoma 6, member B (z | -0.110 | 0.926 | 3.77E-02 | 3.77E+00 | | | |
| 7927307 | NM_001630 | ANXA8L2 | annexin A8-like 2 | -0.110 | 0.926 | 3.17E-02 | 3.17E+00 | | | |
| 7893486 | --- | --- | --- | -0.110 | 0.926 | 6.59E-01 | 6.59E+01 | | | |
| 8179564 | NM_002263 | KIFC1 | kinesin family member C1 | -0.110 | 0.926 | 9.54E-02 | 9.54E+00 | | | |
| 7998629 | --- | --- | --- | -0.110 | 0.926 | 6.39E-02 | 6.39E+00 | | | |
| 7923942 | NM_00112298 | FCAMR | Fc receptor, IgA, IgM, high affinity | -0.110 | 0.926 | 7.74E-02 | 7.74E+00 | | | |
| 8150175 | NM_025115 | C8orf41 | chromosome 8 open reading frame 4 | -0.110 | 0.926 | 4.97E-01 | 4.97E+01 | | | |
| 8092922 | NM_152531 | C3orf21 | chromosome 3 open reading frame 2 | -0.110 | 0.926 | 3.37E-02 | 3.37E+00 | | | |
| 8147543 | BC062359 | C8orf47 | chromosome 8 open reading frame 4 | -0.110 | 0.926 | 6.03E-02 | 6.03E+00 | | | |
| 8006562 | NM_033315 | RASL10B | RAS-like, family 10, member B | -0.110 | 0.926 | 4.63E-02 | 4.63E+00 | | | |
| 7975368 | NM_00103946 | SFRS5 | splicing factor, arginine/serine-rich 5 | -0.110 | 0.926 | 9.29E-02 | 9.29E+00 | | | |
| 8072777 | NR_024038 | MPST | mercaptopyruvate sulfurtransferase | -0.110 | 0.926 | 4.92E-02 | 4.92E+00 | | | |
| 8062064 | NM_020884 | MYH7B | myosin, heavy chain 7B, cardiac mus | -0.110 | 0.926 | 4.24E-02 | 4.24E+00 | | | |
| 7989476 | AK096042 | FLJ38723 | hypothetical FLJ38723 | -0.110 | 0.926 | 6.70E-02 | 6.70E+00 | | | |
| 7933359 | NM_002900 | RBP3 | retinol binding protein 3, interstitial | -0.110 | 0.926 | 7.50E-02 | 7.50E+00 | | | |
| 8026214 | NM_00109862 | NANOS3 | nanos homolog 3 (Drosophila) | -0.110 | 0.926 | 5.24E-02 | 5.24E+00 | | | |
| 8125568 | NM_080680 | COL11A2 | collagen, type XI, alpha 2 | -0.110 | 0.926 | 1.89E-02 | 1.89E+00 | | | |
| 8122717 | NM_032832 | LRP11 | low density lipoprotein receptor-rela | -0.110 | 0.926 | 5.22E-02 | 5.22E+00 | | | |
| 8123609 | NM_004155 | SERPINB9 | serpin peptidase inhibitor, clade B (o | -0.110 | 0.926 | 6.11E-01 | 6.11E+01 | | | |
| 7979574 | NM_030791 | SGPP1 | sphingosine-1-phosphate phosphata | -0.110 | 0.926 | 3.55E-02 | 3.55E+00 | | | |

| | | | | | | | | | | |
|---|---|---|---|---|---|---|---|---|---|---|
| 7994269 | NM_024773 | JMJD5 | jumonji domain containing 5 | -0.111 | 0.926 | 1.26E-01 | 1.26E+01 | | | |
| 7996837 | NM_004360 | CDH1 | cadherin 1, type 1, E-cadherin (epith | -0.111 | 0.926 | 3.68E-02 | 3.68E+00 | | | |
| 8033465 | NM_006351 | TIMM44 | translocase of inner mitochondrial m | -0.111 | 0.926 | 7.46E-02 | 7.46E+00 | | | |
| 8072411 | --- | --- | --- | -0.111 | 0.926 | 1.18E-01 | 1.18E+01 | | | |
| 8040070 | NM_003108 | SOX11 | SRY (sex determining region Y)-box 1 | -0.111 | 0.926 | 1.37E-02 | 1.37E+00 | | | |
| 7981530 | NM_013345 | GPR132 | G protein-coupled receptor 132 | -0.111 | 0.926 | 5.51E-02 | 5.51E+00 | | | |
| 8013109 | --- | --- | --- | -0.111 | 0.926 | 4.19E-01 | 4.19E+01 | | | |
| 8164515 | NM_014908 | DOLK | dolichol kinase | -0.111 | 0.926 | 1.05E-01 | 1.05E+01 | | | |
| 8162479 | NM_017561 | FAM22F | family with sequence similarity 22, m | -0.111 | 0.926 | 2.85E-02 | 2.85E+00 | | | |
| 8002706 | AK129695 | C16orf47 | chromosome 16 open reading frame | -0.111 | 0.926 | 6.20E-02 | 6.20E+00 | | | |
| 8173086 | NM_004463 | FGD1 | FYVE, RhoGEF and PH domain contai | -0.111 | 0.926 | 3.16E-02 | 3.16E+00 | | | |
| 8043522 | NM_144707 | PROM2 | prominin 2 | -0.111 | 0.926 | 6.34E-02 | 6.34E+00 | | | |
| 8075276 | NM_019103 | ZMAT5 | zinc finger, matrin type 5 | -0.111 | 0.926 | 5.92E-02 | 5.92E+00 | | | |
| 7945857 | ENST00000332 | MRGPRG | MAS-related GPR, member G | -0.111 | 0.926 | 1.18E-01 | 1.18E+01 | | | |
| 8022085 | NM_004746 | DLGAP1 | discs, large (Drosophila) homolog-ass | -0.111 | 0.926 | 1.23E-02 | 1.23E+00 | | | |
| 8038443 | NM_025129 | FUZ | fuzzy homolog (Drosophila) | -0.111 | 0.926 | 7.43E-02 | 7.43E+00 | | | |
| 8042896 | NM_016170 | TLX2 | T-cell leukemia homeobox 2 | -0.111 | 0.926 | 6.10E-02 | 6.10E+00 | | | |
| 8035315 | NM_138454 | NXNL1 | nucleoredoxin-like 1 | -0.111 | 0.926 | 4.86E-02 | 4.86E+00 | | | |
| 7897196 | NM_152492 | CCDC27 | coiled-coil domain containing 27 | -0.111 | 0.926 | 3.91E-02 | 3.91E+00 | | | |
| 8010629 | NM_199287 | CCDC137 | coiled-coil domain containing 137 | -0.111 | 0.926 | 3.36E-02 | 3.36E+00 | | | |
| 7975095 | NM_015549 | PLEKHG3 | pleckstrin homology domain contain | -0.111 | 0.926 | 9.80E-02 | 9.80E+00 | | | |
| 8159255 | NM_002297 | LCN1 | lipocalin 1 (tear prealbumin) | -0.111 | 0.926 | 1.15E-01 | 1.15E+01 | | | |
| 8087833 | NM_001947 | DUSP7 | dual specificity phosphatase 7 | -0.111 | 0.926 | 7.57E-02 | 7.57E+00 | | | |
| 8142971 | NM_138693 | KLF14 | Kruppel-like factor 14 | -0.111 | 0.926 | 1.71E-02 | 1.71E+00 | | | |
| 7936144 | NM_000494 | COL17A1 | collagen, type XVII, alpha 1 | -0.111 | 0.926 | 2.72E-02 | 2.72E+00 | | | |
| 7980219 | NM_031464 | RPS6KL1 | ribosomal protein S6 kinase-like 1 | -0.111 | 0.926 | 5.92E-02 | 5.92E+00 | | | |
| 7956737 | --- | --- | --- | -0.111 | 0.926 | 2.23E-01 | 2.23E+01 | | | |
| 8080158 | NM_080865 | GPR62 | G protein-coupled receptor 62 | -0.111 | 0.926 | 1.51E-02 | 1.51E+00 | | | |
| 7973580 | NM_203402 | FITM1 | fat storage-inducing transmembrane | -0.111 | 0.926 | 1.08E-01 | 1.08E+01 | | | |
| 8031102 | NM_015629 | PRPF31 | PRP31 pre-mRNA processing factor 3 | -0.111 | 0.926 | 2.47E-01 | 2.47E+01 | | | |
| 8036956 | NM_198476 | C19orf54 | chromosome 19 open reading frame | -0.111 | 0.926 | 5.13E-02 | 5.13E+00 | | | |
| 8067551 | ENST00000415 | C20orf90 | chromosome 20 open reading frame | -0.111 | 0.926 | 1.31E-01 | 1.31E+01 | | | |
| 8018236 | NM_024417 | FDXR | ferredoxin reductase | -0.111 | 0.926 | 5.76E-02 | 5.76E+00 | | | |
| 8107328 | NR_027706 | FLJ11235 | hypothetical FLJ11235 | -0.111 | 0.926 | 8.30E-02 | 8.30E+00 | | | |
| 8032623 | NM_001060 | TBXA2R | thromboxane A2 receptor | -0.111 | 0.926 | 9.43E-02 | 9.43E+00 | | | |
| 7896437 | --- | --- | --- | -0.111 | 0.926 | 5.62E-01 | 5.62E+01 | | | |
| 8028332 | NM_004823 | KCNK6 | potassium channel, subfamily K, mer | -0.111 | 0.926 | 2.10E-02 | 2.10E+00 | | | |
| 8090823 | NM_005630 | SLCO2A1 | solute carrier organic anion transpor | -0.111 | 0.926 | 3.25E-02 | 3.25E+00 | | | |
| 7915861 | NM_145279 | MOBKL2C | MOB1, Mps One Binder kinase activa | -0.111 | 0.926 | 1.40E-01 | 1.40E+01 | | | |
| 7978132 | NM_024658 | IPO4 | importin 4 | -0.111 | 0.926 | 1.07E-01 | 1.07E+01 | | | |
| 7935361 | NM_003061 | SLIT1 | slit homolog 1 (Drosophila) | -0.111 | 0.926 | 2.81E-02 | 2.81E+00 | | | |
| 8074931 | NM_021916 | ZNF70 | zinc finger protein 70 | -0.111 | 0.926 | 7.24E-02 | 7.24E+00 | | | |
| 7986004 | NM_00100430 | ZNF774 | zinc finger protein 774 | -0.111 | 0.926 | 6.51E-02 | 6.51E+00 | | | |
| 7956229 | NM_00113519 | SLC39A5 | solute carrier family 39 (metal ion tra | -0.111 | 0.926 | 2.91E-02 | 2.91E+00 | | | |
| 7928653 | NM_00109933 | FAM22A | family with sequence similarity 22, m | -0.111 | 0.926 | 2.92E-02 | 2.92E+00 | | | |
| 8003210 | NM_206967 | C16orf74 | chromosome 16 open reading frame | -0.111 | 0.926 | 4.09E-02 | 4.09E+00 | | | |
| 8020308 | NM_181481 | C18orf1 | chromosome 18 open reading frame | -0.111 | 0.926 | 4.84E-02 | 4.84E+00 | | | |
| 8172631 | NM_014009 | FOXP3 | forkhead box P3 | -0.111 | 0.926 | 3.79E-02 | 3.79E+00 | | | |
| 8140028 | NM_178125 | TRIM50 | tripartite motif-containing 50 | -0.111 | 0.926 | 6.41E-02 | 6.41E+00 | | | |
| 7955305 | NM_001652 | AQP6 | aquaporin 6, kidney specific | -0.111 | 0.926 | 9.15E-02 | 9.15E+00 | | | |
| 7896004 | --- | --- | --- | -0.111 | 0.926 | 9.15E-01 | 9.15E+01 | | | |
| 7923635 | NM_014935 | PLEKHA6 | pleckstrin homology domain contain | -0.111 | 0.926 | 8.05E-02 | 8.05E+00 | | | |
| 8112041 | --- | --- | --- | -0.111 | 0.926 | 6.43E-01 | 6.43E+01 | | | |
| 8135990 | NM_001458 | FLNC | filamin C, gamma | -0.111 | 0.926 | 2.26E-02 | 2.26E+00 | | | |
| 7963421 | NM_005554 | KRT6A | keratin 6A | -0.111 | 0.926 | 2.55E-01 | 2.55E+01 | | | |
| 8030299 | NM_144688 | CCDC155 | coiled-coil domain containing 155 | -0.111 | 0.926 | 9.22E-02 | 9.22E+00 | | | |
| 8007250 | NM_000263 | NAGLU | N-acetylglucosaminidase, alpha- | -0.111 | 0.926 | 2.40E-02 | 2.40E+00 | | | |
| 8150099 | NM_00110091 | MBOAT4 | membrane bound O-acyltransferase | -0.111 | 0.926 | 2.05E-01 | 2.05E+01 | | | |
| 7992732 | NM_145252 | ZG16B | zymogen granule protein 16 homolo | -0.111 | 0.926 | 4.50E-02 | 4.50E+00 | | | |
| 7894842 | --- | --- | --- | -0.111 | 0.926 | 6.15E-01 | 6.15E+01 | | | |
| 8111430 | NM_014324 | AMACR | alpha-methylacyl-CoA racemase | -0.111 | 0.926 | 9.51E-02 | 9.51E+00 | | | |
| 8006865 | NM_032192 | PPP1R1B | protein phosphatase 1, regulatory (in | -0.111 | 0.926 | 2.58E-02 | 2.58E+00 | | | |
| 7894148 | --- | --- | --- | -0.111 | 0.926 | 4.67E-01 | 4.67E+01 | | | |
| 7996563 | NM_000196 | HSD11B2 | hydroxysteroid (11-beta) dehydroge | -0.111 | 0.926 | 5.41E-02 | 5.41E+00 | | | |
| 7987792 | NM_178034 | PLA2G4D | phospholipase A2, group IVD (cytoso | -0.111 | 0.926 | 9.55E-02 | 9.55E+00 | | | |
| 8005549 | ENST00000284 | GRAP | GRB2-related adaptor protein | -0.111 | 0.926 | 1.34E-01 | 1.34E+01 | | | |
| 8134869 | NM_002593 | PCOLCE | procollagen C-endopeptidase enhan | -0.111 | 0.926 | 1.21E-01 | 1.21E+01 | | | |
| 8134374 | --- | --- | --- | -0.111 | 0.926 | 5.01E-01 | 5.01E+01 | | | |
| 7915385 | NM_001956 | EDN2 | endothelin 2 | -0.111 | 0.926 | 6.94E-02 | 6.94E+00 | | | |
| 7922326 | --- | --- | --- | -0.111 | 0.926 | 2.59E-01 | 2.59E+01 | | | |
| 8141166 | AK125166 | LOC441268 | hypothetical LOC441268 | -0.112 | 0.926 | 7.45E-02 | 7.45E+00 | | | |
| 8048452 | NM_025216 | WNT10A | wingless-type MMTV integration site | -0.112 | 0.926 | 6.24E-02 | 6.24E+00 | | | |
| 8003635 | NM_172367 | TUSC5 | tumor suppressor candidate 5 | -0.112 | 0.926 | 1.23E-01 | 1.23E+01 | | | |
| 7893818 | --- | --- | --- | -0.112 | 0.926 | 4.01E-01 | 4.01E+01 | | | |
| 8074991 | NM_00109978 | GGT5 | gamma-glutamyltransferase 5 | -0.112 | 0.926 | 9.24E-02 | 9.24E+00 | | | |
| 7917754 | NM_003567 | BCAR3 | breast cancer anti-estrogen resistan | -0.112 | 0.926 | 3.15E-02 | 3.15E+00 | | | |
| 8011808 | NM_00110457 | GPR172B | G protein-coupled receptor 172B | -0.112 | 0.926 | 3.22E-02 | 3.22E+00 | | | |

| | | | | | | | | | | | |
|---|---|---|---|---|---|---|---|---|---|---|---|
| 8008310 | NM_152463 | EME1 | essential meiotic endonuclease 1 ho | -0.112 | 0.926 | 1.58E-01 | 1.58E+01 | | | | |
| 7906417 | NM_021189 | CADM3 | cell adhesion molecule 3 | -0.112 | 0.926 | 3.74E-02 | 3.74E+00 | | | | |
| 7970597 | AK128225 | FLJ46358 | FLJ46358 protein | -0.112 | 0.926 | 7.88E-02 | 7.88E+00 | | | | |
| 8157610 | NM_032552 | DAB2IP | DAB2 interacting protein | -0.112 | 0.926 | 2.51E-02 | 2.51E+00 | | | | |
| 7892665 | --- | --- | --- | -0.112 | 0.926 | 3.15E-01 | 3.15E+01 | | | | |
| 8076978 | NM_002751 | MAPK11 | mitogen-activated protein kinase 11 | -0.112 | 0.926 | 4.49E-02 | 4.49E+00 | | | | |
| 8067242 | ENST00000457 | ANKRD60 | ankyrin repeat domain 60 | -0.112 | 0.926 | 2.27E-02 | 2.27E+00 | | | | |
| 7995829 | NM_005951 | MT1H | metallothionein 1H | -0.112 | 0.926 | 2.63E-01 | 2.63E+01 | | | | |
| 7945536 | NM_016564 | CEND1 | cell cycle exit and neuronal different | -0.112 | 0.926 | 3.41E-02 | 3.41E+00 | | | | |
| 8039477 | BC031875 | FAM71E2 | family with sequence similarity 71, m | -0.112 | 0.926 | 2.77E-02 | 2.77E+00 | | | | |
| 7997726 | NM_001451 | FOXF1 | forkhead box F1 | -0.112 | 0.925 | 2.14E-02 | 2.14E+00 | | | | |
| 7934885 | NM_018363 | RNLS | renalase, FAD-dependent amine oxid | -0.112 | 0.925 | 1.33E-01 | 1.33E+01 | | | | |
| 7914021 | NM_003047 | SLC9A1 | solute carrier family 9 (sodium/hydro | -0.112 | 0.925 | 3.99E-02 | 3.99E+00 | | | | |
| 8048381 | NM_015690 | STK36 | serine/threonine kinase 36, fused ho | -0.112 | 0.925 | 1.12E-01 | 1.12E+01 | | | | |
| 8164632 | NM_032843 | FIBCD1 | fibrinogen C domain containing 1 | -0.112 | 0.925 | 3.23E-02 | 3.23E+00 | | | | |
| 7999903 | BC117562 | C16orf88 | chromosome 16 open reading frame | -0.112 | 0.925 | 6.64E-01 | 6.64E+01 | | | | |
| 7892920 | --- | --- | --- | -0.112 | 0.925 | 8.84E-01 | 8.84E+01 | | | | |
| 8086434 | NM_144719 | CCDC13 | coiled-coil domain containing 13 | -0.112 | 0.925 | 1.73E-02 | 1.73E+00 | | | | |
| 7925749 | NM_00100182 | OR2T34 | olfactory receptor, family 2, subfami | -0.112 | 0.925 | 9.38E-02 | 9.38E+00 | | | | |
| 8173493 | NM_00114279 | CXCR3 | chemokine (C-X-C motif) receptor 3 | -0.112 | 0.925 | 1.05E-01 | 1.05E+01 | | | | |
| 7983145 | AK297603 | STARD9 | StAR-related lipid transfer (START) d | -0.112 | 0.925 | 4.60E-02 | 4.60E+00 | | | | |
| 7895169 | --- | --- | --- | -0.112 | 0.925 | 3.74E-01 | 3.74E+01 | | | | |
| 7895450 | --- | --- | --- | -0.112 | 0.925 | 8.47E-01 | 8.47E+01 | | | | |
| 8039409 | NM_173804 | TMEM86B | transmembrane protein 86B | -0.112 | 0.925 | 7.10E-02 | 7.10E+00 | | | | |
| 8146669 | NM_033058 | TRIM55 | tripartite motif-containing 55 | -0.112 | 0.925 | 3.50E-02 | 3.50E+00 | | | | |
| 7932498 | NM_207371 | C10orf140 | chromosome 10 open reading frame | -0.112 | 0.925 | 6.83E-02 | 6.83E+00 | | | | |
| 8145055 | NM_006129 | BMP1 | bone morphogenetic protein 1 | -0.112 | 0.925 | 4.59E-02 | 4.59E+00 | | | | |
| 8168058 | NM_015686 | FAM155B | family with sequence similarity 155, | -0.112 | 0.925 | 4.84E-02 | 4.84E+00 | | | | |
| 8158939 | NM_004188 | GFI1B | growth factor independent 1B transc | -0.112 | 0.925 | 5.78E-02 | 5.78E+00 | | | | |
| 7896908 | NM_153339 | PUSL1 | pseudouridylate synthase-like 1 | -0.112 | 0.925 | 1.10E-01 | 1.10E+01 | | | | |
| 8131965 | AK056484 | hCG_200992 | hypothetical locus LOC441204 | -0.112 | 0.925 | 1.00E-01 | 1.00E+01 | | | | |
| 7996318 | NM_144601 | CMTM3 | CKLF-like MARVEL transmembrane d | -0.112 | 0.925 | 4.72E-02 | 4.72E+00 | | | | |
| 8137225 | NM_013400 | REPIN1 | replication initiator 1 | -0.112 | 0.925 | 1.05E-01 | 1.05E+01 | | | | |
| 8076302 | NM_138481 | CHADL | chondroadherin-like | -0.112 | 0.925 | 2.69E-02 | 2.69E+00 | | | | |
| 8156846 | --- | --- | --- | -0.112 | 0.925 | 4.69E-02 | 4.69E+00 | | | | |
| 8027152 | NM_004386 | NCAN | neurocan | -0.112 | 0.925 | 8.22E-02 | 8.22E+00 | | | | |
| 7939329 | NM_003477 | PDHX | pyruvate dehydrogenase complex, co | -0.112 | 0.925 | 7.33E-01 | 7.33E+01 | | | | |
| 8019181 | ENST00000456 | KFZp761P02 | hypothetical protein DKFZp761P021 | -0.112 | 0.925 | 6.34E-02 | 6.34E+00 | | | | |
| 7997072 | NM_145059 | FUK | fucokinase | -0.112 | 0.925 | 7.67E-02 | 7.67E+00 | | | | |
| 7967588 | NM_032656 | DHX37 | DEAH (Asp-Glu-Ala-His) box polypept | -0.112 | 0.925 | 4.89E-02 | 4.89E+00 | | | | |
| 7995797 | NM_175617 | MT1E | metallothionein 1E | -0.112 | 0.925 | 1.31E-01 | 1.31E+01 | | | | |
| 7992646 | NM_001694 | ATP6V0C | ATPase, H+ transporting, lysosomal 1 | -0.112 | 0.925 | 3.38E-01 | 3.38E+01 | | | | |
| 8013660 | NM_005165 | ALDOC | aldolase C, fructose-bisphosphate | -0.112 | 0.925 | 3.50E-01 | 3.50E+01 | | | | |
| 7922596 | AK096082 | LOC646976 | hypothetical LOC646976 | -0.112 | 0.925 | 6.61E-02 | 6.61E+00 | | | | |
| 7899096 | NM_015871 | ZNF593 | zinc finger protein 593 | -0.112 | 0.925 | 8.66E-02 | 8.66E+00 | | | | |
| 7919384 | --- | --- | --- | -0.112 | 0.925 | 9.30E-02 | 9.30E+00 | | | | |
| 8171052 | NR_024033 | IL9R | interleukin 9 receptor | -0.112 | 0.925 | 5.24E-02 | 5.24E+00 | | | | |
| 8176972 | NR_024033 | IL9R | interleukin 9 receptor | -0.112 | 0.925 | 5.24E-02 | 5.24E+00 | | | | |
| 7900962 | NM_019100 | DMAP1 | DNA methyltransferase 1 associated | -0.112 | 0.925 | 9.97E-02 | 9.97E+00 | | | | |
| 8093764 | NM_198229 | RGS12 | regulator of G-protein signaling 12 | -0.112 | 0.925 | 3.81E-02 | 3.81E+00 | | | | |
| 8052689 | NM_181784 | SPRED2 | sprouty-related, EVH1 domain conta | -0.112 | 0.925 | 5.57E-02 | 5.57E+00 | | | | |
| 8149646 | NM_024815 | NUDT18 | nudix (nucleoside diphosphate linked | -0.112 | 0.925 | 5.34E-02 | 5.34E+00 | | | | |
| 8145914 | NM_004874 | BAG4 | BCL2-associated athanogene 4 | -0.112 | 0.925 | 1.84E-01 | 1.84E+01 | | | | |
| 7975324 | NM_020692 | GALNTL1 | UDP-N-acetyl-alpha-D-galactosamine | -0.112 | 0.925 | 7.10E-02 | 7.10E+00 | | | | |
| 8073397 | NM_017590 | ZC3H7B | zinc finger CCCH-type containing 7B | -0.112 | 0.925 | 1.22E-01 | 1.22E+01 | | | | |
| 8156476 | NM_005392 | PHF2 | PHD finger protein 2 | -0.112 | 0.925 | 1.11E-01 | 1.11E+01 | | | | |
| 7923798 | NM_022731 | NUCKS1 | nuclear casein kinase and cyclin-depe | -0.112 | 0.925 | 7.84E-01 | 7.84E+01 | | | | |
| 7952453 | NM_019055 | ROBO4 | roundabout homolog 4, magic round | -0.112 | 0.925 | 6.21E-02 | 6.21E+00 | | | | |
| 8070286 | NM_005867 | DSCR4 | Down syndrome critical region gene | -0.112 | 0.925 | 7.62E-02 | 7.62E+00 | | | | |
| 7947599 | NM_003654 | CHST1 | carbohydrate (keratan sulfate Gal-6) | -0.112 | 0.925 | 4.55E-02 | 4.55E+00 | | | | |
| 8016168 | NM_133373 | PLCD3 | phospholipase C, delta 3 | -0.112 | 0.925 | 5.25E-02 | 5.25E+00 | | | | |
| 8031931 | --- | --- | --- | -0.112 | 0.925 | 4.75E-01 | 4.75E+01 | | | | |
| 8153449 | NM_032378 | EEF1D | eukaryotic translation elongation fac | -0.112 | 0.925 | 1.48E-01 | 1.48E+01 | | | | |
| 8029710 | NM_003370 | VASP | vasodilator-stimulated phosphoprot | -0.112 | 0.925 | 1.31E-01 | 1.31E+01 | | | | |
| 8038785 | BC032383 | LOC147645 | hypothetical protein LOC147645 | -0.112 | 0.925 | 3.06E-02 | 3.06E+00 | | | | |
| 7910585 | --- | --- | --- | -0.112 | 0.925 | 8.55E-02 | 8.55E+00 | | | | |
| 7973727 | NM_025081 | KIAA1305 | KIAA1305 | -0.112 | 0.925 | 8.05E-02 | 8.05E+00 | | | | |
| 8116418 | NM_005110 | GFPT2 | glutamine-fructose-6-phosphate trar | -0.112 | 0.925 | 1.12E-01 | 1.12E+01 | | | | |
| 8018400 | NM_004259 | RECQL5 | RecQ protein-like 5 | -0.112 | 0.925 | 6.51E-02 | 6.51E+00 | | | | |
| 8178586 | NM_025261 | LY6G6C | lymphocyte antigen 6 complex, locus | -0.112 | 0.925 | 1.05E-01 | 1.05E+01 | | | | |
| 8129317 | NM_020755 | SERINC1 | serine incorporator 1 | -0.112 | 0.925 | 6.68E-01 | 6.68E+01 | | | | |
| 7943573 | ENST00000299 | LOC643923 | hypothetical protein LOC643923 | -0.112 | 0.925 | 6.07E-02 | 6.07E+00 | | | | |
| 8096528 | NM_005390 | PDHA2 | pyruvate dehydrogenase (lipoamide) | -0.112 | 0.925 | 1.05E-01 | 1.05E+01 | | | | |
| 7894298 | --- | --- | --- | -0.112 | 0.925 | 3.28E-01 | 3.28E+01 | | | | |
| 8034821 | NM_000955 | PTGER1 | prostaglandin E receptor 1 (subtype | -0.112 | 0.925 | 4.27E-02 | 4.27E+00 | | | | |
| 7963545 | NM_175834 | KRT79 | keratin 79 | -0.112 | 0.925 | 7.78E-02 | 7.78E+00 | | | | |
| 8003810 | --- | --- | --- | -0.113 | 0.925 | 1.00E-01 | 1.00E+01 | | | | |

| | | | | | | | | | | |
|---|---|---|---|---|---|---|---|---|---|---|
| 8156309 | NM_006705 | GADD45G | growth arrest and DNA-damage-indu | -0.113 | 0.925 | 1.41E-02 | 1.41E+00 | | | |
| 7931226 | NM_017580 | ZRANB1 | zinc finger, RAN-binding domain con | -0.113 | 0.925 | 6.94E-01 | 6.94E+01 | | | |
| 8128099 | NM_002043 | GABRR2 | gamma-aminobutyric acid (GABA) re | -0.113 | 0.925 | 1.22E-01 | 1.22E+01 | | | |
| 7951535 | NM_153705 | KDELC2 | KDEL (Lys-Asp-Glu-Leu) containing 2 | -0.113 | 0.925 | 4.93E-01 | 4.93E+01 | | | |
| 8056784 | NM_004405 | DLX2 | distal-less homeobox 2 | -0.113 | 0.925 | 4.25E-02 | 4.25E+00 | | | |
| 8003800 | --- | --- | --- | -0.113 | 0.925 | 6.47E-02 | 6.47E+00 | | | |
| 8111099 | --- | --- | --- | -0.113 | 0.925 | 2.26E-01 | 2.26E+01 | | | |
| 8104601 | NM_006317 | BASP1 | brain abundant, membrane attached | -0.113 | 0.925 | 1.09E-01 | 1.09E+01 | | | |
| 8180329 | --- | --- | --- | -0.113 | 0.925 | 5.29E-01 | 5.29E+01 | | | |
| 8020491 | --- | --- | --- | -0.113 | 0.925 | 1.54E-01 | 1.54E+01 | | | |
| 8026350 | NM_207390 | CLEC17A | C-type lectin domain family 17, mem | -0.113 | 0.925 | 2.24E-01 | 2.24E+01 | | | |
| 7901247 | NM_172225 | DMBX1 | diencephalon/mesencephalon home | -0.113 | 0.925 | 5.16E-02 | 5.16E+00 | | | |
| 7905020 | NM_000798 | DRD5 | dopamine receptor D5 | -0.113 | 0.925 | 2.14E-02 | 2.14E+00 | | | |
| 8180232 | --- | --- | --- | -0.113 | 0.925 | 3.77E-01 | 3.77E+01 | | | |
| 8165024 | NM_00104826 | C9orf116 | chromosome 9 open reading frame 1 | -0.113 | 0.925 | 6.89E-02 | 6.89E+00 | | | |
| 8136080 | NM_005631 | SMO | smoothened homolog (Drosophila) | -0.113 | 0.925 | 3.06E-02 | 3.06E+00 | | | |
| 8173287 | NM_007268 | VSIG4 | V-set and immunoglobulin domain co | -0.113 | 0.925 | 1.36E-01 | 1.36E+01 | | | |
| 8021468 | --- | --- | --- | -0.113 | 0.925 | 8.85E-02 | 8.85E+00 | | | |
| 7905510 | NM_178356 | LCE4A | late cornified envelope 4A | -0.113 | 0.925 | 8.40E-02 | 8.40E+00 | | | |
| 8068512 | NM_018962 | DSCR6 | Down syndrome critical region gene | -0.113 | 0.925 | 4.82E-02 | 4.82E+00 | | | |
| 8067270 | NM_153360 | APCDD1L | adenomatosis polyposis coli down-re | -0.113 | 0.925 | 2.32E-02 | 2.32E+00 | | | |
| 7994715 | NM_173618 | INO80E | INO80 complex subunit E | -0.113 | 0.925 | 1.10E-01 | 1.10E+01 | | | |
| 7998749 | NM_022372 | MLST8 | MTOR associated protein, LST8 hom | -0.113 | 0.925 | 6.13E-02 | 6.13E+00 | | | |
| 8028190 | ENST00000418 | OC10013160 | hypothetical protein LOC100131606 | -0.113 | 0.925 | 8.80E-02 | 8.80E+00 | | | |
| 7966810 | NM_173598 | KSR2 | kinase suppressor of ras 2 | -0.113 | 0.925 | 3.68E-02 | 3.68E+00 | | | |
| 7904073 | NM_00100444 | FAM19A3 | family with sequence similarity 19 (c | -0.113 | 0.925 | 4.24E-02 | 4.24E+00 | | | |
| 7960881 | NM_018088 | FAM90A1 | family with sequence similarity 90, m | -0.113 | 0.925 | 1.01E-01 | 1.01E+01 | | | |
| 7911941 | NM_015557 | CHD5 | chromodomain helicase DNA binding | -0.113 | 0.925 | 3.17E-02 | 3.17E+00 | | | |
| 8090591 | NM_015103 | PLXND1 | plexin D1 | -0.113 | 0.925 | 7.06E-03 | 7.06E-01 | | | |
| 7896166 | --- | --- | --- | -0.113 | 0.925 | 6.51E-01 | 6.51E+01 | | | |
| 8053231 | NM_032603 | LOXL3 | lysyl oxidase-like 3 | -0.113 | 0.925 | 6.86E-02 | 6.86E+00 | | | |
| 8109157 | --- | --- | --- | -0.113 | 0.925 | 1.05E-01 | 1.05E+01 | | | |
| 7997128 | NM_152456 | IL34 | interleukin 34 | -0.113 | 0.925 | 2.20E-02 | 2.20E+00 | | | |
| 7977371 | NM_00110091 | PACS2 | phosphofurin acidic cluster sorting p | -0.113 | 0.925 | 1.09E-01 | 1.09E+01 | | | |
| 7947609 | NM_004813 | PEX16 | peroxisomal biogenesis factor 16 | -0.113 | 0.925 | 8.34E-02 | 8.34E+00 | | | |
| 8018803 | NM_003016 | SFRS2 | splicing factor, arginine/serine-rich 2 | -0.113 | 0.925 | 1.59E-01 | 1.59E+01 | | | |
| 8027854 | NM_005303 | FFAR1 | free fatty acid receptor 1 | -0.113 | 0.925 | 8.61E-02 | 8.61E+00 | | | |
| 8117172 | NM_138574 | HDGFL1 | hepatoma derived growth factor-like | -0.113 | 0.925 | 3.36E-02 | 3.36E+00 | | | |
| 7995739 | NM_020988 | GNAO1 | guanine nucleotide binding protein ( | -0.113 | 0.925 | 6.34E-02 | 6.34E+00 | | | |
| 8031962 | NM_017908 | ZNF446 | zinc finger protein 446 | -0.113 | 0.925 | 5.90E-02 | 5.90E+00 | | | |
| 8116033 | NM_000505 | F12 | coagulation factor XII (Hageman fact | -0.113 | 0.925 | 7.66E-02 | 7.66E+00 | | | |
| 8077668 | NM_153480 | IL17RE | interleukin 17 receptor E | -0.113 | 0.925 | 6.91E-02 | 6.91E+00 | | | |
| 7948920 | NM_004790 | SLC22A6 | solute carrier family 22 (organic anio | -0.113 | 0.925 | 6.65E-02 | 6.65E+00 | | | |
| 7941401 | NM_004561 | OVOL1 | ovo-like 1(Drosophila) | -0.113 | 0.925 | 3.37E-02 | 3.37E+00 | | | |
| 8082560 | NM_153833 | H1FOO | H1 histone family, member O, oocyt | -0.113 | 0.925 | 2.65E-02 | 2.65E+00 | | | |
| 7981909 | --- | --- | --- | -0.113 | 0.925 | 1.93E-01 | 1.93E+01 | | | |
| 7914433 | NM_001703 | BAI2 | brain-specific angiogenesis inhibitor | -0.113 | 0.925 | 3.58E-02 | 3.58E+00 | | | |
| 7894144 | --- | --- | --- | -0.113 | 0.925 | 6.30E-01 | 6.30E+01 | | | |
| 7992269 | --- | --- | --- | -0.113 | 0.925 | 6.03E-02 | 6.03E+00 | | | |
| 8008228 | NM_138281 | DLX4 | distal-less homeobox 4 | -0.113 | 0.925 | 7.49E-02 | 7.49E+00 | | | |
| 8136580 | NM_00100874 | RAB19 | RAB19, member RAS oncogene famil | -0.113 | 0.925 | 8.22E-02 | 8.22E+00 | | | |
| 7925741 | NM_00100469 | OR2T33 | olfactory receptor, family 2, subfami | -0.113 | 0.925 | 4.71E-02 | 4.71E+00 | | | |
| 8049906 | NM_032329 | ING5 | inhibitor of growth family, member 5 | -0.113 | 0.925 | 4.45E-02 | 4.45E+00 | | | |
| 8159734 | NM_000718 | CACNA1B | calcium channel, voltage-dependent | -0.113 | 0.925 | 5.15E-03 | 5.15E-01 | | | |
| 8000882 | --- | --- | --- | -0.113 | 0.925 | 3.15E-01 | 3.15E+01 | | | |
| 8097936 | --- | --- | --- | -0.113 | 0.925 | 2.31E-01 | 2.31E+01 | | | |
| 7946087 | NM_00100528 | OR52H1 | olfactory receptor, family 52, subfam | -0.113 | 0.925 | 2.05E-01 | 2.05E+01 | | | |
| 8165890 | NM_00100188 | VCX3B | variable charge, X-linked 3B | -0.113 | 0.925 | 2.57E-02 | 2.57E+00 | | | |
| 8021826 | NM_171999 | SALL3 | sal-like 3 (Drosophila) | -0.113 | 0.925 | 6.16E-02 | 6.16E+00 | | | |
| 8178754 | NM_030651 | PRRT1 | proline-rich transmembrane protein | -0.113 | 0.925 | 2.77E-02 | 2.77E+00 | | | |
| 8179950 | NM_030651 | PRRT1 | proline-rich transmembrane protein | -0.113 | 0.925 | 2.77E-02 | 2.77E+00 | | | |
| 8098730 | NM_033178 | DUX4 | double homeobox, 4 | -0.113 | 0.924 | 2.91E-02 | 2.91E+00 | | | |
| 8098743 | NM_033178 | DUX4 | double homeobox, 4 | -0.113 | 0.924 | 2.91E-02 | 2.91E+00 | | | |
| 8160521 | NM_024761 | MOBKL2B | MOB1, Mps One Binder kinase activa | -0.113 | 0.924 | 8.84E-02 | 8.84E+00 | | | |
| 7958410 | NM_007076 | FICD | FIC domain containing | -0.113 | 0.924 | 5.85E-02 | 5.85E+00 | | | |
| 8164554 | NM_203434 | IER5L | immediate early response 5-like | -0.113 | 0.924 | 2.57E-02 | 2.57E+00 | | | |
| 7959761 | NM_181709 | FAM101A | family with sequence similarity 101, | -0.113 | 0.924 | 9.04E-02 | 9.04E+00 | | | |
| 8055287 | NM_00108536 | FAM128A | family with sequence similarity 128, | -0.113 | 0.924 | 3.94E-02 | 3.94E+00 | | | |
| 8084917 | AK128346 | LOC440993 | hypothetical LOC440993 | -0.113 | 0.924 | 2.19E-01 | 2.19E+01 | | | |
| 8141466 | NM_152742 | GPC2 | glypican 2 | -0.113 | 0.924 | 4.76E-02 | 4.76E+00 | | | |
| 7994541 | NM_014387 | LAT | linker for activation of T cells | -0.113 | 0.924 | 4.74E-01 | 4.74E+01 | | | |
| 7928870 | --- | --- | --- | -0.113 | 0.924 | 2.62E-01 | 2.62E+01 | | | |
| 7985587 | NR_004859 | SCAND2 | SCAN domain containing 2 pseudoge | -0.113 | 0.924 | 1.06E-01 | 1.06E+01 | | | |
| 8169657 | NM_00110557 | ANKRD58 | ankyrin repeat domain 58 | -0.113 | 0.924 | 5.11E-02 | 5.11E+00 | | | |
| 8049752 | NM_00108043 | SNED1 | sushi, nidogen and EGF-like domains | -0.113 | 0.924 | 4.24E-02 | 4.24E+00 | | | |
| 7981447 | NM_00110011 | XRCC3 | X-ray repair complementing defectiv | -0.113 | 0.924 | 1.16E-01 | 1.16E+01 | | | |
| 7936320 | NR_026715 | RPL13AP6 | ribosomal protein L13a pseudogene | -0.113 | 0.924 | 4.82E-01 | 4.82E+01 | | | |

| | | | | | | | | | | |
|---|---|---|---|---|---|---|---|---|---|---|
| 8120004 | NM_00113756( | TMEM151B | transmembrane protein 151B | -0.113 | 0.924 | 1.56E-01 | 1.56E+01 | | | |
| 7963410 | NM_173086 | KRT6C | keratin 6C | -0.113 | 0.924 | 1.88E-02 | 1.88E+00 | | | |
| 7937560 | NM_002457 | MUC2 | mucin 2, oligomeric mucus/gel-form | -0.113 | 0.924 | 7.90E-03 | 7.90E-01 | | | |
| 8033969 | NM_133452 | RAVER1 | ribonucleoprotein, PTB-binding 1 | -0.113 | 0.924 | 2.86E-02 | 2.86E+00 | | | |
| 7998910 | NM_00110317! | CCDC64B | coiled-coil domain containing 64B | -0.113 | 0.924 | 7.24E-02 | 7.24E+00 | | | |
| 8045319 | --- | --- | --- | -0.113 | 0.924 | 1.57E-01 | 1.57E+01 | | | |
| 8055234 | --- | --- | --- | -0.113 | 0.924 | 1.57E-01 | 1.57E+01 | | | |
| 7980338 | NM_024496 | C14orf4 | chromosome 14 open reading frame | -0.113 | 0.924 | 3.48E-02 | 3.48E+00 | | | |
| 8062687 | --- | --- | --- | -0.113 | 0.924 | 1.19E-01 | 1.19E+01 | | | |
| 7998336 | NM_022493 | NARFL | nuclear prelamin A recognition facto | -0.113 | 0.924 | 5.00E-02 | 5.00E+00 | | | |
| 7903884 | NM_032414 | PROK1 | prokineticin 1 | -0.113 | 0.924 | 7.38E-02 | 7.38E+00 | | | |
| 7901102 | NM_015506 | MMACHC | methylmalonic aciduria (cobalamin d | -0.113 | 0.924 | 1.02E-01 | 1.02E+01 | | | |
| 7999596 | NM_138346 | KIAA2013 | KIAA2013 | -0.114 | 0.924 | 4.03E-02 | 4.03E+00 | | | |
| 8119390 | NM_020737 | LRFN2 | leucine rich repeat and fibronectin ty | -0.114 | 0.924 | 6.14E-02 | 6.14E+00 | | | |
| 8092455 | --- | --- | --- | -0.114 | 0.924 | 7.71E-02 | 7.71E+00 | | | |
| 8071809 | NM_000854 | GSTT2 | glutathione S-transferase theta 2 | -0.114 | 0.924 | 6.39E-02 | 6.39E+00 | | | |
| 8074962 | NM_000854 | GSTT2 | glutathione S-transferase theta 2 | -0.114 | 0.924 | 6.39E-02 | 6.39E+00 | | | |
| 8139488 | NM_00101339! | IGFBP3 | insulin-like growth factor binding pro | -0.114 | 0.924 | 3.90E-02 | 3.90E+00 | | | |
| 7917530 | --- | --- | --- | -0.114 | 0.924 | 8.64E-01 | 8.64E+01 | | | |
| 8090559 | NR_003111 | RPL32P3 | ribosomal protein L32 pseudogene 3 | -0.114 | 0.924 | 1.81E-01 | 1.81E+01 | | | |
| 8033445 | NR_026692 | CD209 | CD209 molecule | -0.114 | 0.924 | 7.00E-02 | 7.00E+00 | | | |
| 7922590 | AK125993 | NCRNA0008 | non-protein coding RNA 83 | -0.114 | 0.924 | 7.56E-02 | 7.56E+00 | | | |
| 8115099 | NM_002609 | PDGFRB | platelet-derived growth factor recep | -0.114 | 0.924 | 7.99E-02 | 7.99E+00 | | | |
| 8032347 | NM_020695 | REXO1 | REX1, RNA exonuclease 1 homolog (S | -0.114 | 0.924 | 1.61E-02 | 1.61E+00 | | | |
| 7941565 | NM_022822 | KLC2 | kinesin light chain 2 | -0.114 | 0.924 | 5.43E-02 | 5.43E+00 | | | |
| 8062312 | NM_006097 | MYL9 | myosin, light chain 9, regulatory | -0.114 | 0.924 | 3.00E-01 | 3.00E+01 | | | |
| 7900609 | NM_00101792 | ERMAP | erythroblast membrane-associated p | -0.114 | 0.924 | 1.51E-01 | 1.51E+01 | | | |
| 7938047 | --- | --- | --- | -0.114 | 0.924 | 1.65E-01 | 1.65E+01 | | | |
| 7919299 | AK128227 | OC10013023 | hypothetical LOC100130236 | -0.114 | 0.924 | 1.60E-01 | 1.60E+01 | | | |
| 7919380 | AK128227 | OC10013023 | hypothetical LOC100130236 | -0.114 | 0.924 | 1.60E-01 | 1.60E+01 | | | |
| 8062347 | NM_002895 | RBL1 | retinoblastoma-like 1 (p107) | -0.114 | 0.924 | 7.63E-02 | 7.63E+00 | | | |
| 8003444 | ENST0000033 | LOC146429 | Putative solute carrier family 22 mer | -0.114 | 0.924 | 9.86E-02 | 9.86E+00 | | | |
| 8076757 | NM_014246 | CELSR1 | cadherin, EGF LAG seven-pass G-type | -0.114 | 0.924 | 4.05E-02 | 4.05E+00 | | | |
| 8042530 | --- | --- | --- | -0.114 | 0.924 | 7.50E-02 | 7.50E+00 | | | |
| 8178244 | NM_002931 | RING1 | ring finger protein 1 | -0.114 | 0.924 | 3.31E-01 | 3.31E+01 | | | |
| 8017039 | NM_080415 | 40425 | septin 4 | -0.114 | 0.924 | 5.89E-02 | 5.89E+00 | | | |
| 7996720 | NM_006742 | PSKH1 | protein serine kinase H1 | -0.114 | 0.924 | 4.22E-02 | 4.22E+00 | | | |
| 7953166 | NM_006675 | TSPAN9 | tetraspanin 9 | -0.114 | 0.924 | 2.76E-02 | 2.76E+00 | | | |
| 7992775 | NM_152341 | PAQR4 | progestin and adipoQ receptor famil | -0.114 | 0.924 | 1.86E-02 | 1.86E+00 | | | |
| 8019350 | NM_144998 | STRA13 | stimulated by retinoic acid 13 homol | -0.114 | 0.924 | 6.41E-02 | 6.41E+00 | | | |
| 8175098 | NM_178471 | GPR119 | G protein-coupled receptor 119 | -0.114 | 0.924 | 3.11E-01 | 3.11E+01 | | | |
| 8118137 | NM_000595 | LTA | lymphotoxin alpha (TNF superfamily, | -0.114 | 0.924 | 9.69E-02 | 9.69E+00 | | | |
| 8016147 | NM_00108044 | KIF18B | kinesin family member 18B | -0.114 | 0.924 | 8.60E-02 | 8.60E+00 | | | |
| 8001464 | NM_00114368! | CES7 | carboxylesterase 7 | -0.114 | 0.924 | 8.74E-02 | 8.74E+00 | | | |
| 8017251 | --- | --- | --- | -0.114 | 0.924 | 8.45E-01 | 8.45E+01 | | | |
| 7962349 | NM_173601 | GLT8D3 | glycosyltransferase 8 domain contair | -0.114 | 0.924 | 5.52E-01 | 5.52E+01 | | | |
| 8126269 | NM_178174 | TREML1 | triggering receptor expressed on my | -0.114 | 0.924 | 2.89E-01 | 2.89E+01 | | | |
| 8010036 | NM_180990 | ZACN | zinc activated ligand-gated ion chanr | -0.114 | 0.924 | 1.55E-01 | 1.55E+01 | | | |
| 8071861 | NM_019601 | SUSD2 | sushi domain containing 2 | -0.114 | 0.924 | 2.28E-02 | 2.28E+00 | | | |
| 7970241 | NM_000504 | F10 | coagulation factor X | -0.114 | 0.924 | 3.88E-02 | 3.88E+00 | | | |
| 7949371 | AK124141 | LOC399904 | hypothetical LOC399904 | -0.114 | 0.924 | 1.88E-01 | 1.88E+01 | | | |
| 8141708 | NM_014343 | CLDN15 | claudin 15 | -0.114 | 0.924 | 1.74E-02 | 1.74E+00 | | | |
| 7930168 | --- | --- | --- | -0.114 | 0.924 | 1.21E-01 | 1.21E+01 | | | |
| 7986444 | AK056534 | LOC145757 | hypothetical LOC145757 | -0.114 | 0.924 | 4.58E-02 | 4.58E+00 | | | |
| 7894202 | --- | --- | --- | -0.114 | 0.924 | 8.56E-01 | 8.56E+01 | | | |
| 7961798 | NM_152989 | SOX5 | SRY (sex determining region Y)-box 5 | -0.114 | 0.924 | 1.00E-01 | 1.00E+01 | | | |
| 7911435 | NM_00101498( | FAM132A | family with sequence similarity 132, | -0.114 | 0.924 | 3.35E-02 | 3.35E+00 | | | |
| 7981320 | --- | --- | --- | -0.114 | 0.924 | 1.34E-01 | 1.34E+01 | | | |
| 7895368 | --- | --- | --- | -0.114 | 0.924 | 2.03E-01 | 2.03E+01 | | | |
| 7997839 | NM_030928 | CDT1 | chromatin licensing and DNA replicat | -0.114 | 0.924 | 5.58E-02 | 5.58E+00 | | | |
| 8172197 | NR_002191 | PPP1R2P9 | protein phosphatase 1, regulatory (ir | -0.114 | 0.924 | 6.16E-02 | 6.16E+00 | | | |
| 8126760 | NM_005822 | RCAN2 | regulator of calcineurin 2 | -0.114 | 0.924 | 6.68E-02 | 6.68E+00 | | | |
| 8036479 | NM_033557 | YIF1B | Yip1 interacting factor homolog B (S. | -0.114 | 0.924 | 3.53E-02 | 3.53E+00 | | | |
| 8145829 | NM_025069 | ZNF703 | zinc finger protein 703 | -0.114 | 0.924 | 2.52E-02 | 2.52E+00 | | | |
| 8040514 | --- | --- | --- | -0.114 | 0.924 | 4.68E-01 | 4.68E+01 | | | |
| 7905974 | NM_020897 | HCN3 | hyperpolarization activated cyclic nu | -0.114 | 0.924 | 3.69E-02 | 3.69E+00 | | | |
| 8098848 | NM_213613 | SLC26A1 | solute carrier family 26 (sulfate trans | -0.114 | 0.924 | 1.87E-02 | 1.87E+00 | | | |
| 8016947 | NM_004758 | BZRAP1 | benzodiazapine receptor (peripheral | -0.114 | 0.924 | 3.39E-02 | 3.39E+00 | | | |
| 8027947 | NM_014383 | ZBTB32 | zinc finger and BTB domain containir | -0.114 | 0.924 | 4.24E-02 | 4.24E+00 | | | |
| 7993935 | --- | --- | --- | -0.114 | 0.924 | 8.65E-02 | 8.65E+00 | | | |
| 8062864 | NM_003881 | WISP2 | WNT1 inducible signaling pathway p | -0.114 | 0.924 | 3.91E-02 | 3.91E+00 | | | |
| 8117849 | NM_033229 | TRIM15 | tripartite motif-containing 15 | -0.114 | 0.924 | 5.05E-02 | 5.05E+00 | | | |
| 8177760 | NM_033229 | TRIM15 | tripartite motif-containing 15 | -0.114 | 0.924 | 5.05E-02 | 5.05E+00 | | | |
| 8179070 | NM_033229 | TRIM15 | tripartite motif-containing 15 | -0.114 | 0.924 | 5.05E-02 | 5.05E+00 | | | |
| 8167880 | NM_130467 | PAGE5 | P antigen family, member 5 (prostate | -0.114 | 0.924 | 6.06E-02 | 6.06E+00 | | | |
| 7959312 | NM_00108082! | TMEM120B | transmembrane protein 120B | -0.114 | 0.924 | 8.20E-01 | 8.20E+01 | | | |
| 7995627 | --- | --- | --- | -0.114 | 0.924 | 4.26E-01 | 4.26E+01 | | | |

| | | | | | | | | | | |
|---|---|---|---|---|---|---|---|---|---|---|
| 7917672 | --- | --- | --- | -0.114 | 0.924 | 2.05E-01 | 2.05E+01 | | | |
| 7988414 | NM_001482 | GATM | glycine amidinotransferase (L-arginin | -0.114 | 0.924 | 5.47E-01 | 5.47E+01 | | | |
| 7908812 | NM_004767 | GPR37L1 | G protein-coupled receptor 37 like 1 | -0.114 | 0.924 | 6.67E-02 | 6.67E+00 | | | |
| 8114767 | NM_032420 | PCDH1 | protocadherin 1 | -0.114 | 0.924 | 2.87E-02 | 2.87E+00 | | | |
| 7970577 | NM_005932 | MIPEP | mitochondrial intermediate peptidas | -0.114 | 0.924 | 5.86E-01 | 5.86E+01 | | | |
| 7975696 | NM_182894 | VSX2 | visual system homeobox 2 | -0.114 | 0.924 | 4.48E-02 | 4.48E+00 | | | |
| 7980833 | NM_032560 | SMEK1 | SMEK homolog 1, suppressor of mek | -0.114 | 0.924 | 2.17E-01 | 2.17E+01 | | | |
| 8149307 | NM_173683 | XKR6 | XK, Kell blood group complex subuni | -0.114 | 0.924 | 3.60E-02 | 3.60E+00 | | | |
| 7896547 | --- | --- | --- | -0.114 | 0.924 | 8.01E-01 | 8.01E+01 | | | |
| 8138842 | NM_006092 | NOD1 | nucleotide-binding oligomerization d | -0.114 | 0.924 | 9.22E-02 | 9.22E+00 | | | |
| 8130528 | NM_031924 | RSPH3 | radial spoke 3 homolog (Chlamydom | -0.114 | 0.924 | 4.30E-02 | 4.30E+00 | | | |
| 8038624 | NM_199249 | C19orf48 | chromosome 19 open reading frame | -0.114 | 0.924 | 1.17E-01 | 1.17E+01 | | | |
| 7998494 | NM_00114398 | CCDC154 | coiled-coil domain containing 154 | -0.114 | 0.924 | 6.23E-02 | 6.23E+00 | | | |
| 7910387 | NM_021205 | RHOU | ras homolog gene family, member U | -0.114 | 0.924 | 1.66E-01 | 1.66E+01 | | | |
| 7972001 | --- | --- | --- | -0.114 | 0.924 | 2.89E-01 | 2.89E+01 | | | |
| 8015503 | BC080613 | ZNF385C | zinc finger protein 385C | -0.114 | 0.924 | 6.78E-02 | 6.78E+00 | | | |
| 7987165 | NM_020371 | AVEN | apoptosis, caspase activation inhibito | -0.114 | 0.924 | 8.05E-01 | 8.05E+01 | | | |
| 7999079 | NM_001116 | ADCY9 | adenylate cyclase 9 | -0.114 | 0.924 | 1.37E-01 | 1.37E+01 | | | |
| 7894556 | --- | --- | --- | -0.115 | 0.924 | 3.25E-01 | 3.25E+01 | | | |
| 8153828 | NM_003923 | FOXH1 | forkhead box H1 | -0.115 | 0.924 | 4.87E-02 | 4.87E+00 | | | |
| 8168567 | NM_000307 | POU3F4 | POU class 3 homeobox 4 | -0.115 | 0.924 | 5.28E-02 | 5.28E+00 | | | |
| 8002713 | NM_00101188 | CLEC18B | C-type lectin domain family 18, mem | -0.115 | 0.924 | 4.49E-02 | 4.49E+00 | | | |
| 7961269 | NM_002723 | PRB4 | proline-rich protein BstNI subfamily | -0.115 | 0.924 | 7.62E-02 | 7.62E+00 | | | |
| 8153101 | NM_152888 | COL22A1 | collagen, type XXII, alpha 1 | -0.115 | 0.924 | 2.15E-02 | 2.15E+00 | | | |
| 7971486 | NM_025113 | C13orf18 | chromosome 13 open reading frame | -0.115 | 0.924 | 5.61E-01 | 5.61E+01 | | | |
| 7924758 | --- | --- | --- | -0.115 | 0.924 | 2.28E-01 | 2.28E+01 | | | |
| 8141748 | --- | --- | --- | -0.115 | 0.924 | 1.35E-01 | 1.35E+01 | | | |
| 7915147 | NM_004468 | FHL3 | four and a half LIM domains 3 | -0.115 | 0.924 | 5.58E-02 | 5.58E+00 | | | |
| 8011293 | NM_015229 | KIAA0664 | KIAA0664 | -0.115 | 0.924 | 5.70E-02 | 5.70E+00 | | | |
| 8087596 | NM_003549 | HYAL3 | hyaluronoglucosaminidase 3 | -0.115 | 0.924 | 5.11E-02 | 5.11E+00 | | | |
| 8137483 | AK131514 | FLJ16734 | hypothetical LOC641928 | -0.115 | 0.924 | 2.41E-01 | 2.41E+01 | | | |
| 8079896 | NM_004186 | SEMA3F | sema domain, immunoglobulin doma | -0.115 | 0.924 | 1.44E-02 | 1.44E+00 | | | |
| 7947570 | NM_00107678 | TP53I11 | tumor protein p53 inducible protein | -0.115 | 0.924 | 4.61E-02 | 4.61E+00 | | | |
| 8076894 | NM_015166 | MLC1 | megalencephalic leukoencephalopat | -0.115 | 0.924 | 3.16E-02 | 3.16E+00 | | | |
| 8170622 | NM_013364 | PNMA3 | paraneoplastic antigen MA3 | -0.115 | 0.924 | 1.48E-01 | 1.48E+01 | | | |
| 7893726 | --- | --- | --- | -0.115 | 0.924 | 5.17E-01 | 5.17E+01 | | | |
| 7976755 | NM_207117 | C14orf68 | chromosome 14 open reading frame | -0.115 | 0.924 | 6.58E-02 | 6.58E+00 | | | |
| 8059955 | NM_022449 | RAB17 | RAB17, member RAS oncogene famil | -0.115 | 0.924 | 3.40E-02 | 3.40E+00 | | | |
| 8018849 | NM_003258 | TK1 | thymidine kinase 1, soluble | -0.115 | 0.923 | 1.18E-01 | 1.18E+01 | | | |
| 7929744 | NM_145285 | NKX2-3 | NK2 transcription factor related, locu | -0.115 | 0.923 | 2.86E-02 | 2.86E+00 | | | |
| 7996281 | NM_00113610 | BEAN | brain expressed, associated with Neo | -0.115 | 0.923 | 6.24E-02 | 6.24E+00 | | | |
| 7995714 | NM_001043 | SLC6A2 | solute carrier family 6 (neurotransmi | -0.115 | 0.923 | 4.88E-02 | 4.88E+00 | | | |
| 8044764 | --- | --- | --- | -0.115 | 0.923 | 9.85E-02 | 9.85E+00 | | | |
| 7949344 | NM_005468 | NAALADL1 | N-acetylated alpha-linked acidic dipe | -0.115 | 0.923 | 2.45E-02 | 2.45E+00 | | | |
| 8098500 | NM_017632 | CDKN2AIP | CDKN2A interacting protein | -0.115 | 0.923 | 6.12E-01 | 6.12E+01 | | | |
| 8075005 | AK289871 | ADORA2A | adenosine A2a receptor | -0.115 | 0.923 | 4.50E-02 | 4.50E+00 | | | |
| 8014269 | NM_139285 | GAS2L2 | growth arrest-specific 2 like 2 | -0.115 | 0.923 | 4.13E-02 | 4.13E+00 | | | |
| 8049799 | NM_00100189 | ANO7 | anoctamin 7 | -0.115 | 0.923 | 2.79E-02 | 2.79E+00 | | | |
| 8078619 | NM_002207 | ITGA9 | integrin, alpha 9 | -0.115 | 0.923 | 5.76E-02 | 5.76E+00 | | | |
| 8017378 | NM_001915 | CYB561 | cytochrome b-561 | -0.115 | 0.923 | 1.98E-01 | 1.98E+01 | | | |
| 8098723 | --- | --- | --- | -0.115 | 0.923 | 1.07E-01 | 1.07E+01 | | | |
| 8033257 | NM_000064 | C3 | complement component 3 | -0.115 | 0.923 | 5.11E-02 | 5.11E+00 | | | |
| 8011188 | ENST0000033 | TLCD2 | TLC domain containing 2 | -0.115 | 0.923 | 7.60E-02 | 7.60E+00 | | | |
| 8160879 | NM_006274 | CCL19 | chemokine (C-C motif) ligand 19 | -0.115 | 0.923 | 9.67E-02 | 9.67E+00 | | | |
| 7977965 | NM_016609 | SLC22A17 | solute carrier family 22, member 17 | -0.115 | 0.923 | 4.50E-02 | 4.50E+00 | | | |
| 7941917 | NM_145200 | CABP4 | calcium binding protein 4 | -0.115 | 0.923 | 5.01E-02 | 5.01E+00 | | | |
| 8155942 | NM_00101373 | FOXB2 | forkhead box B2 | -0.115 | 0.923 | 2.24E-02 | 2.24E+00 | | | |
| 8036591 | NM_006149 | LGALS4 | lectin, galactoside-binding, soluble, 4 | -0.115 | 0.923 | 8.50E-02 | 8.50E+00 | | | |
| 7995820 | NM_005947 | MT1B | metallothionein 1B | -0.115 | 0.923 | 7.48E-02 | 7.48E+00 | | | |
| 8070482 | NR_027272 | C21orf129 | chromosome 21 open reading frame | -0.115 | 0.923 | 9.41E-02 | 9.41E+00 | | | |
| 8021344 | AK301799 | BOD1P | biorientation of chromosomes in cell | -0.115 | 0.923 | 2.62E-02 | 2.62E+00 | | | |
| 8010903 | NM_006987 | RPH3AL | rabphilin 3A-like (without C2 domain | -0.115 | 0.923 | 6.03E-02 | 6.03E+00 | | | |
| 8075332 | NM_031937 | TBC1D10A | TBC1 domain family, member 10A | -0.115 | 0.923 | 5.88E-02 | 5.88E+00 | | | |
| 8042811 | NM_144993 | TET3 | tet oncogene family member 3 | -0.115 | 0.923 | 5.52E-02 | 5.52E+00 | | | |
| 8045323 | NM_013310 | C2orf27A | chromosome 2 open reading frame 2 | -0.115 | 0.923 | 8.34E-02 | 8.34E+00 | | | |
| 8142821 | NM_00113591 | KCP | kielin/chordin-like protein | -0.115 | 0.923 | 2.66E-02 | 2.66E+00 | | | |
| 8034514 | NM_024038 | C19orf43 | chromosome 19 open reading frame | -0.115 | 0.923 | 3.00E-01 | 3.00E+01 | | | |
| 7899841 | NM_002143 | HPCA | hippocalcin | -0.115 | 0.923 | 6.07E-02 | 6.07E+00 | | | |
| 8027665 | NM_00108043 | WTIP | Wilms tumor 1 interacting protein | -0.115 | 0.923 | 3.38E-02 | 3.38E+00 | | | |
| 8039474 | NM_00114540 | FAM71E2 | family with sequence similarity 71, m | -0.115 | 0.923 | 1.32E-01 | 1.32E+01 | | | |
| 8072122 | --- | --- | --- | -0.115 | 0.923 | 6.22E-01 | 6.22E+01 | | | |
| 8157802 | --- | --- | --- | -0.115 | 0.923 | 2.26E-01 | 2.26E+01 | | | |
| 8141371 | NM_181538 | GJC3 | gap junction protein, gamma 3, 30.2 | -0.115 | 0.923 | 3.39E-02 | 3.39E+00 | | | |
| 8058866 | NM_003284 | TNP1 | transition protein 1 (during histone t | -0.115 | 0.923 | 7.99E-02 | 7.99E+00 | | | |
| 7904465 | NR_027337 | HIST2H2BA | histone cluster 2, H2ba | -0.115 | 0.923 | 3.58E-01 | 3.58E+01 | | | |
| 8072242 | NM_016418 | NF2 | neurofibromin 2 (merlin) | -0.115 | 0.923 | 1.69E-01 | 1.69E+01 | | | |
| 8055314 | NM_144586 | LYPD1 | LY6/PLAUR domain containing 1 | -0.115 | 0.923 | 5.52E-02 | 5.52E+00 | | | |

| | | | | | | | | | | |
|---|---|---|---|---|---|---|---|---|---|---|
| 7949033 | NM_014067 | MACROD1 | MACRO domain containing 1 | -0.115 | 0.923 | 2.35E-02 | 2.35E+00 | | | |
| 7995377 | --- | --- | --- | -0.115 | 0.923 | 1.99E-01 | 1.99E+01 | | | |
| 7962375 | NM_153026 | PRICKLE1 | prickle homolog 1 (Drosophila) | -0.115 | 0.923 | 1.96E-01 | 1.96E+01 | | | |
| 8066612 | NM_080749 | NEURL2 | neuralized homolog 2 (Drosophila) | -0.115 | 0.923 | 1.15E-01 | 1.15E+01 | | | |
| 8014485 | --- | --- | --- | -0.115 | 0.923 | 4.13E-01 | 4.13E+01 | | | |
| 8071804 | --- | --- | --- | -0.115 | 0.923 | 2.12E-01 | 2.12E+01 | | | |
| 8082380 | NM_172027 | ABTB1 | ankyrin repeat and BTB (POZ) domai | -0.115 | 0.923 | 1.27E-01 | 1.27E+01 | | | |
| 8148385 | NM_003235 | TG | thyroglobulin | -0.115 | 0.923 | 2.48E-02 | 2.48E+00 | | | |
| 8159439 | NM_00108048 | LOC389813 | similar to CG15216-PA | -0.115 | 0.923 | 5.50E-02 | 5.50E+00 | | | |
| 8144315 | BC026965 | ARHGEF10 | Rho guanine nucleotide exchange fa | -0.115 | 0.923 | 7.94E-02 | 7.94E+00 | | | |
| 8059019 | NM_057093 | CRYBA2 | crystallin, beta A2 | -0.115 | 0.923 | 8.51E-02 | 8.51E+00 | | | |
| 8163019 | NM_006686 | ACTL7B | actin-like 7B | -0.115 | 0.923 | 6.63E-02 | 6.63E+00 | | | |
| 8050232 | NM_00103716 | CYS1 | cystin 1 | -0.115 | 0.923 | 5.07E-02 | 5.07E+00 | | | |
| 7917737 | --- | --- | --- | -0.115 | 0.923 | 1.10E-01 | 1.10E+01 | | | |
| 8048782 | NM_000091 | COL4A3 | collagen, type IV, alpha 3 (Goodpastu | -0.115 | 0.923 | 4.54E-02 | 4.54E+00 | | | |
| 7997676 | BC030801 | TMEM148 | transmembrane protein 148 | -0.115 | 0.923 | 4.18E-02 | 4.18E+00 | | | |
| 8011131 | NM_031430 | RILP | Rab interacting lysosomal protein | -0.115 | 0.923 | 5.54E-02 | 5.54E+00 | | | |
| 8125321 | NM_030651 | PRRT1 | proline-rich transmembrane protein | -0.116 | 0.923 | 2.57E-02 | 2.57E+00 | | | |
| 8026090 | NM_000159 | GCDH | glutaryl-Coenzyme A dehydrogenase | -0.116 | 0.923 | 1.02E-01 | 1.02E+01 | | | |
| 8049919 | NM_152783 | D2HGDH | D-2-hydroxyglutarate dehydrogenase | -0.116 | 0.923 | 1.90E-02 | 1.90E+00 | | | |
| 7896921 | NM_152228 | TAS1R3 | taste receptor, type 1, member 3 | -0.116 | 0.923 | 3.58E-02 | 3.58E+00 | | | |
| 8071737 | NM_002415 | MIF | macrophage migration inhibitory fac | -0.116 | 0.923 | 1.63E-01 | 1.63E+01 | | | |
| 7932014 | NR_024256 | FLJ45983 | hypothetical LOC399717 | -0.116 | 0.923 | 3.99E-02 | 3.99E+00 | | | |
| 8166899 | NM_022567 | NYX | nyctalopin | -0.116 | 0.923 | 5.66E-02 | 5.66E+00 | | | |
| 8012883 | NM_006042 | HS3ST3A1 | heparan sulfate (glucosamine) 3-O-s | -0.116 | 0.923 | 4.40E-02 | 4.40E+00 | | | |
| 8125772 | NR_027908 | C6orf227 | chromosome 6 open reading frame 2 | -0.116 | 0.923 | 4.41E-02 | 4.41E+00 | | | |
| 8015223 | NM_031854 | KRTAP4-12 | keratin associated protein 4-12 | -0.116 | 0.923 | 4.03E-02 | 4.03E+00 | | | |
| 8019565 | NM_031854 | KRTAP4-12 | keratin associated protein 4-12 | -0.116 | 0.923 | 4.03E-02 | 4.03E+00 | | | |
| 8167862 | NM_00101503 | PAGE2B | P antigen family, member 2B | -0.116 | 0.923 | 2.89E-01 | 2.89E+01 | | | |
| 7895744 | --- | --- | --- | -0.116 | 0.923 | 4.93E-01 | 4.93E+01 | | | |
| 7958577 | NM_032829 | C12orf34 | chromosome 12 open reading frame | -0.116 | 0.923 | 6.15E-02 | 6.15E+00 | | | |
| 8032608 | NM_174983 | C19orf28 | chromosome 19 open reading frame | -0.116 | 0.923 | 3.99E-02 | 3.99E+00 | | | |
| 7949277 | NM_015104 | ATG2A | ATG2 autophagy related 2 homolog A | -0.116 | 0.923 | 3.86E-02 | 3.86E+00 | | | |
| 8018906 | --- | --- | --- | -0.116 | 0.923 | 1.57E-01 | 1.57E+01 | | | |
| 8039730 | NM_023926 | ZSCAN18 | zinc finger and SCAN domain contair | -0.116 | 0.923 | 3.81E-02 | 3.81E+00 | | | |
| 8144874 | AY176665 | NSAP11 | nervous system abundant protein 11 | -0.116 | 0.923 | 3.46E-02 | 3.46E+00 | | | |
| 7993296 | NM_00114520 | LOC729993 | hypothetical protein LOC729993 | -0.116 | 0.923 | 5.82E-02 | 5.82E+00 | | | |
| 7907882 | NM_020950 | KIAA1614 | KIAA1614 | -0.116 | 0.923 | 5.06E-02 | 5.06E+00 | | | |
| 8111203 | NR_027253 | LOC285696 | hypothetical LOC285696 | -0.116 | 0.923 | 7.51E-02 | 7.51E+00 | | | |
| 8175815 | NM_00103958 | PNCK | pregnancy up-regulated non-ubiquit | -0.116 | 0.923 | 3.07E-02 | 3.07E+00 | | | |
| 8007100 | NM_001552 | IGFBP4 | insulin-like growth factor binding pro | -0.116 | 0.923 | 3.11E-02 | 3.11E+00 | | | |
| 8138450 | NM_152898 | FERD3L | Fer3-like (Drosophila) | -0.116 | 0.923 | 6.38E-02 | 6.38E+00 | | | |
| 7895988 | --- | --- | --- | -0.116 | 0.923 | 1.60E-01 | 1.60E+01 | | | |
| 8153390 | NM_052963 | TOP1MT | topoisomerase (DNA) I, mitochondri | -0.116 | 0.923 | 3.47E-01 | 3.47E+01 | | | |
| 8058516 | --- | --- | --- | -0.116 | 0.923 | 1.65E-01 | 1.65E+01 | | | |
| 7996027 | NM_002996 | CX3CL1 | chemokine (C-X3-C motif) ligand 1 | -0.116 | 0.923 | 4.38E-02 | 4.38E+00 | | | |
| 8137707 | --- | --- | --- | -0.116 | 0.923 | 9.17E-02 | 9.17E+00 | | | |
| 7905483 | NM_178438 | LCE5A | late cornified envelope 5A | -0.116 | 0.923 | 6.67E-02 | 6.67E+00 | | | |
| 7894283 | --- | --- | --- | -0.116 | 0.923 | 8.51E-01 | 8.51E+01 | | | |
| 7892794 | --- | --- | --- | -0.116 | 0.923 | 7.99E-01 | 7.99E+01 | | | |
| 7989491 | AB209041 | TPM1 | tropomyosin 1 (alpha) | -0.116 | 0.923 | 5.63E-02 | 5.63E+00 | | | |
| 8150244 | NM_152413 | GOT1L1 | glutamic-oxaloacetic transaminase 1 | -0.116 | 0.923 | 1.50E-01 | 1.50E+01 | | | |
| 7918048 | --- | --- | --- | -0.116 | 0.923 | 8.14E-01 | 8.14E+01 | | | |
| 7990345 | NM_003612 | SEMA7A | semaphorin 7A, GPI membrane anch | -0.116 | 0.923 | 6.33E-02 | 6.33E+00 | | | |
| 8026405 | NM_00100471 | OR1I1 | olfactory receptor, family 1, subfami | -0.116 | 0.923 | 1.56E-01 | 1.56E+01 | | | |
| 7996185 | NM_002428 | MMP15 | matrix metallopeptidase 15 (membra | -0.116 | 0.923 | 2.77E-02 | 2.77E+00 | | | |
| 7937749 | NM_006757 | TNNT3 | troponin T type 3 (skeletal, fast) | -0.116 | 0.923 | 6.49E-02 | 6.49E+00 | | | |
| 7995310 | ENST0000031 | MGC34800 | hypothetical protein MGC34800 | -0.116 | 0.923 | 3.58E-02 | 3.58E+00 | | | |
| 7902267 | NR_023916 | PIN1L | peptidylprolyl cis/trans isomerase, N | -0.116 | 0.923 | 1.48E-02 | 1.48E+00 | | | |
| 8167835 | NM_00103970 | TRO | trophinin | -0.116 | 0.923 | 1.19E-01 | 1.19E+01 | | | |
| 7980316 | NM_003239 | TGFB3 | transforming growth factor, beta 3 | -0.116 | 0.923 | 1.31E-01 | 1.31E+01 | | | |
| 8066598 | NM_005469 | ACOT8 | acyl-CoA thioesterase 8 | -0.116 | 0.923 | 2.63E-01 | 2.63E+01 | | | |
| 7997491 | NM_002153 | HSD17B2 | hydroxysteroid (17-beta) dehydroge | -0.116 | 0.923 | 4.51E-02 | 4.51E+00 | | | |
| 7906146 | NR_026678 | TMEM79 | transmembrane protein 79 | -0.116 | 0.923 | 5.48E-02 | 5.48E+00 | | | |
| 7945344 | NR_024368 | tcag7.907 | hypothetical LOC402483 | -0.116 | 0.923 | 9.22E-02 | 9.22E+00 | | | |
| 8148621 | NM_052924 | RHPN1 | rhophilin, Rho GTPase binding protei | -0.116 | 0.923 | 5.10E-02 | 5.10E+00 | | | |
| 8066985 | AK299337 | FAM65C | family with sequence similarity 65, m | -0.116 | 0.923 | 1.77E-02 | 1.77E+00 | | | |
| 8036622 | NM_198445 | RINL | Ras and Rab interactor-like | -0.116 | 0.923 | 3.46E-02 | 3.46E+00 | | | |
| 8027053 | NM_00109848 | CRTC1 | CREB regulated transcription coactiv | -0.116 | 0.923 | 5.60E-02 | 5.60E+00 | | | |
| 8036079 | NM_033317 | DMKN | dermokine | -0.116 | 0.923 | 3.80E-02 | 3.80E+00 | | | |
| 7975076 | NM_021979 | HSPA2 | heat shock 70kDa protein 2 | -0.116 | 0.923 | 1.67E-01 | 1.67E+01 | | | |
| 8012079 | NM_004422 | DVL2 | dishevelled, dsh homolog 2 (Drosoph | -0.116 | 0.923 | 6.12E-02 | 6.12E+00 | | | |
| 7998692 | NM_002528 | NTHL1 | nth endonuclease III-like 1 (E. coli) | -0.116 | 0.923 | 2.58E-02 | 2.58E+00 | | | |
| 7905490 | NM_178434 | LCE3C | late cornified envelope 3C | -0.116 | 0.923 | 1.20E-01 | 1.20E+01 | | | |
| 8132694 | NM_000596 | IGFBP1 | insulin-like growth factor binding pro | -0.116 | 0.923 | 6.09E-02 | 6.09E+00 | | | |
| 8032051 | NM_182577 | ODF3L2 | outer dense fiber of sperm tails 3-lik | -0.116 | 0.923 | 4.54E-02 | 4.54E+00 | | | |
| 8065327 | NM_033176 | NKX2-4 | NK2 homeobox 4 | -0.116 | 0.923 | 5.34E-02 | 5.34E+00 | | | |

| | | | | | | | | | | |
|---|---|---|---|---|---|---|---|---|---|---|
| 7916412 | NM_147161 | ACOT11 | acyl-CoA thioesterase 11 | -0.116 | 0.923 | 7.07E-02 | 7.07E+00 | | | |
| 8149685 | NM_139278 | LGI3 | leucine-rich repeat LGI family, memb | -0.116 | 0.923 | 4.77E-02 | 4.77E+00 | | | |
| 8051238 | NM_00102988 | C2orf71 | chromosome 2 open reading frame 7 | -0.116 | 0.923 | 2.91E-02 | 2.91E+00 | | | |
| 8053171 | NM_032779 | CCDC142 | coiled-coil domain containing 142 | -0.116 | 0.923 | 5.62E-02 | 5.62E+00 | | | |
| 7928126 | NM_014431 | KIAA1274 | KIAA1274 | -0.116 | 0.923 | 6.59E-02 | 6.59E+00 | | | |
| 7959402 | NM_030765 | B3GNT4 | UDP-GlcNAc:betaGal beta-1,3-N-ace | -0.116 | 0.923 | 5.24E-02 | 5.24E+00 | | | |
| 8137781 | ENST0000044 | OC10012865 | hypothetical LOC100128653 | -0.116 | 0.923 | 4.20E-02 | 4.20E+00 | | | |
| 8039779 | NM_012254 | SLC27A5 | solute carrier family 27 (fatty acid tra | -0.116 | 0.923 | 4.65E-02 | 4.65E+00 | | | |
| 8137739 | NM_00108045 | INTS1 | integrator complex subunit 1 | -0.116 | 0.922 | 1.72E-02 | 1.72E+00 | | | |
| 8013049 | --- | --- | --- | -0.116 | 0.922 | 2.86E-02 | 2.86E+00 | | | |
| 7929130 | --- | --- | --- | -0.116 | 0.922 | 3.80E-01 | 3.80E+01 | | | |
| 8031374 | NM_002000 | FCAR | Fc fragment of IgA, receptor for | -0.117 | 0.922 | 2.15E-01 | 2.15E+01 | | | |
| 8037947 | NM_198479 | TPRX1 | tetra-peptide repeat homeobox 1 | -0.117 | 0.922 | 1.68E-01 | 1.68E+01 | | | |
| 7892721 | --- | --- | --- | -0.117 | 0.922 | 1.69E-01 | 1.69E+01 | | | |
| 7952893 | BC024764 | IQSEC3 | IQ motif and Sec7 domain 3 | -0.117 | 0.922 | 2.06E-02 | 2.06E+00 | | | |
| 7940358 | NM_207341 | ZP1 | zona pellucida glycoprotein 1 (sperm | -0.117 | 0.922 | 5.15E-02 | 5.15E+00 | | | |
| 8030753 | NM_00103004 | KLK3 | kallikrein-related peptidase 3 | -0.117 | 0.922 | 6.35E-02 | 6.35E+00 | | | |
| 8021963 | NM_001071 | TYMS | thymidylate synthetase | -0.117 | 0.922 | 4.47E-02 | 4.47E+00 | | | |
| 7989718 | NM_016563 | RASL12 | RAS-like, family 12 | -0.117 | 0.922 | 5.04E-02 | 5.04E+00 | | | |
| 8148824 | NM_005526 | HSF1 | heat shock transcription factor 1 | -0.117 | 0.922 | 3.69E-02 | 3.69E+00 | | | |
| 8092162 | ENST0000033 | PP13439 | hypothetical LOC100128046 | -0.117 | 0.922 | 2.86E-02 | 2.86E+00 | | | |
| 8053842 | NM_207328 | GPAT2 | glycerol-3-phosphate acyltransferase | -0.117 | 0.922 | 2.45E-02 | 2.45E+00 | | | |
| 7995342 | --- | --- | --- | -0.117 | 0.922 | 2.13E-01 | 2.13E+01 | | | |
| 7939432 | --- | --- | --- | -0.117 | 0.922 | 6.70E-01 | 6.70E+01 | | | |
| 7999674 | NM_022844 | MYH11 | myosin, heavy chain 11, smooth mus | -0.117 | 0.922 | 1.79E-02 | 1.79E+00 | | | |
| 7963631 | NM_000966 | RARG | retinoic acid receptor, gamma | -0.117 | 0.922 | 8.96E-02 | 8.96E+00 | | | |
| 7894249 | --- | --- | --- | -0.117 | 0.922 | 9.31E-02 | 9.31E+00 | | | |
| 7911796 | NR_024371 | FLJ42875 | hypothetical LOC440556 | -0.117 | 0.922 | 5.26E-02 | 5.26E+00 | | | |
| 8105741 | NM_015183 | MAST4 | microtubule associated serine/threo | -0.117 | 0.922 | 1.26E-02 | 1.26E+00 | | | |
| 8141295 | NM_004889 | ATP5J2 | ATP synthase, H+ transporting, mito | -0.117 | 0.922 | 8.80E-01 | 8.80E+01 | | | |
| 7912764 | NM_198546 | SPATA21 | spermatogenesis associated 21 | -0.117 | 0.922 | 7.67E-02 | 7.67E+00 | | | |
| 7895603 | --- | --- | --- | -0.117 | 0.922 | 5.32E-01 | 5.32E+01 | | | |
| 8032076 | NM_194460 | RNF126 | ring finger protein 126 | -0.117 | 0.922 | 4.82E-02 | 4.82E+00 | | | |
| 7923347 | NM_005558 | LAD1 | ladinin 1 | -0.117 | 0.922 | 2.52E-02 | 2.52E+00 | | | |
| 8137219 | NM_138434 | C7orf29 | chromosome 7 open reading frame 2 | -0.117 | 0.922 | 8.15E-02 | 8.15E+00 | | | |
| 7981919 | NM_022807 | SNRPN | small nuclear ribonucleoprotein poly | -0.117 | 0.922 | 7.38E-01 | 7.38E+01 | | | |
| 8048460 | NM_003936 | CDK5R2 | cyclin-dependent kinase 5, regulator | -0.117 | 0.922 | 3.32E-02 | 3.32E+00 | | | |
| 7974793 | NM_007374 | SIX6 | SIX homeobox 6 | -0.117 | 0.922 | 4.58E-02 | 4.58E+00 | | | |
| 8143635 | NM_00108041 | NOBOX | NOBOX oogenesis homeobox | -0.117 | 0.922 | 3.03E-02 | 3.03E+00 | | | |
| 8066683 | NM_021248 | CDH22 | cadherin-like 22 | -0.117 | 0.922 | 4.73E-02 | 4.73E+00 | | | |
| 7967789 | NM_018663 | PXMP2 | peroxisomal membrane protein 2, 22 | -0.117 | 0.922 | 9.49E-02 | 9.49E+00 | | | |
| 7976560 | NM_000623 | BDKRB2 | bradykinin receptor B2 | -0.117 | 0.922 | 7.71E-02 | 7.71E+00 | | | |
| 7911241 | NM_00100196 | OR2L8 | olfactory receptor, family 2, subfami | -0.117 | 0.922 | 5.66E-01 | 5.66E+01 | | | |
| 7901746 | --- | --- | --- | -0.117 | 0.922 | 7.30E-02 | 7.30E+00 | | | |
| 7994967 | NM_00109978 | FBXL19 | F-box and leucine-rich repeat protei | -0.117 | 0.922 | 3.10E-02 | 3.10E+00 | | | |
| 8180219 | --- | --- | --- | -0.117 | 0.922 | 1.46E-01 | 1.46E+01 | | | |
| 8067773 | NM_020713 | ZNF512B | zinc finger protein 512B | -0.117 | 0.922 | 8.66E-02 | 8.66E+00 | | | |
| 8071758 | NM_005940 | MMP11 | matrix metallopeptidase 11 (stromel | -0.117 | 0.922 | 3.88E-02 | 3.88E+00 | | | |
| 7934896 | --- | --- | --- | -0.117 | 0.922 | 4.24E-01 | 4.24E+01 | | | |
| 7908637 | NM_005298 | GPR25 | G protein-coupled receptor 25 | -0.117 | 0.922 | 2.91E-02 | 2.91E+00 | | | |
| 7981215 | --- | --- | --- | -0.117 | 0.922 | 7.22E-01 | 7.22E+01 | | | |
| 7900538 | --- | --- | --- | -0.117 | 0.922 | 9.94E-02 | 9.94E+00 | | | |
| 8088047 | NM_205853 | MUSTN1 | musculoskeletal, embryonic nuclear | -0.117 | 0.922 | 2.86E-01 | 2.86E+01 | | | |
| 8026198 | NM_023072 | ZSWIM4 | zinc finger, SWIM-type containing 4 | -0.117 | 0.922 | 5.61E-02 | 5.61E+00 | | | |
| 7924465 | NM_024746 | HHIPL2 | HHIP-like 2 | -0.117 | 0.922 | 4.07E-02 | 4.07E+00 | | | |
| 8159491 | NM_000606 | C8G | complement component 8, gamma p | -0.117 | 0.922 | 7.94E-02 | 7.94E+00 | | | |
| 8165406 | NM_015392 | NPDC1 | neural proliferation, differentiation a | -0.117 | 0.922 | 4.02E-02 | 4.02E+00 | | | |
| 8014679 | NM_025248 | SNIP | SNAP25-interacting protein | -0.117 | 0.922 | 3.91E-02 | 3.91E+00 | | | |
| 7921342 | AK057458 | hCG_199489 | hCG1994895 | -0.117 | 0.922 | 5.02E-02 | 5.02E+00 | | | |
| 7901557 | NM_033067 | DMRTB1 | DMRT-like family B with proline-rich | -0.117 | 0.922 | 8.49E-03 | 8.49E-01 | | | |
| 7976635 | BC093661 | C14orf177 | chromosome 14 open reading frame | -0.117 | 0.922 | 4.38E-02 | 4.38E+00 | | | |
| 8001041 | NM_005205 | COX6A2 | cytochrome c oxidase subunit VIa po | -0.117 | 0.922 | 2.18E-02 | 2.18E+00 | | | |
| 8046276 | NM_00100384 | SP5 | Sp5 transcription factor | -0.117 | 0.922 | 5.78E-02 | 5.78E+00 | | | |
| 7931405 | NM_018461 | PPP2R2D | protein phosphatase 2, regulatory su | -0.117 | 0.922 | 6.44E-01 | 6.44E+01 | | | |
| 8085311 | NM_00100133 | ATP2B2 | ATPase, Ca++ transporting, plasma m | -0.117 | 0.922 | 1.82E-02 | 1.82E+00 | | | |
| 8094148 | --- | --- | --- | -0.117 | 0.922 | 5.74E-02 | 5.74E+00 | | | |
| 8116400 | --- | --- | --- | -0.117 | 0.922 | 1.92E-01 | 1.92E+01 | | | |
| 8163936 | NM_014368 | LHX6 | LIM homeobox 6 | -0.117 | 0.922 | 2.25E-02 | 2.25E+00 | | | |
| 8143564 | NM_00103169 | FAM131B | family with sequence similarity 131, | -0.117 | 0.922 | 4.82E-02 | 4.82E+00 | | | |
| 7897737 | NM_198545 | C1orf187 | chromosome 1 open reading frame 1 | -0.117 | 0.922 | 1.11E-01 | 1.11E+01 | | | |
| 8039484 | NM_000641 | IL11 | interleukin 11 | -0.117 | 0.922 | 4.32E-02 | 4.32E+00 | | | |
| 8162664 | AY358791 | UNQ5836 | HSAL5836 | -0.117 | 0.922 | 1.53E-01 | 1.53E+01 | | | |
| 8110518 | NM_00114230 | LOC285679 | hypothetical protein LOC285679 | -0.117 | 0.922 | 5.30E-02 | 5.30E+00 | | | |
| 7895532 | --- | --- | --- | -0.117 | 0.922 | 6.18E-01 | 6.18E+01 | | | |
| 8018107 | NM_012121 | CDC42EP4 | CDC42 effector protein (Rho GTPase | -0.117 | 0.922 | 8.06E-02 | 8.06E+00 | | | |
| 8171240 | NM_016378 | VCX2 | variable charge, X-linked 2 | -0.117 | 0.922 | 4.22E-02 | 4.22E+00 | | | |
| 8051061 | NM_003353 | UCN | urocortin | -0.117 | 0.922 | 4.97E-02 | 4.97E+00 | | | |

| | | | | | | | | | | |
|---|---|---|---|---|---|---|---|---|---|---|
| 7969286 | --- | --- | --- | -0.117 | 0.922 | 9.66E-02 | 9.66E+00 | | | |
| 7906140 | NM_199173 | BGLAP | bone gamma-carboxyglutamate (gla) | -0.117 | 0.922 | 1.37E-01 | 1.37E+01 | | | |
| 7945974 | AF439154 | C11orf40 | chromosome 11 open reading frame | -0.117 | 0.922 | 8.56E-02 | 8.56E+00 | | | |
| 8135969 | NM_022742 | CCDC136 | coiled-coil domain containing 136 | -0.117 | 0.922 | 6.89E-02 | 6.89E+00 | | | |
| 8014738 | NM_017748 | CCDC49 | coiled-coil domain containing 49 | -0.117 | 0.922 | 4.32E-01 | 4.32E+01 | | | |
| 8099965 | NM_007274 | ACOT7 | acyl-CoA thioesterase 7 | -0.117 | 0.922 | 4.13E-01 | 4.13E+01 | | | |
| 7934916 | NM_003956 | CH25H | cholesterol 25-hydroxylase | -0.117 | 0.922 | 3.58E-02 | 3.58E+00 | | | |
| 7905382 | NM_020832 | ZNF687 | zinc finger protein 687 | -0.117 | 0.922 | 3.62E-02 | 3.62E+00 | | | |
| 7964646 | NM_020700 | PPM1H | protein phosphatase 1H (PP2C doma | -0.117 | 0.922 | 1.35E-01 | 1.35E+01 | | | |
| 7919267 | --- | --- | --- | -0.117 | 0.922 | 5.34E-02 | 5.34E+00 | | | |
| 7919347 | --- | --- | --- | -0.117 | 0.922 | 5.34E-02 | 5.34E+00 | | | |
| 8156088 | --- | --- | --- | -0.118 | 0.922 | 1.82E-01 | 1.82E+01 | | | |
| 8131339 | NM_003088 | FSCN1 | fascin homolog 1, actin-bundling pro | -0.118 | 0.922 | 8.46E-02 | 8.46E+00 | | | |
| 8003204 | NM_016095 | GINS2 | GINS complex subunit 2 (Psf2 homol | -0.118 | 0.922 | 7.76E-02 | 7.76E+00 | | | |
| 7896592 | --- | --- | --- | -0.118 | 0.922 | 2.51E-01 | 2.51E+01 | | | |
| 8172030 | --- | --- | --- | -0.118 | 0.922 | 3.68E-02 | 3.68E+00 | | | |
| 7905523 | NM_178352 | LCE1D | late cornified envelope 1D | -0.118 | 0.922 | 4.07E-02 | 4.07E+00 | | | |
| 8060705 | NM_020746 | MAVS | mitochondrial antiviral signaling prot | -0.118 | 0.922 | 5.25E-02 | 5.25E+00 | | | |
| 8010113 | NM_144677 | MGAT5B | mannosyl (alpha-1,6-)-glycoprotein b | -0.118 | 0.922 | 2.91E-02 | 2.91E+00 | | | |
| 8039269 | NM_198988 | LENG9 | leukocyte receptor cluster (LRC) mer | -0.118 | 0.922 | 5.62E-02 | 5.62E+00 | | | |
| 7940749 | NM_080866 | SLC22A9 | solute carrier family 22 (organic anio | -0.118 | 0.922 | 3.52E-02 | 3.52E+00 | | | |
| 8104136 | NM_018942 | HMX1 | H6 family homeobox 1 | -0.118 | 0.922 | 1.54E-02 | 1.54E+00 | | | |
| 8090803 | NM_016577 | RAB6B | RAB6B, member RAS oncogene famil | -0.118 | 0.922 | 3.90E-02 | 3.90E+00 | | | |
| 8158684 | --- | --- | --- | -0.118 | 0.922 | 2.54E-01 | 2.54E+01 | | | |
| 8009713 | NM_178233 | OTOP3 | otopetrin 3 | -0.118 | 0.922 | 4.51E-02 | 4.51E+00 | | | |
| 7893337 | --- | --- | --- | -0.118 | 0.922 | 1.88E-01 | 1.88E+01 | | | |
| 7991695 | NR_027407 | GOLGA8D | golgi autoantigen, golgin subfamily a | -0.118 | 0.922 | 6.23E-02 | 6.23E+00 | | | |
| 7984538 | --- | --- | --- | -0.118 | 0.922 | 1.50E-01 | 1.50E+01 | | | |
| 8012126 | NM_001307 | CLDN7 | claudin 7 | -0.118 | 0.922 | 3.90E-02 | 3.90E+00 | | | |
| 8098870 | NM_012445 | SPON2 | spondin 2, extracellular matrix prote | -0.118 | 0.922 | 1.01E-01 | 1.01E+01 | | | |
| 8155440 | NM_00109927 | FOXD4L2 | forkhead box D4-like 2 | -0.118 | 0.922 | 2.37E-02 | 2.37E+00 | | | |
| 8161583 | NM_00109927 | FOXD4L2 | forkhead box D4-like 2 | -0.118 | 0.922 | 2.37E-02 | 2.37E+00 | | | |
| 8122773 | NM_015440 | MTHFD1L | methylenetetrahydrofolate dehydro | -0.118 | 0.922 | 4.47E-02 | 4.47E+00 | | | |
| 7954692 | NR_026806 | FLJ13224 | hypothetical protein FLJ13224 | -0.118 | 0.922 | 8.86E-02 | 8.86E+00 | | | |
| 8129783 | NM_003980 | MAP7 | microtubule-associated protein 7 | -0.118 | 0.922 | 4.08E-02 | 4.08E+00 | | | |
| 8003611 | NM_024792 | FAM57A | family with sequence similarity 57, m | -0.118 | 0.922 | 7.37E-02 | 7.37E+00 | | | |
| 8003733 | NM_014853 | SGSM2 | small G protein signaling modulator | -0.118 | 0.922 | 6.32E-02 | 6.32E+00 | | | |
| 8024070 | NM_032551 | KISS1R | KISS1 receptor | -0.118 | 0.922 | 9.07E-03 | 9.07E-01 | | | |
| 7953032 | NM_00103902 | LRTM2 | leucine-rich repeats and transmemb | -0.118 | 0.922 | 5.05E-02 | 5.05E+00 | | | |
| 7937228 | NM_00101250 | SPRN | shadow of prion protein homolog (ze | -0.118 | 0.922 | 4.16E-02 | 4.16E+00 | | | |
| 8045083 | NM_032740 | SFT2D3 | SFT2 domain containing 3 | -0.118 | 0.922 | 1.33E-01 | 1.33E+01 | | | |
| 7892878 | --- | --- | --- | -0.118 | 0.922 | 6.86E-01 | 6.86E+01 | | | |
| 8170420 | NM_005491 | MAMLD1 | mastermind-like domain containing | -0.118 | 0.922 | 5.00E-02 | 5.00E+00 | | | |
| 8094068 | --- | --- | --- | -0.118 | 0.922 | 5.82E-01 | 5.82E+01 | | | |
| 8119599 | NM_138296 | PTCRA | pre T-cell antigen receptor alpha | -0.118 | 0.922 | 5.23E-02 | 5.23E+00 | | | |
| 8028030 | NM_052948 | SNX26 | sorting nexin 26 | -0.118 | 0.922 | 4.39E-02 | 4.39E+00 | | | |
| 8051187 | NM_022823 | FNDC4 | fibronectin type III domain containin | -0.118 | 0.922 | 3.18E-02 | 3.18E+00 | | | |
| 8130993 | NM_020223 | FAM20C | family with sequence similarity 20, m | -0.118 | 0.922 | 4.37E-02 | 4.37E+00 | | | |
| 7956261 | NM_002898 | RBMS2 | RNA binding motif, single stranded in | -0.118 | 0.922 | 2.48E-01 | 2.48E+01 | | | |
| 8015196 | NM_030967 | KRTAP1-1 | keratin associated protein 1-1 | -0.118 | 0.922 | 2.11E-02 | 2.11E+00 | | | |
| 8019578 | NM_030967 | KRTAP1-1 | keratin associated protein 1-1 | -0.118 | 0.922 | 2.11E-02 | 2.11E+00 | | | |
| 7913776 | NM_170743 | IL28RA | interleukin 28 receptor, alpha (interf | -0.118 | 0.922 | 2.35E-02 | 2.35E+00 | | | |
| 8074106 | NM_138433 | KLHDC7B | kelch domain containing 7B | -0.118 | 0.922 | 3.65E-02 | 3.65E+00 | | | |
| 7924682 | NM_003240 | LEFTY2 | left-right determination factor 2 | -0.118 | 0.921 | 6.15E-02 | 6.15E+00 | | | |
| 7895146 | --- | --- | --- | -0.118 | 0.921 | 7.96E-01 | 7.96E+01 | | | |
| 8039605 | NM_00100585 | ZNF835 | zinc finger protein 835 | -0.118 | 0.921 | 7.43E-02 | 7.43E+00 | | | |
| 7931553 | NM_003577 | UTF1 | undifferentiated embryonic cell tran | -0.118 | 0.921 | 5.06E-02 | 5.06E+00 | | | |
| 8049598 | NM_194312 | ESPNL | espin-like | -0.118 | 0.921 | 4.20E-02 | 4.20E+00 | | | |
| 7895563 | --- | --- | --- | -0.118 | 0.921 | 8.74E-01 | 8.74E+01 | | | |
| 8076747 | AK123383 | LOC642648 | hypothetical LOC642648 | -0.118 | 0.921 | 5.94E-02 | 5.94E+00 | | | |
| 7990283 | NM_00103864 | GOLGA6 | golgi autoantigen, golgin subfamily a | -0.118 | 0.921 | 1.21E-02 | 1.21E+00 | | | |
| 8061490 | NR_026713 | FAM182A | family with sequence similarity 182, | -0.118 | 0.921 | 1.17E-01 | 1.17E+01 | | | |
| 7939751 | NM_005693 | NR1H3 | nuclear receptor subfamily 1, group | -0.118 | 0.921 | 9.86E-02 | 9.86E+00 | | | |
| 7998843 | NM_031948 | PRSS27 | protease, serine 27 | -0.118 | 0.921 | 3.92E-02 | 3.92E+00 | | | |
| 8000917 | NM_152458 | ZNF785 | zinc finger protein 785 | -0.118 | 0.921 | 3.49E-02 | 3.49E+00 | | | |
| 8114780 | NM_016580 | PCDH12 | protocadherin 12 | -0.118 | 0.921 | 4.47E-02 | 4.47E+00 | | | |
| 8056825 | NM_00114525 | SP9 | Sp9 transcription factor homolog (m | -0.118 | 0.921 | 2.38E-02 | 2.38E+00 | | | |
| 8076749 | NM_207327 | C22orf40 | chromosome 22 open reading frame | -0.118 | 0.921 | 1.77E-01 | 1.77E+01 | | | |
| 8070853 | NR_027129 | C21orf67 | chromosome 21 open reading frame | -0.118 | 0.921 | 4.77E-02 | 4.77E+00 | | | |
| 8024728 | NM_170678 | ITGB1BP3 | integrin beta 1 binding protein 3 | -0.118 | 0.921 | 3.87E-02 | 3.87E+00 | | | |
| 8059985 | NR_026925 | LOC151174 | hypothetical LOC151174 | -0.118 | 0.921 | 1.45E-01 | 1.45E+01 | | | |
| 8170662 | NM_005629 | SLC6A8 | solute carrier family 6 (neurotransmi | -0.118 | 0.921 | 8.03E-02 | 8.03E+00 | | | |
| 8076195 | NM_002608 | PDGFB | platelet-derived growth factor beta p | -0.118 | 0.921 | 4.51E-02 | 4.51E+00 | | | |
| 7956281 | NM_007264 | GPR182 | G protein-coupled receptor 182 | -0.118 | 0.921 | 3.73E-02 | 3.73E+00 | | | |
| 7970455 | NM_015974 | CRYL1 | crystallin, lambda 1 | -0.118 | 0.921 | 2.54E-01 | 2.54E+01 | | | |
| 8014326 | NM_145654 | RDM1 | RAD52 motif 1 | -0.118 | 0.921 | 8.02E-02 | 8.02E+00 | | | |
| 8166661 | --- | --- | --- | -0.118 | 0.921 | 9.81E-02 | 9.81E+00 | | | |

| | | | | | | | | | | |
|---|---|---|---|---|---|---|---|---|---|---|
| 7895912 | --- | --- | --- | -0.118 | 0.921 | 2.05E-01 | 2.05E+01 | | | |
| 7943711 | BC068577 | C11orf87 | chromosome 11 open reading frame | -0.118 | 0.921 | 5.63E-02 | 5.63E+00 | | | |
| 8038427 | NM_021733 | TSKS | testis-specific serine kinase substrate | -0.118 | 0.921 | 6.88E-02 | 6.88E+00 | | | |
| 8180333 | --- | --- | --- | -0.118 | 0.921 | 4.89E-01 | 4.89E+01 | | | |
| 8125731 | NM_005453 | ZBTB22 | zinc finger and BTB domain containin | -0.118 | 0.921 | 6.96E-02 | 6.96E+00 | | | |
| 8171867 | NM_139058 | ARX | aristaless related homeobox | -0.118 | 0.921 | 3.93E-02 | 3.93E+00 | | | |
| 8010638 | NM_004712 | HGS | hepatocyte growth factor-regulated | -0.118 | 0.921 | 2.53E-02 | 2.53E+00 | | | |
| 8122196 | --- | --- | --- | -0.118 | 0.921 | 6.51E-02 | 6.51E+00 | | | |
| 8065948 | NR_024377 | FER1L4 | fer-1-like 4 (C. elegans) | -0.118 | 0.921 | 5.80E-02 | 5.80E+00 | | | |
| 7905862 | NM_152494 | DCST1 | DC-STAMP domain containing 1 | -0.118 | 0.921 | 6.72E-02 | 6.72E+00 | | | |
| 7991877 | NM_005632 | SOLH | small optic lobes homolog (Drosophi | -0.118 | 0.921 | 2.10E-02 | 2.10E+00 | | | |
| 7992811 | NM_022468 | MMP25 | matrix metallopeptidase 25 | -0.118 | 0.921 | 3.05E-02 | 3.05E+00 | | | |
| 8165478 | NM_173691 | C9orf75 | chromosome 9 open reading frame 7 | -0.118 | 0.921 | 2.66E-02 | 2.66E+00 | | | |
| 8027348 | AK131472 | ZNF730 | zinc finger protein 730 | -0.118 | 0.921 | 3.11E-01 | 3.11E+01 | | | |
| 8140909 | NM_014590 | ERVWE1 | endogenous retroviral family W, env | -0.118 | 0.921 | 3.28E-01 | 3.28E+01 | | | |
| 8067506 | BC137326 | C20orf151 | chromosome 20 open reading frame | -0.118 | 0.921 | 2.95E-02 | 2.95E+00 | | | |
| 7905037 | --- | --- | --- | -0.119 | 0.921 | 1.72E-01 | 1.72E+01 | | | |
| 7913582 | NM_00107719 | ZNF436 | zinc finger protein 436 | -0.119 | 0.921 | 3.16E-02 | 3.16E+00 | | | |
| 7927383 | NM_153034 | ZNF488 | zinc finger protein 488 | -0.119 | 0.921 | 8.16E-02 | 8.16E+00 | | | |
| 8131803 | NM_000600 | IL6 | interleukin 6 (interferon, beta 2) | -0.119 | 0.921 | 3.71E-01 | 3.71E+01 | | | |
| 8155554 | NR_002817 | AQP7P1 | aquaporin 7 pseudogene 1 | -0.119 | 0.921 | 2.88E-02 | 2.88E+00 | | | |
| 8118228 | NM_021221 | LY6G5B | lymphocyte antigen 6 complex, locus | -0.119 | 0.921 | 8.22E-01 | 8.22E+01 | | | |
| 8029318 | NM_019612 | IRGC | immunity-related GTPase family, cin | -0.119 | 0.921 | 1.41E-01 | 1.41E+01 | | | |
| 8061019 | NM_024120 | C20orf7 | chromosome 20 open reading frame | -0.119 | 0.921 | 6.83E-01 | 6.83E+01 | | | |
| 8048108 | --- | --- | --- | -0.119 | 0.921 | 2.68E-01 | 2.68E+01 | | | |
| 7952436 | NM_138961 | ESAM | endothelial cell adhesion molecule | -0.119 | 0.921 | 6.91E-02 | 6.91E+00 | | | |
| 7915543 | NM_201649 | SLC6A9 | solute carrier family 6 (neurotransmi | -0.119 | 0.921 | 3.05E-02 | 3.05E+00 | | | |
| 7967698 | --- | --- | --- | -0.119 | 0.921 | 8.25E-01 | 8.25E+01 | | | |
| 7974914 | --- | --- | --- | -0.119 | 0.921 | 5.93E-01 | 5.93E+01 | | | |
| 7937378 | NM_030783 | PTDSS2 | phosphatidylserine synthase 2 | -0.119 | 0.921 | 2.37E-02 | 2.37E+00 | | | |
| 7956009 | NM_152637 | METTL7B | methyltransferase like 7B | -0.119 | 0.921 | 7.45E-02 | 7.45E+00 | | | |
| 7949754 | NM_013246 | CLCF1 | cardiotrophin-like cytokine factor 1 | -0.119 | 0.921 | 2.52E-02 | 2.52E+00 | | | |
| 8059580 | NM_139072 | DNER | delta/notch-like EGF repeat containi | -0.119 | 0.921 | 2.96E-02 | 2.96E+00 | | | |
| 8117614 | NM_003527 | HIST1H2BO | histone cluster 1, H2bo | -0.119 | 0.921 | 6.57E-02 | 6.57E+00 | | | |
| 7966570 | NM_00111132 | DDX54 | DEAD (Asp-Glu-Ala-Asp) box polypep | -0.119 | 0.921 | 6.71E-02 | 6.71E+00 | | | |
| 8151609 | NM_172239 | REXO1L1 | REX1, RNA exonuclease 1 homolog (S | -0.119 | 0.921 | 3.81E-02 | 3.81E+00 | | | |
| 7894080 | --- | --- | --- | -0.119 | 0.921 | 4.56E-01 | 4.56E+01 | | | |
| 8023156 | NM_145653 | TCEB3C | transcription elongation factor B pol | -0.119 | 0.921 | 4.64E-02 | 4.64E+00 | | | |
| 7979963 | NM_012074 | DPF3 | D4, zinc and double PHD fingers, fam | -0.119 | 0.921 | 3.18E-02 | 3.18E+00 | | | |
| 8031489 | NM_033113 | ZNF628 | zinc finger protein 628 | -0.119 | 0.921 | 3.01E-02 | 3.01E+00 | | | |
| 8115014 | NM_014443 | IL17B | interleukin 17B | -0.119 | 0.921 | 4.54E-02 | 4.54E+00 | | | |
| 7997962 | NM_004413 | DPEP1 | dipeptidase 1 (renal) | -0.119 | 0.921 | 3.73E-02 | 3.73E+00 | | | |
| 7998590 | NM_080861 | SPSB3 | splA/ryanodine receptor domain and | -0.119 | 0.921 | 5.90E-02 | 5.90E+00 | | | |
| 7940565 | NM_004265 | FADS2 | fatty acid desaturase 2 | -0.119 | 0.921 | 5.77E-02 | 5.77E+00 | | | |
| 8119435 | NM_004828 | NCR2 | natural cytotoxicity triggering recept | -0.119 | 0.921 | 1.04E-01 | 1.04E+01 | | | |
| 7942717 | NM_000260 | MYO7A | myosin VIIA | -0.119 | 0.921 | 3.00E-02 | 3.00E+00 | | | |
| 7906133 | NM_007221 | PMF1 | polyamine-modulated factor 1 | -0.119 | 0.921 | 8.49E-02 | 8.49E+00 | | | |
| 8033414 | NM_174895 | PCP2 | Purkinje cell protein 2 | -0.119 | 0.921 | 2.42E-02 | 2.42E+00 | | | |
| 7936727 | NM_000982 | RPL21 | ribosomal protein L21 | -0.119 | 0.921 | 4.07E-01 | 4.07E+01 | | | |
| 8021091 | NM_213602 | SIGLEC15 | sialic acid binding Ig-like lectin 15 | -0.119 | 0.921 | 6.10E-02 | 6.10E+00 | | | |
| 8067546 | NR_024470 | hCG_201827 | hypothetical protein LOC100127888 | -0.119 | 0.921 | 4.42E-02 | 4.42E+00 | | | |
| 8024347 | NM_079834 | SCAMP4 | secretory carrier membrane protein | -0.119 | 0.921 | 1.46E-02 | 1.46E+00 | | | |
| 8001576 | NM_033212 | CCDC102A | coiled-coil domain containing 102A | -0.119 | 0.921 | 7.34E-02 | 7.34E+00 | | | |
| 7945688 | NR_003512 | INS-IGF2 | INS-IGF2 readthrough transcript | -0.119 | 0.921 | 2.34E-02 | 2.34E+00 | | | |
| 7914974 | NM_000831 | GRIK3 | glutamate receptor, ionotropic, kain | -0.119 | 0.921 | 2.19E-02 | 2.19E+00 | | | |
| 8087461 | NM_013334 | GMPPB | GDP-mannose pyrophosphorylase B | -0.119 | 0.921 | 2.60E-02 | 2.60E+00 | | | |
| 8075659 | NM_203377 | MB | myoglobin | -0.119 | 0.921 | 2.77E-02 | 2.77E+00 | | | |
| 7911333 | NR_024528 | RPL23AP7 | ribosomal protein L23a pseudogene | -0.119 | 0.921 | 5.86E-01 | 5.86E+01 | | | |
| 8069470 | NR_024528 | RPL23AP7 | ribosomal protein L23a pseudogene | -0.119 | 0.921 | 5.86E-01 | 5.86E+01 | | | |
| 8174026 | DQ145510 | LOC392510 | tyrosine 3/tryptophan 5 -monooxyge | -0.119 | 0.921 | 2.30E-01 | 2.30E+01 | | | |
| 8094144 | NM_030930 | UNC93B1 | unc-93 homolog B1 (C. elegans) | -0.119 | 0.921 | 4.74E-02 | 4.74E+00 | | | |
| 7994074 | NM_000336 | SCNN1B | sodium channel, nonvoltage-gated 1 | -0.119 | 0.921 | 4.96E-02 | 4.96E+00 | | | |
| 8110237 | NM_133369 | UNC5A | unc-5 homolog A (C. elegans) | -0.119 | 0.921 | 2.10E-02 | 2.10E+00 | | | |
| 8043036 | AK293146 | DHFR | dihydrofolate reductase | -0.119 | 0.921 | 7.81E-01 | 7.81E+01 | | | |
| 7958011 | --- | --- | --- | -0.119 | 0.921 | 2.18E-01 | 2.18E+01 | | | |
| 7954645 | NM_00108050 | TSPAN11 | tetraspanin 11 | -0.119 | 0.921 | 7.31E-02 | 7.31E+00 | | | |
| 8023308 | --- | --- | --- | -0.119 | 0.921 | 2.04E-02 | 2.04E+00 | | | |
| 8026361 | --- | --- | --- | -0.119 | 0.921 | 6.37E-01 | 6.37E+01 | | | |
| 8178338 | NM_006778 | TRIM10 | tripartite motif-containing 10 | -0.119 | 0.921 | 7.30E-02 | 7.30E+00 | | | |
| 8111930 | AK075204 | hCG_203914 | hypothetical protein LOC100132356 | -0.119 | 0.921 | 9.69E-02 | 9.69E+00 | | | |
| 8176006 | NM_001586 | TEX28 | testis expressed 28 | -0.119 | 0.921 | 2.78E-02 | 2.78E+00 | | | |
| 8176012 | NM_001586 | TEX28 | testis expressed 28 | -0.119 | 0.921 | 2.78E-02 | 2.78E+00 | | | |
| 8077635 | NM_016819 | OGG1 | 8-oxoguanine DNA glycosylase | -0.119 | 0.921 | 4.96E-02 | 4.96E+00 | | | |
| 8060187 | NM_00108543 | C2orf54 | chromosome 2 open reading frame 5 | -0.119 | 0.921 | 1.00E-01 | 1.00E+01 | | | |
| 7948303 | --- | --- | --- | -0.119 | 0.921 | 6.61E-02 | 6.61E+00 | | | |
| 7991904 | NM_004204 | PIGQ | phosphatidylinositol glycan anchor b | -0.119 | 0.921 | 8.03E-02 | 8.03E+00 | | | |
| 7956867 | NM_003483 | HMGA2 | high mobility group AT-hook 2 | -0.119 | 0.921 | 2.72E-02 | 2.72E+00 | | | |

| | | | | | | | | | | |
|---|---|---|---|---|---|---|---|---|---|---|
| 7899244 | NM_017837 | PIGV | phosphatidylinositol glycan anchor b | -0.119 | 0.921 | 1.02E-01 | 1.02E+01 | | | |
| 8006409 | NM_003885 | CDK5R1 | cyclin-dependent kinase 5, regulator | -0.119 | 0.921 | 4.38E-02 | 4.38E+00 | | | |
| 7896817 | NM_005101 | ISG15 | ISG15 ubiquitin-like modifier | -0.119 | 0.921 | 2.22E-01 | 2.22E+01 | | | |
| 8076509 | --- | --- | --- | -0.119 | 0.921 | 5.25E-02 | 5.25E+00 | | | |
| 8132715 | NM_00110015 | C7orf57 | chromosome 7 open reading frame 5 | -0.119 | 0.921 | 3.68E-02 | 3.68E+00 | | | |
| 7903632 | NM_001408 | CELSR2 | cadherin, EGF LAG seven-pass G-type | -0.119 | 0.921 | 1.92E-02 | 1.92E+00 | | | |
| 8166703 | NM_00101373 | FAM47C | family with sequence similarity 47, m | -0.119 | 0.921 | 6.03E-02 | 6.03E+00 | | | |
| 8096744 | NM_183075 | CYP2U1 | cytochrome P450, family 2, subfamil | -0.119 | 0.921 | 1.85E-01 | 1.85E+01 | | | |
| 8007673 | --- | --- | --- | -0.119 | 0.921 | 8.64E-02 | 8.64E+00 | | | |
| 7943943 | NM_178510 | ANKK1 | ankyrin repeat and kinase domain co | -0.119 | 0.921 | 2.23E-02 | 2.23E+00 | | | |
| 7939569 | NM_020229 | PRDM11 | PR domain containing 11 | -0.119 | 0.921 | 4.92E-02 | 4.92E+00 | | | |
| 7957568 | --- | --- | --- | -0.119 | 0.921 | 3.16E-01 | 3.16E+01 | | | |
| 7997032 | NM_00101188 | CLEC18B | C-type lectin domain family 18, mem | -0.119 | 0.921 | 5.55E-02 | 5.55E+00 | | | |
| 8108321 | NM_00113564 | FAM53C | family with sequence similarity 53, m | -0.119 | 0.921 | 2.26E-01 | 2.26E+01 | | | |
| 8075865 | NM_153609 | TMPRSS6 | transmembrane protease, serine 6 | -0.119 | 0.921 | 4.12E-02 | 4.12E+00 | | | |
| 7970806 | BC027486 | hCG_202017 | hCG2020170 | -0.119 | 0.921 | 2.69E-02 | 2.69E+00 | | | |
| 8043320 | AK098018 | LOC400965 | hypothetical LOC400965 | -0.119 | 0.921 | 1.10E-01 | 1.10E+01 | | | |
| 8154962 | NM_00113500 | DNAJB5 | DnaJ (Hsp40) homolog, subfamily B, | -0.119 | 0.921 | 1.26E-01 | 1.26E+01 | | | |
| 8043909 | NM_002518 | NPAS2 | neuronal PAS domain protein 2 | -0.119 | 0.921 | 5.46E-02 | 5.46E+00 | | | |
| 8155371 | NM_015667 // | 75A7 // FAM | family with sequence similarity 75, m | -0.119 | 0.921 | 1.63E-02 | 1.63E+00 | | | |
| 8165258 | NM_006412 | AGPAT2 | 1-acylglycerol-3-phosphate O-acyltra | -0.120 | 0.921 | 3.00E-02 | 3.00E+00 | | | |
| 8122334 | NM_178445 | CCRL1 | chemokine (C-C motif) receptor-like | -0.120 | 0.920 | 3.40E-01 | 3.40E+01 | | | |
| 8165508 | NM_00100435 | NRARP | NOTCH-regulated ankyrin repeat pro | -0.120 | 0.920 | 6.63E-02 | 6.63E+00 | | | |
| 8034747 | ENST0000034 | C19orf67 | chromosome 19 open reading frame | -0.120 | 0.920 | 2.04E-02 | 2.04E+00 | | | |
| 8003249 | NM_024735 | FBXO31 | F-box protein 31 | -0.120 | 0.920 | 3.84E-02 | 3.84E+00 | | | |
| 8058091 | NM_015265 | SATB2 | SATB homeobox 2 | -0.120 | 0.920 | 2.14E-02 | 2.14E+00 | | | |
| 8053662 | --- | --- | --- | -0.120 | 0.920 | 2.08E-01 | 2.08E+01 | | | |
| 8029117 | NM_00104028 | DMRTC2 | DMRT-like family C2 | -0.120 | 0.920 | 7.60E-02 | 7.60E+00 | | | |
| 8067277 | --- | --- | --- | -0.120 | 0.920 | 1.15E-01 | 1.15E+01 | | | |
| 8002218 | NM_024939 | ESRP2 | epithelial splicing regulatory protein | -0.120 | 0.920 | 2.53E-02 | 2.53E+00 | | | |
| 8068235 | NM_138983 | OLIG1 | oligodendrocyte transcription factor | -0.120 | 0.920 | 2.90E-02 | 2.90E+00 | | | |
| 8115122 | NM_015981 | CAMK2A | calcium/calmodulin-dependent prote | -0.120 | 0.920 | 7.52E-02 | 7.52E+00 | | | |
| 8065254 | --- | --- | --- | -0.120 | 0.920 | 7.18E-01 | 7.18E+01 | | | |
| 8064591 | NM_014731 | ProSAPiP1 | ProSAPiP1 protein | -0.120 | 0.920 | 3.39E-02 | 3.39E+00 | | | |
| 8104693 | NM_178140 | PDZD2 | PDZ domain containing 2 | -0.120 | 0.920 | 4.27E-02 | 4.27E+00 | | | |
| 8154934 | NM_147162 | IL11RA | interleukin 11 receptor, alpha | -0.120 | 0.920 | 4.47E-01 | 4.47E+01 | | | |
| 8104122 | NM_033178 | DUX4 | double homeobox, 4 | -0.120 | 0.920 | 2.14E-02 | 2.14E+00 | | | |
| 8043276 | NR_004378 | SNORD94 | small nucleolar RNA, C/D box 94 | -0.120 | 0.920 | 7.90E-01 | 7.90E+01 | | | |
| 7919669 | NM_00114586 | MTMR11 | myotubularin related protein 11 | -0.120 | 0.920 | 6.17E-02 | 6.17E+00 | | | |
| 7969414 | NM_001730 | KLF5 | Kruppel-like factor 5 (intestinal) | -0.120 | 0.920 | 2.76E-02 | 2.76E+00 | | | |
| 8073842 | NM_017931 | TTC38 | tetratricopeptide repeat domain 38 | -0.120 | 0.920 | 2.30E-01 | 2.30E+01 | | | |
| 7938090 | NM_176875 | CCKBR | cholecystokinin B receptor | -0.120 | 0.920 | 4.30E-02 | 4.30E+00 | | | |
| 8176986 | NM_013239 | PPP2R3B | protein phosphatase 2 (formerly 2A) | -0.120 | 0.920 | 4.39E-02 | 4.39E+00 | | | |
| 7985023 | --- | --- | --- | -0.120 | 0.920 | 4.81E-02 | 4.81E+00 | | | |
| 7997940 | NR_002450 | SNORD68 | small nucleolar RNA, C/D box 68 | -0.120 | 0.920 | 7.22E-01 | 7.22E+01 | | | |
| 8011220 | --- | --- | --- | -0.120 | 0.920 | 6.50E-02 | 6.50E+00 | | | |
| 7915801 | NM_017739 | POMGNT1 | protein O-linked mannose beta1,2-N | -0.120 | 0.920 | 5.76E-02 | 5.76E+00 | | | |
| 8163485 | NM_145051 | RNF183 | ring finger protein 183 | -0.120 | 0.920 | 2.87E-02 | 2.87E+00 | | | |
| 8137783 | NM_00109762 | TMEM184A | transmembrane protein 184A | -0.120 | 0.920 | 5.70E-02 | 5.70E+00 | | | |
| 8049729 | NM_198998 | AQP12A | aquaporin 12A | -0.120 | 0.920 | 2.83E-02 | 2.83E+00 | | | |
| 7987381 | NR_027320 | CSNK1A1P | casein kinase 1, alpha 1 pseudogene | -0.120 | 0.920 | 8.37E-02 | 8.37E+00 | | | |
| 8024909 | NM_015015 | KDM4B | lysine (K)-specific demethylase 4B | -0.120 | 0.920 | 6.62E-02 | 6.62E+00 | | | |
| 7895880 | --- | --- | --- | -0.120 | 0.920 | 5.63E-01 | 5.63E+01 | | | |
| 7981775 | ENST0000043 | KFZP547L11 | hypothetical protein DKFZp547L112 | -0.120 | 0.920 | 5.75E-02 | 5.75E+00 | | | |
| 7900683 | NM_005373 | MPL | myeloproliferative leukemia virus on | -0.120 | 0.920 | 3.72E-02 | 3.72E+00 | | | |
| 7937847 | NR_024627 | KCNQ1DN | KCNQ1 downstream neighbor | -0.120 | 0.920 | 4.71E-02 | 4.71E+00 | | | |
| 8074498 | NM_006440 | TXNRD2 | thioredoxin reductase 2 | -0.120 | 0.920 | 4.77E-02 | 4.77E+00 | | | |
| 8155383 | NM_015667 // | 75A7 // FAM | family with sequence similarity 75, m | -0.120 | 0.920 | 1.83E-02 | 1.83E+00 | | | |
| 8177976 | NM_000595 | LTA | lymphotoxin alpha (TNF superfamily, | -0.120 | 0.920 | 2.90E-02 | 2.90E+00 | | | |
| 8076815 | AY358194 | UNQ6126 | LPEQ6126 | -0.120 | 0.920 | 4.20E-02 | 4.20E+00 | | | |
| 8116658 | NM_012135 | FAM50B | family with sequence similarity 50, m | -0.120 | 0.920 | 4.14E-02 | 4.14E+00 | | | |
| 8021919 | NM_020040 | TUBB4Q | tubulin, beta polypeptide 4, member | -0.120 | 0.920 | 1.08E-02 | 1.08E+00 | | | |
| 8164452 | NM_032799 | ZDHHC12 | zinc finger, DHHC-type containing 12 | -0.120 | 0.920 | 3.09E-02 | 3.09E+00 | | | |
| 8180207 | --- | --- | --- | -0.120 | 0.920 | 3.93E-01 | 3.93E+01 | | | |
| 8018673 | NM_032134 | QRICH2 | glutamine rich 2 | -0.120 | 0.920 | 3.42E-02 | 3.42E+00 | | | |
| 7964555 | NM_006576 | AVIL | advillin | -0.120 | 0.920 | 9.95E-02 | 9.95E+00 | | | |
| 7992136 | --- | --- | --- | -0.120 | 0.920 | 4.52E-02 | 4.52E+00 | | | |
| 8155699 | NM_000144 | FXN | frataxin | -0.120 | 0.920 | 1.89E-01 | 1.89E+01 | | | |
| 8144388 | NM_018088 | FAM90A1 | family with sequence similarity 90, m | -0.120 | 0.920 | 2.00E-02 | 2.00E+00 | | | |
| 7998374 | NM_016541 | GNG13 | guanine nucleotide binding protein ( | -0.120 | 0.920 | 4.39E-02 | 4.39E+00 | | | |
| 7934566 | --- | --- | --- | -0.120 | 0.920 | 2.25E-01 | 2.25E+01 | | | |
| 8036136 | NM_024660 | TMEM149 | transmembrane protein 149 | -0.120 | 0.920 | 1.48E-01 | 1.48E+01 | | | |
| 7944042 | NM_002572 | PAFAH1B2 | platelet-activating factor acetylhydro | -0.120 | 0.920 | 6.22E-01 | 6.22E+01 | | | |
| 8070173 | NM_000219 | KCNE1 | potassium voltage-gated channel, Isk | -0.120 | 0.920 | 3.89E-02 | 3.89E+00 | | | |
| 7945896 | NM_020402 | CHRNA10 | cholinergic receptor, nicotinic, alpha | -0.120 | 0.920 | 6.42E-02 | 6.42E+00 | | | |
| 7893042 | --- | --- | --- | -0.120 | 0.920 | 1.20E-01 | 1.20E+01 | | | |
| 8063729 | NM_207032 | EDN3 | endothelin 3 | -0.120 | 0.920 | 4.40E-02 | 4.40E+00 | | | |

| | | | | | | | | | | |
|---|---|---|---|---|---|---|---|---|---|---|
| 8104307 | AY249325 | C5orf38 | chromosome 5 open reading frame 3 | -0.120 | 0.920 | 6.76E-02 | 6.76E+00 | | | |
| 7927179 | NM_00112337( | TMEM72 | transmembrane protein 72 | -0.120 | 0.920 | 4.22E-02 | 4.22E+00 | | | |
| 8146328 | --- | --- | --- | -0.120 | 0.920 | 6.56E-01 | 6.56E+01 | | | |
| 8064031 | NM_139317 | BIRC7 | baculoviral IAP repeat-containing 7 | -0.120 | 0.920 | 4.02E-02 | 4.02E+00 | | | |
| 7962358 | NM_005748 | YAF2 | YY1 associated factor 2 | -0.120 | 0.920 | 2.23E-01 | 2.23E+01 | | | |
| 8042696 | NM_003124 | SPR | sepiapterin reductase (7,8-dihydrobi | -0.120 | 0.920 | 3.26E-02 | 3.26E+00 | | | |
| 8030113 | NM_006666 | RUVBL2 | RuvB-like 2 (E. coli) | -0.120 | 0.920 | 3.20E-01 | 3.20E+01 | | | |
| 8032637 | NM_00108054: | C19orf29 | chromosome 19 open reading frame | -0.120 | 0.920 | 3.71E-02 | 3.71E+00 | | | |
| 7893043 | --- | --- | --- | -0.120 | 0.920 | 6.89E-01 | 6.89E+01 | | | |
| 8015323 | NM_153490 | KRT13 | keratin 13 | -0.120 | 0.920 | 2.69E-02 | 2.69E+00 | | | |
| 8119670 | NM_201523 | KLC4 | kinesin light chain 4 | -0.120 | 0.920 | 2.31E-02 | 2.31E+00 | | | |
| 8112892 | --- | --- | --- | -0.120 | 0.920 | 1.07E-01 | 1.07E+01 | | | |
| 7967700 | ENST00000415 | OC10012884 | hypothetical protein LOC100128840 | -0.120 | 0.920 | 3.91E-02 | 3.91E+00 | | | |
| 8049530 | NM_00113755( | LRRFIP1 | leucine rich repeat (in FLII) interactin | -0.120 | 0.920 | 8.52E-01 | 8.52E+01 | | | |
| 7949227 | NM_017525 | CDC42BPG | CDC42 binding protein kinase gamm | -0.120 | 0.920 | 3.57E-02 | 3.57E+00 | | | |
| 7892863 | --- | --- | --- | -0.120 | 0.920 | 3.05E-01 | 3.05E+01 | | | |
| 7960280 | NM_152441 | FBXL14 | F-box and leucine-rich repeat protei | -0.120 | 0.920 | 4.15E-02 | 4.15E+00 | | | |
| 7911383 | NM_017891 | C1orf159 | chromosome 1 open reading frame 1 | -0.121 | 0.920 | 1.75E-02 | 1.75E+00 | | | |
| 7965478 | --- | --- | --- | -0.121 | 0.920 | 4.37E-01 | 4.37E+01 | | | |
| 8137183 | NM_198455 | SSPO | SCO-spondin homolog (Bos taurus) | -0.121 | 0.920 | 2.89E-02 | 2.89E+00 | | | |
| 7913206 | NM_019062 | RNF186 | ring finger protein 186 | -0.121 | 0.920 | 7.93E-02 | 7.93E+00 | | | |
| 8072817 | NM_152243 | CDC42EP1 | CDC42 effector protein (Rho GTPase | -0.121 | 0.920 | 2.17E-02 | 2.17E+00 | | | |
| 7966596 | NM_138451 | IQCD | IQ motif containing D | -0.121 | 0.920 | 1.19E-01 | 1.19E+01 | | | |
| 8180335 | --- | --- | --- | -0.121 | 0.920 | 4.78E-01 | 4.78E+01 | | | |
| 8082465 | NM_024768 | CCDC48 | coiled-coil domain containing 48 | -0.121 | 0.920 | 2.27E-02 | 2.27E+00 | | | |
| 8067409 | NM_005560 | LAMA5 | laminin, alpha 5 | -0.121 | 0.920 | 1.63E-02 | 1.63E+00 | | | |
| 8137715 | NM_182924 | MICALL2 | MICAL-like 2 | -0.121 | 0.920 | 1.52E-02 | 1.52E+00 | | | |
| 7973613 | NM_017999 | RNF31 | ring finger protein 31 | -0.121 | 0.920 | 1.28E-01 | 1.28E+01 | | | |
| 7936552 | NM_199131 | VAX1 | ventral anterior homeobox 1 | -0.121 | 0.920 | 4.24E-02 | 4.24E+00 | | | |
| 8125383 | NM_004557 | NOTCH4 | Notch homolog 4 (Drosophila) | -0.121 | 0.920 | 1.78E-02 | 1.78E+00 | | | |
| 7963534 | NM_002272 | KRT4 | keratin 4 | -0.121 | 0.920 | 4.79E-02 | 4.79E+00 | | | |
| 8140386 | NM_016086 | STYXL1 | serine/threonine/tyrosine interactin | -0.121 | 0.920 | 7.10E-01 | 7.10E+01 | | | |
| 8001007 | NM_002773 | PRSS8 | protease, serine, 8 | -0.121 | 0.920 | 1.01E-01 | 1.01E+01 | | | |
| 7956105 | NM_022465 | IKZF4 | IKAROS family zinc finger 4 (Eos) | -0.121 | 0.920 | 3.40E-02 | 3.40E+00 | | | |
| 8077192 | NM_000482 | APOA4 | apolipoprotein A-IV | -0.121 | 0.920 | 2.96E-02 | 2.96E+00 | | | |
| 7903355 | NM_022049 | GPR88 | G protein-coupled receptor 88 | -0.121 | 0.920 | 3.41E-02 | 3.41E+00 | | | |
| 8098549 | NM_020225 | STOX2 | storkhead box 2 | -0.121 | 0.920 | 3.72E-02 | 3.72E+00 | | | |
| 7995668 | NM_005853 | IRX5 | iroquois homeobox 5 | -0.121 | 0.920 | 7.26E-03 | 7.26E-01 | | | |
| 8167360 | NM_002049 | GATA1 | GATA binding protein 1 (globin trans | -0.121 | 0.920 | 5.92E-02 | 5.92E+00 | | | |
| 8016128 | NM_002055 | GFAP | glial fibrillary acidic protein | -0.121 | 0.920 | 3.57E-02 | 3.57E+00 | | | |
| 7977418 | NM_00113487! | C14orf80 | chromosome 14 open reading frame | -0.121 | 0.920 | 2.77E-02 | 2.77E+00 | | | |
| 8143949 | NM_144727 | CRYGN | crystallin, gamma N | -0.121 | 0.920 | 3.22E-02 | 3.22E+00 | | | |
| 8110569 | NM_003900 | SQSTM1 | sequestosome 1 | -0.121 | 0.920 | 2.38E-01 | 2.38E+01 | | | |
| 8012006 | NM_153357 | SLC16A11 | solute carrier family 16, member 11 | -0.121 | 0.920 | 1.83E-02 | 1.83E+00 | | | |
| 8159086 | NM_014694 | ADAMTSL2 | ADAMTS-like 2 | -0.121 | 0.920 | 3.18E-02 | 3.18E+00 | | | |
| 8044109 | NM_006236 | POU3F3 | POU class 3 homeobox 3 | -0.121 | 0.920 | 4.90E-02 | 4.90E+00 | | | |
| 8095376 | NM_005953 | MT2A | metallothionein 2A | -0.121 | 0.920 | 4.87E-02 | 4.87E+00 | | | |
| 8075200 | NM_012265 | RHBDD3 | rhomboid domain containing 3 | -0.121 | 0.920 | 1.98E-02 | 1.98E+00 | | | |
| 8033162 | NM_003685 | KHSRP | KH-type splicing regulatory protein | -0.121 | 0.920 | 8.92E-02 | 8.92E+00 | | | |
| 7995206 | NM_00104245- | TGFB1I1 | transforming growth factor beta 1 in | -0.121 | 0.920 | 2.58E-02 | 2.58E+00 | | | |
| 7926674 | NM_178161 | PTF1A | pancreas specific transcription facto | -0.121 | 0.920 | 4.03E-02 | 4.03E+00 | | | |
| 7937014 | AK127642 | LOC387720 | hypothetical LOC387720 | -0.121 | 0.920 | 4.64E-02 | 4.64E+00 | | | |
| 8035859 | --- | --- | --- | -0.121 | 0.920 | 4.66E-02 | 4.66E+00 | | | |
| 8029950 | NM_014601 | EHD2 | EH-domain containing 2 | -0.121 | 0.920 | 1.04E-02 | 1.04E+00 | | | |
| 7915682 | NM_020883 | ZSWIM5 | zinc finger, SWIM-type containing 5 | -0.121 | 0.920 | 2.23E-02 | 2.23E+00 | | | |
| 7937104 | AK097584 | OC10012883 | hypothetical protein LOC100128830 | -0.121 | 0.920 | 2.29E-01 | 2.29E+01 | | | |
| 7979108 | NM_052978 | TRIM9 | tripartite motif-containing 9 | -0.121 | 0.920 | 4.62E-02 | 4.62E+00 | | | |
| 8013331 | NM_015681 | B9D1 | B9 protein domain 1 | -0.121 | 0.920 | 1.46E-02 | 1.46E+00 | | | |
| 7945641 | NM_004420 | DUSP8 | dual specificity phosphatase 8 | -0.121 | 0.920 | 5.01E-02 | 5.01E+00 | | | |
| 7894997 | --- | --- | --- | -0.121 | 0.920 | 8.30E-01 | 8.30E+01 | | | |
| 7956551 | NM_182947 | GEFT | RhoA/RAC/CDC42 exchange factor | -0.121 | 0.920 | 5.55E-02 | 5.55E+00 | | | |
| 8018522 | NM_00108054: | FBF1 | Fas (TNFRSF6) binding factor 1 | -0.121 | 0.920 | 1.15E-02 | 1.15E+00 | | | |
| 8008237 | NM_002204 | ITGA3 | integrin, alpha 3 (antigen CD49C, alp | -0.121 | 0.920 | 5.40E-02 | 5.40E+00 | | | |
| 8060126 | NM_198998 | AQP12A | aquaporin 12A | -0.121 | 0.920 | 2.45E-02 | 2.45E+00 | | | |
| 8075468 | NM_015715 | PLA2G3 | phospholipase A2, group III | -0.121 | 0.920 | 2.53E-02 | 2.53E+00 | | | |
| 7897469 | NM_00113092- | TMEM201 | transmembrane protein 201 | -0.121 | 0.920 | 3.74E-02 | 3.74E+00 | | | |
| 8061653 | NM_080625 | C20orf160 | chromosome 20 open reading frame | -0.121 | 0.920 | 2.64E-02 | 2.64E+00 | | | |
| 7945228 | ENST00000318 | FLJ34521 | hypothetical protein LOC646383 | -0.121 | 0.920 | 1.68E-01 | 1.68E+01 | | | |
| 8019937 | --- | --- | --- | -0.121 | 0.919 | 7.82E-02 | 7.82E+00 | | | |
| 8015846 | NR_003562 | MPP3 | membrane protein, palmitoylated 3 | -0.121 | 0.919 | 4.90E-02 | 4.90E+00 | | | |
| 8179716 | NM_001264 | CDSN | corneodesmosin | -0.121 | 0.919 | 2.71E-02 | 2.71E+00 | | | |
| 7920506 | NM_00101084( | SHE | Src homology 2 domain containing E | -0.121 | 0.919 | 3.72E-02 | 3.72E+00 | | | |
| 7937612 | NM_002458 | MUC5B | mucin 5B, oligomeric mucus/gel-forr | -0.121 | 0.919 | 1.59E-02 | 1.59E+00 | | | |
| 7968272 | --- | --- | --- | -0.121 | 0.919 | 1.30E-01 | 1.30E+01 | | | |
| 7914361 | NM_001856 | COL16A1 | collagen, type XVI, alpha 1 | -0.121 | 0.919 | 2.25E-02 | 2.25E+00 | | | |
| 8015189 | NM_030966 | KRTAP1-3 | keratin associated protein 1-3 | -0.121 | 0.919 | 1.94E-02 | 1.94E+00 | | | |
| 8019585 | NM_030966 | KRTAP1-3 | keratin associated protein 1-3 | -0.121 | 0.919 | 1.94E-02 | 1.94E+00 | | | |

| | | | | | | | | | | |
|---|---|---|---|---|---|---|---|---|---|---|
| 7892667 | --- | --- | --- | -0.121 | 0.919 | 8.55E-02 | 8.55E+00 | | | |
| 8034043 | NM_030760 | S1PR5 | sphingosine-1-phosphate receptor 5 | -0.121 | 0.919 | 5.31E-03 | 5.31E-01 | | | |
| 7985620 | NM_020778 | ALPK3 | alpha-kinase 3 | -0.121 | 0.919 | 5.90E-02 | 5.90E+00 | | | |
| 8084757 | NM_198485 | TPRG1 | tumor protein p63 regulated 1 | -0.121 | 0.919 | 7.24E-02 | 7.24E+00 | | | |
| 8107408 | NM_021614 | KCNN2 | potassium intermediate/small condu | -0.121 | 0.919 | 1.71E-02 | 1.71E+00 | | | |
| 7936083 | NM_00101166 | PCGF6 | polycomb group ring finger 6 | -0.121 | 0.919 | 3.30E-01 | 3.30E+01 | | | |
| 8004842 | NM_173728 | ARHGEF15 | Rho guanine nucleotide exchange fa | -0.121 | 0.919 | 1.52E-02 | 1.52E+00 | | | |
| 8115849 | --- | --- | --- | -0.121 | 0.919 | 9.60E-02 | 9.60E+00 | | | |
| 8060533 | BC021178 | C20orf141 | chromosome 20 open reading frame | -0.121 | 0.919 | 4.73E-02 | 4.73E+00 | | | |
| 7992181 | NM_003294 | TPSAB1 | tryptase alpha/beta 1 | -0.121 | 0.919 | 1.66E-02 | 1.66E+00 | | | |
| 7997875 | NR_024347 | C16orf81 | chromosome 16 open reading frame | -0.121 | 0.919 | 3.23E-02 | 3.23E+00 | | | |
| 7892938 | --- | --- | --- | -0.121 | 0.919 | 8.61E-01 | 8.61E+01 | | | |
| 8150860 | --- | --- | --- | -0.121 | 0.919 | 8.66E-02 | 8.66E+00 | | | |
| 7953321 | NM_002342 | LTBR | lymphotoxin beta receptor (TNFR su | -0.121 | 0.919 | 2.41E-02 | 2.41E+00 | | | |
| 8094030 | NM_198595 | AFAP1 | actin filament associated protein 1 | -0.121 | 0.919 | 5.27E-02 | 5.27E+00 | | | |
| 7928766 | NM_207373 | C10orf99 | chromosome 10 open reading frame | -0.121 | 0.919 | 5.91E-02 | 5.91E+00 | | | |
| 7980140 | --- | --- | --- | -0.121 | 0.919 | 1.68E-01 | 1.68E+01 | | | |
| 8006119 | --- | --- | --- | -0.121 | 0.919 | 7.16E-02 | 7.16E+00 | | | |
| 7997810 | NM_00101275 | CTU2 | cytosolic thiouridylase subunit 2 hon | -0.121 | 0.919 | 3.71E-02 | 3.71E+00 | | | |
| 8164692 | AF467069 | RAPGEF1 | Rap guanine nucleotide exchange fa | -0.121 | 0.919 | 7.34E-01 | 7.34E+01 | | | |
| 8040549 | --- | --- | --- | -0.121 | 0.919 | 7.55E-02 | 7.55E+00 | | | |
| 8015242 | NM_033062 | KRTAP4-2 | keratin associated protein 4-2 | -0.121 | 0.919 | 2.94E-02 | 2.94E+00 | | | |
| 8040547 | --- | --- | --- | -0.121 | 0.919 | 3.92E-01 | 3.92E+01 | | | |
| 7921088 | NM_006617 | NES | nestin | -0.121 | 0.919 | 1.65E-02 | 1.65E+00 | | | |
| 7919582 | XR_039086 | OC10013307 | hypothetical LOC100133075 | -0.121 | 0.919 | 1.27E-01 | 1.27E+01 | | | |
| 7896865 | NM_153254 | TTLL10 | tubulin tyrosine ligase-like family, me | -0.121 | 0.919 | 3.10E-02 | 3.10E+00 | | | |
| 7895329 | --- | --- | --- | -0.121 | 0.919 | 5.14E-01 | 5.14E+01 | | | |
| 8098958 | NM_181808 | POLN | polymerase (DNA directed) nu | -0.121 | 0.919 | 1.09E-01 | 1.09E+01 | | | |
| 8083887 | NM_005602 | CLDN11 | claudin 11 | -0.121 | 0.919 | 6.28E-02 | 6.28E+00 | | | |
| 8161471 | NM_015667 // | 75A7 // FAM | family with sequence similarity 75, m | -0.121 | 0.919 | 1.66E-02 | 1.66E+00 | | | |
| 7914282 | NM_014654 | SDC3 | syndecan 3 | -0.121 | 0.919 | 3.39E-02 | 3.39E+00 | | | |
| 8174076 | NM_000169 | GLA | galactosidase, alpha | -0.122 | 0.919 | 6.51E-01 | 6.51E+01 | | | |
| 8039144 | NM_00114530 | TMC4 | transmembrane channel-like 4 | -0.122 | 0.919 | 2.69E-02 | 2.69E+00 | | | |
| 8014316 | NM_002985 | CCL5 | chemokine (C-C motif) ligand 5 | -0.122 | 0.919 | 6.76E-01 | 6.76E+01 | | | |
| 7920027 | NM_007185 | TNRC4 | trinucleotide repeat containing 4 | -0.122 | 0.919 | 2.24E-02 | 2.24E+00 | | | |
| 8131056 | NM_017781 | CYP2W1 | cytochrome P450, family 2, subfamil | -0.122 | 0.919 | 4.55E-02 | 4.55E+00 | | | |
| 7975995 | BC113929 | C14orf178 | chromosome 14 open reading frame | -0.122 | 0.919 | 2.07E-01 | 2.07E+01 | | | |
| 8032249 | NM_213604 | ADAMTSL5 | ADAMTS-like 5 | -0.122 | 0.919 | 3.57E-02 | 3.57E+00 | | | |
| 8162920 | NM_147180 | PPP3R2 | protein phosphatase 3 (formerly 2B) | -0.122 | 0.919 | 4.73E-02 | 4.73E+00 | | | |
| 8031622 | NM_033106 | GALP | galanin-like peptide | -0.122 | 0.919 | 3.94E-02 | 3.94E+00 | | | |
| 8049610 | NM_198582 | KLHL30 | kelch-like 30 (Drosophila) | -0.122 | 0.919 | 4.89E-02 | 4.89E+00 | | | |
| 8180240 | --- | --- | --- | -0.122 | 0.919 | 2.46E-01 | 2.46E+01 | | | |
| 7927028 | NM_153368 | GJD4 | gap junction protein, delta 4, 40.1kD | -0.122 | 0.919 | 2.94E-02 | 2.94E+00 | | | |
| 8164440 | NM_052844 | WDR34 | WD repeat domain 34 | -0.122 | 0.919 | 2.65E-02 | 2.65E+00 | | | |
| 8124707 | NM_007028 | TRIM31 | tripartite motif-containing 31 | -0.122 | 0.919 | 3.45E-02 | 3.45E+00 | | | |
| 8109773 | NM_00116166 | WWC1 | WW and C2 domain containing 1 | -0.122 | 0.919 | 2.43E-02 | 2.43E+00 | | | |
| 8170786 | AF514420 | LCAP | lung carcinoma-associated protein 1 | -0.122 | 0.919 | 3.51E-02 | 3.51E+00 | | | |
| 8107098 | --- | --- | --- | -0.122 | 0.919 | 2.80E-01 | 2.80E+01 | | | |
| 8137310 | NM_007188 | ABCB8 | ATP-binding cassette, sub-family B ( | -0.122 | 0.919 | 3.36E-02 | 3.36E+00 | | | |
| 7923332 | NM_000364 | TNNT2 | troponin T type 2 (cardiac) | -0.122 | 0.919 | 5.69E-02 | 5.69E+00 | | | |
| 8161642 | --- | --- | --- | -0.122 | 0.919 | 2.24E-01 | 2.24E+01 | | | |
| 8144810 | --- | --- | --- | -0.122 | 0.919 | 2.66E-01 | 2.66E+01 | | | |
| 8170407 | NM_005365 | MAGEA9 | melanoma antigen family A, 9 | -0.122 | 0.919 | 7.76E-02 | 7.76E+00 | | | |
| 8175611 | NM_005365 | MAGEA9 | melanoma antigen family A, 9 | -0.122 | 0.919 | 7.76E-02 | 7.76E+00 | | | |
| 7962455 | NM_006159 | NELL2 | NEL-like 2 (chicken) | -0.122 | 0.919 | 7.28E-01 | 7.28E+01 | | | |
| 7904433 | NM_006623 | PHGDH | phosphoglycerate dehydrogenase | -0.122 | 0.919 | 2.52E-02 | 2.52E+00 | | | |
| 7977734 | --- | --- | --- | -0.122 | 0.919 | 3.27E-01 | 3.27E+01 | | | |
| 8072039 | NM_032608 | MYO18B | myosin XVIIIB | -0.122 | 0.919 | 1.83E-02 | 1.83E+00 | | | |
| 7991800 | NM_001176 | ARHGDIG | Rho GDP dissociation inhibitor (GDI) | -0.122 | 0.919 | 7.29E-02 | 7.29E+00 | | | |
| 7968260 | NM_145657 | GSX1 | GS homeobox 1 | -0.122 | 0.919 | 2.81E-02 | 2.81E+00 | | | |
| 7945460 | --- | --- | --- | -0.122 | 0.919 | 3.46E-02 | 3.46E+00 | | | |
| 7961820 | ENST0000035 | C12orf67 | chromosome 12 open reading frame | -0.122 | 0.919 | 4.90E-02 | 4.90E+00 | | | |
| 8173486 | NR_002226 | INGX | inhibitor of growth family, X-linked, | -0.122 | 0.919 | 8.29E-02 | 8.29E+00 | | | |
| 8123637 | NM_000904 | NQO2 | NAD(P)H dehydrogenase, quinone 2 | -0.122 | 0.919 | 9.67E-02 | 9.67E+00 | | | |
| 7963845 | NM_053283 | DCD | dermcidin | -0.122 | 0.919 | 2.58E-02 | 2.58E+00 | | | |
| 8149383 | NM_018088 | FAM90A1 | family with sequence similarity 90, m | -0.122 | 0.919 | 5.14E-02 | 5.14E+00 | | | |
| 7992032 | ENST0000039 | FBXL16 | F-box and leucine-rich repeat protei | -0.122 | 0.919 | 4.58E-02 | 4.58E+00 | | | |
| 8064407 | NM_00102987 | RSPO4 | R-spondin family, member 4 | -0.122 | 0.919 | 2.15E-02 | 2.15E+00 | | | |
| 7992379 | NM_00101086 | EME2 | essential meiotic endonuclease 1 ho | -0.122 | 0.919 | 4.55E-02 | 4.55E+00 | | | |
| 8062108 | NM_006404 | PROCR | protein C receptor, endothelial (EPC | -0.122 | 0.919 | 5.14E-02 | 5.14E+00 | | | |
| 8130163 | NM_00100178 | RAET1G | retinoic acid early transcript 1G | -0.122 | 0.919 | 6.26E-02 | 6.26E+00 | | | |
| 7935296 | NM_012465 | TLL2 | tolloid-like 2 | -0.122 | 0.919 | 5.23E-02 | 5.23E+00 | | | |
| 8035639 | NM_023002 | HAPLN4 | hyaluronan and proteoglycan link pr | -0.122 | 0.919 | 5.48E-02 | 5.48E+00 | | | |
| 8041937 | AK128867 | FLJ46838 | FLJ46838 protein | -0.122 | 0.919 | 3.69E-02 | 3.69E+00 | | | |
| 8137847 | BC015632 | C7orf27 | chromosome 7 open reading frame | -0.122 | 0.919 | 9.45E-03 | 9.45E-01 | | | |
| 7914326 | NM_024522 | NKAIN1 | Na+/K+ transporting ATPase interact | -0.122 | 0.919 | 3.56E-02 | 3.56E+00 | | | |
| 8010590 | NM_00108051 | BAHCC1 | BAH domain and coiled-coil containi | -0.122 | 0.919 | 2.28E-02 | 2.28E+00 | | | |

| | | | | | | | | | | |
|---|---|---|---|---|---|---|---|---|---|---|
| 7892862 | --- | --- | --- | -0.122 | 0.919 | 2.46E-01 | 2.46E+01 | | | |
| 8135080 | NM_001283 | AP1S1 | adaptor-related protein complex 1, s | -0.122 | 0.919 | 6.15E-02 | 6.15E+00 | | | |
| 7893613 | --- | --- | --- | -0.122 | 0.919 | 7.30E-02 | 7.30E+00 | | | |
| 8139891 | NM_016038 | SBDS | Shwachman-Bodian-Diamond syndro | -0.122 | 0.919 | 2.29E-01 | 2.29E+01 | | | |
| 7895067 | --- | --- | --- | -0.122 | 0.919 | 8.13E-01 | 8.13E+01 | | | |
| 7939365 | NM_014344 | FJX1 | four jointed box 1 (Drosophila) | -0.122 | 0.919 | 2.49E-02 | 2.49E+00 | | | |
| 8133500 | BC126309 | WBSCR23 | Williams-Beuren syndrome chromos | -0.122 | 0.919 | 9.31E-02 | 9.31E+00 | | | |
| 8038109 | NM_017708 | FAM83E | family with sequence similarity 83, n | -0.122 | 0.919 | 2.02E-02 | 2.02E+00 | | | |
| 7949588 | NM_020404 | CD248 | CD248 molecule, endosialin | -0.122 | 0.919 | 1.44E-01 | 1.44E+01 | | | |
| 8170704 | NM_000033 | ABCD1 | ATP-binding cassette, sub-family D (A | -0.122 | 0.919 | 4.80E-02 | 4.80E+00 | | | |
| 8032137 | NM_00103302( | C19orf6 | chromosome 19 open reading frame | -0.122 | 0.919 | 1.40E-01 | 1.40E+01 | | | |
| 8138997 | NM_00107765 | TBX20 | T-box 20 | -0.122 | 0.919 | 3.30E-02 | 3.30E+00 | | | |
| 8029773 | NM_00100291 | IGFL2 | IGF-like family member 2 | -0.122 | 0.919 | 7.65E-02 | 7.65E+00 | | | |
| 8033571 | NM_198471 | KANK3 | KN motif and ankyrin repeat domain | -0.122 | 0.919 | 5.52E-02 | 5.52E+00 | | | |
| 8042221 | --- | --- | --- | -0.122 | 0.919 | 2.17E-01 | 2.17E+01 | | | |
| 8029624 | ENST0000042 | LRRC68 | leucine rich repeat containing 68 | -0.122 | 0.919 | 4.04E-02 | 4.04E+00 | | | |
| 8067530 | BC093856 | C20orf200 | chromosome 20 open reading frame | -0.122 | 0.919 | 2.48E-02 | 2.48E+00 | | | |
| 8141535 | NM_002319 | LRCH4 | leucine-rich repeats and calponin ho | -0.122 | 0.919 | 3.68E-02 | 3.68E+00 | | | |
| 8031992 | NR_024368 | tcag7.907 | hypothetical LOC402483 | -0.122 | 0.919 | 4.85E-02 | 4.85E+00 | | | |
| 8152648 | BC067796 | C8orf76 | chromosome 8 open reading frame 7 | -0.122 | 0.919 | 6.49E-01 | 6.49E+01 | | | |
| 8039306 | NM_00114597 | RDH13 | retinol dehydrogenase 13 (all-trans/ | -0.122 | 0.919 | 2.35E-02 | 2.35E+00 | | | |
| 8169676 | NM_032498 | RHOXF2 | Rhox homeobox family, member 2 | -0.122 | 0.919 | 7.28E-02 | 7.28E+00 | | | |
| 8174750 | NM_032498 | RHOXF2 | Rhox homeobox family, member 2 | -0.122 | 0.919 | 7.28E-02 | 7.28E+00 | | | |
| 7928429 | NM_002658 | PLAU | plasminogen activator, urokinase | -0.122 | 0.919 | 8.53E-02 | 8.53E+00 | | | |
| 7997942 | NM_014427 | CPNE7 | copine VII | -0.122 | 0.919 | 1.72E-02 | 1.72E+00 | | | |
| 7894309 | --- | --- | --- | -0.122 | 0.919 | 2.93E-01 | 2.93E+01 | | | |
| 7975121 | NM_002028 | FNTB | farnesyltransferase, CAAX box, beta | -0.122 | 0.919 | 1.22E-01 | 1.22E+01 | | | |
| 8110708 | NM_007030 | TPPP | tubulin polymerization promoting pr | -0.123 | 0.919 | 3.58E-02 | 3.58E+00 | | | |
| 8032530 | NM_003021 | SGTA | small glutamine-rich tetratricopeptid | -0.123 | 0.919 | 4.00E-02 | 4.00E+00 | | | |
| 7935930 | NM_014591 | KCNIP2 | Kv channel interacting protein 2 | -0.123 | 0.919 | 5.90E-02 | 5.90E+00 | | | |
| 8169080 | ENST00000243 | H2BFM | H2B histone family, member M | -0.123 | 0.919 | 9.25E-03 | 9.25E-01 | | | |
| 8162472 | NM_021570 | BARX1 | BARX homeobox 1 | -0.123 | 0.919 | 3.17E-02 | 3.17E+00 | | | |
| 7951040 | NM_016540 | GPR83 | G protein-coupled receptor 83 | -0.123 | 0.919 | 7.39E-02 | 7.39E+00 | | | |
| 7911307 | NR_024528 | RPL23AP7 | ribosomal protein L23a pseudogene | -0.123 | 0.919 | 4.76E-01 | 4.76E+01 | | | |
| 8180413 | --- | --- | --- | -0.123 | 0.919 | 5.60E-02 | 5.60E+00 | | | |
| 8071899 | NM_000675 | ADORA2A | adenosine A2a receptor | -0.123 | 0.919 | 1.31E-01 | 1.31E+01 | | | |
| 8015210 | NM_033184 | KRTAP2-4 | keratin associated protein 2-4 | -0.123 | 0.918 | 2.43E-02 | 2.43E+00 | | | |
| 8019576 | NM_033184 | KRTAP2-4 | keratin associated protein 2-4 | -0.123 | 0.918 | 2.43E-02 | 2.43E+00 | | | |
| 7968411 | NR_027062 | EEF1DP3 | eukaryotic translation elongation fac | -0.123 | 0.918 | 3.48E-02 | 3.48E+00 | | | |
| 8167774 | NM_00103174 | RIBC1 | RIB43A domain with coiled-coils 1 | -0.123 | 0.918 | 5.68E-02 | 5.68E+00 | | | |
| 7955852 | NM_173860 | HOXC12 | homeobox C12 | -0.123 | 0.918 | 3.49E-02 | 3.49E+00 | | | |
| 8093104 | NM_138461 | TM4SF19 | transmembrane 4 L six family memb | -0.123 | 0.918 | 1.72E-01 | 1.72E+01 | | | |
| 8149652 | NM_005144 | HR | hairless homolog (mouse) | -0.123 | 0.918 | 2.04E-02 | 2.04E+00 | | | |
| 8015179 | NM_031957 | KRTAP1-5 | keratin associated protein 1-5 | -0.123 | 0.918 | 1.68E-02 | 1.68E+00 | | | |
| 8019588 | NM_031957 | KRTAP1-5 | keratin associated protein 1-5 | -0.123 | 0.918 | 1.68E-02 | 1.68E+00 | | | |
| 8053731 | AK095105 | FLJ37786 | hypothetical LOC642691 | -0.123 | 0.918 | 6.11E-02 | 6.11E+00 | | | |
| 7912004 | NM_207370 | GPR153 | G protein-coupled receptor 153 | -0.123 | 0.918 | 3.70E-02 | 3.70E+00 | | | |
| 7911114 | NM_018012 | KIF26B | kinesin family member 26B | -0.123 | 0.918 | 1.16E-02 | 1.16E+00 | | | |
| 7895531 | --- | --- | --- | -0.123 | 0.918 | 3.19E-01 | 3.19E+01 | | | |
| 8117626 | --- | --- | --- | -0.123 | 0.918 | 7.86E-02 | 7.86E+00 | | | |
| 8180361 | --- | --- | --- | -0.123 | 0.918 | 7.34E-02 | 7.34E+00 | | | |
| 8069269 | NM_001848 | COL6A1 | collagen, type VI, alpha 1 | -0.123 | 0.918 | 2.52E-02 | 2.52E+00 | | | |
| 8156393 | NM_145006 | SUSD3 | sushi domain containing 3 | -0.123 | 0.918 | 8.02E-02 | 8.02E+00 | | | |
| 8049007 | NM_139073 | SPATA3 | spermatogenesis associated 3 | -0.123 | 0.918 | 4.02E-02 | 4.02E+00 | | | |
| 8013071 | NM_144997 | FLCN | folliculin | -0.123 | 0.918 | 2.51E-02 | 2.51E+00 | | | |
| 8036133 | NM_007000 | UPK1A | uroplakin 1A | -0.123 | 0.918 | 1.71E-01 | 1.71E+01 | | | |
| 7992147 | NM_021098 | CACNA1H | calcium channel, voltage-dependent | -0.123 | 0.918 | 2.41E-02 | 2.41E+00 | | | |
| 7921936 | NM_177398 | LMX1A | LIM homeobox transcription factor 1 | -0.123 | 0.918 | 3.20E-02 | 3.20E+00 | | | |
| 7913211 | NM_014589 | PLA2G2E | phospholipase A2, group IIE | -0.123 | 0.918 | 8.97E-02 | 8.97E+00 | | | |
| 8019194 | NM_025161 | C17orf70 | chromosome 17 open reading frame | -0.123 | 0.918 | 4.87E-02 | 4.87E+00 | | | |
| 8078442 | NM_005508 | CCR4 | chemokine (C-C motif) receptor 4 | -0.123 | 0.918 | 3.93E-01 | 3.93E+01 | | | |
| 7903507 | NM_00101088 | FAM102B | family with sequence similarity 102, | -0.123 | 0.918 | 7.50E-01 | 7.50E+01 | | | |
| 7962703 | NM_001844 | COL2A1 | collagen, type II, alpha 1 | -0.123 | 0.918 | 1.34E-02 | 1.34E+00 | | | |
| 8116607 | NR_026855 | KFZP686I152 | hypothetical LOC401232 | -0.123 | 0.918 | 1.74E-02 | 1.74E+00 | | | |
| 8052932 | --- | --- | --- | -0.123 | 0.918 | 9.35E-02 | 9.35E+00 | | | |
| 8074888 | --- | --- | --- | -0.123 | 0.918 | 9.86E-02 | 9.86E+00 | | | |
| 8145027 | NM_022749 | FAM160B2 | family with sequence similarity 160, | -0.123 | 0.918 | 7.22E-02 | 7.22E+00 | | | |
| 7951853 | NM_052968 | APOA5 | apolipoprotein A-V | -0.123 | 0.918 | 7.37E-02 | 7.37E+00 | | | |
| 8038577 | NM_00102465 | ASPDH | aspartate dehydrogenase domain co | -0.123 | 0.918 | 3.46E-02 | 3.46E+00 | | | |
| 7999102 | NM_003223 | TFAP4 | transcription factor AP-4 (activating | -0.123 | 0.918 | 4.58E-02 | 4.58E+00 | | | |
| 7995308 | --- | --- | --- | -0.123 | 0.918 | 1.22E-01 | 1.22E+01 | | | |
| 7944478 | NM_024791 | PDZD3 | PDZ domain containing 3 | -0.123 | 0.918 | 4.26E-02 | 4.26E+00 | | | |
| 8065710 | NM_005225 | E2F1 | E2F transcription factor 1 | -0.123 | 0.918 | 2.38E-02 | 2.38E+00 | | | |
| 8016320 | NM_203400 | RPRML | reprimo-like | -0.123 | 0.918 | 2.12E-02 | 2.12E+00 | | | |
| 7912224 | NM_003039 | SLC2A5 | solute carrier family 2 (facilitated glu | -0.123 | 0.918 | 2.14E-02 | 2.14E+00 | | | |
| 7997502 | --- | --- | --- | -0.123 | 0.918 | 1.04E-01 | 1.04E+01 | | | |
| 7905085 | NM_00100546 | HIST2H3A | histone cluster 2, H3a | -0.123 | 0.918 | 1.71E-02 | 1.71E+00 | | | |

| | | | | | | | | | | |
|---|---|---|---|---|---|---|---|---|---|---|
| 7919614 | NM_00100546 | HIST2H3A | histone cluster 2, H3a | -0.123 | 0.918 | 1.71E-02 | 1.71E+00 | | | |
| 8161571 | NM_00109927 | FOXD4L2 | forkhead box D4-like 2 | -0.123 | 0.918 | 3.39E-02 | 3.39E+00 | | | |
| 8020025 | --- | --- | --- | -0.123 | 0.918 | 1.68E-01 | 1.68E+01 | | | |
| 8073752 | NM_00110459 | FAM118A | family with sequence similarity 118, | -0.123 | 0.918 | 1.80E-01 | 1.80E+01 | | | |
| 8075616 | NM_003490 | SYN3 | synapsin III | -0.123 | 0.918 | 3.68E-02 | 3.68E+00 | | | |
| 7894571 | --- | --- | --- | -0.123 | 0.918 | 4.14E-01 | 4.14E+01 | | | |
| 7955419 | --- | --- | --- | -0.123 | 0.918 | 6.06E-01 | 6.06E+01 | | | |
| 8027117 | NM_033415 | ARMC6 | armadillo repeat containing 6 | -0.123 | 0.918 | 6.03E-02 | 6.03E+00 | | | |
| 8007420 | NM_003734 | AOC3 | amine oxidase, copper containing 3 ( | -0.123 | 0.918 | 1.07E-01 | 1.07E+01 | | | |
| 7912349 | BC126349 | C1orf127 | chromosome 1 open reading frame 1 | -0.123 | 0.918 | 6.00E-02 | 6.00E+00 | | | |
| 8165277 | NM_198946 | LCN6 | lipocalin 6 | -0.123 | 0.918 | 2.78E-02 | 2.78E+00 | | | |
| 8168045 | NM_004429 | EFNB1 | ephrin-B1 | -0.123 | 0.918 | 3.98E-02 | 3.98E+00 | | | |
| 7893650 | --- | --- | --- | -0.123 | 0.918 | 6.66E-01 | 6.66E+01 | | | |
| 8062023 | NM_032514 | MAP1LC3A | microtubule-associated protein 1 ligl | -0.123 | 0.918 | 1.90E-02 | 1.90E+00 | | | |
| 8110158 | NM_173664 | ARL10 | ADP-ribosylation factor-like 10 | -0.123 | 0.918 | 2.37E-02 | 2.37E+00 | | | |
| 8156415 | NM_006648 | WNK2 | WNK lysine deficient protein kinase 2 | -0.123 | 0.918 | 3.05E-02 | 3.05E+00 | | | |
| 8119829 | NM_00114601 | TJAP1 | tight junction associated protein 1 (p | -0.123 | 0.918 | 2.00E-01 | 2.00E+01 | | | |
| 8131661 | NM_000982 | RPL21 | ribosomal protein L21 | -0.124 | 0.918 | 3.84E-01 | 3.84E+01 | | | |
| 8131869 | NR_026676 | RPS2P32 | ribosomal protein S2 pseudogene 32 | -0.124 | 0.918 | 2.85E-02 | 2.85E+00 | | | |
| 7925739 | --- | --- | --- | -0.124 | 0.918 | 1.45E-01 | 1.45E+01 | | | |
| 8146475 | NM_052898 | XKR4 | XK, Kell blood group complex subuni | -0.124 | 0.918 | 2.48E-02 | 2.48E+00 | | | |
| 8147307 | NM_145269 | FAM92A1 | family with sequence similarity 92, m | -0.124 | 0.918 | 3.41E-01 | 3.41E+01 | | | |
| 8069146 | NM_198689 | KRTAP10-7 | keratin associated protein 10-7 | -0.124 | 0.918 | 1.84E-01 | 1.84E+01 | | | |
| 8049876 | NM_032515 | BOK | BCL2-related ovarian killer | -0.124 | 0.918 | 4.37E-02 | 4.37E+00 | | | |
| 8144420 | NR_027000 | LOC349196 | hypothetical LOC349196 | -0.124 | 0.918 | 2.09E-01 | 2.09E+01 | | | |
| 8144494 | NR_027000 | LOC349196 | hypothetical LOC349196 | -0.124 | 0.918 | 2.09E-01 | 2.09E+01 | | | |
| 8161341 | NM_015667 // | 75A7 // FAM | family with sequence similarity 75, m | -0.124 | 0.918 | 1.52E-02 | 1.52E+00 | | | |
| 7995868 | NM_000339 | SLC12A3 | solute carrier family 12 (sodium/chlo | -0.124 | 0.918 | 3.02E-02 | 3.02E+00 | | | |
| 7912133 | ENST00000439 | OC10012947 | hypothetical protein LOC100129476 | -0.124 | 0.918 | 3.06E-02 | 3.06E+00 | | | |
| 8153709 | NM_012162 | FBXL6 | F-box and leucine-rich repeat protein | -0.124 | 0.918 | 5.95E-02 | 5.95E+00 | | | |
| 7923027 | NM_197962 | GLRX2 | glutaredoxin 2 | -0.124 | 0.918 | 2.07E-01 | 2.07E+01 | | | |
| 8015804 | --- | --- | --- | -0.124 | 0.918 | 3.31E-01 | 3.31E+01 | | | |
| 7968650 | NM_203451 | C13orf36 | chromosome 13 open reading frame | -0.124 | 0.918 | 6.64E-02 | 6.64E+00 | | | |
| 7918869 | NM_002506 | NGF | nerve growth factor (beta polypeptid | -0.124 | 0.918 | 2.92E-02 | 2.92E+00 | | | |
| 8159448 | NM_206920 | MAMDC4 | MAM domain containing 4 | -0.124 | 0.918 | 9.62E-03 | 9.62E-01 | | | |
| 8071877 | NR_003714 | POM121L9P | POM121 membrane glycoprotein-lik | -0.124 | 0.918 | 1.87E-01 | 1.87E+01 | | | |
| 8009040 | NM_006039 | MRC2 | mannose receptor, C type 2 | -0.124 | 0.918 | 4.14E-02 | 4.14E+00 | | | |
| 8007848 | NM_005456 | MAPK8IP1 | mitogen-activated protein kinase 8 ir | -0.124 | 0.918 | 6.10E-02 | 6.10E+00 | | | |
| 7937089 | NM_173575 | STK32C | serine/threonine kinase 32C | -0.124 | 0.918 | 1.83E-02 | 1.83E+00 | | | |
| 7934074 | NM_001057 | TACR2 | tachykinin receptor 2 | -0.124 | 0.918 | 2.80E-02 | 2.80E+00 | | | |
| 8024900 | NM_00104820 | UHRF1 | ubiquitin-like with PHD and ring finge | -0.124 | 0.918 | 5.81E-02 | 5.81E+00 | | | |
| 8164243 | NM_130459 | TOR2A | torsin family 2, member A | -0.124 | 0.918 | 4.82E-02 | 4.82E+00 | | | |
| 8013533 | --- | --- | --- | -0.124 | 0.918 | 1.63E-02 | 1.63E+00 | | | |
| 8130997 | BC109246 | PDGFA | platelet-derived growth factor alpha | -0.124 | 0.918 | 4.22E-02 | 4.22E+00 | | | |
| 8138442 | NM_000474 | TWIST1 | twist homolog 1 (Drosophila) | -0.124 | 0.918 | 1.73E-02 | 1.73E+00 | | | |
| 7920744 | NM_000298 | PKLR | pyruvate kinase, liver and RBC | -0.124 | 0.918 | 1.58E-02 | 1.58E+00 | | | |
| 8093440 | NM_00100435 | FGFRL1 | fibroblast growth factor receptor-like | -0.124 | 0.918 | 3.47E-02 | 3.47E+00 | | | |
| 8067125 | NM_003657 | BCAS1 | breast carcinoma amplified sequence | -0.124 | 0.918 | 3.60E-02 | 3.60E+00 | | | |
| 8049689 | NM_018226 | RNPEPL1 | arginyl aminopeptidase (aminopepti | -0.124 | 0.918 | 2.55E-02 | 2.55E+00 | | | |
| 8180282 | --- | --- | --- | -0.124 | 0.918 | 1.39E-01 | 1.39E+01 | | | |
| 7988400 | NM_138356 | SHF | Src homology 2 domain containing F | -0.124 | 0.918 | 2.95E-02 | 2.95E+00 | | | |
| 8076436 | NM_005650 | TCF20 | transcription factor 20 (AR1) | -0.124 | 0.918 | 5.42E-01 | 5.42E+01 | | | |
| 8077036 | BC031069 | FAM116B | family with sequence similarity 116, | -0.124 | 0.918 | 2.97E-02 | 2.97E+00 | | | |
| 8018220 | NM_000835 | GRIN2C | glutamate receptor, ionotropic, N-m | -0.124 | 0.918 | 2.92E-02 | 2.92E+00 | | | |
| 7915078 | NM_173641 | EPHA10 | EPH receptor A10 | -0.124 | 0.918 | 1.81E-02 | 1.81E+00 | | | |
| 8163548 | NM_138424 | KIF12 | kinesin family member 12 | -0.124 | 0.918 | 1.08E-02 | 1.08E+00 | | | |
| 7899595 | NM_002379 | MATN1 | matrilin 1, cartilage matrix protein | -0.124 | 0.918 | 1.12E-01 | 1.12E+01 | | | |
| 7995907 | NM_000078 | CETP | cholesteryl ester transfer protein, pla | -0.124 | 0.918 | 2.23E-02 | 2.23E+00 | | | |
| 8160452 | NM_078487 | CDKN2B | cyclin-dependent kinase inhibitor 2B | -0.124 | 0.918 | 6.28E-02 | 6.28E+00 | | | |
| 7987701 | NM_016642 | SPTBN5 | spectrin, beta, non-erythrocytic 5 | -0.124 | 0.918 | 1.06E-02 | 1.06E+00 | | | |
| 8082965 | NM_012219 | MRAS | muscle RAS oncogene homolog | -0.124 | 0.918 | 7.86E-02 | 7.86E+00 | | | |
| 8077944 | NM_00116249 | CAND2 | cullin-associated and neddylation-dis | -0.124 | 0.918 | 3.46E-02 | 3.46E+00 | | | |
| 7911754 | NM_003820 | TNFRSF14 | tumor necrosis factor receptor super | -0.124 | 0.918 | 7.53E-02 | 7.53E+00 | | | |
| 8029744 | NM_017659 | QPCTL | glutaminyl-peptide cyclotransferase- | -0.124 | 0.918 | 2.21E-02 | 2.21E+00 | | | |
| 8045018 | NM_000312 | PROC | protein C (inactivator of coagulation | -0.124 | 0.918 | 5.50E-02 | 5.50E+00 | | | |
| 8115691 | NM_003062 | SLIT3 | slit homolog 3 (Drosophila) | -0.124 | 0.918 | 9.02E-03 | 9.02E-01 | | | |
| 8040632 | ENST0000040 | FAM59B | family with sequence similarity 59, m | -0.124 | 0.918 | 1.56E-02 | 1.56E+00 | | | |
| 8153550 | NM_178564 | NRBP2 | nuclear receptor binding protein 2 | -0.124 | 0.918 | 1.44E-01 | 1.44E+01 | | | |
| 7991516 | NM_139057 | ADAMTS17 | ADAM metallopeptidase with throm | -0.124 | 0.918 | 3.01E-02 | 3.01E+00 | | | |
| 7920659 | NM_173852 | KRTCAP2 | keratinocyte associated protein 2 | -0.124 | 0.918 | 7.32E-01 | 7.32E+01 | | | |
| 8166613 | NM_002365 | MAGEB3 | melanoma antigen family B, 3 | -0.124 | 0.917 | 3.49E-02 | 3.49E+00 | | | |
| 7896691 | --- | --- | --- | -0.124 | 0.917 | 3.91E-01 | 3.91E+01 | | | |
| 8075886 | NM_000878 | IL2RB | interleukin 2 receptor, beta | -0.124 | 0.917 | 3.91E-01 | 3.91E+01 | | | |
| 7952577 | NM_032531 | KIRREL3 | kin of IRRE like 3 (Drosophila) | -0.124 | 0.917 | 3.64E-02 | 3.64E+00 | | | |
| 8125805 | NM_000841 | GRM4 | glutamate receptor, metabotropic 4 | -0.124 | 0.917 | 3.28E-02 | 3.28E+00 | | | |
| 8120880 | NM_006670 | TPBG | trophoblast glycoprotein | -0.124 | 0.917 | 1.13E-01 | 1.13E+01 | | | |
| 8075637 | NM_004737 | LARGE | like-glycosyltransferase | -0.124 | 0.917 | 4.83E-02 | 4.83E+00 | | | |

| | | | | | | | | | | |
|---|---|---|---|---|---|---|---|---|---|---|
| 7897714 | NM_00101476 | FBXO44 | F-box protein 44 | -0.124 | 0.917 | 2.85E-02 | 2.85E+00 | | | |
| 7974194 | --- | --- | --- | -0.124 | 0.917 | 2.48E-01 | 2.48E+01 | | | |
| 8112740 | NM_032367 | ZBED3 | zinc finger, BED-type containing 3 | -0.124 | 0.917 | 1.18E-02 | 1.18E+00 | | | |
| 8013135 | NM_00100529 | SREBF1 | sterol regulatory element binding tra | -0.124 | 0.917 | 2.58E-02 | 2.58E+00 | | | |
| 8139460 | NM_00114633 | NACAD | NAC alpha domain containing | -0.124 | 0.917 | 1.34E-02 | 1.34E+00 | | | |
| 8052376 | NM_00108043 | CCDC85A | coiled-coil domain containing 85A | -0.124 | 0.917 | 5.24E-02 | 5.24E+00 | | | |
| 8075971 | NM_138797 | ANKRD54 | ankyrin repeat domain 54 | -0.124 | 0.917 | 2.92E-02 | 2.92E+00 | | | |
| 8027778 | NM_144779 | FXYD5 | FXYD domain containing ion transpo | -0.124 | 0.917 | 5.06E-01 | 5.06E+01 | | | |
| 7894266 | --- | --- | --- | -0.124 | 0.917 | 3.61E-01 | 3.61E+01 | | | |
| 7905220 | NM_004425 | ECM1 | extracellular matrix protein 1 | -0.124 | 0.917 | 4.57E-02 | 4.57E+00 | | | |
| 8035539 | NM_001492 | GDF1 | growth differentiation factor 1 | -0.124 | 0.917 | 2.25E-02 | 2.25E+00 | | | |
| 8165453 | NM_00101365 | LRRC26 | leucine rich repeat containing 26 | -0.124 | 0.917 | 2.15E-02 | 2.15E+00 | | | |
| 7899101 | NM_006314 | CNKSR1 | connector enhancer of kinase suppre | -0.124 | 0.917 | 3.09E-02 | 3.09E+00 | | | |
| 7894714 | --- | --- | --- | -0.124 | 0.917 | 3.32E-01 | 3.32E+01 | | | |
| 8109305 | NM_007286 | SYNPO | synaptopodin | -0.124 | 0.917 | 4.39E-02 | 4.39E+00 | | | |
| 8024623 | NM_205843 | NFIC | nuclear factor I/C (CCAAT-binding tra | -0.125 | 0.917 | 3.03E-02 | 3.03E+00 | | | |
| 8119237 | AK092662 | OC10013200 | hypothetical LOC100132005 | -0.125 | 0.917 | 3.64E-01 | 3.64E+01 | | | |
| 7916425 | NM_017904 | TTC22 | tetratricopeptide repeat domain 22 | -0.125 | 0.917 | 6.25E-02 | 6.25E+00 | | | |
| 7893367 | --- | --- | --- | -0.125 | 0.917 | 8.85E-01 | 8.85E+01 | | | |
| 7894655 | --- | --- | --- | -0.125 | 0.917 | 2.33E-01 | 2.33E+01 | | | |
| 8039524 | NM_152600 | ZNF579 | zinc finger protein 579 | -0.125 | 0.917 | 1.63E-02 | 1.63E+00 | | | |
| 7949882 | NM_000695 | ALDH3B2 | aldehyde dehydrogenase 3 family, m | -0.125 | 0.917 | 4.84E-02 | 4.84E+00 | | | |
| 8075846 | AK075161 | FLJ90680 | FLJ90680 protein | -0.125 | 0.917 | 9.57E-02 | 9.57E+00 | | | |
| 8041582 | NM_138370 | SGK493 | protein kinase-like protein SgK493 | -0.125 | 0.917 | 1.77E-02 | 1.77E+00 | | | |
| 8076937 | NM_032019 | HDAC10 | histone deacetylase 10 | -0.125 | 0.917 | 2.94E-02 | 2.94E+00 | | | |
| 8117176 | ENST0000040 | OC10012930 | UPF0607 protein ENSP00000383144 | -0.125 | 0.917 | 1.22E-01 | 1.22E+01 | | | |
| 8104319 | --- | --- | --- | -0.125 | 0.917 | 2.87E-02 | 2.87E+00 | | | |
| 7893013 | --- | --- | --- | -0.125 | 0.917 | 4.49E-01 | 4.49E+01 | | | |
| 8016878 | NM_017949 | CUEDC1 | CUE domain containing 1 | -0.125 | 0.917 | 2.95E-02 | 2.95E+00 | | | |
| 7978222 | NM_000359 | TGM1 | transglutaminase 1 (K polypeptide ep | -0.125 | 0.917 | 3.27E-02 | 3.27E+00 | | | |
| 8161618 | NM_001163 | APBA1 | amyloid beta (A4) precursor protein- | -0.125 | 0.917 | 2.22E-02 | 2.22E+00 | | | |
| 8137833 | NM_013321 | SNX8 | sorting nexin 8 | -0.125 | 0.917 | 7.06E-02 | 7.06E+00 | | | |
| 8020795 | NM_000371 | TTR | transthyretin | -0.125 | 0.917 | 3.91E-02 | 3.91E+00 | | | |
| 7912968 | NM_152232 | TAS1R2 | taste receptor, type 1, member 2 | -0.125 | 0.917 | 3.99E-02 | 3.99E+00 | | | |
| 8067792 | NM_080621 | SAMD10 | sterile alpha motif domain containin | -0.125 | 0.917 | 4.43E-02 | 4.43E+00 | | | |
| 7994981 | NM_152288 | ORAI3 | ORAI calcium release-activated calci | -0.125 | 0.917 | 1.89E-02 | 1.89E+00 | | | |
| 8095697 | NM_001511 | CXCL1 | chemokine (C-X-C motif) ligand 1 (m | -0.125 | 0.917 | 5.85E-02 | 5.85E+00 | | | |
| 7959850 | NM_007197 | FZD10 | frizzled homolog 10 (Drosophila) | -0.125 | 0.917 | 2.91E-02 | 2.91E+00 | | | |
| 7956426 | NM_031479 | INHBE | inhibin, beta E | -0.125 | 0.917 | 3.39E-02 | 3.39E+00 | | | |
| 8079140 | NM_017719 | SNRK | SNF related kinase | -0.125 | 0.917 | 5.38E-01 | 5.38E+01 | | | |
| 7968637 | NM_003914 | CCNA1 | cyclin A1 | -0.125 | 0.917 | 3.16E-02 | 3.16E+00 | | | |
| 7911683 | NM_182533 | C1orf86 | chromosome 1 open reading frame 8 | -0.125 | 0.917 | 2.32E-02 | 2.32E+00 | | | |
| 8153405 | NR_026785 | C8orf51 | chromosome 8 open reading frame 5 | -0.125 | 0.917 | 1.54E-02 | 1.54E+00 | | | |
| 8008982 | NM_018488 | TBX4 | T-box 4 | -0.125 | 0.917 | 1.00E-01 | 1.00E+01 | | | |
| 8152924 | NM_00108039 | OC90 | otoconin 90 | -0.125 | 0.917 | 4.23E-02 | 4.23E+00 | | | |
| 8009430 | NR_027283 | LOC440461 | SH3 domain containing 20 pseudoge | -0.125 | 0.917 | 4.14E-02 | 4.14E+00 | | | |
| 7965038 | --- | --- | --- | -0.125 | 0.917 | 9.30E-02 | 9.30E+00 | | | |
| 7914216 | NR_003035 | SNORA16A | small nucleolar RNA, H/ACA box 16A | -0.125 | 0.917 | 7.17E-01 | 7.17E+01 | | | |
| 8074867 | NR_024591 | POM121L1P | POM121 membrane glycoprotein-lik | -0.125 | 0.917 | 6.30E-02 | 6.30E+00 | | | |
| 8069872 | NM_175858 | KRTAP11-1 | keratin associated protein 11-1 | -0.125 | 0.917 | 7.10E-02 | 7.10E+00 | | | |
| 7985349 | NM_022566 | MESDC1 | mesoderm development candidate 1 | -0.125 | 0.917 | 1.72E-02 | 1.72E+00 | | | |
| 8157777 | NM_00116180 | GPR144 | G protein-coupled receptor 144 | -0.125 | 0.917 | 1.38E-02 | 1.38E+00 | | | |
| 7894097 | --- | --- | --- | -0.125 | 0.917 | 5.39E-01 | 5.39E+01 | | | |
| 8153488 | NM_198488 | FAM83H | family with sequence similarity 83, m | -0.125 | 0.917 | 3.68E-02 | 3.68E+00 | | | |
| 8008016 | NM_014726 | TBKBP1 | TBK1 binding protein 1 | -0.125 | 0.917 | 1.79E-02 | 1.79E+00 | | | |
| 8090503 | --- | --- | --- | -0.125 | 0.917 | 2.66E-01 | 2.66E+01 | | | |
| 8039766 | NM_00112973 | ZNF837 | zinc finger protein 837 | -0.125 | 0.917 | 5.26E-02 | 5.26E+00 | | | |
| 7946903 | NM_153676 | USH1C | Usher syndrome 1C (autosomal rece | -0.125 | 0.917 | 3.22E-02 | 3.22E+00 | | | |
| 7896077 | --- | --- | --- | -0.125 | 0.917 | 2.42E-01 | 2.42E+01 | | | |
| 7982845 | NM_020857 | VPS18 | vacuolar protein sorting 18 homolog | -0.125 | 0.917 | 1.57E-01 | 1.57E+01 | | | |
| 8010747 | NM_212492 | GPS1 | G protein pathway suppressor 1 | -0.125 | 0.917 | 1.77E-02 | 1.77E+00 | | | |
| 7985233 | NM_002891 | RASGRF1 | Ras protein-specific guanine nucleot | -0.125 | 0.917 | 1.38E-01 | 1.38E+01 | | | |
| 7912854 | NR_026567 | ESPNP | espin pseudogene | -0.125 | 0.917 | 2.19E-01 | 2.19E+01 | | | |
| 7894301 | --- | --- | --- | -0.125 | 0.917 | 2.20E-01 | 2.20E+01 | | | |
| 8048205 | NM_000597 | IGFBP2 | insulin-like growth factor binding pro | -0.125 | 0.917 | 1.50E-02 | 1.50E+00 | | | |
| 8143803 | NM_173681 | ATG9B | ATG9 autophagy related 9 homolog | -0.125 | 0.917 | 1.85E-02 | 1.85E+00 | | | |
| 8072143 | NM_172002 | HSCB | HscB iron-sulfur cluster co-chaperon | -0.125 | 0.917 | 1.99E-01 | 1.99E+01 | | | |
| 8178445 | NM_014069 | PSORS1C2 | psoriasis susceptibility 1 candidate 2 | -0.125 | 0.917 | 3.30E-02 | 3.30E+00 | | | |
| 7893061 | --- | --- | --- | -0.125 | 0.917 | 4.31E-01 | 4.31E+01 | | | |
| 7987466 | --- | --- | --- | -0.125 | 0.917 | 2.08E-02 | 2.08E+00 | | | |
| 8037767 | NM_018215 | PNMAL1 | PNMA-like 1 | -0.125 | 0.917 | 6.24E-02 | 6.24E+00 | | | |
| 8002878 | NM_145254 | TMEM170A | transmembrane protein 170A | -0.125 | 0.917 | 3.65E-01 | 3.65E+01 | | | |
| 8142446 | --- | --- | --- | -0.125 | 0.917 | 3.84E-01 | 3.84E+01 | | | |
| 8013013 | --- | --- | --- | -0.125 | 0.917 | 8.06E-01 | 8.06E+01 | | | |
| 7942613 | NM_032564 | DGAT2 | diacylglycerol O-acyltransferase hom | -0.125 | 0.917 | 4.03E-02 | 4.03E+00 | | | |
| 8011975 | NM_177550 | SLC13A5 | solute carrier family 13 (sodium-dep | -0.125 | 0.917 | 5.72E-03 | 5.72E-01 | | | |
| 7938559 | NM_00108052 | RASSF10 | Ras association (RalGDS/AF-6) doma | -0.125 | 0.917 | 7.53E-02 | 7.53E+00 | | | |

| | | | | | | | | | | |
|---|---|---|---|---|---|---|---|---|---|---|
| 8175782 | NM_207107 | HAUS7 | HAUS augmin-like complex, subunit | -0.125 | 0.917 | 2.14E-02 | 2.14E+00 | | | |
| 8016139 | NM_00108044 | KIF18B | kinesin family member 18B | -0.125 | 0.917 | 4.47E-02 | 4.47E+00 | | | |
| 7913385 | NM_00114565 | RAP1GAP | RAP1 GTPase activating protein | -0.125 | 0.917 | 1.79E-02 | 1.79E+00 | | | |
| 7927277 | NM_014696 | GPRIN2 | G protein regulated inducer of neuri | -0.125 | 0.917 | 4.09E-02 | 4.09E+00 | | | |
| 7937498 | NM_173584 | EFCAB4A | EF-hand calcium binding domain 4A | -0.125 | 0.917 | 3.28E-02 | 3.28E+00 | | | |
| 8037888 | NM_015063 | SLC8A2 | solute carrier family 8 (sodium/calci | -0.126 | 0.917 | 2.23E-02 | 2.23E+00 | | | |
| 8032525 | NM_213568 | SLC39A3 | solute carrier family 39 (zinc transpo | -0.126 | 0.917 | 2.03E-02 | 2.03E+00 | | | |
| 7946781 | NM_175058 | PLEKHA7 | pleckstrin homology domain contain | -0.126 | 0.917 | 4.73E-02 | 4.73E+00 | | | |
| 7978710 | --- | --- | --- | -0.126 | 0.917 | 1.67E-01 | 1.67E+01 | | | |
| 7984932 | NM_138967 | SCAMP5 | secretory carrier membrane protein | -0.126 | 0.917 | 1.99E-02 | 1.99E+00 | | | |
| 8062842 | --- | --- | --- | -0.126 | 0.917 | 2.85E-01 | 2.85E+01 | | | |
| 8130824 | AB016902 | HGC6.3 | similar to HGC6.3 | -0.126 | 0.917 | 1.07E-01 | 1.07E+01 | | | |
| 7893234 | --- | --- | --- | -0.126 | 0.917 | 4.69E-01 | 4.69E+01 | | | |
| 8065485 | NM_025176 | NINL | ninein-like | -0.126 | 0.917 | 2.41E-02 | 2.41E+00 | | | |
| 8088415 | NM_007177 | FAM107A | family with sequence similarity 107, | -0.126 | 0.917 | 3.34E-02 | 3.34E+00 | | | |
| 8139887 | AK090474 | LOC441245 | hypothetical LOC441245 | -0.126 | 0.917 | 9.96E-02 | 9.96E+00 | | | |
| 8004152 | NM_015253 | WSCD1 | WSC domain containing 1 | -0.126 | 0.917 | 1.88E-02 | 1.88E+00 | | | |
| 7934145 | NM_207119 | LRRC20 | leucine rich repeat containing 20 | -0.126 | 0.917 | 3.99E-02 | 3.99E+00 | | | |
| 8022243 | --- | --- | --- | -0.126 | 0.917 | 1.78E-01 | 1.78E+01 | | | |
| 7977299 | NM_00111272 | KIAA0284 | KIAA0284 | -0.126 | 0.917 | 2.86E-02 | 2.86E+00 | | | |
| 7904863 | --- | --- | --- | -0.126 | 0.917 | 4.37E-02 | 4.37E+00 | | | |
| 8149289 | NM_031439 | SOX7 | SRY (sex determining region Y)-box 7 | -0.126 | 0.917 | 3.94E-02 | 3.94E+00 | | | |
| 7981425 | --- | --- | --- | -0.126 | 0.916 | 1.67E-01 | 1.67E+01 | | | |
| 8015208 | NM_00112338 | KRTAP2-1 | keratin associated protein 2-1 | -0.126 | 0.916 | 2.23E-02 | 2.23E+00 | | | |
| 7955613 | NM_005556 | KRT7 | keratin 7 | -0.126 | 0.916 | 3.57E-02 | 3.57E+00 | | | |
| 8123584 | NM_00101241 | MYLK4 | myosin light chain kinase family, me | -0.126 | 0.916 | 2.97E-02 | 2.97E+00 | | | |
| 8003812 | NM_012373 | OR3A3 | olfactory receptor, family 3, subfami | -0.126 | 0.916 | 1.78E-01 | 1.78E+01 | | | |
| 7896779 | NM_198317 | KLHL17 | kelch-like 17 (Drosophila) | -0.126 | 0.916 | 2.21E-02 | 2.21E+00 | | | |
| 8016400 | --- | --- | --- | -0.126 | 0.916 | 1.02E-01 | 1.02E+01 | | | |
| 8032699 | NM_015174 | ZFR2 | zinc finger RNA binding protein 2 | -0.126 | 0.916 | 2.37E-02 | 2.37E+00 | | | |
| 8093386 | NM_002477 | MYL5 | myosin, light chain 5, regulatory | -0.126 | 0.916 | 3.39E-01 | 3.39E+01 | | | |
| 8060660 | NM_052970 | HSPA12B | heat shock 70kD protein 12B | -0.126 | 0.916 | 1.08E-02 | 1.08E+00 | | | |
| 8149250 | NM_00108082 | PRAGMIN | homolog of rat pragma of Rnd2 | -0.126 | 0.916 | 4.50E-03 | 4.50E-01 | | | |
| 8049271 | NM_030803 | ATG16L1 | ATG16 autophagy related 16-like 1 ( | -0.126 | 0.916 | 5.32E-01 | 5.32E+01 | | | |
| 7895993 | --- | --- | --- | -0.126 | 0.916 | 3.57E-01 | 3.57E+01 | | | |
| 8027032 | NM_00110041 | C19orf60 | chromosome 19 open reading frame | -0.126 | 0.916 | 7.25E-02 | 7.25E+00 | | | |
| 8070567 | NM_003226 | TFF3 | trefoil factor 3 (intestinal) | -0.126 | 0.916 | 2.97E-02 | 2.97E+00 | | | |
| 8131195 | NM_133463 | AMZ1 | archaelysin family metallopeptidase | -0.126 | 0.916 | 2.20E-02 | 2.20E+00 | | | |
| 7896243 | --- | --- | --- | -0.126 | 0.916 | 1.66E-01 | 1.66E+01 | | | |
| 7931184 | NM_005519 | HMX2 | H6 family homeobox 2 | -0.126 | 0.916 | 4.14E-02 | 4.14E+00 | | | |
| 7892750 | --- | --- | --- | -0.126 | 0.916 | 8.06E-01 | 8.06E+01 | | | |
| 8049684 | NM_00103357 | DUSP28 | dual specificity phosphatase 28 | -0.126 | 0.916 | 1.88E-01 | 1.88E+01 | | | |
| 8121085 | --- | --- | --- | -0.126 | 0.916 | 8.12E-01 | 8.12E+01 | | | |
| 8114518 | NM_194296 | LOC202051 | hypothetical protein LOC202051 | -0.126 | 0.916 | 9.85E-02 | 9.85E+00 | | | |
| 7984662 | NM_018652 | GOLGA6B | golgi autoantigen, golgin subfamily a | -0.126 | 0.916 | 1.08E-02 | 1.08E+00 | | | |
| 8158204 | NM_00104001 | C9orf119 | chromosome 9 open reading frame | -0.126 | 0.916 | 1.23E-01 | 1.23E+01 | | | |
| 8055941 | NM_019845 | RPRM | reprimo, TP53 dependent G2 arrest | -0.126 | 0.916 | 7.01E-02 | 7.01E+00 | | | |
| 8141423 | --- | --- | --- | -0.126 | 0.916 | 1.15E-01 | 1.15E+01 | | | |
| 7904106 | NM_152900 | MAGI3 | membrane associated guanylate kin | -0.126 | 0.916 | 2.76E-02 | 2.76E+00 | | | |
| 7984253 | NM_00110136 | hCG_164572 | hCG1645727 | -0.126 | 0.916 | 5.92E-02 | 5.92E+00 | | | |
| 8003939 | NM_003963 | TM4SF5 | transmembrane 4 L six family memb | -0.126 | 0.916 | 1.62E-02 | 1.62E+00 | | | |
| 8035903 | --- | --- | --- | -0.126 | 0.916 | 2.04E-01 | 2.04E+01 | | | |
| 8118634 | NM_002931 | RING1 | ring finger protein 1 | -0.126 | 0.916 | 2.55E-01 | 2.55E+01 | | | |
| 8092834 | --- | --- | --- | -0.126 | 0.916 | 1.55E-01 | 1.55E+01 | | | |
| 7941732 | NM_005133 | RCE1 | RCE1 homolog, prenyl protein peptid | -0.126 | 0.916 | 8.69E-02 | 8.69E+00 | | | |
| 8074845 | NM_080764 | ZNF280B | zinc finger protein 280B | -0.126 | 0.916 | 2.58E-02 | 2.58E+00 | | | |
| 8162777 | NM_005458 | GABBR2 | gamma-aminobutyric acid (GABA) B | -0.126 | 0.916 | 4.20E-02 | 4.20E+00 | | | |
| 8063417 | NM_017843 | BCAS4 | breast carcinoma amplified sequenc | -0.126 | 0.916 | 4.48E-02 | 4.48E+00 | | | |
| 8090469 | NM_032638 | GATA2 | GATA binding protein 2 | -0.126 | 0.916 | 2.23E-02 | 2.23E+00 | | | |
| 8159627 | NM_080877 | SLC34A3 | solute carrier family 34 (sodium pho | -0.126 | 0.916 | 1.84E-02 | 1.84E+00 | | | |
| 8153497 | NM_182706 | SCRIB | scribbled homolog (Drosophila) | -0.126 | 0.916 | 2.01E-02 | 2.01E+00 | | | |
| 8162140 | --- | --- | --- | -0.126 | 0.916 | 5.29E-02 | 5.29E+00 | | | |
| 7917599 | NM_020063 | BARHL2 | BarH-like homeobox 2 | -0.126 | 0.916 | 2.67E-02 | 2.67E+00 | | | |
| 7998407 | NM_207419 | C1QTNF8 | C1q and tumor necrosis factor relate | -0.126 | 0.916 | 1.26E-02 | 1.26E+00 | | | |
| 7938154 | NM_004517 | ILK | integrin-linked kinase | -0.126 | 0.916 | 5.17E-01 | 5.17E+01 | | | |
| 8035398 | NM_002866 | RAB3A | RAB3A, member RAS oncogene fami | -0.126 | 0.916 | 1.99E-02 | 1.99E+00 | | | |
| 7997740 | NM_022818 | MAP1LC3B | microtubule-associated protein 1 lig | -0.126 | 0.916 | 2.20E-01 | 2.20E+01 | | | |
| 8014349 | NR_027921 | CCL14-CCL15 | chemokine ligand 14, chemokine liga | -0.126 | 0.916 | 3.94E-02 | 3.94E+00 | | | |
| 8132031 | NM_175887 | PRR15 | proline rich 15 | -0.126 | 0.916 | 1.93E-02 | 1.93E+00 | | | |
| 8042925 | NM_004263 | SEMA4F | sema domain, immunoglobulin doma | -0.126 | 0.916 | 2.09E-02 | 2.09E+00 | | | |
| 8139763 | NM_00114571 | LOC389493 | hypothetical protein LOC389493 | -0.126 | 0.916 | 1.06E-02 | 1.06E+00 | | | |
| 7981911 | NM_005664 | MKRN3 | makorin ring finger protein 3 | -0.126 | 0.916 | 1.18E-02 | 1.18E+00 | | | |
| 7918416 | NM_006492 | ALX3 | ALX homeobox 3 | -0.126 | 0.916 | 3.37E-02 | 3.37E+00 | | | |
| 7898084 | NM_015209 | RP1-21O18.1 | kazrin | -0.126 | 0.916 | 2.15E-02 | 2.15E+00 | | | |
| 7892898 | --- | --- | --- | -0.126 | 0.916 | 3.46E-01 | 3.46E+01 | | | |
| 8010583 | NR_027255 | LOC388428 | hypothetical LOC388428 | -0.126 | 0.916 | 1.78E-02 | 1.78E+00 | | | |
| 7955112 | NM_181788 | H1FNT | H1 histone family, member N, testis- | -0.126 | 0.916 | 2.77E-02 | 2.77E+00 | | | |

| | | | | | | | | | | |
|---|---|---|---|---|---|---|---|---|---|---|
| 7941122 | NM_013299 | SAC3D1 | SAC3 domain containing 1 | -0.126 | 0.916 | 3.24E-02 | 3.24E+00 | | | |
| 7909561 | NR_026761 | C1orf97 | chromosome 1 open reading frame 9 | -0.126 | 0.916 | 2.57E-01 | 2.57E+01 | | | |
| 7921492 | NM_00113505 | IGSF9 | immunoglobulin superfamily, memb | -0.126 | 0.916 | 1.88E-02 | 1.88E+00 | | | |
| 7894882 | --- | --- | --- | -0.127 | 0.916 | 8.38E-01 | 8.38E+01 | | | |
| 8098762 | NM_007100 | ATP5I | ATP synthase, H+ transporting, mito | -0.127 | 0.916 | 7.65E-01 | 7.65E+01 | | | |
| 8007154 | NM_021939 | FKBP10 | FK506 binding protein 10, 65 kDa | -0.127 | 0.916 | 1.45E-02 | 1.45E+00 | | | |
| 8015247 | NM_033060 | KRTAP4-1 | keratin associated protein 4-1 | -0.127 | 0.916 | 6.12E-02 | 6.12E+00 | | | |
| 8048541 | NM_001927 | DES | desmin | -0.127 | 0.916 | 1.90E-02 | 1.90E+00 | | | |
| 7992140 | --- | --- | --- | -0.127 | 0.916 | 1.75E-02 | 1.75E+00 | | | |
| 7966189 | NM_213596 | FOXN4 | forkhead box N4 | -0.127 | 0.916 | 1.87E-02 | 1.87E+00 | | | |
| 7910897 | --- | --- | --- | -0.127 | 0.916 | 5.62E-02 | 5.62E+00 | | | |
| 7931561 | NM_145806 | ZNF511 | zinc finger protein 511 | -0.127 | 0.916 | 4.56E-02 | 4.56E+00 | | | |
| 8132849 | --- | --- | --- | -0.127 | 0.916 | 4.42E-01 | 4.42E+01 | | | |
| 7892982 | --- | --- | --- | -0.127 | 0.916 | 8.86E-01 | 8.86E+01 | | | |
| 8013771 | NM_138463 | TLCD1 | TLC domain containing 1 | -0.127 | 0.916 | 4.85E-02 | 4.85E+00 | | | |
| 7893373 | --- | --- | --- | -0.127 | 0.916 | 5.45E-01 | 5.45E+01 | | | |
| 8080896 | NM_173471 | SLC25A26 | solute carrier family 25, member 26 | -0.127 | 0.916 | 7.04E-01 | 7.04E+01 | | | |
| 7963664 | NM_152860 | SP7 | Sp7 transcription factor | -0.127 | 0.916 | 9.54E-02 | 9.54E+00 | | | |
| 7899343 | NM_005281 | GPR3 | G protein-coupled receptor 3 | -0.127 | 0.916 | 6.89E-02 | 6.89E+00 | | | |
| 8028158 | --- | --- | --- | -0.127 | 0.916 | 5.74E-02 | 5.74E+00 | | | |
| 8163509 | NM_000031 | ALAD | aminolevulinate, delta-, dehydratase | -0.127 | 0.916 | 5.17E-02 | 5.17E+00 | | | |
| 7927597 | --- | --- | --- | -0.127 | 0.916 | 1.03E-01 | 1.03E+01 | | | |
| 8133335 | NM_003508 | FZD9 | frizzled homolog 9 (Drosophila) | -0.127 | 0.916 | 1.39E-02 | 1.39E+00 | | | |
| 8092501 | NM_000460 | THPO | thrombopoietin | -0.127 | 0.916 | 3.68E-02 | 3.68E+00 | | | |
| 8034199 | NM_012466 | TSPAN16 | tetraspanin 16 | -0.127 | 0.916 | 1.28E-01 | 1.28E+01 | | | |
| 7928046 | NM_012339 | TSPAN15 | tetraspanin 15 | -0.127 | 0.916 | 4.38E-02 | 4.38E+00 | | | |
| 7948879 | --- | --- | --- | -0.127 | 0.916 | 8.51E-01 | 8.51E+01 | | | |
| 7937698 | NM_00100592 | KRTAP5-1 | keratin associated protein 5-1 | -0.127 | 0.916 | 4.67E-02 | 4.67E+00 | | | |
| 8156257 | NM_00114512 | 75C1 // FAM | family with sequence similarity 75, m | -0.127 | 0.916 | 4.04E-02 | 4.04E+00 | | | |
| 8019711 | NM_145651 | SCGB1C1 | secretoglobin, family 1C, member 1 | -0.127 | 0.916 | 5.15E-02 | 5.15E+00 | | | |
| 7921882 | NM_015441 | OLFML2B | olfactomedin-like 2B | -0.127 | 0.916 | 1.76E-02 | 1.76E+00 | | | |
| 8036170 | ENST00000443 | PRODH2 | proline dehydrogenase (oxidase) 2 | -0.127 | 0.916 | 9.14E-02 | 9.14E+00 | | | |
| 8061666 | --- | --- | --- | -0.127 | 0.916 | 9.64E-02 | 9.64E+00 | | | |
| 8073511 | --- | --- | --- | -0.127 | 0.916 | 5.72E-02 | 5.72E+00 | | | |
| 7990736 | NM_014272 | ADAMTS7 | ADAM metallopeptidase with throm | -0.127 | 0.916 | 6.13E-02 | 6.13E+00 | | | |
| 8075285 | NM_032204 | ASCC2 | activating signal cointegrator 1 comp | -0.127 | 0.916 | 2.65E-01 | 2.65E+01 | | | |
| 7907368 | --- | --- | --- | -0.127 | 0.916 | 3.36E-01 | 3.36E+01 | | | |
| 7898115 | NM_00113621 | TMEM51 | transmembrane protein 51 | -0.127 | 0.916 | 3.95E-02 | 3.95E+00 | | | |
| 8023995 | NM_005860 | FSTL3 | follistatin-like 3 (secreted glycoprote | -0.127 | 0.916 | 1.06E-01 | 1.06E+01 | | | |
| 7995806 | NM_005946 | MT1A | metallothionein 1A | -0.127 | 0.916 | 1.18E-01 | 1.18E+01 | | | |
| 8098758 | NM_133474 | ZNF721 | zinc finger protein 721 | -0.127 | 0.916 | 7.31E-01 | 7.31E+01 | | | |
| 8006594 | NM_002988 | CCL18 | chemokine (C-C motif) ligand 18 (pul | -0.127 | 0.916 | 3.64E-02 | 3.64E+00 | | | |
| 8071339 | NM_013373 | ZDHHC8 | zinc finger, DHHC-type containing 8 | -0.127 | 0.916 | 4.47E-02 | 4.47E+00 | | | |
| 8048628 | NM_00100520 | TMEM198 | transmembrane protein 198 | -0.127 | 0.916 | 4.27E-02 | 4.27E+00 | | | |
| 8039453 | NM_00108548 | TMEM150B | transmembrane protein 150B | -0.127 | 0.916 | 3.72E-02 | 3.72E+00 | | | |
| 8060061 | NR_026664 | MGC16025 | hypothetical LOC85009 | -0.127 | 0.916 | 4.17E-02 | 4.17E+00 | | | |
| 8085300 | NR_024272 | SEC13 | SEC13 homolog (S. cerevisiae) | -0.127 | 0.916 | 5.83E-01 | 5.83E+01 | | | |
| 8121209 | NM_005604 | POU3F2 | POU class 3 homeobox 2 | -0.127 | 0.916 | 3.79E-02 | 3.79E+00 | | | |
| 8024687 | NM_014428 | TJP3 | tight junction protein 3 (zona occlud | -0.127 | 0.916 | 5.81E-02 | 5.81E+00 | | | |
| 8027258 | AK298173 | ZNF90 | zinc finger protein 90 | -0.127 | 0.916 | 5.63E-01 | 5.63E+01 | | | |
| 8158930 | NM_018956 | C9orf9 | chromosome 9 open reading frame 9 | -0.127 | 0.916 | 1.01E-01 | 1.01E+01 | | | |
| 8180332 | --- | --- | --- | -0.127 | 0.916 | 4.74E-01 | 4.74E+01 | | | |
| 7900001 | NM_178548 | TFAP2E | transcription factor AP-2 epsilon (act | -0.127 | 0.916 | 2.89E-02 | 2.89E+00 | | | |
| 7994345 | NM_00102440 | SBK1 | SH3-binding domain kinase 1 | -0.127 | 0.916 | 3.14E-03 | 3.14E-01 | | | |
| 7898537 | NM_002584 | PAX7 | paired box 7 | -0.127 | 0.916 | 3.61E-02 | 3.61E+00 | | | |
| 7986092 | NM_002569 | FURIN | furin (paired basic amino acid cleavi | -0.127 | 0.916 | 1.52E-02 | 1.52E+00 | | | |
| 8098244 | --- | --- | --- | -0.127 | 0.916 | 1.02E-01 | 1.02E+01 | | | |
| 8153835 | NM_032902 | PPP1R16A | protein phosphatase 1, regulatory (i | -0.127 | 0.916 | 6.07E-02 | 6.07E+00 | | | |
| 8180281 | --- | --- | --- | -0.127 | 0.916 | 2.51E-02 | 2.51E+00 | | | |
| 8064766 | NM_00113433 | RNF24 | ring finger protein 24 | -0.127 | 0.916 | 5.64E-02 | 5.64E+00 | | | |
| 8024829 | NM_032868 | MPND | MPN domain containing | -0.127 | 0.915 | 5.31E-02 | 5.31E+00 | | | |
| 8159008 | NM_017503 | SURF2 | surfeit 2 | -0.127 | 0.915 | 3.01E-01 | 3.01E+01 | | | |
| 8064100 | NM_024299 | PPDPF | pancreatic progenitor cell differentia | -0.127 | 0.915 | 1.49E-01 | 1.49E+01 | | | |
| 8114287 | NM_004598 | SPOCK1 | sparc/osteonectin, cwcv and kazal-li | -0.127 | 0.915 | 1.66E-02 | 1.66E+00 | | | |
| 8081779 | NM_173570 | ZDHHC23 | zinc finger, DHHC-type containing 23 | -0.127 | 0.915 | 1.14E-02 | 1.14E+00 | | | |
| 8000616 | NM_024816 | RABEP2 | rabaptin, RAB GTPase binding effect | -0.127 | 0.915 | 2.19E-02 | 2.19E+00 | | | |
| 8013515 | AK096256 | C17orf51 | chromosome 17 open reading frame | -0.127 | 0.915 | 6.50E-02 | 6.50E+00 | | | |
| 8153269 | NM_005293 | GPR20 | G protein-coupled receptor 20 | -0.127 | 0.915 | 3.68E-02 | 3.68E+00 | | | |
| 8009639 | NM_022036 | GPRC5C | G protein-coupled receptor, family C | -0.127 | 0.915 | 3.05E-02 | 3.05E+00 | | | |
| 7950885 | NM_012193 | FZD4 | frizzled homolog 4 (Drosophila) | -0.127 | 0.915 | 1.53E-02 | 1.53E+00 | | | |
| 7912283 | NM_020248 | CTNNBIP1 | catenin, beta interacting protein 1 | -0.127 | 0.915 | 2.96E-02 | 2.96E+00 | | | |
| 8016646 | NM_000088 | COL1A1 | collagen, type I, alpha 1 | -0.127 | 0.915 | 1.12E-02 | 1.12E+00 | | | |
| 7928789 | NM_002921 | RGR | retinal G protein coupled receptor | -0.127 | 0.915 | 3.29E-02 | 3.29E+00 | | | |
| 8039805 | NM_003969 | UBE2M | ubiquitin-conjugating enzyme E2M ( | -0.128 | 0.915 | 5.76E-02 | 5.76E+00 | | | |
| 8149612 | NM_021020 | LZTS1 | leucine zipper, putative tumor suppr | -0.128 | 0.915 | 1.44E-02 | 1.44E+00 | | | |
| 7941694 | NM_006328 | RBM14 | RNA binding motif protein 14 | -0.128 | 0.915 | 2.39E-01 | 2.39E+01 | | | |
| 7998886 | NM_182687 | PKMYT1 | protein kinase, membrane associate | -0.128 | 0.915 | 2.92E-02 | 2.92E+00 | | | |

| | | | | | | | | | | |
|---|---|---|---|---|---|---|---|---|---|---|
| 8009096 | NM_000789 | ACE | angiotensin I converting enzyme (pe | -0.128 | 0.915 | 9.02E-03 | 9.02E-01 | | | |
| 7928189 | NM_170744 | UNC5B | unc-5 homolog B (C. elegans) | -0.128 | 0.915 | 2.19E-02 | 2.19E+00 | | | |
| 8155083 | NM_001216 | CA9 | carbonic anhydrase IX | -0.128 | 0.915 | 6.56E-02 | 6.56E+00 | | | |
| 8102984 | --- | --- | --- | -0.128 | 0.915 | 9.37E-02 | 9.37E+00 | | | |
| 7893533 | --- | --- | --- | -0.128 | 0.915 | 4.17E-01 | 4.17E+01 | | | |
| 8164293 | NM_000476 | AK1 | adenylate kinase 1 | -0.128 | 0.915 | 5.89E-01 | 5.89E+01 | | | |
| 8180347 | --- | --- | --- | -0.128 | 0.915 | 1.28E-02 | 1.28E+00 | | | |
| 8110382 | NM_030567 | PRR7 | proline rich 7 (synaptic) | -0.128 | 0.915 | 8.20E-03 | 8.20E-01 | | | |
| 7950501 | NM_030792 | GDPD5 | glycerophosphodiester phosphodies | -0.128 | 0.915 | 1.95E-02 | 1.95E+00 | | | |
| 8051949 | NM_016932 | SIX2 | SIX homeobox 2 | -0.128 | 0.915 | 4.15E-02 | 4.15E+00 | | | |
| 7912155 | --- | --- | --- | -0.128 | 0.915 | 2.56E-01 | 2.56E+01 | | | |
| 8145603 | NM_006228 | PNOC | prepronociceptin | -0.128 | 0.915 | 9.00E-02 | 9.00E+00 | | | |
| 7952290 | NM_012101 | TRIM29 | tripartite motif-containing 29 | -0.128 | 0.915 | 1.52E-02 | 1.52E+00 | | | |
| 8140220 | NM_030798 | WBSCR16 | Williams-Beuren syndrome chromos | -0.128 | 0.915 | 1.62E-02 | 1.62E+00 | | | |
| 7893929 | --- | --- | --- | -0.128 | 0.915 | 3.23E-01 | 3.23E+01 | | | |
| 7892563 | --- | --- | --- | -0.128 | 0.915 | 2.52E-01 | 2.52E+01 | | | |
| 7934690 | NM_153367 | ZCCHC24 | zinc finger, CCHC domain containing | -0.128 | 0.915 | 3.40E-02 | 3.40E+00 | | | |
| 8178330 | NM_007028 | TRIM31 | tripartite motif-containing 31 | -0.128 | 0.915 | 3.95E-02 | 3.95E+00 | | | |
| 8175808 | NM_080701 | TREX2 | three prime repair exonuclease 2 | -0.128 | 0.915 | 6.14E-02 | 6.14E+00 | | | |
| 8069348 | NM_006031 | PCNT | pericentrin | -0.128 | 0.915 | 4.35E-02 | 4.35E+00 | | | |
| 7893530 | --- | --- | --- | -0.128 | 0.915 | 6.69E-01 | 6.69E+01 | | | |
| 8015214 | NM_033184 | KRTAP2-4 | keratin associated protein 2-4 | -0.128 | 0.915 | 1.24E-02 | 1.24E+00 | | | |
| 8019574 | NM_033184 | KRTAP2-4 | keratin associated protein 2-4 | -0.128 | 0.915 | 1.24E-02 | 1.24E+00 | | | |
| 8175256 | NR_024607 | MGC16121 | hypothetical protein MGC16121 | -0.128 | 0.915 | 1.22E-02 | 1.22E+00 | | | |
| 8163015 | --- | --- | --- | -0.128 | 0.915 | 1.36E-01 | 1.36E+01 | | | |
| 8153380 | NM_00113565 | LY6H | lymphocyte antigen 6 complex, locus | -0.128 | 0.915 | 3.47E-02 | 3.47E+00 | | | |
| 7899265 | NM_006142 | SFN | stratifin | -0.128 | 0.915 | 1.74E-02 | 1.74E+00 | | | |
| 7893336 | --- | --- | --- | -0.128 | 0.915 | 1.59E-01 | 1.59E+01 | | | |
| 8044295 | NM_00109928 | SH3RF3 | SH3 domain containing ring finger 3 | -0.128 | 0.915 | 6.10E-02 | 6.10E+00 | | | |
| 7995033 | NM_014699 | ZNF646 | zinc finger protein 646 | -0.128 | 0.915 | 5.26E-02 | 5.26E+00 | | | |
| 8015681 | NM_016602 | CCR10 | chemokine (C-C motif) receptor 10 | -0.128 | 0.915 | 1.02E-01 | 1.02E+01 | | | |
| 8031550 | NM_00113007 | EPN1 | epsin 1 | -0.128 | 0.915 | 2.00E-02 | 2.00E+00 | | | |
| 7963826 | NM_006741 | PPP1R1A | protein phosphatase 1, regulatory (i | -0.128 | 0.915 | 1.18E-02 | 1.18E+00 | | | |
| 7994961 | NM_001330 | CTF1 | cardiotrophin 1 | -0.128 | 0.915 | 3.24E-02 | 3.24E+00 | | | |
| 7990902 | NR_026811 | LOC80154 | hypothetical LOC80154 | -0.128 | 0.915 | 4.43E-03 | 4.43E-01 | | | |
| 8015737 | --- | --- | --- | -0.128 | 0.915 | 3.36E-01 | 3.36E+01 | | | |
| 8137208 | NM_145230 | ATP6V0E2 | ATPase, H+ transporting V0 subunit | -0.128 | 0.915 | 2.01E-02 | 2.01E+00 | | | |
| 8012841 | --- | --- | --- | -0.128 | 0.915 | 6.65E-01 | 6.65E+01 | | | |
| 7981051 | NM_016186 | SERPINA10 | serpin peptidase inhibitor, clade A (a | -0.128 | 0.915 | 1.12E-02 | 1.12E+00 | | | |
| 8107594 | NM_005460 | SNCAIP | synuclein, alpha interacting protein | -0.128 | 0.915 | 3.21E-02 | 3.21E+00 | | | |
| 7893703 | --- | --- | --- | -0.128 | 0.915 | 8.04E-01 | 8.04E+01 | | | |
| 7975412 | NM_015351 | TTC9 | tetratricopeptide repeat domain 9 | -0.128 | 0.915 | 2.57E-02 | 2.57E+00 | | | |
| 8016382 | NM_199262 | SP6 | Sp6 transcription factor | -0.128 | 0.915 | 4.84E-02 | 4.84E+00 | | | |
| 8043552 | NM_013434 | KCNIP3 | Kv channel interacting protein 3, cals | -0.128 | 0.915 | 3.20E-02 | 3.20E+00 | | | |
| 8130394 | NM_012419 | RGS17 | regulator of G-protein signaling 17 | -0.128 | 0.915 | 8.75E-02 | 8.75E+00 | | | |
| 8166266 | NM_198270 | NHS | Nance-Horan syndrome (congenital | -0.128 | 0.915 | 1.75E-02 | 1.75E+00 | | | |
| 7925705 | NM_032752 | ZNF496 | zinc finger protein 496 | -0.128 | 0.915 | 1.84E-02 | 1.84E+00 | | | |
| 8141241 | NM_020429 | SMURF1 | SMAD specific E3 ubiquitin protein li | -0.128 | 0.915 | 1.48E-01 | 1.48E+01 | | | |
| 8024682 | NM_021231 | C19orf29 | chromosome 19 open reading frame | -0.128 | 0.915 | 4.57E-02 | 4.57E+00 | | | |
| 8046542 | NM_014213 | HOXD9 | homeobox D9 | -0.128 | 0.915 | 3.26E-02 | 3.26E+00 | | | |
| 8029507 | NM_002856 | PVRL2 | poliovirus receptor-related 2 (herpes | -0.128 | 0.915 | 1.59E-02 | 1.59E+00 | | | |
| 8006681 | NM_005568 | LHX1 | LIM homeobox 1 | -0.128 | 0.915 | 3.56E-02 | 3.56E+00 | | | |
| 7904000 | NM_007204 | DDX20 | DEAD (Asp-Glu-Ala-Asp) box polypep | -0.128 | 0.915 | 4.30E-01 | 4.30E+01 | | | |
| 7955797 | NM_020547 | AMHR2 | anti-Mullerian hormone receptor, ty | -0.128 | 0.915 | 2.33E-02 | 2.33E+00 | | | |
| 8168115 | --- | --- | --- | -0.128 | 0.915 | 4.43E-01 | 4.43E+01 | | | |
| 8034907 | NM_005071 | SLC1A6 | solute carrier family 1 (high affinity a | -0.128 | 0.915 | 2.29E-02 | 2.29E+00 | | | |
| 8026877 | NM_000453 | SLC5A5 | solute carrier family 5 (sodium iodide | -0.128 | 0.915 | 1.80E-02 | 1.80E+00 | | | |
| 8054831 | NM_001426 | EN1 | engrailed homeobox 1 | -0.128 | 0.915 | 1.48E-02 | 1.48E+00 | | | |
| 7994804 | NM_013292 | MYLPF | myosin light chain, phosphorylatable | -0.128 | 0.915 | 3.40E-02 | 3.40E+00 | | | |
| 8140129 | NR_026912 | ABHD11 | abhydrolase domain containing 11 | -0.128 | 0.915 | 1.88E-02 | 1.88E+00 | | | |
| 8133459 | NM_003388 | CLIP2 | CAP-GLY domain containing linker pr | -0.128 | 0.915 | 1.94E-02 | 1.94E+00 | | | |
| 8105063 | --- | --- | --- | -0.128 | 0.915 | 6.42E-02 | 6.42E+00 | | | |
| 7901714 | --- | --- | --- | -0.128 | 0.915 | 5.71E-02 | 5.71E+00 | | | |
| 7896168 | --- | --- | --- | -0.128 | 0.915 | 3.81E-01 | 3.81E+01 | | | |
| 8135341 | NM_152750 | FLJ23834 | hypothetical protein FLJ23834 | -0.128 | 0.915 | 7.97E-02 | 7.97E+00 | | | |
| 8032009 | NM_017550 | MIER2 | mesoderm induction early response | -0.128 | 0.915 | 5.42E-02 | 5.42E+00 | | | |
| 8081053 | --- | --- | --- | -0.128 | 0.915 | 6.86E-02 | 6.86E+00 | | | |
| 8023984 | NM_001194 | HCN2 | hyperpolarization activated cyclic nu | -0.128 | 0.915 | 8.08E-02 | 8.08E+00 | | | |
| 7991772 | NM_005331 | HBQ1 | hemoglobin, theta 1 | -0.128 | 0.915 | 4.70E-02 | 4.70E+00 | | | |
| 8079107 | NM_152393 | KBTBD5 | kelch repeat and BTB (POZ) domain | -0.128 | 0.915 | 1.82E-02 | 1.82E+00 | | | |
| 8109315 | NM_00112285 | MYOZ3 | myozenin 3 | -0.128 | 0.915 | 5.58E-02 | 5.58E+00 | | | |
| 8059672 | --- | --- | --- | -0.128 | 0.915 | 5.84E-01 | 5.84E+01 | | | |
| 8023218 | --- | --- | --- | -0.128 | 0.915 | 1.17E-01 | 1.17E+01 | | | |
| 8116313 | NM_00114230 | LOC285679 | hypothetical protein LOC285679 | -0.128 | 0.915 | 8.96E-02 | 8.96E+00 | | | |
| 8076739 | NM_018280 | C22orf26 | chromosome 22 open reading frame | -0.128 | 0.915 | 3.30E-02 | 3.30E+00 | | | |
| 8003926 | NM_00113604 | ZMYND15 | zinc finger, MYND-type containing 1 | -0.129 | 0.915 | 1.89E-02 | 1.89E+00 | | | |
| 8005726 | NM_021012 | KCNJ12 | potassium inwardly-rectifying chann | -0.129 | 0.915 | 2.77E-02 | 2.77E+00 | | | |

| | | | | | | | | | | |
|---|---|---|---|---|---|---|---|---|---|---|
| 8001531 | NM_005950 | MT1G | metallothionein 1G | -0.129 | 0.915 | 7.54E-02 | 7.54E+00 | | | |
| 7958466 | NM_001093 | ACACB | acetyl-Coenzyme A carboxylase beta | -0.129 | 0.915 | 4.81E-02 | 4.81E+00 | | | |
| 8013112 | NM_016084 | RASD1 | RAS, dexamethasone-induced 1 | -0.129 | 0.915 | 4.66E-02 | 4.66E+00 | | | |
| 8132858 | --- | --- | --- | -0.129 | 0.915 | 1.42E-01 | 1.42E+01 | | | |
| 8139883 | --- | --- | --- | -0.129 | 0.915 | 1.53E-01 | 1.53E+01 | | | |
| 8098769 | NM_032219 | MFSD7 | major facilitator superfamily domain | -0.129 | 0.915 | 2.13E-02 | 2.13E+00 | | | |
| 8129089 | NM_021648 | TSPYL4 | TSPY-like 4 | -0.129 | 0.915 | 7.04E-02 | 7.04E+00 | | | |
| 7941039 | NM_003942 | RPS6KA4 | ribosomal protein S6 kinase, 90kDa, | -0.129 | 0.915 | 1.48E-02 | 1.48E+00 | | | |
| 8138147 | NM_030930 | UNC93B1 | unc-93 homolog B1 (C. elegans) | -0.129 | 0.915 | 4.03E-02 | 4.03E+00 | | | |
| 8066498 | NM_003833 | MATN4 | matrilin 4 | -0.129 | 0.915 | 2.10E-02 | 2.10E+00 | | | |
| 7959827 | NM_00113610 | TMEM132C | transmembrane protein 132C | -0.129 | 0.915 | 1.59E-02 | 1.59E+00 | | | |
| 8076586 | NM_173050 | SCUBE1 | signal peptide, CUB domain, EGF-like | -0.129 | 0.915 | 2.90E-02 | 2.90E+00 | | | |
| 8105132 | AK123995 | LOC153684 | hypothetical LOC153684 | -0.129 | 0.915 | 4.41E-02 | 4.41E+00 | | | |
| 7905198 | NM_025150 | TARS2 | threonyl-tRNA synthetase 2, mitocho | -0.129 | 0.915 | 6.06E-02 | 6.06E+00 | | | |
| 7894672 | --- | --- | --- | -0.129 | 0.915 | 4.89E-01 | 4.89E+01 | | | |
| 7979743 | NM_016026 | RDH11 | retinol dehydrogenase 11 (all-trans/9 | -0.129 | 0.915 | 6.06E-01 | 6.06E+01 | | | |
| 8076744 | BC009388 | LOC554174 | hypothetical LOC554174 | -0.129 | 0.915 | 7.49E-02 | 7.49E+00 | | | |
| 7924144 | NR_026911 | RPL21P28 | ribosomal protein L21 pseudogene 2 | -0.129 | 0.915 | 3.57E-01 | 3.57E+01 | | | |
| 8079532 | NM_015466 | PTPN23 | protein tyrosine phosphatase, non-re | -0.129 | 0.915 | 1.39E-02 | 1.39E+00 | | | |
| 7906264 | NM_00108047 | PEAR1 | platelet endothelial aggregation rece | -0.129 | 0.915 | 2.57E-02 | 2.57E+00 | | | |
| 8089970 | NM_175924 | ILDR1 | immunoglobulin-like domain contain | -0.129 | 0.915 | 2.86E-02 | 2.86E+00 | | | |
| 7896261 | --- | --- | --- | -0.129 | 0.915 | 3.12E-01 | 3.12E+01 | | | |
| 8133874 | --- | --- | --- | -0.129 | 0.915 | 1.83E-01 | 1.83E+01 | | | |
| 7894692 | --- | --- | --- | -0.129 | 0.915 | 4.37E-01 | 4.37E+01 | | | |
| 7924166 | NM_018664 | BATF3 | basic leucine zipper transcription fac | -0.129 | 0.915 | 9.26E-02 | 9.26E+00 | | | |
| 7923386 | NM_012134 | LMOD1 | leiomodin 1 (smooth muscle) | -0.129 | 0.915 | 5.31E-02 | 5.31E+00 | | | |
| 8074773 | NM_00101796 | YDJC | YdjC homolog (bacterial) | -0.129 | 0.915 | 1.94E-02 | 1.94E+00 | | | |
| 7898616 | NM_022819 | PLA2G2F | phospholipase A2, group IIF | -0.129 | 0.915 | 2.12E-02 | 2.12E+00 | | | |
| 7944537 | NM_014352 | POU2F3 | POU class 2 homeobox 3 | -0.129 | 0.915 | 5.80E-03 | 5.80E-01 | | | |
| 8148655 | NM_032862 | TIGD5 | tigger transposable element derived | -0.129 | 0.915 | 4.04E-02 | 4.04E+00 | | | |
| 7953418 | NM_002286 | LAG3 | lymphocyte-activation gene 3 | -0.129 | 0.915 | 3.38E-02 | 3.38E+00 | | | |
| 7984799 | --- | --- | --- | -0.129 | 0.915 | 4.46E-02 | 4.46E+00 | | | |
| 8016155 | NM_006688 | C1QL1 | complement component 1, q subcom | -0.129 | 0.915 | 3.92E-02 | 3.92E+00 | | | |
| 7912239 | NM_024980 | GPR157 | G protein-coupled receptor 157 | -0.129 | 0.914 | 3.99E-02 | 3.99E+00 | | | |
| 8012140 | NM_015982 | YBX2 | Y box binding protein 2 | -0.129 | 0.914 | 1.44E-02 | 1.44E+00 | | | |
| 8167287 | NM_022825 | PORCN | porcupine homolog (Drosophila) | -0.129 | 0.914 | 5.83E-02 | 5.83E+00 | | | |
| 8050924 | NM_00114516 | GPR113 | G protein-coupled receptor 113 | -0.129 | 0.914 | 6.45E-02 | 6.45E+00 | | | |
| 8180401 | --- | --- | --- | -0.129 | 0.914 | 6.34E-02 | 6.34E+00 | | | |
| 7922684 | --- | --- | --- | -0.129 | 0.914 | 1.09E-01 | 1.09E+01 | | | |
| 8027760 | NM_005031 | FXYD1 | FXYD domain containing ion transpo | -0.129 | 0.914 | 4.13E-02 | 4.13E+00 | | | |
| 8023061 | --- | --- | --- | -0.129 | 0.914 | 1.11E-01 | 1.11E+01 | | | |
| 8105146 | NM_153361 | MGC42105 | serine/threonine-protein kinase NIM | -0.129 | 0.914 | 4.08E-02 | 4.08E+00 | | | |
| 8044154 | NM_172099 | CD8B | CD8b molecule | -0.129 | 0.914 | 3.81E-01 | 3.81E+01 | | | |
| 7895269 | --- | --- | --- | -0.129 | 0.914 | 1.48E-01 | 1.48E+01 | | | |
| 8073129 | NM_002409 | MGAT3 | mannosyl (beta-1,4-)-glycoprotein be | -0.129 | 0.914 | 6.03E-02 | 6.03E+00 | | | |
| 8036936 | AK096566 | OC10013071 | hypothetical LOC100130713 | -0.129 | 0.914 | 1.64E-01 | 1.64E+01 | | | |
| 8174644 | --- | --- | --- | -0.129 | 0.914 | 1.65E-02 | 1.65E+00 | | | |
| 8018258 | NM_173477 | USH1G | Usher syndrome 1G (autosomal rece | -0.129 | 0.914 | 3.51E-02 | 3.51E+00 | | | |
| 8070233 | NM_144492 | CLDN14 | claudin 14 | -0.129 | 0.914 | 5.67E-02 | 5.67E+00 | | | |
| 8005751 | --- | --- | --- | -0.129 | 0.914 | 6.55E-02 | 6.55E+00 | | | |
| 8150032 | --- | --- | --- | -0.129 | 0.914 | 3.80E-02 | 3.80E+00 | | | |
| 8021914 | NM_014913 | ADNP2 | ADNP homeobox 2 | -0.129 | 0.914 | 4.24E-01 | 4.24E+01 | | | |
| 7962083 | --- | --- | --- | -0.129 | 0.914 | 3.96E-01 | 3.96E+01 | | | |
| 8180412 | --- | --- | --- | -0.129 | 0.914 | 5.52E-02 | 5.52E+00 | | | |
| 7982333 | AK127052 | LOC283711 | hypothetical protein LOC283711 | -0.129 | 0.914 | 4.39E-02 | 4.39E+00 | | | |
| 8067208 | NM_080618 | CTCFL | CCCTC-binding factor (zinc finger pro | -0.129 | 0.914 | 8.66E-02 | 8.66E+00 | | | |
| 8090366 | NM_144639 | UROC1 | urocanase domain containing 1 | -0.129 | 0.914 | 2.34E-02 | 2.34E+00 | | | |
| 7905018 | AK095105 | FLJ37786 | hypothetical LOC642691 | -0.129 | 0.914 | 3.95E-02 | 3.95E+00 | | | |
| 8073824 | --- | --- | --- | -0.129 | 0.914 | 9.54E-02 | 9.54E+00 | | | |
| 7915500 | NM_182517 | C1orf210 | chromosome 1 open reading frame 2 | -0.129 | 0.914 | 3.74E-02 | 3.74E+00 | | | |
| 8110114 | NM_006650 | CPLX2 | complexin 2 | -0.129 | 0.914 | 1.81E-02 | 1.81E+00 | | | |
| 8106923 | NM_005654 | NR2F1 | nuclear receptor subfamily 2, group | -0.129 | 0.914 | 4.72E-02 | 4.72E+00 | | | |
| 8053728 | NM_000798 | DRD5 | dopamine receptor D5 | -0.129 | 0.914 | 1.27E-02 | 1.27E+00 | | | |
| 8024660 | NM_006339 | HMG20B | high-mobility group 20B | -0.129 | 0.914 | 1.82E-02 | 1.82E+00 | | | |
| 8082133 | NM_006810 | PDIA5 | protein disulfide isomerase family A, | -0.129 | 0.914 | 2.91E-01 | 2.91E+01 | | | |
| 8070933 | NM_006657 | FTCD | formiminotransferase cyclodeaminas | -0.129 | 0.914 | 1.27E-02 | 1.27E+00 | | | |
| 7988380 | NM_144565 | DUOXA1 | dual oxidase maturation factor 1 | -0.130 | 0.914 | 1.72E-02 | 1.72E+00 | | | |
| 8126482 | --- | --- | --- | -0.130 | 0.914 | 1.60E-01 | 1.60E+01 | | | |
| 8153320 | --- | --- | --- | -0.130 | 0.914 | 4.45E-02 | 4.45E+00 | | | |
| 7900183 | NM_00103174 | MANEAL | mannosidase, endo-alpha-like | -0.130 | 0.914 | 1.00E-01 | 1.00E+01 | | | |
| 8038643 | NM_017509 | KLK15 | kallikrein-related peptidase 15 | -0.130 | 0.914 | 1.41E-02 | 1.41E+00 | | | |
| 8114443 | NM_004730 | ETF1 | eukaryotic translation termination fa | -0.130 | 0.914 | 6.32E-01 | 6.32E+01 | | | |
| 7892931 | --- | --- | --- | -0.130 | 0.914 | 7.48E-01 | 7.48E+01 | | | |
| 8022608 | --- | --- | --- | -0.130 | 0.914 | 7.04E-02 | 7.04E+00 | | | |
| 8136535 | NM_197964 | C7orf55 | chromosome 7 open reading frame 5 | -0.130 | 0.914 | 3.46E-01 | 3.46E+01 | | | |
| 8123334 | NR_027284 | LOC441177 | hypothetical LOC441177 | -0.130 | 0.914 | 2.15E-02 | 2.15E+00 | | | |
| 8043157 | NM_198482 | SH2D6 | SH2 domain containing 6 | -0.130 | 0.914 | 3.35E-02 | 3.35E+00 | | | |

| | | | | | | | | | | |
|---|---|---|---|---|---|---|---|---|---|---|
| 8138592 | AB052759 | TRA2A | transformer 2 alpha homolog (Droso | -0.130 | 0.914 | 6.76E-01 | 6.76E+01 | | | |
| 7935251 | NM_015631 | TCTN3 | tectonic family member 3 | -0.130 | 0.914 | 4.07E-02 | 4.07E+00 | | | |
| 8115594 | NM_031908 | C1QTNF2 | C1q and tumor necrosis factor relate | -0.130 | 0.914 | 1.38E-02 | 1.38E+00 | | | |
| 8149629 | NM_001495 | GFRA2 | GDNF family receptor alpha 2 | -0.130 | 0.914 | 5.28E-02 | 5.28E+00 | | | |
| 8075009 | NM_031444 | C22orf13 | chromosome 22 open reading frame | -0.130 | 0.914 | 1.63E-01 | 1.63E+01 | | | |
| 8003467 | NM_00108331 | CHMP1A | chromatin modifying protein 1A | -0.130 | 0.914 | 4.15E-02 | 4.15E+00 | | | |
| 8006298 | NM_032932 | RAB11FIP4 | RAB11 family interacting protein 4 (c | -0.130 | 0.914 | 5.84E-02 | 5.84E+00 | | | |
| 8116162 | NM_006261 | PROP1 | PROP paired-like homeobox 1 | -0.130 | 0.914 | 3.29E-02 | 3.29E+00 | | | |
| 8138799 | NM_014817 | TRIL | TLR4 interactor with leucine rich rep | -0.130 | 0.914 | 1.72E-02 | 1.72E+00 | | | |
| 8024605 | NM_021938 | BRUNOL5 | bruno-like 5, RNA binding protein (D | -0.130 | 0.914 | 1.47E-02 | 1.47E+00 | | | |
| 8007188 | NM_033133 | CNP | 2',3'-cyclic nucleotide 3' phosphodie | -0.130 | 0.914 | 2.21E-02 | 2.21E+00 | | | |
| 7906496 | NM_004983 | KCNJ9 | potassium inwardly-rectifying chann | -0.130 | 0.914 | 4.78E-02 | 4.78E+00 | | | |
| 7936904 | NM_022802 | CTBP2 | C-terminal binding protein 2 | -0.130 | 0.914 | 6.17E-02 | 6.17E+00 | | | |
| 8130142 | NM_032832 | LRP11 | low density lipoprotein receptor-rela | -0.130 | 0.914 | 1.79E-02 | 1.79E+00 | | | |
| 8064418 | NM_080489 | SDCBP2 | syndecan binding protein (syntenin) | -0.130 | 0.914 | 4.66E-02 | 4.66E+00 | | | |
| 7981242 | NM_138576 | BCL11B | B-cell CLL/lymphoma 11B (zinc finge | -0.130 | 0.914 | 8.42E-02 | 8.42E+00 | | | |
| 8093413 | NM_032326 | TMEM175 | transmembrane protein 175 | -0.130 | 0.914 | 3.45E-02 | 3.45E+00 | | | |
| 8078435 | NM_00103911 | TRIM71 | tripartite motif-containing 71 | -0.130 | 0.914 | 1.94E-02 | 1.94E+00 | | | |
| 7897753 | NM_005957 | MTHFR | 5,10-methylenetetrahydrofolate red | -0.130 | 0.914 | 1.70E-02 | 1.70E+00 | | | |
| 8013486 | NM_015276 | USP22 | ubiquitin specific peptidase 22 | -0.130 | 0.914 | 4.61E-01 | 4.61E+01 | | | |
| 8160849 | NM_00101736 | ARID3C | AT rich interactive domain 3C (BRIGH | -0.130 | 0.914 | 3.21E-02 | 3.21E+00 | | | |
| 7895837 | --- | --- | --- | -0.130 | 0.914 | 2.71E-01 | 2.71E+01 | | | |
| 7925687 | NM_207401 | C1orf229 | chromosome 1 open reading frame 2 | -0.130 | 0.914 | 8.93E-02 | 8.93E+00 | | | |
| 7915846 | NM_003684 | MKNK1 | MAP kinase interacting serine/threo | -0.130 | 0.914 | 1.13E-01 | 1.13E+01 | | | |
| 8064384 | NM_033129 | SCRT2 | scratch homolog 2, zinc finger protei | -0.130 | 0.914 | 3.78E-02 | 3.78E+00 | | | |
| 7899615 | NM_178865 | SERINC2 | serine incorporator 2 | -0.130 | 0.914 | 2.00E-02 | 2.00E+00 | | | |
| 8032751 | --- | --- | --- | -0.130 | 0.914 | 4.34E-02 | 4.34E+00 | | | |
| 8131296 | NR_015343 | LOC389458 | hypothetical LOC389458 | -0.130 | 0.914 | 1.52E-02 | 1.52E+00 | | | |
| 7981157 | NR_026779 | C14orf139 | chromosome 14 open reading frame | -0.130 | 0.914 | 7.43E-02 | 7.43E+00 | | | |
| 8012054 | NM_001365 | DLG4 | discs, large homolog 4 (Drosophila) | -0.130 | 0.914 | 4.58E-02 | 4.58E+00 | | | |
| 7901577 | NM_052940 | LRRC42 | leucine rich repeat containing 42 | -0.130 | 0.914 | 2.40E-01 | 2.40E+01 | | | |
| 8133027 | AK128836 | OC10013221 | similar to hCG1793472 | -0.130 | 0.914 | 1.20E-02 | 1.20E+00 | | | |
| 8063011 | NM_080753 | WFDC10A | WAP four-disulfide core domain 10A | -0.130 | 0.914 | 2.29E-01 | 2.29E+01 | | | |
| 8060850 | NM_001200 | BMP2 | bone morphogenetic protein 2 | -0.130 | 0.914 | 3.16E-02 | 3.16E+00 | | | |
| 8026063 | NM_014975 | MAST1 | microtubule associated serine/threo | -0.130 | 0.914 | 3.59E-02 | 3.59E+00 | | | |
| 8010778 | NM_001893 | CSNK1D | casein kinase 1, delta | -0.130 | 0.914 | 2.94E-01 | 2.94E+01 | | | |
| 8062852 | NM_181805 | PKIG | protein kinase (cAMP-dependent, ca | -0.130 | 0.914 | 3.25E-01 | 3.25E+01 | | | |
| 7975562 | NM_173462 | PAPLN | papilin, proteoglycan-like sulfated gl | -0.130 | 0.914 | 3.94E-02 | 3.94E+00 | | | |
| 8005695 | NR_002211 | MEIS3P1 | Meis homeobox 3 pseudogene 1 | -0.130 | 0.914 | 9.13E-03 | 9.13E-01 | | | |
| 8151906 | NM_00100155 | GDF6 | growth differentiation factor 6 | -0.130 | 0.914 | 2.22E-02 | 2.22E+00 | | | |
| 8026915 | NM_015683 | ARRDC2 | arrestin domain containing 2 | -0.130 | 0.914 | 2.69E-02 | 2.69E+00 | | | |
| 7937073 | ENST0000034 | FLJ46300 | FLJ46300 protein | -0.130 | 0.914 | 2.26E-02 | 2.26E+00 | | | |
| 8152946 | NM_004519 | KCNQ3 | potassium voltage-gated channel, KC | -0.130 | 0.914 | 1.88E-02 | 1.88E+00 | | | |
| 8021528 | NM_003839 | TNFRSF11A | tumor necrosis factor receptor super | -0.130 | 0.914 | 1.06E-02 | 1.06E+00 | | | |
| 8114964 | NM_003122 | SPINK1 | serine peptidase inhibitor, Kazal type | -0.130 | 0.914 | 5.22E-02 | 5.22E+00 | | | |
| 7958989 | NM_173542 | PLBD2 | phospholipase B domain containing | -0.130 | 0.914 | 4.44E-02 | 4.44E+00 | | | |
| 7922108 | NM_153832 | GPR161 | G protein-coupled receptor 161 | -0.130 | 0.914 | 9.56E-03 | 9.56E-01 | | | |
| 8011062 | NM_130766 | INPP5K | inositol polyphosphate-5-phosphata | -0.131 | 0.914 | 2.04E-01 | 2.04E+01 | | | |
| 7896239 | --- | --- | --- | -0.131 | 0.913 | 8.48E-01 | 8.48E+01 | | | |
| 7893114 | --- | --- | --- | -0.131 | 0.913 | 5.15E-01 | 5.15E+01 | | | |
| 7894830 | --- | --- | --- | -0.131 | 0.913 | 2.49E-01 | 2.49E+01 | | | |
| 7998297 | NM_153350 | FBXL16 | F-box and leucine-rich repeat protei | -0.131 | 0.913 | 2.23E-02 | 2.23E+00 | | | |
| 7999936 | NM_003361 | UMOD | uromodulin | -0.131 | 0.913 | 3.98E-02 | 3.98E+00 | | | |
| 7905517 | NM_178354 | LCE1F | late cornified envelope 1F | -0.131 | 0.913 | 1.40E-02 | 1.40E+00 | | | |
| 8164630 | NM_198180 | QRFP | pyroglutamylated RFamide peptide | -0.131 | 0.913 | 1.22E-02 | 1.22E+00 | | | |
| 8039569 | NM_00100283 | ZNF787 | zinc finger protein 787 | -0.131 | 0.913 | 5.11E-03 | 5.11E-01 | | | |
| 7941594 | NM_153266 | TMEM151A | transmembrane protein 151A | -0.131 | 0.913 | 2.15E-02 | 2.15E+00 | | | |
| 8004133 | NR_027680 | RPAIN | RPA interacting protein | -0.131 | 0.913 | 5.88E-01 | 5.88E+01 | | | |
| 8069037 | NM_000383 | AIRE | autoimmune regulator | -0.131 | 0.913 | 2.77E-02 | 2.77E+00 | | | |
| 8103743 | --- | --- | --- | -0.131 | 0.913 | 2.62E-01 | 2.62E+01 | | | |
| 7895735 | --- | --- | --- | -0.131 | 0.913 | 2.32E-01 | 2.32E+01 | | | |
| 8104072 | ENST0000042 | FLJ38576 | hypothetical LOC651430 | -0.131 | 0.913 | 3.80E-02 | 3.80E+00 | | | |
| 7959891 | --- | --- | --- | -0.131 | 0.913 | 5.84E-02 | 5.84E+00 | | | |
| 8109486 | --- | --- | --- | -0.131 | 0.913 | 6.16E-02 | 6.16E+00 | | | |
| 8063620 | NM_004738 | VAPB | VAMP (vesicle-associated membrane | -0.131 | 0.913 | 1.64E-01 | 1.64E+01 | | | |
| 8092468 | NM_033259 | CAMK2N2 | calcium/calmodulin-dependent prot | -0.131 | 0.913 | 2.04E-02 | 2.04E+00 | | | |
| 7969830 | NM_007129 | ZIC2 | Zic family member 2 (odd-paired hor | -0.131 | 0.913 | 1.06E-02 | 1.06E+00 | | | |
| 8147566 | NM_020697 | KCNS2 | potassium voltage-gated channel, de | -0.131 | 0.913 | 1.80E-02 | 1.80E+00 | | | |
| 8021884 | NM_004715 | CTDP1 | CTD (carboxy-terminal domain, RNA | -0.131 | 0.913 | 1.65E-02 | 1.65E+00 | | | |
| 8076569 | NM_015140 | TTLL12 | tubulin tyrosine ligase-like family, m | -0.131 | 0.913 | 1.08E-02 | 1.08E+00 | | | |
| 7997767 | NM_153813 | ZFPM1 | zinc finger protein, multitype 1 | -0.131 | 0.913 | 2.50E-02 | 2.50E+00 | | | |
| 8121418 | NM_003080 | SMPD2 | sphingomyelin phosphodiesterase 2, | -0.131 | 0.913 | 7.53E-02 | 7.53E+00 | | | |
| 8026735 | NM_133644 | GTPBP3 | GTP binding protein 3 (mitochondria | -0.131 | 0.913 | 1.89E-02 | 1.89E+00 | | | |
| 8086391 | NM_000729 | CCK | cholecystokinin | -0.131 | 0.913 | 1.59E-02 | 1.59E+00 | | | |
| 7913271 | NM_020816 | KIF17 | kinesin family member 17 | -0.131 | 0.913 | 7.49E-03 | 7.49E-01 | | | |
| 7945595 | NM_005961 | MUC6 | mucin 6, oligomeric mucus/gel-form | -0.131 | 0.913 | 3.75E-02 | 3.75E+00 | | | |
| 8133902 | --- | --- | --- | -0.131 | 0.913 | 2.15E-01 | 2.15E+01 | | | |

| | | | | | | | | | | |
|---|---|---|---|---|---|---|---|---|---|---|
| 7921155 | NM_014215 | INSRR | insulin receptor-related receptor | -0.131 | 0.913 | 4.32E-02 | 4.32E+00 | | | |
| 8135033 | ENST0000037 | MUC12 | mucin 12, cell surface associated | -0.131 | 0.913 | 5.09E-01 | 5.09E+01 | | | |
| 8151054 | --- | --- | --- | -0.131 | 0.913 | 5.65E-02 | 5.65E+00 | | | |
| 8012403 | NM_004217 | AURKB | aurora kinase B | -0.131 | 0.913 | 2.50E-02 | 2.50E+00 | | | |
| 7981742 | ENST00000390 | LOC90925 | hypothetical protein LOC90925 | -0.131 | 0.913 | 4.09E-02 | 4.09E+00 | | | |
| 7992121 | NM_00101363 | LOC388199 | hypothetical LOC388199 | -0.131 | 0.913 | 4.24E-02 | 4.24E+00 | | | |
| 8026265 | NM_080864 | RLN3 | relaxin 3 | -0.131 | 0.913 | 3.97E-02 | 3.97E+00 | | | |
| 7932548 | --- | --- | --- | -0.131 | 0.913 | 8.27E-01 | 8.27E+01 | | | |
| 8016433 | NM_002144 | HOXB1 | homeobox B1 | -0.131 | 0.913 | 4.38E-02 | 4.38E+00 | | | |
| 7923372 | NM_012396 | PHLDA3 | pleckstrin homology-like domain, far | -0.131 | 0.913 | 5.20E-02 | 5.20E+00 | | | |
| 8024056 | NM_001972 | ELANE | elastase, neutrophil expressed | -0.131 | 0.913 | 4.77E-02 | 4.77E+00 | | | |
| 8170716 | NM_005393 | PLXNB3 | plexin B3 | -0.131 | 0.913 | 1.88E-02 | 1.88E+00 | | | |
| 7895716 | --- | --- | --- | -0.131 | 0.913 | 2.19E-01 | 2.19E+01 | | | |
| 8009693 | NM_017728 | TMEM104 | transmembrane protein 104 | -0.131 | 0.913 | 1.08E-01 | 1.08E+01 | | | |
| 8175763 | NM_00110315 | PNMA5 | paraneoplastic antigen like 5 | -0.131 | 0.913 | 3.64E-02 | 3.64E+00 | | | |
| 8180331 | --- | --- | --- | -0.131 | 0.913 | 4.71E-01 | 4.71E+01 | | | |
| 8026568 | NM_032207 | C19orf44 | chromosome 19 open reading frame | -0.131 | 0.913 | 2.86E-02 | 2.86E+00 | | | |
| 8108558 | NM_080670 | SLC35A4 | solute carrier family 35, member A4 | -0.131 | 0.913 | 1.25E-01 | 1.25E+01 | | | |
| 8071981 | NM_00114520 | KIAA1671 | KIAA1671 | -0.131 | 0.913 | 2.85E-02 | 2.85E+00 | | | |
| 7966600 | NM_024959 | SLC24A6 | solute carrier family 24 (sodium/pota | -0.131 | 0.913 | 2.91E-01 | 2.91E+01 | | | |
| 7940451 | NM_015533 | DAK | dihydroxyacetone kinase 2 homolog | -0.131 | 0.913 | 3.12E-02 | 3.12E+00 | | | |
| 8163678 | NM_198186 | ASTN2 | astrotactin 2 | -0.131 | 0.913 | 1.85E-02 | 1.85E+00 | | | |
| 8141896 | --- | --- | --- | -0.131 | 0.913 | 9.30E-02 | 9.30E+00 | | | |
| 8036069 | NM_152481 | FAM187B | family with sequence similarity 187, | -0.131 | 0.913 | 7.21E-02 | 7.21E+00 | | | |
| 7925743 | NM_00100469 | OR2T12 | olfactory receptor, family 2, subfami | -0.131 | 0.913 | 2.03E-02 | 2.03E+00 | | | |
| 8176018 | NM_001586 | TEX28 | testis expressed 28 | -0.131 | 0.913 | 1.12E-02 | 1.12E+00 | | | |
| 7931469 | NM_138499 | PWWP2B | PWWP domain containing 2B | -0.131 | 0.913 | 2.50E-02 | 2.50E+00 | | | |
| 7940904 | NM_003377 | VEGFB | vascular endothelial growth factor B | -0.131 | 0.913 | 7.33E-02 | 7.33E+00 | | | |
| 8032088 | NM_214710 | PRSSL1 | protease, serine-like 1 | -0.131 | 0.913 | 2.42E-02 | 2.42E+00 | | | |
| 7965423 | NM_001731 | BTG1 | B-cell translocation gene 1, anti-prol | -0.131 | 0.913 | 3.75E-01 | 3.75E+01 | | | |
| 8027139 | NM_003721 | RFXANK | regulatory factor X-associated ankyri | -0.131 | 0.913 | 2.19E-01 | 2.19E+01 | | | |
| 7946749 | NM_001741 | CALCA | calcitonin-related polypeptide alpha | -0.131 | 0.913 | 6.36E-02 | 6.36E+00 | | | |
| 8062395 | NM_005386 | NNAT | neuronatin | -0.131 | 0.913 | 1.78E-02 | 1.78E+00 | | | |
| 8140864 | NM_000786 | CYP51A1 | cytochrome P450, family 51, subfam | -0.131 | 0.913 | 2.38E-01 | 2.38E+01 | | | |
| 8121884 | --- | --- | --- | -0.131 | 0.913 | 6.45E-01 | 6.45E+01 | | | |
| 8151209 | --- | --- | --- | -0.131 | 0.913 | 5.83E-01 | 5.83E+01 | | | |
| 7986789 | NM_024490 | ATP10A | ATPase, class V, type 10A | -0.131 | 0.913 | 4.25E-02 | 4.25E+00 | | | |
| 8024768 | NM_133475 | ANKRD24 | ankyrin repeat domain 24 | -0.131 | 0.913 | 1.12E-02 | 1.12E+00 | | | |
| 8138353 | NM_005924 | MEOX2 | mesenchyme homeobox 2 | -0.131 | 0.913 | 2.31E-02 | 2.31E+00 | | | |
| 7959484 | NM_201435 | CCDC62 | coiled-coil domain containing 62 | -0.131 | 0.913 | 2.25E-02 | 2.25E+00 | | | |
| 8033564 | NM_005001 | NDUFA7 | NADH dehydrogenase (ubiquinone) : | -0.131 | 0.913 | 3.05E-02 | 3.05E+00 | | | |
| 8149699 | NM_00109933 | PHYHIP | phytanoyl-CoA 2-hydroxylase interac | -0.131 | 0.913 | 3.05E-02 | 3.05E+00 | | | |
| 8148799 | NM_016458 | C8orf30A | chromosome 8 open reading frame 3 | -0.131 | 0.913 | 1.16E-02 | 1.16E+00 | | | |
| 7942820 | --- | --- | --- | -0.131 | 0.913 | 1.90E-01 | 1.90E+01 | | | |
| 8133273 | --- | --- | --- | -0.131 | 0.913 | 3.73E-02 | 3.73E+00 | | | |
| 8124394 | NM_021062 | HIST1H2BB | histone cluster 1, H2bb | -0.131 | 0.913 | 9.38E-02 | 9.38E+00 | | | |
| 8030498 | NM_030973 | MED25 | mediator complex subunit 25 | -0.131 | 0.913 | 1.12E-02 | 1.12E+00 | | | |
| 7975645 | NM_021188 | ZNF410 | zinc finger protein 410 | -0.131 | 0.913 | 5.94E-01 | 5.94E+01 | | | |
| 8076221 | NR_000027 | SNORD83A | small nucleolar RNA, C/D box 83A | -0.131 | 0.913 | 5.48E-01 | 5.48E+01 | | | |
| 8006900 | NM_002686 | PNMT | phenylethanolamine N-methyltransf | -0.131 | 0.913 | 2.60E-02 | 2.60E+00 | | | |
| 7973966 | --- | --- | --- | -0.132 | 0.913 | 4.12E-02 | 4.12E+00 | | | |
| 8000028 | NM_173475 | DCUN1D3 | DCN1, defective in cullin neddylation | -0.132 | 0.913 | 8.80E-02 | 8.80E+00 | | | |
| 7970676 | NM_00100753 | SHISA2 | shisa homolog 2 (Xenopus laevis) | -0.132 | 0.913 | 3.73E-02 | 3.73E+00 | | | |
| 8055130 | NM_004807 | HS6ST1 | heparan sulfate 6-O-sulfotransferase | -0.132 | 0.913 | 1.23E-02 | 1.23E+00 | | | |
| 7992049 | NM_207112 | HAGHL | hydroxyacylglutathione hydrolase-lik | -0.132 | 0.913 | 1.16E-02 | 1.16E+00 | | | |
| 8064751 | NM_015417 | SPEF1 | sperm flagellar 1 | -0.132 | 0.913 | 2.57E-02 | 2.57E+00 | | | |
| 7968128 | NM_030979 | PABPC3 | poly(A) binding protein, cytoplasmic | -0.132 | 0.913 | 1.94E-02 | 1.94E+00 | | | |
| 8178988 | NM_005453 | ZBTB22 | zinc finger and BTB domain containir | -0.132 | 0.913 | 5.41E-02 | 5.41E+00 | | | |
| 8120208 | --- | --- | --- | -0.132 | 0.913 | 1.67E-02 | 1.67E+00 | | | |
| 8078999 | NM_175888 | ZNF620 | zinc finger protein 620 | -0.132 | 0.913 | 6.58E-02 | 6.58E+00 | | | |
| 8042874 | NM_022492 | TTC31 | tetratricopeptide repeat domain 31 | -0.132 | 0.913 | 1.45E-01 | 1.45E+01 | | | |
| 8074082 | NM_152299 | NCAPH2 | non-SMC condensin II complex, subu | -0.132 | 0.913 | 6.34E-02 | 6.34E+00 | | | |
| 8071272 | L20860 | GP1BB | glycoprotein Ib (platelet), beta polyp | -0.132 | 0.913 | 4.13E-01 | 4.13E+01 | | | |
| 8122740 | --- | --- | --- | -0.132 | 0.913 | 1.66E-01 | 1.66E+01 | | | |
| 7955963 | NM_058173 | MUCL1 | mucin-like 1 | -0.132 | 0.913 | 3.94E-02 | 3.94E+00 | | | |
| 8028730 | NM_182704 | SELV | selenoprotein V | -0.132 | 0.913 | 2.70E-02 | 2.70E+00 | | | |
| 7968226 | NM_000982 | RPL21 | ribosomal protein L21 | -0.132 | 0.913 | 3.57E-01 | 3.57E+01 | | | |
| 7895306 | --- | --- | --- | -0.132 | 0.913 | 1.87E-01 | 1.87E+01 | | | |
| 8158424 | NM_00112724 | LRRC8A | leucine rich repeat containing 8 fami | -0.132 | 0.913 | 4.93E-02 | 4.93E+00 | | | |
| 8020549 | --- | --- | --- | -0.132 | 0.913 | 1.02E-01 | 1.02E+01 | | | |
| 7949046 | NM_00103367 | TRPT1 | tRNA phosphotransferase 1 | -0.132 | 0.913 | 7.50E-02 | 7.50E+00 | | | |
| 7969861 | NM_004791 | ITGBL1 | integrin, beta-like 1 (with EGF-like re | -0.132 | 0.913 | 1.29E-02 | 1.29E+00 | | | |
| 7947740 | --- | --- | --- | -0.132 | 0.913 | 2.89E-01 | 2.89E+01 | | | |
| 8059989 | NM_018645 | HES6 | hairy and enhancer of split 6 (Drosop | -0.132 | 0.913 | 2.60E-02 | 2.60E+00 | | | |
| 7997569 | NM_139174 | ADAD2 | adenosine deaminase domain contai | -0.132 | 0.913 | 2.15E-02 | 2.15E+00 | | | |
| 8007130 | NM_031963 | KRTAP9-8 | keratin associated protein 9-8 | -0.132 | 0.913 | 1.21E-01 | 1.21E+01 | | | |
| 8070771 | NM_198691 | KRTAP10-1 | keratin associated protein 10-1 | -0.132 | 0.913 | 4.76E-02 | 4.76E+00 | | | |

| | | | | | | | | | | |
|---|---|---|---|---|---|---|---|---|---|---|
| 7894131 | --- | --- | --- | -0.132 | 0.913 | 5.16E-01 | 5.16E+01 | | | |
| 8064866 | NR_015406 | RP5-1022P6.( | hypothetical LOC149837 | -0.132 | 0.913 | 2.31E-01 | 2.31E+01 | | | |
| 7960850 | NM_153449 | SLC2A14 | solute carrier family 2 (facilitated glu | -0.132 | 0.913 | 2.31E-01 | 2.31E+01 | | | |
| 8153486 | NM_00116291 | OC10013027 | coiled-coil domain containing 121-lik | -0.132 | 0.913 | 2.45E-02 | 2.45E+00 | | | |
| 7904812 | NM_006099 | PIAS3 | protein inhibitor of activated STAT, 3 | -0.132 | 0.913 | 6.54E-02 | 6.54E+00 | | | |
| 7959087 | NM_194286 | KIAA1853 | KIAA1853 | -0.132 | 0.913 | 5.37E-02 | 5.37E+00 | | | |
| 8038540 | NM_004977 | KCNC3 | potassium voltage-gated channel, Sh | -0.132 | 0.913 | 1.99E-02 | 1.99E+00 | | | |
| 8159373 | NM_152421 | FAM69B | family with sequence similarity 69, m | -0.132 | 0.913 | 1.68E-02 | 1.68E+00 | | | |
| 7900531 | NM_007102 | GUCA2B | guanylate cyclase activator 2B (urogu | -0.132 | 0.913 | 2.51E-02 | 2.51E+00 | | | |
| 7951703 | NM_000795 | DRD2 | dopamine receptor D2 | -0.132 | 0.913 | 1.28E-02 | 1.28E+00 | | | |
| 7992414 | NM_006453 | TBL3 | transducin (beta)-like 3 | -0.132 | 0.913 | 7.03E-02 | 7.03E+00 | | | |
| 8051814 | NM_006887 | ZFP36L2 | zinc finger protein 36, C3H type-like | -0.132 | 0.913 | 2.75E-02 | 2.75E+00 | | | |
| 8005444 | ENST00000407 | FLJ35934 | FLJ35934 protein | -0.132 | 0.913 | 7.39E-02 | 7.39E+00 | | | |
| 7901140 | NM_015112 | MAST2 | microtubule associated serine/threo | -0.132 | 0.913 | 8.84E-03 | 8.84E-01 | | | |
| 8144528 | NM_003747 | TNKS | tankyrase, TRF1-interacting ankyrin-r | -0.132 | 0.913 | 4.83E-01 | 4.83E+01 | | | |
| 8075981 | AF324466 | C22orf23 | chromosome 22 open reading frame | -0.132 | 0.913 | 5.13E-02 | 5.13E+00 | | | |
| 8165773 | NR_027383 | SFRS17A | splicing factor, arginine/serine-rich 1 | -0.132 | 0.913 | 6.00E-02 | 6.00E+00 | | | |
| 8176342 | NR_027383 | SFRS17A | splicing factor, arginine/serine-rich 1 | -0.132 | 0.913 | 6.00E-02 | 6.00E+00 | | | |
| 8093792 | NM_001528 | HGFAC | HGF activator | -0.132 | 0.913 | 2.45E-02 | 2.45E+00 | | | |
| 8016487 | NM_006361 | HOXB13 | homeobox B13 | -0.132 | 0.913 | 2.91E-02 | 2.91E+00 | | | |
| 7925174 | NM_014765 | TOMM20 | translocase of outer mitochondrial m | -0.132 | 0.913 | 6.99E-01 | 6.99E+01 | | | |
| 8044574 | NM_173842 | IL1RN | interleukin 1 receptor antagonist | -0.132 | 0.913 | 1.73E-01 | 1.73E+01 | | | |
| 8125042 | NM_025261 | LY6G6C | lymphocyte antigen 6 complex, locus | -0.132 | 0.913 | 5.38E-02 | 5.38E+00 | | | |
| 8052798 | NM_014911 | AAK1 | AP2 associated kinase 1 | -0.132 | 0.913 | 6.39E-01 | 6.39E+01 | | | |
| 8039791 | NM_032792 | ZBTB45 | zinc finger and BTB domain containin | -0.132 | 0.913 | 1.83E-02 | 1.83E+00 | | | |
| 7990952 | NR_026811 | LOC80154 | hypothetical LOC80154 | -0.132 | 0.913 | 4.84E-03 | 4.84E-01 | | | |
| 8161488 | NR_002817 | AQP7P1 | aquaporin 7 pseudogene 1 | -0.132 | 0.912 | 2.72E-02 | 2.72E+00 | | | |
| 7964642 | BC136288 | C12orf61 | chromosome 12 open reading frame | -0.132 | 0.912 | 1.04E-02 | 1.04E+00 | | | |
| 8037723 | NM_015649 | IRF2BP1 | interferon regulatory factor 2 binding | -0.132 | 0.912 | 1.96E-02 | 1.96E+00 | | | |
| 8038587 | NM_032298 | SYT3 | synaptotagmin III | -0.132 | 0.912 | 3.89E-02 | 3.89E+00 | | | |
| 8167430 | NM_033626 | CCDC120 | coiled-coil domain containing 120 | -0.132 | 0.912 | 1.43E-02 | 1.43E+00 | | | |
| 8167013 | NM_014735 | PHF16 | PHD finger protein 16 | -0.132 | 0.912 | 9.23E-02 | 9.23E+00 | | | |
| 7930919 | --- | --- | --- | -0.132 | 0.912 | 5.83E-02 | 5.83E+00 | | | |
| 7947828 | NM_000256 | MYBPC3 | myosin binding protein C, cardiac | -0.132 | 0.912 | 1.00E-02 | 1.00E+00 | | | |
| 8076007 | NM_025045 | BAIAP2L2 | BAI1-associated protein 2-like 2 | -0.132 | 0.912 | 4.13E-02 | 4.13E+00 | | | |
| 8117565 | --- | --- | --- | -0.132 | 0.912 | 1.39E-01 | 1.39E+01 | | | |
| 7999532 | NM_002094 | GSPT1 | G1 to S phase transition 1 | -0.132 | 0.912 | 6.67E-01 | 6.67E+01 | | | |
| 8024306 | NM_005883 | APC2 | adenomatosis polyposis coli 2 | -0.132 | 0.912 | 3.18E-02 | 3.18E+00 | | | |
| 8039378 | NM_003180 | SYT5 | synaptotagmin V | -0.132 | 0.912 | 7.82E-02 | 7.82E+00 | | | |
| 7925585 | NM_032328 | EFCAB2 | EF-hand calcium binding domain 2 | -0.132 | 0.912 | 7.32E-02 | 7.32E+00 | | | |
| 7942261 | NM_005553 | KRTAP5-9 | keratin associated protein 5-9 | -0.132 | 0.912 | 1.18E-02 | 1.18E+00 | | | |
| 7894696 | --- | --- | --- | -0.132 | 0.912 | 2.17E-01 | 2.17E+01 | | | |
| 8001971 | NM_012163 | LRRC29 | leucine rich repeat containing 29 | -0.132 | 0.912 | 3.44E-02 | 3.44E+00 | | | |
| 8053311 | --- | --- | --- | -0.132 | 0.912 | 8.67E-02 | 8.67E+00 | | | |
| 7931439 | NM_006426 | DPYSL4 | dihydropyrimidinase-like 4 | -0.132 | 0.912 | 2.10E-02 | 2.10E+00 | | | |
| 8098604 | NM_181726 | ANKRD37 | ankyrin repeat domain 37 | -0.132 | 0.912 | 1.61E-01 | 1.61E+01 | | | |
| 8064261 | NM_182647 | OPRL1 | opiate receptor-like 1 | -0.132 | 0.912 | 2.58E-02 | 2.58E+00 | | | |
| 8034249 | NM_145045 | CCDC151 | coiled-coil domain containing 151 | -0.132 | 0.912 | 4.89E-02 | 4.89E+00 | | | |
| 8091037 | NM_00113465 | PRR23A | proline rich 23A | -0.132 | 0.912 | 1.34E-01 | 1.34E+01 | | | |
| 7917885 | NM_001839 | CNN3 | calponin 3, acidic | -0.132 | 0.912 | 3.86E-02 | 3.86E+00 | | | |
| 8136065 | NR_002144 // | LOC407835 | mitogen-activated protein kinase kin | -0.132 | 0.912 | 2.03E-02 | 2.03E+00 | | | |
| 8164940 | NM_00113470 | SARDH | sarcosine dehydrogenase | -0.132 | 0.912 | 2.35E-02 | 2.35E+00 | | | |
| 8015206 | NM_00112338 | KRTAP2-1 | keratin associated protein 2-1 | -0.132 | 0.912 | 2.06E-02 | 2.06E+00 | | | |
| 7959249 | --- | --- | --- | -0.132 | 0.912 | 1.10E-01 | 1.10E+01 | | | |
| 8131205 | NM_152744 | SDK1 | sidekick homolog 1, cell adhesion mo | -0.132 | 0.912 | 1.01E-02 | 1.01E+00 | | | |
| 8158094 | NM_144965 | TTC16 | tetratricopeptide repeat domain 16 | -0.132 | 0.912 | 1.42E-02 | 1.42E+00 | | | |
| 8148194 | --- | --- | --- | -0.132 | 0.912 | 3.77E-01 | 3.77E+01 | | | |
| 8139885 | --- | --- | --- | -0.133 | 0.912 | 1.80E-01 | 1.80E+01 | | | |
| 8045224 | NM_152698 | FAM123C | family with sequence similarity 123C | -0.133 | 0.912 | 5.76E-02 | 5.76E+00 | | | |
| 7930524 | NM_145206 | VTI1A | vesicle transport through interaction | -0.133 | 0.912 | 6.97E-01 | 6.97E+01 | | | |
| 8158009 | NM_014580 | SLC2A8 | solute carrier family 2 (facilitated glu | -0.133 | 0.912 | 1.08E-02 | 1.08E+00 | | | |
| 8029551 | NM_000483 | APOC2 | apolipoprotein C-II | -0.133 | 0.912 | 2.77E-02 | 2.77E+00 | | | |
| 7978056 | BC144336 | ZFHX2 | zinc finger homeobox 2 | -0.133 | 0.912 | 7.33E-03 | 7.33E-01 | | | |
| 8076225 | NM_194326 | RPS19BP1 | ribosomal protein S19 binding protei | -0.133 | 0.912 | 7.17E-02 | 7.17E+00 | | | |
| 8143887 | NM_00114245 | ASB10 | ankyrin repeat and SOCS box-contain | -0.133 | 0.912 | 3.40E-02 | 3.40E+00 | | | |
| 7991893 | BC016613 | C16orf11 | chromosome 16 open reading frame | -0.133 | 0.912 | 1.22E-02 | 1.22E+00 | | | |
| 8074617 | NM_153334 | SCARF2 | scavenger receptor class F, member | -0.133 | 0.912 | 1.08E-02 | 1.08E+00 | | | |
| 7896255 | --- | --- | --- | -0.133 | 0.912 | 5.60E-01 | 5.60E+01 | | | |
| 8118302 | BC137541 | C6orf26 | chromosome 6 open reading frame 2 | -0.133 | 0.912 | 1.16E-02 | 1.16E+00 | | | |
| 8039759 | NM_198458 | ZNF497 | zinc finger protein 497 | -0.133 | 0.912 | 2.13E-02 | 2.13E+00 | | | |
| 8146480 | BC043417 | TMEM68 | transmembrane protein 68 | -0.133 | 0.912 | 1.76E-01 | 1.76E+01 | | | |
| 7942123 | NM_053056 | CCND1 | cyclin D1 | -0.133 | 0.912 | 1.33E-02 | 1.33E+00 | | | |
| 7970033 | NM_001846 | COL4A2 | collagen, type IV, alpha 2 | -0.133 | 0.912 | 1.17E-02 | 1.17E+00 | | | |
| 8124695 | NM_025236 | RNF39 | ring finger protein 39 | -0.133 | 0.912 | 2.58E-02 | 2.58E+00 | | | |
| 8178326 | NM_025236 | RNF39 | ring finger protein 39 | -0.133 | 0.912 | 2.58E-02 | 2.58E+00 | | | |
| 7893361 | --- | --- | --- | -0.133 | 0.912 | 3.18E-01 | 3.18E+01 | | | |
| 8042859 | NM_031288 | INO80B | INO80 complex subunit B | -0.133 | 0.912 | 3.11E-02 | 3.11E+00 | | | |

| | | | | | | | | | | |
|---|---|---|---|---|---|---|---|---|---|---|
| 8102875 | --- | --- | --- | -0.133 | 0.912 | 5.98E-02 | 5.98E+00 | | | |
| 8044813 | NM_183240 | TMEM37 | transmembrane protein 37 | -0.133 | 0.912 | 7.76E-02 | 7.76E+00 | | | |
| 8006453 | NM_005623 | CCL8 | chemokine (C-C motif) ligand 8 | -0.133 | 0.912 | 6.28E-02 | 6.28E+00 | | | |
| 8139919 | --- | --- | --- | -0.133 | 0.912 | 4.87E-02 | 4.87E+00 | | | |
| 7955170 | NM_005430 | WNT1 | wingless-type MMTV integration site | -0.133 | 0.912 | 2.16E-02 | 2.16E+00 | | | |
| 7961757 | NM_003034 | ST8SIA1 | ST8 alpha-N-acetyl-neuraminide alph | -0.133 | 0.912 | 1.85E-01 | 1.85E+01 | | | |
| 7949444 | NM_005714 | KCNK7 | potassium channel, subfamily K, mer | -0.133 | 0.912 | 1.02E-02 | 1.02E+00 | | | |
| 8073585 | NR_027786 | SERHL | serine hydrolase-like | -0.133 | 0.912 | 4.64E-02 | 4.64E+00 | | | |
| 7945125 | BC113593 | PRR10 | proline rich 10 | -0.133 | 0.912 | 1.44E-02 | 1.44E+00 | | | |
| 7985431 | NR_026811 | LOC80154 | hypothetical LOC80154 | -0.133 | 0.912 | 4.41E-03 | 4.41E-01 | | | |
| 7958942 | NM_00114487 | CCDC42B | coiled-coil domain containing 42B | -0.133 | 0.912 | 2.80E-02 | 2.80E+00 | | | |
| 8071168 | NR_024593 | POM121L10P | POM121 membrane glycoprotein-lik | -0.133 | 0.912 | 1.38E-02 | 1.38E+00 | | | |
| 8038117 | NM_001352 | DBP | D site of albumin promoter (albumin | -0.133 | 0.912 | 5.55E-02 | 5.55E+00 | | | |
| 8119689 | NM_002821 | PTK7 | PTK7 protein tyrosine kinase 7 | -0.133 | 0.912 | 2.43E-02 | 2.43E+00 | | | |
| 8053183 | NM_00100981 | LBX2 | ladybird homeobox 2 | -0.133 | 0.912 | 3.80E-02 | 3.80E+00 | | | |
| 7972805 | NM_017817 | RAB20 | RAB20, member RAS oncogene famil | -0.133 | 0.912 | 1.53E-01 | 1.53E+01 | | | |
| 7900352 | NM_022120 | OXCT2 | 3-oxoacid CoA transferase 2 | -0.133 | 0.912 | 1.90E-02 | 1.90E+00 | | | |
| 7915257 | NM_022120 | OXCT2 | 3-oxoacid CoA transferase 2 | -0.133 | 0.912 | 1.90E-02 | 1.90E+00 | | | |
| 8148674 | NM_139021 | MAPK15 | mitogen-activated protein kinase 15 | -0.133 | 0.912 | 4.21E-03 | 4.21E-01 | | | |
| 8158380 | NM_013355 | PKN3 | protein kinase N3 | -0.133 | 0.912 | 2.10E-02 | 2.10E+00 | | | |
| 7920575 | NM_020524 | PBXIP1 | pre-B-cell leukemia homeobox intera | -0.133 | 0.912 | 7.24E-02 | 7.24E+00 | | | |
| 7928216 | --- | --- | --- | -0.133 | 0.912 | 4.59E-01 | 4.59E+01 | | | |
| 8164833 | NM_021996 | GBGT1 | globoside alpha-1,3-N-acetylgalactos | -0.133 | 0.912 | 5.01E-02 | 5.01E+00 | | | |
| 8157818 | NM_00104547 | WDR38 | WD repeat domain 38 | -0.133 | 0.912 | 1.37E-02 | 1.37E+00 | | | |
| 7999727 | NM_001171 | ABCC6 | ATP-binding cassette, sub-family C (C | -0.133 | 0.912 | 3.64E-02 | 3.64E+00 | | | |
| 8158513 | NM_020438 | DOLPP1 | dolichyl pyrophosphate phosphatase | -0.133 | 0.912 | 2.62E-02 | 2.62E+00 | | | |
| 8075924 | NM_002405 | MFNG | MFNG O-fucosylpeptide 3-beta-N-ac | -0.133 | 0.912 | 4.72E-02 | 4.72E+00 | | | |
| 8144505 | NM_194284 | CLDN23 | claudin 23 | -0.133 | 0.912 | 1.59E-02 | 1.59E+00 | | | |
| 7986336 | ENST0000044 | LOC728800 | similar to FLJ00402 protein | -0.133 | 0.912 | 3.73E-02 | 3.73E+00 | | | |
| 8073816 | NR_027033 | LOC400931 | hypothetical LOC400931 | -0.133 | 0.912 | 5.26E-02 | 5.26E+00 | | | |
| 8134821 | NM_019606 | MEPCE | methylphosphate capping enzyme | -0.133 | 0.912 | 7.84E-02 | 7.84E+00 | | | |
| 7976669 | NM_00112725 | HHIPL1 | HHIP-like 1 | -0.133 | 0.912 | 2.37E-02 | 2.37E+00 | | | |
| 7897132 | NM_022114 | PRDM16 | PR domain containing 16 | -0.133 | 0.912 | 6.88E-03 | 6.88E-01 | | | |
| 7939657 | NM_00110554 | DGKZ | diacylglycerol kinase, zeta 104kDa | -0.133 | 0.912 | 9.06E-02 | 9.06E+00 | | | |
| 7991815 | NM_006849 | PDIA2 | protein disulfide isomerase family A, | -0.133 | 0.912 | 3.38E-02 | 3.38E+00 | | | |
| 7996448 | NM_003946 | NOL3 | nucleolar protein 3 (apoptosis repres | -0.133 | 0.912 | 2.06E-02 | 2.06E+00 | | | |
| 8066260 | NR_003017 | SNORA71C | small nucleolar RNA, H/ACA box 71C | -0.133 | 0.912 | 6.50E-01 | 6.50E+01 | | | |
| 7903490 | NM_00114398 | NBPF6 | neuroblastoma breakpoint family, m | -0.133 | 0.912 | 6.20E-03 | 6.20E-01 | | | |
| 8125426 | NM_019602 | BTNL2 | butyrophilin-like 2 (MHC class II asso | -0.133 | 0.912 | 6.23E-02 | 6.23E+00 | | | |
| 8178795 | NM_019602 | BTNL2 | butyrophilin-like 2 (MHC class II asso | -0.133 | 0.912 | 6.23E-02 | 6.23E+00 | | | |
| 8179996 | NM_019602 | BTNL2 | butyrophilin-like 2 (MHC class II asso | -0.133 | 0.912 | 6.23E-02 | 6.23E+00 | | | |
| 8169235 | ENST0000027€ | FRMPD3 | FERM and PDZ domain containing 3 | -0.133 | 0.912 | 4.06E-01 | 4.06E+01 | | | |
| 8127532 | --- | --- | --- | -0.133 | 0.912 | 5.90E-02 | 5.90E+00 | | | |
| 8036252 | NM_015526 | CLIP3 | CAP-GLY domain containing linker pr | -0.133 | 0.912 | 8.37E-03 | 8.37E-01 | | | |
| 7893167 | --- | --- | --- | -0.134 | 0.912 | 7.11E-01 | 7.11E+01 | | | |
| 8063177 | NM_030777 | SLC2A10 | solute carrier family 2 (facilitated glu | -0.134 | 0.912 | 3.02E-02 | 3.02E+00 | | | |
| 8113094 | NM_153216 | POU5F2 | POU domain class 5, transcription fa | -0.134 | 0.912 | 2.19E-02 | 2.19E+00 | | | |
| 8123332 | --- | --- | --- | -0.134 | 0.912 | 7.03E-02 | 7.03E+00 | | | |
| 7971723 | NR_027047 | RP11-327P2.4 | hypothetical LOC283521 | -0.134 | 0.912 | 6.88E-02 | 6.88E+00 | | | |
| 8153790 | NM_013432 | NFKBIL2 | nuclear factor of kappa light polypep | -0.134 | 0.912 | 1.81E-02 | 1.81E+00 | | | |
| 8155340 | NM_015667 // | 75A7 // FAM | family with sequence similarity 75, m | -0.134 | 0.912 | 9.69E-03 | 9.69E-01 | | | |
| 7928882 | NM_006829 | C10orf116 | chromosome 10 open reading frame | -0.134 | 0.912 | 3.14E-02 | 3.14E+00 | | | |
| 8033458 | NM_007260 | LYPLA2 | lysophospholipase II | -0.134 | 0.912 | 2.98E-01 | 2.98E+01 | | | |
| 7984436 | NM_00103180 | LBXCOR1 | Lbxcor1 homolog (mouse) | -0.134 | 0.912 | 3.86E-02 | 3.86E+00 | | | |
| 8166355 | NM_014927 | CNKSR2 | connector enhancer of kinase suppre | -0.134 | 0.912 | 7.50E-02 | 7.50E+00 | | | |
| 8043639 | NM_00107740 | FER1L5 | fer-1-like 5 (C. elegans) | -0.134 | 0.912 | 2.30E-02 | 2.30E+00 | | | |
| 8092473 | NM_004366 | CLCN2 | chloride channel 2 | -0.134 | 0.912 | 1.00E-02 | 1.00E+00 | | | |
| 7949172 | NM_004579 | MAP4K2 | mitogen-activated protein kinase kin | -0.134 | 0.912 | 6.28E-02 | 6.28E+00 | | | |
| 8155490 | NR_003670 | KGFLP2 | keratinocyte growth factor-like prote | -0.134 | 0.912 | 1.51E-02 | 1.51E+00 | | | |
| 7894800 | --- | --- | --- | -0.134 | 0.911 | 6.54E-01 | 6.54E+01 | | | |
| 8019090 | NM_00108039 | AATK | apoptosis-associated tyrosine kinase | -0.134 | 0.911 | 1.35E-02 | 1.35E+00 | | | |
| 7894849 | --- | --- | --- | -0.134 | 0.911 | 7.49E-02 | 7.49E+00 | | | |
| 8076826 | NR_026997 | C22orf34 | chromosome 22 open reading frame | -0.134 | 0.911 | 6.66E-02 | 6.66E+00 | | | |
| 7991034 | NM_199330 | HOMER2 | homer homolog 2 (Drosophila) | -0.134 | 0.911 | 1.58E-02 | 1.58E+00 | | | |
| 8071268 | NM_000407 | GP1BB | glycoprotein Ib (platelet), beta polyp | -0.134 | 0.911 | 4.02E-02 | 4.02E+00 | | | |
| 8036010 | NM_000285 | PEPD | peptidase D | -0.134 | 0.911 | 1.56E-01 | 1.56E+01 | | | |
| 8079677 | NM_177938 | P4HTM | prolyl 4-hydroxylase, transmembran | -0.134 | 0.911 | 1.52E-02 | 1.52E+00 | | | |
| 8011200 | NM_052928 | SMYD4 | SET and MYND domain containing 4 | -0.134 | 0.911 | 1.05E-01 | 1.05E+01 | | | |
| 7893881 | --- | --- | --- | -0.134 | 0.911 | 5.61E-01 | 5.61E+01 | | | |
| 7895340 | --- | --- | --- | -0.134 | 0.911 | 8.59E-01 | 8.59E+01 | | | |
| 8148270 | NM_005005 | NDUFB9 | NADH dehydrogenase (ubiquinone) 1 | -0.134 | 0.911 | 6.43E-01 | 6.43E+01 | | | |
| 7980152 | NM_000428 | LTBP2 | latent transforming growth factor be | -0.134 | 0.911 | 7.30E-03 | 7.30E-01 | | | |
| 8061539 | NR_024358 | NCRNA00028 | non-protein coding RNA 28 | -0.134 | 0.911 | 2.48E-02 | 2.48E+00 | | | |
| 7937852 | NM_002555 | SLC22A18 | solute carrier family 22, member 18 | -0.134 | 0.911 | 1.12E-02 | 1.12E+00 | | | |
| 7978544 | NM_022073 | EGLN3 | egl nine homolog 3 (C. elegans) | -0.134 | 0.911 | 3.46E-02 | 3.46E+00 | | | |
| 7936494 | NM_005264 | GFRA1 | GDNF family receptor alpha 1 | -0.134 | 0.911 | 2.54E-02 | 2.54E+00 | | | |
| 7931181 | NM_00110557 | HMX3 | H6 family homeobox 3 | -0.134 | 0.911 | 2.03E-02 | 2.03E+00 | | | |

| | | | | | | | | | | |
|---|---|---|---|---|---|---|---|---|---|---|
| 7902269 | NM_004768 | SFRS11 | splicing factor, arginine/serine-rich 1 | -0.134 | 0.911 | 5.68E-01 | 5.68E+01 | | | |
| 7930311 | NM_183239 | GSTO2 | glutathione S-transferase omega 2 | -0.134 | 0.911 | 2.30E-02 | 2.30E+00 | | | |
| 7998434 | NM_003294 | TPSAB1 | tryptase alpha/beta 1 | -0.134 | 0.911 | 7.81E-03 | 7.81E-01 | | | |
| 7990309 | NM_022369 | STRA6 | stimulated by retinoic acid gene 6 ho | -0.134 | 0.911 | 2.92E-02 | 2.92E+00 | | | |
| 8025421 | NM_016496 | 40239 | membrane-associated ring finger (C3 | -0.134 | 0.911 | 9.82E-02 | 9.82E+00 | | | |
| 8115756 | NM_004137 | KCNMB1 | potassium large conductance calcium | -0.134 | 0.911 | 4.96E-02 | 4.96E+00 | | | |
| 8142747 | NM_006193 | PAX4 | paired box 4 | -0.134 | 0.911 | 7.17E-03 | 7.17E-01 | | | |
| 8109593 | --- | --- | --- | -0.134 | 0.911 | 4.13E-01 | 4.13E+01 | | | |
| 8066754 | NM_080721 | C20orf123 | chromosome 20 open reading frame | -0.134 | 0.911 | 2.77E-02 | 2.77E+00 | | | |
| 7904131 | NM_022836 | DCLRE1B | DNA cross-link repair 1B (PSO2 homo | -0.134 | 0.911 | 9.72E-02 | 9.72E+00 | | | |
| 8112274 | NM_024930 | ELOVL7 | ELOVL family member 7, elongation | -0.134 | 0.911 | 7.91E-01 | 7.91E+01 | | | |
| 7895530 | --- | --- | --- | -0.134 | 0.911 | 8.57E-01 | 8.57E+01 | | | |
| 8177120 | NM_00103351 | OC10013228 | hypothetical protein LOC100132288 | -0.134 | 0.911 | 1.07E-01 | 1.07E+01 | | | |
| 8029642 | NM_031417 | MARK4 | MAP/microtubule affinity-regulating | -0.134 | 0.911 | 3.60E-02 | 3.60E+00 | | | |
| 8075992 | NM_006941 | SOX10 | SRY (sex determining region Y)-box 1 | -0.134 | 0.911 | 1.87E-02 | 1.87E+00 | | | |
| 8149361 | NM_018088 | FAM90A1 | family with sequence similarity 90, n | -0.134 | 0.911 | 2.83E-02 | 2.83E+00 | | | |
| 7995242 | BC021555 | C16orf67 | chromosome 16 open reading frame | -0.134 | 0.911 | 1.53E-01 | 1.53E+01 | | | |
| 7991927 | NM_053284 | WFIKKN1 | WAP, follistatin/kazal, immunoglobu | -0.134 | 0.911 | 2.04E-02 | 2.04E+00 | | | |
| 8060334 | NM_006943 | SOX12 | SRY (sex determining region Y)-box 1 | -0.134 | 0.911 | 2.36E-02 | 2.36E+00 | | | |
| 7897422 | --- | --- | --- | -0.134 | 0.911 | 8.80E-02 | 8.80E+00 | | | |
| 8153328 | NM_003724 | JRK | jerky homolog (mouse) | -0.134 | 0.911 | 2.92E-02 | 2.92E+00 | | | |
| 8064277 | NM_004535 | MYT1 | myelin transcription factor 1 | -0.134 | 0.911 | 1.45E-02 | 1.45E+00 | | | |
| 7896125 | --- | --- | --- | -0.134 | 0.911 | 8.29E-01 | 8.29E+01 | | | |
| 7992584 | NM_001374 | DNASE1L2 | deoxyribonuclease I-like 2 | -0.134 | 0.911 | 4.41E-02 | 4.41E+00 | | | |
| 8053797 | ENST00000456 | LOC400986 | protein immuno-reactive with anti-P | -0.134 | 0.911 | 7.83E-01 | 7.83E+01 | | | |
| 7928602 | NM_005411 | SFTPA1 | surfactant protein A1 | -0.134 | 0.911 | 2.86E-02 | 2.86E+00 | | | |
| 7928632 | NM_005411 | SFTPA1 | surfactant protein A1 | -0.134 | 0.911 | 2.86E-02 | 2.86E+00 | | | |
| 8180330 | --- | --- | --- | -0.134 | 0.911 | 4.58E-01 | 4.58E+01 | | | |
| 8034108 | NM_024029 | YIPF2 | Yip1 domain family, member 2 | -0.134 | 0.911 | 6.61E-02 | 6.61E+00 | | | |
| 8027049 | NM_018316 | KLHL26 | kelch-like 26 (Drosophila) | -0.134 | 0.911 | 1.39E-02 | 1.39E+00 | | | |
| 8054020 | BC038583 | FAM178B | family with sequence similarity 178, | -0.134 | 0.911 | 1.45E-02 | 1.45E+00 | | | |
| 7938309 | NR_027713 | FLJ46111 | keratin 8 pseudogene | -0.134 | 0.911 | 2.23E-02 | 2.23E+00 | | | |
| 8000779 | NM_004608 | TBX6 | T-box 6 | -0.134 | 0.911 | 1.98E-02 | 1.98E+00 | | | |
| 8139031 | --- | --- | --- | -0.135 | 0.911 | 1.64E-01 | 1.64E+01 | | | |
| 8042038 | NR_002229 | RPL23AP32 | ribosomal protein L23a pseudogene | -0.135 | 0.911 | 1.34E-01 | 1.34E+01 | | | |
| 7908161 | NM_030806 | C1orf21 | chromosome 1 open reading frame 2 | -0.135 | 0.911 | 4.48E-01 | 4.48E+01 | | | |
| 7893662 | --- | --- | --- | -0.135 | 0.911 | 6.11E-01 | 6.11E+01 | | | |
| 7991110 | NR_003246 | FLJ40113 | golgi autoantigen, golgin subfamily a | -0.135 | 0.911 | 3.84E-01 | 3.84E+01 | | | |
| 8104613 | ENST00000330 | LOC391742 | TBP-associated factor 11 pseudogene | -0.135 | 0.911 | 1.31E-01 | 1.31E+01 | | | |
| 8088480 | NR_026582 | ID2B | inhibitor of DNA binding 2B, domina | -0.135 | 0.911 | 5.25E-03 | 5.25E-01 | | | |
| 8040473 | NM_004040 | RHOB | ras homolog gene family, member B | -0.135 | 0.911 | 7.49E-02 | 7.49E+00 | | | |
| 7933310 | --- | --- | --- | -0.135 | 0.911 | 2.87E-02 | 2.87E+00 | | | |
| 7911108 | --- | --- | --- | -0.135 | 0.911 | 5.64E-01 | 5.64E+01 | | | |
| 7905507 | NM_178428 | LCE2A | late cornified envelope 2A | -0.135 | 0.911 | 4.57E-02 | 4.57E+00 | | | |
| 8160295 | NR_003009 | SCARNA8 | small Cajal body-specific RNA 8 | -0.135 | 0.911 | 3.28E-01 | 3.28E+01 | | | |
| 8110546 | NM_014757 | MAML1 | mastermind-like 1 (Drosophila) | -0.135 | 0.911 | 1.14E-02 | 1.14E+00 | | | |
| 8099524 | NM_00113083 | LDB2 | LIM domain binding 2 | -0.135 | 0.911 | 5.01E-02 | 5.01E+00 | | | |
| 7986329 | NM_021005 | NR2F2 | nuclear receptor subfamily 2, group | -0.135 | 0.911 | 4.41E-02 | 4.41E+00 | | | |
| 7979455 | NM_021136 | RTN1 | reticulon 1 | -0.135 | 0.911 | 1.84E-01 | 1.84E+01 | | | |
| 7897860 | NM_001243 | TNFRSF8 | tumor necrosis factor receptor super | -0.135 | 0.911 | 1.52E-02 | 1.52E+00 | | | |
| 7990436 | NM_004255 | COX5A | cytochrome c oxidase subunit Va | -0.135 | 0.911 | 6.23E-01 | 6.23E+01 | | | |
| 8002072 | BC008284 | C16orf48 | chromosome 16 open reading frame | -0.135 | 0.911 | 3.86E-02 | 3.86E+00 | | | |
| 8038670 | NM_012427 | KLK5 | kallikrein-related peptidase 5 | -0.135 | 0.911 | 3.99E-02 | 3.99E+00 | | | |
| 8038126 | NM_001217 | CA11 | carbonic anhydrase XI | -0.135 | 0.911 | 1.03E-02 | 1.03E+00 | | | |
| 7959986 | NM_025215 | PUS1 | pseudouridylate synthase 1 | -0.135 | 0.911 | 9.12E-02 | 9.12E+00 | | | |
| 8124998 | NM_033177 | BAT4 | HLA-B associated transcript 4 | -0.135 | 0.911 | 8.04E-02 | 8.04E+00 | | | |
| 7987310 | NM_020660 | GJD2 | gap junction protein, delta 2, 36kDa | -0.135 | 0.911 | 2.21E-02 | 2.21E+00 | | | |
| 8001680 | --- | --- | --- | -0.135 | 0.911 | 1.44E-01 | 1.44E+01 | | | |
| 8033825 | NM_015719 | COL5A3 | collagen, type V, alpha 3 | -0.135 | 0.911 | 8.47E-03 | 8.47E-01 | | | |
| 8061894 | NM_033197 | C20orf114 | chromosome 20 open reading frame | -0.135 | 0.911 | 2.03E-02 | 2.03E+00 | | | |
| 7992789 | NM_016639 | TNFRSF12A | tumor necrosis factor receptor super | -0.135 | 0.911 | 1.01E-02 | 1.01E+00 | | | |
| 8034130 | NM_00113619 | KANK2 | KN motif and ankyrin repeat domain | -0.135 | 0.911 | 4.25E-02 | 4.25E+00 | | | |
| 7939587 | BX640618 | KFZp779M06 | hypothetical DKFZp779M0652 | -0.135 | 0.911 | 2.20E-02 | 2.20E+00 | | | |
| 7910899 | --- | --- | --- | -0.135 | 0.911 | 2.10E-02 | 2.10E+00 | | | |
| 7895718 | --- | --- | --- | -0.135 | 0.911 | 8.98E-01 | 8.98E+01 | | | |
| 7906969 | --- | --- | --- | -0.135 | 0.911 | 5.04E-02 | 5.04E+00 | | | |
| 8098995 | NM_006454 | MXD4 | MAX dimerization protein 4 | -0.135 | 0.911 | 7.29E-03 | 7.29E-01 | | | |
| 8127692 | NM_000863 | HTR1B | 5-hydroxytryptamine (serotonin) rec | -0.135 | 0.911 | 2.28E-02 | 2.28E+00 | | | |
| 7980859 | NM_00108011 | PP8961 | hypothetical protein LOC650662 | -0.135 | 0.911 | 2.31E-02 | 2.31E+00 | | | |
| 7997352 | NM_016373 | WWOX | WW domain containing oxidoreduct | -0.135 | 0.911 | 9.74E-03 | 9.74E-01 | | | |
| 8027719 | NM_001037 | SCN1B | sodium channel, voltage-gated, type | -0.135 | 0.911 | 1.81E-02 | 1.81E+00 | | | |
| 7984011 | NM_012182 | FOXB1 | forkhead box B1 | -0.135 | 0.911 | 4.12E-02 | 4.12E+00 | | | |
| 7946004 | NM_00100475 | OR51S1 | olfactory receptor, family 51, subfam | -0.135 | 0.911 | 1.25E-02 | 1.25E+00 | | | |
| 8011797 | NM_213726 | INCA1 | inhibitor of CDK, cyclin A1 interacting | -0.135 | 0.911 | 1.29E-02 | 1.29E+00 | | | |
| 7989245 | NR_002927 | HSP90AB4P | heat shock protein 90kDa alpha (cyto | -0.135 | 0.911 | 6.54E-02 | 6.54E+00 | | | |
| 8101253 | NM_032693 | ARD1B | ARD1 homolog B (S. cerevisiae) | -0.135 | 0.911 | 7.70E-02 | 7.70E+00 | | | |
| 8036883 | NM_144685 | HIPK4 | homeodomain interacting protein ki | -0.135 | 0.911 | 3.10E-02 | 3.10E+00 | | | |

| | | | | | | | | | | |
|---|---|---|---|---|---|---|---|---|---|---|
| 8171786 | NM_153270 | KLHL34 | kelch-like 34 (Drosophila) | -0.135 | 0.911 | 2.07E-02 | 2.07E+00 | | | |
| 7897068 | NM_003036 | SKI | v-ski sarcoma viral oncogene homolo | -0.135 | 0.911 | 1.35E-02 | 1.35E+00 | | | |
| 7903478 | NM_00114398 | NBPF4 | neuroblastoma breakpoint family, m | -0.135 | 0.911 | 1.16E-02 | 1.16E+00 | | | |
| 7896055 | --- | --- | --- | -0.135 | 0.911 | 1.57E-01 | 1.57E+01 | | | |
| 8065990 | --- | --- | --- | -0.135 | 0.911 | 8.08E-01 | 8.08E+01 | | | |
| 7965510 | NM_020698 | TMCC3 | transmembrane and coiled-coil dom | -0.135 | 0.911 | 3.68E-02 | 3.68E+00 | | | |
| 8034276 | NM_138783 | ZNF653 | zinc finger protein 653 | -0.135 | 0.911 | 1.37E-02 | 1.37E+00 | | | |
| 8027884 | --- | --- | --- | -0.135 | 0.911 | 1.06E-01 | 1.06E+01 | | | |
| 7977296 | NR_024396 | MGC23270 | hypothetical LOC196872 | -0.135 | 0.911 | 1.35E-02 | 1.35E+00 | | | |
| 8016033 | NM_198475 | FAM171A2 | family with sequence similarity 171, | -0.135 | 0.911 | 3.63E-02 | 3.63E+00 | | | |
| 8124446 | --- | --- | --- | -0.135 | 0.910 | 8.18E-02 | 8.18E+00 | | | |
| 8165416 | NM_203468 | ENTPD2 | ectonucleoside triphosphate diphos | -0.135 | 0.910 | 2.90E-02 | 2.90E+00 | | | |
| 8030035 | NM_031485 | GRWD1 | glutamate-rich WD repeat containing | -0.135 | 0.910 | 1.06E-02 | 1.06E+00 | | | |
| 8117422 | NM_003540 | HIST1H4F | histone cluster 1, H4f | -0.135 | 0.910 | 3.72E-01 | 3.72E+01 | | | |
| 7972177 | NM_006237 | POU4F1 | POU class 4 homeobox 1 | -0.135 | 0.910 | 1.57E-02 | 1.57E+00 | | | |
| 7934122 | NM_00114264 | SAR1A | SAR1 homolog A (S. cerevisiae) | -0.135 | 0.910 | 7.08E-01 | 7.08E+01 | | | |
| 7948588 | NM_004200 | SYT7 | synaptotagmin VII | -0.135 | 0.910 | 2.03E-02 | 2.03E+00 | | | |
| 7949545 | NM_032325 | EIF1AD | eukaryotic translation initiation facto | -0.135 | 0.910 | 5.59E-01 | 5.59E+01 | | | |
| 7977241 | NM_022489 | INF2 | inverted formin, FH2 and WH2 doma | -0.135 | 0.910 | 5.80E-02 | 5.80E+00 | | | |
| 7969776 | NR_026644 | UBAC2 | UBA domain containing 2 | -0.135 | 0.910 | 2.52E-01 | 2.52E+01 | | | |
| 8007808 | NM_00114514 | CRHR1 | corticotropin releasing hormone rec | -0.136 | 0.910 | 1.16E-02 | 1.16E+00 | | | |
| 7929945 | --- | --- | --- | -0.136 | 0.910 | 2.25E-02 | 2.25E+00 | | | |
| 7898773 | NM_020526 | EPHA8 | EPH receptor A8 | -0.136 | 0.910 | 1.79E-02 | 1.79E+00 | | | |
| 8013923 | NM_00108545 | GIT1 | G protein-coupled receptor kinase in | -0.136 | 0.910 | 3.72E-02 | 3.72E+00 | | | |
| 7927099 | NR_026827 | LOC84856 | hypothetical LOC84856 | -0.136 | 0.910 | 1.34E-02 | 1.34E+00 | | | |
| 7902223 | --- | --- | --- | -0.136 | 0.910 | 1.52E-02 | 1.52E+00 | | | |
| 8095688 | NM_002993 | CXCL6 | chemokine (C-X-C motif) ligand 6 (gr | -0.136 | 0.910 | 7.27E-03 | 7.27E-01 | | | |
| 7955290 | NM_000486 | AQP2 | aquaporin 2 (collecting duct) | -0.136 | 0.910 | 1.63E-02 | 1.63E+00 | | | |
| 8175016 | NM_017413 | APLN | apelin | -0.136 | 0.910 | 1.12E-02 | 1.12E+00 | | | |
| 8038347 | NM_003598 | TEAD2 | TEA domain family member 2 | -0.136 | 0.910 | 2.53E-02 | 2.53E+00 | | | |
| 8006161 | NM_015594 | TBC1D29 | TBC1 domain family, member 29 | -0.136 | 0.910 | 1.68E-02 | 1.68E+00 | | | |
| 8023914 | NM_025078 | PQLC1 | PQ loop repeat containing 1 | -0.136 | 0.910 | 9.11E-03 | 9.11E-01 | | | |
| 8144625 | NM_001715 | BLK | B lymphoid tyrosine kinase | -0.136 | 0.910 | 1.33E-01 | 1.33E+01 | | | |
| 7991296 | NM_198525 | KIF7 | kinesin family member 7 | -0.136 | 0.910 | 1.62E-02 | 1.62E+00 | | | |
| 8148591 | NM_178172 | GPIHBP1 | glycosylphosphatidylinositol anchore | -0.136 | 0.910 | 3.68E-02 | 3.68E+00 | | | |
| 8007323 | NM_003632 | CNTNAP1 | contactin associated protein 1 | -0.136 | 0.910 | 6.42E-02 | 6.42E+00 | | | |
| 8003824 | NM_004937 | CTNS | cystinosis, nephropathic | -0.136 | 0.910 | 1.76E-01 | 1.76E+01 | | | |
| 8118981 | BC033033 | C6orf81 | chromosome 6 open reading frame 8 | -0.136 | 0.910 | 2.28E-02 | 2.28E+00 | | | |
| 8028325 | NM_033520 | C19orf33 | chromosome 19 open reading frame | -0.136 | 0.910 | 6.01E-03 | 6.01E-01 | | | |
| 7901187 | NM_00101361 | C1orf190 | chromosome 1 open reading frame 1 | -0.136 | 0.910 | 3.97E-02 | 3.97E+00 | | | |
| 8042537 | NM_001692 | ATP6V1B1 | ATPase, H+ transporting, lysosomal 5 | -0.136 | 0.910 | 8.35E-03 | 8.35E-01 | | | |
| 7980003 | --- | --- | --- | -0.136 | 0.910 | 6.56E-02 | 6.56E+00 | | | |
| 7978706 | NM_004496 | FOXA1 | forkhead box A1 | -0.136 | 0.910 | 2.26E-02 | 2.26E+00 | | | |
| 8094093 | NM_080819 | GPR78 | G protein-coupled receptor 78 | -0.136 | 0.910 | 2.71E-02 | 2.71E+00 | | | |
| 7984606 | NM_014249 | NR2E3 | nuclear receptor subfamily 2, group | -0.136 | 0.910 | 2.40E-02 | 2.40E+00 | | | |
| 7894174 | --- | --- | --- | -0.136 | 0.910 | 4.72E-01 | 4.72E+01 | | | |
| 8180275 | --- | --- | --- | -0.136 | 0.910 | 1.16E-01 | 1.16E+01 | | | |
| 8068413 | NM_001236 | CBR3 | carbonyl reductase 3 | -0.136 | 0.910 | 2.56E-02 | 2.56E+00 | | | |
| 8125072 | NM_025258 | C6orf27 | chromosome 6 open reading frame 2 | -0.136 | 0.910 | 1.04E-02 | 1.04E+00 | | | |
| 8049435 | NM_014521 | SH3BP4 | SH3-domain binding protein 4 | -0.136 | 0.910 | 3.38E-02 | 3.38E+00 | | | |
| 8111772 | NM_001343 | DAB2 | disabled homolog 2, mitogen-respon | -0.136 | 0.910 | 3.57E-01 | 3.57E+01 | | | |
| 7964400 | NM_145064 | STAC3 | SH3 and cysteine rich domain 3 | -0.136 | 0.910 | 2.21E-02 | 2.21E+00 | | | |
| 8176644 | NM_004202 | TMSB4Y | thymosin beta 4, Y-linked | -0.136 | 0.910 | 1.62E-01 | 1.62E+01 | | | |
| 8074003 | NM_025204 | TRABD | TraB domain containing | -0.136 | 0.910 | 3.69E-02 | 3.69E+00 | | | |
| 8139917 | NM_022479 | WBSCR17 | Williams-Beuren syndrome chromos | -0.136 | 0.910 | 4.09E-02 | 4.09E+00 | | | |
| 7942611 | --- | --- | --- | -0.136 | 0.910 | 7.22E-02 | 7.22E+00 | | | |
| 7991779 | NM_032039 | ITFG3 | integrin alpha FG-GAP repeat contai | -0.136 | 0.910 | 8.81E-03 | 8.81E-01 | | | |
| 7966389 | NM_144671 | FAM109A | family with sequence similarity 109, | -0.136 | 0.910 | 6.72E-03 | 6.72E-01 | | | |
| 8046522 | --- | --- | --- | -0.136 | 0.910 | 1.11E-01 | 1.11E+01 | | | |
| 8007348 | NM_005854 | RAMP2 | receptor (G protein-coupled) activity | -0.136 | 0.910 | 2.41E-02 | 2.41E+00 | | | |
| 8150592 | NM_005195 | CEBPD | CCAAT/enhancer binding protein (C/ | -0.136 | 0.910 | 8.31E-02 | 8.31E+00 | | | |
| 8131087 | NM_00108046 | UNCX | UNC homeobox | -0.136 | 0.910 | 2.07E-02 | 2.07E+00 | | | |
| 7995324 | --- | --- | --- | -0.136 | 0.910 | 7.70E-02 | 7.70E+00 | | | |
| 8022007 | --- | --- | --- | -0.136 | 0.910 | 1.02E-01 | 1.02E+01 | | | |
| 8159624 | BC052297 | C9orf169 | chromosome 9 open reading frame 1 | -0.136 | 0.910 | 7.98E-03 | 7.98E-01 | | | |
| 8068549 | --- | --- | --- | -0.136 | 0.910 | 1.10E-01 | 1.10E+01 | | | |
| 7911718 | NR_024489 | OC10012953 | small nuclear ribonucleoprotein poly | -0.136 | 0.910 | 1.01E-01 | 1.01E+01 | | | |
| 8031196 | NM_002288 | LAIR2 | leukocyte-associated immunoglobuli | -0.136 | 0.910 | 6.51E-02 | 6.51E+00 | | | |
| 8032553 | NM_003260 | TLE2 | transducin-like enhancer of split 2 (E | -0.136 | 0.910 | 2.44E-02 | 2.44E+00 | | | |
| 8150864 | --- | --- | --- | -0.136 | 0.910 | 4.21E-02 | 4.21E+00 | | | |
| 7895614 | --- | --- | --- | -0.136 | 0.910 | 6.30E-01 | 6.30E+01 | | | |
| 7899284 | NM_00101364 | TRNP1 | TMF1-regulated nuclear protein 1 | -0.136 | 0.910 | 2.36E-02 | 2.36E+00 | | | |
| 8055239 | NM_032545 | CFC1 | cripto, FRL-1, cryptic family 1 | -0.136 | 0.910 | 1.23E-02 | 1.23E+00 | | | |
| 8174246 | NM_080879 | RAB40A | RAB40A, member RAS oncogene fam | -0.136 | 0.910 | 4.91E-02 | 4.91E+00 | | | |
| 8087513 | NM_005879 | TRAIP | TRAF interacting protein | -0.137 | 0.910 | 7.72E-03 | 7.72E-01 | | | |
| 7895727 | --- | --- | --- | -0.137 | 0.910 | 3.48E-01 | 3.48E+01 | | | |
| 8168359 | NM_00101362 | NHSL2 | NHS-like 2 | -0.137 | 0.910 | 1.22E-02 | 1.22E+00 | | | |

| | | | | | | | | | | |
|---|---|---|---|---|---|---|---|---|---|---|
| 7966631 | NM_022363 | LHX5 | LIM homeobox 5 | -0.137 | 0.910 | 4.87E-02 | 4.87E+00 | | | |
| 8066641 | NM_022095 | ZNF335 | zinc finger protein 335 | -0.137 | 0.910 | 2.52E-02 | 2.52E+00 | | | |
| 8079759 | NM_003458 | BSN | bassoon (presynaptic cytomatrix pro | -0.137 | 0.910 | 1.14E-02 | 1.14E+00 | | | |
| 8038707 | NM_144505 | KLK8 | kallikrein-related peptidase 8 | -0.137 | 0.910 | 8.18E-03 | 8.18E-01 | | | |
| 7993024 | NM_139170 | C16orf71 | chromosome 16 open reading frame | -0.137 | 0.910 | 1.63E-02 | 1.63E+00 | | | |
| 8071234 | NM_002688 | 40426 | septin 5 | -0.137 | 0.910 | 4.57E-03 | 4.57E-01 | | | |
| 7940441 | NM_014224 | PGA5 | pepsinogen 5, group I (pepsinogen A | -0.137 | 0.910 | 2.06E-02 | 2.06E+00 | | | |
| 8160823 | NM_147164 | CNTFR | ciliary neurotrophic factor receptor | -0.137 | 0.910 | 9.66E-03 | 9.66E-01 | | | |
| 8042532 | NM_012476 | VAX2 | ventral anterior homeobox 2 | -0.137 | 0.910 | 1.44E-02 | 1.44E+00 | | | |
| 8076513 | --- | --- | --- | -0.137 | 0.910 | 1.36E-02 | 1.36E+00 | | | |
| 8007826 | NM_175882 | IMP5 | intramembrane protease 5 | -0.137 | 0.910 | 1.78E-02 | 1.78E+00 | | | |
| 7938291 | NR_002580 | SNORA3 | small nucleolar RNA, H/ACA box 3 | -0.137 | 0.910 | 7.02E-01 | 7.02E+01 | | | |
| 7937485 | NM_020376 | PNPLA2 | patatin-like phospholipase domain co | -0.137 | 0.910 | 2.21E-02 | 2.21E+00 | | | |
| 8004325 | NM_00114376( | EIF5A | eukaryotic translation initiation facto | -0.137 | 0.910 | 4.06E-02 | 4.06E+00 | | | |
| 7941269 | ENST00000309 | OC10029185 | similar to Putative ubiquitin-like prot | -0.137 | 0.910 | 3.12E-02 | 3.12E+00 | | | |
| 8172713 | XM_293416 | LOC347549 | hypothetical LOC347549 | -0.137 | 0.910 | 2.98E-02 | 2.98E+00 | | | |
| 8007637 | NM_001466 | FZD2 | frizzled homolog 2 (Drosophila) | -0.137 | 0.910 | 1.61E-02 | 1.61E+00 | | | |
| 8090162 | NM_002213 | ITGB5 | integrin, beta 5 | -0.137 | 0.910 | 6.35E-02 | 6.35E+00 | | | |
| 8163618 | NM_005118 | TNFSF15 | tumor necrosis factor (ligand) superf | -0.137 | 0.910 | 2.61E-02 | 2.61E+00 | | | |
| 8017393 | NM_030576 | LIMD2 | LIM domain containing 2 | -0.137 | 0.910 | 2.07E-02 | 2.07E+00 | | | |
| 7915032 | NM_00103863 | RSPO1 | R-spondin homolog (Xenopus laevis) | -0.137 | 0.910 | 1.73E-02 | 1.73E+00 | | | |
| 7896017 | --- | --- | --- | -0.137 | 0.910 | 3.49E-01 | 3.49E+01 | | | |
| 8088680 | NM_173654 | C3orf64 | chromosome 3 open reading frame 6 | -0.137 | 0.910 | 1.62E-01 | 1.62E+01 | | | |
| 7992782 | NM_020982 | CLDN9 | claudin 9 | -0.137 | 0.910 | 2.02E-02 | 2.02E+00 | | | |
| 8093425 | NM_000203 | IDUA | iduronidase, alpha-L- | -0.137 | 0.910 | 2.47E-02 | 2.47E+00 | | | |
| 8001373 | NM_153337 | SNX20 | sorting nexin 20 | -0.137 | 0.909 | 5.44E-02 | 5.44E+00 | | | |
| 7980051 | NM_194278 | C14orf43 | chromosome 14 open reading frame | -0.137 | 0.909 | 6.03E-02 | 6.03E+00 | | | |
| 8075126 | NM_002430 | MN1 | meningioma (disrupted in balanced t | -0.137 | 0.909 | 9.22E-03 | 9.22E-01 | | | |
| 7896024 | --- | --- | --- | -0.137 | 0.909 | 1.71E-01 | 1.71E+01 | | | |
| 7908993 | NM_005686 | SOX13 | SRY (sex determining region Y)-box 1 | -0.137 | 0.909 | 1.45E-02 | 1.45E+00 | | | |
| 8000772 | NM_031478 | FAM57B | family with sequence similarity 57, m | -0.137 | 0.909 | 2.24E-02 | 2.24E+00 | | | |
| 7950921 | NM_00114383 | GRM5 | glutamate receptor, metabotropic 5 | -0.137 | 0.909 | 1.66E-02 | 1.66E+00 | | | |
| 7896882 | NM_00113041 | SCNN1D | sodium channel, nonvoltage-gated 1 | -0.137 | 0.909 | 1.92E-02 | 1.92E+00 | | | |
| 7905536 | NM_173080 | SPRR4 | small proline-rich protein 4 | -0.137 | 0.909 | 7.38E-02 | 7.38E+00 | | | |
| 8005833 | NR_003064 | PYY2 | peptide YY, 2 (seminalplasmin) | -0.137 | 0.909 | 1.63E-02 | 1.63E+00 | | | |
| 8013606 | NM_000638 | VTN | vitronectin | -0.137 | 0.909 | 5.32E-02 | 5.32E+00 | | | |
| 7956285 | AF529371 | HBCBP | HBcAg-binding protein | -0.137 | 0.909 | 3.91E-02 | 3.91E+00 | | | |
| 8012166 | NM_032442 | NEURL4 | neuralized homolog 4 (Drosophila) | -0.137 | 0.909 | 8.87E-02 | 8.87E+00 | | | |
| 8180377 | --- | --- | --- | -0.137 | 0.909 | 4.41E-01 | 4.41E+01 | | | |
| 8036707 | NM_020862 | LRFN1 | leucine rich repeat and fibronectin ty | -0.137 | 0.909 | 3.05E-02 | 3.05E+00 | | | |
| 8035600 | NM_017814 | TMEM161A | transmembrane protein 161A | -0.137 | 0.909 | 2.06E-02 | 2.06E+00 | | | |
| 7962918 | NM_003394 | WNT10B | wingless-type MMTV integration site | -0.137 | 0.909 | 7.18E-02 | 7.18E+00 | | | |
| 7998457 | NM_023076 | UNKL | unkempt homolog (Drosophila)-like | -0.137 | 0.909 | 3.89E-02 | 3.89E+00 | | | |
| 7929649 | NM_024954 | UBTD1 | ubiquitin domain containing 1 | -0.137 | 0.909 | 1.61E-02 | 1.61E+00 | | | |
| 7942300 | NM_173042 | IL18BP | interleukin 18 binding protein | -0.137 | 0.909 | 2.50E-02 | 2.50E+00 | | | |
| 8086985 | NM_000094 | COL7A1 | collagen, type VII, alpha 1 | -0.137 | 0.909 | 1.30E-02 | 1.30E+00 | | | |
| 7945742 | NM_014555 | TRPM5 | transient receptor potential cation cl | -0.137 | 0.909 | 1.40E-02 | 1.40E+00 | | | |
| 8087481 | NM_00100754( | CDH29 | cadherin-like 29 | -0.137 | 0.909 | 2.63E-02 | 2.63E+00 | | | |
| 7971345 | ENST00000400 | MGC5590 | hypothetical protein MGC5590 | -0.137 | 0.909 | 3.84E-02 | 3.84E+00 | | | |
| 8004946 | --- | --- | --- | -0.137 | 0.909 | 6.70E-02 | 6.70E+00 | | | |
| 7895478 | --- | --- | --- | -0.137 | 0.909 | 5.64E-01 | 5.64E+01 | | | |
| 8098822 | NM_001347 | DGKQ | diacylglycerol kinase, theta 110kDa | -0.137 | 0.909 | 3.34E-03 | 3.34E-01 | | | |
| 8004024 | NM_000173 | GP1BA | glycoprotein Ib (platelet), alpha poly | -0.137 | 0.909 | 1.86E-01 | 1.86E+01 | | | |
| 8033319 | NM_005490 | SH2D3A | SH2 domain containing 3A | -0.137 | 0.909 | 3.99E-02 | 3.99E+00 | | | |
| 7915252 | NM_001720 | BMP8B | bone morphogenetic protein 8b | -0.137 | 0.909 | 7.82E-02 | 7.82E+00 | | | |
| 7893675 | --- | --- | --- | -0.137 | 0.909 | 1.04E-01 | 1.04E+01 | | | |
| 8065603 | NR_002781 | TSPYL3 | TSPY-like 3 (pseudogene) | -0.137 | 0.909 | 4.35E-02 | 4.35E+00 | | | |
| 7937433 | NM_000797 | DRD4 | dopamine receptor D4 | -0.137 | 0.909 | 2.89E-02 | 2.89E+00 | | | |
| 7892783 | --- | --- | --- | -0.138 | 0.909 | 2.62E-01 | 2.62E+01 | | | |
| 8101609 | NM_197965 | SLC10A6 | solute carrier family 10 (sodium/bile | -0.138 | 0.909 | 1.79E-02 | 1.79E+00 | | | |
| 7916432 | NM_014762 | DHCR24 | 24-dehydrocholesterol reductase | -0.138 | 0.909 | 3.91E-02 | 3.91E+00 | | | |
| 8090770 | --- | --- | --- | -0.138 | 0.909 | 5.47E-02 | 5.47E+00 | | | |
| 8117286 | --- | --- | --- | -0.138 | 0.909 | 3.49E-01 | 3.49E+01 | | | |
| 7991367 | NM_182616 | C15orf38 | chromosome 15 open reading frame | -0.138 | 0.909 | 4.43E-02 | 4.43E+00 | | | |
| 8140144 | NM_152559 | WBSCR27 | Williams Beuren syndrome chromoso | -0.138 | 0.909 | 1.83E-02 | 1.83E+00 | | | |
| 8102368 | NM_024019 | NEUROG2 | neurogenin 2 | -0.138 | 0.909 | 1.66E-02 | 1.66E+00 | | | |
| 7953516 | NR_002803 | RPL13P5 | ribosomal protein L13 pseudogene 5 | -0.138 | 0.909 | 5.20E-02 | 5.20E+00 | | | |
| 8054740 | NM_003466 | PAX8 | paired box 8 | -0.138 | 0.909 | 6.00E-03 | 6.00E-01 | | | |
| 7997801 | NM_013278 | IL17C | interleukin 17C | -0.138 | 0.909 | 1.38E-02 | 1.38E+00 | | | |
| 8116591 | NM_001453 | FOXC1 | forkhead box C1 | -0.138 | 0.909 | 8.03E-03 | 8.03E-01 | | | |
| 8116818 | NM_001718 | BMP6 | bone morphogenetic protein 6 | -0.138 | 0.909 | 1.82E-01 | 1.82E+01 | | | |
| 7926706 | --- | --- | --- | -0.138 | 0.909 | 2.00E-01 | 2.00E+01 | | | |
| 8070579 | NM_003225 | TFF1 | trefoil factor 1 | -0.138 | 0.909 | 3.62E-02 | 3.62E+00 | | | |
| 8155349 | NM_015667 // | 75A7 // FAM | family with sequence similarity 75, m | -0.138 | 0.909 | 6.72E-03 | 6.72E-01 | | | |
| 7940654 | NM_003357 | SCGB1A1 | secretoglobin, family 1A, member 1 ( | -0.138 | 0.909 | 1.96E-02 | 1.96E+00 | | | |
| 8062782 | NM_032883 | TOX2 | TOX high mobility group box family m | -0.138 | 0.909 | 2.55E-02 | 2.55E+00 | | | |
| 7996647 | NM_016948 | PARD6A | par-6 partitioning defective 6 homol | -0.138 | 0.909 | 5.86E-02 | 5.86E+00 | | | |

| | | | | | | | | | | |
|---|---|---|---|---|---|---|---|---|---|---|
| 8019622 | NM_145041 | TMEM106A | transmembrane protein 106A | -0.138 | 0.909 | 1.14E-01 | 1.14E+01 | | | |
| 8123642 | --- | --- | --- | -0.138 | 0.909 | 7.73E-01 | 7.73E+01 | | | |
| 8007118 | ENST00000318 | KRTAP9-2 | keratin associated protein 9-2 | -0.138 | 0.909 | 7.51E-02 | 7.51E+00 | | | |
| 8019939 | NM_170695 | TGIF1 | TGFB-induced factor homeobox 1 | -0.138 | 0.909 | 4.83E-02 | 4.83E+00 | | | |
| 8131944 | NM_004289 | NFE2L3 | nuclear factor (erythroid-derived 2)- | -0.138 | 0.909 | 5.62E-01 | 5.62E+01 | | | |
| 7935990 | NM_002779 | PSD | pleckstrin and Sec7 domain containi | -0.138 | 0.909 | 4.15E-02 | 4.15E+00 | | | |
| 8123891 | --- | --- | --- | -0.138 | 0.909 | 3.34E-01 | 3.34E+01 | | | |
| 8148018 | --- | --- | --- | -0.138 | 0.909 | 3.47E-01 | 3.47E+01 | | | |
| 8120215 | NM_133367 | PAQR8 | progestin and adipoQ receptor famil | -0.138 | 0.909 | 1.01E-02 | 1.01E+00 | | | |
| 8098163 | --- | --- | --- | -0.138 | 0.909 | 4.65E-01 | 4.65E+01 | | | |
| 7895971 | --- | --- | --- | -0.138 | 0.909 | 8.88E-01 | 8.88E+01 | | | |
| 7919598 | --- | --- | --- | -0.138 | 0.909 | 1.27E-01 | 1.27E+01 | | | |
| 7937404 | BC039077 | C11orf35 | chromosome 11 open reading frame | -0.138 | 0.909 | 4.71E-02 | 4.71E+00 | | | |
| 8101000 | ENST00000437 | LOC541467 | hypothetical LOC541467 | -0.138 | 0.909 | 3.81E-02 | 3.81E+00 | | | |
| 8153838 | NM_004260 | RECQL4 | RecQ protein-like 4 | -0.138 | 0.909 | 1.02E-02 | 1.02E+00 | | | |
| 8031768 | NM_00101087 | ZIK1 | zinc finger protein interacting with K | -0.138 | 0.909 | 5.00E-02 | 5.00E+00 | | | |
| 8152333 | --- | --- | --- | -0.138 | 0.909 | 4.24E-01 | 4.24E+01 | | | |
| 8049128 | NM_031313 | ALPPL2 | alkaline phosphatase, placental-like | -0.138 | 0.909 | 5.33E-02 | 5.33E+00 | | | |
| 7898167 | NM_007272 | CTRC | chymotrypsin C (caldecrin) | -0.138 | 0.909 | 5.08E-02 | 5.08E+00 | | | |
| 8043621 | NM_212481 | ARID5A | AT rich interactive domain 5A (MRF1 | -0.138 | 0.909 | 2.00E-02 | 2.00E+00 | | | |
| 7950420 | NM_00114486 | LOC387787 | lipoyl(octanoyl) transferase | -0.138 | 0.909 | 4.37E-02 | 4.37E+00 | | | |
| 7998307 | NM_00103173 | CCDC78 | coiled-coil domain containing 78 | -0.138 | 0.909 | 1.27E-02 | 1.27E+00 | | | |
| 8011626 | NM_003342 | UBE2G1 | ubiquitin-conjugating enzyme E2G 1 | -0.138 | 0.909 | 7.35E-01 | 7.35E+01 | | | |
| 7983252 | --- | --- | --- | -0.138 | 0.909 | 1.00E-01 | 1.00E+01 | | | |
| 8178554 | NM_025262 | LY6G5C | lymphocyte antigen 6 complex, locu | -0.138 | 0.909 | 3.00E-01 | 3.00E+01 | | | |
| 7947784 | NM_032389 | ARFGAP2 | ADP-ribosylation factor GTPase activ | -0.138 | 0.909 | 1.27E-01 | 1.27E+01 | | | |
| 7904463 | --- | --- | --- | -0.138 | 0.909 | 5.04E-02 | 5.04E+00 | | | |
| 7933501 | NM_00108052 | DRGX | dorsal root ganglia homeobox | -0.138 | 0.909 | 2.20E-02 | 2.20E+00 | | | |
| 8003145 | NM_139174 | ADAD2 | adenosine deaminase domain contai | -0.138 | 0.909 | 1.92E-01 | 1.92E+01 | | | |
| 8015301 | NM_002280 | KRT35 | keratin 35 | -0.138 | 0.909 | 5.63E-02 | 5.63E+00 | | | |
| 8141140 | NM_005221 | DLX5 | distal-less homeobox 5 | -0.138 | 0.909 | 3.21E-02 | 3.21E+00 | | | |
| 8150881 | NM_002655 | PLAG1 | pleiomorphic adenoma gene 1 | -0.138 | 0.909 | 1.65E-01 | 1.65E+01 | | | |
| 7899075 | NM_004455 | EXTL1 | exostoses (multiple)-like 1 | -0.138 | 0.909 | 1.71E-02 | 1.71E+00 | | | |
| 8116439 | NM_052863 | SCGB3A1 | secretoglobin, family 3A, member 1 | -0.138 | 0.909 | 2.04E-02 | 2.04E+00 | | | |
| 8086352 | NM_017886 | ULK4 | unc-51-like kinase 4 (C. elegans) | -0.138 | 0.909 | 3.75E-01 | 3.75E+01 | | | |
| 8024655 | NM_00113558 | C19orf71 | chromosome 19 open reading frame | -0.138 | 0.909 | 2.23E-02 | 2.23E+00 | | | |
| 8180334 | --- | --- | --- | -0.138 | 0.909 | 4.55E-01 | 4.55E+01 | | | |
| 8029671 | NM_177417 | KLC3 | kinesin light chain 3 | -0.138 | 0.909 | 1.04E-02 | 1.04E+00 | | | |
| 8150901 | NM_006211 | PENK | proenkephalin | -0.138 | 0.909 | 1.64E-02 | 1.64E+00 | | | |
| 8180234 | --- | --- | --- | -0.138 | 0.909 | 4.39E-02 | 4.39E+00 | | | |
| 7920077 | NM_00100443 | LINGO4 | leucine rich repeat and Ig domain co | -0.138 | 0.909 | 2.65E-02 | 2.65E+00 | | | |
| 8037563 | NM_006663 | PPP1R13L | protein phosphatase 1, regulatory (i | -0.138 | 0.909 | 1.52E-02 | 1.52E+00 | | | |
| 7981377 | NM_152326 | ANKRD9 | ankyrin repeat domain 9 | -0.138 | 0.909 | 1.89E-02 | 1.89E+00 | | | |
| 8025506 | NM_006221 | PIN1 | peptidylprolyl cis/trans isomerase, N | -0.138 | 0.909 | 3.41E-02 | 3.41E+00 | | | |
| 8037079 | NM_152296 | ATP1A3 | ATPase, Na+/K+ transporting, alpha | -0.138 | 0.909 | 3.86E-02 | 3.86E+00 | | | |
| 8095758 | NM_015436 | RCHY1 | ring finger and CHY zinc finger doma | -0.138 | 0.909 | 8.70E-01 | 8.70E+01 | | | |
| 8053869 | NM_000682 | ADRA2B | adrenergic, alpha-2B-, receptor | -0.138 | 0.909 | 7.22E-03 | 7.22E-01 | | | |
| 8011114 | NM_145351 | SCARF1 | scavenger receptor class F, member | -0.138 | 0.908 | 5.87E-03 | 5.87E-01 | | | |
| 8062427 | NM_080607 | VSTM2L | V-set and transmembrane domain c | -0.138 | 0.908 | 1.31E-02 | 1.31E+00 | | | |
| 7951612 | AK293317 | LAYN | layilin | -0.138 | 0.908 | 2.62E-02 | 2.62E+00 | | | |
| 7894520 | --- | --- | --- | -0.138 | 0.908 | 7.02E-01 | 7.02E+01 | | | |
| 8060805 | NM_001819 | CHGB | chromogranin B (secretogranin 1) | -0.138 | 0.908 | 4.10E-02 | 4.10E+00 | | | |
| 8010512 | NM_020761 | RPTOR | regulatory associated protein of MT | -0.138 | 0.908 | 9.60E-02 | 9.60E+00 | | | |
| 8047692 | NM_005214 | CTLA4 | cytotoxic T-lymphocyte-associated p | -0.139 | 0.908 | 4.88E-01 | 4.88E+01 | | | |
| 8158123 | NM_004957 | FPGS | folylpolyglutamate synthase | -0.139 | 0.908 | 5.47E-03 | 5.47E-01 | | | |
| 8109201 | NM_014983 | HMGXB3 | HMG box domain containing 3 | -0.139 | 0.908 | 3.16E-02 | 3.16E+00 | | | |
| 8155442 | NR_002817 | AQP7P1 | aquaporin 7 pseudogene 1 | -0.139 | 0.908 | 2.09E-02 | 2.09E+00 | | | |
| 7905512 | NM_00102467 | C1orf68 | chromosome 1 open reading frame | -0.139 | 0.908 | 1.78E-02 | 1.78E+00 | | | |
| 8061519 | NM_153324 | DEFB123 | defensin, beta 123 | -0.139 | 0.908 | 1.29E-02 | 1.29E+00 | | | |
| 7972745 | NM_003749 | IRS2 | insulin receptor substrate 2 | -0.139 | 0.908 | 1.69E-02 | 1.69E+00 | | | |
| 8125149 | NM_025257 | SLC44A4 | solute carrier family 44, member 4 | -0.139 | 0.908 | 2.56E-02 | 2.56E+00 | | | |
| 8178653 | NM_025257 | SLC44A4 | solute carrier family 44, member 4 | -0.139 | 0.908 | 2.56E-02 | 2.56E+00 | | | |
| 8179861 | NM_025257 | SLC44A4 | solute carrier family 44, member 4 | -0.139 | 0.908 | 2.56E-02 | 2.56E+00 | | | |
| 7931683 | NM_014974 | DIP2C | DIP2 disco-interacting protein 2 hom | -0.139 | 0.908 | 5.86E-03 | 5.86E-01 | | | |
| 8038852 | NM_053003 | SIGLEC12 | sialic acid binding Ig-like lectin 12 | -0.139 | 0.908 | 3.38E-02 | 3.38E+00 | | | |
| 7911799 | NM_001409 | MEGF6 | multiple EGF-like-domains 6 | -0.139 | 0.908 | 1.82E-02 | 1.82E+00 | | | |
| 8024792 | NM_005755 | EBI3 | Epstein-Barr virus induced 3 | -0.139 | 0.908 | 3.43E-02 | 3.43E+00 | | | |
| 8070896 | AF427488 | C21orf93 | chromosome 21 open reading frame | -0.139 | 0.908 | 2.77E-02 | 2.77E+00 | | | |
| 8093074 | NM_00103961 | ZDHHC19 | zinc finger, DHHC-type containing 19 | -0.139 | 0.908 | 6.19E-03 | 6.19E-01 | | | |
| 8010354 | NM_000152 | GAA | glucosidase, alpha; acid | -0.139 | 0.908 | 1.91E-02 | 1.91E+00 | | | |
| 8034263 | NM_001420 | ELAVL3 | ELAV (embryonic lethal, abnormal vi | -0.139 | 0.908 | 4.58E-02 | 4.58E+00 | | | |
| 8007263 | NM_000413 | HSD17B1 | hydroxysteroid (17-beta) dehydroge | -0.139 | 0.908 | 3.65E-02 | 3.65E+00 | | | |
| 8029301 | NM_174945 | ZNF575 | zinc finger protein 575 | -0.139 | 0.908 | 2.91E-02 | 2.91E+00 | | | |
| 8144317 | NM_014867 | KBTBD11 | kelch repeat and BTB (POZ) domain | -0.139 | 0.908 | 1.92E-02 | 1.92E+00 | | | |
| 8118655 | NM_003782 | B3GALT4 | UDP-Gal:betaGlcNAc beta 1,3-galact | -0.139 | 0.908 | 4.48E-02 | 4.48E+00 | | | |
| 8178264 | NM_003782 | B3GALT4 | UDP-Gal:betaGlcNAc beta 1,3-galact | -0.139 | 0.908 | 4.48E-02 | 4.48E+00 | | | |
| 8179555 | NM_003782 | B3GALT4 | UDP-Gal:betaGlcNAc beta 1,3-galact | -0.139 | 0.908 | 4.48E-02 | 4.48E+00 | | | |

| | | | | | | | | | | | |
|---|---|---|---|---|---|---|---|---|---|---|---|
| 8011320 | NM_014566 | OR1D5 | olfactory receptor, family 1, subfami | -0.139 | 0.908 | 1.04E-01 | 1.04E+01 | | | | |
| 7999112 | NM_016069 | Magmas | mitochondria-associated protein inv | -0.139 | 0.908 | 5.19E-01 | 5.19E+01 | | | | |
| 7941761 | NM_014578 | RHOD | ras homolog gene family, member D | -0.139 | 0.908 | 2.02E-02 | 2.02E+00 | | | | |
| 8112176 | NR_023344 // | RNU6ATAC | RNA, U6atac small nuclear (U12-dep | -0.139 | 0.908 | 5.32E-01 | 5.32E+01 | | | | |
| 7894984 | --- | --- | --- | -0.139 | 0.908 | 7.02E-01 | 7.02E+01 | | | | |
| 8137381 | NM_031946 | AGAP3 | ArfGAP with GTPase domain, ankyrin | -0.139 | 0.908 | 2.47E-02 | 2.47E+00 | | | | |
| 8180370 | --- | --- | --- | -0.139 | 0.908 | 5.27E-02 | 5.27E+00 | | | | |
| 8030158 | NR_027882 | BAX | BCL2-associated X protein | -0.139 | 0.908 | 9.03E-02 | 9.03E+00 | | | | |
| 7963121 | NM_00103780 | NCKAP5L | NCK-associated protein 5-like | -0.139 | 0.908 | 1.32E-02 | 1.32E+00 | | | | |
| 7911854 | NM_207306 | KIAA0495 | KIAA0495 | -0.139 | 0.908 | 8.82E-02 | 8.82E+00 | | | | |
| 8065795 | NR_002165 | HMGB3L1 | high-mobility group box 3-like 1 | -0.139 | 0.908 | 5.25E-02 | 5.25E+00 | | | | |
| 8126296 | NR_027256 | TREML3 | triggering receptor expressed on my | -0.139 | 0.908 | 1.22E-01 | 1.22E+01 | | | | |
| 7982529 | ENST0000045 | LOC441722 | U2 small nuclear RNA auxiliary factor | -0.139 | 0.908 | 3.74E-01 | 3.74E+01 | | | | |
| 8086595 | NM_005283 | XCR1 | chemokine (C motif) receptor 1 | -0.139 | 0.908 | 1.71E-02 | 1.71E+00 | | | | |
| 7966052 | NM_004075 | CRY1 | cryptochrome 1 (photolyase-like) | -0.139 | 0.908 | 3.39E-01 | 3.39E+01 | | | | |
| 7924863 | NM_052843 | OBSCN | obscurin, cytoskeletal calmodulin an | -0.139 | 0.908 | 5.63E-02 | 5.63E+00 | | | | |
| 8039362 | NM_178837 | C19orf51 | chromosome 19 open reading frame | -0.139 | 0.908 | 2.17E-02 | 2.17E+00 | | | | |
| 8149228 | NR_027000 | LOC349196 | hypothetical LOC349196 | -0.139 | 0.908 | 3.09E-01 | 3.09E+01 | | | | |
| 7908694 | NM_020443 | NAV1 | neuron navigator 1 | -0.139 | 0.908 | 1.76E-02 | 1.76E+00 | | | | |
| 8009517 | NM_000346 | SOX9 | SRY (sex determining region Y)-box 9 | -0.139 | 0.908 | 9.61E-03 | 9.61E-01 | | | | |
| 8151627 | 0 | 0 | 0 | -0.139 | 0.908 | 4.28E-02 | 4.28E+00 | | | | |
| 8081686 | NM_033254 | BOC | Boc homolog (mouse) | -0.139 | 0.908 | 2.76E-02 | 2.76E+00 | | | | |
| 7896005 | --- | --- | --- | -0.139 | 0.908 | 8.39E-01 | 8.39E+01 | | | | |
| 8024754 | NM_032607 | CREB3L3 | cAMP responsive element binding pr | -0.139 | 0.908 | 1.71E-02 | 1.71E+00 | | | | |
| 8173755 | NM_004867 | ITM2A | integral membrane protein 2A | -0.139 | 0.908 | 6.62E-01 | 6.62E+01 | | | | |
| 7895972 | --- | --- | --- | -0.139 | 0.908 | 4.59E-01 | 4.59E+01 | | | | |
| 8124865 | NM_014069 | PSORS1C2 | psoriasis susceptibility 1 candidate 2 | -0.139 | 0.908 | 1.63E-02 | 1.63E+00 | | | | |
| 8064894 | NM_152611 | LRRN4 | leucine rich repeat neuronal 4 | -0.139 | 0.908 | 1.62E-02 | 1.62E+00 | | | | |
| 8116272 | NM_014244 | ADAMTS2 | ADAM metallopeptidase with throm | -0.139 | 0.908 | 7.68E-03 | 7.68E-01 | | | | |
| 8013858 | ENST0000043 | LOC10013079 | NADH dehydrogenase (ubiquinone) | -0.139 | 0.908 | 1.44E-01 | 1.44E+01 | | | | |
| 8025328 | NM_00115994 | EVI5L | ecotropic viral integration site 5-like | -0.139 | 0.908 | 1.28E-02 | 1.28E+00 | | | | |
| 8077970 | NM_00100401 | FBLN2 | fibulin 2 | -0.139 | 0.908 | 5.80E-03 | 5.80E-01 | | | | |
| 7947076 | NM_006906 | PTPN5 | protein tyrosine phosphatase, non-re | -0.139 | 0.908 | 1.53E-02 | 1.53E+00 | | | | |
| 7943998 | NM_006169 | NNMT | nicotinamide N-methyltransferase | -0.139 | 0.908 | 1.44E-02 | 1.44E+00 | | | | |
| 8083324 | NM_014779 | TSC22D2 | TSC22 domain family, member 2 | -0.139 | 0.908 | 2.37E-02 | 2.37E+00 | | | | |
| 7949570 | NM_033036 | GAL3ST3 | galactose-3-O-sulfotransferase 3 | -0.139 | 0.908 | 1.30E-02 | 1.30E+00 | | | | |
| 7991540 | --- | --- | --- | -0.140 | 0.908 | 1.91E-01 | 1.91E+01 | | | | |
| 7940662 | NM_000327 | ROM1 | retinal outer segment membrane pro | -0.140 | 0.908 | 3.66E-02 | 3.66E+00 | | | | |
| 8076584 | AK056490 | FLJ30901 | hypothetical protein FLJ30901 | -0.140 | 0.908 | 2.82E-02 | 2.82E+00 | | | | |
| 7959921 | AK098523 | LOC338797 | hypothetical LOC338797 | -0.140 | 0.908 | 4.47E-02 | 4.47E+00 | | | | |
| 7894169 | --- | --- | --- | -0.140 | 0.908 | 4.58E-01 | 4.58E+01 | | | | |
| 7992716 | NM_00113508 | TESSP1 | testis serine protease 1 | -0.140 | 0.908 | 1.82E-02 | 1.82E+00 | | | | |
| 8077490 | NM_014583 | LMCD1 | LIM and cysteine-rich domains 1 | -0.140 | 0.908 | 2.79E-02 | 2.79E+00 | | | | |
| 8062658 | NM_022896 | LPIN3 | lipin 3 | -0.140 | 0.908 | 1.54E-02 | 1.54E+00 | | | | |
| 7998643 | NM_005061 | RPL3L | ribosomal protein L3-like | -0.140 | 0.908 | 2.03E-02 | 2.03E+00 | | | | |
| 8134712 | NM_004722 | AP4M1 | adaptor-related protein complex 4, r | -0.140 | 0.908 | 1.41E-02 | 1.41E+00 | | | | |
| 7986446 | NM_000693 | ALDH1A3 | aldehyde dehydrogenase 1 family, m | -0.140 | 0.908 | 2.34E-02 | 2.34E+00 | | | | |
| 8016615 | NM_174920 | SAMD14 | sterile alpha motif domain containin | -0.140 | 0.908 | 8.88E-03 | 8.88E-01 | | | | |
| 7950003 | NM_198923 | MRGPRD | MAS-related GPR, member D | -0.140 | 0.908 | 5.62E-02 | 5.62E+00 | | | | |
| 8001046 | NM_00113650 | ZNF843 | zinc finger protein 843 | -0.140 | 0.908 | 2.54E-02 | 2.54E+00 | | | | |
| 8157798 | --- | --- | --- | -0.140 | 0.908 | 4.50E-02 | 4.50E+00 | | | | |
| 7981917 | NM_018958 | C15orf2 | chromosome 15 open reading frame | -0.140 | 0.908 | 2.27E-02 | 2.27E+00 | | | | |
| 8015456 | NM_018143 | KLHL11 | kelch-like 11 (Drosophila) | -0.140 | 0.908 | 3.27E-01 | 3.27E+01 | | | | |
| 7938420 | --- | --- | --- | -0.140 | 0.908 | 2.68E-01 | 2.68E+01 | | | | |
| 8143714 | NM_015694 | ZNF777 | zinc finger protein 777 | -0.140 | 0.908 | 1.74E-01 | 1.74E+01 | | | | |
| 7966929 | NM_006861 | RAB35 | RAB35, member RAS oncogene famil | -0.140 | 0.908 | 1.51E-01 | 1.51E+01 | | | | |
| 8163960 | NM_00100445 | OR1L8 | olfactory receptor, family 1, subfami | -0.140 | 0.908 | 2.63E-02 | 2.63E+00 | | | | |
| 8079988 | BC028000 | C3orf45 | chromosome 3 open reading frame 4 | -0.140 | 0.908 | 3.33E-02 | 3.33E+00 | | | | |
| 8027510 | NM_152266 | C19orf40 | chromosome 19 open reading frame | -0.140 | 0.908 | 4.50E-02 | 4.50E+00 | | | | |
| 7999253 | NM_002705 | PPL | periplakin | -0.140 | 0.908 | 2.05E-02 | 2.05E+00 | | | | |
| 8041592 | NM_019063 | EML4 | echinoderm microtubule associated | -0.140 | 0.908 | 5.84E-01 | 5.84E+01 | | | | |
| 8116585 | --- | --- | --- | -0.140 | 0.908 | 5.59E-02 | 5.59E+00 | | | | |
| 7926158 | NM_153256 | C10orf47 | chromosome 10 open reading frame | -0.140 | 0.908 | 1.59E-02 | 1.59E+00 | | | | |
| 7925128 | --- | --- | --- | -0.140 | 0.908 | 1.95E-01 | 1.95E+01 | | | | |
| 8178080 | BC137541 | C6orf26 | chromosome 6 open reading frame 2 | -0.140 | 0.908 | 1.00E-02 | 1.00E+00 | | | | |
| 7998538 | NM_00101365 | C16orf38 | chromosome 16 open reading frame | -0.140 | 0.908 | 1.36E-02 | 1.36E+00 | | | | |
| 8033960 | NM_00103173 | FDX1L | ferredoxin 1-like | -0.140 | 0.908 | 1.81E-02 | 1.81E+00 | | | | |
| 8014214 | NM_00101444 | NLE1 | notchless homolog 1 (Drosophila) | -0.140 | 0.908 | 2.90E-02 | 2.90E+00 | | | | |
| 8033635 | NM_030957 | ADAMTS10 | ADAM metallopeptidase with throm | -0.140 | 0.908 | 1.05E-02 | 1.05E+00 | | | | |
| 8076493 | AF092923 | ATP5L2 | ATP synthase, H+ transporting, mito | -0.140 | 0.908 | 2.47E-01 | 2.47E+01 | | | | |
| 7928529 | --- | --- | --- | -0.140 | 0.908 | 1.76E-02 | 1.76E+00 | | | | |
| 8073449 | NM_014460 | CSDC2 | cold shock domain containing C2, RN | -0.140 | 0.908 | 1.87E-02 | 1.87E+00 | | | | |
| 8030078 | NM_020126 | SPHK2 | sphingosine kinase 2 | -0.140 | 0.907 | 4.28E-02 | 4.28E+00 | | | | |
| 8027920 | NM_014209 | ETV2 | ets variant 2 | -0.140 | 0.907 | 2.10E-02 | 2.10E+00 | | | | |
| 8138920 | --- | --- | --- | -0.140 | 0.907 | 5.93E-02 | 5.93E+00 | | | | |
| 8005097 | NM_006041 | HS3ST3B1 | heparan sulfate (glucosamine) 3-O-s | -0.140 | 0.907 | 1.31E-01 | 1.31E+01 | | | | |
| 8034920 | NM_006844 | ILVBL | ilvB (bacterial acetolactate synthase) | -0.140 | 0.907 | 4.66E-02 | 4.66E+00 | | | | |

| | | | | | | | | | | |
|---|---|---|---|---|---|---|---|---|---|---|
| 8007750 | NM_144608 | HEXIM2 | hexamthylene bis-acetamide inducib | -0.140 | 0.907 | 2.85E-02 | 2.85E+00 | | | |
| 8039520 | NM_00110140 | SBK2 | SH3-binding domain kinase family, m | -0.140 | 0.907 | 4.98E-02 | 4.98E+00 | | | |
| 8173174 | NM_201286 | USP51 | ubiquitin specific peptidase 51 | -0.140 | 0.907 | 2.20E-02 | 2.20E+00 | | | |
| 8010918 | NM_00112815 | VPS53 | vacuolar protein sorting 53 homolog | -0.140 | 0.907 | 6.92E-01 | 6.92E+01 | | | |
| 8060528 | NM_080739 | C20orf141 | chromosome 20 open reading frame | -0.140 | 0.907 | 3.12E-02 | 3.12E+00 | | | |
| 7990442 | NM_017793 | RPP25 | ribonuclease P/MRP 25kDa subunit | -0.140 | 0.907 | 5.86E-02 | 5.86E+00 | | | |
| 8028546 | NM_002307 | LGALS7 | lectin, galactoside-binding, soluble, 7 | -0.140 | 0.907 | 2.54E-02 | 2.54E+00 | | | |
| 8141076 | NM_000305 | PON2 | paraoxonase 2 | -0.140 | 0.907 | 1.25E-01 | 1.25E+01 | | | |
| 8062823 | NM_000457 | HNF4A | hepatocyte nuclear factor 4, alpha | -0.140 | 0.907 | 5.08E-03 | 5.08E-01 | | | |
| 8037166 | NM_006494 | ERF | Ets2 repressor factor | -0.140 | 0.907 | 1.21E-01 | 1.21E+01 | | | |
| 8077226 | NM_032329 | ING5 | inhibitor of growth family, member 5 | -0.140 | 0.907 | 2.37E-02 | 2.37E+00 | | | |
| 7986248 | AY358245 | UNQ6190 | AGVR6190 | -0.140 | 0.907 | 5.30E-02 | 5.30E+00 | | | |
| 7953564 | NM_138425 | C12orf57 | chromosome 12 open reading frame | -0.140 | 0.907 | 5.69E-01 | 5.69E+01 | | | |
| 7998700 | NM_00100994 | PKD1 | polycystic kidney disease 1 (autosom | -0.140 | 0.907 | 4.00E-02 | 4.00E+00 | | | |
| 8136161 | --- | --- | --- | -0.140 | 0.907 | 2.50E-01 | 2.50E+01 | | | |
| 8126382 | ENST0000034 | C6orf132 | chromosome 6 open reading frame 1 | -0.140 | 0.907 | 1.64E-02 | 1.64E+00 | | | |
| 8086051 | NM_015551 | SUSD5 | sushi domain containing 5 | -0.140 | 0.907 | 3.22E-02 | 3.22E+00 | | | |
| 7977978 | NM_005864 | EFS | embryonal Fyn-associated substrate | -0.140 | 0.907 | 1.27E-02 | 1.27E+00 | | | |
| 8158637 | AL834139 | HMCN2 | hemicentin 2 | -0.140 | 0.907 | 1.54E-02 | 1.54E+00 | | | |
| 7896372 | --- | --- | --- | -0.140 | 0.907 | 8.79E-01 | 8.79E+01 | | | |
| 8011193 | NM_032895 | C17orf91 | chromosome 17 open reading frame | -0.140 | 0.907 | 1.15E-01 | 1.15E+01 | | | |
| 8005247 | NM_020201 | NT5M | 5',3'-nucleotidase, mitochondrial | -0.141 | 0.907 | 7.15E-03 | 7.15E-01 | | | |
| 8011262 | NM_020310 | MNT | MAX binding protein | -0.141 | 0.907 | 2.08E-02 | 2.08E+00 | | | |
| 7932177 | NM_00102995 | CDNF | cerebral dopamine neurotrophic fact | -0.141 | 0.907 | 1.53E-01 | 1.53E+01 | | | |
| 8058973 | NM_00110553 | ZNF142 | zinc finger protein 142 | -0.141 | 0.907 | 2.91E-02 | 2.91E+00 | | | |
| 7923907 | NM_000572 | IL10 | interleukin 10 | -0.141 | 0.907 | 1.47E-01 | 1.47E+01 | | | |
| 7956044 | --- | --- | --- | -0.141 | 0.907 | 8.66E-02 | 8.66E+00 | | | |
| 7916654 | NM_181712 | KANK4 | KN motif and ankyrin repeat domain | -0.141 | 0.907 | 1.23E-02 | 1.23E+00 | | | |
| 8108900 | NM_021182 | HMHB1 | histocompatibility (minor) HB-1 | -0.141 | 0.907 | 1.27E-02 | 1.27E+00 | | | |
| 7912252 | --- | --- | --- | -0.141 | 0.907 | 2.55E-01 | 2.55E+01 | | | |
| 8156247 | NM_178828 | C9orf79 | chromosome 9 open reading frame 7 | -0.141 | 0.907 | 2.27E-02 | 2.27E+00 | | | |
| 8151540 | --- | --- | --- | -0.141 | 0.907 | 3.92E-01 | 3.92E+01 | | | |
| 8003298 | NM_003486 | SLC7A5 | solute carrier family 7 (cationic amin | -0.141 | 0.907 | 8.38E-02 | 8.38E+00 | | | |
| 7924832 | NM_003395 | WNT9A | wingless-type MMTV integration site | -0.141 | 0.907 | 5.36E-03 | 5.36E-01 | | | |
| 8030015 | NM_012451 | SYNGR4 | synaptogyrin 4 | -0.141 | 0.907 | 3.09E-02 | 3.09E+00 | | | |
| 8054467 | NM_144710 | 40431 | septin 10 | -0.141 | 0.907 | 1.09E-01 | 1.09E+01 | | | |
| 8075211 | NM_00100727 | RASL10A | RAS-like, family 10, member A | -0.141 | 0.907 | 3.14E-02 | 3.14E+00 | | | |
| 7951966 | NM_00112748 | FXYD2 | FXYD domain containing ion transpo | -0.141 | 0.907 | 5.83E-02 | 5.83E+00 | | | |
| 7935983 | NM_005029 | PITX3 | paired-like homeodomain 3 | -0.141 | 0.907 | 1.85E-02 | 1.85E+00 | | | |
| 8061366 | NM_001052 | SSTR4 | somatostatin receptor 4 | -0.141 | 0.907 | 3.39E-02 | 3.39E+00 | | | |
| 7985571 | NR_026811 | LOC80154 | hypothetical LOC80154 | -0.141 | 0.907 | 1.35E-02 | 1.35E+00 | | | |
| 8029870 | NM_002517 | NPAS1 | neuronal PAS domain protein 1 | -0.141 | 0.907 | 1.20E-02 | 1.20E+00 | | | |
| 8061706 | NM_015352 | POFUT1 | protein O-fucosyltransferase 1 | -0.141 | 0.907 | 2.08E-02 | 2.08E+00 | | | |
| 8008544 | NM_032559 | KIF2B | kinesin family member 2B | -0.141 | 0.907 | 3.04E-02 | 3.04E+00 | | | |
| 7940491 | --- | --- | --- | -0.141 | 0.907 | 1.45E-01 | 1.45E+01 | | | |
| 8003560 | NM_024043 | DBNDD1 | dysbindin (dystrobrevin binding prot | -0.141 | 0.907 | 1.18E-02 | 1.18E+00 | | | |
| 7970441 | NM_004004 | GJB2 | gap junction protein, beta 2, 26kDa | -0.141 | 0.907 | 9.39E-02 | 9.39E+00 | | | |
| 8032926 | NM_002850 | PTPRS | protein tyrosine phosphatase, recept | -0.141 | 0.907 | 1.15E-02 | 1.15E+00 | | | |
| 8009844 | NM_00103180 | LLGL2 | lethal giant larvae homolog 2 (Droso | -0.141 | 0.907 | 2.39E-02 | 2.39E+00 | | | |
| 7950555 | NM_00112892 | LRRC32 | leucine rich repeat containing 32 | -0.141 | 0.907 | 3.17E-02 | 3.17E+00 | | | |
| 8122242 | NM_000288 | PEX7 | peroxisomal biogenesis factor 7 | -0.141 | 0.907 | 1.87E-01 | 1.87E+01 | | | |
| 8076734 | NM_058238 | WNT7B | wingless-type MMTV integration site | -0.141 | 0.907 | 9.98E-02 | 9.98E+00 | | | |
| 8065410 | NM_001899 | CST4 | cystatin S | -0.141 | 0.907 | 4.82E-02 | 4.82E+00 | | | |
| 8086540 | NM_020208 | SLC6A20 | solute carrier family 6 (proline IMINO | -0.141 | 0.907 | 8.71E-03 | 8.71E-01 | | | |
| 7943969 | NM_213621 | HTR3A | 5-hydroxytryptamine (serotonin) rec | -0.141 | 0.907 | 1.44E-02 | 1.44E+00 | | | |
| 8072216 | NM_006478 | GAS2L1 | growth arrest-specific 2 like 1 | -0.141 | 0.907 | 1.63E-02 | 1.63E+00 | | | |
| 7932308 | NM_00101090 | C1QL3 | complement component 1, q subcon | -0.141 | 0.907 | 1.62E-02 | 1.62E+00 | | | |
| 8068510 | --- | --- | --- | -0.141 | 0.907 | 9.24E-02 | 9.24E+00 | | | |
| 8124684 | NM_00110980 | ZFP57 | zinc finger protein 57 homolog (mou | -0.141 | 0.907 | 9.13E-03 | 9.13E-01 | | | |
| 7900792 | NM_002840 | PTPRF | protein tyrosine phosphatase, recept | -0.141 | 0.907 | 9.57E-03 | 9.57E-01 | | | |
| 8170794 | NM_00114615 | AVPR2 | arginine vasopressin receptor 2 | -0.141 | 0.907 | 2.43E-02 | 2.43E+00 | | | |
| 8151125 | ENST0000034C | TCF24 | transcription factor 24 | -0.141 | 0.907 | 1.85E-02 | 1.85E+00 | | | |
| 7897277 | BC030279 | C1orf211 | chromosome 1 open reading frame 2 | -0.141 | 0.907 | 5.26E-02 | 5.26E+00 | | | |
| 8060501 | NR_002981 | SNORA51 | small nucleolar RNA, H/ACA box 51 | -0.141 | 0.907 | 3.97E-01 | 3.97E+01 | | | |
| 8168892 | NM_080390 | TCEAL2 | transcription elongation factor A (SII | -0.141 | 0.907 | 7.57E-02 | 7.57E+00 | | | |
| 8036763 | NM_004714 | DYRK1B | dual-specificity tyrosine-(Y)-phospho | -0.141 | 0.907 | 4.14E-02 | 4.14E+00 | | | |
| 8144557 | NM_012331 | MSRA | methionine sulfoxide reductase A | -0.141 | 0.907 | 2.48E-01 | 2.48E+01 | | | |
| 7954090 | NM_001423 | EMP1 | epithelial membrane protein 1 | -0.141 | 0.907 | 4.82E-02 | 4.82E+00 | | | |
| 7992954 | --- | --- | --- | -0.141 | 0.907 | 6.48E-02 | 6.48E+00 | | | |
| 8045198 | NM_00107953 | CFC1B | cripto, FRL-1, cryptic family 1B | -0.141 | 0.907 | 1.14E-02 | 1.14E+00 | | | |
| 8066513 | NM_002999 | SDC4 | syndecan 4 | -0.141 | 0.907 | 5.32E-02 | 5.32E+00 | | | |
| 8148932 | NM_213605 | ZNF517 | zinc finger protein 517 | -0.142 | 0.907 | 5.94E-03 | 5.94E-01 | | | |
| 8141676 | NM_178176 | MOGAT3 | monoacylglycerol O-acyltransferase | -0.142 | 0.907 | 1.54E-02 | 1.54E+00 | | | |
| 8026139 | NM_002501 | NFIX | nuclear factor I/X (CCAAT-binding tra | -0.142 | 0.907 | 1.07E-02 | 1.07E+00 | | | |
| 7896172 | --- | --- | --- | -0.142 | 0.907 | 3.78E-01 | 3.78E+01 | | | |
| 7924908 | --- | --- | --- | -0.142 | 0.907 | 3.05E-02 | 3.05E+00 | | | |
| 7899310 | NM_032125 | TMEM222 | transmembrane protein 222 | -0.142 | 0.907 | 1.25E-01 | 1.25E+01 | | | |

| | | | | | | | | | | |
|---|---|---|---|---|---|---|---|---|---|---|
| 8134777 | NM_024070 | PVRIG | poliovirus receptor related immunog | -0.142 | 0.907 | 8.47E-02 | 8.47E+00 | | | |
| 7906197 | NM_021817 | HAPLN2 | hyaluronan and proteoglycan link pr | -0.142 | 0.906 | 2.52E-02 | 2.52E+00 | | | |
| 7908591 | --- | --- | --- | -0.142 | 0.906 | 4.53E-02 | 4.53E+00 | | | |
| 7913547 | NM_030761 | WNT4 | wingless-type MMTV integration site | -0.142 | 0.906 | 1.45E-02 | 1.45E+00 | | | |
| 8075668 | ENST00000457 | LOC284912 | hypothetical LOC284912 | -0.142 | 0.906 | 1.36E-02 | 1.36E+00 | | | |
| 8065427 | NM_178311 | GGTLC1 | gamma-glutamyltransferase light cha | -0.142 | 0.906 | 3.54E-02 | 3.54E+00 | | | |
| 7892686 | --- | --- | --- | -0.142 | 0.906 | 7.86E-02 | 7.86E+00 | | | |
| 8002270 | --- | --- | --- | -0.142 | 0.906 | 1.76E-01 | 1.76E+01 | | | |
| 7903694 | NM_031936 | GPR61 | G protein-coupled receptor 61 | -0.142 | 0.906 | 2.50E-02 | 2.50E+00 | | | |
| 7977018 | NM_145725 | TRAF3 | TNF receptor-associated factor 3 | -0.142 | 0.906 | 6.13E-01 | 6.13E+01 | | | |
| 7933073 | NM_153368 | GJD4 | gap junction protein, delta 4, 40.1kD | -0.142 | 0.906 | 2.10E-02 | 2.10E+00 | | | |
| 8028552 | NM_002503 | NFKBIB | nuclear factor of kappa light polypep | -0.142 | 0.906 | 4.28E-02 | 4.28E+00 | | | |
| 7905092 | NM_016074 | BOLA1 | bolA homolog 1 (E. coli) | -0.142 | 0.906 | 8.41E-02 | 8.41E+00 | | | |
| 8134737 | NM_203397 | MBLAC1 | metallo-beta-lactamase domain cont | -0.142 | 0.906 | 3.56E-02 | 3.56E+00 | | | |
| 8023184 | AY669508 | FUSSEL18 | functional smad suppressing elemen | -0.142 | 0.906 | 1.53E-02 | 1.53E+00 | | | |
| 7939524 | NM_000401 | EXT2 | exostoses (multiple) 2 | -0.142 | 0.906 | 2.90E-01 | 2.90E+01 | | | |
| 7960134 | NM_019591 | ZNF26 | zinc finger protein 26 | -0.142 | 0.906 | 5.04E-01 | 5.04E+01 | | | |
| 7922095 | NR_026550 | BRP44 | brain protein 44 | -0.142 | 0.906 | 3.76E-01 | 3.76E+01 | | | |
| 8026982 | NM_032683 | MPV17L2 | MPV17 mitochondrial membrane pro | -0.142 | 0.906 | 7.81E-02 | 7.81E+00 | | | |
| 8062319 | NM_021809 | TGIF2 | TGFB-induced factor homeobox 2 | -0.142 | 0.906 | 3.17E-02 | 3.17E+00 | | | |
| 7975457 | --- | --- | --- | -0.142 | 0.906 | 6.91E-01 | 6.91E+01 | | | |
| 7901050 | NR_001456 | SNORD38A | small nucleolar RNA, C/D box 38A | -0.142 | 0.906 | 8.11E-01 | 8.11E+01 | | | |
| 8002830 | NM_153486 | LDHD | lactate dehydrogenase D | -0.142 | 0.906 | 1.90E-02 | 1.90E+00 | | | |
| 8044640 | NM_021965 | PGM5 | phosphoglucomutase 5 | -0.142 | 0.906 | 3.99E-02 | 3.99E+00 | | | |
| 8180376 | --- | --- | --- | -0.142 | 0.906 | 2.34E-02 | 2.34E+00 | | | |
| 7937257 | NM_145651 | SCGB1C1 | secretoglobin, family 1C, member 1 | -0.142 | 0.906 | 3.84E-02 | 3.84E+00 | | | |
| 7893037 | --- | --- | --- | -0.142 | 0.906 | 1.29E-01 | 1.29E+01 | | | |
| 8019754 | NM_207368 | LOC348262 | hypothetical protein LOC348262 | -0.142 | 0.906 | 2.65E-02 | 2.65E+00 | | | |
| 7982256 | NR_024074 | GOLGA9P | golgi autoantigen, golgin subfamily a | -0.142 | 0.906 | 4.59E-01 | 4.59E+01 | | | |
| 8166948 | --- | --- | --- | -0.142 | 0.906 | 2.53E-01 | 2.53E+01 | | | |
| 7894833 | --- | --- | --- | -0.142 | 0.906 | 3.77E-01 | 3.77E+01 | | | |
| 8119159 | --- | --- | --- | -0.142 | 0.906 | 4.90E-02 | 4.90E+00 | | | |
| 8059868 | NM_212556 | ASB18 | ankyrin repeat and SOCS box-contai | -0.142 | 0.906 | 1.68E-02 | 1.68E+00 | | | |
| 8072941 | NM_003614 | GALR3 | galanin receptor 3 | -0.142 | 0.906 | 1.35E-02 | 1.35E+00 | | | |
| 7908966 | BC000988 | C1orf217 | chromosome 1 open reading frame 2 | -0.142 | 0.906 | 4.81E-02 | 4.81E+00 | | | |
| 8043782 | NM_001298 | CNGA3 | cyclic nucleotide gated channel alpha | -0.142 | 0.906 | 1.10E-02 | 1.10E+00 | | | |
| 8098916 | NM_00112726 | TMEM129 | transmembrane protein 129 | -0.142 | 0.906 | 1.73E-02 | 1.73E+00 | | | |
| 8110362 | NM_00100410 | GRK6 | G protein-coupled receptor kinase 6 | -0.142 | 0.906 | 1.97E-01 | 1.97E+01 | | | |
| 8142830 | NM_199349 | KCP | kielin/chordin-like protein | -0.142 | 0.906 | 2.06E-02 | 2.06E+00 | | | |
| 7942064 | NM_015973 | GAL | galanin prepropeptide | -0.142 | 0.906 | 6.55E-03 | 6.55E-01 | | | |
| 8171336 | --- | --- | --- | -0.142 | 0.906 | 1.60E-01 | 1.60E+01 | | | |
| 8055478 | NM_007226 | NXPH2 | neurexophilin 2 | -0.142 | 0.906 | 2.28E-02 | 2.28E+00 | | | |
| 8153346 | NM_023946 | LYNX1 | Ly6/neurotoxin 1 | -0.142 | 0.906 | 1.63E-02 | 1.63E+00 | | | |
| 7997832 | ENST0000033 | FLJ40448 | hypothetical protein FLJ40448 | -0.142 | 0.906 | 9.13E-03 | 9.13E-01 | | | |
| 7906177 | NM_00110566 | TTC24 | tetratricopeptide repeat domain 24 | -0.142 | 0.906 | 3.51E-02 | 3.51E+00 | | | |
| 7934789 | NM_017551 | GRID1 | glutamate receptor, ionotropic, delta | -0.142 | 0.906 | 9.59E-03 | 9.59E-01 | | | |
| 8034521 | NM_013312 | HOOK2 | hook homolog 2 (Drosophila) | -0.142 | 0.906 | 3.40E-02 | 3.40E+00 | | | |
| 8071134 | ENST00000444 | C22orf37 | chromosome 22 open reading frame | -0.142 | 0.906 | 8.35E-02 | 8.35E+00 | | | |
| 8072584 | --- | --- | --- | -0.142 | 0.906 | 1.62E-01 | 1.62E+01 | | | |
| 7968252 | --- | --- | --- | -0.142 | 0.906 | 7.27E-02 | 7.27E+00 | | | |
| 7992271 | NM_016111 | TELO2 | TEL2, telomere maintenance 2, hom | -0.142 | 0.906 | 5.09E-02 | 5.09E+00 | | | |
| 7917468 | --- | --- | --- | -0.142 | 0.906 | 5.12E-01 | 5.12E+01 | | | |
| 7922351 | AK130460 | LOC646870 | hypothetical protein LOC646870 | -0.142 | 0.906 | 1.90E-01 | 1.90E+01 | | | |
| 7907911 | NM_000721 | CACNA1E | calcium channel, voltage-dependent | -0.142 | 0.906 | 7.71E-03 | 7.71E-01 | | | |
| 7980115 | NM_005050 | ABCD4 | ATP-binding cassette, sub-family D ( | -0.142 | 0.906 | 4.39E-02 | 4.39E+00 | | | |
| 8161615 | NM_002732 | PRKACG | protein kinase, cAMP-dependent, ca | -0.142 | 0.906 | 3.82E-02 | 3.82E+00 | | | |
| 8137906 | NM_018059 | RADIL | Ras association and DIL domains | -0.142 | 0.906 | 2.90E-02 | 2.90E+00 | | | |
| 7894163 | --- | --- | --- | -0.142 | 0.906 | 7.60E-01 | 7.60E+01 | | | |
| 7987928 | NM_138477 | CDAN1 | congenital dyserythropoietic anemia | -0.142 | 0.906 | 1.42E-02 | 1.42E+00 | | | |
| 7934141 | NM_022146 | NPFFR1 | neuropeptide FF receptor 1 | -0.142 | 0.906 | 3.15E-02 | 3.15E+00 | | | |
| 7914758 | NM_00108041 | DLGAP3 | discs, large (Drosophila) homolog-as | -0.142 | 0.906 | 1.45E-02 | 1.45E+00 | | | |
| 8155572 | NR_024060 | FAM27A | family with sequence similarity 27, m | -0.142 | 0.906 | 1.29E-02 | 1.29E+00 | | | |
| 8015376 | NM_005557 | KRT16 | keratin 16 | -0.142 | 0.906 | 1.79E-02 | 1.79E+00 | | | |
| 7898799 | NM_00111410 | C1QC | complement component 1, q subcom | -0.142 | 0.906 | 1.95E-02 | 1.95E+00 | | | |
| 8074384 | NM_005315 | GSC2 | goosecoid homeobox 2 | -0.142 | 0.906 | 2.86E-02 | 2.86E+00 | | | |
| 8001898 | NM_004062 | CDH16 | cadherin 16, KSP-cadherin | -0.142 | 0.906 | 1.67E-02 | 1.67E+00 | | | |
| 8023259 | NR_002571 | SNORD58A | small nucleolar RNA, C/D box 58A | -0.143 | 0.906 | 8.82E-01 | 8.82E+01 | | | |
| 7988177 | NM_153700 | STRC | stereocilin | -0.143 | 0.906 | 1.92E-02 | 1.92E+00 | | | |
| 8118990 | NM_207409 | C6orf126 | chromosome 6 open reading frame 1 | -0.143 | 0.906 | 9.51E-03 | 9.51E-01 | | | |
| 7949532 | NM_005438 | FOSL1 | FOS-like antigen 1 | -0.143 | 0.906 | 2.13E-02 | 2.13E+00 | | | |
| 8170648 | NM_001711 | BGN | biglycan | -0.143 | 0.906 | 1.74E-02 | 1.74E+00 | | | |
| 7901091 | NM_025077 | TOE1 | target of EGR1, member 1 (nuclear) | -0.143 | 0.906 | 1.56E-01 | 1.56E+01 | | | |
| 7971813 | NM_018676 | THSD1 | thrombospondin, type I, domain con | -0.143 | 0.906 | 4.47E-02 | 4.47E+00 | | | |
| 8030768 | NM_00100223 | KLK2 | kallikrein-related peptidase 2 | -0.143 | 0.906 | 1.61E-02 | 1.61E+00 | | | |
| 7958015 | --- | --- | --- | -0.143 | 0.906 | 6.60E-01 | 6.60E+01 | | | |
| 8110990 | NM_001332 | CTNND2 | catenin (cadherin-associated protein | -0.143 | 0.906 | 1.76E-02 | 1.76E+00 | | | |
| 7896521 | --- | --- | --- | -0.143 | 0.906 | 7.92E-01 | 7.92E+01 | | | |

| | | | | | | | | | | |
|---|---|---|---|---|---|---|---|---|---|---|
| 7934553 | NM_032772 | ZNF503 | zinc finger protein 503 | -0.143 | 0.906 | 1.69E-02 | 1.69E+00 | | | |
| 8166999 | NM_019886 | CHST7 | carbohydrate (N-acetylglucosamine ( | -0.143 | 0.906 | 1.07E-02 | 1.07E+00 | | | |
| 8015554 | NM_012285 | KCNH4 | potassium voltage-gated channel, su | -0.143 | 0.906 | 1.85E-02 | 1.85E+00 | | | |
| 7938139 | NM_144666 | DNHD1 | dynein heavy chain domain 1 | -0.143 | 0.906 | 1.79E-02 | 1.79E+00 | | | |
| 8023948 | NM_033513 | C19orf20 | chromosome 19 open reading frame | -0.143 | 0.906 | 1.23E-02 | 1.23E+00 | | | |
| 7949206 | NM_130802 | MEN1 | multiple endocrine neoplasia I | -0.143 | 0.906 | 4.96E-02 | 4.96E+00 | | | |
| 8155148 | NM_016446 | TMEM8B | transmembrane protein 8B | -0.143 | 0.906 | 1.16E-02 | 1.16E+00 | | | |
| 8002792 | NM_024306 | FA2H | fatty acid 2-hydroxylase | -0.143 | 0.906 | 1.25E-02 | 1.25E+00 | | | |
| 7989770 | NM_004884 | IGDCC3 | immunoglobulin superfamily, DCC su | -0.143 | 0.906 | 8.54E-03 | 8.54E-01 | | | |
| 8033662 | NM_178525 | ACTL9 | actin-like 9 | -0.143 | 0.906 | 2.56E-02 | 2.56E+00 | | | |
| 7998136 | NM_022450 | RHBDF1 | rhomboid 5 homolog 1 (Drosophila) | -0.143 | 0.906 | 5.06E-03 | 5.06E-01 | | | |
| 7896330 | --- | --- | --- | -0.143 | 0.906 | 7.91E-01 | 7.91E+01 | | | |
| 8175593 | NM_000202 | IDS | iduronate 2-sulfatase | -0.143 | 0.906 | 5.03E-01 | 5.03E+01 | | | |
| 8046564 | NM_024501 | HOXD1 | homeobox D1 | -0.143 | 0.906 | 1.08E-02 | 1.08E+00 | | | |
| 8148867 | NM_145754 | KIFC2 | kinesin family member C2 | -0.143 | 0.906 | 1.60E-02 | 1.60E+00 | | | |
| 8062873 | NM_022358 | KCNK15 | potassium channel, subfamily K, mer | -0.143 | 0.906 | 1.80E-02 | 1.80E+00 | | | |
| 8031441 | NM_032430 | BRSK1 | BR serine/threonine kinase 1 | -0.143 | 0.906 | 2.60E-02 | 2.60E+00 | | | |
| 8035646 | NM_023002 | HAPLN4 | hyaluronan and proteoglycan link pro | -0.143 | 0.906 | 1.78E-02 | 1.78E+00 | | | |
| 8026579 | NM_024074 | TMEM38A | transmembrane protein 38A | -0.143 | 0.906 | 7.99E-03 | 7.99E-01 | | | |
| 8170965 | NM_139250 | CTAG1A | cancer/testis antigen 1A | -0.143 | 0.906 | 1.63E-02 | 1.63E+00 | | | |
| 8176149 | NM_139250 | CTAG1A | cancer/testis antigen 1A | -0.143 | 0.906 | 1.63E-02 | 1.63E+00 | | | |
| 7983979 | --- | --- | --- | -0.143 | 0.906 | 1.30E-01 | 1.30E+01 | | | |
| 7950064 | ENST00000331 | FLJ42102 | FLJ42102 protein | -0.143 | 0.906 | 5.84E-02 | 5.84E+00 | | | |
| 8156759 | --- | --- | --- | -0.143 | 0.906 | 5.97E-01 | 5.97E+01 | | | |
| 8090462 | --- | --- | --- | -0.143 | 0.906 | 6.25E-02 | 6.25E+00 | | | |
| 8152902 | NM_001115 | ADCY8 | adenylate cyclase 8 (brain) | -0.143 | 0.906 | 3.64E-02 | 3.64E+00 | | | |
| 8026830 | NM_015122 | FCHO1 | FCH domain only 1 | -0.143 | 0.906 | 1.16E-02 | 1.16E+00 | | | |
| 8044440 | NM_153214 | FBLN7 | fibulin 7 | -0.143 | 0.906 | 1.98E-02 | 1.98E+00 | | | |
| 8003243 | AK026130 | FBXO31 | F-box protein 31 | -0.143 | 0.906 | 8.77E-02 | 8.77E+00 | | | |
| 7960635 | NR_003012 | SCARNA11 | small Cajal body-specific RNA 11 | -0.143 | 0.905 | 3.39E-02 | 3.39E+00 | | | |
| 7953469 | NM_002075 | GNB3 | guanine nucleotide binding protein ( | -0.143 | 0.905 | 1.32E-02 | 1.32E+00 | | | |
| 8161265 | NM_00100756 | IGFBPL1 | insulin-like growth factor binding pro | -0.143 | 0.905 | 7.72E-03 | 7.72E-01 | | | |
| 8174968 | NM_00101362 | DCAF12L2 | DDB1 and CUL4 associated factor 12 | -0.143 | 0.905 | 3.04E-02 | 3.04E+00 | | | |
| 8066637 | AK097925 | FLJ40606 | hypothetical protein LOC643549 | -0.143 | 0.905 | 2.58E-02 | 2.58E+00 | | | |
| 8021565 | NM_194449 | PHLPP1 | PH domain and leucine rich repeat p | -0.143 | 0.905 | 2.23E-02 | 2.23E+00 | | | |
| 8039316 | NM_017607 | PPP1R12C | protein phosphatase 1, regulatory (in | -0.143 | 0.905 | 9.46E-03 | 9.46E-01 | | | |
| 8048523 | NM_006736 | DNAJB2 | DnaJ (Hsp40) homolog, subfamily B, | -0.143 | 0.905 | 6.89E-02 | 6.89E+00 | | | |
| 7922717 | NM_002928 | RGS16 | regulator of G-protein signaling 16 | -0.143 | 0.905 | 1.52E-02 | 1.52E+00 | | | |
| 7897089 | NM_014638 | PLCH2 | phospholipase C, eta 2 | -0.143 | 0.905 | 4.10E-03 | 4.10E-01 | | | |
| 7986744 | BC129821 | LOC283685 | golgi autoantigen, golgin subfamily a | -0.143 | 0.905 | 1.74E-02 | 1.74E+00 | | | |
| 8020455 | NM_005257 | GATA6 | GATA binding protein 6 | -0.143 | 0.905 | 1.12E-02 | 1.12E+00 | | | |
| 8174675 | NM_001152 | SLC25A5 | solute carrier family 25 (mitochondri | -0.143 | 0.905 | 1.05E-01 | 1.05E+01 | | | |
| 7895295 | --- | --- | --- | -0.143 | 0.905 | 3.82E-01 | 3.82E+01 | | | |
| 8088425 | NM_138805 | FAM3D | family with sequence similarity 3, me | -0.143 | 0.905 | 2.52E-02 | 2.52E+00 | | | |
| 7973694 | NM_019839 | LTB4R2 | leukotriene B4 receptor 2 | -0.143 | 0.905 | 2.35E-02 | 2.35E+00 | | | |
| 8162645 | NR_002894 | HIATL2 | hippocampus abundant transcript-lik | -0.143 | 0.905 | 2.77E-01 | 2.77E+01 | | | |
| 7972663 | --- | --- | --- | -0.143 | 0.905 | 6.99E-02 | 6.99E+00 | | | |
| 7912473 | NM_012168 | FBXO2 | F-box protein 2 | -0.143 | 0.905 | 1.44E-02 | 1.44E+00 | | | |
| 7893714 | --- | --- | --- | -0.144 | 0.905 | 3.17E-01 | 3.17E+01 | | | |
| 8027002 | NM_004864 | GDF15 | growth differentiation factor 15 | -0.144 | 0.905 | 3.68E-02 | 3.68E+00 | | | |
| 8015173 | NM_031958 | KRTAP3-1 | keratin associated protein 3-1 | -0.144 | 0.905 | 1.33E-02 | 1.33E+00 | | | |
| 8019593 | NM_031958 | KRTAP3-1 | keratin associated protein 3-1 | -0.144 | 0.905 | 1.33E-02 | 1.33E+00 | | | |
| 7990922 | NR_003246 | FLJ40113 | golgi autoantigen, golgin subfamily a | -0.144 | 0.905 | 3.05E-01 | 3.05E+01 | | | |
| 8167210 | NM_205856 | SPACA5 | sperm acrosome associated 5 | -0.144 | 0.905 | 4.84E-02 | 4.84E+00 | | | |
| 7991088 | NR_027001 | LOC388152 | hypothetical LOC388152 | -0.144 | 0.905 | 7.24E-04 | 7.24E-02 | | | |
| 8144982 | NM_182795 | NPM2 | nucleophosmin/nucleoplasmin 2 | -0.144 | 0.905 | 5.51E-02 | 5.51E+00 | | | |
| 8119515 | NM_000409 | GUCA1A | guanylate cyclase activator 1A (retina | -0.144 | 0.905 | 5.86E-02 | 5.86E+00 | | | |
| 7892584 | --- | --- | --- | -0.144 | 0.905 | 2.94E-01 | 2.94E+01 | | | |
| 8068761 | NM_207627 | ABCG1 | ATP-binding cassette, sub-family G (W | -0.144 | 0.905 | 4.16E-02 | 4.16E+00 | | | |
| 7910217 | NM_033131 | WNT3A | wingless-type MMTV integration site | -0.144 | 0.905 | 1.68E-02 | 1.68E+00 | | | |
| 8040265 | NM_002236 | KCNF1 | potassium voltage-gated channel, su | -0.144 | 0.905 | 1.58E-02 | 1.58E+00 | | | |
| 8045146 | AK024408 | FAM128B | family with sequence similarity 128, | -0.144 | 0.905 | 6.38E-02 | 6.38E+00 | | | |
| 7904209 | --- | --- | --- | -0.144 | 0.905 | 2.60E-02 | 2.60E+00 | | | |
| 8149151 | NR_027000 | LOC349196 | hypothetical LOC349196 | -0.144 | 0.905 | 7.71E-02 | 7.71E+00 | | | |
| 8149214 | NR_027000 | LOC349196 | hypothetical LOC349196 | -0.144 | 0.905 | 7.71E-02 | 7.71E+00 | | | |
| 8165498 | NM_00100171 | FAM166A | family with sequence similarity 166, | -0.144 | 0.905 | 1.36E-02 | 1.36E+00 | | | |
| 8003077 | NM_00108044 | SLC38A8 | solute carrier family 38, member 8 | -0.144 | 0.905 | 2.27E-02 | 2.27E+00 | | | |
| 8091676 | --- | --- | --- | -0.144 | 0.905 | 2.74E-01 | 2.74E+01 | | | |
| 8169836 | NM_003399 | XPNPEP2 | X-prolyl aminopeptidase (aminopept | -0.144 | 0.905 | 2.29E-02 | 2.29E+00 | | | |
| 8037535 | --- | --- | --- | -0.144 | 0.905 | 1.49E-01 | 1.49E+01 | | | |
| 8006063 | NM_152345 | ANKRD13B | ankyrin repeat domain 13B | -0.144 | 0.905 | 1.18E-02 | 1.18E+00 | | | |
| 7983132 | AK122666 | LOC645106 | similar to kinesin-like motor protein | -0.144 | 0.905 | 4.38E-02 | 4.38E+00 | | | |
| 8107644 | --- | --- | --- | -0.144 | 0.905 | 8.79E-02 | 8.79E+00 | | | |
| 7922807 | NM_015101 | GLT25D2 | glycosyltransferase 25 domain conta | -0.144 | 0.905 | 1.71E-02 | 1.71E+00 | | | |
| 8104996 | --- | --- | --- | -0.144 | 0.905 | 1.30E-01 | 1.30E+01 | | | |
| 7937713 | NM_138567 | SYT8 | synaptotagmin VIII | -0.144 | 0.905 | 4.43E-03 | 4.43E-01 | | | |
| 8147123 | NM_005181 | CA3 | carbonic anhydrase III, muscle specif | -0.144 | 0.905 | 1.08E-02 | 1.08E+00 | | | |

| | | | | | | | | | | | |
|---|---|---|---|---|---|---|---|---|---|---|---|
| 7971419 | NM_198849 | SIAH3 | seven in absentia homolog 3 (Drosop | -0.144 | 0.905 | 5.40E-02 | 5.40E+00 | | | | |
| 8076865 | NM_00100169 | IL17REL | interleukin 17 receptor E-like | -0.144 | 0.905 | 1.71E-02 | 1.71E+00 | | | | |
| 7952752 | NM_007037 | ADAMTS8 | ADAM metallopeptidase with throm | -0.144 | 0.905 | 1.25E-02 | 1.25E+00 | | | | |
| 8092686 | NM_00100431 | RTP2 | receptor (chemosensory) transporte | -0.144 | 0.905 | 2.29E-02 | 2.29E+00 | | | | |
| 8036031 | --- | --- | --- | -0.144 | 0.905 | 1.27E-01 | 1.27E+01 | | | | |
| 8036584 | NM_002307 | LGALS7 | lectin, galactoside-binding, soluble, 7 | -0.144 | 0.905 | 2.27E-02 | 2.27E+00 | | | | |
| 7894891 | --- | --- | --- | -0.144 | 0.905 | 4.20E-01 | 4.20E+01 | | | | |
| 7915614 | NM_003738 | PTCH2 | patched homolog 2 (Drosophila) | -0.144 | 0.905 | 1.15E-02 | 1.15E+00 | | | | |
| 8032106 | NM_005481 | MED16 | mediator complex subunit 16 | -0.144 | 0.905 | 1.71E-02 | 1.71E+00 | | | | |
| 8038192 | NM_000148 | FUT1 | fucosyltransferase 1 (galactoside 2-a | -0.144 | 0.905 | 7.30E-03 | 7.30E-01 | | | | |
| 8064581 | NM_021826 | FASTKD5 | FAST kinase domains 5 | -0.144 | 0.905 | 3.24E-01 | 3.24E+01 | | | | |
| 7896563 | --- | --- | --- | -0.144 | 0.905 | 2.31E-01 | 2.31E+01 | | | | |
| 8130624 | NM_005922 | MAP3K4 | mitogen-activated protein kinase kin | -0.144 | 0.905 | 1.74E-02 | 1.74E+00 | | | | |
| 8148888 | NM_032902 | PPP1R16A | protein phosphatase 1, regulatory (in | -0.144 | 0.905 | 2.44E-02 | 2.44E+00 | | | | |
| 7903786 | NM_000757 | CSF1 | colony stimulating factor 1 (macroph | -0.144 | 0.905 | 1.18E-02 | 1.18E+00 | | | | |
| 7931500 | NM_00108390 | GPR123 | G protein-coupled receptor 123 | -0.144 | 0.905 | 1.36E-02 | 1.36E+00 | | | | |
| 8066231 | NM_00102986 | KIAA1755 | KIAA1755 | -0.144 | 0.905 | 1.38E-02 | 1.38E+00 | | | | |
| 8175420 | NM_002139 | RBMX | RNA binding motif protein, X-linked | -0.144 | 0.905 | 5.27E-01 | 5.27E+01 | | | | |
| 7900129 | NM_024676 | C1orf113 | chromosome 1 open reading frame 1 | -0.144 | 0.905 | 2.10E-02 | 2.10E+00 | | | | |
| 7911681 | --- | --- | --- | -0.144 | 0.905 | 2.34E-02 | 2.34E+00 | | | | |
| 8099172 | NM_001313 | CRMP1 | collapsin response mediator protein | -0.144 | 0.905 | 2.15E-02 | 2.15E+00 | | | | |
| 7899927 | NM_153212 | GJB4 | gap junction protein, beta 4, 30.3kDa | -0.144 | 0.905 | 2.13E-02 | 2.13E+00 | | | | |
| 8014282 | NM_024302 | MMP28 | matrix metallopeptidase 28 | -0.144 | 0.905 | 6.00E-03 | 6.00E-01 | | | | |
| 8036686 | AK021860 | FBXO17 | F-box protein 17 | -0.144 | 0.905 | 4.59E-02 | 4.59E+00 | | | | |
| 7952004 | NM_174934 | SCN4B | sodium channel, voltage-gated, type | -0.145 | 0.905 | 1.51E-02 | 1.51E+00 | | | | |
| 8114455 | NM_004134 | HSPA9 | heat shock 70kDa protein 9 (mortalin | -0.145 | 0.905 | 4.50E-01 | 4.50E+01 | | | | |
| 8113699 | --- | --- | --- | -0.145 | 0.905 | 3.02E-02 | 3.02E+00 | | | | |
| 7937020 | NM_002417 | MKI67 | antigen identified by monoclonal ant | -0.145 | 0.905 | 3.96E-01 | 3.96E+01 | | | | |
| 7945712 | NM_199292 | TH | tyrosine hydroxylase | -0.145 | 0.905 | 1.21E-02 | 1.21E+00 | | | | |
| 8052882 | NM_017488 | ADD2 | adducin 2 (beta) | -0.145 | 0.905 | 1.06E-02 | 1.06E+00 | | | | |
| 7894975 | --- | --- | --- | -0.145 | 0.905 | 6.69E-02 | 6.69E+00 | | | | |
| 8178059 | NM_021221 | LY6G5B | lymphocyte antigen 6 complex, locus | -0.145 | 0.905 | 8.11E-01 | 8.11E+01 | | | | |
| 7996264 | NM_001795 | CDH5 | cadherin 5, type 2 (vascular endothe | -0.145 | 0.905 | 1.93E-02 | 1.93E+00 | | | | |
| 8119765 | NM_032538 | TTBK1 | tau tubulin kinase 1 | -0.145 | 0.905 | 1.60E-02 | 1.60E+00 | | | | |
| 8000346 | NM_033266 | ERN2 | endoplasmic reticulum to nucleus sig | -0.145 | 0.905 | 2.86E-02 | 2.86E+00 | | | | |
| 7892958 | --- | --- | --- | -0.145 | 0.905 | 4.84E-01 | 4.84E+01 | | | | |
| 7995193 | NM_024742 | ARMC5 | armadillo repeat containing 5 | -0.145 | 0.905 | 7.33E-03 | 7.33E-01 | | | | |
| 7988132 | NM_153700 | STRC | stereocilin | -0.145 | 0.905 | 1.52E-02 | 1.52E+00 | | | | |
| 7923837 | NM_152491 | PM20D1 | peptidase M20 domain containing 1 | -0.145 | 0.905 | 6.78E-02 | 6.78E+00 | | | | |
| 8178086 | NM_005346 | HSPA1B | heat shock 70kDa protein 1B | -0.145 | 0.905 | 2.21E-01 | 2.21E+01 | | | | |
| 8179324 | NM_005346 | HSPA1B | heat shock 70kDa protein 1B | -0.145 | 0.905 | 2.21E-01 | 2.21E+01 | | | | |
| 8077877 | AK094424 | OC10013092 | hypothetical LOC100130924 | -0.145 | 0.905 | 3.71E-01 | 3.71E+01 | | | | |
| 8073546 | NR_024355 | LOC339674 | hypothetical LOC339674 | -0.145 | 0.905 | 2.74E-02 | 2.74E+00 | | | | |
| 7910790 | --- | --- | --- | -0.145 | 0.905 | 4.28E-01 | 4.28E+01 | | | | |
| 7892823 | --- | --- | --- | -0.145 | 0.905 | 2.72E-01 | 2.72E+01 | | | | |
| 8037315 | NM_198850 | PHLDB3 | pleckstrin homology-like domain, fan | -0.145 | 0.904 | 7.06E-03 | 7.06E-01 | | | | |
| 8153876 | NM_025251 | KIAA1688 | KIAA1688 protein | -0.145 | 0.904 | 6.23E-03 | 6.23E-01 | | | | |
| 8033462 | NM_206833 | CTXN1 | cortexin 1 | -0.145 | 0.904 | 1.65E-02 | 1.65E+00 | | | | |
| 7972899 | BX647655 | KFZp451A21 | DKFZp451A211 protein | -0.145 | 0.904 | 9.70E-03 | 9.70E-01 | | | | |
| 8010901 | NM_003585 | DOC2B | double C2-like domains, beta | -0.145 | 0.904 | 1.92E-01 | 1.92E+01 | | | | |
| 8039273 | NM_145057 | CDC42EP5 | CDC42 effector protein (Rho GTPase | -0.145 | 0.904 | 1.44E-02 | 1.44E+00 | | | | |
| 8131098 | ENST0000040 | OC10012837 | hypothetical LOC100128374 | -0.145 | 0.904 | 2.06E-02 | 2.06E+00 | | | | |
| 8071355 | NR_026919 | LOC150197 | hypothetical LOC150197 | -0.145 | 0.904 | 1.78E-02 | 1.78E+00 | | | | |
| 8026751 | NM_138401 | FAM125A | family with sequence similarity 125, | -0.145 | 0.904 | 2.12E-02 | 2.12E+00 | | | | |
| 7992038 | NM_024042 | METRN | meteorin, glial cell differentiation reg | -0.145 | 0.904 | 1.77E-02 | 1.77E+00 | | | | |
| 8006423 | NM_173847 | SPACA3 | sperm acrosome associated 3 | -0.145 | 0.904 | 4.74E-03 | 4.74E-01 | | | | |
| 8146429 | NM_005285 | NPBWR1 | neuropeptides B/W receptor 1 | -0.145 | 0.904 | 2.17E-02 | 2.17E+00 | | | | |
| 8074131 | NM_00108042 | SHANK3 | SH3 and multiple ankyrin repeat dom | -0.145 | 0.904 | 1.05E-02 | 1.05E+00 | | | | |
| 7895503 | --- | --- | --- | -0.145 | 0.904 | 5.88E-01 | 5.88E+01 | | | | |
| 8037728 | NM_00101264 | MYPOP | Myb-related transcription factor, par | -0.145 | 0.904 | 2.60E-02 | 2.60E+00 | | | | |
| 7949873 | NM_080658 | ACY3 | aspartoacylase (aminocyclase) 3 | -0.145 | 0.904 | 2.35E-02 | 2.35E+00 | | | | |
| 8028991 | NM_030622 | CYP2S1 | cytochrome P450, family 2, subfamil | -0.145 | 0.904 | 7.92E-03 | 7.92E-01 | | | | |
| 7907601 | NM_021165 | FAM5B | family with sequence similarity 5, me | -0.145 | 0.904 | 2.87E-02 | 2.87E+00 | | | | |
| 7910142 | NM_00100366 | C1orf95 | chromosome 1 open reading frame 9 | -0.145 | 0.904 | 2.37E-02 | 2.37E+00 | | | | |
| 8092165 | NM_198407 | GHSR | growth hormone secretagogue recep | -0.145 | 0.904 | 6.48E-03 | 6.48E-01 | | | | |
| 8081820 | --- | --- | --- | -0.145 | 0.904 | 4.22E-02 | 4.22E+00 | | | | |
| 7894443 | --- | --- | --- | -0.145 | 0.904 | 5.23E-01 | 5.23E+01 | | | | |
| 7998466 | NM_00103712 | UNKL | unkempt homolog (Drosophila)-like | -0.145 | 0.904 | 6.03E-03 | 6.03E-01 | | | | |
| 8039706 | NM_005773 | ZNF256 | zinc finger protein 256 | -0.145 | 0.904 | 1.10E-01 | 1.10E+01 | | | | |
| 8007607 | NM_006695 | RUNDC3A | RUN domain containing 3A | -0.145 | 0.904 | 3.41E-02 | 3.41E+00 | | | | |
| 8143885 | NM_00109883 | GBX1 | gastrulation brain homeobox 1 | -0.145 | 0.904 | 2.37E-02 | 2.37E+00 | | | | |
| 8132660 | NM_005856 | RAMP3 | receptor (G protein-coupled) activity | -0.145 | 0.904 | 1.44E-02 | 1.44E+00 | | | | |
| 7992092 | NM_022092 | CHTF18 | CTF18, chromosome transmission fid | -0.145 | 0.904 | 1.04E-02 | 1.04E+00 | | | | |
| 8048505 | NR_026909 | STK16 | serine/threonine kinase 16 | -0.145 | 0.904 | 2.46E-01 | 2.46E+01 | | | | |
| 8038314 | NM_018111 | C19orf73 | chromosome 19 open reading frame | -0.145 | 0.904 | 7.25E-02 | 7.25E+00 | | | | |
| 7991714 | 0 | 0 | 0 | -0.145 | 0.904 | 3.53E-03 | 3.53E-01 | | | | |
| 8079099 | NM_145166 | ZBTB47 | zinc finger and BTB domain containin | -0.145 | 0.904 | 9.66E-03 | 9.66E-01 | | | | |

| | | | | | | | | | | |
|---|---|---|---|---|---|---|---|---|---|---|
| 8086981 | NM_033199 | UCN2 | urocortin 2 | -0.145 | 0.904 | 4.81E-02 | 4.81E+00 | | | |
| 7909610 | NM_00104061 | ATF3 | activating transcription factor 3 | -0.145 | 0.904 | 5.56E-02 | 5.56E+00 | | | |
| 7953547 | NM_00100702 | ATN1 | atrophin 1 | -0.145 | 0.904 | 8.35E-03 | 8.35E-01 | | | |
| 7942830 | ENST00000391 | GCRG224 | GCRG-P224 | -0.145 | 0.904 | 6.80E-01 | 6.80E+01 | | | |
| 8044541 | NM_019618 | IL1F9 | interleukin 1 family, member 9 | -0.145 | 0.904 | 1.15E-02 | 1.15E+00 | | | |
| 8027222 | NM_153221 | CILP2 | cartilage intermediate layer protein | -0.145 | 0.904 | 1.18E-02 | 1.18E+00 | | | |
| 8006459 | NM_005408 | CCL13 | chemokine (C-C motif) ligand 13 | -0.145 | 0.904 | 2.53E-02 | 2.53E+00 | | | |
| 8164931 | NM_00108048 | TMEM8C | transmembrane protein 8C | -0.145 | 0.904 | 1.75E-02 | 1.75E+00 | | | |
| 8134263 | NM_000089 | COL1A2 | collagen, type I, alpha 2 | -0.145 | 0.904 | 6.88E-03 | 6.88E-01 | | | |
| 7894976 | --- | --- | --- | -0.145 | 0.904 | 2.82E-01 | 2.82E+01 | | | |
| 7900540 | NM_173642 | RIMKLA | ribosomal modification protein rimK | -0.145 | 0.904 | 1.69E-02 | 1.69E+00 | | | |
| 8126855 | NM_00101373 | C6orf138 | chromosome 6 open reading frame 1 | -0.146 | 0.904 | 4.03E-02 | 4.03E+00 | | | |
| 8119088 | NM_078467 | CDKN1A | cyclin-dependent kinase inhibitor 1A | -0.146 | 0.904 | 4.04E-02 | 4.04E+00 | | | |
| 7896100 | --- | --- | --- | -0.146 | 0.904 | 8.37E-01 | 8.37E+01 | | | |
| 8136930 | NM_177437 | TAS2R60 | taste receptor, type 2, member 60 | -0.146 | 0.904 | 6.95E-02 | 6.95E+00 | | | |
| 8070757 | NM_144991 | C21orf29 | chromosome 21 open reading frame | -0.146 | 0.904 | 1.55E-02 | 1.55E+00 | | | |
| 8099581 | NM_153686 | LCORL | ligand dependent nuclear receptor c | -0.146 | 0.904 | 3.64E-01 | 3.64E+01 | | | |
| 7893455 | --- | --- | --- | -0.146 | 0.904 | 4.05E-01 | 4.05E+01 | | | |
| 8065550 | --- | --- | --- | -0.146 | 0.904 | 1.22E-01 | 1.22E+01 | | | |
| 8142979 | --- | --- | --- | -0.146 | 0.904 | 5.33E-01 | 5.33E+01 | | | |
| 8099253 | --- | --- | --- | -0.146 | 0.904 | 6.93E-02 | 6.93E+00 | | | |
| 7945445 | BC039077 | C11orf35 | chromosome 11 open reading frame | -0.146 | 0.904 | 1.63E-02 | 1.63E+00 | | | |
| 8168578 | NR_024062 | UBE2DNL | ubiquitin-conjugating enzyme E2D N | -0.146 | 0.904 | 9.00E-03 | 9.00E-01 | | | |
| 8076561 | AB051441 | KIAA1654 | KIAA1654 protein | -0.146 | 0.904 | 8.97E-02 | 8.97E+00 | | | |
| 8067820 | BC024764 | IQSEC3 | IQ motif and Sec7 domain 3 | -0.146 | 0.904 | 1.03E-02 | 1.03E+00 | | | |
| 7897745 | NM_020350 | AGTRAP | angiotensin II receptor-associated pr | -0.146 | 0.904 | 7.13E-02 | 7.13E+00 | | | |
| 8019877 | NM_015295 | SMCHD1 | structural maintenance of chromoso | -0.146 | 0.904 | 4.37E-01 | 4.37E+01 | | | |
| 8053480 | --- | --- | --- | -0.146 | 0.904 | 3.51E-02 | 3.51E+00 | | | |
| 7986160 | NM_00103967 | UNC45A | unc-45 homolog A (C. elegans) | -0.146 | 0.904 | 1.02E-02 | 1.02E+00 | | | |
| 8111214 | XM_00113113 | LOC729724 | similar to TBP-associated factor 11 | -0.146 | 0.904 | 7.37E-02 | 7.37E+00 | | | |
| 8073992 | NM_052839 | PANX2 | pannexin 2 | -0.146 | 0.904 | 1.64E-02 | 1.64E+00 | | | |
| 8121273 | --- | --- | --- | -0.146 | 0.904 | 5.59E-01 | 5.59E+01 | | | |
| 8060505 | NM_00111051 | EBF4 | early B-cell factor 4 | -0.146 | 0.904 | 4.75E-03 | 4.75E-01 | | | |
| 8067652 | NM_001958 | EEF1A2 | eukaryotic translation elongation fac | -0.146 | 0.904 | 6.72E-03 | 6.72E-01 | | | |
| 8028973 | NM_000766 | CYP2A13 | cytochrome P450, family 2, subfamil | -0.146 | 0.904 | 8.48E-03 | 8.48E-01 | | | |
| 7958726 | NM_015267 | CUX2 | cut-like homeobox 2 | -0.146 | 0.904 | 1.57E-02 | 1.57E+00 | | | |
| 7952733 | --- | --- | --- | -0.146 | 0.904 | 1.02E-01 | 1.02E+01 | | | |
| 8125316 | NM_022110 | FKBPL | FK506 binding protein like | -0.146 | 0.904 | 3.14E-02 | 3.14E+00 | | | |
| 8178749 | NM_022110 | FKBPL | FK506 binding protein like | -0.146 | 0.904 | 3.14E-02 | 3.14E+00 | | | |
| 7895464 | --- | --- | --- | -0.146 | 0.904 | 1.90E-01 | 1.90E+01 | | | |
| 8005628 | --- | --- | --- | -0.146 | 0.904 | 1.31E-01 | 1.31E+01 | | | |
| 7896053 | --- | --- | --- | -0.146 | 0.904 | 3.46E-01 | 3.46E+01 | | | |
| 8066590 | NM_003279 | TNNC2 | troponin C type 2 (fast) | -0.146 | 0.904 | 4.24E-02 | 4.24E+00 | | | |
| 8142740 | NM_024523 | GCC1 | GRIP and coiled-coil domain contain | -0.146 | 0.904 | 2.36E-01 | 2.36E+01 | | | |
| 8042501 | --- | --- | --- | -0.146 | 0.904 | 7.72E-01 | 7.72E+01 | | | |
| 8063796 | NM_001794 | CDH4 | cadherin 4, type 1, R-cadherin (retin | -0.146 | 0.904 | 2.08E-02 | 2.08E+00 | | | |
| 7990244 | NM_005477 | HCN4 | hyperpolarization activated cyclic nu | -0.146 | 0.904 | 6.32E-03 | 6.32E-01 | | | |
| 8170921 | NM_017514 | PLXNA3 | plexin A3 | -0.146 | 0.904 | 1.08E-02 | 1.08E+00 | | | |
| 8039771 | NM_003433 | ZNF132 | zinc finger protein 132 | -0.146 | 0.904 | 4.39E-02 | 4.39E+00 | | | |
| 8093807 | NM_173660 | DOK7 | docking protein 7 | -0.146 | 0.904 | 1.55E-02 | 1.55E+00 | | | |
| 8167040 | NR_000041 | RNU12 | RNA, U12 small nuclear | -0.146 | 0.904 | 5.76E-02 | 5.76E+00 | | | |
| 7952813 | NM_014987 | IGSF9B | immunoglobulin superfamily, memb | -0.146 | 0.904 | 7.21E-03 | 7.21E-01 | | | |
| 7994874 | NM_00110507 | FBRS | fibrosin | -0.146 | 0.904 | 1.80E-02 | 1.80E+00 | | | |
| 8013384 | NM_000691 | ALDH3A1 | aldehyde dehydrogenase 3 family, m | -0.146 | 0.904 | 7.72E-03 | 7.72E-01 | | | |
| 8110706 | --- | --- | --- | -0.146 | 0.904 | 1.72E-02 | 1.72E+00 | | | |
| 7959946 | NM_016155 | MMP17 | matrix metallopeptidase 17 (membr | -0.146 | 0.904 | 9.12E-03 | 9.12E-01 | | | |
| 8026292 | --- | --- | --- | -0.146 | 0.904 | 7.66E-02 | 7.66E+00 | | | |
| 8003553 | NM_145039 | CENPBD1 | CENPB DNA-binding domains contai | -0.146 | 0.904 | 2.28E-02 | 2.28E+00 | | | |
| 8146305 | --- | --- | --- | -0.146 | 0.904 | 5.45E-01 | 5.45E+01 | | | |
| 7919898 | NM_030913 | SEMA6C | sema domain, transmembrane doma | -0.146 | 0.904 | 1.10E-02 | 1.10E+00 | | | |
| 8012830 | NM_00100431 | TMEM220 | transmembrane protein 220 | -0.146 | 0.904 | 2.37E-02 | 2.37E+00 | | | |
| 8072484 | ENST0000042 | MGC15705 | hypothetical protein MGC15705 | -0.146 | 0.904 | 3.83E-01 | 3.83E+01 | | | |
| 8031956 | NM_014347 | ZNF324 | zinc finger protein 324 | -0.146 | 0.903 | 5.11E-02 | 5.11E+00 | | | |
| 7895757 | --- | --- | --- | -0.146 | 0.903 | 3.62E-01 | 3.62E+01 | | | |
| 7898112 | --- | --- | --- | -0.146 | 0.903 | 3.29E-01 | 3.29E+01 | | | |
| 8026388 | NM_012377 | OR7C2 | olfactory receptor, family 7, subfami | -0.146 | 0.903 | 3.47E-02 | 3.47E+00 | | | |
| 8119993 | NM_007355 | HSP90AB1 | heat shock protein 90kDa alpha (cyt | -0.146 | 0.903 | 5.70E-01 | 5.70E+01 | | | |
| 7893986 | --- | --- | --- | -0.146 | 0.903 | 6.13E-01 | 6.13E+01 | | | |
| 7934651 | --- | --- | --- | -0.146 | 0.903 | 1.93E-01 | 1.93E+01 | | | |
| 8030582 | NM_00114580 | MYH14 | myosin, heavy chain 14 | -0.146 | 0.903 | 6.65E-03 | 6.65E-01 | | | |
| 8049737 | NM_000030 | AGXT | alanine-glyoxylate aminotransferase | -0.146 | 0.903 | 5.91E-03 | 5.91E-01 | | | |
| 8122734 | NM_030949 | PPP1R14C | protein phosphatase 1, regulatory (i | -0.147 | 0.903 | 6.65E-03 | 6.65E-01 | | | |
| 8007584 | NM_080863 | ASB16 | ankyrin repeat and SOCS box-contai | -0.147 | 0.903 | 6.62E-03 | 6.62E-01 | | | |
| 7990943 | NR_003246 | FLJ40113 | golgi autoantigen, golgin subfamily a | -0.147 | 0.903 | 3.14E-01 | 3.14E+01 | | | |
| 7910618 | NM_173508 | SLC35F3 | solute carrier family 35, member F3 | -0.147 | 0.903 | 8.91E-03 | 8.91E-01 | | | |
| 8149264 | NM_024607 | PPP1R3B | protein phosphatase 1, regulatory (i | -0.147 | 0.903 | 1.63E-02 | 1.63E+00 | | | |
| 7945503 | NM_021008 | DEAF1 | deformed epidermal autoregulatory | -0.147 | 0.903 | 1.78E-02 | 1.78E+00 | | | |

| | | | | | | | | | | |
|---|---|---|---|---|---|---|---|---|---|---|
| 8115948 | NM_00100150: | SNCB | synuclein, beta | -0.147 | 0.903 | 2.58E-02 | 2.58E+00 | | | |
| 8001387 | NM_002968 | SALL1 | sal-like 1 (Drosophila) | -0.147 | 0.903 | 5.71E-03 | 5.71E-01 | | | |
| 7998249 | --- | --- | --- | -0.147 | 0.903 | 3.84E-01 | 3.84E+01 | | | |
| 8127364 | NR_003660 | GUSBL2 | glucuronidase, beta-like 2 | -0.147 | 0.903 | 3.82E-01 | 3.82E+01 | | | |
| 8070538 | NM_015500 | C2CD2 | C2 calcium-dependent domain conta | -0.147 | 0.903 | 1.49E-02 | 1.49E+00 | | | |
| 7894817 | --- | --- | --- | -0.147 | 0.903 | 9.85E-02 | 9.85E+00 | | | |
| 8180241 | --- | --- | --- | -0.147 | 0.903 | 2.27E-02 | 2.27E+00 | | | |
| 8118314 | NM_005346 | HSPA1B | heat shock 70kDa protein 1B | -0.147 | 0.903 | 2.29E-01 | 2.29E+01 | | | |
| 8113502 | --- | --- | --- | -0.147 | 0.903 | 5.43E-02 | 5.43E+00 | | | |
| 8069168 | NM_198699 | KRTAP10-12 | keratin associated protein 10-12 | -0.147 | 0.903 | 2.59E-02 | 2.59E+00 | | | |
| 7991159 | NR_027001 | LOC388152 | hypothetical LOC388152 | -0.147 | 0.903 | 1.31E-03 | 1.31E-01 | | | |
| 7945357 | NM_012239 | SIRT3 | sirtuin (silent mating type informatio | -0.147 | 0.903 | 5.51E-02 | 5.51E+00 | | | |
| 8028600 | NM_00100141 | NCCRP1 | non-specific cytotoxic cell receptor p | -0.147 | 0.903 | 7.96E-03 | 7.96E-01 | | | |
| 8171848 | NM_004845 | PCYT1B | phosphate cytidylyltransferase 1, cho | -0.147 | 0.903 | 1.77E-02 | 1.77E+00 | | | |
| 8077786 | NM_001570 | IRAK2 | interleukin-1 receptor-associated kin | -0.147 | 0.903 | 1.83E-02 | 1.83E+00 | | | |
| 8063410 | NM_032521 | PARD6B | par-6 partitioning defective 6 homol | -0.147 | 0.903 | 1.00E-01 | 1.00E+01 | | | |
| 8029299 | --- | --- | --- | -0.147 | 0.903 | 4.34E-02 | 4.34E+00 | | | |
| 8119926 | NM_018426 | TMEM63B | transmembrane protein 63B | -0.147 | 0.903 | 1.02E-01 | 1.02E+01 | | | |
| 8156599 | --- | --- | --- | -0.147 | 0.903 | 4.20E-01 | 4.20E+01 | | | |
| 8078759 | NM_004256 | SLC22A13 | solute carrier family 22 (organic anio | -0.147 | 0.903 | 1.48E-02 | 1.48E+00 | | | |
| 7995326 | --- | --- | --- | -0.147 | 0.903 | 3.93E-02 | 3.93E+00 | | | |
| 7894798 | --- | --- | --- | -0.147 | 0.903 | 2.96E-01 | 2.96E+01 | | | |
| 8059067 | NM_002181 | IHH | Indian hedgehog homolog (Drosophi | -0.147 | 0.903 | 1.44E-02 | 1.44E+00 | | | |
| 8073924 | uc003bil.1 | LOC388915 | hypothetical LOC388915 | -0.147 | 0.903 | 1.12E-02 | 1.12E+00 | | | |
| 7896822 | NM_198576 | AGRN | agrin | -0.147 | 0.903 | 5.27E-03 | 5.27E-01 | | | |
| 8159646 | NM_00100435 | LOC441476 | hypothetical protein LOC441476 | -0.147 | 0.903 | 6.33E-03 | 6.33E-01 | | | |
| 7951928 | NM_020693 | DSCAML1 | Down syndrome cell adhesion molec | -0.147 | 0.903 | 5.31E-03 | 5.31E-01 | | | |
| 7923958 | NM_023938 | C1orf116 | chromosome 1 open reading frame 1 | -0.147 | 0.903 | 6.30E-02 | 6.30E+00 | | | |
| 7962197 | --- | --- | --- | -0.147 | 0.903 | 1.81E-01 | 1.81E+01 | | | |
| 8148968 | BC037297 | FAM87A | family with sequence similarity 87, m | -0.147 | 0.903 | 6.71E-02 | 6.71E+00 | | | |
| 8070847 | NR_027128 | C21orf67 | chromosome 21 open reading frame | -0.147 | 0.903 | 1.56E-02 | 1.56E+00 | | | |
| 8161526 | AK127288 | LOC440896 | hypothetical LOC440896 | -0.147 | 0.903 | 5.12E-02 | 5.12E+00 | | | |
| 8141643 | NM_00101507 | UFSP1 | UFM1-specific peptidase 1 (non-func | -0.147 | 0.903 | 1.66E-02 | 1.66E+00 | | | |
| 8126147 | BC022007 | C6orf64 | chromosome 6 open reading frame 6 | -0.147 | 0.903 | 9.33E-02 | 9.33E+00 | | | |
| 7906838 | NM_014697 | NOS1AP | nitric oxide synthase 1 (neuronal) ad | -0.147 | 0.903 | 1.98E-02 | 1.98E+00 | | | |
| 8021542 | NM_017742 | ZCCHC2 | zinc finger, CCHC domain containing | -0.147 | 0.903 | 2.40E-03 | 2.40E-01 | | | |
| 8100714 | NM_00103173 | YTHDC1 | YTH domain containing 1 | -0.147 | 0.903 | 5.31E-01 | 5.31E+01 | | | |
| 8008662 | NR_002818 | RNF126P1 | ring finger protein 126 pseudogene 1 | -0.147 | 0.903 | 1.38E-02 | 1.38E+00 | | | |
| 7940431 | NM_00107980 | PGA4 | pepsinogen 4, group I (pepsinogen A | -0.147 | 0.903 | 2.09E-02 | 2.09E+00 | | | |
| 8112666 | --- | --- | --- | -0.147 | 0.903 | 1.19E-01 | 1.19E+01 | | | |
| 7992614 | NM_025108 | C16orf59 | chromosome 16 open reading frame | -0.147 | 0.903 | 1.44E-02 | 1.44E+00 | | | |
| 8162216 | NM_016848 | SHC3 | SHC (Src homology 2 domain contair | -0.147 | 0.903 | 6.26E-03 | 6.26E-01 | | | |
| 8007550 | NM_153006 | NAGS | N-acetylglutamate synthase | -0.147 | 0.903 | 7.22E-03 | 7.22E-01 | | | |
| 7919193 | NR_002212 | NUDT4P1 | nudix (nucleoside diphosphate linked | -0.147 | 0.903 | 7.10E-01 | 7.10E+01 | | | |
| 8027574 | NM_022467 | CHST8 | carbohydrate (N-acetylgalactosamine | -0.147 | 0.903 | 9.25E-03 | 9.25E-01 | | | |
| 8066294 | NM_052846 | EMILIN3 | elastin microfibril interfacer 3 | -0.147 | 0.903 | 1.76E-02 | 1.76E+00 | | | |
| 7952321 | NM_00109816 | BSX | brain-specific homeobox | -0.147 | 0.903 | 2.85E-02 | 2.85E+00 | | | |
| 8150960 | --- | --- | --- | -0.147 | 0.903 | 2.42E-01 | 2.42E+01 | | | |
| 7898209 | --- | --- | --- | -0.147 | 0.903 | 2.61E-01 | 2.61E+01 | | | |
| 7969093 | NM_001507 | MLNR | motilin receptor | -0.147 | 0.903 | 4.71E-02 | 4.71E+00 | | | |
| 7928291 | NM_004273 | CHST3 | carbohydrate (chondroitin 6) sulfotra | -0.147 | 0.903 | 4.33E-02 | 4.33E+00 | | | |
| 8085272 | AK022260 | LOC401052 | hypothetical LOC401052 | -0.147 | 0.903 | 1.01E-02 | 1.01E+00 | | | |
| 7935903 | NM_006993 | NPM3 | nucleophosmin/nucleoplasmin 3 | -0.147 | 0.903 | 3.38E-02 | 3.38E+00 | | | |
| 7959002 | NM_138432 | SDSL | serine dehydratase-like | -0.147 | 0.903 | 5.31E-03 | 5.31E-01 | | | |
| 8150197 | NM_024025 | DUSP26 | dual specificity phosphatase 26 (puta | -0.147 | 0.903 | 5.40E-03 | 5.40E-01 | | | |
| 7894720 | --- | --- | --- | -0.147 | 0.903 | 7.49E-01 | 7.49E+01 | | | |
| 8008372 | NM_017957 | EPN3 | epsin 3 | -0.147 | 0.903 | 1.23E-02 | 1.23E+00 | | | |
| 8045309 | NM_138770 | CCDC74A | coiled-coil domain containing 74A | -0.148 | 0.903 | 2.65E-02 | 2.65E+00 | | | |
| 8032037 | NM_012435 | SHC2 | SHC (Src homology 2 domain contair | -0.148 | 0.903 | 1.45E-02 | 1.45E+00 | | | |
| 8179617 | NM_007028 | TRIM31 | tripartite motif-containing 31 | -0.148 | 0.903 | 1.18E-02 | 1.18E+00 | | | |
| 8076393 | NM_024053 | CENPM | centromere protein M | -0.148 | 0.903 | 1.33E-02 | 1.33E+00 | | | |
| 8131427 | BC001076 | C7orf26 | chromosome 7 open reading frame 2 | -0.148 | 0.903 | 3.81E-01 | 3.81E+01 | | | |
| 8049243 | NM_005383 | NEU2 | sialidase 2 (cytosolic sialidase) | -0.148 | 0.903 | 1.18E-02 | 1.18E+00 | | | |
| 8080466 | NM_002217 | ITIH3 | inter-alpha (globulin) inhibitor H3 | -0.148 | 0.903 | 1.29E-02 | 1.29E+00 | | | |
| 7929779 | NM_000392 | ABCC2 | ATP-binding cassette, sub-family C (C | -0.148 | 0.903 | 1.35E-02 | 1.35E+00 | | | |
| 8062813 | NM_178491 | R3HDML | R3H domain containing-like | -0.148 | 0.903 | 3.29E-03 | 3.29E-01 | | | |
| 8036270 | NM_152658 | THAP8 | THAP domain containing 8 | -0.148 | 0.903 | 3.46E-02 | 3.46E+00 | | | |
| 7905999 | NM_00110520 | RUSC1 | RUN and SH3 domain containing 1 | -0.148 | 0.903 | 1.39E-02 | 1.39E+00 | | | |
| 7919301 | AK128227 | OC10013023 | hypothetical LOC100130236 | -0.148 | 0.903 | 7.38E-02 | 7.38E+00 | | | |
| 7892533 | --- | --- | --- | -0.148 | 0.903 | 3.09E-01 | 3.09E+01 | | | |
| 8167786 | --- | --- | --- | -0.148 | 0.903 | 1.12E-01 | 1.12E+01 | | | |
| 8019308 | NM_032711 | MAFG | v-maf musculoaponeurotic fibrosarc | -0.148 | 0.903 | 3.98E-03 | 3.98E-01 | | | |
| 8087453 | NM_198722 | AMIGO3 | adhesion molecule with Ig-like doma | -0.148 | 0.903 | 1.33E-02 | 1.33E+00 | | | |
| 8038093 | NM_00108043 | LMTK3 | lemur tyrosine kinase 3 | -0.148 | 0.903 | 1.31E-02 | 1.31E+00 | | | |
| 7955702 | NM_003578 | SOAT2 | sterol O-acyltransferase 2 | -0.148 | 0.903 | 9.87E-03 | 9.87E-01 | | | |
| 7991828 | --- | --- | --- | -0.148 | 0.903 | 9.68E-03 | 9.68E-01 | | | |
| 7950423 | --- | --- | --- | -0.148 | 0.903 | 6.78E-02 | 6.78E+00 | | | |

| | | | | | | | | | | |
|---|---|---|---|---|---|---|---|---|---|---|
| 7911506 | NM_004421 | DVL1 | dishevelled, dsh homolog 1 (Drosoph | -0.148 | 0.903 | 5.44E-03 | 5.44E-01 | | | |
| 8059177 | NM_006000 | TUBA4A | tubulin, alpha 4a | -0.148 | 0.903 | 3.46E-01 | 3.46E+01 | | | |
| 7931571 | NM_145202 | PRAP1 | proline-rich acidic protein 1 | -0.148 | 0.903 | 1.25E-02 | 1.25E+00 | | | |
| 7985409 | NR_003246 | FLJ40113 | golgi autoantigen, golgin subfamily a | -0.148 | 0.902 | 2.99E-01 | 2.99E+01 | | | |
| 7985450 | NR_003246 | FLJ40113 | golgi autoantigen, golgin subfamily a | -0.148 | 0.902 | 2.99E-01 | 2.99E+01 | | | |
| 8165512 | NM_017820 | EXD3 | exonuclease 3'-5' domain containing | -0.148 | 0.902 | 1.34E-02 | 1.34E+00 | | | |
| 8139769 | AK125790 | LOC401357 | hypothetical LOC401357 | -0.148 | 0.902 | 3.37E-02 | 3.37E+00 | | | |
| 8109149 | NM_024028 | PCYOX1L | prenylcysteine oxidase 1 like | -0.148 | 0.902 | 2.48E-01 | 2.48E+01 | | | |
| 8125007 | NM_025262 | LY6G5C | lymphocyte antigen 6 complex, locus | -0.148 | 0.902 | 2.39E-01 | 2.39E+01 | | | |
| 8139816 | --- | --- | --- | -0.148 | 0.902 | 6.67E-01 | 6.67E+01 | | | |
| 7996908 | NM_006750 | SNTB2 | syntrophin, beta 2 (dystrophin-assoc | -0.148 | 0.902 | 9.81E-02 | 9.81E+00 | | | |
| 8069933 | NM_018944 | C21orf45 | chromosome 21 open reading frame | -0.148 | 0.902 | 4.72E-01 | 4.72E+01 | | | |
| 7896546 | --- | --- | --- | -0.148 | 0.902 | 6.68E-01 | 6.68E+01 | | | |
| 7896022 | --- | --- | --- | -0.148 | 0.902 | 6.80E-01 | 6.80E+01 | | | |
| 8064014 | NM_022082 | SLC17A9 | solute carrier family 17, member 9 | -0.148 | 0.902 | 1.05E-02 | 1.05E+00 | | | |
| 8025375 | NM_005624 | CCL25 | chemokine (C-C motif) ligand 25 | -0.148 | 0.902 | 9.59E-03 | 9.59E-01 | | | |
| 8125780 | NM_054111 | IP6K3 | inositol hexakisphosphate kinase 3 | -0.148 | 0.902 | 1.61E-02 | 1.61E+00 | | | |
| 8175977 | NM_001569 | IRAK1 | interleukin-1 receptor-associated kin | -0.148 | 0.902 | 4.19E-03 | 4.19E-01 | | | |
| 8063970 | NM_001853 | COL9A3 | collagen, type IX, alpha 3 | -0.148 | 0.902 | 1.54E-02 | 1.54E+00 | | | |
| 7950012 | NM_053056 | CCND1 | cyclin D1 | -0.148 | 0.902 | 1.93E-02 | 1.93E+00 | | | |
| 8017361 | --- | --- | --- | -0.148 | 0.902 | 5.68E-01 | 5.68E+01 | | | |
| 7925413 | AF333388 | MT1P2 | metallothionein 1 pseudogene 2 | -0.148 | 0.902 | 4.20E-02 | 4.20E+00 | | | |
| 8002618 | NM_017530 | ZNF821 | zinc finger protein 821 | -0.148 | 0.902 | 9.72E-02 | 9.72E+00 | | | |
| 8147573 | NM_00114246 | OSR2 | odd-skipped related 2 (Drosophila) | -0.148 | 0.902 | 7.65E-03 | 7.65E-01 | | | |
| 8172345 | NM_00111412 | ELK1 | ELK1, member of ETS oncogene fami | -0.148 | 0.902 | 7.33E-03 | 7.33E-01 | | | |
| 8153359 | NM_003695 | LY6D | lymphocyte antigen 6 complex, locus | -0.148 | 0.902 | 9.69E-03 | 9.69E-01 | | | |
| 8029006 | NM_021913 | AXL | AXL receptor tyrosine kinase | -0.148 | 0.902 | 1.89E-02 | 1.89E+00 | | | |
| 8008297 | NM_022167 | XYLT2 | xylosyltransferase II | -0.148 | 0.902 | 1.52E-02 | 1.52E+00 | | | |
| 7984686 | NM_033028 | BBS4 | Bardet-Biedl syndrome 4 | -0.148 | 0.902 | 3.22E-01 | 3.22E+01 | | | |
| 8093916 | NM_015274 | MAN2B2 | mannosidase, alpha, class 2B, memb | -0.148 | 0.902 | 9.25E-03 | 9.25E-01 | | | |
| 8031076 | NM_031896 | CACNG7 | calcium channel, voltage-dependent | -0.148 | 0.902 | 4.71E-03 | 4.71E-01 | | | |
| 8011375 | NM_080704 | TRPV1 | transient receptor potential cation c | -0.148 | 0.902 | 1.06E-02 | 1.06E+00 | | | |
| 7940790 | BC056402 | C11orf84 | chromosome 11 open reading frame | -0.148 | 0.902 | 9.15E-03 | 9.15E-01 | | | |
| 8126379 | ENST0000034 | C6orf132 | chromosome 6 open reading frame 1 | -0.149 | 0.902 | 3.85E-02 | 3.85E+00 | | | |
| 8019334 | NM_178493 | NOTUM | notum pectinacetylesterase homolog | -0.149 | 0.902 | 1.85E-02 | 1.85E+00 | | | |
| 8030848 | NR_024330 | NCRNA0008 | non-protein coding RNA 85 | -0.149 | 0.902 | 5.94E-03 | 5.94E-01 | | | |
| 8153373 | NM_000498 | CYP11B2 | cytochrome P450, family 11, subfam | -0.149 | 0.902 | 4.50E-03 | 4.50E-01 | | | |
| 8088776 | NM_032682 | FOXP1 | forkhead box P1 | -0.149 | 0.902 | 4.53E-01 | 4.53E+01 | | | |
| 8021900 | NM_012283 | KCNG2 | potassium voltage-gated channel, su | -0.149 | 0.902 | 1.79E-02 | 1.79E+00 | | | |
| 7901229 | NM_001441 | FAAH | fatty acid amide hydrolase | -0.149 | 0.902 | 9.21E-03 | 9.21E-01 | | | |
| 7896602 | --- | --- | --- | -0.149 | 0.902 | 4.84E-02 | 4.84E+00 | | | |
| 7941746 | AK026328 | C11orf86 | chromosome 11 open reading frame | -0.149 | 0.902 | 1.30E-02 | 1.30E+00 | | | |
| 8151223 | NM_030958 | SLCO5A1 | solute carrier organic anion transpor | -0.149 | 0.902 | 1.72E-02 | 1.72E+00 | | | |
| 7923442 | NM_177402 | SYT2 | synaptotagmin II | -0.149 | 0.902 | 2.22E-02 | 2.22E+00 | | | |
| 8041804 | NM_00114505 | LOC388946 | hypothetical LOC388946 | -0.149 | 0.902 | 2.67E-02 | 2.67E+00 | | | |
| 8075160 | --- | --- | --- | -0.149 | 0.902 | 1.33E-01 | 1.33E+01 | | | |
| 8027398 | NM_024310 | PLEKHF1 | pleckstrin homology domain contain | -0.149 | 0.902 | 4.13E-02 | 4.13E+00 | | | |
| 7897006 | NM_006983 | MMP23B | matrix metallopeptidase 23B | -0.149 | 0.902 | 5.33E-03 | 5.33E-01 | | | |
| 7939665 | NM_00101233 | MDK | midkine (neurite growth-promoting | -0.149 | 0.902 | 1.11E-02 | 1.11E+00 | | | |
| 8131101 | NM_198949 | NUDT1 | nudix (nucleoside diphosphate linked | -0.149 | 0.902 | 1.21E-01 | 1.21E+01 | | | |
| 7991809 | NM_001176 | ARHGDIG | Rho GDP dissociation inhibitor (GDI) | -0.149 | 0.902 | 2.29E-02 | 2.29E+00 | | | |
| 7998585 | NM_023936 | MRPS34 | mitochondrial ribosomal protein S34 | -0.149 | 0.902 | 9.82E-02 | 9.82E+00 | | | |
| 8060447 | NM_198994 | TGM6 | transglutaminase 6 | -0.149 | 0.902 | 9.39E-03 | 9.39E-01 | | | |
| 8048551 | NM_005876 | SPEG | SPEG complex locus | -0.149 | 0.902 | 9.39E-03 | 9.39E-01 | | | |
| 7910372 | NM_00101085 | RNF187 | ring finger protein 187 | -0.149 | 0.902 | 1.86E-02 | 1.86E+00 | | | |
| 7893740 | --- | --- | --- | -0.149 | 0.902 | 2.05E-01 | 2.05E+01 | | | |
| 7954055 | NM_00113041 | APOLD1 | apolipoprotein L domain containing | -0.149 | 0.902 | 3.08E-01 | 3.08E+01 | | | |
| 8010320 | NM_005189 | CBX2 | chromobox homolog 2 (Pc class hom | -0.149 | 0.902 | 1.47E-02 | 1.47E+00 | | | |
| 7951859 | NM_000482 | APOA4 | apolipoprotein A-IV | -0.149 | 0.902 | 7.26E-03 | 7.26E-01 | | | |
| 7895608 | --- | --- | --- | -0.149 | 0.902 | 6.31E-01 | 6.31E+01 | | | |
| 8020495 | NM_138375 | CABLES1 | Cdk5 and Abl enzyme substrate 1 | -0.149 | 0.902 | 2.62E-02 | 2.62E+00 | | | |
| 8108912 | NM_152550 | SH3RF2 | SH3 domain containing ring finger 2 | -0.149 | 0.902 | 1.40E-02 | 1.40E+00 | | | |
| 7892572 | --- | --- | --- | -0.149 | 0.902 | 4.50E-01 | 4.50E+01 | | | |
| 7966760 | NM_033624 | FBXO21 | F-box protein 21 | -0.149 | 0.902 | 1.11E-01 | 1.11E+01 | | | |
| 7894235 | --- | --- | --- | -0.149 | 0.902 | 1.21E-01 | 1.21E+01 | | | |
| 7989885 | NM_00103172 | DNAJB14 | DnaJ (Hsp40) homolog, subfamily B, | -0.149 | 0.902 | 7.68E-01 | 7.68E+01 | | | |
| 7979572 | --- | --- | --- | -0.149 | 0.902 | 6.04E-01 | 6.04E+01 | | | |
| 8176655 | NM_014893 | NLGN4Y | neuroligin 4, Y-linked | -0.149 | 0.902 | 2.17E-02 | 2.17E+00 | | | |
| 7915827 | NM_006369 | LRRC41 | leucine rich repeat containing 41 | -0.149 | 0.902 | 2.09E-02 | 2.09E+00 | | | |
| 7966746 | NM_003806 | HRK | harakiri, BCL2 interacting protein (co | -0.149 | 0.902 | 1.27E-02 | 1.27E+00 | | | |
| 7970139 | BC029889 | C13orf16 | chromosome 13 open reading frame | -0.149 | 0.902 | 3.15E-02 | 3.15E+00 | | | |
| 7913667 | NM_000403 | GALE | UDP-galactose-4-epimerase | -0.149 | 0.902 | 2.34E-02 | 2.34E+00 | | | |
| 8138007 | --- | --- | --- | -0.149 | 0.902 | 2.54E-02 | 2.54E+00 | | | |
| 7895465 | --- | --- | --- | -0.149 | 0.902 | 6.20E-01 | 6.20E+01 | | | |
| 8062337 | --- | --- | --- | -0.149 | 0.902 | 1.59E-02 | 1.59E+00 | | | |
| 7899167 | NM_024674 | LIN28 | lin-28 homolog (C. elegans) | -0.149 | 0.902 | 2.95E-02 | 2.95E+00 | | | |
| 8149216 | NR_027000 | LOC349196 | hypothetical LOC349196 | -0.149 | 0.902 | 1.22E-01 | 1.22E+01 | | | |

| | | | | | | | | | | |
|---|---|---|---|---|---|---|---|---|---|---|
| 7892860 | --- | --- | --- | -0.149 | 0.902 | 3.20E-01 | 3.20E+01 | | | |
| 8155569 | BC119675 | FAM27E3 | family with sequence similarity 27, m | -0.149 | 0.902 | 1.06E-02 | 1.06E+00 | | | |
| 8161426 | BC119675 | FAM27E3 | family with sequence similarity 27, m | -0.149 | 0.902 | 1.06E-02 | 1.06E+00 | | | |
| 8161437 | BC119675 | FAM27E3 | family with sequence similarity 27, m | -0.149 | 0.902 | 1.06E-02 | 1.06E+00 | | | |
| 8026712 | NM_152363 | ANKLE1 | ankyrin repeat and LEM domain cont | -0.149 | 0.902 | 1.34E-02 | 1.34E+00 | | | |
| 8117239 | --- | --- | --- | -0.149 | 0.902 | 7.46E-02 | 7.46E+00 | | | |
| 8165458 | --- | --- | --- | -0.149 | 0.902 | 7.60E-02 | 7.60E+00 | | | |
| 7967193 | NM_173855 | MORN3 | MORN repeat containing 3 | -0.149 | 0.902 | 2.65E-01 | 2.65E+01 | | | |
| 7906284 | NM_144702 | C1orf92 | chromosome 1 open reading frame 9 | -0.150 | 0.902 | 4.90E-03 | 4.90E-01 | | | |
| 7893137 | --- | --- | --- | -0.150 | 0.902 | 2.96E-01 | 2.96E+01 | | | |
| 8030212 | NM_022165 | LIN7B | lin-7 homolog B (C. elegans) | -0.150 | 0.902 | 1.61E-02 | 1.61E+00 | | | |
| 8067358 | AK097866 | P11-429E11. | hypothetical LOC100128310 | -0.150 | 0.902 | 3.24E-02 | 3.24E+00 | | | |
| 8150578 | --- | --- | --- | -0.150 | 0.902 | 2.75E-02 | 2.75E+00 | | | |
| 8117339 | NM_003531 | HIST1H3C | histone cluster 1, H3c | -0.150 | 0.902 | 7.33E-02 | 7.33E+00 | | | |
| 8065920 | NR_024377 | FER1L4 | fer-1-like 4 (C. elegans) | -0.150 | 0.902 | 1.26E-03 | 1.26E-01 | | | |
| 7992347 | NM_015133 | MAPK8IP3 | mitogen-activated protein kinase 8 in | -0.150 | 0.902 | 1.33E-02 | 1.33E+00 | | | |
| 8091485 | NM_005067 | SIAH2 | seven in absentia homolog 2 (Drosop | -0.150 | 0.901 | 1.61E-02 | 1.61E+00 | | | |
| 7930627 | NM_000684 | ADRB1 | adrenergic, beta-1-, receptor | -0.150 | 0.901 | 5.11E-03 | 5.11E-01 | | | |
| 8098167 | BC022381 | C4orf39 | chromosome 4 open reading frame 3 | -0.150 | 0.901 | 6.15E-02 | 6.15E+00 | | | |
| 7912701 | NM_182623 | FAM131C | family with sequence similarity 131, | -0.150 | 0.901 | 6.32E-02 | 6.32E+00 | | | |
| 7895046 | --- | --- | --- | -0.150 | 0.901 | 6.19E-01 | 6.19E+01 | | | |
| 8037651 | NM_175875 | SIX5 | SIX homeobox 5 | -0.150 | 0.901 | 8.63E-03 | 8.63E-01 | | | |
| 7970329 | NM_000820 | GAS6 | growth arrest-specific 6 | -0.150 | 0.901 | 3.16E-02 | 3.16E+00 | | | |
| 7955974 | NM_021191 | NEUROD4 | neurogenic differentiation 4 | -0.150 | 0.901 | 1.57E-02 | 1.57E+00 | | | |
| 7981982 | NR_003332 | SNORD116-1 | small nucleolar RNA, C/D box 116-17 | -0.150 | 0.901 | 8.39E-01 | 8.39E+01 | | | |
| 7981986 | NR_003332 | SNORD116-1 | small nucleolar RNA, C/D box 116-17 | -0.150 | 0.901 | 8.39E-01 | 8.39E+01 | | | |
| 7972570 | NM_033110 | A2LD1 | AIG2-like domain 1 | -0.150 | 0.901 | 1.24E-02 | 1.24E+00 | | | |
| 8134981 | NM_003302 | TRIP6 | thyroid hormone receptor interactor | -0.150 | 0.901 | 1.58E-02 | 1.58E+00 | | | |
| 8031939 | NM_173548 | ZNF584 | zinc finger protein 584 | -0.150 | 0.901 | 5.71E-02 | 5.71E+00 | | | |
| 8038716 | NM_012315 | KLK9 | kallikrein-related peptidase 9 | -0.150 | 0.901 | 2.36E-02 | 2.36E+00 | | | |
| 7993054 | NM_014692 | SEC14L5 | SEC14-like 5 (S. cerevisiae) | -0.150 | 0.901 | 5.27E-03 | 5.27E-01 | | | |
| 8134880 | NM_023948 | MOSPD3 | motile sperm domain containing 3 | -0.150 | 0.901 | 5.47E-02 | 5.47E+00 | | | |
| 8014865 | NM_006160 | NEUROD2 | neurogenic differentiation 2 | -0.150 | 0.901 | 7.46E-03 | 7.46E-01 | | | |
| 8118724 | --- | --- | --- | -0.150 | 0.901 | 2.49E-01 | 2.49E+01 | | | |
| 7969368 | --- | --- | --- | -0.150 | 0.901 | 4.83E-02 | 4.83E+00 | | | |
| 8133345 | NM_017528 | WBSCR22 | Williams Beuren syndrome chromoso | -0.150 | 0.901 | 4.09E-01 | 4.09E+01 | | | |
| 8004763 | NM_000180 | GUCY2D | guanylate cyclase 2D, membrane (re | -0.150 | 0.901 | 4.89E-03 | 4.89E-01 | | | |
| 7949320 | NM_130769 | GPHA2 | glycoprotein hormone alpha 2 | -0.150 | 0.901 | 1.57E-02 | 1.57E+00 | | | |
| 8061564 | NM_181353 | ID1 | inhibitor of DNA binding 1, dominant | -0.150 | 0.901 | 9.93E-03 | 9.93E-01 | | | |
| 8056959 | NM_00108045 | EVX2 | even-skipped homeobox 2 | -0.150 | 0.901 | 2.16E-02 | 2.16E+00 | | | |
| 8169603 | NM_00103185 | LONRF3 | LON peptidase N-terminal domain ar | -0.150 | 0.901 | 2.31E-02 | 2.31E+00 | | | |
| 8032478 | NM_00110139 | LINGO3 | leucine rich repeat and Ig domain co | -0.150 | 0.901 | 1.63E-02 | 1.63E+00 | | | |
| 8044844 | NM_00110519 | TMEM177 | transmembrane protein 177 | -0.150 | 0.901 | 3.02E-02 | 3.02E+00 | | | |
| 8050557 | NM_00100694 | SDC1 | syndecan 1 | -0.150 | 0.901 | 1.12E-02 | 1.12E+00 | | | |
| 7977782 | ENST0000312 | OR6J1 | olfactory receptor, family 6, subfami | -0.150 | 0.901 | 5.08E-03 | 5.08E-01 | | | |
| 8028806 | NM_020971 | SPTBN4 | spectrin, beta, non-erythrocytic 4 | -0.150 | 0.901 | 5.49E-03 | 5.49E-01 | | | |
| 8095362 | NM_005953 | MT2A | metallothionein 2A | -0.150 | 0.901 | 1.44E-02 | 1.44E+00 | | | |
| 7981326 | AF305836 | DIO3OS | DIO3 opposite strand (non-protein c | -0.150 | 0.901 | 2.07E-02 | 2.07E+00 | | | |
| 8145865 | NM_032777 | GPR124 | G protein-coupled receptor 124 | -0.150 | 0.901 | 1.59E-02 | 1.59E+00 | | | |
| 7894671 | --- | --- | --- | -0.150 | 0.901 | 2.35E-01 | 2.35E+01 | | | |
| 7920217 | NM_00101429 | SPRR2G | small proline-rich protein 2G | -0.150 | 0.901 | 2.47E-02 | 2.47E+00 | | | |
| 8112072 | NM_021147 | CCNO | cyclin O | -0.150 | 0.901 | 2.40E-02 | 2.40E+00 | | | |
| 8106689 | NM_001825 | CKMT2 | creatine kinase, mitochondrial 2 (sar | -0.150 | 0.901 | 9.19E-03 | 9.19E-01 | | | |
| 8024238 | NM_001280 | CIRBP | cold inducible RNA binding protein | -0.150 | 0.901 | 4.89E-01 | 4.89E+01 | | | |
| 8058524 | NM_020989 | CRYGC | crystallin, gamma C | -0.150 | 0.901 | 4.07E-02 | 4.07E+00 | | | |
| 8116335 | NM_016175 | C5orf45 | chromosome 5 open reading frame 4 | -0.150 | 0.901 | 3.09E-02 | 3.09E+00 | | | |
| 8149161 | NR_027000 | LOC349196 | hypothetical LOC349196 | -0.150 | 0.901 | 6.38E-02 | 6.38E+00 | | | |
| 8149210 | NR_027000 | LOC349196 | hypothetical LOC349196 | -0.150 | 0.901 | 6.38E-02 | 6.38E+00 | | | |
| 7912743 | NM_030907 | C1orf89 | chromosome 1 open reading frame 8 | -0.150 | 0.901 | 1.34E-02 | 1.34E+00 | | | |
| 7985767 | NM_022767 | AEN | apoptosis enhancing nuclease | -0.150 | 0.901 | 3.14E-02 | 3.14E+00 | | | |
| 7896092 | --- | --- | --- | -0.150 | 0.901 | 2.53E-01 | 2.53E+01 | | | |
| 8020226 | NM_00114240 | SLMO1 | slowmo homolog 1 (Drosophila) | -0.150 | 0.901 | 3.42E-03 | 3.42E-01 | | | |
| 8049950 | AK094921 | OC10013176 | hypothetical LOC100131763 | -0.150 | 0.901 | 1.20E-02 | 1.20E+00 | | | |
| 8173232 | NM_152424 | FAM123B | family with sequence similarity 123B | -0.151 | 0.901 | 1.08E-02 | 1.08E+00 | | | |
| 8012558 | NM_00114263 | PIK3R5 | phosphoinositide-3-kinase, regulator | -0.151 | 0.901 | 6.57E-02 | 6.57E+00 | | | |
| 8117165 | NM_003107 | SOX4 | SRY (sex determining region Y)-box 4 | -0.151 | 0.901 | 8.61E-03 | 8.61E-01 | | | |
| 8133339 | NM_00107762 | VPS37D | vacuolar protein sorting 37 homolog | -0.151 | 0.901 | 2.28E-02 | 2.28E+00 | | | |
| 8082663 | NR_027766 | NUDT16P | nudix (nucleoside diphosphate linked | -0.151 | 0.901 | 1.86E-02 | 1.86E+00 | | | |
| 8072346 | NM_016498 | MTP18 | mitochondrial protein 18 kDa | -0.151 | 0.901 | 2.16E-02 | 2.16E+00 | | | |
| 8087433 | NM_032316 | NICN1 | nicolin 1 | -0.151 | 0.901 | 4.47E-02 | 4.47E+00 | | | |
| 8001163 | NM_182493 | MYLK3 | myosin light chain kinase 3 | -0.151 | 0.901 | 8.48E-03 | 8.48E-01 | | | |
| 8167924 | NM_013444 | UBQLN2 | ubiquilin 2 | -0.151 | 0.901 | 2.48E-02 | 2.48E+00 | | | |
| 7970858 | NM_002128 | HMGB1 | high-mobility group box 1 | -0.151 | 0.901 | 3.12E-01 | 3.12E+01 | | | |
| 8128667 | --- | --- | --- | -0.151 | 0.901 | 5.74E-02 | 5.74E+00 | | | |
| 8019478 | NM_006137 | CD7 | CD7 molecule | -0.151 | 0.901 | 2.30E-02 | 2.30E+00 | | | |
| 8112435 | --- | --- | --- | -0.151 | 0.901 | 1.77E-02 | 1.77E+00 | | | |
| 7981978 | NR_003330 | SNORD116-1 | small nucleolar RNA, C/D box 116-15 | -0.151 | 0.901 | 7.12E-01 | 7.12E+01 | | | |

| | | | | | | | | | | |
|---|---|---|---|---|---|---|---|---|---|---|
| 8075390 | NM_174977 | SEC14L4 | SEC14-like 4 (S. cerevisiae) | -0.151 | 0.901 | 3.05E-02 | 3.05E+00 | | | |
| 8035408 | NM_000923 | PDE4C | phosphodiesterase 4C, cAMP-specifi | -0.151 | 0.901 | 7.72E-03 | 7.72E-01 | | | |
| 8072931 | NM_014291 | GCAT | glycine C-acetyltransferase (2-amino | -0.151 | 0.901 | 2.10E-02 | 2.10E+00 | | | |
| 8046408 | NM_002610 | PDK1 | pyruvate dehydrogenase kinase, isoz | -0.151 | 0.901 | 6.10E-01 | 6.10E+01 | | | |
| 8069764 | --- | --- | --- | -0.151 | 0.901 | 1.21E-01 | 1.21E+01 | | | |
| 8026390 | NM_173482 | CCDC105 | coiled-coil domain containing 105 | -0.151 | 0.901 | 1.04E-02 | 1.04E+00 | | | |
| 8037594 | NM_005619 | RTN2 | reticulon 2 | -0.151 | 0.901 | 1.25E-02 | 1.25E+00 | | | |
| 7896917 | NM_00102988! | GLTPD1 | glycolipid transfer protein domain co | -0.151 | 0.901 | 5.36E-03 | 5.36E-01 | | | |
| 7896761 | NM_152486 | SAMD11 | sterile alpha motif domain containin | -0.151 | 0.901 | 2.34E-02 | 2.34E+00 | | | |
| 7912031 | NM_019089 | HES2 | hairy and enhancer of split 2 (Drosop | -0.151 | 0.901 | 4.52E-03 | 4.52E-01 | | | |
| 8125775 | AY568085 | C6orf125 | chromosome 6 open reading frame 1 | -0.151 | 0.900 | 5.41E-01 | 5.41E+01 | | | |
| 8148517 | NM_001702 | BAI1 | brain-specific angiogenesis inhibitor | -0.151 | 0.900 | 3.23E-03 | 3.23E-01 | | | |
| 8026900 | NM_002248 | KCNN1 | potassium intermediate/small condu | -0.151 | 0.900 | 2.30E-02 | 2.30E+00 | | | |
| 8104568 | AY358688 | UNQ1870 | GALI1870 | -0.151 | 0.900 | 1.14E-01 | 1.14E+01 | | | |
| 8027886 | NM_015302 | HAUS5 | HAUS augmin-like complex, subunit 5 | -0.151 | 0.900 | 2.34E-03 | 2.34E-01 | | | |
| 8180231 | --- | --- | --- | -0.151 | 0.900 | 3.22E-02 | 3.22E+00 | | | |
| 7994260 | NM_006040 | HS3ST4 | heparan sulfate (glucosamine) 3-O-su | -0.151 | 0.900 | 2.75E-03 | 2.75E-01 | | | |
| 8001938 | NM_003789 | TRADD | TNFRSF1A-associated via death dom | -0.151 | 0.900 | 1.57E-02 | 1.57E+00 | | | |
| 8144951 | --- | --- | --- | -0.151 | 0.900 | 4.02E-02 | 4.02E+00 | | | |
| 8014956 | NM_021724 | NR1D1 | nuclear receptor subfamily 1, group | -0.151 | 0.900 | 1.82E-02 | 1.82E+00 | | | |
| 7995803 | AF348994 | MT1JP | metallothionein 1J (pseudogene) | -0.151 | 0.900 | 2.58E-01 | 2.58E+01 | | | |
| 7917875 | NM_001993 | F3 | coagulation factor III (thromboplasti | -0.151 | 0.900 | 1.04E-02 | 1.04E+00 | | | |
| 8014666 | NM_00100433 | GPR179 | G protein-coupled receptor 179 | -0.151 | 0.900 | 8.13E-03 | 8.13E-01 | | | |
| 7894678 | --- | --- | --- | -0.151 | 0.900 | 3.11E-01 | 3.11E+01 | | | |
| 7995787 | NM_176870 | MT1M | metallothionein 1M | -0.152 | 0.900 | 1.28E-01 | 1.28E+01 | | | |
| 8029759 | NM_00108040 | CCDC61 | coiled-coil domain containing 61 | -0.152 | 0.900 | 2.74E-02 | 2.74E+00 | | | |
| 7896230 | --- | --- | --- | -0.152 | 0.900 | 7.73E-01 | 7.73E+01 | | | |
| 8010071 | NM_001088 | AANAT | arylalkylamine N-acetyltransferase | -0.152 | 0.900 | 3.80E-02 | 3.80E+00 | | | |
| 8144412 | NR_027000 | LOC349196 | hypothetical LOC349196 | -0.152 | 0.900 | 9.00E-02 | 9.00E+00 | | | |
| 8144414 | NR_027000 | LOC349196 | hypothetical LOC349196 | -0.152 | 0.900 | 9.00E-02 | 9.00E+00 | | | |
| 7978059 | BC131533 | ZFHX2 | zinc finger homeobox 2 | -0.152 | 0.900 | 3.07E-02 | 3.07E+00 | | | |
| 8095562 | --- | --- | --- | -0.152 | 0.900 | 4.39E-02 | 4.39E+00 | | | |
| 7920616 | NM_144622 | DCST2 | DC-STAMP domain containing 2 | -0.152 | 0.900 | 1.39E-02 | 1.39E+00 | | | |
| 8021363 | --- | --- | --- | -0.152 | 0.900 | 6.41E-02 | 6.41E+00 | | | |
| 7989924 | NM_006049 | SNAPC5 | small nuclear RNA activating comple | -0.152 | 0.900 | 5.63E-01 | 5.63E+01 | | | |
| 7980700 | --- | --- | --- | -0.152 | 0.900 | 5.59E-02 | 5.59E+00 | | | |
| 8019010 | NM_020649 | CBX8 | chromobox homolog 8 (Pc class hom | -0.152 | 0.900 | 2.58E-02 | 2.58E+00 | | | |
| 8088369 | --- | --- | --- | -0.152 | 0.900 | 7.86E-02 | 7.86E+00 | | | |
| 8023175 | NM_016097 | IER3IP1 | immediate early response 3 interacti | -0.152 | 0.900 | 6.90E-01 | 6.90E+01 | | | |
| 8076563 | NM_173467 | MCAT | malonyl CoA:ACP acyltransferase (m | -0.152 | 0.900 | 1.45E-02 | 1.45E+00 | | | |
| 8032034 | NM_00113626 | FAM148C | family with sequence similarity 148, | -0.152 | 0.900 | 2.34E-02 | 2.34E+00 | | | |
| 8116582 | NM_001452 | FOXF2 | forkhead box F2 | -0.152 | 0.900 | 1.38E-02 | 1.38E+00 | | | |
| 7992956 | NM_032575 | GLIS2 | GLIS family zinc finger 2 | -0.152 | 0.900 | 1.08E-02 | 1.08E+00 | | | |
| 7956257 | NM_207344 | SPRYD4 | SPRY domain containing 4 | -0.152 | 0.900 | 1.77E-01 | 1.77E+01 | | | |
| 8135099 | NM_133457 | EMID2 | EMI domain containing 2 | -0.152 | 0.900 | 8.34E-03 | 8.34E-01 | | | |
| 7937443 | NM_022772 | EPS8L2 | EPS8-like 2 | -0.152 | 0.900 | 8.95E-03 | 8.95E-01 | | | |
| 8148435 | NM_003882 | WISP1 | WNT1 inducible signaling pathway p | -0.152 | 0.900 | 4.98E-03 | 4.98E-01 | | | |
| 8099008 | NM_020972 | ZFYVE28 | zinc finger, FYVE domain containing 2 | -0.152 | 0.900 | 1.12E-02 | 1.12E+00 | | | |
| 8168375 | NM_033053 | DMRTC1 | DMRT-like family C1 | -0.152 | 0.900 | 2.66E-02 | 2.66E+00 | | | |
| 7896618 | --- | --- | --- | -0.152 | 0.900 | 4.74E-01 | 4.74E+01 | | | |
| 7940372 | NM_024092 | TMEM109 | transmembrane protein 109 | -0.152 | 0.900 | 2.18E-02 | 2.18E+00 | | | |
| 8003639 | BC092513 | WDR81 | WD repeat domain 81 | -0.152 | 0.900 | 1.04E-02 | 1.04E+00 | | | |
| 8098193 | --- | --- | --- | -0.152 | 0.900 | 7.97E-01 | 7.97E+01 | | | |
| 7959294 | NM_032790 | ORAI1 | ORAI calcium release-activated calciu | -0.152 | 0.900 | 2.51E-02 | 2.51E+00 | | | |
| 7943803 | NM_00103795 | DIXDC1 | DIX domain containing 1 | -0.152 | 0.900 | 4.03E-02 | 4.03E+00 | | | |
| 7934842 | NM_024756 | MMRN2 | multimerin 2 | -0.152 | 0.900 | 1.02E-02 | 1.02E+00 | | | |
| 8022952 | NM_020180 | BRUNOL4 | bruno-like 4, RNA binding protein (D | -0.152 | 0.900 | 1.91E-02 | 1.91E+00 | | | |
| 8034605 | NM_052850 | GADD45GIP1 | growth arrest and DNA-damage-indu | -0.152 | 0.900 | 1.00E-02 | 1.00E+00 | | | |
| 8029446 | NM_00112789 | CEACAM19 | carcinoembryonic antigen-related ce | -0.152 | 0.900 | 1.34E-02 | 1.34E+00 | | | |
| 7910200 | NM_053052 | SNAP47 | synaptosomal-associated protein, 47 | -0.152 | 0.900 | 2.44E-02 | 2.44E+00 | | | |
| 7945418 | --- | --- | --- | -0.152 | 0.900 | 3.45E-02 | 3.45E+00 | | | |
| 7993019 | NM_032349 | NUDT16L1 | nudix (nucleoside diphosphate linked | -0.152 | 0.900 | 2.62E-02 | 2.62E+00 | | | |
| 8037614 | NM_005282 | GPR4 | G protein-coupled receptor 4 | -0.152 | 0.900 | 1.75E-02 | 1.75E+00 | | | |
| 8070386 | AF426265 | C21orf87 | chromosome 21 open reading frame | -0.152 | 0.900 | 1.94E-02 | 1.94E+00 | | | |
| 8063914 | NM_178463 | C20orf166 | chromosome 20 open reading frame | -0.152 | 0.900 | 4.52E-03 | 4.52E-01 | | | |
| 7963689 | NM_003717 | NPFF | neuropeptide FF-amide peptide prec | -0.152 | 0.900 | 7.72E-01 | 7.72E+01 | | | |
| 7932086 | --- | --- | --- | -0.152 | 0.900 | 5.41E-02 | 5.41E+00 | | | |
| 7953763 | --- | --- | --- | -0.153 | 0.900 | 2.54E-02 | 2.54E+00 | | | |
| 8008208 | NM_007225 | NXPH3 | neurexophilin 3 | -0.153 | 0.900 | 5.47E-03 | 5.47E-01 | | | |
| 8159142 | NM_000093 | COL5A1 | collagen, type V, alpha 1 | -0.153 | 0.900 | 4.84E-03 | 4.84E-01 | | | |
| 8096070 | NM_001201 | BMP3 | bone morphogenetic protein 3 | -0.153 | 0.900 | 2.48E-02 | 2.48E+00 | | | |
| 8032650 | NM_012398 | PIP5K1C | phosphatidylinositol-4-phosphate 5- | -0.153 | 0.900 | 2.94E-03 | 2.94E-01 | | | |
| 8052022 | AY779046 | RPL18A | ribosomal protein L18a | -0.153 | 0.900 | 1.30E-01 | 1.30E+01 | | | |
| 7904361 | NM_017709 | FAM46C | family with sequence similarity 46, m | -0.153 | 0.900 | 4.25E-01 | 4.25E+01 | | | |
| 8012309 | NM_001139 | ALOX12B | arachidonate 12-lipoxygenase, 12R t | -0.153 | 0.900 | 1.00E-02 | 1.00E+00 | | | |
| 8142136 | NM_175884 | FLJ36031 | hypothetical protein FLJ36031 | -0.153 | 0.900 | 4.26E-03 | 4.26E-01 | | | |
| 8070777 | NM_198693 | KRTAP10-2 | keratin associated protein 10-2 | -0.153 | 0.900 | 2.83E-02 | 2.83E+00 | | | |

| | | | | | | | | | | | |
|---|---|---|---|---|---|---|---|---|---|---|---|
| 8033598 | NM_032370 | ZNF414 | zinc finger protein 414 | -0.153 | 0.900 | 9.37E-02 | 9.37E+00 | | | | |
| 7976783 | NM_003836 | DLK1 | delta-like 1 homolog (Drosophila) | -0.153 | 0.900 | 2.55E-02 | 2.55E+00 | | | | |
| 8111216 | ENST0000042б | LOC391769 | histone cluster 2, H3c pseudogene | -0.153 | 0.900 | 1.07E-02 | 1.07E+00 | | | | |
| 7894547 | --- | --- | --- | -0.153 | 0.900 | 2.44E-01 | 2.44E+01 | | | | |
| 8140461 | NM_00114526ะ | FAM185A | family with sequence similarity 185, | -0.153 | 0.900 | 7.09E-02 | 7.09E+00 | | | | |
| 8098342 | NM_003864 | SAP30 | Sin3A-associated protein, 30kDa | -0.153 | 0.900 | 3.54E-02 | 3.54E+00 | | | | |
| 7914525 | NM_023009 | MARCKSL1 | MARCKS-like 1 | -0.153 | 0.900 | 6.65E-03 | 6.65E-01 | | | | |
| 8072825 | NM_013365 | GGA1 | golgi associated, gamma adaptin ear | -0.153 | 0.899 | 1.28E-02 | 1.28E+00 | | | | |
| 7942562 | NM_006656 | NEU3 | sialidase 3 (membrane sialidase) | -0.153 | 0.899 | 1.19E-01 | 1.19E+01 | | | | |
| 7910676 | --- | --- | --- | -0.153 | 0.899 | 6.41E-03 | 6.41E-01 | | | | |
| 8059739 | NM_024409 | NPPC | natriuretic peptide precursor C | -0.153 | 0.899 | 9.69E-03 | 9.69E-01 | | | | |
| 8131666 | NM_002214 | ITGB8 | integrin, beta 8 | -0.153 | 0.899 | 1.96E-02 | 1.96E+00 | | | | |
| 8117987 | NM_00116137( | C6orf136 | chromosome 6 open reading frame 1 | -0.153 | 0.899 | 5.23E-02 | 5.23E+00 | | | | |
| 8012896 | NM_000304 | PMP22 | peripheral myelin protein 22 | -0.153 | 0.899 | 4.98E-02 | 4.98E+00 | | | | |
| 8148796 | NM_00100827: | SCXA | scleraxis homolog A (mouse) | -0.153 | 0.899 | 1.46E-02 | 1.46E+00 | | | | |
| 8148821 | NM_00100827: | SCXA | scleraxis homolog A (mouse) | -0.153 | 0.899 | 1.46E-02 | 1.46E+00 | | | | |
| 8099746 | NM_000730 | CCKAR | cholecystokinin A receptor | -0.153 | 0.899 | 1.61E-02 | 1.61E+00 | | | | |
| 8093826 | NM_000683 | ADRA2C | adrenergic, alpha-2C-, receptor | -0.153 | 0.899 | 5.94E-03 | 5.94E-01 | | | | |
| 7903867 | NM_004978 | KCNC4 | potassium voltage-gated channel, Sh | -0.153 | 0.899 | 7.04E-03 | 7.04E-01 | | | | |
| 7923792 | NM_033102 | SLC45A3 | solute carrier family 45, member 3 | -0.153 | 0.899 | 1.44E-02 | 1.44E+00 | | | | |
| 7906307 | NM_018240 | KIRREL | kin of IRRE like (Drosophila) | -0.153 | 0.899 | 4.84E-03 | 4.84E-01 | | | | |
| 8136235 | NM_001868 | CPA1 | carboxypeptidase A1 (pancreatic) | -0.153 | 0.899 | 1.81E-02 | 1.81E+00 | | | | |
| 8066031 | NM_016558 | SCAND1 | SCAN domain containing 1 | -0.153 | 0.899 | 8.43E-03 | 8.43E-01 | | | | |
| 7893185 | --- | --- | --- | -0.153 | 0.899 | 6.21E-01 | 6.21E+01 | | | | |
| 8054377 | NM_201555 | FHL2 | four and a half LIM domains 2 | -0.153 | 0.899 | 7.22E-03 | 7.22E-01 | | | | |
| 8080144 | NM_00100393: | PARP3 | poly (ADP-ribose) polymerase family | -0.153 | 0.899 | 5.78E-02 | 5.78E+00 | | | | |
| 7892676 | --- | --- | --- | -0.153 | 0.899 | 8.86E-01 | 8.86E+01 | | | | |
| 8000567 | NM_145659 | IL27 | interleukin 27 | -0.153 | 0.899 | 4.13E-02 | 4.13E+00 | | | | |
| 7894484 | --- | --- | --- | -0.153 | 0.899 | 5.94E-02 | 5.94E+00 | | | | |
| 7906072 | NM_014017 | ROBLD3 | roadblock domain containing 3 | -0.153 | 0.899 | 2.98E-01 | 2.98E+01 | | | | |
| 8174317 | NM_138382 | RIPPLY1 | ripply1 homolog (zebrafish) | -0.153 | 0.899 | 1.18E-02 | 1.18E+00 | | | | |
| 8098895 | NM_00101362: | FAM53A | family with sequence similarity 53, n | -0.153 | 0.899 | 1.48E-02 | 1.48E+00 | | | | |
| 8109507 | NM_005546 | ITK | IL2-inducible T-cell kinase | -0.153 | 0.899 | 6.23E-01 | 6.23E+01 | | | | |
| 8006834 | --- | --- | --- | -0.153 | 0.899 | 1.27E-01 | 1.27E+01 | | | | |
| 7928679 | NM_00101297: | PLAC9 | placenta-specific 9 | -0.153 | 0.899 | 1.94E-02 | 1.94E+00 | | | | |
| 7976182 | NM_022054 | KCNK13 | potassium channel, subfamily K, mer | -0.153 | 0.899 | 1.72E-02 | 1.72E+00 | | | | |
| 7901309 | NM_012186 | FOXE3 | forkhead box E3 | -0.153 | 0.899 | 7.07E-03 | 7.07E-01 | | | | |
| 7934269 | --- | --- | --- | -0.153 | 0.899 | 2.77E-02 | 2.77E+00 | | | | |
| 7971511 | --- | --- | --- | -0.153 | 0.899 | 4.97E-01 | 4.97E+01 | | | | |
| 8031973 | NR_026052 | MGC2752 | hypothetical LOC65996 | -0.153 | 0.899 | 4.03E-02 | 4.03E+00 | | | | |
| 8158771 | NM_031426 | AIF1L | allograft inflammatory factor 1-like | -0.153 | 0.899 | 3.37E-03 | 3.37E-01 | | | | |
| 8163569 | NM_030767 | AKNA | AT-hook transcription factor | -0.153 | 0.899 | 4.74E-03 | 4.74E-01 | | | | |
| 8032157 | NM_014963 | SBNO2 | strawberry notch homolog 2 (Drosop | -0.153 | 0.899 | 3.32E-03 | 3.32E-01 | | | | |
| 8060205 | NM_015148 | PASK | PAS domain containing serine/threo | -0.153 | 0.899 | 5.12E-02 | 5.12E+00 | | | | |
| 8004741 | NM_00103714 | CNTROB | centrobin, centrosomal BRCA2 intera | -0.154 | 0.899 | 1.71E-02 | 1.71E+00 | | | | |
| 8030022 | NM_000836 | GRIN2D | glutamate receptor, ionotropic, N-m | -0.154 | 0.899 | 1.21E-02 | 1.21E+00 | | | | |
| 7982703 | NM_033510 | DISP2 | dispatched homolog 2 (Drosophila) | -0.154 | 0.899 | 4.21E-03 | 4.21E-01 | | | | |
| 7951589 | --- | --- | --- | -0.154 | 0.899 | 4.15E-02 | 4.15E+00 | | | | |
| 8074274 | NM_015241 | MICAL3 | microtubule associated monoxygena | -0.154 | 0.899 | 1.01E-02 | 1.01E+00 | | | | |
| 7996934 | NM_016101 | NIP7 | nuclear import 7 homolog (S. cerevis | -0.154 | 0.899 | 7.55E-01 | 7.55E+01 | | | | |
| 8056566 | --- | --- | --- | -0.154 | 0.899 | 4.99E-01 | 4.99E+01 | | | | |
| 8165462 | NM_013366 | ANAPC2 | anaphase promoting complex subun | -0.154 | 0.899 | 3.79E-03 | 3.79E-01 | | | | |
| 8113124 | NM_032290 | ANKRD32 | ankyrin repeat domain 32 | -0.154 | 0.899 | 8.69E-01 | 8.69E+01 | | | | |
| 7972902 | NM_138430 | ADPRHL1 | ADP-ribosylhydrolase like 1 | -0.154 | 0.899 | 2.29E-02 | 2.29E+00 | | | | |
| 8172531 | NM_007213 | PRAF2 | PRA1 domain family, member 2 | -0.154 | 0.899 | 5.07E-02 | 5.07E+00 | | | | |
| 8139456 | NR_002952 | SNORA9 | small nucleolar RNA, H/ACA box 9 | -0.154 | 0.899 | 8.02E-01 | 8.02E+01 | | | | |
| 7987565 | NM_017726 | PPP1R14D | protein phosphatase 1, regulatory (ir | -0.154 | 0.899 | 4.07E-02 | 4.07E+00 | | | | |
| 7950726 | NM_175885 | FAM181B | family with sequence similarity 181, | -0.154 | 0.899 | 9.87E-03 | 9.87E-01 | | | | |
| 7894377 | --- | --- | --- | -0.154 | 0.899 | 8.54E-01 | 8.54E+01 | | | | |
| 8118242 | NM_021246 | LY6G6D | lymphocyte antigen 6 complex, locus | -0.154 | 0.899 | 7.04E-03 | 7.04E-01 | | | | |
| 8150428 | NM_003012 | SFRP1 | secreted frizzled-related protein 1 | -0.154 | 0.899 | 1.04E-02 | 1.04E+00 | | | | |
| 8111925 | NM_00101427! | C5orf39 | chromosome 5 open reading frame 3 | -0.154 | 0.899 | 4.02E-01 | 4.02E+01 | | | | |
| 7940377 | NM_017870 | TMEM132A | transmembrane protein 132A | -0.154 | 0.899 | 3.90E-03 | 3.90E-01 | | | | |
| 8154870 | --- | --- | --- | -0.154 | 0.899 | 2.76E-02 | 2.76E+00 | | | | |
| 8019357 | NM_016286 | DCXR | dicarbonyl/L-xylulose reductase | -0.154 | 0.899 | 1.42E-01 | 1.42E+01 | | | | |
| 8104461 | --- | --- | --- | -0.154 | 0.899 | 4.14E-02 | 4.14E+00 | | | | |
| 8037290 | NM_002784 | PSG9 | pregnancy specific beta-1-glycoprote | -0.154 | 0.899 | 2.43E-02 | 2.43E+00 | | | | |
| 8043630 | AY358167 | UNQ5814 | RFVG5814 | -0.154 | 0.899 | 6.19E-03 | 6.19E-01 | | | | |
| 7962884 | NM_014470 | RND1 | Rho family GTPase 1 | -0.154 | 0.899 | 2.26E-02 | 2.26E+00 | | | | |
| 8061982 | NM_001672 | ASIP | agouti signaling protein, nonagouti h | -0.154 | 0.899 | 1.94E-02 | 1.94E+00 | | | | |
| 8037283 | NM_002780 | PSG4 | pregnancy specific beta-1-glycoprote | -0.154 | 0.899 | 9.86E-02 | 9.86E+00 | | | | |
| 7915578 | NM_024587 | TMEM53 | transmembrane protein 53 | -0.154 | 0.899 | 9.66E-03 | 9.66E-01 | | | | |
| 8024467 | NM_182973 | TMPRSS9 | transmembrane protease, serine 9 | -0.154 | 0.899 | 5.31E-03 | 5.31E-01 | | | | |
| 8117408 | NM_021052 | HIST1H2AE | histone cluster 1, H2ae | -0.154 | 0.899 | 7.72E-01 | 7.72E+01 | | | | |
| 7895310 | --- | --- | --- | -0.154 | 0.899 | 1.05E-01 | 1.05E+01 | | | | |
| 8173457 | NM_201599 | ZMYM3 | zinc finger, MYM-type 3 | -0.154 | 0.899 | 5.46E-02 | 5.46E+00 | | | | |
| 8170196 | --- | --- | --- | -0.154 | 0.899 | 6.08E-01 | 6.08E+01 | | | | |

| | | | | | | | | | | |
|---|---|---|---|---|---|---|---|---|---|---|
| 7936023 | BC126459 | C10orf95 | chromosome 10 open reading frame | -0.154 | 0.899 | 6.05E-03 | 6.05E-01 | | | |
| 8051422 | NR_003143 | MYADML | myeloid-associated differentiation m | -0.154 | 0.899 | 1.79E-02 | 1.79E+00 | | | |
| 7941621 | NM_005700 | DPP3 | dipeptidyl-peptidase 3 | -0.154 | 0.899 | 3.45E-02 | 3.45E+00 | | | |
| 7950195 | --- | --- | --- | -0.154 | 0.899 | 4.34E-02 | 4.34E+00 | | | |
| 7976556 | NR_023938 | C14orf132 | chromosome 14 open reading frame | -0.154 | 0.899 | 5.11E-02 | 5.11E+00 | | | |
| 8144121 | NM_002847 | PTPRN2 | protein tyrosine phosphatase, recept | -0.154 | 0.899 | 2.83E-02 | 2.83E+00 | | | |
| 8054356 | NM_032718 | MFSD9 | major facilitator superfamily domain | -0.154 | 0.899 | 1.95E-02 | 1.95E+00 | | | |
| 8132897 | NM_018697 | LANCL2 | LanC lantibiotic synthetase compone | -0.154 | 0.899 | 4.02E-01 | 4.02E+01 | | | |
| 7940989 | NM_004451 | ESRRA | estrogen-related receptor alpha | -0.154 | 0.899 | 3.80E-02 | 3.80E+00 | | | |
| 8126442 | NM_003192 | TBCC | tubulin folding cofactor C | -0.154 | 0.899 | 3.44E-02 | 3.44E+00 | | | |
| 8029437 | NM_006505 | PVR | poliovirus receptor | -0.154 | 0.899 | 1.21E-02 | 1.21E+00 | | | |
| 8062948 | NM_014276 | RBPJL | recombination signal binding protein | -0.154 | 0.899 | 1.12E-02 | 1.12E+00 | | | |
| 7982805 | NM_005258 | GCHFR | GTP cyclohydrolase I feedback regula | -0.154 | 0.899 | 9.70E-02 | 9.70E+00 | | | |
| 8176566 | --- | --- | --- | -0.154 | 0.899 | 1.16E-01 | 1.16E+01 | | | |
| 8065903 | --- | --- | --- | -0.154 | 0.898 | 5.89E-01 | 5.89E+01 | | | |
| 8171917 | NM_031894 | FTHL17 | ferritin, heavy polypeptide-like 17 | -0.154 | 0.898 | 2.28E-02 | 2.28E+00 | | | |
| 8036862 | AY358191 | UNQ6167 | NINP6167 | -0.155 | 0.898 | 1.93E-01 | 1.93E+01 | | | |
| 8007414 | NM_009590 | AOC2 | amine oxidase, copper containing 2 ( | -0.155 | 0.898 | 4.96E-02 | 4.96E+00 | | | |
| 8036981 | NM_000764 | CYP2A7 | cytochrome P450, family 2, subfamil | -0.155 | 0.898 | 2.52E-02 | 2.52E+00 | | | |
| 7974425 | NM_015589 | SAMD4A | sterile alpha motif domain containin | -0.155 | 0.898 | 2.18E-02 | 2.18E+00 | | | |
| 8151619 | NR_003594 | REXO1L2P | REX1, RNA exonuclease 1 homolog (S | -0.155 | 0.898 | 3.32E-02 | 3.32E+00 | | | |
| 8170803 | NM_003492 | TMEM187 | transmembrane protein 187 | -0.155 | 0.898 | 5.72E-03 | 5.72E-01 | | | |
| 8001547 | NM_015993 | PLLP | plasma membrane proteolipid (plasm | -0.155 | 0.898 | 2.33E-02 | 2.33E+00 | | | |
| 8019367 | NM_002917 | RFNG | RFNG O-fucosylpeptide 3-beta-N-ace | -0.155 | 0.898 | 1.23E-02 | 1.23E+00 | | | |
| 8029076 | NM_00109850( | CEACAM21 | carcinoembryonic antigen-related ce | -0.155 | 0.898 | 1.42E-01 | 1.42E+01 | | | |
| 8010897 | NM_00100443 | METRNL | meteorin, glial cell differentiation re | -0.155 | 0.898 | 1.17E-01 | 1.17E+01 | | | |
| 8166351 | --- | --- | --- | -0.155 | 0.898 | 2.77E-02 | 2.77E+00 | | | |
| 8128322 | NM_014165 | NDUFAF4 | NADH dehydrogenase (ubiquinone) 1 | -0.155 | 0.898 | 8.04E-01 | 8.04E+01 | | | |
| 7949058 | NM_005528 | DNAJC4 | DnaJ (Hsp40) homolog, subfamily C, | -0.155 | 0.898 | 6.29E-03 | 6.29E-01 | | | |
| 8012220 | NM_006942 | SOX15 | SRY (sex determining region Y)-box 1 | -0.155 | 0.898 | 7.65E-03 | 7.65E-01 | | | |
| 8174937 | NM_014253 | ODZ1 | odz, odd Oz/ten-m homolog 1(Droso | -0.155 | 0.898 | 2.94E-02 | 2.94E+00 | | | |
| 8144703 | --- | --- | --- | -0.155 | 0.898 | 8.54E-02 | 8.54E+00 | | | |
| 7912675 | NM_003443 | ZBTB17 | zinc finger and BTB domain containin | -0.155 | 0.898 | 1.17E-02 | 1.17E+00 | | | |
| 8134407 | NM_005222 | DLX6 | distal-less homeobox 6 | -0.155 | 0.898 | 8.48E-03 | 8.48E-01 | | | |
| 7956423 | NM_005538 | INHBC | inhibin, beta C | -0.155 | 0.898 | 2.27E-02 | 2.27E+00 | | | |
| 7919226 | NM_006468 | POLR3C | polymerase (RNA) III (DNA directed) | -0.155 | 0.898 | 6.91E-01 | 6.91E+01 | | | |
| 8110399 | NM_007255 | B4GALT7 | xylosylprotein beta 1,4-galactosyltra | -0.155 | 0.898 | 1.81E-02 | 1.81E+00 | | | |
| 8025237 | NM_00108042 | KIAA1543 | KIAA1543 | -0.155 | 0.898 | 6.57E-03 | 6.57E-01 | | | |
| 7950136 | NM_005169 | PHOX2A | paired-like homeobox 2a | -0.155 | 0.898 | 9.15E-03 | 9.15E-01 | | | |
| 7944978 | NM_004879 | EI24 | etoposide induced 2.4 mRNA | -0.155 | 0.898 | 6.73E-01 | 6.73E+01 | | | |
| 8170402 | NM_016153 | HSFX1 | heat shock transcription factor famil | -0.155 | 0.898 | 1.27E-02 | 1.27E+00 | | | |
| 8175616 | NM_016153 | HSFX1 | heat shock transcription factor famil | -0.155 | 0.898 | 1.27E-02 | 1.27E+00 | | | |
| 7959786 | NM_023928 | AACS | acetoacetyl-CoA synthetase | -0.155 | 0.898 | 6.71E-02 | 6.71E+00 | | | |
| 7897128 | NM_080431 | ACTRT2 | actin-related protein T2 | -0.155 | 0.898 | 3.45E-03 | 3.45E-01 | | | |
| 8168264 | NM_018977 | NLGN3 | neuroligin 3 | -0.155 | 0.898 | 6.43E-03 | 6.43E-01 | | | |
| 7915404 | NM_033553 | GUCA2A | guanylate cyclase activator 2A (guan | -0.155 | 0.898 | 3.44E-02 | 3.44E+00 | | | |
| 7976724 | --- | --- | --- | -0.155 | 0.898 | 1.48E-02 | 1.48E+00 | | | |
| 8069083 | AB209578 | C21orf2 | chromosome 21 open reading frame | -0.155 | 0.898 | 1.15E-02 | 1.15E+00 | | | |
| 8124406 | NM_003526 | HIST1H2BC | histone cluster 1, H2bc | -0.155 | 0.898 | 6.22E-01 | 6.22E+01 | | | |
| 8053139 | NM_00114505 | C2orf81 | chromosome 2 open reading frame 8 | -0.155 | 0.898 | 2.01E-02 | 2.01E+00 | | | |
| 7992889 | NM_012368 | OR2C1 | olfactory receptor, family 2, subfami | -0.155 | 0.898 | 5.24E-02 | 5.24E+00 | | | |
| 7898677 | NM_004807 | HS6ST1 | heparan sulfate 6-O-sulfotransferase | -0.155 | 0.898 | 1.37E-02 | 1.37E+00 | | | |
| 8165094 | NM_181701 | QSOX2 | quiescin Q6 sulfhydryl oxidase 2 | -0.155 | 0.898 | 6.38E-02 | 6.38E+00 | | | |
| 8032312 | NM_138813 | ATP8B3 | ATPase, class I, type 8B, member 3 | -0.155 | 0.898 | 1.28E-02 | 1.28E+00 | | | |
| 8024250 | NM_001405 | EFNA2 | ephrin-A2 | -0.155 | 0.898 | 9.35E-03 | 9.35E-01 | | | |
| 8132248 | --- | --- | --- | -0.155 | 0.898 | 1.70E-01 | 1.70E+01 | | | |
| 8096301 | NM_00104005 | SPP1 | secreted phosphoprotein 1 | -0.155 | 0.898 | 1.89E-02 | 1.89E+00 | | | |
| 8053458 | NM_00101364 | C2orf68 | chromosome 2 open reading frame 6 | -0.155 | 0.898 | 5.86E-03 | 5.86E-01 | | | |
| 8072413 | NM_134269 | SMTN | smoothelin | -0.155 | 0.898 | 9.02E-03 | 9.02E-01 | | | |
| 8177851 | NM_00116137 | C6orf136 | chromosome 6 open reading frame 1 | -0.155 | 0.898 | 8.95E-02 | 8.95E+00 | | | |
| 8179167 | NM_00116137 | C6orf136 | chromosome 6 open reading frame 1 | -0.155 | 0.898 | 8.95E-02 | 8.95E+00 | | | |
| 7982927 | NM_002220 | ITPKA | inositol 1,4,5-trisphosphate 3-kinase | -0.155 | 0.898 | 1.22E-02 | 1.22E+00 | | | |
| 8064245 | NM_198723 | TCEA2 | transcription elongation factor A (SII | -0.155 | 0.898 | 4.33E-02 | 4.33E+00 | | | |
| 7910134 | NM_031944 | MIXL1 | Mix1 homeobox-like 1 (Xenopus laev | -0.155 | 0.898 | 1.46E-02 | 1.46E+00 | | | |
| 8069987 | NM_144659 | TCP10L | t-complex 10 (mouse)-like | -0.155 | 0.898 | 6.96E-03 | 6.96E-01 | | | |
| 8141283 | NM_015545 | PTCD1 | pentatricopeptide repeat domain 1 | -0.155 | 0.898 | 4.52E-03 | 4.52E-01 | | | |
| 7896352 | --- | --- | --- | -0.155 | 0.898 | 5.35E-02 | 5.35E+00 | | | |
| 7915444 | NM_022356 | LEPRE1 | leucine proline-enriched proteoglyca | -0.155 | 0.898 | 5.72E-03 | 5.72E-01 | | | |
| 8069153 | NM_181688 | KRTAP10-10 | keratin associated protein 10-10 | -0.155 | 0.898 | 2.10E-02 | 2.10E+00 | | | |
| 7932495 | NM_00101091 | C10orf114 | chromosome 10 open reading frame | -0.155 | 0.898 | 1.15E-02 | 1.15E+00 | | | |
| 8178070 | NM_021246 | LY6G6D | lymphocyte antigen 6 complex, locus | -0.155 | 0.898 | 4.56E-02 | 4.56E+00 | | | |
| 8179309 | NM_021246 | LY6G6D | lymphocyte antigen 6 complex, locus | -0.155 | 0.898 | 4.56E-02 | 4.56E+00 | | | |
| 8028778 | NM_002446 | MAP3K10 | mitogen-activated protein kinase kin | -0.155 | 0.898 | 3.29E-03 | 3.29E-01 | | | |
| 8026363 | --- | --- | --- | -0.155 | 0.898 | 1.67E-01 | 1.67E+01 | | | |
| 8104727 | --- | --- | --- | -0.155 | 0.898 | 4.28E-01 | 4.28E+01 | | | |
| 8076455 | NM_015703 | RRP7A | ribosomal RNA processing 7 homolog | -0.155 | 0.898 | 9.54E-02 | 9.54E+00 | | | |
| 8032509 | NM_052847 | GNG7 | guanine nucleotide binding protein ( | -0.155 | 0.898 | 9.69E-02 | 9.69E+00 | | | |

| | | | | | | | | | | | |
|---|---|---|---|---|---|---|---|---|---|---|---|
| 8148703 | NM_198572 | SPATC1 | spermatogenesis and centriole assoc | -0.156 | 0.898 | 2.79E-03 | 2.79E-01 | | | | |
| 7914103 | NM_00102988 | AHDC1 | AT hook, DNA binding motif, contain | -0.156 | 0.898 | 6.91E-03 | 6.91E-01 | | | | |
| 8064059 | NM_020882 | COL20A1 | collagen, type XX, alpha 1 | -0.156 | 0.898 | 9.46E-03 | 9.46E-01 | | | | |
| 8134907 | NM_000799 | EPO | erythropoietin | -0.156 | 0.898 | 4.84E-03 | 4.84E-01 | | | | |
| 8159096 | NM_000787 | DBH | dopamine beta-hydroxylase (dopami | -0.156 | 0.898 | 8.61E-03 | 8.61E-01 | | | | |
| 8172561 | NM_006150 | PRICKLE3 | prickle homolog 3 (Drosophila) | -0.156 | 0.898 | 3.42E-02 | 3.42E+00 | | | | |
| 8097256 | NM_002006 | FGF2 | fibroblast growth factor 2 (basic) | -0.156 | 0.898 | 8.96E-03 | 8.96E-01 | | | | |
| 7979510 | NM_017420 | SIX4 | SIX homeobox 4 | -0.156 | 0.898 | 1.12E-02 | 1.12E+00 | | | | |
| 7894149 | --- | --- | --- | -0.156 | 0.898 | 7.36E-01 | 7.36E+01 | | | | |
| 7967727 | NM_021808 | GALNT9 | UDP-N-acetyl-alpha-D-galactosamine | -0.156 | 0.898 | 8.20E-03 | 8.20E-01 | | | | |
| 7935425 | NM_015179 | RRP12 | ribosomal RNA processing 12 homolo | -0.156 | 0.898 | 7.93E-03 | 7.93E-01 | | | | |
| 7896597 | --- | --- | --- | -0.156 | 0.898 | 7.72E-01 | 7.72E+01 | | | | |
| 8039769 | --- | --- | --- | -0.156 | 0.898 | 2.06E-02 | 2.06E+00 | | | | |
| 7960702 | NM_031299 | CDCA3 | cell division cycle associated 3 | -0.156 | 0.898 | 1.36E-02 | 1.36E+00 | | | | |
| 8117720 | --- | --- | --- | -0.156 | 0.898 | 4.25E-02 | 4.25E+00 | | | | |
| 7976239 | AF170294 | PTMAP7 | prothymosin, alpha pseudogene 7 | -0.156 | 0.898 | 4.42E-02 | 4.42E+00 | | | | |
| 8095216 | NM_020722 | KIAA1211 | KIAA1211 | -0.156 | 0.898 | 5.51E-03 | 5.51E-01 | | | | |
| 7982868 | NM_024111 | CHAC1 | ChaC, cation transport regulator hon | -0.156 | 0.898 | 1.43E-02 | 1.43E+00 | | | | |
| 7895337 | --- | --- | --- | -0.156 | 0.898 | 1.18E-01 | 1.18E+01 | | | | |
| 7893586 | --- | --- | --- | -0.156 | 0.898 | 1.38E-01 | 1.38E+01 | | | | |
| 8061940 | DQ080434 | C20orf144 | chromosome 20 open reading frame | -0.156 | 0.897 | 7.26E-03 | 7.26E-01 | | | | |
| 8064739 | NM_00103914 | C20orf27 | chromosome 20 open reading frame | -0.156 | 0.897 | 2.94E-02 | 2.94E+00 | | | | |
| 7925500 | NM_001821 | CHML | choroideremia-like (Rab escort prote | -0.156 | 0.897 | 2.19E-01 | 2.19E+01 | | | | |
| 7905817 | NM_000748 | CHRNB2 | cholinergic receptor, nicotinic, beta 2 | -0.156 | 0.897 | 5.87E-03 | 5.87E-01 | | | | |
| 7938685 | --- | --- | --- | -0.156 | 0.897 | 1.64E-02 | 1.64E+00 | | | | |
| 8166580 | --- | --- | --- | -0.156 | 0.897 | 4.71E-02 | 4.71E+00 | | | | |
| 8024429 | NM_000479 | AMH | anti-Mullerian hormone | -0.156 | 0.897 | 1.72E-03 | 1.72E-01 | | | | |
| 8104348 | --- | --- | --- | -0.156 | 0.897 | 4.36E-01 | 4.36E+01 | | | | |
| 7978239 | NM_004581 | RABGGTA | Rab geranylgeranyltransferase, alpha | -0.156 | 0.897 | 1.36E-02 | 1.36E+00 | | | | |
| 8025456 | NM_00100445 | OR1M1 | olfactory receptor, family 1, subfami | -0.156 | 0.897 | 1.61E-02 | 1.61E+00 | | | | |
| 8008201 | NM_002507 | NGFR | nerve growth factor receptor (TNFR s | -0.156 | 0.897 | 9.17E-03 | 9.17E-01 | | | | |
| 8150689 | --- | --- | --- | -0.156 | 0.897 | 1.04E-01 | 1.04E+01 | | | | |
| 7892553 | --- | --- | --- | -0.156 | 0.897 | 8.79E-01 | 8.79E+01 | | | | |
| 7892544 | --- | --- | --- | -0.156 | 0.897 | 2.80E-01 | 2.80E+01 | | | | |
| 8094701 | NR_026854 | LOC401127 | WD repeat domain 5 pseudogene | -0.156 | 0.897 | 5.72E-02 | 5.72E+00 | | | | |
| 7892859 | --- | --- | --- | -0.156 | 0.897 | 4.94E-01 | 4.94E+01 | | | | |
| 7911750 | NM_00101092 | HES5 | hairy and enhancer of split 5 (Drosop | -0.156 | 0.897 | 1.09E-02 | 1.09E+00 | | | | |
| 8037309 | NM_198850 | PHLDB3 | pleckstrin homology-like domain, far | -0.156 | 0.897 | 2.12E-02 | 2.12E+00 | | | | |
| 8116113 | NR_024019 | FLJ10404 | hypothetical protein FLJ10404 | -0.156 | 0.897 | 6.50E-03 | 6.50E-01 | | | | |
| 7893869 | --- | --- | --- | -0.157 | 0.897 | 7.79E-01 | 7.79E+01 | | | | |
| 8158059 | NM_003165 | STXBP1 | syntaxin binding protein 1 | -0.157 | 0.897 | 1.42E-02 | 1.42E+00 | | | | |
| 7906069 | NM_181885 | RXFP4 | relaxin/insulin-like family peptide rec | -0.157 | 0.897 | 1.75E-02 | 1.75E+00 | | | | |
| 8037144 | NM_133328 | DEDD2 | death effector domain containing 2 | -0.157 | 0.897 | 9.53E-02 | 9.53E+00 | | | | |
| 8164062 | AY567967 | LOC613206 | myeloproliferative disease associated | -0.157 | 0.897 | 5.63E-02 | 5.63E+00 | | | | |
| 8174513 | NM_145234 | CHRDL1 | chordin-like 1 | -0.157 | 0.897 | 1.82E-02 | 1.82E+00 | | | | |
| 7896798 | NM_032129 | PLEKHN1 | pleckstrin homology domain contain | -0.157 | 0.897 | 2.56E-03 | 2.56E-01 | | | | |
| 8136576 | --- | --- | --- | -0.157 | 0.897 | 5.16E-02 | 5.16E+00 | | | | |
| 7922610 | NM_007314 | ABL2 | v-abl Abelson murine leukemia viral | -0.157 | 0.897 | 9.16E-02 | 9.16E+00 | | | | |
| 7936871 | NM_000274 | OAT | ornithine aminotransferase (gyrate a | -0.157 | 0.897 | 4.49E-01 | 4.49E+01 | | | | |
| 8065580 | NM_080611 | DUSP15 | dual specificity phosphatase 15 | -0.157 | 0.897 | 2.70E-03 | 2.70E-01 | | | | |
| 7932862 | --- | --- | --- | -0.157 | 0.897 | 1.16E-01 | 1.16E+01 | | | | |
| 8072705 | NM_014310 | RASD2 | RASD family, member 2 | -0.157 | 0.897 | 1.59E-02 | 1.59E+00 | | | | |
| 8165077 | NM_144653 | NACC2 | NACC family member 2, BEN and BTB | -0.157 | 0.897 | 3.79E-02 | 3.79E+00 | | | | |
| 8060330 | NM_033089 | ZCCHC3 | zinc finger, CCHC domain containing | -0.157 | 0.897 | 2.10E-02 | 2.10E+00 | | | | |
| 8034744 | NM_00114502 | PALM3 | Paralemmin-3 | -0.157 | 0.897 | 2.93E-02 | 2.93E+00 | | | | |
| 8094372 | NM_003102 | SOD3 | superoxide dismutase 3, extracellula | -0.157 | 0.897 | 1.53E-02 | 1.53E+00 | | | | |
| 7918620 | AK093435 | FLJ36116 | hypothetical locus LOC388666 | -0.157 | 0.897 | 1.81E-01 | 1.81E+01 | | | | |
| 8161865 | NM_015225 | PRUNE2 | prune homolog 2 (Drosophila) | -0.157 | 0.897 | 8.81E-03 | 8.81E-01 | | | | |
| 8159583 | NM_007327 | GRIN1 | glutamate receptor, ionotropic, N-m | -0.157 | 0.897 | 8.97E-03 | 8.97E-01 | | | | |
| 8070681 | AF426261 | C21orf84 | chromosome 21 open reading frame | -0.157 | 0.897 | 1.87E-02 | 1.87E+00 | | | | |
| 8035297 | NM_031310 | PLVAP | plasmalemma vesicle associated pro | -0.157 | 0.897 | 5.28E-03 | 5.28E-01 | | | | |
| 7944147 | NM_001680 | FXYD2 | FXYD domain containing ion transpo | -0.157 | 0.897 | 4.27E-02 | 4.27E+00 | | | | |
| 8110721 | NM_024786 | ZDHHC11 | zinc finger, DHHC-type containing 11 | -0.157 | 0.897 | 3.86E-02 | 3.86E+00 | | | | |
| 7952897 | NM_015232 | IQSEC3 | IQ motif and Sec7 domain 3 | -0.157 | 0.897 | 1.74E-02 | 1.74E+00 | | | | |
| 8134463 | NM_002523 | NPTX2 | neuronal pentraxin II | -0.157 | 0.897 | 7.25E-03 | 7.25E-01 | | | | |
| 8000467 | NM_00110976 | GSG1L | GSG1-like | -0.157 | 0.897 | 8.98E-03 | 8.98E-01 | | | | |
| 8022434 | --- | --- | --- | -0.157 | 0.897 | 1.04E-01 | 1.04E+01 | | | | |
| 7960682 | AK289648 | C12orf53 | chromosome 12 open reading frame | -0.157 | 0.897 | 1.96E-02 | 1.96E+00 | | | | |
| 7945204 | NM_021978 | ST14 | suppression of tumorigenicity 14 (co | -0.157 | 0.897 | 7.30E-03 | 7.30E-01 | | | | |
| 8114593 | NM_133174 | APBB3 | amyloid beta (A4) precursor protein- | -0.157 | 0.897 | 1.49E-02 | 1.49E+00 | | | | |
| 7938416 | ENST00000324 | OC10012982 | hypothetical protein LOC100129827 | -0.157 | 0.897 | 1.12E-02 | 1.12E+00 | | | | |
| 8054295 | --- | --- | --- | -0.157 | 0.897 | 1.66E-01 | 1.66E+01 | | | | |
| 8098740 | NM_033178 | DUX4 | double homeobox, 4 | -0.157 | 0.897 | 1.69E-03 | 1.69E-01 | | | | |
| 7958950 | BC022092 | C12orf52 | chromosome 12 open reading frame | -0.157 | 0.897 | 1.36E-02 | 1.36E+00 | | | | |
| 7899795 | --- | --- | --- | -0.157 | 0.897 | 1.39E-01 | 1.39E+01 | | | | |
| 7942400 | NM_176072 | P2RY2 | purinergic receptor P2Y, G-protein co | -0.157 | 0.897 | 2.15E-02 | 2.15E+00 | | | | |
| 8162421 | NM_022755 | IPPK | inositol 1,3,4,5,6-pentakisphosphate | -0.158 | 0.897 | 1.17E-02 | 1.17E+00 | | | | |

| | | | | | | | | | | | |
|---|---|---|---|---|---|---|---|---|---|---|---|
| 7894240 | --- | --- | --- | -0.158 | 0.897 | 6.96E-01 | 6.96E+01 | | | | |
| 8003478 | NM_152339 | SPATA2L | spermatogenesis associated 2-like | -0.158 | 0.897 | 8.94E-03 | 8.94E-01 | | | | |
| 8105504 | NM_152789 | FAM133B | family with sequence similarity 133, | -0.158 | 0.897 | 7.62E-01 | 7.62E+01 | | | | |
| 8071259 | NM_002688 | 40426 | septin 5 | -0.158 | 0.897 | 1.36E-02 | 1.36E+00 | | | | |
| 8047738 | NM_201266 | NRP2 | neuropilin 2 | -0.158 | 0.897 | 1.21E-02 | 1.21E+00 | | | | |
| 7945498 | NM_021920 | SCT | secretin | -0.158 | 0.897 | 6.28E-03 | 6.28E-01 | | | | |
| 7999360 | NM_000982 | RPL21 | ribosomal protein L21 | -0.158 | 0.897 | 4.12E-01 | 4.12E+01 | | | | |
| 8017825 | --- | --- | --- | -0.158 | 0.897 | 3.96E-01 | 3.96E+01 | | | | |
| 7991932 | BC001912 | C16orf14 | chromosome 16 open reading frame | -0.158 | 0.896 | 1.74E-02 | 1.74E+00 | | | | |
| 8079867 | --- | --- | --- | -0.158 | 0.896 | 4.33E-02 | 4.33E+00 | | | | |
| 7998835 | --- | --- | --- | -0.158 | 0.896 | 2.43E-01 | 2.43E+01 | | | | |
| 7897034 | NM_000815 | GABRD | gamma-aminobutyric acid (GABA) A | -0.158 | 0.896 | 7.40E-03 | 7.40E-01 | | | | |
| 7970232 | NM_000131 | F7 | coagulation factor VII (serum prothro | -0.158 | 0.896 | 1.20E-02 | 1.20E+00 | | | | |
| 8113489 | --- | --- | --- | -0.158 | 0.896 | 3.17E-02 | 3.17E+00 | | | | |
| 7894366 | --- | --- | --- | -0.158 | 0.896 | 4.94E-01 | 4.94E+01 | | | | |
| 8003719 | NM_00109820 | HIC1 | hypermethylated in cancer 1 | -0.158 | 0.896 | 1.21E-02 | 1.21E+00 | | | | |
| 7901073 | NM_000374 | UROD | uroporphyrinogen decarboxylase | -0.158 | 0.896 | 3.17E-01 | 3.17E+01 | | | | |
| 8119338 | NM_002062 | GLP1R | glucagon-like peptide 1 receptor | -0.158 | 0.896 | 8.37E-03 | 8.37E-01 | | | | |
| 8130565 | --- | --- | --- | -0.158 | 0.896 | 5.65E-01 | 5.65E+01 | | | | |
| 7992219 | NM_003933 | BAIAP3 | BAI1-associated protein 3 | -0.158 | 0.896 | 1.60E-02 | 1.60E+00 | | | | |
| 7895879 | --- | --- | --- | -0.158 | 0.896 | 5.87E-02 | 5.87E+00 | | | | |
| 8151615 | 0 | 0 | 0 | -0.158 | 0.896 | 2.94E-02 | 2.94E+00 | | | | |
| 8067585 | NM_080606 | BHLHE23 | basic helix-loop-helix family, membe | -0.158 | 0.896 | 1.19E-02 | 1.19E+00 | | | | |
| 7893717 | --- | --- | --- | -0.158 | 0.896 | 8.68E-01 | 8.68E+01 | | | | |
| 8117330 | NM_003529 | HIST1H3A | histone cluster 1, H3a | -0.158 | 0.896 | 5.47E-01 | 5.47E+01 | | | | |
| 7983021 | NR_015346 | MJD7-PLA2G4 | JMJD7-PLA2G4B readthrough transc | -0.158 | 0.896 | 5.12E-03 | 5.12E-01 | | | | |
| 8142580 | NM_00102461 | FEZF1 | FEZ family zinc finger 1 | -0.158 | 0.896 | 8.12E-03 | 8.12E-01 | | | | |
| 7894160 | --- | --- | --- | -0.158 | 0.896 | 2.52E-01 | 2.52E+01 | | | | |
| 8060094 | NM_148961 | OTOS | otospiralin | -0.158 | 0.896 | 2.03E-02 | 2.03E+00 | | | | |
| 7892773 | --- | --- | --- | -0.158 | 0.896 | 1.70E-01 | 1.70E+01 | | | | |
| 7900146 | NM_025079 | ZC3H12A | zinc finger CCCH-type containing 12A | -0.158 | 0.896 | 9.45E-03 | 9.45E-01 | | | | |
| 8158976 | NM_001807 | CEL | carboxyl ester lipase (bile salt-stimul | -0.158 | 0.896 | 4.52E-03 | 4.52E-01 | | | | |
| 8133741 | NM_030570 | UPK3B | uroplakin 3B | -0.158 | 0.896 | 1.12E-02 | 1.12E+00 | | | | |
| 7949503 | NM_016938 | EFEMP2 | EGF-containing fibulin-like extracellu | -0.158 | 0.896 | 7.54E-03 | 7.54E-01 | | | | |
| 7906993 | --- | --- | --- | -0.158 | 0.896 | 5.84E-02 | 5.84E+00 | | | | |
| 7924842 | BC002843 | C1orf35 | chromosome 1 open reading frame 3 | -0.158 | 0.896 | 1.68E-02 | 1.68E+00 | | | | |
| 8036207 | NM_139239 | NFKBID | nuclear factor of kappa light polypep | -0.158 | 0.896 | 1.23E-02 | 1.23E+00 | | | | |
| 7966223 | --- | --- | --- | -0.158 | 0.896 | 6.14E-01 | 6.14E+01 | | | | |
| 7911351 | NR_024321 | NCRNA0011 | non-protein coding RNA 115 | -0.158 | 0.896 | 8.88E-03 | 8.88E-01 | | | | |
| 7897416 | NM_00108039 | SLC45A1 | solute carrier family 45, member 1 | -0.158 | 0.896 | 3.51E-03 | 3.51E-01 | | | | |
| 8130785 | NM_005299 | GPR31 | G protein-coupled receptor 31 | -0.158 | 0.896 | 8.84E-03 | 8.84E-01 | | | | |
| 8150214 | --- | --- | --- | -0.159 | 0.896 | 2.19E-02 | 2.19E+00 | | | | |
| 8090343 | NM_014079 | KLF15 | Kruppel-like factor 15 | -0.159 | 0.896 | 1.99E-02 | 1.99E+00 | | | | |
| 8167228 | NM_205856 | SPACA5 | sperm acrosome associated 5 | -0.159 | 0.896 | 2.66E-02 | 2.66E+00 | | | | |
| 8074063 | NM_024866 | ADM2 | adrenomedullin 2 | -0.159 | 0.896 | 9.80E-03 | 9.80E-01 | | | | |
| 8063526 | NM_00101297 | C20orf106 | chromosome 20 open reading frame | -0.159 | 0.896 | 8.48E-02 | 8.48E+00 | | | | |
| 8158406 | NM_018201 | TBC1D13 | TBC1 domain family, member 13 | -0.159 | 0.896 | 7.39E-02 | 7.39E+00 | | | | |
| 8033190 | NM_024103 | SLC25A23 | solute carrier family 25 (mitochondri | -0.159 | 0.896 | 1.87E-02 | 1.87E+00 | | | | |
| 7952737 | --- | --- | --- | -0.159 | 0.896 | 7.16E-01 | 7.16E+01 | | | | |
| 7996160 | ENST0000033 | LOC388282 | hypothetical LOC388282 | -0.159 | 0.896 | 1.64E-02 | 1.64E+00 | | | | |
| 8020842 | NR_003558 // | 11P1 // WBF | WW domain binding protein 11 pseu | -0.159 | 0.896 | 1.93E-01 | 1.93E+01 | | | | |
| 8123658 | NM_015482 | SLC22A23 | solute carrier family 22, member 23 | -0.159 | 0.896 | 1.69E-02 | 1.69E+00 | | | | |
| 8019798 | NM_032711 | MAFG | v-maf musculoaponeurotic fibrosarc | -0.159 | 0.896 | 7.69E-03 | 7.69E-01 | | | | |
| 8177901 | NM_020442 | VARS2 | valyl-tRNA synthetase 2, mitochondr | -0.159 | 0.896 | 1.27E-02 | 1.27E+00 | | | | |
| 8059014 | NM_017521 | FEV | FEV (ETS oncogene family) | -0.159 | 0.896 | 1.11E-02 | 1.11E+00 | | | | |
| 8125234 | NM_019105 | TNXB | tenascin XB | -0.159 | 0.896 | 5.08E-03 | 5.08E-01 | | | | |
| 8067394 | NM_007232 | HRH3 | histamine receptor H3 | -0.159 | 0.896 | 9.07E-03 | 9.07E-01 | | | | |
| 7945892 | --- | --- | --- | -0.159 | 0.896 | 2.92E-02 | 2.92E+00 | | | | |
| 7960878 | AF268617 // | F1P3 // POU | POU class 5 homeobox 1 pseudogene | -0.159 | 0.896 | 4.58E-02 | 4.58E+00 | | | | |
| 8118395 | NR_026717 | STK19 | serine/threonine kinase 19 | -0.159 | 0.896 | 1.31E-01 | 1.31E+01 | | | | |
| 8178164 | NR_026717 | STK19 | serine/threonine kinase 19 | -0.159 | 0.896 | 1.31E-01 | 1.31E+01 | | | | |
| 7902493 | --- | --- | --- | -0.159 | 0.896 | 7.86E-02 | 7.86E+00 | | | | |
| 7986820 | --- | --- | --- | -0.159 | 0.896 | 2.84E-02 | 2.84E+00 | | | | |
| 8137081 | --- | --- | --- | -0.159 | 0.896 | 4.24E-02 | 4.24E+00 | | | | |
| 7939546 | NM_002231 | CD82 | CD82 molecule | -0.159 | 0.896 | 2.28E-02 | 2.28E+00 | | | | |
| 7896141 | --- | --- | --- | -0.159 | 0.896 | 1.15E-01 | 1.15E+01 | | | | |
| 8016311 | NM_030753 | WNT3 | wingless-type MMTV integration site | -0.159 | 0.896 | 1.63E-02 | 1.63E+00 | | | | |
| 7931623 | NR_002934 | LOC619207 | scavenger receptor protein family m | -0.159 | 0.896 | 1.79E-02 | 1.79E+00 | | | | |
| 8084971 | NM_152699 | SENP5 | SUMO1/sentrin specific peptidase 5 | -0.159 | 0.896 | 7.04E-01 | 7.04E+01 | | | | |
| 8039748 | NM_130786 | A1BG | alpha-1-B glycoprotein | -0.159 | 0.896 | 1.22E-02 | 1.22E+00 | | | | |
| 8069252 | NM_020528 | PCBP3 | poly(rC) binding protein 3 | -0.159 | 0.896 | 4.52E-03 | 4.52E-01 | | | | |
| 8131096 | NM_00112863 | ELFN1 | extracellular leucine-rich repeat and | -0.159 | 0.896 | 1.06E-02 | 1.06E+00 | | | | |
| 8054297 | NM_004854 | CHST10 | carbohydrate sulfotransferase 10 | -0.159 | 0.896 | 1.99E-02 | 1.99E+00 | | | | |
| 8122256 | NM_00100878 | SLC35D3 | solute carrier family 35, member D3 | -0.159 | 0.896 | 1.15E-02 | 1.15E+00 | | | | |
| 8170300 | --- | --- | --- | -0.159 | 0.896 | 1.21E-02 | 1.21E+00 | | | | |
| 8151603 | NR_003594 | REXO1L2P | REX1, RNA exonuclease 1 homolog (S | -0.159 | 0.895 | 3.34E-02 | 3.34E+00 | | | | |
| 8151607 | NR_003594 | REXO1L2P | REX1, RNA exonuclease 1 homolog (S | -0.159 | 0.895 | 3.34E-02 | 3.34E+00 | | | | |

| | | | | | | | | | | |
|---|---|---|---|---|---|---|---|---|---|---|
| 8151623 | NR_003594 | REXO1L2P | REX1, RNA exonuclease 1 homolog (S | -0.159 | 0.895 | 3.34E-02 | 3.34E+00 | | | |
| 8140085 | NM_032951 | MLXIPL | MLX interacting protein-like | -0.159 | 0.895 | 9.80E-03 | 9.80E-01 | | | |
| 8149279 | --- | --- | --- | -0.159 | 0.895 | 3.07E-02 | 3.07E+00 | | | |
| 7893071 | --- | --- | --- | -0.159 | 0.895 | 7.19E-01 | 7.19E+01 | | | |
| 8155497 | NR_027421 | FAM27C | family with sequence similarity 27, m | -0.159 | 0.895 | 8.61E-03 | 8.61E-01 | | | |
| 7998601 | NM_00114600( | IGFALS | insulin-like growth factor binding pro | -0.159 | 0.895 | 9.15E-03 | 9.15E-01 | | | |
| 8165430 | NM_178448 | C9orf140 | chromosome 9 open reading frame 1 | -0.159 | 0.895 | 1.02E-02 | 1.02E+00 | | | |
| 8152215 | NM_005655 | KLF10 | Kruppel-like factor 10 | -0.159 | 0.895 | 2.44E-01 | 2.44E+01 | | | |
| 8053998 | NM_016466 | ANKRD39 | ankyrin repeat domain 39 | -0.159 | 0.895 | 1.82E-02 | 1.82E+00 | | | |
| 7967969 | NM_138284 | IL17D | interleukin 17D | -0.159 | 0.895 | 4.84E-03 | 4.84E-01 | | | |
| 8025766 | NM_199141 | CARM1 | coactivator-associated arginine meth | -0.159 | 0.895 | 8.05E-03 | 8.05E-01 | | | |
| 8138922 | NM_015483 | KBTBD2 | kelch repeat and BTB (POZ) domain c | -0.159 | 0.895 | 7.59E-01 | 7.59E+01 | | | |
| 8176159 | NM_020994 | CTAG2 | cancer/testis antigen 2 | -0.159 | 0.895 | 1.01E-02 | 1.01E+00 | | | |
| 8148772 | NM_016458 | C8orf30A | chromosome 8 open reading frame 3 | -0.159 | 0.895 | 1.47E-03 | 1.47E-01 | | | |
| 8022912 | --- | --- | --- | -0.160 | 0.895 | 3.22E-02 | 3.22E+00 | | | |
| 8170553 | NM_175868 | MAGEA6 | melanoma antigen family A, 6 | -0.160 | 0.895 | 8.76E-03 | 8.76E-01 | | | |
| 8088895 | NR_024241 | FAM86D | family with sequence similarity 86, m | -0.160 | 0.895 | 6.88E-02 | 6.88E+00 | | | |
| 8046546 | NM_019558 | HOXD8 | homeobox D8 | -0.160 | 0.895 | 2.98E-02 | 2.98E+00 | | | |
| 8149986 | NM_018660 | ZNF395 | zinc finger protein 395 | -0.160 | 0.895 | 1.94E-02 | 1.94E+00 | | | |
| 7925950 | NM_053049 | UCN3 | urocortin 3 (stresscopin) | -0.160 | 0.895 | 2.83E-02 | 2.83E+00 | | | |
| 7983826 | --- | --- | --- | -0.160 | 0.895 | 3.68E-02 | 3.68E+00 | | | |
| 8051028 | --- | --- | --- | -0.160 | 0.895 | 1.82E-01 | 1.82E+01 | | | |
| 8164627 | ENST00000358 | FLJ46836 | FLJ46836 protein | -0.160 | 0.895 | 7.33E-03 | 7.33E-01 | | | |
| 7995350 | --- | --- | --- | -0.160 | 0.895 | 4.34E-02 | 4.34E+00 | | | |
| 8061169 | --- | --- | --- | -0.160 | 0.895 | 3.29E-02 | 3.29E+00 | | | |
| 7963265 | NR_026893 | POU6F1 | POU class 6 homeobox 1 | -0.160 | 0.895 | 3.79E-03 | 3.79E-01 | | | |
| 7895189 | --- | --- | --- | -0.160 | 0.895 | 2.52E-01 | 2.52E+01 | | | |
| 8086462 | NM_032806 | C3orf39 | chromosome 3 open reading frame 3 | -0.160 | 0.895 | 2.13E-02 | 2.13E+00 | | | |
| 8010696 | NM_148896 | NPB | neuropeptide B | -0.160 | 0.895 | 1.01E-02 | 1.01E+00 | | | |
| 8019693 | NM_148896 | NPB | neuropeptide B | -0.160 | 0.895 | 1.01E-02 | 1.01E+00 | | | |
| 8138466 | NM_182762 | MACC1 | metastasis associated in colon cance | -0.160 | 0.895 | 1.45E-02 | 1.45E+00 | | | |
| 8010946 | NM_015721 | GEMIN4 | gem (nuclear organelle) associated p | -0.160 | 0.895 | 5.81E-02 | 5.81E+00 | | | |
| 7892851 | --- | --- | --- | -0.160 | 0.895 | 1.52E-01 | 1.52E+01 | | | |
| 7966234 | NM_021625 | TRPV4 | transient receptor potential cation cl | -0.160 | 0.895 | 2.29E-03 | 2.29E-01 | | | |
| 7893939 | --- | --- | --- | -0.160 | 0.895 | 3.20E-02 | 3.20E+00 | | | |
| 8036890 | NM_020956 | PRX | periaxin | -0.160 | 0.895 | 2.73E-03 | 2.73E-01 | | | |
| 8161255 | NM_003028 | SHB | Src homology 2 domain containing a | -0.160 | 0.895 | 2.68E-03 | 2.68E-01 | | | |
| 8108822 | NM_173828 | RELL2 | RELT-like 2 | -0.160 | 0.895 | 4.21E-03 | 4.21E-01 | | | |
| 8131140 | AK024457 | FLJ00049 | FLJ00049 protein | -0.160 | 0.895 | 1.75E-02 | 1.75E+00 | | | |
| 8098732 | NM_033178 | DUX4 | double homeobox, 4 | -0.160 | 0.895 | 9.66E-04 | 9.66E-02 | | | |
| 7990825 | --- | --- | --- | -0.160 | 0.895 | 1.69E-01 | 1.69E+01 | | | |
| 7923621 | NM_002256 | KISS1 | KiSS-1 metastasis-suppressor | -0.160 | 0.895 | 2.13E-02 | 2.13E+00 | | | |
| 7933298 | NM_031912 | SYT15 | synaptotagmin XV | -0.160 | 0.895 | 3.55E-02 | 3.55E+00 | | | |
| 8004372 | NM_003985 | TNK1 | tyrosine kinase, non-receptor, 1 | -0.160 | 0.895 | 1.21E-02 | 1.21E+00 | | | |
| 7970325 | NM_002929 | GRK1 | G protein-coupled receptor kinase 1 | -0.160 | 0.895 | 1.05E-02 | 1.05E+00 | | | |
| 7895508 | --- | --- | --- | -0.160 | 0.895 | 8.19E-02 | 8.19E+00 | | | |
| 7987554 | NM_018163 | DNAJC17 | DnaJ (Hsp40) homolog, subfamily C, | -0.160 | 0.895 | 2.71E-01 | 2.71E+01 | | | |
| 7921144 | NM_00116144 | SH2D2A | SH2 domain protein 2A | -0.160 | 0.895 | 4.82E-02 | 4.82E+00 | | | |
| 8093576 | NR_003004 | SCARNA22 | small Cajal body-specific RNA 22 | -0.160 | 0.895 | 2.61E-01 | 2.61E+01 | | | |
| 7931387 | --- | --- | --- | -0.160 | 0.895 | 3.96E-02 | 3.96E+00 | | | |
| 8148315 | NM_00115954 | POU5F1B | POU class 5 homeobox 1B | -0.160 | 0.895 | 4.34E-02 | 4.34E+00 | | | |
| 8044927 | NM_002193 | INHBB | inhibin, beta B | -0.160 | 0.895 | 1.07E-02 | 1.07E+00 | | | |
| 7995813 | NR_027781 | MT1DP | metallothionein 1D (pseudogene) | -0.160 | 0.895 | 1.90E-02 | 1.90E+00 | | | |
| 8032197 | NM_152769 | C19orf26 | chromosome 19 open reading frame | -0.160 | 0.895 | 1.01E-02 | 1.01E+00 | | | |
| 7932983 | --- | --- | --- | -0.160 | 0.895 | 6.00E-02 | 6.00E+00 | | | |
| 8050441 | --- | --- | --- | -0.160 | 0.895 | 2.13E-02 | 2.13E+00 | | | |
| 8116253 | NM_000843 | GRM6 | glutamate receptor, metabotropic 6 | -0.160 | 0.895 | 4.55E-03 | 4.55E-01 | | | |
| 7937305 | NM_138329 | NLRP6 | NLR family, pyrin domain containing | -0.160 | 0.895 | 2.15E-02 | 2.15E+00 | | | |
| 7916493 | NM_003713 | PPAP2B | phosphatidic acid phosphatase type | -0.160 | 0.895 | 1.04E-02 | 1.04E+00 | | | |
| 8161418 | NM_015234 | GPR116 | G protein-coupled receptor 116 | -0.160 | 0.895 | 2.16E-01 | 2.16E+01 | | | |
| 7898249 | NM_207348 | SLC25A34 | solute carrier family 25, member 34 | -0.160 | 0.895 | 4.44E-03 | 4.44E-01 | | | |
| 7896681 | --- | --- | --- | -0.160 | 0.895 | 4.52E-01 | 4.52E+01 | | | |
| 7945859 | NM_00103916 | MRGPRE | MAS-related GPR, member E | -0.160 | 0.895 | 6.78E-03 | 6.78E-01 | | | |
| 8050095 | AK124409 | FLJ42418 | FLJ42418 protein | -0.160 | 0.895 | 1.22E-02 | 1.22E+00 | | | |
| 7895832 | --- | --- | --- | -0.160 | 0.895 | 8.45E-01 | 8.45E+01 | | | |
| 7985786 | NM_013227 | ACAN | aggrecan | -0.160 | 0.895 | 6.43E-03 | 6.43E-01 | | | |
| 8137637 | --- | --- | --- | -0.161 | 0.895 | 7.21E-03 | 7.21E-01 | | | |
| 7894860 | --- | --- | --- | -0.161 | 0.895 | 4.80E-01 | 4.80E+01 | | | |
| 8156838 | NM_006808 | SEC61B | Sec61 beta subunit | -0.161 | 0.895 | 4.81E-02 | 4.81E+00 | | | |
| 8038319 | NM_002152 | HRC | histidine rich calcium binding protein | -0.161 | 0.895 | 1.31E-02 | 1.31E+00 | | | |
| 7991234 | NM_005928 | MFGE8 | milk fat globule-EGF factor 8 protein | -0.161 | 0.895 | 1.17E-01 | 1.17E+01 | | | |
| 8002845 | NM_00102520( | CTRB2 | chymotrypsinogen B2 | -0.161 | 0.895 | 1.04E-02 | 1.04E+00 | | | |
| 7896585 | --- | --- | --- | -0.161 | 0.895 | 4.39E-01 | 4.39E+01 | | | |
| 7969533 | NM_00104015 | SLAIN1 | SLAIN motif family, member 1 | -0.161 | 0.895 | 2.88E-01 | 2.88E+01 | | | |
| 7920297 | NM_020672 | S100A14 | S100 calcium binding protein A14 | -0.161 | 0.895 | 1.23E-02 | 1.23E+00 | | | |
| 7924230 | NM_00113011 | FAM108A1 | family with sequence similarity 108, | -0.161 | 0.895 | 1.79E-02 | 1.79E+00 | | | |
| 8114215 | NM_002653 | PITX1 | paired-like homeodomain 1 | -0.161 | 0.895 | 8.94E-03 | 8.94E-01 | | | |

| | | | | | | | | | | | |
|---|---|---|---|---|---|---|---|---|---|---|---|
| 7987511 | NM_207380 | C15orf52 | chromosome 15 open reading frame | -0.161 | 0.895 | 5.06E-03 | 5.06E-01 | | | | |
| 8142104 | NM_152749 | ATXN7L1 | ataxin 7-like 1 | -0.161 | 0.895 | 2.02E-01 | 2.02E+01 | | | | |
| 8139085 | --- | --- | --- | -0.161 | 0.895 | 2.65E-02 | 2.65E+00 | | | | |
| 7992678 | NR_015441 | PRO0461 | hypothetical LOC652276 | -0.161 | 0.895 | 3.87E-02 | 3.87E+00 | | | | |
| 8019108 | NM_014984 | AZI1 | 5-azacytidine induced 1 | -0.161 | 0.895 | 9.13E-03 | 9.13E-01 | | | | |
| 8067671 | NM_080823 | SRMS | src-related kinase lacking C-terminal | -0.161 | 0.895 | 8.96E-03 | 8.96E-01 | | | | |
| 7950492 | NM_00103954 | KLHL35 | kelch-like 35 (Drosophila) | -0.161 | 0.895 | 3.45E-03 | 3.45E-01 | | | | |
| 7895962 | --- | --- | --- | -0.161 | 0.895 | 1.73E-01 | 1.73E+01 | | | | |
| 7896231 | --- | --- | --- | -0.161 | 0.894 | 7.05E-01 | 7.05E+01 | | | | |
| 8072645 | NR_027780 | HMGXB4 | HMG box domain containing 4 | -0.161 | 0.894 | 3.54E-01 | 3.54E+01 | | | | |
| 7893255 | --- | --- | --- | -0.161 | 0.894 | 9.05E-01 | 9.05E+01 | | | | |
| 8027006 | NM_032627 | SSBP4 | single stranded DNA binding protein | -0.161 | 0.894 | 8.44E-03 | 8.44E-01 | | | | |
| 8038565 | ENST00000430 | C19orf63 | chromosome 19 open reading frame | -0.161 | 0.894 | 2.04E-02 | 2.04E+00 | | | | |
| 8137334 | NM_007188 | ABCB8 | ATP-binding cassette, sub-family B (M | -0.161 | 0.894 | 1.80E-02 | 1.80E+00 | | | | |
| 8034940 | NM_000435 | NOTCH3 | Notch homolog 3 (Drosophila) | -0.161 | 0.894 | 3.28E-03 | 3.28E-01 | | | | |
| 7900340 | NM_181809 | BMP8A | bone morphogenetic protein 8a | -0.161 | 0.894 | 2.94E-02 | 2.94E+00 | | | | |
| 7892816 | --- | --- | --- | -0.161 | 0.894 | 1.03E-01 | 1.03E+01 | | | | |
| 7896338 | --- | --- | --- | -0.161 | 0.894 | 4.90E-01 | 4.90E+01 | | | | |
| 7982348 | ENST00000416 | OC10012997 | similar to FLJ00287 protein | -0.161 | 0.894 | 7.26E-03 | 7.26E-01 | | | | |
| 8175747 | NM_005362 | MAGEA3 | melanoma antigen family A, 3 | -0.161 | 0.894 | 2.26E-03 | 2.26E-01 | | | | |
| 8099037 | NM_00114606 | MFSD10 | major facilitator superfamily domain | -0.161 | 0.894 | 4.13E-03 | 4.13E-01 | | | | |
| 7913990 | NM_022078 | GPATCH3 | G patch domain containing 3 | -0.161 | 0.894 | 5.65E-02 | 5.65E+00 | | | | |
| 8003348 | NM_178841 | RNF166 | ring finger protein 166 | -0.161 | 0.894 | 2.86E-02 | 2.86E+00 | | | | |
| 8030321 | NM_153329 | ALDH16A1 | aldehyde dehydrogenase 16 family, | -0.161 | 0.894 | 1.81E-03 | 1.81E-01 | | | | |
| 7895783 | --- | --- | --- | -0.161 | 0.894 | 6.10E-02 | 6.10E+00 | | | | |
| 8028908 | NM_025194 | ITPKC | inositol 1,4,5-trisphosphate 3-kinase | -0.161 | 0.894 | 1.23E-02 | 1.23E+00 | | | | |
| 8147516 | NM_002380 | MATN2 | matrilin 2 | -0.161 | 0.894 | 3.07E-02 | 3.07E+00 | | | | |
| 7995525 | NM_033119 | NKD1 | naked cuticle homolog 1 (Drosophila | -0.161 | 0.894 | 3.97E-03 | 3.97E-01 | | | | |
| 8097017 | NM_00112817 | UGT8 | UDP glycosyltransferase 8 | -0.161 | 0.894 | 1.94E-02 | 1.94E+00 | | | | |
| 8024331 | NR_026557 | PLK5P | polo-like kinase 5 pseudogene | -0.161 | 0.894 | 2.94E-03 | 2.94E-01 | | | | |
| 8010702 | NM_024083 | ASPSCR1 | alveolar soft part sarcoma chromoso | -0.161 | 0.894 | 9.65E-03 | 9.65E-01 | | | | |
| 8104781 | NM_016568 | RXFP3 | relaxin/insulin-like family peptide re | -0.161 | 0.894 | 4.11E-03 | 4.11E-01 | | | | |
| 8009832 | NM_207346 | TSEN54 | tRNA splicing endonuclease 54 homo | -0.161 | 0.894 | 3.30E-02 | 3.30E+00 | | | | |
| 8132036 | NM_00108052 | WIPF3 | WAS/WASL interacting protein famil | -0.161 | 0.894 | 9.88E-03 | 9.88E-01 | | | | |
| 8076441 | NM_145912 | NFAM1 | NFAT activating protein with ITAM m | -0.161 | 0.894 | 7.68E-03 | 7.68E-01 | | | | |
| 7907430 | NM_000639 | FASLG | Fas ligand (TNF superfamily, membe | -0.161 | 0.894 | 7.67E-02 | 7.67E+00 | | | | |
| 8091588 | NM_00103870 | GPR149 | G protein-coupled receptor 149 | -0.162 | 0.894 | 1.22E-02 | 1.22E+00 | | | | |
| 7986385 | NM_145728 | SYNM | synemin, intermediate filament prot | -0.162 | 0.894 | 9.13E-03 | 9.13E-01 | | | | |
| 8067798 | NM_018419 | SOX18 | SRY (sex determining region Y)-box 1 | -0.162 | 0.894 | 1.54E-02 | 1.54E+00 | | | | |
| 7893853 | --- | --- | --- | -0.162 | 0.894 | 1.48E-01 | 1.48E+01 | | | | |
| 8149707 | NM_018688 | BIN3 | bridging integrator 3 | -0.162 | 0.894 | 1.14E-01 | 1.14E+01 | | | | |
| 8019954 | NR_024101 | FLJ35776 | hypothetical LOC649446 | -0.162 | 0.894 | 2.81E-01 | 2.81E+01 | | | | |
| 8137464 | NM_004577 | PSPH | phosphoserine phosphatase | -0.162 | 0.894 | 7.01E-01 | 7.01E+01 | | | | |
| 8174283 | NM_00100291 | H2BFWT | H2B histone family, member W, test | -0.162 | 0.894 | 6.38E-03 | 6.38E-01 | | | | |
| 8032232 | NM_017573 | PCSK4 | proprotein convertase subtilisin/kex | -0.162 | 0.894 | 1.04E-02 | 1.04E+00 | | | | |
| 8032365 | NM_031918 | KLF16 | Kruppel-like factor 16 | -0.162 | 0.894 | 7.01E-03 | 7.01E-01 | | | | |
| 8108470 | --- | --- | --- | -0.162 | 0.894 | 4.13E-02 | 4.13E+00 | | | | |
| 7894845 | --- | --- | --- | -0.162 | 0.894 | 6.54E-01 | 6.54E+01 | | | | |
| 8167234 | NM_006743 | RBM3 | RNA binding motif (RNP1, RRM) prot | -0.162 | 0.894 | 1.29E-01 | 1.29E+01 | | | | |
| 7908639 | NM_018265 | C1orf106 | chromosome 1 open reading frame 1 | -0.162 | 0.894 | 4.61E-03 | 4.61E-01 | | | | |
| 8144078 | NM_000193 | SHH | sonic hedgehog homolog (Drosophila | -0.162 | 0.894 | 5.71E-03 | 5.71E-01 | | | | |
| 8179364 | NM_006929 | SKIV2L | superkiller viralicidic activity 2-like (S | -0.162 | 0.894 | 2.09E-02 | 2.09E+00 | | | | |
| 8176091 | BC009467 | LOC158960 | hypothetical protein BC009467 | -0.162 | 0.894 | 1.23E-02 | 1.23E+00 | | | | |
| 8131917 | --- | --- | --- | -0.162 | 0.894 | 2.90E-02 | 2.90E+00 | | | | |
| 7896615 | --- | --- | --- | -0.162 | 0.894 | 2.73E-01 | 2.73E+01 | | | | |
| 8066384 | NM_176791 | GTSF1L | gametocyte specific factor 1-like | -0.162 | 0.894 | 4.09E-01 | 4.09E+01 | | | | |
| 7895663 | --- | --- | --- | -0.162 | 0.894 | 4.28E-01 | 4.28E+01 | | | | |
| 8126948 | J04755 | FTHP1 | ferritin, heavy polypeptide pseudoge | -0.162 | 0.894 | 4.48E-02 | 4.48E+00 | | | | |
| 8026212 | --- | --- | --- | -0.162 | 0.894 | 5.29E-02 | 5.29E+00 | | | | |
| 8003619 | NR_024120 | ELP2P | endozepine-like peptide 2 pseudoge | -0.162 | 0.894 | 2.65E-02 | 2.65E+00 | | | | |
| 8067033 | NM_002237 | KCNG1 | potassium voltage-gated channel, su | -0.162 | 0.894 | 1.27E-02 | 1.27E+00 | | | | |
| 7905548 | NM_005416 | SPRR3 | small proline-rich protein 3 | -0.162 | 0.894 | 8.18E-03 | 8.18E-01 | | | | |
| 7942774 | NM_173039 | AQP11 | aquaporin 11 | -0.162 | 0.894 | 5.11E-02 | 5.11E+00 | | | | |
| 8137468 | ENST00000021 | CCT8L1 | chaperonin containing TCP1, subunit | -0.162 | 0.894 | 1.45E-02 | 1.45E+00 | | | | |
| 8077851 | NM_00109821 | HRH1 | histamine receptor H1 | -0.162 | 0.894 | 7.26E-03 | 7.26E-01 | | | | |
| 7896103 | --- | --- | --- | -0.162 | 0.894 | 7.13E-02 | 7.13E+00 | | | | |
| 7947245 | M30627 | HSP90AA2 | heat shock protein 90kDa alpha (cyto | -0.162 | 0.894 | 5.47E-01 | 5.47E+01 | | | | |
| 8038326 | NM_014037 | SLC6A16 | solute carrier family 6, member 16 | -0.162 | 0.894 | 1.76E-02 | 1.76E+00 | | | | |
| 7892991 | --- | --- | --- | -0.162 | 0.894 | 1.00E-01 | 1.00E+01 | | | | |
| 7894291 | --- | --- | --- | -0.162 | 0.894 | 4.15E-02 | 4.15E+00 | | | | |
| 7988685 | --- | --- | --- | -0.162 | 0.894 | 4.35E-02 | 4.35E+00 | | | | |
| 7928872 | NM_003087 | SNCG | synuclein, gamma (breast cancer-spe | -0.162 | 0.894 | 1.01E-02 | 1.01E+00 | | | | |
| 8045695 | --- | --- | --- | -0.162 | 0.894 | 2.43E-01 | 2.43E+01 | | | | |
| 8109120 | NM_152406 | AFAP1L1 | actin filament associated protein 1-li | -0.162 | 0.894 | 8.67E-04 | 8.67E-02 | | | | |
| 7896574 | --- | --- | --- | -0.162 | 0.893 | 2.54E-01 | 2.54E+01 | | | | |
| 8000897 | --- | --- | --- | -0.163 | 0.893 | 1.14E-01 | 1.14E+01 | | | | |
| 8031714 | NM_006635 | ZNF460 | zinc finger protein 460 | -0.163 | 0.893 | 3.28E-01 | 3.28E+01 | | | | |

| | | | | | | | | | | |
|---|---|---|---|---|---|---|---|---|---|---|
| 7935834 | NM_024895 | PDZD7 | PDZ domain containing 7 | -0.163 | 0.893 | 2.06E-02 | 2.06E+00 | | | |
| 7972973 | AF287261 | SETD8 | SET domain containing (lysine methy | -0.163 | 0.893 | 3.95E-02 | 3.95E+00 | | | |
| 7898296 | BC017946 | C1orf64 | chromosome 1 open reading frame 6 | -0.163 | 0.893 | 1.82E-02 | 1.82E+00 | | | |
| 7948937 | NM_004254 | SLC22A8 | solute carrier family 22 (organic anio | -0.163 | 0.893 | 2.04E-02 | 2.04E+00 | | | |
| 8001529 | --- | --- | --- | -0.163 | 0.893 | 4.64E-01 | 4.64E+01 | | | |
| 7948470 | NM_004778 | GPR44 | G protein-coupled receptor 44 | -0.163 | 0.893 | 6.50E-03 | 6.50E-01 | | | |
| 8153903 | NM_000973 | RPL8 | ribosomal protein L8 | -0.163 | 0.893 | 2.72E-01 | 2.72E+01 | | | |
| 7995681 | NM_004530 | MMP2 | matrix metallopeptidase 2 (gelatinas | -0.163 | 0.893 | 1.81E-03 | 1.81E-01 | | | |
| 7893790 | --- | --- | --- | -0.163 | 0.893 | 6.69E-01 | 6.69E+01 | | | |
| 7956668 | --- | --- | --- | -0.163 | 0.893 | 5.97E-02 | 5.97E+00 | | | |
| 8038584 | NM_00108045 | LRRC4B | leucine rich repeat containing 4B | -0.163 | 0.893 | 1.59E-02 | 1.59E+00 | | | |
| 8004880 | NM_004822 | NTN1 | netrin 1 | -0.163 | 0.893 | 2.75E-03 | 2.75E-01 | | | |
| 8026294 | NR_024282 | LOC113230 | hypothetical protein LOC113230 | -0.163 | 0.893 | 2.01E-02 | 2.01E+00 | | | |
| 8059744 | BC067110 | ECEL1P2 | endothelin converting enzyme-like 1 | -0.163 | 0.893 | 1.26E-02 | 1.26E+00 | | | |
| 8161166 | NM_019897 | OR2S2 | olfactory receptor, family 2, subfami | -0.163 | 0.893 | 3.60E-03 | 3.60E-01 | | | |
| 7997746 | NM_020655 | JPH3 | junctophilin 3 | -0.163 | 0.893 | 7.15E-03 | 7.15E-01 | | | |
| 8153625 | NM_017570 | OPLAH | 5-oxoprolinase (ATP-hydrolysing) | -0.163 | 0.893 | 9.28E-03 | 9.28E-01 | | | |
| 8024420 | NM_007165 | SF3A2 | splicing factor 3a, subunit 2, 66kDa | -0.163 | 0.893 | 1.03E-02 | 1.03E+00 | | | |
| 7895806 | --- | --- | --- | -0.163 | 0.893 | 5.85E-01 | 5.85E+01 | | | |
| 8153411 | NM_015117 | ZC3H3 | zinc finger CCCH-type containing 3 | -0.163 | 0.893 | 2.69E-03 | 2.69E-01 | | | |
| 8061562 | --- | --- | --- | -0.163 | 0.893 | 1.58E-02 | 1.58E+00 | | | |
| 7931556 | NM_014468 | VENTX | VENT homeobox homolog (Xenopus | -0.163 | 0.893 | 1.25E-02 | 1.25E+00 | | | |
| 8027247 | NM_031218 | ZNF93 | zinc finger protein 93 | -0.163 | 0.893 | 2.22E-01 | 2.22E+01 | | | |
| 8068157 | NR_023342 | KRTAP20-4 | keratin associated protein 20-4 | -0.163 | 0.893 | 2.87E-02 | 2.87E+00 | | | |
| 7898725 | NM_005747 | CELA3A | chymotrypsin-like elastase family, m | -0.163 | 0.893 | 4.66E-02 | 4.66E+00 | | | |
| 8070689 | NM_007031 | HSF2BP | heat shock transcription factor 2 bin | -0.163 | 0.893 | 3.79E-03 | 3.79E-01 | | | |
| 8032214 | NM_000156 | GAMT | guanidinoacetate N-methyltransfera | -0.163 | 0.893 | 2.33E-02 | 2.33E+00 | | | |
| 8039090 | --- | --- | --- | -0.163 | 0.893 | 1.78E-02 | 1.78E+00 | | | |
| 7950128 | NM_014042 | C11orf51 | chromosome 11 open reading frame | -0.163 | 0.893 | 4.54E-01 | 4.54E+01 | | | |
| 7929840 | NM_003987 | PAX2 | paired box 2 | -0.163 | 0.893 | 2.73E-03 | 2.73E-01 | | | |
| 8144416 | NR_027000 | LOC349196 | hypothetical LOC349196 | -0.163 | 0.893 | 5.65E-02 | 5.65E+00 | | | |
| 8144418 | NR_027000 | LOC349196 | hypothetical LOC349196 | -0.163 | 0.893 | 5.65E-02 | 5.65E+00 | | | |
| 8144490 | NR_027000 | LOC349196 | hypothetical LOC349196 | -0.163 | 0.893 | 5.65E-02 | 5.65E+00 | | | |
| 8144492 | NR_027000 | LOC349196 | hypothetical LOC349196 | -0.163 | 0.893 | 5.65E-02 | 5.65E+00 | | | |
| 8059838 | NM_018410 | HJURP | Holliday junction recognition protein | -0.163 | 0.893 | 6.43E-03 | 6.43E-01 | | | |
| 8098707 | AY956760 | HSP90AA4P | heat shock protein 90kDa alpha (cyto | -0.163 | 0.893 | 1.55E-01 | 1.55E+01 | | | |
| 8098571 | BC140795 | HELT | HES/HEY-like transcription factor | -0.163 | 0.893 | 2.37E-02 | 2.37E+00 | | | |
| 8073148 | NM_001675 | ATF4 | activating transcription factor 4 (tax- | -0.163 | 0.893 | 3.00E-01 | 3.00E+01 | | | |
| 7895020 | --- | --- | --- | -0.163 | 0.893 | 1.37E-01 | 1.37E+01 | | | |
| 8035445 | NM_005354 | JUND | jun D proto-oncogene | -0.163 | 0.893 | 2.75E-03 | 2.75E-01 | | | |
| 8165639 | BC052297 | C9orf169 | chromosome 9 open reading frame 1 | -0.163 | 0.893 | 2.92E-03 | 2.92E-01 | | | |
| 7950862 | --- | --- | --- | -0.163 | 0.893 | 6.48E-01 | 6.48E+01 | | | |
| 8046755 | --- | --- | --- | -0.163 | 0.893 | 1.47E-01 | 1.47E+01 | | | |
| 8040419 | NM_005378 | MYCN | v-myc myelocytomatosis viral relate | -0.163 | 0.893 | 1.13E-02 | 1.13E+00 | | | |
| 8035007 | NM_005858 | AKAP8 | A kinase (PRKA) anchor protein 8 | -0.164 | 0.893 | 7.54E-02 | 7.54E+00 | | | |
| 7895007 | --- | --- | --- | -0.164 | 0.893 | 5.27E-02 | 5.27E+00 | | | |
| 8153336 | NM_020427 | SLURP1 | secreted LY6/PLAUR domain contain | -0.164 | 0.893 | 1.36E-02 | 1.36E+00 | | | |
| 8158625 | AK125285 | OC10012978 | hypothetical protein LOC100129785 | -0.164 | 0.893 | 6.70E-02 | 6.70E+00 | | | |
| 8063923 | NM_016354 | SLCO4A1 | solute carrier organic anion transpor | -0.164 | 0.893 | 1.27E-02 | 1.27E+00 | | | |
| 8154512 | NM_00104027 | ADAMTSL1 | ADAMTS-like 1 | -0.164 | 0.893 | 1.22E-02 | 1.22E+00 | | | |
| 8028112 | NM_024509 | LRFN3 | leucine rich repeat and fibronectin ty | -0.164 | 0.893 | 5.14E-03 | 5.14E-01 | | | |
| 8027385 | NM_00114633 | VSTM2B | V-set and transmembrane domain co | -0.164 | 0.893 | 3.90E-03 | 3.90E-01 | | | |
| 7896062 | --- | --- | --- | -0.164 | 0.893 | 3.20E-01 | 3.20E+01 | | | |
| 8117630 | NM_003447 | ZNF165 | zinc finger protein 165 | -0.164 | 0.893 | 1.06E-02 | 1.06E+00 | | | |
| 7937483 | NR_002585 | SNORA52 | small nucleolar RNA, H/ACA box 52 | -0.164 | 0.893 | 1.05E-01 | 1.05E+01 | | | |
| 8004545 | NM_001678 | ATP1B2 | ATPase, Na+/K+ transporting, beta 2 | -0.164 | 0.893 | 5.87E-03 | 5.87E-01 | | | |
| 7959016 | NR_027345 | NCRNA0017 | non-protein coding RNA 173 | -0.164 | 0.893 | 1.92E-02 | 1.92E+00 | | | |
| 8085195 | NM_198560 | LHFPL4 | lipoma HMGIC fusion partner-like 4 | -0.164 | 0.893 | 1.10E-02 | 1.10E+00 | | | |
| 8006999 | NM_172220 | CSF3 | colony stimulating factor 3 (granuloc | -0.164 | 0.893 | 1.04E-02 | 1.04E+00 | | | |
| 8030641 | NM_002691 | POLD1 | polymerase (DNA directed), delta 1, | -0.164 | 0.893 | 1.28E-02 | 1.28E+00 | | | |
| 7894775 | --- | --- | --- | -0.164 | 0.893 | 8.43E-01 | 8.43E+01 | | | |
| 8071036 | NM_006272 | S100B | S100 calcium binding protein B | -0.164 | 0.893 | 7.40E-01 | 7.40E+01 | | | |
| 8077204 | NM_000040 | APOC3 | apolipoprotein C-III | -0.164 | 0.893 | 9.39E-03 | 9.39E-01 | | | |
| 8155121 | NM_003995 | NPR2 | natriuretic peptide receptor B/guany | -0.164 | 0.893 | 6.22E-03 | 6.22E-01 | | | |
| 8009585 | NM_023036 | DNAI2 | dynein, axonemal, intermediate chai | -0.164 | 0.893 | 8.70E-03 | 8.70E-01 | | | |
| 7894155 | --- | --- | --- | -0.164 | 0.893 | 4.90E-01 | 4.90E+01 | | | |
| 8036172 | ENST0000044 | PRODH2 | proline dehydrogenase (oxidase) 2 | -0.164 | 0.893 | 2.25E-02 | 2.25E+00 | | | |
| 7913216 | NM_000300 | PLA2G2A | phospholipase A2, group IIA (platele | -0.164 | 0.893 | 1.83E-02 | 1.83E+00 | | | |
| 8133704 | NM_00111019 | FLJ37078 | hypothetical protein FLJ37078 | -0.164 | 0.893 | 1.92E-03 | 1.92E-01 | | | |
| 7925448 | --- | --- | --- | -0.164 | 0.892 | 3.69E-01 | 3.69E+01 | | | |
| 8025515 | NM_015725 | RDH8 | retinol dehydrogenase 8 (all-trans) | -0.164 | 0.892 | 3.79E-03 | 3.79E-01 | | | |
| 8133221 | --- | --- | --- | -0.164 | 0.892 | 6.20E-02 | 6.20E+00 | | | |
| 8034342 | AK292455 | ZNF625 | zinc finger protein 625 | -0.164 | 0.892 | 7.02E-01 | 7.02E+01 | | | |
| 8143365 | --- | --- | --- | -0.164 | 0.892 | 3.68E-01 | 3.68E+01 | | | |
| 8098725 | NM_033178 | DUX4 | double homeobox, 4 | -0.164 | 0.892 | 1.69E-03 | 1.69E-01 | | | |
| 7893557 | --- | --- | --- | -0.164 | 0.892 | 5.32E-01 | 5.32E+01 | | | |
| 7977646 | NM_016423 | ZNF219 | zinc finger protein 219 | -0.164 | 0.892 | 1.45E-02 | 1.45E+00 | | | |

| | | | | | | | | | | |
|---|---|---|---|---|---|---|---|---|---|---|
| 8015387 | NM_000422 | KRT17 | keratin 17 | -0.164 | 0.892 | 1.04E-02 | 1.04E+00 | | | |
| 8029182 | NM_022752 | ZNF574 | zinc finger protein 574 | -0.164 | 0.892 | 1.73E-02 | 1.73E+00 | | | |
| 7892832 | --- | --- | --- | -0.164 | 0.892 | 5.66E-01 | 5.66E+01 | | | |
| 8005435 | NM_148886 | SMCR7 | Smith-Magenis syndrome chromoso | -0.164 | 0.892 | 4.43E-03 | 4.43E-01 | | | |
| 8060988 | NM_014962 | BTBD3 | BTB (POZ) domain containing 3 | -0.164 | 0.892 | 2.58E-02 | 2.58E+00 | | | |
| 7997909 | NM_003119 | SPG7 | spastic paraplegia 7 (pure and compl | -0.164 | 0.892 | 1.77E-02 | 1.77E+00 | | | |
| 8016232 | NM_174919 | SH3D20 | SH3 domain containing 20 | -0.165 | 0.892 | 5.27E-03 | 5.27E-01 | | | |
| 7981106 | NM_173849 | GSC | goosecoid homeobox | -0.165 | 0.892 | 1.14E-02 | 1.14E+00 | | | |
| 8121216 | NM_021620 | PRDM13 | PR domain containing 13 | -0.165 | 0.892 | 7.90E-03 | 7.90E-01 | | | |
| 8104268 | NM_00100384 | SLC6A19 | solute carrier family 6 (neutral amin | -0.165 | 0.892 | 2.20E-03 | 2.20E-01 | | | |
| 8126058 | NM_016059 | PPIL1 | peptidylprolyl isomerase (cyclophilin | -0.165 | 0.892 | 2.77E-02 | 2.77E+00 | | | |
| 8090044 | NM_00103170 | SEMA5B | sema domain, seven thrombospondi | -0.165 | 0.892 | 1.88E-03 | 1.88E-01 | | | |
| 8114245 | NM_006161 | NEUROG1 | neurogenin 1 | -0.165 | 0.892 | 7.21E-03 | 7.21E-01 | | | |
| 7908347 | NM_022375 | OCLM | oculomedin | -0.165 | 0.892 | 8.18E-01 | 8.18E+01 | | | |
| 8000945 | --- | --- | --- | -0.165 | 0.892 | 1.59E-02 | 1.59E+00 | | | |
| 7949373 | NM_014205 | ZNHIT2 | zinc finger, HIT type 2 | -0.165 | 0.892 | 2.02E-02 | 2.02E+00 | | | |
| 8133089 | NM_152626 | ZNF92 | zinc finger protein 92 | -0.165 | 0.892 | 2.93E-01 | 2.93E+01 | | | |
| 7982829 | NM_181642 | SPINT1 | serine peptidase inhibitor, Kunitz typ | -0.165 | 0.892 | 9.31E-03 | 9.31E-01 | | | |
| 8160792 | NM_032596 | C9orf24 | chromosome 9 open reading frame 2 | -0.165 | 0.892 | 7.21E-03 | 7.21E-01 | | | |
| 7893150 | --- | --- | --- | -0.165 | 0.892 | 7.95E-01 | 7.95E+01 | | | |
| 7896000 | --- | --- | --- | -0.165 | 0.892 | 8.31E-01 | 8.31E+01 | | | |
| 8032601 | NM_032753 | RAX2 | retina and anterior neural fold home | -0.165 | 0.892 | 1.59E-02 | 1.59E+00 | | | |
| 8061227 | NM_020689 | SLC24A3 | solute carrier family 24 (sodium/pot | -0.165 | 0.892 | 1.95E-02 | 1.95E+00 | | | |
| 8069135 | NR_026547 | C21orf90 | chromosome 21 open reading frame | -0.165 | 0.892 | 2.36E-02 | 2.36E+00 | | | |
| 7950032 | NM_002007 | FGF4 | fibroblast growth factor 4 | -0.165 | 0.892 | 6.43E-03 | 6.43E-01 | | | |
| 8022418 | --- | --- | --- | -0.165 | 0.892 | 3.32E-01 | 3.32E+01 | | | |
| 7944970 | --- | --- | --- | -0.165 | 0.892 | 1.13E-01 | 1.13E+01 | | | |
| 7944463 | NM_024618 | NLRX1 | NLR family member X1 | -0.165 | 0.892 | 4.06E-03 | 4.06E-01 | | | |
| 7991451 | --- | --- | --- | -0.165 | 0.892 | 3.55E-02 | 3.55E+00 | | | |
| 8030749 | ENST0000032 | MGC45922 | hypothetical LOC284365 | -0.165 | 0.892 | 1.04E-02 | 1.04E+00 | | | |
| 8000974 | NM_024706 | ZNF668 | zinc finger protein 668 | -0.165 | 0.892 | 1.31E-02 | 1.31E+00 | | | |
| 8000632 | --- | --- | --- | -0.165 | 0.892 | 1.21E-01 | 1.21E+01 | | | |
| 8065596 | NM_030815 | PDRG1 | p53 and DNA-damage regulated 1 | -0.165 | 0.892 | 3.51E-02 | 3.51E+00 | | | |
| 8012116 | NM_015343 | DULLARD | dullard homolog (Xenopus laevis) | -0.165 | 0.892 | 3.93E-02 | 3.93E+00 | | | |
| 8071444 | NM_144704 | AIFM3 | apoptosis-inducing factor, mitochon | -0.165 | 0.892 | 1.06E-02 | 1.06E+00 | | | |
| 7937700 | NM_00100148 | KRTAP5-5 | keratin associated protein 5-5 | -0.165 | 0.892 | 5.26E-02 | 5.26E+00 | | | |
| 8025828 | NM_000527 | LDLR | low density lipoprotein receptor | -0.165 | 0.892 | 1.25E-02 | 1.25E+00 | | | |
| 8106532 | --- | --- | --- | -0.166 | 0.892 | 1.45E-01 | 1.45E+01 | | | |
| 8119386 | DQ168992 | TDRG1 | testis development related protein 1 | -0.166 | 0.892 | 7.20E-03 | 7.20E-01 | | | |
| 8112724 | --- | --- | --- | -0.166 | 0.891 | 2.65E-01 | 2.65E+01 | | | |
| 7950036 | NM_005247 | FGF3 | fibroblast growth factor 3 (murine m | -0.166 | 0.891 | 6.89E-03 | 6.89E-01 | | | |
| 7977373 | NM_004689 | MTA1 | metastasis associated 1 | -0.166 | 0.891 | 9.28E-02 | 9.28E+00 | | | |
| 8156610 | NM_014282 | HABP4 | hyaluronan binding protein 4 | -0.166 | 0.891 | 4.04E-01 | 4.04E+01 | | | |
| 7981427 | NM_001823 | CKB | creatine kinase, brain | -0.166 | 0.891 | 7.26E-03 | 7.26E-01 | | | |
| 8002711 | --- | --- | --- | -0.166 | 0.891 | 3.70E-01 | 3.70E+01 | | | |
| 8006668 | NM_024864 | MRM1 | mitochondrial rRNA methyltransfera | -0.166 | 0.891 | 9.01E-03 | 9.01E-01 | | | |
| 8172379 | NM_00103773 | ZNF630 | zinc finger protein 630 | -0.166 | 0.891 | 3.84E-01 | 3.84E+01 | | | |
| 7961889 | --- | --- | --- | -0.166 | 0.891 | 6.08E-02 | 6.08E+00 | | | |
| 8162214 | NM_00114512 | FAM75C1 | family with sequence similarity 75, m | -0.166 | 0.891 | 8.10E-03 | 8.10E-01 | | | |
| 8172317 | NM_006950 | SYN1 | synapsin I | -0.166 | 0.891 | 4.43E-03 | 4.43E-01 | | | |
| 8074371 | NM_022719 | DGCR14 | DiGeorge syndrome critical region ge | -0.166 | 0.891 | 2.95E-02 | 2.95E+00 | | | |
| 7904879 | --- | --- | --- | -0.166 | 0.891 | 4.51E-02 | 4.51E+00 | | | |
| 7929114 | --- | --- | --- | -0.166 | 0.891 | 5.24E-01 | 5.24E+01 | | | |
| 7945162 | NM_003658 | BARX2 | BARX homeobox 2 | -0.166 | 0.891 | 8.31E-03 | 8.31E-01 | | | |
| 8064676 | NM_145762 | GFRA4 | GDNF family receptor alpha 4 | -0.166 | 0.891 | 3.60E-03 | 3.60E-01 | | | |
| 7913965 | NM_00111475 | ZNF683 | zinc finger protein 683 | -0.166 | 0.891 | 4.36E-02 | 4.36E+00 | | | |
| 8054939 | --- | --- | --- | -0.166 | 0.891 | 1.58E-02 | 1.58E+00 | | | |
| 8143941 | NM_198285 | WDR86 | WD repeat domain 86 | -0.166 | 0.891 | 8.48E-03 | 8.48E-01 | | | |
| 8165575 | NM_00109853 | PNPLA7 | patatin-like phospholipase domain c | -0.166 | 0.891 | 4.74E-03 | 4.74E-01 | | | |
| 8030671 | NM_003121 | SPIB | Spi-B transcription factor (Spi-1/PU.1 | -0.166 | 0.891 | 2.52E-02 | 2.52E+00 | | | |
| 7991138 | NM_021077 | NMB | neuromedin B | -0.166 | 0.891 | 6.10E-02 | 6.10E+00 | | | |
| 7918359 | NM_020703 | AMIGO1 | adhesion molecule with Ig-like doma | -0.166 | 0.891 | 1.71E-02 | 1.71E+00 | | | |
| 8004938 | ENST0000042 | OC10012967 | hypothetical protein LOC100129677 | -0.167 | 0.891 | 1.01E-01 | 1.01E+01 | | | |
| 8008110 | ENST0000037 | LOC404266 | hypothetical LOC404266 | -0.167 | 0.891 | 3.38E-03 | 3.38E-01 | | | |
| 7980889 | --- | --- | --- | -0.167 | 0.891 | 8.76E-02 | 8.76E+00 | | | |
| 8062971 | NM_00104822 | DBNDD2 | dysbindin (dystrobrevin binding prot | -0.167 | 0.891 | 1.23E-02 | 1.23E+00 | | | |
| 8133752 | --- | --- | --- | -0.167 | 0.891 | 5.81E-02 | 5.81E+00 | | | |
| 8033023 | NM_00113431 | PRR22 | proline rich 22 | -0.167 | 0.891 | 3.73E-03 | 3.73E-01 | | | |
| 7955348 | NM_005276 | GPD1 | glycerol-3-phosphate dehydrogenase | -0.167 | 0.891 | 1.59E-02 | 1.59E+00 | | | |
| 8123372 | BX647686 | CCR6 | chemokine (C-C motif) receptor 6 | -0.167 | 0.891 | 4.96E-01 | 4.96E+01 | | | |
| 7895934 | --- | --- | --- | -0.167 | 0.891 | 5.03E-01 | 5.03E+01 | | | |
| 8047518 | NM_015934 | NOP58 | NOP58 ribonucleoprotein homolog ( | -0.167 | 0.891 | 7.27E-01 | 7.27E+01 | | | |
| 7960124 | NM_138575 | PGAM5 | phosphoglycerate mutase family me | -0.167 | 0.891 | 5.83E-02 | 5.83E+00 | | | |
| 8071530 | NR_024591 | POM121L1P | POM121 membrane glycoprotein-lik | -0.167 | 0.891 | 1.37E-01 | 1.37E+01 | | | |
| 8074712 | NR_024591 | POM121L1P | POM121 membrane glycoprotein-lik | -0.167 | 0.891 | 1.37E-01 | 1.37E+01 | | | |
| 7899029 | NM_020379 | MAN1C1 | mannosidase, alpha, class 1C, memb | -0.167 | 0.891 | 7.81E-03 | 7.81E-01 | | | |
| 8043407 | NM_152670 | C2orf51 | chromosome 2 open reading frame 5 | -0.167 | 0.891 | 1.48E-02 | 1.48E+00 | | | |

| | | | | | | | | | | |
|---|---|---|---|---|---|---|---|---|---|---|
| 7927389 | NM_002750 | MAPK8 | mitogen-activated protein kinase 8 | -0.167 | 0.891 | 4.58E-01 | 4.58E+01 | | | |
| 7894945 | --- | --- | --- | -0.167 | 0.891 | 1.39E-01 | 1.39E+01 | | | |
| 7895795 | --- | --- | --- | -0.167 | 0.891 | 1.53E-01 | 1.53E+01 | | | |
| 8029530 | NM_000041 | APOE | apolipoprotein E | -0.167 | 0.891 | 6.38E-03 | 6.38E-01 | | | |
| 7892703 | --- | --- | --- | -0.167 | 0.891 | 1.35E-01 | 1.35E+01 | | | |
| 7911413 | NM_003327 | TNFRSF4 | tumor necrosis factor receptor super | -0.167 | 0.891 | 1.09E-02 | 1.09E+00 | | | |
| 7928147 | NM_139155 | ADAMTS14 | ADAM metallopeptidase with thromb | -0.167 | 0.891 | 1.68E-02 | 1.68E+00 | | | |
| 7973105 | NM_002935 | RNASE3 | ribonuclease, RNase A family, 3 (eosi | -0.167 | 0.891 | 8.69E-02 | 8.69E+00 | | | |
| 7934101 | NM_032797 | AIFM2 | apoptosis-inducing factor, mitochon | -0.167 | 0.891 | 3.14E-02 | 3.14E+00 | | | |
| 8035517 | NM_000095 | COMP | cartilage oligomeric matrix protein | -0.167 | 0.891 | 9.21E-03 | 9.21E-01 | | | |
| 8174642 | --- | --- | --- | -0.167 | 0.891 | 8.70E-03 | 8.70E-01 | | | |
| 7915084 | NM_024640 | YRDC | yrdC domain containing (E. coli) | -0.167 | 0.891 | 1.14E-01 | 1.14E+01 | | | |
| 8032972 | --- | --- | --- | -0.167 | 0.891 | 4.30E-01 | 4.30E+01 | | | |
| 7893827 | --- | --- | --- | -0.167 | 0.891 | 6.55E-01 | 6.55E+01 | | | |
| 8018264 | NM_030630 | C17orf28 | chromosome 17 open reading frame | -0.167 | 0.891 | 1.10E-02 | 1.10E+00 | | | |
| 8067812 | BC036837 | C20orf201 | chromosome 20 open reading frame | -0.167 | 0.890 | 8.89E-03 | 8.89E-01 | | | |
| 7914003 | BC033143 | C1orf172 | chromosome 1 open reading frame 1 | -0.167 | 0.890 | 1.35E-02 | 1.35E+00 | | | |
| 8140140 | NM_001306 | CLDN3 | claudin 3 | -0.167 | 0.890 | 1.06E-02 | 1.06E+00 | | | |
| 8063476 | NM_019888 | MC3R | melanocortin 3 receptor | -0.167 | 0.890 | 1.47E-02 | 1.47E+00 | | | |
| 8147990 | --- | --- | --- | -0.167 | 0.890 | 6.18E-01 | 6.18E+01 | | | |
| 8114546 | --- | --- | --- | -0.167 | 0.890 | 2.39E-01 | 2.39E+01 | | | |
| 8074558 | NM_023004 | RTN4R | reticulon 4 receptor | -0.167 | 0.890 | 3.29E-03 | 3.29E-01 | | | |
| 8031145 | NM_024075 | TSEN34 | tRNA splicing endonuclease 34 homo | -0.167 | 0.890 | 9.01E-03 | 9.01E-01 | | | |
| 8131044 | NM_015949 | C7orf20 | chromosome 7 open reading frame 2 | -0.167 | 0.890 | 4.39E-02 | 4.39E+00 | | | |
| 7971663 | NM_198989 | DLEU7 | deleted in lymphocytic leukemia, 7 | -0.167 | 0.890 | 8.03E-03 | 8.03E-01 | | | |
| 7895584 | --- | --- | --- | -0.168 | 0.890 | 8.77E-01 | 8.77E+01 | | | |
| 7899287 | --- | --- | --- | -0.168 | 0.890 | 1.05E-02 | 1.05E+00 | | | |
| 8039607 | NM_006210 | PEG3 | paternally expressed 3 | -0.168 | 0.890 | 1.27E-02 | 1.27E+00 | | | |
| 7945158 | --- | --- | --- | -0.168 | 0.890 | 6.45E-02 | 6.45E+00 | | | |
| 8039504 | NM_00114517( | CG_203914( | UPF0626 protein A | -0.168 | 0.890 | 2.97E-03 | 2.97E-01 | | | |
| 8019088 | AK096606 | OC10013117 | hypothetical LOC100131170 | -0.168 | 0.890 | 1.38E-02 | 1.38E+00 | | | |
| 7989985 | NM_00100443 | ITGA11 | integrin, alpha 11 | -0.168 | 0.890 | 2.18E-03 | 2.18E-01 | | | |
| 7978093 | NM_032452 | JPH4 | junctophilin 4 | -0.168 | 0.890 | 2.94E-03 | 2.94E-01 | | | |
| 7998878 | NM_022119 | PRSS22 | protease, serine, 22 | -0.168 | 0.890 | 6.43E-03 | 6.43E-01 | | | |
| 8115391 | NM_004821 | HAND1 | heart and neural crest derivatives ex | -0.168 | 0.890 | 1.22E-02 | 1.22E+00 | | | |
| 7988327 | NM_00114511 | PATL2 | protein associated with topoisomera | -0.168 | 0.890 | 2.13E-01 | 2.13E+01 | | | |
| 8125903 | NM_003322 | TULP1 | tubby like protein 1 | -0.168 | 0.890 | 5.02E-03 | 5.02E-01 | | | |
| 7984620 | --- | --- | --- | -0.168 | 0.890 | 5.09E-01 | 5.09E+01 | | | |
| 8118367 | NM_006929 | SKIV2L | superkiller viralicidic activity 2-like (S | -0.168 | 0.890 | 1.42E-02 | 1.42E+00 | | | |
| 8178136 | NM_006929 | SKIV2L | superkiller viralicidic activity 2-like (S | -0.168 | 0.890 | 1.42E-02 | 1.42E+00 | | | |
| 8164580 | NM_004878 | PTGES | prostaglandin E synthase | -0.168 | 0.890 | 9.27E-03 | 9.27E-01 | | | |
| 8162455 | NM_004148 | NINJ1 | ninjurin 1 | -0.168 | 0.890 | 4.38E-02 | 4.38E+00 | | | |
| 8171876 | NM_005371 | METTL1 | methyltransferase like 1 | -0.168 | 0.890 | 3.62E-01 | 3.62E+01 | | | |
| 7978366 | NM_004131 | GZMB | granzyme B (granzyme 2, cytotoxic T | -0.168 | 0.890 | 4.33E-01 | 4.33E+01 | | | |
| 8175685 | NM_021049 | MAGEA5 | melanoma antigen family A, 5 | -0.168 | 0.890 | 8.39E-03 | 8.39E-01 | | | |
| 7913237 | NM_018584 | CAMK2N1 | calcium/calmodulin-dependent prot | -0.168 | 0.890 | 5.85E-02 | 5.85E+00 | | | |
| 8051135 | NM_015662 | IFT172 | intraflagellar transport 172 homolog | -0.168 | 0.890 | 2.88E-02 | 2.88E+00 | | | |
| 7960099 | NM_170683 | P2RX2 | purinergic receptor P2X, ligand-gated | -0.168 | 0.890 | 9.39E-03 | 9.39E-01 | | | |
| 8130859 | NM_214462 | DACT2 | dapper, antagonist of beta-catenin, h | -0.168 | 0.890 | 8.48E-03 | 8.48E-01 | | | |
| 8004671 | NM_00108042 | KDM6B | lysine (K)-specific demethylase 6B | -0.168 | 0.890 | 5.15E-03 | 5.15E-01 | | | |
| 8031526 | NM_013301 | CCDC106 | coiled-coil domain containing 106 | -0.168 | 0.890 | 3.98E-03 | 3.98E-01 | | | |
| 8104499 | ENST0000029( | ANKRD33B | ankyrin repeat domain 33B | -0.168 | 0.890 | 5.87E-03 | 5.87E-01 | | | |
| 8113467 | --- | --- | --- | -0.168 | 0.890 | 1.37E-02 | 1.37E+00 | | | |
| 8023939 | --- | --- | --- | -0.168 | 0.890 | 7.21E-02 | 7.21E+00 | | | |
| 8034643 | NM_00112722 | CACNA1A | calcium channel, voltage-dependent, | -0.168 | 0.890 | 7.77E-04 | 7.77E-02 | | | |
| 8038048 | NM_144577 | CCDC114 | coiled-coil domain containing 114 | -0.168 | 0.890 | 5.94E-03 | 5.94E-01 | | | |
| 7895106 | --- | --- | --- | -0.168 | 0.890 | 7.83E-02 | 7.83E+00 | | | |
| 8030044 | NM_170720 | KCNJ14 | potassium inwardly-rectifying chann | -0.168 | 0.890 | 9.51E-03 | 9.51E-01 | | | |
| 8118028 | NM_020442 | VARS2 | valyl-tRNA synthetase 2, mitochondr | -0.168 | 0.890 | 8.76E-03 | 8.76E-01 | | | |
| 8043491 | NM_144705 | TEKT4 | tektin 4 | -0.169 | 0.890 | 1.86E-02 | 1.86E+00 | | | |
| 8060604 | NM_000915 | OXT | oxytocin, prepropeptide | -0.169 | 0.890 | 1.26E-02 | 1.26E+00 | | | |
| 8076882 | NM_00108044 | TTLL8 | tubulin tyrosine ligase-like family, me | -0.169 | 0.890 | 1.28E-02 | 1.28E+00 | | | |
| 8049965 | AK302544 | FAM110C | family with sequence similarity 110, | -0.169 | 0.890 | 1.88E-03 | 1.88E-01 | | | |
| 8149218 | NR_027000 | LOC349196 | hypothetical LOC349196 | -0.169 | 0.890 | 4.84E-02 | 4.84E+00 | | | |
| 8149220 | NR_027000 | LOC349196 | hypothetical LOC349196 | -0.169 | 0.890 | 4.84E-02 | 4.84E+00 | | | |
| 8149222 | NR_027000 | LOC349196 | hypothetical LOC349196 | -0.169 | 0.890 | 4.84E-02 | 4.84E+00 | | | |
| 8149224 | NR_027000 | LOC349196 | hypothetical LOC349196 | -0.169 | 0.890 | 4.84E-02 | 4.84E+00 | | | |
| 8149226 | NR_027000 | LOC349196 | hypothetical LOC349196 | -0.169 | 0.890 | 4.84E-02 | 4.84E+00 | | | |
| 8168524 | NM_014499 | P2RY10 | purinergic receptor P2Y, G-protein co | -0.169 | 0.890 | 6.01E-01 | 6.01E+01 | | | |
| 8065527 | NR_026713 | FAM182A | family with sequence similarity 182, | -0.169 | 0.890 | 1.62E-02 | 1.62E+00 | | | |
| 7895522 | --- | --- | --- | -0.169 | 0.890 | 1.40E-01 | 1.40E+01 | | | |
| 8169806 | --- | --- | --- | -0.169 | 0.890 | 5.29E-02 | 5.29E+00 | | | |
| 8086419 | NR_027753 | HHATL | hedgehog acyltransferase-like | -0.169 | 0.890 | 7.65E-03 | 7.65E-01 | | | |
| 8137798 | NM_032302 | PSMG3 | proteasome (prosome, macropain) a | -0.169 | 0.890 | 1.93E-01 | 1.93E+01 | | | |
| 7981013 | NM_178013 | PRIMA1 | proline rich membrane anchor 1 | -0.169 | 0.889 | 3.45E-03 | 3.45E-01 | | | |
| 7896135 | --- | --- | --- | -0.169 | 0.889 | 1.41E-01 | 1.41E+01 | | | |
| 8125220 | NM_005510 | DOM3Z | dom-3 homolog Z (C. elegans) | -0.169 | 0.889 | 1.10E-01 | 1.10E+01 | | | |

| | | | | | | | | | | |
|---|---|---|---|---|---|---|---|---|---|---|
| 8178699 | NM_005510 | DOM3Z | dom-3 homolog Z (C. elegans) | -0.169 | 0.889 | 1.10E-01 | 1.10E+01 | | | |
| 8018494 | NM_033452 | TRIM47 | tripartite motif-containing 47 | -0.169 | 0.889 | 1.79E-03 | 1.79E-01 | | | |
| 7938368 | --- | --- | --- | -0.169 | 0.889 | 6.98E-02 | 6.98E+00 | | | |
| 8016718 | NM_001267 | CHAD | chondroadherin | -0.169 | 0.889 | 1.22E-02 | 1.22E+00 | | | |
| 7930482 | NM_000681 | ADRA2A | adrenergic, alpha-2A-, receptor | -0.169 | 0.889 | 7.06E-03 | 7.06E-01 | | | |
| 7893019 | --- | --- | --- | -0.169 | 0.889 | 7.88E-01 | 7.88E+01 | | | |
| 7966150 | NM_00116133( | SSH1 | slingshot homolog 1 (Drosophila) | -0.169 | 0.889 | 1.77E-02 | 1.77E+00 | | | |
| 8015187 | NM_030967 | KRTAP1-1 | keratin associated protein 1-1 | -0.169 | 0.889 | 2.27E-02 | 2.27E+00 | | | |
| 8053248 | BC025997 | C2orf65 | chromosome 2 open reading frame 6 | -0.169 | 0.889 | 1.09E-03 | 1.09E-01 | | | |
| 7924884 | NM_003493 | HIST3H3 | histone cluster 3, H3 | -0.169 | 0.889 | 2.23E-02 | 2.23E+00 | | | |
| 8171295 | --- | --- | --- | -0.169 | 0.889 | 1.79E-01 | 1.79E+01 | | | |
| 7894052 | --- | --- | --- | -0.169 | 0.889 | 5.50E-01 | 5.50E+01 | | | |
| 7991106 | AB067507 | KIAA1920 | KIAA1920 protein | -0.169 | 0.889 | 2.63E-02 | 2.63E+00 | | | |
| 7959638 | NM_024809 | TCTN2 | tectonic family member 2 | -0.169 | 0.889 | 2.85E-02 | 2.85E+00 | | | |
| 8126119 | --- | --- | --- | -0.169 | 0.889 | 8.01E-01 | 8.01E+01 | | | |
| 8087691 | NM_00100550! | CACNA2D2 | calcium channel, voltage-dependent | -0.169 | 0.889 | 1.57E-02 | 1.57E+00 | | | |
| 7892669 | --- | --- | --- | -0.169 | 0.889 | 1.77E-01 | 1.77E+01 | | | |
| 8174226 | --- | --- | --- | -0.169 | 0.889 | 6.57E-02 | 6.57E+00 | | | |
| 8037762 | NM_032040 | CCDC8 | coiled-coil domain containing 8 | -0.169 | 0.889 | 9.52E-03 | 9.52E-01 | | | |
| 7986822 | NM_000814 | GABRB3 | gamma-aminobutyric acid (GABA) A | -0.170 | 0.889 | 9.01E-03 | 9.01E-01 | | | |
| 7923705 | --- | --- | --- | -0.170 | 0.889 | 2.03E-02 | 2.03E+00 | | | |
| 8035271 | NM_024527 | ABHD8 | abhydrolase domain containing 8 | -0.170 | 0.889 | 1.25E-03 | 1.25E-01 | | | |
| 8117118 | --- | --- | --- | -0.170 | 0.889 | 2.87E-02 | 2.87E+00 | | | |
| 8007302 | NM_001070 | TUBG1 | tubulin, gamma 1 | -0.170 | 0.889 | 3.61E-02 | 3.61E+00 | | | |
| 8033385 | NM_080662 | PEX11G | peroxisomal biogenesis factor 11 gar | -0.170 | 0.889 | 5.94E-03 | 5.94E-01 | | | |
| 7973327 | NM_181304 | MRPL52 | mitochondrial ribosomal protein L52 | -0.170 | 0.889 | 1.40E-01 | 1.40E+01 | | | |
| 7947129 | NM_00102986 | DBX1 | developing brain homeobox 1 | -0.170 | 0.889 | 1.06E-02 | 1.06E+00 | | | |
| 8144112 | NM_005515 | MNX1 | motor neuron and pancreas homeob | -0.170 | 0.889 | 4.84E-03 | 4.84E-01 | | | |
| 8064242 | NR_027686 | NCRNA0017( | non-protein coding RNA 176 | -0.170 | 0.889 | 1.56E-02 | 1.56E+00 | | | |
| 7920191 | NM_178431 | LCE3A | late cornified envelope 3A | -0.170 | 0.889 | 6.22E-03 | 6.22E-01 | | | |
| 7950282 | --- | --- | --- | -0.170 | 0.889 | 1.62E-01 | 1.62E+01 | | | |
| 7947563 | NM_021926 | ALX4 | ALX homeobox 4 | -0.170 | 0.889 | 7.65E-03 | 7.65E-01 | | | |
| 8176117 | NM_021806 | FAM3A | family with sequence similarity 3, me | -0.170 | 0.889 | 2.56E-03 | 2.56E-01 | | | |
| 8055350 | NM_030923 | TMEM163 | transmembrane protein 163 | -0.170 | 0.889 | 1.82E-02 | 1.82E+00 | | | |
| 8049825 | NM_005336 | HDLBP | high density lipoprotein binding prot | -0.170 | 0.889 | 1.47E-01 | 1.47E+01 | | | |
| 7898809 | NM_017449 | EPHB2 | EPH receptor B2 | -0.170 | 0.889 | 1.38E-02 | 1.38E+00 | | | |
| 7921895 | NM_182581 | C1orf111 | chromosome 1 open reading frame 1 | -0.170 | 0.889 | 1.16E-02 | 1.16E+00 | | | |
| 7911376 | NM_00114246 | HES4 | hairy and enhancer of split 4 (Drosop | -0.170 | 0.889 | 7.37E-03 | 7.37E-01 | | | |
| 8042251 | NM_014562 | OTX1 | orthodenticle homeobox 1 | -0.170 | 0.889 | 1.90E-02 | 1.90E+00 | | | |
| 8176109 | NM_019848 | SLC10A3 | solute carrier family 10 (sodium/bile | -0.170 | 0.889 | 8.30E-03 | 8.30E-01 | | | |
| 7969241 | --- | --- | --- | -0.170 | 0.889 | 4.91E-01 | 4.91E+01 | | | |
| 8141625 | NM_004444 | EPHB4 | EPH receptor B4 | -0.170 | 0.889 | 2.01E-02 | 2.01E+00 | | | |
| 8031843 | NM_025027 | ZNF606 | zinc finger protein 606 | -0.170 | 0.889 | 1.83E-02 | 1.83E+00 | | | |
| 8151631 | NR_003594 | REXO1L2P | REX1, RNA exonuclease 1 homolog (S | -0.170 | 0.889 | 2.02E-02 | 2.02E+00 | | | |
| 8016371 | NM_00114502 | SCRN2 | secernin 2 | -0.170 | 0.889 | 1.12E-02 | 1.12E+00 | | | |
| 8153322 | NM_015193 | ARC | activity-regulated cytoskeleton-assoc | -0.170 | 0.889 | 1.29E-03 | 1.29E-01 | | | |
| 7952268 | NM_006288 | THY1 | Thy-1 cell surface antigen | -0.171 | 0.889 | 8.21E-03 | 8.21E-01 | | | |
| 8046426 | --- | --- | --- | -0.171 | 0.889 | 2.80E-02 | 2.80E+00 | | | |
| 8049942 | NM_080741 | NEU4 | sialidase 4 | -0.171 | 0.888 | 3.60E-03 | 3.60E-01 | | | |
| 7912804 | BC036435 | OC10013214 | hypothetical LOC100132147 | -0.171 | 0.888 | 3.92E-02 | 3.92E+00 | | | |
| 8104930 | NM_004172 | SLC1A3 | solute carrier family 1 (glial high affir | -0.171 | 0.888 | 1.85E-02 | 1.85E+00 | | | |
| 7893067 | --- | --- | --- | -0.171 | 0.888 | 1.90E-01 | 1.90E+01 | | | |
| 8027728 | NM_182983 | HPN | hepsin | -0.171 | 0.888 | 6.57E-03 | 6.57E-01 | | | |
| 7893729 | --- | --- | --- | -0.171 | 0.888 | 7.07E-01 | 7.07E+01 | | | |
| 8113433 | NM_001962 | EFNA5 | ephrin-A5 | -0.171 | 0.888 | 7.07E-03 | 7.07E-01 | | | |
| 8097096 | --- | --- | --- | -0.171 | 0.888 | 2.37E-02 | 2.37E+00 | | | |
| 8070557 | NM_00109840 | ZNF295 | zinc finger protein 295 | -0.171 | 0.888 | 1.38E-01 | 1.38E+01 | | | |
| 8063531 | BC105792 | C20orf107 | chromosome 20 open reading frame | -0.171 | 0.888 | 4.72E-01 | 4.72E+01 | | | |
| 8162462 | NM_014612 | FAM120A | family with sequence similarity 120A | -0.171 | 0.888 | 1.37E-01 | 1.37E+01 | | | |
| 8085276 | NR_002786 | CIDECP | cell death-inducing DFFA-like effecto | -0.171 | 0.888 | 3.64E-02 | 3.64E+00 | | | |
| 8068022 | NR_001458 | MIR155HG | MIR155 host gene (non-protein codi | -0.171 | 0.888 | 2.85E-02 | 2.85E+00 | | | |
| 8037688 | NM_030785 | RSHL1 | radial spokehead-like 1 | -0.171 | 0.888 | 1.73E-03 | 1.73E-01 | | | |
| 8158568 | NM_006676 | USP20 | ubiquitin specific peptidase 20 | -0.171 | 0.888 | 2.54E-02 | 2.54E+00 | | | |
| 7959195 | NM_00103367 | CABP1 | calcium binding protein 1 | -0.171 | 0.888 | 4.43E-03 | 4.43E-01 | | | |
| 8080958 | NM_018971 | GPR27 | G protein-coupled receptor 27 | -0.171 | 0.888 | 2.44E-03 | 2.44E-01 | | | |
| 8126301 | --- | --- | --- | -0.171 | 0.888 | 1.06E-02 | 1.06E+00 | | | |
| 8037513 | NM_138568 | EXOC3L2 | exocyst complex component 3-like 2 | -0.171 | 0.888 | 4.43E-03 | 4.43E-01 | | | |
| 8149165 | NR_027000 | LOC349196 | hypothetical LOC349196 | -0.171 | 0.888 | 4.37E-02 | 4.37E+00 | | | |
| 8149167 | NR_027000 | LOC349196 | hypothetical LOC349196 | -0.171 | 0.888 | 4.37E-02 | 4.37E+00 | | | |
| 8069208 | NM_030582 | COL18A1 | collagen, type XVIII, alpha 1 | -0.171 | 0.888 | 1.40E-03 | 1.40E-01 | | | |
| 7952243 | NM_015645 | C1QTNF5 | C1q and tumor necrosis factor relate | -0.171 | 0.888 | 3.51E-03 | 3.51E-01 | | | |
| 8016159 | NM_024819 | DCAKD | dephospho-CoA kinase domain conta | -0.171 | 0.888 | 1.96E-01 | 1.96E+01 | | | |
| 8129937 | NM_006079 | CITED2 | Cbp/p300-interacting transactivator, | -0.171 | 0.888 | 4.43E-02 | 4.43E+00 | | | |
| 7989489 | --- | --- | --- | -0.171 | 0.888 | 6.62E-02 | 6.62E+00 | | | |
| 8179235 | NR_026791 | HCG27 | HLA complex group 27 | -0.171 | 0.888 | 6.40E-02 | 6.40E+00 | | | |
| 7997755 | NM_079837 | BANP | BTG3 associated nuclear protein | -0.171 | 0.888 | 2.23E-02 | 2.23E+00 | | | |
| 8180280 | --- | --- | --- | -0.171 | 0.888 | 2.02E-02 | 2.02E+00 | | | |

| | | | | | | | | | | |
|---|---|---|---|---|---|---|---|---|---|---|
| 8111151 | --- | --- | --- | -0.171 | 0.888 | 1.30E-01 | 1.30E+01 | | | |
| 8149153 | NR_027000 | LOC349196 | hypothetical LOC349196 | -0.172 | 0.888 | 3.64E-02 | 3.64E+00 | | | |
| 8149157 | NR_027000 | LOC349196 | hypothetical LOC349196 | -0.172 | 0.888 | 3.64E-02 | 3.64E+00 | | | |
| 7895586 | --- | --- | --- | -0.172 | 0.888 | 1.15E-01 | 1.15E+01 | | | |
| 8159249 | NM_016034 | MRPS2 | mitochondrial ribosomal protein S2 | -0.172 | 0.888 | 4.20E-02 | 4.20E+00 | | | |
| 8008716 | NR_002307 | MSX2P1 | msh homeobox 2 pseudogene 1 | -0.172 | 0.888 | 1.42E-02 | 1.42E+00 | | | |
| 8164696 | --- | --- | --- | -0.172 | 0.888 | 5.62E-01 | 5.62E+01 | | | |
| 8074714 | NR_024591 | POM121L1P | POM121 membrane glycoprotein-lik | -0.172 | 0.888 | 1.51E-01 | 1.51E+01 | | | |
| 7982745 | NM_130468 | CHST14 | carbohydrate (N-acetylgalactosamin | -0.172 | 0.888 | 3.29E-03 | 3.29E-01 | | | |
| 8036494 | NM_152657 | GGN | gametogenetin | -0.172 | 0.888 | 4.84E-03 | 4.84E-01 | | | |
| 7941203 | NM_00100432( | SLC22A20 | solute carrier family 22, member 20 | -0.172 | 0.888 | 4.89E-03 | 4.89E-01 | | | |
| 8044760 | --- | --- | --- | -0.172 | 0.888 | 7.65E-03 | 7.65E-01 | | | |
| 8137959 | NM_00108049! | TNRC18 | trinucleotide repeat containing 18 | -0.172 | 0.888 | 7.26E-03 | 7.26E-01 | | | |
| 7893915 | --- | --- | --- | -0.172 | 0.888 | 8.15E-01 | 8.15E+01 | | | |
| 8070489 | NM_020639 | RIPK4 | receptor-interacting serine-threonin | -0.172 | 0.888 | 6.57E-03 | 6.57E-01 | | | |
| 8152701 | --- | --- | --- | -0.172 | 0.887 | 1.31E-01 | 1.31E+01 | | | |
| 8172447 | NM_013271 | PCSK1N | proprotein convertase subtilisin/kex | -0.172 | 0.887 | 8.47E-03 | 8.47E-01 | | | |
| 8114645 | ENST00000319 | OC10028810 | hypothetical protein LOC100288105 | -0.172 | 0.887 | 3.19E-02 | 3.19E+00 | | | |
| 8002181 | NM_022357 | DPEP3 | dipeptidase 3 | -0.172 | 0.887 | 5.57E-03 | 5.57E-01 | | | |
| 8069838 | NM_181611 | KRTAP19-5 | keratin associated protein 19-5 | -0.172 | 0.887 | 2.14E-02 | 2.14E+00 | | | |
| 7928556 | --- | --- | --- | -0.172 | 0.887 | 2.61E-02 | 2.61E+00 | | | |
| 8154527 | --- | --- | --- | -0.172 | 0.887 | 3.39E-01 | 3.39E+01 | | | |
| 8030251 | NM_017636 | TRPM4 | transient receptor potential cation c | -0.173 | 0.887 | 5.87E-03 | 5.87E-01 | | | |
| 8071593 | --- | --- | --- | -0.173 | 0.887 | 5.44E-01 | 5.44E+01 | | | |
| 8130622 | --- | --- | --- | -0.173 | 0.887 | 5.44E-01 | 5.44E+01 | | | |
| 8115144 | NM_00101230 | ARSI | arylsulfatase family, member I | -0.173 | 0.887 | 3.13E-02 | 3.13E+00 | | | |
| 7895941 | --- | --- | --- | -0.173 | 0.887 | 1.66E-01 | 1.66E+01 | | | |
| 8067029 | NM_002237 | KCNG1 | potassium voltage-gated channel, su | -0.173 | 0.887 | 1.06E-02 | 1.06E+00 | | | |
| 8158539 | NM_00101271! | C9orf106 | chromosome 9 open reading frame 1 | -0.173 | 0.887 | 1.72E-02 | 1.72E+00 | | | |
| 8069811 | NM_181624 | KRTAP23-1 | keratin associated protein 23-1 | -0.173 | 0.887 | 1.12E-02 | 1.12E+00 | | | |
| 7961998 | --- | --- | --- | -0.173 | 0.887 | 2.74E-02 | 2.74E+00 | | | |
| 7893348 | --- | --- | --- | -0.173 | 0.887 | 8.95E-03 | 8.95E-01 | | | |
| 8178897 | NM_080680 | COL11A2 | collagen, type XI, alpha 2 | -0.173 | 0.887 | 1.83E-02 | 1.83E+00 | | | |
| 8180105 | NM_080680 | COL11A2 | collagen, type XI, alpha 2 | -0.173 | 0.887 | 1.83E-02 | 1.83E+00 | | | |
| 7958453 | --- | --- | --- | -0.173 | 0.887 | 2.07E-01 | 2.07E+01 | | | |
| 8015821 | NM_004527 | MEOX1 | mesenchyme homeobox 1 | -0.173 | 0.887 | 1.04E-02 | 1.04E+00 | | | |
| 8070900 | ENST00000418 | C21orf111 | chromosome 21 open reading frame | -0.173 | 0.887 | 7.26E-03 | 7.26E-01 | | | |
| 8134856 | NM_033506 | FBXO24 | F-box protein 24 | -0.173 | 0.887 | 5.24E-03 | 5.24E-01 | | | |
| 7893364 | --- | --- | --- | -0.173 | 0.887 | 8.01E-01 | 8.01E+01 | | | |
| 8119444 | NM_00101242( | FOXP4 | forkhead box P4 | -0.173 | 0.887 | 5.55E-03 | 5.55E-01 | | | |
| 8010673 | NM_012140 | SLC25A10 | solute carrier family 25 (mitochondri | -0.173 | 0.887 | 4.74E-03 | 4.74E-01 | | | |
| 7893132 | --- | --- | --- | -0.173 | 0.887 | 7.81E-01 | 7.81E+01 | | | |
| 7901466 | NM_00104269 | FAM159A | family with sequence similarity 159, | -0.173 | 0.887 | 2.77E-02 | 2.77E+00 | | | |
| 7991459 | NM_020211 | RGMA | RGM domain family, member A | -0.173 | 0.887 | 5.77E-03 | 5.77E-01 | | | |
| 8175609 | 0 | 0 | 0 | -0.173 | 0.887 | 7.54E-02 | 7.54E+00 | | | |
| 8114583 | NM_00103523! | SRA1 | steroid receptor RNA activator 1 | -0.173 | 0.887 | 2.25E-01 | 2.25E+01 | | | |
| 7954697 | --- | --- | --- | -0.173 | 0.887 | 1.20E-01 | 1.20E+01 | | | |
| 7964976 | NR_026837 | LOC283392 | hypothetical LOC283392 | -0.173 | 0.887 | 7.23E-03 | 7.23E-01 | | | |
| 8142981 | NM_00101811 | PODXL | podocalyxin-like | -0.174 | 0.887 | 6.43E-03 | 6.43E-01 | | | |
| 8071662 | NM_199127 | GGTLC2 | gamma-glutamyltransferase light cha | -0.174 | 0.887 | 3.65E-02 | 3.65E+00 | | | |
| 8017521 | NM_000334 | SCN4A | sodium channel, voltage-gated, type | -0.174 | 0.887 | 6.33E-03 | 6.33E-01 | | | |
| 8102321 | NM_030821 | PLA2G12A | phospholipase A2, group XIIA | -0.174 | 0.887 | 4.65E-02 | 4.65E+00 | | | |
| 8139201 | --- | --- | --- | -0.174 | 0.887 | 1.01E-02 | 1.01E+00 | | | |
| 7895249 | --- | --- | --- | -0.174 | 0.886 | 6.06E-02 | 6.06E+00 | | | |
| 8137280 | NM_000603 | NOS3 | nitric oxide synthase 3 (endothelial c | -0.174 | 0.886 | 4.55E-03 | 4.55E-01 | | | |
| 8163916 | NM_00113944 | TTLL11 | tubulin tyrosine ligase-like family, me | -0.174 | 0.886 | 7.23E-04 | 7.23E-02 | | | |
| 8066431 | NM_000022 | ADA | adenosine deaminase | -0.174 | 0.886 | 8.37E-02 | 8.37E+00 | | | |
| 8148302 | --- | --- | --- | -0.174 | 0.886 | 7.21E-01 | 7.21E+01 | | | |
| 8024798 | NM_018074 | CCDC94 | coiled-coil domain containing 94 | -0.174 | 0.886 | 2.17E-02 | 2.17E+00 | | | |
| 8074307 | AK094477 | OC10012911 | hypothetical protein LOC100129113 | -0.174 | 0.886 | 1.58E-02 | 1.58E+00 | | | |
| 7973943 | NM_032594 | INSM2 | insulinoma-associated 2 | -0.174 | 0.886 | 7.69E-03 | 7.69E-01 | | | |
| 8037495 | NM_145288 | ZNF296 | zinc finger protein 296 | -0.174 | 0.886 | 5.31E-03 | 5.31E-01 | | | |
| 8080878 | NM_00112814! | ATXN7 | ataxin 7 | -0.174 | 0.886 | 3.26E-01 | 3.26E+01 | | | |
| 8032465 | NM_144616 | JSRP1 | junctional sarcoplasmic reticulum pr | -0.174 | 0.886 | 2.14E-03 | 2.14E-01 | | | |
| 7966666 | --- | --- | --- | -0.174 | 0.886 | 7.37E-02 | 7.37E+00 | | | |
| 8002590 | --- | --- | --- | -0.174 | 0.886 | 9.76E-02 | 9.76E+00 | | | |
| 8082774 | NM_003571 | BFSP2 | beaded filament structural protein 2 | -0.174 | 0.886 | 1.23E-02 | 1.23E+00 | | | |
| 7965484 | --- | --- | --- | -0.174 | 0.886 | 2.64E-01 | 2.64E+01 | | | |
| 8049567 | NM_005855 | RAMP1 | receptor (G protein-coupled) activity | -0.174 | 0.886 | 1.75E-02 | 1.75E+00 | | | |
| 8006786 | NM_020876 | ARHGAP23 | Rho GTPase activating protein 23 | -0.174 | 0.886 | 6.78E-02 | 6.78E+00 | | | |
| 7964388 | NM_020142 | NDUFA4L2 | NADH dehydrogenase (ubiquinone) 1 | -0.174 | 0.886 | 9.65E-03 | 9.65E-01 | | | |
| 7999177 | NM_133450 | ANKS3 | ankyrin repeat and sterile alpha mot | -0.174 | 0.886 | 7.26E-03 | 7.26E-01 | | | |
| 7991722 | NR_003246 | FLJ40113 | golgi autoantigen, golgin subfamily a | -0.174 | 0.886 | 3.74E-01 | 3.74E+01 | | | |
| 7994445 | NM_015503 | SH2B1 | SH2B adaptor protein 1 | -0.174 | 0.886 | 1.58E-02 | 1.58E+00 | | | |
| 7999304 | NM_201400 | FAM86A | family with sequence similarity 86, m | -0.174 | 0.886 | 3.37E-03 | 3.37E-01 | | | |
| 8137472 | --- | --- | --- | -0.174 | 0.886 | 5.83E-02 | 5.83E+00 | | | |
| 8010963 | --- | --- | --- | -0.174 | 0.886 | 1.07E-01 | 1.07E+01 | | | |

| | | | | | | | | | | |
|---|---|---|---|---|---|---|---|---|---|---|
| 8006896 | NM_003673 | TCAP | titin-cap (telethonin) | -0.175 | 0.886 | 7.60E-03 | 7.60E-01 | | | |
| 8049620 | ENST00000408 | FAM132B | family with sequence similarity 132, | -0.175 | 0.886 | 6.08E-03 | 6.08E-01 | | | |
| 7912784 | NR_023386 | CROCCL2 | ciliary rootlet coiled-coil, rootletin-lik | -0.175 | 0.886 | 5.99E-04 | 5.99E-02 | | | |
| 7892913 | --- | --- | --- | -0.175 | 0.886 | 6.08E-01 | 6.08E+01 | | | |
| 8117608 | NM_003511 | HIST1H2AL | histone cluster 1, H2al | -0.175 | 0.886 | 1.51E-01 | 1.51E+01 | | | |
| 8159692 | NM_152285 | ARRDC1 | arrestin domain containing 1 | -0.175 | 0.886 | 1.78E-02 | 1.78E+00 | | | |
| 7915347 | NM_133467 | CITED4 | Cbp/p300-interacting transactivator, | -0.175 | 0.886 | 6.05E-03 | 6.05E-01 | | | |
| 7916304 | NM_147193 | GLIS1 | GLIS family zinc finger 1 | -0.175 | 0.886 | 1.81E-03 | 1.81E-01 | | | |
| 8076732 | --- | --- | --- | -0.175 | 0.886 | 1.52E-02 | 1.52E+00 | | | |
| 7944035 | NM_000040 | APOC3 | apolipoprotein C-III | -0.175 | 0.886 | 4.81E-03 | 4.81E-01 | | | |
| 8180277 | --- | --- | --- | -0.175 | 0.886 | 2.03E-02 | 2.03E+00 | | | |
| 7973414 | NM_015514 | NGDN | neuroguidin, EIF4E binding protein | -0.175 | 0.886 | 4.62E-01 | 4.62E+01 | | | |
| 8168458 | NM_020932 | MAGEE1 | melanoma antigen family E, 1 | -0.175 | 0.886 | 1.61E-02 | 1.61E+00 | | | |
| 8050352 | NM_012344 | NTSR2 | neurotensin receptor 2 | -0.175 | 0.886 | 4.84E-03 | 4.84E-01 | | | |
| 7997582 | NM_021197 | WFDC1 | WAP four-disulfide core domain 1 | -0.175 | 0.886 | 1.26E-03 | 1.26E-01 | | | |
| 7983051 | NM_016642 | SPTBN5 | spectrin, beta, non-erythrocytic 5 | -0.175 | 0.886 | 7.34E-01 | 7.34E+01 | | | |
| 8083030 | --- | --- | --- | -0.175 | 0.886 | 2.22E-01 | 2.22E+01 | | | |
| 8027320 | NR_024523 | LOC641367 | cyclin Y-like pseudogene | -0.175 | 0.886 | 2.46E-01 | 2.46E+01 | | | |
| 8031847 | NM_182572 | ZSCAN1 | zinc finger and SCAN domain contain | -0.175 | 0.886 | 5.31E-03 | 5.31E-01 | | | |
| 8180218 | --- | --- | --- | -0.175 | 0.886 | 5.51E-01 | 5.51E+01 | | | |
| 8076185 | NM_175709 | CBX7 | chromobox homolog 7 | -0.175 | 0.886 | 7.93E-03 | 7.93E-01 | | | |
| 7893910 | --- | --- | --- | -0.175 | 0.886 | 6.06E-01 | 6.06E+01 | | | |
| 7984892 | NM_021819 | LMAN1L | lectin, mannose-binding, 1 like | -0.175 | 0.886 | 1.60E-03 | 1.60E-01 | | | |
| 8060698 | BC043344 | C20orf29 | chromosome 20 open reading frame | -0.175 | 0.886 | 8.70E-03 | 8.70E-01 | | | |
| 8061357 | NM_006192 | PAX1 | paired box 1 | -0.175 | 0.886 | 6.56E-03 | 6.56E-01 | | | |
| 7892808 | --- | --- | --- | -0.175 | 0.886 | 8.01E-02 | 8.01E+00 | | | |
| 8162179 | NM_002048 | GAS1 | growth arrest-specific 1 | -0.175 | 0.886 | 1.70E-03 | 1.70E-01 | | | |
| 7897154 | NM_014448 | ARHGEF16 | Rho guanine exchange factor (GEF) 1 | -0.175 | 0.886 | 1.79E-03 | 1.79E-01 | | | |
| 7947531 | NM_014186 | COMMD9 | COMM domain containing 9 | -0.175 | 0.886 | 2.96E-01 | 2.96E+01 | | | |
| 8093128 | --- | --- | --- | -0.175 | 0.886 | 2.06E-01 | 2.06E+01 | | | |
| 8158987 | NR_001275 | CELP | carboxyl ester lipase pseudogene | -0.175 | 0.886 | 2.52E-02 | 2.52E+00 | | | |
| 8032094 | NM_024888 | PRG2 | plasticity-related gene 2 | -0.175 | 0.886 | 4.19E-03 | 4.19E-01 | | | |
| 7896122 | --- | --- | --- | -0.175 | 0.885 | 4.52E-03 | 4.52E-01 | | | |
| 7896637 | --- | --- | --- | -0.175 | 0.885 | 6.37E-03 | 6.37E-01 | | | |
| 8024323 | NM_138393 | REEP6 | receptor accessory protein 6 | -0.176 | 0.885 | 1.73E-03 | 1.73E-01 | | | |
| 7913242 | NM_024544 | MUL1 | mitochondrial E3 ubiquitin ligase 1 | -0.176 | 0.885 | 9.49E-02 | 9.49E+00 | | | |
| 8074842 | NM_00113011 | FAM108A1 | family with sequence similarity 108, | -0.176 | 0.885 | 1.11E-02 | 1.11E+00 | | | |
| 8070720 | NM_015259 | ICOSLG | inducible T-cell co-stimulator ligand | -0.176 | 0.885 | 7.26E-03 | 7.26E-01 | | | |
| 7894237 | --- | --- | --- | -0.176 | 0.885 | 1.70E-01 | 1.70E+01 | | | |
| 7895472 | --- | --- | --- | -0.176 | 0.885 | 6.09E-01 | 6.09E+01 | | | |
| 8044301 | NM_023016 | ANKRD57 | ankyrin repeat domain 57 | -0.176 | 0.885 | 2.23E-02 | 2.23E+00 | | | |
| 7980908 | NM_006329 | FBLN5 | fibulin 5 | -0.176 | 0.885 | 5.70E-03 | 5.70E-01 | | | |
| 8049682 | --- | --- | --- | -0.176 | 0.885 | 1.23E-02 | 1.23E+00 | | | |
| 7894862 | --- | --- | --- | -0.176 | 0.885 | 6.57E-03 | 6.57E-01 | | | |
| 8110430 | NM_015111 | N4BP3 | Nedd4 binding protein 3 | -0.176 | 0.885 | 2.54E-03 | 2.54E-01 | | | |
| 8034722 | NM_002918 | RFX1 | regulatory factor X, 1 (influences HLA | -0.176 | 0.885 | 2.52E-04 | 2.52E-02 | | | |
| 7895868 | --- | --- | --- | -0.176 | 0.885 | 1.25E-01 | 1.25E+01 | | | |
| 7911229 | NM_00100195 | OR2W3 | olfactory receptor, family 2, subfami | -0.176 | 0.885 | 3.50E-02 | 3.50E+00 | | | |
| 7911231 | NM_00100195 | OR2W3 | olfactory receptor, family 2, subfami | -0.176 | 0.885 | 3.50E-02 | 3.50E+00 | | | |
| 8042701 | NM_004097 | EMX1 | empty spiracles homeobox 1 | -0.176 | 0.885 | 5.08E-03 | 5.08E-01 | | | |
| 8117426 | NM_003524 | HIST1H2BH | histone cluster 1, H2bh | -0.176 | 0.885 | 4.21E-01 | 4.21E+01 | | | |
| 7895776 | --- | --- | --- | -0.176 | 0.885 | 8.41E-01 | 8.41E+01 | | | |
| 7965040 | NM_007350 | PHLDA1 | pleckstrin homology-like domain, far | -0.176 | 0.885 | 7.73E-03 | 7.73E-01 | | | |
| 7909104 | NM_212503 | PCTK3 | PCTAIRE protein kinase 3 | -0.176 | 0.885 | 5.94E-03 | 5.94E-01 | | | |
| 8065013 | --- | --- | --- | -0.176 | 0.885 | 2.90E-02 | 2.90E+00 | | | |
| 8072870 | NM_020315 | PDXP | pyridoxal (pyridoxine, vitamin B6) ph | -0.176 | 0.885 | 4.56E-03 | 4.56E-01 | | | |
| 8069519 | --- | --- | --- | -0.176 | 0.885 | 4.89E-03 | 4.89E-01 | | | |
| 7912887 | NM_002403 | MFAP2 | microfibrillar-associated protein 2 | -0.176 | 0.885 | 8.92E-03 | 8.92E-01 | | | |
| 7941172 | NM_00100877 | SPDYC | speedy homolog C (Xenopus laevis) | -0.176 | 0.885 | 2.94E-03 | 2.94E-01 | | | |
| 7896360 | --- | --- | --- | -0.177 | 0.885 | 1.27E-01 | 1.27E+01 | | | |
| 8038782 | NM_145232 | CTU1 | cytosolic thiouridylase subunit 1 hon | -0.177 | 0.885 | 1.52E-02 | 1.52E+00 | | | |
| 8066391 | --- | --- | --- | -0.177 | 0.885 | 4.50E-02 | 4.50E+00 | | | |
| 7896713 | --- | --- | --- | -0.177 | 0.885 | 4.81E-03 | 4.81E-01 | | | |
| 8114625 | NM_194249 | DND1 | dead end homolog 1 (zebrafish) | -0.177 | 0.885 | 1.19E-01 | 1.19E+01 | | | |
| 8013500 | NM_003876 | TMEM11 | transmembrane protein 11 | -0.177 | 0.885 | 3.36E-02 | 3.36E+00 | | | |
| 8132980 | NM_00108049 | TNRC18 | trinucleotide repeat containing 18 | -0.177 | 0.885 | 1.03E-02 | 1.03E+00 | | | |
| 7895854 | --- | --- | --- | -0.177 | 0.885 | 7.77E-02 | 7.77E+00 | | | |
| 8093852 | NM_002448 | MSX1 | msh homeobox 1 | -0.177 | 0.884 | 1.04E-02 | 1.04E+00 | | | |
| 8148783 | NM_032450 | HEATR7A | HEAT repeat containing 7A | -0.177 | 0.884 | 1.75E-02 | 1.75E+00 | | | |
| 8037872 | NM_00112724 | BBC3 | BCL2 binding component 3 | -0.177 | 0.884 | 4.74E-03 | 4.74E-01 | | | |
| 7938066 | NM_00100344 | OR56A3 | olfactory receptor, family 56, subfam | -0.177 | 0.884 | 6.33E-03 | 6.33E-01 | | | |
| 8106528 | --- | --- | --- | -0.177 | 0.884 | 2.18E-01 | 2.18E+01 | | | |
| 8158492 | BC009114 | FAM73B | family with sequence similarity 73, m | -0.177 | 0.884 | 6.41E-03 | 6.41E-01 | | | |
| 8133233 | NM_015570 | AUTS2 | autism susceptibility candidate 2 | -0.177 | 0.884 | 1.94E-02 | 1.94E+00 | | | |
| 7958200 | NM_00100839 | EID3 | EP300 interacting inhibitor of differe | -0.177 | 0.884 | 3.21E-01 | 3.21E+01 | | | |
| 8037785 | NM_033258 | GNG8 | guanine nucleotide binding protein ( | -0.177 | 0.884 | 3.22E-03 | 3.22E-01 | | | |
| 8179391 | NR_026717 | STK19 | serine/threonine kinase 19 | -0.177 | 0.884 | 7.63E-02 | 7.63E+00 | | | |

| | | | | | | | | | | |
|---|---|---|---|---|---|---|---|---|---|---|
| 7998280 | NM_032259 | WDR24 | WD repeat domain 24 | -0.177 | 0.884 | 5.06E-03 | 5.06E-01 | | | |
| 7902265 | --- | --- | --- | -0.177 | 0.884 | 6.14E-02 | 6.14E+00 | | | |
| 7907904 | NM_016545 | IER5 | immediate early response 5 | -0.177 | 0.884 | 4.52E-03 | 4.52E-01 | | | |
| 8038760 | NM_015596 | KLK13 | kallikrein-related peptidase 13 | -0.178 | 0.884 | 7.65E-03 | 7.65E-01 | | | |
| 8157922 | NM_002316 | LMX1B | LIM homeobox transcription factor 1 | -0.178 | 0.884 | 5.31E-03 | 5.31E-01 | | | |
| 8055265 | --- | --- | --- | -0.178 | 0.884 | 1.44E-02 | 1.44E+00 | | | |
| 8116502 | --- | --- | --- | -0.178 | 0.884 | 2.33E-02 | 2.33E+00 | | | |
| 8092390 | --- | --- | --- | -0.178 | 0.884 | 4.33E-01 | 4.33E+01 | | | |
| 8178727 | NM_004381 | ATF6B | activating transcription factor 6 beta | -0.178 | 0.884 | 1.41E-02 | 1.41E+00 | | | |
| 8025601 | NM_000201 | ICAM1 | intercellular adhesion molecule 1 | -0.178 | 0.884 | 4.06E-02 | 4.06E+00 | | | |
| 7896609 | --- | --- | --- | -0.178 | 0.884 | 1.94E-01 | 1.94E+01 | | | |
| 8150089 | NM_016127 | TMEM66 | transmembrane protein 66 | -0.178 | 0.884 | 1.18E-01 | 1.18E+01 | | | |
| 8073007 | NM_012323 | MAFF | v-maf musculoaponeurotic fibrosarc | -0.178 | 0.884 | 1.58E-02 | 1.58E+00 | | | |
| 8019177 | NM_178520 | TMEM105 | transmembrane protein 105 | -0.178 | 0.884 | 2.77E-03 | 2.77E-01 | | | |
| 8149774 | NM_002318 | LOXL2 | lysyl oxidase-like 2 | -0.178 | 0.884 | 1.74E-03 | 1.74E-01 | | | |
| 7904086 | NM_014813 | LRIG2 | leucine-rich repeats and immunoglol | -0.178 | 0.884 | 2.43E-01 | 2.43E+01 | | | |
| 7896185 | --- | --- | --- | -0.178 | 0.884 | 7.34E-01 | 7.34E+01 | | | |
| 7926152 | NM_024693 | ECHDC3 | enoyl Coenzyme A hydratase domair | -0.178 | 0.884 | 8.47E-03 | 8.47E-01 | | | |
| 8124413 | NM_003539 | HIST1H4D | histone cluster 1, H4d | -0.178 | 0.884 | 4.05E-01 | 4.05E+01 | | | |
| 8104609 | --- | --- | --- | -0.178 | 0.884 | 2.52E-02 | 2.52E+00 | | | |
| 8159283 | NM_020822 | KCNT1 | potassium channel, subfamily T, mer | -0.178 | 0.884 | 3.45E-03 | 3.45E-01 | | | |
| 8065016 | --- | --- | --- | -0.178 | 0.884 | 4.11E-02 | 4.11E+00 | | | |
| 7895050 | --- | --- | --- | -0.178 | 0.884 | 3.93E-01 | 3.93E+01 | | | |
| 7970381 | NR_003366 | ANKRD20B | ankyrin repeat domain 20B | -0.178 | 0.884 | 7.09E-01 | 7.09E+01 | | | |
| 7998015 | NM_032451 | SPIRE2 | spire homolog 2 (Drosophila) | -0.178 | 0.884 | 1.80E-03 | 1.80E-01 | | | |
| 8026541 | AF151902 | FAM32A | family with sequence similarity 32, n | -0.178 | 0.884 | 5.73E-01 | 5.73E+01 | | | |
| 8036151 | NM_144617 | HSPB6 | heat shock protein, alpha-crystallin-r | -0.178 | 0.884 | 1.93E-02 | 1.93E+00 | | | |
| 8075863 | --- | --- | --- | -0.179 | 0.884 | 8.65E-03 | 8.65E-01 | | | |
| 8136631 | NM_003143 | SSBP1 | single-stranded DNA binding protein | -0.179 | 0.884 | 5.10E-01 | 5.10E+01 | | | |
| 8165334 | NM_004669 | CLIC3 | chloride intracellular channel 3 | -0.179 | 0.884 | 1.22E-02 | 1.22E+00 | | | |
| 7942228 | BC004224 | C11orf76 | chromosome 11 open reading frame | -0.179 | 0.884 | 1.09E-02 | 1.09E+00 | | | |
| 8027701 | NM_020895 | GRAMD1A | GRAM domain containing 1A | -0.179 | 0.884 | 8.22E-03 | 8.22E-01 | | | |
| 7896685 | --- | --- | --- | -0.179 | 0.884 | 7.82E-01 | 7.82E+01 | | | |
| 8144410 | NR_027000 | LOC349196 | hypothetical LOC349196 | -0.179 | 0.884 | 4.43E-02 | 4.43E+00 | | | |
| 7915468 | NM_199342 | CCDC23 | coiled-coil domain containing 23 | -0.179 | 0.883 | 5.27E-01 | 5.27E+01 | | | |
| 7945631 | NM_053005 | HCCA2 | HCCA2 protein | -0.179 | 0.883 | 1.34E-02 | 1.34E+00 | | | |
| 7894292 | --- | --- | --- | -0.179 | 0.883 | 5.07E-01 | 5.07E+01 | | | |
| 7893188 | --- | --- | --- | -0.179 | 0.883 | 7.85E-01 | 7.85E+01 | | | |
| 8159786 | NR_027156 | TUBBP5 | tubulin, beta pseudogene 5 | -0.179 | 0.883 | 7.72E-03 | 7.72E-01 | | | |
| 8149720 | NM_004430 | EGR3 | early growth response 3 | -0.179 | 0.883 | 2.16E-01 | 2.16E+01 | | | |
| 7974229 | NM_014315 | KLHDC2 | kelch domain containing 2 | -0.179 | 0.883 | 6.74E-01 | 6.74E+01 | | | |
| 8153426 | NM_00110087 | C8orf73 | chromosome 8 open reading frame 7 | -0.179 | 0.883 | 2.58E-02 | 2.58E+00 | | | |
| 7915156 | NM_002699 | POU3F1 | POU class 3 homeobox 1 | -0.179 | 0.883 | 1.28E-02 | 1.28E+00 | | | |
| 8164853 | NM_020469 | ABO | ABO blood group (transferase A, alph | -0.179 | 0.883 | 2.08E-03 | 2.08E-01 | | | |
| 8032265 | NM_203304 | MEX3D | mex-3 homolog D (C. elegans) | -0.179 | 0.883 | 9.79E-04 | 9.79E-02 | | | |
| 7892788 | --- | --- | --- | -0.179 | 0.883 | 3.22E-01 | 3.22E+01 | | | |
| 7995834 | NR_003669 | MT1IP | metallothionein 1I (pseudogene) | -0.179 | 0.883 | 7.54E-03 | 7.54E-01 | | | |
| 7953665 | NM_199286 | DPPA3 | developmental pluripotency associat | -0.179 | 0.883 | 3.50E-02 | 3.50E+00 | | | |
| 7985030 | ENST00000428 | DNM1P35 | DNM1 pseudogene 35 | -0.179 | 0.883 | 9.02E-03 | 9.02E-01 | | | |
| 8009334 | NM_014405 | CACNG4 | calcium channel, voltage-dependent | -0.180 | 0.883 | 3.75E-03 | 3.75E-01 | | | |
| 7893513 | --- | --- | --- | -0.180 | 0.883 | 2.61E-01 | 2.61E+01 | | | |
| 8090314 | NM_012190 | ALDH1L1 | aldehyde dehydrogenase 1 family, m | -0.180 | 0.883 | 5.72E-03 | 5.72E-01 | | | |
| 8025612 | NM_022377 | ICAM4 | intercellular adhesion molecule 4 (La | -0.180 | 0.883 | 1.47E-01 | 1.47E+01 | | | |
| 7980983 | NM_022151 | MOAP1 | modulator of apoptosis 1 | -0.180 | 0.883 | 4.40E-02 | 4.40E+00 | | | |
| 7992191 | NM_012217 | TPSD1 | tryptase delta 1 | -0.180 | 0.883 | 3.75E-03 | 3.75E-01 | | | |
| 7985482 | NM_00108043 | WHAMM | WAS protein homolog associated wit | -0.180 | 0.883 | 1.56E-01 | 1.56E+01 | | | |
| 7896652 | --- | --- | --- | -0.180 | 0.883 | 4.38E-02 | 4.38E+00 | | | |
| 8032595 | NM_031304 | DOHH | deoxyhypusine hydroxylase/monoox | -0.180 | 0.883 | 3.42E-03 | 3.42E-01 | | | |
| 7938329 | NR_002962 | SNORA23 | small nucleolar RNA, H/ACA box 23 | -0.180 | 0.883 | 6.40E-01 | 6.40E+01 | | | |
| 7945950 | --- | --- | --- | -0.180 | 0.883 | 6.19E-01 | 6.19E+01 | | | |
| 8063347 | --- | --- | --- | -0.180 | 0.883 | 2.23E-02 | 2.23E+00 | | | |
| 7940421 | NM_00107980 | PGA3 | pepsinogen 3, group I (pepsinogen A | -0.180 | 0.883 | 3.90E-03 | 3.90E-01 | | | |
| 8024228 | NM_177401 | MIDN | midnolin | -0.180 | 0.883 | 7.26E-03 | 7.26E-01 | | | |
| 7894379 | --- | --- | --- | -0.180 | 0.883 | 7.43E-01 | 7.43E+01 | | | |
| 7903092 | NM_00102494 | FNBP1L | formin binding protein 1-like | -0.180 | 0.883 | 1.21E-01 | 1.21E+01 | | | |
| 7895213 | --- | --- | --- | -0.180 | 0.883 | 2.95E-01 | 2.95E+01 | | | |
| 7999406 | --- | --- | --- | -0.180 | 0.883 | 7.93E-03 | 7.93E-01 | | | |
| 7895927 | --- | --- | --- | -0.180 | 0.883 | 3.02E-01 | 3.02E+01 | | | |
| 7913824 | NM_020317 | C1orf63 | chromosome 1 open reading frame 6 | -0.180 | 0.883 | 4.30E-01 | 4.30E+01 | | | |
| 8061815 | NM_182658 | C20orf185 | chromosome 20 open reading frame | -0.180 | 0.883 | 1.08E-02 | 1.08E+00 | | | |
| 7973393 | NM_022789 | IL25 | interleukin 25 | -0.180 | 0.883 | 8.34E-03 | 8.34E-01 | | | |
| 8174375 | NM_031273 | TEX13B | testis expressed 13B | -0.180 | 0.883 | 5.36E-03 | 5.36E-01 | | | |
| 7998399 | --- | --- | --- | -0.180 | 0.882 | 1.16E-02 | 1.16E+00 | | | |
| 7894015 | --- | --- | --- | -0.180 | 0.882 | 1.03E-01 | 1.03E+01 | | | |
| 8034871 | --- | --- | --- | -0.180 | 0.882 | 2.72E-01 | 2.72E+01 | | | |
| 7897280 | NM_00102459 | HES3 | hairy and enhancer of split 3 (Drosop | -0.181 | 0.882 | 1.23E-02 | 1.23E+00 | | | |
| 7899939 | NM_002060 | GJA4 | gap junction protein, alpha 4, 37kDa | -0.181 | 0.882 | 1.56E-02 | 1.56E+00 | | | |

| | | | | | | | | | | |
|---|---|---|---|---|---|---|---|---|---|---|
| 7992967 | NM_138440 | VASN | vasorin | -0.181 | 0.882 | 5.20E-03 | 5.20E-01 | | | |
| 7925504 | NM_00100434 | MAP1LC3C | microtubule-associated protein 1 lig | -0.181 | 0.882 | 1.33E-02 | 1.33E+00 | | | |
| 8147461 | NM_002998 | SDC2 | syndecan 2 | -0.181 | 0.882 | 5.04E-03 | 5.04E-01 | | | |
| 8095299 | --- | --- | --- | -0.181 | 0.882 | 5.08E-01 | 5.08E+01 | | | |
[truncated: 168,629 more chars]
