# Supplementary material for: Exposure-Dependent Control of Malaria-Induced Inflammation in Children
Source: PLoS Pathog. 2014 Apr 17;10(4):e1004079. doi: 10.1371/journal.ppat.1004079 (PMC3990727; doi:10.1371/journal.ppat.1004079)
Supplement: Table S3 — Differentially expressed genes in response to in vitro P. falciparum stimulation from before to after malaria. Transcripts are significant if DR-adjusted p-value<0.05 and absolute fold change >1.25. Transcript ID is the Affymetrix accession number. (PDF) [file ppat.1004079.s005.pdf]

Table S3. Differentially expressed genes in response to *in vitro P. falciparum* stimulation from before to after malaria. Transcripts are significant if FDR-adjusted p-value < 0.05 and absolute fold change > 1.25. Transcript ID is the Affymetrix accession number.

Transcript ID	Acession ID	Gene Symbol	Gene Description	log^2 Fold Change	Fold Change	p-value	FDR-adjusted p-value	Regulation relative to baseline	Figure 1D
8146934	NM_015364	LY96	lymphocyte antigen 96	0.599	1.515	1.36E-03	1.36E-01	Up	Anti-microbial
8157524	NR_024168	TLR4	toll-like receptor 4	1.262	2.398	2.02E-03	2.02E-01	Up	Anti-microbial
8166059	NM_016562	TLR7	toll-like receptor 7	0.613	1.530	4.16E-03	4.16E-01	Up	Anti-microbial
8011826	AY358809	C17orf87	chromosome 17 open reading fran	1.138	2.201	6.00E-05	6.00E-03	Up	Antigen presentation/co-stimulation
8063156	NM_001250	CD40	CD40 molecule, TNF receptor supe	0.384	1.305	2.35E-02	2.35E+00	Up	Antigen presentation/co-stimulation
8082035	NM_175862	CD86	CD86 molecule	0.724	1.652	2.11E-03	2.11E-01	Up	Antigen presentation/co-stimulation
8115147	NM_00102515	CD74	CD74 molecule, major histocompa	0.435	1.351	1.16E-03	1.16E-01	Up	Antigen presentation/co-stimulation
8118548	NM_019111	HLA-DRA	major histocompatibility complex,	0.547	1.461	1.43E-03	1.43E-01	Up	Antigen presentation/co-stimulation
8125530	NM_002118	HLA-DMB	major histocompatibility complex,	0.719	1.645	3.25E-04	3.25E-02	Up	Antigen presentation/co-stimulation
8125537	NM_006120	HLA-DMA	major histocompatibility complex,	0.707	1.632	1.45E-04	1.45E-02	Up	Antigen presentation/co-stimulation
8125556	NM_033554	HLA-DPA1	major histocompatibility complex,	0.630	1.548	2.48E-03	2.48E-01	Up	Antigen presentation/co-stimulation
8178193	NM_019111	HLA-DRA	major histocompatibility complex,	0.523	1.437	2.41E-03	2.41E-01	Up	Antigen presentation/co-stimulation
8178884	NM_006120	HLA-DMA	major histocompatibility complex,	0.728	1.656	1.45E-04	1.45E-02	Up	Antigen presentation/co-stimulation
8178891	NM_033554	HLA-DPA1	major histocompatibility complex,	0.630	1.548	2.48E-03	2.48E-01	Up	Antigen presentation/co-stimulation
8179481	NM_019111	HLA-DRA	major histocompatibility complex,	0.540	1.454	1.94E-03	1.94E-01	Up	Antigen presentation/co-stimulation
8180078	NM_002118	HLA-DMB	major histocompatibility complex,	0.708	1.633	3.48E-04	3.48E-02	Up	Antigen presentation/co-stimulation
8180086	NM_006120	HLA-DMA	major histocompatibility complex,	0.728	1.656	1.45E-04	1.45E-02	Up	Antigen presentation/co-stimulation
8180100	NM_033554	HLA-DPA1	major histocompatibility complex,	0.651	1.570	1.03E-03	1.03E-01	Up	Antigen presentation/co-stimulation
7905047	NM_000566	FCGR1A	Fc fragment of IgG, high affinity Ia	2.129	4.374	2.86E-04	2.86E-02	Up	Chemotaxis/Phagocytosis/killing
7905060	NM_000566	FCGR1A	Fc fragment of IgG, high affinity Ia	1.775	3.423	5.39E-04	5.39E-02	Up	Chemotaxis/Phagocytosis/killing
7909371	NM_000573	CR1	complement component (3b/4b) r	0.369	1.292	2.11E-02	2.11E+00	Up	Chemotaxis/Phagocytosis/killing
7919133	NM_00101798	FCGR1B	Fc fragment of IgG, high affinity Ib	1.826	3.547	3.15E-04	3.15E-02	Up	Chemotaxis/Phagocytosis/killing
7919800	NM_004079	CTSS	cathepsin S	0.651	1.571	6.64E-03	6.64E-01	Up	Chemotaxis/Phagocytosis/killing
7921868	NM_000569	FCGR3A	Fc fragment of IgG, low affinity IIIa	1.584	2.998	1.29E-04	1.29E-02	Up	Chemotaxis/Phagocytosis/killing
7921873	NM_000569	FCGR3A	Fc fragment of IgG, low affinity IIIa	1.552	2.933	6.00E-05	6.00E-03	Up	Chemotaxis/Phagocytosis/killing
7926410	NM_002438	MRC1	mannose receptor, C type 1	2.160	4.468	1.06E-03	1.06E-01	Up	Chemotaxis/Phagocytosis/killing
7926451	NM_002438	MRC1	mannose receptor, C type 1	2.160	4.468	1.06E-03	1.06E-01	Up	Chemotaxis/Phagocytosis/killing
7945666	NM_001909	CTSD	cathepsin D	0.433	1.350	2.56E-02	2.56E+00	Up	Chemotaxis/Phagocytosis/killing
7950906	NM_001814	CTSC	cathepsin C	0.836	1.785	2.28E-03	2.28E-01	Up	Chemotaxis/Phagocytosis/killing
7970287	NM_005561	LAMP1	lysosomal-associated membrane p	0.391	1.312	1.16E-03	1.16E-01	Up	Chemotaxis/Phagocytosis/killing
7980958	NM_005606	LGMN	legumain	1.416	2.668	6.17E-05	6.17E-03	Up	Chemotaxis/Phagocytosis/killing
8026971	NM_006332	IFI30	interferon, gamma-inducible prote	0.599	1.515	2.04E-02	2.04E+00	Up	Chemotaxis/Phagocytosis/killing
8072744	NM_013416	NCF4	neutrophil cytosolic factor 4, 40kD	0.477	1.392	1.89E-02	1.89E+00	Up	Chemotaxis/Phagocytosis/killing
8079401	NM_000579	CCR5	chemokine (C-C motif) receptor 5	0.556	1.470	1.56E-02	1.56E+00	Up	Chemotaxis/Phagocytosis/killing
8086600	NM_001295	CCR1	chemokine (C-C motif) receptor 1	0.885	1.847	2.34E-02	2.34E+00	Up	Chemotaxis/Phagocytosis/killing
8093298	NM_000579	CCR5	chemokine (C-C motif) receptor 5	0.556	1.470	1.56E-02	1.56E+00	Up	Chemotaxis/Phagocytosis/killing
8133314	NM_000265	NCF1	neutrophil cytosolic factor 1	0.388	1.309	1.87E-02	1.87E+00	Up	Chemotaxis/Phagocytosis/killing
8133518	NM_000265	NCF1	neutrophil cytosolic factor 1	0.362	1.285	2.11E-02	2.11E+00	Up	Chemotaxis/Phagocytosis/killing
8133876	NM_00100154	CD36	CD36 molecule (thrombospondin r	2.304	4.939	7.31E-05	7.31E-03	Up	Chemotaxis/Phagocytosis/killing
8140227	NM_000265	NCF1	neutrophil cytosolic factor 1	0.390	1.310	1.75E-02	1.75E+00	Up	Chemotaxis/Phagocytosis/killing
8149330	NM_147780	CTSB	cathepsin B	0.786	1.724	1.78E-02	1.78E+00	Up	Chemotaxis/Phagocytosis/killing
8166730	NM_000397	CYBB	cytochrome b-245, beta polypepti	1.132	2.191	1.52E-03	1.52E-01	Up	Chemotaxis/Phagocytosis/killing
8174779	NM_013995	LAMP2	lysosomal-associated membrane p	0.365	1.288	3.02E-02	3.02E+00	Up	Chemotaxis/Phagocytosis/killing
7909250	NM_153758	IL19	interleukin 19	-1.334	0.397	5.48E-06	5.48E-04	Down	Pro-inflammatory mediator
7909271	NM_006850	IL24	interleukin 24	-1.501	0.353	1.76E-08	1.76E-06	Down	Pro-inflammatory mediator
7996022	NM_002990	CCL22	chemokine (C-C motif) ligand 22	-0.967	0.512	1.94E-04	1.94E-02	Down	Pro-inflammatory mediator
8014369	NM_002983	CCL3	chemokine (C-C motif) ligand 3	-1.298	0.407	6.17E-05	6.17E-03	Down	Pro-inflammatory mediator
8014391	NM_021006	CCL3L1	chemokine (C-C motif) ligand 3-lik	-1.314	0.402	6.17E-05	6.17E-03	Down	Pro-inflammatory mediator
8014414	NM_021006	CCL3L1	chemokine (C-C motif) ligand 3-lik	-1.314	0.402	6.17E-05	6.17E-03	Down	Pro-inflammatory mediator
8019731	NM_021006	CCL3L1	chemokine (C-C motif) ligand 3-lik	-1.314	0.402	6.17E-05	6.17E-03	Down	Pro-inflammatory mediator
8044541	NM_019618	IL1F9	interleukin 1 family, member 9	-1.347	0.393	3.98E-05	3.98E-03	Down	Pro-inflammatory mediator
8048864	NM_004591	CCL20	chemokine (C-C motif) ligand 20	-0.882	0.543	9.41E-03	9.41E-01	Down	Pro-inflammatory mediator
8054722	NM_000576	IL1B	interleukin 1, beta	-2.357	0.195	2.70E-05	2.70E-03	Down	Pro-inflammatory mediator
8095680	NM_000584	IL8	interleukin 8	-1.217	0.430	7.31E-05	7.31E-03	Down	Pro-inflammatory mediator
8095688	NM_002993	CXCL6	chemokine (C-X-C motif) ligand 6 (	-0.466	0.724	1.16E-02	1.16E+00	Down	Pro-inflammatory mediator
8095697	NM_001511	CXCL1	chemokine (C-X-C motif) ligand 1 (	-0.718	0.608	1.58E-04	1.58E-02	Down	Pro-inflammatory mediator
8100984	NM_002090	CXCL3	chemokine (C-X-C motif) ligand 3	-0.931	0.524	1.18E-03	1.18E-01	Down	Pro-inflammatory mediator
8100994	NM_002089	CXCL2	chemokine (C-X-C motif) ligand 2	-0.969	0.511	1.20E-03	1.20E-01	Down	Pro-inflammatory mediator
8129618	NM_004666	VNN1	vanin 1	-1.259	0.418	2.72E-04	2.72E-02	Down	Pro-inflammatory mediator
8131803	NM_000600	IL6	interleukin 6 (interferon, beta 2)	-3.101	0.117	5.48E-06	5.48E-04	Down	Pro-inflammatory mediator
7906377	NM_002432	MNDA	myeloid cell nuclear differentiatio	1.880	3.682	7.31E-05	7.31E-03	Up	Regulator
7942300	NM_173042	IL18BP	interleukin 18 binding protein	0.358	1.282	2.93E-03	2.93E-01	Up	Regulator
7950473	NM_004041	ARRB1	arrestin, beta 1	0.464	1.379	1.27E-02	1.27E+00	Up	Regulator
7966089	NM_00114234	CMKLR1	chemokine-like receptor 1	1.448	2.728	5.14E-04	5.14E-02	Up	Regulator
8043981	NM_004633	IL1R2	interleukin 1 receptor, type II	0.339	1.265	6.79E-04	6.79E-02	Up	Regulator
8047692	NM_005214	CTLA4	cytotoxic T-lymphocyte-associated	0.368	1.290	2.06E-02	2.06E+00	Up	Regulator
8066117	NM_015474	SAMHD1	SAM domain and HD domain 1	0.498	1.412	8.11E-04	8.11E-02	Up	Regulator
8089519	NM_181780	BTLA	B and T lymphocyte associated	0.372	1.294	7.79E-03	7.79E-01	Up	Regulator
8092169	NM_003810	TNFSF10	tumor necrosis factor (ligand) supe	0.568	1.483	1.87E-02	1.87E+00	Up	Regulator
8096301	NM_00104005	SPP1	secreted phosphoprotein 1	1.618	3.068	1.88E-02	1.88E+00	Up	Regulator
8100231	NM_003215	TEC	tec protein tyrosine kinase	0.385	1.306	2.12E-02	2.12E+00	Up	Regulator

8112428	NM_005582	CD180	CD180 molecule	0.973	1.963	6.17E-05	6.17E-03	Up	Regulator
8118158	NM_004847	AIF1	allograft inflammatory factor 1	1.211	2.316	2.39E-04	2.39E-02	Up	Regulator
8154178	NM_004972	JAK2	Janus kinase 2	0.673	1.594	1.38E-03	1.38E-01	Up	Regulator
8156321	NM_003177	SYK	spleen tyrosine kinase	0.458	1.373	3.64E-03	3.64E-01	Up	Regulator
8177996	NM_004847	AIF1	allograft inflammatory factor 1	1.211	2.316	2.39E-04	2.39E-02	Up	Regulator
8179276	NM_004847	AIF1	allograft inflammatory factor 1	1.211	2.316	2.39E-04	2.39E-02	Up	Regulator
7922976	NM_000963	PTGS2	prostaglandin-endoperoxide synth	-2.726	0.151	2.95E-05	2.95E-03	Down	Regulator
7924450	NM_007207	DUSP10	dual specificity phosphatase 10	-0.348	0.786	2.46E-02	2.46E+00	Down	Regulator
7968344	NM_001629	ALOX5AP	arachidonate 5-lipoxygenase-activ	-0.426	0.744	1.72E-02	1.72E+00	Down	Regulator
7978644	NM_020529	NFKBIA	nuclear factor of kappa light polyp	-0.377	0.770	2.93E-03	2.93E-01	Down	Regulator
8096635	NM_003998	NFKB1	nuclear factor of kappa light polyp	-0.329	0.796	1.59E-02	1.59E+00	Down	Regulator
8115210	NM_006058	TNIP1	TNFAIP3 interacting protein 1	-0.366	0.776	2.35E-03	2.35E-01	Down	Regulator
8122265	NM_006290	TNFAIP3	tumor necrosis factor, alpha-induc	-0.354	0.783	1.90E-02	1.90E+00	Down	Regulator
8126303	NM_018643	TREM1	triggering receptor expressed on r	-1.418	0.374	1.00E-03	1.00E-01	Down	Regulator
7963770	NM_020370	GPR84	G protein-coupled receptor 84	-0.424	0.745	6.71E-02	6.71E+00		
7994131	NM_002738	PRKCB	protein kinase C, beta	0.306	1.237	9.18E-04	9.18E-02		
8116559	NM_002460	IRF4	interferon regulatory factor 4	-0.303	0.810	6.30E-02	6.30E+00		
8150076	NM_001394	DUSP4	dual specificity phosphatase 4	-0.316	0.803	6.08E-02	6.08E+00		
8100977	NM_002994	CXCL5	chemokine (C-X-C motif) ligand 5	-0.966	0.512	5.58E-02	5.58E+00		
8160879	NM_006274	CCL19	chemokine (C-C motif) ligand 19	-0.578	0.670	5.55E-02	5.55E+00		
7911178	NM_004895	NLRP3	NLR family, pyrin domain containi	-0.339	0.791	6.13E-02	6.13E+00		
7893228	---	---	---	0.486	1.400	1.96E-02	1.96E+00	Up	
7895116	---	---	---	0.405	1.324	1.11E-02	1.11E+00	Up	
7895482	---	---	---	0.515	1.429	2.02E-02	2.02E+00	Up	
7895909	---	---	---	0.404	1.323	4.10E-02	4.10E+00	Up	
7896689	---	---	---	0.487	1.401	3.17E-03	3.17E-01	Up	
7896690	---	---	---	0.486	1.400	1.57E-02	1.57E+00	Up	
7896697	---	---	---	0.450	1.366	3.35E-02	3.35E+00	Up	
7896700	---	---	---	0.751	1.683	2.93E-03	2.93E-01	Up	
7896705	---	---	---	0.585	1.501	8.87E-03	8.87E-01	Up	
7896706	---	---	---	1.847	3.597	7.60E-05	7.60E-03	Up	
7896707	---	---	---	0.537	1.451	8.53E-03	8.53E-01	Up	
7896714	---	---	---	0.669	1.590	3.28E-02	3.28E+00	Up	
7896718	---	---	---	0.719	1.646	5.22E-03	5.22E-01	Up	
7896729	---	---	---	0.477	1.392	3.55E-02	3.55E+00	Up	
7898124	NM_052929	FHAD1	forkhead-associated (FHA) phosph	0.372	1.294	2.02E-03	2.02E-01	Up	
7898799	NM_00111410	C1QC	complement component 1, q subc	0.553	1.467	9.34E-04	9.34E-02	Up	
7898805	NM_000491	C1QB	complement component 1, q subc	0.481	1.396	3.68E-03	3.68E-01	Up	
7901110	NM_006066	AKR1A1	aldo-keto reductase family 1, mem	0.500	1.414	6.74E-04	6.74E-02	Up	
7903980	BC017973	C1orf162	chromosome 1 open reading fram	0.827	1.774	3.40E-04	3.40E-02	Up	
7904726	NM_006472	TXNIP	thioredoxin interacting protein	0.374	1.296	3.72E-03	3.72E-01	Up	
7905147	BC017761	C1orf54	chromosome 1 open reading fram	0.809	1.752	3.06E-03	3.06E-01	Up	
7906486	NM_020125	SLAMF8	SLAM family member 8	0.786	1.725	3.72E-03	3.72E-01	Up	
7906564	NM_003768	PEA15	phosphoprotein enriched in astroc	0.369	1.291	1.19E-02	1.19E+00	Up	
7907657	NM_152663	RALGPS2	Ral GEF with PH domain and SH3 b	0.356	1.280	6.84E-03	6.84E-01	Up	
7908003	NM_030769	NPL	N-acetylneuraminate pyruvate lya	0.888	1.851	3.72E-02	3.72E+00	Up	
7908125	NM_015149	RGL1	ral guanine nucleotide dissociatio	1.053	2.076	2.42E-03	2.42E-01	Up	
7909446	NM_005525	HSD11B1	hydroxysteroid (11-beta) dehydro	1.112	2.161	2.39E-04	2.39E-02	Up	
7913694	NM_000147	FUCA1	fucosidase, alpha-L- 1, tissue	1.596	3.024	1.94E-04	1.94E-02	Up	
7918474	NM_178454	DRAM2	DNA-damage regulated autophagy	0.338	1.264	1.24E-03	1.24E-01	Up	
7920271	NM_019554	S100A4	S100 calcium binding protein A4	0.499	1.413	2.93E-02	2.93E+00	Up	
7921298	NM_030764	FCRL2	Fc receptor-like 2	0.555	1.469	2.41E-03	2.41E-01	Up	
7921319	NM_052938	FCRL1	Fc receptor-like 1	0.451	1.367	1.52E-03	1.52E-01	Up	
7921637	NM_003874	CD84	CD84 molecule	0.907	1.875	2.34E-05	2.34E-03	Up	
7921882	NM_015441	OLFML2B	olfactomedin-like 2B	0.691	1.615	1.80E-02	1.80E+00	Up	
7922689	NM_002065	GLUL	glutamate-ammonia ligase (glutan	0.736	1.666	1.68E-03	1.68E-01	Up	
7926545	NM_032812	PLXDC2	plexin domain containing 2	1.008	2.011	7.35E-03	7.35E-01	Up	
7927215	NM_000698	ALOX5	arachidonate 5-lipoxygenase	0.382	1.303	1.89E-02	1.89E+00	Up	
7932023	NM_014688	USP6NL	USP6 N-terminal like	0.603	1.519	5.92E-04	5.92E-02	Up	
7933872	NM_000399	EGR2	early growth response 2	0.799	1.740	8.69E-03	8.69E-01	Up	
7934196	NM_002778	PSAP	prosaposin	0.518	1.432	3.64E-02	3.64E+00	Up	
7934920	NM_00112760	LIPA	lipase A, lysosomal acid, cholester	1.278	2.425	5.48E-06	5.48E-04	Up	
7935058	NM_013451	MYOF	myoferlin	1.670	3.182	3.51E-04	3.51E-02	Up	
7935270	NM_013314	BLNK	B-cell linker	0.475	1.390	1.61E-02	1.61E+00	Up	
7937802	NM_004356	CD81	CD81 molecule	0.344	1.270	5.39E-03	5.39E-01	Up	
7940028	NM_000062	SERPING1	serpin peptidase inhibitor, clade G	0.881	1.842	4.21E-02	4.21E+00	Up	
7942830	ENST00000391	GCRG224	GCRG-P224	0.425	1.343	1.13E-02	1.13E+00	Up	
7944722	NM_032873	UBASH3B	ubiquitin associated and SH3 dom	0.474	1.389	3.48E-04	3.48E-02	Up	
7945371	NM_021034	IFITM3	interferon induced transmembran	0.766	1.701	2.08E-02	2.08E+00	Up	
7948364	NM_00103939	MPEG1	macrophage expressed 1	1.174	2.256	4.30E-05	4.30E-03	Up	
7948455	NM_152852	MS4A6A	membrane-spanning 4-domains, s	1.059	2.084	1.12E-02	1.12E+00	Up	
7950578	NM_00112862	PAK1	p21 protein (Cdc42/Rac)-activated	0.605	1.521	3.49E-03	3.49E-01	Up	
7951004	NM_020179	C11orf75	chromosome 11 open reading frar	0.488	1.403	4.10E-03	4.10E-01	Up	
7952319	---	---	---	0.465	1.380	1.83E-03	1.83E-01	Up	
7953291	NM_001769	CD9	CD9 molecule	0.636	1.554	2.53E-03	2.53E-01	Up	
7953901	NM_138337	CLEC12A	C-type lectin domain family 12, me	1.085	2.122	1.35E-02	1.35E+00	Up	
7958207	---	---	---	0.327	1.254	1.26E-02	1.26E+00	Up	

7959251	NM_002562	P2RX7	purinergic receptor P2X, ligand-ga	0.857	1.812	3.29E-02	3.29E+00	Up	
7960771	NM_174941	CD163L1	CD163 molecule-like 1	0.430	1.347	2.82E-03	2.82E-01	Up	
7960794	NM_004244	CD163	CD163 molecule	1.640	3.116	9.67E-03	9.67E-01	Up	
7961059	NM_002258	KLRB1	killer cell lectin-like receptor subfa	0.501	1.415	4.06E-02	4.06E+00	Up	
7961440	NM_024829	PLBD1	phospholipase B domain containin	0.842	1.792	4.44E-04	4.44E-02	Up	
7961891	NM_030762	BHLHE41	basic helix-loop-helix family, mem	0.643	1.562	1.27E-03	1.27E-01	Up	
7964834	NM_001874	CPM	carboxypeptidase M	0.754	1.686	6.41E-03	6.41E-01	Up	
7965627	NM_000895	LTA4H	leukotriene A4 hydrolase	0.642	1.560	4.33E-03	4.33E-01	Up	
7971573	NM_001268	RCBTB2	regulator of chromosome condens	0.897	1.862	9.18E-04	9.18E-02	Up	
7974461	NR_003225	LGALS3	lectin, galactoside-binding, soluble	0.485	1.400	3.68E-03	3.68E-01	Up	
7974816	NM_153811	SLC38A6	solute carrier family 38, member 6	0.551	1.465	2.46E-02	2.46E+00	Up	
7975799	NM_017791	FLVCR2	feline leukemia virus subgroup C c	0.586	1.501	1.94E-03	1.94E-01	Up	
7975813	---	---	---	0.446	1.363	2.59E-02	2.59E+00	Up	
7977933	NM_012244	SLC7A8	solute carrier family 7 (cationic am	0.750	1.682	2.41E-03	2.41E-01	Up	
7979455	NM_021136	RTN1	reticulon 1	0.398	1.317	2.66E-02	2.66E+00	Up	
7980146	NM_006432	NPC2	Niemann-Pick disease, type C2	0.599	1.515	3.64E-02	3.64E+00	Up	
7981183	NM_021966	TCL1A	T-cell leukemia/lymphoma 1A	0.522	1.436	2.73E-02	2.73E+00	Up	
7981718	BC020240 // B	IGHM // IGHM	immunoglobulin heavy constant m	0.814	1.758	7.31E-05	7.31E-03	Up	
7981720	---	---	---	0.378	1.300	3.15E-04	3.15E-02	Up	
7981722	AK128476	IGHA1	immunoglobulin heavy constant a	0.555	1.469	9.49E-05	9.49E-03	Up	
7981724	BC021276	IGHD	immunoglobulin heavy constant d	0.815	1.759	2.16E-04	2.16E-02	Up	
7981728	ENST00000390	LOC100293211	similar to hCG2042717	0.794	1.734	1.20E-04	1.20E-02	Up	
7981730	AB001736	IGLJ3	immunoglobulin lambda joining 3	0.719	1.646	6.05E-05	6.05E-03	Up	
7981732	ENST00000338	VSIG6	V-set and immunoglobulin domain	0.707	1.633	3.15E-04	3.15E-02	Up	
7981737	AF067420 // A	IGHA1 // IGHA1	immunoglobulin heavy constant a	0.350	1.274	5.88E-03	5.88E-01	Up	
7981740	BC073771 // B	IGHA1 // IGHA1	immunoglobulin heavy constant a	0.568	1.482	3.40E-04	3.40E-02	Up	
7982564	NM_152594	SPRED1	sprouty-related, EVH1 domain con	0.969	1.957	1.58E-02	1.58E+00	Up	
7985809	NM_007011	ABHD2	abhydrolase domain containing 2	0.334	1.261	1.44E-02	1.44E+00	Up	
7989277	NM_004998	MYO1E	myosin IE	0.529	1.443	3.68E-03	3.68E-01	Up	
7989309	---	---	---	0.516	1.430	6.02E-03	6.02E-01	Up	
7995096	NM_00114580	ITGAM	integrin, alpha M (complement co	0.571	1.486	3.00E-03	3.00E-01	Up	
7995263	AF067420	IGHA1	immunoglobulin heavy constant a	0.429	1.346	2.68E-04	2.68E-02	Up	
7995697	NM_017839	LPCAT2	lysophosphatidylcholine acyltransf	1.425	2.685	2.16E-04	2.16E-02	Up	
7997712	NM_002163	IRF8	interferon regulatory factor 8	0.402	1.321	1.26E-03	1.26E-01	Up	
8000375	NM_00100663	ARHGAP17	Rho GTPase activating protein 17	0.343	1.268	9.40E-05	9.40E-03	Up	
8001104	ENST00000390	LOC100290146	hypothetical protein LOC1002901	0.423	1.341	7.43E-05	7.43E-03	Up	
8002667	NM_031293	PMFBP1	polyamine modulated factor 1 bin	0.522	1.436	1.15E-03	1.15E-01	Up	
8004938	ENST00000420	LOC100129677	hypothetical protein LOC1001296	0.627	1.545	1.45E-04	1.45E-02	Up	
8005458	NM_00104007	LGALS9C	lectin, galactoside-binding, soluble	0.689	1.612	1.26E-04	1.26E-02	Up	
8006214	NM_018404	ADAP2	ArfGAP with dual PH domains 2	0.633	1.551	1.24E-02	1.24E+00	Up	
8006440	NM_006273	CCL7	chemokine (C-C motif) ligand 7	1.362	2.571	9.05E-03	9.05E-01	Up	
8006459	NM_005408	CCL13	chemokine (C-C motif) ligand 13	1.080	2.114	8.12E-04	8.12E-02	Up	
8007228	NM_00113002	ATP6V0A1	ATPase, H+ transporting, lysosoma	0.801	1.742	2.59E-03	2.59E-01	Up	
8007483	NM_145041	TMEM106A	transmembrane protein 106A	0.399	1.318	1.11E-02	1.11E+00	Up	
8008646	NM_021626	SCPEP1	serine carboxypeptidase 1	0.887	1.849	1.26E-03	1.26E-01	Up	
8013450	NM_00104268	LGALS9B	lectin, galactoside-binding, soluble	0.745	1.676	1.30E-04	1.30E-02	Up	
8017210	NM_003916	AP1S2	adaptor-related protein complex 1	0.375	1.296	3.63E-02	3.63E+00	Up	
8017599	NM_000442	PECAM1	platelet/endothelial cell adhesion	0.760	1.694	1.66E-03	1.66E-01	Up	
8019622	NM_145041	TMEM106A	transmembrane protein 106A	0.369	1.292	2.26E-02	2.26E+00	Up	
8020668	NM_018439	IMPACT	Impact homolog (mouse)	0.362	1.286	9.34E-04	9.34E-02	Up	
8021301	NM_004163	RAB27B	RAB27B, member RAS oncogene fa	0.324	1.252	1.56E-02	1.56E+00	Up	
8021727	NM_018235	CNDP2	CNDP dipeptidase 2 (metallopepti	0.351	1.275	1.90E-02	1.90E+00	Up	
8022572	NM_080597	OSBPL1A	oxysterol binding protein-like 1A	0.628	1.545	2.40E-03	2.40E-01	Up	
8026106	NM_004343	CALR	calreticulin	0.337	1.263	4.48E-03	4.48E-01	Up	
8030866	NM_002030	FPR3	formyl peptide receptor 3	2.126	4.364	1.45E-04	1.45E-02	Up	
8031238	NM_006847	LILRB4	leukocyte immunoglobulin-like rec	0.428	1.346	3.99E-02	3.99E+00	Up	
8033332	NR_024075	EMR4P	egf-like module containing, mucin	0.334	1.260	3.41E-02	3.41E+00	Up	
8036224	NM_003332	TYROBP	TYRO protein tyrosine kinase bind	0.462	1.378	3.53E-02	3.53E+00	Up	
8039212	NM_005874	LILRB2	leukocyte immunoglobulin-like rec	0.529	1.443	1.44E-02	1.44E+00	Up	
8042391	NM_002664	PLEK	pleckstrin	0.630	1.547	3.07E-02	3.07E+00	Up	
8042468	NM_001153	ANXA4	annexin A4	0.768	1.703	1.26E-03	1.26E-01	Up	
8042576	NM_017567	NAGK	N-acetylglucosamine kinase	0.503	1.417	6.90E-03	6.90E-01	Up	
8042830	NR_027405	MTHFD2	methylenetetrahydrofolate dehyd	0.382	1.303	7.02E-03	7.02E-01	Up	
8043197	NM_003761	VAMP8	vesicle-associated membrane prot	0.426	1.343	1.25E-02	1.25E+00	Up	
8043203	NM_006634	VAMP5	vesicle-associated membrane prot	0.692	1.616	5.06E-03	5.06E-01	Up	
8043360	BC030813	IGK@	immunoglobulin kappa locus	0.438	1.355	9.74E-04	9.74E-02	Up	
8043431	AF113887 // A	IGKC // IGKC	immunoglobulin kappa constant /	0.561	1.475	3.35E-03	3.35E-01	Up	
8043436	BC073772 // B	IGKC // IGKC	immunoglobulin kappa constant /	0.548	1.462	1.07E-02	1.07E+00	Up	
8043449	BC030813	IGK@	immunoglobulin kappa locus	0.468	1.383	1.32E-03	1.32E-01	Up	
8043459	ENST00000390	IGKC	immunoglobulin kappa constant	0.385	1.306	2.40E-03	2.40E-01	Up	
8043465	BC029444 // B	IGKC // IGKC	immunoglobulin kappa constant /	0.376	1.297	7.68E-04	7.68E-02	Up	
8043468	---	---	---	0.629	1.547	2.17E-03	2.17E-01	Up	
8043470	---	---	---	0.512	1.426	3.86E-03	3.86E-01	Up	
8043476	ENST00000390	IGKC	immunoglobulin kappa constant	0.543	1.457	9.29E-03	9.29E-01	Up	
8043480	---	---	---	0.372	1.294	2.86E-03	2.86E-01	Up	
8044391	NM_006343	MERTK	c-mer proto-oncogene tyrosine kin	1.022	2.031	1.75E-02	1.75E+00	Up	
8046333	NM_024843	CYBRD1	cytochrome b reductase 1	0.445	1.361	1.07E-02	1.07E+00	Up	

8050278	NM_005742	PDIA6	protein disulfide isomerase family	0.338	1.264	5.68E-03	5.68E-01	Up	
8051762	NM_021097	SLC8A1	solute carrier family 8 (sodium/cal	1.502	2.832	2.37E-04	2.37E-02	Up	
8053484	NM_003896	ST3GAL5	ST3 beta-galactoside alpha-2,3-sia	0.533	1.447	9.34E-04	9.34E-02	Up	
8053690	BC032451	IGK@	immunoglobulin kappa locus	0.609	1.525	6.17E-05	6.17E-03	Up	
8055639	---	---	---	0.471	1.386	2.41E-02	2.41E+00	Up	
8056343	NM_014900	COBLL1	COBL-like 1	0.402	1.321	1.86E-02	1.86E+00	Up	
8058498	NM_003468	FZD5	frizzled homolog 5 (Drosophila)	0.395	1.315	3.68E-03	3.68E-01	Up	
8058552	NM_005896	IDH1	isocitrate dehydrogenase 1 (NADP	0.646	1.565	3.30E-04	3.30E-02	Up	
8061247	NM_018993	RIN2	Ras and Rab interactor 2	0.438	1.354	4.68E-02	4.68E+00	Up	
8064471	NM_006065	SIRPB1	signal-regulatory protein beta 1	0.400	1.319	1.43E-03	1.43E-01	Up	
8064808	NM_005116	SLC23A2	solute carrier family 23 (nucleobas	0.503	1.417	2.97E-04	2.97E-02	Up	
8066266	NM_005461	MAFB	v-maf musculoaponeurotic fibrosa	0.523	1.437	2.16E-03	2.16E-01	Up	
8067279	NM_001336	CTSZ	cathepsin Z	0.389	1.310	3.45E-02	3.45E+00	Up	
8070826	NM_000211	ITGB2	integrin, beta 2 (complement com	0.434	1.351	2.86E-03	2.86E-01	Up	
8072015	NM_005160	ADRBK2	adrenergic, beta, receptor kinase 2	0.540	1.454	1.81E-03	1.81E-01	Up	
8073056	NM_145699	APOBEC3A	apolipoprotein B mRNA editing en	1.001	2.002	1.66E-03	1.66E-01	Up	
8074237	NM_017424	CECR1	cat eye syndrome chromosome re	0.644	1.563	3.48E-04	3.48E-02	Up	
8075057	---	---	---	0.755	1.688	1.44E-02	1.44E+00	Up	
8075709	NM_030643	APOL4	apolipoprotein L, 4	0.417	1.335	2.17E-02	2.17E+00	Up	
8075956	NM_006498	LGALS2	lectin, galactoside-binding, soluble	1.276	2.422	2.04E-02	2.04E+00	Up	
8078252	NM_152653	UBE2E2	ubiquitin-conjugating enzyme E2E	0.581	1.496	5.89E-04	5.89E-02	Up	
8078729	NM_002468	MYD88	myeloid differentiation primary re	0.331	1.258	2.63E-03	2.63E-01	Up	
8079392	NM_00112339	CCR2	chemokine (C-C motif) receptor 2	0.611	1.528	7.51E-03	7.51E-01	Up	
8080938	NM_198159	MITF	microphthalmia-associated transc	0.612	1.529	1.56E-02	1.56E+00	Up	
8081431	NM_001627	ALCAM	activated leukocyte cell adhesion	0.614	1.530	1.92E-03	1.92E-01	Up	
8081503	NM_014648	DZIP3	DAZ interacting protein 3, zinc fing	0.326	1.254	7.66E-03	7.66E-01	Up	
8081740	NM_001690	ATP6V1A	ATPase, H+ transporting, lysosoma	0.361	1.285	4.22E-02	4.22E+00	Up	
8082003	NM_018456	EAF2	ELL associated factor 2	0.495	1.410	2.56E-04	2.56E-02	Up	
8082012	NM_021082	SLC15A2	solute carrier family 15 (H+/peptic	0.407	1.326	7.80E-04	7.80E-02	Up	
8082965	NM_012219	MRAS	muscle RAS oncogene homolog	0.571	1.485	3.66E-03	3.66E-01	Up	
8083656	NM_022736	MFSD1	major facilitator superfamily doma	0.926	1.900	4.38E-04	4.38E-02	Up	
8091511	NM_014879	P2RY14	purinergic receptor P2Y, G-protein	0.750	1.682	1.72E-03	1.72E-01	Up	
8091954	NM_014498	GOLIM4	golgi integral membrane protein 4	0.341	1.267	2.41E-02	2.41E+00	Up	
8092177	NM_00114627	NCEH1	neutral cholesterol ester hydrolase	0.655	1.574	2.46E-02	2.46E+00	Up	
8092251	NM_021629	GNB4	guanine nucleotide binding protei	0.498	1.412	2.84E-02	2.84E+00	Up	
8093294	NM_00112304	CCR2	chemokine (C-C motif) receptor 2	0.912	1.882	3.05E-03	3.05E-01	Up	
8094259	NM_015907	LAP3	leucine aminopeptidase 3	0.669	1.590	2.58E-02	2.58E+00	Up	
8094911	NM_020453	ATP10D	ATPase, class V, type 10D	0.362	1.285	1.67E-02	1.67E+00	Up	
8095343	NM_012108	STAP1	signal transducing adaptor family	0.498	1.412	1.46E-02	1.46E+00	Up	
8096617	NM_017935	BANK1	B-cell scaffold protein with ankyrin	0.449	1.365	2.84E-02	2.84E+00	Up	
8096733	NM_00113625	SGMS2	sphingomyelin synthase 2	0.446	1.362	2.02E-02	2.02E+00	Up	
8096917	---	---	---	0.347	1.272	4.55E-02	4.55E+00	Up	
8097586	NM_207123	GAB1	GRB2-associated binding protein 1	0.414	1.333	3.99E-02	3.99E+00	Up	
8100827	NM_144646	IGJ	immunoglobulin J polypeptide, lin	1.465	2.761	3.40E-04	3.40E-02	Up	
8101158	NM_005506	SCARB2	scavenger receptor class B, memb	1.035	2.050	1.92E-03	1.92E-01	Up	
8101304	NM_152545	RASGEF1B	RasGEF domain family, member 1B	0.742	1.673	2.42E-04	2.42E-02	Up	
8101322	AB014771	MOP-1	MOP-1	0.631	1.549	2.83E-02	2.83E+00	Up	
8102362	NM_052864	TIFA	TRAF-interacting protein with fork	0.460	1.375	9.74E-04	9.74E-02	Up	
8102720	NM_020337	ANKRD50	ankyrin repeat domain 50	0.494	1.408	2.58E-02	2.58E+00	Up	
8103399	NM_016205	PDGFC	platelet derived growth factor C	0.539	1.453	1.32E-02	1.32E+00	Up	
8103508	NM_017923	40238	membrane-associated ring finger (	0.652	1.572	7.31E-05	7.31E-03	Up	
8105340	NM_006144	GZMA	granzyme A (granzyme 1, cytotoxi	0.658	1.578	2.58E-02	2.58E+00	Up	
8105481	NM_152687	GAPT	GRB2-binding adaptor protein, tra	0.525	1.439	3.15E-02	3.15E+00	Up	
8105663	NM_020726	NLN	neurolysin (metallopeptidase M3 f	0.966	1.953	1.04E-04	1.04E-02	Up	
8107532	NM_000414	HSD17B4	hydroxysteroid (17-beta) dehydro	0.381	1.302	2.32E-02	2.32E+00	Up	
8107613	NM_003100	SNX2	sorting nexin 2	0.328	1.255	9.85E-04	9.85E-02	Up	
8108217	NM_000358	TGFBI	transforming growth factor, beta-i	1.530	2.888	3.63E-05	3.63E-03	Up	
8109350	NM_078483	SLC36A1	solute carrier family 36 (proton/ar	0.465	1.380	2.39E-02	2.39E+00	Up	
8112409	NM_019072	SGTB	small glutamine-rich tetratricopep	0.475	1.390	2.34E-03	2.34E-01	Up	
8114225	NM_004893	H2AFY	H2A histone family, member Y	0.354	1.278	2.49E-03	2.49E-01	Up	
8115076	NM_005211	CSF1R	colony stimulating factor 1 recepto	0.951	1.933	1.52E-03	1.52E-01	Up	
8115464	NM_032782	HAVCR2	hepatitis A virus cellular receptor 2	0.618	1.535	1.58E-02	1.58E+00	Up	
8117286	---	---	---	0.741	1.671	1.56E-02	1.56E+00	Up	
8118149	NM_007161	LST1	leukocyte specific transcript 1	0.527	1.441	2.38E-03	2.38E-01	Up	
8118324	NM_000063	C2	complement component 2	0.541	1.455	4.35E-03	4.35E-01	Up	
8118594	NM_002121	HLA-DPB1	major histocompatibility complex,	0.493	1.408	6.46E-03	6.46E-01	Up	
8123181	NM_000876	IGF2R	insulin-like growth factor 2 recepto	0.390	1.311	3.45E-02	3.45E+00	Up	
8123315	NM_206855	QKI	quaking homolog, KH domain RNA	0.342	1.268	4.27E-02	4.27E+00	Up	
8124134	NM_000367	TPMT	thiopurine S-methyltransferase	0.474	1.389	5.61E-03	5.61E-01	Up	
8128123	NM_021244	RRAGD	Ras-related GTP binding D	0.497	1.411	1.42E-02	1.42E+00	Up	
8129254	NM_005907	MAN1A1	mannosidase, alpha, class 1A, mer	0.338	1.264	3.27E-03	3.27E-01	Up	
8129458	NM_033515	ARHGAP18	Rho GTPase activating protein 18	1.047	2.067	1.14E-03	1.14E-01	Up	
8129677	NM_00114367	SGK1	serum/glucocorticoid regulated ki	0.575	1.490	2.38E-03	2.38E-01	Up	
8129974	NM_032020	FUCA2	fucosidase, alpha-L- 2, plasma	0.509	1.423	7.02E-03	7.02E-01	Up	
8131179	NM_025250	TTYH3	tweety homolog 3 (Drosophila)	0.356	1.280	7.92E-03	7.92E-01	Up	
8137264	NM_018487	TMEM176A	transmembrane protein 176A	0.632	1.550	2.62E-02	2.62E+00	Up	
8138489	NM_018719	CDCA7L	cell division cycle associated 7-like	0.400	1.320	2.46E-02	2.46E+00	Up	

8138689	NM_003930	SKAP2	src kinase associated phosphoprot	0.455	1.371	1.88E-02	1.88E+00	Up		
8139500	NM_022748	TNS3	tensin 3	0.628	1.545	3.25E-03	3.25E-01	Up		
8140367	NM_002991	CCL24	chemokine (C-C motif) ligand 24	1.495	2.818	1.03E-02	1.03E+00	Up		
8140463	NM_006682	FGL2	fibrinogen-like 2	2.098	4.281	5.48E-06	5.48E-04	Up		
8140814	NM_018843	SLC25A40	solute carrier family 25, member 4	0.362	1.286	1.18E-02	1.18E+00	Up		
8141094	NM_002612	PDK4	pyruvate dehydrogenase kinase, is	0.759	1.692	1.58E-02	1.58E+00	Up		
8141107	NM_00116021	SLC25A13	solute carrier family 25, member 1	0.399	1.318	6.55E-03	6.55E-01	Up		
8143790	NM_014020	TMEM176B	transmembrane protein 176B	0.840	1.790	5.85E-03	5.85E-01	Up		
8144917	NM_000237	LPL	lipoprotein lipase	0.793	1.733	3.63E-02	3.63E+00	Up		
8145293	NM_014265	ADAM28	ADAM metallopeptidase domain 2	0.592	1.507	7.80E-03	7.80E-01	Up		
8145470	NM_001386	DPYSL2	dihydropyrimidinase-like 2	0.531	1.445	1.53E-03	1.53E-01	Up		
8147132	NM_000067	CA2	carbonic anhydrase II	0.487	1.401	1.68E-02	1.68E+00	Up		
8147724	NM_001695	ATP6V1C1	ATPase, H+ transporting, lysosoma	0.364	1.287	2.55E-02	2.55E+00	Up		
8148194	---	---	---	0.401	1.321	2.75E-02	2.75E+00	Up		
8149534	NM_004315	ASAH1	N-acylsphingosine amidohydrolase	0.510	1.424	2.96E-02	2.96E+00	Up		
8152522	NM_006209	ENPP2	ectonucleotide pyrophosphatase/	1.860	3.629	6.04E-06	6.04E-04	Up		
8154207	---	---	---	0.594	1.510	3.68E-03	3.68E-01	Up		
8154209	---	---	---	0.472	1.387	1.92E-02	1.92E+00	Up		
8154245	NM_025239	PDCD1LG2	programmed cell death 1 ligand 2	0.729	1.657	4.51E-02	4.51E+00	Up		
8154951	NM_002065	GLUL	glutamate-ammonia ligase (glutan	0.995	1.993	1.37E-03	1.37E-01	Up		
8155192	NM_022343	GLIPR2	GLI pathogenesis-related 2	0.397	1.317	4.14E-02	4.14E+00	Up		
8155930	NM_001490	GCNT1	glucosaminyl (N-acetyl) transferas	0.364	1.287	3.33E-02	3.33E+00	Up		
8157270	NM_001859	SLC31A1	solute carrier family 31 (copper tra	0.803	1.745	1.16E-02	1.16E+00	Up		
8157761	NM_00114500	NEK6	NIMA (never in mitosis gene a)-rel	0.324	1.252	2.99E-03	2.99E-01	Up		
8161004	NM_001782	CD72	CD72 molecule	0.514	1.428	2.65E-03	2.65E-01	Up		
8162502	NM_000507	FBP1	fructose-1,6-bisphosphatase 1	1.006	2.008	7.75E-04	7.75E-02	Up		
8163452	NM_015258	FKBP15	FK506 binding protein 15, 133kDa	0.391	1.311	2.04E-02	2.04E+00	Up		
8165011	NM_002003	FCN1	ficolin (collagen/fibrinogen domai	0.658	1.578	1.21E-03	1.21E-01	Up		
8166805	NM_005765	ATP6AP2	ATPase, H+ transporting, lysosoma	0.357	1.281	3.68E-03	3.68E-01	Up		
8173162	---	---	---	0.525	1.439	1.89E-02	1.89E+00	Up		
8174051	NM_000061	BTK	Bruton agammaglobulinemia tyros	0.467	1.382	5.20E-03	5.20E-01	Up		
8176174	NM_002436	MPP1	membrane protein, palmitoylated	1.036	2.050	2.65E-03	2.65E-01	Up		
8177988	NM_007161	LST1	leukocyte specific transcript 1	0.527	1.441	2.38E-03	2.38E-01	Up		
8178095	NM_000063	C2	complement component 2	0.553	1.467	3.68E-03	3.68E-01	Up		
8178220	NM_002121	HLA-DPB1	major histocompatibility complex,	0.573	1.487	5.06E-03	5.06E-01	Up		
8179268	NM_007161	LST1	leukocyte specific transcript 1	0.527	1.441	2.38E-03	2.38E-01	Up		
8179331	NM_000063	C2	complement component 2	0.553	1.467	3.68E-03	3.68E-01	Up		
8179519	NM_002121	HLA-DPB1	major histocompatibility complex,	0.541	1.455	1.46E-02	1.46E+00	Up		
7895527	---	---	---	-0.381	0.768	2.62E-02	2.62E+00	Down		
7896695	---	---	---	-0.489	0.713	7.02E-03	7.02E-01	Down		
7896712	---	---	---	-0.607	0.657	2.84E-02	2.84E+00	Down		
7896721	---	---	---	-0.444	0.735	1.48E-02	1.48E+00	Down		
7898375	NR_004421	RNU1A	RNA, U1A small nuclear	-0.670	0.629	2.56E-04	2.56E-02	Down		
7898411	NR_004421	RNU1A	RNA, U1A small nuclear	-0.670	0.629	2.56E-04	2.56E-02	Down		
7899502	NR_004407	RNU11	RNA, U11 small nuclear	-0.413	0.751	1.75E-02	1.75E+00	Down		
7902227	NM_001924	GADD45A	growth arrest and DNA-damage-in	-0.359	0.780	1.14E-03	1.14E-01	Down		
7907092	NM_003953	MPZL1	myelin protein zero-like 1	-0.366	0.776	1.96E-03	1.96E-01	Down		
7908161	NM_030806	C1orf21	chromosome 1 open reading fram	-0.419	0.748	2.51E-02	2.51E+00	Down		
7908388	NM_002922	RGS1	regulator of G-protein signaling 1	-0.649	0.638	2.42E-04	2.42E-02	Down		
7908409	NM_002923	RGS2	regulator of G-protein signaling 2,	-0.357	0.781	2.03E-02	2.03E+00	Down		
7912800	NR_004421	RNU1A	RNA, U1A small nuclear	-0.670	0.629	2.56E-04	2.56E-02	Down		
7912850	NR_004421	RNU1A	RNA, U1A small nuclear	-0.670	0.629	2.56E-04	2.56E-02	Down		
7919151	---	---	---	-0.610	0.655	3.40E-04	3.40E-02	Down		
7919155	---	---	---	-0.476	0.719	2.84E-02	2.84E+00	Down		
7919269	NR_004421	RNU1A	RNA, U1A small nuclear	-0.675	0.627	5.39E-04	5.39E-02	Down		
7919349	NR_004421	RNU1A	RNA, U1A small nuclear	-0.675	0.627	5.39E-04	5.39E-02	Down		
7919392	---	---	---	-0.610	0.655	3.40E-04	3.40E-02	Down		
7919405	---	---	---	-0.476	0.719	2.84E-02	2.84E+00	Down		
7919556	---	---	---	-0.517	0.699	3.92E-02	3.92E+00	Down		
7919576	NR_004421	RNU1A	RNA, U1A small nuclear	-0.670	0.629	2.56E-04	2.56E-02	Down		
7920238	NM_005621	S100A12	S100 calcium binding protein A12	-0.931	0.524	3.63E-02	3.63E+00	Down		
7921228	NM_00114531	ETV3	ets variant 3	-0.347	0.786	3.68E-03	3.68E-01	Down		
7922418	NR_002579	SNORD74	small nucleolar RNA, C/D box 74	-0.563	0.677	3.68E-03	3.68E-01	Down		
7925929	NM_003739	AKR1C3	aldo-keto reductase family 1, men	-0.640	0.642	2.28E-03	2.28E-01	Down		
7926037	NM_004566	PFKFB3	6-phosphofructo-2-kinase/fructos	-0.618	0.652	2.97E-04	2.97E-02	Down		
7928308	NM_019058	DDIT4	DNA-damage-inducible transcript	-0.554	0.681	4.28E-04	4.28E-02	Down		
7928589	NM_005729	PPIF	peptidylprolyl isomerase F	-0.398	0.759	1.37E-03	1.37E-01	Down		
7931914	NM_000417	IL2RA	interleukin 2 receptor, alpha	-1.032	0.489	1.26E-03	1.26E-01	Down		
7933084	NM_005746	NAMPT	nicotinamide phosphoribosyltrans	-0.707	0.613	1.37E-03	1.37E-01	Down		
7938396	NM_000480	AMPD3	adenosine monophosphate deami	-0.361	0.778	2.99E-03	2.99E-01	Down		
7938485	NM_014632	MICAL2	microtubule associated monoxyge	-0.329	0.796	9.41E-03	9.41E-01	Down		
7951271	NM_002421	MMP1	matrix metallopeptidase 1 (interst	-1.742	0.299	1.20E-04	1.20E-02	Down		
7955963	NM_058173	MUCL1	mucin-like 1	-0.629	0.647	2.74E-03	2.74E-01	Down		
7961798	NM_152989	SOX5	SRY (sex determining region Y)-bo	-0.627	0.647	1.37E-03	1.37E-01	Down		
7970441	NM_004004	GJB2	gap junction protein, beta 2, 26kD	-0.474	0.720	5.72E-03	5.72E-01	Down		
7972557	NM_004951	GPR183	G protein-coupled receptor 183	-0.379	0.769	4.69E-04	4.69E-02	Down		
7973336	NM_004995	MMP14	matrix metallopeptidase 14 (mem	-0.784	0.581	1.06E-03	1.06E-01	Down		

7973896	NR_004421	RNU1A	RNA, U1A small nuclear	-0.663	0.632	2.56E-04	2.56E-02	Down	
7975076	NM_021979	HSPA2	heat shock 70kDa protein 2	-0.416	0.750	7.92E-03	7.92E-01	Down	
7978568	NR_004421	RNU1A	RNA, U1A small nuclear	-0.663	0.632	2.56E-04	2.56E-02	Down	
7979131	NM_053064	GNG2	guanine nucleotide binding protei	-0.396	0.760	5.00E-02	5.00E+00	Down	
7983490	NR_022014	C15orf21	Dresden prostate cancer 2	-0.707	0.613	3.48E-04	3.48E-02	Down	
7988426	NM_013309	SLC30A4	solute carrier family 30 (zinc trans	-0.552	0.682	5.52E-03	5.52E-01	Down	
7989501	NM_001218	CA12	carbonic anhydrase XII	-0.894	0.538	3.68E-03	3.68E-01	Down	
7993833	NM_016025	METTL9	methyltransferase like 9	-0.370	0.774	1.83E-03	1.83E-01	Down	
7995787	NM_176870	MT1M	metallothionein 1M	-0.679	0.625	2.04E-02	2.04E+00	Down	
7995793	NR_001447	MT1L	metallothionein 1L (gene/pseudog	-0.634	0.644	6.17E-03	6.17E-01	Down	
7995825	NM_005949	MT1F	metallothionein 1F	-0.400	0.758	4.45E-02	4.45E+00	Down	
7996837	NM_004360	CDH1	cadherin 1, type 1, E-cadherin (epi	-0.387	0.765	3.93E-04	3.93E-02	Down	
7999387	NM_001424	EMP2	epithelial membrane protein 2	-0.398	0.759	1.62E-03	1.62E-01	Down	
8002303	NM_000903	NQO1	NAD(P)H dehydrogenase, quinone	-0.782	0.582	1.48E-04	1.48E-02	Down	
8005475	NM_00103733	TRIM16L	tripartite motif-containing 16-like	-0.396	0.760	4.85E-03	4.85E-01	Down	
8007931	NM_000212	ITGB3	integrin, beta 3 (platelet glycoprot	-1.155	0.449	4.98E-05	4.98E-03	Down	
8016519	---	---	---	-0.332	0.794	1.18E-03	1.18E-01	Down	
8017096	---	---	---	-0.880	0.543	3.57E-03	3.57E-01	Down	
8017098	---	---	---	-0.875	0.545	2.27E-03	2.27E-01	Down	
8019796	NM_002359	MAFG	v-maf musculoaponeurotic fibrosa	-0.377	0.770	2.82E-03	2.82E-01	Down	
8020164	NM_182978	GNAL	guanine nucleotide binding protei	-0.330	0.796	2.38E-02	2.38E+00	Down	
8021635	NM_00114381	SERPINB2	serpin peptidase inhibitor, clade B	-1.471	0.361	7.43E-05	7.43E-03	Down	
8025058	NM_004240	TRIP10	thyroid hormone receptor interact	-0.374	0.772	1.09E-02	1.09E+00	Down	
8029693	NM_006732	FOSB	FBJ murine osteosarcoma viral onc	-0.423	0.746	1.52E-03	1.52E-01	Down	
8030128	NM_014330	PPP1R15A	protein phosphatase 1, regulatory	-0.507	0.704	5.70E-05	5.70E-03	Down	
8031374	NM_002000	FCAR	Fc fragment of IgA, receptor for	-0.551	0.682	1.40E-02	1.40E+00	Down	
8033257	NM_000064	C3	complement component 3	-0.838	0.560	2.53E-03	2.53E-01	Down	
8034851	NM_032571	EMR3	egf-like module containing, mucin	-0.816	0.568	1.06E-03	1.06E-01	Down	
8037374	NM_002659	PLAUR	plasminogen activator, urokinase	-0.713	0.610	1.44E-02	1.44E+00	Down	
8037387	---	---	---	-0.974	0.509	4.97E-02	4.97E+00	Down	
8045695	---	---	---	-0.537	0.689	3.21E-02	3.21E+00	Down	
8046861	NM_002210	ITGAV	integrin, alpha V (vitronectin recep	-0.443	0.736	4.01E-02	4.01E+00	Down	
8052872	NM_003236	TGFA	transforming growth factor, alpha	-0.340	0.790	6.17E-05	6.17E-03	Down	
8059578	---	---	---	-1.231	0.426	5.39E-03	5.39E-01	Down	
8065248	AK097497	LOC100130264	hypothetical LOC100130264	-0.424	0.745	2.12E-02	2.12E+00	Down	
8065353	NM_000361	THBD	thrombomodulin	-0.377	0.770	5.20E-04	5.20E-02	Down	
8065359	NM_012072	CD93	CD93 molecule	-0.931	0.525	3.35E-05	3.35E-03	Down	
8068353	NM_006933	SLC5A3	solute carrier family 5 (sodium/my	-0.344	0.788	5.48E-06	5.48E-04	Down	
8068361	NM_006933	SLC5A3	solute carrier family 5 (sodium/my	-0.456	0.729	5.48E-06	5.48E-04	Down	
8068593	NM_005239	ETS2	v-ets erythroblastosis virus E26 on	-0.463	0.725	1.61E-02	1.61E+00	Down	
8072229	NM_021076	NEFH	neurofilament, heavy polypeptide	-0.602	0.659	1.75E-04	1.75E-02	Down	
8083569	NM_015508	TIPARP	TCDD-inducible poly(ADP-ribose) p	-0.452	0.731	5.65E-04	5.65E-02	Down	
8091411	NM_014220	TM4SF1	transmembrane 4 L six family men	-0.839	0.559	5.48E-06	5.48E-04	Down	
8092348	NM_014398	LAMP3	lysosomal-associated membrane p	-0.475	0.719	3.68E-03	3.68E-01	Down	
8092849	NM_024524	ATP13A3	ATPase type 13A3	-0.459	0.728	1.23E-02	1.23E+00	Down	
8095728	NM_001432	EREG	epiregulin	-1.621	0.325	2.95E-05	2.95E-03	Down	
8097256	NM_002006	FGF2	fibroblast growth factor 2 (basic)	-0.339	0.791	1.02E-04	1.02E-02	Down	
8102787	---	---	---	-0.637	0.643	8.16E-04	8.16E-02	Down	
8105229	NM_015946	PELO	pelota homolog (Drosophila)	-0.374	0.771	5.51E-03	5.51E-01	Down	
8106818	---	---	---	-0.433	0.741	1.75E-02	1.75E+00	Down	
8113433	NM_001962	EFNA5	ephrin-A5	-0.341	0.789	1.87E-02	1.87E+00	Down	
8114572	NM_001945	HBEGF	heparin-binding EGF-like growth fa	-1.059	0.480	2.00E-04	2.00E-02	Down	
8115490	NM_033274	ADAM19	ADAM metallopeptidase domain 1	-0.364	0.777	1.91E-02	1.91E+00	Down	
8115831	NM_004417	DUSP1	dual specificity phosphatase 1	-0.542	0.687	9.92E-05	9.92E-03	Down	
8116952	---	---	---	-0.443	0.736	3.41E-02	3.41E+00	Down	
8118314	NM_005346	HSPA1B	heat shock 70kDa protein 1B	-0.331	0.795	4.64E-03	4.64E-01	Down	
8119088	NM_078467	CDKN1A	cyclin-dependent kinase inhibitor	-0.339	0.791	1.37E-03	1.37E-01	Down	
8124848	NM_003897	IER3	immediate early response 3	-0.327	0.797	2.02E-03	2.02E-01	Down	
8130556	NM_00102446	SOD2	superoxide dismutase 2, mitochon	-0.369	0.774	4.60E-02	4.60E+00	Down	
8130628	NM_020133	AGPAT4	1-acylglycerol-3-phosphate O-acyl	-0.399	0.759	1.51E-03	1.51E-01	Down	
8130641	NR_024277	C6orf59	chromosome 6 open reading fram	-0.396	0.760	1.72E-02	1.72E+00	Down	
8131666	NM_002214	ITGB8	integrin, beta 8	-1.715	0.305	2.29E-05	2.29E-03	Down	
8132725	NM_003364	UPP1	uridine phosphorylase 1	-0.403	0.756	1.68E-03	1.68E-01	Down	
8134257	NM_004126	GNG11	guanine nucleotide binding protei	-0.675	0.626	4.72E-04	4.72E-02	Down	
8135943	---	---	---	-0.666	0.630	7.00E-03	7.00E-01	Down	
8142120	NM_005746	NAMPT	nicotinamide phosphoribosyltrans	-0.671	0.628	9.99E-04	9.99E-02	Down	
8145281	NM_016612	SLC25A37	solute carrier family 25, member 3	-1.355	0.391	7.82E-06	7.82E-04	Down	
8145291	AF495725	SLC25A37	solute carrier family 25, member 3	-1.368	0.388	4.30E-05	4.30E-03	Down	
8147503	NM_018407	LAPTM4B	lysosomal protein transmembrane	-0.323	0.799	3.99E-02	3.99E+00	Down	
8151334	NM_005098	MSC	musculin (activated B-cell factor-1	-0.443	0.735	2.83E-02	2.83E+00	Down	
8151457	NM_012258	HEY1	hairy/enhancer-of-split related wi	-0.323	0.800	2.46E-02	2.46E+00	Down	
8153002	NM_00113524	NDRG1	N-myc downstream regulated 1	-0.479	0.718	3.40E-04	3.40E-02	Down	
8156848	NM_173198	NR4A3	nuclear receptor subfamily 4, grou	-0.362	0.778	3.60E-03	3.60E-01	Down	
8156897	BC008993	C9orf30	chromosome 9 open reading fram	-0.491	0.711	3.15E-04	3.15E-02	Down	
8159900	NM_152629	GLIS3	GLIS family zinc finger 3	-0.501	0.707	3.42E-03	3.42E-01	Down	
8161526	AK127288	LOC440896	hypothetical LOC440896	-0.401	0.757	1.61E-02	1.61E+00	Down	
8163185	NM_003329	TXN	thioredoxin	-0.360	0.779	1.65E-02	1.65E+00	Down	

8167449	NM_002668	PLP2	proteolipid protein 2 (colonic epitl	-0.408	0.753	3.13E-03	3.13E-01	Down	
8169634	---	---	---	-0.580	0.669	6.70E-04	6.70E-02	Down	
8178086	NM_005346	HSPA1B	heat shock 70kDa protein 1B	-0.335	0.793	3.68E-03	3.68E-01	Down	
8178435	NM_003897	IER3	immediate early response 3	-0.354	0.782	2.23E-03	2.23E-01	Down	
8179324	NM_005346	HSPA1B	heat shock 70kDa protein 1B	-0.335	0.793	3.68E-03	3.68E-01	Down	
8179704	NM_003897	IER3	immediate early response 3	-0.327	0.797	2.02E-03	2.02E-01	Down	
8180376	---	---	---	-0.894	0.538	3.48E-04	3.48E-02	Down	
7892501	---	---	---	-0.184	0.880	7.48E-01	7.48E+01		
7892504	---	---	---	-0.068	0.954	7.56E-01	7.56E+01		
7892507	---	---	---	0.018	1.013	9.77E-01	9.77E+01		
7892509	---	---	---	-0.022	0.985	9.53E-01	9.53E+01		
7892512	---	---	---	-0.032	0.978	9.55E-01	9.55E+01		
7892514	---	---	---	0.041	1.029	8.91E-01	8.91E+01		
7892515	---	---	---	0.085	1.061	5.31E-01	5.31E+01		
7892516	---	---	---	-0.127	0.915	5.49E-01	5.49E+01		
7892517	---	---	---	-0.275	0.827	6.56E-01	6.56E+01		
7892519	---	---	---	-0.108	0.928	6.70E-01	6.70E+01		
7892520	---	---	---	0.032	1.022	8.47E-01	8.47E+01		
7892521	---	---	---	-0.195	0.874	6.94E-01	6.94E+01		
7892522	---	---	---	0.086	1.061	7.75E-01	7.75E+01		
7892524	---	---	---	0.041	1.029	9.44E-01	9.44E+01		
7892525	---	---	---	0.172	1.127	3.50E-01	3.50E+01		
7892526	---	---	---	0.061	1.043	9.35E-01	9.35E+01		
7892527	---	---	---	0.035	1.025	9.36E-01	9.36E+01		
7892529	---	---	---	0.148	1.108	4.68E-01	4.68E+01		
7892530	---	---	---	0.002	1.001	9.91E-01	9.91E+01		
7892531	---	---	---	0.403	1.322	2.36E-01	2.36E+01		
7892533	---	---	---	0.019	1.013	9.23E-01	9.23E+01		
7892535	---	---	---	0.251	1.190	7.09E-01	7.09E+01		
7892536	---	---	---	0.051	1.036	9.10E-01	9.10E+01		
7892538	---	---	---	-0.211	0.864	4.15E-01	4.15E+01		
7892539	---	---	---	-0.177	0.885	6.16E-01	6.16E+01		
7892540	---	---	---	0.175	1.129	6.91E-01	6.91E+01		
7892541	---	---	---	-0.066	0.955	8.06E-01	8.06E+01		
7892542	---	---	---	-0.052	0.964	9.17E-01	9.17E+01		
7892543	---	---	---	-0.082	0.945	8.31E-01	8.31E+01		
7892544	---	---	---	0.234	1.176	4.12E-01	4.12E+01		
7892545	---	---	---	0.100	1.072	7.61E-01	7.61E+01		
7892547	---	---	---	0.047	1.033	9.44E-01	9.44E+01		
7892548	---	---	---	-0.043	0.970	9.32E-01	9.32E+01		
7892549	---	---	---	0.029	1.020	9.38E-01	9.38E+01		
7892550	---	---	---	-0.024	0.984	9.51E-01	9.51E+01		
7892551	---	---	---	-0.026	0.982	8.81E-01	8.81E+01		
7892553	---	---	---	0.128	1.093	7.09E-01	7.09E+01		
7892554	---	---	---	0.215	1.161	3.87E-01	3.87E+01		
7892555	---	---	---	0.069	1.049	8.04E-01	8.04E+01		
7892556	---	---	---	-0.016	0.989	8.64E-01	8.64E+01		
7892559	---	---	---	-0.103	0.931	5.91E-01	5.91E+01		
7892560	---	---	---	0.074	1.053	9.23E-01	9.23E+01		
7892561	---	---	---	0.121	1.087	4.23E-01	4.23E+01		
7892562	---	---	---	0.009	1.006	9.74E-01	9.74E+01		
7892563	---	---	---	-0.121	0.920	5.55E-01	5.55E+01		
7892564	---	---	---	0.018	1.013	9.72E-01	9.72E+01		
7892565	---	---	---	-0.056	0.962	8.20E-01	8.20E+01		
7892567	---	---	---	0.117	1.084	7.48E-01	7.48E+01		
7892568	---	---	---	-0.031	0.979	8.73E-01	8.73E+01		
7892569	---	---	---	-0.306	0.809	1.48E-01	1.48E+01		
7892570	---	---	---	-0.111	0.926	8.50E-01	8.50E+01		
7892572	---	---	---	0.024	1.017	9.85E-01	9.85E+01		
7892573	---	---	---	0.122	1.088	7.17E-01	7.17E+01		
7892574	---	---	---	0.167	1.122	5.79E-01	5.79E+01		
7892575	---	---	---	-0.009	0.994	9.79E-01	9.79E+01		
7892576	---	---	---	-0.002	0.999	9.96E-01	9.96E+01		
7892578	---	---	---	0.121	1.087	7.51E-01	7.51E+01		
7892579	---	---	---	-0.012	0.992	9.73E-01	9.73E+01		
7892583	---	---	---	-0.133	0.912	7.51E-01	7.51E+01		
7892584	---	---	---	-0.135	0.911	4.81E-01	4.81E+01		
7892587	---	---	---	-0.123	0.918	7.38E-01	7.38E+01		
7892589	---	---	---	0.065	1.046	9.53E-01	9.53E+01		
7892592	---	---	---	-0.038	0.974	9.43E-01	9.43E+01		
7892594	---	---	---	0.034	1.024	9.64E-01	9.64E+01		
7892596	---	---	---	-0.033	0.978	9.66E-01	9.66E+01		
7892597	---	---	---	-0.114	0.924	7.92E-01	7.92E+01		
7892598	---	---	---	0.214	1.160	5.09E-01	5.09E+01		
7892599	---	---	---	-0.160	0.895	5.78E-01	5.78E+01		
7892604	---	---	---	0.215	1.161	6.92E-01	6.92E+01		
7892605	---	---	---	0.036	1.025	9.53E-01	9.53E+01		

7892606	---	---	---	0.018	1.013	8.84E-01	8.84E+01		
7892608	---	---	---	0.073	1.052	5.72E-01	5.72E+01		
7892610	---	---	---	-0.064	0.957	8.48E-01	8.48E+01		
7892613	---	---	---	0.056	1.040	7.50E-01	7.50E+01		
7892615	---	---	---	0.230	1.173	5.03E-01	5.03E+01		
7892617	---	---	---	0.025	1.018	7.91E-01	7.91E+01		
7892618	---	---	---	0.277	1.212	3.14E-01	3.14E+01		
7892619	---	---	---	0.208	1.155	7.63E-01	7.63E+01		
7892620	---	---	---	-0.047	0.968	9.09E-01	9.09E+01		
7892621	---	---	---	0.045	1.032	7.68E-01	7.68E+01		
7892622	---	---	---	0.002	1.001	9.98E-01	9.98E+01		
7892623	---	---	---	-0.073	0.950	8.14E-01	8.14E+01		
7892625	---	---	---	0.046	1.032	8.35E-01	8.35E+01		
7892627	---	---	---	-0.133	0.912	3.58E-01	3.58E+01		
7892628	---	---	---	-0.207	0.866	5.72E-01	5.72E+01		
7892629	---	---	---	-0.059	0.960	9.50E-01	9.50E+01		
7892630	---	---	---	0.134	1.098	5.15E-01	5.15E+01		
7892633	---	---	---	0.193	1.144	4.89E-01	4.89E+01		
7892634	---	---	---	0.010	1.007	9.67E-01	9.67E+01		
7892635	---	---	---	-0.174	0.886	3.72E-01	3.72E+01		
7892636	---	---	---	0.093	1.067	5.05E-01	5.05E+01		
7892637	---	---	---	-0.140	0.908	7.27E-01	7.27E+01		
7892639	---	---	---	0.148	1.108	7.91E-01	7.91E+01		
7892640	---	---	---	-0.110	0.927	5.15E-01	5.15E+01		
7892641	---	---	---	0.150	1.109	7.94E-01	7.94E+01		
7892642	---	---	---	-0.004	0.997	9.87E-01	9.87E+01		
7892643	---	---	---	0.028	1.019	9.20E-01	9.20E+01		
7892644	---	---	---	-0.009	0.994	9.81E-01	9.81E+01		
7892647	---	---	---	0.054	1.038	9.30E-01	9.30E+01		
7892648	---	---	---	-0.072	0.951	6.91E-01	6.91E+01		
7892650	---	---	---	-0.043	0.970	7.95E-01	7.95E+01		
7892652	---	---	---	-0.072	0.951	8.36E-01	8.36E+01		
7892654	---	---	---	0.029	1.021	8.67E-01	8.67E+01		
7892656	---	---	---	-0.293	0.816	5.18E-01	5.18E+01		
7892661	---	---	---	0.043	1.031	9.30E-01	9.30E+01		
7892662	---	---	---	-0.274	0.827	7.24E-02	7.24E+00		
7892663	---	---	---	0.065	1.046	8.34E-01	8.34E+01		
7892665	---	---	---	-0.155	0.898	4.59E-01	4.59E+01		
7892667	---	---	---	-0.049	0.967	8.04E-01	8.04E+01		
7892668	---	---	---	0.178	1.132	3.49E-01	3.49E+01		
7892669	---	---	---	-0.004	0.997	9.90E-01	9.90E+01		
7892670	---	---	---	0.068	1.048	7.95E-01	7.95E+01		
7892672	---	---	---	0.229	1.172	5.04E-01	5.04E+01		
7892673	---	---	---	-0.003	0.998	9.91E-01	9.91E+01		
7892674	---	---	---	-0.114	0.924	7.98E-01	7.98E+01		
7892675	---	---	---	0.232	1.175	3.86E-01	3.86E+01		
7892676	---	---	---	0.150	1.110	7.34E-01	7.34E+01		
7892677	---	---	---	-0.026	0.982	8.50E-01	8.50E+01		
7892678	---	---	---	0.059	1.042	9.23E-01	9.23E+01		
7892680	---	---	---	0.009	1.006	9.76E-01	9.76E+01		
7892681	---	---	---	-0.183	0.881	1.68E-01	1.68E+01		
7892682	---	---	---	0.007	1.005	9.89E-01	9.89E+01		
7892683	---	---	---	0.026	1.018	9.82E-01	9.82E+01		
7892684	---	---	---	0.137	1.100	5.61E-01	5.61E+01		
7892686	---	---	---	-0.018	0.988	9.25E-01	9.25E+01		
7892688	---	---	---	-0.121	0.920	8.35E-01	8.35E+01		
7892689	---	---	---	0.015	1.010	9.79E-01	9.79E+01		
7892692	---	---	---	-0.036	0.975	9.16E-01	9.16E+01		
7892693	---	---	---	-0.096	0.936	7.27E-01	7.27E+01		
7892694	---	---	---	-0.130	0.914	5.55E-01	5.55E+01		
7892695	---	---	---	0.028	1.020	9.49E-01	9.49E+01		
7892696	---	---	---	-0.049	0.967	8.93E-01	8.93E+01		
7892697	---	---	---	0.088	1.063	6.14E-01	6.14E+01		
7892699	---	---	---	-0.032	0.978	8.92E-01	8.92E+01		
7892701	---	---	---	0.087	1.062	5.52E-01	5.52E+01		
7892702	---	---	---	0.109	1.078	8.64E-01	8.64E+01		
7892703	---	---	---	0.023	1.016	9.34E-01	9.34E+01		
7892704	---	---	---	0.045	1.032	8.52E-01	8.52E+01		
7892706	---	---	---	0.042	1.030	9.73E-01	9.73E+01		
7892707	---	---	---	-0.002	0.999	9.92E-01	9.92E+01		
7892711	---	---	---	-0.097	0.935	9.23E-01	9.23E+01		
7892712	---	---	---	0.055	1.039	7.89E-01	7.89E+01		
7892713	---	---	---	-0.021	0.985	9.20E-01	9.20E+01		
7892719	---	---	---	0.066	1.047	6.89E-01	6.89E+01		
7892720	---	---	---	-0.043	0.970	7.97E-01	7.97E+01		
7892721	---	---	---	0.008	1.005	9.75E-01	9.75E+01		
7892726	---	---	---	0.000	1.000	1.00E+00	1.00E+02		

7892729	---	---	---	-0.038	0.974	9.60E-01	9.60E+01		
7892732	---	---	---	-0.035	0.976	9.77E-01	9.77E+01		
7892733	---	---	---	0.041	1.029	9.56E-01	9.56E+01		
7892735	---	---	---	-0.039	0.973	9.50E-01	9.50E+01		
7892736	---	---	---	-0.102	0.932	7.38E-01	7.38E+01		
7892738	---	---	---	-0.004	0.997	9.90E-01	9.90E+01		
7892740	---	---	---	-0.078	0.947	9.43E-01	9.43E+01		
7892742	---	---	---	0.091	1.065	6.66E-01	6.66E+01		
7892744	---	---	---	-0.141	0.907	7.75E-01	7.75E+01		
7892745	---	---	---	-0.125	0.917	4.75E-01	4.75E+01		
7892747	---	---	---	-0.140	0.907	3.95E-01	3.95E+01		
7892748	---	---	---	0.013	1.009	9.51E-01	9.51E+01		
7892749	---	---	---	0.200	1.148	6.97E-01	6.97E+01		
7892750	---	---	---	-0.014	0.991	9.83E-01	9.83E+01		
7892751	---	---	---	-0.010	0.993	9.66E-01	9.66E+01		
7892753	---	---	---	0.032	1.022	7.75E-01	7.75E+01		
7892754	---	---	---	0.001	1.000	1.00E+00	1.00E+02		
7892757	---	---	---	0.055	1.039	9.22E-01	9.22E+01		
7892758	---	---	---	-0.205	0.867	7.17E-01	7.17E+01		
7892759	---	---	---	0.152	1.111	5.62E-01	5.62E+01		
7892760	---	---	---	0.021	1.015	9.67E-01	9.67E+01		
7892761	---	---	---	0.214	1.160	3.04E-01	3.04E+01		
7892762	---	---	---	0.088	1.063	4.59E-01	4.59E+01		
7892763	---	---	---	0.052	1.037	8.41E-01	8.41E+01		
7892764	---	---	---	-0.167	0.891	3.99E-01	3.99E+01		
7892765	---	---	---	0.086	1.062	6.42E-01	6.42E+01		
7892766	---	---	---	0.109	1.079	6.05E-01	6.05E+01		
7892769	---	---	---	0.625	1.543	1.98E-01	1.98E+01		
7892770	---	---	---	-0.031	0.978	9.30E-01	9.30E+01		
7892771	---	---	---	0.081	1.058	8.67E-01	8.67E+01		
7892773	---	---	---	0.028	1.019	9.32E-01	9.32E+01		
7892774	---	---	---	0.070	1.050	8.78E-01	8.78E+01		
7892775	---	---	---	0.028	1.019	9.74E-01	9.74E+01		
7892776	---	---	---	-0.018	0.988	9.37E-01	9.37E+01		
7892777	---	---	---	0.021	1.015	9.72E-01	9.72E+01		
7892778	---	---	---	-0.067	0.955	7.94E-01	7.94E+01		
7892780	---	---	---	0.130	1.094	5.91E-01	5.91E+01		
7892781	---	---	---	0.144	1.105	7.43E-01	7.43E+01		
7892782	---	---	---	0.011	1.008	9.49E-01	9.49E+01		
7892783	---	---	---	0.082	1.059	4.43E-01	4.43E+01		
7892784	---	---	---	-0.207	0.866	7.69E-01	7.69E+01		
7892785	---	---	---	-0.060	0.959	9.24E-01	9.24E+01		
7892786	---	---	---	-0.011	0.993	9.86E-01	9.86E+01		
7892787	---	---	---	-0.263	0.833	8.25E-01	8.25E+01		
7892788	---	---	---	-0.017	0.988	9.55E-01	9.55E+01		
7892789	---	---	---	-0.058	0.960	9.51E-01	9.51E+01		
7892791	---	---	---	0.026	1.018	8.57E-01	8.57E+01		
7892792	---	---	---	0.082	1.058	7.92E-01	7.92E+01		
7892793	---	---	---	0.163	1.120	5.26E-01	5.26E+01		
7892794	---	---	---	0.235	1.177	5.89E-01	5.89E+01		
7892795	---	---	---	0.080	1.057	7.37E-01	7.37E+01		
7892796	---	---	---	0.009	1.006	9.54E-01	9.54E+01		
7892797	---	---	---	0.003	1.002	9.91E-01	9.91E+01		
7892802	---	---	---	0.199	1.148	6.76E-01	6.76E+01		
7892803	---	---	---	-0.017	0.988	9.07E-01	9.07E+01		
7892807	---	---	---	0.066	1.047	8.91E-01	8.91E+01		
7892808	---	---	---	-0.033	0.977	9.23E-01	9.23E+01		
7892812	---	---	---	-0.013	0.991	9.84E-01	9.84E+01		
7892813	---	---	---	0.173	1.128	8.07E-01	8.07E+01		
7892814	---	---	---	0.038	1.026	8.06E-01	8.06E+01		
7892816	---	---	---	0.048	1.034	7.62E-01	7.62E+01		
7892817	---	---	---	0.073	1.052	6.39E-01	6.39E+01		
7892819	---	---	---	0.597	1.513	1.58E-01	1.58E+01		
7892820	---	---	---	-0.004	0.997	9.81E-01	9.81E+01		
7892823	---	---	---	0.052	1.037	9.03E-01	9.03E+01		
7892824	---	---	---	0.006	1.004	9.77E-01	9.77E+01		
7892825	---	---	---	0.033	1.023	9.15E-01	9.15E+01		
7892826	---	---	---	0.086	1.061	8.85E-01	8.85E+01		
7892827	---	---	---	0.023	1.016	8.61E-01	8.61E+01		
7892829	---	---	---	0.024	1.017	8.78E-01	8.78E+01		
7892830	---	---	---	0.131	1.095	5.36E-01	5.36E+01		
7892831	---	---	---	-0.013	0.991	9.68E-01	9.68E+01		
7892832	---	---	---	-0.059	0.960	8.25E-01	8.25E+01		
7892835	---	---	---	-0.108	0.928	6.47E-01	6.47E+01		
7892836	---	---	---	0.111	1.080	2.82E-01	2.82E+01		
7892837	---	---	---	-0.085	0.943	8.41E-01	8.41E+01		
7892838	---	---	---	0.018	1.012	9.50E-01	9.50E+01		

7892840	---	---	---	0.215	1.160	6.44E-01	6.44E+01		
7892841	---	---	---	-0.095	0.936	7.03E-01	7.03E+01		
7892844	---	---	---	0.021	1.014	9.66E-01	9.66E+01		
7892845	---	---	---	0.016	1.011	9.57E-01	9.57E+01		
7892846	---	---	---	0.029	1.020	7.87E-01	7.87E+01		
7892847	---	---	---	-0.085	0.943	7.69E-01	7.69E+01		
7892848	---	---	---	-0.140	0.907	4.29E-01	4.29E+01		
7892850	---	---	---	0.138	1.101	5.87E-01	5.87E+01		
7892851	---	---	---	-0.174	0.886	5.52E-01	5.52E+01		
7892852	---	---	---	-0.080	0.946	7.52E-01	7.52E+01		
7892853	---	---	---	-0.010	0.993	9.27E-01	9.27E+01		
7892855	---	---	---	0.093	1.067	8.32E-01	8.32E+01		
7892856	---	---	---	-0.288	0.819	5.29E-01	5.29E+01		
7892857	---	---	---	0.003	1.002	9.91E-01	9.91E+01		
7892859	---	---	---	-0.133	0.912	4.97E-01	4.97E+01		
7892860	---	---	---	-0.058	0.961	8.04E-01	8.04E+01		
7892861	---	---	---	0.109	1.079	4.37E-01	4.37E+01		
7892862	---	---	---	0.089	1.063	4.62E-01	4.62E+01		
7892863	---	---	---	0.055	1.039	8.36E-01	8.36E+01		
7892864	---	---	---	0.219	1.164	4.70E-01	4.70E+01		
7892865	---	---	---	0.090	1.064	4.81E-01	4.81E+01		
7892868	---	---	---	0.051	1.036	8.45E-01	8.45E+01		
7892869	---	---	---	-0.145	0.905	5.46E-01	5.46E+01		
7892870	---	---	---	-0.052	0.965	9.00E-01	9.00E+01		
7892871	---	---	---	0.076	1.054	8.33E-01	8.33E+01		
7892872	---	---	---	-0.157	0.897	6.39E-01	6.39E+01		
7892873	---	---	---	0.085	1.061	9.49E-01	9.49E+01		
7892875	---	---	---	-0.163	0.893	5.36E-01	5.36E+01		
7892876	---	---	---	-0.025	0.982	9.53E-01	9.53E+01		
7892878	---	---	---	0.272	1.208	5.64E-01	5.64E+01		
7892880	---	---	---	0.172	1.127	2.63E-01	2.63E+01		
7892882	---	---	---	-0.099	0.933	6.40E-01	6.40E+01		
7892884	---	---	---	-0.113	0.925	5.15E-01	5.15E+01		
7892886	---	---	---	0.103	1.074	8.23E-01	8.23E+01		
7892889	---	---	---	0.067	1.048	5.53E-01	5.53E+01		
7892890	---	---	---	-0.098	0.934	5.28E-01	5.28E+01		
7892891	---	---	---	0.012	1.008	9.62E-01	9.62E+01		
7892893	---	---	---	-0.092	0.938	5.11E-01	5.11E+01		
7892894	---	---	---	0.041	1.029	9.05E-01	9.05E+01		
7892896	---	---	---	-0.033	0.977	8.63E-01	8.63E+01		
7892897	---	---	---	0.026	1.018	9.49E-01	9.49E+01		
7892898	---	---	---	-0.067	0.955	7.12E-01	7.12E+01		
7892899	---	---	---	0.009	1.006	9.67E-01	9.67E+01		
7892900	---	---	---	0.038	1.027	8.27E-01	8.27E+01		
7892901	---	---	---	0.095	1.068	6.28E-01	6.28E+01		
7892902	---	---	---	-0.017	0.988	8.84E-01	8.84E+01		
7892903	---	---	---	-0.107	0.928	7.91E-01	7.91E+01		
7892905	---	---	---	-0.312	0.806	5.52E-01	5.52E+01		
7892906	---	---	---	-0.064	0.957	7.15E-01	7.15E+01		
7892907	---	---	---	0.007	1.005	9.75E-01	9.75E+01		
7892908	---	---	---	-0.157	0.897	6.57E-01	6.57E+01		
7892909	---	---	---	-0.093	0.938	7.55E-01	7.55E+01		
7892910	---	---	---	0.320	1.248	3.44E-01	3.44E+01		
7892911	---	---	---	0.078	1.056	7.50E-01	7.50E+01		
7892913	---	---	---	0.192	1.143	5.98E-01	5.98E+01		
7892915	---	---	---	0.177	1.131	6.59E-01	6.59E+01		
7892916	---	---	---	-0.054	0.963	6.47E-01	6.47E+01		
7892918	---	---	---	0.067	1.047	8.88E-01	8.88E+01		
7892919	---	---	---	-0.139	0.908	6.11E-01	6.11E+01		
7892920	---	---	---	0.145	1.106	5.59E-01	5.59E+01		
7892922	---	---	---	0.081	1.057	7.98E-01	7.98E+01		
7892924	---	---	---	-0.014	0.990	9.55E-01	9.55E+01		
7892925	---	---	---	0.056	1.039	8.05E-01	8.05E+01		
7892926	---	---	---	0.061	1.043	7.41E-01	7.41E+01		
7892928	---	---	---	0.051	1.036	6.06E-01	6.06E+01		
7892930	---	---	---	0.025	1.017	8.91E-01	8.91E+01		
7892931	---	---	---	-0.009	0.994	9.86E-01	9.86E+01		
7892932	---	---	---	-0.059	0.960	8.42E-01	8.42E+01		
7892933	---	---	---	-0.125	0.917	6.91E-01	6.91E+01		
7892934	---	---	---	0.002	1.002	9.97E-01	9.97E+01		
7892935	---	---	---	0.016	1.011	9.54E-01	9.54E+01		
7892936	---	---	---	-0.140	0.907	8.69E-01	8.69E+01		
7892937	---	---	---	0.105	1.075	6.66E-01	6.66E+01		
7892938	---	---	---	-0.093	0.938	7.92E-01	7.92E+01		
7892939	---	---	---	0.121	1.087	5.97E-01	5.97E+01		
7892940	---	---	---	0.008	1.006	9.69E-01	9.69E+01		
7892941	---	---	---	-0.003	0.998	9.90E-01	9.90E+01		

7892944	---	---	---	-0.183	0.881	4.47E-01	4.47E+01		
7892947	---	---	---	0.022	1.015	9.53E-01	9.53E+01		
7892948	---	---	---	0.023	1.016	9.09E-01	9.09E+01		
7892949	---	---	---	-0.040	0.973	9.60E-01	9.60E+01		
7892951	---	---	---	0.021	1.015	9.55E-01	9.55E+01		
7892952	---	---	---	0.091	1.065	8.78E-01	8.78E+01		
7892953	---	---	---	0.065	1.046	7.47E-01	7.47E+01		
7892954	---	---	---	0.089	1.063	7.33E-01	7.33E+01		
7892955	---	---	---	0.015	1.011	9.50E-01	9.50E+01		
7892956	---	---	---	0.027	1.019	8.13E-01	8.13E+01		
7892957	---	---	---	-0.024	0.984	9.56E-01	9.56E+01		
7892958	---	---	---	0.128	1.093	8.45E-01	8.45E+01		
7892959	---	---	---	-0.058	0.961	7.94E-01	7.94E+01		
7892960	---	---	---	0.063	1.045	5.71E-01	5.71E+01		
7892961	---	---	---	-0.045	0.969	8.78E-01	8.78E+01		
7892962	---	---	---	0.445	1.361	4.86E-01	4.86E+01		
7892966	---	---	---	0.305	1.235	7.17E-02	7.17E+00		
7892967	---	---	---	-0.233	0.851	5.15E-01	5.15E+01		
7892968	---	---	---	-0.128	0.915	5.71E-01	5.71E+01		
7892969	---	---	---	-0.002	0.998	9.90E-01	9.90E+01		
7892972	---	---	---	0.294	1.226	3.04E-01	3.04E+01		
7892973	---	---	---	0.101	1.072	8.71E-01	8.71E+01		
7892977	---	---	---	-0.061	0.958	6.05E-01	6.05E+01		
7892978	---	---	---	-0.052	0.965	9.18E-01	9.18E+01		
7892979	---	---	---	0.003	1.002	9.87E-01	9.87E+01		
7892981	---	---	---	0.087	1.062	8.82E-01	8.82E+01		
7892982	---	---	---	0.125	1.090	9.18E-01	9.18E+01		
7892983	---	---	---	-0.004	0.997	9.90E-01	9.90E+01		
7892984	---	---	---	-0.194	0.874	6.08E-01	6.08E+01		
7892985	---	---	---	0.149	1.109	6.20E-01	6.20E+01		
7892986	---	---	---	0.055	1.039	6.83E-01	6.83E+01		
7892987	---	---	---	0.079	1.057	4.10E-01	4.10E+01		
7892989	---	---	---	-0.074	0.950	8.27E-01	8.27E+01		
7892990	---	---	---	0.176	1.129	8.16E-01	8.16E+01		
7892991	---	---	---	-0.005	0.997	9.85E-01	9.85E+01		
7892992	---	---	---	0.024	1.017	8.55E-01	8.55E+01		
7892993	---	---	---	0.028	1.020	8.84E-01	8.84E+01		
7892996	---	---	---	0.096	1.069	6.18E-01	6.18E+01		
7892998	---	---	---	0.089	1.064	8.45E-01	8.45E+01		
7893000	---	---	---	0.076	1.054	8.59E-01	8.59E+01		
7893001	---	---	---	-0.091	0.939	7.09E-01	7.09E+01		
7893002	---	---	---	-0.032	0.978	8.39E-01	8.39E+01		
7893003	---	---	---	-0.096	0.935	6.78E-01	6.78E+01		
7893004	---	---	---	0.081	1.058	8.71E-01	8.71E+01		
7893005	---	---	---	-0.081	0.946	9.14E-01	9.14E+01		
7893007	---	---	---	-0.043	0.971	7.30E-01	7.30E+01		
7893010	---	---	---	-0.037	0.975	8.93E-01	8.93E+01		
7893012	---	---	---	0.003	1.002	9.88E-01	9.88E+01		
7893013	---	---	---	0.111	1.080	5.46E-01	5.46E+01		
7893014	---	---	---	0.155	1.114	7.45E-01	7.45E+01		
7893015	---	---	---	-0.042	0.971	8.33E-01	8.33E+01		
7893017	---	---	---	0.151	1.110	5.05E-01	5.05E+01		
7893018	---	---	---	0.069	1.049	9.38E-01	9.38E+01		
7893019	---	---	---	-0.040	0.973	9.52E-01	9.52E+01		
7893023	---	---	---	0.025	1.018	9.49E-01	9.49E+01		
7893025	---	---	---	0.060	1.042	8.13E-01	8.13E+01		
7893026	---	---	---	-0.068	0.954	4.25E-01	4.25E+01		
7893027	---	---	---	0.031	1.022	9.61E-01	9.61E+01		
7893028	---	---	---	0.152	1.111	7.48E-01	7.48E+01		
7893029	---	---	---	-0.105	0.930	5.30E-01	5.30E+01		
7893030	---	---	---	-0.156	0.897	5.96E-01	5.96E+01		
7893031	---	---	---	-0.178	0.884	5.67E-01	5.67E+01		
7893032	---	---	---	-0.170	0.889	2.96E-01	2.96E+01		
7893033	---	---	---	-0.002	0.999	9.92E-01	9.92E+01		
7893034	---	---	---	0.088	1.063	7.83E-01	7.83E+01		
7893035	---	---	---	0.140	1.102	6.51E-01	6.51E+01		
7893036	---	---	---	0.004	1.003	9.91E-01	9.91E+01		
7893037	---	---	---	-0.003	0.998	9.88E-01	9.88E+01		
7893039	---	---	---	0.005	1.003	9.86E-01	9.86E+01		
7893040	---	---	---	0.222	1.166	3.22E-01	3.22E+01		
7893042	---	---	---	-0.010	0.993	9.62E-01	9.62E+01		
7893043	---	---	---	-0.067	0.954	8.16E-01	8.16E+01		
7893046	---	---	---	0.306	1.236	1.76E-01	1.76E+01		
7893047	---	---	---	0.377	1.298	3.56E-01	3.56E+01		
7893048	---	---	---	-0.006	0.996	9.86E-01	9.86E+01		
7893050	---	---	---	-0.004	0.997	9.85E-01	9.85E+01		
7893051	---	---	---	-0.005	0.997	9.95E-01	9.95E+01		

7893052	---	---	---	0.137	1.100	7.14E-01	7.14E+01		
7893055	---	---	---	0.450	1.366	1.52E-01	1.52E+01		
7893056	---	---	---	-0.098	0.934	8.23E-01	8.23E+01		
7893058	---	---	---	-0.058	0.961	7.30E-01	7.30E+01		
7893059	---	---	---	-0.081	0.945	7.62E-01	7.62E+01		
7893060	---	---	---	0.362	1.286	3.64E-01	3.64E+01		
7893061	---	---	---	0.196	1.145	3.93E-01	3.93E+01		
7893062	---	---	---	-0.113	0.925	8.48E-01	8.48E+01		
7893063	---	---	---	0.051	1.036	9.19E-01	9.19E+01		
7893064	---	---	---	0.002	1.002	9.98E-01	9.98E+01		
7893065	---	---	---	0.223	1.167	5.29E-01	5.29E+01		
7893067	---	---	---	-0.166	0.891	4.72E-01	4.72E+01		
7893068	---	---	---	-0.092	0.939	6.80E-01	6.80E+01		
7893069	---	---	---	0.044	1.031	8.39E-01	8.39E+01		
7893070	---	---	---	-0.163	0.893	2.56E-01	2.56E+01		
7893071	---	---	---	-0.141	0.907	5.78E-01	5.78E+01		
7893072	---	---	---	0.005	1.003	9.89E-01	9.89E+01		
7893073	---	---	---	-0.069	0.954	7.45E-01	7.45E+01		
7893074	---	---	---	0.242	1.182	2.66E-01	2.66E+01		
7893075	---	---	---	0.039	1.028	9.43E-01	9.43E+01		
7893077	---	---	---	0.054	1.038	4.15E-01	4.15E+01		
7893079	---	---	---	0.042	1.030	8.43E-01	8.43E+01		
7893081	---	---	---	-0.011	0.992	9.59E-01	9.59E+01		
7893082	---	---	---	-0.048	0.967	9.33E-01	9.33E+01		
7893084	---	---	---	-0.016	0.989	9.87E-01	9.87E+01		
7893085	---	---	---	0.041	1.029	8.17E-01	8.17E+01		
7893086	---	---	---	-0.151	0.901	6.38E-01	6.38E+01		
7893087	---	---	---	-0.108	0.928	7.34E-01	7.34E+01		
7893089	---	---	---	-0.120	0.920	6.93E-01	6.93E+01		
7893090	---	---	---	-0.024	0.984	9.17E-01	9.17E+01		
7893091	---	---	---	-0.035	0.976	7.94E-01	7.94E+01		
7893094	---	---	---	-0.054	0.963	7.15E-01	7.15E+01		
7893096	---	---	---	0.056	1.039	6.87E-01	6.87E+01		
7893097	---	---	---	0.079	1.056	8.17E-01	8.17E+01		
7893098	---	---	---	0.005	1.003	9.89E-01	9.89E+01		
7893099	---	---	---	-0.194	0.874	6.09E-01	6.09E+01		
7893100	---	---	---	-0.134	0.912	3.76E-01	3.76E+01		
7893101	---	---	---	0.042	1.029	5.79E-01	5.79E+01		
7893102	---	---	---	0.323	1.251	6.40E-01	6.40E+01		
7893103	---	---	---	0.053	1.037	8.09E-01	8.09E+01		
7893104	---	---	---	0.288	1.221	5.49E-01	5.49E+01		
7893105	---	---	---	0.063	1.045	6.34E-01	6.34E+01		
7893106	---	---	---	-0.083	0.944	6.80E-01	6.80E+01		
7893108	---	---	---	-0.230	0.853	8.29E-01	8.29E+01		
7893111	---	---	---	0.252	1.191	3.80E-01	3.80E+01		
7893112	---	---	---	0.194	1.144	2.83E-01	2.83E+01		
7893113	---	---	---	0.189	1.140	9.17E-02	9.17E+00		
7893114	---	---	---	0.118	1.085	5.82E-01	5.82E+01		
7893115	---	---	---	-0.085	0.943	8.88E-01	8.88E+01		
7893116	---	---	---	0.031	1.022	8.62E-01	8.62E+01		
7893118	---	---	---	-0.018	0.988	9.76E-01	9.76E+01		
7893119	---	---	---	-0.025	0.983	9.54E-01	9.54E+01		
7893120	---	---	---	0.176	1.130	3.29E-01	3.29E+01		
7893121	---	---	---	-0.052	0.964	7.83E-01	7.83E+01		
7893122	---	---	---	-0.145	0.904	2.45E-01	2.45E+01		
7893123	---	---	---	0.051	1.036	8.13E-01	8.13E+01		
7893125	---	---	---	-0.126	0.917	2.26E-01	2.26E+01		
7893128	---	---	---	0.130	1.095	7.32E-01	7.32E+01		
7893129	---	---	---	0.057	1.040	8.74E-01	8.74E+01		
7893130	---	---	---	0.045	1.032	8.34E-01	8.34E+01		
7893131	---	---	---	0.124	1.090	5.49E-01	5.49E+01		
7893132	---	---	---	0.033	1.023	9.53E-01	9.53E+01		
7893133	---	---	---	0.013	1.009	9.64E-01	9.64E+01		
7893134	---	---	---	-0.001	0.999	9.99E-01	9.99E+01		
7893136	---	---	---	0.061	1.043	9.00E-01	9.00E+01		
7893137	---	---	---	0.068	1.048	8.41E-01	8.41E+01		
7893138	---	---	---	-0.045	0.969	8.10E-01	8.10E+01		
7893139	---	---	---	0.086	1.061	4.49E-01	4.49E+01		
7893140	---	---	---	0.019	1.013	9.07E-01	9.07E+01		
7893141	---	---	---	0.102	1.074	7.78E-01	7.78E+01		
7893142	---	---	---	-0.085	0.943	8.27E-01	8.27E+01		
7893143	---	---	---	0.078	1.056	8.09E-01	8.09E+01		
7893145	---	---	---	-0.107	0.929	8.41E-01	8.41E+01		
7893146	---	---	---	0.322	1.250	2.80E-01	2.80E+01		
7893147	---	---	---	0.040	1.028	6.43E-01	6.43E+01		
7893148	---	---	---	0.068	1.048	6.40E-01	6.40E+01		
7893150	---	---	---	0.093	1.066	8.66E-01	8.66E+01		

7893151	---	---	---	0.000	1.000	1.00E+00	1.00E+02		
7893152	---	---	---	0.025	1.017	8.40E-01	8.40E+01		
7893153	---	---	---	-0.103	0.931	5.64E-01	5.64E+01		
7893154	---	---	---	0.055	1.039	9.20E-01	9.20E+01		
7893155	---	---	---	0.029	1.020	9.61E-01	9.61E+01		
7893156	---	---	---	0.064	1.045	7.56E-01	7.56E+01		
7893158	---	---	---	0.125	1.091	5.10E-01	5.10E+01		
7893159	---	---	---	-0.172	0.887	6.00E-01	6.00E+01		
7893160	---	---	---	-0.047	0.968	8.41E-01	8.41E+01		
7893161	---	---	---	0.070	1.050	9.49E-01	9.49E+01		
7893164	---	---	---	-0.040	0.973	8.95E-01	8.95E+01		
7893165	---	---	---	0.079	1.056	8.57E-01	8.57E+01		
7893166	---	---	---	-0.004	0.997	9.96E-01	9.96E+01		
7893167	---	---	---	-0.164	0.892	5.80E-01	5.80E+01		
7893168	---	---	---	0.379	1.301	9.43E-02	9.43E+00		
7893169	---	---	---	-0.017	0.988	9.77E-01	9.77E+01		
7893171	---	---	---	0.024	1.016	9.76E-01	9.76E+01		
7893172	---	---	---	-0.138	0.909	6.67E-01	6.67E+01		
7893173	---	---	---	-0.063	0.957	7.68E-01	7.68E+01		
7893175	---	---	---	-0.317	0.803	4.54E-01	4.54E+01		
7893177	---	---	---	-0.028	0.981	9.64E-01	9.64E+01		
7893179	---	---	---	-0.010	0.993	9.88E-01	9.88E+01		
7893180	---	---	---	0.030	1.021	9.57E-01	9.57E+01		
7893181	---	---	---	-0.043	0.971	8.52E-01	8.52E+01		
7893182	---	---	---	-0.028	0.981	9.37E-01	9.37E+01		
7893184	---	---	---	0.022	1.016	8.85E-01	8.85E+01		
7893185	---	---	---	-0.124	0.918	8.37E-01	8.37E+01		
7893187	---	---	---	0.157	1.115	5.99E-01	5.99E+01		
7893188	---	---	---	-0.232	0.851	3.27E-01	3.27E+01		
7893189	---	---	---	0.033	1.023	6.16E-01	6.16E+01		
7893190	---	---	---	-0.225	0.856	4.27E-01	4.27E+01		
7893191	---	---	---	-0.062	0.958	6.34E-01	6.34E+01		
7893194	---	---	---	0.035	1.024	7.72E-01	7.72E+01		
7893196	---	---	---	0.025	1.017	9.11E-01	9.11E+01		
7893201	---	---	---	0.008	1.006	9.85E-01	9.85E+01		
7893202	---	---	---	-0.205	0.867	4.63E-01	4.63E+01		
7893205	---	---	---	-0.129	0.915	3.46E-01	3.46E+01		
7893206	---	---	---	0.067	1.048	8.32E-01	8.32E+01		
7893207	---	---	---	-0.020	0.986	9.57E-01	9.57E+01		
7893208	---	---	---	0.002	1.002	9.89E-01	9.89E+01		
7893209	---	---	---	-0.182	0.881	5.43E-01	5.43E+01		
7893210	---	---	---	-0.045	0.969	8.82E-01	8.82E+01		
7893212	---	---	---	0.057	1.040	8.26E-01	8.26E+01		
7893213	---	---	---	0.179	1.132	8.10E-01	8.10E+01		
7893214	---	---	---	-0.384	0.766	4.01E-01	4.01E+01		
7893215	---	---	---	0.107	1.077	9.00E-01	9.00E+01		
7893216	---	---	---	0.055	1.039	9.19E-01	9.19E+01		
7893217	---	---	---	0.060	1.043	8.20E-01	8.20E+01		
7893218	---	---	---	0.012	1.008	9.17E-01	9.17E+01		
7893219	---	---	---	-0.084	0.943	8.69E-01	8.69E+01		
7893220	---	---	---	0.072	1.051	6.51E-01	6.51E+01		
7893222	---	---	---	0.100	1.072	8.80E-01	8.80E+01		
7893225	---	---	---	0.158	1.116	5.40E-01	5.40E+01		
7893226	---	---	---	0.145	1.106	5.47E-01	5.47E+01		
7893227	---	---	---	-0.105	0.930	7.27E-01	7.27E+01		
7893229	---	---	---	0.032	1.023	9.27E-01	9.27E+01		
7893230	---	---	---	-0.274	0.827	8.25E-01	8.25E+01		
7893231	---	---	---	0.049	1.034	7.71E-01	7.71E+01		
7893234	---	---	---	-0.191	0.876	3.54E-01	3.54E+01		
7893235	---	---	---	-0.068	0.954	7.68E-01	7.68E+01		
7893236	---	---	---	-0.019	0.987	9.38E-01	9.38E+01		
7893237	---	---	---	0.030	1.021	8.04E-01	8.04E+01		
7893238	---	---	---	-0.043	0.971	9.06E-01	9.06E+01		
7893239	---	---	---	0.120	1.087	7.51E-01	7.51E+01		
7893240	---	---	---	-0.057	0.961	8.97E-01	8.97E+01		
7893241	---	---	---	-0.029	0.980	9.56E-01	9.56E+01		
7893242	---	---	---	0.028	1.019	8.27E-01	8.27E+01		
7893243	---	---	---	0.150	1.109	7.24E-01	7.24E+01		
7893244	---	---	---	-0.028	0.981	8.34E-01	8.34E+01		
7893245	---	---	---	0.087	1.062	7.93E-01	7.93E+01		
7893246	---	---	---	0.013	1.009	9.84E-01	9.84E+01		
7893247	---	---	---	0.035	1.024	8.41E-01	8.41E+01		
7893248	---	---	---	0.131	1.095	5.24E-01	5.24E+01		
7893249	---	---	---	0.039	1.027	8.78E-01	8.78E+01		
7893251	---	---	---	0.230	1.173	3.57E-01	3.57E+01		
7893253	---	---	---	-0.021	0.986	9.73E-01	9.73E+01		
7893255	---	---	---	-0.053	0.964	8.37E-01	8.37E+01		

7893256	---	---	---	0.455	1.370	2.41E-01	2.41E+01			
7893257	---	---	---	-0.082	0.945	8.18E-01	8.18E+01			
7893258	---	---	---	0.045	1.032	9.16E-01	9.16E+01			
7893259	---	---	---	-0.001	1.000	9.98E-01	9.98E+01			
7893261	---	---	---	-0.053	0.964	8.06E-01	8.06E+01			
7893262	---	---	---	-0.043	0.971	8.05E-01	8.05E+01			
7893263	---	---	---	-0.013	0.991	9.81E-01	9.81E+01			
7893264	---	---	---	-0.012	0.992	9.53E-01	9.53E+01			
7893265	---	---	---	-0.199	0.871	7.89E-01	7.89E+01			
7893266	---	---	---	-0.002	0.999	9.91E-01	9.91E+01			
7893268	---	---	---	0.090	1.064	7.95E-01	7.95E+01			
7893269	---	---	---	-0.008	0.994	9.86E-01	9.86E+01			
7893270	---	---	---	0.047	1.033	7.60E-01	7.60E+01			
7893271	---	---	---	-0.214	0.862	1.13E-01	1.13E+01			
7893272	---	---	---	0.077	1.055	6.13E-01	6.13E+01			
7893273	---	---	---	0.123	1.089	6.10E-01	6.10E+01			
7893278	---	---	---	-0.012	0.992	9.85E-01	9.85E+01			
7893279	---	---	---	-0.019	0.987	9.77E-01	9.77E+01			
7893281	---	---	---	0.085	1.061	3.42E-01	3.42E+01			
7893283	---	---	---	0.010	1.007	9.53E-01	9.53E+01			
7893285	---	---	---	0.082	1.058	7.51E-01	7.51E+01			
7893286	---	---	---	0.045	1.031	9.27E-01	9.27E+01			
7893287	---	---	---	-0.110	0.926	8.16E-01	8.16E+01			
7893288	---	---	---	-0.056	0.962	8.30E-01	8.30E+01			
7893289	---	---	---	0.064	1.045	5.74E-01	5.74E+01			
7893290	---	---	---	-0.096	0.935	8.33E-01	8.33E+01			
7893291	---	---	---	-0.155	0.898	7.39E-01	7.39E+01			
7893294	---	---	---	-0.024	0.984	9.37E-01	9.37E+01			
7893297	---	---	---	0.192	1.142	2.20E-01	2.20E+01			
7893298	---	---	---	0.012	1.008	9.68E-01	9.68E+01			
7893299	---	---	---	0.152	1.111	8.05E-01	8.05E+01			
7893300	---	---	---	-0.055	0.963	8.78E-01	8.78E+01			
7893302	---	---	---	0.123	1.089	7.29E-01	7.29E+01			
7893304	---	---	---	-0.036	0.975	9.10E-01	9.10E+01			
7893305	---	---	---	-0.028	0.981	9.61E-01	9.61E+01			
7893306	---	---	---	0.023	1.016	9.66E-01	9.66E+01			
7893308	---	---	---	0.053	1.037	9.55E-01	9.55E+01			
7893309	---	---	---	0.112	1.081	7.66E-01	7.66E+01			
7893311	---	---	---	0.028	1.020	9.30E-01	9.30E+01			
7893313	---	---	---	0.012	1.008	9.64E-01	9.64E+01			
7893314	---	---	---	0.111	1.080	6.39E-01	6.39E+01			
7893316	---	---	---	-0.028	0.981	8.53E-01	8.53E+01			
7893318	---	---	---	-0.073	0.951	9.23E-01	9.23E+01			
7893319	---	---	---	0.172	1.127	4.25E-01	4.25E+01			
7893320	---	---	---	0.331	1.258	4.84E-01	4.84E+01			
7893321	---	---	---	0.070	1.050	4.88E-01	4.88E+01			
7893322	---	---	---	0.345	1.271	6.33E-01	6.33E+01			
7893325	---	---	---	0.027	1.019	9.36E-01	9.36E+01			
7893329	---	---	---	-0.079	0.947	6.82E-01	6.82E+01			
7893331	---	---	---	0.050	1.036	9.47E-01	9.47E+01			
7893332	---	---	---	0.020	1.014	9.37E-01	9.37E+01			
7893333	---	---	---	0.051	1.036	9.35E-01	9.35E+01			
7893334	---	---	---	-0.030	0.979	9.55E-01	9.55E+01			
7893335	---	---	---	0.072	1.051	8.08E-01	8.08E+01			
7893336	---	---	---	0.049	1.034	8.37E-01	8.37E+01			
7893337	---	---	---	-0.007	0.995	9.78E-01	9.78E+01			
7893338	---	---	---	0.057	1.040	6.66E-01	6.66E+01			
7893339	---	---	---	-0.065	0.956	8.33E-01	8.33E+01			
7893340	---	---	---	-0.009	0.994	9.77E-01	9.77E+01			
7893341	---	---	---	0.065	1.046	8.06E-01	8.06E+01			
7893343	---	---	---	-0.179	0.883	4.26E-01	4.26E+01			
7893344	---	---	---	-0.001	1.000	9.99E-01	9.99E+01			
7893345	---	---	---	0.263	1.200	7.47E-02	7.47E+00			
7893346	---	---	---	0.034	1.024	9.49E-01	9.49E+01			
7893348	---	---	---	-0.047	0.968	7.73E-01	7.73E+01			
7893349	---	---	---	-0.169	0.889	6.74E-01	6.74E+01			
7893350	---	---	---	-0.340	0.790	2.33E-01	2.33E+01			
7893351	---	---	---	0.004	1.002	9.89E-01	9.89E+01			
7893352	---	---	---	0.026	1.018	9.74E-01	9.74E+01			
7893353	---	---	---	-0.044	0.970	8.45E-01	8.45E+01			
7893354	---	---	---	-0.094	0.937	5.22E-01	5.22E+01			
7893355	---	---	---	0.486	1.401	3.39E-01	3.39E+01			
7893356	---	---	---	-0.044	0.970	8.62E-01	8.62E+01			
7893357	---	---	---	-0.086	0.942	4.44E-01	4.44E+01			
7893358	---	---	---	0.006	1.004	9.97E-01	9.97E+01			
7893359	---	---	---	-0.192	0.875	7.81E-01	7.81E+01			
7893360	---	---	---	-0.237	0.848	7.27E-01	7.27E+01			

7893361	---	---	---	-0.095	0.936	6.10E-01	6.10E+01		
7893362	---	---	---	0.183	1.135	1.45E-01	1.45E+01		
7893363	---	---	---	-0.253	0.839	2.09E-01	2.09E+01		
7893364	---	---	---	-0.077	0.948	8.06E-01	8.06E+01		
7893365	---	---	---	0.132	1.096	8.33E-01	8.33E+01		
7893367	---	---	---	-0.024	0.984	9.52E-01	9.52E+01		
7893369	---	---	---	0.004	1.003	9.93E-01	9.93E+01		
7893371	---	---	---	0.020	1.014	8.87E-01	8.87E+01		
7893372	---	---	---	-0.105	0.930	9.31E-01	9.31E+01		
7893373	---	---	---	-0.071	0.952	7.94E-01	7.94E+01		
7893374	---	---	---	0.068	1.048	4.99E-01	4.99E+01		
7893380	---	---	---	0.271	1.207	5.06E-01	5.06E+01		
7893382	---	---	---	-0.012	0.992	9.27E-01	9.27E+01		
7893383	---	---	---	0.131	1.095	4.13E-01	4.13E+01		
7893384	---	---	---	0.004	1.003	9.91E-01	9.91E+01		
7893385	---	---	---	-0.071	0.952	6.91E-01	6.91E+01		
7893386	---	---	---	-0.011	0.992	9.56E-01	9.56E+01		
7893388	---	---	---	0.165	1.121	8.08E-01	8.08E+01		
7893390	---	---	---	-0.010	0.993	9.64E-01	9.64E+01		
7893391	---	---	---	0.008	1.006	9.74E-01	9.74E+01		
7893392	---	---	---	-0.100	0.933	7.01E-01	7.01E+01		
7893393	---	---	---	-0.189	0.877	1.87E-01	1.87E+01		
7893394	---	---	---	-0.051	0.965	8.09E-01	8.09E+01		
7893395	---	---	---	0.116	1.084	6.78E-01	6.78E+01		
7893396	---	---	---	0.068	1.048	8.81E-01	8.81E+01		
7893397	---	---	---	-0.210	0.865	6.42E-01	6.42E+01		
7893399	---	---	---	-0.076	0.949	8.66E-01	8.66E+01		
7893402	---	---	---	0.058	1.041	8.22E-01	8.22E+01		
7893403	---	---	---	-0.161	0.894	6.64E-01	6.64E+01		
7893405	---	---	---	0.031	1.022	9.60E-01	9.60E+01		
7893407	---	---	---	-0.054	0.963	8.33E-01	8.33E+01		
7893408	---	---	---	0.049	1.034	9.23E-01	9.23E+01		
7893410	---	---	---	-0.030	0.980	8.61E-01	8.61E+01		
7893412	---	---	---	-0.005	0.997	9.95E-01	9.95E+01		
7893413	---	---	---	-0.019	0.987	9.35E-01	9.35E+01		
7893414	---	---	---	0.020	1.014	8.58E-01	8.58E+01		
7893415	---	---	---	-0.137	0.909	8.34E-01	8.34E+01		
7893416	---	---	---	0.136	1.099	8.47E-01	8.47E+01		
7893418	---	---	---	0.038	1.026	8.49E-01	8.49E+01		
7893419	---	---	---	-0.014	0.990	9.62E-01	9.62E+01		
7893420	---	---	---	-0.035	0.976	7.89E-01	7.89E+01		
7893421	---	---	---	0.110	1.079	7.95E-01	7.95E+01		
7893422	---	---	---	-0.135	0.911	6.38E-01	6.38E+01		
7893424	---	---	---	0.057	1.041	7.45E-01	7.45E+01		
7893427	---	---	---	-0.203	0.869	2.69E-01	2.69E+01		
7893428	---	---	---	-0.060	0.959	7.03E-01	7.03E+01		
7893432	---	---	---	0.431	1.348	2.00E-01	2.00E+01		
7893435	---	---	---	0.008	1.006	9.70E-01	9.70E+01		
7893436	---	---	---	-0.019	0.987	9.73E-01	9.73E+01		
7893437	---	---	---	0.153	1.112	7.85E-01	7.85E+01		
7893438	---	---	---	-0.024	0.983	9.22E-01	9.22E+01		
7893439	---	---	---	-0.023	0.984	9.14E-01	9.14E+01		
7893441	---	---	---	-0.062	0.958	9.06E-01	9.06E+01		
7893442	---	---	---	0.201	1.149	5.34E-01	5.34E+01		
7893443	---	---	---	0.027	1.019	9.53E-01	9.53E+01		
7893444	---	---	---	0.016	1.011	9.76E-01	9.76E+01		
7893445	---	---	---	0.003	1.002	9.99E-01	9.99E+01		
7893447	---	---	---	-0.098	0.934	6.69E-01	6.69E+01		
7893448	---	---	---	0.030	1.021	8.83E-01	8.83E+01		
7893449	---	---	---	-0.031	0.979	7.73E-01	7.73E+01		
7893454	---	---	---	0.024	1.017	9.19E-01	9.19E+01		
7893455	---	---	---	-0.024	0.984	9.24E-01	9.24E+01		
7893458	---	---	---	-0.107	0.929	5.78E-01	5.78E+01		
7893459	---	---	---	-0.151	0.900	8.35E-01	8.35E+01		
7893460	---	---	---	-0.025	0.983	8.47E-01	8.47E+01		
7893461	---	---	---	-0.119	0.921	7.09E-01	7.09E+01		
7893462	---	---	---	-0.075	0.949	8.47E-01	8.47E+01		
7893463	---	---	---	0.062	1.044	7.50E-01	7.50E+01		
7893464	---	---	---	0.036	1.025	8.13E-01	8.13E+01		
7893466	---	---	---	0.053	1.038	5.68E-01	5.68E+01		
7893467	---	---	---	0.043	1.030	8.23E-01	8.23E+01		
7893468	---	---	---	0.050	1.035	9.13E-01	9.13E+01		
7893469	---	---	---	0.034	1.024	8.06E-01	8.06E+01		
7893470	---	---	---	0.094	1.067	6.80E-01	6.80E+01		
7893471	---	---	---	-0.023	0.984	9.55E-01	9.55E+01		
7893472	---	---	---	-0.079	0.947	8.52E-01	8.52E+01		
7893473	---	---	---	-0.056	0.962	9.15E-01	9.15E+01		

7893474	---	---	---	-0.091	0.939	8.91E-01	8.91E+01		
7893475	---	---	---	0.095	1.068	7.38E-01	7.38E+01		
7893476	---	---	---	-0.175	0.886	3.04E-01	3.04E+01		
7893477	---	---	---	0.297	1.229	4.68E-01	4.68E+01		
7893478	---	---	---	0.239	1.180	1.61E-01	1.61E+01		
7893480	---	---	---	-0.124	0.917	5.89E-01	5.89E+01		
7893481	---	---	---	-0.041	0.972	9.26E-01	9.26E+01		
7893482	---	---	---	0.300	1.232	5.77E-01	5.77E+01		
7893483	---	---	---	0.172	1.127	1.17E-01	1.17E+01		
7893484	---	---	---	-0.084	0.943	9.37E-01	9.37E+01		
7893485	---	---	---	0.005	1.003	9.91E-01	9.91E+01		
7893486	---	---	---	-0.025	0.983	9.16E-01	9.16E+01		
7893487	---	---	---	0.093	1.066	8.27E-01	8.27E+01		
7893488	---	---	---	0.243	1.184	3.95E-01	3.95E+01		
7893491	---	---	---	0.043	1.031	8.05E-01	8.05E+01		
7893492	---	---	---	0.740	1.670	2.48E-01	2.48E+01		
7893493	---	---	---	-0.244	0.845	4.93E-01	4.93E+01		
7893494	---	---	---	-0.146	0.904	7.23E-01	7.23E+01		
7893496	---	---	---	-0.227	0.854	6.74E-01	6.74E+01		
7893497	---	---	---	-0.040	0.972	9.10E-01	9.10E+01		
7893498	---	---	---	-0.004	0.997	9.85E-01	9.85E+01		
7893500	---	---	---	-0.090	0.940	8.36E-01	8.36E+01		
7893503	---	---	---	0.013	1.009	9.73E-01	9.73E+01		
7893504	---	---	---	-0.162	0.894	6.58E-01	6.58E+01		
7893506	---	---	---	-0.130	0.914	5.91E-01	5.91E+01		
7893507	---	---	---	0.024	1.017	8.13E-01	8.13E+01		
7893508	---	---	---	-0.069	0.953	8.78E-01	8.78E+01		
7893511	---	---	---	0.038	1.027	8.49E-01	8.49E+01		
7893513	---	---	---	0.023	1.016	9.32E-01	9.32E+01		
7893514	---	---	---	-0.259	0.836	7.53E-01	7.53E+01		
7893515	---	---	---	0.049	1.034	7.44E-01	7.44E+01		
7893516	---	---	---	-0.026	0.982	9.22E-01	9.22E+01		
7893517	---	---	---	-0.081	0.946	7.64E-01	7.64E+01		
7893518	---	---	---	0.010	1.007	9.77E-01	9.77E+01		
7893519	---	---	---	0.080	1.057	8.29E-01	8.29E+01		
7893524	---	---	---	0.153	1.112	5.81E-02	5.81E+00		
7893525	---	---	---	-0.262	0.834	3.18E-01	3.18E+01		
7893527	---	---	---	0.247	1.187	1.75E-01	1.75E+01		
7893530	---	---	---	0.013	1.009	9.56E-01	9.56E+01		
7893533	---	---	---	0.063	1.045	7.83E-01	7.83E+01		
7893534	---	---	---	-0.122	0.919	5.81E-01	5.81E+01		
7893536	---	---	---	0.022	1.015	8.64E-01	8.64E+01		
7893538	---	---	---	-0.077	0.948	8.46E-01	8.46E+01		
7893539	---	---	---	-0.078	0.947	7.09E-01	7.09E+01		
7893540	---	---	---	0.041	1.029	7.62E-01	7.62E+01		
7893542	---	---	---	-0.100	0.933	7.12E-01	7.12E+01		
7893543	---	---	---	-0.015	0.990	9.64E-01	9.64E+01		
7893544	---	---	---	-0.080	0.946	7.51E-01	7.51E+01		
7893545	---	---	---	0.056	1.039	7.50E-01	7.50E+01		
7893546	---	---	---	-0.009	0.994	9.77E-01	9.77E+01		
7893547	---	---	---	-0.027	0.981	9.03E-01	9.03E+01		
7893548	---	---	---	-0.005	0.997	9.95E-01	9.95E+01		
7893549	---	---	---	0.051	1.036	9.30E-01	9.30E+01		
7893550	---	---	---	-0.024	0.984	9.09E-01	9.09E+01		
7893551	---	---	---	0.032	1.022	9.16E-01	9.16E+01		
7893552	---	---	---	0.184	1.136	5.34E-01	5.34E+01		
7893554	---	---	---	-0.066	0.955	8.01E-01	8.01E+01		
7893555	---	---	---	-0.053	0.964	8.50E-01	8.50E+01		
7893556	---	---	---	0.043	1.030	9.21E-01	9.21E+01		
7893557	---	---	---	-0.237	0.848	2.21E-01	2.21E+01		
7893558	---	---	---	-0.006	0.996	9.92E-01	9.92E+01		
7893559	---	---	---	0.030	1.021	9.25E-01	9.25E+01		
7893560	---	---	---	0.049	1.035	8.93E-01	8.93E+01		
7893561	---	---	---	-0.034	0.977	8.61E-01	8.61E+01		
7893562	---	---	---	0.119	1.086	9.23E-01	9.23E+01		
7893564	---	---	---	-0.039	0.973	7.75E-01	7.75E+01		
7893565	---	---	---	-0.164	0.892	5.44E-01	5.44E+01		
7893567	---	---	---	-0.074	0.950	9.19E-01	9.19E+01		
7893570	---	---	---	-0.376	0.770	6.54E-01	6.54E+01		
7893571	---	---	---	-0.004	0.998	9.88E-01	9.88E+01		
7893572	---	---	---	0.010	1.007	9.86E-01	9.86E+01		
7893573	---	---	---	-0.124	0.918	6.08E-01	6.08E+01		
7893574	---	---	---	0.106	1.076	6.87E-01	6.87E+01		
7893575	---	---	---	0.162	1.119	5.43E-01	5.43E+01		
7893576	---	---	---	-0.025	0.983	9.66E-01	9.66E+01		
7893578	---	---	---	0.177	1.130	7.51E-01	7.51E+01		
7893581	---	---	---	0.254	1.193	6.16E-01	6.16E+01		

7893582	---	---	---	-0.038	0.974	9.07E-01	9.07E+01		
7893583	---	---	---	0.079	1.056	7.27E-01	7.27E+01		
7893585	---	---	---	-0.107	0.929	5.89E-01	5.89E+01		
7893586	---	---	---	0.001	1.000	1.00E+00	1.00E+02		
7893587	---	---	---	-0.065	0.956	8.86E-01	8.86E+01		
7893588	---	---	---	0.201	1.150	6.94E-01	6.94E+01		
7893590	---	---	---	0.137	1.100	7.78E-01	7.78E+01		
7893591	---	---	---	-0.103	0.931	3.43E-01	3.43E+01		
7893592	---	---	---	0.154	1.113	1.38E-01	1.38E+01		
7893593	---	---	---	0.080	1.057	8.61E-01	8.61E+01		
7893594	---	---	---	-0.193	0.875	8.49E-01	8.49E+01		
7893596	---	---	---	0.302	1.233	4.67E-01	4.67E+01		
7893598	---	---	---	0.012	1.008	9.64E-01	9.64E+01		
7893599	---	---	---	0.009	1.006	9.67E-01	9.67E+01		
7893600	---	---	---	-0.121	0.920	5.70E-01	5.70E+01		
7893601	---	---	---	0.078	1.055	7.27E-01	7.27E+01		
7893602	---	---	---	0.059	1.042	6.99E-01	6.99E+01		
7893603	---	---	---	0.060	1.042	6.74E-01	6.74E+01		
7893604	---	---	---	-0.047	0.968	9.53E-01	9.53E+01		
7893605	---	---	---	0.032	1.022	8.05E-01	8.05E+01		
7893606	---	---	---	0.146	1.107	7.60E-01	7.60E+01		
7893608	---	---	---	-0.120	0.920	7.73E-01	7.73E+01		
7893609	---	---	---	-0.058	0.960	9.16E-01	9.16E+01		
7893610	---	---	---	0.136	1.099	6.33E-01	6.33E+01		
7893613	---	---	---	0.030	1.021	9.16E-01	9.16E+01		
7893615	---	---	---	-0.051	0.965	9.67E-01	9.67E+01		
7893616	---	---	---	-0.138	0.909	5.96E-01	5.96E+01		
7893619	---	---	---	0.343	1.268	2.60E-01	2.60E+01		
7893622	---	---	---	0.423	1.340	5.98E-01	5.98E+01		
7893627	---	---	---	-0.230	0.853	6.91E-01	6.91E+01		
7893628	---	---	---	0.109	1.078	7.30E-01	7.30E+01		
7893630	---	---	---	-0.157	0.897	1.11E-01	1.11E+01		
7893631	---	---	---	0.002	1.002	9.97E-01	9.97E+01		
7893632	---	---	---	0.124	1.090	1.88E-01	1.88E+01		
7893633	---	---	---	0.059	1.042	3.96E-01	3.96E+01		
7893634	---	---	---	-0.025	0.983	8.87E-01	8.87E+01		
7893637	---	---	---	0.116	1.083	7.92E-01	7.92E+01		
7893639	---	---	---	0.095	1.068	6.59E-01	6.59E+01		
7893640	---	---	---	-0.038	0.974	8.68E-01	8.68E+01		
7893641	---	---	---	0.126	1.091	3.05E-01	3.05E+01		
7893642	---	---	---	0.118	1.085	2.25E-01	2.25E+01		
7893644	---	---	---	0.136	1.099	5.56E-01	5.56E+01		
7893645	---	---	---	0.426	1.344	2.10E-01	2.10E+01		
7893646	---	---	---	-0.120	0.920	5.26E-01	5.26E+01		
7893647	---	---	---	0.098	1.071	5.55E-01	5.55E+01		
7893649	---	---	---	-0.090	0.939	6.33E-01	6.33E+01		
7893650	---	---	---	-0.080	0.946	6.38E-01	6.38E+01		
7893651	---	---	---	-0.029	0.980	7.51E-01	7.51E+01		
7893653	---	---	---	0.241	1.182	8.15E-01	8.15E+01		
7893654	---	---	---	0.119	1.086	7.10E-01	7.10E+01		
7893655	---	---	---	-0.269	0.830	3.67E-01	3.67E+01		
7893657	---	---	---	0.019	1.014	9.64E-01	9.64E+01		
7893658	---	---	---	-0.006	0.996	9.86E-01	9.86E+01		
7893660	---	---	---	0.000	1.000	9.99E-01	9.99E+01		
7893661	---	---	---	-0.015	0.990	9.45E-01	9.45E+01		
7893662	---	---	---	-0.022	0.985	8.75E-01	8.75E+01		
7893663	---	---	---	0.169	1.124	5.67E-01	5.67E+01		
7893664	---	---	---	-0.007	0.995	9.87E-01	9.87E+01		
7893665	---	---	---	0.083	1.059	8.46E-01	8.46E+01		
7893667	---	---	---	0.081	1.058	9.00E-01	9.00E+01		
7893668	---	---	---	-0.250	0.841	7.27E-01	7.27E+01		
7893670	---	---	---	0.031	1.021	8.43E-01	8.43E+01		
7893672	---	---	---	0.274	1.209	2.92E-01	2.92E+01		
7893674	---	---	---	0.299	1.230	3.75E-01	3.75E+01		
7893675	---	---	---	-0.020	0.986	9.16E-01	9.16E+01		
7893676	---	---	---	-0.186	0.879	6.52E-02	6.52E+00		
7893677	---	---	---	0.021	1.015	8.16E-01	8.16E+01		
7893682	---	---	---	0.105	1.075	7.72E-01	7.72E+01		
7893683	---	---	---	0.012	1.008	9.85E-01	9.85E+01		
7893684	---	---	---	0.031	1.022	9.41E-01	9.41E+01		
7893685	---	---	---	-0.044	0.970	8.90E-01	8.90E+01		
7893686	---	---	---	-0.137	0.909	8.15E-01	8.15E+01		
7893688	---	---	---	0.000	1.000	1.00E+00	1.00E+02		
7893689	---	---	---	0.014	1.010	9.23E-01	9.23E+01		
7893690	---	---	---	0.049	1.034	9.03E-01	9.03E+01		
7893692	---	---	---	0.000	1.000	1.00E+00	1.00E+02		
7893695	---	---	---	0.063	1.044	7.59E-01	7.59E+01		

7893696	---	---	---	-0.024	0.983	9.59E-01	9.59E+01		
7893697	---	---	---	-0.306	0.809	3.67E-01	3.67E+01		
7893698	---	---	---	0.077	1.055	8.09E-01	8.09E+01		
7893699	---	---	---	0.124	1.090	7.05E-01	7.05E+01		
7893700	---	---	---	0.206	1.154	1.27E-01	1.27E+01		
7893701	---	---	---	0.058	1.041	5.73E-01	5.73E+01		
7893703	---	---	---	-0.051	0.965	9.49E-01	9.49E+01		
7893704	---	---	---	0.181	1.134	4.49E-01	4.49E+01		
7893705	---	---	---	-0.016	0.989	9.57E-01	9.57E+01		
7893707	---	---	---	-0.006	0.996	9.86E-01	9.86E+01		
7893708	---	---	---	-0.030	0.980	8.24E-01	8.24E+01		
7893709	---	---	---	0.000	1.000	1.00E+00	1.00E+02		
7893710	---	---	---	0.027	1.019	8.31E-01	8.31E+01		
7893711	---	---	---	-0.104	0.930	5.86E-01	5.86E+01		
7893713	---	---	---	-0.014	0.990	9.63E-01	9.63E+01		
7893714	---	---	---	0.007	1.005	9.87E-01	9.87E+01		
7893715	---	---	---	0.084	1.060	6.79E-01	6.79E+01		
7893717	---	---	---	-0.070	0.953	8.62E-01	8.62E+01		
7893718	---	---	---	0.020	1.014	9.82E-01	9.82E+01		
7893720	---	---	---	0.132	1.096	3.09E-01	3.09E+01		
7893721	---	---	---	0.073	1.052	6.73E-01	6.73E+01		
7893722	---	---	---	-0.078	0.947	7.61E-01	7.61E+01		
7893724	---	---	---	0.153	1.112	4.39E-01	4.39E+01		
7893725	---	---	---	0.034	1.024	7.83E-01	7.83E+01		
7893726	---	---	---	0.085	1.060	6.93E-01	6.93E+01		
7893727	---	---	---	-0.003	0.998	9.86E-01	9.86E+01		
7893728	---	---	---	0.079	1.056	6.78E-01	6.78E+01		
7893729	---	---	---	0.108	1.078	8.23E-01	8.23E+01		
7893731	---	---	---	0.001	1.001	9.95E-01	9.95E+01		
7893732	---	---	---	0.178	1.131	1.48E-02	1.48E+00		
7893733	---	---	---	0.134	1.097	2.26E-01	2.26E+01		
7893734	---	---	---	0.004	1.003	9.89E-01	9.89E+01		
7893736	---	---	---	0.190	1.141	7.00E-01	7.00E+01		
7893737	---	---	---	0.177	1.131	5.01E-01	5.01E+01		
7893738	---	---	---	0.057	1.040	8.32E-01	8.32E+01		
7893739	---	---	---	-0.015	0.990	9.51E-01	9.51E+01		
7893740	---	---	---	0.123	1.089	4.15E-01	4.15E+01		
7893743	---	---	---	0.055	1.039	8.62E-01	8.62E+01		
7893744	---	---	---	0.053	1.038	7.48E-01	7.48E+01		
7893745	---	---	---	-0.007	0.995	9.89E-01	9.89E+01		
7893747	---	---	---	0.054	1.038	8.81E-01	8.81E+01		
7893748	---	---	---	0.040	1.028	9.26E-01	9.26E+01		
7893750	---	---	---	0.073	1.052	8.72E-01	8.72E+01		
7893751	---	---	---	0.197	1.147	6.35E-01	6.35E+01		
7893752	---	---	---	-0.095	0.936	8.31E-01	8.31E+01		
7893754	---	---	---	-0.006	0.996	9.89E-01	9.89E+01		
7893757	---	---	---	-0.095	0.937	7.99E-01	7.99E+01		
7893759	---	---	---	0.043	1.030	8.97E-01	8.97E+01		
7893761	---	---	---	-0.031	0.979	9.61E-01	9.61E+01		
7893762	---	---	---	-0.019	0.987	8.85E-01	8.85E+01		
7893763	---	---	---	0.004	1.003	9.89E-01	9.89E+01		
7893764	---	---	---	0.155	1.113	4.68E-01	4.68E+01		
7893765	---	---	---	0.227	1.171	3.57E-01	3.57E+01		
7893766	---	---	---	0.085	1.061	7.64E-01	7.64E+01		
7893767	---	---	---	0.051	1.036	8.76E-01	8.76E+01		
7893768	---	---	---	0.168	1.124	7.42E-01	7.42E+01		
7893769	---	---	---	-0.039	0.973	7.63E-01	7.63E+01		
7893771	---	---	---	0.017	1.012	9.64E-01	9.64E+01		
7893773	---	---	---	0.042	1.029	5.98E-01	5.98E+01		
7893774	---	---	---	-0.231	0.852	1.76E-01	1.76E+01		
7893776	---	---	---	0.225	1.169	7.23E-01	7.23E+01		
7893777	---	---	---	0.131	1.095	5.34E-01	5.34E+01		
7893778	---	---	---	0.071	1.050	8.29E-01	8.29E+01		
7893779	---	---	---	0.024	1.017	8.69E-01	8.69E+01		
7893781	---	---	---	-0.024	0.984	9.03E-01	9.03E+01		
7893782	---	---	---	0.182	1.134	4.18E-01	4.18E+01		
7893783	---	---	---	-0.020	0.986	8.96E-01	8.96E+01		
7893784	---	---	---	-0.014	0.990	9.81E-01	9.81E+01		
7893785	---	---	---	-0.039	0.973	7.63E-01	7.63E+01		
7893786	---	---	---	0.321	1.249	2.83E-01	2.83E+01		
7893788	---	---	---	-0.059	0.960	7.23E-01	7.23E+01		
7893789	---	---	---	-0.073	0.951	8.13E-01	8.13E+01		
7893790	---	---	---	-0.049	0.967	8.14E-01	8.14E+01		
7893791	---	---	---	0.100	1.072	8.00E-01	8.00E+01		
7893792	---	---	---	0.030	1.021	8.75E-01	8.75E+01		
7893793	---	---	---	-0.069	0.953	7.97E-01	7.97E+01		
7893794	---	---	---	-0.067	0.955	9.19E-01	9.19E+01		

7893796	---	---	---	-0.020	0.986	9.63E-01	9.63E+01		
7893797	---	---	---	0.167	1.123	7.56E-01	7.56E+01		
7893798	---	---	---	-0.058	0.961	8.82E-01	8.82E+01		
7893800	---	---	---	0.086	1.061	6.60E-01	6.60E+01		
7893802	---	---	---	0.062	1.044	5.70E-01	5.70E+01		
7893804	---	---	---	0.300	1.231	4.43E-01	4.43E+01		
7893805	---	---	---	0.032	1.023	9.22E-01	9.22E+01		
7893806	---	---	---	0.018	1.013	9.64E-01	9.64E+01		
7893807	---	---	---	-0.085	0.943	7.41E-01	7.41E+01		
7893808	---	---	---	0.068	1.048	8.25E-01	8.25E+01		
7893809	---	---	---	-0.021	0.986	9.55E-01	9.55E+01		
7893810	---	---	---	-0.099	0.934	7.79E-01	7.79E+01		
7893812	---	---	---	-0.052	0.964	7.64E-01	7.64E+01		
7893813	---	---	---	0.111	1.080	8.06E-01	8.06E+01		
7893814	---	---	---	0.372	1.294	6.25E-01	6.25E+01		
7893815	---	---	---	0.068	1.048	4.04E-01	4.04E+01		
7893816	---	---	---	0.077	1.055	8.15E-01	8.15E+01		
7893817	---	---	---	0.151	1.110	8.04E-01	8.04E+01		
7893818	---	---	---	-0.090	0.939	7.34E-01	7.34E+01		
7893819	---	---	---	0.257	1.195	4.96E-01	4.96E+01		
7893820	---	---	---	-0.033	0.977	8.69E-01	8.69E+01		
7893821	---	---	---	0.155	1.114	5.92E-01	5.92E+01		
7893822	---	---	---	-0.048	0.967	8.91E-01	8.91E+01		
7893823	---	---	---	-0.558	0.679	6.30E-02	6.30E+00		
7893824	---	---	---	0.036	1.025	8.80E-01	8.80E+01		
7893825	---	---	---	0.179	1.132	6.36E-01	6.36E+01		
7893827	---	---	---	-0.148	0.902	3.43E-01	3.43E+01		
7893828	---	---	---	-0.045	0.969	9.24E-01	9.24E+01		
7893830	---	---	---	-0.147	0.903	8.53E-01	8.53E+01		
7893831	---	---	---	-0.189	0.877	3.19E-01	3.19E+01		
7893832	---	---	---	-0.166	0.891	8.56E-01	8.56E+01		
7893833	---	---	---	0.094	1.067	7.08E-01	7.08E+01		
7893834	---	---	---	0.003	1.002	9.97E-01	9.97E+01		
7893835	---	---	---	0.010	1.007	9.72E-01	9.72E+01		
7893836	---	---	---	0.025	1.017	8.56E-01	8.56E+01		
7893838	---	---	---	0.016	1.011	9.16E-01	9.16E+01		
7893839	---	---	---	0.125	1.091	8.53E-01	8.53E+01		
7893840	---	---	---	-0.084	0.943	4.97E-01	4.97E+01		
7893841	---	---	---	-0.159	0.896	4.03E-01	4.03E+01		
7893842	---	---	---	-0.177	0.884	5.04E-01	5.04E+01		
7893843	---	---	---	-0.072	0.951	9.26E-01	9.26E+01		
7893848	---	---	---	0.168	1.124	7.75E-01	7.75E+01		
7893849	---	---	---	-0.059	0.960	9.32E-01	9.32E+01		
7893850	---	---	---	0.006	1.004	9.85E-01	9.85E+01		
7893853	---	---	---	0.080	1.057	6.75E-01	6.75E+01		
7893855	---	---	---	-0.091	0.939	5.97E-01	5.97E+01		
7893856	---	---	---	-0.089	0.940	7.12E-01	7.12E+01		
7893857	---	---	---	-0.028	0.981	9.49E-01	9.49E+01		
7893861	---	---	---	0.066	1.046	7.24E-01	7.24E+01		
7893862	---	---	---	0.340	1.266	5.53E-01	5.53E+01		
7893866	---	---	---	-0.079	0.946	5.51E-01	5.51E+01		
7893867	---	---	---	-0.032	0.978	9.46E-01	9.46E+01		
7893868	---	---	---	0.107	1.077	8.24E-01	8.24E+01		
7893869	---	---	---	0.049	1.034	9.18E-01	9.18E+01		
7893870	---	---	---	0.017	1.012	9.74E-01	9.74E+01		
7893871	---	---	---	-0.098	0.934	6.92E-01	6.92E+01		
7893872	---	---	---	-0.134	0.912	5.53E-01	5.53E+01		
7893874	---	---	---	0.005	1.004	9.92E-01	9.92E+01		
7893875	---	---	---	-0.006	0.996	9.83E-01	9.83E+01		
7893876	---	---	---	-0.075	0.949	5.29E-01	5.29E+01		
7893877	---	---	---	0.032	1.023	9.67E-01	9.67E+01		
7893878	---	---	---	0.080	1.057	8.43E-01	8.43E+01		
7893879	---	---	---	-0.181	0.882	7.13E-01	7.13E+01		
7893880	---	---	---	0.098	1.070	4.27E-01	4.27E+01		
7893881	---	---	---	0.253	1.191	7.76E-02	7.76E+00		
7893883	---	---	---	0.180	1.133	4.96E-01	4.96E+01		
7893884	---	---	---	0.021	1.015	9.52E-01	9.52E+01		
7893886	---	---	---	0.133	1.096	7.44E-01	7.44E+01		
7893889	---	---	---	-0.010	0.993	9.75E-01	9.75E+01		
7893890	---	---	---	0.138	1.101	8.01E-01	8.01E+01		
7893892	---	---	---	-0.187	0.878	4.20E-01	4.20E+01		
7893893	---	---	---	0.074	1.052	6.02E-01	6.02E+01		
7893894	---	---	---	0.033	1.023	9.53E-01	9.53E+01		
7893896	---	---	---	-0.046	0.968	9.12E-01	9.12E+01		
7893898	---	---	---	0.353	1.278	4.12E-01	4.12E+01		
7893900	---	---	---	0.028	1.019	9.40E-01	9.40E+01		
7893901	---	---	---	0.028	1.020	9.09E-01	9.09E+01		

7893902	---	---	---	0.015	1.011	9.83E-01	9.83E+01		
7893904	---	---	---	-0.020	0.986	9.53E-01	9.53E+01		
7893905	---	---	---	-0.071	0.952	7.75E-01	7.75E+01		
7893906	---	---	---	-0.027	0.982	9.64E-01	9.64E+01		
7893908	---	---	---	0.058	1.041	9.34E-01	9.34E+01		
7893910	---	---	---	-0.138	0.909	4.12E-01	4.12E+01		
7893913	---	---	---	0.078	1.055	7.61E-01	7.61E+01		
7893914	---	---	---	0.030	1.021	8.31E-01	8.31E+01		
7893915	---	---	---	0.382	1.303	4.99E-01	4.99E+01		
7893916	---	---	---	-0.092	0.939	5.52E-01	5.52E+01		
7893919	---	---	---	-0.020	0.986	9.24E-01	9.24E+01		
7893920	---	---	---	-0.228	0.854	7.16E-01	7.16E+01		
7893921	---	---	---	-0.333	0.794	1.11E-01	1.11E+01		
7893922	---	---	---	0.094	1.067	6.11E-01	6.11E+01		
7893923	---	---	---	0.041	1.029	6.94E-01	6.94E+01		
7893924	---	---	---	0.343	1.268	6.06E-01	6.06E+01		
7893925	---	---	---	0.244	1.184	1.86E-01	1.86E+01		
7893926	---	---	---	-0.094	0.937	4.51E-01	4.51E+01		
7893928	---	---	---	-0.004	0.997	9.87E-01	9.87E+01		
7893929	---	---	---	0.015	1.010	9.62E-01	9.62E+01		
7893932	---	---	---	-0.140	0.908	4.04E-01	4.04E+01		
7893933	---	---	---	0.147	1.107	4.26E-01	4.26E+01		
7893934	---	---	---	-0.069	0.953	7.72E-01	7.72E+01		
7893936	---	---	---	0.049	1.035	9.15E-01	9.15E+01		
7893937	---	---	---	0.069	1.049	6.54E-01	6.54E+01		
7893938	---	---	---	-0.045	0.970	8.26E-01	8.26E+01		
7893939	---	---	---	0.064	1.045	8.06E-01	8.06E+01		
7893940	---	---	---	0.123	1.089	8.10E-01	8.10E+01		
7893941	---	---	---	-0.072	0.951	7.13E-01	7.13E+01		
7893942	---	---	---	-0.058	0.961	8.60E-01	8.60E+01		
7893943	---	---	---	-0.263	0.833	5.17E-01	5.17E+01		
7893946	---	---	---	0.093	1.067	5.55E-01	5.55E+01		
7893947	---	---	---	0.054	1.038	9.03E-01	9.03E+01		
7893948	---	---	---	0.035	1.025	8.96E-01	8.96E+01		
7893949	---	---	---	0.038	1.026	8.27E-01	8.27E+01		
7893950	---	---	---	0.237	1.178	8.06E-01	8.06E+01		
7893952	---	---	---	-0.030	0.979	9.03E-01	9.03E+01		
7893953	---	---	---	0.043	1.030	7.67E-01	7.67E+01		
7893954	---	---	---	0.088	1.063	5.96E-01	5.96E+01		
7893955	---	---	---	0.076	1.054	8.74E-01	8.74E+01		
7893956	---	---	---	0.025	1.018	8.06E-01	8.06E+01		
7893957	---	---	---	-0.042	0.972	9.53E-01	9.53E+01		
7893958	---	---	---	-0.116	0.922	4.32E-01	4.32E+01		
7893959	---	---	---	-0.040	0.973	9.28E-01	9.28E+01		
7893960	---	---	---	0.023	1.016	9.12E-01	9.12E+01		
7893961	---	---	---	-0.163	0.893	2.56E-01	2.56E+01		
7893962	---	---	---	0.047	1.033	9.32E-01	9.32E+01		
7893965	---	---	---	0.030	1.021	9.64E-01	9.64E+01		
7893966	---	---	---	-0.016	0.989	9.53E-01	9.53E+01		
7893967	---	---	---	-0.006	0.996	9.90E-01	9.90E+01		
7893968	---	---	---	-0.023	0.984	9.27E-01	9.27E+01		
7893969	---	---	---	-0.004	0.997	9.96E-01	9.96E+01		
7893972	---	---	---	-0.012	0.992	9.64E-01	9.64E+01		
7893973	---	---	---	0.129	1.093	5.41E-01	5.41E+01		
7893976	---	---	---	0.057	1.041	8.57E-01	8.57E+01		
7893978	---	---	---	0.128	1.093	7.97E-01	7.97E+01		
7893979	---	---	---	-0.092	0.938	6.50E-01	6.50E+01		
7893980	---	---	---	0.003	1.002	9.97E-01	9.97E+01		
7893981	---	---	---	-0.059	0.960	8.34E-01	8.34E+01		
7893982	---	---	---	0.228	1.171	6.87E-01	6.87E+01		
7893983	---	---	---	0.059	1.041	9.50E-01	9.50E+01		
7893985	---	---	---	0.045	1.032	9.27E-01	9.27E+01		
7893986	---	---	---	0.181	1.134	5.38E-01	5.38E+01		
7893989	---	---	---	-0.131	0.913	8.62E-01	8.62E+01		
7893991	---	---	---	-0.139	0.908	4.97E-01	4.97E+01		
7893992	---	---	---	-0.171	0.888	7.52E-01	7.52E+01		
7893993	---	---	---	0.014	1.010	8.99E-01	8.99E+01		
7893997	---	---	---	-0.048	0.968	9.36E-01	9.36E+01		
7893998	---	---	---	0.176	1.130	6.17E-01	6.17E+01		
7894000	---	---	---	0.034	1.024	7.53E-01	7.53E+01		
7894001	---	---	---	-0.123	0.918	8.31E-01	8.31E+01		
7894002	---	---	---	0.129	1.094	7.75E-01	7.75E+01		
7894005	---	---	---	-0.050	0.966	7.23E-01	7.23E+01		
7894007	---	---	---	0.017	1.012	9.12E-01	9.12E+01		
7894008	---	---	---	-0.083	0.944	8.68E-01	8.68E+01		
7894009	---	---	---	0.081	1.058	8.33E-01	8.33E+01		
7894010	---	---	---	-0.100	0.933	7.63E-01	7.63E+01		

7894011	---	---	---	0.048	1.034	8.58E-01	8.58E+01		
7894012	---	---	---	-0.077	0.948	6.48E-01	6.48E+01		
7894013	---	---	---	-0.058	0.960	9.36E-01	9.36E+01		
7894015	---	---	---	-0.057	0.961	7.53E-01	7.53E+01		
7894016	---	---	---	0.057	1.040	9.16E-01	9.16E+01		
7894017	---	---	---	-0.100	0.933	8.52E-01	8.52E+01		
7894018	---	---	---	0.220	1.165	5.03E-01	5.03E+01		
7894019	---	---	---	0.043	1.030	8.78E-01	8.78E+01		
7894020	---	---	---	-0.063	0.957	8.52E-01	8.52E+01		
7894024	---	---	---	-0.201	0.870	6.77E-01	6.77E+01		
7894026	---	---	---	0.218	1.163	4.16E-01	4.16E+01		
7894027	---	---	---	0.089	1.064	8.52E-01	8.52E+01		
7894028	---	---	---	0.100	1.072	8.04E-01	8.04E+01		
7894029	---	---	---	-0.110	0.926	3.56E-01	3.56E+01		
7894030	---	---	---	0.036	1.025	9.23E-01	9.23E+01		
7894031	---	---	---	0.019	1.013	9.27E-01	9.27E+01		
7894033	---	---	---	0.036	1.025	7.95E-01	7.95E+01		
7894036	---	---	---	0.034	1.024	8.15E-01	8.15E+01		
7894037	---	---	---	-0.026	0.982	8.67E-01	8.67E+01		
7894041	---	---	---	-0.090	0.939	6.37E-01	6.37E+01		
7894042	---	---	---	0.020	1.014	9.53E-01	9.53E+01		
7894043	---	---	---	-0.040	0.973	7.10E-01	7.10E+01		
7894044	---	---	---	0.148	1.108	6.52E-01	6.52E+01		
7894045	---	---	---	0.131	1.095	6.07E-01	6.07E+01		
7894046	---	---	---	-0.033	0.977	9.35E-01	9.35E+01		
7894048	---	---	---	0.047	1.033	7.99E-01	7.99E+01		
7894049	---	---	---	-0.073	0.951	8.01E-01	8.01E+01		
7894050	---	---	---	0.226	1.170	4.53E-01	4.53E+01		
7894051	---	---	---	-0.006	0.996	9.86E-01	9.86E+01		
7894052	---	---	---	-0.032	0.978	9.53E-01	9.53E+01		
7894055	---	---	---	0.007	1.005	9.84E-01	9.84E+01		
7894056	---	---	---	-0.083	0.944	7.46E-01	7.46E+01		
7894057	---	---	---	-0.044	0.970	8.49E-01	8.49E+01		
7894058	---	---	---	-0.037	0.974	8.73E-01	8.73E+01		
7894060	---	---	---	-0.151	0.901	5.17E-01	5.17E+01		
7894061	---	---	---	0.018	1.013	9.35E-01	9.35E+01		
7894062	---	---	---	0.099	1.071	6.34E-01	6.34E+01		
7894063	---	---	---	0.009	1.006	9.70E-01	9.70E+01		
7894064	---	---	---	-0.106	0.929	8.08E-01	8.08E+01		
7894067	---	---	---	-0.053	0.964	9.28E-01	9.28E+01		
7894068	---	---	---	-0.096	0.935	7.82E-01	7.82E+01		
7894069	---	---	---	0.169	1.124	6.91E-01	6.91E+01		
7894070	---	---	---	0.004	1.003	9.90E-01	9.90E+01		
7894072	---	---	---	0.004	1.003	9.85E-01	9.85E+01		
7894073	---	---	---	-0.182	0.882	7.38E-01	7.38E+01		
7894074	---	---	---	0.073	1.052	6.09E-01	6.09E+01		
7894075	---	---	---	0.051	1.036	9.28E-01	9.28E+01		
7894076	---	---	---	-0.080	0.946	7.51E-01	7.51E+01		
7894078	---	---	---	-0.051	0.965	8.53E-01	8.53E+01		
7894080	---	---	---	0.122	1.088	6.89E-01	6.89E+01		
7894081	---	---	---	0.170	1.125	7.56E-01	7.56E+01		
7894083	---	---	---	0.076	1.054	6.59E-01	6.59E+01		
7894085	---	---	---	0.037	1.026	8.64E-01	8.64E+01		
7894086	---	---	---	-0.013	0.991	9.51E-01	9.51E+01		
7894087	---	---	---	-0.048	0.967	7.27E-01	7.27E+01		
7894088	---	---	---	0.056	1.039	7.50E-01	7.50E+01		
7894089	---	---	---	-0.038	0.974	9.16E-01	9.16E+01		
7894091	---	---	---	-0.094	0.937	8.94E-01	8.94E+01		
7894092	---	---	---	0.100	1.072	5.48E-01	5.48E+01		
7894093	---	---	---	0.134	1.098	8.16E-01	8.16E+01		
7894094	---	---	---	0.126	1.091	4.23E-01	4.23E+01		
7894096	---	---	---	-0.148	0.902	6.24E-01	6.24E+01		
7894097	---	---	---	0.067	1.047	6.84E-01	6.84E+01		
7894098	---	---	---	-0.026	0.982	8.92E-01	8.92E+01		
7894100	---	---	---	-0.070	0.953	7.87E-01	7.87E+01		
7894102	---	---	---	0.025	1.018	8.86E-01	8.86E+01		
7894103	---	---	---	-0.105	0.930	7.15E-01	7.15E+01		
7894104	---	---	---	-0.041	0.972	7.36E-01	7.36E+01		
7894106	---	---	---	-0.060	0.960	8.91E-01	8.91E+01		
7894107	---	---	---	0.098	1.070	6.06E-01	6.06E+01		
7894110	---	---	---	0.054	1.038	7.49E-01	7.49E+01		
7894111	---	---	---	0.004	1.002	9.96E-01	9.96E+01		
7894112	---	---	---	-0.419	0.748	4.15E-01	4.15E+01		
7894114	---	---	---	0.024	1.017	9.27E-01	9.27E+01		
7894115	---	---	---	0.010	1.007	9.80E-01	9.80E+01		
7894116	---	---	---	0.068	1.048	8.87E-01	8.87E+01		
7894117	---	---	---	-0.060	0.959	8.42E-01	8.42E+01		

7894118	---	---	---	-0.055	0.963	8.82E-01	8.82E+01			
7894119	---	---	---	-0.080	0.946	5.83E-01	5.83E+01			
7894120	---	---	---	0.271	1.206	4.70E-01	4.70E+01			
7894122	---	---	---	0.027	1.019	8.05E-01	8.05E+01			
7894124	---	---	---	0.049	1.035	9.50E-01	9.50E+01			
7894125	---	---	---	-0.004	0.997	9.86E-01	9.86E+01			
7894126	---	---	---	0.106	1.076	2.94E-01	2.94E+01			
7894128	---	---	---	0.252	1.191	3.41E-01	3.41E+01			
7894129	---	---	---	0.125	1.090	8.07E-01	8.07E+01			
7894131	---	---	---	0.113	1.082	3.60E-01	3.60E+01			
7894132	---	---	---	-0.042	0.971	8.41E-01	8.41E+01			
7894134	---	---	---	-0.138	0.909	1.59E-01	1.59E+01			
7894135	---	---	---	-0.072	0.951	4.48E-01	4.48E+01			
7894136	---	---	---	0.196	1.146	1.99E-01	1.99E+01			
7894138	---	---	---	-0.144	0.905	5.67E-01	5.67E+01			
7894140	---	---	---	-0.027	0.981	9.64E-01	9.64E+01			
7894141	---	---	---	0.029	1.021	9.37E-01	9.37E+01			
7894142	---	---	---	0.075	1.053	8.06E-01	8.06E+01			
7894144	---	---	---	0.026	1.018	9.57E-01	9.57E+01			
7894145	---	---	---	-0.001	0.999	9.94E-01	9.94E+01			
7894146	---	---	---	0.039	1.028	9.03E-01	9.03E+01			
7894148	---	---	---	0.106	1.076	4.97E-01	4.97E+01			
7894149	---	---	---	0.088	1.063	8.84E-01	8.84E+01			
7894151	---	---	---	0.243	1.183	5.54E-01	5.54E+01			
7894152	---	---	---	0.185	1.137	4.43E-01	4.43E+01			
7894153	---	---	---	0.213	1.159	5.51E-01	5.51E+01			
7894154	---	---	---	0.085	1.061	8.47E-01	8.47E+01			
7894155	---	---	---	-0.005	0.996	9.89E-01	9.89E+01			
7894156	---	---	---	-0.005	0.997	9.90E-01	9.90E+01			
7894158	---	---	---	-0.049	0.967	8.13E-01	8.13E+01			
7894159	---	---	---	0.011	1.008	9.77E-01	9.77E+01			
7894160	---	---	---	-0.144	0.905	5.19E-01	5.19E+01			
7894161	---	---	---	-0.028	0.981	9.02E-01	9.02E+01			
7894162	---	---	---	0.097	1.069	8.98E-01	8.98E+01			
7894163	---	---	---	0.146	1.107	5.60E-01	5.60E+01			
7894165	---	---	---	0.105	1.075	8.09E-01	8.09E+01			
7894167	---	---	---	-0.001	1.000	1.00E+00	1.00E+02			
7894169	---	---	---	0.098	1.070	7.89E-01	7.89E+01			
7894170	---	---	---	0.206	1.153	6.89E-01	6.89E+01			
7894171	---	---	---	0.017	1.012	9.49E-01	9.49E+01			
7894173	---	---	---	-0.210	0.865	3.65E-01	3.65E+01			
7894174	---	---	---	-0.126	0.917	5.81E-01	5.81E+01			
7894176	---	---	---	0.121	1.087	7.85E-01	7.85E+01			
7894177	---	---	---	0.071	1.051	7.49E-01	7.49E+01			
7894178	---	---	---	0.114	1.082	7.26E-01	7.26E+01			
7894180	---	---	---	-0.197	0.873	5.79E-01	5.79E+01			
7894181	---	---	---	0.203	1.151	1.11E-01	1.11E+01			
7894182	---	---	---	0.143	1.104	6.00E-01	6.00E+01			
7894183	---	---	---	-0.003	0.998	9.96E-01	9.96E+01			
7894184	---	---	---	0.031	1.022	9.65E-01	9.65E+01			
7894185	---	---	---	0.061	1.043	7.16E-01	7.16E+01			
7894186	---	---	---	-0.039	0.973	8.78E-01	8.78E+01			
7894188	---	---	---	0.087	1.062	8.78E-01	8.78E+01			
7894189	---	---	---	-0.144	0.905	4.02E-01	4.02E+01			
7894191	---	---	---	-0.294	0.815	4.16E-01	4.16E+01			
7894192	---	---	---	0.038	1.027	7.49E-01	7.49E+01			
7894193	---	---	---	0.272	1.208	4.39E-01	4.39E+01			
7894194	---	---	---	-0.171	0.888	6.11E-01	6.11E+01			
7894195	---	---	---	0.047	1.033	8.63E-01	8.63E+01			
7894196	---	---	---	-0.110	0.926	8.62E-01	8.62E+01			
7894200	---	---	---	0.014	1.010	9.78E-01	9.78E+01			
7894201	---	---	---	0.034	1.024	9.61E-01	9.61E+01			
7894202	---	---	---	0.199	1.148	8.29E-01	8.29E+01			
7894203	---	---	---	0.244	1.185	5.70E-01	5.70E+01			
7894204	---	---	---	-0.065	0.956	9.35E-01	9.35E+01			
7894205	---	---	---	-0.010	0.993	9.65E-01	9.65E+01			
7894207	---	---	---	0.002	1.001	9.92E-01	9.92E+01			
7894210	---	---	---	0.150	1.110	7.09E-01	7.09E+01			
7894211	---	---	---	0.101	1.073	4.41E-01	4.41E+01			
7894212	---	---	---	0.160	1.117	3.91E-01	3.91E+01			
7894213	---	---	---	-0.002	0.999	9.91E-01	9.91E+01			
7894214	---	---	---	0.019	1.013	8.96E-01	8.96E+01			
7894215	---	---	---	0.025	1.017	9.33E-01	9.33E+01			
7894216	---	---	---	0.155	1.114	7.37E-01	7.37E+01			
7894217	---	---	---	-0.049	0.967	8.81E-01	8.81E+01			
7894218	---	---	---	-0.081	0.945	3.89E-01	3.89E+01			
7894219	---	---	---	-0.259	0.835	3.79E-01	3.79E+01			

7894220	---	---	---	0.169	1.125	6.04E-01	6.04E+01		
7894221	---	---	---	0.184	1.136	7.69E-01	7.69E+01		
7894223	---	---	---	0.030	1.021	8.33E-01	8.33E+01		
7894224	---	---	---	-0.090	0.940	8.68E-01	8.68E+01		
7894225	---	---	---	0.086	1.061	7.75E-01	7.75E+01		
7894226	---	---	---	-0.069	0.953	8.96E-01	8.96E+01		
7894227	---	---	---	0.291	1.223	5.96E-01	5.96E+01		
7894229	---	---	---	-0.042	0.972	9.30E-01	9.30E+01		
7894231	---	---	---	-0.054	0.963	8.71E-01	8.71E+01		
7894232	---	---	---	0.029	1.020	8.76E-01	8.76E+01		
7894234	---	---	---	0.181	1.133	4.43E-01	4.43E+01		
7894235	---	---	---	0.025	1.017	9.09E-01	9.09E+01		
7894236	---	---	---	0.034	1.024	5.56E-01	5.56E+01		
7894237	---	---	---	-0.020	0.986	9.66E-01	9.66E+01		
7894238	---	---	---	0.602	1.518	2.95E-01	2.95E+01		
7894239	---	---	---	-0.016	0.989	8.78E-01	8.78E+01		
7894240	---	---	---	-0.256	0.838	6.67E-01	6.67E+01		
7894242	---	---	---	-0.002	0.999	9.97E-01	9.97E+01		
7894246	---	---	---	-0.031	0.979	9.14E-01	9.14E+01		
7894247	---	---	---	0.011	1.007	9.83E-01	9.83E+01		
7894249	---	---	---	-0.002	0.999	9.95E-01	9.95E+01		
7894251	---	---	---	0.136	1.099	7.09E-01	7.09E+01		
7894252	---	---	---	0.143	1.104	2.21E-01	2.21E+01		
7894254	---	---	---	0.086	1.061	8.48E-01	8.48E+01		
7894256	---	---	---	0.031	1.021	8.68E-01	8.68E+01		
7894257	---	---	---	0.049	1.035	9.19E-01	9.19E+01		
7894258	---	---	---	-0.046	0.969	8.38E-01	8.38E+01		
7894262	---	---	---	-0.064	0.957	8.29E-01	8.29E+01		
7894263	---	---	---	0.009	1.006	9.37E-01	9.37E+01		
7894264	---	---	---	0.068	1.049	8.52E-01	8.52E+01		
7894266	---	---	---	0.020	1.014	9.33E-01	9.33E+01		
7894267	---	---	---	0.010	1.007	9.68E-01	9.68E+01		
7894268	---	---	---	-0.348	0.785	5.03E-01	5.03E+01		
7894269	---	---	---	0.049	1.035	7.92E-01	7.92E+01		
7894270	---	---	---	0.387	1.308	5.53E-01	5.53E+01		
7894271	---	---	---	-0.209	0.865	8.32E-01	8.32E+01		
7894272	---	---	---	0.196	1.145	5.49E-01	5.49E+01		
7894273	---	---	---	0.078	1.055	7.49E-01	7.49E+01		
7894274	---	---	---	0.226	1.169	3.75E-01	3.75E+01		
7894276	---	---	---	0.148	1.108	7.75E-01	7.75E+01		
7894277	---	---	---	0.013	1.009	9.77E-01	9.77E+01		
7894278	---	---	---	0.074	1.052	4.17E-01	4.17E+01		
7894280	---	---	---	0.148	1.108	8.25E-01	8.25E+01		
7894281	---	---	---	0.056	1.039	8.95E-01	8.95E+01		
7894282	---	---	---	0.098	1.070	3.72E-01	3.72E+01		
7894283	---	---	---	0.014	1.010	9.84E-01	9.84E+01		
7894284	---	---	---	0.112	1.080	5.41E-01	5.41E+01		
7894285	---	---	---	0.064	1.045	9.28E-01	9.28E+01		
7894286	---	---	---	0.155	1.114	5.98E-01	5.98E+01		
7894288	---	---	---	0.065	1.046	5.98E-01	5.98E+01		
7894289	---	---	---	0.229	1.172	1.14E-01	1.14E+01		
7894290	---	---	---	-0.049	0.966	8.96E-01	8.96E+01		
7894291	---	---	---	-0.167	0.891	3.18E-01	3.18E+01		
7894292	---	---	---	0.278	1.212	7.09E-01	7.09E+01		
7894293	---	---	---	0.008	1.006	9.80E-01	9.80E+01		
7894294	---	---	---	-0.015	0.989	9.50E-01	9.50E+01		
7894295	---	---	---	-0.017	0.988	9.58E-01	9.58E+01		
7894296	---	---	---	-0.150	0.901	5.82E-01	5.82E+01		
7894297	---	---	---	0.208	1.155	5.34E-01	5.34E+01		
7894298	---	---	---	0.112	1.081	4.27E-01	4.27E+01		
7894299	---	---	---	0.007	1.005	9.81E-01	9.81E+01		
7894300	---	---	---	0.092	1.066	8.83E-01	8.83E+01		
7894301	---	---	---	-0.174	0.887	5.04E-01	5.04E+01		
7894303	---	---	---	-0.066	0.955	8.34E-01	8.34E+01		
7894304	---	---	---	0.108	1.077	5.81E-01	5.81E+01		
7894305	---	---	---	-0.026	0.982	9.53E-01	9.53E+01		
7894306	---	---	---	-0.054	0.964	7.98E-01	7.98E+01		
7894307	---	---	---	-0.016	0.989	8.81E-01	8.81E+01		
7894308	---	---	---	0.047	1.033	7.51E-01	7.51E+01		
7894309	---	---	---	0.012	1.008	9.68E-01	9.68E+01		
7894310	---	---	---	-0.117	0.922	9.19E-01	9.19E+01		
7894312	---	---	---	-0.074	0.950	8.41E-01	8.41E+01		
7894314	---	---	---	0.074	1.053	8.84E-01	8.84E+01		
7894315	---	---	---	0.024	1.017	9.57E-01	9.57E+01		
7894316	---	---	---	0.027	1.019	9.52E-01	9.52E+01		
7894318	---	---	---	0.233	1.175	6.56E-01	6.56E+01		
7894320	---	---	---	-0.038	0.974	7.82E-01	7.82E+01		

7894321	---	---	---	0.113	1.081	7.53E-01	7.53E+01		
7894324	---	---	---	0.041	1.029	8.34E-01	8.34E+01		
7894328	---	---	---	-0.053	0.964	9.56E-01	9.56E+01		
7894330	---	---	---	-0.094	0.937	9.30E-01	9.30E+01		
7894331	---	---	---	0.104	1.075	7.63E-01	7.63E+01		
7894332	---	---	---	0.332	1.259	4.37E-01	4.37E+01		
7894336	---	---	---	0.163	1.119	1.75E-01	1.75E+01		
7894337	---	---	---	0.130	1.095	3.86E-01	3.86E+01		
7894341	---	---	---	0.011	1.008	9.87E-01	9.87E+01		
7894343	---	---	---	-0.207	0.867	3.18E-01	3.18E+01		
7894345	---	---	---	-0.141	0.907	8.09E-01	8.09E+01		
7894346	---	---	---	0.131	1.095	5.14E-01	5.14E+01		
7894350	---	---	---	-0.111	0.926	3.97E-01	3.97E+01		
7894351	---	---	---	0.033	1.023	9.44E-01	9.44E+01		
7894352	---	---	---	-0.129	0.915	5.95E-01	5.95E+01		
7894354	---	---	---	0.273	1.208	6.25E-01	6.25E+01		
7894355	---	---	---	0.154	1.113	5.82E-01	5.82E+01		
7894356	---	---	---	0.089	1.064	7.89E-01	7.89E+01		
7894360	---	---	---	0.023	1.016	9.72E-01	9.72E+01		
7894361	---	---	---	0.137	1.099	4.48E-01	4.48E+01		
7894362	---	---	---	-0.168	0.890	8.25E-01	8.25E+01		
7894363	---	---	---	-0.470	0.722	6.33E-01	6.33E+01		
7894364	---	---	---	0.067	1.047	9.18E-01	9.18E+01		
7894366	---	---	---	-0.090	0.939	5.60E-01	5.60E+01		
7894367	---	---	---	0.035	1.024	8.04E-01	8.04E+01		
7894369	---	---	---	-0.048	0.968	7.53E-01	7.53E+01		
7894370	---	---	---	0.080	1.057	9.09E-01	9.09E+01		
7894371	---	---	---	-0.025	0.983	9.57E-01	9.57E+01		
7894372	---	---	---	-0.006	0.996	9.73E-01	9.73E+01		
7894373	---	---	---	-0.051	0.965	8.62E-01	8.62E+01		
7894374	---	---	---	-0.155	0.898	6.05E-01	6.05E+01		
7894375	---	---	---	0.079	1.056	6.33E-01	6.33E+01		
7894376	---	---	---	0.013	1.009	9.18E-01	9.18E+01		
7894377	---	---	---	0.220	1.165	7.92E-01	7.92E+01		
7894379	---	---	---	0.131	1.095	4.56E-01	4.56E+01		
7894380	---	---	---	-0.049	0.967	7.20E-01	7.20E+01		
7894381	---	---	---	-0.265	0.832	7.74E-01	7.74E+01		
7894382	---	---	---	0.038	1.027	7.05E-01	7.05E+01		
7894383	---	---	---	0.053	1.038	9.53E-01	9.53E+01		
7894384	---	---	---	0.036	1.025	9.37E-01	9.37E+01		
7894385	---	---	---	0.134	1.097	7.72E-01	7.72E+01		
7894386	---	---	---	0.044	1.031	8.05E-01	8.05E+01		
7894387	---	---	---	0.037	1.026	9.77E-01	9.77E+01		
7894388	---	---	---	-0.086	0.942	7.48E-01	7.48E+01		
7894389	---	---	---	0.088	1.063	7.13E-01	7.13E+01		
7894391	---	---	---	-0.167	0.891	5.95E-01	5.95E+01		
7894392	---	---	---	0.035	1.024	7.97E-01	7.97E+01		
7894395	---	---	---	0.242	1.183	1.18E-02	1.18E+00		
7894396	---	---	---	-0.007	0.995	9.90E-01	9.90E+01		
7894398	---	---	---	0.121	1.088	9.00E-01	9.00E+01		
7894399	---	---	---	-0.006	0.996	9.85E-01	9.85E+01		
7894400	---	---	---	0.053	1.037	8.54E-01	8.54E+01		
7894402	---	---	---	0.028	1.020	9.00E-01	9.00E+01		
7894403	---	---	---	-0.085	0.943	8.10E-01	8.10E+01		
7894404	---	---	---	0.002	1.001	9.87E-01	9.87E+01		
7894405	---	---	---	0.144	1.105	6.98E-01	6.98E+01		
7894406	---	---	---	0.008	1.006	9.56E-01	9.56E+01		
7894408	---	---	---	0.028	1.019	9.77E-01	9.77E+01		
7894409	---	---	---	0.052	1.037	9.30E-01	9.30E+01		
7894411	---	---	---	0.144	1.105	2.66E-01	2.66E+01		
7894412	---	---	---	-0.138	0.909	4.01E-01	4.01E+01		
7894413	---	---	---	0.055	1.039	7.99E-01	7.99E+01		
7894415	---	---	---	0.245	1.185	7.45E-01	7.45E+01		
7894416	---	---	---	-0.072	0.951	8.18E-01	8.18E+01		
7894418	---	---	---	0.031	1.022	8.52E-01	8.52E+01		
7894419	---	---	---	-0.057	0.961	8.75E-01	8.75E+01		
7894420	---	---	---	0.036	1.025	9.53E-01	9.53E+01		
7894421	---	---	---	-0.055	0.963	7.73E-01	7.73E+01		
7894422	---	---	---	0.012	1.008	9.85E-01	9.85E+01		
7894427	---	---	---	-0.095	0.936	8.11E-01	8.11E+01		
7894428	---	---	---	-0.015	0.990	9.65E-01	9.65E+01		
7894429	---	---	---	-0.063	0.957	8.74E-01	8.74E+01		
7894430	---	---	---	0.209	1.156	7.52E-01	7.52E+01		
7894431	---	---	---	-0.079	0.947	7.50E-01	7.50E+01		
7894432	---	---	---	-0.022	0.985	9.49E-01	9.49E+01		
7894433	---	---	---	0.074	1.053	8.03E-01	8.03E+01		
7894434	---	---	---	0.082	1.059	6.11E-01	6.11E+01		

7894435	---	---	---	0.009	1.006	9.83E-01	9.83E+01		
7894436	---	---	---	-0.057	0.961	8.77E-01	8.77E+01		
7894438	---	---	---	-0.130	0.914	6.71E-01	6.71E+01		
7894439	---	---	---	0.025	1.017	8.27E-01	8.27E+01		
7894440	---	---	---	0.201	1.149	7.35E-01	7.35E+01		
7894442	---	---	---	0.201	1.150	6.78E-01	6.78E+01		
7894443	---	---	---	0.063	1.045	8.14E-01	8.14E+01		
7894444	---	---	---	0.054	1.038	6.66E-01	6.66E+01		
7894445	---	---	---	0.267	1.204	2.66E-01	2.66E+01		
7894446	---	---	---	0.162	1.118	5.71E-01	5.71E+01		
7894447	---	---	---	0.031	1.022	8.80E-01	8.80E+01		
7894449	---	---	---	-0.024	0.983	9.24E-01	9.24E+01		
7894451	---	---	---	-0.161	0.894	3.29E-01	3.29E+01		
7894452	---	---	---	0.205	1.153	5.99E-01	5.99E+01		
7894453	---	---	---	0.130	1.095	6.06E-01	6.06E+01		
7894454	---	---	---	-0.118	0.921	8.22E-01	8.22E+01		
7894456	---	---	---	0.090	1.064	4.14E-01	4.14E+01		
7894457	---	---	---	-0.163	0.893	7.45E-01	7.45E+01		
7894459	---	---	---	0.146	1.107	6.05E-01	6.05E+01		
7894460	---	---	---	0.033	1.023	7.64E-01	7.64E+01		
7894461	---	---	---	0.008	1.006	9.83E-01	9.83E+01		
7894462	---	---	---	-0.256	0.837	3.66E-01	3.66E+01		
7894464	---	---	---	0.074	1.053	7.33E-01	7.33E+01		
7894465	---	---	---	-0.127	0.916	8.75E-01	8.75E+01		
7894466	---	---	---	-0.105	0.930	7.52E-01	7.52E+01		
7894467	---	---	---	-0.014	0.990	9.76E-01	9.76E+01		
7894469	---	---	---	-0.064	0.957	7.03E-01	7.03E+01		
7894470	---	---	---	-0.173	0.887	1.06E-01	1.06E+01		
7894471	---	---	---	0.036	1.025	6.69E-01	6.69E+01		
7894473	---	---	---	0.227	1.171	7.22E-01	7.22E+01		
7894474	---	---	---	0.029	1.021	8.95E-01	8.95E+01		
7894475	---	---	---	0.047	1.033	8.07E-01	8.07E+01		
7894476	---	---	---	-0.049	0.967	8.63E-01	8.63E+01		
7894478	---	---	---	0.075	1.053	6.73E-01	6.73E+01		
7894479	---	---	---	0.106	1.076	8.08E-01	8.08E+01		
7894480	---	---	---	-0.050	0.966	7.38E-01	7.38E+01		
7894481	---	---	---	0.015	1.011	9.07E-01	9.07E+01		
7894483	---	---	---	-0.085	0.943	8.56E-01	8.56E+01		
7894484	---	---	---	-0.077	0.948	8.01E-01	8.01E+01		
7894485	---	---	---	0.027	1.019	7.17E-01	7.17E+01		
7894486	---	---	---	-0.221	0.858	5.34E-01	5.34E+01		
7894489	---	---	---	-0.030	0.980	9.23E-01	9.23E+01		
7894490	---	---	---	0.084	1.060	6.09E-01	6.09E+01		
7894491	---	---	---	-0.075	0.949	9.23E-01	9.23E+01		
7894492	---	---	---	-0.046	0.969	8.08E-01	8.08E+01		
7894493	---	---	---	0.128	1.093	4.84E-01	4.84E+01		
7894494	---	---	---	-0.026	0.982	9.64E-01	9.64E+01		
7894495	---	---	---	0.044	1.031	7.97E-01	7.97E+01		
7894496	---	---	---	0.072	1.051	7.40E-01	7.40E+01		
7894497	---	---	---	-0.013	0.991	9.85E-01	9.85E+01		
7894498	---	---	---	-0.094	0.937	6.38E-01	6.38E+01		
7894499	---	---	---	0.010	1.007	9.58E-01	9.58E+01		
7894501	---	---	---	0.144	1.105	2.42E-01	2.42E+01		
7894503	---	---	---	-0.042	0.972	8.62E-01	8.62E+01		
7894504	---	---	---	-0.037	0.974	8.62E-01	8.62E+01		
7894505	---	---	---	0.241	1.181	4.82E-01	4.82E+01		
7894506	---	---	---	-0.128	0.915	6.89E-01	6.89E+01		
7894508	---	---	---	-0.084	0.943	9.11E-01	9.11E+01		
7894509	---	---	---	0.076	1.054	8.56E-01	8.56E+01		
7894510	---	---	---	0.075	1.053	9.44E-01	9.44E+01		
7894511	---	---	---	-0.030	0.979	9.50E-01	9.50E+01		
7894512	---	---	---	-0.139	0.908	6.91E-01	6.91E+01		
7894513	---	---	---	-0.039	0.973	9.17E-01	9.17E+01		
7894515	---	---	---	0.169	1.124	2.35E-01	2.35E+01		
7894516	---	---	---	0.005	1.003	9.86E-01	9.86E+01		
7894517	---	---	---	-0.041	0.972	9.54E-01	9.54E+01		
7894518	---	---	---	-0.018	0.987	9.18E-01	9.18E+01		
7894519	---	---	---	-0.032	0.978	8.82E-01	8.82E+01		
7894520	---	---	---	0.122	1.089	4.36E-01	4.36E+01		
7894522	---	---	---	0.042	1.030	6.97E-01	6.97E+01		
7894523	---	---	---	-0.031	0.979	8.64E-01	8.64E+01		
7894525	---	---	---	0.311	1.240	2.95E-02	2.95E+00		
7894526	---	---	---	-0.038	0.974	9.12E-01	9.12E+01		
7894527	---	---	---	0.100	1.072	4.25E-01	4.25E+01		
7894531	---	---	---	-0.321	0.801	2.95E-01	2.95E+01		
7894532	---	---	---	0.118	1.085	9.01E-01	9.01E+01		
7894535	---	---	---	0.017	1.012	8.69E-01	8.69E+01		

7894536	---	---	---	0.032	1.022	8.54E-01	8.54E+01		
7894537	---	---	---	-0.239	0.848	2.06E-01	2.06E+01		
7894538	---	---	---	0.139	1.101	6.93E-01	6.93E+01		
7894540	---	---	---	0.017	1.012	8.63E-01	8.63E+01		
7894542	---	---	---	-0.004	0.997	9.89E-01	9.89E+01		
7894543	---	---	---	0.014	1.010	9.86E-01	9.86E+01		
7894544	---	---	---	-0.022	0.985	9.76E-01	9.76E+01		
7894546	---	---	---	0.008	1.006	9.81E-01	9.81E+01		
7894547	---	---	---	0.142	1.103	7.23E-01	7.23E+01		
7894548	---	---	---	-0.094	0.937	3.95E-01	3.95E+01		
7894550	---	---	---	0.034	1.024	8.52E-01	8.52E+01		
7894551	---	---	---	0.030	1.021	9.20E-01	9.20E+01		
7894552	---	---	---	0.068	1.049	6.65E-01	6.65E+01		
7894555	---	---	---	-0.102	0.932	6.42E-01	6.42E+01		
7894556	---	---	---	0.082	1.058	5.82E-01	5.82E+01		
7894557	---	---	---	-0.114	0.924	3.41E-01	3.41E+01		
7894558	---	---	---	-0.079	0.947	6.38E-01	6.38E+01		
7894560	---	---	---	-0.162	0.894	7.55E-01	7.55E+01		
7894561	---	---	---	0.044	1.031	8.43E-01	8.43E+01		
7894562	---	---	---	0.354	1.278	2.56E-01	2.56E+01		
7894563	---	---	---	0.149	1.109	4.47E-01	4.47E+01		
7894565	---	---	---	-0.053	0.964	8.77E-01	8.77E+01		
7894566	---	---	---	0.209	1.156	4.29E-01	4.29E+01		
7894568	---	---	---	-0.011	0.992	9.81E-01	9.81E+01		
7894569	---	---	---	0.271	1.207	4.28E-01	4.28E+01		
7894570	---	---	---	-0.115	0.923	8.08E-01	8.08E+01		
7894571	---	---	---	-0.034	0.977	9.53E-01	9.53E+01		
7894573	---	---	---	0.076	1.054	7.41E-01	7.41E+01		
7894574	---	---	---	0.163	1.120	5.41E-01	5.41E+01		
7894575	---	---	---	-0.030	0.979	9.53E-01	9.53E+01		
7894577	---	---	---	0.105	1.075	5.48E-01	5.48E+01		
7894578	---	---	---	-0.039	0.973	6.93E-01	6.93E+01		
7894579	---	---	---	0.007	1.005	9.83E-01	9.83E+01		
7894581	---	---	---	-0.119	0.921	5.55E-01	5.55E+01		
7894582	---	---	---	-0.240	0.847	5.63E-01	5.63E+01		
7894583	---	---	---	0.014	1.010	9.68E-01	9.68E+01		
7894584	---	---	---	-0.024	0.984	9.47E-01	9.47E+01		
7894590	---	---	---	0.128	1.093	8.97E-01	8.97E+01		
7894592	---	---	---	-0.051	0.965	6.99E-01	6.99E+01		
7894595	---	---	---	0.008	1.006	9.82E-01	9.82E+01		
7894596	---	---	---	0.024	1.017	9.51E-01	9.51E+01		
7894597	---	---	---	0.064	1.045	8.16E-01	8.16E+01		
7894598	---	---	---	-0.095	0.936	8.63E-01	8.63E+01		
7894599	---	---	---	0.046	1.033	8.32E-01	8.32E+01		
7894600	---	---	---	-0.276	0.826	7.50E-01	7.50E+01		
7894601	---	---	---	-0.223	0.857	6.08E-01	6.08E+01		
7894604	---	---	---	-0.074	0.950	6.66E-01	6.66E+01		
7894605	---	---	---	-0.024	0.984	8.76E-01	8.76E+01		
7894606	---	---	---	-0.002	0.999	9.98E-01	9.98E+01		
7894607	---	---	---	0.121	1.087	6.85E-01	6.85E+01		
7894609	---	---	---	0.044	1.031	8.10E-01	8.10E+01		
7894610	---	---	---	-0.060	0.960	7.95E-01	7.95E+01		
7894611	---	---	---	0.006	1.004	9.65E-01	9.65E+01		
7894612	---	---	---	0.048	1.034	7.03E-01	7.03E+01		
7894613	---	---	---	0.052	1.036	7.51E-01	7.51E+01		
7894614	---	---	---	0.096	1.069	8.91E-01	8.91E+01		
7894617	---	---	---	-0.076	0.949	5.42E-01	5.42E+01		
7894619	---	---	---	0.083	1.059	9.05E-01	9.05E+01		
7894620	---	---	---	-0.031	0.979	9.57E-01	9.57E+01		
7894621	---	---	---	0.008	1.006	9.74E-01	9.74E+01		
7894623	---	---	---	0.355	1.279	2.84E-01	2.84E+01		
7894624	---	---	---	0.003	1.002	9.91E-01	9.91E+01		
7894626	---	---	---	0.012	1.008	9.57E-01	9.57E+01		
7894628	---	---	---	0.032	1.022	9.63E-01	9.63E+01		
7894632	---	---	---	0.112	1.081	6.04E-01	6.04E+01		
7894633	---	---	---	0.045	1.032	8.52E-01	8.52E+01		
7894634	---	---	---	-0.006	0.996	9.77E-01	9.77E+01		
7894635	---	---	---	-0.055	0.963	8.79E-01	8.79E+01		
7894637	---	---	---	-0.006	0.996	9.89E-01	9.89E+01		
7894638	---	---	---	-0.023	0.984	9.50E-01	9.50E+01		
7894639	---	---	---	0.120	1.087	8.57E-01	8.57E+01		
7894640	---	---	---	0.031	1.021	7.68E-01	7.68E+01		
7894641	---	---	---	-0.115	0.924	8.38E-01	8.38E+01		
7894642	---	---	---	0.054	1.038	7.36E-01	7.36E+01		
7894644	---	---	---	0.155	1.113	4.43E-01	4.43E+01		
7894645	---	---	---	0.050	1.035	7.54E-01	7.54E+01		
7894647	---	---	---	0.056	1.040	3.71E-01	3.71E+01		

7894648	---	---	---	0.210	1.157	5.63E-01	5.63E+01		
7894649	---	---	---	0.212	1.158	5.55E-01	5.55E+01		
7894650	---	---	---	-0.140	0.907	8.29E-01	8.29E+01		
7894652	---	---	---	-0.152	0.900	7.13E-01	7.13E+01		
7894653	---	---	---	0.030	1.021	7.56E-01	7.56E+01		
7894654	---	---	---	0.167	1.123	8.03E-01	8.03E+01		
7894655	---	---	---	0.003	1.002	9.95E-01	9.95E+01		
7894656	---	---	---	0.301	1.232	2.98E-01	2.98E+01		
7894657	---	---	---	-0.083	0.944	8.17E-01	8.17E+01		
7894658	---	---	---	-0.033	0.978	9.63E-01	9.63E+01		
7894661	---	---	---	0.111	1.080	4.02E-01	4.02E+01		
7894662	---	---	---	-0.135	0.911	7.02E-01	7.02E+01		
7894664	---	---	---	0.133	1.097	7.53E-01	7.53E+01		
7894665	---	---	---	0.071	1.050	7.53E-01	7.53E+01		
7894666	---	---	---	-0.075	0.949	5.43E-01	5.43E+01		
7894668	---	---	---	0.133	1.096	5.04E-01	5.04E+01		
7894669	---	---	---	-0.193	0.875	7.58E-01	7.58E+01		
7894670	---	---	---	0.066	1.047	8.91E-01	8.91E+01		
7894671	---	---	---	0.033	1.023	9.00E-01	9.00E+01		
7894672	---	---	---	0.010	1.007	9.77E-01	9.77E+01		
7894673	---	---	---	-0.146	0.903	5.93E-01	5.93E+01		
7894674	---	---	---	0.068	1.049	6.09E-01	6.09E+01		
7894675	---	---	---	-0.089	0.940	4.25E-01	4.25E+01		
7894676	---	---	---	0.162	1.119	8.55E-01	8.55E+01		
7894678	---	---	---	0.015	1.010	9.69E-01	9.69E+01		
7894679	---	---	---	0.107	1.077	8.04E-01	8.04E+01		
7894680	---	---	---	0.210	1.157	1.39E-01	1.39E+01		
7894681	---	---	---	-0.104	0.930	1.53E-01	1.53E+01		
7894682	---	---	---	0.003	1.002	9.87E-01	9.87E+01		
7894683	---	---	---	0.048	1.034	9.50E-01	9.50E+01		
7894685	---	---	---	-0.027	0.981	9.12E-01	9.12E+01		
7894687	---	---	---	0.087	1.062	3.95E-01	3.95E+01		
7894688	---	---	---	-0.136	0.910	7.52E-01	7.52E+01		
7894689	---	---	---	-0.081	0.946	7.99E-01	7.99E+01		
7894691	---	---	---	0.094	1.067	8.48E-01	8.48E+01		
7894692	---	---	---	0.033	1.023	9.64E-01	9.64E+01		
7894693	---	---	---	0.061	1.043	8.88E-01	8.88E+01		
7894694	---	---	---	0.096	1.069	8.00E-01	8.00E+01		
7894696	---	---	---	-0.001	1.000	9.98E-01	9.98E+01		
7894697	---	---	---	-0.066	0.955	7.77E-01	7.77E+01		
7894698	---	---	---	0.069	1.049	8.47E-01	8.47E+01		
7894699	---	---	---	-0.023	0.984	9.54E-01	9.54E+01		
7894700	---	---	---	0.086	1.061	5.72E-01	5.72E+01		
7894704	---	---	---	0.118	1.085	7.56E-01	7.56E+01		
7894706	---	---	---	0.091	1.065	7.89E-01	7.89E+01		
7894707	---	---	---	0.034	1.024	9.12E-01	9.12E+01		
7894708	---	---	---	-0.092	0.938	5.90E-01	5.90E+01		
7894709	---	---	---	0.042	1.030	8.73E-01	8.73E+01		
7894711	---	---	---	0.037	1.026	8.94E-01	8.94E+01		
7894712	---	---	---	-0.063	0.957	8.26E-01	8.26E+01		
7894714	---	---	---	-0.009	0.994	9.83E-01	9.83E+01		
7894716	---	---	---	0.073	1.052	6.23E-01	6.23E+01		
7894717	---	---	---	-0.018	0.987	9.76E-01	9.76E+01		
7894719	---	---	---	0.061	1.043	8.79E-01	8.79E+01		
7894720	---	---	---	0.131	1.095	6.16E-01	6.16E+01		
7894722	---	---	---	-0.001	1.000	9.98E-01	9.98E+01		
7894723	---	---	---	-0.013	0.991	9.44E-01	9.44E+01		
7894725	---	---	---	0.120	1.087	5.29E-01	5.29E+01		
7894727	---	---	---	0.119	1.086	6.05E-01	6.05E+01		
7894729	---	---	---	0.098	1.070	8.52E-01	8.52E+01		
7894732	---	---	---	-0.043	0.971	9.25E-01	9.25E+01		
7894733	---	---	---	-0.047	0.968	8.69E-01	8.69E+01		
7894734	---	---	---	0.109	1.078	5.51E-01	5.51E+01		
7894736	---	---	---	0.073	1.052	9.13E-01	9.13E+01		
7894737	---	---	---	0.039	1.028	8.99E-01	8.99E+01		
7894740	---	---	---	0.052	1.037	6.76E-01	6.76E+01		
7894741	---	---	---	0.082	1.058	6.35E-01	6.35E+01		
7894742	---	---	---	0.068	1.048	7.94E-01	7.94E+01		
7894744	---	---	---	-0.047	0.968	8.50E-01	8.50E+01		
7894745	---	---	---	0.057	1.041	9.04E-01	9.04E+01		
7894746	---	---	---	0.088	1.063	6.22E-01	6.22E+01		
7894747	---	---	---	0.081	1.058	5.46E-01	5.46E+01		
7894748	---	---	---	0.130	1.094	8.05E-01	8.05E+01		
7894752	---	---	---	-0.050	0.966	8.74E-01	8.74E+01		
7894753	---	---	---	-0.078	0.947	8.06E-01	8.06E+01		
7894754	---	---	---	0.115	1.083	8.70E-01	8.70E+01		
7894755	---	---	---	0.145	1.106	8.07E-01	8.07E+01		

7894756	---	---	---	-0.037	0.975	9.27E-01	9.27E+01			
7894757	---	---	---	-0.042	0.972	8.76E-01	8.76E+01			
7894758	---	---	---	-0.009	0.994	9.65E-01	9.65E+01			
7894760	---	---	---	-0.024	0.984	9.27E-01	9.27E+01			
7894761	---	---	---	-0.088	0.941	7.78E-01	7.78E+01			
7894763	---	---	---	0.153	1.112	6.89E-02	6.89E+00			
7894764	---	---	---	0.033	1.023	9.53E-01	9.53E+01			
7894765	---	---	---	0.085	1.061	7.68E-01	7.68E+01			
7894766	---	---	---	0.534	1.448	2.91E-01	2.91E+01			
7894767	---	---	---	-0.039	0.974	9.23E-01	9.23E+01			
7894768	---	---	---	0.057	1.040	6.54E-01	6.54E+01			
7894769	---	---	---	-0.046	0.969	7.17E-01	7.17E+01			
7894770	---	---	---	0.034	1.024	6.14E-01	6.14E+01			
7894771	---	---	---	0.336	1.263	1.29E-01	1.29E+01			
7894772	---	---	---	0.136	1.099	4.70E-01	4.70E+01			
7894774	---	---	---	0.086	1.061	6.81E-01	6.81E+01			
7894775	---	---	---	0.172	1.127	7.72E-01	7.72E+01			
7894776	---	---	---	0.134	1.097	7.62E-01	7.62E+01			
7894777	---	---	---	-0.279	0.824	2.63E-01	2.63E+01			
7894779	---	---	---	0.054	1.038	7.10E-01	7.10E+01			
7894781	---	---	---	-0.205	0.868	7.11E-01	7.11E+01			
7894782	---	---	---	-0.215	0.862	5.46E-01	5.46E+01			
7894783	---	---	---	-0.236	0.849	1.91E-01	1.91E+01			
7894784	---	---	---	-0.043	0.971	8.30E-01	8.30E+01			
7894787	---	---	---	0.074	1.052	6.05E-01	6.05E+01			
7894788	---	---	---	0.007	1.005	9.88E-01	9.88E+01			
7894789	---	---	---	0.042	1.029	6.20E-01	6.20E+01			
7894790	---	---	---	0.065	1.046	7.28E-01	7.28E+01			
7894791	---	---	---	-0.167	0.891	5.09E-01	5.09E+01			
7894795	---	---	---	0.010	1.007	9.78E-01	9.78E+01			
7894796	---	---	---	-0.080	0.946	5.61E-01	5.61E+01			
7894797	---	---	---	0.368	1.290	5.55E-01	5.55E+01			
7894798	---	---	---	-0.069	0.953	7.39E-01	7.39E+01			
7894799	---	---	---	0.075	1.053	5.29E-01	5.29E+01			
7894800	---	---	---	0.271	1.206	2.39E-01	2.39E+01			
7894801	---	---	---	-0.035	0.976	8.28E-01	8.28E+01			
7894802	---	---	---	0.033	1.023	9.16E-01	9.16E+01			
7894803	---	---	---	0.441	1.357	9.19E-02	9.19E+00			
7894804	---	---	---	-0.166	0.891	7.27E-01	7.27E+01			
7894805	---	---	---	0.613	1.530	5.53E-02	5.53E+00			
7894806	---	---	---	-0.007	0.995	9.70E-01	9.70E+01			
7894807	---	---	---	-0.016	0.989	9.83E-01	9.83E+01			
7894808	---	---	---	0.037	1.026	8.75E-01	8.75E+01			
7894809	---	---	---	-0.173	0.887	6.93E-01	6.93E+01			
7894810	---	---	---	0.237	1.179	2.74E-01	2.74E+01			
7894812	---	---	---	-0.018	0.987	9.85E-01	9.85E+01			
7894813	---	---	---	-0.035	0.976	7.09E-01	7.09E+01			
7894815	---	---	---	-0.082	0.945	8.95E-01	8.95E+01			
7894816	---	---	---	0.058	1.041	9.37E-01	9.37E+01			
7894817	---	---	---	0.091	1.065	5.17E-01	5.17E+01			
7894818	---	---	---	-0.102	0.932	8.00E-01	8.00E+01			
7894819	---	---	---	0.141	1.102	7.13E-01	7.13E+01			
7894820	---	---	---	0.122	1.089	5.98E-01	5.98E+01			
7894821	---	---	---	0.243	1.184	3.41E-01	3.41E+01			
7894822	---	---	---	0.077	1.055	8.43E-01	8.43E+01			
7894823	---	---	---	-0.310	0.807	1.57E-01	1.57E+01			
7894824	---	---	---	-0.035	0.976	8.63E-01	8.63E+01			
7894825	---	---	---	-0.039	0.973	9.64E-01	9.64E+01			
7894826	---	---	---	0.160	1.117	1.74E-01	1.74E+01			
7894827	---	---	---	0.064	1.045	7.75E-01	7.75E+01			
7894830	---	---	---	0.056	1.040	8.49E-01	8.49E+01			
7894832	---	---	---	0.069	1.049	8.79E-01	8.79E+01			
7894833	---	---	---	0.035	1.024	9.24E-01	9.24E+01			
7894834	---	---	---	0.019	1.013	9.19E-01	9.19E+01			
7894836	---	---	---	0.087	1.062	7.01E-01	7.01E+01			
7894837	---	---	---	-0.089	0.940	5.43E-01	5.43E+01			
7894838	---	---	---	0.061	1.043	8.33E-01	8.33E+01			
7894840	---	---	---	0.076	1.054	5.15E-01	5.15E+01			
7894842	---	---	---	0.107	1.077	6.76E-01	6.76E+01			
7894843	---	---	---	-0.180	0.882	4.62E-01	4.62E+01			
7894844	---	---	---	-0.041	0.972	8.32E-01	8.32E+01			
7894845	---	---	---	0.034	1.024	8.17E-01	8.17E+01			
7894846	---	---	---	-0.025	0.983	9.34E-01	9.34E+01			
7894848	---	---	---	0.027	1.019	9.63E-01	9.63E+01			
7894849	---	---	---	0.020	1.014	9.50E-01	9.50E+01			
7894850	---	---	---	0.041	1.029	9.27E-01	9.27E+01			
7894852	---	---	---	0.143	1.104	7.37E-01	7.37E+01			

7894853	---	---	---	0.031	1.022	8.76E-01	8.76E+01		
7894854	---	---	---	-0.031	0.979	8.48E-01	8.48E+01		
7894856	---	---	---	-0.173	0.887	2.83E-01	2.83E+01		
7894858	---	---	---	0.048	1.034	8.23E-01	8.23E+01		
7894859	---	---	---	-0.009	0.994	9.88E-01	9.88E+01		
7894860	---	---	---	0.037	1.026	9.17E-01	9.17E+01		
7894861	---	---	---	-0.157	0.897	5.81E-01	5.81E+01		
7894862	---	---	---	-0.036	0.975	8.11E-01	8.11E+01		
7894863	---	---	---	-0.063	0.958	8.05E-01	8.05E+01		
7894864	---	---	---	0.048	1.034	8.92E-01	8.92E+01		
7894865	---	---	---	-0.101	0.932	7.61E-01	7.61E+01		
7894866	---	---	---	0.117	1.084	6.71E-01	6.71E+01		
7894867	---	---	---	-0.088	0.941	9.07E-01	9.07E+01		
7894869	---	---	---	0.176	1.130	7.05E-01	7.05E+01		
7894870	---	---	---	0.361	1.284	4.43E-01	4.43E+01		
7894872	---	---	---	-0.013	0.991	9.50E-01	9.50E+01		
7894873	---	---	---	0.049	1.035	8.96E-01	8.96E+01		
7894876	---	---	---	0.075	1.053	8.75E-01	8.75E+01		
7894877	---	---	---	-0.001	0.999	9.97E-01	9.97E+01		
7894878	---	---	---	0.214	1.160	3.28E-01	3.28E+01		
7894879	---	---	---	0.138	1.100	8.06E-01	8.06E+01		
7894880	---	---	---	-0.141	0.907	4.75E-01	4.75E+01		
7894881	---	---	---	-0.072	0.951	3.66E-01	3.66E+01		
7894882	---	---	---	0.268	1.204	6.39E-01	6.39E+01		
7894883	---	---	---	0.252	1.191	2.28E-01	2.28E+01		
7894884	---	---	---	-0.099	0.934	6.43E-01	6.43E+01		
7894885	---	---	---	0.111	1.080	6.70E-01	6.70E+01		
7894886	---	---	---	-0.102	0.931	7.75E-01	7.75E+01		
7894888	---	---	---	0.270	1.206	1.38E-01	1.38E+01		
7894889	---	---	---	-0.037	0.975	8.48E-01	8.48E+01		
7894890	---	---	---	0.016	1.011	9.86E-01	9.86E+01		
7894891	---	---	---	-0.084	0.943	7.05E-01	7.05E+01		
7894894	---	---	---	-0.024	0.984	9.14E-01	9.14E+01		
7894895	---	---	---	0.205	1.153	7.45E-01	7.45E+01		
7894896	---	---	---	-0.004	0.997	9.84E-01	9.84E+01		
7894898	---	---	---	0.083	1.059	8.13E-01	8.13E+01		
7894899	---	---	---	0.213	1.159	5.04E-01	5.04E+01		
7894900	---	---	---	-0.017	0.988	9.78E-01	9.78E+01		
7894901	---	---	---	0.176	1.130	4.02E-01	4.02E+01		
7894903	---	---	---	0.127	1.092	7.00E-01	7.00E+01		
7894904	---	---	---	-0.055	0.963	9.10E-01	9.10E+01		
7894905	---	---	---	0.040	1.028	9.44E-01	9.44E+01		
7894906	---	---	---	-0.133	0.912	6.48E-01	6.48E+01		
7894907	---	---	---	0.046	1.033	9.40E-01	9.40E+01		
7894908	---	---	---	0.133	1.097	2.92E-01	2.92E+01		
7894909	---	---	---	0.014	1.010	8.27E-01	8.27E+01		
7894911	---	---	---	-0.095	0.936	9.07E-01	9.07E+01		
7894914	---	---	---	0.044	1.031	8.88E-01	8.88E+01		
7894915	---	---	---	-0.007	0.995	9.88E-01	9.88E+01		
7894916	---	---	---	-0.004	0.997	9.85E-01	9.85E+01		
7894917	---	---	---	-0.022	0.985	9.61E-01	9.61E+01		
7894919	---	---	---	-0.007	0.995	9.65E-01	9.65E+01		
7894925	---	---	---	-0.021	0.985	9.84E-01	9.84E+01		
7894926	---	---	---	-0.010	0.993	9.76E-01	9.76E+01		
7894927	---	---	---	0.014	1.010	9.84E-01	9.84E+01		
7894928	---	---	---	0.020	1.014	9.66E-01	9.66E+01		
7894930	---	---	---	0.195	1.145	6.66E-01	6.66E+01		
7894931	---	---	---	-0.051	0.966	9.20E-01	9.20E+01		
7894934	---	---	---	0.031	1.022	8.67E-01	8.67E+01		
7894937	---	---	---	-0.168	0.890	8.48E-01	8.48E+01		
7894938	---	---	---	0.233	1.175	5.76E-01	5.76E+01		
7894939	---	---	---	0.309	1.239	5.91E-01	5.91E+01		
7894940	---	---	---	-0.078	0.947	9.14E-01	9.14E+01		
7894941	---	---	---	0.024	1.017	8.92E-01	8.92E+01		
7894945	---	---	---	0.013	1.009	9.64E-01	9.64E+01		
7894946	---	---	---	-0.323	0.799	5.73E-01	5.73E+01		
7894947	---	---	---	0.108	1.078	7.69E-01	7.69E+01		
7894949	---	---	---	0.113	1.081	7.67E-01	7.67E+01		
7894950	---	---	---	-0.004	0.997	9.90E-01	9.90E+01		
7894951	---	---	---	0.051	1.036	8.45E-01	8.45E+01		
7894952	---	---	---	0.219	1.164	5.56E-01	5.56E+01		
7894953	---	---	---	-0.013	0.991	9.84E-01	9.84E+01		
7894954	---	---	---	-0.048	0.968	8.66E-01	8.66E+01		
7894955	---	---	---	0.031	1.022	9.67E-01	9.67E+01		
7894956	---	---	---	0.113	1.082	7.83E-01	7.83E+01		
7894957	---	---	---	-0.093	0.938	8.24E-01	8.24E+01		
7894958	---	---	---	0.042	1.029	9.56E-01	9.56E+01		

7894959	---	---	---	0.166	1.122	7.78E-01	7.78E+01			
7894960	---	---	---	0.032	1.022	9.00E-01	9.00E+01			
7894961	---	---	---	0.010	1.007	9.83E-01	9.83E+01			
7894963	---	---	---	-0.014	0.991	9.87E-01	9.87E+01			
7894964	---	---	---	0.004	1.003	9.86E-01	9.86E+01			
7894965	---	---	---	-0.056	0.962	8.57E-01	8.57E+01			
7894968	---	---	---	0.180	1.133	1.07E-01	1.07E+01			
7894969	---	---	---	0.005	1.003	9.89E-01	9.89E+01			
7894970	---	---	---	0.016	1.011	8.95E-01	8.95E+01			
7894971	---	---	---	-0.011	0.993	9.44E-01	9.44E+01			
7894973	---	---	---	-0.016	0.989	9.77E-01	9.77E+01			
7894974	---	---	---	-0.031	0.979	9.44E-01	9.44E+01			
7894975	---	---	---	0.053	1.038	8.05E-01	8.05E+01			
7894976	---	---	---	0.072	1.051	6.63E-01	6.63E+01			
7894977	---	---	---	0.013	1.009	9.78E-01	9.78E+01			
7894978	---	---	---	0.075	1.053	7.12E-01	7.12E+01			
7894979	---	---	---	0.025	1.018	9.16E-01	9.16E+01			
7894980	---	---	---	-0.135	0.911	3.92E-01	3.92E+01			
7894981	---	---	---	0.270	1.206	2.30E-01	2.30E+01			
7894982	---	---	---	-0.025	0.983	8.66E-01	8.66E+01			
7894984	---	---	---	-0.065	0.956	6.78E-01	6.78E+01			
7894986	---	---	---	-0.367	0.776	3.52E-01	3.52E+01			
7894988	---	---	---	-0.058	0.961	7.45E-01	7.45E+01			
7894990	---	---	---	-0.022	0.985	9.09E-01	9.09E+01			
7894991	---	---	---	0.015	1.011	8.87E-01	8.87E+01			
7894992	---	---	---	-0.003	0.998	9.87E-01	9.87E+01			
7894993	---	---	---	-0.055	0.963	9.36E-01	9.36E+01			
7894994	---	---	---	0.140	1.102	6.07E-01	6.07E+01			
7894995	---	---	---	0.096	1.069	7.94E-01	7.94E+01			
7894996	---	---	---	-0.083	0.944	8.62E-01	8.62E+01			
7894997	---	---	---	-0.132	0.912	7.98E-01	7.98E+01			
7894999	---	---	---	0.015	1.011	8.71E-01	8.71E+01			
7895000	---	---	---	0.068	1.049	7.96E-01	7.96E+01			
7895001	---	---	---	-0.028	0.981	9.43E-01	9.43E+01			
7895003	---	---	---	-0.067	0.955	7.32E-01	7.32E+01			
7895004	---	---	---	0.075	1.053	5.29E-01	5.29E+01			
7895005	---	---	---	-0.053	0.964	8.90E-01	8.90E+01			
7895006	---	---	---	0.151	1.110	8.38E-01	8.38E+01			
7895007	---	---	---	0.082	1.059	6.45E-01	6.45E+01			
7895009	---	---	---	0.072	1.051	6.94E-01	6.94E+01			
7895011	---	---	---	0.093	1.067	7.20E-01	7.20E+01			
7895012	---	---	---	0.010	1.007	9.60E-01	9.60E+01			
7895014	---	---	---	-0.079	0.947	6.35E-01	6.35E+01			
7895015	---	---	---	0.035	1.025	9.44E-01	9.44E+01			
7895016	---	---	---	0.178	1.131	5.43E-01	5.43E+01			
7895017	---	---	---	0.109	1.079	9.99E-02	9.99E+00			
7895019	---	---	---	0.178	1.131	6.73E-01	6.73E+01			
7895020	---	---	---	0.003	1.002	9.90E-01	9.90E+01			
7895022	---	---	---	-0.001	0.999	9.99E-01	9.99E+01			
7895023	---	---	---	0.056	1.040	8.95E-01	8.95E+01			
7895024	---	---	---	0.277	1.211	6.25E-02	6.25E+00			
7895026	---	---	---	0.016	1.011	8.86E-01	8.86E+01			
7895030	---	---	---	0.402	1.321	1.45E-01	1.45E+01			
7895032	---	---	---	0.101	1.073	7.14E-01	7.14E+01			
7895033	---	---	---	-0.104	0.931	4.74E-01	4.74E+01			
7895034	---	---	---	-0.065	0.956	7.46E-01	7.46E+01			
7895035	---	---	---	0.082	1.059	8.49E-01	8.49E+01			
7895036	---	---	---	0.068	1.048	7.14E-01	7.14E+01			
7895037	---	---	---	0.151	1.111	6.70E-01	6.70E+01			
7895038	---	---	---	0.304	1.234	3.21E-02	3.21E+00			
7895039	---	---	---	-0.009	0.994	9.69E-01	9.69E+01			
7895040	---	---	---	-0.017	0.988	9.81E-01	9.81E+01			
7895041	---	---	---	-0.119	0.921	4.96E-01	4.96E+01			
7895042	---	---	---	0.015	1.010	9.57E-01	9.57E+01			
7895044	---	---	---	0.078	1.055	8.70E-01	8.70E+01			
7895045	---	---	---	-0.007	0.995	9.85E-01	9.85E+01			
7895046	---	---	---	-0.151	0.901	7.03E-01	7.03E+01			
7895047	---	---	---	0.032	1.022	8.49E-01	8.49E+01			
7895048	---	---	---	0.082	1.059	8.33E-01	8.33E+01			
7895050	---	---	---	-0.294	0.816	9.26E-02	9.26E+00			
7895052	---	---	---	-0.050	0.966	7.23E-01	7.23E+01			
7895053	---	---	---	-0.138	0.909	7.90E-01	7.90E+01			
7895054	---	---	---	0.221	1.165	7.50E-01	7.50E+01			
7895055	---	---	---	-0.011	0.992	9.78E-01	9.78E+01			
7895056	---	---	---	-0.173	0.887	4.04E-01	4.04E+01			
7895057	---	---	---	-0.002	0.998	9.87E-01	9.87E+01			
7895059	---	---	---	0.289	1.221	6.31E-01	6.31E+01			

7895060	---	---	---	0.259	1.197	4.33E-01	4.33E+01		
7895061	---	---	---	0.068	1.048	9.02E-01	9.02E+01		
7895062	---	---	---	-0.053	0.964	8.10E-01	8.10E+01		
7895063	---	---	---	0.001	1.001	9.98E-01	9.98E+01		
7895066	---	---	---	0.000	1.000	1.00E+00	1.00E+02		
7895067	---	---	---	-0.112	0.925	4.21E-01	4.21E+01		
7895068	---	---	---	-0.050	0.966	8.29E-01	8.29E+01		
7895069	---	---	---	-0.044	0.970	9.37E-01	9.37E+01		
7895070	---	---	---	0.049	1.034	7.97E-01	7.97E+01		
7895072	---	---	---	0.035	1.025	8.62E-01	8.62E+01		
7895073	---	---	---	-0.253	0.839	6.79E-01	6.79E+01		
7895074	---	---	---	-0.250	0.841	5.15E-01	5.15E+01		
7895077	---	---	---	-0.051	0.965	7.78E-01	7.78E+01		
7895080	---	---	---	0.154	1.112	7.08E-01	7.08E+01		
7895081	---	---	---	0.047	1.033	9.13E-01	9.13E+01		
7895082	---	---	---	-0.017	0.988	9.14E-01	9.14E+01		
7895084	---	---	---	0.014	1.009	9.58E-01	9.58E+01		
7895085	---	---	---	-0.055	0.963	9.46E-01	9.46E+01		
7895087	---	---	---	0.079	1.056	8.63E-01	8.63E+01		
7895088	---	---	---	-0.038	0.974	9.29E-01	9.29E+01		
7895089	---	---	---	0.024	1.017	9.49E-01	9.49E+01		
7895090	---	---	---	0.178	1.131	3.90E-01	3.90E+01		
7895091	---	---	---	0.020	1.014	9.76E-01	9.76E+01		
7895093	---	---	---	0.057	1.040	8.82E-01	8.82E+01		
7895094	---	---	---	0.104	1.075	7.57E-01	7.57E+01		
7895095	---	---	---	-0.078	0.947	7.68E-01	7.68E+01		
7895097	---	---	---	0.441	1.357	1.09E-01	1.09E+01		
7895098	---	---	---	-0.079	0.946	8.68E-01	8.68E+01		
7895099	---	---	---	0.066	1.047	9.37E-01	9.37E+01		
7895100	---	---	---	0.027	1.019	7.48E-01	7.48E+01		
7895101	---	---	---	0.070	1.050	8.06E-01	8.06E+01		
7895103	---	---	---	0.205	1.153	7.44E-01	7.44E+01		
7895104	---	---	---	0.053	1.037	7.94E-01	7.94E+01		
7895105	---	---	---	-0.263	0.833	4.27E-01	4.27E+01		
7895106	---	---	---	0.026	1.018	8.97E-01	8.97E+01		
7895107	---	---	---	-0.090	0.940	7.57E-01	7.57E+01		
7895110	---	---	---	0.017	1.012	9.27E-01	9.27E+01		
7895112	---	---	---	-0.004	0.997	9.88E-01	9.88E+01		
7895113	---	---	---	-0.151	0.900	3.09E-01	3.09E+01		
7895114	---	---	---	-0.031	0.979	9.40E-01	9.40E+01		
7895115	---	---	---	0.058	1.041	7.49E-01	7.49E+01		
7895118	---	---	---	0.000	1.000	1.00E+00	1.00E+02		
7895119	---	---	---	-0.048	0.967	7.77E-01	7.77E+01		
7895121	---	---	---	0.073	1.052	7.79E-01	7.79E+01		
7895122	---	---	---	0.020	1.014	8.89E-01	8.89E+01		
7895123	---	---	---	0.028	1.020	8.04E-01	8.04E+01		
7895124	---	---	---	0.042	1.030	9.49E-01	9.49E+01		
7895125	---	---	---	-0.045	0.969	9.33E-01	9.33E+01		
7895126	---	---	---	0.024	1.017	9.07E-01	9.07E+01		
7895130	---	---	---	0.048	1.034	7.73E-01	7.73E+01		
7895131	---	---	---	-0.081	0.946	8.63E-01	8.63E+01		
7895132	---	---	---	0.400	1.319	3.07E-01	3.07E+01		
7895133	---	---	---	0.183	1.135	2.10E-01	2.10E+01		
7895134	---	---	---	-0.121	0.919	9.09E-01	9.09E+01		
7895137	---	---	---	-0.099	0.934	6.23E-01	6.23E+01		
7895139	---	---	---	0.013	1.009	9.48E-01	9.48E+01		
7895141	---	---	---	0.195	1.145	5.41E-01	5.41E+01		
7895142	---	---	---	0.933	1.909	6.76E-02	6.76E+00		
7895143	---	---	---	-0.034	0.977	9.00E-01	9.00E+01		
7895144	---	---	---	-0.027	0.982	8.36E-01	8.36E+01		
7895145	---	---	---	-0.087	0.942	5.37E-01	5.37E+01		
7895146	---	---	---	0.263	1.200	6.38E-01	6.38E+01		
7895147	---	---	---	-0.100	0.933	7.92E-01	7.92E+01		
7895149	---	---	---	0.171	1.125	2.57E-01	2.57E+01		
7895150	---	---	---	0.048	1.034	9.47E-01	9.47E+01		
7895151	---	---	---	0.049	1.035	9.37E-01	9.37E+01		
7895152	---	---	---	-0.036	0.975	9.50E-01	9.50E+01		
7895153	---	---	---	-0.001	1.000	1.00E+00	1.00E+02		
7895154	---	---	---	0.093	1.067	8.78E-01	8.78E+01		
7895155	---	---	---	0.366	1.288	1.86E-01	1.86E+01		
7895156	---	---	---	0.078	1.055	5.63E-01	5.63E+01		
7895157	---	---	---	-0.025	0.983	8.48E-01	8.48E+01		
7895158	---	---	---	0.433	1.350	5.15E-02	5.15E+00		
7895160	---	---	---	0.001	1.001	9.97E-01	9.97E+01		
7895161	---	---	---	0.047	1.033	8.67E-01	8.67E+01		
7895162	---	---	---	-0.014	0.990	9.77E-01	9.77E+01		
7895165	---	---	---	0.002	1.001	9.94E-01	9.94E+01		

7895168	---	---	---	-0.019	0.987	9.51E-01	9.51E+01		
7895169	---	---	---	-0.004	0.997	9.89E-01	9.89E+01		
7895170	---	---	---	0.012	1.008	9.63E-01	9.63E+01		
7895171	---	---	---	-0.132	0.913	4.05E-01	4.05E+01		
7895172	---	---	---	0.199	1.148	7.19E-01	7.19E+01		
7895173	---	---	---	0.060	1.042	8.73E-01	8.73E+01		
7895174	---	---	---	-0.077	0.948	6.99E-01	6.99E+01		
7895175	---	---	---	0.087	1.062	8.93E-01	8.93E+01		
7895177	---	---	---	-0.038	0.974	9.35E-01	9.35E+01		
7895178	---	---	---	-0.005	0.997	9.84E-01	9.84E+01		
7895180	---	---	---	0.018	1.012	9.64E-01	9.64E+01		
7895181	---	---	---	0.163	1.120	3.35E-01	3.35E+01		
7895184	---	---	---	0.105	1.076	8.62E-01	8.62E+01		
7895185	---	---	---	0.027	1.019	9.50E-01	9.50E+01		
7895186	---	---	---	-0.068	0.954	8.63E-01	8.63E+01		
7895187	---	---	---	0.069	1.049	8.13E-01	8.13E+01		
7895188	---	---	---	0.276	1.211	4.49E-01	4.49E+01		
7895189	---	---	---	0.027	1.019	9.27E-01	9.27E+01		
7895192	---	---	---	-0.132	0.913	4.68E-01	4.68E+01		
7895193	---	---	---	-0.002	0.999	9.94E-01	9.94E+01		
7895194	---	---	---	0.237	1.178	4.32E-01	4.32E+01		
7895196	---	---	---	0.008	1.005	9.72E-01	9.72E+01		
7895197	---	---	---	0.555	1.469	8.96E-02	8.96E+00		
7895198	---	---	---	-0.039	0.973	9.78E-01	9.78E+01		
7895202	---	---	---	-0.151	0.900	5.75E-01	5.75E+01		
7895203	---	---	---	0.017	1.012	9.88E-01	9.88E+01		
7895204	---	---	---	-0.004	0.997	9.94E-01	9.94E+01		
7895205	---	---	---	-0.027	0.981	9.76E-01	9.76E+01		
7895206	---	---	---	-0.060	0.959	7.56E-01	7.56E+01		
7895208	---	---	---	0.136	1.099	7.40E-01	7.40E+01		
7895209	---	---	---	0.109	1.078	8.20E-01	8.20E+01		
7895210	---	---	---	0.059	1.042	5.56E-01	5.56E+01		
7895211	---	---	---	-0.211	0.864	6.31E-01	6.31E+01		
7895212	---	---	---	0.154	1.112	5.50E-01	5.50E+01		
7895213	---	---	---	-0.156	0.898	6.47E-01	6.47E+01		
7895214	---	---	---	0.006	1.004	9.72E-01	9.72E+01		
7895215	---	---	---	0.054	1.038	6.78E-01	6.78E+01		
7895216	---	---	---	0.029	1.020	7.92E-01	7.92E+01		
7895217	---	---	---	-0.182	0.882	4.28E-01	4.28E+01		
7895219	---	---	---	0.179	1.132	6.18E-01	6.18E+01		
7895220	---	---	---	0.040	1.028	8.73E-01	8.73E+01		
7895221	---	---	---	0.057	1.040	7.27E-01	7.27E+01		
7895223	---	---	---	0.060	1.043	7.83E-01	7.83E+01		
7895224	---	---	---	-0.068	0.954	7.27E-01	7.27E+01		
7895225	---	---	---	0.028	1.019	8.55E-01	8.55E+01		
7895226	---	---	---	-0.063	0.957	6.86E-01	6.86E+01		
7895227	---	---	---	0.006	1.004	9.84E-01	9.84E+01		
7895228	---	---	---	0.045	1.032	9.33E-01	9.33E+01		
7895230	---	---	---	0.017	1.012	8.78E-01	8.78E+01		
7895231	---	---	---	0.179	1.132	6.35E-01	6.35E+01		
7895232	---	---	---	-0.099	0.934	6.80E-01	6.80E+01		
7895233	---	---	---	0.120	1.087	5.34E-01	5.34E+01		
7895234	---	---	---	-0.013	0.991	9.81E-01	9.81E+01		
7895235	---	---	---	0.198	1.147	1.72E-01	1.72E+01		
7895237	---	---	---	-0.020	0.986	9.49E-01	9.49E+01		
7895238	---	---	---	0.153	1.112	3.85E-02	3.85E+00		
7895239	---	---	---	0.018	1.012	9.31E-01	9.31E+01		
7895240	---	---	---	0.271	1.207	1.42E-01	1.42E+01		
7895241	---	---	---	-0.141	0.907	5.89E-01	5.89E+01		
7895246	---	---	---	0.018	1.012	9.76E-01	9.76E+01		
7895247	---	---	---	0.234	1.176	1.78E-01	1.78E+01		
7895248	---	---	---	0.038	1.026	8.69E-01	8.69E+01		
7895249	---	---	---	0.045	1.031	8.33E-01	8.33E+01		
7895252	---	---	---	0.061	1.043	7.27E-01	7.27E+01		
7895253	---	---	---	0.037	1.026	9.10E-01	9.10E+01		
7895254	---	---	---	-0.010	0.993	9.88E-01	9.88E+01		
7895255	---	---	---	0.783	1.720	2.50E-01	2.50E+01		
7895256	---	---	---	0.003	1.002	9.90E-01	9.90E+01		
7895257	---	---	---	0.181	1.133	4.55E-01	4.55E+01		
7895258	---	---	---	0.098	1.070	7.19E-01	7.19E+01		
7895259	---	---	---	0.130	1.094	5.07E-01	5.07E+01		
7895260	---	---	---	-0.090	0.940	8.01E-01	8.01E+01		
7895261	---	---	---	-0.152	0.900	7.94E-01	7.94E+01		
7895262	---	---	---	-0.229	0.853	3.70E-01	3.70E+01		
7895263	---	---	---	0.155	1.113	1.56E-01	1.56E+01		
7895264	---	---	---	0.344	1.269	1.51E-01	1.51E+01		
7895267	---	---	---	-0.016	0.989	9.61E-01	9.61E+01		

7895269	---	---	---	0.011	1.008	9.64E-01	9.64E+01		
7895271	---	---	---	0.221	1.166	2.99E-02	2.99E+00		
7895272	---	---	---	0.032	1.022	7.94E-01	7.94E+01		
7895273	---	---	---	-0.009	0.994	9.46E-01	9.46E+01		
7895274	---	---	---	-0.025	0.983	9.04E-01	9.04E+01		
7895275	---	---	---	-0.040	0.972	7.75E-01	7.75E+01		
7895276	---	---	---	0.297	1.228	3.40E-02	3.40E+00		
7895277	---	---	---	0.067	1.048	6.78E-01	6.78E+01		
7895278	---	---	---	0.137	1.100	7.16E-01	7.16E+01		
7895279	---	---	---	0.077	1.055	9.09E-01	9.09E+01		
7895280	---	---	---	-0.023	0.984	9.38E-01	9.38E+01		
7895282	---	---	---	-0.041	0.972	8.90E-01	8.90E+01		
7895283	---	---	---	0.139	1.101	5.64E-01	5.64E+01		
7895284	---	---	---	0.005	1.003	9.91E-01	9.91E+01		
7895286	---	---	---	0.240	1.181	6.00E-01	6.00E+01		
7895287	---	---	---	0.155	1.114	6.14E-01	6.14E+01		
7895288	---	---	---	-0.101	0.932	6.67E-01	6.67E+01		
7895289	---	---	---	-0.093	0.938	7.25E-01	7.25E+01		
7895290	---	---	---	-0.046	0.968	8.03E-01	8.03E+01		
7895291	---	---	---	0.098	1.071	7.02E-01	7.02E+01		
7895292	---	---	---	0.050	1.035	9.28E-01	9.28E+01		
7895293	---	---	---	0.020	1.014	9.64E-01	9.64E+01		
7895294	---	---	---	-0.103	0.931	8.76E-01	8.76E+01		
7895295	---	---	---	0.120	1.086	5.40E-01	5.40E+01		
7895296	---	---	---	0.194	1.144	6.24E-01	6.24E+01		
7895297	---	---	---	0.070	1.050	8.54E-01	8.54E+01		
7895298	---	---	---	0.039	1.028	8.81E-01	8.81E+01		
7895299	---	---	---	0.160	1.117	6.03E-01	6.03E+01		
7895300	---	---	---	-0.003	0.998	9.97E-01	9.97E+01		
7895301	---	---	---	0.013	1.009	9.87E-01	9.87E+01		
7895303	---	---	---	0.096	1.069	7.16E-01	7.16E+01		
7895304	---	---	---	-0.016	0.989	9.67E-01	9.67E+01		
7895305	---	---	---	-0.004	0.997	9.90E-01	9.90E+01		
7895306	---	---	---	0.015	1.010	9.44E-01	9.44E+01		
7895308	---	---	---	-0.040	0.973	8.70E-01	8.70E+01		
7895310	---	---	---	0.040	1.028	7.78E-01	7.78E+01		
7895312	---	---	---	-0.037	0.975	9.37E-01	9.37E+01		
7895316	---	---	---	-0.172	0.888	4.13E-01	4.13E+01		
7895317	---	---	---	-0.035	0.976	8.41E-01	8.41E+01		
7895318	---	---	---	0.201	1.150	5.77E-01	5.77E+01		
7895319	---	---	---	-0.217	0.861	5.17E-01	5.17E+01		
7895320	---	---	---	0.077	1.055	7.67E-01	7.67E+01		
7895322	---	---	---	-0.019	0.987	9.43E-01	9.43E+01		
7895323	---	---	---	0.032	1.022	8.31E-01	8.31E+01		
7895324	---	---	---	0.016	1.011	9.61E-01	9.61E+01		
7895325	---	---	---	-0.056	0.962	7.87E-01	7.87E+01		
7895326	---	---	---	0.032	1.023	8.17E-01	8.17E+01		
7895328	---	---	---	-0.066	0.955	7.01E-01	7.01E+01		
7895329	---	---	---	-0.122	0.919	6.05E-01	6.05E+01		
7895330	---	---	---	0.011	1.007	9.73E-01	9.73E+01		
7895332	---	---	---	-0.083	0.944	8.62E-01	8.62E+01		
7895333	---	---	---	0.037	1.026	6.57E-01	6.57E+01		
7895334	---	---	---	0.229	1.172	1.95E-01	1.95E+01		
7895336	---	---	---	-0.002	0.999	9.91E-01	9.91E+01		
7895337	---	---	---	0.078	1.056	7.91E-01	7.91E+01		
7895340	---	---	---	-0.075	0.949	8.88E-01	8.88E+01		
7895342	---	---	---	0.027	1.019	8.50E-01	8.50E+01		
7895343	---	---	---	0.007	1.005	9.64E-01	9.64E+01		
7895344	---	---	---	-0.223	0.857	7.03E-01	7.03E+01		
7895345	---	---	---	0.152	1.111	6.62E-01	6.62E+01		
7895346	---	---	---	-0.210	0.864	6.19E-01	6.19E+01		
7895347	---	---	---	0.056	1.039	9.48E-01	9.48E+01		
7895348	---	---	---	0.011	1.008	9.85E-01	9.85E+01		
7895349	---	---	---	0.094	1.067	9.35E-01	9.35E+01		
7895350	---	---	---	0.019	1.013	9.33E-01	9.33E+01		
7895351	---	---	---	0.118	1.085	7.95E-01	7.95E+01		
7895354	---	---	---	0.047	1.033	5.93E-01	5.93E+01		
7895356	---	---	---	-0.120	0.920	7.01E-01	7.01E+01		
7895357	---	---	---	0.063	1.045	7.89E-01	7.89E+01		
7895358	---	---	---	0.044	1.031	7.25E-01	7.25E+01		
7895359	---	---	---	-0.068	0.954	8.00E-01	8.00E+01		
7895360	---	---	---	0.038	1.027	8.70E-01	8.70E+01		
7895361	---	---	---	0.029	1.020	9.56E-01	9.56E+01		
7895362	---	---	---	0.040	1.028	8.89E-01	8.89E+01		
7895365	---	---	---	-0.017	0.989	9.57E-01	9.57E+01		
7895368	---	---	---	-0.093	0.938	5.35E-01	5.35E+01		
7895370	---	---	---	0.046	1.033	9.05E-01	9.05E+01		

7895373	---	---	---	-0.037	0.975	7.75E-01	7.75E+01		
7895374	---	---	---	0.046	1.032	6.59E-01	6.59E+01		
7895375	---	---	---	0.044	1.031	9.12E-01	9.12E+01		
7895376	---	---	---	0.001	1.001	9.92E-01	9.92E+01		
7895379	---	---	---	0.004	1.003	9.88E-01	9.88E+01		
7895382	---	---	---	0.015	1.011	9.77E-01	9.77E+01		
7895387	---	---	---	-0.131	0.913	6.70E-01	6.70E+01		
7895388	---	---	---	0.046	1.032	9.18E-01	9.18E+01		
7895390	---	---	---	-0.132	0.913	6.94E-01	6.94E+01		
7895391	---	---	---	0.012	1.008	9.36E-01	9.36E+01		
7895392	---	---	---	0.082	1.059	2.68E-01	2.68E+01		
7895393	---	---	---	0.017	1.012	9.27E-01	9.27E+01		
7895395	---	---	---	0.042	1.030	9.24E-01	9.24E+01		
7895397	---	---	---	0.121	1.087	7.70E-01	7.70E+01		
7895398	---	---	---	0.058	1.041	6.78E-01	6.78E+01		
7895399	---	---	---	-0.099	0.934	8.23E-01	8.23E+01		
7895400	---	---	---	-0.197	0.873	2.05E-01	2.05E+01		
7895403	---	---	---	-0.054	0.963	8.56E-01	8.56E+01		
7895404	---	---	---	-0.106	0.929	8.77E-01	8.77E+01		
7895405	---	---	---	0.039	1.028	9.52E-01	9.52E+01		
7895406	---	---	---	-0.026	0.982	8.36E-01	8.36E+01		
7895408	---	---	---	-0.040	0.973	9.49E-01	9.49E+01		
7895409	---	---	---	0.290	1.223	4.33E-01	4.33E+01		
7895410	---	---	---	0.141	1.103	6.70E-01	6.70E+01		
7895411	---	---	---	0.025	1.018	9.36E-01	9.36E+01		
7895412	---	---	---	0.156	1.114	5.53E-01	5.53E+01		
7895414	---	---	---	-0.113	0.925	2.15E-01	2.15E+01		
7895416	---	---	---	0.069	1.049	7.82E-01	7.82E+01		
7895418	---	---	---	-0.094	0.937	7.76E-01	7.76E+01		
7895419	---	---	---	0.044	1.031	8.43E-01	8.43E+01		
7895421	---	---	---	-0.021	0.986	9.76E-01	9.76E+01		
7895422	---	---	---	-0.058	0.960	9.35E-01	9.35E+01		
7895424	---	---	---	0.295	1.227	3.63E-01	3.63E+01		
7895425	---	---	---	-0.100	0.933	7.04E-01	7.04E+01		
7895427	---	---	---	-0.037	0.975	8.67E-01	8.67E+01		
7895428	---	---	---	-0.054	0.963	9.54E-01	9.54E+01		
7895429	---	---	---	-0.059	0.960	9.09E-01	9.09E+01		
7895432	---	---	---	-0.016	0.989	9.86E-01	9.86E+01		
7895433	---	---	---	-0.176	0.885	5.26E-01	5.26E+01		
7895434	---	---	---	-0.037	0.974	9.51E-01	9.51E+01		
7895435	---	---	---	0.013	1.009	9.58E-01	9.58E+01		
7895437	---	---	---	0.044	1.031	9.50E-01	9.50E+01		
7895439	---	---	---	-0.147	0.903	5.68E-01	5.68E+01		
7895440	---	---	---	-0.009	0.994	9.72E-01	9.72E+01		
7895441	---	---	---	0.138	1.100	5.63E-01	5.63E+01		
7895442	---	---	---	0.329	1.257	7.73E-02	7.73E+00		
7895444	---	---	---	0.093	1.067	5.70E-01	5.70E+01		
7895445	---	---	---	0.118	1.085	8.09E-01	8.09E+01		
7895446	---	---	---	-0.092	0.939	8.05E-01	8.05E+01		
7895447	---	---	---	0.146	1.106	5.18E-01	5.18E+01		
7895449	---	---	---	0.173	1.128	4.13E-01	4.13E+01		
7895450	---	---	---	-0.009	0.993	9.89E-01	9.89E+01		
7895451	---	---	---	0.047	1.033	7.14E-01	7.14E+01		
7895452	---	---	---	0.053	1.037	8.78E-01	8.78E+01		
7895453	---	---	---	0.203	1.151	7.68E-01	7.68E+01		
7895456	---	---	---	0.074	1.052	9.54E-01	9.54E+01		
7895457	---	---	---	0.437	1.353	4.51E-01	4.51E+01		
7895458	---	---	---	0.033	1.023	9.70E-01	9.70E+01		
7895460	---	---	---	0.292	1.224	4.04E-01	4.04E+01		
7895461	---	---	---	-0.035	0.976	9.00E-01	9.00E+01		
7895463	---	---	---	0.004	1.003	9.84E-01	9.84E+01		
7895464	---	---	---	0.020	1.014	9.24E-01	9.24E+01		
7895465	---	---	---	-0.111	0.926	7.55E-01	7.55E+01		
7895467	---	---	---	-0.272	0.828	3.57E-01	3.57E+01		
7895470	---	---	---	0.025	1.017	9.49E-01	9.49E+01		
7895471	---	---	---	0.092	1.066	8.13E-01	8.13E+01		
7895472	---	---	---	-0.045	0.969	8.16E-01	8.16E+01		
7895475	---	---	---	0.097	1.070	3.94E-01	3.94E+01		
7895476	---	---	---	-0.062	0.958	6.80E-01	6.80E+01		
7895477	---	---	---	0.086	1.061	7.45E-01	7.45E+01		
7895478	---	---	---	0.091	1.065	7.52E-01	7.52E+01		
7895480	---	---	---	-0.468	0.723	2.33E-01	2.33E+01		
7895481	---	---	---	-0.018	0.988	9.53E-01	9.53E+01		
7895483	---	---	---	0.001	1.001	9.96E-01	9.96E+01		
7895484	---	---	---	0.113	1.081	7.15E-01	7.15E+01		
7895486	---	---	---	-0.044	0.970	8.69E-01	8.69E+01		
7895488	---	---	---	0.044	1.031	9.03E-01	9.03E+01		

7895489	---	---	---	-0.058	0.960	8.73E-01	8.73E+01		
7895490	---	---	---	-0.107	0.929	4.99E-01	4.99E+01		
7895491	---	---	---	0.187	1.139	8.57E-01	8.57E+01		
7895492	---	---	---	0.273	1.208	1.97E-01	1.97E+01		
7895494	---	---	---	0.426	1.343	5.79E-01	5.79E+01		
7895495	---	---	---	0.005	1.003	9.88E-01	9.88E+01		
7895496	---	---	---	0.028	1.020	8.46E-01	8.46E+01		
7895497	---	---	---	0.008	1.006	9.65E-01	9.65E+01		
7895498	---	---	---	0.024	1.017	9.43E-01	9.43E+01		
7895499	---	---	---	0.088	1.063	8.50E-01	8.50E+01		
7895501	---	---	---	-0.053	0.964	9.21E-01	9.21E+01		
7895503	---	---	---	-0.055	0.963	9.15E-01	9.15E+01		
7895506	---	---	---	-0.016	0.989	9.53E-01	9.53E+01		
7895507	---	---	---	-0.014	0.990	9.86E-01	9.86E+01		
7895508	---	---	---	-0.044	0.970	7.96E-01	7.96E+01		
7895509	---	---	---	-0.048	0.967	8.66E-01	8.66E+01		
7895510	---	---	---	0.014	1.010	9.62E-01	9.62E+01		
7895511	---	---	---	0.163	1.120	7.41E-01	7.41E+01		
7895512	---	---	---	-0.155	0.898	6.93E-01	6.93E+01		
7895514	---	---	---	0.036	1.025	6.35E-01	6.35E+01		
7895515	---	---	---	-0.010	0.993	9.76E-01	9.76E+01		
7895517	---	---	---	0.042	1.029	6.99E-01	6.99E+01		
7895518	---	---	---	-0.033	0.978	9.01E-01	9.01E+01		
7895519	---	---	---	-0.001	0.999	9.97E-01	9.97E+01		
7895520	---	---	---	-0.012	0.992	8.85E-01	8.85E+01		
7895521	---	---	---	-0.012	0.992	9.47E-01	9.47E+01		
7895522	---	---	---	0.021	1.015	9.39E-01	9.39E+01		
7895525	---	---	---	-0.107	0.928	3.95E-01	3.95E+01		
7895528	---	---	---	0.046	1.033	9.25E-01	9.25E+01		
7895530	---	---	---	0.094	1.067	8.66E-01	8.66E+01		
7895531	---	---	---	0.079	1.057	8.06E-01	8.06E+01		
7895532	---	---	---	-0.159	0.896	4.88E-01	4.88E+01		
7895536	---	---	---	0.004	1.003	9.88E-01	9.88E+01		
7895537	---	---	---	0.145	1.105	7.72E-01	7.72E+01		
7895538	---	---	---	-0.032	0.978	7.59E-01	7.59E+01		
7895539	---	---	---	-0.039	0.973	9.08E-01	9.08E+01		
7895540	---	---	---	-0.007	0.995	9.88E-01	9.88E+01		
7895541	---	---	---	-0.096	0.936	6.94E-01	6.94E+01		
7895542	---	---	---	0.021	1.015	9.53E-01	9.53E+01		
7895544	---	---	---	0.111	1.080	6.89E-01	6.89E+01		
7895545	---	---	---	-0.092	0.938	3.46E-01	3.46E+01		
7895547	---	---	---	0.284	1.218	2.10E-01	2.10E+01		
7895548	---	---	---	-0.033	0.977	9.55E-01	9.55E+01		
7895550	---	---	---	0.029	1.021	9.63E-01	9.63E+01		
7895551	---	---	---	-0.034	0.977	8.31E-01	8.31E+01		
7895552	---	---	---	0.458	1.373	4.19E-01	4.19E+01		
7895554	---	---	---	-0.046	0.968	6.25E-01	6.25E+01		
7895555	---	---	---	-0.064	0.957	9.50E-01	9.50E+01		
7895556	---	---	---	-0.054	0.963	7.79E-01	7.79E+01		
7895557	---	---	---	-0.010	0.993	9.50E-01	9.50E+01		
7895558	---	---	---	0.188	1.139	4.60E-01	4.60E+01		
7895559	---	---	---	-0.029	0.980	9.10E-01	9.10E+01		
7895561	---	---	---	-0.078	0.947	7.40E-01	7.40E+01		
7895562	---	---	---	0.280	1.215	5.63E-01	5.63E+01		
7895563	---	---	---	-0.038	0.974	9.09E-01	9.09E+01		
7895565	---	---	---	0.110	1.079	8.76E-01	8.76E+01		
7895566	---	---	---	0.038	1.027	9.53E-01	9.53E+01		
7895570	---	---	---	0.021	1.014	9.77E-01	9.77E+01		
7895571	---	---	---	0.008	1.006	9.89E-01	9.89E+01		
7895574	---	---	---	0.002	1.001	9.94E-01	9.94E+01		
7895575	---	---	---	0.107	1.077	8.47E-01	8.47E+01		
7895576	---	---	---	0.116	1.084	7.57E-01	7.57E+01		
7895577	---	---	---	0.017	1.012	9.61E-01	9.61E+01		
7895578	---	---	---	0.065	1.046	8.10E-01	8.10E+01		
7895579	---	---	---	0.057	1.040	7.03E-01	7.03E+01		
7895583	---	---	---	-0.042	0.971	9.42E-01	9.42E+01		
7895584	---	---	---	-0.417	0.749	5.02E-01	5.02E+01		
7895585	---	---	---	-0.044	0.970	7.97E-01	7.97E+01		
7895586	---	---	---	0.093	1.066	7.87E-01	7.87E+01		
7895587	---	---	---	0.036	1.025	6.70E-01	6.70E+01		
7895588	---	---	---	0.098	1.070	5.89E-01	5.89E+01		
7895589	---	---	---	-0.192	0.875	8.02E-01	8.02E+01		
7895590	---	---	---	0.034	1.024	8.35E-01	8.35E+01		
7895592	---	---	---	0.182	1.135	6.14E-01	6.14E+01		
7895595	---	---	---	0.034	1.024	8.31E-01	8.31E+01		
7895596	---	---	---	-0.077	0.948	5.02E-01	5.02E+01		
7895597	---	---	---	-0.173	0.887	5.25E-01	5.25E+01		

7895598	---	---	---	0.079	1.056	6.24E-01	6.24E+01		
7895599	---	---	---	-0.030	0.979	8.86E-01	8.86E+01		
7895600	---	---	---	0.240	1.181	5.12E-01	5.12E+01		
7895601	---	---	---	0.000	1.000	1.00E+00	1.00E+02		
7895602	---	---	---	0.053	1.038	8.30E-01	8.30E+01		
7895603	---	---	---	0.106	1.076	7.69E-01	7.69E+01		
7895604	---	---	---	0.021	1.015	9.64E-01	9.64E+01		
7895605	---	---	---	0.219	1.164	4.25E-01	4.25E+01		
7895606	---	---	---	-0.037	0.975	8.06E-01	8.06E+01		
7895608	---	---	---	0.055	1.039	8.68E-01	8.68E+01		
7895609	---	---	---	0.072	1.051	8.08E-01	8.08E+01		
7895610	---	---	---	0.015	1.010	9.73E-01	9.73E+01		
7895611	---	---	---	0.316	1.245	4.87E-01	4.87E+01		
7895612	---	---	---	0.009	1.006	9.90E-01	9.90E+01		
7895614	---	---	---	0.203	1.151	3.47E-01	3.47E+01		
7895616	---	---	---	-0.091	0.939	5.06E-01	5.06E+01		
7895618	---	---	---	0.106	1.076	8.60E-01	8.60E+01		
7895619	---	---	---	0.054	1.038	8.65E-01	8.65E+01		
7895620	---	---	---	0.009	1.006	9.84E-01	9.84E+01		
7895621	---	---	---	0.022	1.015	8.66E-01	8.66E+01		
7895622	---	---	---	0.034	1.024	8.63E-01	8.63E+01		
7895624	---	---	---	-0.119	0.921	8.69E-01	8.69E+01		
7895625	---	---	---	0.018	1.013	9.78E-01	9.78E+01		
7895626	---	---	---	-0.056	0.962	7.51E-01	7.51E+01		
7895627	---	---	---	-0.048	0.967	7.30E-01	7.30E+01		
7895629	---	---	---	0.051	1.036	8.15E-01	8.15E+01		
7895630	---	---	---	0.080	1.057	7.63E-01	7.63E+01		
7895631	---	---	---	0.167	1.123	7.62E-01	7.62E+01		
7895632	---	---	---	0.066	1.047	7.61E-01	7.61E+01		
7895637	---	---	---	-0.133	0.912	7.55E-01	7.55E+01		
7895638	---	---	---	-0.219	0.859	7.73E-01	7.73E+01		
7895640	---	---	---	0.025	1.017	9.33E-01	9.33E+01		
7895641	---	---	---	-0.134	0.911	6.59E-01	6.59E+01		
7895642	---	---	---	0.028	1.020	9.34E-01	9.34E+01		
7895643	---	---	---	-0.062	0.958	8.05E-01	8.05E+01		
7895645	---	---	---	0.080	1.057	8.10E-01	8.10E+01		
7895647	---	---	---	-0.002	0.999	9.95E-01	9.95E+01		
7895649	---	---	---	0.125	1.090	5.41E-01	5.41E+01		
7895650	---	---	---	-0.066	0.955	9.24E-01	9.24E+01		
7895651	---	---	---	-0.151	0.901	3.09E-01	3.09E+01		
7895652	---	---	---	0.070	1.050	5.89E-01	5.89E+01		
7895654	---	---	---	0.019	1.013	9.57E-01	9.57E+01		
7895656	---	---	---	0.033	1.023	8.45E-01	8.45E+01		
7895657	---	---	---	0.051	1.036	7.52E-01	7.52E+01		
7895658	---	---	---	0.062	1.044	8.84E-01	8.84E+01		
7895659	---	---	---	0.077	1.055	4.71E-01	4.71E+01		
7895661	---	---	---	-0.038	0.974	7.56E-01	7.56E+01		
7895662	---	---	---	0.074	1.052	6.63E-01	6.63E+01		
7895663	---	---	---	0.139	1.101	6.14E-01	6.14E+01		
7895664	---	---	---	0.071	1.051	8.48E-01	8.48E+01		
7895665	---	---	---	-0.005	0.996	9.85E-01	9.85E+01		
7895666	---	---	---	-0.097	0.935	8.53E-01	8.53E+01		
7895669	---	---	---	0.063	1.044	8.42E-01	8.42E+01		
7895672	---	---	---	0.066	1.047	8.91E-01	8.91E+01		
7895674	---	---	---	-0.005	0.997	9.84E-01	9.84E+01		
7895675	---	---	---	0.008	1.006	9.87E-01	9.87E+01		
7895676	---	---	---	-0.049	0.967	7.87E-01	7.87E+01		
7895677	---	---	---	-0.125	0.917	8.04E-01	8.04E+01		
7895680	---	---	---	0.025	1.017	8.69E-01	8.69E+01		
7895681	---	---	---	0.103	1.074	7.78E-01	7.78E+01		
7895682	---	---	---	-0.100	0.933	9.11E-01	9.11E+01		
7895684	---	---	---	0.043	1.030	9.04E-01	9.04E+01		
7895685	---	---	---	-0.110	0.927	5.12E-01	5.12E+01		
7895686	---	---	---	0.154	1.113	7.02E-01	7.02E+01		
7895687	---	---	---	-0.040	0.973	6.92E-01	6.92E+01		
7895688	---	---	---	0.044	1.031	8.35E-01	8.35E+01		
7895690	---	---	---	0.026	1.019	8.74E-01	8.74E+01		
7895691	---	---	---	0.126	1.091	1.55E-01	1.55E+01		
7895692	---	---	---	0.049	1.035	8.21E-01	8.21E+01		
7895693	---	---	---	0.098	1.070	4.75E-01	4.75E+01		
7895694	---	---	---	0.062	1.044	7.64E-01	7.64E+01		
7895695	---	---	---	-0.010	0.993	9.80E-01	9.80E+01		
7895697	---	---	---	0.053	1.037	8.09E-01	8.09E+01		
7895704	---	---	---	-0.043	0.971	7.94E-01	7.94E+01		
7895705	---	---	---	0.063	1.045	5.13E-01	5.13E+01		
7895706	---	---	---	-0.108	0.928	7.96E-01	7.96E+01		
7895707	---	---	---	-0.037	0.975	9.53E-01	9.53E+01		

7895708	---	---	---	0.058	1.041	9.12E-01	9.12E+01		
7895710	---	---	---	0.063	1.045	4.59E-01	4.59E+01		
7895711	---	---	---	0.064	1.045	8.62E-01	8.62E+01		
7895712	---	---	---	0.056	1.040	7.40E-01	7.40E+01		
7895713	---	---	---	-0.106	0.929	9.13E-01	9.13E+01		
7895714	---	---	---	0.028	1.020	8.35E-01	8.35E+01		
7895715	---	---	---	0.059	1.042	8.76E-01	8.76E+01		
7895716	---	---	---	0.019	1.013	9.49E-01	9.49E+01		
7895717	---	---	---	0.013	1.009	9.56E-01	9.56E+01		
7895718	---	---	---	0.064	1.046	8.57E-01	8.57E+01		
7895719	---	---	---	0.017	1.012	9.56E-01	9.56E+01		
7895721	---	---	---	0.081	1.058	6.76E-01	6.76E+01		
7895722	---	---	---	-0.036	0.975	9.64E-01	9.64E+01		
7895723	---	---	---	0.103	1.074	8.02E-01	8.02E+01		
7895724	---	---	---	0.044	1.031	8.88E-01	8.88E+01		
7895726	---	---	---	-0.102	0.932	4.20E-01	4.20E+01		
7895727	---	---	---	0.148	1.108	5.20E-01	5.20E+01		
7895728	---	---	---	-0.113	0.925	7.77E-01	7.77E+01		
7895729	---	---	---	0.116	1.084	6.31E-01	6.31E+01		
7895731	---	---	---	-0.238	0.848	5.64E-01	5.64E+01		
7895732	---	---	---	-0.044	0.970	8.34E-01	8.34E+01		
7895733	---	---	---	0.057	1.040	8.78E-01	8.78E+01		
7895734	---	---	---	0.096	1.069	8.15E-01	8.15E+01		
7895735	---	---	---	-0.188	0.878	5.82E-01	5.82E+01		
7895736	---	---	---	-0.074	0.950	6.95E-01	6.95E+01		
7895737	---	---	---	-0.158	0.896	5.76E-01	5.76E+01		
7895738	---	---	---	-0.585	0.667	5.18E-02	5.18E+00		
7895739	---	---	---	0.016	1.011	9.86E-01	9.86E+01		
7895740	---	---	---	-0.194	0.874	4.04E-01	4.04E+01		
7895742	---	---	---	0.055	1.039	9.27E-01	9.27E+01		
7895743	---	---	---	0.016	1.011	9.78E-01	9.78E+01		
7895744	---	---	---	0.081	1.058	7.72E-01	7.72E+01		
7895745	---	---	---	-0.007	0.995	9.78E-01	9.78E+01		
7895748	---	---	---	0.072	1.051	8.45E-01	8.45E+01		
7895751	---	---	---	0.019	1.013	9.44E-01	9.44E+01		
7895752	---	---	---	-0.049	0.967	9.00E-01	9.00E+01		
7895753	---	---	---	0.101	1.073	7.94E-01	7.94E+01		
7895754	---	---	---	0.245	1.185	3.04E-01	3.04E+01		
7895755	---	---	---	0.245	1.185	4.62E-01	4.62E+01		
7895757	---	---	---	0.154	1.113	5.92E-01	5.92E+01		
7895758	---	---	---	-0.024	0.984	8.99E-01	8.99E+01		
7895759	---	---	---	0.225	1.169	4.16E-01	4.16E+01		
7895760	---	---	---	-0.088	0.941	7.04E-01	7.04E+01		
7895761	---	---	---	-0.081	0.946	7.73E-01	7.73E+01		
7895762	---	---	---	-0.003	0.998	9.86E-01	9.86E+01		
7895766	---	---	---	0.019	1.014	9.09E-01	9.09E+01		
7895767	---	---	---	-0.025	0.983	8.71E-01	8.71E+01		
7895768	---	---	---	-0.209	0.865	6.16E-01	6.16E+01		
7895769	---	---	---	0.008	1.006	9.91E-01	9.91E+01		
7895770	---	---	---	0.014	1.010	9.77E-01	9.77E+01		
7895772	---	---	---	0.064	1.045	8.38E-01	8.38E+01		
7895774	---	---	---	0.133	1.097	5.48E-01	5.48E+01		
7895775	---	---	---	-0.007	0.995	9.88E-01	9.88E+01		
7895776	---	---	---	-0.014	0.990	9.88E-01	9.88E+01		
7895777	---	---	---	-0.009	0.994	9.77E-01	9.77E+01		
7895778	---	---	---	0.043	1.031	6.38E-01	6.38E+01		
7895779	---	---	---	-0.020	0.986	8.69E-01	8.69E+01		
7895780	---	---	---	-0.014	0.990	9.19E-01	9.19E+01		
7895782	---	---	---	-0.044	0.970	8.55E-01	8.55E+01		
7895783	---	---	---	-0.078	0.948	7.16E-01	7.16E+01		
7895784	---	---	---	-0.059	0.960	8.07E-01	8.07E+01		
7895785	---	---	---	0.086	1.062	9.09E-01	9.09E+01		
7895786	---	---	---	0.208	1.155	6.52E-01	6.52E+01		
7895787	---	---	---	-0.009	0.994	9.72E-01	9.72E+01		
7895788	---	---	---	-0.102	0.932	9.22E-01	9.22E+01		
7895789	---	---	---	-0.112	0.925	8.08E-01	8.08E+01		
7895790	---	---	---	-0.008	0.995	9.66E-01	9.66E+01		
7895791	---	---	---	-0.091	0.939	7.74E-01	7.74E+01		
7895792	---	---	---	0.232	1.175	3.58E-01	3.58E+01		
7895794	---	---	---	0.009	1.006	9.79E-01	9.79E+01		
7895795	---	---	---	0.016	1.011	9.40E-01	9.40E+01		
7895796	---	---	---	0.201	1.149	5.79E-01	5.79E+01		
7895797	---	---	---	-0.171	0.888	7.56E-01	7.56E+01		
7895801	---	---	---	-0.168	0.890	5.10E-01	5.10E+01		
7895802	---	---	---	0.061	1.044	7.05E-01	7.05E+01		
7895805	---	---	---	0.060	1.042	8.57E-01	8.57E+01		
7895806	---	---	---	-0.110	0.927	9.15E-01	9.15E+01		

7895808	---	---	---	-0.019	0.987	9.51E-01	9.51E+01		
7895809	---	---	---	0.108	1.078	7.77E-01	7.77E+01		
7895810	---	---	---	0.067	1.048	8.49E-01	8.49E+01		
7895813	---	---	---	-0.032	0.978	9.24E-01	9.24E+01		
7895814	---	---	---	0.123	1.089	8.05E-01	8.05E+01		
7895815	---	---	---	0.058	1.041	6.60E-01	6.60E+01		
7895816	---	---	---	-0.079	0.947	8.08E-01	8.08E+01		
7895817	---	---	---	0.216	1.162	3.59E-01	3.59E+01		
7895818	---	---	---	0.007	1.005	9.80E-01	9.80E+01		
7895819	---	---	---	0.065	1.046	4.35E-01	4.35E+01		
7895820	---	---	---	-0.021	0.986	9.81E-01	9.81E+01		
7895821	---	---	---	0.001	1.000	1.00E+00	1.00E+02		
7895824	---	---	---	-0.036	0.975	7.52E-01	7.52E+01		
7895826	---	---	---	0.047	1.033	6.93E-01	6.93E+01		
7895827	---	---	---	-0.115	0.924	5.37E-01	5.37E+01		
7895829	---	---	---	-0.138	0.909	7.83E-01	7.83E+01		
7895831	---	---	---	-0.193	0.875	4.80E-01	4.80E+01		
7895832	---	---	---	0.136	1.099	7.71E-01	7.71E+01		
7895833	---	---	---	-0.184	0.880	8.73E-01	8.73E+01		
7895834	---	---	---	-0.199	0.871	7.30E-01	7.30E+01		
7895835	---	---	---	-0.027	0.982	9.50E-01	9.50E+01		
7895836	---	---	---	0.262	1.199	3.07E-01	3.07E+01		
7895837	---	---	---	-0.094	0.937	7.55E-01	7.55E+01		
7895839	---	---	---	0.360	1.283	5.04E-01	5.04E+01		
7895840	---	---	---	-0.112	0.925	7.87E-01	7.87E+01		
7895841	---	---	---	-0.118	0.922	5.58E-01	5.58E+01		
7895842	---	---	---	0.049	1.035	8.46E-01	8.46E+01		
7895844	---	---	---	0.009	1.006	9.75E-01	9.75E+01		
7895845	---	---	---	-0.008	0.994	9.86E-01	9.86E+01		
7895846	---	---	---	0.094	1.067	7.52E-01	7.52E+01		
7895847	---	---	---	-0.275	0.826	2.99E-01	2.99E+01		
7895848	---	---	---	0.066	1.047	8.78E-01	8.78E+01		
7895850	---	---	---	0.299	1.231	6.30E-01	6.30E+01		
7895853	---	---	---	-0.076	0.949	8.22E-01	8.22E+01		
7895854	---	---	---	0.109	1.078	5.53E-01	5.53E+01		
7895855	---	---	---	0.255	1.194	4.45E-01	4.45E+01		
7895857	---	---	---	-0.102	0.932	6.17E-01	6.17E+01		
7895862	---	---	---	0.082	1.058	7.86E-01	7.86E+01		
7895863	---	---	---	-0.182	0.881	7.76E-01	7.76E+01		
7895865	---	---	---	0.181	1.134	6.83E-01	6.83E+01		
7895866	---	---	---	0.012	1.008	9.72E-01	9.72E+01		
7895868	---	---	---	0.107	1.077	6.84E-01	6.84E+01		
7895869	---	---	---	-0.074	0.950	6.78E-01	6.78E+01		
7895870	---	---	---	0.091	1.065	9.12E-01	9.12E+01		
7895872	---	---	---	0.018	1.013	9.35E-01	9.35E+01		
7895873	---	---	---	-0.167	0.891	3.65E-01	3.65E+01		
7895874	---	---	---	0.053	1.038	9.56E-01	9.56E+01		
7895878	---	---	---	0.014	1.010	9.72E-01	9.72E+01		
7895879	---	---	---	-0.089	0.940	7.52E-01	7.52E+01		
7895880	---	---	---	0.087	1.062	5.62E-01	5.62E+01		
7895881	---	---	---	-0.040	0.973	9.05E-01	9.05E+01		
7895882	---	---	---	0.062	1.044	7.42E-01	7.42E+01		
7895883	---	---	---	0.024	1.017	9.23E-01	9.23E+01		
7895884	---	---	---	-0.050	0.966	9.49E-01	9.49E+01		
7895885	---	---	---	0.127	1.092	4.96E-01	4.96E+01		
7895886	---	---	---	-0.048	0.968	8.73E-01	8.73E+01		
7895888	---	---	---	-0.028	0.981	9.53E-01	9.53E+01		
7895889	---	---	---	-0.099	0.934	6.63E-01	6.63E+01		
7895890	---	---	---	0.080	1.057	7.96E-01	7.96E+01		
7895894	---	---	---	0.398	1.318	6.51E-01	6.51E+01		
7895896	---	---	---	0.034	1.024	9.53E-01	9.53E+01		
7895898	---	---	---	0.065	1.046	8.25E-01	8.25E+01		
7895899	---	---	---	-0.025	0.983	8.68E-01	8.68E+01		
7895900	---	---	---	-0.106	0.929	8.98E-01	8.98E+01		
7895901	---	---	---	0.135	1.098	4.03E-01	4.03E+01		
7895903	---	---	---	-0.102	0.932	8.54E-01	8.54E+01		
7895904	---	---	---	0.326	1.254	4.04E-01	4.04E+01		
7895907	---	---	---	0.051	1.036	6.04E-01	6.04E+01		
7895908	---	---	---	-0.097	0.935	7.59E-01	7.59E+01		
7895910	---	---	---	0.422	1.340	7.05E-02	7.05E+00		
7895911	---	---	---	0.088	1.063	5.73E-01	5.73E+01		
7895912	---	---	---	0.083	1.059	4.48E-01	4.48E+01		
7895913	---	---	---	0.030	1.021	9.65E-01	9.65E+01		
7895914	---	---	---	0.146	1.106	7.26E-01	7.26E+01		
7895916	---	---	---	0.079	1.056	7.56E-01	7.56E+01		
7895917	---	---	---	0.017	1.012	9.27E-01	9.27E+01		
7895918	---	---	---	-0.024	0.983	9.28E-01	9.28E+01		

7895919	---	---	---	0.017	1.012	9.63E-01	9.63E+01		
7895920	---	---	---	0.015	1.011	8.84E-01	8.84E+01		
7895921	---	---	---	0.236	1.178	5.38E-01	5.38E+01		
7895923	---	---	---	-0.059	0.960	7.27E-01	7.27E+01		
7895924	---	---	---	0.016	1.011	9.36E-01	9.36E+01		
7895925	---	---	---	0.148	1.108	8.74E-01	8.74E+01		
7895926	---	---	---	-0.002	0.998	9.90E-01	9.90E+01		
7895927	---	---	---	0.159	1.117	8.03E-01	8.03E+01		
7895928	---	---	---	-0.038	0.974	8.84E-01	8.84E+01		
7895929	---	---	---	0.071	1.051	8.74E-01	8.74E+01		
7895930	---	---	---	0.032	1.022	9.36E-01	9.36E+01		
7895931	---	---	---	0.029	1.020	9.66E-01	9.66E+01		
7895932	---	---	---	-0.091	0.939	6.34E-01	6.34E+01		
7895933	---	---	---	0.337	1.263	5.47E-01	5.47E+01		
7895934	---	---	---	0.098	1.070	7.44E-01	7.44E+01		
7895936	---	---	---	0.042	1.029	7.56E-01	7.56E+01		
7895937	---	---	---	0.017	1.012	9.57E-01	9.57E+01		
7895938	---	---	---	0.050	1.035	8.05E-01	8.05E+01		
7895939	---	---	---	0.085	1.061	6.37E-01	6.37E+01		
7895941	---	---	---	0.182	1.135	4.04E-01	4.04E+01		
7895942	---	---	---	0.071	1.051	6.01E-01	6.01E+01		
7895943	---	---	---	-0.053	0.964	8.84E-01	8.84E+01		
7895944	---	---	---	-0.044	0.970	7.52E-01	7.52E+01		
7895945	---	---	---	0.056	1.040	7.56E-01	7.56E+01		
7895946	---	---	---	0.016	1.011	9.78E-01	9.78E+01		
7895947	---	---	---	0.022	1.015	9.11E-01	9.11E+01		
7895948	---	---	---	-0.088	0.941	6.19E-01	6.19E+01		
7895952	---	---	---	-0.008	0.995	9.77E-01	9.77E+01		
7895953	---	---	---	-0.080	0.946	7.53E-01	7.53E+01		
7895954	---	---	---	-0.136	0.910	4.06E-01	4.06E+01		
7895955	---	---	---	-0.136	0.910	6.42E-01	6.42E+01		
7895956	---	---	---	-0.016	0.989	9.48E-01	9.48E+01		
7895957	---	---	---	0.071	1.050	7.96E-01	7.96E+01		
7895959	---	---	---	0.062	1.044	9.01E-01	9.01E+01		
7895960	---	---	---	0.079	1.057	8.77E-01	8.77E+01		
7895961	---	---	---	0.123	1.089	6.04E-01	6.04E+01		
7895962	---	---	---	-0.185	0.880	1.72E-01	1.72E+01		
7895963	---	---	---	0.051	1.036	7.16E-01	7.16E+01		
7895964	---	---	---	0.015	1.011	9.56E-01	9.56E+01		
7895965	---	---	---	0.144	1.105	6.76E-01	6.76E+01		
7895966	---	---	---	-0.102	0.932	8.33E-01	8.33E+01		
7895967	---	---	---	0.520	1.434	3.79E-01	3.79E+01		
7895969	---	---	---	0.030	1.021	8.98E-01	8.98E+01		
7895971	---	---	---	0.117	1.084	7.89E-01	7.89E+01		
7895972	---	---	---	-0.012	0.992	9.70E-01	9.70E+01		
7895973	---	---	---	-0.065	0.956	5.56E-01	5.56E+01		
7895974	---	---	---	0.032	1.023	8.69E-01	8.69E+01		
7895976	---	---	---	-0.017	0.988	9.81E-01	9.81E+01		
7895979	---	---	---	0.183	1.135	4.27E-01	4.27E+01		
7895981	---	---	---	0.057	1.040	8.78E-01	8.78E+01		
7895982	---	---	---	0.094	1.067	6.85E-01	6.85E+01		
7895983	---	---	---	-0.055	0.963	8.81E-01	8.81E+01		
7895985	---	---	---	0.119	1.086	7.03E-01	7.03E+01		
7895987	---	---	---	0.046	1.033	9.25E-01	9.25E+01		
7895988	---	---	---	0.002	1.001	9.96E-01	9.96E+01		
7895990	---	---	---	-0.051	0.965	8.78E-01	8.78E+01		
7895992	---	---	---	0.068	1.048	9.19E-01	9.19E+01		
7895993	---	---	---	0.006	1.004	9.86E-01	9.86E+01		
7895994	---	---	---	0.000	1.000	1.00E+00	1.00E+02		
7895995	---	---	---	-0.048	0.967	8.46E-01	8.46E+01		
7895996	---	---	---	0.092	1.066	7.13E-01	7.13E+01		
7895997	---	---	---	-0.102	0.932	6.09E-01	6.09E+01		
7895998	---	---	---	-0.084	0.944	9.25E-01	9.25E+01		
7895999	---	---	---	-0.158	0.896	5.27E-01	5.27E+01		
7896000	---	---	---	-0.085	0.943	8.76E-01	8.76E+01		
7896001	---	---	---	-0.007	0.995	9.90E-01	9.90E+01		
7896004	---	---	---	-0.107	0.928	7.00E-01	7.00E+01		
7896005	---	---	---	-0.030	0.980	9.21E-01	9.21E+01		
7896006	---	---	---	0.048	1.034	9.49E-01	9.49E+01		
7896007	---	---	---	0.005	1.003	9.86E-01	9.86E+01		
7896008	---	---	---	0.167	1.123	5.53E-01	5.53E+01		
7896009	---	---	---	-0.102	0.932	9.22E-01	9.22E+01		
7896010	---	---	---	0.062	1.044	9.01E-01	9.01E+01		
7896011	---	---	---	-0.452	0.731	3.71E-01	3.71E+01		
7896014	---	---	---	-0.069	0.953	9.14E-01	9.14E+01		
7896017	---	---	---	0.106	1.076	4.49E-01	4.49E+01		
7896018	---	---	---	0.133	1.096	4.66E-01	4.66E+01		

7896019	---	---	---	-0.221	0.858	5.90E-01	5.90E+01		
7896020	---	---	---	-0.006	0.996	9.88E-01	9.88E+01		
7896021	---	---	---	0.167	1.123	7.75E-01	7.75E+01		
7896022	---	---	---	-0.012	0.992	9.78E-01	9.78E+01		
7896024	---	---	---	-0.030	0.979	8.77E-01	8.77E+01		
7896025	---	---	---	0.042	1.029	8.57E-01	8.57E+01		
7896026	---	---	---	0.016	1.011	9.81E-01	9.81E+01		
7896027	---	---	---	-0.097	0.935	6.35E-01	6.35E+01		
7896028	---	---	---	0.062	1.044	8.08E-01	8.08E+01		
7896029	---	---	---	-0.050	0.966	8.03E-01	8.03E+01		
7896030	---	---	---	-0.039	0.973	8.87E-01	8.87E+01		
7896034	---	---	---	0.036	1.025	9.00E-01	9.00E+01		
7896035	---	---	---	0.007	1.005	9.89E-01	9.89E+01		
7896036	---	---	---	-0.032	0.978	8.26E-01	8.26E+01		
7896037	---	---	---	-0.158	0.896	7.14E-01	7.14E+01		
7896038	---	---	---	0.008	1.005	9.71E-01	9.71E+01		
7896039	---	---	---	0.047	1.033	9.56E-01	9.56E+01		
7896040	---	---	---	-0.020	0.986	9.60E-01	9.60E+01		
7896041	---	---	---	0.043	1.030	8.04E-01	8.04E+01		
7896043	---	---	---	0.045	1.032	8.05E-01	8.05E+01		
7896044	---	---	---	0.029	1.020	9.09E-01	9.09E+01		
7896045	---	---	---	-0.004	0.997	9.86E-01	9.86E+01		
7896046	---	---	---	0.063	1.044	9.24E-01	9.24E+01		
7896047	---	---	---	-0.007	0.995	9.85E-01	9.85E+01		
7896049	---	---	---	0.043	1.030	8.76E-01	8.76E+01		
7896050	---	---	---	0.215	1.161	2.12E-02	2.12E+00		
7896051	---	---	---	-0.163	0.893	4.48E-01	4.48E+01		
7896052	---	---	---	-0.124	0.918	8.79E-01	8.79E+01		
7896053	---	---	---	-0.117	0.922	6.96E-01	6.96E+01		
7896054	---	---	---	-0.027	0.982	9.27E-01	9.27E+01		
7896055	---	---	---	0.066	1.046	8.41E-01	8.41E+01		
7896057	---	---	---	-0.146	0.904	6.11E-01	6.11E+01		
7896058	---	---	---	0.090	1.065	8.03E-01	8.03E+01		
7896059	---	---	---	-0.029	0.980	8.81E-01	8.81E+01		
7896060	---	---	---	0.159	1.116	1.86E-01	1.86E+01		
7896061	---	---	---	-0.101	0.933	7.45E-01	7.45E+01		
7896062	---	---	---	0.045	1.032	8.76E-01	8.76E+01		
7896063	---	---	---	-0.066	0.956	8.58E-01	8.58E+01		
7896064	---	---	---	-0.031	0.979	8.68E-01	8.68E+01		
7896065	---	---	---	-0.028	0.981	9.01E-01	9.01E+01		
7896067	---	---	---	0.106	1.076	3.67E-01	3.67E+01		
7896068	---	---	---	0.119	1.086	7.08E-01	7.08E+01		
7896069	---	---	---	0.084	1.060	7.63E-01	7.63E+01		
7896070	---	---	---	-0.118	0.921	5.30E-01	5.30E+01		
7896073	---	---	---	-0.025	0.983	8.72E-01	8.72E+01		
7896074	---	---	---	0.030	1.021	9.56E-01	9.56E+01		
7896076	---	---	---	-0.042	0.971	7.38E-01	7.38E+01		
7896077	---	---	---	0.073	1.052	7.27E-01	7.27E+01		
7896078	---	---	---	0.043	1.030	6.70E-01	6.70E+01		
7896080	---	---	---	0.041	1.029	8.80E-01	8.80E+01		
7896081	---	---	---	0.200	1.149	6.81E-01	6.81E+01		
7896082	---	---	---	-0.048	0.967	9.07E-01	9.07E+01		
7896084	---	---	---	0.024	1.017	9.30E-01	9.30E+01		
7896085	---	---	---	-0.085	0.943	6.68E-01	6.68E+01		
7896086	---	---	---	0.344	1.269	4.53E-01	4.53E+01		
7896087	---	---	---	0.063	1.045	6.93E-01	6.93E+01		
7896088	---	---	---	0.046	1.033	7.76E-01	7.76E+01		
7896089	---	---	---	-0.107	0.928	6.76E-01	6.76E+01		
7896090	---	---	---	-0.169	0.889	2.11E-01	2.11E+01		
7896091	---	---	---	0.036	1.026	9.27E-01	9.27E+01		
7896092	---	---	---	0.048	1.034	8.67E-01	8.67E+01		
7896094	---	---	---	0.009	1.007	9.53E-01	9.53E+01		
7896095	---	---	---	-0.039	0.973	9.14E-01	9.14E+01		
7896098	---	---	---	-0.090	0.940	6.05E-01	6.05E+01		
7896099	---	---	---	0.056	1.040	7.75E-01	7.75E+01		
7896100	---	---	---	-0.259	0.835	8.08E-01	8.08E+01		
7896101	---	---	---	0.125	1.091	8.33E-01	8.33E+01		
7896102	---	---	---	0.021	1.015	9.66E-01	9.66E+01		
7896103	---	---	---	0.011	1.008	9.57E-01	9.57E+01		
7896106	---	---	---	0.098	1.070	8.78E-01	8.78E+01		
7896107	---	---	---	0.081	1.058	6.67E-01	6.67E+01		
7896108	---	---	---	0.049	1.035	8.62E-01	8.62E+01		
7896110	---	---	---	0.226	1.170	6.70E-01	6.70E+01		
7896111	---	---	---	-0.030	0.979	7.79E-01	7.79E+01		
7896112	---	---	---	-0.051	0.965	8.02E-01	8.02E+01		
7896113	---	---	---	0.260	1.197	2.95E-01	2.95E+01		
7896115	---	---	---	0.040	1.028	6.54E-01	6.54E+01		

7896119	---	---	---	0.089	1.064	8.10E-01	8.10E+01			
7896120	---	---	---	0.021	1.014	9.58E-01	9.58E+01			
7896121	---	---	---	0.017	1.012	9.50E-01	9.50E+01			
7896122	---	---	---	-0.014	0.990	9.56E-01	9.56E+01			
7896123	---	---	---	-0.079	0.947	7.42E-01	7.42E+01			
7896124	---	---	---	0.197	1.147	1.50E-01	1.50E+01			
7896125	---	---	---	0.107	1.077	8.78E-01	8.78E+01			
7896126	---	---	---	0.090	1.064	7.95E-01	7.95E+01			
7896128	---	---	---	-0.198	0.871	3.04E-01	3.04E+01			
7896130	---	---	---	-0.093	0.938	4.65E-01	4.65E+01			
7896132	---	---	---	0.070	1.050	5.39E-01	5.39E+01			
7896133	---	---	---	-0.018	0.988	9.41E-01	9.41E+01			
7896135	---	---	---	-0.115	0.924	7.99E-01	7.99E+01			
7896136	---	---	---	0.068	1.048	6.01E-01	6.01E+01			
7896137	---	---	---	-0.047	0.968	7.83E-01	7.83E+01			
7896138	---	---	---	-0.018	0.987	9.74E-01	9.74E+01			
7896139	---	---	---	0.041	1.028	9.24E-01	9.24E+01			
7896140	---	---	---	0.035	1.024	9.19E-01	9.19E+01			
7896141	---	---	---	-0.031	0.978	9.33E-01	9.33E+01			
7896142	---	---	---	0.139	1.101	7.31E-02	7.31E+00			
7896143	---	---	---	0.147	1.108	5.67E-01	5.67E+01			
7896144	---	---	---	0.055	1.039	6.00E-01	6.00E+01			
7896145	---	---	---	0.167	1.122	4.96E-01	4.96E+01			
7896146	---	---	---	-0.005	0.997	9.92E-01	9.92E+01			
7896148	---	---	---	-0.025	0.983	8.80E-01	8.80E+01			
7896149	---	---	---	0.008	1.006	9.73E-01	9.73E+01			
7896150	---	---	---	0.052	1.037	7.33E-01	7.33E+01			
7896151	---	---	---	0.022	1.015	8.52E-01	8.52E+01			
7896153	---	---	---	0.217	1.162	3.07E-01	3.07E+01			
7896154	---	---	---	0.071	1.051	8.59E-01	8.59E+01			
7896155	---	---	---	-0.143	0.906	8.63E-01	8.63E+01			
7896156	---	---	---	-0.059	0.960	8.49E-01	8.49E+01			
7896157	---	---	---	-0.014	0.991	9.28E-01	9.28E+01			
7896158	---	---	---	-0.282	0.822	7.41E-02	7.41E+00			
7896159	---	---	---	-0.028	0.981	8.63E-01	8.63E+01			
7896160	---	---	---	0.034	1.024	9.01E-01	9.01E+01			
7896161	---	---	---	-0.227	0.854	7.05E-01	7.05E+01			
7896163	---	---	---	0.032	1.022	9.53E-01	9.53E+01			
7896164	---	---	---	0.135	1.098	5.94E-01	5.94E+01			
7896166	---	---	---	0.010	1.007	9.77E-01	9.77E+01			
7896167	---	---	---	-0.007	0.995	9.87E-01	9.87E+01			
7896168	---	---	---	0.008	1.005	9.76E-01	9.76E+01			
7896169	---	---	---	-0.054	0.963	7.87E-01	7.87E+01			
7896170	---	---	---	0.014	1.010	9.48E-01	9.48E+01			
7896171	---	---	---	0.087	1.062	8.35E-01	8.35E+01			
7896172	---	---	---	-0.113	0.924	4.88E-01	4.88E+01			
7896173	---	---	---	-0.056	0.962	7.95E-01	7.95E+01			
7896174	---	---	---	-0.026	0.982	9.25E-01	9.25E+01			
7896175	---	---	---	0.003	1.002	9.89E-01	9.89E+01			
7896176	---	---	---	0.059	1.042	6.76E-01	6.76E+01			
7896178	---	---	---	0.104	1.075	4.74E-01	4.74E+01			
7896181	---	---	---	0.144	1.105	6.97E-01	6.97E+01			
7896183	---	---	---	0.037	1.026	7.78E-01	7.78E+01			
7896184	---	---	---	-0.019	0.987	9.16E-01	9.16E+01			
7896185	---	---	---	-0.034	0.977	9.07E-01	9.07E+01			
7896186	---	---	---	-0.128	0.915	4.38E-01	4.38E+01			
7896188	---	---	---	0.061	1.043	9.07E-01	9.07E+01			
7896189	---	---	---	0.207	1.154	5.36E-01	5.36E+01			
7896190	---	---	---	0.359	1.282	4.97E-01	4.97E+01			
7896191	---	---	---	-0.055	0.963	8.41E-01	8.41E+01			
7896192	---	---	---	0.044	1.031	7.14E-01	7.14E+01			
7896193	---	---	---	-0.032	0.978	9.01E-01	9.01E+01			
7896194	---	---	---	-0.022	0.985	9.09E-01	9.09E+01			
7896197	---	---	---	0.373	1.295	3.48E-01	3.48E+01			
7896198	---	---	---	-0.010	0.993	9.71E-01	9.71E+01			
7896200	---	---	---	0.009	1.006	9.76E-01	9.76E+01			
7896201	---	---	---	0.194	1.144	1.63E-01	1.63E+01			
7896204	---	---	---	0.064	1.045	5.25E-01	5.25E+01			
7896205	---	---	---	0.078	1.055	8.13E-01	8.13E+01			
7896206	---	---	---	0.148	1.108	6.64E-01	6.64E+01			
7896210	---	---	---	-0.027	0.981	8.97E-01	8.97E+01			
7896212	---	---	---	0.017	1.012	9.49E-01	9.49E+01			
7896213	---	---	---	-0.041	0.972	8.80E-01	8.80E+01			
7896217	---	---	---	0.095	1.068	9.01E-01	9.01E+01			
7896218	---	---	---	-0.051	0.966	8.91E-01	8.91E+01			
7896219	---	---	---	-0.139	0.908	8.01E-01	8.01E+01			
7896221	---	---	---	-0.021	0.985	9.49E-01	9.49E+01			

7896222	---	---	---	-0.038	0.974	8.54E-01	8.54E+01		
7896223	---	---	---	0.116	1.084	6.55E-01	6.55E+01		
7896225	---	---	---	0.053	1.037	9.18E-01	9.18E+01		
7896227	---	---	---	0.025	1.017	8.45E-01	8.45E+01		
7896228	---	---	---	-0.088	0.941	5.80E-01	5.80E+01		
7896230	---	---	---	-0.173	0.887	1.65E-01	1.65E+01		
7896231	---	---	---	0.059	1.042	7.72E-01	7.72E+01		
7896232	---	---	---	-0.108	0.928	7.91E-01	7.91E+01		
7896235	---	---	---	0.041	1.029	7.95E-01	7.95E+01		
7896236	---	---	---	-0.039	0.973	9.33E-01	9.33E+01		
7896239	---	---	---	-0.057	0.961	8.85E-01	8.85E+01		
7896240	---	---	---	-0.058	0.960	7.05E-01	7.05E+01		
7896241	---	---	---	0.111	1.080	7.50E-01	7.50E+01		
7896243	---	---	---	0.037	1.026	8.13E-01	8.13E+01		
7896244	---	---	---	0.432	1.349	1.20E-01	1.20E+01		
7896245	---	---	---	0.086	1.061	8.25E-01	8.25E+01		
7896246	---	---	---	0.197	1.146	7.12E-01	7.12E+01		
7896247	---	---	---	0.047	1.033	7.53E-01	7.53E+01		
7896249	---	---	---	0.032	1.023	8.96E-01	8.96E+01		
7896250	---	---	---	-0.002	0.999	9.95E-01	9.95E+01		
7896251	---	---	---	0.334	1.261	3.56E-01	3.56E+01		
7896252	---	---	---	0.058	1.041	6.16E-01	6.16E+01		
7896253	---	---	---	-0.013	0.991	9.63E-01	9.63E+01		
7896254	---	---	---	0.096	1.069	7.95E-01	7.95E+01		
7896255	---	---	---	-0.053	0.964	8.80E-01	8.80E+01		
7896256	---	---	---	-0.036	0.975	9.16E-01	9.16E+01		
7896257	---	---	---	-0.281	0.823	5.08E-01	5.08E+01		
7896258	---	---	---	-0.150	0.901	6.45E-01	6.45E+01		
7896259	---	---	---	0.017	1.012	9.70E-01	9.70E+01		
7896261	---	---	---	0.051	1.036	8.71E-01	8.71E+01		
7896262	---	---	---	0.096	1.069	8.38E-01	8.38E+01		
7896263	---	---	---	-0.024	0.984	9.53E-01	9.53E+01		
7896266	---	---	---	0.017	1.012	9.30E-01	9.30E+01		
7896268	---	---	---	-0.083	0.944	8.70E-01	8.70E+01		
7896269	---	---	---	0.203	1.151	5.15E-01	5.15E+01		
7896270	---	---	---	0.139	1.101	6.35E-01	6.35E+01		
7896271	---	---	---	0.303	1.234	9.54E-02	9.54E+00		
7896274	---	---	---	-0.184	0.880	6.81E-01	6.81E+01		
7896275	---	---	---	0.181	1.134	7.92E-01	7.92E+01		
7896276	---	---	---	-0.064	0.957	9.23E-01	9.23E+01		
7896277	---	---	---	-0.005	0.997	9.87E-01	9.87E+01		
7896278	---	---	---	0.101	1.073	5.69E-01	5.69E+01		
7896279	---	---	---	0.184	1.136	7.17E-01	7.17E+01		
7896280	---	---	---	-0.087	0.941	6.81E-01	6.81E+01		
7896281	---	---	---	-0.047	0.968	8.78E-01	8.78E+01		
7896282	---	---	---	0.031	1.022	7.95E-01	7.95E+01		
7896283	---	---	---	-0.091	0.939	9.49E-01	9.49E+01		
7896285	---	---	---	0.198	1.147	3.89E-01	3.89E+01		
7896286	---	---	---	-0.164	0.893	8.84E-01	8.84E+01		
7896287	---	---	---	0.042	1.029	9.16E-01	9.16E+01		
7896288	---	---	---	0.267	1.203	2.16E-01	2.16E+01		
7896289	---	---	---	0.305	1.235	5.36E-01	5.36E+01		
7896290	---	---	---	0.051	1.036	9.00E-01	9.00E+01		
7896291	---	---	---	0.056	1.039	8.96E-01	8.96E+01		
7896292	---	---	---	-0.028	0.981	8.98E-01	8.98E+01		
7896293	---	---	---	0.093	1.067	8.04E-01	8.04E+01		
7896294	---	---	---	-0.011	0.992	9.88E-01	9.88E+01		
7896295	---	---	---	0.026	1.018	9.71E-01	9.71E+01		
7896297	---	---	---	-0.219	0.859	5.88E-01	5.88E+01		
7896298	---	---	---	-0.005	0.997	9.89E-01	9.89E+01		
7896300	---	---	---	0.002	1.001	9.94E-01	9.94E+01		
7896301	---	---	---	0.022	1.015	9.27E-01	9.27E+01		
7896302	---	---	---	0.040	1.028	9.26E-01	9.26E+01		
7896308	---	---	---	0.071	1.050	5.89E-01	5.89E+01		
7896309	---	---	---	0.059	1.042	8.62E-01	8.62E+01		
7896310	---	---	---	0.110	1.079	6.29E-01	6.29E+01		
7896311	---	---	---	-0.187	0.878	4.68E-01	4.68E+01		
7896312	---	---	---	0.119	1.086	7.68E-01	7.68E+01		
7896314	---	---	---	0.212	1.158	3.56E-01	3.56E+01		
7896318	---	---	---	0.210	1.156	4.25E-01	4.25E+01		
7896319	---	---	---	-0.085	0.943	4.50E-01	4.50E+01		
7896320	---	---	---	0.138	1.100	5.80E-01	5.80E+01		
7896321	---	---	---	0.152	1.111	2.72E-01	2.72E+01		
7896323	---	---	---	0.016	1.011	9.77E-01	9.77E+01		
7896324	---	---	---	0.139	1.101	5.62E-01	5.62E+01		
7896325	---	---	---	0.052	1.037	8.49E-01	8.49E+01		
7896328	---	---	---	0.358	1.282	3.95E-01	3.95E+01		

7896329	---	---	---	0.046	1.032	8.67E-01	8.67E+01			
7896330	---	---	---	0.041	1.029	9.22E-01	9.22E+01			
7896331	---	---	---	0.116	1.084	5.66E-01	5.66E+01			
7896333	---	---	---	-0.003	0.998	9.86E-01	9.86E+01			
7896335	---	---	---	0.038	1.026	8.46E-01	8.46E+01			
7896336	---	---	---	-0.137	0.909	8.41E-01	8.41E+01			
7896337	---	---	---	-0.131	0.913	7.47E-01	7.47E+01			
7896338	---	---	---	0.038	1.027	8.63E-01	8.63E+01			
7896339	---	---	---	-0.133	0.912	4.69E-01	4.69E+01			
7896340	---	---	---	-0.084	0.943	8.59E-01	8.59E+01			
7896341	---	---	---	0.140	1.102	5.10E-01	5.10E+01			
7896342	---	---	---	-0.071	0.952	8.80E-01	8.80E+01			
7896343	---	---	---	-0.048	0.967	8.05E-01	8.05E+01			
7896345	---	---	---	0.061	1.043	7.83E-01	7.83E+01			
7896346	---	---	---	0.000	1.000	1.00E+00	1.00E+02			
7896347	---	---	---	0.013	1.009	9.81E-01	9.81E+01			
7896348	---	---	---	-0.019	0.987	9.62E-01	9.62E+01			
7896349	---	---	---	-0.056	0.962	7.94E-01	7.94E+01			
7896350	---	---	---	0.037	1.026	9.57E-01	9.57E+01			
7896351	---	---	---	-0.071	0.952	8.66E-01	8.66E+01			
7896352	---	---	---	0.013	1.009	9.66E-01	9.66E+01			
7896355	---	---	---	-0.471	0.721	3.12E-01	3.12E+01			
7896356	---	---	---	-0.074	0.950	7.06E-01	7.06E+01			
7896358	---	---	---	-0.037	0.975	9.00E-01	9.00E+01			
7896359	---	---	---	0.088	1.063	7.23E-01	7.23E+01			
7896360	---	---	---	-0.040	0.973	9.18E-01	9.18E+01			
7896361	---	---	---	-0.056	0.962	8.32E-01	8.32E+01			
7896362	---	---	---	0.128	1.093	4.04E-01	4.04E+01			
7896363	---	---	---	0.049	1.034	8.46E-01	8.46E+01			
7896364	---	---	---	0.124	1.089	7.64E-01	7.64E+01			
7896365	---	---	---	-0.122	0.919	8.15E-01	8.15E+01			
7896366	---	---	---	0.034	1.024	8.56E-01	8.56E+01			
7896367	---	---	---	-0.025	0.983	8.49E-01	8.49E+01			
7896368	---	---	---	0.101	1.072	7.98E-01	7.98E+01			
7896369	---	---	---	0.015	1.010	9.71E-01	9.71E+01			
7896370	---	---	---	-0.063	0.957	8.73E-01	8.73E+01			
7896371	---	---	---	-0.042	0.972	8.05E-01	8.05E+01			
7896372	---	---	---	0.045	1.031	9.24E-01	9.24E+01			
7896375	---	---	---	0.052	1.037	9.00E-01	9.00E+01			
7896376	---	---	---	0.129	1.093	2.54E-01	2.54E+01			
7896378	---	---	---	0.018	1.013	9.68E-01	9.68E+01			
7896380	---	---	---	-0.003	0.998	9.87E-01	9.87E+01			
7896381	---	---	---	0.049	1.034	7.73E-01	7.73E+01			
7896383	---	---	---	0.038	1.027	9.53E-01	9.53E+01			
7896384	---	---	---	-0.047	0.968	7.69E-01	7.69E+01			
7896385	---	---	---	-0.008	0.995	9.75E-01	9.75E+01			
7896387	---	---	---	-0.039	0.973	8.27E-01	8.27E+01			
7896388	---	---	---	-0.037	0.975	9.00E-01	9.00E+01			
7896390	---	---	---	-0.019	0.987	9.70E-01	9.70E+01			
7896391	---	---	---	-0.080	0.946	8.23E-01	8.23E+01			
7896392	---	---	---	-0.223	0.857	7.40E-01	7.40E+01			
7896393	---	---	---	-0.042	0.971	9.26E-01	9.26E+01			
7896394	---	---	---	0.156	1.114	6.95E-01	6.95E+01			
7896395	---	---	---	0.026	1.019	9.01E-01	9.01E+01			
7896397	---	---	---	-0.110	0.927	4.47E-01	4.47E+01			
7896398	---	---	---	-0.025	0.983	9.72E-01	9.72E+01			
7896401	---	---	---	0.118	1.085	7.98E-01	7.98E+01			
7896403	---	---	---	0.039	1.027	6.45E-01	6.45E+01			
7896404	---	---	---	0.014	1.009	9.64E-01	9.64E+01			
7896405	---	---	---	0.104	1.074	6.76E-01	6.76E+01			
7896406	---	---	---	0.031	1.022	9.63E-01	9.63E+01			
7896412	---	---	---	0.016	1.011	9.23E-01	9.23E+01			
7896413	---	---	---	-0.146	0.904	2.94E-01	2.94E+01			
7896415	---	---	---	0.293	1.225	1.38E-01	1.38E+01			
7896416	---	---	---	-0.030	0.979	9.06E-01	9.06E+01			
7896417	---	---	---	0.040	1.028	8.93E-01	8.93E+01			
7896418	---	---	---	0.058	1.041	7.71E-01	7.71E+01			
7896420	---	---	---	-0.015	0.989	9.75E-01	9.75E+01			
7896421	---	---	---	-0.077	0.948	6.68E-01	6.68E+01			
7896422	---	---	---	0.044	1.031	8.02E-01	8.02E+01			
7896423	---	---	---	-0.066	0.955	6.33E-01	6.33E+01			
7896424	---	---	---	-0.074	0.950	6.90E-01	6.90E+01			
7896425	---	---	---	-0.078	0.947	5.88E-01	5.88E+01			
7896426	---	---	---	0.080	1.057	6.57E-01	6.57E+01			
7896427	---	---	---	-0.036	0.975	7.57E-01	7.57E+01			
7896430	---	---	---	0.283	1.217	1.09E-01	1.09E+01			
7896431	---	---	---	-0.082	0.945	8.27E-01	8.27E+01			

7896432	---	---	---	0.092	1.066	6.57E-01	6.57E+01		
7896434	---	---	---	-0.022	0.985	9.54E-01	9.54E+01		
7896437	---	---	---	0.219	1.164	3.09E-01	3.09E+01		
7896438	---	---	---	-0.033	0.978	8.92E-01	8.92E+01		
7896439	---	---	---	0.225	1.169	3.46E-01	3.46E+01		
7896442	---	---	---	-0.017	0.988	9.78E-01	9.78E+01		
7896443	---	---	---	0.005	1.004	9.82E-01	9.82E+01		
7896444	---	---	---	-0.014	0.991	9.74E-01	9.74E+01		
7896445	---	---	---	0.341	1.267	3.70E-01	3.70E+01		
7896446	---	---	---	-0.012	0.992	9.63E-01	9.63E+01		
7896447	---	---	---	0.080	1.057	8.66E-01	8.66E+01		
7896448	---	---	---	0.013	1.009	9.39E-01	9.39E+01		
7896450	---	---	---	0.074	1.053	7.75E-01	7.75E+01		
7896453	---	---	---	0.078	1.055	8.59E-01	8.59E+01		
7896456	---	---	---	-0.221	0.858	1.13E-01	1.13E+01		
7896457	---	---	---	-0.109	0.927	8.16E-01	8.16E+01		
7896458	---	---	---	0.008	1.006	9.81E-01	9.81E+01		
7896459	---	---	---	-0.249	0.841	6.93E-01	6.93E+01		
7896460	---	---	---	0.175	1.129	7.83E-01	7.83E+01		
7896461	---	---	---	0.377	1.299	3.75E-01	3.75E+01		
7896464	---	---	---	0.053	1.037	5.96E-01	5.96E+01		
7896466	---	---	---	0.023	1.016	9.49E-01	9.49E+01		
7896467	---	---	---	-0.091	0.939	6.91E-01	6.91E+01		
7896468	---	---	---	-0.055	0.963	8.53E-01	8.53E+01		
7896472	---	---	---	0.019	1.013	9.75E-01	9.75E+01		
7896474	---	---	---	-0.140	0.907	5.99E-01	5.99E+01		
7896476	---	---	---	-0.096	0.935	6.94E-01	6.94E+01		
7896477	---	---	---	0.068	1.048	8.26E-01	8.26E+01		
7896479	---	---	---	-0.172	0.888	2.53E-01	2.53E+01		
7896481	---	---	---	0.043	1.030	7.93E-01	7.93E+01		
7896482	---	---	---	-0.043	0.971	8.27E-01	8.27E+01		
7896483	---	---	---	-0.066	0.955	5.49E-01	5.49E+01		
7896484	---	---	---	-0.103	0.931	6.54E-01	6.54E+01		
7896485	---	---	---	0.045	1.032	6.43E-01	6.43E+01		
7896488	---	---	---	0.009	1.006	9.78E-01	9.78E+01		
7896489	---	---	---	-0.114	0.924	7.98E-01	7.98E+01		
7896490	---	---	---	-0.076	0.949	7.57E-01	7.57E+01		
7896491	---	---	---	0.032	1.022	8.03E-01	8.03E+01		
7896492	---	---	---	0.365	1.288	2.88E-01	2.88E+01		
7896493	---	---	---	-0.004	0.997	9.88E-01	9.88E+01		
7896494	---	---	---	0.044	1.031	7.72E-01	7.72E+01		
7896496	---	---	---	0.009	1.006	9.53E-01	9.53E+01		
7896497	---	---	---	0.031	1.022	8.88E-01	8.88E+01		
7896498	---	---	---	0.013	1.009	9.76E-01	9.76E+01		
7896500	---	---	---	-0.059	0.960	8.78E-01	8.78E+01		
7896501	---	---	---	0.019	1.013	9.20E-01	9.20E+01		
7896502	---	---	---	-0.011	0.993	9.88E-01	9.88E+01		
7896505	---	---	---	0.058	1.041	9.30E-01	9.30E+01		
7896506	---	---	---	-0.226	0.855	2.33E-02	2.33E+00		
7896508	---	---	---	-0.127	0.916	2.58E-01	2.58E+01		
7896511	---	---	---	0.013	1.009	9.16E-01	9.16E+01		
7896512	---	---	---	0.110	1.079	5.73E-01	5.73E+01		
7896517	---	---	---	0.028	1.020	8.99E-01	8.99E+01		
7896519	---	---	---	0.144	1.105	6.98E-01	6.98E+01		
7896520	---	---	---	-0.028	0.981	9.58E-01	9.58E+01		
7896521	---	---	---	0.157	1.115	5.53E-01	5.53E+01		
7896522	---	---	---	-0.011	0.992	9.78E-01	9.78E+01		
7896523	---	---	---	0.097	1.069	9.04E-01	9.04E+01		
7896525	---	---	---	-0.026	0.982	9.53E-01	9.53E+01		
7896526	---	---	---	0.170	1.125	8.39E-01	8.39E+01		
7896528	---	---	---	-0.006	0.996	9.70E-01	9.70E+01		
7896529	---	---	---	-0.027	0.981	9.37E-01	9.37E+01		
7896530	---	---	---	-0.025	0.983	9.77E-01	9.77E+01		
7896531	---	---	---	0.225	1.169	2.59E-03	2.59E-01		
7896533	---	---	---	-0.078	0.948	7.11E-01	7.11E+01		
7896534	---	---	---	-0.185	0.880	5.45E-01	5.45E+01		
7896537	---	---	---	0.143	1.104	7.87E-01	7.87E+01		
7896540	---	---	---	0.003	1.002	9.96E-01	9.96E+01		
7896541	---	---	---	0.011	1.008	9.33E-01	9.33E+01		
7896542	---	---	---	0.142	1.104	2.84E-01	2.84E+01		
7896544	---	---	---	-0.087	0.941	7.51E-01	7.51E+01		
7896546	---	---	---	0.020	1.014	9.74E-01	9.74E+01		
7896547	---	---	---	-0.091	0.939	7.28E-01	7.28E+01		
7896548	---	---	---	-0.050	0.966	9.12E-01	9.12E+01		
7896549	---	---	---	0.040	1.028	5.15E-01	5.15E+01		
7896550	---	---	---	-0.034	0.977	9.75E-01	9.75E+01		
7896552	---	---	---	0.107	1.077	7.77E-01	7.77E+01		

7896553	---	---	---	0.318	1.246	7.99E-02	7.99E+00		
7896554	---	---	---	0.012	1.008	9.48E-01	9.48E+01		
7896559	---	---	---	-0.208	0.866	6.54E-01	6.54E+01		
7896561	---	---	---	0.115	1.083	8.21E-01	8.21E+01		
7896562	---	---	---	-0.059	0.960	8.41E-01	8.41E+01		
7896563	---	---	---	0.064	1.045	8.34E-01	8.34E+01		
7896565	---	---	---	-0.094	0.937	8.04E-01	8.04E+01		
7896566	---	---	---	-0.015	0.989	9.85E-01	9.85E+01		
7896567	---	---	---	-0.002	0.998	9.98E-01	9.98E+01		
7896569	---	---	---	0.219	1.164	5.91E-01	5.91E+01		
7896570	---	---	---	0.284	1.217	3.92E-01	3.92E+01		
7896572	---	---	---	-0.054	0.963	9.16E-01	9.16E+01		
7896574	---	---	---	0.121	1.088	5.10E-01	5.10E+01		
7896575	---	---	---	0.091	1.065	8.50E-01	8.50E+01		
7896576	---	---	---	0.218	1.163	4.52E-01	4.52E+01		
7896578	---	---	---	0.141	1.103	7.47E-01	7.47E+01		
7896580	---	---	---	-0.195	0.874	5.89E-01	5.89E+01		
7896582	---	---	---	-0.031	0.979	9.02E-01	9.02E+01		
7896584	---	---	---	0.024	1.017	9.51E-01	9.51E+01		
7896585	---	---	---	-0.087	0.941	7.78E-01	7.78E+01		
7896586	---	---	---	-0.013	0.991	9.66E-01	9.66E+01		
7896588	---	---	---	-0.138	0.909	6.71E-01	6.71E+01		
7896589	---	---	---	0.054	1.038	9.33E-01	9.33E+01		
7896592	---	---	---	-0.004	0.997	9.86E-01	9.86E+01		
7896594	---	---	---	-0.035	0.976	7.27E-01	7.27E+01		
7896596	---	---	---	0.060	1.042	8.97E-01	8.97E+01		
7896597	---	---	---	-0.096	0.936	8.50E-01	8.50E+01		
7896598	---	---	---	-0.152	0.900	8.32E-01	8.32E+01		
7896599	---	---	---	-0.171	0.888	2.26E-01	2.26E+01		
7896600	---	---	---	0.014	1.010	9.78E-01	9.78E+01		
7896601	---	---	---	-0.065	0.956	9.00E-01	9.00E+01		
7896602	---	---	---	-0.055	0.963	6.99E-01	6.99E+01		
7896603	---	---	---	-0.012	0.992	9.30E-01	9.30E+01		
7896604	---	---	---	0.083	1.059	8.47E-01	8.47E+01		
7896605	---	---	---	0.179	1.132	3.95E-01	3.95E+01		
7896606	---	---	---	-0.026	0.982	8.63E-01	8.63E+01		
7896608	---	---	---	0.096	1.069	6.20E-01	6.20E+01		
7896609	---	---	---	0.046	1.033	8.26E-01	8.26E+01		
7896611	---	---	---	0.130	1.094	6.66E-01	6.66E+01		
7896612	---	---	---	-0.044	0.970	8.31E-01	8.31E+01		
7896613	---	---	---	-0.259	0.836	4.47E-01	4.47E+01		
7896614	---	---	---	-0.233	0.851	2.44E-01	2.44E+01		
7896615	---	---	---	0.161	1.118	3.80E-01	3.80E+01		
7896616	---	---	---	-0.012	0.992	9.56E-01	9.56E+01		
7896618	---	---	---	0.006	1.004	9.87E-01	9.87E+01		
7896619	---	---	---	-0.097	0.935	8.13E-01	8.13E+01		
7896620	---	---	---	0.020	1.014	9.03E-01	9.03E+01		
7896621	---	---	---	-0.018	0.988	9.57E-01	9.57E+01		
7896622	---	---	---	0.032	1.022	8.54E-01	8.54E+01		
7896623	---	---	---	-0.093	0.937	7.07E-01	7.07E+01		
7896624	---	---	---	-0.021	0.985	9.16E-01	9.16E+01		
7896627	---	---	---	-0.020	0.986	9.64E-01	9.64E+01		
7896628	---	---	---	-0.067	0.955	8.71E-01	8.71E+01		
7896629	---	---	---	0.106	1.076	7.91E-01	7.91E+01		
7896630	---	---	---	0.025	1.018	9.61E-01	9.61E+01		
7896631	---	---	---	0.009	1.006	9.44E-01	9.44E+01		
7896632	---	---	---	-0.010	0.993	9.76E-01	9.76E+01		
7896633	---	---	---	-0.247	0.843	5.91E-01	5.91E+01		
7896634	---	---	---	0.093	1.067	9.18E-01	9.18E+01		
7896637	---	---	---	-0.063	0.957	5.77E-01	5.77E+01		
7896639	---	---	---	0.047	1.033	5.15E-01	5.15E+01		
7896640	---	---	---	-0.063	0.957	8.42E-01	8.42E+01		
7896642	---	---	---	-0.007	0.995	9.77E-01	9.77E+01		
7896643	---	---	---	0.096	1.069	9.04E-01	9.04E+01		
7896644	---	---	---	0.000	1.000	1.00E+00	1.00E+02		
7896646	---	---	---	0.216	1.162	5.73E-01	5.73E+01		
7896647	---	---	---	-0.031	0.979	8.25E-01	8.25E+01		
7896648	---	---	---	0.002	1.001	9.97E-01	9.97E+01		
7896650	---	---	---	0.359	1.283	3.92E-01	3.92E+01		
7896651	---	---	---	0.159	1.116	4.34E-01	4.34E+01		
7896652	---	---	---	-0.078	0.947	4.96E-01	4.96E+01		
7896653	---	---	---	0.040	1.028	9.24E-01	9.24E+01		
7896654	---	---	---	0.129	1.093	8.48E-01	8.48E+01		
7896655	---	---	---	0.093	1.067	8.66E-01	8.66E+01		
7896656	---	---	---	0.114	1.082	5.43E-01	5.43E+01		
7896657	---	---	---	0.176	1.130	4.47E-01	4.47E+01		
7896659	---	---	---	-0.037	0.975	9.56E-01	9.56E+01		

7896660	---	---	---	0.060	1.042	8.66E-01	8.66E+01		
7896661	---	---	---	0.190	1.141	4.06E-01	4.06E+01		
7896662	---	---	---	-0.003	0.998	9.96E-01	9.96E+01		
7896664	---	---	---	0.057	1.041	8.31E-01	8.31E+01		
7896668	---	---	---	0.106	1.077	8.50E-01	8.50E+01		
7896669	---	---	---	0.094	1.067	7.75E-01	7.75E+01		
7896670	---	---	---	0.037	1.026	7.72E-01	7.72E+01		
7896671	---	---	---	-0.030	0.979	9.79E-01	9.79E+01		
7896673	---	---	---	-0.112	0.925	5.53E-01	5.53E+01		
7896675	---	---	---	-0.030	0.979	8.74E-01	8.74E+01		
7896676	---	---	---	-0.002	0.999	9.96E-01	9.96E+01		
7896677	---	---	---	-0.024	0.983	9.28E-01	9.28E+01		
7896679	---	---	---	0.020	1.014	9.09E-01	9.09E+01		
7896681	---	---	---	0.212	1.159	1.67E-01	1.67E+01		
7896682	---	---	---	-0.020	0.986	9.78E-01	9.78E+01		
7896683	---	---	---	-0.082	0.945	3.76E-01	3.76E+01		
7896684	---	---	---	0.061	1.043	7.53E-01	7.53E+01		
7896685	---	---	---	-0.166	0.891	5.27E-01	5.27E+01		
7896687	---	---	---	0.061	1.043	8.30E-01	8.30E+01		
7896688	---	---	---	0.045	1.032	8.60E-01	8.60E+01		
7896691	---	---	---	-0.170	0.889	3.39E-01	3.39E+01		
7896692	---	---	---	-0.033	0.978	7.32E-01	7.32E+01		
7896693	---	---	---	-0.266	0.832	1.17E-01	1.17E+01		
7896694	---	---	---	0.129	1.093	2.79E-01	2.79E+01		
7896696	---	---	---	-0.041	0.972	7.14E-01	7.14E+01		
7896698	---	---	---	0.033	1.023	8.46E-01	8.46E+01		
7896699	---	---	---	-0.234	0.850	3.16E-02	3.16E+00		
7896701	---	---	---	0.028	1.020	9.49E-01	9.49E+01		
7896702	---	---	---	0.028	1.020	9.39E-01	9.39E+01		
7896703	---	---	---	0.075	1.053	7.72E-01	7.72E+01		
7896704	---	---	---	0.226	1.170	3.70E-01	3.70E+01		
7896708	---	---	---	-0.218	0.860	2.84E-01	2.84E+01		
7896709	---	---	---	0.088	1.063	8.20E-01	8.20E+01		
7896710	---	---	---	-0.140	0.908	5.30E-01	5.30E+01		
7896711	---	---	---	0.124	1.090	8.02E-01	8.02E+01		
7896713	---	---	---	-0.033	0.977	8.13E-01	8.13E+01		
7896715	---	---	---	0.403	1.322	8.93E-02	8.93E+00		
7896716	---	---	---	-0.018	0.987	9.46E-01	9.46E+01		
7896717	---	---	---	-0.258	0.836	2.59E-02	2.59E+00		
7896719	---	---	---	0.058	1.041	8.91E-01	8.91E+01		
7896720	---	---	---	0.040	1.028	9.29E-01	9.29E+01		
7896722	---	---	---	-0.173	0.887	7.49E-01	7.49E+01		
7896723	---	---	---	0.029	1.021	8.36E-01	8.36E+01		
7896724	---	---	---	0.737	1.667	5.59E-02	5.59E+00		
7896726	---	---	---	0.197	1.146	1.15E-01	1.15E+01		
7896727	---	---	---	0.431	1.348	1.33E-01	1.33E+01		
7896728	---	---	---	-0.235	0.850	7.02E-03	7.02E-01		
7896736	---	---	---	0.012	1.009	9.77E-01	9.77E+01		
7896742	NM_207366	40435	septin 14	-0.030	0.979	9.19E-01	9.19E+01		
7896746	---	---	---	-0.123	0.919	7.11E-01	7.11E+01		
7896748	---	---	---	0.318	1.247	4.42E-01	4.42E+01		
7896752	---	---	---	-0.042	0.971	8.97E-01	8.97E+01		
7896756	BC037297	FAM87A	family with sequence similarity 87	-0.086	0.942	4.88E-01	4.88E+01		
7896759	AK096570	LOC643837	hypothetical LOC643837	0.018	1.012	9.61E-01	9.61E+01		
7896761	NM_152486	SAMD11	sterile alpha motif domain contain	-0.044	0.970	6.91E-01	6.91E+01		
7896779	NM_198317	KLHL17	kelch-like 17 (Drosophila)	-0.032	0.978	7.34E-01	7.34E+01		
7896798	NM_032129	PLEKHN1	pleckstrin homology domain conta	0.003	1.002	9.82E-01	9.82E+01		
7896817	NM_005101	ISG15	ISG15 ubiquitin-like modifier	0.126	1.092	6.69E-01	6.69E+01		
7896822	NM_198576	AGRN	agrin	-0.078	0.947	2.71E-01	2.71E+01		
7896859	---	---	---	-0.039	0.973	8.29E-01	8.29E+01		
7896865	NM_153254	TTLL10	tubulin tyrosine ligase-like family,	-0.019	0.987	8.87E-01	8.87E+01		
7896878	NM_080605	B3GALT6	UDP-Gal:betaGal beta 1,3-galactos	-0.007	0.995	9.68E-01	9.68E+01		
7896882	NM_00113041	SCNN1D	sodium channel, nonvoltage-gated	0.024	1.017	8.57E-01	8.57E+01		
7896908	NM_153339	PUSL1	pseudouridylate synthase-like 1	-0.090	0.939	2.94E-01	2.94E+01		
7896917	NM_00102988	GLTPD1	glycolipid transfer protein domain	-0.078	0.948	4.44E-01	4.44E+01		
7896921	NM_152228	TAS1R3	taste receptor, type 1, member 3	-0.005	0.997	9.78E-01	9.78E+01		
7896929	NM_022834	VWA1	von Willebrand factor A domain co	-0.007	0.995	9.57E-01	9.57E+01		
7896952	NM_018188	ATAD3A	ATPase family, AAA domain contai	-0.084	0.944	3.62E-01	3.62E+01		
7896961	NM_031921	ATAD3B	ATPase family, AAA domain contai	-0.038	0.974	7.38E-01	7.38E+01		
7896985	NM_080875	MIB2	mindbomb homolog 2 (Drosophila	-0.046	0.969	6.33E-01	6.33E+01		
7897006	NM_006983	MMP23B	matrix metallopeptidase 23B	0.006	1.004	9.70E-01	9.70E+01		
7897034	NM_000815	GABRD	gamma-aminobutyric acid (GABA)	0.079	1.056	3.66E-01	3.66E+01		
7897044	NM_002744	PRKCZ	protein kinase C, zeta	-0.088	0.941	4.52E-01	4.52E+01		
7897066	NR_024445	LOC100128003	hypothetical protein LOC1001280(	-0.056	0.962	5.72E-01	5.72E+01		
7897068	NM_003036	SKI	v-ski sarcoma viral oncogene hom	-0.098	0.934	1.21E-01	1.21E+01		
7897076	---	---	---	-0.114	0.924	3.05E-01	3.05E+01		
7897078	NM_007033	RER1	RER1 retention in endoplasmic ret	0.005	1.004	9.82E-01	9.82E+01		

7897089	NM_014638	PLCH2	phospholipase C, eta 2	-0.093	0.938	5.48E-02	5.48E+00		
7897119	AF425266	C1orf93	chromosome 1 open reading fram	-0.048	0.967	5.15E-01	5.15E+01		
7897128	NM_080431	ACTRT2	actin-related protein T2	-0.078	0.947	4.29E-01	4.29E+01		
7897132	NM_022114	PRDM16	PR domain containing 16	-0.045	0.969	6.28E-01	6.28E+01		
7897154	NM_014448	ARHGEF16	Rho guanine exchange factor (GEF	-0.032	0.978	7.87E-01	7.87E+01		
7897172	NM_182752	TPRG1L	tumor protein p63 regulated 1-like	-0.161	0.894	2.67E-02	2.67E+00		
7897179	NM_00112624	TP73	tumor protein p73	-0.036	0.975	7.38E-01	7.38E+01		
7897196	NM_152492	CCDC27	coiled-coil domain containing 27	-0.007	0.995	9.64E-01	9.64E+01		
7897210	NM_004402	DFFB	DNA fragmentation factor, 40kDa,	0.021	1.015	8.85E-01	8.85E+01		
7897236	NM_003636	KCNAB2	potassium voltage-gated channel,	0.023	1.016	8.84E-01	8.84E+01		
7897257	NM_207396	RNF207	ring finger protein 207	0.002	1.002	9.89E-01	9.89E+01		
7897263	NM_207396	RNF207	ring finger protein 207	-0.009	0.994	9.62E-01	9.62E+01		
7897277	BC030279	C1orf211	chromosome 1 open reading fram	-0.031	0.979	8.47E-01	8.47E+01		
7897280	NM_00102459	HES3	hairy and enhancer of split 3 (Dros	0.001	1.001	9.93E-01	9.93E+01		
7897286	AY358179	UNQ6193	GSQS6193	0.013	1.009	9.44E-01	9.44E+01		
7897288	NM_031475	ESPN	espin	0.084	1.060	3.35E-01	3.35E+01		
7897293	---	---	---	0.049	1.035	7.37E-01	7.37E+01		
7897295	NM_138697	TAS1R1	taste receptor, type 1, member 1	-0.014	0.990	9.51E-01	9.51E+01		
7897305	NM_005341	ZBTB48	zinc finger and BTB domain contai	-0.033	0.978	7.52E-01	7.52E+01		
7897322	NM_153812	PHF13	PHD finger protein 13	-0.032	0.978	7.75E-01	7.75E+01		
7897329	NM_138350	THAP3	THAP domain containing, apoptos	0.035	1.025	8.63E-01	8.63E+01		
7897339	NM_015215	CAMTA1	calmodulin binding transcription a	-0.025	0.983	8.63E-01	8.63E+01		
7897370	NM_004781	VAMP3	vesicle-associated membrane prot	0.129	1.093	4.23E-01	4.23E+01		
7897378	NM_016831	PER3	period homolog 3 (Drosophila)	0.043	1.030	7.97E-01	7.97E+01		
7897404	NM_007262	PARK7	Parkinson disease (autosomal rece	0.036	1.025	8.02E-01	8.02E+01		
7897416	NM_00108039	SLC45A1	solute carrier family 45, member 1	-0.023	0.984	9.01E-01	9.01E+01		
7897422	---	---	---	-0.031	0.979	8.54E-01	8.54E+01		
7897424	---	---	---	0.001	1.001	9.96E-01	9.96E+01		
7897426	NM_001215	CA6	carbonic anhydrase VI	0.019	1.013	9.56E-01	9.56E+01		
7897439	---	---	---	0.019	1.013	9.48E-01	9.48E+01		
7897449	NM_025106	SPSB1	splA/ryanodine receptor domain a	-0.168	0.890	1.65E-01	1.65E+01		
7897460	NM_032315	SLC25A33	solute carrier family 25, member 3	0.095	1.068	5.29E-01	5.29E+01		
7897469	NM_00113092	TMEM201	transmembrane protein 201	-0.029	0.980	8.09E-01	8.09E+01		
7897482	NM_005026	PIK3CD	phosphoinositide-3-kinase, catalyt	0.046	1.032	6.60E-01	6.60E+01		
7897527	NM_00110556	UBE4B	ubiquitination factor E4B (UFD2 ho	-0.018	0.987	9.01E-01	9.01E+01		
7897561	NM_015074	KIF1B	kinesin family member 1B	0.163	1.120	3.18E-01	3.18E+01		
7897620	NM_002631	PGD	phosphogluconate dehydrogenase	0.003	1.002	9.97E-01	9.97E+01		
7897632	NM_198544	APITD1	apoptosis-inducing, TAF9-like dom	-0.088	0.941	5.48E-01	5.48E+01		
7897648	NM_004565	PEX14	peroxisomal biogenesis factor 14	-0.043	0.971	6.71E-01	6.71E+01		
7897661	---	---	---	0.030	1.021	8.39E-01	8.39E+01		
7897663	NM_007375	TARDBP	TAR DNA binding protein	-0.007	0.995	9.44E-01	9.44E+01		
7897685	NM_013319	UBIAD1	UbiA prenyltransferase domain co	0.015	1.010	9.27E-01	9.27E+01		
7897691	NM_020780	PTCHD2	patched domain containing 2	0.036	1.025	8.16E-01	8.16E+01		
7897714	NM_00101476	FBXO44	F-box protein 44	-0.023	0.984	8.47E-01	8.47E+01		
7897728	NM_018438	FBXO6	F-box protein 6	0.097	1.070	3.95E-01	3.95E+01		
7897737	NM_198545	C1orf187	chromosome 1 open reading fram	-0.028	0.981	7.70E-01	7.70E+01		
7897745	NM_020350	AGTRAP	angiotensin II receptor-associated	0.000	1.000	1.00E+00	1.00E+02		
7897753	NM_005957	MTHFR	5,10-methylenetetrahydrofolate re	-0.036	0.976	7.71E-01	7.71E+01		
7897774	NM_001286	CLCN6	chloride channel 6	0.060	1.043	7.45E-01	7.45E+01		
7897803	NM_000302	PLOD1	procollagen-lysine 1, 2-oxoglutara	0.131	1.095	2.65E-01	2.65E+01		
7897824	NM_014874	MFN2	mitofusin 2	0.062	1.044	5.23E-01	5.23E+01		
7897849	NM_021933	MIIP	migration and invasion inhibitory p	-0.023	0.984	8.69E-01	8.69E+01		
7897860	NM_001243	TNFRSF8	tumor necrosis factor receptor sup	-0.049	0.966	6.44E-01	6.44E+01		
7897877	NM_001066	TNFRSF1B	tumor necrosis factor receptor sup	-0.274	0.827	3.47E-02	3.47E+00		
7897890	NM_015378	VPS13D	vacuolar protein sorting 13 homol	-0.015	0.990	9.27E-01	9.27E+01		
7897991	NM_00101227	PRAMEF7	PRAME family member 7	-0.052	0.965	6.34E-01	6.34E+01		
7897997	NM_00101369	PRAMEF3	PRAME family member 3	-0.067	0.955	6.31E-01	6.31E+01		
7898030	NM_00109985	PRAMEF20	PRAME family member 20	0.064	1.045	6.79E-01	6.79E+01		
7898052	NM_00109985	PRAMEF20	PRAME family member 20	0.096	1.069	4.43E-01	4.43E+01		
7898057	NM_006474	PDPN	podoplanin	-0.144	0.905	6.73E-01	6.73E+01		
7898070	NM_012231	PRDM2	PR domain containing 2, with ZNF	-0.115	0.924	8.70E-02	8.70E+00		
7898084	NM_015209	RP1-21O18.1	kazrin	-0.033	0.977	8.32E-01	8.32E+01		
7898102	NM_201628	RP1-21O18.1	kazrin	-0.030	0.980	7.96E-01	7.96E+01		
7898112	---	---	---	-0.143	0.906	2.84E-01	2.84E+01		
7898115	NM_00113621	TMEM51	transmembrane protein 51	0.342	1.267	1.07E-01	1.07E+01		
7898161	NM_024329	EFHD2	EF-hand domain family, member D	0.082	1.058	6.76E-01	6.76E+01		
7898167	NM_007272	CTRC	chymotrypsin C (caldecrin)	-0.068	0.954	5.17E-01	5.17E+01		
7898176	NM_033440	CELA2A	chymotrypsin-like elastase family,	0.020	1.014	9.26E-01	9.26E+01		
7898192	NM_015291	DNAJC16	DnaJ (Hsp40) homolog, subfamily	0.068	1.049	6.30E-01	6.30E+01		
7898209	---	---	---	0.014	1.010	9.50E-01	9.50E+01		
7898211	NM_032341	DDI2	DDI1, DNA-damage inducible 1, ho	-0.070	0.953	6.92E-01	6.92E+01		
7898227	NM_015164	PLEKHM2	pleckstrin homology domain conta	0.062	1.044	7.74E-01	7.74E+01		
7898249	NM_207348	SLC25A34	solute carrier family 25, member 3	-0.065	0.956	4.03E-01	4.03E+01		
7898256	NM_00101364	TMEM82	transmembrane protein 82	-0.059	0.960	5.59E-01	5.59E+01		
7898276	---	---	---	-0.012	0.992	9.53E-01	9.53E+01		
7898278	NM_015001	SPEN	spen homolog, transcriptional regu	-0.027	0.982	8.45E-01	8.45E+01		
7898296	BC017946	C1orf64	chromosome 1 open reading fram	0.003	1.002	9.87E-01	9.87E+01		

7898300	NM_004070	CLCNKA	chloride channel Ka	0.032	1.023	8.32E-01	8.32E+01		
7898314	NM_000085	CLCNKB	chloride channel Kb	-0.019	0.987	8.84E-01	8.84E+01		
7898328	NM_00111460	C1orf144	chromosome 1 open reading fram	0.074	1.052	5.67E-01	5.67E+01		
7898337	NM_018090	NECAP2	NECAP endocytosis associated 2	0.094	1.068	3.06E-01	3.06E+01		
7898350	BC126340 // E	32147 // LOC10	hypothetical LOC100132147 // hy	0.003	1.002	9.90E-01	9.90E+01		
7898353	NR_027002	LOC388692	hypothetical LOC388692	-0.117	0.922	6.53E-01	6.53E+01		
7898371	ENST0000036	944634 // LOC64	similar to UPF0627 protein ENSP0	0.002	1.001	9.90E-01	9.90E+01		
7898377	NM_014675	CROCC	ciliary rootlet coiled-coil, rootletin	-0.037	0.975	8.08E-01	8.08E+01		
7898407	BC036435	LOC100132147	hypothetical LOC100132147	0.014	1.009	9.65E-01	9.65E+01		
7898431	NM_016233	PADI3	peptidyl arginine deiminase, type	-0.017	0.988	9.16E-01	9.16E+01		
7898483	NM_018125	ARHGEF10L	Rho guanine nucleotide exchange	0.012	1.009	9.32E-01	9.32E+01		
7898521	NM_032880	IGSF21	immunoglobin superfamily, memb	-0.021	0.985	8.97E-01	8.97E+01		
7898533	NM_152375	KLHDC7A	kelch domain containing 7A	-0.063	0.958	4.69E-01	4.69E+01		
7898537	NM_002584	PAX7	paired box 7	0.036	1.026	8.06E-01	8.06E+01		
7898549	NM_016183	MRTO4	mRNA turnover 4 homolog (S. cere	0.008	1.005	9.82E-01	9.82E+01		
7898556	NM_00104012	PQLC2	PQ loop repeat containing 2	0.025	1.018	8.43E-01	8.43E+01		
7898568	AK096102	LOC100130193	hypothetical protein LOC10013019	0.139	1.101	2.74E-01	2.74E+01		
7898570	---	---	---	-0.177	0.885	1.80E-01	1.80E+01		
7898574	NM_00103236	C1orf151	chromosome 1 open reading fram	0.007	1.005	9.64E-01	9.64E+01		
7898582	BC072682	RPS14P3	ribosomal protein S14 pseudogene	0.062	1.044	7.38E-01	7.38E+01		
7898585	NM_182744	NBL1	neuroblastoma, suppression of tu	-0.081	0.946	4.21E-01	4.21E+01		
7898594	NM_000871	HTR6	5-hydroxytryptamine (serotonin) r	-0.097	0.935	3.37E-01	3.37E+01		
7898602	NM_015207	OTUD3	OTU domain containing 3	-0.029	0.980	8.78E-01	8.78E+01		
7898607	---	---	---	-0.020	0.986	9.27E-01	9.27E+01		
7898616	NM_022819	PLA2G2F	phospholipase A2, group IIF	-0.011	0.992	9.50E-01	9.50E+01		
7898627	NM_00103950	VWA5B1	von Willebrand factor A domain co	0.018	1.013	9.24E-01	9.24E+01		
7898653	NM_207334	FAM43B	family with sequence similarity 43	-0.006	0.996	9.75E-01	9.75E+01		
7898655	NM_001785	CDA	cytidine deaminase	-0.042	0.971	6.92E-01	6.92E+01		
7898663	NM_032409	PINK1	PTEN induced putative kinase 1	-0.185	0.880	3.33E-02	3.33E+00		
7898673	---	---	---	0.038	1.027	8.76E-01	8.76E+01		
7898677	NM_004807	HS6ST1	heparan sulfate 6-O-sulfotransfera	-0.027	0.981	8.53E-01	8.53E+01		
7898679	NM_032264	NBPF3	neuroblastoma breakpoint family,	-0.030	0.980	8.52E-01	8.52E+01		
7898707	NM_00101369	LDLRAD2	low density lipoprotein receptor c	0.021	1.015	8.78E-01	8.78E+01		
7898713	NM_007352	CELA3B	chymotrypsin-like elastase family,	-0.028	0.981	8.25E-01	8.25E+01		
7898723	---	---	---	0.069	1.049	5.81E-01	5.81E+01		
7898725	NM_005747	CELA3A	chymotrypsin-like elastase family,	0.040	1.028	7.80E-01	7.80E+01		
7898736	NR_023918	HSPC157	hypothetical LOC29092	0.029	1.020	9.18E-01	9.18E+01		
7898739	NM_044472	CDC42	cell division cycle 42 (GTP binding	0.109	1.078	1.31E-01	1.31E+01		
7898750	NM_00108362	ZBTB40	zinc finger and BTB domain contai	-0.038	0.974	7.97E-01	7.97E+01		
7898773	NM_020526	EPHA8	EPH receptor A8	-0.050	0.966	5.39E-01	5.39E+01		
7898809	NM_017449	EPHB2	EPH receptor B2	0.054	1.038	7.00E-01	7.00E+01		
7898833	NM_00100999	KDM1	lysine (K)-specific demethylase 1	-0.118	0.922	2.45E-01	2.45E+01		
7898856	---	---	---	0.034	1.024	8.54E-01	8.54E+01		
7898869	ENST00000374	MDS2	myelodysplastic syndrome 2 trans	0.031	1.021	8.50E-01	8.50E+01		
7898875	NM_000975	RPL11	ribosomal protein L11	-0.072	0.951	4.76E-01	4.76E+01		
7898881	NM_003198	TCEB3	transcription elongation factor B (	-0.010	0.993	9.77E-01	9.77E+01		
7898894	NM_020362	C1orf128	chromosome 1 open reading fram	0.081	1.058	7.16E-01	7.16E+01		
7898902	NM_007260	LYPLA2	lysophospholipase II	0.020	1.014	9.13E-01	9.13E+01		
7898910	NM_017761	PNRC2	proline-rich nuclear receptor coac	0.018	1.012	8.85E-01	8.85E+01		
7898916	NM_198173	GRHL3	grainyhead-like 3 (Drosophila)	-0.019	0.987	8.90E-01	8.90E+01		
7898939	NM_020448	NIPAL3	NIPA-like domain containing 3	-0.062	0.958	7.40E-01	7.40E+01		
7898957	NM_013441	RCAN3	RCAN family member 3	-0.055	0.962	7.97E-01	7.97E+01		
7898967	NM_00101098	C1orf130	chromosome 1 open reading fram	-0.051	0.965	6.27E-01	6.27E+01		
7898975	NM_005839	SRRM1	serine/arginine repetitive matrix 1	0.020	1.014	8.86E-01	8.86E+01		
7898988	NM_013943	CLIC4	chloride intracellular channel 4	0.123	1.089	6.90E-01	6.90E+01		
7898998	NM_016124	RHD	Rh blood group, D antigen	-0.075	0.949	8.06E-01	8.06E+01		
7899005	NM_014313	TMEM50A	transmembrane protein 50A	-0.173	0.887	1.12E-02	1.12E+00		
7899016	NM_003002	SDHD	succinate dehydrogenase complex	0.123	1.089	3.20E-01	3.20E+01		
7899018	NM_018202	TMEM57	transmembrane protein 57	-0.003	0.998	9.91E-01	9.91E+01		
7899023	NM_015627	LDLRAP1	low density lipoprotein receptor a	-0.014	0.991	9.64E-01	9.64E+01		
7899029	NM_020379	MAN1C1	mannosidase, alpha, class 1C, mer	-0.085	0.943	4.43E-01	4.43E+01		
7899043	NM_020451	SEPN1	selenoprotein N, 1	0.058	1.041	6.16E-01	6.16E+01		
7899057	NM_019557	FAM54B	family with sequence similarity 54	-0.028	0.981	8.85E-01	8.85E+01		
7899069	---	---	---	0.029	1.020	8.46E-01	8.46E+01		
7899071	---	---	---	0.120	1.087	7.26E-01	7.26E+01		
7899075	NM_004455	EXTL1	exostoses (multiple)-like 1	-0.047	0.968	6.66E-01	6.66E+01		
7899087	NR_026686	PDIK1L	PDLIM1 interacting kinase 1 like	0.118	1.086	3.90E-01	3.90E+01		
7899093	NM_024869	GRRP1	glycine/arginine rich protein 1	-0.055	0.963	5.16E-01	5.16E+01		
7899096	NM_015871	ZNF593	zinc finger protein 593	-0.020	0.986	9.21E-01	9.21E+01		
7899101	NM_006314	CNKSR1	connector enhancer of kinase sup	-0.014	0.990	9.22E-01	9.22E+01		
7899134	NM_022778	CCDC21	coiled-coil domain containing 21	0.029	1.021	8.95E-01	8.95E+01		
7899153	NM_031286	SH3BGRL3	SH3 domain binding glutamic acid-	0.116	1.084	3.11E-01	3.11E+01		
7899160	NM_001803	CD52	CD52 molecule	0.306	1.237	2.12E-02	2.12E+00		
7899167	NM_024674	LIN28	lin-28 homolog (C. elegans)	-0.051	0.966	6.92E-01	6.92E+01		
7899173	NM_024887	DHDDS	dehydrodolichyl diphosphate synt	0.121	1.087	2.31E-01	2.31E+01		
7899187	NM_005517	HMGN2	high-mobility group nucleosomal b	0.062	1.044	6.90E-01	6.90E+01		
7899192	NM_002953	RPS6KA1	ribosomal protein S6 kinase, 90kD	0.062	1.044	6.14E-01	6.14E+01		

7899220	NM_006015	ARID1A	AT rich interactive domain 1A (SW	-0.013	0.991	9.21E-01	9.21E+01		
7899244	NM_017837	PIGV	phosphatidylinositol glycan ancho	-0.037	0.975	8.16E-01	8.16E+01		
7899253	NM_032283	ZDHHC18	zinc finger, DHHC-type containing	0.031	1.022	8.39E-01	8.39E+01		
7899265	NM_006142	SFN	stratifin	-0.040	0.972	6.84E-01	6.84E+01		
7899273	NM_006600	NUDC	nuclear distribution gene C homol	-0.093	0.938	4.44E-01	4.44E+01		
7899284	NM_00101364	TRNP1	TMF1-regulated nuclear protein 1	-0.087	0.941	2.78E-01	2.78E+01		
7899287	---	---	---	-0.009	0.994	9.53E-01	9.53E+01		
7899289	NM_015023	WDTC1	WD and tetratricopeptide repeats	-0.094	0.937	5.77E-01	5.77E+01		
7899310	NM_032125	TMEM222	transmembrane protein 222	-0.040	0.973	8.50E-01	8.50E+01		
7899323	NM_032872	SYTL1	synaptotagmin-like 1	0.017	1.012	8.87E-01	8.87E+01		
7899343	NM_005281	GPR3	G protein-coupled receptor 3	-0.043	0.970	7.50E-01	7.50E+01		
7899346	NM_015933	CCDC72	coiled-coil domain containing 72	-0.026	0.982	8.31E-01	8.31E+01		
7899348	---	---	---	0.061	1.043	4.59E-01	4.59E+01		
7899350	NM_00114391	FAM76A	family with sequence similarity 76	0.107	1.077	5.08E-01	5.08E+01		
7899361	NM_177424	STX12	syntaxin 12	0.039	1.028	8.69E-01	8.69E+01		
7899377	NM_138558	PPP1R8	protein phosphatase 1, regulatory	-0.008	0.995	9.77E-01	9.77E+01		
7899394	NM_00110555	C1orf38	chromosome 1 open reading fram	0.167	1.123	5.10E-01	5.10E+01		
7899407	NM_014474	SMPDL3B	sphingomyelin phosphodiesterase	0.002	1.002	9.89E-01	9.89E+01		
7899417	NM_018053	XKR8	XK, Kell blood group complex subu	-0.076	0.949	5.41E-01	5.41E+01		
7899422	NM_014280	DNAJC8	DnaJ (Hsp40) homolog, subfamily	-0.034	0.977	8.11E-01	8.11E+01		
7899424	NM_178191	ATPIF1	ATPase inhibitory factor 1	-0.042	0.971	6.97E-01	6.97E+01		
7899448	NM_017638	MED18	mediator complex subunit 18	0.055	1.039	7.58E-01	7.58E+01		
7899455	NM_00104818	PHACTR4	phosphatase and actin regulator 4	-0.035	0.976	8.71E-01	8.71E+01		
7899462	NM_00104819	RCC1	regulator of chromosome conden	-0.073	0.950	5.43E-01	5.43E+01		
7899480	NR_002907	SNORA73A	small nucleolar RNA, H/ACA box 7	-0.041	0.972	9.46E-01	9.46E+01		
7899484	---	---	---	-0.069	0.953	9.24E-01	9.24E+01		
7899486	NR_003109	TRNAU1AP	tRNA selenocysteine 1 associated	-0.020	0.986	9.34E-01	9.34E+01		
7899504	NM_006582	GMEB1	glucocorticoid modulatory elemen	-0.067	0.955	7.41E-01	7.41E+01		
7899519	NM_016258	YTHDF2	YTH domain family, member 2	0.004	1.003	9.85E-01	9.85E+01		
7899528	NM_000911	OPRD1	opioid receptor, delta 1	-0.003	0.998	9.86E-01	9.86E+01		
7899534	NM_203342	EPB41	erythrocyte membrane protein ba	-0.102	0.932	4.43E-01	4.43E+01		
7899560	---	---	---	-0.167	0.891	6.08E-01	6.08E+01		
7899562	NM_133178	PTPRU	protein tyrosine phosphatase, rec	0.018	1.012	9.14E-01	9.14E+01		
7899595	NM_002379	MATN1	matrilin 1, cartilage matrix protein	0.041	1.029	7.45E-01	7.45E+01		
7899602	---	---	---	0.081	1.058	6.99E-01	6.99E+01		
7899604	NM_016505	ZCCHC17	zinc finger, CCHC domain containi	-0.073	0.950	6.42E-01	6.42E+01		
7899615	NM_178865	SERINC2	serine incorporator 2	-0.017	0.988	9.09E-01	9.09E+01		
7899627	NM_022164	TINAGL1	tubulointerstitial nephritis antigen	-0.008	0.994	9.61E-01	9.61E+01		
7899643	NM_001525	HCRTR1	hypocretin (orexin) receptor 1	0.015	1.010	9.28E-01	9.28E+01		
7899654	NM_006559	KHDRBS1	KH domain containing, RNA bindin	0.023	1.016	8.69E-01	8.69E+01		
7899675	NM_018056	TMEM39B	transmembrane protein 39B	-0.050	0.966	7.52E-01	7.52E+01		
7899688	NM_012316	KPNA6	karyopherin alpha 6 (importin alp	-0.029	0.980	8.96E-01	8.96E+01		
7899703	NM_175852	TXLNA	taxilin alpha	0.067	1.048	4.66E-01	4.66E+01		
7899719	NM_024296	CCDC28B	coiled-coil domain containing 28B	0.024	1.017	8.92E-01	8.92E+01		
7899727	NM_00116004	IQCC	IQ motif containing C	-0.032	0.978	8.35E-01	8.35E+01		
7899737	NM_003757	EIF3I	eukaryotic translation initiation fa	-0.023	0.984	9.16E-01	9.16E+01		
7899750	NM_032648	FAM167B	family with sequence similarity 16	-0.031	0.979	8.18E-01	8.18E+01		
7899753	NM_005356	LCK	lymphocyte-specific protein tyrosi	-0.056	0.962	6.74E-01	6.74E+01		
7899774	NM_004964	HDAC1	histone deacetylase 1	-0.046	0.969	7.77E-01	7.77E+01		
7899790	NM_052841	TSSK3	testis-specific serine kinase 3	-0.004	0.997	9.90E-01	9.90E+01		
7899795	---	---	---	0.093	1.067	3.44E-01	3.44E+01		
7899813	NM_005610	RBBP4	retinoblastoma binding protein 4	0.113	1.081	2.82E-01	2.82E+01		
7899821	NM_020888	KIAA1522	KIAA1522	0.019	1.013	8.67E-01	8.67E+01		
7899829	NM_022753	S100PBP	S100P binding protein	-0.125	0.917	2.56E-01	2.56E+01		
7899841	NM_002143	HPCA	hippocalcin	0.036	1.025	8.09E-01	8.09E+01		
7899849	---	---	---	-0.006	0.996	9.75E-01	9.75E+01		
7899851	NM_052998	ADC	arginine decarboxylase	-0.006	0.996	9.77E-01	9.77E+01		
7899870	NM_152493	ZNF362	zinc finger protein 362	-0.033	0.978	8.19E-01	8.19E+01		
7899909	---	---	---	-0.119	0.921	6.33E-01	6.33E+01		
7899927	NM_153212	GJB4	gap junction protein, beta 4, 30.3k	0.049	1.035	7.45E-01	7.45E+01		
7899939	NM_002060	GJA4	gap junction protein, alpha 4, 37k	-0.031	0.979	8.13E-01	8.13E+01		
7899955	---	---	---	-0.108	0.928	4.56E-01	4.56E+01		
7899957	NM_005095	ZMYM4	zinc finger, MYM-type 4	0.010	1.007	9.53E-01	9.53E+01		
7899990	NM_014284	NCDN	neurochondrin	-0.030	0.979	8.21E-01	8.21E+01		
7900001	NM_178548	TFAP2E	transcription factor AP-2 epsilon (	-0.062	0.958	3.90E-01	3.90E+01		
7900009	NM_017629	EIF2C4	eukaryotic translation initiation fa	0.035	1.024	8.85E-01	8.85E+01		
7900030	NM_012199	EIF2C1	eukaryotic translation initiation fa	0.047	1.033	7.48E-01	7.48E+01		
7900051	NM_024852	EIF2C3	eukaryotic translation initiation fa	-0.026	0.982	8.93E-01	8.93E+01		
7900076	NM_014466	TEKT2	tektin 2 (testicular)	-0.045	0.970	8.33E-01	8.33E+01		
7900087	NM_017825	ADPRHL2	ADP-ribosylhydrolase like 2	-0.018	0.988	9.02E-01	9.02E+01		
7900095	NM_018067	MAP7D1	MAP7 domain containing 1	-0.053	0.964	4.65E-01	4.65E+01		
7900119	NM_005119	THRAP3	thyroid hormone receptor associa	-0.005	0.997	9.81E-01	9.81E+01		
7900129	NM_024676	C1orf113	chromosome 1 open reading fram	-0.004	0.997	9.84E-01	9.84E+01		
7900146	NM_025079	ZC3H12A	zinc finger CCCH-type containing 1	-0.164	0.893	3.35E-02	3.35E+00		
7900157	NM_001030	RPS27	ribosomal protein S27	-0.031	0.979	8.55E-01	8.55E+01		
7900167	NM_018101	CDCA8	cell division cycle associated 8	0.075	1.053	5.68E-01	5.68E+01		
7900183	NM_00103174	MANEAL	mannosidase, endo-alpha-like	-0.110	0.926	2.73E-01	2.73E+01		

7900192	NM_198446	C1orf122	chromosome 1 open reading fram	-0.028	0.980	8.62E-01	8.62E+01		
7900216	NM_024595	AKIRIN1	akirin 1	-0.048	0.967	5.99E-01	5.99E+01		
7900228	NM_004552	NDUFS5	NADH dehydrogenase (ubiquinone	-0.077	0.948	4.43E-01	4.43E+01		
7900235	NM_012090	MACF1	microtubule-actin crosslinking fact	0.130	1.094	2.04E-01	2.04E+01		
7900336	---	---	---	0.269	1.205	1.78E-01	1.78E+01		
7900340	NM_181809	BMP8A	bone morphogenetic protein 8a	-0.018	0.988	9.51E-01	9.51E+01		
7900352	NM_022120	OXCT2	3-oxoacid CoA transferase 2	-0.036	0.975	7.09E-01	7.09E+01		
7900354	NM_006112	PPIE	peptidylprolyl isomerase E (cyclop	-0.056	0.962	7.82E-01	7.82E+01		
7900365	NM_00113649	MFSD2	major facilitator superfamily doma	-0.067	0.955	5.04E-01	5.04E+01		
7900382	NM_006367	CAP1	CAP, adenylate cyclase-associated	0.027	1.019	8.85E-01	8.85E+01		
7900395	NM_012421	RLF	rearranged L-myc fusion	-0.054	0.963	7.87E-01	7.87E+01		
7900413	NM_005857	ZMPSTE24	zinc metallopeptidase (STE24 hom	0.186	1.138	1.73E-01	1.73E+01		
7900426	NM_022733	SMAP2	small ArfGAP2	0.114	1.082	1.89E-01	1.89E+01		
7900438	NM_023070	ZNF643	zinc finger protein 643	-0.302	0.811	2.89E-02	2.89E+00		
7900446	NM_198494	ZNF642	zinc finger protein 642	0.053	1.037	7.92E-01	7.92E+01		
7900454	NM_022774	DEM1	defects in morphology 1 homolog	-0.029	0.980	9.26E-01	9.26E+01		
7900468	NM_00114258	NFYC	nuclear transcription factor Y, gam	0.020	1.014	9.00E-01	9.00E+01		
7900510	NM_001905	CTPS	CTP synthase	-0.047	0.968	8.24E-01	8.24E+01		
7900531	NM_007102	GUCA2B	guanylate cyclase activator 2B (urc	0.002	1.001	9.91E-01	9.91E+01		
7900538	---	---	---	-0.005	0.996	9.77E-01	9.77E+01		
7900540	NM_173642	RIMKLA	ribosomal modification protein rin	-0.077	0.948	4.94E-01	4.94E+01		
7900546	NM_024664	PPCS	phosphopantothenoylcysteine syn	0.048	1.034	6.70E-01	6.70E+01		
7900576	NM_006347	PPIH	peptidylprolyl isomerase H (cyclop	-0.074	0.950	6.80E-01	6.80E+01		
7900585	NM_004559	YBX1	Y box binding protein 1	0.047	1.033	4.49E-01	4.49E+01		
7900597	BC001508	C1orf50	chromosome 1 open reading fram	-0.073	0.951	7.60E-01	7.60E+01		
7900603	AK299874	C1orf50	chromosome 1 open reading fram	-0.037	0.974	7.97E-01	7.97E+01		
7900609	NM_00101792	ERMAP	erythroblast membrane-associatec	0.003	1.002	9.88E-01	9.88E+01		
7900624	NM_015911	ZNF691	zinc finger protein 691	-0.106	0.929	3.38E-01	3.38E+01		
7900654	NM_144626	TMEM125	transmembrane protein 125	-0.018	0.987	9.01E-01	9.01E+01		
7900683	NM_005373	MPL	myeloproliferative leukemia virus	0.038	1.027	7.02E-01	7.02E+01		
7900699	NM_001255	CDC20	cell division cycle 20 homolog (S. c	0.053	1.038	7.45E-01	7.45E+01		
7900710	NM_00101296	C1orf84	chromosome 1 open reading fram	0.041	1.028	4.96E-01	4.96E+01		
7900792	NM_002840	PTPRF	protein tyrosine phosphatase, rece	-0.013	0.991	9.05E-01	9.05E+01		
7900833	NM_014663	KDM4A	lysine (K)-specific demethylase 4A	0.004	1.002	9.88E-01	9.88E+01		
7900857	NM_174963	ST3GAL3	ST3 beta-galactoside alpha-2,3-sia	-0.010	0.993	9.57E-01	9.57E+01		
7900878	NM_057091	ARTN	artemin	0.004	1.003	9.81E-01	9.81E+01		
7900888	NM_014652	IPO13	importin 13	0.011	1.008	9.57E-01	9.57E+01		
7900922	NM_004047	ATP6V0B	ATPase, H+ transporting, lysosoma	0.171	1.126	4.25E-01	4.25E+01		
7900931	NM_003780	B4GALT2	UDP-Gal:betaGlcNAc beta 1,4- gala	-0.075	0.949	3.68E-01	3.68E+01		
7900944	NM_152499	CCDC24	coiled-coil domain containing 24	-0.090	0.940	3.04E-01	3.04E+01		
7900957	NM_173484	KLF17	Kruppel-like factor 17	0.003	1.002	9.89E-01	9.89E+01		
7900962	NM_019100	DMAP1	DNA methyltransferase 1 associate	-0.024	0.984	8.64E-01	8.64E+01		
7900979	NM_018150	RNF220	ring finger protein 220	-0.022	0.985	8.73E-01	8.73E+01		
7900999	NM_00114563	C1orf228	chromosome 1 open reading fram	0.030	1.021	8.19E-01	8.19E+01		
7901038	NM_001012	RPS8	ribosomal protein S8	0.022	1.015	9.07E-01	9.07E+01		
7901046	NR_000015	SNORD55	small nucleolar RNA, C/D box 55	0.073	1.052	6.82E-01	6.82E+01		
7901048	NR_000024	SNORD46	small nucleolar RNA, C/D box 46	0.131	1.095	6.47E-01	6.47E+01		
7901050	NR_001456	SNORD38A	small nucleolar RNA, C/D box 38A	-0.080	0.946	7.93E-01	7.93E+01		
7901052	NR_001457	SNORD38B	small nucleolar RNA, C/D box 38B	0.050	1.036	9.13E-01	9.13E+01		
7901054	NM_004073	PLK3	polo-like kinase 3 (Drosophila)	-0.152	0.900	1.04E-01	1.04E+01		
7901073	NM_000374	UROD	uroporphyrinogen decarboxylase	-0.026	0.982	9.03E-01	9.03E+01		
7901091	NM_025077	TOE1	target of EGR1, member 1 (nuclea	-0.057	0.961	6.94E-01	6.94E+01		
7901102	NM_015506	MMACHC	methylmalonic aciduria (cobalami	0.057	1.040	6.73E-01	6.73E+01		
7901123	NM_172164	NASP	nuclear autoantigenic sperm prote	-0.014	0.991	9.53E-01	9.53E+01		
7901135	NM_016486	TMEM69	transmembrane protein 69	0.049	1.035	8.05E-01	8.05E+01		
7901140	NM_015112	MAST2	microtubule associated serine/thr	0.012	1.008	9.43E-01	9.43E+01		
7901187	NM_00101361	C1orf190	chromosome 1 open reading fram	0.026	1.018	8.64E-01	8.64E+01		
7901212	ENST00000311	UQCRH	ubiquinol-cytochrome c reductase	-0.071	0.952	7.44E-01	7.44E+01		
7901219	NM_199044	NSUN4	NOL1/NOP2/Sun domain family, m	0.120	1.086	2.79E-01	2.79E+01		
7901227	---	---	---	0.066	1.047	8.63E-01	8.63E+01		
7901229	NM_001441	FAAH	fatty acid amide hydrolase	-0.043	0.971	6.93E-01	6.93E+01		
7901247	NM_172225	DMBX1	diencephalon/mesencephalon hor	-0.021	0.985	8.34E-01	8.34E+01		
7901253	BC144026	C1orf223	chromosome 1 open reading fram	-0.137	0.909	5.19E-01	5.19E+01		
7901299	NM_016308	CMPK1	cytidine monophosphate (UMP-CN	0.057	1.041	6.35E-01	6.35E+01		
7901309	NM_012186	FOXE3	forkhead box E3	-0.002	0.999	9.90E-01	9.90E+01		
7901311	NM_004474	FOXD2	forkhead box D2	-0.040	0.973	7.58E-01	7.58E+01		
7901314	---	---	---	-0.102	0.932	4.51E-01	4.51E+01		
7901336	NM_018571	STRADB	STE20-related kinase adaptor beta	-0.005	0.997	9.91E-01	9.91E+01		
7901357	---	---	---	-0.012	0.992	9.49E-01	9.49E+01		
7901376	NM_014372	RNF11	ring finger protein 11	-0.019	0.987	9.27E-01	9.27E+01		
7901385	NM_024586	OSBPL9	oxysterol binding protein-like 9	0.040	1.028	7.57E-01	7.57E+01		
7901418	NM_152265	BTF3L4	basic transcription factor 3-like 4	0.151	1.111	3.19E-01	3.19E+01		
7901443	---	---	---	-0.002	0.998	9.90E-01	9.90E+01		
7901445	---	---	---	-0.066	0.955	5.41E-01	5.41E+01		
7901447	NM_032864	PRPF38A	PRP38 pre-mRNA processing facto	-0.015	0.989	9.49E-01	9.49E+01		
7901460	NM_015696	GPX7	glutathione peroxidase 7	-0.052	0.965	7.16E-01	7.16E+01		
7901466	NM_00104269	FAM159A	family with sequence similarity 15	0.094	1.067	4.96E-01	4.96E+01		

7901477	---	---	---	-0.028	0.980	8.69E-01	8.69E+01		
7901479	NM_024646	ZYG11B	zyg-11 homolog B (C. elegans)	0.016	1.011	9.53E-01	9.53E+01		
7901513	NM_002979	SCP2	sterol carrier protein 2	0.170	1.125	1.52E-01	1.52E+01		
7901535	NM_153703	PODN	podocan	-0.039	0.973	7.05E-01	7.05E+01		
7901549	NM_000098	CPT2	carnitine palmitoyltransferase 2	0.069	1.049	4.87E-01	4.87E+01		
7901557	NM_033067	DMRTB1	DMRT-like family B with proline-ri	0.001	1.000	9.98E-01	9.98E+01		
7901565	NM_000792	DIO1	deiodinase, iodothyronine, type I	0.038	1.027	9.00E-01	9.00E+01		
7901577	NM_052940	LRRC42	leucine rich repeat containing 42	-0.046	0.969	7.68E-01	7.68E+01		
7901592	BC035374	C1orf83	chromosome 1 open reading fram	0.026	1.018	9.18E-01	9.18E+01		
7901601	NM_016491	MRPL37	mitochondrial ribosomal protein L	-0.020	0.986	9.27E-01	9.27E+01		
7901611	ENST00000361	C1orf191	chromosome 1 open reading fram	0.050	1.035	6.75E-01	6.75E+01		
7901613	NM_147161	ACOT11	acyl-CoA thioesterase 11	-0.076	0.948	4.69E-01	4.69E+01		
7901662	NM_004623	TTC4	tetratricopeptide repeat domain 4	-0.014	0.990	9.56E-01	9.56E+01		
7901687	NM_182532	TMEM61	transmembrane protein 61	-0.081	0.945	4.03E-01	4.03E+01		
7901691	NM_057176	BSND	Bartter syndrome, infantile, with s	0.011	1.008	9.53E-01	9.53E+01		
7901696	NM_174936	PCSK9	proprotein convertase subtilisin/k	-0.029	0.980	8.05E-01	8.05E+01		
7901714	---	---	---	-0.016	0.989	9.28E-01	9.28E+01		
7901744	---	---	---	-0.015	0.990	9.52E-01	9.52E+01		
7901746	---	---	---	-0.035	0.976	7.91E-01	7.91E+01		
7901748	NM_00111341	FGGY	FGGY carbohydrate kinase domair	0.071	1.050	7.68E-01	7.68E+01		
7901804	NM_176877	INADL	InaD-like (Drosophila)	-0.026	0.982	9.50E-01	9.50E+01		
7901852	---	---	---	0.144	1.105	5.47E-01	5.47E+01		
7901856	---	---	---	0.065	1.046	6.08E-01	6.08E+01		
7901858	---	---	---	0.033	1.023	8.84E-01	8.84E+01		
7901867	NM_003368	USP1	ubiquitin specific peptidase 1	0.024	1.017	8.73E-01	8.73E+01		
7901915	NM_013339	ALG6	asparagine-linked glycosylation 6,	0.053	1.038	7.74E-01	7.74E+01		
7901951	NM_002633	PGM1	phosphoglucomutase 1	-0.044	0.970	7.23E-01	7.23E+01		
7901969	NM_005012	ROR1	receptor tyrosine kinase-like orpha	0.013	1.009	9.56E-01	9.56E+01		
7902036	---	---	---	-0.059	0.960	5.73E-01	5.73E+01		
7902038	NM_00100535	AK3L1	adenylate kinase 3-like 1	0.022	1.015	9.12E-01	9.12E+01		
7902074	NM_002303	LEPR	leptin receptor	0.172	1.126	4.28E-01	4.28E+01		
7902104	NM_002600	PDE4B	phosphodiesterase 4B, cAMP-spec	-0.211	0.864	2.46E-02	2.46E+00		
7902166	NM_020948	MIER1	mesoderm induction early respons	0.037	1.026	7.03E-01	7.03E+01		
7902205	NM_001559	IL12RB2	interleukin 12 receptor, beta 2	-0.130	0.914	7.17E-01	7.17E+01		
7902223	---	---	---	0.029	1.021	9.49E-01	9.49E+01		
7902265	---	---	---	-0.012	0.992	9.53E-01	9.53E+01		
7902267	NR_023916	PIN1L	peptidylprolyl cis/trans isomerase,	0.047	1.033	5.93E-01	5.93E+01		
7902269	NM_004768	SFRS11	splicing factor, arginine/serine-rich	-0.065	0.956	4.05E-01	4.05E+01		
7902282	NM_00103664	HHLA3	HERV-H LTR-associating 3	-0.047	0.968	9.08E-01	9.08E+01		
7902345	NM_138467	TYW3	tRNA-yW synthesizing protein 3 hc	0.044	1.031	7.52E-01	7.52E+01		
7902367	NM_000016	ACADM	acyl-Coenzyme A dehydrogenase,	0.100	1.072	2.97E-01	2.97E+01		
7902382	NM_004582	RABGGTB	Rab geranylgeranyltransferase, be	0.000	1.000	1.00E+00	1.00E+02		
7902396	NR_003042	SNORD45C	small nucleolar RNA, C/D box 45C	-0.129	0.914	5.64E-01	5.64E+01		
7902398	NR_002749	SNORD45A	small nucleolar RNA, C/D box 45A	0.137	1.100	6.48E-01	6.48E+01		
7902400	NR_002748	SNORD45B	small nucleolar RNA, C/D box 45B	-0.088	0.941	8.24E-01	8.24E+01		
7902435	---	---	---	0.017	1.012	9.48E-01	9.48E+01		
7902448	NM_00103500	RPL17	ribosomal protein L17	-0.020	0.986	8.95E-01	8.95E+01		
7902472	---	---	---	0.068	1.048	7.08E-01	7.08E+01		
7902474	---	---	---	0.198	1.147	2.51E-01	2.51E+01		
7902476	BX537792	FAM73A	family with sequence similarity 73	0.070	1.050	6.65E-01	6.65E+01		
7902493	---	---	---	0.019	1.013	9.09E-01	9.09E+01		
7902541	NM_006820	IFI44L	interferon-induced protein 44-like	0.416	1.334	4.83E-01	4.83E+01		
7902553	NM_006417	IFI44	interferon-induced protein 44	0.272	1.208	5.54E-01	5.54E+01		
7902592	NM_006465	ARID3B	AT rich interactive domain 3B (BRI	-0.132	0.912	2.16E-01	2.16E+01		
7902594	NM_182948	PRKACB	protein kinase, cAMP-dependent,	0.082	1.058	6.00E-01	6.00E+01		
7902611	---	---	---	0.043	1.030	8.32E-01	8.32E+01		
7902634	NM_025065	BXDC5	brix domain containing 5	0.000	1.000	9.99E-01	9.99E+01		
7902700	---	---	---	-0.091	0.939	8.06E-01	8.06E+01		
7902771	NM_016009	SH3GLB1	SH3-domain GRB2-like endophilin	0.001	1.001	9.97E-01	9.97E+01		
7902789	NM_012262	HS2ST1	heparan sulfate 2-O-sulfotransfera	-0.076	0.949	6.90E-01	6.90E+01		
7902822	NM_006256	PKN2	protein kinase N2	0.042	1.030	7.49E-01	7.49E+01		
7902848	---	---	---	0.131	1.095	6.35E-01	6.35E+01		
7902861	NM_00113447	LRRC8B	leucine rich repeat containing 8 fa	0.137	1.100	2.63E-01	2.63E+01		
7902874	NM_032270	LRRC8C	leucine rich repeat containing 8 fa	0.071	1.050	6.05E-01	6.05E+01		
7902883	NM_00113447	LRRC8D	leucine rich repeat containing 8 fa	-0.076	0.949	5.93E-01	5.93E+01		
7902891	NM_182976	ZNF326	zinc finger protein 326	0.023	1.016	9.13E-01	9.13E+01		
7902909	---	---	---	0.034	1.024	8.16E-01	8.16E+01		
7902911	---	---	---	0.011	1.008	9.49E-01	9.49E+01		
7902913	NM_003503	CDC7	cell division cycle 7 homolog (S. ce	-0.069	0.953	7.83E-01	7.83E+01		
7902930	NR_003130	HSP90B3P	heat shock protein 90kDa beta (Gr	0.127	1.092	1.76E-01	1.76E+01		
7902992	NM_024813	RPAP2	RNA polymerase II associated prot	-0.002	0.999	9.94E-01	9.94E+01		
7903010	NM_000969	RPL5	ribosomal protein L5	0.005	1.003	9.78E-01	9.78E+01		
7903022	NR_000006	SNORD21	small nucleolar RNA, C/D box 21	-0.055	0.962	8.98E-01	8.98E+01		
7903032	NM_007358	MTF2	metal response element binding tr	0.027	1.019	8.76E-01	8.76E+01		
7903079	NM_001938	DR1	down-regulator of transcription 1,	-0.066	0.955	5.09E-01	5.09E+01		
7903092	NM_00102494	FNBP1L	formin binding protein 1-like	-0.082	0.945	8.41E-01	8.41E+01		
7903119	NM_002858	ABCD3	ATP-binding cassette, sub-family D	0.044	1.031	8.27E-01	8.27E+01		

7903171	NM_015485	RWDD3	RWD domain containing 3	0.062	1.044	7.12E-01	7.12E+01		
7903183	---	---	---	-0.007	0.995	9.72E-01	9.72E+01		
7903188	NM_021190	PTBP2	polypyrimidine tract binding prote	-0.031	0.979	8.73E-01	8.73E+01		
7903281	NM_012243	SLC35A3	solute carrier family 35 (UDP-N-ac	0.081	1.058	6.57E-01	6.57E+01		
7903294	NM_033055	HIAT1	hippocampus abundant transcript	0.009	1.006	9.72E-01	9.72E+01		
7903308	NM_019083	CCDC76	coiled-coil domain containing 76	0.041	1.029	8.27E-01	8.27E+01		
7903321	NM_00113084	RTCD1	RNA terminal phosphate cyclase d	0.053	1.038	6.60E-01	6.60E+01		
7903334	NM_003672	CDC14A	CDC14 cell division cycle 14 homol	-0.063	0.957	7.45E-01	7.45E+01		
7903355	NM_022049	GPR88	G protein-coupled receptor 88	-0.048	0.968	6.62E-01	6.62E+01		
7903358	NM_001078	VCAM1	vascular cell adhesion molecule 1	0.046	1.032	9.17E-01	9.17E+01		
7903369	NM_133496	SLC30A7	solute carrier family 30 (zinc trans	0.050	1.036	7.01E-01	7.01E+01		
7903389	---	---	---	-0.041	0.972	9.09E-01	9.09E+01		
7903393	NM_001400	S1PR1	sphingosine-1-phosphate receptor	-0.131	0.913	2.58E-01	2.58E+01		
7903404	NM_017619	RNPC3	RNA-binding region (RNP1, RRM) c	-0.054	0.963	7.87E-01	7.87E+01		
7903407	NM_020978	AMY2B	amylase, alpha 2B (pancreatic)	-0.010	0.993	9.79E-01	9.79E+01		
7903414	NM_000699	AMY2A	amylase, alpha 2A (pancreatic)	0.067	1.048	8.53E-01	8.53E+01		
7903425	NM_004038	AMY1A	amylase, alpha 1A (salivary)	0.070	1.049	8.13E-01	8.13E+01		
7903440	NM_004038	AMY1A	amylase, alpha 1A (salivary)	0.070	1.049	8.13E-01	8.13E+01		
7903474	---	---	---	0.063	1.044	6.16E-01	6.16E+01		
7903478	NM_00114398	NBPF4	neuroblastoma breakpoint family,	0.025	1.017	8.78E-01	8.78E+01		
7903490	NM_00114398	NBPF6	neuroblastoma breakpoint family,	0.051	1.036	6.08E-01	6.08E+01		
7903507	NM_00101088	FAM102B	family with sequence similarity 10	-0.024	0.984	9.49E-01	9.49E+01		
7903519	NM_018061	PRPF38B	PRP38 pre-mRNA processing facto	-0.044	0.970	6.64E-01	6.64E+01		
7903541	NM_007269	STXBP3	syntaxin binding protein 3	-0.002	0.998	9.92E-01	9.92E+01		
7903582	NM_00103500	RPL17	ribosomal protein L17	-0.030	0.980	8.32E-01	8.32E+01		
7903586	NM_020141	TMEM167B	transmembrane protein 167B	0.002	1.002	9.92E-01	9.92E+01		
7903592	NM_020775	KIAA1324	KIAA1324	-0.018	0.988	9.42E-01	9.42E+01		
7903619	NM_006513	SARS	seryl-tRNA synthetase	0.018	1.012	9.24E-01	9.24E+01		
7903632	NM_001408	CELSR2	cadherin, EGF LAG seven-pass G-ty	-0.033	0.977	7.22E-01	7.22E+01		
7903667	NM_00104070	SYPL2	synaptophysin-like 2	0.055	1.039	4.48E-01	4.48E+01		
7903676	NM_153340	ATXN7L2	ataxin 7-like 2	-0.034	0.977	7.09E-01	7.09E+01		
7903688	NM_182580	CYB561D1	cytochrome b-561 domain contair	0.096	1.069	2.58E-01	2.58E+01		
7903694	NM_031936	GPR61	G protein-coupled receptor 61	-0.046	0.969	6.40E-01	6.40E+01		
7903703	NM_006496	GNAI3	guanine nucleotide binding protei	0.039	1.027	8.41E-01	8.41E+01		
7903717	---	---	---	-0.095	0.937	1.88E-01	1.88E+01		
7903719	NM_004037	AMPD2	adenosine monophosphate deami	-0.100	0.933	3.78E-01	3.78E+01		
7903742	NM_000850	GSTM4	glutathione S-transferase mu 4	0.147	1.107	7.15E-01	7.15E+01		
7903753	NM_000848	GSTM2	glutathione S-transferase mu 2 (m	0.277	1.212	5.88E-02	5.88E+00		
7903765	NM_000561	GSTM1	glutathione S-transferase mu 1	0.052	1.037	9.24E-01	9.24E+01		
7903786	NM_000757	CSF1	colony stimulating factor 1 (macro	0.020	1.014	9.63E-01	9.63E+01		
7903803	NM_006621	AHCYL1	adenosylhomocysteinase-like 1	0.082	1.058	5.45E-01	5.45E+01		
7903827	NM_033088	FAM40A	family with sequence similarity 40	0.052	1.037	7.16E-01	7.16E+01		
7903854	NM_00101089	SLC6A17	solute carrier family 6, member 17	-0.054	0.963	7.51E-01	7.51E+01		
7903867	NM_004978	KCNC4	potassium voltage-gated channel,	-0.039	0.973	7.53E-01	7.53E+01		
7903878	NM_022768	RBM15	RNA binding motif protein 15	0.044	1.031	6.24E-01	6.24E+01		
7903884	NM_032414	PROK1	prokineticin 1	0.023	1.016	8.51E-01	8.51E+01		
7903893	NM_000560	CD53	CD53 molecule	0.095	1.068	1.65E-01	1.65E+01		
7903908	NM_006090	CEPT1	choline/ethanolamine phosphotra	0.224	1.168	1.41E-03	1.41E-01		
7903920	NM_00102519	CHI3L2	chitinase 3-like 2	-0.141	0.907	5.07E-01	5.07E+01		
7903970	---	---	---	0.054	1.038	8.72E-01	8.72E+01		
7903972	NM_001688	ATP5F1	ATP synthase, H+ transporting, mi	0.194	1.144	3.67E-02	3.67E+00		
7903988	NM_00101093	RAP1A	RAP1A, member of RAS oncogene	0.115	1.083	2.11E-01	2.11E+01		
7904000	NM_007204	DDX20	DEAD (Asp-Glu-Ala-Asp) box polyp	-0.035	0.976	8.78E-01	8.78E+01		
7904018	NM_018704	CTTNBP2NL	CTTNBP2 N-terminal like	0.076	1.054	8.67E-01	8.67E+01		
7904036	NM_006135	CAPZA1	capping protein (actin filament) m	0.146	1.106	8.25E-03	8.25E-01		
7904048	---	---	---	0.149	1.109	3.60E-01	3.60E+01		
7904050	NM_020963	MOV10	Mov10, Moloney leukemia virus 1	0.014	1.009	9.56E-01	9.56E+01		
7904073	NM_00100444	FAM19A3	family with sequence similarity 19	-0.135	0.911	9.93E-02	9.93E+00		
7904084	NR_002796	AFARP1	AKR7 family pseudogene	-0.005	0.997	9.85E-01	9.85E+01		
7904086	NM_014813	LRIG2	leucine-rich repeats and immunog	0.134	1.097	2.25E-01	2.25E+01		
7904106	NM_152900	MAGI3	membrane associated guanylate k	-0.209	0.865	6.78E-03	6.78E-01		
7904131	NM_022836	DCLRE1B	DNA cross-link repair 1B (PSO2 ho	0.304	1.235	4.40E-02	4.40E+00		
7904137	NM_198268	HIPK1	homeodomain interacting protein	-0.042	0.971	7.89E-01	7.89E+01		
7904164	---	---	---	-0.138	0.908	7.08E-01	7.08E+01		
7904166	---	---	---	0.104	1.075	7.22E-01	7.22E+01		
7904209	---	---	---	-0.056	0.962	6.14E-01	6.14E+01		
7904226	NM_018420	SLC22A15	solute carrier family 22, member 1	-0.005	0.997	9.90E-01	9.90E+01		
7904254	NM_000701	ATP1A1	ATPase, Na+/K+ transporting, alph	0.054	1.038	7.15E-01	7.15E+01		
7904287	NM_001767	CD2	CD2 molecule	-0.147	0.903	1.73E-01	1.73E+01		
7904303	NM_004258	IGSF2	immunoglobulin superfamily, mer	0.253	1.192	7.05E-02	7.05E+00		
7904314	NM_003594	TTF2	transcription termination factor, R	0.000	1.000	1.00E+00	1.00E+02		
7904340	NM_006699	MAN1A2	mannosidase, alpha, class 1A, mer	0.059	1.042	5.54E-01	5.54E+01		
7904361	NM_017709	FAM46C	family with sequence similarity 46	-0.020	0.986	9.44E-01	9.44E+01		
7904394	---	---	---	0.143	1.104	3.47E-01	3.47E+01		
7904433	NM_006623	PHGDH	phosphoglycerate dehydrogenase	-0.010	0.993	9.61E-01	9.61E+01		
7904448	---	---	---	-0.024	0.984	8.50E-01	8.50E+01		
7904463	---	---	---	-0.063	0.957	5.86E-01	5.86E+01		

7904465	NR_027337	HIST2H2BA	histone cluster 2, H2ba	0.075	1.054	6.00E-01	6.00E+01		
7904469	NM_015326	SRGAP2	SLIT-ROBO Rho GTPase activating	0.168	1.124	3.26E-01	3.26E+01		
7904478	AF172850	LOC51152	melanoma antigen	0.079	1.056	8.84E-01	8.84E+01		
7904480	---	---	---	-0.142	0.906	7.24E-01	7.24E+01		
7904482	NM_015326	SRGAP2	SLIT-ROBO Rho GTPase activating	0.059	1.042	8.31E-01	8.31E+01		
7904486	AK294414	NBPF20	neuroblastoma breakpoint family,	-0.017	0.988	8.78E-01	8.78E+01		
7904500	ENST00000436	NBPF12	neuroblastoma breakpoint family,	0.002	1.002	9.86E-01	9.86E+01		
7904506	AK095030	NBPF20	neuroblastoma breakpoint family,	-0.001	0.999	9.95E-01	9.95E+01		
7904562	AK294944	NBPF14	neuroblastoma breakpoint family,	-0.003	0.998	9.81E-01	9.81E+01		
7904569	---	---	---	0.016	1.011	8.70E-01	8.70E+01		
7904572	---	---	---	-0.042	0.972	9.50E-01	9.50E+01		
7904574	NM_00103970	NBPF10	neuroblastoma breakpoint family,	-0.059	0.960	5.01E-01	5.01E+01		
7904693	---	---	---	-0.283	0.822	1.76E-01	1.76E+01		
7904695	NM_004892	SEC22B	SEC22 vesicle trafficking protein h	0.010	1.007	9.73E-01	9.73E+01		
7904702	NM_203458	NOTCH2NL	Notch homolog 2 (Drosophila) N-t	-0.018	0.988	9.29E-01	9.29E+01		
7904713	---	---	---	0.076	1.054	7.61E-01	7.61E+01		
7904715	---	---	---	-0.261	0.835	1.55E-01	1.55E+01		
7904737	NM_00103988	ANKRD34A	ankyrin repeat domain 34A	-0.031	0.979	8.09E-01	8.09E+01		
7904742	NM_153713	LIX1L	Lix1 homolog (mouse)-like	-0.034	0.977	8.10E-01	8.10E+01		
7904751	BC017770	RBM8A	RNA binding motif protein 8A	-0.177	0.885	1.02E-01	1.02E+01		
7904755	NM_003846	PEX11B	peroxisomal biogenesis factor 11	0.039	1.028	7.91E-01	7.91E+01		
7904812	NM_006099	PIAS3	protein inhibitor of activated STAT	-0.044	0.970	7.97E-01	7.97E+01		
7904830	NM_014455	RNF115	ring finger protein 115	-0.035	0.976	8.39E-01	8.39E+01		
7904853	NM_00109761	GPR89A	G protein-coupled receptor 89A	0.008	1.005	9.77E-01	9.77E+01		
7904863	---	---	---	0.025	1.018	8.58E-01	8.58E+01		
7904869	NM_00113011	FAM108A1	family with sequence similarity 10	-0.011	0.992	9.37E-01	9.37E+01		
7904874	NM_183372	RP11-94I2.2	neuroblastoma breakpoint family,	-0.006	0.996	9.66E-01	9.66E+01		
7904879	---	---	---	-0.006	0.996	9.77E-01	9.77E+01		
7904881	NR_002305	PDIA3P	protein disulfide isomerase family	0.262	1.199	1.98E-02	1.98E+00		
7904883	NM_004284	CHD1L	chromodomain helicase DNA bind	-0.020	0.986	9.50E-01	9.50E+01		
7904907	NM_004326	BCL9	B-cell CLL/lymphoma 9	0.009	1.006	9.70E-01	9.70E+01		
7904930	NM_016334	GPR89B	G protein-coupled receptor 89B	0.013	1.009	9.57E-01	9.57E+01		
7904948	NM_00113011	FAM108A1	family with sequence similarity 10	-0.011	0.992	9.37E-01	9.37E+01		
7904955	---	---	---	0.015	1.011	9.19E-01	9.19E+01		
7904959	---	---	---	-0.143	0.906	7.39E-01	7.39E+01		
7904963	---	---	---	-0.142	0.906	7.24E-01	7.24E+01		
7904965	AB042555	PDE4DIP	phosphodiesterase 4D interacting	-0.161	0.894	1.03E-01	1.03E+01		
7904969	NM_030796	VOPP1	vesicular, overexpressed in cancer	0.031	1.022	8.45E-01	8.45E+01		
7904974	NR_003242	C1orf152	profilin 1 pseudogene	0.127	1.092	6.94E-02	6.94E+00		
7904976	NM_00110266	NBPF16	neuroblastoma breakpoint family,	-0.060	0.959	4.37E-01	4.37E+01		
7904997	NM_00114388	PPIAL4B	peptidylprolyl isomerase A (cyclop	-0.042	0.972	7.09E-01	7.09E+01		
7904999	NM_00110266	NBPF16	neuroblastoma breakpoint family,	-0.050	0.966	5.59E-01	5.59E+01		
7905016	NM_00114388	PPIAL4B	peptidylprolyl isomerase A (cyclop	-0.042	0.972	7.09E-01	7.09E+01		
7905018	AK095105	FLJ37786	hypothetical LOC642691	-0.060	0.959	5.17E-01	5.17E+01		
7905020	NM_000798	DRD5	dopamine receptor D5	0.043	1.030	7.20E-01	7.20E+01		
7905028	CR541728	LSP1	lymphocyte-specific protein 1	0.052	1.037	7.94E-01	7.94E+01		
7905035	---	---	---	-0.018	0.988	8.86E-01	8.86E+01		
7905037	---	---	---	-0.046	0.968	7.97E-01	7.97E+01		
7905043	NR_027002	LOC388692	hypothetical LOC388692	-0.031	0.979	8.44E-01	8.44E+01		
7905051	NM_00114388	PPIAL4B	peptidylprolyl isomerase A (cyclop	0.032	1.022	8.41E-01	8.41E+01		
7905054	---	---	---	-0.311	0.806	1.28E-01	1.28E+01		
7905058	NR_027002	LOC388692	hypothetical LOC388692	0.027	1.019	9.08E-01	9.08E+01		
7905067	NM_003548	HIST2H4A	histone cluster 2, H4a	-0.111	0.926	6.16E-01	6.16E+01		
7905079	NM_003516	HIST2H2AA3	histone cluster 2, H2aa3	-0.194	0.874	2.82E-01	2.82E+01		
7905085	NM_00100546	HIST2H3A	histone cluster 2, H3a	-0.052	0.965	6.82E-01	6.82E+01		
7905088	NM_003517	HIST2H2AC	histone cluster 2, H2ac	-0.022	0.985	9.06E-01	9.06E+01		
7905092	NM_016074	BOLA1	bolA homolog 1 (E. coli)	-0.077	0.948	4.51E-01	4.51E+01		
7905099	NM_007259	VPS45	vacuolar protein sorting 45 homol	0.105	1.075	6.04E-01	6.04E+01		
7905116	NM_016274	PLEKHO1	pleckstrin homology domain conta	0.162	1.119	3.47E-01	3.47E+01		
7905127	---	---	---	0.013	1.009	9.63E-01	9.63E+01		
7905163	NM_018997	MRPS21	mitochondrial ribosomal protein S	-0.023	0.984	9.31E-01	9.31E+01		
7905171	NM_004698	PRPF3	PRP3 pre-mRNA processing factor	-0.035	0.976	7.56E-01	7.56E+01		
7905185	NM_015203	RPRD2	regulation of nuclear pre-mRNA d	-0.121	0.920	1.45E-01	1.45E+01		
7905198	NM_025150	TARS2	threonyl-tRNA synthetase 2, mito	0.023	1.016	8.80E-01	8.80E+01		
7905220	NM_004425	ECM1	extracellular matrix protein 1	0.080	1.057	5.44E-01	5.44E+01		
7905233	NM_019032	ADAMTSL4	ADAMTS-like 4	0.015	1.011	9.34E-01	9.34E+01		
7905258	NM_00114541	SETDB1	SET domain, bifurcated 1	0.041	1.029	7.87E-01	7.87E+01		
7905283	NM_003568	ANXA9	annexin A9	-0.071	0.952	4.67E-01	4.67E+01		
7905299	NM_021222	PRUNE	prune homolog (Drosophila)	-0.109	0.927	5.04E-01	5.04E+01		
7905308	NM_00115964	BNIPL	BCL2/adenovirus E1B 19kD intera	-0.034	0.977	7.90E-01	7.90E+01		
7905324	BC002469	C1orf56	chromosome 1 open reading fram	-0.043	0.970	6.05E-01	6.05E+01		
7905329	NM_006818	MLLT11	myeloid/lymphoid or mixed-lineag	0.019	1.013	9.57E-01	9.57E+01		
7905339	NM_144618	GABPB2	GA binding protein transcription f	-0.047	0.968	8.13E-01	8.13E+01		
7905349	NM_024575	TNFAIP8L2	tumor necrosis factor, alpha-induc	0.068	1.048	6.36E-01	6.36E+01		
7905355	NM_024041	SCNM1	sodium channel modifier 1	0.039	1.028	8.04E-01	8.04E+01		
7905365	NM_00113563	PIP5K1A	phosphatidylinositol-4-phosphate	-0.026	0.982	8.73E-01	8.73E+01		
7905374	NM_002810	PSMD4	proteasome (prosome, macropain	0.053	1.037	7.51E-01	7.51E+01		

7905382	NM_020832	ZNF687	zinc finger protein 687	0.008	1.006	9.65E-01	9.65E+01		
7905395	NM_002796	PSMB4	proteasome (prosome, macropain	-0.060	0.959	6.33E-01	6.33E+01		
7905406	NM_020770	CGN	cingulin	-0.042	0.971	6.20E-01	6.20E+01		
7905444	NM_030918	SNX27	sorting nexin family member 27	0.105	1.075	4.62E-01	4.62E+01		
7905483	NM_178438	LCE5A	late cornified envelope 5A	-0.058	0.961	5.17E-01	5.17E+01		
7905490	NM_178434	LCE3C	late cornified envelope 3C	0.040	1.028	8.71E-01	8.71E+01		
7905492	NM_178433	LCE3B	late cornified envelope 3B	0.009	1.006	9.59E-01	9.59E+01		
7905500	NM_178430	LCE2D	late cornified envelope 2D	-0.065	0.956	6.00E-01	6.00E+01		
7905505	NM_014357	LCE2B	late cornified envelope 2B	-0.036	0.975	7.76E-01	7.76E+01		
7905507	NM_178428	LCE2A	late cornified envelope 2A	-0.023	0.984	8.53E-01	8.53E+01		
7905510	NM_178356	LCE4A	late cornified envelope 4A	-0.027	0.981	8.79E-01	8.79E+01		
7905512	NM_00102467	C1orf68	chromosome 1 open reading fram	-0.013	0.991	9.41E-01	9.41E+01		
7905517	NM_178354	LCE1F	late cornified envelope 1F	-0.071	0.952	4.15E-01	4.15E+01		
7905519	NM_178353	LCE1E	late cornified envelope 1E	-0.021	0.986	8.98E-01	8.98E+01		
7905523	NM_178352	LCE1D	late cornified envelope 1D	-0.054	0.963	6.44E-01	6.44E+01		
7905528	NM_178348	LCE1A	late cornified envelope 1A	-0.040	0.972	6.91E-01	6.91E+01		
7905536	NM_173080	SPRR4	small proline-rich protein 4	-0.085	0.943	3.79E-01	3.79E+01		
7905544	NM_005987	SPRR1A	small proline-rich protein 1A	-0.066	0.956	5.79E-01	5.79E+01		
7905548	NM_005416	SPRR3	small proline-rich protein 3	0.004	1.003	9.81E-01	9.81E+01		
7905553	NM_003125	SPRR1B	small proline-rich protein 1B (corn	0.035	1.024	7.87E-01	7.87E+01		
7905559	NM_00101085	LELP1	late cornified envelope-like prolin	-0.005	0.996	9.77E-01	9.77E+01		
7905571	NM_002965	S100A9	S100 calcium binding protein A9	-0.294	0.816	4.49E-01	4.49E+01		
7905581	NM_006271	S100A1	S100 calcium binding protein A1	-0.065	0.956	7.33E-01	7.33E+01		
7905589	ENST00000368	C1orf77	chromosome 1 open reading fram	-0.059	0.960	5.52E-01	5.52E+01		
7905598	NM_012437	SNAPIN	SNAP-associated protein	0.041	1.029	8.32E-01	8.32E+01		
7905631	NM_023015	INTS3	integrator complex subunit 3	0.038	1.027	7.97E-01	7.97E+01		
7905664	NM_024330	SLC27A3	solute carrier family 27 (fatty acid	0.041	1.029	7.97E-01	7.97E+01		
7905677	NM_130898	CREB3L4	cAMP responsive element binding	0.054	1.038	6.27E-01	6.27E+01		
7905691	NM_001030	RPS27	ribosomal protein S27	-0.026	0.982	8.85E-01	8.85E+01		
7905700	NM_014847	UBAP2L	ubiquitin associated protein 2-like	0.039	1.027	6.70E-01	6.70E+01		
7905733	NM_006118	HAX1	HCLS1 associated protein X-1	0.000	1.000	1.00E+00	1.00E+02		
7905754	NM_020452	ATP8B2	ATPase, class I, type 8B, member 2	-0.050	0.966	7.52E-01	7.52E+01		
7905789	NM_000565	IL6R	interleukin 6 receptor	0.139	1.101	1.40E-01	1.40E+01		
7905817	NM_000748	CHRNB2	cholinergic receptor, nicotinic, bet	0.003	1.002	9.86E-01	9.86E+01		
7905826	NR_024163	CKS1B	CDC28 protein kinase regulatory s	0.092	1.066	5.72E-01	5.72E+01		
7905831	NM_025207	FLAD1	FAD1 flavin adenine dinucleotide s	0.016	1.011	9.27E-01	9.27E+01		
7905848	NM_018655	LENEP	lens epithelial protein	-0.008	0.994	9.67E-01	9.67E+01		
7905854	NM_015872	ZBTB7B	zinc finger and BTB domain contai	0.068	1.048	5.25E-01	5.25E+01		
7905862	NM_152494	DCST1	DC-STAMP domain containing 1	-0.010	0.993	9.61E-01	9.61E+01		
7905881	NM_207196	ADAM15	ADAM metallopeptidase domain 1	-0.055	0.962	5.10E-01	5.10E+01		
7905938	NM_018845	RAG1AP1	recombination activating gene 1 a	0.128	1.093	3.04E-01	3.04E+01		
7905951	NM_025058	TRIM46	tripartite motif-containing 46	-0.008	0.994	9.56E-01	9.56E+01		
7905968	NM_002455	MTX1	metaxin 1	0.052	1.037	7.40E-01	7.40E+01		
7905974	NM_020897	HCN3	hyperpolarization activated cyclic	0.000	1.000	1.00E+00	1.00E+02		
7905986	NM_002004	FDPS	farnesyl diphosphate synthase (far	-0.066	0.956	6.75E-01	6.75E+01		
7905999	NM_00110520	RUSC1	RUN and SH3 domain containing 1	-0.038	0.974	7.59E-01	7.59E+01		
7906015	---	---	---	0.298	1.230	1.16E-01	1.16E+01		
7906017	AF268613	POU5F1P4	POU class 5 homeobox 1 pseudoge	0.002	1.001	9.94E-01	9.94E+01		
7906021	NM_018116	MSTO1	misato homolog 1 (Drosophila)	-0.023	0.984	9.03E-01	9.03E+01		
7906041	NM_033657	DAP3	death associated protein 3	0.010	1.007	9.62E-01	9.62E+01		
7906056	NR_024117	MSTO2P	misato homolog 2 pseudogene	0.005	1.003	9.86E-01	9.86E+01		
7906061	NM_152280	SYT11	synaptotagmin XI	-0.087	0.942	7.00E-01	7.00E+01		
7906069	NM_181885	RXFP4	relaxin/insulin-like family peptide	0.017	1.012	9.19E-01	9.19E+01		
7906072	NM_014017	ROBLD3	roadblock domain containing 3	0.107	1.077	5.35E-01	5.35E+01		
7906085	NM_170707	LMNA	lamin A/C	0.059	1.042	6.70E-01	6.70E+01		
7906107	NM_022367	SEMA4A	sema domain, immunoglobulin do	0.152	1.111	5.09E-01	5.09E+01		
7906128	NM_014655	SLC25A44	solute carrier family 25, member 4	0.020	1.014	9.44E-01	9.44E+01		
7906133	NM_007221	PMF1	polyamine-modulated factor 1	-0.022	0.985	8.72E-01	8.72E+01		
7906140	NM_199173	BGLAP	bone gamma-carboxyglutamate (g	-0.016	0.989	9.40E-01	9.40E+01		
7906146	NR_026678	TMEM79	transmembrane protein 79	-0.053	0.964	5.90E-01	5.90E+01		
7906163	NR_026549	RHBG	Rh family, B glycoprotein (gene/ps	-0.041	0.972	7.28E-01	7.28E+01		
7906177	NM_00110566	TTC24	tetratricopeptide repeat domain 2	-0.100	0.933	2.00E-01	2.00E+01		
7906185	NM_144772	APOA1BP	apolipoprotein A-I binding protein	-0.012	0.992	9.64E-01	9.64E+01		
7906194	AK055377	GPATCH4	G patch domain containing 4	-0.058	0.961	7.80E-01	7.80E+01		
7906197	NM_021817	HAPLN2	hyaluronan and proteoglycan link	-0.058	0.961	4.32E-01	4.32E+01		
7906205	NM_021948	BCAN	brevican	0.004	1.003	9.86E-01	9.86E+01		
7906223	NM_015997	C1orf66	chromosome 1 open reading fram	0.049	1.034	6.57E-01	6.57E+01		
7906235	NM_005973	PRCC	papillary renal cell carcinoma (tran	-0.013	0.991	9.36E-01	9.36E+01		
7906244	NM_00100779	NTRK1	neurotrophic tyrosine kinase, rece	-0.022	0.985	7.91E-01	7.91E+01		
7906264	NM_00108047	PEAR1	platelet endothelial aggregation re	0.002	1.001	9.94E-01	9.94E+01		
7906284	NM_144702	C1orf92	chromosome 1 open reading fram	-0.020	0.986	8.84E-01	8.84E+01		
7906303	---	---	---	0.108	1.078	4.20E-01	4.20E+01		
7906305	---	---	---	0.031	1.022	9.43E-01	9.43E+01		
7906307	NM_018240	KIRREL	kin of IRRE like (Drosophila)	0.004	1.003	9.83E-01	9.83E+01		
7906330	NM_001766	CD1D	CD1d molecule	0.032	1.023	7.30E-01	7.30E+01		
7906348	NM_001765	CD1C	CD1c molecule	0.344	1.269	2.22E-01	2.22E+01		
7906386	NM_152501	PYHIN1	pyrin and HIN domain family, men	0.142	1.104	6.57E-01	6.57E+01		

7906400	NM_005531	IFI16	interferon, gamma-inducible prote	0.134	1.097	7.91E-02	7.91E+00		
7906417	NM_021189	CADM3	cell adhesion molecule 3	-0.041	0.972	7.89E-01	7.89E+01		
7906433	---	---	---	-0.021	0.986	9.53E-01	9.53E+01		
7906435	NM_002036	DARC	Duffy blood group, chemokine rec	0.007	1.005	9.66E-01	9.66E+01		
7906467	---	---	---	-0.041	0.972	7.92E-01	7.92E+01		
7906469	NM_017823	DUSP23	dual specificity phosphatase 23	0.081	1.058	3.24E-01	3.24E+01		
7906475	NM_00100431	FCRL6	Fc receptor-like 6	0.059	1.042	8.69E-01	8.69E+01		
7906496	NM_004983	KCNJ9	potassium inwardly-rectifying char	-0.029	0.980	8.61E-01	8.61E+01		
7906552	NM_001231	CASQ1	calsequestrin 1 (fast-twitch, skelet	-0.005	0.997	9.85E-01	9.85E+01		
7906576	NM_015331	NCSTN	nicastrin	0.140	1.102	2.14E-01	2.14E+01		
7906613	NM_021181	SLAMF7	SLAM family member 7	0.248	1.187	6.05E-01	6.05E+01		
7906622	NM_002348	LY9	lymphocyte antigen 9	0.061	1.043	7.07E-01	7.07E+01		
7906652	NM_005600	NIT1	nitrilase 1	0.016	1.011	9.53E-01	9.53E+01		
7906662	NM_016406	UFC1	ubiquitin-fold modifier conjugating	0.005	1.003	9.80E-01	9.80E+01		
7906671	NM_00101444	USP21	ubiquitin specific peptidase 21	-0.019	0.987	9.16E-01	9.16E+01		
7906703	NM_004550	NDUFS2	NADH dehydrogenase (ubiquinone	0.021	1.015	8.96E-01	8.96E+01		
7906720	NM_004106	FCER1G	Fc fragment of IgE, high affinity I, r	0.154	1.113	7.62E-01	7.62E+01		
7906728	NM_032174	TOMM40L	translocase of outer mitochondria	-0.024	0.984	8.62E-01	8.62E+01		
7906742	NM_00110256	PCP4L1	Purkinje cell protein 4 like 1	-0.049	0.967	7.10E-01	7.10E+01		
7906746	NM_003001	SDHC	succinate dehydrogenase complex	0.002	1.001	9.95E-01	9.95E+01		
7906753	---	---	---	0.022	1.015	9.64E-01	9.64E+01		
7906757	NM_00113621	FCGR2A	Fc fragment of IgG, low affinity IIa,	0.386	1.307	5.44E-01	5.44E+01		
7906764	NM_002155	HSPA6	heat shock 70kDa protein 6 (HSP7	-0.206	0.867	1.56E-01	1.56E+01		
7906767	NM_201563	FCGR2C	Fc fragment of IgG, low affinity IIc,	0.391	1.311	2.57E-01	2.57E+01		
7906775	NR_024151	HSPA7	heat shock 70kDa protein 7 (HSP7	-0.113	0.925	6.44E-01	6.44E+01		
7906777	NM_004001	FCGR2B	Fc fragment of IgG, low affinity IIb	0.231	1.174	2.74E-01	2.74E+01		
7906786	NM_032738	FCRLA	Fc receptor-like A	0.138	1.100	4.60E-01	4.60E+01		
7906797	NM_00100290	FCRLB	Fc receptor-like B	0.015	1.011	9.40E-01	9.40E+01		
7906810	NM_007240	DUSP12	dual specificity phosphatase 12	-0.070	0.953	5.20E-01	5.20E+01		
7906819	NM_007348	ATF6	activating transcription factor 6	0.044	1.031	8.30E-01	8.30E+01		
7906838	NM_014697	NOS1AP	nitric oxide synthase 1 (neuronal)	-0.079	0.947	3.09E-01	3.09E+01		
7906852	NM_175866	UHMK1	U2AF homology motif (UHM) kina	-0.018	0.988	9.06E-01	9.06E+01		
7906863	NM_003115	UAP1	UDP-N-acteylglucosamine pyroph	-0.071	0.952	6.66E-01	6.66E+01		
7906898	---	---	---	-0.006	0.996	9.82E-01	9.82E+01		
7906904	NM_016371	HSD17B7	hydroxysteroid (17-beta) dehydro	0.007	1.005	9.84E-01	9.84E+01		
7906948	---	---	---	0.048	1.034	9.24E-01	9.24E+01		
7906954	NM_002585	PBX1	pre-B-cell leukemia homeobox 1	0.030	1.021	8.41E-01	8.41E+01		
7906969	---	---	---	0.010	1.007	9.70E-01	9.70E+01		
7906978	NM_004528	MGST3	microsomal glutathione S-transfer	-0.035	0.976	9.19E-01	9.19E+01		
7906993	---	---	---	0.047	1.033	7.08E-01	7.08E+01		
7906995	NM_012474	UCK2	uridine-cytidine kinase 2	-0.010	0.993	9.65E-01	9.65E+01		
7907008	---	---	---	-0.068	0.954	6.91E-01	6.91E+01		
7907010	---	---	---	-0.107	0.929	6.44E-01	6.44E+01		
7907024	NM_017542	POGK	pogo transposable element with K	0.087	1.062	5.26E-01	5.26E+01		
7907049	---	---	---	0.037	1.026	8.23E-01	8.23E+01		
7907058	NM_002697	POU2F1	POU class 2 homeobox 1	0.019	1.013	8.81E-01	8.81E+01		
7907079	NM_052862	RCSD1	RCSD domain containing 1	0.131	1.095	1.63E-01	1.63E+01		
7907090	AY194294	LOC100128751	INM04	0.246	1.186	1.52E-02	1.52E+00		
7907104	NM_018442	DCAF6	DDB1 and CUL4 associated factor	-0.092	0.939	2.37E-01	2.37E+01		
7907124	NM_152902	TIPRL	TIP41, TOR signaling pathway regu	-0.011	0.992	9.35E-01	9.35E+01		
7907135	NM_199344	SFT2D2	SFT2 domain containing 2	0.195	1.145	1.72E-01	1.72E+01		
7907146	NM_005149	TBX19	T-box 19	0.034	1.024	8.05E-01	8.05E+01		
7907211	---	---	---	0.018	1.013	9.20E-01	9.20E+01		
7907213	NM_152281	GORAB	golgin, RAB6-interacting	0.004	1.003	9.87E-01	9.87E+01		
7907310	NM_015172	BAT2D1	BAT2 domain containing 1	-0.086	0.942	1.16E-01	1.16E+01		
7907349	---	---	---	0.048	1.033	8.78E-01	8.78E+01		
7907351	---	---	---	0.019	1.013	9.25E-01	9.25E+01		
7907353	NM_015935	METTL13	methyltransferase like 13	-0.097	0.935	5.79E-01	5.79E+01		
7907368	---	---	---	-0.046	0.969	8.98E-01	8.98E+01		
7907370	NM_015569	DNM3	dynamin 3	-0.112	0.925	4.87E-01	4.87E+01		
7907404	NM_014283	C1orf9	chromosome 1 open reading fram	-0.049	0.967	7.45E-01	7.45E+01		
7907430	NM_000639	FASLG	Fas ligand (TNF superfamily, mem	-0.050	0.966	8.83E-01	8.83E+01		
7907439	NM_004905	PRDX6	peroxiredoxin 6	-0.103	0.931	3.10E-01	3.10E+01		
7907445	NM_014458	KLHL20	kelch-like 20 (Drosophila)	-0.041	0.972	7.89E-01	7.89E+01		
7907464	---	---	---	-0.033	0.978	9.19E-01	9.19E+01		
7907466	NM_018122	DARS2	aspartyl-tRNA synthetase 2, mitoc	0.001	1.000	9.98E-01	9.98E+01		
7907486	NM_032522	ZBTB37	zinc finger and BTB domain contai	0.086	1.062	5.71E-01	5.71E+01		
7907492	NM_014857	RABGAP1L	RAB GTPase activating protein 1-li	0.030	1.021	8.51E-01	8.51E+01		
7907531	NM_005684	GPR52	G protein-coupled receptor 52	0.130	1.094	4.43E-01	4.43E+01		
7907537	NM_014412	CACYBP	calcyclin binding protein	-0.070	0.953	4.17E-01	4.17E+01		
7907601	NM_021165	FAM5B	family with sequence similarity 5,	-0.059	0.960	7.35E-01	7.35E+01		
7907611	NM_170692	RASAL2	RAS protein activator like 2	0.308	1.238	2.17E-01	2.17E+01		
7907655	---	---	---	-0.040	0.973	9.22E-01	9.22E+01		
7907680	NM_014864	FAM20B	family with sequence similarity 20	-0.086	0.942	3.73E-01	3.73E+01		
7907690	NM_022371	TOR3A	torsin family 3, member A	0.004	1.003	9.85E-01	9.85E+01		
7907700	---	---	---	0.028	1.020	9.45E-01	9.45E+01		
7907702	NM_003101	SOAT1	sterol O-acyltransferase 1	0.173	1.127	2.41E-01	2.41E+01		

7907773	NM_015602	TOR1AIP1	torsin A interacting protein 1	0.058	1.041	6.39E-01	6.39E+01		
7907788	---	---	---	0.033	1.023	9.47E-01	9.47E+01		
7907790	NM_014810	CEP350	centrosomal protein 350kDa	-0.061	0.959	5.54E-01	5.54E+01		
7907830	NM_002826	QSOX1	quiescin Q6 sulfhydryl oxidase 1	-0.079	0.947	6.22E-01	6.22E+01		
7907847	---	---	---	-0.126	0.916	2.29E-01	2.29E+01		
7907849	NM_033343	LHX4	LIM homeobox 4	0.041	1.029	7.63E-01	7.63E+01		
7907859	---	---	---	0.160	1.117	2.10E-01	2.10E+01		
7907861	NM_004736	XPR1	xenotropic and polytropic retrovir	-0.024	0.984	8.93E-01	8.93E+01		
7907882	NM_020950	KIAA1614	KIAA1614	-0.085	0.943	1.96E-01	1.96E+01		
7907904	NM_016545	IER5	immediate early response 5	-0.056	0.962	6.87E-01	6.87E+01		
7907911	NM_000721	CACNA1E	calcium channel, voltage-depende	-0.103	0.931	5.23E-01	5.23E+01		
7907962	---	---	---	-0.018	0.988	9.24E-01	9.24E+01		
7907966	---	---	---	-0.009	0.994	9.80E-01	9.80E+01		
7907968	---	---	---	-0.043	0.971	6.57E-01	6.57E+01		
7907970	---	---	---	-0.010	0.993	9.76E-01	9.76E+01		
7908022	NM_001357	DHX9	DEAH (Asp-Glu-Ala-His) box polype	-0.047	0.968	7.45E-01	7.45E+01		
7908041	NM_002293	LAMC1	laminin, gamma 1 (formerly LAMB	-0.087	0.941	7.52E-01	7.52E+01		
7908097	NM_173156	SMG7	Smg-7 homolog, nonsense mediat	0.043	1.030	7.29E-01	7.29E+01		
7908147	NR_023349	TSEN15	tRNA splicing endonuclease 15 ho	0.013	1.009	9.56E-01	9.56E+01		
7908157	---	---	---	-0.047	0.968	6.84E-01	6.84E+01		
7908169	NM_007212	RNF2	ring finger protein 2	0.021	1.014	9.12E-01	9.12E+01		
7908328	---	---	---	-0.141	0.907	4.36E-01	4.36E+01		
7908330	AY854248	C1orf27	chromosome 1 open reading fram	0.071	1.050	7.19E-01	7.19E+01		
7908347	NM_022375	OCLM	oculomedin	0.127	1.092	5.52E-01	5.52E+01		
7908376	NM_130782	RGS18	regulator of G-protein signaling 18	0.461	1.377	1.73E-01	1.73E+01		
7908407	NM_002954	RPS27A	ribosomal protein S27a	0.008	1.006	9.35E-01	9.35E+01		
7908421	NM_004600	TROVE2	TROVE domain family, member 2	-0.055	0.963	6.92E-01	6.92E+01		
7908437	NM_024529	CDC73	cell division cycle 73, Paf1/RNA po	-0.006	0.996	9.66E-01	9.66E+01		
7908543	NM_133494	NEK7	NIMA (never in mitosis gene a)-rel	0.059	1.042	7.03E-01	7.03E+01		
7908553	NM_002838	PTPRC	protein tyrosine phosphatase, rec	0.023	1.016	8.08E-01	8.08E+01		
7908591	---	---	---	-0.014	0.990	9.53E-01	9.53E+01		
7908612	NM_00110551	FAM58B	family with sequence similarity 58	0.065	1.046	6.69E-01	6.69E+01		
7908635	---	---	---	0.017	1.012	9.20E-01	9.20E+01		
7908637	NM_005298	GPR25	G protein-coupled receptor 25	0.022	1.016	8.78E-01	8.78E+01		
7908639	NM_018265	C1orf106	chromosome 1 open reading fram	0.039	1.028	7.99E-01	7.99E+01		
7908650	NM_178275	IGFN1	immunoglobulin-like and fibronect	-0.028	0.981	8.70E-01	8.70E+01		
7908692	NR_026667	RPS10P7	ribosomal protein S10 pseudogene	-0.038	0.974	8.37E-01	8.37E+01		
7908694	NM_020443	NAV1	neuron navigator 1	-0.145	0.904	3.16E-01	3.16E+01		
7908732	NM_018085	IPO9	importin 9	0.029	1.021	8.47E-01	8.47E+01		
7908758	NM_198149	SHISA4	shisa homolog 4 (Xenopus laevis)	0.017	1.012	9.11E-01	9.11E+01		
7908766	NM_006335	TIMM17A	translocase of inner mitochondria	0.058	1.041	6.90E-01	6.90E+01		
7908777	---	---	---	-0.030	0.980	9.00E-01	9.00E+01		
7908779	NM_020216	RNPEP	arginyl aminopeptidase (aminopep	0.042	1.029	8.27E-01	8.27E+01		
7908812	NM_004767	GPR37L1	G protein-coupled receptor 37 like	-0.056	0.962	5.31E-01	5.31E+01		
7908841	NM_032105	PPP1R12B	protein phosphatase 1, regulatory	-0.037	0.975	8.15E-01	8.15E+01		
7908861	AF314543	OCR1	ovarian cancer-related protein 1	-0.043	0.971	9.19E-01	9.19E+01		
7908867	NM_138391	TMEM183A	transmembrane protein 183A	-0.004	0.997	9.86E-01	9.86E+01		
7908879	NM_015053	PPFIA4	protein tyrosine phosphatase, rec	-0.069	0.953	2.31E-01	2.31E+01		
7908907	NM_000674	ADORA1	adenosine A1 receptor	-0.019	0.987	9.00E-01	9.00E+01		
7908917	NM_006763	BTG2	BTG family, member 2	-0.081	0.946	4.16E-01	4.16E+01		
7908924	NM_002725	PRELP	proline/arginine-rich end leucine-r	-0.085	0.943	4.43E-01	4.43E+01		
7908940	NM_00100139	ATP2B4	ATPase, Ca++ transporting, plasma	-0.144	0.905	3.70E-01	3.70E+01		
7908966	BC000988	C1orf217	chromosome 1 open reading fram	0.012	1.008	9.79E-01	9.79E+01		
7908968	NM_017773	LAX1	lymphocyte transmembrane adap	-0.051	0.966	8.49E-01	8.49E+01		
7908978	NM_014827	ZC3H11A	zinc finger CCCH-type containing 1	-0.024	0.983	8.84E-01	8.84E+01		
7908988	NM_003094	SNRPE	small nuclear ribonucleoprotein p	-0.145	0.904	3.35E-01	3.35E+01		
7908993	NM_005686	SOX13	SRY (sex determining region Y)-bo	0.031	1.022	8.13E-01	8.13E+01		
7909011	NM_002393	MDM4	Mdm4 p53 binding protein homol	0.106	1.077	1.85E-01	1.85E+01		
7909027	NM_00100538	NFASC	neurofascin homolog (chicken)	0.029	1.020	7.67E-01	7.67E+01		
7909064	NM_005076	CNTN2	contactin 2 (axonal)	-0.002	0.998	9.90E-01	9.90E+01		
7909102	---	---	---	0.016	1.011	9.49E-01	9.49E+01		
7909104	NM_212503	PCTK3	PCTAIRE protein kinase 3	-0.083	0.944	5.34E-01	5.34E+01		
7909127	NM_181644	MFSD4	major facilitator superfamily doma	-0.037	0.974	7.54E-01	7.54E+01		
7909140	---	---	---	0.235	1.177	5.25E-01	5.25E+01		
7909142	NM_022731	NUCKS1	nuclear casein kinase and cyclin-d	0.134	1.098	1.55E-01	1.55E+01		
7909144	AK094426	LOC284581	hypothetical protein LOC284581	-0.031	0.979	8.48E-01	8.48E+01		
7909175	NM_015326	SRGAP2	SLIT-ROBO Rho GTPase activating	0.257	1.195	1.01E-01	1.01E+01		
7909188	NM_014002	IKBKE	inhibitor of kappa light polypeptid	-0.056	0.962	6.09E-01	6.09E+01		
7909214	NM_182663	RASSF5	Ras association (RalGDS/AF-6) dor	-0.005	0.996	9.78E-01	9.78E+01		
7909236	NM_004759	MAPKAPK2	mitogen-activated protein kinase-	-0.068	0.954	6.66E-01	6.66E+01		
7909283	---	---	---	-0.017	0.988	9.64E-01	9.64E+01		
7909332	NM_00111475	CD55	CD55 molecule, decay accelerating	-0.121	0.919	2.25E-01	2.25E+01		
7909400	NM_002389	CD46	CD46 molecule, complement regu	0.034	1.024	7.78E-01	7.78E+01		
7909418	NR_026817	LOC148696	hypothetical LOC148696	0.014	1.009	9.63E-01	9.63E+01		
7909441	NM_015714	G0S2	G0/G1switch 2	-0.210	0.864	3.12E-01	3.12E+01		
7909455	NM_025228	TRAF3IP3	TRAF3 interacting protein 3	0.234	1.176	3.92E-02	3.92E+00		
7909478	NM_014388	C1orf107	chromosome 1 open reading fram	0.001	1.001	9.96E-01	9.96E+01		

7909510	NM_018194	HHAT	hedgehog acyltransferase	0.052	1.037	7.97E-01	7.97E+01		
7909529	NM_00113622	RCOR3	REST corepressor 3	0.066	1.047	5.52E-01	5.52E+01		
7909545	NM_145759	TRAF5	TNF receptor-associated factor 5	0.067	1.048	6.88E-01	6.88E+01		
7909561	NR_026761	C1orf97	chromosome 1 open reading fram	0.045	1.032	8.63E-01	8.63E+01		
7909586	NM_006243	PPP2R5A	protein phosphatase 2, regulatory	0.037	1.026	7.81E-01	7.81E+01		
7909601	NR_004389	SNORA16B	small nucleolar RNA, H/ACA box 1	0.293	1.225	4.48E-01	4.48E+01		
7909603	NM_013349	NENF	neuron derived neurotrophic facto	0.036	1.025	8.50E-01	8.50E+01		
7909610	NM_00104061	ATF3	activating transcription factor 3	0.113	1.081	4.87E-01	4.87E+01		
7909628	NM_014053	FLVCR1	feline leukemia virus subgroup C c	-0.005	0.996	9.86E-01	9.86E+01		
7909642	NM_024749	VASH2	vasohibin 2	0.082	1.059	2.30E-01	2.30E+01		
7909661	NM_012424	RPS6KC1	ribosomal protein S6 kinase, 52kD	0.094	1.067	5.52E-01	5.52E+01		
7909689	NM_020197	SMYD2	SET and MYND domain containing	-0.086	0.942	6.22E-01	6.22E+01		
7909782	NM_016052	RRP15	ribosomal RNA processing 15 hom	0.036	1.025	9.05E-01	9.05E+01		
7909815	NM_018060	IARS2	isoleucyl-tRNA synthetase 2, mito	-0.042	0.971	7.38E-01	7.38E+01		
7909862	NM_024709	C1orf115	chromosome 1 open reading fram	-0.041	0.972	7.32E-01	7.32E+01		
7909890	NM_021958	HLX	H2.0-like homeobox	-0.082	0.945	3.44E-01	3.44E+01		
7909896	---	---	---	-0.057	0.961	5.81E-01	5.81E+01		
7909898	NM_198551	MIA3	melanoma inhibitory activity famil	-0.021	0.986	8.78E-01	8.78E+01		
7909931	NM_144695	C1orf58	chromosome 1 open reading fram	-0.027	0.981	8.88E-01	8.88E+01		
7909965	NM_152610	C1orf65	chromosome 1 open reading fram	-0.033	0.977	7.94E-01	7.94E+01		
7909967	NM_001748	CAPN2	calpain 2, (m/II) large subunit	0.070	1.050	7.34E-01	7.34E+01		
7909990	AK290103	LOC100287934	similar to hCG2042721	0.036	1.025	9.29E-01	9.29E+01		
7909992	NM_015176	FBXO28	F-box protein 28	0.116	1.084	4.01E-01	4.01E+01		
7910001	NM_003676	DEGS1	degenerative spermatocyte homo	0.068	1.048	5.76E-01	5.76E+01		
7910014	NM_014184	CNIH4	cornichon homolog 4 (Drosophila)	-0.013	0.991	9.68E-01	9.68E+01		
7910096	---	---	---	0.030	1.021	9.01E-01	9.01E+01		
7910099	NM_00113044	SRP9	signal recognition particle 9kDa	0.065	1.046	4.91E-01	4.91E+01		
7910111	NM_000120	EPHX1	epoxide hydrolase 1, microsomal (	0.069	1.049	6.38E-01	6.38E+01		
7910124	NM_002107 //	H3F3A // H3F3A	H3 histone, family 3A // H3 histor	0.100	1.072	1.59E-01	1.59E+01		
7910134	NM_031944	MIXL1	Mix1 homeobox-like 1 (Xenopus la	-0.053	0.964	7.33E-01	7.33E+01		
7910142	NM_00100366	C1orf95	chromosome 1 open reading fram	-0.048	0.967	7.36E-01	7.36E+01		
7910146	NM_000447	PSEN2	presenilin 2 (Alzheimer disease 4)	0.011	1.008	9.66E-01	9.66E+01		
7910164	NM_020247	CABC1	chaperone, ABC1 activity of bc1 co	-0.075	0.949	3.33E-01	3.33E+01		
7910198	BC007286	ZNF678	zinc finger protein 678	0.035	1.025	8.93E-01	8.93E+01		
7910200	NM_053052	SNAP47	synaptosomal-associated protein,	-0.019	0.987	8.78E-01	8.78E+01		
7910217	NM_033131	WNT3A	wingless-type MMTV integration s	-0.068	0.954	3.39E-01	3.39E+01		
7910229	NM_00102422	ARF1	ADP-ribosylation factor 1	0.046	1.033	7.64E-01	7.64E+01		
7910241	NM_000858	GUK1	guanylate kinase 1	-0.001	1.000	9.98E-01	9.98E+01		
7910257	NM_020435	GJC2	gap junction protein, gamma 2, 47	-0.076	0.949	3.95E-01	3.95E+01		
7910261	NM_00101086	C1orf69	chromosome 1 open reading fram	0.010	1.007	9.61E-01	9.61E+01		
7910265	NM_00109862	OBSCN	obscurin, cytoskeletal calmodulin	-0.004	0.997	9.75E-01	9.75E+01		
7910372	NM_00101085	RNF187	ring finger protein 187	0.024	1.017	8.56E-01	8.56E+01		
7910377	---	---	---	0.006	1.004	9.93E-01	9.93E+01		
7910385	---	---	---	-0.004	0.997	9.90E-01	9.90E+01		
7910387	NM_021205	RHOU	ras homolog gene family, member	-0.052	0.964	8.96E-01	8.96E+01		
7910398	NM_004578	RAB4A	RAB4A, member RAS oncogene fa	0.142	1.104	3.16E-01	3.16E+01		
7910414	---	---	---	0.025	1.017	9.27E-01	9.27E+01		
7910416	NM_014777	URB2	URB2 ribosome biogenesis 2 homo	-0.004	0.997	9.85E-01	9.85E+01		
7910427	NM_004481	GALNT2	UDP-N-acetyl-alpha-D-galactosam	-0.124	0.917	2.21E-01	2.21E+01		
7910446	NM_007357	COG2	component of oligomeric golgi co	-0.013	0.991	9.61E-01	9.61E+01		
7910492	---	---	---	0.037	1.026	9.02E-01	9.02E+01		
7910494	NM_022786	ARV1	ARV1 homolog (S. cerevisiae)	0.140	1.102	2.99E-01	2.99E+01		
7910503	NM_00100434	TRIM67	tripartite motif-containing 67	-0.048	0.967	6.14E-01	6.14E+01		
7910520	NM_014236	GNPAT	glyceronephosphate O-acyltransfe	0.034	1.024	7.94E-01	7.94E+01		
7910539	NM_00101098	C1orf124	chromosome 1 open reading fram	-0.076	0.948	5.74E-01	5.74E+01		
7910550	NM_005999	TSNAX	translin-associated factor X	0.040	1.028	8.33E-01	8.33E+01		
7910559	NM_018662	DISC1	disrupted in schizophrenia 1	0.117	1.084	3.28E-02	3.28E+00		
7910585	---	---	---	0.021	1.015	8.70E-01	8.70E+01		
7910589	NM_019090	KIAA1383	KIAA1383	0.103	1.074	1.75E-01	1.75E+01		
7910591	NM_032324	C1orf57	chromosome 1 open reading fram	-0.003	0.998	9.92E-01	9.92E+01		
7910611	NM_002245	KCNK1	potassium channel, subfamily K, m	0.072	1.051	3.09E-01	3.09E+01		
7910618	NM_173508	SLC35F3	solute carrier family 35, member F	-0.030	0.979	7.77E-01	7.77E+01		
7910630	BC116455	C1orf31	chromosome 1 open reading fram	0.182	1.134	5.86E-02	5.86E+00		
7910636	---	---	---	-0.116	0.923	6.34E-01	6.34E+01		
7910638	---	---	---	0.020	1.014	9.12E-01	9.12E+01		
7910640	NM_004837	GGPS1	geranylgeranyl diphosphate synth	0.003	1.002	9.88E-01	9.88E+01		
7910676	---	---	---	0.012	1.008	9.54E-01	9.54E+01		
7910680	NM_003272	GPR137B	G protein-coupled receptor 137B	-0.087	0.942	7.70E-01	7.70E+01		
7910694	NM_080738	EDARADD	EDAR-associated death domain	-0.051	0.966	7.51E-01	7.51E+01		
7910706	NM_006499	LGALS8	lectin, galactoside-binding, soluble	-0.143	0.906	9.39E-02	9.39E+00		
7910752	NM_000254	MTR	5-methyltetrahydrofolate-homocy	0.033	1.023	8.46E-01	8.46E+01		
7910790	---	---	---	0.029	1.020	8.78E-01	8.78E+01		
7910897	---	---	---	-0.043	0.970	7.38E-01	7.38E+01		
7910899	---	---	---	-0.109	0.928	2.34E-01	2.34E+01		
7910901	NR_027247	LOC100130331	actin, gamma-like	0.065	1.046	6.57E-01	6.57E+01		
7910913	---	---	---	0.027	1.019	8.06E-01	8.06E+01		
7911017	NM_006642	SDCCAG8	serologically defined colon cancer	-0.062	0.958	7.25E-01	7.25E+01		

7911038	NM_205768	ZNF238	zinc finger protein 238	0.005	1.004	9.88E-01	9.88E+01		
7911047	---	---	---	-0.009	0.994	9.64E-01	9.64E+01		
7911078	NM_016076	PPPDE1	PPPDE peptidase domain containi	-0.037	0.975	8.57E-01	8.57E+01		
7911085	NM_198076	FAM36A	family with sequence similarity 36	-0.026	0.982	8.32E-01	8.32E+01		
7911092	---	---	---	-0.003	0.998	9.89E-01	9.89E+01		
7911108	---	---	---	-0.022	0.985	9.52E-01	9.52E+01		
7911110	---	---	---	0.082	1.058	6.08E-01	6.08E+01		
7911114	NM_018012	KIF26B	kinesin family member 26B	-0.002	0.999	9.91E-01	9.91E+01		
7911136	---	---	---	-0.067	0.955	9.24E-01	9.24E+01		
7911138	NM_152609	C1orf71	chromosome 1 open reading fram	0.032	1.022	8.69E-01	8.69E+01		
7911155	NM_016002	SCCPDH	saccharopine dehydrogenase (put	0.044	1.031	8.70E-01	8.70E+01		
7911176	---	---	---	-0.164	0.893	5.98E-01	5.98E+01		
7911218	NM_015431	TRIM58	tripartite motif-containing 58	0.004	1.003	9.81E-01	9.81E+01		
7911229	NM_00100195	OR2W3	olfactory receptor, family 2, subfa	-0.026	0.982	8.86E-01	8.86E+01		
7911231	NM_00100195	OR2W3	olfactory receptor, family 2, subfa	-0.026	0.982	8.86E-01	8.86E+01		
7911241	NM_00100196	OR2L8	olfactory receptor, family 2, subfa	0.223	1.167	3.94E-01	3.94E+01		
7911263	NM_00100469	OR2M5	olfactory receptor, family 2, subfa	0.020	1.014	9.25E-01	9.25E+01		
7911278	NM_030904	OR2T1	olfactory receptor, family 2, subfa	-0.036	0.975	8.38E-01	8.38E+01		
7911283	NM_00100549	OR2T3	olfactory receptor, family 2, subfa	0.093	1.067	5.20E-01	5.20E+01		
7911289	NM_024836	ZNF672	zinc finger protein 672	0.051	1.036	6.34E-01	6.34E+01		
7911307	NR_024528	RPL23AP7	ribosomal protein L23a pseudoger	0.016	1.011	9.33E-01	9.33E+01		
7911309	NR_024540	WASH5P	WAS protein family homolog 5 pse	-0.014	0.990	9.07E-01	9.07E+01		
7911329	NM_207366	40435	septin 14	-0.026	0.982	9.22E-01	9.22E+01		
7911331	---	---	---	0.267	1.203	4.25E-01	4.25E+01		
7911333	NR_024528	RPL23AP7	ribosomal protein L23a pseudoger	0.012	1.008	9.49E-01	9.49E+01		
7911335	---	---	---	0.060	1.043	8.78E-01	8.78E+01		
7911337	---	---	---	0.189	1.140	7.09E-01	7.09E+01		
7911339	---	---	---	0.288	1.221	4.48E-01	4.48E+01		
7911341	---	---	---	0.087	1.062	8.03E-01	8.03E+01		
7911343	AF284753	UIMC1	ubiquitin interaction motif contair	0.033	1.023	8.45E-01	8.45E+01		
7911347	NM_207366	40435	septin 14	-0.030	0.979	9.19E-01	9.19E+01		
7911351	NR_024321	NCRNA00115	non-protein coding RNA 115	-0.104	0.931	3.00E-01	3.00E+01		
7911355	NM_177987	RP11-631M21.2	tubulin, beta 8	-0.089	0.940	3.56E-01	3.56E+01		
7911359	NM_015658	NOC2L	nucleolar complex associated 2 ho	0.005	1.004	9.84E-01	9.84E+01		
7911371	NR_027693	C1orf170	chromosome 1 open reading fram	0.007	1.005	9.67E-01	9.67E+01		
7911376	NM_00114246	HES4	hairy and enhancer of split 4 (Dros	-0.001	0.999	9.94E-01	9.94E+01		
7911383	NM_017891	C1orf159	chromosome 1 open reading fram	-0.035	0.976	8.05E-01	8.05E+01		
7911403	NM_004195	TNFRSF18	tumor necrosis factor receptor sup	-0.049	0.967	6.70E-01	6.70E+01		
7911413	NM_003327	TNFRSF4	tumor necrosis factor receptor sup	0.005	1.004	9.80E-01	9.80E+01		
7911422	NM_016547	SDF4	stromal cell derived factor 4	-0.067	0.955	5.01E-01	5.01E+01		
7911435	NM_00101498	FAM132A	family with sequence similarity 13	-0.081	0.945	3.56E-01	3.56E+01		
7911444	NM_194458	UBE2J2	ubiquitin-conjugating enzyme E2,	-0.029	0.980	8.46E-01	8.46E+01		
7911458	NM_030649	ACAP3	ArfGAP with coiled-coil, ankyrin re	-0.005	0.996	9.74E-01	9.74E+01		
7911486	NM_017871	CPSF3L	cleavage and polyadenylation spec	-0.062	0.958	6.23E-01	6.23E+01		
7911506	NM_004421	DVL1	dishevelled, dsh homolog 1 (Droso	-0.007	0.995	9.64E-01	9.64E+01		
7911529	NM_032348	MXRA8	matrix-remodelling associated 8	-0.132	0.913	9.26E-02	9.26E+00		
7911532	NM_017900	AURKAIP1	aurora kinase A interacting protei	-0.003	0.998	9.88E-01	9.88E+01		
7911539	NM_00103957	CCNL2	cyclin L2	-0.023	0.984	8.83E-01	8.83E+01		
7911559	NM_017971	MRPL20	mitochondrial ribosomal protein L	0.023	1.016	8.95E-01	8.95E+01		
7911566	---	---	---	0.147	1.107	4.51E-01	4.51E+01		
7911568	NM_014188	SSU72	SSU72 RNA polymerase II CTD pho	-0.038	0.974	7.94E-01	7.94E+01		
7911578	NM_024011	CDC2L2	cell division cycle 2-like 2 (PITSLRE	-0.110	0.927	2.78E-01	2.78E+01		
7911591	NM_00111078	RP11-345P4.4	similar to solute carrier family 35,	0.007	1.005	9.68E-01	9.68E+01		
7911600	NM_023018	NADK	NAD kinase	-0.028	0.981	8.68E-01	8.68E+01		
7911619	NM_002074	GNB1	guanine nucleotide binding protei	0.102	1.073	2.57E-01	2.57E+01		
7911634	NM_178545	TMEM52	transmembrane protein 52	-0.152	0.900	7.35E-03	7.35E-01		
7911657	AK094642	hCG_1647286	hypothetical protein LOC728690	0.043	1.030	7.53E-01	7.53E+01		
7911676	---	---	---	-0.032	0.978	8.36E-01	8.36E+01		
7911681	---	---	---	0.065	1.046	6.09E-01	6.09E+01		
7911683	NM_182533	C1orf86	chromosome 1 open reading fram	-0.017	0.989	8.94E-01	8.94E+01		
7911699	AK024162	FLJ14100	hypothetical protein FLJ14100	0.011	1.008	9.64E-01	9.64E+01		
7911701	NM_024848	MORN1	MORN repeat containing 1	-0.024	0.984	8.61E-01	8.61E+01		
7911718	NR_024489	LOC100129534	small nuclear ribonucleoprotein pe	-0.128	0.915	2.11E-01	2.11E+01		
7911720	NM_153818	PEX10	peroxisomal biogenesis factor 10	-0.059	0.960	6.49E-01	6.49E+01		
7911730	NM_018216	PANK4	pantothenate kinase 4	-0.031	0.979	8.02E-01	8.02E+01		
7911750	NM_00101092	HES5	hairy and enhancer of split 5 (Dros	-0.051	0.965	5.95E-01	5.95E+01		
7911754	NM_003820	TNFRSF14	tumor necrosis factor receptor sup	0.011	1.008	9.56E-01	9.56E+01		
7911793	NR_015440	FLJ42875	hypothetical LOC440556	0.013	1.009	9.75E-01	9.75E+01		
7911796	NR_024371	FLJ42875	hypothetical LOC440556	0.041	1.028	7.93E-01	7.93E+01		
7911799	NM_001409	MEGF6	multiple EGF-like-domains 6	-0.052	0.965	5.78E-01	5.78E+01		
7911854	NM_207306	KIAA0495	KIAA0495	-0.020	0.986	9.55E-01	9.55E+01		
7911862	NM_020710	LRRC47	leucine rich repeat containing 47	-0.042	0.971	7.58E-01	7.58E+01		
7911870	NM_014704	KIAA0562	KIAA0562	-0.093	0.938	1.34E-01	1.34E+01		
7911897	NM_207356	C1orf174	chromosome 1 open reading fram	-0.083	0.944	5.86E-01	5.86E+01		
7911902	NM_015102	NPHP4	nephronophthisis 4	0.011	1.007	9.45E-01	9.45E+01		
7911941	NM_015557	CHD5	chromodomain helicase DNA bind	0.000	1.000	9.99E-01	9.99E+01		
7911989	NM_000983	RPL22	ribosomal protein L22	0.116	1.084	5.72E-01	5.72E+01		

7911993	NM_012405	ICMT	isoprenylcysteine carboxyl methyl	-0.084	0.943	3.47E-01	3.47E+01		
7912004	NM_207370	GPR153	G protein-coupled receptor 153	-0.043	0.971	6.47E-01	6.47E+01		
7912012	NM_007274	ACOT7	acyl-CoA thioesterase 7	-0.029	0.980	7.23E-01	7.23E+01		
7912031	NM_019089	HES2	hairy and enhancer of split 2 (Dros	-0.102	0.931	2.16E-01	2.16E+01		
7912040	NM_148965	TNFRSF25	tumor necrosis factor receptor sup	-0.055	0.963	5.78E-01	5.78E+01		
7912056	NM_198681	PLEKHG5	pleckstrin homology domain conta	-0.009	0.994	9.64E-01	9.64E+01		
7912086	NM_024654	NOL9	nucleolar protein 9	0.078	1.055	7.24E-01	7.24E+01		
7912112	NM_018198	DNAJC11	DnaJ (Hsp40) homolog, subfamily	0.086	1.061	4.02E-01	4.02E+01		
7912133	ENST00000439	LOC100129476	hypothetical protein LOC1001294	0.003	1.002	9.87E-01	9.87E+01		
7912145	NM_001561	TNFRSF9	tumor necrosis factor receptor sup	-0.295	0.815	4.37E-01	4.37E+01		
7912155	---	---	---	0.025	1.018	9.53E-01	9.53E+01		
7912162	---	---	---	0.020	1.014	8.75E-01	8.75E+01		
7912166	NM_012102	RERE	arginine-glutamic acid dipeptide (	0.112	1.081	7.26E-02	7.26E+00		
7912194	BC029383	DKFZp566H082	hypothetical LOC54744	0.172	1.127	1.84E-01	1.84E+01		
7912196	---	---	---	0.041	1.029	9.35E-01	9.35E+01		
7912198	NM_001428	ENO1	enolase 1, (alpha)	-0.051	0.965	8.53E-01	8.53E+01		
7912211	NM_207420	SLC2A7	solute carrier family 2 (facilitated	0.004	1.003	9.85E-01	9.85E+01		
7912224	NM_003039	SLC2A5	solute carrier family 2 (facilitated	0.160	1.117	4.89E-02	4.89E+00		
7912239	NM_024980	GPR157	G protein-coupled receptor 157	-0.035	0.976	7.88E-01	7.88E+01		
7912250	EF592573	MIR34A	microRNA 34a	-0.015	0.990	9.15E-01	9.15E+01		
7912252	---	---	---	0.050	1.036	7.97E-01	7.97E+01		
7912254	NR_027045	C1orf200	chromosome 1 open reading fram	0.028	1.020	8.41E-01	8.41E+01		
7912257	NM_00100956	CLSTN1	calsyntenin 1	0.021	1.015	8.87E-01	8.87E+01		
7912283	NM_020248	CTNNBIP1	catenin, beta interacting protein 1	-0.027	0.982	8.27E-01	8.27E+01		
7912292	NM_032368	LZIC	leucine zipper and CTNNBIP1 dom	0.064	1.045	7.43E-01	7.43E+01		
7912303	NM_213566	DFFA	DNA fragmentation factor, 45kDa,	-0.083	0.944	6.04E-01	6.04E+01		
7912316	NM_00107984	CASZ1	castor zinc finger 1	-0.067	0.955	3.64E-01	3.64E+01		
7912343	NM_00107984	CASZ1	castor zinc finger 1	-0.072	0.951	7.04E-01	7.04E+01		
7912347	NM_00107984	CASZ1	castor zinc finger 1	-0.132	0.913	5.98E-01	5.98E+01		
7912349	BC126349	C1orf127	chromosome 1 open reading fram	-0.073	0.951	4.32E-01	4.32E+01		
7912361	NM_006610	MASP2	mannan-binding lectin serine pept	0.022	1.015	8.73E-01	8.73E+01		
7912374	NM_003132	SRM	spermidine synthase	0.042	1.030	7.73E-01	7.73E+01		
7912385	NM_00100199	EXOSC10	exosome component 10	-0.052	0.964	6.20E-01	6.20E+01		
7912412	NM_004958	MTOR	mechanistic target of rapamycin (s	-0.048	0.967	7.46E-01	7.46E+01		
7912473	NM_012168	FBXO2	F-box protein 2	-0.086	0.942	3.13E-01	3.13E+01		
7912481	NM_00112732	MAD2L2	MAD2 mitotic arrest deficient-like	-0.087	0.942	4.10E-01	4.10E+01		
7912496	NM_005957	MTHFR	5,10-methylenetetrahydrofolate r	0.087	1.062	6.11E-01	6.11E+01		
7912511	NM_138346	KIAA2013	KIAA2013	-0.132	0.912	1.90E-01	1.90E+01		
7912520	NM_002521	NPPB	natriuretic peptide precursor B	0.030	1.021	8.48E-01	8.48E+01		
7912535	---	---	---	0.236	1.177	5.97E-01	5.97E+01		
7912537	NM_004753	DHRS3	dehydrogenase/reductase (SDR fa	0.018	1.013	9.53E-01	9.53E+01		
7912585	NM_00101369	PRAMEF3	PRAME family member 3	-0.041	0.972	7.94E-01	7.94E+01		
7912591	NM_00101227	PRAMEF7	PRAME family member 7	-0.052	0.965	6.34E-01	6.34E+01		
7912606	NM_00101227	PRAMEF7	PRAME family member 7	-0.052	0.965	6.34E-01	6.34E+01		
7912622	ENST00000376	LRRC38	leucine rich repeat containing 38	-0.155	0.898	1.53E-01	1.53E+01		
7912646	NM_001229	CASP9	caspase 9, apoptosis-related cyste	-0.080	0.946	4.94E-01	4.94E+01		
7912659	NM_024758	AGMAT	agmatine ureohydrolase (agmatin	0.104	1.074	3.41E-01	3.41E+01		
7912670	NM_006004	UQCRH	ubiquinol-cytochrome c reductase	0.016	1.011	9.46E-01	9.46E+01		
7912672	NR_024279	hCG_2008140	hypothetical LOC729614	0.049	1.035	7.92E-01	7.92E+01		
7912675	NM_003443	ZBTB17	zinc finger and BTB domain contai	-0.055	0.963	6.23E-01	6.23E+01		
7912701	NM_182623	FAM131C	family with sequence similarity 13	0.117	1.085	3.91E-01	3.91E+01		
7912706	NM_004431	EPHA2	EPH receptor A2	-0.076	0.948	1.54E-01	1.54E+01		
7912724	NM_153213	ARHGEF19	Rho guanine nucleotide exchange	-0.062	0.958	4.77E-01	4.77E+01		
7912743	NM_030907	C1orf89	chromosome 1 open reading fram	0.021	1.014	9.08E-01	9.08E+01		
7912750	NM_018994	FBXO42	F-box protein 42	-0.020	0.986	9.09E-01	9.09E+01		
7912762	---	---	---	0.025	1.017	9.34E-01	9.34E+01		
7912764	NM_198546	SPATA21	spermatogenesis associated 21	-0.016	0.989	9.16E-01	9.16E+01		
7912784	NR_023386	CROCCL2	ciliary rootlet coiled-coil, rootletin	-0.012	0.992	9.53E-01	9.53E+01		
7912802	BC036435	LOC100132147	hypothetical LOC100132147	0.036	1.025	9.13E-01	9.13E+01		
7912804	BC036435	LOC100132147	hypothetical LOC100132147	-0.024	0.984	9.28E-01	9.28E+01		
7912808	NM_00110266	NBPF16	neuroblastoma breakpoint family,	-0.073	0.951	3.24E-01	3.24E+01		
7912839	NR_026752	CROCCL1	ciliary rootlet coiled-coil, rootletin	-0.106	0.929	4.52E-01	4.52E+01		
7912852	NM_001412	EIF1AX	eukaryotic translation initiation fa	-0.033	0.978	8.80E-01	8.80E+01		
7912854	NR_026567	ESPNP	espin pseudogene	-0.016	0.989	9.51E-01	9.51E+01		
7912861	ENST0000036	44634 // LOC64	similar to UPF0627 protein ENSP0	0.002	1.001	9.90E-01	9.90E+01		
7912863	NR_002729	MSTP9	macrophage stimulating, pseudog	-0.074	0.950	3.79E-01	3.79E+01		
7912884	BC126340 //	32147 // LOC10	hypothetical LOC100132147 // hy	0.003	1.002	9.90E-01	9.90E+01		
7912887	NM_002403	MFAP2	microfibrillar-associated protein 2	-0.043	0.971	7.67E-01	7.67E+01		
7912898	NM_022089	ATP13A2	ATPase type 13A2	0.023	1.016	8.73E-01	8.73E+01		
7912928	NM_003000	SDHB	succinate dehydrogenase complex	0.133	1.097	5.74E-01	5.74E+01		
7912956	NM_00113620	RCC2	regulator of chromosome conden	0.054	1.038	5.43E-01	5.43E+01		
7912968	NM_152232	TAS1R2	taste receptor, type 1, member 2	0.015	1.011	9.28E-01	9.28E+01		
7912994	NM_00113626	IFFO2	intermediate filament family orph	-0.037	0.975	8.73E-01	8.73E+01		
7913001	NM_020765	UBR4	ubiquitin protein ligase E3 compo	-0.138	0.909	2.84E-02	2.84E+00		
7913110	BC034589	KIAA0090	KIAA0090	-0.016	0.989	9.45E-01	9.45E+01		
7913146	NM_012067	AKR7A3	aldo-keto reductase family 7, mem	-0.078	0.947	5.01E-01	5.01E+01		
7913156	NM_003689	AKR7A2	aldo-keto reductase family 7, mem	0.083	1.059	2.95E-01	2.95E+01		

7913169	NM_004930	CAPZB	capping protein (actin filament) m	0.199	1.148	3.08E-02	3.08E+00		
7913185	---	---	---	-0.080	0.946	8.16E-01	8.16E+01		
7913187	NM_181719	TMCO4	transmembrane and coiled-coil do	0.008	1.006	9.63E-01	9.63E+01		
7913206	NM_019062	RNF186	ring finger protein 186	-0.078	0.947	3.41E-01	3.41E+01		
7913211	NM_014589	PLA2G2E	phospholipase A2, group IIE	0.033	1.023	8.13E-01	8.13E+01		
7913216	NM_000300	PLA2G2A	phospholipase A2, group IIA (plate	-0.063	0.957	5.94E-01	5.94E+01		
7913226	NM_012400	PLA2G2D	phospholipase A2, group IID	-0.057	0.961	4.93E-01	4.93E+01		
7913235	NM_00110557	PLA2G2C	phospholipase A2, group IIC	-0.024	0.984	8.97E-01	8.97E+01		
7913237	NM_018584	CAMK2N1	calcium/calmodulin-dependent pr	-0.260	0.835	1.62E-02	1.62E+00		
7913242	NM_024544	MUL1	mitochondrial E3 ubiquitin ligase 1	-0.093	0.937	2.27E-01	2.27E+01		
7913249	NM_032409	PINK1	PTEN induced putative kinase 1	-0.120	0.920	5.87E-01	5.87E+01		
7913252	NM_032409	PINK1	PTEN induced putative kinase 1	-0.003	0.998	9.89E-01	9.89E+01		
7913256	NM_005216	DDOST	dolichyl-diphosphooligosaccharide	0.113	1.081	2.93E-01	2.93E+01		
7913271	NM_020816	KIF17	kinesin family member 17	-0.085	0.943	3.94E-01	3.94E+01		
7913290	NM_00110316	SH2D5	SH2 domain containing 5	-0.051	0.965	6.82E-01	6.82E+01		
7913300	NM_016287	HP1BP3	heterochromatin protein 1, bindin	-0.020	0.986	8.73E-01	8.73E+01		
7913319	NM_003760	EIF4G3	eukaryotic translation initiation fa	0.089	1.064	5.19E-01	5.19E+01		
7913357	NM_001397	ECE1	endothelin converting enzyme 1	-0.178	0.884	2.81E-01	2.81E+01		
7913380	NM_032264	NBPF3	neuroblastoma breakpoint family,	-0.006	0.996	9.61E-01	9.61E+01		
7913385	NM_00114565	RAP1GAP	RAP1 GTPase activating protein	0.060	1.042	5.64E-01	5.64E+01		
7913415	NM_032236	USP48	ubiquitin specific peptidase 48	0.025	1.018	8.61E-01	8.61E+01		
7913450	NM_005529	HSPG2	heparan sulfate proteoglycan 2	-0.010	0.993	9.24E-01	9.24E+01		
7913547	NM_030761	WNT4	wingless-type MMTV integration s	-0.021	0.986	8.68E-01	8.68E+01		
7913558	NM_033631	LUZP1	leucine zipper protein 1	-0.066	0.955	5.58E-01	5.58E+01		
7913566	NM_000864	HTR1D	5-hydroxytryptamine (serotonin) r	-0.024	0.984	8.79E-01	8.79E+01		
7913571	NM_00110239	HNRNPR	heterogeneous nuclear ribonucleo	0.018	1.012	8.55E-01	8.55E+01		
7913582	NM_00107719	ZNF436	zinc finger protein 436	-0.108	0.928	1.65E-01	1.65E+01		
7913593	NM_003196	TCEA3	transcription elongation factor A (	-0.169	0.889	4.13E-01	4.13E+01		
7913609	NM_017707	ASAP3	ArfGAP with SH3 domain, ankyrin	-0.073	0.951	3.42E-01	3.42E+01		
7913644	NM_004091	E2F2	E2F transcription factor 2	0.056	1.039	5.56E-01	5.56E+01		
7913665	---	---	---	-0.042	0.971	7.81E-01	7.81E+01		
7913667	NM_000403	GALE	UDP-galactose-4-epimerase	-0.074	0.950	3.70E-01	3.70E+01		
7913682	NM_000191	HMGCL	3-hydroxymethyl-3-methylglutaryl	-0.003	0.998	9.89E-01	9.89E+01		
7913705	NM_001841	CNR2	cannabinoid receptor 2 (macropha	-0.006	0.996	9.84E-01	9.84E+01		
7913712	NM_006625	FUSIP1	FUS interacting protein (serine/arg	0.020	1.014	9.08E-01	9.08E+01		
7913776	NM_170743	IL28RA	interleukin 28 receptor, alpha (inte	0.104	1.075	2.40E-01	2.40E+01		
7913803	---	---	---	0.081	1.057	9.23E-01	9.23E+01		
7913805	NM_00103168	RUNX3	runt-related transcription factor 3	-0.229	0.853	2.58E-02	2.58E+00		
7913814	NM_015484	SYF2	SYF2 homolog, RNA splicing factor	-0.026	0.982	8.32E-01	8.32E+01		
7913824	NM_020317	C1orf63	chromosome 1 open reading fram	-0.018	0.988	9.44E-01	9.44E+01		
7913850	NM_019557	FAM54B	family with sequence similarity 54	-0.021	0.986	8.52E-01	8.52E+01		
7913864	NM_00114545	STMN1	stathmin 1	-0.010	0.993	9.53E-01	9.53E+01		
7913869	NM_203401	STMN1	stathmin 1	-0.049	0.967	8.34E-01	8.34E+01		
7913883	NM_000437	PAFAH2	platelet-activating factor acetylhy	0.027	1.019	8.78E-01	8.78E+01		
7913896	NM_00100443	SLC30A2	solute carrier family 30 (zinc trans	-0.033	0.978	8.74E-01	8.74E+01		
7913918	NM_183008	UBXN11	UBX domain protein 11	0.019	1.013	9.36E-01	9.36E+01		
7913938	NM_00103977	AIM1L	absent in melanoma 1-like	-0.012	0.992	9.24E-01	9.24E+01		
7913965	NM_00111475	ZNF683	zinc finger protein 683	-0.040	0.972	8.04E-01	8.04E+01		
7913978	NM_018066	GPN2	GPN-loop GTPase 2	-0.130	0.914	1.00E-01	1.00E+01		
7913990	NM_022078	GPATCH3	G patch domain containing 3	-0.076	0.949	4.51E-01	4.51E+01		
7914000	NM_021969	NR0B2	nuclear receptor subfamily 0, grou	-0.074	0.950	6.09E-01	6.09E+01		
7914003	BC033143	C1orf172	chromosome 1 open reading fram	-0.072	0.951	2.84E-01	2.84E+01		
7914015	NM_052943	FAM46B	family with sequence similarity 46	-0.025	0.983	8.61E-01	8.61E+01		
7914021	NM_003047	SLC9A1	solute carrier family 9 (sodium/hy	-0.035	0.976	7.87E-01	7.87E+01		
7914040	---	---	---	-0.013	0.991	9.44E-01	9.44E+01		
7914042	NM_004672	MAP3K6	mitogen-activated protein kinase k	-0.065	0.956	4.50E-01	4.50E+01		
7914075	NM_003665	FCN3	ficolin (collagen/fibrinogen domai	0.045	1.032	6.70E-01	6.70E+01		
7914084	NM_207397	CD164L2	CD164 sialomucin-like 2	0.070	1.050	4.69E-01	4.69E+01		
7914094	NM_006990	WASF2	WAS protein family, member 2	0.059	1.041	6.01E-01	6.01E+01		
7914103	NM_00102988	AHDC1	AT hook, DNA binding motif, conta	0.025	1.017	8.54E-01	8.54E+01		
7914112	NM_005248	FGR	Gardner-Rasheed feline sarcoma v	0.161	1.118	2.89E-01	2.89E+01		
7914127	NM_002038	IFI6	interferon, alpha-inducible proteir	0.270	1.206	3.04E-01	3.04E+01		
7914139	NM_014110	PPP1R8	protein phosphatase 1, regulatory	-0.074	0.950	7.91E-01	7.91E+01		
7914141	NM_002946	RPA2	replication protein A2, 32kDa	-0.045	0.969	7.77E-01	7.77E+01		
7914153	NM_001990	EYA3	eyes absent homolog 3 (Drosophil	0.015	1.011	9.56E-01	9.56E+01		
7914178	---	---	---	-0.024	0.984	9.21E-01	9.21E+01		
7914180	NM_014752	SPCS2	signal peptidase complex subunit 2	0.216	1.162	4.12E-02	4.12E+00		
7914184	NM_000952	PTAFR	platelet-activating factor receptor	-0.045	0.969	8.67E-01	8.67E+01		
7914194	NM_014280	DNAJC8	DnaJ (Hsp40) homolog, subfamily	0.002	1.002	9.88E-01	9.88E+01		
7914202	AY277594	SNHG12	small nucleolar RNA host gene 12	-0.076	0.949	7.17E-01	7.17E+01		
7914212	NR_002987	SNORA61	small nucleolar RNA, H/ACA box 6	-0.136	0.910	7.03E-01	7.03E+01		
7914214	NR_002976	SNORA44	small nucleolar RNA, H/ACA box 4	0.108	1.078	4.93E-01	4.93E+01		
7914216	NR_003035	SNORA16A	small nucleolar RNA, H/ACA box 1	-0.058	0.960	8.97E-01	8.97E+01		
7914218	NM_00113521	TAF12	TAF12 RNA polymerase II, TATA bo	-0.001	0.999	9.94E-01	9.94E+01		
7914232	NM_00100368	TMEM200B	transmembrane protein 200B	-0.036	0.975	7.27E-01	7.27E+01		
7914235	NM_005626	SFRS4	splicing factor, arginine/serine-rich	0.002	1.001	9.90E-01	9.90E+01		
7914245	NM_00102473	MECR	mitochondrial trans-2-enoyl-CoA r	0.064	1.046	5.55E-01	5.55E+01		

7914270	NM_006762	LAPTM5	lysosomal protein transmembrane	0.188	1.139	1.68E-01	1.68E+01		
7914282	NM_014654	SDC3	syndecan 3	0.215	1.160	7.90E-03	7.90E-01		
7914296	NM_00102065	PUM1	pumilio homolog 1 (Drosophila)	0.018	1.013	8.89E-01	8.89E+01		
7914326	NM_024522	NKAIN1	Na+/K+ transporting ATPase intera	-0.059	0.960	4.31E-01	4.31E+01		
7914334	NM_004814	SNRNP40	small nuclear ribonucleoprotein 40	-0.007	0.995	9.82E-01	9.82E+01		
7914342	NM_004102	FABP3	fatty acid binding protein 3, muscl	-0.014	0.991	9.64E-01	9.64E+01		
7914350	NM_178865	SERINC2	serine incorporator 2	-0.059	0.960	7.29E-01	7.29E+01		
7914354	NM_012392	PEF1	penta-EF-hand domain containing	-0.137	0.910	3.69E-01	3.69E+01		
7914361	NM_001856	COL16A1	collagen, type XVI, alpha 1	-0.058	0.960	4.27E-01	4.27E+01		
7914433	NM_001703	BAI2	brain-specific angiogenesis inhibito	0.007	1.005	9.68E-01	9.68E+01		
7914467	NM_144569	SPOCD1	SPOC domain containing 1	-0.020	0.987	9.19E-01	9.19E+01		
7914489	NM_080391	PTP4A2	protein tyrosine phosphatase type	0.057	1.040	3.09E-01	3.09E+01		
7914500	AF258548	C1orf91	chromosome 1 open reading fram	-0.075	0.949	4.29E-01	4.29E+01		
7914516	NR_026850	MTMR9L	myotubularin related protein 9-lik	-0.044	0.970	6.55E-01	6.55E+01		
7914525	NM_023009	MARCKSL1	MARCKS-like 1	-0.115	0.923	8.46E-02	8.46E+00		
7914530	NM_00114388	BSDC1	BSD domain containing 1	-0.118	0.922	1.72E-01	1.72E+01		
7914550	NM_178547	ZBTB8OS	zinc finger and BTB domain contai	-0.027	0.981	9.38E-01	9.38E+01		
7914555	---	---	---	0.141	1.102	5.67E-01	5.67E+01		
7914557	NM_030786	SYNC	syncoilin, intermediate filament pr	-0.039	0.973	8.15E-01	8.15E+01		
7914563	NM_003680	YARS	tyrosyl-tRNA synthetase	-0.109	0.927	4.48E-01	4.48E+01		
7914580	NM_153756	FNDC5	fibronectin type III domain contair	-0.076	0.949	3.89E-01	3.89E+01		
7914592	NM_033504	TMEM54	transmembrane protein 54	-0.033	0.977	7.94E-01	7.94E+01		
7914603	NM_153341	RNF19B	ring finger protein 19B	-0.241	0.846	3.69E-01	3.69E+01		
7914617	NM_013411	AK2	adenylate kinase 2	0.112	1.080	2.70E-01	2.70E+01		
7914628	---	---	---	0.122	1.089	5.15E-01	5.15E+01		
7914630	NM_018207	TRIM62	tripartite motif-containing 62	0.046	1.032	6.75E-01	6.75E+01		
7914648	NM_198040	PHC2	polyhomeotic homolog 2 (Drosoph	0.004	1.003	9.85E-01	9.85E+01		
7914665	---	---	---	-0.218	0.860	1.84E-01	1.84E+01		
7914750	BC034598	C1orf212	chromosome 1 open reading fram	0.020	1.014	9.15E-01	9.15E+01		
7914758	NM_00108041	DLGAP3	discs, large (Drosophila) homolog-	0.007	1.005	9.76E-01	9.76E+01		
7914764	NM_007167	ZMYM6	zinc finger, MYM-type 6	0.003	1.002	9.85E-01	9.85E+01		
7914791	NM_005066	SFPQ	splicing factor proline/glutamine-r	-0.038	0.974	7.49E-01	7.49E+01		
7914809	NM_024874	KIAA0319L	KIAA0319-like	-0.117	0.922	1.81E-01	1.81E+01		
7914834	NM_002794	PSMB2	proteasome (prosome, macropain	0.063	1.045	6.57E-01	6.57E+01		
7914847	BC026909	C1orf216	chromosome 1 open reading fram	-0.004	0.997	9.82E-01	9.82E+01		
7914880	NM_005202	COL8A2	collagen, type VIII, alpha 2	0.013	1.009	9.49E-01	9.49E+01		
7914887	NM_014408	TRAPPC3	trafficking protein particle comple	-0.020	0.986	9.24E-01	9.24E+01		
7914898	ENST00000312	C1orf113	chromosome 1 open reading fram	-0.037	0.975	7.75E-01	7.75E+01		
7914904	NM_032017	STK40	serine/threonine kinase 40	-0.055	0.963	7.56E-01	7.56E+01		
7914917	NM_032881	LSM10	LSM10, U7 small nuclear RNA asso	0.089	1.064	4.95E-01	4.95E+01		
7914921	---	---	---	0.021	1.015	8.97E-01	8.97E+01		
7914923	NM_145047	OSCP1	organic solute carrier partner 1	0.090	1.065	4.04E-01	4.04E+01		
7914940	NM_031280	MRPS15	mitochondrial ribosomal protein S	0.048	1.034	7.56E-01	7.56E+01		
7914974	NM_000831	GRIK3	glutamate receptor, ionotropic, ka	-0.090	0.940	2.43E-01	2.43E+01		
7914996	BX538212	MEAF6	MYST/Esa1-associated factor 6	-0.066	0.956	6.44E-01	6.44E+01		
7915008	NM_024700	SNIP1	Smad nuclear interacting protein 1	-0.058	0.961	5.48E-01	5.48E+01		
7915015	NM_013285	GNL2	guanine nucleotide binding protei	0.038	1.027	8.33E-01	8.33E+01		
7915032	NM_00103863	RSPO1	R-spondin homolog (Xenopus laev	0.027	1.019	8.42E-01	8.42E+01		
7915078	NM_173641	EPHA10	EPH receptor A10	-0.013	0.991	9.36E-01	9.36E+01		
7915084	NM_024640	YRDC	yrdC domain containing (E. coli)	-0.072	0.951	6.84E-01	6.84E+01		
7915091	NM_005955	MTF1	metal-regulatory transcription fac	-0.361	0.778	9.38E-02	9.38E+00		
7915101	NM_005540	INPP5B	inositol polyphosphate-5-phospha	-0.021	0.986	9.10E-01	9.10E+01		
7915130	NM_006802	SF3A3	splicing factor 3a, subunit 3, 60kDa	-0.014	0.990	9.53E-01	9.53E+01		
7915147	NM_004468	FHL3	four and a half LIM domains 3	-0.041	0.972	7.48E-01	7.48E+01		
7915156	NM_002699	POU3F1	POU class 3 homeobox 1	-0.054	0.964	7.47E-01	7.47E+01		
7915160	NM_022157	RRAGC	Ras-related GTP binding C	-0.007	0.995	9.82E-01	9.82E+01		
7915170	NM_012333	MYCBP	c-myc binding protein	-0.054	0.963	6.97E-01	6.97E+01		
7915200	---	---	---	0.134	1.098	4.81E-01	4.81E+01		
7915202	---	---	---	0.018	1.013	9.40E-01	9.40E+01		
7915207	NM_00113565	PABPC4	poly(A) binding protein, cytoplasm	0.028	1.020	8.38E-01	8.38E+01		
7915227	NR_002983	SNORA55	small nucleolar RNA, H/ACA box 5	0.043	1.030	8.22E-01	8.22E+01		
7915229	NM_014571	HEYL	hairy/enhancer-of-split related wi	-0.092	0.938	2.98E-01	2.98E+01		
7915238	NM_032526	NT5C1A	5'-nucleotidase, cytosolic IA	0.013	1.009	9.29E-01	9.29E+01		
7915245	NM_016257	HPCAL4	hippocalcin like 4	-0.013	0.991	9.68E-01	9.68E+01		
7915252	NM_001720	BMP8B	bone morphogenetic protein 8b	-0.118	0.922	3.62E-01	3.62E+01		
7915257	NM_022120	OXCT2	3-oxoacid CoA transferase 2	-0.036	0.975	7.09E-01	7.09E+01		
7915261	NM_017646	TRIT1	tRNA isopentenyltransferase 1	0.043	1.030	8.50E-01	8.50E+01		
7915277	NM_005376	MYCL1	v-myc myelocytomatosis viral onc	-0.002	0.999	9.92E-01	9.92E+01		
7915284	---	---	---	-0.129	0.915	7.20E-01	7.20E+01		
7915286	NM_000310	PPT1	palmitoyl-protein thioesterase 1	0.343	1.269	9.99E-02	9.99E+00		
7915297	NM_001852	COL9A2	collagen, type IX, alpha 2	0.013	1.009	9.35E-01	9.35E+01		
7915329	---	---	---	0.067	1.048	6.36E-01	6.36E+01		
7915333	NM_014747	RIMS3	regulating synaptic membrane exo	-0.037	0.974	7.87E-01	7.87E+01		
7915345	NM_00114258	NFYC	nuclear transcription factor Y, gam	-0.083	0.944	3.97E-01	3.97E+01		
7915347	NM_133467	CITED4	Cbp/p300-interacting transactivat	-0.099	0.933	2.46E-01	2.46E+01		
7915351	NM_144990	SLFNL1	schlafen-like 1	-0.031	0.979	8.25E-01	8.25E+01		
7915363	NM_00103169	SCMH1	sex comb on midleg homolog 1 (D	-0.066	0.955	6.83E-01	6.83E+01		

7915385	NM_001956	EDN2	endothelin 2	-0.092	0.938	1.85E-01	1.85E+01		
7915392	NM_024503	HIVEP3	human immunodeficiency virus ty	0.145	1.106	6.89E-02	6.89E+00		
7915404	NM_033553	GUCA2A	guanylate cyclase activator 2A (gu	-0.077	0.948	3.22E-01	3.22E+01		
7915408	NM_014947	FOXJ3	forkhead box J3	-0.045	0.969	6.58E-01	6.58E+01		
7915435	NM_00112339	CLDN19	claudin 19	-0.019	0.987	8.96E-01	8.96E+01		
7915444	NM_022356	LEPRE1	leucine proline-enriched proteogly	-0.067	0.955	5.99E-01	5.99E+01		
7915466	---	---	---	-0.056	0.962	7.25E-01	7.25E+01		
7915468	NM_199342	CCDC23	coiled-coil domain containing 23	0.194	1.144	8.96E-02	8.96E+00		
7915472	NM_006516	SLC2A1	solute carrier family 2 (facilitated g	-0.134	0.911	1.26E-01	1.26E+01		
7915485	NM_006824	EBNA1BP2	EBNA1 binding protein 2	-0.058	0.960	8.13E-01	8.13E+01		
7915500	NM_182517	C1orf210	chromosome 1 open reading fram	-0.027	0.982	8.85E-01	8.85E+01		
7915504	NM_022821	ELOVL1	elongation of very long chain fatty	0.122	1.089	3.89E-01	3.89E+01		
7915516	NM_201542	MED8	mediator complex subunit 8	0.125	1.091	5.51E-01	5.51E+01		
7915529	NM_031207	HYI	hydroxypyruvate isomerase homo	-0.024	0.984	8.79E-01	8.79E+01		
7915543	NM_201649	SLC6A9	solute carrier family 6 (neurotrans	-0.043	0.970	7.09E-01	7.09E+01		
7915563	---	---	---	0.103	1.074	6.10E-01	6.10E+01		
7915567	NM_024066	ERI3	exoribonuclease 3	0.101	1.072	2.37E-01	2.37E+01		
7915578	NM_024587	TMEM53	transmembrane protein 53	-0.017	0.988	9.24E-01	9.24E+01		
7915594	---	---	---	0.021	1.015	9.44E-01	9.44E+01		
7915614	NM_003738	PTCH2	patched homolog 2 (Drosophila)	-0.035	0.976	8.21E-01	8.21E+01		
7915640	NM_020365	EIF2B3	eukaryotic translation initiation fa	0.161	1.118	2.35E-01	2.35E+01		
7915659	NM_024602	HECTD3	HECT domain containing 3	0.033	1.023	8.51E-01	8.51E+01		
7915682	NM_020883	ZSWIM5	zinc finger, SWIM-type containing	0.026	1.018	8.16E-01	8.16E+01		
7915695	NM_012222	MUTYH	mutY homolog (E. coli)	-0.088	0.941	2.72E-01	2.72E+01		
7915718	NM_007170	TESK2	testis-specific kinase 2	0.086	1.062	6.44E-01	6.44E+01		
7915733	NM_002574	PRDX1	peroxiredoxin 1	0.318	1.247	1.42E-02	1.42E+00		
7915743	NM_00111493	CCDC17	coiled-coil domain containing 17	-0.010	0.993	9.56E-01	9.56E+01		
7915758	NM_021639	GPBP1L1	GC-rich promoter binding protein	-0.031	0.979	7.99E-01	7.99E+01		
7915787	NM_003629	PIK3R3	phosphoinositide-3-kinase, regula	-0.159	0.895	4.62E-01	4.62E+01		
7915801	NM_017739	POMGNT1	protein O-linked mannose beta1,2	-0.015	0.990	9.44E-01	9.44E+01		
7915827	NM_006369	LRRC41	leucine rich repeat containing 41	-0.031	0.979	7.84E-01	7.84E+01		
7915841	NM_00109761	KNCN	kinocilin	-0.020	0.987	8.79E-01	8.79E+01		
7915846	NM_003684	MKNK1	MAP kinase interacting serine/thre	0.316	1.245	7.49E-03	7.49E-01		
7915861	NM_145279	MOBKL2C	MOB1, Mps One Binder kinase act	0.153	1.112	1.35E-01	1.35E+01		
7915870	NM_022745	ATPAF1	ATP synthase mitochondrial F1 cor	0.071	1.050	6.81E-01	6.81E+01		
7915882	NM_014774	KIAA0494	KIAA0494	0.017	1.012	8.91E-01	8.91E+01		
7915910	NM_005764	PDZK1IP1	PDZK1 interacting protein 1	-0.047	0.968	6.00E-01	6.00E+01		
7915949	AK090467	LOC284542	hypothetical protein LOC284542	0.005	1.003	9.81E-01	9.81E+01		
7915991	NM_032110	DMRTA2	DMRT-like family A2	-0.061	0.959	5.38E-01	5.38E+01		
7915995	NM_007051	FAF1	Fas (TNFRSF6) associated factor 1	0.043	1.030	8.06E-01	8.06E+01		
7916022	---	---	---	-0.040	0.972	7.08E-01	7.08E+01		
7916045	NM_001981	EPS15	epidermal growth factor receptor	0.012	1.008	9.49E-01	9.49E+01		
7916077	NM_002525	NRD1	nardilysin (N-arginine dibasic conv	0.010	1.007	9.63E-01	9.63E+01		
7916120	NM_015913	TXNDC12	thioredoxin domain containing 12	0.179	1.132	1.88E-01	1.88E+01		
7916130	NM_138417	KTI12	KTI12 homolog, chromatin associa	-0.081	0.945	4.58E-01	4.58E+01		
7916135	NM_032449	CC2D1B	coiled-coil and C2 domain contain	-0.052	0.964	6.09E-01	6.09E+01		
7916185	NM_00100988	ZCCHC11	zinc finger, CCHC domain containi	-0.059	0.960	6.55E-01	6.55E+01		
7916219	BC015313	C1orf163	chromosome 1 open reading fram	0.196	1.146	7.64E-02	7.64E+00		
7916225	---	---	---	-0.007	0.995	9.83E-01	9.83E+01		
7916229	NM_018281	ECHDC2	enoyl Coenzyme A hydratase dom	-0.040	0.973	7.55E-01	7.55E+01		
7916246	---	---	---	0.155	1.113	3.02E-01	3.02E+01		
7916262	BC010908	C1orf123	chromosome 1 open reading fram	0.015	1.011	9.33E-01	9.33E+01		
7916274	NM_002370	MAGOH	mago-nashi homolog, proliferatior	-0.014	0.990	9.56E-01	9.56E+01		
7916282	NM_004631	LRP8	low density lipoprotein receptor-r	-0.150	0.901	9.45E-02	9.45E+00		
7916304	NM_147193	GLIS1	GLIS family zinc finger 1	-0.105	0.929	1.89E-01	1.89E+01		
7916316	NM_018087	TMEM48	transmembrane protein 48	0.039	1.027	8.41E-01	8.41E+01		
7916343	NM_018982	YIPF1	Yip1 domain family, member 1	0.089	1.064	5.48E-01	5.48E+01		
7916356	NM_016126	HSPB11	heat shock protein family B (small	0.034	1.024	9.13E-01	9.13E+01		
7916364	NM_00101097	LDLRAD1	low density lipoprotein receptor c	0.022	1.015	8.59E-01	8.59E+01		
7916372	NM_004872	TMEM59	transmembrane protein 59	0.078	1.056	4.06E-01	4.06E+01		
7916393	NM_00103167	CYB5RL	cytochrome b5 reductase-like	0.134	1.097	3.47E-02	3.47E+00		
7916403	NM_145716	SSBP3	single stranded DNA binding prote	-0.093	0.937	5.72E-01	5.72E+01		
7916412	NM_147161	ACOT11	acyl-CoA thioesterase 11	-0.002	0.998	9.89E-01	9.89E+01		
7916422	NM_152268	PARS2	prolyl-tRNA synthetase 2, mitocho	-0.084	0.943	7.94E-02	7.94E+00		
7916425	NM_017904	TTC22	tetratricopeptide repeat domain 2	-0.057	0.961	7.02E-01	7.02E+01		
7916432	NM_014762	DHCR24	24-dehydrocholesterol reductase	0.014	1.009	9.55E-01	9.55E+01		
7916443	NM_015306	USP24	ubiquitin specific peptidase 24	0.025	1.018	8.16E-01	8.16E+01		
7916489	---	---	---	-0.006	0.996	9.76E-01	9.76E+01		
7916493	NM_003713	PPAP2B	phosphatidic acid phosphatase typ	0.026	1.018	8.56E-01	8.56E+01		
7916562	NM_002136	HNRNPA1	heterogeneous nuclear ribonucleo	-0.023	0.984	8.78E-01	8.78E+01		
7916564	---	---	---	-0.025	0.983	9.50E-01	9.50E+01		
7916568	AF263547	DAB1	disabled homolog 1 (Drosophila)	0.029	1.020	9.29E-01	9.29E+01		
7916570	NM_145243	OMA1	OMA1 homolog, zinc metallopepti	0.127	1.092	5.55E-01	5.55E+01		
7916590	NM_001625	AK2	adenylate kinase 2	0.115	1.083	3.05E-01	3.05E+01		
7916592	NM_00108548	MYSM1	Myb-like, SWIRM and MPN domai	0.042	1.029	7.44E-01	7.44E+01		
7916609	NM_002228	JUN	jun oncogene	-0.248	0.842	3.80E-02	3.80E+00		
7916643	NM_032027	TM2D1	TM2 domain containing 1	-0.038	0.974	8.17E-01	8.17E+01		

7916654	NM_181712	KANK4	KN motif and ankyrin repeat doma	-0.009	0.994	9.56E-01	9.56E+01		
7916667	---	---	---	0.161	1.118	2.65E-01	2.65E+01		
7916727	NM_014288	ITGB3BP	integrin beta 3 binding protein (be	0.024	1.017	9.47E-01	9.47E+01		
7916743	---	---	---	0.070	1.050	6.15E-01	6.15E+01		
7916747	NM_002227	JAK1	Janus kinase 1	-0.006	0.996	9.75E-01	9.75E+01		
7916808	NM_015139	SLC35D1	solute carrier family 35 (UDP-glucu	0.003	1.002	9.93E-01	9.93E+01		
7916836	NM_00101806	SERBP1	SERPINE1 mRNA binding protein 1	0.179	1.132	3.90E-02	3.90E+00		
7916928	NM_030816	ANKRD13C	ankyrin repeat domain 13C	-0.015	0.990	9.30E-01	9.30E+01		
7916969	NM_005455	ZRANB2	zinc finger, RAN-binding domain co	0.009	1.006	9.52E-01	9.52E+01		
7917000	---	---	---	0.047	1.033	6.37E-01	6.37E+01		
7917037	NM_00113004	CRYZ	crystallin, zeta (quinone reductase	-0.080	0.946	8.06E-01	8.06E+01		
7917080	S72422 // S72	DLSTP // DLSTP	dihydrolipoamide S-succinyltransfe	-0.015	0.989	9.60E-01	9.60E+01		
7917088	NM_005482	PIGK	phosphatidylinositol glycan ancho	0.043	1.030	8.34E-01	8.34E+01		
7917103	NM_015534	ZZZ3	zinc finger, ZZ-type containing 3	-0.064	0.956	7.15E-01	7.15E+01		
7917120	NM_015017	USP33	ubiquitin specific peptidase 33	0.025	1.018	8.91E-01	8.91E+01		
7917148	NM_022831	AIDA	axin interactor, dorsalization assoc	0.002	1.001	9.91E-01	9.91E+01		
7917151	ENST00000334	NEXN	nexilin (F actin binding protein)	-0.088	0.941	2.41E-01	2.41E+01		
7917156	NM_003902	FUBP1	far upstream element (FUSE) bind	0.036	1.025	7.01E-01	7.01E+01		
7917232	NM_005274	GNG5	guanine nucleotide binding protei	0.123	1.089	2.23E-01	2.23E+01		
7917240	NM_004388	CTBS	chitobiase, di-N-acetyl-	0.127	1.092	2.78E-01	2.78E+01		
7917283	NM_153259	MCOLN2	mucolipin 2	-0.213	0.863	5.26E-01	5.26E+01		
7917331	NR_024113	C1orf52	chromosome 1 open reading fram	-0.037	0.975	8.53E-01	8.53E+01		
7917338	NM_003921	BCL10	B-cell CLL/lymphoma 10	0.081	1.058	5.53E-01	5.53E+01		
7917359	NM_017953	ZNHIT6	zinc finger, HIT type 6	0.071	1.050	7.54E-01	7.54E+01		
7917433	NM_020729	ODF2L	outer dense fiber of sperm tails 2-	-0.081	0.945	6.82E-01	6.82E+01		
7917455	NM_004261	40436	15 kDa selenoprotein	0.063	1.044	4.60E-01	4.60E+01		
7917466	NR_026988	LOC339524	hypothetical LOC339524	0.005	1.004	9.80E-01	9.80E+01		
7917468	---	---	---	-0.208	0.866	4.81E-01	4.81E+01		
7917470	---	---	---	-0.051	0.965	6.93E-01	6.93E+01		
7917472	NM_001514	GTF2B	general transcription factor IIB	0.036	1.025	8.64E-01	8.64E+01		
7917482	NM_00100866	CCBL2	cysteine conjugate-beta lyase 2	-0.005	0.996	9.81E-01	9.81E+01		
7917503	NM_018284	GBP3	guanylate binding protein 3	-0.015	0.989	9.64E-01	9.64E+01		
7917516	NM_002053	GBP1	guanylate binding protein 1, interf	0.474	1.389	8.43E-02	8.43E+00		
7917528	---	---	---	0.172	1.126	4.04E-01	4.04E+01		
7917530	---	---	---	0.081	1.057	7.00E-01	7.00E+01		
7917532	NM_004120	GBP2	guanylate binding protein 2, interf	0.087	1.062	5.34E-01	5.34E+01		
7917561	NM_052941	GBP4	guanylate binding protein 4	0.216	1.162	3.41E-01	3.41E+01		
7917576	NM_052942	GBP5	guanylate binding protein 5	0.276	1.211	3.83E-01	3.83E+01		
7917591	---	---	---	0.009	1.006	9.81E-01	9.81E+01		
7917599	NM_020063	BARHL2	BarH-like homeobox 2	0.009	1.006	9.57E-01	9.57E+01		
7917604	NM_201269	ZNF644	zinc finger protein 644	0.016	1.011	9.39E-01	9.39E+01		
7917645	NR_003287	LOC100008589	28S ribosomal RNA	0.015	1.011	9.16E-01	9.16E+01		
7917649	NM_003243	TGFBR3	transforming growth factor, beta r	-0.099	0.934	8.05E-01	8.05E+01		
7917670	---	---	---	-0.030	0.980	8.63E-01	8.63E+01		
7917672	---	---	---	0.035	1.025	8.23E-01	8.23E+01		
7917674	NM_00112282	SET	SET nuclear oncogene	-0.026	0.982	8.93E-01	8.93E+01		
7917697	NM_005263	GFI1	growth factor independent 1 trans	-0.018	0.988	9.12E-01	9.12E+01		
7917707	NM_005665	EVI5	ecotropic viral integration site 5	0.323	1.251	2.71E-01	2.71E+01		
7917737	---	---	---	-0.038	0.974	8.16E-01	8.16E+01		
7917741	NM_016040	TMED5	transmembrane emp24 protein tra	0.059	1.042	6.83E-01	6.83E+01		
7917752	---	---	---	-0.016	0.989	9.49E-01	9.49E+01		
7917754	NM_003567	BCAR3	breast cancer anti-estrogen resista	-0.065	0.956	7.10E-01	7.10E+01		
7917771	NM_014597	DNTTIP2	deoxynucleotidyltransferase, term	-0.110	0.926	2.64E-01	2.64E+01		
7917875	NM_001993	F3	coagulation factor III (thromboplas	-0.346	0.787	5.45E-02	5.45E+00		
7917885	NM_001839	CNN3	calponin 3, acidic	0.111	1.080	5.71E-01	5.71E+01		
7917906	NM_000971	RPL7	ribosomal protein L7	-0.028	0.981	8.63E-01	8.63E+01		
7917912	NM_000110	DPYD	dihydropyrimidine dehydrogenase	0.312	1.241	4.32E-02	4.32E+00		
7917974	---	---	---	0.417	1.335	2.81E-01	2.81E+01		
7918034	NM_00107739	DPH5	DPH5 homolog (S. cerevisiae)	0.012	1.008	9.70E-01	9.70E+01		
7918048	---	---	---	-0.011	0.993	9.89E-01	9.89E+01		
7918050	NM_002295	RPSA	ribosomal protein SA	-0.048	0.968	8.05E-01	8.05E+01		
7918134	NM_004038	AMY1A	amylase, alpha 1A (salivary)	0.070	1.049	8.13E-01	8.13E+01		
7918149	---	---	---	-0.028	0.981	8.91E-01	8.91E+01		
7918153	---	---	---	-0.041	0.972	6.96E-01	6.96E+01		
7918157	NM_006113	VAV3	vav 3 guanine nucleotide exchange	0.291	1.223	2.88E-01	2.88E+01		
7918188	NM_013386	SLC25A24	solute carrier family 25 (mitochon	0.177	1.131	4.82E-01	4.82E+01		
7918203	NM_00114398	NBPF4	neuroblastoma breakpoint family,	0.028	1.020	7.94E-01	7.94E+01		
7918223	NM_144584	C1orf59	chromosome 1 open reading fram	0.092	1.066	6.00E-01	6.00E+01		
7918255	NM_00104821	CLCC1	chloride channel CLIC-like 1	0.176	1.129	2.78E-01	2.78E+01		
7918275	NM_00114255	WDR47	WD repeat domain 47	-0.120	0.920	5.29E-01	5.29E+01		
7918284	NM_005645	TAF13	TAF13 RNA polymerase II, TATA bo	0.042	1.030	8.62E-01	8.62E+01		
7918294	NM_00112296	C1orf194	chromosome 1 open reading fram	0.041	1.029	7.64E-01	7.64E+01		
7918300	NM_00103229	PSRC1	proline/serine-rich coiled-coil 1	-0.034	0.977	7.45E-01	7.45E+01		
7918323	NM_002959	SORT1	sortilin 1	0.525	1.439	5.36E-02	5.36E+00		
7918345	NM_002790	PSMA5	proteasome (prosome, macropain	0.044	1.031	8.06E-01	8.06E+01		
7918359	NM_020703	AMIGO1	adhesion molecule with Ig-like dor	0.014	1.010	9.37E-01	9.37E+01		
7918365	---	---	---	0.108	1.078	7.44E-01	7.44E+01		

7918367	NM_005272	GNAT2	guanine nucleotide binding protei	0.071	1.050	4.96E-01	4.96E+01		
7918394	NM_139053	EPS8L3	EPS8-like 3	-0.002	0.999	9.87E-01	9.87E+01		
7918416	NM_006492	ALX3	ALX homeobox 3	-0.071	0.952	4.14E-01	4.14E+01		
7918424	---	---	---	0.171	1.125	3.99E-01	3.99E+01		
7918426	NM_004696	SLC16A4	solute carrier family 16, member 4	0.173	1.127	1.54E-01	1.54E+01		
7918437	NM_006402	HBXIP	hepatitis B virus x interacting prot	0.144	1.105	5.88E-02	5.88E+00		
7918444	NM_005549	KCNA10	potassium voltage-gated channel,	0.000	1.000	1.00E+00	1.00E+02		
7918457	NM_002232	KCNA3	potassium voltage-gated channel,	0.062	1.044	6.54E-01	6.54E+01		
7918487	NM_024901	DENND2D	DENN/MADD domain containing 2	0.158	1.116	1.25E-01	1.25E+01		
7918517	NM_024102	WDR77	WD repeat domain 77	0.116	1.084	3.03E-01	3.03E+01		
7918550	---	---	---	0.191	1.142	2.39E-01	2.39E+01		
7918552	NM_019099	C1orf183	chromosome 1 open reading fram	0.000	1.000	1.00E+00	1.00E+02		
7918569	NM_017744	ST7L	suppression of tumorigenicity 7 lik	0.049	1.035	7.36E-01	7.36E+01		
7918593	NM_175744	RHOC	ras homolog gene family, member	0.009	1.006	9.68E-01	9.68E+01		
7918606	NM_005167	PPM1J	protein phosphatase 1J (PP2C dom	0.011	1.008	9.45E-01	9.45E+01		
7918620	AK093435	FLJ36116	hypothetical locus LOC388666	-0.041	0.972	9.07E-01	9.07E+01		
7918634	NM_006608	PHTF1	putative homeodomain transcripti	-0.148	0.903	2.91E-01	2.91E+01		
7918657	NM_015967	PTPN22	protein tyrosine phosphatase, nor	0.032	1.023	9.36E-01	9.36E+01		
7918681	NM_018364	RSBN1	round spermatid basic protein 1	-0.068	0.954	6.63E-01	6.63E+01		
7918703	NM_006594	AP4B1	adaptor-related protein complex 4	-0.006	0.996	9.78E-01	9.78E+01		
7918725	NM_015906	TRIM33	tripartite motif-containing 33	-0.004	0.997	9.81E-01	9.81E+01		
7918749	BC063894	DENND2C	DENN/MADD domain containing 2	0.003	1.002	9.86E-01	9.86E+01		
7918755	BC063894	DENND2C	DENN/MADD domain containing 2	-0.264	0.833	4.70E-01	4.70E+01		
7918759	NM_005872	BCAS2	breast carcinoma amplified seque	-0.050	0.966	8.05E-01	8.05E+01		
7918792	BC063894	DENND2C	DENN/MADD domain containing 2	0.020	1.014	8.93E-01	8.93E+01		
7918813	NM_002524	NRAS	neuroblastoma RAS viral (v-ras) or	-0.019	0.987	9.43E-01	9.43E+01		
7918825	NM_00100755	CSDE1	cold shock domain containing E1,	0.017	1.012	8.57E-01	8.57E+01		
7918847	NM_00110239	SIKE1	suppressor of IKBKE 1	0.043	1.031	8.26E-01	8.26E+01		
7918869	NM_002506	NGF	nerve growth factor (beta polypep	-0.033	0.978	7.75E-01	7.75E+01		
7918876	---	---	---	-0.041	0.972	8.05E-01	8.05E+01		
7918900	---	---	---	-0.064	0.957	9.04E-01	9.04E+01		
7918911	---	---	---	-0.105	0.930	3.90E-01	3.90E+01		
7918913	NM_001542	IGSF3	immunoglobulin superfamily, men	-0.045	0.970	6.82E-01	6.82E+01		
7918925	NM_025188	TRIM45	tripartite motif-containing 45	-0.073	0.950	4.51E-01	4.51E+01		
7918936	NM_024626	VTCN1	V-set domain containing T cell acti	-0.011	0.992	9.53E-01	9.53E+01		
7918955	NM_00113558	GDAP2	ganglioside induced differentiatio	0.069	1.049	5.55E-01	5.55E+01		
7919038	NM_201263	WARS2	tryptophanyl tRNA synthetase 2, n	-0.059	0.960	7.54E-01	7.54E+01		
7919051	NM_00108047	ZNF697	zinc finger protein 697	0.012	1.009	9.56E-01	9.56E+01		
7919095	NM_024408	NOTCH2	Notch homolog 2 (Drosophila)	0.106	1.076	6.33E-01	6.33E+01		
7919129	---	---	---	0.076	1.054	7.61E-01	7.61E+01		
7919131	---	---	---	0.207	1.154	1.57E-01	1.57E+01		
7919153	---	---	---	-0.110	0.926	9.61E-01	9.61E+01		
7919157	NM_00114388	PPIAL4B	peptidylprolyl isomerase A (cyclop	-0.017	0.988	9.01E-01	9.01E+01		
7919160	---	---	---	-0.317	0.803	8.70E-02	8.70E+00		
7919162	NM_00114388	PPIAL4B	peptidylprolyl isomerase A (cyclop	0.032	1.022	8.41E-01	8.41E+01		
7919166	---	---	---	-0.317	0.803	8.70E-02	8.70E+00		
7919168	NM_022359	PDE4DIP	phosphodiesterase 4D interacting	-0.127	0.916	5.69E-01	5.69E+01		
7919193	NR_002212	NUDT4P1	nudix (nucleoside diphosphate link	0.070	1.050	6.20E-01	6.20E+01		
7919197	NM_032305	POLR3GL	polymerase (RNA) III (DNA directe	0.019	1.014	9.41E-01	9.41E+01		
7919208	NR_002328	GNRHR2	gonadotropin-releasing hormone (	0.010	1.007	9.63E-01	9.63E+01		
7919217	NM_00101275	NUDT17	nudix (nucleoside diphosphate link	-0.015	0.990	9.27E-01	9.27E+01		
7919226	NM_006468	POLR3C	polymerase (RNA) III (DNA directe	0.101	1.072	4.75E-01	4.75E+01		
7919251	NM_016334	GPR89B	G protein-coupled receptor 89B	0.010	1.007	9.67E-01	9.67E+01		
7919267	---	---	---	-0.016	0.989	9.36E-01	9.36E+01		
7919271	NM_183372	RP11-94I2.2	neuroblastoma breakpoint family,	-0.056	0.962	5.04E-01	5.04E+01		
7919299	AK128227	LOC100130236	hypothetical LOC100130236	0.007	1.005	9.78E-01	9.78E+01		
7919301	AK128227	LOC100130236	hypothetical LOC100130236	-0.026	0.982	8.75E-01	8.75E+01		
7919305	NM_005399	PRKAB2	protein kinase, AMP-activated, be	-0.169	0.889	5.64E-02	5.64E+00		
7919314	NM_001461	FMO5	flavin containing monooxygenase	0.092	1.066	6.31E-01	6.31E+01		
7919326	NM_016361	ACP6	acid phosphatase 6, lysophosphati	0.003	1.002	9.89E-01	9.89E+01		
7919347	---	---	---	-0.016	0.989	9.36E-01	9.36E+01		
7919351	NM_183372	RP11-94I2.2	neuroblastoma breakpoint family,	-0.054	0.964	5.29E-01	5.29E+01		
7919380	AK128227	LOC100130236	hypothetical LOC100130236	0.007	1.005	9.78E-01	9.78E+01		
7919384	---	---	---	-0.020	0.986	9.49E-01	9.49E+01		
7919386	---	---	---	-0.121	0.920	3.55E-01	3.55E+01		
7919394	NR_024510	LOC728855	hypothetical LOC728855	-0.021	0.985	9.50E-01	9.50E+01		
7919403	---	---	---	-0.110	0.926	9.61E-01	9.61E+01		
7919407	NM_00114388	PPIAL4B	peptidylprolyl isomerase A (cyclop	0.040	1.028	7.75E-01	7.75E+01		
7919412	NM_00110266	NBPF16	neuroblastoma breakpoint family,	-0.047	0.968	5.78E-01	5.78E+01		
7919436	NM_178230	PPIAL4A	peptidylprolyl isomerase A (cyclop	-0.057	0.962	4.75E-01	4.75E+01		
7919438	NM_00110266	NBPF16	neuroblastoma breakpoint family,	-0.052	0.965	5.42E-01	5.42E+01		
7919560	---	---	---	-0.506	0.704	5.71E-02	5.71E+00		
7919564	---	---	---	0.048	1.034	6.60E-01	6.60E+01		
7919566	---	---	---	-0.008	0.994	9.65E-01	9.65E+01		
7919568	---	---	---	-0.063	0.958	7.63E-01	7.63E+01		
7919578	---	---	---	-0.080	0.946	8.70E-01	8.70E+01		
7919580	AK125737 // A	40570 // LOC44	hypothetical LOC440570 // hypot	0.074	1.053	7.54E-01	7.54E+01		

7919582	XR_039086	LOC100133075	hypothetical LOC100133075	-0.079	0.947	4.34E-01	4.34E+01		
7919584	NM_00102459	HIST2H2BF	histone cluster 2, H2bf	-0.029	0.980	9.24E-01	9.24E+01		
7919589	NM_00112337	HIST2H3D	histone cluster 2, H3d	-0.018	0.987	9.12E-01	9.12E+01		
7919596	AK125737 // A	40570 // LOC44	hypothetical LOC440570 // hypot	0.074	1.053	7.54E-01	7.54E+01		
7919598	---	---	---	-0.158	0.896	3.90E-01	3.90E+01		
7919600	---	---	---	-0.051	0.966	8.63E-01	8.63E+01		
7919604	AK092418	HIST2H2BF	histone cluster 2, H2bf	0.062	1.044	6.76E-01	6.76E+01		
7919606	NM_00102459	HIST2H2BF	histone cluster 2, H2bf	-0.038	0.974	8.76E-01	8.76E+01		
7919612	NM_00112337	HIST2H3D	histone cluster 2, H3d	-0.042	0.971	7.59E-01	7.59E+01		
7919614	NM_00100546	HIST2H3A	histone cluster 2, H3a	-0.052	0.965	6.82E-01	6.82E+01		
7919619	NM_003516	HIST2H2AA3	histone cluster 2, H2aa3	-0.194	0.874	2.82E-01	2.82E+01		
7919625	NM_003528 //	// HIST2H2BE //	histone cluster 2, H2be // histone	0.045	1.031	7.39E-01	7.39E+01		
7919627	NM_003548	HIST2H4A	histone cluster 2, H4a	-0.111	0.926	6.16E-01	6.16E+01		
7919637	NM_003528	HIST2H2BE	histone cluster 2, H2be	-0.016	0.989	9.67E-01	9.67E+01		
7919642	NM_175065	HIST2H2AB	histone cluster 2, H2ab	0.016	1.011	9.64E-01	9.64E+01		
7919659	NM_005850	SF3B4	splicing factor 3b, subunit 4, 49kD	-0.050	0.966	5.63E-01	5.63E+01		
7919669	NM_00114586	MTMR11	myotubularin related protein 11	-0.090	0.939	2.06E-01	2.06E+01		
7919715	NM_030920	ANP32E	acidic (leucine-rich) nuclear phosp	-0.048	0.967	7.14E-01	7.14E+01		
7919729	NM_016022	APH1A	anterior pharynx defective 1 homo	0.110	1.079	5.04E-01	5.04E+01		
7919743	NM_019032	ADAMTSL4	ADAMTS-like 4	0.067	1.048	7.15E-01	7.15E+01		
7919747	---	---	---	-0.010	0.993	9.85E-01	9.85E+01		
7919749	---	---	---	-0.049	0.967	9.24E-01	9.24E+01		
7919751	NM_021960	MCL1	myeloid cell leukemia sequence 1	-0.034	0.977	7.60E-01	7.60E+01		
7919761	---	---	---	-0.013	0.991	9.80E-01	9.80E+01		
7919763	NM_207042	ENSA	endosulfine alpha	0.040	1.028	7.88E-01	7.88E+01		
7919780	NM_018178	GOLPH3L	golgi phosphoprotein 3-like	-0.091	0.939	5.48E-01	5.48E+01		
7919815	NM_000396	CTSK	cathepsin K	0.145	1.106	4.87E-01	4.87E+01		
7919825	NM_001668	ARNT	aryl hydrocarbon receptor nuclear	-0.011	0.992	9.58E-01	9.58E+01		
7919854	---	---	---	-0.039	0.973	9.18E-01	9.18E+01		
7919856	NM_181746	LASS2	LAG1 homolog, ceramide synthase	-0.050	0.966	7.07E-01	7.07E+01		
7919888	NM_00103870	CDC42SE1	CDC42 small effector 1	0.033	1.023	8.27E-01	8.27E+01		
7919898	NM_030913	SEMA6C	sema domain, transmembrane do	-0.019	0.987	8.91E-01	8.91E+01		
7919923	NM_212551	LYSMD1	LysM, putative peptidoglycan-bind	-0.008	0.994	9.77E-01	9.77E+01		
7919929	NM_013353	TMOD4	tropomodulin 4 (muscle)	-0.043	0.970	8.50E-01	8.50E+01		
7919940	NM_005997	VPS72	vacuolar protein sorting 72 homol	0.007	1.005	9.69E-01	9.69E+01		
7919950	NM_002651	PI4KB	phosphatidylinositol 4-kinase, cata	-0.003	0.998	9.85E-01	9.85E+01		
7919971	NM_000449	RFX5	regulatory factor X, 5 (influences H	0.153	1.112	1.89E-01	1.89E+01		
7919984	NM_003944	SELENBP1	selenium binding protein 1	-0.055	0.962	5.73E-01	5.73E+01		
7920000	NM_015100	POGZ	pogo transposable element with Z	-0.053	0.964	5.56E-01	5.56E+01		
7920025	---	---	---	0.055	1.039	6.00E-01	6.00E+01		
7920027	NM_007185	TNRC4	trinucleotide repeat containing 4	-0.073	0.951	3.03E-01	3.03E+01		
7920047	NM_031420	MRPL9	mitochondrial ribosomal protein L	-0.101	0.933	4.84E-01	4.84E+01		
7920057	NM_00108396	TDRKH	tudor and KH domain containing	-0.017	0.988	9.55E-01	9.55E+01		
7920077	NM_00100443	LINGO4	leucine rich repeat and Ig domain	-0.027	0.981	8.40E-01	8.40E+01		
7920082	NM_005060	RORC	RAR-related orphan receptor C	-0.014	0.991	9.35E-01	9.35E+01		
7920114	NM_053055	THEM4	thioesterase superfamily member	-0.102	0.932	7.53E-01	7.53E+01		
7920123	NM_002966	S100A10	S100 calcium binding protein A10	0.111	1.080	6.31E-01	6.31E+01		
7920128	NM_005620	S100A11	S100 calcium binding protein A11	0.105	1.075	7.08E-01	7.08E+01		
7920141	NM_007113	TCHH	trichohyalin	-0.050	0.966	6.78E-01	6.78E+01		
7920178	NM_016190	CRNN	cornulin	-0.046	0.969	7.00E-01	7.00E+01		
7920182	NM_178435	LCE3E	late cornified envelope 3E	-0.015	0.990	9.37E-01	9.37E+01		
7920185	NM_032563	LCE3D	late cornified envelope 3D	0.005	1.003	9.85E-01	9.85E+01		
7920191	NM_178431	LCE3A	late cornified envelope 3A	-0.019	0.987	9.00E-01	9.00E+01		
7920193	NM_178351	LCE1C	late cornified envelope 1C	-0.118	0.922	3.29E-01	3.29E+01		
7920217	NM_00101429	SPRR2G	small proline-rich protein 2G	-0.069	0.953	5.63E-01	5.63E+01		
7920228	NM_020393	PGLYRP4	peptidoglycan recognition protein	-0.015	0.990	9.24E-01	9.24E+01		
7920244	NM_002964	S100A8	S100 calcium binding protein A8	-0.100	0.933	9.17E-01	9.17E+01		
7920258	NM_014624	S100A6	S100 calcium binding protein A6	0.292	1.224	1.52E-02	1.52E+00		
7920264	NM_002962	S100A5	S100 calcium binding protein A5	0.037	1.026	8.05E-01	8.05E+01		
7920297	NM_020672	S100A14	S100 calcium binding protein A14	0.033	1.023	8.29E-01	8.29E+01		
7920317	NM_004515	ILF2	interleukin enhancer binding facto	-0.015	0.989	9.37E-01	9.37E+01		
7920333	---	---	---	0.028	1.019	9.53E-01	9.53E+01		
7920335	---	---	---	-0.082	0.945	8.54E-01	8.54E+01		
7920337	NM_023015	INTS3	integrator complex subunit 3	-0.006	0.996	9.83E-01	9.83E+01		
7920341	NM_020699	GATAD2B	GATA zinc finger domain containir	-0.103	0.931	2.13E-01	2.13E+01		
7920354	NM_014856	DENND4B	DENN/MADD domain containing 4	0.065	1.046	3.71E-01	3.71E+01		
7920382	NM_181715	CRTC2	CREB regulated transcription coact	-0.093	0.938	3.56E-01	3.56E+01		
7920401	NM_014437	SLC39A1	solute carrier family 39 (zinc trans	-0.039	0.974	8.34E-01	8.34E+01		
7920409	NM_006694	JTB	jumping translocation breakpoint	-0.067	0.954	6.58E-01	6.58E+01		
7920422	NM_002870	RAB13	RAB13, member RAS oncogene fa	-0.233	0.851	5.24E-01	5.24E+01		
7920472	NM_152263	TPM3	tropomyosin 3	0.041	1.029	7.83E-01	7.83E+01		
7920492	NM_00109861	C1orf43	chromosome 1 open reading fram	0.018	1.012	9.40E-01	9.40E+01		
7920506	NM_00101084	SHE	Src homology 2 domain containing	0.032	1.022	8.08E-01	8.08E+01		
7920515	NM_017582	UBE2Q1	ubiquitin-conjugating enzyme E2Q	-0.026	0.982	8.54E-01	8.54E+01		
7920531	NM_001111	ADAR	adenosine deaminase, RNA-specif	0.103	1.074	5.13E-01	5.13E+01		
7920552	NM_002249	KCNN3	potassium intermediate/small con	-0.015	0.990	9.14E-01	9.14E+01		
7920567	NM_006556	PMVK	phosphomevalonate kinase	0.000	1.000	1.00E+00	1.00E+02		

7920575	NM_020524	PBXIP1	pre-B-cell leukemia homeobox int	-0.041	0.972	7.05E-01	7.05E+01		
7920594	NM_138300	PYGO2	pygopus homolog 2 (Drosophila)	-0.048	0.968	7.07E-01	7.07E+01		
7920600	NM_183001	SHC1	SHC (Src homology 2 domain cont	-0.026	0.982	8.78E-01	8.78E+01		
7920616	NM_144622	DCST2	DC-STAMP domain containing 2	0.036	1.025	8.05E-01	8.05E+01		
7920633	NM_018973	DPM3	dolichyl-phosphate mannosyltrans	-0.102	0.932	2.15E-01	2.15E+01		
7920642	NM_00101801	MUC1	mucin 1, cell surface associated	0.007	1.005	9.77E-01	9.77E+01		
7920659	NM_173852	KRTCAP2	keratinocyte associated protein 2	-0.049	0.966	7.66E-01	7.66E+01		
7920664	NM_007112	THBS3	thrombospondin 3	0.037	1.026	8.69E-01	8.69E+01		
7920697	NM_000157	GBA	glucosidase, beta; acid (includes g	-0.013	0.991	9.81E-01	9.81E+01		
7920707	NM_006589	FAM189B	family with sequence similarity 18	-0.004	0.997	9.83E-01	9.83E+01		
7920725	NM_005698	SCAMP3	secretory carrier membrane prote	0.074	1.053	3.38E-01	3.38E+01		
7920737	NM_003993	CLK2	CDC-like kinase 2	-0.074	0.950	4.11E-01	4.11E+01		
7920744	NM_000298	PKLR	pyruvate kinase, liver and RBC	-0.085	0.943	4.03E-01	4.03E+01		
7920757	NM_002004	FDPS	farnesyl diphosphate synthase (far	-0.026	0.982	8.91E-01	8.91E+01		
7920766	NM_018489	ASH1L	ash1 (absent, small, or homeotic)-	-0.031	0.979	7.95E-01	7.95E+01		
7920799	NM_139118	YY1AP1	YY1 associated protein 1	0.011	1.007	9.48E-01	9.48E+01		
7920810	NM_00103753	GON4L	gon-4-like (C. elegans)	-0.029	0.980	8.41E-01	8.41E+01		
7920839	NM_006912	RIT1	Ras-like without CAAX 1	-0.024	0.984	9.44E-01	9.44E+01		
7920852	BC062637	KIAA0907	KIAA0907	-0.054	0.963	7.03E-01	7.03E+01		
7920873	NR_002974	SNORA42	small nucleolar RNA, H/ACA box 4	-0.155	0.898	2.56E-01	2.56E+01		
7920877	NM_004723	ARHGEF2	Rho/Rac guanine nucleotide excha	0.026	1.018	9.15E-01	9.15E+01		
7920903	NM_003145	SSR2	signal sequence receptor, beta (tr	0.056	1.040	6.93E-01	6.93E+01		
7920912	NM_020131	UBQLN4	ubiquilin 4	-0.032	0.978	7.89E-01	7.89E+01		
7920928	NM_024897	PAQR6	progestin and adipoQ receptor fan	-0.030	0.980	7.40E-01	7.40E+01		
7920944	NM_015327	SMG5	Smg-5 homolog, nonsense mediat	0.118	1.085	2.01E-01	2.01E+01		
7920971	NM_144580	C1orf85	chromosome 1 open reading fram	0.335	1.262	6.04E-02	6.04E+00		
7920984	NM_005998	CCT3	chaperonin containing TCP1, subu	-0.031	0.979	8.80E-01	8.80E+01		
7921014	NM_005920	MEF2D	myocyte enhancer factor 2D	-0.052	0.965	6.83E-01	6.83E+01		
7921031	---	---	---	-0.185	0.880	6.54E-01	6.54E+01		
7921076	NM_182679	GPATCH4	G patch domain containing 4	-0.083	0.944	6.32E-01	6.32E+01		
7921088	NM_006617	NES	nestin	-0.078	0.947	3.42E-01	3.42E+01		
7921110	NM_030980	ISG20L2	interferon stimulated exonuclease	-0.045	0.969	8.18E-01	8.18E+01		
7921121	NM_145729	MRPL24	mitochondrial ribosomal protein L	-0.119	0.921	5.38E-01	5.38E+01		
7921133	NM_004494	HDGF	hepatoma-derived growth factor (	-0.035	0.976	8.04E-01	8.04E+01		
7921144	NM_00116144	SH2D2A	SH2 domain protein 2A	-0.167	0.891	3.06E-01	3.06E+01		
7921155	NM_014215	INSRR	insulin receptor-related receptor	0.018	1.013	9.00E-01	9.00E+01		
7921179	NM_198236	ARHGEF11	Rho guanine nucleotide exchange	0.309	1.239	1.08E-01	1.08E+01		
7921222	NM_00100434	ETV3L	ets variant 3-like	-0.122	0.919	2.07E-01	2.07E+01		
7921275	NM_052939	FCRL3	Fc receptor-like 3	0.236	1.178	3.02E-01	3.02E+01		
7921332	NM_005894	CD5L	CD5 molecule-like	0.079	1.057	3.38E-01	3.38E+01		
7921342	AK057458	hCG_1994895	hCG1994895	-0.067	0.955	6.05E-01	6.05E+01		
7921344	NM_012081	ELL2	elongation factor, RNA polymerase	-0.065	0.956	8.55E-01	8.55E+01		
7921360	NM_00100518	OR6Y1	olfactory receptor, family 6, subfa	-0.070	0.953	5.84E-01	5.84E+01		
7921434	NM_004833	AIM2	absent in melanoma 2	0.178	1.132	4.15E-01	4.15E+01		
7921462	NM_00101366	VSIG8	V-set and immunoglobulin domair	0.049	1.035	7.39E-01	7.39E+01		
7921487	NM_003564	TAGLN2	transgelin 2	0.066	1.047	7.89E-01	7.89E+01		
7921492	NM_00113505	IGSF9	immunoglobulin superfamily, men	-0.115	0.923	1.65E-01	1.65E+01		
7921526	NM_145167	PIGM	phosphatidylinositol glycan ancho	0.111	1.080	5.48E-01	5.48E+01		
7921538	NM_052868	IGSF8	immunoglobulin superfamily, men	-0.013	0.991	9.27E-01	9.27E+01		
7921552	NM_015726	DCAF8	DDB1 and CUL4 associated factor	-0.022	0.985	8.94E-01	8.94E+01		
7921571	NM_002857	PEX19	peroxisomal biogenesis factor 19	-0.133	0.912	8.79E-02	8.79E+00		
7921583	NM_00109839	COPA	coatomer protein complex, subun	0.083	1.059	6.84E-01	6.84E+01		
7921621	---	---	---	0.207	1.154	5.53E-01	5.53E+01		
7921625	NM_052931	SLAMF6	SLAM family member 6	0.283	1.217	2.04E-02	2.04E+00		
7921652	NM_003037	SLAMF1	signaling lymphocytic activation m	-0.303	0.811	2.55E-01	2.55E+01		
7921667	NM_001778	CD48	CD48 molecule	-0.023	0.984	9.37E-01	9.37E+01		
7921677	NM_016382	CD244	CD244 molecule, natural killer cell	0.052	1.037	9.22E-01	9.22E+01		
7921713	NM_016946	F11R	F11 receptor	-0.103	0.931	4.84E-01	4.84E+01		
7921738	NM_007122	USF1	upstream transcription factor 1	0.163	1.120	1.17E-01	1.17E+01		
7921752	NM_00102559	ARHGAP30	Rho GTPase activating protein 30	-0.004	0.997	9.85E-01	9.85E+01		
7921773	NM_030916	PVRL4	poliovirus receptor-related 4	-0.003	0.998	9.86E-01	9.86E+01		
7921786	NM_012394	PFDN2	prefoldin subunit 2	-0.060	0.959	5.92E-01	5.92E+01		
7921793	NM_032998	DEDD	death effector domain containing	-0.001	0.999	9.97E-01	9.97E+01		
7921806	NM_003779	B4GALT3	UDP-Gal:betaGlcNAc beta 1,4- gal	0.006	1.004	9.74E-01	9.74E+01		
7921834	NM_001643	APOA2	apolipoprotein A-II	0.056	1.040	8.13E-01	8.13E+01		
7921840	NM_00107748	NR1I3	nuclear receptor subfamily 1, grou	0.071	1.050	6.18E-01	6.18E+01		
7921895	NM_182581	C1orf111	chromosome 1 open reading fram	0.023	1.016	8.88E-01	8.88E+01		
7921900	NM_053282	SH2D1B	SH2 domain containing 1B	0.150	1.109	8.08E-01	8.08E+01		
7921930	---	---	---	0.063	1.045	7.58E-01	7.58E+01		
7921936	NM_177398	LMX1A	LIM homeobox transcription facto	-0.036	0.976	7.56E-01	7.56E+01		
7921970	NM_000696	ALDH9A1	aldehyde dehydrogenase 9 family,	0.016	1.011	9.04E-01	9.04E+01		
7921987	NM_019026	TMCO1	transmembrane and coiled-coil do	0.045	1.032	7.92E-01	7.92E+01		
7922001	BC114214	FAM78B	family with sequence similarity 78	-0.022	0.985	8.32E-01	8.32E+01		
7922008	NM_053053	TADA1L	transcriptional adaptor 1 (HFI1 ho	-0.029	0.980	9.27E-01	9.27E+01		
7922029	NM_005814	GPA33	glycoprotein A33 (transmembrane	0.159	1.117	2.64E-01	2.64E+01		
7922040	NM_198053	CD247	CD247 molecule	-0.044	0.970	8.73E-01	8.73E+01		
7922051	NM_003851	CREG1	cellular repressor of E1A-stimulate	0.133	1.096	7.72E-01	7.72E+01		

7922095	NR_026550	BRP44	brain protein 44	-0.058	0.961	6.80E-01	6.80E+01		
7922104	NM_004483	GCSH	glycine cleavage system protein H	0.055	1.039	8.06E-01	8.06E+01		
7922108	NM_153832	GPR161	G protein-coupled receptor 161	-0.009	0.994	9.57E-01	9.57E+01		
7922121	NR_026844	ANKRD36BL1	ankyrin repeat domain 36B-like 1 (	0.258	1.196	7.10E-02	7.10E+00		
7922162	NM_006996	SLC19A2	solute carrier family 19 (thiamine	-0.053	0.964	8.23E-01	8.23E+01		
7922174	NM_000130	F5	coagulation factor V (proaccelerin,	-0.011	0.992	9.86E-01	9.86E+01		
7922219	NM_000655	SELL	selectin L	0.059	1.042	8.38E-01	8.38E+01		
7922250	NM_181093	SCYL3	SCY1-like 3 (S. cerevisiae)	0.064	1.046	6.78E-01	6.78E+01		
7922268	NM_014970	KIFAP3	kinesin-associated protein 3	-0.070	0.953	5.68E-01	5.68E+01		
7922299	---	---	---	-0.068	0.954	6.33E-01	6.33E+01		
7922309	NM_003762	VAMP4	vesicle-associated membrane prot	-0.031	0.979	8.63E-01	8.63E+01		
7922326	---	---	---	0.009	1.006	9.67E-01	9.67E+01		
7922328	---	---	---	-0.097	0.935	4.29E-01	4.29E+01		
7922330	NM_153747	PIGC	phosphatidylinositol glycan ancho	0.061	1.043	5.98E-01	5.98E+01		
7922351	AK130460	LOC646870	hypothetical protein LOC646870	0.047	1.033	8.86E-01	8.86E+01		
7922400	---	---	---	-0.102	0.932	6.80E-01	6.80E+01		
7922402	NR_002746	SNORD47	small nucleolar RNA, C/D box 47	-0.054	0.963	8.45E-01	8.45E+01		
7922404	NR_003940	SNORD80	small nucleolar RNA, C/D box 80	-0.095	0.936	7.56E-01	7.56E+01		
7922406	NR_003939	SNORD79	small nucleolar RNA, C/D box 79	0.065	1.046	8.17E-01	8.17E+01		
7922408	NR_003944	SNORD78	small nucleolar RNA, C/D box 78	-0.015	0.989	9.78E-01	9.78E+01		
7922410	NR_002750	SNORD44	small nucleolar RNA, C/D box 44	0.034	1.024	9.55E-01	9.55E+01		
7922414	NR_003942	SNORD76	small nucleolar RNA, C/D box 76	-0.046	0.969	8.45E-01	8.45E+01		
7922416	NR_003941	SNORD75	small nucleolar RNA, C/D box 75	-0.395	0.760	1.11E-01	1.11E+01		
7922432	NM_172071	RC3H1	ring finger and CCCH-type zinc fing	0.015	1.010	9.30E-01	9.30E+01		
7922456	---	---	---	-0.197	0.872	2.45E-01	2.45E+01		
7922460	---	---	---	0.156	1.115	7.19E-01	7.19E+01		
7922462	NM_022100	MRPS14	mitochondrial ribosomal protein S	0.072	1.051	6.59E-01	6.59E+01		
7922474	NM_014656	KIAA0040	KIAA0040	0.229	1.172	6.46E-02	6.46E+00		
7922504	NM_022457	RFWD2	ring finger and WD repeat domain	0.080	1.057	5.36E-01	5.36E+01		
7922523	---	---	---	0.062	1.044	7.75E-01	7.75E+01		
7922525	NM_004319	ASTN1	astrotactin 1	-0.011	0.993	9.50E-01	9.50E+01		
7922590	AK125993	NCRNA00083	non-protein coding RNA 83	-0.014	0.990	9.30E-01	9.30E+01		
7922596	AK096082	LOC646976	hypothetical LOC646976	0.086	1.062	5.57E-01	5.57E+01		
7922610	NM_007314	ABL2	v-abl Abelson murine leukemia vir	0.026	1.018	9.30E-01	9.30E+01		
7922637	NM_145034	TOR1AIP2	torsin A interacting protein 2	0.007	1.005	9.81E-01	9.81E+01		
7922646	NM_022347	IFRG15	interferon responsive gene 15	-0.015	0.990	9.62E-01	9.62E+01		
7922648	NM_015602	TOR1AIP1	torsin A interacting protein 1	-0.038	0.974	7.56E-01	7.56E+01		
7922652	---	---	---	-0.104	0.931	3.90E-01	3.90E+01		
7922656	NM_032360	ACBD6	acyl-Coenzyme A binding domain c	0.057	1.040	7.52E-01	7.52E+01		
7922669	NM_005819	STX6	syntaxin 6	0.122	1.088	4.97E-01	4.97E+01		
7922684	---	---	---	-0.034	0.977	8.41E-01	8.41E+01		
7922686	NM_00100999	ZNF648	zinc finger protein 648	-0.120	0.920	7.81E-02	7.81E+00		
7922707	NM_021133	RNASEL	ribonuclease L (2',5'-oligoisoadeny	0.114	1.082	4.35E-01	4.35E+01		
7922717	NM_002928	RGS16	regulator of G-protein signaling 16	-0.050	0.966	6.60E-01	6.60E+01		
7922727	NM_00110245	RGS8	regulator of G-protein signaling 8	-0.024	0.984	8.77E-01	8.77E+01		
7922752	---	---	---	0.104	1.075	5.35E-01	5.35E+01		
7922773	NM_000433	NCF2	neutrophil cytosolic factor 2	0.441	1.357	3.02E-01	3.02E+01		
7922793	NM_005717	ARPC5	actin related protein 2/3 complex,	0.073	1.052	5.53E-01	5.53E+01		
7922807	NM_015101	GLT25D2	glycosyltransferase 25 domain cor	-0.002	0.999	9.97E-01	9.97E+01		
7922823	NM_025191	EDEM3	ER degradation enhancer, mannos	0.023	1.016	9.25E-01	9.25E+01		
7922846	NM_052966	FAM129A	family with sequence similarity 12	-0.305	0.810	2.83E-02	2.83E+00		
7922870	NM_030934	C1orf25	chromosome 1 open reading fram	0.072	1.051	6.05E-01	6.05E+01		
7922887	---	---	---	0.172	1.127	7.09E-01	7.09E+01		
7922889	NM_006469	IVNS1ABP	influenza virus NS1A binding prote	-0.057	0.961	6.62E-01	6.62E+01		
7922912	NM_003292	TPR	translocated promoter region (to a	-0.018	0.988	9.00E-01	9.00E+01		
7923007	NM_015984	UCHL5	ubiquitin carboxyl-terminal hydrol	0.012	1.008	9.53E-01	9.53E+01		
7923027	NM_197962	GLRX2	glutaredoxin 2	0.053	1.038	8.10E-01	8.10E+01		
7923119	NM_194314	ZBTB41	zinc finger and BTB domain contai	0.019	1.013	9.29E-01	9.29E+01		
7923131	NM_00114279	DENND1B	DENN/MADD domain containing 1	0.111	1.080	6.09E-01	6.09E+01		
7923141	NM_144977	DENND1B	DENN/MADD domain containing 1	0.178	1.131	2.17E-01	2.17E+01		
7923183	NM_012482	ZNF281	zinc finger protein 281	-0.055	0.963	5.53E-01	5.53E+01		
7923221	NM_00103172	DDX59	DEAD (Asp-Glu-Ala-Asp) box polyp	0.012	1.009	9.64E-01	9.64E+01		
7923233	NM_017596	KIF21B	kinesin family member 21B	-0.047	0.968	5.99E-01	5.99E+01		
7923319	NM_016456	TMEM9	transmembrane protein 9	-0.093	0.937	3.24E-01	3.24E+01		
7923332	NM_000364	TNNT2	troponin T type 2 (cardiac)	-0.110	0.926	3.46E-01	3.46E+01		
7923347	NM_005558	LAD1	ladinin 1	-0.063	0.957	6.47E-01	6.47E+01		
7923360	NM_003281	TNNI1	troponin I type 1 (skeletal, slow)	-0.025	0.983	7.95E-01	7.95E+01		
7923372	NM_012396	PHLDA3	pleckstrin homology-like domain, f	-0.047	0.968	7.45E-01	7.45E+01		
7923378	NM_004078	CSRP1	cysteine and glycine-rich protein 1	-0.006	0.996	9.82E-01	9.82E+01		
7923386	NM_012134	LMOD1	leiomodin 1 (smooth muscle)	0.051	1.036	6.42E-01	6.42E+01		
7923394	NM_138795	ARL8A	ADP-ribosylation factor-like 8A	0.073	1.052	6.75E-01	6.75E+01		
7923406	NM_080588	PTPN7	protein tyrosine phosphatase, non	0.033	1.023	8.29E-01	8.29E+01		
7923426	NM_014176	UBE2T	ubiquitin-conjugating enzyme E2T	0.096	1.069	7.49E-01	7.49E+01		
7923438	NM_001030	RPS27	ribosomal protein S27	-0.035	0.976	8.31E-01	8.31E+01		
7923440	---	---	---	0.037	1.026	9.53E-01	9.53E+01		
7923442	NM_177402	SYT2	synaptotagmin II	-0.119	0.921	1.76E-01	1.76E+01		
7923453	NM_006618	KDM5B	lysine (K)-specific demethylase 5B	-0.136	0.910	2.01E-01	2.01E+01		

7923483	NM_002871	RABIF	RAB interacting factor	0.045	1.032	7.83E-01	7.83E+01		
7923489	NM_021633	KLHL12	kelch-like 12 (Drosophila)	0.104	1.075	4.27E-01	4.27E+01		
7923501	---	---	---	0.116	1.084	5.51E-01	5.51E+01		
7923503	NM_015999	ADIPOR1	adiponectin receptor 1	0.097	1.069	6.00E-01	6.00E+01		
7923516	NM_016243	CYB5R1	cytochrome b5 reductase 1	0.049	1.035	7.63E-01	7.63E+01		
7923528	NM_002479	MYOG	myogenin (myogenic factor 4)	-0.004	0.998	9.85E-01	9.85E+01		
7923534	NM_004997	MYBPH	myosin binding protein H	0.034	1.024	7.50E-01	7.50E+01		
7923547	NM_001276	CHI3L1	chitinase 3-like 1 (cartilage glycopr	0.395	1.315	2.72E-01	2.72E+01		
7923582	---	---	---	-0.025	0.983	9.47E-01	9.47E+01		
7923584	NR_027902	C1orf157	chromosome 1 open reading fram	-0.010	0.993	9.33E-01	9.33E+01		
7923621	NM_002256	KISS1	KiSS-1 metastasis-suppressor	-0.058	0.960	4.56E-01	4.56E+01		
7923628	NM_198447	GOLT1A	golgi transport 1 homolog A (S. ce	0.011	1.007	9.64E-01	9.64E+01		
7923635	NM_014935	PLEKHA6	pleckstrin homology domain conta	0.030	1.021	7.97E-01	7.97E+01		
7923659	NM_032833	PPP1R15B	protein phosphatase 1, regulatory	-0.147	0.903	6.92E-02	6.92E+00		
7923662	NM_002646	PIK3C2B	phosphoinositide-3-kinase, class 2	-0.042	0.972	8.33E-01	8.33E+01		
7923698	---	---	---	0.080	1.057	7.03E-01	7.03E+01		
7923700	NM_006338	LRRN2	leucine rich repeat neuronal 2	-0.010	0.993	9.56E-01	9.56E+01		
7923705	---	---	---	-0.004	0.997	9.84E-01	9.84E+01		
7923707	NM_203376	TMEM81	transmembrane protein 81	-0.059	0.960	7.76E-01	7.76E+01		
7923712	NM_005057	RBBP5	retinoblastoma binding protein 5	-0.083	0.944	4.42E-01	4.42E+01		
7923731	NM_015375	DSTYK	dual serine/threonine and tyrosine	0.007	1.005	9.67E-01	9.67E+01		
7923753	NM_030952	NUAK2	NUAK family, SNF1-like kinase, 2	-0.004	0.997	9.87E-01	9.87E+01		
7923762	NM_018203	KLHDC8A	kelch domain containing 8A	0.008	1.005	9.66E-01	9.66E+01		
7923778	NM_001973	ELK4	ELK4, ETS-domain protein (SRF acc	-0.021	0.986	8.64E-01	8.64E+01		
7923792	NM_033102	SLC45A3	solute carrier family 45, member 3	0.000	1.000	9.99E-01	9.99E+01		
7923798	NM_022731	NUCKS1	nuclear casein kinase and cyclin-de	0.014	1.010	9.30E-01	9.30E+01		
7923810	---	---	---	0.093	1.066	8.27E-01	8.27E+01		
7923812	NM_003929	RAB7L1	RAB7, member RAS oncogene fam	0.002	1.001	9.96E-01	9.96E+01		
7923824	NM_173854	SLC41A1	solute carrier family 41, member 1	-0.113	0.925	2.71E-01	2.71E+01		
7923837	NM_152491	PM20D1	peptidase M20 domain containing	0.015	1.011	9.27E-01	9.27E+01		
7923885	NM_014002	IKBKE	inhibitor of kappa light polypeptid	0.018	1.012	9.51E-01	9.51E+01		
7923889	NM_006893	LGTN	ligatin	-0.019	0.987	9.27E-01	9.27E+01		
7923905	---	---	---	-0.265	0.832	4.70E-01	4.70E+01		
7923907	NM_000572	IL10	interleukin 10	-0.304	0.810	3.41E-01	3.41E+01		
7923917	NM_005449	FAIM3	Fas apoptotic inhibitory molecule	0.167	1.122	1.12E-01	1.12E+01		
7923942	NM_00112298	FCAMR	Fc receptor, IgA, IgM, high affinity	0.071	1.050	4.06E-01	4.06E+01		
7923956	---	---	---	-0.097	0.935	3.60E-01	3.60E+01		
7923958	NM_023938	C1orf116	chromosome 1 open reading fram	-0.028	0.981	8.14E-01	8.14E+01		
7923967	NM_018566	YOD1	YOD1 OTU deubiquinating enzyme	-0.005	0.997	9.84E-01	9.84E+01		
7923974	---	---	---	-0.090	0.940	7.52E-01	7.52E+01		
7923991	NM_025179	PLXNA2	plexin A2	-0.003	0.998	9.87E-01	9.87E+01		
7924092	NM_021194	SLC30A1	solute carrier family 30 (zinc trans	0.221	1.166	2.91E-01	2.91E+01		
7924107	NM_014873	LPGAT1	lysophosphatidylglycerol acyltrans	0.112	1.081	4.29E-01	4.29E+01		
7924144	NR_026911	RPL21P28	ribosomal protein L21 pseudogene	-0.020	0.986	9.14E-01	9.14E+01		
7924150	NM_018252	TMEM206	transmembrane protein 206	0.030	1.021	8.88E-01	8.88E+01		
7924166	NM_018664	BATF3	basic leucine zipper transcription f	-0.035	0.976	8.53E-01	8.53E+01		
7924190	NM_144567	ANGEL2	angel homolog 2 (Drosophila)	0.061	1.043	7.29E-01	7.29E+01		
7924230	NM_00113011	FAM108A1	family with sequence similarity 10	0.005	1.003	9.75E-01	9.75E+01		
7924327	NM_018040	GPATCH2	G patch domain containing 2	0.060	1.043	7.06E-01	7.06E+01		
7924351	NM_004446	EPRS	glutamyl-prolyl-tRNA synthetase	0.032	1.023	8.48E-01	8.48E+01		
7924386	---	---	---	-0.011	0.993	9.53E-01	9.53E+01		
7924388	NM_006085	BPNT1	3'(2'), 5'-bisphosphate nucleotidas	0.014	1.010	9.66E-01	9.66E+01		
7924405	NM_012414	RAB3GAP2	RAB3 GTPase activating protein su	0.053	1.038	7.64E-01	7.64E+01		
7924463	---	---	---	0.267	1.203	4.25E-01	4.25E+01		
7924465	NM_024746	HHIPL2	HHIP-like 2	-0.022	0.985	8.66E-01	8.66E+01		
7924491	BC043142	AIDA	axin interactor, dorsalization assoc	-0.172	0.888	3.15E-01	3.15E+01		
7924508	NM_017982	SUSD4	sushi domain containing 4	-0.026	0.982	8.64E-01	8.64E+01		
7924526	NM_005426	TP53BP2	tumor protein p53 binding protein	-0.201	0.870	1.60E-02	1.60E+00		
7924549	BC032332	PCMTD2	protein-L-isoaspartate (D-aspartat	-0.016	0.989	9.61E-01	9.61E+01		
7924553	ENST00000415	DEGS1	degenerative spermatocyte homo	0.034	1.024	8.04E-01	8.04E+01		
7924558	NM_002533	NVL	nuclear VCP-like	0.022	1.015	9.28E-01	9.28E+01		
7924582	NM_025160	WDR26	WD repeat domain 26	-0.038	0.974	7.42E-01	7.42E+01		
7924603	NM_002296	LBR	lamin B receptor	0.060	1.043	5.92E-01	5.92E+01		
7924636	NM_014698	TMEM63A	transmembrane protein 63A	0.003	1.002	9.89E-01	9.89E+01		
7924663	NM_020997	LEFTY1	left-right determination factor 1	0.001	1.001	9.97E-01	9.97E+01		
7924669	NM_013328	PYCR2	pyrroline-5-carboxylate reductase	-0.031	0.979	7.94E-01	7.94E+01		
7924682	NM_003240	LEFTY2	left-right determination factor 2	-0.115	0.923	1.72E-01	1.72E+01		
7924686	NM_152608	C1orf55	chromosome 1 open reading fram	0.044	1.031	7.56E-01	7.56E+01		
7924701	NM_022735	ACBD3	acyl-Coenzyme A binding domain c	0.045	1.031	5.71E-01	5.71E+01		
7924733	NM_001618	PARP1	poly (ADP-ribose) polymerase 1	0.025	1.017	9.01E-01	9.01E+01		
7924756	---	---	---	0.024	1.017	8.73E-01	8.73E+01		
7924758	---	---	---	0.025	1.018	9.50E-01	9.50E+01		
7924760	NM_002221	ITPKB	inositol 1,4,5-trisphosphate 3-kina	-0.051	0.966	7.08E-01	7.08E+01		
7924819	---	---	---	0.043	1.030	7.85E-01	7.85E+01		
7924823	NM_023007	JMJD4	jumonji domain containing 4	-0.049	0.967	5.27E-01	5.27E+01		
7924832	NM_003395	WNT9A	wingless-type MMTV integration s	-0.046	0.969	6.01E-01	6.01E+01		
7924840	---	---	---	-0.009	0.994	9.77E-01	9.77E+01		

7924842	BC002843	C1orf35	chromosome 1 open reading fram	-0.016	0.989	9.22E-01	9.22E+01		
7924853	NM_181462	MRPL55	mitochondrial ribosomal protein L	-0.014	0.990	9.19E-01	9.19E+01		
7924863	NM_052843	OBSCN	obscurin, cytoskeletal calmodulin	-0.018	0.988	9.44E-01	9.44E+01		
7924868	NM_145214	TRIM11	tripartite motif-containing 11	-0.019	0.987	8.73E-01	8.73E+01		
7924874	NM_016102	TRIM17	tripartite motif-containing 17	-0.012	0.992	9.53E-01	9.53E+01		
7924884	NM_003493	HIST3H3	histone cluster 3, H3	-0.023	0.984	8.48E-01	8.48E+01		
7924893	---	---	---	0.012	1.008	9.37E-01	9.37E+01		
7924897	NM_145257	C1orf96	chromosome 1 open reading fram	0.003	1.002	9.89E-01	9.89E+01		
7924908	---	---	---	0.011	1.007	9.37E-01	9.37E+01		
7924910	NM_001100	ACTA1	actin, alpha 1, skeletal muscle	-0.026	0.982	7.77E-01	7.77E+01		
7924923	NM_018230	NUP133	nucleoporin 133kDa	0.098	1.070	4.03E-01	4.03E+01		
7924956	NM_012089	ABCB10	ATP-binding cassette, sub-family B	-0.012	0.992	9.49E-01	9.49E+01		
7924969	NM_014409	TAF5L	TAF5-like RNA polymerase II, p300	0.014	1.010	9.50E-01	9.50E+01		
7924996	NM_032800	C1orf198	chromosome 1 open reading fram	-0.076	0.949	5.39E-01	5.39E+01		
7925003	NM_024525	TTC13	tetratricopeptide repeat domain 1	0.087	1.062	3.94E-01	3.94E+01		
7925028	NM_198552	FAM89A	family with sequence similarity 89	0.039	1.028	7.42E-01	7.42E+01		
7925033	NM_152379	C1orf131	chromosome 1 open reading fram	0.161	1.118	2.05E-01	2.05E+01		
7925043	NM_175876	EXOC8	exocyst complex component 8	-0.021	0.986	8.83E-01	8.83E+01		
7925048	NM_022051	EGLN1	egl nine homolog 1 (C. elegans)	0.038	1.027	7.72E-01	7.72E+01		
7925062	NM_020808	SIPA1L2	signal-induced proliferation-associ	0.083	1.059	7.90E-01	7.90E+01		
7925089	NM_014801	PCNXL2	pecanex-like 2 (Drosophila)	-0.087	0.942	5.17E-01	5.17E+01		
7925128	---	---	---	-0.050	0.966	7.03E-01	7.03E+01		
7925130	NM_005646	TARBP1	TAR (HIV-1) RNA binding protein 1	0.055	1.039	7.68E-01	7.68E+01		
7925161	NM_182972	IRF2BP2	interferon regulatory factor 2 bind	-0.022	0.985	8.69E-01	8.69E+01		
7925174	NM_014765	TOMM20	translocase of outer mitochondria	0.018	1.013	9.49E-01	9.49E+01		
7925184	NM_015014	RBM34	RNA binding motif protein 34	-0.065	0.956	3.64E-01	3.64E+01		
7925201	NM_016374	ARID4B	AT rich interactive domain 4B (RBI	-0.029	0.980	8.41E-01	8.41E+01		
7925229	NM_152490	B3GALNT2	beta-1,3-N-acetylgalactosaminyltr	0.009	1.006	9.76E-01	9.76E+01		
7925244	---	---	---	0.014	1.010	9.49E-01	9.49E+01		
7925250	NM_00109872	GNG4	guanine nucleotide binding protei	-0.067	0.955	5.79E-01	5.79E+01		
7925257	NM_000081	LYST	lysosomal trafficking regulator	0.000	1.000	1.00E+00	1.00E+02		
7925318	---	---	---	0.386	1.307	1.11E-01	1.11E+01		
7925361	NM_006499	LGALS8	lectin, galactoside-binding, soluble	-0.010	0.993	9.66E-01	9.66E+01		
7925364	NM_018072	HEATR1	HEAT repeat containing 1	0.056	1.039	7.69E-01	7.69E+01		
7925413	AF333388	MT1P2	metallothionein 1 pseudogene 2	-0.189	0.877	8.70E-02	8.70E+00		
7925448	---	---	---	-0.019	0.987	9.53E-01	9.53E+01		
7925480	NM_000143	FH	fumarate hydratase	0.015	1.010	9.57E-01	9.57E+01		
7925492	NM_014322	OPN3	opsin 3	0.139	1.101	3.36E-01	3.36E+01		
7925500	NM_001821	CHML	choroideremia-like (Rab escort pro	0.043	1.030	8.60E-01	8.60E+01		
7925504	NM_00100434	MAP1LC3C	microtubule-associated protein 1 l	-0.051	0.965	6.16E-01	6.16E+01		
7925509	---	---	---	0.025	1.017	9.02E-01	9.02E+01		
7925531	NM_181690	AKT3	v-akt murine thymoma viral oncog	-0.150	0.902	1.58E-01	1.58E+01		
7925550	NM_001126	ADSS	adenylosuccinate synthase	-0.088	0.941	6.20E-01	6.20E+01		
7925561	NM_198076	FAM36A	family with sequence similarity 36	0.002	1.001	9.93E-01	9.93E+01		
7925565	NM_031844	HNRNPU	heterogeneous nuclear ribonucleo	-0.013	0.991	8.96E-01	8.96E+01		
7925585	NM_032328	EFCAB2	EF-hand calcium binding domain 2	0.051	1.036	6.06E-01	6.06E+01		
7925587	---	---	---	-0.102	0.931	5.18E-01	5.18E+01		
7925589	NM_022743	SMYD3	SET and MYND domain containing	0.052	1.037	8.30E-01	8.30E+01		
7925609	---	---	---	0.024	1.017	9.69E-01	9.69E+01		
7925622	NM_015446	AHCTF1	AT hook containing transcription f	-0.045	0.969	7.51E-01	7.51E+01		
7925677	NM_024804	ZNF669	zinc finger protein 669	0.031	1.021	8.58E-01	8.58E+01		
7925687	NM_207401	C1orf229	chromosome 1 open reading fram	-0.034	0.977	7.89E-01	7.89E+01		
7925691	NM_003431	ZNF124	zinc finger protein 124	0.245	1.185	5.53E-02	5.53E+00		
7925701	---	---	---	-0.019	0.987	9.73E-01	9.73E+01		
7925705	NM_032752	ZNF496	zinc finger protein 496	0.024	1.017	8.05E-01	8.05E+01		
7925728	NM_00100528	OR6F1	olfactory receptor, family 6, subfa	-0.049	0.966	7.83E-01	7.83E+01		
7925737	NM_00100195	OR11L1	olfactory receptor, family 11, subf	-0.025	0.983	8.22E-01	8.22E+01		
7925739	---	---	---	-0.053	0.964	7.52E-01	7.52E+01		
7925741	NM_00100469	OR2T33	olfactory receptor, family 2, subfa	-0.048	0.967	6.03E-01	6.03E+01		
7925743	NM_00100469	OR2T12	olfactory receptor, family 2, subfa	-0.035	0.976	7.19E-01	7.19E+01		
7925749	NM_00100182	OR2T34	olfactory receptor, family 2, subfa	0.049	1.035	7.92E-01	7.92E+01		
7925763	NM_030645	SH3BP5L	SH3-binding domain protein 5-like	-0.030	0.979	8.55E-01	8.55E+01		
7925773	NM_017865	ZNF692	zinc finger protein 692	-0.081	0.945	2.98E-01	2.98E+01		
7925792	NM_006624	ZMYND11	zinc finger, MYND domain contain	-0.114	0.924	2.26E-01	2.26E+01		
7925813	NR_027152	C10orf108	chromosome 10 open reading fran	-0.024	0.983	8.46E-01	8.46E+01		
7925821	---	---	---	0.051	1.036	8.43E-01	8.43E+01		
7925823	NM_012341	GTPBP4	GTP binding protein 4	0.048	1.034	6.97E-01	6.97E+01		
7925851	NM_014023	WDR37	WD repeat domain 37	0.069	1.049	5.26E-01	5.26E+01		
7925870	NR_015376	NCRNA00200	non-protein coding RNA 200	-0.034	0.976	8.64E-01	8.64E+01		
7925876	NM_002627	PFKP	phosphofructokinase, platelet	-0.091	0.939	5.26E-01	5.26E+01		
7925904	NM_00104017	AKR1CL2	aldo-keto reductase family 1, mem	-0.027	0.982	9.49E-01	9.49E+01		
7925918	NM_001353	AKR1C1	aldo-keto reductase family 1, mem	-0.246	0.843	9.30E-02	9.30E+00		
7925950	NM_053049	UCN3	urocortin 3 (stresscopin)	-0.014	0.990	9.23E-01	9.23E+01		
7925954	NM_00104716	NET1	neuroepithelial cell transforming 1	0.004	1.003	9.90E-01	9.90E+01		
7925972	NM_005185	CALML3	calmodulin-like 3	-0.069	0.953	3.54E-01	3.54E+01		
7925978	NM_017782	C10orf18	chromosome 10 open reading fran	0.052	1.036	7.08E-01	7.08E+01		
7925996	NM_032807	FBXO18	F-box protein, helicase, 18	0.011	1.007	9.54E-01	9.54E+01		

7926021	NM_00114554	RBM17	RNA binding motif protein 17	-0.105	0.930	7.90E-02	7.90E+00		
7926059	---	---	---	-0.194	0.874	4.31E-01	4.31E+01		
7926084	NM_00100197	ATP5C1	ATP synthase, H+ transporting, mi	0.022	1.016	9.01E-01	9.01E+01		
7926096	NM_031923	TAF3	TAF3 RNA polymerase II, TATA box	-0.071	0.952	5.09E-01	5.09E+01		
7926105	NM_00100229	GATA3	GATA binding protein 3	-0.097	0.935	5.35E-01	5.35E+01		
7926127	NM_00102507	CUGBP2	CUG triplet repeat, RNA binding p	0.214	1.160	2.16E-04	2.16E-02		
7926150	NM_014688	USP6NL	USP6 N-terminal like	-0.018	0.987	9.04E-01	9.04E+01		
7926152	NM_024693	ECHDC3	enoyl Coenzyme A hydratase dom	0.000	1.000	1.00E+00	1.00E+02		
7926158	NM_153256	C10orf47	chromosome 10 open reading fra	-0.032	0.978	8.58E-01	8.58E+01		
7926170	NM_018706	DHTKD1	dehydrogenase E1 and transketol	0.144	1.105	1.31E-01	1.31E+01		
7926207	NM_006023	CDC123	cell division cycle 123 homolog (S.	-0.027	0.981	8.95E-01	8.95E+01		
7926223	NM_153498	CAMK1D	calcium/calmodulin-dependent pr	0.059	1.042	6.96E-01	6.96E+01		
7926239	NM_00100821	OPTN	optineurin	-0.129	0.914	1.78E-01	1.78E+01		
7926283	NM_003675	PRPF18	PRP18 pre-mRNA processing facto	0.013	1.009	9.53E-01	9.53E+01		
7926297	---	---	---	-0.026	0.982	9.18E-01	9.18E+01		
7926299	NM_016299	HSPA14	heat shock 70kDa protein 14	0.012	1.009	9.53E-01	9.53E+01		
7926319	NM_024670	SUV39H2	suppressor of variegation 3-9 hom	0.027	1.019	8.93E-01	8.93E+01		
7926356	NM_00100148	PTER	phosphotriesterase related	0.052	1.037	7.87E-01	7.87E+01		
7926368	NM_003380	VIM	vimentin	0.025	1.017	9.00E-01	9.00E+01		
7926385	NM_003473	STAM	signal transducing adaptor molecu	-0.118	0.921	1.82E-01	1.82E+01		
7926403	NM_00109884	FAM23A	family with sequence similarity 23	0.213	1.159	5.38E-02	5.38E+00		
7926445	NM_00109884	FAM23A	family with sequence similarity 23	0.213	1.159	5.38E-02	5.38E+00		
7926506	NM_201596	CACNB2	calcium channel, voltage-depende	0.049	1.034	7.42E-01	7.42E+01		
7926531	NM_178815	ARL5B	ADP-ribosylation factor-like 5B	0.081	1.057	5.31E-01	5.31E+01		
7926565	NM_004641	MLLT10	myeloid/lymphoid or mixed-lineag	-0.043	0.971	7.89E-01	7.89E+01		
7926596	NM_012071	COMMD3	COMM domain containing 3	-0.059	0.960	6.96E-01	6.96E+01		
7926609	NM_005180	BMI1	BMI1 polycomb ring finger oncoge	-0.064	0.956	6.95E-01	6.95E+01		
7926659	---	---	---	-0.055	0.962	7.35E-01	7.35E+01		
7926661	NM_012228	MSRB2	methionine sulfoxide reductase B2	0.122	1.088	2.69E-01	2.69E+01		
7926670	---	---	---	0.017	1.012	9.00E-01	9.00E+01		
7926674	NM_178161	PTF1A	pancreas specific transcription fac	-0.016	0.989	9.01E-01	9.01E+01		
7926677	NM_00114537	OTUD1	OTU domain containing 1	0.060	1.042	8.41E-01	8.41E+01		
7926706	---	---	---	-0.063	0.957	7.57E-01	7.57E+01		
7926786	NM_019043	APBB1IP	amyloid beta (A4) precursor prote	0.044	1.031	7.82E-01	7.82E+01		
7926805	---	---	---	0.106	1.076	7.47E-01	7.47E+01		
7926807	NM_014317	PDSS1	prenyl (decaprenyl) diphosphate s	-0.123	0.918	7.14E-01	7.14E+01		
7926817	---	---	---	0.027	1.019	9.37E-01	9.37E+01		
7926836	NM_021252	RAB18	RAB18, member RAS oncogene fa	0.092	1.066	5.56E-01	5.56E+01		
7926851	NR_024557	WAC	WW domain containing adaptor w	-0.018	0.988	9.03E-01	9.03E+01		
7926896	NM_001826	CKS1B	CDC28 protein kinase regulatory s	0.164	1.121	5.97E-01	5.97E+01		
7926900	NM_005204	MAP3K8	mitogen-activated protein kinase	-0.011	0.993	9.76E-01	9.76E+01		
7926916	NR_024285	ZEB1	zinc finger E-box binding homeob	-0.136	0.910	5.39E-01	5.39E+01		
7926936	NM_024688	C10orf68	chromosome 10 open reading fra	0.049	1.034	7.75E-01	7.75E+01		
7926979	---	---	---	0.177	1.131	3.91E-01	3.91E+01		
7926983	NM_183013	CREM	cAMP responsive element modula	-0.243	0.845	3.63E-02	3.63E+00		
7927008	NM_181698	CCNY	cyclin Y	0.062	1.044	5.48E-01	5.48E+01		
7927028	NM_153368	GJD4	gap junction protein, delta 4, 40.1	0.032	1.022	7.78E-01	7.78E+01		
7927062	NM_006954	ZNF33A	zinc finger protein 33A	-0.005	0.997	9.87E-01	9.87E+01		
7927071	NM_00100709	ZNF37A	zinc finger protein 37A	0.075	1.054	5.56E-01	5.56E+01		
7927082	NR_003086	HSD17B7P2	hydroxysteroid (17-beta) dehydro	-0.041	0.972	9.12E-01	9.12E+01		
7927089	---	---	---	0.267	1.203	4.25E-01	4.25E+01		
7927091	NR_024497	LOC399744	hypothetical LOC399744	0.018	1.012	9.20E-01	9.20E+01		
7927095	BC032332	PCMTD2	protein-L-isoaspartate (D-asparta	-0.019	0.987	9.53E-01	9.53E+01		
7927099	NR_026827	LOC84856	hypothetical LOC84856	-0.031	0.979	8.06E-01	8.06E+01		
7927106	---	---	---	0.037	1.026	9.38E-01	9.38E+01		
7927108	NM_014753	BMS1	BMS1 homolog, ribosome assemb	-0.126	0.916	3.36E-01	3.36E+01		
7927146	NM_018590	CSGALNACT2	chondroitin sulfate N-acetylgalact	0.092	1.066	6.97E-01	6.97E+01		
7927153	---	---	---	-0.053	0.964	7.60E-01	7.60E+01		
7927155	NM_173160	FXYD4	FXYD domain containing ion trans	0.019	1.013	9.00E-01	9.00E+01		
7927167	---	---	---	0.013	1.009	9.60E-01	9.60E+01		
7927173	NM_145312	ZNF485	zinc finger protein 485	0.007	1.005	9.86E-01	9.86E+01		
7927179	NM_00112337	TMEM72	transmembrane protein 72	-0.041	0.972	7.87E-01	7.87E+01		
7927186	NM_032023	RASSF4	Ras association (RalGDS/AF-6) do	0.131	1.095	6.57E-01	6.57E+01		
7927202	NM_006963	ZNF22	zinc finger protein 22 (KOX 15)	0.016	1.011	9.24E-01	9.24E+01		
7927231	NM_00114400	AGAP5	ArfGAP with GTPase domain, anky	-0.033	0.978	8.53E-01	8.53E+01		
7927233	NM_015262	FAM21C	family with sequence similarity 21	0.062	1.044	6.64E-01	6.64E+01		
7927267	NM_019054	FAM35A	family with sequence similarity 35	0.222	1.167	1.31E-02	1.31E+00		
7927277	NM_014696	GPRIN2	G protein regulated inducer of ne	0.027	1.019	8.29E-01	8.29E+01		
7927285	NM_005614	RHEB	Ras homolog enriched in brain	0.180	1.133	2.04E-01	2.04E+01		
7927288	NM_019054	FAM35A	family with sequence similarity 35	0.269	1.205	3.60E-03	3.60E-01		
7927305	NM_00114400	AGAP5	ArfGAP with GTPase domain, anky	0.001	1.001	9.95E-01	9.95E+01		
7927307	NM_001630	ANXA8L2	annexin A8-like 2	-0.029	0.980	7.63E-01	7.63E+01		
7927323	NM_018232	FAM21B	family with sequence similarity 21	0.065	1.046	6.34E-01	6.34E+01		
7927353	NM_00114400	AGAP5	ArfGAP with GTPase domain, anky	0.044	1.031	8.01E-01	8.01E+01		
7927363	NM_00113755	FAM25B	family with sequence similarity 25	-0.033	0.977	8.18E-01	8.18E+01		
7927367	NM_00104008	ANXA8	annexin A8	-0.032	0.978	7.26E-01	7.26E+01		
7927383	NM_153034	ZNF488	zinc finger protein 488	-0.093	0.938	3.18E-01	3.18E+01		

7927389	NM_002750	MAPK8	mitogen-activated protein kinase	-0.105	0.930	4.91E-01	4.91E+01				
7927405	NM_020945	WDFY4	WDFY family member 4	0.136	1.099	2.85E-01	2.85E+01				
7927425	NM_020945	WDFY4	WDFY family member 4	0.065	1.046	5.68E-01	5.68E+01				
7927482	NM_00114293	CHAT	choline acetyltransferase	0.012	1.008	9.47E-01	9.47E+01				
7927502	NM_003055	SLC18A3	solute carrier family 18 (vesicular	-0.062	0.958	5.88E-01	5.88E+01				
7927513	NM_015262	FAM21C	family with sequence similarity 21	0.088	1.063	5.23E-01	5.23E+01				
7927519	NM_006327	TIMM23	translocase of inner mitochondrial	0.004	1.002	9.87E-01	9.87E+01				
7927527	---	---	---	-0.066	0.955	7.81E-01	7.81E+01				
7927536	NM_005437	NCOA4	nuclear receptor coactivator 4	0.173	1.128	4.94E-01	4.94E+01				
7927548	NM_006327	TIMM23	translocase of inner mitochondrial	0.016	1.011	9.60E-01	9.60E+01				
7927552	NM_00107766	AGAP6	ArfGAP with GTPase domain, anky	0.057	1.040	6.92E-01	6.92E+01				
7927560	NM_00100575	FAM21A	family with sequence similarity 21	0.070	1.050	5.78E-01	5.78E+01				
7927595	---	---	---	0.027	1.019	9.28E-01	9.28E+01				
7927597	---	---	---	0.037	1.026	8.92E-01	8.92E+01				
7927643	---	---	---	-0.029	0.980	8.80E-01	8.80E+01				
7927645	---	---	---	0.012	1.008	9.73E-01	9.73E+01				
7927649	NM_018464	CISD1	CDGSH iron sulfur domain 1	0.006	1.004	9.88E-01	9.88E+01				
7927658	NM_003338	UBE2D1	ubiquitin-conjugating enzyme E2D	0.182	1.135	3.85E-01	3.85E+01				
7927669	NM_003201	TFAM	transcription factor A, mitochondr	0.011	1.008	9.55E-01	9.55E+01				
7927706	---	---	---	-0.203	0.869	7.47E-01	7.47E+01				
7927732	NM_032199	ARID5B	AT rich interactive domain 5B (MR	0.055	1.039	5.53E-01	5.53E+01				
7927767	NM_032804	ADO	2-aminoethanethiol (cysteamine)	0.188	1.139	9.78E-02	9.78E+00				
7927775	NM_030759	NRBF2	nuclear receptor binding factor 2	0.066	1.047	6.20E-01	6.20E+01				
7927784	NM_032776	JMJD1C	jumonji domain containing 1C	0.047	1.033	7.51E-01	7.51E+01				
7927786	NM_00100133	REEP3	receptor accessory protein 3	0.184	1.136	2.65E-01	2.65E+01				
7927799	ENST00000298	REEP3	receptor accessory protein 3	0.212	1.158	2.75E-01	2.75E+01				
7927801	NR_001446	ANXA2P3	annexin A2 pseudogene 3	0.129	1.094	2.23E-01	2.23E+01				
7927814	NM_012238	SIRT1	sirtuin (silent mating type informa	-0.040	0.973	7.56E-01	7.56E+01				
7927854	NM_012207	HNRNPH3	heterogeneous nuclear ribonucleo	0.028	1.020	7.73E-01	7.73E+01				
7927872	---	---	---	0.000	1.000	1.00E+00	1.00E+02				
7927889	NM_018237	CCAR1	cell division cycle and apoptosis re	0.032	1.023	7.64E-01	7.64E+01				
7927926	NM_024045	DDX50	DEAD (Asp-Glu-Ala-Asp) box polyp	0.043	1.030	7.56E-01	7.56E+01				
7927936	NM_004728	DDX21	DEAD (Asp-Glu-Ala-Asp) box polyp	0.066	1.047	6.48E-01	6.48E+01				
7927955	NM_015634	KIAA1279	KIAA1279	-0.067	0.955	7.88E-01	7.88E+01				
7927964	NM_002727	SRGN	serglycin	-0.029	0.980	9.23E-01	9.23E+01				
7927972	NM_004896	VPS26A	vacuolar protein sorting 26 homol	0.119	1.086	4.41E-01	4.41E+01				
7927981	NM_003171	SUPV3L1	suppressor of var1, 3-like 1 (S. cere	-0.077	0.948	7.25E-01	7.25E+01				
7928019	NM_033500	HK1	hexokinase 1	0.100	1.072	3.19E-01	3.19E+01				
7928046	NM_012339	TSPAN15	tetraspanin 15	-0.127	0.916	2.59E-01	2.59E+01				
7928064	NM_145306	C10orf35	chromosome 10 open reading frar	0.010	1.007	9.58E-01	9.58E+01				
7928069	NM_005203	COL13A1	collagen, type XIII, alpha 1	0.002	1.001	9.90E-01	9.90E+01				
7928107	NM_018649	H2AFY2	H2A histone family, member Y2	0.029	1.020	8.81E-01	8.81E+01				
7928119	NM_004096	EIF4EBP2	eukaryotic translation initiation fa	0.059	1.042	7.87E-01	7.87E+01				
7928126	NM_014431	KIAA1274	KIAA1274	0.081	1.057	3.31E-01	3.31E+01				
7928147	NM_139155	ADAMTS14	ADAM metallopeptidase with thro	0.033	1.023	8.06E-01	8.06E+01				
7928171	NM_003901	SGPL1	sphingosine-1-phosphate lyase 1	0.315	1.244	4.01E-02	4.01E+00				
7928189	NM_170744	UNC5B	unc-5 homolog B (C. elegans)	0.004	1.003	9.77E-01	9.77E+01				
7928208	NM_018344	SLC29A3	solute carrier family 29 (nucleosid	0.036	1.025	8.30E-01	8.30E+01				
7928216	---	---	---	0.002	1.001	9.92E-01	9.92E+01				
7928291	NM_004273	CHST3	carbohydrate (chondroitin 6) sulfo	-0.084	0.943	3.47E-01	3.47E+01				
7928300	NM_173473	C10orf104	chromosome 10 open reading frar	0.054	1.038	6.76E-01	6.76E+01				
7928306	---	---	---	0.148	1.108	5.91E-01	5.91E+01				
7928318	NM_138357	CCDC109A	coiled-coil domain containing 109	-0.010	0.993	9.58E-01	9.58E+01				
7928342	NM_015901	NUDT13	nudix (nucleoside diphosphate lin	0.068	1.048	7.68E-01	7.68E+01				
7928369	NM_004922	SEC24C	SEC24 family, member C (S. cerevi	0.075	1.053	4.83E-01	4.83E+01				
7928395	NM_173540	FUT11	fucosyltransferase 11 (alpha (1,3)	0.023	1.016	9.22E-01	9.22E+01				
7928401	NM_203298	CHCHD1	coiled-coil-helix-coiled-coil-helix d	-0.017	0.988	9.54E-01	9.54E+01				
7928408	NM_015037	KIAA0913	KIAA0913	0.010	1.007	9.62E-01	9.62E+01				
7928411	NM_015037	KIAA0913	KIAA0913	-0.007	0.995	9.67E-01	9.67E+01				
7928429	NM_002658	PLAU	plasminogen activator, urokinase	-0.012	0.992	9.66E-01	9.66E+01				
7928444	NM_014000	VCL	vinculin	0.099	1.071	4.29E-01	4.29E+01				
7928471	NM_006721	ADK	adenosine kinase	0.058	1.041	7.72E-01	7.72E+01				
7928489	---	---	---	0.006	1.004	9.87E-01	9.87E+01				
7928491	NM_012330	MYST4	MYST histone acetyltransferase (m	0.070	1.049	6.74E-01	6.74E+01				
7928516	NM_144660	SAMD8	sterile alpha motif domain contair	0.037	1.026	8.50E-01	8.50E+01				
7928524	NM_003375	VDAC2	voltage-dependent anion channel	0.004	1.003	9.88E-01	9.88E+01				
7928529	---	---	---	-0.036	0.975	8.49E-01	8.49E+01				
7928531	NR_024421	C10orf41	chromosome 10 open reading frar	-0.033	0.977	9.28E-01	9.28E+01				
7928547	---	---	---	-0.007	0.995	9.66E-01	9.66E+01				
7928551	NM_033022	RPS24	ribosomal protein S24	0.014	1.010	9.44E-01	9.44E+01				
7928556	---	---	---	-0.017	0.989	9.49E-01	9.49E+01				
7928558	NM_020338	ZMIZ1	zinc finger, MIZ-type containing 1	0.005	1.003	9.87E-01	9.87E+01				
7928600	NM_00109969	EIF5AL1	eukaryotic translation initiation fa	-0.059	0.960	7.88E-01	7.88E+01				
7928602	NM_005411	SFTPA1	surfactant protein A1	-0.066	0.955	2.45E-01	2.45E+01				
7928619	NM_00109933	FAM22A	family with sequence similarity 22	-0.044	0.970	5.37E-01	5.37E+01				
7928630	0	0	0	-0.080	0.946	6.91E-01	6.91E+01				
7928632	NM_005411	SFTPA1	surfactant protein A1	-0.066	0.955	2.45E-01	2.45E+01				

7928645	NM_00109933	FAM22A	family with sequence similarity 22	-0.070	0.952	2.51E-01	2.51E+01		
7928653	NM_00109933	FAM22A	family with sequence similarity 22	-0.059	0.960	3.78E-01	3.78E+01		
7928679	NM_00101297	PLAC9	placenta-specific 9	-0.047	0.968	8.05E-01	8.05E+01		
7928695	NM_032333	C10orf58	chromosome 10 open reading fran	0.159	1.116	4.27E-01	4.27E+01		
7928705	NM_030927	TSPAN14	tetraspanin 14	0.056	1.040	7.52E-01	7.52E+01		
7928752	NM_014394	GHITM	growth hormone inducible transm	-0.011	0.992	9.63E-01	9.63E+01		
7928766	NM_207373	C10orf99	chromosome 10 open reading fran	-0.077	0.948	4.04E-01	4.04E+01		
7928789	NM_002921	RGR	retinal G protein coupled receptor	0.023	1.016	8.78E-01	8.78E+01		
7928800	NM_018999	FAM190B	family with sequence similarity 19	-0.179	0.883	9.48E-02	9.48E+00		
7928821	---	---	---	0.202	1.150	5.39E-01	5.39E+01		
7928823	NM_00103001	OPN4	opsin 4	-0.002	0.998	9.86E-01	9.86E+01		
7928838	NM_007078	LDB3	LIM domain binding 3	-0.074	0.950	3.46E-01	3.46E+01		
7928870	---	---	---	-0.101	0.932	4.15E-01	4.15E+01		
7928872	NM_003087	SNCG	synuclein, gamma (breast cancer-s	-0.049	0.967	4.87E-01	4.87E+01		
7928882	NM_006829	C10orf116	chromosome 10 open reading fran	-0.112	0.925	8.28E-02	8.28E+00		
7928890	NM_133447	AGAP11	ankyrin repeat and GTPase domai	0.033	1.023	7.15E-01	7.15E+01		
7928907	---	---	---	0.378	1.299	1.07E-01	1.07E+01		
7928909	NM_019054	FAM35A	family with sequence similarity 35	0.257	1.195	4.07E-02	4.07E+00		
7928915	NM_00109933	FAM22A	family with sequence similarity 22	-0.038	0.974	6.45E-01	6.45E+01		
7928937	NM_004897	MINPP1	multiple inositol polyphosphate hi	0.211	1.157	3.73E-01	3.73E+01		
7928944	NM_004670	PAPSS2	3'-phosphoadenosine 5'-phospho	-0.336	0.792	3.50E-01	3.50E+01		
7928959	NM_000314	PTEN	phosphatase and tensin homolog	0.048	1.034	6.66E-01	6.66E+01		
7929012	NM_020799	STAMBPL1	STAM binding protein-like 1	0.230	1.173	4.68E-02	4.68E+00		
7929026	NM_00114194	ACTA2	actin, alpha 2, smooth muscle, aor	0.106	1.076	4.67E-01	4.67E+01		
7929032	NM_000043	FAS	Fas (TNF receptor superfamily, me	0.009	1.006	9.78E-01	9.78E+01		
7929047	NM_001547	IFIT2	interferon-induced protein with te	0.380	1.301	3.75E-01	3.75E+01		
7929052	NM_00103168	IFIT3	interferon-induced protein with te	0.716	1.643	1.09E-01	1.09E+01		
7929065	NM_001548	IFIT1	interferon-induced protein with te	0.596	1.511	4.36E-01	4.36E+01		
7929072	NM_012420	IFIT5	interferon-induced protein with te	0.200	1.149	5.34E-01	5.34E+01		
7929114	---	---	---	-0.041	0.972	7.67E-01	7.67E+01		
7929116	NM_006413	RPP30	ribonuclease P/MRP 30kDa subuni	0.112	1.081	4.35E-01	4.35E+01		
7929130	---	---	---	0.117	1.085	3.70E-01	3.70E+01		
7929132	NM_032373	PCGF5	polycomb group ring finger 5	0.089	1.064	2.65E-01	2.65E+01		
7929168	NM_025235	TNKS2	tankyrase, TRF1-interacting ankyri	0.064	1.046	6.14E-01	6.14E+01		
7929201	NM_003972	BTAF1	BTAF1 RNA polymerase II, B-TFIID	0.002	1.001	9.91E-01	9.91E+01		
7929243	NM_017838	NHP2	NHP2 ribonucleoprotein homolog	0.016	1.011	9.42E-01	9.42E+01		
7929247	NM_017824	40242	membrane-associated ring finger (	0.006	1.004	9.81E-01	9.81E+01		
7929256	---	---	---	0.126	1.091	7.27E-01	7.27E+01		
7929282	NM_002729	HHEX	hematopoietically expressed hom	0.310	1.240	8.99E-04	8.99E-02		
7929288	NM_019053	EXOC6	exocyst complex component 6	0.129	1.093	3.70E-01	3.70E+01		
7929315	NM_183374	CYP26C1	cytochrome P450, family 26, subfa	-0.019	0.987	8.88E-01	8.88E+01		
7929383	NM_00113465	TMEM20	transmembrane protein 20	-0.018	0.987	9.53E-01	9.53E+01		
7929511	NM_001776	ENTPD1	ectonucleoside triphosphate dipho	0.219	1.164	4.66E-01	4.66E+01		
7929550	NM_00113437	CCNJ	cyclin J	-0.007	0.995	9.76E-01	9.76E+01		
7929562	NM_014803	ZNF518A	zinc finger protein 518A	0.159	1.116	2.30E-01	2.30E+01		
7929589	---	---	---	0.039	1.027	8.62E-01	8.62E+01		
7929593	NR_026712	RPL13AP5	ribosomal protein L13a pseudoger	-0.008	0.994	9.64E-01	9.64E+01		
7929596	NM_032440	LCOR	ligand dependent nuclear recepto	-0.038	0.974	7.67E-01	7.67E+01		
7929609	BC024315	C10orf12	chromosome 10 open reading fran	-0.027	0.981	8.92E-01	8.92E+01		
7929616	NM_005479	FRAT1	frequently rearranged in advanced	-0.001	0.999	9.98E-01	9.98E+01		
7929624	NM_002629	PGAM1	phosphoglycerate mutase 1 (brain	-0.015	0.990	9.46E-01	9.46E+01		
7929634	NM_198046	ZDHHC16	zinc finger, DHHC-type containing	-0.117	0.922	4.02E-01	4.02E+01		
7929649	NM_024954	UBTD1	ubiquitin domain containing 1	0.082	1.059	4.02E-01	4.02E+01		
7929653	NM_020349	ANKRD2	ankyrin repeat domain 2 (stretch r	-0.007	0.995	9.63E-01	9.63E+01		
7929664	NM_138413	DHDPSL	dihydrodipicolinate synthase-like,	0.042	1.030	6.80E-01	6.80E+01		
7929677	NM_018425	PI4K2A	phosphatidylinositol 4-kinase type	0.204	1.152	6.71E-02	6.71E+00		
7929689	NR_026753	MARVELD1	MARVEL domain containing 1	0.069	1.049	4.97E-01	4.97E+01		
7929692	NM_00100226	ZFYVE27	zinc finger, FYVE domain containin	0.003	1.002	9.88E-01	9.88E+01		
7929711	NM_00101091	GOLGA7B	golgi autoantigen, golgin subfamily	0.037	1.026	8.24E-01	8.24E+01		
7929719	NM_014472	C10orf28	chromosome 10 open reading fran	-0.018	0.987	9.34E-01	9.34E+01		
7929744	NM_145285	NKX2-3	NK2 transcription factor related, lo	-0.067	0.954	4.83E-01	4.83E+01		
7929750	NM_020354	ENTPD7	ectonucleoside triphosphate dipho	-0.172	0.888	4.33E-02	4.33E+00		
7929768	NM_015960	CUTC	cutC copper transporter homolog	-0.024	0.983	8.93E-01	8.93E+01		
7929779	NM_000392	ABCC2	ATP-binding cassette, sub-family C	-0.045	0.969	7.87E-01	7.87E+01		
7929816	NM_005063	SCD	stearoyl-CoA desaturase (delta-9-c	0.149	1.109	4.45E-01	4.45E+01		
7929822	NM_003393	WNT8B	wingless-type MMTV integration s	-0.043	0.971	8.38E-01	8.38E+01		
7929840	NM_003987	PAX2	paired box 2	-0.035	0.976	6.57E-01	6.57E+01		
7929858	NM_018121	FAM178A	family with sequence similarity 17	0.135	1.098	7.05E-02	7.05E+00		
7929882	NM_017893	SEMA4G	sema domain, immunoglobulin do	-0.049	0.967	6.58E-01	6.58E+01		
7929901	NM_021830	C10orf2	chromosome 10 open reading fran	-0.025	0.983	9.33E-01	9.33E+01		
7929911	NM_032429	LZTS2	leucine zipper, putative tumor sup	-0.044	0.970	5.52E-01	5.52E+01		
7929919	NM_030971	SFXN3	sideroflexin 3	0.053	1.038	7.56E-01	7.56E+01		
7929932	NM_030929	KAZALD1	Kazal-type serine peptidase inhibit	0.012	1.008	9.54E-01	9.54E+01		
7929945	---	---	---	-0.021	0.986	9.00E-01	9.00E+01		
7929958	NM_033637	BTRC	beta-transducin repeat containing	0.007	1.005	9.77E-01	9.77E+01		
7929976	NM_015448	RP11-529I10.4	deleted in a mouse model of prim	-0.079	0.947	7.03E-01	7.03E+01		
7929988	NM_024747	HPS6	Hermansky-Pudlak syndrome 6	-0.095	0.936	3.66E-01	3.66E+01		

7929990	NM_015062	PPRC1	peroxisome proliferator-activated	0.013	1.009	9.47E-01	9.47E+01		
7930008	NM_004741	NOLC1	nucleolar and coiled-body phosph	-0.062	0.958	7.44E-01	7.44E+01		
7930031	NM_004193	GBF1	golgi-specific brefeldin A resistant	-0.022	0.985	9.09E-01	9.09E+01		
7930074	NM_002502	NFKB2	nuclear factor of kappa light polyp	-0.148	0.903	1.63E-01	1.63E+01		
7930099	NM_024326	FBXL15	F-box and leucine-rich repeat prot	0.001	1.001	9.96E-01	9.96E+01		
7930106	NM_024789	TMEM180	transmembrane protein 180	-0.025	0.983	8.56E-01	8.56E+01		
7930120	NM_016169	SUFU	suppressor of fused homolog (Dro	-0.022	0.985	8.57E-01	8.57E+01		
7930139	NM_030912	TRIM8	tripartite motif-containing 8	-0.052	0.964	7.27E-01	7.27E+01		
7930148	NM_178858	SFXN2	sideroflexin 2	0.007	1.005	9.78E-01	9.78E+01		
7930162	NM_017787	C10orf26	chromosome 10 open reading fran	0.141	1.103	1.91E-01	1.91E+01		
7930168	---	---	---	0.078	1.055	7.06E-01	7.06E+01		
7930170	NM_00113620	C10orf32	chromosome 10 open reading fran	0.068	1.048	7.25E-01	7.25E+01		
7930179	---	---	---	0.008	1.006	9.91E-01	9.91E+01		
7930181	NM_020682	AS3MT	arsenic (+3 oxidation state) methy	0.001	1.001	9.99E-01	9.99E+01		
7930194	NM_017649	CNNM2	cyclin M2	0.004	1.003	9.84E-01	9.84E+01		
7930205	NM_00114390	hCG_2024410	rcRPE	0.059	1.041	8.10E-01	8.10E+01		
7930213	NM_006951	TAF5	TAF5 RNA polymerase II, TATA box	0.002	1.002	9.90E-01	9.90E+01		
7930226	NM_014976	PDCD11	programmed cell death 11	-0.011	0.993	9.57E-01	9.57E+01		
7930264	NM_004210	NEURL	neuralized homolog (Drosophila)	-0.027	0.982	7.99E-01	7.99E+01		
7930276	NM_014720	SLK	STE20-like kinase (yeast)	0.015	1.010	9.20E-01	9.20E+01		
7930304	NM_004832	GSTO1	glutathione S-transferase omega 1	-0.101	0.932	7.09E-01	7.09E+01		
7930311	NM_183239	GSTO2	glutathione S-transferase omega 2	-0.034	0.977	8.52E-01	8.52E+01		
7930341	NM_014978	SORCS3	sortilin-related VPS10 domain con	-0.012	0.992	9.47E-01	9.47E+01		
7930376	---	---	---	-0.027	0.981	8.78E-01	8.78E+01		
7930380	NM_016824	ADD3	adducin 3 (gamma)	0.055	1.039	6.75E-01	6.75E+01		
7930398	NM_005962	MXI1	MAX interactor 1	-0.143	0.906	1.05E-01	1.05E+01		
7930413	NM_004419	DUSP5	dual specificity phosphatase 5	-0.314	0.804	7.94E-03	7.94E-01		
7930422	NM_005445	SMC3	structural maintenance of chromo	-0.010	0.993	9.48E-01	9.48E+01		
7930450	---	---	---	0.124	1.090	6.78E-01	6.78E+01		
7930454	NM_145341	PDCD4	programmed cell death 4 (neoplas	-0.143	0.906	2.04E-02	2.04E+00		
7930470	NM_007373	SHOC2	soc-2 suppressor of clear homolog	0.073	1.052	5.35E-01	5.35E+01		
7930482	NM_000681	ADRA2A	adrenergic, alpha-2A-, receptor	0.015	1.010	9.10E-01	9.10E+01		
7930498	NM_016234	ACSL5	acyl-CoA synthetase long-chain fa	-0.092	0.938	6.20E-01	6.20E+01		
7930524	NM_145206	VTI1A	vesicle transport through interacti	0.017	1.012	9.51E-01	9.51E+01		
7930537	NM_00114627	TCF7L2	transcription factor 7-like 2 (T-cell	0.289	1.222	3.64E-01	3.64E+01		
7930559	---	---	---	0.022	1.015	9.50E-01	9.50E+01		
7930561	NM_004132	HABP2	hyaluronan binding protein 2	0.025	1.017	8.37E-01	8.37E+01		
7930577	NM_033338	CASP7	caspase 7, apoptosis-related cyste	0.221	1.166	1.63E-01	1.63E+01		
7930614	NM_198514	NHLRC2	NHL repeat containing 2	0.023	1.016	8.85E-01	8.85E+01		
7930627	NM_000684	ADRB1	adrenergic, beta-1-, receptor	-0.016	0.989	9.24E-01	9.24E+01		
7930663	NM_198496	VWA2	von Willebrand factor A domain co	0.000	1.000	1.00E+00	1.00E+02		
7930682	NM_020940	FAM160B1	family with sequence similarity 16	-0.091	0.939	4.38E-01	4.38E+01		
7930703	NM_139169	TRUB1	TruB pseudouridine (psi) synthase	0.008	1.006	9.76E-01	9.76E+01		
7930790	NM_006229	PNLIPRP1	pancreatic lipase-related protein 1	-0.001	0.999	9.93E-01	9.93E+01		
7930870	NM_020243	TOMM22	translocase of outer mitochondria	0.164	1.121	6.06E-01	6.06E+01		
7930872	NM_199461	NANOS1	nanos homolog 1 (Drosophila)	0.025	1.017	8.54E-01	8.54E+01		
7930880	---	---	---	0.032	1.023	9.16E-01	9.16E+01		
7930882	NM_207009	FAM45A	family with sequence similarity 45	-0.221	0.858	7.53E-02	7.53E+00		
7930894	NM_005308	GRK5	G protein-coupled receptor kinase	-0.167	0.890	3.07E-01	3.07E+01		
7930915	---	---	---	0.042	1.030	9.53E-01	9.53E+01		
7930919	---	---	---	0.031	1.022	8.58E-01	8.58E+01		
7930921	NM_004281	BAG3	BCL2-associated athanogene 3	-0.009	0.994	9.66E-01	9.66E+01		
7930927	NM_014937	INPP5F	inositol polyphosphate-5-phospha	0.099	1.071	7.10E-01	7.10E+01		
7930956	NM_007190	SEC23IP	SEC23 interacting protein	0.124	1.090	9.25E-02	9.25E+00		
7930994	BC108662	C10orf85	chromosome 10 open reading fran	-0.023	0.984	8.62E-01	8.62E+01		
7930996	NM_018117	BRWD2	bromodomain and WD repeat dor	0.238	1.180	3.90E-02	3.90E+00		
7931081	NM_021622	PLEKHA1	pleckstrin homology domain conta	0.064	1.045	7.60E-01	7.60E+01		
7931097	NM_002775	HTRA1	HtrA serine peptidase 1	0.010	1.007	9.53E-01	9.53E+01		
7931140	NR_003570	FLJ46361	deleted in malignant brain tumors	-0.007	0.995	9.76E-01	9.76E+01		
7931159	NM_153336	PSTK	phosphoseryl-tRNA kinase	-0.094	0.937	4.51E-01	4.51E+01		
7931181	NM_00110557	HMX3	H6 family homeobox 3	-0.108	0.928	2.75E-01	2.75E+01		
7931184	NM_005519	HMX2	H6 family homeobox 2	-0.039	0.974	7.32E-01	7.32E+01		
7931187	NM_004725	BUB3	budding uninhibited by benzimida	-0.001	0.999	9.98E-01	9.98E+01		
7931204	NM_022126	LHPP	phospholysine phosphohistidine i	0.006	1.004	9.78E-01	9.78E+01		
7931216	NM_032182	FAM175B	family with sequence similarity 17	0.054	1.038	7.03E-01	7.03E+01		
7931226	NM_017580	ZRANB1	zinc finger, RAN-binding domain c	-0.023	0.984	9.10E-01	9.10E+01		
7931239	NM_015608	C10orf137	chromosome 10 open reading fran	0.009	1.006	9.58E-01	9.58E+01		
7931268	NM_016567	BCCIP	BRCA2 and CDKN1A interacting pr	0.005	1.003	9.85E-01	9.85E+01		
7931348	NM_207426	FOXI2	forkhead box I2	-0.025	0.983	8.62E-01	8.62E+01		
7931353	NM_006504	PTPRE	protein tyrosine phosphatase, rec	0.199	1.148	5.15E-01	5.15E+01		
7931379	NM_002412	MGMT	O-6-methylguanine-DNA methyltr	-0.062	0.958	4.42E-01	4.42E+01		
7931387	---	---	---	-0.057	0.961	7.83E-01	7.83E+01		
7931393	NM_006541	GLRX3	glutaredoxin 3	-0.019	0.987	9.48E-01	9.48E+01		
7931405	NM_018461	PPP2R2D	protein phosphatase 2, regulatory	-0.012	0.991	9.51E-01	9.51E+01		
7931417	NM_00110552	JAKMIP3	Janus kinase and microtubule inte	-0.043	0.971	6.15E-01	6.15E+01		
7931439	NM_006426	DPYSL4	dihydropyrimidinase-like 4	0.002	1.001	9.91E-01	9.91E+01		
7931455	NM_00114375	LRRC27	leucine rich repeat containing 27	-0.030	0.980	8.62E-01	8.62E+01		

7931469	NM_138499	PWWP2B	PWWP domain containing 2B	0.017	1.012	8.96E-01	8.96E+01		
7931479	NM_005539	INPP5A	inositol polyphosphate-5-phospha	-0.060	0.959	6.58E-01	6.58E+01		
7931500	NM_00108390	GPR123	G protein-coupled receptor 123	-0.018	0.988	9.15E-01	9.15E+01		
7931519	NM_152643	KNDC1	kinase non-catalytic C-lobe domai	-0.050	0.966	5.59E-01	5.59E+01		
7931553	NM_003577	UTF1	undifferentiated embryonic cell tr	-0.041	0.972	6.49E-01	6.49E+01		
7931556	NM_014468	VENTX	VENT homeobox homolog (Xenop	0.019	1.014	9.16E-01	9.16E+01		
7931561	NM_145806	ZNF511	zinc finger protein 511	-0.025	0.983	9.11E-01	9.11E+01		
7931571	NM_145202	PRAP1	proline-rich acidic protein 1	-0.032	0.978	7.94E-01	7.94E+01		
7931582	NM_152911	PAOX	polyamine oxidase (exo-N4-amino	-0.013	0.991	9.21E-01	9.21E+01		
7931607	NM_138384	MTG1	mitochondrial GTPase 1 homolog (	0.006	1.004	9.80E-01	9.80E+01		
7931623	NR_002934	LOC619207	scavenger receptor protein family	-0.021	0.985	8.93E-01	8.93E+01		
7931643	NM_000773	CYP2E1	cytochrome P450, family 2, subfar	0.030	1.021	8.30E-01	8.30E+01		
7931656	NM_033178	DUX4	double homeobox, 4	-0.010	0.993	9.56E-01	9.56E+01		
7931659	NM_033178	DUX4	double homeobox, 4	-0.010	0.993	9.56E-01	9.56E+01		
7931662	NM_033178	DUX4	double homeobox, 4	-0.010	0.993	9.56E-01	9.56E+01		
7931665	NM_033178	DUX4	double homeobox, 4	-0.017	0.989	9.16E-01	9.16E+01		
7931668	NM_033178	DUX4	double homeobox, 4	-0.010	0.993	9.56E-01	9.56E+01		
7931671	NM_033178	DUX4	double homeobox, 4	-0.010	0.993	9.56E-01	9.56E+01		
7931681	---	---	---	-0.109	0.927	7.32E-01	7.32E+01		
7931683	NM_014974	DIP2C	DIP2 disco-interacting protein 2 ho	-0.004	0.997	9.80E-01	9.80E+01		
7931726	---	---	---	0.144	1.105	5.74E-01	5.74E+01		
7931728	NM_015155	LARP4B	La ribonucleoprotein domain fami	0.169	1.124	1.44E-02	1.44E+00		
7931754	NM_004508	IDI1	isopentenyl-diphosphate delta iso	-0.022	0.985	9.17E-01	9.17E+01		
7931764	NM_018702	ADARB2	adenosine deaminase, RNA-specif	0.008	1.006	9.63E-01	9.63E+01		
7931778	NM_014889	PITRM1	pitrilysin metallopeptidase 1	-0.103	0.931	2.39E-01	2.39E+01		
7931810	NM_001300	KLF6	Kruppel-like factor 6	-0.053	0.964	7.71E-01	7.71E+01		
7931859	NM_017422	CALML5	calmodulin-like 5	-0.030	0.979	8.06E-01	8.06E+01		
7931863	NM_024701	ASB13	ankyrin repeat and SOCS box-cont	-0.011	0.992	9.56E-01	9.56E+01		
7931873	NM_001494	GDI2	GDP dissociation inhibitor 2	0.129	1.094	8.70E-02	8.70E+00		
7931888	NM_019046	ANKRD16	ankyrin repeat domain 16	0.034	1.024	8.47E-01	8.47E+01		
7931899	NM_002189	IL15RA	interleukin 15 receptor, alpha	-0.094	0.937	6.51E-01	6.51E+01		
7931928	---	---	---	-0.504	0.705	9.26E-02	9.26E+00		
7931930	NM_006257	PRKCQ	protein kinase C, theta	-0.107	0.928	4.81E-01	4.81E+01		
7931951	NM_00102988	SFMBT2	Scm-like with four mbt domains 2	-0.008	0.995	9.78E-01	9.78E+01		
7931977	NM_030569	ITIH5	inter-alpha (globulin) inhibitor H5	-0.002	0.999	9.90E-01	9.90E+01		
7932014	NR_024256	FLJ45983	hypothetical LOC399717	-0.072	0.951	5.55E-01	5.55E+01		
7932019	ENST00000379	C10orf31	chromosome 10 open reading fran	0.148	1.108	7.77E-02	7.77E+00		
7932041	NM_080599	UPF2	UPF2 regulator of nonsense transc	0.020	1.014	8.83E-01	8.83E+01		
7932086	---	---	---	-0.049	0.966	7.59E-01	7.59E+01		
7932094	NM_006214	PHYH	phytanoyl-CoA 2-hydroxylase	0.116	1.084	4.03E-01	4.03E+01		
7932109	NM_012247	SEPHS1	selenophosphate synthetase 1	0.032	1.023	8.92E-01	8.92E+01		
7932160	BC072452	FAM107B	family with sequence similarity 10	0.055	1.039	5.36E-01	5.36E+01		
7932177	NM_00102995	CDNF	cerebral dopamine neurotrophic f	-0.021	0.986	9.29E-01	9.29E+01		
7932186	NM_00103385	DCLRE1C	DNA cross-link repair 1C (PSO2 ho	0.231	1.174	1.11E-02	1.11E+00		
7932209	---	---	---	0.014	1.010	9.68E-01	9.68E+01		
7932221	BC029034	C10orf111	chromosome 10 open reading fran	-0.097	0.935	1.63E-01	1.63E+01		
7932227	NM_004808	NMT2	N-myristoyltransferase 2	0.104	1.075	6.24E-01	6.24E+01		
7932243	NM_00101092	FAM171A1	family with sequence similarity 17	0.012	1.008	9.53E-01	9.53E+01		
7932285	NM_024948	FAM188A	family with sequence similarity 18	0.010	1.007	9.57E-01	9.57E+01		
7932308	NM_00101090	C1QL3	complement component 1, q subc	0.003	1.002	9.89E-01	9.89E+01		
7932311	NM_012425	RSU1	Ras suppressor protein 1	0.099	1.071	4.49E-01	4.49E+01		
7932326	NM_001081	CUBN	cubilin (intrinsic factor-cobalamin	-0.019	0.987	9.70E-01	9.70E+01		
7932433	NM_182543	NSUN6	NOL1/NOP2/Sun domain family, m	0.157	1.115	3.10E-01	3.10E+01		
7932495	NM_00101091	C10orf114	chromosome 10 open reading fran	-0.050	0.966	7.38E-01	7.38E+01		
7932498	NM_207371	C10orf140	chromosome 10 open reading fran	-0.009	0.994	9.54E-01	9.54E+01		
7932510	---	---	---	0.129	1.093	7.07E-01	7.07E+01		
7932512	NM_022365	DNAJC1	DnaJ (Hsp40) homolog, subfamily	-0.095	0.937	4.21E-01	4.21E+01		
7932530	NM_005028	PIP4K2A	phosphatidylinositol-5-phosphate	0.073	1.052	5.15E-01	5.15E+01		
7932548	---	---	---	0.020	1.014	9.54E-01	9.54E+01		
7932554	NM_020824	ARHGAP21	Rho GTPase activating protein 21	0.183	1.136	1.02E-01	1.02E+01		
7932612	---	---	---	0.053	1.037	7.15E-01	7.15E+01		
7932616	NM_005470	ABI1	abl-interactor 1	0.102	1.073	3.68E-01	3.68E+01		
7932635	---	---	---	0.103	1.074	6.37E-01	6.37E+01		
7932678	NM_139312	YME1L1	YME1-like 1 (S. cerevisiae)	-0.019	0.987	8.80E-01	8.80E+01		
7932703	NM_145698	ACBD5	acyl-Coenzyme A binding domain (	0.105	1.076	4.67E-01	4.67E+01		
7932765	NM_173496	MPP7	membrane protein, palmitoylated	-0.087	0.941	6.91E-01	6.91E+01		
7932788	---	---	---	-0.006	0.996	9.78E-01	9.78E+01		
7932792	---	---	---	0.096	1.069	3.44E-01	3.44E+01		
7932794	---	---	---	0.004	1.002	9.88E-01	9.88E+01		
7932796	NM_021738	SVIL	supervillin	-0.073	0.951	5.29E-01	5.29E+01		
7932834	NM_018109	MTPAP	mitochondrial poly(A) polymerase	0.082	1.058	2.56E-01	2.56E+01		
7932860	---	---	---	-0.058	0.960	6.87E-01	6.87E+01		
7932862	---	---	---	0.026	1.018	8.88E-01	8.88E+01		
7932867	NM_00114377	ZNF438	zinc finger protein 438	0.352	1.276	2.34E-01	2.34E+01		
7932881	AK094743	LOC390414	hypothetical LOC390414	-0.004	0.997	9.85E-01	9.85E+01		
7932883	---	---	---	-0.075	0.949	6.57E-01	6.57E+01		
7932885	NM_018287	ARHGAP12	Rho GTPase activating protein 12	0.012	1.009	9.64E-01	9.64E+01		

7932911	NM_004521	KIF5B	kinesin family member 5B	0.033	1.023	7.90E-01	7.90E+01		
7932938	NM_025209	EPC1	enhancer of polycomb homolog 1	-0.034	0.977	8.31E-01	8.31E+01		
7932964	NM_006333	C1D	C1D nuclear receptor co-repressor	0.071	1.050	7.32E-01	7.32E+01		
7932966	NM_033666	ITGB1	integrin, beta 1 (fibronectin recept	-0.093	0.938	5.91E-01	5.91E+01		
7932983	---	---	---	0.005	1.003	9.85E-01	9.85E+01		
7932985	NM_003873	NRP1	neuropilin 1	0.083	1.059	9.14E-01	9.14E+01		
7933008	---	---	---	0.209	1.156	5.25E-01	5.25E+01		
7933010	NM_019619	PARD3	par-3 partitioning defective 3 hom	-0.086	0.942	6.73E-01	6.73E+01		
7933047	NM_003591	CUL2	cullin 2	0.074	1.053	6.83E-01	6.83E+01		
7933071	---	---	---	0.064	1.045	8.25E-01	8.25E+01		
7933073	NM_153368	GJD4	gap junction protein, delta 4, 40.1	0.010	1.007	9.63E-01	9.63E+01		
7933075	NM_031866	FZD8	frizzled homolog 8 (Drosophila)	-0.036	0.975	7.57E-01	7.57E+01		
7933080	---	---	---	0.083	1.059	7.28E-01	7.28E+01		
7933113	---	---	---	0.067	1.048	8.06E-01	8.06E+01		
7933115	NR_027269	CDC10L	CDC10 cell division cycle 10 homol	-0.027	0.981	7.94E-01	7.94E+01		
7933129	NR_026777	ZNF37B	zinc finger protein 37B (pseudoger	-0.026	0.982	9.16E-01	9.16E+01		
7933145	---	---	---	0.062	1.044	8.43E-01	8.43E+01		
7933149	NM_145313	RASGEF1A	RasGEF domain family, member 1A	-0.064	0.957	4.44E-01	4.44E+01		
7933164	NM_004966	HNRNPF	heterogeneous nuclear ribonucleo	0.020	1.014	8.92E-01	8.92E+01		
7933178	---	---	---	-0.063	0.957	7.36E-01	7.36E+01		
7933180	NM_00109928	ZNF239	zinc finger protein 239	-0.048	0.967	8.00E-01	8.00E+01		
7933186	NM_006973	ZNF32	zinc finger protein 32	0.015	1.011	9.63E-01	9.63E+01		
7933192	NR_002726	HNRNPA3P1	heterogeneous nuclear ribonucleo	0.002	1.001	9.90E-01	9.90E+01		
7933204	NM_007021	C10orf10	chromosome 10 open reading fran	-0.010	0.993	9.72E-01	9.72E+01		
7933209	NM_006963	ZNF22	zinc finger protein 22 (KOX 15)	-0.014	0.990	9.53E-01	9.53E+01		
7933228	NM_00100226	40245	membrane-associated ring finger (	0.045	1.032	7.75E-01	7.75E+01		
7933237	NM_174890	ANUBL1	AN1, ubiquitin-like, homolog (Xen	0.128	1.093	2.89E-01	2.89E+01		
7933252	NM_133446	AGAP4	ArfGAP with GTPase domain, anky	0.065	1.046	6.39E-01	6.39E+01		
7933290	NR_003611	BMS1P5	BMS1 pseudogene 5	0.048	1.034	8.31E-01	8.31E+01		
7933298	NM_031912	SYT15	synaptotagmin XV	0.026	1.018	8.66E-01	8.66E+01		
7933310	---	---	---	0.011	1.008	9.56E-01	9.56E+01		
7933312	NM_00109884	ANXA8L1	annexin A8-like 1	-0.084	0.944	2.52E-01	2.52E+01		
7933327	NM_00113755	FAM25B	family with sequence similarity 25	-0.033	0.977	8.18E-01	8.18E+01		
7933331	NM_00114400	AGAP5	ArfGAP with GTPase domain, anky	0.044	1.031	8.01E-01	8.01E+01		
7933359	NM_002900	RBP3	retinol binding protein 3, interstiti	0.085	1.061	2.78E-01	2.78E+01		
7933366	NM_016204	GDF2	growth differentiation factor 2	-0.045	0.969	7.10E-01	7.10E+01		
7933372	NM_004962	GDF10	growth differentiation factor 10	-0.020	0.986	8.63E-01	8.63E+01		
7933405	NM_133446	AGAP4	ArfGAP with GTPase domain, anky	0.058	1.041	6.66E-01	6.66E+01		
7933413	NR_003611	BMS1P5	BMS1 pseudogene 5	0.034	1.024	8.93E-01	8.93E+01		
7933423	NM_00113755	FAM25B	family with sequence similarity 25	-0.033	0.977	8.18E-01	8.18E+01		
7933427	NM_00114400	AGAP5	ArfGAP with GTPase domain, anky	0.053	1.037	6.54E-01	6.54E+01		
7933469	NM_021226	ARHGAP22	Rho GTPase activating protein 22	0.114	1.082	3.45E-01	3.45E+01		
7933484	NM_00100693	LRRC18	leucine rich repeat containing 18	0.124	1.090	4.92E-01	4.92E+01		
7933498	ENST00000311	FAM170B	family with sequence similarity 17	0.005	1.003	9.77E-01	9.77E+01		
7933501	NM_00108052	DRGX	dorsal root ganglia homeobox	0.003	1.002	9.88E-01	9.88E+01		
7933509	NM_000124	ERCC6	excision repair cross-complementi	-0.080	0.946	5.48E-01	5.48E+01		
7933537	NM_018245	OGDHL	oxoglutarate dehydrogenase-like	-0.070	0.953	5.55E-01	5.55E+01		
7933561	NM_003631	PARG	poly (ADP-ribose) glycohydrolase	0.062	1.044	8.00E-01	8.00E+01		
7933574	NM_00107768	AGAP7	ArfGAP with GTPase domain, anky	0.063	1.045	6.15E-01	6.15E+01		
7933582	NM_006327	TIMM23	translocase of inner mitochondria	0.023	1.016	9.09E-01	9.09E+01		
7933593	---	---	---	-0.066	0.955	7.81E-01	7.81E+01		
7933619	NM_147156	SGMS1	sphingomyelin synthase 1	-0.029	0.980	9.09E-01	9.09E+01		
7933632	---	---	---	0.034	1.024	8.96E-01	8.96E+01		
7933634	---	---	---	0.101	1.072	7.59E-01	7.59E+01		
7933659	NM_015235	CSTF2T	cleavage stimulation factor, 3' pre	0.084	1.060	5.26E-01	5.26E+01		
7933707	NM_032997	ZWINT	ZW10 interactor	0.028	1.020	8.33E-01	8.33E+01		
7933723	NM_152230	IPMK	inositol polyphosphate multikinase	0.128	1.093	5.78E-01	5.78E+01		
7933760	NM_005436	CCDC6	coiled-coil domain containing 6	0.072	1.051	5.71E-01	5.71E+01		
7933772	NM_020987	ANK3	ankyrin 3, node of Ranvier (ankyri	-0.206	0.867	5.16E-01	5.16E+01		
7933877	NM_004241	JMJD1C	jumonji domain containing 1C	-0.134	0.911	1.74E-01	1.74E+01		
7933931	---	---	---	-0.037	0.975	8.34E-01	8.34E+01		
7933945	---	---	---	0.005	1.004	9.81E-01	9.81E+01		
7933947	NM_022079	HERC4	hect domain and RLD 4	-0.015	0.990	9.34E-01	9.34E+01		
7933976	---	---	---	-0.135	0.911	2.35E-01	2.35E+01		
7933999	NM_017987	RUFY2	RUN and FYVE domain containing	-0.065	0.956	6.48E-01	6.48E+01		
7934074	NM_001057	TACR2	tachykinin receptor 2	-0.060	0.959	5.68E-01	5.68E+01		
7934083	NM_020999	NEUROG3	neurogenin 3	-0.071	0.952	4.39E-01	4.39E+01		
7934101	NM_032797	AIFM2	apoptosis-inducing factor, mitocho	-0.035	0.976	8.95E-01	8.95E+01		
7934114	NM_173555	TYSND1	trypsin domain containing 1	-0.073	0.950	4.66E-01	4.66E+01		
7934122	NM_00114264	SAR1A	SAR1 homolog A (S. cerevisiae)	0.011	1.008	9.55E-01	9.55E+01		
7934133	NM_021129	PPA1	pyrophosphatase (inorganic) 1	-0.107	0.929	7.07E-01	7.07E+01		
7934141	NM_022146	NPFFR1	neuropeptide FF receptor 1	0.021	1.015	9.01E-01	9.01E+01		
7934145	NM_207119	LRRC20	leucine rich repeat containing 20	-0.010	0.993	9.60E-01	9.60E+01		
7934154	---	---	---	-0.061	0.959	8.01E-01	8.01E+01		
7934161	NM_005041	PRF1	perforin 1 (pore forming protein)	0.141	1.103	6.69E-01	6.69E+01		
7934178	NM_000281	PCBD1	pterin-4 alpha-carbinolamine dehy	0.132	1.096	5.10E-01	5.10E+01		
7934185	NM_022153	C10orf54	chromosome 10 open reading fran	0.106	1.076	1.94E-01	1.94E+01		

7934215	NM_014767	SPOCK2	sparc/osteonectin, cwcv and kazal	-0.173	0.887	3.21E-02	3.21E+00		
7934244	NM_00100276	DNAJB12	DnaJ (Hsp40) homolog, subfamily	-0.070	0.953	5.30E-01	5.30E+01		
7934255	NM_006077	CBARA1	calcium binding atopy-related auto	0.247	1.186	6.76E-02	6.76E+00		
7934269	---	---	---	-0.150	0.901	1.13E-01	1.13E+01		
7934271	NM_032562	PLA2G12B	phospholipase A2, group XIIB	-0.009	0.994	9.61E-01	9.61E+01		
7934278	NM_000917	P4HA1	prolyl 4-hydroxylase, alpha polype	-0.068	0.954	8.41E-01	8.41E+01		
7934297	---	---	---	-0.187	0.879	3.70E-01	3.70E+01		
7934299	NM_015901	NUDT13	nudix (nucleoside diphosphate link	0.014	1.010	9.68E-01	9.68E+01		
7934301	NM_007265	ECD	ecdysoneless homolog (Drosophila	-0.070	0.953	6.01E-01	6.01E+01		
7934320	NM_015190	DNAJC9	DnaJ (Hsp40) homolog, subfamily	-0.153	0.899	2.66E-01	2.66E+01		
7934326	NM_016065	MRPS16	mitochondrial ribosomal protein S	0.059	1.042	6.66E-01	6.66E+01		
7934367	NM_004034	ANXA7	annexin A7	-0.111	0.926	2.03E-01	2.03E+01		
7934384	NM_00102459	ZMYND17	zinc finger, MYND-type containing	-0.073	0.951	5.98E-01	5.98E+01		
7934393	NM_00114235	PPP3CB	protein phosphatase 3 (formerly 2	0.021	1.015	9.01E-01	9.01E+01		
7934411	NM_152586	USP54	ubiquitin specific peptidase 54	-0.007	0.995	9.78E-01	9.78E+01		
7934451	NM_00114400	AGAP5	ArfGAP with GTPase domain, anky	0.054	1.038	6.50E-01	6.50E+01		
7934459	NM_003635	NDST2	N-deacetylase/N-sulfotransferase	-0.055	0.962	6.33E-01	6.33E+01		
7934477	NM_172171	CAMK2G	calcium/calmodulin-dependent pr	-0.054	0.963	6.44E-01	6.44E+01		
7934513	NM_207012	AP3M1	adaptor-related protein complex 3	0.085	1.061	2.74E-01	2.74E+01		
7934527	NM_00100389	DUPD1	dual specificity phosphatase and p	-0.041	0.972	8.10E-01	8.10E+01		
7934533	NM_00100727	DUSP13	dual specificity phosphatase 13	-0.067	0.955	3.66E-01	3.66E+01		
7934544	NM_144589	COMTD1	catechol-O-methyltransferase dom	-0.072	0.952	2.57E-01	2.57E+01		
7934553	NM_032772	ZNF503	zinc finger protein 503	0.015	1.010	9.26E-01	9.26E+01		
7934566	---	---	---	0.088	1.063	5.09E-01	5.09E+01		
7934615	NM_004747	DLG5	discs, large homolog 5 (Drosophila	-0.090	0.940	3.75E-01	3.75E+01		
7934651	---	---	---	0.179	1.132	1.03E-01	1.03E+01		
7934653	NM_007055	POLR3A	polymerase (RNA) III (DNA directe	0.027	1.019	8.75E-01	8.75E+01		
7934690	NM_153367	ZCCHC24	zinc finger, CCHC domain containi	-0.052	0.965	6.01E-01	6.01E+01		
7934698	NM_00109866	SFTPA2	surfactant protein A2	-0.118	0.921	6.89E-02	6.89E+00		
7934706	---	---	---	0.025	1.017	9.51E-01	9.51E+01		
7934708	NM_00109866	SFTPA2	surfactant protein A2	-0.118	0.921	6.89E-02	6.89E+00		
7934717	0	0	0	0.025	1.017	9.51E-01	9.51E+01		
7934719	NM_003019	SFTPD	surfactant protein D	-0.087	0.941	3.63E-01	3.63E+01		
7934731	NM_006333	C1D	C1D nuclear receptor co-repressor	0.145	1.106	3.35E-01	3.35E+01		
7934733	NM_145869	ANXA11	annexin A11	0.120	1.086	1.42E-01	1.42E+01		
7934753	NM_00109969	EIF5AL1	eukaryotic translation initiation fa	-0.081	0.945	6.76E-01	6.76E+01		
7934783	NM_015613	LRIT1	leucine-rich repeat, immunoglobu	-0.074	0.950	5.15E-01	5.15E+01		
7934789	NM_017551	GRID1	glutamate receptor, ionotropic, de	0.006	1.004	9.73E-01	9.73E+01		
7934810	---	---	---	0.062	1.044	5.34E-01	5.34E+01		
7934812	NM_015045	WAPAL	wings apart-like homolog (Drosop	-0.018	0.988	8.79E-01	8.79E+01		
7934842	NM_024756	MMRN2	multimerin 2	-0.001	0.999	9.95E-01	9.95E+01		
7934850	---	---	---	0.010	1.007	9.66E-01	9.66E+01		
7934852	NM_005271	GLUD1	glutamate dehydrogenase 1	0.184	1.136	1.51E-02	1.51E+00		
7934870	NM_032810	ATAD1	ATPase family, AAA domain contai	0.021	1.015	9.19E-01	9.19E+01		
7934885	NM_018363	RNLS	renalase, FAD-dependent amine o	0.117	1.084	1.27E-01	1.27E+01		
7934896	---	---	---	0.094	1.067	7.56E-01	7.56E+01		
7934906	NM_00114194	ACTA2	actin, alpha 2, smooth muscle, aor	0.116	1.084	3.69E-01	3.69E+01		
7934916	NM_003956	CH25H	cholesterol 25-hydroxylase	0.066	1.047	7.11E-01	7.11E+01		
7934945	NM_148977	PANK1	pantothenate kinase 1	-0.016	0.989	9.05E-01	9.05E+01		
7934959	---	---	---	0.050	1.035	8.05E-01	8.05E+01		
7934966	---	---	---	0.004	1.003	9.81E-01	9.81E+01		
7935002	NM_003133	SRP9	signal recognition particle 9kDa	0.069	1.049	5.26E-01	5.26E+01		
7935006	NM_152429	FGFBP3	fibroblast growth factor binding pr	-0.044	0.970	7.94E-01	7.94E+01		
7935011	NM_014912	CPEB3	cytoplasmic polyadenylation elem	0.185	1.137	7.00E-02	7.00E+00		
7935027	NM_004969	IDE	insulin-degrading enzyme	0.077	1.055	5.71E-01	5.71E+01		
7935054	---	---	---	0.154	1.113	2.93E-01	2.93E+01		
7935123	NM_145246	C10orf4	chromosome 10 open reading fran	-0.053	0.964	7.16E-01	7.16E+01		
7935139	NR_002319	PIPSL	PIP5K1A and PSMD4-like	-0.086	0.942	5.11E-01	5.11E+01		
7935146	NM_022451	NOC3L	nucleolar complex associated 3 ho	0.052	1.037	8.39E-01	8.39E+01		
7935180	NM_020992	PDLIM1	PDZ and LIM domain 1	0.281	1.215	6.41E-02	6.41E+00		
7935230	NM_002860	ALDH18A1	aldehyde dehydrogenase 18 family	0.049	1.035	8.27E-01	8.27E+01		
7935251	NM_015631	TCTN3	tectonic family member 3	0.001	1.001	9.97E-01	9.97E+01		
7935296	NM_012465	TLL2	tolloid-like 2	-0.011	0.992	9.57E-01	9.57E+01		
7935320	NM_020123	TM9SF3	transmembrane 9 superfamily me	0.021	1.015	8.71E-01	8.71E+01		
7935337	NM_152309	PIK3AP1	phosphoinositide-3-kinase adapto	0.144	1.105	7.16E-01	7.16E+01		
7935359	---	---	---	0.061	1.043	8.36E-01	8.36E+01		
7935361	NM_003061	SLIT1	slit homolog 1 (Drosophila)	-0.008	0.994	9.65E-01	9.65E+01		
7935403	NM_032900	ARHGAP19	Rho GTPase activating protein 19	-0.010	0.993	9.74E-01	9.74E+01		
7935421	NM_012083	FRAT2	frequently rearranged in advanced	0.100	1.071	2.41E-01	2.41E+01		
7935425	NM_015179	RRP12	ribosomal RNA processing 12 hom	-0.064	0.957	5.49E-01	5.49E+01		
7935462	NM_016046	EXOSC1	exosome component 1	-0.049	0.967	8.17E-01	8.17E+01		
7935474	NM_022362	MMS19	MMS19 nucleotide excision repair	-0.045	0.969	6.89E-01	6.89E+01		
7935521	NM_021732	AVPI1	arginine vasopressin-induced 1	0.109	1.079	2.35E-01	2.35E+01		
7935528	NM_003015	SFRP5	secreted frizzled-related protein 5	-0.003	0.998	9.86E-01	9.86E+01		
7935553	NM_032211	LOXL4	lysyl oxidase-like 4	0.015	1.011	8.81E-01	8.81E+01		
7935572	NM_032709	PYROXD2	pyridine nucleotide-disulphide oxi	-0.062	0.958	6.54E-01	6.54E+01		
7935588	NM_000195	HPS1	Hermansky-Pudlak syndrome 1	0.025	1.017	8.52E-01	8.52E+01		

7935627	NM_002079	GOT1	glutamic-oxaloacetic transaminase	0.009	1.006	9.81E-01	9.81E+01		
7935639	NM_031212	SLC25A28	solute carrier family 25, member 2	-0.015	0.990	9.44E-01	9.44E+01		
7935647	NM_078470	COX15	COX15 homolog, cytochrome c oxi	0.014	1.010	9.56E-01	9.56E+01		
7935660	NM_015221	DNMBP	dynamin binding protein	0.087	1.062	3.75E-01	3.75E+01		
7935692	NM_00110062	ERLIN1	ER lipid raft associated 1	0.049	1.034	8.46E-01	8.46E+01		
7935707	NM_001278	CHUK	conserved helix-loop-helix ubiquit	0.142	1.103	3.04E-01	3.04E+01		
7935730	NM_018294	CWF19L1	CWF19-like 1, cell cycle control (S.	0.014	1.010	9.54E-01	9.54E+01		
7935746	NM_00100134	BLOC1S2	biogenesis of lysosomal organelles	0.145	1.106	2.08E-01	2.08E+01		
7935776	NM_005063	SCD	stearoyl-CoA desaturase (delta-9-c	0.318	1.246	2.10E-01	2.10E+01		
7935780	NM_015490	SEC31B	SEC31 homolog B (S. cerevisiae)	-0.005	0.996	9.85E-01	9.85E+01		
7935810	NM_005004	NDUFB8	NADH dehydrogenase (ubiquinone	-0.004	0.997	9.85E-01	9.85E+01		
7935819	NM_032112	MRPL43	mitochondrial ribosomal protein L	-0.015	0.990	9.24E-01	9.24E+01		
7935834	NM_024895	PDZD7	PDZ domain containing 7	-0.036	0.976	7.62E-01	7.62E+01		
7935855	NM_006562	LBX1	ladybird homeobox 1	-0.100	0.933	2.09E-01	2.09E+01		
7935861	---	---	---	0.126	1.091	6.56E-01	6.56E+01		
7935863	---	---	---	0.090	1.064	6.87E-01	6.87E+01		
7935865	NM_013274	POLL	polymerase (DNA directed), lambd	0.041	1.029	7.40E-01	7.40E+01		
7935882	NM_022039	FBXW4	F-box and WD repeat domain cont	-0.104	0.931	2.68E-01	2.68E+01		
7935892	NM_033163	FGF8	fibroblast growth factor 8 (androg	-0.051	0.965	7.52E-01	7.52E+01		
7935903	NM_006993	NPM3	nucleophosmin/nucleoplasmin 3	-0.052	0.965	7.72E-01	7.72E+01		
7935910	NM_012215	MGEA5	meningioma expressed antigen 5 (	0.009	1.007	9.58E-01	9.58E+01		
7935930	NM_014591	KCNIP2	Kv channel interacting protein 2	0.076	1.054	6.56E-01	6.56E+01		
7935951	NM_024541	C10orf76	chromosome 10 open reading fran	0.090	1.065	3.84E-01	3.84E+01		
7935968	NM_003893	LDB1	LIM domain binding 1	-0.104	0.930	3.48E-01	3.48E+01		
7935983	NM_005029	PITX3	paired-like homeodomain 3	0.004	1.002	9.86E-01	9.86E+01		
7935990	NM_002779	PSD	pleckstrin and Sec7 domain contai	-0.084	0.943	3.58E-01	3.58E+01		
7936011	NM_024040	CUEDC2	CUE domain containing 2	-0.043	0.971	7.87E-01	7.87E+01		
7936023	BC126459	C10orf95	chromosome 10 open reading fran	-0.024	0.983	8.81E-01	8.81E+01		
7936028	NM_005736	ACTR1A	ARP1 actin-related protein 1 homo	0.017	1.012	9.54E-01	9.54E+01		
7936041	NM_004311	ARL3	ADP-ribosylation factor-like 3	-0.112	0.925	3.69E-01	3.69E+01		
7936062	---	---	---	0.053	1.037	7.30E-01	7.30E+01		
7936064	NM_012229	NT5C2	5'-nucleotidase, cytosolic II	0.248	1.188	1.07E-01	1.07E+01		
7936083	NM_00101166	PCGF6	polycomb group ring finger 6	-0.071	0.952	7.15E-01	7.15E+01		
7936091	NM_032747	USMG5	up-regulated during skeletal musc	-0.071	0.952	5.62E-01	5.62E+01		
7936096	NM_014976	PDCD11	programmed cell death 11	-0.028	0.981	8.50E-01	8.50E+01		
7936100	NM_015916	CALHM2	calcium homeostasis modulator 2	0.070	1.049	4.99E-01	4.99E+01		
7936108	NM_00100141	CALHM1	calcium homeostasis modulator 1	-0.069	0.954	4.05E-01	4.05E+01		
7936115	NM_014631	SH3PXD2A	SH3 and PX domains 2A	-0.064	0.957	5.33E-01	5.33E+01		
7936134	NM_024928	OBFC1	oligonucleotide/oligosaccharide-b	0.052	1.037	8.30E-01	8.30E+01		
7936144	NM_000494	COL17A1	collagen, type XVII, alpha 1	-0.034	0.977	7.41E-01	7.41E+01		
7936242	NM_033397	ITPRIP	inositol 1,4,5-triphosphate recepto	-0.044	0.970	6.81E-01	6.81E+01		
7936284	NM_020383	XPNPEP1	X-prolyl aminopeptidase (aminope	0.108	1.078	4.85E-01	4.85E+01		
7936307	NM_005871	SMNDC1	survival motor neuron domain cor	0.015	1.011	9.33E-01	9.33E+01		
7936320	NR_026715	RPL13AP6	ribosomal protein L13a pseudoger	0.014	1.010	9.56E-01	9.56E+01		
7936322	NM_020918	GPAM	glycerol-3-phosphate acyltransfera	0.057	1.040	7.62E-01	7.62E+01		
7936346	NM_022494	ZDHHC6	zinc finger, DHHC-type containing	0.035	1.024	7.70E-01	7.70E+01		
7936406	---	---	---	0.130	1.094	5.49E-01	5.49E+01		
7936419	NM_018017	C10orf118	chromosome 10 open reading fran	-0.051	0.965	7.10E-01	7.10E+01		
7936463	NM_002313	ABLIM1	actin binding LIM protein 1	-0.195	0.873	1.52E-01	1.52E+01		
7936494	NM_005264	GFRA1	GDNF family receptor alpha 1	-0.037	0.975	7.81E-01	7.81E+01		
7936507	BC021737	C10orf82	chromosome 10 open reading fran	0.090	1.064	2.24E-01	2.24E+01		
7936552	NM_199131	VAX1	ventral anterior homeobox 1	-0.054	0.963	5.99E-01	5.99E+01		
7936559	NM_173791	PDZD8	PDZ domain containing 8	-0.031	0.979	8.18E-01	8.18E+01		
7936567	NM_014904	RAB11FIP2	RAB11 family interacting protein 2	0.089	1.064	4.20E-01	4.20E+01		
7936578	NM_022063	C10orf84	chromosome 10 open reading fran	0.010	1.007	9.63E-01	9.63E+01		
7936596	NM_153810	C10orf46	chromosome 10 open reading fran	0.095	1.068	3.04E-01	3.04E+01		
7936614	NM_003750	EIF3A	eukaryotic translation initiation fa	0.053	1.037	4.15E-01	4.15E+01		
7936637	NR_002917	SNORA19	small nucleolar RNA, H/ACA box 1	0.265	1.202	6.16E-02	6.16E+00		
7936639	---	---	---	0.222	1.166	2.46E-01	2.46E+01		
7936641	NM_213649	SFXN4	sideroflexin 4	0.089	1.064	7.70E-01	7.70E+01		
7936661	NM_006793	PRDX3	peroxiredoxin 3	0.018	1.013	9.50E-01	9.50E+01		
7936673	NM_00100533	RGS10	regulator of G-protein signaling 10	-0.032	0.978	8.64E-01	8.64E+01		
7936683	NM_00103392	TIAL1	TIA1 cytotoxic granule-associated	0.028	1.020	8.16E-01	8.16E+01		
7936706	NM_024834	C10orf119	chromosome 10 open reading fran	0.081	1.058	3.56E-01	3.56E+01		
7936727	NM_000982	RPL21	ribosomal protein L21	-0.015	0.990	9.37E-01	9.37E+01		
7936762	NM_00100197	ATE1	arginyltransferase 1	0.040	1.028	8.39E-01	8.39E+01		
7936777	NM_017615	NSMCE4A	non-SMC element 4 homolog A (S.	-0.007	0.995	9.74E-01	9.74E+01		
7936809	NM_152644	FAM24B	family with sequence similarity 24	0.095	1.068	7.23E-01	7.23E+01		
7936826	NM_022466	IKZF5	IKAROS family zinc finger 5 (Pegas	0.033	1.023	8.97E-01	8.97E+01		
7936833	---	---	---	0.059	1.042	7.63E-01	7.63E+01		
7936856	NM_015892	CHST15	carbohydrate (N-acetylgalactosam	-0.037	0.974	9.16E-01	9.16E+01		
7936871	NM_000274	OAT	ornithine aminotransferase (gyrate	-0.102	0.932	6.58E-01	6.58E+01		
7936882	NM_00114634	NKX1-2	NK1 homeobox 2	-0.016	0.989	9.16E-01	9.16E+01		
7936884	NM_014661	FAM53B	family with sequence similarity 53	-0.080	0.946	6.58E-01	6.58E+01		
7936891	NM_212554	METTL10	methyltransferase like 10	0.037	1.026	8.19E-01	8.19E+01		
7936902	---	---	---	0.164	1.121	7.39E-01	7.39E+01		
7936904	NM_022802	CTBP2	C-terminal binding protein 2	-0.071	0.952	4.84E-01	4.84E+01		

7936923	M21191	ALDOAP2	aldolase A, fructose-bisphosphate	0.023	1.016	8.79E-01	8.79E+01		
7936928	NM_147191	MMP21	matrix metallopeptidase 21	-0.108	0.928	3.04E-01	3.04E+01		
7936937	NM_000375	UROS	uroporphyrinogen III synthase	0.060	1.043	7.68E-01	7.68E+01		
7936949	NM_018180	DHX32	DEAH (Asp-Glu-Ala-His) box polype	0.155	1.114	4.24E-01	4.24E+01		
7937014	AK127642	LOC387720	hypothetical LOC387720	-0.098	0.934	3.42E-01	3.42E+01		
7937020	NM_002417	MKI67	antigen identified by monoclonal a	0.315	1.244	1.45E-01	1.45E+01		
7937039	NM_00100546	EBF3	early B-cell factor 3	-0.018	0.988	9.02E-01	9.02E+01		
7937059	NM_174937	TCERG1L	transcription elongation regulator	-0.004	0.997	9.78E-01	9.78E+01		
7937073	ENST00000341	FLJ46300	FLJ46300 protein	-0.008	0.995	9.63E-01	9.63E+01		
7937079	NM_004052	BNIP3	BCL2/adenovirus E1B 19kDa intera	-0.035	0.976	9.19E-01	9.19E+01		
7937089	NM_173575	STK32C	serine/threonine kinase 32C	-0.018	0.987	9.01E-01	9.01E+01		
7937104	AK097584	LOC100128830	hypothetical protein LOC1001288	0.001	1.000	9.99E-01	9.99E+01		
7937106	NM_177400	NKX6-2	NK6 homeobox 2	0.018	1.013	9.04E-01	9.04E+01		
7937112	NM_017609	C10orf92	chromosome 10 open reading fran	-0.115	0.923	4.87E-02	4.87E+00		
7937150	NM_001109	ADAM8	ADAM metallopeptidase domain 8	-0.076	0.949	3.00E-01	3.00E+01		
7937175	NM_006659	TUBGCP2	tubulin, gamma complex associate	-0.056	0.962	7.15E-01	7.15E+01		
7937198	---	---	---	-0.056	0.962	6.16E-01	6.16E+01		
7937200	NM_015722	CALY	calcyon neuron-specific vesicular p	-0.079	0.946	4.69E-01	4.69E+01		
7937208	NM_198472	C10orf125	chromosome 10 open reading fran	0.017	1.012	9.14E-01	9.14E+01		
7937217	NM_004092	ECHS1	enoyl Coenzyme A hydratase, shor	0.074	1.053	5.29E-01	5.29E+01		
7937228	NM_00101250	SPRN	shadow of prion protein homolog	-0.050	0.966	6.98E-01	6.98E+01		
7937247	NM_130784	SYCE1	synaptonemal complex central ele	-0.049	0.967	6.99E-01	6.99E+01		
7937257	NM_145651	SCGB1C1	secretoglobin, family 1C, member	0.002	1.002	9.88E-01	9.88E+01		
7937275	NM_021932	RIC8A	resistance to inhibitors of cholines	0.008	1.005	9.74E-01	9.74E+01		
7937287	NM_002817	PSMD13	proteasome (prosome, macropain	-0.041	0.972	8.09E-01	8.09E+01		
7937305	NM_138329	NLRP6	NLR family, pyrin domain containi	0.037	1.026	7.85E-01	7.85E+01		
7937314	NM_025092	ATHL1	ATH1, acid trehalase-like 1 (yeast)	-0.108	0.928	5.61E-01	5.61E+01		
7937330	NM_006435	IFITM2	interferon induced transmembran	0.236	1.178	1.56E-02	1.56E+00		
7937335	NM_003641	IFITM1	interferon induced transmembran	0.036	1.026	9.16E-01	9.16E+01		
7937341	NM_178537	B4GALNT4	beta-1,4-N-acetyl-galactosaminyl t	0.031	1.022	8.45E-01	8.45E+01		
7937363	NM_007183	PKP3	plakophilin 3	-0.008	0.994	9.62E-01	9.62E+01		
7937378	NM_030783	PTDSS2	phosphatidylserine synthase 2	-0.050	0.966	5.72E-01	5.72E+01		
7937391	NM_198075	LRRC56	leucine rich repeat containing 56	0.033	1.023	8.34E-01	8.34E+01		
7937404	BC039077	C11orf35	chromosome 11 open reading fran	-0.088	0.941	5.39E-01	5.39E+01		
7937407	NM_003475	RASSF7	Ras association (RalGDS/AF-6) dor	-0.021	0.986	8.79E-01	8.79E+01		
7937415	NM_020901	PHRF1	PHD and ring finger domains 1	-0.062	0.958	5.74E-01	5.74E+01		
7937433	NM_000797	DRD4	dopamine receptor D4	0.008	1.006	9.65E-01	9.65E+01		
7937438	NM_174940	TMEM80	transmembrane protein 80	-0.086	0.942	3.85E-01	3.85E+01		
7937443	NM_022772	EPS8L2	EPS8-like 2	-0.002	0.999	9.90E-01	9.90E+01		
7937465	NM_006755	TALDO1	transaldolase 1	-0.055	0.962	8.69E-01	8.69E+01		
7937474	AF435951	NS3BP	NS3BP	-0.079	0.947	6.07E-01	6.07E+01		
7937476	NM_001004	RPLP2	ribosomal protein, large, P2	0.019	1.013	9.53E-01	9.53E+01		
7937483	NR_002585	SNORA52	small nucleolar RNA, H/ACA box 5	-0.069	0.953	6.06E-01	6.06E+01		
7937485	NM_020376	PNPLA2	patatin-like phospholipase domair	0.027	1.019	8.15E-01	8.15E+01		
7937498	NM_173584	EFCAB4A	EF-hand calcium binding domain 4	-0.020	0.986	8.87E-01	8.87E+01		
7937508	NM_004357	CD151	CD151 molecule (Raph blood grou	0.053	1.037	7.76E-01	7.76E+01		
7937518	NM_00102523	TSPAN4	tetraspanin 4	-0.073	0.951	3.91E-01	3.91E+01		
7937533	NM_012305	AP2A2	adaptor-related protein complex 2	0.102	1.073	3.09E-01	3.09E+01		
7937560	NM_002457	MUC2	mucin 2, oligomeric mucus/gel-for	-0.026	0.982	7.56E-01	7.56E+01		
7937612	NM_002458	MUC5B	mucin 5B, oligomeric mucus/gel-fo	-0.036	0.975	7.55E-01	7.55E+01		
7937667	NM_003957	BRSK2	BR serine/threonine kinase 2	-0.037	0.974	7.44E-01	7.44E+01		
7937696	NM_00100432	KRTAP5-2	keratin associated protein 5-2	-0.072	0.952	7.51E-01	7.51E+01		
7937698	NM_00100592	KRTAP5-1	keratin associated protein 5-1	-0.035	0.976	7.82E-01	7.82E+01		
7937700	NM_00100148	KRTAP5-5	keratin associated protein 5-5	-0.050	0.966	7.51E-01	7.51E+01		
7937707	NR_026643	FAM99A	family with sequence similarity 99	-0.166	0.891	3.97E-01	3.97E+01		
7937709	NM_00101241	KRTAP5-6	keratin associated protein 5-6	-0.075	0.949	5.57E-01	5.57E+01		
7937713	NM_138567	SYT8	synaptotagmin VIII	-0.018	0.987	8.63E-01	8.63E+01		
7937728	NM_00114582	TNNI2	troponin I type 2 (skeletal, fast)	-0.043	0.971	6.39E-01	6.39E+01		
7937735	NM_002339	LSP1	lymphocyte-specific protein 1	0.017	1.012	9.10E-01	9.10E+01		
7937749	NM_006757	TNNT3	troponin T type 3 (skeletal, fast)	-0.078	0.948	3.86E-01	3.86E+01		
7937763	NM_021134	MRPL23	mitochondrial ribosomal protein L	0.009	1.006	9.56E-01	9.56E+01		
7937774	NM_016412	IGF2AS	insulin-like growth factor 2 antiser	0.022	1.016	8.73E-01	8.73E+01		
7937782	NM_139022	TSPAN32	tetraspanin 32	0.023	1.016	8.33E-01	8.33E+01		
7937798	---	---	---	0.079	1.056	6.93E-01	6.93E+01		
7937813	NM_005706	TSSC4	tumor suppressing subtransferable	0.023	1.016	8.80E-01	8.80E+01		
7937823	NM_000218	KCNQ1	potassium voltage-gated channel,	0.039	1.027	7.68E-01	7.68E+01		
7937847	NR_024627	KCNQ1DN	KCNQ1 downstream neighbor	0.018	1.013	9.09E-01	9.09E+01		
7937852	NM_002555	SLC22A18	solute carrier family 22, member 1	-0.058	0.961	5.71E-01	5.71E+01		
7937866	---	---	---	-0.004	0.998	9.91E-01	9.91E+01		
7937876	---	---	---	0.001	1.001	9.97E-01	9.97E+01		
7937882	NM_004314	ART1	ADP-ribosyltransferase 1	0.037	1.026	7.97E-01	7.97E+01		
7937892	NR_027015	PGAP2	post-GPI attachment to proteins 2	-0.043	0.971	7.62E-01	7.62E+01		
7937900	NM_003156	STIM1	stromal interaction molecule 1	0.016	1.011	9.50E-01	9.50E+01		
7937913	---	---	---	0.065	1.046	8.13E-01	8.13E+01		
7937915	NM_001033	RRM1	ribonucleotide reductase M1	0.106	1.076	5.26E-01	5.26E+01		
7937936	---	---	---	0.039	1.027	8.64E-01	8.64E+01		
7937944	NM_00100413	OR52M1	olfactory receptor, family 52, subf	-0.019	0.987	9.27E-01	9.27E+01		

7938010	NM_00100516	OR52B6	olfactory receptor, family 52, subf	0.133	1.097	6.58E-01	6.58E+01		
7938035	NM_006074	TRIM22	tripartite motif-containing 22	0.056	1.040	7.97E-01	7.97E+01		
7938047	---	---	---	-0.063	0.957	7.17E-01	7.17E+01		
7938066	NM_00100344	OR56A3	olfactory receptor, family 56, subf	0.033	1.023	8.33E-01	8.33E+01		
7938076	NM_00100517	OR52W1	olfactory receptor, family 52, subf	-0.019	0.987	9.30E-01	9.30E+01		
7938090	NM_176875	CCKBR	cholecystokinin B receptor	0.006	1.004	9.77E-01	9.77E+01		
7938100	NM_000543	SMPD1	sphingomyelin phosphodiesterase	0.065	1.046	7.16E-01	7.16E+01		
7938111	NM_012192	FXC1	fracture callus 1 homolog (rat)	-0.097	0.935	3.27E-01	3.27E+01		
7938118	NM_173589	DNHD1	dynein heavy chain domain 1	0.060	1.043	6.60E-01	6.60E+01		
7938128	NM_144666	DNHD1	dynein heavy chain domain 1	0.019	1.013	9.55E-01	9.55E+01		
7938133	NM_144666	DNHD1	dynein heavy chain domain 1	-0.110	0.927	3.77E-01	3.77E+01		
7938139	NM_144666	DNHD1	dynein heavy chain domain 1	-0.008	0.994	9.65E-01	9.65E+01		
7938154	NM_004517	ILK	integrin-linked kinase	-0.040	0.973	8.28E-01	8.28E+01		
7938170	NM_00100448	OR2AG1	olfactory receptor, family 2, subfa	-0.029	0.980	8.29E-01	8.29E+01		
7938179	NM_207186	OR10A4	olfactory receptor, family 10, subf	0.143	1.104	2.03E-01	2.03E+01		
7938208	NM_014469	RBMXL2	RNA binding motif protein, X-linke	0.042	1.030	7.04E-01	7.04E+01		
7938231	NM_003621	PPFIBP2	PTPRF interacting protein, binding	0.039	1.027	8.58E-01	8.58E+01		
7938263	NM_003754	EIF3F	eukaryotic translation initiation fa	-0.065	0.956	7.03E-01	7.03E+01		
7938269	NM_003320	TUB	tubby homolog (mouse)	0.032	1.022	7.65E-01	7.65E+01		
7938286	NM_000990	RPL27A	ribosomal protein L27a	0.038	1.027	8.22E-01	8.22E+01		
7938291	NR_002580	SNORA3	small nucleolar RNA, H/ACA box 3	-0.016	0.989	9.74E-01	9.74E+01		
7938293	NR_002977	SNORA45	small nucleolar RNA, H/ACA box 4	-0.082	0.945	7.55E-01	7.55E+01		
7938295	NM_000990	RPL27A	ribosomal protein L27a	0.014	1.010	9.52E-01	9.52E+01		
7938301	NM_020642	C11orf17	chromosome 11 open reading fran	0.058	1.041	8.63E-01	8.63E+01		
7938309	NR_027713	FLJ46111	keratin 8 pseudogene	-0.080	0.946	2.98E-01	2.98E+01		
7938313	NM_006391	IPO7	importin 7	0.059	1.042	6.82E-01	6.82E+01		
7938329	NR_002962	SNORA23	small nucleolar RNA, H/ACA box 2	0.051	1.036	9.09E-01	9.09E+01		
7938331	NM_003442	ZNF143	zinc finger protein 143	0.036	1.025	8.63E-01	8.63E+01		
7938348	NM_003390	WEE1	WEE1 homolog (S. pombe)	-0.013	0.991	9.64E-01	9.64E+01		
7938364	BX641032	WEE1	WEE1 homolog (S. pombe)	0.110	1.079	6.38E-01	6.38E+01		
7938366	BX641032	WEE1	WEE1 homolog (S. pombe)	-0.024	0.984	9.77E-01	9.77E+01		
7938368	---	---	---	-0.049	0.967	8.25E-01	8.25E+01		
7938370	NM_015055	SWAP70	SWAP switching B-cell complex 70	0.093	1.067	6.56E-01	6.56E+01		
7938388	---	---	---	-0.035	0.976	8.99E-01	8.99E+01		
7938390	NM_001124	ADM	adrenomedullin	-0.104	0.931	8.33E-02	8.33E+00		
7938416	ENST00000324	LOC100129827	hypothetical protein LOC1001298	-0.048	0.967	6.93E-01	6.93E+01		
7938420	---	---	---	-0.026	0.982	9.14E-01	9.14E+01		
7938422	NM_014633	CTR9	Ctr9, Paf1/RNA polymerase II com	-0.017	0.988	9.27E-01	9.27E+01		
7938448	NM_017944	USP47	ubiquitin specific peptidase 47	-0.075	0.949	5.16E-01	5.16E+01		
7938519	NM_032867	MICALCL	MICAL C-terminal like	-0.101	0.932	3.04E-01	3.04E+01		
7938528	NM_018222	PARVA	parvin, alpha	-0.054	0.963	5.43E-01	5.43E+01		
7938559	NM_00108052	RASSF10	Ras association (RalGDS/AF-6) dor	0.003	1.002	9.87E-01	9.87E+01		
7938563	NM_001178	ARNTL	aryl hydrocarbon receptor nuclear	0.024	1.017	9.35E-01	9.35E+01		
7938592	NM_032228	FAR1	fatty acyl CoA reductase 1	0.274	1.209	4.28E-02	4.28E+00		
7938608	NM_006108	SPON1	spondin 1, extracellular matrix pro	-0.143	0.906	5.86E-01	5.86E+01		
7938625	---	---	---	0.042	1.030	8.31E-01	8.31E+01		
7938629	NM_000922	PDE3B	phosphodiesterase 3B, cGMP-inhi	-0.228	0.854	7.05E-02	7.05E+00		
7938669	NM_014267	C11orf58	chromosome 11 open reading fran	0.013	1.009	9.32E-01	9.32E+01		
7938681	---	---	---	-0.025	0.982	9.02E-01	9.02E+01		
7938683	NR_002822	MGC72080	MGC72080 pseudogene	-0.046	0.969	8.16E-01	8.16E+01		
7938685	---	---	---	-0.103	0.931	1.42E-01	1.42E+01		
7938687	NM_005013	NUCB2	nucleobindin 2	0.137	1.100	1.85E-01	1.85E+01		
7938702	NR_026750	KFZp686O2416	hypothetical protein DKFZp686O2	0.120	1.086	7.15E-01	7.15E+01		
7938738	NM_004976	KCNC1	potassium voltage-gated channel,	-0.005	0.996	9.78E-01	9.78E+01		
7938748	---	---	---	-0.056	0.962	7.40E-01	7.40E+01		
7938756	AF512499	FAM10A5	family with sequence similarity 10	-0.085	0.943	2.85E-01	2.85E+01		
7938762	NM_005316	GTF2H1	general transcription factor IIH, po	0.143	1.104	1.71E-01	1.71E+01		
7938777	NM_005566	LDHA	lactate dehydrogenase A	-0.195	0.874	8.46E-02	8.46E+00		
7938812	NM_153347	TMEM86A	transmembrane protein 86A	0.132	1.096	1.58E-01	1.58E+01		
7938880	NM_00109852	HTATIP2	HIV-1 Tat interactive protein 2, 30	-0.210	0.864	4.59E-02	4.59E+00		
7938890	NM_005788	PRMT3	protein arginine methyltransferase	0.022	1.015	9.37E-01	9.37E+01		
7939005	---	---	---	0.008	1.005	9.78E-01	9.78E+01		
7939068	NM_001028	RPS25	ribosomal protein S25	-0.045	0.969	7.35E-01	7.35E+01		
7939072	NM_152636	METT5D1	methyltransferase 5 domain conta	0.099	1.071	5.53E-01	5.53E+01		
7939087	BC047775	C11orf46	chromosome 11 open reading fran	0.124	1.090	4.06E-01	4.06E+01		
7939120	NM_002901	RCN1	reticulocalbin 1, EF-hand calcium b	0.123	1.089	6.20E-01	6.20E+01		
7939137	NM_006360	EIF3M	eukaryotic translation initiation fa	0.034	1.024	8.78E-01	8.78E+01		
7939150	NM_024081	PRRG4	proline rich Gla (G-carboxyglutami	0.163	1.120	3.42E-01	3.42E+01		
7939158	NM_00107678	QSER1	glutamine and serine rich 1	0.206	1.153	3.68E-02	3.68E+00		
7939184	NM_018393	TCP11L1	t-complex 11 (mouse)-like 1	-0.058	0.961	7.57E-01	7.57E+01		
7939197	NM_005734	HIPK3	homeodomain interacting protein	0.065	1.046	7.15E-01	7.15E+01		
7939242	NM_005898	CAPRIN1	cell cycle associated protein 1	0.022	1.016	8.66E-01	8.66E+01		
7939265	NM_024662	NAT10	N-acetyltransferase 10 (GCN5-rela	0.098	1.070	5.24E-01	5.24E+01		
7939298	NM_001752	CAT	catalase	0.096	1.069	6.14E-01	6.14E+01		
7939329	NM_003477	PDHX	pyruvate dehydrogenase complex,	0.038	1.027	8.76E-01	8.76E+01		
7939341	NM_000610	CD44	CD44 molecule (Indian blood grou	0.014	1.010	9.58E-01	9.58E+01		
7939365	NM_014344	FJX1	four jointed box 1 (Drosophila)	-0.073	0.951	5.63E-01	5.63E+01		

7939368	NM_017583	TRIM44	tripartite motif-containing 44	-0.003	0.998	9.87E-01	9.87E+01		
7939376	NM_174902	LDLRAD3	low density lipoprotein receptor c	0.104	1.075	1.66E-01	1.66E+01		
7939383	NM_024841	PRR5L	proline rich 5 like	-0.186	0.879	4.16E-01	4.16E+01		
7939424	NR_024625	API5	apoptosis inhibitor 5	0.010	1.007	9.61E-01	9.61E+01		
7939432	---	---	---	-0.091	0.939	7.17E-01	7.17E+01		
7939434	NM_018259	TTC17	tetratricopeptide repeat domain 1	0.046	1.032	6.88E-01	6.88E+01		
7939465	NM_016142	HSD17B12	hydroxysteroid (17-beta) dehydro	0.058	1.041	8.20E-01	8.20E+01		
7939477	NM_139178	ALKBH3	alkB, alkylation repair homolog 3 (	0.086	1.062	4.77E-01	4.77E+01		
7939492	NM_00114503	LOC387763	hypothetical protein LOC387763	0.022	1.015	9.00E-01	9.00E+01		
7939507	NM_032592	ACCS	1-aminocyclopropane-1-carboxyla	0.033	1.023	8.07E-01	8.07E+01		
7939524	NM_000401	EXT2	exostoses (multiple) 2	0.039	1.027	7.90E-01	7.90E+01		
7939546	NM_002231	CD82	CD82 molecule	-0.196	0.873	2.99E-02	2.99E+00		
7939559	NM_130783	TSPAN18	tetraspanin 18	-0.075	0.949	5.34E-01	5.34E+01		
7939569	NM_020229	PRDM11	PR domain containing 11	-0.005	0.997	9.80E-01	9.80E+01		
7939587	BX640618	DKFZp779M065	hypothetical DKFZp779M0652	-0.065	0.956	6.40E-01	6.40E+01		
7939590	NM_018389	SLC35C1	solute carrier family 35, member C	0.029	1.020	9.09E-01	9.09E+01		
7939595	NM_021117	CRY2	cryptochrome 2 (photolyase-like)	0.026	1.019	7.72E-01	7.72E+01		
7939613	NM_005456	MAPK8IP1	mitogen-activated protein kinase 8	0.073	1.052	5.41E-01	5.41E+01		
7939620	NM_152312	GYLTL1B	glycosyltransferase-like 1B	-0.053	0.964	6.15E-01	6.15E+01		
7939657	NM_00110554	DGKZ	diacylglycerol kinase, zeta 104kDa	-0.170	0.889	1.25E-01	1.25E+01		
7939665	NM_00101233	MDK	midkine (neurite growth-promotin	-0.053	0.964	6.34E-01	6.34E+01		
7939676	NR_024587	KIAA0652	KIAA0652	-0.021	0.986	9.20E-01	9.20E+01		
7939723	NM_00100367	C11orf49	chromosome 11 open reading fran	-0.049	0.967	7.83E-01	7.83E+01		
7939738	NM_000107	DDB2	damage-specific DNA binding prot	-0.126	0.916	3.71E-01	3.71E+01		
7939751	NM_005693	NR1H3	nuclear receptor subfamily 1, grou	0.333	1.259	6.30E-02	6.30E+00		
7939767	NM_003682	MADD	MAP-kinase activating death doma	-0.038	0.974	8.21E-01	8.21E+01		
7939805	NM_00112822	SLC39A13	solute carrier family 39 (zinc trans	0.003	1.002	9.87E-01	9.87E+01		
7939818	NM_175732	PTPMT1	protein tyrosine phosphatase, mit	0.050	1.035	5.91E-01	5.91E+01		
7939825	NM_004551	NDUFS3	NADH dehydrogenase (ubiquinone	-0.014	0.990	9.60E-01	9.60E+01		
7939835	ENST0000035€	FAM180B	family with sequence similarity 18	-0.068	0.954	5.35E-01	5.35E+01		
7939839	NM_002843	PTPRJ	protein tyrosine phosphatase, rec	0.136	1.099	4.94E-01	4.94E+01		
7939902	NR_024504	LOC646813	hypothetical LOC646813	-0.016	0.989	9.60E-01	9.60E+01		
7939996	---	---	---	0.039	1.028	7.51E-01	7.51E+01		
7940002	NM_00100521	LRRC55	leucine rich repeat containing 55	-0.017	0.988	9.12E-01	9.12E+01		
7940020	---	---	---	-0.046	0.968	8.05E-01	8.05E+01		
7940022	NM_178570	RTN4RL2	reticulon 4 receptor-like 2	-0.088	0.941	5.15E-01	5.15E+01		
7940046	NM_006831	CLP1	CLP1, cleavage and polyadenylatio	-0.043	0.971	7.69E-01	7.69E+01		
7940051	NM_015457	ZDHHC5	zinc finger, DHHC-type containing	0.050	1.035	6.91E-01	6.91E+01		
7940066	NM_015959	TMX2	thioredoxin-related transmembra	0.084	1.060	7.11E-01	7.11E+01		
7940070	NM_170746	C11orf31	chromosome 11 open reading fran	0.044	1.031	7.60E-01	7.60E+01		
7940079	NM_00108545	CTNND1	catenin (cadherin-associated prote	0.220	1.164	4.14E-01	4.14E+01		
7940112	NM_00100528	OR9Q2	olfactory receptor, family 9, subfa	-0.085	0.943	5.10E-01	5.10E+01		
7940118	NR_024091	ZFP91-CNTF	ZFP91-CNTF readthrough transcrip	0.009	1.007	9.65E-01	9.65E+01		
7940135	NM_080661	GLYATL1	glycine-N-acyltransferase-like 1	0.202	1.150	2.01E-01	2.01E+01		
7940153	NM_022074	FAM111A	family with sequence similarity 11	0.141	1.103	1.97E-01	1.97E+01		
7940160	NM_015177	DTX4	deltex homolog 4 (Drosophila)	0.064	1.046	6.11E-01	6.11E+01		
7940182	NM_00100470	OR4D10	olfactory receptor, family 4, subfa	-0.047	0.968	8.16E-01	8.16E+01		
7940191	NM_004177	STX3	syntaxin 3	-0.058	0.961	8.21E-01	8.21E+01		
7940259	NM_021201	MS4A7	membrane-spanning 4-domains, s	0.442	1.358	3.66E-01	3.66E+01		
7940287	NM_152866	MS4A1	membrane-spanning 4-domains, s	0.301	1.232	1.70E-01	1.70E+01		
7940333	NM_00109883	MS4A15	membrane-spanning 4-domains, s	0.013	1.009	9.53E-01	9.53E+01		
7940349	NM_024098	CCDC86	coiled-coil domain containing 86	-0.092	0.938	4.44E-01	4.44E+01		
7940358	NM_207341	ZP1	zona pellucida glycoprotein 1 (spe	0.013	1.009	9.33E-01	9.33E+01		
7940372	NM_024092	TMEM109	transmembrane protein 109	0.091	1.065	3.88E-01	3.88E+01		
7940377	NM_017870	TMEM132A	transmembrane protein 132A	-0.014	0.990	9.20E-01	9.20E+01		
7940391	NM_006725	CD6	CD6 molecule	-0.132	0.912	3.04E-01	3.04E+01		
7940409	NM_014207	CD5	CD5 molecule	-0.087	0.941	6.36E-01	6.36E+01		
7940421	NM_00107980	PGA3	pepsinogen 3, group I (pepsinoger	0.090	1.065	2.72E-01	2.72E+01		
7940431	NM_00107980	PGA4	pepsinogen 4, group I (pepsinoger	0.087	1.062	2.65E-01	2.65E+01		
7940441	NM_014224	PGA5	pepsinogen 5, group I (pepsinoger	0.064	1.045	3.97E-01	3.97E+01		
7940451	NM_015533	DAK	dihydroxyacetone kinase 2 homol	-0.085	0.943	2.26E-01	2.26E+01		
7940473	NM_016464	TMEM138	transmembrane protein 138	0.169	1.124	5.61E-01	5.61E+01		
7940486	BC002331	SDHAF2	succinate dehydrogenase complex	0.035	1.025	8.86E-01	8.86E+01		
7940491	---	---	---	0.074	1.053	6.20E-01	6.20E+01		
7940493	NM_145017	C11orf66	chromosome 11 open reading fran	0.005	1.004	9.77E-01	9.77E+01		
7940530	NM_00112739	C11orf9	chromosome 11 open reading fran	-0.075	0.949	3.50E-01	3.50E+01		
7940561	NM_004111	FEN1	flap structure-specific endonuclea	-0.041	0.972	8.21E-01	8.21E+01		
7940565	NM_004265	FADS2	fatty acid desaturase 2	-0.103	0.931	3.27E-01	3.27E+01		
7940580	---	---	---	0.055	1.039	7.70E-01	7.70E+01		
7940582	NM_004183	BEST1	bestrophin 1	-0.047	0.968	8.04E-01	8.04E+01		
7940598	AK093779	LOC399900	hypothetical LOC399900	0.008	1.005	9.77E-01	9.77E+01		
7940600	NM_00104069	INCENP	inner centromere protein antigens	-0.046	0.969	6.95E-01	6.95E+01		
7940626	NM_002407	SCGB2A1	secretoglobin, family 2A, member	0.016	1.011	9.29E-01	9.29E+01		
7940643	NM_00108392	ASRGL1	asparaginase like 1	0.228	1.171	8.54E-02	8.54E+00		
7940654	NM_003357	SCGB1A1	secretoglobin, family 1A, member	-0.074	0.950	4.42E-01	4.42E+01		
7940660	---	---	---	0.000	1.000	1.00E+00	1.00E+02		
7940662	NM_000327	ROM1	retinal outer segment membrane	-0.029	0.980	8.78E-01	8.78E+01		

7940667	NR_004390	SNORA57	small nucleolar RNA, H/ACA box 5	-0.279	0.824	1.40E-01	1.40E+01		
7940669	NM_00108537	C11orf83	chromosome 11 open reading fra	-0.117	0.922	2.80E-01	2.80E+01		
7940673	NM_012202	GNG3	guanine nucleotide binding protei	0.054	1.038	7.38E-01	7.38E+01		
7940679	NM_173810	TTC9C	tetratricopeptide repeat domain 9	-0.002	0.999	9.94E-01	9.94E+01		
7940688	NM_002696	POLR2G	polymerase (RNA) II (DNA directed	0.031	1.021	8.60E-01	8.60E+01		
7940698	NM_006473	TAF6L	TAF6-like RNA polymerase II, p300	0.013	1.009	9.46E-01	9.46E+01		
7940711	NM_199337	TMEM179B	transmembrane protein 179B	-0.016	0.989	9.37E-01	9.37E+01		
7940717	NM_00101266	SLC3A2	solute carrier family 3 (activators	-0.229	0.853	1.16E-01	1.16E+01		
7940749	NM_080866	SLC22A9	solute carrier family 22 (organic a	0.020	1.014	9.03E-01	9.03E+01		
7940762	NM_00114253	LGALS12	lectin, galactoside-binding, soluble	-0.001	0.999	9.94E-01	9.94E+01		
7940775	NM_004585	RARRES3	retinoic acid receptor responder (	0.184	1.136	2.81E-01	2.81E+01		
7940781	NM_201428	RTN3	reticulon 3	0.061	1.043	7.54E-01	7.54E+01		
7940790	BC056402	C11orf84	chromosome 11 open reading fra	0.023	1.016	9.00E-01	9.00E+01		
7940798	NM_017490	MARK2	MAP/microtubule affinity-regulati	0.010	1.007	9.60E-01	9.60E+01		
7940822	---	---	---	-0.013	0.991	9.83E-01	9.83E+01		
7940824	NM_024771	NAT11	N-acetyltransferase 11 (GCN5-rela	-0.070	0.953	6.60E-01	6.60E+01		
7940835	NM_004074	COX8A	cytochrome c oxidase subunit 8A (	0.115	1.083	4.45E-01	4.45E+01		
7940840	NM_017670	OTUB1	OTU domain, ubiquitin aldehyde b	-0.074	0.950	6.54E-01	6.54E+01		
7940851	NM_013280	FLRT1	fibronectin leucine rich transmem	0.068	1.048	4.04E-01	4.04E+01		
7940857	NM_006819	STIP1	stress-induced-phosphoprotein 1	-0.045	0.969	7.47E-01	7.47E+01		
7940869	NM_178443	FERMT3	fermitin family homolog 3 (Drosop	0.006	1.004	9.85E-01	9.85E+01		
7940884	NM_032344	NUDT22	nudix (nucleoside diphosphate lin	0.044	1.031	7.08E-01	7.08E+01		
7940891	NM_005528	DNAJC4	DnaJ (Hsp40) homolog, subfamily	0.019	1.014	9.09E-01	9.09E+01		
7940904	NM_003377	VEGFB	vascular endothelial growth factor	-0.099	0.934	2.91E-01	2.91E+01		
7940914	NM_004470	FKBP2	FK506 binding protein 2, 13kDa	0.017	1.012	9.26E-01	9.26E+01		
7940924	NM_000932	PLCB3	phospholipase C, beta 3 (phospha	-0.073	0.951	2.50E-01	2.50E+01		
7940959	NM_020155	GPR137	G protein-coupled receptor 137	-0.087	0.942	2.76E-01	2.76E+01		
7940971	NM_033310	KCNK4	potassium channel, subfamily K, m	-0.024	0.984	8.47E-01	8.47E+01		
7940989	NM_004451	ESRRA	estrogen-related receptor alpha	-0.012	0.992	9.64E-01	9.64E+01		
7940996	NM_012094	PRDX5	peroxiredoxin 5	-0.001	1.000	9.98E-01	9.98E+01		
7941004	NM_032251	CCDC88B	coiled-coil domain containing 88B	-0.041	0.972	6.85E-01	6.85E+01		
7941039	NM_003942	RPS6KA4	ribosomal protein S6 kinase, 90kD	-0.008	0.995	9.58E-01	9.58E+01		
7941059	ENST00000316	LOC439914	hypothetical gene supported by N	0.038	1.027	8.47E-01	8.47E+01		
7941062	NM_018484	SLC22A11	solute carrier family 22 (organic a	0.018	1.012	8.88E-01	8.88E+01		
7941074	NM_144585	SLC22A12	solute carrier family 22 (organic a	0.028	1.019	8.33E-01	8.33E+01		
7941087	NM_006244	PPP2R5B	protein phosphatase 2, regulatory	-0.033	0.978	8.06E-01	8.06E+01		
7941104	NM_001667	ARL2	ADP-ribosylation factor-like 2	-0.070	0.953	5.31E-01	5.31E+01		
7941111	NM_013306	SNX15	sorting nexin 15	0.040	1.028	7.82E-01	7.82E+01		
7941122	NM_013299	SAC3D1	SAC3 domain containing 1	-0.015	0.990	9.09E-01	9.09E+01		
7941127	NM_006782	ZFPL1	zinc finger protein-like 1	-0.057	0.961	6.75E-01	6.75E+01		
7941136	NM_013265	C11orf2	chromosome 11 open reading fra	-0.042	0.971	7.15E-01	7.15E+01		
7941148	NM_003273	TM7SF2	transmembrane 7 superfamily me	-0.015	0.990	9.17E-01	9.17E+01		
7941164	NM_004927	MRPL49	mitochondrial ribosomal protein L	0.002	1.002	9.92E-01	9.92E+01		
7941172	NM_00100877	SPDYC	speedy homolog C (Xenopus laevis	-0.062	0.958	4.18E-01	4.18E+01		
7941179	NM_005186	CAPN1	calpain 1, (mu/I) large subunit	0.065	1.046	6.28E-01	6.28E+01		
7941203	NM_00100432	SLC22A20	solute carrier family 22, member 2	-0.105	0.930	2.65E-01	2.65E+01		
7941214	NM_002689	POLA2	polymerase (DNA directed), alpha	0.052	1.037	7.79E-01	7.79E+01		
7941236	NM_006779	CDC42EP2	CDC42 effector protein (Rho GTPa	-0.150	0.901	2.01E-02	2.01E+00		
7941243	NM_006268	DPF2	D4, zinc and double PHD fingers fa	-0.079	0.947	5.52E-01	5.52E+01		
7941255	NM_145719	TIGD3	tigger transposable element deriv	-0.025	0.983	8.61E-01	8.61E+01		
7941260	NM_031904	FRMD8	FERM domain containing 8	0.043	1.030	7.54E-01	7.54E+01		
7941269	ENST00000309	LOC100291851	similar to Putative ubiquitin-like p	-0.004	0.997	9.85E-01	9.85E+01		
7941272	NR_002819	MALAT1	metastasis associated lung adeno	0.015	1.010	8.77E-01	8.77E+01		
7941274	NM_020680	SCYL1	SCY1-like 1 (S. cerevisiae)	-0.137	0.909	3.50E-02	3.50E+00		
7941296	NM_006396	SSSCA1	Sjogren syndrome/scleroderma au	-0.035	0.976	8.53E-01	8.53E+01		
7941302	NM_00109878	FAM89B	family with sequence similarity 89	-0.035	0.976	8.16E-01	8.16E+01		
7941311	NM_153253	SIPA1	signal-induced proliferation-assoc	0.023	1.016	8.08E-01	8.08E+01		
7941350	NM_032223	PCNXL3	pecanex-like 3 (Drosophila)	-0.040	0.973	6.54E-01	6.54E+01		
7941364	NM_032223	PCNXL3	pecanex-like 3 (Drosophila)	-0.024	0.984	8.68E-01	8.68E+01		
7941380	---	---	---	0.034	1.024	8.15E-01	8.15E+01		
7941382	NM_182710	KAT5	K(lysine) acetyltransferase 5	-0.072	0.951	4.94E-01	4.94E+01		
7941401	NM_004561	OVOL1	ovo-like 1(Drosophila)	0.000	1.000	9.99E-01	9.99E+01		
7941425	NM_025128	MUS81	MUS81 endonuclease homolog (S.	-0.100	0.933	2.51E-01	2.51E+01		
7941444	NM_001335	CTSW	cathepsin W	-0.029	0.980	9.54E-01	9.54E+01		
7941457	NM_006848	CCDC85B	coiled-coil domain containing 85B	-0.001	1.000	9.97E-01	9.97E+01		
7941460	NM_006442	DRAP1	DR1-associated protein 1 (negativ	0.029	1.021	8.88E-01	8.88E+01		
7941469	NM_152762	TSGA10IP	testis specific, 10 interacting prote	-0.075	0.949	2.51E-01	2.51E+01		
7941478	NM_005146	SART1	squamous cell carcinoma antigen	0.000	1.000	1.00E+00	1.00E+02		
7941501	NM_003860	BANF1	barrier to autointegration factor 1	-0.100	0.933	5.15E-01	5.15E+01		
7941505	NM_001323	CST6	cystatin E/M	-0.004	0.997	9.85E-01	9.85E+01		
7941511	NM_006842	SF3B2	splicing factor 3b, subunit 2, 145k	-0.093	0.938	2.55E-01	2.55E+01		
7941537	NM_018026	PACS1	phosphofurin acidic cluster sorting	-0.048	0.967	7.38E-01	7.38E+01		
7941563	---	---	---	-0.027	0.982	9.49E-01	9.49E+01		
7941565	NM_022822	KLC2	kinesin light chain 2	-0.075	0.950	3.90E-01	3.90E+01		
7941583	NM_030981	RAB1B	RAB1B, member RAS oncogene fa	-0.007	0.995	9.82E-01	9.82E+01		
7941587	NM_182553	CNIH2	cornichon homolog 2 (Drosophila)	-0.044	0.970	6.76E-01	6.76E+01		
7941594	NM_153266	TMEM151A	transmembrane protein 151A	0.007	1.005	9.73E-01	9.73E+01		

7941608	---	---	---	-0.060	0.959	6.91E-01	6.91E+01		
7941610	NM_145065	PELI3	pellino homolog 3 (Drosophila)	0.025	1.018	8.33E-01	8.33E+01		
7941621	NM_005700	DPP3	dipeptidyl-peptidase 3	0.005	1.003	9.85E-01	9.85E+01		
7941639	NM_024649	BBS1	Bardet-Biedl syndrome 1	-0.022	0.985	9.13E-01	9.13E+01		
7941662	NM_001104	ACTN3	actinin, alpha 3	0.067	1.047	6.82E-01	6.82E+01		
7941685	NM_005125	CCS	copper chaperone for superoxide	-0.049	0.967	7.44E-01	7.44E+01		
7941694	NM_006328	RBM14	RNA binding motif protein 14	0.046	1.033	7.91E-01	7.91E+01		
7941702	NM_006328	RBM14	RNA binding motif protein 14	-0.049	0.966	7.42E-01	7.42E+01		
7941707	NM_006328	RBM14	RNA binding motif protein 14	-0.064	0.957	7.43E-01	7.43E+01		
7941709	NM_002896	RBM4	RNA binding motif protein 4	-0.033	0.977	8.46E-01	8.46E+01		
7941714	NM_024650	C11orf80	chromosome 11 open reading fran	0.014	1.009	9.55E-01	9.55E+01		
7941732	NM_005133	RCE1	RCE1 homolog, prenyl protein pep	-0.083	0.944	3.78E-01	3.78E+01		
7941743	NM_024036	LRFN4	leucine rich repeat and fibronectir	-0.072	0.951	5.25E-01	5.25E+01		
7941746	AK026328	C11orf86	chromosome 11 open reading fran	-0.063	0.957	5.31E-01	5.31E+01		
7941749	NM_177963	SYT12	synaptotagmin XII	0.006	1.004	9.77E-01	9.77E+01		
7941761	NM_014578	RHOD	ras homolog gene family, member	-0.005	0.996	9.77E-01	9.77E+01		
7941769	NM_012308	KDM2A	lysine (K)-specific demethylase 2A	-0.013	0.991	9.40E-01	9.40E+01		
7941795	---	---	---	0.092	1.066	3.47E-01	3.47E+01		
7941797	NM_001619	ADRBK1	adrenergic, beta, receptor kinase 1	-0.049	0.967	7.06E-01	7.06E+01		
7941822	NM_207354	ANKRD13D	ankyrin repeat domain 13 family, r	-0.040	0.973	6.60E-01	6.60E+01		
7941843	NM_017857	SSH3	slingshot homolog 3 (Drosophila)	-0.017	0.988	9.17E-01	9.17E+01		
7941863	---	---	---	-0.076	0.949	6.75E-01	6.75E+01		
7941865	NM_004584	RAD9A	RAD9 homolog A (S. pombe)	-0.039	0.974	6.17E-01	6.17E+01		
7941879	NM_198517	TBC1D10C	TBC1 domain family, member 10C	-0.021	0.985	8.62E-01	8.62E+01		
7941890	NM_020811	ATPGD1	ATP-grasp domain containing 1	-0.041	0.972	6.54E-01	6.54E+01		
7941900	NM_003952	RPS6KB2	ribosomal protein S6 kinase, 70kD	-0.028	0.981	8.20E-01	8.20E+01		
7941917	NM_145200	CABP4	calcium binding protein 4	-0.066	0.955	5.71E-01	5.71E+01		
7941927	NM_003977	AIP	aryl hydrocarbon receptor interact	-0.011	0.992	9.64E-01	9.64E+01		
7941936	NM_000852	GSTP1	glutathione S-transferase pi 1	-0.162	0.894	2.09E-01	2.09E+01		
7941946	NM_007103	NDUFV1	NADH dehydrogenase (ubiquinone	-0.033	0.978	8.75E-01	8.75E+01		
7941961	NM_000694	ALDH3B1	aldehyde dehydrogenase 3 family,	-0.031	0.979	7.83E-01	7.83E+01		
7941976	NM_002496	NDUFS8	NADH dehydrogenase (ubiquinone	0.021	1.015	8.95E-01	8.95E+01		
7941985	NM_006019	TCIRG1	T-cell, immune regulator 1, ATPase	0.047	1.033	7.54E-01	7.54E+01		
7942032	NM_018312	SAPS3	SAPS domain family, member 3	-0.012	0.992	9.23E-01	9.23E+01		
7942064	NM_015973	GAL	galanin prepropeptide	0.034	1.024	8.21E-01	8.21E+01		
7942073	NM_002180	IGHMBP2	immunoglobulin mu binding prote	-0.020	0.986	8.76E-01	8.76E+01		
7942091	NM_139075	TPCN2	two pore segment channel 2	-0.003	0.998	9.86E-01	9.86E+01		
7942118	NM_138768	MYEOV	myeloma overexpressed (in a subs	-0.050	0.966	6.30E-01	6.30E+01		
7942123	NM_053056	CCND1	cyclin D1	-0.075	0.950	3.86E-01	3.86E+01		
7942168	NM_003824	FADD	Fas (TNFRSF6)-associated via deat	-0.055	0.962	5.97E-01	5.97E+01		
7942174	NM_003626	PPFIA1	protein tyrosine phosphatase, rece	0.069	1.049	6.12E-01	6.12E+01		
7942228	BC004224	C11orf76	chromosome 11 open reading fran	0.042	1.029	7.19E-01	7.19E+01		
7942232	NM_018161	NADSYN1	NAD synthetase 1	0.011	1.008	9.56E-01	9.56E+01		
7942255	NM_00101250	KRTAP5-7	keratin associated protein 5-7	-0.004	0.997	9.86E-01	9.86E+01		
7942261	NM_005553	KRTAP5-9	keratin associated protein 5-9	-0.097	0.935	2.74E-01	2.74E+01		
7942267	NM_00101271	KRTAP5-10	keratin associated protein 5-10	0.043	1.030	6.74E-01	6.74E+01		
7942274	ENST00000329	OR7E87P	olfactory receptor, family 7, subfa	-0.045	0.969	8.35E-01	8.35E+01		
7942279	NM_018172	FAM86C	family with sequence similarity 86	-0.011	0.992	9.61E-01	9.61E+01		
7942284	NM_00100203	DEFB108B	defensin, beta 108B	0.013	1.009	9.74E-01	9.74E+01		
7942287	---	---	---	-0.161	0.894	5.53E-01	5.53E+01		
7942289	NM_018320	RNF121	ring finger protein 121	0.018	1.013	9.30E-01	9.30E+01		
7942315	NM_00114530	LRTOMT	leucine rich transmembrane and O	-0.008	0.995	9.66E-01	9.66E+01		
7942328	NM_000804	FOLR3	folate receptor 3 (gamma)	0.112	1.081	3.45E-01	3.45E+01		
7942342	NM_001567	INPPL1	inositol polyphosphate phosphata	-0.027	0.981	8.48E-01	8.48E+01		
7942381	NM_033388	ATG16L2	ATG16 autophagy related 16-like 2	-0.068	0.954	4.73E-01	4.73E+01		
7942400	NM_176072	P2RY2	purinergic receptor P2Y, G-protein	-0.039	0.974	8.03E-01	8.03E+01		
7942409	NM_176796	P2RY6	pyrimidinergic receptor P2Y, G-pro	0.184	1.136	6.18E-02	6.18E+00		
7942417	NM_014786	ARHGEF17	Rho guanine nucleotide exchange	0.041	1.029	7.55E-01	7.55E+01		
7942439	NM_032871	RELT	RELT tumor necrosis factor recepto	0.001	1.001	9.96E-01	9.96E+01		
7942453	NM_021200	PLEKHB1	pleckstrin homology domain conta	-0.027	0.982	8.76E-01	8.76E+01		
7942465	NM_016055	MRPL48	mitochondrial ribosomal protein L	-0.007	0.995	9.75E-01	9.75E+01		
7942476	NM_025155	PAAF1	proteasomal ATPase-associated fa	0.084	1.060	6.60E-01	6.60E+01		
7942503	NM_016147	PPME1	protein phosphatase methylestera	0.057	1.040	7.54E-01	7.54E+01		
7942520	---	---	---	-0.122	0.919	4.53E-01	4.53E+01		
7942527	NM_006591	POLD3	polymerase (DNA-directed), delta	0.182	1.134	1.52E-01	1.52E+01		
7942544	NM_00109863	RNF169	ring finger protein 169	0.038	1.027	8.08E-01	8.08E+01		
7942551	---	---	---	0.006	1.004	9.88E-01	9.88E+01		
7942553	NM_014752	SPCS2	signal peptidase complex subunit 2	0.201	1.149	3.45E-02	3.45E+00		
7942562	NM_006656	NEU3	sialidase 3 (membrane sialidase)	0.158	1.116	1.25E-01	1.25E+01		
7942586	NM_001005	RPS3	ribosomal protein S3	-0.020	0.986	8.79E-01	8.79E+01		
7942592	NR_000005	SNORD15A	small nucleolar RNA, C/D box 15A	-0.086	0.942	8.50E-01	8.50E+01		
7942594	NR_000025	SNORD15B	small nucleolar RNA, C/D box 15B	0.117	1.084	7.25E-01	7.25E+01		
7942596	NM_001235	SERPINH1	serpin peptidase inhibitor, clade H	0.078	1.056	5.39E-01	5.39E+01		
7942611	---	---	---	0.011	1.008	9.59E-01	9.59E+01		
7942613	NM_032564	DGAT2	diacylglycerol O-acyltransferase ho	-0.073	0.950	4.70E-01	4.70E+01		
7942626	NM_003369	UVRAG	UV radiation resistance associated	0.108	1.078	2.81E-01	2.81E+01		
7942650	NM_020193	C11orf30	chromosome 11 open reading fran	-0.115	0.923	3.95E-01	3.95E+01		

7942674	NM_015516	TSKU	tsukushin	-0.076	0.949	5.35E-01	5.35E+01		
7942679	NM_018367	ACER3	alkaline ceramidase 3	0.111	1.080	7.46E-01	7.46E+01		
7942693	NM_138706	B3GNT6	UDP-GlcNAc:betaGal beta-1,3-N-a	0.000	1.000	9.99E-01	9.99E+01		
7942697	NM_004055	CAPN5	calpain 5	-0.031	0.979	7.42E-01	7.42E+01		
7942715	NM_006189	OMP	olfactory marker protein	-0.018	0.987	9.02E-01	9.02E+01		
7942717	NM_000260	MYO7A	myosin VIIA	-0.023	0.984	8.30E-01	8.30E+01		
7942769	AL834516	DKFZp434E111	hypothetical protein DKFZp434E11	-0.179	0.883	6.15E-02	6.15E+00		
7942771	AK096213	FLJ38894	hypothetical protein LOC646029	0.009	1.006	9.74E-01	9.74E+01		
7942774	NM_173039	AQP11	aquaporin 11	0.090	1.064	4.06E-01	4.06E+01		
7942779	---	---	---	0.177	1.130	5.35E-01	5.35E+01		
7942783	BC002752	C11orf67	chromosome 11 open reading fra	-0.068	0.954	7.71E-01	7.71E+01		
7942791	NR_003287	LOC100008589	28S ribosomal RNA	0.016	1.011	8.33E-01	8.33E+01		
7942793	NM_003251	THRSP	thyroid hormone responsive (SPO	-0.082	0.945	4.24E-01	4.24E+01		
7942796	NM_00102985	KCTD21	potassium channel tetramerisatio	-0.103	0.931	5.80E-01	5.80E+01		
7942812	---	---	---	-0.099	0.934	5.49E-01	5.49E+01		
7942820	---	---	---	0.055	1.039	7.03E-01	7.03E+01		
7942824	NM_001031	RPS28	ribosomal protein S28	0.073	1.052	6.04E-01	6.04E+01		
7942839	NM_015885	PCF11	PCF11, cleavage and polyadenylat	-0.050	0.966	5.89E-01	5.89E+01		
7942858	NM_182603	ANKRD42	ankyrin repeat domain 42	-0.054	0.963	8.10E-01	8.10E+01		
7942875	---	---	---	-0.004	0.997	9.85E-01	9.85E+01		
7942877	---	---	---	0.056	1.040	8.82E-01	8.82E+01		
7942879	NM_018480	TMEM126B	transmembrane protein 126B	0.040	1.028	7.42E-01	7.42E+01		
7942889	NM_032273	TMEM126A	transmembrane protein 126A	-0.020	0.986	9.36E-01	9.36E+01		
7942912	---	---	---	0.128	1.093	5.16E-01	5.16E+01		
7942914	NM_152991	EED	embryonic ectoderm developmen	-0.012	0.992	9.53E-01	9.53E+01		
7942932	NM_016401	C11orf73	chromosome 11 open reading fra	-0.004	0.997	9.88E-01	9.88E+01		
7942957	NM_007173	PRSS23	protease, serine, 23	0.189	1.140	2.28E-01	2.28E+01		
7942964	NM_022918	TMEM135	transmembrane protein 135	0.090	1.064	5.98E-01	5.98E+01		
7943098	NM_005959	MTNR1B	melatonin receptor 1B	0.041	1.029	7.44E-01	7.44E+01		
7943124	---	---	---	-0.008	0.995	9.74E-01	9.74E+01		
7943126	NM_033395	KIAA1731	KIAA1731	-0.015	0.990	9.53E-01	9.53E+01		
7943158	NR_002569	SCARNA9	small Cajal body-specific RNA 9	0.154	1.113	1.35E-01	1.35E+01		
7943160	NR_002569	SCARNA9	small Cajal body-specific RNA 9	-0.029	0.980	8.73E-01	8.73E+01		
7943162	BC012298	C11orf54	chromosome 11 open reading fra	0.100	1.072	6.37E-01	6.37E+01		
7943176	NM_004268	MED17	mediator complex subunit 17	0.033	1.023	8.59E-01	8.59E+01		
7943193	---	---	---	0.292	1.224	2.79E-01	2.79E+01		
7943218	NM_015368	PANX1	pannexin 1	0.056	1.040	8.76E-01	8.76E+01		
7943226	NM_00108048	FOLR4	folate receptor 4 (delta) homolog	-0.035	0.976	7.46E-01	7.46E+01		
7943231	NM_017704	ANKRD49	ankyrin repeat domain 49	0.026	1.018	8.90E-01	8.90E+01		
7943236	NM_002033	FUT4	fucosyltransferase 4 (alpha (1,3) fu	0.010	1.007	9.63E-01	9.63E+01		
7943288	NM_032102	SFRS2B	splicing factor, arginine/serine-ric	-0.077	0.948	5.16E-01	5.16E+01		
7943293	NM_015036	ENDOD1	endonuclease domain containing	0.005	1.004	9.86E-01	9.86E+01		
7943297	NM_014679	CEP57	centrosomal protein 57kDa	-0.069	0.953	6.29E-01	6.29E+01		
7943347	---	---	---	-0.018	0.988	9.20E-01	9.20E+01		
7943398	NM_00113014	YAP1	Yes-associated protein 1, 65kDa	0.004	1.003	9.84E-01	9.84E+01		
7943413	NM_001165	BIRC3	baculoviral IAP repeat-containing	-0.246	0.843	1.48E-02	1.48E+00		
7943424	NM_001166	BIRC2	baculoviral IAP repeat-containing	-0.231	0.852	1.36E-02	1.36E+00		
7943552	NM_015423	AASDHPPT	aminoadipate-semialdehyde dehy	0.054	1.038	6.35E-01	6.35E+01		
7943573	ENST00000299	LOC643923	hypothetical protein LOC643923	-0.010	0.993	9.61E-01	9.61E+01		
7943580	NM_003478	CUL5	cullin 5	0.258	1.196	2.38E-03	2.38E-01		
7943605	NM_000019	ACAT1	acetyl-Coenzyme A acetyltransfera	0.070	1.049	7.47E-01	7.47E+01		
7943620	NM_000051	ATM	ataxia telangiectasia mutated	0.087	1.062	5.10E-01	5.10E+01		
7943690	NM_004398	DDX10	DEAD (Asp-Glu-Ala-Asp) box polyp	-0.036	0.975	8.77E-01	8.77E+01		
7943711	BC068577	C11orf87	chromosome 11 open reading fra	-0.043	0.971	7.85E-01	7.85E+01		
7943721	NM_004109	FDX1	ferredoxin 1	0.158	1.116	4.84E-02	4.84E+00		
7943760	NM_015191	SIK2	salt-inducible kinase 2	-0.039	0.973	8.13E-01	8.13E+01		
7943795	NM_080659	C11orf52	chromosome 11 open reading fra	-0.072	0.951	4.46E-01	4.46E+01		
7943803	NM_00103795	DIXDC1	DIX domain containing 1	-0.039	0.973	8.80E-01	8.80E+01		
7943827	NM_001931	DLAT	dihydrolipoamide S-acetyltransfer	-0.040	0.972	8.16E-01	8.16E+01		
7943853	NM_003002	SDHD	succinate dehydrogenase complex	0.092	1.066	4.18E-01	4.18E+01		
7943882	NM_000317	PTS	6-pyruvoyltetrahydropterin syntha	0.058	1.041	7.52E-01	7.52E+01		
7943890	ENST00000416	LOC100132686	hypothetical LOC100132686	0.111	1.080	6.76E-01	6.76E+01		
7943892	NM_181351	NCAM1	neural cell adhesion molecule 1	0.231	1.174	2.43E-01	2.43E+01		
7943919	NM_017868	TTC12	tetratricopeptide repeat domain 1	-0.057	0.961	7.87E-01	7.87E+01		
7943943	NM_178510	ANKK1	ankyrin repeat and kinase domain	0.027	1.019	7.92E-01	7.92E+01		
7943954	---	---	---	0.080	1.057	7.59E-01	7.59E+01		
7943956	---	---	---	0.123	1.089	7.72E-01	7.72E+01		
7943969	NM_213621	HTR3A	5-hydroxytryptamine (serotonin) r	-0.017	0.988	9.22E-01	9.22E+01		
7943984	NM_006006	ZBTB16	zinc finger and BTB domain contai	-0.079	0.947	4.82E-01	4.82E+01		
7943998	NM_006169	NNMT	nicotinamide N-methyltransferase	-0.059	0.960	5.21E-01	5.21E+01		
7944006	NM_016090	RBM7	RNA binding motif protein 7	0.068	1.049	6.71E-01	6.71E+01		
7944011	NM_015523	REXO2	REX2, RNA exonuclease 2 homolog	0.029	1.020	8.75E-01	8.75E+01		
7944035	NM_000040	APOC3	apolipoprotein C-III	-0.002	0.999	9.90E-01	9.90E+01		
7944042	NM_002572	PAFAH1B2	platelet-activating factor acetylhy	0.031	1.022	8.55E-01	8.55E+01		
7944049	NM_00104045	SIDT2	SID1 transmembrane family, mem	0.280	1.214	1.17E-01	1.17E+01		
7944096	NM_207343	RNF214	ring finger protein 214	0.060	1.043	6.29E-01	6.29E+01		
7944113	NM_014956	CEP164	centrosomal protein 164kDa	0.005	1.003	9.76E-01	9.76E+01		

7944147	NM_001680	FXYD2	FXYD domain containing ion transp	-0.051	0.965	6.38E-01	6.38E+01		
7944152	NM_001558	IL10RA	interleukin 10 receptor, alpha	0.129	1.093	2.79E-01	2.79E+01		
7944179	NM_000733	CD3E	CD3e molecule, epsilon (CD3-TCR	-0.101	0.933	5.95E-01	5.95E+01		
7944185	NM_000073	CD3G	CD3g molecule, gamma (CD3-TCR	-0.096	0.936	6.05E-01	6.05E+01		
7944195	NM_004788	UBE4A	ubiquitination factor E4A (UFD2 ho	0.062	1.044	5.98E-01	5.98E+01		
7944216	NM_006476	ATP5L	ATP synthase, H+ transporting, mi	-0.001	0.999	9.95E-01	9.95E+01		
7944223	NM_005933	MLL	myeloid/lymphoid or mixed-lineag	-0.109	0.927	1.73E-01	1.73E+01		
7944262	---	---	---	-0.104	0.930	4.19E-01	4.19E+01		
7944271	NM_00108044	TTC36	tetratricopeptide repeat domain 3	-0.033	0.978	8.37E-01	8.37E+01		
7944275	NM_032780	TMEM25	transmembrane protein 25	-0.036	0.975	8.13E-01	8.13E+01		
7944285	NM_001655	ARCN1	archain 1	0.013	1.009	9.44E-01	9.44E+01		
7944335	NM_001716	CXCR5	chemokine (C-X-C motif) receptor	-0.035	0.976	9.36E-01	9.36E+01		
7944339	---	---	---	-0.087	0.942	7.44E-01	7.44E+01		
7944341	NM_006760	UPK2	uroplakin 2	-0.076	0.949	4.19E-01	4.19E+01		
7944365	NM_198489	CCDC84	coiled-coil domain containing 84	-0.124	0.918	2.72E-01	2.72E+01		
7944375	NM_016146	TRAPPC4	trafficking protein particle comple	-0.128	0.915	5.27E-01	5.27E+01		
7944382	NM_021729	VPS11	vacuolar protein sorting 11 homol	0.020	1.014	9.24E-01	9.24E+01		
7944401	NM_000190	HMBS	hydroxymethylbilane synthase	-0.057	0.961	7.22E-01	7.22E+01		
7944418	NM_014807	C2CD2L	C2CD2-like	0.011	1.008	9.33E-01	9.33E+01		
7944435	NM_015517	HINFP	histone H4 transcription factor	-0.089	0.940	3.63E-01	3.63E+01		
7944447	NM_022169	ABCG4	ATP-binding cassette, sub-family G	-0.024	0.984	8.31E-01	8.31E+01		
7944463	NM_024618	NLRX1	NLR family member X1	-0.113	0.925	1.17E-01	1.17E+01		
7944478	NM_024791	PDZD3	PDZ domain containing 3	0.056	1.039	6.55E-01	6.55E+01		
7944493	NM_005188	CBL	Cas-Br-M (murine) ecotropic retro	0.078	1.055	5.63E-01	5.63E+01		
7944510	NM_032015	RNF26	ring finger protein 26	-0.058	0.960	6.49E-01	6.49E+01		
7944516	---	---	---	-0.058	0.960	5.88E-01	5.88E+01		
7944525	---	---	---	0.125	1.090	2.57E-01	2.57E+01		
7944530	NM_178507	OAF	OAF homolog (Drosophila)	-0.029	0.980	8.26E-01	8.26E+01		
7944537	NM_014352	POU2F3	POU class 2 homeobox 3	0.010	1.007	9.50E-01	9.50E+01		
7944560	NM_015313	ARHGEF12	Rho guanine nucleotide exchange	0.109	1.078	6.52E-01	6.52E+01		
7944603	NM_014619	GRIK4	glutamate receptor, ionotropic, ka	-0.011	0.993	9.51E-01	9.51E+01		
7944623	NM_152715	TBCEL	tubulin folding cofactor E-like	0.174	1.128	1.58E-01	1.58E+01		
7944667	NM_003105	SORL1	sortilin-related receptor, L(DLR cla	-0.074	0.950	7.44E-01	7.44E+01		
7944765	---	---	---	0.005	1.003	9.77E-01	9.77E+01		
7944769	NM_020716	GRAMD1B	GRAM domain containing 1B	-0.210	0.865	2.59E-01	2.59E+01		
7944791	---	---	---	-0.067	0.955	7.03E-01	7.03E+01		
7944795	NM_00100196	OR4D5	olfactory receptor, family 4, subfa	-0.026	0.982	8.68E-01	8.68E+01		
7944803	NM_00113014	VWA5A	von Willebrand factor A domain co	0.219	1.164	8.43E-02	8.43E+00		
7944825	X64981	OR10D1P	olfactory receptor, family 10, subf	0.030	1.021	8.26E-01	8.26E+01		
7944850	NM_032811	TBRG1	transforming growth factor beta re	-0.034	0.977	8.36E-01	8.36E+01		
7944867	NM_170601	SIAE	sialic acid acetylesterase	-0.031	0.978	9.19E-01	9.19E+01		
7944876	NM_006176	NRGN	neurogranin (protein kinase C subs	-0.003	0.998	9.90E-01	9.90E+01		
7944882	NM_022370	ROBO3	roundabout, axon guidance recept	-0.081	0.946	2.84E-01	2.84E+01		
7944931	NM_198277	SLC37A2	solute carrier family 37 (glycerol-3	0.259	1.197	8.14E-02	8.14E+00		
7944955	NM_022062	PKNOX2	PBX/knotted 1 homeobox 2	-0.015	0.990	9.02E-01	9.02E+01		
7944970	---	---	---	-0.011	0.992	9.73E-01	9.73E+01		
7944978	NM_004879	EI24	etoposide induced 2.4 mRNA	-0.011	0.992	9.68E-01	9.68E+01		
7944991	NM_152713	STT3A	STT3, subunit of the oligosaccharyl	0.186	1.137	9.97E-02	9.97E+00		
7945014	NM_001274	CHEK1	CHK1 checkpoint homolog (S. pom	0.217	1.162	5.08E-01	5.08E+01		
7945058	NM_024556	FAM118B	family with sequence similarity 11	0.059	1.042	6.30E-01	6.30E+01		
7945069	---	---	---	-0.115	0.924	5.72E-01	5.72E+01		
7945071	NM_017547	FOXRED1	FAD-dependent oxidoreductase do	-0.015	0.989	9.33E-01	9.33E+01		
7945084	---	---	---	0.054	1.038	9.18E-01	9.18E+01		
7945086	NM_00103966	TIRAP	toll-interleukin 1 receptor (TIR) do	-0.022	0.985	9.27E-01	9.27E+01		
7945101	NM_014026	DCPS	decapping enzyme, scavenger	-0.003	0.998	9.92E-01	9.92E+01		
7945125	BC113593	PRR10	proline rich 10	-0.043	0.971	6.20E-01	6.20E+01		
7945130	---	---	---	-0.061	0.959	5.22E-01	5.22E+01		
7945132	NM_002017	FLI1	Friend leukemia virus integration 1	0.194	1.144	1.74E-03	1.74E-01		
7945146	NM_000890	KCNJ5	potassium inwardly-rectifying chan	0.011	1.007	9.35E-01	9.35E+01		
7945156	---	---	---	0.326	1.254	3.75E-01	3.75E+01		
7945158	---	---	---	0.001	1.000	9.99E-01	9.99E+01		
7945162	NM_003658	BARX2	BARX homeobox 2	0.033	1.023	7.74E-01	7.74E+01		
7945169	NM_138788	TMEM45B	transmembrane protein 45B	-0.106	0.929	7.40E-01	7.40E+01		
7945182	NM_001642	APLP2	amyloid beta (A4) precursor-like p	0.100	1.072	8.26E-01	8.26E+01		
7945204	NM_021978	ST14	suppression of tumorigenicity 14 (	0.046	1.033	7.13E-01	7.13E+01		
7945226	---	---	---	0.092	1.066	7.36E-01	7.36E+01		
7945228	ENST00000318	FLJ34521	hypothetical protein LOC646383	0.025	1.018	9.16E-01	9.16E+01		
7945232	NM_139055	ADAMTS15	ADAM metallopeptidase with thro	-0.016	0.989	9.50E-01	9.50E+01		
7945241	AK096377	C11orf44	chromosome 11 open reading fran	-0.017	0.988	9.38E-01	9.38E+01		
7945245	NM_016522	NTM	neurotrimin	0.023	1.016	8.96E-01	8.96E+01		
7945262	NM_032801	JAM3	junctional adhesion molecule 3	-0.114	0.924	4.86E-01	4.86E+01		
7945275	NM_052875	VPS26B	vacuolar protein sorting 26 homol	0.005	1.003	9.85E-01	9.85E+01		
7945283	NM_014384	ACAD8	acyl-Coenzyme A dehydrogenase f	-0.062	0.958	6.56E-01	6.56E+01		
7945342	NM_207366	40435	septin 14	-0.030	0.979	9.19E-01	9.19E+01		
7945344	NR_024368	tcag7.907	hypothetical LOC402483	-0.021	0.985	8.67E-01	8.67E+01		
7945347	---	---	---	0.267	1.203	4.25E-01	4.25E+01		
7945349	NM_016526	BET1L	blocked early in transport 1 homo	-0.031	0.979	8.75E-01	8.75E+01		

7945357	NM_012239	SIRT3	sirtuin (silent mating type informa	-0.114	0.924	1.27E-01	1.27E+01		
7945368	NM_00102529	IFITM5	interferon induced transmembran	-0.143	0.906	2.50E-01	2.50E+01		
7945377	NM_00113505	SIGIRR	single immunoglobulin and toll-int	-0.054	0.963	5.28E-01	5.28E+01		
7945394	NM_00101230	ANO9	anoctamin 9	0.058	1.041	6.28E-01	6.28E+01		
7945418	---	---	---	-0.061	0.958	5.09E-01	5.09E+01		
7945420	NM_002939	RNH1	ribonuclease/angiogenin inhibitor	0.043	1.030	7.23E-01	7.23E+01		
7945436	NM_176795	HRAS	v-Ha-ras Harvey rat sarcoma viral	-0.014	0.990	9.46E-01	9.46E+01		
7945445	BC039077	C11orf35	chromosome 11 open reading fran	0.004	1.003	9.74E-01	9.74E+01		
7945460	---	---	---	0.027	1.019	7.39E-01	7.39E+01		
7945462	NM_004031	IRF7	interferon regulatory factor 7	-0.047	0.968	6.67E-01	6.67E+01		
7945475	NM_021924	MUPCDH	mucin-like protocadherin	-0.057	0.961	5.27E-01	5.27E+01		
7945498	NM_021920	SCT	secretin	0.011	1.008	9.49E-01	9.49E+01		
7945503	NM_021008	DEAF1	deformed epidermal autoregulato	-0.056	0.962	7.05E-01	7.05E+01		
7945521	NM_182612	PDDC1	Parkinson disease 7 domain conta	0.026	1.018	8.44E-01	8.44E+01		
7945536	NM_016564	CEND1	cell cycle exit and neuronal differe	0.023	1.016	8.85E-01	8.85E+01		
7945539	NM_024698	SLC25A22	solute carrier family 25 (mitochon	-0.008	0.994	9.64E-01	9.64E+01		
7945550	NM_018494	LRDD	leucine-rich repeats and death do	-0.009	0.994	9.49E-01	9.49E+01		
7945573	NM_021128	POLR2L	polymerase (RNA) II (DNA directed	-0.012	0.992	9.57E-01	9.57E+01		
7945579	NM_00114267	CHID1	chitinase domain containing 1	0.014	1.010	9.27E-01	9.27E+01		
7945595	NM_005961	MUC6	mucin 6, oligomeric mucus/gel-for	-0.063	0.957	6.48E-01	6.48E+01		
7945620	NM_019009	TOLLIP	toll interacting protein	-0.013	0.991	9.28E-01	9.28E+01		
7945631	NM_053005	HCCA2	HCCA2 protein	-0.057	0.961	5.53E-01	5.53E+01		
7945641	NM_004420	DUSP8	dual specificity phosphatase 8	-0.088	0.941	4.01E-01	4.01E+01		
7945645	NM_00100592	KRTAP5-1	keratin associated protein 5-1	-0.075	0.949	4.48E-01	4.48E+01		
7945648	NM_00100432	KRTAP5-2	keratin associated protein 5-2	-0.010	0.993	9.53E-01	9.53E+01		
7945652	NM_00101270	KRTAP5-3	keratin associated protein 5-3	-0.019	0.987	9.12E-01	9.12E+01		
7945657	NM_00101270	KRTAP5-4	keratin associated protein 5-4	-0.110	0.926	3.43E-01	3.43E+01		
7945663	---	---	---	-0.025	0.983	8.78E-01	8.78E+01		
7945678	ENST00000391	C11orf89	chromosome 11 open reading fran	-0.071	0.952	4.83E-01	4.83E+01		
7945680	NR_002196	H19	H19, imprinted maternally express	0.017	1.012	9.12E-01	9.12E+01		
7945688	NR_003512	INS-IGF2	INS-IGF2 readthrough transcript	-0.034	0.977	6.51E-01	6.51E+01		
7945712	NM_199292	TH	tyrosine hydroxylase	-0.085	0.943	1.41E-01	1.41E+01		
7945730	NM_005170	ASCL2	achaete-scute complex homolog 2	0.234	1.176	1.37E-01	1.37E+01		
7945734	AB029488	C11orf21	chromosome 11 open reading fran	0.050	1.035	7.19E-01	7.19E+01		
7945740	NM_016093	RPL26L1	ribosomal protein L26-like 1	0.026	1.018	9.08E-01	9.08E+01		
7945742	NM_014555	TRPM5	transient receptor potential cation	-0.074	0.950	5.82E-01	5.82E+01		
7945767	NM_000076	CDKN1C	cyclin-dependent kinase inhibitor	-0.009	0.994	9.50E-01	9.50E+01		
7945781	NM_003311	PHLDA2	pleckstrin homology-like domain,	-0.091	0.939	3.36E-01	3.36E+01		
7945786	NM_005969	NAP1L4	nucleosome assembly protein 1-lil	0.021	1.015	9.04E-01	9.04E+01		
7945803	NM_00101443	CARS	cysteinyl-tRNA synthetase	0.027	1.019	8.27E-01	8.27E+01		
7945829	---	---	---	0.127	1.092	8.16E-01	8.16E+01		
7945857	ENST00000332	MRGPRG	MAS-related GPR, member G	-0.070	0.953	5.62E-01	5.62E+01		
7945859	NM_00103916	MRGPRE	MAS-related GPR, member E	-0.013	0.991	9.49E-01	9.49E+01		
7945864	NM_00113052	ZNF195	zinc finger protein 195	-0.002	0.999	9.95E-01	9.95E+01		
7945873	NR_002822	MGC72080	MGC72080 pseudogene	-0.044	0.970	8.55E-01	8.55E+01		
7945875	NM_00109965	M86C // FAM8	family with sequence similarity 86	0.092	1.066	6.70E-01	6.70E+01		
7945892	---	---	---	-0.045	0.969	8.94E-01	8.94E+01		
7945894	---	---	---	0.048	1.034	9.12E-01	9.12E+01		
7945896	NM_020402	CHRNA10	cholinergic receptor, nicotinic, alp	-0.030	0.979	8.63E-01	8.63E+01		
7945902	NM_016320	NUP98	nucleoporin 98kDa	-0.088	0.941	4.32E-01	4.32E+01		
7945942	---	---	---	0.155	1.114	6.44E-01	6.44E+01		
7945944	NM_001665	RHOG	ras homolog gene family, member	-0.006	0.996	9.76E-01	9.76E+01		
7945950	---	---	---	0.071	1.050	7.92E-01	7.92E+01		
7945958	NM_00100516	OR52B4	olfactory receptor, family 52, subf	0.086	1.062	5.59E-01	5.59E+01		
7945962	NM_003141	TRIM21	tripartite motif-containing 21	0.047	1.033	8.67E-01	8.67E+01		
7945974	AF439154	C11orf40	chromosome 11 open reading fran	0.088	1.063	2.44E-01	2.44E+01		
7945989	---	---	---	-0.144	0.905	1.35E-01	1.35E+01		
7946004	NM_00100475	OR51S1	olfactory receptor, family 51, subf	-0.068	0.954	5.57E-01	5.57E+01		
7946033	NM_000518	HBB	hemoglobin, beta	0.488	1.402	6.78E-01	6.78E+01		
7946041	NM_000519	HBD	hemoglobin, delta	0.243	1.183	2.82E-01	2.82E+01		
7946048	NM_000559	HBG1	hemoglobin, gamma A	0.018	1.013	9.18E-01	9.18E+01		
7946054	NM_000559	HBG1	hemoglobin, gamma A	0.018	1.013	9.18E-01	9.18E+01		
7946069	NM_033180	OR51B2	olfactory receptor, family 51, subf	0.046	1.032	7.33E-01	7.33E+01		
7946087	NM_00100528	OR52H1	olfactory receptor, family 52, subf	0.070	1.049	8.31E-01	8.31E+01		
7946089	NM_033034	TRIM5	tripartite motif-containing 5	0.105	1.076	5.13E-01	5.13E+01		
7946128	NM_032127	FAM160A2	family with sequence similarity 16	-0.050	0.966	6.29E-01	6.29E+01		
7946149	NM_001164	APBB1	amyloid beta (A4) precursor prote	-0.029	0.980	8.84E-01	8.84E+01		
7946180	NM_006458	TRIM3	tripartite motif-containing 3	-0.044	0.970	6.77E-01	6.77E+01		
7946201	NM_012402	ARFIP2	ADP-ribosylation factor interacting	-0.075	0.949	6.00E-01	6.00E+01		
7946211	NM_015324	RRP8	ribosomal RNA processing 8, meth	-0.030	0.980	9.10E-01	9.10E+01		
7946221	NM_006284	TAF10	TAF10 RNA polymerase II, TATA bo	0.036	1.025	7.51E-01	7.51E+01		
7946228	NM_000391	TPP1	tripeptidyl peptidase I	0.155	1.113	4.29E-01	4.29E+01		
7946245	NM_003737	DCHS1	dachsous 1 (Drosophila)	-0.028	0.981	8.38E-01	8.38E+01		
7946267	NM_022061	MRPL17	mitochondrial ribosomal protein L	0.024	1.017	9.04E-01	9.04E+01		
7946275	NR_003945	GVIN1	GTPase, very large interferon indu	0.051	1.036	8.25E-01	8.25E+01		
7946340	NM_024557	RIC3	resistance to inhibitors of cholines	0.062	1.044	7.94E-01	7.94E+01		
7946354	NM_002315	LMO1	LIM domain only 1 (rhombotin 1)	0.033	1.023	7.94E-01	7.94E+01		

7946380	NM_014818	TRIM66	tripartite motif-containing 66	-0.018	0.988	9.19E-01	9.19E+01		
7946426	---	---	---	0.012	1.008	9.78E-01	9.78E+01		
7946428	NM_020643	C11orf16	chromosome 11 open reading fran	-0.030	0.979	8.32E-01	8.32E+01		
7946439	NM_020644	TMEM9B	TMEM9 domain family, member B	0.016	1.011	9.44E-01	9.44E+01		
7946446	NM_020645	NRIP3	nuclear receptor interacting prote	-0.161	0.894	6.35E-01	6.35E+01		
7946478	NM_015213	DENND5A	DENN/MADD domain containing 5	-0.182	0.882	1.95E-01	1.95E+01		
7946504	NM_015012	TMEM41B	transmembrane protein 41B	0.053	1.038	7.48E-01	7.48E+01		
7946516	NM_030962	SBF2	SET binding factor 2	0.138	1.101	5.23E-01	5.23E+01		
7946559	NM_00101799	GNG10	guanine nucleotide binding protei	0.224	1.168	1.53E-01	1.53E+01		
7946563	---	---	---	-0.096	0.935	4.70E-01	4.70E+01		
7946565	---	---	---	0.050	1.036	2.32E-01	2.32E+01		
7946567	---	---	---	-0.118	0.921	4.20E-01	4.20E+01		
7946569	NM_016422	RNF141	ring finger protein 141	0.072	1.051	6.72E-01	6.72E+01		
7946589	NM_130385	MRVI1	murine retrovirus integration site	0.065	1.046	7.10E-01	7.10E+01		
7946610	NM_001418	EIF4G2	eukaryotic translation initiation fa	0.011	1.007	9.53E-01	9.53E+01		
7946635	NM_021211	ZBED5	zinc finger, BED-type containing 5	-0.002	0.999	9.92E-01	9.92E+01		
7946655	NR_002207	CSNK2A1P	casein kinase 2, alpha 1 polypeptic	0.035	1.025	8.92E-01	8.92E+01		
7946659	---	---	---	0.025	1.018	8.02E-01	8.02E+01		
7946661	NM_015881	DKK3	dickkopf homolog 3 (Xenopus laev	-0.071	0.952	4.35E-01	4.35E+01		
7946676	---	---	---	-0.059	0.960	7.53E-01	7.53E+01		
7946680	NM_032320	BTBD10	BTB (POZ) domain containing 10	0.113	1.081	3.70E-01	3.70E+01		
7946697	NM_012250	RRAS2	related RAS viral (r-ras) oncogene	-0.039	0.973	7.73E-01	7.73E+01		
7946703	NM_016451	COPB1	coatomer protein complex, subun	-0.031	0.979	8.68E-01	8.68E+01		
7946728	NM_148976	PSMA1	proteasome (prosome, macropain	-0.057	0.961	7.15E-01	7.15E+01		
7946742	NM_024514	CYP2R1	cytochrome P450, family 2, subfar	-0.138	0.909	4.75E-01	4.75E+01		
7946749	NM_001741	CALCA	calcitonin-related polypeptide alp	-0.084	0.943	4.01E-01	4.01E+01		
7946781	NM_175058	PLEKHA7	pleckstrin homology domain conta	0.191	1.141	2.01E-02	2.01E+00		
7946807	NM_021029	RPL36A	ribosomal protein L36a	0.010	1.007	9.56E-01	9.56E+01		
7946812	NM_001017	RPS13	ribosomal protein S13	-0.004	0.997	9.88E-01	9.88E+01		
7946849	---	---	---	0.009	1.006	9.83E-01	9.83E+01		
7946853	NM_000525	KCNJ11	potassium inwardly-rectifying char	-0.045	0.970	8.06E-01	8.06E+01		
7946860	NM_000352	ABCC8	ATP-binding cassette, sub-family C	0.003	1.002	9.87E-01	9.87E+01		
7946903	NM_153676	USH1C	Usher syndrome 1C (autosomal re	-0.068	0.954	4.19E-01	4.19E+01		
7946933	NM_012139	SERGEF	secretion regulating guanine nucle	-0.091	0.939	4.96E-01	4.96E+01		
7946957	NM_138421	SAAL1	serum amyloid A-like 1	-0.027	0.982	9.11E-01	9.11E+01		
7947015	NM_006292	TSG101	tumor susceptibility gene 101	0.035	1.025	8.52E-01	8.52E+01		
7947027	NM_00104069	UEVLD	UEV and lactate/malate dehyroger	0.124	1.090	5.41E-01	5.41E+01		
7947040	NM_194285	SPTY2D1	SPT2, Suppressor of Ty, domain co	0.087	1.062	4.49E-01	4.49E+01		
7947076	NM_006906	PTPN5	protein tyrosine phosphatase, non	-0.076	0.949	4.05E-01	4.05E+01		
7947129	NM_00102986	DBX1	developing brain homeobox 1	0.027	1.019	8.71E-01	8.71E+01		
7947138	NM_022725	FANCF	Fanconi anemia, complementation	0.056	1.040	7.72E-01	7.72E+01		
7947147	NM_148893	SVIP	small VCP/p97-interacting protein	-0.047	0.968	8.33E-01	8.33E+01		
7947189	NM_030771	CCDC34	coiled-coil domain containing 34	0.073	1.052	6.95E-01	6.95E+01		
7947221	NM_018362	LIN7C	lin-7 homolog C (C. elegans)	0.114	1.082	3.56E-01	3.56E+01		
7947245	M30627	HSP90AA2	heat shock protein 90kDa alpha (c	-0.055	0.963	7.24E-01	7.24E+01		
7947270	NM_002233	KCNA4	potassium voltage-gated channel,	0.027	1.019	8.41E-01	8.41E+01		
7947358	NM_002901	RCN1	reticulocalbin 1, EF-hand calcium b	0.022	1.015	9.30E-01	9.30E+01		
7947396	NM_001326	CSTF3	cleavage stimulation factor, 3' pre	0.037	1.026	7.59E-01	7.59E+01		
7947421	---	---	---	-0.017	0.988	9.60E-01	9.60E+01		
7947423	---	---	---	-0.004	0.997	9.93E-01	9.93E+01		
7947425	NM_203330	CD59	CD59 molecule, complement regu	-0.196	0.873	4.60E-01	4.60E+01		
7947434	NM_033406	FBXO3	F-box protein 3	0.047	1.033	8.35E-01	8.35E+01		
7947450	NM_005574	LMO2	LIM domain only 2 (rhombotin-like	0.074	1.053	4.02E-01	4.02E+01		
7947462	NM_145804	ABTB2	ankyrin repeat and BTB (POZ) dom	-0.133	0.912	1.29E-01	1.29E+01		
7947481	NM_198381	ELF5	E74-like factor 5 (ets domain trans	-0.055	0.963	6.29E-01	6.29E+01		
7947490	NM_015957	APIP	APAF1 interacting protein	-0.204	0.868	2.58E-01	2.58E+01		
7947526	---	---	---	-0.067	0.955	5.20E-01	5.20E+01		
7947528	NM_014186	COMMD9	COMM domain containing 9	-0.059	0.960	7.51E-01	7.51E+01		
7947531	NM_014186	COMMD9	COMM domain containing 9	0.092	1.066	5.61E-01	5.61E+01		
7947540	NM_145803	TRAF6	TNF receptor-associated factor 6	-0.010	0.993	9.63E-01	9.63E+01		
7947563	NM_021926	ALX4	ALX homeobox 4	-0.099	0.934	1.95E-01	1.95E+01		
7947570	NM_00107678	TP53I11	tumor protein p53 inducible prote	-0.044	0.970	7.04E-01	7.04E+01		
7947599	NM_003654	CHST1	carbohydrate (keratan sulfate Gal-	-0.030	0.979	7.94E-01	7.94E+01		
7947609	NM_004813	PEX16	peroxisomal biogenesis factor 16	-0.026	0.982	8.22E-01	8.22E+01		
7947624	NM_00110180	PHF21A	PHD finger protein 21A	-0.017	0.988	9.01E-01	9.01E+01		
7947649	NM_000741	CHRM4	cholinergic receptor, muscarinic 4	-0.041	0.972	7.72E-01	7.72E+01		
7947652	NM_017749	AMBRA1	autophagy/beclin-1 regulator 1	-0.088	0.941	2.94E-01	2.94E+01		
7947674	---	---	---	-0.025	0.983	9.54E-01	9.54E+01		
7947676	NM_173811	HARBI1	harbinger transposase derived 1	0.074	1.052	6.08E-01	6.08E+01		
7947681	NM_004308	ARHGAP1	Rho GTPase activating protein 1	0.027	1.019	9.11E-01	9.11E+01		
7947694	NM_00100893	CKAP5	cytoskeleton associated protein 5	-0.006	0.996	9.81E-01	9.81E+01		
7947740	---	---	---	0.045	1.032	7.61E-01	7.61E+01		
7947742	---	---	---	0.049	1.035	7.90E-01	7.90E+01		
7947784	NM_032389	ARFGAP2	ADP-ribosylation factor GTPase ac	-0.004	0.997	9.84E-01	9.84E+01		
7947801	NM_016223	PACSIN3	protein kinase C and casein kinase	-0.047	0.968	7.16E-01	7.16E+01		
7947815	NM_001610	ACP2	acid phosphatase 2, lysosomal	0.379	1.301	8.58E-02	8.58E+00		
7947828	NM_000256	MYBPC3	myosin binding protein C, cardiac	0.033	1.023	7.70E-01	7.70E+01		

7947861	NM_00108054	SPI1	spleen focus forming virus (SFFV) p	0.101	1.073	5.24E-01	5.24E+01		
7947867	NM_002804	PSMC3	proteasome (prosome, macropain	-0.064	0.957	7.05E-01	7.05E+01		
7947881	NM_005055	RAPSN	receptor-associated protein of the	-0.035	0.976	7.63E-01	7.63E+01		
7947892	---	---	---	-0.167	0.891	5.94E-01	5.94E+01		
7947894	NM_198700	CUGBP1	CUG triplet repeat, RNA binding pr	0.022	1.015	8.62E-01	8.62E+01		
7947915	---	---	---	0.008	1.006	9.71E-01	9.71E+01		
7947917	NM_016506	KBTBD4	kelch repeat and BTB (POZ) domai	-0.050	0.966	6.94E-01	6.94E+01		
7947928	NM_031909	C1QTNF4	C1q and tumor necrosis factor rela	-0.103	0.931	1.73E-01	1.73E+01		
7947934	NM_014342	MTCH2	mitochondrial carrier homolog 2 (	0.068	1.048	6.76E-01	6.76E+01		
7947947	NM_024783	AGBL2	ATP/GTP binding protein-like 2	-0.021	0.986	9.54E-01	9.54E+01		
7947969	NM_015308	FNBP4	formin binding protein 4	-0.028	0.981	8.67E-01	8.67E+01		
7947989	---	---	---	0.040	1.028	9.07E-01	9.07E+01		
7947991	NM_015231	NUP160	nucleoporin 160kDa	0.033	1.023	8.18E-01	8.18E+01		
7948033	---	---	---	0.157	1.115	6.05E-01	6.05E+01		
7948037	---	---	---	0.092	1.066	9.00E-01	9.00E+01		
7948054	---	---	---	0.000	1.000	1.00E+00	1.00E+02		
7948088	NM_032315	SLC25A33	solute carrier family 25, member 3	0.105	1.075	6.00E-01	6.00E+01		
7948092	NR_003034	LOC441601	septin 7 pseudogene	-0.059	0.960	6.40E-01	6.40E+01		
7948113	NR_027688	OR7E5P	olfactory receptor, family 7, subfa	-0.007	0.995	9.82E-01	9.82E+01		
7948176	NM_033396	TNKS1BP1	tankyrase 1 binding protein 1, 182	0.001	1.000	9.97E-01	9.97E+01		
7948192	NM_003146	SSRP1	structure specific recognition prot	-0.127	0.916	2.73E-01	2.73E+01		
7948211	AB209132	SSRP1	structure specific recognition prot	-0.055	0.963	8.92E-01	8.92E+01		
7948213	NM_006093	PRG3	proteoglycan 3	-0.016	0.989	9.28E-01	9.28E+01		
7948229	NM_017611	SLC43A3	solute carrier family 43, member 3	-0.031	0.979	9.51E-01	9.51E+01		
7948249	NM_003627	SLC43A1	solute carrier family 43, member 1	-0.044	0.970	6.88E-01	6.88E+01		
7948267	NM_012456	TIMM10	translocase of inner mitochondrial	0.023	1.016	9.42E-01	9.42E+01		
7948274	NM_004223	UBE2L6	ubiquitin-conjugating enzyme E2L	0.149	1.109	2.44E-01	2.44E+01		
7948282	NM_145008	YPEL4	yippee-like 4 (Drosophila)	0.070	1.050	6.01E-01	6.01E+01		
7948293	NM_153450	MED19	mediator complex subunit 19	0.063	1.044	5.71E-01	5.71E+01		
7948303	---	---	---	-0.061	0.959	5.53E-01	5.53E+01		
7948306	---	---	---	0.122	1.088	7.11E-01	7.11E+01		
7948314	NM_00100447	OR10Q1	olfactory receptor, family 10, subf	0.086	1.061	4.74E-01	4.74E+01		
7948332	NM_004811	LPXN	leupaxin	-0.119	0.921	1.71E-01	1.71E+01		
7948367	---	---	---	-0.026	0.982	8.98E-01	8.98E+01		
7948379	NM_002556	OSBP	oxysterol binding protein	-0.012	0.992	9.32E-01	9.32E+01		
7948397	---	---	---	-0.040	0.973	9.46E-01	9.46E+01		
7948399	NM_152716	PATL1	protein associated with topoisome	0.067	1.047	5.95E-01	5.95E+01		
7948420	NM_001444	FABP5	fatty acid binding protein 5 (psoria	0.273	1.208	5.65E-02	5.65E+00		
7948424	NM_017840	MRPL16	mitochondrial ribosomal protein L	0.017	1.012	9.33E-01	9.33E+01		
7948470	NM_004778	GPR44	G protein-coupled receptor 44	-0.074	0.950	3.62E-01	3.62E+01		
7948476	NM_014502	PRPF19	PRP19/PSO4 pre-mRNA processing	0.074	1.053	5.43E-01	5.43E+01		
7948493	NM_016582	SLC15A3	solute carrier family 15, member 3	0.128	1.093	2.99E-01	2.99E+01		
7948502	---	---	---	-0.220	0.859	5.07E-01	5.07E+01		
7948504	NM_017966	VPS37C	vacuolar protein sorting 37 homol	0.019	1.013	8.98E-01	8.98E+01		
7948511	NM_152718	VWCE	von Willebrand factor C and EGF d	-0.052	0.964	5.30E-01	5.30E+01		
7948534	NM_001923	DDB1	damage-specific DNA binding prot	0.059	1.042	6.09E-01	6.09E+01		
7948565	NM_00116145	CYBASC3	cytochrome b, ascorbate depende	-0.011	0.992	9.64E-01	9.64E+01		
7948574	NM_024811	CPSF7	cleavage and polyadenylation spec	-0.060	0.959	5.63E-01	5.63E+01		
7948588	NM_004200	SYT7	synaptotagmin VII	-0.037	0.975	7.56E-01	7.56E+01		
7948599	ENST00000244	DKFZP434K028	hypothetical LOC26070	-0.011	0.993	9.51E-01	9.51E+01		
7948606	NM_014206	C11orf10	chromosome 11 open reading fran	0.050	1.035	7.31E-01	7.31E+01		
7948612	NM_013402	FADS1	fatty acid desaturase 1	-0.087	0.941	6.19E-01	6.19E+01		
7948630	NM_021727	FADS3	fatty acid desaturase 3	-0.042	0.972	6.85E-01	6.85E+01		
7948643	NM_013401	RAB3IL1	RAB3A interacting protein (rabin3)	0.005	1.004	9.74E-01	9.74E+01		
7948656	NM_002032	FTH1	ferritin, heavy polypeptide 1	-0.021	0.985	9.64E-01	9.64E+01		
7948667	NM_001620	AHNAK	AHNAK nucleoprotein	0.145	1.105	2.05E-01	2.05E+01		
7948679	NM_001404	EEF1G	eukaryotic translation elongation f	0.007	1.005	9.81E-01	9.81E+01		
7948685	NM_022830	TUT1	terminal uridylyl transferase 1, U6	-0.029	0.980	8.34E-01	8.34E+01		
7948696	NM_004739	MTA2	metastasis associated 1 family, me	0.003	1.002	9.88E-01	9.88E+01		
7948718	NM_153265	EML3	echinoderm microtubule associate	-0.002	0.999	9.91E-01	9.91E+01		
7948741	NM_012200	B3GAT3	beta-1,3-glucuronyltransferase 3 (	-0.084	0.943	6.90E-01	6.90E+01		
7948744	NM_198335	GANAB	glucosidase, alpha; neutral AB	0.039	1.027	7.52E-01	7.52E+01		
7948772	NM_030628	INTS5	integrator complex subunit 5	0.005	1.003	9.81E-01	9.81E+01		
7948775	NM_024099	C11orf48	chromosome 11 open reading fran	-0.038	0.974	8.23E-01	8.23E+01		
7948782	NM_015853	UBXN1	UBX domain protein 1	-0.175	0.886	5.88E-02	5.88E+00		
7948798	NM_00113070	BSCL2	Berardinelli-Seip congenital lipody	-0.021	0.985	9.22E-01	9.22E+01		
7948814	NM_00107955	HNRNPUL2	heterogeneous nuclear ribonucleo	-0.040	0.972	7.50E-01	7.50E+01		
7948829	NM_024784	ZBTB3	zinc finger and BTB domain contai	-0.087	0.941	2.47E-01	2.47E+01		
7948834	NM_006473	TAF6L	TAF6-like RNA polymerase II, p300	0.012	1.009	9.47E-01	9.47E+01		
7948836	NM_00108050	TMEM223	transmembrane protein 223	-0.045	0.969	8.16E-01	8.16E+01		
7948839	NM_006362	NXF1	nuclear RNA export factor 1	-0.144	0.905	6.30E-02	6.30E+00		
7948864	NM_003164	STX5	syntaxin 5	-0.023	0.984	9.01E-01	9.01E+01		
7948879	---	---	---	-0.088	0.941	6.66E-01	6.66E+01		
7948881	NM_018093	WDR74	WD repeat domain 74	-0.145	0.905	1.81E-01	1.81E+01		
7948894	NR_002716	RNU2-1	RNA, U2 small nuclear 1	-0.248	0.842	4.98E-01	4.98E+01		
7948898	NR_002560	SNORD31	small nucleolar RNA, C/D box 31	0.024	1.017	9.67E-01	9.67E+01		
7948900	NR_002561	SNORD30	small nucleolar RNA, C/D box 30	-0.292	0.817	2.30E-01	2.30E+01		

7948902	NR_002559	SNORD29	small nucleolar RNA, C/D box 29	-0.144	0.905	6.24E-01	6.24E+01		
7948904	NR_002562	SNORD28	small nucleolar RNA, C/D box 28	-0.158	0.896	6.91E-01	6.91E+01		
7948906	NR_002563	SNORD27	small nucleolar RNA, C/D box 27	-0.229	0.853	5.55E-01	5.55E+01		
7948908	NR_002564	SNORD26	small nucleolar RNA, C/D box 26	-0.059	0.960	8.64E-01	8.64E+01		
7948910	NR_002565	SNORD25	small nucleolar RNA, C/D box 25	-0.048	0.967	9.37E-01	9.37E+01		
7948912	NM_000738	CHRM1	cholinergic receptor, muscarinic 1	-0.012	0.992	9.53E-01	9.53E+01		
7948918	---	---	---	-0.006	0.996	9.75E-01	9.75E+01		
7948920	NM_004790	SLC22A6	solute carrier family 22 (organic ar	0.015	1.010	9.17E-01	9.17E+01		
7948937	NM_004254	SLC22A8	solute carrier family 22 (organic ar	-0.117	0.922	3.47E-01	3.47E+01		
7948973	NM_054108	HRASLS5	HRAS-like suppressor family, mem	-0.044	0.970	7.54E-01	7.54E+01		
7948987	NM_007069	PLA2G16	phospholipase A2, group XVI	-0.125	0.917	5.55E-01	5.55E+01		
7948995	ENST00000398	ATL3	atlastin GTPase 3	-0.012	0.991	9.56E-01	9.56E+01		
7948997	NM_015459	ATL3	atlastin GTPase 3	-0.074	0.950	5.83E-01	5.83E+01		
7949015	NM_00114493	LOC65998	hypothetical protein LOC65998	-0.058	0.960	6.27E-01	6.27E+01		
7949033	NM_014067	MACROD1	MACRO domain containing 1	-0.058	0.960	4.06E-01	4.06E+01		
7949046	NM_00103367	TRPT1	tRNA phosphotransferase 1	-0.050	0.966	7.42E-01	7.42E+01		
7949058	NM_005528	DNAJC4	DnaJ (Hsp40) homolog, subfamily	-0.074	0.950	5.57E-01	5.57E+01		
7949060	NM_138689 //	// PPP1R14B //	protein phosphatase 1, regulatory	-0.229	0.853	3.48E-03	3.48E-01		
7949067	NM_004322	BAD	BCL2-associated agonist of cell dea	-0.071	0.952	4.12E-01	4.12E+01		
7949075	NM_016404	TRMT112	tRNA methyltransferase 11-2 hom	-0.042	0.971	7.87E-01	7.87E+01		
7949080	NM_015080	NRXN2	neurexin 2	-0.037	0.975	7.88E-01	7.88E+01		
7949104	NM_00109867	RASGRP2	RAS guanyl releasing protein 2 (ca	0.066	1.047	5.48E-01	5.48E+01		
7949146	NM_004630	SF1	splicing factor 1	-0.009	0.994	9.53E-01	9.53E+01		
7949172	NM_004579	MAP4K2	mitogen-activated protein kinase	-0.068	0.954	5.93E-01	5.93E+01		
7949206	NM_130802	MEN1	multiple endocrine neoplasia I	0.003	1.002	9.86E-01	9.86E+01		
7949227	NM_017525	CDC42BPG	CDC42 binding protein kinase gam	-0.037	0.975	7.15E-01	7.15E+01		
7949264	NM_006795	EHD1	EH-domain containing 1	-0.156	0.897	9.17E-02	9.17E+00		
7949273	---	---	---	-0.022	0.985	9.02E-01	9.02E+01		
7949275	---	---	---	-0.014	0.990	9.40E-01	9.40E+01		
7949277	NM_015104	ATG2A	ATG2 autophagy related 2 homolo	-0.063	0.957	5.29E-01	5.29E+01		
7949320	NM_130769	GPHA2	glycoprotein hormone alpha 2	0.014	1.009	9.49E-01	9.49E+01		
7949340	NM_138456	BATF2	basic leucine zipper transcription f	-0.017	0.988	9.43E-01	9.43E+01		
7949344	NM_005468	NAALADL1	N-acetylated alpha-linked acidic di	0.024	1.017	8.26E-01	8.26E+01		
7949364	NM_080668	CDCA5	cell division cycle associated 5	0.012	1.008	9.49E-01	9.49E+01		
7949371	AK124141	LOC399904	hypothetical LOC399904	-0.035	0.976	8.24E-01	8.24E+01		
7949373	NM_014205	ZNHIT2	zinc finger, HIT type 2	-0.113	0.924	3.66E-01	3.66E+01		
7949377	NM_001997	FAU	Finkel-Biskis-Reilly murine sarcom	0.049	1.035	6.57E-01	6.57E+01		
7949383	NM_032431	SYVN1	synovial apoptosis inhibitor 1, syn	0.093	1.066	2.68E-01	2.68E+01		
7949410	BC018448	MALAT1	metastasis associated lung adeno	0.020	1.014	9.11E-01	9.11E+01		
7949412	NM_00113014	LTBP3	latent transforming growth factor	-0.044	0.970	7.13E-01	7.13E+01		
7949440	NM_00109940	EHBP1L1	EH domain binding protein 1-like 1	0.020	1.014	9.02E-01	9.02E+01		
7949444	NM_005714	KCNK7	potassium channel, subfamily K, m	-0.026	0.982	7.67E-01	7.67E+01		
7949454	NM_002419	MAP3K11	mitogen-activated protein kinase	0.057	1.040	5.49E-01	5.49E+01		
7949465	NM_021975	RELA	v-rel reticuloendotheliosis viral on	-0.114	0.924	1.25E-01	1.25E+01		
7949482	NM_032193	RNASEH2C	ribonuclease H2, subunit C	-0.013	0.991	9.61E-01	9.61E+01		
7949490	NM_138368	DKFZp761E198	DKFZp761E198 protein	-0.061	0.959	6.07E-01	6.07E+01		
7949496	NM_005507	CFL1	cofilin 1 (non-muscle)	0.131	1.095	2.92E-01	2.92E+01		
7949503	NM_016938	EFEMP2	EGF-containing fibulin-like extrace	-0.060	0.960	5.91E-01	5.91E+01		
7949518	NM_198897	FIBP	fibroblast growth factor (acidic) in	0.000	1.000	1.00E+00	1.00E+02		
7949532	NM_005438	FOSL1	FOS-like antigen 1	-0.078	0.947	3.94E-01	3.94E+01		
7949540	NM_00113563	C11orf68	chromosome 11 open reading fran	-0.120	0.920	3.03E-01	3.03E+01		
7949545	NM_032325	EIF1AD	eukaryotic translation initiation fa	0.066	1.047	5.40E-01	5.40E+01		
7949553	NM_053054	CATSPER1	cation channel, sperm associated	0.047	1.033	6.00E-01	6.00E+01		
7949568	AK090761	LOC100131232	hypothetical LOC100131232	0.005	1.004	9.82E-01	9.82E+01		
7949570	NM_033036	GAL3ST3	galactose-3-O-sulfotransferase 3	0.003	1.002	9.86E-01	9.86E+01		
7949577	NM_020470	YIF1A	Yip1 interacting factor homolog A	0.041	1.029	7.40E-01	7.40E+01		
7949588	NM_020404	CD248	CD248 molecule, endosialin	0.026	1.018	9.24E-01	9.24E+01		
7949592	NM_004292	RIN1	Ras and Rab interactor 1	-0.003	0.998	9.86E-01	9.86E+01		
7949603	NM_015399	BRMS1	breast cancer metastasis suppress	-0.026	0.982	8.59E-01	8.59E+01		
7949619	NM_001532	SLC29A2	solute carrier family 29 (nucleosid	-0.017	0.988	8.92E-01	8.92E+01		
7949637	NM_170739	MRPL11	mitochondrial ribosomal protein L	-0.106	0.929	2.29E-01	2.29E+01		
7949645	NM_207340	ZDHHC24	zinc finger, DHHC-type containing	-0.058	0.961	4.09E-01	4.09E+01		
7949650	NM_003793	CTSF	cathepsin F	-0.058	0.961	4.42E-01	4.42E+01		
7949674	NM_031492	RBM4B	RNA binding motif protein 4B	-0.019	0.987	9.00E-01	9.00E+01		
7949679	NM_006946	SPTBN2	spectrin, beta, non-erythrocytic 2	-0.028	0.981	7.77E-01	7.77E+01		
7949717	---	---	---	0.008	1.005	9.65E-01	9.65E+01		
7949744	---	---	---	0.039	1.027	9.42E-01	9.42E+01		
7949746	NM_021173	POLD4	polymerase (DNA-directed), delta	0.047	1.033	5.25E-01	5.25E+01		
7949754	NM_013246	CLCF1	cardiotrophin-like cytokine factor	-0.061	0.958	3.79E-01	3.79E+01		
7949765	NM_00100870	PPP1CA	protein phosphatase 1, catalytic su	0.002	1.001	9.94E-01	9.94E+01		
7949776	NM_020441	CORO1B	coronin, actin binding protein, 1B	0.013	1.009	9.18E-01	9.18E+01		
7949792	NM_005608	PTPRCAP	protein tyrosine phosphatase, rec	0.041	1.029	7.14E-01	7.14E+01		
7949798	NM_025124	TMEM134	transmembrane protein 134	-0.005	0.997	9.78E-01	9.78E+01		
7949808	NM_004910	PITPNM1	phosphatidylinositol transfer prot	0.001	1.001	9.97E-01	9.97E+01		
7949836	NM_005851	CDK2AP2	cyclin-dependent kinase 2 associa	0.031	1.021	8.50E-01	8.50E+01		
7949843	NM_016366	CABP2	calcium binding protein 2	-0.013	0.991	9.30E-01	9.30E+01		
7949857	NM_181843	NUDT8	nudix (nucleoside diphosphate lin	-0.012	0.991	9.46E-01	9.46E+01		

7949863	NM_005995	TBX10	T-box 10	-0.026	0.982	8.30E-01	8.30E+01		
7949873	NM_080658	ACY3	aspartoacylase (aminocyclase) 3	-0.045	0.970	6.16E-01	6.16E+01		
7949882	NM_000695	ALDH3B2	aldehyde dehydrogenase 3 family,	-0.046	0.968	7.35E-01	7.35E+01		
7949894	---	---	---	0.073	1.052	7.51E-01	7.51E+01		
7949896	ENST00000454	// OR7E87P //	olfactory receptor, family 7, subfa	0.017	1.012	9.49E-01	9.49E+01		
7949898	ENST00000319	LOC645332	family with sequence similarity 86	-0.072	0.951	6.38E-01	6.38E+01		
7949904	NM_030930	UNC93B1	unc-93 homolog B1 (C. elegans)	0.094	1.067	5.35E-01	5.35E+01		
7949916	NM_001277	CHKA	choline kinase alpha	0.073	1.052	6.62E-01	6.62E+01		
7949931	NM_017635	SUV420H1	suppressor of variegation 4-20 hor	-0.030	0.979	8.32E-01	8.32E+01		
7949948	NM_022338	C11orf24	chromosome 11 open reading fra	0.048	1.034	8.34E-01	8.34E+01		
7949954	---	---	---	-0.186	0.879	6.60E-01	6.60E+01		
7949956	NM_004923	MTL5	metallothionein-like 5, testis-speci	-0.027	0.982	8.34E-01	8.34E+01		
7949971	NM_001876	CPT1A	carnitine palmitoyltransferase 1A	0.055	1.039	8.55E-01	8.55E+01		
7949995	NM_181515	MRPL21	mitochondrial ribosomal protein L	0.089	1.064	6.80E-01	6.80E+01		
7950003	NM_198923	MRGPRD	MAS-related GPR, member D	-0.061	0.959	7.45E-01	7.45E+01		
7950012	NM_053056	CCND1	cyclin D1	-0.009	0.994	9.61E-01	9.61E+01		
7950016	NM_153451	ORAOV1	oral cancer overexpressed 1	0.086	1.062	5.67E-01	5.67E+01		
7950023	NM_005117	FGF19	fibroblast growth factor 19	0.004	1.002	9.85E-01	9.85E+01		
7950032	NM_002007	FGF4	fibroblast growth factor 4	-0.004	0.997	9.74E-01	9.74E+01		
7950036	NM_005247	FGF3	fibroblast growth factor 3 (murine	-0.102	0.932	2.20E-01	2.20E+01		
7950062	---	---	---	0.082	1.058	7.99E-01	7.99E+01		
7950064	ENST00000331	FLJ42102	FLJ42102 protein	-0.055	0.963	6.51E-01	6.51E+01		
7950067	NM_001360	DHCR7	7-dehydrocholesterol reductase	-0.103	0.931	5.95E-01	5.95E+01		
7950078	NM_00100540	KRTAP5-11	keratin associated protein 5-11	-0.071	0.952	4.51E-01	4.51E+01		
7950086	NM_006185	NUMA1	nuclear mitotic apparatus protein	-0.076	0.949	3.97E-01	3.97E+01		
7950119	BC001706	C11orf59	chromosome 11 open reading fra	0.109	1.078	2.09E-01	2.09E+01		
7950128	NM_014042	C11orf51	chromosome 11 open reading fra	0.039	1.027	8.05E-01	8.05E+01		
7950136	NM_005169	PHOX2A	paired-like homeobox 2a	-0.019	0.987	8.95E-01	8.95E+01		
7950140	AF229166	LOC220077	docking protein 1-like protein	0.018	1.013	8.90E-01	8.90E+01		
7950142	NM_030813	CLPB	ClpB caseinolytic peptidase B hom	-0.093	0.937	3.35E-01	3.35E+01		
7950195	---	---	---	-0.058	0.961	5.73E-01	5.73E+01		
7950197	NM_015242	ARAP1	ArfGAP with RhoGAP domain, ank	0.022	1.015	9.16E-01	9.16E+01		
7950235	NM_006645	STARD10	StAR-related lipid transfer (START)	-0.120	0.920	3.98E-01	3.98E+01		
7950248	NM_014824	FCHSD2	FCH and double SH3 domains 2	0.228	1.171	2.91E-03	2.91E-01		
7950269	---	---	---	0.233	1.175	4.16E-01	4.16E+01		
7950271	EF363480	FAM168A	family with sequence similarity 16	-0.006	0.996	9.77E-01	9.77E+01		
7950282	---	---	---	-0.049	0.967	9.09E-01	9.09E+01		
7950284	NM_002869	RAB6A	RAB6A, member RAS oncogene fa	-0.016	0.989	9.22E-01	9.22E+01		
7950294	NM_016565	CHCHD8	coiled-coil-helix-coiled-coil-helix d	-0.024	0.983	8.89E-01	8.89E+01		
7950299	NM_153614	DNAJB13	DnaJ (Hsp40) related, subfamily B,	-0.003	0.998	9.86E-01	9.86E+01		
7950307	NM_003355	UCP2	uncoupling protein 2 (mitochondr	0.362	1.285	6.22E-02	6.22E+00		
7950321	NM_003356	UCP3	uncoupling protein 3 (mitochondr	-0.030	0.980	8.49E-01	8.49E+01		
7950332	BC035599	C2CD3	C2 calcium-dependent domain co	0.117	1.084	4.44E-01	4.44E+01		
7950336	NM_015531	C2CD3	C2 calcium-dependent domain co	-0.090	0.940	4.27E-01	4.27E+01		
7950370	---	---	---	-0.053	0.964	8.58E-01	8.58E+01		
7950372	---	---	---	0.120	1.087	6.57E-01	6.57E+01		
7950374	NM_182904	P4HA3	prolyl 4-hydroxylase, alpha polype	0.003	1.002	9.88E-01	9.88E+01		
7950391	NM_173582	PGM2L1	phosphoglucomutase 2-like 1	-0.151	0.900	2.40E-01	2.40E+01		
7950409	NM_005472	KCNE3	potassium voltage-gated channel,	0.129	1.093	4.43E-01	4.43E+01		
7950420	NM_00114486	LOC387787	lipoyl(octanoyl) transferase	0.007	1.005	9.78E-01	9.78E+01		
7950423	---	---	---	0.056	1.040	8.21E-01	8.21E+01		
7950425	NM_015424	CHRDL2	chordin-like 2	-0.069	0.953	3.91E-01	3.91E+01		
7950442	---	---	---	-0.101	0.933	4.05E-01	4.05E+01		
7950444	---	---	---	-0.075	0.949	7.01E-01	7.01E+01		
7950447	NM_182969	XRRA1	X-ray radiation resistance associat	0.025	1.018	9.04E-01	9.04E+01		
7950469	---	---	---	0.114	1.082	5.07E-01	5.07E+01		
7950471	NM_00100528	OR2AT4	olfactory receptor, family 2, subfa	-0.003	0.998	9.88E-01	9.88E+01		
7950490	---	---	---	-0.060	0.959	6.25E-01	6.25E+01		
7950492	NM_00103954	KLHL35	kelch-like 35 (Drosophila)	-0.031	0.978	7.43E-01	7.43E+01		
7950501	NM_030792	GDPD5	glycerophosphodiester phosphodi	-0.049	0.967	6.62E-01	6.62E+01		
7950534	NM_004626	WNT11	wingless-type MMTV integration s	-0.097	0.935	3.40E-01	3.40E+01		
7950544	NM_004705	PRKRIR	protein-kinase, interferon-inducib	0.030	1.021	8.62E-01	8.62E+01		
7950555	NM_00112892	LRRC32	leucine rich repeat containing 32	0.072	1.051	4.97E-01	4.97E+01		
7950597	NM_001293	CLNS1A	chloride channel, nucleotide-sensi	0.008	1.005	9.77E-01	9.77E+01		
7950606	NM_016578	RSF1	remodeling and spacing factor 1	-0.107	0.928	1.59E-01	1.59E+01		
7950626	BC071734	RPS20P27	ribosomal protein S20 pseudogen	0.041	1.029	8.15E-01	8.15E+01		
7950628	NM_033547	INTS4	integrator complex subunit 4	-0.024	0.984	9.27E-01	9.27E+01		
7950641	NM_023930	KCTD14	potassium channel tetramerisatio	0.063	1.045	6.76E-01	6.76E+01		
7950644	NM_004549	NDUFC2	NADH dehydrogenase (ubiquinone	-0.004	0.997	9.86E-01	9.86E+01		
7950654	NM_024079	ALG8	asparagine-linked glycosylation 8,	0.060	1.042	8.28E-01	8.28E+01		
7950669	NM_00102985	KCTD21	potassium channel tetramerisatio	-0.138	0.909	1.61E-01	1.61E+01		
7950671	NM_080491	GAB2	GRB2-associated binding protein 2	-0.207	0.866	1.20E-01	1.20E+01		
7950701	NM_00109881	ODZ4	odz, odd Oz/ten-m homolog 4 (Dr	-0.018	0.988	8.73E-01	8.73E+01		
7950726	NM_175885	FAM181B	family with sequence similarity 18	-0.081	0.946	3.75E-01	3.75E+01		
7950743	NM_014488	RAB30	RAB30, member RAS oncogene fa	0.217	1.162	1.64E-01	1.64E+01		
7950751	---	---	---	0.178	1.131	5.89E-01	5.89E+01		
7950753	NM_021825	CCDC90B	coiled-coil domain containing 90B	-0.006	0.996	9.83E-01	9.83E+01		

7950762	---	---	---	-0.075	0.950	6.37E-01	6.37E+01		
7950796	NM_00103961	CREBZF	CREB/ATF bZIP transcription facto	-0.026	0.982	8.63E-01	8.63E+01		
7950810	NM_206927	SYTL2	synaptotagmin-like 2	-0.025	0.983	9.53E-01	9.53E+01		
7950838	NM_007166	PICALM	phosphatidylinositol binding clath	0.161	1.118	1.92E-01	1.92E+01		
7950862	---	---	---	0.071	1.051	8.86E-01	8.86E+01		
7950864	NM_00101481	ME3	malic enzyme 3, NADP(+)-depende	-0.016	0.989	9.00E-01	9.00E+01		
7950885	NM_012193	FZD4	frizzled homolog 4 (Drosophila)	-0.001	1.000	9.97E-01	9.97E+01		
7950899	NM_022337	RAB38	RAB38, member RAS oncogene fa	-0.066	0.955	6.80E-01	6.80E+01		
7950921	NM_00114383	GRM5	glutamate receptor, metabotropi	-0.046	0.969	6.59E-01	6.59E+01		
7950983	NM_012124	CHORDC1	cysteine and histidine-rich domain	-0.080	0.946	6.08E-01	6.08E+01		
7950990	NM_152313	SLC36A4	solute carrier family 36 (proton/a	0.007	1.005	9.84E-01	9.84E+01		
7951008	NM_024116	TAF1D	TATA box binding protein (TBP)-as	-0.037	0.975	8.24E-01	8.24E+01		
7951030	NR_003036	SNORD6	small nucleolar RNA, C/D box 6	0.021	1.015	9.61E-01	9.61E+01		
7951032	NR_003026	SNORA1	small nucleolar RNA, H/ACA box 1	0.000	1.000	1.00E+00	1.00E+02		
7951034	NR_002920	SNORA8	small nucleolar RNA, H/ACA box 8	0.190	1.141	2.26E-01	2.26E+01		
7951036	NR_003033	SNORD5	small nucleolar RNA, C/D box 5	0.054	1.038	8.18E-01	8.18E+01		
7951038	NR_002973	SNORA40	small nucleolar RNA, H/ACA box 4	0.018	1.013	9.76E-01	9.76E+01		
7951040	NM_016540	GPR83	G protein-coupled receptor 83	-0.094	0.937	3.02E-01	3.02E+01		
7951046	NM_005591	MRE11A	MRE11 meiotic recombination 11	-0.019	0.987	9.36E-01	9.36E+01		
7951068	NM_016403	CWC15	CWC15 spliceosome-associated p	-0.038	0.974	8.40E-01	8.40E+01		
7951077	NM_144665	SESN3	sestrin 3	-0.040	0.973	9.00E-01	9.00E+01		
7951089	---	---	---	-0.023	0.984	9.16E-01	9.16E+01		
7951091	---	---	---	0.021	1.015	9.68E-01	9.68E+01		
7951093	NM_144664	FAM76B	family with sequence similarity 76	-0.029	0.980	8.44E-01	8.44E+01		
7951108	---	---	---	-0.022	0.985	9.44E-01	9.44E+01		
7951112	NR_023356	MTMR2	myotubularin related protein 2	-0.046	0.969	8.37E-01	8.37E+01		
7951131	---	---	---	0.106	1.076	7.27E-01	7.27E+01		
7951133	NM_032427	MAML2	mastermind-like 2 (Drosophila)	0.009	1.006	9.68E-01	9.68E+01		
7951140	AY358248	UNQ6228	hypothetical LOC100131541	0.134	1.097	5.53E-01	5.53E+01		
7951144	NM_024725	CCDC82	coiled-coil domain containing 82	-0.103	0.931	2.25E-01	2.25E+01		
7951157	AK313893	CCDC82	coiled-coil domain containing 82	-0.092	0.938	7.01E-01	7.01E+01		
7951159	---	---	---	-0.026	0.982	9.44E-01	9.44E+01		
7951207	NM_052932	TMEM123	transmembrane protein 123	0.003	1.002	9.89E-01	9.89E+01		
7951325	NM_032299	DCUN1D5	DCN1, defective in cullin neddylati	-0.027	0.981	9.06E-01	9.06E+01		
7951372	NM_033306	CASP4	caspase 4, apoptosis-related cyste	0.099	1.071	7.08E-01	7.08E+01		
7951397	NM_033292	CASP1	caspase 1, apoptosis-related cyste	0.221	1.166	4.96E-01	4.96E+01		
7951408	NM_052889	CARD16	caspase recruitment domain famil	0.084	1.060	7.89E-01	7.89E+01		
7951422	NM_032424	KIAA1826	KIAA1826	0.034	1.024	8.43E-01	8.43E+01		
7951435	---	---	---	0.053	1.037	7.94E-01	7.94E+01		
7951447	NM_152434	CWF19L2	CWF19-like 2, cell cycle control (S.	-0.059	0.960	6.39E-01	6.39E+01		
7951485	NM_017515	SLC35F2	solute carrier family 35, member F	-0.060	0.960	7.78E-01	7.78E+01		
7951497	NM_002519	NPAT	nuclear protein, ataxia-telangiecta	-0.037	0.975	8.79E-01	8.79E+01		
7951535	NM_153705	KDELC2	KDEL (Lys-Asp-Glu-Leu) containing	0.182	1.134	1.35E-01	1.35E+01		
7951554	NM_002906	RDX	radixin	-0.077	0.948	8.05E-01	8.05E+01		
7951589	---	---	---	-0.012	0.992	9.65E-01	9.65E+01		
7951596	NM_006235	POU2AF1	POU class 2 associating factor 1	0.154	1.113	3.33E-02	3.33E+00		
7951612	AK293317	LAYN	layilin	-0.043	0.971	7.15E-01	7.15E+01		
7951614	NM_002716	PPP2R1B	protein phosphatase 2 (formerly 2	-0.006	0.996	9.74E-01	9.74E+01		
7951633	NM_024740	ALG9	asparagine-linked glycosylation 9,	-0.086	0.942	5.72E-01	5.72E+01		
7951660	ENST00000317	RPL37AP8	ribosomal protein L37a pseudoge	-0.042	0.971	7.35E-01	7.35E+01		
7951668	---	---	---	-0.053	0.964	9.10E-01	9.10E+01		
7951679	NM_012459	TIMM8B	translocase of inner mitochondria	0.012	1.009	9.64E-01	9.64E+01		
7951695	NM_00114502	C11orf34	chromosome 11 open reading fra	0.138	1.101	6.06E-01	6.06E+01		
7951701	---	---	---	0.066	1.047	8.62E-01	8.62E+01		
7951703	NM_000795	DRD2	dopamine receptor D2	-0.081	0.945	1.68E-01	1.68E+01		
7951734	NM_004724	ZW10	ZW10, kinetochore associated, ho	0.042	1.029	8.53E-01	8.53E+01		
7951752	NM_020886	USP28	ubiquitin specific peptidase 28	0.235	1.177	1.56E-01	1.56E+01		
7951781	BC071695	C11orf71	chromosome 11 open reading fra	0.058	1.041	6.71E-01	6.71E+01		
7951807	NM_014333	CADM1	cell adhesion molecule 1	-0.053	0.964	9.27E-01	9.27E+01		
7951824	---	---	---	0.026	1.018	8.95E-01	8.95E+01		
7951826	NM_032725	BUD13	BUD13 homolog (S. cerevisiae)	0.033	1.023	8.09E-01	8.09E+01		
7951838	NM_003904	ZNF259	zinc finger protein 259	0.027	1.019	9.00E-01	9.00E+01		
7951853	NM_052968	APOA5	apolipoprotein A-V	0.040	1.028	7.01E-01	7.01E+01		
7951859	NM_000482	APOA4	apolipoprotein A-IV	-0.002	0.999	9.86E-01	9.86E+01		
7951865	NM_000039	APOA1	apolipoprotein A-I	0.029	1.021	6.97E-01	6.97E+01		
7951873	NM_025164	QSK	serine/threonine-protein kinase Q	-0.076	0.949	5.18E-01	5.18E+01		
7951896	NM_004716	PCSK7	proprotein convertase subtilisin/k	-0.091	0.939	5.23E-01	5.23E+01		
7951910	NM_012104	BACE1	beta-site APP-cleaving enzyme 1	0.016	1.011	9.25E-01	9.25E+01		
7951928	NM_020693	DSCAML1	Down syndrome cell adhesion mo	-0.007	0.995	9.64E-01	9.64E+01		
7951966	NM_00112748	FXYD2	FXYD domain containing ion trans	-0.018	0.987	8.62E-01	8.62E+01		
7951977	NM_022003	FXYD6	FXYD domain containing ion trans	0.010	1.007	9.60E-01	9.60E+01		
7951987	NM_00107726	TMPRSS13	transmembrane protease, serine 1	0.028	1.020	8.95E-01	8.95E+01		
7952004	NM_174934	SCN4B	sodium channel, voltage-gated, ty	-0.005	0.996	9.79E-01	9.79E+01		
7952011	NM_004588	SCN2B	sodium channel, voltage-gated, ty	0.011	1.008	9.49E-01	9.49E+01		
7952022	NM_00109852	AMICA1	adhesion molecule, interacts with	-0.319	0.801	3.11E-02	3.11E+00		
7952036	NM_198275	MPZL3	myelin protein zero-like 3	-0.268	0.830	5.65E-02	5.65E+00		
7952056	NM_000732	CD3D	CD3d molecule, delta (CD3-TCR co	-0.207	0.867	3.88E-02	3.88E+00		

7952069	NM_020153	C11orf60	chromosome 11 open reading fran	-0.017	0.989	9.64E-01	9.64E+01		
7952086	NM_007180	TREH	trehalase (brush-border membran	0.049	1.035	6.53E-01	6.53E+01		
7952103	NM_004397	DDX6	DEAD (Asp-Glu-Ala-Asp) box polyp	-0.045	0.969	5.53E-01	5.53E+01		
7952116	NM_182557	BCL9L	B-cell CLL/lymphoma 9-like	-0.079	0.946	3.62E-01	3.62E+01		
7952126	NR_003040	RPL23AP64	ribosomal protein L23a pseudoger	0.016	1.011	9.38E-01	9.38E+01		
7952129	NM_001028	RPS25	ribosomal protein S25	-0.014	0.990	9.40E-01	9.40E+01		
7952132	NM_001467	SLC37A4	solute carrier family 37 (glucose-6	-0.013	0.991	9.34E-01	9.34E+01		
7952145	NM_006389	HYOU1	hypoxia up-regulated 1	0.112	1.081	4.63E-01	4.63E+01		
7952179	NM_002105	H2AFX	H2A histone family, member X	-0.132	0.912	6.89E-02	6.89E+00		
7952185	NM_001382	DPAGT1	dolichyl-phosphate (UDP-N-acetyl	0.120	1.086	2.11E-01	2.11E+01		
7952200	NM_00114501	CCDC153	coiled-coil domain containing 153	0.048	1.033	7.45E-01	7.45E+01		
7952205	NM_006500	MCAM	melanoma cell adhesion molecule	-0.035	0.976	7.51E-01	7.51E+01		
7952227	NM_015645	C1QTNF5	C1q and tumor necrosis factor rela	-0.025	0.983	8.18E-01	8.18E+01		
7952243	NM_015645	C1QTNF5	C1q and tumor necrosis factor rela	-0.002	0.999	9.90E-01	9.90E+01		
7952268	NM_006288	THY1	Thy-1 cell surface antigen	0.024	1.017	8.62E-01	8.62E+01		
7952290	NM_012101	TRIM29	tripartite motif-containing 29	0.008	1.006	9.63E-01	9.63E+01		
7952321	NM_00109816	BSX	brain-specific homeobox	0.055	1.039	4.88E-01	4.88E+01		
7952325	NM_006597	HSPA8	heat shock 70kDa protein 8	-0.008	0.994	9.61E-01	9.61E+01		
7952335	NR_003125	SNORD14E	small nucleolar RNA, C/D box 14E	-0.090	0.939	8.42E-01	8.42E+01		
7952339	NR_001453	SNORD14C	small nucleolar RNA, C/D box 14C	-0.088	0.941	7.90E-01	7.90E+01		
7952341	NM_024769	ASAM	adipocyte-specific adhesion molec	0.009	1.006	9.73E-01	9.73E+01		
7952350	NM_018400	SCN3B	sodium channel, voltage-gated, ty	-0.062	0.958	6.35E-01	6.35E+01		
7952361	NM_003455	ZNF202	zinc finger protein 202	-0.087	0.942	3.79E-01	3.79E+01		
7952384	NM_00100447	OR10S1	olfactory receptor, family 10, subf	-0.065	0.956	7.09E-01	7.09E+01		
7952386	ENST00000307	OR10G6	olfactory receptor, family 10, subf	-0.049	0.967	6.52E-01	6.52E+01		
7952388	NM_00100446	OR10G7	olfactory receptor, family 10, subf	0.066	1.047	6.71E-01	6.71E+01		
7952394	NM_00100291	OR8D2	olfactory receptor, family 8, subfa	0.010	1.007	9.66E-01	9.66E+01		
7952408	NM_170601	SIAE	sialic acid acetylesterase	0.090	1.065	5.71E-01	5.71E+01		
7952426	NM_014312	VSIG2	V-set and immunoglobulin domair	0.009	1.006	9.54E-01	9.54E+01		
7952436	NM_138961	ESAM	endothelial cell adhesion molecule	-0.040	0.972	7.14E-01	7.14E+01		
7952445	NM_024631	C11orf61	chromosome 11 open reading fran	-0.089	0.940	5.40E-01	5.40E+01		
7952453	NM_019055	ROBO4	roundabout homolog 4, magic rou	-0.128	0.915	6.65E-02	6.65E+00		
7952475	NM_152722	HEPACAM	hepatocyte cell adhesion molecule	-0.006	0.996	9.76E-01	9.76E+01		
7952484	NM_00108054	TMEM218	transmembrane protein 218	0.002	1.002	9.95E-01	9.95E+01		
7952488	---	---	---	0.022	1.015	8.86E-01	8.86E+01		
7952549	NM_032795	RPUSD4	RNA pseudouridylate synthase dor	0.076	1.054	6.28E-01	6.28E+01		
7952557	NM_003139	SRPR	signal recognition particle recepto	-0.022	0.985	8.97E-01	8.97E+01		
7952577	NM_032531	KIRREL3	kin of IRRE like 3 (Drosophila)	-0.040	0.972	7.91E-01	7.91E+01		
7952601	NM_00114382	ETS1	v-ets erythroblastosis virus E26 on	-0.011	0.993	9.62E-01	9.62E+01		
7952641	NM_00114268	RICS	Rho GTPase-activating protein	-0.017	0.989	9.44E-01	9.44E+01		
7952671	---	---	---	0.044	1.031	7.89E-01	7.89E+01		
7952677	NM_006165	NFRKB	nuclear factor related to kappaB b	-0.042	0.971	7.30E-01	7.30E+01		
7952707	NM_020228	PRDM10	PR domain containing 10	-0.069	0.953	4.54E-01	4.54E+01		
7952733	---	---	---	0.003	1.002	9.88E-01	9.88E+01		
7952737	---	---	---	0.193	1.143	4.97E-01	4.97E+01		
7952739	NM_014155	ZBTB44	zinc finger and BTB domain contai	0.008	1.006	9.74E-01	9.74E+01		
7952752	NM_007037	ADAMTS8	ADAM metallopeptidase with thro	0.015	1.011	9.15E-01	9.15E+01		
7952766	---	---	---	-0.080	0.946	2.65E-01	2.65E+01		
7952768	NM_014758	SNX19	sorting nexin 19	0.038	1.027	7.94E-01	7.94E+01		
7952795	---	---	---	-0.036	0.975	7.94E-01	7.94E+01		
7952805	NR_024344	LOC283174	hypothetical LOC283174	-0.167	0.891	3.67E-01	3.67E+01		
7952813	NM_014987	IGSF9B	immunoglobulin superfamily, men	-0.040	0.973	7.51E-01	7.51E+01		
7952830	NM_015261	NCAPD3	non-SMC condensin II complex, su	-0.035	0.976	8.90E-01	8.90E+01		
7952869	NM_014174	THYN1	thymocyte nuclear protein 1	-0.098	0.934	3.90E-01	3.90E+01		
7952884	NM_054025	B3GAT1	beta-1,3-glucuronyltransferase 1 (	-0.017	0.988	9.27E-01	9.27E+01		
7952893	BC024764	IQSEC3	IQ motif and Sec7 domain 3	-0.050	0.966	4.35E-01	4.35E+01		
7952897	NM_015232	IQSEC3	IQ motif and Sec7 domain 3	-0.022	0.985	8.31E-01	8.31E+01		
7952914	NM_032358	CCDC77	coiled-coil domain containing 77	-0.006	0.996	9.86E-01	9.86E+01		
7952927	NM_173593	B4GALNT3	beta-1,4-N-acetyl-galactosaminyl t	-0.055	0.963	6.38E-01	6.38E+01		
7952950	ENST00000318	LOC100049716	hypothetical protein LOC1000497	0.162	1.119	3.60E-01	3.60E+01		
7952953	NM_018979	WNK1	WNK lysine deficient protein kinas	0.006	1.004	9.74E-01	9.74E+01		
7952984	---	---	---	0.011	1.008	9.82E-01	9.82E+01		
7952986	NM_213655	HSN2	hereditary sensory neuropathy, ty	-0.011	0.992	9.76E-01	9.76E+01		
7952988	NR_027948	ERC1	ELKS/RAB6-interacting/CAST famil	-0.164	0.892	2.17E-02	2.17E+00		
7953021	NM_024551	ADIPOR2	adiponectin receptor 2	0.062	1.044	5.83E-01	5.83E+01		
7953032	NM_00103902	LRTM2	leucine-rich repeats and transmen	0.029	1.020	8.52E-01	8.52E+01		
7953100	NM_002014	FKBP4	FK506 binding protein 4, 59kDa	-0.226	0.855	9.22E-02	9.22E+00		
7953111	NM_018463	ITFG2	integrin alpha FG-GAP repeat cont	0.047	1.033	7.52E-01	7.52E+01		
7953130	NR_027363	C12orf32	chromosome 12 open reading fran	0.112	1.081	2.68E-01	2.68E+01		
7953135	NM_003324	TULP3	tubby like protein 3	-0.155	0.898	1.67E-01	1.67E+01		
7953150	NM_003213	TEAD4	TEA domain family member 4	-0.031	0.978	7.51E-01	7.51E+01		
7953166	NM_006675	TSPAN9	tetraspanin 9	-0.097	0.935	2.63E-01	2.63E+01		
7953200	NM_001759	CCND2	cyclin D2	-0.060	0.959	6.88E-01	6.88E+01		
7953243	NM_005002	NDUFA9	NADH dehydrogenase (ubiquinone	0.076	1.054	5.11E-01	5.11E+01		
7953264	NM_002235	KCNA6	potassium voltage-gated channel,	-0.163	0.893	7.05E-02	7.05E+00		
7953274	NM_000217	KCNA1	potassium voltage-gated channel,	-0.081	0.945	2.66E-01	2.66E+01		
7953303	NM_018173	PLEKHG6	pleckstrin homology domain conta	0.023	1.016	8.54E-01	8.54E+01		

7953321	NM_002342	LTBR	lymphotoxin beta receptor (TNFR	0.106	1.076	1.61E-01	1.61E+01			
7953333	NM_001242	CD27	CD27 molecule	0.011	1.008	9.69E-01	9.69E+01			
7953341	NM_018009	TAPBPL	TAP binding protein-like	-0.021	0.986	8.99E-01	8.99E+01			
7953351	NM_014865	NCAPD2	non-SMC condensin I complex, sul	0.000	1.000	1.00E+00	1.00E+02			
7953383	NR_004387	SCARNA10	small Cajal body-specific RNA 10	-0.368	0.775	8.78E-02	8.78E+00			
7953385	NM_002046	GAPDH	glyceraldehyde-3-phosphate dehy	-0.201	0.870	5.59E-02	5.59E+00			
7953390	NM_00103991	ZNF384	zinc finger protein 384	-0.062	0.958	5.97E-01	5.97E+01			
7953395	NM_016319	COPS7A	COP9 constitutive photomorphoge	-0.002	0.998	9.92E-01	9.92E+01			
7953409	NM_002824	PTMS	parathymosin	0.054	1.038	8.03E-01	8.03E+01			
7953418	NM_002286	LAG3	lymphocyte-activation gene 3	0.049	1.034	7.57E-01	7.57E+01			
7953428	NM_000616	CD4	CD4 molecule	0.261	1.199	1.47E-02	1.47E+00			
7953450	NM_014262	LEPREL2	leprecan-like 2	-0.066	0.955	3.12E-01	3.12E+01			
7953469	NM_002075	GNB3	guanine nucleotide binding protei	0.002	1.001	9.91E-01	9.91E+01			
7953483	NM_00109853	USP5	ubiquitin specific peptidase 5 (isop	0.042	1.029	7.72E-01	7.72E+01			
7953508	NM_000365	TPI1	triosephosphate isomerase 1	-0.005	0.997	9.88E-01	9.88E+01			
7953516	NR_002803	RPL13P5	ribosomal protein L13 pseudogene	-0.058	0.960	6.95E-01	6.95E+01			
7953518	U72518	LOC171220	destrin-2 pseudogene	-0.085	0.943	7.15E-01	7.15E+01			
7953520	NM_201650	LRRC23	leucine rich repeat containing 23	-0.064	0.957	6.55E-01	6.55E+01			
7953532	NM_001975	ENO2	enolase 2 (gamma, neuronal)	-0.220	0.859	5.29E-02	5.29E+00			
7953547	NM_00100702	ATN1	atrophin 1	-0.040	0.972	7.09E-01	7.09E+01			
7953564	NM_138425	C12orf57	chromosome 12 open reading fram	0.025	1.017	9.53E-01	9.53E+01			
7953569	NM_080549	PTPN6	protein tyrosine phosphatase, nor	0.304	1.234	1.49E-02	1.49E+00			
7953594	NM_006331	EMG1	EMG1 nucleolar protein homolog	-0.028	0.981	9.18E-01	9.18E+01			
7953622	AY358730	UNQ2963	hypothetical LOC283314	-0.009	0.994	9.77E-01	9.77E+01			
7953626	NM_014718	CLSTN3	calsyntenin 3	-0.032	0.978	8.49E-01	8.49E+01			
7953651	NM_00113102	PEX5	peroxisomal biogenesis factor 5	0.030	1.021	8.71E-01	8.71E+01			
7953665	NM_199286	DPPA3	developmental pluripotency assoc	0.024	1.017	9.50E-01	9.50E+01			
7953675	NM_024865	NANOG	Nanog homeobox	-0.031	0.979	8.49E-01	8.49E+01			
7953697	---	---	---	-0.180	0.883	3.09E-01	3.09E+01			
7953699	NM_018416	FOXJ2	forkhead box J2	-0.034	0.977	7.58E-01	7.58E+01			
7953715	NR_024260	NECAP1	NECAP endocytosis associated 1	-0.066	0.955	6.80E-01	6.80E+01			
7953723	NM_016184	CLEC4A	C-type lectin domain family 4, mer	0.251	1.190	6.40E-01	6.40E+01			
7953735	NR_027000	LOC349196	hypothetical LOC349196	-0.124	0.918	3.93E-01	3.93E+01			
7953737	NM_00100703	CLEC6A	C-type lectin domain family 6, mer	0.776	1.712	1.14E-01	1.14E+01			
7953749	NM_080387	CLEC4D	C-type lectin domain family 4, mer	0.366	1.289	3.52E-01	3.52E+01			
7953763	---	---	---	-0.015	0.990	9.17E-01	9.17E+01			
7953765	NM_020734	RIMKLB	ribosomal modification protein rin	-0.074	0.950	7.70E-01	7.70E+01			
7953812	NM_004426	PHC1	polyhomeotic homolog 1 (Drosoph	-0.003	0.998	9.85E-01	9.85E+01			
7953835	NM_005810	KLRG1	killer cell lectin-like receptor subfa	0.204	1.152	6.64E-01	6.64E+01			
7953844	NM_004400	DDX12	DEAD/H (Asp-Glu-Ala-Asp/His) box	0.011	1.007	9.78E-01	9.78E+01			
7953878	NM_00100441	CLEC2D	C-type lectin domain family 2, mer	-0.087	0.942	4.12E-01	4.12E+01			
7953892	NM_016523	KLRF1	killer cell lectin-like receptor subfa	0.403	1.322	5.09E-01	5.09E+01			
7953943	NM_031412	GABARAPL1	GABA(A) receptor-associated prot	-0.273	0.827	6.50E-03	6.50E-01			
7953949	NM_002262	KLRD1	killer cell lectin-like receptor subfa	0.133	1.096	7.97E-01	7.97E+01			
7953977	---	---	---	0.188	1.139	8.88E-01	8.88E+01			
7953981	NM_001987	ETV6	ets variant 6	0.109	1.079	5.62E-01	5.62E+01			
7953993	NM_030766	BCL2L14	BCL2-like 14 (apoptosis facilitator)	0.078	1.055	6.33E-01	6.33E+01			
7954006	---	---	---	0.007	1.005	9.64E-01	9.64E+01			
7954008	---	---	---	0.074	1.053	6.66E-01	6.66E+01			
7954012	NM_058169	LOH12CR1	loss of heterozygosity, 12, chromo	0.021	1.015	9.09E-01	9.09E+01			
7954021	NM_001310	CREBL2	cAMP responsive element binding	0.265	1.201	2.11E-02	2.11E+00			
7954029	NM_004064	CDKN1B	cyclin-dependent kinase inhibitor	-0.138	0.909	1.00E-01	1.00E+01			
7954036	NM_016355	DDX47	DEAD (Asp-Glu-Ala-Asp) box polyp	0.027	1.019	8.91E-01	8.91E+01			
7954055	NM_00113041	APOLD1	apolipoprotein L domain containir	-0.022	0.985	9.49E-01	9.49E+01			
7954061	---	---	---	-0.019	0.987	9.18E-01	9.18E+01			
7954063	NR_003932	RPL13AP20	ribosomal protein L13a pseudoger	-0.043	0.970	8.05E-01	8.05E+01			
7954071	NR_002774	HTR7P	5-hydroxytryptamine (serotonin) r	-0.043	0.971	7.77E-01	7.77E+01			
7954077	NM_020853	KIAA1467	KIAA1467	0.046	1.033	6.92E-01	6.92E+01			
7954090	NM_001423	EMP1	epithelial membrane protein 1	0.453	1.368	1.82E-01	1.82E+01			
7954104	NM_018179	ATF7IP	activating transcription factor 7 in	0.010	1.007	9.56E-01	9.56E+01			
7954124	NM_177925	H2AFJ	H2A histone family, member J	-0.031	0.979	8.53E-01	8.53E+01			
7954173	NM_007178	STRAP	serine/threonine kinase receptor a	-0.019	0.987	9.22E-01	9.22E+01			
7954185	NM_015954	DERA	2-deoxyribose-5-phosphate aldola	0.059	1.042	7.94E-01	7.94E+01			
7954243	---	---	---	-0.028	0.981	9.04E-01	9.04E+01			
7954277	---	---	---	-0.033	0.977	8.09E-01	8.09E+01			
7954279	NM_153207	AEBP2	AE binding protein 2	-0.017	0.988	8.96E-01	8.96E+01			
7954310	NR_003286	LOC100008588	18S ribosomal RNA	-0.010	0.993	9.51E-01	9.51E+01			
7954382	NM_024854	PYROXD1	pyridine nucleotide-disulphide oxi	0.105	1.075	5.77E-01	5.77E+01			
7954388	NM_016072	GOLT1B	golgi transport 1 homolog B (S. cer	0.106	1.076	2.98E-01	2.98E+01			
7954407	NM_018686	CMAS	cytidine monophosphate N-acetyl	0.034	1.024	8.37E-01	8.37E+01			
7954419	NM_018638	ETNK1	ethanolamine kinase 1	0.167	1.123	1.37E-03	1.37E-01			
7954434	---	---	---	-0.123	0.918	2.25E-01	2.25E+01			
7954436	NM_006152	LRMP	lymphoid-restricted membrane pr	0.240	1.181	2.07E-02	2.07E+00			
7954460	NM_00100166	LYRM5	LYR motif containing 5	-0.059	0.960	7.25E-01	7.25E+01			
7954465	---	---	---	-0.142	0.907	1.60E-01	1.60E+01			
7954469	NM_007211	RASSF8	Ras association (RalGDS/AF-6) dor	-0.033	0.977	8.78E-01	8.78E+01			
7954481	NM_005086	SSPN	sarcospan (Kras oncogene-associa	0.005	1.004	9.88E-01	9.88E+01			

7954492	NM_015633	FGFR1OP2	FGFR1 oncogene partner 2	0.128	1.093	1.17E-01	1.17E+01		
7954503	NM_004264	MED21	mediator complex subunit 21	-0.055	0.962	7.41E-01	7.41E+01		
7954511	NM_015000	STK38L	serine/threonine kinase 38 like	0.201	1.150	1.52E-01	1.52E+01		
7954559	NM_003622	PPFIBP1	PTPRF interacting protein, binding	-0.196	0.873	2.22E-01	2.22E+01		
7954591	NM_021821	MRPS35	mitochondrial ribosomal protein S	0.160	1.117	3.10E-01	3.10E+01		
7954602	---	---	---	0.153	1.112	8.13E-01	8.13E+01		
7954604	NM_020782	KLHDC5	kelch domain containing 5	-0.002	0.999	9.92E-01	9.92E+01		
7954613	NM_018318	CCDC91	coiled-coil domain containing 91	0.000	1.000	1.00E+00	1.00E+02		
7954631	NM_018099	FAR2	fatty acyl CoA reductase 2	0.027	1.019	9.27E-01	9.27E+01		
7954645	NM_00108050	TSPAN11	tetraspanin 11	-0.037	0.975	7.69E-01	7.69E+01		
7954653	NM_00108050	TSPAN11	tetraspanin 11	0.004	1.003	9.84E-01	9.84E+01		
7954655	NM_030653	DDX11	DEAD/H (Asp-Glu-Ala-Asp/His) box	0.009	1.006	9.77E-01	9.77E+01		
7954690	---	---	---	-0.068	0.954	7.72E-01	7.72E+01		
7954692	NR_026806	FLJ13224	hypothetical protein FLJ13224	-0.100	0.933	4.32E-01	4.32E+01		
7954697	---	---	---	0.083	1.059	7.03E-01	7.03E+01		
7954711	NM_018169	C12orf35	chromosome 12 open reading fran	-0.079	0.947	3.27E-01	3.27E+01		
7954717	NM_001714	BICD1	bicaudal D homolog 1 (Drosophila	-0.062	0.958	6.63E-01	6.63E+01		
7954752	NM_012062	DNM1L	dynamin 1-like	-0.036	0.975	8.09E-01	8.09E+01		
7954787	---	---	---	-0.051	0.965	5.96E-01	5.96E+01		
7954810	NM_198578	LRRK2	leucine-rich repeat kinase 2	0.444	1.360	2.25E-01	2.25E+01		
7954940	NM_016488	PPHLN1	periphilin 1	0.000	1.000	1.00E+00	1.00E+02		
7954961	---	---	---	0.023	1.016	9.05E-01	9.05E+01		
7954963	---	---	---	0.064	1.045	5.48E-01	5.48E+01		
7954969	NM_00111418	IRAK4	interleukin-1 receptor-associated	0.143	1.104	3.73E-01	3.73E+01		
7954997	NM_00102535	ANO6	anoctamin 6	-0.032	0.978	9.22E-01	9.22E+01		
7955019	NM_152641	ARID2	AT rich interactive domain 2 (ARID	-0.080	0.946	5.34E-01	5.34E+01		
7955043	---	---	---	-0.064	0.957	8.93E-01	8.93E+01		
7955045	BC008360	FAM113B	family with sequence similarity 11	0.135	1.098	2.29E-01	2.29E+01		
7955055	NM_017842	SLC48A1	solute carrier family 48 (heme tran	-0.047	0.968	7.15E-01	7.15E+01		
7955063	NM_00114384	TMEM106C	transmembrane protein 106C	-0.056	0.962	8.06E-01	8.06E+01		
7955076	---	---	---	-0.056	0.962	7.32E-01	7.32E+01		
7955078	NM_000289	PFKM	phosphofructokinase, muscle	0.025	1.018	9.19E-01	9.19E+01		
7955107	NM_00101363	C12orf68	chromosome 12 open reading fran	-0.083	0.944	5.03E-01	5.03E+01		
7955110	ENST00000454	DKFZP779L185	hypothetical LOC643162	-0.027	0.982	9.22E-01	9.22E+01		
7955112	NM_181788	H1FNT	H1 histone family, member N, test	0.000	1.000	1.00E+00	1.00E+02		
7955142	NM_000725	CACNB3	calcium channel, voltage-depende	-0.080	0.946	5.17E-01	5.17E+01		
7955156	NM_033124	CCDC65	coiled-coil domain containing 65	0.030	1.021	9.09E-01	9.09E+01		
7955170	NM_005430	WNT1	wingless-type MMTV integration s	-0.012	0.992	9.34E-01	9.34E+01		
7955179	NM_032704	TUBA1C	tubulin, alpha 1c	-0.005	0.997	9.86E-01	9.86E+01		
7955184	NM_006262	PRPH	peripherin	-0.049	0.967	7.16E-01	7.16E+01		
7955195	NM_005480	TROAP	trophinin associated protein (tasti	-0.026	0.982	8.48E-01	8.48E+01		
7955231	NM_012284	KCNH3	potassium voltage-gated channel,	-0.061	0.959	4.84E-01	4.84E+01		
7955250	NM_012272	PRPF40B	PRP40 pre-mRNA processing facto	-0.103	0.931	1.46E-01	1.46E+01		
7955277	NM_003217	TMBIM6	transmembrane BAX inhibitor mot	0.048	1.034	7.23E-01	7.23E+01		
7955290	NM_000486	AQP2	aquaporin 2 (collecting duct)	-0.027	0.981	7.26E-01	7.26E+01		
7955297	NM_001651	AQP5	aquaporin 5	0.015	1.011	9.22E-01	9.22E+01		
7955305	NM_001652	AQP6	aquaporin 6, kidney specific	-0.014	0.990	9.19E-01	9.19E+01		
7955331	NM_003076	SMARCD1	SWI/SNF related, matrix associate	-0.050	0.966	6.66E-01	6.66E+01		
7955348	NM_005276	GPD1	glycerol-3-phosphate dehydrogen	-0.044	0.970	6.99E-01	6.99E+01		
7955358	BC007849	C12orf62	chromosome 12 open reading fran	0.017	1.012	9.49E-01	9.49E+01		
7955361	NM_052879	LARP4	La ribonucleoprotein domain fami	0.142	1.103	1.19E-01	1.19E+01		
7955376	NM_173602	DIP2B	DIP2 disco-interacting protein 2 hc	0.131	1.095	2.35E-01	2.35E+01		
7955419	---	---	---	0.188	1.139	3.80E-01	3.80E+01		
7955425	NM_005171	ATF1	activating transcription factor 1	-0.017	0.988	9.47E-01	9.47E+01		
7955450	NM_015416	LETMD1	LETM1 domain containing 1	0.009	1.006	9.77E-01	9.77E+01		
7955462	---	---	---	0.294	1.226	2.84E-01	2.84E+01		
7955464	NM_014764	DAZAP2	DAZ associated protein 2	0.038	1.027	7.92E-01	7.92E+01		
7955469	NM_00103996	SLC4A8	solute carrier family 4, sodium bic	0.102	1.073	5.91E-01	5.91E+01		
7955535	NM_004302	ACVR1B	activin A receptor, type IB	0.072	1.051	7.25E-01	7.25E+01		
7955562	NM_000020	ACVRL1	activin A receptor type II-like 1	0.042	1.029	7.96E-01	7.96E+01		
7955578	NM_181711	GRASP	GRP1 (general receptor for phospl	-0.098	0.934	3.36E-01	3.36E+01		
7955589	NM_002135	NR4A1	nuclear receptor subfamily 4, grou	-0.085	0.943	3.18E-01	3.18E+01		
7955606	NM_021934	C12orf44	chromosome 12 open reading fran	0.082	1.058	4.59E-01	4.59E+01		
7955613	NM_005556	KRT7	keratin 7	0.019	1.014	9.04E-01	9.04E+01		
7955624	NM_002284	KRT86	keratin 86	-0.016	0.989	9.16E-01	9.16E+01		
7955637	NM_199187	KRT18	keratin 18	0.006	1.004	9.79E-01	9.79E+01		
7955646	NM_001417	EIF4B	eukaryotic translation initiation fa	-0.016	0.989	9.30E-01	9.30E+01		
7955663	NM_170754	TENC1	tensin like C1 domain containing p	-0.026	0.982	8.16E-01	8.16E+01		
7955694	NM_002178	IGFBP6	insulin-like growth factor binding p	-0.019	0.987	9.03E-01	9.03E+01		
7955702	NM_003578	SOAT2	sterol O-acyltransferase 2	0.073	1.052	4.19E-01	4.19E+01		
7955719	NM_00109966	HIGD1A	HIG1 hypoxia inducible domain fan	0.010	1.007	9.65E-01	9.65E+01		
7955721	NM_00100430	ZNF740	zinc finger protein 740	0.023	1.016	8.98E-01	8.98E+01		
7955729	NM_032889	MFSD5	major facilitator superfamily doma	0.168	1.124	2.37E-01	2.37E+01		
7955768	NM_002624	PFDN5	prefoldin subunit 5	0.061	1.043	6.14E-01	6.14E+01		
7955777	NM_021640	C12orf10	chromosome 12 open reading fran	0.048	1.034	7.40E-01	7.40E+01		
7955787	NM_138473	SP1	Sp1 transcription factor	0.031	1.022	7.89E-01	7.89E+01		
7955797	NM_020547	AMHR2	anti-Mullerian hormone receptor,	-0.067	0.955	3.40E-01	3.40E+01		

7955810	NM_018457	PRR13	proline rich 13	0.043	1.030	7.61E-01	7.61E+01		
7955817	NM_005016	PCBP2	poly(rC) binding protein 2	-0.024	0.984	8.30E-01	8.30E+01		
7955829	NM_134323	TARBP2	TAR (HIV-1) RNA binding protein 2	-0.032	0.978	7.42E-01	7.42E+01		
7955845	NM_017410	HOXC13	homeobox C13	0.015	1.010	9.20E-01	9.20E+01		
7955852	NM_173860	HOXC12	homeobox C12	-0.022	0.985	8.85E-01	8.85E+01		
7955890	NM_031157	HNRNPA1	heterogeneous nuclear ribonucleo	-0.094	0.937	3.90E-01	3.90E+01		
7955896	NM_016057	COPZ1	coatomer protein complex, subuni	-0.039	0.973	8.93E-01	8.93E+01		
7955908	NM_005337	NCKAP1L	NCK-associated protein 1-like	0.187	1.139	1.02E-01	1.02E+01		
7955943	NM_000924	PDE1B	phosphodiesterase 1B, calmodulin	0.017	1.012	9.17E-01	9.17E+01		
7955974	NM_021191	NEUROD4	neurogenic differentiation 4	-0.037	0.974	7.23E-01	7.23E+01		
7956007	NM_206899	OR10P1	olfactory receptor, family 10, subf	-0.082	0.945	4.41E-01	4.41E+01		
7956009	NM_152637	METTL7B	methyltransferase like 7B	0.220	1.165	5.45E-02	5.45E+00		
7956013	NM_001487	BLOC1S1	biogenesis of lysosomal organelles	0.123	1.089	4.27E-01	4.27E+01		
7956018	NM_002905	RDH5	retinol dehydrogenase 5 (11-cis/9-	-0.007	0.995	9.73E-01	9.73E+01		
7956026	NM_005811	GDF11	growth differentiation factor 11	-0.048	0.968	7.52E-01	7.52E+01		
7956031	NM_014182	ORMDL2	ORM1-like 2 (S. cerevisiae)	0.073	1.052	7.09E-01	7.09E+01		
7956038	NM_002429	MMP19	matrix metallopeptidase 19	0.082	1.058	5.95E-01	5.95E+01		
7956044	---	---	---	-0.045	0.969	7.75E-01	7.75E+01		
7956046	NM_201444	DGKA	diacylglycerol kinase, alpha 80kDa	-0.061	0.959	7.97E-01	7.97E+01		
7956076	NM_001798	CDK2	cyclin-dependent kinase 2	-0.121	0.920	4.70E-01	4.70E+01		
7956088	NM_002868	RAB5B	RAB5B, member RAS oncogene fan	-0.080	0.946	5.55E-01	5.55E+01		
7956097	NM_000456	SUOX	sulfite oxidase	-0.077	0.948	6.61E-01	6.61E+01		
7956105	NM_022465	IKZF4	IKAROS family zinc finger 4 (Eos)	0.064	1.046	6.42E-01	6.42E+01		
7956114	NM_001029	RPS26	ribosomal protein S26	0.014	1.010	9.74E-01	9.74E+01		
7956152	NM_006191	PA2G4	proliferation-associated 2G4, 38kD	-0.009	0.994	9.63E-01	9.63E+01		
7956159	NM_00103526	RPL41	ribosomal protein L41	0.011	1.008	9.75E-01	9.75E+01		
7956162	NM_032786	ZC3H10	zinc finger CCCH-type containing 1	-0.149	0.902	3.53E-02	3.53E+00		
7956166	NM_015292	ESYT1	extended synaptotagmin-like prot	0.028	1.020	8.44E-01	8.44E+01		
7956200	NM_002475	MYL6B	myosin, light chain 6B, alkali, smoo	0.013	1.009	9.44E-01	9.44E+01		
7956211	NM_021019	MYL6	myosin, light chain 6, alkali, smoot	-0.008	0.994	9.65E-01	9.65E+01		
7956220	NM_024068	OBFC2B	oligonucleotide/oligosaccharide-b	0.051	1.036	8.46E-01	8.46E+01		
7956229	NM_00113519	SLC39A5	solute carrier family 39 (metal ion	-0.036	0.975	7.94E-01	7.94E+01		
7956242	NM_144576	COQ10A	coenzyme Q10 homolog A (S. cere	-0.088	0.941	5.45E-01	5.45E+01		
7956251	NM_016584	IL23A	interleukin 23, alpha subunit p19	-0.086	0.942	6.78E-01	6.78E+01		
7956257	NM_207344	SPRYD4	SPRY domain containing 4	0.050	1.035	8.24E-01	8.24E+01		
7956261	NM_002898	RBMS2	RNA binding motif, single stranded	0.163	1.120	3.41E-01	3.41E+01		
7956269	---	---	---	0.150	1.110	7.32E-01	7.32E+01		
7956281	NM_007264	GPR182	G protein-coupled receptor 182	0.040	1.028	7.38E-01	7.38E+01		
7956285	AF529371	HBCBP	HBcAg-binding protein	-0.012	0.992	9.40E-01	9.40E+01		
7956287	NM_005967	NAB2	NGFI-A binding protein 2 (EGR1 bi	0.037	1.026	6.28E-01	6.28E+01		
7956301	NM_002332	LRP1	low density lipoprotein-related pro	0.318	1.247	1.51E-01	1.51E+01		
7956395	NM_007224	NXPH4	neurexophilin 4	-0.057	0.961	6.76E-01	6.76E+01		
7956401	NM_005412	SHMT2	serine hydroxymethyltransferase 2	-0.006	0.996	9.86E-01	9.86E+01		
7956417	---	---	---	0.261	1.198	3.13E-01	3.13E+01		
7956419	---	---	---	0.039	1.027	8.68E-01	8.68E+01		
7956423	NM_005538	INHBC	inhibin, beta C	-0.029	0.980	8.49E-01	8.49E+01		
7956426	NM_031479	INHBE	inhibin, beta E	0.052	1.037	7.00E-01	7.00E+01		
7956430	NM_005269	GLI1	GLI family zinc finger 1	0.040	1.028	8.16E-01	8.16E+01		
7956443	NM_004990	MARS	methionyl-tRNA synthetase	-0.051	0.966	6.78E-01	6.78E+01		
7956470	NM_052897	MBD6	methyl-CpG binding domain prote	-0.089	0.940	4.58E-01	4.58E+01		
7956488	NM_004984	KIF5A	kinesin family member 5A	-0.028	0.981	7.94E-01	7.94E+01		
7956522	AF063608	KIF5A	kinesin family member 5A	0.043	1.030	8.49E-01	8.49E+01		
7956524	NM_024779	PIP4K2C	phosphatidylinositol-5-phosphate	-0.047	0.968	8.16E-01	8.16E+01		
7956539	NM_178502	DTX3	deltex homolog 3 (Drosophila)	-0.014	0.990	9.50E-01	9.50E+01		
7956551	NM_182947	GEFT	RhoA/RAC/CDC42 exchange factor	-0.090	0.939	3.32E-01	3.32E+01		
7956593	NM_006812	OS9	osteosarcoma amplified 9, endopl	0.120	1.087	5.34E-01	5.34E+01		
7956613	NM_005981	TSPAN31	tetraspanin 31	0.023	1.016	9.11E-01	9.11E+01		
7956623	NM_138396	40246	membrane-associated ring finger (	0.018	1.013	9.24E-01	9.24E+01		
7956639	NM_005726	TSFM	Ts translation elongation factor, m	-0.064	0.956	7.05E-01	7.05E+01		
7956658	NM_004731	SLC16A7	solute carrier family 16, member 7	-0.205	0.867	2.98E-01	2.98E+01		
7956668	---	---	---	0.035	1.025	8.06E-01	8.06E+01		
7956670	NM_006313	USP15	ubiquitin specific peptidase 15	0.118	1.085	6.08E-02	6.08E+00		
7956697	NM_015026	MON2	MON2 homolog (S. cerevisiae)	0.045	1.032	7.11E-01	7.11E+01		
7956737	---	---	---	-0.080	0.946	5.69E-01	5.69E+01		
7956743	NM_00103499	RPL14	ribosomal protein L14	-0.027	0.982	8.62E-01	8.62E+01		
7956749	NM_014254	TMEM5	transmembrane protein 5	0.031	1.022	8.38E-01	8.38E+01		
7956785	NM_007235	XPOT	exportin, tRNA (nuclear export rec	-0.026	0.982	8.86E-01	8.86E+01		
7956795	NM_013254	TBK1	TANK-binding kinase 1	-0.101	0.932	6.91E-01	6.91E+01		
7956819	NM_178169	RASSF3	Ras association (RalGDS/AF-6) dom	0.070	1.050	7.05E-01	7.05E+01		
7956842	NM_014319	LEMD3	LEM domain containing 3	-0.026	0.982	8.69E-01	8.69E+01		
7956867	NM_003483	HMGA2	high mobility group AT-hook 2	-0.041	0.972	7.35E-01	7.35E+01		
7956876	NM_032338	LLPH	LLP homolog, long-term synaptic f	0.015	1.010	9.53E-01	9.53E+01		
7956878	NM_007199	IRAK3	interleukin-1 receptor-associated	-0.321	0.800	5.35E-01	5.35E+01		
7956894	NM_033647	HELB	helicase (DNA) B	0.038	1.027	8.50E-01	8.50E+01		
7956908	---	---	---	0.028	1.020	9.28E-01	9.28E+01		
7956910	NM_018448	CAND1	cullin-associated and neddylation-	0.005	1.003	9.78E-01	9.78E+01		
7956930	NM_006482	DYRK2	dual-specificity tyrosine-(Y)-phosp	0.072	1.051	3.41E-01	3.41E+01		

7956937	NM_015646	RAP1B	RAP1B, member of RAS oncogene	0.078	1.056	4.81E-01	4.81E+01		
7956949	NM_020401	NUP107	nucleoporin 107kDa	-0.026	0.982	9.09E-01	9.09E+01		
7956978	NM_018656	SLC35E3	solute carrier family 35, member E	0.006	1.004	9.82E-01	9.82E+01		
7956989	NM_002392	MDM2	Mdm2 p53 binding protein homol	-0.068	0.954	4.62E-01	4.62E+01		
7957008	NM_007007	CPSF6	cleavage and polyadenylation spec	-0.046	0.969	6.91E-01	6.91E+01		
7957023	NM_000239	LYZ	lysozyme (renal amyloidosis)	0.523	1.437	3.16E-01	3.16E+01		
7957032	NM_006530	YEATS4	YEATS domain containing 4	0.034	1.024	8.87E-01	8.87E+01		
7957043	NM_006654	FRS2	fibroblast growth factor receptor s	0.042	1.030	7.72E-01	7.72E+01		
7957052	NM_006431	CCT2	chaperonin containing TCP1, subu	-0.009	0.994	9.75E-01	9.75E+01		
7957106	NM_014515	CNOT2	CCR4-NOT transcription complex,	0.025	1.017	8.78E-01	8.78E+01		
7957126	NM_014505	KCNMB4	potassium large conductance calci	-0.071	0.952	4.70E-01	4.70E+01		
7957167	NM_018279	TMEM19	transmembrane protein 19	0.151	1.111	1.52E-01	1.52E+01		
7957177	NM_014999	RAB21	RAB21, member RAS oncogene fa	-0.062	0.958	7.26E-01	7.26E+01		
7957186	NM_022771	TBC1D15	TBC1 domain family, member 15	-0.007	0.995	9.77E-01	9.77E+01		
7957242	NM_00113626	LOC552889	hypothetical protein LOC552889	0.031	1.022	8.14E-01	8.14E+01		
7957260	NM_006851	GLIPR1	GLI pathogenesis-related 1	0.067	1.047	7.73E-01	7.73E+01		
7957271	---	---	---	0.049	1.034	8.07E-01	8.07E+01		
7957275	---	---	---	-0.098	0.934	3.71E-01	3.71E+01		
7957277	NM_015336	ZDHHC17	zinc finger, DHHC-type containing	0.023	1.016	8.95E-01	8.95E+01		
7957404	BC029120	C12orf26	chromosome 12 open reading fra	0.022	1.016	9.18E-01	9.18E+01		
7957417	NM_152588	TMTC2	transmembrane and tetratricopep	0.076	1.054	7.30E-01	7.30E+01		
7957467	NM_00100989	C12orf29	chromosome 12 open reading fra	0.064	1.045	8.04E-01	8.04E+01		
7957530	NM_021104	RPL41	ribosomal protein L41	0.009	1.006	9.10E-01	9.10E+01		
7957536	uc001tcn.1	NUDT4	nudix (nucleoside diphosphate lin	0.082	1.059	5.56E-01	5.56E+01		
7957540	NM_014050	MRPL42	mitochondrial ribosomal protein L	0.098	1.070	3.92E-01	3.92E+01		
7957549	---	---	---	0.063	1.045	8.06E-01	8.06E+01		
7957551	NM_003877	SOCS2	suppressor of cytokine signaling 2	-0.151	0.900	2.80E-01	2.80E+01		
7957560	NM_003805	CRADD	CASP2 and RIPK1 domain containi	-0.028	0.981	8.45E-01	8.45E+01		
7957568	---	---	---	-0.068	0.954	6.10E-01	6.10E+01		
7957570	NM_005761	PLXNC1	plexin C1	0.378	1.299	1.67E-01	1.67E+01		
7957604	---	---	---	-0.041	0.972	7.85E-01	7.85E+01		
7957606	---	---	---	0.039	1.028	8.25E-01	8.25E+01		
7957611	---	---	---	0.056	1.039	6.58E-01	6.58E+01		
7957613	NM_017599	VEZT	vezatin, adherens junctions transn	-0.003	0.998	9.88E-01	9.88E+01		
7957633	NM_006838	METAP2	methionyl aminopeptidase 2	-0.021	0.985	8.90E-01	8.90E+01		
7957649	NM_003095	SNRPF	small nuclear ribonucleoprotein p	0.012	1.008	9.68E-01	9.68E+01		
7957654	NM_152435	AMDHD1	amidohydrolase domain containin	0.000	1.000	1.00E+00	1.00E+02		
7957665	NM_005230	ELK3	ELK3, ETS-domain protein (SRF ac	-0.211	0.864	1.49E-02	1.49E+00		
7957715	NM_00113517	NEDD1	neural precursor cell expressed, d	0.028	1.019	8.50E-01	8.50E+01		
7957737	NM_00103228	TMPO	thymopoietin	0.060	1.043	6.58E-01	6.58E+01		
7957746	NM_213611	SLC25A3	solute carrier family 25 (mitochon	-0.027	0.981	8.36E-01	8.36E+01		
7957759	NM_181861	APAF1	apoptotic peptidase activating fac	0.049	1.035	7.97E-01	7.97E+01		
7957793	NM_022496	ACTR6	ARP6 actin-related protein 6 hom	0.080	1.057	6.24E-01	6.24E+01		
7957806	NM_017988	SCYL2	SCY1-like 2 (S. cerevisiae)	0.078	1.055	6.38E-01	6.38E+01		
7958000	NM_020244	CHPT1	choline phosphotransferase 1	0.252	1.191	2.99E-02	2.99E+00		
7958011	---	---	---	0.170	1.125	2.66E-01	2.66E+01		
7958015	---	---	---	0.179	1.132	6.12E-01	6.12E+01		
7958019	NM_018370	DRAM1	DNA-damage regulated autophagy	0.615	1.531	1.79E-01	1.79E+01		
7958051	NM_004316	ASCL1	achaete-scute complex homolog 1	-0.050	0.966	6.92E-01	6.92E+01		
7958130	NM_003299	HSP90B1	heat shock protein 90kDa beta (G	0.181	1.133	4.66E-03	4.66E-01		
7958147	NM_003211	TDG	thymine-DNA glycosylase	0.037	1.026	8.62E-01	8.62E+01		
7958152	NM_002629	PGAM1	phosphoglycerate mutase 1 (brain	-0.010	0.993	9.64E-01	9.64E+01		
7958158	NM_013320	HCFC2	host cell factor C2	0.131	1.095	4.44E-01	4.44E+01		
7958174	NM_003330	TXNRD1	thioredoxin reductase 1	-0.139	0.908	5.10E-01	5.10E+01		
7958197	NR_001593	RPL18AP3	ribosomal protein L18a pseudoge	-0.009	0.994	9.74E-01	9.74E+01		
7958200	NM_00100839	EID3	EP300 interacting inhibitor of diff	0.004	1.003	9.86E-01	9.86E+01		
7958202	NM_018413	CHST11	carbohydrate (chondroitin 4) sulfo	0.233	1.175	5.63E-02	5.63E+00		
7958211	NM_152318	C12orf45	chromosome 12 open reading fra	0.015	1.010	9.60E-01	9.60E+01		
7958216	NM_015275	KIAA1033	KIAA1033	0.109	1.079	1.67E-01	1.67E+01		
7958253	NM_00114519	C12orf75	chromosome 12 open reading fra	-0.039	0.974	9.16E-01	9.16E+01		
7958262	NM_152772	TCP11L2	t-complex 11 (mouse)-like 2	-0.125	0.917	5.85E-01	5.85E+01		
7958273	---	---	---	-0.269	0.830	9.17E-02	9.17E+00		
7958275	NM_018082	POLR3B	polymerase (RNA) III (DNA direct	0.010	1.007	9.77E-01	9.77E+01		
7958331	NM_018157	RIC8B	resistance to inhibitors of choline	-0.074	0.950	6.34E-01	6.34E+01		
7958346	NM_152261	C12orf23	chromosome 12 open reading fra	-0.069	0.953	7.09E-01	7.09E+01		
7958352	NM_00101807	BTBD11	BTB (POZ) domain containing 11	-0.025	0.983	9.25E-01	9.25E+01		
7958375	---	---	---	-0.198	0.872	2.08E-01	2.08E+01		
7958379	NM_007062	PWP1	PWP1 homolog (S. cerevisiae)	-0.060	0.959	6.34E-01	6.34E+01		
7958398	NM_014653	WSCD2	WSC domain containing 2	0.014	1.010	9.27E-01	9.27E+01		
7958410	NM_007076	FICD	FIC domain containing	0.028	1.020	8.86E-01	8.86E+01		
7958414	NM_014301	ISCU	iron-sulfur cluster scaffold homol	-0.113	0.925	4.12E-01	4.12E+01		
7958439	NM_032663	USP30	ubiquitin specific peptidase 30	0.050	1.036	8.26E-01	8.26E+01		
7958453	---	---	---	0.026	1.018	9.05E-01	9.05E+01		
7958455	NM_003362	UNG	uracil-DNA glycosylase	-0.006	0.996	9.83E-01	9.83E+01		
7958466	NM_001093	ACACB	acetyl-Coenzyme A carboxylase be	-0.028	0.981	8.33E-01	8.33E+01		
7958532	NM_130466	UBE3B	ubiquitin protein ligase E3B	-0.038	0.974	7.09E-01	7.09E+01		
7958565	NM_000431	MVK	mevalonate kinase	0.050	1.036	5.98E-01	5.98E+01		

7958577	NM_032829	C12orf34	chromosome 12 open reading fran	-0.094	0.937	1.40E-01	1.40E+01		
7958582	NM_032300	TCHP	trichoplein, keratin filament bindi	0.001	1.000	9.97E-01	9.97E+01		
7958600	NM_033121	ANKRD13A	ankyrin repeat domain 13A	0.210	1.157	9.25E-03	9.25E-01		
7958644	NM_170665	ATP2A2	ATPase, Ca++ transporting, cardiac	0.297	1.229	5.83E-03	5.83E-01		
7958692	NM_00108253	TCTN1	tectonic family member 1	0.143	1.104	1.03E-01	1.03E+01		
7958724	ENST00000331	LOC100131138	similar to hCG2040918	-0.011	0.993	9.53E-01	9.53E+01		
7958726	NM_015267	CUX2	cut-like homeobox 2	0.002	1.001	9.89E-01	9.89E+01		
7958749	NM_005475	SH2B3	SH2B adaptor protein 3	0.001	1.001	9.92E-01	9.92E+01		
7958759	---	---	---	0.066	1.047	7.68E-01	7.68E+01		
7958761	NM_00113653	ACAD10	acyl-Coenzyme A dehydrogenase f	0.006	1.004	9.78E-01	9.78E+01		
7958784	NM_000690	ALDH2	aldehyde dehydrogenase 2 family	0.019	1.013	9.60E-01	9.60E+01		
7958800	NM_003668	MAPKAPK5	mitogen-activated protein kinase-	0.080	1.057	4.81E-01	4.81E+01		
7958819	NM_006817	ERP29	endoplasmic reticulum protein 29	0.013	1.009	9.51E-01	9.51E+01		
7958826	---	---	---	0.089	1.064	8.47E-01	8.47E+01		
7958828	NM_00114390	TRAFD1	TRAF-type zinc finger domain cont	0.139	1.101	5.15E-01	5.15E+01		
7958846	NM_002834	PTPN11	protein tyrosine phosphatase, non	-0.051	0.965	7.01E-01	7.01E+01		
7958884	NM_016816	OAS1	2',5'-oligoadenylate synthetase 1,	0.551	1.466	1.03E-01	1.03E+01		
7958895	NM_006187	OAS3	2'-5'-oligoadenylate synthetase 3,	0.250	1.189	4.27E-01	4.27E+01		
7958913	NM_002535	OAS2	2'-5'-oligoadenylate synthetase 2,	0.297	1.229	2.53E-01	2.53E+01		
7958931	NM_004416	DTX1	deltex homolog 1 (Drosophila)	0.031	1.022	7.82E-01	7.82E+01		
7958942	NM_00114487	CCDC42B	coiled-coil domain containing 42B	-0.048	0.967	6.95E-01	6.95E+01		
7958948	NM_00114487	CCDC42B	coiled-coil domain containing 42B	-0.041	0.972	7.08E-01	7.08E+01		
7958950	BC022092	C12orf52	chromosome 12 open reading fran	0.012	1.008	9.38E-01	9.38E+01		
7958960	NM_00114381	TPCN1	two pore segment channel 1	0.181	1.134	2.91E-01	2.91E+01		
7958989	NM_173542	PLBD2	phospholipase B domain containin	0.033	1.023	8.70E-01	8.70E+01		
7959002	NM_138432	SDSL	serine dehydratase-like	0.087	1.062	3.29E-01	3.29E+01		
7959014	---	---	---	0.106	1.076	7.53E-01	7.53E+01		
7959016	NR_027345	NCRNA00173	non-protein coding RNA 173	-0.054	0.963	6.00E-01	6.00E+01		
7959023	NM_00108548	MAP1LC3B2	microtubule-associated protein 1 l	-0.052	0.964	8.64E-01	8.64E+01		
7959025	NM_00110990	RNFT2	ring finger protein, transmembran	0.020	1.014	8.97E-01	8.97E+01		
7959039	NM_153348	FBXW8	F-box and WD repeat domain cont	0.044	1.031	7.30E-01	7.30E+01		
7959052	NM_181578	RFC5	replication factor C (activator 1) 5,	0.196	1.145	1.51E-02	1.51E+00		
7959070	NM_002567	PEBP1	phosphatidylethanolamine binding	-0.095	0.936	3.41E-01	3.41E+01		
7959078	---	---	---	0.050	1.035	6.13E-01	6.13E+01		
7959080	NM_022491	SUDS3	suppressor of defective silencing 3	-0.029	0.980	8.27E-01	8.27E+01		
7959087	NM_194286	KIAA1853	KIAA1853	0.066	1.047	5.57E-01	5.57E+01		
7959100	---	---	---	0.073	1.052	5.90E-01	5.90E+01		
7959123	NM_006253	PRKAB1	protein kinase, AMP-activated, be	0.027	1.019	8.74E-01	8.74E+01		
7959131	NM_207311	CCDC64	coiled-coil domain containing 64	-0.163	0.893	2.63E-01	2.63E+01		
7959144	---	---	---	-0.261	0.835	4.39E-01	4.39E+01		
7959146	NR_001577	NME2P1	non-metastatic cells 2, protein (NN	0.008	1.005	9.78E-01	9.78E+01		
7959148	NM_012240	SIRT4	sirtuin (silent mating type informa	-0.122	0.919	5.54E-01	5.54E+01		
7959153	NM_004373	COX6A1	cytochrome c oxidase subunit VIa	0.031	1.022	8.63E-01	8.63E+01		
7959157	NM_176818	GATC	glutamyl-tRNA(Gln) amidotransfer	0.030	1.021	7.98E-01	7.98E+01		
7959164	NM_00103749	DYNLL1	dynein, light chain, LC8-type 1	0.020	1.014	9.29E-01	9.29E+01		
7959173	NM_014868	RNF10	ring finger protein 10	0.007	1.005	9.69E-01	9.69E+01		
7959195	NM_00103367	CABP1	calcium binding protein 1	-0.013	0.991	9.42E-01	9.42E+01		
7959205	NM_014730	MLEC	malectin	0.045	1.032	7.38E-01	7.38E+01		
7959212	NM_00108053	UNC119B	unc-119 homolog B (C. elegans)	-0.103	0.931	2.99E-01	2.99E+01		
7959220	NM_000017	ACADS	acyl-Coenzyme A dehydrogenase,	-0.031	0.979	8.40E-01	8.40E+01		
7959232	---	---	---	0.010	1.007	9.68E-01	9.68E+01		
7959234	NM_000545	HNF1A	HNF1 homeobox A	0.055	1.039	6.80E-01	6.80E+01		
7959249	---	---	---	0.027	1.019	8.97E-01	8.97E+01		
7959267	NM_002560	P2RX4	purinergic receptor P2X, ligand-ga	0.315	1.244	1.11E-01	1.11E+01		
7959282	NM_194271	RNF34	ring finger protein 34	-0.015	0.989	9.48E-01	9.48E+01		
7959294	NM_032790	ORAI1	ORAI calcium release-activated cal	-0.061	0.959	5.89E-01	5.89E+01		
7959298	NM_00108082	TMEM120B	transmembrane protein 120B	-0.103	0.931	4.10E-01	4.10E+01		
7959312	NM_00108082	TMEM120B	transmembrane protein 120B	-0.246	0.843	9.22E-02	9.22E+00		
7959314	NM_015048	SETD1B	SET domain containing 1B	0.011	1.008	9.53E-01	9.53E+01		
7959322	NM_002813	PSMD9	proteasome (prosome, macropain	0.010	1.007	9.64E-01	9.64E+01		
7959330	NM_144668	WDR66	WD repeat domain 66	-0.019	0.987	9.13E-01	9.13E+01		
7959354	NM_020993	BCL7A	B-cell CLL/lymphoma 7A	-0.041	0.972	8.12E-01	8.12E+01		
7959361	NM_014938	MLXIP	MLX interacting protein	0.056	1.040	7.76E-01	7.76E+01		
7959402	NM_030765	B3GNT4	UDP-GlcNAc:betaGal beta-1,3-N-a	-0.006	0.996	9.74E-01	9.74E+01		
7959473	NM_003677	DENR	density-regulated protein	0.062	1.044	4.29E-01	4.29E+01		
7959484	NM_201435	CCDC62	coiled-coil domain containing 62	-0.016	0.989	9.56E-01	9.56E+01		
7959500	NM_003959	HIP1R	huntingtin interacting protein 1 re	-0.059	0.960	5.23E-01	5.23E+01		
7959535	NM_024623	OGFOD2	2-oxoglutarate and iron-depender	-0.020	0.986	8.97E-01	8.97E+01		
7959549	NM_00100225	ARL6IP4	ADP-ribosylation-like factor 6 inte	0.026	1.018	8.58E-01	8.58E+01		
7959563	NM_152269	C12orf65	chromosome 12 open reading fran	-0.079	0.947	4.41E-01	4.41E+01		
7959574	NM_020382	SETD8	SET domain containing (lysine met	0.053	1.038	6.98E-01	6.98E+01		
7959586	NM_180699	SNRNP35	small nuclear ribonucleoprotein 35	0.012	1.009	9.63E-01	9.63E+01		
7959597	NM_006815	TMED2	transmembrane emp24 domain tr	0.104	1.075	1.49E-01	1.49E+01		
7959604	NM_020936	DDX55	DEAD (Asp-Glu-Ala-Asp) box polyp	0.006	1.004	9.80E-01	9.80E+01		
7959621	---	---	---	-0.018	0.988	9.27E-01	9.27E+01		
7959623	NM_001516	GTF2H3	general transcription factor IIH, po	0.126	1.091	4.82E-01	4.82E+01		
7959638	NM_024809	TCTN2	tectonic family member 2	-0.114	0.924	5.34E-01	5.34E+01		

7959657	NM_012463	ATP6V0A2	ATPase, H+ transporting, lysosoma	0.085	1.060	3.69E-01	3.69E+01				
7959696	NM_207437	DNAH10	dynein, axonemal, heavy chain 10	0.014	1.010	9.02E-01	9.02E+01				
7959751	NM_152437	ZNF664	zinc finger protein 664	-0.059	0.960	4.25E-01	4.25E+01				
7959761	NM_181709	FAM101A	family with sequence similarity 10	-0.077	0.948	3.41E-01	3.41E+01				
7959772	NM_006312	NCOR2	nuclear receptor co-repressor 2	-0.019	0.987	9.51E-01	9.51E+01				
7959777	NM_080626	BRI3BP	BRI3 binding protein	0.025	1.017	8.64E-01	8.64E+01				
7959786	NM_023928	AACS	acetoacetyl-CoA synthetase	-0.011	0.992	9.73E-01	9.73E+01				
7959827	NM_00113610	TMEM132C	transmembrane protein 132C	-0.011	0.993	9.56E-01	9.56E+01				
7959834	NM_144669	GLT1D1	glycosyltransferase 1 domain cont	0.063	1.045	9.02E-01	9.02E+01				
7959848	---	---	---	0.010	1.007	9.62E-01	9.62E+01				
7959850	NM_007197	FZD10	frizzled homolog 10 (Drosophila)	-0.066	0.955	5.05E-01	5.05E+01				
7959880	---	---	---	-0.094	0.937	5.15E-01	5.15E+01				
7959882	NM_006325	RAN	RAN, member RAS oncogene fami	-0.025	0.983	8.65E-01	8.65E+01				
7959891	---	---	---	0.039	1.028	8.84E-01	8.84E+01				
7959921	AK098523	LOC338797	hypothetical LOC338797	-0.090	0.940	3.09E-01	3.09E+01				
7959927	NM_004592	SFRS8	splicing factor, arginine/serine-rich	-0.051	0.965	6.18E-01	6.18E+01				
7959946	NM_016155	MMP17	matrix metallopeptidase 17 (mem	-0.055	0.962	6.48E-01	6.48E+01				
7959957	NM_003565	ULK1	unc-51-like kinase 1 (C. elegans)	-0.039	0.973	7.81E-01	7.81E+01				
7959986	NM_025215	PUS1	pseudouridylate synthase 1	-0.037	0.974	8.08E-01	8.08E+01				
7959995	NM_015409	EP400	E1A binding protein p400	-0.035	0.976	6.76E-01	6.76E+01				
7960054	NR_003290	EP400NL	EP400 N-terminal like	-0.005	0.996	9.81E-01	9.81E+01				
7960068	NM_024078	NOC4L	nucleolar complex associated 4 ho	0.089	1.064	3.44E-01	3.44E+01				
7960088	NM_00114264	FBRSL1	fibrosin-like 1	-0.059	0.960	6.33E-01	6.33E+01				
7960099	NM_170683	P2RX2	purinergic receptor P2X, ligand-ga	-0.002	0.999	9.88E-01	9.88E+01				
7960117	NM_018663	PXMP2	peroxisomal membrane protein 2,	0.005	1.004	9.83E-01	9.83E+01				
7960124	NM_138575	PGAM5	phosphoglycerate mutase family n	-0.004	0.997	9.84E-01	9.84E+01				
7960131	---	---	---	-0.008	0.994	9.53E-01	9.53E+01				
7960134	NM_019591	ZNF26	zinc finger protein 26	0.026	1.018	9.22E-01	9.22E+01				
7960143	NM_003428	ZNF84	zinc finger protein 84	-0.002	0.999	9.93E-01	9.93E+01				
7960165	NM_003415	ZNF268	zinc finger protein 268	-0.045	0.969	7.63E-01	7.63E+01				
7960177	NM_003044	SLC6A12	solute carrier family 6 (neurotrans	0.046	1.032	7.98E-01	7.98E+01				
7960221	NM_005056	KDM5A	lysine (K)-specific demethylase 5A	-0.039	0.973	7.45E-01	7.45E+01				
7960253	NM_016533	NINJ2	ninjurin 2	-0.010	0.993	9.53E-01	9.53E+01				
7960259	---	---	---	0.246	1.186	1.76E-01	1.76E+01				
7960261	NM_134424	RAD52	RAD52 homolog (S. cerevisiae)	-0.100	0.933	3.64E-01	3.64E+01				
7960280	NM_152441	FBXL14	F-box and leucine-rich repeat prot	-0.084	0.943	4.84E-01	4.84E+01				
7960283	NM_172364	CACNA2D4	calcium channel, voltage-depende	-0.065	0.956	3.32E-01	3.32E+01				
7960320	NM_152640	DCP1B	DCP1 decapping enzyme homolog	-0.123	0.918	2.75E-01	2.75E+01				
7960331	NM_031474	NRIP2	nuclear receptor interacting prote	-0.016	0.989	9.30E-01	9.30E+01				
7960338	---	---	---	-0.031	0.979	9.01E-01	9.01E+01				
7960357	---	---	---	-0.043	0.971	6.94E-01	6.94E+01				
7960359	---	---	---	0.018	1.013	9.60E-01	9.60E+01				
7960362	AY358109	UNQ3104	ACAH3104	0.043	1.030	7.94E-01	7.94E+01				
7960370	NM_032680	EFCAB4B	EF-hand calcium binding domain 4	0.017	1.012	9.40E-01	9.40E+01				
7960381	CR627161	EFCAB4B	EF-hand calcium binding domain 4	0.052	1.037	8.08E-01	8.08E+01				
7960397	NM_020638	FGF23	fibroblast growth factor 23	-0.074	0.950	4.86E-01	4.86E+01				
7960407	NM_020996	FGF6	fibroblast growth factor 6	-0.039	0.973	7.45E-01	7.45E+01				
7960512	---	---	---	-0.063	0.957	5.65E-01	5.65E+01				
7960518	NM_001065	TNFRSF1A	tumor necrosis factor receptor sup	0.140	1.102	4.83E-01	4.83E+01				
7960544	NM_199245	VAMP1	vesicle-associated membrane prot	-0.042	0.971	8.11E-01	8.11E+01				
7960553	NM_016497	MRPL51	mitochondrial ribosomal protein L	0.002	1.001	9.94E-01	9.94E+01				
7960559	NM_080730	IFFO1	intermediate filament family orph	-0.028	0.981	8.67E-01	8.67E+01				
7960575	NM_00103371	NOP2	NOP2 nucleolar protein homolog (	-0.088	0.941	4.10E-01	4.10E+01				
7960594	NM_001273	CHD4	chromodomain helicase DNA bind	-0.129	0.915	1.35E-01	1.35E+01				
7960635	NR_003012	SCARNA11	small Cajal body-specific RNA 11	0.041	1.029	8.04E-01	8.04E+01				
7960637	NM_020400	LPAR5	lysophosphatidic acid receptor 5	-0.002	0.998	9.91E-01	9.91E+01				
7960642	NM_032489	ACRBP	acrosin binding protein	-0.037	0.975	7.30E-01	7.30E+01				
7960654	NM_016162	ING4	inhibitor of growth family, membe	0.029	1.021	8.62E-01	8.62E+01				
7960666	NM_133476	ZNF384	zinc finger protein 384	-0.082	0.945	2.01E-01	2.01E+01				
7960682	AK289648	C12orf53	chromosome 12 open reading fran	-0.049	0.967	6.19E-01	6.19E+01				
7960689	NR_026581	MLF2	myeloid leukemia factor 2	-0.033	0.978	8.44E-01	8.44E+01				
7960702	NM_031299	CDCA3	cell division cycle associated 3	-0.016	0.989	9.35E-01	9.35E+01				
7960709	NM_00114631	SPSB2	splA/ryanodine receptor domain a	-0.040	0.973	6.93E-01	6.93E+01				
7960716	NM_00114483	PHB2	prohibitin 2	-0.031	0.979	8.78E-01	8.78E+01				
7960728	NR_003010	SCARNA12	small Cajal body-specific RNA 12	0.142	1.103	4.28E-01	4.28E+01				
7960730	NM_005768	LPCAT3	lysophosphatidylcholine acyltransf	0.137	1.099	4.35E-01	4.35E+01				
7960744	NM_001733	C1R	complement component 1, r subco	-0.001	0.999	9.95E-01	9.95E+01				
7960757	NM_016546	C1RL	complement component 1, r subco	0.048	1.034	7.47E-01	7.47E+01				
7960764	NM_031491	RBP5	retinol binding protein 5, cellular	-0.053	0.964	7.03E-01	7.03E+01				
7960850	NM_153449	SLC2A14	solute carrier family 2 (facilitated g	-0.120	0.920	3.48E-01	3.48E+01				
7960863	---	---	---	-0.009	0.994	9.75E-01	9.75E+01				
7960865	NM_006931	SLC2A3	solute carrier family 2 (facilitated g	-0.167	0.891	1.24E-01	1.24E+01				
7960874	NM_004054	C3AR1	complement component 3a recep	0.751	1.683	1.79E-01	1.79E+01				
7960878	AF268617 //	5F1P3 // POU5	POU class 5 homeobox 1 pseudoge	-0.046	0.968	8.02E-01	8.02E+01				
7960881	NM_018088	FAM90A1	family with sequence similarity 90	0.031	1.022	8.08E-01	8.08E+01				
7960889	NR_024254	LOC653113	family with sequence similarity 86	-0.054	0.963	8.01E-01	8.01E+01				
7960894	NR_024420	LOC389634	hypothetical LOC389634	-0.049	0.967	6.36E-01	6.36E+01				

7960896	NM_00104007	LOC650293	seven transmembrane helix recep	0.037	1.026	9.08E-01	9.08E+01			
7960898	---	---	---	0.060	1.042	7.56E-01	7.56E+01			
7960900	NM_014358	CLEC4E	C-type lectin domain family 4, mer	0.064	1.046	9.53E-01	9.53E+01			
7960933	NM_002355	M6PR	mannose-6-phosphate receptor (c	0.268	1.204	5.80E-02	5.80E+00			
7960947	NM_000014	A2M	alpha-2-macroglobulin	0.200	1.148	6.30E-01	6.30E+01			
7960984	NM_002864	PZP	pregnancy-zone protein	0.165	1.121	6.58E-01	6.58E+01			
7961022	BC051265	PTMA	prothymosin, alpha	-0.003	0.998	9.86E-01	9.86E+01			
7961031	NM_004400	DDX12	DEAD/H (Asp-Glu-Ala-Asp/His) box	-0.001	0.999	9.97E-01	9.97E+01			
7961075	NM_001781	CD69	CD69 molecule	-0.266	0.832	1.17E-01	1.17E+01			
7961083	NM_005127	CLEC2B	C-type lectin domain family 2, mer	0.068	1.048	7.51E-01	7.51E+01			
7961142	NM_002543	OLR1	oxidized low density lipoprotein (l	0.268	1.205	7.86E-01	7.86E+01			
7961151	NM_007360	KLRK1	killer cell lectin-like receptor subfa	0.029	1.021	9.24E-01	9.24E+01			
7961166	NM_013431	KLRC4	killer cell lectin-like receptor subfa	0.056	1.040	9.43E-01	9.43E+01			
7961173	---	---	---	-0.003	0.998	9.97E-01	9.97E+01			
7961175	NM_002261	KLRC3	killer cell lectin-like receptor subfa	0.045	1.032	9.61E-01	9.61E+01			
7961182	NM_002260	KLRC2	killer cell lectin-like receptor subfa	-0.025	0.983	9.81E-01	9.81E+01			
7961187	NM_002259	KLRC1	killer cell lectin-like receptor subfa	-0.027	0.982	9.73E-01	9.73E+01			
7961208	NM_018048	MAGOHB	mago-nashi homolog B (Drosophil	0.002	1.001	9.96E-01	9.96E+01			
7961230	NM_003651	CSDA	cold shock domain protein A	0.152	1.111	5.08E-01	5.08E+01			
7961252	NM_007244	PRR4	proline rich 4 (lacrimal)	0.011	1.008	9.65E-01	9.65E+01			
7961269	NM_002723	PRB4	proline-rich protein BstNI subfami	0.032	1.022	8.62E-01	8.62E+01			
7961285	NM_176889	TAS2R20	taste receptor, type 2, member 20	0.045	1.032	9.13E-01	9.13E+01			
7961293	NM_176887	TAS2R46	taste receptor, type 2, member 46	-0.036	0.976	9.56E-01	9.56E+01			
7961300	NM_006249	PRB3	proline-rich protein BstNI subfami	-0.033	0.977	7.57E-01	7.57E+01			
7961306	NM_005039	PRB1	proline-rich protein BstNI subfami	0.011	1.007	9.57E-01	9.57E+01			
7961320	NM_006248	PRB2	proline-rich protein BstNI subfami	0.020	1.014	9.00E-01	9.00E+01			
7961339	NM_002336	LRP6	low density lipoprotein receptor-r	0.100	1.071	7.54E-01	7.54E+01			
7961363	---	---	---	-0.059	0.960	7.17E-01	7.17E+01			
7961371	NM_030640	DUSP16	dual specificity phosphatase 16	-0.182	0.881	9.22E-02	9.22E+00			
7961390	NM_015987	HEBP1	heme binding protein 1	0.163	1.119	4.34E-01	4.34E+01			
7961453	---	---	---	0.055	1.039	7.44E-01	7.44E+01			
7961483	NM_175054	HIST4H4	histone cluster 4, H4	0.130	1.095	5.40E-01	5.40E+01			
7961489	NM_016312	WBP11	WW domain binding protein 11	-0.077	0.948	4.61E-01	4.61E+01			
7961532	NM_001175	ARHGDIB	Rho GDP dissociation inhibitor (GD	0.082	1.059	1.88E-01	1.88E+01			
7961569	---	---	---	0.026	1.018	9.12E-01	9.12E+01			
7961654	NM_002907	RECQL	RecQ protein-like (DNA helicase Q	0.099	1.071	2.72E-01	2.72E+01			
7961693	NM_002300	LDHB	lactate dehydrogenase B	-0.008	0.995	9.77E-01	9.77E+01			
7961757	NM_003034	ST8SIA1	ST8 alpha-N-acetyl-neuraminide a	-0.087	0.942	8.69E-01	8.69E+01			
7961767	AK295862	KIAA0528	KIAA0528	0.137	1.100	1.97E-01	1.97E+01			
7961820	ENST00000358	C12orf67	chromosome 12 open reading fran	0.052	1.037	7.08E-01	7.08E+01			
7961865	NM_033360	KRAS	v-Ki-ras2 Kirsten rat sarcoma viral	-0.108	0.928	1.94E-01	1.94E+01			
7961887	---	---	---	0.054	1.038	6.37E-01	6.37E+01			
7961889	---	---	---	-0.070	0.952	5.82E-01	5.82E+01			
7961900	NM_002223	ITPR2	inositol 1,4,5-triphosphate recepto	0.163	1.119	2.65E-01	2.65E+01			
7961960	---	---	---	0.209	1.156	6.93E-01	6.93E+01			
7961962	---	---	---	0.227	1.170	4.62E-01	4.62E+01			
7961983	NM_016551	TM7SF3	transmembrane 7 superfamily me	0.163	1.119	7.78E-02	7.78E+00			
7961998	---	---	---	0.046	1.032	8.15E-01	8.15E+01			
7962013	NM_016570	ERGIC2	ERGIC and golgi 2	0.035	1.025	8.67E-01	8.67E+01			
7962083	---	---	---	-0.021	0.986	8.88E-01	8.88E+01			
7962085	NM_006390	IPO8	importin 8	-0.021	0.985	9.24E-01	9.24E+01			
7962112	NM_00100225	CAPRIN2	caprin family member 2	-0.146	0.903	2.37E-01	2.37E+01			
7962146	NM_00113581	FAM60A	family with sequence similarity 60	-0.288	0.819	6.20E-02	6.20E+00			
7962151	NM_144973	DENND5B	DENN/MADD domain containing 5	0.221	1.166	1.63E-01	1.63E+01			
7962185	NR_004854	AMN1	antagonist of mitotic exit network	-0.048	0.967	8.33E-01	8.33E+01			
7962194	NM_00101369	H3F3C	H3 histone, family 3C	-0.026	0.982	9.30E-01	9.30E+01			
7962197	---	---	---	-0.192	0.875	7.27E-01	7.27E+01			
7962212	NM_004572	PKP2	plakophilin 2	0.043	1.030	7.25E-01	7.25E+01			
7962226	NM_198992	SYT10	synaptotagmin X	-0.008	0.995	9.65E-01	9.65E+01			
7962238	---	---	---	-0.021	0.985	8.49E-01	8.49E+01			
7962240	---	---	---	-0.029	0.980	8.67E-01	8.67E+01			
7962312	NM_005164	ABCD2	ATP-binding cassette, sub-family D	0.201	1.150	3.72E-01	3.72E+01			
7962327	NM_052885	SLC2A13	solute carrier family 2 (facilitated g	0.023	1.016	9.12E-01	9.12E+01			
7962349	NM_173601	GLT8D3	glycosyltransferase 8 domain cont	-0.028	0.981	9.11E-01	9.11E+01			
7962358	NM_005748	YAF2	YY1 associated factor 2	-0.019	0.987	9.44E-01	9.44E+01			
7962367	NM_033114	ZCRB1	zinc finger CCHC-type and RNA bin	0.079	1.056	6.20E-01	6.20E+01			
7962375	NM_153026	PRICKLE1	prickle homolog 1 (Drosophila)	0.144	1.105	2.37E-01	2.37E+01			
7962441	NM_002822	TWF1	twinfilin, actin-binding protein, ho	0.028	1.019	8.87E-01	8.87E+01			
7962455	NM_006159	NELL2	NEL-like 2 (chicken)	-0.274	0.827	4.03E-01	4.03E+01			
7962489	NM_015899	PLEKHA9	pleckstrin homology domain conta	0.019	1.013	9.55E-01	9.55E+01			
7962499	NM_004719	SFRS2IP	splicing factor, arginine/serine-rich	0.064	1.046	5.11E-01	5.11E+01			
7962516	NM_030674	SLC38A1	solute carrier family 38, member 1	-0.149	0.902	1.17E-01	1.17E+01			
7962537	NM_018976	SLC38A2	solute carrier family 38, member 2	-0.058	0.961	6.23E-01	6.23E+01			
7962557	---	---	---	0.069	1.049	8.10E-01	8.10E+01			
7962579	NM_00114366	AMIGO2	adhesion molecule with Ig-like dor	0.014	1.010	9.49E-01	9.49E+01			
7962590	NM_024604	RPAP3	RNA polymerase II associated prot	0.110	1.079	3.67E-01	3.67E+01			
7962623	NM_00109853	RAPGEF3	Rap guanine nucleotide exchange	0.026	1.018	8.88E-01	8.88E+01			

7962625	NM_00109853	RAPGEF3	Rap guanine nucleotide exchange	-0.070	0.952	3.51E-01	3.51E+01		
7962659	NM_015401	HDAC7	histone deacetylase 7	-0.004	0.997	9.82E-01	9.82E+01		
7962689	NM_00101753	VDR	vitamin D (1,25- dihydroxyvitamin	-0.005	0.996	9.87E-01	9.87E+01		
7962703	NM_001844	COL2A1	collagen, type II, alpha 1	0.040	1.028	5.98E-01	5.98E+01		
7962760	NM_014554	SENP1	SUMO1/sentrin specific peptidase	-0.102	0.932	4.44E-01	4.44E+01		
7962783	NM_024095	ASB8	ankyrin repeat and SOCS box-cont	0.012	1.008	9.63E-01	9.63E+01		
7962792	NM_00100413	OR10AD1	olfactory receptor, family 10, subf	0.099	1.071	7.53E-01	7.53E+01		
7962794	NM_152320	ZNF641	zinc finger protein 641	0.119	1.086	2.92E-01	2.92E+01		
7962811	NM_017822	C12orf41	chromosome 12 open reading fran	-0.069	0.953	6.39E-01	6.39E+01		
7962831	NM_001240	CCNT1	cyclin T1	-0.009	0.994	9.76E-01	9.76E+01		
7962842	NM_015270	ADCY6	adenylate cyclase 6	-0.054	0.963	6.10E-01	6.10E+01		
7962869	NM_004818	DDX23	DEAD (Asp-Glu-Ala-Asp) box polyp	0.064	1.046	6.01E-01	6.01E+01		
7962884	NM_014470	RND1	Rho family GTPase 1	-0.167	0.891	3.72E-01	3.72E+01		
7962893	---	---	---	0.027	1.019	9.29E-01	9.29E+01		
7962895	NM_016594	FKBP11	FK506 binding protein 11, 19 kDa	0.043	1.030	8.02E-01	8.02E+01		
7962904	NM_001659	ARF3	ADP-ribosylation factor 3	0.012	1.008	9.67E-01	9.67E+01		
7962918	NM_003394	WNT10B	wingless-type MMTV integration s	-0.085	0.943	4.32E-01	4.32E+01		
7962930	NM_015086	DDN	dendrin	-0.026	0.982	8.05E-01	8.05E+01		
7962935	NM_212461	PRKAG1	protein kinase, AMP-activated, ga	0.048	1.034	7.98E-01	7.98E+01		
7962951	NM_003482	MLL2	myeloid/lymphoid or mixed-lineag	-0.019	0.987	8.66E-01	8.66E+01		
7963020	NM_021044	DHH	desert hedgehog homolog (Drosop	0.029	1.020	8.31E-01	8.31E+01		
7963024	NM_018113	LMBR1L	limb region 1 homolog (mouse)-lik	-0.021	0.986	8.93E-01	8.93E+01		
7963046	NM_006082	TUBA1B	tubulin, alpha 1b	0.073	1.052	7.40E-01	7.40E+01		
7963052	---	---	---	0.019	1.013	9.16E-01	9.16E+01		
7963054	NM_006009	TUBA1A	tubulin, alpha 1a	-0.011	0.993	9.73E-01	9.73E+01		
7963061	NM_00100822	C1QL4	complement component 1, q subc	-0.039	0.974	7.51E-01	7.51E+01		
7963064	NM_006337	MCRS1	microspherule protein 1	0.010	1.007	9.53E-01	9.53E+01		
7963092	NM_175736	FMNL3	formin-like 3	-0.141	0.907	2.56E-01	2.56E+01		
7963121	NM_00103780	NCKAP5L	NCK-associated protein 5-like	-0.061	0.959	4.78E-01	4.78E+01		
7963134	NM_00103780	NCKAP5L	NCK-associated protein 5-like	0.024	1.017	9.16E-01	9.16E+01		
7963137	---	---	---	-0.224	0.856	3.92E-01	3.92E+01		
7963139	NM_181708	BCDIN3D	BCDIN3 domain containing	0.027	1.019	8.78E-01	8.78E+01		
7963142	NM_012306	FAIM2	Fas apoptotic inhibitory molecule	-0.028	0.981	8.13E-01	8.13E+01		
7963157	NM_013277	RACGAP1	Rac GTPase activating protein 1	0.114	1.082	2.99E-01	2.99E+01		
7963174	NM_147190	LASS5	LAG1 homolog, ceramide synthase	0.002	1.002	9.92E-01	9.92E+01		
7963187	NM_00111354	LIMA1	LIM domain and actin binding 1	0.094	1.067	4.05E-01	4.05E+01		
7963212	NM_000617	SLC11A2	solute carrier family 11 (proton-co	0.058	1.041	8.46E-01	8.46E+01		
7963235	NM_030809	CSRNP2	cysteine-serine-rich nuclear protei	-0.009	0.994	9.73E-01	9.73E+01		
7963244	NM_005653	TFCP2	transcription factor CP2	-0.029	0.980	8.96E-01	8.96E+01		
7963261	AK292677	LOC494150	prohibitin pseudogene	0.117	1.084	5.35E-01	5.35E+01		
7963263	---	---	---	0.016	1.011	9.64E-01	9.64E+01		
7963265	NR_026893	POU6F1	POU class 6 homeobox 1	-0.033	0.977	7.11E-01	7.11E+01		
7963280	NM_00103387	SMAGP	small trans-membrane and glycosy	-0.030	0.980	8.38E-01	8.38E+01		
7963289	NM_016293	BIN2	bridging integrator 2	0.243	1.183	7.00E-02	7.00E+00		
7963304	NM_001971	CELA1	chymotrypsin-like elastase family,	0.089	1.063	3.29E-01	3.29E+01		
7963313	NM_007210	GALNT6	UDP-N-acetyl-alpha-D-galactosam	-0.140	0.908	1.01E-01	1.01E+01		
7963328	NM_00101369	FIGNL2	fidgetin-like 2	-0.162	0.894	2.78E-01	2.78E+01		
7963353	NM_002281	KRT81	keratin 81	-0.050	0.966	8.13E-01	8.13E+01		
7963359	NM_002282	KRT83	keratin 83	-0.062	0.958	5.93E-01	5.93E+01		
7963366	NM_002283	KRT85	keratin 85	-0.017	0.988	9.06E-01	9.06E+01		
7963375	NM_033045	KRT84	keratin 84	0.022	1.015	9.00E-01	9.00E+01		
7963406	NM_005555	KRT6B	keratin 6B	-0.182	0.881	2.88E-01	2.88E+01		
7963410	NM_173086	KRT6C	keratin 6C	-0.041	0.972	7.14E-01	7.14E+01		
7963421	NM_005554	KRT6A	keratin 6A	0.013	1.009	9.75E-01	9.75E+01		
7963438	NM_033448	KRT71	keratin 71	-0.067	0.955	6.39E-01	6.39E+01		
7963459	NM_080747	KRT72	keratin 72	0.114	1.082	4.02E-01	4.02E+01		
7963502	NM_175078	KRT77	keratin 77	0.006	1.004	9.74E-01	9.74E+01		
7963513	NM_015848	KRT76	keratin 76	-0.014	0.990	9.53E-01	9.53E+01		
7963534	NM_002272	KRT4	keratin 4	-0.052	0.965	5.81E-01	5.81E+01		
7963545	NM_175834	KRT79	keratin 79	-0.073	0.950	4.32E-01	4.32E+01		
7963555	NM_173352	KRT78	keratin 78	-0.024	0.984	8.56E-01	8.56E+01		
7963567	NM_002273	KRT8	keratin 8	-0.019	0.987	9.27E-01	9.27E+01		
7963575	NM_001417	EIF4B	eukaryotic translation initiation fa	0.050	1.035	7.93E-01	7.93E+01		
7963577	NM_032840	SPRYD3	SPRY domain containing 3	-0.019	0.987	9.24E-01	9.24E+01		
7963590	NM_015989	CSAD	cysteine sulfinic acid decarboxylas	0.031	1.022	8.31E-01	8.31E+01		
7963614	NM_000889	ITGB7	integrin, beta 7	0.093	1.067	3.39E-01	3.39E+01		
7963631	NM_000966	RARG	retinoic acid receptor, gamma	-0.067	0.955	5.87E-01	5.87E+01		
7963646	NM_015665	AAAS	achalasia, adrenocortical insufficie	-0.059	0.960	7.31E-01	7.31E+01		
7963664	NM_152860	SP7	Sp7 transcription factor	-0.011	0.992	9.53E-01	9.53E+01		
7963670	NM_006301	MAP3K12	mitogen-activated protein kinase k	-0.014	0.990	9.24E-01	9.24E+01		
7963689	NM_003717	NPFF	neuropeptide FF-amide peptide pr	0.067	1.048	7.93E-01	7.93E+01		
7963698	NM_00113005	ATF7	activating transcription factor 7	0.036	1.025	8.52E-01	8.52E+01		
7963713	NM_005176	ATP5G2	ATP synthase, H+ transporting, mi	0.037	1.026	7.36E-01	7.36E+01		
7963721	NM_020898	CALCOCO1	calcium binding and coiled-coil do	-0.083	0.944	4.11E-01	4.11E+01		
7963741	NM_014311	SMUG1	single-strand-selective monofunct	0.046	1.032	7.90E-01	7.90E+01		
7963750	NM_012117	CBX5	chromobox homolog 5 (HP1 alpha	-0.025	0.983	8.80E-01	8.80E+01		
7963774	NM_015481	ZNF385A	zinc finger protein 385A	0.102	1.073	5.10E-01	5.10E+01		

7963786	NM_002205	ITGA5	integrin, alpha 5 (fibronectin recep	-0.194	0.874	1.02E-01	1.02E+01		
7963817	NM_144594	GTSF1	gametocyte specific factor 1	-0.160	0.895	6.93E-01	6.93E+01		
7963826	NM_006741	PPP1R1A	protein phosphatase 1, regulatory	-0.082	0.945	3.57E-01	3.57E+01		
7963845	NM_053283	DCD	dermcidin	0.015	1.011	9.01E-01	9.01E+01		
7963851	NM_00109881	KIAA0748	KIAA0748	0.050	1.035	8.64E-01	8.64E+01		
7963869	NM_004426	PHC1	polyhomeotic homolog 1 (Drosopł	0.003	1.002	9.86E-01	9.86E+01		
7963880	NM_00114499	ITGA7	integrin, alpha 7	-0.010	0.993	9.64E-01	9.64E+01		
7963911	NM_001780	CD63	CD63 molecule	0.166	1.122	1.88E-01	1.88E+01		
7963923	NR_026723	SARNP	SAP domain containing ribonucleo	0.053	1.037	6.78E-01	6.78E+01		
7963935	NM_032364	DNAJC14	DnaJ (Hsp40) homolog, subfamily	-0.014	0.991	9.45E-01	9.45E+01		
7963946	NM_002429	MMP19	matrix metallopeptidase 19	-0.099	0.934	8.52E-01	8.52E+01		
7963965	NM_032345	WIBG	within bgcn homolog (Drosophila)	-0.010	0.993	9.61E-01	9.61E+01		
7963970	NM_006928	SILV	silver homolog (mouse)	0.098	1.071	4.10E-01	4.10E+01		
7963988	NM_003075	SMARCC2	SWI/SNF related, matrix associate	-0.082	0.945	2.26E-01	2.26E+01		
7964021	NM_194358	RNF41	ring finger protein 41	0.031	1.022	8.92E-01	8.92E+01		
7964033	NM_173595	ANKRD52	ankyrin repeat domain 52	-0.079	0.947	4.97E-01	4.97E+01		
7964064	NM_004077	CS	citrate synthase	0.025	1.017	8.56E-01	8.56E+01		
7964076	NM_014255	CNPY2	canopy 2 homolog (zebrafish)	-0.022	0.985	8.92E-01	8.92E+01		
7964089	NM_014871	PAN2	PAN2 poly(A) specific ribonuclease	0.103	1.074	3.33E-01	3.33E+01		
7964119	NM_005419	STAT2	signal transducer and activator of	0.365	1.288	6.29E-02	6.29E+00		
7964145	NM_003920	TIMELESS	timeless homolog (Drosophila)	0.059	1.042	7.03E-01	7.03E+01		
7964177	NM_012064	MIP	major intrinsic protein of lens fibe	-0.024	0.984	9.13E-01	9.13E+01		
7964183	NM_013267	GLS2	glutaminase 2 (liver, mitochondria	0.101	1.072	4.54E-01	4.54E+01		
7964203	NM_013449	BAZ2A	bromodomain adjacent to zinc fing	0.016	1.011	9.01E-01	9.01E+01		
7964234	NM_001686	ATP5B	ATP synthase, H+ transporting, mi	0.002	1.002	9.90E-01	9.90E+01		
7964246	NR_003046	SNORD59B	small nucleolar RNA, C/D box 59B	0.066	1.047	8.84E-01	8.84E+01		
7964250	NM_006601	PTGES3	prostaglandin E synthase 3 (cytoso	0.040	1.028	7.13E-01	7.13E+01		
7964260	---	---	---	0.057	1.040	8.06E-01	8.06E+01		
7964262	NM_00111320	NACA	nascent polypeptide-associated co	0.015	1.011	8.85E-01	8.85E+01		
7964271	NM_000946	PRIM1	primase, DNA, polypeptide 1 (49kD	0.044	1.031	8.74E-01	8.74E+01		
7964300	NM_014830	ZBTB39	zinc finger and BTB domain contai	-0.001	0.999	9.97E-01	9.97E+01		
7964360	NM_003153	STAT6	signal transducer and activator of	0.040	1.028	7.94E-01	7.94E+01		
7964388	NM_020142	NDUFA4L2	NADH dehydrogenase (ubiquinone	-0.062	0.958	5.22E-01	5.22E+01		
7964400	NM_145064	STAC3	SH3 and cysteine rich domain 3	-0.025	0.983	8.43E-01	8.43E+01		
7964413	NM_014925	R3HDM2	R3H domain containing 2	0.048	1.034	5.75E-01	5.75E+01		
7964436	NM_032496	ARHGAP9	Rho GTPase activating protein 9	0.012	1.008	9.58E-01	9.58E+01		
7964460	NM_004083	DDIT3	DNA-damage-inducible transcript	-0.043	0.970	8.41E-01	8.41E+01		
7964466	NM_006400	DCTN2	dynactin 2 (p50)	-0.093	0.938	5.43E-01	5.43E+01		
7964484	NM_001478	B4GALNT1	beta-1,4-N-acetyl-galactosaminyl t	-0.027	0.981	8.44E-01	8.44E+01		
7964499	NM_00112277	AGAP2	ArfGAP with GTPase domain, anky	0.034	1.024	8.28E-01	8.28E+01		
7964522	NM_000075	CDK4	cyclin-dependent kinase 4	0.009	1.006	9.77E-01	9.77E+01		
7964535	NM_000785	CYP27B1	cytochrome P450, family 27, subfa	0.064	1.045	6.52E-01	6.52E+01		
7964555	NM_006576	AVIL	advillin	-0.175	0.886	4.13E-02	4.13E+00		
7964575	AK022448	AVIL	advillin	0.107	1.077	4.02E-01	4.02E+01		
7964577	---	---	---	0.020	1.014	9.53E-01	9.53E+01		
7964579	NM_005730	CTDSP2	CTD (carboxy-terminal domain, RN	0.039	1.028	8.49E-01	8.49E+01		
7964640	---	---	---	0.329	1.256	1.11E-01	1.11E+01		
7964642	BC136288	C12orf61	chromosome 12 open reading fran	-0.080	0.946	3.17E-01	3.17E+01		
7964646	NM_020700	PPM1H	protein phosphatase 1H (PP2C dor	-0.064	0.957	5.15E-01	5.15E+01		
7964658	---	---	---	-0.285	0.821	9.40E-03	9.40E-01		
7964677	NM_152440	C12orf66	chromosome 12 open reading fran	0.026	1.018	8.54E-01	8.54E+01		
7964701	NM_002076	GNS	glucosamine (N-acetyl)-6-sulfatase	0.426	1.343	1.29E-01	1.29E+01		
7964718	---	---	---	-0.338	0.791	1.82E-01	1.82E+01		
7964739	NM_032338	LLPH	LLP homolog, long-term synaptic f	0.041	1.029	7.30E-01	7.30E+01		
7964745	NM_016056	TMBIM4	transmembrane BAX inhibitor mot	0.083	1.059	5.23E-01	5.23E+01		
7964757	NM_016836	RBMS1	RNA binding motif, single strandec	0.097	1.070	7.85E-01	7.85E+01		
7964759	NM_021150	GRIP1	glutamate receptor interacting pro	0.027	1.019	8.82E-01	8.82E+01		
7964810	NM_017440	MDM1	Mdm1 nuclear protein homolog (r	0.028	1.020	8.45E-01	8.45E+01		
7964828	---	---	---	-0.021	0.985	9.39E-01	9.39E+01		
7964830	---	---	---	0.040	1.028	9.04E-01	9.04E+01		
7964832	---	---	---	0.073	1.052	6.05E-01	6.05E+01		
7964868	ENST00000451	LOC100131613	PRO1454	0.156	1.114	3.79E-01	3.79E+01		
7964937	NM_144982	ZFC3H1	zinc finger, C3H1-type containing	0.007	1.005	9.67E-01	9.67E+01		
7964976	NR_026837	LOC283392	hypothetical LOC283392	-0.046	0.969	6.58E-01	6.58E+01		
7965022	NM_007043	KRR1	KRR1, small subunit (SSU) process	0.030	1.021	8.62E-01	8.62E+01		
7965038	---	---	---	0.030	1.021	8.52E-01	8.52E+01		
7965040	NM_007350	PHLDA1	pleckstrin homology-like domain,	-0.064	0.957	7.20E-01	7.20E+01		
7965048	NM_004537	NAP1L1	nucleosome assembly protein 1-lik	-0.021	0.986	8.89E-01	8.89E+01		
7965064	NM_020841	OSBPL8	oxysterol binding protein-like 8	-0.255	0.838	1.03E-02	1.03E+00		
7965090	NM_001321	CSRP2	cysteine and glycine-rich protein 2	-0.122	0.919	2.94E-01	2.94E+01		
7965094	NM_203394	E2F7	E2F transcription factor 7	0.015	1.010	9.21E-01	9.21E+01		
7965123	NM_00114388	PPP1R12A	protein phosphatase 1, regulatory	0.022	1.015	8.92E-01	8.92E+01		
7965152	---	---	---	-0.026	0.982	9.09E-01	9.09E+01		
7965200	NM_014167	CCDC59	coiled-coil domain containing 59	-0.105	0.930	3.04E-01	3.04E+01		
7965335	NM_001946	DUSP6	dual specificity phosphatase 6	0.369	1.291	1.76E-01	1.76E+01		
7965343	NM_172240	WDR51B	WD repeat domain 51B	-0.044	0.970	8.29E-01	8.29E+01		
7965357	NM_003774	GALNT4	UDP-N-acetyl-alpha-D-galactosam	0.032	1.023	9.19E-01	9.19E+01		

7965359	NM_00100132	ATP2B1	ATPase, Ca++ transporting, plasma	-0.018	0.988	9.55E-01	9.55E+01		
7965423	NM_001731	BTG1	B-cell translocation gene 1, anti-pr	-0.093	0.937	4.43E-01	4.43E+01		
7965429	---	---	---	0.028	1.020	9.72E-01	9.72E+01		
7965436	NM_003566	EEA1	early endosome antigen 1	0.225	1.169	1.98E-02	1.98E+00		
7965467	ENST00000358	RPL41	ribosomal protein L41	-0.010	0.993	9.35E-01	9.35E+01		
7965471	NM_003348	UBE2N	ubiquitin-conjugating enzyme E2N	0.083	1.059	4.77E-01	4.77E+01		
7965478	---	---	---	0.027	1.019	9.22E-01	9.22E+01		
7965480	NM_002629	PGAM1	phosphoglycerate mutase 1 (brain	-0.010	0.993	9.67E-01	9.67E+01		
7965484	---	---	---	-0.086	0.942	4.96E-01	4.96E+01		
7965508	---	---	---	-0.045	0.969	9.19E-01	9.19E+01		
7965510	NM_020698	TMCC3	transmembrane and coiled-coil do	-0.172	0.888	5.48E-02	5.48E+00		
7965515	NM_018838	NDUFA12	NADH dehydrogenase (ubiquinone	-0.043	0.971	8.92E-01	8.92E+01		
7965573	NM_021229	NTN4	netrin 4	-0.079	0.947	4.98E-01	4.98E+01		
7965652	NM_002595	PCTK2	PCTAIRE protein kinase 2	-0.140	0.908	1.59E-01	1.59E+01		
7965673	---	---	---	-0.058	0.961	5.52E-01	5.52E+01		
7965675	---	---	---	-0.097	0.935	5.93E-01	5.93E+01		
7965723	NM_015054	UHRF1BP1L	UHRF1 binding protein 1-like	-0.058	0.961	7.18E-01	7.18E+01		
7965789	NM_001177	ARL1	ADP-ribosylation factor-like 1	0.093	1.067	3.95E-01	3.95E+01		
7965812	NM_024312	GNPTAB	N-acetylglucosamine-1-phosphate	0.079	1.056	7.45E-01	7.45E+01		
7965842	---	---	---	0.115	1.083	3.79E-01	3.79E+01		
7965846	NM_016053	CCDC53	coiled-coil domain containing 53	0.056	1.040	8.40E-01	8.40E+01		
7965855	NM_024057	NUP37	nucleoporin 37kDa	0.097	1.069	6.14E-01	6.14E+01		
7965956	NM_006166	NFYB	nuclear transcription factor Y, beta	0.022	1.015	9.18E-01	9.18E+01		
7965976	---	---	---	-0.010	0.993	9.58E-01	9.58E+01		
7966003	NM_018171	APPL2	adaptor protein, phosphotyrosine	0.023	1.016	8.87E-01	8.87E+01		
7966035	NM_006825	CKAP4	cytoskeleton-associated protein 4	-0.235	0.850	5.87E-03	5.87E-01		
7966046	NM_00103305	MTERFD3	MTERF domain containing 3	0.058	1.041	7.57E-01	7.57E+01		
7966052	NM_004075	CRY1	cryptochrome 1 (photolyase-like)	-0.117	0.922	5.84E-01	5.84E+01		
7966072	NM_012406	PRDM4	PR domain containing 4	0.010	1.007	9.57E-01	9.57E+01		
7966098	NM_014706	SART3	squamous cell carcinoma antigen	-0.053	0.964	6.94E-01	6.94E+01		
7966122	NM_181724	TMEM119	transmembrane protein 119	-0.062	0.958	5.52E-01	5.52E+01		
7966127	NM_003006	SELPLG	selectin P ligand	0.140	1.102	3.04E-01	3.04E+01		
7966135	NM_014325	CORO1C	coronin, actin binding protein, 1C	0.402	1.321	2.37E-01	2.37E+01		
7966150	NM_00116133	SSH1	slingshot homolog 1 (Drosophila)	-0.210	0.865	3.80E-02	3.80E+00		
7966172	NM_018711	SVOP	SV2 related protein homolog (rat)	-0.013	0.991	9.51E-01	9.51E+01		
7966183	NM_00114537	ALKBH2	alkB, alkylation repair homolog 2 (	-0.136	0.910	3.32E-01	3.32E+01		
7966189	NM_213596	FOXN4	forkhead box N4	-0.062	0.958	3.46E-01	3.46E+01		
7966202	NM_031954	KCTD10	potassium channel tetramerisation	0.035	1.025	8.48E-01	8.48E+01		
7966213	NM_052845	MMAB	methylmalonic aciduria (cobalami	-0.140	0.908	1.22E-01	1.22E+01		
7966223	---	---	---	-0.217	0.860	5.48E-01	5.48E+01		
7966234	NM_021625	TRPV4	transient receptor potential cation	-0.052	0.964	4.81E-01	4.81E+01		
7966259	NM_016433	GLTP	glycolipid transfer protein	-0.092	0.938	5.59E-01	5.59E+01		
7966266	---	---	---	0.050	1.035	8.55E-01	8.55E+01		
7966268	NM_057169	GIT2	G protein-coupled receptor kinase	0.087	1.062	3.38E-01	3.38E+01		
7966293	BC133010	C12orf76	chromosome 12 open reading fran	0.064	1.045	6.51E-01	6.51E+01		
7966299	---	---	---	-0.112	0.925	4.42E-01	4.42E+01		
7966301	NM_016238	ANAPC7	anaphase promoting complex subu	0.007	1.005	9.76E-01	9.76E+01		
7966315	NM_005719	ARPC3	actin related protein 2/3 complex,	0.266	1.203	1.20E-02	1.20E+00		
7966321	NM_016301	GPN3	GPN-loop GTPase 3	0.090	1.064	6.42E-01	6.42E+01		
7966332	NM_016226	VPS29	vacuolar protein sorting 29 homol	0.075	1.053	5.63E-01	5.63E+01		
7966345	NM_139283	PPTC7	PTC7 protein phosphatase homolo	0.006	1.004	9.77E-01	9.77E+01		
7966356	NM_00104010	HVCN1	hydrogen voltage-gated channel 1	0.265	1.202	1.84E-03	1.84E-01		
7966368	NM_002710	PPP1CC	protein phosphatase 1, catalytic su	0.133	1.097	1.94E-03	1.94E-01		
7966389	NM_144671	FAM109A	family with sequence similarity 10	-0.032	0.978	7.71E-01	7.71E+01		
7966397	NM_002973	ATXN2	ataxin 2	0.019	1.013	9.00E-01	9.00E+01		
7966427	NM_006768	BRAP	BRCA1 associated protein	0.026	1.018	8.06E-01	8.06E+01		
7966441	NR_015404	C12orf47	chromosome 12 open reading fran	0.020	1.014	9.55E-01	9.55E+01		
7966448	NM_138341	TMEM116	transmembrane protein 116	0.163	1.119	3.70E-01	3.70E+01		
7966462	NM_024953	C12orf30	chromosome 12 open reading fran	0.062	1.044	6.70E-01	6.70E+01		
7966488	NM_00110966	C12orf51	chromosome 12 open reading fran	-0.031	0.979	7.78E-01	7.78E+01		
7966517	NM_00110966	C12orf51	chromosome 12 open reading fran	-0.070	0.953	5.60E-01	5.60E+01		
7966534	NM_00102466	RPL6	ribosomal protein L6	-0.009	0.994	9.44E-01	9.44E+01		
7966570	NM_00111132	DDX54	DEAD (Asp-Glu-Ala-Asp) box polyp	-0.031	0.979	8.33E-01	8.33E+01		
7966596	NM_138451	IQCD	IQ motif containing D	-0.017	0.988	9.37E-01	9.37E+01		
7966600	NM_024959	SLC24A6	solute carrier family 24 (sodium/p	0.078	1.056	6.39E-01	6.39E+01		
7966631	NM_022363	LHX5	LIM homeobox 5	-0.056	0.962	5.85E-01	5.85E+01		
7966638	NM_016196	RBM19	RNA binding motif protein 19	-0.028	0.981	8.32E-01	8.32E+01		
7966666	---	---	---	-0.062	0.958	6.89E-01	6.89E+01		
7966690	NM_016569	TBX3	T-box 3	-0.085	0.943	3.74E-01	3.74E+01		
7966706	NM_015335	MED13L	mediator complex subunit 13-like	0.024	1.017	8.72E-01	8.72E+01		
7966738	AK298857	C12orf49	chromosome 12 open reading fran	0.060	1.042	5.60E-01	5.60E+01		
7966746	NM_003806	HRK	harakiri, BCL2 interacting protein (	-0.094	0.937	3.85E-01	3.85E+01		
7966749	NM_017899	TESC	tescalcin	0.029	1.020	9.00E-01	9.00E+01		
7966760	NM_033624	FBXO21	F-box protein 21	-0.027	0.982	8.68E-01	8.68E+01		
7966810	NM_173598	KSR2	kinase suppressor of ras 2	0.050	1.036	6.80E-01	6.80E+01		
7966829	NM_018639	WSB2	WD repeat and SOCS box-containi	0.155	1.113	3.46E-01	3.46E+01		
7966839	NM_019086	FLJ20674	hypothetical protein FLJ20674	-0.048	0.967	6.57E-01	6.57E+01		

7966851	NM_016281	TAOK3	TAO kinase 3	0.097	1.069	3.60E-01	3.60E+01		
7966876	---	---	---	-0.019	0.987	9.28E-01	9.28E+01		
7966929	NM_006861	RAB35	RAB35, member RAS oncogene far	0.055	1.039	8.38E-01	8.38E+01		
7966938	NM_006836	GCN1L1	GCN1 general control of amino-ac	-0.045	0.969	6.69E-01	6.69E+01		
7966996	NM_053275	RPLP0	ribosomal protein, large, P0	-0.132	0.912	2.78E-01	2.78E+01		
7967002	NM_00108085	PXN	paxillin	-0.052	0.965	4.24E-01	4.24E+01		
7967021	BC052611	PXN	paxillin	-0.061	0.959	6.23E-01	6.23E+01		
7967025	---	---	---	-0.031	0.979	8.92E-01	8.92E+01		
7967030	NR_003925	RNU4-1	RNA, U4 small nuclear 1	0.067	1.047	8.70E-01	8.70E+01		
7967032	---	---	---	0.062	1.044	6.04E-01	6.04E+01		
7967039	NM_002442	MSI1	musashi homolog 1 (Drosophila)	0.029	1.020	7.84E-01	7.84E+01		
7967056	NM_016399	TRIAP1	TP53 regulated inhibitor of apopto	-0.010	0.993	9.44E-01	9.44E+01		
7967060	NM_003769	SFRS9	splicing factor, arginine/serine-rich	0.048	1.034	5.68E-01	5.68E+01		
7967067	NM_00103749	DYNLL1	dynein, light chain, LC8-type 1	0.074	1.053	6.62E-01	6.62E+01		
7967072	NM_032314	COQ5	coenzyme Q5 homolog, methyltra	0.035	1.025	9.18E-01	9.18E+01		
7967084	NM_015918	POP5	processing of precursor 5, ribonuc	-0.007	0.995	9.83E-01	9.83E+01		
7967089	---	---	---	0.029	1.020	9.19E-01	9.19E+01		
7967091	NM_139015	UNQ1887	signal peptide peptidase 3	0.084	1.060	4.41E-01	4.41E+01		
7967109	BC014661	C12orf43	chromosome 12 open reading frar	-0.033	0.978	8.63E-01	8.63E+01		
7967117	NM_003733	OASL	2'-5'-oligoadenylate synthetase-lik	-0.187	0.878	3.70E-01	3.70E+01		
7967127	NM_006549	CAMKK2	calcium/calmodulin-dependent pr	0.143	1.104	1.75E-01	1.75E+01		
7967149	NM_016237	ANAPC5	anaphase promoting complex sub	0.129	1.094	3.55E-01	3.55E+01		
7967175	NM_032590	KDM2B	lysine (K)-specific demethylase 2B	0.000	1.000	9.98E-01	9.98E+01		
7967193	NM_173855	MORN3	MORN repeat containing 3	0.035	1.024	8.58E-01	8.58E+01		
7967202	NM_019034	RHOF	ras homolog gene family, member	-0.059	0.960	6.05E-01	6.05E+01		
7967210	NR_002809	LOC338799	hypothetical LOC338799	-0.037	0.975	8.55E-01	8.55E+01		
7967230	NM_019887	DIABLO	diablo homolog (Drosophila)	-0.005	0.997	9.85E-01	9.85E+01		
7967240	NM_022916	VPS33A	vacuolar protein sorting 33 homol	0.019	1.013	9.33E-01	9.33E+01		
7967255	NM_002956	CLIP1	CAP-GLY domain containing linker	-0.065	0.956	6.46E-01	6.46E+01		
7967287	NM_017612	ZCCHC8	zinc finger, CCHC domain containir	-0.024	0.983	9.11E-01	9.11E+01		
7967304	NM_198261	RSRC2	arginine/serine-rich coiled-coil 2	-0.059	0.960	6.39E-01	6.39E+01		
7967318	NM_177551	NIACR1	niacin receptor 1	-0.098	0.934	8.31E-01	8.31E+01		
7967331	NM_024667	VPS37B	vacuolar protein sorting 37 homol	-0.035	0.976	8.40E-01	8.40E+01		
7967358	NM_020845	PITPNM2	phosphatidylinositol transfer prote	0.065	1.046	6.00E-01	6.00E+01		
7967386	NM_022782	MPHOSPH9	M-phase phosphoprotein 9	0.076	1.054	6.60E-01	6.60E+01		
7967412	NM_004642	CDK2AP1	cyclin-dependent kinase 2 associat	-0.071	0.952	4.44E-01	4.44E+01		
7967420	NM_018183	SBNO1	strawberry notch homolog 1 (Dros	0.023	1.016	8.60E-01	8.60E+01		
7967452	---	---	---	0.022	1.015	9.71E-01	9.71E+01		
7967456	NM_145058	RILPL2	Rab interacting lysosomal protein-	-0.279	0.824	7.11E-02	7.11E+00		
7967463	NM_178314	RILPL1	Rab interacting lysosomal protein-	-0.044	0.970	8.23E-01	8.23E+01		
7967473	NM_001414	EIF2B1	eukaryotic translation initiation fa	0.076	1.054	7.36E-01	7.36E+01		
7967486	NM_025140	CCDC92	coiled-coil domain containing 92	-0.063	0.957	7.52E-01	7.52E+01		
7967493	NM_006312	NCOR2	nuclear receptor co-repressor 2	-0.033	0.977	7.09E-01	7.09E+01		
7967544	NM_005505	SCARB1	scavenger receptor class B, memb	0.032	1.022	8.01E-01	8.01E+01		
7967563	NM_021009	UBC	ubiquitin C	-0.029	0.980	8.02E-01	8.02E+01		
7967586	---	---	---	-0.098	0.934	3.94E-01	3.94E+01		
7967588	NM_032656	DHX37	DEAH (Asp-Glu-Ala-His) box polype	0.024	1.016	8.55E-01	8.55E+01		
7967620	---	---	---	0.019	1.013	9.27E-01	9.27E+01		
7967622	---	---	---	-0.010	0.993	9.76E-01	9.76E+01		
7967624	NM_145648	SLC15A4	solute carrier family 15, member 4	0.031	1.022	9.16E-01	9.16E+01		
7967636	NR_026666	MGC16384	hypothetical LOC114130	-0.061	0.959	8.97E-01	8.97E+01		
7967641	NM_133448	TMEM132D	transmembrane protein 132D	-0.057	0.961	5.64E-01	5.64E+01		
7967660	NM_015347	RIMBP2	RIMS binding protein 2	0.009	1.006	9.63E-01	9.63E+01		
7967685	NM_194356	STX2	syntaxin 2	-0.064	0.957	7.27E-01	7.27E+01		
7967698	---	---	---	0.040	1.028	9.09E-01	9.09E+01		
7967700	ENST00000415	LOC100128840	hypothetical protein LOC10012884	-0.036	0.975	7.94E-01	7.94E+01		
7967709	---	---	---	-0.020	0.986	9.16E-01	9.16E+01		
7967711	NM_175066	DDX51	DEAD (Asp-Glu-Ala-Asp) box polyp	-0.059	0.960	5.15E-01	5.15E+01		
7967727	NM_021808	GALNT9	UDP-N-acetyl-alpha-D-galactosam	-0.060	0.959	5.56E-01	5.56E+01		
7967736	NM_006231	POLE	polymerase (DNA directed), epsilo	0.033	1.023	7.68E-01	7.68E+01		
7967789	NM_018663	PXMP2	peroxisomal membrane protein 2,	-0.013	0.991	9.44E-01	9.44E+01		
7967792	---	---	---	0.030	1.021	9.30E-01	9.30E+01		
7967794	NM_015114	ANKLE2	ankyrin repeat and LEM domain co	-0.138	0.909	8.58E-02	8.58E+00		
7967810	NM_005895	GOLGA3	golgi autoantigen, golgin subfamily	-0.029	0.980	7.58E-01	7.58E+01		
7967841	NM_00116134	CHFR	checkpoint with forkhead and ring	0.035	1.024	8.31E-01	8.31E+01		
7967870	---	---	---	-0.040	0.973	8.93E-01	8.93E+01		
7967879	NM_152274	FAM58A	family with sequence similarity 58	0.014	1.010	9.53E-01	9.53E+01		
7967881	NM_017520	MPHOSPH8	M-phase phosphoprotein 8	0.055	1.039	6.58E-01	6.58E+01		
7967896	ENST00000421	PBRM1	polybromo 1	-0.057	0.961	8.40E-01	8.40E+01		
7967898	---	---	---	0.049	1.035	8.44E-01	8.44E+01		
7967900	NM_003453	ZMYM2	zinc finger, MYM-type 2	-0.005	0.997	9.81E-01	9.81E+01		
7967969	NM_138284	IL17D	interleukin 17D	-0.047	0.968	6.59E-01	6.59E+01		
7967976	NM_005870	SAP18	Sin3A-associated protein, 18kDa	-0.060	0.960	5.39E-01	5.39E+01		
7967987	NM_024026	MRP63	mitochondrial ribosomal protein 6	-0.029	0.980	8.88E-01	8.88E+01		
7968015	NM_148957	TNFRSF19	tumor necrosis factor receptor sup	-0.069	0.953	5.50E-01	5.50E+01		
7968035	NM_153023	SPATA13	spermatogenesis associated 13	-0.082	0.945	4.27E-01	4.27E+01		
7968126	NM_002339	LSP1	lymphocyte-specific protein 1	-0.037	0.974	8.87E-01	8.87E+01		

7968128	NM_030979	PABPC3	poly(A) binding protein, cytoplasm	-0.017	0.988	9.24E-01	9.24E+01		
7968132	NM_014089	NUPL1	nucleoporin like 1	0.002	1.002	9.89E-01	9.89E+01		
7968199	NM_001260	CDK8	cyclin-dependent kinase 8	0.018	1.013	9.51E-01	9.51E+01		
7968212	NM_006646	WASF3	WAS protein family, member 3	-0.060	0.959	3.40E-01	3.40E+01		
7968226	NM_000982	RPL21	ribosomal protein L21	-0.018	0.987	9.16E-01	9.16E+01		
7968232	NR_002574	SNORD102	small nucleolar RNA, C/D box 102	-0.011	0.992	9.83E-01	9.83E+01		
7968234	NR_002575	SNORA27	small nucleolar RNA, H/ACA box 27	0.064	1.046	8.52E-01	8.52E+01		
7968242	NM_002097	GTF3A	general transcription factor IIIA	0.040	1.028	8.05E-01	8.05E+01		
7968252	---	---	---	0.051	1.036	5.67E-01	5.67E+01		
7968254	NM_152705	POLR1D	polymerase (RNA) I polypeptide D,	0.052	1.037	7.13E-01	7.13E+01		
7968260	NM_145657	GSX1	GS homeobox 1	-0.076	0.949	4.05E-01	4.05E+01		
7968265	NM_000209	PDX1	pancreatic and duodenal homeobo	-0.028	0.981	8.37E-01	8.37E+01		
7968270	NR_002162 //	// ATP5EP2 //	ATP synthase, H+ transporting, mi	0.250	1.189	4.17E-03	4.17E-01		
7968272	---	---	---	-0.006	0.996	9.77E-01	9.77E+01		
7968274	NM_175854	PAN3	PAN3 poly(A) specific ribonuclease	0.058	1.041	6.94E-01	6.94E+01		
7968295	---	---	---	0.090	1.064	7.87E-01	7.87E+01		
7968297	NM_015932	POMP	proteasome maturation protein	0.057	1.041	7.56E-01	7.56E+01		
7968323	ENST00000400	LOC440131	similar to bA90M5.1 (novel protei	0.055	1.039	5.51E-01	5.51E+01		
7968329	---	---	---	0.117	1.084	6.18E-01	6.18E+01		
7968331	NM_003347	UBE2L3	ubiquitin-conjugating enzyme E2L	0.032	1.023	8.36E-01	8.36E+01		
7968333	NM_005800	USPL1	ubiquitin specific peptidase like 1	0.080	1.057	5.16E-01	5.16E+01		
7968411	NR_027062	EEF1DP3	eukaryotic translation elongation f	-0.051	0.965	7.14E-01	7.14E+01		
7968417	NM_023037	FRY	furry homolog (Drosophila)	0.035	1.025	8.78E-01	8.78E+01		
7968514	---	---	---	-0.018	0.987	9.74E-01	9.74E+01		
7968516	NM_015032	PDS5B	PDS5, regulator of cohesion maint	-0.016	0.989	9.21E-01	9.21E+01		
7968563	NM_002915	RFC3	replication factor C (activator 1) 3,	0.188	1.139	3.51E-01	3.51E+01		
7968637	NM_003914	CCNA1	cyclin A1	-0.062	0.958	6.41E-01	6.41E+01		
7968650	NM_203451	C13orf36	chromosome 13 open reading fran	0.017	1.012	9.24E-01	9.24E+01		
7968658	NM_181503	EXOSC8	exosome component 8	-0.047	0.968	8.44E-01	8.44E+01		
7968670	NM_016617	UFM1	ubiquitin-fold modifier 1	0.059	1.042	7.21E-01	7.21E+01		
7968703	NM_00101275	NHLRC3	NHL repeat containing 3	0.190	1.141	1.40E-01	1.40E+01		
7968711	NR_026745	COG6	component of oligomeric golgi cor	-0.010	0.993	9.73E-01	9.73E+01		
7968734	NM_014252	SLC25A15	solute carrier family 25 (mitochon	0.066	1.047	7.98E-01	7.98E+01		
7968759	---	---	---	0.021	1.015	9.00E-01	9.00E+01		
7968761	NM_024561	NARG1L	NMDA receptor regulated 1-like	0.030	1.021	9.24E-01	9.24E+01		
7968787	NR_002822	MGC72080	MGC72080 pseudogene	-0.028	0.980	9.23E-01	9.23E+01		
7968789	NM_014059	C13orf15	chromosome 13 open reading fran	-0.041	0.972	8.78E-01	8.78E+01		
7968796	---	---	---	0.112	1.081	6.81E-01	6.81E+01		
7968798	---	---	---	0.012	1.008	9.60E-01	9.60E+01		
7968800	NM_178009	DGKH	diacylglycerol kinase, eta	0.133	1.096	3.84E-01	3.84E+01		
7968835	NM_016248	AKAP11	A kinase (PRKA) anchor protein 11	0.128	1.093	1.50E-01	1.50E+01		
7968872	NM_013238	DNAJC15	DnaJ (Hsp40) homolog, subfamily	0.232	1.174	3.10E-02	3.10E+00		
7968890	NM_003646	DGKZ	diacylglycerol kinase, zeta 104kDa	-0.009	0.994	9.48E-01	9.48E+01		
7968898	BC039586	KIAA1704	KIAA1704	0.007	1.005	9.75E-01	9.75E+01		
7968913	---	---	---	0.026	1.018	8.82E-01	8.82E+01		
7968915	NM_004128	GTF2F2	general transcription factor IIF, po	0.091	1.065	3.95E-01	3.95E+01		
7968928	NM_002901	RCN1	reticulocalbin 1, EF-hand calcium b	0.049	1.035	8.31E-01	8.31E+01		
7968931	NM_031431	COG3	component of oligomeric golgi cor	-0.051	0.965	7.17E-01	7.17E+01		
7968972	NM_005694	COX17	COX17 cytochrome c oxidase asse	-0.074	0.950	6.81E-01	6.81E+01		
7968976	NM_015116	LRCH1	leucine-rich repeats and calponin l	-0.043	0.970	7.79E-01	7.79E+01		
7968999	NM_018283	NUDT15	nudix (nucleoside diphosphate link	-0.041	0.972	7.51E-01	7.51E+01		
7969003	NM_021999	ITM2B	integral membrane protein 2B	0.079	1.056	4.82E-01	4.82E+01		
7969017	NM_000321	RB1	retinoblastoma 1	0.268	1.204	2.84E-02	2.84E+00		
7969048	---	---	---	0.059	1.042	7.75E-01	7.75E+01		
7969058	---	---	---	0.046	1.033	7.93E-01	7.93E+01		
7969060	NM_00107967	FNDC3A	fibronectin type III domain contair	0.125	1.090	3.62E-01	3.62E+01		
7969093	NM_001507	MLNR	motilin receptor	-0.055	0.963	7.17E-01	7.17E+01		
7969096	NM_030911	CDADC1	cytidine and dCMP deaminase dor	0.021	1.015	9.12E-01	9.12E+01		
7969114	NM_031915	SETDB2	SET domain, bifurcated 2	0.294	1.226	2.23E-03	2.23E-01		
7969129	NM_00104044	PHF11	PHD finger protein 11	0.056	1.039	7.97E-01	7.97E+01		
7969143	---	---	---	0.006	1.004	9.74E-01	9.74E+01		
7969145	NM_138450	ARL11	ADP-ribosylation factor-like 11	0.174	1.128	2.13E-01	2.13E+01		
7969153	NM_213590	TRIM13	tripartite motif-containing 13	0.134	1.097	9.74E-02	9.74E+00		
7969177	NR_002183	FAM10A4	ST13-like tumor suppressor	-0.044	0.970	6.97E-01	6.97E+01		
7969179	NM_024570	RNASEH2B	ribonuclease H2, subunit B	0.222	1.167	1.18E-03	1.18E-01		
7969202	---	---	---	-0.051	0.965	9.27E-01	9.27E+01		
7969204	NM_052950	WDFY2	WD repeat and FYVE domain conta	0.094	1.067	3.17E-01	3.17E+01		
7969228	NM_00100412	ALG11	asparagine-linked glycosylation 11	0.054	1.038	7.72E-01	7.72E+01		
7969241	---	---	---	0.005	1.004	9.89E-01	9.89E+01		
7969243	NM_018204	CKAP2	cytoskeleton associated protein 2	-0.035	0.976	8.92E-01	8.92E+01		
7969263	NM_00101172	HNRNPA1L2	heterogeneous nuclear ribonucleo	-0.098	0.934	5.10E-01	5.10E+01		
7969271	NM_00113091	SUGT1	SGT1, suppressor of G2 allele of Sk	-0.067	0.955	7.05E-01	7.05E+01		
7969286	---	---	---	-0.001	1.000	9.97E-01	9.97E+01		
7969339	---	---	---	0.220	1.165	4.47E-01	4.47E+01		
7969341	NM_030794	TDRD3	tudor domain containing 3	-0.055	0.962	6.87E-01	6.87E+01		
7969368	---	---	---	0.086	1.061	4.98E-01	4.98E+01		
7969370	---	---	---	-0.070	0.953	6.36E-01	6.36E+01		

7969372	---	---	---	0.006	1.004	9.72E-01	9.72E+01		
7969414	NM_001730	KLF5	Kruppel-like factor 5 (intestinal)	-0.055	0.963	6.07E-01	6.07E+01		
7969428	NM_006002	UCHL3	ubiquitin carboxyl-terminal estera	0.073	1.052	6.36E-01	6.36E+01		
7969472	ENST00000318	FLJ35379	similar to Alu subfamily J sequence	0.000	1.000	1.00E+00	1.00E+02		
7969533	NM_00104015	SLAIN1	SLAIN motif family, member 1	-0.067	0.955	8.05E-01	8.05E+01		
7969559	---	---	---	-0.012	0.992	9.15E-01	9.15E+01		
7969574	NM_000224	KRT18	keratin 18	-0.010	0.993	9.81E-01	9.81E+01		
7969626	NM_180989	GPR180	G protein-coupled receptor 180	0.015	1.011	9.56E-01	9.56E+01		
7969651	NM_006260	DNAJC3	DnaJ (Hsp40) homolog, subfamily	0.130	1.094	5.34E-01	5.34E+01		
7969671	---	---	---	-0.040	0.973	8.28E-01	8.28E+01		
7969677	NM_144778	MBNL2	muscleblind-like 2 (Drosophila)	0.055	1.039	7.85E-01	7.85E+01		
7969693	NM_021033	RAP2A	RAP2A, member of RAS oncogene	0.169	1.124	2.81E-01	2.81E+01		
7969703	NM_002271	IPO5	importin 5	-0.021	0.986	9.33E-01	9.33E+01		
7969770	---	---	---	0.010	1.007	9.85E-01	9.85E+01		
7969772	---	---	---	0.039	1.027	8.05E-01	8.05E+01		
7969776	NR_026644	UBAC2	UBA domain containing 2	-0.038	0.974	7.42E-01	7.42E+01		
7969792	---	---	---	0.020	1.014	8.07E-01	8.07E+01		
7969794	AY358798	UNQ1829	FRSS1829	0.255	1.193	3.25E-01	3.25E+01		
7969796	NM_004800	TM9SF2	transmembrane 9 superfamily me	0.134	1.098	2.22E-01	2.22E+01		
7969815	NM_206808	CLYBL	citrate lyase beta like	0.017	1.012	9.60E-01	9.60E+01		
7969828	---	---	---	-0.040	0.973	7.69E-01	7.69E+01		
7969830	NM_007129	ZIC2	Zic family member 2 (odd-paired h	0.003	1.002	9.86E-01	9.86E+01		
7969861	NM_004791	ITGBL1	integrin, beta-like 1 (with EGF-like	0.002	1.002	9.89E-01	9.89E+01		
7969881	NM_003291	TPP2	tripeptidyl peptidase II	0.084	1.060	4.27E-01	4.27E+01		
7969933	AK126520	BIVM	basic, immunoglobulin-like variabl	0.010	1.007	9.81E-01	9.81E+01		
7969935	NM_000123	ERCC5	excision repair cross-complementi	0.010	1.007	9.57E-01	9.57E+01		
7969975	---	---	---	-0.057	0.961	7.51E-01	7.51E+01		
7969979	NM_032859	ABHD13	abhydrolase domain containing 13	-0.004	0.997	9.81E-01	9.81E+01		
7969986	NM_006573	TNFSF13B	tumor necrosis factor (ligand) sup	0.337	1.263	1.65E-01	1.65E+01		
7970033	NM_001846	COL4A2	collagen, type IV, alpha 2	0.024	1.017	8.11E-01	8.11E+01		
7970084	NM_018210	CARKD	carbohydrate kinase domain conta	0.003	1.002	9.86E-01	9.86E+01		
7970096	NM_005537	ING1	inhibitor of growth family, membe	-0.110	0.927	6.76E-02	6.76E+00		
7970111	NM_003899	ARHGEF7	Rho guanine nucleotide exchange	-0.120	0.920	8.59E-02	8.59E+00		
7970139	BC029889	C13orf16	chromosome 13 open reading fran	-0.040	0.973	7.72E-01	7.72E+01		
7970162	NM_015205	ATP11A	ATPase, class VI, type 11A	0.106	1.076	5.53E-01	5.53E+01		
7970194	NM_00111273	MCF2L	MCF.2 cell line derived transformi	-0.001	0.999	9.95E-01	9.95E+01		
7970232	NM_000131	F7	coagulation factor VII (serum prot	-0.011	0.992	9.57E-01	9.57E+01		
7970241	NM_000504	F10	coagulation factor X	0.023	1.016	8.33E-01	8.33E+01		
7970251	NM_003891	PROZ	protein Z, vitamin K-dependent pl	-0.045	0.970	7.38E-01	7.38E+01		
7970262	NM_00100889	CUL4A	cullin 4A	0.046	1.033	6.35E-01	6.35E+01		
7970301	NM_017905	TMCO3	transmembrane and coiled-coil do	-0.046	0.969	7.99E-01	7.99E+01		
7970317	NR_026580	TFDP1	transcription factor Dp-1	0.092	1.066	5.38E-01	5.38E+01		
7970325	NM_002929	GRK1	G protein-coupled receptor kinase	-0.081	0.946	5.61E-01	5.61E+01		
7970329	NM_000820	GAS6	growth arrest-specific 6	0.027	1.019	9.08E-01	9.08E+01		
7970347	NM_00107864	CDC16	cell division cycle 16 homolog (S. c	0.063	1.045	6.92E-01	6.92E+01		
7970370	NM_023011	UPF3A	UPF3 regulator of nonsense transc	0.055	1.039	6.92E-01	6.92E+01		
7970376	NM_032436	ZNF828	zinc finger protein 828	0.163	1.119	1.78E-02	1.78E+00		
7970381	NR_003366	ANKRD20B	ankyrin repeat domain 20B	-0.125	0.917	7.75E-01	7.75E+01		
7970388	---	---	---	0.027	1.019	9.49E-01	9.49E+01		
7970392	---	---	---	-1.032	0.489	1.73E-01	1.73E+01		
7970395	NM_006001	TUBA3C	tubulin, alpha 3c	0.038	1.027	7.63E-01	7.63E+01		
7970413	NR_003272	PSPC1	paraspeckle component 1	-0.065	0.956	5.53E-01	5.53E+01		
7970426	---	---	---	0.008	1.006	9.86E-01	9.86E+01		
7970428	NM_00103965	ZMYM5	zinc finger, MYM-type 5	-0.081	0.946	5.95E-01	5.95E+01		
7970439	NM_021954	GJA3	gap junction protein, alpha 3, 46kD	-0.083	0.944	2.92E-01	2.92E+01		
7970455	NM_015974	CRYL1	crystallin, lambda 1	-0.004	0.997	9.88E-01	9.88E+01		
7970467	NM_174928	N6AMT2	N-6 adenine-specific DNA methylt	-0.044	0.970	8.86E-01	8.86E+01		
7970473	NM_022459	XPO4	exportin 4	0.019	1.013	9.40E-01	9.40E+01		
7970498	NM_014572	LATS2	LATS, large tumor suppressor, hon	0.038	1.026	7.87E-01	7.87E+01		
7970524	NM_153251	ZDHHC20	zinc finger, DHHC-type containing	0.163	1.119	5.53E-02	5.53E+00		
7970542	---	---	---	0.025	1.018	9.63E-01	9.63E+01		
7970546	NM_152726	EFHA1	EF-hand domain family, member A	0.086	1.061	5.98E-01	5.98E+01		
7970565	---	---	---	0.045	1.031	7.56E-01	7.56E+01		
7970569	NM_014363	SACS	spastic ataxia of Charlevoix-Saguer	-0.155	0.898	3.94E-01	3.94E+01		
7970577	NM_005932	MIPEP	mitochondrial intermediate peptid	0.003	1.002	9.93E-01	9.93E+01		
7970597	AK128225	FLJ46358	FLJ46358 protein	0.048	1.034	7.85E-01	7.85E+01		
7970602	NM_006437	PARP4	poly (ADP-ribose) polymerase fam	0.024	1.017	9.23E-01	9.23E+01		
7970624	NM_018451	CENPJ	centromere protein J	-0.018	0.988	9.51E-01	9.51E+01		
7970648	NM_152704	FAM123A	family with sequence similarity 12	-0.050	0.966	5.16E-01	5.16E+01		
7970655	NM_004685	MTMR6	myotubularin related protein 6	0.069	1.049	6.39E-01	6.39E+01		
7970674	---	---	---	0.009	1.006	9.66E-01	9.66E+01		
7970676	NM_00100753	SHISA2	shisa homolog 2 (Xenopus laevis)	-0.032	0.978	8.32E-01	8.32E+01		
7970681	NM_005977	RNF6	ring finger protein (C3H2C3 type) (	0.019	1.013	9.09E-01	9.09E+01		
7970696	NM_182488	USP12	ubiquitin specific peptidase 12	-0.130	0.914	1.76E-01	1.76E+01		
7970704	NM_152912	MTIF3	mitochondrial translational initiati	0.035	1.025	8.04E-01	8.04E+01		
7970716	NM_153371	LNX2	ligand of numb-protein X 2	-0.060	0.959	7.63E-01	7.63E+01		
7970727	NM_001265	CDX2	caudal type homeobox 2	0.023	1.016	8.09E-01	8.09E+01		

7970735	---	---	---	0.012	1.009	9.54E-01	9.54E+01		
7970737	NM_004119	FLT3	fms-related tyrosine kinase 3	-0.308	0.808	1.39E-01	1.39E+01		
7970793	NM_181785	SLC46A3	solute carrier family 46, member 3	0.269	1.205	5.53E-02	5.53E+00		
7970806	BC027486	hCG_2020170	hCG2020170	-0.051	0.965	6.18E-01	6.18E+01		
7970810	NM_003045	SLC7A1	solute carrier family 7 (cationic am	-0.018	0.988	9.38E-01	9.38E+01		
7970828	AB064667	OK/SW-CL.58	OK/SW-CL.58	0.060	1.042	6.66E-01	6.66E+01		
7970831	NM_007106	UBL3	ubiquitin-like 3	-0.036	0.976	8.25E-01	8.25E+01		
7970842	---	---	---	-0.094	0.937	4.05E-01	4.05E+01		
7970858	NM_002128	HMGB1	high-mobility group box 1	-0.088	0.941	6.13E-01	6.13E+01		
7970864	NM_006644	HSPH1	heat shock 105kDa/110kDa protei	-0.060	0.960	7.60E-01	7.60E+01		
7970892	NM_052818	N4BP2L1	NEDD4 binding protein 2-like 1	0.103	1.074	5.91E-01	5.91E+01		
7970905	---	---	---	-0.079	0.947	7.76E-01	7.76E+01		
7970907	NM_014887	N4BP2L2	NEDD4 binding protein 2-like 2	0.001	1.001	9.95E-01	9.95E+01		
7970922	---	---	---	-0.144	0.905	6.35E-01	6.35E+01		
7970973	---	---	---	0.032	1.022	9.37E-01	9.37E+01		
7970999	NM_015087	SPG20	spastic paraplegia 20 (Troyer synd	0.054	1.038	8.45E-01	8.45E+01		
7971013	---	---	---	-0.225	0.855	1.31E-01	1.31E+01		
7971015	NM_00112721	SMAD9	SMAD family member 9	0.026	1.018	8.63E-01	8.63E+01		
7971027	NM_013338	ALG5	asparagine-linked glycosylation 5,	0.096	1.069	5.24E-01	5.24E+01		
7971039	NM_00101428	FAM48A	family with sequence similarity 48	0.017	1.012	9.15E-01	9.15E+01		
7971071	NM_145203	CSNK1A1L	casein kinase 1, alpha 1-like	-0.003	0.998	9.86E-01	9.86E+01		
7971122	---	---	---	0.026	1.018	9.15E-01	9.15E+01		
7971124	NM_016617	UFM1	ubiquitin-fold modifier 1	0.169	1.124	3.63E-02	3.63E+00		
7971134	NM_025138	C13orf23	chromosome 13 open reading fran	0.050	1.035	6.00E-01	6.00E+01		
7971150	NM_005780	LHFP	lipoma HMGIC fusion partner	-0.094	0.937	3.36E-01	3.36E+01		
7971163	---	---	---	0.052	1.037	6.93E-01	6.93E+01		
7971167	NR_024507	LOC646982	twelve-thirteen translocation leuk	0.074	1.052	4.04E-01	4.04E+01		
7971177	NM_002015	FOXO1	forkhead box O1	0.018	1.012	9.00E-01	9.00E+01		
7971184	NM_005830	MRPS31	mitochondrial ribosomal protein S	0.011	1.007	9.64E-01	9.64E+01		
7971197	NM_172373	ELF1	E74-like factor 1 (ets domain trans	0.055	1.039	4.32E-01	4.32E+01		
7971208	NM_152903	KBTBD6	kelch repeat and BTB (POZ) domai	-0.017	0.988	9.38E-01	9.38E+01		
7971216	---	---	---	0.171	1.126	4.05E-01	4.05E+01		
7971218	NM_032138	KBTBD7	kelch repeat and BTB (POZ) domai	0.036	1.025	8.84E-01	8.84E+01		
7971222	NM_004294	MTRF1	mitochondrial translational release	0.094	1.067	5.15E-01	5.15E+01		
7971239	---	---	---	0.231	1.174	5.53E-01	5.53E+01		
7971241	NR_002822	MGC72080	MGC72080 pseudogene	-0.032	0.978	9.07E-01	9.07E+01		
7971243	---	---	---	-0.056	0.962	4.94E-01	4.94E+01		
7971246	NM_015058	KIAA0564	KIAA0564	0.053	1.037	7.74E-01	7.74E+01		
7971296	NM_00100226	EPSTI1	epithelial stromal interaction 1 (br	0.288	1.221	1.53E-01	1.53E+01		
7971345	ENST00000400	MGC5590	hypothetical protein MGC5590	0.050	1.035	6.95E-01	6.95E+01		
7971350	NM_183422	TSC22D1	TSC22 domain family, member 1	-0.063	0.957	7.17E-01	7.17E+01		
7971361	NM_012345	NUFIP1	nuclear fragile X mental retardatio	-0.052	0.964	8.77E-01	8.77E+01		
7971373	---	---	---	-0.121	0.920	7.77E-01	7.77E+01		
7971375	NM_003295	TPT1	tumor protein, translationally-con	-0.050	0.966	4.84E-01	4.84E+01		
7971386	NR_002967	SNORA31	small nucleolar RNA, H/ACA box 3	-0.038	0.974	8.63E-01	8.63E+01		
7971388	NM_00101087	SLC25A30	solute carrier family 25, member 3	0.233	1.175	5.38E-02	5.38E+00		
7971419	NM_198849	SIAH3	seven in absentia homolog 3 (Dros	-0.073	0.951	7.61E-01	7.61E+01		
7971422	NM_015070	ZC3H13	zinc finger CCCH-type containing 1	-0.051	0.965	6.19E-01	6.19E+01		
7971461	NM_002298	LCP1	lymphocyte cytosolic protein 1 (L-	0.100	1.072	4.42E-01	4.42E+01		
7971482	---	---	---	0.058	1.041	8.26E-01	8.26E+01		
7971486	NM_025113	C13orf18	chromosome 13 open reading fran	0.364	1.287	2.03E-01	2.03E+01		
7971511	---	---	---	0.284	1.217	2.88E-01	2.88E+01		
7971513	NM_001984	ESD	esterase D/formylglutathione hyd	-0.067	0.954	7.13E-01	7.13E+01		
7971541	NM_003850	SUCLA2	succinate-CoA ligase, ADP-forming	0.066	1.047	6.88E-01	6.88E+01		
7971550	NM_014166	MED4	mediator complex subunit 4	0.030	1.021	8.64E-01	8.64E+01		
7971563	NM_020357	PCNP	PEST proteolytic signal containing	0.084	1.060	5.34E-01	5.34E+01		
7971565	NM_005767	LPAR6	lysophosphatidic acid receptor 6	0.217	1.163	1.29E-01	1.29E+01		
7971602	NM_018191	RCBTB1	regulator of chromosome conden	0.107	1.077	4.60E-01	4.60E+01		
7971615	NM_032565	EBPL	emopamil binding protein-like	0.061	1.043	6.83E-01	6.83E+01		
7971620	NM_002267	KPNA3	karyopherin alpha 3 (importin alph	0.047	1.033	7.46E-01	7.46E+01		
7971644	NM_020456	C13orf1	chromosome 13 open reading fran	-0.002	0.998	9.90E-01	9.90E+01		
7971653	NR_002612	DLEU2	deleted in lymphocytic leukemia 2	0.141	1.102	5.30E-01	5.30E+01		
7971663	NM_198989	DLEU7	deleted in lymphocytic leukemia, 7	-0.057	0.961	6.06E-01	6.06E+01		
7971692	NM_012141	INTS6	integrator complex subunit 6	-0.089	0.940	4.47E-01	4.47E+01		
7971711	---	---	---	0.245	1.185	1.63E-01	1.63E+01		
7971713	NM_024705	DHRS12	dehydrogenase/reductase (SDR fa	0.009	1.006	9.65E-01	9.65E+01		
7971723	NR_027047	RP11-327P2.4	hypothetical LOC283521	0.091	1.065	4.25E-01	4.25E+01		
7971813	NM_018676	THSD1	thrombospondin, type I, domain c	-0.038	0.974	8.45E-01	8.45E+01		
7971820	NM_016075	VPS36	vacuolar protein sorting 36 homol	0.042	1.030	7.11E-01	7.11E+01		
7971858	---	---	---	-0.074	0.950	6.47E-01	6.47E+01		
7971862	---	---	---	0.020	1.014	9.02E-01	9.02E+01		
7971864	---	---	---	-0.063	0.958	6.39E-01	6.39E+01		
7971899	---	---	---	-0.061	0.959	6.50E-01	6.50E+01		
7971920	NM_194247	HNRNPA3	heterogeneous nuclear ribonucleo	0.036	1.026	6.15E-01	6.15E+01		
7971967	NM_014953	DIS3	DIS3 mitotic control homolog (S. c	-0.007	0.995	9.70E-01	9.70E+01		
7971996	---	---	---	0.216	1.162	5.36E-01	5.36E+01		
7971998	---	---	---	0.082	1.058	3.60E-01	3.60E+01		

7972001	---	---	---	-0.092	0.938	4.05E-01	4.05E+01		
7972003	NM_007249	KLF12	Kruppel-like factor 12	0.038	1.027	8.75E-01	8.75E+01		
7972021	NM_014832	TBC1D4	TBC1 domain family, member 4	0.098	1.070	4.03E-01	4.03E+01		
7972044	NM_203497	COMMD6	COMM domain containing 6	0.085	1.061	5.02E-01	5.02E+01		
7972055	NM_138444	KCTD12	potassium channel tetramerisation	0.226	1.170	9.52E-02	9.52E+00		
7972062	NM_012158	FBXL3	F-box and leucine-rich repeat prot	0.001	1.001	9.96E-01	9.96E+01		
7972069	NM_015057	MYCBP2	MYC binding protein 2	0.082	1.058	2.29E-01	2.29E+01		
7972177	NM_006237	POU4F1	POU class 4 homeobox 1	-0.009	0.994	9.64E-01	9.64E+01		
7972180	NM_024546	RNF219	ring finger protein 219	0.025	1.018	9.07E-01	9.07E+01		
7972190	NM_022118	RBM26	RNA binding motif protein 26	0.041	1.029	7.51E-01	7.51E+01		
7972215	---	---	---	0.239	1.180	3.27E-01	3.27E+01		
7972247	BC036697	SP3P	Sp3 transcription factor pseudoge	0.015	1.010	8.78E-01	8.78E+01		
7972257	---	---	---	-0.007	0.995	9.83E-01	9.83E+01		
7972297	NM_005845	ABCC4	ATP-binding cassette, sub-family C	0.005	1.004	9.86E-01	9.86E+01		
7972438	NM_178861	RNF113B	ring finger protein 113B	-0.078	0.948	4.32E-01	4.32E+01		
7972442	---	---	---	0.067	1.048	6.37E-01	6.37E+01		
7972444	NM_003576	STK24	serine/threonine kinase 24 (STE20	-0.028	0.981	7.85E-01	7.85E+01		
7972487	NM_015296	DOCK9	dedicator of cytokinesis 9	-0.177	0.884	3.06E-01	3.06E+01		
7972546	---	---	---	0.033	1.023	7.68E-01	7.68E+01		
7972548	NM_005292	GPR18	G protein-coupled receptor 18	0.121	1.087	3.92E-01	3.92E+01		
7972561	---	---	---	0.169	1.125	5.04E-01	5.04E+01		
7972567	NM_033132	ZIC5	Zic family member 5 (odd-paired h	0.029	1.020	8.39E-01	8.39E+01		
7972570	NM_033110	A2LD1	AIG2-like domain 1	0.000	1.000	1.00E+00	1.00E+02		
7972577	NM_001029	RPS26	ribosomal protein S26	0.013	1.009	9.77E-01	9.77E+01		
7972579	NM_032813	TMTC4	transmembrane and tetratricopep	-0.060	0.959	7.78E-01	7.78E+01		
7972663	---	---	---	-0.034	0.977	8.33E-01	8.33E+01		
7972711	---	---	---	0.045	1.032	7.87E-01	7.87E+01		
7972723	NM_018011	ARGLU1	arginine and glutamate rich 1	0.034	1.024	7.55E-01	7.55E+01		
7972737	NM_002312	LIG4	ligase IV, DNA, ATP-dependent	0.008	1.006	9.74E-01	9.74E+01		
7972743	---	---	---	0.429	1.347	9.39E-02	9.39E+00		
7972745	NM_003749	IRS2	insulin receptor substrate 2	-0.079	0.947	3.72E-01	3.72E+01		
7972748	---	---	---	-0.024	0.984	8.97E-01	8.97E+01		
7972805	NM_017817	RAB20	RAB20, member RAS oncogene far	0.168	1.124	2.94E-01	2.94E+01		
7972808	AK093771	CARKD	carbohydrate kinase domain conta	-0.059	0.960	6.03E-01	6.03E+01		
7972810	NM_024537	CARS2	cysteinyl-tRNA synthetase 2, mito	0.136	1.099	1.45E-01	1.45E+01		
7972828	NM_017664	ANKRD10	ankyrin repeat domain 10	-0.007	0.995	9.64E-01	9.64E+01		
7972840	NM_006322	TUBGCP3	tubulin, gamma complex associate	0.079	1.056	6.39E-01	6.39E+01		
7972867	NM_00112720	PCID2	PCI domain containing 2	0.064	1.045	6.69E-01	6.69E+01		
7972888	BC008975	PCID2	PCI domain containing 2	-0.036	0.976	8.92E-01	8.92E+01		
7972890	NM_024719	GRTP1	growth hormone regulated TBC pr	0.018	1.012	8.57E-01	8.57E+01		
7972899	BX647655	DKFZp451A211	DKFZp451A211 protein	-0.031	0.979	8.05E-01	8.05E+01		
7972902	NM_138430	ADPRHL1	ADP-ribosylhydrolase like 1	-0.045	0.970	7.14E-01	7.14E+01		
7972921	---	---	---	-0.094	0.937	8.10E-01	8.10E+01		
7972923	NM_000705	ATP4B	ATPase, H+/K+ exchanging, beta p	0.010	1.007	9.51E-01	9.51E+01		
7972936	NM_182614	FAM70B	family with sequence similarity 70	-0.007	0.995	9.77E-01	9.77E+01		
7972946	NM_007368	RASA3	RAS p21 protein activator 3	-0.114	0.924	5.23E-01	5.23E+01		
7972973	AF287261	SETD8	SET domain containing (lysine met	-0.062	0.958	5.83E-01	5.83E+01		
7972977	---	---	---	-0.036	0.975	8.65E-01	8.65E+01		
7973036	NM_005484	PARP2	poly (ADP-ribose) polymerase 2	0.082	1.059	6.18E-01	6.18E+01		
7973054	---	---	---	-0.067	0.955	7.07E-01	7.07E+01		
7973056	NM_001641	APEX1	APEX nuclease (multifunctional DN	0.011	1.008	9.68E-01	9.68E+01		
7973067	NM_000270	NP	nucleoside phosphorylase	-0.177	0.885	2.84E-01	2.84E+01		
7973080	AK093210	LOC254028	hypothetical LOC254028	-0.048	0.968	7.14E-01	7.14E+01		
7973101	NM_005615	RNASE6	ribonuclease, RNase A family, k6	0.815	1.759	5.26E-02	5.26E+00		
7973105	NM_002935	RNASE3	ribonuclease, RNase A family, 3 (e	0.010	1.007	9.61E-01	9.61E+01		
7973108	ENST00000258	LOC643332	similar to Nonsecretory ribonuclea	0.058	1.041	8.20E-01	8.20E+01		
7973116	NM_00102999	METT11D1	methyltransferase 11 domain cont	0.019	1.013	9.11E-01	9.11E+01		
7973158	NM_018071	FLJ10357	hypothetical protein FLJ10357	0.071	1.050	3.75E-01	3.75E+01		
7973214	NM_014828	TOX4	TOX high mobility group box famil	-0.059	0.960	5.29E-01	5.29E+01		
7973221	AK301287	TRAJ17	T cell receptor alpha joining 17	-0.061	0.959	7.59E-01	7.59E+01		
7973303	X01403	TRA@	T cell receptor alpha locus	-0.242	0.846	5.54E-01	5.54E+01		
7973306	NM_022060	ABHD4	abhydrolase domain containing 4	-0.212	0.863	3.14E-02	3.14E+00		
7973314	NM_005015	OXA1L	oxidase (cytochrome c) assembly 1	0.021	1.015	8.72E-01	8.72E+01		
7973327	NM_181304	MRPL52	mitochondrial ribosomal protein L	-0.123	0.918	3.17E-01	3.17E+01		
7973352	NM_014045	LRP10	low density lipoprotein receptor-r	-0.094	0.937	4.32E-01	4.32E+01		
7973363	NM_173527	REM2	RAS (RAD and GEM)-like GTP bindi	-0.023	0.984	8.80E-01	8.80E+01		
7973371	BC009645	C14orf119	chromosome 14 open reading fran	0.069	1.049	5.16E-01	5.16E+01		
7973377	NM_004050	BCL2L2	BCL2-like 2	-0.044	0.970	8.08E-01	8.08E+01		
7973384	NM_004643	PABPN1	poly(A) binding protein, nuclear 1	-0.018	0.987	9.04E-01	9.04E+01		
7973393	NM_022789	IL25	interleukin 25	-0.062	0.958	5.35E-01	5.35E+01		
7973414	NM_015514	NGDN	neuroguidin, EIF4E binding protein	-0.084	0.943	5.97E-01	5.97E+01		
7973427	NM_024328	THTPA	thiamine triphosphatase	-0.054	0.963	8.05E-01	8.05E+01		
7973448	NM_021004	DHRS4	dehydrogenase/reductase (SDR fa	0.063	1.044	7.57E-01	7.57E+01		
7973458	NM_198083	DHRS4L2	dehydrogenase/reductase (SDR fa	0.113	1.082	5.24E-01	5.24E+01		
7973468	NM_138360	LRRC16B	leucine rich repeat containing 16B	-0.090	0.940	2.57E-01	2.57E+01		
7973510	NM_006032	CPNE6	copine VI (neuronal)	-0.005	0.996	9.75E-01	9.75E+01		
7973530	NM_004563	PCK2	phosphoenolpyruvate carboxykina	0.238	1.179	1.48E-02	1.48E+00		

7973545	NM_025230	DCAF11	DDB1 and CUL4 associated factor	0.069	1.049	5.91E-01	5.91E+01		
7973564	NM_176783	PSME1	proteasome (prosome, macropain	0.033	1.023	8.40E-01	8.40E+01		
7973580	NM_203402	FITM1	fat storage-inducing transmembra	-0.092	0.938	3.53E-01	3.53E+01		
7973584	NM_017999	RNF31	ring finger protein 31	-0.013	0.991	9.27E-01	9.27E+01		
7973611	---	---	---	0.085	1.061	7.45E-01	7.45E+01		
7973613	NM_017999	RNF31	ring finger protein 31	0.022	1.015	9.04E-01	9.04E+01		
7973618	NM_006084	IRF9	interferon regulatory factor 9	0.003	1.002	9.90E-01	9.90E+01		
7973629	NM_00104820	REC8	REC8 homolog (yeast)	-0.082	0.945	2.08E-01	2.08E+01		
7973652	NM_174944	TSSK4	testis-specific serine kinase 4	-0.105	0.930	4.70E-01	4.70E+01		
7973660	NM_016576	GMPR2	guanosine monophosphate reduct	0.016	1.011	9.14E-01	9.14E+01		
7973679	BC025332	C14orf21	chromosome 14 open reading fran	-0.028	0.981	8.63E-01	8.63E+01		
7973694	NM_019839	LTB4R2	leukotriene B4 receptor 2	0.003	1.002	9.85E-01	9.85E+01		
7973702	NM_00114391	LTB4R	leukotriene B4 receptor	0.001	1.000	9.98E-01	9.98E+01		
7973709	NM_004554	NFATC4	nuclear factor of activated T-cells,	-0.044	0.970	7.03E-01	7.03E+01		
7973727	NM_025081	KIAA1305	KIAA1305	0.043	1.031	7.75E-01	7.75E+01		
7973732	NM_015299	KIAA0323	KIAA0323	-0.076	0.949	4.94E-01	4.94E+01		
7973745	NM_005249	FOXG1	forkhead box G1	-0.032	0.978	7.11E-01	7.11E+01		
7973748	NR_026731	C14orf23	chromosome 14 open reading fran	-0.015	0.989	9.19E-01	9.19E+01		
7973770	NM_016106	SCFD1	sec1 family domain containing 1	0.138	1.100	1.49E-01	1.49E+01		
7973797	NM_00113505	COCH	coagulation factor C homolog, coc	-0.001	0.999	9.96E-01	9.96E+01		
7973813	NM_007077	AP4S1	adaptor-related protein complex 4	-0.191	0.876	1.70E-01	1.70E+01		
7973826	NM_025152	NUBPL	nucleotide binding protein-like	0.102	1.073	7.21E-01	7.21E+01		
7973840	NM_00103005	ARHGAP5	Rho GTPase activating protein 5	-0.056	0.962	5.98E-01	5.98E+01		
7973865	---	---	---	-0.011	0.992	9.52E-01	9.52E+01		
7973867	---	---	---	0.042	1.029	9.60E-01	9.60E+01		
7973871	---	---	---	0.189	1.140	7.09E-01	7.09E+01		
7973894	CR595167	RPL23AP71	ribosomal protein L23a pseudoger	-0.118	0.922	1.63E-01	1.63E+01		
7973898	---	---	---	0.016	1.011	9.11E-01	9.11E+01		
7973902	NM_003136	SRP54	signal recognition particle 54kDa	0.043	1.030	7.97E-01	7.97E+01		
7973918	NM_173607	FAM177A1	family with sequence similarity 17	-0.162	0.894	2.95E-01	2.95E+01		
7973924	NM_014672	KIAA0391	KIAA0391	0.027	1.019	8.94E-01	8.94E+01		
7973936	NM_002791	PSMA6	proteasome (prosome, macropain	-0.038	0.974	8.73E-01	8.73E+01		
7973943	NM_032594	INSM2	insulinoma-associated 2	0.021	1.014	8.93E-01	8.93E+01		
7973966	---	---	---	0.011	1.008	9.57E-01	9.57E+01		
7973983	---	---	---	0.059	1.042	6.60E-01	6.60E+01		
7974066	NM_002687	PNN	pinin, desmosome associated prot	-0.046	0.969	6.51E-01	6.51E+01		
7974090	NM_005930	CTAGE5	CTAGE family, member 5	0.065	1.046	6.62E-01	6.62E+01		
7974125	NM_015091	FAM179B	family with sequence similarity 17	0.044	1.031	8.64E-01	8.64E+01		
7974146	NM_017922	PRPF39	PRP39 pre-mRNA processing facto	-0.003	0.998	9.88E-01	9.88E+01		
7974190	NM_145261	DNAJC19	DnaJ (Hsp40) homolog, subfamily	0.015	1.011	9.33E-01	9.33E+01		
7974194	---	---	---	-0.048	0.967	7.89E-01	7.89E+01		
7974198	NM_152329	PPIL5	peptidylprolyl isomerase (cyclophi	0.111	1.080	5.41E-01	5.41E+01		
7974207	NM_002408	MGAT2	mannosyl (alpha-1,6-)-glycoprotei	0.086	1.061	5.91E-01	5.91E+01		
7974214	NM_172193	KLHDC1	kelch domain containing 1	-0.005	0.997	9.89E-01	9.89E+01		
7974229	NM_014315	KLHDC2	kelch domain containing 2	-0.021	0.985	9.22E-01	9.22E+01		
7974245	---	---	---	0.091	1.065	8.27E-01	8.27E+01		
7974247	---	---	---	0.205	1.153	4.55E-01	4.55E+01		
7974249	NM_001663	ARF6	ADP-ribosylation factor 6	-0.054	0.963	6.34E-01	6.34E+01		
7974253	---	---	---	-0.058	0.960	7.05E-01	7.05E+01		
7974255	---	---	---	-0.082	0.945	7.05E-01	7.05E+01		
7974257	NM_015684	ATP5S	ATP synthase, H+ transporting, mi	0.163	1.119	5.45E-02	5.45E+00		
7974303	NM_030755	TMX1	thioredoxin-related transmembra	0.051	1.036	7.21E-01	7.21E+01		
7974314	---	---	---	0.141	1.102	7.52E-01	7.52E+01		
7974339	---	---	---	0.025	1.018	8.71E-01	8.71E+01		
7974341	NM_053064	GNG2	guanine nucleotide binding protei	-0.256	0.838	1.44E-01	1.44E+01		
7974352	NM_016039	C14orf166	chromosome 14 open reading fran	0.001	1.001	9.95E-01	9.95E+01		
7974363	NM_000953	PTGDR	prostaglandin D2 receptor (DP)	0.173	1.128	4.31E-01	4.31E+01		
7974380	NM_002806	PSMC6	proteasome (prosome, macropain	-0.011	0.993	9.53E-01	9.53E+01		
7974387	NM_145251	STYX	serine/threonine/tyrosine interact	0.086	1.062	4.29E-01	4.29E+01		
7974425	NM_015589	SAMD4A	sterile alpha motif domain contair	0.204	1.152	1.48E-02	1.48E+00		
7974455	NM_144578	MAPK1IP1L	mitogen-activated protein kinase	0.089	1.063	2.37E-01	2.37E+01		
7974471	---	---	---	0.055	1.039	8.64E-01	8.64E+01		
7974473	NM_017943	FBXO34	F-box protein 34	-0.086	0.942	3.72E-01	3.72E+01		
7974479	---	---	---	-0.075	0.949	6.54E-01	6.54E+01		
7974481	---	---	---	-0.072	0.951	4.98E-01	4.98E+01		
7974483	NM_182926	KTN1	kinectin 1 (kinesin receptor)	-0.119	0.921	4.06E-02	4.06E+00		
7974531	NR_004844	RPL13AP3	ribosomal protein L13a pseudoger	0.021	1.015	9.15E-01	9.15E+01		
7974533	NM_021255	PELI2	pellino homolog 2 (Drosophila)	0.093	1.067	3.07E-01	3.07E+01		
7974542	NM_017799	C14orf101	chromosome 14 open reading fran	0.122	1.088	1.84E-01	1.84E+01		
7974566	NM_018229	MUDENG	MU-2/AP1M2 domain containing,	0.006	1.004	9.83E-01	9.83E+01		
7974576	NM_00101171	NAT12	N-acetyltransferase 12 (GCN5-rela	-0.083	0.944	4.26E-01	4.26E+01		
7974585	---	---	---	0.075	1.054	4.29E-01	4.29E+01		
7974587	NM_018477	ACTR10	actin-related protein 10 homolog	0.016	1.011	9.41E-01	9.41E+01		
7974603	NM_002788	PSMA3	proteasome (prosome, macropain	-0.011	0.993	9.63E-01	9.63E+01		
7974617	---	---	---	0.093	1.067	6.78E-01	6.78E+01		
7974619	---	---	---	0.140	1.102	3.09E-01	3.09E+01		
7974621	NM_002892	ARID4A	AT rich interactive domain 4A (RBI	0.001	1.001	9.95E-01	9.95E+01		

7974653	NM_014749	KIAA0586	KIAA0586	-0.011	0.993	9.66E-01	9.66E+01		
7974689	NM_016651	DACT1	dapper, antagonist of beta-catenin	0.216	1.161	1.25E-01	1.25E+01		
7974725	NM_016475	JKAMP	JNK1/MAPK8-associated membrar	0.129	1.094	5.02E-01	5.02E+01		
7974771	NM_022495	C14orf135	chromosome 14 open reading frar	0.021	1.014	9.07E-01	9.07E+01		
7974781	NM_021003	PPM1A	protein phosphatase 1A (formerly	-0.030	0.979	8.41E-01	8.41E+01		
7974793	NM_007374	SIX6	SIX homeobox 6	-0.060	0.959	5.49E-01	5.49E+01		
7974799	NM_002431	MNAT1	menage a trois homolog 1, cyclin H	0.005	1.004	9.87E-01	9.87E+01		
7974814	---	---	---	0.213	1.159	3.70E-01	3.70E+01		
7974835	NM_006255	PRKCH	protein kinase C, eta	-0.085	0.943	5.09E-01	5.09E+01		
7974851	NM_001530	HIF1A	hypoxia inducible factor 1, alpha s	-0.277	0.826	1.14E-01	1.14E+01		
7974870	NM_003082	SNAPC1	small nuclear RNA activating comp	-0.036	0.975	9.14E-01	9.14E+01		
7974912	---	---	---	0.140	1.102	8.47E-01	8.47E+01		
7974914	---	---	---	-0.036	0.975	8.73E-01	8.73E+01		
7974916	---	---	---	0.120	1.087	4.03E-01	4.03E+01		
7974920	NM_182914	SYNE2	spectrin repeat containing, nuclea	-0.053	0.964	7.52E-01	7.52E+01		
7975045	NM_005956	MTHFD1	methylenetetrahydrofolate dehyd	0.063	1.044	7.93E-01	7.93E+01		
7975066	NM_004857	AKAP5	A kinase (PRKA) anchor protein 5	-0.030	0.979	9.62E-01	9.62E+01		
7975068	NM_00112332	ZBTB1	zinc finger and BTB domain contai	-0.077	0.948	5.17E-01	5.17E+01		
7975095	NM_015549	PLEKHG3	pleckstrin homology domain conta	0.033	1.023	8.62E-01	8.62E+01		
7975121	NM_002028	FNTB	farnesyltransferase, CAAX box, bet	-0.095	0.937	3.98E-01	3.98E+01		
7975136	NM_178155	FUT8	fucosyltransferase 8 (alpha (1,6) fu	0.115	1.083	3.16E-01	3.16E+01		
7975167	NM_020806	GPHN	gephyrin	-0.011	0.992	9.70E-01	9.70E+01		
7975224	NM_004094	EIF2S1	eukaryotic translation initiation fa	0.112	1.081	2.99E-01	2.99E+01		
7975268	NM_001172	ARG2	arginase, type II	0.016	1.011	9.37E-01	9.37E+01		
7975292	NM_002877	RAD51L1	RAD51-like 1 (S. cerevisiae)	0.071	1.050	8.06E-01	8.06E+01		
7975309	---	---	---	0.071	1.050	8.05E-01	8.05E+01		
7975311	NM_018199	EXD2	exonuclease 3'-5' domain containi	-0.107	0.929	1.85E-01	1.85E+01		
7975324	NM_020692	GALNTL1	UDP-N-acetyl-alpha-D-galactosam	0.025	1.017	8.80E-01	8.80E+01		
7975344	NM_018375	SLC39A9	solute carrier family 39 (zinc trans	0.054	1.038	7.42E-01	7.42E+01		
7975361	NM_014734	KIAA0247	KIAA0247	0.048	1.034	8.17E-01	8.17E+01		
7975368	NM_00103946	SFRS5	splicing factor, arginine/serine-rich	-0.016	0.989	8.71E-01	8.71E+01		
7975386	---	---	---	-0.106	0.929	8.89E-01	8.89E+01		
7975390	NM_00103485	SMOC1	SPARC related modular calcium bi	0.034	1.024	7.71E-01	7.71E+01		
7975410	---	---	---	0.038	1.027	8.47E-01	8.47E+01		
7975412	NM_015351	TTC9	tetratricopeptide repeat domain 9	-0.009	0.994	9.66E-01	9.66E+01		
7975416	NM_014982	PCNX	pecanex homolog (Drosophila)	0.024	1.017	9.10E-01	9.10E+01		
7975453	NR_001276	SNORD56B	small nucleolar RNA, C/D box 56B	0.167	1.123	5.76E-01	5.76E+01		
7975455	---	---	---	0.207	1.154	3.75E-01	3.75E+01		
7975457	---	---	---	0.079	1.056	6.10E-01	6.10E+01		
7975459	NM_015556	SIPA1L1	signal-induced proliferation-associ	0.009	1.006	9.77E-01	9.77E+01		
7975506	NM_015604	DCAF4	DDB1 and CUL4 associated factor 4	-0.016	0.989	9.56E-01	9.56E+01		
7975521	NM_021239	RBM25	RNA binding motif protein 25	-0.027	0.981	8.18E-01	8.18E+01		
7975545	NM_000021	PSEN1	presenilin 1	0.123	1.089	3.64E-01	3.64E+01		
7975562	NM_173462	PAPLN	papilin, proteoglycan-like sulfated	-0.072	0.951	2.98E-01	2.98E+01		
7975595	NM_024644	C14orf169	chromosome 14 open reading frar	-0.003	0.998	9.90E-01	9.90E+01		
7975598	NM_00103716	ACOT1	acyl-CoA thioesterase 1	0.023	1.016	9.44E-01	9.44E+01		
7975602	NM_006821	ACOT2	acyl-CoA thioesterase 2	0.024	1.017	9.25E-01	9.25E+01		
7975626	NM_194278	C14orf43	chromosome 14 open reading frar	-0.059	0.960	6.80E-01	6.80E+01		
7975632	NM_152444	PTGR2	prostaglandin reductase 2	0.163	1.119	4.13E-01	4.13E+01		
7975645	NM_021188	ZNF410	zinc finger protein 410	-0.012	0.992	9.56E-01	9.56E+01		
7975661	NM_182476	COQ6	coenzyme Q6 homolog, monooxyg	0.014	1.010	9.49E-01	9.49E+01		
7975687	NM_00102467	LIN52	lin-52 homolog (C. elegans)	-0.010	0.993	9.74E-01	9.74E+01		
7975696	NM_182894	VSX2	visual system homeobox 2	0.010	1.007	9.47E-01	9.47E+01		
7975705	NM_194279	ISCA2	iron-sulfur cluster assembly 2 hom	0.036	1.025	8.52E-01	8.52E+01		
7975725	NM_019589	YLPM1	YLP motif containing 1	-0.011	0.993	9.53E-01	9.53E+01		
7975747	NM_001933	DLST	dihydrolipoamide S-succinyltransfe	-0.091	0.939	3.71E-01	3.71E+01		
7975760	NM_014239	EIF2B2	eukaryotic translation initiation fa	0.088	1.063	5.53E-01	5.53E+01		
7975779	NM_005252	FOS	FBJ murine osteosarcoma viral onc	-0.276	0.826	3.94E-02	3.94E+00		
7975787	NM_00113504	JDP2	Jun dimerization protein 2	0.005	1.004	9.76E-01	9.76E+01		
7975793	NM_006399	BATF	basic leucine zipper transcription f	-0.004	0.997	9.90E-01	9.90E+01		
7975815	NM_015072	TTLL5	tubulin tyrosine ligase-like family,	-0.107	0.929	2.99E-01	2.99E+01		
7975863	NM_017926	C14orf118	chromosome 14 open reading frar	-0.076	0.949	5.95E-01	5.95E+01		
7975889	NM_014909	VASH1	vasohibin 1	-0.002	0.998	9.90E-01	9.90E+01		
7975924	---	---	---	-0.050	0.966	6.37E-01	6.37E+01		
7975926	NM_033426	KIAA1737	KIAA1737	-0.024	0.984	8.82E-01	8.82E+01		
7975956	NM_001513	GSTZ1	glutathione transferase zeta 1	0.010	1.007	9.64E-01	9.64E+01		
7975976	NM_012111	AHSA1	AHA1, activator of heat shock 90k	-0.012	0.992	9.55E-01	9.55E+01		
7975989	NM_031210	C14orf156	chromosome 14 open reading frar	0.112	1.081	5.77E-01	5.77E+01		
7975995	BC113929	C14orf178	chromosome 14 open reading frar	-0.137	0.909	1.38E-01	1.38E+01		
7976053	---	---	---	0.280	1.215	1.06E-01	1.06E+01		
7976069	---	---	---	-0.038	0.974	8.46E-01	8.46E+01		
7976080	NM_003608	GPR65	G protein-coupled receptor 65	0.292	1.224	8.58E-02	8.58E+00		
7976101	NM_024824	ZC3H14	zinc finger CCCH-type containing 1	0.037	1.026	7.63E-01	7.63E+01		
7976148	NR_024620	PRO1768	PRO1768	0.054	1.038	8.49E-01	8.49E+01		
7976152	---	---	---	0.053	1.037	8.59E-01	8.59E+01		
7976158	---	---	---	-0.102	0.932	4.80E-01	4.80E+01		
7976160	NM_018319	TDP1	tyrosyl-DNA phosphodiesterase 1	-0.053	0.964	8.05E-01	8.05E+01		

7976182	NM_022054	KCNK13	potassium channel, subfamily K, m	-0.040	0.973	7.51E-01	7.51E+01		
7976189	NM_002802	PSMC1	proteasome (prosome, macropain	-0.030	0.979	8.93E-01	8.93E+01		
7976200	NM_006888	CALM1	calmodulin 1 (phosphorylase kinas	0.023	1.016	7.89E-01	7.89E+01		
7976216	NM_00110236	C14orf159	chromosome 14 open reading fran	-0.002	0.999	9.93E-01	9.93E+01		
7976239	AF170294	PTMAP7	prothymosin, alpha pseudogene 7	-0.041	0.972	7.83E-01	7.83E+01		
7976243	NM_017437	CPSF2	cleavage and polyadenylation spec	0.087	1.062	3.41E-01	3.41E+01		
7976292	NM_024832	RIN3	Ras and Rab interactor 3	-0.029	0.980	8.44E-01	8.44E+01		
7976307	NM_005113	GOLGA5	golgi autoantigen, golgin subfamily	0.094	1.067	4.73E-01	4.73E+01		
7976333	NM_015676	C14orf109	chromosome 14 open reading fran	0.058	1.041	7.60E-01	7.60E+01		
7976407	BC009073	FAM181A	family with sequence similarity 18	0.022	1.016	8.45E-01	8.45E+01		
7976425	NM_023112	OTUB2	OTU domain, ubiquitin aldehyde b	-0.062	0.958	7.49E-01	7.49E+01		
7976436	NM_145249	IFI27L1	interferon, alpha-inducible protein	-0.056	0.962	6.45E-01	6.45E+01		
7976443	NM_00113008	IFI27	interferon, alpha-inducible protein	0.309	1.239	4.44E-01	4.44E+01		
7976506	NR_015340	SERPINA13	serpin peptidase inhibitor, clade A	-0.063	0.958	6.34E-01	6.34E+01		
7976512	NR_015415	FLJ45244	hypothetical locus FLJ45244	0.003	1.002	9.89E-01	9.89E+01		
7976515	NM_016417	GLRX5	glutaredoxin 5	-0.008	0.994	9.61E-01	9.61E+01		
7976556	NR_023938	C14orf132	chromosome 14 open reading fran	-0.112	0.926	3.38E-01	3.38E+01		
7976560	NM_000623	BDKRB2	bradykinin receptor B2	-0.047	0.968	7.71E-01	7.71E+01		
7976598	NM_032632	PAPOLA	poly(A) polymerase alpha	0.032	1.022	7.98E-01	7.98E+01		
7976621	NM_003384	VRK1	vaccinia related kinase 1	-0.003	0.998	9.89E-01	9.89E+01		
7976635	BC093661	C14orf177	chromosome 14 open reading fran	0.028	1.019	8.45E-01	8.45E+01		
7976642	---	---	---	0.020	1.014	9.16E-01	9.16E+01		
7976646	---	---	---	0.314	1.243	5.78E-02	5.78E+00		
7976648	NM_00109940	CCNK	cyclin K	0.035	1.025	7.51E-01	7.51E+01		
7976667	---	---	---	-0.032	0.978	8.41E-01	8.41E+01		
7976669	NM_00112725	HHIPL1	HHIP-like 1	-0.074	0.950	2.25E-01	2.25E+01		
7976681	NM_006668	CYP46A1	cytochrome P450, family 46, subfa	-0.073	0.951	3.10E-01	3.10E+01		
7976724	---	---	---	-0.096	0.936	1.51E-01	1.51E+01		
7976726	NM_016337	EVL	Enah/Vasp-like	0.058	1.041	7.04E-01	7.04E+01		
7976744	NM_003403	YY1	YY1 transcription factor	-0.056	0.962	5.17E-01	5.17E+01		
7976755	NM_207117	C14orf68	chromosome 14 open reading fran	-0.021	0.986	8.51E-01	8.51E+01		
7976766	NM_024515	WDR25	WD repeat domain 25	-0.036	0.976	8.45E-01	8.45E+01		
7976778	AK094097	C14orf70	chromosome 14 open reading fran	-0.091	0.939	4.45E-01	4.45E+01		
7976783	NM_003836	DLK1	delta-like 1 homolog (Drosophila)	-0.054	0.963	6.23E-01	6.23E+01		
7976795	NR_003530	MEG3	maternally expressed 3 (non-prote	0.036	1.025	7.73E-01	7.73E+01		
7976804	---	---	---	-0.006	0.996	9.77E-01	9.77E+01		
7976808	---	---	---	-0.060	0.959	7.48E-01	7.48E+01		
7976828	AK123165	FLJ41170	hypothetical LOC440200	-0.031	0.979	8.45E-01	8.45E+01		
7976844	---	---	---	-0.023	0.984	9.06E-01	9.06E+01		
7976863	NM_00116172	PPP2R5C	protein phosphatase 2, regulatory	-0.180	0.883	1.17E-02	1.17E+00		
7976874	---	---	---	-0.249	0.842	3.20E-01	3.20E+01		
7976876	NM_001376	DYNC1H1	dynein, cytoplasmic 1, heavy chair	-0.037	0.975	7.65E-01	7.65E+01		
7976957	NM_181291	WDR20	WD repeat domain 20	-0.062	0.958	5.30E-01	5.30E+01		
7976967	NM_018335	ZNF839	zinc finger protein 839	-0.065	0.956	6.57E-01	6.57E+01		
7976976	NM_014844	TECPR2	tectonin beta-propeller repeat cor	-0.073	0.950	6.63E-01	6.63E+01		
7977003	NM_015156	RCOR1	REST corepressor 1	0.075	1.053	5.53E-01	5.53E+01		
7977018	NM_145725	TRAF3	TNF receptor-associated factor 3	-0.002	0.998	9.90E-01	9.90E+01		
7977033	NM_030943	AMN	amnionless homolog (mouse)	-0.080	0.946	2.77E-01	2.77E+01		
7977046	NM_006291	TNFAIP2	tumor necrosis factor, alpha-induc	0.043	1.030	8.30E-01	8.30E+01		
7977058	NM_001969	EIF5	eukaryotic translation initiation fa	-0.092	0.938	4.31E-01	4.31E+01		
7977077	NM_00112891	MARK3	MAP/microtubule affinity-regulati	-0.020	0.986	8.88E-01	8.88E+01		
7977103	---	---	---	-0.008	0.995	9.87E-01	9.87E+01		
7977105	NM_152307	TRMT61A	tRNA methyltransferase 61 homol	-0.013	0.991	9.23E-01	9.23E+01		
7977119	NM_032374	C14orf153	chromosome 14 open reading fran	0.015	1.011	9.50E-01	9.50E+01		
7977127	NM_00113010	KLC1	kinesin light chain 1	-0.011	0.993	9.50E-01	9.50E+01		
7977149	NM_024071	ZFYVE21	zinc finger, FYVE domain containin	0.032	1.022	8.45E-01	8.45E+01		
7977203	NM_00108046	ASPG	asparaginase homolog (S. cerevisia	-0.056	0.962	5.84E-01	5.84E+01		
7977216	NM_015656	KIF26A	kinesin family member 26A	-0.012	0.992	9.27E-01	9.27E+01		
7977234	BC041103	C14orf180	chromosome 14 open reading fran	-0.051	0.965	5.73E-01	5.73E+01		
7977241	NM_022489	INF2	inverted formin, FH2 and WH2 dor	-0.054	0.963	7.39E-01	7.39E+01		
7977249	NM_022489	INF2	inverted formin, FH2 and WH2 dor	-0.018	0.988	9.07E-01	9.07E+01		
7977270	AK131040	LOC388022	hypothetical gene supported by A	0.263	1.200	6.06E-01	6.06E+01		
7977273	NM_152328	ADSSL1	adenylosuccinate synthase like 1	0.000	1.000	9.98E-01	9.98E+01		
7977288	NM_006427	SIVA1	SIVA1, apoptosis-inducing factor	-0.062	0.958	5.01E-01	5.01E+01		
7977294	NM_00113760	ZBTB42	zinc finger and BTB domain contai	0.001	1.001	9.97E-01	9.97E+01		
7977296	NR_024396	MGC23270	hypothetical LOC196872	0.030	1.021	8.71E-01	8.71E+01		
7977299	NM_00111272	KIAA0284	KIAA0284	-0.040	0.973	7.81E-01	7.81E+01		
7977319	NM_138790	PLD4	phospholipase D family, member 4	0.002	1.002	9.88E-01	9.88E+01		
7977344	NM_00110091	PACS2	phosphofurin acidic cluster sorting	0.051	1.036	7.02E-01	7.02E+01		
7977371	NM_00110091	PACS2	phosphofurin acidic cluster sorting	0.007	1.005	9.72E-01	9.72E+01		
7977373	NM_004689	MTA1	metastasis associated 1	-0.120	0.920	3.00E-01	3.00E+01		
7977397	NM_001312	CRIP2	cysteine-rich protein 2	0.029	1.021	7.78E-01	7.78E+01		
7977409	NM_001311	CRIP1	cysteine-rich protein 1 (intestinal)	-0.040	0.973	6.74E-01	6.74E+01		
7977418	NM_00113487	C14orf80	chromosome 14 open reading fran	-0.032	0.978	7.92E-01	7.92E+01		
7977432	NM_025268	TMEM121	transmembrane protein 121	-0.017	0.989	8.93E-01	8.93E+01		
7977435	---	---	---	-0.008	0.995	9.77E-01	9.77E+01		
7977438	---	---	---	-0.076	0.949	5.52E-01	5.52E+01		

7977440	NR_026800	KIAA0125	KIAA0125	0.059	1.042	7.25E-01	7.25E+01		
7977445	NR_026800	KIAA0125	KIAA0125	0.108	1.077	5.05E-01	5.05E+01		
7977452	ENST00000400	FLJ39632	hypothetical LOC642477	0.036	1.025	7.94E-01	7.94E+01		
7977454	NM_00108353	POTEE	POTE ankyrin domain family, mem	0.091	1.065	5.24E-01	5.24E+01		
7977482	NM_138376	TTC5	tetratricopeptide repeat domain 5	0.077	1.055	6.34E-01	6.34E+01		
7977497	NM_021178	CCNB1IP1	cyclin B1 interacting protein 1	-0.029	0.980	9.09E-01	9.09E+01		
7977507	NR_002312	RPPH1	ribonuclease P RNA component H1	-0.018	0.988	9.74E-01	9.74E+01		
7977511	NM_007110	TEP1	telomerase-associated protein 1	0.109	1.078	3.21E-02	3.21E+00		
7977567	BC146951	KLHL33	kelch-like 33 (Drosophila)	-0.105	0.930	2.09E-01	2.09E+01		
7977571	NM_017807	OSGEP	O-sialoglycoprotein endopeptidase	0.203	1.151	6.92E-02	6.92E+00		
7977584	NM_00110081	TMEM55B	transmembrane protein 55B	-0.038	0.974	7.72E-01	7.72E+01		
7977615	NM_198232	RNASE1	ribonuclease, RNase A family, 1 (p	0.520	1.434	5.82E-02	5.82E+00		
7977621	NM_201540	NDRG2	NDRG family member 2	-0.047	0.968	6.88E-01	6.88E+01		
7977646	NM_016423	ZNF219	zinc finger protein 219	0.011	1.008	9.56E-01	9.56E+01		
7977653	---	---	---	0.088	1.063	2.27E-01	2.27E+01		
7977655	NM_00100473	OR5AU1	olfactory receptor, family 5, subfa	0.065	1.046	5.34E-01	5.34E+01		
7977657	NM_031314	HNRNPC	heterogeneous nuclear ribonucleo	0.007	1.005	9.62E-01	9.62E+01		
7977674	NM_007192	SUPT16H	suppressor of Ty 16 homolog (S. ce	-0.016	0.989	9.44E-01	9.44E+01		
7977693	NM_020920	CHD8	chromodomain helicase DNA bind	-0.068	0.954	4.47E-01	4.47E+01		
7977732	NR_002916	SNORD8	small nucleolar RNA, C/D box 8	-0.013	0.991	9.73E-01	9.73E+01		
7977734	---	---	---	0.073	1.052	4.43E-01	4.43E+01		
7977736	NM_032846	RAB2B	RAB2B, member RAS oncogene fa	-0.017	0.989	9.39E-01	9.39E+01		
7977749	NM_019852	METTL3	methyltransferase like 3	-0.032	0.978	8.01E-01	8.01E+01		
7977761	NM_005407	SALL2	sal-like 2 (Drosophila)	-0.055	0.962	7.07E-01	7.07E+01		
7977773	---	---	---	0.000	1.000	1.00E+00	1.00E+02		
7977775	NM_001344	DAD1	defender against cell death 1	0.017	1.012	9.61E-01	9.61E+01		
7977780	NM_022060	ABHD4	abhydrolase domain containing 4	-0.007	0.995	9.70E-01	9.70E+01		
7977782	ENST00000312	OR6J1	olfactory receptor, family 6, subfa	-0.026	0.982	7.92E-01	7.92E+01		
7977786	NM_003982	SLC7A7	solute carrier family 7 (cationic am	-0.178	0.884	7.08E-01	7.08E+01		
7977803	NM_00107735	RBM23	RNA binding motif protein 23	-0.096	0.935	4.81E-01	4.81E+01		
7977820	NM_00103961	PRMT5	protein arginine methyltransferase	-0.053	0.964	8.36E-01	8.36E+01		
7977841	NM_017815	HAUS4	HAUS augmin-like complex, subun	0.029	1.020	8.98E-01	8.98E+01		
7977868	NM_021944	C14orf93	chromosome 14 open reading fra	0.067	1.048	5.59E-01	5.59E+01		
7977879	NM_00114493	PSMB5	proteasome (prosome, macropain	-0.048	0.967	8.06E-01	8.06E+01		
7977888	NM_022478	CDH24	cadherin-like 24	0.022	1.016	8.58E-01	8.58E+01		
7977906	NM_014977	ACIN1	apoptotic chromatin condensation	0.038	1.026	6.57E-01	6.57E+01		
7977928	NM_001805	CEBPE	CCAAT/enhancer binding protein (	0.072	1.051	5.73E-01	5.73E+01		
7977955	NR_026862	PPP1R3E	protein phosphatase 1, regulatory	-0.024	0.984	8.50E-01	8.50E+01		
7977965	NM_016609	SLC22A17	solute carrier family 22, member 1	0.005	1.004	9.73E-01	9.73E+01		
7977978	NM_005864	EFS	embryonal Fyn-associated substra	-0.049	0.967	6.08E-01	6.08E+01		
7977987	NM_002471	MYH6	myosin, heavy chain 6, cardiac mu	-0.054	0.963	6.19E-01	6.19E+01		
7978054	---	---	---	-0.007	0.995	9.76E-01	9.76E+01		
7978056	BC144336	ZFHX2	zinc finger homeobox 2	-0.067	0.955	4.27E-01	4.27E+01		
7978059	BC131533	ZFHX2	zinc finger homeobox 2	0.034	1.024	8.45E-01	8.45E+01		
7978064	---	---	---	0.014	1.010	9.53E-01	9.53E+01		
7978066	NM_003917	AP1G2	adaptor-related protein complex 1	-0.078	0.947	3.61E-01	3.61E+01		
7978093	NM_032452	JPH4	junctophilin 4	-0.044	0.970	7.22E-01	7.22E+01		
7978107	NM_006177	NRL	neural retina leucine zipper	-0.048	0.968	7.36E-01	7.36E+01		
7978114	BC002491	FAM158A	family with sequence similarity 15	0.010	1.007	9.67E-01	9.67E+01		
7978123	NM_002818	PSME2	proteasome (prosome, macropain	0.155	1.113	4.14E-01	4.14E+01		
7978132	NM_024658	IPO4	importin 4	-0.102	0.932	2.78E-01	2.78E+01		
7978166	NM_006405	TM9SF1	transmembrane 9 superfamily me	-0.115	0.923	3.10E-01	3.10E+01		
7978174	NM_014169	CHMP4A	chromatin modifying protein 4A	0.030	1.021	8.03E-01	8.03E+01		
7978187	NM_014169	CHMP4A	chromatin modifying protein 4A	0.058	1.041	5.04E-01	5.04E+01		
7978192	NM_138476	MDP1	magnesium-dependent phosphata	-0.027	0.981	9.22E-01	9.22E+01		
7978201	NM_006156	NEDD8	neural precursor cell expressed, de	0.030	1.021	8.46E-01	8.46E+01		
7978208	NM_012461	TINF2	TERF1 (TRF1)-interacting nuclear f	0.050	1.035	7.06E-01	7.06E+01		
7978222	NM_000359	TGM1	transglutaminase 1 (K polypeptide	-0.039	0.973	7.59E-01	7.59E+01		
7978239	NM_004581	RABGGTA	Rab geranylgeranyltransferase, alp	-0.066	0.956	4.06E-01	4.06E+01		
7978260	NM_00113605	DHRS1	dehydrogenase/reductase (SDR fa	0.029	1.020	9.24E-01	9.24E+01		
7978272	NM_014430	CIDEB	cell death-inducing DFFA-like effec	0.004	1.003	9.85E-01	9.85E+01		
7978285	NM_139247	ADCY4	adenylate cyclase 4	-0.035	0.976	7.95E-01	7.95E+01		
7978312	NM_006871	RIPK3	receptor-interacting serine-threon	-0.037	0.975	8.40E-01	8.40E+01		
7978331	NM_00103977	CBLN3	cerebellin 3 precursor	-0.123	0.918	3.92E-01	3.92E+01		
7978335	NM_020195	SDR39U1	short chain dehydrogenase/reduct	0.001	1.001	9.97E-01	9.97E+01		
7978360	NM_033423	GZMH	granzyme H (cathepsin G-like 2, pr	0.004	1.003	9.92E-01	9.92E+01		
7978366	NM_004131	GZMB	granzyme B (granzyme 2, cytotoxi	-0.241	0.846	5.73E-01	5.73E+01		
7978389	---	---	---	0.002	1.001	9.94E-01	9.94E+01		
7978428	NM_00108389	STRN3	striatin, calmodulin binding protei	-0.056	0.962	6.41E-01	6.41E+01		
7978449	NM_015382	HECTD1	HECT domain containing 1	0.016	1.011	9.28E-01	9.28E+01		
7978492	NM_015473	HEATR5A	HEAT repeat containing 5A	0.245	1.185	6.43E-02	6.43E+00		
7978527	NM_080664	C14orf126	chromosome 14 open reading fra	0.124	1.090	3.47E-01	3.47E+01		
7978534	---	---	---	0.146	1.106	6.57E-01	6.57E+01		
7978538	NR_027263	C14orf128	chromosome 14 open reading fra	-0.046	0.969	7.79E-01	7.79E+01		
7978542	NM_020696	KIAA1143	KIAA1143	0.020	1.014	9.49E-01	9.49E+01		
7978544	NM_022073	EGLN3	egl nine homolog 3 (C. elegans)	-0.018	0.988	9.28E-01	9.28E+01		
7978553	NM_138288	C14orf147	chromosome 14 open reading fra	0.200	1.148	1.37E-01	1.37E+01		

7978558	NM_018453	EAPP	E2F-associated phosphoprotein	0.038	1.026	8.21E-01	8.21E+01		
7978570	NM_021249	SNX6	sorting nexin 6	0.267	1.203	4.66E-03	4.66E-01		
7978595	NM_013448	BAZ1A	bromodomain adjacent to zinc fing	0.139	1.101	4.39E-02	4.39E+00		
7978626	---	---	---	0.178	1.131	8.12E-01	8.12E+01		
7978628	NM_017917	PPP2R3C	protein phosphatase 2 (formerly 2	-0.002	0.999	9.94E-01	9.94E+01		
7978653	NM_014990	GARNL1	GTPase activating Rap/RanGAP do	-0.143	0.906	4.18E-01	4.18E+01		
7978662	---	---	---	0.011	1.007	9.85E-01	9.85E+01		
7978666	NM_016586	MBIP	MAP3K12 binding inhibitory prote	0.072	1.051	6.31E-01	6.31E+01		
7978706	NM_004496	FOXA1	forkhead box A1	0.018	1.013	9.00E-01	9.00E+01		
7978710	---	---	---	0.145	1.105	1.27E-01	1.27E+01		
7978718	NM_006364	SEC23A	Sec23 homolog A (S. cerevisiae)	0.047	1.033	7.73E-01	7.73E+01		
7978739	NM_00107953	TRAPPC6B	trafficking protein particle comple	-0.100	0.933	4.42E-01	4.42E+01		
7978748	NM_203301	FBXO33	F-box protein 33	-0.119	0.921	1.32E-01	1.32E+01		
7978754	NM_014267	C11orf58	chromosome 11 open reading fran	0.032	1.022	7.76E-01	7.76E+01		
7978760	NM_017658	KLHL28	kelch-like 28 (Drosophila)	0.038	1.027	8.39E-01	8.39E+01		
7978766	NM_002013	FKBP3	FK506 binding protein 3, 25kDa	-0.034	0.976	7.68E-01	7.68E+01		
7978776	NM_018353	C14orf106	chromosome 14 open reading fran	0.223	1.167	5.36E-02	5.36E+00		
7978795	NM_080746	RPL10L	ribosomal protein L10-like	-0.050	0.966	6.12E-01	6.12E+01		
7978824	NM_00103000	RPS29	ribosomal protein S29	0.033	1.023	8.49E-01	8.49E+01		
7978831	NM_152329	PPIL5	peptidylprolyl isomerase (cyclophi	0.041	1.029	8.16E-01	8.16E+01		
7978833	NM_001001	RPL36AL	ribosomal protein L36a-like	-0.077	0.948	3.05E-01	3.05E+01		
7978838	NM_018139	C14orf104	chromosome 14 open reading fran	-0.016	0.989	9.65E-01	9.65E+01		
7978866	NM_004713	SDCCAG1	serologically defined colon cancer	0.005	1.003	9.84E-01	9.84E+01		
7978905	---	---	---	-0.045	0.969	8.66E-01	8.66E+01		
7978909	---	---	---	0.062	1.044	7.12E-01	7.12E+01		
7978911	NM_00101270	C14orf182	chromosome 14 open reading fran	-0.165	0.892	3.67E-01	3.67E+01		
7978917	NM_00101483	LOC196913	hypothetical protein LOC196913	-0.131	0.913	4.43E-01	4.43E+01		
7978932	NM_006939	SOS2	son of sevenless homolog 2 (Drosc	-0.086	0.942	5.03E-01	5.03E+01		
7978956	NM_024884	L2HGDH	L-2-hydroxyglutarate dehydrogena	0.067	1.048	5.48E-01	5.48E+01		
7978970	NM_004196	CDKL1	cyclin-dependent kinase-like 1 (CD	0.021	1.015	9.16E-01	9.16E+01		
7978997	NM_198794	MAP4K5	mitogen-activated protein kinase l	-0.032	0.978	8.47E-01	8.47E+01		
7979033	NM_021818	SAV1	salvador homolog 1 (Drosophila)	-0.031	0.979	9.19E-01	9.19E+01		
7979044	NM_020921	NIN	ninein (GSK3B interacting protein)	0.055	1.039	5.83E-01	5.83E+01		
7979085	NM_002863	PYGL	phosphorylase, glycogen, liver	0.271	1.206	5.53E-01	5.53E+01		
7979108	NM_052978	TRIM9	tripartite motif-containing 9	-0.057	0.961	5.34E-01	5.34E+01		
7979127	---	---	---	-0.084	0.943	5.16E-01	5.16E+01		
7979179	NM_014584	ERO1L	ERO1-like (S. cerevisiae)	0.067	1.048	7.86E-01	7.86E+01		
7979196	NM_198066	GNPNAT1	glucosamine-phosphate N-acetyltr	0.073	1.052	5.53E-01	5.53E+01		
7979223	NM_00116014	DDHD1	DDHD domain containing 1	0.098	1.071	3.62E-01	3.62E+01		
7979250	NM_005776	CNIH	cornichon homolog (Drosophila)	-0.034	0.977	8.50E-01	8.50E+01		
7979260	NM_004124	GMFB	glia maturation factor, beta	0.207	1.154	1.11E-01	1.11E+01		
7979269	NM_000161	GCH1	GTP cyclohydrolase 1	0.152	1.111	6.51E-01	6.51E+01		
7979328	NM_014924	KIAA0831	KIAA0831	-0.078	0.947	6.48E-01	6.48E+01		
7979351	NR_027123	C14orf33	chromosome 14 open reading fran	-0.053	0.964	6.95E-01	6.95E+01		
7979355	---	---	---	-0.009	0.994	9.54E-01	9.54E+01		
7979367	NM_006544	EXOC5	exocyst complex component 5	0.051	1.036	7.73E-01	7.73E+01		
7979412	---	---	---	0.188	1.139	4.75E-01	4.75E+01		
7979426	NM_022571	GPR135	G protein-coupled receptor 135	0.044	1.031	6.60E-01	6.60E+01		
7979437	NM_144581	C14orf149	chromosome 14 open reading fran	0.035	1.025	9.19E-01	9.19E+01		
7979473	NM_016029	DHRS7	dehydrogenase/reductase (SDR fa	-0.218	0.860	1.74E-01	1.74E+01		
7979510	NM_017420	SIX4	SIX homeobox 4	0.035	1.024	7.57E-01	7.57E+01		
7979516	NM_020810	TRMT5	TRM5 tRNA methyltransferase 5 h	0.135	1.098	1.36E-01	1.36E+01		
7979548	NM_145171	GPHB5	glycoprotein hormone beta 5	-0.015	0.990	9.34E-01	9.34E+01		
7979551	NM_006246	PPP2R5E	protein phosphatase 2, regulatory	0.011	1.008	9.53E-01	9.53E+01		
7979565	NM_080666	WDR89	WD repeat domain 89	0.032	1.023	8.78E-01	8.78E+01		
7979572	---	---	---	0.098	1.070	6.15E-01	6.15E+01		
7979574	NM_030791	SGPP1	sphingosine-1-phosphate phospha	0.048	1.034	6.07E-01	6.07E+01		
7979611	NM_006977	ZBTB25	zinc finger and BTB domain contai	-0.086	0.942	5.83E-01	5.83E+01		
7979658	NM_002083	GPX2	glutathione peroxidase 2 (gastroin	-0.004	0.998	9.88E-01	9.88E+01		
7979663	NM_198686	RAB15	RAB15, member RAS onocogene fa	-0.036	0.975	8.05E-01	8.05E+01		
7979671	NM_145113	MAX	MYC associated factor X	0.102	1.073	1.55E-01	1.55E+01		
7979694	---	---	---	0.086	1.062	2.29E-01	2.29E+01		
7979698	NM_015994	ATP6V1D	ATPase, H+ transporting, lysosoma	0.109	1.079	7.09E-01	7.09E+01		
7979725	NM_004569	PIGH	phosphatidylinositol glycan ancho	-0.071	0.952	7.89E-01	7.89E+01		
7979732	NM_006370	VTI1B	vesicle transport through interacti	0.052	1.037	6.51E-01	6.51E+01		
7979741	---	---	---	-0.137	0.910	2.35E-01	2.35E+01		
7979743	NM_016026	RDH11	retinol dehydrogenase 11 (all-tran	-0.009	0.994	9.74E-01	9.74E+01		
7979757	NM_015346	ZFYVE26	zinc finger, FYVE domain containin	0.022	1.015	9.01E-01	9.01E+01		
7979802	---	---	---	0.020	1.014	9.73E-01	9.73E+01		
7979804	---	---	---	0.165	1.121	5.77E-01	5.77E+01		
7979808	---	---	---	0.042	1.030	8.10E-01	8.10E+01		
7979813	NM_004926	ZFP36L1	zinc finger protein 36, C3H type-lik	0.107	1.077	1.13E-01	1.13E+01		
7979820	NM_207442	C14orf181	chromosome 14 open reading fran	0.054	1.038	5.91E-01	5.91E+01		
7979824	NM_00113000	ACTN1	actinin, alpha 1	-0.132	0.912	5.11E-01	5.11E+01		
7979849	NM_003861	DCAF5	DDB1 and CUL4 associated factor !	-0.008	0.995	9.74E-01	9.74E+01		
7979862	---	---	---	-0.026	0.982	9.09E-01	9.09E+01		
7979864	NM_004450	ERH	enhancer of rudimentary homolog	0.037	1.026	8.07E-01	8.07E+01		

7979876	ENST00000413	LOC100130174	hypothetical LOC100130174	0.004	1.002	9.80E-01	9.80E+01		
7979878	NM_003049	SLC10A1	solute carrier family 10 (sodium/b	-0.007	0.995	9.88E-01	9.88E+01		
7979886	---	---	---	-0.034	0.976	9.00E-01	9.00E+01		
7979906	NM_016468	COX16	COX16 cytochrome c oxidase asse	0.111	1.080	2.94E-01	2.94E+01		
7979916	NM_018373	SYNJ2BP	synaptojanin 2 binding protein	0.046	1.032	8.87E-01	8.87E+01		
7979927	NM_003814	ADAM20	ADAM metallopeptidase domain 2	-0.015	0.990	9.53E-01	9.53E+01		
7979931	NM_005466	MED6	mediator complex subunit 6	-0.022	0.985	9.00E-01	9.00E+01		
7979943	NM_033141	MAP3K9	mitogen-activated protein kinase l	-0.012	0.992	9.50E-01	9.50E+01		
7979963	NM_012074	DPF3	D4, zinc and double PHD fingers, f	-0.016	0.989	9.16E-01	9.16E+01		
7979980	---	---	---	-0.006	0.996	9.81E-01	9.81E+01		
7979982	---	---	---	-0.090	0.940	4.62E-01	4.62E+01		
7979984	NM_021260	ZFYVE1	zinc finger, FYVE domain containin	0.006	1.004	9.77E-01	9.77E+01		
7980001	---	---	---	0.030	1.021	9.31E-01	9.31E+01		
7980003	---	---	---	-0.301	0.812	6.30E-02	6.30E+00		
7980005	NM_00100574	NUMB	numb homolog (Drosophila)	0.000	1.000	1.00E+00	1.00E+02		
7980024	NM_203309	HEATR4	HEAT repeat containing 4	-0.009	0.994	9.75E-01	9.75E+01		
7980044	NM_006029	PNMA1	paraneoplastic antigen MA1	-0.232	0.851	6.13E-03	6.13E-01		
7980051	NM_194278	C14orf43	chromosome 14 open reading fra	-0.037	0.975	7.14E-01	7.14E+01		
7980069	NM_182480	COQ6	coenzyme Q6 homolog, monooxyg	-0.041	0.972	7.16E-01	7.16E+01		
7980080	NM_001249	ENTPD5	ectonucleoside triphosphate dipho	0.154	1.112	3.28E-01	3.28E+01		
7980098	NM_005589	ALDH6A1	aldehyde dehydrogenase 6 family,	0.066	1.047	6.34E-01	6.34E+01		
7980115	NM_005050	ABCD4	ATP-binding cassette, sub-family D	-0.006	0.996	9.73E-01	9.73E+01		
7980140	---	---	---	0.018	1.012	9.50E-01	9.50E+01		
7980152	NM_000428	LTBP2	latent transforming growth factor	-0.049	0.967	6.79E-01	6.79E+01		
7980189	NM_00103947	KIAA0317	KIAA0317	0.000	1.000	1.00E+00	1.00E+02		
7980219	NM_031464	RPS6KL1	ribosomal protein S6 kinase-like 1	-0.057	0.961	5.60E-01	5.60E+01		
7980233	NM_002632	PGF	placental growth factor	-0.019	0.987	9.19E-01	9.19E+01		
7980246	NM_00104010	MLH3	mutL homolog 3 (E. coli)	0.044	1.031	7.75E-01	7.75E+01		
7980265	NM_203488	ACYP1	acylphosphatase 1, erythrocyte (c	0.024	1.016	9.24E-01	9.24E+01		
7980271	NM_033116	NEK9	NIMA (never in mitosis gene a)- re	0.074	1.053	4.29E-01	4.29E+01		
7980296	NM_006827	TMED10	transmembrane emp24-like traffic	0.218	1.163	2.75E-02	2.75E+00		
7980309	NM_007176	C14orf1	chromosome 14 open reading fra	-0.097	0.935	5.93E-01	5.93E+01		
7980316	NM_003239	TGFB3	transforming growth factor, beta 3	-0.017	0.988	9.44E-01	9.44E+01		
7980327	NM_015305	ANGEL1	angel homolog 1 (Drosophila)	0.084	1.060	2.78E-01	2.78E+01		
7980338	NM_024496	C14orf4	chromosome 14 open reading fra	0.056	1.040	6.57E-01	6.57E+01		
7980352	NM_021257	NGB	neuroglobin	-0.033	0.977	7.97E-01	7.97E+01		
7980358	NM_013382	POMT2	protein-O-mannosyltransferase 2	-0.064	0.957	5.40E-01	5.40E+01		
7980381	NM_213601	TMED8	transmembrane emp24 protein tra	0.144	1.105	1.55E-01	1.55E+01		
7980390	NM_00111347	C14orf148	chromosome 14 open reading fra	0.003	1.002	9.92E-01	9.92E+01		
7980403	BC015054	C14orf133	chromosome 14 open reading fra	0.056	1.040	8.10E-01	8.10E+01		
7980425	NM_199296	ISM2	isthmin 2 homolog (zebrafish)	-0.038	0.974	7.77E-01	7.77E+01		
7980438	NM_004863	SPTLC2	serine palmitoyltransferase, long c	0.032	1.022	9.00E-01	9.00E+01		
7980452	---	---	---	-0.003	0.998	9.91E-01	9.91E+01		
7980454	NM_006020	ALKBH1	alkB, alkylation repair homolog 1 (	0.076	1.054	7.92E-01	7.92E+01		
7980463	NM_012245	SNW1	SNW domain containing 1	-0.028	0.981	8.62E-01	8.62E+01		
7980477	---	---	---	0.047	1.033	8.84E-01	8.84E+01		
7980496	NM_152446	C14orf145	chromosome 14 open reading fra	-0.037	0.974	8.97E-01	8.97E+01		
7980523	NM_015859	GTF2A1	general transcription factor IIA, 1,	0.053	1.037	6.31E-01	6.31E+01		
7980535	NM_00103749	DYNLL1	dynein, light chain, LC8-type 1	0.053	1.037	8.10E-01	8.10E+01		
7980547	NM_005065	SEL1L	sel-1 suppressor of lin-12-like (C. e	0.192	1.142	6.99E-02	6.99E+00		
7980580	NM_000153	GALC	galactosylceramidase	0.222	1.166	3.17E-01	3.17E+01		
7980680	NM_00108547	FOXN3	forkhead box N3	0.243	1.183	3.25E-04	3.25E-02		
7980700	---	---	---	0.049	1.034	7.31E-01	7.31E+01		
7980702	NM_017970	C14orf102	chromosome 14 open reading fra	-0.010	0.993	9.65E-01	9.65E+01		
7980720	NM_00101085	TTC7B	tetratricopeptide repeat domain 7	0.139	1.101	2.93E-01	2.93E+01		
7980742	ENST00000433	LOC283588	hypothetical LOC283588	-0.226	0.855	3.95E-01	3.95E+01		
7980744	NM_004755	RPS6KA5	ribosomal protein S6 kinase, 90kD	0.043	1.030	8.47E-01	8.47E+01		
7980765	NM_003485	GPR68	G protein-coupled receptor 68	-0.213	0.863	6.89E-02	6.89E+00		
7980773	NM_00108041	CCDC88C	coiled-coil domain containing 88C	-0.062	0.958	7.14E-01	7.14E+01		
7980828	NM_00108041	CCDC88C	coiled-coil domain containing 88C	-0.037	0.975	9.36E-01	9.36E+01		
7980833	NM_032560	SMEK1	SMEK homolog 1, suppressor of m	-0.035	0.976	7.50E-01	7.50E+01		
7980859	NM_00108011	PP8961	hypothetical protein LOC650662	-0.020	0.986	8.96E-01	8.96E+01		
7980889	---	---	---	-0.028	0.980	8.50E-01	8.50E+01		
7980891	NM_00112859	TC2N	tandem C2 domains, nuclear	-0.004	0.997	9.89E-01	9.89E+01		
7980906	---	---	---	-0.041	0.972	9.20E-01	9.20E+01		
7980908	NM_006329	FBLN5	fibulin 5	0.080	1.057	4.31E-01	4.31E+01		
7980923	NM_004239	TRIP11	thyroid hormone receptor interact	-0.060	0.959	6.93E-01	6.93E+01		
7980940	NM_004993	ATXN3	ataxin 3	0.020	1.014	9.09E-01	9.09E+01		
7980955	NM_004545	NDUFB1	NADH dehydrogenase (ubiquinone	0.008	1.006	9.68E-01	9.68E+01		
7980970	NM_014216	ITPK1	inositol 1,3,4-triphosphate 5/6 kin	-0.037	0.975	8.27E-01	8.27E+01		
7980983	NM_022151	MOAP1	modulator of apoptosis 1	-0.007	0.995	9.75E-01	9.75E+01		
7980990	NM_032490	C14orf142	chromosome 14 open reading fra	0.016	1.011	9.46E-01	9.46E+01		
7980998	NM_00100286	BTBD7	BTB (POZ) domain containing 7	0.068	1.048	6.85E-01	6.85E+01		
7981013	NM_178013	PRIMA1	proline rich membrane anchor 1	0.025	1.017	8.69E-01	8.69E+01		
7981032	NM_020414	DDX24	DEAD (Asp-Glu-Ala-Asp) box polyp	-0.070	0.953	4.93E-01	4.93E+01		
7981046	NM_032036	IFI27L2	interferon, alpha-inducible protei	0.090	1.065	4.49E-01	4.49E+01		
7981051	NM_016186	SERPINA10	serpin peptidase inhibitor, clade A	-0.013	0.991	9.49E-01	9.49E+01		

7981068	NM_00100223	SERPINA1	serpin peptidase inhibitor, clade A	-0.450	0.732	3.45E-01	3.45E+01		
7981078	NM_00108045	SERPINA11	serpin peptidase inhibitor, clade A	0.022	1.015	8.71E-01	8.71E+01		
7981106	NM_173849	GSC	goosecoid homeobox	0.048	1.034	7.17E-01	7.17E+01		
7981111	NM_177438	DICER1	dicer 1, ribonuclease type III	0.236	1.178	1.85E-02	1.85E+00		
7981142	NM_024734	CLMN	calmin (calponin-like, transmembr	0.319	1.247	2.39E-01	2.39E+01		
7981157	NR_026779	C14orf139	chromosome 14 open reading fram	0.101	1.073	2.00E-01	2.00E+01		
7981161	NM_152592	C14orf49	chromosome 14 open reading fram	0.005	1.003	9.75E-01	9.75E+01		
7981181	NR_003002	SCARNA13	small Cajal body-specific RNA 13	0.058	1.041	9.09E-01	9.09E+01		
7981192	NM_018036	ATG2B	ATG2 autophagy related 2 homolo	-0.040	0.973	8.50E-01	8.50E+01		
7981215	---	---	---	0.139	1.101	5.51E-01	5.51E+01		
7981217	NM_018036	ATG2B	ATG2 autophagy related 2 homolo	-0.018	0.988	9.50E-01	9.50E+01		
7981242	NM_138576	BCL11B	B-cell CLL/lymphoma 11B (zinc fing	-0.032	0.978	8.16E-01	8.16E+01		
7981249	NM_032233	SETD3	SET domain containing 3	0.094	1.067	4.04E-01	4.04E+01		
7981266	NM_00114499	CCDC85C	coiled-coil domain containing 85C	-0.033	0.977	7.45E-01	7.45E+01		
7981273	NM_00114499	CCDC85C	coiled-coil domain containing 85C	-0.036	0.975	8.66E-01	8.66E+01		
7981278	NM_206918	DEGS2	degenerative spermatocyte homo	-0.062	0.958	4.94E-01	4.94E+01		
7981283	NM_00103935	SLC25A29	solute carrier family 25, member 2	0.014	1.010	9.51E-01	9.51E+01		
7981290	NM_004184	WARS	tryptophanyl-tRNA synthetase	0.462	1.377	7.17E-02	7.17E+00		
7981317	---	---	---	-0.010	0.993	9.56E-01	9.56E+01		
7981320	---	---	---	0.031	1.022	8.73E-01	8.73E+01		
7981326	AF305836	DIO3OS	DIO3 opposite strand (non-protein	-0.009	0.994	9.55E-01	9.55E+01		
7981333	---	---	---	-0.529	0.693	1.40E-01	1.40E+01		
7981335	NM_00101796	HSP90AA1	heat shock protein 90kDa alpha (c	-0.028	0.981	8.13E-01	8.13E+01		
7981364	NM_032630	CINP	cyclin-dependent kinase 2 interact	0.069	1.049	6.91E-01	6.91E+01		
7981377	NM_152326	ANKRD9	ankyrin repeat domain 9	0.015	1.011	9.37E-01	9.37E+01		
7981383	---	---	---	-0.009	0.994	9.73E-01	9.73E+01		
7981385	---	---	---	0.056	1.039	8.41E-01	8.41E+01		
7981387	NM_006035	CDC42BPB	CDC42 binding protein kinase beta	-0.032	0.978	8.79E-01	8.79E+01		
7981425	---	---	---	-0.006	0.996	9.74E-01	9.74E+01		
7981427	NM_001823	CKB	creatine kinase, brain	-0.070	0.953	6.66E-01	6.66E+01		
7981439	NM_00101504	BAG5	BCL2-associated athanogene 5	-0.044	0.970	7.52E-01	7.52E+01		
7981447	NM_00110011	XRCC3	X-ray repair complementing defec	-0.045	0.969	7.69E-01	7.69E+01		
7981460	NM_015316	PPP1R13B	protein phosphatase 1, regulatory	-0.246	0.843	6.11E-04	6.11E-02		
7981481	NM_004894	C14orf2	chromosome 14 open reading fram	0.017	1.012	9.28E-01	9.28E+01		
7981488	AF462446	LOC100288144	hypothetical protein LOC1002881	-0.069	0.954	4.23E-01	4.23E+01		
7981490	NM_207379	TMEM179	transmembrane protein 179	0.036	1.025	7.60E-01	7.60E+01		
7981494	NM_00101443	AKT1	v-akt murine thymoma viral oncog	-0.049	0.966	6.14E-01	6.14E+01		
7981512	---	---	---	-0.050	0.966	7.62E-01	7.62E+01		
7981523	---	---	---	0.044	1.031	8.14E-01	8.14E+01		
7981525	NM_017955	CDCA4	cell division cycle associated 4	-0.133	0.912	1.08E-01	1.08E+01		
7981530	NM_013345	GPR132	G protein-coupled receptor 132	-0.137	0.910	2.43E-01	2.43E+01		
7981538	NM_002226	JAG2	jagged 2	0.003	1.002	9.84E-01	9.84E+01		
7981566	NM_177533	NUDT14	nudix (nucleoside diphosphate link	0.029	1.020	8.16E-01	8.16E+01		
7981573	NM_145685	BRF1	BRF1 homolog, subunit of RNA pol	-0.003	0.998	9.84E-01	9.84E+01		
7981601	AK301335	IGHV4-31	immunoglobulin heavy variable 4-	0.294	1.226	7.12E-04	7.12E-02		
7981708	ENST00000390	IGHE	immunoglobulin heavy constant e	-0.042	0.971	8.66E-01	8.66E+01		
7981714	---	---	---	-0.014	0.990	9.61E-01	9.61E+01		
7981742	ENST00000390	LOC90925	hypothetical protein LOC90925	0.020	1.014	9.37E-01	9.37E+01		
7981750	AK127783	LOC400968	hypothetical LOC400968	0.015	1.010	9.50E-01	9.50E+01		
7981752	NR_027407	GOLGA8D	golgi autoantigen, golgin subfamily	-0.046	0.969	4.73E-01	4.73E+01		
7981771	---	---	---	-0.018	0.988	9.01E-01	9.01E+01		
7981773	---	---	---	0.137	1.099	6.09E-01	6.09E+01		
7981775	ENST00000435	DKFZP547L112	hypothetical protein DKFZp547L11	-0.028	0.981	8.88E-01	8.88E+01		
7981777	AK127783	LOC400968	hypothetical LOC400968	-0.078	0.947	5.60E-01	5.60E+01		
7981787	NM_00100141	LOC283767	golgi autoantigen, golgin subfamily	0.020	1.014	9.13E-01	9.13E+01		
7981824	NM_014608	CYFIP1	cytoplasmic FMR1 interacting prot	0.427	1.345	8.71E-02	8.71E+00		
7981859	NR_024074	GOLGA9P	golgi autoantigen, golgin subfamily	-0.017	0.988	9.16E-01	9.16E+01		
7981872	---	---	---	-0.010	0.993	9.61E-01	9.61E+01		
7981874	NM_00101242	GOLGA8E	golgi autoantigen, golgin subfamily	-0.027	0.981	7.15E-01	7.15E+01		
7981893	---	---	---	-0.018	0.988	9.01E-01	9.01E+01		
7981895	NM_00101242	GOLGA8E	golgi autoantigen, golgin subfamily	-0.026	0.982	8.27E-01	8.27E+01		
7981909	---	---	---	-0.007	0.995	9.77E-01	9.77E+01		
7981911	NM_005664	MKRN3	makorin ring finger protein 3	-0.009	0.994	9.77E-01	9.77E+01		
7981917	NM_018958	C15orf2	chromosome 15 open reading fram	-0.067	0.955	4.45E-01	4.45E+01		
7981919	NM_022807	SNRPN	small nuclear ribonucleoprotein po	-0.083	0.944	5.53E-01	5.53E+01		
7981943	NR_022008	PAR5	Prader-Willi/Angelman syndrome-	-0.082	0.945	8.64E-01	8.64E+01		
7981949	NR_003316	SNORD116-1	small nucleolar RNA, C/D box 116-	0.407	1.326	2.31E-01	2.31E+01		
7981951	NR_003317	SNORD116-2	small nucleolar RNA, C/D box 116-	0.097	1.070	7.24E-01	7.24E+01		
7981953	NR_003318	SNORD116-3	small nucleolar RNA, C/D box 116-	0.316	1.245	3.18E-01	3.18E+01		
7981955	NR_003319	SNORD116-4	small nucleolar RNA, C/D box 116-	0.205	1.153	5.42E-01	5.42E+01		
7981958	NR_003320	SNORD116-5	small nucleolar RNA, C/D box 116-	0.307	1.237	3.64E-01	3.64E+01		
7981960	NR_003321	SNORD116-6	small nucleolar RNA, C/D box 116-	0.092	1.066	7.87E-01	7.87E+01		
7981962	NR_003320	SNORD116-5	small nucleolar RNA, C/D box 116-	0.307	1.237	3.64E-01	3.64E+01		
7981964	NR_003323	SNORD116-8	small nucleolar RNA, C/D box 116-	0.347	1.272	2.84E-01	2.84E+01		
7981966	NR_003318	SNORD116-3	small nucleolar RNA, C/D box 116-	0.316	1.245	3.18E-01	3.18E+01		
7981976	NR_003329	SNORD116-14	small nucleolar RNA, C/D box 116-	0.229	1.172	6.08E-01	6.08E+01		
7981978	NR_003330	SNORD116-15	small nucleolar RNA, C/D box 116-	0.069	1.049	8.38E-01	8.38E+01		

7981982	NR_003332	SNORD116-17	small nucleolar RNA, C/D box 116-	0.011	1.008	9.86E-01	9.86E+01			
7981986	NR_003332	SNORD116-17	small nucleolar RNA, C/D box 116-	0.011	1.008	9.86E-01	9.86E+01			
7981988	NR_003334	SNORD116-20	small nucleolar RNA, C/D box 116-	0.022	1.016	9.74E-01	9.74E+01			
7981990	NR_003335	SNORD116-21	small nucleolar RNA, C/D box 116-	-0.100	0.933	8.77E-01	8.77E+01			
7982100	---	---	---	0.399	1.319	2.19E-01	2.19E+01			
7982129	ENST00000358	RPL41	ribosomal protein L41	-0.010	0.993	9.35E-01	9.35E+01			
7982131	NM_00101242	GOLGA8G	golgi autoantigen, golgin subfamily	-0.049	0.967	4.29E-01	4.29E+01			
7982152	---	---	---	0.041	1.029	8.06E-01	8.06E+01			
7982154	NR_002824	HERC2P2	hect domain and RLD 2 pseudoger	-0.009	0.994	9.71E-01	9.71E+01			
7982187	NM_005503	APBA2	amyloid beta (A4) precursor prote	0.047	1.033	8.58E-01	8.58E+01			
7982204	NM_005517	HMGN2	high-mobility group nucleosomal b	0.069	1.049	6.25E-01	6.25E+01			
7982206	NR_024074	GOLGA9P	golgi autoantigen, golgin subfamily	0.017	1.012	9.07E-01	9.07E+01			
7982228	---	---	---	-0.041	0.972	8.09E-01	8.09E+01			
7982230	NR_024074	GOLGA9P	golgi autoantigen, golgin subfamily	0.019	1.013	9.25E-01	9.25E+01			
7982250	---	---	---	-0.041	0.972	8.09E-01	8.09E+01			
7982254	---	---	---	0.031	1.021	8.35E-01	8.35E+01			
7982256	NR_024074	GOLGA9P	golgi autoantigen, golgin subfamily	-0.038	0.974	8.66E-01	8.66E+01			
7982267	---	---	---	-0.042	0.971	8.07E-01	8.07E+01			
7982271	NR_024074	GOLGA9P	golgi autoantigen, golgin subfamily	0.062	1.044	7.44E-01	7.44E+01			
7982282	---	---	---	-0.033	0.978	8.49E-01	8.49E+01			
7982284	ENST00000314	LOC728047	similar to Golgin subfamily A mem	-0.107	0.928	3.29E-01	3.29E+01			
7982287	NM_00103984	ARHGAP11B	Rho GTPase activating protein 11B	0.019	1.013	9.74E-01	9.74E+01			
7982290	---	---	---	0.060	1.042	9.23E-01	9.23E+01			
7982307	---	---	---	-0.033	0.978	8.49E-01	8.49E+01			
7982309	NM_014967	MTMR15	myotubularin related protein 15	0.120	1.086	5.57E-01	5.57E+01			
7982326	NM_015995	KLF13	Kruppel-like factor 13	-0.051	0.965	6.35E-01	6.35E+01			
7982333	AK127052	LOC283711	hypothetical protein LOC283711	-0.024	0.983	8.98E-01	8.98E+01			
7982339	AK097050	LOC100130857	hypothetical protein LOC1001308	-0.074	0.950	6.42E-01	6.42E+01			
7982341	NM_000746	CHRNA7	cholinergic receptor, nicotinic, alp	0.014	1.009	9.64E-01	9.64E+01			
7982348	ENST00000416	LOC100129979	similar to FLJ00287 protein	-0.044	0.970	6.94E-01	6.94E+01			
7982350	NR_024074	GOLGA9P	golgi autoantigen, golgin subfamily	-0.065	0.956	7.87E-01	7.87E+01			
7982356	---	---	---	-0.041	0.972	8.25E-01	8.25E+01			
7982377	NM_013372	GREM1	gremlin 1, cysteine knot superfam	0.081	1.058	7.15E-01	7.15E+01			
7982388	---	---	---	0.025	1.018	9.80E-01	9.80E+01			
7982497	NM_012125	CHRM5	cholinergic receptor, muscarinic 5	0.041	1.029	8.05E-01	8.05E+01			
7982504	NM_152595	PGBD4	piggyBac transposable element de	0.065	1.046	6.71E-01	6.71E+01			
7982507	NM_016454	TMEM85	transmembrane protein 85	-0.146	0.904	5.53E-02	5.53E+00			
7982514	---	---	---	0.203	1.151	4.25E-01	4.25E+01			
7982527	NM_014106	ZNF770	zinc finger protein 770	0.001	1.001	9.98E-01	9.98E+01			
7982529	ENST00000454	LOC441722	U2 small nuclear RNA auxiliary fac	-0.061	0.958	6.93E-01	6.93E+01			
7982531	NM_006305 //	// ANP32A //	acidic (leucine-rich) nuclear phosp	-0.006	0.996	9.68E-01	9.68E+01			
7982535	NM_032499	C15orf41	chromosome 15 open reading fran	-0.108	0.928	4.01E-01	4.01E+01			
7982587	NM_207444	C15orf53	chromosome 15 open reading fran	-0.061	0.959	6.44E-01	6.44E+01			
7982597	NM_003246	THBS1	thrombospondin 1	-0.182	0.882	9.04E-01	9.04E+01			
7982620	NM_00101370	EIF2AK4	eukaryotic translation initiation fa	0.011	1.008	9.64E-01	9.64E+01			
7982688	NM_020168	PAK6	p21 protein (Cdc42/Rac)-activated	-0.040	0.973	7.51E-01	7.51E+01			
7982703	NM_033510	DISP2	dispatched homolog 2 (Drosophila	0.042	1.030	6.25E-01	6.25E+01			
7982712	NM_033286	C15orf23	chromosome 15 open reading fran	0.088	1.063	6.54E-01	6.54E+01			
7982723	NM_002225	IVD	isovaleryl Coenzyme A dehydroger	0.027	1.019	8.78E-01	8.78E+01			
7982738	NM_014952	BAHD1	bromo adjacent homology domair	-0.083	0.944	3.91E-01	3.91E+01			
7982745	NM_130468	CHST14	carbohydrate (N-acetylgalactosam	-0.022	0.985	9.08E-01	9.08E+01			
7982753	NM_152260	RPUSD2	RNA pseudouridylate synthase dor	-0.001	0.999	9.95E-01	9.95E+01			
7982805	NM_005258	GCHFR	GTP cyclohydrolase I feedback reg	-0.012	0.992	9.50E-01	9.50E+01			
7982810	NM_00113044	C15orf62	chromosome 15 open reading fran	-0.012	0.992	9.49E-01	9.49E+01			
7982812	NM_00107726	ZFYVE19	zinc finger, FYVE domain containin	-0.029	0.980	8.07E-01	8.07E+01			
7982829	NM_181642	SPINT1	serine peptidase inhibitor, Kunitz t	-0.060	0.960	6.18E-01	6.18E+01			
7982845	NM_020857	VPS18	vacuolar protein sorting 18 homol	0.052	1.037	7.56E-01	7.56E+01			
7982868	NM_024111	CHAC1	ChaC, cation transport regulator h	-0.040	0.973	7.68E-01	7.68E+01			
7982878	NM_007236	CHP	calcium binding protein P22	0.054	1.038	8.05E-01	8.05E+01			
7982904	NM_015138	RTF1	Rtf1, Paf1/RNA polymerase II com	-0.013	0.991	9.47E-01	9.47E+01			
7982927	NM_002220	ITPKA	inositol 1,4,5-trisphosphate 3-kina	-0.068	0.954	4.68E-01	4.68E+01			
7982935	---	---	---	0.105	1.075	5.41E-01	5.41E+01			
7982957	NM_00108054	MGA	MAX gene associated	0.012	1.009	9.53E-01	9.53E+01			
7982985	NM_014994	MAPKBP1	mitogen-activated protein kinase I	-0.020	0.986	9.04E-01	9.04E+01			
7983021	NR_015346	JMJD7-PLA2G4E	JMJD7-PLA2G4B readthrough tran	-0.035	0.976	7.99E-01	7.99E+01			
7983051	NM_016642	SPTBN5	spectrin, beta, non-erythrocytic 5	-0.140	0.908	7.38E-01	7.38E+01			
7983054	NR_027911	CAPN3	calpain 3, (p94)	0.178	1.131	2.95E-02	2.95E+00			
7983111	NM_003825	SNAP23	synaptosomal-associated protein,	0.149	1.109	1.18E-01	1.18E+01			
7983123	NM_018097	HAUS2	HAUS augmin-like complex, subun	0.044	1.031	8.87E-01	8.87E+01			
7983132	AK122666	LOC645106	similar to kinesin-like motor prote	-0.019	0.987	9.53E-01	9.53E+01			
7983145	AK297603	STARD9	StAR-related lipid transfer (START)	0.004	1.003	9.86E-01	9.86E+01			
7983157	NM_024956	TMEM62	transmembrane protein 62	0.184	1.136	1.85E-01	1.85E+01			
7983173	NM_012142	CCNDBP1	cyclin D-type binding-protein 1	-0.025	0.983	8.56E-01	8.56E+01			
7983206	NM_014444	TUBGCP4	tubulin, gamma complex associate	0.066	1.047	7.01E-01	7.01E+01			
7983252	---	---	---	0.041	1.029	7.01E-01	7.01E+01			
7983274	NM_005313	PDIA3	protein disulfide isomerase family	0.244	1.185	2.37E-02	2.37E+00			
7983290	NM_00101810	SERF2	small EDRK-rich factor 2	0.092	1.066	3.29E-01	3.29E+01			

7983298	NM_016400	C15orf63	chromosome 15 open reading fran	-0.013	0.991	9.44E-01	9.44E+01		
7983306	NM_024908	WDR76	WD repeat domain 76	0.014	1.010	9.68E-01	9.68E+01		
7983321	NM_138423	CASC4	cancer susceptibility candidate 4	-0.042	0.971	7.15E-01	7.15E+01		
7983335	NM_016396	CTDSPL2	CTD (carboxy-terminal domain, RN	0.011	1.007	9.60E-01	9.60E+01		
7983350	NM_003758	EIF3J	eukaryotic translation initiation fa	0.015	1.010	9.28E-01	9.28E+01		
7983360	NM_004048	B2M	beta-2-microglobulin	0.031	1.022	6.79E-01	6.79E+01		
7983365	NM_182985	TRIM69	tripartite motif-containing 69	-0.029	0.980	8.73E-01	8.73E+01		
7983393	NM_003104	SORD	sorbitol dehydrogenase	0.067	1.047	6.98E-01	6.98E+01		
7983469	NR_027635	SPATA5L1	spermatogenesis associated 5-like	0.060	1.042	7.85E-01	7.85E+01		
7983502	NM_012388	PLDN	pallidin homolog (mouse)	0.148	1.108	7.05E-02	7.05E+00		
7983512	NM_021199	SQRDL	sulfide quinone reductase-like (ye	-0.016	0.989	9.76E-01	9.76E+01		
7983553	NM_205850	SLC24A5	solute carrier family 24, member 5	-0.024	0.984	9.13E-01	9.13E+01		
7983594	NM_00102524	DUT	deoxyuridine triphosphatase	0.044	1.031	7.91E-01	7.91E+01		
7983606	NM_014335	EID1	EP300 interacting inhibitor of diffe	-0.029	0.980	8.25E-01	8.25E+01		
7983616	NM_00100155	GALK2	galactokinase 2	0.130	1.095	1.69E-01	1.69E+01		
7983648	---	---	---	-0.176	0.885	7.09E-01	7.09E+01		
7983663	NM_005154	USP8	ubiquitin specific peptidase 8	0.073	1.052	6.54E-01	6.54E+01		
7983677	---	---	---	0.016	1.011	9.49E-01	9.49E+01		
7983679	NM_007347	AP4E1	adaptor-related protein complex 4	0.051	1.036	7.97E-01	7.97E+01		
7983734	NM_014548	TMOD2	tropomodulin 2 (neuronal)	-0.044	0.970	8.31E-01	8.31E+01		
7983744	NM_014547	TMOD3	tropomodulin 3 (ubiquitous)	-0.035	0.976	8.46E-01	8.46E+01		
7983761	---	---	---	-0.079	0.947	8.43E-01	8.43E+01		
7983763	NM_002748	MAPK6	mitogen-activated protein kinase	0.061	1.044	7.41E-01	7.41E+01		
7983771	---	---	---	0.222	1.166	4.60E-01	4.60E+01		
7983773	---	---	---	0.063	1.045	7.38E-01	7.38E+01		
7983811	NM_004855	PIGB	phosphatidylinositol glycan ancho	0.116	1.084	3.76E-01	3.76E+01		
7983826	---	---	---	-0.105	0.930	5.33E-01	5.33E+01		
7983843	NM_207036	TCF12	transcription factor 12	0.014	1.010	9.23E-01	9.23E+01		
7983910	NM_020980	AQP9	aquaporin 9	-0.664	0.631	2.53E-01	2.53E+01		
7983938	---	---	---	-0.019	0.987	9.10E-01	9.10E+01		
7983940	NM_00104045	FAM63B	family with sequence similarity 63	-0.158	0.896	4.64E-01	4.64E+01		
7983951	---	---	---	0.094	1.067	7.84E-01	7.84E+01		
7983953	NM_017610	RNF111	ring finger protein 111	0.053	1.038	6.89E-01	6.89E+01		
7983979	---	---	---	0.151	1.110	2.95E-01	2.95E+01		
7983987	---	---	---	0.059	1.042	6.99E-01	6.99E+01		
7984008	---	---	---	-0.108	0.928	7.49E-01	7.49E+01		
7984011	NM_012182	FOXB1	forkhead box B1	-0.038	0.974	7.92E-01	7.92E+01		
7984014	---	---	---	-0.086	0.942	5.08E-01	5.08E+01		
7984016	NM_207322	FAM148A	family with sequence similarity 14	0.006	1.004	9.75E-01	9.75E+01		
7984079	NM_000366	TPM1	tropomyosin 1 (alpha)	0.043	1.030	7.78E-01	7.78E+01		
7984103	NM_032857	LACTB	lactamase, beta	0.094	1.068	6.74E-01	6.74E+01		
7984112	NM_016530	RAB8B	RAB8B, member RAS oncogene fa	0.013	1.009	9.56E-01	9.56E+01		
7984124	NM_031301	APH1B	anterior pharynx defective 1 hom	-0.129	0.914	5.26E-01	5.26E+01		
7984132	NM_006537	USP3	ubiquitin specific peptidase 3	-0.045	0.970	7.68E-01	7.68E+01		
7984155	NM_003099	SNX1	sorting nexin 1	0.181	1.134	1.02E-01	1.02E+01		
7984174	NM_024798	SNX22	sorting nexin 22	-0.045	0.969	6.89E-01	6.89E+01		
7984188	---	---	---	0.065	1.046	8.10E-01	8.10E+01		
7984190	NM_016213	TRIP4	thyroid hormone receptor interact	0.091	1.065	6.68E-01	6.68E+01		
7984203	NM_015042	ZNF609	zinc finger protein 609	-0.065	0.956	6.98E-01	6.98E+01		
7984217	NM_025201	PLEKHO2	pleckstrin homology domain conta	0.082	1.059	6.02E-01	6.02E+01		
7984227	NM_182703	ANKDD1A	ankyrin repeat and death domain	0.037	1.026	7.23E-01	7.23E+01		
7984245	NM_178859	OSTbeta	organic solute transporter beta	-0.078	0.947	3.36E-01	3.36E+01		
7984253	NM_00110136	hCG_1645727	hCG1645727	0.000	1.000	1.00E+00	1.00E+02		
7984263	NM_016395	PTPLAD1	protein tyrosine phosphatase-like	-0.040	0.972	8.11E-01	8.11E+01		
7984276	NM_004727	SLC24A1	solute carrier family 24 (sodium/p	0.019	1.013	9.22E-01	9.22E+01		
7984289	NM_004663	RAB11A	RAB11A, member RAS oncogene f	0.037	1.026	7.62E-01	7.62E+01		
7984298	NM_133375	DIS3L	DIS3 mitotic control homolog (S. c	0.062	1.044	7.59E-01	7.59E+01		
7984319	NM_002755	MAP2K1	mitogen-activated protein kinase	-0.122	0.919	1.66E-01	1.66E+01		
7984353	NR_027654	SMAD6	SMAD family member 6	-0.016	0.989	8.80E-01	8.80E+01		
7984364	NM_005902	SMAD3	SMAD family member 3	-0.137	0.910	3.60E-01	3.60E+01		
7984405	NM_00114393	C15orf61	chromosome 15 open reading fran	0.020	1.014	9.53E-01	9.53E+01		
7984408	NM_145160	MAP2K5	mitogen-activated protein kinase	-0.052	0.965	7.86E-01	7.86E+01		
7984436	NM_00103180	LBXCOR1	Lbxcor1 homolog (mouse)	0.016	1.011	9.08E-01	9.08E+01		
7984453	NM_016166	PIAS1	protein inhibitor of activated STAT	0.064	1.045	6.51E-01	6.51E+01		
7984470	NM_015322	FEM1B	fem-1 homolog b (C. elegans)	0.144	1.105	1.23E-02	1.23E+00		
7984517	NM_015554	GLCE	glucuronic acid epimerase	0.054	1.038	8.33E-01	8.33E+01		
7984538	---	---	---	0.008	1.006	9.65E-01	9.65E+01		
7984540	NM_138555	KIF23	kinesin family member 23	0.230	1.173	2.39E-01	2.39E+01		
7984562	NM_001003	RPLP1	ribosomal protein, large, P1	-0.050	0.966	7.56E-01	7.56E+01		
7984606	NM_014249	NR2E3	nuclear receptor subfamily 2, grou	-0.019	0.987	8.94E-01	8.94E+01		
7984620	---	---	---	-0.061	0.958	8.02E-01	8.02E+01		
7984624	---	---	---	-0.208	0.866	5.24E-01	5.24E+01		
7984641	NM_005744	ARIH1	ariadne homolog, ubiquitin-conjug	-0.060	0.959	6.31E-01	6.31E+01		
7984660	---	---	---	-0.141	0.907	2.26E-01	2.26E+01		
7984662	NM_018652	GOLGA6B	golgi autoantigen, golgin subfamil	-0.036	0.975	6.78E-01	6.78E+01		
7984686	NM_033028	BBS4	Bardet-Biedl syndrome 4	0.093	1.067	6.52E-01	6.52E+01		
7984704	NM_002499	NEO1	neogenin homolog 1 (chicken)	-0.037	0.975	8.93E-01	8.93E+01		

7984743	NM_00102473	CD276	CD276 molecule	0.079	1.057	6.30E-01	6.30E+01		
7984759	NM_153356	TBC1D21	TBC1 domain family, member 21	0.006	1.004	9.77E-01	9.77E+01		
7984771	NM_005576	LOXL1	lysyl oxidase-like 1	0.001	1.001	9.97E-01	9.97E+01		
7984779	NM_033240	PML	promyelocytic leukemia	0.033	1.023	8.62E-01	8.62E+01		
7984799	---	---	---	0.004	1.003	9.85E-01	9.85E+01		
7984819	NM_025055	CCDC33	coiled-coil domain containing 33	0.010	1.007	9.27E-01	9.27E+01		
7984843	NM_006465	ARID3B	AT rich interactive domain 3B (BRI	-0.118	0.921	5.92E-01	5.92E+01		
7984846	NM_00113002	CLK3	CDC-like kinase 3	-0.006	0.996	9.78E-01	9.78E+01		
7984862	NM_000761	CYP1A2	cytochrome P450, family 1, subfar	-0.011	0.992	9.44E-01	9.44E+01		
7984871	NM_004383	CSK	c-src tyrosine kinase	0.074	1.053	4.36E-01	4.36E+01		
7984892	NM_021819	LMAN1L	lectin, mannose-binding, 1 like	-0.056	0.962	4.88E-01	4.88E+01		
7984922	NM_002435	MPI	mannose phosphate isomerase	0.047	1.033	7.46E-01	7.46E+01		
7984932	NM_138967	SCAMP5	secretory carrier membrane prote	-0.031	0.978	8.71E-01	8.71E+01		
7984952	NM_015492	C15orf39	chromosome 15 open reading frar	0.018	1.012	8.93E-01	8.93E+01		
7984961	NM_018652	GOLGA6B	golgi autoantigen, golgin subfamily	-0.038	0.974	6.57E-01	6.57E+01		
7984985	NM_00103864	GOLGA6	golgi autoantigen, golgin subfamily	-0.057	0.961	8.37E-01	8.37E+01		
7984989	NM_017828	COMMD4	COMM domain containing 4	0.051	1.036	7.27E-01	7.27E+01		
7985001	NM_024608	NEIL1	nei endonuclease VIII-like 1 (E. col	-0.079	0.947	3.98E-01	3.98E+01		
7985023	---	---	---	-0.004	0.997	9.86E-01	9.86E+01		
7985030	ENST00000428	DNM1P35	DNM1 pseudogene 35	-0.052	0.964	7.16E-01	7.16E+01		
7985034	---	---	---	-0.038	0.974	9.01E-01	9.01E+01		
7985044	NM_173469	UBE2Q2	ubiquitin-conjugating enzyme E2Q	-0.060	0.959	5.80E-01	5.80E+01		
7985053	NM_147188	FBXO22	F-box protein 22	-0.037	0.974	8.33E-01	8.33E+01		
7985066	NM_152335	C15orf27	chromosome 15 open reading frar	0.006	1.004	9.74E-01	9.74E+01		
7985080	NM_145805	ISL2	ISL LIM homeobox 2	-0.049	0.966	6.60E-01	6.60E+01		
7985089	NM_002902	RCN2	reticulocalbin 2, EF-hand calcium b	-0.070	0.953	5.48E-01	5.48E+01		
7985099	NM_003978	PSTPIP1	proline-serine-threonine phosphat	-0.059	0.960	5.74E-01	5.74E+01		
7985117	---	---	---	0.032	1.022	8.27E-01	8.27E+01		
7985119	NM_018200	HMG20A	high-mobility group 20A	0.006	1.004	9.81E-01	9.81E+01		
7985134	NM_005530	IDH3A	isocitrate dehydrogenase 3 (NAD+	0.053	1.038	7.48E-01	7.48E+01		
7985147	NM_018602	DNAJA4	DnaJ (Hsp40) homolog, subfamily	-0.063	0.957	7.27E-01	7.27E+01		
7985166	NM_004136	IREB2	iron-responsive element binding p	0.127	1.092	2.26E-01	2.26E+01		
7985202	NM_002789	PSMA4	proteasome (prosome, macropain	0.101	1.073	5.88E-01	5.88E+01		
7985221	---	---	---	-0.143	0.906	2.99E-01	2.99E+01		
7985224	NM_144572	TBC1D2B	TBC1 domain family, member 2B	-0.061	0.958	8.92E-01	8.92E+01		
7985226	NM_206839	MORF4L1	mortality factor 4 like 1	-0.016	0.989	9.00E-01	9.00E+01		
7985233	NM_002891	RASGRF1	Ras protein-specific guanine nucle	-0.050	0.966	8.13E-01	8.13E+01		
7985240	NM_007364	TMED3	transmembrane emp24 protein tr	-0.008	0.995	9.64E-01	9.64E+01		
7985248	NM_015206	KIAA1024	KIAA1024	-0.055	0.963	7.44E-01	7.44E+01		
7985253	BC101201	C15orf37	chromosome 15 open reading frar	-0.041	0.972	7.87E-01	7.87E+01		
7985257	---	---	---	-0.019	0.987	9.14E-01	9.14E+01		
7985259	NM_019006	ZFAND6	zinc finger, AN1-type domain 6	0.016	1.011	9.49E-01	9.49E+01		
7985266	---	---	---	0.056	1.040	7.73E-01	7.73E+01		
7985268	NM_000137	FAH	fumarylacetoacetate hydrolase (fu	-0.094	0.937	5.26E-01	5.26E+01		
7985285	NM_014862	ARNT2	aryl-hydrocarbon receptor nuclear	0.268	1.204	2.74E-01	2.74E+01		
7985310	NM_021214	FAM108C1	family with sequence similarity 10	-0.029	0.980	8.96E-01	8.96E+01		
7985317	NM_018689	KIAA1199	KIAA1199	0.113	1.082	8.72E-01	8.72E+01		
7985349	NM_022566	MESDC1	mesoderm development candidate	-0.060	0.959	6.57E-01	6.57E+01		
7985364	NM_172217	IL16	interleukin 16 (lymphocyte chemo	-0.010	0.993	9.40E-01	9.40E+01		
7985402	NR_024474	LOC100133144	hypothetical protein LOC1001331	-0.095	0.936	5.48E-01	5.48E+01		
7985409	NR_003246	FLJ40113	golgi autoantigen, golgin subfamily	-0.116	0.923	2.28E-01	2.28E+01		
7985418	ENST00000342	LOC440297	chondroitin sulfate proteoglycan 4	-0.052	0.964	4.03E-01	4.03E+01		
7985431	NR_026811	LOC80154	hypothetical LOC80154	-0.055	0.962	3.80E-01	3.80E+01		
7985444	NR_003661	FLJ43276	similar to ubiquitin-conjugating en	-0.095	0.936	5.15E-01	5.15E+01		
7985450	NR_003246	FLJ40113	golgi autoantigen, golgin subfamily	-0.116	0.923	2.28E-01	2.28E+01		
7985459	ENST00000342	LOC440297	chondroitin sulfate proteoglycan 4	-0.052	0.964	4.03E-01	4.03E+01		
7985472	NR_003246	FLJ40113	golgi autoantigen, golgin subfamily	-0.116	0.922	3.38E-01	3.38E+01		
7985482	NM_00108043	WHAMM	WAS protein homolog associated	0.004	1.002	9.86E-01	9.86E+01		
7985488	BC112329	FAM103A1	family with sequence similarity 10	0.048	1.034	5.99E-01	5.99E+01		
7985493	NM_023003	TM6SF1	transmembrane 6 superfamily me	0.460	1.376	5.45E-02	5.45E+00		
7985562	NR_003246	FLJ40113	golgi autoantigen, golgin subfamily	-0.117	0.922	3.41E-01	3.41E+01		
7985571	NR_026811	LOC80154	hypothetical LOC80154	-0.028	0.981	8.29E-01	8.29E+01		
7985577	NM_181877	ZSCAN2	zinc finger and SCAN domain conta	-0.115	0.923	1.73E-01	1.73E+01		
7985587	NR_004859	SCAND2	SCAN domain containing 2 pseudo	0.039	1.027	8.08E-01	8.08E+01		
7985605	NM_014630	ZNF592	zinc finger protein 592	-0.020	0.986	9.09E-01	9.09E+01		
7985620	NM_020778	ALPK3	alpha-kinase 3	-0.063	0.957	5.35E-01	5.35E+01		
7985636	NM_004213	SLC28A1	solute carrier family 28 (sodium-co	-0.010	0.993	9.40E-01	9.40E+01		
7985662	NM_002605	PDE8A	phosphodiesterase 8A	-0.061	0.958	7.08E-01	7.08E+01		
7985690	BX648930	LOC440300	chondroitin sulfate proteoglycan 4	-0.032	0.978	7.69E-01	7.69E+01		
7985695	NM_006738	AKAP13	A kinase (PRKA) anchor protein 13	-0.056	0.962	5.09E-01	5.09E+01		
7985757	NM_022839	MRPS11	mitochondrial ribosomal protein S	-0.084	0.943	4.48E-01	4.48E+01		
7985765	---	---	---	0.001	1.001	9.95E-01	9.95E+01		
7985767	NM_022767	AEN	apoptosis enhancing nuclease	0.048	1.034	7.04E-01	7.04E+01		
7985777	NM_002201	ISG20	interferon stimulated exonuclease	0.009	1.006	9.81E-01	9.81E+01		
7985786	NM_013227	ACAN	aggrecan	-0.091	0.939	2.93E-01	2.93E+01		
7985918	---	---	---	-0.007	0.995	9.80E-01	9.80E+01		
7985920	NM_00103995	MESP2	mesoderm posterior 2 homolog (m	-0.044	0.970	7.07E-01	7.07E+01		

7985924	NM_198526	ZNF710	zinc finger protein 710	0.026	1.018	8.21E-01	8.21E+01				
7985930	---	---	---	0.034	1.024	8.18E-01	8.18E+01				
7985932	---	---	---	0.020	1.014	8.81E-01	8.81E+01				
7985934	NM_020210	SEMA4B	sema domain, immunoglobulin do	-0.040	0.972	7.25E-01	7.25E+01				
7985983	NM_016645	NGRN	neugrin, neurite outgrowth associ	0.001	1.001	9.94E-01	9.94E+01				
7985997	---	---	---	-0.090	0.940	6.82E-01	6.82E+01				
7986001	ENST00000338	LOC728477	similar to hCG27445	-0.023	0.984	8.84E-01	8.84E+01				
7986004	NM_00100430	ZNF774	zinc finger protein 774	0.020	1.014	9.05E-01	9.05E+01				
7986010	NM_003870	IQGAP1	IQ motif containing GTPase activat	0.068	1.048	7.41E-01	7.41E+01				
7986049	NM_022769	CRTC3	CREB regulated transcription coac	0.054	1.038	5.53E-01	5.53E+01				
7986068	NM_000057	BLM	Bloom syndrome, RecQ helicase-li	-0.029	0.980	9.51E-01	9.51E+01				
7986092	NM_002569	FURIN	furin (paired basic amino acid clea	-0.137	0.909	3.02E-01	3.02E+01				
7986110	NM_002005	FES	feline sarcoma oncogene	-0.054	0.963	5.34E-01	5.34E+01				
7986132	NM_006122	MAN2A2	mannosidase, alpha, class 2A, mer	-0.037	0.975	7.58E-01	7.58E+01				
7986160	NM_00103967	UNC45A	unc-45 homolog A (C. elegans)	-0.006	0.996	9.75E-01	9.75E+01				
7986186	NM_033544	RCCD1	RCC1 domain containing 1	0.018	1.013	9.16E-01	9.16E+01				
7986214	NM_013272	SLCO3A1	solute carrier organic anion transp	0.157	1.115	3.18E-01	3.18E+01				
7986246	---	---	---	0.003	1.002	9.91E-01	9.91E+01				
7986248	AY358245	UNQ6190	AGVR6190	-0.011	0.993	9.57E-01	9.57E+01				
7986250	NM_001271	CHD2	chromodomain helicase DNA bind	-0.105	0.930	8.87E-02	8.87E+00				
7986293	NM_018349	MCTP2	multiple C2 domains, transmembr	-0.049	0.967	8.78E-01	8.78E+01				
7986323	NM_015710	GLTSCR2	glioma tumor suppressor candidat	-0.030	0.980	9.19E-01	9.19E+01				
7986329	NM_021005	NR2F2	nuclear receptor subfamily 2, grou	-0.072	0.952	5.55E-01	5.55E+01				
7986336	ENST00000444	LOC728800	similar to FLJ00402 protein	0.043	1.031	7.17E-01	7.17E+01				
7986350	NM_183376	ARRDC4	arrestin domain containing 4	0.178	1.131	6.35E-01	6.35E+01				
7986359	NM_000875	IGF1R	insulin-like growth factor 1 recept	-0.013	0.991	9.72E-01	9.72E+01				
7986383	NM_000875	IGF1R	insulin-like growth factor 1 recept	-0.016	0.989	9.67E-01	9.67E+01				
7986385	NM_145728	SYNM	synemin, intermediate filament pr	-0.134	0.911	5.65E-01	5.65E+01				
7986394	NM_144598	LRRC28	leucine rich repeat containing 28	0.057	1.040	7.89E-01	7.89E+01				
7986411	NM_005587	MEF2A	myocyte enhancer factor 2A	0.202	1.150	6.75E-03	6.75E-01				
7986428	AK127420	LOC400464	similar to FLJ43276 protein	0.016	1.011	9.30E-01	9.30E+01				
7986433	NM_198243	ASB7	ankyrin repeat and SOCS box-cont	-0.091	0.939	4.49E-01	4.49E+01				
7986442	---	---	---	-0.154	0.899	4.20E-01	4.20E+01				
7986444	AK056534	LOC145757	hypothetical LOC145757	-0.008	0.994	9.67E-01	9.67E+01				
7986446	NM_000693	ALDH1A3	aldehyde dehydrogenase 1 family,	-0.004	0.997	9.85E-01	9.85E+01				
7986463	NM_024652	LRRK1	leucine-rich repeat kinase 1	0.130	1.095	7.63E-02	7.63E+00				
7986501	---	---	---	0.079	1.056	8.82E-01	8.82E+01				
7986503	NR_003260	C15orf51	dynamin 1 pseudogene	-0.085	0.943	5.82E-01	5.82E+01				
7986509	ENST00000423	C15orf51	dynamin 1 pseudogene	0.000	1.000	1.00E+00	1.00E+02				
7986512	ENST00000426	LOC100289668	similar to dynamin 1	0.000	1.000	1.00E+00	1.00E+02				
7986515	ENST00000426	LOC100289668	similar to dynamin 1	0.120	1.087	7.61E-01	7.61E+01				
7986517	ENST00000423	C15orf51	dynamin 1 pseudogene	-0.033	0.978	9.58E-01	9.58E+01				
7986520	AK302717	C15orf51	dynamin 1 pseudogene	-0.013	0.991	9.85E-01	9.85E+01				
7986522	ENST00000423	C15orf51	dynamin 1 pseudogene	-0.033	0.978	9.58E-01	9.58E+01				
7986525	ENST00000426	LOC100289668	similar to dynamin 1	0.120	1.087	7.61E-01	7.61E+01				
7986527	AK125787	C15orf51	dynamin 1 pseudogene	0.000	1.000	1.00E+00	1.00E+02				
7986541	NR_003659	WASH3P	WAS protein family homolog 3 pse	-0.049	0.966	6.27E-01	6.27E+01				
7986561	ENST00000338	VSIG7	V-set and immunoglobulin domair	-0.002	0.999	9.94E-01	9.94E+01				
7986565	XM_00112627	LOC648913	similar to a disintegrin and metallo	0.095	1.068	2.70E-01	2.70E+01				
7986567	---	---	---	0.114	1.082	4.25E-01	4.25E+01				
7986569	NR_002824	HERC2P2	hect domain and RLD 2 pseudoger	-0.006	0.996	9.80E-01	9.80E+01				
7986598	NM_00100141	LOC283767	golgi autoantigen, golgin subfamily	0.045	1.031	8.32E-01	8.32E+01				
7986633	---	---	---	0.114	1.082	4.25E-01	4.25E+01				
7986639	ENST00000338	VSIG6	V-set and immunoglobulin domair	0.062	1.044	7.73E-01	7.73E+01				
7986642	NR_027407	GOLGA8D	golgi autoantigen, golgin subfamily	-0.036	0.975	6.48E-01	6.48E+01				
7986661	---	---	---	0.041	1.029	8.06E-01	8.06E+01				
7986665	NM_030922	NIPA2	non imprinted in Prader-Willi/Ang	0.140	1.102	5.53E-02	5.53E+00				
7986675	NM_144599	NIPA1	non imprinted in Prader-Willi/Ang	-0.146	0.904	2.26E-01	2.26E+01				
7986687	NR_003521	WHAMML1	WAS protein homolog associated	-0.071	0.952	7.33E-01	7.33E+01				
7986701	NR_002824	HERC2P2	hect domain and RLD 2 pseudoger	-0.022	0.985	9.16E-01	9.16E+01				
7986736	NM_00100141	LOC283767	golgi autoantigen, golgin subfamily	0.018	1.013	9.49E-01	9.49E+01				
7986741	NM_00100141	LOC283767	golgi autoantigen, golgin subfamily	0.036	1.026	8.70E-01	8.70E+01				
7986744	BC129821	LOC283685	golgi autoantigen, golgin subfamily	0.031	1.022	8.48E-01	8.48E+01				
7986765	NM_000969	RPL5	ribosomal protein L5	-0.042	0.972	7.25E-01	7.25E+01				
7986767	BC069077	C15orf49	chromosome 15 open reading frar	-0.064	0.957	5.97E-01	5.97E+01				
7986769	NM_130839	UBE3A	ubiquitin protein ligase E3A	0.046	1.032	7.15E-01	7.15E+01				
7986789	NM_024490	ATP10A	ATPase, class V, type 10A	0.110	1.079	2.61E-01	2.61E+01				
7986820	---	---	---	-0.099	0.934	3.87E-01	3.87E+01				
7986822	NM_000814	GABRB3	gamma-aminobutyric acid (GABA)	-0.075	0.949	5.41E-01	5.41E+01				
7986838	NM_000275	OCA2	oculocutaneous albinism II	0.041	1.029	6.95E-01	6.95E+01				
7986863	NM_004667	HERC2	hect domain and RLD 2	-0.023	0.984	8.82E-01	8.82E+01				
7986922	NM_00101242	GOLGA8G	golgi autoantigen, golgin subfamily	-0.049	0.967	4.29E-01	4.29E+01				
7986943	---	---	---	0.041	1.029	8.06E-01	8.06E+01				
7986945	---	---	---	-0.034	0.977	8.52E-01	8.52E+01				
7986947	NR_024074	GOLGA9P	golgi autoantigen, golgin subfamily	-0.015	0.989	9.36E-01	9.36E+01				
7986960	NM_015307	FAM189A1	family with sequence similarity 18	-0.055	0.962	7.11E-01	7.11E+01				
7986969	NM_138704	NDNL2	necdin-like 2	-0.081	0.945	4.24E-01	4.24E+01				

7987012	NM_139320	CHRFAM7A	CHRNA7 (cholinergic receptor, nic	0.039	1.028	7.75E-01	7.75E+01		
7987027	NR_024074	GOLGA9P	golgi autoantigen, golgin subfamily	0.013	1.009	9.53E-01	9.53E+01		
7987046	---	---	---	-0.041	0.972	8.25E-01	8.25E+01		
7987048	NM_017762	MTMR10	myotubularin related protein 10	0.094	1.068	5.29E-01	5.29E+01		
7987097	---	---	---	0.002	1.001	9.90E-01	9.90E+01		
7987099	NM_130901	OTUD7A	OTU domain containing 7A	-0.048	0.967	7.26E-01	7.26E+01		
7987110	---	---	---	0.031	1.021	8.35E-01	8.35E+01		
7987112	---	---	---	-0.041	0.972	8.09E-01	8.09E+01		
7987114	NR_024074	GOLGA9P	golgi autoantigen, golgin subfamily	0.044	1.031	7.64E-01	7.64E+01		
7987137	---	---	---	-0.041	0.972	8.25E-01	8.25E+01		
7987139	NR_024074	GOLGA9P	golgi autoantigen, golgin subfamily	-0.065	0.956	7.87E-01	7.87E+01		
7987145	NM_00110318	FMN1	formin 1	0.047	1.033	9.35E-01	9.35E+01		
7987163	ENST00000414	FMN1	formin 1	0.099	1.071	8.18E-01	8.18E+01		
7987165	NM_020371	AVEN	apoptosis, caspase activation inhib	-0.103	0.931	5.54E-01	5.54E+01		
7987172	NM_020154	C15orf24	chromosome 15 open reading frar	0.052	1.037	8.05E-01	8.05E+01		
7987192	NM_00104249	SLC12A6	solute carrier family 12 (potassium	0.027	1.019	9.00E-01	9.00E+01		
7987225	NM_018648	NOP10	NOP10 ribonucleoprotein homolo	0.149	1.108	3.47E-01	3.47E+01		
7987230	NM_153613	LPCAT4	lysophosphatidylcholine acyltrans	0.012	1.009	9.42E-01	9.42E+01		
7987248	NR_027410	GOLGA8B	golgi autoantigen, golgin subfamily	-0.082	0.945	5.17E-01	5.17E+01		
7987279	NR_027410	GOLGA8B	golgi autoantigen, golgin subfamily	-0.071	0.952	5.69E-01	5.69E+01		
7987310	NM_020660	GJD2	gap junction protein, delta 2, 36kD	0.009	1.006	9.61E-01	9.61E+01		
7987325	NM_014691	AQR	aquarius homolog (mouse)	-0.004	0.998	9.86E-01	9.86E+01		
7987361	NM_014106	ZNF770	zinc finger protein 770	0.027	1.019	8.94E-01	8.94E+01		
7987365	NM_024865	NANOG	Nanog homeobox	-0.001	0.999	9.96E-01	9.96E+01		
7987381	NR_027320	CSNK1A1P	casein kinase 1, alpha 1 pseudoge	0.088	1.063	4.27E-01	4.27E+01		
7987405	NM_005739	RASGRP1	RAS guanyl releasing protein 1 (ca	-0.112	0.925	4.97E-01	4.97E+01		
7987439	NM_007223	GPR176	G protein-coupled receptor 176	-0.005	0.996	9.74E-01	9.74E+01		
7987449	NM_003134	SRP14	signal recognition particle 14kDa (	-0.019	0.987	9.25E-01	9.25E+01		
7987454	NM_00100394	BMF	Bcl2 modifying factor	-0.201	0.870	2.29E-02	2.29E+00		
7987466	---	---	---	0.062	1.044	6.01E-01	6.01E+01		
7987472	AK095915	C15orf56	chromosome 15 open reading frar	-0.047	0.968	6.98E-01	6.98E+01		
7987475	NM_004573	PLCB2	phospholipase C, beta 2	0.042	1.029	7.85E-01	7.85E+01		
7987511	NM_207380	C15orf52	chromosome 15 open reading frar	-0.057	0.961	4.43E-01	4.43E+01		
7987526	NM_00108079	C15orf57	chromosome 15 open reading frar	0.032	1.022	9.22E-01	9.22E+01		
7987536	NM_018145	FAM82A2	family with sequence similarity 82	0.046	1.032	6.89E-01	6.89E+01		
7987554	NM_018163	DNAJC17	DnaJ (Hsp40) homolog, subfamily	-0.064	0.957	7.53E-01	7.53E+01		
7987565	NM_017726	PPP1R14D	protein phosphatase 1, regulatory	0.038	1.027	7.53E-01	7.53E+01		
7987572	---	---	---	-0.073	0.951	6.69E-01	6.69E+01		
7987574	NM_133639	RHOV	ras homolog gene family, member	-0.024	0.983	8.15E-01	8.15E+01		
7987584	NM_017553	INO80	INO80 homolog (S. cerevisiae)	0.025	1.018	8.52E-01	8.52E+01		
7987650	NM_002344	LTK	leukocyte receptor tyrosine kinase	-0.019	0.987	9.19E-01	9.19E+01		
7987675	NM_015540	RPAP1	RNA polymerase II associated prot	-0.033	0.977	7.95E-01	7.95E+01		
7987701	NM_016642	SPTBN5	spectrin, beta, non-erythrocytic 5	-0.013	0.991	9.16E-01	9.16E+01		
7987772	NM_139265	EHD4	EH-domain containing 4	-0.064	0.956	6.70E-01	6.70E+01		
7987792	NM_178034	PLA2G4D	phospholipase A2, group IVD (cyto	-0.059	0.960	6.82E-01	6.82E+01		
7987840	NM_015289	VPS39	vacuolar protein sorting 39 homol	0.046	1.033	7.37E-01	7.37E+01		
7987869	NM_015497	TMEM87A	transmembrane protein 87A	0.065	1.046	5.25E-01	5.25E+01		
7987892	NM_022473	ZFP106	zinc finger protein 106 homolog (r	0.061	1.043	6.97E-01	6.97E+01		
7987916	NM_153260	LRRC57	leucine rich repeat containing 57	-0.045	0.969	7.35E-01	7.35E+01		
7987926	---	---	---	-0.348	0.785	5.83E-01	5.83E+01		
7987928	NM_138477	CDAN1	congenital dyserythropoietic anem	-0.027	0.981	8.45E-01	8.45E+01		
7987960	NM_173500	TTBK2	tau tubulin kinase 2	-0.065	0.956	5.73E-01	5.73E+01		
7987981	NM_174916	UBR1	ubiquitin protein ligase E3 compor	-0.010	0.993	9.56E-01	9.56E+01		
7988031	NM_001006	RPS3A	ribosomal protein S3A	0.008	1.006	9.53E-01	9.53E+01		
7988033	NM_000119	EPB42	erythrocyte membrane protein ba	-0.075	0.949	5.10E-01	5.10E+01		
7988050	NM_201631	TGM5	transglutaminase 5	-0.039	0.974	7.97E-01	7.97E+01		
7988077	NM_014793	LCMT2	leucine carboxyl methyltransferas	0.075	1.053	6.37E-01	6.37E+01		
7988082	NM_152455	ZSCAN29	zinc finger and SCAN domain conta	-0.098	0.934	5.35E-01	5.35E+01		
7988091	---	---	---	0.090	1.064	7.25E-01	7.25E+01		
7988093	NM_00114198	TP53BP1	tumor protein p53 binding proteir	-0.172	0.888	8.66E-02	8.66E+00		
7988124	NM_014659	HISPPD2A	histidine acid phosphatase domair	0.022	1.015	9.28E-01	9.28E+01		
7988132	NM_153700	STRC	stereocilin	-0.035	0.976	7.97E-01	7.97E+01		
7988177	NM_153700	STRC	stereocilin	-0.038	0.974	7.83E-01	7.83E+01		
7988208	NR_002318	CATSPER2P1	cation channel, sperm associated 2	0.033	1.023	9.13E-01	9.13E+01		
7988212	NM_025165	ELL3	elongation factor RNA polymerase	0.049	1.034	8.45E-01	8.45E+01		
7988227	NM_00103351	SERINC4	serine incorporator 4	-0.014	0.990	9.14E-01	9.14E+01		
7988245	NM_005926	MFAP1	microfibrillar-associated protein 1	-0.037	0.975	7.82E-01	7.82E+01		
7988281	---	---	---	-0.002	0.999	9.92E-01	9.92E+01		
7988283	ENST00000313	LOC645212	hypothetical LOC645212	-0.047	0.968	5.76E-01	5.76E+01		
7988286	NM_025137	SPG11	spastic paraplegia 11 (autosomal r	0.090	1.064	3.67E-01	3.67E+01		
7988327	NM_00114511	PATL2	protein associated with topoisome	0.199	1.148	2.70E-01	2.70E+01		
7988344	---	---	---	0.073	1.052	7.01E-01	7.01E+01		
7988346	---	---	---	-0.076	0.949	4.83E-01	4.83E+01		
7988380	NM_144565	DUOXA1	dual oxidase maturation factor 1	-0.010	0.993	9.41E-01	9.41E+01		
7988400	NM_138356	SHF	Src homology 2 domain containing	0.051	1.036	7.15E-01	7.15E+01		
7988414	NM_001482	GATM	glycine amidinotransferase (L-argi	0.089	1.063	7.72E-01	7.72E+01		
7988424	---	---	---	0.134	1.097	4.49E-01	4.49E+01		

7988444	NM_016132	MYEF2	myelin expression factor 2	0.053	1.037	8.43E-01	8.43E+01		
7988581	NM_014701	SECISBP2L	SECIS binding protein 2-like	0.027	1.019	8.41E-01	8.41E+01		
7988605	NM_004236	COPS2	COP9 constitutive photomorphoge	0.074	1.052	5.70E-01	5.70E+01		
7988623	---	---	---	-0.055	0.962	8.66E-01	8.66E+01		
7988644	NM_024837	ATP8B4	ATPase, class I, type 8B, member 4	-0.065	0.956	9.03E-01	9.03E+01		
7988685	---	---	---	-0.027	0.981	8.69E-01	8.69E+01		
7988687	NM_005254	GABPB1	GA binding protein transcription fa	-0.021	0.986	9.48E-01	9.48E+01		
7988713	NM_017672	TRPM7	transient receptor potential cation	0.018	1.013	9.10E-01	9.10E+01		
7988753	NM_032802	SPPL2A	signal peptide peptidase-like 2A	0.150	1.110	4.14E-01	4.14E+01		
7988763	NM_207381	TNFAIP8L3	tumor necrosis factor, alpha-induc	-0.035	0.976	7.97E-01	7.97E+01		
7988789	NM_015263	DMXL2	Dmx-like 2	0.410	1.329	3.56E-01	3.56E+01		
7988833	NM_153374	LYSMD2	LysM, putative peptidoglycan-bind	0.049	1.034	6.73E-01	6.73E+01		
7988838	NM_138792	LEO1	Leo1, Paf1/RNA polymerase II com	-0.048	0.967	8.13E-01	8.13E+01		
7988859	NM_006578	GNB5	guanine nucleotide binding protei	-0.071	0.952	4.50E-01	4.50E+01		
7988921	NM_000259	MYO5A	myosin VA (heavy chain 12, myoxi	0.091	1.065	5.87E-01	5.87E+01		
7988963	NM_006628	ARPP19	cAMP-regulated phosphoprotein,	-0.012	0.992	9.44E-01	9.44E+01		
7988970	NM_019600	KIAA1370	KIAA1370	-0.078	0.947	6.15E-01	6.15E+01		
7988987	NM_004498	ONECUT1	one cut homeobox 1	-0.068	0.954	5.15E-01	5.15E+01		
7989013	NM_016304	RSL24D1	ribosomal L24 domain containing	0.031	1.021	7.97E-01	7.97E+01		
7989023	NM_004580	RAB27A	RAB27A, member RAS oncogene fa	-0.193	0.875	1.50E-01	1.50E+01		
7989037	NM_004748	CCPG1	cell cycle progression 1	0.048	1.034	8.80E-01	8.80E+01		
7989128	NM_144571	CNOT6L	CCR4-NOT transcription complex,	-0.059	0.960	6.68E-01	6.68E+01		
7989132	NM_022841	RFX7	regulatory factor X, 7	0.018	1.013	9.37E-01	9.37E+01		
7989144	---	---	---	0.208	1.155	2.75E-01	2.75E+01		
7989159	NM_017661	ZNF280D	zinc finger protein 280D	0.070	1.049	6.31E-01	6.31E+01		
7989193	---	---	---	-0.022	0.985	9.61E-01	9.61E+01		
7989195	---	---	---	0.135	1.098	7.08E-01	7.08E+01		
7989218	---	---	---	-0.242	0.845	4.01E-01	4.01E+01		
7989224	NM_001110	ADAM10	ADAM metallopeptidase domain 1	0.074	1.052	7.34E-01	7.34E+01		
7989243	---	---	---	-0.057	0.962	8.76E-01	8.76E+01		
7989245	NR_002927	HSP90AB4P	heat shock protein 90kDa alpha (c	0.013	1.009	9.53E-01	9.53E+01		
7989251	---	---	---	0.024	1.017	9.00E-01	9.00E+01		
7989253	NM_024755	SLTM	SAFB-like, transcription modulator	-0.023	0.984	8.15E-01	8.15E+01		
7989315	NM_004492	GTF2A2	general transcription factor IIA, 2,	0.082	1.059	6.68E-01	6.68E+01		
7989323	NM_004330	BNIP2	BCL2/adenovirus E1B 19kDa intera	0.066	1.047	6.11E-01	6.11E+01		
7989335	NM_00100285	ANXA2	annexin A2	0.205	1.152	3.33E-01	3.33E+01		
7989347	NM_024611	NARG2	NMDA receptor regulated 2	0.086	1.062	3.99E-01	3.99E+01		
7989365	NM_134260	RORA	RAR-related orphan receptor A	-0.168	0.890	1.83E-01	1.83E+01		
7989387	NM_020821	VPS13C	vacuolar protein sorting 13 homol	0.143	1.105	5.37E-02	5.37E+00		
7989473	NM_00100759	FAM148B	family with sequence similarity 14	0.022	1.016	9.09E-01	9.09E+01		
7989476	AK096042	FLJ38723	hypothetical FLJ38723	-0.036	0.976	7.82E-01	7.82E+01		
7989489	---	---	---	-0.052	0.965	8.47E-01	8.47E+01		
7989491	AB209041	TPM1	tropomyosin 1 (alpha)	-0.014	0.990	9.42E-01	9.42E+01		
7989493	NM_015920	RPS27L	ribosomal protein S27-like	-0.012	0.992	9.64E-01	9.64E+01		
7989516	NM_003922	HERC1	hect (homologous to the E6-AP (U	0.001	1.001	9.97E-01	9.97E+01		
7989596	NM_014326	DAPK2	death-associated protein kinase 2	-0.037	0.975	7.68E-01	7.68E+01		
7989611	NM_032231	FAM96A	family with sequence similarity 96	0.262	1.199	6.73E-02	6.73E+00		
7989617	---	---	---	0.056	1.040	8.55E-01	8.55E+01		
7989619	NM_000942	PPIB	peptidylprolyl isomerase B (cyclop	0.093	1.067	4.43E-01	4.43E+01		
7989628	NM_022048	CSNK1G1	casein kinase 1, gamma 1	-0.002	0.998	9.90E-01	9.90E+01		
7989659	---	---	---	0.170	1.125	6.60E-01	6.60E+01		
7989661	NM_002537	OAZ2	ornithine decarboxylase antizyme	0.100	1.072	5.84E-01	5.84E+01		
7989670	NM_194272	RBPMS2	RNA binding protein with multiple	0.011	1.007	9.55E-01	9.55E+01		
7989680	NM_025049	PIF1	PIF1 5'-to-3' DNA helicase homolo	0.029	1.021	7.55E-01	7.55E+01		
7989696	NM_00112788	SPG21	spastic paraplegia 21 (autosomal r	0.255	1.193	1.95E-02	1.95E+00		
7989718	NM_016563	RASL12	RAS-like, family 12	-0.006	0.996	9.75E-01	9.75E+01		
7989725	NM_005707	PDCD7	programmed cell death 7	-0.016	0.989	9.16E-01	9.16E+01		
7989735	NM_006660	CLPX	ClpX caseinolytic peptidase X hom	0.002	1.001	9.91E-01	9.91E+01		
7989759	NM_017851	PARP16	poly (ADP-ribose) polymerase fam	0.044	1.031	8.16E-01	8.16E+01		
7989770	NM_004884	IGDCC3	immunoglobulin superfamily, DCC	0.000	1.000	9.99E-01	9.99E+01		
7989785	NM_020962	IGDCC4	immunoglobulin superfamily, DCC	-0.010	0.993	9.64E-01	9.64E+01		
7989806	NM_197960	DPP8	dipeptidyl-peptidase 8	0.083	1.059	5.43E-01	5.43E+01		
7989834	AK296134	C15orf44	chromosome 15 open reading fran	-0.069	0.953	6.34E-01	6.34E+01		
7989849	NM_00114482	DENND4A	DENN/MADD domain containing 4	0.023	1.016	9.05E-01	9.05E+01		
7989883	---	---	---	0.122	1.088	7.51E-01	7.51E+01		
7989885	NM_00103172	DNAJB14	DnaJ (Hsp40) homolog, subfamily	0.058	1.041	7.87E-01	7.87E+01		
7989887	NM_032445	MEGF11	multiple EGF-like-domains 11	-0.029	0.980	7.91E-01	7.91E+01		
7989924	NM_006049	SNAPC5	small nuclear RNA activating comp	0.001	1.001	9.98E-01	9.98E+01		
7989932	AK125772	RPL4	ribosomal protein L4	0.012	1.008	9.68E-01	9.68E+01		
7989953	NM_024666	AAGAB	alpha- and gamma-adaptin binding	-0.082	0.945	6.39E-01	6.39E+01		
7989968	NM_033429	CALML4	calmodulin-like 4	0.159	1.117	2.69E-01	2.69E+01		
7989975	NM_017882	CLN6	ceroid-lipofuscinosis, neuronal 6, l	0.137	1.100	1.65E-01	1.65E+01		
7989985	NM_00100443	ITGA11	integrin, alpha 11	-0.005	0.997	9.76E-01	9.76E+01		
7990020	NM_006305	ANP32A	acidic (leucine-rich) nuclear phosp	-0.003	0.998	9.85E-01	9.85E+01		
7990027	NR_026808	C15orf28	chromosome 15 open reading fran	-0.089	0.940	7.38E-01	7.38E+01		
7990029	---	---	---	0.012	1.009	9.75E-01	9.75E+01		
7990033	NM_005078	TLE3	transducin-like enhancer of split 3	-0.087	0.941	3.35E-01	3.35E+01		

7990090	---	---	---	-0.015	0.989	9.50E-01	9.50E+01		
7990092	NM_006901	MYO9A	myosin IXA	-0.068	0.954	6.11E-01	6.11E+01		
7990151	NM_182470	PKM2	pyruvate kinase, muscle	-0.007	0.995	9.86E-01	9.86E+01		
7990165	NM_020214	PARP6	poly (ADP-ribose) polymerase fam	0.029	1.020	8.71E-01	8.71E+01		
7990191	NM_052840	BRUNOL6	bruno-like 6, RNA binding protein	-0.060	0.959	4.25E-01	4.25E+01		
7990211	NM_000520	HEXA	hexosaminidase A (alpha polypept	0.088	1.063	4.91E-01	4.91E+01		
7990231	NR_023318	ADPGK	ADP-dependent glucokinase	-0.010	0.993	9.77E-01	9.77E+01		
7990244	NM_005477	HCN4	hyperpolarization activated cyclic	-0.012	0.991	9.36E-01	9.36E+01		
7990253	NM_012428	NPTN	neuroplastin	0.035	1.025	8.68E-01	8.68E+01		
7990269	BC111368	C15orf59	chromosome 15 open reading fran	-0.087	0.941	3.41E-01	3.41E+01		
7990273	NM_004809	STOML1	stomatin (EPB72)-like 1	-0.023	0.984	8.97E-01	8.97E+01		
7990283	NM_00103864	GOLGA6	golgi autoantigen, golgin subfamily	-0.037	0.975	6.37E-01	6.37E+01		
7990309	NM_022369	STRA6	stimulated by retinoic acid gene 6	-0.065	0.956	6.05E-01	6.05E+01		
7990345	NM_003612	SEMA7A	semaphorin 7A, GPI membrane an	-0.106	0.929	4.25E-01	4.25E+01		
7990361	NM_032907	UBL7	ubiquitin-like 7 (bone marrow stro	0.070	1.049	4.93E-01	4.93E+01		
7990379	NM_00114244	EDC3	enhancer of mRNA decapping 3 ho	0.099	1.071	4.70E-01	4.70E+01		
7990400	NM_00109943	ULK3	unc-51-like kinase 3 (C. elegans)	0.004	1.003	9.85E-01	9.85E+01		
7990417	NM_005697	SCAMP2	secretory carrier membrane prote	0.040	1.028	8.29E-01	8.29E+01		
7990429	NM_020447	C15orf17	chromosome 15 open reading fran	0.032	1.022	8.71E-01	8.71E+01		
7990436	NM_004255	COX5A	cytochrome c oxidase subunit Va	0.098	1.070	2.56E-01	2.56E+01		
7990442	NM_017793	RPP25	ribonuclease P/MRP 25kDa subuni	0.031	1.022	8.02E-01	8.02E+01		
7990452	---	---	---	-0.042	0.972	8.05E-01	8.05E+01		
7990457	NM_006715	MAN2C1	mannosidase, alpha, class 2C, mer	-0.048	0.967	6.54E-01	6.54E+01		
7990487	NM_015477	SIN3A	SIN3 homolog A, transcription reg	-0.091	0.939	3.79E-01	3.79E+01		
7990511	NM_002833	PTPN9	protein tyrosine phosphatase, nor	0.087	1.062	6.27E-01	6.27E+01		
7990528	NM_005701	SNUPN	snurportin 1	0.037	1.026	8.63E-01	8.63E+01		
7990540	NM_018285	IMP3	IMP3, U3 small nucleolar ribonucle	-0.021	0.986	8.72E-01	8.72E+01		
7990564	---	---	---	-0.162	0.894	5.75E-01	5.75E+01		
7990566	NM_000126	ETFA	electron-transfer-flavoprotein, alp	0.041	1.029	8.29E-01	8.29E+01		
7990582	NM_020843	SCAPER	S-phase cyclin A-associated protei	-0.007	0.995	9.74E-01	9.74E+01		
7990618	---	---	---	-0.046	0.969	6.89E-01	6.89E+01		
7990620	NM_005724	TSPAN3	tetraspanin 3	-0.026	0.982	9.15E-01	9.15E+01		
7990632	NM_024776	SGK269	NKF3 kinase family member	0.100	1.072	8.27E-01	8.27E+01		
7990643	NM_032808	LINGO1	leucine rich repeat and Ig domain	0.019	1.013	9.10E-01	9.10E+01		
7990654	---	---	---	0.016	1.011	9.64E-01	9.64E+01		
7990657	NM_144572	TBC1D2B	TBC1 domain family, member 2B	0.018	1.013	9.60E-01	9.60E+01		
7990700	NM_025234	WDR61	WD repeat domain 61	0.016	1.011	9.56E-01	9.56E+01		
7990729	NM_000750	CHRNB4	cholinergic receptor, nicotinic, bet	0.093	1.067	4.61E-01	4.61E+01		
7990736	NM_014272	ADAMTS7	ADAM metallopeptidase with thro	0.015	1.010	9.30E-01	9.30E+01		
7990757	NM_004390	CTSH	cathepsin H	-0.129	0.914	7.08E-01	7.08E+01		
7990810	NM_006441	MTHFS	5,10-methenyltetrahydrofolate sy	-0.081	0.945	7.31E-01	7.31E+01		
7990815	NM_00110088	ST20	suppressor of tumorigenicity 20	-0.070	0.953	8.14E-01	8.14E+01		
7990818	NM_00111473	BCL2A1	BCL2-related protein A1	-0.120	0.920	6.69E-01	6.69E+01		
7990825	---	---	---	0.003	1.002	9.88E-01	9.88E+01		
7990827	ENST00000447	LOC100133746	hypothetical LOC100133746	-0.041	0.972	7.53E-01	7.53E+01		
7990830	NM_015154	MESDC2	mesoderm development candidate	0.052	1.037	6.75E-01	6.75E+01		
7990839	NM_181900	STARD5	StAR-related lipid transfer (START)	-0.174	0.886	2.83E-01	2.83E+01		
7990873	NM_032246	MEX3B	mex-3 homolog B (C. elegans)	0.029	1.020	8.23E-01	8.23E+01		
7990879	NM_024580	EFTUD1	elongation factor Tu GTP binding c	-0.144	0.905	4.20E-01	4.20E+01		
7990894	NR_003246	FLJ40113	golgi autoantigen, golgin subfamily	-0.069	0.953	5.24E-01	5.24E+01		
7990898	NM_000661	RPL9	ribosomal protein L9	0.007	1.005	9.63E-01	9.63E+01		
7990902	NR_026811	LOC80154	hypothetical LOC80154	-0.061	0.959	3.63E-01	3.63E+01		
7990916	NM_001021	RPS17	ribosomal protein S17	-0.011	0.992	9.16E-01	9.16E+01		
7990922	NR_003246	FLJ40113	golgi autoantigen, golgin subfamily	-0.121	0.919	2.54E-01	2.54E+01		
7990928	ENST00000342	LOC440297	chondroitin sulfate proteoglycan 4	-0.052	0.964	4.03E-01	4.03E+01		
7990943	NR_003246	FLJ40113	golgi autoantigen, golgin subfamily	-0.122	0.919	2.70E-01	2.70E+01		
7990949	NM_000661	RPL9	ribosomal protein L9	0.005	1.004	9.75E-01	9.75E+01		
7990952	NR_026811	LOC80154	hypothetical LOC80154	-0.053	0.964	4.06E-01	4.06E+01		
7990965	NM_001021	RPS17	ribosomal protein S17	-0.011	0.992	9.16E-01	9.16E+01		
7991017	BC020234	LOC283693	actin, gamma pseudogene	0.016	1.011	9.47E-01	9.47E+01		
7991034	NM_199330	HOMER2	homer homolog 2 (Drosophila)	-0.010	0.993	9.64E-01	9.64E+01		
7991047	AK097109	LOC100131860	hypothetical protein LOC1001318	0.128	1.092	7.73E-01	7.73E+01		
7991049	NM_144597	C15orf40	chromosome 15 open reading fran	0.003	1.002	9.90E-01	9.90E+01		
7991057	NM_025238	BTBD1	BTB (POZ) domain containing 1	0.147	1.107	1.36E-02	1.36E+00		
7991070	NM_016073	HDGFRP3	hepatoma-derived growth factor,	-0.034	0.976	9.49E-01	9.49E+01		
7991088	NR_027001	LOC388152	hypothetical LOC388152	-0.037	0.975	6.07E-01	6.07E+01		
7991106	AB067507	KIAA1920	KIAA1920 protein	-0.081	0.945	6.58E-01	6.58E+01		
7991110	NR_003246	FLJ40113	golgi autoantigen, golgin subfamily	-0.115	0.923	2.53E-01	2.53E+01		
7991120	NR_003661	FLJ43276	similar to ubiquitin-conjugating en	-0.124	0.917	2.41E-01	2.41E+01		
7991126	NM_032856	WDR73	WD repeat domain 73	-0.021	0.986	9.36E-01	9.36E+01		
7991138	NM_021077	NMB	neuromedin B	-0.067	0.955	6.38E-01	6.38E+01		
7991143	NM_014300	SEC11A	SEC11 homolog A (S. cerevisiae)	0.041	1.029	5.99E-01	5.99E+01		
7991159	NR_027001	LOC388152	hypothetical LOC388152	-0.029	0.980	6.91E-01	6.91E+01		
7991171	---	---	---	-0.114	0.924	8.20E-01	8.20E+01		
7991173	NM_022480	KLHL25	kelch-like 25 (Drosophila)	0.006	1.004	9.73E-01	9.73E+01		
7991209	NM_022163	MRPL46	mitochondrial ribosomal protein L	-0.019	0.987	9.45E-01	9.45E+01		
7991214	---	---	---	0.301	1.232	5.34E-01	5.34E+01		

7991224	NM_178232	HAPLN3	hyaluronan and proteoglycan link	-0.081	0.945	6.51E-01	6.51E+01		
7991234	NM_005928	MFGE8	milk fat globule-EGF factor 8 prote	0.013	1.009	9.53E-01	9.53E+01		
7991258	NM_002693	POLG	polymerase (DNA directed), gamm	-0.041	0.972	6.83E-01	6.83E+01		
7991296	NM_198525	KIF7	kinesin family member 7	0.026	1.018	8.59E-01	8.59E+01		
7991313	NM_002666	PLIN1	perilipin 1	-0.008	0.995	9.68E-01	9.68E+01		
7991323	NM_003847	PEX11A	peroxisomal biogenesis factor 11 a	0.036	1.025	8.32E-01	8.32E+01		
7991332	NM_018670	MESP1	mesoderm posterior 1 homolog (m	0.014	1.010	9.07E-01	9.07E+01		
7991335	NM_001150	ANPEP	alanyl (membrane) aminopeptidas	-0.342	0.789	4.28E-01	4.28E+01		
7991357	NR_023361	AP3S2	adaptor-related protein complex 3	0.104	1.075	6.34E-01	6.34E+01		
7991367	NM_182616	C15orf38	chromosome 15 open reading fran	-0.032	0.978	8.63E-01	8.63E+01		
7991374	NM_002168	IDH2	isocitrate dehydrogenase 2 (NADP	0.248	1.188	3.21E-02	3.21E+00		
7991386	NM_006384	CIB1	calcium and integrin binding 1 (cal	-0.096	0.935	1.57E-01	1.57E+01		
7991401	NM_198527	HDDC3	HD domain containing 3	0.005	1.003	9.86E-01	9.86E+01		
7991406	NM_003981	PRC1	protein regulator of cytokinesis 1	0.100	1.072	5.75E-01	5.75E+01		
7991427	NM_018668	VPS33B	vacuolar protein sorting 33 homol	0.028	1.020	8.57E-01	8.57E+01		
7991451	---	---	---	0.067	1.048	7.43E-01	7.43E+01		
7991453	NM_207446	FAM174B	family with sequence similarity 17	-0.055	0.963	2.53E-01	2.53E+01		
7991459	NM_020211	RGMA	RGM domain family, member A	-0.037	0.975	8.01E-01	8.01E+01		
7991465	---	---	---	-0.097	0.935	8.01E-01	8.01E+01		
7991478	NM_00110261	LOC145814	pyroglutamyl-peptidase 1-like	-0.078	0.947	5.31E-01	5.31E+01		
7991501	---	---	---	0.357	1.281	9.54E-02	9.54E+00		
7991503	NM_152449	LYSMD4	LysM, putative peptidoglycan-binc	-0.002	0.998	9.91E-01	9.91E+01		
7991512	NR_003260	C15orf51	dynamin 1 pseudogene	0.016	1.011	9.44E-01	9.44E+01		
7991516	NM_139057	ADAMTS17	ADAM metallopeptidase with thro	-0.016	0.989	9.24E-01	9.24E+01		
7991540	---	---	---	-0.048	0.967	8.32E-01	8.32E+01		
7991546	NM_178842	LASS3	LAG1 homolog, ceramide synthase	0.014	1.010	9.73E-01	9.73E+01		
7991562	---	---	---	0.115	1.083	8.48E-01	8.48E+01		
7991566	NM_018148	LINS1	lines homolog 1 (Drosophila)	0.077	1.055	7.15E-01	7.15E+01		
7991581	NM_014918	CHSY1	chondroitin sulfate synthase 1	0.068	1.048	5.01E-01	5.01E+01		
7991587	NM_203472	SELS	selenoprotein S	0.022	1.016	9.33E-01	9.33E+01		
7991598	BC067846	SNRPA1	small nuclear ribonucleoprotein po	-0.023	0.984	9.49E-01	9.49E+01		
7991630	NM_078474	TM2D3	TM2 domain containing 3	0.013	1.009	9.44E-01	9.44E+01		
7991640	NM_152334	TARSL2	threonyl-tRNA synthetase-like 2	0.038	1.027	8.62E-01	8.62E+01		
7991668	0	0	0	-0.050	0.966	4.82E-01	4.82E+01		
7991695	NR_027407	GOLGA8D	golgi autoantigen, golgin subfamily	-0.053	0.964	4.05E-01	4.05E+01		
7991714	0	0	0	-0.061	0.959	3.47E-01	3.47E+01		
7991722	NR_003246	FLJ40113	golgi autoantigen, golgin subfamily	-0.137	0.909	1.65E-01	1.65E+01		
7991731	BC032901	LOC100132169	similar to hCG1742852	-0.001	0.999	9.97E-01	9.97E+01		
7991735	NM_024571	SNRNP25	small nuclear ribonucleoprotein 25	-0.017	0.988	9.63E-01	9.63E+01		
7991742	NM_002434	MPG	N-methylpurine-DNA glycosylase	-0.019	0.987	8.55E-01	8.55E+01		
7991758	NM_00100393	HBM	hemoglobin, mu	-0.060	0.959	4.99E-01	4.99E+01		
7991762	NM_000558	HBA1	hemoglobin, alpha 1	0.149	1.109	8.15E-01	8.15E+01		
7991766	NM_000558	HBA1	hemoglobin, alpha 1	0.149	1.109	8.15E-01	8.15E+01		
7991772	NM_005331	HBQ1	hemoglobin, theta 1	-0.106	0.929	1.69E-01	1.69E+01		
7991779	NM_032039	ITFG3	integrin alpha FG-GAP repeat cont	-0.076	0.949	1.79E-01	1.79E+01		
7991800	NM_001176	ARHGDIG	Rho GDP dissociation inhibitor (GD	-0.036	0.976	7.89E-01	7.89E+01		
7991809	NM_001176	ARHGDIG	Rho GDP dissociation inhibitor (GD	-0.036	0.976	8.04E-01	8.04E+01		
7991815	NM_006849	PDIA2	protein disulfide isomerase family	-0.009	0.994	9.54E-01	9.54E+01		
7991828	---	---	---	0.005	1.004	9.74E-01	9.74E+01		
7991835	---	---	---	-0.038	0.974	7.62E-01	7.62E+01		
7991837	NM_005009	NME4	non-metastatic cells 4, protein exp	-0.099	0.934	1.90E-01	1.90E+01		
7991846	NM_020664	DECR2	2,4-dienoyl CoA reductase 2, pero	0.016	1.011	9.05E-01	9.05E+01		
7991860	NM_014700	RAB11FIP3	RAB11 family interacting protein 3	-0.051	0.965	5.15E-01	5.15E+01		
7991877	NM_005632	SOLH	small optic lobes homolog (Drosop	0.000	1.000	1.00E+00	1.00E+02		
7991893	BC016613	C16orf11	chromosome 16 open reading fran	-0.051	0.965	6.30E-01	6.30E+01		
7991898	ENST00000409	PIGQ	phosphatidylinositol glycan ancho	-0.020	0.986	9.09E-01	9.09E+01		
7991904	NM_004204	PIGQ	phosphatidylinositol glycan ancho	0.013	1.009	9.41E-01	9.41E+01		
7991917	NM_021168	RAB40C	RAB40C, member RAS oncogene fa	-0.092	0.938	2.89E-01	2.89E+01		
7991927	NM_053284	WFIKKN1	WAP, follistatin/kazal, immunoglo	0.022	1.016	8.50E-01	8.50E+01		
7991932	BC001912	C16orf14	chromosome 16 open reading fran	-0.001	0.999	9.97E-01	9.97E+01		
7991939	NM_145294	WDR90	WD repeat domain 90	-0.008	0.995	9.49E-01	9.49E+01		
7991989	NM_138769	RHOT2	ras homolog gene family, member	-0.071	0.952	4.27E-01	4.27E+01		
7992010	NM_003961	RHBDL1	rhomboid, veinlet-like 1 (Drosophi	0.026	1.018	8.33E-01	8.33E+01		
7992021	NM_005861	STUB1	STIP1 homology and U-box contain	-0.051	0.966	6.63E-01	6.63E+01		
7992032	ENST00000397	FBXL16	F-box and leucine-rich repeat prot	-0.018	0.987	9.13E-01	9.13E+01		
7992038	NM_024042	METRN	meteorin, glial cell differentiation	-0.059	0.960	5.34E-01	5.34E+01		
7992043	BC001181	FAM173A	family with sequence similarity 17	0.011	1.008	9.49E-01	9.49E+01		
7992049	NM_207112	HAGHL	hydroxyacylglutathione hydrolase-	-0.039	0.973	5.91E-01	5.91E+01		
7992067	---	---	---	0.038	1.027	8.04E-01	8.04E+01		
7992092	NM_022092	CHTF18	CTF18, chromosome transmission	-0.004	0.997	9.78E-01	9.78E+01		
7992121	NM_00101363	LOC388199	hypothetical LOC388199	-0.014	0.990	9.36E-01	9.36E+01		
7992125	NM_014587	SOX8	SRY (sex determining region Y)-box	-0.079	0.946	5.03E-01	5.03E+01		
7992131	---	---	---	0.011	1.008	9.63E-01	9.63E+01		
7992136	---	---	---	0.020	1.014	9.02E-01	9.02E+01		
7992140	---	---	---	-0.030	0.980	8.05E-01	8.05E+01		
7992145	NM_001053	SSTR5	somatostatin receptor 5	-0.064	0.957	6.51E-01	6.51E+01		
7992147	NM_021098	CACNA1H	calcium channel, voltage-depende	-0.062	0.958	3.64E-01	3.64E+01		

7992181	NM_003294	TPSAB1	tryptase alpha/beta 1	-0.050	0.966	5.98E-01	5.98E+01		
7992191	NM_012217	TPSD1	tryptase delta 1	-0.005	0.997	9.84E-01	9.84E+01		
7992197	---	---	---	0.021	1.015	8.81E-01	8.81E+01		
7992205	NM_194259	UBE2I	ubiquitin-conjugating enzyme E2I	0.027	1.019	8.52E-01	8.52E+01		
7992219	NM_003933	BAIAP3	BAI1-associated protein 3	-0.047	0.968	6.09E-01	6.09E+01		
7992255	NM_032520	GNPTG	N-acetylglucosamine-1-phosphate	0.024	1.017	9.03E-01	9.03E+01		
7992269	---	---	---	-0.066	0.955	5.72E-01	5.72E+01		
7992271	NM_016111	TELO2	TEL2, telomere maintenance 2, ho	-0.058	0.960	6.62E-01	6.62E+01		
7992293	NM_024600	TMEM204	transmembrane protein 204	0.022	1.016	9.13E-01	9.13E+01		
7992302	NM_020825	CRAMP1L	Crm, cramped-like (Drosophila)	0.030	1.021	6.10E-01	6.10E+01		
7992337	NM_144570	HN1L	hematological and neurological ex	-0.003	0.998	9.85E-01	9.85E+01		
7992347	NM_015133	MAPK8IP3	mitogen-activated protein kinase 8	-0.041	0.972	6.95E-01	6.95E+01		
7992379	NM_00101086	EME2	essential meiotic endonuclease 1 h	-0.026	0.982	8.48E-01	8.48E+01		
7992388	NM_012225	NUBP2	nucleotide binding protein 2 (MinD	-0.040	0.973	7.67E-01	7.67E+01		
7992402	NM_004548	NDUFB10	NADH dehydrogenase (ubiquinone	-0.039	0.973	8.35E-01	8.35E+01		
7992409	NM_174903	RNF151	ring finger protein 151	0.019	1.013	9.22E-01	9.22E+01		
7992414	NM_006453	TBL3	transducin (beta)-like 3	-0.074	0.950	4.71E-01	4.71E+01		
7992439	NM_005262	GFER	growth factor, augmenter of liver	-0.087	0.941	3.72E-01	3.72E+01		
7992447	NM_004209	SYNGR3	synaptogyrin 3	-0.063	0.958	6.78E-01	6.78E+01		
7992458	---	---	---	0.035	1.024	8.47E-01	8.47E+01		
7992460	NM_00109945	NPW	neuropeptide W	0.026	1.018	8.70E-01	8.70E+01		
7992463	NM_00113001	SLC9A3R2	solute carrier family 9 (sodium/hy	-0.024	0.983	8.16E-01	8.16E+01		
7992474	NM_000548	TSC2	tuberous sclerosis 2	-0.001	0.999	9.94E-01	9.94E+01		
7992518	NM_014353	RAB26	RAB26, member RAS oncogene fa	-0.039	0.973	7.36E-01	7.36E+01		
7992529	NM_032271	TRAF7	TNF receptor-associated factor 7	-0.016	0.989	9.16E-01	9.16E+01		
7992552	NM_022372	MLST8	MTOR associated protein, LST8 ho	0.013	1.009	9.15E-01	9.15E+01		
7992568	NM_004424	E4F1	E4F transcription factor 1	0.016	1.011	9.20E-01	9.20E+01		
7992584	NM_001374	DNASE1L2	deoxyribonuclease I-like 2	-0.135	0.911	9.60E-02	9.60E+00		
7992594	NM_001761	CCNF	cyclin F	-0.009	0.994	9.58E-01	9.58E+01		
7992614	NM_025108	C16orf59	chromosome 16 open reading fra	-0.028	0.981	7.91E-01	7.91E+01		
7992632	NM_006181	NTN3	netrin 3	-0.025	0.983	8.54E-01	8.54E+01		
7992639	NM_020705	TBC1D24	TBC1 domain family, member 24	0.055	1.039	6.51E-01	6.51E+01		
7992646	NM_001694	ATP6V0C	ATPase, H+ transporting, lysosoma	0.158	1.115	3.62E-01	3.62E+01		
7992656	NM_015944	AMDHD2	amidohydrolase domain containin	0.051	1.036	6.31E-01	6.31E+01		
7992670	NM_002613	PDPK1	3-phosphoinositide dependent pro	0.014	1.010	9.36E-01	9.36E+01		
7992678	NR_015441	PRO0461	hypothetical LOC652276	0.039	1.028	8.17E-01	8.17E+01		
7992682	AK293900	PDPK1	3-phosphoinositide dependent pro	0.159	1.116	1.37E-01	1.37E+01		
7992685	NM_018992	KCTD5	potassium channel tetramerisation	0.037	1.026	8.44E-01	8.44E+01		
7992692	NM_016333	SRRM2	serine/arginine repetitive matrix 2	-0.019	0.987	8.64E-01	8.64E+01		
7992716	NM_00113508	TESSP1	testis serine protease 1	-0.100	0.933	2.69E-01	2.69E+01		
7992722	NM_006799	PRSS21	protease, serine, 21 (testisin)	-0.028	0.981	8.04E-01	8.04E+01		
7992732	NM_145252	ZG16B	zymogen granule protein 16 homo	-0.073	0.951	4.13E-01	4.13E+01		
7992737	NM_138439	FLYWCH2	FLYWCH family member 2	-0.114	0.924	1.55E-01	1.55E+01		
7992744	NM_032296	FLYWCH1	FLYWCH-type zinc finger 1	-0.032	0.978	7.97E-01	7.97E+01		
7992758	NM_172229	KREMEN2	kringle containing transmembrane	-0.088	0.941	2.14E-01	2.14E+01		
7992775	NM_152341	PAQR4	progestin and adipoQ receptor fa	-0.019	0.987	8.68E-01	8.68E+01		
7992782	NM_020982	CLDN9	claudin 9	0.023	1.016	9.09E-01	9.09E+01		
7992789	NM_016639	TNFRSF12A	tumor necrosis factor receptor sup	-0.059	0.960	5.40E-01	5.40E+01		
7992795	NM_024339	THOC6	THO complex 6 homolog (Drosoph	0.013	1.009	9.49E-01	9.49E+01		
7992811	NM_022468	MMP25	matrix metallopeptidase 25	-0.012	0.992	9.49E-01	9.49E+01		
7992828	NM_00101263	IL32	interleukin 32	-0.020	0.986	9.53E-01	9.53E+01		
7992841	---	---	---	0.108	1.078	6.04E-01	6.04E+01		
7992843	---	---	---	-0.002	0.999	9.95E-01	9.95E+01		
7992845	NM_003456	ZNF205	zinc finger protein 205	0.020	1.014	8.67E-01	8.67E+01		
7992854	NM_004220	ZNF213	zinc finger protein 213	-0.031	0.979	7.91E-01	7.91E+01		
7992863	NM_012360	OR1F1	olfactory receptor, family 1, subfa	-0.052	0.965	6.66E-01	6.66E+01		
7992865	NR_002169 //	R1F2P // OR1F1	olfactory receptor, family 1, subfa	-0.213	0.863	3.00E-01	3.00E+01		
7992867	AK096958	FLJ39639	hypothetical protein FLJ39639	-0.086	0.942	5.99E-01	5.99E+01		
7992870	NM_005741	ZNF263	zinc finger protein 263	-0.074	0.950	4.59E-01	4.59E+01		
7992877	NM_153028	ZNF75A	zinc finger protein 75a	0.009	1.006	9.80E-01	9.80E+01		
7992887	AK093979	ZNF75A	zinc finger protein 75a	0.110	1.080	4.96E-01	4.96E+01		
7992889	NM_012368	OR2C1	olfactory receptor, family 2, subfa	0.009	1.006	9.64E-01	9.64E+01		
7992893	---	---	---	0.146	1.107	7.35E-01	7.35E+01		
7992895	NM_017810	ZNF434	zinc finger protein 434	0.021	1.015	9.33E-01	9.33E+01		
7992897	NM_003450	ZNF174	zinc finger protein 174	-0.095	0.936	5.55E-01	5.55E+01		
7992905	NM_00108360	NAT15	N-acetyltransferase 15 (GCN5-rela	-0.034	0.976	7.98E-01	7.98E+01		
7992917	NM_015041	CLUAP1	clusterin associated protein 1	-0.078	0.948	7.47E-01	7.47E+01		
7992934	NM_005223	DNASE1	deoxyribonuclease I	-0.005	0.996	9.79E-01	9.79E+01		
7992954	---	---	---	-0.015	0.990	9.39E-01	9.39E+01		
7992956	NM_032575	GLIS2	GLIS family zinc finger 2	-0.017	0.988	9.07E-01	9.07E+01		
7992967	NM_138440	VASN	vasorin	0.003	1.002	9.88E-01	9.88E+01		
7992973	NM_005147	DNAJA3	DnaJ (Hsp40) homolog, subfamily	0.048	1.034	7.30E-01	7.30E+01		
7992987	NM_00112720	HMOX2	heme oxygenase (decycling) 2	0.062	1.044	6.80E-01	6.80E+01		
7992996	AY358225	UNQ3118	GRTR3118	0.190	1.141	3.91E-01	3.91E+01		
7992998	NM_00114229	MGRN1	mahogunin, ring finger 1	0.058	1.041	4.23E-01	4.23E+01		
7993017	---	---	---	-0.010	0.993	9.74E-01	9.74E+01		
7993019	NM_032349	NUDT16L1	nudix (nucleoside diphosphate lin	0.006	1.004	9.67E-01	9.67E+01		

7993024	NM_139170	C16orf71	chromosome 16 open reading frar	-0.022	0.985	8.62E-01	8.62E+01		
7993035	NM_016936	UBN1	ubinuclein 1	-0.086	0.942	4.07E-01	4.07E+01		
7993054	NM_014692	SEC14L5	SEC14-like 5 (S. cerevisiae)	-0.010	0.993	9.50E-01	9.50E+01		
7993071	NM_019109	ALG1	asparagine-linked glycosylation 1,	0.100	1.072	4.27E-01	4.27E+01		
7993108	AK094332	LOC440337	hypothetical LOC440337	-0.042	0.972	7.40E-01	7.40E+01		
7993114	NM_024109	C16orf68	chromosome 16 open reading frar	-0.077	0.948	5.52E-01	5.52E+01		
7993126	NM_020686	ABAT	4-aminobutyrate aminotransferas	-0.018	0.988	9.35E-01	9.35E+01		
7993148	NM_000303	PMM2	phosphomannomutase 2	0.127	1.092	3.27E-01	3.27E+01		
7993159	NM_014117	C16orf72	chromosome 16 open reading frar	-0.087	0.942	2.31E-01	2.31E+01		
7993165	---	---	---	-0.003	0.998	9.88E-01	9.88E+01		
7993167	NM_024997	ATF7IP2	activating transcription factor 7 in	0.206	1.153	3.42E-01	3.42E+01		
7993185	NM_002484	NUBP1	nucleotide binding protein 1 (Min	0.147	1.107	3.85E-01	3.85E+01		
7993195	NM_000246	CIITA	class II, major histocompatibility c	0.198	1.147	9.72E-03	9.72E-01		
7993223	NM_015226	CLEC16A	C-type lectin domain family 16, m	0.079	1.056	2.56E-01	2.56E+01		
7993248	NM_152308	C16orf75	chromosome 16 open reading frar	-0.016	0.989	9.19E-01	9.19E+01		
7993259	NM_003498	SNN	stannin	0.069	1.049	4.43E-01	4.43E+01		
7993265	NM_015914	TXNDC11	thioredoxin domain containing 11	-0.036	0.975	8.78E-01	8.78E+01		
7993274	NM_032167	RUNDC2A	RUN domain containing 2A	0.109	1.079	4.67E-01	4.67E+01		
7993281	NM_00108053	SNX29	sorting nexin 29	0.145	1.106	1.76E-01	1.76E+01		
7993296	NM_00114520	LOC729993	hypothetical protein LOC729993	0.019	1.013	9.13E-01	9.13E+01		
7993310	NM_014048	MKL2	MKL/myocardin-like 2	0.073	1.052	4.00E-01	4.00E+01		
7993339	ENST00000391	LOC100129038	PRO2812	0.169	1.124	3.02E-01	3.02E+01		
7993341	NM_016561	BFAR	bifunctional apoptosis regulator	-0.005	0.997	9.84E-01	9.84E+01		
7993349	NM_006985	NPIP	nuclear pore complex interacting	-0.021	0.985	8.71E-01	8.71E+01		
7993359	NM_006985	NPIP	nuclear pore complex interacting	-0.021	0.985	8.70E-01	8.70E+01		
7993369	---	---	---	-0.023	0.984	8.95E-01	8.95E+01		
7993371	NM_00100406	NOMO3	NODAL modulator 3	0.000	1.000	1.00E+00	1.00E+02		
7993404	NM_006985	NPIP	nuclear pore complex interacting	-0.026	0.982	7.78E-01	7.78E+01		
7993433	NM_015027	PDXDC1	pyridoxal-dependent decarboxylas	0.088	1.063	3.95E-01	3.95E+01		
7993451	---	---	---	0.023	1.016	9.74E-01	9.74E+01		
7993458	NM_033201	C16orf45	chromosome 16 open reading frar	-0.018	0.988	9.50E-01	9.50E+01		
7993467	NM_00114397	NDE1	nudE nuclear distribution gene E h	-0.076	0.949	6.66E-01	6.66E+01		
7993478	NM_004996	ABCC1	ATP-binding cassette, sub-family C	0.117	1.084	2.84E-01	2.84E+01		
7993511	NM_00100406	NOMO3	NODAL modulator 3	0.000	1.000	1.00E+00	1.00E+02		
7993546	NM_006985	NPIP	nuclear pore complex interacting	-0.028	0.980	7.44E-01	7.44E+01		
7993580	NM_006985	NPIP	nuclear pore complex interacting	-0.028	0.981	7.43E-01	7.43E+01		
7993586	---	---	---	0.023	1.016	9.74E-01	9.74E+01		
7993606	---	---	---	0.122	1.088	4.98E-01	4.98E+01		
7993608	NM_016138	COQ7	coenzyme Q7 homolog, ubiquinon	0.042	1.030	8.33E-01	8.33E+01		
7993624	NM_016524	SYT17	synaptotagmin XVII	0.128	1.093	2.79E-01	2.79E+01		
7993680	BC050464	C16orf62	chromosome 16 open reading frar	0.045	1.032	8.26E-01	8.26E+01		
7993726	NM_017888	ACSM5	acyl-CoA synthetase medium-chai	-0.030	0.979	8.53E-01	8.53E+01		
7993774	---	---	---	0.219	1.164	3.35E-01	3.35E+01		
7993800	NM_00112830	LYRM1	LYR motif containing 1	-0.003	0.998	9.92E-01	9.92E+01		
7993807	NM_020422	TMEM159	transmembrane protein 159	-0.081	0.946	6.51E-01	6.51E+01		
7993825	NM_00101239	RUNDC2B	RUN domain containing 2B	0.064	1.046	8.41E-01	8.41E+01		
7993846	---	---	---	-0.222	0.857	1.63E-01	1.63E+01		
7993848	NM_144672	OTOA	otoancorin	-0.008	0.994	9.65E-01	9.65E+01		
7993872	NM_003366	UQCRC2	ubiquinol-cytochrome c reductase	0.012	1.008	9.63E-01	9.63E+01		
7993935	---	---	---	-0.025	0.983	8.96E-01	8.96E+01		
7993946	NM_013302	EEF2K	eukaryotic elongation factor-2 kin	0.121	1.087	2.69E-01	2.69E+01		
7993973	NM_018119	POLR3E	polymerase (RNA) III (DNA directe	-0.050	0.966	6.42E-01	6.42E+01		
7993999	NM_015092	SMG1	SMG1 homolog, phosphatidylinosi	0.041	1.029	7.16E-01	7.16E+01		
7994006	NM_015092	SMG1	SMG1 homolog, phosphatidylinosi	-0.009	0.994	9.51E-01	9.51E+01		
7994026	NM_130464	NPIPL3	nuclear pore complex interacting	0.011	1.008	9.24E-01	9.24E+01		
7994052	NM_006043	HS3ST2	heparan sulfate (glucosamine) 3-O	-0.113	0.924	2.28E-01	2.28E+01		
7994074	NM_000336	SCNN1B	sodium channel, nonvoltage-gated	0.019	1.013	8.98E-01	8.98E+01		
7994095	NM_019116	UBFD1	ubiquitin family domain containing	-0.027	0.982	9.07E-01	9.07E+01		
7994102	NM_032486	DCTN5	dynactin 5 (p25)	0.008	1.005	9.77E-01	9.77E+01		
7994109	NM_005030	PLK1	polo-like kinase 1 (Drosophila)	0.034	1.024	8.78E-01	8.78E+01		
7994161	NM_006910	RBBP6	retinoblastoma binding protein 6	-0.002	0.998	9.88E-01	9.88E+01		
7994187	NM_014494	TNRC6A	trinucleotide repeat containing 6A	0.044	1.031	7.59E-01	7.59E+01		
7994216	NM_052944	SLC5A11	solute carrier family 5 (sodium/glu	0.013	1.009	9.30E-01	9.30E+01		
7994235	ENST00000426	LOC554206	hypothetical LOC554206	-0.046	0.968	8.10E-01	8.10E+01		
7994237	NM_016309	LCMT1	leucine carboxyl methyltransferas	0.025	1.018	9.00E-01	9.00E+01		
7994252	NM_001169	AQP8	aquaporin 8	-0.032	0.978	8.06E-01	8.06E+01		
7994260	NM_006040	HS3ST4	heparan sulfate (glucosamine) 3-O	-0.027	0.981	8.38E-01	8.38E+01		
7994265	---	---	---	-0.082	0.945	8.36E-01	8.36E+01		
7994267	NM_00114554	C16orf82	chromosome 16 open reading frar	-0.050	0.966	5.77E-01	5.77E+01		
7994269	NM_024773	JMJD5	jumonji domain containing 5	0.041	1.029	8.05E-01	8.05E+01		
7994280	NM_000418	IL4R	interleukin 4 receptor	-0.002	0.999	9.93E-01	9.93E+01		
7994292	NM_181078	IL21R	interleukin 21 receptor	-0.016	0.989	9.26E-01	9.26E+01		
7994308	NM_015202	KIAA0556	KIAA0556	0.007	1.005	9.74E-01	9.74E+01		
7994341	---	---	---	0.022	1.015	9.74E-01	9.74E+01		
7994343	---	---	---	0.091	1.065	8.23E-01	8.23E+01		
7994345	NM_00102440	SBK1	SH3-binding domain kinase 1	-0.023	0.984	8.79E-01	8.79E+01		
7994350	---	---	---	-0.062	0.958	4.70E-01	4.70E+01		

7994353	NM_018690	APOB48R	apolipoprotein B48 receptor	-0.033	0.977	9.20E-01	9.20E+01		
7994362	NM_138414	CCDC101	coiled-coil domain containing 101	0.011	1.007	9.67E-01	9.67E+01		
7994371	ENST00000446	NPIPL3	nuclear pore complex interacting p	-0.004	0.997	9.75E-01	9.75E+01		
7994386	NM_007245	ATXN2L	ataxin 2-like	-0.042	0.972	7.02E-01	7.02E+01		
7994415	NM_00103780	EIF3C	eukaryotic translation initiation fa	-0.004	0.997	9.86E-01	9.86E+01		
7994445	NM_015503	SH2B1	SH2B adaptor protein 1	0.006	1.004	9.84E-01	9.84E+01		
7994487	NM_001770	CD19	CD19 molecule	0.025	1.017	9.02E-01	9.02E+01		
7994506	NM_032815	NFATC2IP	nuclear factor of activated T-cells,	-0.079	0.947	4.83E-01	4.83E+01		
7994518	NM_032038	SPNS1	spinster homolog 1 (Drosophila)	0.014	1.010	9.33E-01	9.33E+01		
7994535	NM_014387	LAT	linker for activation of T cells	-0.011	0.992	9.63E-01	9.63E+01		
7994541	NM_014387	LAT	linker for activation of T cells	-0.090	0.940	4.94E-01	4.94E+01		
7994559	NR_003610	PDXDC2	pyridoxal-dependent decarboxylas	-0.003	0.998	9.83E-01	9.83E+01		
7994565	NM_018427	RRN3	RRN3 RNA polymerase I transcript	0.124	1.090	4.10E-01	4.10E+01		
7994570	---	---	---	-0.093	0.938	7.15E-01	7.15E+01		
7994572	NR_002939	RUNDC2C	RUN domain containing 2C	-0.120	0.920	6.00E-01	6.00E+01		
7994576	NR_002939	RUNDC2C	RUN domain containing 2C	0.078	1.055	7.72E-01	7.72E+01		
7994582	NM_003166	SULT1A3	sulfotransferase family, cytosolic,	-0.033	0.978	7.94E-01	7.94E+01		
7994603	NM_00103028	SPN	sialophorin	0.026	1.018	8.67E-01	8.67E+01		
7994609	NM_014298	QPRT	quinolinate phosphoribosyltransfe	-0.063	0.957	5.09E-01	5.09E+01		
7994615	NM_152338	ZG16	zymogen granule protein 16 homo	0.006	1.004	9.71E-01	9.71E+01		
7994620	NM_007317	KIF22	kinesin family member 22	0.013	1.009	9.44E-01	9.44E+01		
7994637	NM_00104253	MAZ	MYC-associated zinc finger protein	-0.088	0.941	3.41E-01	3.41E+01		
7994647	NM_145239	PRRT2	proline-rich transmembrane prote	-0.012	0.992	9.49E-01	9.49E+01		
7994655	NM_024516	C16orf53	chromosome 16 open reading fran	-0.015	0.990	9.36E-01	9.36E+01		
7994659	NM_017458	MVP	major vault protein	0.030	1.021	8.64E-01	8.64E+01		
7994675	NM_181718	ASPHD1	aspartate beta-hydroxylase domai	0.035	1.025	7.89E-01	7.89E+01		
7994683	NM_00108361	TMEM219	transmembrane protein 219	0.037	1.026	8.62E-01	8.62E+01		
7994692	NM_016151	TAOK2	TAO kinase 2	-0.057	0.962	5.60E-01	5.60E+01		
7994715	NM_173618	INO80E	INO80 complex subunit E	-0.040	0.973	7.82E-01	7.82E+01		
7994737	NM_000034	ALDOA	aldolase A, fructose-bisphosphate	0.001	1.001	9.98E-01	9.98E+01		
7994756	NM_002720	PPP4C	protein phosphatase 4 (formerly X	-0.048	0.967	7.11E-01	7.11E+01		
7994769	NM_007074	CORO1A	coronin, actin binding protein, 1A	-0.039	0.973	8.00E-01	8.00E+01		
7994781	NM_003166	SULT1A3	sulfotransferase family, cytosolic,	-0.037	0.974	7.48E-01	7.48E+01		
7994804	NM_013292	MYLPF	myosin light chain, phosphorylatal	-0.042	0.971	6.66E-01	6.66E+01		
7994817	NM_016643	ZNF771	zinc finger protein 771	-0.130	0.914	1.90E-01	1.90E+01		
7994826	NM_002209	ITGAL	integrin, alpha L (antigen CD11A (p	0.002	1.001	9.94E-01	9.94E+01		
7994858	NM_024031	PRR14	proline rich 14	-0.079	0.947	1.65E-01	1.65E+01		
7994874	NM_00110507	FBRS	fibrosin	-0.063	0.957	5.73E-01	5.73E+01		
7994889	NM_006662	SRCAP	Snf2-related CREBBP activator pro	-0.056	0.962	5.39E-01	5.39E+01		
7994926	NR_002966	SNORA30	small nucleolar RNA, H/ACA box 3	-0.075	0.949	8.24E-01	8.24E+01		
7994928	NM_000294	PHKG2	phosphorylase kinase, gamma 2 (t	-0.014	0.990	9.55E-01	9.55E+01		
7994939	NM_014771	RNF40	ring finger protein 40	-0.046	0.969	7.97E-01	7.97E+01		
7994961	NM_001330	CTF1	cardiotrophin 1	0.038	1.027	7.07E-01	7.07E+01		
7994967	NM_00109978	FBXL19	F-box and leucine-rich repeat prot	0.002	1.001	9.89E-01	9.89E+01		
7994981	NM_152288	ORAI3	ORAI calcium release-activated cal	-0.023	0.984	8.45E-01	8.45E+01		
7994985	NM_014712	SETD1A	SET domain containing 1A	0.002	1.001	9.89E-01	9.89E+01		
7995007	NM_025193	HSD3B7	hydroxy-delta-5-steroid dehydrog	0.039	1.028	7.14E-01	7.14E+01		
7995017	NM_004604	STX4	syntaxin 4	0.114	1.082	5.06E-01	5.06E+01		
7995030	NM_024706	ZNF668	zinc finger protein 668	0.039	1.027	8.00E-01	8.00E+01		
7995033	NM_014699	ZNF646	zinc finger protein 646	-0.058	0.961	5.38E-01	5.38E+01		
7995040	NM_00112295	BCKDK	branched chain ketoacid dehydrog	0.053	1.038	7.73E-01	7.73E+01		
7995055	NM_182958	MYST1	MYST histone acetyltransferase 1	-0.015	0.989	9.33E-01	9.33E+01		
7995069	NM_004960	FUS	fusion (involved in t(12;16) in mali	0.012	1.008	9.22E-01	9.22E+01		
7995088	NM_00100827	TRIM72	tripartite motif-containing 72	-0.075	0.949	5.57E-01	5.57E+01		
7995128	NM_000887	ITGAX	integrin, alpha X (complement cor	0.130	1.094	7.64E-01	7.64E+01		
7995161	NM_005353	ITGAD	integrin, alpha D	0.040	1.028	6.89E-01	6.89E+01		
7995193	NM_024742	ARMC5	armadillo repeat containing 5	-0.064	0.957	4.03E-01	4.03E+01		
7995206	NM_00104245	TGFB1I1	transforming growth factor beta 1	-0.049	0.967	5.63E-01	5.63E+01		
7995222	NM_003041	SLC5A2	solute carrier family 5 (sodium/glu	-0.007	0.995	9.74E-01	9.74E+01		
7995242	BC021555	C16orf67	chromosome 16 open reading fran	-0.100	0.933	2.81E-01	2.81E+01		
7995252	NM_00113091	ZNF720	zinc finger protein 720	0.170	1.125	3.91E-01	3.91E+01		
7995258	NM_003414	ZNF267	zinc finger protein 267	-0.096	0.936	2.67E-01	2.67E+01		
7995292	NM_005629	SLC6A8	solute carrier family 6 (neurotrans	0.072	1.051	3.95E-01	3.95E+01		
7995306	---	---	---	0.017	1.012	9.00E-01	9.00E+01		
7995308	---	---	---	0.018	1.012	9.44E-01	9.44E+01		
7995310	ENST00000319	MGC34800	hypothetical protein MGC34800	-0.020	0.986	8.67E-01	8.67E+01		
7995320	---	---	---	-0.101	0.932	3.26E-01	3.26E+01		
7995322	---	---	---	-0.030	0.980	8.35E-01	8.35E+01		
7995324	---	---	---	-0.047	0.968	7.36E-01	7.36E+01		
7995326	---	---	---	-0.004	0.997	9.86E-01	9.86E+01		
7995328	---	---	---	-0.129	0.914	1.94E-01	1.94E+01		
7995330	---	---	---	-0.038	0.974	8.05E-01	8.05E+01		
7995332	---	---	---	-0.021	0.986	9.09E-01	9.09E+01		
7995334	---	---	---	-0.008	0.994	9.66E-01	9.66E+01		
7995336	---	---	---	-0.047	0.968	7.44E-01	7.44E+01		
7995338	---	---	---	-0.036	0.975	7.81E-01	7.81E+01		
7995340	---	---	---	-0.071	0.952	5.21E-01	5.21E+01		

7995342	---	---	---	-0.097	0.935	2.93E-01	2.93E+01		
7995344	---	---	---	-0.073	0.951	5.73E-01	5.73E+01		
7995346	---	---	---	-0.064	0.956	6.59E-01	6.59E+01		
7995348	---	---	---	-0.072	0.951	5.25E-01	5.25E+01		
7995350	---	---	---	-0.010	0.993	9.54E-01	9.54E+01		
7995352	---	---	---	-0.097	0.935	3.95E-01	3.95E+01		
7995354	NM_014321	ORC6L	origin recognition complex, subun	0.144	1.105	6.12E-01	6.12E+01		
7995377	---	---	---	0.034	1.024	8.62E-01	8.62E+01		
7995379	BC013044	DNAJA2	DnaJ (Hsp40) homolog, subfamily	-0.027	0.982	9.05E-01	9.05E+01		
7995382	NM_00103183	PHKB	phosphorylase kinase, beta	0.187	1.138	9.11E-02	9.11E+00		
7995417	---	---	---	0.039	1.028	7.47E-01	7.47E+01		
7995421	NM_031490	LONP2	lon peptidase 2, peroxisomal	0.033	1.023	7.85E-01	7.85E+01		
7995438	---	---	---	0.142	1.103	9.93E-02	9.93E+00		
7995448	NM_153261	TMEM188	transmembrane protein 188	0.203	1.151	2.09E-01	2.09E+01		
7995456	NM_182922	HEATR3	HEAT repeat containing 3	0.159	1.116	1.69E-01	1.69E+01		
7995477	---	---	---	0.014	1.010	9.58E-01	9.58E+01		
7995479	NM_00104028	PAPD5	PAP associated domain containing	0.016	1.011	9.54E-01	9.54E+01		
7995492	NM_001114	ADCY7	adenylate cyclase 7	0.157	1.115	5.88E-02	5.88E+00		
7995525	NM_033119	NKD1	naked cuticle homolog 1 (Drosoph	-0.047	0.968	6.70E-01	6.70E+01		
7995539	NM_022162	NOD2	nucleotide-binding oligomerizatio	-0.097	0.935	5.44E-01	5.44E+01		
7995552	NM_015247	CYLD	cylindromatosis (turban tumor syn	-0.057	0.962	6.61E-01	6.61E+01		
7995574	NM_002136	HNRNPA1	heterogeneous nuclear ribonucleo	-0.027	0.981	8.09E-01	8.09E+01		
7995580	---	---	---	0.087	1.062	3.30E-01	3.30E+01		
7995583	NM_025134	CHD9	chromodomain helicase DNA bind	0.271	1.207	6.70E-04	6.70E-02		
7995627	---	---	---	0.074	1.053	6.73E-01	6.73E+01		
7995629	---	---	---	0.018	1.013	9.49E-01	9.49E+01		
7995631	NM_005611	RBL2	retinoblastoma-like 2 (p130)	0.131	1.095	5.05E-01	5.05E+01		
7995655	NM_00108043	FTO	fat mass and obesity associated	-0.048	0.967	7.95E-01	7.95E+01		
7995668	NM_005853	IRX5	iroquois homeobox 5	-0.022	0.985	8.37E-01	8.37E+01		
7995674	NM_024335	IRX6	iroquois homeobox 6	0.017	1.012	9.07E-01	9.07E+01		
7995681	NM_004530	MMP2	matrix metallopeptidase 2 (gelatin	0.023	1.016	9.35E-01	9.35E+01		
7995714	NM_001043	SLC6A2	solute carrier family 6 (neurotrans	0.009	1.006	9.61E-01	9.61E+01		
7995739	NM_020988	GNAO1	guanine nucleotide binding protei	-0.197	0.872	9.61E-02	9.61E+00		
7995755	NM_018233	OGFOD1	2-oxoglutarate and iron-depender	0.023	1.016	9.23E-01	9.23E+01		
7995772	NM_032935	MT4	metallothionein 4	-0.083	0.944	3.10E-01	3.10E+01		
7995776	NM_005954	MT3	metallothionein 3	-0.029	0.980	8.10E-01	8.10E+01		
7995783	NM_005953	MT2A	metallothionein 2A	-0.341	0.789	4.04E-01	4.04E+01		
7995797	NM_175617	MT1E	metallothionein 1E	-0.412	0.752	9.52E-02	9.52E+00		
7995803	AF348994	MT1JP	metallothionein 1J (pseudogene)	-0.202	0.870	2.20E-01	2.20E+01		
7995806	NM_005946	MT1A	metallothionein 1A	-0.331	0.795	1.08E-01	1.08E+01		
7995813	NR_027781	MT1DP	metallothionein 1D (pseudogene)	-0.158	0.896	2.54E-01	2.54E+01		
7995820	NM_005947	MT1B	metallothionein 1B	-0.169	0.889	2.25E-01	2.25E+01		
7995829	NM_005951	MT1H	metallothionein 1H	-0.525	0.695	1.74E-01	1.74E+01		
7995834	NR_003669	MT1IP	metallothionein 1I (pseudogene)	-0.154	0.899	1.45E-01	1.45E+01		
7995838	NM_005952	MT1X	metallothionein 1X	-0.304	0.810	3.96E-01	3.96E+01		
7995843	NM_014669	NUP93	nucleoporin 93kDa	0.010	1.007	9.57E-01	9.57E+01		
7995868	NM_000339	SLC12A3	solute carrier family 12 (sodium/cl	-0.040	0.973	7.51E-01	7.51E+01		
7995895	NM_014685	HERPUD1	homocysteine-inducible, endoplas	0.211	1.157	5.95E-03	5.95E-01		
7995907	NM_000078	CETP	cholesteryl ester transfer protein,	-0.054	0.964	5.39E-01	5.39E+01		
7995926	NM_032206	NLRC5	NLR family, CARD domain containi	-0.028	0.981	7.97E-01	7.97E+01		
7995976	NM_152727	CPNE2	copine II	-0.085	0.943	4.88E-01	4.88E+01		
7995994	NM_133368	RSPRY1	ring finger and SPRY domain conta	-0.046	0.969	8.06E-01	8.06E+01		
7996012	NM_012106	ARL2BP	ADP-ribosylation factor-like 2 bind	-0.088	0.941	6.11E-01	6.11E+01		
7996027	NM_002996	CX3CL1	chemokine (C-X3-C motif) ligand 1	-0.031	0.979	8.29E-01	8.29E+01		
7996034	NM_002987	CCL17	chemokine (C-C motif) ligand 17	0.032	1.022	7.70E-01	7.70E+01		
7996041	NM_020312	COQ9	coenzyme Q9 homolog (S. cerevisi	-0.087	0.942	4.41E-01	4.41E+01		
7996051	NM_032940	POLR2C	polymerase (RNA) II (DNA directed	0.021	1.014	9.36E-01	9.36E+01		
7996064	NM_153837	GPR114	G protein-coupled receptor 114	-0.021	0.986	9.01E-01	9.01E+01		
7996081	NM_201524	GPR56	G protein-coupled receptor 56	0.116	1.084	4.68E-01	4.68E+01		
7996100	NM_170776	GPR97	G protein-coupled receptor 97	-0.090	0.940	5.98E-01	5.98E+01		
7996137	NM_005886	KATNB1	katanin p80 (WD repeat containin	0.016	1.011	9.03E-01	9.03E+01		
7996160	ENST00000335	LOC388282	hypothetical LOC388282	0.040	1.028	7.95E-01	7.95E+01		
7996164	NM_199046	TEPP	testis, prostate and placenta expre	-0.057	0.961	5.73E-01	5.73E+01		
7996174	NM_024598	C16orf57	chromosome 16 open reading fran	0.016	1.011	9.25E-01	9.25E+01		
7996185	NM_002428	MMP15	matrix metallopeptidase 15 (mem	0.009	1.006	9.49E-01	9.49E+01		
7996219	NM_020465	NDRG4	NDRG family member 4	-0.030	0.980	8.02E-01	8.02E+01		
7996241	NM_00116030	SETD6	SET domain containing 6	-0.031	0.979	8.36E-01	8.36E+01		
7996256	---	---	---	-0.045	0.969	7.87E-01	7.87E+01		
7996264	NM_001795	CDH5	cadherin 5, type 2 (vascular endot	0.015	1.010	9.07E-01	9.07E+01		
7996281	NM_00113610	BEAN	brain expressed, associated with N	-0.074	0.950	2.89E-01	2.89E+01		
7996286	NM_004614	TK2	thymidine kinase 2, mitochondrial	0.023	1.016	9.03E-01	9.03E+01		
7996290	NM_052999	CMTM1	CKLF-like MARVEL transmembrane	0.019	1.013	9.29E-01	9.29E+01		
7996313	NM_144673	CMTM2	CKLF-like MARVEL transmembrane	0.025	1.017	8.50E-01	8.50E+01		
7996318	NM_144601	CMTM3	CKLF-like MARVEL transmembrane	0.030	1.021	8.52E-01	8.52E+01		
7996341	NM_020786	PDP2	pyruvate dehyrogenase phosphata	0.008	1.005	9.84E-01	9.84E+01		
7996345	NM_003869	CES2	carboxylesterase 2 (intestine, liver	0.005	1.004	9.82E-01	9.82E+01		
7996377	NM_173815	CES8	carboxylesterase 8 (putative)	0.021	1.015	8.69E-01	8.69E+01		

7996393	NM_001755	CBFB	core-binding factor, beta subunit	0.068	1.048	4.03E-01	4.03E+01		
7996403	NM_025187	C16orf70	chromosome 16 open reading fran	0.051	1.036	8.15E-01	8.15E+01		
7996423	NM_018378	FBXL8	F-box and leucine-rich repeat prot	0.022	1.016	8.15E-01	8.15E+01		
7996430	NM_00104066	HSF4	heat shock transcription factor 4	-0.030	0.979	7.45E-01	7.45E+01		
7996448	NM_003946	NOL3	nucleolar protein 3 (apoptosis rep	-0.053	0.964	5.91E-01	5.91E+01		
7996455	NM_001950	E2F4	E2F transcription factor 4, p107/p1	0.066	1.047	5.29E-01	5.29E+01		
7996468	NM_024712	ELMO3	engulfment and cell motility 3	-0.037	0.975	7.19E-01	7.19E+01		
7996490	NM_014187	TMEM208	transmembrane protein 208	0.082	1.058	5.84E-01	5.84E+01		
7996563	NM_000196	HSD11B2	hydroxysteroid (11-beta) dehydrog	0.030	1.021	8.63E-01	8.63E+01		
7996571	NM_024519	FAM65A	family with sequence similarity 65	-0.014	0.991	9.39E-01	9.39E+01		
7996593	NM_006565	CTCF	CCCTC-binding factor (zinc finger p	0.004	1.003	9.84E-01	9.84E+01		
7996608	NM_00101383	RLTPR	RGD motif, leucine rich repeats, tr	-0.038	0.974	6.62E-01	6.62E+01		
7996647	NM_016948	PARD6A	par-6 partitioning defective 6 hom	-0.074	0.950	5.82E-01	5.82E+01		
7996651	NM_00101298	C16orf86	chromosome 16 open reading fran	-0.061	0.958	5.15E-01	5.15E+01		
7996675	NM_020457	THAP11	THAP domain containing 11	-0.060	0.959	5.75E-01	5.75E+01		
7996677	NM_005796	NUTF2	nuclear transport factor 2	0.066	1.047	7.36E-01	7.36E+01		
7996685	NM_014329	EDC4	enhancer of mRNA decapping 4	-0.015	0.990	9.24E-01	9.24E+01		
7996715	NM_198443	NRN1L	neuritin 1-like	-0.005	0.997	9.77E-01	9.77E+01		
7996720	NM_006742	PSKH1	protein serine kinase H1	-0.063	0.957	5.08E-01	5.08E+01		
7996725	NM_017803	DUS2L	dihydrouridine synthase 2-like, SM	0.118	1.085	2.65E-01	2.65E+01		
7996744	NM_173163	NFATC3	nuclear factor of activated T-cells,	0.087	1.062	3.72E-01	3.72E+01		
7996759	---	---	---	0.074	1.053	8.90E-01	8.90E+01		
7996761	NM_012320	PLA2G15	phospholipase A2, group XV	0.058	1.041	7.13E-01	7.13E+01		
7996772	NM_003983	SLC7A6	solute carrier family 7 (cationic am	0.029	1.020	9.11E-01	9.11E+01		
7996785	NM_019023	PRMT7	protein arginine methyltransferase	-0.020	0.986	8.93E-01	8.93E+01		
7996807	NM_133458	ZFP90	zinc finger protein 90 homolog (m	-0.049	0.966	8.34E-01	8.34E+01		
7996860	NM_024562	TMCO7	transmembrane and coiled-coil do	0.121	1.088	3.20E-01	3.20E+01		
7996883	NM_005329	HAS3	hyaluronan synthase 3	-0.023	0.984	8.45E-01	8.45E+01		
7996891	NM_032830	CIRH1A	cirrhosis, autosomal recessive 1A (	0.113	1.081	5.17E-01	5.17E+01		
7996908	NM_006750	SNTB2	syntrophin, beta 2 (dystrophin-ass	0.147	1.108	3.06E-01	3.06E+01		
7996919	NM_013245	VPS4A	vacuolar protein sorting 4 homolo	0.009	1.006	9.74E-01	9.74E+01		
7996934	NM_016101	NIP7	nuclear import 7 homolog (S. cere	-0.069	0.954	7.85E-01	7.85E+01		
7996947	NM_030579	CYB5B	cytochrome b5 type B (outer mito	-0.048	0.967	7.75E-01	7.75E+01		
7996954	NM_138714	NFAT5	nuclear factor of activated T-cells 5	-0.120	0.920	8.61E-02	8.61E+00		
7996976	NM_007014	WWP2	WW domain containing E3 ubiquit	0.016	1.011	9.36E-01	9.36E+01		
7997010	NM_182619	CLEC18A	C-type lectin domain family 18, me	-0.027	0.981	8.34E-01	8.34E+01		
7997025	NM_017990	PDPR	pyruvate dehydrogenase phospha	-0.130	0.914	5.71E-01	5.71E+01		
7997032	NM_00101188	CLEC18B	C-type lectin domain family 18, me	-0.020	0.986	8.69E-01	8.69E+01		
7997048	NM_007242	DDX19B	DEAD (Asp-Glu-Ala-As) box polype	0.116	1.084	4.24E-01	4.24E+01		
7997059	NM_018332	DDX19A	DEAD (Asp-Glu-Ala-As) box polype	0.031	1.022	8.99E-01	8.99E+01		
7997072	NM_145059	FUK	fucokinase	0.016	1.011	9.19E-01	9.19E+01		
7997099	NM_012426	SF3B3	splicing factor 3b, subunit 3, 130kD	-0.007	0.995	9.75E-01	9.75E+01		
7997128	NM_152456	IL34	interleukin 34	0.007	1.005	9.64E-01	9.64E+01		
7997135	NM_138383	MTSS1L	metastasis suppressor 1-like	-0.010	0.993	9.50E-01	9.50E+01		
7997139	NM_001740	CALB2	calbindin 2	0.118	1.085	4.39E-02	4.39E+00		
7997158	NM_00101796	MARVELD3	MARVEL domain containing 3	-0.005	0.996	9.80E-01	9.80E+01		
7997164	---	---	---	0.074	1.053	7.94E-01	7.94E+01		
7997168	NM_014761	KIAA0174	KIAA0174	0.055	1.039	6.46E-01	6.46E+01		
7997179	NM_001361	DHODH	dihydroorotate dehydrogenase	-0.022	0.985	8.97E-01	8.97E+01		
7997192	NM_020995	HPR	haptoglobin-related protein	-0.021	0.986	9.67E-01	9.67E+01		
7997197	NM_014003	DHX38	DEAH (Asp-Glu-Ala-His) box polype	-0.121	0.920	1.61E-01	1.61E+01		
7997226	---	---	---	0.121	1.088	6.65E-01	6.65E+01		
7997230	NM_002811	PSMD7	proteasome (prosome, macropain	-0.041	0.972	7.68E-01	7.68E+01		
7997239	NR_003610	PDXDC2	pyridoxal-dependent decarboxylas	-0.017	0.988	8.85E-01	8.85E+01		
7997245	---	---	---	0.001	1.001	9.95E-01	9.95E+01		
7997247	NM_032268	ZNRF1	zinc and ring finger 1	-0.060	0.959	5.28E-01	5.28E+01		
7997257	NM_153688	ZFP1	zinc finger protein 1 homolog (mo	-0.038	0.974	8.68E-01	8.68E+01		
7997264	NM_001906	CTRB1	chymotrypsinogen B1	-0.010	0.993	9.54E-01	9.54E+01		
7997272	NM_007285	GABARAPL2	GABA(A) receptor-associated prot	-0.012	0.992	9.53E-01	9.53E+01		
7997281	NM_018975	TERF2IP	telomeric repeat binding factor 2,	0.018	1.012	8.92E-01	8.92E+01		
7997319	---	---	---	-0.054	0.963	6.29E-01	6.29E+01		
7997321	NM_014940	MON1B	MON1 homolog B (yeast)	-0.017	0.989	9.43E-01	9.43E+01		
7997332	NM_00110566	NUDT7	nudix (nucleoside diphosphate link	-0.089	0.940	7.00E-01	7.00E+01		
7997352	NM_016373	WWOX	WW domain containing oxidoredu	-0.040	0.973	6.80E-01	6.80E+01		
7997396	NM_015251	ATMIN	ATM interactor	-0.064	0.957	5.14E-01	5.14E+01		
7997414	NM_022041	GAN	gigaxonin	0.043	1.030	7.82E-01	7.82E+01		
7997427	NM_198390	CMIP	c-Maf-inducing protein	0.042	1.029	8.92E-01	8.92E+01		
7997453	NM_002661	PLCG2	phospholipase C, gamma 2 (phosp	0.240	1.181	1.21E-01	1.21E+01		
7997489	---	---	---	0.059	1.042	5.93E-01	5.93E+01		
7997491	NM_002153	HSD17B2	hydroxysteroid (17-beta) dehydrog	-0.003	0.998	9.86E-01	9.86E+01		
7997498	---	---	---	-0.054	0.963	7.88E-01	7.88E+01		
7997502	---	---	---	-0.027	0.982	8.53E-01	8.53E+01		
7997520	NM_001537	HSBP1	heat shock factor binding protein 1	0.247	1.186	1.84E-02	1.84E+00		
7997525	NM_012213	MLYCD	malonyl-CoA decarboxylase	-0.030	0.979	9.00E-01	9.00E+01		
7997533	NM_013370	OSGIN1	oxidative stress induced growth in	0.016	1.011	8.96E-01	8.96E+01		
7997542	NM_019065	NECAB2	N-terminal EF-hand calcium bindir	-0.071	0.952	5.17E-01	5.17E+01		
7997556	NM_178452	LRRC50	leucine rich repeat containing 50	-0.017	0.988	8.87E-01	8.87E+01		

7997569	NM_139174	ADAD2	adenosine deaminase domain con	-0.036	0.975	6.68E-01	6.68E+01		
7997582	NM_021197	WFDC1	WAP four-disulfide core domain 1	-0.017	0.988	9.01E-01	9.01E+01		
7997626	NM_024731	KLHL36	kelch-like 36 (Drosophila)	0.055	1.039	7.02E-01	7.02E+01		
7997633	NM_005153	USP10	ubiquitin specific peptidase 10	0.029	1.020	9.05E-01	9.05E+01		
7997662	NM_014732	KIAA0513	KIAA0513	0.061	1.043	7.46E-01	7.46E+01		
7997676	BC030801	TMEM148	transmembrane protein 148	-0.016	0.989	9.33E-01	9.33E+01		
7997680	NM_014615	KIAA0182	KIAA0182	0.013	1.009	9.42E-01	9.42E+01		
7997702	NM_001861	COX4I1	cytochrome c oxidase subunit IV is	0.007	1.005	9.67E-01	9.67E+01		
7997726	NM_001451	FOXF1	forkhead box F1	0.011	1.008	9.53E-01	9.53E+01		
7997733	NM_005251	FOXC2	forkhead box C2 (MFH-1, mesench	0.013	1.009	9.29E-01	9.29E+01		
7997735	NM_005250	FOXL1	forkhead box L1	-0.099	0.933	2.16E-01	2.16E+01		
7997740	NM_022818	MAP1LC3B	microtubule-associated protein 1 l	-0.022	0.985	9.20E-01	9.20E+01		
7997746	NM_020655	JPH3	junctophilin 3	-0.021	0.986	8.90E-01	8.90E+01		
7997755	NM_079837	BANP	BTG3 associated nuclear protein	-0.108	0.928	2.91E-01	2.91E+01		
7997765	NM_00112746	ZNF469	zinc finger protein 469	0.021	1.015	9.08E-01	9.08E+01		
7997767	NM_153813	ZFPM1	zinc finger protein, multitype 1	-0.087	0.942	3.74E-01	3.74E+01		
7997779	NM_144604	ZC3H18	zinc finger CCCH-type containing 1	-0.067	0.955	5.29E-01	5.29E+01		
7997801	NM_013278	IL17C	interleukin 17C	-0.073	0.951	2.00E-01	2.00E+01		
7997808	ENST00000435	MGC23284	hypothetical LOC197187	0.055	1.039	8.41E-01	8.41E+01		
7997810	NM_00101275	CTU2	cytosolic thiouridylase subunit 2 h	-0.018	0.988	8.91E-01	8.91E+01		
7997827	NM_00114286	FAM38A	family with sequence similarity 38	-0.017	0.988	9.22E-01	9.22E+01		
7997832	ENST00000333	FLJ40448	hypothetical protein FLJ40448	-0.026	0.982	8.62E-01	8.62E+01		
7997839	NM_030928	CDT1	chromatin licensing and DNA repli	-0.023	0.984	8.61E-01	8.61E+01		
7997852	NM_016209	TRAPPC2L	trafficking protein particle comple	-0.010	0.993	9.62E-01	9.62E+01		
7997861	ENST00000452	LOC642452	hypothetical LOC642452	0.067	1.048	6.59E-01	6.59E+01		
7997863	NM_174917	ACSF3	acyl-CoA synthetase family membe	-0.070	0.953	5.48E-01	5.48E+01		
7997875	NR_024347	C16orf81	chromosome 16 open reading fran	-0.039	0.973	7.55E-01	7.55E+01		
7997896	NM_182531	ZNF778	zinc finger protein 778	0.000	1.000	1.00E+00	1.00E+02		
7997904	AK295122	ZNF778	zinc finger protein 778	0.245	1.185	7.07E-01	7.07E+01		
7997907	NM_013275	ANKRD11	ankyrin repeat domain 11	-0.052	0.965	6.61E-01	6.61E+01		
7997909	NM_003119	SPG7	spastic paraplegia 7 (pure and com	-0.009	0.994	9.58E-01	9.58E+01		
7997933	NM_033251	RPL13	ribosomal protein L13	-0.072	0.951	7.09E-01	7.09E+01		
7997940	NR_002450	SNORD68	small nucleolar RNA, C/D box 68	-0.022	0.985	9.73E-01	9.73E+01		
7997942	NM_014427	CPNE7	copine VII	-0.008	0.994	9.53E-01	9.53E+01		
7997962	NM_004413	DPEP1	dipeptidase 1 (renal)	-0.030	0.980	8.36E-01	8.36E+01		
7997976	AK303024	C16orf55	chromosome 16 open reading fran	-0.086	0.942	4.45E-01	4.45E+01		
7997982	NM_052988	CDK10	cyclin-dependent kinase 10	-0.046	0.969	7.15E-01	7.15E+01		
7998002	NM_152287	ZNF276	zinc finger protein 276	-0.061	0.959	5.98E-01	5.98E+01		
7998015	NM_032451	SPIRE2	spire homolog 2 (Drosophila)	-0.037	0.975	7.56E-01	7.56E+01		
7998033	NM_014972	TCF25	transcription factor 25 (basic helix	-0.115	0.923	1.27E-01	1.27E+01		
7998053	---	---	---	-0.086	0.942	6.27E-01	6.27E+01		
7998055	NM_002386	MC1R	melanocortin 1 receptor (alpha me	-0.075	0.949	4.70E-01	4.70E+01		
7998063	NM_006086	TUBB3	tubulin, beta 3	-0.082	0.945	2.43E-01	2.43E+01		
7998072	NM_207514	DEF8	differentially expressed in FDCP 8	0.007	1.005	9.78E-01	9.78E+01		
7998083	NR_003228	AFG3L1	AFG3 ATPase family gene 3-like 1 (	0.027	1.019	8.10E-01	8.10E+01		
7998103	NM_001481	GAS8	growth arrest-specific 8	-0.072	0.951	6.47E-01	6.47E+01		
7998115	---	---	---	0.267	1.203	4.25E-01	4.25E+01		
7998117	NM_207366	40435	septin 14	0.013	1.009	9.74E-01	9.74E+01		
7998119	NR_024540	WASH5P	WAS protein family homolog 5 pse	-0.068	0.954	5.58E-01	5.58E+01		
7998129	NM_016310	POLR3K	polymerase (RNA) III (DNA directe	0.023	1.016	9.03E-01	9.03E+01		
7998136	NM_022450	RHBDF1	rhomboid 5 homolog 1 (Drosophila	-0.028	0.981	8.13E-01	8.13E+01		
7998157	NM_00107735	C16orf35	chromosome 16 open reading fran	-0.067	0.954	4.07E-01	4.07E+01		
7998174	NM_018032	LUC7L	LUC7-like (S. cerevisiae)	-0.048	0.967	7.08E-01	7.08E+01		
7998211	NM_003502	AXIN1	axin 1	-0.035	0.976	7.68E-01	7.68E+01		
7998222	NM_006428	MRPL28	mitochondrial ribosomal protein L	-0.050	0.966	7.44E-01	7.44E+01		
7998233	NM_021259	TMEM8A	transmembrane protein 8A	0.002	1.001	9.89E-01	9.89E+01		
7998249	---	---	---	-0.161	0.895	6.93E-01	6.93E+01		
7998251	NM_032366	C16orf13	chromosome 16 open reading fran	0.019	1.013	8.89E-01	8.89E+01		
7998265	AK097604	LOC100130285	hypothetical LOC100130285	-0.014	0.990	9.12E-01	9.12E+01		
7998267	NM_00100592	JMJD8	jumonji domain containing 8	0.015	1.010	9.49E-01	9.49E+01		
7998280	NM_032259	WDR24	WD repeat domain 24	-0.003	0.998	9.85E-01	9.85E+01		
7998297	NM_153350	FBXL16	F-box and leucine-rich repeat prot	-0.066	0.956	5.13E-01	5.13E+01		
7998307	NM_00103173	CCDC78	coiled-coil domain containing 78	0.034	1.024	7.64E-01	7.64E+01		
7998336	NM_022493	NARFL	nuclear prelamin A recognition fac	-0.020	0.986	9.16E-01	9.16E+01		
7998351	NM_00102519	MSLNL	mesothelin-like	-0.038	0.974	6.99E-01	6.99E+01		
7998367	NM_058192	RPUSD1	RNA pseudouridylate synthase dom	-0.038	0.974	7.87E-01	7.87E+01		
7998374	NM_016541	GNG13	guanine nucleotide binding protei	-0.028	0.981	8.24E-01	8.24E+01		
7998381	NM_022773	LMF1	lipase maturation factor 1	-0.017	0.988	9.34E-01	9.34E+01		
7998392	---	---	---	-0.006	0.996	9.71E-01	9.71E+01		
7998399	---	---	---	-0.033	0.977	7.92E-01	7.92E+01		
7998405	NR_027242	LOC146336	hypothetical LOC146336	0.005	1.003	9.85E-01	9.85E+01		
7998407	NM_207419	C1QTNF8	C1q and tumor necrosis factor rela	-0.030	0.979	8.22E-01	8.22E+01		
7998414	---	---	---	-0.045	0.969	6.69E-01	6.69E+01		
7998427	NM_012467	TPSG1	tryptase gamma 1	-0.013	0.991	9.36E-01	9.36E+01		
7998434	NM_003294	TPSAB1	tryptase alpha/beta 1	-0.046	0.969	6.52E-01	6.52E+01		
7998444	ENST00000440	PRSS29P	protease, serine, 29 pseudogene	-0.019	0.987	8.78E-01	8.78E+01		
7998449	NM_00100141	C16orf42	chromosome 16 open reading fran	-0.065	0.956	6.23E-01	6.23E+01		

7998457	NM_023076	UNKL	unkempt homolog (Drosophila)-lik	-0.051	0.965	6.40E-01	6.40E+01		
7998466	NM_00103712	UNKL	unkempt homolog (Drosophila)-lik	-0.042	0.971	6.92E-01	6.92E+01		
7998485	BC092497	C16orf91	chromosome 16 open reading fram	-0.002	0.999	9.90E-01	9.90E+01		
7998494	NM_00114398	CCDC154	coiled-coil domain containing 154	-0.009	0.994	9.53E-01	9.53E+01		
7998510	NM_001287	CLCN7	chloride channel 7	0.087	1.062	5.60E-01	5.60E+01		
7998538	NM_00101365	C16orf38	chromosome 16 open reading fran	-0.021	0.985	8.79E-01	8.79E+01		
7998542	NM_014714	IFT140	intraflagellar transport 140 homol	-0.006	0.996	9.77E-01	9.77E+01		
7998577	NM_002513	NME3	non-metastatic cells 3, protein exp	-0.033	0.977	8.31E-01	8.31E+01		
7998585	NM_023936	MRPS34	mitochondrial ribosomal protein S	0.015	1.011	9.34E-01	9.34E+01		
7998590	NM_080861	SPSB3	splA/ryanodine receptor domain a	-0.068	0.954	4.26E-01	4.26E+01		
7998601	NM_00114600	IGFALS	insulin-like growth factor binding p	0.083	1.059	3.28E-01	3.28E+01		
7998604	NM_005326	HAGH	hydroxyacylglutathione hydrolase	-0.059	0.960	6.56E-01	6.56E+01		
7998629	---	---	---	-0.031	0.979	7.40E-01	7.40E+01		
7998634	NM_00100960	HS3ST6	heparan sulfate (glucosamine) 3-O	-0.082	0.945	5.41E-01	5.41E+01		
7998637	NM_016332	SEPX1	selenoprotein X, 1	-0.101	0.932	4.05E-01	4.05E+01		
7998643	NM_005061	RPL3L	ribosomal protein L3-like	-0.093	0.937	2.39E-01	2.39E+01		
7998655	NM_002952	RPS2	ribosomal protein S2	-0.005	0.996	9.77E-01	9.77E+01		
7998664	NR_002327	SNORA10	small nucleolar RNA, H/ACA box 1(	-0.099	0.934	5.38E-01	5.38E+01		
7998668	NM_144603	NOXO1	NADPH oxidase organizer 1	-0.021	0.986	8.53E-01	8.53E+01		
7998679	NM_178167	ZNF598	zinc finger protein 598	-0.050	0.966	6.81E-01	6.81E+01		
7998692	NM_002528	NTHL1	nth endonuclease III-like 1 (E. coli)	0.029	1.020	8.63E-01	8.63E+01		
7998700	NM_00100994	PKD1	polycystic kidney disease 1 (autoso	-0.036	0.975	8.05E-01	8.05E+01		
7998724	NM_020764	CASKIN1	CASK interacting protein 1	-0.072	0.951	5.25E-01	5.25E+01		
7998749	NM_022372	MLST8	MTOR associated protein, LST8 ho	-0.019	0.987	9.14E-01	9.14E+01		
7998759	NM_00104237	PGP	phosphoglycolate phosphatase	-0.089	0.940	3.42E-01	3.42E+01		
7998762	NM_001919	DCI	dodecenoyl-Coenzyme A delta isor	-0.107	0.928	6.04E-02	6.04E+00		
7998774	NM_080594	RNPS1	RNA binding protein S1, serine-ricl	0.008	1.005	9.54E-01	9.54E+01		
7998817	NM_00104821	CEMP1	cementum protein 1	0.010	1.007	9.51E-01	9.51E+01		
7998820	AK293900	PDPK1	3-phosphoinositide dependent pro	0.147	1.107	2.27E-01	2.27E+01		
7998823	NM_002613	PDPK1	3-phosphoinositide dependent pro	0.117	1.084	2.56E-01	2.56E+01		
7998825	NM_002613	PDPK1	3-phosphoinositide dependent pro	0.197	1.147	1.52E-01	1.52E+01		
7998835	---	---	---	-0.004	0.997	9.85E-01	9.85E+01		
7998841	NM_002613	PDPK1	3-phosphoinositide dependent pro	0.117	1.084	2.56E-01	2.56E+01		
7998843	NM_031948	PRSS27	protease, serine 27	-0.036	0.975	7.63E-01	7.63E+01		
7998852	NM_007108	TCEB2	transcription elongation factor B (S	0.062	1.044	6.90E-01	6.90E+01		
7998861	NM_152891	PRSS33	protease, serine, 33	-0.083	0.944	2.11E-01	2.11E+01		
7998872	NR_026864	TMPRSS8	transmembrane protease, serine 8	0.015	1.011	9.05E-01	9.05E+01		
7998878	NM_022119	PRSS22	protease, serine, 22	-0.053	0.964	6.24E-01	6.24E+01		
7998886	NM_182687	PKMYT1	protein kinase, membrane associa	-0.052	0.965	5.96E-01	5.96E+01		
7998902	NM_017885	HCFC1R1	host cell factor C1 regulator 1 (XPO	-0.011	0.993	9.49E-01	9.49E+01		
7998910	NM_00110317	CCDC64B	coiled-coil domain containing 64B	-0.107	0.928	3.64E-01	3.64E+01		
7998921	NM_032805	ZSCAN10	zinc finger and SCAN domain conta	-0.048	0.967	7.27E-01	7.27E+01		
7998927	---	---	---	-0.019	0.987	9.77E-01	9.77E+01		
7998931	NM_198088	ZNF200	zinc finger protein 200	0.013	1.009	9.49E-01	9.49E+01		
7998940	NM_000243	MEFV	Mediterranean fever	0.011	1.008	9.80E-01	9.80E+01		
7998952	NM_033208	TIGD7	tigger transposable element derive	-0.027	0.982	9.34E-01	9.34E+01		
7998967	NM_017810	ZNF434	zinc finger protein 434	-0.025	0.983	8.76E-01	8.76E+01		
7998983	NM_178844	NLRC3	NLR family, CARD domain containi	-0.102	0.932	3.05E-01	3.05E+01		
7999008	NM_032444	BTBD12	BTB (POZ) domain containing 12	-0.007	0.995	9.68E-01	9.68E+01		
7999023	---	---	---	-0.100	0.933	6.34E-01	6.34E+01		
7999025	NM_016292	TRAP1	TNF receptor-associated protein 1	0.046	1.032	7.98E-01	7.98E+01		
7999044	NM_004380	CREBBP	CREB binding protein	-0.062	0.958	3.31E-01	3.31E+01		
7999079	NM_001116	ADCY9	adenylate cyclase 9	0.028	1.020	9.11E-01	9.11E+01		
7999102	NM_003223	TFAP4	transcription factor AP-4 (activatir	-0.011	0.992	9.56E-01	9.56E+01		
7999112	NM_016069	Magmas	mitochondria-associated protein i	0.001	1.000	9.99E-01	9.99E+01		
7999120	NM_024535	CORO7	coronin 7	-0.046	0.968	5.89E-01	5.89E+01		
7999149	NM_020677	NMRAL1	NmrA-like family domain containir	0.004	1.003	9.86E-01	9.86E+01		
7999160	NM_013399	C16orf5	chromosome 16 open reading fran	-0.035	0.976	8.27E-01	8.27E+01		
7999173	NM_145253	FAM100A	family with sequence similarity 10	0.039	1.028	8.05E-01	8.05E+01		
7999177	NM_133450	ANKS3	ankyrin repeat and sterile alpha m	-0.021	0.986	9.03E-01	9.03E+01		
7999196	NM_021646	ZNF500	zinc finger protein 500	0.001	1.001	9.95E-01	9.95E+01		
7999217	NM_024589	ROGDI	rogdi homolog (Drosophila)	0.016	1.011	9.16E-01	9.16E+01		
7999233	NM_032569	GLYR1	glyoxylate reductase 1 homolog (A	0.047	1.033	6.35E-01	6.35E+01		
7999253	NM_002705	PPL	periplakin	-0.074	0.950	4.50E-01	4.50E+01		
7999279	NM_016256	NAGPA	N-acetylglucosamine-1-phosphodi	-0.079	0.947	4.27E-01	4.27E+01		
7999291	NM_152459	C16orf89	chromosome 16 open reading fran	-0.023	0.985	8.64E-01	8.64E+01		
7999304	NM_201400	FAM86A	family with sequence similarity 86	-0.007	0.995	9.68E-01	9.68E+01		
7999317	NM_015421	TMEM186	transmembrane protein 186	0.021	1.015	9.51E-01	9.51E+01		
7999319	NM_014316	CARHSP1	calcium regulated heat stable prot	-0.016	0.989	9.16E-01	9.16E+01		
7999326	NM_003470	USP7	ubiquitin specific peptidase 7 (her	-0.013	0.991	9.27E-01	9.27E+01		
7999356	---	---	---	-0.113	0.925	5.36E-01	5.36E+01		
7999360	NM_000982	RPL21	ribosomal protein L21	-0.018	0.988	9.33E-01	9.33E+01		
7999362	---	---	---	-0.004	0.997	9.94E-01	9.94E+01		
7999382	---	---	---	-0.026	0.982	8.78E-01	8.78E+01		
7999384	---	---	---	0.162	1.119	5.67E-01	5.67E+01		
7999406	---	---	---	0.067	1.047	6.66E-01	6.66E+01		
7999412	NM_00107951	FAM18A	family with sequence similarity 18	-0.258	0.836	2.83E-02	2.83E+00		

7999419	NM_014015	DEXI	Dexi homolog (mouse)	-0.018	0.988	9.56E-01	9.56E+01		
7999423	NM_003745	SOCS1	suppressor of cytokine signaling 1	-0.131	0.913	7.17E-02	7.17E+00		
7999427	NM_005425	TNP2	transition protein 2 (during histon	0.037	1.026	7.73E-01	7.73E+01		
7999431	NM_021247	PRM3	protamine 3	-0.060	0.959	6.68E-01	6.68E+01		
7999433	---	---	---	-0.024	0.983	8.51E-01	8.51E+01		
7999435	NM_002762	PRM2	protamine 2	-0.012	0.992	9.50E-01	9.50E+01		
7999447	ENST00000344	LOC400499	hypothetical LOC400499	-0.028	0.981	8.63E-01	8.63E+01		
7999468	NR_024320	LITAF	lipopolysaccharide-induced TNF fa	0.198	1.147	2.93E-01	2.93E+01		
7999476	---	---	---	0.218	1.163	6.39E-01	6.39E+01		
7999478	NM_015914	TXNDC11	thioredoxin domain containing 11	0.137	1.100	2.76E-02	2.76E+00		
7999496	NM_014153	ZC3H7A	zinc finger CCCH-type containing 7	0.024	1.017	9.00E-01	9.00E+01		
7999520	NM_015659	RSL1D1	ribosomal L1 domain containing 1	0.008	1.006	9.69E-01	9.69E+01		
7999532	NM_002094	GSPT1	G1 to S phase transition 1	0.007	1.005	9.74E-01	9.74E+01		
7999553	NM_018340	CPPED1	calcineurin-like phosphoesterase d	0.208	1.155	3.76E-01	3.76E+01		
7999562	NM_002582	PARN	poly(A)-specific ribonuclease (dea	-0.015	0.990	9.22E-01	9.22E+01		
7999591	NR_023387	ABCC6P2	ATP-binding cassette, sub-family C	-0.083	0.944	3.26E-01	3.26E+01		
7999594	---	---	---	0.023	1.016	9.74E-01	9.74E+01		
7999596	NM_138346	KIAA2013	KIAA2013	-0.033	0.977	7.93E-01	7.93E+01		
7999598	NM_173474	NTAN1	N-terminal asparagine amidase	0.233	1.175	1.50E-01	1.50E+01		
7999608	NM_018427	RRN3	RRN3 RNA polymerase I transcript	0.044	1.031	8.84E-01	8.84E+01		
7999614	NM_006985	NPIP	nuclear pore complex interacting p	-0.045	0.969	7.11E-01	7.11E+01		
7999634	NM_006985	NPIP	nuclear pore complex interacting p	-0.023	0.984	9.12E-01	9.12E+01		
7999642	NM_014647	KIAA0430	KIAA0430	-0.009	0.994	9.57E-01	9.57E+01		
7999674	NM_022844	MYH11	myosin, heavy chain 11, smooth m	-0.101	0.933	2.96E-01	2.96E+01		
7999718	AY507846	C16orf63	chromosome 16 open reading fran	0.071	1.051	6.89E-01	6.89E+01		
7999725	---	---	---	0.047	1.033	7.57E-01	7.57E+01		
7999727	NM_001171	ABCC6	ATP-binding cassette, sub-family C	-0.032	0.978	7.89E-01	7.89E+01		
7999750	---	---	---	0.023	1.016	9.74E-01	9.74E+01		
7999752	NM_020466	LYRM2	LYR motif containing 2	0.090	1.064	7.10E-01	7.10E+01		
7999754	NM_022166	XYLT1	xylosyltransferase I	-0.073	0.951	3.94E-01	3.94E+01		
7999766	NM_006985	NPIP	nuclear pore complex interacting p	-0.021	0.985	8.04E-01	8.04E+01		
7999769	NM_006985	NPIP	nuclear pore complex interacting p	-0.032	0.978	7.18E-01	7.18E+01		
7999791	NM_00100406	NOMO3	NODAL modulator 3	0.000	1.000	1.00E+00	1.00E+02		
7999827	NM_001019	RPS15A	ribosomal protein S15a	0.055	1.039	6.81E-01	6.81E+01		
7999834	NM_015161	ARL6IP1	ADP-ribosylation factor-like 6 inte	0.086	1.061	5.76E-01	5.76E+01		
7999841	NM_015092	SMG1	SMG1 homolog, phosphatidylinosi	0.030	1.021	8.48E-01	8.48E+01		
7999884	---	---	---	0.036	1.025	9.23E-01	9.23E+01		
7999889	NM_016641	GDE1	glycerophosphodiester phosphodi	0.116	1.084	4.97E-01	4.97E+01		
7999903	BC117562	C16orf88	chromosome 16 open reading fran	0.106	1.076	5.79E-01	5.79E+01		
7999916	NM_00100291	GPR139	G protein-coupled receptor 139	-0.050	0.966	6.57E-01	6.57E+01		
7999936	NM_003361	UMOD	uromodulin	-0.029	0.980	8.32E-01	8.32E+01		
7999999	---	---	---	0.662	1.583	1.14E-01	1.14E+01		
8000003	NM_017736	THUMPD1	THUMP domain containing 1	0.054	1.038	6.06E-01	6.06E+01		
8000028	NM_173475	DCUN1D3	DCN1, defective in cullin neddylati	-0.163	0.893	3.32E-01	3.32E+01		
8000117	NM_001888	CRYM	crystallin, mu	0.038	1.027	7.40E-01	7.40E+01		
8000131	NM_130464	NPIPL3	nuclear pore complex interacting p	0.014	1.010	9.05E-01	9.05E+01		
8000156	ENST00000446	SMG1	SMG1 homolog, phosphatidylinosi	0.005	1.003	9.76E-01	9.76E+01		
8000167	NM_015092	SMG1	SMG1 homolog, phosphatidylinosi	0.002	1.002	9.86E-01	9.86E+01		
8000184	NM_005849	IGSF6	immunoglobulin superfamily, men	0.337	1.263	3.51E-01	3.51E+01		
8000192	ENST00000198	RRN3	RRN3 RNA polymerase I transcript	0.140	1.102	1.66E-01	1.66E+01		
8000200	NM_018427	RRN3	RRN3 RNA polymerase I transcript	0.136	1.099	3.25E-01	3.25E+01		
8000205	NM_130464	NPIPL3	nuclear pore complex interacting p	0.014	1.010	8.94E-01	8.94E+01		
8000217	NM_015092	SMG1	SMG1 homolog, phosphatidylinosi	-0.026	0.982	8.18E-01	8.18E+01		
8000222	NM_015092	SMG1	SMG1 homolog, phosphatidylinosi	0.041	1.029	7.16E-01	7.16E+01		
8000236	NM_001802	CDR2	cerebellar degeneration-related p	-0.080	0.946	7.44E-01	7.44E+01		
8000244	NM_020718	USP31	ubiquitin specific peptidase 31	-0.046	0.968	7.25E-01	7.25E+01		
8000263	NM_153603	COG7	component of oligomeric golgi cor	-0.009	0.993	9.66E-01	9.66E+01		
8000284	NM_015044	GGA2	golgi associated, gamma adaptin e	0.056	1.040	7.63E-01	7.63E+01		
8000310	NM_00108361	EARS2	glutamyl-tRNA synthetase 2, mito	-0.029	0.980	8.47E-01	8.47E+01		
8000323	NM_005003	NDUFAB1	NADH dehydrogenase (ubiquinone	0.141	1.103	3.40E-01	3.40E+01		
8000346	NM_033266	ERN2	endoplasmic reticulum to nucleus	0.049	1.034	7.74E-01	7.74E+01		
8000399	NM_00101298	ZKSCAN2	zinc finger with KRAB and SCAN do	-0.024	0.983	9.20E-01	9.20E+01		
8000409	NM_005517	HMGN2	high-mobility group nucleosomal b	0.068	1.048	6.34E-01	6.34E+01		
8000411	NM_00114534	JMJD5	jumonji domain containing 5	0.031	1.022	9.12E-01	9.12E+01		
8000413	NM_145080	NSMCE1	non-SMC element 1 homolog (S. c	-0.030	0.979	9.02E-01	9.02E+01		
8000425	NM_001520	GTF3C1	general transcription factor IIIC, po	-0.098	0.935	1.70E-01	1.70E+01		
8000467	NM_00110976	GSG1L	GSG1-like	-0.010	0.993	9.56E-01	9.56E+01		
8000480	---	---	---	0.013	1.009	9.64E-01	9.64E+01		
8000482	NM_015171	XPO6	exportin 6	0.073	1.052	5.51E-01	5.51E+01		
8000501	NR_003610	PDXDC2	pyridoxal-dependent decarboxylas	-0.025	0.983	8.27E-01	8.27E+01		
8000507	NM_00103780	EIF3C	eukaryotic translation initiation fa	-0.004	0.997	9.86E-01	9.86E+01		
8000537	NR_003610	PDXDC2	pyridoxal-dependent decarboxylas	-0.017	0.989	8.86E-01	8.86E+01		
8000543	NM_000086	CLN3	ceroid-lipofuscinosis, neuronal 3	0.022	1.015	9.03E-01	9.03E+01		
8000567	NM_145659	IL27	interleukin 27	0.075	1.053	4.73E-01	4.73E+01		
8000574	NM_00104248	NUPR1	nuclear protein, transcriptional reg	0.102	1.073	5.91E-01	5.91E+01		
8000582	NM_001054	SULT1A2	sulfotransferase family, cytosolic,	0.080	1.057	6.76E-01	6.76E+01		
8000590	NM_177534	SULT1A1	sulfotransferase family, cytosolic,	0.056	1.040	7.44E-01	7.44E+01		

8000600	---	---	---	-0.059	0.960	5.24E-01	5.24E+01		
8000603	NM_003321	TUFM	Tu translation elongation factor, m	0.001	1.001	9.94E-01	9.94E+01		
8000616	NM_024816	RABEP2	rabaptin, RAB GTPase binding effe	-0.071	0.952	3.95E-01	3.95E+01		
8000632	---	---	---	0.102	1.073	4.88E-01	4.88E+01		
8000636	NM_130464	NPIPL3	nuclear pore complex interacting p	-0.005	0.996	9.81E-01	9.81E+01		
8000638	NM_015092	SMG1	SMG1 homolog, phosphatidylinosi	-0.006	0.996	9.72E-01	9.72E+01		
8000649	---	---	---	0.005	1.004	9.85E-01	9.85E+01		
8000651	NM_015092	SMG1	SMG1 homolog, phosphatidylinosi	-0.013	0.991	8.96E-01	8.96E+01		
8000676	NM_130464	NPIPL3	nuclear pore complex interacting p	0.009	1.006	9.44E-01	9.44E+01		
8000687	NM_015092	SMG1	SMG1 homolog, phosphatidylinosi	-0.014	0.990	9.60E-01	9.60E+01		
8000690	---	---	---	0.005	1.004	9.85E-01	9.85E+01		
8000692	NM_00103182	BOLA2	bolA homolog 2 (E. coli)	-0.028	0.981	7.48E-01	7.48E+01		
8000702	NM_175900	C16orf54	chromosome 16 open reading frar	0.097	1.069	1.76E-01	1.76E+01		
8000706	NM_006319	CDIPT	CDP-diacylglycerol--inositol 3-phos	-0.011	0.992	9.63E-01	9.63E+01		
8000716	NM_012410	SEZ6L2	seizure related 6 homolog (mouse	-0.023	0.984	8.50E-01	8.50E+01		
8000738	NM_178863	KCTD13	potassium channel tetramerisation	-0.043	0.971	7.63E-01	7.63E+01		
8000748	NM_003609	HIRIP3	HIRA interacting protein 3	-0.001	1.000	9.98E-01	9.98E+01		
8000757	NM_003586	DOC2A	double C2-like domains, alpha	-0.013	0.991	9.27E-01	9.27E+01		
8000772	NM_031478	FAM57B	family with sequence similarity 57	-0.047	0.968	5.76E-01	5.76E+01		
8000779	NM_004608	TBX6	T-box 6	-0.011	0.992	9.49E-01	9.49E+01		
8000791	NM_031477	YPEL3	yippee-like 3 (Drosophila)	-0.023	0.984	9.43E-01	9.43E+01		
8000799	NM_024307	GDPD3	glycerophosphodiester phosphodi	-0.033	0.977	9.01E-01	9.01E+01		
8000811	NM_00104005	MAPK3	mitogen-activated protein kinase 3	0.020	1.014	9.26E-01	9.26E+01		
8000823	NM_015092	SMG1	SMG1 homolog, phosphatidylinosi	-0.009	0.994	9.53E-01	9.53E+01		
8000834	NM_015092	SMG1	SMG1 homolog, phosphatidylinosi	-0.004	0.997	9.76E-01	9.76E+01		
8000848	NM_006110	CD2BP2	CD2 (cytoplasmic tail) binding prot	-0.056	0.962	6.94E-01	6.94E+01		
8000856	NM_015527	TBC1D10B	TBC1 domain family, member 10B	-0.035	0.976	7.77E-01	7.77E+01		
8000869	NM_052838	40422	septin 1	0.061	1.044	7.03E-01	7.03E+01		
8000882	---	---	---	-0.046	0.968	7.52E-01	7.52E+01		
8000884	NM_024096	DCTPP1	dCTP pyrophosphatase 1	-0.038	0.974	7.78E-01	7.78E+01		
8000890	NM_012248	SEPHS2	selenophosphate synthetase 2	0.007	1.005	9.63E-01	9.63E+01		
8000897	---	---	---	-0.119	0.921	5.12E-01	5.12E+01		
8000899	NM_024671	ZNF768	zinc finger protein 768	-0.004	0.997	9.85E-01	9.85E+01		
8000903	NM_023931	ZNF747	zinc finger protein 747	-0.033	0.978	7.87E-01	7.87E+01		
8000906	NM_033410	ZNF764	zinc finger protein 764	-0.041	0.972	8.38E-01	8.38E+01		
8000910	NM_145271	ZNF688	zinc finger protein 688	-0.026	0.982	8.71E-01	8.71E+01		
8000917	NM_152458	ZNF785	zinc finger protein 785	0.039	1.028	7.61E-01	7.61E+01		
8000924	NM_138447	ZNF689	zinc finger protein 689	-0.009	0.994	9.72E-01	9.72E+01		
8000930	---	---	---	0.400	1.320	5.30E-01	5.30E+01		
8000932	NM_00101497	C16orf93	chromosome 16 open reading frar	-0.038	0.974	7.73E-01	7.73E+01		
8000941	NM_00108041	ZNF629	zinc finger protein 629	-0.086	0.942	3.56E-01	3.56E+01		
8000945	---	---	---	0.148	1.108	2.95E-01	2.95E+01		
8000948	NM_004765	BCL7C	B-cell CLL/lymphoma 7C	-0.009	0.994	9.53E-01	9.53E+01		
8000963	NM_052874	STX1B	syntaxin 1B	-0.025	0.983	9.00E-01	9.00E+01		
8000974	NM_024706	ZNF668	zinc finger protein 668	-0.053	0.964	6.93E-01	6.93E+01		
8000978	NM_00103950	POL3S	polyserase 3	-0.074	0.950	3.70E-01	3.70E+01		
8000998	NM_024006	VKORC1	vitamin K epoxide reductase comp	0.019	1.013	9.48E-01	9.48E+01		
8001007	NM_002773	PRSS8	protease, serine, 8	0.020	1.014	9.02E-01	9.02E+01		
8001014	NM_173502	PRSS36	protease, serine, 36	-0.063	0.957	4.02E-01	4.02E+01		
8001030	NM_013258	PYCARD	PYD and CARD domain containing	0.099	1.071	4.14E-01	4.14E+01		
8001041	NM_005205	COX6A2	cytochrome c oxidase subunit VIa	-0.028	0.981	7.97E-01	7.97E+01		
8001046	NM_00113650	ZNF843	zinc finger protein 843	-0.017	0.988	9.28E-01	9.28E+01		
8001048	NM_003041	SLC5A2	solute carrier family 5 (sodium/glu	0.006	1.004	9.74E-01	9.74E+01		
8001067	NR_002827	HERC2P4	hect domain and RLD 2 pseudoger	0.005	1.004	9.86E-01	9.86E+01		
8001082	NM_005629	SLC6A8	solute carrier family 6 (neurotrans	0.070	1.050	3.67E-01	3.67E+01		
8001099	NR_002827	HERC2P4	hect domain and RLD 2 pseudoger	-0.011	0.992	9.73E-01	9.73E+01		
8001102	---	---	---	0.248	1.188	3.68E-03	3.68E-01		
8001108	AK128823 // A	46121 // FLJ46	similar to Ig heavy chain V-I region	-0.007	0.995	9.68E-01	9.68E+01		
8001147	NM_198490	RAB43	RAB43, member RAS oncogene far	-0.009	0.994	9.73E-01	9.73E+01		
8001149	NM_018206	VPS35	vacuolar protein sorting 35 homol	0.125	1.090	2.88E-01	2.88E+01		
8001163	NM_182493	MYLK3	myosin light chain kinase 3	-0.044	0.970	6.74E-01	6.74E+01		
8001178	BC056676	C16orf87	chromosome 16 open reading frar	0.135	1.098	3.10E-01	3.10E+01		
8001185	NM_005880	DNAJA2	DnaJ (Hsp40) homolog, subfamily	0.010	1.007	9.49E-01	9.49E+01		
8001211	NM_030790	ITFG1	integrin alpha FG-GAP repeat cont	0.278	1.212	6.80E-03	6.80E-01		
8001271	NM_033151	ABCC11	ATP-binding cassette, sub-family C	0.048	1.034	6.97E-01	6.97E+01		
8001306	NM_00100661	SIAH1	seven in absentia homolog 1 (Dros	0.023	1.016	8.78E-01	8.78E+01		
8001317	NM_153029	N4BP1	NEDD4 binding protein 1	-0.118	0.921	5.23E-01	5.23E+01		
8001348	---	---	---	0.031	1.022	9.10E-01	9.10E+01		
8001350	NM_013263	BRD7	bromodomain containing 7	-0.069	0.953	4.74E-01	4.74E+01		
8001371	---	---	---	0.108	1.078	7.18E-01	7.18E+01		
8001373	NM_153337	SNX20	sorting nexin 20	0.089	1.064	5.05E-01	5.05E+01		
8001387	NM_002968	SALL1	sal-like 1 (Drosophila)	0.031	1.022	8.25E-01	8.25E+01		
8001410	NM_00101239	AKTIP	AKT interacting protein	0.156	1.114	4.68E-01	4.68E+01		
8001449	NM_024336	IRX3	iroquois homeobox 3	-0.031	0.979	7.97E-01	7.97E+01		
8001455	---	---	---	-0.007	0.995	9.76E-01	9.76E+01		
8001457	NM_00102519	CES1	carboxylesterase 1 (monocyte/ma	-0.527	0.694	1.09E-01	1.09E+01		
8001464	NM_00114368	CES7	carboxylesterase 7	0.016	1.011	9.37E-01	9.37E+01		

8001477	NM_001144	AMFR	autocrine motility factor receptor	0.028	1.020	8.47E-01	8.47E+01		
8001496	NM_007006	NUDT21	nudix (nucleoside diphosphate link	0.050	1.035	6.98E-01	6.98E+01		
8001507	NM_031885	BBS2	Bardet-Biedl syndrome 2	0.049	1.034	8.59E-01	8.59E+01		
8001529	---	---	---	-0.040	0.973	9.16E-01	9.16E+01		
8001531	NM_005950	MT1G	metallothionein 1G	-0.768	0.587	6.04E-02	6.04E+00		
8001537	NM_024946	FAM192A	family with sequence similarity 19	0.014	1.010	9.49E-01	9.49E+01		
8001547	NM_015993	PLLP	plasma membrane proteolipid (pla	-0.005	0.997	9.85E-01	9.85E+01		
8001552	NM_020313	CIAPIN1	cytokine induced apoptosis inhibit	0.139	1.101	3.93E-01	3.93E+01		
8001564	NM_018110	DOK4	docking protein 4	-0.012	0.992	9.56E-01	9.56E+01		
8001576	NM_033212	CCDC102A	coiled-coil domain containing 102A	-0.028	0.980	8.04E-01	8.04E+01		
8001587	NM_005550	KIFC3	kinesin family member C3	0.036	1.026	7.15E-01	7.15E+01		
8001615	NM_001297	CNGB1	cyclic nucleotide gated channel be	-0.021	0.986	8.63E-01	8.63E+01		
8001651	NM_020807	ZNF319	zinc finger protein 319	-0.065	0.956	6.42E-01	6.42E+01		
8001658	NM_013242	C16orf80	chromosome 16 open reading fran	-0.056	0.962	7.99E-01	7.99E+01		
8001666	NM_001896	CSNK2A2	casein kinase 2, alpha prime polyp	-0.047	0.968	8.04E-01	8.04E+01		
8001680	---	---	---	-0.006	0.996	9.77E-01	9.77E+01		
8001682	NM_00108049	KLKBL4	plasma kallikrein-like protein 4	0.015	1.011	9.28E-01	9.28E+01		
8001693	NM_016284	CNOT1	CCR4-NOT transcription complex,	0.032	1.022	7.60E-01	7.60E+01		
8001748	NR_002980	SNORA50	small nucleolar RNA, H/ACA box 5	0.347	1.272	1.99E-01	1.99E+01		
8001750	NM_018231	SLC38A7	solute carrier family 38, member 7	0.085	1.060	4.59E-01	4.59E+01		
8001764	NM_002080	GOT2	glutamic-oxaloacetic transaminase	-0.077	0.948	6.45E-01	6.45E+01		
8001776	---	---	---	-0.041	0.972	8.36E-01	8.36E+01		
8001782	NM_002954	RPS27A	ribosomal protein S27a	-0.005	0.997	9.73E-01	9.73E+01		
8001816	ENST00000456	FLJ27243	FLJ27243 protein	0.061	1.043	7.90E-01	7.90E+01		
8001818	NM_004614	TK2	thymidine kinase 2, mitochondrial	0.061	1.043	8.27E-01	8.27E+01		
8001830	NM_181521	CMTM4	CKLF-like MARVEL transmembrane	-0.003	0.998	9.88E-01	9.88E+01		
8001841	NM_006141	DYNC1LI2	dynein, cytoplasmic 1, light interm	-0.022	0.985	8.82E-01	8.82E+01		
8001876	NM_00101815	NAE1	NEDD8 activating enzyme E1 subu	-0.096	0.936	5.06E-01	5.06E+01		
8001898	NM_004062	CDH16	cadherin 16, KSP-cadherin	-0.068	0.954	4.68E-01	4.68E+01		
8001924	NR_024525	FAM96B	family with sequence similarity 96	-0.032	0.978	8.49E-01	8.49E+01		
8001932	NM_033309	B3GNT9	UDP-GlcNAc:betaGal beta-1,3-N-a	-0.065	0.956	5.97E-01	5.97E+01		
8001938	NM_003789	TRADD	TNFRSF1A-associated via death do	-0.078	0.947	5.16E-01	5.16E+01		
8001953	NM_178516	EXOC3L	exocyst complex component 3-like	-0.088	0.941	2.91E-01	2.91E+01		
8001971	NM_012163	LRRC29	leucine rich repeat containing 29	-0.037	0.974	7.89E-01	7.89E+01		
8001981	NM_013241	FHOD1	formin homology 2 domain contai	-0.046	0.969	6.69E-01	6.69E+01		
8002020	NM_016140	TPPP3	tubulin polymerization-promoting	0.025	1.018	8.54E-01	8.54E+01		
8002029	NM_013304	ZDHHC1	zinc finger, DHHC-type containing	-0.081	0.946	3.75E-01	3.75E+01		
8002041	NM_004691	ATP6V0D1	ATPase, H+ transporting, lysosoma	0.276	1.210	1.41E-01	1.41E+01		
8002051	NM_001138	AGRP	agouti related protein homolog (m	0.039	1.027	7.08E-01	7.08E+01		
8002057	NM_00108248	ACD	adrenocortical dysplasia homolog	-0.081	0.945	3.70E-01	3.70E+01		
8002072	BC008284	C16orf48	chromosome 16 open reading fran	-0.057	0.961	5.51E-01	5.51E+01		
8002087	NM_020850	RANBP10	RAN binding protein 10	0.076	1.054	5.29E-01	5.29E+01		
8002102	---	---	---	0.044	1.031	8.69E-01	8.69E+01		
8002104	NM_025082	CENPT	centromere protein T	-0.061	0.959	5.01E-01	5.01E+01		
8002121	NM_001907	CTRL	chymotrypsin-like	-0.035	0.976	7.44E-01	7.44E+01		
8002133	NM_002801	PSMB10	proteasome (prosome, macropain	0.062	1.044	6.67E-01	6.67E+01		
8002143	NM_000229	LCAT	lecithin-cholesterol acyltransferas	-0.032	0.978	7.57E-01	7.57E+01		
8002152	NM_005072	SLC12A4	solute carrier family 12 (potassium	0.127	1.092	1.41E-01	1.41E+01		
8002181	NM_022357	DPEP3	dipeptidase 3	-0.008	0.995	9.64E-01	9.64E+01		
8002194	NM_022355	DPEP2	dipeptidase 2	0.163	1.120	1.34E-01	1.34E+01		
8002209	---	---	---	0.013	1.009	9.37E-01	9.37E+01		
8002211	NM_018380	DDX28	DEAD (Asp-Glu-Ala-Asp) box polyp	0.096	1.069	7.19E-01	7.19E+01		
8002216	---	---	---	0.117	1.085	6.23E-01	6.23E+01		
8002218	NM_024939	ESRP2	epithelial splicing regulatory prote	-0.032	0.978	8.44E-01	8.44E+01		
8002237	NM_032178	SLC7A6OS	solute carrier family 7, member 6	-0.077	0.948	4.35E-01	4.35E+01		
8002245	---	---	---	-0.098	0.934	7.57E-01	7.57E+01		
8002249	NM_018667	SMPD3	sphingomyelin phosphodiesterase	-0.041	0.972	7.51E-01	7.51E+01		
8002262	---	---	---	0.006	1.004	9.76E-01	9.76E+01		
8002266	NM_00103969	CHTF8	CTF8, chromosome transmission fi	-0.001	0.999	9.95E-01	9.95E+01		
8002270	---	---	---	-0.136	0.910	5.33E-01	5.33E+01		
8002272	NM_032382	COG8	component of oligomeric golgi cor	-0.028	0.981	8.17E-01	8.17E+01		
8002289	NM_005652	TERF2	telomeric repeat binding factor 2	0.003	1.002	9.88E-01	9.88E+01		
8002301	---	---	---	-0.047	0.968	8.79E-01	8.79E+01		
8002312	NM_014062	NOB1	NIN1/RPN12 binding protein 1 hor	-0.083	0.944	7.13E-01	7.13E+01		
8002322	NR_003610	PDXDC2	pyridoxal-dependent decarboxylas	-0.004	0.997	9.88E-01	9.88E+01		
8002333	NM_015092	SMG1	SMG1 homolog, phosphatidylinosi	0.002	1.001	9.89E-01	9.89E+01		
8002342	---	---	---	-0.014	0.990	9.64E-01	9.64E+01		
8002344	NM_058219	EXOSC6	exosome component 6	-0.022	0.985	8.47E-01	8.47E+01		
8002347	NM_001605	AARS	alanyl-tRNA synthetase	-0.007	0.995	9.73E-01	9.73E+01		
8002370	NM_006927	ST3GAL2	ST3 beta-galactoside alpha-2,3-sia	-0.038	0.974	7.94E-01	7.94E+01		
8002379	---	---	---	0.031	1.021	9.56E-01	9.56E+01		
8002381	NM_015386	COG4	component of oligomeric golgi cor	0.059	1.042	7.95E-01	7.95E+01		
8002403	NM_138383	MTSS1L	metastasis suppressor 1-like	0.000	1.000	1.00E+00	1.00E+02		
8002421	NM_018052	VAC14	Vac14 homolog (S. cerevisiae)	-0.165	0.892	1.28E-01	1.28E+01		
8002444	AK128439	VAC14	Vac14 homolog (S. cerevisiae)	0.015	1.011	9.41E-01	9.41E+01		
8002523	NM_018348	FTSJD1	FtsJ methyltransferase domain cor	0.003	1.002	9.87E-01	9.87E+01		
8002533	NM_145911	ZNF23	zinc finger protein 23 (KOX 16)	-0.057	0.961	5.99E-01	5.99E+01		

8002547	NM_006961	ZNF19	zinc finger protein 19	0.006	1.004	9.77E-01	9.77E+01		
8002571	NM_015020	PHLPP2	PH domain and leucine rich repeat	-0.040	0.973	8.63E-01	8.63E+01		
8002590	---	---	---	-0.067	0.954	6.05E-01	6.05E+01		
8002592	NM_00103000	AP1G1	adaptor-related protein complex 1	0.030	1.021	8.63E-01	8.63E+01		
8002618	NM_017530	ZNF821	zinc finger protein 821	-0.052	0.965	7.05E-01	7.05E+01		
8002660	NM_017853	TXNL4B	thioredoxin-like 4B	0.067	1.048	5.87E-01	5.87E+01		
8002692	NM_006885	ZFHX3	zinc finger homeobox 3	-0.008	0.994	9.53E-01	9.53E+01		
8002706	AK129695	C16orf47	chromosome 16 open reading fran	-0.059	0.960	6.10E-01	6.10E+01		
8002711	---	---	---	0.013	1.009	9.63E-01	9.63E+01		
8002713	NM_00101188	CLEC18B	C-type lectin domain family 18, me	-0.031	0.979	7.73E-01	7.73E+01		
8002729	NR_027264	GLG1	golgi apparatus protein 1	0.040	1.028	7.33E-01	7.33E+01		
8002758	---	---	---	0.085	1.061	8.75E-01	8.75E+01		
8002760	ENST00000412	LOC100132346	similar to heat shock 10 kDa prote	-0.103	0.931	5.66E-01	5.66E+01		
8002762	NM_018124	RFWD3	ring finger and WD repeat domain	-0.016	0.989	9.29E-01	9.29E+01		
8002778	NM_152649	MLKL	mixed lineage kinase domain-like	-0.234	0.850	2.57E-01	2.57E+01		
8002792	NM_024306	FA2H	fatty acid 2-hydroxylase	-0.065	0.956	4.42E-01	4.42E+01		
8002802	NM_030581	WDR59	WD repeat domain 59	-0.060	0.959	7.27E-01	7.27E+01		
8002830	NM_153486	LDHD	lactate dehydrogenase D	-0.006	0.996	9.77E-01	9.77E+01		
8002845	NM_00102520	CTRB2	chymotrypsinogen B2	-0.042	0.972	6.30E-01	6.30E+01		
8002854	NM_014567	BCAR1	breast cancer anti-estrogen resista	0.069	1.049	3.72E-01	3.72E+01		
8002865	NM_006324	CFDP1	craniofacial development protein	0.045	1.032	6.51E-01	6.51E+01		
8002878	NM_145254	TMEM170A	transmembrane protein 170A	-0.035	0.976	8.42E-01	8.42E+01		
8002891	NM_024533	CHST5	carbohydrate (N-acetylglucosamir	-0.043	0.971	8.05E-01	8.05E+01		
8002904	NM_012091	ADAT1	adenosine deaminase, tRNA-speci	0.020	1.014	9.54E-01	9.54E+01		
8002919	NM_00113008	KARS	lysyl-tRNA synthetase	0.117	1.084	2.78E-01	2.78E+01		
8002937	---	---	---	-0.064	0.956	5.10E-01	5.10E+01		
8002967	---	---	---	-0.123	0.918	7.10E-01	7.10E+01		
8002969	NM_00103180	MAF	v-maf musculoaponeurotic fibrosa	0.193	1.143	1.06E-01	1.06E+01		
8002975	NM_152342	CDYL2	chromodomain protein, Y-like 2	-0.149	0.902	8.70E-02	8.70E+00		
8002987	NM_020188	C16orf61	chromosome 16 open reading fran	0.121	1.087	3.17E-01	3.17E+01		
8002999	NM_004483	GCSH	glycine cleavage system protein H	0.057	1.040	8.02E-01	8.02E+01		
8003056	AF220234	GAFA2	FGF-2 activity-associated protein 2	0.009	1.006	9.73E-01	9.73E+01		
8003066	---	---	---	-0.052	0.964	8.88E-01	8.88E+01		
8003075	---	---	---	-0.084	0.943	5.87E-01	5.87E+01		
8003077	NM_00108044	SLC38A8	solute carrier family 38, member 8	-0.029	0.980	8.52E-01	8.52E+01		
8003087	---	---	---	0.182	1.134	8.19E-02	8.19E+00		
8003089	NM_003791	MBTPS1	membrane-bound transcription fa	0.031	1.022	8.47E-01	8.47E+01		
8003116	NM_031463	HSDL1	hydroxysteroid dehydrogenase like	0.047	1.033	7.75E-01	7.75E+01		
8003125	NM_005679	TAF1C	TATA box binding protein (TBP)-as	-0.041	0.972	7.64E-01	7.64E+01		
8003145	NM_139174	ADAD2	adenosine deaminase domain con	0.180	1.133	2.93E-01	2.93E+01		
8003149	NM_172347	KCNG4	potassium voltage-gated channel,	0.004	1.003	9.78E-01	9.78E+01		
8003156	---	---	---	-0.008	0.994	9.72E-01	9.72E+01		
8003158	NM_020947	KIAA1609	KIAA1609	-0.008	0.995	9.73E-01	9.73E+01		
8003171	NM_021149	COTL1	coactosin-like 1 (Dictyostelium)	0.291	1.223	2.68E-03	2.68E-01		
8003180	NM_00114554	ZDHHC7	zinc finger, DHHC-type containing	0.100	1.072	6.17E-01	6.17E+01		
8003193	BC093665	FAM92B	family with sequence similarity 92	-0.057	0.962	5.25E-01	5.25E+01		
8003204	NM_016095	GINS2	GINS complex subunit 2 (Psf2 hom	0.074	1.053	6.70E-01	6.70E+01		
8003210	NM_206967	C16orf74	chromosome 16 open reading fran	-0.037	0.975	7.27E-01	7.27E+01		
8003217	NM_006067	COX4NB	COX4 neighbor	0.035	1.025	8.84E-01	8.84E+01		
8003226	---	---	---	-0.082	0.945	6.66E-01	6.66E+01		
8003233	NR_027490	MTHFSD	methenyltetrahydrofolate synthet	-0.038	0.974	8.48E-01	8.48E+01		
8003243	AK026130	FBXO31	F-box protein 31	0.010	1.007	9.56E-01	9.56E+01		
8003249	NM_024735	FBXO31	F-box protein 31	-0.045	0.969	7.61E-01	7.61E+01		
8003263	NM_015144	ZCCHC14	zinc finger, CCHC domain containi	0.037	1.026	8.80E-01	8.80E+01		
8003283	NM_017566	KLHDC4	kelch domain containing 4	-0.117	0.922	2.12E-01	2.12E+01		
8003298	NM_003486	SLC7A5	solute carrier family 7 (cationic am	-0.296	0.814	1.83E-03	1.83E-01		
8003322	NM_000101	CYBA	cytochrome b-245, alpha polypept	0.036	1.025	8.41E-01	8.41E+01		
8003332	NM_002461	MVD	mevalonate (diphospho) decarbox	-0.091	0.939	2.05E-01	2.05E+01		
8003344	NM_178310	SNAI3	snail homolog 3 (Drosophila)	-0.019	0.987	9.00E-01	9.00E+01		
8003348	NM_178841	RNF166	ring finger protein 166	-0.053	0.964	7.42E-01	7.42E+01		
8003357	NM_00114286	FAM38A	family with sequence similarity 38	-0.064	0.957	3.27E-01	3.27E+01		
8003401	NM_000485	APRT	adenine phosphoribosyltransferas	-0.072	0.951	4.74E-01	4.74E+01		
8003410	NM_000512	GALNS	galactosamine (N-acetyl)-6-sulfate	0.061	1.043	5.51E-01	5.51E+01		
8003425	NM_005187	CBFA2T3	core-binding factor, runt domain,	0.003	1.002	9.85E-01	9.85E+01		
8003444	ENST00000333	LOC146429	Putative solute carrier family 22 m	0.040	1.028	8.34E-01	8.34E+01		
8003448	NM_013275	ANKRD11	ankyrin repeat domain 11	-0.108	0.928	1.99E-01	1.99E+01		
8003465	---	---	---	0.012	1.008	9.58E-01	9.58E+01		
8003467	NM_00108331	CHMP1A	chromatin modifying protein 1A	-0.012	0.992	9.51E-01	9.51E+01		
8003478	NM_152339	SPATA2L	spermatogenesis associated 2-like	-0.052	0.965	6.92E-01	6.92E+01		
8003484	NM_004913	C16orf7	chromosome 16 open reading fran	-0.016	0.989	9.22E-01	9.22E+01		
8003503	NM_000135	FANCA	Fanconi anemia, complementatior	-0.011	0.992	9.37E-01	9.37E+01		
8003553	NM_145039	CENPBD1	CENPB DNA-binding domains cont	-0.006	0.996	9.86E-01	9.86E+01		
8003560	NM_024043	DBNDD1	dysbindin (dystrobrevin binding pr	-0.043	0.971	7.03E-01	7.03E+01		
8003583	NM_007317	KIF22	kinesin family member 22	0.017	1.012	9.16E-01	9.16E+01		
8003601	NM_175900	C16orf54	chromosome 16 open reading fran	0.097	1.069	1.76E-01	1.76E+01		
8003605	---	---	---	-0.131	0.913	6.78E-01	6.78E+01		
8003607	NM_00101367	C17orf97	chromosome 17 open reading fran	-0.128	0.915	4.88E-01	4.88E+01		

8003611	NM_024792	FAM57A	family with sequence similarity 57	-0.118	0.921	2.16E-01	2.16E+01		
8003619	NR_024120	ELP2P	endozepine-like peptide 2 pseudo	-0.050	0.966	7.94E-01	7.94E+01		
8003621	NM_018146	RNMTL1	RNA methyltransferase like 1	-0.037	0.975	8.27E-01	8.27E+01		
8003627	NM_013337	TIMM22	translocase of inner mitochondria	0.055	1.039	7.60E-01	7.60E+01		
8003633	ENST00000391	BHLHA9	basic helix-loop-helix family, mem	-0.047	0.968	8.46E-01	8.46E+01		
8003635	NM_172367	TUSC5	tumor suppressor candidate 5	-0.007	0.995	9.76E-01	9.76E+01		
8003639	BC092513	WDR81	WD repeat domain 81	0.040	1.028	7.51E-01	7.51E+01		
8003656	NM_000934	SERPINF2	serpin peptidase inhibitor, clade F	-0.006	0.996	9.77E-01	9.77E+01		
8003679	NM_002945	RPA1	replication protein A1, 70kDa	-0.039	0.974	7.97E-01	7.97E+01		
8003700	NM_001383	DPH1	DPH1 homolog (S. cerevisiae)	-0.027	0.982	8.30E-01	8.30E+01		
8003719	NM_00109820	HIC1	hypermethylated in cancer 1	-0.066	0.956	5.13E-01	5.13E+01		
8003722	NM_021947	SRR	serine racemase	-0.016	0.989	9.37E-01	9.37E+01		
8003733	NM_014853	SGSM2	small G protein signaling modulato	-0.004	0.997	9.77E-01	9.77E+01		
8003758	NM_000430	PAFAH1B1	platelet-activating factor acetylhy	-0.030	0.979	8.06E-01	8.06E+01		
8003769	---	---	---	0.147	1.107	7.27E-01	7.27E+01		
8003771	---	---	---	-0.051	0.965	7.55E-01	7.55E+01		
8003773	NM_015085	GARNL4	GTPase activating Rap/RanGAP do	0.011	1.007	9.52E-01	9.52E+01		
8003800	---	---	---	-0.066	0.955	5.89E-01	5.89E+01		
8003804	NM_014565	OR1A1	olfactory receptor, family 1, subfa	-0.100	0.933	4.20E-01	4.20E+01		
8003806	NM_003552	OR1D4	olfactory receptor, family 1, subfa	0.000	1.000	1.00E+00	1.00E+02		
8003810	---	---	---	-0.145	0.904	1.59E-01	1.59E+01		
8003812	NM_012373	OR3A3	olfactory receptor, family 3, subfa	-0.032	0.978	9.11E-01	9.11E+01		
8003824	NM_004937	CTNS	cystinosis, nephropathic	0.311	1.240	1.42E-01	1.42E+01		
8003840	NM_00101476	TMEM93	transmembrane protein 93	-0.046	0.968	7.15E-01	7.15E+01		
8003844	NM_031965	GSG2	germ cell associated 2 (haspin)	0.032	1.023	9.04E-01	9.04E+01		
8003846	NM_015113	ZZEF1	zinc finger, ZZ-type with EF-hand d	-0.015	0.989	9.24E-01	9.24E+01		
8003848	---	---	---	0.039	1.027	9.60E-01	9.60E+01		
8003850	NM_144611	CYB5D2	cytochrome b5 domain containing	-0.059	0.960	6.11E-01	6.11E+01		
8003857	---	---	---	0.142	1.103	4.27E-01	4.27E+01		
8003859	---	---	---	0.064	1.045	8.86E-01	8.86E+01		
8003861	NM_182538	SPNS3	spinster homolog 3 (Drosophila)	-0.015	0.989	9.31E-01	9.31E+01		
8003875	NM_00112475	SPNS2	spinster homolog 2 (Drosophila)	-0.049	0.967	6.38E-01	6.38E+01		
8003892	NM_00111497	SMTNL2	smoothelin-like 2	-0.042	0.972	6.52E-01	6.52E+01		
8003903	NM_004313	ARRB2	arrestin, beta 2	0.204	1.151	1.76E-01	1.76E+01		
8003922	NM_00100168	MED11	mediator complex subunit 11	0.018	1.013	9.49E-01	9.49E+01		
8003926	NM_00113604	ZMYND15	zinc finger, MYND-type containing	0.027	1.019	8.63E-01	8.63E+01		
8003939	NM_003963	TM4SF5	transmembrane 4 L six family men	-0.043	0.971	6.99E-01	6.99E+01		
8003948	NM_00101498	GLTPD2	glycolipid transfer protein domain	-0.102	0.932	2.01E-01	2.01E+01		
8003953	NM_002798	PSMB6	proteasome (prosome, macropain	0.011	1.007	9.75E-01	9.75E+01		
8003962	NM_002663	PLD2	phospholipase D2	-0.168	0.890	9.17E-02	9.17E+00		
8003991	NM_153827	MINK1	misshapen-like kinase 1 (zebrafish	-0.033	0.978	8.44E-01	8.44E+01		
8004024	NM_000173	GP1BA	glycoprotein Ib (platelet), alpha po	0.031	1.021	8.98E-01	8.98E+01		
8004030	NM_015528	RNF167	ring finger protein 167	-0.050	0.966	7.50E-01	7.50E+01		
8004043	NM_001976	ENO3	enolase 3 (beta, muscle)	-0.018	0.988	9.54E-01	9.54E+01		
8004057	NM_006612	KIF1C	kinesin family member 1C	0.036	1.025	8.46E-01	8.46E+01		
8004081	NM_153018	ZFP3	zinc finger protein 3 homolog (mo	0.075	1.054	6.59E-01	6.59E+01		
8004111	NM_004703	RABEP1	rabaptin, RAB GTPase binding effe	0.028	1.019	8.47E-01	8.47E+01		
8004133	NR_027680	RPAIN	RPA interacting protein	-0.001	0.999	9.98E-01	9.98E+01		
8004144	NM_024039	MIS12	MIS12, MIND kinetochore comple	-0.077	0.948	4.00E-01	4.00E+01		
8004152	NM_015253	WSCD1	WSC domain containing 1	0.012	1.008	9.36E-01	9.36E+01		
8004175	NM_032731	TXNDC17	thioredoxin domain containing 17	0.095	1.068	5.67E-01	5.67E+01		
8004184	NM_017523	XAF1	XIAP associated factor 1	0.174	1.128	5.97E-01	5.97E+01		
8004195	NM_153230	FBXO39	F-box protein 39	-0.019	0.987	9.46E-01	9.46E+01		
8004201	NR_002710	ALOX12P2	arachidonate 12-lipoxygenase pse	-0.055	0.962	6.06E-01	6.06E+01		
8004219	---	---	---	0.111	1.080	8.65E-01	8.65E+01		
8004221	NM_000697	ALOX12	arachidonate 12-lipoxygenase	0.017	1.012	9.43E-01	9.43E+01		
8004237	NM_00100433	RNASEK	ribonuclease, RNase K	0.091	1.065	5.66E-01	5.66E+01		
8004241	NM_00100433	RNASEK	ribonuclease, RNase K	0.013	1.009	9.46E-01	9.46E+01		
8004247	NM_00114279	C17orf49	chromosome 17 open reading fran	-0.198	0.872	3.47E-02	3.47E+00		
8004255	NM_181844	BCL6B	B-cell CLL/lymphoma 6, member B	-0.021	0.985	9.21E-01	9.21E+01		
8004266	NM_201566	SLC16A13	solute carrier family 16, member 1	0.057	1.040	7.61E-01	7.61E+01		
8004271	NM_000018	ACADVL	acyl-Coenzyme A dehydrogenase,	-0.043	0.971	8.79E-01	8.79E+01		
8004293	NM_203413	C17orf81	chromosome 17 open reading fran	0.025	1.017	8.53E-01	8.53E+01		
8004309	NM_001042	SLC2A4	solute carrier family 2 (facilitated g	0.049	1.034	7.07E-01	7.07E+01		
8004325	NM_00114376	EIF5A	eukaryotic translation initiation fa	-0.045	0.969	8.31E-01	8.31E+01		
8004331	NM_014716	ACAP1	ArfGAP with coiled-coil, ankyrin re	-0.022	0.985	8.77E-01	8.77E+01		
8004360	NM_00100291	KCTD11	potassium channel tetramerisation	-0.014	0.991	9.54E-01	9.54E+01		
8004372	NM_003985	TNK1	tyrosine kinase, non-receptor, 1	-0.058	0.961	6.31E-01	6.31E+01		
8004385	NM_020795	NLGN2	neuroligin 2	-0.033	0.977	7.87E-01	7.87E+01		
8004394	NM_199339	SPEM1	spermatid maturation 1	0.017	1.012	9.44E-01	9.44E+01		
8004400	NM_175734	C17orf74	chromosome 17 open reading fran	-0.062	0.958	5.69E-01	5.69E+01		
8004404	NM_178518	TMEM102	transmembrane protein 102	0.003	1.002	9.90E-01	9.90E+01		
8004416	NM_000747	CHRNB1	cholinergic receptor, nicotinic, bet	0.001	1.001	9.96E-01	9.96E+01		
8004428	NM_00110261	AMAC1L3	acyl-malonyl condensing enzyme 1	-0.012	0.992	9.40E-01	9.40E+01		
8004431	NM_000937	POLR2A	polymerase (RNA) II (DNA directed	-0.114	0.924	2.35E-01	2.35E+01		
8004464	NM_172089	NFSF12-TNFSF1	TNFSF12-TNFSF13 readthrough tra	-0.094	0.937	2.75E-01	2.75E+01		
8004485	NM_015670	SENP3	SUMO1/sentrin/SMT3 specific pep	-0.026	0.982	8.64E-01	8.64E+01		

8004497	NM_001416	EIF4A1	eukaryotic translation initiation fa	0.004	1.002	9.89E-01	9.89E+01		
8004506	NR_002918	SNORA48	small nucleolar RNA, H/ACA box 4	-0.054	0.964	7.98E-01	7.98E+01		
8004508	NR_002912	SNORA67	small nucleolar RNA, H/ACA box 6	-0.131	0.913	5.63E-01	5.63E+01		
8004510	NM_001251	CD68	CD68 molecule	0.532	1.446	7.22E-02	7.22E+00		
8004521	NR_024603	MPDU1	mannose-P-dolichol utilization def	0.121	1.087	5.53E-01	5.53E+01		
8004534	NM_001040	SHBG	sex hormone-binding globulin	0.045	1.032	7.56E-01	7.56E+01		
8004545	NM_001678	ATP1B2	ATPase, Na+/K+ transporting, beta	-0.094	0.937	3.38E-01	3.38E+01		
8004556	NM_018081	WRAP53	WD repeat containing, antisense t	-0.094	0.937	3.70E-01	3.70E+01		
8004671	NM_00108042	KDM6B	lysine (K)-specific demethylase 6B	-0.150	0.901	4.01E-02	4.01E+00		
8004691	NM_203411	TMEM88	transmembrane protein 88	-0.046	0.969	7.68E-01	7.68E+01		
8004694	NM_144607	CYB5D1	cytochrome b5 domain containing	-0.105	0.930	5.46E-01	5.46E+01		
8004699	NM_00100527	CHD3	chromodomain helicase DNA bind	-0.110	0.926	3.77E-01	3.77E+01		
8004741	NM_00103714	CNTROB	centrobin, centrosomal BRCA2 int	-0.068	0.954	5.12E-01	5.12E+01		
8004763	NM_000180	GUCY2D	guanylate cyclase 2D, membrane (	-0.060	0.959	4.29E-01	4.29E+01		
8004784	NM_001141	ALOX15B	arachidonate 15-lipoxygenase, typ	-0.049	0.967	6.38E-01	6.38E+01		
8004802	NM_017622	C17orf59	chromosome 17 open reading frar	-0.073	0.951	5.10E-01	5.10E+01		
8004804	NM_012393	PFAS	phosphoribosylformylglycinamidir	-0.066	0.955	4.62E-01	4.62E+01		
8004832	NM_201520	SLC25A35	solute carrier family 25, member 3	-0.042	0.971	7.61E-01	7.61E+01		
8004842	NM_173728	ARHGEF15	Rho guanine nucleotide exchange	-0.009	0.994	9.49E-01	9.49E+01		
8004867	NM_00102557	NDEL1	nudE nuclear distribution gene E h	-0.015	0.990	9.28E-01	9.28E+01		
8004880	NM_004822	NTN1	netrin 1	-0.010	0.993	9.56E-01	9.56E+01		
8004905	NM_153210	USP43	ubiquitin specific peptidase 43	-0.050	0.966	6.43E-01	6.43E+01		
8004940	NM_020233	C17orf48	chromosome 17 open reading frar	0.125	1.091	5.29E-01	5.29E+01		
8004946	---	---	---	-0.047	0.968	6.99E-01	6.99E+01		
8005029	NM_003010	MAP2K4	mitogen-activated protein kinase	0.087	1.062	5.67E-01	5.67E+01		
8005089	NM_001303	COX10	COX10 homolog, cytochrome c oxi	-0.012	0.992	9.54E-01	9.54E+01		
8005097	NM_006041	HS3ST3B1	heparan sulfate (glucosamine) 3-O	-0.360	0.779	1.58E-01	1.58E+01		
8005110	NM_020652	ZNF286A	zinc finger protein 286A	-0.091	0.939	5.08E-01	5.08E+01		
8005117	NM_178571	TBC1D26	TBC1 domain family, member 26	0.011	1.008	9.63E-01	9.63E+01		
8005132	NR_002211	MEIS3P1	Meis homeobox 3 pseudogene 1	0.011	1.008	9.50E-01	9.50E+01		
8005134	NM_000676	ADORA2B	adenosine A2b receptor	0.064	1.045	6.82E-01	6.82E+01		
8005141	NM_017775	TTC19	tetratricopeptide repeat domain 1	-0.001	0.999	9.98E-01	9.98E+01		
8005155	---	---	---	0.018	1.012	9.57E-01	9.57E+01		
8005157	NM_004278	PIGL	phosphatidylinositol glycan ancho	0.037	1.026	8.63E-01	8.63E+01		
8005166	NM_018955	UBB	ubiquitin B	0.081	1.058	5.01E-01	5.01E+01		
8005171	NM_016113	TRPV2	transient receptor potential cation	0.122	1.088	3.37E-01	3.37E+01		
8005191	BC027986	NCRNA00188	non-protein coding RNA 188	0.006	1.004	9.83E-01	9.83E+01		
8005200	NR_003043	SNORD49B	small nucleolar RNA, C/D box 49B	0.077	1.055	8.50E-01	8.50E+01		
8005202	NR_002744	SNORD49A	small nucleolar RNA, C/D box 49A	0.051	1.036	9.00E-01	9.00E+01		
8005231	NR_026809	FAM106A	family with sequence similarity 10	0.036	1.025	9.10E-01	9.10E+01		
8005235	NM_201274	MPRIP	myosin phosphatase Rho interacti	-0.060	0.959	7.42E-01	7.42E+01		
8005245	---	---	---	0.084	1.060	6.35E-01	6.35E+01		
8005247	NM_020201	NT5M	5',3'-nucleotidase, mitochondrial	-0.055	0.963	5.72E-01	5.72E+01		
8005260	NM_018019	MED9	mediator complex subunit 9	-0.036	0.975	6.72E-01	6.72E+01		
8005267	NM_030665	RAI1	retinoic acid induced 1	-0.031	0.979	7.73E-01	7.73E+01		
8005289	NM_00113009	LRRC48	leucine rich repeat containing 48	-0.069	0.953	7.19E-01	7.19E+01		
8005305	NM_024052	C17orf39	chromosome 17 open reading frar	-0.019	0.987	9.48E-01	9.48E+01		
8005313	NM_001388	DRG2	developmentally regulated GTP bi	0.058	1.041	6.35E-01	6.35E+01		
8005328	NM_016239	MYO15A	myosin XVA	-0.008	0.995	9.53E-01	9.53E+01		
8005399	NM_017758	ALKBH5	alkB, alkylation repair homolog 5 (	0.015	1.010	9.09E-01	9.09E+01		
8005407	NM_004140	LLGL1	lethal giant larvae homolog 1 (Dro	-0.048	0.967	5.90E-01	5.90E+01		
8005433	---	---	---	-0.021	0.986	9.03E-01	9.03E+01		
8005435	NM_148886	SMCR7	Smith-Magenis syndrome chromos	-0.059	0.960	3.79E-01	3.79E+01		
8005441	NM_144775	SMCR8	Smith-Magenis syndrome chromos	-0.025	0.983	8.78E-01	8.78E+01		
8005444	ENST00000407	FLJ35934	FLJ35934 protein	-0.105	0.930	6.23E-01	6.23E+01		
8005446	AK127974	LOC339240	keratin pseudogene	0.020	1.014	9.42E-01	9.42E+01		
8005449	NM_000422	KRT17	keratin 17	-0.022	0.985	8.79E-01	8.79E+01		
8005471	NM_001031	RPS28	ribosomal protein S28	0.079	1.056	6.19E-01	6.19E+01		
8005473	NM_006451	PAIP1	poly(A) binding protein interacting	0.073	1.052	8.04E-01	8.04E+01		
8005501	NM_00113503	FAM18B2	family with sequence similarity 18	0.030	1.021	8.84E-01	8.84E+01		
8005512	NM_002767	PRPSAP2	phosphoribosyl pyrophosphate sy	0.128	1.093	3.41E-01	3.41E+01		
8005529	NM_152351	SLC5A10	solute carrier family 5 (sodium/glu	-0.041	0.972	6.66E-01	6.66E+01		
8005547	NR_006880	SNORD3A	small nucleolar RNA, C/D box 3A	-0.508	0.703	8.15E-02	8.15E+00		
8005549	ENST00000284	GRAP	GRB2-related adaptor protein	0.005	1.003	9.86E-01	9.86E+01		
8005553	NR_006880	SNORD3A	small nucleolar RNA, C/D box 3A	-0.508	0.703	8.15E-02	8.15E+00		
8005555	AK026312	LOC79999	hypothetical LOC79999	-0.049	0.966	6.31E-01	6.31E+01		
8005557	NM_014964	EPN2	epsin 2	-0.074	0.950	4.42E-01	4.42E+01		
8005574	---	---	---	-0.047	0.968	8.58E-01	8.58E+01		
8005576	NM_139033	MAPK7	mitogen-activated protein kinase 7	-0.004	0.997	9.85E-01	9.85E+01		
8005601	---	---	---	-0.079	0.947	3.50E-01	3.50E+01		
8005603	NM_018242	SLC47A1	solute carrier family 47, member 1	-0.009	0.994	9.63E-01	9.63E+01		
8005628	---	---	---	-0.061	0.959	7.94E-01	7.94E+01		
8005638	NM_00103180	ALDH3A2	aldehyde dehydrogenase 3 family,	0.061	1.043	7.11E-01	7.11E+01		
8005657	---	---	---	0.077	1.054	8.21E-01	8.21E+01		
8005661	NM_00103355	CYTSB	cytospin B	0.115	1.083	2.69E-01	2.69E+01		
8005679	NR_023380	CCDC144C	coiled-coil domain containing 144C	-0.009	0.994	9.77E-01	9.77E+01		
8005687	NR_026809	FAM106A	family with sequence similarity 10	0.002	1.002	9.96E-01	9.96E+01		

8005689	NR_003554	LOC220594	TL132 protein	0.053	1.037	8.85E-01	8.85E+01		
8005695	NR_002211	MEIS3P1	Meis homeobox 3 pseudogene 1	-0.005	0.997	9.75E-01	9.75E+01		
8005699	NM_015510	DHRS7B	dehydrogenase/reductase (SDR fa	-0.083	0.944	5.38E-01	5.38E+01		
8005707	NM_002756	MAP2K3	mitogen-activated protein kinase	-0.079	0.947	4.04E-01	4.04E+01		
8005726	NM_021012	KCNJ12	potassium inwardly-rectifying cha	-0.032	0.978	7.89E-01	7.89E+01		
8005733	NR_003678	C20orf191	nuclear receptor co-repressor 1 ps	0.062	1.044	7.76E-01	7.76E+01		
8005736	---	---	---	0.025	1.018	8.64E-01	8.64E+01		
8005739	BC031617	FAM27L	family with sequence similarity 27	0.014	1.010	9.52E-01	9.52E+01		
8005751	---	---	---	0.001	1.000	9.99E-01	9.99E+01		
8005753	---	---	---	-0.050	0.966	8.37E-01	8.37E+01		
8005765	NM_015626	WSB1	WD repeat and SOCS box-containi	0.139	1.101	1.36E-01	1.36E+01		
8005777	BC112354	LOC440419	similar to ubiquitin specific protea	-0.020	0.986	8.83E-01	8.83E+01		
8005785	NM_014238	KSR1	kinase suppressor of ras 1	-0.026	0.982	9.08E-01	9.08E+01		
8005809	NM_009587	LGALS9	lectin, galactoside-binding, soluble	0.559	1.473	5.63E-02	5.63E+00		
8005814	NM_016231	NLK	nemo-like kinase	0.116	1.084	1.54E-01	1.54E+01		
8005833	NR_003064	PYY2	peptide YY, 2 (seminalplasmin)	-0.038	0.974	7.40E-01	7.40E+01		
8005839	NM_014573	TMEM97	transmembrane protein 97	0.001	1.001	9.97E-01	9.97E+01		
8005847	NM_021137	TNFAIP1	tumor necrosis factor, alpha-induc	-0.130	0.914	2.61E-01	2.61E+01		
8005857	NM_152464	TMEM199	transmembrane protein 199	0.129	1.094	3.67E-01	3.67E+01		
8005865	NM_015077	SARM1	sterile alpha and TIR motif contain	-0.049	0.967	6.58E-01	6.58E+01		
8005879	NR_027384	SLC13A2	solute carrier family 13 (sodium-d	-0.015	0.989	9.02E-01	9.02E+01		
8005903	NM_003170	SUPT6H	suppressor of Ty 6 homolog (S. cer	-0.114	0.924	1.90E-01	1.90E+01		
8005943	NM_000984	RPL23A	ribosomal protein L23a	-0.020	0.986	8.68E-01	8.68E+01		
8005951	NR_000013	SNORD42B	small nucleolar RNA, C/D box 42B	-0.086	0.942	8.88E-01	8.88E+01		
8005955	NR_000014	SNORD42A	small nucleolar RNA, C/D box 42A	0.045	1.032	9.30E-01	9.30E+01		
8005957	NR_000009	SNORD4B	small nucleolar RNA, C/D box 4B	-0.019	0.987	9.77E-01	9.77E+01		
8005959	NM_178170	NEK8	NIMA (never in mitosis gene a)- re	0.021	1.015	9.02E-01	9.02E+01		
8005978	NM_004295	TRAF4	TNF receptor-associated factor 4	-0.108	0.928	2.60E-02	2.60E+00		
8005994	NM_005702	ERAL1	Era G-protein-like 1 (E. coli)	0.022	1.015	9.27E-01	9.27E+01		
8006005	NM_016518	PIPOX	pipecolic acid oxidase	-0.016	0.989	9.04E-01	9.04E+01		
8006019	NM_005208	CRYBA1	crystallin, beta A1	0.021	1.015	9.36E-01	9.36E+01		
8006028	---	---	---	0.232	1.175	4.71E-01	4.71E+01		
8006030	NM_020791	TAOK1	TAO kinase 1	-0.007	0.995	9.73E-01	9.73E+01		
8006052	NM_138349	TP53I13	tumor protein p53 inducible prote	0.012	1.008	9.33E-01	9.33E+01		
8006063	NM_152345	ANKRD13B	ankyrin repeat domain 13B	-0.038	0.974	8.05E-01	8.05E+01		
8006081	---	---	---	0.133	1.096	5.44E-01	5.44E+01		
8006112	NM_032141	CCDC55	coiled-coil domain containing 55	-0.007	0.995	9.66E-01	9.66E+01		
8006119	---	---	---	0.000	1.000	1.00E+00	1.00E+02		
8006123	NM_001304	CPD	carboxypeptidase D	-0.227	0.855	1.65E-01	1.65E+01		
8006148	NM_004871	GOSR1	golgi SNAP receptor complex mem	0.111	1.080	2.98E-01	2.98E+01		
8006161	NM_015594	TBC1D29	TBC1 domain family, member 29	-0.019	0.987	8.86E-01	8.86E+01		
8006170	NR_015341	LRRC37B2	leucine rich repeat containing 37,	0.036	1.025	8.27E-01	8.27E+01		
8006181	---	---	---	-0.051	0.965	7.98E-01	7.98E+01		
8006183	NR_024187	SUZ12P	suppressor of zeste 12 homolog ps	-0.009	0.994	9.77E-01	9.77E+01		
8006229	NM_032322	RNF135	ring finger protein 135	0.256	1.194	1.06E-03	1.06E-01		
8006237	ENST00000433	LOC400590	hypothetical LOC400590	0.161	1.118	3.80E-01	3.80E+01		
8006239	NM_00104249	NF1	neurofibromin 1	-0.068	0.954	6.96E-01	6.96E+01		
8006298	NM_032932	RAB11FIP4	RAB11 family interacting protein 4	0.042	1.030	6.68E-01	6.68E+01		
8006319	---	---	---	-0.020	0.986	9.16E-01	9.16E+01		
8006321	---	---	---	-0.121	0.920	2.99E-01	2.99E+01		
8006325	NM_015355	SUZ12	suppressor of zeste 12 homolog (D	-0.015	0.990	9.37E-01	9.37E+01		
8006336	NM_052888	LRRC37B	leucine rich repeat containing 37B	0.110	1.079	4.32E-01	4.32E+01		
8006345	NM_00103356	RHOT1	ras homolog gene family, member	0.230	1.173	6.92E-02	6.92E+00		
8006367	NM_138328	RHBDL3	rhomboid, veinlet-like 3 (Drosophi	0.039	1.027	7.50E-01	7.50E+01		
8006377	NM_00109850	ZNF207	zinc finger protein 207	0.030	1.021	8.04E-01	8.04E+01		
8006392	NM_002815	PSMD11	proteasome (prosome, macropain	-0.103	0.931	4.04E-01	4.04E+01		
8006409	NM_003885	CDK5R1	cyclin-dependent kinase 5, regulat	-0.037	0.974	7.94E-01	7.94E+01		
8006415	NM_015544	TMEM98	transmembrane protein 98	0.091	1.065	2.84E-01	2.84E+01		
8006423	NM_173847	SPACA3	sperm acrosome associated 3	-0.003	0.998	9.86E-01	9.86E+01		
8006433	NM_002982	CCL2	chemokine (C-C motif) ligand 2	0.229	1.172	7.05E-01	7.05E+01		
8006445	NM_002986	CCL11	chemokine (C-C motif) ligand 11	-0.039	0.974	7.91E-01	7.91E+01		
8006453	NM_005623	CCL8	chemokine (C-C motif) ligand 8	1.461	2.753	5.07E-02	5.07E+00		
8006466	NM_207313	TMEM132E	transmembrane protein 132E	-0.049	0.967	6.68E-01	6.68E+01		
8006477	NM_052857	ZNF830	zinc finger protein 830	-0.083	0.944	4.81E-01	4.81E+01		
8006479	NM_013975	LIG3	ligase III, DNA, ATP-dependent	-0.019	0.987	9.17E-01	9.17E+01		
8006531	NM_144975	SLFN5	schlafen family member 5	0.014	1.010	9.67E-01	9.67E+01		
8006540	NR_003037	SNORD7	small nucleolar RNA, C/D box 7	-0.232	0.851	5.00E-01	5.00E+01		
8006542	NM_00103000	AP2B1	adaptor-related protein complex 2	-0.036	0.975	7.92E-01	7.92E+01		
8006562	NM_033315	RASL10B	RAS-like, family 10, member B	0.014	1.010	9.10E-01	9.10E+01		
8006569	NM_145272	C17orf50	chromosome 17 open reading fra	-0.063	0.957	4.12E-01	4.12E+01		
8006573	NM_139215	TAF15	TAF15 RNA polymerase II, TATA bo	0.002	1.002	9.86E-01	9.86E+01		
8006590	---	---	---	-0.039	0.973	9.37E-01	9.37E+01		
8006592	---	---	---	-0.050	0.966	8.46E-01	8.46E+01		
8006594	NM_002988	CCL18	chemokine (C-C motif) ligand 18 (p	0.156	1.115	6.48E-01	6.48E+01		
8006602	NM_002984	CCL4	chemokine (C-C motif) ligand 4	-0.425	0.745	1.76E-01	1.76E+01		
8006608	NM_00100143	CCL4L1	chemokine (C-C motif) ligand 4-lik	-0.433	0.740	1.56E-01	1.56E+01		
8006621	NM_00100143	CCL4L1	chemokine (C-C motif) ligand 4-lik	-0.464	0.725	1.49E-01	1.49E+01		

8006627	NM_004773	ZNHIT3	zinc finger, HIT type 3	-0.055	0.963	8.50E-01	8.50E+01		
8006638	NM_024835	GGNBP2	gametogenetin binding protein 2	-0.084	0.944	4.93E-01	4.93E+01		
8006655	NM_024308	DHRS11	dehydrogenase/reductase (SDR fa	-0.081	0.945	5.72E-01	5.72E+01		
8006668	NM_024864	MRM1	mitochondrial rRNA methyltransfe	-0.064	0.957	5.76E-01	5.76E+01		
8006679	---	---	---	-0.006	0.996	9.68E-01	9.68E+01		
8006681	NM_005568	LHX1	LIM homeobox 1	-0.005	0.997	9.77E-01	9.77E+01		
8006690	NM_012138	AATF	apoptosis antagonizing transcripti	0.003	1.002	9.85E-01	9.85E+01		
8006715	NM_001488	TADA2L	transcriptional adaptor 2 (ADA2 ho	0.041	1.029	8.74E-01	8.74E+01		
8006736	NM_007026	DUSP14	dual specificity phosphatase 14	-0.021	0.985	9.19E-01	9.19E+01		
8006746	NM_00112339	TBC1D3	TBC1 domain family, member 3	0.009	1.006	9.77E-01	9.77E+01		
8006762	NM_032351	MRPL45	mitochondrial ribosomal protein L	-0.326	0.798	6.29E-02	6.29E+00		
8006768	NM_014598	SOCS7	suppressor of cytokine signaling 7	-0.052	0.965	5.44E-01	5.44E+01		
8006779	NM_020876	ARHGAP23	Rho GTPase activating protein 23	-0.041	0.972	8.40E-01	8.40E+01		
8006786	NM_020876	ARHGAP23	Rho GTPase activating protein 23	0.000	1.000	1.00E+00	1.00E+02		
8006788	NM_005937	MLLT6	myeloid/lymphoid or mixed-lineag	-0.088	0.941	1.44E-01	1.44E+01		
8006812	NM_002795	PSMB3	proteasome (prosome, macropain	0.092	1.066	6.44E-01	6.44E+01		
8006820	NM_006148	LASP1	LIM and SH3 protein 1	0.029	1.020	8.88E-01	8.88E+01		
8006834	---	---	---	-0.105	0.930	4.33E-01	4.33E+01		
8006845	NM_000981	RPL19	ribosomal protein L19	0.030	1.021	8.22E-01	8.22E+01		
8006850	NM_016507	CRKRS	Cdc2-related kinase, arginine/serin	-0.051	0.965	5.90E-01	5.90E+01		
8006865	NM_032192	PPP1R1B	protein phosphatase 1, regulatory	0.019	1.013	8.85E-01	8.85E+01		
8006877	NM_006804	STARD3	StAR-related lipid transfer (START)	0.035	1.024	8.30E-01	8.30E+01		
8006896	NM_003673	TCAP	titin-cap (telethonin)	-0.029	0.980	8.73E-01	8.73E+01		
8006900	NM_002686	PNMT	phenylethanolamine N-methyltrar	0.039	1.027	7.98E-01	7.98E+01		
8006906	NM_00100586	ERBB2	v-erb-b2 erythroblastic leukemia v	-0.020	0.986	8.88E-01	8.88E+01		
8006968	NM_178171	GSDMA	gasdermin A	0.040	1.028	7.95E-01	7.95E+01		
8006984	NM_002809	PSMD3	proteasome (prosome, macropain	-0.005	0.996	9.85E-01	9.85E+01		
8006999	NM_172220	CSF3	colony stimulating factor 3 (granul	-0.114	0.924	1.11E-01	1.11E+01		
8007008	NM_003250	THRA	thyroid hormone receptor, alpha (	0.029	1.021	8.99E-01	8.99E+01		
8007023	NM_00101224	MSL1	male-specific lethal 1 homolog (Dr	-0.034	0.977	8.92E-01	8.92E+01		
8007028	NM_007359	CASC3	cancer susceptibility candidate 3	-0.066	0.956	4.62E-01	4.62E+01		
8007058	NM_133264	WIPF2	WAS/WASL interacting protein far	-0.012	0.992	9.53E-01	9.53E+01		
8007069	---	---	---	0.005	1.003	9.85E-01	9.85E+01		
8007084	NM_000964	RARA	retinoic acid receptor, alpha	-0.061	0.959	6.59E-01	6.59E+01		
8007100	NM_001552	IGFBP4	insulin-like growth factor binding p	0.023	1.016	8.63E-01	8.63E+01		
8007112	NM_00114604	KRTAP4-9	keratin associated protein 4-9	-0.008	0.994	9.53E-01	9.53E+01		
8007115	NM_00114604	KRTAP4-9	keratin associated protein 4-9	-0.035	0.976	6.92E-01	6.92E+01		
8007118	ENST00000318	KRTAP9-2	keratin associated protein 9-2	-0.154	0.899	1.95E-01	1.95E+01		
8007123	NM_031961	KRTAP9-2	keratin associated protein 9-2	-0.044	0.970	5.04E-01	5.04E+01		
8007127	NM_031962	KRTAP9-3	keratin associated protein 9-3	0.038	1.027	8.05E-01	8.05E+01		
8007130	NM_031963	KRTAP9-8	keratin associated protein 9-8	-0.044	0.970	6.11E-01	6.11E+01		
8007134	NM_033191	KRTAP9-4	keratin associated protein 9-4	-0.024	0.983	7.92E-01	7.92E+01		
8007141	NM_005801	EIF1	eukaryotic translation initiation fa	-0.058	0.961	3.81E-01	3.81E+01		
8007148	NM_000805	GAST	gastrin	-0.030	0.980	9.13E-01	9.13E+01		
8007152	---	---	---	-0.007	0.995	9.78E-01	9.78E+01		
8007154	NM_021939	FKBP10	FK506 binding protein 10, 65 kDa	-0.047	0.968	6.62E-01	6.62E+01		
8007188	NM_033133	CNP	2',3'-cyclic nucleotide 3' phosphod	-0.037	0.974	8.21E-01	8.21E+01		
8007197	NM_017595	NKIRAS2	NFKB inhibitor interacting Ras-like	-0.052	0.965	7.27E-01	7.27E+01		
8007212	NM_003152	STAT5A	signal transducer and activator of	-0.018	0.987	9.27E-01	9.27E+01		
8007250	NM_000263	NAGLU	N-acetylglucosaminidase, alpha-	-0.019	0.987	9.04E-01	9.04E+01		
8007259	---	---	---	-0.029	0.980	7.97E-01	7.97E+01		
8007263	NM_000413	HSD17B1	hydroxysteroid (17-beta) dehydrog	-0.019	0.987	8.98E-01	8.98E+01		
8007272	NM_025233	COASY	Coenzyme A synthase	0.015	1.011	9.27E-01	9.27E+01		
8007290	NM_170607	MLX	MAX-like protein X	-0.139	0.908	1.11E-01	1.11E+01		
8007302	NM_001070	TUBG1	tubulin, gamma 1	-0.133	0.912	2.83E-01	2.83E+01		
8007312	NM_016437	TUBG2	tubulin, gamma 2	-0.197	0.872	1.29E-01	1.29E+01		
8007323	NM_003632	CNTNAP1	contactin associated protein 1	-0.027	0.982	8.74E-01	8.74E+01		
8007348	NM_005854	RAMP2	receptor (G protein-coupled) activ	-0.081	0.945	4.29E-01	4.29E+01		
8007355	NM_032353	VPS25	vacuolar protein sorting 25 homol	0.085	1.060	5.70E-01	5.70E+01		
8007363	NM_032387	WNK4	WNK lysine deficient protein kinas	0.006	1.004	9.75E-01	9.75E+01		
8007397	NM_176863	PSME3	proteasome (prosome, macropain	-0.022	0.985	9.16E-01	9.16E+01		
8007414	NM_009590	AOC2	amine oxidase, copper containing	0.017	1.012	9.33E-01	9.33E+01		
8007420	NM_003734	AOC3	amine oxidase, copper containing	0.039	1.028	8.52E-01	8.52E+01		
8007427	NM_003734	AOC3	amine oxidase, copper containing	-0.068	0.954	6.50E-01	6.50E+01		
8007435	NM_173079	RUNDC1	RUN domain containing 1	-0.052	0.965	7.77E-01	7.77E+01		
8007441	NM_000988	RPL27	ribosomal protein L27	0.045	1.031	7.79E-01	7.79E+01		
8007446	NM_005533	IFI35	interferon-induced protein 35	0.247	1.187	2.12E-01	2.12E+01		
8007462	NR_003108	NBR2	neighbor of BRCA1 gene 2 (non-pr	0.111	1.080	3.75E-01	3.75E+01		
8007493	NM_001661	ARL4D	ADP-ribosylation factor-like 4D	-0.110	0.926	4.06E-01	4.06E+01		
8007505	NM_004941	DHX8	DEAH (Asp-Glu-Ala-His) box polype	-0.018	0.987	9.24E-01	9.24E+01		
8007537	NM_145273	CD300LG	CD300 molecule-like family memb	0.016	1.011	9.22E-01	9.22E+01		
8007548	NR_026770	C17orf88	chromosome 17 open reading frar	-0.072	0.951	6.17E-01	6.17E+01		
8007550	NM_153006	NAGS	N-acetylglutamate synthase	-0.081	0.945	1.59E-01	1.59E+01		
8007561	NM_138387	G6PC3	glucose 6 phosphatase, catalytic, 3	0.027	1.019	8.78E-01	8.78E+01		
8007569	AK291924	C17orf53	chromosome 17 open reading frar	-0.025	0.983	8.81E-01	8.81E+01		
8007584	NM_080863	ASB16	ankyrin repeat and SOCS box-cont	0.005	1.003	9.82E-01	9.82E+01		
8007594	NM_177441	TMUB2	transmembrane and ubiquitin-like	-0.021	0.986	9.13E-01	9.13E+01		

8007607	NM_006695	RUNDC3A	RUN domain containing 3A	-0.098	0.934	3.04E-01	3.04E+01				
8007620	NM_002087	GRN	granulin	0.295	1.227	6.73E-02	6.73E+00				
8007637	NM_001466	FZD2	frizzled homolog 2 (Drosophila)	-0.030	0.980	8.57E-01	8.57E+01				
8007641	ENST00000434	LOC728675	hypothetical LOC728675	-0.043	0.971	7.45E-01	7.45E+01				
8007651	NM_145663	DBF4B	DBF4 homolog B (S. cerevisiae)	0.089	1.063	4.22E-01	4.22E+01				
8007673	---	---	---	0.045	1.031	7.77E-01	7.77E+01				
8007675	NM_002390	ADAM11	ADAM metallopeptidase domain 1	0.021	1.015	8.78E-01	8.78E+01				
8007701	NM_016438	HIGD1B	HIG1 hypoxia inducible domain fa	-0.063	0.957	8.05E-01	8.05E+01				
8007715	NM_021079	NMT1	N-myristoyltransferase 1	-0.022	0.985	8.96E-01	8.96E+01				
8007730	NM_00113570	ACBD4	acyl-Coenzyme A binding domain	-0.025	0.983	8.61E-01	8.61E+01				
8007745	NM_006460	HEXIM1	hexamethylene bis-acetamide ind	-0.127	0.916	1.92E-01	1.92E+01				
8007750	NM_144608	HEXIM2	hexamthylene bis-acetamide indu	0.009	1.006	9.50E-01	9.50E+01				
8007757	NM_005892	FMNL1	formin-like 1	-0.106	0.929	2.90E-01	2.90E+01				
8007790	NM_003954	MAP3K14	mitogen-activated protein kinase	-0.022	0.985	8.87E-01	8.87E+01				
8007794	NM_00103908	ARL17	ADP-ribosylation factor-like 17	-0.006	0.996	9.89E-01	9.89E+01				
8007797	NM_001029 //	RPS26 // RPS26	ribosomal protein S26 // riboson	0.011	1.008	9.81E-01	9.81E+01				
8007799	NR_026680	MGC57346	hypothetical LOC401884	-0.126	0.916	5.76E-01	5.76E+01				
8007803	NR_026905	C17orf69	chromosome 17 open reading fra	-0.001	0.999	9.98E-01	9.98E+01				
8007808	NM_00114514	CRHR1	corticotropin releasing hormone r	-0.091	0.939	1.78E-01	1.78E+01				
8007826	NM_175882	IMP5	intramembrane protease 5	0.019	1.013	8.86E-01	8.86E+01				
8007848	NM_005456	MAPK8IP1	mitogen-activated protein kinase	-0.091	0.939	2.43E-01	2.43E+01				
8007850	NM_014834	LRRC37A	leucine rich repeat containing 37A	0.016	1.011	9.54E-01	9.54E+01				
8007865	---	---	---	0.044	1.031	8.64E-01	8.64E+01				
8007867	NM_00100660	LRRC37A2	leucine rich repeat containing 37,	0.014	1.010	9.58E-01	9.58E+01				
8007883	---	---	---	0.044	1.031	8.64E-01	8.64E+01				
8007885	NM_006178	NSF	N-ethylmaleimide-sensitive factor	0.153	1.112	2.84E-01	2.84E+01				
8007895	NM_003396	WNT9B	wingless-type MMTV integration s	-0.092	0.938	2.99E-01	2.99E+01				
8007904	NM_004287	GOSR2	golgi SNAP receptor complex mem	0.047	1.033	7.77E-01	7.77E+01				
8007917	---	---	---	-0.040	0.973	8.88E-01	8.88E+01				
8007919	NR_002940	LRRC37A4	leucine rich repeat containing 37,	-0.002	0.999	9.97E-01	9.97E+01				
8007921	NM_00100284	MYL4	myosin, light chain 4, alkali; atrial,	-0.067	0.955	5.17E-01	5.17E+01				
8007976	NM_006310	NPEPPS	aminopeptidase puromycin sensiti	0.017	1.012	9.40E-01	9.40E+01				
8007990	---	---	---	0.046	1.032	9.50E-01	9.50E+01				
8007992	NM_002265	KPNB1	karyopherin (importin) beta 1	0.129	1.093	1.25E-01	1.25E+01				
8008016	NM_014726	TBKBP1	TBK1 binding protein 1	0.009	1.006	9.60E-01	9.60E+01				
8008029	NM_013351	TBX21	T-box 21	-0.014	0.991	9.53E-01	9.53E+01				
8008038	---	---	---	-0.009	0.994	9.90E-01	9.90E+01				
8008052	NM_003110	SP2	Sp2 transcription factor	0.007	1.005	9.73E-01	9.73E+01				
8008064	NM_018129	PNPO	pyridoxamine 5'-phosphate oxidas	0.025	1.017	8.50E-01	8.50E+01				
8008074	NM_176096	CDK5RAP3	CDK5 regulatory subunit associate	0.085	1.061	4.31E-01	4.31E+01				
8008087	NM_003204	NFE2L1	nuclear factor (erythroid-derived 2	0.023	1.016	9.44E-01	9.44E+01				
8008096	NM_152244	SNX11	sorting nexin 11	0.007	1.005	9.81E-01	9.81E+01				
8008110	ENST00000376	LOC404266	hypothetical LOC404266	-0.090	0.939	2.12E-01	2.12E+01				
8008113	NM_005831	CALCOCO2	calcium binding and coiled-coil do	0.212	1.158	2.84E-02	2.84E+00				
8008132	NM_005175	ATP5G1	ATP synthase, H+ transporting, mi	0.092	1.066	3.67E-01	3.67E+01				
8008139	NM_023079	UBE2Z	ubiquitin-conjugating enzyme E2Z	0.127	1.092	3.91E-01	3.91E+01				
8008185	NM_016428	ABI3	ABI family, member 3	0.084	1.060	5.06E-01	5.06E+01				
8008201	NM_002507	NGFR	nerve growth factor receptor (TNF	0.028	1.020	7.98E-01	7.98E+01				
8008208	NM_007225	NXPH3	neurexophilin 3	0.031	1.022	8.25E-01	8.25E+01				
8008213	NM_007067	MYST2	MYST histone acetyltransferase 2	-0.051	0.965	7.51E-01	7.51E+01				
8008228	NM_138281	DLX4	distal-less homeobox 4	-0.100	0.933	1.53E-01	1.53E+01				
8008237	NM_002204	ITGA3	integrin, alpha 3 (antigen CD49C, a	-0.078	0.947	4.89E-01	4.89E+01				
8008263	NM_002611	PDK2	pyruvate dehydrogenase kinase, is	-0.035	0.976	8.78E-01	8.78E+01				
8008277	NM_000023	SGCA	sarcoglycan, alpha (50kDa dystrop	-0.043	0.971	7.27E-01	7.27E+01				
8008289	NM_153229	TMEM92	transmembrane protein 92	-0.046	0.968	6.65E-01	6.65E+01				
8008297	NM_022167	XYLT2	xylosyltransferase II	-0.074	0.950	4.47E-01	4.47E+01				
8008310	NM_152463	EME1	essential meiotic endonuclease 1	-0.025	0.983	8.91E-01	8.91E+01				
8008339	NM_018346	RSAD1	radical S-adenosyl methionine dor	-0.142	0.906	8.62E-02	8.62E+00				
8008372	NM_017957	EPN3	epsin 3	-0.071	0.952	3.31E-01	3.31E+01				
8008409	NM_018896	CACNA1G	calcium channel, voltage-depende	-0.016	0.989	8.41E-01	8.41E+01				
8008454	NM_003786	ABCC3	ATP-binding cassette, sub-family C	0.089	1.063	5.21E-01	5.21E+01				
8008493	NM_016424	LUC7L3	LUC7-like 3 (S. cerevisiae)	-0.033	0.977	7.68E-01	7.68E+01				
8008511	NM_175575	WFIKKN2	WAP, follistatin/kazal, immunoglo	0.010	1.007	9.56E-01	9.56E+01				
8008517	NM_198175	NME1	non-metastatic cells 1, protein (NM	0.049	1.034	8.81E-01	8.81E+01				
8008530	NM_016001	UTP18	UTP18, small subunit (SSU) proces	0.184	1.136	3.33E-02	3.33E+00				
8008544	NM_032559	KIF2B	kinesin family member 2B	-0.016	0.989	9.09E-01	9.09E+01				
8008588	NM_002126	HLF	hepatic leukemia factor	-0.053	0.964	7.73E-01	7.73E+01				
8008598	NM_00110240	PCTP	phosphatidylcholine transfer prote	-0.007	0.996	9.88E-01	9.88E+01				
8008627	NM_005450	NOG	noggin	-0.111	0.926	5.30E-01	5.30E+01				
8008629	NM_003647	DGKE	diacylglycerol kinase, epsilon 64kD	-0.039	0.973	8.63E-01	8.63E+01				
8008644	---	---	---	-0.013	0.991	9.61E-01	9.61E+01				
8008662	NR_002818	RNF126P1	ring finger protein 126 pseudogen	0.010	1.007	9.55E-01	9.55E+01				
8008664	NM_003488	AKAP1	A kinase (PRKA) anchor protein 1	0.011	1.008	9.60E-01	9.60E+01				
8008682	NM_138962	MSI2	musashi homolog 2 (Drosophila)	0.028	1.020	9.07E-01	9.07E+01				
8008700	AK021772	FLJ11710	hypothetical protein FLJ11710	-0.010	0.993	9.67E-01	9.67E+01				
8008702	---	---	---	-0.005	0.997	9.78E-01	9.78E+01				
8008704	---	---	---	-0.014	0.990	9.49E-01	9.49E+01				

8008706	NM_080677	DYNLL2	dynein, light chain, LC8-type 2	-0.124	0.918	4.25E-01	4.25E+01		
8008716	NR_002307	MSX2P1	msh homeobox 2 pseudogene 1	-0.057	0.961	5.98E-01	5.98E+01		
8008736	NM_006151	LPO	lactoperoxidase	-0.013	0.991	9.29E-01	9.29E+01		
8008819	NM_00100540	YPEL2	yippee-like 2 (Drosophila)	-0.048	0.967	8.43E-01	8.43E+01		
8008825	NM_024612	DHX40	DEAH (Asp-Glu-Ala-His) box polype	-0.089	0.940	6.34E-01	6.34E+01		
8008834	NM_004859	CLTC	clathrin, heavy chain (Hc)	0.133	1.097	3.31E-01	3.31E+01		
8008870	NM_030938	TMEM49	transmembrane protein 49	0.062	1.044	7.56E-01	7.56E+01		
8008885	---	---	---	0.047	1.033	8.58E-01	8.58E+01		
8008887	NM_003161	RPS6KB1	ribosomal protein S6 kinase, 70kD	-0.001	0.999	9.93E-01	9.93E+01		
8008900	NM_000717	CA4	carbonic anhydrase IV	-0.009	0.993	9.50E-01	9.50E+01		
8008914	NM_181707	C17orf64	chromosome 17 open reading fran	-0.004	0.997	9.85E-01	9.85E+01		
8008922	NM_003620	PPM1D	protein phosphatase 1D magnesiu	-0.017	0.988	9.49E-01	9.49E+01		
8008933	NM_00109943	BCAS3	breast carcinoma amplified sequer	-0.038	0.974	8.05E-01	8.05E+01		
8008965	---	---	---	0.126	1.091	7.81E-01	7.81E+01		
8008969	NM_005994	TBX2	T-box 2	-0.062	0.958	3.35E-01	3.35E+01		
8008980	BC046200	C17orf82	chromosome 17 open reading fran	-0.049	0.966	7.22E-01	7.22E+01		
8008982	NM_018488	TBX4	T-box 4	-0.028	0.981	8.32E-01	8.32E+01		
8008991	---	---	---	0.111	1.080	7.89E-01	7.89E+01		
8009008	NM_181725	METTL2A	methyltransferase like 2A	-0.047	0.968	7.97E-01	7.97E+01		
8009014	NM_006852	TLK2	tousled-like kinase 2	-0.086	0.942	2.13E-01	2.13E+01		
8009040	NM_006039	MRC2	mannose receptor, C type 2	-0.041	0.972	6.44E-01	6.44E+01		
8009075	NM_025185	TANC2	tetratricopeptide repeat, ankyrin r	-0.050	0.966	7.78E-01	7.78E+01		
8009094	ENST00000416	LOC342541	similar to TRIMCyp	0.026	1.018	8.87E-01	8.87E+01		
8009096	NM_000789	ACE	angiotensin I converting enzyme (p	0.070	1.050	3.85E-01	3.85E+01		
8009145	NM_030779	KCNH6	potassium voltage-gated channel,	-0.019	0.987	8.27E-01	8.27E+01		
8009164	NM_005828	DCAF7	DDB1 and CUL4 associated factor 7	0.058	1.041	5.58E-01	5.58E+01		
8009176	NM_016360	TACO1	translational activator of mitochor	0.069	1.049	6.56E-01	6.56E+01		
8009183	NM_203351	MAP3K3	mitogen-activated protein kinase k	0.051	1.036	6.98E-01	6.98E+01		
8009205	NM_007372	DDX42	DEAD (Asp-Glu-Ala-Asp) box polyp	0.037	1.026	7.56E-01	7.56E+01		
8009227	NM_002805	PSMC5	proteasome (prosome, macropain	-0.036	0.975	7.68E-01	7.68E+01		
8009241	NR_004380	SNORD104	small nucleolar RNA, C/D box 104	-0.186	0.879	5.02E-01	5.02E+01		
8009253	---	---	---	0.236	1.178	3.62E-01	3.62E+01		
8009255	NM_138363	CCDC45	coiled-coil domain containing 45	-0.023	0.984	9.00E-01	9.00E+01		
8009277	NM_003835	RGS9	regulator of G-protein signaling 9	-0.064	0.957	7.86E-01	7.86E+01		
8009301	NM_002737	PRKCA	protein kinase C, alpha	0.129	1.093	6.05E-01	6.05E+01		
8009326	NM_145811	CACNG5	calcium channel, voltage-depende	-0.004	0.997	9.83E-01	9.83E+01		
8009334	NM_014405	CACNG4	calcium channel, voltage-depende	-0.046	0.969	7.01E-01	7.01E+01		
8009349	---	---	---	0.015	1.011	9.56E-01	9.56E+01		
8009351	---	---	---	0.151	1.111	1.14E-01	1.14E+01		
8009353	NM_181671	PITPNC1	phosphatidylinositol transfer prote	-0.204	0.868	1.58E-01	1.58E+01		
8009366	NM_015462	NOL11	nucleolar protein 11	0.040	1.028	8.47E-01	8.47E+01		
8009380	NR_003706	SNORA38B	small nucleolar RNA, H/ACA box 38	-0.038	0.974	8.80E-01	8.80E+01		
8009382	NM_004459	BPTF	bromodomain PHD finger transcrip	-0.059	0.960	5.45E-01	5.45E+01		
8009417	NM_002266	KPNA2	karyopherin alpha 2 (RAG cohort 1	0.024	1.017	9.53E-01	9.53E+01		
8009430	NR_027283	LOC440461	SH3 domain containing 20 pseudo	-0.055	0.963	5.16E-01	5.16E+01		
8009432	NM_016627	AMZ2	archaelysin family metallopeptidas	0.028	1.019	8.71E-01	8.71E+01		
8009443	NM_014960	ARSG	arylsulfatase G	0.060	1.043	3.85E-01	3.85E+01		
8009457	NM_212472	PRKAR1A	protein kinase, cAMP-dependent,	0.001	1.001	9.94E-01	9.94E+01		
8009476	NM_002758	MAP2K6	mitogen-activated protein kinase k	0.169	1.125	3.40E-01	3.40E+01		
8009502	NM_000891	KCNJ2	potassium inwardly-rectifying char	0.390	1.311	9.28E-02	9.28E+00		
8009513	---	---	---	0.003	1.002	9.89E-01	9.89E+01		
8009517	NM_000346	SOX9	SRY (sex determining region Y)-box	-0.041	0.972	6.20E-01	6.20E+01		
8009526	NM_001050	SSTR2	somatostatin receptor 2	-0.131	0.913	1.80E-01	1.80E+01		
8009533	NM_018714	COG1	component of oligomeric golgi cor	-0.075	0.949	4.13E-01	4.13E+01		
8009552	NM_017941	C17orf80	chromosome 17 open reading fran	0.008	1.005	9.74E-01	9.74E+01		
8009561	NM_000999	RPL38	ribosomal protein L38	0.035	1.024	7.51E-01	7.51E+01		
8009568	NM_032646	TTYH2	tweety homolog 2 (Drosophila)	-0.037	0.974	7.07E-01	7.07E+01		
8009585	NM_023036	DNAI2	dynein, axonemal, intermediate ch	-0.009	0.994	9.61E-01	9.61E+01		
8009602	NM_153209	KIF19	kinesin family member 19	0.020	1.014	8.77E-01	8.77E+01		
8009631	NM_181790	GPR142	G protein-coupled receptor 142	-0.001	0.999	9.94E-01	9.94E+01		
8009639	NM_022036	GPRC5C	G protein-coupled receptor, family	-0.062	0.958	5.15E-01	5.15E+01		
8009653	NM_007261	CD300A	CD300a molecule	0.041	1.029	8.90E-01	8.90E+01		
8009666	NM_175738	RAB37	RAB37, member RAS oncogene fan	0.174	1.128	1.80E-02	1.80E+00		
8009685	NM_004252	SLC9A3R1	solute carrier family 9 (sodium/hy	0.001	1.001	9.96E-01	9.96E+01		
8009693	NM_017728	TMEM104	transmembrane protein 104	0.030	1.021	8.94E-01	8.94E+01		
8009705	NM_178160	OTOP2	otopetrin 2	-0.014	0.991	9.33E-01	9.33E+01		
8009713	NM_178233	OTOP3	otopetrin 3	-0.051	0.965	6.24E-01	6.24E+01		
8009727	NM_001545	ICT1	immature colon carcinoma transcr	0.024	1.017	9.04E-01	9.04E+01		
8009737	NM_015353	KCTD2	potassium channel tetramerisation	0.032	1.023	8.08E-01	8.08E+01		
8009746	NM_004695	SLC16A5	solute carrier family 16, member 5	0.056	1.039	6.08E-01	6.08E+01		
8009755	NM_024585	ARMC7	armadillo repeat containing 7	0.039	1.028	7.97E-01	7.97E+01		
8009761	NM_024844	NUP85	nucleoporin 85kDa	0.060	1.043	6.05E-01	6.05E+01		
8009784	NM_015971	MRPS7	mitochondrial ribosomal protein S	0.052	1.037	7.44E-01	7.44E+01		
8009792	---	---	---	0.201	1.150	5.55E-01	5.55E+01		
8009796	NM_014738	KIAA0195	KIAA0195	-0.073	0.951	2.35E-01	2.35E+01		
8009832	NM_207346	TSEN54	tRNA splicing endonuclease 54 ho	-0.070	0.953	3.51E-01	3.51E+01		
8009844	NM_00103180	LLGL2	lethal giant larvae homolog 2 (Dro	-0.029	0.980	8.31E-01	8.31E+01		

8009873	NR_003587	MYO15B	myosin XVB pseudogene	-0.035	0.976	8.95E-01	8.95E+01		
8009875	NR_003587	MYO15B	myosin XVB pseudogene	-0.020	0.986	8.29E-01	8.29E+01		
8009913	NR_003587	MYO15B	myosin XVB pseudogene	-0.018	0.988	9.28E-01	9.28E+01		
8009929	NM_00116299	LOC643008	hypothetical protein LOC643008	-0.065	0.956	7.05E-01	7.05E+01		
8009932	NM_013260	SAP30BP	SAP30 binding protein	-0.085	0.943	3.57E-01	3.57E+01		
8009995	NM_00108041	UNK	unkempt homolog (Drosophila)	0.033	1.023	8.64E-01	8.64E+01		
8010012	NM_00111332	LOC100134934	hypothetical protein LOC1001349	-0.021	0.986	8.94E-01	8.94E+01		
8010017	NM_001258	CDK3	cyclin-dependent kinase 3	-0.032	0.978	7.90E-01	7.90E+01		
8010021	NM_001258	CDK3	cyclin-dependent kinase 3	0.035	1.025	7.86E-01	7.86E+01		
8010030	NM_003857	GALR2	galanin receptor 2	-0.057	0.961	6.54E-01	6.54E+01		
8010036	NM_180990	ZACN	zinc activated ligand-gated ion cha	-0.037	0.974	7.91E-01	7.91E+01		
8010050	NM_182565	FAM100B	family with sequence similarity 10	-0.009	0.994	9.70E-01	9.70E+01		
8010061	NM_182965	SPHK1	sphingosine kinase 1	-0.022	0.985	9.44E-01	9.44E+01		
8010071	NM_001088	AANAT	arylalkylamine N-acetyltransferase	-0.014	0.990	9.37E-01	9.37E+01		
8010078	NR_004397	SNORD1C	small nucleolar RNA, C/D box 1C	-0.081	0.945	8.03E-01	8.03E+01		
8010084	---	---	---	-0.006	0.996	9.85E-01	9.85E+01		
8010086	NM_00108051	C17orf95	chromosome 17 open reading fran	-0.162	0.894	1.59E-01	1.59E+01		
8010092	NM_024311	MFSD11	major facilitator superfamily doma	-0.038	0.974	8.57E-01	8.57E+01		
8010113	NM_144677	MGAT5B	mannosyl (alpha-1,6-)-glycoprotei	-0.002	0.999	9.89E-01	9.89E+01		
8010137	NR_003013	SCARNA16	small Cajal body-specific RNA 16	-0.015	0.990	9.43E-01	9.43E+01		
8010139	NM_00114399	SEC14L1	SEC14-like 1 (S. cerevisiae)	0.156	1.114	2.49E-01	2.49E+01		
8010161	NM_006640	40430	septin 9	-0.053	0.964	7.36E-01	7.36E+01		
8010184	---	---	---	0.222	1.166	5.32E-01	5.32E+01		
8010188	NM_00114264	TNRC6C	trinucleotide repeat containing 6C	-0.170	0.889	1.29E-01	1.29E+01		
8010212	NM_152468	TMC8	transmembrane channel-like 8	0.000	1.000	1.00E+00	1.00E+02		
8010237	ENST00000340	C17orf99	chromosome 17 open reading fran	0.028	1.020	8.18E-01	8.18E+01		
8010243	NM_004710	SYNGR2	synaptogyrin 2	0.121	1.087	5.21E-01	5.21E+01		
8010248	NM_00101098	AFMID	arylformamidase	0.023	1.016	8.96E-01	8.96E+01		
8010260	NM_001168	BIRC5	baculoviral IAP repeat-containing	0.098	1.070	2.63E-01	2.63E+01		
8010271	NM_024419	PGS1	phosphatidylglycerophosphate syn	-0.121	0.919	3.47E-01	3.47E+01		
8010287	NM_030968	C1QTNF1	C1q and tumor necrosis factor rela	-0.157	0.897	2.41E-02	2.41E+00		
8010295	NM_00104257	ENGASE	endo-beta-N-acetylglucosaminidas	0.017	1.012	9.09E-01	9.09E+01		
8010313	NM_178543	ENPP7	ectonucleotide pyrophosphatase/	-0.010	0.993	9.73E-01	9.73E+01		
8010320	NM_005189	CBX2	chromobox homolog 2 (Pc class ho	-0.023	0.984	9.04E-01	9.04E+01		
8010328	NM_017950	CCDC40	coiled-coil domain containing 40	0.005	1.003	9.78E-01	9.78E+01		
8010354	NM_000152	GAA	glucosidase, alpha; acid	0.007	1.005	9.80E-01	9.80E+01		
8010379	NM_024110	CARD14	caspase recruitment domain famil	-0.071	0.952	3.18E-01	3.18E+01		
8010405	NM_173626	SLC26A11	solute carrier family 26, member 1	-0.080	0.946	3.09E-01	3.09E+01		
8010426	NM_020954	KIAA1618	KIAA1618	0.048	1.034	8.07E-01	8.07E+01		
8010454	NM_020914	RNF213	ring finger protein 213	0.055	1.039	7.98E-01	7.98E+01		
8010499	AK096344	FLJ35220	hypothetical protein FLJ35220	-0.061	0.959	5.89E-01	5.89E+01		
8010512	NM_020761	RPTOR	regulatory associated protein of M	-0.105	0.930	2.60E-01	2.60E+01		
8010562	NM_006340	BAIAP2	BAI1-associated protein 2	-0.037	0.975	7.78E-01	7.78E+01		
8010583	NR_027255	LOC388428	hypothetical LOC388428	-0.053	0.964	5.69E-01	5.69E+01		
8010590	NM_00108051	BAHCC1	BAH domain and coiled-coil contai	-0.021	0.986	8.48E-01	8.48E+01		
8010614	NM_012418	FSCN2	fascin homolog 2, actin-bundling p	0.063	1.044	7.51E-01	7.51E+01		
8010624	NM_031945	TSPAN10	tetraspanin 10	-0.059	0.960	5.48E-01	5.48E+01		
8010629	NM_199287	CCDC137	coiled-coil domain containing 137	0.021	1.015	8.75E-01	8.75E+01		
8010638	NM_004712	HGS	hepatocyte growth factor-regulate	0.038	1.026	7.46E-01	7.46E+01		
8010664	NM_002949	MRPL12	mitochondrial ribosomal protein L	0.001	1.000	9.98E-01	9.98E+01		
8010673	NM_012140	SLC25A10	solute carrier family 25 (mitochon	0.015	1.010	9.34E-01	9.34E+01		
8010686	NM_00100224	ANAPC11	anaphase promoting complex sub	-0.006	0.996	9.83E-01	9.83E+01		
8010696	NM_148896	NPB	neuropeptide B	-0.080	0.946	3.28E-01	3.28E+01		
8010702	NM_024083	ASPSCR1	alveolar soft part sarcoma chromo	-0.011	0.992	9.55E-01	9.55E+01		
8010719	NM_144999	LRRC45	leucine rich repeat containing 45	-0.073	0.950	3.70E-01	3.70E+01		
8010737	NM_005052	RAC3	ras-related C3 botulinum toxin sub	-0.045	0.969	7.16E-01	7.16E+01		
8010747	NM_212492	GPS1	G protein pathway suppressor 1	-0.036	0.976	7.23E-01	7.23E+01		
8010766	---	---	---	0.114	1.082	7.87E-01	7.87E+01		
8010768	---	---	---	-0.018	0.987	9.65E-01	9.65E+01		
8010770	NM_00104242	SLC16A3	solute carrier family 16, member 3	-0.079	0.946	4.44E-01	4.44E+01		
8010778	NM_001893	CSNK1D	casein kinase 1, delta	-0.057	0.961	7.29E-01	7.29E+01		
8010783	NM_018949	UTS2R	urotensin 2 receptor	-0.030	0.979	8.20E-01	8.20E+01		
8010787	NM_173620	HEXDC	hexosaminidase (glycosyl hydrolas	-0.030	0.979	7.97E-01	7.97E+01		
8010804	NM_00103861	NARF	nuclear prelamin A recognition fac	-0.104	0.931	1.65E-01	1.65E+01		
8010820	NM_004514	FOXK2	forkhead box K2	0.002	1.001	9.89E-01	9.89E+01		
8010832	NM_024619	FN3KRP	fructosamine 3 kinase related prot	-0.014	0.991	9.61E-01	9.61E+01		
8010841	NM_022158	FN3K	fructosamine 3 kinase	-0.112	0.926	4.19E-01	4.19E+01		
8010848	NM_005993	TBCD	tubulin folding cofactor D	-0.137	0.909	6.47E-02	6.47E+00		
8010897	NM_00100443	METRNL	meteorin, glial cell differentiation	0.055	1.039	7.89E-01	7.89E+01		
8010901	NM_003585	DOC2B	double C2-like domains, beta	-0.182	0.882	1.83E-01	1.83E+01		
8010903	NM_006987	RPH3AL	rabphilin 3A-like (without C2 doma	0.076	1.054	4.02E-01	4.02E+01		
8010915	NM_182705	FAM101B	family with sequence similarity 10	0.014	1.010	9.47E-01	9.47E+01		
8010918	NM_00112815	VPS53	vacuolar protein sorting 53 homol	-0.044	0.970	8.10E-01	8.10E+01		
8010924	NM_00112815	VPS53	vacuolar protein sorting 53 homol	0.006	1.004	9.76E-01	9.76E+01		
8010946	NM_015721	GEMIN4	gem (nuclear organelle) associated	-0.015	0.990	9.51E-01	9.51E+01		
8010949	NM_016080	GLOD4	glyoxalase domain containing 4	0.030	1.021	8.46E-01	8.46E+01		
8010963	---	---	---	0.180	1.133	1.82E-01	1.82E+01		

8010978	ENST00000435	LOC100130876	hypothetical LOC100130876	-0.098	0.934	7.79E-01	7.79E+01		
8010983	NM_021962	ABR	active BCR-related gene	0.190	1.140	3.36E-01	3.36E+01		
8011011	NR_024058	YWHAE	tyrosine 3-monooxygenase/trypto	0.030	1.021	7.39E-01	7.39E+01		
8011018	NM_016823	CRK	v-crk sarcoma virus CT10 oncogen	0.123	1.089	1.75E-01	1.75E+01		
8011027	NM_00108077	MYO1C	myosin IC	-0.040	0.973	7.55E-01	7.55E+01		
8011062	NM_130766	INPP5K	inositol polyphosphate-5-phospha	-0.072	0.951	5.34E-01	5.34E+01		
8011077	NM_006224	PITPNA	phosphatidylinositol transfer prot	0.110	1.079	6.06E-01	6.06E+01		
8011093	NM_152346	SLC43A2	solute carrier family 43, member 2	0.008	1.005	9.80E-01	9.80E+01		
8011110	---	---	---	-0.164	0.892	4.96E-01	4.96E+01		
8011112	---	---	---	-0.141	0.907	7.61E-01	7.61E+01		
8011114	NM_145351	SCARF1	scavenger receptor class F, memb	-0.054	0.963	4.93E-01	4.93E+01		
8011131	NM_031430	RILP	Rab interacting lysosomal protein	-0.047	0.968	7.38E-01	7.38E+01		
8011141	NM_006445	PRPF8	PRP8 pre-mRNA processing factor	-0.077	0.948	3.55E-01	3.55E+01		
8011188	ENST00000330	TLCD2	TLC domain containing 2	0.024	1.017	8.23E-01	8.23E+01		
8011193	NM_032895	C17orf91	chromosome 17 open reading fra	-0.080	0.946	6.78E-01	6.78E+01		
8011200	NM_052928	SMYD4	SET and MYND domain containing	-0.041	0.972	7.73E-01	7.73E+01		
8011212	NM_003001	SDHC	succinate dehydrogenase complex	-0.001	1.000	1.00E+00	1.00E+02		
8011220	---	---	---	-0.119	0.921	1.75E-01	1.75E+01		
8011222	NM_017575	SMG6	Smg-6 homolog, nonsense mediat	-0.009	0.994	9.62E-01	9.62E+01		
8011245	NM_018128	TSR1	TSR1, 20S rRNA accumulation, hor	-0.005	0.997	9.84E-01	9.84E+01		
8011262	NM_020310	MNT	MAX binding protein	-0.004	0.998	9.78E-01	9.78E+01		
8011270	ENST00000381	LOC284009	hypothetical protein LOC284009	-0.086	0.942	4.83E-01	4.83E+01		
8011291	---	---	---	0.062	1.044	6.73E-01	6.73E+01		
8011293	NM_015229	KIAA0664	KIAA0664	0.045	1.032	6.89E-01	6.89E+01		
8011320	NM_014566	OR1D5	olfactory receptor, family 1, subfa	-0.120	0.920	4.21E-01	4.21E+01		
8011328	---	---	---	-0.047	0.968	8.33E-01	8.33E+01		
8011331	NM_002551	OR3A2	olfactory receptor, family 3, subfa	0.024	1.017	8.99E-01	8.99E+01		
8011375	NM_080704	TRPV1	transient receptor potential cation	-0.083	0.944	4.78E-01	4.78E+01		
8011396	NM_013276	SHPK	sedoheptulokinase	-0.122	0.919	3.04E-01	3.04E+01		
8011407	NM_014604	TAX1BP3	Tax1 (human T-cell leukemia virus	-0.116	0.923	1.20E-01	1.20E+01		
8011415	NM_002561	P2RX5	purinergic receptor P2X, ligand-ga	0.003	1.002	9.92E-01	9.92E+01		
8011430	NM_002208	ITGAE	integrin, alpha E (antigen CD103, h	-0.106	0.929	3.01E-01	3.01E+01		
8011462	NM_018553	C17orf85	chromosome 17 open reading fra	-0.251	0.841	8.90E-03	8.90E-01		
8011499	NM_002558	P2RX1	purinergic receptor P2X, ligand-ga	0.094	1.067	1.24E-01	1.24E+01		
8011516	NM_174954	ATP2A3	ATPase, Ca++ transporting, ubiqui	-0.017	0.988	8.83E-01	8.83E+01		
8011542	NM_015113	ZZEF1	zinc finger, ZZ-type with EF-hand d	0.080	1.057	4.09E-01	4.09E+01		
8011599	NM_016376	ANKFY1	ankyrin repeat and FYVE domain c	0.036	1.025	8.04E-01	8.04E+01		
8011626	NM_003342	UBE2G1	ubiquitin-conjugating enzyme E2G	0.139	1.101	6.74E-02	6.74E+00		
8011640	NM_014520	MYBBP1A	MYB binding protein (P160) 1a	0.034	1.024	8.06E-01	8.06E+01		
8011671	NM_00112289	GGT6	gamma-glutamyltransferase 6	-0.012	0.992	9.53E-01	9.53E+01		
8011692	NM_014389	PELP1	proline, glutamate and leucine ric	-0.052	0.964	6.78E-01	6.78E+01		
8011713	NM_022059	CXCL16	chemokine (C-X-C motif) ligand 16	0.406	1.325	4.04E-01	4.04E+01		
8011734	NM_000080	CHRNE	cholinergic receptor, nicotinic, eps	0.011	1.008	9.44E-01	9.44E+01		
8011747	NM_003562	SLC25A11	solute carrier family 25 (mitochon	0.067	1.047	7.51E-01	7.51E+01		
8011759	NM_005022	PFN1	profilin 1	0.075	1.054	4.25E-01	4.25E+01		
8011765	NM_004890	SPAG7	sperm associated antigen 7	-0.098	0.934	3.75E-01	3.75E+01		
8011774	NM_015099	CAMTA2	calmodulin binding transcription a	0.054	1.038	6.04E-01	6.04E+01		
8011797	NM_213726	INCA1	inhibitor of CDK, cyclin A1 interact	0.000	1.000	9.99E-01	9.99E+01		
8011808	NM_00110457	GPR172B	G protein-coupled receptor 172B	-0.051	0.965	5.52E-01	5.52E+01		
8011823	NM_032530	ZNF594	zinc finger protein 594	0.154	1.113	4.99E-01	4.99E+01		
8011832	NM_002532	NUP88	nucleoporin 88kDa	0.047	1.033	7.68E-01	7.68E+01		
8011850	NM_001212	C1QBP	complement component 1, q subc	-0.104	0.931	3.38E-01	3.38E+01		
8011861	NM_020162	DHX33	DEAH (Asp-Glu-Ala-His) box polype	0.042	1.029	8.19E-01	8.19E+01		
8011875	NM_016041	DERL2	Der1-like domain family, member	0.056	1.040	6.13E-01	6.13E+01		
8011884	NM_033004	NLRP1	NLR family, pyrin domain containi	-0.036	0.975	8.55E-01	8.55E+01		
8011912	NM_014336	AIPL1	aryl hydrocarbon receptor interac	-0.006	0.996	9.75E-01	9.75E+01		
8011924	NM_031220	PITPNM3	PITPNM family member 3	0.011	1.008	9.36E-01	9.36E+01		
8011945	NM_014804	KIAA0753	KIAA0753	-0.195	0.873	2.99E-02	2.99E+00		
8011968	NM_016060	MED31	mediator complex subunit 31	-0.009	0.994	9.71E-01	9.71E+01		
8011975	NM_177550	SLC13A5	solute carrier family 13 (sodium-d	-0.025	0.983	8.30E-01	8.30E+01		
8012000	NM_00100433	RNASEK	ribonuclease, RNase K	0.073	1.052	5.84E-01	5.84E+01		
8012004	---	---	---	0.019	1.013	8.97E-01	8.97E+01		
8012006	NM_153357	SLC16A11	solute carrier family 16, member 1	-0.049	0.967	6.06E-01	6.06E+01		
8012028	NM_080912	ASGR2	asialoglycoprotein receptor 2	-0.006	0.996	9.76E-01	9.76E+01		
8012043	NM_001671	ASGR1	asialoglycoprotein receptor 1	0.004	1.003	9.84E-01	9.84E+01		
8012054	NM_001365	DLG4	discs, large homolog 4 (Drosophila	0.013	1.009	9.66E-01	9.66E+01		
8012079	NM_004422	DVL2	dishevelled, dsh homolog 2 (Droso	-0.045	0.970	6.44E-01	6.44E+01		
8012099	NM_024297	PHF23	PHD finger protein 23	0.031	1.021	8.99E-01	8.99E+01		
8012110	NM_007278	GABARAP	GABA(A) receptor-associated prot	0.248	1.188	1.40E-01	1.40E+01		
8012116	NM_015343	DULLARD	dullard homolog (Xenopus laevis)	-0.030	0.979	8.57E-01	8.57E+01		
8012126	NM_001307	CLDN7	claudin 7	0.035	1.025	7.82E-01	7.82E+01		
8012140	NM_015982	YBX2	Y box binding protein 2	-0.041	0.972	7.04E-01	7.04E+01		
8012150	NM_004489	GPS2	G protein pathway suppressor 2	-0.042	0.971	7.03E-01	7.03E+01		
8012166	NM_032442	NEURL4	neuralized homolog 4 (Drosophila)	-0.037	0.975	7.97E-01	7.97E+01		
8012197	NM_020360	PLSCR3	phospholipid scramblase 3	0.004	1.002	9.85E-01	9.85E+01		
8012207	BC030270	C17orf61	chromosome 17 open reading fra	-0.068	0.954	6.82E-01	6.82E+01		
8012212	NM_020899	ZBTB4	zinc finger and BTB domain contai	-0.041	0.972	7.04E-01	7.04E+01		

8012218	---	---	---	-0.013	0.991	9.67E-01	9.67E+01		
8012220	NM_006942	SOX15	SRY (sex determining region Y)-box	-0.019	0.987	9.04E-01	9.04E+01		
8012226	NM_004860	FXR2	fragile X mental retardation, autos	-0.023	0.984	8.68E-01	8.68E+01		
8012247	NM_133491	SAT2	spermidine/spermine N1-acetyltra	-0.033	0.977	8.71E-01	8.71E+01		
8012257	NM_000546	TP53	tumor protein p53	-0.029	0.980	8.56E-01	8.56E+01		
8012270	---	---	---	0.194	1.144	5.87E-01	5.87E+01		
8012274	NM_032356	LSMD1	LSM domain containing 1	0.014	1.010	9.49E-01	9.49E+01		
8012304	NM_021210	TRAPPC1	trafficking protein particle comple	0.066	1.047	6.93E-01	6.93E+01		
8012309	NM_001139	ALOX12B	arachidonate 12-lipoxygenase, 12R	-0.017	0.988	9.18E-01	9.18E+01		
8012326	NM_021628	ALOXE3	arachidonate lipoxygenase 3	0.041	1.029	6.99E-01	6.99E+01		
8012344	NM_032580	HES7	hairy and enhancer of split 7 (Dros	-0.043	0.970	5.56E-01	5.56E+01		
8012349	NM_002616	PER1	period homolog 1 (Drosophila)	-0.050	0.966	6.09E-01	6.09E+01		
8012376	NM_014232	VAMP2	vesicle-associated membrane prot	-0.081	0.945	6.25E-01	6.25E+01		
8012383	NM_032354	TMEM107	transmembrane protein 107	0.102	1.073	4.29E-01	4.29E+01		
8012397	NM_017622	C17orf59	chromosome 17 open reading fran	-0.044	0.970	8.05E-01	8.05E+01		
8012403	NM_004217	AURKB	aurora kinase B	-0.038	0.974	8.04E-01	8.04E+01		
8012416	NR_026951	C17orf44	chromosome 17 open reading fran	0.008	1.006	9.80E-01	9.80E+01		
8012423	NM_025099	C17orf68	chromosome 17 open reading fran	-0.028	0.981	8.24E-01	8.24E+01		
8012450	NM_201520	SLC25A35	solute carrier family 25, member 3	0.053	1.037	6.75E-01	6.75E+01		
8012464	NR_024447	LOC100128288	hypothetical protein LOC1001282	0.171	1.126	2.00E-01	2.00E+01		
8012466	NM_213597	KRBA2	KRAB-A domain containing 2	-0.089	0.940	4.97E-01	4.97E+01		
8012469	NM_000987	RPL26	ribosomal protein L26	-0.034	0.977	8.08E-01	8.08E+01		
8012473	NM_00114668	RNF222	ring finger protein 222	-0.017	0.988	8.95E-01	8.95E+01		
8012475	NM_005964	MYH10	myosin, heavy chain 10, non-musc	0.000	1.000	1.00E+00	1.00E+02		
8012528	NM_00112807	SPDYE4	speedy homolog E4 (Xenopus laev	-0.080	0.946	5.32E-01	5.32E+01		
8012539	NM_00101085	PIK3R6	phosphoinositide-3-kinase, regula	0.035	1.025	8.36E-01	8.36E+01		
8012558	NM_00114263	PIK3R5	phosphoinositide-3-kinase, regula	0.077	1.055	6.58E-01	6.58E+01		
8012581	NM_004853	STX8	syntaxin 8	0.086	1.062	6.83E-01	6.83E+01		
8012605	NM_201433	GAS7	growth arrest-specific 7	0.257	1.195	6.71E-02	6.71E+00		
8012787	NM_002470	MYH3	myosin, heavy chain 3, skeletal mu	-0.056	0.962	6.60E-01	6.60E+01		
8012823	NM_004589	SCO1	SCO cytochrome oxidase deficient	0.093	1.067	4.83E-01	4.83E+01		
8012830	NM_00100431	TMEM220	transmembrane protein 220	0.006	1.004	9.82E-01	9.82E+01		
8012837	NM_00110138	hCG_1776018	phosphoinositide-interacting regu	-0.018	0.987	9.37E-01	9.37E+01		
8012841	---	---	---	0.287	1.220	2.25E-01	2.25E+01		
8012843	NM_144680	ZNF18	zinc finger protein 18	0.007	1.005	9.78E-01	9.78E+01		
8012852	---	---	---	-0.017	0.988	9.68E-01	9.68E+01		
8012856	NM_018127	ELAC2	elaC homolog 2 (E. coli)	-0.001	0.999	9.97E-01	9.97E+01		
8012883	NM_006042	HS3ST3A1	heparan sulfate (glucosamine) 3-O	0.089	1.064	2.70E-01	2.70E+01		
8012887	NM_00100753	CDRT15	CMT1A duplicated region transcrip	-0.010	0.993	9.58E-01	9.58E+01		
8012891	---	---	---	0.044	1.031	8.57E-01	8.57E+01		
8012896	NM_000304	PMP22	peripheral myelin protein 22	0.259	1.197	7.34E-01	7.34E+01		
8012918	NM_173622	CDRT4	CMT1A duplicated region transcrip	-0.035	0.976	9.25E-01	9.25E+01		
8012924	NM_145301	FAM18B2	family with sequence similarity 18	-0.069	0.953	6.95E-01	6.95E+01		
8012931	NM_006382	CDRT1	CMT1A duplicated region transcrip	0.018	1.012	9.45E-01	9.45E+01		
8012951	NM_006382	CDRT1	CMT1A duplicated region transcrip	-0.374	0.772	1.21E-01	1.21E+01		
8012953	NM_006470	TRIM16	tripartite motif-containing 16	-0.096	0.935	6.27E-01	6.27E+01		
8012958	NM_014501	UBE2S	ubiquitin-conjugating enzyme E2S	-0.107	0.929	3.94E-01	3.94E+01		
8012961	NM_006311	NCOR1	nuclear receptor co-repressor 1	-0.020	0.986	8.97E-01	8.97E+01		
8013013	---	---	---	0.098	1.070	6.14E-01	6.14E+01		
8013015	NM_181716	CENPV	centromere protein V	0.013	1.009	9.54E-01	9.54E+01		
8013022	NM_00111356	C17orf76	chromosome 17 open reading fran	0.053	1.037	5.81E-01	5.81E+01		
8013026	NM_020653	ZNF287	zinc finger protein 287	0.019	1.014	9.53E-01	9.53E+01		
8013035	NM_020787	ZNF624	zinc finger protein 624	-0.044	0.970	8.13E-01	8.13E+01		
8013042	NM_000422	KRT17	keratin 17	-0.010	0.993	9.44E-01	9.44E+01		
8013049	---	---	---	-0.002	0.999	9.91E-01	9.91E+01		
8013061	NM_012452	TNFRSF13B	tumor necrosis factor receptor sup	0.006	1.004	9.75E-01	9.75E+01		
8013068	NM_178836	PLD6	phospholipase D family, member 6	0.050	1.035	6.45E-01	6.45E+01		
8013071	NM_144997	FLCN	folliculin	0.075	1.053	5.55E-01	5.55E+01		
8013094	NM_003653	COPS3	COP9 constitutive photomorphoge	0.064	1.045	7.68E-01	7.68E+01		
8013109	---	---	---	-0.112	0.926	6.20E-01	6.20E+01		
8013112	NM_016084	RASD1	RAS, dexamethasone-induced 1	-0.146	0.904	4.01E-02	4.01E+00		
8013135	NM_00100529	SREBF1	sterol regulatory element binding	-0.045	0.970	6.42E-01	6.42E+01		
8013157	NM_00103355	TOM1L2	target of myb1-like 2 (chicken)	0.013	1.009	9.56E-01	9.56E+01		
8013159	NM_00108296	TOM1L2	target of myb1-like 2 (chicken)	-0.004	0.997	9.85E-01	9.85E+01		
8013179	NM_145691	ATPAF2	ATP synthase mitochondrial F1 cor	0.007	1.005	9.74E-01	9.74E+01		
8013191	NM_002018	FLII	flightless I homolog (Drosophila)	0.023	1.016	9.16E-01	9.16E+01		
8013222	NM_004618	TOP3A	topoisomerase (DNA) III alpha	0.056	1.039	7.35E-01	7.35E+01		
8013243	NM_004169	SHMT1	serine hydroxymethyltransferase 1	0.004	1.003	9.85E-01	9.85E+01		
8013259	---	---	---	-0.026	0.982	7.96E-01	7.96E+01		
8013262	NM_032582	USP32	ubiquitin specific peptidase 32	0.024	1.017	9.28E-01	9.28E+01		
8013268	NR_026809	FAM106A	family with sequence similarity 10	0.041	1.029	9.11E-01	9.11E+01		
8013272	NM_014695	CCDC144A	coiled-coil domain containing 144A	0.097	1.069	8.17E-01	8.17E+01		
8013294	NM_00103939	TBC1D28	TBC1 domain family, member 28	-0.023	0.984	9.02E-01	9.02E+01		
8013307	NR_026718	FOXO3B	forkhead box O3B pseudogene	-0.226	0.855	5.58E-02	5.58E+00		
8013323	NR_006880	SNORD3A	small nucleolar RNA, C/D box 3A	-0.508	0.703	8.15E-02	8.15E+00		
8013325	NR_006880	SNORD3A	small nucleolar RNA, C/D box 3A	-0.508	0.703	8.15E-02	8.15E+00		
8013327	AK026312	LOC79999	hypothetical LOC79999	-0.049	0.966	6.31E-01	6.31E+01		

8013329	NR_006880	SNORD3A	small nucleolar RNA, C/D box 3A	-0.508	0.703	8.15E-02	8.15E+00		
8013331	NM_015681	B9D1	B9 protein domain 1	-0.006	0.996	9.70E-01	9.70E+01		
8013348	NM_002952	RPS2	ribosomal protein S2	0.006	1.004	9.76E-01	9.76E+01		
8013354	---	---	---	0.003	1.002	9.95E-01	9.95E+01		
8013384	NM_000691	ALDH3A1	aldehyde dehydrogenase 3 family,	-0.035	0.976	6.94E-01	6.94E+01		
8013399	NM_014683	ULK2	unc-51-like kinase 2 (C. elegans)	-0.075	0.950	5.52E-01	5.52E+01		
8013431	NM_007202	AKAP10	A kinase (PRKA) anchor protein 10	0.136	1.099	1.51E-01	1.51E+01		
8013465	NM_005557	KRT16	keratin 16	-0.049	0.967	5.72E-01	5.72E+01		
8013473	AK127974	LOC339240	keratin pseudogene	-0.007	0.995	9.78E-01	9.78E+01		
8013479	NM_00100430	CCDC144NL	coiled-coil domain containing 144	0.000	1.000	9.99E-01	9.99E+01		
8013486	NM_015276	USP22	ubiquitin specific peptidase 22	-0.069	0.953	4.80E-01	4.80E+01		
8013500	NM_003876	TMEM11	transmembrane protein 11	-0.047	0.968	8.13E-01	8.13E+01		
8013507	---	---	---	0.068	1.048	6.14E-01	6.14E+01		
8013509	NM_152914	C17orf103	chromosome 17 open reading fran	-0.101	0.932	2.35E-01	2.35E+01		
8013515	AK096256	C17orf51	chromosome 17 open reading fran	-0.165	0.892	2.91E-01	2.91E+01		
8013521	---	---	---	0.156	1.114	5.53E-01	5.53E+01		
8013533	---	---	---	0.006	1.004	9.76E-01	9.76E+01		
8013536	NM_000625	NOS2	nitric oxide synthase 2, inducible	0.044	1.031	6.66E-01	6.66E+01		
8013574	AK097823	FLJ40504	keratin 18 pseudogene	-0.003	0.998	9.89E-01	9.89E+01		
8013581	NM_174887	IFT20	intraflagellar transport 20 homolo	-0.018	0.988	9.33E-01	9.33E+01		
8013588	NM_015584	POLDIP2	polymerase (DNA-directed), delta	0.009	1.007	9.73E-01	9.73E+01		
8013606	NM_000638	VTN	vitronectin	0.005	1.003	9.80E-01	9.80E+01		
8013616	NM_080669	SLC46A1	solute carrier family 46 (folate trar	0.150	1.109	1.82E-01	1.82E+01		
8013622	NM_144610	FLJ25006	uncharacterized serine/threonine-	0.017	1.012	9.17E-01	9.17E+01		
8013633	NM_054035	UNC119	unc-119 homolog (C. elegans)	-0.107	0.929	3.93E-01	3.93E+01		
8013641	NM_033198	PIGS	phosphatidylinositol glycan ancho	0.027	1.019	8.69E-01	8.69E+01		
8013660	NM_005165	ALDOC	aldolase C, fructose-bisphosphate	0.047	1.033	8.47E-01	8.47E+01		
8013696	NM_014680	KIAA0100	KIAA0100	0.118	1.085	1.28E-01	1.28E+01		
8013741	NM_006923	SDF2	stromal cell-derived factor 2	0.007	1.005	9.82E-01	9.82E+01		
8013747	NM_152465	PROCA1	protein interacting with cyclin A1	0.020	1.014	9.09E-01	9.09E+01		
8013753	NM_031934	RAB34	RAB34, member RAS oncogene fai	-0.043	0.970	8.63E-01	8.63E+01		
8013771	NM_138463	TLCD1	TLC domain containing 1	-0.138	0.909	3.49E-01	3.49E+01		
8013776	NM_018182	C17orf63	chromosome 17 open reading fran	-0.087	0.942	3.96E-01	3.96E+01		
8013788	NM_004475	FLOT2	flotillin 2	-0.055	0.963	7.62E-01	7.62E+01		
8013804	NM_144683	DHRS13	dehydrogenase/reductase (SDR fa	-0.024	0.983	8.73E-01	8.73E+01		
8013812	NM_00103356	PHF12	PHD finger protein 12	-0.084	0.943	5.06E-01	5.06E+01		
8013833	NM_178860	SEZ6	seizure related 6 homolog (mouse	0.003	1.002	9.89E-01	9.89E+01		
8013858	ENST00000437	LOC100130794	NADH dehydrogenase (ubiquinone	-0.117	0.922	3.31E-01	3.31E+01		
8013860	NM_078471	MYO18A	myosin XVIIIA	-0.060	0.959	5.06E-01	5.06E+01		
8013906	NM_002822	TWF1	twinfilin, actin-binding protein, ho	0.030	1.021	9.01E-01	9.01E+01		
8013908	NM_020772	NUFIP2	nuclear fragile X mental retardatio	0.022	1.016	8.26E-01	8.26E+01		
8013915	---	---	---	0.051	1.036	7.47E-01	7.47E+01		
8013919	NM_198147	ABHD15	abhydrolase domain containing 15	0.023	1.016	9.00E-01	9.00E+01		
8013923	NM_00108545	GIT1	G protein-coupled receptor kinase	-0.032	0.978	8.45E-01	8.45E+01		
8013947	NM_032854	CORO6	coronin 6	0.043	1.030	7.51E-01	7.51E+01		
8013965	NM_033389	SSH2	slingshot homolog 2 (Drosophila)	0.050	1.035	6.78E-01	6.78E+01		
8013985	---	---	---	0.012	1.008	9.68E-01	9.68E+01		
8014008	NM_000386	BLMH	bleomycin hydrolase	-0.171	0.888	9.22E-02	9.22E+00		
8014035	---	---	---	0.037	1.026	7.88E-01	7.88E+01		
8014037	NM_015986	CRLF3	cytokine receptor-like factor 3	0.121	1.088	1.09E-01	1.09E+01		
8014063	NM_006495	EVI2B	ecotropic viral integration site 2B	0.092	1.066	4.77E-01	4.77E+01		
8014066	NM_00100392	EVI2A	ecotropic viral integration site 2A	0.043	1.030	8.43E-01	8.43E+01		
8014073	NM_018405	C17orf79	chromosome 17 open reading fran	0.034	1.024	8.39E-01	8.39E+01		
8014081	NM_018428	UTP6	UTP6, small subunit (SSU) process	0.108	1.078	3.62E-01	3.62E+01		
8014100	NM_022344	C17orf75	chromosome 17 open reading fran	-0.148	0.903	4.84E-01	4.84E+01		
8014140	---	---	---	-0.152	0.900	6.80E-01	6.80E+01		
8014160	NM_002981	CCL1	chemokine (C-C motif) ligand 1	-0.251	0.840	2.17E-01	2.17E+01		
8014189	NM_057178	RFFL	ring finger and FYVE-like domain c	-0.121	0.919	1.57E-01	1.57E+01		
8014197	---	---	---	-0.131	0.913	8.04E-01	8.04E+01		
8014199	NM_002878	RAD51L3	RAD51-like 3 (S. cerevisiae)	-0.022	0.985	9.00E-01	9.00E+01		
8014214	NM_00101444	NLE1	notchless homolog 1 (Drosophila)	0.070	1.050	4.45E-01	4.45E+01		
8014230	NM_152462	AMAC1	acyl-malonyl condensing enzyme 1	-0.047	0.968	8.63E-01	8.63E+01		
8014233	NM_00110458	SLFN11	schlafen family member 11	0.282	1.216	1.94E-01	1.94E+01		
8014241	NM_018042	SLFN12	schlafen family member 12	0.123	1.089	5.38E-01	5.38E+01		
8014248	NM_144682	SLFN13	schlafen family member 13	-0.077	0.948	8.29E-01	8.29E+01		
8014257	NM_00114502	SLFN12L	schlafen family member 12-like	0.011	1.008	9.82E-01	9.82E+01		
8014264	NM_000286	PEX12	peroxisomal biogenesis factor 12	-0.050	0.966	7.46E-01	7.46E+01		
8014269	NM_139285	GAS2L2	growth arrest-specific 2 like 2	-0.033	0.977	7.38E-01	7.38E+01		
8014282	NM_024302	MMP28	matrix metallopeptidase 28	-0.058	0.961	5.34E-01	5.34E+01		
8014298	NM_152781	C17orf66	chromosome 17 open reading fran	-0.094	0.937	6.31E-01	6.31E+01		
8014316	NM_002985	CCL5	chemokine (C-C motif) ligand 5	-0.010	0.993	9.85E-01	9.85E+01		
8014326	NM_145654	RDM1	RAD52 motif 1	-0.058	0.961	7.91E-01	7.91E+01		
8014349	NR_027921	CCL14-CCL15	chemokine ligand 14, chemokine l	-0.010	0.993	9.69E-01	9.69E+01		
8014376	NM_00100141	TBC1D3B	TBC1 domain family, member 3B	-0.003	0.998	9.88E-01	9.88E+01		
8014397	NM_00100141	TBC1D3C	TBC1 domain family, member 3C	0.010	1.007	9.70E-01	9.70E+01		
8014420	NM_00112339	TBC1D3D	TBC1 domain family, member 3D	0.017	1.012	9.51E-01	9.51E+01		
8014437	NM_00104028	TBC1D3G	TBC1 domain family, member 3G	0.011	1.008	9.66E-01	9.66E+01		

8014452	---	---	---	0.034	1.024	9.44E-01	9.44E+01		
8014454	NM_025109	MYO19	myosin XIX	0.010	1.007	9.75E-01	9.75E+01		
8014485	---	---	---	0.070	1.050	7.43E-01	7.43E+01		
8014487	NM_198839	ACACA	acetyl-Coenzyme A carboxylase al	0.031	1.021	8.54E-01	8.54E+01		
8014551	NM_007247	SYNRG	synergin, gamma	0.070	1.050	4.35E-01	4.35E+01		
8014574	NM_007010	DDX52	DEAD (Asp-Glu-Ala-Asp) box polyp	0.023	1.016	9.20E-01	9.20E+01		
8014603	NM_00112339	TBC1D3	TBC1 domain family, member 3	0.008	1.006	9.77E-01	9.77E+01		
8014633	NM_00112339	TBC1D3D	TBC1 domain family, member 3D	0.012	1.009	9.64E-01	9.64E+01		
8014650	NM_006310	NPEPPS	aminopeptidase puromycin sensiti	-0.017	0.989	9.22E-01	9.22E+01		
8014664	---	---	---	0.026	1.019	9.67E-01	9.67E+01		
8014666	NM_00100433	GPR179	G protein-coupled receptor 179	-0.019	0.987	8.63E-01	8.63E+01		
8014679	NM_025248	SNIP	SNAP25-interacting protein	0.020	1.014	9.07E-01	9.07E+01		
8014702	NM_005937	MLLT6	myeloid/lymphoid or mixed-lineag	0.101	1.073	6.94E-01	6.94E+01		
8014704	---	---	---	-0.063	0.957	8.63E-01	8.63E+01		
8014706	NM_007144	PCGF2	polycomb group ring finger 2	0.042	1.029	7.30E-01	7.30E+01		
8014723	NM_003559	PIP4K2B	phosphatidylinositol-5-phosphate	-0.095	0.936	3.07E-01	3.07E+01		
8014738	NM_017748	CCDC49	coiled-coil domain containing 49	-0.155	0.898	7.90E-02	7.90E+00		
8014749	NM_000978	RPL23	ribosomal protein L23	0.023	1.016	9.02E-01	9.02E+01		
8014755	NR_002576	SNORA21	small nucleolar RNA, H/ACA box 2	-0.163	0.893	5.89E-01	5.89E+01		
8014768	NM_020405	PLXDC1	plexin domain containing 1	0.008	1.005	9.72E-01	9.72E+01		
8014794	NM_199247	CACNB1	calcium channel, voltage-depende	0.032	1.022	8.48E-01	8.48E+01		
8014812	NM_198993	STAC2	SH3 and cysteine rich domain 2	-0.008	0.994	9.64E-01	9.64E+01		
8014825	NM_032875	FBXL20	F-box and leucine-rich repeat prot	-0.150	0.901	1.95E-01	1.95E+01		
8014841	NM_004774	MED1	mediator complex subunit 1	-0.039	0.973	7.97E-01	7.97E+01		
8014861	---	---	---	0.430	1.347	3.56E-01	3.56E+01		
8014865	NM_006160	NEUROD2	neurogenic differentiation 2	0.048	1.034	6.29E-01	6.29E+01		
8014882	NM_032339	C17orf37	chromosome 17 open reading fran	0.037	1.026	7.91E-01	7.91E+01		
8014891	NM_012481	IKZF3	IKAROS family zinc finger 3 (Aiolos	-0.123	0.918	4.23E-01	4.23E+01		
8014903	NM_00104247	GSDMB	gasdermin B	-0.129	0.914	5.94E-01	5.94E+01		
8014916	NM_139280	ORMDL3	ORM1-like 3 (S. cerevisiae)	-0.089	0.940	4.95E-01	4.95E+01		
8014925	NM_014815	MED24	mediator complex subunit 24	0.057	1.040	5.90E-01	5.90E+01		
8014956	NM_021724	NR1D1	nuclear receptor subfamily 1, grou	0.051	1.036	7.57E-01	7.57E+01		
8014969	NM_152219	GJD3	gap junction protein, delta 3, 31.9	0.011	1.007	9.52E-01	9.52E+01		
8014974	NM_001067	TOP2A	topoisomerase (DNA) II alpha 170k	0.413	1.331	8.00E-02	8.00E+00		
8015016	NM_032865	TNS4	tensin 4	-0.060	0.959	3.95E-01	3.95E+01		
8015031	NM_001838	CCR7	chemokine (C-C motif) receptor 7	-0.277	0.825	1.31E-02	1.31E+00		
8015039	NM_003079	SMARCE1	SWI/SNF related, matrix associate	-0.062	0.958	4.13E-01	4.13E+01		
8015173	NM_031958	KRTAP3-1	keratin associated protein 3-1	-0.007	0.995	9.56E-01	9.56E+01		
8015179	NM_031957	KRTAP1-5	keratin associated protein 1-5	-0.030	0.979	7.70E-01	7.70E+01		
8015187	NM_030967	KRTAP1-1	keratin associated protein 1-1	0.026	1.018	8.69E-01	8.69E+01		
8015189	NM_030966	KRTAP1-3	keratin associated protein 1-3	-0.003	0.998	9.82E-01	9.82E+01		
8015196	NM_030967	KRTAP1-1	keratin associated protein 1-1	0.005	1.004	9.63E-01	9.63E+01		
8015206	NM_00112338	KRTAP2-1	keratin associated protein 2-1	-0.043	0.971	4.82E-01	4.82E+01		
8015208	NM_00112338	KRTAP2-1	keratin associated protein 2-1	-0.049	0.967	4.32E-01	4.32E+01		
8015210	NM_033184	KRTAP2-4	keratin associated protein 2-4	-0.036	0.975	5.54E-01	5.54E+01		
8015214	NM_033184	KRTAP2-4	keratin associated protein 2-4	-0.028	0.981	6.76E-01	6.76E+01		
8015218	NM_031960	KRTAP4-8	keratin associated protein 4-8	0.003	1.002	9.83E-01	9.83E+01		
8015221	NM_033059	KRTAP4-11	keratin associated protein 4-11	0.000	1.000	1.00E+00	1.00E+02		
8015223	NM_031854	KRTAP4-12	keratin associated protein 4-12	0.002	1.001	9.88E-01	9.88E+01		
8015230	NM_033059	KRTAP4-11	keratin associated protein 4-11	-0.003	0.998	9.84E-01	9.84E+01		
8015232	NM_033188	KRTAP4-5	keratin associated protein 4-5	-0.032	0.978	8.20E-01	8.20E+01		
8015236	NM_032524	KRTAP4-4	keratin associated protein 4-4	-0.012	0.992	9.43E-01	9.43E+01		
8015240	NM_033187	KRTAP4-3	keratin associated protein 4-3	0.031	1.022	8.33E-01	8.33E+01		
8015242	NM_033062	KRTAP4-2	keratin associated protein 4-2	-0.041	0.972	6.58E-01	6.58E+01		
8015247	NM_033060	KRTAP4-1	keratin associated protein 4-1	0.086	1.061	3.56E-01	3.56E+01		
8015252	NM_031964	KRTAP17-1	keratin associated protein 17-1	-0.049	0.966	7.27E-01	7.27E+01		
8015257	NM_004138	KRT33A	keratin 33A	-0.030	0.980	8.61E-01	8.61E+01		
8015262	NM_002279	KRT33B	keratin 33B	-0.057	0.961	6.80E-01	6.80E+01		
8015301	NM_002280	KRT35	keratin 35	-0.045	0.969	7.68E-01	7.68E+01		
8015323	NM_153490	KRT13	keratin 13	-0.045	0.969	7.02E-01	7.02E+01		
8015349	NM_002276	KRT19	keratin 19	-0.020	0.986	9.15E-01	9.15E+01		
8015366	NM_000526	KRT14	keratin 14	-0.121	0.920	2.52E-01	2.52E+01		
8015376	NM_005557	KRT16	keratin 16	-0.074	0.950	3.37E-01	3.37E+01		
8015387	NM_000422	KRT17	keratin 17	-0.008	0.995	9.62E-01	9.62E+01		
8015396	NM_177977	HAP1	huntingtin-associated protein 1	-0.045	0.969	7.26E-01	7.26E+01		
8015410	---	---	---	-0.031	0.979	8.27E-01	8.27E+01		
8015412	NM_002230	JUP	junction plakoglobin	0.061	1.043	3.54E-01	3.54E+01		
8015445	NM_052935	NT5C3L	5'-nucleotidase, cytosolic III-like	-0.037	0.975	8.90E-01	8.90E+01		
8015456	NM_018143	KLHL11	kelch-like 11 (Drosophila)	-0.083	0.944	6.19E-01	6.19E+01		
8015460	NM_001096	ACLY	ATP citrate lyase	-0.024	0.984	8.65E-01	8.65E+01		
8015490	NM_003315	DNAJC7	DnaJ (Hsp40) homolog, subfamily	-0.031	0.979	8.80E-01	8.80E+01		
8015503	BC080613	ZNF385C	zinc finger protein 385C	-0.013	0.991	9.53E-01	9.53E+01		
8015511	NM_024119	DHX58	DEXH (Asp-Glu-X-His) box polypep	0.028	1.019	8.81E-01	8.81E+01		
8015526	NM_021078	KAT2A	K(lysine) acetyltransferase 2A	0.062	1.044	6.50E-01	6.50E+01		
8015545	NM_201434	RAB5C	RAB5C, member RAS oncogene fa	0.173	1.127	3.94E-01	3.94E+01		
8015554	NM_012285	KCNH4	potassium voltage-gated channel,	-0.029	0.980	8.06E-01	8.06E+01		
8015572	NM_001524	HCRT	hypocretin (orexin) neuropeptide	-0.004	0.997	9.81E-01	9.81E+01		

8015575	NR_024573	GHDC	GH3 domain containing	-0.025	0.983	8.26E-01	8.26E+01		
8015590	NM_012448	STAT5B	signal transducer and activator of	-0.118	0.921	2.46E-01	2.46E+01		
8015607	NM_139276	STAT3	signal transducer and activator of	0.026	1.018	8.93E-01	8.93E+01		
8015642	NM_016556	PSMC3IP	PSMC3 interacting protein	0.049	1.034	8.08E-01	8.08E+01		
8015655	NM_178126	FAM134C	family with sequence similarity 13	-0.040	0.973	7.77E-01	7.77E+01		
8015665	NM_024927	PLEKHH3	pleckstrin homology domain conta	-0.091	0.939	2.72E-01	2.72E+01		
8015681	NM_016602	CCR10	chemokine (C-C motif) receptor 10	-0.020	0.986	9.27E-01	9.27E+01		
8015685	NM_001991	EZH1	enhancer of zeste homolog 1 (Dro	-0.082	0.945	4.59E-01	4.59E+01		
8015706	NR_024461	LOC100190938	hypothetical LOC100190938	-0.061	0.959	5.38E-01	5.38E+01		
8015712	NM_00104043	CCDC56	coiled-coil domain containing 56	-0.009	0.994	9.77E-01	9.77E+01		
8015715	NM_003766	BECN1	beclin 1, autophagy related	-0.018	0.988	9.26E-01	9.26E+01		
8015730	NR_027254	LOC388387	hypothetical LOC388387	0.011	1.008	9.53E-01	9.53E+01		
8015737	---	---	---	0.007	1.005	9.73E-01	9.73E+01		
8015741	NM_00113604	AARSD1	alanyl-tRNA synthetase domain co	0.066	1.047	6.76E-01	6.76E+01		
8015757	---	---	---	0.052	1.037	8.55E-01	8.55E+01		
8015759	NM_006373	VAT1	vesicle amine transport protein 1	-0.010	0.993	9.65E-01	9.65E+01		
8015796	---	---	---	-0.525	0.695	9.72E-02	9.72E+00		
8015798	NR_027413	LOC100130581	hypothetical LOC100130581	0.015	1.011	9.72E-01	9.72E+01		
8015804	---	---	---	-0.014	0.990	9.53E-01	9.53E+01		
8015821	NM_004527	MEOX1	mesenchyme homeobox 1	0.032	1.022	7.91E-01	7.91E+01		
8015827	NM_025237	SOST	sclerosteosis	0.005	1.003	9.84E-01	9.84E+01		
8015835	NM_004090	DUSP3	dual specificity phosphatase 3	0.268	1.204	1.11E-01	1.11E+01		
8015846	NR_003562	MPP3	membrane protein, palmitoylated	-0.024	0.983	8.81E-01	8.81E+01		
8015890	NM_004160	PYY	peptide YY	0.011	1.008	9.60E-01	9.60E+01		
8015900	NM_032376	TMEM101	transmembrane protein 101	-0.027	0.981	8.34E-01	8.34E+01		
8015908	NM_152344	LSM12	LSM12 homolog (S. cerevisiae)	0.059	1.042	5.06E-01	5.06E+01		
8015914	NM_005474	HDAC5	histone deacetylase 5	-0.013	0.991	9.01E-01	9.01E+01		
8015946	NM_178542	C17orf65	chromosome 17 open reading frar	-0.036	0.975	8.18E-01	8.18E+01		
8015955	NM_020218	ATXN7L3	ataxin 7-like 3	-0.036	0.975	8.32E-01	8.32E+01		
8015969	NM_014233	UBTF	upstream binding transcription fac	0.057	1.041	5.30E-01	5.30E+01		
8016016	---	---	---	0.030	1.021	8.77E-01	8.77E+01		
8016018	NM_00114378	SLC25A39	solute carrier family 25, member 3	-0.031	0.979	8.32E-01	8.32E+01		
8016033	NM_198475	FAM171A2	family with sequence similarity 17	-0.103	0.931	2.29E-01	2.29E+01		
8016044	NM_000419	ITGA2B	integrin, alpha 2b (platelet glycopr	-0.005	0.997	9.76E-01	9.76E+01		
8016077	NM_00100290	GPATCH8	G patch domain containing 8	-0.074	0.950	4.07E-01	4.07E+01		
8016086	---	---	---	-0.005	0.997	9.85E-01	9.85E+01		
8016088	NM_144609	CCDC43	coiled-coil domain containing 43	0.106	1.076	5.89E-01	5.89E+01		
8016094	NM_005497	GJC1	gap junction protein, gamma 1, 45	-0.009	0.994	9.61E-01	9.61E+01		
8016099	NM_004247	EFTUD2	elongation factor Tu GTP binding d	0.137	1.099	1.45E-01	1.45E+01		
8016128	NM_002055	GFAP	glial fibrillary acidic protein	-0.012	0.992	9.35E-01	9.35E+01		
8016139	NM_00108044	KIF18B	kinesin family member 18B	0.012	1.008	9.54E-01	9.54E+01		
8016147	NM_00108044	KIF18B	kinesin family member 18B	-0.021	0.986	9.07E-01	9.07E+01		
8016155	NM_006688	C1QL1	complement component 1, q subc	-0.060	0.959	5.38E-01	5.38E+01		
8016159	NM_024819	DCAKD	dephospho-CoA kinase domain co	-0.104	0.930	4.44E-01	4.44E+01		
8016168	NM_133373	PLCD3	phospholipase C, delta 3	-0.082	0.945	1.97E-01	1.97E+01		
8016187	NM_152343	C17orf46	chromosome 17 open reading frar	-0.018	0.987	9.33E-01	9.33E+01		
8016194	NM_003954	MAP3K14	mitogen-activated protein kinase	-0.027	0.981	8.31E-01	8.31E+01		
8016213	---	---	---	0.084	1.060	7.87E-01	7.87E+01		
8016215	NM_199282	ARHGAP27	Rho GTPase activating protein 27	0.059	1.042	4.49E-01	4.49E+01		
8016232	NM_174919	SH3D20	SH3 domain containing 20	-0.004	0.997	9.84E-01	9.84E+01		
8016239	NR_027774	PLEKHM1	pleckstrin homology domain conta	-0.065	0.956	7.97E-01	7.97E+01		
8016245	NR_002940	LRRC37A4	leucine rich repeat containing 37,	-0.018	0.987	9.55E-01	9.55E+01		
8016257	---	---	---	0.003	1.002	9.90E-01	9.90E+01		
8016259	NM_00100660	LRRC37A2	leucine rich repeat containing 37,	-0.034	0.977	8.88E-01	8.88E+01		
8016261	NM_005456	MAPK8IP1	mitogen-activated protein kinase 8	-0.087	0.941	1.95E-01	1.95E+01		
8016266	NM_015443	KIAA1267	KIAA1267	-0.038	0.974	7.34E-01	7.34E+01		
8016285	NM_00111373	ARL17P1	ADP-ribosylation factor-like 17 pse	0.015	1.010	9.70E-01	9.70E+01		
8016300	NM_00111373	ARL17P1	ADP-ribosylation factor-like 17 pse	0.013	1.009	9.77E-01	9.77E+01		
8016311	NM_030753	WNT3	wingless-type MMTV integration s	0.033	1.023	8.57E-01	8.57E+01		
8016320	NM_203400	RPRML	reprimo-like	-0.092	0.938	2.82E-01	2.82E+01		
8016324	NM_00111409	CDC27	cell division cycle 27 homolog (S. c	0.108	1.078	3.29E-01	3.29E+01		
8016336	NM_145798	OSBPL7	oxysterol binding protein-like 7	-0.071	0.952	4.29E-01	4.29E+01		
8016366	NM_145255	MRPL10	mitochondrial ribosomal protein L	0.025	1.017	8.99E-01	8.99E+01		
8016371	NM_00114502	SCRN2	secernin 2	-0.005	0.997	9.78E-01	9.78E+01		
8016382	NM_199262	SP6	Sp6 transcription factor	-0.081	0.946	4.04E-01	4.04E+01		
8016390	NM_016429	COPZ2	coatomer protein complex, subun	0.049	1.034	7.27E-01	7.27E+01		
8016400	---	---	---	-0.039	0.973	8.12E-01	8.12E+01		
8016402	NM_00112722	CBX1	chromobox homolog 1 (HP1 beta h	-0.009	0.994	9.63E-01	9.63E+01		
8016412	---	---	---	0.086	1.061	7.65E-01	7.65E+01		
8016414	NM_003726	SKAP1	src kinase associated phosphoprot	0.042	1.029	8.96E-01	8.96E+01		
8016429	---	---	---	0.106	1.076	6.31E-01	6.31E+01		
8016433	NM_002144	HOXB1	homeobox B1	-0.068	0.954	5.34E-01	5.34E+01		
8016444	NM_002146	HOXB3	homeobox B3	-0.107	0.928	3.19E-01	3.19E+01		
8016452	NM_024015	HOXB4	homeobox B4	-0.021	0.985	9.13E-01	9.13E+01		
8016468	NM_004502	HOXB7	homeobox B7	0.063	1.045	4.96E-01	4.96E+01		
8016482	---	---	---	0.028	1.020	8.39E-01	8.39E+01		
8016487	NM_006361	HOXB13	homeobox B13	0.058	1.041	5.47E-01	5.47E+01		

8016508	NM_007241	SNF8	SNF8, ESCRT-II complex subunit, h	0.062	1.044	6.75E-01	6.75E+01		
8016532	NM_031498	GNGT2	guanine nucleotide binding protei	0.173	1.127	4.47E-01	4.47E+01		
8016540	NM_00114380	PHOSPHO1	phosphatase, orphan 1	-0.084	0.944	4.19E-01	4.19E+01		
8016546	NM_014897	ZNF652	zinc finger protein 652	-0.040	0.973	7.86E-01	7.86E+01		
8016552	NM_002634	PHB	prohibitin	-0.038	0.974	8.85E-01	8.85E+01		
8016562	NM_00100723	SPOP	speckle-type POZ protein	-0.005	0.997	9.81E-01	9.81E+01		
8016578	NM_005827	SLC35B1	solute carrier family 35, member E	0.220	1.165	2.84E-02	2.84E+00		
8016590	NM_030802	FAM117A	family with sequence similarity 11	-0.003	0.998	9.87E-01	9.87E+01		
8016600	NM_170685	TAC4	tachykinin 4 (hemokinin)	0.021	1.015	9.00E-01	9.00E+01		
8016607	---	---	---	-0.047	0.968	8.36E-01	8.36E+01		
8016615	NM_174920	SAMD14	sterile alpha motif domain contair	-0.017	0.989	9.02E-01	9.02E+01		
8016628	NM_032595	PPP1R9B	protein phosphatase 1, regulatory	0.126	1.091	2.11E-01	2.11E+01		
8016640	NR_024193	HILS1	histone linker H1 domain, spermat	-0.028	0.981	8.71E-01	8.71E+01		
8016646	NM_000088	COL1A1	collagen, type I, alpha 1	-0.056	0.962	3.24E-01	3.24E+01		
8016699	NM_016504	MRPL27	mitochondrial ribosomal protein L	0.091	1.065	4.39E-01	4.39E+01		
8016708	NM_018509	LRRC59	leucine rich repeat containing 59	0.073	1.052	4.92E-01	4.92E+01		
8016718	NM_001267	CHAD	chondroadherin	-0.084	0.944	4.70E-01	4.70E+01		
8016725	NM_052855	ANKRD40	ankyrin repeat domain 40	-0.066	0.955	6.35E-01	6.35E+01		
8016733	NM_016424	LUC7L3	LUC7-like 3 (S. cerevisiae)	-0.020	0.986	9.19E-01	9.19E+01		
8016739	NM_005749	TOB1	transducer of ERBB2, 1	-0.019	0.987	9.44E-01	9.44E+01		
8016745	NM_00113052	SPAG9	sperm associated antigen 9	0.028	1.020	8.78E-01	8.78E+01		
8016787	---	---	---	-0.003	0.998	9.89E-01	9.89E+01		
8016789	NM_017643	MBTD1	mbt domain containing 1	0.100	1.072	3.56E-01	3.56E+01		
8016806	---	---	---	0.016	1.011	9.49E-01	9.49E+01		
8016832	NM_012329	MMD	monocyte to macrophage differen	0.037	1.026	8.66E-01	8.66E+01		
8016847	NM_005082	TRIM25	tripartite motif-containing 25	-0.024	0.983	9.50E-01	9.50E+01		
8016858	NM_004645	COIL	coilin	0.065	1.046	7.33E-01	7.33E+01		
8016870	NM_016070	MRPS23	mitochondrial ribosomal protein S	-0.009	0.994	9.77E-01	9.77E+01		
8016878	NM_017949	CUEDC1	CUE domain containing 1	0.010	1.007	9.51E-01	9.51E+01		
8016891	NM_007146	VEZF1	vascular endothelial zinc finger 1	-0.041	0.972	7.58E-01	7.58E+01		
8016898	NM_00107816	SFRS1	splicing factor, arginine/serine-rich	-0.021	0.985	8.47E-01	8.47E+01		
8016947	NM_004758	BZRAP1	benzodiazapine receptor (periphe	-0.101	0.932	1.48E-01	1.48E+01		
8016980	---	---	---	-0.014	0.990	9.83E-01	9.83E+01		
8016982	NM_003168	SUPT4H1	suppressor of Ty 4 homolog 1 (S. c	0.015	1.010	9.16E-01	9.16E+01		
8016994	NM_017763	RNF43	ring finger protein 43	-0.006	0.996	9.78E-01	9.78E+01		
8017010	NM_00108043	HSF5	heat shock transcription factor fan	0.110	1.079	2.26E-01	2.26E+01		
8017019	NM_004687	MTMR4	myotubularin related protein 4	0.063	1.045	6.30E-01	6.30E+01		
8017039	NM_080415	40425	septin 4	0.104	1.075	1.85E-01	1.85E+01		
8017102	---	---	---	-0.056	0.962	6.89E-01	6.89E+01		
8017106	NM_015294	TRIM37	tripartite motif-containing 37	0.009	1.006	9.76E-01	9.76E+01		
8017133	NM_182620	SKA2	spindle and kinetochore associate	0.234	1.176	2.29E-01	2.29E+01		
8017143	NM_016077	PTRH2	peptidyl-tRNA hydrolase 2	0.026	1.018	9.10E-01	9.10E+01		
8017162	NM_016125	RNFT1	ring finger protein, transmembran	0.096	1.069	4.84E-01	4.84E+01		
8017173	NR_027486	TBC1D3P2	TBC1 domain family, member 3 ps	-0.013	0.991	9.45E-01	9.45E+01		
8017186	NM_022070	HEATR6	HEAT repeat containing 6	0.059	1.042	7.96E-01	7.96E+01		
8017212	NM_032582	USP32	ubiquitin specific peptidase 32	0.009	1.006	9.77E-01	9.77E+01		
8017235	NM_006380	APPBP2	amyloid beta precursor protein (cy	0.050	1.035	7.94E-01	7.94E+01		
8017251	---	---	---	0.082	1.058	7.83E-01	7.83E+01		
8017253	---	---	---	-0.020	0.986	9.40E-01	9.40E+01		
8017257	---	---	---	0.291	1.223	5.30E-01	5.30E+01		
8017283	NR_026641	INTS2	integrator complex subunit 2	-0.015	0.990	9.62E-01	9.62E+01		
8017312	NM_005121	MED13	mediator complex subunit 13	-0.092	0.938	2.12E-01	2.12E+01		
8017344	AK123839	LOC100129112	hypothetical protein LOC1001291	0.014	1.010	9.77E-01	9.77E+01		
8017346	NR_027486	TBC1D3P2	TBC1 domain family, member 3 ps	-0.019	0.987	9.00E-01	9.00E+01		
8017361	---	---	---	0.020	1.014	9.49E-01	9.49E+01		
8017378	NM_001915	CYB561	cytochrome b-561	-0.005	0.997	9.84E-01	9.84E+01		
8017393	NM_030576	LIMD2	LIM domain containing 2	0.025	1.017	8.18E-01	8.18E+01		
8017402	NM_153335	STRADA	STE20-related kinase adaptor alph	-0.003	0.998	9.85E-01	9.85E+01		
8017421	NM_020198	CCDC47	coiled-coil domain containing 47	0.085	1.061	6.70E-01	6.70E+01		
8017437	NM_017647	FTSJ3	FtsJ homolog 3 (E. coli)	-0.120	0.920	2.93E-01	2.93E+01		
8017460	NM_00109842	SMARCD2	SWI/SNF related, matrix associate	-0.036	0.975	8.05E-01	8.05E+01		
8017476	NM_022644	CSH2	chorionic somatomammotropin ho	-0.132	0.912	2.36E-01	2.36E+01		
8017488	NM_022640	CSH1	chorionic somatomammotropin ho	0.000	1.000	1.00E+00	1.00E+02		
8017499	NM_022579	CSHL1	chorionic somatomammotropin ho	0.028	1.019	7.81E-01	7.81E+01		
8017511	NM_000626	CD79B	CD79b molecule, immunoglobulin	0.154	1.112	2.56E-02	2.56E+00		
8017521	NM_000334	SCN4A	sodium channel, voltage-gated, ty	0.048	1.034	6.87E-01	6.87E+01		
8017547	NM_00109978	ICAM2	intercellular adhesion molecule 2	0.035	1.025	8.36E-01	8.36E+01		
8017555	NM_001433	ERN1	endoplasmic reticulum to nucleus	-0.114	0.924	8.87E-02	8.87E+00		
8017582	NM_018469	TEX2	testis expressed 2	0.014	1.009	9.53E-01	9.53E+01		
8017621	NM_007215	POLG2	polymerase (DNA directed), gamm	0.058	1.041	6.89E-01	6.89E+01		
8017634	NM_004396	DDX5	DEAD (Asp-Glu-Ala-Asp) box polyp	-0.002	0.999	9.90E-01	9.90E+01		
8017651	NM_022739	SMURF2	SMAD specific E3 ubiquitin proteir	0.070	1.050	5.77E-01	5.77E+01		
8017671	NR_026899	LOC146880	hypothetical LOC146880	-0.023	0.984	8.98E-01	8.98E+01		
8017675	NR_024386	PLEKHM1P	pleckstrin homology domain conta	-0.023	0.984	9.03E-01	9.03E+01		
8017688	NM_199340	LRRC37A3	leucine rich repeat containing 37,	0.016	1.011	9.52E-01	9.52E+01		
8017702	---	---	---	0.057	1.041	8.62E-01	8.62E+01		
8017704	NR_026903	FLJ32065	hypothetical protein FLJ32065	0.034	1.024	9.36E-01	9.36E+01		

8017711	NM_006572	GNA13	guanine nucleotide binding protei	0.106	1.076	4.04E-01	4.04E+01		
8017718	NM_004655	AXIN2	axin 2	-0.034	0.977	8.21E-01	8.21E+01		
8017776	NM_014877	HELZ	helicase with zinc finger	0.059	1.042	5.16E-01	5.16E+01		
8017810	NM_002816	PSMD12	proteasome (prosome, macropain	-0.066	0.955	5.27E-01	5.27E+01		
8017825	---	---	---	0.141	1.103	6.73E-01	6.73E+01		
8017829	---	---	---	-0.232	0.851	1.91E-01	1.91E+01		
8017840	ENST0000044З	LRRC37A4	leucine rich repeat containing 37,	0.030	1.021	9.17E-01	9.17E+01		
8017843	NM_004694	SLC16A6	solute carrier family 16, member 6	0.100	1.072	9.07E-01	9.07E+01		
8017850	NM_017983	WIPI1	WD repeat domain, phosphoinosit	0.096	1.069	7.23E-01	7.23E+01		
8017867	NM_017565	FAM20A	family with sequence similarity 20	0.298	1.229	1.07E-01	1.07E+01		
8018082	NM_00115977	SLC39A11	solute carrier family 39 (metal ion	0.097	1.070	7.70E-01	7.70E+01		
8018097	NM_00109883	FAM104A	family with sequence similarity 10	-0.061	0.959	4.29E-01	4.29E+01		
8018107	NM_012121	CDC42EP4	CDC42 effector protein (Rho GTPa	-0.032	0.978	8.39E-01	8.39E+01		
8018164	BC093631	KIF19	kinesin family member 19	-0.002	0.998	9.89E-01	9.89E+01		
8018169	NM_174892	CD300LB	CD300 molecule-like family memb	0.183	1.135	7.87E-02	7.87E+00		
8018177	NM_006678	CD300C	CD300c molecule	0.072	1.051	6.28E-01	6.28E+01		
8018189	NM_181449	CD300E	CD300e molecule	-0.011	0.993	9.83E-01	9.83E+01		
8018196	NM_139018	CD300LF	CD300 molecule-like family memb	0.212	1.158	1.62E-01	1.62E+01		
8018209	NM_015654	NAT9	N-acetyltransferase 9 (GCN5-relat	0.039	1.027	8.45E-01	8.45E+01		
8018220	NM_000835	GRIN2C	glutamate receptor, ionotropic, N-	-0.012	0.991	9.23E-01	9.23E+01		
8018236	NM_024417	FDXR	ferredoxin reductase	-0.066	0.956	3.20E-01	3.20E+01		
8018251	NM_178128	FADS6	fatty acid desaturase domain fami	0.053	1.038	6.75E-01	6.75E+01		
8018258	NM_173477	USH1G	Usher syndrome 1G (autosomal re	-0.012	0.992	9.49E-01	9.49E+01		
8018264	NM_030630	C17orf28	chromosome 17 open reading fra	-0.006	0.996	9.77E-01	9.77E+01		
8018288	NM_006356	ATP5H	ATP synthase, H+ transporting, mi	0.006	1.004	9.80E-01	9.80E+01		
8018297	NM_014595	NT5C	5', 3'-nucleotidase, cytosolic	-0.021	0.985	9.00E-01	9.00E+01		
8018305	NM_016185	HN1	hematological and neurological ex	0.045	1.032	7.38E-01	7.38E+01		
8018315	NM_006937	SUMO2	SMT3 suppressor of mif two 3 hon	-0.014	0.990	9.23E-01	9.23E+01		
8018324	NM_138619	GGA3	golgi associated, gamma adaptin e	0.015	1.011	8.97E-01	8.97E+01		
8018343	NM_020679	MIF4GD	MIF4G domain containing	-0.023	0.984	8.78E-01	8.78E+01		
8018352	NM_00112612	SLC25A19	solute carrier family 25 (mitochon	0.211	1.157	6.92E-02	6.92E+00		
8018364	NM_002086	GRB2	growth factor receptor-bound pro	0.233	1.175	5.67E-02	5.67E+00		
8018377	---	---	---	-0.049	0.967	7.87E-01	7.87E+01		
8018379	NM_020753	CASKIN2	CASK interacting protein 2	0.009	1.006	9.55E-01	9.55E+01		
8018400	NM_004259	RECQL5	RecQ protein-like 5	-0.040	0.973	6.84E-01	6.84E+01		
8018428	NM_000154	GALK1	galactokinase 1	-0.030	0.980	7.83E-01	7.83E+01		
8018439	NM_005324	H3F3B	H3 histone, family 3B (H3.3B)	-0.088	0.941	4.19E-01	4.19E+01		
8018445	NM_00108041	UNK	unkempt homolog (Drosophila)	-0.026	0.982	8.86E-01	8.86E+01		
8018449	NM_199242	UNC13D	unc-13 homolog D (C. elegans)	0.011	1.008	9.35E-01	9.35E+01		
8018482	NM_012478	WBP2	WW domain binding protein 2	0.005	1.003	9.86E-01	9.86E+01		
8018494	NM_033452	TRIM47	tripartite motif-containing 47	-0.001	1.000	9.97E-01	9.97E+01		
8018502	NM_173547	TRIM65	tripartite motif-containing 65	-0.079	0.947	3.16E-01	3.16E+01		
8018511	NM_032478	MRPL38	mitochondrial ribosomal protein L	-0.041	0.972	7.44E-01	7.44E+01		
8018522	NM_00108054	FBF1	Fas (TNFRSF6) binding factor 1	-0.002	0.998	9.85E-01	9.85E+01		
8018558	NM_004035	ACOX1	acyl-Coenzyme A oxidase 1, palmi	0.090	1.064	7.20E-01	7.20E+01		
8018579	NM_001988	EVPL	envoplakin	0.005	1.004	9.81E-01	9.81E+01		
8018600	NM_014230	SRP68	signal recognition particle 68kDa	-0.055	0.962	6.86E-01	6.86E+01		
8018620	NM_00114529	EXOC7	exocyst complex component 7	-0.060	0.959	6.07E-01	6.07E+01		
8018646	NM_001454	FOXJ1	forkhead box J1	-0.042	0.972	7.83E-01	7.83E+01		
8018652	NM_052916	RNF157	ring finger protein 157	0.116	1.083	5.83E-01	5.83E+01		
8018673	NM_032134	QRICH2	glutamine rich 2	-0.093	0.938	3.62E-01	3.62E+01		
8018694	NM_002766	PRPSAP1	phosphoribosyl pyrophosphate sy	-0.136	0.910	2.72E-01	2.72E+01		
8018708	NM_022066	UBE2O	ubiquitin-conjugating enzyme E2O	-0.114	0.924	1.05E-01	1.05E+01		
8018731	NM_024599	RHBDF2	rhomboid 5 homolog 2 (Drosophil	0.007	1.005	9.64E-01	9.64E+01		
8018754	NM_134268	CYGB	cytoglobin	0.009	1.006	9.53E-01	9.53E+01		
8018761	NM_006456	ST6GALNAC2	ST6 (alpha-N-acetyl-neuraminyl-2,	-0.118	0.921	4.10E-01	4.10E+01		
8018774	NM_018414	ST6GALNAC1	ST6 (alpha-N-acetyl-neuraminyl-2,	0.026	1.018	8.73E-01	8.73E+01		
8018786	NM_00100852	MXRA7	matrix-remodelling associated 7	-0.197	0.872	1.23E-01	1.23E+01		
8018791	---	---	---	-0.294	0.816	3.70E-01	3.70E+01		
8018793	NM_00108146	JMJD6	jumonji domain containing 6	-0.253	0.839	7.17E-03	7.17E-01		
8018803	NM_003016	SFRS2	splicing factor, arginine/serine-ricl	-0.094	0.937	1.33E-01	1.33E+01		
8018814	---	---	---	0.260	1.197	3.02E-01	3.02E+01		
8018816	---	---	---	-0.001	0.999	9.95E-01	9.95E+01		
8018823	NM_00112719	TMC6	transmembrane channel-like 6	-0.065	0.956	4.96E-01	4.96E+01		
8018849	NM_003258	TK1	thymidine kinase 1, soluble	0.078	1.056	5.98E-01	5.98E+01		
8018864	NM_003955	SOCS3	suppressor of cytokine signaling 3	-0.219	0.859	1.86E-01	1.86E+01		
8018906	---	---	---	-0.007	0.995	9.68E-01	9.68E+01		
8018922	NM_004762	CYTH1	cytohesin 1	-0.026	0.982	8.77E-01	8.77E+01		
8018935	---	---	---	0.029	1.020	9.13E-01	9.13E+01		
8018937	NM_025090	USP36	ubiquitin specific peptidase 36	-0.096	0.935	2.85E-01	2.85E+01		
8018966	NM_003255	TIMP2	TIMP metallopeptidase inhibitor 2	0.626	1.544	5.53E-02	5.53E+00		
8018972	AK057217	TIMP2	TIMP metallopeptidase inhibitor 2	0.114	1.082	4.60E-01	4.60E+01		
8018975	NM_005567	LGALS3BP	lectin, galactoside-binding, soluble	-0.051	0.965	7.34E-01	7.34E+01		
8018982	NM_138793	CANT1	calcium activated nucleotidase 1	-0.011	0.992	9.63E-01	9.63E+01		
8019005	NM_00108257	hCG_1776007	hexaribonucleotide binding protei	0.008	1.006	9.65E-01	9.65E+01		
8019010	NM_020649	CBX8	chromobox homolog 8 (Pc class ho	0.007	1.005	9.66E-01	9.66E+01		
8019018	NM_003655	CBX4	chromobox homolog 4 (Pc class ho	-0.065	0.956	5.73E-01	5.73E+01		

8019031	NM_019020	TBC1D16	TBC1 domain family, member 16	0.005	1.004	9.77E-01	9.77E+01		
8019046	NM_014740	EIF4A3	eukaryotic translation initiation fa	0.033	1.023	9.13E-01	9.13E+01		
8019061	NM_000199	SGSH	N-sulfoglucosamine sulfohydrolase	-0.027	0.982	8.76E-01	8.76E+01		
8019071	NM_020914	RNF213	ring finger protein 213	-0.055	0.963	6.51E-01	6.51E+01		
8019074	NM_002522	NPTX1	neuronal pentraxin I	-0.086	0.942	4.26E-01	4.26E+01		
8019081	---	---	---	0.014	1.009	9.64E-01	9.64E+01		
8019083	NR_026857	FLJ90757	hypothetical LOC440465	-0.005	0.997	9.85E-01	9.85E+01		
8019088	AK096606	LOC100131170	hypothetical LOC100131170	-0.011	0.992	9.61E-01	9.61E+01		
8019090	NM_00108039	AATK	apoptosis-associated tyrosine kina	-0.034	0.977	7.43E-01	7.43E+01		
8019108	NM_014984	AZI1	5-azacytidine induced 1	0.041	1.029	6.94E-01	6.94E+01		
8019136	BC064483	C17orf56	chromosome 17 open reading fram	-0.031	0.978	6.90E-01	6.90E+01		
8019149	NM_00103798	SLC38A10	solute carrier family 38, member 1	-0.027	0.982	8.21E-01	8.21E+01		
8019170	BC108932	C17orf55	chromosome 17 open reading fram	-0.033	0.978	7.94E-01	7.94E+01		
8019177	NM_178520	TMEM105	transmembrane protein 105	0.007	1.005	9.69E-01	9.69E+01		
8019181	ENST00000456	DKFZp761P0212	hypothetical protein DKFZp761P02	-0.055	0.962	5.53E-01	5.53E+01		
8019183	NM_001614	ACTG1	actin, gamma 1	0.020	1.014	9.21E-01	9.21E+01		
8019194	NM_025161	C17orf70	chromosome 17 open reading fram	-0.019	0.987	8.87E-01	8.87E+01		
8019211	NM_017921	NPLOC4	nuclear protein localization 4 hom	-0.013	0.991	9.44E-01	9.44E+01		
8019231	NM_002602	PDE6G	phosphodiesterase 6G, cGMP-spec	-0.033	0.978	8.93E-01	8.93E+01		
8019238	BC090923	C17orf90	chromosome 17 open reading fram	-0.045	0.969	7.10E-01	7.10E+01		
8019250	NM_000918	P4HB	prolyl 4-hydroxylase, beta polypep	0.038	1.027	8.88E-01	8.88E+01		
8019263	NM_004309	ARHGDIA	Rho GDP dissociation inhibitor (GD	-0.021	0.986	9.35E-01	9.35E+01		
8019273	NM_005782	THOC4	THO complex 4	-0.011	0.992	9.61E-01	9.61E+01		
8019280	NM_002861	PCYT2	phosphate cytidylyltransferase 2, e	-0.011	0.993	9.53E-01	9.53E+01		
8019296	NM_016538	SIRT7	sirtuin (silent mating type informa	-0.067	0.955	4.50E-01	4.50E+01		
8019308	NM_032711	MAFG	v-maf musculoaponeurotic fibrosa	-0.160	0.895	1.75E-02	1.75E+00		
8019316	NM_006907	PYCR1	pyrroline-5-carboxylate reductase	-0.058	0.961	1.98E-01	1.98E+01		
8019331	NM_00114511	MYADML2	myeloid-associated differentiation	-0.016	0.989	8.85E-01	8.85E+01		
8019334	NM_178493	NOTUM	notum pectinacetylesterase homo	-0.056	0.962	5.25E-01	5.25E+01		
8019347	---	---	---	-0.031	0.979	8.73E-01	8.73E+01		
8019350	NM_144998	STRA13	stimulated by retinoic acid 13 hom	0.003	1.002	9.88E-01	9.88E+01		
8019357	NM_016286	DCXR	dicarbonyl/L-xylulose reductase	0.065	1.046	5.06E-01	5.06E+01		
8019367	NM_002917	RFNG	RFNG O-fucosylpeptide 3-beta-N-a	0.017	1.012	9.15E-01	9.15E+01		
8019376	NM_022156	DUS1L	dihydrouridine synthase 1-like (S. c	-0.061	0.959	6.53E-01	6.53E+01		
8019392	NM_004104	FASN	fatty acid synthase	-0.076	0.949	3.71E-01	3.71E+01		
8019437	NM_198082	CCDC57	coiled-coil domain containing 57	-0.040	0.972	6.00E-01	6.00E+01		
8019463	NM_139062	CSNK1D	casein kinase 1, delta	-0.071	0.952	5.72E-01	5.72E+01		
8019478	NM_006137	CD7	CD7 molecule	-0.048	0.968	6.20E-01	6.20E+01		
8019486	NM_003004	SECTM1	secreted and transmembrane 1	0.307	1.237	5.36E-02	5.36E+00		
8019494	BC008897	C17orf101	chromosome 17 open reading fram	-0.059	0.960	6.60E-01	6.60E+01		
8019507	NM_00110040	C17orf62	chromosome 17 open reading fram	0.002	1.001	9.90E-01	9.90E+01		
8019517	---	---	---	0.140	1.102	4.75E-01	4.75E+01		
8019521	NM_004514	FOXK2	forkhead box K2	0.010	1.007	9.83E-01	9.83E+01		
8019523	NM_019613	WDR45L	WDR45-like	0.079	1.056	6.17E-01	6.17E+01		
8019532	NM_006822	RAB40B	RAB40B, member RAS oncogene fa	-0.047	0.968	6.77E-01	6.77E+01		
8019559	AK126018	B3GNTL1	UDP-GlcNAc:betaGal beta-1,3-N-a	-0.097	0.935	4.43E-01	4.43E+01		
8019563	NR_003682	MGC70870	C-terminal binding protein 2 pseud	0.041	1.029	9.07E-01	9.07E+01		
8019565	NM_031854	KRTAP4-12	keratin associated protein 4-12	0.002	1.001	9.88E-01	9.88E+01		
8019570	NM_033059	KRTAP4-11	keratin associated protein 4-11	0.010	1.007	9.36E-01	9.36E+01		
8019574	NM_033184	KRTAP2-4	keratin associated protein 2-4	-0.028	0.981	6.76E-01	6.76E+01		
8019576	NM_033184	KRTAP2-4	keratin associated protein 2-4	-0.036	0.975	5.54E-01	5.54E+01		
8019578	NM_030967	KRTAP1-1	keratin associated protein 1-1	0.005	1.004	9.63E-01	9.63E+01		
8019585	NM_030966	KRTAP1-3	keratin associated protein 1-3	-0.003	0.998	9.82E-01	9.82E+01		
8019588	NM_031957	KRTAP1-5	keratin associated protein 1-5	-0.030	0.979	7.70E-01	7.70E+01		
8019593	NM_031958	KRTAP3-1	keratin associated protein 3-1	-0.007	0.995	9.56E-01	9.56E+01		
8019631	0	0	0	0.002	1.001	9.98E-01	9.98E+01		
8019633	0	0	0	0.002	1.001	9.98E-01	9.98E+01		
8019635	0	0	0	0.002	1.001	9.98E-01	9.98E+01		
8019637	0	0	0	0.002	1.001	9.98E-01	9.98E+01		
8019639	0	0	0	0.002	1.001	9.98E-01	9.98E+01		
8019641	0	0	0	0.002	1.001	9.98E-01	9.98E+01		
8019643	0	0	0	0.175	1.129	2.60E-01	2.60E+01		
8019649	---	---	---	0.026	1.019	9.67E-01	9.67E+01		
8019651	NM_00100143	CCL4L1	chemokine (C-C motif) ligand 4-like	-0.464	0.725	1.49E-01	1.49E+01		
8019655	NM_00100141	TBC1D3B	TBC1 domain family, member 3B	-0.012	0.992	9.63E-01	9.63E+01		
8019669	NM_000160	GCGR	glucagon receptor	-0.004	0.997	9.78E-01	9.78E+01		
8019687	NM_00100224	ANAPC11	anaphase promoting complex subu	0.005	1.004	9.85E-01	9.85E+01		
8019693	NM_148896	NPB	neuropeptide B	-0.080	0.946	3.28E-01	3.28E+01		
8019699	NM_033059	KRTAP4-11	keratin associated protein 4-11	-0.038	0.974	5.50E-01	5.50E+01		
8019703	0	0	0	0.002	1.001	9.98E-01	9.98E+01		
8019705	0	0	0	0.002	1.001	9.98E-01	9.98E+01		
8019707	0	0	0	0.002	1.001	9.98E-01	9.98E+01		
8019709	0	0	0	-0.006	0.996	9.90E-01	9.90E+01		
8019711	NM_145651	SCGB1C1	secretoglobin, family 1C, member	-0.003	0.998	9.86E-01	9.86E+01		
8019716	NM_00104028	TBC1D3G	TBC1 domain family, member 3G	0.004	1.002	9.89E-01	9.89E+01		
8019737	NM_002266	KPNA2	karyopherin alpha 2 (RAG cohort 1	0.025	1.018	9.49E-01	9.49E+01		
8019754	NM_207368	LOC348262	hypothetical protein LOC348262	0.006	1.004	9.74E-01	9.74E+01		

8019762	---	---	---	0.050	1.036	8.96E-01	8.96E+01		
8019765	NM_004309	ARHGDIA	Rho GDP dissociation inhibitor (GD	-0.033	0.977	9.29E-01	9.29E+01		
8019772	NM_005782	THOC4	THO complex 4	0.016	1.011	9.53E-01	9.53E+01		
8019778	NM_002861	PCYT2	phosphate cytidylyltransferase 2, e	-0.034	0.977	8.14E-01	8.14E+01		
8019798	NM_032711	MAFG	v-maf musculoaponeurotic fibrosa	-0.042	0.971	7.77E-01	7.77E+01		
8019802	0	0	0	-0.047	0.968	9.16E-01	9.16E+01		
8019807	NM_005151	USP14	ubiquitin specific peptidase 14 (tR	0.020	1.014	9.22E-01	9.22E+01		
8019842	NM_001071	TYMS	thymidylate synthetase	0.264	1.201	4.04E-01	4.04E+01		
8019877	NM_015295	SMCHD1	structural maintenance of chromo	-0.036	0.975	7.34E-01	7.34E+01		
8019885	NM_015295	SMCHD1	structural maintenance of chromo	-0.005	0.997	9.78E-01	9.78E+01		
8019908	---	---	---	0.121	1.087	6.38E-01	6.38E+01		
8019910	---	---	---	0.066	1.047	8.55E-01	8.55E+01		
8019912	NM_032048	EMILIN2	elastin microfibril interfacer 2	0.100	1.072	6.51E-01	6.51E+01		
8019924	NM_006471	MYL12A	myosin, light chain 12A, regulatory	0.017	1.012	9.33E-01	9.33E+01		
8019930	NM_033546	MYL12B	myosin, light chain 12B, regulatory	-0.029	0.980	8.72E-01	8.72E+01		
8019937	---	---	---	0.024	1.017	8.68E-01	8.68E+01		
8019939	NM_170695	TGIF1	TGFB-induced factor homeobox 1	-0.182	0.881	3.03E-02	3.03E+00		
8019954	NR_024101	FLJ35776	hypothetical LOC649446	-0.071	0.952	7.51E-01	7.51E+01		
8019962	---	---	---	0.081	1.057	4.49E-01	4.49E+01		
8019988	NM_00110524	PTPRM	protein tyrosine phosphatase, rec	0.040	1.028	9.47E-01	9.47E+01		
8020025	---	---	---	-0.029	0.980	8.87E-01	8.87E+01		
8020029	NM_00102530	RAB12	RAB12, member RAS oncogene fa	0.038	1.027	8.05E-01	8.05E+01		
8020058	NM_021074	NDUFV2	NADH dehydrogenase (ubiquinone	-0.131	0.913	2.08E-01	2.08E+01		
8020068	NM_015208	ANKRD12	ankyrin repeat domain 12	-0.080	0.946	2.51E-01	2.51E+01		
8020086	---	---	---	-0.098	0.934	7.95E-01	7.95E+01		
8020088	---	---	---	-0.119	0.921	8.13E-01	8.13E+01		
8020100	NM_006788	RALBP1	ralA binding protein 1	-0.120	0.920	4.39E-02	4.39E+00		
8020110	NM_006868	RAB31	RAB31, member RAS oncogene fa	-0.058	0.961	9.22E-01	9.22E+01		
8020123	NM_00109852	TXNDC2	thioredoxin domain containing 2 (	-0.047	0.968	7.63E-01	7.63E+01		
8020129	NM_003574	VAPA	VAMP (vesicle-associated membra	0.099	1.071	3.17E-01	3.17E+01		
8020139	---	---	---	0.100	1.072	5.98E-01	5.98E+01		
8020149	NM_003826	NAPG	N-ethylmaleimide-sensitive factor	-0.076	0.948	6.38E-01	6.38E+01		
8020162	NM_054028	AMAC1L2	acyl-malonyl condensing enzyme 1	0.003	1.002	9.85E-01	9.85E+01		
8020179	NM_020412	CHMP1B	chromatin modifying protein 1B	-0.039	0.973	7.27E-01	7.27E+01		
8020183	NM_014214	IMPA2	inositol(myo)-1(or 4)-monophosph	0.023	1.016	9.05E-01	9.05E+01		
8020220	NM_032525	TUBB6	tubulin, beta 6	-0.029	0.980	8.73E-01	8.73E+01		
8020226	NM_00114240	SLMO1	slowmo homolog 1 (Drosophila)	0.048	1.034	6.76E-01	6.76E+01		
8020241	NM_020232	PSMG2	proteasome (prosome, macropain	0.128	1.093	1.82E-01	1.82E+01		
8020254	NM_031216	SEH1L	SEH1-like (S. cerevisiae)	0.170	1.125	7.17E-02	7.17E+00		
8020267	NM_032142	CEP192	centrosomal protein 192kDa	0.015	1.010	9.64E-01	9.64E+01		
8020308	NM_181481	C18orf1	chromosome 18 open reading fra	-0.168	0.890	3.33E-03	3.33E-01		
8020321	---	---	---	-0.181	0.882	2.53E-01	2.53E+01		
8020323	NM_003799	RNMT	RNA (guanine-7-) methyltransfera	0.014	1.009	9.58E-01	9.58E+01		
8020349	NR_003366	ANKRD20B	ankyrin repeat domain 20B	0.169	1.124	7.12E-01	7.12E+01		
8020382	NM_005406	ROCK1	Rho-associated, coiled-coil contai	0.070	1.050	7.17E-01	7.17E+01		
8020411	NM_006938	SNRPD1	small nuclear ribonucleoprotein D	0.098	1.070	5.86E-01	5.86E+01		
8020419	---	---	---	0.084	1.060	6.82E-01	6.82E+01		
8020423	NM_020774	MIB1	mindbomb homolog 1 (Drosophila	-0.001	1.000	9.98E-01	9.98E+01		
8020449	---	---	---	0.056	1.040	7.12E-01	7.12E+01		
8020455	NM_005257	GATA6	GATA binding protein 6	-0.066	0.955	4.97E-01	4.97E+01		
8020491	---	---	---	0.007	1.005	9.83E-01	9.83E+01		
8020495	NM_138375	CABLES1	Cdk5 and Abl enzyme substrate 1	0.070	1.050	6.48E-01	6.48E+01		
8020508	NM_003831	RIOK3	RIO kinase 3 (yeast)	0.148	1.108	1.57E-01	1.57E+01		
8020527	NM_013326	C18orf8	chromosome 18 open reading fra	0.150	1.110	7.09E-02	7.09E+00		
8020549	---	---	---	-0.045	0.969	7.35E-01	7.35E+01		
8020630	NM_153211	TTC39C	tetratricopeptide repeat domain 3	-0.095	0.936	4.21E-01	4.21E+01		
8020647	NR_024232	TTC39C	tetratricopeptide repeat domain 3	-0.051	0.965	6.80E-01	6.80E+01		
8020650	---	---	---	-0.085	0.943	7.14E-01	7.14E+01		
8020653	NM_012189	CABYR	calcium binding tyrosine-(Y)-phosp	0.013	1.009	9.40E-01	9.40E+01		
8020760	---	---	---	-0.009	0.994	9.57E-01	9.57E+01		
8020795	NM_000371	TTR	transthyretin	0.009	1.006	9.65E-01	9.65E+01		
8020804	---	---	---	0.362	1.285	3.21E-01	3.21E+01		
8020806	NM_017831	RNF125	ring finger protein 125	0.163	1.120	5.14E-01	5.14E+01		
8020814	NM_016271	RNF138	ring finger protein 138	0.089	1.064	4.46E-01	4.46E+01		
8020825	---	---	---	0.075	1.053	7.54E-01	7.54E+01		
8020842	NR_003558 //	P11P1 // WBP1	WW domain binding protein 11 ps	-0.050	0.966	6.28E-01	6.28E+01		
8020878	NM_014268	MAPRE2	microtubule-associated protein, R	-0.034	0.977	8.09E-01	8.09E+01		
8020889	NM_00113517	ZNF397	zinc finger protein 397	0.142	1.103	1.11E-01	1.11E+01		
8020903	NM_020474	GALNT1	UDP-N-acetyl-alpha-D-galactosam	0.089	1.064	7.27E-01	7.27E+01		
8020919	NM_031446	C18orf21	chromosome 18 open reading fra	0.047	1.033	8.04E-01	8.04E+01		
8020930	NM_018255	ELP2	elongation protein 2 homolog (S. c	0.049	1.034	7.75E-01	7.75E+01		
8021001	NM_020776	KIAA1328	KIAA1328	-0.034	0.977	8.43E-01	8.43E+01		
8021015	NM_002647	PIK3C3	phosphoinositide-3-kinase, class 3	-0.008	0.994	9.63E-01	9.63E+01		
8021047	NM_015559	SETBP1	SET binding protein 1	0.185	1.137	3.60E-02	3.60E+00		
8021091	NM_213602	SIGLEC15	sialic acid binding Ig-like lectin 15	-0.053	0.964	6.70E-01	6.70E+01		
8021113	NM_145055	C18orf25	chromosome 18 open reading fra	0.047	1.033	7.30E-01	7.30E+01		
8021129	BC093850	C18orf23	chromosome 18 open reading fra	0.070	1.050	6.96E-01	6.96E+01		

8021147	NM_016097	IER3IP1	immediate early response 3 intera	-0.039	0.974	8.88E-01	8.88E+01		
8021154	NM_00114239	KIAA0427	KIAA0427	-0.011	0.993	9.51E-01	9.51E+01		
8021169	NM_006033	LIPG	lipase, endothelial	-0.055	0.963	5.49E-01	5.49E+01		
8021181	NR_003003	SCARNA17	small Cajal body-specific RNA 17	-0.182	0.882	4.32E-01	4.32E+01		
8021183	NR_003003	SCARNA17	small Cajal body-specific RNA 17	-0.059	0.960	8.56E-01	8.56E+01		
8021208	NM_002396	ME2	malic enzyme 2, NAD(+)-depender	0.180	1.133	2.73E-02	2.73E+00		
8021222	NM_018696	ELAC1	elaC homolog 1 (E. coli)	-0.063	0.957	6.89E-01	6.89E+01		
8021228	NM_005359	SMAD4	SMAD family member 4	-0.038	0.974	7.97E-01	7.97E+01		
8021275	NM_007195	POLI	polymerase (DNA directed) iota	0.004	1.003	9.88E-01	9.88E+01		
8021312	NM_015285	WDR7	WD repeat domain 7	0.160	1.117	1.08E-01	1.08E+01		
8021344	AK301799	BOD1P	biorientation of chromosomes in c	0.032	1.022	7.61E-01	7.61E+01		
8021357	NM_004852	ONECUT2	one cut homeobox 2	-0.038	0.974	7.35E-01	7.35E+01		
8021363	---	---	---	0.028	1.020	9.03E-01	9.03E+01		
8021365	NM_005603	ATP8B1	ATPase, class I, type 8B, member 1	0.144	1.105	5.15E-01	5.15E+01		
8021368	---	---	---	0.501	1.415	5.46E-02	5.46E+00		
8021372	---	---	---	0.112	1.081	6.20E-01	6.20E+01		
8021418	NM_006785	MALT1	mucosa associated lymphoid tissu	-0.057	0.961	6.11E-01	6.11E+01		
8021442	NM_018181	ZNF532	zinc finger protein 532	-0.019	0.987	9.40E-01	9.40E+01		
8021453	NM_033280	SEC11C	SEC11 homolog C (S. cerevisiae)	0.131	1.095	2.00E-01	2.00E+01		
8021461	NM_002091	GRP	gastrin-releasing peptide	-0.055	0.962	6.34E-01	6.34E+01		
8021468	---	---	---	0.044	1.031	6.69E-01	6.69E+01		
8021470	NM_021127	PMAIP1	phorbol-12-myristate-13-acetate-i	-0.321	0.800	2.96E-02	2.96E+00		
8021478	---	---	---	-0.026	0.982	9.42E-01	9.42E+01		
8021496	NM_020854	KIAA1468	KIAA1468	0.078	1.055	5.02E-01	5.02E+01		
8021528	NM_003839	TNFRSF11A	tumor necrosis factor receptor sup	-0.042	0.971	6.93E-01	6.93E+01		
8021542	NM_017742	ZCCHC2	zinc finger, CCHC domain containi	-0.041	0.972	7.51E-01	7.51E+01		
8021546	NM_017742	ZCCHC2	zinc finger, CCHC domain containi	0.061	1.044	7.93E-01	7.93E+01		
8021563	---	---	---	-0.017	0.988	9.53E-01	9.53E+01		
8021565	NM_194449	PHLPP1	PH domain and leucine rich repeat	-0.097	0.935	1.97E-01	1.97E+01		
8021653	NM_002640	SERPINB8	serpin peptidase inhibitor, clade B	-0.179	0.883	3.53E-01	3.53E+01		
8021707	NM_004232	SOCS6	suppressor of cytokine signaling 6	0.279	1.213	7.51E-02	7.51E+00		
8021712	---	---	---	-0.063	0.957	6.57E-01	6.57E+01		
8021714	---	---	---	0.040	1.028	7.33E-01	7.33E+01		
8021716	NM_014177	C18orf55	chromosome 18 open reading fran	0.070	1.049	8.01E-01	8.01E+01		
8021725	---	---	---	-0.025	0.982	8.75E-01	8.75E+01		
8021756	NM_017757	ZNF407	zinc finger protein 407	-0.022	0.985	9.16E-01	9.16E+01		
8021768	NM_005786	TSHZ1	teashirt zinc finger homeobox 1	0.060	1.043	6.98E-01	6.98E+01		
8021774	ENST00000405	FLJ44313	FLJ44313 protein	-0.047	0.968	6.57E-01	6.57E+01		
8021783	NM_007345	ZNF236	zinc finger protein 236	-0.055	0.963	5.79E-01	5.79E+01		
8021824	---	---	---	0.159	1.116	3.25E-01	3.25E+01		
8021826	NM_171999	SALL3	sal-like 3 (Drosophila)	0.000	1.000	9.98E-01	9.98E+01		
8021832	NM_198531	ATP9B	ATPase, class II, type 9B	0.043	1.030	7.95E-01	7.95E+01		
8021866	NM_172387	NFATC1	nuclear factor of activated T-cells,	0.062	1.044	4.42E-01	4.42E+01		
8021884	NM_004715	CTDP1	CTD (carboxy-terminal domain, RN	-0.043	0.970	5.06E-01	5.06E+01		
8021900	NM_012283	KCNG2	potassium voltage-gated channel,	-0.093	0.938	4.05E-01	4.05E+01		
8021905	BC014195	C18orf22	chromosome 18 open reading fran	-0.034	0.977	8.30E-01	8.30E+01		
8021914	NM_014913	ADNP2	ADNP homeobox 2	0.020	1.014	9.41E-01	9.41E+01		
8021919	NM_020040	TUBB4Q	tubulin, beta polypeptide 4, memb	-0.050	0.966	5.53E-01	5.53E+01		
8021924	NM_005131	THOC1	THO complex 1	0.079	1.056	6.00E-01	6.00E+01		
8021963	NM_001071	TYMS	thymidylate synthetase	-0.033	0.977	8.06E-01	8.06E+01		
8021966	NM_017512	ENOSF1	enolase superfamily member 1	-0.133	0.912	4.53E-01	4.53E+01		
8021984	NM_005433	YES1	v-yes-1 Yamaguchi sarcoma viral o	0.010	1.007	9.81E-01	9.81E+01		
8022007	---	---	---	0.015	1.011	9.69E-01	9.69E+01		
8022009	NM_022840	METTL4	methyltransferase like 4	0.052	1.037	7.86E-01	7.86E+01		
8022022	NM_014646	LPIN2	lipin 2	-0.032	0.978	8.06E-01	8.06E+01		
8022043	---	---	---	-0.028	0.981	8.66E-01	8.66E+01		
8022085	NM_004746	DLGAP1	discs, large (Drosophila) homolog-	-0.046	0.968	5.61E-01	5.61E+01		
8022110	NM_00114382	ZFP161	zinc finger protein 161 homolog (r	-0.043	0.970	7.91E-01	7.91E+01		
8022118	NM_012307	EPB41L3	erythrocyte membrane protein ba	-0.150	0.901	7.60E-01	7.60E+01		
8022145	NM_173464	L3MBTL4	l(3)mbt-like 4 (Drosophila)	0.168	1.124	1.58E-01	1.58E+01		
8022170	NM_00102466	RPL6	ribosomal protein L6	-0.019	0.987	8.60E-01	8.60E+01		
8022243	---	---	---	-0.060	0.959	5.34E-01	5.34E+01		
8022251	NM_00104238	PPP4R1	protein phosphatase 4, regulatory	-0.159	0.896	1.29E-01	1.29E+01		
8022277	---	---	---	-0.075	0.949	7.98E-01	7.98E+01		
8022279	---	---	---	0.048	1.034	9.49E-01	9.49E+01		
8022320	ENST00000446	NPIPL3	nuclear pore complex interacting p	0.011	1.008	9.39E-01	9.39E+01		
8022326	NM_023075	MPPE1	metallophosphoesterase 1	-0.022	0.985	9.33E-01	9.33E+01		
8022342	NM_006796	AFG3L2	AFG3 ATPase family gene 3-like 2 (	0.097	1.070	3.27E-01	3.27E+01		
8022356	NM_00112862	SPIRE1	spire homolog 1 (Drosophila)	0.110	1.079	6.47E-01	6.47E+01		
8022380	NM_024899	CEP76	centrosomal protein 76kDa	-0.050	0.966	7.72E-01	7.72E+01		
8022393	NM_002828	PTPN2	protein tyrosine phosphatase, nor	0.127	1.092	3.63E-01	3.63E+01		
8022410	---	---	---	-0.093	0.937	6.54E-01	6.54E+01		
8022418	---	---	---	0.017	1.012	9.33E-01	9.33E+01		
8022426	ENST00000342	LOC646359	similar to telomeric repeat binding	-0.142	0.906	5.38E-01	5.38E+01		
8022434	---	---	---	0.028	1.020	8.29E-01	8.29E+01		
8022441	NM_005406	ROCK1	Rho-associated, coiled-coil contai	0.023	1.016	8.63E-01	8.63E+01		
8022473	NM_052911	ESCO1	establishment of cohesion 1 homo	0.037	1.026	8.27E-01	8.27E+01		

8022488	NM_138340	ABHD3	abhydrolase domain containing 3	0.051	1.036	7.82E-01	7.82E+01			
8022506	---	---	---	0.146	1.107	6.82E-01	6.82E+01			
8022514	NM_032933	C18orf45	chromosome 18 open reading fram	0.117	1.084	5.29E-01	5.29E+01			
8022531	NM_000271	NPC1	Niemann-Pick disease, type C1	0.270	1.206	1.65E-01	1.65E+01			
8022557	---	---	---	0.123	1.089	6.75E-01	6.75E+01			
8022606	---	---	---	0.169	1.124	5.65E-01	5.65E+01			
8022608	---	---	---	-0.046	0.968	7.69E-01	7.69E+01			
8022623	---	---	---	0.036	1.025	7.29E-01	7.29E+01			
8022625	NM_00100755	SS18	synovial sarcoma translocation, ch	-0.075	0.949	3.80E-01	3.80E+01			
8022653	NR_024259	LOC728606	hypothetical LOC728606	-0.002	0.999	9.91E-01	9.91E+01			
8022711	NM_024422	DSC2	desmocollin 2	-0.002	0.999	9.99E-01	9.99E+01			
8022767	NM_014939	KIAA1012	KIAA1012	0.087	1.062	4.29E-01	4.29E+01			
8022814	NM_002136	HNRNPA1	heterogeneous nuclear ribonucleo	-0.026	0.982	8.58E-01	8.58E+01			
8022870	---	---	---	-0.054	0.963	9.05E-01	9.05E+01			
8022882	NM_006965	ZNF24	zinc finger protein 24	-0.005	0.997	9.78E-01	9.78E+01			
8022902	NM_00109881	INO80C	INO80 complex subunit C	-0.049	0.967	7.99E-01	7.99E+01			
8022912	---	---	---	-0.009	0.993	9.58E-01	9.58E+01			
8022914	NM_018170	RPRD1A	regulation of nuclear pre-mRNA do	0.050	1.035	7.73E-01	7.73E+01			
8022927	NM_012319	SLC39A6	solute carrier family 39 (zinc trans	-0.070	0.953	6.51E-01	6.51E+01			
8022941	NM_015476	C18orf10	chromosome 18 open reading fram	-0.027	0.982	8.89E-01	8.89E+01			
8022952	NM_020180	BRUNOL4	bruno-like 4, RNA binding protein	-0.005	0.997	9.77E-01	9.77E+01			
8022970	---	---	---	0.009	1.006	9.56E-01	9.56E+01			
8022972	NM_000972	RPL7A	ribosomal protein L7a	0.026	1.018	9.05E-01	9.05E+01			
8022996	NM_020964	KIAA1632	KIAA1632	0.132	1.096	3.30E-01	3.30E+01			
8023059	---	---	---	0.265	1.202	1.61E-01	1.61E+01			
8023061	---	---	---	-0.046	0.969	8.74E-01	8.74E+01			
8023063	NM_00100193	ATP5A1	ATP synthase, H+ transporting, mi	0.026	1.018	8.69E-01	8.69E+01			
8023121	NM_013305	ST8SIA5	ST8 alpha-N-acetyl-neuraminide a	-0.022	0.985	8.73E-01	8.73E+01			
8023133	NM_004671	PIAS2	protein inhibitor of activated STAT	0.076	1.054	5.96E-01	5.96E+01			
8023156	NM_145653	TCEB3C	transcription elongation factor B p	-0.098	0.934	2.25E-01	2.25E+01			
8023165	NM_032124	HDHD2	haloacid dehalogenase-like hydrol	0.077	1.055	6.54E-01	6.54E+01			
8023175	NM_016097	IER3IP1	immediate early response 3 intera	-0.049	0.967	7.15E-01	7.15E+01			
8023184	AY669508	FUSSEL18	functional smad suppressing elem	-0.100	0.933	3.79E-01	3.79E+01			
8023191	NM_005901	SMAD2	SMAD family member 2	0.138	1.101	3.42E-01	3.42E+01			
8023195	NM_005901	SMAD2	SMAD family member 2	0.039	1.027	8.10E-01	8.10E+01			
8023212	NM_00103936	ZBTB7C	zinc finger and BTB domain contai	-0.047	0.968	5.67E-01	5.67E+01			
8023218	---	---	---	-0.075	0.950	6.58E-01	6.58E+01			
8023220	NM_005904	SMAD7	SMAD family member 7	0.026	1.018	9.09E-01	9.09E+01			
8023228	NM_017653	DYM	dymeclin	0.062	1.044	6.52E-01	6.52E+01			
8023246	BC093004	C18orf32	chromosome 18 open reading fram	0.150	1.109	3.41E-01	3.41E+01			
8023252	NM_000985	RPL17	ribosomal protein L17	-0.062	0.958	7.30E-01	7.30E+01			
8023259	NR_002571	SNORD58A	small nucleolar RNA, C/D box 58A	-0.103	0.931	7.45E-01	7.45E+01			
8023261	NM_006111	ACAA2	acetyl-Coenzyme A acyltransferase	0.119	1.086	3.87E-01	3.87E+01			
8023308	---	---	---	0.063	1.044	5.87E-01	5.87E+01			
8023323	NM_015846	MBD1	methyl-CpG binding domain prote	-0.073	0.950	4.04E-01	4.04E+01			
8023344	NM_00110165	CXXC1	CXXC finger 1 (PHD domain)	-0.040	0.972	8.16E-01	8.16E+01			
8023377	NM_016626	MEX3C	mex-3 homolog C (C. elegans)	0.030	1.021	8.44E-01	8.44E+01			
8023382	NM_003927	MBD2	methyl-CpG binding domain prote	0.104	1.075	3.22E-01	3.22E+01			
8023392	NR_002970	SNORA37	small nucleolar RNA, H/ACA box 3	-0.091	0.939	5.63E-01	5.63E+01			
8023415	NM_00108396	TCF4	transcription factor 4	0.274	1.209	1.79E-01	1.79E+01			
8023450	NR_024546	TXNL1	thioredoxin-like 1	-0.026	0.982	8.38E-01	8.38E+01			
8023466	NM_00101251	FECH	ferrochelatase (protoporphyria)	0.031	1.022	8.81E-01	8.81E+01			
8023481	NM_004539	NARS	asparaginyl-tRNA synthetase	0.139	1.101	4.98E-02	4.98E+00			
8023526	ENST00000446	LOC100132992	similar to hCG1979072	-0.085	0.943	6.70E-01	6.70E+01			
8023549	NM_013435	RAX	retina and anterior neural fold hon	-0.062	0.958	3.85E-01	3.85E+01			
8023561	NM_005570	LMAN1	lectin, mannose-binding, 1	0.002	1.001	9.90E-01	9.90E+01			
8023646	NM_000633	BCL2	B-cell CLL/lymphoma 2	0.037	1.026	8.30E-01	8.30E+01			
8023656	NM_002035	KDSR	3-ketodihydrosphingosine reducta	-0.093	0.937	6.31E-01	6.31E+01			
8023672	NM_004869	VPS4B	vacuolar protein sorting 4 homolo	-0.015	0.990	9.22E-01	9.22E+01			
8023708	---	---	---	-0.028	0.981	9.16E-01	9.16E+01			
8023735	NM_019022	TMX3	thioredoxin-related transmembran	0.083	1.059	4.84E-01	4.84E+01			
8023757	NM_006566	CD226	CD226 molecule	-0.025	0.983	9.48E-01	9.48E+01			
8023766	NM_173630	RTTN	rotatin	-0.045	0.969	8.04E-01	8.04E+01			
8023855	NM_148923	CYB5A	cytochrome b5 type A (microsoma	-0.035	0.976	8.98E-01	8.98E+01			
8023862	---	---	---	0.025	1.018	9.11E-01	9.11E+01			
8023868	NR_024484	LOC400657	hypothetical LOC400657	0.130	1.094	1.80E-01	1.80E+01			
8023871	NM_175907	ZADH2	zinc binding alcohol dehydrogenas	0.013	1.009	9.63E-01	9.63E+01			
8023882	NM_014643	ZNF516	zinc finger protein 516	-0.068	0.954	4.04E-01	4.04E+01			
8023889	NM_00102510	MBP	myelin basic protein	-0.097	0.935	2.03E-01	2.03E+01			
8023914	NM_025078	PQLC1	PQ loop repeat containing 1	0.026	1.018	8.49E-01	8.49E+01			
8023920	NM_006701	TXNL4A	thioredoxin-like 4A	0.045	1.032	8.84E-01	8.84E+01			
8023933	---	---	---	0.012	1.009	9.77E-01	9.77E+01			
8023939	---	---	---	-0.036	0.975	8.57E-01	8.57E+01			
8023941	NM_130760	MADCAM1	mucosal vascular addressin cell ad	-0.019	0.987	8.78E-01	8.78E+01			
8023948	NM_033513	C19orf20	chromosome 19 open reading fram	0.006	1.004	9.66E-01	9.66E+01			
8023955	NM_001728	BSG	basigin (Ok blood group)	-0.024	0.983	8.78E-01	8.78E+01			
8023968	NM_004359	CDC34	cell division cycle 34 homolog (S. c	-0.016	0.989	9.57E-01	9.57E+01			

8023977	NM_005317	GZMM	granzyme M (lymphocyte met-ase	-0.027	0.981	8.66E-01	8.66E+01		
8023984	NM_001194	HCN2	hyperpolarization activated cyclic	0.008	1.005	9.65E-01	9.65E+01		
8023990	NM_020637	FGF22	fibroblast growth factor 22	-0.036	0.975	7.86E-01	7.86E+01		
8023995	NM_005860	FSTL3	follistatin-like 3 (secreted glycopro	-0.060	0.959	6.00E-01	6.00E+01		
8024001	---	---	---	-0.046	0.968	7.87E-01	7.87E+01		
8024003	NM_002579	PALM	paralemmin	-0.020	0.986	8.52E-01	8.52E+01		
8024013	BC052236	C19orf21	chromosome 19 open reading fra	-0.026	0.982	8.84E-01	8.84E+01		
8024019	NM_002819	PTBP1	polypyrimidine tract binding prote	0.003	1.002	9.86E-01	9.86E+01		
8024038	NM_001700	AZU1	azurocidin 1	-0.088	0.941	1.59E-01	1.59E+01		
8024048	NM_002777	PRTN3	proteinase 3	-0.049	0.967	6.78E-01	6.78E+01		
8024056	NM_001972	ELANE	elastase, neutrophil expressed	-0.030	0.979	7.56E-01	7.56E+01		
8024062	NM_001928	CFD	complement factor D (adipsin)	0.099	1.071	1.56E-01	1.56E+01		
8024070	NM_032551	KISS1R	KISS1 receptor	-0.029	0.980	8.37E-01	8.37E+01		
8024078	NM_005224	ARID3A	AT rich interactive domain 3A (BRI	-0.029	0.980	7.91E-01	7.91E+01		
8024089	NM_024100	WDR18	WD repeat domain 18	0.039	1.028	7.15E-01	7.15E+01		
8024100	NM_138690	GRIN3B	glutamate receptor, ionotropic, N-	0.009	1.006	9.49E-01	9.49E+01		
8024111	NM_004368	CNN2	calponin 2	0.105	1.076	5.57E-01	5.57E+01		
8024120	NM_019112	ABCA7	ATP-binding cassette, sub-family A	-0.073	0.950	2.91E-01	2.91E+01		
8024170	NM_012292	HMHA1	histocompatibility (minor) HA-1	-0.041	0.972	7.94E-01	7.94E+01		
8024194	NM_002085	GPX4	glutathione peroxidase 4 (phospho	0.018	1.013	9.61E-01	9.61E+01		
8024204	NM_000455	STK11	serine/threonine kinase 11	0.006	1.004	9.78E-01	9.78E+01		
8024219	NM_001687	ATP5D	ATP synthase, H+ transporting, mi	-0.044	0.970	7.23E-01	7.23E+01		
8024228	NM_177401	MIDN	midnolin	-0.065	0.956	3.28E-01	3.28E+01		
8024238	NM_001280	CIRBP	cold inducible RNA binding protein	0.000	1.000	9.98E-01	9.98E+01		
8024246	NM_017914	C19orf24	chromosome 19 open reading fra	-0.020	0.986	8.99E-01	8.99E+01		
8024250	NM_001405	EFNA2	ephrin-A2	-0.017	0.988	9.18E-01	9.18E+01		
8024255	NR_024247	MUM1	melanoma associated antigen (mu	-0.106	0.929	2.91E-01	2.91E+01		
8024273	NM_024407	NDUFS7	NADH dehydrogenase (ubiquinone	0.035	1.024	8.45E-01	8.45E+01		
8024282	NM_170711	DAZAP1	DAZ associated protein 1	-0.055	0.963	4.27E-01	4.27E+01		
8024299	NM_001018	RPS15	ribosomal protein S15	-0.024	0.984	9.09E-01	9.09E+01		
8024304	---	---	---	-0.043	0.971	7.97E-01	7.97E+01		
8024306	NM_005883	APC2	adenomatosis polyposis coli 2	0.004	1.003	9.80E-01	9.80E+01		
8024323	NM_138393	REEP6	receptor accessory protein 6	-0.031	0.978	7.74E-01	7.74E+01		
8024331	NR_026557	PLK5P	polo-like kinase 5 pseudogene	0.000	1.000	1.00E+00	1.00E+02		
8024347	NM_079834	SCAMP4	secretory carrier membrane prote	-0.013	0.991	9.25E-01	9.25E+01		
8024356	NM_138422	ADAT3	adenosine deaminase, tRNA-speci	-0.041	0.972	7.34E-01	7.34E+01		
8024358	NM_001319	CSNK1G2	casein kinase 1, gamma 2	-0.042	0.972	8.05E-01	8.05E+01		
8024373	NM_00103984	C19orf36	chromosome 19 open reading fra	-0.041	0.972	6.51E-01	6.51E+01		
8024391	NM_032482	DOT1L	DOT1-like, histone H3 methyltrans	0.009	1.006	9.63E-01	9.63E+01		
8024420	NM_007165	SF3A2	splicing factor 3a, subunit 2, 66kDa	-0.023	0.984	8.73E-01	8.73E+01		
8024429	NM_000479	AMH	anti-Mullerian hormone	0.000	1.000	9.98E-01	9.98E+01		
8024436	NM_004152	OAZ1	ornithine decarboxylase antizyme	0.038	1.027	8.42E-01	8.42E+01		
8024444	AY358234	UNQ6493	EPWW6493	-0.044	0.970	7.41E-01	7.41E+01		
8024446	NM_00107723	SPPL2B	signal peptide peptidase-like 2B	-0.093	0.938	3.95E-01	3.95E+01		
8024467	NM_182973	TMPRSS9	transmembrane protease, serine 9	0.012	1.008	9.42E-01	9.42E+01		
8024485	NM_015675	GADD45B	growth arrest and DNA-damage-in	-0.163	0.893	3.05E-01	3.05E+01		
8024497	NM_003249	THOP1	thimet oligopeptidase 1	-0.137	0.910	5.58E-02	5.58E+00		
8024518	NM_152791	ZNF555	zinc finger protein 555	0.146	1.107	2.50E-01	2.50E+01		
8024532	NM_173480	ZNF57	zinc finger protein 57	0.009	1.006	9.81E-01	9.81E+01		
8024572	NM_002068	GNA15	guanine nucleotide binding protei	-0.228	0.854	3.66E-01	3.66E+01		
8024582	NM_003775	S1PR4	sphingosine-1-phosphate receptor	0.059	1.042	6.20E-01	6.20E+01		
8024584	NM_020170	NCLN	nicalin homolog (zebrafish)	-0.023	0.984	8.62E-01	8.62E+01		
8024605	NM_021938	BRUNOL5	bruno-like 5, RNA binding protein	-0.088	0.941	1.60E-01	1.60E+01		
8024623	NM_205843	NFIC	nuclear factor I/C (CCAAT-binding	-0.043	0.971	7.32E-01	7.32E+01		
8024637	NM_00113619	FZR1	fizzy/cell division cycle 20 related	-0.033	0.977	7.54E-01	7.54E+01		
8024655	NM_00113558	C19orf71	chromosome 19 open reading fra	0.017	1.012	9.21E-01	9.21E+01		
8024660	NM_006339	HMG20B	high-mobility group 20B	-0.007	0.995	9.66E-01	9.66E+01		
8024676	NM_133261	GIPC3	GIPC PDZ domain containing famil	-0.127	0.915	1.57E-01	1.57E+01		
8024682	NM_021231	C19orf29	chromosome 19 open reading fra	-0.024	0.983	8.62E-01	8.62E+01		
8024687	NM_014428	TJP3	tight junction protein 3 (zona occlu	-0.001	1.000	9.97E-01	9.97E+01		
8024708	NM_172251	MRPL54	mitochondrial ribosomal protein L	-0.018	0.988	9.44E-01	9.44E+01		
8024728	NM_170678	ITGB1BP3	integrin beta 1 binding protein 3	-0.064	0.957	5.23E-01	5.23E+01		
8024740	NM_015897	PIAS4	protein inhibitor of activated STAT	-0.017	0.988	8.84E-01	8.84E+01		
8024754	NM_032607	CREB3L3	cAMP responsive element binding	-0.084	0.943	2.56E-01	2.56E+01		
8024768	NM_133475	ANKRD24	ankyrin repeat domain 24	-0.045	0.969	6.87E-01	6.87E+01		
8024792	NM_005755	EBI3	Epstein-Barr virus induced 3	-0.225	0.856	8.90E-03	8.90E-01		
8024798	NM_018074	CCDC94	coiled-coil domain containing 94	-0.059	0.960	6.82E-01	6.82E+01		
8024816	NM_024333	FSD1	fibronectin type III and SPRY doma	-0.002	0.999	9.93E-01	9.93E+01		
8024829	NM_032868	MPND	MPN domain containing	-0.016	0.989	9.02E-01	9.02E+01		
8024843	NM_005483	CHAF1A	chromatin assembly factor 1, subu	0.018	1.012	9.44E-01	9.44E+01		
8024864	NM_00100152	HDGF2	hepatoma-derived growth factor-r	-0.081	0.945	2.86E-01	2.86E+01		
8024882	---	---	---	-0.045	0.969	8.22E-01	8.22E+01		
8024896	---	---	---	-0.064	0.957	5.89E-01	5.89E+01		
8024898	NM_018708	FEM1A	fem-1 homolog a (C. elegans)	-0.017	0.988	9.44E-01	9.44E+01		
8024900	NM_00104820	UHRF1	ubiquitin-like with PHD and ring fi	-0.041	0.972	7.95E-01	7.95E+01		
8024909	NM_015015	KDM4B	lysine (K)-specific demethylase 4B	-0.042	0.972	7.15E-01	7.15E+01		
8024936	NM_002967	SAFB	scaffold attachment factor B	0.000	1.000	1.00E+00	1.00E+02		

8024956	NM_198706	HSD11B1L	hydroxysteroid (11-beta) dehydrog	-0.047	0.968	7.45E-01	7.45E+01		
8024966	NM_015414	RPL36	ribosomal protein L36	-0.020	0.987	9.02E-01	9.02E+01		
8024995	NM_004558	NRTN	neurturin	-0.020	0.986	8.96E-01	8.96E+01		
8025000	NM_00101792	VMAC	vimentin-type intermediate filame	-0.021	0.986	8.85E-01	8.85E+01		
8025004	NM_004058	CAPS	calcyphosine	-0.039	0.973	7.09E-01	7.09E+01		
8025028	NM_006012	CLPP	ClpP caseinolytic peptidase, ATP-d	-0.067	0.955	5.13E-01	5.13E+01		
8025035	NM_032306	ALKBH7	alkB, alkylation repair homolog 7 (	0.057	1.040	6.33E-01	6.33E+01		
8025041	NM_139161	CRB3	crumbs homolog 3 (Drosophila)	-0.034	0.977	7.89E-01	7.89E+01		
8025051	NM_006087	TUBB4	tubulin, beta 4	-0.116	0.923	3.30E-01	3.30E+01		
8025053	NM_003811	TNFSF9	tumor necrosis factor (ligand) supe	-0.132	0.912	5.90E-02	5.90E+00		
8025076	NM_005428	VAV1	vav 1 guanine nucleotide exchange	0.129	1.093	3.17E-01	3.17E+01		
8025103	NM_001974	EMR1	egf-like module containing, mucin	-0.128	0.915	7.89E-01	7.89E+01		
8025126	NM_144614	MBD3L2	methyl-CpG binding domain prote	-0.022	0.985	8.44E-01	8.44E+01		
8025132	NM_024341	ZNF557	zinc finger protein 557	-0.024	0.983	9.09E-01	9.09E+01		
8025142	NM_00113095	ARHGEF18	Rho/Rac guanine nucleotide excha	0.080	1.057	4.04E-01	4.04E+01		
8025179	NM_018083	ZNF358	zinc finger protein 358	0.053	1.038	7.38E-01	7.38E+01		
8025183	NM_020533	MCOLN1	mucolipin 1	0.238	1.179	6.99E-02	6.99E+00		
8025199	NM_006702	PNPLA6	patatin-like phospholipase domain	0.005	1.003	9.77E-01	9.77E+01		
8025237	NM_00108042	KIAA1543	KIAA1543	-0.034	0.977	7.49E-01	7.49E+01		
8025255	NM_006949	STXBP2	syntaxin binding protein 2	0.065	1.046	6.33E-01	6.33E+01		
8025278	NM_020415	RETN	resistin	-0.042	0.971	8.46E-01	8.46E+01		
8025285	NM_174918	C19orf59	chromosome 19 open reading fran	-0.180	0.882	5.84E-01	5.84E+01		
8025296	NM_174894	TRAPPC5	trafficking protein particle comple	0.073	1.052	5.76E-01	5.76E+01		
8025301	NR_026692	CD209	CD209 molecule	0.047	1.033	8.90E-01	8.90E+01		
8025321	NR_002931	CLEC4GP1	C-type lectin domain family 4, mer	-0.036	0.976	7.97E-01	7.97E+01		
8025328	NM_00115994	EVI5L	ecotropic viral integration site 5-li	-0.024	0.983	8.78E-01	8.78E+01		
8025351	NM_145185	MAP2K7	mitogen-activated protein kinase k	-0.019	0.987	9.13E-01	9.13E+01		
8025368	NM_003083	SNAPC2	small nuclear RNA activating comp	0.042	1.029	7.45E-01	7.45E+01		
8025375	NM_005624	CCL25	chemokine (C-C motif) ligand 25	-0.017	0.989	9.09E-01	9.09E+01		
8025382	NM_024552	LASS4	LAG1 homolog, ceramide synthase	0.031	1.022	8.78E-01	8.78E+01		
8025395	NM_001031	RPS28	ribosomal protein S28	0.075	1.053	6.07E-01	6.07E+01		
8025414	NM_004218	RAB11B	RAB11B, member RAS oncogene fa	-0.036	0.975	8.75E-01	8.75E+01		
8025421	NM_016496	40239	membrane-associated ring finger (	0.022	1.015	8.93E-01	8.93E+01		
8025429	NM_005968	HNRNPM	heterogeneous nuclear ribonucleo	-0.032	0.978	7.14E-01	7.14E+01		
8025450	NM_00100469	OR2Z1	olfactory receptor, family 2, subfa	-0.047	0.968	8.04E-01	8.04E+01		
8025456	NM_00100445	OR1M1	olfactory receptor, family 1, subfa	-0.040	0.973	8.49E-01	8.49E+01		
8025458	NM_020933	ZNF317	zinc finger protein 317	0.001	1.001	9.95E-01	9.95E+01		
8025478	NM_032497	ZNF559	zinc finger protein 559	-0.052	0.965	8.15E-01	8.15E+01		
8025488	NM_003451	ZNF177	zinc finger protein 177	-0.014	0.990	9.54E-01	9.54E+01		
8025498	NR_000011 //	)RA70 // SNOR	small nucleolar RNA, H/ACA box 7(	-0.027	0.981	9.13E-01	9.13E+01		
8025500	NM_024292	UBL5	ubiquitin-like 5	0.003	1.002	9.88E-01	9.88E+01		
8025506	NM_006221	PIN1	peptidylprolyl cis/trans isomerase,	-0.043	0.971	7.46E-01	7.46E+01		
8025515	NM_015725	RDH8	retinol dehydrogenase 8 (all-trans	-0.048	0.968	6.63E-01	6.63E+01		
8025551	NM_018381	C19orf66	chromosome 19 open reading fran	-0.007	0.995	9.74E-01	9.74E+01		
8025563	NM_00104066	PPAN-P2RY11	PPAN-P2RY11 readthrough transcr	-0.041	0.972	7.05E-01	7.05E+01		
8025586	NM_146388	MRPL4	mitochondrial ribosomal protein L	-0.016	0.989	9.27E-01	9.27E+01		
8025601	NM_000201	ICAM1	intercellular adhesion molecule 1	0.057	1.041	8.77E-01	8.77E+01		
8025612	NM_022377	ICAM4	intercellular adhesion molecule 4 (	0.026	1.018	8.97E-01	8.97E+01		
8025621	NM_003259	ICAM5	intercellular adhesion molecule 5,	-0.041	0.972	6.92E-01	6.92E+01		
8025633	NM_00111130	PDE4A	phosphodiesterase 4A, cAMP-spec	-0.091	0.939	4.06E-01	4.06E+01		
8025659	NM_032885	ATG4D	ATG4 autophagy related 4 homolo	-0.047	0.968	6.58E-01	6.58E+01		
8025672	NM_020428	SLC44A2	solute carrier family 44, member 2	0.041	1.029	8.34E-01	8.34E+01		
8025697	NM_012218	ILF3	interleukin enhancer binding facto	-0.086	0.942	4.88E-01	4.88E+01		
8025728	NM_031209	QTRT1	queuine tRNA-ribosyltransferase 1	0.003	1.002	9.87E-01	9.87E+01		
8025741	NM_00100536	DNM2	dynamin 2	-0.112	0.925	1.93E-01	1.93E+01		
8025766	NM_199141	CARM1	coactivator-associated arginine me	0.062	1.044	6.44E-01	6.44E+01		
8025783	BC011833	C19orf52	chromosome 19 open reading fran	-0.022	0.985	9.16E-01	9.16E+01		
8025788	NM_00112884	SMARCA4	SWI/SNF related, matrix associate	-0.092	0.938	3.70E-01	3.70E+01		
8025828	NM_000527	LDLR	low density lipoprotein receptor	-0.093	0.938	3.35E-01	3.35E+01		
8025850	NM_020812	DOCK6	dedicator of cytokinesis 6	-0.052	0.964	5.92E-01	5.92E+01		
8025877	NM_022737	LPPR2	lipid phosphate phosphatase-relat	0.001	1.001	9.97E-01	9.97E+01		
8025888	NM_175871	C19orf39	chromosome 19 open reading fran	0.006	1.004	9.73E-01	9.73E+01		
8025895	NM_002743	PRKCSH	protein kinase C substrate 80K-H	-0.042	0.972	7.43E-01	7.43E+01		
8025918	NM_001299	CNN1	calponin 1, basic, smooth muscle	-0.022	0.985	9.05E-01	9.05E+01		
8025927	NM_145295	ZNF627	zinc finger protein 627	0.089	1.064	5.15E-01	5.15E+01		
8025964	NM_152262	ZNF439	zinc finger protein 439	0.062	1.044	8.27E-01	8.27E+01		
8025968	NM_021915	ZNF69	zinc finger protein 69	-0.010	0.993	9.73E-01	9.73E+01		
8025973	NM_144566	ZNF700	zinc finger protein 700	0.194	1.144	3.06E-02	3.06E+00		
8025998	NM_003437	ZNF136	zinc finger protein 136	-0.025	0.982	9.07E-01	9.07E+01		
8026007	NM_153358	ZNF791	zinc finger protein 791	-0.025	0.983	9.00E-01	9.00E+01		
8026013	NM_00109973	MORG1	mitogen-activated protein kinase o	0.066	1.047	5.47E-01	5.47E+01		
8026024	NM_004317	ASNA1	arsA arsenite transporter, ATP-bin	-0.016	0.989	9.49E-01	9.49E+01		
8026047	NM_002229	JUNB	jun B proto-oncogene	-0.102	0.932	3.64E-01	3.64E+01		
8026051	NM_006397	RNASEH2A	ribonuclease H2, subunit A	0.001	1.001	9.98E-01	9.98E+01		
8026063	NM_014975	MAST1	microtubule associated serine/thr	0.018	1.012	9.18E-01	9.18E+01		
8026090	NM_000159	GCDH	glutaryl-Coenzyme A dehydrogena	0.037	1.026	7.90E-01	7.90E+01		
8026122	NM_005053	RAD23A	RAD23 homolog A (S. cerevisiae)	-0.048	0.967	7.20E-01	7.20E+01		

8026133	NM_152654	DAND5	DAN domain family, member 5	-0.055	0.963	6.51E-01	6.51E+01		
8026139	NM_002501	NFIX	nuclear factor I/X (CCAAT-binding	-0.053	0.964	5.91E-01	5.91E+01		
8026155	NM_052876	NACC1	nucleus accumbens associated 1, E	-0.008	0.994	9.68E-01	9.68E+01		
8026163	NM_004907	IER2	immediate early response 2	-0.086	0.942	4.83E-01	4.83E+01		
8026170	NM_030818	CCDC130	coiled-coil domain containing 130	-0.072	0.952	6.12E-01	6.12E+01		
8026182	NM_00103172	MRI1	methylthioribose-1-phosphate iso	-0.034	0.977	8.06E-01	8.06E+01		
8026193	NM_014047	C19orf53	chromosome 19 open reading fran	-0.061	0.959	6.15E-01	6.15E+01		
8026198	NM_023072	ZSWIM4	zinc finger, SWIM-type containing	-0.041	0.972	6.54E-01	6.54E+01		
8026212	---	---	---	-0.052	0.965	7.28E-01	7.28E+01		
8026214	NM_00109862	NANOS3	nanos homolog 3 (Drosophila)	0.047	1.033	6.01E-01	6.01E+01		
8026217	NM_017721	CC2D1A	coiled-coil and C2 domain contain	-0.022	0.985	8.18E-01	8.18E+01		
8026250	NM_138353	DCAF15	DDB1 and CUL4 associated factor	-0.033	0.977	8.03E-01	8.03E+01		
8026265	NM_080864	RLN3	relaxin 3	0.018	1.013	9.08E-01	9.08E+01		
8026272	NM_004843	IL27RA	interleukin 27 receptor, alpha	-0.019	0.987	9.15E-01	9.15E+01		
8026292	---	---	---	-0.113	0.925	1.75E-01	1.75E+01		
8026294	NR_024282	LOC113230	hypothetical protein LOC113230	-0.051	0.965	6.66E-01	6.66E+01		
8026298	---	---	---	0.037	1.026	7.88E-01	7.88E+01		
8026300	NM_078481	CD97	CD97 molecule	0.055	1.039	7.73E-01	7.73E+01		
8026315	NM_213560	PKN1	protein kinase N1	-0.042	0.971	8.05E-01	8.05E+01		
8026339	NM_003096	SNRPG	small nuclear ribonucleoprotein p	-0.016	0.989	9.42E-01	9.42E+01		
8026341	NM_138501	TECR	trans-2,3-enoyl-CoA reductase	-0.054	0.963	7.91E-01	7.91E+01		
8026350	NM_207390	CLEC17A	C-type lectin domain family 17, m	0.000	1.000	1.00E+00	1.00E+02		
8026361	---	---	---	-0.006	0.996	9.88E-01	9.88E+01		
8026363	---	---	---	0.068	1.048	8.24E-01	8.24E+01		
8026388	NM_012377	OR7C2	olfactory receptor, family 7, subfa	-0.060	0.959	7.23E-01	7.23E+01		
8026390	NM_173482	CCDC105	coiled-coil domain containing 105	-0.026	0.982	8.48E-01	8.48E+01		
8026405	NM_00100471	OR1I1	olfactory receptor, family 1, subfa	0.060	1.043	6.16E-01	6.16E+01		
8026420	---	---	---	0.205	1.152	2.15E-01	2.15E+01		
8026422	---	---	---	0.017	1.012	9.59E-01	9.59E+01		
8026440	NM_000984	RPL23A	ribosomal protein L23a	-0.011	0.993	9.36E-01	9.36E+01		
8026488	NM_00100446	OR10H5	olfactory receptor, family 10, subf	-0.068	0.954	7.24E-01	7.24E+01		
8026503	NR_024335	FLJ25328	hypothetical LOC148231	0.013	1.009	9.56E-01	9.56E+01		
8026513	NM_00114516	TPM4	tropomyosin 4	-0.022	0.985	8.67E-01	8.67E+01		
8026520	NM_005370	RAB8A	RAB8A, member RAS oncogene fa	0.165	1.121	2.35E-01	2.35E+01		
8026533	NM_032855	HSH2D	hematopoietic SH2 domain contai	0.061	1.043	7.27E-01	7.27E+01		
8026541	AF151902	FAM32A	family with sequence similarity 32	0.098	1.071	5.15E-01	5.15E+01		
8026548	NM_00113052	AP1M1	adaptor-related protein complex 1	-0.090	0.939	3.03E-01	3.03E+01		
8026564	NM_016270	KLF2	Kruppel-like factor 2 (lung)	-0.013	0.991	9.43E-01	9.43E+01		
8026568	NM_032207	C19orf44	chromosome 19 open reading fran	-0.017	0.988	9.22E-01	9.22E+01		
8026579	NM_024074	TMEM38A	transmembrane protein 38A	-0.040	0.972	7.57E-01	7.57E+01		
8026610	NM_015260	SIN3B	SIN3 homolog B, transcription reg	-0.047	0.968	7.50E-01	7.50E+01		
8026638	NM_004145	MYO9B	myosin IXB	0.059	1.042	6.82E-01	6.82E+01		
8026679	NM_018467	USE1	unconventional SNARE in the ER 1	-0.062	0.958	6.24E-01	6.24E+01		
8026687	NM_024578	OCEL1	occludin/ELL domain containing 1	-0.017	0.988	9.11E-01	9.11E+01		
8026698	NM_00103354	C19orf62	chromosome 19 open reading fran	-0.046	0.969	7.11E-01	7.11E+01		
8026712	NM_152363	ANKLE1	ankyrin repeat and LEM domain co	-0.069	0.953	4.34E-01	4.34E+01		
8026724	NM_023937	MRPL34	mitochondrial ribosomal protein L	-0.067	0.955	5.84E-01	5.84E+01		
8026729	NM_024050	DDA1	DET1 and DDB1 associated 1	-0.006	0.996	9.80E-01	9.80E+01		
8026735	NM_133644	GTPBP3	GTP binding protein 3 (mitochond	0.007	1.005	9.75E-01	9.75E+01		
8026751	NM_138401	FAM125A	family with sequence similarity 12	-0.010	0.993	9.56E-01	9.56E+01		
8026787	NM_173544	FAM129C	family with sequence similarity 12	0.262	1.199	9.54E-03	9.54E-01		
8026806	NM_024656	GLT25D1	glycosyltransferase 25 domain cor	0.144	1.105	2.49E-01	2.49E+01		
8026821	NM_018174	MAP1S	microtubule-associated protein 1S	-0.028	0.980	7.56E-01	7.56E+01		
8026830	NM_015122	FCHO1	FCH domain only 1	-0.010	0.993	9.57E-01	9.57E+01		
8026861	NM_014256	B3GNT3	UDP-GlcNAc:betaGal beta-1,3-N-a	-0.039	0.973	6.93E-01	6.93E+01		
8026868	NM_000980	RPL18A	ribosomal protein L18a	-0.001	0.999	9.97E-01	9.97E+01		
8026877	NM_000453	SLC5A5	solute carrier family 5 (sodium iod	0.018	1.013	8.95E-01	8.95E+01		
8026895	NM_138442	CCDC124	coiled-coil domain containing 124	0.018	1.013	8.71E-01	8.71E+01		
8026900	NM_002248	KCNN1	potassium intermediate/small cor	-0.001	1.000	9.98E-01	9.98E+01		
8026915	NM_015683	ARRDC2	arrestin domain containing 2	-0.108	0.928	2.50E-01	2.50E+01		
8026926	NM_015016	MAST3	microtubule associated serine/thr	-0.106	0.929	2.81E-01	2.81E+01		
8026954	NM_005027	PIK3R2	phosphoinositide-3-kinase, regula	-0.019	0.987	9.17E-01	9.17E+01		
8026982	NM_032683	MPV17L2	MPV17 mitochondrial membrane	-0.002	0.999	9.89E-01	9.89E+01		
8026991	NM_017712	PGPEP1	pyroglutamyl-peptidase I	-0.026	0.982	8.51E-01	8.51E+01		
8027002	NM_004864	GDF15	growth differentiation factor 15	-0.042	0.972	8.05E-01	8.05E+01		
8027006	NM_032627	SSBP4	single stranded DNA binding prote	0.050	1.035	6.54E-01	6.54E+01		
8027018	BC001080	C19orf50	chromosome 19 open reading fran	-0.043	0.971	6.10E-01	6.10E+01		
8027024	NM_00103393	UBA52	ubiquitin A-52 residue ribosomal p	-0.002	0.999	9.93E-01	9.93E+01		
8027032	NM_00110041	C19orf60	chromosome 19 open reading fran	-0.067	0.955	3.75E-01	3.75E+01		
8027040	NM_012109	TMEM59L	transmembrane protein 59-like	0.006	1.004	9.73E-01	9.73E+01		
8027049	NM_018316	KLHL26	kelch-like 26 (Drosophila)	-0.009	0.994	9.63E-01	9.63E+01		
8027053	NM_00109848	CRTC1	CREB regulated transcription coac	0.022	1.016	8.92E-01	8.92E+01		
8027074	NM_002911	UPF1	UPF1 regulator of nonsense transc	-0.046	0.968	6.96E-01	6.96E+01		
8027100	NM_019070	DDX49	DEAD (Asp-Glu-Ala-Asp) box polyp	0.055	1.039	4.29E-01	4.29E+01		
8027117	NM_033415	ARMC6	armadillo repeat containing 6	-0.036	0.975	7.72E-01	7.72E+01		
8027128	NM_178526	SLC25A42	solute carrier family 25, member 4	-0.030	0.980	7.90E-01	7.90E+01		
8027139	NM_003721	RFXANK	regulatory factor X-associated ank	0.078	1.056	5.89E-01	5.89E+01		

8027152	NM_004386	NCAN	neurocan	-0.027	0.982	8.80E-01	8.80E+01		
8027169	NM_017660	GATAD2A	GATA zinc finger domain containin	-0.023	0.984	8.05E-01	8.05E+01		
8027184	NM_015329	KIAA0892	KIAA0892	-0.049	0.966	6.24E-01	6.24E+01		
8027205	NM_015965	NDUFA13	NADH dehydrogenase (ubiquinone	0.099	1.071	5.59E-01	5.59E+01		
8027213	NM_198537	YJEFN3	YjeF N-terminal domain containing	-0.046	0.968	6.80E-01	6.80E+01		
8027222	NM_153221	CILP2	cartilage intermediate layer protei	-0.003	0.998	9.85E-01	9.85E+01		
8027233	NM_033204	ZNF101	zinc finger protein 101	0.031	1.022	8.92E-01	8.92E+01		
8027247	NM_031218	ZNF93	zinc finger protein 93	0.196	1.146	1.11E-01	1.11E+01		
8027258	AK298173	ZNF90	zinc finger protein 90	-0.079	0.947	6.41E-01	6.41E+01		
8027268	BC067843	ZNF66	zinc finger protein 66	-0.100	0.933	5.23E-01	5.23E+01		
8027272	NM_003429	ZNF85	zinc finger protein 85	0.098	1.070	3.80E-01	3.80E+01		
8027279	NM_025189	ZNF430	zinc finger protein 430	0.052	1.037	7.75E-01	7.75E+01		
8027285	NM_182515	ZNF714	zinc finger protein 714	0.042	1.030	8.16E-01	8.16E+01		
8027297	NR_027130	ZNF738	zinc finger protein 738	0.008	1.005	9.78E-01	9.78E+01		
8027320	NR_024523	LOC641367	cyclin Y-like pseudogene	0.052	1.037	8.31E-01	8.31E+01		
8027330	NM_00101166	PCGF6	polycomb group ring finger 6	0.048	1.034	8.77E-01	8.77E+01		
8027343	AK302042	LOC440518	similar to Golgin subfamily A mem	0.009	1.006	9.75E-01	9.75E+01		
8027345	NM_020855	ZNF492	zinc finger protein 492	-0.042	0.971	9.01E-01	9.01E+01		
8027348	AK131472	ZNF730	zinc finger protein 730	0.153	1.112	5.24E-01	5.24E+01		
8027352	---	---	---	-0.009	0.994	9.75E-01	9.75E+01		
8027368	NM_203282	ZNF254	zinc finger protein 254	0.060	1.042	6.66E-01	6.66E+01		
8027377	---	---	---	-0.077	0.948	6.50E-01	6.50E+01		
8027381	---	---	---	-0.029	0.980	7.73E-01	7.73E+01		
8027385	NM_00114633	VSTM2B	V-set and transmembrane domain	-0.010	0.993	9.45E-01	9.45E+01		
8027390	NM_006627	POP4	processing of precursor 4, ribonuc	0.072	1.051	7.09E-01	7.09E+01		
8027398	NM_024310	PLEKHF1	pleckstrin homology domain conta	-0.063	0.958	5.76E-01	5.76E+01		
8027414	---	---	---	-0.023	0.984	9.27E-01	9.27E+01		
8027416	NM_003796	C19orf2	chromosome 19 open reading fran	-0.062	0.958	6.45E-01	6.45E+01		
8027429	---	---	---	0.063	1.045	8.47E-01	8.47E+01		
8027437	---	---	---	0.010	1.007	9.83E-01	9.83E+01		
8027439	NM_00113615	ZNF507	zinc finger protein 507	0.009	1.006	9.78E-01	9.78E+01		
8027473	NM_004708	PDCD5	programmed cell death 5	-0.027	0.981	8.45E-01	8.45E+01		
8027510	NM_152266	C19orf40	chromosome 19 open reading fran	0.009	1.006	9.63E-01	9.63E+01		
8027521	NM_018025	GPATCH1	G patch domain containing 1	0.024	1.017	9.17E-01	9.17E+01		
8027556	NM_002333	LRP3	low density lipoprotein receptor-r	-0.092	0.938	2.65E-01	2.65E+01		
8027566	NM_001806	CEBPG	CCAAT/enhancer binding protein (	0.018	1.013	9.17E-01	9.17E+01		
8027574	NM_022467	CHST8	carbohydrate (N-acetylgalactosam	-0.041	0.972	6.89E-01	6.89E+01		
8027584	NM_024076	KCTD15	potassium channel tetramerisation	0.038	1.027	7.72E-01	7.72E+01		
8027592	NM_00111409	LSM14A	LSM14A, SCD6 homolog A (S. cere	0.009	1.006	9.44E-01	9.44E+01		
8027604	NM_014686	KIAA0355	KIAA0355	-0.032	0.978	8.48E-01	8.48E+01		
8027621	NM_000175	GPI	glucose phosphate isomerase	0.112	1.080	4.74E-01	4.74E+01		
8027642	NM_032346	PDCD2L	programmed cell death 2-like	-0.085	0.943	6.46E-01	6.46E+01		
8027650	NM_005499	UBA2	ubiquitin-like modifier activating e	0.027	1.019	8.68E-01	8.68E+01		
8027665	NM_00108043	WTIP	Wilms tumor 1 interacting protein	-0.011	0.992	9.47E-01	9.47E+01		
8027674	NM_018443	ZNF302	zinc finger protein 302	-0.013	0.991	9.58E-01	9.58E+01		
8027685	NM_00102999	ZNF181	zinc finger protein 181	-0.061	0.958	7.97E-01	7.97E+01		
8027701	NM_020895	GRAMD1A	GRAM domain containing 1A	-0.222	0.857	2.37E-02	2.37E+00		
8027719	NM_001037	SCN1B	sodium channel, voltage-gated, ty	-0.069	0.953	5.17E-01	5.17E+01		
8027728	NM_182983	HPN	hepsin	-0.050	0.966	5.75E-01	5.75E+01		
8027748	NM_021910	FXYD3	FXYD domain containing ion trans	-0.044	0.970	7.36E-01	7.36E+01		
8027760	NM_005031	FXYD1	FXYD domain containing ion trans	-0.018	0.988	9.09E-01	9.09E+01		
8027770	NM_022006	FXYD7	FXYD domain containing ion trans	0.001	1.001	9.96E-01	9.96E+01		
8027778	NM_144779	FXYD5	FXYD domain containing ion trans	-0.014	0.991	9.63E-01	9.63E+01		
8027793	NM_205834	LSR	lipolysis stimulated lipoprotein rec	-0.071	0.952	2.98E-01	2.98E+01		
8027805	NM_003367	USF2	upstream transcription factor 2, c-	-0.036	0.975	7.97E-01	7.97E+01		
8027819	NM_021175	HAMP	hepcidin antimicrobial peptide	0.002	1.001	9.92E-01	9.92E+01		
8027837	NM_001771	CD22	CD22 molecule	0.274	1.209	1.35E-01	1.35E+01		
8027854	NM_005303	FFAR1	free fatty acid receptor 1	-0.048	0.967	6.57E-01	6.57E+01		
8027856	NM_005304	FFAR3	free fatty acid receptor 3	-0.056	0.962	5.95E-01	5.95E+01		
8027876	NM_032635	TMEM147	transmembrane protein 147	0.061	1.043	6.23E-01	6.23E+01		
8027884	---	---	---	-0.039	0.973	8.78E-01	8.78E+01		
8027886	NM_015302	HAUS5	HAUS augmin-like complex, subun	-0.026	0.982	8.08E-01	8.08E+01		
8027908	NM_024321	RBM42	RNA binding motif protein 42	-0.081	0.945	5.53E-01	5.53E+01		
8027920	NM_014209	ETV2	ets variant 2	-0.005	0.996	9.77E-01	9.77E+01		
8027932	NM_001863	COX6B1	cytochrome c oxidase subunit Vib	-0.002	0.999	9.91E-01	9.91E+01		
8027947	NM_014383	ZBTB32	zinc finger and BTB domain contai	0.006	1.004	9.76E-01	9.76E+01		
8027956	NM_014727	MLL4	myeloid/lymphoid or mixed-lineag	-0.005	0.997	9.75E-01	9.75E+01		
8027996	NM_172341	PSENEN	presenilin enhancer 2 homolog (C.	0.041	1.029	8.13E-01	8.13E+01		
8028002	---	---	---	-0.175	0.886	6.66E-01	6.66E+01		
8028004	NM_019104	LIN37	lin-37 homolog (C. elegans)	-0.065	0.956	5.86E-01	5.86E+01		
8028016	NM_00103988	C19orf55	chromosome 19 open reading fran	-0.024	0.983	8.71E-01	8.71E+01		
8028030	NM_052948	SNX26	sorting nexin 26	-0.070	0.953	4.41E-01	4.41E+01		
8028058	NM_199180	KIRREL2	kin of IRRE like 2 (Drosophila)	0.060	1.043	6.78E-01	6.78E+01		
8028102	---	---	---	-0.021	0.985	9.24E-01	9.24E+01		
8028104	NM_014266	HCST	hematopoietic cell signal transduc	0.260	1.198	1.43E-02	1.43E+00		
8028112	NM_024509	LRFN3	leucine rich repeat and fibronectin	-0.019	0.987	9.33E-01	9.33E+01		
8028117	---	---	---	-0.156	0.898	2.41E-01	2.41E+01		

8028119	NM_00108396	WDR62	WD repeat domain 62	-0.088	0.941	7.90E-02	7.90E+00		
8028158	---	---	---	0.006	1.004	9.76E-01	9.76E+01		
8028162	NM_001281	TBCB	tubulin folding cofactor B	-0.042	0.971	7.52E-01	7.52E+01		
8028172	NM_001749	CAPNS1	calpain, small subunit 1	-0.081	0.946	6.70E-01	6.70E+01		
8028186	NM_007145	ZNF146	zinc finger protein 146	-0.020	0.986	9.14E-01	9.14E+01		
8028190	ENST00000418	LOC100131606	hypothetical protein LOC10013160	-0.115	0.923	3.94E-01	3.94E+01		
8028192	---	---	---	-0.103	0.931	3.16E-01	3.16E+01		
8028219	NM_144689	ZNF420	zinc finger protein 420	0.010	1.007	9.74E-01	9.74E+01		
8028233	NM_181786	HKR1	GLI-Kruppel family member HKR1	-0.005	0.997	9.85E-01	9.85E+01		
8028241	NM_032453	ZNF527	zinc finger protein 527	-0.044	0.970	7.87E-01	7.87E+01		
8028248	NM_144694	ZNF570	zinc finger protein 570	0.000	1.000	1.00E+00	1.00E+02		
8028286	NM_015073	SIPA1L3	signal-induced proliferation-associ	-0.075	0.950	3.89E-01	3.89E+01		
8028309	---	---	---	0.052	1.037	8.92E-01	8.92E+01		
8028311	NM_021102	SPINT2	serine peptidase inhibitor, Kunitz t	0.030	1.021	8.78E-01	8.78E+01		
8028323	---	---	---	0.009	1.006	9.74E-01	9.74E+01		
8028325	NM_033520	C19orf33	chromosome 19 open reading fran	-0.049	0.966	6.44E-01	6.44E+01		
8028332	NM_004823	KCNK6	potassium channel, subfamily K, m	0.090	1.065	3.09E-01	3.09E+01		
8028341	NM_021185	CATSPERG	cation channel, sperm-associated,	-0.061	0.959	5.24E-01	5.24E+01		
8028380	NM_002812	PSMD8	proteasome (prosome, macropain	0.030	1.021	8.91E-01	8.91E+01		
8028389	NM_00104252	SPRED3	sprouty-related, EVH1 domain cor	-0.087	0.941	4.48E-01	4.48E+01		
8028397	NM_174905	FAM98C	family with sequence similarity 98	-0.107	0.928	2.02E-01	2.02E+01		
8028407	NM_000540	RYR1	ryanodine receptor 1 (skeletal)	0.020	1.014	8.08E-01	8.08E+01		
8028514	NM_013234	EIF3K	eukaryotic translation initiation fa	0.010	1.007	9.67E-01	9.67E+01		
8028524	NM_004924	ACTN4	actinin, alpha 4	-0.054	0.963	7.94E-01	7.94E+01		
8028546	NM_002307	LGALS7	lectin, galactoside-binding, soluble	-0.012	0.992	9.30E-01	9.30E+01		
8028552	NM_002503	NFKBIB	nuclear factor of kappa light polyp	-0.056	0.962	5.48E-01	5.48E+01		
8028583	NM_00101483	PAK4	p21 protein (Cdc42/Rac)-activated	-0.020	0.986	8.63E-01	8.63E+01		
8028600	NM_00100141	NCCRP1	non-specific cytotoxic cell recepto	-0.058	0.961	3.98E-01	3.98E+01		
8028607	NM_172138	IL28A	interleukin 28A (interferon, lambd	-0.034	0.977	8.68E-01	8.68E+01		
8028613	NM_172140	IL29	interleukin 29 (interferon, lambda	0.016	1.011	9.20E-01	9.20E+01		
8028624	NM_018028	SAMD4B	sterile alpha motif domain contair	-0.089	0.940	2.82E-01	2.82E+01		
8028643	---	---	---	-0.039	0.973	8.16E-01	8.16E+01		
8028645	NM_017592	MED29	mediator complex subunit 29	-0.015	0.989	9.34E-01	9.34E+01		
8028652	NM_003407	ZFP36	zinc finger protein 36, C3H type, h	-0.108	0.928	3.54E-01	3.54E+01		
8028656	NM_022835	PLEKHG2	pleckstrin homology domain conta	-0.093	0.938	5.30E-01	5.30E+01		
8028674	NM_003169	SUPT5H	suppressor of Ty 5 homolog (S. cer	-0.188	0.878	3.13E-03	3.13E-01		
8028705	NM_00100156	TIMM50	translocase of inner mitochondria	-0.008	0.994	9.70E-01	9.70E+01		
8028730	NM_182704	SELV	selenoprotein V	-0.005	0.996	9.72E-01	9.72E+01		
8028750	NM_203471	LGALS14	lectin, galactoside-binding, soluble	-0.038	0.974	7.97E-01	7.97E+01		
8028756	NM_006503	PSMC4	proteasome (prosome, macropain	-0.068	0.954	6.88E-01	6.88E+01		
8028778	NM_002446	MAP3K10	mitogen-activated protein kinase k	-0.048	0.967	4.73E-01	4.73E+01		
8028791	NM_012268	PLD3	phospholipase D family, member 3	0.260	1.198	6.01E-03	6.01E-01		
8028806	NM_020971	SPTBN4	spectrin, beta, non-erythrocytic 4	-0.052	0.964	5.84E-01	5.84E+01		
8028851	NM_138392	SHKBP1	SH3KBP1 binding protein 1	-0.048	0.967	6.90E-01	6.90E+01		
8028872	NM_00104254	LTBP4	latent transforming growth factor	0.006	1.004	9.66E-01	9.66E+01		
8028908	NM_025194	ITPKC	inositol 1,4,5-trisphosphate 3-kina	-0.063	0.957	5.55E-01	5.55E+01		
8028916	NM_004596	SNRPA	small nuclear ribonucleoprotein p	0.130	1.094	2.65E-01	2.65E+01		
8028924	NM_006533	MIA	melanoma inhibitory activity	-0.006	0.996	9.76E-01	9.76E+01		
8028930	NM_016154	RAB4B	RAB4B, member RAS oncogene fa	-0.006	0.996	9.79E-01	9.79E+01		
8028940	NM_080732	EGLN2	egl nine homolog 2 (C. elegans)	0.003	1.002	9.88E-01	9.88E+01		
8028973	NM_000766	CYP2A13	cytochrome P450, family 2, subfar	-0.046	0.969	6.24E-01	6.24E+01		
8028984	NM_000774	CYP2F1	cytochrome P450, family 2, subfar	0.022	1.015	9.49E-01	9.49E+01		
8028991	NM_030622	CYP2S1	cytochrome P450, family 2, subfar	0.063	1.044	5.84E-01	5.84E+01		
8029006	NM_021913	AXL	AXL receptor tyrosine kinase	-0.017	0.988	8.93E-01	8.93E+01		
8029029	NM_007040	HNRNPUL1	heterogeneous nuclear ribonucleo	-0.087	0.941	2.68E-01	2.68E+01		
8029050	NM_052848	CCDC97	coiled-coil domain containing 97	-0.104	0.931	2.63E-01	2.63E+01		
8029056	NM_00104259	TMEM91	transmembrane protein 91	-0.018	0.988	9.02E-01	9.02E+01		
8029065	NM_000709	BCKDHA	branched chain keto acid dehydro	-0.008	0.995	9.74E-01	9.74E+01		
8029076	NM_00109850	CEACAM21	carcinoembryonic antigen-related	0.125	1.090	3.10E-01	3.10E+01		
8029107	NM_001815	CEACAM3	carcinoembryonic antigen-related	-0.031	0.979	7.95E-01	7.95E+01		
8029117	NM_00104028	DMRTC2	DMRT-like family C2	-0.020	0.986	8.97E-01	8.97E+01		
8029129	NM_001022	RPS19	ribosomal protein S19	-0.081	0.945	4.49E-01	4.49E+01		
8029136	NM_001783	CD79A	CD79a molecule, immunoglobulin-	0.259	1.196	7.19E-02	7.19E+00		
8029147	NM_199002	ARHGEF1	Rho guanine nucleotide exchange	-0.120	0.920	2.12E-01	2.12E+01		
8029182	NM_022752	ZNF574	zinc finger protein 574	-0.035	0.976	8.48E-01	8.48E+01		
8029188	NM_133444	ZNF526	zinc finger protein 526	-0.030	0.979	8.54E-01	8.54E+01		
8029193	NM_015125	CIC	capicua homolog (Drosophila)	-0.038	0.974	7.07E-01	7.07E+01		
8029219	NM_173633	TMEM145	transmembrane protein 145	-0.005	0.997	9.85E-01	9.85E+01		
8029236	NM_001410	MEGF8	multiple EGF-like-domains 8	-0.025	0.982	8.31E-01	8.31E+01		
8029273	NM_001410	MEGF8	multiple EGF-like-domains 8	-0.018	0.988	9.54E-01	9.54E+01		
8029280	NM_020406	CD177	CD177 molecule	-0.008	0.994	9.73E-01	9.73E+01		
8029299	---	---	---	0.012	1.008	9.61E-01	9.61E+01		
8029301	NM_174945	ZNF575	zinc finger protein 575	-0.028	0.981	8.13E-01	8.13E+01		
8029311	NM_024327	ZNF576	zinc finger protein 576	-0.146	0.904	1.78E-01	1.78E+01		
8029318	NM_019612	IRGC	immunity-related GTPase family, c	0.006	1.004	9.77E-01	9.77E+01		
8029340	NM_003445	ZNF155	zinc finger protein 155	-0.043	0.971	8.88E-01	8.88E+01		
8029375	---	---	---	-0.038	0.974	8.35E-01	8.35E+01		

8029377	NM_013398	ZNF224	zinc finger protein 224	0.014	1.009	9.49E-01	9.49E+01		
8029385	NM_013362	ZNF225	zinc finger protein 225	0.075	1.053	6.51E-01	6.51E+01		
8029392	NM_006630	ZNF234	zinc finger protein 234	0.096	1.068	5.91E-01	5.91E+01		
8029399	NM_00114622	ZNF226	zinc finger protein 226	-0.055	0.962	7.01E-01	7.01E+01		
8029413	NM_182490	ZNF227	zinc finger protein 227	-0.033	0.977	8.80E-01	8.80E+01		
8029423	NM_181756	ZNF233	zinc finger protein 233	0.059	1.042	7.45E-01	7.45E+01		
8029437	NM_006505	PVR	poliovirus receptor	-0.135	0.911	1.82E-01	1.82E+01		
8029446	NM_00112789	CEACAM19	carcinoembryonic antigen-related	-0.028	0.981	8.31E-01	8.31E+01		
8029458	NM_00103921	CEACAM16	carcinoembryonic antigen-related	-0.018	0.987	8.82E-01	8.82E+01		
8029465	NM_005178	BCL3	B-cell CLL/lymphoma 3	0.014	1.010	9.47E-01	9.47E+01		
8029476	NM_012116	CBLC	Cas-Br-M (murine) ecotropic retro	-0.024	0.983	8.63E-01	8.63E+01		
8029507	NM_002856	PVRL2	poliovirus receptor-related 2 (herp	0.025	1.018	8.89E-01	8.89E+01		
8029521	NM_00112891	TOMM40	translocase of outer mitochondria	-0.005	0.996	9.78E-01	9.78E+01		
8029530	NM_000041	APOE	apolipoprotein E	0.038	1.027	6.75E-01	6.75E+01		
8029541	NM_001646	APOC4	apolipoprotein C-IV	0.081	1.058	1.08E-01	1.08E+01		
8029551	NM_000483	APOC2	apolipoprotein C-II	0.015	1.010	9.01E-01	9.01E+01		
8029560	NM_001294	CLPTM1	cleft lip and palate associated tran	0.034	1.024	7.83E-01	7.83E+01		
8029580	NM_006509	RELB	v-rel reticuloendotheliosis viral on	-0.056	0.962	7.25E-01	7.25E+01		
8029592	NM_007056	SFRS16	splicing factor, arginine/serine-rich	0.021	1.015	9.33E-01	9.33E+01		
8029615	---	---	---	0.316	1.245	2.44E-01	2.44E+01		
8029624	ENST00000421	LRRC68	leucine rich repeat containing 68	0.038	1.027	6.58E-01	6.58E+01		
8029640	NM_212550	BLOC1S3	biogenesis of lysosomal organelles	-0.085	0.942	2.63E-01	2.63E+01		
8029642	NM_031417	MARK4	MAP/microtubule affinity-regulati	-0.004	0.997	9.82E-01	9.82E+01		
8029671	NM_177417	KLC3	kinesin light chain 3	-0.051	0.965	4.32E-01	4.32E+01		
8029688	NM_012099	CD3EAP	CD3e molecule, epsilon associated	-0.088	0.941	2.45E-01	2.45E+01		
8029701	NM_00108040	FLJ40125	protein phosphatase 1B-like	0.082	1.058	7.07E-01	7.07E+01		
8029710	NM_003370	VASP	vasodilator-stimulated phosphopr	-0.039	0.973	7.68E-01	7.68E+01		
8029728	NM_000164	GIPR	gastric inhibitory polypeptide rece	-0.052	0.964	5.97E-01	5.97E+01		
8029744	NM_017659	QPCTL	glutaminyl-peptide cyclotransferas	0.003	1.002	9.86E-01	9.86E+01		
8029754	NM_004497	FOXA3	forkhead box A3	-0.037	0.975	7.64E-01	7.64E+01		
8029759	NM_00108040	CCDC61	coiled-coil domain containing 61	-0.076	0.949	5.21E-01	5.21E+01		
8029773	NM_00100291	IGFL2	IGF-like family member 2	-0.052	0.965	6.68E-01	6.68E+01		
8029779	NM_198541	IGFL1	IGF-like family member 1	-0.070	0.953	4.47E-01	4.47E+01		
8029784	NM_152794	HIF3A	hypoxia inducible factor 3, alpha s	-0.060	0.959	5.52E-01	5.52E+01		
8029814	NM_006247	PPP5C	protein phosphatase 5, catalytic su	0.061	1.043	6.70E-01	6.70E+01		
8029831	NM_005184	CALM3	calmodulin 3 (phosphorylase kinas	0.072	1.051	5.09E-01	5.09E+01		
8029845	---	---	---	-0.019	0.987	9.65E-01	9.65E+01		
8029847	NM_00103988	FKRP	fukutin related protein	-0.015	0.990	9.16E-01	9.16E+01		
8029854	NM_005628	SLC1A5	solute carrier family 1 (neutral am	0.042	1.029	6.81E-01	6.81E+01		
8029856	NM_004491	GRLF1	glucocorticoid receptor DNA bindi	-0.028	0.981	8.98E-01	8.98E+01		
8029870	NM_002517	NPAS1	neuronal PAS domain protein 1	0.023	1.016	8.56E-01	8.56E+01		
8029884	NR_027280	SAE1	SUMO1 activating enzyme subunit	-0.033	0.977	8.03E-01	8.03E+01		
8029894	NM_015603	CCDC9	coiled-coil domain containing 9	-0.082	0.944	3.84E-01	3.84E+01		
8029907	NM_001736	C5AR1	complement component 5a recep	0.225	1.169	4.93E-01	4.93E+01		
8029918	NM_014681	DHX34	DEAH (Asp-Glu-Ala-His) box polype	-0.076	0.949	4.64E-01	4.64E+01		
8029937	---	---	---	0.024	1.017	8.78E-01	8.78E+01		
8029939	NM_015711	GLTSCR1	glioma tumor suppressor candidat	0.023	1.016	8.27E-01	8.27E+01		
8029950	NM_014601	EHD2	EH-domain containing 2	-0.027	0.981	8.13E-01	8.13E+01		
8029958	NM_015710	GLTSCR2	glioma tumor suppressor candidat	-0.016	0.989	9.36E-01	9.36E+01		
8029969	NM_003009	SEPW1	selenoprotein W, 1	0.024	1.017	9.07E-01	9.07E+01		
8029996	BC043386	C19orf68	chromosome 19 open reading fran	-0.033	0.977	7.83E-01	7.83E+01		
8030007	NM_001425	EMP3	epithelial membrane protein 3	0.118	1.086	6.51E-01	6.51E+01		
8030015	NM_012451	SYNGR4	synaptogyrin 4	0.012	1.008	9.44E-01	9.44E+01		
8030022	NM_000836	GRIN2D	glutamate receptor, ionotropic, N-	-0.112	0.925	2.08E-01	2.08E+01		
8030035	NM_031485	GRWD1	glutamate-rich WD repeat contain	-0.018	0.987	9.01E-01	9.01E+01		
8030044	NM_170720	KCNJ14	potassium inwardly-rectifying char	0.005	1.004	9.77E-01	9.77E+01		
8030049	NM_017457	CYTH2	cytohesin 2	-0.238	0.848	2.50E-02	2.50E+00		
8030064	NM_017457	CYTH2	cytohesin 2	-0.141	0.907	5.11E-01	5.11E+01		
8030067	NM_004605	SULT2B1	sulfotransferase family, cytosolic,	-0.037	0.974	7.61E-01	7.61E+01		
8030078	NM_020126	SPHK2	sphingosine kinase 2	-0.008	0.994	9.73E-01	9.73E+01		
8030092	NR_004401	SEC1	secretory blood group 1	-0.115	0.923	3.79E-01	3.79E+01		
8030094	NM_000511	FUT2	fucosyltransferase 2 (secretor stat	-0.068	0.954	7.03E-01	7.03E+01		
8030113	NM_006666	RUVBL2	RuvB-like 2 (E. coli)	-0.123	0.918	3.04E-01	3.04E+01		
8030133	NM_006184	NUCB1	nucleobindin 1	0.116	1.084	3.60E-01	3.60E+01		
8030148	NM_014475	DHDH	dihydrodiol dehydrogenase (dimer	0.018	1.013	9.23E-01	9.23E+01		
8030158	NR_027882	BAX	BCL2-associated X protein	-0.076	0.949	4.88E-01	4.88E+01		
8030171	NM_000146	FTL	ferritin, light polypeptide	0.263	1.200	2.74E-01	2.74E+01		
8030181	NM_033378	CGB2	chorionic gonadotropin, beta poly	-0.084	0.943	2.79E-01	2.79E+01		
8030187	NM_033043	CGB5	chorionic gonadotropin, beta poly	-0.066	0.955	2.01E-01	2.01E+01		
8030199	NM_003089	SNRNP70	small nuclear ribonucleoprotein 70	0.000	1.000	9.99E-01	9.99E+01		
8030212	NM_022165	LIN7B	lin-7 homolog B (C. elegans)	-0.117	0.922	4.48E-01	4.48E+01		
8030251	NM_017636	TRPM4	transient receptor potential cation	-0.003	0.998	9.86E-01	9.86E+01		
8030277	NM_001774	CD37	CD37 molecule	0.233	1.175	2.95E-02	2.95E+00		
8030299	NM_144688	CCDC155	coiled-coil domain containing 155	-0.040	0.973	7.52E-01	7.52E+01		
8030321	NM_153329	ALDH16A1	aldehyde dehydrogenase 16 family	-0.053	0.964	5.36E-01	5.36E+01		
8030339	NM_001459	FLT3LG	fms-related tyrosine kinase 3 ligan	0.055	1.039	5.20E-01	5.20E+01		
8030351	NM_012423	RPL13A	ribosomal protein L13a	-0.035	0.976	7.85E-01	7.85E+01		

8030360	NR_000021	SNORD32A	small nucleolar RNA, C/D box 32A	0.153	1.112	7.31E-01	7.31E+01		
8030362	NR_000020	SNORD33	small nucleolar RNA, C/D box 33	0.092	1.066	8.05E-01	8.05E+01		
8030368	NM_001015	RPS11	ribosomal protein S11	0.010	1.007	9.36E-01	9.36E+01		
8030374	NM_004107	FCGRT	Fc fragment of IgG, receptor, trans	0.145	1.106	2.83E-01	2.83E+01		
8030383	NM_020650	RCN3	reticulocalbin 3, EF-hand calcium b	0.023	1.016	9.14E-01	9.14E+01		
8030391	---	---	---	-0.088	0.941	5.77E-01	5.77E+01		
8030403	NM_020719	PRR12	proline rich 12	-0.095	0.936	2.11E-01	2.11E+01		
8030416	NM_021228	SCAF1	SR-related CTD-associated factor 1	0.069	1.049	4.57E-01	4.57E+01		
8030429	NM_138639	BCL2L12	BCL2-like 12 (proline rich)	-0.133	0.912	1.58E-01	1.58E+01		
8030437	NM_198319	PRMT1	protein arginine methyltransferase	-0.089	0.940	5.60E-01	5.60E+01		
8030470	NM_014203	AP2A1	adaptor-related protein complex 2	0.142	1.103	4.79E-02	4.79E+00		
8030498	NM_030973	MED25	mediator complex subunit 25	-0.016	0.989	8.97E-01	8.97E+01		
8030525	NM_017432	PTOV1	prostate tumor overexpressed 1	0.031	1.022	7.95E-01	7.95E+01		
8030539	NM_024682	TBC1D17	TBC1 domain family, member 17	-0.075	0.949	2.93E-01	2.93E+01		
8030557	NM_012068	ATF5	activating transcription factor 5	0.159	1.116	4.04E-01	4.04E+01		
8030569	NM_015428	ZNF473	zinc finger protein 473	-0.119	0.921	4.61E-01	4.61E+01		
8030582	NM_00114580	MYH14	myosin, heavy chain 14	-0.008	0.994	9.46E-01	9.46E+01		
8030641	NM_002691	POLD1	polymerase (DNA directed), delta	0.036	1.026	7.73E-01	7.73E+01		
8030671	NM_003121	SPIB	Spi-B transcription factor (Spi-1/PU	-0.015	0.990	9.29E-01	9.29E+01		
8030706	NM_175063	C19orf63	chromosome 19 open reading fran	0.034	1.024	8.24E-01	8.24E+01		
8030721	NM_002975	CLEC11A	C-type lectin domain family 11, me	-0.012	0.992	9.49E-01	9.49E+01		
8030736	NM_033068	ACPT	acid phosphatase, testicular	-0.011	0.993	9.49E-01	9.49E+01		
8030749	ENST00000326	MGC45922	hypothetical LOC284365	0.015	1.011	9.52E-01	9.52E+01		
8030753	NM_00103004	KLK3	kallikrein-related peptidase 3	-0.013	0.991	9.22E-01	9.22E+01		
8030768	NM_00100223	KLK2	kallikrein-related peptidase 2	-0.013	0.991	9.38E-01	9.38E+01		
8030782	NM_014441	SIGLEC9	sialic acid binding Ig-like lectin 9	0.058	1.041	8.32E-01	8.32E+01		
8030789	NM_014385	SIGLEC7	sialic acid binding Ig-like lectin 7	0.008	1.005	9.77E-01	9.77E+01		
8030804	NM_001772	CD33	CD33 molecule	0.112	1.081	3.12E-01	3.12E+01		
8030831	NM_007147	ZNF175	zinc finger protein 175	-0.031	0.979	8.81E-01	8.81E+01		
8030838	ENST00000301	FLJ30403	hypothetical protein LOC729975	-0.101	0.933	4.71E-01	4.71E+01		
8030842	---	---	---	0.028	1.020	8.96E-01	8.96E+01		
8030848	NR_024330	NCRNA00085	non-protein coding RNA 85	-0.089	0.940	3.39E-01	3.39E+01		
8030860	NM_001462	FPR2	formyl peptide receptor 2	0.343	1.269	6.54E-01	6.54E+01		
8030881	NM_014225	PPP2R1A	protein phosphatase 2 (formerly 2	-0.041	0.972	6.66E-01	6.66E+01		
8030899	NM_00101085	ZNF766	zinc finger protein 766	0.126	1.091	3.00E-01	3.00E+01		
8030908	NM_144684	ZNF480	zinc finger protein 480	-0.048	0.967	8.16E-01	8.16E+01		
8030925	NM_00114543	LOC400713	zinc finger protein LOC400713	0.044	1.031	8.89E-01	8.89E+01		
8030931	NM_032423	ZNF528	zinc finger protein 528	0.101	1.073	7.38E-01	7.38E+01		
8030946	NM_00103988	ZNF808	zinc finger protein 808	0.167	1.122	1.30E-01	1.30E+01		
8030974	ENST00000338	LOC646508	hypothetical LOC646508	-0.120	0.920	3.82E-01	3.82E+01		
8030978	NM_138374	ZNF845	zinc finger protein 845	0.124	1.090	6.42E-01	6.42E+01		
8030980	NR_003699	ZNF525	zinc finger protein 525	0.045	1.031	8.07E-01	8.07E+01		
8030982	NM_00104018	ZNF765	zinc finger protein 765	0.012	1.008	9.33E-01	9.33E+01		
8030991	NR_003148 //	47804 // LOC14	tropomyosin 3 pseudogene // tro	0.016	1.011	9.05E-01	9.05E+01		
8030993	NM_00100840	ZNF761	zinc finger protein 761	0.123	1.089	5.32E-01	5.32E+01		
8030999	NM_018555	ZNF331	zinc finger protein 331	-0.095	0.936	5.74E-01	5.74E+01		
8031011	---	---	---	-0.035	0.976	7.94E-01	7.94E+01		
8031047	NM_00102081	MYADM	myeloid-associated differentiation	-0.097	0.935	6.42E-01	6.42E+01		
8031076	NM_031896	CACNG7	calcium channel, voltage-depende	-0.003	0.998	9.87E-01	9.87E+01		
8031084	NM_031895	CACNG8	calcium channel, voltage-depende	0.014	1.010	9.12E-01	9.12E+01		
8031090	NM_145814	CACNG6	calcium channel, voltage-depende	-0.051	0.966	5.98E-01	5.98E+01		
8031097	NM_004542	NDUFA3	NADH dehydrogenase (ubiquinone	0.037	1.026	7.97E-01	7.97E+01		
8031102	NM_015629	PRPF31	PRP31 pre-mRNA processing facto	-0.036	0.975	8.32E-01	8.32E+01		
8031122	NM_014516	CNOT3	CCR4-NOT transcription complex,	-0.020	0.986	8.76E-01	8.76E+01		
8031145	NM_024075	TSEN34	tRNA splicing endonuclease 34 ho	-0.068	0.954	6.05E-01	6.05E+01		
8031152	NM_001013	RPS9	ribosomal protein S9	-0.006	0.996	9.85E-01	9.85E+01		
8031157	NM_020659	TTYH1	tweety homolog 1 (Drosophila)	-0.041	0.972	6.00E-01	6.00E+01		
8031176	NM_052925	LENG8	leukocyte receptor cluster (LRC) m	-0.029	0.980	8.25E-01	8.25E+01		
8031196	NM_002288	LAIR2	leukocyte-associated immunoglob	-0.072	0.951	6.38E-01	6.38E+01		
8031207	NM_00113091	LILRA2	leukocyte immunoglobulin-like rec	-0.026	0.982	9.54E-01	9.54E+01		
8031213	NM_006863	LILRA1	leukocyte immunoglobulin-like rec	0.283	1.217	2.08E-01	2.08E+01		
8031223	NM_006669	LILRB1	leukocyte immunoglobulin-like rec	0.308	1.238	1.27E-01	1.27E+01		
8031253	NR_003061	LILRP2	leukocyte immunoglobulin-like rec	0.046	1.032	7.57E-01	7.57E+01		
8031260	NM_153443	KIR3DL3	killer cell immunoglobulin-like rece	0.034	1.024	8.87E-01	8.87E+01		
8031277	NM_015868	KIR2DL3	killer cell immunoglobulin-like rece	0.071	1.051	8.76E-01	8.76E+01		
8031293	NM_014511	KIR2DL3	killer cell immunoglobulin-like rece	0.171	1.126	7.90E-01	7.90E+01		
8031297	NM_014218	KIR2DL1	killer cell immunoglobulin-like rece	0.111	1.080	7.92E-01	7.92E+01		
8031311	NM_013289	KIR3DL1	killer cell immunoglobulin-like rece	0.007	1.005	9.85E-01	9.85E+01		
8031328	NM_006737	KIR3DL2	killer cell immunoglobulin-like rece	0.015	1.010	9.65E-01	9.65E+01		
8031344	AY366242	KIR2DL2	killer cell immunoglobulin-like rece	0.008	1.005	9.85E-01	9.85E+01		
8031346	NM_002255	KIR2DL4	killer cell immunoglobulin-like rece	0.086	1.061	7.57E-01	7.57E+01		
8031358	NM_012314	KIR2DS4	killer cell immunoglobulin-like rece	0.100	1.072	8.04E-01	8.04E+01		
8031387	NM_004829	NCR1	natural cytotoxicity triggering rece	0.021	1.015	9.32E-01	9.32E+01		
8031441	NM_032430	BRSK1	BR serine/threonine kinase 1	-0.075	0.950	3.15E-01	3.15E+01		
8031466	NM_032701	SUV420H2	suppressor of variegation 4-20 ho	0.015	1.010	9.09E-01	9.09E+01		
8031475	NM_139172	TMEM190	transmembrane protein 190	-0.004	0.997	9.82E-01	9.82E+01		
8031483	NM_00113613	RPL28	ribosomal protein L28	-0.074	0.950	6.30E-01	6.30E+01		

8031489	NM_033113	ZNF628	zinc finger protein 628	-0.039	0.973	7.03E-01	7.03E+01		
8031495	NM_020378	NAT14	N-acetyltransferase 14 (GCN5-rela	-0.059	0.960	6.62E-01	6.62E+01		
8031499	AF086519	LOC284297	hypothetical LOC284297	-0.023	0.984	8.20E-01	8.20E+01		
8031511	NM_153219	ZNF524	zinc finger protein 524	0.008	1.006	9.61E-01	9.61E+01		
8031514	XM_00171840(	LOC100133142	similar to zinc finger protein 208	0.018	1.013	9.60E-01	9.60E+01		
8031516	NM_016202	ZNF580	zinc finger protein 580	-0.064	0.957	4.91E-01	4.91E+01		
8031522	NM_016535	ZNF581	zinc finger protein 581	-0.039	0.973	6.86E-01	6.86E+01		
8031526	NM_013301	CCDC106	coiled-coil domain containing 106	-0.031	0.979	7.99E-01	7.99E+01		
8031536	NM_007279	U2AF2	U2 small nuclear RNA auxiliary fac	-0.061	0.958	5.74E-01	5.74E+01		
8031550	NM_00113007	EPN1	epsin 1	-0.025	0.983	8.65E-01	8.65E+01		
8031568	---	---	---	-0.001	0.999	9.96E-01	9.96E+01		
8031617	NM_018337	ZNF444	zinc finger protein 444	-0.004	0.997	9.82E-01	9.82E+01		
8031622	NM_033106	GALP	galanin-like peptide	-0.024	0.984	9.08E-01	9.08E+01		
8031629	ENST00000376	ZSCAN5C	zinc finger and SCAN domain conta	0.047	1.033	8.49E-01	8.49E+01		
8031640	NM_152478	ZNF583	zinc finger protein 583	-0.025	0.983	9.28E-01	9.28E+01		
8031646	AK302988	LOC100288114	hypothetical protein LOC1002881	-0.132	0.912	8.08E-01	8.08E+01		
8031659	NM_020828	ZFP28	zinc finger protein 28 homolog (m	0.014	1.009	9.56E-01	9.56E+01		
8031669	NM_00100166	ZNF470	zinc finger protein 470	-0.013	0.991	9.65E-01	9.65E+01		
8031675	NM_021216	ZNF71	zinc finger protein 71	0.035	1.024	7.51E-01	7.51E+01		
8031690	NM_003417	ZNF264	zinc finger protein 264	-0.006	0.996	9.75E-01	9.75E+01		
8031700	NM_00101587	AURKC	aurora kinase C	-0.019	0.987	8.78E-01	8.78E+01		
8031714	NM_006635	ZNF460	zinc finger protein 460	-0.061	0.959	7.49E-01	7.49E+01		
8031720	NM_213598	ZNF543	zinc finger protein 543	-0.023	0.984	9.35E-01	9.35E+01		
8031726	NM_020657	ZNF304	zinc finger protein 304	-0.159	0.895	4.51E-01	4.51E+01		
8031732	NM_173631	ZNF547	zinc finger protein 547	0.016	1.011	9.53E-01	9.53E+01		
8031737	NM_152909	ZNF548	zinc finger protein 548	-0.035	0.976	8.67E-01	8.67E+01		
8031744	NM_006959	ZNF17	zinc finger protein 17	0.110	1.080	3.07E-01	3.07E+01		
8031750	NM_00109849	ZNF419	zinc finger protein 419	-0.033	0.978	8.13E-01	8.13E+01		
8031759	NM_198542	ZNF773	zinc finger protein 773	-0.036	0.975	8.55E-01	8.55E+01		
8031768	NM_00101087	ZIK1	zinc finger protein interacting with	0.013	1.009	9.54E-01	9.54E+01		
8031778	NM_020880	ZNF530	zinc finger protein 530	-0.066	0.955	5.33E-01	5.33E+01		
8031784	NM_003435	ZNF134	zinc finger protein 134	-0.033	0.977	8.93E-01	8.93E+01		
8031792	NM_006385	ZNF211	zinc finger protein 211	-0.087	0.941	6.95E-01	6.95E+01		
8031807	NM_138347	ZNF551	zinc finger protein 551	0.016	1.011	9.53E-01	9.53E+01		
8031815	NM_173632	ZNF776	zinc finger protein 776	-0.019	0.987	9.37E-01	9.37E+01		
8031821	NM_017652	ZNF586	zinc finger protein 586	-0.043	0.970	7.70E-01	7.70E+01		
8031825	AK299091	LOC100288322	similar to zinc finger protein 587	0.099	1.071	6.88E-01	6.88E+01		
8031827	NM_032828	ZNF587	zinc finger protein 587	0.097	1.069	4.06E-01	4.06E+01		
8031837	AF294842	ZNF587	zinc finger protein 587	-0.013	0.991	9.73E-01	9.73E+01		
8031843	NM_025027	ZNF606	zinc finger protein 606	-0.032	0.978	8.82E-01	8.82E+01		
8031847	NM_182572	ZSCAN1	zinc finger and SCAN domain conta	-0.056	0.962	4.44E-01	4.44E+01		
8031857	NM_003436	ZNF135	zinc finger protein 135	-0.111	0.926	3.26E-01	3.26E+01		
8031865	---	---	---	-0.025	0.983	8.54E-01	8.54E+01		
8031867	---	---	---	0.111	1.080	6.70E-01	6.70E+01		
8031871	NM_133502	ZNF274	zinc finger protein 274	-0.005	0.996	9.74E-01	9.74E+01		
8031884	NM_014480	ZNF544	zinc finger protein 544	-0.038	0.974	8.33E-01	8.33E+01		
8031893	NM_021089	ZNF8	zinc finger protein 8	-0.079	0.947	4.36E-01	4.36E+01		
8031899	---	---	---	-0.001	0.999	9.97E-01	9.97E+01		
8031908	NM_181846	ZSCAN22	zinc finger and SCAN domain conta	-0.066	0.955	5.15E-01	5.15E+01		
8031913	NM_005762	TRIM28	tripartite motif-containing 28	0.005	1.003	9.81E-01	9.81E+01		
8031931	---	---	---	0.009	1.006	9.82E-01	9.82E+01		
8031933	NM_001009	RPS5	ribosomal protein S5	-0.034	0.977	9.11E-01	9.11E+01		
8031939	NM_173548	ZNF584	zinc finger protein 584	-0.060	0.959	7.15E-01	7.15E+01		
8031949	NM_207395	ZNF324B	zinc finger protein 324B	-0.070	0.953	3.40E-01	3.40E+01		
8031956	NM_014347	ZNF324	zinc finger protein 324	-0.030	0.979	8.88E-01	8.88E+01		
8031962	NM_017908	ZNF446	zinc finger protein 446	-0.110	0.926	4.14E-02	4.14E+00		
8031973	NR_026052	MGC2752	hypothetical LOC65996	0.017	1.012	9.29E-01	9.29E+01		
8031981	NR_003659	WASH3P	WAS protein family homolog 3 pse	0.029	1.020	8.79E-01	8.79E+01		
8031990	NM_207366	40435	septin 14	-0.030	0.979	9.19E-01	9.19E+01		
8031992	NR_024368	tcag7.907	hypothetical LOC402483	-0.025	0.983	7.64E-01	7.64E+01		
8031997	---	---	---	0.267	1.203	4.25E-01	4.25E+01		
8031999	NM_003712	PPAP2C	phosphatidic acid phosphatase typ	-0.065	0.956	5.31E-01	5.31E+01		
8032009	NM_017550	MIER2	mesoderm induction early respons	0.039	1.027	7.82E-01	7.82E+01		
8032034	NM_00113626	FAM148C	family with sequence similarity 14	-0.023	0.984	8.88E-01	8.88E+01		
8032037	NM_012435	SHC2	SHC (Src homology 2 domain conta	-0.060	0.959	4.97E-01	4.97E+01		
8032051	NM_182577	ODF3L2	outer dense fiber of sperm tails 3-	-0.015	0.990	9.27E-01	9.27E+01		
8032057	NM_005035	POLRMT	polymerase (RNA) mitochondrial (	-0.005	0.996	9.77E-01	9.77E+01		
8032070	ENST00000358	FLJ45684	FLJ45684 locus	-0.054	0.963	7.52E-01	7.52E+01		
8032076	NM_194460	RNF126	ring finger protein 126	-0.063	0.957	5.99E-01	5.99E+01		
8032088	NM_214710	PRSSL1	protease, serine-like 1	-0.034	0.977	6.99E-01	6.99E+01		
8032094	NM_024888	PRG2	plasticity-related gene 2	-0.124	0.918	1.95E-01	1.95E+01		
8032106	NM_005481	MED16	mediator complex subunit 16	0.035	1.024	7.57E-01	7.57E+01		
8032127	NM_138774	C19orf22	chromosome 19 open reading fran	0.019	1.013	9.07E-01	9.07E+01		
8032137	NM_00103302	C19orf6	chromosome 19 open reading fran	-0.013	0.991	9.41E-01	9.41E+01		
8032149	NM_002695	POLR2E	polymerase (RNA) II (DNA directec	-0.013	0.991	9.49E-01	9.49E+01		
8032157	NM_014963	SBNO2	strawberry notch homolog 2 (Dros	-0.060	0.959	4.88E-01	4.88E+01		
8032197	NM_152769	C19orf26	chromosome 19 open reading fran	-0.066	0.955	5.20E-01	5.20E+01		

8032212	AK127759	NDUFS7	NADH dehydrogenase (ubiquinone	-0.077	0.948	5.15E-01	5.15E+01		
8032214	NM_000156	GAMT	guanidinoacetate N-methyltransfe	-0.031	0.979	8.17E-01	8.17E+01		
8032224	NM_152482	C19orf25	chromosome 19 open reading fran	-0.056	0.962	6.19E-01	6.19E+01		
8032232	NM_017573	PCSK4	proprotein convertase subtilisin/k	-0.076	0.949	3.54E-01	3.54E+01		
8032249	NM_213604	ADAMTSL5	ADAMTS-like 5	-0.095	0.936	4.13E-01	4.13E+01		
8032265	NM_203304	MEX3D	mex-3 homolog D (C. elegans)	0.002	1.001	9.91E-01	9.91E+01		
8032275	NM_003926	MBD3	methyl-CpG binding domain prote	-0.041	0.972	7.64E-01	7.64E+01		
8032284	NM_006830	UQCR	ubiquinol-cytochrome c reductase	-0.093	0.937	5.97E-01	5.97E+01		
8032290	NM_003200	TCF3	transcription factor 3 (E2A immun	0.047	1.033	6.82E-01	6.82E+01		
8032312	NM_138813	ATP8B3	ATPase, class I, type 8B, member 3	-0.014	0.990	9.19E-01	9.19E+01		
8032347	NM_020695	REXO1	REX1, RNA exonuclease 1 homolog	0.010	1.007	9.54E-01	9.54E+01		
8032365	NM_031918	KLF16	Kruppel-like factor 16	-0.045	0.969	6.14E-01	6.14E+01		
8032371	NM_031213	FAM108A1	family with sequence similarity 10	0.044	1.031	8.39E-01	8.39E+01		
8032375	NM_152771	C19orf34	chromosome 19 open reading fran	-0.056	0.962	5.29E-01	5.29E+01		
8032380	NM_017797	BTBD2	BTB (POZ) domain containing 2	-0.042	0.971	7.14E-01	7.14E+01		
8032392	NM_199054	MKNK2	MAP kinase interacting serine/thre	-0.060	0.959	6.54E-01	6.54E+01		
8032410	NM_130807	MOBKL2A	MOB1, Mps One Binder kinase act	0.029	1.021	8.07E-01	8.07E+01		
8032418	NM_003938	AP3D1	adaptor-related protein complex 3	0.061	1.043	6.25E-01	6.25E+01		
8032455	NM_018049	PLEKHJ1	pleckstrin homology domain conta	-0.022	0.985	8.64E-01	8.64E+01		
8032465	NM_144616	JSRP1	junctional sarcoplasmic reticulum	0.013	1.009	9.16E-01	9.16E+01		
8032473	NM_198532	C19orf35	chromosome 19 open reading fran	-0.040	0.973	7.47E-01	7.47E+01		
8032478	NM_00110139	LINGO3	leucine rich repeat and Ig domain	-0.045	0.969	7.67E-01	7.67E+01		
8032480	NM_016199	LSM7	LSM7 homolog, U6 small nuclear R	0.037	1.026	8.77E-01	8.77E+01		
8032484	NM_012458	TIMM13	translocase of inner mitochondrial	0.114	1.082	4.25E-01	4.25E+01		
8032491	NM_032737	LMNB2	lamin B2	-0.044	0.970	7.03E-01	7.03E+01		
8032509	NM_052847	GNG7	guanine nucleotide binding protei	0.129	1.094	3.48E-01	3.48E+01		
8032518	NM_145173	DIRAS1	DIRAS family, GTP-binding RAS-like	-0.045	0.970	6.61E-01	6.61E+01		
8032525	NM_213568	SLC39A3	solute carrier family 39 (zinc trans	-0.087	0.942	4.04E-01	4.04E+01		
8032530	NM_003021	SGTA	small glutamine-rich tetratricopep	0.002	1.001	9.92E-01	9.92E+01		
8032544	NM_021217	ZNF77	zinc finger protein 77	-0.026	0.982	9.01E-01	9.01E+01		
8032553	NM_003260	TLE2	transducin-like enhancer of split 2	-0.010	0.993	9.50E-01	9.50E+01		
8032576	NM_198969	AES	amino-terminal enhancer of split	-0.010	0.993	9.77E-01	9.77E+01		
8032588	NM_00113650	LOC284422	similar to HSPC323	-0.003	0.998	9.88E-01	9.88E+01		
8032595	NM_031304	DOHH	deoxyhypusine hydroxylase/mono	-0.076	0.949	3.62E-01	3.62E+01		
8032601	NM_032753	RAX2	retina and anterior neural fold hor	-0.059	0.960	6.06E-01	6.06E+01		
8032608	NM_174983	C19orf28	chromosome 19 open reading fran	-0.005	0.996	9.75E-01	9.75E+01		
8032623	NM_001060	TBXA2R	thromboxane A2 receptor	-0.023	0.984	9.00E-01	9.00E+01		
8032637	NM_00108054	C19orf29	chromosome 19 open reading fran	-0.002	0.999	9.92E-01	9.92E+01		
8032650	NM_012398	PIP5K1C	phosphatidylinositol-4-phosphate	-0.028	0.981	8.08E-01	8.08E+01		
8032682	NM_139355	MATK	megakaryocyte-associated tyrosin	0.017	1.012	9.02E-01	9.02E+01		
8032699	NM_015174	ZFR2	zinc finger RNA binding protein 2	-0.083	0.944	2.40E-01	2.40E+01		
8032718	NM_001348	DAPK3	death-associated protein kinase 3	-0.053	0.964	7.03E-01	7.03E+01		
8032730	NM_001961	EEF2	eukaryotic translation elongation f	-0.083	0.944	5.92E-01	5.92E+01		
8032751	---	---	---	-0.025	0.983	8.83E-01	8.83E+01		
8032755	NM_015898	ZBTB7A	zinc finger and BTB domain contai	-0.067	0.955	5.23E-01	5.23E+01		
8032761	NM_030662	MAP2K2	mitogen-activated protein kinase k	-0.133	0.912	1.81E-01	1.81E+01		
8032770	NM_016539	SIRT6	sirtuin (silent mating type informa	-0.006	0.996	9.67E-01	9.67E+01		
8032782	NM_144615	TMIGD2	transmembrane and immunoglobu	-0.020	0.986	9.00E-01	9.00E+01		
8032804	NM_003025	SH3GL1	SH3-domain GRB2-like 1	-0.044	0.970	7.40E-01	7.40E+01		
8032815	NM_025241	UBXN6	UBX domain protein 6	-0.022	0.985	8.86E-01	8.86E+01		
8032829	NM_00108040	PLIN4	perilipin 4	-0.094	0.937	2.47E-01	2.47E+01		
8032834	NM_052972	LRG1	leucine-rich alpha-2-glycoprotein 1	-0.010	0.993	9.56E-01	9.56E+01		
8032839	NM_032108	SEMA6B	sema domain, transmembrane do	-0.010	0.993	9.64E-01	9.64E+01		
8032863	NM_019107	C19orf10	chromosome 19 open reading fran	0.041	1.029	8.16E-01	8.16E+01		
8032871	NM_139159	DPP9	dipeptidyl-peptidase 9	-0.055	0.962	7.72E-01	7.72E+01		
8032899	NM_182919	TICAM1	toll-like receptor adaptor molecule	-0.050	0.966	6.43E-01	6.43E+01		
8032909	NM_005817	PLIN3	perilipin 3	-0.030	0.980	8.79E-01	8.79E+01		
8032922	BC137009	C19orf31	chromosome 19 open reading fran	-0.028	0.980	8.01E-01	8.01E+01		
8032926	NM_002850	PTPRS	protein tyrosine phosphatase, rece	-0.040	0.973	7.09E-01	7.09E+01		
8032972	---	---	---	0.047	1.033	7.52E-01	7.52E+01		
8032974	NM_014649	SAFB2	scaffold attachment factor B2	-0.068	0.954	5.53E-01	5.53E+01		
8032996	NM_205767	C19orf70	chromosome 19 open reading fran	0.008	1.006	9.62E-01	9.62E+01		
8033002	NM_004793	LONP1	lon peptidase 1, mitochondrial	-0.042	0.971	8.26E-01	8.26E+01		
8033023	NM_00113431	PRR22	proline rich 22	-0.057	0.961	5.10E-01	5.10E+01		
8033025	NM_020175	DUS3L	dihydrouridine synthase 3-like (S. c	-0.050	0.966	7.04E-01	7.04E+01		
8033043	NM_000150	FUT6	fucosyltransferase 6 (alpha (1,3) fu	-0.030	0.980	7.97E-01	7.97E+01		
8033064	NM_002034	FUT5	fucosyltransferase 5 (alpha (1,3) fu	-0.037	0.975	8.27E-01	8.27E+01		
8033069	NM_175614	NDUFA11	NADH dehydrogenase (ubiquinone	0.032	1.022	8.47E-01	8.47E+01		
8033075	NM_003624	RANBP3	RAN binding protein 3	-0.087	0.941	4.51E-01	4.51E+01		
8033097	NM_000635	RFX2	regulatory factor X, 2 (influences H	-0.018	0.987	9.04E-01	9.04E+01		
8033118	NM_005934	MLLT1	myeloid/lymphoid or mixed-lineag	-0.055	0.963	4.96E-01	4.96E+01		
8033135	NM_002096	GTF2F1	general transcription factor IIF, po	-0.058	0.960	6.87E-01	6.87E+01		
8033159	NM_004158	PSPN	persephin	-0.019	0.987	9.06E-01	9.06E+01		
8033162	NM_003685	KHSRP	KH-type splicing regulatory protein	-0.048	0.967	7.12E-01	7.12E+01		
8033179	NM_173637	SLC25A41	solute carrier family 25, member 4	0.005	1.003	9.78E-01	9.78E+01		
8033190	NM_024103	SLC25A23	solute carrier family 25 (mitochon	0.038	1.026	7.28E-01	7.28E+01		
8033207	NM_024898	DENND1C	DENN/MADD domain containing 1	-0.080	0.946	4.28E-01	4.28E+01		

8033233	NM_006087	TUBB4	tubulin, beta 4	-0.074	0.950	7.65E-01	7.65E+01		
8033241	NM_001252	CD70	CD70 molecule	-0.036	0.975	8.05E-01	8.05E+01		
8033248	NM_003807	TNFSF14	tumor necrosis factor (ligand) supe	-0.115	0.924	3.02E-01	3.02E+01		
8033300	NM_00108045	GPR108	G protein-coupled receptor 108	0.025	1.017	9.24E-01	9.24E+01		
8033319	NM_005490	SH2D3A	SH2 domain containing 3A	-0.125	0.917	1.41E-01	1.41E+01		
8033330	---	---	---	-0.182	0.881	6.43E-01	6.43E+01		
8033352	NM_144614	MBD3L2	methyl-CpG binding domain prote	-0.053	0.964	5.60E-01	5.60E+01		
8033356	NM_144614	MBD3L2	methyl-CpG binding domain prote	-0.031	0.979	7.87E-01	7.87E+01		
8033359	NM_144614	MBD3L2	methyl-CpG binding domain prote	-0.031	0.979	7.87E-01	7.87E+01		
8033385	NM_080662	PEX11G	peroxisomal biogenesis factor 11 g	-0.032	0.978	8.32E-01	8.32E+01		
8033392	NM_020196	XAB2	XPA binding protein 2	0.008	1.006	9.70E-01	9.70E+01		
8033414	NM_174895	PCP2	Purkinje cell protein 2	-0.009	0.994	9.56E-01	9.56E+01		
8033433	NM_198492	CLEC4G	C-type lectin domain family 4, mer	0.092	1.066	1.38E-01	1.38E+01		
8033445	NR_026692	CD209	CD209 molecule	0.218	1.163	3.09E-01	3.09E+01		
8033455	ENST00000327	FLJ22184	hypothetical protein FLJ22184	-0.062	0.958	5.61E-01	5.61E+01		
8033458	NM_007260	LYPLA2	lysophospholipase II	-0.002	0.999	9.95E-01	9.95E+01		
8033460	---	---	---	0.137	1.100	5.53E-01	5.53E+01		
8033462	NM_206833	CTXN1	cortexin 1	-0.038	0.974	7.82E-01	7.82E+01		
8033465	NM_006351	TIMM44	translocase of inner mitochondrial	0.026	1.018	8.31E-01	8.31E+01		
8033479	NM_001419	ELAVL1	ELAV (embryonic lethal, abnormal	-0.024	0.983	8.69E-01	8.69E+01		
8033487	NM_032447	FBN3	fibrillin 3	-0.004	0.997	9.77E-01	9.77E+01		
8033554	NM_016579	CD320	CD320 molecule	-0.031	0.979	8.46E-01	8.46E+01		
8033564	NM_005001	NDUFA7	NADH dehydrogenase (ubiquinone	-0.059	0.960	6.57E-01	6.57E+01		
8033571	NM_198471	KANK3	KN motif and ankyrin repeat doma	-0.120	0.920	1.52E-01	1.52E+01		
8033587	NM_032152	PRAM1	PML-RARA regulated adaptor mole	0.057	1.040	6.21E-01	6.21E+01		
8033598	NM_032370	ZNF414	zinc finger protein 414	-0.009	0.994	9.49E-01	9.49E+01		
8033605	NM_012335	MYO1F	myosin IF	-0.007	0.995	9.86E-01	9.86E+01		
8033635	NM_030957	ADAMTS10	ADAM metallopeptidase with thro	0.029	1.021	8.05E-01	8.05E+01		
8033662	NM_178525	ACTL9	actin-like 9	-0.077	0.948	3.66E-01	3.66E+01		
8033667	NM_144693	ZNF558	zinc finger protein 558	-0.099	0.934	5.61E-01	5.61E+01		
8033674	NM_024690	MUC16	mucin 16, cell surface associated	-0.011	0.992	9.40E-01	9.40E+01		
8033740	NM_00100519	OR7D4	olfactory receptor, family 7, subfa	-0.008	0.995	9.72E-01	9.72E+01		
8033754	NM_006631	ZNF266	zinc finger protein 266	-0.008	0.994	9.81E-01	9.81E+01		
8033780	NM_024106	ZNF426	zinc finger protein 426	-0.156	0.898	3.69E-01	3.69E+01		
8033789	NM_00100872	ZNF121	zinc finger protein 121	0.060	1.042	7.56E-01	7.56E+01		
8033795	NM_152289	ZNF561	zinc finger protein 561	0.038	1.026	8.22E-01	8.22E+01		
8033801	NM_00113003	ZNF562	zinc finger protein 562	0.062	1.044	7.51E-01	7.51E+01		
8033813	NM_017703	FBXL12	F-box and leucine-rich repeat prot	0.064	1.046	6.67E-01	6.67E+01		
8033818	NM_058164	OLFM2	olfactomedin 2	-0.054	0.963	5.89E-01	5.89E+01		
8033825	NM_015719	COL5A3	collagen, type V, alpha 3	-0.022	0.985	7.89E-01	7.89E+01		
8033892	NM_031917	ANGPTL6	angiopoietin-like 6	-0.073	0.951	4.75E-01	4.75E+01		
8033899	NM_003755	EIF3G	eukaryotic translation initiation fa	-0.010	0.993	9.50E-01	9.50E+01		
8033912	NM_00113082	DNMT1	DNA (cytosine-5-)-methyltransfera	0.228	1.172	2.25E-03	2.25E-01		
8033956	NM_004230	S1PR2	sphingosine-1-phosphate receptor	0.038	1.027	7.52E-01	7.52E+01		
8033960	NM_00103173	FDX1L	ferredoxin 1-like	-0.029	0.980	8.47E-01	8.47E+01		
8033969	NM_133452	RAVER1	ribonucleoprotein, PTB-binding 1	-0.029	0.980	7.85E-01	7.85E+01		
8033987	NM_002162	ICAM3	intercellular adhesion molecule 3	0.128	1.093	2.38E-01	2.38E+01		
8033996	NM_003331	TYK2	tyrosine kinase 2	-0.015	0.990	9.15E-01	9.15E+01		
8034021	NM_007065	CDC37	cell division cycle 37 homolog (S. c	-0.109	0.927	4.35E-01	4.35E+01		
8034034	NM_203500	KEAP1	kelch-like ECH-associated protein	-0.031	0.979	8.33E-01	8.33E+01		
8034043	NM_030760	S1PR5	sphingosine-1-phosphate receptor	0.008	1.006	9.61E-01	9.61E+01		
8034055	NM_023008	KRI1	KRI1 homolog (S. cerevisiae)	0.081	1.058	5.73E-01	5.73E+01		
8034075	NM_001800	CDKN2D	cyclin-dependent kinase inhibitor	-0.033	0.977	7.68E-01	7.68E+01		
8034097	NR_024333	LOC147727	hypothetical LOC147727	-0.017	0.989	9.60E-01	9.60E+01		
8034101	NM_006858	TMED1	transmembrane emp24 protein tra	-0.074	0.950	4.42E-01	4.42E+01		
8034108	NM_024029	YIPF2	Yip1 domain family, member 2	-0.003	0.998	9.85E-01	9.85E+01		
8034130	NM_00113619	KANK2	KN motif and ankyrin repeat doma	-0.101	0.932	3.28E-01	3.28E+01		
8034151	NM_020812	DOCK6	dedicator of cytokinesis 6	-0.092	0.939	1.41E-01	1.41E+01		
8034199	NM_012466	TSPAN16	tetraspanin 16	-0.086	0.942	6.62E-01	6.62E+01		
8034202	NM_004283	RAB3D	RAB3D, member RAS oncogene fa	-0.032	0.978	8.26E-01	8.26E+01		
8034210	NM_198536	TMEM205	transmembrane protein 205	-0.020	0.987	9.39E-01	9.39E+01		
8034217	NM_000121	EPOR	erythropoietin receptor	0.047	1.033	7.91E-01	7.91E+01		
8034249	NM_145045	CCDC151	coiled-coil domain containing 151	-0.070	0.953	5.15E-01	5.15E+01		
8034263	NM_001420	ELAVL3	ELAV (embryonic lethal, abnormal	-0.029	0.980	8.25E-01	8.25E+01		
8034276	NM_138783	ZNF653	zinc finger protein 653	-0.082	0.945	2.51E-01	2.51E+01		
8034286	NM_016581	ECSIT	ECSIT homolog (Drosophila)	-0.023	0.984	8.61E-01	8.61E+01		
8034299	NM_032377	ELOF1	elongation factor 1 homolog (S. ce	-0.037	0.974	8.55E-01	8.55E+01		
8034304	NM_00111103	ACP5	acid phosphatase 5, tartrate resist	0.311	1.241	6.80E-03	6.80E-01		
8034313	NR_002944	HNRPA1L-2	heterogeneous nuclear ribonucleo	-0.021	0.985	8.73E-01	8.73E+01		
8034315	NM_00108049	ZNF823	zinc finger protein 823	0.067	1.047	8.16E-01	8.16E+01		
8034320	NM_00108041	ZNF433	zinc finger protein 433	0.097	1.070	5.27E-01	5.27E+01		
8034342	AK292455	ZNF625	zinc finger protein 625	0.092	1.066	7.44E-01	7.44E+01		
8034349	NM_016264	ZNF44	zinc finger protein 44	0.113	1.081	6.79E-01	6.79E+01		
8034379	NM_030824	ZNF442	zinc finger protein 442	0.061	1.043	6.52E-01	6.52E+01		
8034408	NM_020714	ZNF490	zinc finger protein 490	0.158	1.116	2.30E-01	2.30E+01		
8034416	NM_006013	RPL10	ribosomal protein L10	-0.046	0.968	8.06E-01	8.06E+01		
8034420	NM_000528	MAN2B1	mannosidase, alpha, class 2B, mer	0.179	1.132	2.08E-01	2.08E+01		

8034448	NM_016145	C19orf56	chromosome 19 open reading fran	-0.050	0.966	8.03E-01	8.03E+01		
8034454	NM_001930	DHPS	deoxyhypusine synthase	-0.024	0.983	9.12E-01	9.12E+01		
8034469	NM_032301	FBXW9	F-box and WD repeat domain cont	-0.020	0.987	8.84E-01	8.84E+01		
8034482	NM_00113619	TNPO2	transportin 2	-0.094	0.937	2.60E-01	2.60E+01		
8034512	NR_002751	SNORD41	small nucleolar RNA, C/D box 41	0.049	1.034	9.39E-01	9.39E+01		
8034514	NM_024038	C19orf43	chromosome 19 open reading fran	-0.014	0.991	9.29E-01	9.29E+01		
8034521	NM_013312	HOOK2	hook homolog 2 (Drosophila)	-0.073	0.950	5.51E-01	5.51E+01		
8034544	NM_005809	PRDX2	peroxiredoxin 2	0.032	1.022	8.03E-01	8.03E+01		
8034565	NM_001375	DNASE2	deoxyribonuclease II, lysosomal	0.271	1.207	5.53E-02	5.53E+00		
8034578	NM_006563	KLF1	Kruppel-like factor 1 (erythroid)	0.045	1.031	7.45E-01	7.45E+01		
8034583	NM_00110557	SYCE2	synaptonemal complex central ele	0.051	1.036	7.57E-01	7.57E+01		
8034589	NM_004461	FARSA	phenylalanyl-tRNA synthetase, alp	-0.004	0.997	9.85E-01	9.85E+01		
8034605	NM_052850	GADD45GIP1	growth arrest and DNA-damage-in	-0.021	0.986	8.98E-01	8.98E+01		
8034608	NM_005583	LYL1	lymphoblastic leukemia derived se	-0.019	0.987	8.82E-01	8.82E+01		
8034615	NM_017722	TRMT1	TRM1 tRNA methyltransferase 1 h	-0.046	0.969	7.41E-01	7.41E+01		
8034631	NM_003765	STX10	syntaxin 10	0.010	1.007	9.50E-01	9.50E+01		
8034643	NM_00112722	CACNA1A	calcium channel, voltage-depende	-0.040	0.973	5.34E-01	5.34E+01		
8034696	---	---	---	-0.063	0.957	6.70E-01	6.70E+01		
8034698	---	---	---	-0.014	0.990	9.53E-01	9.53E+01		
8034700	BC119719	C19orf57	chromosome 19 open reading fran	-0.043	0.970	6.81E-01	6.81E+01		
8034712	NM_024825	PODNL1	podocan-like 1	-0.014	0.990	9.48E-01	9.48E+01		
8034722	NM_002918	RFX1	regulatory factor X, 1 (influences H	-0.055	0.963	4.56E-01	4.56E+01		
8034744	NM_00114502	PALM3	Paralemmin-3	-0.034	0.977	7.93E-01	7.93E+01		
8034747	ENST00000343	C19orf67	chromosome 19 open reading fran	-0.038	0.974	6.70E-01	6.70E+01		
8034754	NM_138352	SAMD1	sterile alpha motif domain contair	-0.094	0.937	3.87E-01	3.87E+01		
8034762	NM_002730	PRKACA	protein kinase, cAMP-dependent,	0.080	1.057	5.98E-01	5.98E+01		
8034783	NM_00100870	LPHN1	latrophilin 1	-0.043	0.971	7.12E-01	7.12E+01		
8034806	NM_005804	DDX39	DEAD (Asp-Glu-Ala-Asp) box polyp	-0.068	0.954	6.78E-01	6.78E+01		
8034821	NM_000955	PTGER1	prostaglandin E receptor 1 (subtyp	-0.067	0.955	4.48E-01	4.48E+01		
8034827	NM_005716	GIPC1	GIPC PDZ domain containing famil	-0.007	0.995	9.61E-01	9.61E+01		
8034837	NM_006145	DNAJB1	DnaJ (Hsp40) homolog, subfamily	-0.202	0.869	2.14E-01	2.14E+01		
8034843	NM_004146	NDUFB7	NADH dehydrogenase (ubiquinone	-0.019	0.987	9.19E-01	9.19E+01		
8034871	---	---	---	-0.060	0.959	6.57E-01	6.57E+01		
8034873	NM_013447	EMR2	egf-like module containing, mucin	0.404	1.323	1.02E-01	1.02E+01		
8034907	NM_005071	SLC1A6	solute carrier family 1 (high affinity	-0.007	0.995	9.74E-01	9.74E+01		
8034920	NM_006844	ILVBL	ilvB (bacterial acetolactate synthas	0.030	1.021	8.46E-01	8.46E+01		
8034940	NM_000435	NOTCH3	Notch homolog 3 (Drosophila)	0.018	1.013	8.67E-01	8.67E+01		
8034983	NM_058243	BRD4	bromodomain containing 4	-0.041	0.972	6.60E-01	6.60E+01		
8035007	NM_005858	AKAP8	A kinase (PRKA) anchor protein 8	-0.047	0.968	5.85E-01	5.85E+01		
8035023	NM_014371	AKAP8L	A kinase (PRKA) anchor protein 8-l	-0.019	0.987	9.12E-01	9.12E+01		
8035040	NM_021241	WIZ	widely interspaced zinc finger mot	-0.001	1.000	9.98E-01	9.98E+01		
8035050	NM_022904	RASAL3	RAS protein activator like 3	-0.092	0.938	4.35E-01	4.35E+01		
8035120	NM_021235	EPS15L1	epidermal growth factor receptor	-0.180	0.882	3.17E-02	3.17E+00		
8035144	---	---	---	0.033	1.023	9.51E-01	9.51E+01		
8035156	NM_006387	CHERP	calcium homeostasis endoplasmic	-0.052	0.965	6.14E-01	6.14E+01		
8035175	---	---	---	-0.023	0.984	9.29E-01	9.29E+01		
8035177	NM_024881	SLC35E1	solute carrier family 35, member E	-0.030	0.979	8.56E-01	8.56E+01		
8035187	NM_004831	MED26	mediator complex subunit 26	0.013	1.009	9.43E-01	9.43E+01		
8035193	NM_024104	C19orf42	chromosome 19 open reading fran	0.121	1.088	3.57E-01	3.57E+01		
8035201	NM_015692	CPAMD8	C3 and PZP-like, alpha-2-macroglo	-0.045	0.969	6.87E-01	6.87E+01		
8035232	---	---	---	0.118	1.085	1.50E-01	1.50E+01		
8035234	---	---	---	-0.057	0.961	7.84E-01	7.84E+01		
8035236	NM_033417	HAUS8	HAUS augmin-like complex, subun	-0.044	0.970	7.58E-01	7.58E+01		
8035249	NM_005234	NR2F6	nuclear receptor subfamily 2, grou	-0.014	0.990	9.32E-01	9.32E+01		
8035254	NM_031941	USHBP1	Usher syndrome 1C binding protei	-0.040	0.973	6.92E-01	6.92E+01		
8035271	NM_024527	ABHD8	abhydrolase domain containing 8	-0.051	0.965	5.78E-01	5.78E+01		
8035278	NM_020959	ANO8	anoctamin 8	0.029	1.021	8.06E-01	8.06E+01		
8035297	NM_031310	PLVAP	plasmalemma vesicle associated p	0.004	1.003	9.78E-01	9.78E+01		
8035304	NM_004335	BST2	bone marrow stromal cell antigen	0.157	1.115	6.39E-01	6.39E+01		
8035311	ENST00000341	TMEM221	transmembrane protein 221	-0.050	0.966	7.03E-01	7.03E+01		
8035315	NM_138454	NXNL1	nucleoredoxin-like 1	-0.063	0.957	5.25E-01	5.25E+01		
8035345	NM_005543	INSL3	insulin-like 3 (Leydig cell)	-0.056	0.962	5.72E-01	5.72E+01		
8035351	NM_000215	JAK3	Janus kinase 3	0.019	1.013	9.15E-01	9.15E+01		
8035380	NM_005535	IL12RB1	interleukin 12 receptor, beta 1	0.102	1.073	2.31E-01	2.31E+01		
8035398	NM_002866	RAB3A	RAB3A, member RAS oncogene fa	-0.044	0.970	7.37E-01	7.37E+01		
8035408	NM_000923	PDE4C	phosphodiesterase 4C, cAMP-spec	-0.052	0.965	6.02E-01	6.02E+01		
8035435	NM_00114530	KIAA1683	KIAA1683	-0.045	0.969	6.31E-01	6.31E+01		
8035445	NM_005354	JUND	jun D proto-oncogene	-0.086	0.942	2.25E-01	2.25E+01		
8035449	NM_012321	LSM4	LSM4 homolog, U6 small nuclear R	-0.060	0.959	3.55E-01	3.55E+01		
8035456	NM_145256	LRRC25	leucine rich repeat containing 25	0.068	1.049	7.51E-01	7.51E+01		
8035465	NM_016368	ISYNA1	inositol-3-phosphate synthase 1	0.023	1.016	8.92E-01	8.92E+01		
8035477	NM_006532	ELL	elongation factor RNA polymerase	-0.107	0.928	1.21E-01	1.21E+01		
8035494	NM_012181	FKBP8	FK506 binding protein 8, 38kDa	-0.015	0.990	9.09E-01	9.09E+01		
8035506	NM_004750	CRLF1	cytokine receptor-like factor 1	-0.095	0.936	3.01E-01	3.01E+01		
8035517	NM_000095	COMP	cartilage oligomeric matrix protei	-0.018	0.987	9.16E-01	9.16E+01		
8035539	NM_001492	GDF1	growth differentiation factor 1	-0.021	0.986	8.67E-01	8.67E+01		
8035551	BC004943	MGC10814	hypothetical protein MGC10814	0.045	1.032	6.94E-01	6.94E+01		

8035553	NM_007263	COPE	coatomer protein complex, subun	0.046	1.032	7.04E-01	7.04E+01		
8035566	NM_004838	HOMER3	homer homolog 3 (Drosophila)	-0.008	0.994	9.58E-01	9.58E+01		
8035581	NM_00101739	SFRS14	splicing factor, arginine/serine-rich	-0.050	0.966	7.34E-01	7.34E+01		
8035600	NM_017814	TMEM161A	transmembrane protein 161A	-0.048	0.967	6.84E-01	6.84E+01		
8035612	NM_00114578	MEF2B	myocyte enhancer factor 2B	-0.075	0.949	4.34E-01	4.34E+01		
8035628	NM_176880	NR2C2AP	nuclear receptor 2C2-associated p	-0.067	0.954	7.24E-01	7.24E+01		
8035639	NM_023002	HAPLN4	hyaluronan and proteoglycan link	-0.031	0.979	8.54E-01	8.54E+01		
8035646	NM_023002	HAPLN4	hyaluronan and proteoglycan link	-0.072	0.951	5.28E-01	5.28E+01		
8035648	NM_00100152	TM6SF2	transmembrane 6 superfamily me	0.003	1.002	9.81E-01	9.81E+01		
8035666	NM_172231	SF4	splicing factor 4	-0.099	0.934	2.78E-01	2.78E+01		
8035682	---	---	---	0.028	1.020	8.65E-01	8.65E+01		
8035694	NM_025245	PBX4	pre-B-cell leukemia homeobox 4	-0.113	0.924	2.50E-01	2.50E+01		
8035703	NM_004720	LPAR2	lysophosphatidic acid receptor 2	-0.030	0.980	8.53E-01	8.53E+01		
8035714	NM_016573	GMIP	GEM interacting protein	-0.033	0.978	8.14E-01	8.14E+01		
8035737	NM_020410	ATP13A1	ATPase type 13A1	-0.021	0.986	8.81E-01	8.81E+01		
8035765	NM_021030	ZNF14	zinc finger protein 14	-0.037	0.975	9.16E-01	9.16E+01		
8035773	NM_00109926	ZNF506	zinc finger protein 506	-0.003	0.998	9.91E-01	9.91E+01		
8035779	NM_00107667	ZNF626	zinc finger protein 626	-0.090	0.940	7.53E-01	7.53E+01		
8035787	---	---	---	0.048	1.034	8.94E-01	8.94E+01		
8035789	NM_00103988	ZNF826	zinc finger protein 826	0.107	1.077	6.38E-01	6.38E+01		
8035793	NM_00115929	ZNF737	zinc finger protein 737	0.085	1.061	7.44E-01	7.44E+01		
8035795	NM_145297	ZNF626	zinc finger protein 626	0.095	1.068	6.27E-01	6.27E+01		
8035801	---	---	---	-0.116	0.923	3.53E-01	3.53E+01		
8035803	NM_021269	ZNF708	zinc finger protein 708	0.077	1.055	4.69E-01	4.69E+01		
8035808	NM_173531	ZNF100	zinc finger protein 100	0.106	1.076	8.33E-01	8.33E+01		
8035829	NM_000995	RPL34	ribosomal protein L34	0.028	1.019	7.72E-01	7.72E+01		
8035842	NM_003430	ZNF91	zinc finger protein 91	0.053	1.037	8.16E-01	8.16E+01		
8035859	---	---	---	0.005	1.004	9.82E-01	9.82E+01		
8035880	NM_006003	UQCRFS1	ubiquinol-cytochrome c reductase	0.036	1.025	9.04E-01	9.04E+01		
8035884	---	---	---	0.032	1.022	8.09E-01	8.09E+01		
8035886	NM_031448	C19orf12	chromosome 19 open reading fran	-0.033	0.977	8.40E-01	8.40E+01		
8035896	NM_020856	TSHZ3	teashirt zinc finger homeobox 3	0.282	1.216	6.65E-02	6.65E+00		
8035903	---	---	---	-0.100	0.933	3.03E-01	3.03E+01		
8035905	NM_032139	ANKRD27	ankyrin repeat domain 27 (VPS9 d	-0.084	0.944	5.78E-01	5.78E+01		
8035939	---	---	---	-0.121	0.920	4.11E-01	4.11E+01		
8035941	NM_014270	SLC7A9	solute carrier family 7 (cationic am	0.027	1.019	8.66E-01	8.66E+01		
8035956	---	---	---	0.018	1.012	9.28E-01	9.28E+01		
8035958	NM_032816	CCDC123	coiled-coil domain containing 123	-0.090	0.940	5.29E-01	5.29E+01		
8035993	NM_019849	SLC7A10	solute carrier family 7, (neutral am	-0.057	0.961	4.74E-01	4.74E+01		
8036004	NM_004364	CEBPA	CCAAT/enhancer binding protein (	0.032	1.022	8.33E-01	8.33E+01		
8036010	NM_000285	PEPD	peptidase D	0.037	1.026	8.36E-01	8.36E+01		
8036031	---	---	---	-0.024	0.984	8.93E-01	8.93E+01		
8036045	NM_175872	ZNF792	zinc finger protein 792	-0.041	0.972	8.15E-01	8.15E+01		
8036069	NM_152481	FAM187B	family with sequence similarity 18	0.022	1.015	8.29E-01	8.29E+01		
8036079	NM_033317	DMKN	dermokine	-0.017	0.989	9.07E-01	9.07E+01		
8036103	NM_198538	SBSN	suprabasin	-0.018	0.988	9.16E-01	9.16E+01		
8036133	NM_007000	UPK1A	uroplakin 1A	-0.015	0.990	9.47E-01	9.47E+01		
8036136	NM_024660	TMEM149	transmembrane protein 149	0.089	1.064	3.56E-01	3.56E+01		
8036143	NM_00104042	U2AF1L4	U2 small nuclear RNA auxiliary fac	0.015	1.011	9.73E-01	9.73E+01		
8036151	NM_144617	HSPB6	heat shock protein, alpha-crystalli	-0.135	0.911	1.74E-01	1.74E+01		
8036170	ENST00000443	PRODH2	proline dehydrogenase (oxidase) 2	0.017	1.012	9.36E-01	9.36E+01		
8036172	ENST00000443	PRODH2	proline dehydrogenase (oxidase) 2	-0.025	0.982	8.57E-01	8.57E+01		
8036207	NM_139239	NFKBID	nuclear factor of kappa light polyp	-0.007	0.995	9.70E-01	9.70E+01		
8036232	NM_00103987	C19orf46	chromosome 19 open reading fran	0.024	1.017	8.69E-01	8.69E+01		
8036242	NM_032878	ALKBH6	alkB, alkylation repair homolog 6 (	-0.050	0.966	7.46E-01	7.46E+01		
8036252	NM_015526	CLIP3	CAP-GLY domain containing linker	-0.082	0.945	3.94E-01	3.94E+01		
8036270	NM_152658	THAP8	THAP domain containing 8	-0.022	0.985	8.93E-01	8.93E+01		
8036276	NM_006233	POLR2I	polymerase (RNA) II (DNA directed	0.014	1.010	9.68E-01	9.68E+01		
8036291	NM_00104247	ZNF565	zinc finger protein 565	0.035	1.024	8.47E-01	8.47E+01		
8036324	NM_00101275	ZNF260	zinc finger protein 260	-0.034	0.977	8.78E-01	8.78E+01		
8036351	BC052603	ZNF850P	zinc finger protein 850 pseudogen	-0.055	0.962	7.39E-01	7.39E+01		
8036357	NM_206894	ZNF790	zinc finger protein 790	0.038	1.026	9.22E-01	9.22E+01		
8036363	---	---	---	0.022	1.015	9.84E-01	9.84E+01		
8036365	NM_00103723	ZNF829	zinc finger protein 829	-0.077	0.948	6.57E-01	6.57E+01		
8036389	NM_152279	ZNF585B	zinc finger protein 585B	-0.034	0.977	9.19E-01	9.19E+01		
8036420	NM_014898	ZFP30	zinc finger protein 30 homolog (m	0.008	1.006	9.80E-01	9.80E+01		
8036460	NM_004647	DPF1	D4, zinc and double PHD fingers fa	-0.058	0.961	6.21E-01	6.21E+01		
8036473	NM_033256	PPP1R14A	protein phosphatase 1, regulatory	0.095	1.068	7.11E-01	7.11E+01		
8036479	NM_033557	YIF1B	Yip1 interacting factor homolog B	-0.084	0.943	1.29E-01	1.29E+01		
8036483	NM_033557	YIF1B	Yip1 interacting factor homolog B	-0.011	0.993	9.51E-01	9.51E+01		
8036494	NM_152657	GGN	gametogenetin	-0.042	0.971	6.94E-01	6.94E+01		
8036503	NM_170604	RASGRP4	RAS guanyl releasing protein 4	0.017	1.012	9.32E-01	9.32E+01		
8036525	NM_00104260	MAP4K1	mitogen-activated protein kinase k	0.060	1.042	7.45E-01	7.45E+01		
8036557	---	---	---	-0.041	0.972	8.50E-01	8.50E+01		
8036563	NM_144691	CAPN12	calpain 12	0.041	1.029	7.27E-01	7.27E+01		
8036584	NM_002307	LGALS7	lectin, galactoside-binding, soluble	-0.006	0.996	9.67E-01	9.67E+01		
8036591	NM_006149	LGALS4	lectin, galactoside-binding, soluble	-0.004	0.997	9.88E-01	9.88E+01		

8036602	NM_001398	ECH1	enoyl Coenzyme A hydratase 1, pe	-0.060	0.959	6.56E-01	6.56E+01		
8036613	NM_001533	HNRNPL	heterogeneous nuclear ribonucleo	0.061	1.043	5.71E-01	5.71E+01		
8036622	NM_198445	RINL	Ras and Rab interactor-like	-0.048	0.967	5.74E-01	5.74E+01		
8036636	NM_012237	SIRT2	sirtuin (silent mating type informa	-0.005	0.997	9.80E-01	9.80E+01		
8036656	NM_017827	SARS2	seryl-tRNA synthetase 2, mitochor	-0.024	0.983	8.37E-01	8.37E+01		
8036676	NM_024907	FBXO17	F-box protein 17	-0.015	0.989	9.27E-01	9.27E+01		
8036686	AK021860	FBXO17	F-box protein 17	-0.033	0.977	7.90E-01	7.90E+01		
8036699	NM_00108046	SYCN	syncollin	-0.040	0.973	8.36E-01	8.36E+01		
8036702	NM_172139	IL28B	interleukin 28B (interferon, lambd	0.062	1.044	6.06E-01	6.06E+01		
8036707	NM_020862	LRFN1	leucine rich repeat and fibronectir	0.058	1.041	6.31E-01	6.31E+01		
8036710	NM_004877	GMFG	glia maturation factor, gamma	0.041	1.029	7.49E-01	7.49E+01		
8036720	NM_019088	PAF1	Paf1, RNA polymerase II associatec	-0.085	0.943	5.48E-01	5.48E+01		
8036737	NM_001020	RPS16	ribosomal protein S16	-0.052	0.965	7.33E-01	7.33E+01		
8036749	NM_153232	EID2	EP300 interacting inhibitor of diffe	-0.024	0.984	8.44E-01	8.44E+01		
8036763	NM_004714	DYRK1B	dual-specificity tyrosine-(Y)-phosp	-0.049	0.967	5.34E-01	5.34E+01		
8036777	NM_001436	FBL	fibrillarin	0.003	1.002	9.90E-01	9.90E+01		
8036787	NM_003890	FCGBP	Fc fragment of IgG binding proteir	-0.171	0.888	6.46E-02	6.46E+00		
8036808	NM_018457	PRR13	proline rich 13	0.039	1.027	7.81E-01	7.81E+01		
8036813	NM_00100585	ZNF780B	zinc finger protein 780B	0.091	1.065	6.38E-01	6.38E+01		
8036820	NM_00114257	ZNF780A	zinc finger protein 780A	-0.064	0.957	6.35E-01	6.35E+01		
8036830	NM_152479	TTC9B	tetratricopeptide repeat domain 9	0.055	1.039	6.88E-01	6.88E+01		
8036835	NM_024877	CNTD2	cyclin N-terminal domain containi	-0.050	0.966	7.53E-01	7.53E+01		
8036840	NM_001626	AKT2	v-akt murine thymoma viral oncog	-0.017	0.989	9.11E-01	9.11E+01		
8036862	AY358191	UNQ6167	NINP6167	0.092	1.066	2.86E-01	2.86E+01		
8036867	BC027935	C19orf47	chromosome 19 open reading fran	0.068	1.048	4.46E-01	4.46E+01		
8036883	NM_144685	HIPK4	homeodomain interacting protein	0.039	1.028	7.33E-01	7.33E+01		
8036890	NM_020956	PRX	periaxin	-0.049	0.967	5.53E-01	5.53E+01		
8036902	NM_013376	SERTAD1	SERTA domain containing 1	-0.103	0.931	2.15E-01	2.15E+01		
8036908	NM_013368	SERTAD3	SERTA domain containing 3	0.119	1.086	6.23E-01	6.23E+01		
8036913	NM_000713	BLVRB	biliverdin reductase B (flavin reduc	0.237	1.179	5.90E-02	5.90E+00		
8036923	NM_004756	NUMBL	numb homolog (Drosophila)-like	-0.037	0.975	7.09E-01	7.09E+01		
8036936	AK096566	LOC100130713	hypothetical LOC100130713	-0.219	0.859	6.19E-02	6.19E+00		
8036938	NM_024876	ADCK4	aarF domain containing kinase 4	-0.016	0.989	9.00E-01	9.00E+01		
8036956	NM_198476	C19orf54	chromosome 19 open reading fran	-0.006	0.996	9.72E-01	9.72E+01		
8036969	NM_000762	CYP2A6	cytochrome P450, family 2, subfar	-0.002	0.998	9.89E-01	9.89E+01		
8036981	NM_000764	CYP2A7	cytochrome P450, family 2, subfar	-0.002	0.999	9.94E-01	9.94E+01		
8036989	---	---	---	-0.009	0.994	9.68E-01	9.68E+01		
8037003	---	---	---	-0.029	0.980	9.36E-01	9.36E+01		
8037005	NM_000660	TGFB1	transforming growth factor, beta 1	-0.042	0.971	8.62E-01	8.62E+01		
8037018	NM_030578	B9D2	B9 protein domain 2	-0.088	0.941	3.41E-01	3.41E+01		
8037023	NM_020158	EXOSC5	exosome component 5	0.008	1.006	9.64E-01	9.64E+01		
8037032	NM_198540	B3GNT8	UDP-GlcNAc:betaGal beta-1,3-N-a	0.048	1.034	6.76E-01	6.76E+01		
8037037	NM_018035	ATP5SL	ATP5S-like	-0.001	0.999	9.98E-01	9.98E+01		
8037043	---	---	---	0.025	1.018	8.62E-01	8.62E+01		
8037045	NM_001817	CEACAM4	carcinoembryonic antigen-related	0.039	1.028	6.89E-01	6.89E+01		
8037061	NM_173506	LYPD4	LY6/PLAUR domain containing 4	-0.012	0.992	9.49E-01	9.49E+01		
8037071	NM_006423	RABAC1	Rab acceptor 1 (prenylated)	-0.103	0.931	3.56E-01	3.56E+01		
8037079	NM_152296	ATP1A3	ATPase, Na+/K+ transporting, alph	-0.018	0.987	9.14E-01	9.14E+01		
8037103	NM_002088	GRIK5	glutamate receptor, ionotropic, ka	0.024	1.017	8.87E-01	8.87E+01		
8037123	NM_002698	POU2F2	POU class 2 homeobox 2	0.054	1.038	6.00E-01	6.00E+01		
8037144	NM_133328	DEDD2	death effector domain containing	-0.043	0.971	7.52E-01	7.52E+01		
8037152	NM_019884	GSK3A	glycogen synthase kinase 3 alpha	-0.011	0.993	9.57E-01	9.57E+01		
8037166	NM_006494	ERF	Ets2 repressor factor	-0.022	0.985	9.13E-01	9.13E+01		
8037170	NM_002573	PAFAH1B3	platelet-activating factor acetylhy	-0.060	0.959	4.27E-01	4.27E+01		
8037179	NM_032488	CNFN	cornifelin	0.009	1.006	9.58E-01	9.58E+01		
8037186	NM_005357	LIPE	lipase, hormone-sensitive	-0.033	0.978	8.10E-01	8.10E+01		
8037197	NM_198477	CXCL17	chemokine (C-X-C motif) ligand 17	0.010	1.007	9.67E-01	9.67E+01		
8037283	NM_002780	PSG4	pregnancy specific beta-1-glycopro	-0.052	0.964	7.71E-01	7.71E+01		
8037290	NM_002784	PSG9	pregnancy specific beta-1-glycopro	-0.038	0.974	7.61E-01	7.61E+01		
8037301	NM_014400	LYPD3	LY6/PLAUR domain containing 3	-0.030	0.980	8.27E-01	8.27E+01		
8037309	NM_198850	PHLDB3	pleckstrin homology-like domain, f	-0.032	0.978	8.47E-01	8.47E+01		
8037315	NM_198850	PHLDB3	pleckstrin homology-like domain, f	-0.006	0.996	9.77E-01	9.77E+01		
8037322	NM_014297	ETHE1	ethylmalonic encephalopathy 1	0.023	1.016	9.25E-01	9.25E+01		
8037331	NM_006297	XRCC1	X-ray repair complementing defec	-0.123	0.918	2.56E-01	2.56E+01		
8037352	NM_00100756	IRGQ	immunity-related GTPase family, Q	-0.122	0.919	2.56E-01	2.56E+01		
8037355	NM_182498	ZNF428	zinc finger protein 428	-0.064	0.957	6.27E-01	6.27E+01		
8037363	NM_145296	CADM4	cell adhesion molecule 4	-0.065	0.956	5.06E-01	5.06E+01		
8037389	NM_019108	C19orf61	chromosome 19 open reading fran	0.068	1.048	5.73E-01	5.73E+01		
8037408	NM_002250	KCNN4	potassium intermediate/small con	-0.029	0.980	7.98E-01	7.98E+01		
8037422	NM_00103174	LYPD5	LY6/PLAUR domain containing 5	-0.005	0.997	9.81E-01	9.81E+01		
8037474	NM_013256	ZNF180	zinc finger protein 180	-0.036	0.976	8.35E-01	8.35E+01		
8037495	NM_145288	ZNF296	zinc finger protein 296	-0.034	0.977	7.82E-01	7.82E+01		
8037502	NM_00113106	NKPD1	NTPase, KAP family P-loop domair	-0.005	0.996	9.76E-01	9.76E+01		
8037505	NM_024108	TRAPPC6A	trafficking protein particle comple	-0.050	0.966	6.52E-01	6.52E+01		
8037513	NM_138568	EXOC3L2	exocyst complex component 3-like	0.011	1.007	9.45E-01	9.45E+01		
8037535	---	---	---	-0.028	0.981	8.80E-01	8.80E+01		
8037537	NM_000400	ERCC2	excision repair cross-complementi	-0.017	0.988	9.09E-01	9.09E+01		

8037563	NM_006663	PPP1R13L	protein phosphatase 1, regulatory	-0.047	0.968	5.89E-01	5.89E+01		
8037579	NM_202001	ERCC1	excision repair cross-complementi	-0.168	0.890	6.92E-02	6.92E+00		
8037594	NM_005619	RTN2	reticulon 2	-0.052	0.965	6.05E-01	6.05E+01		
8037608	NM_00101798	OPA3	optic atrophy 3 (autosomal recess	0.055	1.039	5.91E-01	5.91E+01		
8037614	NM_005282	GPR4	G protein-coupled receptor 4	-0.059	0.960	5.57E-01	5.57E+01		
8037621	NM_012155	EML2	echinoderm microtubule associate	0.042	1.030	7.96E-01	7.96E+01		
8037640	---	---	---	0.010	1.007	9.56E-01	9.56E+01		
8037642	NM_177542	SNRPD2	small nuclear ribonucleoprotein D	-0.018	0.988	9.09E-01	9.09E+01		
8037647	NM_00108046	FBXO46	F-box protein 46	-0.077	0.948	4.70E-01	4.70E+01		
8037651	NM_175875	SIX5	SIX homeobox 5	-0.016	0.989	8.89E-01	8.89E+01		
8037657	NM_004409	DMPK	dystrophia myotonica-protein kina	-0.040	0.973	6.37E-01	6.37E+01		
8037679	NM_004943	DMWD	dystrophia myotonica, WD repeat	-0.083	0.944	2.54E-01	2.54E+01		
8037688	NM_030785	RSHL1	radial spokehead-like 1	-0.026	0.982	8.47E-01	8.47E+01		
8037695	NM_004819	SYMPK	symplekin	-0.002	0.998	9.91E-01	9.91E+01		
8037723	NM_015649	IRF2BP1	interferon regulatory factor 2 binc	-0.032	0.978	7.94E-01	7.94E+01		
8037728	NM_00101264	MYPOP	Myb-related transcription factor, p	-0.072	0.951	3.94E-01	3.94E+01		
8037732	NM_00102986	NANOS2	nanos homolog 2 (Drosophila)	0.043	1.030	7.53E-01	7.53E+01		
8037737	NM_002516	NOVA2	neuro-oncological ventral antigen	0.005	1.003	9.76E-01	9.76E+01		
8037742	NM_005091	PGLYRP1	peptidoglycan recognition protein	-0.054	0.963	7.31E-01	7.31E+01		
8037762	NM_032040	CCDC8	coiled-coil domain containing 8	-0.046	0.969	7.35E-01	7.35E+01		
8037767	NM_018215	PNMAL1	PNMA-like 1	0.024	1.017	8.46E-01	8.46E+01		
8037775	NM_000960	PTGIR	prostaglandin I2 (prostacyclin) rec	-0.200	0.871	5.18E-02	5.18E+00		
8037785	NM_033258	GNG8	guanine nucleotide binding protei	-0.109	0.928	4.08E-01	4.08E+01		
8037788	NM_145056	DACT3	dapper, antagonist of beta-catenir	-0.095	0.936	3.11E-01	3.11E+01		
8037794	NM_016457	PRKD2	protein kinase D2	-0.007	0.995	9.82E-01	9.82E+01		
8037814	---	---	---	0.059	1.042	7.38E-01	7.38E+01		
8037816	NM_013403	STRN4	striatin, calmodulin binding protei	0.028	1.020	8.08E-01	8.08E+01		
8037835	NM_005628	SLC1A5	solute carrier family 1 (neutral am	0.113	1.082	3.81E-01	3.81E+01		
8037847	NM_004069	AP2S1	adaptor-related protein complex 2	0.054	1.038	8.52E-01	8.52E+01		
8037853	NM_017854	TMEM160	transmembrane protein 160	-0.028	0.981	7.98E-01	7.98E+01		
8037856	NM_015168	ZC3H4	zinc finger CCCH-type containing 4	-0.033	0.978	7.97E-01	7.97E+01		
8037872	NM_00112724	BBC3	BCL2 binding component 3	-0.030	0.979	8.31E-01	8.31E+01		
8037878	NM_020160	MEIS3	Meis homeobox 3	0.018	1.013	9.26E-01	9.26E+01		
8037888	NM_015063	SLC8A2	solute carrier family 8 (sodium/cal	-0.032	0.978	7.82E-01	7.82E+01		
8037913	NM_003827	NAPA	N-ethylmaleimide-sensitive factor	0.139	1.101	2.69E-01	2.69E+01		
8037931	NM_00110141	ZNF541	zinc finger protein 541	-0.081	0.945	2.72E-01	2.72E+01		
8037947	NM_198479	TPRX1	tetra-peptide repeat homeobox 1	0.055	1.039	8.59E-01	8.59E+01		
8037962	NM_019855	CABP5	calcium binding protein 5	-0.008	0.994	9.73E-01	9.73E+01		
8037970	NM_003706	PLA2G4C	phospholipase A2, group IVC (cyto	0.151	1.111	5.98E-01	5.98E+01		
8037991	NM_000234	LIG1	ligase I, DNA, ATP-dependent	-0.059	0.960	5.76E-01	5.76E+01		
8038023	---	---	---	0.052	1.037	8.23E-01	8.23E+01		
8038029	NM_014959	CARD8	caspase recruitment domain famil	-0.022	0.985	9.02E-01	9.02E+01		
8038048	NM_144577	CCDC114	coiled-coil domain containing 114	0.011	1.008	9.56E-01	9.56E+01		
8038069	NM_018273	TMEM143	transmembrane protein 143	-0.010	0.993	9.62E-01	9.62E+01		
8038078	NM_006801	KDELR1	KDEL (Lys-Asp-Glu-Leu) endoplasm	-0.021	0.985	9.12E-01	9.12E+01		
8038086	NM_000979	RPL18	ribosomal protein L18	-0.004	0.997	9.86E-01	9.86E+01		
8038093	NM_00108043	LMTK3	lemur tyrosine kinase 3	-0.073	0.951	5.50E-01	5.50E+01		
8038109	NM_017708	FAM83E	family with sequence similarity 83	-0.151	0.900	1.36E-02	1.36E+00		
8038117	NM_001352	DBP	D site of albumin promoter (album	-0.047	0.968	6.98E-01	6.98E+01		
8038126	NM_001217	CA11	carbonic anhydrase XI	0.057	1.040	5.50E-01	5.50E+01		
8038139	NM_145807	NTN5	netrin 5	-0.059	0.960	6.34E-01	6.34E+01		
8038150	NM_182574	MAMSTR	MEF2 activating motif and SAP do	-0.115	0.924	1.42E-01	1.42E+01		
8038192	NM_000148	FUT1	fucosyltransferase 1 (galactoside 2	-0.027	0.981	8.62E-01	8.62E+01		
8038200	---	---	---	0.125	1.091	1.59E-01	1.59E+01		
8038225	NM_020904	PLEKHA4	pleckstrin homology domain conta	-0.074	0.950	3.85E-01	3.85E+01		
8038261	NM_002103	GYS1	glycogen synthase 1 (muscle)	-0.074	0.950	3.93E-01	3.93E+01		
8038278	NM_000894	LHB	luteinizing hormone beta polypep	-0.026	0.982	8.89E-01	8.89E+01		
8038280	NM_000737	CGB	chorionic gonadotropin, beta poly	-0.078	0.947	1.38E-01	1.38E+01		
8038288	NM_033377	CGB1	chorionic gonadotropin, beta poly	-0.085	0.943	2.11E-01	2.11E+01		
8038292	NM_033043	CGB5	chorionic gonadotropin, beta poly	-0.070	0.953	2.35E-01	2.35E+01		
8038299	NM_033142	CGB7	chorionic gonadotropin, beta poly	-0.041	0.972	6.54E-01	6.54E+01		
8038314	NM_018111	C19orf73	chromosome 19 open reading frar	-0.028	0.981	8.67E-01	8.67E+01		
8038319	NM_002152	HRC	histidine rich calcium binding prot	0.044	1.031	7.73E-01	7.73E+01		
8038326	NM_014037	SLC6A16	solute carrier family 6, member 16	0.133	1.097	4.83E-01	4.83E+01		
8038343	---	---	---	0.127	1.092	3.20E-01	3.20E+01		
8038347	NM_003598	TEAD2	TEA domain family member 2	-0.054	0.963	6.02E-01	6.02E+01		
8038362	NM_178449	PTH2	parathyroid hormone 2	-0.057	0.961	5.40E-01	5.40E+01		
8038382	NM_017916	PIH1D1	PIH1 domain containing 1	-0.053	0.964	7.18E-01	7.18E+01		
8038393	---	---	---	-0.065	0.956	3.94E-01	3.94E+01		
8038395	NM_015953	NOSIP	nitric oxide synthase interacting pi	-0.053	0.964	7.00E-01	7.00E+01		
8038407	NM_006270	RRAS	related RAS viral (r-ras) oncogene	-0.048	0.967	6.14E-01	6.14E+01		
8038416	NM_001571	IRF3	interferon regulatory factor 3	-0.075	0.949	4.04E-01	4.04E+01		
8038427	NM_021733	TSKS	testis-specific serine kinase substra	-0.035	0.976	7.81E-01	7.81E+01		
8038443	NM_025129	FUZ	fuzzy homolog (Drosophila)	-0.039	0.973	7.23E-01	7.23E+01		
8038458	NM_007254	PNKP	polynucleotide kinase 3'-phosphat	-0.042	0.972	6.95E-01	6.95E+01		
8038477	NM_032375	AKT1S1	AKT1 substrate 1 (proline-rich)	-0.050	0.966	7.40E-01	7.40E+01		
8038487	NM_172374	IL4I1	interleukin 4 induced 1	0.155	1.113	2.30E-01	2.30E+01		

8038505	NM_052884	SIGLEC11	sialic acid binding Ig-like lectin 11	0.075	1.054	4.04E-01	4.04E+01		
8038515	NM_016440	VRK3	vaccinia related kinase 3	-0.030	0.979	8.08E-01	8.08E+01		
8038540	NM_004977	KCNC3	potassium voltage-gated channel,	-0.004	0.998	9.85E-01	9.85E+01		
8038547	NR_002798	NAPSB	napsin B aspartic peptidase pseud	0.040	1.028	8.25E-01	8.25E+01		
8038556	NM_004851	NAPSA	napsin A aspartic peptidase	0.028	1.020	8.51E-01	8.51E+01		
8038565	ENST00000430	C19orf63	chromosome 19 open reading fran	-0.071	0.952	4.73E-01	4.73E+01		
8038571	NM_138334	JOSD2	Josephin domain containing 2	-0.019	0.987	9.09E-01	9.09E+01		
8038577	NM_00102465	ASPDH	aspartate dehydrogenase domain	-0.031	0.978	7.89E-01	7.89E+01		
8038584	NM_00108045	LRRC4B	leucine rich repeat containing 4B	0.096	1.069	2.35E-01	2.35E+01		
8038587	NM_032298	SYT3	synaptotagmin III	-0.040	0.973	7.45E-01	7.45E+01		
8038598	NM_016148	SHANK1	SH3 and multiple ankyrin repeat d	-0.012	0.991	9.46E-01	9.46E+01		
8038624	NM_199249	C19orf48	chromosome 19 open reading fran	-0.005	0.997	9.85E-01	9.85E+01		
8038633	NM_002257	KLK1	kallikrein 1	0.012	1.008	9.67E-01	9.67E+01		
8038643	NM_017509	KLK15	kallikrein-related peptidase 15	0.007	1.005	9.63E-01	9.63E+01		
8038670	NM_012427	KLK5	kallikrein-related peptidase 5	-0.057	0.961	5.48E-01	5.48E+01		
8038683	NM_002774	KLK6	kallikrein-related peptidase 6	-0.003	0.998	9.88E-01	9.88E+01		
8038707	NM_144505	KLK8	kallikrein-related peptidase 8	-0.079	0.946	1.16E-01	1.16E+01		
8038716	NM_012315	KLK9	kallikrein-related peptidase 9	-0.025	0.983	8.46E-01	8.46E+01		
8038725	NM_002776	KLK10	kallikrein-related peptidase 10	-0.036	0.975	7.08E-01	7.08E+01		
8038735	NM_144947	KLK11	kallikrein-related peptidase 11	-0.037	0.975	8.19E-01	8.19E+01		
8038747	NM_145894	KLK12	kallikrein-related peptidase 12	-0.056	0.962	6.44E-01	6.44E+01		
8038760	NM_015596	KLK13	kallikrein-related peptidase 13	0.002	1.002	9.90E-01	9.90E+01		
8038770	NM_022046	KLK14	kallikrein-related peptidase 14	0.016	1.011	9.27E-01	9.27E+01		
8038782	NM_145232	CTU1	cytosolic thiouridylase subunit 1 h	-0.029	0.980	8.85E-01	8.85E+01		
8038785	BC032383	LOC147645	hypothetical protein LOC147645	-0.017	0.988	9.19E-01	9.19E+01		
8038792	NM_001985	ETFB	electron-transfer-flavoprotein, bet	-0.003	0.998	9.89E-01	9.89E+01		
8038804	NM_152353	CLDND2	claudin domain containing 2	0.001	1.001	9.97E-01	9.97E+01		
8038809	NM_005601	NKG7	natural killer cell group 7 sequenc	0.145	1.106	7.33E-01	7.33E+01		
8038815	NM_030657	LIM2	lens intrinsic membrane protein 2,	0.011	1.008	9.51E-01	9.51E+01		
8038824	NM_033130	SIGLEC10	sialic acid binding Ig-like lectin 10	0.186	1.137	2.26E-01	2.26E+01		
8038839	NM_014442	SIGLEC8	sialic acid binding Ig-like lectin 8	0.035	1.025	7.71E-01	7.71E+01		
8038852	NM_053003	SIGLEC12	sialic acid binding Ig-like lectin 12	-0.074	0.950	4.25E-01	4.25E+01		
8038861	NM_001245	SIGLEC6	sialic acid binding Ig-like lectin 6	0.092	1.066	4.16E-01	4.16E+01		
8038877	NM_003830	SIGLEC5	sialic acid binding Ig-like lectin 5	-0.007	0.995	9.85E-01	9.85E+01		
8038890	NM_001523	HAS1	hyaluronan synthase 1	-0.087	0.942	2.22E-01	2.22E+01		
8038899	NM_002029	FPR1	formyl peptide receptor 1	0.590	1.505	7.03E-02	7.03E+00		
8038904	NM_032679	ZNF577	zinc finger protein 577	0.161	1.118	3.74E-01	3.74E+01		
8038913	NM_023074	ZNF649	zinc finger protein 649	0.015	1.010	9.56E-01	9.56E+01		
8038919	NM_021632	ZNF350	zinc finger protein 350	0.044	1.031	8.44E-01	8.44E+01		
8038942	NM_014650	ZNF432	zinc finger protein 432	-0.110	0.927	5.04E-01	5.04E+01		
8038954	NM_178523	ZNF616	zinc finger protein 616	0.041	1.029	8.99E-01	8.99E+01		
8038962	NM_00110265 //	ZNF836 //	zinc finger protein 836 // zinc fing	0.032	1.022	8.21E-01	8.21E+01		
8038967	NM_00110554	ZNF83	zinc finger protein 83	0.148	1.108	4.71E-01	4.71E+01		
8038989	NM_198457	ZNF600	zinc finger protein 600	-0.114	0.924	5.14E-01	5.14E+01		
8038993	NM_006969	ZNF28	zinc finger protein 28	0.125	1.091	5.98E-01	5.98E+01		
8038998	NM_199132	ZNF468	zinc finger protein 468	0.089	1.064	7.77E-01	7.77E+01		
8039010	NM_018260	ZNF701	zinc finger protein 701	0.021	1.015	8.91E-01	8.91E+01		
8039013	NM_203307	ZNF321	zinc finger protein 321	0.035	1.025	8.70E-01	8.70E+01		
8039034	NM_00110260	ZNF160	zinc finger protein 160	0.036	1.025	8.67E-01	8.67E+01		
8039068	NM_021074	NDUFV2	NADH dehydrogenase (ubiquinone	-0.134	0.911	1.56E-01	1.56E+01		
8039090	---	---	---	-0.026	0.982	8.93E-01	8.93E+01		
8039120	NM_206818	OSCAR	osteoclast associated, immunoglo	-0.059	0.960	4.79E-01	4.79E+01		
8039131	NM_013342	TFPT	TCF3 (E2A) fusion partner (in child	-0.034	0.977	8.03E-01	8.03E+01		
8039139	NM_024316	LENG1	leukocyte receptor cluster (LRC) m	0.156	1.114	1.69E-01	1.69E+01		
8039144	NM_00114530	TMC4	transmembrane channel-like 4	0.015	1.011	9.22E-01	9.22E+01		
8039166	NM_024298	MBOAT7	membrane bound O-acyltransfera	-0.007	0.995	9.64E-01	9.64E+01		
8039180	NM_00108144	LILRB5	leukocyte immunoglobulin-like rec	0.114	1.082	1.59E-01	1.59E+01		
8039196	NM_00108145	LILRB3	leukocyte immunoglobulin-like rec	0.270	1.206	3.18E-01	3.18E+01		
8039226	NM_006865	LILRA3	leukocyte immunoglobulin-like rec	0.255	1.193	6.11E-01	6.11E+01		
8039246	NM_012276	LILRA4	leukocyte immunoglobulin-like rec	0.097	1.070	3.27E-01	3.27E+01		
8039257	NM_002287	LAIR1	leukocyte-associated immunoglob	0.227	1.171	3.16E-01	3.16E+01		
8039269	NM_198988	LENG9	leukocyte receptor cluster (LRC) m	-0.036	0.975	7.52E-01	7.52E+01		
8039273	NM_145057	CDC42EP5	CDC42 effector protein (Rho GTPa	-0.015	0.990	8.97E-01	8.97E+01		
8039280	NM_00112725	NLRP7	NLR family, pyrin domain containi	-0.160	0.895	2.94E-01	2.94E+01		
8039294	NM_00108389	GP6	glycoprotein VI (platelet)	-0.097	0.935	3.21E-01	3.21E+01		
8039306	NM_00114597	RDH13	retinol dehydrogenase 13 (all-tran	-0.153	0.899	5.53E-02	5.53E+00		
8039316	NM_017607	PPP1R12C	protein phosphatase 1, regulatory	0.000	1.000	9.99E-01	9.99E+01		
8039340	NM_003283	TNNT1	troponin T type 1 (skeletal, slow)	0.134	1.097	5.04E-01	5.04E+01		
8039362	NM_178837	C19orf51	chromosome 19 open reading fran	-0.045	0.970	7.00E-01	7.00E+01		
8039378	NM_003180	SYT5	synaptotagmin V	0.015	1.010	9.18E-01	9.18E+01		
8039409	NM_173804	TMEM86B	transmembrane protein 86B	-0.017	0.989	9.03E-01	9.03E+01		
8039413	NM_014931	SAPS1	SAPS domain family, member 1	-0.029	0.980	8.48E-01	8.48E+01		
8039440	NM_012267	HSPBP1	HSPA (heat shock 70kDa) binding p	-0.029	0.980	8.47E-01	8.47E+01		
8039453	NM_00108548	TMEM150B	transmembrane protein 150B	0.021	1.015	9.00E-01	9.00E+01		
8039474	NM_00114540	FAM71E2	family with sequence similarity 71	-0.001	1.000	9.98E-01	9.98E+01		
8039477	BC031875	FAM71E2	family with sequence similarity 71	-0.029	0.980	7.97E-01	7.97E+01		
8039484	NM_000641	IL11	interleukin 11	-0.022	0.985	8.75E-01	8.75E+01		

8039491	NM_014501	UBE2S	ubiquitin-conjugating enzyme E2S	-0.145	0.905	1.67E-01	1.67E+01		
8039504	NM_00114517	hCG_2039146	UPF0626 protein A	-0.040	0.972	7.27E-01	7.27E+01		
8039511	NM_024710	ISOC2	isochorismatase domain containin	-0.022	0.985	8.78E-01	8.78E+01		
8039520	NM_00110140	SBK2	SH3-binding domain kinase family,	-0.096	0.935	4.13E-01	4.13E+01		
8039524	NM_152600	ZNF579	zinc finger protein 579	-0.047	0.968	5.77E-01	5.77E+01		
8039526	NM_032836	FIZ1	FLT3-interacting zinc finger 1	-0.020	0.986	9.02E-01	9.02E+01		
8039530	NM_203374	ZNF784	zinc finger protein 784	-0.026	0.982	8.06E-01	8.06E+01		
8039569	NM_00100283	ZNF787	zinc finger protein 787	-0.049	0.966	6.75E-01	6.75E+01		
8039605	NM_00100585	ZNF835	zinc finger protein 835	-0.061	0.959	4.10E-01	4.10E+01		
8039607	NM_006210	PEG3	paternally expressed 3	-0.048	0.967	6.51E-01	6.51E+01		
8039655	NM_00103965	ZNF550	zinc finger protein 550	-0.021	0.985	9.12E-01	9.12E+01		
8039664	NM_017879	ZNF416	zinc finger protein 416	-0.041	0.972	8.33E-01	8.33E+01		
8039674	NM_00108538	ZNF154	zinc finger protein 154	-0.016	0.989	9.66E-01	9.66E+01		
8039680	NM_024833	ZNF671	zinc finger protein 671	0.001	1.001	9.96E-01	9.96E+01		
8039692	NM_00114498	ZNF814	zinc finger protein 814	0.062	1.044	8.78E-01	8.78E+01		
8039695	NM_152475	ZNF417	zinc finger protein 417	-0.075	0.949	8.31E-01	8.31E+01		
8039698	NM_133460	ZNF418	zinc finger protein 418	0.010	1.007	9.78E-01	9.78E+01		
8039706	NM_005773	ZNF256	zinc finger protein 256	0.016	1.011	9.58E-01	9.58E+01		
8039719	NM_025027	ZNF606	zinc finger protein 606	0.050	1.035	6.37E-01	6.37E+01		
8039730	NM_023926	ZSCAN18	zinc finger and SCAN domain conta	-0.032	0.978	7.88E-01	7.88E+01		
8039740	NM_024620	ZNF329	zinc finger protein 329	-0.042	0.971	9.06E-01	9.06E+01		
8039748	NM_130786	A1BG	alpha-1-B glycoprotein	-0.037	0.975	7.27E-01	7.27E+01		
8039759	NM_198458	ZNF497	zinc finger protein 497	-0.070	0.952	4.02E-01	4.02E+01		
8039766	NM_00112973	ZNF837	zinc finger protein 837	-0.032	0.978	7.96E-01	7.96E+01		
8039769	---	---	---	-0.020	0.986	9.12E-01	9.12E+01		
8039771	NM_003433	ZNF132	zinc finger protein 132	0.062	1.044	6.60E-01	6.60E+01		
8039779	NM_012254	SLC27A5	solute carrier family 27 (fatty acid	-0.006	0.996	9.73E-01	9.73E+01		
8039791	NM_032792	ZBTB45	zinc finger and BTB domain contai	-0.004	0.997	9.85E-01	9.85E+01		
8039796	NM_014453	CHMP2A	chromatin modifying protein 2A	0.033	1.023	8.47E-01	8.47E+01		
8039805	NM_003969	UBE2M	ubiquitin-conjugating enzyme E2M	-0.071	0.952	5.29E-01	5.29E+01		
8039809	NM_198055	MZF1	myeloid zinc finger 1	0.021	1.015	8.96E-01	8.96E+01		
8039820	NM_153443	KIR3DL3	killer cell immunoglobulin-like rec	0.008	1.005	9.83E-01	9.83E+01		
8039829	NM_014219	KIR2DL2	killer cell immunoglobulin-like rec	0.072	1.051	8.75E-01	8.75E+01		
8039842	NM_012312	KIR2DS2	killer cell immunoglobulin-like rec	0.105	1.075	8.07E-01	8.07E+01		
8039859	NM_002255	KIR2DL4	killer cell immunoglobulin-like rec	0.090	1.065	7.53E-01	7.53E+01		
8039871	NM_00108353	KIR3DS1	killer cell immunoglobulin-like rec	0.075	1.053	8.47E-01	8.47E+01		
8039896	NM_014512	KIR2DS1	killer cell immunoglobulin-like rec	0.104	1.075	7.77E-01	7.77E+01		
8039905	NM_020141	TMEM167B	transmembrane protein 167B	0.002	1.002	9.92E-01	9.92E+01		
8039909	NM_00103957	CCNL2	cyclin L2	-0.024	0.983	8.80E-01	8.80E+01		
8039923	NM_017900	AURKAIP1	aurora kinase A interacting protei	-0.031	0.979	8.24E-01	8.24E+01		
8039933	NM_017761	PNRC2	proline-rich nuclear receptor coac	0.018	1.012	8.85E-01	8.85E+01		
8039939	NM_017971	MRPL20	mitochondrial ribosomal protein L	0.023	1.016	8.95E-01	8.95E+01		
8039945	---	---	---	0.147	1.107	4.51E-01	4.51E+01		
8039947	NM_006625	FUSIP1	FUS interacting protein (serine/arg	0.012	1.008	9.53E-01	9.53E+01		
8039961	NR_024080	ACP1	acid phosphatase 1, soluble	0.074	1.053	4.74E-01	4.74E+01		
8040018	NM_016030	TTC15	tetratricopeptide repeat domain 1	0.130	1.094	2.47E-01	2.47E+01		
8040036	NM_001011	RPS7	ribosomal protein S7	-0.012	0.992	9.56E-01	9.56E+01		
8040045	NM_199235	COLEC11	collectin sub-family member 11	0.011	1.008	9.28E-01	9.28E+01		
8040070	NM_003108	SOX11	SRY (sex determining region Y)-bo	0.002	1.001	9.88E-01	9.88E+01		
8040080	NM_080657	RSAD2	radical S-adenosyl methionine dor	0.472	1.387	5.35E-01	5.35E+01		
8040090	NM_014746	RNF144A	ring finger protein 144A	-0.107	0.928	5.53E-01	5.53E+01		
8040101	---	---	---	0.032	1.022	8.08E-01	8.08E+01		
8040103	NM_002166	ID2	inhibitor of DNA binding 2, domina	-0.151	0.901	3.41E-01	3.41E+01		
8040142	NM_016207	CPSF3	cleavage and polyadenylation spe	0.004	1.003	9.89E-01	9.89E+01		
8040163	NM_00103961	IAH1	isoamyl acetate-hydrolyzing ester	-0.140	0.907	1.50E-01	1.50E+01		
8040171	---	---	---	-0.142	0.906	5.72E-01	5.72E+01		
8040211	NM_003597	KLF11	Kruppel-like factor 11	0.186	1.137	3.07E-01	3.07E+01		
8040221	AF504647	LOC100131506	hypothetical LOC100131506	-0.057	0.961	6.66E-01	6.66E+01		
8040231	BC093999	C2orf48	chromosome 2 open reading fram	-0.055	0.962	6.60E-01	6.60E+01		
8040238	NM_134421	HPCAL1	hippocalcin-like 1	0.050	1.035	6.81E-01	6.81E+01		
8040249	NM_005742	PDIA6	protein disulfide isomerase family	0.163	1.119	5.80E-02	5.80E+00		
8040265	NM_002236	KCNF1	potassium voltage-gated channel,	-0.050	0.966	6.54E-01	6.54E+01		
8040278	NM_152391	PQLC3	PQ loop repeat containing 3	0.210	1.157	2.31E-02	2.31E+00		
8040290	---	---	---	-0.057	0.961	6.82E-01	6.82E+01		
8040334	---	---	---	0.067	1.048	7.18E-01	7.18E+01		
8040338	---	---	---	0.024	1.017	9.40E-01	9.40E+01		
8040340	NM_145693	LPIN1	lipin 1	-0.013	0.991	9.60E-01	9.60E+01		
8040362	XR_038318	LOC729992	similar to heat shock 70kD protein	-0.080	0.946	6.66E-01	6.66E+01		
8040365	NM_021643	TRIB2	tribbles homolog 2 (Drosophila)	0.142	1.104	4.31E-01	4.31E+01		
8040374	NM_145175	FAM84A	family with sequence similarity 84	-0.084	0.944	3.75E-01	3.75E+01		
8040386	NM_004939	DDX1	DEAD (Asp-Glu-Ala-Asp) box polyp	-0.007	0.995	9.67E-01	9.67E+01		
8040415	---	---	---	0.001	1.001	9.95E-01	9.95E+01		
8040419	NM_005378	MYCN	v-myc myelocytomatosis viral rela	-0.032	0.978	8.37E-01	8.37E+01		
8040456	NM_00110556	MSGN1	mesogenin 1	-0.045	0.969	6.57E-01	6.57E+01		
8040465	---	---	---	-0.101	0.932	5.45E-01	5.45E+01		
8040467	---	---	---	0.070	1.050	6.92E-01	6.92E+01		
8040469	AY189285	LOC100131373	hypothetical LOC100131373	0.075	1.053	4.54E-01	4.54E+01		

8040473	NM_004040	RHOB	ras homolog gene family, member	-0.002	0.998	9.89E-01	9.89E+01		
8040477	---	---	---	0.060	1.043	7.86E-01	7.86E+01		
8040479	NM_182828	GDF7	growth differentiation factor 7	-0.095	0.936	1.35E-01	1.35E+01		
8040486	---	---	---	-0.021	0.985	9.01E-01	9.01E+01		
8040514	---	---	---	-0.033	0.978	9.04E-01	9.04E+01		
8040530	NM_054033	FKBP1B	FK506 binding protein 1B, 12.6 kD	-0.062	0.958	2.51E-01	2.51E+01		
8040547	---	---	---	0.076	1.054	6.60E-01	6.60E+01		
8040549	---	---	---	0.039	1.028	7.53E-01	7.53E+01		
8040552	NM_147223	NCOA1	nuclear receptor coactivator 1	-0.074	0.950	6.40E-01	6.40E+01		
8040578	NM_024322	CENPO	centromere protein O	0.117	1.085	4.66E-01	4.66E+01		
8040587	NM_014971	EFR3B	EFR3 homolog B (S. cerevisiae)	-0.012	0.991	9.30E-01	9.30E+01		
8040614	---	---	---	0.034	1.024	8.47E-01	8.47E+01		
8040618	NM_016131	RAB10	RAB10, member RAS oncogene fa	0.119	1.086	2.91E-01	2.91E+01		
8040630	---	---	---	0.152	1.111	6.76E-01	6.76E+01		
8040632	ENST00000407	FAM59B	family with sequence similarity 59	-0.076	0.949	4.42E-01	4.42E+01		
8040639	NM_000183	HADHB	hydroxyacyl-Coenzyme A dehydro	0.116	1.084	3.99E-01	3.99E+01		
8040655	NM_033505	SELI	selenoprotein I	0.135	1.098	3.12E-01	3.12E+01		
8040690	NM_00110551	C2orf70	chromosome 2 open reading fram	0.010	1.007	9.69E-01	9.69E+01		
8040695	NM_002246	KCNK3	potassium channel, subfamily K, m	0.020	1.014	9.16E-01	9.16E+01		
8040698	NM_017877	C2orf18	chromosome 2 open reading fram	0.136	1.099	1.53E-01	1.53E+01		
8040712	NM_001809	CENPA	centromere protein A	0.033	1.023	8.47E-01	8.47E+01		
8040753	NM_017727	TMEM214	transmembrane protein 214	0.033	1.023	8.52E-01	8.52E+01		
8040774	NM_021831	AGBL5	ATP/GTP binding protein-like 5	-0.106	0.929	1.32E-01	1.32E+01		
8040792	NM_007046	EMILIN1	elastin microfibril interfacer 1	-0.003	0.998	9.86E-01	9.86E+01		
8040802	NM_000221	KHK	ketohexokinase (fructokinase)	-0.054	0.963	7.19E-01	7.19E+01		
8040815	NM_032604	ABHD1	abhydrolase domain containing 1	-0.036	0.976	8.33E-01	8.33E+01		
8040827	NM_175769	TCF23	transcription factor 23	-0.023	0.984	8.73E-01	8.73E+01		
8040831	NM_016085	C2orf28	chromosome 2 open reading fram	-0.038	0.974	8.06E-01	8.06E+01		
8040843	NM_004341	CAD	carbamoyl-phosphate synthetase	-0.060	0.960	5.30E-01	5.30E+01		
8040908	NM_014748	SNX17	sorting nexin 17	0.058	1.041	6.83E-01	6.83E+01		
8040927	NM_013392	NRBP1	nuclear receptor binding protein 1	-0.053	0.964	7.09E-01	7.09E+01		
8040949	NM_173853	KRTCAP3	keratinocyte associated protein 3	-0.007	0.995	9.80E-01	9.80E+01		
8040985	NM_032434	ZNF512	zinc finger protein 512	0.117	1.084	3.16E-01	3.16E+01		
8041000	NM_007266	GPN1	GPN-loop GTPase 1	-0.013	0.991	9.60E-01	9.60E+01		
8041015	NM_018158	SLC4A1AP	solute carrier family 4 (anion exch	-0.107	0.928	4.44E-01	4.44E+01		
8041027	NM_004891	MRPL33	mitochondrial ribosomal protein L	-0.060	0.959	4.97E-01	4.97E+01		
8041031	NM_199193	BRE	brain and reproductive organ-expr	0.053	1.037	7.05E-01	7.05E+01		
8041048	NM_005253	FOSL2	FOS-like antigen 2	-0.256	0.838	2.02E-03	2.02E-01		
8041122	NM_002709	PPP1CB	protein phosphatase 1, catalytic su	0.022	1.015	8.30E-01	8.30E+01		
8041149	NM_015131	WDR43	WD repeat domain 43	0.072	1.051	7.34E-01	7.34E+01		
8041168	NR_002741	SNORD53	small nucleolar RNA, C/D box 53	0.016	1.011	9.76E-01	9.76E+01		
8041170	---	---	---	0.122	1.088	8.06E-01	8.06E+01		
8041172	NM_199280	FAM179A	family with sequence similarity 17	0.019	1.013	9.19E-01	9.19E+01		
8041179	NM_024692	CLIP4	CAP-GLY domain containing linker	0.123	1.089	3.39E-01	3.39E+01		
8041197	NM_00112740	YPEL5	yippee-like 5 (Drosophila)	-0.085	0.943	3.38E-01	3.38E+01		
8041204	NR_002327	SNORA10	small nucleolar RNA, H/ACA box 1(	-0.099	0.934	5.38E-01	5.38E+01		
8041206	NM_030915	LBH	limb bud and heart development h	-0.016	0.989	9.53E-01	9.53E+01		
8041212	NM_182551	LCLAT1	lysocardiolipin acyltransferase 1	0.104	1.075	5.81E-01	5.81E+01		
8041223	---	---	---	0.018	1.012	9.50E-01	9.50E+01		
8041225	NM_014600	EHD3	EH-domain containing 3	-0.023	0.984	9.19E-01	9.19E+01		
8041236	NM_014946	SPAST	spastin	-0.010	0.993	9.70E-01	9.70E+01		
8041257	NM_017964	SLC30A6	solute carrier family 30 (zinc trans	-0.023	0.984	9.12E-01	9.12E+01		
8041273	NM_032312	YIPF4	Yip1 domain family, member 4	0.009	1.007	9.62E-01	9.62E+01		
8041281	---	---	---	0.039	1.027	8.54E-01	8.54E+01		
8041283	NM_016252	BIRC6	baculoviral IAP repeat-containing (	0.016	1.011	9.03E-01	9.03E+01		
8041360	NM_017735	TTC27	tetratricopeptide repeat domain 2	0.010	1.007	9.72E-01	9.72E+01		
8041422	NM_170672	RASGRP3	RAS guanyl releasing protein 3 (ca	0.327	1.254	7.18E-02	7.18E+00		
8041447	NM_016441	CRIM1	cysteine rich transmembrane BMP	-0.165	0.892	5.28E-01	5.28E+01		
8041487	NM_174931	CCDC75	coiled-coil domain containing 75	0.073	1.052	5.65E-01	5.65E+01		
8041495	NM_144736	C2orf56	chromosome 2 open reading fram	0.016	1.011	9.49E-01	9.49E+01		
8041508	NM_012413	QPCT	glutaminyl-peptide cyclotransfera	0.523	1.437	1.51E-01	1.51E+01		
8041542	NM_138801	GALM	galactose mutarotase (aldose 1-ep	0.059	1.042	8.00E-01	8.00E+01		
8041553	NM_024775	GEMIN6	gem (nuclear organelle) associated	0.100	1.072	5.51E-01	5.51E+01		
8041570	NM_152390	TMEM178	transmembrane protein 178	-0.014	0.990	8.74E-01	8.74E+01		
8041582	NM_138370	SGK493	protein kinase-like protein SgK493	-0.007	0.995	9.61E-01	9.61E+01		
8041592	NM_019063	EML4	echinoderm microtubule associate	-0.042	0.971	7.53E-01	7.53E+01		
8041617	NM_020744	MTA3	metastasis associated 1 family, me	0.008	1.005	9.77E-01	9.77E+01		
8041713	NM_177968	PPM1B	protein phosphatase 1B (formerly	0.027	1.019	8.50E-01	8.50E+01		
8041727	NM_000341	SLC3A1	solute carrier family 3 (cystine, dib	0.077	1.055	6.92E-01	6.92E+01		
8041745	NM_024766	C2orf34	chromosome 2 open reading fram	-0.036	0.975	8.92E-01	8.92E+01		
8041763	NM_005400	PRKCE	protein kinase C, epsilon	0.143	1.104	1.53E-01	1.53E+01		
8041781	NM_001430	EPAS1	endothelial PAS domain protein 1	0.176	1.130	8.38E-02	8.38E+00		
8041804	NM_00114505	LOC388946	hypothetical LOC388946	-0.040	0.973	8.05E-01	8.05E+01		
8041808	NM_012249	RHOQ	ras homolog gene family, member	0.030	1.021	8.78E-01	8.78E+01		
8041813	NM_014171	CRIPT	cysteine-rich PDZ-binding protein	-0.032	0.978	8.44E-01	8.44E+01		
8041820	NM_014011	SOCS5	suppressor of cytokine signaling 5	-0.009	0.994	9.63E-01	9.63E+01		
8041826	NM_020458	TTC7A	tetratricopeptide repeat domain 7	0.019	1.013	8.76E-01	8.76E+01		

8041867	NM_000251	MSH2	mutS homolog 2, colon cancer, no	0.034	1.024	8.94E-01	8.94E+01		
8041886	AK026773	LOC440863	hypothetical gene supported by A	0.081	1.058	6.59E-01	6.59E+01		
8041888	NM_000179	MSH6	mutS homolog 6 (E. coli)	-0.007	0.995	9.75E-01	9.75E+01		
8041902	NM_002158	FOXN2	forkhead box N2	0.152	1.111	1.07E-01	1.07E+01		
8041913	NR_024188	KLRAQ1	KLRAQ motif containing 1	0.124	1.090	2.30E-01	2.30E+01		
8041937	AK128867	FLJ46838	FLJ46838 protein	0.017	1.012	9.19E-01	9.19E+01		
8041967	NM_015701	ERLEC1	endoplasmic reticulum lectin 1	0.009	1.006	9.65E-01	9.65E+01		
8041982	NM_138448	ACYP2	acylphosphatase 2, muscle type	-0.009	0.994	9.78E-01	9.78E+01		
8041995	NM_003128	SPTBN1	spectrin, beta, non-erythrocytic 1	-0.014	0.990	9.68E-01	9.68E+01		
8042038	NR_002229	RPL23AP32	ribosomal protein L23a pseudoger	-0.053	0.964	7.09E-01	7.09E+01		
8042040	NM_00103975	EML6	echinoderm microtubule associate	0.036	1.025	8.46E-01	8.46E+01		
8042052	NM_002954	RPS27A	ribosomal protein S27a	-0.002	0.998	9.86E-01	9.86E+01		
8042059	NR_027258	NCRNA00117	non-protein coding RNA 117	0.091	1.065	5.01E-01	5.01E+01		
8042066	NM_080667	CCDC104	coiled-coil domain containing 104	-0.234	0.850	1.24E-02	1.24E+00		
8042086	NM_006296	VRK2	vaccinia related kinase 2	0.188	1.139	3.69E-01	3.69E+01		
8042107	---	---	---	-0.109	0.927	4.44E-01	4.44E+01		
8042109	---	---	---	-0.049	0.966	8.36E-01	8.36E+01		
8042119	NM_022894	PAPOLG	poly(A) polymerase gamma	-0.066	0.955	5.86E-01	5.86E+01		
8042144	NM_002908	REL	v-rel reticuloendotheliosis viral on	-0.060	0.960	7.48E-01	7.48E+01		
8042157	---	---	---	-0.054	0.963	9.16E-01	9.16E+01		
8042161	NM_002618	PEX13	peroxisomal biogenesis factor 13	0.055	1.039	8.27E-01	8.27E+01		
8042195	NM_152392	AHSA2	AHA1, activator of heat shock 90k	0.047	1.033	7.09E-01	7.09E+01		
8042207	NM_152516	COMMD1	copper metabolism (Murr1) doma	0.115	1.083	5.58E-01	5.58E+01		
8042211	NM_006577	B3GNT2	UDP-GlcNAc:betaGal beta-1,3-N-a	0.134	1.097	1.56E-01	1.56E+01		
8042219	---	---	---	0.097	1.069	8.37E-01	8.37E+01		
8042221	---	---	---	0.053	1.038	6.80E-01	6.80E+01		
8042251	NM_014562	OTX1	orthodenticle homeobox 1	-0.063	0.957	5.60E-01	5.60E+01		
8042257	---	---	---	-0.121	0.919	5.60E-01	5.60E+01		
8042270	NM_00100152	UGP2	UDP-glucose pyrophosphorylase 2	0.007	1.005	9.80E-01	9.80E+01		
8042283	NM_014181	HSPC159	galectin-related protein	0.040	1.028	9.27E-01	9.27E+01		
8042291	NM_203437	AFTPH	aftiphilin	0.074	1.052	2.16E-01	2.16E+01		
8042306	---	---	---	0.280	1.214	2.37E-02	2.37E+00		
8042310	NM_003038	SLC1A4	solute carrier family 1 (glutamate/	-0.002	0.999	9.95E-01	9.95E+01		
8042326	NM_015147	CEP68	centrosomal protein 68kDa	-0.068	0.954	5.15E-01	5.15E+01		
8042333	---	---	---	0.003	1.002	9.90E-01	9.90E+01		
8042335	NM_003375	VDAC2	voltage-dependent anion channel	-0.046	0.968	7.28E-01	7.28E+01		
8042337	NM_00100538	ACTR2	ARP2 actin-related protein 2 homo	0.092	1.066	3.04E-01	3.04E+01		
8042381	NM_020143	PNO1	partner of NOB1 homolog (S. cerev	0.081	1.058	5.49E-01	5.49E+01		
8042413	NM_138964	PROKR1	prokineticin receptor 1	0.011	1.008	9.54E-01	9.54E+01		
8042416	NM_014882	ARHGAP25	Rho GTPase activating protein 25	0.233	1.175	6.04E-02	6.04E+00		
8042487	NM_178439	GMCL1	germ cell-less homolog 1 (Drosoph	-0.050	0.966	6.84E-01	6.84E+01		
8042495	NM_006857	SNRNP27	small nuclear ribonucleoprotein 2	0.020	1.014	9.00E-01	9.00E+01		
8042501	---	---	---	0.128	1.093	6.59E-01	6.59E+01		
8042503	NM_002357	MXD1	MAX dimerization protein 1	-0.030	0.979	9.60E-01	9.60E+01		
8042513	---	---	---	0.024	1.017	8.41E-01	8.41E+01		
8042515	NM_006196	PCBP1	poly(rC) binding protein 1	0.040	1.028	7.25E-01	7.25E+01		
8042519	NM_016297	PCYOX1	prenylcysteine oxidase 1	-0.183	0.881	1.84E-01	1.84E+01		
8042530	---	---	---	0.004	1.003	9.86E-01	9.86E+01		
8042532	NM_012476	VAX2	ventral anterior homeobox 2	-0.029	0.980	8.24E-01	8.24E+01		
8042537	NM_001692	ATP6V1B1	ATPase, H+ transporting, lysosoma	-0.027	0.981	7.44E-01	7.44E+01		
8042574	NR_002822	MGC72080	MGC72080 pseudogene	-0.078	0.947	6.51E-01	6.51E+01		
8042588	NM_005791	MPHOSPH10	M-phase phosphoprotein 10 (U3 s	-0.038	0.974	8.30E-01	8.30E+01		
8042601	NM_014497	ZNF638	zinc finger protein 638	-0.031	0.979	8.17E-01	8.17E+01		
8042696	NM_003124	SPR	sepiapterin reductase (7,8-dihydro	-0.066	0.955	6.77E-01	6.77E+01		
8042701	NM_004097	EMX1	empty spiracles homeobox 1	-0.018	0.988	8.78E-01	8.78E+01		
8042705	NM_006062	SMYD5	SMYD family member 5	-0.002	0.999	9.92E-01	9.92E+01		
8042720	NM_006429	CCT7	chaperonin containing TCP1, subu	-0.094	0.937	6.20E-01	6.20E+01		
8042737	NM_015120	ALMS1	Alstrom syndrome 1	-0.044	0.970	8.21E-01	8.21E+01		
8042772	NM_213622	STAMBP	STAM binding protein	-0.028	0.981	8.84E-01	8.84E+01		
8042801	NM_080916	DGUOK	deoxyguanosine kinase	-0.049	0.967	7.62E-01	7.62E+01		
8042811	NM_144993	TET3	tet oncogene family member 3	0.018	1.012	9.21E-01	9.21E+01		
8042825	---	---	---	0.036	1.025	7.56E-01	7.56E+01		
8042843	NM_032118	WDR54	WD repeat domain 54	-0.067	0.955	7.08E-01	7.08E+01		
8042859	NM_031288	INO80B	INO80 complex subunit B	-0.049	0.966	7.34E-01	7.34E+01		
8042867	NM_012477	WBP1	WW domain binding protein 1	-0.093	0.938	2.97E-01	2.97E+01		
8042874	NM_022492	TTC31	tetratricopeptide repeat domain 3	0.016	1.011	9.44E-01	9.44E+01		
8042896	NM_016170	TLX2	T-cell leukemia homeobox 2	-0.079	0.947	3.01E-01	3.01E+01		
8042905	NM_013247	HTRA2	HtrA serine peptidase 2	-0.020	0.986	9.12E-01	9.12E+01		
8042917	NM_001381	DOK1	docking protein 1, 62kDa (downstr	0.099	1.071	3.07E-01	3.07E+01		
8042925	NM_004263	SEMA4F	sema domain, immunoglobulin do	-0.028	0.980	7.92E-01	7.92E+01		
8042942	NM_000189	HK2	hexokinase 2	-0.177	0.885	5.71E-01	5.71E+01		
8042953	NM_019896	POLE4	polymerase (DNA-directed), epsilo	0.133	1.097	8.62E-02	8.62E+00		
8042962	NM_014763	MRPL19	mitochondrial ribosomal protein L	0.014	1.010	9.37E-01	9.37E+01		
8042972	---	---	---	-0.116	0.923	6.38E-01	6.38E+01		
8043018	---	---	---	-0.043	0.971	7.93E-01	7.93E+01		
8043034	---	---	---	0.198	1.147	1.25E-01	1.25E+01		
8043036	AK293146	DHFR	dihydrofolate reductase	0.454	1.370	1.38E-01	1.38E+01		

8043055	NM_001370	DNAH6	dynein, axonemal, heavy chain 6	-0.045	0.969	7.83E-01	7.83E+01		
8043100	NM_021103	TMSB10	thymosin beta 10	0.199	1.148	3.63E-02	3.63E+00		
8043105	NM_020122	KCMF1	potassium channel modulatory fac	0.013	1.009	9.23E-01	9.23E+01		
8043114	NM_031283	TCF7L1	transcription factor 7-like 1 (T-cell	0.001	1.001	9.93E-01	9.93E+01		
8043131	NM_00113502	ELMOD3	ELMO/CED-12 domain containing	-0.013	0.991	9.55E-01	9.55E+01		
8043155	---	---	---	-0.146	0.904	2.74E-01	2.74E+01		
8043157	NM_198482	SH2D6	SH2 domain containing 6	-0.086	0.942	2.28E-01	2.28E+01		
8043187	NM_005911	MAT2A	methionine adenosyltransferase II	0.139	1.101	9.91E-03	9.91E-01		
8043209	NM_016494	RNF181	ring finger protein 181	0.097	1.069	2.91E-01	2.91E+01		
8043218	NM_006590	USP39	ubiquitin specific peptidase 39	-0.060	0.959	6.37E-01	6.37E+01		
8043236	NM_012483	GNLY	granulysin	0.182	1.135	6.78E-01	6.78E+01		
8043251	NM_017952	PTCD3	Pentatricopeptide repeat domain	-0.057	0.961	7.54E-01	7.54E+01		
8043276	NR_004378	SNORD94	small nucleolar RNA, C/D box 94	0.141	1.103	5.73E-01	5.73E+01		
8043278	NM_016622	MRPL35	mitochondrial ribosomal protein L	0.056	1.040	7.89E-01	7.89E+01		
8043283	NM_018433	KDM3A	lysine (K)-specific demethylase 3A	-0.087	0.942	5.33E-01	5.33E+01		
8043310	NM_022780	RMND5A	required for meiotic nuclear divisi	-0.070	0.953	7.16E-01	7.16E+01		
8043320	AK098018	LOC400965	hypothetical LOC400965	-0.108	0.928	3.56E-01	3.56E+01		
8043322	NM_022662	ANAPC1	anaphase promoting complex subi	-0.057	0.961	8.32E-01	8.32E+01		
8043324	NM_00102445	RGPD1	RANBP2-like and GRIP domain con	-0.080	0.946	5.29E-01	5.29E+01		
8043347	---	---	---	-0.016	0.989	9.64E-01	9.64E+01		
8043349	NM_022662	ANAPC1	anaphase promoting complex subi	-0.058	0.960	8.63E-01	8.63E+01		
8043363	NR_024204	NCRNA00152	non-protein coding RNA 152	0.005	1.004	9.87E-01	9.87E+01		
8043375	---	---	---	-0.054	0.963	8.40E-01	8.40E+01		
8043377	NM_012477	WBP1	WW domain binding protein 1	-0.108	0.928	4.59E-01	4.59E+01		
8043393	NM_018271	THNSL2	threonine synthase-like 2 (S. cerev	-0.010	0.993	9.61E-01	9.61E+01		
8043407	NM_152670	C2orf51	chromosome 2 open reading fram	0.024	1.017	8.66E-01	8.66E+01		
8043413	NM_144563	RPIA	ribose 5-phosphate isomerase A	-0.055	0.963	8.02E-01	8.02E+01		
8043426	---	---	---	0.024	1.017	8.92E-01	8.92E+01		
8043433	---	---	---	0.236	1.177	2.69E-03	2.69E-01		
8043438	BC093097	IGKC	immunoglobulin kappa constant	0.317	1.246	4.39E-03	4.39E-01		
8043443	---	---	---	0.157	1.115	3.32E-01	3.32E+01		
8043474	ENST00000390	LOC652493	similar to Ig kappa chain V-I region	0.046	1.032	7.52E-01	7.52E+01		
8043484	---	---	---	0.205	1.153	2.29E-02	2.29E+00		
8043491	NM_144705	TEKT4	tektin 4	0.004	1.003	9.83E-01	9.83E+01		
8043502	---	---	---	-0.155	0.898	8.63E-01	8.63E+01		
8043504	NM_002371	MAL	mal, T-cell differentiation protein	0.152	1.111	5.10E-01	5.10E+01		
8043512	NM_021088	ZNF2	zinc finger protein 2	0.010	1.007	9.63E-01	9.63E+01		
8043522	NM_144707	PROM2	prominin 2	-0.049	0.967	7.03E-01	7.03E+01		
8043552	NM_013434	KCNIP3	Kv channel interacting protein 3, c	-0.041	0.972	7.05E-01	7.05E+01		
8043564	NM_016044	FAHD2A	fumarylacetoacetate hydrolase do	-0.045	0.970	8.37E-01	8.37E+01		
8043570	---	---	---	-0.082	0.945	2.72E-01	2.72E+01		
8043585	NM_004804	CIAO1	cytosolic iron-sulfur protein assem	-0.057	0.961	6.44E-01	6.44E+01		
8043597	NM_178495	ITPRIPL1	inositol 1,4,5-triphosphate recepto	-0.097	0.935	2.77E-01	2.77E+01		
8043602	NM_015341	NCAPH	non-SMC condensin I complex, sul	-0.117	0.922	6.48E-01	6.48E+01		
8043621	NM_212481	ARID5A	AT rich interactive domain 5A (MR	-0.090	0.940	2.30E-01	2.30E+01		
8043630	AY358167	UNQ5814	RFVG5814	-0.075	0.949	6.05E-01	6.05E+01		
8043632	NM_00111338	FER1L5	fer-1-like 5 (C. elegans)	-0.043	0.971	7.19E-01	7.19E+01		
8043639	NM_00107740	FER1L5	fer-1-like 5 (C. elegans)	-0.082	0.945	4.77E-01	4.77E+01		
8043657	NM_020184	CNNM4	cyclin M4	-0.067	0.955	6.56E-01	6.56E+01		
8043666	NM_017623	CNNM3	cyclin M3	-0.032	0.978	8.45E-01	8.45E+01		
8043682	---	---	---	-0.087	0.941	5.72E-01	5.72E+01		
8043685	---	---	---	0.038	1.026	8.93E-01	8.93E+01		
8043687	AK304740	ANKRD36	ankyrin repeat domain 36	0.025	1.018	9.50E-01	9.50E+01		
8043697	NM_025190	ANKRD36B	ankyrin repeat domain 36B	-0.020	0.986	9.44E-01	9.44E+01		
8043718	NM_001862	COX5B	cytochrome c oxidase subunit Vb	0.055	1.039	7.00E-01	7.00E+01		
8043725	NM_001079	ZAP70	zeta-chain (TCR) associated protei	0.041	1.028	7.80E-01	7.80E+01		
8043743	---	---	---	-0.225	0.856	9.35E-02	9.35E+00		
8043745	ENST00000429	TMEM131	transmembrane protein 131	-0.026	0.982	8.80E-01	8.80E+01		
8043782	NM_001298	CNGA3	cyclic nucleotide gated channel alp	0.021	1.015	8.97E-01	8.97E+01		
8043791	NM_00113422	INPP4A	inositol polyphosphate-4-phospha	0.102	1.073	3.38E-01	3.38E+01		
8043820	NM_014044	UNC50	unc-50 homolog (C. elegans)	-0.050	0.966	7.75E-01	7.75E+01		
8043848	NM_145212	MRPL30	mitochondrial ribosomal protein L	-0.023	0.984	9.48E-01	9.48E+01		
8043861	NM_015904	EIF5B	eukaryotic translation initiation fa	-0.010	0.993	9.58E-01	9.58E+01		
8043890	NM_00101171	NMS	neuromedin S	-0.024	0.984	8.77E-01	8.77E+01		
8043900	---	---	---	-0.023	0.984	9.53E-01	9.53E+01		
8043902	NM_024065	PDCL3	phosducin-like 3	-0.037	0.975	8.36E-01	8.36E+01		
8043909	NM_002518	NPAS2	neuronal PAS domain protein 2	-0.068	0.954	4.50E-01	4.50E+01		
8043932	NM_00109969	RPL31	ribosomal protein L31	-0.003	0.998	9.90E-01	9.90E+01		
8043937	NM_017546	C2orf29	chromosome 2 open reading fram	0.038	1.027	7.56E-01	7.56E+01		
8043945	NM_145686	MAP4K4	mitogen-activated protein kinase I	-0.172	0.887	3.03E-02	3.03E+00		
8044049	NM_003853	IL18RAP	interleukin 18 receptor accessory	0.139	1.101	7.93E-01	7.93E+01		
8044109	NM_006236	POU3F3	POU class 3 homeobox 3	-0.012	0.992	9.40E-01	9.40E+01		
8044111	NM_182640	MRPS9	mitochondrial ribosomal protein S	-0.108	0.928	3.93E-01	3.93E+01		
8044127	NM_024093	C2orf49	chromosome 2 open reading fram	0.102	1.073	4.34E-01	4.34E+01		
8044133	NM_003581	NCK2	NCK adaptor protein 2	-0.124	0.917	3.80E-01	3.80E+01		
8044154	NM_172099	CD8B	CD8b molecule	-0.106	0.929	4.34E-01	4.34E+01		
8044161	NM_182588	RGPD4	RANBP2-like and GRIP domain con	-0.068	0.954	6.00E-01	6.00E+01		

8044189	---	---	---	-0.016	0.989	9.12E-01	9.12E+01		
8044236	NM_181453	GCC2	GRIP and coiled-coil domain conta	-0.040	0.973	7.77E-01	7.77E+01		
8044258	NM_004987	LIMS1	LIM and senescent cell antigen-like	0.062	1.044	7.90E-01	7.90E+01		
8044263	NM_006267	RANBP2	RAN binding protein 2	-0.001	0.999	9.95E-01	9.95E+01		
8044295	NM_00109928	SH3RF3	SH3 domain containing ring finger	0.049	1.034	6.56E-01	6.56E+01		
8044301	NM_023016	ANKRD57	ankyrin repeat domain 57	-0.204	0.868	1.20E-01	1.20E+01		
8044304	NM_00112336	RGPD6	RANBP2-like and GRIP domain con	-0.088	0.941	4.68E-01	4.68E+01		
8044333	NM_004987	LIMS1	LIM and senescent cell antigen-like	0.195	1.145	3.04E-01	3.04E+01		
8044351	CR590757	RPL22P15	ribosomal protein L22 pseudogene	-0.054	0.963	6.72E-01	6.72E+01		
8044375	NM_138621	BCL2L11	BCL2-like 11 (apoptosis facilitator)	0.061	1.043	7.64E-01	7.64E+01		
8044415	---	---	---	0.134	1.097	4.05E-01	4.05E+01		
8044417	NM_032824	TMEM87B	transmembrane protein 87B	0.200	1.149	1.54E-01	1.54E+01		
8044440	NM_153214	FBLN7	fibulin 7	-0.065	0.956	4.63E-01	4.63E+01		
8044450	NM_198581	ZC3H6	zinc finger CCCH-type containing 6	0.025	1.017	9.18E-01	9.18E+01		
8044462	NM_153712	TTL	tubulin tyrosine ligase	0.109	1.079	3.09E-01	3.09E+01		
8044491	NM_032309	CHCHD5	coiled-coil-helix-coiled-coil-helix d	-0.114	0.924	2.96E-01	2.96E+01		
8044499	NM_005415	SLC20A1	solute carrier family 20 (phosphate	0.035	1.025	8.60E-01	8.60E+01		
8044548	NM_014440	IL1F6	interleukin 1 family, member 6 (ep	0.065	1.046	5.87E-01	5.87E+01		
8044552	NM_173170	IL1F5	interleukin 1 family, member 5 (de	-0.070	0.952	4.35E-01	4.35E+01		
8044563	NM_032556	IL1F10	interleukin 1 family, member 10 (t	-0.006	0.996	9.80E-01	9.80E+01		
8044574	NM_173842	IL1RN	interleukin 1 receptor antagonist	-0.245	0.844	6.61E-01	6.61E+01		
8044584	NM_012455	PSD4	pleckstrin and Sec7 domain contai	-0.053	0.964	6.33E-01	6.33E+01		
8044613	NM_00114535	CBWD1	COBW domain containing 1	0.139	1.101	1.29E-01	1.29E+01		
8044634	NM_012184	FOXD4L1	forkhead box D4-like 1	-0.062	0.958	3.18E-01	3.18E+01		
8044640	NM_021965	PGM5	phosphoglucomutase 5	-0.005	0.997	9.85E-01	9.85E+01		
8044649	NR_024077	WASH2P	WAS protein family homolog 2 pse	-0.020	0.986	8.88E-01	8.88E+01		
8044669	NM_013412	RABL2A	RAB, member of RAS oncogene fa	0.032	1.022	8.63E-01	8.63E+01		
8044682	---	---	---	0.061	1.043	8.03E-01	8.03E+01		
8044686	NM_005721	ACTR3	ARP3 actin-related protein 3 homo	0.087	1.062	3.63E-01	3.63E+01		
8044731	---	---	---	-0.049	0.967	8.18E-01	8.18E+01		
8044745	NM_006773	DDX18	DEAD (Asp-Glu-Ala-Asp) box polyp	-0.005	0.996	9.76E-01	9.76E+01		
8044760	---	---	---	-0.031	0.979	8.33E-01	8.33E+01		
8044764	---	---	---	0.067	1.048	5.53E-01	5.53E+01		
8044766	NM_016133	INSIG2	insulin induced gene 2	-0.178	0.884	3.32E-01	3.32E+01		
8044773	NM_006770	MARCO	macrophage receptor with collage	0.074	1.053	5.15E-01	5.15E+01		
8044793	NM_182915	STEAP3	STEAP family member 3	-0.023	0.984	8.62E-01	8.62E+01		
8044804	NM_020548	DBI	diazepam binding inhibitor (GABA	0.008	1.006	9.78E-01	9.78E+01		
8044813	NM_183240	TMEM37	transmembrane protein 37	-0.041	0.972	7.29E-01	7.29E+01		
8044844	NM_00110519	TMEM177	transmembrane protein 177	0.022	1.015	8.99E-01	8.99E+01		
8044849	NM_002830	PTPN4	protein tyrosine phosphatase, nor	0.003	1.002	9.91E-01	9.91E+01		
8044882	NM_020909	EPB41L5	erythrocyte membrane protein ba	-0.131	0.913	3.35E-01	3.35E+01		
8044909	---	---	---	-0.030	0.980	9.77E-01	9.77E+01		
8044913	---	---	---	0.179	1.132	6.90E-01	6.90E+01		
8044915	---	---	---	-0.037	0.974	9.27E-01	9.27E+01		
8044919	NM_002881	RALB	v-ral simian leukemia viral oncoge	0.277	1.212	1.61E-01	1.61E+01		
8044927	NM_002193	INHBB	inhibin, beta B	-0.059	0.960	6.13E-01	6.13E+01		
8044933	NM_005270	GLI2	GLI family zinc finger 2	-0.047	0.968	6.89E-01	6.89E+01		
8044961	NR_023343	RNU4ATAC	RNA, U4atac small nuclear (U12-de	-0.206	0.867	3.06E-01	3.06E+01		
8044963	---	---	---	-0.003	0.998	9.95E-01	9.95E+01		
8044965	NM_004622	TSN	translin	0.059	1.042	6.92E-01	6.92E+01		
8045009	NM_002101	GYPC	glycophorin C (Gerbich blood grou	0.000	1.000	1.00E+00	1.00E+02		
8045018	NM_000312	PROC	protein C (inactivator of coagulatio	-0.009	0.994	9.53E-01	9.53E+01		
8045030	NM_00108052	MYO7B	myosin VIIB	0.002	1.001	9.87E-01	9.87E+01		
8045083	NM_032740	SFT2D3	SFT2 domain containing 3	0.072	1.051	3.66E-01	3.66E+01		
8045086	---	---	---	-0.069	0.953	6.33E-01	6.33E+01		
8045088	---	---	---	0.109	1.079	7.00E-01	7.00E+01		
8045090	NM_020120	UGGT1	UDP-glucose glycoprotein glucosyl	0.057	1.040	6.14E-01	6.14E+01		
8045136	NM_032144	RAB6C	RAB6C, member RAS oncogene fa	-0.002	0.998	9.88E-01	9.88E+01		
8045142	NM_025029	FAM128B	family with sequence similarity 12	-0.102	0.932	3.94E-01	3.94E+01		
8045146	AK024408	FAM128B	family with sequence similarity 12	0.025	1.017	8.66E-01	8.66E+01		
8045148	ENST00000427	FAM128A	family with sequence similarity 12	0.011	1.008	9.63E-01	9.63E+01		
8045167	---	---	---	0.128	1.093	2.94E-01	2.94E+01		
8045171	NM_033416	IMP4	IMP4, U3 small nucleolar ribonucle	-0.065	0.956	7.83E-01	7.83E+01		
8045182	NM_014369	PTPN18	protein tyrosine phosphatase, nor	0.066	1.047	6.46E-01	6.46E+01		
8045198	NM_00107953	CFC1B	cripto, FRL-1, cryptic family 1B	-0.065	0.956	4.16E-01	4.16E+01		
8045208	NM_00108353	POTEE	POTE ankyrin domain family, mem	0.003	1.002	9.85E-01	9.85E+01		
8045216	NM_207364	GPR148	G protein-coupled receptor 148	-0.020	0.986	9.12E-01	9.12E+01		
8045224	NM_152698	FAM123C	family with sequence similarity 12	-0.005	0.996	9.81E-01	9.81E+01		
8045229	NM_032995	ARHGEF4	Rho guanine nucleotide exchange	-0.075	0.949	3.56E-01	3.56E+01		
8045247	NM_017958	PLEKHB2	pleckstrin homology domain conta	0.106	1.076	6.71E-01	6.71E+01		
8045279	---	---	---	-0.015	0.990	9.53E-01	9.53E+01		
8045287	---	---	---	-0.048	0.967	9.24E-01	9.24E+01		
8045289	NM_012249	RHOQ	ras homolog gene family, member	0.165	1.121	3.44E-01	3.44E+01		
8045291	NM_080386	TUBA3D	tubulin, alpha 3d	-0.001	0.999	9.95E-01	9.95E+01		
8045301	NM_017751	SMPD4	sphingomyelin phosphodiesterase	-0.084	0.944	4.19E-01	4.19E+01		
8045309	NM_138770	CCDC74A	coiled-coil domain containing 74A	-0.042	0.972	8.10E-01	8.10E+01		
8045319	---	---	---	-0.062	0.958	8.05E-01	8.05E+01		

8045321	NM_00109977	POTEF	POTE ankyrin domain family, mem	0.035	1.024	8.18E-01	8.18E+01		
8045323	NM_013310	C2orf27A	chromosome 2 open reading fram	-0.019	0.987	9.10E-01	9.10E+01		
8045332	---	---	---	-0.105	0.930	4.73E-01	4.73E+01		
8045347	---	---	---	0.241	1.182	6.43E-01	6.43E+01		
8045349	NM_002410	MGAT5	mannosyl (alpha-1,6-)-glycoprotei	0.017	1.012	9.56E-01	9.56E+01		
8045381	NM_058241	CCNT2	cyclin T2	0.045	1.032	7.23E-01	7.23E+01		
8045398	NM_012233	RAB3GAP1	RAB3 GTPase activating protein su	0.117	1.085	3.75E-01	3.75E+01		
8045423	NR_002973	SNORA40	small nucleolar RNA, H/ACA box 4	0.156	1.114	4.76E-01	4.76E+01		
8045425	NM_015361	R3HDM1	R3H domain containing 1	-0.019	0.987	9.15E-01	9.15E+01		
8045453	---	---	---	0.036	1.026	9.07E-01	9.07E+01		
8045455	NM_014607	UBXN4	UBX domain protein 4	-0.022	0.985	8.50E-01	8.50E+01		
8045514	NM_00100166	SPOPL	speckle-type POZ protein-like	0.069	1.049	8.24E-01	8.24E+01		
8045533	---	---	---	0.245	1.185	7.99E-01	7.99E+01		
8045539	NM_003937	KYNU	kynureninase (L-kynurenine hydro	-0.418	0.748	6.46E-02	6.46E+00		
8045563	NM_018460	ARHGAP15	Rho GTPase activating protein 15	0.106	1.076	5.09E-01	5.09E+01		
8045604	NM_018328	MBD5	methyl-CpG binding domain prote	0.002	1.001	9.91E-01	9.91E+01		
8045619	NM_015630	EPC2	enhancer of polycomb homolog 2	-0.040	0.973	7.54E-01	7.54E+01		
8045637	NM_004522	KIF5C	kinesin family member 5C	-0.218	0.860	4.37E-01	4.37E+01		
8045664	NM_177964	LYPD6B	LY6/PLAUR domain containing 6B	0.013	1.009	9.25E-01	9.25E+01		
8045674	NM_194317	LYPD6	LY6/PLAUR domain containing 6	0.080	1.057	2.64E-01	2.64E+01		
8045697	NM_018151	RIF1	RAP1 interacting factor homolog (	-0.096	0.935	3.74E-01	3.74E+01		
8045736	NM_052905	FMNL2	formin-like 2	0.153	1.112	7.87E-01	7.87E+01		
8045768	NM_152522	ARL6IP6	ADP-ribosylation-like factor 6 inte	-0.007	0.995	9.82E-01	9.82E+01		
8045804	---	---	---	0.118	1.085	8.72E-01	8.72E+01		
8045814	---	---	---	0.020	1.014	9.33E-01	9.33E+01		
8045816	NM_00108311	GPD2	glycerol-3-phosphate dehydrogen	0.102	1.073	8.29E-01	8.29E+01		
8045846	---	---	---	0.078	1.056	7.56E-01	7.56E+01		
8045857	BC021739	LOC554201	hypothetical LOC554201	-0.053	0.964	7.53E-01	7.53E+01		
8045860	NM_003628	PKP4	plakophilin 4	-0.018	0.988	9.54E-01	9.54E+01		
8045882	NM_00101792	DAPL1	death associated protein-like 1	0.031	1.022	8.10E-01	8.10E+01		
8045887	NR_002822	MGC72080	MGC72080 pseudogene	-0.034	0.977	9.04E-01	9.04E+01		
8045919	NM_022826	40244	membrane-associated ring finger (	-0.021	0.986	9.00E-01	9.00E+01		
8045933	NM_004180	TANK	TRAF family member-associated N	-0.134	0.911	3.24E-01	3.24E+01		
8045946	NM_005805	PSMD14	proteasome (prosome, macropain	-0.031	0.979	9.09E-01	9.09E+01		
8045974	NM_022058	SLC4A10	solute carrier family 4, sodium bic	-0.108	0.928	7.89E-01	7.89E+01		
8046003	NM_012198	GCA	grancalcin, EF-hand calcium bindi	0.359	1.283	1.10E-01	1.10E+01		
8046048	NM_024969	CSRNP3	cysteine-serine-rich nuclear protei	-0.034	0.977	8.34E-01	8.34E+01		
8046086	NM_203463	LASS6	LAG1 homolog, ceramide synthase	0.234	1.176	8.27E-02	8.27E+00		
8046169	NM_004792	PPIG	peptidylprolyl isomerase G (cyclop	-0.012	0.992	9.54E-01	9.54E+01		
8046201	NM_003142	SSB	Sjogren syndrome antigen B (auto	0.062	1.044	5.34E-01	5.34E+01		
8046213	NM_172070	UBR3	ubiquitin protein ligase E3 compo	-0.069	0.953	6.78E-01	6.78E+01		
8046276	NM_00100384	SP5	Sp5 transcription factor	-0.078	0.947	3.20E-01	3.20E+01		
8046279	---	---	---	-0.010	0.993	9.67E-01	9.67E+01		
8046306	NM_015530	GORASP2	golgi reassembly stacking protein	0.038	1.027	7.61E-01	7.61E+01		
8046346	NM_003642	HAT1	histone acetyltransferase 1	-0.022	0.985	9.05E-01	9.05E+01		
8046373	NM_178120	DLX1	distal-less homeobox 1	-0.035	0.976	7.29E-01	7.29E+01		
8046380	NM_000210	ITGA6	integrin, alpha 6	0.398	1.318	1.99E-01	1.99E+01		
8046408	NM_002610	PDK1	pyruvate dehydrogenase kinase, is	0.216	1.162	3.12E-01	3.12E+01		
8046426	---	---	---	-0.006	0.996	9.77E-01	9.77E+01		
8046461	NM_133646	ZAK	sterile alpha motif and leucine zip	-0.396	0.760	1.23E-01	1.23E+01		
8046500	---	---	---	-0.025	0.982	8.70E-01	8.70E+01		
8046502	NM_024583	SCRN3	secernin 3	0.218	1.163	1.78E-01	1.78E+01		
8046515	NM_002107 //	H3F3A // H3F3A	H3 histone, family 3A // H3 histor	0.096	1.068	2.12E-01	2.12E+01		
8046522	---	---	---	0.008	1.006	9.85E-01	9.85E+01		
8046524	NM_000523	HOXD13	homeobox D13	-0.022	0.985	8.67E-01	8.67E+01		
8046542	NM_014213	HOXD9	homeobox D9	-0.022	0.985	8.30E-01	8.30E+01		
8046546	NM_019558	HOXD8	homeobox D8	-0.058	0.961	6.91E-01	6.91E+01		
8046555	NM_014621	HOXD4	homeobox D4	-0.023	0.984	8.70E-01	8.70E+01		
8046564	NM_024501	HOXD1	homeobox D1	-0.027	0.981	8.18E-01	8.18E+01		
8046573	NR_027850	MTX2	metaxin 2	0.062	1.044	8.08E-01	8.08E+01		
8046590	NM_194247	HNRNPA3	heterogeneous nuclear ribonucle	0.028	1.020	7.17E-01	7.17E+01		
8046604	NM_003659	AGPS	alkylglycerone phosphate synthas	0.145	1.106	3.41E-01	3.41E+01		
8046628	NM_152945	RBM45	RNA binding motif protein 45	0.047	1.033	7.68E-01	7.68E+01		
8046680	NM_019091	PLEKHA3	pleckstrin homology domain conta	-0.009	0.994	9.78E-01	9.78E+01		
8046685	NM_182678	UBE2E3	ubiquitin-conjugating enzyme E2E	-0.001	0.999	9.98E-01	9.98E+01		
8046695	NM_000885	ITGA4	integrin, alpha 4 (antigen CD49D,	0.294	1.226	5.48E-06	5.48E-04		
8046726	NM_00113044	SSFA2	sperm specific antigen 2	0.179	1.132	1.54E-01	1.54E+01		
8046755	---	---	---	-0.002	0.999	9.93E-01	9.93E+01		
8046759	NM_018981	DNAJC10	DnaJ (Hsp40) homolog, subfamily	0.062	1.044	7.59E-01	7.59E+01		
8046804	NM_138285	NUP35	nucleoporin 35kDa	-0.013	0.991	9.65E-01	9.65E+01		
8046848	NM_018471	ZC3H15	zinc finger CCCH-type containing 1	0.026	1.018	8.62E-01	8.62E+01		
8046895	NM_177454	FAM171B	family with sequence similarity 17	-0.047	0.968	7.78E-01	7.78E+01		
8046922	NM_000090	COL3A1	collagen, type III, alpha 1	-0.064	0.956	3.92E-01	3.92E+01		
8046975	NM_032168	WDR75	WD repeat domain 75	0.018	1.013	9.55E-01	9.55E+01		
8046997	NM_019048	ASNSD1	asparagine synthetase domain co	0.025	1.018	9.07E-01	9.07E+01		
8047038	NM_000534	PMS1	PMS1 postmeiotic segregation inc	0.065	1.046	7.71E-01	7.71E+01		
8047059	NM_031314	HNRNPC	heterogeneous nuclear ribonucle	-0.050	0.966	5.98E-01	5.98E+01		

8047062	NM_00104251	C2orf88	chromosome 2 open reading fram	0.152	1.111	1.50E-01	1.50E+01		
8047078	NM_017694	MFSD6	major facilitator superfamily doma	0.004	1.002	9.86E-01	9.86E+01		
8047086	NM_005966	NAB1	NGFI-A binding protein 1 (EGR1 bi	-0.031	0.979	9.17E-01	9.17E+01		
8047097	NM_014905	GLS	glutaminase	-0.001	0.999	9.95E-01	9.95E+01		
8047125	---	---	---	0.158	1.115	7.27E-01	7.27E+01		
8047161	NM_00103171	OBFC2A	oligonucleotide/oligosaccharide-b	-0.002	0.999	9.96E-01	9.96E+01		
8047174	NM_00112725	SLC39A10	solute carrier family 39 (zinc trans	-0.071	0.952	7.00E-01	7.00E+01		
8047213	---	---	---	0.113	1.081	8.33E-01	8.33E+01		
8047215	---	---	---	0.116	1.084	6.51E-01	6.51E+01		
8047217	NM_025147	COQ10B	coenzyme Q10 homolog B (S. cere	-0.011	0.992	9.63E-01	9.63E+01		
8047223	NM_002157	HSPE1	heat shock 10kDa protein 1 (chape	-0.045	0.970	8.32E-01	8.32E+01		
8047228	NM_199482	MOBKL3	MOB1, Mps One Binder kinase act	-0.010	0.993	9.63E-01	9.63E+01		
8047241	---	---	---	0.011	1.008	9.85E-01	9.85E+01		
8047243	NM_138395	MARS2	methionyl-tRNA synthetase 2, mit	-0.008	0.994	9.71E-01	9.71E+01		
8047248	NM_00111466	PLCL1	phospholipase C-like 1	0.080	1.057	8.15E-01	8.15E+01		
8047262	NM_153689	C2orf69	chromosome 2 open reading fram	0.149	1.109	7.17E-02	7.17E+00		
8047272	NM_015535	SPATS2L	spermatogenesis associated, serin	0.263	1.200	4.91E-01	4.91E+01		
8047286	---	---	---	-0.039	0.973	8.89E-01	8.89E+01		
8047339	NR_026584	BZW1L1	basic leucine zipper and W2 doma	-0.125	0.917	2.63E-01	2.63E+01		
8047356	NM_021824	NIF3L1	NIF3 NGG1 interacting factor 3-lik	-0.199	0.871	5.56E-02	5.56E+00		
8047372	NM_002491	NDUFB3	NADH dehydrogenase (ubiquinone	0.082	1.058	6.83E-01	6.83E+01		
8047379	---	---	---	-0.084	0.943	6.56E-01	6.56E+01		
8047381	NM_003879	CFLAR	CASP8 and FADD-like apoptosis re	-0.081	0.945	6.64E-01	6.64E+01		
8047401	---	---	---	0.000	1.000	1.00E+00	1.00E+02		
8047403	NM_032977	CASP10	caspase 10, apoptosis-related cyst	0.309	1.239	3.06E-03	3.06E-01		
8047419	NM_001228	CASP8	caspase 8, apoptosis-related cyste	-0.014	0.990	9.53E-01	9.53E+01		
8047441	---	---	---	0.115	1.083	7.30E-01	7.30E+01		
8047443	NM_018571	STRADB	STE20-related kinase adaptor beta	0.052	1.037	8.47E-01	8.47E+01		
8047518	NM_015934	NOP58	NOP58 ribonucleoprotein homolo	-0.118	0.922	4.16E-01	4.16E+01		
8047538	NM_001204	BMPR2	bone morphogenetic protein rece	-0.093	0.938	6.16E-01	6.16E+01		
8047565	NM_173511	FAM117B	family with sequence similarity 11	0.107	1.077	5.79E-01	5.79E+01		
8047635	NM_000976	RPL12	ribosomal protein L12	0.020	1.014	9.27E-01	9.27E+01		
8047641	NM_177538	CYP20A1	cytochrome P450, family 20, subfa	0.069	1.049	5.83E-01	5.83E+01		
8047659	NM_005759	ABI2	abl-interactor 2	-0.099	0.933	4.32E-01	4.32E+01		
8047677	NM_006139	CD28	CD28 molecule	0.083	1.060	8.30E-01	8.30E+01		
8047702	NM_012092	ICOS	inducible T-cell co-stimulator	-0.204	0.868	5.15E-01	5.15E+01		
8047736	---	---	---	-0.031	0.979	8.04E-01	8.04E+01		
8047738	NM_201266	NRP2	neuropilin 2	0.291	1.223	3.04E-01	3.04E+01		
8047771	NM_001959	EEF1B2	eukaryotic translation elongation	0.030	1.021	8.69E-01	8.69E+01		
8047778	NR_002589	SNORD51	small nucleolar RNA, C/D box 51	-0.155	0.898	4.06E-01	4.06E+01		
8047784	NM_020923	ZDBF2	zinc finger, DBF-type containing 2	-0.152	0.900	7.10E-01	7.10E+01		
8047815	NM_00113619	FASTKD2	FAST kinase domains 2	0.043	1.030	8.24E-01	8.24E+01		
8047839	NM_004379	CREB1	cAMP responsive element binding	0.002	1.002	9.90E-01	9.90E+01		
8047854	NM_00114230	CCNYL1	cyclin Y-like 1	0.245	1.185	1.09E-01	1.09E+01		
8047865	NM_015040	PIKFYVE	phosphoinositide kinase, FYVE fing	0.123	1.089	3.07E-01	3.07E+01		
8047952	NM_182587	UNC80	unc-80 homolog (C. elegans)	-0.003	0.998	9.88E-01	9.88E+01		
8047963	---	---	---	0.052	1.037	6.55E-01	6.55E+01		
8048014	NM_199229	RPE	ribulose-5-phosphate-3-epimerase	0.147	1.107	6.19E-02	6.19E+00		
8048075	---	---	---	-0.088	0.941	7.38E-01	7.38E+01		
8048108	---	---	---	0.037	1.026	8.48E-01	8.48E+01		
8048116	---	---	---	0.045	1.032	8.31E-01	8.31E+01		
8048120	NM_004044	ATIC	5-aminoimidazole-4-carboxamide	0.073	1.052	7.14E-01	7.14E+01		
8048141	NM_00114231	TMEM169	transmembrane protein 169	0.001	1.000	9.98E-01	9.98E+01		
8048146	NM_021141	XRCC5	X-ray repair complementing defec	-0.016	0.989	9.10E-01	9.10E+01		
8048171	AK125369	DKFZp434H141	hypothetical protein DKFZp434H1	-0.070	0.952	7.59E-01	7.59E+01		
8048175	NM_014140	SMARCAL1	SWI/SNF related, matrix associate	0.007	1.005	9.75E-01	9.75E+01		
8048195	NM_000998	RPL37A	ribosomal protein L37a	-0.039	0.974	7.53E-01	7.53E+01		
8048205	NM_000597	IGFBP2	insulin-like growth factor binding	-0.049	0.967	6.38E-01	6.38E+01		
8048211	NM_198483	RUFY4	RUN and FYVE domain containing	0.006	1.004	9.77E-01	9.77E+01		
8048234	NM_152862	ARPC2	actin related protein 2/3 complex,	0.030	1.021	8.59E-01	8.59E+01		
8048249	NM_00107719	GPBAR1	G protein-coupled bile acid recept	-0.038	0.974	7.46E-01	7.46E+01		
8048257	NM_015488	PNKD	paroxysmal nonkinesigenic dyskin	-0.070	0.953	5.39E-01	5.39E+01		
8048272	BC052750	C2orf62	chromosome 2 open reading fram	-0.045	0.969	6.31E-01	6.31E+01		
8048283	NM_000578	SLC11A1	solute carrier family 11 (proton-co	-0.304	0.810	4.97E-01	4.97E+01		
8048304	NM_021198	CTDSP1	CTD (carboxy-terminal domain, RN	-0.026	0.982	7.97E-01	7.97E+01		
8048340	NM_005444	RQCD1	RCD1 required for cell differentiat	0.007	1.005	9.78E-01	9.78E+01		
8048350	NM_032726	PLCD4	phospholipase C, delta 4	-0.077	0.948	5.96E-01	5.96E+01		
8048370	NM_004328	BCS1L	BCS1-like (yeast)	-0.017	0.988	9.53E-01	9.53E+01		
8048381	NM_015690	STK36	serine/threonine kinase 36, fused	-0.080	0.946	3.98E-01	3.98E+01		
8048411	NM_014640	TTLL4	tubulin tyrosine ligase-like family,	-0.082	0.945	7.75E-01	7.75E+01		
8048432	NM_000784	CYP27A1	cytochrome P450, family 27, subfa	0.307	1.237	5.69E-01	5.69E+01		
8048445	NM_006522	WNT6	wingless-type MMTV integration s	0.006	1.004	9.75E-01	9.75E+01		
8048452	NM_025216	WNT10A	wingless-type MMTV integration s	-0.009	0.994	9.54E-01	9.54E+01		
8048460	NM_003936	CDK5R2	cyclin-dependent kinase 5, regulat	0.006	1.004	9.76E-01	9.76E+01		
8048466	---	---	---	-0.056	0.962	8.83E-01	8.83E+01		
8048468	NM_024293	FAM134A	family with sequence similarity 13	-0.036	0.975	6.93E-01	6.93E+01		
8048478	NM_138802	ZFAND2B	zinc finger, AN1-type domain 2B	-0.024	0.984	8.21E-01	8.21E+01		

8048489	NM_018089	ANKZF1	ankyrin repeat and zinc finger dom	0.040	1.028	7.96E-01	7.96E+01		
8048505	NR_026909	STK16	serine/threonine kinase 16	-0.029	0.980	8.75E-01	8.75E+01		
8048518	NR_003063	TUBA4B	tubulin, alpha 4b (pseudogene)	0.073	1.052	4.09E-01	4.09E+01		
8048523	NM_006736	DNAJB2	DnaJ (Hsp40) homolog, subfamily	-0.053	0.964	6.70E-01	6.70E+01		
8048541	NM_001927	DES	desmin	0.008	1.005	9.52E-01	9.52E+01		
8048551	NM_005876	SPEG	SPEG complex locus	-0.016	0.989	8.82E-01	8.82E+01		
8048595	NM_013335	GMPPA	GDP-mannose pyrophosphorylase	0.096	1.069	4.43E-01	4.43E+01		
8048628	NM_00100520	TMEM198	transmembrane protein 198	-0.014	0.990	9.30E-01	9.30E+01		
8048639	NM_002191	INHA	inhibin, alpha	-0.018	0.988	8.93E-01	8.93E+01		
8048647	NM_052902	STK11IP	serine/threonine kinase 11 interac	-0.015	0.989	9.23E-01	9.23E+01		
8048673	NM_201574	SLC4A3	solute carrier family 4, anion exch	-0.001	0.999	9.95E-01	9.95E+01		
8048699	---	---	---	-0.039	0.974	7.76E-01	7.76E+01		
8048703	NM_032338	LLPH	LLP homolog, long-term synaptic f	0.039	1.027	7.87E-01	7.87E+01		
8048712	---	---	---	-0.058	0.961	5.03E-01	5.03E+01		
8048717	NM_152386	SGPP2	sphingosine-1-phosphate phospho	-0.052	0.964	8.27E-01	8.27E+01		
8048752	NM_022915	MRPL44	mitochondrial ribosomal protein L	0.131	1.095	4.18E-01	4.18E+01		
8048761	---	---	---	0.108	1.077	7.47E-01	7.47E+01		
8048772	NM_032276	RHBDD1	rhomboid domain containing 1	0.051	1.036	7.97E-01	7.97E+01		
8048782	NM_000091	COL4A3	collagen, type IV, alpha 3 (Goodpa	0.005	1.003	9.76E-01	9.76E+01		
8048835	NM_020194	MFF	mitochondrial fission factor	0.051	1.036	6.48E-01	6.48E+01		
8048847	NM_00113518	AGFG1	ArfGAP with FG repeats 1	-0.016	0.989	9.43E-01	9.43E+01		
8048898	NM_007237	SP140	SP140 nuclear body protein	0.116	1.084	4.80E-01	4.80E+01		
8048926	NM_138402	SP140L	SP140 nuclear body protein-like	0.050	1.035	7.53E-01	7.53E+01		
8048940	NM_00108039	SP100	SP100 nuclear antigen	0.024	1.017	9.15E-01	9.15E+01		
8048976	---	---	---	-0.063	0.957	8.36E-01	8.36E+01		
8048980	NM_016289	CAB39	calcium binding protein 39	0.001	1.001	9.97E-01	9.97E+01		
8048995	NM_030926	ITM2C	integral membrane protein 2C	-0.135	0.911	2.40E-01	2.40E+01		
8049007	NM_139073	SPATA3	spermatogenesis associated 3	0.026	1.018	8.63E-01	8.63E+01		
8049016	NM_002807	PSMD1	proteasome (prosome, macropain	-0.096	0.936	5.75E-01	5.75E+01		
8049042	---	---	---	-0.150	0.901	7.38E-01	7.38E+01		
8049071	---	---	---	-0.101	0.933	3.95E-01	3.95E+01		
8049073	---	---	---	0.037	1.026	8.52E-01	8.52E+01		
8049075	NM_145236	B3GNT7	UDP-GlcNAc:betaGal beta-1,3-N-a	0.042	1.030	7.93E-01	7.93E+01		
8049083	---	---	---	-0.031	0.979	8.53E-01	8.53E+01		
8049088	NM_022730	COPS7B	COP9 constitutive photomorphoge	-0.070	0.953	6.02E-01	6.02E+01		
8049097	NM_152383	DIS3L2	DIS3 mitotic control homolog (S. c	-0.097	0.935	8.58E-02	8.58E+00		
8049128	NM_031313	ALPPL2	alkaline phosphatase, placental-lik	-0.035	0.976	8.48E-01	8.48E+01		
8049137	NM_001631	ALPI	alkaline phosphatase, intestinal	-0.070	0.952	4.41E-01	4.41E+01		
8049152	NM_000751	CHRND	cholinergic receptor, nicotinic, del	-0.049	0.966	6.57E-01	6.57E+01		
8049166	NM_005199	CHRNG	cholinergic receptor, nicotinic, gar	-0.044	0.970	5.99E-01	5.99E+01		
8049180	NM_004846	EIF4E2	eukaryotic translation initiation fa	0.144	1.105	3.14E-01	3.14E+01		
8049187	NM_025202	EFHD1	EF-hand domain family, member D	0.013	1.009	9.49E-01	9.49E+01		
8049195	---	---	---	0.000	1.000	1.00E+00	1.00E+02		
8049199	NM_00110314	GIGYF2	GRB10 interacting GYF protein 2	-0.002	0.998	9.87E-01	9.87E+01		
8049239	AY358535	C2orf82	chromosome 2 open reading fram	-0.032	0.978	7.61E-01	7.61E+01		
8049243	NM_005383	NEU2	sialidase 2 (cytosolic sialidase)	-0.069	0.953	3.80E-01	3.80E+01		
8049246	NM_00101791	INPP5D	inositol polyphosphate-5-phospha	0.029	1.020	8.80E-01	8.80E+01		
8049271	NM_030803	ATG16L1	ATG16 autophagy related 16-like 1	0.039	1.028	8.41E-01	8.41E+01		
8049297	NR_003008	SCARNA5	small Cajal body-specific RNA 5	-0.041	0.972	8.98E-01	8.98E+01		
8049299	NR_003006	SCARNA6	small Cajal body-specific RNA 6	0.076	1.054	7.68E-01	7.68E+01		
8049317	NM_152879	DGKD	diacylglycerol kinase, delta 130kDa	0.081	1.058	6.04E-01	6.04E+01		
8049435	NM_014521	SH3BP4	SH3-domain binding protein 4	-0.018	0.987	9.02E-01	9.02E+01		
8049448	NM_00103713	AGAP1	ArfGAP with GTPase domain, anky	-0.065	0.956	7.45E-01	7.45E+01		
8049478	NM_198189	COPS8	COP9 constitutive photomorphoge	-0.013	0.991	9.64E-01	9.64E+01		
8049487	NM_024101	MLPH	melanophilin	0.037	1.026	8.01E-01	8.01E+01		
8049509	NM_015893	PRLH	prolactin releasing hormone	-0.012	0.992	9.43E-01	9.43E+01		
8049512	NM_00113755	LRRFIP1	leucine rich repeat (in FLII) interac	0.003	1.002	9.88E-01	9.88E+01		
8049528	NM_00113755	LRRFIP1	leucine rich repeat (in FLII) interac	-0.003	0.998	9.91E-01	9.91E+01		
8049530	NM_00113755	LRRFIP1	leucine rich repeat (in FLII) interac	-0.060	0.960	7.73E-01	7.73E+01		
8049532	NM_00113755	LRRFIP1	leucine rich repeat (in FLII) interac	0.043	1.030	9.44E-01	9.44E+01		
8049534	NM_00113755	LRRFIP1	leucine rich repeat (in FLII) interac	0.145	1.105	6.18E-01	6.18E+01		
8049536	NM_00113755	LRRFIP1	leucine rich repeat (in FLII) interac	-0.002	0.999	9.90E-01	9.90E+01		
8049538	NM_00113755	LRRFIP1	leucine rich repeat (in FLII) interac	-0.031	0.978	7.60E-01	7.60E+01		
8049540	NM_00113755	LRRFIP1	leucine rich repeat (in FLII) interac	0.271	1.206	3.19E-01	3.19E+01		
8049542	NM_00113755	LRRFIP1	leucine rich repeat (in FLII) interac	0.028	1.020	9.63E-01	9.63E+01		
8049544	NM_00113755	LRRFIP1	leucine rich repeat (in FLII) interac	0.144	1.105	3.42E-01	3.42E+01		
8049567	NM_005855	RAMP1	receptor (G protein-coupled) activ	-0.016	0.989	9.16E-01	9.16E+01		
8049574	NM_080678	UBE2F	ubiquitin-conjugating enzyme E2F	0.196	1.146	2.65E-01	2.65E+01		
8049582	NM_016510	SCLY	selenocysteine lyase	0.021	1.015	8.61E-01	8.61E+01		
8049598	NM_194312	ESPNL	espin-like	-0.014	0.990	9.24E-01	9.24E+01		
8049610	NM_198582	KLHL30	kelch-like 30 (Drosophila)	-0.045	0.969	7.52E-01	7.52E+01		
8049620	ENST00000408	FAM132B	family with sequence similarity 13	0.000	1.000	1.00E+00	1.00E+02		
8049635	NM_015650	TRAF3IP1	TNF receptor-associated factor 3 i	-0.166	0.891	2.06E-02	2.06E+00		
8049657	NM_00104044	ASB1	ankyrin repeat and SOCS box-cont	0.003	1.002	9.87E-01	9.87E+01		
8049670	NM_002081	GPC1	glypican 1	-0.053	0.964	6.27E-01	6.27E+01		
8049682	---	---	---	-0.008	0.994	9.67E-01	9.67E+01		
8049684	NM_00103357	DUSP28	dual specificity phosphatase 28	0.091	1.065	3.30E-01	3.30E+01		

8049689	NM_018226	RNPEPL1	arginyl aminopeptidase (aminope	0.002	1.001	9.91E-01	9.91E+01		
8049702	NM_023083	CAPN10	calpain 10	-0.007	0.995	9.52E-01	9.52E+01		
8049722	NM_005301	GPR35	G protein-coupled receptor 35	-0.061	0.959	5.74E-01	5.74E+01		
8049729	NM_198998	AQP12A	aquaporin 12A	-0.014	0.990	8.99E-01	8.99E+01		
8049737	NM_000030	AGXT	alanine-glyoxylate aminotransfera	-0.016	0.989	9.18E-01	9.18E+01		
8049752	NM_00108043	SNED1	sushi, nidogen and EGF-like domai	-0.073	0.951	6.19E-01	6.19E+01		
8049784	NM_002712	PPP1R7	protein phosphatase 1, regulatory	0.022	1.016	9.11E-01	9.11E+01		
8049799	NM_00100189	ANO7	anoctamin 7	-0.015	0.990	9.10E-01	9.10E+01		
8049825	NM_005336	HDLBP	high density lipoprotein binding pr	0.023	1.016	9.44E-01	9.44E+01		
8049827	NM_00100849	40423	septin 2	-0.011	0.992	9.57E-01	9.57E+01		
8049847	NM_014808	FARP2	FERM, RhoGEF and pleckstrin dom	0.050	1.035	6.94E-01	6.94E+01		
8049876	NM_032515	BOK	BCL2-related ovarian killer	0.002	1.001	9.90E-01	9.90E+01		
8049888	NM_013325	ATG4B	ATG4 autophagy related 4 homolo	0.014	1.010	9.49E-01	9.49E+01		
8049906	NM_032329	ING5	inhibitor of growth family, membe	0.003	1.002	9.86E-01	9.86E+01		
8049919	NM_152783	D2HGDH	D-2-hydroxyglutarate dehydrogen	-0.069	0.954	2.61E-01	2.61E+01		
8049934	NM_022134	GAL3ST2	galactose-3-O-sulfotransferase 2	-0.011	0.993	9.16E-01	9.16E+01		
8049942	NM_080741	NEU4	sialidase 4	-0.020	0.986	8.06E-01	8.06E+01		
8049950	AK094921	LOC100131763	hypothetical LOC100131763	-0.078	0.947	4.88E-01	4.88E+01		
8049952	NM_173821	C2orf85	chromosome 2 open reading fram	-0.052	0.965	5.49E-01	5.49E+01		
8049959	AK123321	FLJ41327	FLJ41327 protein	-0.079	0.947	8.34E-01	8.34E+01		
8049961	NM_183421	FBXO25	F-box protein 25	0.029	1.020	9.04E-01	9.04E+01		
8049963	BC032332	PCMTD2	protein-L-isoaspartate (D-aspartat	-0.016	0.989	9.61E-01	9.61E+01		
8049965	AK302544	FAM110C	family with sequence similarity 11	-0.105	0.930	1.27E-01	1.27E+01		
8049969	NM_015677	SH3YL1	SH3 domain containing, Ysc84-like	0.022	1.015	9.24E-01	9.24E+01		
8049990	NM_00100291	FAM150B	family with sequence similarity 15	-0.046	0.969	6.38E-01	6.38E+01		
8049998	NM_152834	TMEM18	transmembrane protein 18	-0.029	0.980	8.98E-01	8.98E+01		
8050007	NM_012293	PXDN	peroxidasin homolog (Drosophila)	-0.055	0.963	6.80E-01	6.80E+01		
8050031	NM_015025	MYT1L	myelin transcription factor 1-like	-0.051	0.965	5.23E-01	5.23E+01		
8050060	NM_003310	TSSC1	tumor suppressing subtransferabl	0.008	1.005	9.77E-01	9.77E+01		
8050071	NM_018269	ADI1	acireductone dioxygenase 1	0.037	1.026	8.88E-01	8.88E+01		
8050079	NM_002936	RNASEH1	ribonuclease H1	0.025	1.018	9.08E-01	9.08E+01		
8050089	BC113076	TMSL2	thymosin-like 2 (pseudogene)	-0.028	0.981	7.62E-01	7.62E+01		
8050093	---	---	---	-0.047	0.968	7.97E-01	7.97E+01		
8050095	AK124409	FLJ42418	FLJ42418 protein	-0.097	0.935	6.24E-01	6.24E+01		
8050102	NM_207315	CMPK2	cytidine monophosphate (UMP-CM	-0.037	0.974	9.47E-01	9.47E+01		
8050113	AK125905	LOC100129581	hypothetical LOC100129581	0.015	1.010	9.14E-01	9.14E+01		
8050115	AK127578	C2orf46	chromosome 2 open reading fram	0.125	1.091	7.47E-01	7.47E+01		
8050128	NM_020738	KIDINS220	kinase D-interacting substrate, 22(	0.122	1.088	4.04E-01	4.04E+01		
8050176	NM_004763	ITGB1BP1	integrin beta 1 binding protein 1	0.011	1.008	9.64E-01	9.64E+01		
8050190	NM_003183	ADAM17	ADAM metallopeptidase domain 1	0.009	1.006	9.77E-01	9.77E+01		
8050213	---	---	---	0.017	1.012	9.73E-01	9.73E+01		
8050215	NM_006826	YWHAQ	tyrosine 3-monooxygenase/trypto	-0.009	0.993	9.57E-01	9.57E+01		
8050232	NM_00103716	CYS1	cystin 1	-0.087	0.942	2.09E-01	2.09E+01		
8050240	NM_002539	ODC1	ornithine decarboxylase 1	-0.012	0.992	9.77E-01	9.77E+01		
8050253	---	---	---	-0.046	0.969	7.27E-01	7.27E+01		
8050255	NM_024894	NOL10	nucleolar protein 10	0.071	1.051	6.16E-01	6.16E+01		
8050302	NM_004850	ROCK2	Rho-associated, coiled-coil contai	-0.005	0.997	9.78E-01	9.78E+01		
8050336	NR_003094	E2F6	E2F transcription factor 6	0.193	1.143	1.51E-01	1.51E+01		
8050350	---	---	---	0.150	1.109	4.36E-01	4.36E+01		
8050352	NM_012344	NTSR2	neurotensin receptor 2	-0.003	0.998	9.86E-01	9.86E+01		
8050367	NM_015909	NBAS	neuroblastoma amplified sequenc	-0.040	0.973	7.93E-01	7.93E+01		
8050425	---	---	---	-0.010	0.993	9.56E-01	9.56E+01		
8050427	NM_030797	FAM49A	family with sequence similarity 49	0.036	1.025	9.44E-01	9.44E+01		
8050441	---	---	---	-0.085	0.943	5.23E-01	5.23E+01		
8050443	NM_00114228	SMC6	structural maintenance of chromo	0.161	1.118	2.07E-01	2.07E+01		
8050474	NM_020905	RDH14	retinol dehydrogenase 14 (all-tran	0.068	1.048	4.46E-01	4.46E+01		
8050497	NM_145260	OSR1	odd-skipped related 1 (Drosophila	0.046	1.033	6.68E-01	6.68E+01		
8050503	NM_00100823	TTC32	tetratricopeptide repeat domain 3	-0.048	0.968	8.79E-01	8.79E+01		
8050548	NM_014713	LAPTM4A	lysosomal protein transmembrane	0.198	1.147	2.12E-01	2.12E+01		
8050557	NM_00100694	SDC1	syndecan 1	0.033	1.023	7.64E-01	7.64E+01		
8050565	NM_015317	PUM2	pumilio homolog 2 (Drosophila)	-0.002	0.999	9.90E-01	9.90E+01		
8050591	NM_174889	NDUFAF2	NADH dehydrogenase (ubiquinone	0.030	1.021	9.09E-01	9.09E+01		
8050594	NM_022460	HS1BP3	HCLS1 binding protein 3	-0.004	0.997	9.85E-01	9.85E+01		
8050658	NM_017552	ATAD2B	ATPase family, AAA domain contai	0.032	1.022	7.99E-01	7.99E+01		
8050687	---	---	---	-0.014	0.990	9.77E-01	9.77E+01		
8050689	NM_025203	C2orf44	chromosome 2 open reading fram	0.060	1.043	7.93E-01	7.93E+01		
8050695	NM_016047	SF3B14	splicing factor 3B, 14 kDa subunit	0.044	1.031	8.35E-01	8.35E+01		
8050702	NM_004881	TP53I3	tumor protein p53 inducible prote	0.005	1.003	9.86E-01	9.86E+01		
8050713	NM_199346	PFN4	profilin family, member 4	0.001	1.000	9.99E-01	9.99E+01		
8050719	NM_006277	ITSN2	intersectin 2	-0.046	0.969	5.46E-01	5.46E+01		
8050761	---	---	---	0.033	1.023	9.53E-01	9.53E+01		
8050763	ENST00000380	CENPO	centromere protein O	0.122	1.089	3.79E-01	3.79E+01		
8050766	NM_004036	ADCY3	adenylate cyclase 3	-0.063	0.957	5.97E-01	5.97E+01		
8050790	NM_016544	DNAJC27	DnaJ (Hsp40) homolog, subfamily	-0.088	0.941	6.66E-01	6.66E+01		
8050813	NM_175629	DNMT3A	DNA (cytosine-5-)-methyltransfera	-0.002	0.999	9.89E-01	9.89E+01		
8050846	NM_021907	DTNB	dystrobrevin, beta	0.075	1.053	4.14E-01	4.14E+01		
8050869	---	---	---	0.165	1.122	4.76E-01	4.76E+01		

8050875	NM_018263	ASXL2	additional sex combs like 2 (Droso	-0.045	0.969	6.02E-01	6.02E+01		
8050894	NM_002254	KIF3C	kinesin family member 3C	-0.017	0.988	9.07E-01	9.07E+01		
8050908	NM_000182	HADHA	hydroxyacyl-Coenzyme A dehydro	0.096	1.069	4.64E-01	4.64E+01		
8050924	NM_00114516	GPR113	G protein-coupled receptor 113	0.019	1.013	8.80E-01	8.80E+01		
8051012	NM_013388	PREB	prolactin regulatory element bindi	0.046	1.032	5.95E-01	5.95E+01		
8051024	NM_178553	C2orf53	chromosome 2 open reading fram	-0.019	0.987	8.91E-01	8.91E+01		
8051028	---	---	---	-0.132	0.913	6.91E-01	6.91E+01		
8051030	NM_021095	SLC5A6	solute carrier family 5 (sodium-de	-0.018	0.987	9.33E-01	9.33E+01		
8051050	NM_003459	SLC30A3	solute carrier family 30 (zinc trans	0.035	1.025	7.71E-01	7.71E+01		
8051061	NM_003353	UCN	urocortin	-0.065	0.956	5.31E-01	5.31E+01		
8051066	NM_002437	MPV17	MpV17 mitochondrial inner memb	0.069	1.049	6.20E-01	6.20E+01		
8051075	NM_001521	GTF3C2	general transcription factor IIIC, pc	0.075	1.054	3.90E-01	3.90E+01		
8051097	NM_00103411	EIF2B4	eukaryotic translation initiation fa	-0.011	0.993	9.61E-01	9.61E+01		
8051113	NM_144631	ZNF513	zinc finger protein 513	-0.079	0.947	3.44E-01	3.44E+01		
8051119	NM_177983	PPM1G	protein phosphatase 1G (formerly	-0.035	0.976	7.84E-01	7.84E+01		
8051133	NR_002201	FTHL3P	ferritin, heavy polypeptide-like 3 p	0.057	1.040	6.55E-01	6.55E+01		
8051135	NM_015662	IFT172	intraflagellar transport 172 homol	0.010	1.007	9.70E-01	9.70E+01		
8051187	NM_022823	FNDC4	fibronectin type III domain contair	-0.009	0.993	9.56E-01	9.56E+01		
8051197	NM_024584	CCDC121	coiled-coil domain containing 121	0.087	1.062	5.95E-01	5.95E+01		
8051204	NM_014860	SUPT7L	suppressor of Ty 7 (S. cerevisiae)-l	-0.076	0.949	5.27E-01	5.27E+01		
8051215	NM_022128	RBKS	ribokinase	-0.062	0.958	6.37E-01	6.37E+01		
8051226	NM_017910	TRMT61B	tRNA methyltransferase 61 homol	0.066	1.047	8.04E-01	8.04E+01		
8051238	NM_00102988	C2orf71	chromosome 2 open reading fram	0.031	1.022	7.52E-01	7.52E+01		
8051241	NM_004304	ALK	anaplastic lymphoma receptor tyr	0.015	1.010	9.35E-01	9.35E+01		
8051298	NM_024572	GALNT14	UDP-N-acetyl-alpha-D-galactosam	-0.017	0.988	9.00E-01	9.00E+01		
8051370	---	---	---	0.085	1.061	5.22E-01	5.22E+01		
8051372	NM_015955	MEMO1	mediator of cell motility 1	0.138	1.100	1.14E-01	1.14E+01		
8051387	NM_032574	DPY30	dpy-30 homolog (C. elegans)	-0.022	0.985	9.28E-01	9.28E+01		
8051394	---	---	---	0.053	1.038	8.40E-01	8.40E+01		
8051396	NM_021209	NLRC4	NLR family, CARD domain containi	0.332	1.259	5.17E-01	5.17E+01		
8051413	NM_015475	FAM98A	family with sequence similarity 98	0.138	1.100	3.18E-01	3.18E+01		
8051422	NR_003143	MYADML	myeloid-associated differentiation	-0.040	0.973	7.51E-01	7.51E+01		
8051427	NM_005102	FEZ2	fasciculation and elongation prote	0.076	1.054	6.04E-01	6.04E+01		
8051443	NM_003162	STRN	striatin, calmodulin binding protei	0.015	1.011	9.23E-01	9.23E+01		
8051462	---	---	---	-0.089	0.940	8.03E-01	8.03E+01		
8051464	NM_019024	HEATR5B	HEAT repeat containing 5B	0.068	1.048	5.48E-01	5.48E+01		
8051501	NM_002759	EIF2AK2	eukaryotic translation initiation fa	0.179	1.132	5.97E-01	5.97E+01		
8051528	NM_005760	CEBPZ	CCAAT/enhancer binding protein (	-0.027	0.981	8.88E-01	8.88E+01		
8051547	NM_005813	PRKD3	protein kinase D3	0.004	1.003	9.87E-01	9.87E+01		
8051573	NM_006449	CDC42EP3	CDC42 effector protein (Rho GTPa	-0.158	0.896	7.00E-02	7.00E+00		
8051583	NM_000104	CYP1B1	cytochrome P450, family 1, subfar	0.271	1.207	5.02E-01	5.02E+01		
8051589	NM_022374	ATL2	atlastin GTPase 2	0.049	1.035	7.64E-01	7.64E+01		
8051605	NM_138394	HNRPLL	heterogeneous nuclear ribonucleo	0.110	1.079	4.47E-01	4.47E+01		
8051622	NM_00103168	SFRS7	splicing factor, arginine/serine-ricl	0.009	1.006	9.65E-01	9.65E+01		
8051637	NM_198963	DHX57	DEAH (Asp-Glu-Ala-Asp/His) box p	0.019	1.013	9.24E-01	9.24E+01		
8051670	NM_005633	SOS1	son of sevenless homolog 1 (Droso	-0.074	0.950	4.96E-01	4.96E+01		
8051773	---	---	---	-0.058	0.960	6.99E-01	6.99E+01		
8051777	NM_004718	COX7A2L	cytochrome c oxidase subunit VIIa	0.021	1.015	8.49E-01	8.49E+01		
8051793	NM_148962	OXER1	oxoeicosanoid (OXE) receptor 1	0.010	1.007	9.64E-01	9.64E+01		
8051799	NM_012205	HAAO	3-hydroxyanthranilate 3,4-dioxyge	-0.003	0.998	9.85E-01	9.85E+01		
8051812	---	---	---	0.186	1.137	2.97E-01	2.97E+01		
8051814	NM_006887	ZFP36L2	zinc finger protein 36, C3H type-lik	-0.041	0.972	6.46E-01	6.46E+01		
8051820	NM_022065	THADA	thyroid adenoma associated	-0.022	0.985	9.18E-01	9.18E+01		
8051882	NM_133259	LRPPRC	leucine-rich PPR-motif containing	0.079	1.057	5.39E-01	5.39E+01		
8051926	---	---	---	-0.051	0.965	8.50E-01	8.50E+01		
8051928	NM_006036	PREPL	prolyl endopeptidase-like	-0.046	0.968	8.41E-01	8.41E+01		
8051949	NM_016932	SIX2	SIX homeobox 2	-0.009	0.994	9.66E-01	9.66E+01		
8051963	NM_018079	SRBD1	S1 RNA binding domain 1	0.080	1.057	5.81E-01	5.81E+01		
8051985	---	---	---	-0.004	0.997	9.82E-01	9.82E+01		
8051998	NM_139279	MCFD2	multiple coagulation factor deficie	-0.073	0.950	6.80E-01	6.80E+01		
8052010	NM_001743	CALM2	calmodulin 2 (phosphorylase kinas	0.194	1.144	2.59E-03	2.59E-01		
8052016	---	---	---	-0.047	0.968	7.87E-01	7.87E+01		
8052018	NM_022055	KCNK12	potassium channel, subfamily K, m	-0.119	0.921	8.62E-02	8.62E+00		
8052022	AY779046	RPL18A	ribosomal protein L18a	-0.036	0.975	8.64E-01	8.64E+01		
8052024	NM_025133	FBXO11	F-box protein 11	0.083	1.059	4.35E-01	4.35E+01		
8052087	---	---	---	-0.010	0.993	9.72E-01	9.72E+01		
8052125	NM_016115	ASB3	ankyrin repeat and SOCS box-cont	0.011	1.008	9.64E-01	9.64E+01		
8052139	---	---	---	-0.048	0.967	8.41E-01	8.41E+01		
8052141	---	---	---	-0.152	0.900	5.91E-01	5.91E+01		
8052143	NM_006794	GPR75	G protein-coupled receptor 75	-0.059	0.960	6.89E-01	6.89E+01		
8052149	NM_014614	PSME4	proteasome (prosome, macropain	-0.051	0.965	7.78E-01	7.78E+01		
8052200	NM_00100393	TSPYL6	TSPY-like 6	0.011	1.007	9.63E-01	9.63E+01		
8052204	NM_020532	RTN4	reticulon 4	0.161	1.118	6.22E-02	6.22E+00		
8052250	NM_00100536	MTIF2	mitochondrial translational initiati	0.040	1.028	8.60E-01	8.60E+01		
8052269	NM_00113559	CCDC88A	coiled-coil domain containing 88A	0.295	1.227	2.70E-01	2.70E+01		
8052307	NM_00112296	SMEK2	SMEK homolog 2, suppressor of m	0.009	1.006	9.61E-01	9.61E+01		
8052331	NM_033109	PNPT1	polyribonucleotide nucleotidyltran	0.107	1.077	7.38E-01	7.38E+01		

8052370	---	---	---	-0.045	0.969	7.72E-01	7.72E+01		
8052376	NM_00108043	CCDC85A	coiled-coil domain containing 85A	0.021	1.015	8.81E-01	8.81E+01		
8052380	---	---	---	-0.081	0.945	3.20E-01	3.20E+01		
8052382	NM_00111463	FANCL	Fanconi anemia, complementation	0.248	1.188	1.65E-01	1.65E+01		
8052397	---	---	---	0.012	1.008	9.49E-01	9.49E+01		
8052399	NM_022893	BCL11A	B-cell CLL/lymphoma 11A (zinc fin	0.185	1.137	3.94E-01	3.94E+01		
8052443	NM_014709	USP34	ubiquitin specific peptidase 34	-0.022	0.985	8.71E-01	8.71E+01		
8052526	NM_003400	XPO1	exportin 1 (CRM1 homolog, yeast)	0.005	1.004	9.81E-01	9.81E+01		
8052562	NM_006430	CCT4	chaperonin containing TCP1, subu	0.035	1.025	8.07E-01	8.07E+01		
8052583	---	---	---	-0.044	0.970	7.93E-01	7.93E+01		
8052598	NM_015910	C2orf86	chromosome 2 open reading fram	0.145	1.106	4.24E-01	4.24E+01		
8052622	NR_003131	LOC388955	PX19 protein pseudogene	-0.067	0.955	5.95E-01	5.95E+01		
8052624	AK094167	FLJ36848	hypothetical LOC647115	-0.045	0.969	7.09E-01	7.09E+01		
8052654	NM_020651	PELI1	pellino homolog 1 (Drosophila)	-0.018	0.988	9.36E-01	9.36E+01		
8052667	---	---	---	-0.003	0.998	9.88E-01	9.88E+01		
8052669	NM_014755	SERTAD2	SERTA domain containing 2	-0.057	0.961	7.12E-01	7.12E+01		
8052676	---	---	---	-0.015	0.990	9.53E-01	9.53E+01		
8052680	NM_004161	RAB1A	RAB1A, member RAS oncogene fa	0.179	1.132	1.11E-01	1.11E+01		
8052689	NM_181784	SPRED2	sprouty-related, EVH1 domain con	-0.238	0.848	3.59E-02	3.59E+00		
8052698	NM_006333	C1D	C1D nuclear receptor co-repressor	0.068	1.048	6.87E-01	6.87E+01		
8052703	NM_138458	WDR92	WD repeat domain 92	0.092	1.066	5.55E-01	5.55E+01		
8052721	NM_000945	PPP3R1	protein phosphatase 3 (formerly 2	0.114	1.082	1.48E-01	1.48E+01		
8052731	---	---	---	-0.264	0.833	2.20E-01	2.20E+01		
8052735	NM_015463	CNRIP1	cannabinoid receptor interacting p	-0.072	0.951	4.63E-01	4.63E+01		
8052762	NM_002056	GFPT1	glutamine-fructose-6-phosphate t	-0.046	0.969	7.92E-01	7.92E+01		
8052784	NM_015700	NFU1	NFU1 iron-sulfur cluster scaffold h	-0.052	0.965	7.38E-01	7.38E+01		
8052798	NM_014911	AAK1	AP2 associated kinase 1	-0.040	0.973	8.74E-01	8.74E+01		
8052803	NM_014911	AAK1	AP2 associated kinase 1	-0.026	0.982	8.75E-01	8.75E+01		
8052834	BC005079	C2orf42	chromosome 2 open reading fram	-0.012	0.992	9.57E-01	9.57E+01		
8052845	NM_022173	TIA1	TIA1 cytotoxic granule-associated	-0.013	0.991	9.12E-01	9.12E+01		
8052861	NM_003096	SNRPG	small nuclear ribonucleoprotein po	-0.038	0.974	8.31E-01	8.31E+01		
8052866	NM_032822	FAM136A	family with sequence similarity 13	-0.024	0.983	9.00E-01	9.00E+01		
8052882	NM_017488	ADD2	adducin 2 (beta)	-0.052	0.965	7.86E-01	7.86E+01		
8052908	NM_173535	CLEC4F	C-type lectin domain family 4, mer	-0.011	0.993	9.58E-01	9.58E+01		
8052925	NM_144582	TEX261	testis expressed 261	0.255	1.193	7.11E-02	7.11E+00		
8052932	---	---	---	-0.053	0.964	6.38E-01	6.38E+01		
8052956	NM_015189	EXOC6B	exocyst complex component 6B	-0.128	0.915	3.56E-01	3.56E+01		
8052979	NM_144579	SFXN5	sideroflexin 5	0.025	1.017	9.14E-01	9.14E+01		
8052994	NM_015470	RAB11FIP5	RAB11 family interacting protein 5	-0.039	0.974	7.51E-01	7.51E+01		
8053004	NM_032319	C2orf7	chromosome 2 open reading fram	-0.131	0.913	3.39E-01	3.39E+01		
8053009	NM_00108041	FBXO41	F-box protein 41	-0.049	0.967	5.16E-01	5.16E+01		
8053022	NM_001965	EGR4	early growth response 4	-0.139	0.908	5.53E-02	5.53E+00		
8053030	NM_016347	NAT8B	N-acetyltransferase 8B (GCN5-rela	0.003	1.002	9.86E-01	9.86E+01		
8053036	NM_016058	TPRKB	TP53RK binding protein	-0.023	0.984	9.42E-01	9.42E+01		
8053046	NM_003584	DUSP11	dual specificity phosphatase 11 (R	-0.012	0.992	9.49E-01	9.49E+01		
8053057	---	---	---	0.041	1.029	8.75E-01	8.75E+01		
8053059	NM_212552	BOLA3	bolA homolog 3 (E. coli)	-0.011	0.993	9.73E-01	9.73E+01		
8053064	NM_018221	MOBKL1B	MOB1, Mps One Binder kinase act	0.187	1.138	1.79E-02	1.79E+00		
8053073	NM_133478	SLC4A5	solute carrier family 4, sodium bic	0.008	1.006	9.75E-01	9.75E+01		
8053107	NM_004082	DCTN1	dynactin 1 (p150, glued homolog,	-0.069	0.953	4.47E-01	4.47E+01		
8053139	NM_00114505	C2orf81	chromosome 2 open reading fram	0.033	1.023	7.92E-01	7.92E+01		
8053142	NM_033046	RTKN	rhotekin	-0.007	0.995	9.64E-01	9.64E+01		
8053158	NM_006302	MOGS	mannosyl-oligosaccharide glucosid	0.011	1.007	9.66E-01	9.66E+01		
8053165	NM_053050	MRPL53	mitochondrial ribosomal protein L	-0.043	0.971	8.47E-01	8.47E+01		
8053171	NM_032779	CCDC142	coiled-coil domain containing 142	0.020	1.014	8.93E-01	8.93E+01		
8053183	NM_00100981	LBX2	ladybird homeobox 2	-0.011	0.992	9.59E-01	9.59E+01		
8053187	NM_032673	PCGF1	polycomb group ring finger 1	0.060	1.042	6.93E-01	6.93E+01		
8053200	NM_133637	DQX1	DEAQ box RNA-dependent ATPase	0.016	1.011	9.16E-01	9.16E+01		
8053214	NM_181575	AUP1	ancient ubiquitous protein 1	-0.039	0.974	7.78E-01	7.78E+01		
8053231	NM_032603	LOXL3	lysyl oxidase-like 3	0.030	1.021	8.01E-01	8.01E+01		
8053248	BC025997	C2orf65	chromosome 2 open reading fram	-0.006	0.996	9.69E-01	9.69E+01		
8053263	---	---	---	0.043	1.030	7.81E-01	7.81E+01		
8053311	---	---	---	0.004	1.003	9.86E-01	9.86E+01		
8053366	NM_003849	SUCLG1	succinate-CoA ligase, alpha subuni	0.147	1.107	9.01E-02	9.01E+00		
8053379	AK296608	LOC129293	hypothetical protein LOC129293	0.045	1.032	8.46E-01	8.46E+01		
8053386	---	---	---	0.081	1.058	5.24E-01	5.24E+01		
8053388	NM_006464	TGOLN2	trans-golgi network protein 2	0.023	1.016	9.07E-01	9.07E+01		
8053406	NM_017750	RETSAT	retinol saturase (all-trans-retinol 1	-0.113	0.925	3.94E-01	3.94E+01		
8053417	NM_001747	CAPG	capping protein (actin filament), g	0.140	1.102	6.83E-01	6.83E+01		
8053427	---	---	---	-0.016	0.989	9.74E-01	9.74E+01		
8053429	NM_000821	GGCX	gamma-glutamyl carboxylase	0.048	1.034	8.33E-01	8.33E+01		
8053449	NM_00103173	TMEM150A	transmembrane protein 150A	0.006	1.004	9.78E-01	9.78E+01		
8053458	NM_00101364	C2orf68	chromosome 2 open reading fram	-0.015	0.990	9.30E-01	9.30E+01		
8053467	NM_000542	SFTPB	surfactant protein B	-0.017	0.989	8.91E-01	8.91E+01		
8053480	---	---	---	-0.131	0.913	9.94E-02	9.94E+00		
8053496	NM_015425	POLR1A	polymerase (RNA) I polypeptide A,	0.064	1.045	5.53E-01	5.53E+01		
8053533	NM_006839	IMMT	inner membrane protein, mitocho	0.078	1.056	4.39E-01	4.39E+01		

8053551	NM_022912	REEP1	receptor accessory protein 1	0.068	1.048	5.32E-01	5.32E+01		
8053562	NM_016079	VPS24	vacuolar protein sorting 24 homol	0.139	1.101	1.74E-01	1.74E+01		
8053576	NM_005667	RNF103	ring finger protein 103	-0.130	0.914	2.86E-01	2.86E+01		
8053584	NR_027353	CD8A	CD8a molecule	-0.158	0.896	2.91E-01	2.91E+01		
8053599	NM_012477	WBP1	WW domain binding protein 1	-0.101	0.932	5.09E-01	5.09E+01		
8053610	NM_022662	ANAPC1	anaphase promoting complex sub	-0.033	0.977	8.62E-01	8.62E+01		
8053622	NM_00102445	RGPD1	RANBP2-like and GRIP domain cor	-0.080	0.946	5.29E-01	5.29E+01		
8053662	---	---	---	-0.209	0.865	1.61E-01	1.61E+01		
8053668	NM_004836	EIF2AK3	eukaryotic translation initiation fa	0.158	1.116	6.90E-03	6.90E-01		
8053710	---	---	---	0.026	1.018	8.41E-01	8.41E+01		
8053713	---	---	---	0.308	1.238	1.24E-02	1.24E+00		
8053722	NR_027238	LOC654342	lymphocyte-specific protein 1 pse	-0.039	0.973	8.64E-01	8.64E+01		
8053728	NM_000798	DRD5	dopamine receptor D5	0.035	1.024	7.68E-01	7.68E+01		
8053731	AK095105	FLJ37786	hypothetical LOC642691	-0.004	0.997	9.85E-01	9.85E+01		
8053733	NM_020382	SETD8	SET domain containing (lysine met	0.066	1.047	6.43E-01	6.43E+01		
8053735	---	---	---	0.311	1.240	5.11E-03	5.11E-01		
8053761	---	---	---	-0.028	0.981	8.88E-01	8.88E+01		
8053763	NM_031902	MRPS5	mitochondrial ribosomal protein S	0.023	1.016	8.92E-01	8.92E+01		
8053775	NM_032788	ZNF514	zinc finger protein 514	-0.023	0.984	9.16E-01	9.16E+01		
8053797	ENST00000456	LOC400986	protein immuno-reactive with ant	-0.127	0.916	6.58E-01	6.58E+01		
8053801	NM_025190	ANKRD36B	ankyrin repeat domain 36B	-0.044	0.970	8.86E-01	8.86E+01		
8053834	NM_025190	ANKRD36B	ankyrin repeat domain 36B	-0.113	0.924	4.73E-01	4.73E+01		
8053840	---	---	---	-0.095	0.936	3.28E-01	3.28E+01		
8053842	NM_207328	GPAT2	glycerol-3-phosphate acyltransfera	-0.053	0.964	5.15E-01	5.15E+01		
8053869	NM_000682	ADRA2B	adrenergic, alpha-2B-, receptor	-0.005	0.997	9.81E-01	9.81E+01		
8053872	NM_00100203	ASTL	astacin-like metallo-endopeptidas	-0.057	0.961	5.34E-01	5.34E+01		
8053882	NM_004418	DUSP2	dual specificity phosphatase 2	-0.043	0.971	7.77E-01	7.77E+01		
8053890	NM_020151	STARD7	StAR-related lipid transfer (START)	0.097	1.070	2.99E-01	2.99E+01		
8053901	NM_017849	TMEM127	transmembrane protein 127	0.014	1.010	9.56E-01	9.56E+01		
8053909	NM_014014	SNRNP200	small nuclear ribonucleoprotein 20	-0.070	0.953	5.94E-01	5.94E+01		
8053944	NR_026875	NEURL3	neuralized homolog 3 (Drosophila)	-0.018	0.987	9.08E-01	9.08E+01		
8053949	NM_017991	KIAA1310	KIAA1310	0.051	1.036	6.38E-01	6.38E+01		
8053975	NM_00114229	LMAN2L	lectin, mannose-binding 2-like	0.060	1.042	6.80E-01	6.80E+01		
8053984	NM_144994	ANKRD23	ankyrin repeat domain 23	-0.043	0.971	7.10E-01	7.10E+01		
8053998	NM_016466	ANKRD39	ankyrin repeat domain 39	-0.070	0.953	4.05E-01	4.05E+01		
8054004	NM_017789	SEMA4C	sema domain, immunoglobulin do	-0.100	0.933	4.85E-01	4.85E+01		
8054020	BC038583	FAM178B	family with sequence similarity 17	-0.063	0.957	5.01E-01	5.01E+01		
8054045	NM_199336	FAHD2B	fumarylacetoacetate hydrolase do	0.026	1.018	8.59E-01	8.59E+01		
8054054	NM_025190	ANKRD36B	ankyrin repeat domain 36B	-0.041	0.972	8.59E-01	8.59E+01		
8054064	NM_025190	ANKRD36B	ankyrin repeat domain 36B	0.032	1.023	9.30E-01	9.30E+01		
8054077	NM_005735	ACTR1B	ARP1 actin-related protein 1 homo	-0.083	0.944	6.30E-01	6.30E+01		
8054090	---	---	---	-0.036	0.975	9.28E-01	9.28E+01		
8054092	NM_015348	TMEM131	transmembrane protein 131	0.061	1.043	6.82E-01	6.82E+01		
8054131	BC047722	C2orf64	chromosome 2 open reading fram	0.026	1.018	8.75E-01	8.75E+01		
8054135	NM_012214	MGAT4A	mannosyl (alpha-1,3-)-glycoprotei	0.064	1.046	7.08E-01	7.08E+01		
8054154	NM_207362	C2orf55	chromosome 2 open reading fram	-0.088	0.941	3.85E-01	3.85E+01		
8054192	NM_138798	MITD1	MIT, microtubule interacting and t	0.035	1.024	8.25E-01	8.25E+01		
8054209	NM_174898	LYG1	lysozyme G-like 1	-0.086	0.942	7.11E-01	7.11E+01		
8054217	NM_005783	TXNDC9	thioredoxin domain containing 9	-0.056	0.962	7.72E-01	7.72E+01		
8054227	NM_016316	REV1	REV1 homolog (S. cerevisiae)	0.028	1.020	8.47E-01	8.47E+01		
8054254	NM_002285	AFF3	AF4/FMR2 family, member 3	0.315	1.244	3.08E-02	3.08E+00		
8054281	NM_198461	LONRF2	LON peptidase N-terminal domain	0.000	1.000	9.99E-01	9.99E+01		
8054295	---	---	---	-0.012	0.992	9.64E-01	9.64E+01		
8054297	NM_004854	CHST10	carbohydrate sulfotransferase 10	-0.098	0.934	4.01E-01	4.01E+01		
8054308	NM_00110242	TBC1D8	TBC1 domain family, member 8 (w	-0.055	0.962	7.72E-01	7.72E+01		
8054329	NM_173647	RNF149	ring finger protein 149	-0.031	0.979	8.04E-01	8.04E+01		
8054354	---	---	---	0.037	1.026	9.22E-01	9.22E+01		
8054356	NM_032718	MFSD9	major facilitator superfamily doma	0.031	1.022	8.49E-01	8.49E+01		
8054364	NM_004257	TGFBRAP1	transforming growth factor, beta r	0.009	1.006	9.77E-01	9.77E+01		
8054377	NM_201555	FHL2	four and a half LIM domains 2	-0.007	0.995	9.76E-01	9.76E+01		
8054393	---	---	---	-0.244	0.845	3.61E-01	3.61E+01		
8054395	NM_025076	UXS1	UDP-glucuronate decarboxylase 1	0.072	1.051	6.11E-01	6.11E+01		
8054414	NM_00114401	RGPD3	RANBP2-like and GRIP domain cor	-0.083	0.944	5.01E-01	5.01E+01		
8054437	NM_022662	ANAPC1	anaphase promoting complex sub	-0.058	0.961	8.35E-01	8.35E+01		
8054449	AK095987	hCG_1993592	hypothetical LOC644903	-0.003	0.998	9.90E-01	9.90E+01		
8054465	ENST00000429	hCG_1732469	hCG1732469	0.006	1.004	9.81E-01	9.81E+01		
8054467	NM_144710	40431	septin 10	-0.283	0.822	2.56E-01	2.56E+01		
8054477	CR590757	RPL22P15	ribosomal protein L22 pseudogene	-0.032	0.978	8.21E-01	8.21E+01		
8054479	NM_005434	MALL	mal, T-cell differentiation protein-	0.025	1.018	8.40E-01	8.40E+01		
8054519	NM_004987	LIMS1	LIM and senescent cell antigen-like	0.195	1.145	3.04E-01	3.04E+01		
8054532	0	0	0	-0.088	0.941	4.91E-01	4.91E+01		
8054557	0	0	0	-0.098	0.934	3.95E-01	3.95E+01		
8054611	NR_024204	NCRNA00152	non-protein coding RNA 152	-0.020	0.986	9.53E-01	9.53E+01		
8054614	NM_022662	ANAPC1	anaphase promoting complex sub	-0.072	0.951	5.77E-01	5.77E+01		
8054664	NM_032494	ZC3H8	zinc finger CCCH-type containing 8	-0.130	0.914	3.72E-01	3.72E+01		
8054676	NM_005054	RGPD5	RANBP2-like and GRIP domain cor	-0.088	0.941	4.88E-01	4.88E+01		
8054740	NM_003466	PAX8	paired box 8	0.048	1.034	6.47E-01	6.47E+01		

8054758	NR_024005	MGC13005	hypothetical LOC84771	0.087	1.062	6.04E-01	6.04E+01		
8054762	NR_024528	RPL23AP7	ribosomal protein L23a pseudoger	-0.004	0.997	9.78E-01	9.78E+01		
8054766	NM_001097	ACR	acrosin	0.028	1.019	8.11E-01	8.11E+01		
8054771	NM_025181	SLC35F5	solute carrier family 35, member F	0.130	1.094	2.76E-01	2.76E+01		
8054796	---	---	---	-0.061	0.959	5.74E-01	5.74E+01		
8054804	NM_019044	CCDC93	coiled-coil domain containing 93	-0.068	0.954	7.22E-01	7.22E+01		
8054831	NM_001426	EN1	engrailed homeobox 1	-0.009	0.994	9.52E-01	9.52E+01		
8054862	NR_000034	TMEM185B	transmembrane protein 185B (pse	-0.069	0.953	5.99E-01	5.99E+01		
8054870	---	---	---	-0.075	0.950	8.04E-01	8.04E+01		
8054888	NM_015282	CLASP1	cytoplasmic linker associated prot	-0.192	0.875	1.03E-02	1.03E+00		
8054930	NM_032390	MKI67IP	MKI67 (FHA domain) interacting n	0.046	1.033	7.50E-01	7.50E+01		
8054939	---	---	---	0.108	1.078	1.29E-01	1.29E+01		
8054943	---	---	---	0.253	1.191	6.46E-01	6.46E+01		
8054945	NM_139343	BIN1	bridging integrator 1	-0.091	0.939	3.18E-01	3.18E+01		
8054978	NM_000122	ERCC3	excision repair cross-complementi	-0.124	0.917	1.68E-01	1.68E+01		
8054997	NM_006609	MAP3K2	mitogen-activated protein kinase k	0.006	1.004	9.83E-01	9.83E+01		
8055016	---	---	---	-0.125	0.917	5.17E-01	5.17E+01		
8055021	NM_017969	IWS1	IWS1 homolog (S. cerevisiae)	-0.013	0.991	9.43E-01	9.43E+01		
8055038	NM_017980	LIMS2	LIM and senescent cell antigen-like	-0.061	0.959	4.67E-01	4.67E+01		
8055060	NM_018383	WDR33	WD repeat domain 33	-0.034	0.977	7.91E-01	7.91E+01		
8055085	---	---	---	0.078	1.055	5.84E-01	5.84E+01		
8055089	NM_004805	POLR2D	polymerase (RNA) II (DNA directed	0.089	1.064	4.68E-01	4.68E+01		
8055104	NM_00114592	SAP130	Sin3A-associated protein, 130kDa	0.058	1.041	5.17E-01	5.17E+01		
8055130	NM_004807	HS6ST1	heparan sulfate 6-O-sulfotransfera	-0.055	0.963	3.81E-01	3.81E+01		
8055135	---	---	---	0.120	1.087	7.49E-01	7.49E+01		
8055139	AK056598	LOC151121	hypothetical LOC151121	-0.059	0.960	6.79E-01	6.79E+01		
8055151	NM_00109977	POTEF	POTE ankyrin domain family, mem	0.100	1.071	4.83E-01	4.83E+01		
8055171	---	---	---	-0.095	0.936	6.83E-01	6.83E+01		
8055183	NM_017751	SMPD4	sphingomyelin phosphodiesterase	-0.051	0.965	7.97E-01	7.97E+01		
8055194	NM_207312	TUBA3E	tubulin, alpha 3e	-0.075	0.949	7.03E-01	7.03E+01		
8055202	---	---	---	0.171	1.126	6.16E-01	6.16E+01		
8055204	---	---	---	-0.028	0.981	8.86E-01	8.86E+01		
8055208	NM_032357	CCDC115	coiled-coil domain containing 115	-0.058	0.961	6.60E-01	6.60E+01		
8055220	NM_00108353	POTEE	POTE ankyrin domain family, mem	0.059	1.042	7.52E-01	7.52E+01		
8055234	---	---	---	-0.062	0.958	8.05E-01	8.05E+01		
8055239	NM_032545	CFC1	cripto, FRL-1, cryptic family 1	-0.078	0.948	3.42E-01	3.42E+01		
8055247	---	---	---	-0.048	0.967	7.15E-01	7.15E+01		
8055249	NM_00100999	FAM168B	family with sequence similarity 16	-0.064	0.957	6.34E-01	6.34E+01		
8055261	NM_032144	RAB6C	RAB6C, member RAS oncogene fa	-0.012	0.992	9.53E-01	9.53E+01		
8055265	---	---	---	0.020	1.014	9.09E-01	9.09E+01		
8055281	NR_002826	LOC401010	nucleolar complex associated 2 ho	-0.081	0.946	3.90E-01	3.90E+01		
8055284	ENST00000427	FAM128A	family with sequence similarity 12	0.011	1.008	9.63E-01	9.63E+01		
8055287	NM_00108536	FAM128A	family with sequence similarity 12	-0.019	0.987	8.80E-01	8.80E+01		
8055291	BC043584	C2orf27B	chromosome 2 open reading fram	-0.142	0.906	7.56E-02	7.56E+00		
8055309	---	---	---	-0.030	0.980	7.84E-01	7.84E+01		
8055314	NM_144586	LYPD1	LY6/PLAUR domain containing 1	0.017	1.012	9.15E-01	9.15E+01		
8055350	NM_030923	TMEM163	transmembrane protein 163	0.062	1.044	6.87E-01	6.87E+01		
8055404	NM_014607	UBXN4	UBX domain protein 4	-0.087	0.941	3.94E-01	3.94E+01		
8055406	NM_002299	LCT	lactase	0.051	1.036	6.75E-01	6.75E+01		
8055426	NM_005915	MCM6	minichromosome maintenance co	-0.176	0.885	2.24E-01	2.24E+01		
8055445	NM_001349	DARS	aspartyl-tRNA synthetase	-0.060	0.959	6.53E-01	6.53E+01		
8055465	NM_00100854	CXCR4	chemokine (C-X-C motif) receptor	-0.202	0.869	7.06E-03	7.06E-01		
8055476	NR_024058	YWHAE	tyrosine 3-monooxygenase/trypto	0.067	1.048	6.76E-01	6.76E+01		
8055478	NM_007226	NXPH2	neurexophilin 2	0.003	1.002	9.82E-01	9.82E+01		
8055486	---	---	---	-0.137	0.910	7.07E-01	7.07E+01		
8055592	---	---	---	0.112	1.081	5.90E-01	5.90E+01		
8055606	NM_00100663	GTDC1	glycosyltransferase-like domain co	-0.100	0.933	5.97E-01	5.97E+01		
8055624	NM_014795	ZEB2	zinc finger E-box binding homeobo	0.254	1.193	2.66E-01	2.66E+01		
8055664	---	---	---	0.054	1.038	8.15E-01	8.15E+01		
8055672	NM_015702	MMADHC	methylmalonic aciduria (cobalami	-0.047	0.968	7.68E-01	7.68E+01		
8055702	NM_004688	NMI	N-myc (and STAT) interactor	0.118	1.085	5.17E-01	5.17E+01		
8055711	NM_004543	NEB	nebulin	-0.021	0.986	9.24E-01	9.24E+01		
8055862	NM_012097	ARL5A	ADP-ribosylation factor-like 5A	0.045	1.032	6.66E-01	6.66E+01		
8055890	NM_005843	STAM2	signal transducing adaptor molecu	0.097	1.070	5.57E-01	5.57E+01		
8055911	ENST00000428	LOC100129449	PRO2055	-0.039	0.973	9.53E-01	9.53E+01		
8055913	NM_017892	PRPF40A	PRP40 pre-mRNA processing facto	0.027	1.019	8.31E-01	8.31E+01		
8055941	NM_019845	RPRM	reprimo, TP53 dependent G2 arres	0.027	1.019	8.84E-01	8.84E+01		
8055952	NM_006186	NR4A2	nuclear receptor subfamily 4, grou	-0.492	0.711	6.47E-02	6.47E+00		
8055967	---	---	---	0.018	1.012	9.30E-01	9.30E+01		
8055978	NM_152789	FAM133B	family with sequence similarity 13	0.056	1.039	6.35E-01	6.35E+01		
8055980	NM_004288	CYTIP	cytohesin 1 interacting protein	-0.225	0.856	9.72E-03	9.72E-01		
8055992	NM_145259	ACVR1C	activin A receptor, type IC	-0.114	0.924	6.88E-01	6.88E+01		
8056005	NM_001105	ACVR1	activin A receptor, type I	-0.044	0.970	8.37E-01	8.37E+01		
8056041	---	---	---	-0.041	0.972	7.51E-01	7.51E+01		
8056045	---	---	---	0.051	1.036	8.43E-01	8.43E+01		
8056047	NM_00112821	WDSUB1	WD repeat, sterile alpha motif and	-0.022	0.985	9.33E-01	9.33E+01		
8056060	NM_013450	BAZ2B	bromodomain adjacent to zinc fing	0.225	1.169	2.01E-01	2.01E+01		

8056102	NM_014880	CD302	CD302 molecule	0.102	1.074	6.57E-01	6.57E+01		
8056113	NM_002349	LY75	lymphocyte antigen 75	0.017	1.012	9.53E-01	9.53E+01		
8056201	NM_016836	RBMS1	RNA binding motif, single strandec	0.193	1.143	2.84E-01	2.84E+01		
8056206	NM_016836	RBMS1	RNA binding motif, single strandec	0.115	1.083	2.89E-01	2.89E+01		
8056215	---	---	---	0.102	1.073	6.37E-01	6.37E+01		
8056217	NM_198530	MXRA7	matrix-remodelling associated 7	-0.300	0.812	5.59E-02	5.59E+00		
8056220	NM_015446	AHCTF1	AT hook containing transcription f	-0.059	0.960	7.32E-01	7.32E+01		
8056222	NM_001935	DPP4	dipeptidyl-peptidase 4	-0.377	0.770	8.71E-02	8.71E+00		
8056285	NM_022168	IFIH1	interferon induced with helicase C	0.221	1.166	3.65E-01	3.65E+01		
8056426	NM_024753	TTC21B	tetratricopeptide repeat domain 2	-0.026	0.982	9.40E-01	9.40E+01		
8056545	NM_013233	STK39	serine threonine kinase 39 (STE20,	-0.057	0.961	7.40E-01	7.40E+01		
8056566	---	---	---	0.137	1.100	3.69E-01	3.69E+01		
8056716	NM_014168	METTL5	methyltransferase like 5	-0.097	0.935	3.69E-01	3.69E+01		
8056728	---	---	---	0.018	1.013	9.24E-01	9.24E+01		
8056734	NM_012290	TLK1	tousled-like kinase 1	-0.022	0.985	9.09E-01	9.09E+01		
8056753	NM_024770	METTL8	methyltransferase like 8	-0.097	0.935	3.98E-01	3.98E+01		
8056763	---	---	---	-0.002	0.998	9.91E-01	9.91E+01		
8056766	NM_003705	SLC25A12	solute carrier family 25 (mitochon	0.075	1.054	6.33E-01	6.33E+01		
8056784	NM_004405	DLX2	distal-less homeobox 2	-0.044	0.970	7.29E-01	7.29E+01		
8056792	---	---	---	0.079	1.057	7.70E-01	7.70E+01		
8056798	NM_003111	SP3	Sp3 transcription factor	-0.021	0.986	8.58E-01	8.58E+01		
8056810	AK095037	LOC100129312	hypothetical LOC100129312	0.011	1.007	9.56E-01	9.56E+01		
8056812	NM_013341	OLA1	Obg-like ATPase 1	-0.052	0.965	7.87E-01	7.87E+01		
8056821	---	---	---	0.029	1.020	9.00E-01	9.00E+01		
8056825	NM_00114525	SP9	Sp9 transcription factor homolog (	-0.054	0.963	6.51E-01	6.51E+01		
8056829	NM_004882	CIR1	corepressor interacting with RBPJ,	0.096	1.069	5.35E-01	5.35E+01		
8056837	NM_00103304	GPR155	G protein-coupled receptor 155	-0.042	0.971	8.83E-01	8.83E+01		
8056860	NM_003387	WIPF1	WAS/WASL interacting protein far	0.053	1.038	6.27E-01	6.27E+01		
8056909	NM_001880	ATF2	activating transcription factor 2	0.022	1.015	8.80E-01	8.80E+01		
8056930	NM_001689	ATP5G3	ATP synthase, H+ transporting, mi	0.050	1.035	5.98E-01	5.98E+01		
8056959	NM_00108045	EVX2	even-skipped homeobox 2	-0.027	0.981	8.38E-01	8.38E+01		
8056966	---	---	---	-0.017	0.988	9.53E-01	9.53E+01		
8056972	---	---	---	0.060	1.043	4.63E-01	4.63E+01		
8056977	NM_006164	NFE2L2	nuclear factor (erythroid-derived 2	-0.100	0.933	4.34E-01	4.34E+01		
8056993	---	---	---	0.061	1.043	8.84E-01	8.84E+01		
8057034	NM_003690	PRKRA	protein kinase, interferon-inducibl	-0.033	0.978	8.78E-01	8.78E+01		
8057056	NM_133378	TTN	titin	0.074	1.052	6.40E-01	6.40E+01		
8057377	NM_173648	CCDC141	coiled-coil domain containing 141	0.115	1.083	5.99E-01	5.99E+01		
8057394	NM_178123	SESTD1	SEC14 and spectrin domains 1	0.109	1.079	7.15E-01	7.15E+01		
8057439	---	---	---	0.008	1.005	9.77E-01	9.77E+01		
8057441	NM_020943	CWC22	CWC22 spliceosome-associated pr	0.001	1.001	9.94E-01	9.94E+01		
8057463	NM_201548	CERKL	ceramide kinase-like	0.116	1.084	1.91E-01	1.91E+01		
8057554	NM_014412	CACYBP	calcyclin binding protein	-0.118	0.922	3.90E-01	3.90E+01		
8057561	NM_018471	ZC3H15	zinc finger CCCH-type containing 1	0.005	1.003	9.83E-01	9.83E+01		
8057613	---	---	---	0.009	1.006	9.74E-01	9.74E+01		
8057689	NM_022353	OSGEPL1	O-sialoglycoprotein endopeptidase	0.116	1.084	4.65E-01	4.65E+01		
8057700	NM_016467	ORMDL1	ORM1-like 1 (S. cerevisiae)	-0.036	0.975	8.32E-01	8.32E+01		
8057719	NM_014362	HIBCH	3-hydroxyisobutyryl-Coenzyme A h	-0.006	0.996	9.85E-01	9.85E+01		
8057732	NM_00114264	TMEM194B	transmembrane protein 194B	0.066	1.046	8.24E-01	8.24E+01		
8057744	NM_007315	STAT1	signal transducer and activator of	0.270	1.206	1.01E-01	1.01E+01		
8057771	NM_003151	STAT4	signal transducer and activator of	-0.140	0.908	4.04E-01	4.04E+01		
8057887	NM_004226	STK17B	serine/threonine kinase 17b	0.010	1.007	9.56E-01	9.56E+01		
8057931	---	---	---	0.041	1.029	8.10E-01	8.10E+01		
8057933	NM_012086	GTF3C3	general transcription factor IIIC, po	0.052	1.037	7.59E-01	7.59E+01		
8057990	NM_153697	ANKRD44	ankyrin repeat domain 44	0.194	1.144	6.53E-03	6.53E-01		
8058024	NM_012433	SF3B1	splicing factor 3b, subunit 1, 155kD	-0.004	0.997	9.77E-01	9.77E+01		
8058052	NM_002156	HSPD1	heat shock 60kDa protein 1 (chape	-0.072	0.952	6.38E-01	6.38E+01		
8058091	NM_015265	SATB2	SATB homeobox 2	0.000	1.000	1.00E+00	1.00E+02		
8058118	NM_152387	KCTD18	potassium channel tetramerisation	0.015	1.011	9.51E-01	9.51E+01		
8058127	NR_027856	CLK1	CDC-like kinase 1	-0.078	0.947	4.29E-01	4.29E+01		
8058145	---	---	---	-0.133	0.912	3.97E-01	3.97E+01		
8058147	NM_131916	PPIL3	peptidylprolyl isomerase (cyclophi	0.044	1.031	8.79E-01	8.79E+01		
8058161	NM_006190	ORC2L	origin recognition complex, subun	-0.028	0.981	8.53E-01	8.53E+01		
8058182	NM_173822	FAM126B	family with sequence similarity 12	0.061	1.043	7.63E-01	7.63E+01		
8058197	---	---	---	0.280	1.214	2.59E-01	2.59E+01		
8058201	---	---	---	-0.076	0.949	8.07E-01	8.07E+01		
8058203	NM_139163	ALS2CR12	amyotrophic lateral sclerosis 2 (juv	-0.178	0.884	1.29E-01	1.29E+01		
8058221	NM_015049	TRAK2	trafficking protein, kinesin binding	0.011	1.008	9.67E-01	9.67E+01		
8058273	NM_033066	MPP4	membrane protein, palmitoylated	0.051	1.036	7.44E-01	7.44E+01		
8058333	---	---	---	0.016	1.011	9.35E-01	9.35E+01		
8058335	NM_003352	SUMO1	SMT3 suppressor of mif two 3 hon	0.020	1.014	9.39E-01	9.39E+01		
8058340	---	---	---	-0.023	0.984	9.01E-01	9.01E+01		
8058342	---	---	---	0.017	1.012	9.71E-01	9.71E+01		
8058348	---	---	---	0.317	1.246	5.47E-01	5.47E+01		
8058373	NM_018256	WDR12	WD repeat domain 12	0.024	1.017	9.44E-01	9.44E+01		
8058388	---	---	---	0.124	1.090	8.61E-01	8.61E+01		
8058390	NM_213589	RAPH1	Ras association (RalGDS/AF-6) and	0.299	1.231	5.85E-02	5.85E+00		

8058415	NM_017759	INO80D	INO80 complex subunit D	-0.081	0.946	5.38E-01	5.38E+01		
8058428	NM_005006	NDUFS1	NADH dehydrogenase (ubiquinone	0.032	1.023	8.51E-01	8.51E+01		
8058458	---	---	---	-0.023	0.984	9.30E-01	9.30E+01		
8058477	NM_003709	KLF7	Kruppel-like factor 7 (ubiquitous)	-0.058	0.961	8.27E-01	8.27E+01		
8058486	NM_145280	FAM119A	family with sequence similarity 11	0.010	1.007	9.56E-01	9.56E+01		
8058496	---	---	---	0.076	1.054	8.18E-01	8.18E+01		
8058509	NM_00108047	PLEKHM3	pleckstrin homology domain conta	-0.089	0.940	7.99E-01	7.99E+01		
8058512	NM_00108047	PLEKHM3	pleckstrin homology domain conta	-0.198	0.872	1.47E-01	1.47E+01		
8058514	---	---	---	0.109	1.079	6.76E-01	6.76E+01		
8058516	---	---	---	-0.074	0.950	6.74E-01	6.74E+01		
8058518	---	---	---	-0.038	0.974	8.92E-01	8.92E+01		
8058524	NM_020989	CRYGC	crystallin, gamma C	0.006	1.004	9.76E-01	9.76E+01		
8058614	NM_006055	LANCL1	LanC lantibiotic synthetase compo	-0.027	0.982	9.19E-01	9.19E+01		
8058664	---	---	---	0.077	1.055	8.48E-01	8.48E+01		
8058670	NM_016260	IKZF2	IKAROS family zinc finger 2 (Helios	-0.010	0.993	9.84E-01	9.84E+01		
8058837	NM_018441	PECR	peroxisomal trans-2-enoyl-CoA red	-0.053	0.964	8.45E-01	8.45E+01		
8058849	NM_020814	40241	membrane-associated ring finger (	-0.048	0.967	4.93E-01	4.93E+01		
8058855	---	---	---	0.130	1.094	1.01E-01	1.01E+01		
8058866	NM_003284	TNP1	transition protein 1 (during histon	0.055	1.039	6.95E-01	6.95E+01		
8058869	NM_022648	TNS1	tensin 1	0.142	1.103	1.85E-01	1.85E+01		
8058914	NM_001087	AAMP	angio-associated, migratory cell pr	0.003	1.002	9.87E-01	9.87E+01		
8058927	NM_022152	TMBIM1	transmembrane BAX inhibitor mot	0.119	1.086	6.89E-01	6.89E+01		
8058940	NM_020935	USP37	ubiquitin specific peptidase 37	0.026	1.018	9.22E-01	9.22E+01		
8058969	---	---	---	0.057	1.040	9.05E-01	9.05E+01		
8058973	NM_00110553	ZNF142	zinc finger protein 142	-0.097	0.935	2.96E-01	2.96E+01		
8058985	NM_022453	RNF25	ring finger protein 25	-0.074	0.950	6.74E-01	6.74E+01		
8058997	NM_017431	PRKAG3	protein kinase, AMP-activated, ga	-0.031	0.979	8.03E-01	8.03E+01		
8059012	---	---	---	0.133	1.096	1.48E-01	1.48E+01		
8059014	NM_017521	FEV	FEV (ETS oncogene family)	-0.074	0.950	4.83E-01	4.83E+01		
8059019	NM_057093	CRYBA2	crystallin, beta A2	-0.004	0.997	9.84E-01	9.84E+01		
8059026	---	---	---	-0.037	0.974	8.11E-01	8.11E+01		
8059028	NM_194302	CCDC108	coiled-coil domain containing 108	-0.006	0.996	9.58E-01	9.58E+01		
8059067	NM_002181	IHH	Indian hedgehog homolog (Drosop	-0.039	0.973	8.37E-01	8.37E+01		
8059071	NM_024782	NHEJ1	nonhomologous end-joining facto	-0.066	0.955	7.05E-01	7.05E+01		
8059081	NM_00114488	SLC23A3	solute carrier family 23 (nucleobas	-0.105	0.930	3.39E-01	3.39E+01		
8059097	NM_015680	C2orf24	chromosome 2 open reading fram	-0.092	0.938	4.04E-01	4.04E+01		
8059111	NM_005689	ABCB6	ATP-binding cassette, sub-family B	-0.027	0.982	8.67E-01	8.67E+01		
8059139	NM_00107719	ATG9A	ATG9 autophagy related 9 homolo	0.036	1.025	8.05E-01	8.05E+01		
8059158	NM_024506	GLB1L	galactosidase, beta 1-like	0.120	1.086	4.43E-01	4.43E+01		
8059177	NM_006000	TUBA4A	tubulin, alpha 4a	-0.029	0.980	9.05E-01	9.05E+01		
8059186	NM_002846	PTPRN	protein tyrosine phosphatase, rece	0.012	1.008	9.48E-01	9.48E+01		
8059222	NM_012100	DNPEP	aspartyl aminopeptidase	0.024	1.017	8.52E-01	8.52E+01		
8059244	NM_024536	CHPF	chondroitin polymerizing factor	-0.028	0.981	8.66E-01	8.66E+01		
8059279	NM_004438	EPHA4	EPH receptor A4	-0.095	0.936	6.63E-01	6.63E+01		
8059319	NM_005687	FARSB	phenylalanyl-tRNA synthetase, bet	0.085	1.061	7.51E-01	7.51E+01		
8059339	---	---	---	-0.030	0.979	8.78E-01	8.78E+01		
8059361	NM_020830	WDFY1	WD repeat and FYVE domain conta	0.237	1.178	3.84E-02	3.84E+00		
8059376	NM_00113652	SERPINE2	serpin peptidase inhibitor, clade E	-0.009	0.994	9.86E-01	9.86E+01		
8059387	NM_024785	FAM124B	family with sequence similarity 12	0.009	1.006	9.64E-01	9.64E+01		
8059393	NM_003590	CUL3	cullin 3	0.026	1.018	8.30E-01	8.30E+01		
8059413	NM_014689	DOCK10	dedicator of cytokinesis 10	0.198	1.147	1.37E-03	1.37E-01		
8059475	---	---	---	0.124	1.090	3.92E-01	3.92E+01		
8059565	NM_017933	PID1	phosphotyrosine interaction doma	-0.834	0.561	5.88E-02	5.88E+00		
8059576	---	---	---	-0.007	0.995	9.73E-01	9.73E+01		
8059580	NM_139072	DNER	delta/notch-like EGF repeat contai	-0.180	0.883	5.28E-02	5.28E+00		
8059596	NM_004238	TRIP12	thyroid hormone receptor interact	-0.046	0.969	6.74E-01	6.74E+01		
8059648	---	---	---	-0.007	0.995	9.91E-01	9.91E+01		
8059650	NM_080424	SP110	SP110 nuclear body protein	0.179	1.132	8.71E-02	8.71E+00		
8059672	---	---	---	-0.145	0.904	3.04E-01	3.04E+01		
8059674	NM_005683	GPR55	G protein-coupled receptor 55	0.064	1.045	6.43E-01	6.43E+01		
8059680	NM_000867	HTR2B	5-hydroxytryptamine (serotonin) r	-0.132	0.913	5.04E-01	5.04E+01		
8059687	---	---	---	0.200	1.149	5.38E-01	5.38E+01		
8059689	NM_005381	NCL	nucleolin	-0.037	0.975	6.58E-01	6.58E+01		
8059708	NR_002921	SNORA75	small nucleolar RNA, H/ACA box 7	0.024	1.017	9.71E-01	9.71E+01		
8059712	NR_004398	SNORD82	small nucleolar RNA, C/D box 82	-0.133	0.912	6.14E-01	6.14E+01		
8059716	BC033054	C2orf52	chromosome 2 open reading fram	-0.088	0.941	7.06E-01	7.06E+01		
8059720	NM_006056	NMUR1	neuromedin U receptor 1	0.011	1.008	9.28E-01	9.28E+01		
8059731	NM_002601	PDE6D	phosphodiesterase 6D, cGMP-spe	-0.086	0.942	5.65E-01	5.65E+01		
8059739	NM_024409	NPPC	natriuretic peptide precursor C	0.035	1.025	7.97E-01	7.97E+01		
8059744	BC067110	ECEL1P2	endothelin converting enzyme-like	-0.055	0.963	5.58E-01	5.58E+01		
8059748	NM_004826	ECEL1	endothelin converting enzyme-like	-0.017	0.988	9.00E-01	9.00E+01		
8059770	NM_145702	TIGD1	tigger transposable element deriv	-0.058	0.961	8.29E-01	8.29E+01		
8059776	NM_002242	KCNJ13	potassium inwardly-rectifying cha	0.078	1.055	7.14E-01	7.14E+01		
8059783	NM_019850	NGEF	neuronal guanine nucleotide exch	-0.033	0.977	7.75E-01	7.75E+01		
8059799	---	---	---	0.153	1.112	5.83E-01	5.83E+01		
8059838	NM_018410	HJURP	Holliday junction recognition prot	0.160	1.117	2.54E-01	2.54E+01		
8059852	NR_024322	MSL3L2	male-specific lethal 3-like 2 (Dros	0.041	1.028	9.24E-01	9.24E+01		

8059854	NM_005737	ARL4C	ADP-ribosylation factor-like 4C	-0.101	0.933	4.13E-01	4.13E+01		
8059864	NM_001485	GBX2	gastrulation brain homeobox 2	0.011	1.008	9.52E-01	9.52E+01		
8059868	NM_212556	ASB18	ankyrin repeat and SOCS box-cont	-0.059	0.960	5.67E-01	5.67E+01		
8059900	---	---	---	0.120	1.086	2.65E-01	2.65E+01		
8059955	NM_022449	RAB17	RAB17, member RAS oncogene fai	0.055	1.039	6.59E-01	6.59E+01		
8059969	NM_030768	ILKAP	integrin-linked kinase-associated s	-0.009	0.994	9.77E-01	9.77E+01		
8059985	NR_026925	LOC151174	hypothetical LOC151174	-0.077	0.948	4.51E-01	4.51E+01		
8059989	NM_018645	HES6	hairy and enhancer of split 6 (Dros	0.043	1.030	7.23E-01	7.23E+01		
8059996	NM_022817	PER2	period homolog 2 (Drosophila)	0.094	1.067	3.94E-01	3.94E+01		
8060030	NM_006037	HDAC4	histone deacetylase 4	-0.054	0.963	6.29E-01	6.29E+01		
8060061	NR_026664	MGC16025	hypothetical LOC85009	-0.031	0.979	8.41E-01	8.41E+01		
8060063	NM_004544	NDUFA10	NADH dehydrogenase (ubiquinone	-0.055	0.962	6.04E-01	6.04E+01		
8060082	NM_00108083	PRR21	proline rich 21	-0.034	0.976	7.45E-01	7.45E+01		
8060086	AF487338	MYEOV2	myeloma overexpressed 2	-0.046	0.969	7.23E-01	7.23E+01		
8060094	NM_148961	OTOS	otospiralin	-0.069	0.953	3.76E-01	3.76E+01		
8060101	AK092442	PP14571	similar to hCG1777210	-0.040	0.973	7.27E-01	7.27E+01		
8060103	NM_016552	ANKMY1	ankyrin repeat and MYND domain	0.029	1.020	8.27E-01	8.27E+01		
8060126	NM_198998	AQP12A	aquaporin 12A	0.032	1.023	7.09E-01	7.09E+01		
8060187	NM_00108543	C2orf54	chromosome 2 open reading fram	-0.050	0.966	7.14E-01	7.14E+01		
8060196	NM_182501	MTERFD2	MTERF domain containing 2	-0.058	0.961	8.26E-01	8.26E+01		
8060205	NM_015148	PASK	PAS domain containing serine/thre	-0.102	0.932	5.63E-01	5.63E+01		
8060225	NM_203346	HDLBP	high density lipoprotein binding pr	-0.046	0.969	7.92E-01	7.92E+01		
8060257	NM_006374	STK25	serine/threonine kinase 25 (STE20	-0.047	0.968	6.66E-01	6.66E+01		
8060274	NM_015963	THAP4	THAP domain containing 4	0.000	1.000	1.00E+00	1.00E+02		
8060286	NM_012145	DTYMK	deoxythymidylate kinase (thymidy	-0.032	0.978	8.87E-01	8.87E+01		
8060294	NM_005018	PDCD1	programmed cell death 1	-0.033	0.977	7.69E-01	7.69E+01		
8060325	NM_207469	DEFB132	defensin, beta 132	0.021	1.015	8.87E-01	8.87E+01		
8060330	NM_033089	ZCCHC3	zinc finger, CCHC domain containi	-0.063	0.958	4.73E-01	4.73E+01		
8060334	NM_006943	SOX12	SRY (sex determining region Y)-bo	-0.093	0.937	2.52E-01	2.52E+01		
8060339	NM_024958	NRSN2	neurensin 2	0.021	1.015	9.07E-01	9.07E+01		
8060344	NM_021158	TRIB3	tribbles homolog 3 (Drosophila)	-0.024	0.983	8.55E-01	8.55E+01		
8060353	NM_031229	RBCK1	RanBP-type and C3HC4-type zinc f	0.041	1.028	7.52E-01	7.52E+01		
8060370	NM_031424	FAM110A	family with sequence similarity 11	-0.105	0.930	3.15E-01	3.15E+01		
8060379	NM_178578	PSMF1	proteasome (prosome, macropain	0.129	1.093	1.39E-01	1.39E+01		
8060418	NM_00104002	SIRPA	signal-regulatory protein alpha	0.269	1.205	3.41E-01	3.41E+01		
8060427	NM_080836	STK35	serine/threonine kinase 35	-0.113	0.925	2.37E-01	2.37E+01		
8060447	NM_198994	TGM6	transglutaminase 6	-0.002	0.999	9.90E-01	9.90E+01		
8060484	NR_027700	NOP56	NOP56 ribonucleoprotein homolo	0.013	1.009	9.66E-01	9.66E+01		
8060501	NR_002981	SNORA51	small nucleolar RNA, H/ACA box 5	0.006	1.004	9.85E-01	9.85E+01		
8060503	NR_002738	SNORD57	small nucleolar RNA, C/D box 57	0.087	1.062	7.59E-01	7.59E+01		
8060505	NM_00111051	EBF4	early B-cell factor 4	-0.056	0.962	5.34E-01	5.34E+01		
8060528	NM_080739	C20orf141	chromosome 20 open reading fra	0.018	1.013	8.99E-01	8.99E+01		
8060533	BC021178	C20orf141	chromosome 20 open reading fra	-0.023	0.984	8.81E-01	8.81E+01		
8060539	NM_002836	PTPRA	protein tyrosine phosphatase, rec	-0.005	0.996	9.65E-01	9.65E+01		
8060594	NM_001501	GNRH2	gonadotropin-releasing hormone	0.011	1.008	9.44E-01	9.44E+01		
8060599	NM_030811	MRPS26	mitochondrial ribosomal protein S	-0.052	0.965	5.68E-01	5.68E+01		
8060604	NM_000915	OXT	oxytocin, prepropeptide	-0.115	0.923	1.30E-01	1.30E+01		
8060609	---	---	---	0.021	1.014	8.47E-01	8.47E+01		
8060611	NM_033453	ITPA	inosine triphosphatase (nucleosid	0.142	1.104	1.22E-01	1.22E+01		
8060627	NM_139321	ATRN	attractin	0.127	1.092	2.15E-01	2.15E+01		
8060660	NM_052970	HSPA12B	heat shock 70kD protein 12B	-0.039	0.973	7.03E-01	7.03E+01		
8060675	NM_021873	CDC25B	cell division cycle 25 homolog B (S	0.091	1.065	5.26E-01	5.26E+01		
8060698	BC043344	C20orf29	chromosome 20 open reading fra	-0.129	0.914	1.58E-01	1.58E+01		
8060705	NM_020746	MAVS	mitochondrial antiviral signaling p	-0.061	0.959	4.96E-01	4.96E+01		
8060722	NM_153638	PANK2	pantothenate kinase 2	0.040	1.028	7.19E-01	7.19E+01		
8060734	---	---	---	-0.055	0.963	8.76E-01	8.76E+01		
8060736	---	---	---	0.165	1.121	7.05E-02	7.05E+00		
8060738	---	---	---	0.282	1.216	6.76E-02	6.76E+00		
8060745	NM_175839	SMOX	spermine oxidase	-0.203	0.869	9.26E-02	9.26E+00		
8060758	NM_000311	PRNP	prion protein	-0.036	0.975	8.10E-01	8.10E+01		
8060765	NM_012409	PRND	prion protein 2 (dublet)	-0.009	0.994	9.64E-01	9.64E+01		
8060772	NM_003818	CDS2	CDP-diacylglycerol synthase (phos	0.001	1.001	9.96E-01	9.96E+01		
8060792	AK125948	LOC643406	hypothetical protein LOC643406	-0.006	0.996	9.81E-01	9.81E+01		
8060796	BC035800	C20orf196	chromosome 20 open reading fra	0.109	1.079	3.85E-01	3.85E+01		
8060803	---	---	---	0.087	1.062	3.79E-01	3.79E+01		
8060805	NM_001819	CHGB	chromogranin B (secretogranin 1)	-0.219	0.859	4.96E-01	4.96E+01		
8060813	NM_032485	MCM8	minichromosome maintenance co	-0.025	0.983	9.49E-01	9.49E+01		
8060837	---	---	---	0.023	1.016	9.49E-01	9.49E+01		
8060839	NM_019095	CRLS1	cardiolipin synthase 1	0.101	1.073	4.79E-01	4.79E+01		
8060850	NM_001200	BMP2	bone morphogenetic protein 2	-0.013	0.991	9.53E-01	9.53E+01		
8060854	NM_182734	PLCB1	phospholipase C, beta 1 (phospho	-0.008	0.994	9.83E-01	9.83E+01		
8060895	NR_004386	RNU105B	RNA, U105B small nucleolar	0.047	1.033	8.42E-01	8.42E+01		
8060949	NM_022096	ANKRD5	ankyrin repeat domain 5	-0.083	0.944	7.33E-01	7.33E+01		
8060977	NM_00100960	C20orf94	chromosome 20 open reading fra	0.089	1.063	7.52E-01	7.52E+01		
8060988	NM_014962	BTBD3	BTB (POZ) domain containing 3	0.099	1.071	6.76E-01	6.76E+01		
8061019	NM_024120	C20orf7	chromosome 20 open reading fra	0.024	1.017	9.18E-01	9.18E+01		
8061035	NM_080676	MACROD2	MACRO domain containing 2	0.030	1.021	8.70E-01	8.70E+01		

8061073	---	---	---	0.135	1.098	3.83E-01	3.83E+01		
8061075	NM_003092	SNRPB2	small nuclear ribonucleoprotein p	-0.055	0.962	8.21E-01	8.21E+01		
8061112	---	---	---	0.030	1.021	8.84E-01	8.84E+01		
8061114	NM_00101154	DSTN	destrin (actin depolymerizing facto	-0.089	0.940	5.70E-01	5.70E+01		
8061129	BC016869	C20orf72	chromosome 20 open reading fra	0.240	1.181	8.56E-02	8.56E+00		
8061136	NM_002823	PTMA	prothymosin, alpha	-0.017	0.988	9.45E-01	9.45E+01		
8061138	NM_020536	CSRP2BP	CSRP2 binding protein	0.086	1.062	4.81E-01	4.81E+01		
8061154	NM_003434	ZNF133	zinc finger protein 133	0.041	1.028	7.45E-01	7.45E+01		
8061169	---	---	---	0.003	1.002	9.88E-01	9.88E+01		
8061184	---	---	---	-0.037	0.975	8.10E-01	8.10E+01		
8061186	NM_006363	SEC23B	Sec23 homolog B (S. cerevisiae)	0.066	1.047	5.98E-01	5.98E+01		
8061211	NM_080820	DTD1	D-tyrosyl-tRNA deacylase 1 homol	-0.055	0.962	6.64E-01	6.64E+01		
8061222	---	---	---	0.062	1.044	6.67E-01	6.67E+01		
8061227	NM_020689	SLC24A3	solute carrier family 24 (sodium/p	-0.163	0.893	2.00E-01	2.00E+01		
8061262	NM_016100	NAT5	N-acetyltransferase 5 (GCN5-relat	0.052	1.037	7.47E-01	7.47E+01		
8061303	NM_002196	INSM1	insulinoma-associated 1	-0.037	0.975	7.54E-01	7.54E+01		
8061305	NM_018474	C20orf19	chromosome 20 open reading fra	-0.001	0.999	9.97E-01	9.97E+01		
8061324	NM_012255	XRN2	5'-3' exoribonuclease 2	0.102	1.073	2.70E-01	2.70E+01		
8061357	NM_006192	PAX1	paired box 1	-0.015	0.989	9.20E-01	9.20E+01		
8061364	NM_021104	RPL41	ribosomal protein L41	0.007	1.005	9.24E-01	9.24E+01		
8061366	NM_001052	SSTR4	somatostatin receptor 4	-0.074	0.950	4.50E-01	4.50E+01		
8061368	NM_013248	NXT1	NTF2-like export factor 1	-0.074	0.950	4.95E-01	4.95E+01		
8061373	NM_022482	GZF1	GDNF-inducible zinc finger protei	-0.087	0.941	4.49E-01	4.49E+01		
8061414	---	---	---	0.022	1.015	8.99E-01	8.99E+01		
8061416	NM_003650	CST7	cystatin F (leukocystatin)	0.028	1.020	9.56E-01	9.56E+01		
8061426	---	---	---	0.179	1.132	5.38E-01	5.38E+01		
8061428	NM_001247	ENTPD6	ectonucleoside triphosphate diph	-0.037	0.974	8.32E-01	8.32E+01		
8061445	---	---	---	0.060	1.043	6.46E-01	6.46E+01		
8061447	NM_002862	PYGB	phosphorylase, glycogen; brain	-0.038	0.974	7.99E-01	7.99E+01		
8061483	NR_004846	CTD-2514C3.1	hypothetical LOC100134868	0.105	1.075	4.09E-01	4.09E+01		
8061490	NR_026713	FAM182A	family with sequence similarity 18	0.001	1.001	9.94E-01	9.94E+01		
8061497	NR_003579	FRG1B	FSHD region gene 1 family, memb	-0.031	0.978	9.07E-01	9.07E+01		
8061519	NM_153324	DEFB123	defensin, beta 123	0.047	1.033	6.51E-01	6.51E+01		
8061529	NM_014012	REM1	RAS (RAD and GEM)-like GTP-bind	0.006	1.004	9.80E-01	9.80E+01		
8061539	NR_024358	NCRNA00028	non-protein coding RNA 28	-0.031	0.979	8.51E-01	8.51E+01		
8061542	NM_178580	HM13	histocompatibility (minor) 13	-0.027	0.982	8.48E-01	8.48E+01		
8061562	---	---	---	-0.021	0.986	9.36E-01	9.36E+01		
8061564	NM_181353	ID1	inhibitor of DNA binding 1, domin	-0.049	0.967	6.73E-01	6.73E+01		
8061572	NM_032609	COX4I2	cytochrome c oxidase subunit IV i	-0.031	0.979	8.47E-01	8.47E+01		
8061605	NM_033118	MYLK2	myosin light chain kinase 2	0.047	1.033	6.66E-01	6.66E+01		
8061620	NM_00100840	TTLL9	tubulin tyrosine ligase-like family,	0.021	1.014	9.30E-01	9.30E+01		
8061647	NM_00101171	XKR7	XK, Kell blood group complex sub	-0.029	0.980	8.30E-01	8.30E+01		
8061653	NM_080625	C20orf160	chromosome 20 open reading fra	-0.032	0.978	7.29E-01	7.29E+01		
8061666	---	---	---	-0.042	0.971	8.08E-01	8.08E+01		
8061668	NM_002110	HCK	hemopoietic cell kinase	-0.048	0.967	9.36E-01	9.36E+01		
8061685	NM_014742	TM9SF4	transmembrane 9 superfamily pro	-0.210	0.864	6.92E-02	6.92E+00		
8061706	NM_015352	POFUT1	protein O-fucosyltransferase 1	-0.019	0.987	9.29E-01	9.29E+01		
8061715	NM_004798	KIF3B	kinesin family member 3B	-0.264	0.833	2.41E-03	2.41E-01		
8061725	NM_015338	ASXL1	additional sex combs like 1 (Droso	-0.092	0.938	3.94E-01	3.94E+01		
8061772	NM_012325	MAPRE1	microtubule-associated protein, R	0.079	1.056	5.48E-01	5.48E+01		
8061780	NM_025227	BPIL1	bactericidal/permeability-increasi	-0.040	0.972	7.03E-01	7.03E+01		
8061815	NM_182658	C20orf185	chromosome 20 open reading fra	-0.066	0.955	5.02E-01	5.02E+01		
8061831	NM_182519	C20orf186	chromosome 20 open reading fra	0.042	1.029	7.61E-01	7.61E+01		
8061869	NM_178466	C20orf71	chromosome 20 open reading fra	0.059	1.042	5.58E-01	5.58E+01		
8061881	---	---	---	-0.039	0.973	7.35E-01	7.35E+01		
8061883	NM_130852	PLUNC	palate, lung and nasal epithelium	-0.023	0.984	8.91E-01	8.91E+01		
8061894	NM_033197	C20orf114	chromosome 20 open reading fra	0.063	1.045	4.51E-01	4.51E+01		
8061912	---	---	---	-0.169	0.889	3.57E-01	3.57E+01		
8061919	NM_005093	CBFA2T2	core-binding factor, runt domain,	-0.038	0.974	7.86E-01	7.86E+01		
8061940	DQ080434	C20orf144	chromosome 20 open reading fra	-0.080	0.946	4.25E-01	4.25E+01		
8061944	BC137111	C20orf134	chromosome 20 open reading fra	-0.071	0.952	4.82E-01	4.82E+01		
8061946	NM_032819	ZNF341	zinc finger protein 341	-0.001	0.999	9.92E-01	9.92E+01		
8061958	NM_176812	CHMP4B	chromatin modifying protein 4B	-0.050	0.966	5.68E-01	5.68E+01		
8061966	NM_016732	RALY	RNA binding protein, autoantigeni	-0.014	0.990	9.37E-01	9.37E+01		
8061982	NM_001672	ASIP	agouti signaling protein, nonagou	-0.018	0.988	9.19E-01	9.19E+01		
8061986	NM_031483	ITCH	itchy E3 ubiquitin protein ligase h	0.062	1.044	5.23E-01	5.23E+01		
8062016	NM_014183	DYNLRB1	dynein, light chain, roadblock-type	-0.003	0.998	9.86E-01	9.86E+01		
8062023	NM_032514	MAP1LC3A	microtubule-associated protein 1	-0.089	0.940	3.78E-01	3.78E+01		
8062034	NM_021202	TP53INP2	tumor protein p53 inducible nucle	-0.133	0.912	5.46E-02	5.46E+00		
8062041	NM_018677	ACSS2	acyl-CoA synthetase short-chain f	-0.040	0.973	8.52E-01	8.52E+01		
8062064	NM_020884	MYH7B	myosin, heavy chain 7B, cardiac m	-0.007	0.995	9.56E-01	9.56E+01		
8062108	NM_006404	PROCR	protein C receptor, endothelial (E	-0.031	0.979	8.73E-01	8.73E+01		
8062117	---	---	---	0.097	1.070	8.23E-01	8.23E+01		
8062119	AF348994 //	MT1JP // MT1J	metallothionein 1J (pseudogene)	-0.193	0.875	3.18E-01	3.18E+01		
8062123	NM_006690	MMP24	matrix metallopeptidase 24 (mem	-0.044	0.970	6.75E-01	6.75E+01		
8062137	NM_007186	CEP250	centrosomal protein 250kDa	-0.035	0.976	7.30E-01	7.30E+01		
8062174	NM_198398	ERGIC3	ERGIC and golgi 3	-0.065	0.956	5.36E-01	5.36E+01		

8062204	---	---	---	0.017	1.012	9.60E-01	9.60E+01		
8062206	NM_080748	ROMO1	reactive oxygen species modulato	0.116	1.084	2.47E-01	2.47E+01		
8062211	NM_032194	BXDC1	brix domain containing 1	0.167	1.123	2.27E-01	2.27E+01		
8062213	NM_016436	PHF20	PHD finger protein 20	-0.126	0.916	1.58E-01	1.58E+01		
8062251	NM_012156	EPB41L1	erythrocyte membrane protein ba	-0.099	0.934	8.90E-02	8.90E+00		
8062286	BC019311	C20orf4	chromosome 20 open reading fra	-0.026	0.982	8.73E-01	8.73E+01		
8062293	NM_014902	DLGAP4	discs, large (Drosophila) homolog-	-0.042	0.972	6.34E-01	6.34E+01		
8062312	NM_006097	MYL9	myosin, light chain 9, regulatory	-0.013	0.991	9.50E-01	9.50E+01		
8062319	NM_021809	TGIF2	TGFB-induced factor homeobox 2	-0.030	0.979	8.30E-01	8.30E+01		
8062326	NR_026562	C20orf24	chromosome 20 open reading fra	0.009	1.006	9.76E-01	9.76E+01		
8062337	---	---	---	-0.007	0.996	9.66E-01	9.66E+01		
8062339	BC130646	C20orf118	chromosome 20 open reading fra	0.147	1.107	6.73E-02	6.73E+00		
8062347	NM_002895	RBL1	retinoblastoma-like 1 (p107)	0.010	1.007	9.64E-01	9.64E+01		
8062349	NM_002951	RPN2	ribophorin II	0.131	1.095	1.65E-01	1.65E+01		
8062371	NM_022077	MANBAL	mannosidase, beta A, lysosomal-li	-0.031	0.979	8.58E-01	8.58E+01		
8062377	NM_005417	SRC	v-src sarcoma (Schmidt-Ruppin A-	0.010	1.007	9.60E-01	9.60E+01		
8062395	NM_005386	NNAT	neuronatin	0.028	1.020	8.74E-01	8.74E+01		
8062404	---	---	---	-0.031	0.979	8.74E-01	8.74E+01		
8062409	NM_030877	CTNNBL1	catenin, beta like 1	-0.008	0.994	9.77E-01	9.77E+01		
8062427	NM_080607	VSTM2L	V-set and transmembrane domain	-0.034	0.977	7.98E-01	7.98E+01		
8062433	NM_021215	RPRD1B	regulation of nuclear pre-mRNA d	0.002	1.002	9.90E-01	9.90E+01		
8062480	NR_003239	SNHG11	small nucleolar RNA host gene 11	-0.019	0.987	8.63E-01	8.63E+01		
8062490	NR_002986	SNORA60	small nucleolar RNA, H/ACA box 6	-0.021	0.986	9.16E-01	9.16E+01		
8062492	NM_020336	KIAA1219	KIAA1219	-0.061	0.958	4.23E-01	4.23E+01		
8062527	NM_00101808	ADIG	adipogenin	-0.026	0.982	8.78E-01	8.78E+01		
8062545	NM_024855	ACTR5	ARP5 actin-related protein 5 hom	-0.086	0.942	5.32E-01	5.32E+01		
8062555	---	---	---	0.064	1.045	7.73E-01	7.73E+01		
8062557	NM_015568	PPP1R16B	protein phosphatase 1, regulatory	-0.131	0.913	2.12E-01	2.12E+01		
8062569	---	---	---	-0.039	0.973	8.48E-01	8.48E+01		
8062576	NM_021931	DHX35	DEAH (Asp-Glu-Ala-His) box polyp	0.070	1.050	7.16E-01	7.16E+01		
8062603	NM_003286	TOP1	topoisomerase (DNA) I	0.043	1.030	6.42E-01	6.42E+01		
8062623	NM_002660	PLCG1	phospholipase C, gamma 1	-0.005	0.997	9.85E-01	9.85E+01		
8062658	NM_022896	LPIN3	lipin 3	-0.055	0.963	5.41E-01	5.41E+01		
8062681	---	---	---	-0.064	0.957	8.07E-01	8.07E+01		
8062687	---	---	---	0.095	1.068	5.15E-01	5.15E+01		
8062695	NM_006275	SFRS6	splicing factor, arginine/serine-ric	-0.018	0.988	9.16E-01	9.16E+01		
8062705	NM_032107	L3MBTL	l(3)mbt-like (Drosophila)	0.038	1.027	7.51E-01	7.51E+01		
8062766	NM_002466	MYBL2	v-myb myeloblastosis viral oncog	-0.027	0.981	8.52E-01	8.52E+01		
8062782	NM_032883	TOX2	TOX high mobility group box famil	-0.082	0.944	3.04E-01	3.04E+01		
8062794	---	---	---	0.058	1.041	7.53E-01	7.53E+01		
8062796	NM_024034	GDAP1L1	ganglioside-induced differentiatio	-0.042	0.971	7.54E-01	7.54E+01		
8062813	NM_178491	R3HDML	R3H domain containing-like	-0.049	0.967	6.20E-01	6.20E+01		
8062823	NM_000457	HNF4A	hepatocyte nuclear factor 4, alpha	0.005	1.003	9.72E-01	9.72E+01		
8062842	---	---	---	-0.050	0.966	7.98E-01	7.98E+01		
8062844	NM_024331	TTPAL	tocopherol (alpha) transfer protei	-0.104	0.930	4.13E-01	4.13E+01		
8062852	NM_181805	PKIG	protein kinase (cAMP-dependent,	0.003	1.002	9.90E-01	9.90E+01		
8062864	NM_003881	WISP2	WNT1 inducible signaling pathway	-0.081	0.946	2.28E-01	2.28E+01		
8062873	NM_022358	KCNK15	potassium channel, subfamily K, m	0.006	1.004	9.77E-01	9.77E+01		
8062880	NM_003404	YWHAB	tyrosine 3-monooxygenase/trypto	0.063	1.045	4.26E-01	4.26E+01		
8062890	NM_00112475	PABPC1L	poly(A) binding protein, cytoplasm	-0.065	0.956	4.32E-01	4.32E+01		
8062908	NM_006282	STK4	serine/threonine kinase 4	-0.072	0.951	1.79E-01	1.79E+01		
8062948	NM_014276	RBPJL	recombination signal binding prot	-0.053	0.964	6.34E-01	6.34E+01		
8062962	---	---	---	-0.015	0.990	9.53E-01	9.53E+01		
8062964	NM_033542	SYS1	SYS1 Golgi-localized integral mem	0.049	1.034	7.45E-01	7.45E+01		
8062971	NM_00104822	DBNDD2	dysbindin (dystrobrevin binding p	-0.018	0.988	9.07E-01	9.07E+01		
8062981	NM_015937	PIGT	phosphatidylinositol glycan ancho	-0.026	0.982	8.72E-01	8.72E+01		
8063000	NM_006103	WFDC2	WAP four-disulfide core domain 2	0.025	1.018	8.14E-01	8.14E+01		
8063011	NM_080753	WFDC10A	WAP four-disulfide core domain 1	0.026	1.018	8.72E-01	8.72E+01		
8063014	NM_172005	WFDC13	WAP four-disulfide core domain 1	-0.007	0.995	9.68E-01	9.68E+01		
8063028	NM_052951	DNTTIP1	deoxynucleotidyltransferase, term	-0.016	0.989	9.12E-01	9.12E+01		
8063043	NM_181802	UBE2C	ubiquitin-conjugating enzyme E2C	0.107	1.077	1.95E-01	1.95E+01		
8063057	NM_033421	SNX21	sorting nexin family member 21	-0.052	0.964	4.96E-01	4.96E+01		
8063071	NM_080752	ZSWIM3	zinc finger, SWIM-type containing	-0.073	0.951	5.02E-01	5.02E+01		
8063074	NM_080603	ZSWIM1	zinc finger, SWIM-type containing	0.022	1.015	8.91E-01	8.91E+01		
8063078	NM_00112769	CTSA	cathepsin A	0.244	1.185	1.68E-01	1.68E+01		
8063097	NM_022104	PCIF1	PDX1 C-terminal inhibiting factor 1	-0.104	0.930	1.61E-01	1.61E+01		
8063115	NM_004994	MMP9	matrix metallopeptidase 9 (gelati	0.317	1.246	6.24E-01	6.24E+01		
8063174	ENST0000044	ZNF840P	zinc finger protein 840 (pseudoge	-0.147	0.903	6.17E-01	6.17E+01		
8063177	NM_030777	SLC2A10	solute carrier family 2 (facilitated	0.006	1.004	9.73E-01	9.73E+01		
8063187	NM_172111	EYA2	eyes absent homolog 2 (Drosophil	-0.068	0.954	5.15E-01	5.15E+01		
8063211	NM_181659	NCOA3	nuclear receptor coactivator 3	0.021	1.015	8.94E-01	8.94E+01		
8063240	---	---	---	0.068	1.048	6.92E-01	6.92E+01		
8063242	NM_006420	ARFGEF2	ADP-ribosylation factor guanine n	0.035	1.025	7.89E-01	7.89E+01		
8063283	NM_001316	CSE1L	CSE1 chromosome segregation 1-l	-0.034	0.977	8.49E-01	8.49E+01		
8063315	NM_017895	DDX27	DEAD (Asp-Glu-Ala-Asp) box polyp	-0.077	0.948	6.34E-01	6.34E+01		
8063337	NR_003605	C20orf199	chromosome 20 open reading fra	-0.020	0.986	8.91E-01	8.91E+01		
8063345	NR_002433	SNORD12C	small nucleolar RNA, C/D box 12C	-0.301	0.812	4.91E-01	4.91E+01		

8063347	---	---	---	-0.005	0.997	9.84E-01	9.84E+01		
8063351	NM_015266	SLC9A8	solute carrier family 9 (sodium/hy	0.074	1.052	5.77E-01	5.77E+01		
8063369	NM_018683	RNF114	ring finger protein 114	0.181	1.133	3.26E-02	3.26E+00		
8063386	NM_005194	CEBPB	CCAAT/enhancer binding protein (	0.015	1.011	9.49E-01	9.49E+01		
8063389	AK090605	LOC284751	hypothetical LOC284751	-0.002	0.998	9.90E-01	9.90E+01		
8063394	NM_002827	PTPN1	protein tyrosine phosphatase, nor	-0.083	0.944	5.12E-01	5.12E+01		
8063408	---	---	---	-0.112	0.925	7.04E-01	7.04E+01		
8063410	NM_032521	PARD6B	par-6 partitioning defective 6 hom	-0.081	0.946	5.52E-01	5.52E+01		
8063417	NM_017843	BCAS4	breast carcinoma amplified seque	-0.055	0.963	5.98E-01	5.98E+01		
8063427	NM_014484	MOCS3	molybdenum cofactor synthesis 3	-0.129	0.915	3.41E-01	3.41E+01		
8063437	NM_173485	TSHZ2	teashirt zinc finger homeobox 2	-0.031	0.979	9.57E-01	9.57E+01		
8063444	ENST00000371	TSHZ2	teashirt zinc finger homeobox 2	-0.042	0.971	9.57E-01	9.57E+01		
8063449	---	---	---	0.055	1.039	7.18E-01	7.18E+01		
8063453	NM_002623	PFDN4	prefoldin subunit 4	0.004	1.002	9.91E-01	9.91E+01		
8063473	NM_000976	RPL12	ribosomal protein L12	0.021	1.015	9.22E-01	9.22E+01		
8063476	NM_019888	MC3R	melanocortin 3 receptor	0.091	1.065	4.16E-01	4.16E+01		
8063478	NM_080821	C20orf108	chromosome 20 open reading fra	-0.002	0.999	9.90E-01	9.90E+01		
8063484	NM_00103352	CSTF1	cleavage stimulation factor, 3' pre	-0.049	0.967	6.98E-01	6.98E+01		
8063497	NM_020356	CASS4	Cas scaffolding protein family me	0.163	1.120	1.53E-01	1.53E+01		
8063509	NM_016407	C20orf43	chromosome 20 open reading fra	0.101	1.073	1.57E-01	1.57E+01		
8063522	---	---	---	-0.106	0.929	8.18E-01	8.18E+01		
8063526	NM_00101297	C20orf106	chromosome 20 open reading fra	0.051	1.036	7.63E-01	7.63E+01		
8063531	BC105792	C20orf107	chromosome 20 open reading fra	0.027	1.019	9.36E-01	9.36E+01		
8063547	---	---	---	-0.059	0.960	7.27E-01	7.27E+01		
8063549	---	---	---	-0.061	0.959	6.44E-01	6.44E+01		
8063566	NM_003610	RAE1	RAE1 RNA export 1 homolog (S. p	0.076	1.054	6.78E-01	6.78E+01		
8063583	NM_017495	RBM38	RNA binding motif protein 38	-0.120	0.920	1.58E-01	1.58E+01		
8063607	NM_020673	RAB22A	RAB22A, member RAS oncogene f	-0.022	0.985	8.82E-01	8.82E+01		
8063620	NM_004738	VAPB	VAMP (vesicle-associated membra	-0.072	0.952	4.84E-01	4.84E+01		
8063634	ENST00000419	MGC4294	hypothetical protein MGC4294	0.011	1.007	9.56E-01	9.56E+01		
8063636	NM_00100143	STX16	syntaxin 16	-0.006	0.996	9.77E-01	9.77E+01		
8063650	NM_024663	NPEPL1	aminopeptidase-like 1	-0.065	0.956	5.88E-01	5.88E+01		
8063668	NM_000516	GNAS	GNAS complex locus	0.027	1.019	7.51E-01	7.51E+01		
8063697	NM_198976	TH1L	TH1-like (Drosophila)	0.113	1.081	7.31E-02	7.31E+00		
8063716	NM_030773	TUBB1	tubulin, beta 1	-0.001	0.999	9.98E-01	9.98E+01		
8063723	NM_178457	ZNF831	zinc finger protein 831	0.053	1.037	7.56E-01	7.56E+01		
8063729	NM_207032	EDN3	endothelin 3	-0.004	0.997	9.81E-01	9.81E+01		
8063739	NM_080672	PHACTR3	phosphatase and actin regulator 3	-0.039	0.973	6.42E-01	6.42E+01		
8063755	BC054002	C20orf177	chromosome 20 open reading fra	0.025	1.017	9.22E-01	9.22E+01		
8063761	NM_177980	CDH26	cadherin-like 26	0.044	1.031	7.16E-01	7.16E+01		
8063785	AY358539	C20orf197	chromosome 20 open reading fra	0.006	1.004	9.81E-01	9.81E+01		
8063796	NM_001794	CDH4	cadherin 4, type 1, R-cadherin (ret	-0.084	0.944	2.95E-01	2.95E+01		
8063814	NM_144703	LSM14B	LSM14B, SCD6 homolog B (S. cere	0.020	1.014	8.79E-01	8.79E+01		
8063835	NM_144703	LSM14B	LSM14B, SCD6 homolog B (S. cere	0.007	1.005	9.85E-01	9.85E+01		
8063839	NM_198935	SS18L1	synovial sarcoma translocation ge	-0.048	0.968	5.47E-01	5.47E+01		
8063857	NM_015666	GTPBP5	GTP binding protein 5 (putative)	0.022	1.015	8.88E-01	8.88E+01		
8063869	AK126744	FLJ44790	hypothetical FLJ44790	-0.038	0.974	7.92E-01	7.92E+01		
8063873	NM_144498	OSBPL2	oxysterol binding protein-like 2	0.010	1.007	9.65E-01	9.65E+01		
8063893	NM_007002	ADRM1	adhesion regulating molecule 1	-0.033	0.977	8.59E-01	8.59E+01		
8063903	NM_001024	RPS21	ribosomal protein S21	0.041	1.029	7.63E-01	7.63E+01		
8063914	NM_178463	C20orf166	chromosome 20 open reading fra	-0.027	0.981	8.70E-01	8.70E+01		
8063923	NM_016354	SLCO4A1	solute carrier organic anion transp	-0.153	0.899	2.73E-01	2.73E+01		
8063949	NM_018270	C20orf20	chromosome 20 open reading fra	0.053	1.037	7.60E-01	7.60E+01		
8063955	NM_007346	OGFR	opioid growth factor receptor	-0.009	0.994	9.65E-01	9.65E+01		
8063970	NM_001853	COL9A3	collagen, type IX, alpha 3	-0.083	0.944	1.69E-01	1.69E+01		
8064003	BC046933	TCFL5	transcription factor-like 5 (basic h	-0.042	0.971	8.66E-01	8.66E+01		
8064007	NM_017896	C20orf11	chromosome 20 open reading fra	0.038	1.027	8.47E-01	8.47E+01		
8064014	NM_022082	SLC17A9	solute carrier family 17, member 9	-0.054	0.963	5.40E-01	5.40E+01		
8064031	NM_139317	BIRC7	baculoviral IAP repeat-containing	0.020	1.014	8.48E-01	8.48E+01		
8064042	NM_175609	ARFGAP1	ADP-ribosylation factor GTPase ac	-0.022	0.985	8.70E-01	8.70E+01		
8064059	NM_020882	COL20A1	collagen, type XX, alpha 1	-0.028	0.981	7.96E-01	7.96E+01		
8064100	NM_024299	PPDPF	pancreatic progenitor cell differen	0.028	1.020	8.58E-01	8.58E+01		
8064108	BC000912	C20orf195	chromosome 20 open reading fra	-0.051	0.965	5.15E-01	5.15E+01		
8064111	NM_032957	RTEL1	regulator of telomere elongation h	-0.015	0.990	8.75E-01	8.75E+01		
8064156	NM_032527	ZGPAT	zinc finger, CCCH-type with G patc	-0.036	0.975	7.16E-01	7.16E+01		
8064168	NM_017806	LIME1	Lck interacting transmembrane ad	-0.020	0.986	8.92E-01	8.92E+01		
8064175	NM_020062	SLC2A4RG	SLC2A4 regulator	-0.029	0.980	8.09E-01	8.09E+01		
8064188	---	---	---	-0.061	0.959	5.89E-01	5.89E+01		
8064191	NM_199360	TPD52L2	tumor protein D52-like 2	0.061	1.043	8.15E-01	8.15E+01		
8064203	NM_080622	C20orf135	chromosome 20 open reading fra	-0.025	0.983	8.77E-01	8.77E+01		
8064208	NM_025219	DNAJC5	DnaJ (Hsp40) homolog, subfamily	0.068	1.048	6.32E-01	6.32E+01		
8064218	NM_012469	PRPF6	PRP6 pre-mRNA processing factor	-0.021	0.986	9.01E-01	9.01E+01		
8064242	NR_027686	NCRNA00176	non-protein coding RNA 176	-0.067	0.955	5.91E-01	5.91E+01		
8064245	NM_198723	TCEA2	transcription elongation factor A (	0.039	1.027	8.03E-01	8.03E+01		
8064261	NM_182647	OPRL1	opiate receptor-like 1	-0.026	0.982	8.53E-01	8.53E+01		
8064277	NM_004535	MYT1	myelin transcription factor 1	0.000	1.000	1.00E+00	1.00E+02		
8064302	NM_018257	PCMTD2	protein-L-isoaspartate (D-aspartat	0.020	1.014	9.57E-01	9.57E+01		

8064322	BC134417	C20orf96	chromosome 20 open reading frar	-0.074	0.950	7.34E-01	7.34E+01		
8064336	NM_144628	TBC1D20	TBC1 domain family, member 20	-0.041	0.972	7.40E-01	7.40E+01		
8064351	NM_177559	CSNK2A1	casein kinase 2, alpha 1 polypeptic	-0.001	1.000	9.97E-01	9.97E+01		
8064370	NM_004609	TCF15	transcription factor 15 (basic helix	-0.028	0.981	8.09E-01	8.09E+01		
8064382	AK125343	SRXN1	sulfiredoxin 1 homolog (S. cerevisi	-0.003	0.998	9.89E-01	9.89E+01		
8064384	NM_033129	SCRT2	scratch homolog 2, zinc finger pro	0.020	1.014	9.02E-01	9.02E+01		
8064388	NM_033409	C20orf54	chromosome 20 open reading frar	0.039	1.027	6.91E-01	6.91E+01		
8064394	ENST00000217	RPS10P5	ribosomal protein S10 pseudogene	-0.058	0.960	5.47E-01	5.47E+01		
8064396	NM_015985	ANGPT4	angiopoietin 4	0.012	1.008	9.54E-01	9.54E+01		
8064407	NM_00102987	RSPO4	R-spondin family, member 4	-0.020	0.986	8.85E-01	8.85E+01		
8064415	ENST00000381	C20orf46	chromosome 20 open reading frar	0.042	1.030	8.73E-01	8.73E+01		
8064418	NM_080489	SDCBP2	syndecan binding protein (synteni	-0.066	0.955	5.01E-01	5.01E+01		
8064430	---	---	---	0.011	1.008	9.57E-01	9.57E+01		
8064432	NM_000801	FKBP1A	FK506 binding protein 1A, 12kDa	0.038	1.027	8.71E-01	8.71E+01		
8064438	NM_016143	NSFL1C	NSFL1 (p97) cofactor (p47)	0.026	1.018	9.08E-01	9.08E+01		
8064451	NM_00112296	SIRPB2	signal-regulatory protein beta 2	-0.020	0.987	9.57E-01	9.57E+01		
8064485	NM_018556	SIRPG	signal-regulatory protein gamma	0.130	1.094	1.53E-01	1.53E+01		
8064502	NM_003091	SNRPB	small nuclear ribonucleoprotein p	-0.030	0.980	8.92E-01	8.92E+01		
8064522	NM_174856	IDH3B	isocitrate dehydrogenase 3 (NAD+	-0.068	0.954	6.06E-01	6.06E+01		
8064557	AK293638	FAM113A	family with sequence similarity 11	0.038	1.027	7.32E-01	7.32E+01		
8064575	NM_000490	AVP	arginine vasopressin	-0.018	0.988	9.11E-01	9.11E+01		
8064581	NM_021826	FASTKD5	FAST kinase domains 5	-0.032	0.978	8.48E-01	8.48E+01		
8064591	NM_014731	ProSAPiP1	ProSAPiP1 protein	-0.013	0.991	9.33E-01	9.33E+01		
8064601	NM_023935	DDRGK1	DDRGK domain containing 1	-0.140	0.907	1.87E-01	1.87E+01		
8064613	NM_032034	SLC4A11	solute carrier family 4, sodium bor	-0.045	0.969	6.02E-01	6.02E+01		
8064637	NM_00100998	C20orf194	chromosome 20 open reading frar	0.047	1.033	8.27E-01	8.27E+01		
8064676	NM_145762	GFRA4	GDNF family receptor alpha 4	-0.030	0.980	7.92E-01	7.92E+01		
8064686	NM_025220	ADAM33	ADAM metallopeptidase domain 3	-0.027	0.981	8.32E-01	8.32E+01		
8064716	NM_023068	SIGLEC1	sialic acid binding Ig-like lectin 1, s	0.107	1.077	2.29E-01	2.29E+01		
8064739	NM_00103914	C20orf27	chromosome 20 open reading frar	-0.015	0.990	9.16E-01	9.16E+01		
8064751	NM_015417	SPEF1	sperm flagellar 1	-0.067	0.954	5.41E-01	5.41E+01		
8064762	NM_001810	CENPB	centromere protein B, 80kDa	-0.063	0.958	4.84E-01	4.84E+01		
8064766	NM_00113433	RNF24	ring finger protein 24	-0.044	0.970	7.57E-01	7.57E+01		
8064779	NM_000678	ADRA1D	adrenergic, alpha-1D-, receptor	-0.032	0.978	7.45E-01	7.45E+01		
8064790	NM_014737	RASSF2	Ras association (RalGDS/AF-6) dor	-0.020	0.986	9.69E-01	9.69E+01		
8064831	---	---	---	-0.080	0.946	6.37E-01	6.37E+01		
8064833	NM_00100992	C20orf30	chromosome 20 open reading frar	-0.039	0.974	7.84E-01	7.84E+01		
8064842	---	---	---	0.004	1.003	9.85E-01	9.85E+01		
8064844	NM_002592	PCNA	proliferating cell nuclear antigen	0.036	1.026	9.08E-01	9.08E+01		
8064857	---	---	---	-0.053	0.964	8.06E-01	8.06E+01		
8064866	NR_015406	RP5-1022P6.6	hypothetical LOC149837	-0.086	0.942	7.52E-01	7.52E+01		
8064868	NM_019593	RP5-1022P6.2	hypothetical protein KIAA1434	0.221	1.166	5.64E-02	5.64E+00		
8064879	NM_015939	TRMT6	tRNA methyltransferase 6 homolo	0.023	1.016	9.28E-01	9.28E+01		
8064894	NM_152611	LRRN4	leucine rich repeat neuronal 4	-0.064	0.956	4.96E-01	4.96E+01		
8064939	NM_021156	TMX4	thioredoxin-related transmembrar	0.137	1.100	4.14E-01	4.14E+01		
8064976	NM_015190	DNAJC9	DnaJ (Hsp40) homolog, subfamily	-0.078	0.948	5.63E-01	5.63E+01		
8064978	NM_000214	JAG1	jagged 1 (Alagille syndrome)	-0.014	0.990	9.44E-01	9.44E+01		
8065013	---	---	---	-0.094	0.937	5.09E-01	5.09E+01		
8065016	---	---	---	0.012	1.008	9.49E-01	9.49E+01		
8065018	NM_017714	TASP1	taspase, threonine aspartase, 1	-0.008	0.994	9.70E-01	9.70E+01		
8065032	NM_016649	ESF1	ESF1, nucleolar pre-rRNA processi	0.047	1.033	7.41E-01	7.41E+01		
8065084	AK125594	LOC613266	hypothetical LOC613266	0.043	1.030	7.99E-01	7.99E+01		
8065089	NM_024704	KIF16B	kinesin family member 16B	0.031	1.022	9.18E-01	9.18E+01		
8065122	---	---	---	-0.080	0.946	5.56E-01	5.56E+01		
8065136	NM_00104257	RRBP1	ribosome binding protein 1 homol	0.069	1.049	6.75E-01	6.75E+01		
8065165	NM_152227	SNX5	sorting nexin 5	0.175	1.129	4.65E-03	4.65E-01		
8065185	NM_021220	OVOL2	ovo-like 2 (Drosophila)	-0.063	0.957	3.67E-01	3.67E+01		
8065194	---	---	---	-0.020	0.986	9.61E-01	9.61E+01		
8065196	---	---	---	-0.083	0.944	5.93E-01	5.93E+01		
8065202	NM_00109940	C20orf12	chromosome 20 open reading frar	-0.115	0.923	3.31E-01	3.31E+01		
8065242	AF161557	HSPC072	hypothetical LOC29075	-0.170	0.889	5.38E-01	5.38E+01		
8065252	---	---	---	-0.050	0.966	7.47E-01	7.47E+01		
8065254	---	---	---	0.052	1.037	8.52E-01	8.52E+01		
8065256	NM_016652	CRNKL1	crooked neck pre-mRNA splicing fa	-0.011	0.992	9.50E-01	9.50E+01		
8065280	NM_020343	C20orf74	chromosome 20 open reading frar	0.016	1.011	9.44E-01	9.44E+01		
8065325	---	---	---	0.047	1.033	8.21E-01	8.21E+01		
8065327	NM_033176	NKX2-4	NK2 homeobox 4	-0.010	0.993	9.53E-01	9.53E+01		
8065330	---	---	---	-0.089	0.940	4.04E-01	4.04E+01		
8065344	NM_021784	FOXA2	forkhead box A2	-0.028	0.981	8.44E-01	8.44E+01		
8065363	AF258576	LOC200261	hypothetical LOC200261	-0.105	0.929	2.02E-01	2.02E+01		
8065370	---	---	---	0.041	1.029	8.47E-01	8.47E+01		
8065372	NM_022080	NAPB	N-ethylmaleimide-sensitive factor	-0.003	0.998	9.87E-01	9.87E+01		
8065403	NM_000099	CST3	cystatin C	0.187	1.138	9.87E-02	9.87E+00		
8065410	NM_001899	CST4	cystatin S	-0.011	0.992	9.62E-01	9.62E+01		
8065416	NM_001322	CST2	cystatin SA	-0.064	0.957	5.67E-01	5.67E+01		
8065421	NM_001900	CST5	cystatin D	-0.049	0.967	6.08E-01	6.08E+01		
8065427	NM_178311	GGTLC1	gamma-glutamyltransferase light c	-0.001	0.999	9.95E-01	9.95E+01		

8065433	NM_020531	C20orf3	chromosome 20 open reading fran	0.033	1.023	9.12E-01	9.12E+01		
8065444	NM_032501	ACSS1	acyl-CoA synthetase short-chain fa	0.019	1.014	9.01E-01	9.01E+01		
8065460	NM_014588	VSX1	visual system homeobox 1	0.039	1.027	6.94E-01	6.94E+01		
8065469	NM_00104247	ABHD12	abhydrolase domain containing 12	0.213	1.159	1.42E-01	1.42E+01		
8065485	NM_025176	NINL	ninein-like	0.028	1.020	7.73E-01	7.73E+01		
8065510	NM_152667	NANP	N-acetylneuraminic acid phosphat	0.124	1.090	3.88E-01	3.88E+01		
8065517	NM_015655	ZNF337	zinc finger protein 337	-0.097	0.935	3.04E-01	3.04E+01		
8065527	NR_026713	FAM182A	family with sequence similarity 18	0.050	1.035	5.63E-01	5.63E+01		
8065541	NR_003678	C20orf191	nuclear receptor co-repressor 1 ps	0.025	1.017	9.47E-01	9.47E+01		
8065550	---	---	---	-0.053	0.964	7.60E-01	7.60E+01		
8065566	NM_00103750	DEFB124	defensin, beta 124	-0.056	0.962	6.89E-01	6.89E+01		
8065569	NM_138578	BCL2L1	BCL2-like 1	0.073	1.052	7.51E-01	7.51E+01		
8065576	NM_004118	FOXS1	forkhead box S1	-0.061	0.959	5.08E-01	5.08E+01		
8065580	NM_080611	DUSP15	dual specificity phosphatase 15	-0.028	0.981	7.92E-01	7.92E+01		
8065596	NM_030815	PDRG1	p53 and DNA-damage regulated 1	0.021	1.014	9.19E-01	9.19E+01		
8065603	NR_002781	TSPYL3	TSPY-like 3 (pseudogene)	-0.051	0.965	6.91E-01	6.91E+01		
8065607	NM_002657	PLAGL2	pleiomorphic adenoma gene-like 2	-0.258	0.836	2.06E-02	2.06E+00		
8065612	NM_080616	C20orf112	chromosome 20 open reading fran	-0.134	0.912	6.75E-02	6.75E+00		
8065633	---	---	---	0.042	1.029	8.89E-01	8.89E+01		
8065637	NM_053041	COMMD7	COMM domain containing 7	-0.063	0.957	6.10E-01	6.10E+01		
8065668	NM_016408	CDK5RAP1	CDK5 regulatory subunit associate	0.016	1.011	9.53E-01	9.53E+01		
8065683	NM_003098	SNTA1	syntrophin, alpha 1 (dystrophin-as	-0.011	0.993	9.53E-01	9.53E+01		
8065693	NM_031232	NECAB3	N-terminal EF-hand calcium bindir	-0.073	0.950	2.66E-01	2.66E+01		
8065710	NM_005225	E2F1	E2F transcription factor 1	-0.081	0.945	2.45E-01	2.45E+01		
8065730	NM_003908	EIF2S2	eukaryotic translation initiation fa	-0.040	0.973	8.15E-01	8.15E+01		
8065738	NM_000687	AHCY	adenosylhomocysteinase	0.080	1.057	4.03E-01	4.03E+01		
8065752	---	---	---	0.155	1.114	6.27E-01	6.27E+01		
8065756	---	---	---	0.088	1.063	6.60E-01	6.60E+01		
8065762	NM_080476	PIGU	phosphatidylinositol glycan ancho	-0.096	0.936	5.33E-01	5.33E+01		
8065776	NM_014071	NCOA6	nuclear receptor coactivator 6	-0.044	0.970	7.09E-01	7.09E+01		
8065795	NR_002165	HMGB3L1	high-mobility group box 3-like 1	0.032	1.022	8.89E-01	8.89E+01		
8065798	NM_178026	GGT7	gamma-glutamyltransferase 7	-0.043	0.971	7.53E-01	7.53E+01		
8065817	NM_000178	GSS	glutathione synthetase	0.020	1.014	9.19E-01	9.19E+01		
8065832	NM_015638	TRPC4AP	transient receptor potential catior	0.037	1.026	7.67E-01	7.67E+01		
8065855	NR_026728	EDEM2	ER degradation enhancer, mannos	0.201	1.150	6.30E-02	6.30E+00		
8065868	NM_002212	EIF6	eukaryotic translation initiation fa	-0.084	0.944	4.25E-01	4.25E+01		
8065880	NM_178468	FAM83C	family with sequence similarity 83	-0.011	0.992	9.51E-01	9.51E+01		
8065889	NM_018244	UQCC	ubiquinol-cytochrome c reductase	-0.033	0.978	8.84E-01	8.84E+01		
8065903	---	---	---	-0.023	0.984	9.67E-01	9.67E+01		
8065920	NR_024377	FER1L4	fer-1-like 4 (C. elegans)	-0.021	0.986	8.73E-01	8.73E+01		
8065948	NR_024377	FER1L4	fer-1-like 4 (C. elegans)	0.015	1.011	9.40E-01	9.40E+01		
8065963	NM_152930	CPNE1	copine I	-0.024	0.983	8.27E-01	8.27E+01		
8065990	---	---	---	0.007	1.005	9.91E-01	9.91E+01		
8065992	NM_021100	NFS1	NFS1 nitrogen fixation 1 homolog	0.006	1.004	9.74E-01	9.74E+01		
8066009	NM_184234	RBM39	RNA binding motif protein 39	-0.015	0.990	8.88E-01	8.88E+01		
8066031	NM_016558	SCAND1	SCAN domain containing 1	-0.019	0.987	8.89E-01	8.89E+01		
8066038	NM_032214	SLA2	Src-like-adaptor 2	-0.090	0.939	7.38E-01	7.38E+01		
8066051	NM_032013	NDRG3	NDRG family member 3	-0.069	0.953	7.53E-01	7.53E+01		
8066091	BC113405	C20orf117	chromosome 20 open reading fran	0.078	1.056	4.02E-01	4.02E+01		
8066136	NM_002895	RBL1	retinoblastoma-like 1 (p107)	0.033	1.023	8.68E-01	8.68E+01		
8066189	NM_021081	GHRH	growth hormone releasing hormo	0.014	1.010	9.38E-01	9.38E+01		
8066195	NM_006698	BLCAP	bladder cancer associated protein	0.048	1.034	6.04E-01	6.04E+01		
8066198	---	---	---	-0.022	0.985	9.00E-01	9.00E+01		
8066200	BC013755	KIAA0406	KIAA0406	0.035	1.025	8.21E-01	8.21E+01		
8066210	---	---	---	0.432	1.349	6.21E-01	6.21E+01		
8066212	---	---	---	0.002	1.001	9.96E-01	9.96E+01		
8066214	NM_004613	TGM2	transglutaminase 2 (C polypeptide	-0.013	0.991	9.70E-01	9.70E+01		
8066231	NM_00102986	KIAA1755	KIAA1755	0.027	1.019	8.46E-01	8.46E+01		
8066247	NR_027241	LOC388796	hypothetical LOC388796	-0.018	0.988	9.51E-01	9.51E+01		
8066254	BC012894	LOC388796	hypothetical LOC388796	0.154	1.113	4.94E-01	4.94E+01		
8066256	NR_002910	SNORA71B	small nucleolar RNA, H/ACA box 7	-0.040	0.973	8.70E-01	8.70E+01		
8066260	NR_003017	SNORA71C	small nucleolar RNA, H/ACA box 7	0.017	1.012	9.23E-01	9.23E+01		
8066262	NR_003018	SNORA71D	small nucleolar RNA, H/ACA box 7	0.084	1.060	7.83E-01	7.83E+01		
8066275	---	---	---	0.266	1.203	2.78E-01	2.78E+01		
8066279	NM_015035	ZHX3	zinc fingers and homeoboxes 3	-0.051	0.965	6.16E-01	6.16E+01		
8066294	NM_052846	EMILIN3	elastin microfibril interfacer 3	-0.030	0.980	8.00E-01	8.00E+01		
8066303	NM_032221	CHD6	chromodomain helicase DNA bind	-0.159	0.895	7.74E-02	7.74E+00		
8066384	NM_176791	GTSF1L	gametocyte specific factor 1-like	-0.050	0.966	7.92E-01	7.92E+01		
8066391	---	---	---	0.008	1.006	9.80E-01	9.80E+01		
8066393	NM_020433	JPH2	junctophilin 2	-0.026	0.982	8.56E-01	8.56E+01		
8066402	NM_016470	C20orf111	chromosome 20 open reading fran	-0.072	0.952	7.25E-01	7.25E+01		
8066407	NM_00108047	FITM2	fat storage-inducing transmembra	0.057	1.040	7.78E-01	7.78E+01		
8066417	NM_006811	SERINC3	serine incorporator 3	-0.065	0.956	5.46E-01	5.46E+01		
8066431	NM_000022	ADA	adenosine deaminase	-0.072	0.952	7.52E-01	7.52E+01		
8066444	---	---	---	-0.057	0.961	8.36E-01	8.36E+01		
8066451	NM_182970	RIMS4	regulating synaptic membrane exo	-0.023	0.984	8.67E-01	8.67E+01		
8066459	---	---	---	-0.011	0.992	9.77E-01	9.77E+01		

8066461	NM_006809	TOMM34	translocase of outer mitochondria	-0.048	0.967	8.56E-01	8.56E+01		
8066482	NM_145652	WFDC5	WAP four-disulfide core domain 5	0.008	1.006	9.66E-01	9.66E+01		
8066489	NM_080869	WFDC12	WAP four-disulfide core domain 1	0.014	1.010	9.51E-01	9.51E+01		
8066498	NM_003833	MATN4	matrilin 4	-0.086	0.942	3.01E-01	3.01E+01		
8066513	NM_002999	SDC4	syndecan 4	-0.040	0.973	8.92E-01	8.92E+01		
8066521	NM_014477	TP53TG5	TP53 target 5	0.049	1.034	7.18E-01	7.18E+01		
8066528	ENST00000425	PIGT	phosphatidylinositol glycan ancho	-0.134	0.911	4.49E-01	4.49E+01		
8066536	NM_080827	WFDC6	WAP four-disulfide core domain 6	0.053	1.038	5.94E-01	5.94E+01		
8066557	---	---	---	0.041	1.029	8.48E-01	8.48E+01		
8066567	---	---	---	-0.026	0.982	8.16E-01	8.16E+01		
8066574	NM_172006	WFDC10B	WAP four-disulfide core domain 1	-0.026	0.982	8.64E-01	8.64E+01		
8066579	NM_080614	WFDC3	WAP four-disulfide core domain 3	-0.086	0.942	6.00E-01	6.00E+01		
8066590	NM_003279	TNNC2	troponin C type 2 (fast)	-0.073	0.950	4.69E-01	4.69E+01		
8066598	NM_005469	ACOT8	acyl-CoA thioesterase 8	-0.077	0.948	4.35E-01	4.35E+01		
8066609	NM_080608	C20orf165	chromosome 20 open reading fran	-0.023	0.984	8.66E-01	8.66E+01		
8066612	NM_080749	NEURL2	neuralized homolog 2 (Drosophila)	0.003	1.002	9.89E-01	9.89E+01		
8066619	NM_006227	PLTP	phospholipid transfer protein	0.127	1.092	1.55E-01	1.55E+01		
8066637	AK097925	FLJ40606	hypothetical protein LOC643549	-0.066	0.956	5.73E-01	5.73E+01		
8066641	NM_022095	ZNF335	zinc finger protein 335	-0.051	0.965	5.14E-01	5.14E+01		
8066668	NM_020967	NCOA5	nuclear receptor coactivator 5	0.013	1.009	9.42E-01	9.42E+01		
8066683	NM_021248	CDH22	cadherin-like 22	0.044	1.031	7.15E-01	7.15E+01		
8066697	NM_173179	SLC35C2	solute carrier family 35, member C	0.006	1.004	9.76E-01	9.76E+01		
8066716	NM_133171	ELMO2	engulfment and cell motility 2	0.123	1.089	4.09E-01	4.09E+01		
8066754	NM_080721	C20orf123	chromosome 20 open reading fran	-0.009	0.994	9.61E-01	9.61E+01		
8066757	NM_022829	SLC13A3	solute carrier family 13 (sodium-de	0.002	1.002	9.87E-01	9.87E+01		
8066776	NM_033550	TP53RK	TP53 regulating kinase	0.022	1.015	9.16E-01	9.16E+01		
8066786	NM_183047	ZMYND8	zinc finger, MYND-type containing	0.113	1.082	2.10E-01	2.10E+01		
8066820	---	---	---	0.075	1.053	6.10E-01	6.10E+01		
8066822	NM_018837	SULF2	sulfatase 2	0.022	1.015	9.47E-01	9.47E+01		
8066848	NM_020820	PREX1	phosphatidylinositol-3,4,5-trisphos	-0.046	0.969	7.77E-01	7.77E+01		
8066889	NM_017453	STAU1	staufen, RNA binding protein, hom	-0.003	0.998	9.86E-01	9.86E+01		
8066905	NM_021035	ZNFX1	zinc finger, NFX1-type containing 1	0.033	1.023	8.63E-01	8.63E+01		
8066939	NM_004776	B4GALT5	UDP-Gal:betaGlcNAc beta 1,4- gala	0.273	1.209	2.84E-01	2.84E+01		
8066953	NM_006038	SPATA2	spermatogenesis associated 2	-0.018	0.987	9.33E-01	9.33E+01		
8066964	NM_199129	TMEM189	transmembrane protein 189	-0.107	0.929	1.62E-01	1.62E+01		
8066983	---	---	---	0.054	1.038	8.53E-01	8.53E+01		
8066985	AK299337	FAM65C	family with sequence similarity 65	-0.006	0.996	9.74E-01	9.74E+01		
8067007	NM_021109	TMSB4X	thymosin beta 4, X-linked	-0.029	0.980	7.59E-01	7.59E+01		
8067011	NM_015339	ADNP	activity-dependent neuroprotecto	-0.002	0.999	9.92E-01	9.92E+01		
8067017	NM_003859	DPM1	dolichyl-phosphate mannosyltrans	-0.057	0.961	6.73E-01	6.73E+01		
8067029	NM_002237	KCNG1	potassium voltage-gated channel,	0.003	1.002	9.86E-01	9.86E+01		
8067033	NM_002237	KCNG1	potassium voltage-gated channel,	0.017	1.012	9.00E-01	9.00E+01		
8067040	NM_012340	NFATC2	nuclear factor of activated T-cells,	-0.180	0.883	9.19E-02	9.19E+00		
8067113	NM_006526	ZNF217	zinc finger protein 217	0.007	1.005	9.73E-01	9.73E+01		
8067121	NR_002189	SUMO1P1	SUMO1 pseudogene 1	0.005	1.003	9.86E-01	9.86E+01		
8067125	NM_003657	BCAS1	breast carcinoma amplified sequer	-0.074	0.950	6.64E-01	6.64E+01		
8067167	NM_198433	AURKA	aurora kinase A	0.086	1.062	7.77E-01	7.77E+01		
8067178	NM_080615	GCNT7	glucosaminyl (N-acetyl) transferas	-0.047	0.968	7.86E-01	7.86E+01		
8067183	---	---	---	0.068	1.048	4.47E-01	4.47E+01		
8067199	---	---	---	0.218	1.164	6.66E-01	6.66E+01		
8067201	---	---	---	0.150	1.109	5.29E-01	5.29E+01		
8067203	ENST00000371	RBM38	RNA binding motif protein 38	-0.076	0.949	5.96E-01	5.96E+01		
8067206	NM_00100873	HMGB1L1	high-mobility group box 1-like 1	0.033	1.023	8.66E-01	8.66E+01		
8067208	NM_080618	CTCFL	CCCTC-binding factor (zinc finger p	-0.038	0.974	7.57E-01	7.57E+01		
8067221	NM_030776	ZBP1	Z-DNA binding protein 1	0.040	1.028	8.77E-01	8.77E+01		
8067233	NM_020182	PMEPA1	prostate transmembrane protein,	-0.281	0.823	1.45E-04	1.45E-02		
8067242	ENST00000457	ANKRD60	ankyrin repeat domain 60	-0.067	0.954	3.95E-01	3.95E+01		
8067248	NR_003505	PPP4R1L	protein phosphatase 4, regulatory	0.002	1.001	9.96E-01	9.96E+01		
8067270	NM_153360	APCDD1L	adenomatosis polyposis coli down	-0.043	0.971	5.83E-01	5.83E+01		
8067277	---	---	---	-0.091	0.939	2.96E-01	2.96E+01		
8067288	NM_00100197	ATP5E	ATP synthase, H+ transporting, mi	0.141	1.103	1.05E-01	1.05E+01		
8067295	NM_016045	SLMO2	slowmo homolog 2 (Drosophila)	0.033	1.023	7.42E-01	7.42E+01		
8067303	---	---	---	-0.004	0.997	9.87E-01	9.87E+01		
8067351	NM_006242	PPP1R3D	protein phosphatase 1, regulatory	-0.002	0.998	9.88E-01	9.88E+01		
8067358	AK097866	RP11-429E11.3	hypothetical LOC100128310	-0.044	0.970	8.47E-01	8.47E+01		
8067361	NM_003185	TAF4	TAF4 RNA polymerase II, TATA box	-0.034	0.977	7.61E-01	7.61E+01		
8067380	NM_144703	LSM14B	LSM14B, SCD6 homolog B (S. cere	-0.114	0.924	6.80E-01	6.80E+01		
8067382	NM_002792	PSMA7	proteasome (prosome, macropain	-0.038	0.974	8.05E-01	8.05E+01		
8067394	NM_007232	HRH3	histamine receptor H3	0.015	1.011	9.07E-01	9.07E+01		
8067409	NM_005560	LAMA5	laminin, alpha 5	-0.038	0.974	5.48E-01	5.48E+01		
8067495	NM_031215	CABLES2	Cdk5 and Abl enzyme substrate 2	0.075	1.053	6.05E-01	6.05E+01		
8067506	BC137326	C20orf151	chromosome 20 open reading fran	0.033	1.023	7.83E-01	7.83E+01		
8067521	NM_080473	GATA5	GATA binding protein 5	0.024	1.017	7.58E-01	7.58E+01		
8067530	BC093856	C20orf200	chromosome 20 open reading fran	0.003	1.002	9.86E-01	9.86E+01		
8067543	ENST00000370	RP11-93B14.6	hypothetical protein FLJ32154	0.035	1.024	7.97E-01	7.97E+01		
8067546	NR_024470	hCG_2018279	hypothetical protein LOC1001278	-0.056	0.962	6.11E-01	6.11E+01		
8067551	ENST00000415	C20orf90	chromosome 20 open reading fran	-0.003	0.998	9.86E-01	9.86E+01		

8067554	NM_006602	TCFL5	transcription factor-like 5 (basic he	-0.025	0.983	8.63E-01	8.63E+01		
8067563	NM_033081	DIDO1	death inducer-obliterator 1	-0.035	0.976	6.92E-01	6.92E+01		
8067585	NM_080606	BHLHE23	basic helix-loop-helix family, mem	-0.033	0.977	8.08E-01	8.08E+01		
8067593	NM_017798	YTHDF1	YTH domain family, member 1	0.012	1.008	9.45E-01	9.45E+01		
8067602	NM_152864	NKAIN4	Na+/K+ transporting ATPase intera	-0.092	0.938	4.25E-01	4.25E+01		
8067652	NM_001958	EEF1A2	eukaryotic translation elongation f	-0.062	0.958	3.64E-01	3.64E+01		
8067662	NM_005975	PTK6	PTK6 protein tyrosine kinase 6	-0.010	0.993	9.50E-01	9.50E+01		
8067671	NM_080823	SRMS	src-related kinase lacking C-termin	-0.079	0.947	3.83E-01	3.83E+01		
8067680	NM_00103733	PRIC285	peroxisomal proliferator-activated	-0.021	0.986	8.67E-01	8.67E+01		
8067709	NM_012384	GMEB2	glucocorticoid modulatory elemen	-0.068	0.954	4.85E-01	4.85E+01		
8067722	NM_015894	STMN3	stathmin-like 3	-0.045	0.969	7.82E-01	7.82E+01		
8067727	NM_003224	ARFRP1	ADP-ribosylation factor related pr	-0.059	0.960	5.55E-01	5.55E+01		
8067743	NM_025224	ZBTB46	zinc finger and BTB domain contai	0.005	1.003	9.82E-01	9.82E+01		
8067754	U63828	C20orf181	chromosome 20 open reading fran	-0.049	0.967	6.89E-01	6.89E+01		
8067756	NM_017859	UCKL1	uridine-cytidine kinase 1-like 1	-0.007	0.995	9.73E-01	9.73E+01		
8067773	NM_020713	ZNF512B	zinc finger protein 512B	0.027	1.019	8.65E-01	8.65E+01		
8067792	NM_080621	SAMD10	sterile alpha motif domain contair	-0.050	0.966	6.92E-01	6.92E+01		
8067798	NM_018419	SOX18	SRY (sex determining region Y)-bo	-0.001	1.000	9.98E-01	9.98E+01		
8067802	NM_00103946	RGS19	regulator of G-protein signaling 19	-0.007	0.995	9.63E-01	9.63E+01		
8067812	BC036837	C20orf201	chromosome 20 open reading fran	-0.038	0.974	7.78E-01	7.78E+01		
8067820	BC024764	IQSEC3	IQ motif and Sec7 domain 3	-0.039	0.973	6.25E-01	6.25E+01		
8067864	NR_003366	ANKRD20B	ankyrin repeat domain 20B	-0.173	0.887	3.38E-01	3.38E+01		
8067867	---	---	---	-0.109	0.927	2.08E-01	2.08E+01		
8067903	NM_013396	USP25	ubiquitin specific peptidase 25	0.053	1.038	5.67E-01	5.67E+01		
8067955	NM_001338	CXADR	coxsackie virus and adenovirus rec	-0.020	0.986	8.85E-01	8.85E+01		
8067978	NM_021130	PPIA	peptidylprolyl isomerase A (cyclop	-0.035	0.976	6.80E-01	6.80E+01		
8068022	NR_001458	MIR155HG	MIR155 host gene (non-protein co	-0.336	0.792	3.82E-01	3.82E+01		
8068039	NM_002040	GABPA	GA binding protein transcription fa	0.015	1.011	9.24E-01	9.24E+01		
8068062	NM_006447	USP16	ubiquitin specific peptidase 16	-0.015	0.990	9.22E-01	9.22E+01		
8068083	NM_020152	C21orf7	chromosome 21 open reading fran	0.221	1.165	8.01E-02	8.01E+00		
8068105	NR_027655	BACH1	BTB and CNC homology 1, basic le	-0.155	0.898	5.29E-01	5.29E+01		
8068136	NM_181600	KRTAP13-4	keratin associated protein 13-4	0.021	1.015	8.94E-01	8.94E+01		
8068145	NM_181605	KRTAP6-3	keratin associated protein 6-3	-0.047	0.968	6.89E-01	6.89E+01		
8068157	NR_023342	KRTAP20-4	keratin associated protein 20-4	-0.043	0.971	8.06E-01	8.06E+01		
8068168	NM_000454	SOD1	superoxide dismutase 1, soluble	-0.073	0.951	4.27E-01	4.27E+01		
8068192	NM_178817	MRAP	melanocortin 2 receptor accessory	-0.045	0.970	7.51E-01	7.51E+01		
8068200	NR_026845	C21orf119	chromosome 21 open reading fran	-0.051	0.965	6.51E-01	6.51E+01		
8068202	NM_058187	C21orf63	chromosome 21 open reading fran	-0.095	0.936	5.34E-01	5.34E+01		
8068214	---	---	---	-0.194	0.874	6.53E-01	6.53E+01		
8068218	---	---	---	-0.115	0.924	6.92E-01	6.92E+01		
8068235	NM_138983	OLIG1	oligodendrocyte transcription fact	-0.038	0.974	6.18E-01	6.18E+01		
8068238	NM_207585	IFNAR2	interferon (alpha, beta and omega	0.251	1.190	2.82E-02	2.82E+00		
8068254	NM_000628	IL10RB	interleukin 10 receptor, beta	0.164	1.120	4.39E-01	4.39E+01		
8068266	NM_000629	IFNAR1	interferon (alpha, beta and omega	0.107	1.077	6.19E-01	6.19E+01		
8068280	NM_005534	IFNGR2	interferon gamma receptor 2 (inte	0.305	1.235	1.02E-01	1.02E+01		
8068289	NM_138927	SON	SON DNA binding protein	0.002	1.002	9.86E-01	9.86E+01		
8068305	NM_003024	ITSN1	intersectin 1 (SH3 domain protein)	0.058	1.041	7.98E-01	7.98E+01		
8068350	AK127913	FLJ46020	FLJ46020 protein	0.051	1.036	8.69E-01	8.69E+01		
8068363	NR_027267	C21orf82	chromosome 21 open reading fran	-0.176	0.885	3.09E-01	3.09E+01		
8068375	NM_058182	FAM165B	family with sequence similarity 16	0.033	1.023	8.57E-01	8.57E+01		
8068397	---	---	---	0.041	1.029	9.09E-01	9.09E+01		
8068401	NM_001757	CBR1	carbonyl reductase 1	0.101	1.072	5.38E-01	5.38E+01		
8068410	NM_015955	MEMO1	mediator of cell motility 1	0.116	1.084	1.57E-01	1.57E+01		
8068413	NM_001236	CBR3	carbonyl reductase 3	-0.096	0.936	6.34E-01	6.34E+01		
8068422	NM_005128	DOPEY2	dopey family member 2	0.129	1.094	3.28E-01	3.28E+01		
8068460	NM_015358	MORC3	MORC family CW-type zinc finger 3	0.013	1.009	9.39E-01	9.39E+01		
8068478	NM_005441	CHAF1B	chromatin assembly factor 1, subu	0.021	1.015	9.42E-01	9.42E+01		
8068494	---	---	---	-0.074	0.950	6.41E-01	6.41E+01		
8068496	NM_009586	SIM2	single-minded homolog 2 (Drosop	-0.046	0.968	5.83E-01	5.83E+01		
8068510	---	---	---	-0.024	0.983	8.99E-01	8.99E+01		
8068512	NM_018962	DSCR6	Down syndrome critical region ger	-0.059	0.960	5.90E-01	5.90E+01		
8068522	NM_003316	TTC3	tetratricopeptide repeat domain 3	0.019	1.013	9.42E-01	9.42E+01		
8068549	---	---	---	0.104	1.075	4.13E-01	4.13E+01		
8068551	NM_101395	DYRK1A	dual-specificity tyrosine-(Y)-phosp	-0.028	0.981	8.13E-01	8.13E+01		
8068583	NM_002243	KCNJ15	potassium inwardly-rectifying char	0.352	1.276	4.25E-01	4.25E+01		
8068610	---	---	---	0.139	1.101	6.91E-01	6.91E+01		
8068612	NM_004627	WRB	tryptophan rich basic protein	-0.124	0.918	5.04E-01	5.04E+01		
8068651	NM_006198	PCP4	Purkinje cell protein 4	0.021	1.015	8.67E-01	8.67E+01		
8068671	NM_012105	BACE2	beta-site APP-cleaving enzyme 2	0.033	1.023	7.73E-01	7.73E+01		
8068697	NM_002463	MX2	myxovirus (influenza virus) resista	0.245	1.185	5.05E-01	5.05E+01		
8068713	NM_002462	MX1	myxovirus (influenza virus) resista	0.373	1.295	4.42E-01	4.42E+01		
8068761	NM_207627	ABCG1	ATP-binding cassette, sub-family G	-0.007	0.995	9.77E-01	9.77E+01		
8068788	NM_018961	UBASH3A	ubiquitin associated and SH3 dom	0.049	1.035	8.47E-01	8.47E+01		
8068810	NM_018964	SLC37A1	solute carrier family 37 (glycerol-3	0.017	1.012	9.37E-01	9.37E+01		
8068833	NM_002606	PDE9A	phosphodiesterase 9A	0.040	1.028	8.76E-01	8.76E+01		
8068857	NM_021075	NDUFV3	NADH dehydrogenase (ubiquinone	0.075	1.053	5.24E-01	5.24E+01		
8068866	NM_004571	PKNOX1	PBX/knotted 1 homeobox 1	-0.043	0.970	7.85E-01	7.85E+01		

8068883	NM_000394	CRYAA	crystallin, alpha A	0.003	1.002	9.83E-01	9.83E+01		
8068891	AK123727	FLJ41733	FLJ41733 protein	0.019	1.013	9.28E-01	9.28E+01		
8068898	NM_080593	HIST1H2BK	histone cluster 1, H2bk	0.065	1.046	6.20E-01	6.20E+01		
8068902	NM_015056	RRP1B	ribosomal RNA processing 1 homo	0.045	1.031	7.19E-01	7.19E+01		
8068919	NM_003681	PDXK	pyridoxal (pyridoxine, vitamin B6)	0.108	1.078	5.66E-01	5.66E+01		
8068938	NM_003683	RRP1	ribosomal RNA processing 1 homo	-0.070	0.953	4.19E-01	4.19E+01		
8068952	NM_020132	AGPAT3	1-acylglycerol-3-phosphate O-acyl	0.044	1.031	7.97E-01	7.97E+01		
8068974	NM_003274	TRAPPC10	trafficking protein particle comple	-0.077	0.948	5.04E-01	5.04E+01		
8069003	NM_005049	PWP2	PWP2 periodic tryptophan protein	-0.122	0.919	9.26E-02	9.26E+00		
8069026	NM_004649	C21orf33	chromosome 21 open reading fran	0.009	1.006	9.50E-01	9.50E+01		
8069037	NM_000383	AIRE	autoimmune regulator	-0.035	0.976	7.27E-01	7.27E+01		
8069057	NR_024108	PFKL	phosphofructokinase, liver	0.031	1.022	8.18E-01	8.18E+01		
8069083	AB209578	C21orf2	chromosome 21 open reading fran	-0.003	0.998	9.83E-01	9.83E+01		
8069085	NM_003307	TRPM2	transient receptor potential cation	-0.048	0.967	7.56E-01	7.56E+01		
8069122	NM_030891	LRRC3	leucine rich repeat containing 3	-0.047	0.968	6.83E-01	6.83E+01		
8069131	ENST00000443	C21orf30	chromosome 21 open reading fran	0.049	1.035	7.87E-01	7.87E+01		
8069135	NR_026547	C21orf90	chromosome 21 open reading fran	-0.073	0.950	4.73E-01	4.73E+01		
8069142	NM_198687	KRTAP10-4	keratin associated protein 10-4	-0.080	0.946	5.15E-01	5.15E+01		
8069146	NM_198689	KRTAP10-7	keratin associated protein 10-7	-0.090	0.940	4.48E-01	4.48E+01		
8069148	NM_198695	KRTAP10-8	keratin associated protein 10-8	-0.036	0.975	6.60E-01	6.60E+01		
8069150	NM_198690	KRTAP10-9	keratin associated protein 10-9	-0.057	0.961	8.08E-01	8.08E+01		
8069153	NM_181688	KRTAP10-10	keratin associated protein 10-10	-0.006	0.996	9.67E-01	9.67E+01		
8069156	NM_198692	KRTAP10-11	keratin associated protein 10-11	-0.032	0.978	8.25E-01	8.25E+01		
8069161	NM_198697	KRTAP12-3	keratin associated protein 12-3	-0.079	0.947	4.59E-01	4.59E+01		
8069168	NM_198699	KRTAP10-12	keratin associated protein 10-12	0.004	1.003	9.82E-01	9.82E+01		
8069174	AF391113	C21orf70	chromosome 21 open reading fran	0.081	1.057	6.70E-01	6.70E+01		
8069178	NR_027673	ADARB1	adenosine deaminase, RNA-specif	-0.152	0.900	1.61E-01	1.61E+01		
8069200	AF426268	C21orf89	chromosome 21 open reading fran	0.036	1.025	9.00E-01	9.00E+01		
8069208	NM_030582	COL18A1	collagen, type XVIII, alpha 1	-0.045	0.969	5.57E-01	5.57E+01		
8069252	NM_020528	PCBP3	poly(rC) binding protein 3	-0.067	0.955	5.89E-01	5.89E+01		
8069269	NM_001848	COL6A1	collagen, type VI, alpha 1	-0.046	0.969	6.93E-01	6.93E+01		
8069301	NM_001849	COL6A2	collagen, type VI, alpha 2	-0.006	0.996	9.68E-01	9.68E+01		
8069332	NR_002776	MCM3APAS	MCM3AP antisense RNA (non-pro	0.009	1.006	9.57E-01	9.57E+01		
8069340	NM_058181	C21orf57	chromosome 21 open reading fran	-0.036	0.976	8.75E-01	8.75E+01		
8069348	NM_006031	PCNT	pericentrin	-0.086	0.942	2.93E-01	2.93E+01		
8069399	NM_015151	DIP2A	DIP2 disco-interacting protein 2 ho	-0.054	0.963	6.80E-01	6.80E+01		
8069446	---	---	---	0.045	1.032	9.01E-01	9.01E+01		
8069448	---	---	---	0.017	1.012	9.83E-01	9.83E+01		
8069450	NM_206962	PRMT2	protein arginine methyltransferase	0.031	1.022	8.98E-01	8.98E+01		
8069470	NR_024528	RPL23AP7	ribosomal protein L23a pseudoger	0.012	1.008	9.49E-01	9.49E+01		
8069503	AK125677	LOC441956	similar to cDNA sequence BC0215	-0.065	0.956	7.52E-01	7.52E+01		
8069508	ENST00000333	CCDC29	coiled-coil domain containing 29	0.067	1.047	9.24E-01	9.24E+01		
8069511	NR_027270	C21orf81	ankyrin repeat domain 20 family, r	-0.046	0.969	8.70E-01	8.70E+01		
8069517	---	---	---	0.033	1.023	9.48E-01	9.48E+01		
8069519	---	---	---	0.046	1.032	7.51E-01	7.51E+01		
8069532	NM_006948	HSPA13	heat shock protein 70kDa family, r	-0.037	0.975	8.72E-01	8.72E+01		
8069541	NM_022136	SAMSN1	SAM domain, SH3 domain and nuc	-0.281	0.823	4.56E-02	4.56E+00		
8069553	NM_003489	NRIP1	nuclear receptor interacting prote	0.126	1.092	3.76E-01	3.76E+01		
8069561	NM_152265	BTF3L4	basic transcription factor 3-like 4	0.158	1.116	2.99E-01	2.99E+01		
8069565	NM_00113091	BTG3	BTG family, member 3	-0.173	0.887	1.06E-01	1.06E+01		
8069574	NM_00110042	C21orf91	chromosome 21 open reading fran	0.109	1.078	4.96E-01	4.96E+01		
8069620	NM_080794	MRPL39	mitochondrial ribosomal protein L	0.030	1.021	8.96E-01	8.96E+01		
8069633	NM_00100370	ATP5J	ATP synthase, H+ transporting, mi	-0.008	0.994	9.63E-01	9.63E+01		
8069644	NM_000484	APP	amyloid beta (A4) precursor prote	0.147	1.107	5.77E-01	5.77E+01		
8069666	---	---	---	0.055	1.039	5.45E-01	5.45E+01		
8069700	NM_013240	N6AMT1	N-6 adenine-specific DNA methylt	0.024	1.017	9.27E-01	9.27E+01		
8069711	NM_015565	RNF160	ring finger protein 160	0.086	1.061	4.47E-01	4.47E+01		
8069744	NM_016940	RWDD2B	RWD domain containing 2B	0.163	1.119	4.48E-01	4.48E+01		
8069753	NM_006585	CCT8	chaperonin containing TCP1, subu	-0.030	0.979	7.73E-01	7.73E+01		
8069764	---	---	---	0.110	1.080	6.80E-01	6.80E+01		
8069766	---	---	---	0.015	1.011	9.74E-01	9.74E+01		
8069768	---	---	---	-0.151	0.900	6.60E-01	6.60E+01		
8069811	NM_181624	KRTAP23-1	keratin associated protein 23-1	0.034	1.024	8.18E-01	8.18E+01		
8069838	NM_181611	KRTAP19-5	keratin associated protein 19-5	-0.058	0.961	7.15E-01	7.15E+01		
8069852	NM_181602	KRTAP6-1	keratin associated protein 6-1	0.023	1.016	8.93E-01	8.93E+01		
8069863	NM_175857	KRTAP8-1	keratin associated protein 8-1	-0.012	0.992	9.49E-01	9.49E+01		
8069872	NM_175858	KRTAP11-1	keratin associated protein 11-1	-0.072	0.952	3.80E-01	3.80E+01		
8069880	NM_003253	TIAM1	T-cell lymphoma invasion and met	0.129	1.093	4.48E-01	4.48E+01		
8069910	NM_020706	SFRS15	splicing factor, arginine/serine-ric	-0.003	0.998	9.84E-01	9.84E+01		
8069933	NM_018944	C21orf45	chromosome 21 open reading fran	-0.150	0.902	1.76E-01	1.76E+01		
8069943	NM_014825	URB1	URB1 ribosome biogenesis 1 hom	0.015	1.010	9.14E-01	9.14E+01		
8069985	NR_002996	SNORA80	small nucleolar RNA, H/ACA box 8	-0.025	0.982	8.88E-01	8.88E+01		
8069987	NM_144659	TCP10L	t-complex 10 (mouse)-like	-0.065	0.956	5.19E-01	5.19E+01		
8069998	AF282851	C21orf59	chromosome 21 open reading fran	-0.050	0.966	6.83E-01	6.83E+01		
8070010	NM_003895	SYNJ1	synaptojanin 1	0.305	1.236	7.65E-03	7.65E-01		
8070046	NR_027873	C21orf66	chromosome 21 open reading fran	-0.008	0.995	9.73E-01	9.73E+01		
8070081	---	---	---	0.030	1.021	8.32E-01	8.32E+01		

8070083	NM_006134	TMEM50B	transmembrane protein 50B	-0.001	1.000	9.99E-01	9.99E+01		
8070102	NM_000819	GART	phosphoribosylglycinamide formy	0.106	1.076	2.79E-01	2.79E+01		
8070129	NM_017613	DONSON	downstream neighbor of SON	0.139	1.101	1.21E-01	1.21E+01		
8070141	NM_145858	CRYZL1	crystallin, zeta (quinone reductase	-0.013	0.991	9.70E-01	9.70E+01		
8070160	NM_001697	ATP5O	ATP synthase, H+ transporting, mi	0.058	1.041	6.91E-01	6.91E+01		
8070169	---	---	---	0.088	1.063	5.23E-01	5.23E+01		
8070171	---	---	---	0.131	1.095	3.39E-01	3.39E+01		
8070173	NM_000219	KCNE1	potassium voltage-gated channel,	0.094	1.068	5.47E-01	5.47E+01		
8070182	NM_004414	RCAN1	regulator of calcineurin 1	-0.027	0.981	8.81E-01	8.81E+01		
8070194	NM_00100189	RUNX1	runt-related transcription factor 1	-0.101	0.932	3.37E-01	3.37E+01		
8070215	NM_017438	SETD4	SET domain containing 4	-0.036	0.976	8.84E-01	8.84E+01		
8070233	NM_144492	CLDN14	claudin 14	0.057	1.040	5.98E-01	5.98E+01		
8070239	NM_000411	HLCS	holocarboxylase synthetase (bioti	0.070	1.050	7.10E-01	7.10E+01		
8070257	NM_153681	PIGP	phosphatidylinositol glycan ancho	0.059	1.042	6.95E-01	6.95E+01		
8070269	NM_006052	DSCR3	Down syndrome critical region ge	0.074	1.053	7.87E-01	7.87E+01		
8070286	NM_005867	DSCR4	Down syndrome critical region ge	-0.003	0.998	9.85E-01	9.85E+01		
8070330	NM_003720	PSMG1	proteasome (prosome, macropain	-0.004	0.997	9.87E-01	9.87E+01		
8070341	NM_033656	BRWD1	bromodomain and WD repeat dor	-0.055	0.963	6.93E-01	6.93E+01		
8070386	AF426265	C21orf87	chromosome 21 open reading frar	0.086	1.061	7.77E-01	7.77E+01		
8070389	NM_004965	HMGN1	high-mobility group nucleosome b	0.021	1.015	8.78E-01	8.78E+01		
8070482	NR_027272	C21orf129	chromosome 21 open reading frar	-0.063	0.958	4.86E-01	4.86E+01		
8070489	NM_020639	RIPK4	receptor-interacting serine-threon	-0.097	0.935	2.56E-01	2.56E+01		
8070503	NM_00104042	PRDM15	PR domain containing 15	-0.024	0.983	8.32E-01	8.32E+01		
8070538	NM_015500	C2CD2	C2 calcium-dependent domain cor	0.072	1.051	6.24E-01	6.24E+01		
8070557	NM_00109840	ZNF295	zinc finger protein 295	-0.072	0.951	6.89E-01	6.89E+01		
8070563	NR_027243	C21orf128	chromosome 21 open reading frar	-0.006	0.996	9.78E-01	9.78E+01		
8070567	NM_003226	TFF3	trefoil factor 3 (intestinal)	0.058	1.041	5.55E-01	5.55E+01		
8070579	NM_003225	TFF1	trefoil factor 1	-0.008	0.995	9.65E-01	9.65E+01		
8070615	NM_018669	WDR4	WD repeat domain 4	-0.040	0.973	7.54E-01	7.54E+01		
8070629	BC005107	C21orf105	chromosome 21 open reading frar	-0.007	0.995	9.82E-01	9.82E+01		
8070632	NM_000071	CBS	cystathionine-beta-synthase	0.035	1.024	7.62E-01	7.62E+01		
8070655	NM_00102520	U2AF1	U2 small nuclear RNA auxiliary fac	0.017	1.012	9.09E-01	9.09E+01		
8070665	NM_173354	SIK1	salt-inducible kinase 1	-0.011	0.993	9.53E-01	9.53E+01		
8070681	AF426261	C21orf84	chromosome 21 open reading frar	-0.001	0.999	9.95E-01	9.95E+01		
8070689	NM_007031	HSF2BP	heat shock transcription factor 2 b	-0.042	0.971	7.98E-01	7.98E+01		
8070701	NM_000100	CSTB	cystatin B (stefin B)	-0.015	0.990	9.40E-01	9.40E+01		
8070708	NR_026961	LOC284837	hypothetical LOC284837	-0.068	0.954	5.91E-01	5.91E+01		
8070712	---	---	---	0.105	1.075	6.31E-01	6.31E+01		
8070716	ENST00000430	C21orf32	chromosome 21 open reading frar	-0.085	0.943	4.70E-01	4.70E+01		
8070720	NM_015259	ICOSLG	inducible T-cell co-stimulator ligar	-0.054	0.963	6.91E-01	6.91E+01		
8070744	NM_004928	C21orf2	chromosome 21 open reading frar	-0.043	0.970	6.91E-01	6.91E+01		
8070757	NM_144991	C21orf29	chromosome 21 open reading frar	-0.025	0.983	8.73E-01	8.73E+01		
8070771	NM_198691	KRTAP10-1	keratin associated protein 10-1	-0.038	0.974	7.45E-01	7.45E+01		
8070777	NM_198693	KRTAP10-2	keratin associated protein 10-2	0.067	1.048	5.52E-01	5.52E+01		
8070780	NM_198696	KRTAP10-3	keratin associated protein 10-3	-0.118	0.922	2.21E-01	2.21E+01		
8070782	NM_198694	KRTAP10-5	keratin associated protein 10-5	-0.071	0.952	7.49E-01	7.49E+01		
8070786	NM_198688	KRTAP10-6	keratin associated protein 10-6	-0.021	0.986	8.41E-01	8.41E+01		
8070789	NM_198698	KRTAP12-4	keratin associated protein 12-4	0.015	1.011	8.99E-01	8.99E+01		
8070791	NM_181684	KRTAP12-2	keratin associated protein 12-2	0.031	1.022	7.89E-01	7.89E+01		
8070795	NM_181686 //	AP12-1 // KRTAI	keratin associated protein 12-1 //	0.090	1.064	4.81E-01	4.81E+01		
8070799	NM_182688	UBE2G2	ubiquitin-conjugating enzyme E2G	0.036	1.025	8.21E-01	8.21E+01		
8070811	NM_006936	SUMO3	SMT3 suppressor of mif two 3 hor	0.082	1.059	4.04E-01	4.04E+01		
8070819	NM_004339	PTTG1IP	pituitary tumor-transforming 1 int	0.176	1.130	2.61E-01	2.61E+01		
8070847	NR_027128	C21orf67	chromosome 21 open reading frar	0.016	1.011	9.16E-01	9.16E+01		
8070853	NR_027129	C21orf67	chromosome 21 open reading frar	0.014	1.010	9.16E-01	9.16E+01		
8070863	BC004343	C21orf122	chromosome 21 open reading frar	-0.015	0.990	9.36E-01	9.36E+01		
8070867	ENST00000416	C21orf122	chromosome 21 open reading frar	-0.034	0.977	8.06E-01	8.06E+01		
8070876	NM_015227	POFUT2	protein O-fucosyltransferase 2	-0.011	0.993	9.63E-01	9.63E+01		
8070891	AF426264	C21orf86	chromosome 21 open reading frar	-0.001	0.999	9.97E-01	9.97E+01		
8070896	AF427488	C21orf93	chromosome 21 open reading frar	-0.046	0.968	6.38E-01	6.38E+01		
8070900	ENST00000418	C21orf111	chromosome 21 open reading frar	-0.003	0.998	9.89E-01	9.89E+01		
8070912	NM_194255	SLC19A1	solute carrier family 19 (folate trar	0.036	1.025	7.38E-01	7.38E+01		
8070925	---	---	---	-0.081	0.946	4.06E-01	4.06E+01		
8070930	---	---	---	-0.065	0.956	7.08E-01	7.08E+01		
8070933	NM_006657	FTCD	formiminotransferase cyclodeami	-0.024	0.983	8.41E-01	8.41E+01		
8070953	NM_00114285	C21orf56	chromosome 21 open reading frar	-0.063	0.958	4.67E-01	4.67E+01		
8070961	NM_002340	LSS	lanosterol synthase (2,3-oxidosqu	-0.052	0.964	7.14E-01	7.14E+01		
8070988	NM_003906	MCM3AP	minichromosome maintenance co	0.021	1.015	8.78E-01	8.78E+01		
8071020	NM_058180	C21orf58	chromosome 21 open reading frar	-0.029	0.980	8.18E-01	8.18E+01		
8071036	NM_006272	S100B	S100 calcium binding protein B	0.471	1.386	3.85E-01	3.85E+01		
8071044	0	0	0	-0.069	0.953	7.85E-01	7.85E+01		
8071049	0	0	0	0.131	1.095	7.97E-01	7.97E+01		
8071051	AK126241 //	44253 // FLJ44	hypothetical gene supported by A	-0.029	0.980	9.26E-01	9.26E+01		
8071061	AK097082	psiTPTE22	TPTE pseudogene	-0.010	0.993	9.75E-01	9.75E+01		
8071069	NM_014339	IL17RA	interleukin 17 receptor A	0.106	1.076	4.82E-01	4.82E+01		
8071084	NR_024483	CECR4	cat eye syndrome chromosome re	0.064	1.045	6.00E-01	6.00E+01		
8071086	NM_031413	CECR2	cat eye syndrome chromosome re	0.050	1.035	6.91E-01	6.91E+01		

8071107	NM_031481	SLC25A18	solute carrier family 25 (mitochon	0.004	1.003	9.86E-01	9.86E+01		
8071119	NM_015367	BCL2L13	BCL2-like 13 (apoptosis facilitator)	-0.003	0.998	9.88E-01	9.88E+01		
8071134	ENST00000444	C22orf37	chromosome 22 open reading fran	-0.059	0.960	7.22E-01	7.22E+01		
8071136	NM_00112764	PEX26	peroxisomal biogenesis factor 26	0.022	1.016	8.63E-01	8.63E+01		
8071147	NM_018943	TUBA8	tubulin, alpha 8	-0.090	0.940	4.73E-01	4.73E+01		
8071155	NM_017414	USP18	ubiquitin specific peptidase 18	0.074	1.052	7.85E-01	7.85E+01		
8071168	NR_024593	POM121L10P	POM121 membrane glycoprotein-	-0.019	0.987	8.70E-01	8.70E+01		
8071170	NR_024593	POM121L10P	POM121 membrane glycoprotein-	-0.002	0.999	9.90E-01	9.90E+01		
8071185	NR_002733	DGCR5	DiGeorge syndrome critical region	-0.045	0.970	6.37E-01	6.37E+01		
8071194	---	---	---	-0.074	0.950	7.83E-01	7.83E+01		
8071206	NM_003776	MRPL40	mitochondrial ribosomal protein L	0.020	1.014	9.35E-01	9.35E+01		
8071234	NM_002688	40426	septin 5	-0.004	0.997	9.66E-01	9.66E+01		
8071259	NM_002688	40426	septin 5	-0.061	0.958	4.25E-01	4.25E+01		
8071268	NM_000407	GP1BB	glycoprotein Ib (platelet), beta pol	-0.052	0.964	6.32E-01	6.32E+01		
8071272	L20860	GP1BB	glycoprotein Ib (platelet), beta pol	-0.116	0.923	2.79E-01	2.79E+01		
8071274	L20860	GP1BB	glycoprotein Ib (platelet), beta pol	-0.066	0.956	8.03E-01	8.03E+01		
8071276	NM_080647	TBX1	T-box 1	-0.047	0.968	6.04E-01	6.04E+01		
8071289	NM_000754	COMT	catechol-O-methyltransferase	-0.023	0.984	8.31E-01	8.31E+01		
8071301	NM_152906	C22orf25	chromosome 22 open reading fran	0.001	1.001	9.92E-01	9.92E+01		
8071312	---	---	---	-0.047	0.968	6.91E-01	6.91E+01		
8071314	NM_022720	DGCR8	DiGeorge syndrome critical region	-0.018	0.988	9.12E-01	9.12E+01		
8071332	NM_002882	RANBP1	RAN binding protein 1	0.000	1.000	1.00E+00	1.00E+02		
8071339	NM_013373	ZDHHC8	zinc finger, DHHC-type containing	-0.034	0.977	7.52E-01	7.52E+01		
8071355	NR_026919	LOC150197	hypothetical LOC150197	0.023	1.016	8.83E-01	8.83E+01		
8071357	AB051440	LOC440792	proline dehydrogenase (oxidase) 1	-0.051	0.965	7.38E-01	7.38E+01		
8071368	NR_026815	TMEM191A	transmembrane protein 191A	0.007	1.005	9.81E-01	9.81E+01		
8071382	NM_003426	ZNF74	zinc finger protein 74	-0.015	0.990	9.20E-01	9.20E+01		
8071390	---	---	---	0.183	1.135	5.54E-01	5.54E+01		
8071392	NM_00100389	MED15	mediator complex subunit 15	-0.091	0.939	3.36E-01	3.36E+01		
8071411	NR_024592	POM121L4P	POM121 membrane glycoprotein-	0.040	1.028	8.58E-01	8.58E+01		
8071414	NR_026815	TMEM191A	transmembrane protein 191A	-0.019	0.987	9.44E-01	9.44E+01		
8071420	NM_000185	SERPIND1	serpin peptidase inhibitor, clade D	0.035	1.025	9.02E-01	9.02E+01		
8071426	NM_004782	SNAP29	synaptosomal-associated protein,	0.113	1.081	3.35E-01	3.35E+01		
8071434	NM_005207	CRKL	v-crk sarcoma virus CT10 oncogen	-0.108	0.928	1.01E-01	1.01E+01		
8071444	NM_144704	AIFM3	apoptosis-inducing factor, mitocho	0.004	1.003	9.81E-01	9.81E+01		
8071466	NM_006767	LZTR1	leucine-zipper-like transcription re	-0.007	0.995	9.74E-01	9.74E+01		
8071516	NM_004327	BCR	breakpoint cluster region	-0.048	0.967	6.81E-01	6.81E+01		
8071530	NR_024591	POM121L1P	POM121 membrane glycoprotein-	-0.043	0.971	8.41E-01	8.41E+01		
8071532	NM_015672	RIMBP3	RIMS binding protein 3	-0.056	0.962	6.90E-01	6.90E+01		
8071536	NM_015094	HIC2	hypermethylated in cancer 2	-0.097	0.935	1.53E-01	1.53E+01		
8071541	NR_026815	TMEM191A	transmembrane protein 191A	0.055	1.039	7.45E-01	7.45E+01		
8071547	NM_198157	UBE2L3	ubiquitin-conjugating enzyme E2L	0.019	1.014	9.24E-01	9.24E+01		
8071554	NM_152612	CCDC116	coiled-coil domain containing 116	-0.018	0.988	9.11E-01	9.11E+01		
8071559	NM_022044	SDF2L1	stromal cell-derived factor 2-like 1	0.193	1.143	3.34E-03	3.34E-01		
8071564	---	---	---	0.012	1.009	9.60E-01	9.60E+01		
8071566	NM_014337	PPIL2	peptidylprolyl isomerase (cyclophi	0.024	1.017	8.74E-01	8.74E+01		
8071593	---	---	---	0.001	1.001	9.98E-01	9.98E+01		
8071595	---	---	---	0.089	1.063	7.50E-01	7.50E+01		
8071597	BC032452	IGL@	immunoglobulin lambda locus	0.051	1.036	5.98E-01	5.98E+01		
8071642	ENST00000390	IGLV6-57	immunoglobulin lambda variable 6	0.060	1.043	7.46E-01	7.46E+01		
8071649	BC043345	BMS1	BMS1 homolog, ribosome assemb	-0.031	0.979	9.31E-01	9.31E+01		
8071655	---	---	---	0.003	1.002	9.88E-01	9.88E+01		
8071662	NM_199127	GGTLC2	gamma-glutamyltransferase light c	0.032	1.022	9.00E-01	9.00E+01		
8071671	NM_002073	GNAZ	guanine nucleotide binding protei	0.039	1.027	6.74E-01	6.74E+01		
8071676	NM_004914	RAB36	RAB36, member RAS oncogene fa	-0.032	0.978	8.46E-01	8.46E+01		
8071691	NM_004327	BCR	breakpoint cluster region	-0.171	0.888	6.90E-02	6.90E+00		
8071713	AF174606	FBXW4P1	F-box and WD repeat domain cont	-0.043	0.971	7.97E-01	7.97E+01		
8071717	NM_153615	RGL4	ral guanine nucleotide dissociatio	-0.002	0.999	9.90E-01	9.90E+01		
8071737	NM_002415	MIF	macrophage migration inhibitory f	0.023	1.016	9.10E-01	9.10E+01		
8071745	NM_182520	C22orf15	chromosome 22 open reading fran	-0.012	0.992	9.50E-01	9.50E+01		
8071758	NM_005940	MMP11	matrix metallopeptidase 11 (strom	-0.019	0.987	8.77E-01	8.77E+01		
8071768	NM_003073	SMARCB1	SWI/SNF related, matrix associate	0.021	1.015	9.27E-01	9.27E+01		
8071783	NM_030807	SLC2A11	solute carrier family 2 (facilitated	-0.033	0.977	7.64E-01	7.64E+01		
8071804	---	---	---	0.036	1.025	8.84E-01	8.84E+01		
8071806	NM_001355	DDT	D-dopachrome tautomerase	0.025	1.018	9.04E-01	9.04E+01		
8071809	NM_000854	GSTT2	glutathione S-transferase theta 2	-0.069	0.954	4.27E-01	4.27E+01		
8071819	---	---	---	-0.099	0.933	4.46E-01	4.46E+01		
8071823	NM_012295	CABIN1	calcineurin binding protein 1	-0.061	0.959	4.78E-01	4.78E+01		
8071861	NM_019601	SUSD2	sushi domain containing 2	0.022	1.016	8.54E-01	8.54E+01		
8071877	NR_003714	POM121L9P	POM121 membrane glycoprotein-	-0.024	0.984	8.99E-01	8.99E+01		
8071881	NM_015330	CYTSA	cytospin A	-0.094	0.937	3.76E-01	3.76E+01		
8071899	NM_000675	ADORA2A	adenosine A2a receptor	-0.174	0.887	5.36E-02	5.36E+00		
8071920	NM_004175	SNRPD3	small nuclear ribonucleoprotein D	0.002	1.001	9.91E-01	9.91E+01		
8071927	NM_005265	GGT1	gamma-glutamyltransferase 1	-0.067	0.955	1.82E-01	1.82E+01		
8071951	---	---	---	-0.062	0.958	4.93E-01	4.93E+01		
8071981	NM_00114520	KIAA1671	KIAA1671	-0.136	0.910	8.93E-02	8.93E+00		
8071989	NM_004076	CRYBB3	crystallin, beta B3	-0.124	0.918	4.72E-02	4.72E+00		

8071997	NM_000496	CRYBB2	crystallin, beta B2	-0.040	0.972	8.06E-01	8.06E+01		
8072004	NM_00101361	IGLL3	immunoglobulin lambda-like polyp	0.079	1.056	2.04E-01	2.04E+01		
8072009	BC047380	CRYBB2P1	crystallin, beta B2 pseudogene 1	0.054	1.038	7.23E-01	7.23E+01		
8072039	NM_032608	MYO18B	myosin XVIIIB	0.001	1.001	9.94E-01	9.94E+01		
8072108	NM_020437	ASPHD2	aspartate beta-hydroxylase domai	0.186	1.137	1.67E-01	1.67E+01		
8072113	NM_00101369	SRRD	SRR1 domain containing	0.021	1.015	9.37E-01	9.37E+01		
8072122	---	---	---	0.009	1.006	9.61E-01	9.61E+01		
8072124	NM_001886	CRYBA4	crystallin, beta A4	-0.067	0.955	6.34E-01	6.34E+01		
8072133	NR_026963	LOC284900	hypothetical LOC284900	-0.020	0.986	9.18E-01	9.18E+01		
8072143	NM_172002	HSCB	HscB iron-sulfur cluster co-chaper	0.040	1.028	8.21E-01	8.21E+01		
8072153	NM_173510	CCDC117	coiled-coil domain containing 117	0.033	1.023	7.65E-01	7.65E+01		
8072170	NM_00103957	KREMEN1	kringle containing transmembrane	-0.220	0.859	1.59E-01	1.59E+01		
8072189	NM_133455	EMID1	EMI domain containing 1	-0.008	0.994	9.64E-01	9.64E+01		
8072206	NM_005243	EWSR1	Ewing sarcoma breakpoint region	0.010	1.007	9.50E-01	9.50E+01		
8072216	NM_006478	GAS2L1	growth arrest-specific 2 like 1	-0.023	0.984	8.25E-01	8.25E+01		
8072242	NM_016418	NF2	neurofibromin 2 (merlin)	-0.047	0.968	7.51E-01	7.51E+01		
8072266	NM_182527	CABP7	calcium binding protein 7	-0.025	0.983	9.02E-01	9.02E+01		
8072272	---	---	---	0.027	1.019	9.34E-01	9.34E+01		
8072274	NM_00100368	UCRC	ubiquinol-cytochrome c reductase	0.068	1.049	6.35E-01	6.35E+01		
8072279	NM_153050	MTMR3	myotubularin related protein 3	-0.071	0.952	5.89E-01	5.89E+01		
8072302	NM_153050	MTMR3	myotubularin related protein 3	-0.021	0.986	9.39E-01	9.39E+01		
8072316	NM_00101743	CCDC157	coiled-coil domain containing 157	-0.019	0.987	8.95E-01	8.95E+01		
8072328	NM_012429	SEC14L2	SEC14-like 2 (S. cerevisiae)	0.023	1.016	9.30E-01	9.30E+01		
8072344	---	---	---	-0.060	0.959	8.66E-01	8.66E+01		
8072346	NM_016498	MTP18	mitochondrial protein 18 kDa	-0.047	0.968	7.38E-01	7.38E+01		
8072353	AY358802	UNQ1945	VLGN1945	0.067	1.048	5.78E-01	5.78E+01		
8072374	NM_00100147	SLC35E4	solute carrier family 35, member E	-0.037	0.974	7.93E-01	7.93E+01		
8072382	NM_030758	OSBP2	oxysterol binding protein 2	-0.056	0.962	5.94E-01	5.94E+01		
8072407	NR_002323	TUG1	taurine upregulated 1 (non-protei	0.013	1.009	9.33E-01	9.33E+01		
8072411	---	---	---	0.060	1.042	6.90E-01	6.90E+01		
8072413	NM_134269	SMTN	smoothelin	-0.012	0.992	9.44E-01	9.44E+01		
8072436	NM_00100283	INPP5J	inositol polyphosphate-5-phospha	-0.001	0.999	9.96E-01	9.96E+01		
8072454	NR_024210	RNF185	ring finger protein 185	0.069	1.049	7.27E-01	7.27E+01		
8072461	NM_016733	LIMK2	LIM domain kinase 2	-0.300	0.812	6.06E-02	6.06E+00		
8072484	ENST00000425	MGC15705	hypothetical protein MGC15705	0.066	1.047	7.63E-01	7.63E+01		
8072488	NM_004147	DRG1	developmentally regulated GTP bi	0.077	1.055	5.29E-01	5.29E+01		
8072494	NM_00100746	SFI1	Sfi1 homolog, spindle assembly as	-0.034	0.976	7.85E-01	7.85E+01		
8072529	NM_014662	DEPDC5	DEP domain containing 5	0.114	1.082	1.50E-01	1.50E+01		
8072577	NM_003405	YWHAH	tyrosine 3-monooxygenase/trypto	0.045	1.032	8.22E-01	8.22E+01		
8072582	---	---	---	0.111	1.080	7.07E-01	7.07E+01		
8072584	---	---	---	-0.075	0.949	6.16E-01	6.16E+01		
8072610	NM_012179	FBXO7	F-box protein 7	0.081	1.057	3.67E-01	3.67E+01		
8072626	NM_000362	TIMP3	TIMP metallopeptidase inhibitor 3	0.021	1.014	9.02E-01	9.02E+01		
8072636	NM_00100849	ISX	intestine-specific homeobox	-0.032	0.978	7.57E-01	7.57E+01		
8072645	NR_027780	HMGXB4	HMG box domain containing 4	-0.066	0.955	5.59E-01	5.59E+01		
8072659	NR_024194	TOM1	target of myb1 (chicken)	-0.140	0.908	3.63E-01	3.63E+01		
8072678	NM_002133	HMOX1	heme oxygenase (decycling) 1	0.425	1.342	8.96E-02	8.96E+00		
8072687	NM_006739	MCM5	minichromosome maintenance co	-0.051	0.965	8.40E-01	8.40E+01		
8072705	NM_014310	RASD2	RASD family, member 2	-0.081	0.945	3.36E-01	3.36E+01		
8072710	NM_030641	APOL6	apolipoprotein L, 6	0.257	1.195	1.09E-01	1.09E+01		
8072729	---	---	---	0.119	1.086	6.51E-01	6.51E+01		
8072735	NM_145343	APOL1	apolipoprotein L, 1	0.236	1.178	4.14E-01	4.14E+01		
8072757	NM_000395	CSF2RB	colony stimulating factor 2 recept	0.230	1.173	3.85E-01	3.85E+01		
8072777	NR_024038	MPST	mercaptopyruvate sulfurtransfera	-0.018	0.987	8.90E-01	8.90E+01		
8072784	NM_024681	KCTD17	potassium channel tetramerisation	-0.036	0.975	7.41E-01	7.41E+01		
8072796	NM_153609	TMPRSS6	transmembrane protease, serine 6	-0.022	0.985	9.24E-01	9.24E+01		
8072798	NM_013385	CYTH4	cytohesin 4	0.085	1.060	6.85E-01	6.85E+01		
8072817	NM_152243	CDC42EP1	CDC42 effector protein (Rho GTPa	-0.039	0.973	7.27E-01	7.27E+01		
8072825	NM_013365	GGA1	golgi associated, gamma adaptin e	0.142	1.104	6.71E-02	6.71E+00		
8072847	NM_018957	SH3BP1	SH3-domain binding protein 1	-0.022	0.985	8.78E-01	8.78E+01		
8072870	NM_020315	PDXP	pyridoxal (pyridoxine, vitamin B6)	0.018	1.013	9.07E-01	9.07E+01		
8072876	NM_002305	LGALS1	lectin, galactoside-binding, soluble	0.111	1.080	6.48E-01	6.48E+01		
8072883	NM_024313	NOL12	nucleolar protein 12	0.019	1.013	9.13E-01	9.13E+01		
8072894	NM_00103914	TRIOBP	TRIO and F-actin binding protein	-0.027	0.982	8.32E-01	8.32E+01		
8072926	NM_005318	H1F0	H1 histone family, member 0	-0.143	0.906	4.13E-01	4.13E+01		
8072931	NM_014291	GCAT	glycine C-acetyltransferase (2-ami	0.078	1.056	6.23E-01	6.23E+01		
8072941	NM_003614	GALR3	galanin receptor 3	-0.085	0.943	3.62E-01	3.62E+01		
8072946	NM_016091	EIF3L	eukaryotic translation initiation fa	0.010	1.007	9.74E-01	9.74E+01		
8072962	NM_033386	MICALL1	MICAL-like 1	-0.053	0.964	4.78E-01	4.78E+01		
8072979	NM_021974	POLR2F	polymerase (RNA) II (DNA directed	-0.083	0.944	5.39E-01	5.39E+01		
8072989	NM_012407	PICK1	protein interacting with PRKCA 1	-0.014	0.991	9.29E-01	9.29E+01		
8073007	NM_012323	MAFF	v-maf musculoaponeurotic fibrosa	-0.147	0.903	1.69E-01	1.69E+01		
8073013	---	---	---	0.003	1.002	9.92E-01	9.92E+01		
8073015	NM_006855	KDELR3	KDEL (Lys-Asp-Glu-Leu) endoplasm	-0.043	0.971	8.69E-01	8.69E+01		
8073022	NM_00100288	CBY1	chibby homolog 1 (Drosophila)	-0.120	0.920	3.64E-01	3.64E+01		
8073032	NM_020243	TOMM22	translocase of outer mitochondria	-0.051	0.965	8.20E-01	8.20E+01		
8073039	NM_004286	GTPBP1	GTP binding protein 1	-0.081	0.945	4.70E-01	4.70E+01		

8073062	NM_004900	APOBEC3B	apolipoprotein B mRNA editing en	0.214	1.160	1.14E-01	1.14E+01		
8073068	NM_014508	APOBEC3C	apolipoprotein B mRNA editing en	0.036	1.025	9.16E-01	9.16E+01		
8073072	NM_152426	APOBEC3D	apolipoprotein B mRNA editing en	0.052	1.037	7.78E-01	7.78E+01		
8073081	NM_145298	APOBEC3F	apolipoprotein B mRNA editing en	-0.073	0.950	8.00E-01	8.00E+01		
8073088	NM_021822	APOBEC3G	apolipoprotein B mRNA editing en	-0.218	0.860	4.14E-01	4.14E+01		
8073096	NM_181773	APOBEC3H	apolipoprotein B mRNA editing en	-0.197	0.873	5.02E-01	5.02E+01		
8073114	NM_006116	MAP3K7IP1	mitogen-activated protein kinase k	-0.066	0.955	3.75E-01	3.75E+01		
8073129	NM_002409	MGAT3	mannosyl (beta-1,4-)-glycoprotein	-0.007	0.995	9.66E-01	9.66E+01		
8073135	NM_019008	SMCR7L	Smith-Magenis syndrome chromos	-0.059	0.960	5.97E-01	5.97E+01		
8073148	NM_001675	ATF4	activating transcription factor 4 (ta	-0.140	0.907	8.90E-02	8.90E+00		
8073152	NM_021096	CACNA1I	calcium channel, voltage-depende	-0.010	0.993	9.44E-01	9.44E+01		
8073192	NM_006003	UQCRFS1	ubiquinol-cytochrome c reductase	0.093	1.067	5.78E-01	5.78E+01		
8073194	NM_004810	GRAP2	GRB2-related adaptor protein 2	-0.174	0.886	1.83E-01	1.83E+01		
8073207	NM_138435	FAM83F	family with sequence similarity 83	-0.002	0.999	9.95E-01	9.95E+01		
8073214	NM_00102484	TNRC6B	trinucleotide repeat containing 6B	-0.075	0.949	4.06E-01	4.06E+01		
8073242	NM_000026	ADSL	adenylosuccinate lyase	0.045	1.032	7.88E-01	7.88E+01		
8073259	NM_015705	SGSM3	small G protein signaling modulato	-0.007	0.995	9.70E-01	9.70E+01		
8073293	---	---	---	0.067	1.047	5.94E-01	5.94E+01		
8073296	NM_005297	MCHR1	melanin-concentrating hormone r	-0.053	0.964	6.66E-01	6.66E+01		
8073309	---	---	---	0.114	1.082	2.70E-01	2.70E+01		
8073311	NM_022098	XPNPEP3	X-prolyl aminopeptidase (aminope	0.032	1.023	8.52E-01	8.52E+01		
8073334	NM_014248	RBX1	ring-box 1	0.094	1.067	4.46E-01	4.46E+01		
8073343	---	---	---	0.000	1.000	1.00E+00	1.00E+02		
8073345	NM_001429	EP300	E1A binding protein p300	-0.008	0.995	9.55E-01	9.55E+01		
8073379	NM_031488	L3MBTL2	l(3)mbt-like 2 (Drosophila)	-0.025	0.983	9.00E-01	9.00E+01		
8073397	NM_017590	ZC3H7B	zinc finger CCCH-type containing 7	0.012	1.008	9.37E-01	9.37E+01		
8073422	NM_003216	TEF	thyrotrophic embryonic factor	-0.049	0.966	6.40E-01	6.40E+01		
8073430	NM_001098	ACO2	aconitase 2, mitochondrial	-0.004	0.998	9.86E-01	9.86E+01		
8073449	NM_014460	CSDC2	cold shock domain containing C2,	0.021	1.014	8.56E-01	8.56E+01		
8073457	NM_001469	XRCC6	X-ray repair complementing defec	-0.015	0.990	9.23E-01	9.23E+01		
8073470	NM_00114296	CTA-216E10.6	hypothetical FLJ23584	-0.034	0.977	8.11E-01	8.11E+01		
8073511	---	---	---	0.014	1.010	9.53E-01	9.53E+01		
8073513	NM_024821	CCDC134	coiled-coil domain containing 134	-0.100	0.933	2.48E-01	2.48E+01		
8073522	NM_004599	SREBF2	sterol regulatory element binding	-0.016	0.989	9.37E-01	9.37E+01		
8073546	NR_024355	LOC339674	hypothetical LOC339674	-0.021	0.985	9.06E-01	9.06E+01		
8073573	NM_00100203	FAM109B	family with sequence similarity 10	-0.013	0.991	9.49E-01	9.49E+01		
8073578	BC024237	C22orf32	chromosome 22 open reading fran	0.044	1.031	8.79E-01	8.79E+01		
8073582	BC039496	LOC388906	hypothetical protein LOC388906	-0.038	0.974	7.58E-01	7.58E+01		
8073585	NR_027786	SERHL	serine hydrolase-like	-0.018	0.988	9.10E-01	9.10E+01		
8073603	J04119	RNU12P	RNA, U12 small nuclear pseudoger	-0.276	0.826	2.62E-02	2.62E+00		
8073612	NM_000714	TSPO	translocator protein (18kDa)	0.048	1.034	6.52E-01	6.52E+01		
8073623	NM_00104437	MPPED1	metallophosphoesterase domain c	-0.048	0.967	7.25E-01	7.25E+01		
8073645	NM_015380	SAMM50	sorting and assembly machinery co	-0.029	0.980	8.84E-01	8.84E+01		
8073662	NM_00100382	PARVB	parvin, beta	0.054	1.038	6.06E-01	6.06E+01		
8073682	NM_022141	PARVG	parvin, gamma	0.221	1.165	2.30E-02	2.30E+00		
8073698	NM_00101298	RP3-474I12.5	hypothetical LOC388910	0.000	1.000	9.99E-01	9.99E+01		
8073705	NM_181333	PRR5	proline rich 5 (renal)	0.008	1.005	9.53E-01	9.53E+01		
8073733	NM_153645	NUP50	nucleoporin 50kDa	0.027	1.019	9.12E-01	9.12E+01		
8073743	NM_006953	UPK3A	uroplakin 3A	0.013	1.009	9.49E-01	9.49E+01		
8073752	NM_00110459	FAM118A	family with sequence similarity 11	-0.098	0.934	6.71E-01	6.71E+01		
8073775	NM_006486	FBLN1	fibulin 1	0.102	1.073	2.01E-01	2.01E+01		
8073799	NM_013236	ATXN10	ataxin 10	-0.010	0.993	9.65E-01	9.65E+01		
8073816	NR_027033	LOC400931	hypothetical LOC400931	-0.025	0.983	8.75E-01	8.75E+01		
8073824	---	---	---	-0.066	0.955	6.92E-01	6.92E+01		
8073826	NM_005036	PPARA	peroxisome proliferator-activated	-0.036	0.975	7.56E-01	7.56E+01		
8073842	NM_017931	TTC38	tetratricopeptide repeat domain 3	0.105	1.076	1.38E-01	1.38E+01		
8073875	NM_018006	TRMU	tRNA 5-methylaminomethyl-2-thio	-0.034	0.977	8.26E-01	8.26E+01		
8073890	NM_015124	GRAMD4	GRAM domain containing 4	0.081	1.058	5.09E-01	5.09E+01		
8073909	NM_014346	TBC1D22A	TBC1 domain family, member 22A	-0.046	0.969	6.91E-01	6.91E+01		
8073924	uc003bil.1	LOC388915	hypothetical LOC388915	-0.001	0.999	9.94E-01	9.94E+01		
8073929	NM_015381	FAM19A5	family with sequence similarity 19	-0.006	0.996	9.64E-01	9.64E+01		
8073941	---	---	---	-0.019	0.987	9.00E-01	9.00E+01		
8073943	NM_014838	ZBED4	zinc finger, BED-type containing 4	0.012	1.008	9.60E-01	9.60E+01		
8073949	NM_00113510	CRELD2	cysteine-rich with EGF-like domain	0.124	1.089	2.15E-01	2.15E+01		
8073960	NM_00100185	PIM3	pim-3 oncogene	-0.018	0.988	9.01E-01	9.01E+01		
8073992	NM_052839	PANX2	pannexin 2	-0.045	0.970	6.84E-01	6.84E+01		
8074003	NM_025204	TRABD	TraB domain containing	-0.037	0.975	6.92E-01	6.92E+01		
8074020	NM_031454	RP3-402G11.5	selenoprotein O	-0.058	0.960	5.46E-01	5.46E+01		
8074030	NM_014678	SAPS2	SAPS domain family, member 2	0.004	1.003	9.81E-01	9.81E+01		
8074057	---	---	---	-0.076	0.949	6.93E-01	6.93E+01		
8074061	---	---	---	-0.067	0.954	5.15E-01	5.15E+01		
8074063	NM_024866	ADM2	adrenomedullin 2	-0.041	0.972	6.93E-01	6.93E+01		
8074070	NM_017584	MIOX	myo-inositol oxygenase	0.021	1.015	9.03E-01	9.03E+01		
8074082	NM_152299	NCAPH2	non-SMC condensin II complex, su	0.045	1.032	6.99E-01	6.99E+01		
8074106	NM_138433	KLHDC7B	kelch domain containing 7B	0.025	1.017	8.46E-01	8.46E+01		
8074108	NM_012324	MAPK8IP2	mitogen-activated protein kinase 8	-0.090	0.939	1.29E-01	1.29E+01		
8074131	NM_00108042	SHANK3	SH3 and multiple ankyrin repeat d	-0.040	0.972	6.02E-01	6.02E+01		

8074153	NM_001097	ACR	acrosin	0.022	1.015	9.09E-01	9.09E+01		
8074157	NR_026982	RPL23AP82	ribosomal protein L23a pseudoger	-0.004	0.997	9.79E-01	9.79E+01		
8074168	ENST00000400	FLJ39632	hypothetical LOC642477	0.036	1.025	7.94E-01	7.94E+01		
8074192	---	---	---	-0.079	0.947	6.54E-01	6.54E+01		
8074196	NM_014406	CCT8L2	chaperonin containing TCP1, subu	-0.030	0.979	7.95E-01	7.95E+01		
8074212	NM_00103781	GAB4	GRB2-associated binding protein f	-0.026	0.982	8.06E-01	8.06E+01		
8074223	NM_031890	CECR6	cat eye syndrome chromosome re	-0.048	0.967	5.91E-01	5.91E+01		
8074227	NM_033070	CECR5	cat eye syndrome chromosome re	0.010	1.007	9.63E-01	9.63E+01		
8074251	NM_001696	ATP6V1E1	ATPase, H+ transporting, lysosoma	-0.029	0.980	9.09E-01	9.09E+01		
8074261	NM_197966	BID	BH3 interacting domain death ago	0.005	1.004	9.76E-01	9.76E+01		
8074274	NM_015241	MICAL3	microtubule associated monoxyge	-0.028	0.981	8.45E-01	8.45E+01		
8074286	NM_015241	MICAL3	microtubule associated monoxyge	-0.022	0.985	9.42E-01	9.42E+01		
8074307	AK094477	LOC100129113	hypothetical protein LOC10012911	-0.082	0.945	4.36E-01	4.36E+01		
8074316	NR_003267	GGT3P	gamma-glutamyltransferase 3 pse	-0.065	0.956	2.41E-01	2.41E+01		
8074349	NM_005137	DGCR2	DiGeorge syndrome critical region	0.039	1.027	7.51E-01	7.51E+01		
8074371	NM_022719	DGCR14	DiGeorge syndrome critical region	-0.035	0.976	7.57E-01	7.57E+01		
8074384	NM_005315	GSC2	goosecoid homeobox 2	-0.071	0.952	5.51E-01	5.51E+01		
8074388	NM_005984	SLC25A1	solute carrier family 25 (mitochon	-0.037	0.974	7.39E-01	7.39E+01		
8074399	NM_007098	CLTCL1	clathrin, heavy chain-like 1	0.006	1.004	9.67E-01	9.67E+01		
8074432	NM_003325	HIRA	HIR histone cell cycle regulation de	-0.005	0.997	9.81E-01	9.81E+01		
8074458	BC062599	C22orf39	chromosome 22 open reading fran	0.026	1.018	8.73E-01	8.73E+01		
8074464	NM_005659	UFD1L	ubiquitin fusion degradation 1 like	-0.076	0.949	6.66E-01	6.66E+01		
8074473	NM_00113086	CLDN5	claudin 5	-0.018	0.988	8.72E-01	8.72E+01		
8074481	NM_053004	GNB1L	guanine nucleotide binding protei	-0.053	0.964	5.86E-01	5.86E+01		
8074498	NM_006440	TXNRD2	thioredoxin reductase 2	-0.029	0.980	8.06E-01	8.06E+01		
8074522	NM_001670	ARVCF	armadillo repeat gene deletes in v	0.079	1.056	4.42E-01	4.42E+01		
8074541	NM_022727	TRMT2A	TRM2 tRNA methyltransferase 2 h	-0.008	0.994	9.53E-01	9.53E+01		
8074558	NM_023004	RTN4R	reticulon 4 receptor	-0.075	0.949	2.88E-01	2.88E+01		
8074565	NM_033257	DGCR6L	DiGeorge syndrome critical region	-0.043	0.971	8.04E-01	8.04E+01		
8074569	NR_003267 // P	// GGT3P // G	gamma-glutamyltransferase 3 pse	-0.053	0.964	4.10E-01	4.10E+01		
8074577	NR_003700	PI4KAP2	phosphatidylinositol 4-kinase, cata	-0.024	0.984	9.24E-01	9.24E+01		
8074593	NM_015672	RIMBP3	RIMS binding protein 3	-0.012	0.992	9.49E-01	9.49E+01		
8074606	NM_017414	USP18	ubiquitin specific peptidase 18	0.091	1.065	8.53E-01	8.53E+01		
8074615	---	---	---	-0.003	0.998	9.86E-01	9.86E+01		
8074617	NM_153334	SCARF2	scavenger receptor class F, membe	-0.016	0.989	9.16E-01	9.16E+01		
8074632	NM_032775	KLHL22	kelch-like 22 (Drosophila)	0.063	1.044	5.23E-01	5.23E+01		
8074640	---	---	---	0.072	1.052	8.32E-01	8.32E+01		
8074642	---	---	---	0.045	1.031	9.22E-01	9.22E+01		
8074644	---	---	---	-0.054	0.963	7.42E-01	7.42E+01		
8074647	NM_058004	PI4KA	phosphatidylinositol 4-kinase, cata	0.020	1.014	9.10E-01	9.10E+01		
8074688	NM_030573	THAP7	THAP domain containing 7	0.008	1.006	9.72E-01	9.72E+01		
8074695	NR_003608	MGC16703	tubulin, alpha pseudogene	-0.006	0.996	9.81E-01	9.81E+01		
8074701	NM_004173	SLC7A4	solute carrier family 7 (cationic am	-0.016	0.989	9.05E-01	9.05E+01		
8074712	NR_024591	POM121L1P	POM121 membrane glycoprotein-	-0.043	0.971	8.41E-01	8.41E+01		
8074714	NR_024591	POM121L1P	POM121 membrane glycoprotein-	-0.012	0.992	9.53E-01	9.53E+01		
8074716	NR_003267	GGT3P	gamma-glutamyltransferase 3 pse	-0.059	0.960	2.64E-01	2.64E+01		
8074748	NR_003700	PI4KAP2	phosphatidylinositol 4-kinase, cata	-0.007	0.995	9.82E-01	9.82E+01		
8074771	NM_015672	RIMBP3	RIMS binding protein 3	0.007	1.005	9.68E-01	9.68E+01		
8074773	NM_00101796	YDJC	YdjC homolog (bacterial)	-0.071	0.952	4.25E-01	4.25E+01		
8074780	NM_013313	YPEL1	yippee-like 1 (Drosophila)	-0.018	0.988	9.62E-01	9.62E+01		
8074791	NM_002745	MAPK1	mitogen-activated protein kinase 1	-0.012	0.992	9.53E-01	9.53E+01		
8074806	NM_014634	PPM1F	protein phosphatase 1F (PP2C dor	-0.066	0.955	6.16E-01	6.16E+01		
8074817	NM_003935	TOP3B	topoisomerase (DNA) III beta	0.041	1.029	8.33E-01	8.33E+01		
8074842	NM_00113011	FAM108A1	family with sequence similarity 10	0.011	1.007	9.38E-01	9.38E+01		
8074845	NM_080764	ZNF280B	zinc finger protein 280B	-0.100	0.933	4.32E-01	4.32E+01		
8074867	NR_024591	POM121L1P	POM121 membrane glycoprotein-	-0.032	0.978	7.63E-01	7.63E+01		
8074878	---	---	---	0.019	1.013	9.16E-01	9.16E+01		
8074880	NM_004914	RAB36	RAB36, member RAS oncogene fai	-0.010	0.993	9.61E-01	9.61E+01		
8074888	---	---	---	-0.028	0.981	8.46E-01	8.46E+01		
8074890	---	---	---	-0.023	0.984	9.46E-01	9.46E+01		
8074892	NR_003950	ZDHHC8P	zinc finger, DHHC-type containing	-0.020	0.986	9.14E-01	9.14E+01		
8074909	NM_020070	IGLL1	immunoglobulin lambda-like polyp	0.004	1.003	9.79E-01	9.79E+01		
8074925	NR_024448	LOC91316	glucuronidase, beta/ immunoglob	0.017	1.012	9.63E-01	9.63E+01		
8074927	NR_024448	LOC91316	glucuronidase, beta/ immunoglob	-0.006	0.996	9.85E-01	9.85E+01		
8074931	NM_021916	ZNF70	zinc finger protein 70	0.039	1.027	7.94E-01	7.94E+01		
8074934	NM_013378	VPREB3	pre-B lymphocyte 3	0.079	1.056	4.06E-01	4.06E+01		
8074939	NM_213720	CHCHD10	coiled-coil-helix-coiled-coil-helix d	0.016	1.011	9.35E-01	9.35E+01		
8074944	NM_198440	DERL3	Der1-like domain family, member	-0.028	0.981	8.13E-01	8.13E+01		
8074962	NM_000854	GSTT2	glutathione S-transferase theta 2	-0.069	0.954	4.27E-01	4.27E+01		
8074969	NM_001355	DDT	D-dopachrome tautomerase	0.036	1.025	9.24E-01	9.24E+01		
8074980	NM_000853	GSTT1	glutathione S-transferase theta 1	-0.017	0.988	9.44E-01	9.44E+01		
8074991	NM_00109978	GGT5	gamma-glutamyltransferase 5	-0.034	0.977	7.90E-01	7.90E+01		
8075005	AK289871	ADORA2A	adenosine A2a receptor	-0.025	0.983	8.48E-01	8.48E+01		
8075009	NM_031444	C22orf13	chromosome 22 open reading fran	0.003	1.002	9.87E-01	9.87E+01		
8075016	NM_207644	C22orf36	chromosome 22 open reading fran	0.025	1.018	8.06E-01	8.06E+01		
8075024	NR_024593	POM121L10P	POM121 membrane glycoprotein-	-0.007	0.995	9.70E-01	9.70E+01		
8075052	NM_182492	LRP5L	low density lipoprotein receptor-r	0.064	1.045	5.25E-01	5.25E+01		

8075063	NM_022081	HPS4	Hermansky-Pudlak syndrome 4	-0.037	0.975	8.22E-01	8.22E+01		
8075089	NM_00100869	TFIP11	tuftelin interacting protein 11	-0.137	0.910	1.72E-01	1.72E+01		
8075106	NM_003595	TPST2	tyrosylprotein sulfotransferase 2	0.143	1.104	3.50E-02	3.50E+00		
8075118	NM_001887	CRYBB1	crystallin, beta B1	-0.015	0.990	9.09E-01	9.09E+01		
8075126	NM_002430	MN1	meningioma (disrupted in balance	-0.081	0.946	1.69E-01	1.69E+01		
8075130	NM_012399	PITPNB	phosphatidylinositol transfer prot	0.017	1.012	8.97E-01	8.97E+01		
8075160	---	---	---	0.002	1.001	9.94E-01	9.94E+01		
8075182	NM_005080	XBP1	X-box binding protein 1	0.037	1.026	8.07E-01	8.07E+01		
8075200	NM_012265	RHBDD3	rhomboid domain containing 3	0.008	1.005	9.65E-01	9.65E+01		
8075211	NM_00100727	RASL10A	RAS-like, family 10, member A	-0.049	0.966	7.43E-01	7.43E+01		
8075217	NM_001127	AP1B1	adaptor-related protein complex 1	0.143	1.104	1.39E-01	1.39E+01		
8075239	NM_00100287	THOC5	THO complex 5	-0.009	0.994	9.72E-01	9.72E+01		
8075263	NM_003634	NIPSNAP1	nipsnap homolog 1 (C. elegans)	-0.015	0.990	9.53E-01	9.53E+01		
8075276	NM_019103	ZMAT5	zinc finger, matrin type 5	-0.036	0.975	7.83E-01	7.83E+01		
8075285	NM_032204	ASCC2	activating signal cointegrator 1 co	0.055	1.039	7.35E-01	7.35E+01		
8075306	---	---	---	0.115	1.083	6.96E-01	6.96E+01		
8075316	NM_020530	OSM	oncostatin M	-0.057	0.961	5.72E-01	5.72E+01		
8075322	NM_00103766	GATSL3	GATS protein-like 3	-0.023	0.984	8.85E-01	8.85E+01		
8075332	NM_031937	TBC1D10A	TBC1 domain family, member 10A	0.021	1.015	9.11E-01	9.11E+01		
8075343	NM_005877	SF3A1	splicing factor 3a, subunit 1, 120kD	-0.036	0.976	7.91E-01	7.91E+01		
8075365	NM_00101798	RNF215	ring finger protein 215	-0.008	0.995	9.64E-01	9.64E+01		
8075390	NM_174977	SEC14L4	SEC14-like 4 (S. cerevisiae)	0.045	1.032	7.36E-01	7.36E+01		
8075401	NM_004861	GAL3ST1	galactose-3-O-sulfotransferase 1	-0.008	0.994	9.56E-01	9.56E+01		
8075406	NM_014303	PES1	pescadillo homolog 1, containing	-0.003	0.998	9.90E-01	9.90E+01		
8075423	NM_152511	DUSP18	dual specificity phosphatase 18	-0.099	0.934	6.59E-01	6.59E+01		
8075430	NM_014941	MORC2	MORC family CW-type zinc finger	-0.056	0.962	7.01E-01	7.01E+01		
8075458	NM_134270	SMTN	smoothelin	-0.006	0.996	9.77E-01	9.77E+01		
8075462	NM_080430	SELM	selenoprotein M	0.012	1.008	9.53E-01	9.53E+01		
8075468	NM_015715	PLA2G3	phospholipase A2, group III	-0.073	0.951	5.55E-01	5.55E+01		
8075477	NR_024209	RNF185	ring finger protein 185	-0.027	0.981	9.60E-01	9.60E+01		
8075481	---	---	---	-0.318	0.802	2.59E-01	2.59E+01		
8075483	NM_052880	PIK3IP1	phosphoinositide-3-kinase interac	-0.169	0.890	3.27E-01	3.27E+01		
8075493	NM_014323	PATZ1	POZ (BTB) and AT hook containing	-0.062	0.958	5.51E-01	5.51E+01		
8075507	NM_019843	EIF4ENIF1	eukaryotic translation initiation fa	-0.050	0.966	7.36E-01	7.36E+01		
8075529	NM_014338	PISD	phosphatidylserine decarboxylase	-0.103	0.931	3.60E-01	3.60E+01		
8075542	NM_173566	C22orf30	chromosome 22 open reading fra	0.017	1.012	9.01E-01	9.01E+01		
8075550	AK294232	C22orf24	chromosome 22 open reading fra	-0.082	0.945	5.06E-01	5.06E+01		
8075555	NM_00101085	C22orf42	chromosome 22 open reading fra	0.089	1.063	2.99E-01	2.99E+01		
8075585	NM_014306	C22orf28	chromosome 22 open reading fra	0.177	1.131	2.11E-01	2.11E+01		
8075616	NM_003490	SYN3	synapsin III	-0.009	0.994	9.55E-01	9.55E+01		
8075633	---	---	---	-0.162	0.894	1.26E-01	1.26E+01		
8075637	NM_004737	LARGE	like-glycosyltransferase	0.089	1.063	4.41E-01	4.41E+01		
8075657	---	---	---	0.029	1.020	8.31E-01	8.31E+01		
8075659	NM_203377	MB	myoglobin	-0.054	0.963	7.09E-01	7.09E+01		
8075668	ENST00000457	LOC284912	hypothetical LOC284912	-0.037	0.975	8.13E-01	8.13E+01		
8075691	NM_021104	RPL41	ribosomal protein L41	0.006	1.004	9.44E-01	9.44E+01		
8075695	NR_027833	APOL3	apolipoprotein L, 3	0.082	1.058	6.90E-01	6.90E+01		
8075705	---	---	---	-0.065	0.956	6.89E-01	6.89E+01		
8075720	NM_030882	APOL2	apolipoprotein L, 2	0.098	1.071	5.98E-01	5.98E+01		
8075728	NM_002473	MYH9	myosin, heavy chain 9, non-muscl	-0.147	0.903	5.53E-02	5.53E+00		
8075798	NM_003753	EIF3D	eukaryotic translation initiation fa	0.117	1.085	1.39E-01	1.39E+01		
8075817	---	---	---	-0.085	0.943	3.72E-01	3.72E+01		
8075828	NM_006860	RABL4	RAB, member of RAS oncogene fa	0.022	1.015	9.16E-01	9.16E+01		
8075846	AK075161	FLJ90680	FLJ90680 protein	-0.068	0.954	5.49E-01	5.49E+01		
8075857	NM_003312	TST	thiosulfate sulfurtransferase (rho	-0.045	0.969	8.59E-01	8.59E+01		
8075863	---	---	---	0.070	1.050	5.77E-01	5.77E+01		
8075865	NM_153609	TMPRSS6	transmembrane protease, serine	-0.059	0.960	5.29E-01	5.29E+01		
8075886	NM_000878	IL2RB	interleukin 2 receptor, beta	-0.170	0.889	3.39E-01	3.39E+01		
8075897	NM_031910	C1QTNF6	C1q and tumor necrosis factor rel	0.013	1.009	9.45E-01	9.45E+01		
8075906	NM_001051	SSTR3	somatostatin receptor 3	-0.039	0.973	7.45E-01	7.45E+01		
8075910	NM_002872	RAC2	ras-related C3 botulinum toxin sub	-0.078	0.947	6.07E-01	6.07E+01		
8075921	NM_052906	ELFN2	extracellular leucine-rich repeat a	-0.080	0.946	3.95E-01	3.95E+01		
8075924	NM_002405	MFNG	MFNG O-fucosylpeptide 3-beta-N-	0.063	1.044	6.80E-01	6.80E+01		
8075934	NM_014550	CARD10	caspase recruitment domain fami	-0.067	0.955	3.99E-01	3.99E+01		
8075963	AY364241	SH3BP1	SH3-domain binding protein 1	0.000	1.000	1.00E+00	1.00E+02		
8075969	---	---	---	-0.052	0.965	7.94E-01	7.94E+01		
8075971	NM_138797	ANKRD54	ankyrin repeat domain 54	-0.074	0.950	4.47E-01	4.47E+01		
8075981	AF324466	C22orf23	chromosome 22 open reading fra	0.006	1.004	9.75E-01	9.75E+01		
8075992	NM_006941	SOX10	SRY (sex determining region Y)-bo	-0.004	0.997	9.78E-01	9.78E+01		
8076002	NM_013356	SLC16A8	solute carrier family 16, member	0.039	1.027	6.94E-01	6.94E+01		
8076007	NM_025045	BAIAP2L2	BAI1-associated protein 2-like 2	0.031	1.022	7.54E-01	7.54E+01		
8076025	NM_003560	PLA2G6	phospholipase A2, group VI (cytos	-0.008	0.995	9.72E-01	9.72E+01		
8076046	NM_012264	TMEM184B	transmembrane protein 184B	-0.137	0.910	3.76E-01	3.76E+01		
8076056	NM_001894	CSNK1E	casein kinase 1, epsilon	-0.218	0.860	4.14E-03	4.14E-01		
8076072	NM_152868	KCNJ4	potassium inwardly-rectifying cha	-0.009	0.993	9.52E-01	9.52E+01		
8076077	NM_006386	DDX17	DEAD (Asp-Glu-Ala-Asp) box polyp	-0.003	0.998	9.80E-01	9.80E+01		
8076128	NM_014876	JOSD1	Josephin domain containing 1	-0.271	0.829	6.30E-02	6.30E+00		

8076137	NM_015374	UNC84B	unc-84 homolog B (C. elegans)	0.023	1.016	8.62E-01	8.62E+01		
8076169	NM_014293	NPTXR	neuronal pentraxin receptor	0.014	1.010	9.22E-01	9.22E+01		
8076185	NM_175709	CBX7	chromobox homolog 7	-0.188	0.878	5.26E-02	5.26E+00		
8076193	AK074445	FLJ23865	hypothetical protein FLJ23865	0.027	1.019	9.19E-01	9.19E+01		
8076195	NM_002608	PDGFB	platelet-derived growth factor bet	-0.035	0.976	7.77E-01	7.77E+01		
8076209	NM_000967	RPL3	ribosomal protein L3	-0.010	0.993	9.57E-01	9.57E+01		
8076219	NR_000028	SNORD83B	small nucleolar RNA, C/D box 83B	-0.060	0.959	7.44E-01	7.44E+01		
8076221	NR_000027	SNORD83A	small nucleolar RNA, C/D box 83A	-0.002	0.998	9.93E-01	9.93E+01		
8076223	NR_002439	SNORD43	small nucleolar RNA, C/D box 43	-0.282	0.823	2.81E-01	2.81E+01		
8076225	NM_194326	RPS19BP1	ribosomal protein S19 binding pro	0.056	1.039	6.81E-01	6.81E+01		
8076241	NM_020831	MKL1	megakaryoblastic leukemia (transl	-0.072	0.951	2.81E-01	2.81E+01		
8076260	NM_006358	SLC25A17	solute carrier family 25 (mitochon	-0.010	0.993	9.65E-01	9.65E+01		
8076272	NM_003932	ST13	suppression of tumorigenicity 13 (	-0.026	0.982	8.23E-01	8.23E+01		
8076298	---	---	---	-0.005	0.997	9.83E-01	9.83E+01		
8076302	NM_138481	CHADL	chondroadherin-like	-0.049	0.967	5.63E-01	5.63E+01		
8076307	NM_002883	RANGAP1	Ran GTPase activating protein 1	0.025	1.018	8.84E-01	8.84E+01		
8076331	NM_016272	TOB2	transducer of ERBB2, 2	-0.060	0.959	6.74E-01	6.74E+01		
8076339	NM_032758	PHF5A	PHD finger protein 5A	-0.003	0.998	9.92E-01	9.92E+01		
8076344	NM_00101805	POLR3H	polymerase (RNA) III (DNA directe	-0.064	0.956	6.10E-01	6.10E+01		
8076355	NM_002676	PMM1	phosphomannomutase 1	0.003	1.002	9.88E-01	9.88E+01		
8076365	NM_015704	PPPDE2	PPPDE peptidase domain containi	-0.073	0.951	5.78E-01	5.78E+01		
8076374	NM_005008	NHP2L1	NHP2 non-histone chromosome p	-0.031	0.979	8.70E-01	8.70E+01		
8076384	---	---	---	-0.065	0.956	7.39E-01	7.39E+01		
8076387	NM_052945	TNFRSF13C	tumor necrosis factor receptor sup	-0.039	0.974	7.05E-01	7.05E+01		
8076393	NM_024053	CENPM	centromere protein M	-0.026	0.982	8.44E-01	8.44E+01		
8076403	NM_000262	NAGA	N-acetylgalactosaminidase, alpha-	0.237	1.178	9.57E-02	9.57E+00		
8076417	NM_002490	NDUFA6	NADH dehydrogenase (ubiquinone	-0.082	0.945	5.09E-01	5.09E+01		
8076424	NM_000106	CYP2D6	cytochrome P450, family 2, subfar	0.043	1.030	8.73E-01	8.73E+01		
8076431	NR_002570	CYP2D7P1	cytochrome P450, family 2, subfar	-0.071	0.952	6.71E-01	6.71E+01		
8076436	NM_005650	TCF20	transcription factor 20 (AR1)	-0.070	0.953	6.82E-01	6.82E+01		
8076441	NM_145912	NFAM1	NFAT activating protein with ITAM	0.057	1.040	5.34E-01	5.34E+01		
8076449	NR_002184	RRP7B	ribosomal RNA processing 7 homo	0.016	1.011	9.28E-01	9.28E+01		
8076455	NM_015703	RRP7A	ribosomal RNA processing 7 homo	0.081	1.058	6.01E-01	6.01E+01		
8076461	---	---	---	0.070	1.049	7.59E-01	7.59E+01		
8076463	BC014647	RRP7B	ribosomal RNA processing 7 homo	0.102	1.074	7.73E-01	7.73E+01		
8076465	NM_032311	POLDIP3	polymerase (DNA-directed), delta	0.003	1.002	9.88E-01	9.88E+01		
8076481	NM_000398	CYB5R3	cytochrome b5 reductase 3	-0.147	0.903	2.27E-01	2.27E+01		
8076493	AF092923	ATP5L2	ATP synthase, H+ transporting, mi	-0.030	0.980	9.07E-01	9.07E+01		
8076497	NM_017436	A4GALT	alpha 1,4-galactosyltransferase	-0.142	0.906	7.17E-02	7.17E+00		
8076509	---	---	---	-0.115	0.923	3.81E-01	3.81E+01		
8076511	NM_000969	RPL5	ribosomal protein L5	-0.001	0.999	9.96E-01	9.96E+01		
8076513	---	---	---	-0.033	0.977	7.87E-01	7.87E+01		
8076515	NM_014570	ARFGAP3	ADP-ribosylation factor GTPase ac	0.119	1.086	4.39E-01	4.39E+01		
8076533	NM_007229	PACSIN2	protein kinase C and casein kinase	0.039	1.027	8.47E-01	8.47E+01		
8076547	NR_027779	TTLL1	tubulin tyrosine ligase-like family,	-0.003	0.998	9.87E-01	9.87E+01		
8076561	AB051441	KIAA1654	KIAA1654 protein	0.026	1.018	8.54E-01	8.54E+01		
8076563	NM_173467	MCAT	malonyl CoA:ACP acyltransferase (	-0.022	0.985	8.97E-01	8.97E+01		
8076569	NM_015140	TTLL12	tubulin tyrosine ligase-like family,	-0.050	0.966	6.31E-01	6.31E+01		
8076584	AK056490	FLJ30901	hypothetical protein FLJ30901	-0.022	0.985	8.71E-01	8.71E+01		
8076586	NM_173050	SCUBE1	signal peptide, CUB domain, EGF-l	-0.050	0.966	7.47E-01	7.47E+01		
8076668	BC104183	KIAA1644	KIAA1644	-0.035	0.976	8.37E-01	8.37E+01		
8076673	NM_032287	LDOC1L	leucine zipper, down-regulated in	-0.030	0.979	8.67E-01	8.67E+01		
8076677	NM_138415	PHF21B	PHD finger protein 21B	0.011	1.007	9.47E-01	9.47E+01		
8076690	NM_00100988	C22orf9	chromosome 22 open reading frar	0.254	1.192	5.20E-02	5.20E+00		
8076732	---	---	---	-0.029	0.980	8.18E-01	8.18E+01		
8076734	NM_058238	WNT7B	wingless-type MMTV integration s	-0.112	0.925	2.71E-01	2.71E+01		
8076739	NM_018280	C22orf26	chromosome 22 open reading frar	0.013	1.009	9.53E-01	9.53E+01		
8076744	BC009388	LOC554174	hypothetical LOC554174	-0.074	0.950	5.38E-01	5.38E+01		
8076747	AK123383	LOC642648	hypothetical LOC642648	0.008	1.006	9.67E-01	9.67E+01		
8076749	NM_207327	C22orf40	chromosome 22 open reading frar	-0.008	0.994	9.77E-01	9.77E+01		
8076757	NM_014246	CELSR1	cadherin, EGF LAG seven-pass G-ty	-0.082	0.945	3.82E-01	3.82E+01		
8076792	NM_022766	CERK	ceramide kinase	-0.108	0.928	3.31E-01	3.31E+01		
8076813	BC041833	FLJ32756	hypothetical LOC642757	-0.008	0.995	9.77E-01	9.77E+01		
8076815	AY358194	UNQ6126	LPEQ6126	-0.039	0.973	7.70E-01	7.70E+01		
8076826	NR_026997	C22orf34	chromosome 22 open reading frar	-0.040	0.973	8.07E-01	8.07E+01		
8076836	NM_014577	BRD1	bromodomain containing 1	-0.094	0.937	2.99E-01	2.99E+01		
8076851	NM_024105	ALG12	asparagine-linked glycosylation 12	-0.008	0.995	9.49E-01	9.49E+01		
8076865	NM_00100169	IL17REL	interleukin 17 receptor E-like	-0.036	0.976	7.59E-01	7.59E+01		
8076882	NM_00108044	TTLL8	tubulin tyrosine ligase-like family,	0.074	1.053	5.55E-01	5.55E+01		
8076894	NM_015166	MLC1	megalencephalic leukoencephalop	0.115	1.083	2.52E-01	2.52E+01		
8076909	NM_020461	TUBGCP6	tubulin, gamma complex associate	-0.049	0.967	6.34E-01	6.34E+01		
8076937	NM_032019	HDAC10	histone deacetylase 10	-0.064	0.957	5.09E-01	5.09E+01		
8076962	NM_002969	MAPK12	mitogen-activated protein kinase 1	0.011	1.008	9.53E-01	9.53E+01		
8076978	NM_002751	MAPK11	mitogen-activated protein kinase 1	-0.043	0.971	6.62E-01	6.62E+01		
8076998	NM_012401	PLXNB2	plexin B2	0.102	1.074	5.34E-01	5.34E+01		
8077036	BC031069	FAM116B	family with sequence similarity 11	-0.040	0.973	6.35E-01	6.35E+01		
8077042	NM_002972	SBF1	SET binding factor 1	-0.035	0.976	7.78E-01	7.78E+01		

8077082	NM_033200	LMF2	lipase maturation factor 2	0.003	1.002	9.85E-01	9.85E+01		
8077099	NM_005138	SCO2	SCO cytochrome oxidase deficient	0.004	1.002	9.87E-01	9.87E+01		
8077103	NM_00111375	TYMP	thymidine phosphorylase	0.135	1.098	2.79E-01	2.79E+01		
8077116	NM_00101444	ODF3B	outer dense fiber of sperm tails 3E	-0.041	0.972	8.43E-01	8.43E+01		
8077123	NR_027928	CHKB-CPT1B	choline kinase-like, carnitine palm	-0.019	0.987	9.00E-01	9.00E+01		
8077160	NM_000487	ARSA	arylsulfatase A	-0.044	0.970	7.95E-01	7.95E+01		
8077171	NM_00113092	RABL2B	RAB, member of RAS oncogene fa	0.035	1.024	8.41E-01	8.41E+01		
8077185	NM_000039	APOA1	apolipoprotein A-I	0.015	1.010	8.86E-01	8.86E+01		
8077192	NM_000482	APOA4	apolipoprotein A-IV	0.014	1.010	8.87E-01	8.87E+01		
8077204	NM_000040	APOC3	apolipoprotein C-III	-0.010	0.993	9.51E-01	9.51E+01		
8077211	NM_013325	ATG4B	ATG4 autophagy related 4 homolo	0.004	1.003	9.86E-01	9.86E+01		
8077226	NM_032329	ING5	inhibitor of growth family, membe	0.078	1.056	6.80E-01	6.80E+01		
8077238	NM_152783	D2HGDH	D-2-hydroxyglutarate dehydrogen	-0.060	0.959	5.21E-01	5.21E+01		
8077250	NM_015963	THAP4	THAP domain containing 4	0.000	1.000	1.00E+00	1.00E+02		
8077262	NM_012145	DTYMK	deoxythymidylate kinase (thymidy	-0.032	0.978	8.87E-01	8.87E+01		
8077351	---	---	---	-0.020	0.986	9.24E-01	9.24E+01		
8077353	NM_182916	TRNT1	tRNA nucleotidyl transferase, CCA	-0.022	0.985	9.32E-01	9.32E+01		
8077370	NM_006515	SETMAR	SET domain and mariner transpos	-0.014	0.990	9.61E-01	9.61E+01		
8077376	NM_00109995	ITPR1	inositol 1,4,5-triphosphate recept	0.074	1.053	4.87E-01	4.87E+01		
8077441	NM_003670	BHLHE40	basic helix-loop-helix family, mem	-0.280	0.824	6.52E-02	6.52E+00		
8077450	NM_018184	ARL8B	ADP-ribosylation factor-like 8B	0.023	1.016	9.36E-01	9.36E+01		
8077458	NM_014674	EDEM1	ER degradation enhancer, mannos	0.035	1.024	8.25E-01	8.25E+01		
8077490	NM_014583	LMCD1	LIM and cysteine-rich domains 1	0.009	1.006	9.51E-01	9.51E+01		
8077513	NM_015453	THUMPD3	THUMP domain containing 3	0.052	1.037	7.60E-01	7.60E+01		
8077526	---	---	---	-0.063	0.957	7.51E-01	7.51E+01		
8077528	NM_00108051	SETD5	SET domain containing 5	-0.102	0.932	7.53E-02	7.53E+00		
8077550	NM_00107752	MTMR14	myotubularin related protein 14	0.091	1.065	4.02E-01	4.02E+01		
8077595	NM_00100369	BRPF1	bromodomain and PHD finger con	0.043	1.030	6.76E-01	6.76E+01		
8077612	NM_00102593	TTLL3	tubulin tyrosine ligase-like family,	0.097	1.069	3.66E-01	3.66E+01		
8077635	NM_016819	OGG1	8-oxoguanine DNA glycosylase	0.086	1.062	3.67E-01	3.67E+01		
8077652	NM_005718	ARPC4	actin related protein 2/3 complex,	0.150	1.110	2.48E-01	2.48E+01		
8077663	NM_032492	JAGN1	jagunal homolog 1 (Drosophila)	-0.002	0.998	9.93E-01	9.93E+01		
8077668	NM_153480	IL17RE	interleukin 17 receptor E	-0.051	0.965	6.66E-01	6.66E+01		
8077688	NM_153461	IL17RC	interleukin 17 receptor C	-0.079	0.947	2.98E-01	2.98E+01		
8077712	NM_00103171	CRELD1	cysteine-rich with EGF-like domair	-0.006	0.996	9.77E-01	9.77E+01		
8077728	AK092352	LOC442075	hypothetical LOC442075	-0.052	0.965	8.36E-01	8.36E+01		
8077770	NM_018462	C3orf10	chromosome 3 open reading fram	-0.050	0.966	8.11E-01	8.11E+01		
8077777	---	---	---	-0.049	0.967	9.31E-01	9.31E+01		
8077779	---	---	---	-0.063	0.957	6.92E-01	6.92E+01		
8077781	NM_000551	VHL	von Hippel-Lindau tumor suppress	0.083	1.059	5.43E-01	5.43E+01		
8077786	NM_001570	IRAK2	interleukin-1 receptor-associated	-0.213	0.863	1.03E-02	1.03E+00		
8077804	NM_014760	TATDN2	TatD DNase domain containing 2	-0.016	0.989	9.50E-01	9.50E+01		
8077815	NR_026829	C3orf42	chromosome 3 open reading fram	0.005	1.004	9.88E-01	9.88E+01		
8077851	NM_00109821	HRH1	histamine receptor H1	-0.070	0.952	5.91E-01	5.91E+01		
8077858	NM_006395	ATG7	ATG7 autophagy related 7 homolo	0.083	1.059	7.83E-01	7.83E+01		
8077877	AK094424	LOC100130924	hypothetical LOC100130924	-0.065	0.956	7.72E-01	7.72E+01		
8077879	NM_133625	SYN2	synapsin II	-0.001	1.000	9.97E-01	9.97E+01		
8077914	NM_025265	TSEN2	tRNA splicing endonuclease 2 hom	-0.021	0.986	9.53E-01	9.53E+01		
8077929	---	---	---	0.028	1.020	9.54E-01	9.54E+01		
8077931	NM_014160	MKRN2	makorin ring finger protein 2	-0.015	0.990	9.49E-01	9.49E+01		
8077942	---	---	---	0.185	1.136	6.05E-01	6.05E+01		
8077944	NM_00116249	CAND2	cullin-associated and neddylation-	-0.081	0.945	4.92E-01	4.92E+01		
8077970	NM_00100401	FBLN2	fibulin 2	-0.018	0.988	9.07E-01	9.07E+01		
8077989	NR_002223	TPRXL	tetra-peptide repeat homeobox-lil	0.049	1.035	6.36E-01	6.36E+01		
8077993	NM_024334	TMEM43	transmembrane protein 43	-0.039	0.973	7.94E-01	7.94E+01		
8078008	NM_014463	LSM3	LSM3 homolog, U6 small nuclear R	0.086	1.061	4.51E-01	4.51E+01		
8078014	NM_003043	SLC6A6	solute carrier family 6 (neurotrans	-0.155	0.898	3.89E-01	3.89E+01		
8078033	NM_016474	C3orf19	chromosome 3 open reading fram	-0.019	0.987	9.13E-01	9.13E+01		
8078091	NM_003298	NR2C2	nuclear receptor subfamily 2, grou	-0.105	0.930	1.71E-01	1.71E+01		
8078110	NM_014296	CAPN7	calpain 7	-0.048	0.967	7.48E-01	7.48E+01		
8078136	---	---	---	0.061	1.043	5.94E-01	5.94E+01		
8078138	NM_033083	EAF1	ELL associated factor 1	-0.050	0.966	7.43E-01	7.43E+01		
8078147	NM_000060	BTD	biotinidase	-0.113	0.925	3.06E-01	3.06E+01		
8078153	---	---	---	-0.144	0.905	3.76E-01	3.76E+01		
8078173	NM_138381	OXNAD1	oxidoreductase NAD-binding doma	0.041	1.029	8.74E-01	8.74E+01		
8078187	NM_015184	PLCL2	phospholipase C-like 2	0.088	1.063	6.59E-01	6.59E+01		
8078214	NM_004162	RAB5A	RAB5A, member RAS oncogene fa	0.142	1.103	6.65E-02	6.65E+00		
8078227	NM_003884	KAT2B	K(lysine) acetyltransferase 2B	0.144	1.105	1.34E-01	1.34E+01		
8078248	NM_002128	HMGB1	high-mobility group box 1	-0.006	0.996	9.66E-01	9.66E+01		
8078260	---	---	---	0.095	1.068	5.91E-01	5.91E+01		
8078262	NM_003341	UBE2E1	ubiquitin-conjugating enzyme E2E	0.206	1.153	3.02E-02	3.02E+00		
8078270	NM_002948	RPL15	ribosomal protein L15	0.011	1.008	9.63E-01	9.63E+01		
8078272	NM_005126	NR1D2	nuclear receptor subfamily 1, grou	0.009	1.006	9.74E-01	9.74E+01		
8078300	NM_017897	OXSM	3-oxoacyl-ACP synthase, mitochor	0.080	1.057	5.77E-01	5.77E+01		
8078305	NM_052953	LRRC3B	leucine rich repeat containing 3B	-0.036	0.975	7.69E-01	7.69E+01		
8078312	NM_182523	CMC1	COX assembly mitochondrial prote	0.114	1.082	5.23E-01	5.23E+01		
8078350	NM_00102484	TGFBR2	transforming growth factor, beta r	0.069	1.049	6.34E-01	6.34E+01		

8078360	NM_178862	STT3B	STT3, subunit of the oligosacchary	0.167	1.123	1.83E-02	1.83E+00		
8078378	---	---	---	0.037	1.026	9.09E-01	9.09E+01		
8078397	NM_178868	CMTM8	CKLF-like MARVEL transmembrane	-0.007	0.995	9.86E-01	9.86E+01		
8078405	NM_138410	CMTM7	CKLF-like MARVEL transmembrane	0.036	1.025	8.51E-01	8.51E+01		
8078412	NM_015442	CNOT10	CCR4-NOT transcription complex,	0.064	1.045	6.23E-01	6.23E+01		
8078435	NM_00103911	TRIM71	tripartite motif-containing 71	-0.034	0.977	7.97E-01	7.97E+01		
8078442	NM_005508	CCR4	chemokine (C-C motif) receptor 4	-0.303	0.811	2.11E-01	2.11E+01		
8078450	NM_006371	CRTAP	cartilage associated protein	0.020	1.014	9.18E-01	9.18E+01		
8078479	NM_013374	PDCD6IP	programmed cell death 6 interacti	0.023	1.016	9.10E-01	9.10E+01		
8078544	NM_000249	MLH1	mutL homolog 1, colon cancer, no	0.077	1.055	5.81E-01	5.81E+01		
8078567	---	---	---	0.036	1.025	8.39E-01	8.39E+01		
8078569	NM_002078	GOLGA4	golgi autoantigen, golgin subfamily	-0.031	0.978	8.06E-01	8.06E+01		
8078600	NM_006756	TCEA1	transcription elongation factor A (	0.014	1.010	9.27E-01	9.27E+01		
8078603	---	---	---	0.054	1.038	8.78E-01	8.78E+01		
8078605	NM_178339	C3orf35	chromosome 3 open reading fram	-0.119	0.921	2.56E-01	2.56E+01		
8078619	NM_002207	ITGA9	integrin, alpha 9	-0.074	0.950	5.50E-01	5.50E+01		
8078650	NM_00100839	CTDSPL	CTD (carboxy-terminal domain, RN	0.009	1.006	9.51E-01	9.51E+01		
8078665	NM_015873	VILL	villin-like	-0.072	0.951	5.01E-01	5.01E+01		
8078688	---	---	---	0.132	1.096	7.09E-01	7.09E+01		
8078690	NM_007335	DLEC1	deleted in lung and esophageal ca	-0.041	0.972	7.52E-01	7.52E+01		
8078738	NM_005109	OXSR1	oxidative-stress responsive 1	-0.111	0.926	4.14E-01	4.14E+01		
8078759	NM_004256	SLC22A13	solute carrier family 22 (organic ar	0.015	1.010	9.38E-01	9.38E+01		
8078818	NM_005107	EXOG	endo/exonuclease (5'-3'), endonuc	0.088	1.063	5.72E-01	5.72E+01		
8078832	---	---	---	0.148	1.108	3.57E-01	3.57E+01		
8078834	NM_020839	WDR48	WD repeat domain 48	-0.043	0.971	8.10E-01	8.10E+01		
8078857	NM_00110551	TTC21A	tetratricopeptide repeat domain 2	0.057	1.040	5.79E-01	5.79E+01		
8078898	NM_017875	SLC25A38	solute carrier family 25, member 3	-0.069	0.953	7.07E-01	7.07E+01		
8078905	NM_002295	RPSA	ribosomal protein SA	-0.038	0.974	8.54E-01	8.54E+01		
8078916	NR_002325	SNORA6	small nucleolar RNA, H/ACA box 6	-0.002	0.999	9.97E-01	9.97E+01		
8078918	NR_002324	SNORA62	small nucleolar RNA, H/ACA box 6	0.085	1.061	8.69E-01	8.69E+01		
8078933	NM_015460	MYRIP	myosin VIIA and Rab interacting p	0.010	1.007	9.51E-01	9.51E+01		
8078958	---	---	---	-0.062	0.958	7.17E-01	7.17E+01		
8078960	---	---	---	0.034	1.024	8.24E-01	8.24E+01		
8078962	NM_005875	EIF1B	eukaryotic translation initiation fa	-0.067	0.954	6.07E-01	6.07E+01		
8078984	NM_003973	RPL14	ribosomal protein L14	-0.056	0.962	5.55E-01	5.55E+01		
8078999	NM_175888	ZNF620	zinc finger protein 620	0.011	1.008	9.65E-01	9.65E+01		
8079005	NM_198484	ZNF621	zinc finger protein 621	0.046	1.033	7.93E-01	7.93E+01		
8079019	NM_001030	RPS27	ribosomal protein S27	-0.025	0.983	8.90E-01	8.90E+01		
8079021	NM_001904	CTNNB1	catenin (cadherin-associated prote	0.049	1.035	7.51E-01	7.51E+01		
8079037	NM_00104264	TRAK1	trafficking protein, kinesin binding	0.044	1.031	7.75E-01	7.75E+01		
8079060	NM_004624	VIPR1	vasoactive intestinal peptide recep	0.052	1.037	6.20E-01	6.20E+01		
8079074	NM_016305	SS18L2	synovial sarcoma translocation ge	-0.019	0.987	9.22E-01	9.22E+01		
8079079	NM_005385	NKTR	natural killer-tumor recognition se	-0.060	0.959	6.53E-01	6.53E+01		
8079099	NM_145166	ZBTB47	zinc finger and BTB domain contai	-0.030	0.980	7.77E-01	7.77E+01		
8079107	NM_152393	KBTBD5	kelch repeat and BTB (POZ) domai	-0.053	0.964	6.15E-01	6.15E+01		
8079131	NM_00112990	LOC729085	hypothetical protein LOC729085	-0.005	0.996	9.79E-01	9.79E+01		
8079140	NM_017719	SNRK	SNF related kinase	0.025	1.017	9.00E-01	9.00E+01		
8079149	---	---	---	0.055	1.039	8.47E-01	8.47E+01		
8079153	NM_016006	ABHD5	abhydrolase domain containing 5	0.045	1.032	9.12E-01	9.12E+01		
8079165	---	---	---	-0.037	0.974	7.61E-01	7.61E+01		
8079167	NM_00114503	C3orf77	chromosome 3 open reading fram	-0.034	0.977	8.21E-01	8.21E+01		
8079187	AK131507	ZNF852	zinc finger protein 852	0.040	1.028	9.35E-01	9.35E+01		
8079189	NM_018651	ZNF167	zinc finger protein 167	0.022	1.015	9.34E-01	9.34E+01		
8079217	NM_003420	ZNF35	zinc finger protein 35	-0.001	1.000	9.99E-01	9.99E+01		
8079274	NM_144638	TMEM42	transmembrane protein 42	-0.042	0.971	7.64E-01	7.64E+01		
8079279	NM_003241	TGM4	transglutaminase 4 (prostate)	0.007	1.005	9.66E-01	9.66E+01		
8079294	NM_015004	EXOSC7	exosome component 7	0.042	1.030	8.12E-01	8.12E+01		
8079305	NM_003278	CLEC3B	C-type lectin domain family 3, mer	-0.045	0.970	7.07E-01	7.07E+01		
8079311	NM_015340	LARS2	leucyl-tRNA synthetase 2, mitocho	0.037	1.026	8.47E-01	8.47E+01		
8079334	NM_014240	LIMD1	LIM domains containing 1	0.159	1.116	1.12E-01	1.12E+01		
8079346	NM_014016	SACM1L	SAC1 suppressor of actin mutation	0.093	1.067	3.42E-01	3.42E+01		
8079368	---	---	---	0.108	1.077	7.54E-01	7.54E+01		
8079377	NM_006564	CXCR6	chemokine (C-X-C motif) receptor	-0.022	0.985	9.53E-01	9.53E+01		
8079426	NM_147196	TMIE	transmembrane inner ear	-0.023	0.984	9.09E-01	9.09E+01		
8079462	NM_015175	NBEAL2	neurobeachin-like 2	-0.070	0.953	3.29E-01	3.29E+01		
8079517	NM_025010	KLHL18	kelch-like 18 (Drosophila)	-0.029	0.980	8.29E-01	8.29E+01		
8079532	NM_015466	PTPN23	protein tyrosine phosphatase, nor	-0.043	0.971	6.05E-01	6.05E+01		
8079563	NM_138615	DHX30	DEAH (Asp-Glu-Ala-His) box polype	-0.120	0.920	1.42E-01	1.42E+01		
8079590	NM_004345	CAMP	cathelicidin antimicrobial peptide	0.024	1.017	8.64E-01	8.64E+01		
8079598	NM_016089	ZNF589	zinc finger protein 589	0.042	1.029	8.05E-01	8.05E+01		
8079630	NM_015933	CCDC72	coiled-coil domain containing 72	-0.032	0.978	7.32E-01	7.32E+01		
8079637	NM_130384	ATRIP	ATR interacting protein	-0.057	0.961	5.99E-01	5.99E+01		
8079662	NM_006321	ARIH2	ariadne homolog 2 (Drosophila)	-0.033	0.977	8.05E-01	8.05E+01		
8079677	NM_177938	P4HTM	prolyl 4-hydroxylase, transmembra	0.001	1.001	9.94E-01	9.94E+01		
8079693	NM_018031	WDR6	WD repeat domain 6	0.004	1.003	9.83E-01	9.83E+01		
8079707	NM_199417	NDUFAF3	NADH dehydrogenase (ubiquinone	-0.049	0.967	6.99E-01	6.99E+01		
8079733	NM_173546	KLHDC8B	kelch domain containing 8B	0.130	1.094	2.30E-01	2.30E+01		

8079742	---	---	---	0.273	1.208	3.27E-01	3.27E+01		
8079746	NM_022171	TCTA	T-cell leukemia translocation alter	0.008	1.006	9.58E-01	9.58E+01		
8079753	NM_004393	DAG1	dystroglycan 1 (dystrophin-associa	0.006	1.004	9.77E-01	9.77E+01		
8079759	NM_003458	BSN	bassoon (presynaptic cytomatrix p	0.029	1.020	8.64E-01	8.64E+01		
8079772	NM_001640	APEH	N-acylaminoacyl-peptide hydrolas	0.014	1.010	9.36E-01	9.36E+01		
8079796	NM_022064	RNF123	ring finger protein 123	-0.019	0.987	8.98E-01	8.98E+01		
8079842	NM_005777	RBM6	RNA binding motif protein 6	0.005	1.003	9.78E-01	9.78E+01		
8079867	---	---	---	-0.085	0.942	7.45E-01	7.45E+01		
8079869	NM_005778	RBM5	RNA binding motif protein 5	-0.021	0.985	8.68E-01	8.68E+01		
8079896	NM_004186	SEMA3F	sema domain, immunoglobulin do	-0.021	0.986	8.75E-01	8.75E+01		
8079919	NM_144499	GNAT1	guanine nucleotide binding protei	-0.011	0.992	9.49E-01	9.49E+01		
8079950	NM_002070	GNAI2	guanine nucleotide binding protei	0.007	1.005	9.86E-01	9.86E+01		
8079964	---	---	---	-0.066	0.955	6.67E-01	6.67E+01		
8079988	BC028000	C3orf45	chromosome 3 open reading fram	-0.012	0.991	9.50E-01	9.50E+01		
8079993	NM_007022	CYB561D2	cytochrome b-561 domain contair	-0.015	0.990	9.34E-01	9.34E+01		
8080001	NM_016173	HEMK1	HemK methyltransferase family m	-0.039	0.973	7.23E-01	7.23E+01		
8080013	NM_004635	MAPKAPK3	mitogen-activated protein kinase-	0.021	1.015	9.49E-01	9.49E+01		
8080084	NM_006010	MANF	mesencephalic astrocyte-derived r	0.097	1.069	4.25E-01	4.25E+01		
8080093	NM_013286	RBM15B	RNA binding motif protein 15B	-0.050	0.966	6.95E-01	6.95E+01		
8080100	NM_015106	RAD54L2	RAD54-like 2 (S. cerevisiae)	-0.085	0.943	3.95E-01	3.95E+01		
8080121	NM_015926	TEX264	testis expressed 264	0.030	1.021	8.09E-01	8.09E+01		
8080144	NM_00100393	PARP3	poly (ADP-ribose) polymerase fam	0.007	1.005	9.73E-01	9.73E+01		
8080158	NM_080865	GPR62	G protein-coupled receptor 62	-0.020	0.986	8.59E-01	8.59E+01		
8080162	NM_015407	ABHD14A	abhydrolase domain containing 14	-0.060	0.959	4.35E-01	4.35E+01		
8080168	NM_000666	ACY1	aminoacylase 1	0.008	1.005	9.64E-01	9.64E+01		
8080184	NM_000688	ALAS1	aminolevulinate, delta-, synthase 1	0.090	1.064	7.12E-01	7.12E+01		
8080198	NM_144641	PPM1M	protein phosphatase 1M (PP2C do	0.059	1.042	6.39E-01	6.39E+01		
8080212	NM_145262	GLYCTK	glycerate kinase	-0.032	0.978	7.47E-01	7.47E+01		
8080226	NM_015512	DNAH1	dynein, axonemal, heavy chain 1	-0.048	0.968	5.17E-01	5.17E+01		
8080320	NM_007184	NISCH	nischarin	0.034	1.024	7.67E-01	7.67E+01		
8080344	NM_015136	STAB1	stabilin 1	0.176	1.130	5.36E-02	5.36E+00		
8080416	NM_00112476	LOC440957	similar to CG32736-PA	0.122	1.088	3.98E-01	3.98E+01		
8080419	NM_206825	GNL3	guanine nucleotide binding protei	-0.040	0.973	8.73E-01	8.73E+01		
8080438	NM_014041	SPCS1	signal peptidase complex subunit 1	-0.002	0.998	9.87E-01	9.87E+01		
8080466	NM_002217	ITIH3	inter-alpha (globulin) inhibitor H3	-0.032	0.978	7.63E-01	7.63E+01		
8080487	NM_006254	PRKCD	protein kinase C, delta	0.051	1.036	9.04E-01	9.04E+01		
8080619	---	---	---	0.061	1.043	6.35E-01	6.35E+01		
8080621	NR_024460	CCDC66	coiled-coil domain containing 66	0.060	1.042	7.72E-01	7.72E+01		
8080645	NM_012096	APPL1	adaptor protein, phosphotyrosine	0.023	1.016	8.93E-01	8.93E+01		
8080676	NM_177966	PDE12	phosphodiesterase 12	-0.016	0.989	9.53E-01	9.53E+01		
8080685	NM_007159	SLMAP	sarcolemma associated protein	-0.024	0.984	8.91E-01	8.91E+01		
8080714	NM_001457	FLNB	filamin B, beta	-0.035	0.976	8.19E-01	8.19E+01		
8080762	NM_020676	ABHD6	abhydrolase domain containing 6	-0.008	0.995	9.81E-01	9.81E+01		
8080773	NM_00109878	RPP14	ribonuclease P/MRP 14kDa subuni	0.083	1.059	6.87E-01	6.87E+01		
8080781	NM_017771	PXK	PX domain containing serine/threo	0.042	1.029	8.63E-01	8.63E+01		
8080878	NM_00112814	ATXN7	ataxin 7	0.019	1.013	8.63E-01	8.63E+01		
8080896	NM_173471	SLC25A26	solute carrier family 25, member 2	-0.112	0.925	4.81E-01	4.81E+01		
8080909	---	---	---	0.005	1.003	9.88E-01	9.88E+01		
8080911	NM_032505	KBTBD8	kelch repeat and BTB (POZ) domai	0.077	1.055	6.58E-01	6.58E+01		
8080923	NM_002802	PSMC1	proteasome (prosome, macropain	-0.033	0.978	8.80E-01	8.80E+01		
8080926	NM_006407	ARL6IP5	ADP-ribosylation-like factor 6 inte	0.069	1.049	5.91E-01	5.91E+01		
8080958	NM_018971	GPR27	G protein-coupled receptor 27	-0.020	0.986	8.98E-01	8.98E+01		
8080960	---	---	---	0.043	1.030	7.95E-01	7.95E+01		
8080973	NM_174907	PPP4R2	protein phosphatase 4, regulatory	-0.060	0.959	6.93E-01	6.93E+01		
8080980	AY036895	FLJ10213	hypothetical protein FLJ10213	-0.065	0.956	7.35E-01	7.35E+01		
8080991	NM_194247	HNRNPA3	heterogeneous nuclear ribonucleo	0.035	1.024	6.17E-01	6.17E+01		
8080998	ENST00000455	FRG2C	FSHD region gene 2 family, membe	-0.069	0.953	6.14E-01	6.14E+01		
8081028	---	---	---	0.115	1.083	4.21E-02	4.21E+00		
8081053	---	---	---	0.022	1.016	8.74E-01	8.74E+01		
8081055	NM_014043	CHMP2B	chromatin modifying protein 2B	0.064	1.045	7.05E-01	7.05E+01		
8081069	NM_018293	ZNF654	zinc finger protein 654	-0.098	0.935	6.09E-01	6.09E+01		
8081073	NM_173824	C3orf38	chromosome 3 open reading fram	0.134	1.097	2.59E-01	2.59E+01		
8081115	NM_182896	ARL13B	ADP-ribosylation factor-like 13B	-0.095	0.936	4.99E-01	4.99E+01		
8081128	NM_022072	NSUN3	NOL1/NOP2/Sun domain family, m	0.010	1.007	9.78E-01	9.78E+01		
8081135	NM_00113442	CDV3	CDV3 homolog (mouse)	0.081	1.058	3.29E-01	3.29E+01		
8081171	NM_153605	CRYBG3	beta-gamma crystallin domain cor	0.234	1.176	2.84E-01	2.84E+01		
8081214	NM_005290	GPR15	G protein-coupled receptor 15	-0.389	0.764	2.19E-01	2.19E+01		
8081241	BC006512	C3orf26	chromosome 3 open reading fram	-0.072	0.951	8.21E-01	8.21E+01		
8081254	---	---	---	0.096	1.069	6.80E-01	6.80E+01		
8081256	NM_018309	TBC1D23	TBC1 domain family, member 23	0.062	1.044	7.83E-01	7.83E+01		
8081277	NM_020202	NIT2	nitrilase family, member 2	0.003	1.002	9.92E-01	9.92E+01		
8081316	NM_006070	TFG	TRK-fused gene	0.016	1.011	9.42E-01	9.42E+01		
8081335	ENST00000426	LOC100287852	similar to CG5323	-0.099	0.933	6.88E-01	6.88E+01		
8081337	---	---	---	0.008	1.006	9.86E-01	9.86E+01		
8081343	NM_017819	RG9MTD1	RNA (guanine-9-) methyltransfera	-0.040	0.973	8.47E-01	8.47E+01		
8081348	NM_020357	PCNP	PEST proteolytic signal containing	-0.073	0.951	5.04E-01	5.04E+01		
8081358	NM_014415	ZBTB11	zinc finger and BTB domain contai	-0.199	0.871	2.19E-01	2.19E+01		

8081362	NM_024548	CEP97	centrosomal protein 97kDa	0.019	1.013	9.46E-01	9.46E+01		
8081375	NM_145037	FAM55C	family with sequence similarity 55	-0.058	0.960	7.87E-01	7.87E+01		
8081386	NM_031419	NFKBIZ	nuclear factor of kappa light polyp	-0.054	0.963	6.74E-01	6.74E+01		
8081465	NM_00114256	BBX	bobby sox homolog (Drosophila)	-0.068	0.954	5.61E-01	5.61E+01		
8081537	NM_016388	TRAT1	T cell receptor associated transme	-0.198	0.872	3.62E-01	3.62E+01		
8081548	NM_015480	PVRL3	poliovirus receptor-related 3	-0.287	0.820	2.91E-02	2.91E+00		
8081564	NM_198196	CD96	CD96 molecule	-0.258	0.836	6.44E-02	6.44E+00		
8081582	NM_153268	PLCXD2	phosphatidylinositol-specific phos	-0.142	0.906	3.97E-01	3.97E+01		
8081590	NM_00113443	PHLDB2	pleckstrin homology-like domain,	-0.280	0.824	1.69E-01	1.69E+01		
8081612	NM_018394	ABHD10	abhydrolase domain containing 10	0.202	1.150	1.87E-02	1.87E+00		
8081645	NM_024616	C3orf52	chromosome 3 open reading fram	-0.004	0.997	9.88E-01	9.88E+01		
8081657	NM_00100419	CD200	CD200 molecule	-0.167	0.891	4.32E-01	4.32E+01		
8081686	NM_033254	BOC	Boc homolog (mouse)	0.025	1.017	8.55E-01	8.55E+01		
8081710	NM_017699	SIDT1	SID1 transmembrane family, mem	-0.015	0.989	9.61E-01	9.61E+01		
8081758	NM_017577	GRAMD1C	GRAM domain containing 1C	0.217	1.162	3.70E-01	3.70E+01		
8081779	NM_173570	ZDHHC23	zinc finger, DHHC-type containing	-0.021	0.986	9.12E-01	9.12E+01		
8081799	NM_173799	TIGIT	T cell immunoreceptor with Ig and	-0.034	0.977	9.29E-01	9.29E+01		
8081818	---	---	---	-0.073	0.951	5.77E-01	5.77E+01		
8081820	---	---	---	0.069	1.049	6.87E-01	6.87E+01		
8081838	NM_020754	CDGAP	Cdc42 GTPase-activating protein	0.256	1.194	2.61E-01	2.61E+01		
8081945	NM_004547	NDUFB4	NADH dehydrogenase (ubiquinone	-0.050	0.966	7.45E-01	7.45E+01		
8081953	NM_005513	GTF2E1	general transcription factor IIE, po	0.063	1.045	7.72E-01	7.72E+01		
8082058	NM_005213	CSTA	cystatin A (stefin A)	-0.078	0.947	8.35E-01	8.35E+01		
8082066	NM_014367	FAM162A	family with sequence similarity 16	-0.022	0.985	9.35E-01	9.35E+01		
8082075	NM_138287	DTX3L	deltex 3-like (Drosophila)	0.158	1.115	4.96E-01	4.96E+01		
8082086	NM_00111352	PARP15	poly (ADP-ribose) polymerase fam	-0.065	0.956	7.53E-01	7.53E+01		
8082100	NM_017554	PARP14	poly (ADP-ribose) polymerase fam	0.222	1.167	1.76E-01	1.76E+01		
8082118	---	---	---	-0.014	0.990	9.80E-01	9.80E+01		
8082120	NM_032839	DIRC2	disrupted in renal carcinoma 2	-0.037	0.974	8.93E-01	8.93E+01		
8082133	NM_006810	PDIA5	protein disulfide isomerase family	0.092	1.066	5.43E-01	5.43E+01		
8082163	---	---	---	-0.037	0.975	8.06E-01	8.06E+01		
8082165	NM_00102466	KALRN	kalirin, RhoGEF kinase	0.008	1.006	9.42E-01	9.42E+01		
8082229	NM_000373	UMPS	uridine monophosphate synthetas	-0.046	0.968	8.10E-01	8.10E+01		
8082244	NR_002822	MGC72080	MGC72080 pseudogene	-0.008	0.994	9.80E-01	9.80E+01		
8082246	---	---	---	-0.011	0.993	9.53E-01	9.53E+01		
8082248	NR_002822	MGC72080	MGC72080 pseudogene	-0.016	0.989	9.63E-01	9.63E+01		
8082250	---	---	---	0.024	1.017	9.27E-01	9.27E+01		
8082252	---	---	---	-0.098	0.934	6.77E-01	6.77E+01		
8082254	NR_024251	LOC100125556	family with sequence similarity 86	-0.068	0.954	5.82E-01	5.82E+01		
8082289	NM_152889	CHST13	carbohydrate (chondroitin 4) sulfo	-0.056	0.962	6.06E-01	6.06E+01		
8082305	NM_032343	CHCHD6	coiled-coil-helix-coiled-coil-helix d	-0.013	0.991	9.56E-01	9.56E+01		
8082314	NM_032242	PLXNA1	plexin A1	0.037	1.026	7.73E-01	7.73E+01		
8082350	NM_004526	MCM2	minichromosome maintenance co	-0.049	0.967	7.25E-01	7.25E+01		
8082368	NM_015720	PODXL2	podocalyxin-like 2	-0.047	0.968	5.76E-01	5.76E+01		
8082380	NM_172027	ABTB1	ankyrin repeat and BTB (POZ) dom	-0.011	0.992	9.33E-01	9.33E+01		
8082406	---	---	---	0.072	1.051	6.76E-01	6.76E+01		
8082408	NM_013336	SEC61A1	Sec61 alpha 1 subunit (S. cerevisia	0.177	1.130	2.33E-01	2.33E+01		
8082422	NM_021937	EEFSEC	eukaryotic elongation factor, seler	-0.014	0.990	9.01E-01	9.01E+01		
8082431	NM_004637	RAB7A	RAB7A, member RAS oncogene fa	0.096	1.068	5.16E-01	5.16E+01		
8082442	---	---	---	0.148	1.108	6.10E-01	6.10E+01		
8082444	NM_014049	ACAD9	acyl-Coenzyme A dehydrogenase f	-0.011	0.992	9.70E-01	9.70E+01		
8082465	NM_024768	CCDC48	coiled-coil domain containing 48	-0.058	0.961	5.37E-01	5.37E+01		
8082473	NM_000174	GP9	glycoprotein IX (platelet)	-0.056	0.962	6.21E-01	6.21E+01		
8082478	NM_016128	COPG	coatomer protein complex, subun	0.003	1.002	9.90E-01	9.90E+01		
8082504	NM_00100610	C3orf37	chromosome 3 open reading fram	-0.076	0.949	6.16E-01	6.16E+01		
8082517	NR_026991	C3orf47	chromosome 3 open reading fram	0.041	1.029	8.03E-01	8.03E+01		
8082523	NM_052985	IFT122	intraflagellar transport 122 homol	0.133	1.097	2.93E-01	2.93E+01		
8082560	NM_153833	H1FOO	H1 histone family, member O, ooc	-0.022	0.985	8.93E-01	8.93E+01		
8082570	---	---	---	0.342	1.267	2.63E-01	2.63E+01		
8082574	NM_007117	TRH	thyrotropin-releasing hormone	0.037	1.026	7.85E-01	7.85E+01		
8082605	---	---	---	-0.040	0.973	8.27E-01	8.27E+01		
8082607	NM_014382	ATP2C1	ATPase, Ca++ transporting, type 2	0.027	1.019	8.87E-01	8.87E+01		
8082663	NR_027766	NUDT16P	nudix (nucleoside diphosphate lin	-0.051	0.965	6.30E-01	6.30E+01		
8082667	NM_152395	NUDT16	nudix (nucleoside diphosphate lin	0.029	1.020	8.69E-01	8.69E+01		
8082688	NM_015268	DNAJC13	DnaJ (Hsp40) homolog, subfamily	0.173	1.127	5.46E-02	5.46E+00		
8082750	NM_024818	UBA5	ubiquitin-like modifier activating e	-0.034	0.977	8.74E-01	8.74E+01		
8082774	NM_003571	BFSP2	beaded filament structural protei	0.001	1.001	9.95E-01	9.95E+01		
8082788	NM_00113442	CDV3	CDV3 homolog (mouse)	0.038	1.027	6.58E-01	6.58E+01		
8082816	NM_021203	SRPRB	signal recognition particle recepto	0.051	1.036	7.97E-01	7.97E+01		
8082827	NM_025180	CEP63	centrosomal protein 63kDa	0.091	1.065	4.81E-01	4.81E+01		
8082886	NM_000532	PCCB	propionyl Coenzyme A carboxylase	0.030	1.021	8.63E-01	8.63E+01		
8082905	NM_025246	TMEM22	transmembrane protein 22	0.041	1.029	7.09E-01	7.09E+01		
8082911	NM_006153	NCK1	NCK adaptor protein 1	0.144	1.105	1.40E-01	1.40E+01		
8082916	NM_144717	IL20RB	interleukin 20 receptor beta	0.106	1.076	5.61E-01	5.61E+01		
8082940	NM_015396	ARMC8	armadillo repeat containing 8	0.053	1.038	7.35E-01	7.35E+01		
8083000	NM_00103303	FAIM	Fas apoptotic inhibitory molecule	-0.037	0.975	8.83E-01	8.83E+01		
8083011	NM_020191	MRPS22	mitochondrial ribosomal protein S	-0.008	0.994	9.65E-01	9.65E+01		

8083030	---	---	---	0.075	1.053	6.11E-01	6.11E+01		
8083032	---	---	---	0.051	1.036	7.39E-01	7.39E+01		
8083034	NM_022131	CLSTN2	calsyntenin 2	-0.056	0.962	5.91E-01	5.91E+01		
8083063	NM_00110464	SLC25A36	solute carrier family 25, member 3	0.021	1.014	9.43E-01	9.43E+01		
8083071	NM_080862	SPSB4	splA/ryanodine receptor domain a	-0.068	0.954	5.64E-01	5.64E+01		
8083075	NM_152282	ACPL2	acid phosphatase-like 2	-0.074	0.950	5.97E-01	5.97E+01		
8083090	NM_00108041	ZBTB38	zinc finger and BTB domain contai	-0.041	0.972	8.67E-01	8.67E+01		
8083092	NM_00108041	ZBTB38	zinc finger and BTB domain contai	-0.043	0.970	8.61E-01	8.61E+01		
8083094	NM_006506	RASA2	RAS p21 protein activator 2	0.042	1.029	8.31E-01	8.31E+01		
8083119	NM_014245	RNF7	ring finger protein 7	-0.018	0.988	9.34E-01	9.34E+01		
8083136	NM_001679	ATP1B3	ATPase, Na+/K+ transporting, beta	0.085	1.061	7.61E-01	7.61E+01		
8083144	---	---	---	0.210	1.156	6.51E-01	6.51E+01		
8083164	---	---	---	0.005	1.003	9.83E-01	9.83E+01		
8083183	NM_00108041	SR140	U2-associated SR140 protein	0.040	1.028	6.87E-01	6.87E+01		
8083214	NM_004267	CHST2	carbohydrate (N-acetylglucosamin	-0.008	0.995	9.53E-01	9.53E+01		
8083221	NM_002586	PBX2	pre-B-cell leukemia homeobox 2	0.004	1.003	9.85E-01	9.85E+01		
8083223	NM_173552	C3orf58	chromosome 3 open reading fram	0.149	1.109	2.27E-01	2.27E+01		
8083272	NM_004130	GYG1	glycogenin 1	0.250	1.190	7.89E-02	7.89E+00		
8083282	NM_032383	HPS3	Hermansky-Pudlak syndrome 3	0.105	1.075	5.77E-01	5.77E+01		
8083310	NM_007282	RNF13	ring finger protein 13	0.142	1.103	5.81E-01	5.81E+01		
8083324	NM_014779	TSC22D2	TSC22 domain family, member 2	-0.074	0.950	4.31E-01	4.31E+01		
8083333	NM_032025	EIF2A	eukaryotic translation initiation fa	0.076	1.054	3.52E-01	3.52E+01		
8083352	NM_016275	SELT	selenoprotein T	0.077	1.055	4.24E-01	4.24E+01		
8083429	NM_021038	MBNL1	muscleblind-like (Drosophila)	0.080	1.057	2.25E-01	2.25E+01		
8083445	---	---	---	0.083	1.059	7.16E-01	7.16E+01		
8083457	NM_002886	RAP2B	RAP2B, member of RAS oncogene	0.254	1.193	2.25E-03	2.25E-01		
8083469	---	---	---	0.001	1.000	9.99E-01	9.99E+01		
8083523	NM_003875	GMPS	guanine monphosphate synthetas	0.069	1.049	4.85E-01	4.85E+01		
8083546	NM_003471	KCNAB1	potassium voltage-gated channel,	-0.039	0.973	7.03E-01	7.03E+01		
8083592	---	---	---	0.160	1.117	3.95E-01	3.95E+01		
8083605	NM_016625	RSRC1	arginine/serine-rich coiled-coil 1	0.095	1.068	4.83E-01	4.83E+01		
8083630	NM_024996	GFM1	G elongation factor, mitochondria	0.189	1.140	2.19E-02	2.19E+00		
8083704	NM_013263	BRD7	bromodomain containing 7	0.033	1.023	8.78E-01	8.78E+01		
8083709	NM_005496	SMC4	structural maintenance of chromo	0.000	1.000	1.00E+00	1.00E+02		
8083749	NM_139245	PPM1L	protein phosphatase 1 (formerly 2	-0.042	0.971	8.72E-01	8.72E+01		
8083757	NM_015938	NMD3	NMD3 homolog (S. cerevisiae)	0.118	1.085	3.51E-01	3.51E+01		
8083775	---	---	---	0.000	1.000	1.00E+00	1.00E+02		
8083777	ENST00000407	LOC131055	peptidylprolyl isomerase A pseudo	0.009	1.006	9.65E-01	9.65E+01		
8083792	---	---	---	0.043	1.031	7.09E-01	7.09E+01		
8083794	NM_018657	MYNN	myoneurin	-0.086	0.942	5.25E-01	5.25E+01		
8083826	NM_003262	SEC62	SEC62 homolog (S. cerevisiae)	-0.019	0.987	9.09E-01	9.09E+01		
8083850	---	---	---	0.052	1.037	8.04E-01	8.04E+01		
8083854	NM_002740	PRKCI	protein kinase C, iota	-0.112	0.925	3.45E-01	3.45E+01		
8083876	NM_005414	SKIL	SKI-like oncogene	-0.174	0.887	6.20E-02	6.20E+00		
8083887	NM_005602	CLDN11	claudin 11	-0.096	0.935	3.69E-01	3.69E+01		
8083893	---	---	---	0.014	1.010	9.71E-01	9.71E+01		
8083901	NM_022763	FNDC3B	fibronectin type III domain contair	-0.292	0.817	3.83E-01	3.83E+01		
8083933	---	---	---	-0.057	0.961	8.56E-01	8.56E+01		
8084012	---	---	---	0.050	1.035	7.44E-01	7.44E+01		
8084016	NM_006218	PIK3CA	phosphoinositide-3-kinase, catalyt	0.021	1.015	9.16E-01	9.16E+01		
8084035	NM_016331	ZNF639	zinc finger protein 639	-0.014	0.990	9.49E-01	9.49E+01		
8084045	NM_033540	MFN1	mitofusin 1	0.049	1.034	6.59E-01	6.59E+01		
8084064	NM_006636	MTHFD2	methylenetetrahydrofolate dehyd	0.211	1.157	3.30E-01	3.30E+01		
8084067	NM_178042	ACTL6A	actin-like 6A	-0.034	0.977	8.40E-01	8.40E+01		
8084092	NM_002492	NDUFB5	NADH dehydrogenase (ubiquinone	0.084	1.060	5.15E-01	5.15E+01		
8084122	---	---	---	-0.024	0.984	9.13E-01	9.13E+01		
8084128	NM_133462	TTC14	tetratricopeptide repeat domain 1	0.041	1.029	7.51E-01	7.51E+01		
8084146	NM_00101343	FXR1	fragile X mental retardation, autos	-0.044	0.970	6.25E-01	6.25E+01		
8084173	NM_014616	ATP11B	ATPase, class VI, type 11B	-0.015	0.990	9.44E-01	9.44E+01		
8084206	NM_032047	B3GNT5	UDP-GlcNAc:betaGal beta-1,3-N-a	0.632	1.550	8.11E-02	8.11E+00		
8084213	---	---	---	0.088	1.063	8.19E-01	8.19E+01		
8084215	---	---	---	0.010	1.007	9.70E-01	9.70E+01		
8084219	NM_017644	KLHL24	kelch-like 24 (Drosophila)	0.004	1.003	9.86E-01	9.86E+01		
8084232	NM_018023	YEATS2	YEATS domain containing 2	0.067	1.047	5.55E-01	5.55E+01		
8084266	NM_182537	HTR3D	5-hydroxytryptamine (serotonin) r	-0.008	0.994	9.64E-01	9.64E+01		
8084303	NM_003907	EIF2B5	eukaryotic translation initiation fa	-0.011	0.993	9.64E-01	9.64E+01		
8084323	NM_004423	DVL3	dishevelled, dsh homolog 3 (Droso	-0.132	0.913	4.71E-02	4.71E+00		
8084345	NM_004068	AP2M1	adaptor-related protein complex 2	0.068	1.048	6.40E-01	6.40E+01		
8084360	NM_018358	ABCF3	ATP-binding cassette, sub-family F	-0.008	0.995	9.63E-01	9.63E+01		
8084382	NM_138345	VWA5B2	von Willebrand factor A domain co	-0.025	0.983	8.66E-01	8.66E+01		
8084397	NM_014693	ECE2	endothelin converting enzyme 2	0.001	1.001	9.92E-01	9.92E+01		
8084423	NM_002808	PSMD2	proteasome (prosome, macropain	-0.012	0.991	9.64E-01	9.64E+01		
8084439	NM_182917	EIF4G1	eukaryotic translation initiation fa	0.006	1.004	9.70E-01	9.70E+01		
8084478	BC037299	FAM131A	family with sequence similarity 13	0.008	1.005	9.77E-01	9.77E+01		
8084488	NM_006232	POLR2H	polymerase (RNA) II (DNA directed	0.030	1.021	8.47E-01	8.47E+01		
8084496	NM_003741	CHRD	chordin	-0.047	0.968	6.97E-01	6.97E+01		
8084524	NM_004443	EPHB3	EPH receptor B3	-0.019	0.987	9.04E-01	9.04E+01		

8084541	NM_00100992	VPS8	vacuolar protein sorting 8 homolo	0.061	1.043	7.70E-01	7.70E+01		
8084607	NM_021627	SENP2	SUMO1/sentrin/SMT3 specific pep	-0.046	0.968	7.91E-01	7.91E+01		
8084630	---	---	---	-0.265	0.832	3.29E-01	3.29E+01		
8084634	NM_016306	DNAJB11	DnaJ (Hsp40) homolog, subfamily	0.230	1.173	3.53E-02	3.53E+00		
8084694	NM_001967	EIF4A2	eukaryotic translation initiation fa	-0.018	0.987	8.78E-01	8.78E+01		
8084704	AB209021	EIF4A2	eukaryotic translation initiation fa	-0.076	0.949	8.32E-01	8.32E+01		
8084708	NR_002588	SNORA4	small nucleolar RNA, H/ACA box 4	-0.114	0.924	6.76E-01	6.76E+01		
8084715	---	---	---	-0.039	0.973	8.31E-01	8.31E+01		
8084717	NM_173216	ST6GAL1	ST6 beta-galactosamide alpha-2,6-	0.124	1.090	2.79E-01	2.79E+01		
8084726	NM_153708	RTP1	receptor (chemosensory) transpor	0.028	1.020	8.48E-01	8.48E+01		
8084732	NM_022147	RTP4	receptor (chemosensory) transpor	0.130	1.095	6.71E-01	6.71E+01		
8084739	NR_024413	FLJ42393	hypothetical LOC401105	-0.107	0.928	4.45E-01	4.45E+01		
8084742	NM_005578	LPP	LIM domain containing preferred t	0.109	1.078	5.89E-01	5.89E+01		
8084757	NM_198485	TPRG1	tumor protein p63 regulated 1	0.172	1.127	4.35E-01	4.35E+01		
8084794	NM_002182	IL1RAP	interleukin 1 receptor accessory p	-0.254	0.838	1.04E-01	1.04E+01		
8084818	NM_178335	CCDC50	coiled-coil domain containing 50	0.025	1.017	9.22E-01	9.22E+01		
8084844	NM_130837	OPA1	optic atrophy 1 (autosomal domin	0.032	1.022	8.46E-01	8.46E+01		
8084878	---	---	---	0.121	1.087	8.16E-01	8.16E+01		
8084880	NM_005524	HES1	hairy and enhancer of split 1, (Dro	-0.154	0.899	3.56E-02	3.56E+00		
8084887	---	---	---	0.041	1.029	7.09E-01	7.09E+01		
8084891	NM_153690	FAM43A	family with sequence similarity 43	-0.013	0.991	9.36E-01	9.36E+01		
8084895	NM_152673	MUC20	mucin 20, cell surface associated	-0.002	0.999	9.94E-01	9.94E+01		
8084904	NR_003265	SDHALP2	succinate dehydrogenase complex	-0.095	0.936	5.79E-01	5.79E+01		
8084912	NR_003265	SDHALP2	succinate dehydrogenase complex	-0.012	0.992	9.64E-01	9.64E+01		
8084917	AK128346	LOC440993	hypothetical LOC440993	0.008	1.006	9.85E-01	9.85E+01		
8084921	AK094115	LOC100128262	hypothetical LOC100128262	-0.048	0.968	8.09E-01	8.09E+01		
8084929	NM_152672	OSTalpha	organic solute transporter alpha	-0.063	0.957	5.82E-01	5.82E+01		
8084945	---	---	---	0.017	1.012	9.50E-01	9.50E+01		
8084947	NM_00110557	FBXO45	F-box protein 45	0.137	1.099	3.66E-01	3.66E+01		
8084951	NM_198565	LRRC33	leucine rich repeat containing 33	0.077	1.055	5.47E-01	5.47E+01		
8084963	NM_002577	PAK2	p21 protein (Cdc42/Rac)-activated	0.103	1.074	3.62E-01	3.62E+01		
8084971	NM_152699	SENP5	SUMO1/sentrin specific peptidase	-0.058	0.960	7.15E-01	7.15E+01		
8084982	NR_024388	LOC152217	hypothetical LOC152217	0.089	1.063	5.81E-01	5.81E+01		
8084986	NM_032288	FYTTD1	forty-two-three domain containing	-0.005	0.996	9.74E-01	9.74E+01		
8085000	NM_032773	LRCH3	leucine-rich repeats and calponin	-0.069	0.954	3.93E-01	3.93E+01		
8085026	NM_000996	RPL35A	ribosomal protein L35a	-0.005	0.996	9.64E-01	9.64E+01		
8085052	---	---	---	0.024	1.017	9.63E-01	9.63E+01		
8085054	NM_207366	40435	septin 14	-0.030	0.979	9.19E-01	9.19E+01		
8085058	---	---	---	-0.010	0.993	9.68E-01	9.68E+01		
8085081	NM_016302	CRBN	cereblon	0.016	1.011	9.33E-01	9.33E+01		
8085094	NM_182760	SUMF1	sulfatase modifying factor 1	0.162	1.119	1.09E-01	1.09E+01		
8085112	---	---	---	-0.083	0.944	7.89E-01	7.89E+01		
8085122	AB024705	C3orf32	chromosome 3 open reading fram	-0.062	0.958	4.54E-01	4.54E+01		
8085138	NM_000916	OXTR	oxytocin receptor	-0.085	0.943	2.82E-01	2.82E+01		
8085145	NM_020165	RAD18	RAD18 homolog (S. cerevisiae)	0.006	1.004	9.85E-01	9.85E+01		
8085195	NM_198560	LHFPL4	lipoma HMGIC fusion partner-like	-0.020	0.986	8.76E-01	8.76E+01		
8085206	NM_003656	CAMK1	calcium/calmodulin-dependent pr	0.212	1.158	2.75E-02	2.75E+00		
8085220	NM_133480	TADA3L	transcriptional adaptor 3 (NGG1 h	0.026	1.018	8.84E-01	8.84E+01		
8085233	NM_173659	RPUSD3	RNA pseudouridylate synthase do	-0.043	0.971	7.26E-01	7.26E+01		
8085244	NM_022094	CIDEC	cell death-inducing DFFA-like effe	-0.049	0.967	7.43E-01	7.43E+01		
8085263	NM_018447	TMEM111	transmembrane protein 111	-0.057	0.961	8.10E-01	8.10E+01		
8085272	AK022260	LOC401052	hypothetical LOC401052	-0.018	0.987	9.20E-01	9.20E+01		
8085276	NR_002786	CIDECP	cell death-inducing DFFA-like effe	-0.131	0.913	3.49E-01	3.49E+01		
8085283	NM_173472	C3orf24	chromosome 3 open reading fram	0.045	1.032	7.45E-01	7.45E+01		
8085287	NM_018462	C3orf10	chromosome 3 open reading fram	-0.019	0.987	9.41E-01	9.41E+01		
8085293	NR_024138	GHRL	ghrelin/obestatin prepropeptide	-0.055	0.963	6.67E-01	6.67E+01		
8085300	NR_024272	SEC13	SEC13 homolog (S. cerevisiae)	0.069	1.049	6.78E-01	6.78E+01		
8085311	NM_00100133	ATP2B2	ATPase, Ca++ transporting, plasma	-0.051	0.965	7.04E-01	7.04E+01		
8085340	NM_014667	VGLL4	vestigial like 4 (Drosophila)	0.074	1.053	5.52E-01	5.52E+01		
8085350	NM_138807	C3orf31	chromosome 3 open reading fram	0.044	1.031	8.36E-01	8.36E+01		
8085358	---	---	---	-0.059	0.960	7.64E-01	7.64E+01		
8085360	NM_003256	TIMP4	TIMP metallopeptidase inhibitor 4	0.032	1.022	7.96E-01	7.96E+01		
8085370	NR_003112	GSTM1L	glutathione S-transferase mu 1-lik	0.002	1.001	9.94E-01	9.94E+01		
8085372	---	---	---	0.081	1.058	8.85E-01	8.85E+01		
8085374	NM_002880	RAF1	v-raf-1 murine leukemia viral onco	0.026	1.018	8.33E-01	8.33E+01		
8085393	NM_018306	TMEM40	transmembrane protein 40	-0.028	0.981	8.52E-01	8.52E+01		
8085407	NM_00100707	RPL32	ribosomal protein L32	-0.084	0.944	5.41E-01	5.41E+01		
8085412	NM_00113438	IQSEC1	IQ motif and Sec7 domain 1	0.134	1.097	9.03E-02	9.03E+00		
8085429	AY358240	UNQ6487	LMNE6487	-0.017	0.988	9.53E-01	9.53E+01		
8085431	NM_024923	NUP210	nucleoporin 210kDa	-0.017	0.988	9.23E-01	9.23E+01		
8085475	NM_004625	WNT7A	wingless-type MMTV integration s	-0.055	0.962	6.52E-01	6.52E+01		
8085481	NM_144636	CHCHD4	coiled-coil-helix-coiled-coil-helix d	-0.004	0.997	9.88E-01	9.88E+01		
8085486	NM_004628	XPC	xeroderma pigmentosum, comple	-0.072	0.951	5.25E-01	5.25E+01		
8085504	NM_00108042	GRIP2	glutamate receptor interacting pro	0.000	1.000	1.00E+00	1.00E+02		
8085531	NM_022497	MRPS25	mitochondrial ribosomal protein S	0.074	1.052	5.03E-01	5.03E+01		
8085537	NM_022340	ZFYVE20	zinc finger, FYVE domain containin	0.136	1.099	4.59E-01	4.59E+01		
8085554	---	---	---	-0.024	0.984	9.33E-01	9.33E+01		

8085556	NM_004844	SH3BP5	SH3-domain binding protein 5 (BT	-0.159	0.896	1.72E-01	1.72E+01		
8085579	---	---	---	0.229	1.172	8.37E-01	8.37E+01		
8085581	NM_005677	COLQ	collagen-like tail subunit (single st	0.017	1.012	9.20E-01	9.20E+01		
8085606	---	---	---	0.043	1.030	7.27E-01	7.27E+01		
8085608	NM_012260	HACL1	2-hydroxyacyl-CoA lyase 1	-0.095	0.936	5.20E-01	5.20E+01		
8085628	NM_015199	ANKRD28	ankyrin repeat domain 28	-0.005	0.997	9.86E-01	9.86E+01		
8085660	NM_206831	DPH3	DPH3, KTI11 homolog (S. cerevisia	0.059	1.042	7.37E-01	7.37E+01		
8085665	NM_015150	RFTN1	raftlin, lipid raft linker 1	-0.230	0.853	2.38E-03	2.38E-01		
8085689	NM_014744	TBC1D5	TBC1 domain family, member 5	0.143	1.104	1.55E-01	1.55E+01		
8085714	---	---	---	0.224	1.168	1.57E-01	1.57E+01		
8085716	NM_002971	SATB1	SATB homeobox 1	-0.097	0.935	4.20E-01	4.20E+01		
8085770	---	---	---	0.077	1.055	7.05E-01	7.05E+01		
8085815	NM_001068	TOP2B	topoisomerase (DNA) II beta 180k	-0.015	0.990	9.36E-01	9.36E+01		
8085852	NM_018297	NGLY1	N-glycanase 1	0.093	1.066	2.96E-01	2.96E+01		
8085914	NM_003615	SLC4A7	solute carrier family 4, sodium bic	0.186	1.137	1.75E-01	1.75E+01		
8085946	NM_005442	EOMES	eomesodermin homolog (Xenopus	-0.038	0.974	9.11E-01	9.11E+01		
8085984	NM_017784	OSBPL10	oxysterol binding protein-like 10	0.146	1.107	2.70E-01	2.70E+01		
8085999	NM_017801	CMTM6	CKLF-like MARVEL transmembrane	0.168	1.124	1.61E-01	1.61E+01		
8086008	NM_016141	DYNC1LI1	dynein, cytoplasmic 1, light interm	0.069	1.049	7.47E-01	7.47E+01		
8086048	NM_00103977	TMPPE	transmembrane protein with meta	0.029	1.020	8.74E-01	8.74E+01		
8086051	NM_015551	SUSD5	sushi domain containing 5	-0.049	0.966	7.31E-01	7.31E+01		
8086057	NM_014517	UBP1	upstream binding protein 1 (LBP-1	0.019	1.013	8.74E-01	8.74E+01		
8086077	NM_015097	CLASP2	cytoplasmic linker associated prot	0.048	1.034	6.99E-01	6.99E+01		
8086125	NM_014831	LBA1	lupus brain antigen 1	-0.125	0.917	2.82E-01	2.82E+01		
8086141	NM_014805	EPM2AIP1	EPM2A (laforin) interacting protei	-0.073	0.950	6.05E-01	6.05E+01		
8086148	NM_000992	RPL29	ribosomal protein L29	-0.045	0.969	8.38E-01	8.38E+01		
8086150	NM_006309	LRRFIP2	leucine rich repeat (in FLII) interac	-0.058	0.961	7.63E-01	7.63E+01		
8086185	NM_006225	PLCD1	phospholipase C, delta 1	-0.065	0.956	6.11E-01	6.11E+01		
8086201	NM_001607	ACAA1	acetyl-Coenzyme A acyltransferase	0.079	1.056	4.75E-01	4.75E+01		
8086317	NM_031899	GORASP1	golgi reassembly stacking protein	0.031	1.021	7.38E-01	7.38E+01		
8086330	NM_033027	CSRNP1	cysteine-serine-rich nuclear protei	-0.173	0.887	1.05E-02	1.05E+00		
8086341	NM_194293	XIRP1	xin actin-binding repeat containing	-0.002	0.998	9.88E-01	9.88E+01		
8086352	NM_017886	ULK4	unc-51-like kinase 4 (C. elegans)	-0.051	0.966	7.73E-01	7.73E+01		
8086391	NM_000729	CCK	cholecystokinin	0.001	1.001	9.95E-01	9.95E+01		
8086406	NM_004206	SEC22C	SEC22 vesicle trafficking protein h	-0.061	0.959	7.99E-01	7.99E+01		
8086417	---	---	---	-0.012	0.992	9.53E-01	9.53E+01		
8086419	NR_027753	HHATL	hedgehog acyltransferase-like	-0.083	0.944	4.97E-01	4.97E+01		
8086434	NM_144719	CCDC13	coiled-coil domain containing 13	-0.012	0.992	9.28E-01	9.28E+01		
8086451	NM_00109966	HIGD1A	HIG1 hypoxia inducible domain fa	0.001	1.001	9.98E-01	9.98E+01		
8086462	NM_032806	C3orf39	chromosome 3 open reading fram	-0.017	0.988	9.28E-01	9.28E+01		
8086467	NM_018075	ANO10	anoctamin 10	0.086	1.061	7.51E-01	7.51E+01		
8086482	NM_181489	ZNF445	zinc finger protein 445	-0.019	0.987	9.21E-01	9.21E+01		
8086498	NM_020696	KIAA1143	KIAA1143	0.020	1.014	9.43E-01	9.43E+01		
8086503	NM_00113517	ZDHHC3	zinc finger, DHHC-type containing	-0.062	0.958	7.63E-01	7.63E+01		
8086505	NM_016598	ZDHHC3	zinc finger, DHHC-type containing	-0.065	0.956	6.82E-01	6.82E+01		
8086517	NM_022842	CDCP1	CUB domain containing protein 1	0.357	1.281	2.08E-01	2.08E+01		
8086530	NM_015444	TMEM158	transmembrane protein 158	0.076	1.054	2.81E-01	2.81E+01		
8086538	BC047037	LOC644714	hypothetical protein LOC644714	0.198	1.147	5.67E-01	5.67E+01		
8086540	NM_020208	SLC6A20	solute carrier family 6 (proline IMI	-0.031	0.979	8.40E-01	8.40E+01		
8086572	NM_024513	FYCO1	FYVE and coiled-coil domain conta	-0.084	0.944	4.57E-01	4.57E+01		
8086595	NM_005283	XCR1	chemokine (C motif) receptor 1	0.176	1.130	8.24E-02	8.24E+00		
8086607	NM_002343	LTF	lactotransferrin	0.001	1.001	9.98E-01	9.98E+01		
8086627	NM_147129	ALS2CL	ALS2 C-terminal like	-0.070	0.953	4.83E-01	4.83E+01		
8086669	NM_199183	TESSP5	testis serine protease 5	-0.055	0.963	4.49E-01	4.49E+01		
8086683	NM_182702	TESSP2	testis serine protease 2	-0.030	0.980	7.73E-01	7.73E+01		
8086689	NM_000258	MYL3	myosin, light chain 3, alkali; ventri	0.021	1.015	9.18E-01	9.18E+01		
8086698	NM_144716	CCDC12	coiled-coil domain containing 12	-0.071	0.952	6.62E-01	6.62E+01		
8086706	NM_014159	SETD2	SET domain containing 2	-0.106	0.929	1.57E-01	1.57E+01		
8086752	NR_003041	SNORD13	small nucleolar RNA, C/D box 13	0.309	1.239	4.13E-01	4.13E+01		
8086754	NM_012235	SCAP	SREBF chaperone	0.001	1.001	9.96E-01	9.96E+01		
8086810	NM_003074	SMARCC1	SWI/SNF related, matrix associate	0.004	1.003	9.80E-01	9.80E+01		
8086842	NM_002375	MAP4	microtubule-associated protein 4	0.010	1.007	9.53E-01	9.53E+01		
8086876	---	---	---	-0.051	0.965	8.81E-01	8.81E+01		
8086908	NM_00113008	PLXNB1	plexin B1	-0.011	0.993	9.23E-01	9.23E+01		
8086949	NM_024661	CCDC51	coiled-coil domain containing 51	0.082	1.058	5.35E-01	5.35E+01		
8086953	NM_016479	SHISA5	shisa homolog 5 (Xenopus laevis)	0.141	1.103	1.62E-01	1.62E+01		
8086981	NM_033199	UCN2	urocortin 2	0.038	1.026	8.43E-01	8.43E+01		
8086985	NM_000094	COL7A1	collagen, type VII, alpha 1	-0.081	0.945	1.71E-01	1.71E+01		
8087100	NM_003365	UQCRC1	ubiquinol-cytochrome c reductase	0.203	1.151	1.15E-02	1.15E+00		
8087116	NM_00100826	TMEM89	transmembrane protein 89	-0.051	0.966	6.60E-01	6.60E+01		
8087119	NM_022911	SLC26A6	solute carrier family 26, member 6	-0.057	0.961	5.97E-01	5.97E+01		
8087145	NM_001407	CELSR3	cadherin, EGF LAG seven-pass G-ty	0.004	1.002	9.85E-01	9.85E+01		
8087182	NM_016453	NCKIPSD	NCK interacting protein with SH3 c	0.001	1.001	9.94E-01	9.94E+01		
8087201	NM_016291	IP6K2	inositol hexakisphosphate kinase 2	-0.087	0.942	4.47E-01	4.47E+01		
8087210	NM_004157	PRKAR2A	protein kinase, cAMP-dependent,	-0.062	0.958	6.79E-01	6.79E+01		
8087224	NM_000387	SLC25A20	solute carrier family 25 (carnitine/	0.038	1.026	8.71E-01	8.71E+01		
8087234	NM_006321	ARIH2	ariadne homolog 2 (Drosophila)	-0.150	0.901	1.25E-01	1.25E+01		

8087236	NM_00100999	DALRD3	DALR anticodon binding domain c	-0.069	0.954	3.04E-01	3.04E+01		
8087250	---	---	---	-0.137	0.910	4.27E-01	4.27E+01		
8087252	---	---	---	-0.017	0.988	9.19E-01	9.19E+01		
8087254	NM_000884	IMPDH2	IMP (inosine monophosphate) deh	-0.073	0.951	7.52E-01	7.52E+01		
8087271	NM_017730	QRICH1	glutamine-rich 1	-0.024	0.984	8.94E-01	8.94E+01		
8087283	NM_005051	QARS	glutaminyl-tRNA synthetase	-0.050	0.966	6.57E-01	6.57E+01		
8087308	NM_006677	USP19	ubiquitin specific peptidase 19	0.020	1.014	8.97E-01	8.97E+01		
8087337	NM_002292	LAMB2	laminin, beta 2 (laminin S)	-0.027	0.981	7.88E-01	7.88E+01		
8087372	NM_022903	CCDC71	coiled-coil domain containing 71	0.039	1.027	8.10E-01	8.10E+01		
8087374	NM_198562	C3orf62	chromosome 3 open reading fram	0.021	1.015	9.35E-01	9.35E+01		
8087380	NM_003363	USP4	ubiquitin specific peptidase 4 (pro	0.036	1.025	8.45E-01	8.45E+01		
8087405	NM_201397	GPX1	glutathione peroxidase 1	-0.036	0.975	8.34E-01	8.34E+01		
8087409	NM_001664	RHOA	ras homolog gene family, member	0.087	1.062	4.78E-01	4.78E+01		
8087419	NM_000481	AMT	aminomethyltransferase	-0.020	0.986	9.24E-01	9.24E+01		
8087433	NM_032316	NICN1	nicolin 1	0.058	1.041	5.38E-01	5.38E+01		
8087447	NM_020998	MST1	macrophage stimulating 1 (hepato	0.157	1.115	1.78E-01	1.78E+01		
8087453	NM_198722	AMIGO3	adhesion molecule with Ig-like do	0.016	1.011	9.02E-01	9.02E+01		
8087461	NM_013334	GMPPB	GDP-mannose pyrophosphorylase	-0.050	0.966	6.37E-01	6.37E+01		
8087473	NM_153273	IP6K1	inositol hexakisphosphate kinase 1	-0.082	0.945	4.51E-01	4.51E+01		
8087481	NM_00100754	CDH29	cadherin-like 29	0.017	1.012	9.16E-01	9.16E+01		
8087485	NM_003335	UBA7	ubiquitin-like modifier activating e	0.056	1.040	6.77E-01	6.77E+01		
8087513	NM_005879	TRAIP	TRAF interacting protein	0.011	1.008	9.53E-01	9.53E+01		
8087576	NM_006764	IFRD2	interferon-related developmental	-0.017	0.988	8.98E-01	8.98E+01		
8087596	NM_003549	HYAL3	hyaluronoglucosaminidase 3	-0.077	0.948	2.02E-01	2.02E+01		
8087611	NM_007312	HYAL1	hyaluronoglucosaminidase 1	-0.096	0.936	1.13E-01	1.13E+01		
8087624	NM_033158	HYAL2	hyaluronoglucosaminidase 2	-0.047	0.968	6.66E-01	6.66E+01		
8087634	NM_007275	TUSC2	tumor suppressor candidate 2	-0.012	0.992	9.49E-01	9.49E+01		
8087640	NM_007182	RASSF1	Ras association (RalGDS/AF-6) do	-0.017	0.989	9.27E-01	9.27E+01		
8087653	NM_015896	ZMYND10	zinc finger, MYND-type containing	-0.079	0.946	2.93E-01	2.93E+01		
8087669	NM_006545	TUSC4	tumor suppressor candidate 4	-0.075	0.950	6.30E-01	6.30E+01		
8087685	NM_007024	TMEM115	transmembrane protein 115	-0.149	0.902	1.36E-02	1.36E+00		
8087691	NM_00100550	CACNA2D2	calcium channel, voltage-depende	0.013	1.009	9.35E-01	9.35E+01		
8087731	BC034766	C3orf18	chromosome 3 open reading fram	-0.011	0.992	9.57E-01	9.57E+01		
8087739	NM_013324	CISH	cytokine inducible SH2-containing	-0.060	0.960	5.48E-01	5.48E+01		
8087746	---	---	---	0.016	1.011	9.53E-01	9.53E+01		
8087748	NM_014703	VPRBP	Vpr (HIV-1) binding protein	-0.012	0.992	9.50E-01	9.50E+01		
8087780	---	---	---	-0.143	0.906	2.32E-01	2.32E+01		
8087790	NM_004704	RRP9	ribosomal RNA processing 9, small	-0.049	0.966	6.92E-01	6.92E+01		
8087806	NM_020418	PCBP4	poly(rC) binding protein 4	-0.056	0.962	4.77E-01	4.77E+01		
8087825	NM_032750	ABHD14B	abhydrolase domain containing 14	-0.009	0.994	9.61E-01	9.61E+01		
8087830	NM_000992	RPL29	ribosomal protein L29	-0.046	0.969	8.07E-01	8.07E+01		
8087833	NM_001947	DUSP7	dual specificity phosphatase 7	0.010	1.007	9.53E-01	9.53E+01		
8087852	NM_017442	TLR9	toll-like receptor 9	0.008	1.005	9.67E-01	9.67E+01		
8087860	NM_007284	TWF2	twinfilin, actin-binding protein, ho	0.040	1.028	7.63E-01	7.63E+01		
8087874	NM_025222	WDR82	WD repeat domain 82	0.030	1.021	8.68E-01	8.68E+01		
8087881	---	---	---	-0.004	0.997	9.93E-01	9.93E+01		
8087885	NM_004656	BAP1	BRCA1 associated protein-1 (ubiqu	0.016	1.011	9.16E-01	9.16E+01		
8087907	NM_020163	SEMA3G	sema domain, immunoglobulin do	-0.038	0.974	7.01E-01	7.01E+01		
8087925	NM_003280	TNNC1	troponin C type 1 (slow)	-0.005	0.996	9.84E-01	9.84E+01		
8087935	NM_022908	NT5DC2	5'-nucleotidase domain containing	0.056	1.039	5.47E-01	5.47E+01		
8087951	NM_018165	PBRM1	polybromo 1	0.103	1.074	2.12E-01	2.12E+01		
8087985	NM_00101098	GLT8D1	glycosyltransferase 8 domain cont	-0.050	0.966	7.40E-01	7.40E+01		
8088001	NM_003157	NEK4	NIMA (never in mitosis gene a)-rel	-0.128	0.915	4.21E-01	4.21E+01		
8088020	NM_002218	ITIH4	inter-alpha (globulin) inhibitor H4	-0.049	0.966	7.04E-01	7.04E+01		
8088047	NM_205853	MUSTN1	musculoskeletal, embryonic nucle	-0.128	0.915	3.09E-01	3.09E+01		
8088054	NM_198563	TMEM110	transmembrane protein 110	-0.024	0.984	9.00E-01	9.00E+01		
8088065	NM_00100515	SFMBT1	Scm-like with four mbt domains 1	0.033	1.023	8.89E-01	8.89E+01		
8088090	NM_052859	RFT1	RFT1 homolog (S. cerevisiae)	-0.056	0.962	8.33E-01	8.33E+01		
8088092	NM_052859	RFT1	RFT1 homolog (S. cerevisiae)	0.071	1.050	6.22E-01	6.22E+01		
8088106	NM_00113505	TKT	transketolase	-0.017	0.988	9.49E-01	9.49E+01		
8088126	---	---	---	0.020	1.014	9.73E-01	9.73E+01		
8088128	NM_018403	DCP1A	DCP1 decapping enzyme homolog	-0.011	0.993	9.58E-01	9.58E+01		
8088142	NM_018397	CHDH	choline dehydrogenase	-0.046	0.968	7.94E-01	7.94E+01		
8088151	NM_022899	ACTR8	ARP8 actin-related protein 8 homo	0.037	1.026	8.23E-01	8.23E+01		
8088167	NM_021237	SELK	selenoprotein K	-0.038	0.974	8.10E-01	8.10E+01		
8088172	---	---	---	-0.007	0.995	9.65E-01	9.65E+01		
8088219	NM_00111273	C3orf63	chromosome 3 open reading fram	0.088	1.063	1.30E-01	1.30E+01		
8088247	NM_00112861	ARHGEF3	Rho guanine nucleotide exchange	0.094	1.068	3.67E-01	3.67E+01		
8088292	NM_130387	ASB14	ankyrin repeat and SOCS box-cont	0.061	1.043	7.57E-01	7.57E+01		
8088339	NM_001660	ARF4	ADP-ribosylation factor 4	0.027	1.019	9.00E-01	9.00E+01		
8088348	NM_152678	FAM116A	family with sequence similarity 11	0.098	1.070	1.94E-01	1.94E+01		
8088369	---	---	---	-0.048	0.968	6.51E-01	6.51E+01		
8088384	NM_000925	PDHB	pyruvate dehydrogenase (lipoamid	0.025	1.017	8.95E-01	8.95E+01		
8088415	NM_007177	FAM107A	family with sequence similarity 10	-0.120	0.920	6.47E-02	6.47E+00		
8088425	NM_138805	FAM3D	family with sequence similarity 3,	-0.106	0.929	1.00E-01	1.00E+01		
8088458	NM_002012	FHIT	fragile histidine triad gene	0.118	1.085	5.93E-01	5.93E+01		
8088468	AF134979	NPCDR1	nasopharyngeal carcinoma, down-	0.049	1.035	9.27E-01	9.27E+01		

8088474	---	---	---	-0.098	0.934	6.23E-01	6.23E+01		
8088478	---	---	---	-0.020	0.986	9.27E-01	9.27E+01		
8088480	NR_026582	ID2B	inhibitor of DNA binding 2B, domi	0.004	1.003	9.75E-01	9.75E+01		
8088526	NM_025075	THOC7	THO complex 7 homolog (Drosoph	0.035	1.025	8.72E-01	8.72E+01		
8088535	NM_014814	PSMD6	proteasome (prosome, macropain	-0.047	0.968	7.38E-01	7.38E+01		
8088642	NM_015541	LRIG1	leucine-rich repeats and immunog	-0.126	0.917	2.49E-01	2.49E+01		
8088664	NM_003848	SUCLG2	succinate-CoA ligase, GDP-forming	0.092	1.066	6.38E-01	6.38E+01		
8088680	NM_173654	C3orf64	chromosome 3 open reading fram	-0.082	0.945	6.98E-01	6.98E+01		
8088700	NM_007114	TMF1	TATA element modulatory factor 1	-0.053	0.964	6.57E-01	6.57E+01		
8088718	NM_003968	UBA3	ubiquitin-like modifier activating e	0.040	1.028	8.29E-01	8.29E+01		
8088768	---	---	---	0.653	1.573	5.45E-02	5.45E+00		
8088776	NM_032682	FOXP1	forkhead box P1	-0.014	0.990	9.54E-01	9.54E+01		
8088803	NM_173359	EIF4E3	eukaryotic translation initiation fa	-0.034	0.977	8.78E-01	8.78E+01		
8088813	NM_00112612	PROK2	prokineticin 2	-0.060	0.959	8.05E-01	8.05E+01		
8088820	NM_012234	RYBP	RING1 and YY1 binding protein	-0.101	0.932	3.90E-01	3.90E+01		
8088830	NM_018130	SHQ1	SHQ1 homolog (S. cerevisiae)	0.154	1.113	1.67E-01	1.67E+01		
8088846	---	---	---	0.073	1.052	5.01E-01	5.01E+01		
8088889	---	---	---	-0.003	0.998	9.87E-01	9.87E+01		
8088893	---	---	---	0.018	1.013	9.37E-01	9.37E+01		
8088895	NR_024241	FAM86D	family with sequence similarity 86	0.089	1.063	5.43E-01	5.43E+01		
8088905	ENST00000442	LOC100287293	similar to hCG2036843	-0.013	0.991	9.53E-01	9.53E+01		
8088911	NM_00112822	ZNF717	zinc finger protein 717	0.004	1.003	9.90E-01	9.90E+01		
8088919	NM_133631	ROBO1	roundabout, axon guidance recept	-0.202	0.870	8.23E-02	8.23E+00		
8088958	NM_000158	GBE1	glucan (1,4-alpha-), branching enz	-0.039	0.973	9.01E-01	9.01E+01		
8089000	NM_00100839	CGGBP1	CGG triplet repeat binding protein	0.039	1.027	7.53E-01	7.53E+01		
8089029	NM_176815	DHFRL1	dihydrofolate reductase-like 1	0.067	1.047	5.98E-01	5.98E+01		
8089036	---	---	---	0.001	1.000	9.99E-01	9.99E+01		
8089038	---	---	---	-0.089	0.940	6.11E-01	6.11E+01		
8089040	NM_00104253	MINA	MYC induced nuclear antigen	0.118	1.086	4.38E-01	4.38E+01		
8089062	NM_019895	CLDND1	claudin domain containing 1	-0.276	0.826	5.83E-04	5.83E-02		
8089072	NM_000097	CPOX	coproporphyrinogen oxidase	-0.206	0.867	1.59E-02	1.59E+00		
8089082	NM_080927	DCBLD2	discoidin, CUB and LCCL domain co	-0.055	0.962	8.50E-01	8.50E+01		
8089112	NM_182909	FILIP1L	filamin A interacting protein 1-like	0.070	1.050	7.61E-01	7.61E+01		
8089128	NM_014820	TOMM70A	translocase of outer mitochondria	0.066	1.047	5.48E-01	5.48E+01		
8089203	NM_020654	SENP7	SUMO1/sentrin specific peptidase	0.004	1.003	9.87E-01	9.87E+01		
8089230	---	---	---	-0.007	0.995	9.76E-01	9.76E+01		
8089234	NM_014415	ZBTB11	zinc finger and BTB domain contai	-0.036	0.975	8.01E-01	8.01E+01		
8089247	---	---	---	-0.058	0.960	9.64E-01	9.64E+01		
8089249	NM_000986	RPL24	ribosomal protein L24	-0.005	0.997	9.81E-01	9.81E+01		
8089261	NM_170662	CBLB	Cas-Br-M (murine) ecotropic retro	0.069	1.049	6.34E-01	6.34E+01		
8089285	---	---	---	0.147	1.108	5.55E-01	5.55E+01		
8089295	---	---	---	0.066	1.047	7.85E-01	7.85E+01		
8089299	NM_001777	CD47	CD47 molecule	0.181	1.134	5.85E-03	5.85E-01		
8089314	NM_018010	IFT57	intraflagellar transport 57 homolo	0.029	1.020	9.05E-01	9.05E+01		
8089467	NM_024508	ZBED2	zinc finger, BED-type containing 2	0.015	1.011	9.68E-01	9.68E+01		
8089478	NM_00100875	GCET2	germinal center expressed transcr	0.092	1.066	6.36E-01	6.36E+01		
8089527	NM_022488	ATG3	ATG3 autophagy related 3 homolo	0.275	1.210	5.83E-03	5.83E-01		
8089584	NM_015412	C3orf17	chromosome 3 open reading fram	-0.054	0.963	6.51E-01	6.51E+01		
8089596	ENST00000393	WDR52	WD repeat domain 52	-0.137	0.909	6.08E-01	6.08E+01		
8089647	NM_00100989	KIAA2018	KIAA2018	0.031	1.022	8.55E-01	8.55E+01		
8089652	NM_025146	NAT13	N-acetyltransferase 13 (GCN5-rela	0.051	1.036	7.08E-01	7.08E+01		
8089659	AK302488	KIAA1407	KIAA1407	-0.020	0.986	9.28E-01	9.28E+01		
8089677	---	---	---	0.021	1.015	9.50E-01	9.50E+01		
8089694	NM_007136	ZNF80	zinc finger protein 80	0.032	1.022	9.48E-01	9.48E+01		
8089701	NM_015642	ZBTB20	zinc finger and BTB domain contai	-0.184	0.881	5.26E-02	5.26E+00		
8089723	---	---	---	-0.086	0.942	4.97E-01	4.97E+01		
8089743	NM_212543	B4GALT4	UDP-Gal:betaGlcNAc beta 1,4- gal	0.030	1.021	9.33E-01	9.33E+01		
8089759	NM_018266	TMEM39A	transmembrane protein 39A	-0.028	0.980	8.96E-01	8.96E+01		
8089771	NM_005191	CD80	CD80 molecule	-0.162	0.894	6.97E-01	6.97E+01		
8089795	NM_005694	COX17	COX17 cytochrome c oxidase asse	-0.075	0.950	6.94E-01	6.94E+01		
8089801	NM_002093	GSK3B	glycogen synthase kinase 3 beta	0.005	1.004	9.83E-01	9.83E+01		
8089830	NM_00109967	LRRC58	leucine rich repeat containing 58	0.029	1.020	8.71E-01	8.71E+01		
8089849	---	---	---	-0.017	0.988	9.53E-01	9.53E+01		
8089867	NM_173825	RABL3	RAB, member of RAS oncogene fa	0.098	1.071	5.13E-01	5.13E+01		
8089911	NM_005335	HCLS1	hematopoietic cell-specific Lyn sub	0.079	1.056	3.81E-01	3.81E+01		
8089926	---	---	---	0.103	1.074	5.90E-01	5.90E+01		
8089928	---	---	---	0.087	1.062	7.61E-01	7.61E+01		
8089930	NM_004487	GOLGB1	golgin B1, golgi integral membran	-0.164	0.893	5.07E-02	5.07E+00		
8089954	NM_00102357	IQCB1	IQ motif containing B1	0.087	1.062	5.29E-01	5.29E+01		
8089970	NM_175924	ILDR1	immunoglobulin-like domain conta	0.092	1.066	2.24E-01	2.24E+01		
8089988	NM_00101792	CCDC58	coiled-coil domain containing 58	-0.045	0.969	8.52E-01	8.52E+01		
8089993	NM_019069	WDR5B	WD repeat domain 5B	-0.003	0.998	9.90E-01	9.90E+01		
8089999	NR_026698	KPNA1	karyopherin alpha 1 (importin alph	-0.055	0.963	6.93E-01	6.93E+01		
8090018	NM_031458	PARP9	poly (ADP-ribose) polymerase fam	0.151	1.111	4.67E-01	4.67E+01		
8090030	NM_024610	HSPBAP1	HSPB (heat shock 27kDa) associate	0.162	1.119	1.28E-01	1.28E+01		
8090044	NM_00103170	SEMA5B	sema domain, seven thrombospor	0.015	1.011	9.17E-01	9.17E+01		
8090070	NM_183357	ADCY5	adenylate cyclase 5	-0.049	0.966	7.40E-01	7.40E+01		

8090091	NM_198402	PTPLB	protein tyrosine phosphatase-like	0.102	1.073	4.25E-01	4.25E+01		
8090098	NM_053025	MYLK	myosin light chain kinase	-0.076	0.949	2.76E-01	2.76E+01		
8090133	NM_022757	CCDC14	coiled-coil domain containing 14	0.099	1.071	5.28E-01	5.28E+01		
8090162	NM_002213	ITGB5	integrin, beta 5	-0.023	0.984	8.63E-01	8.63E+01		
8090193	NM_020733	HEG1	HEG homolog 1 (zebrafish)	0.032	1.023	9.16E-01	9.16E+01		
8090214	NM_024628	SLC12A8	solute carrier family 12 (potassium	0.012	1.008	9.57E-01	9.57E+01		
8090237	NM_021964	ZNF148	zinc finger protein 148	0.051	1.036	7.05E-01	7.05E+01		
8090256	NM_003794	SNX4	sorting nexin 4	0.020	1.014	9.44E-01	9.44E+01		
8090277	NM_022776	OSBPL11	oxysterol binding protein-like 11	0.250	1.190	1.51E-01	1.51E+01		
8090295	NM_017836	SLC41A3	solute carrier family 41, member 3	-0.092	0.938	4.39E-01	4.39E+01		
8090314	NM_012190	ALDH1L1	aldehyde dehydrogenase 1 family,	-0.039	0.974	7.88E-01	7.88E+01		
8090343	NM_014079	KLF15	Kruppel-like factor 15	0.012	1.008	9.52E-01	9.52E+01		
8090349	---	---	---	-0.041	0.972	7.83E-01	7.83E+01		
8090351	NM_025112	ZXDC	ZXD family zinc finger C	-0.086	0.942	2.81E-01	2.81E+01		
8090364	AK093796	ZXDC	ZXD family zinc finger C	-0.024	0.984	9.19E-01	9.19E+01		
8090366	NM_144639	UROC1	urocanase domain containing 1	0.055	1.039	5.14E-01	5.14E+01		
8090388	BC032025	C3orf22	chromosome 3 open reading fram	-0.013	0.991	9.50E-01	9.50E+01		
8090420	NM_016372	TPRA1	transmembrane protein, adipocyt	-0.008	0.994	9.49E-01	9.49E+01		
8090433	NM_007283	MGLL	monoglyceride lipase	0.232	1.174	1.66E-01	1.66E+01		
8090448	NM_003707	RUVBL1	RuvB-like 1 (E. coli)	0.034	1.024	8.77E-01	8.77E+01		
8090462	---	---	---	0.043	1.030	7.39E-01	7.39E+01		
8090464	NM_153330	DNAJB8	DnaJ (Hsp40) homolog, subfamily	-0.055	0.963	5.91E-01	5.91E+01		
8090469	NM_032638	GATA2	GATA binding protein 2	-0.033	0.977	8.25E-01	8.25E+01		
8090485	NM_007354	C3orf27	chromosome 3 open reading fram	-0.016	0.989	8.85E-01	8.85E+01		
8090490	NM_002950	RPN1	ribophorin I	0.140	1.102	3.04E-01	3.04E+01		
8090503	---	---	---	0.016	1.011	9.49E-01	9.49E+01		
8090505	---	---	---	0.003	1.002	9.82E-01	9.82E+01		
8090507	---	---	---	-0.006	0.996	9.90E-01	9.90E+01		
8090509	NM_004637	RAB7A	RAB7A, member RAS oncogene fa	0.069	1.049	7.38E-01	7.38E+01		
8090533	NM_020701	ISY1	ISY1 splicing factor homolog (S. ce	-0.109	0.927	4.32E-01	4.32E+01		
8090546	NM_00112719	CNBP	CCHC-type zinc finger, nucleic acid	0.045	1.032	4.54E-01	4.54E+01		
8090555	NM_006026	H1FX	H1 histone family, member X	-0.048	0.967	7.09E-01	7.09E+01		
8090559	NR_003111	RPL32P3	ribosomal protein L32 pseudogene	-0.040	0.973	8.20E-01	8.20E+01		
8090565	NR_002992	SNORA7B	small nucleolar RNA, H/ACA box 7	-0.016	0.989	9.67E-01	9.67E+01		
8090577	NM_003925	MBD4	methyl-CpG binding domain prote	0.058	1.041	7.42E-01	7.42E+01		
8090591	NM_015103	PLXND1	plexin D1	0.113	1.081	3.46E-01	3.46E+01		
8090630	NM_00101739	TMCC1	transmembrane and coiled-coil do	0.046	1.033	8.30E-01	8.30E+01		
8090637	---	---	---	0.072	1.051	6.16E-01	6.16E+01		
8090639	NM_014602	PIK3R4	phosphoinositide-3-kinase, regula	-0.046	0.968	7.95E-01	7.95E+01		
8090662	---	---	---	0.002	1.001	9.97E-01	9.97E+01		
8090678	NM_007208	MRPL3	mitochondrial ribosomal protein L	0.031	1.022	8.47E-01	8.47E+01		
8090737	NM_153240	NPHP3	nephronophthisis 3 (adolescent)	-0.006	0.996	9.83E-01	9.83E+01		
8090770	---	---	---	0.010	1.007	9.53E-01	9.53E+01		
8090772	NM_007027	TOPBP1	topoisomerase (DNA) II binding pr	0.036	1.026	8.23E-01	8.23E+01		
8090803	NM_016577	RAB6B	RAB6B, member RAS oncogene fa	-0.013	0.991	9.17E-01	9.17E+01		
8090823	NM_005630	SLCO2A1	solute carrier organic anion transp	-0.018	0.987	8.84E-01	8.84E+01		
8090840	NM_00100586	RYK	RYK receptor-like tyrosine kinase	0.033	1.023	8.53E-01	8.53E+01		
8090852	NM_016201	AMOTL2	angiomotin like 2	-0.075	0.949	4.12E-01	4.12E+01		
8090866	NR_024400	ANAPC13	anaphase promoting complex sub	0.130	1.094	3.19E-01	3.19E+01		
8090872	NM_178554	KY	kyphoscoliosis peptidase	0.061	1.043	5.54E-01	5.54E+01		
8090891	NM_004441	EPHB1	EPH receptor B1	-0.038	0.974	7.71E-01	7.71E+01		
8090893	NM_018133	MSL2	male-specific lethal 2 homolog (Dr	-0.090	0.940	5.56E-01	5.56E+01		
8090898	NM_005862	STAG1	stromal antigen 1	0.012	1.008	9.50E-01	9.50E+01		
8090934	---	---	---	-0.148	0.902	6.66E-01	6.66E+01		
8090936	---	---	---	-0.078	0.948	3.03E-01	3.03E+01		
8090938	NM_173543	DZIP1L	DAZ interacting protein 1-like	0.006	1.004	9.80E-01	9.80E+01		
8090960	NM_016216	DBR1	debranching enzyme homolog 1 (S	0.127	1.092	2.37E-01	2.37E+01		
8090972	NM_178130	TXNDC6	thioredoxin domain containing 6	0.009	1.006	9.56E-01	9.56E+01		
8091009	NM_006219	PIK3CB	phosphoinositide-3-kinase, catalyt	0.046	1.033	8.82E-01	8.82E+01		
8091037	NM_00113465	PRR23A	proline rich 23A	-0.081	0.946	5.77E-01	5.77E+01		
8091048	NR_023350	COPB2	coatomer protein complex, subun	0.060	1.042	6.83E-01	6.83E+01		
8091071	NM_004164	RBP2	retinol binding protein 2, cellular	-0.028	0.981	9.00E-01	9.00E+01		
8091078	NM_002899	RBP1	retinol binding protein 1, cellular	0.183	1.135	1.13E-01	1.13E+01		
8091087	NM_178177	NMNAT3	nicotinamide nucleotide adenylylt	0.006	1.004	9.74E-01	9.74E+01		
8091095	---	---	---	-0.045	0.969	7.95E-01	7.95E+01		
8091103	NM_006286	TFDP2	transcription factor Dp-2 (E2F dim	0.017	1.012	9.56E-01	9.56E+01		
8091118	---	---	---	0.096	1.069	6.66E-01	6.66E+01		
8091120	NM_00103954	GK5	glycerol kinase 5 (putative)	-0.057	0.961	8.62E-01	8.62E+01		
8091141	NM_019001	XRN1	5'-3' exoribonuclease 1	0.047	1.033	7.51E-01	7.51E+01		
8091190	NM_001184	ATR	ataxia telangiectasia and Rad3 rela	0.017	1.012	9.29E-01	9.29E+01		
8091241	---	---	---	-0.120	0.920	7.92E-01	7.92E+01		
8091260	NM_173653	SLC9A9	solute carrier family 9 (sodium/hy	0.120	1.086	3.96E-01	3.96E+01		
8091327	NM_021105	PLSCR1	phospholipid scramblase 1	0.349	1.274	3.60E-01	3.60E+01		
8091352	NM_020131	UBQLN4	ubiquilin 4	-0.018	0.988	9.58E-01	9.58E+01		
8091432	NM_016094	COMMD2	COMM domain containing 2	-0.047	0.968	8.67E-01	8.67E+01		
8091444	---	---	---	0.098	1.071	6.77E-01	6.77E+01		
8091452	NM_00107980	TMEM183B	transmembrane protein 183B	0.001	1.001	9.94E-01	9.94E+01		

8091458	NM_014445	SERP1	stress-associated endoplasmic reti	0.094	1.067	2.94E-01	2.94E+01		
8091485	NM_005067	SIAH2	seven in absentia homolog 2 (Dros	-0.019	0.987	8.62E-01	8.62E+01		
8091503	NM_013308	GPR171	G protein-coupled receptor 171	-0.259	0.836	1.56E-02	1.56E+00		
8091546	NM_00112322	TMEM14E	transmembrane protein 14E	0.129	1.093	4.27E-01	4.27E+01		
8091550	NM_020776	KIAA1328	KIAA1328	-0.032	0.978	9.54E-01	9.54E+01		
8091552	---	---	---	0.081	1.058	3.17E-01	3.17E+01		
8091554	---	---	---	0.015	1.011	9.53E-01	9.53E+01		
8091562	NM_020865	DHX36	DEAH (Asp-Glu-Ala-His) box polype	0.034	1.024	7.57E-01	7.57E+01		
8091588	NM_00103870	GPR149	G protein-coupled receptor 149	-0.061	0.958	6.83E-01	6.83E+01		
8091595	AK128002	FLJ46120	hypothetical LOC647008	0.018	1.013	8.84E-01	8.84E+01		
8091637	NM_004733	SLC33A1	solute carrier family 33 (acetyl-Co/	0.167	1.123	4.47E-02	4.47E+00		
8091648	NM_007107	SSR3	signal sequence receptor, gamma	0.077	1.055	6.52E-01	6.52E+01		
8091656	NM_00111352	METT5D1	methyltransferase 5 domain conta	0.099	1.071	7.52E-01	7.52E+01		
8091658	NM_020307	CCNL1	cyclin L1	-0.039	0.974	7.73E-01	7.73E+01		
8091676	---	---	---	-0.024	0.984	9.21E-01	9.21E+01		
8091696	---	---	---	-0.041	0.972	7.34E-01	7.34E+01		
8091764	NM_002268	KPNA4	karyopherin alpha 4 (importin alpl	0.028	1.020	8.47E-01	8.47E+01		
8091778	NR_003001	SCARNA7	small Cajal body-specific RNA 7	0.064	1.046	8.78E-01	8.78E+01		
8091780	NM_00103862	B3GALNT1	beta-1,3-N-acetylgalactosaminyltr	0.037	1.026	8.31E-01	8.31E+01		
8091806	NM_000984	RPL23A	ribosomal protein L23a	-0.016	0.989	8.96E-01	8.96E+01		
8091809	---	---	---	-0.072	0.952	6.66E-01	6.66E+01		
8091941	NM_007217	PDCD10	programmed cell death 10	-0.003	0.998	9.88E-01	9.88E+01		
8092000	NR_001566	TERC	telomerase RNA component	-0.106	0.929	6.46E-01	6.46E+01		
8092035	NM_024947	PHC3	polyhomeotic homolog 3 (Drosopl	0.034	1.024	8.16E-01	8.16E+01		
8092065	---	---	---	-0.114	0.924	2.72E-01	2.72E+01		
8092067	NM_00109964	RPL22L1	ribosomal protein L22-like 1	0.195	1.145	3.41E-01	3.41E+01		
8092073	NM_020390	EIF5A2	eukaryotic translation initiation fa	-0.010	0.993	9.66E-01	9.66E+01		
8092081	---	---	---	0.021	1.015	9.27E-01	9.27E+01		
8092095	NM_015028	TNIK	TRAF2 and NCK interacting kinase	0.110	1.080	3.69E-01	3.69E+01		
8092132	---	---	---	0.000	1.000	1.00E+00	1.00E+02		
8092162	ENST00000331	PP13439	hypothetical LOC100128046	-0.259	0.836	1.31E-02	1.31E+00		
8092165	NM_198407	GHSR	growth hormone secretagogue rec	0.054	1.038	6.60E-01	6.60E+01		
8092201	NM_024665	TBL1XR1	transducin (beta)-like 1 X-linked re	0.071	1.051	4.90E-01	4.90E+01		
8092218	---	---	---	0.061	1.043	8.36E-01	8.36E+01		
8092228	---	---	---	0.062	1.044	6.11E-01	6.11E+01		
8092230	NM_022470	ZMAT3	zinc finger, matrin type 3	-0.028	0.981	8.91E-01	8.91E+01		
8092239	---	---	---	0.069	1.049	7.78E-01	7.78E+01		
8092241	NM_171830	KCNMB3	potassium large conductance calci	-0.040	0.973	8.24E-01	8.24E+01		
8092265	NM_020409	MRPL47	mitochondrial ribosomal protein L	-0.034	0.977	8.79E-01	8.79E+01		
8092314	NM_145261	DNAJC19	DnaJ (Hsp40) homolog, subfamily	0.040	1.028	7.98E-01	7.98E+01		
8092321	NM_020640	DCUN1D1	DCN1, defective in cullin neddylati	0.046	1.033	7.97E-01	7.97E+01		
8092328	NM_020166	MCCC1	methylcrotonoyl-Coenzyme A carb	0.073	1.052	6.42E-01	6.42E+01		
8092358	NM_015078	MCF2L2	MCF.2 cell line derived transformi	-0.036	0.976	8.80E-01	8.80E+01		
8092390	---	---	---	0.237	1.179	2.38E-01	2.38E+01		
8092392	NM_130446	KLHL6	kelch-like 6 (Drosophila)	0.049	1.034	7.32E-01	7.32E+01		
8092404	NM_024871	MAP6D1	MAP6 domain containing 1	0.038	1.026	7.15E-01	7.15E+01		
8092409	NM_018622	PARL	presenilin associated, rhomboid-li	0.101	1.073	2.83E-01	2.83E+01		
8092418	NM_005688	ABCC5	ATP-binding cassette, sub-family C	0.122	1.088	4.04E-01	4.04E+01		
8092455	---	---	---	-0.011	0.993	9.56E-01	9.56E+01		
8092457	NM_005787	ALG3	asparagine-linked glycosylation 3,	0.044	1.031	6.57E-01	6.57E+01		
8092468	NM_033259	CAMK2N2	calcium/calmodulin-dependent pr	-0.048	0.967	4.85E-01	4.85E+01		
8092473	NM_004366	CLCN2	chloride channel 2	-0.035	0.976	7.54E-01	7.54E+01		
8092501	NM_000460	THPO	thrombopoietin	0.020	1.014	8.63E-01	8.63E+01		
8092514	NM_022149	MAGEF1	melanoma antigen family F, 1	0.055	1.039	5.60E-01	5.60E+01		
8092532	---	---	---	-0.047	0.968	7.88E-01	7.88E+01		
8092534	NM_080652	TMEM41A	transmembrane protein 41A	0.051	1.036	7.36E-01	7.36E+01		
8092552	NM_006548	IGF2BP2	insulin-like growth factor 2 mRNA	0.045	1.031	8.74E-01	8.74E+01		
8092564	NM_004593	TRA2B	transformer 2 beta homolog (Dros	-0.037	0.974	7.87E-01	7.87E+01		
8092596	NM_001346	DGKG	diacylglycerol kinase, gamma 90kD	0.005	1.004	9.83E-01	9.83E+01		
8092621	NM_017541	CRYGS	crystallin, gamma S	0.042	1.029	8.77E-01	8.77E+01		
8092627	NM_018138	TBCCD1	TBCC domain containing 1	0.029	1.020	8.83E-01	8.83E+01		
8092640	NM_002916	RFC4	replication factor C (activator 1) 4,	0.012	1.009	9.70E-01	9.70E+01		
8092661	NM_001879	MASP1	mannan-binding lectin serine pept	-0.056	0.962	6.46E-01	6.46E+01		
8092682	NM_001048	SST	somatostatin	-0.009	0.994	9.57E-01	9.57E+01		
8092686	NM_00100431	RTP2	receptor (chemosensory) transpor	-0.049	0.966	7.14E-01	7.14E+01		
8092691	NM_001706	BCL6	B-cell CLL/lymphoma 6	0.017	1.012	9.53E-01	9.53E+01		
8092702	AF315716	LOC100132319	hypothetical LOC100132319	0.051	1.036	9.27E-01	9.27E+01		
8092765	NM_178496	C3orf59	chromosome 3 open reading fram	-0.253	0.839	2.64E-01	2.64E+01		
8092834	---	---	---	0.076	1.054	4.63E-01	4.63E+01		
8092836	NM_00108051	CPN2	carboxypeptidase N, polypeptide 2	0.042	1.030	7.27E-01	7.27E+01		
8092839	NM_00113505	LRRC15	leucine rich repeat containing 15	-0.011	0.992	9.63E-01	9.63E+01		
8092845	NM_004488	GP5	glycoprotein V (platelet)	0.010	1.007	9.55E-01	9.55E+01		
8092883	---	---	---	-0.265	0.832	6.30E-02	6.30E+00		
8092888	NM_138399	TMEM44	transmembrane protein 44	-0.028	0.981	8.17E-01	8.17E+01		
8092905	NM_018385	LSG1	large subunit GTPase 1 homolog (S	0.011	1.008	9.64E-01	9.64E+01		
8092922	NM_152531	C3orf21	chromosome 3 open reading fram	0.002	1.001	9.89E-01	9.89E+01		
8092931	---	---	---	0.029	1.021	8.56E-01	8.56E+01		

8092933	NM_012287	ACAP2	ArfGAP with coiled-coil, ankyrin re	0.151	1.110	8.35E-02	8.35E+00		
8092957	---	---	---	0.096	1.069	7.16E-01	7.16E+01		
8092959	NM_006241	PPP1R2	protein phosphatase 1, regulatory	-0.027	0.981	8.55E-01	8.55E+01		
8092968	---	---	---	0.002	1.002	9.94E-01	9.94E+01		
8092978	NM_018406	MUC4	mucin 4, cell surface associated	0.001	1.001	9.96E-01	9.96E+01		
8093013	NM_005781	TNK2	tyrosine kinase, non-receptor, 2	-0.062	0.958	5.53E-01	5.53E+01		
8093035	---	---	---	-0.040	0.973	8.63E-01	8.63E+01		
8093039	NR_003264	SDHALP1	succinate dehydrogenase complex	-0.052	0.965	7.44E-01	7.44E+01		
8093053	NM_003234	TFRC	transferrin receptor (p90, CD71)	-0.015	0.990	9.72E-01	9.72E+01		
8093074	NM_00103961	ZDHHC19	zinc finger, DHHC-type containing	0.051	1.036	5.99E-01	5.99E+01		
8093086	NM_005017	PCYT1A	phosphate cytidylyltransferase 1, c	0.072	1.051	7.51E-01	7.51E+01		
8093104	NM_138461	TM4SF19	transmembrane 4 L six family mem	-0.334	0.794	1.47E-01	1.47E+01		
8093112	NM_015562	UBXN7	UBX domain protein 7	-0.020	0.986	9.33E-01	9.33E+01		
8093126	---	---	---	-0.076	0.949	8.78E-01	8.78E+01		
8093128	---	---	---	-0.034	0.977	8.79E-01	8.79E+01		
8093130	NM_152617	RNF168	ring finger protein 168	-0.120	0.920	8.33E-02	8.33E+00		
8093141	NM_182627	WDR53	WD repeat domain 53	-0.064	0.957	6.98E-01	6.98E+01		
8093150	---	---	---	-0.007	0.995	9.81E-01	9.81E+01		
8093156	NM_007362	NCBP2	nuclear cap binding protein subun	0.010	1.007	9.63E-01	9.63E+01		
8093171	NM_005929	MFI2	antigen p97 (melanoma associated	-0.078	0.947	3.67E-01	3.67E+01		
8093191	NM_00109842	DLG1	discs, large homolog 1 (Drosophila	-0.005	0.997	9.82E-01	9.82E+01		
8093219	NM_203314	BDH1	3-hydroxybutyrate dehydrogenase	0.001	1.001	9.95E-01	9.95E+01		
8093230	NM_00114564	KIAA0226	KIAA0226	-0.013	0.991	9.54E-01	9.54E+01		
8093256	---	---	---	-0.037	0.975	8.33E-01	8.33E+01		
8093258	NM_032263	IQCG	IQ motif containing G	-0.002	0.999	9.97E-01	9.97E+01		
8093314	NM_004547	NDUFB4	NADH dehydrogenase (ubiquinone	-0.050	0.966	7.45E-01	7.45E+01		
8093330	---	---	---	0.037	1.026	9.49E-01	9.49E+01		
8093336	NM_003441	ZNF141	zinc finger protein 141	0.114	1.083	4.99E-01	4.99E+01		
8093343	NM_00112717	PIGG	phosphatidylinositol glycan ancho	0.060	1.043	5.31E-01	5.31E+01		
8093360	NM_000283	PDE6B	phosphodiesterase 6B, cGMP-spec	-0.055	0.963	6.31E-01	6.31E+01		
8093386	NM_002477	MYL5	myosin, light chain 5, regulatory	-0.075	0.949	5.47E-01	5.47E+01		
8093398	NM_006315	PCGF3	polycomb group ring finger 3	-0.057	0.961	5.09E-01	5.09E+01		
8093413	NM_032326	TMEM175	transmembrane protein 175	0.098	1.070	3.07E-01	3.07E+01		
8093425	NM_000203	IDUA	iduronidase, alpha-L-	-0.025	0.983	8.68E-01	8.68E+01		
8093440	NM_00100435	FGFRL1	fibroblast growth factor receptor-l	-0.089	0.940	2.83E-01	2.83E+01		
8093456	NM_052861	C4orf42	chromosome 4 open reading fram	0.014	1.010	9.74E-01	9.74E+01		
8093462	NM_00101740	MAEA	macrophage erythroblast attacher	-0.091	0.939	3.75E-01	3.75E+01		
8093476	NM_020894	KIAA1530	KIAA1530	-0.011	0.993	9.52E-01	9.52E+01		
8093494	NM_175918	CRIPAK	cysteine-rich PAK1 inhibitor	0.066	1.047	7.44E-01	7.44E+01		
8093500	NM_006342	TACC3	transforming, acidic coiled-coil cor	-0.054	0.963	7.97E-01	7.97E+01		
8093518	NM_000142	FGFR3	fibroblast growth factor receptor 3	-0.017	0.988	9.03E-01	9.03E+01		
8093539	NM_133330	WHSC1	Wolf-Hirschhorn syndrome candid	0.013	1.009	9.22E-01	9.22E+01		
8093576	NR_003004	SCARNA22	small Cajal body-specific RNA 22	0.167	1.123	1.70E-01	1.70E+01		
8093590	NM_002938	RNF4	ring finger protein 4	0.029	1.020	8.05E-01	8.05E+01		
8093601	NM_003704	C4orf8	chromosome 4 open reading fram	-0.050	0.966	6.08E-01	6.08E+01		
8093624	NM_00114585	SH3BP2	SH3-domain binding protein 2	0.048	1.034	7.59E-01	7.59E+01		
8093643	NM_176801	ADD1	adducin 1 (alpha)	-0.017	0.988	9.19E-01	9.19E+01		
8093683	---	---	---	0.213	1.159	3.60E-01	3.60E+01		
8093685	NM_002111	HTT	huntingtin	0.024	1.017	8.78E-01	8.78E+01		
8093764	NM_198229	RGS12	regulator of G-protein signaling 12	0.049	1.034	5.99E-01	5.99E+01		
8093792	NM_001528	HGFAC	HGF activator	0.002	1.001	9.91E-01	9.91E+01		
8093807	NM_173660	DOK7	docking protein 7	-0.032	0.978	7.59E-01	7.59E+01		
8093826	NM_000683	ADRA2C	adrenergic, alpha-2C-, receptor	-0.036	0.976	7.37E-01	7.37E+01		
8093829	NM_145291	ZNF509	zinc finger protein 509	0.077	1.055	7.73E-01	7.73E+01		
8093839	NM_014392	D4S234E	DNA segment on chromosome 4 (u	0.056	1.039	7.87E-01	7.87E+01		
8093852	NM_002448	MSX1	msh homeobox 1	-0.001	1.000	9.97E-01	9.97E+01		
8093872	NM_005750	C4orf6	chromosome 4 open reading fram	0.016	1.011	9.45E-01	9.45E+01		
8093878	NM_153717	EVC	Ellis van Creveld syndrome	-0.031	0.979	8.34E-01	8.34E+01		
8093916	NM_015274	MAN2B2	mannosidase, alpha, class 2B, mer	-0.047	0.968	5.48E-01	5.48E+01		
8093936	NM_033296	MRFAP1	Mof4 family associated protein 1	-0.036	0.975	7.47E-01	7.47E+01		
8093943	AK026375	LOC93622	hypothetical LOC93622	-0.113	0.925	4.02E-01	4.02E+01		
8093950	NM_005980	S100P	S100 calcium binding protein P	0.032	1.022	8.32E-01	8.32E+01		
8093957	NM_018366	CNO	cappuccino homolog (mouse)	-0.021	0.985	9.25E-01	9.25E+01		
8093961	NM_014743	KIAA0232	KIAA0232	-0.037	0.975	8.27E-01	8.27E+01		
8093976	NM_020773	TBC1D14	TBC1 domain family, member 14	0.211	1.158	6.06E-02	6.06E+00		
8093993	NM_152293	TADA2B	transcriptional adaptor 2 (ADA2 ho	-0.027	0.982	8.92E-01	8.92E+01		
8093997	NM_020777	SORCS2	sortilin-related VPS10 domain con	-0.063	0.957	5.53E-01	5.53E+01		
8094026	---	---	---	0.049	1.035	7.57E-01	7.57E+01		
8094028	NM_198595	AFAP1	actin filament associated protein 1	0.002	1.001	9.92E-01	9.92E+01		
8094030	NM_198595	AFAP1	actin filament associated protein 1	0.002	1.001	9.90E-01	9.90E+01		
8094032	NM_018986	SH3TC1	SH3 domain and tetratricopeptide	0.033	1.023	7.81E-01	7.81E+01		
8094056	NM_053044	HTRA3	HtrA serine peptidase 3	-0.003	0.998	9.85E-01	9.85E+01		
8094068	---	---	---	0.006	1.004	9.85E-01	9.85E+01		
8094070	BC035655	C4orf23	chromosome 4 open reading fram	0.027	1.019	8.78E-01	8.78E+01		
8094086	---	---	---	-0.060	0.960	6.78E-01	6.78E+01		
8094093	NM_080819	GPR78	G protein-coupled receptor 78	-0.029	0.980	8.30E-01	8.30E+01		
8094101	NM_00101444	CPZ	carboxypeptidase Z	0.026	1.018	8.58E-01	8.58E+01		

8094116	NM_00110566	USP17	ubiquitin specific peptidase 17	0.000	1.000	1.00E+00	1.00E+02		
8094118	NM_00110566	USP17	ubiquitin specific peptidase 17	0.000	1.000	1.00E+00	1.00E+02		
8094120	NM_00110566	USP17	ubiquitin specific peptidase 17	0.000	1.000	1.00E+00	1.00E+02		
8094122	NM_00110566	USP17	ubiquitin specific peptidase 17	0.003	1.002	9.88E-01	9.88E+01		
8094124	NM_00110566	USP17	ubiquitin specific peptidase 17	0.000	1.000	1.00E+00	1.00E+02		
8094126	NM_00110566	USP17	ubiquitin specific peptidase 17	0.000	1.000	1.00E+00	1.00E+02		
8094128	NM_00110566	USP17	ubiquitin specific peptidase 17	0.000	1.000	1.00E+00	1.00E+02		
8094130	NM_00110566	USP17	ubiquitin specific peptidase 17	0.005	1.003	9.84E-01	9.84E+01		
8094132	NM_00110566	USP17	ubiquitin specific peptidase 17	0.000	1.000	1.00E+00	1.00E+02		
8094134	NR_027279	USP17L6P	ubiquitin specific peptidase 17-like	0.058	1.041	7.40E-01	7.40E+01		
8094136	NM_00100203	DEFB108B	defensin, beta 108B	0.013	1.009	9.74E-01	9.74E+01		
8094144	NM_030930	UNC93B1	unc-93 homolog B1 (C. elegans)	0.000	1.000	9.99E-01	9.99E+01		
8094148	---	---	---	-0.087	0.941	5.56E-01	5.56E+01		
8094154	AF073924	OR7E35P	olfactory receptor, family 7, subfa	-0.068	0.954	6.13E-01	6.13E+01		
8094156	NM_000798	DRD5	dopamine receptor D5	-0.020	0.986	9.09E-01	9.09E+01		
8094165	NR_003132	HSP90AB2P	heat shock protein 90kDa alpha (c	-0.062	0.958	7.16E-01	7.16E+01		
8094169	NM_182485	CPEB2	cytoplasmic polyadenylation elem	0.005	1.004	9.84E-01	9.84E+01		
8094228	NM_004334	BST1	bone marrow stromal cell antigen	0.237	1.179	2.18E-01	2.18E+01		
8094240	NM_001775	CD38	CD38 molecule	0.530	1.443	5.36E-02	5.36E+00		
8094251	NR_027696	FLJ39653	hypothetical FLJ39653	0.028	1.020	8.50E-01	8.50E+01		
8094271	NM_025205	MED28	mediator complex subunit 28	-0.036	0.975	8.34E-01	8.34E+01		
8094342	NM_145048	PACRGL	PARK2 co-regulated-like	0.148	1.108	1.90E-01	1.90E+01		
8094355	---	---	---	0.163	1.120	6.64E-02	6.64E+00		
8094372	NM_003102	SOD3	superoxide dismutase 3, extracellu	-0.106	0.929	1.31E-01	1.31E+01		
8094378	NM_018323	PI4K2B	phosphatidylinositol 4-kinase type	0.068	1.048	5.98E-01	5.98E+01		
8094408	NM_013367	ANAPC4	anaphase promoting complex sub	0.040	1.028	8.21E-01	8.21E+01		
8094456	NM_00114543	LOC389203	hypothetical protein LOC389203	0.090	1.064	7.39E-01	7.39E+01		
8094460	NM_005349	RBPJ	recombination signal binding prot	0.013	1.009	9.53E-01	9.53E+01		
8094476	NM_018317	TBC1D19	TBC1 domain family, member 19	-0.005	0.996	9.88E-01	9.88E+01		
8094499	---	---	---	-0.103	0.931	6.69E-01	6.69E+01		
8094501	NM_020860	STIM2	stromal interaction molecule 2	0.083	1.059	4.69E-01	4.69E+01		
8094533	AK304357	FLJ16686	FLJ16686 protein	0.239	1.180	6.58E-01	6.58E+01		
8094556	NM_018290	PGM2	phosphoglucomutase 2	0.175	1.129	1.07E-01	1.07E+01		
8094574	NM_015173	TBC1D1	TBC1 (tre-2/USP6, BUB2, cdc16) d	0.201	1.149	2.08E-02	2.08E+00		
8094599	NM_016531	KLF3	Kruppel-like factor 3 (basic)	0.077	1.055	5.62E-01	5.62E+01		
8094625	NM_015990	KLHL5	kelch-like 5 (Drosophila)	-0.019	0.987	9.44E-01	9.44E+01		
8094679	NM_175737	KLB	klotho beta	-0.033	0.977	7.76E-01	7.76E+01		
8094701	NR_026854	LOC401127	WD repeat domain 5 pseudogene	-0.021	0.986	9.26E-01	9.26E+01		
8094704	NM_005339	UBE2K	ubiquitin-conjugating enzyme E2K	-0.012	0.992	9.28E-01	9.28E+01		
8094717	---	---	---	0.053	1.037	8.97E-01	8.97E+01		
8094719	NM_018177	N4BP2	NEDD4 binding protein 2	0.039	1.027	8.57E-01	8.57E+01		
8094741	---	---	---	-0.023	0.984	9.53E-01	9.53E+01		
8094743	NM_004310	RHOH	ras homolog gene family, member	-0.217	0.860	5.53E-02	5.53E+00		
8094830	NM_018126	TMEM33	transmembrane protein 33	0.097	1.069	2.50E-01	2.50E+01		
8094848	NM_006345	SLC30A9	solute carrier family 30 (zinc trans	0.036	1.026	8.23E-01	8.23E+01		
8094870	NM_00108050	SHISA3	shisa homolog 3 (Xenopus laevis)	-0.029	0.980	9.16E-01	9.16E+01		
8094872	---	---	---	0.029	1.020	8.33E-01	8.33E+01		
8094874	---	---	---	-0.072	0.951	5.49E-01	5.49E+01		
8094876	NM_021927	GUF1	GUF1 GTPase homolog (S. cerevisi	0.112	1.081	2.37E-01	2.37E+01		
8094938	NM_207330	NIPAL1	NIPA-like domain containing 1	0.067	1.047	2.76E-01	2.76E+01		
8094948	NM_020846	SLAIN2	SLAIN motif family, member 2	-0.086	0.942	2.21E-01	2.21E+01		
8094960	NM_152679	SLC10A4	solute carrier family 10 (sodium/b	-0.030	0.980	8.10E-01	8.10E+01		
8094968	NM_175619	ZAR1	zygote arrest 1	-0.084	0.943	4.19E-01	4.19E+01		
8094974	NM_00107983	OCIAD1	OCIA domain containing 1	-0.038	0.974	7.86E-01	7.86E+01		
8095005	---	---	---	-0.190	0.877	4.35E-01	4.35E+01		
8095009	NM_00104040	DCUN1D4	DCN1, defective in cullin neddylati	0.029	1.020	8.60E-01	8.60E+01		
8095048	NM_030917	FIP1L1	FIP1 like 1 (S. cerevisiae)	-0.022	0.985	8.84E-01	8.84E+01		
8095072	ENST0000031	LOC441016	similar to COMM domain containi	0.009	1.006	9.63E-01	9.63E+01		
8095074	NM_133267	GSX2	GS homeobox 2	-0.027	0.981	8.14E-01	8.14E+01		
8095135	---	---	---	0.141	1.103	8.23E-02	8.23E+00		
8095139	NM_024592	SRD5A3	steroid 5 alpha-reductase 3	0.160	1.117	3.07E-01	3.07E+01		
8095148	NM_018475	TMEM165	transmembrane protein 165	0.029	1.020	8.74E-01	8.74E+01		
8095159	---	---	---	-0.021	0.985	9.53E-01	9.53E+01		
8095163	NM_00102492	EXOC1	exocyst complex component 1	0.085	1.061	5.55E-01	5.55E+01		
8095185	---	---	---	0.131	1.095	6.18E-01	6.18E+01		
8095187	NM_025009	CEP135	centrosomal protein 135kDa	-0.100	0.933	3.67E-01	3.67E+01		
8095214	---	---	---	-0.013	0.991	9.76E-01	9.76E+01		
8095216	NM_020722	KIAA1211	KIAA1211	-0.024	0.983	8.91E-01	8.91E+01		
8095221	NM_00107952	PAICS	phosphoribosylaminoimidazole ca	-0.038	0.974	8.97E-01	8.97E+01		
8095230	NM_006947	SRP72	signal recognition particle 72kDa	0.053	1.037	6.83E-01	6.83E+01		
8095260	---	---	---	-0.069	0.954	6.38E-01	6.38E+01		
8095262	NM_005612	REST	RE1-silencing transcription factor	-0.044	0.970	6.41E-01	6.41E+01		
8095269	NM_000938	POLR2B	polymerase (RNA) II (DNA directed	0.010	1.007	9.57E-01	9.57E+01		
8095295	---	---	---	0.065	1.046	8.84E-01	8.84E+01		
8095299	---	---	---	0.157	1.115	6.81E-01	6.81E+01		
8095333	NM_006544	EXOC5	exocyst complex component 5	0.015	1.011	9.69E-01	9.69E+01		
8095341	---	---	---	0.239	1.180	2.46E-02	2.46E+00		

8095360	---	---	---	0.068	1.049	7.29E-01	7.29E+01		
8095362	NM_005953	MT2A	metallothionein 2A	-0.342	0.789	1.57E-01	1.57E+01		
8095376	NM_005953	MT2A	metallothionein 2A	-0.322	0.800	2.02E-01	2.02E+01		
8095451	NM_214711	C4orf40	chromosome 4 open reading fram	-0.013	0.991	9.01E-01	9.01E+01		
8095539	NM_020368	UTP3	UTP3, small subunit (SSU) process	-0.005	0.997	9.84E-01	9.84E+01		
8095545	NM_00103744	RUFY3	RUN and FYVE domain containing	-0.019	0.987	9.50E-01	9.50E+01		
8095562	---	---	---	0.077	1.055	8.89E-01	8.89E+01		
8095566	NM_173468	MOBKL1A	MOB1, Mps One Binder kinase act	0.082	1.059	6.57E-01	6.57E+01		
8095574	NM_000788	DCK	deoxycytidine kinase	0.162	1.119	8.09E-03	8.09E-01		
8095585	NM_00109848	SLC4A4	solute carrier family 4, sodium bic	0.160	1.117	2.22E-01	2.22E+01		
8095626	---	---	---	0.048	1.034	8.78E-01	8.78E+01		
8095694	NM_002620	PF4V1	platelet factor 4 variant 1	0.126	1.091	2.02E-01	2.02E+01		
8095703	---	---	---	-0.072	0.951	8.07E-01	8.07E+01		
8095736	NM_001657	AREG	amphiregulin	-0.036	0.976	9.16E-01	9.16E+01		
8095758	NM_015436	RCHY1	ring finger and CHY zinc finger dor	0.039	1.027	8.70E-01	8.70E+01		
8095773	NM_003715	USO1	USO1 homolog, vesicle docking pr	0.097	1.070	5.34E-01	5.34E+01		
8095800	---	---	---	-0.049	0.967	6.83E-01	6.83E+01		
8095802	NM_018115	SDAD1	SDA1 domain containing 1	-0.042	0.971	8.67E-01	8.67E+01		
8095826	NM_003943	STBD1	starch binding domain 1	0.081	1.057	6.30E-01	6.30E+01		
8095854	NM_018243	40432	septin 11	-0.006	0.996	9.84E-01	9.84E+01		
8095870	NM_004354	CCNG2	cyclin G2	-0.029	0.980	8.44E-01	8.44E+01		
8095894	NM_020236	MRPL1	mitochondrial ribosomal protein L	0.102	1.074	5.91E-01	5.91E+01		
8096002	---	---	---	-0.005	0.997	9.85E-01	9.85E+01		
8096004	NM_198892	BMP2K	BMP2 inducible kinase	0.188	1.139	1.72E-01	1.72E+01		
8096025	---	---	---	0.073	1.052	7.98E-01	7.98E+01		
8096032	NM_020226	PRDM8	PR domain containing 8	-0.040	0.973	7.68E-01	7.68E+01		
8096070	NM_001201	BMP3	bone morphogenetic protein 3	0.044	1.031	7.49E-01	7.49E+01		
8096077	---	---	---	0.100	1.072	6.06E-01	6.06E+01		
8096079	---	---	---	0.155	1.113	2.36E-01	2.36E+01		
8096081	NM_021204	ENOPH1	enolase-phosphatase 1	-0.014	0.991	9.65E-01	9.65E+01		
8096098	NM_016129	COPS4	COP9 constitutive photomorphoge	-0.068	0.954	6.35E-01	6.35E+01		
8096109	NM_016067	MRPS18C	mitochondrial ribosomal protein S	0.062	1.044	6.95E-01	6.95E+01		
8096116	NM_032717	AGPAT9	1-acylglycerol-3-phosphate O-acyl	-0.059	0.960	9.22E-01	9.22E+01		
8096160	NM_00102561	ARHGAP24	Rho GTPase activating protein 24	0.102	1.074	7.42E-01	7.42E+01		
8096176	NM_080683	PTPN13	protein tyrosine phosphatase, nor	0.111	1.080	7.85E-01	7.85E+01		
8096224	NM_005935	AFF1	AF4/FMR2 family, member 1	0.034	1.024	8.31E-01	8.31E+01		
8096251	NM_024047	NUDT9	nudix (nucleoside diphosphate link	0.070	1.050	5.55E-01	5.55E+01		
8096296	AY956764	HSP90AB3P	heat shock protein 90kDa alpha (c	-0.126	0.916	4.86E-01	4.86E+01		
8096314	NM_000297	PKD2	polycystic kidney disease 2 (autos	-0.023	0.984	9.19E-01	9.19E+01		
8096331	---	---	---	0.103	1.074	5.11E-01	5.11E+01		
8096335	NM_017912	HERC6	hect domain and RLD 6	0.058	1.041	9.32E-01	9.32E+01		
8096361	NM_016323	HERC5	hect domain and RLD 5	0.215	1.161	6.51E-01	6.51E+01		
8096385	NM_014606	HERC3	hect domain and RLD 3	0.153	1.112	1.19E-01	1.19E+01		
8096461	NM_005172	ATOH1	atonal homolog 1 (Drosophila)	-0.018	0.988	9.36E-01	9.36E+01		
8096463	NM_00112842	SMARCAD1	SWI/SNF-related, matrix-associate	-0.039	0.973	8.23E-01	8.23E+01		
8096489	NM_006457	PDLIM5	PDZ and LIM domain 5	0.304	1.234	3.04E-02	3.04E+00		
8096528	NM_005390	PDHA2	pyruvate dehydrogenase (lipoamid	-0.023	0.984	9.30E-01	9.30E+01		
8096533	---	---	---	0.011	1.008	9.62E-01	9.62E+01		
8096538	NM_00110042	RAP1GDS1	RAP1, GTP-GDP dissociation stimu	0.139	1.101	1.85E-01	1.85E+01		
8096556	NM_015143	METAP1	methionyl aminopeptidase 1	0.115	1.083	3.54E-01	3.54E+01		
8096602	NM_014395	DAPP1	dual adaptor of phosphotyrosine a	0.166	1.122	4.62E-01	4.62E+01		
8096661	---	---	---	0.101	1.072	6.99E-01	6.99E+01		
8096663	NM_00100838	CISD2	CDGSH iron sulfur domain 2	0.303	1.234	1.51E-01	1.51E+01		
8096669	NM_017628	TET2	tet oncogene family member 2	0.177	1.131	3.56E-01	3.56E+01		
8096675	NM_00112720	TET2	tet oncogene family member 2	0.190	1.141	2.37E-01	2.37E+01		
8096718	NM_004757	AIMP1	aminoacyl tRNA synthetase compl	0.036	1.025	7.53E-01	7.53E+01		
8096744	NM_183075	CYP2U1	cytochrome P450, family 2, subfar	0.013	1.009	9.67E-01	9.67E+01		
8096753	NM_005327	HADH	hydroxyacyl-Coenzyme A dehydro	0.049	1.034	7.73E-01	7.73E+01		
8096765	NM_033625	RPL34	ribosomal protein L34	-0.018	0.987	9.40E-01	9.40E+01		
8096771	NM_021227	OSTC	oligosaccharyltransferase complex	0.071	1.050	7.02E-01	7.02E+01		
8096777	---	---	---	-0.031	0.979	9.22E-01	9.22E+01		
8096781	NM_006323	SEC24B	SEC24 family, member B (S. cerevi	0.042	1.029	7.14E-01	7.14E+01		
8096808	NM_017918	CCDC109B	coiled-coil domain containing 109	0.096	1.069	5.21E-01	5.21E+01		
8096821	NM_018983	GAR1	GAR1 ribonucleoprotein homolog	-0.003	0.998	9.80E-01	9.80E+01		
8096899	NM_152400	C4orf32	chromosome 4 open reading fram	0.071	1.051	4.25E-01	4.25E+01		
8096905	NM_018569	C4orf16	chromosome 4 open reading fram	0.203	1.151	3.80E-02	3.80E+00		
8096919	NM_025144	ALPK1	alpha-kinase 1	0.230	1.173	1.61E-01	1.61E+01		
8096938	NM_016648	LARP7	La ribonucleoprotein domain fami	0.035	1.024	8.33E-01	8.33E+01		
8096955	---	---	---	0.035	1.024	8.36E-01	8.36E+01		
8097017	NM_00112817	UGT8	UDP glycosyltransferase 8	0.109	1.079	4.94E-01	4.94E+01		
8097030	---	---	---	-0.047	0.968	8.66E-01	8.66E+01		
8097056	NR_002963	SNORA24	small nucleolar RNA, H/ACA box 2	-0.284	0.821	3.72E-01	3.72E+01		
8097064	BC032332	PCMTD2	protein-L-isoaspartate (D-aspartat	-0.033	0.977	8.93E-01	8.93E+01		
8097066	NM_020961	METTL14	methyltransferase like 14	0.063	1.045	5.81E-01	5.81E+01		
8097096	---	---	---	0.078	1.056	5.30E-01	5.30E+01		
8097116	---	---	---	0.045	1.031	9.24E-01	9.24E+01		
8097118	ENST00000415	tcag7.907	hypothetical LOC402483	0.009	1.006	9.73E-01	9.73E+01		

8097128	NM_00103419	EXOSC9	exosome component 9	0.043	1.030	7.72E-01	7.72E+01		
8097146	---	---	---	-0.056	0.962	7.97E-01	7.97E+01		
8097148	NM_015312	KIAA1109	KIAA1109	-0.020	0.986	9.11E-01	9.11E+01		
8097262	NM_145207	SPATA5	spermatogenesis associated 5	-0.009	0.994	9.75E-01	9.75E+01		
8097282	NM_005841	SPRY1	sprouty homolog 1, antagonist of	-0.021	0.985	9.16E-01	9.16E+01		
8097373	BC034253	C4orf29	chromosome 4 open reading fram	0.019	1.013	9.08E-01	9.08E+01		
8097415	---	---	---	-0.026	0.982	8.73E-01	8.73E+01		
8097417	NM_199320	PHF17	PHD finger protein 17	-0.073	0.951	5.55E-01	5.55E+01		
8097443	---	---	---	0.098	1.071	3.04E-01	3.04E+01		
8097447	---	---	---	0.058	1.041	7.23E-01	7.23E+01		
8097449	NM_032961	PCDH10	protocadherin 10	-0.001	0.999	9.96E-01	9.96E+01		
8097461	NM_012118	CCRN4L	CCR4 carbon catabolite repression	-0.032	0.978	8.10E-01	8.10E+01		
8097468	---	---	---	0.055	1.039	7.61E-01	7.61E+01		
8097470	---	---	---	-0.055	0.963	8.16E-01	8.16E+01		
8097472	---	---	---	0.073	1.052	7.43E-01	7.43E+01		
8097476	---	---	---	0.080	1.057	5.41E-01	5.41E+01		
8097480	NM_057175	NARG1	NMDA receptor regulated 1	0.062	1.044	7.10E-01	7.10E+01		
8097507	NM_031296	RAB33B	RAB33B, member RAS oncogene f	-0.018	0.987	9.27E-01	9.27E+01		
8097513	NM_002413	MGST2	microsomal glutathione S-transfer	0.344	1.269	1.09E-01	1.09E+01		
8097521	NM_032547	SCOC	short coiled-coil protein	0.014	1.010	9.52E-01	9.52E+01		
8097543	NM_014487	ZNF330	zinc finger protein 330	0.163	1.119	1.16E-01	1.16E+01		
8097553	NM_172174	IL15	interleukin 15	-0.094	0.937	7.68E-01	7.68E+01		
8097570	NM_032557	USP38	ubiquitin specific peptidase 38	-0.096	0.935	4.67E-01	4.67E+01		
8097600	NM_003601	SMARCA5	SWI/SNF related, matrix associate	-0.026	0.982	7.91E-01	7.91E+01		
8097626	NR_003675	LOC441046	glucuronidase, beta pseudogene	-0.032	0.978	8.62E-01	8.62E+01		
8097647	NM_002940	ABCE1	ATP-binding cassette, sub-family E	0.132	1.096	4.12E-01	4.12E+01		
8097655	---	---	---	-0.132	0.913	3.94E-01	3.94E+01		
8097657	NM_005900	SMAD1	SMAD family member 1	0.030	1.021	9.56E-01	9.56E+01		
8097670	NM_172250	MMAA	methylmalonic aciduria (cobalami	0.072	1.051	7.54E-01	7.54E+01		
8097679	NM_007080	LSM6	LSM6 homolog, U6 small nuclear R	0.121	1.088	1.90E-01	1.90E+01		
8097687	NM_004575	POU4F2	POU class 4 homeobox 2	-0.010	0.993	9.56E-01	9.56E+01		
8097717	NM_024605	ARHGAP10	Rho GTPase activating protein 10	0.459	1.375	6.39E-02	6.39E+00		
8097743	---	---	---	0.252	1.191	2.18E-01	2.18E+01		
8097773	NM_006439	MAB21L2	mab-21-like 2 (C. elegans)	0.051	1.036	8.21E-01	8.21E+01		
8097782	NM_001006	RPS3A	ribosomal protein S3A	0.010	1.007	9.40E-01	9.40E+01		
8097792	NR_000007	SNORD73A	small nucleolar RNA, C/D box 73A	-0.104	0.930	7.71E-01	7.71E+01		
8097811	AL137273	DKFZP434I0714	hypothetical protein DKFZP434I07	0.025	1.017	8.97E-01	8.97E+01		
8097813	NM_00102559	ARFIP1	ADP-ribosylation factor interacting	0.153	1.112	3.52E-01	3.52E+01		
8097827	---	---	---	0.049	1.035	7.53E-01	7.53E+01		
8097867	NM_015196	KIAA0922	KIAA0922	-0.016	0.989	9.38E-01	9.38E+01		
8097903	NM_003264	TLR2	toll-like receptor 2	-0.458	0.728	1.54E-01	1.54E+01		
8097936	---	---	---	-0.074	0.950	5.17E-01	5.17E+01		
8097938	NM_000910	NPY2R	neuropeptide Y receptor Y2	-0.066	0.955	4.27E-01	4.27E+01		
8097955	---	---	---	-0.061	0.959	8.77E-01	8.77E+01		
8097973	NM_000857	GUCY1B3	guanylate cyclase 1, soluble, beta	0.203	1.151	2.91E-01	2.91E+01		
8098103	NM_020840	FNIP2	folliculin interacting protein 2	0.177	1.130	6.80E-01	6.80E+01		
8098121	NM_014247	RAPGEF2	Rap guanine nucleotide exchange	0.197	1.146	2.09E-01	2.09E+01		
8098150	NM_018352	C4orf43	chromosome 4 open reading fram	0.138	1.101	4.44E-01	4.44E+01		
8098163	---	---	---	0.140	1.102	5.34E-01	5.34E+01		
8098165	---	---	---	-0.001	0.999	9.96E-01	9.96E+01		
8098167	BC022381	C4orf39	chromosome 4 open reading fram	0.064	1.045	6.58E-01	6.58E+01		
8098177	NM_007246	KLHL2	kelch-like 2, Mayven (Drosophila)	-0.143	0.905	3.38E-01	3.38E+01		
8098193	---	---	---	0.019	1.013	9.67E-01	9.67E+01		
8098240	---	---	---	0.013	1.009	9.57E-01	9.57E+01		
8098244	---	---	---	-0.061	0.959	6.46E-01	6.46E+01		
8098289	---	---	---	-0.133	0.912	7.51E-01	7.51E+01		
8098291	NM_173872	CLCN3	chloride channel 3	-0.035	0.976	8.53E-01	8.53E+01		
8098326	---	---	---	-0.042	0.972	8.85E-01	8.85E+01		
8098328	NM_017423	GALNT7	UDP-N-acetyl-alpha-D-galactosam	0.110	1.079	4.14E-01	4.14E+01		
8098342	NM_003864	SAP30	Sin3A-associated protein, 30kDa	0.080	1.057	5.55E-01	5.55E+01		
8098414	NM_021928	SPCS3	signal peptidase complex subunit	0.179	1.132	9.64E-02	9.64E+00		
8098421	---	---	---	0.086	1.062	3.49E-01	3.49E+01		
8098470	NM_024949	WWC2	WW and C2 domain containing 2	0.064	1.046	8.64E-01	8.64E+01		
8098500	NM_017632	CDKN2AIP	CDKN2A interacting protein	0.008	1.006	9.73E-01	9.73E+01		
8098508	NM_001564	ING2	inhibitor of growth family, membe	0.091	1.065	6.41E-01	6.41E+01		
8098512	NM_021942	C4orf41	chromosome 4 open reading fram	0.122	1.088	3.17E-01	3.17E+01		
8098547	---	---	---	-0.070	0.953	8.71E-01	8.71E+01		
8098549	NM_020225	STOX2	storkhead box 2	-0.082	0.945	3.93E-01	3.93E+01		
8098571	BC140795	HELT	HES/HEY-like transcription factor	-0.001	1.000	9.97E-01	9.97E+01		
8098576	NM_001151	SLC25A4	solute carrier family 25 (mitochon	-0.145	0.904	3.04E-01	3.04E+01		
8098581	NM_031953	SNX25	sorting nexin 25	-0.221	0.858	5.45E-02	5.45E+00		
8098604	NM_181726	ANKRD37	ankyrin repeat domain 37	-0.034	0.976	8.52E-01	8.52E+01		
8098637	NM_207352	CYP4V2	cytochrome P450, family 4, subfar	0.088	1.063	6.73E-01	6.73E+01		
8098705	NM_019041	MTRF1L	mitochondrial translational releas	0.033	1.023	8.26E-01	8.26E+01		
8098707	AY956760	HSP90AA4P	heat shock protein 90kDa alpha (c	-0.019	0.987	9.37E-01	9.37E+01		
8098714	NM_004477	FRG1	FSHD region gene 1	0.219	1.164	9.17E-02	9.17E+00		
8098723	---	---	---	-0.085	0.943	4.43E-01	4.43E+01		

8098725	NM_033178	DUX4	double homeobox, 4	-0.010	0.993	9.50E-01	9.50E+01		
8098730	NM_033178	DUX4	double homeobox, 4	0.000	1.000	9.99E-01	9.99E+01		
8098732	NM_033178	DUX4	double homeobox, 4	-0.016	0.989	8.91E-01	8.91E+01		
8098740	NM_033178	DUX4	double homeobox, 4	-0.009	0.994	9.50E-01	9.50E+01		
8098743	NM_033178	DUX4	double homeobox, 4	0.000	1.000	9.99E-01	9.99E+01		
8098758	NM_133474	ZNF721	zinc finger protein 721	0.117	1.085	4.25E-01	4.25E+01		
8098762	NM_007100	ATP5I	ATP synthase, H+ transporting, mi	0.027	1.019	8.64E-01	8.64E+01		
8098769	NM_032219	MFSD7	major facilitator superfamily doma	-0.037	0.975	7.72E-01	7.72E+01		
8098782	NM_006651	CPLX1	complexin 1	-0.020	0.986	9.07E-01	9.07E+01		
8098789	NM_005255	GAK	cyclin G associated kinase	0.059	1.041	4.16E-01	4.16E+01		
8098822	NM_001347	DGKQ	diacylglycerol kinase, theta 110kD	-0.066	0.955	3.66E-01	3.66E+01		
8098848	NM_213613	SLC26A1	solute carrier family 26 (sulfate tra	-0.004	0.997	9.84E-01	9.84E+01		
8098870	NM_012445	SPON2	spondin 2, extracellular matrix pro	-0.061	0.959	5.89E-01	5.89E+01		
8098877	NR_024569	LOC100130872	hypothetical LOC100130872	-0.019	0.987	8.89E-01	8.89E+01		
8098880	NM_00101261	CTBP1	C-terminal binding protein 1	0.051	1.036	5.03E-01	5.03E+01		
8098895	NM_00101362	FAM53A	family with sequence similarity 53	-0.050	0.966	6.36E-01	6.36E+01		
8098902	---	---	---	-0.071	0.952	8.10E-01	8.10E+01		
8098904	NM_006527	SLBP	stem-loop binding protein	0.028	1.020	8.57E-01	8.57E+01		
8098916	NM_00112726	TMEM129	transmembrane protein 129	0.009	1.006	9.54E-01	9.54E+01		
8098924	NM_012318	LETM1	leucine zipper-EF-hand containing	-0.070	0.953	6.06E-01	6.06E+01		
8098942	NM_005663	WHSC2	Wolf-Hirschhorn syndrome candid	-0.085	0.943	3.14E-01	3.14E+01		
8098958	NM_181808	POLN	polymerase (DNA directed) nu	-0.054	0.964	7.63E-01	7.63E+01		
8098985	NM_024511	HAUS3	HAUS augmin-like complex, subun	-0.106	0.929	3.36E-01	3.36E+01		
8098995	NM_006454	MXD4	MAX dimerization protein 4	0.024	1.017	8.62E-01	8.62E+01		
8099008	NM_020972	ZFYVE28	zinc finger, FYVE domain containin	0.025	1.018	8.55E-01	8.55E+01		
8099027	---	---	---	0.050	1.035	8.79E-01	8.79E+01		
8099029	NM_024309	TNIP2	TNFAIP3 interacting protein 2	-0.082	0.944	4.77E-01	4.77E+01		
8099037	NM_00114606	MFSD10	major facilitator superfamily doma	-0.047	0.968	6.05E-01	6.05E+01		
8099051	NM_003703	NOP14	NOP14 nucleolar protein homolog	-0.007	0.995	9.77E-01	9.77E+01		
8099073	NM_002337	LRPAP1	low density lipoprotein receptor-r	0.080	1.057	5.82E-01	5.82E+01		
8099082	---	---	---	-0.061	0.958	7.34E-01	7.34E+01		
8099084	NR_024253	LOC348926	family with sequence similarity 86	-0.072	0.952	3.68E-01	3.68E+01		
8099091	---	---	---	-0.110	0.926	1.76E-01	1.76E+01		
8099107	NM_017816	LYAR	Ly1 antibody reactive homolog (m	0.115	1.083	5.55E-01	5.55E+01		
8099121	NM_016930	STX18	syntaxin 18	0.033	1.023	8.74E-01	8.74E+01		
8099130	---	---	---	0.028	1.020	8.97E-01	8.97E+01		
8099132	NM_018659	CYTL1	cytokine-like 1	-0.035	0.976	7.94E-01	7.94E+01		
8099172	NM_001313	CRMP1	collapsin response mediator prote	-0.053	0.964	6.38E-01	6.38E+01		
8099200	NM_144720	JAKMIP1	janus kinase and microtubule inte	-0.033	0.977	9.00E-01	9.00E+01		
8099233	ENST00000446	LOC100133131	hypothetical protein LOC1001331	-0.059	0.960	5.56E-01	5.56E+01		
8099235	NM_203462	MRFAP1L1	Morf4 family associated protein 1-	0.006	1.004	9.80E-01	9.80E+01		
8099240	---	---	---	-0.018	0.988	9.64E-01	9.64E+01		
8099246	NM_025196	GRPEL1	GrpE-like 1, mitochondrial (E. coli)	-0.003	0.998	9.90E-01	9.90E+01		
8099253	---	---	---	-0.022	0.985	8.63E-01	8.63E+01		
8099279	NM_00113008	ABLIM2	actin binding LIM protein family, n	0.001	1.000	9.97E-01	9.97E+01		
8099304	NM_003501	ACOX3	acyl-Coenzyme A oxidase 3, prista	-0.002	0.998	9.90E-01	9.90E+01		
8099340	NM_017491	WDR1	WD repeat domain 1	0.001	1.001	9.97E-01	9.97E+01		
8099362	---	---	---	0.023	1.016	9.53E-01	9.53E+01		
8099364	NM_053042	ZNF518B	zinc finger protein 518B	0.009	1.006	9.74E-01	9.74E+01		
8099388	NM_005114	HS3ST1	heparan sulfate (glucosamine) 3-O	0.025	1.018	8.55E-01	8.55E+01		
8099395	NM_004249	RAB28	RAB28, member RAS oncogene fa	-0.028	0.981	8.86E-01	8.86E+01		
8099406	NM_001189	NKX3-2	NK3 homeobox 2	-0.050	0.966	5.91E-01	5.91E+01		
8099410	NM_148894	BOD1L	biorientation of chromosomes in c	-0.062	0.958	5.23E-01	5.23E+01		
8099449	NM_033535	FBXL5	F-box and leucine-rich repeat prot	0.072	1.051	7.07E-01	7.07E+01		
8099467	NM_005130	FGFBP1	fibroblast growth factor binding p	-0.057	0.961	5.80E-01	5.80E+01		
8099471	NM_031950	FGFBP2	fibroblast growth factor binding p	-0.050	0.966	9.00E-01	9.00E+01		
8099506	NM_153365	TAPT1	transmembrane anterior posterior	-0.010	0.993	9.74E-01	9.74E+01		
8099524	NM_00113083	LDB2	LIM domain binding 2	-0.021	0.985	9.22E-01	9.22E+01		
8099537	---	---	---	-0.015	0.990	9.50E-01	9.50E+01		
8099541	NM_000320	QDPR	quinoid dihydropteridine reductas	0.111	1.080	5.42E-01	5.42E+01		
8099551	NM_015688	FAM184B	family with sequence similarity 18	-0.033	0.978	8.32E-01	8.32E+01		
8099570	BC050697	DCAF16	DDB1 and CUL4 associated factor	0.000	1.000	1.00E+00	1.00E+02		
8099581	NM_153686	LCORL	ligand dependent nuclear recepto	0.127	1.092	4.88E-01	4.88E+01		
8099631	---	---	---	0.088	1.063	3.95E-01	3.95E+01		
8099649	NM_001358	DHX15	DEAH (Asp-Glu-Ala-His) box polyp	0.005	1.003	9.72E-01	9.72E+01		
8099668	---	---	---	0.073	1.052	8.27E-01	8.27E+01		
8099670	NM_00113072	CCDC149	coiled-coil domain containing 149	0.170	1.125	4.78E-01	4.78E+01		
8099696	NM_153825	SEPSECS	Sep (O-phosphoserine) tRNA:Sec (	0.031	1.022	9.09E-01	9.09E+01		
8099721	NM_015187	KIAA0746	KIAA0746 protein	0.121	1.087	2.23E-01	2.23E+01		
8099746	NM_000730	CCKAR	cholecystokinin A receptor	-0.053	0.964	7.75E-01	7.75E+01		
8099760	NM_015230	ARAP2	ArfGAP with RhoGAP domain, ank	-0.083	0.944	6.85E-01	6.85E+01		
8099797	NM_00108539	RELL1	RELT-like 1	-0.025	0.983	8.71E-01	8.71E+01		
8099807	AF220235	GAFA3	FGF-2 activity-associated protein 3	0.086	1.061	6.70E-01	6.70E+01		
8099817	NR_026804	FLJ13197	hypothetical FLJ13197	0.041	1.029	8.78E-01	8.78E+01		
8099826	NM_030956	TLR10	toll-like receptor 10	0.174	1.128	5.21E-01	5.21E+01		
8099841	NM_006068	TLR6	toll-like receptor 6	0.259	1.196	2.78E-01	2.78E+01		
8099860	NM_002913	RFC1	replication factor C (activator 1) 1,	0.020	1.014	8.88E-01	8.88E+01		

8099887	NM_00102492	RPL9	ribosomal protein L9	0.003	1.002	9.85E-01	9.85E+01		
8099897	NM_003359	UGDH	UDP-glucose dehydrogenase	-0.007	0.995	9.84E-01	9.84E+01		
8099912	BC008502	C4orf34	chromosome 4 open reading fram	0.085	1.061	6.90E-01	6.90E+01		
8099918	AK291454	UBE2K	ubiquitin-conjugating enzyme E2K	-0.030	0.979	8.49E-01	8.49E+01		
8099922	---	---	---	-0.023	0.984	9.74E-01	9.74E+01		
8099924	---	---	---	-0.062	0.958	5.91E-01	5.91E+01		
8099926	NM_00110039	PDS5A	PDS5, regulator of cohesion maint	0.009	1.006	9.49E-01	9.49E+01		
8099965	NM_007274	ACOT7	acyl-CoA thioesterase 7	-0.083	0.944	6.74E-01	6.74E+01		
8099967	NM_00109863	RBM47	RNA binding motif protein 47	0.397	1.317	1.07E-01	1.07E+01		
8100001	---	---	---	-0.005	0.997	9.86E-01	9.86E+01		
8100015	NM_207406	BEND4	BEN domain containing 4	0.017	1.012	9.34E-01	9.34E+01		
8100026	NM_006095	ATP8A1	ATPase, aminophospholipid transp	0.049	1.034	8.39E-01	8.39E+01		
8100068	---	---	---	-0.033	0.977	8.57E-01	8.57E+01		
8100070	NM_198353	KCTD8	potassium channel tetramerisation	-0.040	0.973	7.33E-01	7.33E+01		
8100125	---	---	---	-0.042	0.972	9.04E-01	9.04E+01		
8100179	NM_152995	NFXL1	nuclear transcription factor, X-box	0.119	1.086	4.88E-01	4.88E+01		
8100210	NM_003328	TXK	TXK tyrosine kinase	0.205	1.152	3.44E-01	3.44E+01		
8100227	---	---	---	0.035	1.024	9.07E-01	9.07E+01		
8100251	NM_015030	FRYL	FRY-like	-0.031	0.978	8.71E-01	8.71E+01		
8100292	NM_015030	FRYL	FRY-like	0.041	1.029	7.29E-01	7.29E+01		
8100298	NM_00101444	OCIAD2	OCIA domain containing 2	-0.241	0.846	1.66E-01	1.66E+01		
8100308	---	---	---	-0.044	0.970	9.36E-01	9.36E+01		
8100310	---	---	---	-0.240	0.847	6.35E-01	6.35E+01		
8100328	NM_022832	USP46	ubiquitin specific peptidase 46	0.078	1.055	6.04E-01	6.04E+01		
8100347	NM_152540	SCFD2	sec1 family domain containing 2	0.052	1.037	8.46E-01	8.46E+01		
8100362	NM_00112632	LNX1	ligand of numb-protein X 1	0.066	1.046	5.04E-01	5.04E+01		
8100376	---	---	---	-0.068	0.954	6.69E-01	6.69E+01		
8100378	NR_027153	RPL21P44	ribosomal protein L21 pseudogene	-0.089	0.940	6.94E-01	6.94E+01		
8100382	NM_012110	CHIC2	cysteine-rich hydrophobic domain	-0.093	0.938	3.83E-01	3.83E+01		
8100426	---	---	---	-0.110	0.926	4.10E-01	4.10E+01		
8100428	NM_004898	CLOCK	clock homolog (mouse)	0.056	1.040	8.27E-01	8.27E+01		
8100456	---	---	---	0.130	1.095	6.47E-01	6.47E+01		
8100495	NM_002703	PPAT	phosphoribosyl pyrophosphate an	0.130	1.094	4.26E-01	4.26E+01		
8100532	NM_032313	C4orf14	chromosome 4 open reading fram	-0.023	0.984	8.92E-01	8.92E+01		
8100541	NM_001553	IGFBP7	insulin-like growth factor binding	-0.051	0.965	7.27E-01	7.27E+01		
8100603	NM_001812	CENPC1	centromere protein C 1	-0.067	0.954	7.78E-01	7.78E+01		
8100615	NM_018227	UBA6	ubiquitin-like modifier activating e	-0.073	0.951	5.65E-01	5.65E+01		
8100714	NM_00103173	YTHDC1	YTH domain containing 1	-0.083	0.944	2.08E-01	2.08E+01		
8100754	---	---	---	0.039	1.028	8.21E-01	8.21E+01		
8100782	---	---	---	-0.031	0.979	8.13E-01	8.13E+01		
8100834	NM_002092	GRSF1	G-rich RNA sequence binding facto	0.010	1.007	9.54E-01	9.54E+01		
8100851	---	---	---	0.045	1.032	8.05E-01	8.05E+01		
8100893	NM_173827	COX18	COX18 cytochrome c oxidase asse	0.134	1.098	2.93E-01	2.93E+01		
8100902	NM_032217	ANKRD17	ankyrin repeat domain 17	-0.002	0.998	9.86E-01	9.86E+01		
8100941	---	---	---	0.069	1.049	9.04E-01	9.04E+01		
8100962	---	---	---	-0.080	0.946	4.66E-01	4.66E+01		
8100964	---	---	---	-0.093	0.938	5.06E-01	5.06E+01		
8100966	NM_002619	PF4	platelet factor 4	0.179	1.132	7.17E-02	7.17E+00		
8100971	NM_002704	PPBP	pro-platelet basic protein (chemok	0.230	1.173	7.94E-01	7.94E+01		
8101000	ENST00000437	LOC541467	hypothetical LOC541467	-0.011	0.992	9.49E-01	9.49E+01		
8101013	NM_015436	RCHY1	ring finger and CHY zinc finger dor	0.014	1.009	9.53E-01	9.53E+01		
8101043	NM_203505	G3BP2	GTPase activating protein (SH3 do	-0.029	0.980	8.33E-01	8.33E+01		
8101061	---	---	---	0.042	1.030	8.40E-01	8.40E+01		
8101065	NM_006239	PPEF2	protein phosphatase, EF-hand calc	0.066	1.047	6.39E-01	6.39E+01		
8101086	NM_014435	NAAA	N-acylethanolamine acid amidase	0.155	1.114	5.23E-01	5.23E+01		
8101141	---	---	---	0.012	1.008	9.53E-01	9.53E+01		
8101210	---	---	---	-0.294	0.816	6.19E-02	6.19E+00		
8101212	NM_006835	CCNI	cyclin I	0.049	1.035	5.34E-01	5.34E+01		
8101224	---	---	---	0.013	1.009	9.64E-01	9.64E+01		
8101228	NM_144571	CNOT6L	CCR4-NOT transcription complex,	-0.062	0.958	6.57E-01	6.57E+01		
8101253	NM_032693	ARD1B	ARD1 homolog B (S. cerevisiae)	-0.006	0.996	9.83E-01	9.83E+01		
8101260	NM_058172	ANTXR2	anthrax toxin receptor 2	-0.191	0.876	7.47E-02	7.47E+00		
8101324	NM_031370	HNRNPD	heterogeneous nuclear ribonucleo	-0.007	0.995	9.39E-01	9.39E+01		
8101340	NR_003249	HNRPDL	heterogeneous nuclear ribonucleo	-0.008	0.994	9.49E-01	9.49E+01		
8101366	NM_00103758	SCD5	stearoyl-CoA desaturase 5	-0.091	0.939	4.53E-01	4.53E+01		
8101376	NM_014933	SEC31A	SEC31 homolog A (S. cerevisiae)	0.079	1.056	2.75E-01	2.75E+01		
8101429	NM_016619	PLAC8	placenta-specific 8	-0.097	0.935	7.78E-01	7.78E+01		
8101437	NM_015697	COQ2	coenzyme Q2 homolog, prenyltrar	-0.050	0.966	7.55E-01	7.55E+01		
8101449	NM_00109854	HPSE	heparanase	-0.397	0.759	1.27E-01	1.27E+01		
8101489	NM_139076	FAM175A	family with sequence similarity 17	-0.050	0.966	8.36E-01	8.36E+01		
8101507	NM_006168	NKX6-1	NK6 homeobox 1	-0.040	0.973	6.97E-01	6.97E+01		
8101511	NM_014991	WDFY3	WD repeat and FYVE domain conta	0.429	1.347	2.07E-01	2.07E+01		
8101609	NM_197965	SLC10A6	solute carrier family 10 (sodium/b	-0.007	0.995	9.75E-01	9.75E+01		
8101622	NM_138501	TECR	trans-2,3-enoyl-CoA reductase	-0.080	0.946	6.24E-01	6.24E+01		
8101624	NM_020803	KLHL8	kelch-like 8 (Drosophila)	0.128	1.093	1.82E-01	1.82E+01		
8101648	NM_016245	HSD17B11	hydroxysteroid (17-beta) dehydro	0.066	1.047	7.87E-01	7.87E+01		
8101657	---	---	---	-0.061	0.959	7.62E-01	7.62E+01		

8101699	---	---	---	0.112	1.081	6.54E-01	6.54E+01		
8101701	NM_152542	PPM1K	protein phosphatase 1K (PP2C dor	0.033	1.023	8.97E-01	8.97E+01		
8101718	NM_032906	PIGY	phosphatidylinositol glycan ancho	0.061	1.043	5.79E-01	5.79E+01		
8101723	NM_153757	NAP1L5	nucleosome assembly protein 1-lik	0.016	1.011	9.27E-01	9.27E+01		
8101728	NM_014883	FAM13A	family with sequence similarity 13	0.120	1.086	4.48E-01	4.48E+01		
8101757	NM_198281	GPRIN3	GPRIN family member 3	0.040	1.028	8.11E-01	8.11E+01		
8101762	NM_000345	SNCA	synuclein, alpha (non A4 compone	0.296	1.228	2.11E-02	2.11E+00		
8101774	NM_183049	TMSL3	thymosin-like 3	-0.009	0.994	9.22E-01	9.22E+01		
8101828	NM_005723	TSPAN5	tetraspanin 5	-0.235	0.850	2.26E-01	2.26E+01		
8101839	NM_001968	EIF4E	eukaryotic translation initiation fa	0.029	1.020	8.73E-01	8.73E+01		
8101844	NM_000671	ADH5	alcohol dehydrogenase 5 (class III)	0.006	1.004	9.83E-01	9.83E+01		
8101925	NM_021970	MAPKSP1	MAPK scaffold protein 1	0.045	1.032	8.40E-01	8.40E+01		
8101934	NM_00103172	DNAJB14	DnaJ (Hsp40) homolog, subfamily	0.050	1.035	6.73E-01	6.73E+01		
8101945	NM_002106	H2AFZ	H2A histone family, member Z	-0.111	0.926	3.70E-01	3.70E+01		
8101971	NM_000944	PPP3CA	protein phosphatase 3 (formerly 2	0.013	1.009	9.56E-01	9.56E+01		
8101992	NM_022154	SLC39A8	solute carrier family 39 (zinc trans	0.056	1.039	9.56E-01	9.56E+01		
8102006	NM_005908	MANBA	mannosidase, beta A, lysosomal	0.155	1.113	6.04E-01	6.04E+01		
8102024	NM_181886	UBE2D3	ubiquitin-conjugating enzyme E2D	-0.019	0.987	9.19E-01	9.19E+01		
8102037	---	---	---	0.061	1.043	8.89E-01	8.89E+01		
8102050	NM_178833	NHEDC2	Na+/H+ exchanger domain contair	-0.133	0.912	3.94E-01	3.94E+01		
8102171	NM_033115	TBCK	TBC1 domain containing kinase	0.096	1.069	6.13E-01	6.13E+01		
8102214	NM_005443	PAPSS1	3'-phosphoadenosine 5'-phosphos	0.299	1.230	2.41E-01	2.41E+01		
8102232	NM_016269	LEF1	lymphoid enhancer-binding factor	0.021	1.015	9.69E-01	9.69E+01		
8102311	NM_001226	CASP6	caspase 6, apoptosis-related cyste	-0.013	0.991	9.61E-01	9.61E+01		
8102321	NM_030821	PLA2G12A	phospholipase A2, group XIIA	-0.047	0.968	7.71E-01	7.71E+01		
8102350	---	---	---	0.192	1.142	6.47E-02	6.47E+00		
8102368	NM_024019	NEUROG2	neurogenin 2	-0.034	0.977	7.12E-01	7.12E+01		
8102410	NM_001029	RPS26	ribosomal protein S26	0.013	1.009	9.77E-01	9.77E+01		
8102415	NM_001221	CAMK2D	calcium/calmodulin-dependent pr	0.203	1.151	1.84E-01	1.84E+01		
8102468	NM_003619	PRSS12	protease, serine, 12 (neurotrypsin	0.009	1.006	9.61E-01	9.61E+01		
8102482	NM_014822	SEC24D	SEC24 family, member D (S. cerevi	0.106	1.076	5.72E-01	5.72E+01		
8102518	NM_00100170	C4orf3	chromosome 4 open reading fram	0.008	1.005	9.49E-01	9.49E+01		
8102530	---	---	---	0.267	1.203	4.25E-01	4.25E+01		
8102619	NM_001154	ANXA5	annexin A5	0.092	1.066	7.75E-01	7.75E+01		
8102636	NM_152399	TMEM155	transmembrane protein 155	0.002	1.001	9.93E-01	9.93E+01		
8102643	NM_001237	CCNA2	cyclin A2	0.184	1.136	1.05E-01	1.05E+01		
8102695	---	---	---	-0.002	0.999	9.94E-01	9.94E+01		
8102728	---	---	---	0.019	1.013	9.63E-01	9.63E+01		
8102730	NM_152778	MFSD8	major facilitator superfamily doma	-0.023	0.984	9.18E-01	9.18E+01		
8102745	NM_006320	PGRMC2	progesterone receptor membrane	0.004	1.003	9.85E-01	9.85E+01		
8102751	NM_144643	SCLT1	sodium channel and clathrin linker	0.200	1.149	3.54E-02	3.54E+00		
8102781	ENST00000330	LOC100292301	similar to hCG1993567	0.045	1.032	8.87E-01	8.87E+01		
8102783	---	---	---	-0.008	0.994	9.75E-01	9.75E+01		
8102789	NM_017489	TERF1	telomeric repeat binding factor (N	-0.074	0.950	5.91E-01	5.91E+01		
8102817	NM_201999	ELF2	E74-like factor 2 (ets domain trans	-0.047	0.968	7.32E-01	7.32E+01		
8102839	NM_002494	NDUFC1	NADH dehydrogenase (ubiquinone	-0.028	0.981	8.78E-01	8.78E+01		
8102848	NM_030648	SETD7	SET domain containing (lysine met	0.065	1.046	8.18E-01	8.18E+01		
8102860	NM_002107 //	H3F3A // H3F3A	H3 histone, family 3A // H3 histon	0.099	1.071	3.19E-01	3.19E+01		
8102862	NM_018717	MAML3	mastermind-like 3 (Drosophila)	-0.072	0.951	6.23E-01	6.23E+01		
8102869	---	---	---	-0.065	0.956	7.86E-01	7.86E+01		
8102875	---	---	---	0.020	1.014	9.49E-01	9.49E+01		
8102912	NM_015130	TBC1D9	TBC1 domain family, member 9 (w	-0.043	0.971	9.05E-01	9.05E+01		
8102950	NM_003866	INPP4B	inositol polyphosphate-4-phospha	-0.019	0.987	9.66E-01	9.66E+01		
8102979	---	---	---	0.096	1.069	7.38E-01	7.38E+01		
8102982	BC064848	GAB1	GRB2-associated binding protein 1	-0.090	0.939	3.35E-01	3.35E+01		
8102984	---	---	---	-0.005	0.996	9.81E-01	9.81E+01		
8103005	NM_014885	ANAPC10	anaphase promoting complex sub	0.142	1.103	5.44E-01	5.44E+01		
8103011	NM_199324	OTUD4	OTU domain containing 4	0.137	1.100	3.55E-01	3.55E+01		
8103023	---	---	---	-0.071	0.952	7.01E-01	7.01E+01		
8103025	NM_178835	ZNF827	zinc finger protein 827	-0.093	0.937	4.55E-01	4.55E+01		
8103043	NM_00102999	SLC10A7	solute carrier family 10 (sodium/b	0.149	1.108	1.74E-01	1.74E+01		
8103079	NM_138364	PRMT10	protein arginine methyltransferase	-0.087	0.941	7.18E-01	7.18E+01		
8103094	NM_000901	NR3C2	nuclear receptor subfamily 3, grou	-0.038	0.974	8.74E-01	8.74E+01		
8103106	NM_006726	LRBA	LPS-responsive vesicle trafficking,	0.002	1.001	9.93E-01	9.93E+01		
8103188	NM_004564	PET112L	PET112-like (yeast)	0.115	1.083	2.53E-01	2.53E+01		
8103206	NM_033632	FBXW7	F-box and WD repeat domain cont	-0.068	0.954	5.23E-01	5.23E+01		
8103222	---	---	---	0.053	1.037	5.58E-01	5.58E+01		
8103226	NM_152680	TMEM154	transmembrane protein 154	0.279	1.213	8.79E-02	8.79E+00		
8103240	NR_001562	ANXA2P1	annexin A2 pseudogene 1	0.106	1.076	6.03E-01	6.03E+01		
8103244	NM_173662	RNF175	ring finger protein 175	-0.216	0.861	2.91E-01	2.91E+01		
8103289	NM_002669	PLRG1	pleiotropic regulator 1 (PRL1 hom	0.041	1.029	8.05E-01	8.05E+01		
8103309	---	---	---	0.025	1.018	9.58E-01	9.58E+01		
8103389	NM_001334	CTSO	cathepsin O	0.177	1.130	4.59E-01	4.59E+01		
8103413	---	---	---	-0.083	0.944	7.85E-01	7.85E+01		
8103431	NM_00100839	C4orf46	chromosome 4 open reading fram	-0.085	0.943	5.17E-01	5.17E+01		
8103437	NM_005038	PPID	peptidylprolyl isomerase D	-0.002	0.998	9.90E-01	9.90E+01		
8103483	---	---	---	0.369	1.291	3.44E-01	3.44E+01		

8103518	NM_012403	ANP32C	acidic (leucine-rich) nuclear phosp	-0.010	0.993	9.38E-01	9.38E+01		
8103520	NM_00101241	TRIM61	tripartite motif-containing 61	0.068	1.048	7.73E-01	7.73E+01		
8103535	NR_026575	GK3P	glycerol kinase 3 pseudogene	-0.083	0.944	6.34E-01	6.34E+01		
8103563	NM_017631	DDX60	DEAD (Asp-Glu-Ala-Asp) box polyp	0.191	1.141	5.63E-01	5.63E+01		
8103601	NM_00101296	DDX60L	DEAD (Asp-Glu-Ala-Asp) box polyp	0.078	1.056	8.63E-01	8.63E+01		
8103622	NM_032783	CBR4	carbonyl reductase 4	0.071	1.051	6.18E-01	6.18E+01		
8103646	NM_012224	NEK1	NIMA (never in mitosis gene a)-rel	-0.202	0.869	1.07E-01	1.07E+01		
8103684	NM_017867	C4orf27	chromosome 4 open reading fram	-0.036	0.975	8.76E-01	8.76E+01		
8103725	---	---	---	0.032	1.022	9.12E-01	9.12E+01		
8103728	NM_00113068	HMGB2	high-mobility group box 2	-0.054	0.963	8.10E-01	8.10E+01		
8103743	---	---	---	0.029	1.020	8.13E-01	8.13E+01		
8103753	NM_006792	MORF4	mortality factor 4	-0.046	0.968	7.09E-01	7.09E+01		
8103755	NM_012180	FBXO8	F-box protein 8	0.045	1.032	7.99E-01	7.99E+01		
8103822	NM_005429	VEGFC	vascular endothelial growth factor	0.022	1.015	8.68E-01	8.68E+01		
8103834	NM_000027	AGA	aspartylglucosaminidase	0.158	1.115	2.21E-01	2.21E+01		
8103847	---	---	---	-0.049	0.967	9.12E-01	9.12E+01		
8103853	NR_027107	MGC45800	hypothetical LOC90768	-0.023	0.984	8.88E-01	8.88E+01		
8103859	NM_00101273	DCTD	dCMP deaminase	0.023	1.016	9.03E-01	9.03E+01		
8103873	---	---	---	-0.064	0.957	5.74E-01	5.74E+01		
8103875	---	---	---	0.152	1.111	5.07E-01	5.07E+01		
8103881	NM_152682	RWDD4A	RWD domain containing 4A	0.075	1.053	6.59E-01	6.59E+01		
8103911	NM_002199	IRF2	interferon regulatory factor 2	0.047	1.033	7.99E-01	7.99E+01		
8103922	NM_004346	CASP3	caspase 3, apoptosis-related cyste	-0.013	0.991	9.71E-01	9.71E+01		
8103951	NM_001995	ACSL1	acyl-CoA synthetase long-chain fa	-0.282	0.822	3.83E-01	3.83E+01		
8103975	NR_003542	UNQ9368	RTFV9368	-0.218	0.860	2.78E-01	2.78E+01		
8103979	NM_020827	KIAA1430	KIAA1430	-0.035	0.976	8.74E-01	8.74E+01		
8104012	---	---	---	0.155	1.114	6.41E-01	6.41E+01		
8104070	---	---	---	-0.056	0.962	8.06E-01	8.06E+01		
8104072	ENST00000423	FLJ38576	hypothetical LOC651430	-0.007	0.995	9.73E-01	9.73E+01		
8104115	---	---	---	0.041	1.029	7.97E-01	7.97E+01		
8104119	NM_020040	TUBB4Q	tubulin, beta polypeptide 4, memb	0.117	1.084	4.13E-01	4.13E+01		
8104122	NM_033178	DUX4	double homeobox, 4	-0.025	0.983	8.13E-01	8.13E+01		
8104136	NM_018942	HMX1	H6 family homeobox 1	0.017	1.012	8.96E-01	8.96E+01		
8104139	NR_027420 //	89834 // LOC38	ankyrin repeat domain 57 pseudog	-0.015	0.990	9.85E-01	9.85E+01		
8104141	NM_052909	PLEKHG4B	pleckstrin homology domain conta	-0.042	0.971	7.15E-01	7.15E+01		
8104166	NM_004168	SDHA	succinate dehydrogenase complex	-0.021	0.985	9.16E-01	9.16E+01		
8104180	NM_020731	AHRR	aryl-hydrocarbon receptor repress	-0.045	0.969	7.17E-01	7.17E+01		
8104201	NM_007277	EXOC3	exocyst complex component 3	-0.035	0.976	7.86E-01	7.86E+01		
8104220	NM_018140	CEP72	centrosomal protein 72kDa	-0.045	0.969	6.31E-01	6.31E+01		
8104253	NM_033120	NKD2	naked cuticle homolog 2 (Drosoph	0.020	1.014	8.62E-01	8.62E+01		
8104268	NM_00100384	SLC6A19	solute carrier family 6 (neutral am	0.030	1.021	8.41E-01	8.41E+01		
8104281	NM_182632	SLC6A18	solute carrier family 6, member 18	-0.026	0.982	8.36E-01	8.36E+01		
8104298	NM_004553	NDUFS6	NADH dehydrogenase (ubiquinone	-0.023	0.984	8.96E-01	8.96E+01		
8104307	AY249325	C5orf38	chromosome 5 open reading fram	0.040	1.028	7.83E-01	7.83E+01		
8104314	NM_024337	IRX1	iroquois homeobox 1	0.033	1.023	7.82E-01	7.82E+01		
8104319	---	---	---	-0.014	0.990	9.27E-01	9.27E+01		
8104321	NM_139056	ADAMTS16	ADAM metallopeptidase with thro	0.002	1.001	9.90E-01	9.90E+01		
8104348	---	---	---	0.043	1.030	9.03E-01	9.03E+01		
8104350	NM_015325	KIAA0947	KIAA0947	0.030	1.021	8.44E-01	8.44E+01		
8104369	NM_001047	SRD5A1	steroid-5-alpha-reductase, alpha p	-0.026	0.982	8.92E-01	8.92E+01		
8104378	NM_006999	POLS	polymerase (DNA directed) sigma	-0.026	0.982	8.98E-01	8.98E+01		
8104394	NM_020546	ADCY2	adenylate cyclase 2 (brain)	-0.005	0.996	9.73E-01	9.73E+01		
8104422	NM_002454	MTRR	5-methyltetrahydrofolate-homocy	0.043	1.030	7.52E-01	7.52E+01		
8104449	NM_012073	CCT5	chaperonin containing TCP1, subu	0.032	1.022	8.78E-01	8.78E+01		
8104461	---	---	---	-0.019	0.987	9.56E-01	9.56E+01		
8104463	NM_005885	40243	membrane-associated ring finger (	-0.012	0.991	9.44E-01	9.44E+01		
8104499	ENST00000296	ANKRD33B	ankyrin repeat domain 33B	-0.024	0.983	8.43E-01	8.43E+01		
8104506	NM_007118	TRIO	triple functional domain (PTPRF in	-0.021	0.986	9.49E-01	9.49E+01		
8104568	AY358688	UNQ1870	GALI1870	-0.043	0.970	8.70E-01	8.70E+01		
8104570	NM_019018	FAM105A	family with sequence similarity 10	0.296	1.227	4.59E-02	4.59E+00		
8104580	NM_138348	FAM105B	family with sequence similarity 10	-0.205	0.868	3.31E-02	3.31E+00		
8104590	AK129556	LOC100130744	hypothetical LOC100130744	-0.154	0.899	4.69E-02	4.69E+00		
8104592	NM_012304	FBXL7	F-box and leucine-rich repeat prot	-0.028	0.981	8.30E-01	8.30E+01		
8104601	NM_006317	BASP1	brain abundant, membrane attach	-0.228	0.854	5.46E-02	5.46E+00		
8104609	---	---	---	-0.079	0.947	5.30E-01	5.30E+01		
8104613	ENST00000330	// LOC391742 /	TBP-associated factor 11 pseudog	0.028	1.019	8.78E-01	8.78E+01		
8104617	XM_936262 //	91764 // LOC39	similar to TBP-associated factor 11	0.029	1.020	8.33E-01	8.33E+01		
8104621	NR_003660	GUSBL2	glucuronidase, beta-like 2	0.012	1.009	9.76E-01	9.76E+01		
8104693	NM_178140	PDZD2	PDZ domain containing 2	-0.009	0.994	9.63E-01	9.63E+01		
8104725	---	---	---	0.080	1.057	5.12E-01	5.12E+01		
8104727	---	---	---	0.038	1.026	8.11E-01	8.11E+01		
8104731	---	---	---	0.166	1.122	1.24E-01	1.24E+01		
8104738	NM_006713	SUB1	SUB1 homolog (S. cerevisiae)	0.077	1.055	2.39E-01	2.39E+01		
8104760	NM_152295	TARS	threonyl-tRNA synthetase	-0.080	0.946	5.55E-01	5.55E+01		
8104781	NM_016568	RXFP3	relaxin/insulin-like family peptide	-0.029	0.980	7.94E-01	7.94E+01		
8104825	NM_018321	BXDC2	brix domain containing 2	0.021	1.015	9.53E-01	9.53E+01		
8104838	NM_194283	DNAJC21	DnaJ (Hsp40) homolog, subfamily	-0.055	0.962	5.73E-01	5.73E+01		

8104901	NM_002185	IL7R	interleukin 7 receptor	-0.220	0.859	1.89E-02	1.89E+00		
8104912	NM_005983	SKP2	S-phase kinase-associated protein	0.038	1.026	8.68E-01	8.68E+01		
8104930	NM_004172	SLC1A3	solute carrier family 1 (glial high af	0.661	1.581	7.07E-02	7.07E+00		
8104944	NM_015384	NIPBL	Nipped-B homolog (Drosophila)	-0.030	0.979	8.08E-01	8.08E+01		
8104996	---	---	---	0.014	1.009	9.66E-01	9.66E+01		
8104998	NM_018034	WDR70	WD repeat domain 70	0.109	1.079	2.81E-01	2.81E+01		
8105061	NM_001465	FYB	FYN binding protein (FYB-120/130	0.253	1.192	1.90E-02	1.90E+00		
8105063	---	---	---	-0.062	0.958	4.29E-01	4.29E+01		
8105067	NM_000958	PTGER4	prostaglandin E receptor 4 (subtyp	0.008	1.006	9.71E-01	9.71E+01		
8105104	NM_175921	C5orf51	chromosome 5 open reading fram	0.074	1.053	6.62E-01	6.62E+01		
8105132	AK123995	LOC153684	hypothetical LOC153684	0.048	1.034	7.38E-01	7.38E+01		
8105136	NM_003432	ZNF131	zinc finger protein 131	-0.122	0.919	4.67E-01	4.67E+01		
8105144	---	---	---	-0.043	0.971	8.45E-01	8.45E+01		
8105146	NM_153361	MGC42105	serine/threonine-protein kinase N	-0.131	0.913	2.47E-01	2.47E+01		
8105153	NM_012343	NNT	nicotinamide nucleotide transhydr	0.109	1.079	2.99E-01	2.99E+01		
8105181	NM_016640	MRPS30	mitochondrial ribosomal protein S	0.055	1.039	6.83E-01	6.83E+01		
8105189	BC137195	LOC100287671	hypothetical protein LOC10028767	-0.078	0.947	5.61E-01	5.61E+01		
8105191	NM_024615	PARP8	poly (ADP-ribose) polymerase fam	-0.011	0.992	9.65E-01	9.65E+01		
8105311	NM_002495	NDUFS4	NADH dehydrogenase (ubiquinone	0.238	1.179	3.32E-03	3.32E-01		
8105321	---	---	---	-0.050	0.966	9.05E-01	9.05E+01		
8105328	NM_052870	SNX18	sorting nexin 18	-0.018	0.988	9.49E-01	9.49E+01		
8105331	NM_002104	GZMK	granzyme K (granzyme 3; tryptase	0.395	1.315	3.22E-01	3.22E+01		
8105353	NM_015360	SKIV2L2	superkiller viralicidic activity 2-like	0.047	1.033	6.23E-01	6.23E+01		
8105432	NM_021104	RPL41	ribosomal protein L41	0.003	1.002	9.73E-01	9.73E+01		
8105434	---	---	---	-0.013	0.991	9.74E-01	9.74E+01		
8105436	NM_005921	MAP3K1	mitogen-activated protein kinase k	0.103	1.074	4.09E-01	4.09E+01		
8105463	NM_022913	GPBP1	GC-rich promoter binding protein	-0.052	0.964	5.82E-01	5.82E+01		
8105499	NM_174889	NDUFAF2	NADH dehydrogenase (ubiquinone	0.022	1.016	9.27E-01	9.27E+01		
8105504	NM_152789	FAM133B	family with sequence similarity 13	0.051	1.036	6.85E-01	6.85E+01		
8105506	NM_020928	ZSWIM6	zinc finger, SWIM-type containing	0.107	1.077	6.23E-01	6.23E+01		
8105523	NM_004520	KIF2A	kinesin heavy chain member 2A	0.004	1.003	9.81E-01	9.81E+01		
8105612	NM_005869	SDCCAG10	serologically defined colon cancer	0.080	1.057	4.82E-01	4.82E+01		
8105631	---	---	---	-0.081	0.945	2.52E-01	2.52E+01		
8105633	NM_015342	PPWD1	peptidylprolyl isomerase domain a	0.011	1.008	9.55E-01	9.55E+01		
8105647	NM_00109375	C5orf44	chromosome 5 open reading fram	0.110	1.079	4.39E-01	4.39E+01		
8105681	NM_018695	ERBB2IP	erbb2 interacting protein	0.007	1.005	9.73E-01	9.73E+01		
8105714	NM_139168	SFRS12	splicing factor, arginine/serine-rich	-0.017	0.988	9.09E-01	9.09E+01		
8105739	---	---	---	-0.046	0.969	8.02E-01	8.02E+01		
8105741	NM_015183	MAST4	microtubule associated serine/thr	-0.096	0.935	2.44E-01	2.44E+01		
8105778	NM_181523	PIK3R1	phosphoinositide-3-kinase, regula	-0.093	0.937	6.22E-01	6.22E+01		
8105801	NM_022902	SLC30A5	solute carrier family 30 (zinc trans	-0.019	0.987	9.16E-01	9.16E+01		
8105878	NM_133338	RAD17	RAD17 homolog (S. pombe)	0.093	1.067	5.96E-01	5.96E+01		
8105908	NM_002538	OCLN	occludin	-0.149	0.902	7.02E-01	7.02E+01		
8105918	NM_001515	GTF2H2	general transcription factor IIH, po	0.083	1.059	6.95E-01	6.95E+01		
8105935	AK302597	POM121L8P	POM121 membrane glycoprotein-	0.199	1.148	4.09E-01	4.09E+01		
8105937	AK289851	SMA5	glucuronidase, beta pseudogene	-0.019	0.987	9.59E-01	9.59E+01		
8105958	NM_000344	SMN1	survival of motor neuron 1, telome	-0.005	0.996	9.82E-01	9.82E+01		
8105970	NM_001515	GTF2H2	general transcription factor IIH, po	0.083	1.059	7.15E-01	7.15E+01		
8105987	---	---	---	0.123	1.089	3.85E-01	3.85E+01		
8105989	AK302597	POM121L8P	POM121 membrane glycoprotein-	0.192	1.143	4.05E-01	4.05E+01		
8105991	BC171739	GUSBL1	glucuronidase, beta-like 1	-0.007	0.995	9.85E-01	9.85E+01		
8105995	AK289851	SMA5	glucuronidase, beta pseudogene	0.006	1.004	9.87E-01	9.87E+01		
8106006	NM_000344	SMN1	survival of motor neuron 1, telome	0.010	1.007	9.63E-01	9.63E+01		
8106017	---	---	---	-0.025	0.983	9.51E-01	9.51E+01		
8106025	NM_018429	BDP1	B double prime 1, subunit of RNA	-0.065	0.956	5.23E-01	5.23E+01		
8106068	NM_022132	MCCC2	methylcrotonoyl-Coenzyme A carb	0.048	1.034	8.04E-01	8.04E+01		
8106096	---	---	---	0.054	1.038	6.42E-01	6.42E+01		
8106098	NM_005909	MAP1B	microtubule-associated protein 1B	-0.068	0.954	5.84E-01	5.84E+01		
8106107	NM_024754	PTCD2	pentatricopeptide repeat domain	0.082	1.058	6.41E-01	6.41E+01		
8106122	NM_002270	TNPO1	transportin 1	-0.018	0.988	9.18E-01	9.18E+01		
8106181	NM_00103763	BTF3	basic transcription factor 3	0.007	1.005	9.68E-01	9.68E+01		
8106250	---	---	---	-0.007	0.995	9.69E-01	9.69E+01		
8106252	NM_000521	HEXB	hexosaminidase B (beta polypeptic	0.345	1.270	1.10E-01	1.10E+01		
8106271	NM_014886	TINP1	TGF beta-inducible nuclear protein	0.052	1.036	7.61E-01	7.61E+01		
8106278	---	---	---	-0.033	0.978	9.54E-01	9.54E+01		
8106280	NM_000859	HMGCR	3-hydroxy-3-methylglutaryl-Coenz	-0.086	0.942	5.48E-01	5.48E+01		
8106352	XM_00171851	LOC100129566	hypothetical protein LOC1001295(	0.016	1.011	9.49E-01	9.49E+01		
8106354	NM_006633	IQGAP2	IQ motif containing GTPase activat	0.315	1.244	1.48E-02	1.48E+00		
8106393	NM_001992	F2R	coagulation factor II (thrombin) re	-0.096	0.935	7.45E-01	7.45E+01		
8106403	NM_005242	F2RL1	coagulation factor II (thrombin) re	-0.111	0.926	2.42E-01	2.42E+01		
8106429	NM_018046	AGGF1	angiogenic factor with G patch and	0.083	1.059	3.29E-01	3.29E+01		
8106473	---	---	---	-0.015	0.990	9.49E-01	9.49E+01		
8106475	---	---	---	0.077	1.055	6.66E-01	6.66E+01		
8106479	NM_004866	SCAMP1	secretory carrier membrane prote	-0.081	0.945	5.83E-01	5.83E+01		
8106516	NM_152405	JMY	junction mediating and regulatory	-0.259	0.836	1.36E-01	1.36E+01		
8106528	---	---	---	-0.232	0.852	3.55E-01	3.55E+01		
8106530	---	---	---	-0.056	0.962	7.53E-01	7.53E+01		

8106532	---	---	---	0.008	1.005	9.80E-01	9.80E+01		
8106534	NM_00111439	PAPD4	PAP associated domain containing	0.158	1.116	2.36E-02	2.36E+00		
8106573	NM_003248	THBS4	thrombospondin 4	-0.021	0.985	9.18E-01	9.18E+01		
8106633	NM_002439	MSH3	mutS homolog 3 (E. coli)	0.092	1.066	5.55E-01	5.55E+01		
8106660	NM_006909	RASGRF2	Ras protein-specific guanine nucle	0.095	1.068	7.17E-01	7.17E+01		
8106689	NM_001825	CKMT2	creatine kinase, mitochondrial 2 (s	-0.111	0.926	2.53E-01	2.53E+01		
8106702	NM_032280	ZCCHC9	zinc finger, CCHC domain containi	0.004	1.003	9.85E-01	9.85E+01		
8106709	NM_031482	ATG10	ATG10 autophagy related 10 hom	-0.063	0.958	8.66E-01	8.66E+01		
8106722	NM_00101797	ATP6AP1L	ATPase, H+ transporting, lysosoma	0.204	1.152	7.19E-02	7.19E+00		
8106730	NM_022550	XRCC4	X-ray repair complementing defec	0.117	1.085	6.65E-01	6.65E+01		
8106743	NM_004385	VCAN	versican	0.012	1.008	9.94E-01	9.94E+01		
8106761	---	---	---	0.041	1.029	8.05E-01	8.05E+01		
8106769	XR_019029	LOC645181	similar to PDGFA associated prote	0.021	1.015	9.16E-01	9.16E+01		
8106776	NM_001867	COX7C	cytochrome c oxidase subunit VIIc	-0.031	0.979	8.48E-01	8.48E+01		
8106784	NM_002890	RASA1	RAS p21 protein activator (GTPase	0.039	1.027	7.59E-01	7.59E+01		
8106820	NM_006467	POLR3G	polymerase (RNA) III (DNA directe	0.059	1.042	7.94E-01	7.94E+01		
8106923	NM_005654	NR2F1	nuclear receptor subfamily 2, grou	0.049	1.034	7.27E-01	7.27E+01		
8106976	NM_199243	GPR150	G protein-coupled receptor 150	-0.040	0.973	7.07E-01	7.07E+01		
8107005	NM_001750	CAST	calpastatin	0.075	1.053	5.52E-01	5.52E+01		
8107044	NM_022350	ERAP2	endoplasmic reticulum aminopept	-0.013	0.991	9.84E-01	9.84E+01		
8107066	NM_005575	LNPEP	leucyl/cystinyl aminopeptidase	0.052	1.037	5.77E-01	5.77E+01		
8107090	---	---	---	-0.036	0.976	8.27E-01	8.27E+01		
8107094	---	---	---	0.088	1.063	6.57E-01	6.57E+01		
8107096	---	---	---	0.052	1.037	7.52E-01	7.52E+01		
8107098	---	---	---	-0.004	0.997	9.86E-01	9.86E+01		
8107115	---	---	---	-0.022	0.985	8.66E-01	8.66E+01		
8107119	NM_198507	FAM174A	family with sequence similarity 17	-0.091	0.939	7.31E-01	7.31E+01		
8107129	NM_180991	SLCO4C1	solute carrier organic anion transp	0.294	1.226	7.47E-02	7.47E+00		
8107133	NM_000919	PAM	peptidylglycine alpha-amidating m	0.051	1.036	7.34E-01	7.34E+01		
8107164	NM_015216	HISPPD1	histidine acid phosphatase domai	0.277	1.211	7.02E-03	7.02E-01		
8107204	---	---	---	0.013	1.009	9.50E-01	9.50E+01		
8107234	NM_002372	MAN2A1	mannosidase, alpha, class 2A, mer	0.209	1.156	9.20E-03	9.20E-01		
8107259	NM_138773	SLC25A46	solute carrier family 25, member 4	0.087	1.062	3.12E-01	3.12E+01		
8107282	NM_139281	WDR36	WD repeat domain 36	0.095	1.068	5.15E-01	5.15E+01		
8107307	NM_001744	CAMK4	calcium/calmodulin-dependent pr	-0.166	0.891	3.93E-01	3.93E+01		
8107321	AB048207	C5orf26	chromosome 5 open reading fram	-0.032	0.978	9.01E-01	9.01E+01		
8107328	NR_027706	FLJ11235	hypothetical FLJ11235	-0.045	0.969	7.33E-01	7.33E+01		
8107348	---	---	---	0.163	1.120	1.09E-01	1.09E+01		
8107350	NM_003135	SRP19	signal recognition particle 19kDa	-0.029	0.980	8.73E-01	8.73E+01		
8107353	BC104811	ZRSR1	zinc finger (CCCH type), RNA-bindi	-0.044	0.970	7.51E-01	7.51E+01		
8107356	NM_152624	DCP2	DCP2 decapping enzyme homolog	0.028	1.020	7.92E-01	7.92E+01		
8107375	NM_022828	YTHDC2	YTH domain containing 2	0.119	1.086	1.30E-01	1.30E+01		
8107406	---	---	---	-0.064	0.957	6.09E-01	6.09E+01		
8107408	NM_021614	KCNN2	potassium intermediate/small con	-0.038	0.974	6.66E-01	6.66E+01		
8107421	NM_001284	AP3S1	adaptor-related protein complex 3	0.057	1.040	7.17E-01	7.17E+01		
8107468	---	---	---	-0.045	0.969	7.27E-01	7.27E+01		
8107470	NM_002823	PTMA	prothymosin, alpha	-0.010	0.993	9.31E-01	9.31E+01		
8107474	NM_005509	DMXL1	Dmx-like 1	0.278	1.212	1.79E-02	1.79E+00		
8107518	---	---	---	0.250	1.189	2.56E-01	2.56E+01		
8107520	NM_014350	TNFAIP8	tumor necrosis factor, alpha-induc	0.020	1.014	9.13E-01	9.13E+01		
8107530	---	---	---	0.010	1.007	9.67E-01	9.67E+01		
8107578	NM_152546	SRFBP1	serum response factor binding pro	0.161	1.118	1.58E-01	1.58E+01		
8107594	NM_005460	SNCAIP	synuclein, alpha interacting protei	0.053	1.037	6.24E-01	6.24E+01		
8107644	---	---	---	-0.028	0.981	8.86E-01	8.86E+01		
8107646	NM_00113623	PRDM6	PR domain containing 6	-0.001	0.999	9.93E-01	9.93E+01		
8107655	NM_004384	CSNK1G3	casein kinase 1, gamma 3	-0.021	0.986	8.84E-01	8.84E+01		
8107671	---	---	---	-0.066	0.955	6.96E-01	6.96E+01		
8107673	NM_023927	GRAMD3	GRAM domain containing 3	-0.051	0.965	8.17E-01	8.17E+01		
8107691	NM_032177	PHAX	phosphorylated adaptor for RNA e	-0.007	0.995	9.80E-01	9.80E+01		
8107706	NM_005573	LMNB1	lamin B1	0.084	1.060	7.68E-01	7.68E+01		
8107720	AK093561	LOC100131792	hypothetical LOC100131792	-0.148	0.902	4.43E-01	4.43E+01		
8107750	NM_130809	PRRC1	proline-rich coiled-coil 1	0.094	1.067	5.04E-01	5.04E+01		
8107769	NM_001046	SLC12A2	solute carrier family 12 (sodium/p	-0.012	0.992	9.63E-01	9.63E+01		
8107814	NM_016048	ISOC1	isochorismatase domain containin	0.007	1.005	9.81E-01	9.81E+01		
8107855	NR_023344	RNU6ATAC	RNA, U6atac small nuclear (U12-d	-0.093	0.938	5.48E-01	5.48E+01		
8107868	NM_020240	CDC42SE2	CDC42 small effector 2	-0.040	0.973	7.38E-01	7.38E+01		
8107897	NM_003687	PDLIM4	PDZ and LIM domain 4	-0.032	0.978	7.71E-01	7.71E+01		
8107920	NM_003060	SLC22A5	solute carrier family 22 (organic ca	0.164	1.120	1.56E-01	1.56E+01		
8107934	---	---	---	0.037	1.026	8.54E-01	8.54E+01		
8107942	NM_005732	RAD50	RAD50 homolog (S. cerevisiae)	0.065	1.046	5.94E-01	5.94E+01		
8107985	NM_00103978	CCNI2	cyclin I family, member 2	0.002	1.001	9.91E-01	9.91E+01		
8107992	NM_175873	ANKRD43	ankyrin repeat domain 43	0.006	1.004	9.73E-01	9.73E+01		
8107996	---	---	---	-0.072	0.951	7.25E-01	7.25E+01		
8107998	NM_014402	UQCRQ	ubiquinol-cytochrome c reductase	0.043	1.031	7.35E-01	7.35E+01		
8108006	NM_052971	LEAP2	liver expressed antimicrobial pept	-0.080	0.946	6.71E-01	6.71E+01		
8108013	---	---	---	0.013	1.009	9.65E-01	9.65E+01		
8108015	NM_002154	HSPA4	heat shock 70kDa protein 4	0.075	1.053	5.38E-01	5.38E+01		

8108050	NM_003202	TCF7	transcription factor 7 (T-cell specif	-0.020	0.986	9.53E-01	9.53E+01		
8108066	NM_003337	UBE2B	ubiquitin-conjugating enzyme E2B	-0.057	0.962	5.29E-01	5.29E+01		
8108080	NM_015288	PHF15	PHD finger protein 15	-0.022	0.985	9.11E-01	9.11E+01		
8108099	NM_021982	SEC24A	SEC24 family, member A (S. cerevi	0.047	1.033	7.61E-01	7.61E+01		
8108127	NM_001745	CAMLG	calcium modulating ligand	-0.085	0.943	5.27E-01	5.27E+01		
8108134	NM_014829	DDX46	DEAD (Asp-Glu-Ala-Asp) box polyp	0.006	1.004	9.64E-01	9.64E+01		
8108161	---	---	---	-0.024	0.983	9.58E-01	9.58E+01		
8108163	NM_152409	C5orf24	chromosome 5 open reading fram	0.007	1.005	9.74E-01	9.74E+01		
8108166	NM_024715	TXNDC15	thioredoxin domain containing 15	-0.015	0.990	9.07E-01	9.07E+01		
8108174	NM_032151	PCBD2	pterin-4 alpha-carbinolamine dehy	0.075	1.054	7.25E-01	7.25E+01		
8108180	---	---	---	0.007	1.005	9.77E-01	9.77E+01		
8108192	---	---	---	0.025	1.018	8.86E-01	8.86E+01		
8108199	NM_145282	LOC153328	mitochondrial carrier protein-like	-0.059	0.960	5.03E-01	5.03E+01		
8108238	NM_00100141	SMAD5	SMAD family member 5	0.171	1.126	2.85E-01	2.85E+01		
8108321	NM_00113564	FAM53C	family with sequence similarity 53	-0.020	0.987	9.19E-01	9.19E+01		
8108330	NM_016604	KDM3B	lysine (K)-specific demethylase 3B	-0.017	0.988	9.06E-01	9.06E+01		
8108359	NM_016606	REEP2	receptor accessory protein 2	0.035	1.024	7.56E-01	7.56E+01		
8108370	NM_001964	EGR1	early growth response 1	-0.124	0.918	2.35E-01	2.35E+01		
8108376	---	---	---	0.085	1.061	4.48E-01	4.48E+01		
8108378	NM_001903	CTNNA1	catenin (cadherin-associated prote	0.182	1.135	4.83E-01	4.83E+01		
8108399	---	---	---	0.103	1.074	5.52E-01	5.52E+01		
8108403	NM_199189	MATR3	matrin 3	0.000	1.000	1.00E+00	1.00E+02		
8108422	---	---	---	0.066	1.047	7.63E-01	7.63E+01		
8108424	NM_00103311	PAIP2	poly(A) binding protein interacting	-0.050	0.966	5.42E-01	5.42E+01		
8108435	NM_181838	UBE2D2	ubiquitin-conjugating enzyme E2D	0.083	1.059	3.04E-01	3.04E+01		
8108447	NM_016463	CXXC5	CXXC finger 5	-0.028	0.981	8.66E-01	8.66E+01		
8108453	NM_032289	PSD2	pleckstrin and Sec7 domain contai	0.027	1.019	7.87E-01	7.87E+01		
8108470	---	---	---	0.006	1.004	9.77E-01	9.77E+01		
8108472	NM_005859	PURA	purine-rich element binding prote	-0.094	0.937	1.40E-01	1.40E+01		
8108475	NM_00100718	C5orf53	chromosome 5 open reading fram	-0.036	0.975	8.70E-01	8.70E+01		
8108478	NM_032412	C5orf32	chromosome 5 open reading fram	-0.129	0.915	3.32E-01	3.32E+01		
8108483	NM_031467	SLC4A9	solute carrier family 4, sodium bic	-0.048	0.967	6.60E-01	6.60E+01		
8108510	NM_020690	NKHD1-EIF4EBP	ANKHD1-EIF4EBP3 readthrough tr	-0.014	0.990	8.87E-01	8.87E+01		
8108558	NM_080670	SLC35A4	solute carrier family 35, member A	0.053	1.038	7.67E-01	7.67E+01		
8108568	NM_006083	IK	IK cytokine, down-regulator of HLA	-0.069	0.953	4.84E-01	4.84E+01		
8108579	NM_018502	TMCO6	transmembrane and coiled-coil do	-0.056	0.962	5.94E-01	5.94E+01		
8108593	NM_017706	WDR55	WD repeat domain 55	-0.086	0.942	4.93E-01	4.93E+01		
8108603	NM_012208	HARS2	histidyl-tRNA synthetase 2, mitoch	-0.058	0.961	6.33E-01	6.33E+01		
8108620	NM_144723	ZMAT2	zinc finger, matrin type 2	0.050	1.036	6.96E-01	6.96E+01		
8108683	NM_018936	PCDHB2	protocadherin beta 2	-0.030	0.979	8.88E-01	8.88E+01		
8108716	NM_020957	PCDHB16	protocadherin beta 16	-0.063	0.957	6.66E-01	6.66E+01		
8108822	NM_173828	RELL2	RELT-like 2	0.009	1.006	9.63E-01	9.63E+01		
8108832	NM_014773	KIAA0141	KIAA0141	0.005	1.003	9.82E-01	9.82E+01		
8108847	NM_183401	RNF14	ring finger protein 14	0.067	1.048	7.13E-01	7.13E+01		
8108861	NM_030571	NDFIP1	Nedd4 family interacting protein 1	0.036	1.025	8.62E-01	8.62E+01		
8108873	NM_015071	ARHGAP26	Rho GTPase activating protein 26	-0.025	0.983	9.49E-01	9.49E+01		
8108900	NM_021182	HMHB1	histocompatibility (minor) HB-1	0.048	1.034	7.38E-01	7.38E+01		
8108912	NM_152550	SH3RF2	SH3 domain containing ring finger	-0.094	0.937	3.20E-01	3.20E+01		
8108927	NM_018989	RBM27	RNA binding motif protein 27	0.001	1.001	9.95E-01	9.95E+01		
8108949	NM_002700	POU4F3	POU class 4 homeobox 3	0.045	1.032	7.29E-01	7.29E+01		
8108954	NM_006706	TCERG1	transcription elongation regulator	0.003	1.002	9.86E-01	9.86E+01		
8108977	---	---	---	-0.087	0.942	4.29E-01	4.29E+01		
8109062	NM_205836	FBXO38	F-box protein 38	0.063	1.044	5.95E-01	5.95E+01		
8109086	NM_000024	ADRB2	adrenergic, beta-2-, receptor, surf	-0.010	0.993	9.70E-01	9.70E+01		
8109093	NM_014945	ABLIM3	actin binding LIM protein family, n	-0.021	0.986	8.37E-01	8.37E+01		
8109120	NM_152406	AFAP1L1	actin filament associated protein 1	-0.030	0.979	8.18E-01	8.18E+01		
8109141	NM_152407	GRPEL2	GrpE-like 2, mitochondrial (E. coli)	0.008	1.006	9.64E-01	9.64E+01		
8109149	NM_024028	PCYOX1L	prenylcysteine oxidase 1 like	0.055	1.039	6.15E-01	6.15E+01		
8109157	---	---	---	-0.007	0.995	9.73E-01	9.73E+01		
8109179	NM_133263	PPARGC1B	peroxisome proliferator-activated	0.055	1.039	5.38E-01	5.38E+01		
8109201	NM_014983	HMGXB3	HMG box domain containing 3	-0.043	0.971	7.13E-01	7.13E+01		
8109222	NM_000971	RPL7	ribosomal protein L7	-0.035	0.976	8.08E-01	8.08E+01		
8109226	NM_001804	CDX1	caudal type homeobox 1	-0.019	0.987	9.35E-01	9.35E+01		
8109254	NM_00113524	TCOF1	Treacher Collins-Franceschetti syn	-0.012	0.992	9.28E-01	9.28E+01		
8109283	NM_001543	NDST1	N-deacetylase/N-sulfotransferase	-0.144	0.905	3.17E-01	3.17E+01		
8109305	NM_007286	SYNPO	synaptopodin	-0.027	0.981	8.29E-01	8.29E+01		
8109315	NM_00112285	MYOZ3	myozenin 3	-0.013	0.991	9.50E-01	9.50E+01		
8109326	NM_032947	MST150	MSTP150	-0.232	0.851	1.97E-02	1.97E+00		
8109333	NM_002084	GPX3	glutathione peroxidase 3 (plasma)	-0.206	0.867	1.48E-02	1.48E+00		
8109344	NM_000405	GM2A	GM2 ganglioside activator	0.449	1.365	7.17E-02	7.17E+00		
8109368	NM_005754	G3BP1	GTPase activating protein (SH3 do	0.046	1.032	7.69E-01	7.69E+01		
8109407	NM_198321	GALNT10	UDP-N-acetyl-alpha-D-galactosam	-0.036	0.975	7.80E-01	7.80E+01		
8109424	---	---	---	-0.189	0.877	2.25E-01	2.25E+01		
8109426	AK090581	GALNT10	UDP-N-acetyl-alpha-D-galactosam	-0.033	0.977	8.81E-01	8.81E+01		
8109428	NR_024084	SAP30L	SAP30-like	-0.029	0.980	9.01E-01	9.01E+01		
8109438	NM_033551	LARP1	La ribonucleoprotein domain fami	0.068	1.048	3.69E-01	3.69E+01		
8109462	NM_004779	CNOT8	CCR4-NOT transcription complex,	0.072	1.051	5.65E-01	5.65E+01		

8109475	NM_014180	MRPL22	mitochondrial ribosomal protein L	-0.055	0.963	7.87E-01	7.87E+01			
8109486	---	---	---	0.060	1.042	6.71E-01	6.71E+01			
8109505	NR_002168	PPP1R2P3	protein phosphatase 1, regulatory	0.038	1.027	7.38E-01	7.38E+01			
8109507	NM_005546	ITK	IL2-inducible T-cell kinase	-0.011	0.992	9.79E-01	9.79E+01			
8109526	---	---	---	0.030	1.021	9.56E-01	9.56E+01			
8109528	NM_00103733	CYFIP2	cytoplasmic FMR1 interacting prot	-0.072	0.952	6.32E-01	6.32E+01			
8109563	NM_00109928	NIPAL4	NIPA-like domain containing 4	-0.154	0.899	5.71E-02	5.71E+00			
8109576	NM_017872	THG1L	tRNA-histidine guanylyltransferase	-0.018	0.988	9.61E-01	9.61E+01			
8109593	---	---	---	-0.006	0.996	9.87E-01	9.87E+01			
8109597	NM_145049	UBLCP1	ubiquitin-like domain containing C	-0.021	0.986	9.02E-01	9.02E+01			
8109612	NM_000679	ADRA1B	adrenergic, alpha-1B-, receptor	-0.045	0.969	5.91E-01	5.91E+01			
8109620	NM_003314	TTC1	tetratricopeptide repeat domain 1	-0.022	0.985	9.00E-01	9.00E+01			
8109629	NM_00104044	FABP6	fatty acid binding protein 6, ileal	-0.014	0.990	9.51E-01	9.51E+01			
8109639	NM_004219	PTTG1	pituitary tumor-transforming 1	0.104	1.075	6.83E-01	6.83E+01			
8109693	---	---	---	0.023	1.016	8.75E-01	8.75E+01			
8109697	NM_004060	CCNG1	cyclin G1	-0.109	0.927	3.38E-01	3.38E+01			
8109732	NM_013283	MAT2B	methionine adenosyltransferase II	0.066	1.047	4.86E-01	4.86E+01			
8109746	---	---	---	-0.030	0.980	8.37E-01	8.37E+01			
8109750	NM_001002	RPLP0	ribosomal protein, large, P0	-0.100	0.933	5.37E-01	5.37E+01			
8109773	NM_00116166	WWC1	WW and C2 domain containing 1	-0.024	0.983	8.35E-01	8.35E+01			
8109802	NM_002887	RARS	arginyl-tRNA synthetase	-0.010	0.993	9.65E-01	9.65E+01			
8109819	NR_024356	FBLL1	fibrillarin-like 1	0.037	1.026	7.63E-01	7.63E+01			
8109821	NM_006013	RPL10	ribosomal protein L10	-0.030	0.979	8.48E-01	8.48E+01			
8109843	NM_004946	DOCK2	dedicator of cytokinesis 2	0.096	1.069	3.62E-01	3.62E+01			
8109901	NM_012188	FOXI1	forkhead box I1	-0.040	0.973	7.33E-01	7.33E+01			
8109969	NM_021025	TLX3	T-cell leukemia homeobox 3	-0.031	0.979	8.92E-01	8.92E+01			
8109981	NM_003862	FGF18	fibroblast growth factor 18	-0.007	0.995	9.56E-01	9.56E+01			
8109999	NM_00103171	ERGIC1	endoplasmic reticulum-golgi interm	-0.033	0.978	9.12E-01	9.12E+01			
8110022	NM_003945	ATP6V0E1	ATPase, H+ transporting, lysosoma	0.103	1.074	3.49E-01	3.49E+01			
8110032	NM_153607	C5orf41	chromosome 5 open reading fram	-0.059	0.960	6.42E-01	6.42E+01			
8110043	NM_013979	BNIP1	BCL2/adenovirus E1B 19kDa intera	0.011	1.008	9.76E-01	9.76E+01			
8110055	NM_030627	CPEB4	cytoplasmic polyadenylation elem	0.108	1.078	5.37E-01	5.37E+01			
8110090	NM_022754	SFXN1	sideroflexin 1	-0.079	0.947	7.60E-01	7.60E+01			
8110106	NM_00113105	HRH2	histamine receptor H2	0.030	1.021	8.74E-01	8.74E+01			
8110114	NM_006650	CPLX2	complexin 2	0.002	1.001	9.90E-01	9.90E+01			
8110123	NM_00107952	FAM153B	family with sequence similarity 15	0.048	1.034	8.45E-01	8.45E+01			
8110147	NM_198567	C5orf25	chromosome 5 open reading fram	-0.020	0.986	9.53E-01	9.53E+01			
8110158	NM_173664	ARL10	ADP-ribosylation factor-like 10	-0.026	0.982	8.31E-01	8.31E+01			
8110166	NM_138820	HIGD2A	HIG1 hypoxia inducible domain fa	0.019	1.013	9.47E-01	9.47E+01			
8110169	NM_014613	FAF2	Fas associated factor family memb	0.018	1.012	9.56E-01	9.56E+01			
8110183	NM_017675	PCDH24	protocadherin 24	-0.049	0.967	6.31E-01	6.31E+01			
8110218	NM_00109940	EIF4E1B	eukaryotic translation initiation fa	0.037	1.026	7.37E-01	7.37E+01			
8110224	NM_012171	TSPAN17	tetraspanin 17	-0.052	0.964	5.34E-01	5.34E+01			
8110237	NM_133369	UNC5A	unc-5 homolog A (C. elegans)	0.007	1.005	9.72E-01	9.72E+01			
8110253	NM_012279	ZNF346	zinc finger protein 346	-0.045	0.969	8.29E-01	8.29E+01			
8110265	NM_213647	FGFR4	fibroblast growth factor receptor 4	0.024	1.017	8.44E-01	8.44E+01			
8110289	NM_022455	NSD1	nuclear receptor binding SET doma	0.005	1.004	9.78E-01	9.78E+01			
8110318	NM_013237	PRELID1	PRELI domain containing 1	0.087	1.062	6.46E-01	6.46E+01			
8110327	NM_006480	RGS14	regulator of G-protein signaling 14	-0.046	0.969	5.87E-01	5.87E+01			
8110347	NM_003052	SLC34A1	solute carrier family 34 (sodium p	-0.018	0.988	9.27E-01	9.27E+01			
8110362	NM_00100410	GRK6	G protein-coupled receptor kinase	-0.027	0.981	8.44E-01	8.44E+01			
8110382	NM_030567	PRR7	proline rich 7 (synaptic)	-0.064	0.957	4.80E-01	4.80E+01			
8110392	NM_017510	TMED9	transmembrane emp24 protein tra	0.057	1.041	8.06E-01	8.06E+01			
8110399	NM_007255	B4GALT7	xylosylprotein beta 1,4-galactosylt	-0.004	0.997	9.81E-01	9.81E+01			
8110408	NM_032361	THOC3	THO complex 3	-0.002	0.999	9.92E-01	9.92E+01			
8110415	NM_022491	SUDS3	suppressor of defective silencing 3	0.014	1.010	9.46E-01	9.46E+01			
8110417	NM_00107952	FAM153C	family with sequence similarity 15	-0.022	0.985	9.57E-01	9.57E+01			
8110427	---	---	---	-0.029	0.980	8.46E-01	8.46E+01			
8110430	NM_015111	N4BP3	Nedd4 binding protein 3	-0.035	0.976	8.17E-01	8.17E+01			
8110437	NM_022762	RMND5B	required for meiotic nuclear divisi	0.008	1.005	9.73E-01	9.73E+01			
8110450	NM_031266	HNRNPAB	heterogeneous nuclear ribonucleo	0.033	1.023	8.05E-01	8.05E+01			
8110461	---	---	---	0.008	1.005	9.70E-01	9.70E+01			
8110463	NM_058230	ZNF354B	zinc finger protein 354B	-0.073	0.950	6.57E-01	6.57E+01			
8110499	NM_025158	RUFY1	RUN and FYVE domain containing	0.089	1.063	3.72E-01	3.72E+01			
8110518	NM_00114230	LOC285679	hypothetical protein LOC285679	-0.054	0.963	6.94E-01	6.94E+01			
8110522	NM_001746	CANX	calnexin	0.153	1.112	1.96E-01	1.96E+01			
8110546	NM_014757	MAML1	mastermind-like 1 (Drosophila)	-0.027	0.982	8.16E-01	8.16E+01			
8110569	NM_003900	SQSTM1	sequestosome 1	-0.165	0.892	1.32E-01	1.32E+01			
8110589	NM_015455	CNOT6	CCR4-NOT transcription complex,	-0.077	0.948	5.64E-01	5.64E+01			
8110618	NM_006628	ARPP19	cAMP-regulated phosphoprotein,	0.036	1.025	9.01E-01	9.01E+01			
8110631	NM_152547	BTNL9	butyrophilin-like 9	-0.039	0.973	6.59E-01	6.59E+01			
8110649	NM_033549	TRIM41	tripartite motif-containing 41	-0.016	0.989	9.35E-01	9.35E+01			
8110666	NM_032765	TRIM52	tripartite motif-containing 52	-0.077	0.948	7.12E-01	7.12E+01			
8110668	---	---	---	0.189	1.140	1.60E-01	1.60E+01			
8110670	NM_207366	40435	septin 14	-0.030	0.979	9.19E-01	9.19E+01			
8110678	NM_145265	CCDC127	coiled-coil domain containing 127	-0.050	0.966	7.87E-01	7.87E+01			
8110681	NM_138464	C5orf55	chromosome 5 open reading fram	-0.053	0.964	7.27E-01	7.27E+01			

8110685	NR_024158	LOC25845	hypothetical LOC25845	0.048	1.034	7.89E-01	7.89E+01			
8110688	NM_004174	SLC9A3	solute carrier family 9 (sodium/hy	-0.036	0.975	7.31E-01	7.31E+01			
8110706	---	---	---	-0.034	0.977	8.51E-01	8.51E+01			
8110708	NM_007030	TPPP	tubulin polymerization promoting	-0.076	0.949	3.49E-01	3.49E+01			
8110721	NM_024786	ZDHHC11	zinc finger, DHHC-type containing	-0.062	0.958	5.55E-01	5.55E+01			
8110734	NR_027633	BRD9	bromodomain containing 9	-0.055	0.963	7.61E-01	7.61E+01			
8110755	NM_006598	SLC12A7	solute carrier family 12 (potassium	-0.134	0.911	3.99E-02	3.99E+00			
8110783	NM_198253	TERT	telomerase reverse transcriptase	-0.025	0.983	8.04E-01	8.04E+01			
8110803	NM_030782	CLPTM1L	CLPTM1-like	0.165	1.121	5.88E-02	5.88E+00			
8110821	NM_001044	SLC6A3	solute carrier family 6 (neurotrans	-0.021	0.986	8.47E-01	8.47E+01			
8110841	NM_024830	LPCAT1	lysophosphatidylcholine acyltransf	-0.115	0.923	3.80E-01	3.80E+01			
8110858	AK295082	PDCD6	programmed cell death 6	-0.020	0.986	9.49E-01	9.49E+01			
8110861	NM_032479	MRPL36	mitochondrial ribosomal protein L	-0.053	0.964	7.90E-01	7.90E+01			
8110872	NM_033267	IRX2	iroquois homeobox 2	0.045	1.032	7.04E-01	7.04E+01			
8110882	BC132707	FLJ33360	FLJ33360 protein	-0.032	0.978	8.74E-01	8.74E+01			
8110886	NM_032286	MED10	mediator complex subunit 10	-0.005	0.996	9.81E-01	9.81E+01			
8110894	NM_017755	NSUN2	NOL1/NOP2/Sun domain family, m	0.004	1.003	9.86E-01	9.86E+01			
8110914	---	---	---	0.002	1.001	9.89E-01	9.89E+01			
8110916	ENST00000382	LOC442132	similar to hypothetical protein FLJ	-0.104	0.931	5.04E-01	5.04E+01			
8110932	NM_003966	SEMA5A	sema domain, seven thrombospon	0.056	1.040	5.45E-01	5.45E+01			
8110982	NM_004394	DAP	death-associated protein	0.186	1.137	3.67E-02	3.67E+00			
8110990	NM_001332	CTNND2	catenin (cadherin-associated prote	-0.001	0.999	9.93E-01	9.93E+01			
8111099	---	---	---	-0.036	0.975	8.82E-01	8.82E+01			
8111101	NM_054027	ANKH	ankylosis, progressive homolog (m	-0.163	0.893	9.07E-02	9.07E+00			
8111129	NM_033414	ZNF622	zinc finger protein 622	0.078	1.056	6.16E-01	6.16E+01			
8111136	NM_00103485	FAM134B	family with sequence similarity 13	-0.142	0.906	5.02E-01	5.02E+01			
8111151	---	---	---	0.041	1.029	9.53E-01	9.53E+01			
8111203	NR_027253	LOC285696	hypothetical LOC285696	-0.049	0.967	7.17E-01	7.17E+01			
8111210	---	---	---	0.006	1.004	9.83E-01	9.83E+01			
8111214	XM_00113113	LOC729724	similar to TBP-associated factor 11	-0.016	0.989	9.27E-01	9.27E+01			
8111216	ENST00000426	LOC391769	histone cluster 2, H3c pseudogene	-0.013	0.991	9.25E-01	9.25E+01			
8111286	NM_013235	RNASEN	ribonuclease type III, nuclear	-0.108	0.928	3.40E-01	3.40E+01			
8111331	NM_022130	GOLPH3	golgi phosphoprotein 3 (coat-prot	0.034	1.024	7.13E-01	7.13E+01			
8111339	NM_00104044	MTMR12	myotubularin related protein 12	0.025	1.018	9.07E-01	9.07E+01			
8111360	NM_016107	ZFR	zinc finger RNA binding protein	0.046	1.032	5.26E-01	5.26E+01			
8111430	NM_014324	AMACR	alpha-methylacyl-CoA racemase	0.065	1.046	5.67E-01	5.67E+01			
8111443	NM_181435	C1QTNF3	C1q and tumor necrosis factor rela	0.048	1.034	8.32E-01	8.32E+01			
8111455	NR_003504	GUSBL1	glucuronidase, beta-like 1	0.014	1.010	9.71E-01	9.71E+01			
8111457	NM_002853	RAD1	RAD1 homolog (S. pombe)	0.012	1.008	9.60E-01	9.60E+01			
8111552	NM_00108541	C5orf33	chromosome 5 open reading fram	-0.038	0.974	8.44E-01	8.44E+01			
8111584	BC144069	C5orf42	chromosome 5 open reading fram	-0.037	0.975	8.66E-01	8.66E+01			
8111629	NM_153485	NUP155	nucleoporin 155kDa	0.066	1.047	5.48E-01	5.48E+01			
8111666	---	---	---	0.136	1.099	5.06E-01	5.06E+01			
8111698	NM_152756	RICTOR	RPTOR independent companion of	0.010	1.007	9.67E-01	9.67E+01			
8111739	NM_001465	FYB	FYN binding protein (FYB-120/130	0.221	1.166	1.48E-02	1.48E+00			
8111772	NM_001343	DAB2	disabled homolog 2, mitogen-resp	0.435	1.352	2.82E-01	2.82E+01			
8111788	NM_012382	TTC33	tetratricopeptide repeat domain 3	0.041	1.029	8.17E-01	8.17E+01			
8111796	NM_206907	PRKAA1	protein kinase, AMP-activated, alp	-0.032	0.978	8.38E-01	8.38E+01			
8111812	---	---	---	-0.121	0.920	4.39E-01	4.39E+01			
8111814	NM_000997	RPL37	ribosomal protein L37	0.064	1.045	5.86E-01	5.86E+01			
8111892	NM_000436	OXCT1	3-oxoacid CoA transferase 1	-0.128	0.915	3.70E-01	3.70E+01			
8111913	---	---	---	0.046	1.033	8.15E-01	8.15E+01			
8111925	NM_00101427	C5orf39	chromosome 5 open reading fram	-0.050	0.966	8.34E-01	8.34E+01			
8111930	AK075204	hCG_2039148	hypothetical protein LOC1001323	-0.035	0.976	9.12E-01	9.12E+01			
8111941	NM_00109827	HMGCS1	3-hydroxy-3-methylglutaryl-Coenz	-0.065	0.956	7.50E-01	7.50E+01			
8111974	NM_006451	PAIP1	poly(A) binding protein interacting	0.053	1.037	6.38E-01	6.38E+01			
8111989	---	---	---	-0.026	0.982	9.05E-01	9.05E+01			
8112007	NM_198449	EMB	embigin homolog (mouse)	-0.073	0.951	6.78E-01	6.78E+01			
8112020	NM_176806	MOCS2	molybdenum cofactor synthesis 2	0.000	1.000	1.00E+00	1.00E+02			
8112033	NM_019087	ARL15	ADP-ribosylation factor-like 15	0.225	1.169	2.32E-01	2.32E+01			
8112041	---	---	---	-0.021	0.986	9.49E-01	9.49E+01			
8112043	---	---	---	0.164	1.121	4.21E-01	4.21E+01			
8112070	---	---	---	0.135	1.098	3.62E-01	3.62E+01			
8112072	NM_021147	CCNO	cyclin O	-0.125	0.917	1.47E-01	1.47E+01			
8112081	NM_019030	DHX29	DEAH (Asp-Glu-Ala-His) box polype	0.011	1.008	9.53E-01	9.53E+01			
8112139	NM_002184	IL6ST	interleukin 6 signal transducer (gp	0.086	1.062	7.31E-01	7.31E+01			
8112174	---	---	---	-0.177	0.884	1.93E-02	1.93E+00			
8112176	NR_023344 //	// RNU6ATAC /	RNA, U6atac small nuclear (U12-de	-0.208	0.866	3.10E-01	3.10E+01			
8112180	---	---	---	0.030	1.021	8.55E-01	8.55E+01			
8112182	NM_152622	MIER3	mesoderm induction early respons	-0.067	0.955	6.54E-01	6.54E+01			
8112220	NM_00110463	PDE4D	phosphodiesterase 4D, cAMP-spec	-0.169	0.889	1.19E-01	1.19E+01			
8112274	NM_024930	ELOVL7	ELOVL family member 7, elongatio	-0.601	0.659	1.01E-01	1.01E+01			
8112302	NM_00104824	C5orf43	chromosome 5 open reading fram	0.173	1.127	3.63E-02	3.63E+00			
8112312	NM_014473	DIMT1L	DIM1 dimethyladenosine transfera	-0.051	0.965	7.71E-01	7.71E+01			
8112327	NM_001826	CKS1B	CDC28 protein kinase regulatory s	0.176	1.130	5.76E-01	5.76E+01			
8112331	NM_030940	ISCA1	iron-sulfur cluster assembly 1 hom	0.009	1.006	9.78E-01	9.78E+01			
8112335	---	---	---	0.128	1.093	3.56E-01	3.56E+01			

8112342	NM_197941	ADAMTS6	ADAM metallopeptidase with thro	0.013	1.009	9.61E-01	9.61E+01		
8112376	NM_022145	CENPK	centromere protein K	0.145	1.106	8.64E-01	8.64E+01		
8112388	NM_001656	TRIM23	tripartite motif-containing 23	-0.021	0.986	9.30E-01	9.30E+01		
8112433	---	---	---	0.028	1.019	8.07E-01	8.07E+01		
8112435	---	---	---	-0.039	0.974	6.87E-01	6.87E+01		
8112439	NM_176816	CCDC125	coiled-coil domain containing 125	0.071	1.050	7.17E-01	7.17E+01		
8112458	NM_003187	TAF9	TAF9 RNA polymerase II, TATA box	0.046	1.033	7.04E-01	7.04E+01		
8112469	BC171739	GUSBL1	glucuronidase, beta-like 1	-0.002	0.999	9.96E-01	9.96E+01		
8112476	---	---	---	0.137	1.099	4.82E-01	4.82E+01		
8112478	NM_004536	NAIP	NLR family, apoptosis inhibitory pr	0.455	1.371	8.71E-02	8.71E+00		
8112491	AK289851	SMA5	glucuronidase, beta pseudogene	-0.023	0.984	9.53E-01	9.53E+01		
8112519	---	---	---	0.126	1.091	3.91E-01	3.91E+01		
8112521	NM_004536	NAIP	NLR family, apoptosis inhibitory pr	0.462	1.377	7.90E-02	7.90E+00		
8112538	NM_001515	GTF2H2	general transcription factor IIH, po	0.084	1.060	6.91E-01	6.91E+01		
8112558	NR_003504	GUSBL1	glucuronidase, beta-like 1	0.019	1.013	9.61E-01	9.61E+01		
8112560	AK289851	SMA5	glucuronidase, beta pseudogene	-0.005	0.996	9.88E-01	9.88E+01		
8112564	BC171739	GUSBL1	glucuronidase, beta-like 1	-0.005	0.997	9.88E-01	9.88E+01		
8112570	NM_015084	MRPS27	mitochondrial ribosomal protein S	0.020	1.014	9.21E-01	9.21E+01		
8112584	NM_152625	ZNF366	zinc finger protein 366	-0.196	0.873	6.90E-02	6.90E+00		
8112596	NM_023039	ANKRA2	ankyrin repeat, family A (RFXANK-	0.004	1.003	9.90E-01	9.90E+01		
8112607	---	---	---	0.112	1.081	8.73E-01	8.73E+01		
8112613	---	---	---	-0.021	0.986	9.24E-01	9.24E+01		
8112622	NM_032380	GFM2	G elongation factor, mitochondria	-0.013	0.991	9.61E-01	9.61E+01		
8112666	---	---	---	0.064	1.045	8.12E-01	8.12E+01		
8112687	NM_005713	COL4A3BP	collagen, type IV, alpha 3 (Goodpa	0.046	1.033	7.72E-01	7.72E+01		
8112709	NM_00109927	C5orf37	chromosome 5 open reading fram	0.155	1.113	3.16E-01	3.16E+01		
8112724	---	---	---	0.088	1.063	6.00E-01	6.00E+01		
8112728	NM_00108970	hCG_1757335	RAP1B, member of RAS oncogene	0.086	1.061	4.01E-01	4.01E+01		
8112731	NM_004101	F2RL2	coagulation factor II (thrombin) re	0.156	1.114	4.84E-01	4.84E+01		
8112738	---	---	---	-0.020	0.986	9.44E-01	9.44E+01		
8112740	NM_032367	ZBED3	zinc finger, BED-type containing 3	0.040	1.028	7.12E-01	7.12E+01		
8112746	NM_018268	WDR41	WD repeat domain 41	0.275	1.210	3.86E-02	3.86E+00		
8112767	NM_004607	TBCA	tubulin folding cofactor A	-0.007	0.995	9.72E-01	9.72E+01		
8112772	NM_003664	AP3B1	adaptor-related protein complex 3	0.057	1.041	7.53E-01	7.53E+01		
8112803	NM_005779	LHFPL2	lipoma HMGIC fusion partner-like	0.316	1.245	5.45E-02	5.45E+00		
8112807	NM_000046	ARSB	arylsulfatase B	0.219	1.164	1.69E-01	1.69E+01		
8112857	NM_00101089	MTX3	metaxin 3	-0.089	0.940	7.84E-01	7.84E+01		
8112865	NM_178276	SERINC5	serine incorporator 5	0.165	1.121	4.96E-01	4.96E+01		
8112881	---	---	---	0.176	1.129	7.76E-01	7.76E+01		
8112883	NR_004845	LOC644936	cytoplasmic beta-actin pseudogen	0.007	1.005	9.73E-01	9.73E+01		
8112890	NM_004269	MED27	mediator complex subunit 27	-0.020	0.986	9.64E-01	9.64E+01		
8112892	---	---	---	-0.009	0.994	9.65E-01	9.65E+01		
8112894	---	---	---	0.185	1.137	3.02E-01	3.02E+01		
8112914	---	---	---	-0.089	0.940	6.11E-01	6.11E+01		
8112916	---	---	---	0.047	1.033	2.94E-01	2.94E+01		
8112918	---	---	---	-0.073	0.951	5.61E-01	5.61E+01		
8112940	NM_012446	SSBP2	single-stranded DNA binding prote	0.048	1.034	8.41E-01	8.41E+01		
8112959	---	---	---	-0.012	0.991	9.63E-01	9.63E+01		
8112961	NM_001025	RPS23	ribosomal protein S23	-0.026	0.982	9.00E-01	9.00E+01		
8112967	NM_174909	TMEM167A	transmembrane protein 167A	0.211	1.158	1.26E-01	1.26E+01		
8112994	---	---	---	-0.011	0.993	9.83E-01	9.83E+01		
8113006	---	---	---	0.043	1.030	8.78E-01	8.78E+01		
8113008	---	---	---	0.168	1.124	5.41E-01	5.41E+01		
8113010	NM_001239	CCNH	cyclin H	-0.008	0.995	9.76E-01	9.76E+01		
8113023	NM_153354	TMEM161B	transmembrane protein 161B	0.026	1.018	8.97E-01	8.97E+01		
8113035	---	---	---	0.156	1.114	2.87E-01	2.87E+01		
8113039	NM_002397	MEF2C	myocyte enhancer factor 2C	0.350	1.274	9.71E-02	9.71E+00		
8113050	NM_004365	CETN3	centrin, EF-hand protein, 3 (CDC31	0.003	1.002	9.89E-01	9.89E+01		
8113059	NM_203406	MBLAC2	metallo-beta-lactamase domain co	0.097	1.069	5.22E-01	5.22E+01		
8113064	NM_198273	LYSMD3	LysM, putative peptidoglycan-bind	0.035	1.025	8.41E-01	8.41E+01		
8113071	ENST00000440	LOC729040	hypothetical LOC729040	-0.053	0.964	6.64E-01	6.64E+01		
8113073	NM_020801	ARRDC3	arrestin domain containing 3	-0.246	0.843	3.52E-01	3.52E+01		
8113083	NM_032042	FAM172A	family with sequence similarity 17	0.101	1.072	4.13E-01	4.13E+01		
8113094	NM_153216	POU5F2	POU domain class 5, transcription	-0.094	0.937	2.78E-01	2.78E+01		
8113097	NM_00114567	C5orf36	chromosome 5 open reading fram	0.241	1.182	3.99E-01	3.99E+01		
8113120	BC028919	TOB2	transducer of ERBB2, 2	0.012	1.009	9.06E-01	9.06E+01		
8113124	NM_032290	ANKRD32	ankyrin repeat domain 32	-0.001	0.999	9.98E-01	9.98E+01		
8113130	NM_024717	MCTP1	multiple C2 domains, transmembr	0.448	1.364	1.76E-01	1.76E+01		
8113157	NM_014639	TTC37	tetratricopeptide repeat domain 3	0.028	1.019	8.79E-01	8.79E+01		
8113214	NM_002064	GLRX	glutaredoxin (thioltransferase)	0.005	1.004	9.89E-01	9.89E+01		
8113220	NM_012081	ELL2	elongation factor, RNA polymerase	-0.082	0.945	7.52E-01	7.52E+01		
8113250	NM_00104045	ERAP1	endoplasmic reticulum aminopept	0.183	1.135	1.48E-02	1.48E+00		
8113274	---	---	---	-0.028	0.981	9.67E-01	9.67E+01		
8113305	NM_001270	CHD1	chromodomain helicase DNA bind	-0.035	0.976	8.42E-01	8.42E+01		
8113344	---	---	---	0.369	1.291	3.44E-01	3.44E+01		
8113348	---	---	---	0.052	1.036	7.97E-01	7.97E+01		
8113358	NM_005668	ST8SIA4	ST8 alpha-N-acetyl-neuraminide a	0.314	1.243	3.15E-02	3.15E+00		

8113369	NM_180991	SLCO4C1	solute carrier organic anion transp	0.246	1.186	4.19E-01	4.19E+01		
8113421	---	---	---	0.094	1.068	5.38E-01	5.38E+01		
8113443	---	---	---	-0.028	0.981	8.46E-01	8.46E+01		
8113445	BC126144	FBXL17	F-box and leucine-rich repeat prot	0.017	1.012	9.27E-01	9.27E+01		
8113467	---	---	---	-0.062	0.958	6.95E-01	6.95E+01		
8113469	NM_014819	PJA2	praja ring finger 2	0.080	1.057	4.05E-01	4.05E+01		
8113489	---	---	---	0.089	1.064	4.67E-01	4.67E+01		
8113491	NM_139164	STARD4	StAR-related lipid transfer (START)	0.105	1.076	5.22E-01	5.22E+01		
8113502	---	---	---	0.030	1.021	8.78E-01	8.78E+01		
8113504	NM_004772	C5orf13	chromosome 5 open reading fram	0.081	1.058	7.14E-01	7.14E+01		
8113542	NM_005669	REEP5	receptor accessory protein 5	0.062	1.044	7.04E-01	7.04E+01		
8113551	NM_00108537	MCC	mutated in colorectal cancers	0.098	1.071	4.25E-01	4.25E+01		
8113573	NM_032028	TSSK1B	testis-specific serine kinase 1B	-0.024	0.984	8.32E-01	8.32E+01		
8113591	NM_005023	PGGT1B	protein geranylgeranyltransferase	0.268	1.204	4.35E-03	4.35E-01		
8113616	NM_020177	FEM1C	fem-1 homolog c (C. elegans)	0.057	1.040	5.41E-01	5.41E+01		
8113623	NM_021649	TICAM2	toll-like receptor adaptor molecule	0.231	1.173	3.21E-02	3.21E+00		
8113651	NM_004707	ATG12	ATG12 autophagy related 12 hom	0.041	1.029	8.49E-01	8.49E+01		
8113658	AK291655	LVRN	laeverin	0.001	1.001	9.97E-01	9.97E+01		
8113660	---	---	---	-0.026	0.982	8.76E-01	8.76E+01		
8113699	---	---	---	0.018	1.012	9.61E-01	9.61E+01		
8113701	---	---	---	0.194	1.144	3.95E-01	3.95E+01		
8113724	---	---	---	0.126	1.091	6.73E-01	6.73E+01		
8113733	NM_153223	CEP120	centrosomal protein 120kDa	-0.034	0.977	8.27E-01	8.27E+01		
8113761	NM_020747	ZNF608	zinc finger protein 608	0.008	1.006	9.81E-01	9.81E+01		
8113784	---	---	---	-0.014	0.990	9.37E-01	9.37E+01		
8113790	NM_178450	40240	membrane-associated ring finger (	-0.044	0.970	8.50E-01	8.50E+01		
8113873	NM_005340	HINT1	histidine triad nucleotide binding p	-0.143	0.905	6.30E-02	6.30E+00		
8113879	---	---	---	0.111	1.080	4.86E-01	4.86E+01		
8113881	NM_016340	RAPGEF6	Rap guanine nucleotide exchange	0.068	1.048	7.42E-01	7.42E+01		
8113914	NM_133372	FNIP1	folliculin interacting protein 1	0.092	1.066	6.68E-01	6.68E+01		
8113938	NM_015256	ACSL6	acyl-CoA synthetase long-chain fa	0.144	1.105	1.54E-01	1.54E+01		
8113981	NM_004199	P4HA2	prolyl 4-hydroxylase, alpha polype	-0.029	0.980	8.54E-01	8.54E+01		
8114002	ENST00000416	P4HA2	prolyl 4-hydroxylase, alpha polype	-0.002	0.999	9.96E-01	9.96E+01		
8114010	NM_002198	IRF1	interferon regulatory factor 1	0.053	1.038	8.23E-01	8.23E+01		
8114030	NM_007054	KIF3A	kinesin family member 3A	-0.307	0.808	8.89E-03	8.89E-01		
8114050	NM_015146	40429	septin 8	0.012	1.009	9.52E-01	9.52E+01		
8114068	NM_133456	SHROOM1	shroom family member 1	-0.003	0.998	9.85E-01	9.85E+01		
8114083	NM_014423	AFF4	AF4/FMR2 family, member 4	0.017	1.012	9.05E-01	9.05E+01		
8114111	---	---	---	0.000	1.000	1.00E+00	1.00E+02		
8114113	NM_017665	ZCCHC10	zinc finger, CCHC domain containi	0.113	1.082	3.93E-01	3.93E+01		
8114138	NM_020199	C5orf15	chromosome 5 open reading fram	0.037	1.026	8.33E-01	8.33E+01		
8114145	NM_003374	VDAC1	voltage-dependent anion channel	-0.038	0.974	7.87E-01	7.87E+01		
8114152	NM_006930	SKP1	S-phase kinase-associated protein	-0.127	0.916	2.23E-01	2.23E+01		
8114158	NM_002715	PPP2CA	protein phosphatase 2 (formerly 2	-0.020	0.986	8.73E-01	8.73E+01		
8114185	NM_080656	CDKN2AIPNL	CDKN2A interacting protein N-ter	-0.062	0.958	7.48E-01	7.48E+01		
8114193	NM_00103350	SAR1B	SAR1 homolog B (S. cerevisiae)	0.114	1.083	4.44E-01	4.44E+01		
8114207	---	---	---	0.369	1.291	3.44E-01	3.44E+01		
8114209	---	---	---	0.150	1.109	6.95E-01	6.95E+01		
8114211	---	---	---	0.235	1.177	6.66E-01	6.66E+01		
8114213	---	---	---	-0.046	0.968	9.14E-01	9.14E+01		
8114215	NM_002653	PITX1	paired-like homeodomain 1	-0.083	0.944	3.11E-01	3.11E+01		
8114239	NM_130848	C5orf20	chromosome 5 open reading fram	0.088	1.063	2.35E-01	2.35E+01		
8114245	NM_006161	NEUROG1	neurogenin 1	-0.032	0.978	7.89E-01	7.89E+01		
8114287	NM_004598	SPOCK1	sparc/osteonectin, cwcv and kazal	-0.051	0.965	7.09E-01	7.09E+01		
8114300	NM_017415	KLHL3	kelch-like 3 (Drosophila)	0.019	1.013	9.50E-01	9.50E+01		
8114320	NM_006805	HNRNPA0	heterogeneous nuclear ribonucleo	-0.004	0.997	9.77E-01	9.77E+01		
8114326	NM_016603	FAM13B	family with sequence similarity 13	0.014	1.009	9.50E-01	9.50E+01		
8114363	---	---	---	-0.099	0.934	8.70E-01	8.70E+01		
8114365	NM_139199	BRD8	bromodomain containing 8	-0.102	0.932	3.39E-01	3.39E+01		
8114396	NM_004661	CDC23	cell division cycle 23 homolog (S. c	0.017	1.012	9.53E-01	9.53E+01		
8114443	NM_004730	ETF1	eukaryotic translation termination	0.066	1.047	5.01E-01	5.01E+01		
8114455	NM_004134	HSPA9	heat shock 70kDa protein 9 (morta	-0.023	0.984	8.86E-01	8.86E+01		
8114468	NR_002913	SNORD63	small nucleolar RNA, C/D box 63	-0.050	0.966	9.06E-01	9.06E+01		
8114476	NM_00103763	SIL1	SIL1 homolog, endoplasmic reticul	0.135	1.098	2.78E-01	2.78E+01		
8114489	---	---	---	0.141	1.102	5.97E-01	5.97E+01		
8114491	NM_005847	SLC23A1	solute carrier family 23 (nucleoba	-0.012	0.992	9.27E-01	9.27E+01		
8114511	NM_016459	MGC29506	hypothetical protein MGC29506	0.079	1.056	5.06E-01	5.06E+01		
8114518	NM_194296	LOC202051	hypothetical protein LOC202051	0.003	1.002	9.88E-01	9.88E+01		
8114526	NM_152686	DNAJC18	DnaJ (Hsp40) homolog, subfamily	-0.110	0.927	4.56E-01	4.56E+01		
8114536	NM_198282	TMEM173	transmembrane protein 173	0.109	1.078	2.04E-01	2.04E+01		
8114546	---	---	---	-0.139	0.908	1.26E-01	1.26E+01		
8114550	NM_004883	NRG2	neuregulin 2	-0.025	0.983	8.25E-01	8.25E+01		
8114567	NM_002622	PFDN1	prefoldin subunit 1	0.056	1.040	7.87E-01	7.87E+01		
8114583	NM_00103523	SRA1	steroid receptor RNA activator 1	-0.075	0.949	6.60E-01	6.60E+01		
8114593	NM_133174	APBB3	amyloid beta (A4) precursor prote	-0.080	0.946	3.69E-01	3.69E+01		
8114612	NM_000591	CD14	CD14 molecule	-0.513	0.701	2.81E-01	2.81E+01		
8114618	NM_002488	NDUFA2	NADH dehydrogenase (ubiquinone	0.020	1.014	9.53E-01	9.53E+01		

8114625	NM_194249	DND1	dead end homolog 1 (zebrafish)	-0.054	0.964	8.07E-01	8.07E+01		
8114628	NM_002109	HARS	histidyl-tRNA synthetase	-0.143	0.906	2.35E-01	2.35E+01		
8114643	---	---	---	-0.144	0.905	1.61E-01	1.61E+01		
8114645	ENST00000319	LOC100288105	hypothetical protein LOC1002881(	-0.065	0.956	7.22E-01	7.22E+01		
8114647	NM_031947	SLC25A2	solute carrier family 25 (mitochon	-0.077	0.948	7.12E-01	7.12E+01		
8114653	NM_005642	TAF7	TAF7 RNA polymerase II, TATA box	-0.040	0.973	8.01E-01	8.01E+01		
8114658	NM_005219	DIAPH1	diaphanous homolog 1 (Drosophila	0.090	1.064	1.47E-01	1.47E+01		
8114691	NM_003883	HDAC3	histone deacetylase 3	0.055	1.039	7.08E-01	7.08E+01		
8114709	NM_033449	FCHSD1	FCH and double SH3 domains 1	-0.031	0.979	8.41E-01	8.41E+01		
8114733	NM_022481	ARAP3	ArfGAP with RhoGAP domain, ank	0.018	1.013	8.86E-01	8.86E+01		
8114767	NM_032420	PCDH1	protocadherin 1	-0.016	0.989	9.07E-01	9.07E+01		
8114778	NM_004483	GCSH	glycine cleavage system protein H	-0.010	0.993	9.75E-01	9.75E+01		
8114780	NM_016580	PCDH12	protocadherin 12	-0.060	0.959	6.51E-01	6.51E+01		
8114787	NM_005471	GNPDA1	glucosamine-6-phosphate deamin	0.320	1.248	4.39E-03	4.39E-01		
8114814	NM_000176	NR3C1	nuclear receptor subfamily 3, grou	-0.295	0.815	3.25E-04	3.25E-02		
8114829	NM_00102494	YIPF5	Yip1 domain family, member 5	-0.133	0.912	2.50E-01	2.50E+01		
8114856	BC130586	PLAC8L1	PLAC8-like 1	-0.006	0.996	9.90E-01	9.90E+01		
8114861	NM_020117	LARS	leucyl-tRNA synthetase	0.027	1.019	8.86E-01	8.86E+01		
8114900	NM_004576	PPP2R2B	protein phosphatase 2 (formerly 2	-0.031	0.979	9.01E-01	9.01E+01		
8114964	NM_003122	SPINK1	serine peptidase inhibitor, Kazal ty	0.053	1.037	9.53E-01	9.53E+01		
8115014	NM_014443	IL17B	interleukin 17B	-0.001	0.999	9.95E-01	9.95E+01		
8115022	NM_00102510	CSNK1A1	casein kinase 1, alpha 1	0.010	1.007	9.50E-01	9.50E+01		
8115074	---	---	---	0.100	1.072	7.42E-01	7.42E+01		
8115099	NM_002609	PDGFRB	platelet-derived growth factor rec	0.050	1.035	5.77E-01	5.77E+01		
8115122	NM_015981	CAMK2A	calcium/calmodulin-dependent pr	-0.034	0.977	7.95E-01	7.95E+01		
8115144	NM_00101230	ARSI	arylsulfatase family, member I	-0.019	0.987	9.28E-01	9.28E+01		
8115158	NM_00102507	RPS14	ribosomal protein S14	-0.003	0.998	9.88E-01	9.88E+01		
8115164	---	---	---	0.357	1.281	3.14E-01	3.14E+01		
8115168	NM_018047	RBM22	RNA binding motif protein 22	0.002	1.001	9.96E-01	9.96E+01		
8115181	NM_00113564	DCTN4	dynactin 4 (p62)	-0.129	0.914	2.82E-01	2.82E+01		
8115234	NM_001155	ANXA6	annexin A6	-0.092	0.938	4.38E-01	4.38E+01		
8115261	NM_015621	CCDC69	coiled-coil domain containing 69	0.030	1.021	8.27E-01	8.27E+01		
8115327	NM_003118	SPARC	secreted protein, acidic, cysteine-r	-0.009	0.993	9.77E-01	9.77E+01		
8115346	NM_004045	ATOX1	ATX1 antioxidant protein 1 homol	0.244	1.184	2.35E-01	2.35E+01		
8115375	NM_018691	FAM114A2	family with sequence similarity 11	-0.020	0.986	9.49E-01	9.49E+01		
8115391	NM_004821	HAND1	heart and neural crest derivatives	-0.066	0.955	6.03E-01	6.03E+01		
8115397	NM_032385	C5orf4	chromosome 5 open reading fram	0.056	1.040	5.77E-01	5.77E+01		
8115410	NM_015465	GEMIN5	gem (nuclear organelle) associated	-0.008	0.994	9.77E-01	9.77E+01		
8115476	NM_004270	MED7	mediator complex subunit 7	0.102	1.073	5.50E-01	5.50E+01		
8115487	NM_00100134	C5orf40	chromosome 5 open reading fram	0.023	1.016	9.38E-01	9.38E+01		
8115524	NM_014666	CLINT1	clathrin interactor 1	0.009	1.006	9.56E-01	9.56E+01		
8115543	NM_024007	EBF1	early B-cell factor 1	-0.114	0.924	7.23E-01	7.23E+01		
8115562	NM_144726	RNF145	ring finger protein 145	-0.055	0.963	7.48E-01	7.48E+01		
8115580	NM_052927	PWWP2A	PWWP domain containing 2A	-0.031	0.979	8.15E-01	8.15E+01		
8115584	NM_024565	CCNJL	cyclin J-like	0.028	1.020	8.44E-01	8.44E+01		
8115594	NM_031908	C1QTNF2	C1q and tumor necrosis factor rela	-0.012	0.991	9.53E-01	9.53E+01		
8115606	NM_006425	SLU7	SLU7 splicing factor homolog (S. ce	0.034	1.024	8.40E-01	8.40E+01		
8115664	---	---	---	-0.103	0.931	5.10E-01	5.10E+01		
8115666	NM_145266	NUDCD2	NudC domain containing 2	-0.026	0.982	9.10E-01	9.10E+01		
8115681	NM_024594	PANK3	pantothenate kinase 3	-0.033	0.978	7.94E-01	7.94E+01		
8115691	NM_003062	SLIT3	slit homolog 3 (Drosophila)	-0.074	0.950	2.98E-01	2.98E+01		
8115732	AY358216	UNQ9374	VCEW9374	0.115	1.083	3.45E-01	3.45E+01		
8115734	NM_005565	LCP2	lymphocyte cytosolic protein 2 (SH	0.048	1.034	7.72E-01	7.72E+01		
8115756	NM_004137	KCNMB1	potassium large conductance calci	0.213	1.159	4.76E-02	4.76E+00		
8115765	NM_033644	FBXW11	F-box and WD repeat domain cont	0.053	1.037	6.04E-01	6.04E+01		
8115783	NM_005990	STK10	serine/threonine kinase 10	-0.065	0.956	6.85E-01	6.85E+01		
8115806	NM_152277	UBTD2	ubiquitin domain containing 2	-0.083	0.944	6.78E-01	6.78E+01		
8115812	---	---	---	-0.039	0.973	8.25E-01	8.25E+01		
8115814	NM_00101799	SH3PXD2B	SH3 and PX domains 2B	-0.280	0.824	1.72E-01	1.72E+01		
8115840	NM_004387	NKX2-5	NK2 transcription factor related, lo	-0.005	0.996	9.73E-01	9.73E+01		
8115847	---	---	---	0.154	1.113	3.34E-01	3.34E+01		
8115849	---	---	---	-0.057	0.961	5.56E-01	5.56E+01		
8115865	NM_138369	BOD1	biorientation of chromosomes in c	-0.018	0.987	9.16E-01	9.16E+01		
8115871	AK131247	FLJ16171	FLJ16171 protein	-0.174	0.887	5.08E-01	5.08E+01		
8115884	---	---	---	-0.036	0.975	8.41E-01	8.41E+01		
8115886	NM_032361	THOC3	THO complex 3	-0.001	0.999	9.96E-01	9.96E+01		
8115895	NM_020444	KIAA1191	KIAA1191	-0.105	0.930	6.00E-01	6.00E+01		
8115907	NM_016391	NOP16	NOP16 nucleolar protein homolog	-0.067	0.954	8.27E-01	8.27E+01		
8115918	NM_007097	CLTB	clathrin, light chain (Lcb)	0.048	1.034	7.44E-01	7.44E+01		
8115927	NM_014901	RNF44	ring finger protein 44	0.011	1.008	9.56E-01	9.56E+01		
8115939	---	---	---	-0.007	0.995	9.85E-01	9.85E+01		
8115941	NM_052899	GPRIN1	G protein regulated inducer of ne	0.003	1.002	9.86E-01	9.86E+01		
8115948	NM_00100150	SNCB	synuclein, beta	-0.079	0.947	4.54E-01	4.54E+01		
8115978	NM_016290	UIMC1	ubiquitin interaction motif contain	-0.089	0.940	4.32E-01	4.32E+01		
8115997	NM_00103167	RAB24	RAB24, member RAS oncogene fa	0.064	1.046	6.87E-01	6.87E+01		
8116012	NM_031300	MXD3	MAX dimerization protein 3	0.008	1.006	9.50E-01	9.50E+01		
8116020	NM_006816	LMAN2	lectin, mannose-binding 2	0.103	1.074	5.34E-01	5.34E+01		

8116031	NM_00102988	PFN3	profilin 3	0.071	1.051	5.02E-01	5.02E+01		
8116033	NM_000505	F12	coagulation factor XII (Hageman fa	-0.058	0.960	4.78E-01	4.78E+01		
8116051	NM_080881	DBN1	drebrin 1	-0.039	0.973	7.35E-01	7.35E+01		
8116070	NM_005451	PDLIM7	PDZ and LIM domain 7 (enigma)	-0.036	0.976	7.78E-01	7.78E+01		
8116086	NM_024872	DOK3	docking protein 3	-0.075	0.950	6.58E-01	6.58E+01		
8116096	NM_016222	DDX41	DEAD (Asp-Glu-Ala-Asp) box polyp	-0.003	0.998	9.88E-01	9.88E+01		
8116113	NR_024019	FLJ10404	hypothetical protein FLJ10404	-0.017	0.988	8.59E-01	8.59E+01		
8116128	NR_026921	LOC202181	hypothetical protein LOC202181	0.008	1.006	9.77E-01	9.77E+01		
8116130	NM_00107952	FAM153B	family with sequence similarity 15	-0.012	0.991	9.76E-01	9.76E+01		
8116162	NM_006261	PROP1	PROP paired-like homeobox 1	-0.012	0.991	9.44E-01	9.44E+01		
8116168	NM_017838	NHP2	NHP2 ribonucleoprotein homolog	0.000	1.000	1.00E+00	1.00E+02		
8116177	NM_153373	AGXT2L2	alanine-glyoxylate aminotransfera	-0.039	0.973	7.54E-01	7.54E+01		
8116195	NM_173465	COL23A1	collagen, type XXIII, alpha 1	-0.060	0.959	3.95E-01	3.95E+01		
8116227	NM_020666	CLK4	CDC-like kinase 4	-0.084	0.943	4.46E-01	4.46E+01		
8116245	---	---	---	0.100	1.072	6.57E-01	6.57E+01		
8116253	NM_000843	GRM6	glutamate receptor, metabotropic	-0.007	0.995	9.72E-01	9.72E+01		
8116272	NM_014244	ADAMTS2	ADAM metallopeptidase with thro	0.017	1.012	9.16E-01	9.16E+01		
8116297	NM_005520	HNRNPH1	heterogeneous nuclear ribonucle	-0.051	0.966	5.29E-01	5.29E+01		
8116313	NM_00114230	LOC285679	hypothetical protein LOC285679	-0.127	0.916	3.17E-01	3.17E+01		
8116316	NM_014275	MGAT4B	mannosyl (alpha-1,3-)-glycoprotei	-0.093	0.938	1.65E-01	1.65E+01		
8116335	NM_016175	C5orf45	chromosome 5 open reading fram	-0.011	0.992	9.46E-01	9.46E+01		
8116346	---	---	---	-0.010	0.993	9.85E-01	9.85E+01		
8116348	NM_198868	TBC1D9B	TBC1 domain family, member 9B (	0.007	1.005	9.77E-01	9.77E+01		
8116372	NM_018434	RNF130	ring finger protein 130	0.426	1.344	1.42E-01	1.42E+01		
8116384	NM_175062	RASGEF1C	RasGEF domain family, member 1(	0.004	1.002	9.83E-01	9.83E+01		
8116400	---	---	---	-0.029	0.980	9.36E-01	9.36E+01		
8116402	NM_139068	MAPK9	mitogen-activated protein kinase 9	0.031	1.022	8.84E-01	8.84E+01		
8116418	NM_005110	GFPT2	glutamine-fructose-6-phosphate t	-0.247	0.843	2.16E-02	2.16E+00		
8116439	NM_052863	SCGB3A1	secretoglobin, family 3A, member	-0.050	0.966	6.35E-01	6.35E+01		
8116445	NM_182925	FLT4	fms-related tyrosine kinase 4	0.049	1.034	6.35E-01	6.35E+01		
8116484	NM_00111461	MGAT1	mannosyl (alpha-1,3-)-glycoprotei	0.182	1.134	1.80E-01	1.80E+01		
8116494	NM_152283	ZFP62	zinc finger protein 62 homolog (m	0.213	1.159	3.45E-01	3.45E+01		
8116502	---	---	---	-0.009	0.994	9.60E-01	9.60E+01		
8116520	NM_006098	GNB2L1	guanine nucleotide binding protei	-0.055	0.962	6.54E-01	6.54E+01		
8116530	NR_002592	SNORD96A	small nucleolar RNA, C/D box 96A	0.008	1.006	9.82E-01	9.82E+01		
8116532	NR_002591	SNORD95	small nucleolar RNA, C/D box 95	0.026	1.018	9.56E-01	9.56E+01		
8116534	NM_032765	TRIM52	tripartite motif-containing 52	-0.068	0.954	6.57E-01	6.57E+01		
8116548	NM_020185	DUSP22	dual specificity phosphatase 22	0.022	1.015	9.27E-01	9.27E+01		
8116571	---	---	---	0.122	1.089	9.23E-01	9.23E+01		
8116579	NM_033260	FOXQ1	forkhead box Q1	0.012	1.009	9.50E-01	9.50E+01		
8116582	NM_001452	FOXF2	forkhead box F2	-0.005	0.996	9.81E-01	9.81E+01		
8116585	---	---	---	-0.031	0.979	8.42E-01	8.42E+01		
8116591	NM_001453	FOXC1	forkhead box C1	0.017	1.012	8.94E-01	8.94E+01		
8116595	NM_020135	WRNIP1	Werner helicase interacting protei	-0.088	0.941	1.01E-01	1.01E+01		
8116607	NR_026855	DKFZP686I1521	hypothetical LOC401232	0.038	1.027	7.14E-01	7.14E+01		
8116610	NM_000904	NQO2	NAD(P)H dehydrogenase, quinone	-0.099	0.933	5.23E-01	5.23E+01		
8116620	NM_00101298	M136B // FAM1	family with sequence similarity 13	0.036	1.025	8.53E-01	8.53E+01		
8116622	NM_003804	RIPK1	receptor (TNFRSF)-interacting seri	-0.037	0.975	7.97E-01	7.97E+01		
8116649	NM_001069	TUBB2A	tubulin, beta 2A	-0.314	0.804	5.15E-02	5.15E+00		
8116651	---	---	---	-0.141	0.907	6.54E-01	6.54E+01		
8116653	NM_001069	TUBB2A	tubulin, beta 2A	-0.314	0.804	5.15E-02	5.15E+00		
8116655	NM_00112859	PSMG4	proteasome (prosome, macropain	0.009	1.006	9.75E-01	9.75E+01		
8116658	NM_012135	FAM50B	family with sequence similarity 50	-0.025	0.983	8.78E-01	8.78E+01		
8116664	NM_003913	PRPF4B	PRP4 pre-mRNA processing factor	0.024	1.017	8.63E-01	8.63E+01		
8116696	NM_00108540	C6orf201	chromosome 6 open reading fram	0.004	1.003	9.88E-01	9.88E+01		
8116710	NR_026590	CDYL	chromodomain protein, Y-like	-0.060	0.960	5.11E-01	5.11E+01		
8116724	NM_006567	FARS2	phenylalanyl-tRNA synthetase 2, n	0.043	1.030	8.77E-01	8.77E+01		
8116740	NM_00100369	RREB1	ras responsive element binding pr	0.174	1.128	2.00E-01	2.00E+01		
8116760	NM_031480	RIOK1	RIO kinase 1 (yeast)	-0.068	0.954	7.64E-01	7.64E+01		
8116807	NM_152551	SNRNP48	small nuclear ribonucleoprotein 4	0.092	1.066	4.85E-01	4.85E+01		
8116818	NM_001718	BMP6	bone morphogenetic protein 6	-0.143	0.906	7.67E-02	7.67E+00		
8116835	NM_145649	GCNT2	glucosaminyl (N-acetyl) transferas	0.269	1.205	1.59E-01	1.59E+01		
8116859	NM_016462	TMEM14C	transmembrane protein 14C	-0.073	0.951	7.83E-01	7.83E+01		
8116867	NM_030969	TMEM14B	transmembrane protein 14B	-0.025	0.983	9.06E-01	9.06E+01		
8116910	NM_002114	HIVEP1	human immunodeficiency virus ty	-0.060	0.959	6.74E-01	6.74E+01		
8116921	NM_001955	EDN1	endothelin 1	0.024	1.017	9.53E-01	9.53E+01		
8116929	NM_002948	RPL15	ribosomal protein L15	0.000	1.000	1.00E+00	1.00E+02		
8116932	NM_030948	PHACTR1	phosphatase and actin regulator 1	-0.148	0.903	1.41E-01	1.41E+01		
8116956	NM_031244	SIRT5	sirtuin (silent mating type informa	0.034	1.023	8.47E-01	8.47E+01		
8116969	NM_016167	NOL7	nucleolar protein 7, 27kDa	-0.032	0.978	8.06E-01	8.06E+01		
8116983	NM_004233	CD83	CD83 molecule	-0.137	0.909	3.66E-01	3.66E+01		
8116992	---	---	---	-0.007	0.995	9.88E-01	9.88E+01		
8116998	NM_004973	JARID2	jumonji, AT rich interactive domai	-0.051	0.966	7.86E-01	7.86E+01		
8117018	---	---	---	-0.260	0.835	3.49E-01	3.49E+01		
8117020	NM_013262	MYLIP	myosin regulatory light chain inter	0.164	1.120	2.08E-01	2.08E+01		
8117034	NM_006877	GMPR	guanosine monophosphate reduct	0.106	1.076	4.98E-01	4.98E+01		
8117071	NM_016255	FAM8A1	family with sequence similarity 8,	0.131	1.095	8.15E-02	8.15E+00		

8117081	NM_153042	AOF1	amine oxidase (flavin containing) d	0.280	1.215	2.46E-02	2.46E+00		
8117106	NM_182757	RNF144B	ring finger protein 144B	-0.219	0.859	5.38E-01	5.38E+01		
8117118	---	---	---	-0.022	0.985	9.44E-01	9.44E+01		
8117128	NM_001949	E2F3	E2F transcription factor 3	0.010	1.007	9.61E-01	9.61E+01		
8117140	NM_017774	CDKAL1	CDK5 regulatory subunit associate	0.006	1.004	9.80E-01	9.80E+01		
8117165	NM_003107	SOX4	SRY (sex determining region Y)-bo	-0.074	0.950	4.25E-01	4.25E+01		
8117172	NM_138574	HDGFL1	hepatoma derived growth factor-l	-0.034	0.977	7.67E-01	7.67E+01		
8117176	ENST00000400	LOC100129307	UPF0607 protein ENSP0000038314	0.035	1.025	8.62E-01	8.62E+01		
8117187	---	---	---	0.035	1.025	8.00E-01	8.00E+01		
8117194	NM_020662	MRS2	MRS2 magnesium homeostasis fac	0.170	1.125	8.33E-02	8.33E+00		
8117207	NM_170740	ALDH5A1	aldehyde dehydrogenase 5 family,	0.098	1.070	3.17E-01	3.17E+01		
8117219	NM_018473	ACOT13	acyl-CoA thioesterase 13	0.261	1.198	6.65E-02	6.65E+00		
8117237	---	---	---	0.015	1.011	9.27E-01	9.27E+01		
8117239	---	---	---	-0.004	0.997	9.86E-01	9.86E+01		
8117243	NM_017640	LRRC16A	leucine rich repeat containing 16A	0.201	1.150	1.45E-01	1.45E+01		
8117321	NM_006355	TRIM38	tripartite motif-containing 38	0.144	1.105	3.20E-01	3.20E+01		
8117330	NM_003529	HIST1H3A	histone cluster 1, H3a	-0.244	0.844	8.94E-02	8.94E+00		
8117334	NM_003538	HIST1H4A	histone cluster 1, H4a	-0.173	0.887	3.63E-01	3.63E+01		
8117339	NM_003531	HIST1H3C	histone cluster 1, H3c	0.011	1.008	9.49E-01	9.49E+01		
8117368	NM_003542	HIST1H4C	histone cluster 1, H4c	0.192	1.143	4.68E-01	4.68E+01		
8117372	NM_003512	HIST1H2AC	histone cluster 1, H2ac	0.060	1.043	6.26E-01	6.26E+01		
8117377	NM_005321	HIST1H1E	histone cluster 1, H1e	0.050	1.035	7.48E-01	7.48E+01		
8117382	NM_021063	HIST1H2BD	histone cluster 1, H2bd	-0.085	0.943	7.03E-01	7.03E+01		
8117395	NM_003522	HIST1H2BF	histone cluster 1, H2bf	0.225	1.169	1.59E-01	1.59E+01		
8117402	NM_003545	HIST1H4E	histone cluster 1, H4e	0.189	1.140	2.36E-01	2.36E+01		
8117408	NM_021052	HIST1H2AE	histone cluster 1, H2ae	0.073	1.052	7.79E-01	7.79E+01		
8117422	NM_003540	HIST1H4F	histone cluster 1, H4f	0.206	1.153	1.76E-01	1.76E+01		
8117426	NM_003524	HIST1H2BH	histone cluster 1, H2bh	0.108	1.077	6.28E-01	6.28E+01		
8117435	NM_007047	BTN3A2	butyrophilin, subfamily 3, member	-0.062	0.958	5.94E-01	5.94E+01		
8117447	NM_006995	BTN2A2	butyrophilin, subfamily 2, member	0.098	1.070	1.27E-01	1.27E+01		
8117458	NM_00114500	BTN3A1	butyrophilin, subfamily 3, member	-0.023	0.984	9.28E-01	9.28E+01		
8117476	NM_006994	BTN3A3	butyrophilin, subfamily 3, member	-0.050	0.966	7.25E-01	7.25E+01		
8117485	NM_078476	BTN2A1	butyrophilin, subfamily 2, member	-0.047	0.968	7.44E-01	7.44E+01		
8117510	NM_006353	HMGN4	high mobility group nucleosomal b	-0.048	0.967	5.88E-01	5.88E+01		
8117522	NM_013375	ABT1	activator of basal transcription 1	0.101	1.073	2.73E-01	2.73E+01		
8117531	---	---	---	-0.089	0.940	5.79E-01	5.79E+01		
8117535	NM_021064	HIST1H2AG	histone cluster 1, H2ag	-0.108	0.928	4.57E-01	4.57E+01		
8117537	NM_003495	HIST1H4I	histone cluster 1, H4i	0.023	1.016	9.20E-01	9.20E+01		
8117565	---	---	---	-0.063	0.957	5.64E-01	5.64E+01		
8117572	NM_00107678	ZNF391	zinc finger protein 391	-0.038	0.974	9.06E-01	9.06E+01		
8117580	NM_003509	HIST1H2AI	histone cluster 1, H2ai	0.009	1.006	9.68E-01	9.68E+01		
8117583	NM_003509	HIST1H2AI	histone cluster 1, H2ai	0.000	1.000	1.00E+00	1.00E+02		
8117594	NM_003521	HIST1H2BM	histone cluster 1, H2bm	0.233	1.176	4.41E-01	4.41E+01		
8117608	NM_003511	HIST1H2AL	histone cluster 1, H2al	0.011	1.008	9.65E-01	9.65E+01		
8117614	NM_003527	HIST1H2BO	histone cluster 1, H2bo	-0.077	0.948	7.67E-01	7.67E+01		
8117626	---	---	---	0.086	1.061	3.44E-01	3.44E+01		
8117628	---	---	---	-0.033	0.977	9.33E-01	9.33E+01		
8117630	NM_003447	ZNF165	zinc finger protein 165	-0.237	0.848	5.21E-02	5.21E+00		
8117646	NM_006298	ZNF192	zinc finger protein 192	-0.010	0.993	9.74E-01	9.74E+01		
8117653	---	---	---	-0.116	0.923	7.16E-01	7.16E+01		
8117655	NM_006299	ZNF193	zinc finger protein 193	0.028	1.020	8.78E-01	8.78E+01		
8117667	NM_00102356	ZNF187	zinc finger protein 187	-0.010	0.993	9.61E-01	9.61E+01		
8117685	NM_024493	ZKSCAN3	zinc finger with KRAB and SCAN do	0.182	1.135	2.20E-01	2.20E+01		
8117698	---	---	---	0.017	1.012	9.08E-01	9.08E+01		
8117710	---	---	---	-0.046	0.969	7.51E-01	7.51E+01		
8117720	---	---	---	0.000	1.000	1.00E+00	1.00E+02		
8117739	---	---	---	-0.065	0.956	5.87E-01	5.87E+01		
8117741	NM_007160	OR2H2	olfactory receptor, family 2, subfa	-0.013	0.991	9.46E-01	9.46E+01		
8117760	NM_00109847	HLA-F	major histocompatibility complex,	-0.029	0.980	8.62E-01	8.62E+01		
8117773	AK128290	LOC554223	hypothetical LOC554223	0.026	1.018	8.62E-01	8.62E+01		
8117777	NM_002127	HLA-G	major histocompatibility complex,	-0.027	0.981	8.42E-01	8.42E+01		
8117800	NM_002116	HLA-A	major histocompatibility complex,	-0.021	0.986	9.40E-01	9.40E+01		
8117813	NR_024240	HLA-J	major histocompatibility complex,	-0.030	0.979	8.07E-01	8.07E+01		
8117822	NM_170783	ZNRD1	zinc ribbon domain containing 1	-0.004	0.997	9.87E-01	9.87E+01		
8117834	NM_021959	PPP1R11	protein phosphatase 1, regulatory	0.021	1.015	9.35E-01	9.35E+01		
8117840	NM_138700	TRIM40	tripartite motif-containing 40	0.004	1.003	9.86E-01	9.86E+01		
8117849	NM_033229	TRIM15	tripartite motif-containing 15	0.029	1.020	7.47E-01	7.47E+01		
8117861	NR_027822	HLA-L	major histocompatibility complex,	-0.044	0.970	8.03E-01	8.03E+01		
8117869	NM_172016	TRIM39	tripartite motif-containing 39	-0.049	0.967	7.38E-01	7.38E+01		
8117881	NM_024839	RPP21	ribonuclease P/MRP 21kDa subuni	-0.079	0.947	6.37E-01	6.37E+01		
8117888	NM_006325	RAN	RAN, member RAS oncogene fami	-0.081	0.945	6.33E-01	6.33E+01		
8117890	NM_005516	HLA-E	major histocompatibility complex,	0.026	1.018	8.97E-01	8.97E+01		
8117900	NM_013993	DDR1	discoidin domain receptor tyrosin	-0.002	0.998	9.88E-01	9.88E+01		
8117922	NM_025263	PRR3	proline rich 3	-0.057	0.961	7.94E-01	7.94E+01		
8117929	NM_00102509	ABCF1	ATP-binding cassette, sub-family F	-0.130	0.914	1.10E-01	1.10E+01		
8117955	NM_014046	MRPS18B	mitochondrial ribosomal protein S	0.027	1.019	9.07E-01	9.07E+01		
8117965	NM_024909	C6orf134	chromosome 6 open reading fram	-0.122	0.919	4.09E-01	4.09E+01		

8117987	NM_00116137	C6orf136	chromosome 6 open reading fram	-0.070	0.952	6.07E-01	6.07E+01		
8117995	NM_178014	TUBB	tubulin, beta	-0.094	0.937	7.04E-01	7.04E+01		
8118007	NM_001517	GTF2H4	general transcription factor IIH, pc	0.015	1.010	9.27E-01	9.27E+01		
8118023	NM_001517	GTF2H4	general transcription factor IIH, pc	0.011	1.008	9.70E-01	9.70E+01		
8118028	NM_020442	VARS2	valyl-tRNA synthetase 2, mitochor	-0.016	0.989	9.04E-01	9.04E+01		
8118069	NM_00101090	MUC21	mucin 21, cell surface associated	-0.063	0.957	4.87E-01	4.87E+01		
8118086	NM_007109	TCF19	transcription factor 19	-0.040	0.973	8.06E-01	8.06E+01		
8118093	NR_026791	HCG27	HLA complex group 27	0.094	1.067	2.33E-01	2.33E+01		
8118098	---	---	---	0.054	1.038	5.91E-01	5.91E+01		
8118100	NM_000247	MICA	MHC class I polypeptide-related se	-0.130	0.914	3.11E-01	3.11E+01		
8118111	NM_006674	HCP5	HLA complex P5	-0.035	0.976	8.98E-01	8.98E+01		
8118116	NM_005931	MICB	MHC class I polypeptide-related se	0.031	1.021	8.92E-01	8.92E+01		
8118124	NM_00101170	MCCD1	mitochondrial coiled-coil domain 1	0.049	1.035	6.58E-01	6.58E+01		
8118127	NM_005007	NFKBIL1	nuclear factor of kappa light polyp	-0.066	0.955	5.87E-01	5.87E+01		
8118137	NM_000595	LTA	lymphotoxin alpha (TNF superfam	-0.063	0.957	7.54E-01	7.54E+01		
8118142	NM_000594	TNF	tumor necrosis factor (TNF superfa	-0.138	0.909	5.35E-01	5.35E+01		
8118147	---	---	---	0.001	1.001	9.96E-01	9.96E+01		
8118174	NM_080686	BAT2	HLA-B associated transcript 2	-0.004	0.997	9.81E-01	9.81E+01		
8118207	NR_002971	SNORA38	small nucleolar RNA, H/ACA box 3	-0.099	0.934	6.38E-01	6.38E+01		
8118209	NM_019101	APOM	apolipoprotein M	-0.007	0.995	9.76E-01	9.76E+01		
8118218	NM_001320	CSNK2B	casein kinase 2, beta polypeptide	0.090	1.064	3.78E-01	3.78E+01		
8118228	NM_021221	LY6G5B	lymphocyte antigen 6 complex, loc	-0.025	0.983	9.51E-01	9.51E+01		
8118235	NM_00100369	LY6G6F	lymphocyte antigen 6 complex, loc	-0.034	0.977	8.07E-01	8.07E+01		
8118242	NM_021246	LY6G6D	lymphocyte antigen 6 complex, loc	0.024	1.017	8.57E-01	8.57E+01		
8118249	NM_138277	C6orf25	chromosome 6 open reading fram	-0.058	0.961	5.89E-01	5.89E+01		
8118259	NM_025259	MSH5	mutS homolog 5 (E. coli)	0.120	1.087	2.74E-01	2.74E+01		
8118302	BC137541	C6orf26	chromosome 6 open reading fram	-0.033	0.978	8.01E-01	8.01E+01		
8118310	NM_005345	HSPA1A	heat shock 70kDa protein 1A	-0.289	0.818	2.61E-03	2.61E-01		
8118319	---	---	---	0.208	1.155	7.45E-01	7.45E+01		
8118322	NR_002742	SNORD52	small nucleolar RNA, C/D box 52	0.189	1.140	3.95E-01	3.95E+01		
8118345	NM_001710	CFB	complement factor B	-0.245	0.844	6.68E-01	6.68E+01		
8118367	NM_006929	SKIV2L	superkiller viralicidic activity 2-like	-0.058	0.961	6.62E-01	6.62E+01		
8118395	NR_026717	STK19	serine/threonine kinase 19	-0.028	0.981	8.96E-01	8.96E+01		
8118498	NM_000500	CYP21A2	cytochrome P450, family 21, subfa	0.003	1.002	9.88E-01	9.88E+01		
8118509	NM_005155	PPT2	palmitoyl-protein thioesterase 2	-0.035	0.976	7.51E-01	7.51E+01		
8118535	NM_006913	RNF5	ring finger protein 5	-0.032	0.978	8.54E-01	8.54E+01		
8118544	---	---	---	-0.060	0.959	6.97E-01	6.97E+01		
8118556	NM_002122	HLA-DQA1	major histocompatibility complex,	0.257	1.195	7.77E-01	7.77E+01		
8118564	NM_020056	HLA-DQA2	major histocompatibility complex,	0.237	1.178	3.39E-01	3.39E+01		
8118571	NM_002800	PSMB9	proteasome (prosome, macropain	0.138	1.101	3.90E-01	3.90E+01		
8118580	NM_005104	BRD2	bromodomain containing 2	-0.101	0.932	2.26E-01	2.26E+01		
8118613	NM_006979	SLC39A7	solute carrier family 39 (zinc trans	-0.066	0.955	6.81E-01	6.81E+01		
8118622	NM_014234	HSD17B8	hydroxysteroid (17-beta) dehydro	0.041	1.029	8.57E-01	8.57E+01		
8118634	NM_002931	RING1	ring finger protein 1	0.000	1.000	1.00E+00	1.00E+02		
8118644	NM_022551	RPS18	ribosomal protein S18	0.020	1.014	9.23E-01	9.23E+01		
8118655	NM_003782	B3GALT4	UDP-Gal:betaGlcNAc beta 1,3-gala	0.012	1.008	9.54E-01	9.54E+01		
8118661	NM_014260	PFDN6	prefoldin subunit 6	0.032	1.022	8.91E-01	8.91E+01		
8118667	---	---	---	-0.022	0.985	9.10E-01	9.10E+01		
8118669	NM_002263	KIFC1	kinesin family member C1	0.064	1.045	6.93E-01	6.93E+01		
8118682	NR_027692	PHF1	PHD finger protein 1	-0.185	0.880	5.77E-02	5.77E+00		
8118696	NM_006772	SYNGAP1	synaptic Ras GTPase activating pro	-0.055	0.963	6.10E-01	6.10E+01		
8118724	---	---	---	0.021	1.014	9.23E-01	9.23E+01		
8118726	NR_027908	C6orf227	chromosome 6 open reading fram	0.009	1.006	9.61E-01	9.61E+01		
8118734	NM_002224	ITPR3	inositol 1,4,5-triphosphate recepto	-0.075	0.949	3.90E-01	3.90E+01		
8118794	NM_145899	HMGA1	high mobility group AT-hook 1	-0.053	0.964	7.09E-01	7.09E+01		
8118804	NM_020804	PACSIN1	protein kinase C and casein kinase	0.038	1.027	7.07E-01	7.07E+01		
8118820	---	---	---	0.049	1.035	8.90E-01	8.90E+01		
8118824	---	---	---	0.047	1.033	8.16E-01	8.16E+01		
8118826	NM_003093	SNRPC	small nuclear ribonucleoprotein po	0.135	1.098	2.35E-01	2.35E+01		
8118833	NM_017754	UHRF1BP1	UHRF1 binding protein 1	0.208	1.155	1.55E-01	1.55E+01		
8118861	---	---	---	0.228	1.171	1.63E-01	1.63E+01		
8118863	NM_015245	ANKS1A	ankyrin repeat and sterile alpha m	0.054	1.038	7.15E-01	7.15E+01		
8118890	NM_152753	SCUBE3	signal peptide, CUB domain, EGF-l	-0.046	0.968	6.66E-01	6.66E+01		
8118915	NM_003427	ZNF76	zinc finger protein 76 (expressed i	0.018	1.012	9.40E-01	9.40E+01		
8118932	NM_022047	DEF6	differentially expressed in FDCP 6	-0.006	0.996	9.74E-01	9.74E+01		
8118945	NM_006238	PPARD	peroxisome proliferator-activated	0.071	1.050	6.87E-01	6.87E+01		
8118963	NM_021922	FANCE	Fanconi anemia, complementation	-0.059	0.960	6.07E-01	6.07E+01		
8118974	NM_007104	RPL10A	ribosomal protein L10a	-0.068	0.954	6.87E-01	6.87E+01		
8118979	---	---	---	0.274	1.209	4.06E-01	4.06E+01		
8118981	BC033033	C6orf81	chromosome 6 open reading fram	0.002	1.001	9.94E-01	9.94E+01		
8118990	NM_207409	C6orf126	chromosome 6 open reading fram	-0.018	0.988	9.14E-01	9.14E+01		
8118995	NM_182548	LHFPL5	lipoma HMGIC fusion partner-like	-0.057	0.961	6.33E-01	6.33E+01		
8119000	NM_001315	MAPK14	mitogen-activated protein kinase	-0.069	0.954	6.63E-01	6.63E+01		
8119016	NM_002754	MAPK13	mitogen-activated protein kinase	-0.058	0.961	6.80E-01	6.80E+01		
8119034	NM_015695	BRPF3	bromodomain and PHD finger con	-0.154	0.899	2.01E-01	2.01E+01		
8119052	NM_173676	PNPLA1	patatin-like phospholipase domair	-0.095	0.936	2.21E-01	2.21E+01		
8119067	NM_173562	KCTD20	potassium channel tetramerisatior	-0.068	0.954	7.07E-01	7.07E+01		

8119076	---	---	---	0.168	1.123	5.25E-01	5.25E+01		
8119078	---	---	---	0.066	1.047	7.54E-01	7.54E+01		
8119080	NM_003017	SFRS3	splicing factor, arginine/serine-rich	-0.045	0.969	7.01E-01	7.01E+01		
8119107	---	---	---	-0.019	0.987	9.34E-01	9.34E+01		
8119109	NM_152734	C6orf89	chromosome 6 open reading fram	0.010	1.007	9.67E-01	9.67E+01		
8119124	NM_153370	PI16	peptidase inhibitor 16	-0.034	0.977	7.89E-01	7.89E+01		
8119132	NM_173558	FGD2	FYVE, RhoGEF and PH domain con	0.229	1.172	5.92E-04	5.92E-02		
8119153	NM_004373	COX6A1	cytochrome c oxidase subunit VIa	0.028	1.020	8.61E-01	8.61E+01		
8119159	---	---	---	0.007	1.005	9.74E-01	9.74E+01		
8119161	NM_002648	PIM1	pim-1 oncogene	0.091	1.065	3.98E-01	3.98E+01		
8119169	NM_017772	TBC1D22B	TBC1 domain family, member 22B	0.047	1.033	7.71E-01	7.71E+01		
8119184	NM_003958	RNF8	ring finger protein 8	-0.098	0.935	2.63E-01	2.63E+01		
8119196	---	---	---	-0.039	0.974	9.49E-01	9.49E+01		
8119198	NM_015050	FTSJD2	FtsJ methyltransferase domain co	0.019	1.013	9.51E-01	9.51E+01		
8119223	AK127725	FLJ45825	hypothetical protein LOC646888	-0.098	0.934	8.70E-01	8.70E+01		
8119227	NM_021943	ZFAND3	zinc finger, AN1-type domain 3	0.050	1.035	7.44E-01	7.44E+01		
8119237	AK092662	LOC100132005	hypothetical LOC100132005	-0.037	0.975	8.64E-01	8.64E+01		
8119239	---	---	---	0.130	1.095	7.97E-01	7.97E+01		
8119241	---	---	---	-0.103	0.931	8.70E-01	8.70E+01		
8119338	NM_002062	GLP1R	glucagon-like peptide 1 receptor	-0.026	0.982	8.29E-01	8.29E+01		
8119386	DQ168992	TDRG1	testis development related protei	-0.050	0.966	7.51E-01	7.51E+01		
8119390	NM_020737	LRFN2	leucine rich repeat and fibronecti	-0.086	0.942	3.12E-01	3.12E+01		
8119396	NM_00101087	BZRPL1	benzodiazapine receptor (periphe	0.039	1.027	7.83E-01	7.83E+01		
8119403	NM_006789	APOBEC2	apolipoprotein B mRNA editing en	-0.025	0.983	8.88E-01	8.88E+01		
8119408	NM_002505	NFYA	nuclear transcription factor Y, alph	0.002	1.001	9.94E-01	9.94E+01		
8119423	NR_026938	LOC221442	adenylate cyclase 10 pseudogene	0.082	1.059	8.68E-01	8.68E+01		
8119427	NM_198153	TREML4	triggering receptor expressed on r	-0.044	0.970	8.07E-01	8.07E+01		
8119435	NM_004828	NCR2	natural cytotoxicity triggering rece	-0.056	0.962	5.81E-01	5.81E+01		
8119444	NM_00101242	FOXP4	forkhead box P4	0.067	1.047	4.76E-01	4.76E+01		
8119466	NM_005586	MDFI	MyoD family inhibitor	-0.100	0.933	2.51E-01	2.51E+01		
8119492	NM_004053	BYSL	bystin-like	0.029	1.020	8.75E-01	8.75E+01		
8119503	NM_138572	TAF8	TAF8 RNA polymerase II, TATA bo	-0.025	0.983	8.49E-01	8.49E+01		
8119515	NM_000409	GUCA1A	guanylate cyclase activator 1A (ret	-0.035	0.976	7.05E-01	7.05E+01		
8119525	AY465895	HCRP1	hepatocellular carcinoma-related	0.021	1.015	9.53E-01	9.53E+01		
8119529	NM_015255	UBR2	ubiquitin protein ligase E3 compo	-0.015	0.990	9.24E-01	9.24E+01		
8119582	NM_015349	KIAA0240	KIAA0240	0.017	1.012	9.10E-01	9.10E+01		
8119599	NM_138296	PTCRA	pre T-cell antigen receptor alpha	0.028	1.019	7.97E-01	7.97E+01		
8119609	NM_006586	CNPY3	canopy 3 homolog (zebrafish)	-0.019	0.987	9.14E-01	9.14E+01		
8119627	NM_006245	PPP2R5D	protein phosphatase 2, regulatory	-0.100	0.933	2.94E-01	2.94E+01		
8119648	NM_057161	KLHDC3	kelch domain containing 3	0.037	1.026	8.16E-01	8.16E+01		
8119661	BC011933	C6orf153	chromosome 6 open reading fram	-0.015	0.990	9.62E-01	9.62E+01		
8119670	NM_201523	KLC4	kinesin light chain 4	-0.058	0.960	4.68E-01	4.68E+01		
8119689	NM_002821	PTK7	PTK7 protein tyrosine kinase 7	-0.001	0.999	9.96E-01	9.96E+01		
8119712	NM_003131	SRF	serum response factor (c-fos seru	-0.109	0.927	1.59E-01	1.59E+01		
8119722	NM_015089	CUL9	cullin 9	-0.053	0.964	4.93E-01	4.93E+01		
8119765	NM_032538	TTBK1	tau tubulin kinase 1	-0.037	0.974	7.51E-01	7.51E+01		
8119801	NM_033450	ABCC10	ATP-binding cassette, sub-family C	-0.053	0.964	5.15E-01	5.15E+01		
8119829	NM_00114601	TJAP1	tight junction associated protein 1	-0.122	0.919	1.36E-01	1.36E+01		
8119842	NM_203290	POLR1C	polymerase (RNA) I polypeptide C,	-0.031	0.979	8.97E-01	8.97E+01		
8119858	NM_006502	POLH	polymerase (DNA directed), eta	-0.012	0.991	9.54E-01	9.54E+01		
8119874	NM_014628	MAD2L1BP	MAD2L1 binding protein	-0.057	0.961	6.97E-01	6.97E+01		
8119896	---	---	---	-0.030	0.979	7.51E-01	7.51E+01		
8119898	NM_00102536	VEGFA	vascular endothelial growth factor	-0.147	0.903	5.15E-01	5.15E+01		
8119918	NM_153246	C6orf223	chromosome 6 open reading fram	-0.074	0.950	5.45E-01	5.45E+01		
8119926	NM_018426	TMEM63B	transmembrane protein 63B	-0.079	0.947	5.28E-01	5.28E+01		
8119974	NM_00107817	SLC29A1	solute carrier family 29 (nucleosid	-0.004	0.997	9.83E-01	9.83E+01		
8119993	NM_007355	HSP90AB1	heat shock protein 90kDa alpha (c	-0.025	0.983	9.13E-01	9.13E+01		
8120004	NM_00113756	TMEM151B	transmembrane protein 151B	0.024	1.017	8.62E-01	8.62E+01		
8120022	NM_001253	CDC5L	CDC5 cell division cycle 5-like (S. p	0.007	1.005	9.69E-01	9.69E+01		
8120043	NM_00102463	RUNX2	runt-related transcription factor 2	-0.260	0.835	2.64E-02	2.64E+00		
8120061	NM_014936	ENPP4	ectonucleotide pyrophosphatase/	-0.035	0.976	8.87E-01	8.87E+01		
8120102	NM_012120	CD2AP	CD2-associated protein	0.072	1.051	5.81E-01	5.81E+01		
8120208	---	---	---	0.027	1.019	8.92E-01	8.92E+01		
8120210	NM_002190	IL17A	interleukin 17A	-0.043	0.970	7.95E-01	7.95E+01		
8120215	NM_133367	PAQR8	progestin and adipoQ receptor fa	0.048	1.034	7.50E-01	7.50E+01		
8120239	NM_014051	TMEM14A	transmembrane protein 14A	-0.063	0.957	7.69E-01	7.69E+01		
8120249	NR_001445 //	RN7SK // RN7SI	RNA, 7SK small nuclear // RNA, 7S	-0.029	0.980	7.27E-01	7.27E+01		
8120251	NM_033480	FBXO9	F-box protein 9	-0.029	0.980	8.78E-01	8.78E+01		
8120269	AK095315	FBXO9	F-box protein 9	-0.106	0.929	6.13E-01	6.13E+01		
8120273	---	---	---	-0.232	0.851	5.20E-04	5.20E-02		
8120378	NM_020931	KIAA1586	KIAA1586	0.024	1.017	9.42E-01	9.42E+01		
8120382	NM_00103162	ZNF451	zinc finger protein 451	0.019	1.013	9.34E-01	9.34E+01		
8120402	NM_004282	BAG2	BCL2-associated athanogene 2	-0.010	0.993	9.62E-01	9.62E+01		
8120428	NM_000801	FKBP1A	FK506 binding protein 1A, 12kDa	0.231	1.174	8.09E-03	8.09E-01		
8120431	NM_003463	PTP4A1	protein tyrosine phosphatase type	0.014	1.010	9.53E-01	9.53E+01		
8120441	NM_015153	PHF3	PHD finger protein 3	-0.038	0.974	7.51E-01	7.51E+01		
8120501	NM_001858	COL19A1	collagen, type XIX, alpha 1	-0.011	0.992	9.54E-01	9.54E+01		

8120585	NM_00104430	SMAP1	small ArfGAP 1	-0.002	0.999	9.90E-01	9.90E+01		
8120600	---	---	---	0.119	1.086	3.82E-01	3.82E+01		
8120602	NM_024576	OGFRL1	opioid growth factor receptor-like	-0.256	0.838	1.98E-01	1.98E+01		
8120698	NM_133645	MTO1	mitochondrial translation optimiza	0.040	1.028	8.18E-01	8.18E+01		
8120756	---	---	---	0.181	1.134	6.23E-01	6.23E+01		
8120758	NM_015571	SENP6	SUMO1/sentrin specific peptidase	-0.006	0.996	9.77E-01	9.77E+01		
8120833	NM_031469	SH3BGRL2	SH3 domain binding glutamic acid-	0.178	1.131	5.55E-01	5.55E+01		
8120880	NM_006670	TPBG	trophoblast glycoprotein	-0.102	0.932	9.87E-02	9.87E+00		
8120883	NM_015018	DOPEY1	dopey family member 1	0.086	1.061	5.15E-01	5.15E+01		
8120943	NM_016230	CYB5R4	cytochrome b5 reductase 4	0.204	1.152	2.04E-01	2.04E+01		
8120967	NM_002526	NT5E	5'-nucleotidase, ecto (CD73)	-0.118	0.921	8.30E-01	8.30E+01		
8120992	NM_015021	ZNF292	zinc finger protein 292	0.001	1.001	9.96E-01	9.96E+01		
8121002	NM_00104249	C6orf162	chromosome 6 open reading fram	0.055	1.039	8.30E-01	8.30E+01		
8121031	NM_006416	SLC35A1	solute carrier family 35 (CMP-sialic	-0.032	0.978	8.73E-01	8.73E+01		
8121076	NM_006813	PNRC1	proline-rich nuclear receptor coac	-0.098	0.935	1.83E-01	1.83E+01		
8121085	---	---	---	-0.142	0.906	7.03E-01	7.03E+01		
8121087	NM_00101085	PM20D2	peptidase M20 domain containing	-0.037	0.975	8.03E-01	8.03E+01		
8121095	NM_014942	ANKRD6	ankyrin repeat domain 6	-0.069	0.953	5.43E-01	5.43E+01		
8121118	NM_012115	CASP8AP2	caspase 8 associated protein 2	-0.011	0.992	9.63E-01	9.63E+01		
8121161	NM_015323	KIAA0776	KIAA0776	0.124	1.090	3.63E-01	3.63E+01		
8121209	NM_005604	POU3F2	POU class 3 homeobox 2	-0.081	0.945	3.86E-01	3.86E+01		
8121214	---	---	---	0.034	1.024	9.60E-01	9.60E+01		
8121216	NM_021620	PRDM13	PR domain containing 13	-0.003	0.998	9.85E-01	9.85E+01		
8121257	NM_001198	PRDM1	PR domain containing 1, with ZNF	0.102	1.073	7.16E-01	7.16E+01		
8121269	---	---	---	0.100	1.072	6.76E-01	6.76E+01		
8121273	---	---	---	0.023	1.016	9.67E-01	9.67E+01		
8121275	---	---	---	-0.147	0.903	7.46E-01	7.46E+01		
8121277	NM_001624	AIM1	absent in melanoma 1	0.277	1.212	2.48E-02	2.48E+00		
8121298	---	---	---	0.133	1.096	5.71E-01	5.71E+01		
8121300	NM_018292	QRSL1	glutaminyl-tRNA synthase (glutam	0.178	1.131	1.26E-01	1.26E+01		
8121312	NM_016487	C6orf203	chromosome 6 open reading fram	-0.009	0.994	9.77E-01	9.77E+01		
8121365	NM_001455	FOXO3	forkhead box O3	-0.187	0.879	1.91E-01	1.91E+01		
8121370	NM_032131	ARMC2	armadillo repeat containing 2	0.017	1.012	9.49E-01	9.49E+01		
8121416	---	---	---	-0.006	0.996	9.89E-01	9.89E+01		
8121418	NM_003080	SMPD2	sphingomyelin phosphodiesterase	-0.122	0.919	2.77E-01	2.77E+01		
8121429	NM_014845	FIG4	FIG4 homolog (S. cerevisiae)	0.255	1.193	6.02E-02	6.02E+00		
8121454	NM_005284	GPR6	G protein-coupled receptor 6	-0.132	0.912	1.91E-01	1.91E+01		
8121461	NM_015891	CDC40	cell division cycle 40 homolog (S. c	0.106	1.076	1.53E-01	1.53E+01		
8121483	---	---	---	0.115	1.083	7.00E-01	7.00E+01		
8121489	NM_001634	AMD1	adenosylmethionine decarboxylas	0.217	1.162	3.04E-02	3.04E+00		
8121502	NM_138408	GTF3C6	general transcription factor IIIC, po	0.074	1.052	5.61E-01	5.61E+01		
8121510	NM_032194	BXDC1	brix domain containing 1	0.204	1.152	2.09E-01	2.09E+01		
8121515	NM_018593	SLC16A10	solute carrier family 16, member 1	-0.447	0.734	2.07E-01	2.07E+01		
8121525	NM_153369	KIAA1919	KIAA1919	0.081	1.057	5.63E-01	5.63E+01		
8121563	NM_002356	MARCKS	myristoylated alanine-rich protein	-0.137	0.910	3.04E-01	3.04E+01		
8121578	NM_152729	NT5DC1	5'-nucleotidase domain containing	0.014	1.010	9.53E-01	9.53E+01		
8121588	NM_013352	DSE	dermatan sulfate epimerase	0.440	1.356	1.65E-01	1.65E+01		
8121596	BC146842	FAM26F	family with sequence similarity 26	0.296	1.228	1.00E-01	1.00E+01		
8121613	NM_016104	RWDD1	RWD domain containing 1	-0.014	0.991	9.52E-01	9.52E+01		
8121685	NM_173674	DCBLD1	discoidin, CUB and LCCL domain co	-0.063	0.957	7.61E-01	7.61E+01		
8121704	NM_138459	NUS1	nuclear undecaprenyl pyrophosph	-0.019	0.987	8.46E-01	8.46E+01		
8121727	NR_002730	BRD7P3	bromodomain containing 7 pseud	0.000	1.000	1.00E+00	1.00E+02		
8121729	NM_002667	PLN	phospholamban	0.007	1.005	9.83E-01	9.83E+01		
8121734	NM_014034	ASF1A	ASF1 anti-silencing function 1 hom	-0.116	0.923	3.67E-01	3.67E+01		
8121739	---	---	---	-0.003	0.998	9.91E-01	9.91E+01		
8121743	---	---	---	-0.028	0.981	9.07E-01	9.07E+01		
8121755	---	---	---	-0.152	0.900	4.00E-02	4.00E+00		
8121757	NM_004506	HSF2	heat shock transcription factor 2	0.024	1.017	9.27E-01	9.27E+01		
8121782	---	---	---	0.023	1.016	8.92E-01	8.92E+01		
8121861	NM_181782	NCOA7	nuclear receptor coactivator 7	-0.042	0.971	8.07E-01	8.07E+01		
8121884	---	---	---	-0.227	0.854	5.29E-01	5.29E+01		
8121886	NM_138571	HINT3	histidine triad nucleotide binding p	0.071	1.051	7.45E-01	7.45E+01		
8121927	NM_030963	RNF146	ring finger protein 146	0.037	1.026	8.01E-01	8.01E+01		
8122013	NM_032438	L3MBTL3	l(3)mbt-like 3 (Drosophila)	0.156	1.114	5.27E-02	5.27E+00		
8122038	NM_052913	TMEM200A	transmembrane protein 200A	0.017	1.012	9.48E-01	9.48E+01		
8122136	NM_001016	RPS12	ribosomal protein S12	-0.035	0.976	7.51E-01	7.51E+01		
8122142	NR_002434	SNORD101	small nucleolar RNA, C/D box 101	-0.362	0.778	7.51E-02	7.51E+00		
8122144	NR_002436	SNORA33	small nucleolar RNA, H/ACA box 3	-0.144	0.905	4.48E-01	4.48E+01		
8122174	---	---	---	-0.020	0.986	9.24E-01	9.24E+01		
8122182	NM_004865	TBPL1	TBP-like 1	-0.084	0.943	4.81E-01	4.81E+01		
8122194	---	---	---	-0.063	0.958	5.68E-01	5.68E+01		
8122196	---	---	---	-0.044	0.970	7.87E-01	7.87E+01		
8122198	---	---	---	0.011	1.007	9.71E-01	9.71E+01		
8122202	NM_00113017	MYB	v-myb myeloblastosis viral oncoge	0.092	1.066	7.08E-01	7.08E+01		
8122222	NM_018945	PDE7B	phosphodiesterase 7B	-0.076	0.949	8.29E-01	8.29E+01		
8122240	---	---	---	-0.057	0.962	9.18E-01	9.18E+01		
8122242	NM_000288	PEX7	peroxisomal biogenesis factor 7	0.002	1.001	9.90E-01	9.90E+01		

8122256	NM_00100878	SLC35D3	solute carrier family 35, member D	0.070	1.050	5.17E-01	5.17E+01		
8122259	---	---	---	-0.053	0.964	8.43E-01	8.43E+01		
8122261	NM_000416	IFNGR1	interferon gamma receptor 1	0.236	1.177	2.70E-01	2.70E+01		
8122317	NM_014320	HEBP2	heme binding protein 2	-0.090	0.940	8.01E-01	8.01E+01		
8122327	NM_015439	CCDC28A	coiled-coil domain containing 28A	-0.056	0.962	8.26E-01	8.26E+01		
8122334	NM_178445	CCRL1	chemokine (C-C motif) receptor-lik	-0.141	0.907	5.98E-01	5.98E+01		
8122336	NM_021243	C6orf115	chromosome 6 open reading fram	0.129	1.094	2.03E-01	2.03E+01		
8122343	NM_016217	HECA	headcase homolog (Drosophila)	-0.046	0.969	7.72E-01	7.72E+01		
8122348	NM_016485	VTA1	Vps20-associated 1 homolog (S. ce	0.101	1.072	4.27E-01	4.27E+01		
8122426	NM_014721	PHACTR2	phosphatase and actin regulator 2	0.146	1.106	1.56E-01	1.56E+01		
8122440	NM_032860	LTV1	LTV1 homolog (S. cerevisiae)	0.067	1.048	7.05E-01	7.05E+01		
8122457	NM_003764	STX11	syntaxin 11	0.152	1.111	5.29E-01	5.29E+01		
8122464	NM_007124	UTRN	utrophin	0.190	1.141	5.23E-02	5.23E+00		
8122554	NM_006834	RAB32	RAB32, member RAS oncogene fa	0.330	1.257	9.03E-02	9.03E+00		
8122598	---	---	---	0.025	1.018	8.40E-01	8.40E+01		
8122600	NM_00112771	STXBP5	syntaxin binding protein 5 (tomos	-0.207	0.867	4.97E-02	4.97E+00		
8122637	NM_015278	SASH1	SAM and SH3 domain containing 1	0.215	1.161	5.34E-01	5.34E+01		
8122660	NM_005715	UST	uronyl-2-sulfotransferase	0.003	1.002	9.92E-01	9.92E+01		
8122672	NM_015093	MAP3K7IP2	mitogen-activated protein kinase	-0.069	0.953	5.52E-01	5.52E+01		
8122684	NM_00100225	SUMO4	SMT3 suppressor of mif two 3 hon	-0.259	0.836	7.39E-02	7.39E+00		
8122689	AY358952	C6orf72	chromosome 6 open reading fram	-0.166	0.891	2.37E-01	2.37E+01		
8122699	BC071678	RPS18P9	ribosomal protein S18 pseudogen	0.032	1.023	7.92E-01	7.92E+01		
8122701	---	---	---	0.079	1.056	8.47E-01	8.47E+01		
8122703	---	---	---	-0.040	0.973	8.81E-01	8.81E+01		
8122705	NM_005389	PCMT1	protein-L-isoaspartate (D-aspartat	0.098	1.070	5.53E-01	5.53E+01		
8122717	NM_032832	LRP11	low density lipoprotein receptor-r	-0.030	0.979	8.15E-01	8.15E+01		
8122734	NM_030949	PPP1R14C	protein phosphatase 1, regulatory	-0.039	0.973	7.09E-01	7.09E+01		
8122740	---	---	---	0.053	1.037	5.87E-01	5.87E+01		
8122756	NM_00102988	PLEKHG1	pleckstrin homology domain conta	0.200	1.149	2.28E-01	2.28E+01		
8122773	NM_015440	MTHFD1L	methylenetetrahydrofolate dehyd	-0.115	0.923	3.28E-01	3.28E+01		
8122840	X74439	ESR1	estrogen receptor 1	-0.093	0.937	1.96E-01	1.96E+01		
8122909	NM_014892	RBM16	RNA binding motif protein 16	-0.002	0.999	9.90E-01	9.90E+01		
8122933	NM_012454	TIAM2	T-cell lymphoma invasion and met	-0.206	0.867	6.76E-02	6.76E+00		
8122966	NM_00100134	CLDN20	claudin 20	0.170	1.125	3.85E-01	3.85E+01		
8122971	NM_020732	ARID1B	AT rich interactive domain 1B (SW	-0.018	0.988	9.25E-01	9.25E+01		
8122982	NM_024630	ZDHHC14	zinc finger, DHHC-type containing	-0.130	0.914	3.70E-01	3.70E+01		
8122986	NM_016224	SNX9	sorting nexin 9	-0.302	0.811	2.93E-03	2.93E-01		
8123006	NM_003898	SYNJ2	synaptojanin 2	-0.034	0.977	8.38E-01	8.38E+01		
8123044	NM_020245	TULP4	tubby like protein 4	0.014	1.010	9.49E-01	9.49E+01		
8123062	NM_020823	TMEM181	transmembrane protein 181	0.009	1.006	9.74E-01	9.74E+01		
8123080	NM_00100999	SYTL3	synaptotagmin-like 3	-0.212	0.863	3.31E-01	3.31E+01		
8123129	NM_004906	WTAP	Wilms tumor 1 associated protein	-0.157	0.897	6.07E-02	6.07E+00		
8123137	NM_005891	ACAT2	acetyl-Coenzyme A acetyltransfer	-0.154	0.899	2.81E-01	2.81E+01		
8123148	NM_014161	MRPL18	mitochondrial ribosomal protein L	0.013	1.009	9.68E-01	9.68E+01		
8123230	NR_003288	LOC729603	calcium binding protein P22 pseu	0.178	1.131	2.32E-01	2.32E+01		
8123232	NM_003057	SLC22A1	solute carrier family 22 (organic c	-0.027	0.981	9.16E-01	9.16E+01		
8123274	NM_005922	MAP3K4	mitogen-activated protein kinase	-0.179	0.883	4.39E-02	4.39E+00		
8123332	---	---	---	0.049	1.035	8.70E-01	8.70E+01		
8123334	NR_027284	LOC441177	hypothetical LOC441177	-0.002	0.999	9.91E-01	9.91E+01		
8123340	---	---	---	0.044	1.031	7.71E-01	7.71E+01		
8123342	NM_007045	FGFR1OP	FGFR1 oncogene partner	-0.028	0.981	8.89E-01	8.89E+01		
8123362	BX647686	CCR6	chemokine (C-C motif) receptor 6	0.033	1.023	8.76E-01	8.76E+01		
8123364	NM_031409	CCR6	chemokine (C-C motif) receptor 6	0.052	1.037	8.36E-01	8.36E+01		
8123372	BX647686	CCR6	chemokine (C-C motif) receptor 6	-0.008	0.994	9.78E-01	9.78E+01		
8123407	NM_00104000	MLLT4	myeloid/lymphoid or mixed-linea	-0.289	0.819	8.62E-02	8.62E+00		
8123463	NM_018288	PHF10	PHD finger protein 10	0.009	1.006	9.65E-01	9.65E+01		
8123467	NM_018341	C6orf70	chromosome 6 open reading fram	0.081	1.058	6.01E-01	6.01E+01		
8123488	NR_026780	C6orf208	chromosome 6 open reading fram	0.022	1.015	8.38E-01	8.38E+01		
8123494	NM_032448	FAM120B	family with sequence similarity 12	0.001	1.001	9.96E-01	9.96E+01		
8123507	NM_003194	TBP	TATA box binding protein	0.013	1.009	9.51E-01	9.51E+01		
8123520	BC118988	C20orf69	chromosome 20 open reading fra	0.006	1.005	9.82E-01	9.82E+01		
8123524	NM_018303	EXOC2	exocyst complex component 2	-0.048	0.967	7.55E-01	7.55E+01		
8123558	NM_148959	HUS1B	HUS1 checkpoint homolog b (S. po	-0.146	0.903	2.26E-01	2.26E+01		
8123562	NM_001500	GMDS	GDP-mannose 4,6-dehydratase	0.018	1.013	9.26E-01	9.26E+01		
8123584	NM_00101241	MYLK4	myosin light chain kinase family, n	0.124	1.090	3.16E-01	3.16E+01		
8123598	NM_030666	SERPINB1	serpin peptidase inhibitor, clade B	-0.041	0.972	9.05E-01	9.05E+01		
8123606	BC025340	MGC39372	hypothetical protein MGC39372	-0.032	0.978	9.50E-01	9.50E+01		
8123609	NM_004155	SERPINB9	serpin peptidase inhibitor, clade B	-0.300	0.812	6.06E-02	6.06E+00		
8123621	NM_004568	SERPINB6	serpin peptidase inhibitor, clade B	-0.104	0.930	6.00E-01	6.00E+01		
8123637	NM_000904	NQO2	NAD(P)H dehydrogenase, quinone	0.013	1.009	9.56E-01	9.56E+01		
8123642	---	---	---	0.109	1.079	6.37E-01	6.37E+01		
8123644	NM_001069	TUBB2A	tubulin, beta 2A	-0.185	0.880	2.07E-01	2.07E+01		
8123651	NM_178012	TUBB2B	tubulin, beta 2B	-0.191	0.876	2.58E-02	2.58E+00		
8123658	NM_015482	SLC22A23	solute carrier family 22, member 2	-0.004	0.997	9.81E-01	9.81E+01		
8123678	NM_183373	C6orf145	chromosome 6 open reading fram	0.077	1.055	7.38E-01	7.38E+01		
8123695	NM_206836	PECI	peroxisomal D3,D2-enoyl-CoA isor	-0.054	0.963	7.81E-01	7.81E+01		
8123714	---	---	---	0.076	1.054	7.52E-01	7.52E+01		

8123728	NM_020408	LYRM4	LYR motif containing 4	-0.008	0.994	9.80E-01	9.80E+01		
8123737	---	---	---	0.063	1.045	7.89E-01	7.89E+01		
8123760	NR_026970	RP3-398D13.1	hypothetical LOC285780	0.057	1.040	6.80E-01	6.80E+01		
8123763	---	---	---	-0.018	0.987	9.09E-01	9.09E+01		
8123767	NM_003144	SSR1	signal sequence receptor, alpha	0.170	1.125	1.66E-02	1.66E+00		
8123802	NM_201280	MUTED	muted homolog (mouse)	0.090	1.065	3.50E-01	3.50E+01		
8123891	---	---	---	-0.014	0.991	9.57E-01	9.57E+01		
8123893	NM_005906	MAK	male germ cell-associated kinase	-0.072	0.951	6.84E-01	6.84E+01		
8123929	NM_207582	HERV-FRD	HERV-FRD provirus ancestral Env p	-0.120	0.920	2.23E-01	2.23E+01		
8123936	NM_00114239	NEDD9	neural precursor cell expressed, de	0.060	1.043	5.55E-01	5.55E+01		
8123951	NM_00114394	C6orf105	chromosome 6 open reading fram	-0.058	0.961	8.54E-01	8.54E+01		
8123961	NM_016495	TBC1D7	TBC1 domain family, member 7	-0.168	0.890	3.35E-01	3.35E+01		
8123976	NM_018988	GFOD1	glucose-fructose oxidoreductase d	-0.024	0.983	8.92E-01	8.92E+01		
8123985	NM_016167	NOL7	nucleolar protein 7, 27kDa	-0.035	0.976	8.18E-01	8.18E+01		
8123989	NM_005493	RANBP9	RAN binding protein 9	-0.023	0.984	8.86E-01	8.86E+01		
8124008	NM_00103171	CCDC90A	coiled-coil domain containing 90A	0.160	1.118	2.93E-01	2.93E+01		
8124020	---	---	---	0.025	1.017	9.71E-01	9.71E+01		
8124022	NM_183040	DTNBP1	dystrobrevin binding protein 1	0.116	1.084	1.56E-01	1.56E+01		
8124038	---	---	---	0.055	1.039	7.92E-01	7.92E+01		
8124040	NM_000332	ATXN1	ataxin 1	-0.024	0.983	9.25E-01	9.25E+01		
8124055	---	---	---	0.005	1.003	9.81E-01	9.81E+01		
8124059	NM_005124	NUP153	nucleoporin 153kDa	-0.050	0.966	6.76E-01	6.76E+01		
8124082	---	---	---	-0.080	0.946	4.12E-01	4.12E+01		
8124088	NM_022113	KIF13A	kinesin family member 13A	0.100	1.071	8.07E-01	8.07E+01		
8124144	NM_003472	DEK	DEK oncogene	0.012	1.009	9.27E-01	9.27E+01		
8124160	---	---	---	-0.299	0.813	8.52E-01	8.52E+01		
8124166	NM_00108048	MBOAT1	membrane bound O-acyltransfera	0.286	1.220	5.89E-02	5.89E+00		
8124183	---	---	---	0.022	1.015	9.57E-01	9.57E+01		
8124194	---	---	---	-0.063	0.957	6.00E-01	6.00E+01		
8124262	NM_016614	TTRAP	TRAF and TNF receptor associated	0.005	1.003	9.88E-01	9.88E+01		
8124273	NM_030939	C6orf62	chromosome 6 open reading fram	0.063	1.045	5.67E-01	5.67E+01		
8124280	NM_014722	FAM65B	family with sequence similarity 65	0.098	1.070	5.15E-01	5.15E+01		
8124305	---	---	---	-0.062	0.958	7.11E-01	7.11E+01		
8124307	NR_002174	CMAH	cytidine monophosphate-N-acetyl	-0.218	0.860	3.96E-01	3.96E+01		
8124327	---	---	---	0.205	1.153	4.72E-02	4.72E+00		
8124385	NM_003544	HIST1H4B	histone cluster 1, H4b	0.138	1.101	6.39E-01	6.39E+01		
8124391	NM_003513	HIST1H2AB	histone cluster 1, H2ab	-0.125	0.917	7.97E-01	7.97E+01		
8124394	NM_021062	HIST1H2BB	histone cluster 1, H2bb	-0.024	0.983	8.89E-01	8.89E+01		
8124397	NM_005319	HIST1H1C	histone cluster 1, H1c	0.081	1.058	7.74E-01	7.74E+01		
8124406	NM_003526	HIST1H2BC	histone cluster 1, H2bc	0.217	1.162	8.30E-02	8.30E+00		
8124413	NM_003539	HIST1H4D	histone cluster 1, H4d	0.125	1.091	5.97E-01	5.97E+01		
8124416	NM_003530	HIST1H3D	histone cluster 1, H3d	0.002	1.001	9.93E-01	9.93E+01		
8124423	NM_003518	HIST1H2BG	histone cluster 1, H2bg	-0.075	0.950	7.11E-01	7.11E+01		
8124430	NM_005320	HIST1H1D	histone cluster 1, H1d	0.062	1.044	8.04E-01	8.04E+01		
8124437	NM_021018	HIST1H3F	histone cluster 1, H3f	0.005	1.004	9.88E-01	9.88E+01		
8124446	---	---	---	-0.002	0.999	9.91E-01	9.91E+01		
8124448	NM_003543	HIST1H4H	histone cluster 1, H4h	0.215	1.161	6.92E-02	6.92E+00		
8124467	---	---	---	-0.089	0.940	5.79E-01	5.79E+01		
8124469	BC171739	GUSBL1	glucuronidase, beta-like 1	-0.013	0.991	9.71E-01	9.71E+01		
8124492	NM_080593	HIST1H2BK	histone cluster 1, H2bk	0.065	1.046	8.78E-01	8.78E+01		
8124502	NM_007149	ZNF184	zinc finger protein 184	-0.085	0.943	5.60E-01	5.60E+01		
8124524	NM_003510	HIST1H2AK	histone cluster 1, H2ak	-0.076	0.948	5.71E-01	5.71E+01		
8124531	NM_003533	HIST1H3I	histone cluster 1, H3i	-0.124	0.918	6.34E-01	6.34E+01		
8124551	NR_002936	LOC222699	transducer of ERBB2, 2 pseudogen	-0.058	0.961	6.33E-01	6.33E+01		
8124553	NM_019110	ZKSCAN4	zinc finger with KRAB and SCAN do	0.033	1.023	8.55E-01	8.55E+01		
8124610	NM_006510	TRIM27	tripartite motif-containing 27	0.020	1.014	8.79E-01	8.79E+01		
8124650	NM_006398	UBD	ubiquitin D	0.072	1.051	8.74E-01	8.74E+01		
8124654	NM_001470	GABBR1	gamma-aminobutyric acid (GABA)	0.015	1.010	9.37E-01	9.37E+01		
8124684	NM_00110980	ZFP57	zinc finger protein 57 homolog (m	0.033	1.023	9.01E-01	9.01E+01		
8124689	NR_002139	HCG4	HLA complex group 4	0.051	1.036	7.50E-01	7.50E+01		
8124691	AY358246	HCG8	HLA complex group 8	0.004	1.003	9.89E-01	9.89E+01		
8124695	NM_025236	RNF39	ring finger protein 39	-0.032	0.978	7.91E-01	7.91E+01		
8124707	NM_007028	TRIM31	tripartite motif-containing 31	-0.033	0.977	8.01E-01	8.01E+01		
8124716	NM_006778	TRIM10	tripartite motif-containing 10	-0.101	0.933	2.02E-01	2.02E+01		
8124726	NM_003449	TRIM26	tripartite motif-containing 26	0.045	1.032	7.19E-01	7.19E+01		
8124742	NM_005275	GNL1	guanine nucleotide binding protei	-0.143	0.906	1.30E-01	1.30E+01		
8124756	NM_002714	PPP1R10	protein phosphatase 1, regulatory	-0.121	0.919	1.09E-01	1.09E+01		
8124775	NM_003587	DHX16	DEAH (Asp-Glu-Ala-His) box polype	0.040	1.028	6.68E-01	6.68E+01		
8124798	NM_133471	KIAA1949	KIAA1949	-0.036	0.975	8.25E-01	8.25E+01		
8124806	NM_007243	NRM	nurim (nuclear envelope membrar	-0.090	0.940	4.70E-01	4.70E+01		
8124813	NM_014641	MDC1	mediator of DNA-damage checkpo	-0.103	0.931	3.80E-01	3.80E+01		
8124828	NM_005803	FLOT1	flotillin 1	0.030	1.021	9.06E-01	9.06E+01		
8124859	NM_014070	C6orf15	chromosome 6 open reading fram	-0.076	0.949	4.32E-01	4.32E+01		
8124862	NM_001264	CDSN	corneodesmosin	-0.016	0.989	9.16E-01	9.16E+01		
8124865	NM_014069	PSORS1C2	psoriasis susceptibility 1 candidate	0.036	1.025	7.30E-01	7.30E+01		
8124868	NM_00110556	CCHCR1	coiled-coil alpha-helical rod protei	-0.082	0.945	1.82E-01	1.82E+01		
8124889	NM_002701	POU5F1	POU class 5 homeobox 1	-0.020	0.986	8.41E-01	8.41E+01		

8124901	NM_002117 //	HLA-C // HLA-C	major histocompatibility complex,	0.009	1.007	9.60E-01	9.60E+01		
8124911	NM_005514	HLA-B	major histocompatibility complex,	0.003	1.002	9.86E-01	9.86E+01		
8124920	---	---	---	-0.056	0.962	8.08E-01	8.08E+01		
8124926	NM_004640	BAT1	HLA-B associated transcript 1	-0.051	0.965	4.93E-01	4.93E+01		
8124940	NR_003140	SNORD117	small nucleolar RNA, C/D box 117	-0.030	0.979	9.56E-01	9.56E+01		
8124942	NM_130463	ATP6V1G2	ATPase, H+ transporting, lysosoma	0.022	1.016	9.09E-01	9.09E+01		
8124950	NM_002341	LTB	lymphotoxin beta (TNF superfamil	-0.049	0.966	6.99E-01	6.99E+01		
8124955	NM_00114546	NCR3	natural cytotoxicity triggering rece	0.005	1.003	9.78E-01	9.78E+01		
8124967	NM_004639	BAT3	HLA-B associated transcript 3	-0.022	0.985	9.05E-01	9.05E+01		
8124994	NM_021184	C6orf47	chromosome 6 open reading fram	-0.033	0.977	8.48E-01	8.48E+01		
8124998	NM_033177	BAT4	HLA-B associated transcript 4	-0.129	0.914	6.89E-02	6.89E+00		
8125007	NM_025262	LY6G5C	lymphocyte antigen 6 complex, loc	-0.159	0.896	7.33E-02	7.33E+00		
8125017	NM_021160	BAT5	HLA-B associated transcript 5	0.043	1.031	6.29E-01	6.29E+01		
8125038	NR_003673	LY6G6E	lymphocyte antigen 6 complex, loc	-0.046	0.968	6.58E-01	6.58E+01		
8125042	NM_025261	LY6G6C	lymphocyte antigen 6 complex, loc	-0.008	0.994	9.64E-01	9.64E+01		
8125048	NM_013974	DDAH2	dimethylarginine dimethylaminoh	0.142	1.103	8.58E-02	8.58E+00		
8125059	NM_001288	CLIC1	chloride intracellular channel 1	0.213	1.159	2.74E-01	2.74E+01		
8125072	NM_025258	C6orf27	chromosome 6 open reading fram	-0.006	0.996	9.66E-01	9.66E+01		
8125091	NM_006295	VARS	valyl-tRNA synthetase	0.054	1.038	7.03E-01	7.03E+01		
8125123	---	---	---	0.032	1.022	8.47E-01	8.47E+01		
8125125	NM_021177	LSM2	LSM2 homolog, U6 small nuclear F	0.119	1.086	3.35E-01	3.35E+01		
8125134	NM_005527	HSPA1L	heat shock 70kDa protein 1-like	-0.108	0.928	6.09E-01	6.09E+01		
8125139	NM_000434	NEU1	sialidase 1 (lysosomal sialidase)	-0.015	0.990	9.64E-01	9.64E+01		
8125149	NM_025257	SLC44A4	solute carrier family 44, member 4	-0.028	0.981	8.38E-01	8.38E+01		
8125172	NM_006709	EHMT2	euchromatic histone-lysine N-met	-0.134	0.911	2.91E-02	2.91E+00		
8125201	NM_181842	ZBTB12	zinc finger and BTB domain contai	-0.019	0.987	8.38E-01	8.38E+01		
8125204	NM_002904	RDBP	RD RNA binding protein	-0.076	0.948	4.81E-01	4.81E+01		
8125220	NM_005510	DOM3Z	dom-3 homolog Z (C. elegans)	-0.102	0.932	4.34E-01	4.34E+01		
8125234	NM_019105	TNXB	tenascin XB	-0.017	0.988	8.53E-01	8.53E+01		
8125289	NR_001284	TNXA	tenascin XA pseudogene	-0.024	0.983	8.86E-01	8.86E+01		
8125295	NM_004381	ATF6B	activating transcription factor 6 be	-0.059	0.960	6.56E-01	6.56E+01		
8125316	NM_022110	FKBPL	FK506 binding protein like	-0.038	0.974	8.27E-01	8.27E+01		
8125321	NM_030651	PRRT1	proline-rich transmembrane prote	-0.009	0.994	9.64E-01	9.64E+01		
8125328	NM_006411	AGPAT1	1-acylglycerol-3-phosphate O-acyl	-0.040	0.973	7.73E-01	7.73E+01		
8125341	NM_001136	AGER	advanced glycosylation end produ	-0.029	0.980	8.59E-01	8.59E+01		
8125360	NM_002586	PBX2	pre-B-cell leukemia homeobox 2	0.023	1.016	8.72E-01	8.72E+01		
8125373	NM_022107	GPSM3	G-protein signaling modulator 3 (A	-0.003	0.998	9.86E-01	9.86E+01		
8125383	NM_004557	NOTCH4	Notch homolog 4 (Drosophila)	-0.079	0.946	2.94E-01	2.94E+01		
8125426	NM_019602	BTNL2	butyrophilin-like 2 (MHC class II as	-0.064	0.957	5.65E-01	5.65E+01		
8125436	NM_002125	HLA-DRB5	major histocompatibility complex,	0.243	1.184	7.27E-01	7.27E+01		
8125447	NM_002123	HLA-DQB1	major histocompatibility complex,	0.339	1.265	6.29E-01	6.29E+01		
8125461	AK097297	HLA-DQB1	major histocompatibility complex,	0.405	1.324	7.13E-01	7.13E+01		
8125463	NR_003937	HLA-DQB2	major histocompatibility complex,	0.233	1.175	6.06E-02	6.06E+00		
8125470	NM_002120	HLA-DOB	major histocompatibility complex,	-0.028	0.981	9.36E-01	9.36E+01		
8125483	NM_000544	TAP2	transporter 2, ATP-binding cassett	0.046	1.032	8.21E-01	8.21E+01		
8125500	NM_004159	PSMB8	proteasome (prosome, macropain	0.149	1.109	2.29E-01	2.29E+01		
8125512	NM_000593	TAP1	transporter 1, ATP-binding cassett	0.068	1.048	6.66E-01	6.66E+01		
8125527	AF275684 // A	1R2P1 // PPP1F	protein phosphatase 1, regulatory	-0.051	0.965	7.26E-01	7.26E+01		
8125545	NM_002119	HLA-DOA	major histocompatibility complex,	0.243	1.183	1.43E-02	1.43E+00		
8125568	NM_080680	COL11A2	collagen, type XI, alpha 2	-0.027	0.981	7.73E-01	7.73E+01		
8125638	NM_021976	RXRB	retinoid X receptor, beta	0.001	1.001	9.97E-01	9.97E+01		
8125649	NM_022553	VPS52	vacuolar protein sorting 52 homol	0.070	1.050	5.13E-01	5.13E+01		
8125671	NM_005452	WDR46	WD repeat domain 46	-0.062	0.958	6.40E-01	6.40E+01		
8125687	NM_004761	RGL2	ral guanine nucleotide dissociatior	-0.115	0.923	2.50E-01	2.50E+01		
8125713	NM_172208	TAPBP	TAP binding protein (tapasin)	0.042	1.030	7.51E-01	7.51E+01		
8125731	NM_005453	ZBTB22	zinc finger and BTB domain contai	-0.084	0.943	3.09E-01	3.09E+01		
8125734	NM_00114196	DAXX	death-domain associated protein	-0.026	0.982	8.82E-01	8.82E+01		
8125748	NR_001444	LYPLA2P1	lysophospholipase II pseudogene 1	0.027	1.019	9.00E-01	9.00E+01		
8125750	NM_000976	RPL12	ribosomal protein L12	0.033	1.023	8.78E-01	8.78E+01		
8125752	NM_015921	CUTA	cutA divalent cation tolerance hor	0.054	1.038	7.45E-01	7.45E+01		
8125766	NM_001188	BAK1	BCL2-antagonist/killer 1	-0.033	0.978	9.01E-01	9.01E+01		
8125772	NR_027908	C6orf227	chromosome 6 open reading fram	0.016	1.011	9.20E-01	9.20E+01		
8125775	AY568085	C6orf125	chromosome 6 open reading fram	0.116	1.083	5.74E-01	5.74E+01		
8125780	NM_054111	IP6K3	inositol hexakisphosphate kinase 3	-0.038	0.974	7.45E-01	7.45E+01		
8125788	NM_181336	LEMD2	LEM domain containing 2	-0.035	0.976	7.63E-01	7.63E+01		
8125798	NM_002418	MLN	motilin	-0.032	0.978	7.72E-01	7.72E+01		
8125805	NM_000841	GRM4	glutamate receptor, metabotropic	-0.038	0.974	6.28E-01	6.28E+01		
8125818	NM_178508	C6orf1	chromosome 6 open reading fram	-0.087	0.941	3.69E-01	3.69E+01		
8125825	NM_006703	NUDT3	nudix (nucleoside diphosphate linl	0.121	1.087	2.46E-01	2.46E+01		
8125835	NM_001014	RPS10	ribosomal protein S10	-0.005	0.997	9.85E-01	9.85E+01		
8125843	NM_012391	SPDEF	SAM pointed domain containing e	-0.046	0.968	6.09E-01	6.09E+01		
8125850	NM_024294	C6orf106	chromosome 6 open reading fram	0.027	1.019	8.47E-01	8.47E+01		
8125857	---	---	---	0.092	1.066	6.84E-01	6.84E+01		
8125859	NM_005643	TAF11	TAF11 RNA polymerase II, TATA bo	0.050	1.035	7.72E-01	7.72E+01		
8125870	NM_00109372	TCP11	t-complex 11 homolog (mouse)	-0.067	0.955	3.72E-01	3.72E+01		
8125887	NM_003214	TEAD3	TEA domain family member 3	-0.086	0.942	2.03E-01	2.03E+01		
8125903	NM_003322	TULP1	tubby like protein 1	0.036	1.025	7.78E-01	7.78E+01		

8125919	NM_00114577	FKBP5	FK506 binding protein 5	0.183	1.135	1.09E-01	1.09E+01		
8125936	NM_001832	CLPS	colipase, pancreatic	-0.067	0.954	5.01E-01	5.01E+01		
8125941	NM_003137	SRPK1	SFRS protein kinase 1	-0.079	0.947	4.77E-01	4.77E+01		
8125993	NM_016135	ETV7	ets variant 7	0.071	1.051	6.37E-01	6.37E+01		
8126018	NM_007271	STK38	serine/threonine kinase 38	0.067	1.047	5.34E-01	5.34E+01		
8126058	NM_016059	PPIL1	peptidylprolyl isomerase (cyclophi	-0.010	0.993	9.65E-01	9.65E+01		
8126066	NM_014341	MTCH1	mitochondrial carrier homolog 1 (	0.026	1.018	9.02E-01	9.02E+01		
8126095	NM_138493	C6orf129	chromosome 6 open reading fram	0.071	1.051	7.86E-01	7.86E+01		
8126119	---	---	---	-0.061	0.958	8.57E-01	8.57E+01		
8126121	NM_052893	BTBD9	BTB (POZ) domain containing 9	-0.058	0.960	5.72E-01	5.72E+01		
8126147	BC022007	C6orf64	chromosome 6 open reading fram	-0.013	0.991	9.50E-01	9.50E+01		
8126173	NM_00113510	KCNK16	potassium channel, subfamily K, m	-0.033	0.977	7.97E-01	7.97E+01		
8126212	---	---	---	-0.050	0.966	6.00E-01	6.00E+01		
8126240	AK123643	FLJ41649	hypothetical LOC401260	-0.065	0.956	5.27E-01	5.27E+01		
8126248	NM_173561	UNC5CL	unc-5 homolog C (C. elegans)-like	0.054	1.038	6.56E-01	6.56E+01		
8126259	AJ420538	C6orf130	chromosome 6 open reading fram	-0.069	0.954	5.09E-01	5.09E+01		
8126269	NM_178174	TREML1	triggering receptor expressed on r	-0.022	0.985	9.10E-01	9.10E+01		
8126279	NM_018965	TREM2	triggering receptor expressed on r	0.184	1.136	4.78E-02	4.78E+00		
8126288	NM_024807	TREML2	triggering receptor expressed on r	0.016	1.011	9.16E-01	9.16E+01		
8126296	NR_027256	TREML3	triggering receptor expressed on r	-0.039	0.973	8.13E-01	8.13E+01		
8126301	---	---	---	-0.059	0.960	6.68E-01	6.68E+01		
8126312	NM_007162	TFEB	transcription factor EB	-0.015	0.990	9.18E-01	9.18E+01		
8126337	NM_006653	FRS3	fibroblast growth factor receptor s	-0.016	0.989	9.46E-01	9.46E+01		
8126360	NM_004275	MED20	mediator complex subunit 20	-0.088	0.941	5.77E-01	5.77E+01		
8126371	NM_001760	CCND3	cyclin D3	0.002	1.001	9.91E-01	9.91E+01		
8126379	ENST00000341	C6orf132	chromosome 6 open reading fram	-0.010	0.993	9.52E-01	9.52E+01		
8126382	ENST00000341	C6orf132	chromosome 6 open reading fram	0.013	1.009	9.27E-01	9.27E+01		
8126387	NM_002098	GUCA1B	guanylate cyclase activator 1B (ret	-0.059	0.960	5.73E-01	5.73E+01		
8126394	NM_018141	MRPS10	mitochondrial ribosomal protein S	-0.062	0.958	6.91E-01	6.91E+01		
8126402	NM_033502	TRERF1	transcriptional regulating factor 1	0.093	1.066	3.27E-01	3.27E+01		
8126428	NM_033502	TRERF1	transcriptional regulating factor 1	0.182	1.135	4.04E-01	4.04E+01		
8126436	NM_000322	PRPH2	peripherin 2 (retinal degeneration	-0.055	0.962	7.08E-01	7.08E+01		
8126442	NM_003192	TBCC	tubulin folding cofactor C	-0.093	0.938	4.97E-01	4.97E+01		
8126446	NM_00100873	C6orf226	chromosome 6 open reading fram	0.004	1.003	9.90E-01	9.90E+01		
8126450	NM_000986	RPL24	ribosomal protein L24	-0.002	0.999	9.91E-01	9.91E+01		
8126452	NM_000287	PEX6	peroxisomal biogenesis factor 6	-0.061	0.959	4.27E-01	4.27E+01		
8126474	NM_014623	MEA1	male-enhanced antigen 1	-0.097	0.935	3.21E-01	3.21E+01		
8126482	---	---	---	0.095	1.068	2.82E-01	2.82E+01		
8126484	---	---	---	-0.092	0.938	3.41E-01	3.41E+01		
8126486	NM_014780	CUL7	cullin 7	0.012	1.008	9.52E-01	9.52E+01		
8126512	NM_015950	MRPL2	mitochondrial ribosomal protein L	-0.008	0.994	9.76E-01	9.76E+01		
8126524	NM_199184	C6orf108	chromosome 6 open reading fram	-0.091	0.939	3.61E-01	3.61E+01		
8126531	NM_206922	CRIP3	cysteine-rich protein 3	-0.008	0.995	9.64E-01	9.64E+01		
8126542	NM_014345	ZNF318	zinc finger protein 318	0.034	1.024	8.59E-01	8.59E+01		
8126556	NM_023932	DLK2	delta-like 2 homolog (Drosophila)	-0.053	0.964	5.94E-01	5.94E+01		
8126574	NM_015388	YIPF3	Yip1 domain family, member 3	-0.040	0.973	7.94E-01	7.94E+01		
8126588	NM_020750	XPO5	exportin 5	-0.007	0.995	9.77E-01	9.77E+01		
8126629	NM_019096	GTPBP2	GTP binding protein 2	-0.100	0.933	1.65E-01	1.65E+01		
8126646	NM_018135	MRPS18A	mitochondrial ribosomal protein S	0.021	1.015	9.36E-01	9.36E+01		
8126653	NM_032111	MRPL14	mitochondrial ribosomal protein L	-0.062	0.958	5.80E-01	5.80E+01		
8126658	NM_178148	SLC35B2	solute carrier family 35, member B	-0.011	0.992	9.47E-01	9.47E+01		
8126666	NM_004556	NFKBIE	nuclear factor of kappa light polyp	-0.035	0.976	7.69E-01	7.69E+01		
8126681	NM_020745	AARS2	alanyl-tRNA synthetase 2, mitocho	0.065	1.046	5.12E-01	5.12E+01		
8126705	---	---	---	0.262	1.199	3.41E-02	3.41E+00		
8126729	NM_00111408	CLIC5	chloride intracellular channel 5	-0.207	0.867	1.45E-01	1.45E+01		
8126760	NM_005822	RCAN2	regulator of calcineurin 2	0.019	1.013	9.56E-01	9.56E+01		
8126839	NM_014452	TNFRSF21	tumor necrosis factor receptor sup	-0.020	0.986	9.56E-01	9.56E+01		
8126855	NM_00101373	C6orf138	chromosome 6 open reading fram	0.058	1.041	5.76E-01	5.76E+01		
8126860	NM_000255	MUT	methylmalonyl Coenzyme A mutas	0.084	1.060	5.88E-01	5.88E+01		
8126948	J04755	FTHP1	ferritin, heavy polypeptide pseudo	0.039	1.027	8.03E-01	8.03E+01		
8126952	NM_004906	WTAP	Wilms tumor 1 associated protein	-0.160	0.895	4.83E-02	4.83E+00		
8127024	NM_052872	IL17F	interleukin 17F	-0.124	0.918	6.66E-01	6.66E+01		
8127031	NM_002388	MCM3	minichromosome maintenance co	0.103	1.074	5.13E-01	5.13E+01		
8127051	NM_012288	TRAM2	translocation associated membrar	0.090	1.064	4.04E-01	4.04E+01		
8127107	---	---	---	-0.214	0.862	6.11E-01	6.11E+01		
8127109	NM_016513	ICK	intestinal cell (MAK-like) kinase	0.021	1.015	9.38E-01	9.38E+01		
8127128	NM_033480	FBXO9	F-box protein 9	-0.045	0.969	6.34E-01	6.34E+01		
8127145	NM_021814	ELOVL5	ELOVL family member 5, elongatio	0.088	1.063	5.43E-01	5.43E+01		
8127158	NM_001498	GCLC	glutamate-cysteine ligase, catalyti	0.307	1.237	1.23E-01	1.23E+01		
8127360	---	---	---	-0.172	0.888	2.94E-01	2.94E+01		
8127364	NR_003660	GUSBL2	glucuronidase, beta-like 2	0.024	1.017	9.44E-01	9.44E+01		
8127419	---	---	---	0.002	1.001	9.94E-01	9.94E+01		
8127423	---	---	---	-0.271	0.829	1.02E-01	1.02E+01		
8127425	NM_018368	LMBRD1	LMBR1 domain containing 1	0.049	1.034	7.63E-01	7.63E+01		
8127446	NM_001851	COL9A1	collagen, type IX, alpha 1	0.023	1.016	8.72E-01	8.72E+01		
8127484	NM_080742	B3GAT2	beta-1,3-glucuronyltransferase 2 (	-0.041	0.972	7.55E-01	7.55E+01		
8127526	NM_001000 //	RPL39 // RPL39	ribosomal protein L39 // ribosom	-0.004	0.997	9.83E-01	9.83E+01		

8127532	---	---	---	0.033	1.023	8.57E-01	8.57E+01		
8127534	NM_138441	C6orf150	chromosome 6 open reading fram	0.140	1.102	6.61E-01	6.61E+01		
8127542	---	---	---	0.086	1.061	7.64E-01	7.64E+01		
8127544	NM_001402	EEF1A1	eukaryotic translation elongation f	0.000	1.000	1.00E+00	1.00E+02		
8127549	NM_012434	SLC17A5	solute carrier family 17 (anion/sug	0.171	1.126	3.70E-01	3.70E+01		
8127629	NM_001865	COX7A2	cytochrome c oxidase subunit VIIa	0.026	1.018	8.78E-01	8.78E+01		
8127637	NM_018247	TMEM30A	transmembrane protein 30A	0.076	1.054	6.74E-01	6.74E+01		
8127658	---	---	---	-0.050	0.966	6.99E-01	6.99E+01		
8127660	---	---	---	-0.075	0.949	7.72E-01	7.72E+01		
8127692	NM_000863	HTR1B	5-hydroxytryptamine (serotonin) r	0.031	1.021	8.14E-01	8.14E+01		
8127698	NM_017934	PHIP	pleckstrin homology domain intera	-0.034	0.977	7.64E-01	7.64E+01		
8127743	NM_004242	HMGN3	high mobility group nucleosomal b	0.032	1.022	8.51E-01	8.51E+01		
8127778	NM_017633	FAM46A	family with sequence similarity 46	0.120	1.086	3.41E-01	3.41E+01		
8127787	NM_015525	IBTK	inhibitor of Bruton agammaglobul	0.154	1.112	2.26E-01	2.26E+01		
8127822	---	---	---	0.153	1.111	1.94E-01	1.94E+01		
8127824	NM_198920	UBE2CBP	ubiquitin-conjugating enzyme E2C	0.043	1.030	8.47E-01	8.47E+01		
8127943	NM_153816	SNX14	sorting nexin 14	0.093	1.066	5.02E-01	5.02E+01		
8127977	NM_00115967	SYNCRIP	synaptotagmin binding, cytoplasm	0.052	1.036	5.74E-01	5.74E+01		
8127987	NR_002743	SNORD50A	small nucleolar RNA, C/D box 50A	-0.108	0.928	5.26E-01	5.26E+01		
8127989	NR_003044	SNORD50B	small nucleolar RNA, C/D box 50B	-0.218	0.860	3.42E-01	3.42E+01		
8128013	NM_020320	RARS2	arginyl-tRNA synthetase 2, mitoch	0.068	1.048	5.98E-01	5.98E+01		
8128034	NM_018064	AKIRIN2	akirin 2	0.176	1.130	1.64E-01	1.64E+01		
8128052	NM_003800	RNGTT	RNA guanylyltransferase and 5'-ph	-0.035	0.976	8.69E-01	8.69E+01		
8128075	---	---	---	0.067	1.048	9.00E-01	9.00E+01		
8128079	NM_080743	SRrp35	serine-arginine repressor protein (	-0.126	0.917	3.65E-01	3.65E+01		
8128099	NM_002043	GABRR2	gamma-aminobutyric acid (GABA)	0.004	1.003	9.86E-01	9.86E+01		
8128111	NM_016021	UBE2J1	ubiquitin-conjugating enzyme E2,	0.309	1.239	5.36E-02	5.36E+00		
8128133	NM_020466	LYRM2	LYR motif containing 2	0.020	1.014	9.19E-01	9.19E+01		
8128138	NM_014611	MDN1	MDN1, midasin homolog (yeast)	-0.018	0.988	9.27E-01	9.27E+01		
8128247	NM_021813	BACH2	BTB and CNC homology 1, basic le	-0.045	0.969	8.43E-01	8.43E+01		
8128258	---	---	---	0.051	1.036	7.99E-01	7.99E+01		
8128260	NM_145331	MAP3K7	mitogen-activated protein kinase k	0.051	1.036	6.72E-01	6.72E+01		
8128312	---	---	---	-0.040	0.972	7.05E-01	7.05E+01		
8128322	NM_014165	NDUFAF4	NADH dehydrogenase (ubiquinone	0.010	1.007	9.83E-01	9.83E+01		
8128356	NM_012160	FBXL4	F-box and leucine-rich repeat prot	0.092	1.066	5.53E-01	5.53E+01		
8128371	NM_032511	C6orf168	chromosome 6 open reading fram	0.009	1.006	9.64E-01	9.64E+01		
8128394	NM_032870	SFRS18	splicing factor, arginine/serine-rich	-0.005	0.997	9.77E-01	9.77E+01		
8128429	NM_005190	CCNC	cyclin C	0.052	1.037	7.48E-01	7.48E+01		
8128472	NM_006828	ASCC3	activating signal cointegrator 1 co	0.078	1.056	4.90E-01	4.90E+01		
8128522	NM_020771	HACE1	HECT domain and ankyrin repeat c	-0.001	0.999	9.98E-01	9.98E+01		
8128549	---	---	---	0.026	1.018	9.36E-01	9.36E+01		
8128572	NM_002726	PREP	prolyl endopeptidase	0.065	1.046	7.31E-01	7.31E+01		
8128592	NM_004849	ATG5	ATG5 autophagy related 5 homolo	-0.002	0.998	9.90E-01	9.90E+01		
8128604	---	---	---	0.064	1.045	8.69E-01	8.69E+01		
8128606	NM_032730	RTN4IP1	reticulon 4 interacting protein 1	0.238	1.179	1.06E-01	1.06E+01		
8128620	NM_00108045	BEND3	BEN domain containing 3	-0.017	0.988	9.34E-01	9.34E+01		
8128638	NM_198081	SCML4	sex comb on midleg-like 4 (Drosop	0.064	1.045	6.83E-01	6.83E+01		
8128650	NM_007214	SEC63	SEC63 homolog (S. cerevisiae)	0.118	1.085	1.98E-01	1.98E+01		
8128667	---	---	---	-0.128	0.915	1.85E-01	1.85E+01		
8128669	NM_014028	OSTM1	osteopetrosis associated transmen	0.167	1.123	2.00E-01	2.00E+01		
8128683	NM_003795	SNX3	sorting nexin 3	0.095	1.068	4.12E-01	4.12E+01		
8128698	NM_014454	SESN1	sestrin 1	-0.028	0.981	9.01E-01	9.01E+01		
8128712	---	---	---	0.074	1.053	7.01E-01	7.01E+01		
8128716	NM_006016	CD164	CD164 molecule, sialomucin	0.184	1.136	1.23E-01	1.23E+01		
8128737	NM_022765	MICAL1	microtubule associated monoxyge	0.045	1.032	6.76E-01	6.76E+01		
8128767	NM_014797	ZBTB24	zinc finger and BTB domain contai	-0.022	0.985	9.28E-01	9.28E+01		
8128867	NM_015076	CDC2L6	cell division cycle 2-like 6 (CDK8-lik	0.040	1.028	8.45E-01	8.45E+01		
8128886	---	---	---	0.234	1.176	4.01E-01	4.01E+01		
8128892	---	---	---	-0.407	0.754	1.20E-01	1.20E+01		
8128894	NM_002912	REV3L	REV3-like, catalytic subunit of DNA	-0.037	0.975	8.53E-01	8.53E+01		
8128939	NM_147686	TRAF3IP2	TRAF3 interacting protein 2	-0.261	0.834	9.91E-03	9.91E-01		
8128956	NM_002037	FYN	FYN oncogene related to SRC, FGR	-0.241	0.846	4.78E-02	4.78E+00		
8129037	---	---	---	0.023	1.016	9.09E-01	9.09E+01		
8129045	NM_001527	HDAC2	histone deacetylase 2	0.017	1.012	9.11E-01	9.11E+01		
8129069	---	---	---	0.118	1.085	7.98E-01	7.98E+01		
8129082	NM_000493	COL10A1	collagen, type X, alpha 1	-0.033	0.978	7.78E-01	7.78E+01		
8129087	---	---	---	0.085	1.060	8.75E-01	8.75E+01		
8129089	NM_021648	TSPYL4	TSPY-like 4	-0.128	0.915	9.29E-02	9.29E+00		
8129095	---	---	---	-0.060	0.960	8.16E-01	8.16E+01		
8129099	NM_003309	TSPYL1	TSPY-like 1	-0.070	0.952	5.90E-01	5.90E+01		
8129120	NM_00108548	FAM162B	family with sequence similarity 16	-0.005	0.997	9.77E-01	9.77E+01		
8129181	NM_020399	GOPC	golgi associated PDZ and coiled-co	0.039	1.027	7.94E-01	7.94E+01		
8129193	NM_00104247	C6orf204	chromosome 6 open reading fram	0.107	1.077	5.01E-01	5.01E+01		
8129214	AK299076	MCM9	minichromosome maintenance co	-0.080	0.946	8.45E-01	8.45E+01		
8129218	NM_153255	MCM9	minichromosome maintenance co	-0.044	0.970	8.18E-01	8.18E+01		
8129231	NM_024581	FAM184A	family with sequence similarity 18	0.216	1.161	4.43E-01	4.43E+01		
8129317	NM_020755	SERINC1	serine incorporator 1	0.006	1.004	9.83E-01	9.83E+01		

8129363	NM_016063	HDDC2	HD domain containing 2	-0.118	0.921	5.28E-01	5.28E+01		
8129375	---	---	---	-0.004	0.997	9.85E-01	9.85E+01		
8129377	---	---	---	-0.004	0.997	9.84E-01	9.84E+01		
8129379	NM_00100203(	ECHDC1	enoyl Coenzyme A hydratase dom	0.098	1.070	3.19E-01	3.19E+01		
8129390	---	---	---	-0.028	0.981	9.29E-01	9.29E+01		
8129410	NM_00101092	THEMIS	thymocyte selection associated	0.027	1.019	9.51E-01	9.51E+01		
8129454	---	---	---	0.034	1.024	8.32E-01	8.32E+01		
8129482	NM_00101737	SAMD3	sterile alpha motif domain contair	0.077	1.055	8.96E-01	8.96E+01		
8129497	NM_001431	EPB41L2	erythrocyte membrane protein ba	-0.179	0.883	2.84E-01	2.84E+01		
8129522	NM_004830	MED23	mediator complex subunit 23	0.000	1.000	1.00E+00	1.00E+02		
8129562	NM_001901	CTGF	connective tissue growth factor	0.027	1.019	8.57E-01	8.57E+01		
8129590	NM_003569	STX7	syntaxin 7	0.300	1.231	1.01E-02	1.01E+00		
8129637	NM_004665	VNN2	vanin 2	-0.240	0.847	4.48E-01	4.48E+01		
8129649	NM_052831	C6orf192	chromosome 6 open reading fram	0.026	1.018	9.24E-01	9.24E+01		
8129675	---	---	---	-0.062	0.958	6.66E-01	6.66E+01		
8129695	NM_022568	ALDH8A1	aldehyde dehydrogenase 8 family,	-0.057	0.961	7.25E-01	7.25E+01		
8129706	NM_006620	HBS1L	HBS1-like (S. cerevisiae)	-0.050	0.966	7.83E-01	7.83E+01		
8129773	NM_014739	BCLAF1	BCL2-associated transcription fact	-0.022	0.985	8.76E-01	8.76E+01		
8129783	NM_003980	MAP7	microtubule-associated protein 7	0.090	1.065	4.26E-01	4.26E+01		
8129804	NM_005923	MAP3K5	mitogen-activated protein kinase I	-0.037	0.975	8.40E-01	8.40E+01		
8129861	NM_000416	IFNGR1	interferon gamma receptor 1	0.236	1.177	4.18E-01	4.18E+01		
8129880	NM_022121	PERP	PERP, TP53 apoptosis effector	-0.193	0.875	5.73E-02	5.73E+00		
8129901	NM_031922	REPS1	RALBP1 associated Eps domain co	-0.019	0.987	9.33E-01	9.33E+01		
8129924	NM_153235	TXLNB	taxilin beta	0.054	1.038	8.49E-01	8.49E+01		
8129937	NM_006079	CITED2	Cbp/p300-interacting transactivat	-0.069	0.954	4.49E-01	4.49E+01		
8129953	NM_006734	HIVEP2	human immunodeficiency virus ty	-0.199	0.871	8.62E-02	8.62E+00		
8129963	NM_182503	ADAT2	adenosine deaminase, tRNA-speci	0.196	1.145	2.99E-01	2.99E+01		
8129985	NM_006718	PLAGL1	pleiomorphic adenoma gene-like 1	0.227	1.171	1.36E-02	1.36E+00		
8130003	NM_031287	SF3B5	splicing factor 3b, subunit 5, 10kD	-0.008	0.994	9.77E-01	9.77E+01		
8130009	---	---	---	0.080	1.057	6.80E-01	6.80E+01		
8130013	NM_005670	EPM2A	epilepsy, progressive myoclonus ty	-0.033	0.978	8.73E-01	8.73E+01		
8130032	NM_032145	FBXO30	F-box protein 30	-0.025	0.983	9.05E-01	9.05E+01		
8130038	NM_00104268	SHPRH	SNF2 histone linker PHD RING heli	0.040	1.028	8.05E-01	8.05E+01		
8130071	NM_024713	C15orf29	chromosome 15 open reading fran	0.030	1.021	9.33E-01	9.33E+01		
8130073	NM_207360	ZC3H12D	zinc finger CCCH-type containing 1	-0.060	0.960	5.29E-01	5.29E+01		
8130087	NM_139126	PPIL4	peptidylprolyl isomerase (cyclophi	-0.006	0.996	9.77E-01	9.77E+01		
8130102	NM_007044	KATNA1	katanin p60 (ATPase-containing) s	0.083	1.059	5.57E-01	5.57E+01		
8130116	NM_004690	LATS1	LATS, large tumor suppressor, hon	0.003	1.002	9.86E-01	9.86E+01		
8130129	NM_198887	NUP43	nucleoporin 43kDa	0.064	1.045	7.56E-01	7.56E+01		
8130142	NM_032832	LRP11	low density lipoprotein receptor-r	-0.044	0.970	6.81E-01	6.81E+01		
8130163	NM_00100178	RAET1G	retinoic acid early transcript 1G	-0.054	0.963	6.92E-01	6.92E+01		
8130173	NM_130900	RAET1L	retinoic acid early transcript 1L	-0.087	0.942	3.72E-01	3.72E+01		
8130176	NM_024518	ULBP3	UL16 binding protein 3	0.083	1.059	4.99E-01	4.99E+01		
8130185	NM_020861	ZBTB2	zinc finger and BTB domain contai	-0.032	0.978	8.92E-01	8.92E+01		
8130211	NM_182961	SYNE1	spectrin repeat containing, nuclea	0.015	1.010	9.74E-01	9.74E+01		
8130383	NM_019041	MTRF1L	mitochondrial translational releas	0.051	1.036	6.34E-01	6.34E+01		
8130394	NM_012419	RGS17	regulator of G-protein signaling 17	-0.059	0.960	7.15E-01	7.15E+01		
8130403	---	---	---	0.015	1.011	9.22E-01	9.22E+01		
8130408	NM_00113070(	IPCEF1	interaction protein for cytohesin e	-0.034	0.977	9.13E-01	9.13E+01		
8130436	---	---	---	0.051	1.036	8.31E-01	8.31E+01		
8130438	NM_016020	TFB1M	transcription factor B1, mitochond	0.055	1.039	8.53E-01	8.53E+01		
8130464	NM_018452	C6orf35	chromosome 6 open reading fram	0.015	1.010	9.63E-01	9.63E+01		
8130470	---	---	---	-0.023	0.984	8.96E-01	8.96E+01		
8130474	NM_032861	SERAC1	serine active site containing 1	0.027	1.019	8.96E-01	8.96E+01		
8130499	NM_006519	DYNLT1	dynein, light chain, Tctex-type 1	0.162	1.119	3.84E-01	3.84E+01		
8130505	NM_003379	EZR	ezrin	-0.153	0.899	8.43E-02	8.43E+00		
8130528	NM_031924	RSPH3	radial spoke 3 homolog (Chlamydo	0.026	1.018	9.12E-01	9.12E+01		
8130539	NM_054114	TAGAP	T-cell activation RhoGTPase activa	0.171	1.126	8.43E-02	8.43E+00		
8130553	AK130765	FLJ27255	hypothetical LOC401281	-0.112	0.925	7.29E-01	7.29E+01		
8130565	---	---	---	-0.129	0.914	6.48E-01	6.48E+01		
8130567	NM_030752	TCP1	t-complex 1	-0.040	0.972	8.06E-01	8.06E+01		
8130578	NR_002960	SNORA20	small nucleolar RNA, H/ACA box 2(	0.230	1.173	4.70E-01	4.70E+01		
8130580	NR_002965	SNORA29	small nucleolar RNA, H/ACA box 2!	0.070	1.050	9.13E-01	9.13E+01		
8130622	---	---	---	0.001	1.001	9.98E-01	9.98E+01		
8130624	NM_005922	MAP3K4	mitogen-activated protein kinase I	-0.010	0.993	9.54E-01	9.54E+01		
8130626	---	---	---	-0.196	0.873	4.07E-01	4.07E+01		
8130660	---	---	---	0.116	1.084	7.98E-01	7.98E+01		
8130703	NM_003181	T	T, brachyury homolog (mouse)	-0.021	0.986	8.96E-01	8.96E+01		
8130715	NM_175922	PRR18	proline rich 18	-0.084	0.944	2.99E-01	2.99E+01		
8130720	NM_145169	SFT2D1	SFT2 domain containing 1	0.264	1.200	8.70E-02	8.70E+00		
8130732	NM_016098	BRP44L	brain protein 44-like	0.073	1.052	6.48E-01	6.48E+01		
8130739	NM_021135	RPS6KA2	ribosomal protein S6 kinase, 90kD	0.176	1.130	1.98E-01	1.98E+01		
8130765	BC112329	FAM103A1	family with sequence similarity 10	0.046	1.032	6.26E-01	6.26E+01		
8130768	NM_003730	RNASET2	ribonuclease T2	0.112	1.080	5.15E-01	5.15E+01		
8130783	AK000385	RNASET2	ribonuclease T2	0.043	1.030	8.30E-01	8.30E+01		
8130785	NM_005299	GPR31	G protein-coupled receptor 31	-0.080	0.946	5.15E-01	5.15E+01		
8130824	AB016902	HGC6.3	similar to HGC6.3	-0.038	0.974	8.04E-01	8.04E+01		

8130827	AB016899	C6orf54	chromosome 6 open reading fram	-0.038	0.974	7.78E-01	7.78E+01		
8130859	NM_214462	DACT2	dapper, antagonist of beta-catenir	-0.044	0.970	6.85E-01	6.85E+01		
8130867	NM_003247	THBS2	thrombospondin 2	-0.048	0.967	7.02E-01	7.02E+01		
8130891	NM_182552	WDR27	WD repeat domain 27	0.048	1.034	8.13E-01	8.13E+01		
8130916	NM_018288	PHF10	PHD finger protein 10	-0.044	0.970	8.18E-01	8.18E+01		
8130939	NM_005618	DLL1	delta-like 1 (Drosophila)	-0.102	0.931	3.02E-01	3.02E+01		
8130952	NM_002793	PSMB1	proteasome (prosome, macropain	-0.023	0.984	8.75E-01	8.75E+01		
8130962	NM_002598	PDCD2	programmed cell death 2	-0.020	0.986	8.52E-01	8.52E+01		
8130988	NM_018452	C6orf35	chromosome 6 open reading fram	0.030	1.021	9.11E-01	9.11E+01		
8130993	NM_020223	FAM20C	family with sequence similarity 20	0.189	1.140	2.50E-01	2.50E+01		
8130997	BC109246	PDGFA	platelet-derived growth factor alp	-0.031	0.979	8.08E-01	8.08E+01		
8131000	NM_017802	HEATR2	HEAT repeat containing 2	-0.044	0.970	7.07E-01	7.07E+01		
8131013	NM_00113096	UNC84A	unc-84 homolog A (C. elegans)	-0.010	0.993	9.77E-01	9.77E+01		
8131042	AK126112	FLJ44124	hypothetical LOC641737	0.013	1.009	9.62E-01	9.62E+01		
8131044	NM_015949	C7orf20	chromosome 7 open reading fram	0.005	1.004	9.83E-01	9.83E+01		
8131056	NM_017781	CYP2W1	cytochrome P450, family 2, subfar	-0.071	0.952	3.15E-01	3.15E+01		
8131069	NM_00103996	GPER	G protein-coupled estrogen recept	0.027	1.019	8.10E-01	8.10E+01		
8131087	NM_00108046	UNCX	UNC homeobox	-0.006	0.996	9.71E-01	9.71E+01		
8131091	NM_002360	MAFK	v-maf musculoaponeurotic fibrosa	-0.025	0.983	8.55E-01	8.55E+01		
8131096	NM_00112863	ELFN1	extracellular leucine-rich repeat ar	-0.028	0.981	8.31E-01	8.31E+01		
8131098	ENST00000402	LOC100128374	hypothetical LOC100128374	-0.010	0.993	9.60E-01	9.60E+01		
8131101	NM_198949	NUDT1	nudix (nucleoside diphosphate linl	-0.012	0.992	9.50E-01	9.50E+01		
8131111	NM_00103728	EIF3B	eukaryotic translation initiation fa	-0.029	0.980	8.70E-01	8.70E+01		
8131135	NM_018641	CHST12	carbohydrate (chondroitin 4) sulfo	-0.036	0.975	8.41E-01	8.41E+01		
8131140	AK024457	FLJ00049	FLJ00049 protein	0.044	1.031	8.16E-01	8.16E+01		
8131155	NM_152558	IQCE	IQ motif containing E	0.056	1.040	5.04E-01	5.04E+01		
8131195	NM_133463	AMZ1	archaelysin family metallopeptidas	-0.066	0.955	4.70E-01	4.70E+01		
8131205	NM_152744	SDK1	sidekick homolog 1, cell adhesion	-0.006	0.996	9.74E-01	9.74E+01		
8131253	NM_00103716	FOXK1	forkhead box K1	-0.068	0.954	4.27E-01	4.27E+01		
8131263	X58060	RNU13P2	RNA, U13 small nuclear pseudoger	0.094	1.067	6.93E-01	6.93E+01		
8131265	NM_014855	KIAA0415	KIAA0415	0.001	1.001	9.96E-01	9.96E+01		
8131286	NR_023384	RNF216L	ring finger protein 216-like	-0.032	0.978	8.37E-01	8.37E+01		
8131292	NM_021163	RBAK	RB-associated KRAB zinc finger	0.104	1.075	5.92E-01	5.92E+01		
8131296	NR_015343	LOC389458	hypothetical LOC389458	-0.002	0.999	9.88E-01	9.88E+01		
8131303	NM_015610	WIPI2	WD repeat domain, phosphoinosit	0.014	1.010	9.34E-01	9.34E+01		
8131326	NM_00104066	SLC29A4	solute carrier family 29 (nucleosid	0.005	1.003	9.87E-01	9.87E+01		
8131335	---	---	---	-0.101	0.932	7.57E-01	7.57E+01		
8131339	NM_003088	FSCN1	fascin homolog 1, actin-bundling p	-0.155	0.898	7.86E-02	7.86E+00		
8131349	NM_00109762	OCM	oncomodulin	-0.081	0.945	8.63E-01	8.63E+01		
8131356	NM_015622	C7orf28A	chromosome 7 open reading fram	-0.063	0.957	4.36E-01	4.36E+01		
8131374	NM_006303	AIMP2	aminoacyl tRNA synthetase compl	0.077	1.055	5.52E-01	5.52E+01		
8131379	---	---	---	0.029	1.020	8.31E-01	8.31E+01		
8131385	---	---	---	-0.187	0.878	4.06E-01	4.06E+01		
8131387	NM_032172	USP42	ubiquitin specific peptidase 42	-0.015	0.989	9.33E-01	9.33E+01		
8131406	NM_018890	RAC1	ras-related C3 botulinum toxin sub	0.042	1.030	9.07E-01	9.07E+01		
8131414	NM_00113438	ZDHHC4	zinc finger, DHHC-type containing	0.000	1.000	1.00E+00	1.00E+02		
8131427	BC001076	C7orf26	chromosome 7 open reading fram	-0.035	0.976	8.75E-01	8.75E+01		
8131438	---	---	---	-0.060	0.960	6.18E-01	6.18E+01		
8131444	NR_002217	PMS2CL	PMS2 C-terminal like pseudogene	0.024	1.017	9.14E-01	9.14E+01		
8131475	NM_020156	C1GALT1	core 1 synthase, glycoprotein-N-ac	0.182	1.134	1.28E-01	1.28E+01		
8131479	NM_019005	MIOS	missing oocyte, meiosis regulator,	0.106	1.076	5.46E-01	5.46E+01		
8131494	---	---	---	0.012	1.008	9.84E-01	9.84E+01		
8131496	NM_138426	GLCCI1	glucocorticoid induced transcript 1	-0.036	0.976	8.46E-01	8.46E+01		
8131506	NM_152745	NXPH1	neurexophilin 1	-0.067	0.955	3.94E-01	3.94E+01		
8131512	---	---	---	-0.014	0.991	9.64E-01	9.64E+01		
8131519	NM_014660	PHF14	PHD finger protein 14	-0.030	0.979	8.20E-01	8.20E+01		
8131573	NM_212460	ARL4A	ADP-ribosylation factor-like 4A	-0.103	0.931	3.93E-01	3.93E+01		
8131583	NM_00115976	BZW2	basic leucine zipper and W2 doma	0.120	1.086	4.49E-01	4.49E+01		
8131600	NM_014399	TSPAN13	tetraspanin 13	-0.005	0.997	9.90E-01	9.90E+01		
8131614	NM_001621	AHR	aryl hydrocarbon receptor	-0.095	0.936	7.29E-01	7.29E+01		
8131631	NM_178423	HDAC9	histone deacetylase 9	0.112	1.081	3.81E-01	3.81E+01		
8131661	NM_000982	RPL21	ribosomal protein L21	-0.013	0.991	9.50E-01	9.50E+01		
8131705	NR_026673	RPL23P8	ribosomal protein L23 pseudogene	0.047	1.033	7.99E-01	7.99E+01		
8131709	NM_003112	SP4	Sp4 transcription factor	0.012	1.008	9.60E-01	9.60E+01		
8131811	---	---	---	-0.061	0.959	5.98E-01	5.98E+01		
8131813	---	---	---	-0.135	0.911	3.93E-01	3.93E+01		
8131815	NM_018846	KLHL7	kelch-like 7 (Drosophila)	-0.076	0.949	5.98E-01	5.98E+01		
8131831	NM_007342	NUPL2	nucleoporin like 2	0.098	1.071	5.06E-01	5.06E+01		
8131860	BC012331	C7orf30	chromosome 7 open reading fram	0.010	1.007	9.53E-01	9.53E+01		
8131869	NR_026676	RPS2P32	ribosomal protein S2 pseudogene	-0.033	0.977	7.51E-01	7.51E+01		
8131871	NM_138771	CCDC126	coiled-coil domain containing 126	-0.025	0.983	9.16E-01	9.16E+01		
8131881	NM_199136	C7orf46	chromosome 7 open reading fram	0.095	1.068	5.04E-01	5.04E+01		
8131917	---	---	---	-0.108	0.928	1.71E-01	1.71E+01		
8131919	NM_000905	NPY	neuropeptide Y	0.125	1.090	2.82E-01	2.82E+01		
8131927	NM_016447	MPP6	membrane protein, palmitoylated	-0.084	0.944	7.02E-01	7.02E+01		
8131942	---	---	---	-0.052	0.965	8.34E-01	8.34E+01		
8131944	NM_004289	NFE2L3	nuclear factor (erythroid-derived 2	0.030	1.021	8.74E-01	8.74E+01		

8131949	NM_016587	CBX3	chromobox homolog 3 (HP1 gamm	0.029	1.020	7.78E-01	7.78E+01		
8131957	NM_013322	SNX10	sorting nexin 10	0.768	1.703	1.11E-01	1.11E+01		
8131965	AK056484	hCG_2009921	hypothetical locus LOC441204	-0.039	0.973	8.37E-01	8.37E+01		
8131970	NM_001989	EVX1	even-skipped homeobox 1	-0.015	0.990	9.20E-01	9.20E+01		
8131975	NM_006024	TAX1BP1	Tax1 (human T-cell leukemia virus	0.025	1.018	8.47E-01	8.47E+01		
8131996	NM_182898	CREB5	cAMP responsive element binding	-0.062	0.958	7.28E-01	7.28E+01		
8132013	NM_004067	CHN2	chimerin (chimaerin) 2	0.260	1.197	3.04E-02	3.04E+00		
8132031	NM_175887	PRR15	proline rich 15	0.012	1.008	9.27E-01	9.27E+01		
8132036	NM_00108052	WIPF3	WAS/WASL interacting protein far	-0.019	0.987	9.25E-01	9.25E+01		
8132043	---	---	---	0.076	1.054	8.40E-01	8.40E+01		
8132045	NM_032639	PLEKHA8	pleckstrin homology domain conta	0.047	1.033	7.75E-01	7.75E+01		
8132055	NM_152793	C7orf41	chromosome 7 open reading fram	-0.059	0.960	7.23E-01	7.23E+01		
8132062	NM_147128	ZNRF2	zinc and ring finger 2	0.006	1.004	9.78E-01	9.78E+01		
8132070	NM_002047	GARS	glycyl-tRNA synthetase	-0.031	0.978	8.26E-01	8.26E+01		
8132092	NM_006774	INMT	indolethylamine N-methyltransfer	-0.035	0.976	7.36E-01	7.36E+01		
8132097	NM_032222	FAM188B	family with sequence similarity 18	0.021	1.015	9.07E-01	9.07E+01		
8132130	NM_000823	GHRHR	growth hormone releasing hormo	-0.036	0.975	7.54E-01	7.54E+01		
8132151	NM_001118	ADCYAP1R1	adenylate cyclase activating polyp	0.059	1.042	5.51E-01	5.51E+01		
8132188	NM_015060	AVL9	AVL9 homolog (S. cerevisiase)	0.010	1.007	9.67E-01	9.67E+01		
8132209	NR_003502	LOC441208	zinc and ring finger 2 pseudogene	-0.022	0.985	9.07E-01	9.07E+01		
8132218	NM_198428	BBS9	Bardet-Biedl syndrome 9	-0.140	0.908	2.68E-01	2.68E+01		
8132243	---	---	---	-0.278	0.825	2.91E-01	2.91E+01		
8132248	---	---	---	0.020	1.014	9.56E-01	9.56E+01		
8132290	---	---	---	0.003	1.002	9.90E-01	9.90E+01		
8132292	NM_001788	40428	septin 7	-0.005	0.996	9.74E-01	9.74E+01		
8132302	ENST00000381	PP13004	hypothetical LOC402481	-0.104	0.930	2.84E-01	2.84E+01		
8132305	NM_030636	EEPD1	endonuclease/exonuclease/phosp	0.186	1.138	1.51E-01	1.51E+01		
8132376	NM_032016	STARD3NL	STARD3 N-terminal like	0.000	1.000	1.00E+00	1.00E+02		
8132406	NM_005402	RALA	v-ral simian leukemia viral oncoge	-0.177	0.885	7.24E-02	7.24E+00		
8132417	NM_003718	CDC2L5	cell division cycle 2-like 5 (choline	-0.071	0.952	3.81E-01	3.81E+01		
8132458	NM_031903	MRPL32	mitochondrial ribosomal protein L	0.000	1.000	1.00E+00	1.00E+02		
8132501	---	---	---	0.016	1.011	9.77E-01	9.77E+01		
8132503	NM_004760	STK17A	serine/threonine kinase 17a	-0.069	0.953	6.68E-01	6.68E+01		
8132513	---	---	---	-0.103	0.931	6.67E-01	6.67E+01		
8132515	NM_000712	BLVRA	biliverdin reductase A	0.156	1.114	6.51E-01	6.51E+01		
8132523	NM_015983	UBE2D4	ubiquitin-conjugating enzyme E2D	-0.020	0.986	9.22E-01	9.22E+01		
8132531	NM_175064	SPDYE1	speedy homolog E1 (Xenopus laev	0.037	1.026	8.64E-01	8.64E+01		
8132539	NM_014063	DBNL	drebrin-like	0.078	1.056	3.47E-01	3.47E+01		
8132557	NM_001129	AEBP1	AE binding protein 1	0.002	1.002	9.88E-01	9.88E+01		
8132580	NM_006555	YKT6	YKT6 v-SNARE homolog (S. cerevis	0.028	1.020	8.93E-01	8.93E+01		
8132589	---	---	---	-0.016	0.989	9.52E-01	9.52E+01		
8132592	NM_002541	OGDH	oxoglutarate (alpha-ketoglutarate	0.060	1.043	7.08E-01	7.08E+01		
8132617	NM_031449	ZMIZ2	zinc finger, MIZ-type containing 2	-0.070	0.953	5.01E-01	5.01E+01		
8132642	NM_021130	PPIA	peptidylprolyl isomerase A (cyclop	0.014	1.010	9.53E-01	9.53E+01		
8132646	NM_031443	CCM2	cerebral cavernous malformation	0.060	1.042	5.44E-01	5.44E+01		
8132660	NM_005856	RAMP3	receptor (G protein-coupled) activ	-0.017	0.988	8.92E-01	8.92E+01		
8132667	NM_021116	ADCY1	adenylate cyclase 1 (brain)	-0.064	0.957	4.57E-01	4.57E+01		
8132694	NM_000596	IGFBP1	insulin-like growth factor binding	-0.045	0.969	7.19E-01	7.19E+01		
8132715	NM_00110015	C7orf57	chromosome 7 open reading fram	-0.014	0.991	9.35E-01	9.35E+01		
8132805	NM_198570	VWC2	von Willebrand factor C domain co	-0.062	0.958	4.51E-01	4.51E+01		
8132819	NM_006060	IKZF1	IKAROS family zinc finger 1 (Ikaros	-0.010	0.993	9.76E-01	9.76E+01		
8132830	---	---	---	0.137	1.100	5.50E-01	5.50E+01		
8132832	AK057400	LOC100129427	hypothetical protein LOC10012942	0.033	1.023	8.06E-01	8.06E+01		
8132840	NM_182595	POM121L12	POM121 membrane glycoprotein-	-0.043	0.971	7.36E-01	7.36E+01		
8132843	NM_017645	HAUS6	HAUS augmin-like complex, subun	-0.012	0.992	9.78E-01	9.78E+01		
8132849	---	---	---	-0.027	0.982	8.93E-01	8.93E+01		
8132858	---	---	---	0.013	1.009	9.61E-01	9.61E+01		
8132897	NM_018697	LANCL2	LanC lantibiotic synthetase compo	0.018	1.012	9.49E-01	9.49E+01		
8132909	---	---	---	-0.058	0.961	7.52E-01	7.52E+01		
8132911	---	---	---	-0.063	0.957	8.37E-01	8.37E+01		
8132922	NM_015969	MRPS17	mitochondrial ribosomal protein S	0.033	1.023	8.76E-01	8.76E+01		
8132929	NM_001483	GBAS	glioblastoma amplified sequence	0.088	1.063	6.01E-01	6.01E+01		
8132943	NM_001762	CCT6A	chaperonin containing TCP1, subu	0.026	1.018	8.97E-01	8.97E+01		
8132964	NM_015411	SUMF2	sulfatase modifying factor 2	0.016	1.011	9.24E-01	9.24E+01		
8132978	ENST00000447	LOC100287093	similar to Putative uncharacterized	-0.029	0.980	8.49E-01	8.49E+01		
8132980	NM_00108049	TNRC18	trinucleotide repeat containing 18	0.013	1.009	9.50E-01	9.50E+01		
8132992	---	---	---	0.057	1.040	7.99E-01	7.99E+01		
8132998	---	---	---	-0.133	0.912	6.07E-01	6.07E+01		
8133012	---	---	---	0.076	1.054	7.11E-01	7.11E+01		
8133027	AK128836	LOC100132217	similar to hCG1793472	-0.039	0.973	5.97E-01	5.97E+01		
8133030	NM_002040	GABPA	GA binding protein transcription fa	0.066	1.047	6.89E-01	6.89E+01		
8133038	---	---	---	0.203	1.151	5.55E-01	5.55E+01		
8133049	NM_016220	ZNF107	zinc finger protein 107	0.115	1.083	5.42E-01	5.42E+01		
8133062	NR_003099	ZNF273	zinc finger protein 273	0.027	1.019	9.15E-01	9.15E+01		
8133070	---	---	---	0.020	1.014	9.49E-01	9.49E+01		
8133074	NR_027393	INTS4L1	integrator complex subunit 4-like	-0.030	0.980	8.59E-01	8.59E+01		
8133089	NM_152626	ZNF92	zinc finger protein 92	0.049	1.034	7.94E-01	7.94E+01		

8133095	NR_027392	INTS4L2	integrator complex subunit 4-like 2	-0.040	0.972	7.73E-01	7.73E+01		
8133114	NM_173517	VKORC1L1	vitamin K epoxide reductase comp	0.069	1.049	7.01E-01	7.01E+01		
8133119	BC014249	VKORC1L1	vitamin K epoxide reductase comp	0.017	1.012	9.33E-01	9.33E+01		
8133122	NM_00102494	ASL	argininosuccinate lyase	0.041	1.029	7.72E-01	7.72E+01		
8133145	NM_014478	CRCP	CGRP receptor component	0.009	1.006	9.66E-01	9.66E+01		
8133155	NM_003596	TPST1	tyrosylprotein sulfotransferase 1	0.058	1.041	8.49E-01	8.49E+01		
8133167	NM_153033	KCTD7	potassium channel tetramerisation	0.053	1.037	7.68E-01	7.68E+01		
8133176	NM_014504	RABGEF1	RAB guanine nucleotide exchange	-0.165	0.892	1.47E-01	1.47E+01		
8133192	NM_017994	C7orf42	chromosome 7 open reading fram	0.004	1.003	9.80E-01	9.80E+01		
8133202	NM_018264	TYW1	tRNA-yW synthesizing protein 1 h	-0.085	0.943	5.46E-01	5.46E+01		
8133209	NR_003666	SPDYE7P	speedy homolog E7 (Xenopus laev	0.052	1.036	7.60E-01	7.60E+01		
8133215	NM_022906	STAG3L4	stromal antigen 3-like 4	0.065	1.046	8.91E-01	8.91E+01		
8133219	---	---	---	0.005	1.004	9.80E-01	9.80E+01		
8133221	---	---	---	0.159	1.117	3.87E-02	3.87E+00		
8133233	NM_015570	AUTS2	autism susceptibility candidate 2	-0.311	0.806	2.72E-02	2.72E+00		
8133273	---	---	---	0.031	1.021	8.05E-01	8.05E+01		
8133275	NM_172020	POM121	POM121 membrane glycoprotein	-0.098	0.934	1.47E-01	1.47E+01		
8133300	NR_003614	PMS2L2	postmeiotic segregation increased	0.085	1.061	7.08E-01	7.08E+01		
8133309	NR_027775	PMS2L5	postmeiotic segregation increased	0.012	1.008	9.57E-01	9.57E+01		
8133326	NM_172020	POM121	POM121 membrane glycoprotein	-0.133	0.912	3.52E-01	3.52E+01		
8133331	NM_172020	POM121	POM121 membrane glycoprotein	-0.050	0.966	6.66E-01	6.66E+01		
8133335	NM_003508	FZD9	frizzled homolog 9 (Drosophila)	-0.030	0.979	8.21E-01	8.21E+01		
8133339	NM_00107762	VPS37D	vacuolar protein sorting 37 homol	-0.004	0.997	9.80E-01	9.80E+01		
8133345	NM_017528	WBSCR22	Williams Beuren syndrome chrom	-0.064	0.957	5.98E-01	5.98E+01		
8133372	NM_000501	ELN	elastin	-0.018	0.988	8.63E-01	8.63E+01		
8133413	NM_002314	LIMK1	LIM domain kinase 1	0.083	1.059	4.90E-01	4.90E+01		
8133434	NM_022170	EIF4H	eukaryotic translation initiation fa	0.123	1.089	6.57E-02	6.57E+00		
8133442	NM_032464	LAT2	linker for activation of T cells fami	0.067	1.048	5.84E-01	5.84E+01		
8133459	NM_003388	CLIP2	CAP-GLY domain containing linker	-0.004	0.997	9.82E-01	9.82E+01		
8133477	NM_016328	GTF2IRD1	GTF2I repeat domain containing 1	-0.026	0.982	9.01E-01	9.01E+01		
8133500	BC126309	WBSCR23	Williams-Beuren syndrome chrom	0.022	1.015	9.15E-01	9.15E+01		
8133504	NM_032999	GTF2I	general transcription factor IIi	0.058	1.041	6.81E-01	6.81E+01		
8133531	NR_027775	PMS2L5	postmeiotic segregation increased	0.020	1.014	9.35E-01	9.35E+01		
8133540	NM_00114506	GATSL1	GATS protein-like 1	-0.080	0.946	5.51E-01	5.51E+01		
8133549	NM_00100379	GTF2IRD2B	GTF2I repeat domain containing 2	0.003	1.002	9.90E-01	9.90E+01		
8133571	NR_003664	SPDYE8P	speedy homolog E8 (Xenopus laev	0.051	1.036	6.91E-01	6.91E+01		
8133582	NR_003664	SPDYE8P	speedy homolog E8 (Xenopus laev	0.043	1.030	7.88E-01	7.88E+01		
8133590	NR_003664	SPDYE8P	speedy homolog E8 (Xenopus laev	0.049	1.035	7.18E-01	7.18E+01		
8133600	NR_003664	SPDYE8P	speedy homolog E8 (Xenopus laev	0.053	1.037	6.74E-01	6.74E+01		
8133610	NM_00101373	STAG3L3	stromal antigen 3-like 3	-0.031	0.979	8.52E-01	8.52E+01		
8133625	NM_198924	TRIM73	tripartite motif-containing 73	-0.020	0.986	9.11E-01	9.11E+01		
8133633	NM_00103957	NSUN5B	NOL1/NOP2/Sun domain family, n	-0.018	0.988	9.35E-01	9.35E+01		
8133654	NM_00109943	SPDYE5	speedy homolog E5 (Xenopus laev	0.058	1.041	7.43E-01	7.43E+01		
8133662	NM_00104045	RHBDD2	rhomboid domain containing 2	0.107	1.077	5.38E-01	5.38E+01		
8133670	NM_000941	POR	P450 (cytochrome) oxidoreductase	0.086	1.061	5.23E-01	5.23E+01		
8133690	NM_005918	MDH2	malate dehydrogenase 2, NAD (mi	0.027	1.019	8.43E-01	8.43E+01		
8133704	NM_00111019	FLJ37078	hypothetical protein FLJ37078	-0.021	0.986	8.52E-01	8.52E+01		
8133721	NM_001540	HSPB1	heat shock 27kDa protein 1	0.126	1.091	3.52E-01	3.52E+01		
8133736	NM_020892	DTX2	deltex homolog 2 (Drosophila)	0.030	1.021	9.42E-01	9.42E+01		
8133741	NM_030570	UPK3B	uroplakin 3B	-0.028	0.981	8.06E-01	8.06E+01		
8133752	---	---	---	-0.076	0.948	5.43E-01	5.43E+01		
8133754	NR_023383	PMS2L11	postmeiotic segregation increased	-0.002	0.998	9.91E-01	9.91E+01		
8133788	NM_002835	PTPN12	protein tyrosine phosphatase, nor	0.028	1.020	9.54E-01	9.54E+01		
8133809	NM_198467	RSBN1L	round spermatid basic protein 1-li	0.054	1.038	6.89E-01	6.89E+01		
8133818	NM_00112735	PHTF2	putative homeodomain transcripti	0.318	1.246	1.14E-03	1.14E-01		
8133874	---	---	---	0.031	1.022	8.50E-01	8.50E+01		
8133896	---	---	---	-0.015	0.990	9.27E-01	9.27E+01		
8133902	---	---	---	-0.009	0.993	9.78E-01	9.78E+01		
8133914	NR_024549	DMTF1	cyclin D binding myb-like transcrip	0.016	1.011	9.18E-01	9.18E+01		
8134079	NM_033107	GTPBP10	GTP-binding protein 10 (putative)	0.132	1.096	3.45E-01	3.45E+01		
8134098	NM_012395	PFTK1	PFTAIRE protein kinase 1	0.244	1.185	3.59E-01	3.59E+01		
8134117	NM_003505	FZD1	frizzled homolog 1 (Drosophila)	0.037	1.026	8.45E-01	8.45E+01		
8134122	NM_005751	AKAP9	A kinase (PRKA) anchor protein (y	-0.046	0.969	7.51E-01	7.51E+01		
8134180	NM_019004	ANKIB1	ankyrin repeat and IBR domain co	-0.044	0.970	6.34E-01	6.34E+01		
8134201	NM_021167	GATAD1	GATA zinc finger domain containi	0.047	1.033	6.05E-01	6.05E+01		
8134211	NM_032120	C7orf64	chromosome 7 open reading fram	0.018	1.013	9.50E-01	9.50E+01		
8134263	NM_000089	COL1A2	collagen, type I, alpha 2	-0.033	0.977	7.24E-01	7.24E+01		
8134318	NM_022900	CASD1	CAS1 domain containing 1	0.066	1.047	7.58E-01	7.58E+01		
8134349	---	---	---	0.072	1.051	4.77E-01	4.77E+01		
8134374	---	---	---	-0.006	0.996	9.81E-01	9.81E+01		
8134407	NM_005222	DLX6	distal-less homeobox 6	0.003	1.002	9.86E-01	9.86E+01		
8134429	---	---	---	-0.002	0.999	9.97E-01	9.97E+01		
8134431	---	---	---	-0.022	0.985	9.22E-01	9.22E+01		
8134433	---	---	---	0.007	1.005	9.77E-01	9.77E+01		
8134435	NM_014916	LMTK2	lemur tyrosine kinase 2	-0.057	0.961	6.94E-01	6.94E+01		
8134452	NM_177455	BHLHA15	basic helix-loop-helix family, mem	0.035	1.025	8.06E-01	8.06E+01		
8134454	NM_015379	BRI3	brain protein I3	0.244	1.185	2.50E-01	2.50E+01		

8134460	AK054626	FLJ30064	hypothetical protein LOC644975	0.089	1.064	6.79E-01	6.79E+01		
8134463	NM_002523	NPTX2	neuronal pentraxin II	0.019	1.013	8.97E-01	8.97E+01		
8134470	NM_003496	TRRAP	transformation/transcription dom	-0.045	0.969	6.13E-01	6.13E+01		
8134552	NM_005720	ARPC1B	actin related protein 2/3 complex,	0.106	1.076	4.42E-01	4.42E+01		
8134581	NM_006409	ARPC1A	actin related protein 2/3 complex,	0.018	1.013	9.48E-01	9.48E+01		
8134589	NM_003910	BUD31	BUD31 homolog (S. cerevisiae)	-0.101	0.932	5.02E-01	5.02E+01		
8134599	NM_006693	CPSF4	cleavage and polyadenylation spec	-0.142	0.906	6.36E-02	6.36E+00		
8134613	NM_213603	ZNF789	zinc finger protein 789	-0.073	0.951	4.99E-01	4.99E+01		
8134621	NM_014569	ZKSCAN5	zinc finger with KRAB and SCAN do	-0.032	0.978	8.29E-01	8.29E+01		
8134631	NM_138494	ZNF655	zinc finger protein 655	-0.058	0.961	4.35E-01	4.35E+01		
8134644	NM_145115	ZNF498	zinc finger protein 498	-0.026	0.982	8.67E-01	8.67E+01		
8134680	NM_003439	ZKSCAN1	zinc finger with KRAB and SCAN do	0.012	1.009	9.57E-01	9.57E+01		
8134689	NM_145914	ZSCAN21	zinc finger and SCAN domain conta	0.048	1.034	7.40E-01	7.40E+01		
8134699	NM_006833	COPS6	COP9 constitutive photomorphoge	0.022	1.015	9.18E-01	9.18E+01		
8134712	NM_004722	AP4M1	adaptor-related protein complex 4	-0.044	0.970	8.04E-01	8.04E+01		
8134730	NM_152755	CNPY4	canopy 4 homolog (zebrafish)	-0.112	0.925	5.38E-01	5.38E+01		
8134737	NM_203397	MBLAC1	metallo-beta-lactamase domain co	-0.013	0.991	9.39E-01	9.39E+01		
8134740	NM_00100839	C7orf59	chromosome 7 open reading fram	-0.059	0.960	6.28E-01	6.28E+01		
8134745	NM_012447	STAG3	stromal antigen 3	-0.134	0.911	6.76E-02	6.76E+00		
8134777	NM_024070	PVRIG	poliovirus receptor related immun	-0.041	0.972	7.73E-01	7.73E+01		
8134789	NM_013440	PILRB	paired immunoglobin-like type 2 r	-0.022	0.985	8.33E-01	8.33E+01		
8134814	NM_013439	PILRA	paired immunoglobin-like type 2 r	-0.113	0.925	7.91E-01	7.91E+01		
8134821	NM_019606	MEPCE	methylphosphate capping enzyme	-0.064	0.957	5.53E-01	5.53E+01		
8134827	NM_173564	C7orf51	chromosome 7 open reading fram	-0.088	0.941	1.57E-01	1.57E+01		
8134834	NM_006076	AGFG2	ArfGAP with FG repeats 2	-0.005	0.996	9.76E-01	9.76E+01		
8134856	NM_033506	FBXO24	F-box protein 24	-0.036	0.975	7.88E-01	7.88E+01		
8134869	NM_002593	PCOLCE	procollagen C-endopeptidase enha	-0.001	1.000	9.98E-01	9.98E+01		
8134880	NM_023948	MOSPD3	motile sperm domain containing 3	-0.038	0.974	7.13E-01	7.13E+01		
8134890	NM_005273	GNB2	guanine nucleotide binding protei	0.079	1.056	3.38E-01	3.38E+01		
8134904	NM_005837	POP7	processing of precursor 7, ribonuc	0.012	1.008	9.55E-01	9.55E+01		
8134907	NM_000799	EPO	erythropoietin	0.028	1.019	8.06E-01	8.06E+01		
8134915	NM_003386	ZAN	zonadhesin	-0.019	0.987	8.57E-01	8.57E+01		
8134965	NM_020246	SLC12A9	solute carrier family 12 (potassium	0.019	1.013	9.25E-01	9.25E+01		
8134981	NM_003302	TRIP6	thyroid hormone receptor interact	0.002	1.002	9.88E-01	9.88E+01		
8134992	NM_015908	SRRT	serrate RNA effector molecule hor	-0.107	0.929	2.83E-01	2.83E+01		
8135031	ENST00000379	MUC12	mucin 12, cell surface associated	-0.052	0.965	7.75E-01	7.75E+01		
8135033	ENST00000379	MUC12	mucin 12, cell surface associated	0.043	1.030	9.12E-01	9.12E+01		
8135064	NM_030961	TRIM56	tripartite motif-containing 56	0.000	1.000	1.00E+00	1.00E+02		
8135069	NM_000602	SERPINE1	serpin peptidase inhibitor, clade E	0.016	1.011	9.43E-01	9.43E+01		
8135080	NM_001283	AP1S1	adaptor-related protein complex 1	-0.097	0.935	4.29E-01	4.29E+01		
8135089	NM_006349	ZNHIT1	zinc finger, HIT type 1	0.057	1.041	5.61E-01	5.61E+01		
8135099	NM_133457	EMID2	EMI domain containing 2	0.021	1.015	8.88E-01	8.88E+01		
8135114	NM_181552	CUX1	cut-like homeobox 1	-0.093	0.938	2.26E-01	2.26E+01		
8135149	NM_020979	SH2B2	SH2B adaptor protein 2	-0.008	0.994	9.63E-01	9.63E+01		
8135162	NM_024653	PRKRIP1	PRKR interacting protein 1 (IL11 in	-0.026	0.982	9.09E-01	9.09E+01		
8135172	NM_00112634	ORAI2	ORAI calcium release-activated cal	-0.059	0.960	6.41E-01	6.41E+01		
8135181	NM_152892	LRWD1	leucine-rich repeats and WD repea	-0.016	0.989	8.94E-01	8.94E+01		
8135197	NM_00103161	SPDYE2	speedy homolog E2 (Xenopus laev	0.020	1.014	9.53E-01	9.53E+01		
8135204	NM_00103161	SPDYE2	speedy homolog E2 (Xenopus laev	0.032	1.022	9.12E-01	9.12E+01		
8135211	NR_026879	FAM185A	family with sequence similarity 18	0.034	1.024	9.30E-01	9.30E+01		
8135229	NM_031905	ARMC10	armadillo repeat containing 10	-0.017	0.989	9.50E-01	9.50E+01		
8135235	NM_004279	PMPCB	peptidase (mitochondrial processi	-0.079	0.947	6.16E-01	6.16E+01		
8135250	NM_002803	PSMC2	proteasome (prosome, macropain	0.099	1.071	4.96E-01	4.96E+01		
8135268	NM_001417	EIF4B	eukaryotic translation initiation fa	-0.034	0.977	9.09E-01	9.09E+01		
8135271	---	---	---	-0.098	0.934	3.79E-01	3.79E+01		
8135273	---	---	---	-0.049	0.967	7.57E-01	7.57E+01		
8135275	---	---	---	0.025	1.018	9.30E-01	9.30E+01		
8135277	NM_182931	MLL5	myeloid/lymphoid or mixed-lineag	-0.078	0.947	2.35E-01	2.35E+01		
8135319	---	---	---	0.033	1.023	8.78E-01	8.78E+01		
8135321	---	---	---	0.133	1.097	5.50E-01	5.50E+01		
8135323	NM_021930	RINT1	RAD50 interactor 1	0.080	1.057	5.89E-01	5.89E+01		
8135341	NM_152750	FLJ23834	hypothetical protein FLJ23834	-0.218	0.860	4.49E-01	4.49E+01		
8135363	NM_002649	PIK3CG	phosphoinositide-3-kinase, catalyt	0.048	1.034	8.34E-01	8.34E+01		
8135378	NM_002736	PRKAR2B	protein kinase, cAMP-dependent,	0.245	1.185	1.30E-01	1.30E+01		
8135392	NM_012257	HBP1	HMG-box transcription factor 1	-0.021	0.986	9.23E-01	9.23E+01		
8135422	NM_00100840	BCAP29	B-cell receptor-associated protein	0.046	1.033	7.50E-01	7.50E+01		
8135458	NM_024814	CBLL1	Cas-Br-M (murine) ecotropic retro	-0.024	0.984	9.07E-01	9.07E+01		
8135464	NM_000108	DLD	dihydrolipoamide dehydrogenase	-0.031	0.979	8.78E-01	8.78E+01		
8135480	NM_012328	DNAJB9	DnaJ (Hsp40) homolog, subfamily	-0.001	0.999	9.97E-01	9.97E+01		
8135488	NM_00109966	LRRN3	leucine rich repeat neuronal 3	-0.212	0.864	6.94E-01	6.94E+01		
8135497	NM_021994	ZNF277	zinc finger protein 277	-0.072	0.951	6.76E-01	6.76E+01		
8135514	NM_001550	IFRD1	interferon-related developmental	-0.014	0.991	9.58E-01	9.58E+01		
8135568	NM_199072	MDFIC	MyoD family inhibitor domain con	0.145	1.106	4.35E-03	4.35E-01		
8135576	NM_015641	TES	testis derived transcript (3 LIM do	-0.005	0.996	9.77E-01	9.77E+01		
8135625	NM_006136	CAPZA2	capping protein (actin filament) m	0.214	1.160	9.99E-02	9.99E+00		
8135638	NM_018412	ST7	suppression of tumorigenicity 7	-0.078	0.947	6.41E-01	6.41E+01		
8135688	NM_016200	LSM8	LSM8 homolog, U6 small nuclear R	-0.033	0.978	8.49E-01	8.49E+01		

8135718	NM_019071	ING3	inhibitor of growth family, membe	0.030	1.021	8.44E-01	8.44E+01		
8135763	NM_057168	WNT16	wingless-type MMTV integration s	0.004	1.003	9.85E-01	9.85E+01		
8135856	NM_001662	ARF5	ADP-ribosylation factor 5	0.002	1.002	9.88E-01	9.88E+01		
8135876	NM_014390	SND1	staphylococcal nuclease and tudor	0.090	1.065	4.04E-01	4.04E+01		
8135909	NM_000230	LEP	leptin	0.041	1.029	8.03E-01	8.03E+01		
8135915	NM_013332	C7orf68	chromosome 7 open reading fram	-0.028	0.981	8.86E-01	8.86E+01		
8135922	NM_018396	METTL2B	methyltransferase like 2B	-0.037	0.975	8.87E-01	8.87E+01		
8135931	NR_024368	tcag7.907	hypothetical LOC402483	-0.002	0.998	9.90E-01	9.90E+01		
8135955	NM_001219	CALU	calumenin	0.316	1.245	8.58E-02	8.58E+00		
8135967	---	---	---	0.077	1.055	7.87E-01	7.87E+01		
8135969	NM_022742	CCDC136	coiled-coil domain containing 136	-0.098	0.934	4.25E-01	4.25E+01		
8135990	NM_001458	FLNC	filamin C, gamma	-0.012	0.992	9.13E-01	9.13E+01		
8136039	NM_004231	ATP6V1F	ATPase, H+ transporting, lysosoma	0.214	1.160	7.91E-02	7.91E+00		
8136045	NM_002200	IRF5	interferon regulatory factor 5	-0.022	0.985	9.06E-01	9.06E+01		
8136059	---	---	---	0.011	1.007	9.73E-01	9.73E+01		
8136061	NR_002187	tcag7.1015	triosephosphate isomerase 1 pseu	-0.091	0.939	3.14E-01	3.14E+01		
8136065	NR_002144 //	// LOC407835 /	mitogen-activated protein kinase	-0.011	0.992	9.37E-01	9.37E+01		
8136067	NM_178562	TSPAN33	tetraspanin 33	-0.045	0.969	8.74E-01	8.74E+01		
8136078	---	---	---	-0.090	0.939	8.23E-01	8.23E+01		
8136080	NM_005631	SMO	smoothened homolog (Drosophila	-0.010	0.993	9.46E-01	9.46E+01		
8136095	NM_015328	AHCYL2	adenosylhomocysteinase-like 2	-0.001	0.999	9.97E-01	9.97E+01		
8136140	NM_005011	NRF1	nuclear respiratory factor 1	-0.009	0.994	9.63E-01	9.63E+01		
8136159	---	---	---	-0.017	0.988	9.79E-01	9.79E+01		
8136161	---	---	---	0.064	1.046	8.30E-01	8.30E+01		
8136163	NM_014997	KLHDC10	kelch domain containing 10	0.005	1.004	9.80E-01	9.80E+01		
8136177	AF277177	LOC100287552	PNAS-119	-0.050	0.966	8.07E-01	8.07E+01		
8136179	---	---	---	-0.105	0.930	7.87E-01	7.87E+01		
8136235	NM_001868	CPA1	carboxypeptidase A1 (pancreatic)	-0.030	0.979	8.56E-01	8.56E+01		
8136259	NM_013255	MKLN1	muskelin 1, intracellular mediator	-0.008	0.995	9.64E-01	9.64E+01		
8136289	---	---	---	-0.040	0.972	7.56E-01	7.56E+01		
8136293	NM_021807	EXOC4	exocyst complex component 4	0.065	1.046	5.87E-01	5.87E+01		
8136341	NM_199186	BPGM	2,3-bisphosphoglycerate mutase	-0.090	0.939	6.00E-01	6.00E+01		
8136388	NM_018295	TMEM140	transmembrane protein 140	0.027	1.019	9.15E-01	9.15E+01		
8136401	NM_015135	NUP205	nucleoporin 205kDa	-0.023	0.984	9.07E-01	9.07E+01		
8136446	---	---	---	0.120	1.087	6.40E-01	6.40E+01		
8136471	---	---	---	0.006	1.004	9.88E-01	9.88E+01		
8136473	NM_015905	TRIM24	tripartite motif-containing 24	0.084	1.060	3.54E-01	3.54E+01		
8136516	NM_173569	UBN2	ubinuclein 2	-0.010	0.993	9.64E-01	9.64E+01		
8136535	NM_197964	C7orf55	chromosome 7 open reading fram	0.077	1.055	4.16E-01	4.16E+01		
8136539	NM_016019	LUC7L2	LUC7-like 2 (S. cerevisiae)	0.046	1.033	5.88E-01	5.88E+01		
8136557	NM_00113096	TBXAS1	thromboxane A synthase 1 (platel	0.206	1.154	4.35E-01	4.35E+01		
8136576	---	---	---	-0.058	0.961	7.36E-01	7.36E+01		
8136580	NM_00100874	RAB19	RAB19, member RAS oncogene fa	-0.073	0.951	5.33E-01	5.33E+01		
8136589	---	---	---	0.058	1.041	7.51E-01	7.51E+01		
8136591	NM_052853	ADCK2	aarF domain containing kinase 2	0.028	1.019	8.41E-01	8.41E+01		
8136602	NM_004546	NDUFB2	NADH dehydrogenase (ubiquinone	0.059	1.041	4.42E-01	4.42E+01		
8136614	NM_018238	AGK	acylglycerol kinase	-0.007	0.995	9.79E-01	9.79E+01		
8136631	NM_003143	SSBP1	single-stranded DNA binding prote	-0.021	0.986	9.18E-01	9.18E+01		
8136641	NM_016943	TAS2R3	taste receptor, type 2, member 3	-0.002	0.999	9.96E-01	9.96E+01		
8136647	NM_018980	TAS2R5	taste receptor, type 2, member 5	0.123	1.089	4.16E-01	4.16E+01		
8136654	---	---	---	0.144	1.105	7.04E-01	7.04E+01		
8136658	---	---	---	-0.342	0.789	2.91E-01	2.91E+01		
8136727	0	0	0	-0.101	0.932	7.74E-02	7.74E+00		
8136801	NR_001296	TRY6	trypsinogen C	-0.045	0.970	8.78E-01	8.78E+01		
8136807	0	0	0	-0.099	0.934	7.85E-01	7.85E+01		
8136811	NM_004445	EPHB6	EPH receptor B6	0.007	1.005	9.75E-01	9.75E+01		
8136832	NM_178829	C7orf34	chromosome 7 open reading fram	0.061	1.043	6.57E-01	6.57E+01		
8136837	NM_00100166	OR6V1	olfactory receptor, family 6, subfa	-0.077	0.948	3.40E-01	3.40E+01		
8136849	NM_00114367	GSTK1	glutathione S-transferase kappa 1	0.137	1.099	1.48E-01	1.48E+01		
8136863	NM_153345	TMEM139	transmembrane protein 139	-0.025	0.983	8.47E-01	8.47E+01		
8136869	NM_032982	CASP2	caspase 2, apoptosis-related cyste	0.024	1.017	8.63E-01	8.63E+01		
8136889	---	---	---	0.049	1.035	8.00E-01	8.00E+01		
8136893	NM_000083	CLCN1	chloride channel 1, skeletal muscle	-0.016	0.989	9.33E-01	9.33E+01		
8136918	NM_003461	ZYX	zyxin	0.152	1.111	1.79E-01	1.79E+01		
8136930	NM_177437	TAS2R60	taste receptor, type 2, member 60	0.087	1.062	6.80E-01	6.80E+01		
8136934	NM_176883	TAS2R41	taste receptor, type 2, member 41	-0.131	0.913	3.68E-01	3.68E+01		
8136936	NM_178561	CTAGE6	CTAGE family, member 6	-0.114	0.924	6.30E-01	6.30E+01		
8136938	---	---	---	-0.318	0.802	6.33E-01	6.33E+01		
8136940	NM_00113002	FAM115C	family with sequence similarity 11	-0.233	0.851	6.89E-02	6.89E+00		
8136954	NM_00113002	FAM115C	family with sequence similarity 11	-0.199	0.871	1.53E-01	1.53E+01		
8136983	NR_002158	OR2A20P	olfactory receptor, family 2, subfa	0.037	1.026	8.87E-01	8.87E+01		
8136985	NM_00100528	OR2A1	olfactory receptor, family 2, subfa	0.037	1.026	8.45E-01	8.45E+01		
8136987	NM_005435	ARHGEF5	Rho guanine nucleotide exchange	0.016	1.011	9.18E-01	9.18E+01		
8137008	AK290098	C7orf11	chromosome 7 open reading fram	-0.085	0.943	4.56E-01	4.56E+01		
8137044	---	---	---	-0.095	0.936	4.85E-01	4.85E+01		
8137054	NM_003592	CUL1	cullin 1	-0.015	0.989	9.54E-01	9.54E+01		
8137081	---	---	---	0.061	1.043	8.38E-01	8.38E+01		

8137091	NM_170686	ZNF398	zinc finger protein 398	-0.086	0.942	4.60E-01	4.60E+01		
8137101	NM_003575	ZNF282	zinc finger protein 282	-0.024	0.983	8.27E-01	8.27E+01		
8137112	NM_012256	ZNF212	zinc finger protein 212	-0.076	0.948	3.38E-01	3.38E+01		
8137118	NR_015357	ZNF783	zinc finger family member 783	-0.046	0.968	7.17E-01	7.17E+01		
8137131	---	---	---	-0.036	0.976	9.19E-01	9.19E+01		
8137133	NM_032534	KRBA1	KRAB-A domain containing 1	-0.033	0.978	8.34E-01	8.34E+01		
8137151	NM_198455	SSPO	SCO-spondin homolog (Bos taurus	-0.030	0.979	7.19E-01	7.19E+01		
8137183	NM_198455	SSPO	SCO-spondin homolog (Bos taurus	-0.054	0.963	5.52E-01	5.52E+01		
8137202	NM_00109922	ZNF862	zinc finger protein 862	0.048	1.034	7.45E-01	7.45E+01		
8137208	NM_145230	ATP6V0E2	ATPase, H+ transporting V0 subun	-0.027	0.982	8.31E-01	8.31E+01		
8137215	NM_00114292	LRRC61	leucine rich repeat containing 61	-0.037	0.974	7.11E-01	7.11E+01		
8137219	NM_138434	C7orf29	chromosome 7 open reading fram	-0.049	0.966	6.32E-01	6.32E+01		
8137225	NM_013400	REPIN1	replication initiator 1	-0.028	0.980	8.33E-01	8.33E+01		
8137228	NM_173680	ZNF775	zinc finger protein 775	-0.067	0.955	4.26E-01	4.26E+01		
8137232	NM_175571	GIMAP8	GTPase, IMAP family member 8	0.287	1.220	1.03E-02	1.03E+00		
8137240	NM_153236	GIMAP7	GTPase, IMAP family member 7	0.021	1.015	9.07E-01	9.07E+01		
8137244	NM_018326	GIMAP4	GTPase, IMAP family member 4	0.099	1.071	1.59E-01	1.59E+01		
8137250	NM_015660	GIMAP2	GTPase, IMAP family member 2	0.132	1.096	2.26E-01	2.26E+01		
8137252	NM_130759	GIMAP1	GTPase, IMAP family member 1	-0.033	0.978	8.47E-01	8.47E+01		
8137257	NM_018384	GIMAP5	GTPase, IMAP family member 5	0.099	1.071	6.05E-01	6.05E+01		
8137271	NM_001091	ABP1	amiloride binding protein 1 (amine	-0.037	0.975	7.67E-01	7.67E+01		
8137280	NM_000603	NOS3	nitric oxide synthase 3 (endothelia	0.065	1.046	3.90E-01	3.90E+01		
8137310	NM_007188	ABCB8	ATP-binding cassette, sub-family B	-0.027	0.981	7.89E-01	7.89E+01		
8137330	ENST00000356	ABCB8	ATP-binding cassette, sub-family B	-0.065	0.956	4.48E-01	4.48E+01		
8137332	NM_007188	ABCB8	ATP-binding cassette, sub-family B	-0.008	0.995	9.68E-01	9.68E+01		
8137334	NM_007188	ABCB8	ATP-binding cassette, sub-family B	-0.078	0.947	5.48E-01	5.48E+01		
8137336	NM_004769	ACCN3	amiloride-sensitive cation channel	-0.102	0.932	2.71E-01	2.71E+01		
8137352	NM_003040	SLC4A2	solute carrier family 4, anion exch	-0.066	0.956	3.31E-01	3.31E+01		
8137381	NM_031946	AGAP3	ArfGAP with GTPase domain, anky	-0.033	0.977	7.85E-01	7.85E+01		
8137404	NM_019015	CHPF2	chondroitin polymerizing factor 2	-0.109	0.927	1.55E-01	1.55E+01		
8137414	NM_016118	NUB1	negative regulator of ubiquitin-like	-0.029	0.980	8.83E-01	8.83E+01		
8137448	NM_022087	GALNT11	UDP-N-acetyl-alpha-D-galactosam	-0.012	0.992	9.64E-01	9.64E+01		
8137464	NM_004577	PSPH	phosphoserine phosphatase	0.347	1.272	2.31E-01	2.31E+01		
8137466	---	---	---	0.349	1.274	2.08E-01	2.08E+01		
8137468	ENST00000021	CCT8L1	chaperonin containing TCP1, subu	-0.019	0.987	9.16E-01	9.16E+01		
8137470	---	---	---	-0.010	0.993	9.64E-01	9.64E+01		
8137472	---	---	---	-0.046	0.968	8.48E-01	8.48E+01		
8137474	NM_020445	ACTR3B	ARP3 actin-related protein 3 hom	-0.118	0.921	3.55E-01	3.55E+01		
8137483	AK131514	FLJ16734	hypothetical LOC641928	-0.060	0.960	7.72E-01	7.72E+01		
8137526	NM_005542	INSIG1	insulin induced gene 1	-0.040	0.972	8.05E-01	8.05E+01		
8137537	NM_001427	EN2	engrailed homeobox 2	0.015	1.010	9.22E-01	9.22E+01		
8137542	NM_053043	RBM33	RNA binding motif protein 33	-0.072	0.951	4.28E-01	4.28E+01		
8137558	NM_053043	RBM33	RNA binding motif protein 33	-0.017	0.989	9.30E-01	9.30E+01		
8137566	NM_030936	RNF32	ring finger protein 32	-0.021	0.986	9.02E-01	9.02E+01		
8137582	---	---	---	0.071	1.051	8.63E-01	8.63E+01		
8137584	NM_138400	NOM1	nucleolar protein with MIF4G dom	-0.116	0.923	2.87E-01	2.87E+01		
8137596	NM_014671	UBE3C	ubiquitin protein ligase E3C	0.074	1.053	4.08E-01	4.08E+01		
8137627	NM_058246	DNAJB6	DnaJ (Hsp40) homolog, subfamily	-0.163	0.893	2.12E-02	2.12E+00		
8137635	---	---	---	-0.064	0.957	5.94E-01	5.94E+01		
8137637	---	---	---	0.013	1.009	9.43E-01	9.43E+01		
8137639	NM_018051	WDR60	WD repeat domain 60	-0.074	0.950	6.78E-01	6.78E+01		
8137666	NM_207366	40435	septin 14	-0.030	0.979	9.19E-01	9.19E+01		
8137668	---	---	---	0.267	1.203	4.25E-01	4.25E+01		
8137670	NM_002607	PDGFA	platelet-derived growth factor alp	0.048	1.034	8.36E-01	8.36E+01		
8137675	NM_002735	PRKAR1B	protein kinase, cAMP-dependent,	0.031	1.022	8.83E-01	8.83E+01		
8137680	NM_006869	ADAP1	ArfGAP with dual PH domains 1	-0.080	0.946	5.52E-01	5.52E+01		
8137693	NM_00103161	COX19	COX19 cytochrome c oxidase asse	-0.007	0.995	9.78E-01	9.78E+01		
8137700	NM_032350	C7orf50	chromosome 7 open reading fram	0.015	1.010	9.22E-01	9.22E+01		
8137707	---	---	---	0.003	1.002	9.86E-01	9.86E+01		
8137709	NM_182491	ZFAND2A	zinc finger, AN1-type domain 2A	-0.187	0.878	1.61E-01	1.61E+01		
8137715	NM_182924	MICALL2	MICAL-like 2	-0.016	0.989	9.13E-01	9.13E+01		
8137739	NM_00108045	INTS1	integrator complex subunit 1	-0.069	0.953	3.08E-01	3.08E+01		
8137781	ENST00000443	LOC100128653	hypothetical LOC100128653	-0.043	0.970	7.73E-01	7.73E+01		
8137783	NM_00109762	TMEM184A	transmembrane protein 184A	-0.041	0.972	7.05E-01	7.05E+01		
8137798	NM_032302	PSMG3	proteasome (prosome, macropain	0.046	1.033	7.17E-01	7.17E+01		
8137802	ENST00000382	LOC401296	hypothetical LOC401296	0.019	1.014	9.07E-01	9.07E+01		
8137805	NM_003550	MAD1L1	MAD1 mitotic arrest deficient-like	-0.038	0.974	6.50E-01	6.50E+01		
8137826	NM_013393	FTSJ2	FtsJ homolog 2 (E. coli)	0.051	1.036	6.54E-01	6.54E+01		
8137833	NM_013321	SNX8	sorting nexin 8	0.056	1.039	6.79E-01	6.79E+01		
8137847	BC015632	C7orf27	chromosome 7 open reading fram	-0.008	0.995	9.52E-01	9.52E+01		
8137863	---	---	---	0.270	1.206	4.79E-01	4.79E+01		
8137865	NM_007353	GNA12	guanine nucleotide binding protei	-0.103	0.931	4.44E-01	4.44E+01		
8137874	NM_032415	CARD11	caspase recruitment domain famil	0.016	1.011	9.46E-01	9.46E+01		
8137906	NM_018059	RADIL	Ras association and DIL domains	0.004	1.003	9.80E-01	9.80E+01		
8137931	NM_00110060	MMD2	monocyte to macrophage differen	-0.039	0.973	7.80E-01	7.80E+01		
8137942	NM_00108049	TNRC18	trinucleotide repeat containing 18	-0.013	0.991	9.54E-01	9.54E+01		
8137953	NM_00108049	TNRC18	trinucleotide repeat containing 18	-0.017	0.988	9.22E-01	9.22E+01		

8137959	NM_00108049	TNRC18	trinucleotide repeat containing 18	-0.097	0.935	2.29E-01	2.29E+01		
8137962	AK125308	LOC100129484	hypothetical LOC100129484	-0.115	0.923	7.08E-02	7.08E+00		
8137964	NM_024963	FBXL18	F-box and leucine-rich repeat prot	-0.012	0.992	9.44E-01	9.44E+01		
8137979	NM_001101	ACTB	actin, beta	0.097	1.070	5.85E-01	5.85E+01		
8137986	NM_207111	RNF216	ring finger protein 216	-0.082	0.945	3.69E-01	3.69E+01		
8138007	---	---	---	-0.033	0.977	8.26E-01	8.26E+01		
8138030	NR_003085	PMS2	PMS2 postmeiotic segregation inc	0.120	1.087	5.45E-01	5.45E+01		
8138045	NM_014413	EIF2AK1	eukaryotic translation initiation fa	0.006	1.004	9.77E-01	9.77E+01		
8138065	---	---	---	-0.002	0.999	9.93E-01	9.93E+01		
8138067	NM_004227	CYTH3	cytohesin 3	-0.114	0.924	1.37E-01	1.37E+01		
8138088	NM_00103716	C7orf70	chromosome 7 open reading fram	-0.105	0.930	4.47E-01	4.47E+01		
8138091	NM_139179	DAGLB	diacylglycerol lipase, beta	-0.022	0.985	9.30E-01	9.30E+01		
8138108	NM_006854	KDELR2	KDEL (Lys-Asp-Glu-Leu) endoplasm	0.107	1.077	3.27E-01	3.27E+01		
8138116	NM_016265	ZNF12	zinc finger protein 12	-0.015	0.990	9.24E-01	9.24E+01		
8138128	NM_015622	C7orf28A	chromosome 7 open reading fram	-0.058	0.960	5.06E-01	5.06E+01		
8138145	NR_002822	MGC72080	MGC72080 pseudogene	-0.077	0.948	6.80E-01	6.80E+01		
8138147	NM_030930	UNC93B1	unc-93 homolog B1 (C. elegans)	0.008	1.005	9.67E-01	9.67E+01		
8138151	NR_002822	MGC72080	MGC72080 pseudogene	0.003	1.002	9.92E-01	9.92E+01		
8138155	---	---	---	0.026	1.018	9.10E-01	9.10E+01		
8138189	NM_002947	RPA3	replication protein A3, 14kDa	-0.024	0.983	8.55E-01	8.55E+01		
8138224	NM_002489	NDUFA4	NADH dehydrogenase (ubiquinone	0.170	1.125	1.64E-02	1.64E+00		
8138353	NM_005924	MEOX2	mesenchyme homeobox 2	-0.058	0.961	4.21E-01	4.21E+01		
8138361	NM_021029	RPL36A	ribosomal protein L36a	0.033	1.023	8.73E-01	8.73E+01		
8138370	NM_020319	ANKMY2	ankyrin repeat and MYND domain	0.204	1.152	5.24E-02	5.24E+00		
8138401	NM_015132	SNX13	sorting nexin 13	0.126	1.092	1.76E-01	1.76E+01		
8138442	NM_000474	TWIST1	twist homolog 1 (Drosophila)	-0.062	0.958	5.17E-01	5.17E+01		
8138450	NM_152898	FERD3L	Fer3-like (Drosophila)	-0.024	0.984	8.86E-01	8.86E+01		
8138454	NM_00100292	TWISTNB	TWIST neighbor	0.023	1.016	9.20E-01	9.20E+01		
8138466	NM_182762	MACC1	metastasis associated in colon can	0.091	1.065	8.09E-01	8.09E+01		
8138531	NM_001402	EEF1A1	eukaryotic translation elongation f	-0.009	0.994	9.18E-01	9.18E+01		
8138545	---	---	---	0.016	1.011	9.73E-01	9.73E+01		
8138547	NM_019059	TOMM7	translocase of outer mitochondria	0.008	1.006	9.75E-01	9.75E+01		
8138581	NM_013293	TRA2A	transformer 2 alpha homolog (Dro	0.025	1.017	8.32E-01	8.32E+01		
8138592	AB052759	TRA2A	transformer 2 alpha homolog (Dro	-0.014	0.990	9.49E-01	9.49E+01		
8138596	NR_002711 //	CLK2P // CLK2P	CDC-like kinase 2, pseudogene //	-0.041	0.972	8.08E-01	8.08E+01		
8138602	NM_004403	DFNA5	deafness, autosomal dominant 5	-0.044	0.970	8.82E-01	8.82E+01		
8138613	NM_015550	OSBPL3	oxysterol binding protein-like 3	0.191	1.142	1.27E-01	1.27E+01		
8138640	NM_018947	CYCS	cytochrome c, somatic	0.042	1.029	8.25E-01	8.25E+01		
8138670	NM_031243	HNRNPA2B1	heterogeneous nuclear ribonucleo	0.011	1.007	9.12E-01	9.12E+01		
8138718	NM_006735	HOXA2	homeobox A2	0.236	1.178	5.11E-02	5.11E+00		
8138721	NM_153631	HOXA3	homeobox A3	0.013	1.009	9.51E-01	9.51E+01		
8138735	NM_019102	HOXA5	homeobox A5	0.016	1.011	9.27E-01	9.27E+01		
8138741	NM_024014	HOXA6	homeobox A6	-0.093	0.938	3.27E-01	3.27E+01		
8138745	NM_006896	HOXA7	homeobox A7	0.007	1.005	9.73E-01	9.73E+01		
8138749	NM_152739	HOXA9	homeobox A9	0.098	1.070	3.79E-01	3.79E+01		
8138757	NM_018951	HOXA10	homeobox A10	-0.057	0.961	5.41E-01	5.41E+01		
8138770	NM_000522	HOXA13	homeobox A13	-0.048	0.967	6.05E-01	6.05E+01		
8138773	NM_022170	EIF4H	eukaryotic translation initiation fa	0.108	1.078	1.61E-01	1.61E+01		
8138776	NM_152740	HIBADH	3-hydroxyisobutyrate dehydrogen	0.039	1.028	8.76E-01	8.76E+01		
8138789	NM_175061	JAZF1	JAZF zinc finger 1	-0.016	0.989	9.44E-01	9.44E+01		
8138799	NM_014817	TRIL	TLR4 interactor with leucine rich r	0.004	1.003	9.81E-01	9.81E+01		
8138805	NM_019029	CPVL	carboxypeptidase, vitellogenic-like	0.555	1.469	5.65E-02	5.65E+00		
8138824	NM_014766	SCRN1	secernin 1	0.039	1.027	8.87E-01	8.87E+01		
8138842	NM_006092	NOD1	nucleotide-binding oligomerization	0.105	1.076	4.83E-01	4.83E+01		
8138857	NM_024051	GGCT	gamma-glutamyl cyclotransferase	0.173	1.127	8.81E-02	8.81E+00		
8138862	---	---	---	0.092	1.066	7.63E-01	7.63E+01		
8138912	NR_024466	LSM5	LSM5 homolog, U6 small nuclear R	-0.034	0.977	8.93E-01	8.93E+01		
8138920	---	---	---	0.039	1.028	7.97E-01	7.97E+01		
8138922	NM_015483	KBTBD2	kelch repeat and BTB (POZ) domai	0.099	1.071	3.71E-01	3.71E+01		
8138930	NR_003500	RP9P	retinitis pigmentosa 9 pseudogene	0.165	1.121	9.05E-02	9.05E+00		
8138939	---	---	---	-0.032	0.978	9.53E-01	9.53E+01		
8138941	NM_00100200	NT5C3	5'-nucleotidase, cytosolic III	0.077	1.055	7.71E-01	7.71E+01		
8138950	NM_203288	RP9	retinitis pigmentosa 9 (autosomal	0.090	1.065	5.75E-01	5.75E+01		
8138977	NM_015283	DPY19L1	dpy-19-like 1 (C. elegans)	-0.128	0.915	5.04E-01	5.04E+01		
8138997	NM_00107765	TBX20	T-box 20	0.023	1.016	9.16E-01	9.16E+01		
8139003	NM_022373	HERPUD2	HERPUD family member 2	-0.084	0.944	4.03E-01	4.03E+01		
8139015	---	---	---	0.010	1.007	9.50E-01	9.50E+01		
8139031	---	---	---	0.117	1.085	6.05E-01	6.05E+01		
8139033	NM_001637	AOAH	acyloxyacyl hydrolase (neutrophil)	0.010	1.007	9.85E-01	9.85E+01		
8139055	---	---	---	-0.119	0.921	6.58E-01	6.58E+01		
8139057	NM_014800	ELMO1	engulfment and cell motility 1	0.063	1.045	6.33E-01	6.33E+01		
8139083	---	---	---	0.089	1.064	7.51E-01	7.51E+01		
8139085	---	---	---	-0.012	0.992	9.60E-01	9.60E+01		
8139100	M30894	TARP	TCR gamma alternate reading fran	-0.122	0.919	8.05E-01	8.05E+01		
8139107	---	---	---	-0.078	0.947	8.55E-01	8.55E+01		
8139113	---	---	---	-0.054	0.963	9.01E-01	9.01E+01		
8139118	---	---	---	0.062	1.044	9.09E-01	9.09E+01		

8139121	ENST00000390	TRGV3	T cell receptor gamma variable 3	-0.019	0.987	9.73E-01	9.73E+01		
8139125	BC030554	TARP	TCR gamma alternate reading fran	-0.026	0.982	9.56E-01	9.56E+01		
8139128	---	---	---	0.003	1.002	9.97E-01	9.97E+01		
8139158	---	---	---	-0.003	0.998	9.89E-01	9.89E+01		
8139160	NM_00110528	FAM183B	acyloxyacyl hydrolase (neutrophil)	0.066	1.047	8.78E-01	8.78E+01		
8139165	NM_014396	VPS41	vacuolar protein sorting 41 homol	0.163	1.119	2.64E-01	2.64E+01		
8139198	NM_152682	RWDD4A	RWD domain containing 4A	0.100	1.072	5.12E-01	5.12E+01		
8139201	---	---	---	0.040	1.028	8.35E-01	8.35E+01		
8139203	NM_138701	C7orf11	chromosome 7 open reading fram	-0.057	0.961	6.91E-01	6.91E+01		
8139207	NM_002192	INHBA	inhibin, beta A	-0.562	0.677	2.31E-01	2.31E+01		
8139228	NM_00109985	C7orf25	chromosome 7 open reading fram	-0.065	0.956	7.19E-01	7.19E+01		
8139232	NM_002787	PSMA2	proteasome (prosome, macropain	0.085	1.060	6.03E-01	6.03E+01		
8139242	---	---	---	0.045	1.031	7.72E-01	7.72E+01		
8139244	NM_018224	C7orf44	chromosome 7 open reading fram	-0.050	0.966	8.01E-01	8.01E+01		
8139250	NM_032014	MRPS24	mitochondrial ribosomal protein S	-0.063	0.957	6.35E-01	6.35E+01		
8139256	NM_017920	URG4	up-regulated gene 4	-0.157	0.897	4.04E-01	4.04E+01		
8139264	NR_003655	POLR2J4	polymerase (RNA) II (DNA directed	-0.037	0.975	8.90E-01	8.90E+01		
8139270	NM_006989	RASA4	RAS p21 protein activator 4	0.016	1.011	9.60E-01	9.60E+01		
8139276	NM_000290	PGAM2	phosphoglycerate mutase 2 (musc	0.012	1.008	9.58E-01	9.58E+01		
8139281	NM_013284	POLM	polymerase (DNA directed), mu	-0.010	0.993	9.43E-01	9.43E+01		
8139299	NM_00112721	POLD2	polymerase (DNA directed), delta	-0.077	0.948	5.03E-01	5.03E+01		
8139314	NM_033508	GCK	glucokinase (hexokinase 4)	-0.021	0.985	8.89E-01	8.89E+01		
8139356	NM_015332	NUDCD3	NudC domain containing 3	0.040	1.028	7.67E-01	7.67E+01		
8139367	NM_013389	NPC1L1	NPC1 (Niemann-Pick disease, type	-0.028	0.981	8.29E-01	8.29E+01		
8139392	NM_019082	DDX56	DEAD (Asp-Glu-Ala-Asp) box polyp	-0.045	0.969	8.10E-01	8.10E+01		
8139411	NM_182547	TMED4	transmembrane emp24 protein tra	-0.063	0.958	6.77E-01	6.77E+01		
8139421	NM_012412	H2AFV	H2A histone family, member V	-0.105	0.930	1.16E-01	1.16E+01		
8139430	NM_033224	PURB	purine-rich element binding prote	-0.118	0.921	1.01E-02	1.01E+00		
8139433	NM_033054	MYO1G	myosin IG	-0.163	0.893	4.20E-01	4.20E+01		
8139456	NR_002952	SNORA9	small nucleolar RNA, H/ACA box 9	-0.029	0.980	9.53E-01	9.53E+01		
8139458	AK096229	LOC100128364	hypothetical LOC100128364	0.107	1.077	7.08E-01	7.08E+01		
8139460	NM_00114633	NACAD	NAC alpha domain containing	-0.079	0.947	4.39E-01	4.39E+01		
8139468	NM_004749	TBRG4	transforming growth factor beta re	0.032	1.022	8.24E-01	8.24E+01		
8139482	NR_002919	SNORA5A	small nucleolar RNA, H/ACA box 5	-0.032	0.978	9.53E-01	9.53E+01		
8139484	NR_002991	SNORA5C	small nucleolar RNA, H/ACA box 5	0.065	1.046	6.06E-01	6.06E+01		
8139486	NR_002990	SNORA5B	small nucleolar RNA, H/ACA box 5	-0.081	0.945	5.34E-01	5.34E+01		
8139488	NM_00101339	IGFBP3	insulin-like growth factor binding p	0.017	1.012	9.09E-01	9.09E+01		
8139592	NM_004507	HUS1	HUS1 checkpoint homolog (S. pom	-0.014	0.990	9.52E-01	9.52E+01		
8139656	NM_00100155	GRB10	growth factor receptor-bound pro	0.005	1.003	9.85E-01	9.85E+01		
8139706	NM_014302	SEC61G	Sec61 gamma subunit	0.019	1.013	9.16E-01	9.16E+01		
8139712	NM_030796	VOPP1	vesicular, overexpressed in cancer	0.022	1.015	8.96E-01	8.96E+01		
8139723	NR_027342	FKBP9L	FK506 binding protein 9-like	-0.052	0.964	6.50E-01	6.50E+01		
8139737	NM_004577	PSPH	phosphoserine phosphatase	0.045	1.032	7.27E-01	7.27E+01		
8139758	NM_016139	CHCHD2	coiled-coil-helix-coiled-coil-helix d	-0.003	0.998	9.89E-01	9.89E+01		
8139763	NM_00114571	LOC389493	hypothetical protein LOC389493	0.013	1.009	9.24E-01	9.24E+01		
8139769	AK125790	LOC401357	hypothetical LOC401357	0.049	1.034	6.81E-01	6.81E+01		
8139778	---	---	---	-0.096	0.936	5.04E-01	5.04E+01		
8139780	---	---	---	-0.305	0.809	2.96E-01	2.96E+01		
8139796	AK128010	LOC441233	hypothetical LOC441233	-0.179	0.883	5.36E-01	5.36E+01		
8139816	---	---	---	-0.117	0.922	6.65E-01	6.65E+01		
8139828	BC063653	LOC441239	hypothetical protein LOC441239	-0.052	0.965	6.00E-01	6.00E+01		
8139840	NM_00100725	ERV3	endogenous retroviral sequence 3	0.142	1.104	7.23E-01	7.23E+01		
8139859	NM_000181	GUSB	glucuronidase, beta	0.115	1.083	3.31E-01	3.31E+01		
8139879	---	---	---	-0.124	0.918	3.95E-01	3.95E+01		
8139883	---	---	---	0.002	1.001	9.91E-01	9.91E+01		
8139885	---	---	---	-0.104	0.930	6.71E-01	6.71E+01		
8139887	AK090474	LOC441245	hypothetical LOC441245	-0.001	0.999	9.93E-01	9.93E+01		
8139889	---	---	---	-0.110	0.927	5.96E-01	5.96E+01		
8139891	NM_016038	SBDS	Shwachman-Bodian-Diamond synd	-0.024	0.984	9.15E-01	9.15E+01		
8139896	NR_022007	PMS2L4	postmeiotic segregation increased	0.036	1.026	8.67E-01	8.67E+01		
8139907	---	---	---	-0.112	0.925	3.87E-01	3.87E+01		
8139917	NM_022479	WBSCR17	Williams-Beuren syndrome chrom	-0.058	0.961	6.38E-01	6.38E+01		
8139919	---	---	---	-0.005	0.997	9.83E-01	9.83E+01		
8139935	NM_00114544	TYW1B	tRNA-yW synthesizing protein 1 ho	0.022	1.016	9.54E-01	9.54E+01		
8139943	NR_003666	SPDYE7P	speedy homolog E7 (Xenopus laev	0.008	1.006	9.82E-01	9.82E+01		
8139947	NM_032158	NSUN5C	NOL1/NOP2/Sun domain family, m	-0.012	0.992	9.54E-01	9.54E+01		
8139966	NM_198924	TRIM73	tripartite motif-containing 73	-0.016	0.989	9.26E-01	9.26E+01		
8139977	NM_00101373	STAG3L3	stromal antigen 3-like 3	-0.039	0.973	8.06E-01	8.06E+01		
8139996	NR_003664	SPDYE8P	speedy homolog E8 (Xenopus laev	0.048	1.034	6.82E-01	6.82E+01		
8140009	NR_003664	SPDYE8P	speedy homolog E8 (Xenopus laev	0.053	1.037	6.74E-01	6.74E+01		
8140020	NM_018044	NSUN5	NOL1/NOP2/Sun domain family, m	-0.028	0.981	8.37E-01	8.37E+01		
8140028	NM_178125	TRIM50	tripartite motif-containing 50	-0.115	0.923	2.91E-01	2.91E+01		
8140037	NM_032408	BAZ1B	bromodomain adjacent to zinc fing	-0.018	0.988	8.84E-01	8.84E+01		
8140061	NM_001707	BCL7B	B-cell CLL/lymphoma 7B	-0.008	0.995	9.77E-01	9.77E+01		
8140070	NM_012453	TBL2	transducin (beta)-like 2	0.063	1.045	2.27E-01	2.27E+01		
8140085	NM_032951	MLXIPL	MLX interacting protein-like	0.016	1.011	9.23E-01	9.23E+01		
8140107	NM_032317	DNAJC30	DnaJ (Hsp40) homolog, subfamily	0.003	1.002	9.87E-01	9.87E+01		

8140113	NM_004603	STX1A	syntaxin 1A (brain)	-0.024	0.983	9.06E-01	9.06E+01			
8140129	NR_026912	ABHD11	abhydrolase domain containing 11	0.032	1.023	7.87E-01	7.87E+01			
8140140	NM_001306	CLDN3	claudin 3	-0.005	0.997	9.78E-01	9.78E+01			
8140144	NM_152559	WBSCR27	Williams Beuren syndrome chrom	-0.041	0.972	7.05E-01	7.05E+01			
8140151	NM_181471	RFC2	replication factor C (activator 1) 2,	0.088	1.063	7.02E-01	7.02E+01			
8140170	NM_173537	GTF2IRD2	GTF2I repeat domain containing 2	-0.001	1.000	9.99E-01	9.99E+01			
8140196	NM_00102520	STAG3L2	stromal antigen 3-like 2	-0.033	0.977	8.46E-01	8.46E+01			
8140211	NR_003664	SPDYE8P	speedy homolog E8 (Xenopus laev	0.058	1.041	5.59E-01	5.59E+01			
8140220	NM_030798	WBSCR16	Williams-Beuren syndrome chrom	-0.005	0.996	9.77E-01	9.77E+01			
8140239	NR_003613	PMS2L1	postmeiotic segregation increased	0.061	1.043	7.53E-01	7.53E+01			
8140249	NM_00114506	GATSL1	GATS protein-like 1	-0.080	0.946	5.51E-01	5.51E+01			
8140258	NR_027775	PMS2L5	postmeiotic segregation increased	0.045	1.032	8.16E-01	8.16E+01			
8140269	NR_027775	PMS2L5	postmeiotic segregation increased	0.056	1.040	7.79E-01	7.79E+01			
8140280	NR_003614	PMS2L2	postmeiotic segregation increased	0.056	1.040	7.77E-01	7.77E+01			
8140291	NM_003602	FKBP6	FK506 binding protein 6, 36kDa	-0.077	0.948	7.70E-01	7.70E+01			
8140297	NM_00109941	POM121C	POM121 membrane glycoprotein	-0.100	0.933	1.55E-01	1.55E+01			
8140311	NM_005395	PMS2L3	postmeiotic segregation increased	-0.005	0.996	9.86E-01	9.86E+01			
8140319	NM_005338	HIP1	huntingtin interacting protein 1	-0.061	0.959	8.04E-01	8.04E+01			
8140356	---	---	---	-0.006	0.996	9.86E-01	9.86E+01			
8140371	NM_031925	TMEM120A	transmembrane protein 120A	-0.053	0.964	7.03E-01	7.03E+01			
8140386	NM_016086	STYXL1	serine/threonine/tyrosine interact	0.115	1.083	6.98E-01	6.98E+01			
8140398	NM_012479	YWHAG	tyrosine 3-monooxygenase/trypto	0.053	1.037	7.35E-01	7.35E+01			
8140403	NM_080744	SRCRB4D	scavenger receptor cysteine rich d	-0.058	0.961	6.56E-01	6.56E+01			
8140420	NR_003262 //	PSL2A // FDPSL	MGC44478 // MGC44478 // MGC	0.013	1.009	9.65E-01	9.65E+01			
8140424	NM_175064	SPDYE1	speedy homolog E1 (Xenopus laev	0.056	1.039	7.66E-01	7.66E+01			
8140433	NM_012230	POMZP3	POM (POM121 homolog, rat) and	-0.106	0.929	1.79E-01	1.79E+01			
8140443	NR_003262 //	PSL2A // FDPSL	MGC44478 // MGC44478 // MGC	0.032	1.022	8.95E-01	8.95E+01			
8140445	NR_003664	SPDYE8P	speedy homolog E8 (Xenopus laev	0.054	1.038	6.00E-01	6.00E+01			
8140454	NM_175064	SPDYE1	speedy homolog E1 (Xenopus laev	0.055	1.039	7.72E-01	7.72E+01			
8140461	NM_00114526	FAM185A	family with sequence similarity 18	-0.057	0.961	7.44E-01	7.44E+01			
8140468	NM_017439	PION	pigeon homolog (Drosophila)	0.325	1.252	1.17E-01	1.17E+01			
8140478	NM_017439	PION	pigeon homolog (Drosophila)	0.427	1.345	6.36E-02	6.36E+00			
8140500	NM_032936	TMEM60	transmembrane protein 60	0.140	1.102	3.44E-01	3.44E+01			
8140534	NM_006379	SEMA3C	sema domain, immunoglobulin do	-0.095	0.936	8.41E-01	8.41E+01			
8140707	---	---	---	-0.038	0.974	8.78E-01	8.78E+01			
8140709	NM_00114274	KIAA1324L	KIAA1324-like	0.220	1.165	5.29E-01	5.29E+01			
8140730	NM_024315	C7orf23	chromosome 7 open reading fram	-0.094	0.937	5.98E-01	5.98E+01			
8140737	---	---	---	-0.137	0.909	5.71E-01	5.71E+01			
8140782	NM_000927	ABCB1	ATP-binding cassette, sub-family B	0.057	1.040	8.79E-01	8.79E+01			
8140828	NM_003130	SRI	sorcin	0.069	1.049	6.66E-01	6.66E+01			
8140859	NM_006980	MTERF	mitochondrial transcription termi	-0.012	0.991	9.64E-01	9.64E+01			
8140864	NM_000786	CYP51A1	cytochrome P450, family 51, subfa	-0.044	0.970	8.83E-01	8.83E+01			
8140878	NM_194455	KRIT1	KRIT1, ankyrin repeat containing	-0.006	0.996	9.72E-01	9.72E+01			
8140909	NM_014590	ERVWE1	endogenous retroviral family W, e	0.168	1.124	4.70E-01	4.70E+01			
8140915	NM_000466	PEX1	peroxisomal biogenesis factor 1	-0.047	0.968	7.89E-01	7.89E+01			
8140942	NM_00104005	FAM133B	family with sequence similarity 13	0.045	1.032	6.94E-01	6.94E+01			
8140955	NM_001259	CDK6	cyclin-dependent kinase 6	0.073	1.052	8.04E-01	8.04E+01			
8140965	---	---	---	-0.026	0.982	8.88E-01	8.88E+01			
8140967	NM_017654	SAMD9	sterile alpha motif domain contair	0.126	1.091	6.48E-01	6.48E+01			
8140971	NM_152703	SAMD9L	sterile alpha motif domain contair	0.291	1.224	2.00E-01	2.00E+01			
8141024	NM_005868	BET1	blocked early in transport 1 homo	-0.045	0.969	7.88E-01	7.88E+01			
8141050	NM_001006	RPS3A	ribosomal protein S3A	0.006	1.004	9.64E-01	9.64E+01			
8141076	NM_000305	PON2	paraoxonase 2	0.091	1.065	3.33E-01	3.33E+01			
8141133	NM_006304	SHFM1	split hand/foot malformation (ectr	0.031	1.022	7.47E-01	7.47E+01			
8141140	NM_005221	DLX5	distal-less homeobox 5	-0.067	0.955	5.27E-01	5.27E+01			
8141150	NM_133436	ASNS	asparagine synthetase	-0.074	0.950	7.08E-01	7.08E+01			
8141166	AK125166	LOC441268	hypothetical LOC441268	0.008	1.005	9.72E-01	9.72E+01			
8141169	NR_002822	MGC72080	MGC72080 pseudogene	-0.056	0.962	6.35E-01	6.35E+01			
8141173	NM_006188	OCM2	oncomodulin 2	-0.117	0.922	7.64E-01	7.64E+01			
8141180	NM_015395	TECPR1	tectonin beta-propeller repeat cor	-0.009	0.994	9.65E-01	9.65E+01			
8141222	NM_001006	RPS3A	ribosomal protein S3A	0.013	1.009	9.16E-01	9.16E+01			
8141228	NM_00113445	TMEM130	transmembrane protein 130	0.005	1.003	9.77E-01	9.77E+01			
8141241	NM_020429	SMURF1	SMAD specific E3 ubiquitin protein	-0.070	0.952	6.38E-01	6.38E+01			
8141273	NM_014891	PDAP1	PDGFA associated protein 1	-0.015	0.990	9.51E-01	9.51E+01			
8141281	---	---	---	-0.049	0.966	8.84E-01	8.84E+01			
8141283	NM_015545	PTCD1	pentatricopeptide repeat domain	-0.038	0.974	7.85E-01	7.85E+01			
8141295	NM_004889	ATP5J2	ATP synthase, H+ transporting, mi	0.044	1.031	7.94E-01	7.94E+01			
8141303	---	---	---	-0.006	0.996	9.78E-01	9.78E+01			
8141305	NM_032164	ZNF394	zinc finger protein 394	-0.063	0.957	7.62E-01	7.62E+01			
8141363	NM_033017	TRIM4	tripartite motif-containing 4	0.031	1.022	8.95E-01	8.95E+01			
8141371	NM_181538	GJC3	gap junction protein, gamma 3, 30	0.071	1.051	4.73E-01	4.73E+01			
8141380	NM_032924	ZNF3	zinc finger protein 3	0.030	1.021	8.43E-01	8.43E+01			
8141395	NM_005916	MCM7	minichromosome maintenance co	-0.065	0.956	7.78E-01	7.78E+01			
8141419	---	---	---	-0.091	0.939	4.68E-01	4.68E+01			
8141423	---	---	---	0.013	1.009	9.53E-01	9.53E+01			
8141425	NM_005641	TAF6	TAF6 RNA polymerase II, TATA box	-0.041	0.972	7.39E-01	7.39E+01			
8141445	BC015722	C7orf43	chromosome 7 open reading fram	0.000	1.000	1.00E+00	1.00E+02			

8141459	NM_024637	GAL3ST4	galactose-3-O-sulfotransferase 4	-0.189	0.877	2.94E-01	2.94E+01		
8141466	NM_152742	GPC2	glypican 2	-0.059	0.960	4.72E-01	4.72E+01		
8141477	NM_178831	GATS	GATS, stromal antigen 3 opposite	-0.058	0.961	6.70E-01	6.70E+01		
8141490	NR_003613	PMS2L1	postmeiotic segregation increased	0.201	1.150	2.54E-01	2.54E+01		
8141517	BC026269	C7orf47	chromosome 7 open reading fram	-0.040	0.972	8.25E-01	8.25E+01		
8141522	BC031966	C7orf61	chromosome 7 open reading fram	-0.070	0.952	5.20E-01	5.20E+01		
8141526	NM_030935	TSC22D4	TSC22 domain family, member 4	0.025	1.017	8.63E-01	8.63E+01		
8141535	NM_002319	LRCH4	leucine-rich repeats and calponin	0.009	1.007	9.62E-01	9.62E+01		
8141560	NM_003227	TFR2	transferrin receptor 2	-0.040	0.973	7.72E-01	7.72E+01		
8141580	NM_016188	ACTL6B	actin-like 6B	0.018	1.013	8.93E-01	8.93E+01		
8141595	NM_022574	GIGYF1	GRB10 interacting GYF protein 1	-0.031	0.979	7.95E-01	7.95E+01		
8141625	NM_004444	EPHB4	EPH receptor B4	-0.031	0.979	8.55E-01	8.55E+01		
8141643	NM_00101507	UFSP1	UFM1-specific peptidase 1 (non-fu	-0.018	0.988	9.12E-01	9.12E+01		
8141648	NM_015831	ACHE	acetylcholinesterase (Yt blood gro	-0.073	0.951	4.88E-01	4.88E+01		
8141659	---	---	---	0.084	1.060	3.70E-01	3.70E+01		
8141664	NM_003378	VGF	VGF nerve growth factor inducible	-0.060	0.960	6.15E-01	6.15E+01		
8141676	NM_178176	MOGAT3	monoacylglycerol O-acyltransferas	0.008	1.005	9.64E-01	9.64E+01		
8141688	NM_001084	PLOD3	procollagen-lysine, 2-oxoglutarate	0.152	1.111	2.56E-01	2.56E+01		
8141708	NM_014343	CLDN15	claudin 15	-0.047	0.968	6.87E-01	6.87E+01		
8141717	NM_016068	FIS1	fission 1 (mitochondrial outer mer	-0.001	1.000	9.98E-01	9.98E+01		
8141728	NM_022777	RABL5	RAB, member RAS oncogene famil	-0.142	0.906	1.02E-01	1.02E+01		
8141748	---	---	---	0.029	1.020	8.99E-01	8.99E+01		
8141750	NM_00103161	SPDYE2	speedy homolog E2 (Xenopus laev	0.027	1.019	9.27E-01	9.27E+01		
8141757	NM_017621	ALKBH4	alkB, alkylation repair homolog 4 (	-0.040	0.972	7.63E-01	7.63E+01		
8141762	NM_006234	POLR2J	polymerase (RNA) II (DNA directed	0.075	1.053	5.91E-01	5.91E+01		
8141765	---	---	---	0.013	1.009	9.67E-01	9.67E+01		
8141768	NM_006989	RASA4	RAS p21 protein activator 4	0.068	1.048	3.80E-01	3.80E+01		
8141791	NM_00109761	POLR2J3	polymerase (RNA) II (DNA directed	-0.052	0.965	8.78E-01	8.78E+01		
8141795	NM_00109761	POLR2J3	polymerase (RNA) II (DNA directed	0.008	1.006	9.81E-01	9.81E+01		
8141803	NM_006989	RASA4	RAS p21 protein activator 4	0.087	1.062	2.35E-01	2.35E+01		
8141829	NM_032959	POLR2J2	polymerase (RNA) II (DNA directed	-0.019	0.987	9.48E-01	9.48E+01		
8141843	NM_006989	RASA4	RAS p21 protein activator 4	0.131	1.095	5.07E-01	5.07E+01		
8141896	---	---	---	0.070	1.050	6.24E-01	6.24E+01		
8141898	NM_014377	DNAJC2	DnaJ (Hsp40) homolog, subfamily	0.035	1.025	8.02E-01	8.02E+01		
8142017	---	---	---	0.083	1.059	2.81E-01	2.81E+01		
8142036	NM_182691	SRPK2	SFRS protein kinase 2	0.051	1.036	7.27E-01	7.27E+01		
8142059	---	---	---	0.062	1.044	6.04E-01	6.04E+01		
8142084	CR601484	YBX1P2	Y box binding protein 1 pseudoger	0.034	1.024	8.34E-01	8.34E+01		
8142087	NM_020725	ATXN7L1	ataxin 7-like 1	-0.101	0.932	6.55E-01	6.55E+01		
8142096	NM_020725	ATXN7L1	ataxin 7-like 1	-0.078	0.948	8.28E-01	8.28E+01		
8142098	NM_020725	ATXN7L1	ataxin 7-like 1	-0.109	0.927	8.05E-01	8.05E+01		
8142100	NM_020725	ATXN7L1	ataxin 7-like 1	-0.088	0.941	7.50E-01	7.50E+01		
8142102	NM_020725	ATXN7L1	ataxin 7-like 1	-0.057	0.961	8.62E-01	8.62E+01		
8142104	NM_152749	ATXN7L1	ataxin 7-like 1	-0.143	0.906	3.32E-01	3.32E+01		
8142110	NM_006754	SYPL1	synaptophysin-like 1	0.120	1.087	1.66E-01	1.66E+01		
8142136	NM_175884	FLJ36031	hypothetical protein FLJ36031	-0.079	0.947	4.25E-01	4.25E+01		
8142143	NM_006348	COG5	component of oligomeric golgi cor	0.061	1.043	5.95E-01	5.95E+01		
8142230	---	---	---	-0.034	0.977	8.36E-01	8.36E+01		
8142307	NM_015723	PNPLA8	patatin-like phospholipase domair	0.076	1.054	5.25E-01	5.25E+01		
8142324	NM_00113047	THAP5	THAP domain containing 5	-0.019	0.987	9.46E-01	9.46E+01		
8142343	---	---	---	-0.063	0.957	9.30E-01	9.30E+01		
8142345	NM_014705	DOCK4	dedicator of cytokinesis 4	0.066	1.047	9.24E-01	9.24E+01		
8142405	---	---	---	-0.023	0.984	9.53E-01	9.53E+01		
8142407	NM_022484	TMEM168	transmembrane protein 168	0.049	1.034	7.62E-01	7.62E+01		
8142415	NM_152556	C7orf60	chromosome 7 open reading fram	-0.070	0.953	7.26E-01	7.26E+01		
8142446	---	---	---	-0.095	0.936	5.34E-01	5.34E+01		
8142464	---	---	---	0.086	1.062	7.35E-01	7.35E+01		
8142468	NM_153649	TPM3	tropomyosin 3	0.030	1.021	7.46E-01	7.46E+01		
8142540	NM_014888	FAM3C	family with sequence similarity 3,	0.188	1.139	2.37E-02	2.37E+00		
8142554	NM_005763	AASS	aminoadipate-semialdehyde synth	-0.067	0.955	7.30E-01	7.30E+01		
8142580	NM_00102461	FEZF1	FEZ family zinc finger 1	0.054	1.038	6.30E-01	6.30E+01		
8142663	NM_005000	NDUFA5	NADH dehydrogenase (ubiquinone	-0.026	0.982	8.94E-01	8.94E+01		
8142671	NM_003941	WASL	Wiskott-Aldrich syndrome-like	0.019	1.013	9.00E-01	9.00E+01		
8142730	NM_176814	ZNF800	zinc finger protein 800	0.011	1.008	9.50E-01	9.50E+01		
8142740	NM_024523	GCC1	GRIP and coiled-coil domain conta	0.023	1.016	8.90E-01	8.90E+01		
8142747	NM_006193	PAX4	paired box 4	0.004	1.003	9.85E-01	9.85E+01		
8142763	---	---	---	0.175	1.129	2.72E-01	2.72E+01		
8142765	NM_022143	LRRC4	leucine rich repeat containing 4	0.037	1.026	8.06E-01	8.06E+01		
8142770	ENST00000424	MGC27345	hypothetical protein MGC27345	0.114	1.082	5.52E-01	5.52E+01		
8142774	NM_018077	RBM28	RNA binding motif protein 28	-0.018	0.988	9.17E-01	9.17E+01		
8142795	NM_000883	IMPDH1	IMP (inosine monophosphate) deh	-0.027	0.981	8.10E-01	8.10E+01		
8142814	NM_001708	OPN1SW	opsin 1 (cone pigments), short-wa	0.027	1.019	9.33E-01	9.33E+01		
8142821	NM_00113591	KCP	kielin/chordin-like protein	0.028	1.019	7.92E-01	7.92E+01		
8142830	NM_199349	KCP	kielin/chordin-like protein	-0.062	0.958	6.00E-01	6.00E+01		
8142852	NM_012470	TNPO3	transportin 3	0.010	1.007	9.66E-01	9.66E+01		
8142878	NM_139286	CDC26	cell division cycle 26 homolog (S. c	-0.020	0.986	8.67E-01	8.67E+01		
8142886	NM_003344	UBE2H	ubiquitin-conjugating enzyme E2H	-0.076	0.948	5.34E-01	5.34E+01		

8142899	NM_016478	ZC3HC1	zinc finger, C3HC-type containing 1	0.020	1.014	8.82E-01	8.82E+01		
8142912	NM_032842	TMEM209	transmembrane protein 209	-0.009	0.994	9.77E-01	9.77E+01		
8142945	NM_012133	COPG2	coatomer protein complex, subuni	0.036	1.025	8.34E-01	8.34E+01		
8142971	NM_138693	KLF14	Kruppel-like factor 14	0.034	1.023	7.29E-01	7.29E+01		
8142979	---	---	---	-0.061	0.959	8.77E-01	8.77E+01		
8142981	NM_00101811	PODXL	podocalyxin-like	-0.004	0.997	9.84E-01	9.84E+01		
8142993	---	---	---	0.047	1.033	7.82E-01	7.82E+01		
8142997	NM_020911	PLXNA4	plexin A4	0.010	1.007	9.52E-01	9.52E+01		
8143028	NM_017812	CHCHD3	coiled-coil-helix-coiled-coil-helix d	-0.107	0.929	4.92E-01	4.92E+01		
8143035	---	---	---	-0.017	0.988	9.53E-01	9.53E+01		
8143038	---	---	---	-0.109	0.927	6.71E-01	6.71E+01		
8143040	NM_032826	SLC35B4	solute carrier family 35, member B	-0.026	0.982	9.12E-01	9.12E+01		
8143054	NM_001628	AKR1B1	aldo-keto reductase family 1, mem	-0.209	0.865	1.97E-01	1.97E+01		
8143065	NR_024185	C7orf49	chromosome 7 open reading fram	-0.050	0.966	8.49E-01	8.49E+01		
8143070	NM_014149	WDR91	WD repeat domain 91	0.127	1.092	1.76E-01	1.76E+01		
8143088	NM_013316	CNOT4	CCR4-NOT transcription complex,	-0.009	0.994	9.64E-01	9.64E+01		
8143108	---	---	---	0.214	1.160	5.81E-01	5.81E+01		
8143110	NM_012450	SLC13A4	solute carrier family 13 (sodium/su	-0.016	0.989	9.27E-01	9.27E+01		
8143132	NM_00112861	LUZP6	leucine zipper protein 6	0.123	1.089	9.61E-02	9.61E+00		
8143144	NM_002825	PTN	pleiotrophin	-0.021	0.986	8.93E-01	8.93E+01		
8143188	NM_194071	CREB3L2	cAMP responsive element binding	0.123	1.089	1.40E-01	1.40E+01		
8143247	NM_020910	KIAA1549	KIAA1549	-0.042	0.971	7.36E-01	7.36E+01		
8143279	NM_020119	ZC3HAV1	zinc finger CCCH-type, antiviral 1	0.035	1.024	8.12E-01	8.12E+01		
8143296	NM_198508	KLRG2	killer cell lectin-like receptor subfa	-0.093	0.937	2.12E-01	2.12E+01		
8143307	NM_022740	HIPK2	homeodomain interacting protein	-0.127	0.916	1.97E-01	1.97E+01		
8143327	NM_022750	PARP12	poly (ADP-ribose) polymerase fam	0.029	1.020	9.49E-01	9.49E+01		
8143341	NM_030647	JHDM1D	jumonji C domain containing histo	-0.023	0.984	9.28E-01	9.28E+01		
8143365	---	---	---	0.090	1.065	7.18E-01	7.18E+01		
8143367	NM_207113	SLC37A3	solute carrier family 37 (glycerol-3	-0.033	0.977	9.22E-01	9.22E+01		
8143383	---	---	---	0.070	1.049	9.14E-01	9.14E+01		
8143385	---	---	---	0.001	1.001	9.96E-01	9.96E+01		
8143387	NM_013446	MKRN1	makorin ring finger protein 1	0.110	1.079	1.59E-01	1.59E+01		
8143417	NM_004333	BRAF	v-raf murine sarcoma viral oncoge	-0.062	0.958	6.35E-01	6.35E+01		
8143433	NM_016071	MRPS33	mitochondrial ribosomal protein S	0.143	1.104	4.97E-01	4.97E+01		
8143441	BC012493	KIAA1147	KIAA1147	-0.008	0.994	9.76E-01	9.76E+01		
8143459	---	---	---	-0.248	0.842	4.61E-01	4.61E+01		
8143461	---	---	---	-0.083	0.944	7.09E-01	7.09E+01		
8143518	NM_019841	TRPV5	transient receptor potential cation	-0.060	0.959	4.83E-01	4.83E+01		
8143534	NM_000420	KEL	Kell blood group, metallo-endopep	0.082	1.059	5.43E-01	5.43E+01		
8143564	NM_00103169	FAM131B	family with sequence similarity 13	-0.056	0.962	5.15E-01	5.15E+01		
8143575	NM_005232	EPHA1	EPH receptor A1	-0.039	0.974	6.91E-01	6.91E+01		
8143594	---	---	---	-0.080	0.946	3.38E-01	3.38E+01		
8143597	ENST00000307	FAM115A	family with sequence similarity 11	-0.130	0.914	8.52E-01	8.52E+01		
8143599	---	---	---	-0.318	0.802	6.33E-01	6.33E+01		
8143610	NM_005435	ARHGEF5	Rho guanine nucleotide exchange	0.015	1.010	9.29E-01	9.29E+01		
8143627	NM_00100528	OR2A1	olfactory receptor, family 2, subfa	0.022	1.016	9.01E-01	9.01E+01		
8143629	NR_002157	OR2A9P	olfactory receptor, family 2, subfa	0.040	1.028	8.85E-01	8.85E+01		
8143635	NM_00108041	NOBOX	NOBOX oogenesis homeobox	-0.012	0.992	9.57E-01	9.57E+01		
8143661	---	---	---	0.128	1.093	6.87E-01	6.87E+01		
8143663	NM_004456	EZH2	enhancer of zeste homolog 2 (Dro	-0.088	0.941	7.07E-01	7.07E+01		
8143684	NM_004911	PDIA4	protein disulfide isomerase family	0.281	1.215	2.37E-02	2.37E+00		
8143697	NM_152411	ZNF786	zinc finger protein 786	0.049	1.035	7.39E-01	7.39E+01		
8143708	---	---	---	-0.016	0.989	9.07E-01	9.07E+01		
8143714	NM_015694	ZNF777	zinc finger protein 777	-0.015	0.990	9.44E-01	9.44E+01		
8143725	NM_152557	ZNF746	zinc finger protein 746	-0.024	0.983	8.37E-01	8.37E+01		
8143733	NR_027788	ZNF767	zinc finger family member 767	-0.056	0.962	7.73E-01	7.73E+01		
8143749	NM_207336	ZNF467	zinc finger protein 467	-0.033	0.977	7.76E-01	7.76E+01		
8143759	NM_145230	ATP6V0E2	ATPase, H+ transporting V0 subun	-0.076	0.949	6.48E-01	6.48E+01		
8143772	NM_002889	RARRES2	retinoic acid receptor responder (t	-0.053	0.964	5.13E-01	5.13E+01		
8143781	NM_024711	GIMAP6	GTPase, IMAP family member 6	0.136	1.099	1.09E-01	1.09E+01		
8143788	---	---	---	0.028	1.020	8.71E-01	8.71E+01		
8143803	NM_173681	ATG9B	ATG9 autophagy related 9 homolo	-0.054	0.963	5.20E-01	5.20E+01		
8143825	NM_000238	KCNH2	potassium voltage-gated channel,	-0.054	0.963	6.06E-01	6.06E+01		
8143850	NM_004935	CDK5	cyclin-dependent kinase 5	-0.152	0.900	3.06E-01	3.06E+01		
8143863	NM_006712	FASTK	Fas-activated serine/threonine kin	-0.064	0.956	4.31E-01	4.31E+01		
8143879	NM_00113604	TMUB1	transmembrane and ubiquitin-like	-0.031	0.978	8.41E-01	8.41E+01		
8143885	NM_00109883	GBX1	gastrulation brain homeobox 1	0.037	1.026	7.87E-01	7.87E+01		
8143887	NM_00114245	ASB10	ankyrin repeat and SOCS box-cont	0.015	1.010	9.23E-01	9.23E+01		
8143899	---	---	---	0.097	1.070	7.36E-01	7.36E+01		
8143905	NM_007189	ABCF2	ATP-binding cassette, sub-family F	-0.097	0.935	4.06E-01	4.06E+01		
8143919	NM_00100380	SMARCD3	SWI/SNF related, matrix associate	-0.010	0.993	9.53E-01	9.53E+01		
8143937	NM_016118	NUB1	negative regulator of ubiquitin-like	0.014	1.010	9.50E-01	9.50E+01		
8143939	AK127717	LOC401433	hypothetical LOC401433	0.026	1.018	8.75E-01	8.75E+01		
8143941	NM_198285	WDR86	WD repeat domain 86	-0.078	0.947	5.36E-01	5.36E+01		
8143949	NM_144727	CRYGN	crystallin, gamma N	-0.013	0.991	9.53E-01	9.53E+01		
8143955	---	---	---	-0.151	0.901	4.34E-01	4.34E+01		
8143957	NM_005614	RHEB	Ras homolog enriched in brain	0.188	1.139	3.27E-01	3.27E+01		

8143961	NM_016203	PRKAG2	protein kinase, AMP-activated, gar	0.078	1.056	6.00E-01	6.00E+01			
8143984	---	---	---	0.093	1.067	4.32E-01	4.32E+01			
8143986	---	---	---	-0.127	0.916	4.60E-01	4.60E+01			
8143988	NM_170606	MLL3	myeloid/lymphoid or mixed-lineag	-0.008	0.995	9.67E-01	9.67E+01			
8144044	AK124285	FLJ42291	hypothetical LOC346547	0.068	1.049	7.34E-01	7.34E+01			
8144047	NM_007349	PAXIP1	PAX interacting (with transcription	-0.027	0.981	8.70E-01	8.70E+01			
8144078	NM_000193	SHH	sonic hedgehog homolog (Drosoph	-0.047	0.968	7.25E-01	7.25E+01			
8144082	NR_026865	C7orf13	chromosome 7 open reading fram	-0.014	0.990	9.56E-01	9.56E+01			
8144089	NM_022458	LMBR1	limb region 1 homolog (mouse)	-0.032	0.978	8.63E-01	8.63E+01			
8144112	NM_005515	MNX1	motor neuron and pancreas home	0.055	1.039	6.19E-01	6.19E+01			
8144119	---	---	---	-0.054	0.963	6.76E-01	6.76E+01			
8144121	NM_002847	PTPRN2	protein tyrosine phosphatase, rece	-0.035	0.976	8.20E-01	8.20E+01			
8144184	NM_020728	ESYT2	extended synaptotagmin-like prot	0.028	1.020	8.10E-01	8.10E+01			
8144239	NM_183421	FBXO25	F-box protein 25	-0.056	0.962	6.24E-01	6.24E+01			
8144253	NM_004745	DLGAP2	discs, large (Drosophila) homolog-	-0.054	0.963	6.23E-01	6.23E+01			
8144267	NM_018941	CLN8	ceroid-lipofuscinosis, neuronal 8 (	0.132	1.096	2.85E-02	2.85E+00			
8144279	NM_018941	CLN8	ceroid-lipofuscinosis, neuronal 8 (	0.139	1.101	1.51E-01	1.51E+01			
8144315	BC026965	ARHGEF10	Rho guanine nucleotide exchange	-0.023	0.984	9.49E-01	9.49E+01			
8144317	NM_014867	KBTBD11	kelch repeat and BTB (POZ) domai	-0.093	0.937	3.41E-01	3.41E+01			
8144361	NM_024596	MCPH1	microcephalin 1	0.027	1.019	8.74E-01	8.74E+01			
8144378	NM_018361	AGPAT5	1-acylglycerol-3-phosphate O-acyl	0.241	1.182	1.29E-02	1.29E+00			
8144388	NM_018088	FAM90A1	family with sequence similarity 90	-0.053	0.964	6.24E-01	6.24E+01			
8144392	NR_003668	DEFB109P1B	defensin, beta 109, pseudogene 1	0.119	1.086	8.08E-01	8.08E+01			
8144395	NM_201402 //	P17L2 // USP1	ubiquitin specific peptidase 17-like	-0.029	0.980	8.64E-01	8.64E+01			
8144397	NM_201402	USP17L2	ubiquitin specific peptidase 17-like	0.011	1.007	9.60E-01	9.60E+01			
8144410	NR_027000	LOC349196	hypothetical LOC349196	-0.061	0.959	7.35E-01	7.35E+01			
8144412	NR_027000	LOC349196	hypothetical LOC349196	-0.045	0.969	8.26E-01	8.26E+01			
8144414	NR_027000	LOC349196	hypothetical LOC349196	-0.045	0.969	8.26E-01	8.26E+01			
8144416	NR_027000	LOC349196	hypothetical LOC349196	-0.081	0.945	6.16E-01	6.16E+01			
8144418	NR_027000	LOC349196	hypothetical LOC349196	-0.081	0.945	6.16E-01	6.16E+01			
8144420	NR_027000	LOC349196	hypothetical LOC349196	-0.065	0.956	7.11E-01	7.11E+01			
8144422	NM_00104007	LOC650293	seven transmembrane helix recep	-0.032	0.978	7.78E-01	7.78E+01			
8144424	NM_018088	FAM90A1	family with sequence similarity 90	-0.060	0.959	5.81E-01	5.81E+01			
8144428	NM_018088	FAM90A1	family with sequence similarity 90	-0.050	0.966	6.52E-01	6.52E+01			
8144432	NM_018088	FAM90A1	family with sequence similarity 90	-0.050	0.966	6.52E-01	6.52E+01			
8144436	NM_018088	FAM90A1	family with sequence similarity 90	-0.050	0.966	6.52E-01	6.52E+01			
8144440	NM_018088	FAM90A1	family with sequence similarity 90	-0.060	0.959	5.81E-01	5.81E+01			
8144444	NM_018088	FAM90A1	family with sequence similarity 90	-0.050	0.966	6.52E-01	6.52E+01			
8144448	NM_018088	FAM90A1	family with sequence similarity 90	-0.041	0.972	7.49E-01	7.49E+01			
8144488	NR_027000	LOC349196	hypothetical LOC349196	-0.059	0.960	8.59E-01	8.59E+01			
8144490	NR_027000	LOC349196	hypothetical LOC349196	-0.081	0.945	6.16E-01	6.16E+01			
8144492	NR_027000	LOC349196	hypothetical LOC349196	-0.081	0.945	6.16E-01	6.16E+01			
8144494	NR_027000	LOC349196	hypothetical LOC349196	-0.065	0.956	7.11E-01	7.11E+01			
8144496	NM_00104007	LOC650293	seven transmembrane helix recep	-0.032	0.978	8.07E-01	8.07E+01			
8144498	AK304567	hCG_1990547	family with sequence similarity 86	0.038	1.027	9.00E-01	9.00E+01			
8144505	NM_194284	CLDN23	claudin 23	0.003	1.002	9.87E-01	9.87E+01			
8144516	NM_153332	ERI1	exoribonuclease 1	0.091	1.065	5.29E-01	5.29E+01			
8144528	NM_003747	TNKS	tankyrase, TRF1-interacting ankyri	-0.052	0.964	7.58E-01	7.58E+01			
8144557	NM_012331	MSRA	methionine sulfoxide reductase A	0.146	1.107	3.20E-01	3.20E+01			
8144577	NM_00104003	C8orf74	chromosome 8 open reading fram	-0.079	0.947	4.94E-01	4.94E+01			
8144584	---	---	---	0.012	1.008	9.75E-01	9.75E+01			
8144586	NM_015458	MTMR9	myotubularin related protein 9	-0.028	0.981	8.44E-01	8.44E+01			
8144600	NM_054028	AMAC1L2	acyl-malonyl condensing enzyme 1	-0.200	0.870	4.57E-01	4.57E+01			
8144619	---	---	---	-0.010	0.993	9.56E-01	9.56E+01			
8144625	NM_001715	BLK	B lymphoid tyrosine kinase	0.004	1.003	9.87E-01	9.87E+01			
8144643	NM_002052	GATA4	GATA binding protein 4	-0.021	0.986	8.90E-01	8.90E+01			
8144658	NM_145043	NEIL2	nei like 2 (E. coli)	-0.037	0.975	7.45E-01	7.45E+01			
8144667	---	---	---	0.007	1.005	9.80E-01	9.80E+01			
8144669	NM_004462	FDFT1	farnesyl-diphosphate farnesyltrans	-0.027	0.981	8.88E-01	8.88E+01			
8144685	---	---	---	0.093	1.067	4.12E-01	4.12E+01			
8144699	---	---	---	-0.006	0.996	9.92E-01	9.92E+01			
8144703	---	---	---	-0.076	0.949	2.46E-01	2.46E+01			
8144717	---	---	---	0.145	1.105	3.74E-01	3.74E+01			
8144758	NM_016353	ZDHHC2	zinc finger, DHHC-type containing	-0.080	0.946	5.29E-01	5.29E+01			
8144774	NM_152415	VPS37A	vacuolar protein sorting 37 homol	0.004	1.003	9.85E-01	9.85E+01			
8144810	---	---	---	-0.029	0.980	8.66E-01	8.66E+01			
8144812	NM_006197	PCM1	pericentriolar material 1	-0.013	0.991	9.43E-01	9.43E+01			
8144874	AY176665	NSAP11	nervous system abundant protein	-0.083	0.944	5.95E-01	5.95E+01			
8144880	NM_022071	SH2D4A	SH2 domain containing 4A	-0.009	0.994	9.64E-01	9.64E+01			
8144894	NM_018142	INTS10	integrator complex subunit 10	-0.029	0.980	8.64E-01	8.64E+01			
8144931	NM_001693	ATP6V1B2	ATPase, H+ transporting, lysosoma	0.142	1.103	7.05E-01	7.05E+01			
8144949	---	---	---	-0.089	0.940	4.43E-01	4.43E+01			
8144951	---	---	---	0.012	1.008	9.53E-01	9.53E+01			
8144953	NM_00110016	XPO7	exportin 7	0.093	1.067	3.75E-01	3.75E+01			
8144982	NM_182795	NPM2	nucleophosmin/nucleoplasmin 2	-0.011	0.992	9.63E-01	9.63E+01			
8144995	NM_003867	FGF17	fibroblast growth factor 17	-0.032	0.978	8.16E-01	8.16E+01			
8145027	NM_022749	FAM160B2	family with sequence similarity 16	0.010	1.007	9.53E-01	9.53E+01			

8145047	NM_003018	SFTPC	surfactant protein C	-0.001	0.999	9.94E-01	9.94E+01		
8145055	NM_006129	BMP1	bone morphogenetic protein 1	-0.022	0.985	8.78E-01	8.78E+01		
8145085	NM_001722	POLR3D	polymerase (RNA) III (DNA directe	-0.012	0.991	9.54E-01	9.54E+01		
8145134	---	---	---	0.014	1.010	9.57E-01	9.57E+01		
8145136	NM_005605	PPP3CC	protein phosphatase 3 (formerly 2	-0.099	0.933	3.33E-01	3.33E+01		
8145151	NM_005775	SORBS3	sorbin and SH3 domain containing	0.124	1.090	1.85E-01	1.85E+01		
8145175	NM_176871	PDLIM2	PDZ and LIM domain 2 (mystique)	-0.063	0.958	3.75E-01	3.75E+01		
8145193	NM_00101384	C8orf58	chromosome 8 open reading fram	-0.013	0.991	9.23E-01	9.23E+01		
8145201	NM_021174	KIAA1967	KIAA1967	-0.080	0.946	3.62E-01	3.62E+01		
8145225	---	---	---	-0.023	0.984	9.66E-01	9.66E+01		
8145227	NM_00116003	RHOBTB2	Rho-related BTB domain containin	-0.031	0.979	8.01E-01	8.01E+01		
8145259	NM_152272	CHMP7	CHMP family, member 7	0.104	1.075	5.70E-01	5.70E+01		
8145272	NM_00113610	R3HCC1	R3H domain and coiled-coil contai	-0.070	0.952	5.44E-01	5.44E+01		
8145361	NM_005382	NEFM	neurofilament, medium polypepti	-0.168	0.890	2.73E-01	2.73E+01		
8145365	NM_024940	DOCK5	dedicator of cytokinesis 5	0.151	1.110	7.14E-01	7.14E+01		
8145440	NM_002717	PPP2R2A	protein phosphatase 2 (formerly 2	0.048	1.034	6.58E-01	6.58E+01		
8145454	NM_004331	BNIP3L	BCL2/adenovirus E1B 19kDa intera	0.011	1.008	9.67E-01	9.67E+01		
8145490	NM_173174	PTK2B	PTK2B protein tyrosine kinase 2 be	0.033	1.023	8.79E-01	8.79E+01		
8145529	---	---	---	0.086	1.061	7.85E-01	7.85E+01		
8145555	NM_016240	SCARA3	scavenger receptor class A, memb	-0.056	0.962	6.80E-01	6.80E+01		
8145568	---	---	---	-0.011	0.992	9.65E-01	9.65E+01		
8145586	NM_018091	ELP3	elongation protein 3 homolog (S. c	0.137	1.100	2.48E-02	2.48E+00		
8145603	NM_006228	PNOC	prepronociceptin	0.019	1.013	9.16E-01	9.16E+01		
8145622	---	---	---	0.061	1.043	7.42E-01	7.42E+01		
8145624	NM_001440	EXTL3	exostoses (multiple)-like 3	-0.090	0.940	3.43E-01	3.43E+01		
8145636	NM_024567	HMBOX1	homeobox containing 1	-0.106	0.929	4.97E-01	4.97E+01		
8145652	NM_015344	LEPROTL1	leptin receptor overlapping transc	-0.012	0.992	9.63E-01	9.63E+01		
8145660	NM_006571	DCTN6	dynactin 6	-0.122	0.919	1.78E-01	1.78E+01		
8145669	NM_00100871	RBPMS	RNA binding protein with multiple	-0.086	0.942	5.39E-01	5.39E+01		
8145702	NM_000553	WRN	Werner syndrome, RecQ helicase-	0.024	1.017	9.16E-01	9.16E+01		
8145782	NM_032509	MAK16	MAK16 homolog (S. cerevisiae)	0.035	1.025	9.04E-01	9.04E+01		
8145793	NR_003041	SNORD13	small nucleolar RNA, C/D box 13	0.235	1.177	5.54E-01	5.54E+01		
8145795	XM_00234609	LOC100293539	similar to ribosomal protein 10	0.061	1.043	7.60E-01	7.60E+01		
8145799	NM_080872	UNC5D	unc-5 homolog D (C. elegans)	-0.055	0.962	5.30E-01	5.30E+01		
8145829	NM_025069	ZNF703	zinc finger protein 703	0.036	1.026	7.03E-01	7.03E+01		
8145854	NM_007198	PROSC	proline synthetase co-transcribed	0.071	1.051	4.31E-01	4.31E+01		
8145865	NM_032777	GPR124	G protein-coupled receptor 124	-0.023	0.984	8.43E-01	8.43E+01		
8145887	---	---	---	-0.026	0.982	9.19E-01	9.19E+01		
8145889	NM_004095	EIF4EBP1	eukaryotic translation initiation fa	-0.048	0.967	6.58E-01	6.58E+01		
8145894	NM_004674	ASH2L	ash2 (absent, small, or homeotic)-	0.008	1.006	9.68E-01	9.68E+01		
8145914	NM_004874	BAG4	BCL2-associated athanogene 4	0.000	1.000	1.00E+00	1.00E+02		
8145922	NM_015214	DDHD2	DDHD domain containing 2	-0.116	0.923	3.42E-01	3.42E+01		
8145942	NM_144652	LETM2	leucine zipper-EF-hand containing	-0.175	0.886	1.40E-01	1.40E+01		
8145954	NM_006283	TACC1	transforming, acidic coiled-coil con	-0.030	0.980	8.26E-01	8.26E+01		
8145977	NM_021623	PLEKHA2	pleckstrin homology domain conta	0.035	1.025	8.98E-01	8.98E+01		
8145989	NM_153692	HTRA4	HtrA serine peptidase 4	0.236	1.178	8.56E-02	8.56E+00		
8146000	NM_003816	ADAM9	ADAM metallopeptidase domain 9	0.312	1.242	4.81E-01	4.81E+01		
8146118	---	---	---	0.002	1.001	9.96E-01	9.96E+01		
8146122	NM_016099	GOLGA7	golgi autoantigen, golgin subfamily	0.004	1.003	9.84E-01	9.84E+01		
8146142	NM_178819	AGPAT6	1-acylglycerol-3-phosphate O-acyl	-0.210	0.865	2.60E-02	2.60E+00		
8146159	NM_00113429	AP3M2	adaptor-related protein complex 3	-0.173	0.887	2.57E-01	2.57E+01		
8146171	NM_001556	IKBKB	inhibitor of kappa light polypeptid	0.036	1.025	7.65E-01	7.65E+01		
8146198	NM_002690	POLB	polymerase (DNA directed), beta	0.249	1.188	4.71E-02	4.71E+00		
8146216	NM_00113569	VDAC3	voltage-dependent anion channel	-0.013	0.991	9.53E-01	9.53E+01		
8146225	NM_00113567	C8orf40	chromosome 8 open reading fram	-0.045	0.969	8.24E-01	8.24E+01		
8146239	---	---	---	0.009	1.007	9.75E-01	9.75E+01		
8146243	NM_032410	HOOK3	hook homolog 3 (Drosophila)	0.014	1.010	9.53E-01	9.53E+01		
8146268	NM_002027	FNTA	farnesyltransferase, CAAX box, alp	0.036	1.025	8.06E-01	8.06E+01		
8146278	NM_032237	SGK196	protein kinase-like protein SgK196	-0.051	0.965	6.75E-01	6.75E+01		
8146285	NM_152419	HGSNAT	heparan-alpha-glucosaminide N-a	-0.084	0.944	6.11E-01	6.11E+01		
8146305	---	---	---	-0.025	0.983	9.61E-01	9.61E+01		
8146328	---	---	---	0.006	1.004	9.85E-01	9.85E+01		
8146334	---	---	---	0.049	1.034	7.36E-01	7.36E+01		
8146336	AK301677	KIAA0146	KIAA0146	0.096	1.069	6.00E-01	6.00E+01		
8146357	NM_005914	MCM4	minichromosome maintenance co	0.095	1.068	6.90E-01	6.90E+01		
8146379	NM_003350	UBE2V2	ubiquitin-conjugating enzyme E2 v	-0.009	0.994	9.62E-01	9.62E+01		
8146388	AK128232	FLJ46365	hypothetical LOC401459	-0.029	0.980	8.74E-01	8.74E+01		
8146425	---	---	---	0.140	1.102	4.41E-01	4.41E+01		
8146427	NM_052937	PCMTD1	protein-L-isoaspartate (D-aspartat	-0.005	0.997	9.82E-01	9.82E+01		
8146429	NM_005285	NPBWR1	neuropeptides B/W receptor 1	-0.062	0.958	7.03E-01	7.03E+01		
8146435	NM_170587	RGS20	regulator of G-protein signaling 20	-0.041	0.972	8.04E-01	8.04E+01		
8146448	NM_014175	MRPL15	mitochondrial ribosomal protein L	0.136	1.099	5.24E-01	5.24E+01		
8146456	NR_004385	RNU105C	RNA, U105C small nucleolar	-0.154	0.898	4.53E-01	4.53E+01		
8146462	NM_022454	SOX17	SRY (sex determining region Y)-bo	-0.026	0.982	8.81E-01	8.81E+01		
8146475	NM_052898	XKR4	XK, Kell blood group complex subu	-0.040	0.973	6.29E-01	6.29E+01		
8146480	BC043417	TMEM68	transmembrane protein 68	0.048	1.034	8.80E-01	8.80E+01		
8146482	NM_024831	TGS1	trimethylguanosine synthase hom	-0.037	0.975	7.63E-01	7.63E+01		

8146500	NM_002350	LYN	v-yes-1 Yamaguchi sarcoma viral r	0.155	1.113	6.77E-01	6.77E+01		
8146517	NM_00101166	CHCHD7	coiled-coil-helix-coiled-coil-helix d	0.105	1.075	2.39E-01	2.39E+01		
8146544	NM_00107761	UBXN2B	UBX domain protein 2B	0.017	1.012	9.65E-01	9.65E+01		
8146550	NM_005625	SDCBP	syndecan binding protein (synteni	0.316	1.245	2.65E-01	2.65E+01		
8146559	---	---	---	0.054	1.038	6.31E-01	6.31E+01		
8146564	NM_002865	RAB2A	RAB2A, member RAS oncogene fa	0.095	1.068	3.41E-01	3.41E+01		
8146579	NM_017780	CHD7	chromodomain helicase DNA bind	-0.004	0.997	9.88E-01	9.88E+01		
8146637	NM_152758	YTHDF3	YTH domain family, member 3	0.044	1.031	7.27E-01	7.27E+01		
8146645	NM_152414	BHLHE22	basic helix-loop-helix family, mem	-0.017	0.988	8.88E-01	8.88E+01		
8146647	---	---	---	0.149	1.109	6.33E-01	6.33E+01		
8146649	NM_014637	MTFR1	mitochondrial fission regulator 1	0.024	1.017	9.51E-01	9.51E+01		
8146669	NM_033058	TRIM55	tripartite motif-containing 55	0.001	1.001	9.95E-01	9.95E+01		
8146685	NM_015169	RRS1	RRS1 ribosome biogenesis regulat	0.022	1.015	9.26E-01	9.26E+01		
8146717	NM_013257	SGK3	serum/glucocorticoid regulated ki	0.139	1.101	6.33E-01	6.33E+01		
8146756	NM_00107720	CSPP1	centrosome and spindle pole asso	-0.041	0.972	8.33E-01	8.33E+01		
8146892	---	---	---	-0.022	0.985	9.53E-01	9.53E+01		
8146898	---	---	---	0.297	1.229	5.45E-02	5.45E+00		
8146908	NM_004770	KCNB2	potassium voltage-gated channel,	-0.026	0.982	8.31E-01	8.31E+01		
8146914	NM_017489	TERF1	telomeric repeat binding factor (N	-0.104	0.931	3.22E-01	3.22E+01		
8146921	NM_172037	RDH10	retinol dehydrogenase 10 (all-tran	0.061	1.043	8.24E-01	8.24E+01		
8146930	NM_017866	TMEM70	transmembrane protein 70	0.256	1.194	5.14E-04	5.14E-02		
8146941	---	---	---	0.103	1.074	6.35E-01	6.35E+01		
8146945	NM_018972	GDAP1	ganglioside-induced differentiatio	0.235	1.177	8.43E-02	8.43E+00		
8146955	---	---	---	-0.065	0.956	7.16E-01	7.16E+01		
8147040	NM_00110553	ZBTB10	zinc finger and BTB domain contai	-0.075	0.949	5.35E-01	5.35E+01		
8147049	NM_001444	FABP5	fatty acid binding protein 5 (psoria	0.271	1.207	5.45E-02	5.45E+00		
8147057	NM_152284	CHMP4C	chromatin modifying protein 4C	0.019	1.013	8.95E-01	8.95E+01		
8147101	NM_001951	E2F5	E2F transcription factor 5, p130-bi	0.284	1.218	6.90E-02	6.90E+00		
8147112	NM_198584	CA13	carbonic anhydrase XIII	0.032	1.022	9.18E-01	9.18E+01		
8147123	NM_005181	CA3	carbonic anhydrase III, muscle spe	-0.076	0.948	5.24E-01	5.24E+01		
8147156	NM_007013	WWP1	WW domain containing E3 ubiquit	0.070	1.050	6.60E-01	6.60E+01		
8147172	NM_003909	CPNE3	copine III	-0.005	0.996	9.83E-01	9.83E+01		
8147206	NM_003821	RIPK2	receptor-interacting serine-threon	-0.203	0.869	4.14E-01	4.14E+01		
8147221	NM_004337	OSGIN2	oxidative stress induced growth in	-0.258	0.836	4.98E-02	4.98E+00		
8147228	NM_001359	DECR1	2,4-dienoyl CoA reductase 1, mito	0.287	1.220	7.24E-04	7.24E-02		
8147262	NM_016023	OTUD6B	OTU domain containing 6B	0.069	1.049	6.66E-01	6.66E+01		
8147303	---	---	---	0.028	1.020	8.75E-01	8.75E+01		
8147307	NM_145269	FAM92A1	family with sequence similarity 92	0.087	1.062	5.34E-01	5.34E+01		
8147344	NM_00116177	PDP1	pyruvate dehyrogenase phosphata	0.209	1.156	1.16E-02	1.16E+00		
8147371	---	---	---	-0.043	0.970	7.48E-01	7.48E+01		
8147396	NM_017864	INTS8	integrator complex subunit 8	-0.076	0.949	6.48E-01	6.48E+01		
8147439	NM_024613	PLEKHF2	pleckstrin homology domain conta	0.233	1.175	1.12E-02	1.12E+00		
8147447	NM_014754	PTDSS1	phosphatidylserine synthase 1	0.102	1.073	3.91E-01	3.91E+01		
8147461	NM_002998	SDC2	syndecan 2	0.422	1.340	3.22E-01	3.22E+01		
8147469	NM_016134	PGCP	plasma glutamate carboxypeptida	0.418	1.336	7.50E-02	7.50E+00		
8147479	---	---	---	0.043	1.030	7.86E-01	7.86E+01		
8147481	---	---	---	0.013	1.009	9.16E-01	9.16E+01		
8147483	NM_178812	MTDH	metadherin	0.063	1.044	3.50E-01	3.50E+01		
8147516	NM_002380	MATN2	matrilin 2	0.036	1.026	7.68E-01	7.68E+01		
8147543	BC062359	C8orf47	chromosome 8 open reading fram	-0.016	0.989	9.16E-01	9.16E+01		
8147548	NM_00114586	POP1	processing of precursor 1, ribonuc	0.113	1.082	3.28E-01	3.28E+01		
8147566	NM_020697	KCNS2	potassium voltage-gated channel,	0.052	1.037	5.69E-01	5.69E+01		
8147573	NM_00114246	OSR2	odd-skipped related 2 (Drosophila	0.015	1.010	9.27E-01	9.27E+01		
8147580	NM_017890	VPS13B	vacuolar protein sorting 13 homol	0.019	1.013	9.00E-01	9.00E+01		
8147654	NM_005034	POLR2K	polymerase (RNA) II (DNA directed	-0.048	0.967	7.85E-01	7.85E+01		
8147693	NR_002182	NACAP1	nascent-polypeptide-associated co	-0.017	0.988	9.07E-01	9.07E+01		
8147756	NM_024812	BAALC	brain and acute leukemia, cytoplas	-0.293	0.816	1.50E-02	1.50E+00		
8147777	NM_138455	CTHRC1	collagen triple helix repeat contair	-0.001	1.000	9.99E-01	9.99E+01		
8147785	NM_015420	DCAF13	DDB1 and CUL4 associated factor	0.026	1.018	8.59E-01	8.59E+01		
8147848	NM_181354	OXR1	oxidation resistance 1	0.258	1.196	3.68E-03	3.68E-01		
8147864	NM_014673	TTC35	tetratricopeptide repeat domain 3	0.106	1.076	3.28E-01	3.28E+01		
8147883	NM_020189	ENY2	enhancer of yellow 2 homolog (Dr	0.011	1.008	9.56E-01	9.56E+01		
8147970	NM_198120	EBAG9	estrogen receptor binding site ass	0.051	1.036	7.45E-01	7.45E+01		
8147988	---	---	---	0.131	1.095	7.59E-01	7.59E+01		
8147990	---	---	---	0.215	1.160	3.18E-01	3.18E+01		
8147994	NM_032334	UTP23	UTP23, small subunit (SSU) proces	0.104	1.075	5.50E-01	5.50E+01		
8148018	---	---	---	0.024	1.017	9.17E-01	9.17E+01		
8148020	---	---	---	-0.053	0.964	8.24E-01	8.24E+01		
8148022	NM_080651	MED30	mediator complex subunit 30	-0.173	0.887	3.45E-02	3.45E+00		
8148049	NM_002514	NOV	nephroblastoma overexpressed ge	-0.028	0.981	8.43E-01	8.43E+01		
8148057	---	---	---	-0.045	0.969	8.49E-01	8.49E+01		
8148149	NM_014943	ZHX2	zinc fingers and homeoboxes 2	-0.080	0.946	5.76E-01	5.76E+01		
8148158	NM_145647	WDR67	WD repeat domain 67	0.154	1.113	2.37E-01	2.37E+01		
8148184	NM_032899	FAM83A	family with sequence similarity 83	0.072	1.051	3.95E-01	3.95E+01		
8148196	---	---	---	-0.094	0.937	7.29E-01	7.29E+01		
8148208	NM_144963	FAM91A1	family with sequence similarity 91	0.162	1.118	3.38E-01	3.38E+01		
8148261	---	---	---	0.013	1.009	9.51E-01	9.51E+01		

8148263	NM_017956	TRMT12	tRNA methyltransferase 12 homol	-0.079	0.947	7.15E-01	7.15E+01		
8148265	NM_007218	RNF139	ring finger protein 139	0.013	1.009	9.52E-01	9.52E+01		
8148270	NM_005005	NDUFB9	NADH dehydrogenase (ubiquinone	0.008	1.006	9.73E-01	9.73E+01		
8148280	NM_003129	SQLE	squalene epoxidase	-0.230	0.853	2.21E-01	2.21E+01		
8148293	NM_173685	NSMCE2	non-SMC element 2, MMS21 hom	-0.024	0.983	9.35E-01	9.35E+01		
8148302	---	---	---	0.180	1.133	4.01E-01	4.01E+01		
8148304	NM_025195	TRIB1	tribbles homolog 1 (Drosophila)	-0.008	0.994	9.85E-01	9.85E+01		
8148309	---	---	---	0.327	1.254	1.94E-01	1.94E+01		
8148315	NM_00115954	POU5F1B	POU class 5 homeobox 1B	0.091	1.065	5.91E-01	5.91E+01		
8148317	NM_002467	MYC	v-myc myelocytomatosis viral onc	-0.031	0.979	9.20E-01	9.20E+01		
8148325	---	---	---	0.032	1.022	9.39E-01	9.39E+01		
8148329	---	---	---	-0.082	0.945	2.78E-01	2.78E+01		
8148333	NM_015137	EFR3A	EFR3 homolog A (S. cerevisiae)	0.201	1.150	5.64E-02	5.64E+00		
8148358	NM_016018	PHF20L1	PHD finger protein 20-like 1	-0.048	0.968	7.37E-01	7.37E+01		
8148385	NM_003235	TG	thyroglobulin	-0.084	0.944	1.82E-01	1.82E+01		
8148435	NM_003882	WISP1	WNT1 inducible signaling pathway	-0.052	0.965	5.88E-01	5.88E+01		
8148448	NM_006558	KHDRBS3	KH domain containing, RNA bindin	-0.068	0.954	4.58E-01	4.58E+01		
8148467	NM_017444	CHRAC1	chromatin accessibility complex 1	0.045	1.031	7.46E-01	7.46E+01		
8148474	---	---	---	-0.068	0.954	7.19E-01	7.19E+01		
8148476	NM_014957	DENND3	DENN/MADD domain containing 3	-0.070	0.953	6.20E-01	6.20E+01		
8148501	NM_032611	PTP4A3	protein tyrosine phosphatase type	-0.002	0.999	9.91E-01	9.91E+01		
8148512	NM_207414	FLJ43860	FLJ43860 protein	-0.072	0.951	6.05E-01	6.05E+01		
8148517	NM_001702	BAI1	brain-specific angiogenesis inhibit	-0.008	0.994	9.53E-01	9.53E+01		
8148548	NM_005672	PSCA	prostate stem cell antigen	-0.051	0.965	6.51E-01	6.51E+01		
8148559	NM_016647	C8orf55	chromosome 8 open reading fram	-0.042	0.971	7.60E-01	7.60E+01		
8148572	NM_002346	LY6E	lymphocyte antigen 6 complex, lo	0.186	1.138	5.16E-01	5.16E+01		
8148591	NM_178172	GPIHBP1	glycosylphosphatidylinositol anch	-0.001	1.000	9.98E-01	9.98E+01		
8148597	NM_173832	ZFP41	zinc finger protein 41 homolog (m	0.021	1.015	8.85E-01	8.85E+01		
8148607	NM_138465	GLI4	GLI family zinc finger 4	0.002	1.001	9.91E-01	9.91E+01		
8148615	NM_030895	ZNF696	zinc finger protein 696	0.019	1.013	8.92E-01	8.92E+01		
8148619	---	---	---	0.065	1.046	5.61E-01	5.61E+01		
8148621	NM_052924	RHPN1	rhophilin, Rho GTPase binding pro	-0.034	0.977	8.04E-01	8.04E+01		
8148640	---	---	---	-0.084	0.943	7.10E-01	7.10E+01		
8148642	NM_024736	GSDMD	gasdermin D	-0.024	0.983	8.93E-01	8.93E+01		
8148655	NM_032862	TIGD5	tigger transposable element deriv	-0.075	0.949	2.26E-01	2.26E+01		
8148658	NM_014789	ZNF623	zinc finger protein 623	0.044	1.031	8.27E-01	8.27E+01		
8148662	NM_173831	ZNF707	zinc finger protein 707	0.083	1.059	5.24E-01	5.24E+01		
8148674	NM_139021	MAPK15	mitogen-activated protein kinase	-0.020	0.986	8.69E-01	8.69E+01		
8148694	NM_000837	GRINA	glutamate receptor, ionotropic, N-	0.152	1.111	5.52E-01	5.52E+01		
8148703	NM_198572	SPATC1	spermatogenesis and centriole ass	-0.006	0.996	9.75E-01	9.75E+01		
8148710	NM_019037	EXOSC4	exosome component 4	-0.059	0.960	6.73E-01	6.73E+01		
8148715	NM_003801	GPAA1	glycosylphosphatidylinositol anch	-0.053	0.964	5.73E-01	5.73E+01		
8148728	NM_001916	CYC1	cytochrome c-1	-0.026	0.982	8.79E-01	8.79E+01		
8148737	NM_032272	MAF1	MAF1 homolog (S. cerevisiae)	0.001	1.001	9.96E-01	9.96E+01		
8148748	NR_024207	KIAA1875	KIAA1875	-0.029	0.980	7.87E-01	7.87E+01		
8148772	NM_016458	C8orf30A	chromosome 8 open reading fram	0.008	1.005	9.64E-01	9.64E+01		
8148783	NM_032450	HEATR7A	HEAT repeat containing 7A	-0.105	0.930	3.45E-01	3.45E+01		
8148796	NM_00100827	SCXA	scleraxis homolog A (mouse)	-0.040	0.973	7.72E-01	7.72E+01		
8148799	NM_016458	C8orf30A	chromosome 8 open reading fram	-0.004	0.997	9.84E-01	9.84E+01		
8148808	NM_032450	HEATR7A	HEAT repeat containing 7A	0.019	1.014	8.80E-01	8.80E+01		
8148821	NM_00100827	SCXA	scleraxis homolog A (mouse)	-0.040	0.973	7.72E-01	7.72E+01		
8148824	NM_005526	HSF1	heat shock transcription factor 1	-0.057	0.961	5.52E-01	5.52E+01		
8148841	NM_024531	GPR172A	G protein-coupled receptor 172A	-0.017	0.988	9.07E-01	9.07E+01		
8148850	NM_174922	ADCK5	aarF domain containing kinase 5	-0.021	0.985	8.05E-01	8.05E+01		
8148867	NM_145754	KIFC2	kinesin family member C2	-0.074	0.950	4.03E-01	4.03E+01		
8148888	NM_032902	PPP1R16A	protein phosphatase 1, regulatory	-0.034	0.977	7.76E-01	7.76E+01		
8148902	NM_005309	GPT	glutamic-pyruvate transaminase (a	-0.031	0.979	8.35E-01	8.35E+01		
8148917	NM_138431	MFSD3	major facilitator superfamily doma	-0.015	0.990	9.22E-01	9.22E+01		
8148923	NM_014665	LRRC14	leucine rich repeat containing 14	-0.055	0.963	5.60E-01	5.60E+01		
8148930	---	---	---	-0.043	0.971	7.40E-01	7.40E+01		
8148932	NM_213605	ZNF517	zinc finger protein 517	-0.040	0.973	7.14E-01	7.14E+01		
8148941	NM_003416	ZNF7	zinc finger protein 7	0.031	1.022	8.35E-01	8.35E+01		
8148949	NR_002807	TMED10P	transmembrane emp24-like traffic	0.097	1.070	5.95E-01	5.95E+01		
8148951	NR_026974	C8orf77	chromosome 8 open reading fram	-0.129	0.914	6.26E-02	6.26E+00		
8148955	NM_023080	C8orf33	chromosome 8 open reading fram	-0.063	0.957	6.41E-01	6.41E+01		
8148964	NM_207366	40435	septin 14	-0.030	0.979	9.19E-01	9.19E+01		
8148968	BC037297	FAM87A	family with sequence similarity 87	-0.026	0.982	8.62E-01	8.62E+01		
8148982	AK128318	LOC389607	hypothetical LOC389607	0.068	1.048	8.04E-01	8.04E+01		
8148985	NM_207332	ERICH1	glutamate-rich 1	0.080	1.057	3.93E-01	3.93E+01		
8149071	NM_001147	ANGPT2	angiopoietin 2	0.051	1.036	8.47E-01	8.47E+01		
8149104	NM_001926	DEFA6	defensin, alpha 6, Paneth cell-spec	-0.028	0.981	8.76E-01	8.76E+01		
8149116	NM_005217	DEFA3	defensin, alpha 3, neutrophil-spec	0.033	1.023	9.22E-01	9.22E+01		
8149122	AF355799	DEFT1P	defensin, theta 1 pseudogene	-0.004	0.998	9.85E-01	9.85E+01		
8149126	NM_005217	DEFA3	defensin, alpha 3, neutrophil-spec	0.033	1.023	9.22E-01	9.22E+01		
8149132	AF355799	DEFT1P	defensin, theta 1 pseudogene	-0.004	0.998	9.85E-01	9.85E+01		
8149137	NM_005217	DEFA3	defensin, alpha 3, neutrophil-spec	0.033	1.023	9.22E-01	9.22E+01		
8149146	---	---	---	-0.082	0.945	2.17E-01	2.17E+01		

8149148	ENST0000042(	OR7E125P	olfactory receptor, family 7, subfa	0.002	1.001	9.90E-01	9.90E+01		
8149151	NR_027000	LOC349196	hypothetical LOC349196	-0.069	0.953	6.55E-01	6.55E+01		
8149153	NR_027000	LOC349196	hypothetical LOC349196	-0.059	0.960	7.03E-01	7.03E+01		
8149157	NR_027000	LOC349196	hypothetical LOC349196	-0.059	0.960	7.03E-01	7.03E+01		
8149161	NR_027000	LOC349196	hypothetical LOC349196	-0.037	0.974	8.10E-01	8.10E+01		
8149165	NR_027000	LOC349196	hypothetical LOC349196	-0.073	0.951	6.74E-01	6.74E+01		
8149167	NR_027000	LOC349196	hypothetical LOC349196	-0.073	0.951	6.74E-01	6.74E+01		
8149204	NM_018088	FAM90A1	family with sequence similarity 90	-0.039	0.973	7.52E-01	7.52E+01		
8149208	ENST00000420	OR7E125P	olfactory receptor, family 7, subfa	-0.019	0.987	9.36E-01	9.36E+01		
8149210	NR_027000	LOC349196	hypothetical LOC349196	-0.037	0.974	8.10E-01	8.10E+01		
8149214	NR_027000	LOC349196	hypothetical LOC349196	-0.069	0.953	6.55E-01	6.55E+01		
8149216	NR_027000	LOC349196	hypothetical LOC349196	-0.110	0.926	5.24E-01	5.24E+01		
8149218	NR_027000	LOC349196	hypothetical LOC349196	-0.077	0.948	6.76E-01	6.76E+01		
8149220	NR_027000	LOC349196	hypothetical LOC349196	-0.077	0.948	6.76E-01	6.76E+01		
8149222	NR_027000	LOC349196	hypothetical LOC349196	-0.077	0.948	6.76E-01	6.76E+01		
8149224	NR_027000	LOC349196	hypothetical LOC349196	-0.077	0.948	6.76E-01	6.76E+01		
8149226	NR_027000	LOC349196	hypothetical LOC349196	-0.077	0.948	6.76E-01	6.76E+01		
8149228	NR_027000	LOC349196	hypothetical LOC349196	-0.110	0.927	6.42E-01	6.42E+01		
8149241	NM_201402	USP17L2	ubiquitin specific peptidase 17-like	0.011	1.007	9.60E-01	9.60E+01		
8149243	NM_201402 //	P17L2 // USP17	ubiquitin specific peptidase 17-like	-0.026	0.982	8.78E-01	8.78E+01		
8149245	NR_003668	DEFB109P1B	defensin, beta 109, pseudogene 1	0.119	1.086	8.08E-01	8.08E+01		
8149248	---	---	---	0.026	1.018	9.74E-01	9.74E+01		
8149250	NM_00108082	PRAGMIN	homolog of rat pragma of Rnd2	-0.041	0.972	7.02E-01	7.02E+01		
8149256	---	---	---	-0.048	0.967	7.15E-01	7.15E+01		
8149258	NM_004225	MFHAS1	malignant fibrous histiocytoma am	-0.201	0.870	1.28E-01	1.28E+01		
8149264	NM_024607	PPP1R3B	protein phosphatase 1, regulatory	-0.249	0.842	1.68E-03	1.68E-01		
8149273	---	---	---	-0.074	0.950	8.30E-01	8.30E+01		
8149279	---	---	---	0.015	1.011	9.53E-01	9.53E+01		
8149283	NM_178857	RP1L1	retinitis pigmentosa 1-like 1	0.003	1.002	9.87E-01	9.87E+01		
8149289	NM_031439	SOX7	SRY (sex determining region Y)-bo	-0.054	0.963	6.66E-01	6.66E+01		
8149296	NM_017884	PINX1	PIN2-interacting protein 1	-0.009	0.993	9.75E-01	9.75E+01		
8149307	NM_173683	XKR6	XK, Kell blood group complex subu	-0.083	0.944	3.76E-01	3.76E+01		
8149315	BC132953	C8orf15	chromosome 8 open reading fram	0.061	1.043	8.14E-01	8.14E+01		
8149345	---	---	---	0.028	1.020	8.99E-01	8.99E+01		
8149356	NM_201402 //	P17L2 // USP17	ubiquitin specific peptidase 17-like	-0.026	0.982	8.86E-01	8.86E+01		
8149358	NR_003668	DEFB109P1B	defensin, beta 109, pseudogene 1	0.071	1.051	8.99E-01	8.99E+01		
8149361	NM_018088	FAM90A1	family with sequence similarity 90	-0.051	0.965	7.40E-01	7.40E+01		
8149365	NR_003494	FAM86B1	family with sequence similarity 86	0.004	1.003	9.83E-01	9.83E+01		
8149380	NR_003668	DEFB109P1B	defensin, beta 109, pseudogene 1	0.160	1.117	7.58E-01	7.58E+01		
8149383	NM_018088	FAM90A1	family with sequence similarity 90	-0.050	0.966	7.82E-01	7.82E+01		
8149387	NR_003494	FAM86B1	family with sequence similarity 86	0.105	1.075	6.80E-01	6.80E+01		
8149389	NR_003494	FAM86B1	family with sequence similarity 86	-0.014	0.990	9.45E-01	9.45E+01		
8149399	NM_152271	LONRF1	LON peptidase N-terminal domain	0.109	1.079	4.11E-01	4.11E+01		
8149465	NM_019851	FGF20	fibroblast growth factor 20	0.000	1.000	1.00E+00	1.00E+02		
8149475	NM_013354	CNOT7	CCR4-NOT transcription complex,	-0.005	0.997	9.79E-01	9.79E+01		
8149555	NM_015310	PSD3	pleckstrin and Sec7 domain contai	-0.100	0.933	7.38E-01	7.38E+01		
8149574	NM_018371	CSGALNACT1	chondroitin sulfate N-acetylgalact	-0.298	0.813	8.23E-02	8.23E+00		
8149590	---	---	---	-0.324	0.799	1.17E-01	1.17E+01		
8149612	NM_021020	LZTS1	leucine zipper, putative tumor sup	-0.044	0.970	7.51E-01	7.51E+01		
8149629	NM_001495	GFRA2	GDNF family receptor alpha 2	0.134	1.098	3.51E-01	3.51E+01		
8149638	NM_003974	DOK2	docking protein 2, 56kDa	0.316	1.245	4.74E-03	4.74E-01		
8149646	NM_024815	NUDT18	nudix (nucleoside diphosphate lin	-0.033	0.977	7.79E-01	7.79E+01		
8149652	NM_005144	HR	hairless homolog (mouse)	-0.008	0.995	9.53E-01	9.53E+01		
8149673	NM_025232	REEP4	receptor accessory protein 4	0.127	1.092	2.93E-01	2.93E+01		
8149685	NM_139278	LGI3	leucine-rich repeat LGI family, me	-0.003	0.998	9.86E-01	9.86E+01		
8149699	NM_00109933	PHYHIP	phytanoyl-CoA 2-hydroxylase inte	-0.035	0.976	7.99E-01	7.99E+01		
8149705	---	---	---	-0.052	0.964	7.14E-01	7.14E+01		
8149707	NM_018688	BIN3	bridging integrator 3	-0.094	0.937	4.59E-01	4.59E+01		
8149720	NM_004430	EGR3	early growth response 3	0.040	1.028	8.31E-01	8.31E+01		
8149733	NM_003842	TNFRSF10B	tumor necrosis factor receptor su	-0.012	0.992	9.57E-01	9.57E+01		
8149749	NM_003840	TNFRSF10D	tumor necrosis factor receptor su	-0.023	0.984	8.96E-01	8.96E+01		
8149762	NM_003844	TNFRSF10A	tumor necrosis factor receptor su	0.123	1.089	3.81E-01	3.81E+01		
8149774	NM_002318	LOXL2	lysyl oxidase-like 2	-0.062	0.958	5.95E-01	5.95E+01		
8149793	NM_004901	ENTPD4	ectonucleoside triphosphate dipho	-0.045	0.970	7.50E-01	7.50E+01		
8149809	---	---	---	-0.205	0.868	3.76E-02	3.76E+00		
8149811	NM_006167	NKX3-1	NK3 homeobox 1	-0.047	0.968	6.29E-01	6.29E+01		
8149820	NM_00113627	NKX2-6	NK2 transcription factor related, lo	-0.056	0.962	6.11E-01	6.11E+01		
8149833	---	---	---	-0.072	0.951	4.71E-01	4.71E+01		
8149835	NM_006158	NEFL	neurofilament, light polypeptide	-0.041	0.972	7.93E-01	7.93E+01		
8149847	---	---	---	0.007	1.005	9.72E-01	9.72E+01		
8149849	NM_000825	GNRH1	gonadotropin-releasing hormone	-0.075	0.949	6.95E-01	6.95E+01		
8149857	NM_017634	KCTD9	potassium channel tetramerisatio	-0.016	0.989	9.23E-01	9.23E+01		
8149898	NM_030795	STMN4	stathmin-like 4	0.014	1.010	9.27E-01	9.27E+01		
8149907	NM_171982	TRIM35	tripartite motif-containing 35	0.002	1.002	9.88E-01	9.88E+01		
8149918	NM_000742	CHRNA2	cholinergic receptor, nicotinic, alp	-0.014	0.990	9.29E-01	9.29E+01		
8149927	NM_001831	CLU	clusterin	-0.114	0.924	4.91E-01	4.91E+01		
8149942	NM_018246	CCDC25	coiled-coil domain containing 25	0.061	1.043	7.45E-01	7.45E+01		

8149966	NM_173833	SCARA5	scavenger receptor class A, memb	-0.068	0.954	6.39E-01	6.39E+01		
8149986	NM_018660	ZNF395	zinc finger protein 395	-0.061	0.958	5.71E-01	5.71E+01		
8150014	NM_018250	INTS9	integrator complex subunit 9	0.116	1.084	3.16E-01	3.16E+01		
8150032	---	---	---	0.008	1.005	9.79E-01	9.79E+01		
8150034	---	---	---	0.102	1.073	8.46E-01	8.46E+01		
8150036	NM_015254	KIF13B	kinesin family member 13B	0.003	1.002	9.89E-01	9.89E+01		
8150087	---	---	---	-0.040	0.973	8.48E-01	8.48E+01		
8150089	NM_016127	TMEM66	transmembrane protein 66	-0.043	0.971	6.00E-01	6.00E+01		
8150099	NM_00110091	MBOAT4	membrane bound O-acyltransfera	-0.146	0.904	1.70E-01	1.70E+01		
8150101	---	---	---	0.028	1.019	9.63E-01	9.63E+01		
8150103	NM_002095	GTF2E2	general transcription factor IIE, po	0.097	1.069	4.51E-01	4.51E+01		
8150112	NM_000637	GSR	glutathione reductase	0.150	1.109	3.74E-01	3.74E+01		
8150126	NM_00100955	PPP2CB	protein phosphatase 2 (formerly 2	-0.013	0.991	9.63E-01	9.63E+01		
8150149	---	---	---	-0.055	0.963	8.23E-01	8.23E+01		
8150165	NM_032664	FUT10	fucosyltransferase 10 (alpha (1,3)	-0.049	0.967	8.07E-01	8.07E+01		
8150175	NM_025115	C8orf41	chromosome 8 open reading fram	0.003	1.002	9.90E-01	9.90E+01		
8150186	NM_024787	RNF122	ring finger protein 122	0.092	1.066	6.51E-01	6.51E+01		
8150197	NM_024025	DUSP26	dual specificity phosphatase 26 (p	-0.020	0.986	9.12E-01	9.12E+01		
8150204	---	---	---	-0.048	0.967	8.16E-01	8.16E+01		
8150206	NM_152344	LSM12	LSM12 homolog (S. cerevisiae)	0.059	1.042	5.35E-01	5.35E+01		
8150214	---	---	---	-0.031	0.979	8.66E-01	8.66E+01		
8150217	NR_003671	hCG_1640171	hCG1640171	-0.121	0.919	5.98E-01	5.98E+01		
8150219	NM_018310	BRF2	BRF2, subunit of RNA polymerase	-0.010	0.993	9.63E-01	9.63E+01		
8150225	NM_00100281	RAB11FIP1	RAB11 family interacting protein 1	0.001	1.001	9.92E-01	9.92E+01		
8150244	NM_152413	GOT1L1	glutamic-oxaloacetic transaminase	-0.073	0.951	6.48E-01	6.48E+01		
8150249	NM_000025	ADRB3	adrenergic, beta-3-, receptor	-0.021	0.986	9.15E-01	9.15E+01		
8150253	NM_000349	STAR	steroidogenic acute regulatory pro	0.001	1.001	9.95E-01	9.95E+01		
8150266	NM_014462	LSM1	LSM1 homolog, U6 small nuclear R	0.061	1.043	7.57E-01	7.57E+01		
8150274	---	---	---	0.052	1.037	6.76E-01	6.76E+01		
8150276	NM_00110255	PPAPDC1B	phosphatidic acid phosphatase typ	0.031	1.022	9.08E-01	9.08E+01		
8150287	NM_023034	WHSC1L1	Wolf-Hirschhorn syndrome candid	0.009	1.006	9.61E-01	9.61E+01		
8150318	NM_023110	FGFR1	fibroblast growth factor receptor 1	-0.022	0.985	9.23E-01	9.23E+01		
8150356	NR_003129	RNF5P1	ring finger protein 5 pseudogene 1	-0.028	0.981	8.95E-01	8.95E+01		
8150428	NM_003012	SFRP1	secreted frizzled-related protein 1	-0.014	0.990	9.30E-01	9.30E+01		
8150433	NM_152568	NKX6-3	NK6 homeobox 3	0.033	1.023	7.81E-01	7.81E+01		
8150439	NM_020476	ANK1	ankyrin 1, erythrocytic	-0.042	0.971	6.35E-01	6.35E+01		
8150489	---	---	---	0.046	1.032	7.68E-01	7.68E+01		
8150491	NM_00109941	MYST3	MYST histone acetyltransferase (m	-0.122	0.919	2.61E-01	2.61E+01		
8150509	NM_000930	PLAT	plasminogen activator, tissue	-0.070	0.953	4.93E-01	4.93E+01		
8150537	NM_006749	SLC20A2	solute carrier family 20 (phosphat	-0.070	0.952	5.44E-01	5.44E+01		
8150559	NM_018105	THAP1	THAP domain containing, apoptos	0.034	1.024	8.65E-01	8.65E+01		
8150565	NM_00116022	RNF170	ring finger protein 170	0.161	1.118	2.53E-01	2.53E+01		
8150576	---	---	---	0.038	1.027	9.18E-01	9.18E+01		
8150578	---	---	---	0.031	1.022	7.88E-01	7.88E+01		
8150592	NM_005195	CEBPD	CCAAT/enhancer binding protein (	-0.009	0.994	9.61E-01	9.61E+01		
8150599	NM_006904	PRKDC	protein kinase, DNA-activated, cat	0.062	1.044	6.04E-01	6.04E+01		
8150689	---	---	---	-0.056	0.962	8.84E-01	8.84E+01		
8150714	NM_052937	PCMTD1	protein-L-isoaspartate (D-aspartat	-0.011	0.993	9.52E-01	9.52E+01		
8150757	NM_014781	RB1CC1	RB1-inducible coiled-coil 1	-0.037	0.975	8.60E-01	8.60E+01		
8150797	NM_015941	ATP6V1H	ATPase, H+ transporting, lysosoma	0.008	1.006	9.82E-01	9.82E+01		
8150818	NM_006756	TCEA1	transcription elongation factor A (	0.015	1.010	9.00E-01	9.00E+01		
8150830	NM_006330	LYPLA1	lysophospholipase I	0.062	1.044	7.60E-01	7.60E+01		
8150842	---	---	---	-0.272	0.828	1.81E-03	1.81E-01		
8150844	---	---	---	0.008	1.006	9.67E-01	9.67E+01		
8150860	---	---	---	-0.004	0.998	9.86E-01	9.86E+01		
8150862	---	---	---	0.408	1.327	1.07E-01	1.07E+01		
8150864	---	---	---	0.032	1.022	8.45E-01	8.45E+01		
8150866	---	---	---	0.212	1.158	4.71E-01	4.71E+01		
8150870	NM_002806	PSMC6	proteasome (prosome, macropain	0.056	1.039	7.86E-01	7.86E+01		
8150872	NM_001023	RPS20	ribosomal protein S20	-0.016	0.989	8.99E-01	8.99E+01		
8150877	NR_002437	SNORD54	small nucleolar RNA, C/D box 54	-0.139	0.908	7.68E-01	7.68E+01		
8150881	NM_002655	PLAG1	pleiomorphic adenoma gene 1	0.023	1.016	9.49E-01	9.49E+01		
8150901	NM_006211	PENK	proenkephalin	-0.037	0.975	7.73E-01	7.73E+01		
8150906	NM_017813	IMPAD1	inositol monophosphatase domair	0.123	1.089	5.49E-01	5.49E+01		
8150908	NM_017813	IMPAD1	inositol monophosphatase domair	0.074	1.053	5.08E-01	5.08E+01		
8150928	NM_003580	NSMAF	neutral sphingomyelinase (N-SMa	0.081	1.058	6.34E-01	6.34E+01		
8150960	---	---	---	0.018	1.013	9.24E-01	9.24E+01		
8150962	NM_014729	TOX	thymocyte selection-associated hi	0.299	1.231	3.32E-02	3.32E+00		
8150988	NM_004318	ASPH	aspartate beta-hydroxylase	-0.125	0.917	6.78E-01	6.78E+01		
8151054	---	---	---	-0.077	0.948	2.84E-01	2.84E+01		
8151056	NM_004820	CYP7B1	cytochrome P450, family 7, subfar	-0.054	0.964	7.33E-01	7.33E+01		
8151066	NM_018120	ARMC1	armadillo repeat containing 1	0.100	1.072	5.52E-01	5.52E+01		
8151074	NM_002603	PDE7A	phosphodiesterase 7A	0.172	1.126	2.35E-02	2.35E+00		
8151099	---	---	---	0.053	1.037	8.25E-01	8.25E+01		
8151101	NM_00108041	MYBL1	v-myb myeloblastosis viral oncoge	-0.081	0.945	8.82E-01	8.82E+01		
8151118	NM_025054	VCPIP1	valosin containing protein (p97)/p	0.071	1.050	3.51E-01	3.51E+01		
8151123	NR_002734	PTTG3P	pituitary tumor-transforming 3 (ps	0.073	1.052	7.94E-01	7.94E+01		

8151125	ENST00000340	TCF24	transcription factor 24	-0.029	0.980	8.21E-01	8.21E+01		
8151136	NM_006837	COPS5	COP9 constitutive photomorphoge	-0.026	0.982	8.80E-01	8.80E+01		
8151149	NM_006421	ARFGEF1	ADP-ribosylation factor guanine nu	0.000	1.000	1.00E+00	1.00E+02		
8151209	---	---	---	0.153	1.112	5.89E-01	5.89E+01		
8151223	NM_030958	SLCO5A1	solute carrier organic anion transp	-0.179	0.883	1.37E-01	1.37E+01		
8151252	---	---	---	-0.147	0.903	2.74E-01	2.74E+01		
8151254	NM_006540	NCOA2	nuclear receptor coactivator 2	-0.026	0.982	8.98E-01	8.98E+01		
8151281	NM_014294	TRAM1	translocation associated membrar	0.195	1.145	6.78E-03	6.78E-01		
8151296	NM_016027	LACTB2	lactamase, beta 2	0.196	1.146	9.57E-02	9.57E+00		
8151369	NM_153225	C8orf84	chromosome 8 open reading fram	0.031	1.021	8.86E-01	8.86E+01		
8151376	NM_000971	RPL7	ribosomal protein L7	-0.025	0.983	8.82E-01	8.82E+01		
8151384	NM_014393	STAU2	staufen, RNA binding protein, hom	-0.032	0.978	8.13E-01	8.13E+01		
8151411	---	---	---	-0.031	0.979	8.18E-01	8.18E+01		
8151413	NM_005648	TCEB1	transcription elongation factor B (	0.074	1.052	5.48E-01	5.48E+01		
8151421	---	---	---	0.379	1.300	2.99E-01	2.99E+01		
8151436	NM_000318	PXMP3	peroxisomal membrane protein 3,	0.123	1.089	3.58E-01	3.58E+01		
8151471	NM_014018	MRPS28	mitochondrial ribosomal protein S	-0.034	0.976	9.00E-01	9.00E+01		
8151475	NM_00102525	TPD52	tumor protein D52	0.103	1.074	5.08E-01	5.08E+01		
8151512	NM_018440	PAG1	phosphoprotein associated with g	-0.118	0.922	1.97E-01	1.97E+01		
8151540	---	---	---	-0.018	0.988	9.58E-01	9.58E+01		
8151549	NM_005536	IMPA1	inositol(myo)-1(or 4)-monophosph	-0.040	0.972	7.81E-01	7.81E+01		
8151561	NM_024699	ZFAND1	zinc finger, AN1-type domain 1	0.002	1.002	9.95E-01	9.95E+01		
8151603	NR_003594	REXO1L2P	REX1, RNA exonuclease 1 homolog	-0.008	0.994	9.54E-01	9.54E+01		
8151605	AF495523	REXO1L1	REX1, RNA exonuclease 1 homolog	-0.177	0.885	1.78E-01	1.78E+01		
8151607	NR_003594	REXO1L2P	REX1, RNA exonuclease 1 homolog	-0.008	0.994	9.54E-01	9.54E+01		
8151609	NM_172239	REXO1L1	REX1, RNA exonuclease 1 homolog	-0.038	0.974	6.21E-01	6.21E+01		
8151613	AF495523	REXO1L1	REX1, RNA exonuclease 1 homolog	-0.177	0.885	1.78E-01	1.78E+01		
8151615	0	0	0	0.000	1.000	1.00E+00	1.00E+02		
8151617	AF495523 // A	// REXO1L1 //	REX1, RNA exonuclease 1 homolog	-0.177	0.885	1.78E-01	1.78E+01		
8151619	NR_003594	REXO1L2P	REX1, RNA exonuclease 1 homolog	-0.013	0.991	9.19E-01	9.19E+01		
8151621	AF495523	REXO1L1	REX1, RNA exonuclease 1 homolog	-0.177	0.885	1.78E-01	1.78E+01		
8151623	NR_003594	REXO1L2P	REX1, RNA exonuclease 1 homolog	-0.008	0.994	9.54E-01	9.54E+01		
8151625	AF495523	REXO1L1	REX1, RNA exonuclease 1 homolog	-0.177	0.885	1.78E-01	1.78E+01		
8151627	0	0	0	-0.009	0.994	9.58E-01	9.58E+01		
8151629	0	0	0	-0.146	0.904	5.55E-01	5.55E+01		
8151631	NR_003594	REXO1L2P	REX1, RNA exonuclease 1 homolog	0.001	1.001	9.96E-01	9.96E+01		
8151709	---	---	---	0.235	1.177	5.95E-01	5.95E+01		
8151711	NM_002485	NBN	nibrin	0.033	1.023	9.49E-01	9.49E+01		
8151747	NM_00100849	TMEM64	transmembrane protein 64	-0.028	0.981	8.33E-01	8.33E+01		
8151784	---	---	---	-0.013	0.991	9.49E-01	9.49E+01		
8151788	NM_203390	RBM12B	RNA binding motif protein 12B	0.061	1.043	5.34E-01	5.34E+01		
8151816	NM_005261	GEM	GTP binding protein overexpressed	-0.078	0.947	7.54E-01	7.54E+01		
8151842	NM_015496	KIAA1429	KIAA1429	-0.018	0.987	9.30E-01	9.30E+01		
8151869	---	---	---	0.018	1.012	9.49E-01	9.49E+01		
8151890	NM_033285	TP53INP1	tumor protein p53 inducible nucle	0.141	1.103	1.35E-01	1.35E+01		
8151906	NM_00100155	GDF6	growth differentiation factor 6	-0.009	0.994	9.55E-01	9.55E+01		
8151927	---	---	---	0.214	1.160	5.35E-01	5.35E+01		
8151929	---	---	---	-0.059	0.960	8.06E-01	8.06E+01		
8151931	NM_033512	TSPYL5	TSPY-like 5	0.312	1.242	4.45E-02	4.45E+00		
8151935	NM_000989	RPL30	ribosomal protein L30	0.009	1.006	9.74E-01	9.74E+01		
8151987	---	---	---	0.091	1.065	6.58E-01	6.58E+01		
8151991	---	---	---	0.009	1.006	9.89E-01	9.89E+01		
8151993	NM_004374	COX6C	cytochrome c oxidase subunit VIc	0.027	1.019	8.89E-01	8.89E+01		
8152041	NM_183419	RNF19A	ring finger protein 19A	0.006	1.004	9.84E-01	9.84E+01		
8152053	NM_198401	ANKRD46	ankyrin repeat domain 46	0.031	1.021	9.11E-01	9.11E+01		
8152079	NM_002568	PABPC1	poly(A) binding protein, cytoplasm	-0.022	0.985	9.12E-01	9.12E+01		
8152096	NM_145690	YWHAZ	tyrosine 3-monooxygenase/trypto	0.040	1.028	4.27E-01	4.27E+01		
8152111	NM_00104251	ZNF706	zinc finger protein 706	0.211	1.158	7.00E-02	7.00E+00		
8152117	---	---	---	-0.003	0.998	9.88E-01	9.88E+01		
8152119	NM_00104062	NCALD	neurocalcin delta	0.010	1.007	9.78E-01	9.78E+01		
8152133	NM_015713	RRM2B	ribonucleotide reductase M2 B (TP	0.085	1.061	5.53E-01	5.53E+01		
8152148	NM_015902	UBR5	ubiquitin protein ligase E3 compor	-0.052	0.964	6.87E-01	6.87E+01		
8152211	---	---	---	0.166	1.122	6.80E-01	6.80E+01		
8152215	NM_005655	KLF10	Kruppel-like factor 10	-0.038	0.974	8.33E-01	8.33E+01		
8152222	NM_015878	AZIN1	antizyme inhibitor 1	0.056	1.040	7.41E-01	7.41E+01		
8152240	---	---	---	0.087	1.062	7.04E-01	7.04E+01		
8152255	NM_030780	SLC25A32	solute carrier family 25, member 3	0.082	1.058	4.96E-01	4.96E+01		
8152291	NM_139166	ABRA	actin-binding Rho activating prote	0.126	1.091	2.57E-01	2.57E+01		
8152323	NM_001568	EIF3E	eukaryotic translation initiation fa	-0.032	0.978	8.78E-01	8.78E+01		
8152333	---	---	---	-0.108	0.928	6.46E-01	6.46E+01		
8152369	NM_014379	KCNV1	potassium channel, subfamily V, m	-0.071	0.952	3.46E-01	3.46E+01		
8152453	NM_014112	TRPS1	trichorhinophalangeal syndrome I	0.026	1.018	9.49E-01	9.49E+01		
8152465	NM_003756	EIF3H	eukaryotic translation initiation fa	0.020	1.014	8.88E-01	8.88E+01		
8152477	NM_006265	RAD21	RAD21 homolog (S. pombe)	-0.037	0.975	6.57E-01	6.57E+01		
8152491	NM_000127	EXT1	exostoses (multiple) 1	-0.196	0.873	4.36E-02	4.36E+00		
8152553	NM_003184	TAF2	TAF2 RNA polymerase II, TATA box	0.034	1.024	8.49E-01	8.49E+01		
8152606	NM_021021	SNTB1	syntrophin, beta 1 (dystrophin-ass	0.019	1.013	9.16E-01	9.16E+01		

8152626	---	---	---	-0.055	0.963	9.00E-01	9.00E+01		
8152628	NM_024295	DERL1	Der1-like domain family, member	0.050	1.035	6.76E-01	6.76E+01		
8152648	BC067796	C8orf76	chromosome 8 open reading fram	0.170	1.125	6.47E-02	6.47E+00		
8152656	NM_00101792	ZHX1	zinc fingers and homeoboxes 1	0.165	1.121	6.06E-02	6.06E+00		
8152666	---	---	---	0.201	1.150	2.84E-01	2.84E+01		
8152668	NM_014109	ATAD2	ATPase family, AAA domain contai	0.111	1.080	4.51E-01	4.51E+01		
8152701	---	---	---	0.029	1.021	9.17E-01	9.17E+01		
8152703	NM_058229	FBXO32	F-box protein 32	-0.036	0.975	9.16E-01	9.16E+01		
8152750	NM_194291	TMEM65	transmembrane protein 65	0.053	1.037	7.40E-01	7.40E+01		
8152759	NR_027427	TATDN1	TatD DNase domain containing 1	-0.025	0.983	8.89E-01	8.89E+01		
8152764	NM_014751	MTSS1	metastasis suppressor 1	-0.197	0.872	3.28E-01	3.28E+01		
8152782	NM_014846	KIAA0196	KIAA0196	0.133	1.097	4.71E-01	4.71E+01		
8152812	NM_174911	FAM84B	family with sequence similarity 84	0.143	1.104	3.56E-01	3.56E+01		
8152815	BC137383	TMEM75	transmembrane protein 75	-0.065	0.956	7.64E-01	7.64E+01		
8152819	---	---	---	-0.082	0.945	6.57E-01	6.57E+01		
8152845	BC017297	FAM49B	family with sequence similarity 49	0.274	1.209	7.65E-03	7.65E-01		
8152863	---	---	---	0.132	1.096	4.96E-01	4.96E+01		
8152865	---	---	---	0.215	1.161	9.93E-02	9.93E+00		
8152867	NM_018482	ASAP1	ArfGAP with SH3 domain, ankyrin	0.133	1.097	5.23E-01	5.23E+01		
8152900	---	---	---	0.342	1.268	2.67E-01	2.67E+01		
8152902	NM_001115	ADCY8	adenylate cyclase 8 (brain)	-0.021	0.986	9.10E-01	9.10E+01		
8152924	NM_00108039	OC90	otoconin 90	-0.010	0.993	9.55E-01	9.55E+01		
8152938	NM_00114509	HHLA1	HERV-H LTR-associating 1	-0.023	0.984	8.47E-01	8.47E+01		
8152946	NM_004519	KCNQ3	potassium voltage-gated channel,	-0.035	0.976	7.56E-01	7.56E+01		
8152976	NM_144649	TMEM71	transmembrane protein 71	0.115	1.083	6.80E-01	6.80E+01		
8152988	NM_00104555	SLA	Src-like-adaptor	0.048	1.034	8.48E-01	8.48E+01		
8153021	NM_003033	ST3GAL1	ST3 beta-galactoside alpha-2,3-sia	-0.134	0.911	5.87E-01	5.87E+01		
8153039	---	---	---	0.136	1.099	6.56E-01	6.56E+01		
8153041	---	---	---	-0.075	0.949	7.03E-01	7.03E+01		
8153043	NM_020863	ZFAT	zinc finger and AT hook domain co	0.032	1.022	8.65E-01	8.65E+01		
8153101	NM_152888	COL22A1	collagen, type XXII, alpha 1	-0.031	0.979	7.71E-01	7.71E+01		
8153167	NM_016601	KCNK9	potassium channel, subfamily K, m	0.039	1.027	7.85E-01	7.85E+01		
8153175	NM_031466	TRAPPC9	trafficking protein particle comple	-0.073	0.950	5.99E-01	5.99E+01		
8153201	NM_012154	EIF2C2	eukaryotic translation initiation fa	-0.119	0.921	1.52E-01	1.52E+01		
8153223	NM_153831	PTK2	PTK2 protein tyrosine kinase 2	0.020	1.014	9.53E-01	9.53E+01		
8153258	BC033223	SLC45A4	solute carrier family 45, member 4	0.026	1.018	9.56E-01	9.56E+01		
8153269	NM_005293	GPR20	G protein-coupled receptor 20	-0.029	0.980	8.24E-01	8.24E+01		
8153273	NM_207414	FLJ43860	FLJ43860 protein	0.030	1.021	8.27E-01	8.27E+01		
8153304	NM_145003	TSNARE1	t-SNARE domain containing 1	-0.087	0.942	3.59E-01	3.59E+01		
8153320	---	---	---	0.045	1.032	6.58E-01	6.58E+01		
8153322	NM_015193	ARC	activity-regulated cytoskeleton-as	-0.046	0.968	6.62E-01	6.62E+01		
8153328	NM_003724	JRK	jerky homolog (mouse)	-0.022	0.985	9.00E-01	9.00E+01		
8153334	NM_005672	PSCA	prostate stem cell antigen	0.031	1.022	8.36E-01	8.36E+01		
8153336	NM_020427	SLURP1	secreted LY6/PLAUR domain conta	0.041	1.028	7.23E-01	7.23E+01		
8153342	NM_205545	LYPD2	LY6/PLAUR domain containing 2	-0.001	1.000	9.98E-01	9.98E+01		
8153346	NM_023946	LYNX1	Ly6/neurotoxin 1	-0.008	0.994	9.53E-01	9.53E+01		
8153359	NM_003695	LY6D	lymphocyte antigen 6 complex, loc	-0.050	0.966	6.02E-01	6.02E+01		
8153363	NM_000497	CYP11B1	cytochrome P450, family 11, subfa	0.011	1.008	9.53E-01	9.53E+01		
8153373	NM_000498	CYP11B2	cytochrome P450, family 11, subfa	0.014	1.009	9.34E-01	9.34E+01		
8153380	NM_00113565	LY6H	lymphocyte antigen 6 complex, loc	-0.018	0.988	9.19E-01	9.19E+01		
8153390	NM_052963	TOP1MT	topoisomerase (DNA) I, mitochond	0.089	1.064	4.75E-01	4.75E+01		
8153405	NR_026785	C8orf51	chromosome 8 open reading fram	-0.008	0.994	9.67E-01	9.67E+01		
8153409	NM_201589	MAFA	v-maf musculoaponeurotic fibrosa	0.013	1.009	9.53E-01	9.53E+01		
8153411	NM_015117	ZC3H3	zinc finger CCCH-type containing 3	0.101	1.073	1.31E-01	1.31E+01		
8153424	NM_00110087	C8orf73	chromosome 8 open reading fram	-0.085	0.943	7.13E-01	7.13E+01		
8153426	NM_00110087	C8orf73	chromosome 8 open reading fram	-0.057	0.961	8.04E-01	8.04E+01		
8153430	NM_145201	NAPRT1	nicotinate phosphoribosyltransfer	0.015	1.011	9.36E-01	9.36E+01		
8153449	NM_032378	EEF1D	eukaryotic translation elongation f	0.004	1.003	9.83E-01	9.83E+01		
8153457	AY358690	EEF1D	eukaryotic translation elongation f	-0.181	0.882	1.34E-01	1.34E+01		
8153459	NM_023078	PYCRL	pyrroline-5-carboxylate reductase-	-0.029	0.980	7.94E-01	7.94E+01		
8153474	NM_003313	TSTA3	tissue specific transplantation anti	0.019	1.014	9.16E-01	9.16E+01		
8153486	NM_00116291	LOC100130274	coiled-coil domain containing 121-	0.012	1.008	9.56E-01	9.56E+01		
8153488	NM_198488	FAM83H	family with sequence similarity 83	-0.047	0.968	6.16E-01	6.16E+01		
8153497	NM_182706	SCRIB	scribbled homolog (Drosophila)	-0.076	0.949	2.77E-01	2.77E+01		
8153536	NM_00113603	PUF60	poly-U binding splicing factor 60KD	0.018	1.012	8.97E-01	8.97E+01		
8153550	NM_178564	NRBP2	nuclear receptor binding protein 2	-0.056	0.962	6.91E-01	6.91E+01		
8153568	NM_201384	PLEC1	plectin 1, intermediate filament bi	-0.121	0.920	2.86E-03	2.86E-01		
8153609	NM_032789	PARP10	poly (ADP-ribose) polymerase fam	-0.035	0.976	7.40E-01	7.40E+01		
8153625	NM_017570	OPLAH	5-oxoprolinase (ATP-hydrolysing)	-0.043	0.971	6.88E-01	6.88E+01		
8153652	NM_030974	SHARPIN	SHANK-associated RH domain inte	-0.003	0.998	9.83E-01	9.83E+01		
8153664	NM_015201	BOP1	block of proliferation 1	-0.039	0.974	6.84E-01	6.84E+01		
8153678	NM_015201	BOP1	block of proliferation 1	-0.134	0.911	3.21E-02	3.21E+00		
8153684	NM_012079	DGAT1	diacylglycerol O-acyltransferase ho	-0.017	0.988	9.24E-01	9.24E+01		
8153709	NM_012162	FBXL6	F-box and leucine-rich repeat prot	-0.089	0.940	3.48E-01	3.48E+01		
8153727	NM_013291	CPSF1	cleavage and polyadenylation spec	-0.043	0.971	7.18E-01	7.18E+01		
8153762	NM_017767	SLC39A4	solute carrier family 39 (zinc trans	-0.004	0.998	9.82E-01	9.82E+01		
8153776	NM_183057	VPS28	vacuolar protein sorting 28 homol	-0.006	0.996	9.76E-01	9.76E+01		

8153790	NM_013432	NFKBIL2	nuclear factor of kappa light polyp	-0.033	0.978	7.32E-01	7.32E+01		
8153823	NM_032687	CYHR1	cysteine/histidine-rich 1	-0.041	0.972	7.47E-01	7.47E+01		
8153828	NM_003923	FOXH1	forkhead box H1	0.040	1.028	7.97E-01	7.97E+01		
8153835	NM_032902	PPP1R16A	protein phosphatase 1, regulatory	-0.062	0.958	6.27E-01	6.27E+01		
8153838	NM_004260	RECQL4	RecQ protein-like 4	-0.032	0.978	7.78E-01	7.78E+01		
8153862	NM_00102467	LRRC24	leucine rich repeat containing 24	-0.053	0.964	5.87E-01	5.87E+01		
8153868	BC073936	C8orf82	chromosome 8 open reading fram	-0.080	0.946	3.06E-01	3.06E+01		
8153876	NM_025251	KIAA1688	KIAA1688 protein	-0.006	0.996	9.72E-01	9.72E+01		
8153890	NM_138367	ZNF251	zinc finger protein 251	0.004	1.003	9.85E-01	9.85E+01		
8153903	NM_000973	RPL8	ribosomal protein L8	-0.011	0.993	9.66E-01	9.66E+01		
8153911	NM_014066	COMMD5	COMM domain containing 5	-0.059	0.960	4.04E-01	4.04E+01		
8153920	NM_021061	ZNF250	zinc finger protein 250	-0.045	0.970	7.73E-01	7.73E+01		
8153937	NM_031309	SCRT1	scratch homolog 1, zinc finger pro	-0.003	0.998	9.88E-01	9.88E+01		
8153939	NM_012079	DGAT1	diacylglycerol O-acyltransferase ho	-0.017	0.988	9.24E-01	9.24E+01		
8153959	NM_203447	DOCK8	dedicator of cytokinesis 8	0.061	1.043	6.47E-01	6.47E+01		
8154059	NM_003070	SMARCA2	SWI/SNF related, matrix associate	-0.032	0.978	8.03E-01	8.03E+01		
8154100	NM_003383	VLDLR	very low density lipoprotein recep	-0.141	0.907	2.74E-01	2.74E+01		
8154128	DQ246450	DSERG1	Down syndrome encephalopathy r	0.036	1.025	9.23E-01	9.23E+01		
8154130	---	---	---	0.034	1.024	9.05E-01	9.05E+01		
8154151	NM_203453	PPAPDC2	phosphatidic acid phosphatase typ	0.090	1.065	5.09E-01	5.09E+01		
8154153	NM_017913	CDC37L1	cell division cycle 37 homolog (S. c	-0.028	0.981	8.78E-01	8.78E+01		
8154163	NM_005772	RCL1	RNA terminal phosphate cyclase-li	-0.070	0.952	6.80E-01	6.80E+01		
8154211	---	---	---	0.499	1.414	2.45E-01	2.45E+01		
8154254	NM_020829	KIAA1432	KIAA1432	0.195	1.145	9.17E-02	9.17E+00		
8154285	NM_005511	MLANA	melan-A	0.074	1.052	7.09E-01	7.09E+01		
8154305	NM_016275	SELT	selenoprotein T	0.092	1.066	4.82E-01	4.82E+01		
8154316	NM_152896	UHRF2	ubiquitin-like with PHD and ring fi	0.029	1.020	8.69E-01	8.69E+01		
8154333	NM_015061	KDM4C	lysine (K)-specific demethylase 4C	0.025	1.018	8.80E-01	8.80E+01		
8154357	---	---	---	0.006	1.004	9.66E-01	9.66E+01		
8154359	AY779046	RPL18A	ribosomal protein L18a	-0.010	0.993	9.63E-01	9.63E+01		
8154363	NM_001029	RPS26	ribosomal protein S26	0.039	1.027	9.35E-01	9.35E+01		
8154388	---	---	---	0.024	1.017	8.76E-01	8.76E+01		
8154392	---	---	---	0.002	1.001	9.96E-01	9.96E+01		
8154394	NM_00103969	SNAPC3	small nuclear RNA activating comp	0.060	1.042	6.21E-01	6.21E+01		
8154476	NM_003026	SH3GL2	SH3-domain GRB2-like 2	-0.057	0.962	6.43E-01	6.43E+01		
8154512	NM_00104027	ADAMTSL1	ADAMTS-like 1	-0.137	0.910	6.23E-02	6.23E+00		
8154523	NM_006570	RRAGA	Ras-related GTP binding A	0.004	1.002	9.88E-01	9.88E+01		
8154527	---	---	---	0.100	1.071	4.94E-01	4.94E+01		
8154531	NM_017925	DENND4C	DENN/MADD domain containing 4	0.035	1.025	8.66E-01	8.66E+01		
8154572	---	---	---	-0.410	0.753	2.15E-01	2.15E+01		
8154574	NM_017794	KIAA1797	KIAA1797	0.054	1.038	7.50E-01	7.50E+01		
8154620	---	---	---	-0.117	0.922	3.18E-01	3.18E+01		
8154635	NM_002451	MTAP	methylthioadenosine phosphoryla	0.012	1.009	9.60E-01	9.60E+01		
8154654	NR_024274	C9orf53	chromosome 9 open reading fram	0.064	1.046	6.42E-01	6.42E+01		
8154725	NM_000224	KRT18	keratin 18	-0.031	0.979	9.53E-01	9.53E+01		
8154727	AF091236	LOC138412	solute carrier family 25 (mitochon	-0.006	0.996	9.80E-01	9.80E+01		
8154733	NM_002197	ACO1	aconitase 1, soluble	-0.108	0.928	4.04E-01	4.04E+01		
8154760	NM_212558	TMEM215	transmembrane protein 215	0.006	1.004	9.73E-01	9.73E+01		
8154763	---	---	---	-0.039	0.973	9.19E-01	9.19E+01		
8154765	NM_001539	DNAJA1	DnaJ (Hsp40) homolog, subfamily	-0.001	0.999	9.95E-01	9.95E+01		
8154785	NM_016410	CHMP5	chromatin modifying protein 5	0.167	1.122	3.06E-01	3.06E+01		
8154793	NM_002504	NFX1	nuclear transcription factor, X-box	0.047	1.033	7.58E-01	7.58E+01		
8154836	NR_003573 //	// ANXA2P2 //	annexin A2 pseudogene 2 // anne	0.198	1.147	3.04E-01	3.04E+01		
8154838	ENST00000331	TRBV21OR9-2	T cell receptor beta variable 21/O	-0.016	0.989	9.47E-01	9.47E+01		
8154841	ENST00000390	TRBV23OR9-2	T cell receptor beta variable 23/O	-0.091	0.939	6.51E-01	6.51E+01		
8154848	NM_007343	PRSS3	protease, serine, 3	-0.065	0.956	4.71E-01	4.71E+01		
8154856	NM_017811	UBE2R2	ubiquitin-conjugating enzyme E2R	-0.016	0.989	9.53E-01	9.53E+01		
8154866	---	---	---	0.110	1.079	7.03E-01	7.03E+01		
8154868	---	---	---	0.037	1.026	9.24E-01	9.24E+01		
8154870	---	---	---	-0.122	0.919	3.38E-01	3.38E+01		
8154872	NM_016525	UBAP1	ubiquitin associated protein 1	-0.097	0.935	2.92E-01	2.92E+01		
8154885	NM_001161	NUDT2	nudix (nucleoside diphosphate lin	-0.044	0.970	8.33E-01	8.33E+01		
8154916	NM_000155	GALT	galactose-1-phosphate uridylyltra	0.000	1.000	1.00E+00	1.00E+02		
8154934	NM_147162	IL11RA	interleukin 11 receptor, alpha	0.051	1.036	6.81E-01	6.81E+01		
8154962	NM_00113500	DNAJB5	DnaJ (Hsp40) homolog, subfamily	-0.053	0.964	6.60E-01	6.60E+01		
8154973	NM_203299	C9orf131	chromosome 9 open reading fram	-0.104	0.930	2.96E-01	2.96E+01		
8155048	NM_014806	RUSC2	RUN and SH3 domain containing 2	-0.093	0.938	2.61E-01	2.61E+01		
8155062	NM_006285	TESK1	testis-specific kinase 1	0.095	1.068	4.19E-01	4.19E+01		
8155073	NM_174923	CCDC107	coiled-coil domain containing 107	-0.010	0.993	9.53E-01	9.53E+01		
8155083	NM_001216	CA9	carbonic anhydrase IX	-0.085	0.943	3.69E-01	3.69E+01		
8155096	NM_006368	CREB3	cAMP responsive element binding	-0.141	0.907	1.20E-01	1.20E+01		
8155110	NM_00108049	RGP1	RGP1 retrograde golgi transport h	-0.068	0.954	6.16E-01	6.16E+01		
8155121	NM_003995	NPR2	natriuretic peptide receptor B/gua	-0.054	0.963	7.14E-01	7.14E+01		
8155148	NM_016446	TMEM8B	transmembrane protein 8B	-0.066	0.955	4.42E-01	4.42E+01		
8155167	NM_00103979	HRCT1	histidine rich carboxyl terminus 1	-0.004	0.997	9.80E-01	9.80E+01		
8155169	NM_021111	RECK	reversion-inducing-cysteine-rich p	-0.019	0.987	9.35E-01	9.35E+01		
8155203	NM_007096	CLTA	clathrin, light chain (Lca)	0.242	1.182	3.50E-02	3.50E+00		

8155234	NM_032226	ZCCHC7	zinc finger, CCHC domain containi	0.134	1.098	1.57E-01	1.57E+01		
8155246	---	---	---	0.157	1.115	6.16E-01	6.16E+01		
8155248	---	---	---	0.412	1.331	3.81E-01	3.81E+01		
8155250	NM_012203	GRHPR	glyoxylate reductase/hydroxypyru	0.000	1.000	1.00E+00	1.00E+02		
8155268	NM_022490	POLR1E	polymerase (RNA) I polypeptide E,	-0.122	0.919	5.20E-01	5.20E+01		
8155301	NM_144964	RG9MTD3	RNA (guanine-9-) methyltransfera	0.041	1.029	7.73E-01	7.73E+01		
8155312	NM_024345	DCAF10	DDB1 and CUL4 associated factor	0.027	1.019	8.69E-01	8.69E+01		
8155327	NM_000692	ALDH1B1	aldehyde dehydrogenase 1 family,	0.026	1.018	9.23E-01	9.23E+01		
8155332	NM_00103716	C7orf70	chromosome 7 open reading fram	-0.153	0.899	2.34E-01	2.34E+01		
8155340	NM_015667 //	M75A7 // FAM7	family with sequence similarity 75	-0.022	0.985	8.55E-01	8.55E+01		
8155345	NR_026801	FAM74A3	family with sequence similarity 74	-0.005	0.997	9.83E-01	9.83E+01		
8155349	NM_015667 //	M75A7 // FAM7	family with sequence similarity 75	-0.009	0.994	9.56E-01	9.56E+01		
8155371	NM_015667 //	M75A7 // FAM7	family with sequence similarity 75	-0.004	0.998	9.82E-01	9.82E+01		
8155383	NM_015667 //	M75A7 // FAM7	family with sequence similarity 75	-0.010	0.993	9.47E-01	9.47E+01		
8155393	AK292642	LOC554249	hypothetical LOC554249	-0.033	0.977	8.62E-01	8.62E+01		
8155422	NM_201453	CBWD3	COBW domain containing 3	0.140	1.102	2.02E-01	2.02E+01		
8155440	NM_00109927	FOXD4L2	forkhead box D4-like 2	-0.091	0.939	1.50E-01	1.50E+01		
8155442	NR_002817	AQP7P1	aquaporin 7 pseudogene 1	-0.040	0.973	7.04E-01	7.04E+01		
8155453	---	---	---	0.090	1.064	8.00E-01	8.00E+01		
8155455	AK126863	LOC441426	hypothetical gene supported by A	-0.062	0.958	7.98E-01	7.98E+01		
8155458	D14041	RBPJ	recombination signal binding prot	-0.020	0.987	9.43E-01	9.43E+01		
8155490	NR_003670	KGFLP2	keratinocyte growth factor-like pr	-0.047	0.968	6.95E-01	6.95E+01		
8155497	NR_027421	FAM27C	family with sequence similarity 27	0.003	1.002	9.90E-01	9.90E+01		
8155501	---	---	---	-0.022	0.985	8.62E-01	8.62E+01		
8155510	NR_024060	FAM27A	family with sequence similarity 27	0.015	1.010	9.51E-01	9.51E+01		
8155514	BC032035	FAM27E3	family with sequence similarity 27	-0.108	0.928	3.06E-01	3.06E+01		
8155521	NR_024060	FAM27A	family with sequence similarity 27	-0.080	0.946	7.10E-01	7.10E+01		
8155537	---	---	---	-0.024	0.984	8.57E-01	8.57E+01		
8155547	NR_024496	LOC442421	hypothetical LOC442421	-0.089	0.940	2.72E-01	2.72E+01		
8155550	AK292642	LOC554249	hypothetical LOC554249	-0.040	0.973	8.01E-01	8.01E+01		
8155554	NR_002817	AQP7P1	aquaporin 7 pseudogene 1	-0.001	0.999	9.93E-01	9.93E+01		
8155563	ENST00000367	MTHFD1L	methylenetetrahydrofolate dehyd	-0.130	0.914	5.89E-01	5.89E+01		
8155569	BC119675	FAM27E3	family with sequence similarity 27	-0.067	0.954	5.20E-01	5.20E+01		
8155572	NR_024060	FAM27A	family with sequence similarity 27	0.014	1.010	9.53E-01	9.53E+01		
8155598	---	---	---	0.040	1.028	8.71E-01	8.71E+01		
8155600	---	---	---	0.040	1.028	8.71E-01	8.71E+01		
8155630	ENST00000367	MTHFD1L	methylenetetrahydrofolate dehyd	-0.080	0.946	6.83E-01	6.83E+01		
8155636	NM_201453	CBWD3	COBW domain containing 3	0.106	1.077	2.91E-01	2.91E+01		
8155661	NM_199135	FOXD4L3	forkhead box D4-like 3	-0.082	0.945	3.05E-01	3.05E+01		
8155696	NM_138333	FAM122A	family with sequence similarity 12	0.090	1.065	3.35E-01	3.35E+01		
8155699	NM_000144	FXN	frataxin	-0.001	0.999	9.99E-01	9.99E+01		
8155707	NM_004817	TJP2	tight junction protein 2 (zona occl	0.034	1.024	9.17E-01	9.17E+01		
8155770	NM_015110	SMC5	structural maintenance of chromo	-0.069	0.953	4.49E-01	4.49E+01		
8155849	NM_000700	ANXA1	annexin A1	-0.186	0.879	1.42E-01	1.42E+01		
8155883	NM_012383	OSTF1	osteoclast stimulating factor 1	0.027	1.019	9.09E-01	9.09E+01		
8155898	NM_006200	PCSK5	proprotein convertase subtilisin/k	0.082	1.058	4.84E-01	4.84E+01		
8155942	NM_00101373	FOXB2	forkhead box B2	-0.018	0.987	8.58E-01	8.58E+01		
8155946	NM_033305	VPS13A	vacuolar protein sorting 13 homol	-0.077	0.948	5.55E-01	5.55E+01		
8156026	NM_00109880	CEP78	centrosomal protein 78kDa	0.174	1.128	3.70E-01	3.70E+01		
8156043	NM_058179	PSAT1	phosphoserine aminotransferase	-0.039	0.973	8.84E-01	8.84E+01		
8156060	NM_007005	TLE4	transducin-like enhancer of split 4	0.084	1.060	5.98E-01	5.98E+01		
8156088	---	---	---	-0.039	0.973	8.64E-01	8.64E+01		
8156112	---	---	---	0.015	1.011	9.61E-01	9.61E+01		
8156116	NM_00100155	C9orf103	chromosome 9 open reading fram	0.097	1.069	4.05E-01	4.05E+01		
8156160	NM_017576	KIF27	kinesin family member 27	-0.098	0.934	6.62E-01	6.62E+01		
8156164	NM_017576	KIF27	kinesin family member 27	-0.109	0.927	7.95E-01	7.95E+01		
8156167	NM_024635	MAK10	MAK10 homolog, amino-acid N-ac	-0.003	0.998	9.89E-01	9.89E+01		
8156196	NM_00100170	C9orf170	chromosome 9 open reading fram	-0.043	0.971	7.52E-01	7.52E+01		
8156199	NM_004938	DAPK1	death-associated protein kinase 1	0.519	1.433	8.98E-02	8.98E+00		
8156228	NM_001912	CTSL1	cathepsin L1	-0.009	0.994	9.84E-01	9.84E+01		
8156247	NM_178828	C9orf79	chromosome 9 open reading fram	0.018	1.012	9.16E-01	9.16E+01		
8156257	NM_00114512	M75C1 // FAM7	family with sequence similarity 75	-0.027	0.981	7.97E-01	7.97E+01		
8156263	NM_006717	SPIN1	spindlin 1	-0.030	0.979	8.72E-01	8.72E+01		
8156290	NM_001827	CKS2	CDC28 protein kinase regulatory s	-0.016	0.989	9.76E-01	9.76E+01		
8156295	NM_024077	SECISBP2	SECIS binding protein 2	-0.031	0.979	7.91E-01	7.91E+01		
8156309	NM_006705	GADD45G	growth arrest and DNA-damage-in	-0.097	0.935	2.65E-01	2.65E+01		
8156319	NR_002822	MGC72080	MGC72080 pseudogene	-0.071	0.952	7.07E-01	7.07E+01		
8156358	---	---	---	0.228	1.171	6.66E-01	6.66E+01		
8156373	NM_00108353	FGD3	FYVE, RhoGEF and PH domain con	-0.107	0.928	1.11E-01	1.11E+01		
8156393	NM_145006	SUSD3	sushi domain containing 3	-0.061	0.959	6.73E-01	6.73E+01		
8156404	NM_032310	C9orf89	chromosome 9 open reading fram	-0.141	0.907	6.79E-02	6.79E+00		
8156415	NM_006648	WNK2	WNK lysine deficient protein kinas	-0.086	0.942	2.44E-01	2.44E+01		
8156452	NM_014612	FAM120A	family with sequence similarity 12	0.113	1.082	1.49E-01	1.49E+01		
8156476	NM_005392	PHF2	PHD finger protein 2	-0.066	0.955	4.05E-01	4.05E+01		
8156506	NM_152422	PTPDC1	protein tyrosine phosphatase dom	0.034	1.024	7.87E-01	7.87E+01		
8156519	---	---	---	0.150	1.110	2.31E-01	2.31E+01		
8156523	---	---	---	0.115	1.083	2.84E-01	2.84E+01		

8156529	NM_194320	ZNF169	zinc finger protein 169	0.009	1.006	9.72E-01	9.72E+01		
8156538	NM_032558	HIATL1	hippocampus abundant transcript	0.087	1.062	7.57E-01	7.57E+01		
8156549	NM_032823	C9orf3	chromosome 9 open reading fram	0.027	1.019	8.78E-01	8.78E+01		
8156569	---	---	---	-0.051	0.965	7.03E-01	7.03E+01		
8156575	NM_015251	ATMIN	ATM interactor	-0.029	0.980	9.04E-01	9.04E+01		
8156577	---	---	---	-0.099	0.933	4.32E-01	4.32E+01		
8156579	---	---	---	0.269	1.205	4.88E-01	4.88E+01		
8156581	NM_00101089	C9orf102	chromosome 9 open reading fram	0.084	1.060	4.84E-01	4.84E+01		
8156599	---	---	---	0.013	1.009	9.66E-01	9.66E+01		
8156601	AL133663	C9orf102	chromosome 9 open reading fram	0.050	1.035	8.41E-01	8.41E+01		
8156604	BC022957	C9orf102	chromosome 9 open reading fram	0.030	1.021	8.52E-01	8.52E+01		
8156610	NM_014282	HABP4	hyaluronan binding protein 4	-0.035	0.976	8.84E-01	8.84E+01		
8156620	NM_017561	FAM22F	family with sequence similarity 22	-0.025	0.983	8.17E-01	8.17E+01		
8156633	BC144521	KIAA1529	KIAA1529	-0.014	0.990	9.36E-01	9.36E+01		
8156688	NM_014290	TDRD7	tudor domain containing 7	0.094	1.067	7.58E-01	7.58E+01		
8156706	NM_003275	TMOD1	tropomodulin 1	-0.220	0.859	1.08E-01	1.08E+01		
8156718	NM_002486	NCBP1	nuclear cap binding protein subun	-0.007	0.995	9.65E-01	9.65E+01		
8156748	---	---	---	-0.019	0.987	9.57E-01	9.57E+01		
8156750	NM_006401	ANP32B	acidic (leucine-rich) nuclear phosp	-0.017	0.988	9.03E-01	9.03E+01		
8156759	---	---	---	0.099	1.071	8.78E-01	8.78E+01		
8156761	NM_018946	NANS	N-acetylneuraminic acid synthase	0.271	1.207	2.64E-02	2.64E+00		
8156770	NM_024642	GALNT12	UDP-N-acetyl-alpha-D-galactosam	0.013	1.009	9.61E-01	9.61E+01		
8156826	NM_004612	TGFBR1	transforming growth factor, beta r	0.207	1.154	5.15E-02	5.15E+00		
8156838	NM_006808	SEC61B	Sec61 beta subunit	0.045	1.031	7.46E-01	7.46E+01		
8156846	---	---	---	0.028	1.020	8.13E-01	8.13E+01		
8156861	NM_017919	STX17	syntaxin 17	0.138	1.100	3.56E-01	3.56E+01		
8156873	NM_014425	INVS	inversin	-0.013	0.991	9.49E-01	9.49E+01		
8156895	---	---	---	0.093	1.066	8.84E-01	8.84E+01		
8156919	NM_00101811	MURC	muscle-related coiled-coil protein	-0.132	0.913	4.26E-01	4.26E+01		
8156923	NM_207299	RP11-35N6.1	plasticity related gene 3	-0.046	0.968	7.05E-01	7.05E+01		
8156935	NM_003452	ZNF189	zinc finger protein 189	0.048	1.034	7.89E-01	7.89E+01		
8156945	NM_019592	RNF20	ring finger protein 20	0.091	1.065	3.51E-01	3.51E+01		
8156982	NM_00104255	SMC2	structural maintenance of chromo	0.052	1.037	8.57E-01	8.57E+01		
8157021	NM_015469	NIPSNAP3A	nipsnap homolog 3A (C. elegans)	0.172	1.127	4.27E-01	4.27E+01		
8157038	NM_080546	SLC44A1	solute carrier family 44, member 1	0.043	1.031	9.36E-01	9.36E+01		
8157074	NM_00107980	FKTN	fukutin	0.083	1.059	6.99E-01	6.99E+01		
8157092	NM_018112	TMEM38B	transmembrane protein 38B	-0.015	0.990	9.77E-01	9.77E+01		
8157101	---	---	---	0.015	1.011	9.65E-01	9.65E+01		
8157125	NM_002874	RAD23B	RAD23 homolog B (S. cerevisiae)	0.051	1.036	7.20E-01	7.20E+01		
8157139	---	---	---	-0.016	0.989	9.47E-01	9.47E+01		
8157153	NM_007203	PALM2-AKAP2	PALM2-AKAP2 readthrough transc	-0.060	0.959	6.38E-01	6.38E+01		
8157203	NM_00101588	DNAJC25	DnaJ (Hsp40) homolog, subfamily	0.012	1.009	9.50E-01	9.50E+01		
8157214	---	---	---	0.169	1.125	1.90E-01	1.90E+01		
8157216	NM_003358	UGCG	UDP-glucose ceramide glucosyltra	0.010	1.007	9.80E-01	9.80E+01		
8157231	---	---	---	0.053	1.037	8.68E-01	8.68E+01		
8157246	BC105048	KIAA1958	KIAA1958	0.036	1.025	8.05E-01	8.05E+01		
8157253	NM_00101299	SNX30	sorting nexin family member 30	0.232	1.175	2.98E-01	2.98E+01		
8157264	NM_001860	SLC31A2	solute carrier family 31 (copper tra	0.396	1.316	9.03E-02	9.03E+00		
8157283	NM_004697	PRPF4	PRP4 pre-mRNA processing factor	0.072	1.051	5.56E-01	5.56E+01		
8157300	NM_017688	BSPRY	B-box and SPRY domain containing	0.104	1.075	1.53E-01	1.53E+01		
8157324	NM_144488	RGS3	regulator of G-protein signaling 3	0.038	1.027	6.42E-01	6.42E+01		
8157362	NM_133374	ZNF618	zinc finger protein 618	-0.095	0.936	4.37E-01	4.37E+01		
8157383	NM_032888	COL27A1	collagen, type XXVII, alpha 1	-0.057	0.961	4.97E-01	4.97E+01		
8157454	NM_004888	ATP6V1G1	ATPase, H+ transporting, lysosoma	0.243	1.183	5.65E-04	5.65E-02		
8157463	NM_153045	C9orf91	chromosome 9 open reading fram	0.202	1.150	1.07E-02	1.07E+00		
8157516	NM_012210	TRIM32	tripartite motif-containing 32	0.039	1.027	7.89E-01	7.89E+01		
8157534	NM_007018	CEP110	centrosomal protein 110kDa	0.053	1.038	5.91E-01	5.91E+01		
8157582	NM_000177	GSN	gelsolin (amyloidosis, Finnish type	0.229	1.172	1.62E-02	1.62E+00		
8157605	NM_004099	STOM	stomatin	0.299	1.230	3.72E-01	3.72E+01		
8157610	NM_032552	DAB2IP	DAB2 interacting protein	0.001	1.001	9.92E-01	9.92E+01		
8157638	NM_138777	MRRF	mitochondrial ribosome recycling	0.058	1.041	7.91E-01	7.91E+01		
8157650	NM_000962	PTGS1	prostaglandin-endoperoxide synth	0.246	1.186	2.17E-02	2.17E+00		
8157691	NM_182620	SKA2	spindle and kinetochore associate	0.075	1.053	6.63E-01	6.63E+01		
8157696	NM_00100192	OR5C1	olfactory receptor, family 5, subfa	-0.005	0.997	9.83E-01	9.83E+01		
8157698	NM_080859	OR1K1	olfactory receptor, family 1, subfa	0.041	1.029	8.06E-01	8.06E+01		
8157700	NM_012197	RABGAP1	RAB GTPase activating protein 1	0.013	1.009	9.39E-01	9.39E+01		
8157729	---	---	---	0.266	1.203	2.71E-01	2.71E+01		
8157731	NM_173689	CRB2	crumbs homolog 2 (Drosophila)	-0.079	0.947	3.38E-01	3.38E+01		
8157777	NM_00116180	GPR144	G protein-coupled receptor 144	-0.007	0.995	9.61E-01	9.61E+01		
8157798	---	---	---	0.083	1.059	4.63E-01	4.63E+01		
8157802	---	---	---	-0.077	0.948	7.63E-01	7.63E+01		
8157804	NM_182487	OLFML2A	olfactomedin-like 2A	-0.019	0.987	9.00E-01	9.00E+01		
8157818	NM_00104547	WDR38	WD repeat domain 38	-0.072	0.951	5.76E-01	5.76E+01		
8157828	NM_030978	ARPC5L	actin related protein 2/3 complex,	-0.051	0.965	7.64E-01	7.64E+01		
8157843	NM_005833	RABEPK	Rab9 effector protein with kelch m	0.033	1.023	8.78E-01	8.78E+01		
8157858	NM_015635	GAPVD1	GTPase activating protein and VPS	0.072	1.051	6.24E-01	6.24E+01		
8157890	NM_006195	PBX3	pre-B-cell leukemia homeobox 3	0.115	1.083	1.91E-01	1.91E+01		

8157905	NM_033446	FAM125B	family with sequence similarity 12	-0.159	0.896	2.04E-01	2.04E+01		
8157922	NM_002316	LMX1B	LIM homeobox transcription facto	-0.023	0.984	8.69E-01	8.69E+01		
8157933	NM_014007	ZBTB43	zinc finger and BTB domain contai	0.027	1.019	8.91E-01	8.91E+01		
8157939	---	---	---	-0.142	0.906	2.72E-01	2.72E+01		
8157941	NM_00109927	ZBTB34	zinc finger and BTB domain contai	0.148	1.108	2.63E-01	2.63E+01		
8157945	NM_00109927	ZBTB34	zinc finger and BTB domain contai	0.083	1.060	7.40E-01	7.40E+01		
8157947	NM_00109927	ZBTB34	zinc finger and BTB domain contai	0.206	1.154	2.49E-01	2.49E+01		
8157949	NM_014636	RALGPS1	Ral GEF with PH domain and SH3 b	-0.008	0.994	9.60E-01	9.60E+01		
8157976	NM_032293	GARNL3	GTPase activating Rap/RanGAP do	0.067	1.047	5.91E-01	5.91E+01		
8158009	NM_014580	SLC2A8	solute carrier family 2 (facilitated g	-0.032	0.978	7.57E-01	7.57E+01		
8158022	NM_007135	ZNF79	zinc finger protein 79	-0.080	0.946	4.70E-01	4.70E+01		
8158028	NM_138361	LRSAM1	leucine rich repeat and sterile alph	-0.032	0.978	7.92E-01	7.92E+01		
8158059	NM_003165	STXBP1	syntaxin binding protein 1	0.051	1.036	8.11E-01	8.11E+01		
8158094	NM_144965	TTC16	tetratricopeptide repeat domain 1	0.045	1.031	6.22E-01	6.22E+01		
8158112	NM_001261	CDK9	cyclin-dependent kinase 9	-0.043	0.971	7.71E-01	7.71E+01		
8158123	NM_004957	FPGS	folylpolyglutamate synthase	-0.003	0.998	9.85E-01	9.85E+01		
8158145	---	---	---	-0.023	0.984	8.31E-01	8.31E+01		
8158167	NM_005564	LCN2	lipocalin 2	-0.123	0.918	1.50E-01	1.50E+01		
8158177	NM_024112	C9orf16	chromosome 9 open reading fram	-0.117	0.922	3.06E-02	3.06E+00		
8158183	NM_004408	DNM1	dynamin 1	0.040	1.028	7.94E-01	7.94E+01		
8158204	NM_00104001	C9orf119	chromosome 9 open reading fram	0.027	1.019	9.23E-01	9.23E+01		
8158212	---	---	---	-0.054	0.964	8.30E-01	8.30E+01		
8158214	NM_016035	COQ4	coenzyme Q4 homolog (S. cerevisi	-0.146	0.904	1.20E-01	1.20E+01		
8158224	NM_005094	SLC27A4	solute carrier family 27 (fatty acid	-0.044	0.970	7.09E-01	7.09E+01		
8158240	NM_021109	TMSB4X	thymosin beta 4, X-linked	-0.040	0.973	6.57E-01	6.57E+01		
8158242	NM_030914	URM1	ubiquitin related modifier 1 homo	0.038	1.027	8.79E-01	8.79E+01		
8158250	NM_016174	CERCAM	cerebral endothelial cell adhesion	-0.031	0.979	8.41E-01	8.41E+01		
8158269	NM_002540	ODF2	outer dense fiber of sperm tails 2	-0.020	0.986	8.99E-01	8.99E+01		
8158298	NM_00100372	GLE1	GLE1 RNA export mediator homol	-0.080	0.946	3.27E-01	3.27E+01		
8158317	NM_00113043	SPTAN1	spectrin, alpha, non-erythrocytic 1	-0.070	0.952	5.06E-01	5.06E+01		
8158372	NM_003011	SET	SET nuclear oncogene	0.009	1.006	9.37E-01	9.37E+01		
8158380	NM_013355	PKN3	protein kinase N3	-0.043	0.971	7.89E-01	7.89E+01		
8158404	---	---	---	0.076	1.054	8.69E-01	8.69E+01		
8158406	NM_018201	TBC1D13	TBC1 domain family, member 13	-0.081	0.946	6.15E-01	6.15E+01		
8158418	NM_004435	ENDOG	endonuclease G	-0.031	0.979	7.97E-01	7.97E+01		
8158424	NM_00112724	LRRC8A	leucine rich repeat containing 8 fa	-0.069	0.953	3.01E-01	3.01E+01		
8158431	NM_00110087	PHYHD1	phytanoyl-CoA dioxygenase doma	-0.005	0.996	9.78E-01	9.78E+01		
8158446	NM_015354	NUP188	nucleoporin 188kDa	-0.023	0.984	8.62E-01	8.62E+01		
8158492	BC009114	FAM73B	family with sequence similarity 73	-0.009	0.994	9.52E-01	9.52E+01		
8158513	NM_020438	DOLPP1	dolichyl pyrophosphate phosphata	0.020	1.014	9.27E-01	9.27E+01		
8158523	NM_178001	PPP2R4	protein phosphatase 2A activator,	0.112	1.081	5.16E-01	5.16E+01		
8158539	NM_00101271	C9orf106	chromosome 9 open reading fram	0.042	1.029	8.69E-01	8.69E+01		
8158544	NM_014064	METTL11A	methyltransferase like 11A	0.032	1.022	8.40E-01	8.40E+01		
8158554	NM_016307	PRRX2	paired related homeobox 2	0.000	1.000	1.00E+00	1.00E+02		
8158560	NM_014506	TOR1B	torsin family 1, member B (torsin B	0.052	1.037	8.33E-01	8.33E+01		
8158568	NM_006676	USP20	ubiquitin specific peptidase 20	-0.041	0.972	8.14E-01	8.14E+01		
8158597	NM_00113655	GPR107	G protein-coupled receptor 107	0.152	1.111	1.54E-01	1.54E+01		
8158625	AK125285	LOC100129785	hypothetical protein LOC10012978	0.019	1.014	9.54E-01	9.54E+01		
8158627	NM_014286	FREQ	frequenin homolog (Drosophila)	-0.171	0.888	1.67E-01	1.67E+01		
8158637	AL834139	HMCN2	hemicentin 2	-0.005	0.996	9.79E-01	9.79E+01		
8158666	AK128680	HMCN2	hemicentin 2	0.011	1.008	9.49E-01	9.49E+01		
8158671	NM_000050	ASS1	argininosuccinate synthetase 1	-0.061	0.959	5.56E-01	5.56E+01		
8158684	---	---	---	0.078	1.055	5.55E-01	5.55E+01		
8158686	NM_003934	FUBP3	far upstream element (FUSE) bind	-0.056	0.962	5.04E-01	5.04E+01		
8158708	NM_021619	PRDM12	PR domain containing 12	-0.066	0.956	5.15E-01	5.15E+01		
8158714	NM_014285	EXOSC2	exosome component 2	0.054	1.038	8.22E-01	8.22E+01		
8158725	NM_005157	ABL1	c-abl oncogene 1, receptor tyrosin	-0.073	0.950	5.39E-01	5.39E+01		
8158739	NM_006059	LAMC3	laminin, gamma 3	-0.016	0.989	9.16E-01	9.16E+01		
8158771	NM_031426	AIF1L	allograft inflammatory factor 1-like	-0.034	0.977	8.05E-01	8.05E+01		
8158783	NM_005085	NUP214	nucleoporin 214kDa	-0.055	0.963	6.68E-01	6.68E+01		
8158825	NM_032728	PPAPDC3	phosphatidic acid phosphatase typ	-0.037	0.974	7.55E-01	7.55E+01		
8158829	NM_013318	BAT2L	HLA-B associated transcript 2-like	-0.088	0.941	6.39E-01	6.39E+01		
8158839	NM_013318	BAT2L	HLA-B associated transcript 2-like	-0.084	0.944	4.51E-01	4.51E+01		
8158862	NR_002914	SNORD62A	small nucleolar RNA, C/D box 62A	-0.137	0.909	5.89E-01	5.89E+01		
8158864	NR_002914	SNORD62A	small nucleolar RNA, C/D box 62A	-0.137	0.909	5.89E-01	5.89E+01		
8158866	NM_00113611	POMT1	protein-O-mannosyltransferase 1	-0.040	0.973	7.15E-01	7.15E+01		
8158890	NM_032536	NTNG2	netrin G2	0.034	1.024	8.08E-01	8.08E+01		
8158910	---	---	---	-0.044	0.970	8.59E-01	8.59E+01		
8158912	NM_020064	BARHL1	BarH-like homeobox 1	-0.032	0.978	8.07E-01	8.07E+01		
8158918	NM_012204	GTF3C4	general transcription factor IIIC, po	-0.116	0.923	3.04E-01	3.04E+01		
8158930	NM_018956	C9orf9	chromosome 9 open reading fram	0.003	1.002	9.90E-01	9.90E+01		
8158939	NM_004188	GFI1B	growth factor independent 1B tran	0.026	1.018	8.00E-01	8.00E+01		
8158952	NM_001402	EEF1A1	eukaryotic translation elongation f	-0.010	0.993	9.14E-01	9.14E+01		
8158961	NM_00112282	GTF3C5	general transcription factor IIIC, po	-0.092	0.938	3.14E-01	3.14E+01		
8158976	NM_001807	CEL	carboxyl ester lipase (bile salt-stim	-0.034	0.977	8.44E-01	8.44E+01		
8158987	NR_001275	CELP	carboxyl ester lipase pseudogene	-0.040	0.972	7.96E-01	7.96E+01		
8158998	NR_000016	SNORD36C	small nucleolar RNA, C/D box 36C	-0.127	0.916	5.83E-01	5.83E+01		

8159004	NR_002447	SNORD24	small nucleolar RNA, C/D box 24	-0.080	0.946	8.47E-01	8.47E+01		
8159008	NM_017503	SURF2	surfeit 2	-0.040	0.973	8.57E-01	8.57E+01		
8159017	NM_153710	C9orf96	chromosome 9 open reading fram	-0.022	0.985	8.73E-01	8.73E+01		
8159036	NM_139025	ADAMTS13	ADAM metallopeptidase with thro	-0.061	0.959	2.72E-01	2.72E+01		
8159078	NM_017586	C9orf7	chromosome 9 open reading fram	0.009	1.006	9.56E-01	9.56E+01		
8159086	NM_014694	ADAMTSL2	ADAMTS-like 2	-0.057	0.961	6.27E-01	6.27E+01		
8159096	NM_000787	DBH	dopamine beta-hydroxylase (dopa	-0.014	0.990	9.26E-01	9.26E+01		
8159111	NM_017588	WDR5	WD repeat domain 5	-0.031	0.979	8.73E-01	8.73E+01		
8159127	NM_002957	RXRA	retinoid X receptor, alpha	0.002	1.002	9.90E-01	9.90E+01		
8159142	NM_000093	COL5A1	collagen, type V, alpha 1	-0.026	0.982	7.91E-01	7.91E+01		
8159211	NM_004108	FCN2	ficolin (collagen/fibrinogen domai	-0.029	0.980	8.30E-01	8.30E+01		
8159239	BC034752	C9orf62	chromosome 9 open reading fram	-0.001	0.999	9.96E-01	9.96E+01		
8159243	NM_014811	KIAA0649	KIAA0649	-0.010	0.993	9.50E-01	9.50E+01		
8159249	NM_016034	MRPS2	mitochondrial ribosomal protein S	0.040	1.028	8.11E-01	8.11E+01		
8159255	NM_002297	LCN1	lipocalin 1 (tear prealbumin)	0.019	1.013	9.27E-01	9.27E+01		
8159259	NM_014582	OBP2A	odorant binding protein 2A	0.027	1.019	8.74E-01	8.74E+01		
8159265	NM_00101804	PAEP	progestagen-associated endometr	-0.022	0.985	8.35E-01	8.35E+01		
8159283	NM_020822	KCNT1	potassium channel, subfamily T, m	-0.091	0.939	2.08E-01	2.08E+01		
8159318	NM_00114563	GPSM1	G-protein signaling modulator 1 (A	-0.025	0.983	8.23E-01	8.23E+01		
8159337	NM_015160	PMPCA	peptidase (mitochondrial processi	0.009	1.006	9.62E-01	9.62E+01		
8159354	NM_016215	EGFL7	EGF-like-domain, multiple 7	-0.032	0.978	7.56E-01	7.56E+01		
8159373	NM_152421	FAM69B	family with sequence similarity 69	-0.038	0.974	7.48E-01	7.48E+01		
8159379	NM_032928	TMEM141	transmembrane protein 141	0.018	1.013	9.24E-01	9.24E+01		
8159385	NM_00103937	KIAA1984	KIAA1984	-0.042	0.971	6.45E-01	6.45E+01		
8159415	NM_024718	C9orf86	chromosome 9 open reading fram	0.049	1.034	6.27E-01	6.27E+01		
8159439	NM_00108048	LOC389813	similar to CG15216-PA	-0.029	0.980	8.47E-01	8.47E+01		
8159441	NM_00113586	PHPT1	phosphohistidine phosphatase 1	0.031	1.022	8.82E-01	8.82E+01		
8159448	NM_206920	MAMDC4	MAM domain containing 4	-0.041	0.972	5.93E-01	5.93E+01		
8159476	NM_021138	TRAF2	TNF receptor-associated factor 2	-0.113	0.924	7.05E-02	7.05E+00		
8159491	NM_000606	C8G	complement component 8, gamm	-0.066	0.955	5.37E-01	5.37E+01		
8159501	NM_178536	LCN12	lipocalin 12	-0.014	0.990	9.09E-01	9.09E+01		
8159519	ENST00000371	C9orf141	chromosome 9 open reading fram	-0.078	0.947	4.34E-01	4.34E+01		
8159521	NM_000954	PTGDS	prostaglandin D2 synthase 21kDa	0.004	1.003	9.82E-01	9.82E+01		
8159531	NM_207510	LCNL1	lipocalin-like 1	-0.012	0.992	9.34E-01	9.34E+01		
8159541	BC002613	C9orf142	chromosome 9 open reading fram	-0.088	0.941	2.63E-01	2.63E+01		
8159549	NM_004479	FUT7	fucosyltransferase 7 (alpha (1,3) fu	-0.063	0.957	5.53E-01	5.53E+01		
8159554	NM_207309	UAP1L1	UDP-N-acteylglucosamine pyropho	-0.009	0.994	9.63E-01	9.63E+01		
8159566	NM_016219	MAN1B1	mannosidase, alpha, class 1B, mer	0.046	1.032	6.57E-01	6.57E+01		
8159583	NM_007327	GRIN1	glutamate receptor, ionotropic, N-	-0.045	0.969	7.35E-01	7.35E+01		
8159609	NM_003731	SSNA1	Sjogren syndrome nuclear autoan	-0.044	0.970	7.48E-01	7.48E+01		
8159616	NM_00114402	NDOR1	NADPH dependent diflavin oxidore	-0.074	0.950	4.13E-01	4.13E+01		
8159624	BC052297	C9orf169	chromosome 9 open reading fram	-0.010	0.993	9.42E-01	9.42E+01		
8159627	NM_080877	SLC34A3	solute carrier family 34 (sodium p	-0.027	0.982	8.04E-01	8.04E+01		
8159642	NM_006088	TUBB2C	tubulin, beta 2C	-0.145	0.905	3.16E-01	3.16E+01		
8159646	NM_00100435	LOC441476	hypothetical protein LOC441476	-0.026	0.982	8.73E-01	8.73E+01		
8159654	NM_015456	COBRA1	cofactor of BRCA1	-0.033	0.977	7.95E-01	7.95E+01		
8159667	NM_017723	C9orf167	chromosome 9 open reading fram	-0.004	0.997	9.84E-01	9.84E+01		
8159670	NM_006647	NOXA1	NADPH oxidase activator 1	-0.021	0.985	8.29E-01	8.29E+01		
8159687	NM_032477	MRPL41	mitochondrial ribosomal protein L	-0.050	0.966	6.30E-01	6.30E+01		
8159692	NM_152285	ARRDC1	arrestin domain containing 1	-0.089	0.940	5.19E-01	5.19E+01		
8159702	NM_024757	EHMT1	euchromatic histone-lysine N-met	0.004	1.003	9.79E-01	9.79E+01		
8159732	NR_024341	FLJ40292	hypothetical LOC643210	0.008	1.006	9.67E-01	9.67E+01		
8159734	NM_000718	CACNA1B	calcium channel, voltage-depende	-0.054	0.963	4.49E-01	4.49E+01		
8159786	NR_027156	TUBBP5	tubulin, beta pseudogene 5	-0.020	0.986	9.03E-01	9.03E+01		
8159790	NR_024077	WASH2P	WAS protein family homolog 2 pse	-0.047	0.968	6.47E-01	6.47E+01		
8159808	NM_207305	FOXD4	forkhead box D4	-0.064	0.957	2.94E-01	2.94E+01		
8159815	NM_00114535	CBWD1	COBW domain containing 1	0.132	1.096	1.52E-01	1.52E+01		
8159838	NM_152569	C9orf66	chromosome 9 open reading fram	0.052	1.037	8.63E-01	8.63E+01		
8159854	NM_014878	KIAA0020	KIAA0020	0.049	1.034	8.47E-01	8.47E+01		
8159873	---	---	---	-0.049	0.967	8.16E-01	8.16E+01		
8159876	NM_134428	RFX3	regulatory factor X, 3 (influences H	-0.013	0.991	9.63E-01	9.63E+01		
8159945	NM_016282	AK3	adenylate kinase 3	-0.009	0.994	9.67E-01	9.67E+01		
8159957	---	---	---	0.081	1.058	5.71E-01	5.71E+01		
8159961	---	---	---	0.471	1.386	2.94E-01	2.94E+01		
8159965	---	---	---	0.422	1.339	2.70E-01	2.70E+01		
8159981	NM_006911	RLN1	relaxin 1	0.070	1.050	7.39E-01	7.39E+01		
8159992	NM_024896	ERMP1	endoplasmic reticulum metallope	0.138	1.100	3.36E-01	3.36E+01		
8160011	NM_00101796	KIAA2026	KIAA2026	0.031	1.022	8.78E-01	8.78E+01		
8160016	NM_012416	RANBP6	RAN binding protein 6	0.026	1.018	9.06E-01	9.06E+01		
8160024	NM_000170	GLDC	glycine dehydrogenase (decarbox	0.086	1.062	6.84E-01	6.84E+01		
8160033	NM_003094	SNRPE	small nuclear ribonucleoprotein p	0.020	1.014	9.18E-01	9.18E+01		
8160036	AK292632	C9orf123	chromosome 9 open reading fram	0.072	1.051	7.21E-01	7.21E+01		
8160151	NM_178566	ZDHHC21	zinc finger, DHHC-type containing	0.280	1.214	1.24E-01	1.24E+01		
8160213	NM_152574	TTC39B	tetratricopeptide repeat domain 3	0.127	1.092	6.00E-01	6.00E+01		
8160238	NM_033222	PSIP1	PC4 and SFRS1 interacting protein	0.030	1.021	8.49E-01	8.49E+01		
8160284	NM_017645	HAUS6	HAUS augmin-like complex, subun	-0.061	0.959	7.78E-01	7.78E+01		
8160295	NR_003009	SCARNA8	small Cajal body-specific RNA 8	-0.241	0.846	4.10E-01	4.10E+01		

8160297	NM_001122	PLIN2	perilipin 2	-0.076	0.949	8.92E-01	8.92E+01		
8160308	NM_001010	RPS6	ribosomal protein S6	0.006	1.004	9.67E-01	9.67E+01		
8160332	NM_004529	MLLT3	myeloid/lymphoid or mixed-lineag	-0.080	0.946	6.91E-01	6.91E+01		
8160346	NM_00101091	PTPLAD2	protein tyrosine phosphatase-like	0.108	1.078	5.87E-01	5.87E+01		
8160405	NM_018847	KLHL9	kelch-like 9 (Drosophila)	0.034	1.024	8.17E-01	8.17E+01		
8160452	NM_078487	CDKN2B	cyclin-dependent kinase inhibitor	-0.092	0.938	4.00E-01	4.00E+01		
8160472	NM_00100412	TUSC1	tumor suppressor candidate 1	-0.012	0.992	9.57E-01	9.57E+01		
8160478	BC071953	C9orf82	chromosome 9 open reading fram	0.066	1.047	5.60E-01	5.60E+01		
8160487	NM_00103168	PLAA	phospholipase A2-activating prote	0.060	1.042	5.70E-01	5.70E+01		
8160521	NM_024761	MOBKL2B	MOB1, Mps One Binder kinase act	0.192	1.143	2.26E-01	2.26E+01		
8160531	NM_018325	C9orf72	chromosome 9 open reading fram	0.101	1.073	7.64E-01	7.64E+01		
8160557	---	---	---	-0.003	0.998	9.95E-01	9.95E+01		
8160559	NM_014314	DDX58	DEAD (Asp-Glu-Ala-Asp) box polyp	0.046	1.032	8.75E-01	8.75E+01		
8160581	NM_005802	TOPORS	topoisomerase I binding, arginine/	-0.085	0.943	4.65E-01	4.65E+01		
8160587	NM_002493	NDUFB6	NADH dehydrogenase (ubiquinone	0.028	1.019	9.26E-01	9.26E+01		
8160602	NM_175069	APTX	aprataxin	0.052	1.037	6.68E-01	6.68E+01		
8160622	NM_018225	SMU1	smu-1 suppressor of mec-8 and ur	0.050	1.035	7.09E-01	7.09E+01		
8160637	NM_001497	B4GALT1	UDP-Gal:betaGlcNAc beta 1,4- gala	0.048	1.034	8.84E-01	8.84E+01		
8160647	NM_004323	BAG1	BCL2-associated athanogene	0.099	1.071	4.29E-01	4.29E+01		
8160663	NM_001170	AQP7	aquaporin 7	0.107	1.077	5.06E-01	5.06E+01		
8160670	NM_004925	AQP3	aquaporin 3 (Gill blood group)	0.012	1.009	9.48E-01	9.48E+01		
8160682	NM_022917	NOL6	nucleolar protein family 6 (RNA-as	0.006	1.004	9.77E-01	9.77E+01		
8160718	NR_023917	PTENP1	phosphatase and tensin homolog	-0.022	0.985	8.78E-01	8.78E+01		
8160722	NM_018449	UBAP2	ubiquitin associated protein 2	0.007	1.005	9.56E-01	9.56E+01		
8160756	NM_015397	DCAF12	DDB1 and CUL4 associated factor	0.204	1.152	6.25E-02	6.25E+00		
8160767	---	---	---	-0.013	0.991	9.78E-01	9.78E+01		
8160769	---	---	---	0.042	1.029	8.05E-01	8.05E+01		
8160782	---	---	---	-0.068	0.954	6.38E-01	6.38E+01		
8160786	NM_020702	KIAA1161	KIAA1161	0.032	1.022	8.50E-01	8.50E+01		
8160792	NM_032596	C9orf24	chromosome 9 open reading fram	-0.055	0.963	7.10E-01	7.10E+01		
8160805	NM_147202	C9orf25	chromosome 9 open reading fram	-0.026	0.982	8.41E-01	8.41E+01		
8160816	NM_198573	ENHO	energy homeostasis associated	0.011	1.008	9.56E-01	9.56E+01		
8160823	NM_147164	CNTFR	ciliary neurotrophic factor recepto	-0.078	0.947	4.25E-01	4.25E+01		
8160835	NM_148179	C9orf23	chromosome 9 open reading fram	-0.011	0.993	9.54E-01	9.54E+01		
8160839	NM_024348	DCTN3	dynactin 3 (p22)	-0.053	0.964	6.34E-01	6.34E+01		
8160849	NM_00101736	ARID3C	AT rich interactive domain 3C (BRI	0.056	1.040	6.75E-01	6.75E+01		
8160857	NM_005866	SIGMAR1	sigma non-opioid intracellular rece	-0.007	0.995	9.63E-01	9.63E+01		
8160870	NM_006664	CCL27	chemokine (C-C motif) ligand 27	-0.051	0.965	6.20E-01	6.20E+01		
8160889	NM_002989	CCL21	chemokine (C-C motif) ligand 21	-0.024	0.984	8.72E-01	8.72E+01		
8160898	NM_00114191	C9orf144B	hypothetical protein LOC259308	-0.049	0.967	8.01E-01	8.01E+01		
8160912	NM_203299	C9orf131	chromosome 9 open reading fram	-0.232	0.851	1.37E-01	1.37E+01		
8160914	NM_007126	VCP	valosin-containing protein	-0.080	0.946	5.99E-01	5.99E+01		
8160935	NM_004629	FANCG	Fanconi anemia, complementation	-0.046	0.968	6.80E-01	6.80E+01		
8160953	NM_032634	PIGO	phosphatidylinositol glycan ancho	-0.051	0.965	6.01E-01	6.01E+01		
8160968	NM_013442	STOML2	stomatin (EPB72)-like 2	-0.014	0.990	9.50E-01	9.50E+01		
8160981	BC004406	KIAA1539	KIAA1539	-0.007	0.995	9.75E-01	9.75E+01		
8161017	NM_014450	SIT1	signaling threshold regulating tran	-0.030	0.979	8.86E-01	8.86E+01		
8161024	NR_003051	RMRP	RNA component of mitochondrial	0.071	1.050	8.77E-01	8.77E+01		
8161026	NM_174923	CCDC107	coiled-coil domain containing 107	-0.015	0.990	9.00E-01	9.00E+01		
8161044	NM_003289	TPM2	tropomyosin 2 (beta)	-0.019	0.987	9.57E-01	9.57E+01		
8161056	NM_006289	TLN1	talin 1	0.043	1.030	7.94E-01	7.94E+01		
8161114	NM_020944	GBA2	glucosidase, beta (bile acid) 2	0.005	1.004	9.84E-01	9.84E+01		
8161147	NM_032593	HINT2	histidine triad nucleotide binding p	0.092	1.066	6.34E-01	6.34E+01		
8161164	NM_00100448	OR13J1	olfactory receptor, family 13, subf	-0.067	0.955	6.65E-01	6.65E+01		
8161166	NM_019897	OR2S2	olfactory receptor, family 2, subfa	-0.035	0.976	7.44E-01	7.44E+01		
8161169	---	---	---	0.044	1.031	5.71E-01	5.71E+01		
8161174	NM_005476	GNE	glucosamine (UDP-N-acetyl)-2-epi	-0.064	0.957	6.99E-01	6.99E+01		
8161192	NM_194328	RNF38	ring finger protein 38	-0.034	0.977	8.40E-01	8.40E+01		
8161211	NM_016734	PAX5	paired box 5	0.224	1.168	1.89E-01	1.89E+01		
8161229	NM_012166	FBXO10	F-box protein 10	0.018	1.013	9.03E-01	9.03E+01		
8161242	NM_016042	EXOSC3	exosome component 3	-0.067	0.955	6.23E-01	6.23E+01		
8161255	NM_003028	SHB	Src homology 2 domain containing	0.038	1.027	7.75E-01	7.75E+01		
8161265	NM_00100756	IGFBPL1	insulin-like growth factor binding p	0.007	1.005	9.67E-01	9.67E+01		
8161341	NM_015667 //	M75A7 // FAM7	family with sequence similarity 75	-0.014	0.991	9.19E-01	9.19E+01		
8161353	AK292642	LOC554249	hypothetical LOC554249	-0.052	0.965	6.78E-01	6.78E+01		
8161362	NR_003670	KGFLP2	keratinocyte growth factor-like pr	-0.074	0.950	5.91E-01	5.91E+01		
8161373	ENST00000438	LOC100289528	hypothetical protein LOC10028952	-0.130	0.914	7.43E-01	7.43E+01		
8161375	---	---	---	0.090	1.064	8.00E-01	8.00E+01		
8161377	ENST00000367	MTHFD1L	methylenetetrahydrofolate dehyd	-0.080	0.946	6.83E-01	6.83E+01		
8161418	NM_015234	GPR116	G protein-coupled receptor 116	-0.144	0.905	5.04E-01	5.04E+01		
8161421	---	---	---	-0.033	0.978	8.24E-01	8.24E+01		
8161426	BC119675	FAM27E3	family with sequence similarity 27	-0.067	0.954	5.20E-01	5.20E+01		
8161431	---	---	---	0.013	1.009	9.53E-01	9.53E+01		
8161433	---	---	---	0.033	1.023	8.98E-01	8.98E+01		
8161437	BC119675	FAM27E3	family with sequence similarity 27	-0.067	0.954	5.20E-01	5.20E+01		
8161442	---	---	---	-0.154	0.899	3.89E-01	3.89E+01		
8161446	BC032035	FAM27E3	family with sequence similarity 27	-0.096	0.936	3.58E-01	3.58E+01		

8161451	AK292642	LOC554249	hypothetical LOC554249	-0.033	0.977	8.62E-01	8.62E+01		
8161458	NR_003670	KGFLP2	keratinocyte growth factor-like pr	-0.016	0.989	9.22E-01	9.22E+01		
8161471	NM_015667 //	I75A7 // FAM7	family with sequence similarity 75	-0.010	0.993	9.44E-01	9.44E+01		
8161476	---	---	---	-0.026	0.982	9.13E-01	9.13E+01		
8161484	ENST00000367	MTHFD1L	methylenetetrahydrofolate dehyd	-0.123	0.918	6.11E-01	6.11E+01		
8161488	NR_002817	AQP7P1	aquaporin 7 pseudogene 1	-0.043	0.971	7.00E-01	7.00E+01		
8161499	BC032035	FAM27E3	family with sequence similarity 27	-0.096	0.936	3.70E-01	3.70E+01		
8161503	NR_024060	FAM27A	family with sequence similarity 27	0.016	1.011	9.48E-01	9.48E+01		
8161509	NM_004477	FRG1	FSHD region gene 1	0.151	1.110	1.32E-01	1.32E+01		
8161513	NR_002836	PGM5P2	phosphoglucomutase 5 pseudoger	0.032	1.022	8.78E-01	8.78E+01		
8161520	NR_002836	PGM5P2	phosphoglucomutase 5 pseudoger	0.036	1.025	8.73E-01	8.73E+01		
8161533	NM_00108547	FOXD4L6	forkhead box D4-like 6	-0.081	0.945	2.93E-01	2.93E+01		
8161537	NM_201453	CBWD3	COBW domain containing 3	0.106	1.076	3.39E-01	3.39E+01		
8161554	ENST00000438	LOC100289528	hypothetical protein LOC1002895	-0.130	0.914	7.43E-01	7.43E+01		
8161563	---	---	---	0.179	1.132	4.71E-02	4.71E+00		
8161571	NM_00109927	FOXD4L2	forkhead box D4-like 2	-0.106	0.929	1.28E-01	1.28E+01		
8161575	ENST00000377	CBWD5	COBW domain containing 5	0.172	1.127	2.08E-01	2.08E+01		
8161580	---	---	---	0.167	1.123	5.53E-02	5.53E+00		
8161583	NM_00109927	FOXD4L2	forkhead box D4-like 2	-0.091	0.939	1.50E-01	1.50E+01		
8161587	NM_201453	CBWD3	COBW domain containing 3	0.103	1.074	2.78E-01	2.78E+01		
8161615	NM_002732	PRKACG	protein kinase, cAMP-dependent,	-0.021	0.986	9.06E-01	9.06E+01		
8161618	NM_001163	APBA1	amyloid beta (A4) precursor prote	0.006	1.004	9.75E-01	9.75E+01		
8161632	NM_00109966	PTAR1	protein prenyltransferase alpha su	0.007	1.005	9.81E-01	9.81E+01		
8161642	---	---	---	0.038	1.027	7.46E-01	7.46E+01		
8161648	NM_001206	KLF9	Kruppel-like factor 9	0.048	1.034	6.59E-01	6.59E+01		
8161693	---	---	---	-0.035	0.976	8.31E-01	8.31E+01		
8161701	NM_013390	TMEM2	transmembrane protein 2	-0.226	0.855	2.50E-02	2.50E+00		
8161727	NM_016014	FAM108B1	family with sequence similarity 10	-0.063	0.957	6.47E-01	6.47E+01		
8161747	NM_00110242	ZFAND5	zinc finger, AN1-type domain 5	-0.119	0.921	6.02E-01	6.02E+01		
8161824	NM_017998	C9orf40	chromosome 9 open reading fram	-0.046	0.968	7.27E-01	7.27E+01		
8161829	BC034033	C9orf41	chromosome 9 open reading fram	0.048	1.034	7.63E-01	7.63E+01		
8161852	---	---	---	-0.252	0.840	2.99E-01	2.99E+01		
8161857	NM_018339	RFK	riboflavin kinase	0.040	1.028	8.29E-01	8.29E+01		
8161865	NM_015225	PRUNE2	prune homolog 2 (Drosophila)	-0.042	0.971	8.01E-01	8.01E+01		
8161906	NM_002072	GNAQ	guanine nucleotide binding protei	0.156	1.115	5.89E-01	5.89E+01		
8161919	NM_005077	TLE1	transducin-like enhancer of split 1	0.086	1.062	7.10E-01	7.10E+01		
8161938	---	---	---	-0.005	0.997	9.88E-01	9.88E+01		
8161943	BC047037	LOC644714	hypothetical protein LOC644714	0.052	1.037	9.49E-01	9.49E+01		
8161964	NM_174938	FRMD3	FERM domain containing 3	0.108	1.078	3.03E-01	3.03E+01		
8161988	NM_013438	UBQLN1	ubiquilin 1	0.025	1.018	8.18E-01	8.18E+01		
8162019	NM_017576	KIF27	kinesin family member 27	-0.059	0.960	8.27E-01	8.27E+01		
8162047	NM_002140	HNRNPK	heterogeneous nuclear ribonucle	-0.003	0.998	9.85E-01	9.85E+01		
8162059	NM_022127	SLC28A3	solute carrier family 28 (sodium-c	0.340	1.265	3.00E-01	3.00E+01		
8162082	---	---	---	0.024	1.017	9.44E-01	9.44E+01		
8162086	NM_015239	AGTPBP1	ATP/GTP binding protein 1	0.065	1.046	6.59E-01	6.59E+01		
8162117	NM_016548	GOLM1	golgi membrane protein 1	-0.001	1.000	9.98E-01	9.98E+01		
8162140	---	---	---	-0.013	0.991	9.64E-01	9.64E+01		
8162142	NM_030940	ISCA1	iron-sulfur cluster assembly 1 hom	0.048	1.033	8.17E-01	8.17E+01		
8162147	NM_024617	ZCCHC6	zinc finger, CCHC domain containi	0.025	1.017	8.73E-01	8.73E+01		
8162177	---	---	---	0.000	1.000	9.99E-01	9.99E+01		
8162179	NM_002048	GAS1	growth arrest-specific 1	-0.094	0.937	2.75E-01	2.75E+01		
8162194	NM_178432	CCRK	cell cycle related kinase	-0.030	0.979	8.08E-01	8.08E+01		
8162214	NM_00114512	// FAM75C1 //	family with sequence similarity 75	-0.035	0.976	7.51E-01	7.51E+01		
8162216	NM_016848	SHC3	SHC (Src homology 2 domain cont	-0.082	0.944	2.84E-01	2.84E+01		
8162231	NM_00114228	SEMA4D	sema domain, immunoglobulin do	0.055	1.039	7.26E-01	7.26E+01		
8162236	NM_006378	SEMA4D	sema domain, immunoglobulin do	0.029	1.020	8.67E-01	8.67E+01		
8162247	---	---	---	-0.019	0.987	8.91E-01	8.91E+01		
8162254	NM_017594	DIRAS2	DIRAS family, GTP-binding RAS-like	0.087	1.062	4.37E-01	4.37E+01		
8162260	---	---	---	-0.060	0.959	8.18E-01	8.18E+01		
8162264	NM_001698	AUH	AU RNA binding protein/enoyl-Co	0.001	1.001	9.96E-01	9.96E+01		
8162276	NM_005384	NFIL3	nuclear factor, interleukin 3 regula	-0.142	0.906	5.97E-01	5.97E+01		
8162294	NM_006415	SPTLC1	serine palmitoyltransferase, long c	0.070	1.050	6.80E-01	6.80E+01		
8162313	NM_013417	IARS	isoleucyl-tRNA synthetase	-0.064	0.957	7.15E-01	7.15E+01		
8162352	NR_024020	NOL8	nucleolar protein 8	0.033	1.023	8.18E-01	8.18E+01		
8162421	NM_022755	IPPK	inositol 1,3,4,5,6-pentakisphospha	-0.051	0.966	6.66E-01	6.66E+01		
8162438	NM_00100380	BICD2	bicaudal D homolog 2 (Drosophila	-0.007	0.995	9.71E-01	9.71E+01		
8162449	NM_031486	ZNF484	zinc finger protein 484	-0.033	0.977	9.11E-01	9.11E+01		
8162455	NM_004148	NINJ1	ninjurin 1	-0.124	0.918	1.78E-01	1.78E+01		
8162462	NM_014612	FAM120A	family with sequence similarity 12	0.076	1.054	6.75E-01	6.75E+01		
8162466	NM_198841	FAM120AOS	family with sequence similarity 12	-0.056	0.962	6.12E-01	6.12E+01		
8162472	NM_021570	BARX1	BARX homeobox 1	-0.009	0.994	9.60E-01	9.60E+01		
8162479	NM_017561	FAM22F	family with sequence similarity 22	-0.026	0.982	8.41E-01	8.41E+01		
8162490	NM_032558	HIATL1	hippocampus abundant transcript	0.057	1.041	7.42E-01	7.42E+01		
8162529	---	---	---	0.054	1.038	8.97E-01	8.97E+01		
8162531	---	---	---	-0.454	0.730	1.57E-01	1.57E+01		
8162533	NM_00108360	PTCH1	patched homolog 1 (Drosophila)	0.056	1.039	7.41E-01	7.41E+01		
8162562	NR_023389	C9orf130	chromosome 9 open reading fram	0.022	1.016	9.09E-01	9.09E+01		

8162586	NM_007001	SLC35D2	solute carrier family 35, member D	0.073	1.052	7.83E-01	7.83E+01		
8162601	NM_153695	ZNF367	zinc finger protein 367	0.013	1.009	9.53E-01	9.53E+01		
8162610	NM_033331	CDC14B	CDC14 cell division cycle 14 homol	-0.007	0.995	9.73E-01	9.73E+01		
8162631	NM_014930	ZNF510	zinc finger protein 510	-0.010	0.993	9.68E-01	9.68E+01		
8162645	NR_002894	HIATL2	hippocampus abundant transcript	-0.059	0.960	6.83E-01	6.83E+01		
8162664	AY358791	UNQ5836	HSAL5836	-0.160	0.895	6.07E-02	6.07E+00		
8162696	NR_027302	XPA	xeroderma pigmentosum, comple	0.009	1.007	9.62E-01	9.62E+01		
8162706	NM_016481	C9orf156	chromosome 9 open reading fram	-0.058	0.960	6.99E-01	6.99E+01		
8162729	NM_014788	TRIM14	tripartite motif-containing 14	0.037	1.026	7.92E-01	7.92E+01		
8162759	NM_018421	TBC1D2	TBC1 domain family, member 2	-0.075	0.949	7.81E-01	7.81E+01		
8162777	NM_005458	GABBR2	gamma-aminobutyric acid (GABA)	0.047	1.033	6.51E-01	6.51E+01		
8162803	NM_173551	ANKS6	ankyrin repeat and sterile alpha m	0.036	1.025	7.68E-01	7.68E+01		
8162823	---	---	---	-0.103	0.931	6.35E-01	6.35E+01		
8162825	---	---	---	-0.071	0.952	5.91E-01	5.91E+01		
8162827	NR_024532	ALG2	asparagine-linked glycosylation 2,	-0.002	0.999	9.89E-01	9.89E+01		
8162833	NM_015051	ERP44	endoplasmic reticulum protein 44	0.254	1.192	5.88E-02	5.88E+00		
8162848	---	---	---	0.002	1.002	9.91E-01	9.91E+01		
8162850	NM_017746	TEX10	testis expressed 10	-0.015	0.990	9.60E-01	9.60E+01		
8162868	---	---	---	-0.027	0.982	8.50E-01	8.50E+01		
8162880	NM_019051	MRPL50	mitochondrial ribosomal protein L	0.077	1.055	7.39E-01	7.39E+01		
8162920	NM_147180	PPP3R2	protein phosphatase 3 (formerly 2	-0.018	0.987	9.27E-01	9.27E+01		
8162940	NM_005502	ABCA1	ATP-binding cassette, sub-family A	-0.039	0.973	9.64E-01	9.64E+01		
8162998	---	---	---	-0.061	0.959	7.00E-01	7.00E+01		
8163000	---	---	---	-0.081	0.945	7.67E-01	7.67E+01		
8163002	NM_004235	KLF4	Kruppel-like factor 4 (gut)	-0.010	0.993	9.68E-01	9.68E+01		
8163013	---	---	---	0.073	1.052	5.40E-01	5.40E+01		
8163015	---	---	---	-0.020	0.986	9.27E-01	9.27E+01		
8163019	NM_006686	ACTL7B	actin-like 7B	0.000	1.000	1.00E+00	1.00E+02		
8163023	NM_003640	IKBKAP	inhibitor of kappa light polypeptid	0.004	1.002	9.87E-01	9.87E+01		
8163086	NM_032012	C9orf5	chromosome 9 open reading fram	-0.084	0.944	4.70E-01	4.70E+01		
8163105	---	---	---	-0.061	0.959	9.07E-01	9.07E+01		
8163107	---	---	---	0.118	1.085	7.35E-01	7.35E+01		
8163109	NM_014334	C9orf4	chromosome 9 open reading fram	-0.012	0.992	9.50E-01	9.50E+01		
8163147	---	---	---	-0.031	0.979	8.13E-01	8.13E+01		
8163257	NM_057159	LPAR1	lysophosphatidic acid receptor 1	0.163	1.119	7.29E-01	7.29E+01		
8163271	---	---	---	0.137	1.100	4.55E-01	4.55E+01		
8163275	NM_00108039	KIAA0368	KIAA0368	-0.053	0.964	6.89E-01	6.89E+01		
8163326	---	---	---	-0.030	0.979	9.55E-01	9.55E+01		
8163383	NM_022486	SUSD1	sushi domain containing 1	0.083	1.059	8.16E-01	8.16E+01		
8163402	NM_005156	ROD1	ROD1 regulator of differentiation 1	0.030	1.021	8.05E-01	8.05E+01		
8163424	---	---	---	0.196	1.146	3.04E-01	3.04E+01		
8163426	---	---	---	0.006	1.004	9.81E-01	9.81E+01		
8163428	AK000637	C9orf80	chromosome 9 open reading fram	0.050	1.035	8.05E-01	8.05E+01		
8163437	NM_033051	SLC46A2	solute carrier family 46, member 2	0.012	1.008	9.43E-01	9.43E+01		
8163481	NM_139286	CDC26	cell division cycle 26 homolog (S. c	0.035	1.025	8.57E-01	8.57E+01		
8163485	NM_145051	RNF183	ring finger protein 183	-0.022	0.985	9.07E-01	9.07E+01		
8163491	NM_00101236	WDR31	WD repeat domain 31	-0.052	0.964	6.68E-01	6.68E+01		
8163505	NM_031219	HDHD3	haloacid dehalogenase-like hydrol	-0.083	0.944	4.04E-01	4.04E+01		
8163509	NM_000031	ALAD	aminolevulinate, delta-, dehydrata	0.054	1.038	6.59E-01	6.59E+01		
8163525	NR_027261	POLE3	polymerase (DNA directed), epsilo	0.023	1.016	8.89E-01	8.89E+01		
8163533	AK056275	FLJ31713	hypothetical protein FLJ31713	-0.026	0.982	8.79E-01	8.79E+01		
8163535	NM_001633	AMBP	alpha-1-microglobulin/bikunin pre	-0.038	0.974	8.33E-01	8.33E+01		
8163548	NM_138424	KIF12	kinesin family member 12	0.008	1.006	9.58E-01	9.58E+01		
8163569	NM_030767	AKNA	AT-hook transcription factor	-0.080	0.946	3.43E-01	3.43E+01		
8163599	NM_015404	DFNB31	deafness, autosomal recessive 31	-0.038	0.974	7.68E-01	7.68E+01		
8163616	---	---	---	0.264	1.200	7.63E-01	7.63E+01		
8163618	NM_005118	TNFSF15	tumor necrosis factor (ligand) supe	-0.111	0.926	6.05E-01	6.05E+01		
8163629	NM_001244	TNFSF8	tumor necrosis factor (ligand) supe	-0.069	0.953	8.39E-01	8.39E+01		
8163678	NM_198186	ASTN2	astrotactin 2	-0.023	0.984	8.04E-01	8.04E+01		
8163710	NR_003708	SNORA70C	small nucleolar RNA, H/ACA box 70	0.068	1.048	7.36E-01	7.36E+01		
8163714	---	---	---	0.009	1.006	9.81E-01	9.81E+01		
8163729	---	---	---	-0.004	0.997	9.86E-01	9.86E+01		
8163731	---	---	---	-0.054	0.963	8.05E-01	8.05E+01		
8163733	NM_018249	CDK5RAP2	CDK5 regulatory subunit associate	-0.086	0.942	4.54E-01	4.54E+01		
8163775	NM_00108049	MEGF9	multiple EGF-like-domains 9	-0.095	0.937	7.73E-01	7.73E+01		
8163784	NM_012164	FBXW2	F-box and WD repeat domain cont	0.026	1.018	9.09E-01	9.09E+01		
8163795	NM_005047	PSMD5	proteasome (prosome, macropain	0.056	1.040	7.50E-01	7.50E+01		
8163807	NM_015651	PHF19	PHD finger protein 19	-0.055	0.963	6.58E-01	6.58E+01		
8163825	NM_005658	TRAF1	TNF receptor-associated factor 1	-0.310	0.807	5.85E-03	5.85E-01		
8163882	NM_016322	RAB14	RAB14, member RAS oncogene fa	0.125	1.090	7.99E-02	7.99E+00		
8163892	AF220263	C9orf31	chromosome 9 open reading fram	0.248	1.187	4.83E-02	4.83E+00		
8163896	NM_004099	STOM	stomatin	0.236	1.177	3.50E-01	3.50E+01		
8163916	NM_00113944	TTLL11	tubulin tyrosine ligase-like family,	-0.068	0.954	4.16E-01	4.16E+01		
8163930	NM_014222	NDUFA8	NADH dehydrogenase (ubiquinone	-0.097	0.935	6.51E-01	6.51E+01		
8163936	NM_014368	LHX6	LIM homeobox 6	0.052	1.037	7.09E-01	7.09E+01		
8163948	NR_027125	RBM18	RNA binding motif protein 18	0.062	1.044	6.91E-01	6.91E+01		
8163960	NM_00100445	OR1L8	olfactory receptor, family 1, subfa	0.028	1.020	8.42E-01	8.42E+01		

8163962	NM_00100445	OR1B1	olfactory receptor, family 1, subfa	0.046	1.033	7.10E-01	7.10E+01		
8163964	NM_005388	PDCL	phosducin-like	-0.059	0.960	7.73E-01	7.73E+01		
8163972	NM_00110058	RC3H2	ring finger and CCCH-type zinc fing	0.030	1.021	8.77E-01	8.77E+01		
8163999	NM_006626	ZBTB6	zinc finger and BTB domain contai	-0.042	0.972	7.13E-01	7.13E+01		
8164006	---	---	---	0.237	1.179	3.72E-01	3.72E+01		
8164008	NR_026677	C9orf45	chromosome 9 open reading fram	-0.034	0.977	9.05E-01	9.05E+01		
8164013	NM_018387	STRBP	spermatid perinuclear RNA binding	0.062	1.044	7.92E-01	7.92E+01		
8164034	NM_020946	DENND1A	DENN/MADD domain containing 1	0.138	1.100	2.74E-01	2.74E+01		
8164060	---	---	---	-0.055	0.962	7.68E-01	7.68E+01		
8164062	AY567967	LOC613206	myeloproliferative disease associa	0.108	1.077	4.29E-01	4.29E+01		
8164067	NM_002799	PSMB7	proteasome (prosome, macropain	-0.137	0.909	3.40E-01	3.40E+01		
8164077	NM_004959	NR5A1	nuclear receptor subfamily 5, grou	0.031	1.022	8.49E-01	8.49E+01		
8164100	NM_007209	RPL35	ribosomal protein L35	0.001	1.001	9.98E-01	9.98E+01		
8164105	NM_002077	GOLGA1	golgi autoantigen, golgin subfamil	0.043	1.030	8.04E-01	8.04E+01		
8164129	---	---	---	0.060	1.043	8.81E-01	8.81E+01		
8164131	NM_173690	SCAI	suppressor of cancer cell invasion	0.011	1.008	9.71E-01	9.71E+01		
8164153	---	---	---	-0.054	0.963	8.52E-01	8.52E+01		
8164155	NM_00112335	PPP6C	protein phosphatase 6, catalytic su	0.033	1.023	7.75E-01	7.75E+01		
8164165	NM_005347	HSPA5	heat shock 70kDa protein 5 (gluco	0.235	1.177	1.46E-01	1.46E+01		
8164177	NM_00100661	MAPKAP1	mitogen-activated protein kinase a	0.037	1.026	8.29E-01	8.29E+01		
8164200	NM_012098	ANGPTL2	angiopoietin-like 2	-0.039	0.973	6.70E-01	6.70E+01		
8164210	NM_000976	RPL12	ribosomal protein L12	0.115	1.083	6.11E-01	6.11E+01		
8164215	NR_002449	SNORA65	small nucleolar RNA, H/ACA box 6	-0.095	0.936	5.98E-01	5.98E+01		
8164217	NM_022833	FAM129B	family with sequence similarity 12	-0.051	0.965	7.52E-01	7.52E+01		
8164235	NM_00100291	PTRH1	peptidyl-tRNA hydrolase 1 homolo	-0.001	1.000	9.98E-01	9.98E+01		
8164243	NM_130459	TOR2A	torsin family 2, member A	-0.064	0.957	5.08E-01	5.08E+01		
8164252	NM_170600	SH2D3C	SH2 domain containing 3C	-0.018	0.988	9.20E-01	9.20E+01		
8164269	NM_000118	ENG	endoglin	0.137	1.099	4.51E-01	4.51E+01		
8164293	NM_000476	AK1	adenylate kinase 1	-0.186	0.879	3.67E-01	3.67E+01		
8164304	NM_013443	ST6GALNAC6	ST6 (alpha-N-acetyl-neuraminyl-2,	-0.168	0.890	1.60E-01	1.60E+01		
8164314	NM_175039	ST6GALNAC4	ST6 (alpha-N-acetyl-neuraminyl-2,	0.025	1.017	8.58E-01	8.58E+01		
8164336	NM_003863	DPM2	dolichyl-phosphate mannosyltrans	0.008	1.006	9.75E-01	9.75E+01		
8164343	NM_00103525	FAM102A	family with sequence similarity 10	-0.291	0.817	3.33E-02	3.33E+00		
8164362	NM_025072	PTGES2	prostaglandin E synthase 2	-0.052	0.964	5.23E-01	5.23E+01		
8164373	NM_012127	CIZ1	CDKN1A interacting zinc finger pro	-0.041	0.972	5.73E-01	5.73E+01		
8164398	NM_004486	GOLGA2	golgi autoantigen, golgin subfamil	-0.093	0.938	2.19E-01	2.19E+01		
8164428	NM_015679	TRUB2	TruB pseudouridine (psi) synthase	-0.110	0.927	2.34E-01	2.34E+01		
8164440	NM_052844	WDR34	WD repeat domain 34	-0.025	0.983	8.48E-01	8.48E+01		
8164452	NM_032799	ZDHHC12	zinc finger, DHHC-type containing	-0.009	0.994	9.56E-01	9.56E+01		
8164464	NM_006336	ZER1	zer-1 homolog (C. elegans)	-0.185	0.880	1.37E-03	1.37E-01		
8164481	NM_016390	C9orf114	chromosome 9 open reading fram	-0.006	0.996	9.68E-01	9.68E+01		
8164496	NM_004059	CCBL1	cysteine conjugate-beta lyase, cyt	-0.074	0.950	5.06E-01	5.06E+01		
8164515	NM_014908	DOLK	dolichol kinase	0.047	1.033	7.25E-01	7.25E+01		
8164521	NM_020145	SH3GLB2	SH3-domain GRB2-like endophilin	-0.046	0.968	7.34E-01	7.34E+01		
8164535	NM_000755	CRAT	carnitine acetyltransferase	-0.084	0.944	2.84E-01	2.84E+01		
8164554	NM_203434	IER5L	immediate early response 5-like	-0.052	0.964	4.43E-01	4.43E+01		
8164560	---	---	---	0.025	1.018	8.41E-01	8.41E+01		
8164562	NM_199350	C9orf50	chromosome 9 open reading fram	-0.055	0.963	6.23E-01	6.23E+01		
8164572	NM_017873	ASB6	ankyrin repeat and SOCS box-cont	-0.002	0.998	9.89E-01	9.89E+01		
8164580	NM_004878	PTGES	prostaglandin E synthase	-0.223	0.857	2.47E-02	2.47E+00		
8164587	NM_000113	TOR1A	torsin family 1, member A (torsin A	0.092	1.066	5.52E-01	5.52E+01		
8164596	NM_016520	C9orf78	chromosome 9 open reading fram	-0.045	0.969	7.72E-01	7.72E+01		
8164607	NM_015033	FNBP1	formin binding protein 1	-0.085	0.943	1.29E-01	1.29E+01		
8164627	ENST00000358	FLJ46836	FLJ46836 protein	-0.067	0.955	4.32E-01	4.32E+01		
8164630	NM_198180	QRFP	pyroglutamylated RFamide peptid	-0.033	0.977	8.06E-01	8.06E+01		
8164632	NM_032843	FIBCD1	fibrinogen C domain containing 1	-0.041	0.972	7.03E-01	7.03E+01		
8164644	NM_033387	FAM78A	family with sequence similarity 78	0.135	1.098	2.09E-01	2.09E+01		
8164649	NM_013318	BAT2L	HLA-B associated transcript 2-like	-0.048	0.967	7.54E-01	7.54E+01		
8164653	NM_031432	UCK1	uridine-cytidine kinase 1	-0.120	0.920	3.38E-01	3.38E+01		
8164665	NM_005312	RAPGEF1	Rap guanine nucleotide exchange	0.125	1.091	4.50E-01	4.50E+01		
8164692	AF467069	RAPGEF1	Rap guanine nucleotide exchange	0.091	1.065	7.51E-01	7.51E+01		
8164694	---	---	---	-0.039	0.973	8.92E-01	8.92E+01		
8164696	---	---	---	-0.116	0.923	2.49E-01	2.49E+01		
8164698	NM_004269	MED27	mediator complex subunit 27	-0.012	0.992	9.76E-01	9.76E+01		
8164701	NM_015046	SETX	senataxin	0.100	1.072	2.02E-01	2.02E+01		
8164742	NM_022779	DDX31	DEAD (Asp-Glu-Ala-Asp) box polyp	-0.003	0.998	9.89E-01	9.89E+01		
8164766	NM_152572	C9orf98	chromosome 9 open reading fram	-0.191	0.876	3.21E-02	3.21E+00		
8164781	NM_000368	TSC1	tuberous sclerosis 1	0.056	1.040	7.12E-01	7.12E+01		
8164808	---	---	---	-0.018	0.988	9.27E-01	9.27E+01		
8164810	NM_006266	RALGDS	ral guanine nucleotide dissociation	-0.033	0.978	8.74E-01	8.74E+01		
8164833	NM_021996	GBGT1	globoside alpha-1,3-N-acetylgalact	0.017	1.012	9.04E-01	9.04E+01		
8164848	---	---	---	0.091	1.065	6.66E-01	6.66E+01		
8164853	NM_020469	ABO	ABO blood group (transferase A, a	-0.108	0.928	1.71E-01	1.71E+01		
8164862	NM_006753	SURF6	surfeit 6	-0.066	0.955	5.53E-01	5.53E+01		
8164870	---	---	---	-0.006	0.996	9.87E-01	9.87E+01		
8164872	NM_181491	MED22	mediator complex subunit 22	-0.033	0.977	8.67E-01	8.67E+01		
8164883	NM_033161	SURF4	surfeit 4	0.028	1.019	9.12E-01	9.12E+01		

8164896	NM_003172	SURF1	surfeit 1	-0.020	0.986	9.24E-01	9.24E+01		
8164907	NM_020385	REXO4	REX4, RNA exonuclease 4 homolog	0.060	1.042	7.45E-01	7.45E+01		
8164918	NM_017585	SLC2A6	solute carrier family 2 (facilitated g	-0.016	0.989	9.41E-01	9.41E+01		
8164931	NM_00108048	TMEM8C	transmembrane protein 8C	-0.020	0.986	8.89E-01	8.89E+01		
8164937	BC146946	FAM163B	family with sequence similarity 16	-0.027	0.981	8.00E-01	8.00E+01		
8164940	NM_00113470	SARDH	sarcosine dehydrogenase	0.009	1.006	9.53E-01	9.53E+01		
8164967	NM_00113439	VAV2	vav 2 guanine nucleotide exchange	-0.121	0.920	5.06E-01	5.06E+01		
8164995	NM_007371	BRD3	bromodomain containing 3	-0.061	0.959	4.62E-01	4.62E+01		
8165021	---	---	---	-0.009	0.993	9.53E-01	9.53E+01		
8165024	NM_00104826	C9orf116	chromosome 9 open reading fram	-0.066	0.955	5.67E-01	5.67E+01		
8165038	NM_00101241	SOHLH1	spermatogenesis and oogenesis sp	-0.093	0.938	3.32E-01	3.32E+01		
8165046	NM_015447	CAMSAP1	calmodulin regulated spectrin-asso	-0.043	0.971	8.15E-01	8.15E+01		
8165064	NM_016172	UBAC1	UBA domain containing 1	0.159	1.116	2.11E-02	2.11E+00		
8165077	NM_144653	NACC2	NACC family member 2, BEN and B	0.096	1.069	3.70E-01	3.70E+01		
8165083	NM_178138	LHX3	LIM homeobox 3	-0.003	0.998	9.85E-01	9.85E+01		
8165094	NM_181701	QSOX2	quiescin Q6 sulfhydryl oxidase 2	0.043	1.030	8.07E-01	8.07E+01		
8165107	NM_052813	CARD9	caspase recruitment domain famil	-0.073	0.950	3.95E-01	3.95E+01		
8165131	NM_003086	SNAPC4	small nuclear RNA activating comp	-0.026	0.982	8.31E-01	8.31E+01		
8165156	NM_00103970	SDCCAG3	serologically defined colon cancer	-0.004	0.997	9.85E-01	9.85E+01		
8165171	NM_019892	INPP5E	inositol polyphosphate-5-phospha	-0.051	0.965	5.89E-01	5.89E+01		
8165183	NM_014866	SEC16A	SEC16 homolog A (S. cerevisiae)	-0.055	0.963	5.68E-01	5.68E+01		
8165217	NM_017617	NOTCH1	Notch homolog 1, translocation-as	-0.080	0.946	4.21E-01	4.21E+01		
8165255	---	---	---	0.009	1.006	9.54E-01	9.54E+01		
8165258	NM_006412	AGPAT2	1-acylglycerol-3-phosphate O-acyl	-0.010	0.993	9.55E-01	9.55E+01		
8165271	NM_00100171	LCN10	lipocalin 10	-0.028	0.981	8.89E-01	8.89E+01		
8165277	NM_198946	LCN6	lipocalin 6	0.037	1.026	7.42E-01	7.42E+01		
8165285	NM_178469	LCN8	lipocalin 8	-0.059	0.960	7.38E-01	7.38E+01		
8165295	ENST00000392	LCN8	lipocalin 8	-0.130	0.914	2.78E-01	2.78E+01		
8165309	NM_003792	EDF1	endothelial differentiation-related	-0.012	0.991	9.53E-01	9.53E+01		
8165319	NM_018998	FBXW5	F-box and WD repeat domain cont	-0.040	0.973	7.52E-01	7.52E+01		
8165334	NM_004669	CLIC3	chloride intracellular channel 3	-0.044	0.970	7.13E-01	7.13E+01		
8165345	NM_001606	ABCA2	ATP-binding cassette, sub-family A	-0.113	0.925	1.44E-01	1.44E+01		
8165398	NM_004479	FUT7	fucosyltransferase 7 (alpha (1,3) fu	0.013	1.009	9.44E-01	9.44E+01		
8165406	NM_015392	NPDC1	neural proliferation, differentiatio	-0.035	0.976	7.78E-01	7.78E+01		
8165416	NM_203468	ENTPD2	ectonucleoside triphosphate dipho	-0.059	0.960	5.09E-01	5.09E+01		
8165430	NM_178448	C9orf140	chromosome 9 open reading fram	0.021	1.015	8.98E-01	8.98E+01		
8165438	NM_013379	DPP7	dipeptidyl-peptidase 7	-0.051	0.966	5.56E-01	5.56E+01		
8165453	NM_00101365	LRRC26	leucine rich repeat containing 26	-0.053	0.964	5.08E-01	5.08E+01		
8165458	---	---	---	0.030	1.021	8.99E-01	8.99E+01		
8165462	NM_013366	ANAPC2	anaphase promoting complex sub	-0.013	0.991	9.33E-01	9.33E+01		
8165478	NM_173691	C9orf75	chromosome 9 open reading fram	0.005	1.003	9.78E-01	9.78E+01		
8165486	NM_053045	TMEM203	transmembrane protein 203	-0.023	0.984	8.77E-01	8.77E+01		
8165492	NM_031297	RNF208	ring finger protein 208	-0.105	0.930	8.12E-02	8.12E+00		
8165496	NM_006088	TUBB2C	tubulin, beta 2C	-0.216	0.861	3.68E-01	3.68E+01		
8165498	NM_00100171	FAM166A	family with sequence similarity 16	-0.046	0.969	6.68E-01	6.68E+01		
8165508	NM_00100435	NRARP	NOTCH-regulated ankyrin repeat p	-0.014	0.990	9.30E-01	9.30E+01		
8165512	NM_017820	EXD3	exonuclease 3'-5' domain containi	-0.051	0.966	4.91E-01	4.91E+01		
8165538	NM_00103311	ENTPD8	ectonucleoside triphosphate dipho	-0.017	0.988	9.06E-01	9.06E+01		
8165552	NM_00113096	NELF	nasal embryonic LHRH factor	-0.011	0.993	9.61E-01	9.61E+01		
8165575	NM_00109853	PNPLA7	patatin-like phospholipase domain	0.003	1.002	9.84E-01	9.84E+01		
8165610	NM_138778	WDR85	WD repeat domain 85	-0.109	0.927	1.76E-01	1.76E+01		
8165622	NM_138462	ZMYND19	zinc finger, MYND-type containing	-0.050	0.966	7.87E-01	7.87E+01		
8165630	NM_032937	C9orf37	chromosome 9 open reading fram	-0.081	0.946	6.66E-01	6.66E+01		
8165637	NM_031297	RNF208	ring finger protein 208	-0.105	0.930	8.12E-02	8.12E+00		
8165639	BC052297	C9orf169	chromosome 9 open reading fram	0.015	1.011	9.09E-01	9.09E+01		
8165642	NM_053045	TMEM203	transmembrane protein 203	-0.031	0.979	9.10E-01	9.10E+01		
8165644	---	---	---	-0.061	0.959	5.48E-01	5.48E+01		
8165646	---	---	---	0.114	1.082	9.00E-01	9.00E+01		
8165648	AK290098	C7orf11	chromosome 7 open reading fram	-0.072	0.951	4.99E-01	4.99E+01		
8165650	---	---	---	0.007	1.005	9.64E-01	9.64E+01		
8165653	AK293612	HNRNPM	heterogeneous nuclear ribonucleo	-0.039	0.973	5.99E-01	5.99E+01		
8165656	---	---	---	-0.138	0.909	6.80E-01	6.80E+01		
8165658	U18810	VIPR2	vasoactive intestinal peptide recep	0.253	1.191	3.94E-01	3.94E+01		
8165661	---	---	---	0.058	1.041	2.03E-01	2.03E+01		
8165665	---	---	---	-0.012	0.992	9.09E-01	9.09E+01		
8165667	---	---	---	-0.042	0.971	8.97E-01	8.97E+01		
8165669	AB064665	LOC440552	OK/SW-cl.16	-0.009	0.994	9.53E-01	9.53E+01		
8165672	L23320	RFC1	replication factor C (activator 1) 1,	0.063	1.045	9.11E-01	9.11E+01		
8165674	AY423734	SH3KBP1	SH3-domain kinase binding protei	-0.003	0.998	9.81E-01	9.81E+01		
8165676	---	---	---	0.009	1.006	9.53E-01	9.53E+01		
8165680	---	---	---	0.218	1.163	7.65E-01	7.65E+01		
8165682	---	---	---	0.169	1.124	7.57E-01	7.57E+01		
8165684	---	---	---	0.181	1.134	8.02E-01	8.02E+01		
8165686	---	---	---	-0.002	0.999	9.89E-01	9.89E+01		
8165690	---	---	---	-0.012	0.991	8.92E-01	8.92E+01		
8165692	---	---	---	-0.128	0.915	7.97E-01	7.97E+01		
8165694	---	---	---	0.060	1.043	8.78E-01	8.78E+01		

8165696	---	---	---	0.189	1.140	7.09E-01	7.09E+01		
8165698	---	---	---	0.288	1.221	4.48E-01	4.48E+01		
8165700	---	---	---	0.119	1.086	6.80E-01	6.80E+01		
8165703	AF284753	UIMC1	ubiquitin interaction motif contair	0.033	1.023	8.45E-01	8.45E+01		
8165705	---	---	---	0.004	1.003	9.81E-01	9.81E+01		
8165707	BC028919	TOB2	transducer of ERBB2, 2	0.012	1.009	9.06E-01	9.06E+01		
8165709	---	---	---	-0.108	0.928	7.64E-01	7.64E+01		
8165711	NM_018390	PLCXD1	phosphatidylinositol-specific phos	0.053	1.038	7.53E-01	7.53E+01		
8165735	NM_00116153	CSF2RA	colony stimulating factor 2 recepto	-0.194	0.874	4.61E-01	4.61E+01		
8165752	NM_002183	IL3RA	interleukin 3 receptor, alpha (low	-0.283	0.822	6.30E-01	6.30E+01		
8165765	NR_026711	NCRNA00105	non-protein coding RNA 105	-0.056	0.962	6.64E-01	6.64E+01		
8165773	NR_027383	SFRS17A	splicing factor, arginine/serine-ricl	-0.023	0.984	9.06E-01	9.06E+01		
8165794	NM_002414	CD99	CD99 molecule	-0.049	0.967	7.94E-01	7.94E+01		
8165808	NM_00114191	XG	Xg blood group	-0.013	0.991	9.49E-01	9.49E+01		
8165833	NM_00101171	ARSH	arylsulfatase family, member H	-0.010	0.993	9.55E-01	9.55E+01		
8165856	---	---	---	-0.080	0.946	9.33E-01	9.33E+01		
8165881	NM_013452	VCX	variable charge, X-linked	-0.008	0.994	9.53E-01	9.53E+01		
8165890	NM_00100188	VCX3B	variable charge, X-linked 3B	0.010	1.007	9.44E-01	9.44E+01		
8165903	---	---	---	0.067	1.047	5.78E-01	5.78E+01		
8165911	NM_005647	TBL1X	transducin (beta)-like 1X-linked	-0.003	0.998	9.88E-01	9.88E+01		
8165947	NM_015691	WWC3	WWC family member 3	0.058	1.041	6.95E-01	6.95E+01		
8165995	NM_005333	HCCS	holocytochrome c synthase (cytoc	0.115	1.083	3.27E-01	3.27E+01		
8166015	NM_006800	MSL3	male-specific lethal 3 homolog (Dr	-0.033	0.977	8.98E-01	8.98E+01		
8166049	NM_00103909	PRPS2	phosphoribosyl pyrophosphate syı	0.152	1.111	1.67E-01	1.67E+01		
8166065	NM_138636	TLR8	toll-like receptor 8	0.834	1.783	6.64E-02	6.64E+00		
8166072	NM_021109	TMSB4X	thymosin beta 4, X-linked	-0.016	0.989	8.44E-01	8.44E+01		
8166098	NM_004251	RAB9A	RAB9A, member RAS oncogene fa	-0.013	0.991	9.61E-01	9.61E+01		
[truncated: 102,787 more chars]
