# Supplementary material for: Exposure-Dependent Control of Malaria-Induced Inflammation in Children
Source: PLoS Pathog. 2014 Apr 17;10(4):e1004079. doi: 10.1371/journal.ppat.1004079 (PMC3990727; doi:10.1371/journal.ppat.1004079)
Supplement: Table S4 — Microarray Expression and q-RT-PCR values of selected genes from 18 individuals at healthy baseline and day 7 after the first malaria episode with and without P. falciparum in vitro stimulation. (PDF) [file ppat.1004079.s006.pdf]

Table S4. Microarray expression and qRT-PCR values of selected genes from 18 individuals at heathy baseline and day 7 after the first malria episode with and without *P. falciparum in vitro* stimulation.

sample ID	8131803 MicroArray (IL6)	qRT-PCR (IL6)	8097903MicroArray (TLR2)	qRT-PCR (TLR2)	8054722 MicroArray (IL1B)	qRT-PCR (IL1B)	8100977 MicroArray (CXCL5)	qRT-PCR (CXCL5)	7923907 MicroArray (IL10)	qRT-PCR (IL10)	8108217 MicroArray (TGFB1)	qRT-PCR (TGFB1)
kam030d7	8.389997	-1.283	10.496984	0.15	9.559377	0.885	9.659943	2.323	8.819094	-0.295	9.743152	3.476
kam030d7Pf	8.539314	0.864	8.752682	-1.51	9.4624405	8.664	9.827184	3.000	9.258825	-1.293	9.774952	1.505
kam030HB	8.463279	1.368	9.260496	-0.49	13.545498	7.018	9.742528	3.013	9.284801	-0.776	9.698462	2.798
kam030HBPf	7.8641243	-2.017	11.047403	1.55	8.6120205	2.939	9.484722	2.393	8.6452265	-0.336	9.854697	4.851
kam037d7	8.008485	-1.747	9.027215	0.41	8.710117	-0.482	9.66119	1.286	8.540438	-2.955	9.409943	2.188
kam037d7Pf	8.627278	0.000	8.952916	0.00	10.188317	0.000	10.159607	0.000	9.156057	0.000	9.401285	0.000
kam037HB	8.440537	-1.868	10.409264	1.82	9.351778	0.214	9.703073	2.219	8.832004	-2.279	9.758773	4.254
kam037HBPf	8.587777	1.011	8.920889	-0.97	12.967815	7.298	10.039303	2.463	8.645118	0.315	9.841249	2.826
kam042d7	8.564416	-1.388	10.806799	1.18	9.058263	1.896	9.550266	1.722	8.620294	-0.889	9.451335	3.147
kam042d7Pf	8.693871	0.580	9.176298	-1.40	10.862459	5.096	9.636208	2.862	8.595593	-0.902	10.301153	3.166
kam042HB	8.553246	-2.404	9.826215	1.18	8.993108	0.520	9.793538	3.037	9.108786	-1.358	9.722551	2.931
kam042HBPf	8.368288	-1.943	8.26763		9.358124		10.106576	2.665	8.988258	-2.878	9.4859705	1.569
kam054d7	8.142515	0.000	10.021244	-0.56	8.869347	-0.269	9.301519	3.762	8.622308	-2.662	9.538771	3.129
kam054d7Pf	9.076228	1.848	8.215921	-0.29	13.951735	7.988	9.789044	2.732	8.531186	0.385	9.176298	1.761
kam054HB	7.9809394	-0.519	10.533914	1.62	11.567585	2.880	9.573552	2.087	8.7579565	-0.628	9.796364	4.083
kam054HBPf	7.7485285	-2.184	10.615197	1.60	10.276648	3.770	9.637077	1.332	8.624577	-2.411	9.510427	3.653
kam057d7	8.272676	-1.911	10.953433	1.93	9.408564	1.894	9.773881	1.402	8.549014	-1.404	9.853949	2.283
kam057d7Pf	8.255736	1.067	9.9949045	-1.40	12.30365	5.427	10.225207	1.563	8.627169	0.323	9.619615	2.368
kam057HB	8.41096	-1.699	10.011675	0.93	9.2212	0.931	10.656157	3.263	8.767489	-1.077	9.454423	2.346
kam057HBPf	8.702172	0.895	9.092678	-1.06	13.651221	7.684	10.002913	3.410	9.378078	-0.769	9.490068	2.644
kam058d7	9.023172	-1.565	10.101044	1.10	9.269314	0.900	9.624064	1.755	9.0187845	-1.630	10.026343	2.067
kam058d7Pf	7.9763637	2.058	8.176522	-1.47	12.537708	7.052	10.523032	3.487	9.390512	0.123	9.7572565	2.470
kam058HB	8.682924	-0.821	10.182493	0.11	8.973324	-0.092	10.189516	-3.737	8.693766	-3.458	10.011423	1.703
kam058HBPf	8.523719	-7.966	10.745851	1.91	9.588452	1.926	10.0682	4.419	9.050611	-1.462	9.964991	2.568
kam064d7	8.179511	-0.718	10.008261	-0.81	9.684626	2.788	9.937903	4.457	8.59701	-1.796	9.1687975	1.811
kam064d7Pf	8.746111	-0.897	9.538907	-2.64	10.733507	2.209	9.285518	1.763	8.735894	-2.029	9.523699	0.858
kam064HB	8.296962	-0.966	10.718618	3.10	10.999204	6.134	9.767125	2.357	8.696307	-0.808	9.711306	3.688
kam064HBPf	8.889808	0.003	9.893347	0.77	11.55302	4.157	10.315161	3.578	8.6437845	-0.290	9.629138	3.429
kam068d7	8.684818	0.573	8.751076	-1.43	11.931144	6.625	10.211305	4.012	9.361308	0.379	9.582989	2.157
kam068d7Pf	8.582368	2.676	10.743345	0.16	13.208037	8.900	9.935253	3.043	9.239455	0.614	9.983621	3.725
kam068HB	8.327642	-1.816	10.615179	1.85	10.277509	1.331	9.586239	2.173	9.305629	-2.065	9.684696	3.758
kam068HBPf	8.202711	-0.986	8.51112	-1.43	12.403776	5.945	10.089305	2.049	9.144888	-0.173	9.729451	3.634
kam082d7	13.686429	6.757	11.955083	5.00	14.985527	10.962	12.014729	12.008	10.705546	5.801	9.095924	-1.149
kam082d7Pf	8.479861	-1.120	11.106432	2.60	10.738498	3.371	9.64967	3.652	9.4289465	2.352	10.784577	4.888
kam082HB	14.44331	8.245	11.885777	5.05	15.244987	12.107	12.231179	9.777	10.862598	5.962	8.905236	-0.727
kam082HBPf	13.840788	6.690	11.882108	4.42	15.317382	11.443	13.733613	12.711	10.128652	3.587	9.174776	1.226
kam114d7	11.928973	3.586	10.82316	2.62	14.794178	9.163	13.44797	10.711	9.1016865	1.137	8.93313	0.554
kam114d7Pf	13.826327	6.105	12.014582	4.02	15.101566	10.605	12.9615	9.116	10.16453	3.329	8.904243	0.928
kam114HB	12.821764	5.117	11.845944	4.07	14.697885	9.788	13.614678	11.746	8.907492	1.432	9.777469	2.783
kam114HBPf	13.646471	5.619	11.614383	3.40	15.16828	10.812	13.823689	11.744	10.187636	2.070	9.2084055	0.938
kam119d7	11.593414	3.051	11.118792	2.18	14.377522	8.058	13.165561	10.188	9.190121	1.305	9.533934	2.038
kam119d7Pf	10.063664	0.931	10.853029	1.70	12.893976	5.629	12.43739	8.335	9.178515	0.934	10.079632	3.380
kam119HB	7.9609866	-1.552	10.340395	0.77	10.055621	2.019	10.966145	6.186	9.276938	1.607	11.093609	4.749
kam119HBPf	12.615067	4.215	11.505851	2.52	14.789869	10.807	13.928564	11.781	8.902918	1.642	10.785648	4.683
kam123d7	8.437544	-1.245	10.676654	1.58	11.734531	4.076	12.729362	9.296	9.180034	1.072	10.80035	4.327
kam123d7Pf	10.592307	2.367	12.078284	4.93	13.992567	8.677	12.973012	10.186	8.69801	1.362	10.811198	5.450
kam123HB	8.824354	0.116	10.712243	3.49	10.87651	3.782	10.113742	4.687	8.826739	2.051	10.985998	6.011
kam123HBPf	13.499441	5.926	12.179844	4.86	15.03841	10.485	13.56375	11.255	9.2308855	2.981	9.6921625	2.767
kam125d7	11.597903	2.178	11.338425	1.85	14.618011	8.691	12.638929	9.097	8.836776	0.158	9.790626	3.675
kam125d7Pf	13.447704	5.342	12.102806	4.43	15.121053	10.918	13.257775	10.052	9.72603	3.147	9.008961	2.121
kam125HB	12.134872	4.560	11.970764	5.20	14.955926	10.953	13.22295	11.048	9.754303	3.091	9.550131	3.539
kam125HBPf	13.725585	5.471	11.781249	3.31	15.12394	10.541	13.842038	11.891	9.164001	1.352	9.224966	1.014
kam129d7	13.659231	6.388	10.347854	0.67	14.752181	10.835	12.497513	10.174	9.546354	3.332	8.798213	-1.146
kam129d7Pf	13.240211	4.556	11.277573	2.47	14.361446	7.972	12.065874	8.049	8.70328	1.238	9.313382	2.732
kam129HB	8.181898	-2.531	9.439332	-0.71	9.606831	0.954	10.021965	3.595	8.494136	-1.272	9.230332	1.332
kam129HBPf	8.698392	-0.450	10.5103	1.54	11.34704	5.604	10.052745	9.001	9.387285	1.397	10.329987	3.407
kam130d7	10.96925	3.356	11.713735	4.16	13.246757	7.493	13.702423	12.215	8.721168	2.317	10.99342	5.810
kam130d7Pf	13.451468	6.242	11.361889	3.68	14.843763	10.438	13.947596	12.763	9.350541	2.180	9.418443	2.369
kam130HB	13.704904	6.874	10.478011	2.23	14.955772	11.418	10.506228	6.524	11.112563	5.421	8.24103	-1.474
kam130HBPf	14.451719	7.176	11.217982	3.38	14.851555	11.124	13.075598	11.090	9.150636	2.753	8.745405	-0.986
kam136d7	13.386837	5.327	11.133559	2.71	15.029847	9.933	13.698018	11.234	8.7823105	1.250	8.323145	-0.676
kam136d7Pf	13.862986	6.475	11.326581	3.73	14.870279	12.066	13.059504	10.558	9.503229	2.299	9.176547	-0.054
kam136HB	13.503685	6.589	11.640607	4.11	15.111172	12.268	13.552146	13.258	9.474842	2.251	9.43644	1.987
kam136HBPf	12.488001	4.861	10.92384	3.23	14.866194	9.988	13.118201	10.736	9.056421	1.633	9.054875	1.266
kam168d7	9.074248	1.507	11.705499	5.03	13.227531	8.018	13.20558	11.796	8.990842	2.385	11.752297	7.905
kam168d7Pf	9.136273	0.795	11.16173	3.12	12.487815	6.473	13.322244	11.107	8.930028	1.937	11.796117	6.839
kam168HB	10.838078	3.028	11.291165	3.03	14.745407	11.232	13.055076	11.063	9.398081	2.377	9.467606	2.263
kam168HBPf	8.101083	-1.344	10.597383	3.00	10.14817	2.704	11.497677	8.904	9.754152	3.243	11.910759	8.337
