# Supplementary material for: Exposure-Dependent Control of Malaria-Induced Inflammation in Children
Source: PLoS Pathog. 2014 Apr 17;10(4):e1004079. doi: 10.1371/journal.ppat.1004079 (PMC3990727; doi:10.1371/journal.ppat.1004079)
Supplement: Table S5 — Sequences of primers and probes used for q-RT-PCR validation. (PDF) [file ppat.1004079.s007.pdf]

Table S5. Sequences of primers and probes used for qRT-PCR validation

Gene	Forward Primer	Reverse Primer	Probe
IL1B[1]	AACAGGCTGCTCTGGGATTCT	TGGCGAGCTCAGGTACTTCTG	TTCAGCCAATCTTCATTGCTCAAGTGTCTG
TLR2[1]	CCCATTGCTCTTTCACTGCTT	GCCTGATTGGAGGATTCTTCCT	CCCAAGACCCACACCATCCACAAAGTATG
IL6[1]	TGGATGCTTCCAATCTGGATT	TGCACAGCTCTGGCTTGTTC	CCAGTGATGATTTTCACCAGGCAAGTCTC
TGFBI[1]	CATGGACAGACCCTGGAAACTC	TCCCGTACCTCCCCCTCTT	CCTCTGCATTGAGAACAGCTGCATCG
IL10[1]	CATCAAGGCGCATGTGAACT	ACGGCCTTGCTCTTGTTTTC	ACAGCGCCGTAGCCTCAGCCTGA
CXCL5[1]	TTGATCCAGAAGCCCCTTTTC	CCATGCGTGCTCATTTCTCTT	TCAGTTTTCCTTGTTTCCACCGTCCAAA
SFI1[2]	GCGGGCAGCAGAATTCC	AGGTCTGCTCGCATCAGCTT	CATGGCCCAGCTCCTGCGTTG

[1]Probes were labeled with 6 – Carboxyfluorescein (6-FAM) at the 5'-end and with a non-fluorescent black hole quencher BHQ1 at the 3'-end.
[2]Reference gene probe was labeled with CAL Fluor® Gold540 at the 5'-end and BHQ1 at the 3'-end.
